# UVB-Induced Skin Autoinflammation Due to *Nlrp1b* Mutation and Its Inhibition by Anti-IL-1β Antibody

**DOI:** 10.3389/fimmu.2022.876390

**Published:** 2022-06-17

**Authors:** Yuya Murase, Takuya Takeichi, Jun Koseki, Yuki Miyasaka, Yoshinao Muro, Tamio Ohno, Teppei Shimamura, Masashi Akiyama

**Affiliations:** ^1^ Department of Dermatology, Nagoya University Graduate School of Medicine, Nagoya, Japan; ^2^ Division of Systems Biology, Nagoya University Graduate School of Medicine, Nagoya, Japan; ^3^ Division of Experimental Animals, Nagoya University Graduate School of Medicine, Nagoya, Japan

**Keywords:** NLRP1, NLRP1B, inflammasome, UVB, IL-1β, IL-18, autoinflammation, skin - immunology

## Abstract

NLRP1 (NACHT and leucine-rich repeat-containing protein family, pyrin domain-containing protein 1) is an innate immune sensor that is involved in the formation of inflammasome complexes. NLRP1 hyperactivity has been reported to cause inherited autoinflammatory diseases including familial keratosis lichenoides chronica and NLRP1-associated autoinflammation with arthritis and dyskeratosis. We generated *Nlrp1b* (the mouse homologue of human *NLRP1*) gain-of-function knock-in (*Nlrp1b* KI) mice with UVB irradiation-induced autoinflammatory skin lesions. We demonstrated that UVB irradiation induces IL-1β upregulation and IL-1β-dependent inflammation *via* caspase-1 activation in these *Nlrp1b* KI mice. RNA sequencing revealed the upregulation of inflammasome pathway-related genes, keratinocyte stress marker genes, and keratinocyte differentiation marker genes in the *Nlrp1b* KI mice after UVB irradiation. The skin inflammation and hyperkeratosis from UVB irradiation in the *Nlrp1b* KI mice were inhibited by both intraperitoneal and subcutaneous administration of anti-IL-1β antibodies before UVB irradiation. UVB irradiation and the IL-1β pathway are important in the pathogenesis of NLRP1-associated autoinflammatory skin lesions.

## Introduction

The innate immune system is essential for host defense, tissue homeostasis, and tumor immnosurveillance (1). Members of the nucleotide-binding domain (NACHT) and leucine-rich repeat-containing protein family (NLR) are known to be innate immune sensors. This family is reported to include 22 members in humans (2). NLR family pyrin domain-containing 1 (NLRP1), encoded by *NLRP1*, was the first NLR to be shown to form an inflammasome complex (3, 4). NLRP1 senses pathogen-associated molecular patterns (PAMPs) mainly derived from infection, and danger-associated molecular patterns (DAMPs) derived from damaged or dying cells (5). Recognition of the inflammatory ligand results in the formation of inflammasomes (3, 6), which are multi-protein signaling hubs that initiate immune responses by recruiting and activating proteases, such as caspase-1 (Casp-1) (4–7).

**Figure 1:**
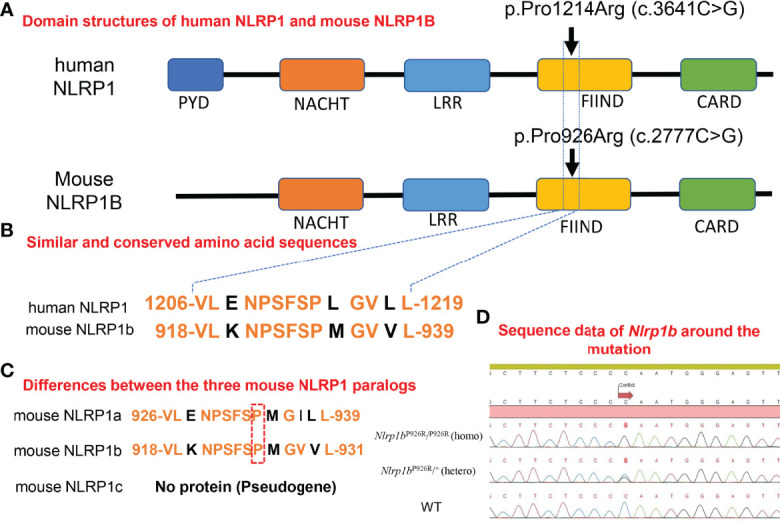
Homology in domain structures and amino acid sequences between human and mouse NLRP1 and the present knock-in mutation. **(A)** Domain structures of human NLRP1 and mouse NLRP1B. The previously reported pathogenic mutation in *NLRP1* in humans, and the missense mutation we inserted into the BALB/c mice in this study. **(B)** Similar and conserved amino acid sequences between human NLRP1 and mouse NLRP1B. **(C)** Differences between the three mouse NLRP1 paralogs. **(D)** Sequence data of *Nlrp1b* around the mutation in *Nlrp1b* KI mice (mutant-*Nlrp1b* ^P926R^/^P926R^: homo, mutant-*Nlrp1b* ^P926R^/^+^: hetero) and WT mice.

NLRP1 consists of a NACHT domain, a function-to-find domain (FIIND) (7, 8), and two effector-recruitment domains: an N-terminal pyrin domain (PYD) and a C-terminal caspase-activating and recruitment domain (CARD) (**Figure 1A**) (7). NLRP1 is known to be activated by various stimuli to form NLRP1 inflammasome complexes (7). Inflammasomes consist of apoptosis-associated speck-like protein containing CARD (ASC), Casp-1, and a sensor protein, such as an NLR family member (9). The inflammasome complex subsequently activates Casp-1, which in turn initiates downstream responses, including the maturation and release of interleukin-1β (IL-1β) and IL-18 (4, 5, 10, 11).

In mice, Masters et al. reported that NLRP1A generates a functional caspase-1-containing inflammasome *in vivo* to drive IL-1β-dependent inflammatory disease (12). Furthermore, it was reported that DPP8/9 inhibitors activate the murine NLRP1B inflammasome (13–16). Zhong et al. reported that DPP9 inhibition causes NLRP1-dependent ASC speck formation and the cleavage of IL-1β in human cells (17). In addition, the cleavage of NLRP1B by anthrax lethal factor (LF) was reported to result in a loss of 44 amino acids from the N-terminus of NLRP1B, leading to the activation of NLRP1B (18–21). The functional degradation model, which predicts mature/assembled NLRP1B inflammasomes that are produced by the cleavage of the NLRP1B N-terminus by LF, consists of the FIIND-CARD fragment and Casp-1 (6). NLRP1 senses UVB radiation, resulting in the activation and secretion of pro-IL-1β and pro-IL-18 (9, 22). There are several reported mechanisms for the activation of the human NLRP1/murine NLRP1 inflammasomes described above. However, no studies have detailed the mechanism behind autoinflammatory skin disorders caused by *NLRP1* mutations.

Zhong et al. including our group previously reported an inherited cutaneous inflammatory disease, familial keratosis lichenoides chronica (FKLC) due to gain-of-function mutations in *NLRP1* (1). Grandmange et al. reported an NLRP1-associated autoinflammatory skin disorder: NLRP1-associated autoinflammation with arthritis and dyskeratosis (NAIAD) (**Figures 1A, B**) (23).

To analyze the pathogenic mechanism behind the autoinflammatory skin syndromes caused by *NLRP1* mutations, we generated *Nlrp1b* mutant knock-in mice (*Nlrp1b* ^P926R^/^P926R^: homo, *Nlrp1b* ^P926R^/^+^: hetero) (*Nlrp1b* KI (homo and hetero) mice) as a novel mouse model of cutaneous autoinflammatory lesions resulting from NLRP1 mutants and we examined the expression of proteins, such as cytokines, and the mRNA expression profile, including inflammasome-related genes in the model lesions. Strikingly, blocking by anti-IL-1β antibody injections prevented the ultraviolet-induced lesional phenotype in the *Nlrp1b* KI mice. In this study, we demonstrate *in vivo* that the *Nlrp1b* gain-of-function mutation induces IL-1β-dependent autoinflammation caused by NLRP1B inflammasome activation.

## Materials and Methods

### Generation of the *Nlrp1b* KI Mice

BALB/c mice were purchased from Japan SLC (Hamamatsu, Japan). All mice were fed a commercial CE-2 diet (CREA Japan, Tokyo) and had ad libitum access to water. The mice were bred in a pathogen-free facility at the Institute for Laboratory Animal Research, Graduate School of Medicine, Nagoya University, and were maintained under a controlled temperature of 23 ± 1°C, a humidity of 55 ± 10%, and a light cycle of 12-h light (from 09:00 to 21:00)/12-h dark (from 21:00 to 09:00). Animal care and all experimental procedures were approved by the Animal Experiment Committee, Graduate School of Medicine, Nagoya University, and were conducted according to the Regulations on Animal Experiments of Nagoya University.

Targeted disruption of the *Nlrp1b* gene on a BALB/c background was carried out by using the CRISPR/Cas9 method as previously described (24). CRISPR RNA (crRNA, 5’- ATT CTC AGT ACA ACT CCC AT -3’) targeting exon 9 was designed using the CRISPOR website (25). The designed crRNA and trans-activating crRNA (tracrRNA) (Genome CraftType CT, FASMAC, Kanagawa, Japan) and Cas9 protein (New England Biolabs, Tokyo, Japan) were mixed and incubated at 37°C for 20 min to form a ribonucleoprotein complex (RNP). The ssODN (5’- AAG CCT GGA TAC ACA GTA CTG AAA AAC CCA AGC TTC TCC CGA ATG GGA GTT GTA CTG AGA ATA ATC CCT GCT GCC CGG CAC-3’) was designed to include missense (non-synonymous) c.2777C>G mutations to avoid cleavage by Cas9, and the target c.2777C>G mutation was obtained from FASMAC. The final concentrations of components in RNP preparation with ssODN were 8 μM guide RNA (crRNA + tracrRNA), 200 ng/μl Cas9 protein, and 250 ng/μl ssODN. The mixture was electroporated into zygotes using a NEPA 21 electroporator (NEPA GENE Co., Ltd., Chiba, Japan) and the embryos were transferred to the oviductal ampullae of pseudo-pregnant ICR mice.

For sequencing and genotyping, genomic DNA was extracted using KAPA Express Extract (Kapa Biosystems, Woburn, MA) from the pinna and tail of the offspring and was used for PCR amplification. The region targeted by the Cas9 nuclease was amplified by using the GoTaq Green Master mix (Promega, Madison, WI, USA) and a primer pair (5’-ATG CTA TTT CCA TCC AAG TTT CGC-3’ and 5’-CTC TGG TGT GAG TGG TCT AGA AGA AG -3’). Mutations in the *Nlrp1b* gene in offspring were confirmed by Sanger sequencing of the PCR products using the Eurofins DNA sequence service (Eurofins Genomics, Tokyo, Japan). Potential off-target cleavage sites predicted by the CRISPOR website [the five regions with the highest Mit off-target scores (**Supplemental Table S1**)] were sequenced, and no mutations were detected in these sites. We subsequently mated male and female founder KI mice without potential off-target cleavage sites to establish *Nlrp1b* KI (homo and hetero) mice (**Supplemental Table S2**).

### UVB Irradiation

To apply UVB irradiation to the back skin, the mice were anaesthetized with isoflurane (3%). Afterwards, the back skin of the *Nlrp1b* KI mice or the WT mice at 5 weeks of age was shaved with an electric animal shaver (E-20-555, Natsume Seisakusho, Japan) and a depilatory cream (Epilat Kracie Sensitive Skin Hair Removal Cream; Kracie, Tokyo, Japan) was applied. The mice were placed below a UVB light source (UV802L, Philips, Villingen-Schwenningen, Germany) at a distance of 3 cm and irradiated for 14 min, equal to a dose of 840 mJ/cm^2^. The UVB dose was confirmed by UV radiometer (UVR-305/365-D DETECTOR, TOKYO OPTICAL CO., LTD., Japan). n≧3.

### Immunohistochemistry

Immunohistochemical analysis of skin samples from the mice was performed as described previously (26), with slight modifications. Thin sections (3 μm) were cut from samples embedded in paraffin blocks. The sections were soaked for 20 min at room temperature in 0.3% H_2_O_2_/methanol to block endogenous peroxidase activity. After washing in PBS with 0.01% Triton X-100, the sections were incubated for 30 min in PBS with 4% BSA followed by incubation overnight with the primary antibodies in PBS containing 1% BSA according to the manufacturer’s instructions. After washing in PBS, the thin sections were stained with the corresponding secondary antibodies for 1 hour at room temperature and washed in PBS. The Vectastain Elite ABC-PO kit (Vector Laboratories, Burlingame, CA) was used for staining. The following polyclonal antibodies were purchased from commercial sources: anti-IL-1beta (ab9722; Abcam, Cambridge, UK), anti-IL-18 (ab71495; Abcam). n≧5.

### Western Blotting

Zirconia balls were added to the proteins extracted from the skin of the *Nlrp1b* KI mice and WT mice at 5 weeks of age (Day 0) before UVB irradiation and at Day 5 after UVB irradiation. Then, the proteins were dissolved in 1 ml sample buffer (NuPAGE LDS sample buffer 250μL, sample reducing agent 100μL, 25×protease inhibiter 40 μL, and water 610 μL) and crushed. After centrifugation at 10,000 rpm for 10 min at 4°C, the supernatant of each sample was subjected to SDS-PAGE. Strips of membrane were incubated with anti-IL-1beta (ab9722; Abcam), anti-pro caspase-1 + p10 + p12 (ab179515; Abcam), or anti-GAPDH (ab9485; Abcam) antibodies. The antibody–antigen complexes were detected with horseradish peroxidase-conjugated goat anti-rabbit IgG (Dako, Glostrup, Denmark) at a dilution of 1:1,000, followed by detection with enhanced chemiluminescence western blotting substrate (GE Healthcare BioSciences, Little Chalfont, UK), as described by the manufacturer. n=2. Each experiment was performed three times.

### RNA Sequencing

Total RNA extracted from the skin of 5-week-old *Nlrp1b* KI (homo and hetero) mice and WT mice both before and after UVB irradiation was purified using the RNeasy Mini Kit (QIAGEN, Hilden, Germany). RNA quality was assessed with a 2100 Bioanalyzer (Agilent Technologies). RNA sequencing was performed by Macrogen Japan Corp. using the TruSeq RNA Library Prep Kit v2 (Tokyo, Japan). Next-generation sequencing was performed using the Illumina Novaseq 6000 platform to obtain 101-bp paired-end reads. n=3.

### RNA-Seq Differential Expression Analysis

The sequence reads were aligned to the GENCODE mouse reference transcriptome (version M24) and transcript-expression levels were calculated using Kallisto (version 0.46.0) (27). We then used the R package DESeq2 to detect differentially expressed genes before and after UVB irradiation for the 5-week-old *Nlrp1b* KI (homo and hetero) mice, and the WT mice. The upregulated and downregulated genes of the *Nlrp1b* KI mice compared with the WT mice after UVB irradiation were identified by the following criteria. (Details of these genes are summarized in **Supplemental Table S3**.)

Upregulated genes: 171 genes satisfying the following criteria

log_2_-fold change (homo after UVB irradiation vs hetero before UVB irradiation) ≥ 2
log_2_-fold change (hetero after UVB irradiation vs hetero before UVB irradiation) ≥ 2
log_2_-fold change (homo after UVB irradiation vs WT after UVB irradiation)
> 0.5 or log_2_-fold change (hetero after UVB irradiation vs WT after UVB irradiation)
> 0.5
FDR of DESeq2 (homo after UVB irradiation vs homo after UVB irradiation) ≤ 0.01
FDR of DESeq2 (hetero after UVB irradiation vs hetero after UVB irradiation) ≤ 0.01
Downregulated genes: 55 genes satisfying the following criteria
log_2_-fold change (homo after UVB irradiation vs homo after UVB irradiation) ≤ -2
log_2_-fold change (hetero after UVB irradiation vs hetero after UVB irradiation) ≤ -2
log_2_-fold change (homo after UVB irradiation vs WT after UVB irradiation)
< -0.5 or log_2_-fold change (hetero after UVB irradiation vs WT after UVB irradiation)
< -0.5
FDR of DESeq2 (homo after UVB irradiation vs homo after UVB irradiation) ≤ 0.01
FDR of DESeq2 (hetero after UVB irradiation vs hetero after UVB irradiation) ≤ 0.01

### Geneset Enrichment Analysis

To identify functionally enriched gene sets between two phenotypes of three conditions (homo before and after UVB irradiation, hetero before and after UVB irradiation, or WT before and after UVB irradiation), gene set variation analysis (28) was performed. We calculated GSVA enrichment scores for each sample and tested whether there was a difference between the enrichment scores for each pair of phenotypes using a simple linear model and moderated t-statistics computed by the limma R package using an empirical Bayes shrinkage method (29). For functional gene sets, we used pre-annotated pathway gene sets (4,588 gene sets) from 12 databases (BioCarta, EHMN, HumanCyc, INOH, KEGG, NEtPath, PharmGKB, PID, Reactome, Signalink, SMPDB, and Wikipathways) and the C5 collection of curated gene sets (14,765 gene sets) that form part of the Molecular Signatures Database (MSigDB) (version 7.2). **Figure 4C** shows bar plots of enriched pathway significance related to inflammasomes for three phenotypes. The details of gene enrichment analysis are summarized in **Supplemental Tables S4, S5**.

### Immune Cell Deconvolution

To estimate immune cell fractions from bulk RNA-seq data, we used CIBERSORT (30), which is available at https://cibersort.stanford.edu/runcibersort.php. **Figure 4D** shows the resulting fractions of 22 immune cell subsets: B cells memory, B cells naïve, dendritic cells activated, dendritic cells resting, eosinophils, macrophages M0, macrophages M1, macrophages M2, mast cells activated, mast cells resting, monocytes, neutrophils, NK cells activated, NK cells resting, plasma cells, T cells CD4 memory activated, T cells memory resting, T cells CD4 naïve, T cells CD8, T cells follicular helper, T cells gamma delta, and Tregs.

### ELISA Analysis

Blood samples from *Nlrp1b* KI mice and WT mice at 5 weeks of age before UVB irradiation and at Day 5 and Day 10 after UVB irradiation were obtained from the facial vein and were collected in a microtainer (365967, BD, USA). Sera were collected after centrifugation at 5,500 rpm for 10 minutes at 4°C. The concentrations of IL-1β and IL-18 were measured by commercially available ELISA kits according to the manufacturers’ protocols (ab197742, ab216165, abcam). n=3-6 each. Each experiment was performed twice. The total amounts of cleaved and pro-form IL-1β and IL-18 were statistically analyzed by two-way Student’s t-test. Significant differences are shown as *P < 0.05.

### Treatments of the Mice With IL-1β Antibody

*Nlrp1b* KI (hetero) mice were treated with an intraperitoneal or subcutaneous injection of 2 mg/kg anti-IL-1β antibody (I-437, Leinco Technologies, Inc., Missouri, USA), which is specific for IL-1β, or 2 mg/kg IgG Isotype Control (I-140, Leinco Technologies, Inc., Missouri, USA) before UVB irradiation at Day 0.

## Results

### We Successfully Generated *Nlrp1b* Gain-of-Function Mutant Knock-in BALB/c Mice (*Nlrp1b* KI Mice).

Mice have three *Nlrp1* paralogs (*Nlrp1a*, *Nlrp1b*, *Nlrp1c*), which differ according to the mouse strain (31). *Nlrp1c* is not translated into protein because *Nlrp1c* is a pseudogene; however, *Nlrp1a* and *Nlrp1b* have very similar sequences in the region where we designed the insertion of a specific mutation (**Figure 1C**) (Ref. Ensembl (https://asia.ensembl.org/index.html)). It was reported that the C57BL/6 strain of mouse, which is commonly used for *in vivo* experiments, expresses all *Nlrp1* paralogs, whereas BALB/c mice express only the *Nlrp1b* paralog (31). Therefore, BALB/c mice are suitable for evaluating the role of mouse NLRP1B molecules by excluding the effects of other *Nlrp1* paralogs (31). To determine the role of molecules in the mouse NLRP1 family, we used a CRISPR-Cas9 gene-targeting approach to generate *Nlrp1b* KI mice. Based on abnormal autoinflammatory and autoimmune cases in humans, in order to generate a novel mouse model of cutaneous autoinflammatory lesions resulting from *NLRP1* mutations, we searched for similar and conserved amino acids sequence between human NLRP1 and mouse NLRP1B. One similar amino acid sequence in mouse NLRP1B was found near the missense mutation c.3641C>G (p.Pro1214Arg) in humans, causing NAIAD (23) (**Figure 1B**). In BALB/c mice, we introduced the mouse missense mutation c.2777C>G (p.Pro926Arg), which corresponds to the human missense mutation c.3641C>G (p.Pro1214Arg) (23), and established *Nlrp1b* KI (homo and hetero) mice by mating *Nlrp1b* KI (hetero) male and female mice (**Figure 1D**).

### UVB Irradiation Induces Autoinflammation and Hyperkeratosis in *Nlrp1b* KI Mice

We observed several *Nlrp1b* KI (homo and hetero) mice and wild-type (*Nlrp1b* ^+^/^+^: WT) mice for 6 months. However, neither spontaneous inflammatory skin lesions nor hyperkeratosis was seen. The fertility and survivability of the *Nlrp1b* KI (homo and hetero) mice were similar to those of the WT mice. In addition, to induce autoinflammatory skin lesions, we shaved back hair and topically applied a commercially available 5% imiquimod (IMQ) cream (Beselna^®^ Cream) to the back skin of the *Nlrp1b* KI mice (homo and hetero) and the WT mice. We adopted this method when we induced autoinflammatory skin lesions in our previously reported “deficiency of interleukin-36 receptor antagonist” (DITRA) mouse model (32). Although inflammatory skin lesions and hyperkeratosis were induced, there were no significant differences in the skin phenotype between the *Nlrp1b* KI mice (homo and hetero) and the WT mice.

**Figure 2:**
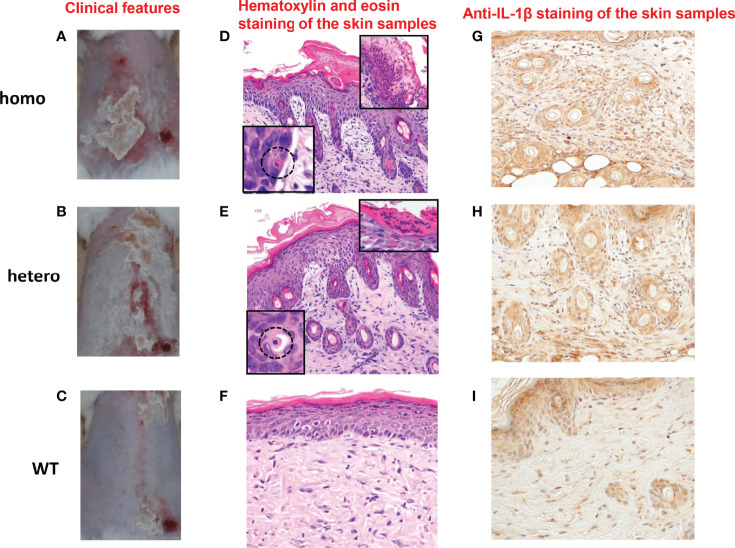
Cutaneous inflammatory lesions induced by UVB irradiation in *Nlrp1b* KI mice. **(A–C)** Clinical features of *Nlrp1b* KI (homo) mice **(A)**, *Nlrp1b* KI (hetero) mice **(B)**, and WT mice **(C)** at 5 weeks of age, 9 days after UVB irradiation. (n≧3) The *Nlrp1b* KI mice **(A, B)** show many scales on the back. **(D–F)** Hematoxylin and eosin staining of the skin samples reveals acanthosis, hyperkeratosis, inflammatory cell infiltration **(D, E)**, and dyskeratosis (**D, E** insert) in the Nlrp1b KI mice, but such features are not seen or are mild in the WT mice (F) (original magnification ×200). **(G–I)** Inflammatory cell infiltration in the epidermis stained positive for IL-1b (original magnification × 400).

UVB radiation from the sun is a major stimulus to the skin. It has been reported that NLRP1 senses UVB radiation, resulting in the activation and secretion of pro-IL-1β and pro-IL-18 in human keratinocytes (9, 22). Thus, we sought to establish UVB-induced autoinflammation and hyperkeratinization in the *Nlrp1b* KI (homo and hetero) mice by applying UVB irradiation to the shaved back skin. Although differences in the roles of inflammasomes between human and murine skin have been reported (33, 34), the UVB irradiation successfully induced skin inflammatory lesions, including erosions, crusts, hyperkeratosis, dyskeratosis, and thickening, that were much more severe in our *Nlrp1b* KI (homo and hetero) mice than in the WT mice (**Figures 2A–C**; **Supplemental Figure 1**). Histopathological examination showed more severe hyperkeratosis, acanthosis, and inflammatory cell infiltration in the *Nlrp1b* KI (homo and hetero) mice than in the WT mice (**Figures 2D–F**), and eosinophilic dyskeratotic cells were observed in the *Nlrp1b* KI (homo and hetero) mice—findings that are consistent with those of skin lesions in patients with NAIAD, multiple self-healing palmoplantar carcinoma, and FKLC (23) (**Figures 2D, E**). In the *Nlrp1b* KI (homo and hetero) mice, the infiltration of various inflammatory cells was seen in the dermis and severe hyperkeratosis with remarkable infiltration of inflammatory cells was observed in the subcorneal areas and in the stratum corneum of the epidermis (**Figures 2D–F**). These findings are similar to those seen in generalized pustular psoriasis, a representative autoinflammatory keratinization disease (AiKD). The inflammatory cells in the epidermis stained positive for IL-1β (**Figures 2G–I**). These findings indicate that UVB irradiation might induce more severe inflammatory responses in *Nlrp1b* KI (homo and hetero) mice than in WT mice. However, the UVB irradiation did not induce any clinical phenotypes, such as arthritis, other than skin lesions.

### UVB Activates IL-1β *via* the Activation of Casp-1 Caused by NLRP1B Inflammasome Activation.

**Figure 3:**
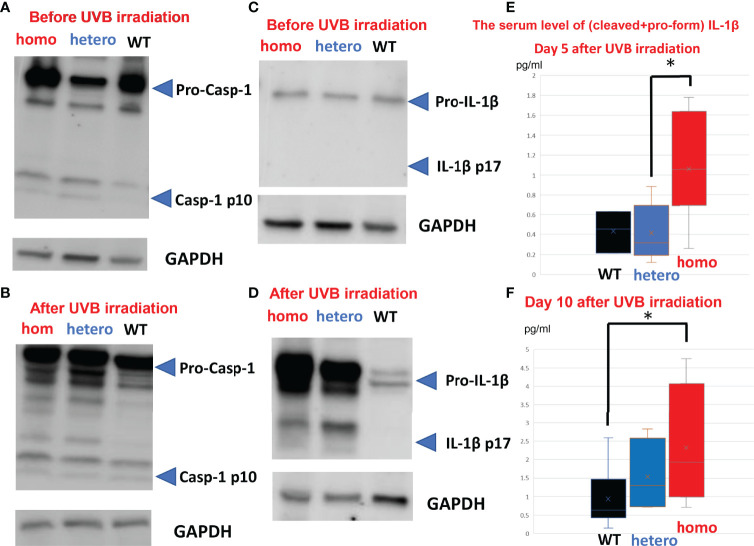
Activation of Casp-1 and conversion from pro-IL-1β to IL-1β in *Nlrp1b* KI mice. **(A)** In the *Nlrp1b* KI (homo) mice and the *Nlrp1b* KI (hetero) mice, Casp-1 p10 (cleaved form) was detected before UVB irradiation, but was not detected in the WT mice. (n = 2) **(B)** In the *Nlrp1b* KI (homo) mice, the *Nlrp1b* KI (hetero) mice, and the WT mice, Casp-1 p10 was detected after UVB irradiation. (n = 2) **(C)** In the *Nlrp1b* KI (homo) mice, the *Nlrp1b* KI (hetero) mice, and the WT mice, activated IL-1β was not detected before UVB irradiation. (n = 2) **(D)** Conversion from pro-IL-1β to activated IL-1β was detected in the *Nlrp1b* KI (homo) mice and the *Nlrp1b* KI (hetero) mice, but not in the WT mice. (n = 2) **(E, F)** The serum level of IL-1β (the total amount of cleaved and pro-form IL-1β) was significantly higher in the *Nlrp1b* KI mice than in the WT mice at Day 5 **(E)** and at Day 10 **(F)** after UVB irradiation. (n = 3-6) The serum level of IL-1β (the total amount of cleaved and pro-form IL-1β) was more greatly increased in the *Nlrp1b* KI (homo and hetero) mice than in the WT mice at Day 10 compared to Day 5 **(E, F)** (n = 3-6). Significant differences are shown as *P < 0.05.

Inflammasomes are thought to be the platforms that initiate innate immunity by recruiting and activating Casp-1 (35). Boucher et al. reported a model of mechanisms whereby full-length Casp-1 p46 is activated to gain protease activity on inflammasomes before finally being converted to Casp-1 p20/p10, which is released from inflammasomes to be deactivated (35). Thus, we monitored the activation of Casp-1 by examining the presence of Casp-1 p10. In the *Nlrp1b* KI (homo and hetero) mice, Casp-1 p10 was detected before UVB irradiation (**Figure 3A**). In the WT mice, however, Casp-1 p10 was not detected before UVB irradiation (**Figure 3A**). After UVB irradiation, in the *Nlrp1b* KI (homo) mice, the *Nlrp1b* KI (hetero) mice, and the WT mice, Casp-1 p10 was detected (**Figure 3B**).

The proinflammatory cytokine IL-1β is converted from pro-IL-1β p34 to activated IL-1β p17 *via* activated Casp-1 (16, 36). Thus, we confirmed the protease activity of Casp-1 by examining the conversion from pro-IL-1β to activated IL-1β. Activated IL-1β was not detected before UVB irradiation in the *Nlrp1b* KI (homo) mice, the *Nlrp1b* KI (hetero) mice, or the WT mice, (**Figure 3C**). In contrast, the conversion from pro-IL-1β to activated IL-1β was detected in the *Nlrp1b* KI (homo) mice and the *Nlrp1b* KI (hetero) mice, but not in the WT mice (**Figure 3D**). IL-1β cleavage does not appear to differ between the KI (homo) mice and the KI (hetero) mice (**Figure 3D**). However, IL-1β cleavage is greater in the KI (homo and hetero) mice than in the WT mice (**Figure 3D**). We planned to examine the cleaved IL-18. Unfortunately, we were unable to find any antibodies that could distinguish between cleaved IL-18 and pro-form IL-18. As Casp-1 cleaves the pro-forms of both IL-1β and IL-18, we speculate that cleaved IL-18 also might be elevated, similarly to cleaved IL-1β. These findings indicate that in the *Nlrp1b* KI (homo and hetero) mice, Casp-1, a key protease in the NLR family of inflammasomes, might be mildly activated without any stimuli, although its activation is insufficient to convert pro-IL-1β to mature IL-1β.

In addition, to analyze the systemic influences of *Nlrp1b* gain of function, we examined the serum level of IL-1β. Our ELISA detects both cleaved and pro-form IL-1β. The total amounts of cleaved and pro-form IL-1β were significantly higher in the *Nlrp1b* KI (homo) mice than in the *Nlrp1b* KI (hetero) mice at Day 5 after UVB irradiation (**Figure 3E**). And the total amounts of cleaved and pro-form IL-1β were significantly higher in the *Nlrp1b* KI (homo) mice than in the WT mice at Day 10 after UVB irradiation (**Figure 3F**). Furthermore, the increase in the serum level of IL-1β (total amounts of cleaved and pro-form IL-1β) from Day 5 to Day 10 was greater in the *Nlrp1b* KI (homo and hetero) mice than in the WT mice (**Figures 3E, F**). Our ELISA also detects both cleaved and pro-form IL-18. The serum level of IL-18 (total amounts of cleaved and pro-form IL-18), which is another substrate of Casp-1, was also higher in the *Nlrp1b* KI (homo) mice than in the *Nlrp1b* KI (hetero) mice or in the WT mice (**Supplemental Figure S2**).

These findings indicate that UVB irradiation might induce more IL-1β production *via* Casp-1 in *Nlrp1b* KI (homo and hetero) mice than in WT mice and that IL-1β production and IL-1β-dependent inflammation might persist longer and be more severe in *Nlrp1b* KI mice than in WT mice.

### Inflammasome Pathway-Related Genes, Keratinocyte Stress Marker Genes, and Keratinocyte Differentiation Marker Genes are Upregulated in *Nlrp1b* KI Mice After UVB Irradiation

**Figure 4:**
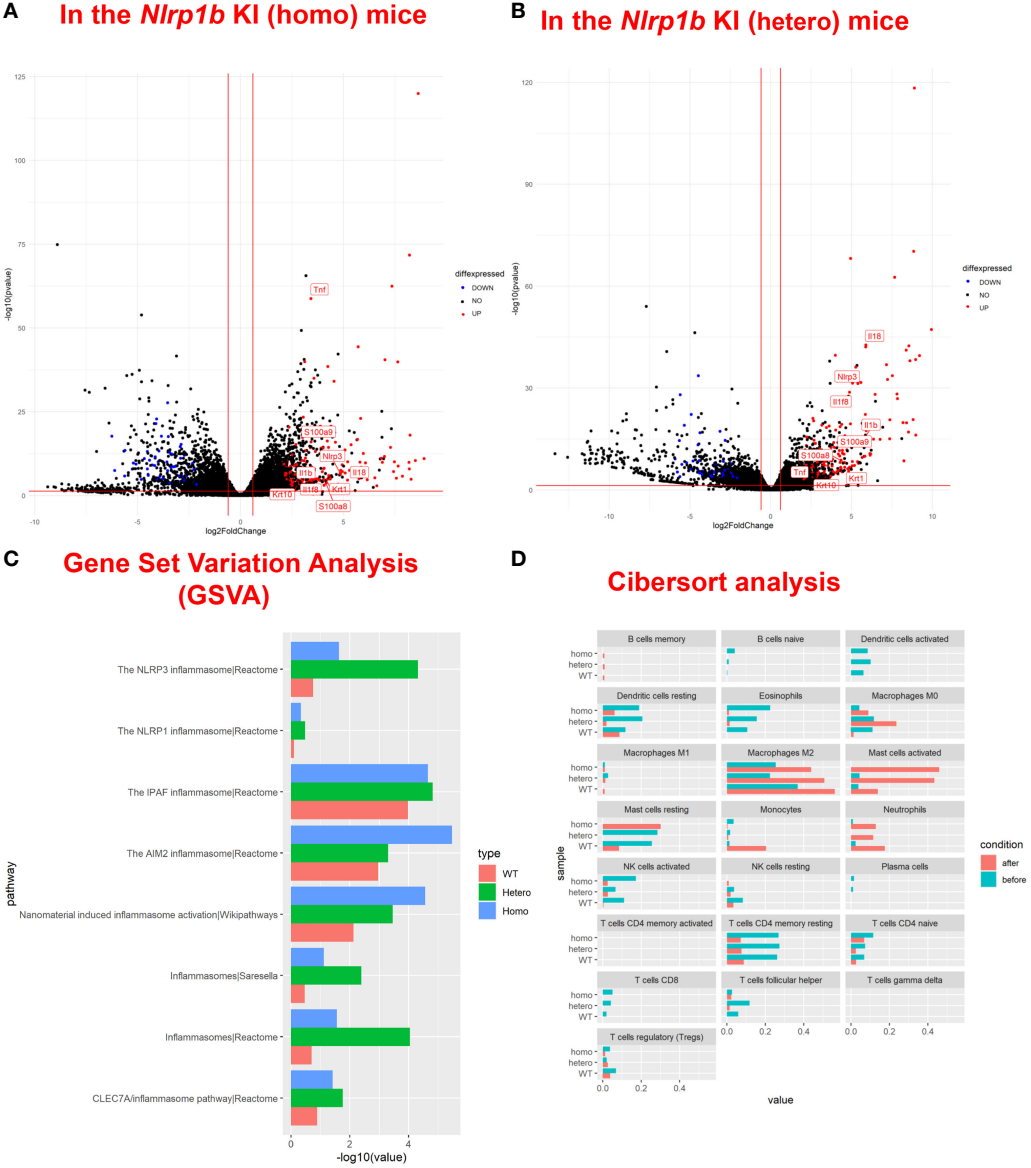
Upregulation of inflammasome pathway-related genes, keratinocyte stress marker genes, and keratinocyte differentiation marker genes in *Nlrp1b* KI mice after UVB irradiation. A volcano plot of upregulated/downregulated genes in the *Nlrp1b* KI (homo) mice **(A)** and in the *Nlrp1b* KI (hetero) mice **(B)** shows that both IL-1β and IL-18, which are final products of the NLRP1B inflammasome pathway, and keratin 1 (K1) and keratin 10 (K10), which are keratinocyte differentiation marker genes, are significantly (padj<0.005) and greatly (log_2_-fold change ≧2) upregulated after UVB irradiation compared with those before UVB irradiation, compared to the WT mice. **(C)** Gene set variation analysis (GSVA) shows that UVB irradiation upregulated the expression of genes related to inflammasomes, including NLRP1, NLRP3, AIM2 and IPAF, more intensively in the *Nlrp1b* KI (homo and hetero) mice than in the WT mice. **(D)** Cibersort analysis shows that fewer M2 macrophages infiltrated into the lesional skin of the *Nlrp1b* KI (homo and hetero) mice than into the lesional skin of the WT mice after UVB irradiation. The Cibersort analysis also shows that more activated mast cells infiltrated into the lesional skin of the *Nlrp1b* KI (homo and hetero) mice than into the lesional skin of the WT mice after UVB irradiation.

To examine the baseline gene expression and the changes of gene expression caused by UVB irradiation in the *Nlrp1b* KI (homo and hetero) mice and the WT mice, we performed RNA sequencing by using mRNA extracted from the skin before UVB-irradiation and after UVB-induced skin inflammatory lesion development for the respective mice. Various inflammasome-related gene products activate and drive pathological inflammation in sterile inflammatory diseases (37). Saresella et al. reported on the mRNA expression of genes involved in the assembly, activation, and downstream signaling of inflammasomes in Alzheimer’s disease (38). We compared the mRNA expression of various genes, including inflammasome-related genes, before and after UVB irradiation in the *Nlrp1b* KI (homo and hetero) mice and the WT mice. The NLRP1B inflammasome is known to activate Casp-1, leading to the activation of IL-1β and IL-18 from the proforms of IL-1β and IL-18, respectively. IL-1β is a strong inflammatory cytokine that induces inflammation. In this study, the mRNA expression levels of IL-1β and IL-18, which are final products of the NLRP1B inflammasome pathway, and of keratin 1 (K1) and keratin 10 (K10), which are keratinocyte differentiation marker genes, were significantly (padj<0.005) and greatly (log_2_-fold change ≧2) upregulated in the *Nlrp1b* KI (homo and hetero) mice after UVB irradiation compared with those before UVB irradiation, indicating the activation of the inflammasome pathway and the highly keratinized nature of the lesional skin in the *Nlrp1b* KI mice (**Figures 4A, B**; **Supplemental Table S3**). In addition, *S100a8/9*, which are keratinocyte stress marker genes (1), were also significantly and greatly upregulated after UVB irradiation (**Figures 4A, B**; **Supplemental Table S3**). Furthermore, the gene expression of *Nlrp3*, which is another NLR family member, and that of *Tnf*, which is also associated with the innate immune system, were also significantly and greatly upregulated after UVB irradiation (**Figures 4A, B**; **Supplemental Table S3**). These data are similar to gene expression profiles previously reported in human cases with *NLRP1* mutations (38) and Alzheimer’s disease (38). Moreover, after UVB irradiation, the mRNA expression levels of *Casp1*, which encodes the key enzyme Casp-1 of the NLRP1B inflammasome pathway, and of *Pycard*, which encodes the adaptor protein ASC, were also significantly, but not greatly, increased compared to those before UVB irradiation, but only in the *Nlrp1b* KI (homo and hetero) mice (**Table 1**). In contrast, in the WT mice, the mRNA expression levels of *Casp-1*, *Pycard*, *K1*, *K10*, and *S100a8/9* were not significantly increased after UVB irradiation (**Figures 4A, B**; **Table 1**). Furthermore, gene set variation analysis (GSVA) showed that UVB irradiation upregulated genes related to various inflammasome pathways, including the NLRP1, NLRP3, AIM2 and IPAF inflammasome pathways, more intensively in the *Nlrp1b* KI (homo and hetero) mice than in the WT mice (**Figure 4C**). Interestingly, in the *Nlrp1b* KI (homo and hetero) mice, NLRP3, AIM2 and IPAF inflammasome-related genes, as well as NLRP1B inflammasome-related genes, were upregulated more intensively after UVB irradiation (**Figure 4C**). These gene expression profiles might provide supportive evidence for the idea of activation of the NLRP1B inflammasome pathway by UVB irradiation in the *Nlrp1b* KI (homo and hetero) mice. Furthermore, our data indicate that the activation of NLRP1B inflammasomes might affect and/or collaborate with other inflammation pathways, including the NLRP3, AIM2, and IPAF inflammasome pathways, and with the TNF-pathway, which is the other innate immune pathway (**Figures 4A–C**; **Table 1**). In addition, the present pathway analysis showed the significant activation of keratinization and the IL-17 signaling pathway, which is known to be related to psoriasis (**Supplemental Figure S3**). These findings might also support idea that the activation of an inflammatory response is accompanied by hyperkeratosis and the highly keratinized nature of the lesional skin in the *Nlrp1b* KI mice.

**Table 1:** Changes of the mRNA expressions of the autoinflammatory lesional skins in KI mice and WT mice after UVB irradiation, compared to before UVB irradiation.

	hetero_baseMean	hetero_log_2-_FoldChange	hetero_padj	homo_baseMean	homo_log_2_-FoldChange	homo_padj	WT_baseMean	WT_log_2-_FoldChange	WT_padj
Casp1	978.2729916	-1.031714732	0.001541566	908.8195708	-1.369189382	0.000833818	855.0807974	-0.809863752	0.2581059
Casp8	924.901517	-1.034061398	0.01061925	814.3166122	-1.136141076	7.74E-07	889.8814677	-1.061061534	0.0314754
Ccl2	422.847902	-1.289895179	0.051621372	242.6608332	-1.81003796	0.001006825	824.3147017	-0.915724507	NA
Cflar	1940.19069	0.490375246	0.400716051	1855.950212	0.753174471	0.002176554	1840.003053	-0.019414876	0.9968535
Cxcl2	3251.795636	-7.293530772	1.40E-07	3118.337765	-8.811021261	2.91E-07	1360.021666	-3.052052034	0.3319967
Hsp90aa1	7286.11275	0.47022588	7821.807797	0.814429946	0.00013146	8123.472544	0.592019957	0.5752833
Il18	863.359312	-2.494408165	2.75E-06	948.0207156	-3.432872965	6.12E-56	826.1510371	-2.600414446	0.0039496
Il1b	2633.724343	-4.037050248	3.64E-07	1887.008252	-4.559818151	0.00177324	2669.678636	-1.514515059	NA
Il33	1007.618389	-2.931069986	0.000933988	446.3339066	-2.183798108	1.24E-06	1606.900225	-3.574548085	0.0327084
Irf1	763.5594619	-0.434949575	0.143260614	610.8835514	-0.717487373	0.03181745	877.4735635	0.261319602	0.9542205
Mapk3	5036.654241	-0.563374177	0.070071088	5409.174008	-0.808514578	0.001492378	4488.344273	-0.669886406	0.6181282
Mefv	86.01210396	-2.249922779	0.00068006	48.55830202	-2.732374968	2.79E-05	155.7283011	-2.841472122	NA
Myd88	1093.351237	-0.906489949	0.004864392	983.1013028	-0.780748606	0.003351202	1287.229587	-1.051406763	0.1815874
Naip1	24.72881218	5.081643613	0.005867367	55.04438677	5.437888476	1.52E-09	39.46719672	5.058191965	0.0090656
Naip2	144.4861488	-2.157314821	2.92E-06	100.3388981	-2.16969138	4.43E-09	175.0530497	-1.974775953	0.1354912
Naip5	44.5945371	-1.992767089	1.42E-05	30.42363325	-2.230339924	0.000145949	51.32375115	-1.522187184	0.4729861
Naip6	24.90272821	-1.699903253	0.036991114	16.72022875	-2.154082332	0.004587738	29.99549119	-1.027071561	0.7483662
Nfkb1	2337.021255	-0.563270625	0.221208296	2241.704827	-0.536630035	0.04580665	2273.008443	-0.431110724	0.4550497
Nlrc4	56.26284184	-1.275962498	0.011526387	42.04457225	-1.597094052	0.000374313	67.94751956	-1.269509705	0.5727335
Nlrc5	826.5583207	-0.271896352	0.846128612	778.0802458	-1.309455948	0.00375267	717.8820894	-0.187522644	0.9672843
Nlrp1b	68.9207564	-0.840608829	0.159387703	54.09385205	-1.595991297	0.002065236	91.44958355	-1.265808423	0.2856722
Nlrp3	398.2609948	-5.356728832	8.17E-34	343.9221186	-5.289026344	6.32E-06	319.138401	-2.683262375	0.2848767
Nod1	737.4518869	-0.364677905	0.469542324	718.6894341	-0.714069215	0.01109103	750.6519924	-0.32033752	0.5606787
Pea15a	2299.056802	-0.650444778	0.232190196	2164.209214	-1.179237436	1.39E-10	2761.823828	-0.809553495	0.5306266
Pycard	1091.515602	-1.085950354	6.41E-07	1190.765073	-1.479217846	1.32E-18	853.3419485	-1.145471836	0.397448
Tnf	759.8853803	-4.962862984	1.28E-26	619.3834863	-4.942913245	0.000314238	234.8853719	-2.65300892	0.0011486
Tnfsf14	39.18960268	-2.242552071	0.002189947	25.24709407	-2.521211788	0.001333554	114.6929862	-3.434447292	NA

padj, p-value adjusted. NA, not analyzed.

In addition, Cibersort analysis surprisingly showed that fewer M2 macrophages infiltrated the lesional skin of the *Nlrp1b* KI (homo and hetero) mice than infiltrated the lesional skin of the WT mice after UVB irradiation (**Figure 4D**). M2 macrophages are known to promote cell proliferation and tissue repair (39), and the present findings indicate that the amount of M2 macrophages in the tissue might be associated with the autoinflammatory skin phenotype resulting from the *Nlrp1b* mutation. Furthermore, the Cibersort analysis surprisingly showed that activated mast cells infiltrated the lesional skin in greater numbers for the *Nlrp1b* KI (homo and hetero) mice than for the WT mice after UVB irradiation (**Figure 4D**). The present findings indicate that the amount of activated mast cells in the tissue also might be associated with autoinflammation caused by the *Nlrp1b* mutation. Furthermore, the Cibersort analysis showed that there was no significant infiltration of neutrophils, which are the main inflammatory effector cells, in the *Nlrp1b* KI mice before UVB irradiation (**Figure 4D**, blue bars). Therefore, we speculate that there might be no spontaneous inflammation in these mice.

### Anti-IL-1β Antibody Inhibits UVB-Induced Skin Autoinflammation and Hyperkeratosis in *Nlrp1b* KI (Hetero) Mice

**Figure 5:**
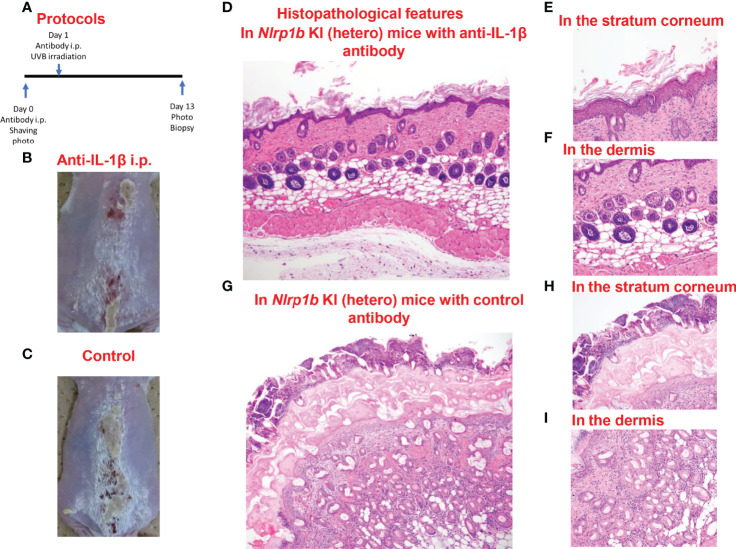
Prevention of UVB-induced cutaneous inflammatory lesions in *Nlrp1b* KI (hetero) mice. **(A)** Protocols for the development of UVB-induced autoinflammatory skin lesions. For prevention by anti-IL-1β antibody, *Nlrp1b* KI (hetero) mice were treated with the intraperitoneal (i.p.) injection of anti-IL-1β antibody on Days 0 and 1. **(B, C)** The skin inflammation and hyperkeratosis induced by UVB irradiation were inhibited in the *Nlrp1b* KI (hetero) mice with anti-IL-1β antibody treatment by i.p. administration before UVB irradiation **(B)**, compared with in the *Nlrp1b* KI (hetero) mice with control IgG antibody treatment by i.p. administration **(C)**. **(D–F)** Histopathological features of skin samples from the treated mice. In the *Nlrp1b* KI (hetero) mice with anti-IL-1β antibody treatment, hyperkeratosis and inflammatory cell infiltration **(D, F)** are very mild, whereas in the *Nlrp1b* KI (hetero) mice with control IgG antibody treatment, severe hyperkeratosis with remarkable inflammatory cell infiltration in subcorneal areas and in the stratum corneum is observed **(G–I)**, and infiltration of various inflammatory cells in the dermis is seen **(I)**.

In the *Nlrp1b* KI (hetero) mice which underwent anti-IL-1β antibody treatment before UVB irradiation, the skin inflammation and hyperkeratosis caused by the irradiation were inhibited compared with those in the *Nlrp1b* KI (hetero) mice with control IgG antibody treatment by intraperitoneal (i.p.) administration (**Figures 5A–C**) and subcutaneous (s.c.) administration (**Supplemental Figure S4**). Histopathologically, the hyperkeratosis and inflammatory cell infiltration were milder in the *Nlrp1b* KI (hetero) mice with anti-IL-1β antibody treatment (**Figures 5D–F**) than in the *Nlrp1b* KI (hetero) mice with control IgG antibody treatment (**Figures 5G–I**). These findings indicate that the skin autoinflammation phenotype resulting from UVB irradiation in the *Nlrp1b* KI mice might be driven predominantly by IL-1β.

## Discussion

NLRP1 is known to be an inflammasome sensor in humans. To our knowledge, certain neuropsychiatric disorders [e.g., multiple sclerosis (40), Alzheimer’s disease (41), autism spectrum disorder (42), schizophrenia (43)], ophthalmic diseases [e.g., uveitis (44), corneal intraepithelial dyskeratosis (45, 46)], colorectal adenomatous polyposis (47), respiratory papillomatosis (48), orthopedic diseases [e.g., rheumatoid arthritis (49)], endocrine disorders [e.g., autoimmune Addison’s disease (50, 51), type 1 diabetes (51)] and skin disorders [e.g., NAIAD (23), multiple self-healing palmoplantar carcinoma (1), keratosis lichenoides chronica (1), vitiligo (50–53)] have been reported as NLRP1-related diseases. Thus, NLRP1 is one of the most important molecules for various systemic disorders, including inflammatory diseases. However, the detailed function of NLRP1 and the pathogenic mechanism behind the autoinflammatory skin lesions caused by *NLRP1* mutations remain unclear. Several phenotypes have been reported for autoinflammatory skin lesions due to *NLRP1* mutations (1, 23, 50–53).

Zhong et al. including our group reported two human autoinflammatory skin disorders with abnormal keratinization caused by gain-of-function mutations in *NLRP1*: familial keratosis lichenoides chronica, and multiple self-healing palmoplantar carcinoma (1). Furthermore, Grandemange reported a different missense mutation, p.Pro1214Arg in *NLRP1*, which caused NAIAD, an autoinflammatory syndrome with abnormal skin keratinization (23). To analyze the function of NLRP1B and pathogenetic mechanisms of autoinflammatory skin lesions caused by the murine *Nlrp1b* gain-of-function mutation, we generated *Nlrp1b* KI mice with autoinflammatory skin lesions resulting from an *Nlrp1b* gain-of-function mutation which was homologous to the human *NLRP1* mutation reported as a cause in an NAIAD family, and we examined the pathogenetic mechanisms and gene expression profiles of autoinflammatory skin lesions in the mice. Our data show that the *Nlrp1b* gain-of-function mutation leads to UVB-induced skin inflammation with hyperkeratosis accompanied by excessive inflammatory cell infiltration.

We have been advocating a new disease category: autoinflammatory keratinization disorders (AiKDs). These consist of various inflammatory keratinization disorders with autoinflammatory mechanisms as their predominant causes. To date, AiKDs are thought to include pustular psoriasis and related diseases, pityriasis rubra pilaris (PRP) type V, familial keratosis lichenoides chronica (FKLC), hidradenitis suppurativa, and porokeratosis (54, 55). Of these, FKLC is an AiKD caused by a gain-of-function mutation in *NLRP1* (1). Interestingly, the present results from histopathological and ELISA analyses of *Nlrp1* KI mice are similar to those of our previously reported cases with AiKD (pustular psoriasis) due to *CARD14* (56) or *IL36RN* mutations (57). Therefore, we consider that the mechanisms behind the cutaneous inflammation in the present *Nlrp1b* KI mice are similar to the autoinflammatory pathomechanisms seen in human AiKDs.

NLRP1B has been reported to form inflammasomes that activate Casp-1, leading to IL-1β-dependent inflammation by several stimuli, such as anthrax lethal factor (LF) (18–21), ATP depletion in cells (58, 59), and the inhibition of DPP8/9 (13–16). Our findings suggest the possibility that UVB irradiation also might activate Casp-1, resulting in the maturation and secretion of IL-1β *in vivo*. In the present study, our *Nlrp1b* KI (homo and hetero) mice showed skin inflammation with epidermal inflammatory cell infiltration, dyskeratosis, and hyperkeratosis accompanied by the infiltration of inflammatory cells in subcorneal areas and in the stratum corneum of the epidermis. Furthermore, our data indicate that, in *Nlrp1b* KI (homo and hetero) mice, Casp-1, which is the key protease of inflammasomes in the NLR family, might be slightly activated even without any stimuli, although its activation is not enough to convert pro-IL-1β to mature IL-1β. We had planned to examine the cleaved IL-18. Unfortunately, we were unable to find any antibodies that could distinguish between cleaved IL-18 and pro-form IL-18. As Casp-1 cleaves the pro-forms of both IL-1β and IL-18, we speculate that cleaved IL-18 also might be elevated, similarly to cleaved IL-1β. Our data also show that in *Nlrp1b* KI (homo and hetero) mice, but not in WT mice, UVB irradiation can induce the activation of Casp-1, resulting in the maturation of IL-1β. These findings indicate that the *Nlrp1b* gain-of-function mutation might induce the inflammatory keratinization phenotype caused by autoinflammatory mechanisms *via* the activation of NLRP1B inflammasomes. LF (18–21), which is an inhibitor of Dpp8/9 (13–16), and ATP depletion (58, 59) have been reported as activators of NLRP1B. This study is the first to indicate that UVB irradiation also might activate NLRP1B directly, or might indirectly activate NLRP1B *via* DAMPs derived from damaged or dying cells caused by UVB irradiation. Our *Nlrp1b* KI mice carrying the *Nlrp1b* mutation, which corresponds to the human *NLRP1* mutation that causes autoinflammatory skin lesions, showed autoinflammatory skin lesions induced by UVB irradiation. Therefore, the present *Nlrp1b* KI mouse model is considered to be a useful tool for investigating the pathogenic mechanisms or exacerbation processes of human NAIAD cases with a skin autoinflammatory phenotype resulting from *NLRP1* mutations. Indeed, from the present findings, we speculate that the autoinflammatory skin lesions in patients with *NLRP1* mutations might be triggered by UVB. Perhaps sunscreen might be an effective preventive measure and/or treatment for cutaneous autoinflammation in patients with *NLRP1* mutations.

In the *Nlrp1b* KI (hetero) mice, anti-IL-1β antibody treatment reduced the severity of the skin phenotype. Therefore, IL-1β, the final product of NLR family inflammasome pathways, is thought to predominantly induce the skin inflammation in our *Nlrp1b* KI mice. In addition, the present study shows that UVB irradiation upregulates inflammatory response-related genes, such as TNF pathway-related genes, and genes related to various inflammasome pathways, including NLRP1, NLRP3, AIM2, and IPAF inflammasome pathways, as well as NLRP1B inflammasome-related genes, more intensively in *Nlrp1b* KI (homo and hetero) mice than in WT mice. Our results suggest that the activation of NLRP1 inflammasomes might affect and/or collaborate with other inflammatory pathways including TNF-pathway and pathways of other inflammasomes, such as AIM2, IPAF and NLRP3 inflammasomes. The present *Nlrp1b* KI mouse model can be used to search for novel effective treatments for autoinflammatory skin lesions resulting from *Nlrp1b* mutations.

In conclusion, we reported a putative mechanism behind NLRP1B inflammasome activation induced by UVB irradiation in *Nlrp1b* KI mice. The findings suggest the possibility that autoinflammatory skin lesions resulting from *NLRP1* mutations might be related to IL-1β-dependent inflammation and UVB irradiation. Our data provide new insights into the mechanism behind NLRP1B/NLRP1 inflammasome activation and the pathogenic mechanisms of autoinflammatory skin lesions caused by *Nlrp1b/NLRP1* gain-of-function mutations.

## Data Availability Statement

The datasets presented in this study can be found in online repositories. The names of the repository/repositories and accession number(s) can be found below: https://www.ncbi.nlm.nih.gov/, GSE172065.

## Ethics Statement

This study was reviewed and approved by the Ethics Committee of the Nagoya University Graduate School of Medicine. All studies were conducted according to the Declaration of Helsinki. Animal care and all experimental procedures were approved by the Animal Experiment Committee, Graduate School of Medicine, Nagoya University and were conducted according to the Regulations on Animal Experiments of Nagoya University.

## Author Contributions

Research design: YMu, TT, MA. Experiments: YMu, TT, YMi. Data acquisition: JK, YMi. Data Analysis: YMu, TT, JK, TS. Collection of samples and information: YMu, TT. Manuscript writing: YMu, TT, MA. Writing assistance: TT, JK, TS, MA. All authors contributed to the article and approved the submitted version.

## Funding

This research was supported by Health and Labor Sciences Research Grant for Research on Intractable Diseases 20FC1052 from the Ministry of Health, Labor and Welfare of Japan to MA. This research was also supported by AMED under Grant Number JP20ek0109488 to TT and MA. This study was supported in part by JSPS KAKENHI Grants Number 18H02832 to MA and 20K08648 to TT. This investigation was also supported in part by the Hori Science and Arts Foundation to TT, by grants from the Maruho Takagi Dermatology Foundation to TT, and by the Japanese Dermatological Association Dermatological Research Fund, supported by ROHTO Pharmaceutical Co., Ltd. to TT, and by the Japanese Dermatological Association Dermatological Research Fund, supported by Shiseido Co., Ltd. to YM. The funders were not involved in the study design, collection, analysis, interpretation of data, the writing of this article or the decision to submit it for publication.

## Conflict of Interest

The authors declare that the research was conducted in the absence of any commercial or financial relationships that could be construed as a potential conflict of interest.

## Publisher’s Note

All claims expressed in this article are solely those of the authors and do not necessarily represent those of their affiliated organizations, or those of the publisher, the editors and the reviewers. Any product that may be evaluated in this article, or claim that may be made by its manufacturer, is not guaranteed or endorsed by the publisher.

## Abbreviations

NLRP1, NACHT and leucine-rich repeat-containing protein family, pyrin domain-containing protein 1.
