# Supplementary material for: UVB-Induced Skin Autoinflammation Due to Nlrp1b Mutation and Its Inhibition by Anti-IL-1β Antibody
Source: Front Immunol. 2022 Jun 17;13:876390. doi: 10.3389/fimmu.2022.876390 (PMC9248282; doi:10.3389/fimmu.2022.876390)
Supplement: Supplementary file 1 [file DataSheet_1.docx]

**Supplemental information**

**UVB-induced skin autoinflammation due to NLRP1 mutation and its inhibition by anti-IL-1β antibody**

**by Murase Y, et al.**

**Supplemental Figures**

**Supplemental Figure S1. Clinical course of autoinflammatory skin lesions on the back of *Nlrp1b* KI and WT mice after UVB irradiation**

Inflammatory lesions with hyperkeratosis are seen on the back, and the inflammation in the KI mice is more severe than in the WT mice.

**Supplemental Figure S2. Serum concentration of IL-18 in *Nlrp1b* KI and WT mice after UVB irradiation**

The serum level of IL-18 (the total amount of cleaved and pro-form IL-1β) is significantly higher in the KI mice (homo) than in the KI mice (hetero) at Day 5, but not at Day 10.

**Supplemental Figure S3. Pathway analysis of upregulated gene sets**

Pathway analysis shows the significant activation of keratinization and the IL-17 signaling pathway.

**Supplemental Figure S4. Prevention of UVB-induced cutaneous inflammatory lesions in *Nlrp1b* KI mice (hetero) mice**

(A) Protocols for the development of UVB-induced autoinflammatory skin lesions. For the prevention of lesions by anti-IL-1β antibody, *Nlrp1b* KI (hetero) mice were treated with subcutaneous (s.c.) injections of anti-IL-1β antibody from Days 0 and 1. (B, C) Skin inflammation and hyperkeratosis induced by UVB irradiation were inhibited in the *Nlrp1b*-KI (hetero) mice with anti-IL-1β antibody treatment by s.c. administration before UVB irradiation (B), compared with in *Nlrp1b* KI (hetero) mice with control IgG antibody treatment by s.c. administration (C). (D-F) Histopathological features of skin samples from the treated mice. In the *Nlrp1b* heterozygous KI (hetero) mice with anti-IL-1β antibody treatment, the hyperkeratosis and inflammatory cell infiltration (E, F) are very mild, whereas in the *Nlrp1b* KI (hetero) mice with control IgG antibody treatment, severe hyperkeratosis with the remarkable infiltration of inflammatory cells in the subcorneal areas and in the stratum corneum is observed (G-I) and the infiltration of various inflammatory cells in the dermis is seen (I).

**Supplemental Tables**

**Supplemental Table S1.** **List of loci and primers for potential off-target cleavage sites predicted by CRISPOR**

| Chrom | Locus | Primer sequence |
| --- | --- | --- |
| Chr9 | intron:6030419C18Rik | AATCTCAAGCAGGGACATGG |
|  |  | GCTCTAGGCAAGCCCCTATT |
| chr12 | intron:Nrxn3 | GGAGCCTGGGAAATTCTAGC |
|  |  | TAAAGGGGAGAGGGGAAATG |
| chr18 | intron:Proc | CCAAGCCTCAAGAGTGGATT |
|  |  | AGGACGCCAAGGAATAAAGG |
| chr9 | intron:Gm16322 | AACTCCTGAGTGCCTGCTGT |
|  |  | TTGCAGGAAGTAGGGGCTTA |
| chr15 | intergenic:Gm24232-Gm23987 | CCATGAAAGGTGGTTCTTTCT |
|  |  | TTCCTTGGCTTCTGCTTTTC |

**Supplemental Table S2. Frequencies of *Nlrp1b* KI genotypes in pups derived from intercrosses between *Nlrp1b* KI mice (*Nlrp1b*^P926R/+^)**

| *Nlrp1b* KI (*Nlrp1b*^P926R/P926R^: homo) | *Nlrp1b* KI (*Nlrp1b*^P926R/+^: hetero) | WT | Total |
| --- | --- | --- | --- |
| 45 | 83 | 58 | 186 |

**Supplemental Table S3. Significantly and greatly upregulated or downregulated genes induced by UVB irradiation in *Nlrp1b* KI mice**

See the attached file.

**Supplemental Table S4. Gene Ontology analysis of lesional skin**

See the attached file.

**Supplemental Table S5 Pathway analysis of lesional skin**

See the attached file.
