# Supplementary material for: UVB-Induced Skin Autoinflammation Due to Nlrp1b Mutation and Its Inhibition by Anti-IL-1β Antibody
Source: Front Immunol. 2022 Jun 17;13:876390. doi: 10.3389/fimmu.2022.876390 (PMC9248282; doi:10.3389/fimmu.2022.876390)
Supplement: Supplementary file 2 [file Image_1.pdf]

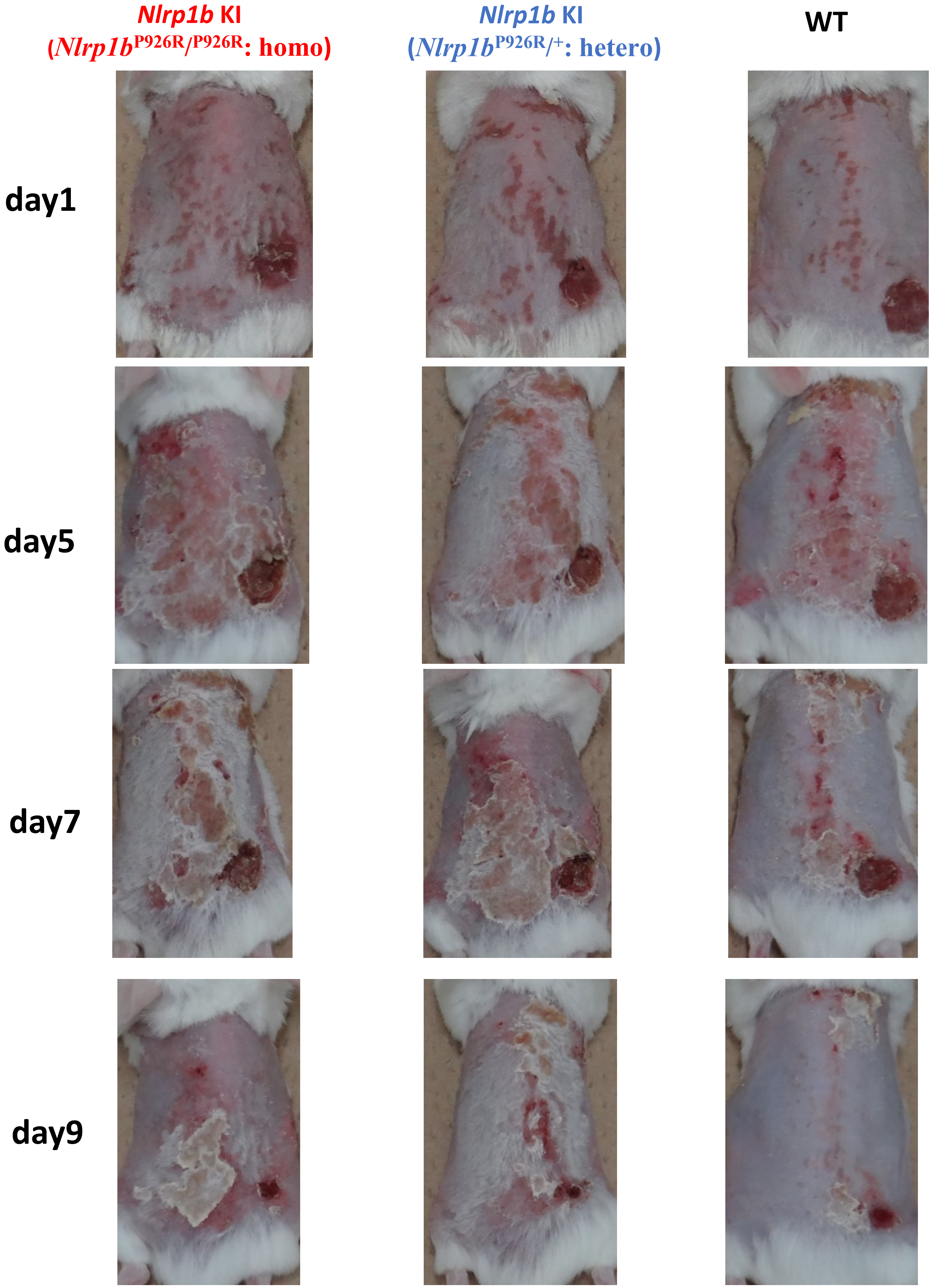

Supplemental Figure S1 Clinical course of autoinflammatory skin lesions on the back of *Nlrp1b* KI and WT mice after UVB irradiation
