# Supplementary material for: UVB-Induced Skin Autoinflammation Due to Nlrp1b Mutation and Its Inhibition by Anti-IL-1β Antibody
Source: Front Immunol. 2022 Jun 17;13:876390. doi: 10.3389/fimmu.2022.876390 (PMC9248282; doi:10.3389/fimmu.2022.876390)
Supplement: Supplementary file 3 [file Image_2.pdf]

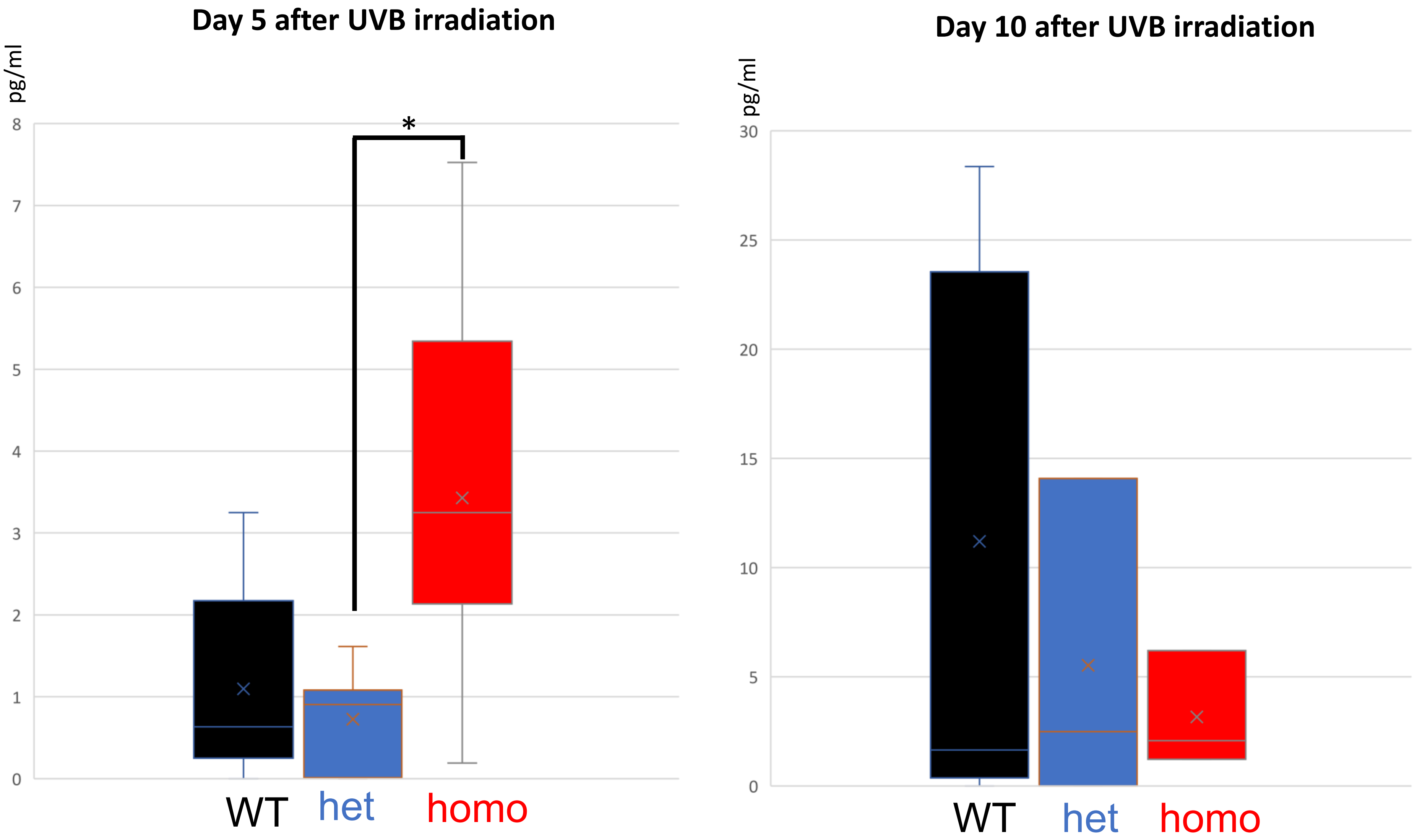

Supplementary Figure S2. Serum concentration of IL-18 (cleaved+pro-form) in *Nlrp1b* KI and WT mice after UVB irradiation
