# Supplementary figures and images for: UVB-Induced Skin Autoinflammation Due to Nlrp1b Mutation and Its Inhibition by Anti-IL-1β Antibody
Source: Front Immunol. 2022 Jun 17;13:876390. doi: 10.3389/fimmu.2022.876390 (PMC9248282; doi:10.3389/fimmu.2022.876390)

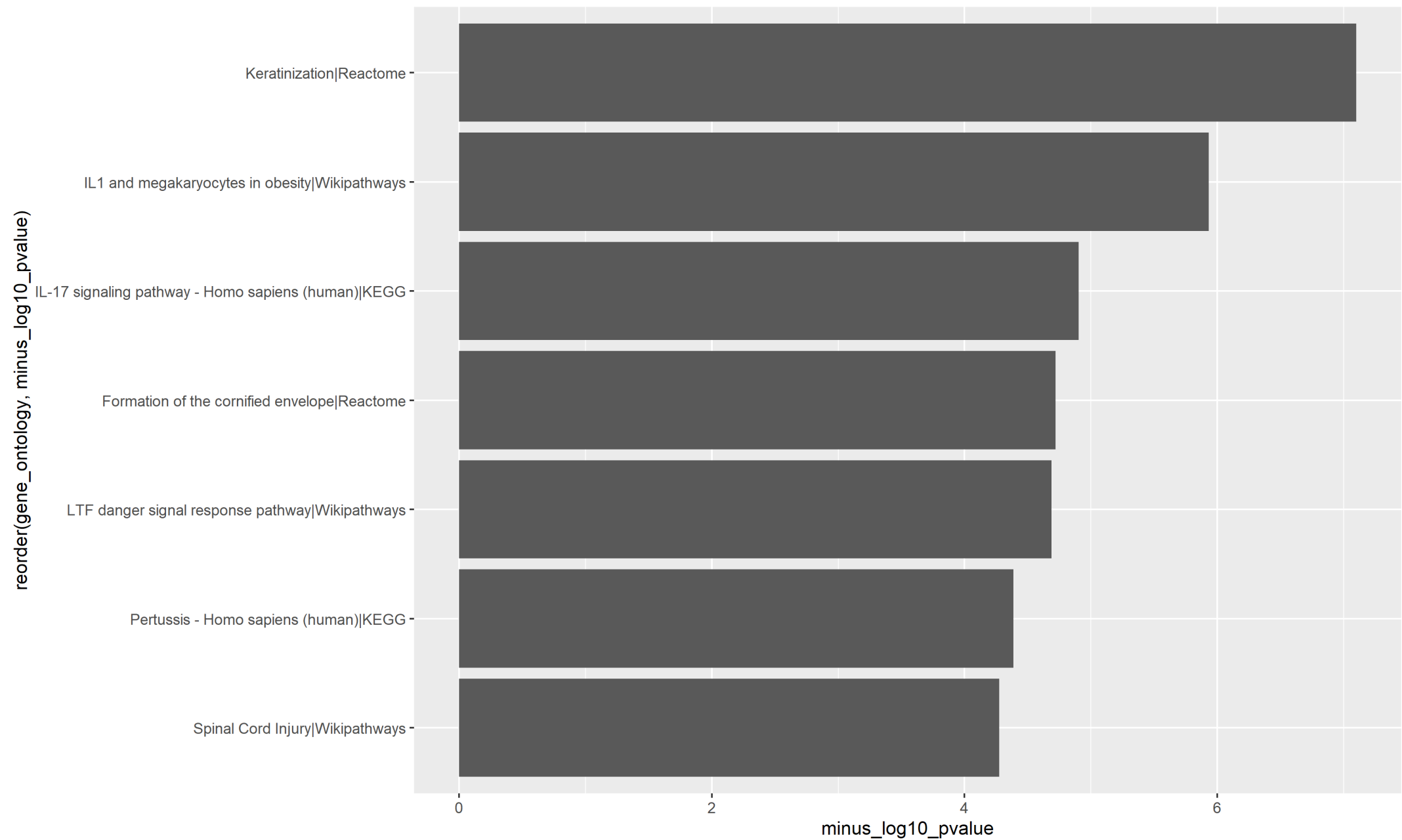

**Supplemental Figure S3 Pathway analysis of upregulated gene sets**

Supplement: Supplementary file 4 [file Image_3.pdf]
