# Supplementary material for: UVB-Induced Skin Autoinflammation Due to Nlrp1b Mutation and Its Inhibition by Anti-IL-1β Antibody
Source: Front Immunol. 2022 Jun 17;13:876390. doi: 10.3389/fimmu.2022.876390 (PMC9248282; doi:10.3389/fimmu.2022.876390)
Supplement: Supplementary file 5 [file Image_4.pdf]

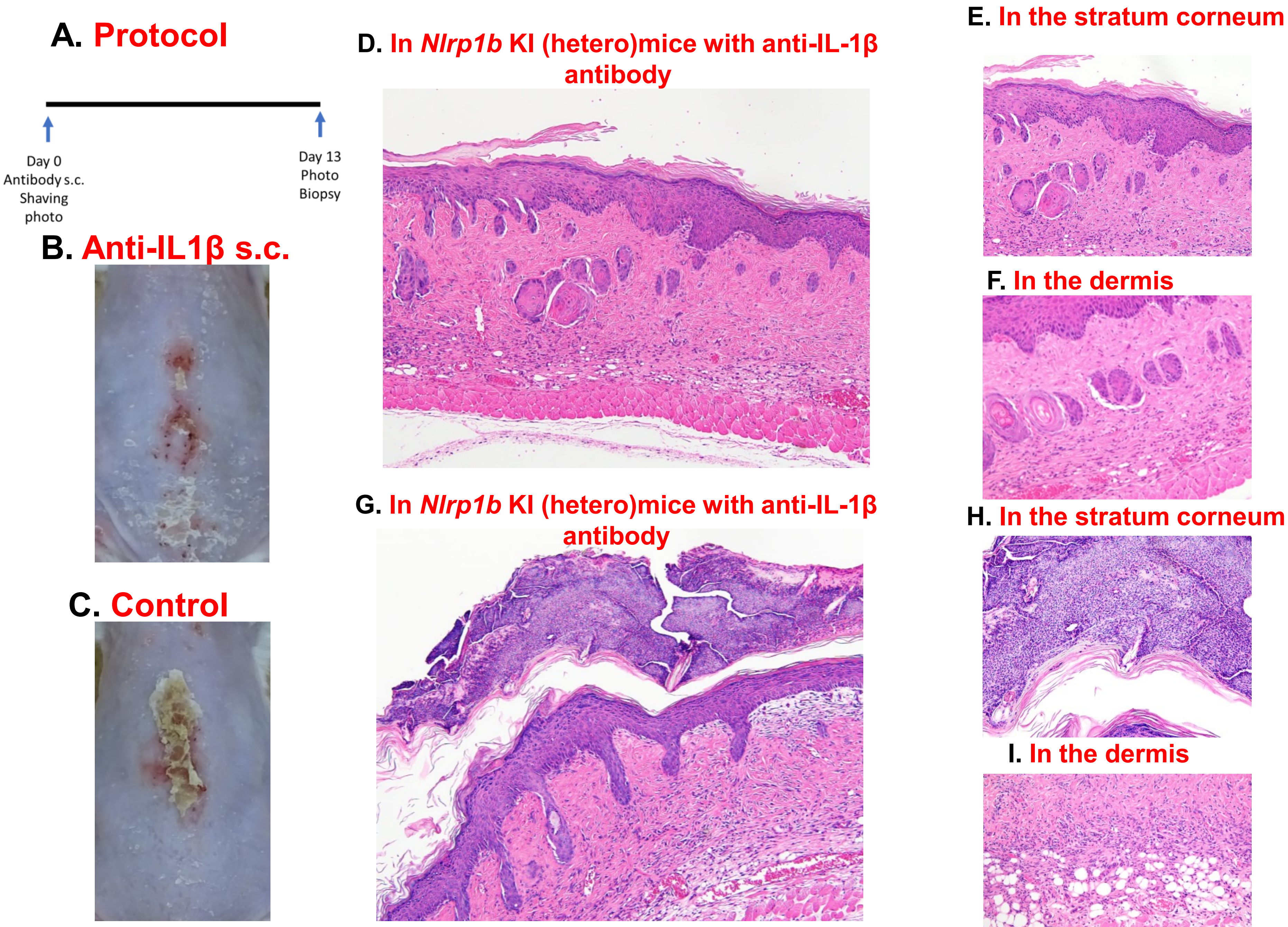

Supplemental Figure S4. Prevention of UVB-induced cutaneous inflammatory lesions in Nlrp1b KI mice (hetero) mice
