# Supplementary material for: UVB-Induced Skin Autoinflammation Due to Nlrp1b Mutation and Its Inhibition by Anti-IL-1β Antibody
Source: Front Immunol. 2022 Jun 17;13:876390. doi: 10.3389/fimmu.2022.876390 (PMC9248282; doi:10.3389/fimmu.2022.876390)
Supplement: Supplementary file 6 [file Table_3.pdf]

Supplemental Table S3 Significantly and greatly up-regulated or down-regulated genes induced by UVB irradiation in *Nlrp1b* KI mice

gene symbol	log2FC (hetero after vs hetero before)	log2FC (homo after vs homo before)	log2FC (WT after vs WT before)	log2FC (hetero after vs WT after)	log2FC (homo after vs WT after)	DESeq2 p-value (homo after vs homo before)	DESeq2 p-value (het	DESeq2 adjusted p-value (homo after vs homo before)	DESeq2 adjuste	significance
2310043M15Ri	2.571198955	2.106322239	1.809718021	0.808399213	0.407541359	0.000220704	3.03E-10	0.001990035	1.95E-08	UP
2610528A11Ri	8.550340592	7.635746084	6.751454214	1.297793007	0.65178154	1.43E-40	3.92E-43	2.01E-37	9.17E-40	UP
4631405J19Rik	5.186847196	5.296189182	3.695263484	1.500934997	1.51058149	2.93E-08	6.16E-08	6.73E-07	2.37E-06	UP
Acod1	8.963215899	6.884296238	5.021034204	1.429362368	0.007839269	1.29E-10	4.20E-39	4.83E-09	5.89E-36	UP
Adam8	2.883640414	3.151199758	1.869424304	0.561140394	-0.031763149	6.99E-12	7.31E-08	3.29E-10	2.75E-06	UP
Agtr2	2.751240529	2.196170278	1.21357525	1.139753457	1.019569235	6.17E-07	1.63E-11	1.08E-05	1.38E-09	UP
Akr1d1	8.80618116	8.232345288	6.652476061	2.437726199	1.918121841	9.84E-19	1.93E-21	1.35E-16	7.52E-19	UP
Ankrd37	2.028364809	2.092177431	1.484249477	1.168578445	0.765083239	3.54E-06	1.40E-06	5.24E-05	3.90E-05	UP
Ankrd66	4.353958542	4.275079805	2.123744753	0.858157816	0.140057959	1.07E-05	6.09E-07	0.000142179	1.90E-05	UP
Apoa1	4.162157768	3.305722467	2.703990198	1.466592951	1.265412501	0.000344079	2.03E-05	0.002881042	0.000404803	UP
Aqp5	3.128638651	4.241582557	2.903365789	2.044393549	1.606833758	3.26E-39	1.90E-10	4.03E-36	1.27E-08	UP
B230303A05Ril	5.37164243	4.852670456	4.520603183	0.851039246	0.332067272	5.30E-07	4.81E-09	9.41E-06	2.34E-07	UP
Calm4	3.260553133	3.07620869	1.584514241	1.685837984	1.400012334	4.01E-11	1.53E-07	1.62E-09	5.44E-06	UP
Casp14	4.94412273	3.63600566	2.634265289	2.241144346	1.801268507	7.79E-14	7.46E-69	5.08E-12	5.24E-65	UP
Ccl3	7.804674128	8.178340614	6.058029112	1.615042341	0.348173092	1.31E-10	6.13E-29	4.89E-09	4.16E-26	UP
Ccl4	7.160368407	7.565641282	6.278308586	1.445745783	0.212086206	6.83E-06	1.37E-37	9.48E-05	1.60E-34	UP
Ccno	3.325392504	2.684309074	1.985882839	0.955035029	-0.402369478	0.001008236	1.64E-06	0.007184581	4.52E-05	UP
Cd14	3.213120711	3.204712127	1.969975982	0.771601945	-0.0666759	0.000143517	1.72E-05	0.001363121	0.000353061	UP
Cd5l	3.19909933	3.921639305	1.335702567	0.737417529	1.316062497	2.07E-14	5.79E-10	1.55E-12	3.51E-08	UP
Cd93	2.616283932	2.017443691	1.613917305	0.505128271	-0.122366922	1.75E-10	9.08E-22	6.32E-09	3.61E-19	UP
Cdhr1	6.007005446	4.966013987	5.638740705	1.15400348	-0.028862118	2.57E-11	6.14E-15	1.09E-09	9.18E-13	UP
Chga	2.451407209	3.04114054	2.397872362	0.204379807	0.808173977	2.12E-05	0.000108427	0.000259013	0.00168397	UP
Clec2e	4.590372327	5.370122451	3.668069538	1.186622772	0.933534172	6.80E-16	1.15E-16	6.21E-14	2.26E-14	UP
Clec4d	6.35388173	5.972048026	4.397131907	0.679418545	-0.364531724	1.49E-08	1.20E-15	3.63E-07	1.94E-13	UP
Clec4e	5.878729753	5.799849892	4.654887858	0.794916679	-0.374257576	1.07E-10	7.96E-43	4.04E-09	1.68E-39	UP
Col10a1	3.321050548	2.562938501	1.836023921	2.432728715	0.85542786	0.000529672	6.33E-08	0.004151964	2.43E-06	UP
Col7a1	2.083474494	3.047139012	1.341225883	0.975937198	0.831266397	4.34E-24	0.000173928	1.36E-21	0.002517466	UP
Csta3	5.62104645	3.255706576	4.92394691	0.726900958	-0.671129873	5.48E-07	6.26E-14	9.67E-06	8.00E-12	UP
Cstdc4	9.946443144	8.919379144	8.241006165	1.009689203	-0.069274635	9.15E-12	5.82E-48	4.24E-10	2.04E-44	UP
Cstdc5	8.882947013	7.357111285	8.803812756	1.039619872	0.503147732	3.29E-63	5.24E-119	1.38E-59	1.10E-114	UP
Cstdc6	6.181702497	6.065311768	5.583225995	1.13556676	0.811118899	1.73E-10	2.55E-11	6.29E-09	2.02E-09	UP
Cxcl2	8.237118745	8.202652687	5.970217628	1.746608098	0.432668164	1.17E-08	2.73E-09	2.91E-07	1.40E-07	UP
Cxcl3	8.614476525	7.865576424	7.206828827	1.204295352	-0.430520674	1.75E-06	9.74E-39	2.77E-05	1.28E-35	UP
Cyp21a1	2.087053925	3.223773095	1.357123557	0.624162174	0.86339479	8.46E-06	0.000545585	0.000114572	0.006524714	UP
Cyp4f18	5.386909328	5.858859855	3.777825708	1.367932302	0.293590579	3.77E-09	4.92E-32	1.05E-07	3.84E-29	UP
D030007L05Ril	3.052107894	3.547190026	2.207093716	0.936172651	0.088664634	9.80E-05	0.000420068	0.000974134	0.005250223	UP
Defb14	4.329056766	3.036315111	3.553113073	0.788901672	-0.029673144	9.24E-07	2.23E-07	1.55E-05	7.68E-06	UP
Degs2	2.947219442	3.086618318	2.292707411	1.279053372	0.795375233	4.25E-11	6.48E-06	1.71E-09	0.000151368	UP
Esm1	3.260464208	2.309855301	2.538908628	0.885438288	0.11491117	0.000300002	1.41E-07	0.002568302	5.05E-06	UP
F10	2.391405593	3.450071167	2.260331783	0.666267814	0.002109427	1.16E-05	0.000209896	0.000151762	0.00295087	UP
Fam71f2	3.816052818	2.98300951	2.075863036	1.483786254	0.844160677	1.28E-05	8.19E-10	0.000164993	4.73E-08	UP
Fetub	3.729448013	3.424640274	3.522112969	0.77113502	-0.160260829	1.02E-16	3.47E-13	1.05E-14	4.08E-11	UP
Fgd3	2.705715883	2.079999767	1.54318496	0.607916053	0.103551114	5.86E-08	6.74E-06	1.26E-06	0.000156778	UP
Flg	5.060087081	4.029035954	2.139107369	3.166542361	2.980317374	2.61E-05	4.00E-32	0.000309427	3.30E-29	UP
Gast	3.586882016	3.317610023	1.595254617	1.991627399	1.722355406	0.000299741	6.86E-05	0.002567114	0.001161042	UP
Gm11427	3.094322569	3.445934728	1.728327223	0.66889885	0.199700846	8.34E-05	0.000463391	0.00085105	0.00571036	UP
Gm17749	3.912873376	3.304150836	1.27211966	2.03767906	0.78150079	0.000288845	1.86E-05	0.002486014	0.000377579	UP
Gm3519	2.291020268	2.782591934	1.678032461	1.1898012	1.257935039	4.32E-08	5.08E-08	9.50E-07	1.99E-06	UP
Gm37277	2.531863942	2.487729246	0.71085397	1.424615174	0.969728104	3.20E-05	1.29E-08	0.000371188	5.71E-07	UP
Gm45694	3.187455443	2.764895842	1.734144352	1.435092607	0.938263354	3.26E-05	1.19E-07	0.000376772	4.34E-06	UP
Gm47128	3.400251625	3.555073703	2.290380721	0.905177576	0.807109113	1.60E-05	0.00010516	0.000202365	0.001645369	UP
Gm47316	2.83948811	2.512993041	1.294740722	1.87287671	1.772283016	2.70E-13	6.30E-11	1.60E-11	4.65E-09	UP
Gm48704	2.896800488	2.772831213	3.504451292	0.709543795	-0.23765772	0.001173509	0.000202507	0.008165572	0.002867306	UP
Gm49428	3.321169598	2.990044725	1.852505663	0.923374828	-0.661769024	0.000938476	2.05E-09	0.006779494	1.08E-07	UP
Gm5478	8.979748317	7.658362596	6.295082206	1.534245705	0.198773326	1.74E-09	7.04E-17	5.15E-08	1.44E-14	UP
Gm6325	3.474016667	4.344489034	2.650078317	0.87254603	1.501434315	1.27E-05	0.000227178	0.000164255	0.003151756	UP
Gm6551	3.306306384	3.016257977	2.241567243	1.385478904	1.015976322	5.01E-18	6.81E-08	5.98E-16	2.59E-06	UP
Gm7463	5.423520311	4.943316784	2.446564429	2.976955882	2.496752355	7.41E-08	1.57E-08	1.57E-06	6.76E-07	UP
Gm8080	3.952945163	2.948076337	2.579105234	1.373839929	0.69686744	0.001425977	9.62E-06	0.009613363	0.000214463	UP
Gm9696	3.000187344	2.257856043	1.746221161	1.28873146	0.947040587	1.74E-08	7.94E-13	4.14E-07	8.80E-11	UP
Grip1	2.414715751	2.224634612	2.058409649	0.730168768	0.459083531	2.97E-08	3.05E-07	6.79E-07	1.02E-05	UP
Gsdmc	7.22457958	8.284721025	6.641686888	1.440814248	0.876351664	1.34E-05	3.10E-33	0.000171376	2.84E-30	UP
Gsdmc2	5.0463529	4.840496626	4.074022383	0.994903962	0.560093684	2.54E-07	4.38E-09	4.82E-06	2.14E-07	UP
Gsdmc4	4.880383486	4.872046356	4.052986383	1.21668716	0.819059973	3.21E-08	2.17E-07	7.30E-07	7.50E-06	UP
Hcar2	2.388770869	2.32804226	1.555493087	1.212338441	0.760463546	4.75E-09	2.79E-09	1.30E-07	1.41E-07	UP
Hdc	3.689948357	3.915427837	2.698112413	0.531078748	-0.151546775	6.85E-15	2.75E-10	5.40E-13	1.79E-08	UP
Htr1b	4.53686201	3.975989486	3.300585404	0.533284897	-0.381705832	8.14E-08	2.81E-11	1.70E-06	2.21E-09	UP
Igfl3	4.092590503	5.832059108	2.300754402	2.311005588	2.344510475	9.79E-24	4.38E-06	2.82E-21	0.00010731	UP
Il18	2.568306254	3.420973254	2.23642162	0.358811267	0.555261634	1.75E-59	7.32E-08	6.12E-56	2.75E-06	UP
Il1b	4.570909446	4.149841802	2.912522529	1.086170146	-0.300791141	0.00019414	7.79E-09	0.00177324	3.64E-07	UP
Il1f5	2.200068526	2.280901785	1.086427266	1.621987987	1.497728233	6.75E-11	8.39E-14	2.62E-09	1.05E-11	UP
Il1f8	4.708399273	4.21357854	3.035463954	1.731529276	1.349358291	5.47E-17	1.64E-12	5.80E-15	1.72E-10	UP
Kcnv1	4.583123945	3.524782911	2.390613449	1.9388382	0.880497165	0.000704059	2.08E-07	0.005314008	7.23E-06	UP
Klk13	4.930531645	2.970655935	4.206123376	1.328268252	0.395415082	3.22E-11	3.51E-20	1.33E-09	1.09E-17	UP
Klk6	3.162558237	2.987192526	2.946766465	1.020691929	0.626716851	7.17E-05	8.37E-20	0.000747845	2.45E-17	UP
Klk8	4.499551732	3.391456225	3.077296333	1.367643516	1.022580553	5.63E-15	9.85E-16	4.51E-13	1.65E-13	UP
Klk9	4.179113859	2.781393695	2.789455595	1.332866919	0.651616895	7.10E-05	2.05E-06	0.00074264	5.54E-05	UP
Krt1	4.769272601	4.168246779	2.021907248	2.497854287	1.981235893	2.42E-05	6.10E-07	0.000289874	1.90E-05	UP
Krt10	2.87898806	2.726511017	0.608274303	2.25278187	1.815301916	0.000763601	5.91E-05	0.005686389	0.001027767	UP
Krt14	2.017625974	2.16498491	1.720971847	0.64933224	0.352763481	2.47E-09	1.07E-09	7.15E-08	5.99E-08	UP
Krt16	4.348851399	3.68278576	3.945100043	1.204434279	-0.006971654	1.08E-05	1.78E-09	0.000142932	9.53E-08	UP
Krt6a	2.65983655	2.548184554	2.231773329	0.893670301	0.090233195	4.33E-07	4.94E-21	7.87E-06	1.79E-18	UP
Krt6b	5.580679973	4.941885338	4.641311959	1.33717323	0.1041423	6.94E-10	2.48E-32	2.25E-08	2.18E-29	UP
Lce1f	3.851992176	3.331256416	2.616083186	1.209654128	1.180082586	0.001133432	3.70E-06	0.007928738	9.29E-05	UP
Lce1g	3.407199358	2.839401248	3.16815765	0.936726565	0.322764214	0.000809782	3.87E-09	0.005971006	1.91E-07	UP
Lce1k	3.265163812	2.657682428	2.997934606	1.161534929	0.729503372	0.001472134	5.74E-07	0.009886416	1.80E-05	UP
Lce3b	4.823633417	2.578147372	3.295966453	1.445459471	0.154380822	5.40E-05	2.83E-06	0.00058559	7.38E-05	UP
Lce3e	7.845103073	5.753786069	7.095143336	1.741278167	0.427091527	9.32E-06	1.29E-27	0.000125164	7.78E-25	UP
Lce3f	5.627749794	3.881097597	5.572600321	1.210794516	0.071390053	2.22E-21	5.95E-16	4.54E-19	1.06E-13	UP
Lilrb4a	4.267456965	4.6070988	2.892230761	0.579411395	-0.146828973	1.23E-16	1.11E-10	1.24E-14	7.90E-09	UP
Mak	3.252859038	2.572734852	0.917442884	1.088094085	0.718607867	0.000606201	3.33E-05	0.004675073	0.000621047	UP
Mest	3.444021329	2.723422499	2.13034049	1.175101895	0.325551176	8.50E-10	4.71E-19	2.70E-08	1.29E-16	UP
Mgarp	3.530170066	4.057597826	2.39072005	0.815791764	0.733804517	7.15E-09	7.11E-08	1.88E-07	2.69E-06	UP
Mmp12	2.575771432	2.488998957	1.382379933	1.128677666	0.107054008	1.07E-07	5.25E-06	2.18E-06	0.000125466	UP
Mmp13	5.857462494	5.683566588	4.586393767	0.799247575	-0.778140393	1.73E-17	5.91E-23	1.96E-15	2.49E-20	UP
Mmp9	4.294481412	4.015131328	3.54311136	0.796954205	-0.348988165	6.02E-15	2.53E-19	4.81E-13	7.10E-17	UP
Mos	2.914825724	2.901775212	1.857330763	0.702235372	0.839410755	0.000586459	0.000323743	0.004544517	0.004227268	UP
Mrgpra2a	5.827840744	4.733084708	3.75994205	0.928401891	-0.166354144	0.000429521	1.28E-10	0.003471881	8.95E-09	UP
Mrgpra2b	6.629156484	6.525674523	5.693520749	0.580866523	-0.501683046	5.16E-06	4.10E-20	7.42E-05	1.25E-17	UP
Ndufa4l2	2.036678031	3.116538286	0.95513612	1.183784257	1.161597715	1.22E-40	3.23E-05	1.83E-37	0.000604688	UP
Nfkbid	3.610833178	3.113289699	1.652877393	1.368048572	0.449998495	3.78E-08	1.47E-19	8.49E-07	4.19E-17	UP
Ngp	6.139356955	7.42786133	2.700883941	0.70886861	1.557477035	7.56E-08	2.07E-11	1.59E-06	1.69E-09	UP
Nlrp3	5.261016588	4.854268857	3.890989879	1.112118674	0.172330876	3.40E-07	7.76E-37	6.32E-06	8.17E-34	UP
Nmu	4.412948658	4.231017313	3.509724677	1.244429545	1.025755224	1.20E-05	4.63E-06	0.000156274	0.000112304	UP
Nos2	4.397029194	3.587163461	3.457140892	1.296118274	0.502683362	1.15E-08	5.48E-20	2.86E-07	1.63E-17	UP
Nxph4	3.76516929	4.39082681	2.178930161	1.279117951	1.23080411	2.04E-21	3.11E-09	4.20E-19	1.57E-07	UP
Olfm4	4.437264606	3.340131525	3.135134193	0.84878561	-0.117678023	3.96E-05	2.13E-12	0.000445097	2.17E-10	UP
Olfr558	2.413690609	2.734578406	1.58060438	0.504428334	-0.148720351	5.28E-10	1.06E-09	1.75E-08	5.97E-08	UP
Osm	4.432631886	5.073069176	3.598081295	0.881332701	0.010626656	2.38E-14	1.61E-11	1.74E-12	1.36E-09	UP
Otop3	2.763110304	2.506649807	2.100234423	1.174065656	0.550577753	5.82E-06	2.18E-14	8.24E-05	3.02E-12	UP
P4ha3	4.799600437	4.271314519	2.934419597	0.578473633	0.192183403	6.68E-14	1.87E-14	4.47E-12	2.67E-12	UP
Pglyrp3	2.112617045	2.188230986	1.323206577	1.286948309	0.925565201	2.15E-07	9.17E-06	4.15E-06	0.000206205	UP
Pitx1	4.447651619	4.53984368	2.703453098	1.511311403	0.99620058	8.75E-35	1.74E-14	6.56E-32	2.51E-12	UP
Pla2g4d	5.93006817	4.346207341	3.999405306	2.378080266	1.003214733	4.07E-06	1.75E-14	5.95E-05	2.51E-12	UP
Plaur	3.054453205	3.21422392	1.635805778	1.074941304	0.409831686	1.31E-10	9.71E-10	4.87E-09	5.48E-08	UP
Prnd	3.251952484	2.990101109	2.736855868	0.663660687	0.193689876	1.29E-08	5.27E-09	3.18E-07	2.55E-07	UP
Prss12	2.228702899	2.154542076	1.543671407	0.791018141	0.555040417	6.89E-07	2.60E-16	1.19E-05	4.80E-14	UP
Prss27	2.184117393	2.05663497	2.561131792	0.590812341	0.321602997	7.09E-06	0.000216003	9.80E-05	0.003020604	UP
Psca	5.660247881	4.853349642	4.713435626	0.849573999	1.157086297	1.07E-13	2.96E-10	6.81E-12	1.91E-08	UP
Ptprn	3.167137762	3.182352417	2.433959154	0.659714411	-0.113265312	2.40E-07	9.90E-12	4.58E-06	8.72E-10	UP
Ptprz1	3.418849008	2.243385664	2.770575472	0.967877964	0.210242934	1.39E-05	3.88E-11	0.000178296	2.98E-09	UP
Rbp2	3.991346211	3.564851526	3.000794851	1.530411442	1.198858691	1.04E-35	2.26E-40	8.75E-33	3.65E-37	UP
Retnlg	6.45894712	7.472464003	5.574525492	1.231008997	0.206580707	8.56E-11	7.35E-29	3.28E-09	4.83E-26	UP
Rgn	2.674230288	2.800558008	2.734321824	0.622544811	1.003739357	0.000122368	7.14E-05	0.001190791	0.001196621	UP
Rptn	4.014213405	3.577796317	1.856726338	2.813528849	1.938757867	1.48E-18	2.35E-18	1.93E-16	5.88E-16	UP
Rsad2	3.284910179	2.605283987	1.582103242	0.646713992	-0.540740449	1.50E-09	1.95E-08	4.51E-08	8.23E-07	UP
Rtp3	2.670176371	2.423507112	1.119299026	0.681284409	0.198197038	0.000248435	4.86E-07	0.002187479	1.54E-05	UP
S100a8	5.784386844	3.791235841	3.862529006	1.056142012	-0.34811551	9.61E-06	7.72E-16	0.000128681	1.34E-13	UP
S100a9	5.891793727	5.084465994	4.401681715	0.852218096	-0.412705874	1.97E-07	2.21E-43	3.83E-06	5.83E-40	UP
Serpina9	3.821773651	2.4983852	2.517135712	2.064865626	1.195896179	0.00103073	8.42E-26	0.00731014	4.22E-23	UP
Serpinb12	8.843869242	8.209950132	6.015913353	1.790675143	1.39167551	1.86E-72	5.85E-71	1.30E-68	6.16E-67	UP
Serpinb3a	8.520505463	6.835540743	7.081450591	1.439054873	0.082308696	7.31E-12	9.68E-18	3.42E-10	2.15E-15	UP
Serpinb3b	4.246619295	4.276230166	3.403150867	0.883640113	0.540018905	4.55E-15	2.50E-12	3.69E-13	2.49E-10	UP
Sh2d3c	2.311679537	2.223987446	1.377120287	0.691364207	0.168825513	3.24E-07	1.98E-09	6.05E-06	1.05E-07	UP
Slc23a4	3.486297417	2.340765137	2.226692022	1.955152207	1.215260778	1.80E-05	3.43E-12	0.000223532	3.33E-10	UP
Slc25a48	2.615267991	2.448772403	1.857656221	0.746832526	0.371191551	2.73E-07	0.000127717	5.14E-06	0.001933656	UP
Slc2a1	2.674827314	2.510423761	1.983910214	0.75258424	0.422431278	7.32E-13	2.44E-15	3.97E-11	3.84E-13	UP
Slc2a3	2.875680177	2.657962239	1.387320198	1.178799195	0.485149304	5.36E-07	6.56E-09	9.51E-06	3.12E-07	UP
Slfn4	7.519071153	6.994526805	5.227488311	1.094069216	0.018391453	2.22E-12	2.41E-34	1.13E-10	2.30E-31	UP
Spp1	5.909984054	5.204764843	4.879063011	0.877003091	-0.271922181	9.05E-11	3.50E-18	3.45E-09	8.48E-16	UP
Sprr1b	4.264554179	2.430069623	5.131208646	0.988952315	-0.059655189	1.79E-09	4.56E-14	5.29E-08	5.96E-12	UP
Sprr2a1	7.360703434	7.288023396	5.700625269	1.660078165	1.587398128	4.31E-15	9.42E-16	3.52E-13	1.60E-13	UP
Sprr2a2	8.402848082	8.630969538	6.286091774	2.531043463	2.149783844	1.18E-120	1.75E-20	2.48E-116	5.74E-18	UP
Sprr2a3	5.728381824	5.717225796	4.482584426	2.122252795	1.674568393	4.38E-45	4.83E-15	1.02E-41	7.32E-13	UP
Sprr2d	8.37932933	6.622562321	6.231817498	2.111611245	1.175847369	0.00120235	6.81E-42	0.008344136	1.30E-38	UP
Sprr2e	6.764747884	4.058290094	5.479325684	1.750339287	0.609639189	0.000895803	1.32E-15	0.006509292	2.13E-13	UP
Sprr2g	3.927951684	2.524243616	2.744570008	1.528634746	0.84337939	0.000112301	2.88E-13	0.001101508	3.42E-11	UP
Sprr2i	4.291915693	2.507645548	4.520757918	0.860463264	-0.420993062	0.000883843	8.43E-09	0.006434605	3.91E-07	UP
Sprr2k	5.041755426	4.053008139	3.240846306	1.682528015	0.411447235	1.67E-05	7.82E-08	0.000210067	2.93E-06	UP
Srgn	2.357304234	2.174037795	1.4182758	0.535262704	-0.142858284	7.84E-07	2.45E-10	1.35E-05	1.61E-08	UP
Stfa1	3.713347694	3.028962015	2.327393433	0.834126208	0.14974053	0.001128959	8.57E-05	0.007901703	0.001385894	UP
Stfa2	7.365366752	4.360389859	6.575308978	1.071932334	0.078831753	1.14E-05	2.22E-21	0.000150185	8.50E-19	UP
Stfa2l1	9.203520233	8.493691639	7.388464476	1.068042058	-0.201456571	3.76E-11	2.93E-40	1.52E-09	4.40E-37	UP
Stfa3	7.66533549	7.0214914	7.408323873	0.673841052	0.306058138	3.35E-41	2.52E-63	5.42E-38	1.33E-59	UP
Teddm3	3.58710198	3.131845237	2.694261528	1.218826666	0.728174055	4.75E-14	3.17E-10	3.32E-12	2.03E-08	UP
Them5	2.533810767	2.417659079	0.979777001	1.790461209	1.376872198	3.68E-07	2.59E-07	6.80E-06	8.84E-06	UP
Tmprss11a	4.18736943	2.537475312	1.595735661	1.947077061	0.772124649	8.29E-05	2.83E-11	0.000847956	2.21E-09	UP
Tmprss11b	8.178757685	5.964397442	5.463930478	2.509105355	0.407259923	2.74E-13	1.51E-20	1.61E-11	5.06E-18	UP
Tmprss11d	3.896755347	3.436580352	3.363352459	2.092803415	0.717510675	4.27E-08	1.13E-14	9.41E-07	1.64E-12	UP
Tmprss11g	3.794427283	2.436617704	2.1618779	1.887586534	0.363244439	3.31E-05	8.75E-08	0.000381707	3.25E-06	UP
Tmprss4	2.042919894	2.428063089	1.391378948	0.842713633	0.809139617	9.12E-08	0.000608948	1.89E-06	0.007148516	UP
Tnf	4.877064822	3.81276411	2.741574533	1.831735471	0.447021173	2.65E-05	1.83E-29	0.000314238	1.28E-26	UP
Tprg	2.05582639	2.688709237	0.489022902	1.959096517	2.132844919	6.55E-16	5.87E-06	6.01E-14	0.000138675	UP
Tpsab1	3.116917767	3.158868409	2.185735687	0.79530071	0.861952882	1.08E-08	6.19E-10	2.71E-07	3.70E-08	UP
Trem1	6.145332852	6.15519779	3.967232127	0.617540043	-0.775187428	1.98E-05	5.54E-11	0.000243494	4.12E-09	UP
Treml4	6.172910611	5.196291934	3.652274687	1.567983943	0.100727818	2.41E-05	1.25E-20	0.000289133	4.31E-18	UP
Trib3	2.192678707	2.103889258	2.095625838	0.595534615	0.134595307	4.48E-10	3.35E-08	1.51E-08	1.34E-06	UP
Ucn2	2.914690245	4.044912132	1.954436267	0.971336077	0.860980705	1.02E-08	3.03E-05	2.56E-07	0.000572671	UP
Umodl1	3.310296413	4.279446212	3.029194637	0.331183009	0.804566945	1.00E-06	4.57E-06	1.68E-05	0.000111045	UP
Wfdc12	4.454941501	3.463671238	3.326543083	0.908324189	0.033697755	3.51E-09	2.02E-11	9.78E-08	1.66E-09	UP
Wfdc5	2.240839286	2.362402946	1.406502268	1.210720539	1.099007659	4.02E-21	0.000107199	7.89E-19	0.001668103	UP
1700001C19Ril	-2.535500634	-3.005111061	-1.862281015	-0.797527382	-0.350202489	6.70E-05	8.77E-05	0.00070822	0.001407062	DOWN

2210407C18Rik	-3.692172364	-3.277035726	-2.410911574	-0.206482023	-0.516889723	0.000327327	0.00017169	0.002757266	0.0024885 DOWN
Alox8	-5.778922688	-6.111625844	-4.68602267	-1.237640237	0.429658361	3.63E-08	5.14E-06	8.17E-07	0.000123165 DOWN
Asphd2	-2.56904579	-3.110267065	-2.154295812	-0.724986136	-1.047912651	1.58E-09	0.000320131	4.73E-08	0.004187551 DOWN
Bmp4	-3.139786512	-2.556963479	-2.663601114	-0.56916856	-0.106688761	8.43E-10	6.01E-18	2.69E-08	1.36E-15 DOWN
Cacna2d3	-3.018866213	-4.002204984	-3.160635419	-0.988980587	-0.014731567	8.52E-11	2.52E-05	3.27E-09	0.000491187 DOWN
Cacng4	-3.473133471	-3.577003507	-2.15690311	-0.675112287	0.076267864	9.96E-11	1.42E-08	3.79E-09	6.18E-07 DOWN
Ccer2	-4.514779866	-3.516055326	-2.906556944	-0.653432838	-0.653432838	0.000104922	1.02E-06	0.001037361	2.96E-05 DOWN
Chrna4	-3.54977809	-4.670676836	-2.984922783	-0.926501611	-1.210605248	9.61E-12	3.77E-06	4.45E-10	9.43E-05 DOWN
Cldn10	-4.377110997	-2.813780667	-3.655180681	-1.143603959	0.284534993	2.30E-06	1.59E-17	3.56E-05	3.41E-15 DOWN
Ctse	-2.809873247	-4.081905264	-2.150435425	-0.502953181	-0.438703275	1.37E-23	3.21E-06	3.88E-21	8.22E-05 DOWN
Cyp2b23	-5.622231251	-3.82825093	-4.950198781	-0.505311975	0.344565781	0.000312353	0.000122653	0.00265456	0.00186908 DOWN
Cyp4f37	-4.245931544	-3.540438589	-3.761970122	-0.596009219	-0.365661484	4.04E-08	1.49E-05	9.02E-07	0.000313845 DOWN
Efhd1	-3.381605436	-2.887884174	-2.838572706	-0.712856464	0.584281923	5.53E-16	2.45E-08	5.12E-14	1.02E-06 DOWN
Gm12496	-4.232171136	-3.981481064	-3.312997755	-0.844175529	-0.167482896	8.23E-06	3.16E-06	0.00011172	8.10E-05 DOWN
Gm13490	-4.992649498	-4.523408605	-5.082772172	-0.505391935	1.193521822	7.30E-14	2.71E-07	4.84E-12	9.18E-06 DOWN
Gm28196	-5.915520695	-4.423817389	-3.954412924	-1.422500042	-0.324360018	2.81E-11	6.99E-10	1.18E-09	4.11E-08 DOWN
Gm30655	-3.782536255	-5.257280288	-3.115037665	0.196779077	-0.52129465	1.26E-08	3.75E-06	3.10E-07	9.40E-05 DOWN
Gm43429	-2.362823168	-2.933763782	-1.941276475	-0.640048327	-0.181970972	2.78E-15	7.34E-05	2.32E-13	0.001224129 DOWN
Gm45774	-2.175713482	-2.203545167	-1.577180049	-1.152859441	-1.640069858	3.21E-05	0.000365146	0.000371557	0.00466318 DOWN
Gm47991	-4.934640932	-4.821123801	-3.313389897	-0.966365077	0.733493848	1.52E-05	7.05E-07	0.000193187	2.16E-05 DOWN
Gm49708	-2.476132697	-2.736755647	-1.630697949	-0.645337532	-0.251265999	9.75E-13	1.69E-08	5.21E-11	7.23E-07 DOWN
Gm5434	-3.218826918	-3.181306842	-2.070391013	-0.654135003	-0.371627482	0.00099825	0.00064448	0.00712551	0.007486344 DOWN
Gm5849	-5.495971608	-4.284674032	-4.887891264	-0.810416466	0.69647487	8.36E-10	3.10E-08	2.67E-08	1.25E-06 DOWN
Gng13	-5.198472665	-5.209043556	-4.410112038	-1.006218976	0.228898799	1.97E-10	9.82E-09	7.07E-09	4.51E-07 DOWN
Gnmt	-2.954900844	-3.248204272	-1.588903798	-0.553111804	0.737827197	2.72E-09	1.26E-05	7.82E-08	0.000271739 DOWN
Gpr143	-2.879331383	-6.25202818	-3.454338278	0.745970881	-0.515268502	2.06E-18	7.08E-05	2.64E-16	0.00119091 DOWN
Gucy2c	-4.122791838	-5.083478239	-3.281933378	-0.519843296	0.323256088	4.33E-10	2.06E-05	1.47E-08	0.000409527 DOWN
H2-M2	-4.918508937	-5.616383557	-4.650289783	-0.11659032	-1.47793371	3.42E-14	6.47E-23	2.45E-12	2.67E-20 DOWN
H2-M5	-5.649420827	-4.528085682	-5.012780361	-0.694752757	-0.353401162	1.19E-08	3.05E-15	2.95E-07	4.73E-13 DOWN
Hsd17b14	-3.103893201	-3.912148295	-2.035900557	-0.929620728	-0.776739025	1.63E-06	1.91E-06	2.61E-05	5.19E-05 DOWN
Hunk	-2.433559497	-3.546856231	-1.743728135	-0.578838006	-0.328078726	2.14E-28	1.62E-05	8.62E-26	0.000335465 DOWN
Lef1os1	-4.395671988	-4.147703716	-3.53958096	-0.958681943	0.997242604	2.96E-12	1.07E-05	1.49E-10	0.000235639 DOWN
Mc1r	-3.017858281	-5.636101979	-1.899683817	-0.649579239	-0.765150659	1.48E-06	0.000398546	2.38E-05	0.005022963 DOWN
Mc5r	-4.475743976	-2.917155222	-3.83050273	-0.615506741	1.217758981	8.03E-12	2.37E-34	3.74E-10	2.30E-31 DOWN
Mtcl1	-2.64770036	-4.116235318	-1.586819314	-0.84330774	-1.435018218	3.67E-22	1.27E-06	8.38E-20	3.59E-05 DOWN
Mucl1	-3.209235731	-2.646182457	-2.569181038	-1.536871683	-1.885502004	0.000841109	1.43E-06	0.006171661	3.99E-05 DOWN
Notum	-4.807609802	-2.620162092	-3.077672264	-1.618270413	0.664517351	1.16E-06	3.27E-09	1.90E-05	1.64E-07 DOWN
Omp	-2.961487204	-3.338176822	-2.508640676	-0.662032842	0.096102803	4.32E-13	7.40E-05	2.44E-11	0.001231211 DOWN
P2rx2	-4.504345323	-4.067124741	-3.803535775	-0.625058516	-0.876373722	1.33E-05	5.44E-14	0.000170116	6.99E-12 DOWN
Scd3	-5.347528048	-3.50770524	-5.07283141	-0.509982008	0.974395455	2.67E-18	9.61E-20	3.34E-16	2.77E-17 DOWN
Slc13a5	-4.245341359	-3.769792647	-2.377639163	-0.796848329	-0.172091984	9.27E-06	2.67E-05	0.000124592	0.000514185 DOWN
Slc39a8	-3.11732654	-3.180381318	-2.272896241	-0.58320851	0.069807383	2.05E-09	8.54E-11	6.03E-08	6.18E-09 DOWN
Slco5a1	-2.444493028	-3.444132113	-2.51476788	-0.314854476	-0.640045015	1.38E-13	1.22E-05	8.63E-12	0.000264266 DOWN
Slmapos2	-2.927512495	-2.339034365	-2.282441425	-0.715682329	-0.391155563	2.55E-10	4.08E-09	8.99E-09	2.01E-07 DOWN
Sv2b	-3.494120147	-3.381666867	-2.221991831	-1.331195308	-0.946303136	4.54E-09	6.27E-06	1.24E-07	0.000147384 DOWN
Tbx4	-2.426368687	-2.143230804	-1.870242372	-0.581807379	-0.612593514	0.000450365	3.38E-05	0.003609795	0.000627363 DOWN
Tmem171	-2.813476529	-2.266466582	-0.979639176	-0.852668318	-0.625359408	1.63E-08	2.69E-15	3.93E-07	4.19E-13 DOWN
Tmem56	-5.594216381	-3.965076522	-4.087814886	-1.106534464	1.042845244	5.34E-14	9.09E-29	3.67E-12	5.80E-26 DOWN
Tnfrsf13c	-4.430967264	-2.987509393	-2.40921861	-1.142941296	0.560891898	1.43E-06	2.38E-09	2.30E-05	1.23E-07 DOWN
Trat1	-2.850897719	-2.738423992	-1.296582069	-1.384288421	-1.549893803	3.09E-05	1.67E-06	0.000359597	4.58E-05 DOWN
Trim71	-3.619899524	-3.440378704	-2.737401449	-0.746366806	-0.110859847	0.000206209	0.000107818	0.001874625	0.001675757 DOWN
Tspan1	-3.722331918	-4.482587434	-2.442206432	-0.121868589	-0.520627516	9.76E-07	6.06E-05	1.63E-05	0.001048906 DOWN
Vat1l	-2.071579097	-4.171817179	-1.401049128	-0.633419368	-1.181866032	1.20E-09	0.00028561	3.67E-08	0.00378358 DOWN
Zfp618	-2.335973679	-3.848187773	-0.631355134	-1.057531915	-0.921331686	2.01E-18	0.000107246	2.58E-16	0.001668103 DOWN

Abbreviation: Log2FC, Log2 Fold Change
