# Supplementary material for: UVB-Induced Skin Autoinflammation Due to Nlrp1b Mutation and Its Inhibition by Anti-IL-1β Antibody
Source: Front Immunol. 2022 Jun 17;13:876390. doi: 10.3389/fimmu.2022.876390 (PMC9248282; doi:10.3389/fimmu.2022.876390)
Supplement: Supplementary file 8 [file Table_5.pdf]

Supplemental Table S5 Pathway analysis of lesional skin

<table>
<tr><th>condition</th><th>ID</th><th>logFC</th><th>AveExpr</th><th>t</th><th>P.Value</th><th>adj.P.Val</th><th>B</th></tr>
<tr><td>homo after UVB irradiation VS homo before UVB irradiation</td><td>α-tocopherol degradation|HumanCyc</td><td>0.823103048</td><td>0.008014414</td><td>3.190471602</td><td>0.01042825</td><td>0.06159816</td><td>-2.71359774</td></tr>
<tr><td>hetero after UVB irradiation VS hetero before UVB irradiation</td><td>α-tocopherol degradation|HumanCyc</td><td>1.059727139</td><td>0.052439807</td><td>5.297803354</td><td>0.00061585</td><td>0.03018301</td><td>0.111307454</td></tr>
<tr><td>WT after UVB irradiation VS WT before UVB irradiation</td><td>α-tocopherol degradation|HumanCyc</td><td>0.963280877</td><td>0.038211938</td><td>3.245077597</td><td>0.00870243</td><td>0.24856491</td><td>-2.3417275</td></tr>
<tr><td>homo after UVB irradiation VS homo before UVB irradiation</td><td>β-alanine degradation|HumanCyc</td><td>-0.43954332</td><td>0.060818477</td><td>-1.5603332</td><td>0.15171745</td><td>0.29821001</td><td>-5.20946935</td></tr>
<tr><td>hetero after UVB irradiation VS hetero before UVB irradiation</td><td>β-alanine degradation|HumanCyc</td><td>-1.14454652</td><td>-0.09610546</td><td>-7.48366026</td><td>5.35E-05</td><td>0.02920766</td><td>2.381264318</td></tr>
<tr><td>WT after UVB irradiation VS WT before UVB irradiation</td><td>β-alanine degradation|HumanCyc</td><td>-1.2197698</td><td>-0.1680384</td><td>-5.19410355</td><td>0.00039415</td><td>0.15645713</td><td>0.290977187</td></tr>
<tr><td>homo after UVB irradiation VS homo before UVB irradiation</td><td>γ-glutamyl cycle|HumanCyc</td><td>-0.13413704</td><td>-0.01753237</td><td>-0.56867854</td><td>0.58291884</td><td>0.70506157</td><td>-6.19769555</td></tr>
<tr><td>hetero after UVB irradiation VS hetero before UVB irradiation</td><td>γ-glutamyl cycle|HumanCyc</td><td>0.203796546</td><td>0.081523536</td><td>0.808552186</td><td>0.44100239</td><td>0.66669615</td><td>-6.00536004</td></tr>
<tr><td>WT after UVB irradiation VS WT before UVB irradiation</td><td>γ-glutamyl cycle|HumanCyc</td><td>0.034078019</td><td>0.046398349</td><td>0.123826312</td><td>0.90388667</td><td>0.97566251</td><td>-5.86138994</td></tr>
<tr><td>homo after UVB irradiation VS homo before UVB irradiation</td><td>γ-linolenate biosynthesis|HumanCyc</td><td>-0.19847932</td><td>0.030667011</td><td>-0.9873104</td><td>0.34824872</td><td>0.50415461</td><td>-5.87086459</td></tr>
<tr><td>hetero after UVB irradiation VS hetero before UVB irradiation</td><td>γ-linolenate biosynthesis|HumanCyc</td><td>-0.14951185</td><td>-0.0200035</td><td>-0.84707573</td><td>0.4203598</td><td>0.64729728</td><td>-5.97344099</td></tr>
<tr><td>WT after UVB irradiation VS WT before UVB irradiation</td><td>γ-linolenate biosynthesis|HumanCyc</td><td>-0.36852688</td><td>0.050187049</td><td>-1.54118793</td><td>0.15404887</td><td>0.55854784</td><td>-4.79813928</td></tr>
<tr><td>homo after UVB irradiation VS homo before UVB irradiation</td><td>(S)-reticuline biosynthesis|HumanCyc</td><td>-1.0234841</td><td>0.007517396</td><td>-5.10903186</td><td>0.00055817</td><td>0.01666204</td><td>0.138640915</td></tr>
<tr><td>hetero after UVB irradiation VS hetero before UVB irradiation</td><td>(S)-reticuline biosynthesis|HumanCyc</td><td>-0.58609196</td><td>-0.02703766</td><td>-1.86848388</td><td>0.0967665</td><td>0.2808394</td><td>-4.77115309</td></tr>
<tr><td>WT after UVB irradiation VS WT before UVB irradiation</td><td>(S)-reticuline biosynthesis|HumanCyc</td><td>-0.849039</td><td>0.021479961</td><td>-2.95394328</td><td>0.01431645</td><td>0.28307166</td><td>-2.77724071</td></tr>
<tr><td>homo after UVB irradiation VS homo before UVB irradiation</td><td><i>myo</i>-inositol <i>de novo</i> biosynthesis|HumanCyc</td><td>0.023261274</td><td>-0.06136417</td><td>0.06082907</td><td>0.95277053</td><td>0.97106032</td><td>-6.36646054</td></tr>
<tr><td>hetero after UVB irradiation VS hetero before UVB irradiation</td><td><i>myo</i>-inositol <i>de novo</i> biosynthesis|HumanCyc</td><td>0.259949355</td><td>-0.04030012</td><td>0.863706974</td><td>0.41165888</td><td>0.63929852</td><td>-5.95927603</td></tr>
<tr><td>WT after UVB irradiation VS WT before UVB irradiation</td><td><i>myo</i>-inositol <i>de novo</i> biosynthesis|HumanCyc</td><td>-0.18646893</td><td>-0.05111066</td><td>-0.66814061</td><td>0.51904603</td><td>0.82992425</td><td>-5.64824396</td></tr>
<tr><td>homo after UVB irradiation VS homo before UVB irradiation</td><td><i>N</i>-acetylglucosamine degradation I|HumanCyc</td><td>0.849210727</td><td>-0.02056256</td><td>3.880417185</td><td>0.00344138</td><td>0.03315055</td><td>-1.63221451</td></tr>
<tr><td>hetero after UVB irradiation VS hetero before UVB irradiation</td><td><i>N</i>-acetylglucosamine degradation I|HumanCyc</td><td>0.931024352</td><td>-0.00021826</td><td>4.750962033</td><td>0.001251</td><td>0.04329482</td><td>-0.57299985</td></tr>
<tr><td>WT after UVB irradiation VS WT before UVB irradiation</td><td><i>N</i>-acetylglucosamine degradation I|HumanCyc</td><td>1.069740278</td><td>0.039931768</td><td>4.55622953</td><td>0.00102645</td><td>0.15645713</td><td>-0.50128875</td></tr>
<tr><td>homo after UVB irradiation VS homo before UVB irradiation</td><td><i>N</i>-acetylglucosamine degradation II|HumanCyc</td><td>0.842801126</td><td>-0.03258971</td><td>3.814939743</td><td>0.00381405</td><td>0.03511082</td><td>-1.73271475</td></tr>
<tr><td>hetero after UVB irradiation VS hetero before UVB irradiation</td><td><i>N</i>-acetylglucosamine degradation II|HumanCyc</td><td>1.004191316</td><td>-0.00065507</td><td>6.427321122</td><td>0.00016215</td><td>0.02920766</td><td>1.372109742</td></tr>
<tr><td>WT after UVB irradiation VS WT before UVB irradiation</td><td><i>N</i>-acetylglucosamine degradation II|HumanCyc</td><td>1.1093572</td><td>0.045258827</td><td>4.84854446</td><td>0.00065722</td><td>0.15645713</td><td>-0.12903144</td></tr>
<tr><td>homo after UVB irradiation VS homo before UVB irradiation</td><td><i>S</i>-methyl-5_-thioadenosine degradation|HumanCyc</td><td>-0.54796223</td><td>-0.01283963</td><td>-1.55686645</td><td>0.15253256</td><td>0.2992968</td><td>-5.21404817</td></tr>
<tr><td>hetero after UVB irradiation VS hetero before UVB irradiation</td><td><i>S</i>-methyl-5_-thioadenosine degradation|HumanCyc</td><td>0.07560607</td><td>-0.00722522</td><td>0.171717809</td><td>0.86771213</td><td>0.94981554</td><td>-6.33057348</td></tr>
<tr><td>WT after UVB irradiation VS WT before UVB irradiation</td><td><i>S</i>-methyl-5_-thioadenosine degradation|HumanCyc</td><td>0.07078435</td><td>-0.04810184</td><td>0.164507989</td><td>0.87258267</td><td>0.96283151</td><td>-5.85546105</td></tr>
<tr><td>homo after UVB irradiation VS homo before UVB irradiation</td><td><i>S</i>-methyl-5-thio-α-D-ribose 1-phosphate degradation|HumanCyc</td><td>0.670950702</td><td>-0.02576823</td><td>2.513321302</td><td>0.03213188</td><td>0.11947408</td><td>-3.79567417</td></tr>
<tr><td>hetero after UVB irradiation VS hetero before UVB irradiation</td><td><i>S</i>-methyl-5-thio-α-D-ribose 1-phosphate degradation|HumanCyc</td><td>0.034298508</td><td>0.053046859</td><td>0.097658473</td><td>0.92448677</td><td>0.97054702</td><td>-6.34137043</td></tr>
<tr><td>WT after UVB irradiation VS WT before UVB irradiation</td><td><i>S</i>-methyl-5-thio-α-D-ribose 1-phosphate degradation|HumanCyc</td><td>0.400422659</td><td>-0.09430216</td><td>1.299654214</td><td>0.22264859</td><td>0.62952955</td><td>-5.08302709</td></tr>
<tr><td>homo after UVB irradiation VS homo before UVB irradiation</td><td><i>trans_ trans</i>-farnesyl diphosphate biosynthesis|HumanCyc</td><td>-0.48837573</td><td>-0.02076243</td><td>-1.3933291</td><td>0.19562198</td><td>0.35165746</td><td>-5.42301838</td></tr>
<tr><td>hetero after UVB irradiation VS hetero before UVB irradiation</td><td><i>trans_ trans</i>-farnesyl diphosphate biosynthesis|HumanCyc</td><td>-0.04249745</td><td>0.069739826</td><td>-0.11566018</td><td>0.91063101</td><td>0.97054702</td><td>-6.33929011</td></tr>
<tr><td>WT after UVB irradiation VS WT before UVB irradiation</td><td><i>trans_ trans</i>-farnesyl diphosphate biosynthesis|HumanCyc</td><td>-0.5122857</td><td>-0.06774275</td><td>-1.83711529</td><td>0.09580766</td><td>0.4928536</td><td>-4.41244606</td></tr>
<tr><td>homo after UVB irradiation VS homo before UVB irradiation</td><td>1_25-dihydroxyvitamin D₃ biosynthesis|HumanCyc</td><td>0.279294037</td><td>-0.0129012</td><td>1.349568141</td><td>0.20879233</td><td>0.36681253</td><td>-5.47643874</td></tr>
<tr><td>hetero after UVB irradiation VS hetero before UVB irradiation</td><td>1_25-dihydroxyvitamin D₃ biosynthesis|HumanCyc</td><td>0.405995944</td><td>-0.0133111</td><td>1.186714477</td><td>0.26771912</td><td>0.4964458</td><td>-5.64179237</td></tr>
<tr><td>WT after UVB irradiation VS WT before UVB irradiation</td><td>1_25-dihydroxyvitamin D₃ biosynthesis|HumanCyc</td><td>0.463256004</td><td>0.035043864</td><td>1.532945787</td><td>0.15604688</td><td>0.5607129</td><td>-4.8083511</td></tr>
<tr><td>homo after UVB irradiation VS homo before UVB irradiation</td><td>11-beta-hydroxylase deficiency (CYP11B1)|SMPDB</td><td>0.235351843</td><td>-0.00442303</td><td>1.337763903</td><td>0.2124708</td><td>0.36957778</td><td>-5.49065548</td></tr>
<tr><td>hetero after UVB irradiation VS hetero before UVB irradiation</td><td>11-beta-hydroxylase deficiency (CYP11B1)|SMPDB</td><td>0.307187072</td><td>0.103522065</td><td>1.723908534</td><td>0.12110487</td><td>0.3132781</td><td>-4.97251635</td></tr>
<tr><td>WT after UVB irradiation VS WT before UVB irradiation</td><td>11-beta-hydroxylase deficiency (CYP11B1)|SMPDB</td><td>0.03938021</td><td>0.054409473</td><td>0.163687683</td><td>0.87321181</td><td>0.96283151</td><td>-5.85559705</td></tr>
<tr><td>homo after UVB irradiation VS homo before UVB irradiation</td><td>17-alpha-hydroxylase deficiency (CYP17)|SMPDB</td><td>0.235351843</td><td>-0.00442303</td><td>1.337763903</td><td>0.2124708</td><td>0.36957778</td><td>-5.49065548</td></tr>
<tr><td>hetero after UVB irradiation VS hetero before UVB irradiation</td><td>17-alpha-hydroxylase deficiency (CYP17)|SMPDB</td><td>0.307187072</td><td>0.103522065</td><td>1.723908534</td><td>0.12110487</td><td>0.3132781</td><td>-4.97251635</td></tr>
<tr><td>WT after UVB irradiation VS WT before UVB irradiation</td><td>17-alpha-hydroxylase deficiency (CYP17)|SMPDB</td><td>0.03938021</td><td>0.054409473</td><td>0.163687683</td><td>0.87321181</td><td>0.96283151</td><td>-5.85559705</td></tr>
<tr><td>homo after UVB irradiation VS homo before UVB irradiation</td><td>17-Beta Hydroxysteroid Dehydrogenase III Deficiency|SMPDB</td><td>0.247630977</td><td>-0.00081892</td><td>1.200495598</td><td>0.25935762</td><td>0.42308928</td><td>-5.64961013</td></tr>
<tr><td>hetero after UVB irradiation VS hetero before UVB irradiation</td><td>17-Beta Hydroxysteroid Dehydrogenase III Deficiency|SMPDB</td><td>0.017963989</td><td>0.013614493</td><td>0.092630718</td><td>0.92836166</td><td>0.97054702</td><td>-6.34188888</td></tr>
<tr><td>WT after UVB irradiation VS WT before UVB irradiation</td><td>17-Beta Hydroxysteroid Dehydrogenase III Deficiency|SMPDB</td><td>-0.31598762</td><td>0.004101129</td><td>-1.31107214</td><td>0.21890754</td><td>0.6260768</td><td>-5.07027776</td></tr>
<tr><td>homo after UVB irradiation VS homo before UVB irradiation</td><td>1D-<i>myo</i>-inositol hexakisphosphate biosynthesis II (mammalian)|HumanCyc</td><td>0.823941548</td><td>-0.02244897</td><td>4.437684789</td><td>0.00146847</td><td>0.02184045</td><td>-0.80045389</td></tr>
<tr><td>hetero after UVB irradiation VS hetero before UVB irradiation</td><td>1D-<i>myo</i>-inositol hexakisphosphate biosynthesis II (mammalian)|HumanCyc</td><td>0.474196254</td><td>0.09256389</td><td>1.668408767</td><td>0.13187509</td><td>0.32858792</td><td>-5.04787862</td></tr>
<tr><td>WT after UVB irradiation VS WT before UVB irradiation</td><td>1D-<i>myo</i>-inositol hexakisphosphate biosynthesis II (mammalian)|HumanCyc</td><td>0.475870066</td><td>0.008430947</td><td>1.787979469</td><td>0.10382269</td><td>0.50425697</td><td>-4.47881071</td></tr>
<tr><td>homo after UVB irradiation VS homo before UVB irradiation</td><td>1D-<i>myo</i>-inositol hexakisphosphate biosynthesis V (from Ins(1_3_4)P3)|HumanCyc</td><td>0.575138361</td><td>-0.03250566</td><td>1.617527651</td><td>0.13881766</td><td>0.28141074</td><td>-5.13310119</td></tr>
<tr><td>hetero after UVB irradiation VS hetero before UVB irradiation</td><td>1D-<i>myo</i>-inositol hexakisphosphate biosynthesis V (from Ins(1_3_4)P3)|HumanCyc</td><td>0.515454422</td><td>-0.08750378</td><td>1.5325017</td><td>0.16204044</td><td>0.37510427</td><td>-5.22716655</td></tr>
<tr><td>WT after UVB irradiation VS WT before UVB irradiation</td><td>1D-<i>myo</i>-inositol hexakisphosphate biosynthesis V (from Ins(1_3_4)P3)|HumanCyc</td><td>0.278814602</td><td>0.023696311</td><td>1.022127574</td><td>0.33061981</td><td>0.71290873</td><td>-5.36754819</td></tr>
<tr><td>homo after UVB irradiation VS homo before UVB irradiation</td><td>2_-deoxy-α-D-ribose 1-phosphate degradation|HumanCyc</td><td>-0.5634533</td><td>-0.00344466</td><td>-2.83631568</td><td>0.01874056</td><td>0.0875711</td><td>-3.28044723</td></tr>
<tr><td>hetero after UVB irradiation VS hetero before UVB irradiation</td><td>2_-deoxy-α-D-ribose 1-phosphate degradation|HumanCyc</td><td>-0.40638875</td><td>-0.00092294</td><td>-2.2970928</td><td>0.04911245</td><td>0.19705504</td><td>-4.14280865</td></tr>
<tr><td>WT after UVB irradiation VS WT before UVB irradiation</td><td>2_-deoxy-α-D-ribose 1-phosphate degradation|HumanCyc</td><td>-0.35491579</td><td>-0.04750508</td><td>-1.27895217</td><td>0.22956607</td><td>0.62952955</td><td>-5.10594692</td></tr>
<tr><td>homo after UVB irradiation VS homo before UVB irradiation</td><td>21-hydroxylase deficiency (CYP21)|SMPDB</td><td>0.235351843</td><td>-0.00442303</td><td>1.337763903</td><td>0.2124708</td><td>0.36957778</td><td>-5.49065548</td></tr>
<tr><td>hetero after UVB irradiation VS hetero before UVB irradiation</td><td>21-hydroxylase deficiency (CYP21)|SMPDB</td><td>0.307187072</td><td>0.103522065</td><td>1.723908534</td><td>0.12110487</td><td>0.3132781</td><td>-4.97251635</td></tr>
<tr><td>WT after UVB irradiation VS WT before UVB irradiation</td><td>21-hydroxylase deficiency (CYP21)|SMPDB</td><td>0.03938021</td><td>0.054409473</td><td>0.163687683</td><td>0.87321181</td><td>0.96283151</td><td>-5.85559705</td></tr>
<tr><td>homo after UVB irradiation VS homo before UVB irradiation</td><td>27-Hydroxylase Deficiency|SMPDB</td><td>0.143425895</td><td>0.01576019</td><td>0.783036509</td><td>0.45290274</td><td>0.6002769</td><td>-6.04960791</td></tr>
<tr><td>hetero after UVB irradiation VS hetero before UVB irradiation</td><td>27-Hydroxylase Deficiency|SMPDB</td><td>-0.01773938</td><td>0.040584313</td><td>-0.07540586</td><td>0.94165152</td><td>0.97054702</td><td>-6.34345771</td></tr>
<tr><td>WT after UVB irradiation VS WT before UVB irradiation</td><td>27-Hydroxylase Deficiency|SMPDB</td><td>0.061518516</td><td>0.026229795</td><td>0.23751225</td><td>0.8170163</td><td>0.94525798</td><td>-5.84065895</td></tr>
<tr><td>homo after UVB irradiation VS homo before UVB irradiation</td><td>2-amino-3-carboxymuconate semialdehyde degradation to glutaryl-CoA|HumanCyc</td><td>0.121115277</td><td>0.064401848</td><td>0.330451082</td><td>0.74831382</td><td>0.83620396</td><td>-6.31012382</td></tr>
<tr><td>hetero after UVB irradiation VS hetero before UVB irradiation</td><td>2-amino-3-carboxymuconate semialdehyde degradation to glutaryl-CoA|HumanCyc</td><td>-0.43482882</td><td>-0.02124553</td><td>-1.29965826</td><td>0.22814527</td><td>0.45170905</td><td>-5.51380086</td></tr>
<tr><td>WT after UVB irradiation VS WT before UVB irradiation</td><td>2-amino-3-carboxymuconate semialdehyde degradation to glutaryl-CoA|HumanCyc</td><td>-0.91897076</td><td>-0.06856898</td><td>-4.2607305</td><td>0.00163074</td><td>0.15689229</td><td>-0.89301676</td></tr>
<tr><td>homo after UVB irradiation VS homo before UVB irradiation</td><td>2-aminoadipic 2-oxoadipic aciduria|SMPDB</td><td>-0.02100802</td><td>-0.06308448</td><td>-0.07524201</td><td>0.941601</td><td>0.96450524</td><td>-6.36540732</td></tr>
<tr><td>hetero after UVB irradiation VS hetero before UVB irradiation</td><td>2-aminoadipic 2-oxoadipic aciduria|SMPDB</td><td>-0.10000854</td><td>-0.05054657</td><td>-0.39990823</td><td>0.69917117</td><td>0.84778456</td><td>-6.26061929</td></tr>
<tr><td>WT after UVB irradiation VS WT before UVB irradiation</td><td>2-aminoadipic 2-oxoadipic aciduria|SMPDB</td><td>-0.11826398</td><td>-0.1134336</td><td>-0.44264387</td><td>0.66736875</td><td>0.87935805</td><td>-5.77091861</td></tr>
<tr><td>homo after UVB irradiation VS homo before UVB irradiation</td><td>2-Hydroxyglutric Aciduria (D And L Form)|SMPDB</td><td>-0.24547682</td><td>-0.00810915</td><td>-1.40068944</td><td>0.19347783</td><td>0.34903982</td><td>-5.41392471</td></tr>
<tr><td>hetero after UVB irradiation VS hetero before UVB irradiation</td><td>2-Hydroxyglutric Aciduria (D And L Form)|SMPDB</td><td>-0.17636771</td><td>-0.04343396</td><td>-1.04504584</td><td>0.32505313</td><td>0.55421891</td><td>-5.79050219</td></tr>
<tr><td>WT after UVB irradiation VS WT before UVB irradiation</td><td>2-Hydroxyglutric Aciduria (D And L Form)|SMPDB</td><td>-0.06328445</td><td>0.007258138</td><td>-0.28249238</td><td>0.78328047</td><td>0.93661609</td><td>-5.82889545</td></tr>
<tr><td>homo after UVB irradiation VS homo before UVB irradiation</td><td>2-ketoglutarate dehydrogenase complex deficiency|SMPDB</td><td>-0.23678174</td><td>0.014544733</td><td>-0.76379894</td><td>0.46374733</td><td>0.6088463</td><td>-6.06461144</td></tr>
<tr><td>hetero after UVB irradiation VS hetero before UVB irradiation</td><td>2-ketoglutarate dehydrogenase complex deficiency|SMPDB</td><td>-0.47814985</td><td>0.004803127</td><td>-1.74993278</td><td>0.11633994</td><td>0.30887493</td><td>-4.93678811</td></tr>
<tr><td>WT after UVB irradiation VS WT before UVB irradiation</td><td>2-ketoglutarate dehydrogenase complex deficiency|SMPDB</td><td>-0.1568339</td><td>-0.12513755</td><td>-0.50250429</td><td>0.62610623</td><td>0.87257466</td><td>-5.74292911</td></tr>
<tr><td>homo after UVB irradiation VS homo before UVB irradiation</td><td>2-LTR circle formation|Reactome</td><td>-0.70661859</td><td>-0.06097203</td><td>-3.24758905</td><td>0.00949577</td><td>0.05835911</td><td>-2.62256921</td></tr>
<tr><td>hetero after UVB irradiation VS hetero before UVB irradiation</td><td>2-LTR circle formation|Reactome</td><td>-0.58617127</td><td>-0.08152719</td><td>-1.90421713</td><td>0.09150452</td><td>0.27391014</td><td>-4.7203795</td></tr>
<tr><td>WT after UVB irradiation VS WT before UVB irradiation</td><td>2-LTR circle formation|Reactome</td><td>-0.11824348</td><td>0.004821076</td><td>-0.36831104</td><td>0.72025682</td><td>0.90672477</td><td>-5.80092395</td></tr>
<tr><td>homo after UVB irradiation VS homo before UVB irradiation</td><td>2-Methyl-3-Hydroxybutryl CoA Dehydrogenase Deficiency|SMPDB</td><td>-0.23657683</td><td>-0.05522798</td><td>-0.8011182</td><td>0.4428632</td><td>0.59091566</td><td>-6.03520901</td></tr>
<tr><td>hetero after UVB irradiation VS hetero before UVB irradiation</td><td>2-Methyl-3-Hydroxybutryl CoA Dehydrogenase Deficiency|SMPDB</td><td>-0.90022729</td><td>-0.04719335</td><td>-5.31014337</td><td>0.00060637</td><td>0.03004176</td><td>0.126180045</td></tr>
<tr><td>WT after UVB irradiation VS WT before UVB irradiation</td><td>2-Methyl-3-Hydroxybutryl CoA Dehydrogenase Deficiency|SMPDB</td><td>-0.36319435</td><td>-0.13086657</td><td>-1.31407353</td><td>0.21793282</td><td>0.62581393</td><td>-5.06691375</td></tr>
<tr><td>homo after UVB irradiation VS homo before UVB irradiation</td><td>2-oxobutanoate degradation|HumanCyc</td><td>-0.27407913</td><td>-0.08475107</td><td>-0.73399032</td><td>0.48088099</td><td>0.62360198</td><td>-6.08720906</td></tr>
<tr><td>hetero after UVB irradiation VS hetero before UVB irradiation</td><td>2-oxobutanoate degradation|HumanCyc</td><td>-1.21286304</td><td>-0.04725294</td><td>-7.37313121</td><td>5.98E-05</td><td>0.02920766</td><td>2.282689985</td></tr>
<tr><td>WT after UVB irradiation VS WT before UVB irradiation</td><td>2-oxobutanoate degradation|HumanCyc</td><td>-0.29533056</td><td>-0.08156191</td><td>-1.02262374</td><td>0.33039623</td><td>0.71290873</td><td>-5.36708625</td></tr>
<tr><td>homo after UVB irradiation VS homo before UVB irradiation</td><td>2-oxoglutarate decarboxylation to succinyl-CoA|HumanCyc</td><td>-0.49785351</td><td>-0.02340332</td><td>-1.79797443</td><td>0.10434504</td><td>0.23665249</td><td>-4.88279089</td></tr>
<tr><td>hetero after UVB irradiation VS hetero before UVB irradiation</td><td>2-oxoglutarate decarboxylation to succinyl-CoA|HumanCyc</td><td>-0.33463823</td><td>-0.0230999</td><td>-0.94295537</td><td>0.37195127</td><td>0.60055044</td><td>-5.88867869</td></tr>
<tr><td>WT after UVB irradiation VS WT before UVB irradiation</td><td>2-oxoglutarate decarboxylation to succinyl-CoA|HumanCyc</td><td>-0.00281095</td><td>-0.0085839</td><td>-0.01260824</td><td>0.99018635</td><td>0.99676022</td><td>-5.86907066</td></tr>
<tr><td>homo after UVB irradiation VS homo before UVB irradiation</td><td>2-oxoisovalerate decarboxylation to isobutanoyl-CoA|HumanCyc</td><td>-0.44595201</td><td>-0.04613575</td><td>-1.05905377</td><td>0.31605879</td><td>0.4705329</td><td>-5.8001292</td></tr>
<tr><td>hetero after UVB irradiation VS hetero before UVB irradiation</td><td>2-oxoisovalerate decarboxylation to isobutanoyl-CoA|HumanCyc</td><td>-1.11061639</td><td>-0.06945064</td><td>-6.03214826</td><td>0.00025381</td><td>0.02988205</td><td>0.953617922</td></tr>
<tr><td>WT after UVB irradiation VS WT before UVB irradiation</td><td>2-oxoisovalerate decarboxylation to isobutanoyl-CoA|HumanCyc</td><td>-0.71343856</td><td>-0.04406239</td><td>-2.84017689</td><td>0.01740963</td><td>0.30353778</td><td>-2.94831979</td></tr>
<tr><td>homo after UVB irradiation VS homo before UVB irradiation</td><td>3-Beta-Hydroxysteroid Dehydrogenase Deficiency|SMPDB</td><td>0.235351843</td><td>-0.00442303</td><td>1.337763903</td><td>0.2124708</td><td>0.36957778</td><td>-5.49065548</td></tr>
<tr><td>hetero after UVB irradiation VS hetero before UVB irradiation</td><td>3-Beta-Hydroxysteroid Dehydrogenase Deficiency|SMPDB</td><td>0.307187072</td><td>0.103522065</td><td>1.723908534</td><td>0.12110487</td><td>0.3132781</td><td>-4.97251635</td></tr>
<tr><td>WT after UVB irradiation VS WT before UVB irradiation</td><td>3-Beta-Hydroxysteroid Dehydrogenase Deficiency|SMPDB</td><td>0.03938021</td><td>0.054409473</td><td>0.163687683</td><td>0.87321181</td><td>0.96283151</td><td>-5.85559705</td></tr>
<tr><td>homo after UVB irradiation VS homo before UVB irradiation</td><td>3-Hydroxy-3-Methylglutaryl-CoA Lyase Deficiency|SMPDB</td><td>-0.23657683</td><td>-0.05522798</td><td>-0.8011182</td><td>0.4428632</td><td>0.59091566</td><td>-6.03520901</td></tr>
<tr><td>hetero after UVB irradiation VS hetero before UVB irradiation</td><td>3-Hydroxy-3-Methylglutaryl-CoA Lyase Deficiency|SMPDB</td><td>-0.90022729</td><td>-0.04719335</td><td>-5.31014337</td><td>0.00060637</td><td>0.03004176</td><td>0.126180045</td></tr>
<tr><td>WT after UVB irradiation VS WT before UVB irradiation</td><td>3-Hydroxy-3-Methylglutaryl-CoA Lyase Deficiency|SMPDB</td><td>-0.36319435</td><td>-0.13086657</td><td>-1.31407353</td><td>0.21793282</td><td>0.62581393</td><td>-5.06691375</td></tr>
<tr><td>homo after UVB irradiation VS homo before UVB irradiation</td><td>3-hydroxyisobutyric acid dehydrogenase deficiency|SMPDB</td><td>-0.23657683</td><td>-0.05522798</td><td>-0.8011182</td><td>0.4428632</td><td>0.59091566</td><td>-6.03520901</td></tr>
<tr><td>hetero after UVB irradiation VS hetero before UVB irradiation</td><td>3-hydroxyisobutyric acid dehydrogenase deficiency|SMPDB</td><td>-0.90022729</td><td>-0.04719335</td><td>-5.31014337</td><td>0.00060637</td><td>0.03004176</td><td>0.126180045</td></tr>
<tr><td>WT after UVB irradiation VS WT before UVB irradiation</td><td>3-hydroxyisobutyric acid dehydrogenase deficiency|SMPDB</td><td>-0.36319435</td><td>-0.13086657</td><td>-1.31407353</td><td>0.21793282</td><td>0.62581393</td><td>-5.06691375</td></tr>
<tr><td>homo after UVB irradiation VS homo before UVB irradiation</td><td>3-hydroxyisobutyric aciduria|SMPDB</td><td>-0.23657683</td><td>-0.05522798</td><td>-0.8011182</td><td>0.4428632</td><td>0.59091566</td><td>-6.03520901</td></tr>
<tr><td>hetero after UVB irradiation VS hetero before UVB irradiation</td><td>3-hydroxyisobutyric aciduria|SMPDB</td><td>-0.90022729</td><td>-0.04719335</td><td>-5.31014337</td><td>0.00060637</td><td>0.03004176</td><td>0.126180045</td></tr>
<tr><td>WT after UVB irradiation VS WT before UVB irradiation</td><td>3-hydroxyisobutyric aciduria|SMPDB</td><td>-0.36319435</td><td>-0.13086657</td><td>-1.31407353</td><td>0.21793282</td><td>0.62581393</td><td>-5.06691375</td></tr>
<tr><td>homo after UVB irradiation VS homo before UVB irradiation</td><td>3-Methylcrotonyl Coa Carboxylase Deficiency Type I|SMPDB</td><td>-0.23657683</td><td>-0.05522798</td><td>-0.8011182</td><td>0.4428632</td><td>0.59091566</td><td>-6.03520901</td></tr>
<tr><td>hetero after UVB irradiation VS hetero before UVB irradiation</td><td>3-Methylcrotonyl Coa Carboxylase Deficiency Type I|SMPDB</td><td>-0.90022729</td><td>-0.04719335</td><td>-5.31014337</td><td>0.00060637</td><td>0.03004176</td><td>0.126180045</td></tr>
<tr><td>WT after UVB irradiation VS WT before UVB irradiation</td><td>3-Methylcrotonyl Coa Carboxylase Deficiency Type I|SMPDB</td><td>-0.36319435</td><td>-0.13086657</td><td>-1.31407353</td><td>0.21793282</td><td>0.62581393</td><td>-5.06691375</td></tr>
<tr><td>homo after UVB irradiation VS homo before UVB irradiation</td><td>3-Methylglutaconic Aciduria Type I|SMPDB</td><td>-0.23657683</td><td>-0.05522798</td><td>-0.8011182</td><td>0.4428632</td><td>0.59091566</td><td>-6.03520901</td></tr>
<tr><td>hetero after UVB irradiation VS hetero before UVB irradiation</td><td>3-Methylglutaconic Aciduria Type I|SMPDB</td><td>-0.90022729</td><td>-0.04719335</td><td>-5.31014337</td><td>0.00060637</td><td>0.03004176</td><td>0.126180045</td></tr>
<tr><td>WT after UVB irradiation VS WT before UVB irradiation</td><td>3-Methylglutaconic Aciduria Type I|SMPDB</td><td>-0.36319435</td><td>-0.13086657</td><td>-1.31407353</td><td>0.21793282</td><td>0.62581393</td><td>-5.06691375</td></tr>
<tr><td>homo after UVB irradiation VS homo before UVB irradiation</td><td>3-Methylglutaconic Aciduria Type III|SMPDB</td><td>-0.23657683</td><td>-0.05522798</td><td>-0.8011182</td><td>0.4428632</td><td>0.59091566</td><td>-6.03520901</td></tr>
<tr><td>hetero after UVB irradiation VS hetero before UVB irradiation</td><td>3-Methylglutaconic Aciduria Type III|SMPDB</td><td>-0.90022729</td><td>-0.04719335</td><td>-5.31014337</td><td>0.00060637</td><td>0.03004176</td><td>0.126180045</td></tr>
<tr><td>WT after UVB irradiation VS WT before UVB irradiation</td><td>3-Methylglutaconic Aciduria Type III|SMPDB</td><td>-0.36319435</td><td>-0.13086657</td><td>-1.31407353</td><td>0.21793282</td><td>0.62581393</td><td>-5.06691375</td></tr>
<tr><td>homo after UVB irradiation VS homo before UVB irradiation</td><td>3-Methylglutaconic Aciduria Type IV|SMPDB</td><td>-0.23657683</td><td>-0.05522798</td><td>-0.8011182</td><td>0.4428632</td><td>0.59091566</td><td>-6.03520901</td></tr>
<tr><td>hetero after UVB irradiation VS hetero before UVB irradiation</td><td>3-Methylglutaconic Aciduria Type IV|SMPDB</td><td>-0.90022729</td><td>-0.04719335</td><td>-5.31014337</td><td>0.00060637</td><td>0.03004176</td><td>0.126180045</td></tr>
<tr><td>WT after UVB irradiation VS WT before UVB irradiation</td><td>3-Methylglutaconic Aciduria Type IV|SMPDB</td><td>-0.36319435</td><td>-0.13086657</td><td>-1.31407353</td><td>0.21793282</td><td>0.62581393</td><td>-5.06691375</td></tr>
<tr><td>homo after UVB irradiation VS homo before UVB irradiation</td><td>3-Methylthiofentanyl Action Pathway|SMPDB</td><td>0.191132361</td><td>0.029768717</td><td>1.061313976</td><td>0.31508276</td><td>0.46923284</td><td>-5.79783739</td></tr>
<tr><td>hetero after UVB irradiation VS hetero before UVB irradiation</td><td>3-Methylthiofentanyl Action Pathway|SMPDB</td><td>-0.07904315</td><td>0.055005373</td><td>-0.44437398</td><td>0.66796482</td><td>0.82651398</td><td>-6.24068873</td></tr>
<tr><td>WT after UVB irradiation VS WT before UVB irradiation</td><td>3-Methylthiofentanyl Action Pathway|SMPDB</td><td>0.022316377</td><td>0.035038618</td><td>0.098344171</td><td>0.92358676</td><td>0.97566251</td><td>-5.86425414</td></tr>
<tr><td>homo after UVB irradiation VS homo before UVB irradiation</td><td>3-oxo-10R-octadecatrienoate beta-oxidation|EHMN</td><td>-0.43062589</td><td>0.009130014</td><td>-1.54102809</td><td>0.15630606</td><td>0.30417351</td><td>-5.23489234</td></tr>
<tr><td>hetero after UVB irradiation VS hetero before UVB irradiation</td><td>3-oxo-10R-octadecatrienoate beta-oxidation|EHMN</td><td>-0.61467347</td><td>0.002455457</td><td>-2.5522554</td><td>0.03269166</td><td>0.15685114</td><td>-3.75575607</td></tr>
<tr><td>WT after UVB irradiation VS WT before UVB irradiation</td><td>3-oxo-10R-octadecatrienoate beta-oxidation|EHMN</td><td>-0.46173471</td><td>-0.01781795</td><td>-1.93189144</td><td>0.08193934</td><td>0.46860292</td><td>-4.28222624</td></tr>
<tr><td>homo after UVB irradiation VS homo before UVB irradiation</td><td>3-Phosphoglycerate dehydrogenase deficiency|SMPDB</td><td>-0.11801782</td><td>0.021953564</td><td>-0.50783827</td><td>0.62328187</td><td>0.73728109</td><td>-6.23179983</td></tr>
<tr><td>hetero after UVB irradiation VS hetero before UVB irradiation</td><td>3-Phosphoglycerate dehydrogenase deficiency|SMPDB</td><td>-0.03882851</td><td>0.032366748</td><td>-0.2244119</td><td>0.82778335</td><td>0.93322694</td><td>-6.31930458</td></tr>
<tr><td>WT after UVB irradiation VS WT before UVB irradiation</td><td>3-Phosphoglycerate dehydrogenase deficiency|SMPDB</td><td>0.143497804</td><td>-0.01134274</td><td>0.603206807</td><td>0.55969543</td><td>0.84911968</td><td>-5.68832383</td></tr>
<tr><td>homo after UVB irradiation VS homo before UVB irradiation</td><td>3-phosphoinositide biosynthesis|HumanCyc</td><td>0.146752893</td><td>-0.04709193</td><td>0.77671872</td><td>0.45644568</td><td>0.60277262</td><td>-6.05457125</td></tr>
<tr><td>hetero after UVB irradiation VS hetero before UVB irradiation</td><td>3-phosphoinositide biosynthesis|HumanCyc</td><td>0.169455814</td><td>-0.00593388</td><td>0.932380372</td><td>0.37708237</td><td>0.60405274</td><td>-5.89839023</td></tr>
<tr><td>WT after UVB irradiation VS WT before UVB irradiation</td><td>3-phosphoinositide biosynthesis|HumanCyc</td><td>0.201433279</td><td>0.006077927</td><td>1.030187614</td><td>0.32700182</td><td>0.71049278</td><td>-5.36002216</td></tr>
<tr><td>homo after UVB irradiation VS homo before UVB irradiation</td><td>3-phosphoinositide degradation|HumanCyc</td><td>0.588105177</td><td>-0.02220197</td><td>3.475696052</td><td>0.00655245</td><td>0.04701822</td><td>-2.26121708</td></tr>
<tr><td>hetero after UVB irradiation VS hetero before UVB irradiation</td><td>3-phosphoinositide degradation|HumanCyc</td><td>0.138392583</td><td>0.060643213</td><td>0.597841101</td><td>0.56566416</td><td>0.76437184</td><td>-6.15676547</td></tr>
<tr><td>WT after UVB irradiation VS WT before UVB irradiation</td><td>3-phosphoinositide degradation|HumanCyc</td><td>0.25139942</td><td>-0.03032739</td><td>1.035811825</td><td>0.32449484</td><td>0.70873647</td><td>-5.35474281</td></tr>
<tr><td>homo after UVB irradiation VS homo before UVB irradiation</td><td>4-aminobutyrate degradation|HumanCyc</td><td>-0.96078212</td><td>-0.06336452</td><td>-4.40813768</td><td>0.00153462</td><td>0.02224473</td><td>-0.84341892</td></tr>
<tr><td>hetero after UVB irradiation VS hetero before UVB irradiation</td><td>4-aminobutyrate degradation|HumanCyc</td><td>-0.24947082</td><td>0.110968118</td><td>-0.57540571</td><td>0.5800548</td><td>0.77397513</td><td>-6.17046785</td></tr>
<tr><td>WT after UVB irradiation VS WT before UVB irradiation</td><td>4-aminobutyrate degradation|HumanCyc</td><td>-0.22371324</td><td>0.065559803</td><td>-0.68008487</td><td>0.51176395</td><td>0.82675132</td><td>-5.64046526</td></tr>
<tr><td>homo after UVB irradiation VS homo before UVB irradiation</td><td>4-hydroxy-2-nonenal detoxification|HumanCyc</td><td>0.246914548</td><td>0.014062312</td><td>0.648349168</td><td>0.53229323</td><td>0.66844084</td><td>-6.14763908</td></tr>
<tr><td>hetero after UVB irradiation VS hetero before UVB irradiation</td><td>4-hydroxy-2-nonenal detoxification|HumanCyc</td><td>0.685242352</td><td>0.033498554</td><td>2.024982708</td><td>0.07566761</td><td>0.24670455</td><td>-4.54626168</td></tr>
<tr><td>WT after UVB irradiation VS WT before UVB irradiation</td><td>4-hydroxy-2-nonenal detoxification|HumanCyc</td><td>0.698500547</td><td>-0.11429349</td><td>2.231475267</td><td>0.04950962</td><td>0.40833376</td><td>-3.85511741</td></tr>
<tr><td>homo after UVB irradiation VS homo before UVB irradiation</td><td>4-hydroxybenzoate biosynthesis|HumanCyc</td><td>0.424826044</td><td>0.046036282</td><td>1.494500289</td><td>0.1678716</td><td>0.3179186</td><td>-5.29539923</td></tr>
<tr><td>homo after UVB irradiation VS homo before UVB irradiation</td><td>4-Hydroxybutyric Aciduria/Succinic Semialdehyde Dehydrogenase Deficiency|SMPDB</td><td>-0.24547682</td><td>-0.00810915</td><td>-1.40068944</td><td>0.19347783</td><td>0.34903982</td><td>-5.41392471</td></tr>
<tr><td>hetero after UVB irradiation VS hetero before UVB irradiation</td><td>4-Hydroxybutyric Aciduria/Succinic Semialdehyde Dehydrogenase Deficiency|SMPDB</td><td>-0.17636771</td><td>-0.04343396</td><td>-1.04504584</td><td>0.32505313</td><td>0.55421891</td><td>-5.79050219</td></tr>
<tr><td>WT after UVB irradiation VS WT before UVB irradiation</td><td>4-Hydroxybutyric Aciduria/Succinic Semialdehyde Dehydrogenase Deficiency|SMPDB</td><td>-0.06328445</td><td>0.007258138</td><td>-0.28249238</td><td>0.78328047</td><td>0.93661609</td><td>-5.82889545</td></tr>
<tr><td>homo after UVB irradiation VS homo before UVB irradiation</td><td>4-hydroxyproline degradation|HumanCyc</td><td>-0.25739676</td><td>-0.05755528</td><td>-0.67426642</td><td>0.51639765</td><td>0.65423742</td><td>-6.13006455</td></tr>
<tr><td>hetero after UVB irradiation VS hetero before UVB irradiation</td><td>4-hydroxyproline degradation|HumanCyc</td><td>-0.44114359</td><td>0.081480998</td><td>-1.16656845</td><td>0.27533807</td><td>0.50543332</td><td>-5.66377327</td></tr>
<tr><td>WT after UVB irradiation VS WT before UVB irradiation</td><td>4-hydroxyproline degradation|HumanCyc</td><td>-0.74660522</td><td>-0.14638831</td><td>-3.22032573</td><td>0.00907676</td><td>0.24856491</td><td>-2.37855015</td></tr>
<tr><td>homo after UVB irradiation VS homo before UVB irradiation</td><td>4-hydroxytamoxifen_ Dexamethasone_ and Retinoic Acids Regulation of p27 Expression|Wikipathways</td><td>0.031104333</td><td>-0.01311158</td><td>0.177022415</td><td>0.86325077</td><td>0.91686509</td><td>-6.35163957</td></tr>
<tr><td>hetero after UVB irradiation VS hetero before UVB irradiation</td><td>4-hydroxytamoxifen_ Dexamethasone_ and Retinoic Acids Regulation of p27 Expression|Wikipathways</td><td>0.023504342</td><td>-0.03897845</td><td>0.101956953</td><td>0.9211756</td><td>0.97054702</td><td>-6.34090551</td></tr>
<tr><td>WT after UVB irradiation VS WT before UVB irradiation</td><td>4-hydroxytamoxifen_ Dexamethasone_ and Retinoic Acids Regulation of p27 Expression|Wikipathways</td><td>-0.11515832</td><td>-0.02711909</td><td>-0.51974478</td><td>0.61446034</td><td>0.87257466</td><td>-5.734244</td></tr>
<tr><td>homo after UVB irradiation VS homo before UVB irradiation</td><td>5-aminoimidazole ribonucleotide biosynthesis|HumanCyc</td><td>-0.97811737</td><td>-0.00351542</td><td>-4.05533475</td><td>0.00262206</td><td>0.02941608</td><td>-1.36644258</td></tr>
<tr><td>hetero after UVB irradiation VS hetero before UVB irradiation</td><td>5-aminoimidazole ribonucleotide biosynthesis|HumanCyc</td><td>-0.53634906</td><td>-0.06789237</td><td>-1.89414876</td><td>0.09295903</td><td>0.27513459</td><td>-4.73472264</td></tr>
<tr><td>WT after UVB irradiation VS WT before UVB irradiation</td><td>5-aminoimidazole ribonucleotide biosynthesis|HumanCyc</td><td>-0.31838672</td><td>-0.03579076</td><td>-0.7858872</td><td>0.4500086</td><td>0.80572091</td><td>-5.56619533</td></tr>
<tr><td>homo after UVB irradiation VS homo before UVB irradiation</td><td>5-oxoprolinase deficiency|SMPDB</td><td>-0.03868231</td><td>-0.05039994</td><td>-0.170337</td><td>0.86835923</td><td>0.9186887</td><td>-6.35288321</td></tr>
</table>

hetero after UVB irradiation VS hetero before UVB irradiation	5-oxoprolinase deficiency|SMPDB	0.017134286	0.027343289	0.102228327	0.92096661	0.97054702	-6.34087549
WT after UVB irradiation VS WT before UVB irradiation	5-oxoprolinase deficiency|SMPDB	0.144724093	-0.01736596	0.615162034	0.55207887	0.84516674	-5.68122724
homo after UVB irradiation VS homo before UVB irradiation	5-Oxoprolinuria|SMPDB	-0.03868231	-0.05039994	-0.170337	0.86835923	0.9186887	-6.35288321
hetero after UVB irradiation VS hetero before UVB irradiation	5-Oxoprolinuria|SMPDB	0.017134286	0.027343289	0.102228327	0.92096661	0.97054702	-6.34087549
WT after UVB irradiation VS WT before UVB irradiation	5-Oxoprolinuria|SMPDB	0.144724093	-0.01736596	0.615162034	0.55207887	0.84516674	-5.68122724
homo after UVB irradiation VS homo before UVB irradiation	5-Phosphoribose 1-diphosphate biosynthesis|Reactome	-0.1437614	0.004937334	-0.52325245	0.61292232	0.72940831	-6.22350209
hetero after UVB irradiation VS hetero before UVB irradiation	5-Phosphoribose 1-diphosphate biosynthesis|Reactome	-0.14064702	-0.04077113	-0.52504472	0.61308388	0.79886687	-6.19945667
WT after UVB irradiation VS WT before UVB irradiation	5-Phosphoribose 1-diphosphate biosynthesis|Reactome	-0.11784593	0.007349038	-0.36310157	0.7240262	0.90882588	-5.80282697
homo after UVB irradiation VS homo before UVB irradiation	7-(3-amino-3-carboxypropyl)-wyosine biosynthesis|HumanCyc	-0.86342711	-0.00962926	-3.135039	0.01142371	0.06544625	-2.80209873
hetero after UVB irradiation VS hetero before UVB irradiation	7-(3-amino-3-carboxypropyl)-wyosine biosynthesis|HumanCyc	0.453158367	0.02426701	1.222381808	0.25465231	0.47937588	-5.60223162
WT after UVB irradiation VS WT before UVB irradiation	7-(3-amino-3-carboxypropyl)-wyosine biosynthesis|HumanCyc	-0.40426788	-0.01375789	-1.06197178	0.31302394	0.69668171	-5.32988958
homo after UVB irradiation VS homo before UVB irradiation	A tetrasaccharide linker sequence is required for GAG synthesis|Reactome	0.441937834	-0.00401106	2.512259323	0.03218893	0.11958881	-3.79735618
hetero after UVB irradiation VS hetero before UVB irradiation	A tetrasaccharide linker sequence is required for GAG synthesis|Reactome	0.239623106	0.022915716	0.958138256	0.36467444	0.59328506	-5.87458209
WT after UVB irradiation VS WT before UVB irradiation	A tetrasaccharide linker sequence is required for GAG synthesis|Reactome	0.35047491	0.00501266	1.432457689	0.18227996	0.59839062	-4.93018713
homo after UVB irradiation VS homo before UVB irradiation	A third proteolytic cleavage releases NICD|Reactome	0.573345879	-0.02373446	3.182870982	0.01055927	0.06213097	-2.72572396
hetero after UVB irradiation VS hetero before UVB irradiation	A third proteolytic cleavage releases NICD|Reactome	0.744674712	0.005535318	4.395743631	0.00202903	0.0502626	-1.04395186
WT after UVB irradiation VS WT before UVB irradiation	A third proteolytic cleavage releases NICD|Reactome	0.515941795	0.024185499	1.774354269	0.10615166	0.50446894	-4.49706479
homo after UVB irradiation VS homo before UVB irradiation	a4b7 Integrin signaling|PID	0.954235033	-0.05670972	4.430892635	0.0014834	0.02191977	-0.81031868
hetero after UVB irradiation VS hetero before UVB irradiation	a4b7 Integrin signaling|PID	0.871088157	0.027095503	4.735208159	0.00127762	0.04345809	-0.59344745
WT after UVB irradiation VS WT before UVB irradiation	a4b7 Integrin signaling|PID	0.660736679	-0.08608104	2.134578944	0.05834862	0.42483181	-3.99544345
homo after UVB irradiation VS homo before UVB irradiation	a6b1 and a6b4 Integrin signaling|PID	0.43377163	-0.04265315	1.867231867	0.09335053	0.21980728	-4.78336344
hetero after UVB irradiation VS hetero before UVB irradiation	a6b1 and a6b4 Integrin signaling|PID	0.419739264	0.027947246	2.265867487	0.05161874	0.20126451	-4.18971536
WT after UVB irradiation VS WT before UVB irradiation	a6b1 and a6b4 Integrin signaling|PID	0.3396583	-0.01863224	1.736279447	0.11291488	0.51512295	-4.54771773
homo after UVB irradiation VS homo before UVB irradiation	Abacavir metabolism|Reactome	-0.19486132	0.03843208	-0.65834985	0.52612551	0.66255701	-6.14093176
hetero after UVB irradiation VS hetero before UVB irradiation	Abacavir metabolism|Reactome	0.062261275	-0.12607566	0.23563294	0.81934412	0.92853568	-6.31652213
WT after UVB irradiation VS WT before UVB irradiation	Abacavir metabolism|Reactome	-0.14796023	0.008272571	-0.52902242	0.60823919	0.87257466	-5.72945588
homo after UVB irradiation VS homo before UVB irradiation	Abacavir Pathway_ Pharmacokinetics/Pharmacodynamics|PharmGKB	0.136423269	0.021525165	0.545084966	0.5984026	0.71899698	-6.2113501
hetero after UVB irradiation VS hetero before UVB irradiation	Abacavir Pathway_ Pharmacokinetics/Pharmacodynamics|PharmGKB	0.157817411	-0.00839267	0.678400739	0.51571331	0.73273731	-6.1036474
WT after UVB irradiation VS WT before UVB irradiation	Abacavir Pathway_ Pharmacokinetics/Pharmacodynamics|PharmGKB	0.050024755	-0.03922029	0.188743489	0.85403904	0.95632621	-5.85113846
homo after UVB irradiation VS homo before UVB irradiation	Abacavir transmembrane transport|Reactome	0.687703602	0.036195595	3.145055168	0.01123685	0.06453768	-2.78609716
hetero after UVB irradiation VS hetero before UVB irradiation	Abacavir transmembrane transport|Reactome	0.115438381	-0.03854305	0.486624696	0.63892986	0.81644023	-6.21990163
WT after UVB irradiation VS WT before UVB irradiation	Abacavir transmembrane transport|Reactome	0.286703511	-0.05009545	0.949378256	0.3646298	0.74545864	-5.43331642
homo after UVB irradiation VS homo before UVB irradiation	Abacavir transport and metabolism|Reactome	0.243389207	0.076378018	1.039641942	0.32453676	0.48049119	-5.81965503
hetero after UVB irradiation VS hetero before UVB irradiation	Abacavir transport and metabolism|Reactome	0.111681289	-0.0819421	0.485523345	0.63967873	0.81716806	-6.22046615
WT after UVB irradiation VS WT before UVB irradiation	Abacavir transport and metabolism|Reactome	0.171051621	0.024691655	0.71407983	0.49137852	0.81710765	-5.61764602
homo after UVB irradiation VS homo before UVB irradiation	Abasic sugar-phosphate removal via the single-nucleotide replacement pathway|Reactome	-0.46777822	0.116978883	-1.51896466	0.1617	0.31074167	-5.26372146
hetero after UVB irradiation VS hetero before UVB irradiation	Abasic sugar-phosphate removal via the single-nucleotide replacement pathway|Reactome	-0.467936	-0.07748278	-1.32397018	0.22030147	0.44184836	-5.4852419
WT after UVB irradiation VS WT before UVB irradiation	Abasic sugar-phosphate removal via the single-nucleotide replacement pathway|Reactome	-0.35734693	-0.07849097	-0.79316208	0.44594746	0.80309229	-5.5607411
homo after UVB irradiation VS homo before UVB irradiation	ABC transporter disorders|Reactome	0.097181515	-0.04093626	0.424768631	0.68057389	0.7845242	-6.2724491
hetero after UVB irradiation VS hetero before UVB irradiation	ABC transporter disorders|Reactome	0.280255404	0.045519023	1.501738893	0.16967558	0.38622363	-5.26661473
WT after UVB irradiation VS WT before UVB irradiation	ABC transporter disorders|Reactome	-0.01049528	0.005990406	-0.04801053	0.9626456	0.98815178	-5.86798363
homo after UVB irradiation VS homo before UVB irradiation	ABC transporters - Homo sapiens (human)|KEGG	0.243330917	-0.0108199	1.392617339	0.19583039	0.35189357	-5.42389613
hetero after UVB irradiation VS hetero before UVB irradiation	ABC transporters - Homo sapiens (human)|KEGG	-0.06675195	-0.00898851	-0.39919364	0.69967784	0.84794778	-6.26092314
WT after UVB irradiation VS WT before UVB irradiation	ABC transporters - Homo sapiens (human)|KEGG	0.067600826	-0.01608202	0.286655852	0.78018025	0.93661609	-5.82770532
homo after UVB irradiation VS homo before UVB irradiation	ABC transporters in lipid homeostasis|Reactome	0.080399926	-0.00491203	0.321546079	0.75483971	0.84063368	-6.31320751
hetero after UVB irradiation VS hetero before UVB irradiation	ABC transporters in lipid homeostasis|Reactome	0.020511139	0.003530736	0.094827556	0.9266683	0.97054702	-6.34166571
WT after UVB irradiation VS WT before UVB irradiation	ABC transporters in lipid homeostasis|Reactome	-0.04899149	-0.04458023	-0.18118643	0.85981186	0.95916804	-5.85254953
homo after UVB irradiation VS homo before UVB irradiation	ABC-family proteins mediated transport|Reactome	0.081300626	0.001501797	0.444918711	0.66645868	0.77195595	-6.26322453
hetero after UVB irradiation VS hetero before UVB irradiation	ABC-family proteins mediated transport|Reactome	-0.10815139	-0.00235151	-0.65396696	0.53057288	0.74479557	-6.12039776
WT after UVB irradiation VS WT before UVB irradiation	ABC-family proteins mediated transport|Reactome	-0.10756907	-0.0378168	-0.51040773	0.62075384	0.87257466	-5.73898204
homo after UVB irradiation VS homo before UVB irradiation	Abciximab Action Pathway|SMPDB	0.250990463	0.059108895	0.688272445	0.50792806	0.64727869	-6.12030765
hetero after UVB irradiation VS hetero before UVB irradiation	Abciximab Action Pathway|SMPDB	0.140468489	-0.09168173	0.363509274	0.72517643	0.86482318	-6.27542943
WT after UVB irradiation VS WT before UVB irradiation	Abciximab Action Pathway|SMPDB	0.031786681	-0.05176445	0.082324754	0.93599995	0.98056728	-5.86571905
homo after UVB irradiation VS homo before UVB irradiation	Abnormal conversion of 2-oxoglutarate to 2-hydroxyglutarate|Reactome	0.75853214	-0.0102717	2.294747761	0.0462596	0.14003669	-4.13896681
hetero after UVB irradiation VS hetero before UVB irradiation	Abnormal conversion of 2-oxoglutarate to 2-hydroxyglutarate|Reactome	0.084135561	0.009875163	0.197001018	0.84849606	0.94443102	-6.32553714
WT after UVB irradiation VS WT before UVB irradiation	Abnormal conversion of 2-oxoglutarate to 2-hydroxyglutarate|Reactome	0.005390724	0.12172669	0.013005468	0.98987719	0.99676022	-5.86906551
homo after UVB irradiation VS homo before UVB irradiation	Abortive elongation of HIV-1 transcript in the absence of Tat|Reactome	-0.71667726	0.001326788	-3.36861896	0.00779395	0.05187376	-2.43035372
hetero after UVB irradiation VS hetero before UVB irradiation	Abortive elongation of HIV-1 transcript in the absence of Tat|Reactome	-0.20323408	-0.03315387	-0.85667093	0.41532435	0.64192892	-5.96529674
WT after UVB irradiation VS WT before UVB irradiation	Abortive elongation of HIV-1 transcript in the absence of Tat|Reactome	-0.21680785	0.040552727	-0.59492513	0.56500594	0.85125516	-5.69316429
homo after UVB irradiation VS homo before UVB irradiation	ACE Inhibitor Pathway_ Pharmacodynamics|PharmGKB	0.149430321	-0.04622985	0.905130419	0.38803163	0.54134863	-5.94693193
hetero after UVB irradiation VS hetero before UVB irradiation	ACE Inhibitor Pathway_ Pharmacodynamics|PharmGKB	0.393058791	0.021985605	2.029212312	0.0751637	0.24558862	-4.54009905
WT after UVB irradiation VS WT before UVB irradiation	ACE Inhibitor Pathway_ Pharmacodynamics|PharmGKB	0.280771856	-0.02298454	1.169614914	0.26906294	0.66278879	-5.22263483
homo after UVB irradiation VS homo before UVB irradiation	ACE Inhibitor Pathway|Wikipathways	0.225438574	-0.03456876	1.182961293	0.26591179	0.42933282	-5.66903033
hetero after UVB irradiation VS hetero before UVB irradiation	ACE Inhibitor Pathway|Wikipathways	0.458097805	0.030153312	2.27306347	0.05103016	0.20064099	-4.178917
WT after UVB irradiation VS WT before UVB irradiation	ACE Inhibitor Pathway|Wikipathways	0.358191516	-0.0329228	1.292020168	0.2251792	0.62952955	-5.09150856
homo after UVB irradiation VS homo before UVB irradiation	Acebutolol Action Pathway|SMPDB	0.396415397	-0.03677376	2.343069613	0.04268583	0.13402181	-4.06364635
hetero after UVB irradiation VS hetero before UVB irradiation	Acebutolol Action Pathway|SMPDB	-0.01555055	-0.01775524	-0.07410913	0.94265284	0.97054702	-6.34356282
WT after UVB irradiation VS WT before UVB irradiation	Acebutolol Action Pathway|SMPDB	0.056685205	-0.03978418	0.223404833	0.82768199	0.94525798	-5.84393438
homo after UVB irradiation VS homo before UVB irradiation	Acenocoumarol Action Pathway|SMPDB	0.538210712	0.026444726	3.007777411	0.01409598	0.07305822	-3.00571236
hetero after UVB irradiation VS hetero before UVB irradiation	Acenocoumarol Action Pathway|SMPDB	0.558085555	0.09913938	2.984394118	0.01649409	0.11352943	-3.09486545
WT after UVB irradiation VS WT before UVB irradiation	Acenocoumarol Action Pathway|SMPDB	0.440879848	0.077377741	1.569633667	0.14732454	0.55622203	-4.76265269
homo after UVB irradiation VS homo before UVB irradiation	Acetaminophen Action Pathway|SMPDB	0.40423582	0.045705158	1.979500697	0.07780079	0.19626432	-4.61879689
hetero after UVB irradiation VS hetero before UVB irradiation	Acetaminophen Action Pathway|SMPDB	0.482496143	0.064876536	3.04499191	0.01500113	0.1130168	-3.00250826
WT after UVB irradiation VS WT before UVB irradiation	Acetaminophen Action Pathway|SMPDB	0.275725339	0.086595857	1.261970114	0.23537138	0.62952955	-5.12455679
homo after UVB irradiation VS homo before UVB irradiation	Acetaminophen Metabolism Pathway|SMPDB	0.010308936	0.018150014	0.052683886	0.95908772	0.97559823	-6.36695715
hetero after UVB irradiation VS hetero before UVB irradiation	Acetaminophen Metabolism Pathway|SMPDB	0.281590945	0.021985974	1.495351804	0.17130034	0.38806509	-5.27474904
WT after UVB irradiation VS WT before UVB irradiation	Acetaminophen Metabolism Pathway|SMPDB	0.39096595	0.011810158	1.62708719	0.13452835	0.54709793	-4.6898666
homo after UVB irradiation VS homo before UVB irradiation	Acetaminophen Pathway_ Pharmacokinetics|PharmGKB	0.254595207	0.043149964	1.540078891	0.1565348	0.30417351	-5.23613761
hetero after UVB irradiation VS hetero before UVB irradiation	Acetaminophen Pathway_ Pharmacokinetics|PharmGKB	0.003198058	-0.00161008	0.012308593	0.99046588	0.9943047	-6.34645911
WT after UVB irradiation VS WT before UVB irradiation	Acetaminophen Pathway_ Pharmacokinetics|PharmGKB	0.177058396	-0.02271735	0.729080397	0.48254529	0.81552033	-5.60725969
homo after UVB irradiation VS homo before UVB irradiation	acetate conversion to acetyl-CoA|HumanCyc	-0.35535207	0.079273981	-0.99995838	0.34240275	0.49837773	-5.85868136
hetero after UVB irradiation VS hetero before UVB irradiation	acetate conversion to acetyl-CoA|HumanCyc	-0.51243692	-0.02089391	-2.16857447	0.06026502	0.22010255	-4.33496501
WT after UVB irradiation VS WT before UVB irradiation	acetate conversion to acetyl-CoA|HumanCyc	-0.42382408	-0.07501019	-1.42445424	0.18452352	0.59839062	-4.93967065
homo after UVB irradiation VS homo before UVB irradiation	acetone degradation I (to methylglyoxal)|HumanCyc	0.411073341	0.036650462	2.124055451	0.0613741	0.16973601	-4.40159622
hetero after UVB irradiation VS hetero before UVB irradiation	acetone degradation I (to methylglyoxal)|HumanCyc	0.495268869	0.000729858	2.135885172	0.06347753	0.22631041	-4.3834192
WT after UVB irradiation VS WT before UVB irradiation	acetone degradation I (to methylglyoxal)|HumanCyc	0.443898147	0.045244784	1.632198567	0.13343926	0.54656243	-4.68332145
homo after UVB irradiation VS homo before UVB irradiation	acetylation and deacetylation of rela in nucleus|BioCarta	-0.05220055	-0.01867059	-0.17051219	0.86822528	0.9186887	-6.35285123
hetero after UVB irradiation VS hetero before UVB irradiation	acetylation and deacetylation of rela in nucleus|BioCarta	0.036601862	-0.03397278	0.216185424	0.83398549	0.93635724	-6.32125929
WT after UVB irradiation VS WT before UVB irradiation	acetylation and deacetylation of rela in nucleus|BioCarta	-0.45161637	-0.05524309	-2.17818926	0.05419743	0.41669978	-3.93250843
homo after UVB irradiation VS homo before UVB irradiation	Acetylation|Reactome	1.081746638	-0.06596393	5.150826459	0.00052668	0.01666204	0.194700228
hetero after UVB irradiation VS hetero before UVB irradiation	Acetylation|Reactome	0.153210101	-0.07413777	0.460909216	0.6565277	0.82651398	-6.2327667
WT after UVB irradiation VS WT before UVB irradiation	Acetylation|Reactome	0.883149676	0.042677932	3.052152663	0.01209688	0.27611764	-2.62983278
homo after UVB irradiation VS homo before UVB irradiation	Acetylcholine binding and downstream events|Reactome	0.106608129	0.018460278	0.48363748	0.63972296	0.75031467	-6.24435373
hetero after UVB irradiation VS hetero before UVB irradiation	Acetylcholine binding and downstream events|Reactome	-0.39673811	-0.00107584	-2.11855793	0.06524756	0.22947406	-4.40902394
WT after UVB irradiation VS WT before UVB irradiation	Acetylcholine binding and downstream events|Reactome	-0.66670677	-0.00479849	-3.09744745	0.01119442	0.26766971	-2.56198852
homo after UVB irradiation VS homo before UVB irradiation	Acetylcholine Neurotransmitter Release Cycle|Reactome	0.237216675	0.010902776	1.329695495	0.21501636	0.37301091	-5.50032479
hetero after UVB irradiation VS hetero before UVB irradiation	Acetylcholine Neurotransmitter Release Cycle|Reactome	-0.14110283	0.030781132	-0.67260548	0.51921445	0.73623731	-6.10767008
WT after UVB irradiation VS WT before UVB irradiation	Acetylcholine Neurotransmitter Release Cycle|Reactome	-0.04820461	0.014726427	-0.17071212	0.86782742	0.96280353	-5.85441062
homo after UVB irradiation VS homo before UVB irradiation	Acetylcholine regulates insulin secretion|Reactome	0.417148012	-0.05708477	2.36699699	0.04101801	0.13402181	-4.02621583
hetero after UVB irradiation VS hetero before UVB irradiation	Acetylcholine regulates insulin secretion|Reactome	0.703805716	0.034204685	3.916170637	0.00401368	0.07009325	-1.71163009
WT after UVB irradiation VS WT before UVB irradiation	Acetylcholine regulates insulin secretion|Reactome	0.398612036	0.080077662	1.679660053	0.12369682	0.52904005	-4.62202462
homo after UVB irradiation VS homo before UVB irradiation	Acetylcholine Synthesis|Wikipathways	-0.26102317	-0.00494724	-0.98580891	0.3489476	0.50461003	-5.87230261
hetero after UVB irradiation VS hetero before UVB irradiation	Acetylcholine Synthesis|Wikipathways	-0.18273819	-0.06673707	-0.59156581	0.56966875	0.76639616	-6.16064658
WT after UVB irradiation VS WT before UVB irradiation	Acetylcholine Synthesis|Wikipathways	-0.13303136	-0.08328467	-0.42192248	0.68193697	0.8867899	-5.77981613
homo after UVB irradiation VS homo before UVB irradiation	acetyl-CoA biosynthesis from citrate|HumanCyc	-0.44565938	-0.02008781	-1.20562374	0.25746559	0.42090507	-5.64389124
hetero after UVB irradiation VS hetero before UVB irradiation	acetyl-CoA biosynthesis from citrate|HumanCyc	0.575740637	0.016023849	1.501210001	0.1698096	0.38622363	-5.26728904
WT after UVB irradiation VS WT before UVB irradiation	acetyl-CoA biosynthesis from citrate|HumanCyc	0.628392528	0.063341005	1.690744125	0.12151568	0.52647129	-4.60757694
homo after UVB irradiation VS homo before UVB irradiation	Acetylsalicylic Acid Action Pathway|SMPDB	0.40423582	0.045705158	1.979500697	0.07780079	0.19626432	-4.61879689
hetero after UVB irradiation VS hetero before UVB irradiation	Acetylsalicylic Acid Action Pathway|SMPDB	0.482496143	0.064876536	3.04499191	0.01500113	0.1130168	-3.00250826
WT after UVB irradiation VS WT before UVB irradiation	Acetylsalicylic Acid Action Pathway|SMPDB	0.275725339	0.086595857	1.261970114	0.23537138	0.62952955	-5.12455679
homo after UVB irradiation VS homo before UVB irradiation	Acrosome Reaction|Reactome	-0.33656395	0.037980451	-0.83167102	0.42623934	0.57741028	-6.01023298
hetero after UVB irradiation VS hetero before UVB irradiation	Acrosome Reaction|Reactome	0.978200112	0.037596009	3.341295662	0.00948248	0.0985871	-2.55449507
WT after UVB irradiation VS WT before UVB irradiation	Acrosome Reaction|Reactome	0.251954137	-0.07121976	0.666544097	0.52002404	0.83040203	-5.64927421
homo after UVB irradiation VS homo before UVB irradiation	Acrylamide Biotransformation and Exposure Biomarkers|Wikipathways	0.388460901	0.020895461	1.066764261	0.31273867	0.46923284	-5.79229523
hetero after UVB irradiation VS hetero before UVB irradiation	Acrylamide Biotransformation and Exposure Biomarkers|Wikipathways	-0.14760988	0.040949837	-0.3635581	0.72514129	0.86482318	-6.27541048
WT after UVB irradiation VS WT before UVB irradiation	Acrylamide Biotransformation and Exposure Biomarkers|Wikipathways	-0.06962327	0.086915055	-0.17823013	0.86207255	0.95916804	-5.85308595
homo after UVB irradiation VS homo before UVB irradiation	actions of nitric oxide in the heart|BioCarta	0.532375688	-0.02587747	3.245729773	0.00952472	0.05835911	-2.62552946
hetero after UVB irradiation VS hetero before UVB irradiation	actions of nitric oxide in the heart|BioCarta	0.348312828	0.016524217	1.746327771	0.11698945	0.30984191	-4.94175172
WT after UVB irradiation VS WT before UVB irradiation	actions of nitric oxide in the heart|BioCarta	0.130735491	-0.00127383	0.498934203	0.62853148	0.87289644	-5.74469293
homo after UVB irradiation VS homo before UVB irradiation	Activated NOTCH1 Transmits Signal to the Nucleus|Reactome	0.32826505	-0.03782381	1.79424516	0.10496983	0.2370387	-4.88809565
hetero after UVB irradiation VS hetero before UVB irradiation	Activated NOTCH1 Transmits Signal to the Nucleus|Reactome	0.370083155	0.023376509	2.240755567	0.05372573	0.20588996	-4.22734219
WT after UVB irradiation VS WT before UVB irradiation	Activated NOTCH1 Transmits Signal to the Nucleus|Reactome	0.137442682	0.005083892	0.597961418	0.56305572	0.85017866	-5.69139684
homo after UVB irradiation VS homo before UVB irradiation	Activated NTRK2 signals through CDK5|Reactome	0.943639172	-0.04470208	4.686915547	0.00101766	0.01843909	-0.44343195
hetero after UVB irradiation VS hetero before UVB irradiation	Activated NTRK2 signals through CDK5|Reactome	0.681839785	0.052898324	2.558124672	0.03238732	0.15685114	-3.74679713
WT after UVB irradiation VS WT before UVB irradiation	Activated NTRK2 signals through CDK5|Reactome	0.712696021	0.055616912	2.608716524	0.02593653	0.34104092	-3.29614837
homo after UVB irradiation VS homo before UVB irradiation	Activated NTRK2 signals through FRS2 and FRS3|Reactome	0.120610564	0.053910613	0.384284233	0.7093267	0.8084837	-6.28973715
hetero after UVB irradiation VS hetero before UVB irradiation	Activated NTRK2 signals through FRS2 and FRS3|Reactome	-0.1153365	0.042411511	-0.54782317	0.59802241	0.78757185	-6.18665017
WT after UVB irradiation VS WT before UVB irradiation	Activated NTRK2 signals through FRS2 and FRS3|Reactome	-0.04345312	0.008922432	-0.18548717	0.85652545	0.95782364	-5.8517535
homo after UVB irradiation VS homo before UVB irradiation	Activated NTRK2 signals through FYN|Reactome	0.188396343	-0.04421919	0.774884908	0.45747746	0.60388612	-6.05600533
hetero after UVB irradiation VS hetero before UVB irradiation	Activated NTRK2 signals through FYN|Reactome	-0.00550288	0.045121233	-0.01865862	0.98554774	0.99273516	-6.34635244
WT after UVB irradiation VS WT before UVB irradiation	Activated NTRK2 signals through FYN|Reactome	0.248346374	-0.03248567	1.047918942	0.3191472	0.70304529	-5.34330107
homo after UVB irradiation VS homo before UVB irradiation	Activated NTRK2 signals through PLCG1|Reactome	0.753853625	0.0055166	2.638860242	0.02605267	0.10678321	-3.59614531
hetero after UVB irradiation VS hetero before UVB irradiation	Activated NTRK2 signals through PLCG1|Reactome	0.388280331	-0.00836801	1.404501095	0.19594179	0.4161797	-5.38826358
WT after UVB irradiation VS WT before UVB irradiation	Activated NTRK2 signals through PLCG1|Reactome	0.516039945	-0.06216238	1.797824106	0.10216915	0.50394202	-4.46558072
homo after UVB irradiation VS homo before UVB irradiation	Activated NTRK2 signals through RAS|Reactome	0.279986724	-0.04020178	1.212354456	0.25499923	0.41874827	-5.63635843
hetero after UVB irradiation VS hetero before UVB irradiation	Activated NTRK2 signals through RAS|Reactome	0.364713965	-0.01011118	2.004070213	0.07820737	0.25169216	-4.57667124
WT after UVB irradiation VS WT before UVB irradiation	Activated NTRK2 signals through RAS|Reactome	0.203150865	0.004844452	0.961411267	0.3588355	0.74322074	-5.42271062
homo after UVB irradiation VS homo before UVB irradiation	Activated PKN1 stimulates transcription of AR (androgen receptor) regulated genes KLK2 and KLK3|Reactome	-0.32832782	0.055163457	-1.61983139	0.13831926	0.28082069	-5.1299932
hetero after UVB irradiation VS hetero before UVB irradiation	Activated PKN1 stimulates transcription of AR (androgen receptor) regulated genes KLK2 and KLK3|Reactome	-0.22540374	-0.011937	-1.19852122	0.26333431	0.4899093	-5.62878724
WT after UVB irradiation VS WT before UVB irradiation	Activated PKN1 stimulates transcription of AR (androgen receptor) regulated genes KLK2 and KLK3|Reactome	-0.32729656	0.028738545	-1.69689355	0.12032059	0.5258413	-4.59954028
homo after UVB irradiation VS homo before UVB irradiation	Activated PKN1 stimulates transcription of AR (androgen receptor) regulated genes KLK2 and KLK3|Wikipathways	1.010892975	-0.02764662	4.27965775	0.00186143	0.023988	-1.03177918
hetero after UVB irradiation VS hetero before UVB irradiation	Activated PKN1 stimulates transcription of AR (androgen receptor) regulated genes KLK2 and KLK3|Wikipathways	0.456162143	0.00273275	1.264035578	0.2400627	0.46268432	-5.55501695
WT after UVB irradiation VS WT before UVB irradiation	Activated PKN1 stimulates transcription of AR (androgen receptor) regulated genes KLK2 and KLK3|Wikipathways	0.36698389	-0.04546868	1.055887625	0.31566397	0.69936155	-5.33571326
homo after UVB irradiation VS homo before UVB irradiation	Activated point mutants of FGFR2|Reactome	-0.31990956	0.058499883	-1.32496308	0.21652131	0.37419996	-5.50597789
hetero after UVB irradiation VS hetero before UVB irradiation	Activated point mutants of FGFR2|Reactome	-0.35814887	0.041228686	-1.74985853	0.11635328	0.30887493	-4.93689038
WT after UVB irradiation VS WT before UVB irradiation	Activated point mutants of FGFR2|Reactome	-0.5602751	0.043099089	-2.25524081	0.04754731	0.401167	-3.82043972
homo after UVB irradiation VS homo before UVB irradiation	activated TAK1 mediates p38 MAPK activation|Reactome	0.342921217	-0.03361983	1.788835078	0.10588236	0.23874511	-4.89578209
hetero after UVB irradiation VS hetero before UVB irradiation	activated TAK1 mediates p38 MAPK activation|Reactome	0.417145268	0.051859796	2.083805781	0.06894367	0.2352135	-4.46020333

WT after UVB irradiation VS WT before UVB irradiation	activated TAK1 mediates p38 MAPK activation|Reactome	0.149390424	-0.00271482	0.672228212	0.516547	0.82895075	-5.64559598
homo after UVB irradiation VS homo before UVB irradiation	Activation and oligomerization of BAK protein|Reactome	0.681346405	-0.05442809	2.236189516	0.05098413	0.14951415	-4.22971
hetero after UVB irradiation VS hetero before UVB irradiation	Activation and oligomerization of BAK protein|Reactome	1.132959839	0.045757474	7.144604751	7.54E-05	0.02920766	2.073858986
WT after UVB irradiation VS WT before UVB irradiation	Activation and oligomerization of BAK protein|Reactome	0.971840718	-0.11025089	3.119364786	0.01078268	0.26333964	-2.52919935
homo after UVB irradiation VS homo before UVB irradiation	Activation of AKT2|Reactome	0.384797869	0.009365815	1.68318929	0.12522802	0.26466492	-5.04358922
hetero after UVB irradiation VS hetero before UVB irradiation	Activation of AKT2|Reactome	0.58540901	0.061134047	2.902069782	0.01877252	0.12051431	-3.22059701
WT after UVB irradiation VS WT before UVB irradiation	Activation of AKT2|Reactome	0.04475142	0.048315971	0.187765117	0.85478591	0.95645482	-5.85132437
homo after UVB irradiation VS homo before UVB irradiation	Activation of AMPA receptors|Reactome	-0.58318917	0.104303216	-2.33074267	0.0435709	0.13524454	-4.08289605
hetero after UVB irradiation VS hetero before UVB irradiation	Activation of AMPA receptors|Reactome	-0.48057535	-0.0228631	-1.85598854	0.09867321	0.28258275	-4.78881994
WT after UVB irradiation VS WT before UVB irradiation	Activation of AMPA receptors|Reactome	-0.79054239	0.08118638	-2.7289343	0.02108544	0.31542987	-3.11564811
homo after UVB irradiation VS homo before UVB irradiation	Activation of anterior HOX genes in hindbrain development during early embryogenesis|Wikipathways	-0.12555892	0.067331119	-0.60724217	0.55808806	0.6849905	-6.17422093
hetero after UVB irradiation VS hetero before UVB irradiation	Activation of anterior HOX genes in hindbrain development during early embryogenesis|Wikipathways	-0.05727777	-0.01037641	-0.20973024	0.83886084	0.93932613	-6.32274259
WT after UVB irradiation VS WT before UVB irradiation	Activation of anterior HOX genes in hindbrain development during early embryogenesis|Wikipathways	0.135098584	0.03057355	0.49794529	0.62920409	0.87289644	-5.74517939
homo after UVB irradiation VS homo before UVB irradiation	Activation of APC/C and APC/C:Cdc20 mediated degradation of mitotic proteins|Reactome	-0.586645	0.063785355	-3.00481877	0.01416522	0.07314252	-3.01045118
hetero after UVB irradiation VS hetero before UVB irradiation	Activation of APC/C and APC/C:Cdc20 mediated degradation of mitotic proteins|Reactome	0.283377899	0.009346832	1.316394697	0.22272066	0.44410029	-5.49417751
WT after UVB irradiation VS WT before UVB irradiation	Activation of APC/C and APC/C:Cdc20 mediated degradation of mitotic proteins|Reactome	-0.06168994	-0.06094803	-0.23267587	0.82066848	0.94525798	-5.84180418
homo after UVB irradiation VS homo before UVB irradiation	Activation of ATR in response to replication stress|Reactome	-0.52538935	-0.04991124	-2.13538706	0.06023761	0.16751767	-4.38434976
hetero after UVB irradiation VS hetero before UVB irradiation	Activation of ATR in response to replication stress|Reactome	0.025883013	-0.02390962	0.088250556	0.93173911	0.97054702	-6.34231826
WT after UVB irradiation VS WT before UVB irradiation	Activation of ATR in response to replication stress|Reactome	-0.02146701	-0.01932521	-0.07213175	0.94390794	0.98295421	-5.86651611
homo after UVB irradiation VS homo before UVB irradiation	Activation of BAD and translocation to mitochondria|Reactome	0.552381703	-0.07878994	2.535704255	0.03095268	0.11718899	-3.76019759
hetero after UVB irradiation VS hetero before UVB irradiation	Activation of BAD and translocation to mitochondria|Reactome	0.56687651	0.004145892	2.734957188	0.02444862	0.13718149	-3.47634217
WT after UVB irradiation VS WT before UVB irradiation	Activation of BAD and translocation to mitochondria|Reactome	0.452017641	-0.0742774	2.026432261	0.06999225	0.44574118	-4.14973387
homo after UVB irradiation VS homo before UVB irradiation	Activation of BH3-only proteins|Reactome	0.306304585	-0.05721665	1.617677669	0.13878516	0.28141074	-5.13289887
hetero after UVB irradiation VS hetero before UVB irradiation	Activation of BH3-only proteins|Reactome	0.33823523	-0.0026056	2.050510888	0.07267504	0.24158558	-4.50900575
WT after UVB irradiation VS WT before UVB irradiation	Activation of BH3-only proteins|Reactome	0.232632645	-0.00021682	1.154878072	0.27477799	0.66570326	-5.23777946
homo after UVB irradiation VS homo before UVB irradiation	Activation of BIM and translocation to mitochondria|Reactome	-0.17432166	-0.03332586	-0.52949188	0.60875426	0.72706564	-6.22007686
hetero after UVB irradiation VS hetero before UVB irradiation	Activation of BIM and translocation to mitochondria|Reactome	0.206349881	-0.00546108	0.610238952	0.55779981	0.76144101	-6.14898759
WT after UVB irradiation VS WT before UVB irradiation	Activation of BIM and translocation to mitochondria|Reactome	0.052833792	-0.04503083	0.235412	0.81860174	0.94525798	-5.84115914
homo after UVB irradiation VS homo before UVB irradiation	Activation of BMF and translocation to mitochondria|Reactome	0.120505749	-0.03448402	0.412796265	0.68902328	0.79106872	-6.27773536
hetero after UVB irradiation VS hetero before UVB irradiation	Activation of BMF and translocation to mitochondria|Reactome	-0.60336432	0.003594715	-1.8598005	0.0980878	0.28242841	-4.78343524
WT after UVB irradiation VS WT before UVB irradiation	Activation of BMF and translocation to mitochondria|Reactome	-0.16432119	0.047248054	-0.54714724	0.59617982	0.86530281	-5.71987198
homo after UVB irradiation VS homo before UVB irradiation	Activation of C3 and C5|Reactome	0.784959851	-0.09999415	2.524127027	0.03155712	0.11811274	-3.7785533
hetero after UVB irradiation VS hetero before UVB irradiation	Activation of C3 and C5|Reactome	0.444494638	0.072511692	1.48989358	0.17269973	0.39026542	-5.28168486
WT after UVB irradiation VS WT before UVB irradiation	Activation of C3 and C5|Reactome	0.636995107	-0.00924932	1.864858877	0.09153796	0.48385863	-4.37461824
homo after UVB irradiation VS homo before UVB irradiation	Activation of CaMK IV|Reactome	1.04315772	-0.01561465	4.566260211	0.001214	0.02014127	-0.61505659
hetero after UVB irradiation VS hetero before UVB irradiation	Activation of CaMK IV|Reactome	0.272819494	0.012877047	0.712034469	0.49568306	0.71474957	-6.07969567
WT after UVB irradiation VS WT before UVB irradiation	Activation of CaMK IV|Reactome	0.201447202	-0.02892331	0.485113481	0.63796376	0.87636259	-5.75140856
homo after UVB irradiation VS homo before UVB irradiation	activation of camp-dependent protein kinase pka|BioCarta	0.379551349	0.013537509	2.16430727	0.05742834	0.1628682	-4.34020288
hetero after UVB irradiation VS hetero before UVB irradiation	activation of camp-dependent protein kinase pka|BioCarta	0.24676012	0.015597779	1.420440659	0.19140657	0.41074913	-5.36865228
WT after UVB irradiation VS WT before UVB irradiation	activation of camp-dependent protein kinase pka|BioCarta	0.149730136	-0.03373106	0.516768889	0.61646267	0.87257466	-5.73576291
homo after UVB irradiation VS homo before UVB irradiation	Activation of Ca-permeable Kainate Receptor|Reactome	-0.4255247	-0.05440509	-1.68269443	0.12532577	0.26466492	-5.04427086
hetero after UVB irradiation VS hetero before UVB irradiation	Activation of Ca-permeable Kainate Receptor|Reactome	-0.32439968	-0.04214232	-1.61046316	0.14405119	0.34739964	-5.12527674
WT after UVB irradiation VS WT before UVB irradiation	Activation of Ca-permeable Kainate Receptor|Reactome	-0.13972547	0.069563579	-0.63121751	0.54194318	0.83916339	-5.67149504
homo after UVB irradiation VS homo before UVB irradiation	Activation of caspases through apoptosome-mediated cleavage|Reactome	0.559708311	0.022472492	1.657754002	0.13034279	0.27177943	-5.07848833
hetero after UVB irradiation VS hetero before UVB irradiation	Activation of caspases through apoptosome-mediated cleavage|Reactome	0.655477124	0.073652291	3.066446317	0.01450676	0.1130168	-2.96985691
WT after UVB irradiation VS WT before UVB irradiation	Activation of caspases through apoptosome-mediated cleavage|Reactome	0.796091303	-0.05990195	3.524175982	0.0054317	0.20968398	-1.93038582
homo after UVB irradiation VS homo before UVB irradiation	activation of csk by camp-dependent protein kinase inhibits signaling through the t cell receptor|BioCarta	0.127892931	0.010766702	0.711923871	0.49382041	0.63443556	-6.10342228
hetero after UVB irradiation VS hetero before UVB irradiation	activation of csk by camp-dependent protein kinase inhibits signaling through the t cell receptor|BioCarta	0.1177184	0.007849057	0.617809163	0.55302896	0.75787914	-6.14416673
WT after UVB irradiation VS WT before UVB irradiation	activation of csk by camp-dependent protein kinase inhibits signaling through the t cell receptor|BioCarta	0.120390661	-0.05814131	0.407169582	0.69239358	0.89503579	-5.78590057
homo after UVB irradiation VS homo before UVB irradiation	Activation of DNA fragmentation factor|Reactome	-0.20152655	-0.03668943	-0.92803906	0.37662748	0.52945846	-5.9262742
hetero after UVB irradiation VS hetero before UVB irradiation	Activation of DNA fragmentation factor|Reactome	0.114619369	-0.02256632	0.569157729	0.58409845	0.77618681	-6.17419784
WT after UVB irradiation VS WT before UVB irradiation	Activation of DNA fragmentation factor|Reactome	0.043030214	-0.04456098	0.172899872	0.8661519	0.96152545	-5.85403099
homo after UVB irradiation VS homo before UVB irradiation	Activation of E2F1 target genes at G1/S|Reactome	-0.53435365	-0.03020835	-1.71266811	0.11952847	0.25817499	-5.00279871
hetero after UVB irradiation VS hetero before UVB irradiation	Activation of E2F1 target genes at G1/S|Reactome	0.395803645	0.055572445	1.337665513	0.2159846	0.43741933	-5.46900423
WT after UVB irradiation VS WT before UVB irradiation	Activation of E2F1 target genes at G1/S|Reactome	0.300773995	-0.00216916	0.991405511	0.34468414	0.7291211	-5.39580072
homo after UVB irradiation VS homo before UVB irradiation	Activation of G protein gated Potassium channels|Reactome	0.227909476	-0.0130962	1.107580443	0.29560878	0.45577992	-5.75009619
hetero after UVB irradiation VS hetero before UVB irradiation	Activation of G protein gated Potassium channels|Reactome	0.111975855	0.033657182	0.713384829	0.49488925	0.71428009	-6.07871261
WT after UVB irradiation VS WT before UVB irradiation	Activation of G protein gated Potassium channels|Reactome	-0.00386805	-0.05146768	-0.01498999	0.98833265	0.99664845	-5.86903736
homo after UVB irradiation VS homo before UVB irradiation	Activation of GABAB receptors|Reactome	0.360763269	-0.02159689	2.006470782	0.07444799	0.19493053	-4.57869567
hetero after UVB irradiation VS hetero before UVB irradiation	Activation of GABAB receptors|Reactome	0.21007887	0.036901545	1.25563105	0.2429491	0.46586538	-5.56462969
WT after UVB irradiation VS WT before UVB irradiation	Activation of GABAB receptors|Reactome	0.113131058	-0.03418705	0.462751502	0.65336902	0.87935805	-5.76189402
homo after UVB irradiation VS homo before UVB irradiation	Activation of gene expression by SREBF (SREBP)|Reactome	-0.33290388	-0.05830563	-1.26385121	0.23675845	0.39787967	-5.57773766
hetero after UVB irradiation VS hetero before UVB irradiation	Activation of gene expression by SREBF (SREBP)|Reactome	-0.12721543	0.026621601	-0.5827205	0.57534057	0.77084136	-6.1660533
WT after UVB irradiation VS WT before UVB irradiation	Activation of gene expression by SREBF (SREBP)|Reactome	-0.00091335	0.024345638	-0.00343685	0.99732485	0.99863683	-5.86914521
homo after UVB irradiation VS homo before UVB irradiation	Activation of gene expression by SREBF (SREBP)|Wikipathways	-0.33348882	-0.05890951	-1.18005454	0.26701105	0.43049875	-5.67222954
hetero after UVB irradiation VS hetero before UVB irradiation	Activation of gene expression by SREBF (SREBP)|Wikipathways	-0.0131577	0.016608144	-0.05320095	0.95881161	0.98102691	-6.34500612
WT after UVB irradiation VS WT before UVB irradiation	Activation of gene expression by SREBF (SREBP)|Wikipathways	-0.11811544	0.042531955	-0.42143741	0.68227967	0.886983	-5.7800195
homo after UVB irradiation VS homo before UVB irradiation	Activation of IRF3/IRF7 mediated by TBK1/IKK epsilon|Reactome	0.547357254	-0.00857642	3.345905502	0.00808737	0.05305596	-2.46634873
hetero after UVB irradiation VS hetero before UVB irradiation	Activation of IRF3/IRF7 mediated by TBK1/IKK epsilon|Reactome	0.536823114	-0.01566501	3.469368694	0.00780083	0.08956216	-2.36328883
WT after UVB irradiation VS WT before UVB irradiation	Activation of IRF3/IRF7 mediated by TBK1/IKK epsilon|Reactome	0.368735785	-0.04692463	1.512276559	0.16115732	0.56269533	-4.83381784
homo after UVB irradiation VS homo before UVB irradiation	Activation of kainate receptors upon glutamate binding|Reactome	0.205413239	-0.00137905	0.947722044	0.36702327	0.52028198	-5.90818478
hetero after UVB irradiation VS hetero before UVB irradiation	Activation of kainate receptors upon glutamate binding|Reactome	0.406598504	0.008858958	2.185403615	0.05867378	0.21639222	-4.30994745
WT after UVB irradiation VS WT before UVB irradiation	Activation of kainate receptors upon glutamate binding|Reactome	0.185483126	-0.00615128	0.842839102	0.41886413	0.78496204	-5.52233321
homo after UVB irradiation VS homo before UVB irradiation	Activation of Matrix Metalloproteinases|Reactome	0.838975788	0.004111768	5.300039204	0.00042894	0.01666204	0.39251599
hetero after UVB irradiation VS hetero before UVB irradiation	Activation of Matrix Metalloproteinases|Reactome	0.692887451	0.110646834	3.074297117	0.01433011	0.1130168	-2.95791557
WT after UVB irradiation VS WT before UVB irradiation	Activation of Matrix Metalloproteinases|Reactome	0.609390609	0.046672445	2.528379845	0.02978216	0.35982837	-3.41636315
homo after UVB irradiation VS homo before UVB irradiation	Activation of Na-permeable kainate receptors|Reactome	-1.10963403	0.014316283	-6.16428292	0.00013883	0.01666204	1.467547009
hetero after UVB irradiation VS hetero before UVB irradiation	Activation of Na-permeable kainate receptors|Reactome	0.104168295	0.06846038	0.277141398	0.7883497	0.90946544	-6.30506805
WT after UVB irradiation VS WT before UVB irradiation	Activation of Na-permeable kainate receptors|Reactome	0.558019765	0.062381067	1.637797608	0.1322553	0.54612111	-4.67613909
homo after UVB irradiation VS homo before UVB irradiation	Activation of NF-kappaB in B cells|Reactome	0.171557956	-0.03042803	0.69799685	0.5020979	0.64181944	-6.11342716
hetero after UVB irradiation VS hetero before UVB irradiation	Activation of NF-kappaB in B cells|Reactome	0.216002365	-0.05867444	1.009879563	0.34067061	0.56961725	-5.82520851
WT after UVB irradiation VS WT before UVB irradiation	Activation of NF-kappaB in B cells|Reactome	-0.07520055	-0.07607125	-0.31567016	0.75869035	0.92423014	-5.81893731
homo after UVB irradiation VS homo before UVB irradiation	Activation of Nicotinic Acetylcholine Receptors|Reactome	0.106608129	0.018460278	0.48363748	0.63972296	0.75031467	-6.24435373
hetero after UVB irradiation VS hetero before UVB irradiation	Activation of Nicotinic Acetylcholine Receptors|Reactome	-0.39673811	-0.00107584	-2.11855793	0.06524756	0.22947406	-4.40902394
WT after UVB irradiation VS WT before UVB irradiation	Activation of Nicotinic Acetylcholine Receptors|Reactome	-0.66670677	-0.00479849	-3.09744745	0.01119442	0.26766971	-2.56198852
homo after UVB irradiation VS homo before UVB irradiation	Activation of NIMA Kinases NEK9_ NEK6_ NEK7|Reactome	-0.13423089	-0.00556231	-0.46937892	0.64950802	0.7575105	-6.2514776
hetero after UVB irradiation VS hetero before UVB irradiation	Activation of NIMA Kinases NEK9_ NEK6_ NEK7|Reactome	0.572162359	-0.01508151	2.404863092	0.04135529	0.17913033	-3.98003268
WT after UVB irradiation VS WT before UVB irradiation	Activation of NIMA Kinases NEK9_ NEK6_ NEK7|Reactome	0.057803139	-0.03610677	0.179103344	0.86140466	0.95916804	-5.85292842
homo after UVB irradiation VS homo before UVB irradiation	Activation of NMDA receptor and postsynaptic events|Reactome	0.068803429	0.008588025	0.389318164	0.7057239	0.80552596	-6.28767851
hetero after UVB irradiation VS hetero before UVB irradiation	Activation of NMDA receptor and postsynaptic events|Reactome	-0.14293068	-9.98E-05	-0.77160776	0.46143606	0.68531299	-6.03477893
WT after UVB irradiation VS WT before UVB irradiation	Activation of NMDA receptor and postsynaptic events|Reactome	-0.04706031	-0.04742563	-0.21386568	0.83491492	0.94951669	-5.84603674
homo after UVB irradiation VS homo before UVB irradiation	Activation of NOXA and translocation to mitochondria|Reactome	-0.38441418	-0.08726211	-1.33814693	0.21235059	0.36957778	-5.49019548
hetero after UVB irradiation VS hetero before UVB irradiation	Activation of NOXA and translocation to mitochondria|Reactome	0.225550956	0.030955755	0.979945714	0.354408	0.58212313	-5.8540219
WT after UVB irradiation VS WT before UVB irradiation	Activation of NOXA and translocation to mitochondria|Reactome	0.228513132	-0.09396121	0.858027794	0.41081047	0.77688258	-5.51019002
homo after UVB irradiation VS homo before UVB irradiation	activation of pkc through g-protein coupled receptors|BioCarta	0.503009958	-0.00270463	2.389454517	0.03951108	0.13139666	-3.99100917
hetero after UVB irradiation VS hetero before UVB irradiation	activation of pkc through g-protein coupled receptors|BioCarta	0.21082698	0.081208633	0.903241699	0.39148762	0.61872419	-5.92469012
WT after UVB irradiation VS WT before UVB irradiation	activation of pkc through g-protein coupled receptors|BioCarta	0.036750458	-0.01260478	0.12913115	0.89979364	0.97566251	-5.8607113
homo after UVB irradiation VS homo before UVB irradiation	Activation of PPARGC1A (PGC-1alpha) by phosphorylation|Reactome	0.04097929	0.000358315	0.198353298	0.84699715	0.90528768	-6.3473539
hetero after UVB irradiation VS hetero before UVB irradiation	Activation of PPARGC1A (PGC-1alpha) by phosphorylation|Reactome	-0.11565325	-0.05761263	-0.39673359	0.70142331	0.84916002	-6.26196517
WT after UVB irradiation VS WT before UVB irradiation	Activation of PPARGC1A (PGC-1alpha) by phosphorylation|Reactome	-0.18746725	-0.00877716	-0.68771488	0.50714453	0.82465997	-5.63543094
homo after UVB irradiation VS homo before UVB irradiation	Activation of PUMA and translocation to mitochondria|Reactome	-0.22469121	-0.03029405	-0.95320813	0.36437832	0.51814121	-5.90308734
hetero after UVB irradiation VS hetero before UVB irradiation	Activation of PUMA and translocation to mitochondria|Reactome	0.024816587	-0.00354771	0.147682653	0.88606628	0.96043665	-6.33472512
WT after UVB irradiation VS WT before UVB irradiation	Activation of PUMA and translocation to mitochondria|Reactome	-0.09453271	0.00998718	-0.3944208	0.70148466	0.90059561	-5.79099007
homo after UVB irradiation VS homo before UVB irradiation	Activation of RAC1|Reactome	0.055148106	0.050547855	0.229005064	0.82377555	0.89258642	-6.34035079
hetero after UVB irradiation VS hetero before UVB irradiation	Activation of RAC1|Reactome	0.178891672	0.036298004	0.799646305	0.44587129	0.67049863	-6.01255953
WT after UVB irradiation VS WT before UVB irradiation	Activation of RAC1|Reactome	0.286667629	0.021521498	1.388045865	0.19502895	0.60625027	-4.9823826
homo after UVB irradiation VS homo before UVB irradiation	Activation of RAS in B cells|Reactome	0.325099882	-0.00619926	1.217555032	0.25310671	0.41631312	-5.63051742
hetero after UVB irradiation VS hetero before UVB irradiation	Activation of RAS in B cells|Reactome	0.569948782	-0.01050752	2.738654627	0.02430567	0.13715811	-3.47068121
WT after UVB irradiation VS WT before UVB irradiation	Activation of RAS in B cells|Reactome	0.079540528	-0.0274378	0.272619409	0.79064792	0.9410709	-5.83164919
homo after UVB irradiation VS homo before UVB irradiation	Activation of SMO|Reactome	-0.01243709	-0.0484912	-0.059391	0.95388562	0.97176299	-6.36655339
hetero after UVB irradiation VS hetero before UVB irradiation	Activation of SMO|Reactome	-0.03487893	-0.0598596	-0.17463816	0.86548756	0.94783573	-6.33002676
WT after UVB irradiation VS WT before UVB irradiation	Activation of SMO|Reactome	0.193856938	0.039066517	0.886442643	0.3960307	0.76476626	-5.48697802
homo after UVB irradiation VS homo before UVB irradiation	Activation of the AP-1 family of transcription factors|Reactome	-0.0015612	0.019086062	-0.00752939	0.99415004	0.99523987	-6.3684178
hetero after UVB irradiation VS hetero before UVB irradiation	Activation of the AP-1 family of transcription factors|Reactome	0.376687426	0.038572852	1.857668473	0.09841481	0.28258275	-4.78644743
WT after UVB irradiation VS WT before UVB irradiation	Activation of the AP-1 family of transcription factors|Reactome	0.129592298	0.038185521	0.630758464	0.54223148	0.83916339	-5.6717765
homo after UVB irradiation VS homo before UVB irradiation	Activation of the mRNA upon binding of the cap-binding complex and eIFs_ and subsequent binding to 43S|Reactome	-0.95794809	0.017958498	-5.35174245	0.00039978	0.01666204	0.460213637
hetero after UVB irradiation VS hetero before UVB irradiation	Activation of the mRNA upon binding of the cap-binding complex and eIFs_ and subsequent binding to 43S|Reactome	-0.26075918	-0.01877883	-0.78861122	0.45195443	0.67696625	-6.02138563
WT after UVB irradiation VS WT before UVB irradiation	Activation of the mRNA upon binding of the cap-binding complex and eIFs_ and subsequent binding to 43S|Reactome	-0.55925773	-0.02212922	-1.83628921	0.09593756	0.4928536	-4.41356855
homo after UVB irradiation VS homo before UVB irradiation	Activation of the phototransduction cascade|Reactome	0.565338863	0.094250767	2.921477347	0.01626539	0.07968643	-3.14398513
hetero after UVB irradiation VS hetero before UVB irradiation	Activation of the phototransduction cascade|Reactome	0.240288042	0.059613728	1.244584657	0.24678671	0.47084715	-5.57719826
WT after UVB irradiation VS WT before UVB irradiation	Activation of the phototransduction cascade|Reactome	-0.19391358	-0.00418846	-0.92038686	0.37886613	0.75122805	-5.45841619
homo after UVB irradiation VS homo before UVB irradiation	Activation of the pre-replicative complex|Reactome	-0.45163616	-0.04267316	-1.48738275	0.16970543	0.32032865	-5.30455739
hetero after UVB irradiation VS hetero before UVB irradiation	Activation of the pre-replicative complex|Reactome	0.145664169	-0.00029121	0.479200464	0.64398642	0.81977783	-6.22368368
WT after UVB irradiation VS WT before UVB irradiation	Activation of the pre-replicative complex|Reactome	0.155307903	-0.02168498	0.54384273	0.59836909	0.86643996	-5.72164191
homo after UVB irradiation VS homo before UVB irradiation	Activation of the TFAP2 (AP-2) family of transcription factors|Reactome	-0.70214375	-0.0318203	-3.43417254	0.0070075	0.04862651	-2.32669336
hetero after UVB irradiation VS hetero before UVB irradiation	Activation of the TFAP2 (AP-2) family of transcription factors|Reactome	-0.25943438	-0.02547774	-0.92949605	0.37849082	0.60574479	-5.90102372
WT after UVB irradiation VS WT before UVB irradiation	Activation of the TFAP2 (AP-2) family of transcription factors|Reactome	-0.37045131	0.011894117	-1.18903757	0.261675	0.65669403	-5.20245827
homo after UVB irradiation VS homo before UVB irradiation	Activation of TRKA receptors|Reactome	0.68692237	0.046672387	3.558478853	0.00573474	0.04393429	-2.13114804
hetero after UVB irradiation VS hetero before UVB irradiation	Activation of TRKA receptors|Reactome	0.254433124	-0.01470203	1.078148162	0.3108623	0.53928344	-5.75700782
WT after UVB irradiation VS WT before UVB irradiation	Activation of TRKA receptors|Reactome	0.342016943	0.00302139	1.206609003	0.25513141	0.64985228	-5.18399547
homo after UVB irradiation VS homo before UVB irradiation	Activation_ myristolyation of BID and translocation to mitochondria|Reactome	0.738287625	-0.07667083	2.631859378	0.02635914	0.10736472	-3.607304
hetero after UVB irradiation VS hetero before UVB irradiation	Activation_ myristolyation of BID and translocation to mitochondria|Reactome	1.052105995	0.018039166	6.494358595	0.00015056	0.02920766	1.440792891
WT after UVB irradiation VS WT before UVB irradiation	Activation_ myristolyation of BID and translocation to mitochondria|Reactome	0.860761212	-0.05767437	3.583989497	0.0049145	0.20968398	-1.84331481
homo after UVB irradiation VS homo before UVB irradiation	Activation_ translocation and oligomerization of BAX|Reactome	1.073583929	-0.01240551	4.644738189	0.00108214	0.01891175	-0.50316513
hetero after UVB irradiation VS hetero before UVB irradiation	Activation_ translocation and oligomerization of BAX|Reactome	1.172196029	0.044386625	6.725364569	0.0001171	0.02920766	1.672484808
WT after UVB irradiation VS WT before UVB irradiation	Activation_ translocation and oligomerization of BAX|Reactome	1.043583221	0.078895368	4.342838475	0.00143217	0.15689229	-0.78265212
homo after UVB irradiation VS homo before UVB irradiation	Acute Intermittent Porphyria|SMPDB	0.388154593	-0.02664649	1.768839848	0.10931886	0.24265581	-4.92409481
hetero after UVB irradiation VS hetero before UVB irradiation	Acute Intermittent Porphyria|SMPDB	0.451496919	0.003377552	2.70023395	0.02583369	0.14017854	-3.52950073
WT after UVB irradiation VS WT before UVB irradiation	Acute Intermittent Porphyria|SMPDB	0.381835405	-0.00693641	1.754704281	0.10959456	0.5076251	-4.52327279
homo after UVB irradiation VS homo before UVB irradiation	Acute myeloid leukemia - Homo sapiens (human)|KEGG	0.398493107	-0.01044436	2.28752908	0.04681805	0.14082424	-4.1501858
hetero after UVB irradiation VS hetero before UVB irradiation	Acute myeloid leukemia - Homo sapiens (human)|KEGG	0.451379091	-0.00341345	2.783424419	0.02264114	0.13437279	-3.40213627
WT after UVB irradiation VS WT before UVB irradiation	Acute myeloid leukemia - Homo sapiens (human)|KEGG	0.341150486	0.018947468	1.702647057	0.11921206	0.5237862	-4.59200754
homo after UVB irradiation VS homo before UVB irradiation	acyl carrier protein metabolism|HumanCyc	-1.05115143	-0.00105616	-4.71051034	0.0009834	0.0182528	-0.41013939
hetero after UVB irradiation VS hetero before UVB irradiation	acyl carrier protein metabolism|HumanCyc	-1.03662298	-0.03612612	-4.27935092	0.00238702	0.05507363	-1.20271485
WT after UVB irradiation VS WT before UVB irradiation	acyl carrier protein metabolism|HumanCyc	-0.66467625	0.033920093	-2.22132686	0.05037119	0.41004423	-3.86989612
homo after UVB irradiation VS homo before UVB irradiation	Acyl chain remodeling of CL|Reactome	-0.35142567	-0.02386388	-1.40324737	0.1927374	0.34894487	-5.41075717
hetero after UVB irradiation VS hetero before UVB irradiation	Acyl chain remodeling of CL|Reactome	-0.44425311	0.023499789	-1.72263002	0.12134356	0.31331055	-4.97426534
WT after UVB irradiation VS WT before UVB irradiation	Acyl chain remodeling of CL|Reactome	-0.48526566	0.02354762	-2.02861612	0.06973671	0.44543716	-4.14664553

homo after UVB irradiation VS homo before UVB irradiation	Acyl chain remodeling of DAG and TAG|Reactome	-0.04917792	-0.04635129	-0.17756175	0.86283894	0.91685035	-6.35153716
hetero after UVB irradiation VS hetero before UVB irradiation	Acyl chain remodeling of DAG and TAG|Reactome	-0.34033591	-0.06919839	-1.2370954	0.24941707	0.47407965	-5.58567666
WT after UVB irradiation VS WT before UVB irradiation	Acyl chain remodeling of DAG and TAG|Reactome	0.063513575	-0.06970884	0.216276929	0.83308507	0.94894798	-5.84551389
homo after UVB irradiation VS homo before UVB irradiation	Acyl chain remodelling of PC|Reactome	-0.08934708	0.038263159	-0.40227004	0.6964897	0.79803563	-6.28226277
hetero after UVB irradiation VS hetero before UVB irradiation	Acyl chain remodelling of PC|Reactome	0.125192929	0.018832463	0.748487258	0.47453853	0.69660117	-6.05258488
WT after UVB irradiation VS WT before UVB irradiation	Acyl chain remodelling of PC|Reactome	-0.14182398	0.049071185	-0.64723628	0.53193833	0.83483909	-5.661556
homo after UVB irradiation VS homo before UVB irradiation	Acyl chain remodelling of PE|Reactome	-0.15383747	0.012738576	-0.71205917	0.49374041	0.63443556	-6.10332421
hetero after UVB irradiation VS hetero before UVB irradiation	Acyl chain remodelling of PE|Reactome	0.043989623	0.033014578	0.290175231	0.77869585	0.90221039	-6.30109577
WT after UVB irradiation VS WT before UVB irradiation	Acyl chain remodelling of PE|Reactome	-0.25155345	0.038151151	-1.08498256	0.30319088	0.68820712	-5.30762859
homo after UVB irradiation VS homo before UVB irradiation	Acyl chain remodelling of PG|Reactome	0.103397706	-0.00237539	0.454289822	0.6599404	0.76688662	-6.25879505
hetero after UVB irradiation VS hetero before UVB irradiation	Acyl chain remodelling of PG|Reactome	0.136234568	0.080715677	0.786347506	0.45320917	0.67817708	-6.0231832
WT after UVB irradiation VS WT before UVB irradiation	Acyl chain remodelling of PG|Reactome	0.067834654	0.001771141	0.340064156	0.74078678	0.91734632	-5.8109261
homo after UVB irradiation VS homo before UVB irradiation	Acyl chain remodelling of PI|Reactome	0.396005162	0.033072841	1.795003606	0.10484249	0.2370387	-4.88701721
hetero after UVB irradiation VS hetero before UVB irradiation	Acyl chain remodelling of PI|Reactome	0.368895231	0.068508015	2.068545172	0.07063028	0.23794	-4.48260071
WT after UVB irradiation VS WT before UVB irradiation	Acyl chain remodelling of PI|Reactome	0.280480917	0.002607378	1.098143145	0.29767445	0.68387495	-5.29473108
homo after UVB irradiation VS homo before UVB irradiation	Acyl chain remodelling of PS|Reactome	0.188805268	0.000561641	0.951604814	0.36514987	0.51891513	-5.90457956
hetero after UVB irradiation VS hetero before UVB irradiation	Acyl chain remodelling of PS|Reactome	0.233213631	0.029748771	1.354395984	0.21080915	0.43289558	-5.44902396
WT after UVB irradiation VS WT before UVB irradiation	Acyl chain remodelling of PS|Reactome	0.007228562	0.019501856	0.027991906	0.97821494	0.99268361	-5.86875427
homo after UVB irradiation VS homo before UVB irradiation	acyl-CoA hydrolysis|HumanCyc	-0.51053305	0.112328632	-1.86908175	0.09307222	0.21939482	-4.78068474
hetero after UVB irradiation VS hetero before UVB irradiation	acyl-CoA hydrolysis|HumanCyc	0.072029965	0.075680981	0.285765607	0.78195751	0.90426031	-6.30245971
WT after UVB irradiation VS WT before UVB irradiation	acyl-CoA hydrolysis|HumanCyc	-0.24529996	0.114141446	-0.7712195	0.45827001	0.80891799	-5.57705826
homo after UVB irradiation VS homo before UVB irradiation	Adaptive Immune System|Reactome	0.306052339	-0.02924265	1.784469441	0.10662405	0.23957183	-4.90197664
hetero after UVB irradiation VS hetero before UVB irradiation	Adaptive Immune System|Reactome	0.33401569	0.010988574	2.124182521	0.06466778	0.22842741	-4.40071856
WT after UVB irradiation VS WT before UVB irradiation	Adaptive Immune System|Reactome	0.203010781	-0.02379929	1.00509481	0.33836361	0.7221059	-5.38329721
homo after UVB irradiation VS homo before UVB irradiation	Adefovir Dipivoxil Metabolism Pathway|SMPDB	0.539315324	0.02806897	1.857872473	0.09477057	0.22145466	-4.79689833
hetero after UVB irradiation VS hetero before UVB irradiation	Adefovir Dipivoxil Metabolism Pathway|SMPDB	0.286565026	0.070696225	1.395274783	0.19860932	0.41868045	-5.39955386
WT after UVB irradiation VS WT before UVB irradiation	Adefovir Dipivoxil Metabolism Pathway|SMPDB	-0.01265505	-0.05306528	-0.05451363	0.95759106	0.98742794	-5.86764596
homo after UVB irradiation VS homo before UVB irradiation	adenine and adenosine salvage I|HumanCyc	1.023277005	-0.00720676	3.862218227	0.00354095	0.03382425	-1.66009431
hetero after UVB irradiation VS hetero before UVB irradiation	adenine and adenosine salvage I|HumanCyc	0.893112229	0.041840051	2.990933128	0.01632583	0.11352943	-3.08489057
WT after UVB irradiation VS WT before UVB irradiation	adenine and adenosine salvage I|HumanCyc	1.135079099	0.037071593	4.782994574	0.00072555	0.15645713	-0.21116413
homo after UVB irradiation VS homo before UVB irradiation	adenine and adenosine salvage II|HumanCyc	-0.43277833	-0.02449884	-1.15414725	0.27696995	0.43713957	-5.70048694
hetero after UVB irradiation VS hetero before UVB irradiation	adenine and adenosine salvage II|HumanCyc	-0.75871064	-0.0312817	-2.35623422	0.04469169	0.18702113	-4.05363706
WT after UVB irradiation VS WT before UVB irradiation	adenine and adenosine salvage II|HumanCyc	-0.51651428	-0.04903485	-1.36103486	0.20314606	0.61688026	-5.01360513
homo after UVB irradiation VS homo before UVB irradiation	adenine and adenosine salvage III|HumanCyc	0.030133192	-0.03496605	0.08666039	0.93276154	0.96074649	-6.36441474
hetero after UVB irradiation VS hetero before UVB irradiation	adenine and adenosine salvage III|HumanCyc	0.136739247	0.21248816	0.372545724	0.7186835	0.86269112	-6.27187992
WT after UVB irradiation VS WT before UVB irradiation	adenine and adenosine salvage III|HumanCyc	0.408213962	-0.02790269	1.390871498	0.19419587	0.60625027	-4.97909328
homo after UVB irradiation VS homo before UVB irradiation	Adenine phosphoribosyltransferase deficiency (APRT)|SMPDB	0.019015587	-0.03943216	0.101997834	0.92090359	0.95153785	-6.36286155
hetero after UVB irradiation VS hetero before UVB irradiation	Adenine phosphoribosyltransferase deficiency (APRT)|SMPDB	-0.02085195	-0.0438046	-0.1098564	0.91509485	0.97054702	-6.33999905
WT after UVB irradiation VS WT before UVB irradiation	Adenine phosphoribosyltransferase deficiency (APRT)|SMPDB	0.165884344	-0.0622813	0.759490799	0.46494629	0.80891799	-5.58561498
homo after UVB irradiation VS homo before UVB irradiation	Adenosine Deaminase Deficiency|SMPDB	0.019015587	-0.03943216	0.101997834	0.92090359	0.95153785	-6.36286155
hetero after UVB irradiation VS hetero before UVB irradiation	Adenosine Deaminase Deficiency|SMPDB	-0.02085195	-0.0438046	-0.1098564	0.91509485	0.97054702	-6.33999905
WT after UVB irradiation VS WT before UVB irradiation	Adenosine Deaminase Deficiency|SMPDB	0.165884344	-0.0622813	0.759490799	0.46494629	0.80891799	-5.58561498
homo after UVB irradiation VS homo before UVB irradiation	adenosine deoxyribonucleotides <i>de novo</i> biosynthesis|HumanCyc	-0.26766388	-0.07651359	-1.13478893	0.2846023	0.44528503	-5.72129654
hetero after UVB irradiation VS hetero before UVB irradiation	adenosine deoxyribonucleotides <i>de novo</i> biosynthesis|HumanCyc	-0.15783943	-0.04297759	-0.59914586	0.56483354	0.76437184	-6.1559538
WT after UVB irradiation VS WT before UVB irradiation	adenosine deoxyribonucleotides <i>de novo</i> biosynthesis|HumanCyc	-0.05360404	-0.09347628	-0.22827277	0.82399739	0.94525798	-5.84282656
homo after UVB irradiation VS homo before UVB irradiation	adenosine nucleotides degradation|HumanCyc	0.40737013	-0.04797213	1.918973072	0.08585265	0.20862332	-4.70801301
hetero after UVB irradiation VS hetero before UVB irradiation	adenosine nucleotides degradation|HumanCyc	0.327294918	0.045923312	2.042581359	0.07359212	0.24223901	-4.52059361
WT after UVB irradiation VS WT before UVB irradiation	adenosine nucleotides degradation|HumanCyc	-0.04558856	-0.00135548	-0.17731169	0.86277515	0.95964299	-5.85325082
homo after UVB irradiation VS homo before UVB irradiation	Adenosine P1 receptors|Reactome	0.226281659	0.006999935	0.702556867	0.49937827	0.63924743	-6.11017085
hetero after UVB irradiation VS hetero before UVB irradiation	Adenosine P1 receptors|Reactome	0.263619566	0.034063731	0.857607863	0.41483493	0.64192892	-5.96449738
WT after UVB irradiation VS WT before UVB irradiation	Adenosine P1 receptors|Reactome	0.372272977	-0.080057	1.19247558	0.26038425	0.65515019	-5.19886141
homo after UVB irradiation VS homo before UVB irradiation	adenosine ribonucleotides <i>de novo</i> biosynthesis|HumanCyc	0.271029457	-0.05705286	1.264181706	0.23664493	0.39783533	-5.57735589
hetero after UVB irradiation VS hetero before UVB irradiation	adenosine ribonucleotides <i>de novo</i> biosynthesis|HumanCyc	0.039922707	0.022354828	0.215526106	0.83448311	0.93635724	-6.32141283
WT after UVB irradiation VS WT before UVB irradiation	adenosine ribonucleotides <i>de novo</i> biosynthesis|HumanCyc	0.186117669	-0.00671248	0.740939105	0.47563312	0.81430085	-5.5989126
homo after UVB irradiation VS homo before UVB irradiation	Adenylate cyclase activating pathway|Reactome	0.405386386	-0.08914101	1.565101924	0.15060252	0.29665708	-5.20316139
hetero after UVB irradiation VS hetero before UVB irradiation	Adenylate cyclase activating pathway|Reactome	0.141746706	0.057989873	0.615280212	0.5546201	0.75937471	-6.14578325
WT after UVB irradiation VS WT before UVB irradiation	Adenylate cyclase activating pathway|Reactome	0.238321232	-0.0129968	0.861294757	0.40909215	0.77620433	-5.50755394
homo after UVB irradiation VS homo before UVB irradiation	Adenylate cyclase inhibitory pathway|Reactome	0.491541476	-0.06690044	2.289704283	0.04664908	0.14068672	-4.14680612
hetero after UVB irradiation VS hetero before UVB irradiation	Adenylate cyclase inhibitory pathway|Reactome	0.314368875	0.055650203	1.346396421	0.21327037	0.43535861	-5.45859701
WT after UVB irradiation VS WT before UVB irradiation	Adenylate cyclase inhibitory pathway|Reactome	0.347682046	-0.02244335	1.322676585	0.21515887	0.62581393	-5.05724231
homo after UVB irradiation VS homo before UVB irradiation	Adenylosuccinate Lyase Deficiency|SMPDB	0.019015587	-0.03943216	0.101997834	0.92090359	0.95153785	-6.36286155
hetero after UVB irradiation VS hetero before UVB irradiation	Adenylosuccinate Lyase Deficiency|SMPDB	-0.02085195	-0.0438046	-0.1098564	0.91509485	0.97054702	-6.33999905
WT after UVB irradiation VS WT before UVB irradiation	Adenylosuccinate Lyase Deficiency|SMPDB	0.165884344	-0.0622813	0.759490799	0.46494629	0.80891799	-5.58561498
homo after UVB irradiation VS homo before UVB irradiation	Adherens junction - Homo sapiens (human)|KEGG	0.02615198	-0.00778165	0.109511708	0.91510162	0.95070625	-6.36200868
hetero after UVB irradiation VS hetero before UVB irradiation	Adherens junction - Homo sapiens (human)|KEGG	0.151120176	-0.01831674	0.801086072	0.44508171	0.67012749	-6.01140023
WT after UVB irradiation VS WT before UVB irradiation	Adherens junction - Homo sapiens (human)|KEGG	0.013685777	-0.01057234	0.059442108	0.9537617	0.98742794	-5.86736154
homo after UVB irradiation VS homo before UVB irradiation	Adherens junctions interactions|Reactome	0.02901512	0.02309855	0.148181246	0.88533342	0.93057836	-6.35666443
hetero after UVB irradiation VS hetero before UVB irradiation	Adherens junctions interactions|Reactome	-0.13588894	0.022049645	-0.68139755	0.51390853	0.73158211	-6.10155513
WT after UVB irradiation VS WT before UVB irradiation	Adherens junctions interactions|Reactome	-0.22445877	0.010263649	-0.90242832	0.38788055	0.76130447	-5.47363913
homo after UVB irradiation VS homo before UVB irradiation	Adipocytokine signaling pathway - Homo sapiens (human)|KEGG	0.349657586	0.020419842	2.115766748	0.06221845	0.17113823	-4.41419263
hetero after UVB irradiation VS hetero before UVB irradiation	Adipocytokine signaling pathway - Homo sapiens (human)|KEGG	0.019078583	-0.01799729	0.09317663	0.92794083	0.97054702	-6.34183391
WT after UVB irradiation VS WT before UVB irradiation	Adipocytokine signaling pathway - Homo sapiens (human)|KEGG	0.095303027	-0.05118003	0.416372698	0.68586244	0.89062661	-5.78212947
homo after UVB irradiation VS homo before UVB irradiation	Adipogenesis|Wikipathways	0.197534648	-0.00363789	1.251947263	0.24087695	0.40257839	-5.59144165
hetero after UVB irradiation VS hetero before UVB irradiation	Adipogenesis|Wikipathways	-0.01807443	-0.00748121	-0.08658139	0.93302656	0.97054702	-6.34247643
WT after UVB irradiation VS WT before UVB irradiation	Adipogenesis|Wikipathways	-0.04010952	-0.02696451	-0.16319281	0.87359139	0.96283151	-5.85567876
homo after UVB irradiation VS homo before UVB irradiation	ADP signalling through P2Y purinoceptor 1|Reactome	0.39856345	-0.00862488	1.957116292	0.08069166	0.20118642	-4.65191982
hetero after UVB irradiation VS hetero before UVB irradiation	ADP signalling through P2Y purinoceptor 1|Reactome	0.678546052	0.031880682	4.001032635	0.00354872	0.06529021	-1.59093564
WT after UVB irradiation VS WT before UVB irradiation	ADP signalling through P2Y purinoceptor 1|Reactome	0.286781642	-0.03867284	1.319412531	0.21620785	0.62581393	-5.06091676
homo after UVB irradiation VS homo before UVB irradiation	ADP signalling through P2Y purinoceptor 12|Reactome	0.519549979	0.006304555	2.321009829	0.04428241	0.13674659	-4.098078
hetero after UVB irradiation VS hetero before UVB irradiation	ADP signalling through P2Y purinoceptor 12|Reactome	0.645850712	0.030960849	3.805647209	0.00471891	0.07419102	-1.87037027
WT after UVB irradiation VS WT before UVB irradiation	ADP signalling through P2Y purinoceptor 12|Reactome	0.329073915	-0.03066936	1.413860736	0.18752934	0.59994616	-4.9521713
homo after UVB irradiation VS homo before UVB irradiation	adp-ribosylation factor|BioCarta	0.633655672	-0.02664282	3.542187747	0.00588681	0.04470579	-2.15669343
hetero after UVB irradiation VS hetero before UVB irradiation	adp-ribosylation factor|BioCarta	0.498677318	0.012761113	2.474141421	0.03702786	0.16802342	-3.87480708
WT after UVB irradiation VS WT before UVB irradiation	adp-ribosylation factor|BioCarta	0.450089742	-0.05657021	2.320169626	0.04256259	0.39022568	-3.7252392
homo after UVB irradiation VS homo before UVB irradiation	Adrenal Hyperplasia Type 3 or Congenital Adrenal Hyperplasia due to 21-hydroxylase Deficiency|SMPDB	0.235351843	-0.00442303	1.337763903	0.2124708	0.36957778	-5.49065548
hetero after UVB irradiation VS hetero before UVB irradiation	Adrenal Hyperplasia Type 3 or Congenital Adrenal Hyperplasia due to 21-hydroxylase Deficiency|SMPDB	0.307187072	0.103522065	1.723908534	0.12110487	0.3132781	-4.97251635
WT after UVB irradiation VS WT before UVB irradiation	Adrenal Hyperplasia Type 3 or Congenital Adrenal Hyperplasia due to 21-hydroxylase Deficiency|SMPDB	0.03938021	0.054409473	0.163687683	0.87321181	0.96283151	-5.85559705
homo after UVB irradiation VS homo before UVB irradiation	Adrenal Hyperplasia Type 5 or Congenital Adrenal Hyperplasia due to 17 Alpha-hydroxylase Deficiency|SMPDB	0.235351843	-0.00442303	1.337763903	0.2124708	0.36957778	-5.49065548
hetero after UVB irradiation VS hetero before UVB irradiation	Adrenal Hyperplasia Type 5 or Congenital Adrenal Hyperplasia due to 17 Alpha-hydroxylase Deficiency|SMPDB	0.307187072	0.103522065	1.723908534	0.12110487	0.3132781	-4.97251635
WT after UVB irradiation VS WT before UVB irradiation	Adrenal Hyperplasia Type 5 or Congenital Adrenal Hyperplasia due to 17 Alpha-hydroxylase Deficiency|SMPDB	0.03938021	0.054409473	0.163687683	0.87321181	0.96283151	-5.85559705
homo after UVB irradiation VS homo before UVB irradiation	Adrenaline signalling through Alpha-2 adrenergic receptor|Reactome	0.468209697	-0.05652327	1.625187848	0.13716662	0.27947468	-5.12275744
hetero after UVB irradiation VS hetero before UVB irradiation	Adrenaline signalling through Alpha-2 adrenergic receptor|Reactome	0.079282637	-0.09601567	0.355959681	0.73061908	0.86896773	-6.27833019
WT after UVB irradiation VS WT before UVB irradiation	Adrenaline signalling through Alpha-2 adrenergic receptor|Reactome	-0.1027384	-0.05828404	-0.36630018	0.72171088	0.90775368	-5.80166164
homo after UVB irradiation VS homo before UVB irradiation	Adrenaline_noradrenaline inhibits insulin secretion|Reactome	0.402292587	-0.00776817	1.963255355	0.07988887	0.19976593	-4.64285039
hetero after UVB irradiation VS hetero before UVB irradiation	Adrenaline_noradrenaline inhibits insulin secretion|Reactome	0.543881397	0.041909617	2.936268181	0.01778893	0.11751009	-3.16833561
WT after UVB irradiation VS WT before UVB irradiation	Adrenaline_noradrenaline inhibits insulin secretion|Reactome	0.26024027	-0.05940747	1.006359334	0.33778412	0.72206121	-5.38213527
homo after UVB irradiation VS homo before UVB irradiation	Adrenergic signaling in cardiomyocytes - Homo sapiens (human)|KEGG	0.386175006	-0.04391155	2.320760091	0.04430082	0.13674659	-4.09846736
hetero after UVB irradiation VS hetero before UVB irradiation	Adrenergic signaling in cardiomyocytes - Homo sapiens (human)|KEGG	0.177964248	0.01363418	0.867650103	0.40961465	0.63634069	-5.955884
WT after UVB irradiation VS WT before UVB irradiation	Adrenergic signaling in cardiomyocytes - Homo sapiens (human)|KEGG	0.160144551	-0.03256509	0.674349515	0.51525294	0.8287185	-5.64421601
homo after UVB irradiation VS homo before UVB irradiation	Adrenoceptors|Reactome	0.292279419	-0.05951494	1.277073716	0.23225193	0.39363857	-5.56240959
hetero after UVB irradiation VS hetero before UVB irradiation	Adrenoceptors|Reactome	-0.35960714	-0.0308612	-1.48440803	0.17411631	0.39158454	-5.28864089
WT after UVB irradiation VS WT before UVB irradiation	Adrenoceptors|Reactome	-0.07959043	-0.07310525	-0.2408919	0.81446687	0.94525798	-5.83984485
homo after UVB irradiation VS homo before UVB irradiation	Adrenoleukodystrophy_ X-linked|SMPDB	0.161860886	-0.05103371	0.630831613	0.54319908	0.67708436	-6.15916189
hetero after UVB irradiation VS hetero before UVB irradiation	Adrenoleukodystrophy_ X-linked|SMPDB	-0.12884308	-0.07596147	-0.45167658	0.66290222	0.82651398	-6.237224
WT after UVB irradiation VS WT before UVB irradiation	Adrenoleukodystrophy_ X-linked|SMPDB	-0.13599861	-0.0440626	-0.59210605	0.56682	0.85125516	-5.69479783
homo after UVB irradiation VS homo before UVB irradiation	Advanced glycosylation endproduct receptor signaling|Reactome	0.538374165	-0.0394424	3.240731427	0.00960302	0.05838531	-2.6334886
hetero after UVB irradiation VS hetero before UVB irradiation	Advanced glycosylation endproduct receptor signaling|Reactome	0.554156665	-0.01027665	2.768573245	0.02317996	0.13628011	-3.42487372
WT after UVB irradiation VS WT before UVB irradiation	Advanced glycosylation endproduct receptor signaling|Reactome	0.507845601	0.087876163	2.302645363	0.04385559	0.39042586	-3.75099658
homo after UVB irradiation VS homo before UVB irradiation	Aflatoxin activation and detoxification|Reactome	-0.01797277	0.005493024	-0.07755363	0.93981076	0.96450524	-6.36521766
hetero after UVB irradiation VS hetero before UVB irradiation	Aflatoxin activation and detoxification|Reactome	-0.01600888	0.011786456	-0.07769689	0.93988267	0.97054702	-6.34326756
WT after UVB irradiation VS WT before UVB irradiation	Aflatoxin activation and detoxification|Reactome	0.083664883	0.023573124	0.37214706	0.71748623	0.90568259	-5.79950581
homo after UVB irradiation VS homo before UVB irradiation	Aflatoxin B1 metabolism|Wikipathways	0.7587514	-0.01817813	3.330383399	0.00829447	0.0539495	-2.49096906
hetero after UVB irradiation VS hetero before UVB irradiation	Aflatoxin B1 metabolism|Wikipathways	-0.26888728	-0.08438617	-0.58474952	0.57403672	0.7705309	-6.16481968
WT after UVB irradiation VS WT before UVB irradiation	Aflatoxin B1 metabolism|Wikipathways	0.553257385	-0.01761506	1.381865264	0.19686172	0.6083714	-4.98956228
homo after UVB irradiation VS homo before UVB irradiation	African trypanosomiasis - Homo sapiens (human)|KEGG	0.766112344	-0.01980208	4.182628592	0.00215692	0.02581999	-1.17565117
hetero after UVB irradiation VS hetero before UVB irradiation	African trypanosomiasis - Homo sapiens (human)|KEGG	0.749774539	0.034517527	4.504662375	0.00174595	0.04958933	-0.89735574
WT after UVB irradiation VS WT before UVB irradiation	African trypanosomiasis - Homo sapiens (human)|KEGG	0.399556507	0.018028446	1.421830905	0.18526401	0.59839062	-4.94277179
homo after UVB irradiation VS homo before UVB irradiation	Agents Acting on the Renin-Angiotensin System Pathway_ Pharmacodynamics|PharmGKB	0.149430321	-0.04622985	0.905130419	0.38803163	0.54134863	-5.94693193
hetero after UVB irradiation VS hetero before UVB irradiation	Agents Acting on the Renin-Angiotensin System Pathway_ Pharmacodynamics|PharmGKB	0.393058791	0.021985605	2.029212312	0.0751637	0.24558862	-4.54009905
WT after UVB irradiation VS WT before UVB irradiation	Agents Acting on the Renin-Angiotensin System Pathway_ Pharmacodynamics|PharmGKB	0.280771856	-0.02298454	1.169614914	0.26906294	0.66278879	-5.22263483
homo after UVB irradiation VS homo before UVB irradiation	AGE-RAGE pathway|Wikipathways	0.561010718	-0.02898082	2.899464109	0.01687141	0.08177799	-3.17926281
hetero after UVB irradiation VS hetero before UVB irradiation	AGE-RAGE pathway|Wikipathways	0.507540345	0.030292988	2.841277291	0.02066168	0.12675092	-3.31358252
WT after UVB irradiation VS WT before UVB irradiation	AGE-RAGE pathway|Wikipathways	0.394596207	-0.02969357	1.751776348	0.11011619	0.50951142	-4.52716581
homo after UVB irradiation VS homo before UVB irradiation	AGE-RAGE signaling pathway in diabetic complications - Homo sapiens (human)|KEGG	0.291123415	-0.03248829	1.772249965	0.1087256	0.24228458	-4.91927693
hetero after UVB irradiation VS hetero before UVB irradiation	AGE-RAGE signaling pathway in diabetic complications - Homo sapiens (human)|KEGG	0.345302237	0.021698816	2.010189808	0.07745578	0.24985383	-4.56778306
WT after UVB irradiation VS WT before UVB irradiation	AGE-RAGE signaling pathway in diabetic complications - Homo sapiens (human)|KEGG	0.221683023	-0.00552635	0.979790488	0.35011477	0.73549869	-5.40630099
homo after UVB irradiation VS homo before UVB irradiation	Agmatine biosynthesis|Reactome	0.019093771	0.017913353	0.045719151	0.96449178	0.97798567	-6.36732529
hetero after UVB irradiation VS hetero before UVB irradiation	Agmatine biosynthesis|Reactome	-0.00443037	-0.01838395	-0.00993425	0.99230495	0.99514323	-6.34648776
WT after UVB irradiation VS WT before UVB irradiation	Agmatine biosynthesis|Reactome	0.689556509	0.005888329	2.161220901	0.05577781	0.41769317	-3.95704096
homo after UVB irradiation VS homo before UVB irradiation	agrin in postsynaptic differentiation|BioCarta	0.35638233	0.013080457	1.710454568	0.1199481	0.25846296	-5.00587418
hetero after UVB irradiation VS hetero before UVB irradiation	agrin in postsynaptic differentiation|BioCarta	0.350734455	0.026848541	2.141809026	0.0628832	0.22462511	-4.37465267
WT after UVB irradiation VS WT before UVB irradiation	agrin in postsynaptic differentiation|BioCarta	0.198242141	-0.00422827	0.962709626	0.35821431	0.74302468	-5.42155972
homo after UVB irradiation VS homo before UVB irradiation	ahr signal transduction pathway|BioCarta	0.156886226	-0.03407549	0.526877103	0.61049919	0.72800713	-6.22151693
hetero after UVB irradiation VS hetero before UVB irradiation	ahr signal transduction pathway|BioCarta	6.68E-05	0.029943568	0.000199329	0.9998456	0.9998456	-6.34654128
WT after UVB irradiation VS WT before UVB irradiation	ahr signal transduction pathway|BioCarta	-0.08177804	-0.13771026	-0.35840984	0.72742759	0.91040977	-5.8045183
homo after UVB irradiation VS homo before UVB irradiation	AICA-Ribosiduria|SMPDB	0.019015587	-0.03943216	0.101997834	0.92090359	0.95153785	-6.36286155
hetero after UVB irradiation VS hetero before UVB irradiation	AICA-Ribosiduria|SMPDB	-0.02085195	-0.0438046	-0.1098564	0.91509485	0.97054702	-6.33999905
WT after UVB irradiation VS WT before UVB irradiation	AICA-Ribosiduria|SMPDB	0.165884344	-0.0622813	0.759490799	0.46494629	0.80891799	-5.58561498
homo after UVB irradiation VS homo before UVB irradiation	akap95 role in mitosis and chromosome dynamics|BioCarta	-0.48390018	-0.00270525	-2.41790117	0.03768046	0.12734936	-3.94631431
hetero after UVB irradiation VS hetero before UVB irradiation	akap95 role in mitosis and chromosome dynamics|BioCarta	-0.07275121	-0.05491664	-0.36663398	0.72292856	0.86482318	-6.27421159
WT after UVB irradiation VS WT before UVB irradiation	akap95 role in mitosis and chromosome dynamics|BioCarta	0.094645764	-0.03800252	0.441797916	0.66796072	0.87935805	-5.77128986
homo after UVB irradiation VS homo before UVB irradiation	AKT phosphorylates targets in the cytosol|Reactome	0.020144271	-0.03128525	0.082024279	0.93634946	0.96227135	-6.36483461
hetero after UVB irradiation VS hetero before UVB irradiation	AKT phosphorylates targets in the cytosol|Reactome	0.093656579	-0.016885	0.429039854	0.67865362	0.83354977	-6.24778871
WT after UVB irradiation VS WT before UVB irradiation	AKT phosphorylates targets in the cytosol|Reactome	0.164654524	-0.01499483	0.792191147	0.44648808	0.80374894	-5.56147158
homo after UVB irradiation VS homo before UVB irradiation	AKT phosphorylates targets in the nucleus|Reactome	-0.00479514	-0.02304418	-0.02502766	0.98055678	0.98676245	-6.36811172
hetero after UVB irradiation VS hetero before UVB irradiation	AKT phosphorylates targets in the nucleus|Reactome	-0.02410826	-0.0295467	-0.1242124	0.90405942	0.97054702	-6.33817916
WT after UVB irradiation VS WT before UVB irradiation	AKT phosphorylates targets in the nucleus|Reactome	-0.10293965	-0.02133688	-0.49917602	0.62836706	0.87289644	-5.74457383
homo after UVB irradiation VS homo before UVB irradiation	akt signaling pathway|BioCarta	0.276283839	-0.04664502	1.104400053	0.29691675	0.45739605	-5.75342793

hetero after UVB irradiation VS hetero before UVB irradiation	akt signaling pathway|BioCarta	0.043303211	-0.03919774	0.20568552	0.84191942	0.94031753	-6.32364932
WT after UVB irradiation VS WT before UVB irradiation	akt signaling pathway|BioCarta	-0.24853175	-0.0675992	-1.21073815	0.2536129	0.64815341	-5.17962832
homo after UVB irradiation VS homo before UVB irradiation	AKT-mediated inactivation of FOXO1A|Reactome	0.634629129	-0.03415062	3.13262261	0.01146927	0.06546084	-2.80595973
hetero after UVB irradiation VS hetero before UVB irradiation	AKT-mediated inactivation of FOXO1A|Reactome	0.42794189	0.054973673	1.53918473	0.16042285	0.37252774	-5.21853851
WT after UVB irradiation VS WT before UVB irradiation	AKT-mediated inactivation of FOXO1A|Reactome	0.316370395	-0.03720487	1.253541651	0.23829687	0.63195092	-5.13372828
homo after UVB irradiation VS homo before UVB irradiation	Alanine and aspartate metabolism|Wikipathways	-0.24791608	0.033340291	-0.97754046	0.35281485	0.50783175	-5.88018992
hetero after UVB irradiation VS hetero before UVB irradiation	Alanine and aspartate metabolism|Wikipathways	-0.4444997	0.01735968	-1.71676183	0.1224447	0.31407031	-4.98228526
WT after UVB irradiation VS WT before UVB irradiation	Alanine and aspartate metabolism|Wikipathways	-0.2493081	-0.00200638	-1.10952877	0.29296473	0.67966352	-5.28347682
homo after UVB irradiation VS homo before UVB irradiation	Alanine Aspartate Asparagine metabolism|INOH	-0.41193078	0.050142962	-2.07402868	0.06664263	0.17952228	-4.47737255
hetero after UVB irradiation VS hetero before UVB irradiation	Alanine Aspartate Asparagine metabolism|INOH	-0.26811224	0.009811395	-1.34464136	0.21381363	0.43624105	-5.46069249
WT after UVB irradiation VS WT before UVB irradiation	Alanine Aspartate Asparagine metabolism|INOH	0.006451114	-0.03180747	0.029326254	0.97717678	0.99265119	-5.86871553
homo after UVB irradiation VS homo before UVB irradiation	alanine biosynthesis/degradation|HumanCyc	0.202381465	-0.11470774	0.546039937	0.59777166	0.71883735	-6.21080793
hetero after UVB irradiation VS hetero before UVB irradiation	alanine biosynthesis/degradation|HumanCyc	-0.08245069	-0.01086968	-0.21034748	0.83839434	0.93915002	-6.32260268
WT after UVB irradiation VS WT before UVB irradiation	alanine biosynthesis/degradation|HumanCyc	0.325492052	-0.03544055	0.81043277	0.43640313	0.79685269	-5.54761739
homo after UVB irradiation VS homo before UVB irradiation	Alanine Metabolism|SMPDB	-0.39210009	0.022178168	-1.25326746	0.24041731	0.40210456	-5.58992632
hetero after UVB irradiation VS hetero before UVB irradiation	Alanine Metabolism|SMPDB	-0.35251865	0.016423987	-1.29321274	0.23026395	0.45170905	-5.52131443
WT after UVB irradiation VS WT before UVB irradiation	Alanine Metabolism|SMPDB	0.415408645	-0.09593973	1.608715996	0.13850837	0.55342759	-4.71329834
homo after UVB irradiation VS homo before UVB irradiation	Alanine_ aspartate and glutamate metabolism - Homo sapiens (human)|KEGG	-0.29559327	0.026271729	-1.53010274	0.15895687	0.30741088	-5.24919841
hetero after UVB irradiation VS hetero before UVB irradiation	Alanine_ aspartate and glutamate metabolism - Homo sapiens (human)|KEGG	-0.23161918	-0.04768271	-1.21633009	0.25683172	0.48214126	-5.60900116
WT after UVB irradiation VS WT before UVB irradiation	Alanine_ aspartate and glutamate metabolism - Homo sapiens (human)|KEGG	0.037675894	-0.00852212	0.178952249	0.86152022	0.95916804	-5.85295573
homo after UVB irradiation VS homo before UVB irradiation	Alcoholism - Homo sapiens (human)|KEGG	0.254338846	0.008974686	1.494968226	0.16775165	0.31782325	-5.29479621
hetero after UVB irradiation VS hetero before UVB irradiation	Alcoholism - Homo sapiens (human)|KEGG	0.207833703	0.0475864	1.307018653	0.22574602	0.44814911	-5.50519097
WT after UVB irradiation VS WT before UVB irradiation	Alcoholism - Homo sapiens (human)|KEGG	0.237335693	-0.00651246	1.17660365	0.26638574	0.66118679	-5.21540302
homo after UVB irradiation VS homo before UVB irradiation	Aldosterone synthesis and secretion - Homo sapiens (human)|KEGG	0.574541734	-0.02661359	3.659220334	0.00488118	0.0397281	-1.97377618
hetero after UVB irradiation VS hetero before UVB irradiation	Aldosterone synthesis and secretion - Homo sapiens (human)|KEGG	0.275949715	0.038654221	1.268769629	0.23844951	0.4612216	-5.54958343
WT after UVB irradiation VS WT before UVB irradiation	Aldosterone synthesis and secretion - Homo sapiens (human)|KEGG	0.260427009	-0.01099159	1.020645907	0.33128815	0.71318926	-5.36892657
homo after UVB irradiation VS homo before UVB irradiation	Aldosterone-regulated sodium reabsorption - Homo sapiens (human)|KEGG	0.25579977	0.005004125	1.263214474	0.23697727	0.39802924	-5.57847298
hetero after UVB irradiation VS hetero before UVB irradiation	Aldosterone-regulated sodium reabsorption - Homo sapiens (human)|KEGG	0.188487315	0.024744378	1.131762971	0.28891299	0.51871614	-5.70111301
WT after UVB irradiation VS WT before UVB irradiation	Aldosterone-regulated sodium reabsorption - Homo sapiens (human)|KEGG	-0.04405707	0.005048357	-0.21062598	0.83737509	0.94951669	-5.84673008
homo after UVB irradiation VS homo before UVB irradiation	Alendronate Action Pathway|SMPDB	-0.19230642	-0.01987665	-0.61925406	0.55047761	0.67913558	-6.16661869
hetero after UVB irradiation VS hetero before UVB irradiation	Alendronate Action Pathway|SMPDB	0.185820603	0.008098609	0.631890782	0.54421801	0.75147406	-6.13505553
WT after UVB irradiation VS WT before UVB irradiation	Alendronate Action Pathway|SMPDB	-0.14894747	0.045069977	-0.50755372	0.62268399	0.87257466	-5.74041409
homo after UVB irradiation VS homo before UVB irradiation	Alfentanil Action Pathway|SMPDB	0.191132361	0.029768717	1.061313976	0.31508276	0.46923284	-5.79783739
hetero after UVB irradiation VS hetero before UVB irradiation	Alfentanil Action Pathway|SMPDB	-0.07904315	0.055005373	-0.44437398	0.66796482	0.82651398	-6.24068873
WT after UVB irradiation VS WT before UVB irradiation	Alfentanil Action Pathway|SMPDB	0.022316377	0.035038618	0.098344171	0.92358676	0.97566251	-5.86425414
homo after UVB irradiation VS homo before UVB irradiation	alk in cardiac myocytes|BioCarta	-0.34686757	-0.019255	-1.63372918	0.13534646	0.277359	-5.1111925
hetero after UVB irradiation VS hetero before UVB irradiation	alk in cardiac myocytes|BioCarta	-0.36207897	-0.01498972	-2.07556646	0.06984937	0.23618207	-4.47230183
WT after UVB irradiation VS WT before UVB irradiation	alk in cardiac myocytes|BioCarta	-0.45257743	-0.01247529	-2.3637923	0.03950308	0.39022568	-3.66093681
homo after UVB irradiation VS homo before UVB irradiation	ALK1 signaling events|PID	0.027645807	-0.01041042	0.121864626	0.90557473	0.94424622	-6.36047522
hetero after UVB irradiation VS hetero before UVB irradiation	ALK1 signaling events|PID	0.232401625	-0.03037488	1.226252924	0.25326621	0.47860174	-5.59788921
WT after UVB irradiation VS WT before UVB irradiation	ALK1 signaling events|PID	-0.10476935	0.038775922	-0.50653396	0.62337437	0.87257466	-5.74092393
homo after UVB irradiation VS homo before UVB irradiation	ALK2 signaling events|PID	-0.48220288	0.055482043	-2.10637337	0.06318884	0.17349384	-4.42844831
hetero after UVB irradiation VS hetero before UVB irradiation	ALK2 signaling events|PID	-0.5376775	-0.02193483	-2.29417707	0.04934128	0.19762528	-4.14719403
WT after UVB irradiation VS WT before UVB irradiation	ALK2 signaling events|PID	-0.76738537	0.033772281	-3.72106074	0.00391305	0.20870715	-1.64546326
homo after UVB irradiation VS homo before UVB irradiation	Alkaptonuria|SMPDB	0.031995945	0.027270645	0.129408983	0.89976401	0.9399608	-6.35945841
hetero after UVB irradiation VS hetero before UVB irradiation	Alkaptonuria|SMPDB	-0.38264835	0.006355022	-1.73471603	0.11910451	0.31235808	-4.9577083
WT after UVB irradiation VS WT before UVB irradiation	Alkaptonuria|SMPDB	0.126407683	0.040850924	0.512443468	0.61937892	0.87257466	-5.73795593
homo after UVB irradiation VS homo before UVB irradiation	ALKBH2 mediated reversal of alkylation damage|Reactome	0.277460239	-0.00031064	0.684782546	0.51003046	0.64923309	-6.12275572
hetero after UVB irradiation VS hetero before UVB irradiation	ALKBH2 mediated reversal of alkylation damage|Reactome	0.965944144	0.022027617	3.813100061	0.00466743	0.07412592	-1.85961228
WT after UVB irradiation VS WT before UVB irradiation	ALKBH2 mediated reversal of alkylation damage|Reactome	0.140739359	0.034479899	0.342427484	0.73906064	0.91673557	-5.81011905
homo after UVB irradiation VS homo before UVB irradiation	ALKBH3 mediated reversal of alkylation damage|Reactome	-0.65366496	-0.06516891	-3.05485636	0.01303965	0.0709643	-2.93033218
hetero after UVB irradiation VS hetero before UVB irradiation	ALKBH3 mediated reversal of alkylation damage|Reactome	-0.29886946	0.012858881	-0.85740167	0.41494261	0.64192892	-5.96467336
WT after UVB irradiation VS WT before UVB irradiation	ALKBH3 mediated reversal of alkylation damage|Reactome	-0.06796248	0.081287925	-0.21224041	0.83614888	0.94951669	-5.84638588
homo after UVB irradiation VS homo before UVB irradiation	Allograft rejection - Homo sapiens (human)|KEGG	0.307255697	-0.07085999	1.150759023	0.27829397	0.43862281	-5.70414814
hetero after UVB irradiation VS hetero before UVB irradiation	Allograft rejection - Homo sapiens (human)|KEGG	0.042400342	-0.02769018	0.138133243	0.89337943	0.96526714	-6.3362021
WT after UVB irradiation VS WT before UVB irradiation	Allograft rejection - Homo sapiens (human)|KEGG	0.195311958	-0.05553016	0.559060866	0.58832209	0.86161188	-5.71340757
homo after UVB irradiation VS homo before UVB irradiation	Allograft Rejection|Wikipathways	0.486289202	-0.025612	2.487049669	0.03357311	0.1222943	-3.83725053
hetero after UVB irradiation VS hetero before UVB irradiation	Allograft Rejection|Wikipathways	0.28366266	0.031611113	1.224294963	0.25396651	0.47932892	-5.60008673
WT after UVB irradiation VS WT before UVB irradiation	Allograft Rejection|Wikipathways	0.407608397	-0.00261106	1.361161222	0.20310744	0.61688026	-5.01346001
homo after UVB irradiation VS homo before UVB irradiation	allopregnanolone biosynthesis|HumanCyc	0.310771295	0.101290561	1.167323762	0.27186847	0.43571479	-5.68617302
hetero after UVB irradiation VS hetero before UVB irradiation	allopregnanolone biosynthesis|HumanCyc	-0.15976276	0.125653213	-0.60811973	0.55913962	0.76190086	-6.15032744
WT after UVB irradiation VS WT before UVB irradiation	allopregnanolone biosynthesis|HumanCyc	-0.28638739	0.08015792	-1.02579059	0.32897187	0.71204479	-5.36413369
homo after UVB irradiation VS homo before UVB irradiation	Alpha 6 Beta 4 signaling pathway|Wikipathways	0.436983796	-0.05104922	1.874359954	0.09228238	0.21775781	-4.77303536
hetero after UVB irradiation VS hetero before UVB irradiation	Alpha 6 Beta 4 signaling pathway|Wikipathways	0.344674759	0.045797352	2.075569744	0.06984901	0.23618207	-4.47229702
WT after UVB irradiation VS WT before UVB irradiation	Alpha 6 Beta 4 signaling pathway|Wikipathways	0.376864886	0.033519571	1.893504772	0.08731481	0.47813759	-4.33530281
homo after UVB irradiation VS homo before UVB irradiation	Alpha Linolenic Acid and Linoleic Acid Metabolism|SMPDB	-0.73214293	-0.04450369	-4.25730954	0.00192545	0.02442117	-1.06479447
hetero after UVB irradiation VS hetero before UVB irradiation	Alpha Linolenic Acid and Linoleic Acid Metabolism|SMPDB	-0.25851598	0.019947972	-1.21481298	0.25738048	0.4829725	-5.61069459
WT after UVB irradiation VS WT before UVB irradiation	Alpha Linolenic Acid and Linoleic Acid Metabolism|SMPDB	-0.5435257	-0.04192064	-2.37836888	0.03852942	0.39022568	-3.63939471
homo after UVB irradiation VS homo before UVB irradiation	Alpha4 beta1 integrin signaling events|PID	0.86279691	-0.03149717	5.088870684	0.00057407	0.01666204	0.111496827
hetero after UVB irradiation VS hetero before UVB irradiation	Alpha4 beta1 integrin signaling events|PID	0.892084934	0.040043188	4.812183588	0.0011531	0.04138456	-0.49392753
WT after UVB irradiation VS WT before UVB irradiation	Alpha4 beta1 integrin signaling events|PID	0.690287158	0.023091798	3.091327463	0.01131223	0.2678418	-2.57114904
homo after UVB irradiation VS homo before UVB irradiation	Alpha6 beta4 integrin-ligand interactions|PID	0.776033287	-0.03550955	3.445608708	0.00687898	0.04810019	-2.3086454
hetero after UVB irradiation VS hetero before UVB irradiation	Alpha6 beta4 integrin-ligand interactions|PID	0.522297283	0.049132892	2.222844281	0.05528016	0.20875475	-4.25412406
WT after UVB irradiation VS WT before UVB irradiation	Alpha6 beta4 integrin-ligand interactions|PID	0.599288286	-0.02058357	2.609341474	0.02590863	0.34104092	-3.29521165
homo after UVB irradiation VS homo before UVB irradiation	Alpha6Beta4Integrin|NetPath	0.27113084	-0.00863804	1.205332503	0.25757274	0.42092954	-5.64421649
hetero after UVB irradiation VS hetero before UVB irradiation	Alpha6Beta4Integrin|NetPath	0.33512697	0.024300505	1.889200364	0.09368191	0.27672034	-4.7417615
WT after UVB irradiation VS WT before UVB irradiation	Alpha6Beta4Integrin|NetPath	0.169385284	0.032185418	0.858911674	0.41034509	0.77688258	-5.50947767
homo after UVB irradiation VS homo before UVB irradiation	Alpha9 beta1 integrin signaling events|PID	0.913098051	-0.02020654	5.785963595	0.00022465	0.01666204	1.011652105
hetero after UVB irradiation VS hetero before UVB irradiation	Alpha9 beta1 integrin signaling events|PID	0.777206094	0.041153589	3.43824779	0.0081783	0.09152359	-2.40959164
WT after UVB irradiation VS WT before UVB irradiation	Alpha9 beta1 integrin signaling events|PID	0.638318106	-0.02872572	2.387566221	0.03792719	0.39022568	-3.6257889
homo after UVB irradiation VS homo before UVB irradiation	Alpha-defensins|Reactome	0.395387381	-0.09111185	1.290396408	0.22778325	0.38735877	-5.54685356
hetero after UVB irradiation VS hetero before UVB irradiation	Alpha-defensins|Reactome	0.510154879	0.175008428	1.552636215	0.15721077	0.36728174	-5.20111041
WT after UVB irradiation VS WT before UVB irradiation	Alpha-defensins|Reactome	0.131528347	-0.08395374	0.361409332	0.72525231	0.90898113	-5.80343947
homo after UVB irradiation VS homo before UVB irradiation	AlphaE beta7 integrin cell surface interactions|PID	0.154453564	-0.02956668	0.481856433	0.64094128	0.75095947	-6.24525466
hetero after UVB irradiation VS hetero before UVB irradiation	AlphaE beta7 integrin cell surface interactions|PID	0.047047058	-0.10357522	0.219876098	0.83120146	0.93531602	-6.32039127
WT after UVB irradiation VS WT before UVB irradiation	AlphaE beta7 integrin cell surface interactions|PID	-0.08500471	-0.13093073	-0.33378113	0.74538314	0.9185045	-5.81304517
homo after UVB irradiation VS homo before UVB irradiation	alpha-linolenic (omega3) and linoleic (omega6) acid metabolism|Reactome	0.200806268	-0.04769281	0.954899812	0.36356553	0.51763025	-5.90151064
hetero after UVB irradiation VS hetero before UVB irradiation	alpha-linolenic (omega3) and linoleic (omega6) acid metabolism|Reactome	0.172604527	-0.0234803	0.680816358	0.51425824	0.73158211	-6.10196154
WT after UVB irradiation VS WT before UVB irradiation	alpha-linolenic (omega3) and linoleic (omega6) acid metabolism|Reactome	0.162942968	-0.07876283	0.722319694	0.48651402	0.81710765	-5.61196465
homo after UVB irradiation VS homo before UVB irradiation	alpha-linolenic acid (ALA) metabolism|Reactome	0.200806268	-0.04769281	0.954899812	0.36356553	0.51763025	-5.90151064
hetero after UVB irradiation VS hetero before UVB irradiation	alpha-linolenic acid (ALA) metabolism|Reactome	0.172604527	-0.0234803	0.680816358	0.51425824	0.73158211	-6.10196154
WT after UVB irradiation VS WT before UVB irradiation	alpha-linolenic acid (ALA) metabolism|Reactome	0.162942968	-0.07876283	0.722319694	0.48651402	0.81710765	-5.61196465
homo after UVB irradiation VS homo before UVB irradiation	alpha-Linolenic acid metabolism - Homo sapiens (human)|KEGG	0.033326893	0.047391803	0.160741093	0.8757028	0.92385836	-6.35458512
hetero after UVB irradiation VS hetero before UVB irradiation	alpha-Linolenic acid metabolism - Homo sapiens (human)|KEGG	0.051610802	0.012778826	0.275586368	0.78950409	0.91010613	-6.30553002
WT after UVB irradiation VS WT before UVB irradiation	alpha-Linolenic acid metabolism - Homo sapiens (human)|KEGG	0.00303381	0.004473124	0.012709013	0.99010792	0.99676022	-5.86906937
homo after UVB irradiation VS homo before UVB irradiation	Alpha-oxidation of phytanate|Reactome	0.538620548	0.048173052	3.179205475	0.01062307	0.06218329	-2.73157302
hetero after UVB irradiation VS hetero before UVB irradiation	Alpha-oxidation of phytanate|Reactome	0.172610613	-0.05197656	0.712854739	0.49520077	0.71428009	-6.07909872
WT after UVB irradiation VS WT before UVB irradiation	Alpha-oxidation of phytanate|Reactome	0.18151304	-0.04758161	0.586878431	0.57019246	0.85351326	-5.69780798
homo after UVB irradiation VS homo before UVB irradiation	alpha-synuclein and parkin-mediated proteolysis in parkinson`s disease|BioCarta	-0.33091752	-0.03810342	-0.91711356	0.38203597	0.53514858	-5.93617969
hetero after UVB irradiation VS hetero before UVB irradiation	alpha-synuclein and parkin-mediated proteolysis in parkinson`s disease|BioCarta	-0.70883624	-0.07825692	-2.48924602	0.03614642	0.16681566	-3.85181629
WT after UVB irradiation VS WT before UVB irradiation	alpha-synuclein and parkin-mediated proteolysis in parkinson`s disease|BioCarta	0.015036105	-0.0017745	0.036107243	0.97190175	0.99045333	-5.86849077
homo after UVB irradiation VS homo before UVB irradiation	Alpha-synuclein signaling|PID	0.514130334	-0.04275068	3.103373457	0.01203577	0.0671005	-2.8527134
hetero after UVB irradiation VS hetero before UVB irradiation	Alpha-synuclein signaling|PID	0.70294525	0.049001124	4.268090185	0.0024251	0.05526805	-1.21818906
WT after UVB irradiation VS WT before UVB irradiation	Alpha-synuclein signaling|PID	0.572546394	0.030700193	2.552262442	0.02858339	0.35176669	-3.38067039
homo after UVB irradiation VS homo before UVB irradiation	Alprenolol Action Pathway|SMPDB	0.396415397	-0.03677376	2.343069613	0.04268583	0.13402181	-4.06364635
hetero after UVB irradiation VS hetero before UVB irradiation	Alprenolol Action Pathway|SMPDB	-0.01555055	-0.01775524	-0.07410913	0.94265284	0.97054702	-6.34356282
WT after UVB irradiation VS WT before UVB irradiation	Alprenolol Action Pathway|SMPDB	0.056685205	-0.03978418	0.223404833	0.82768199	0.94525798	-5.84393438
homo after UVB irradiation VS homo before UVB irradiation	Alteplase Action Pathway|SMPDB	0.538210712	0.026444726	3.007777411	0.01409598	0.07305822	-3.00571236
hetero after UVB irradiation VS hetero before UVB irradiation	Alteplase Action Pathway|SMPDB	0.558085555	0.09913938	2.984394118	0.01649409	0.11352943	-3.09486545
WT after UVB irradiation VS WT before UVB irradiation	Alteplase Action Pathway|SMPDB	0.440879848	0.077377741	1.569633667	0.14732454	0.55622203	-4.76265269
homo after UVB irradiation VS homo before UVB irradiation	Alternative complement activation|Reactome	0.517855573	-0.02287163	1.700084792	0.12193186	0.26055457	-5.02025491
hetero after UVB irradiation VS hetero before UVB irradiation	Alternative complement activation|Reactome	0.058744861	0.034932993	0.177718605	0.8631424	0.94663211	-6.32944015
WT after UVB irradiation VS WT before UVB irradiation	Alternative complement activation|Reactome	0.568975038	0.050735306	1.655091719	0.12865735	0.53955751	-4.65387123
homo after UVB irradiation VS homo before UVB irradiation	alternative complement pathway|BioCarta	0.825765362	-0.04742243	3.362035523	0.00787784	0.05213077	-2.4407828
hetero after UVB irradiation VS hetero before UVB irradiation	alternative complement pathway|BioCarta	0.280941904	0.08257082	0.969449451	0.35932214	0.5876535	-5.86396362
WT after UVB irradiation VS WT before UVB irradiation	alternative complement pathway|BioCarta	0.451476913	0.011414872	1.481165394	0.16912406	0.5755511	-4.87175969
homo after UVB irradiation VS homo before UVB irradiation	Alternative NF-kappaB pathway|PID	0.760124338	-0.02413666	3.428097386	0.00707679	0.04876599	-2.33628535
hetero after UVB irradiation VS hetero before UVB irradiation	Alternative NF-kappaB pathway|PID	0.267188469	-0.09652473	0.856183736	0.415579	0.64210478	-5.96571211
WT after UVB irradiation VS WT before UVB irradiation	Alternative NF-kappaB pathway|PID	0.569453393	-0.07176458	1.721162701	0.11570701	0.51627893	-4.56767839
homo after UVB irradiation VS homo before UVB irradiation	Alvimopan Action Pathway|SMPDB	0.191132361	0.029768717	1.061313976	0.31508276	0.46923284	-5.79783739
hetero after UVB irradiation VS hetero before UVB irradiation	Alvimopan Action Pathway|SMPDB	-0.07904315	0.055005373	-0.44437398	0.66796482	0.82651398	-6.24068873
WT after UVB irradiation VS WT before UVB irradiation	Alvimopan Action Pathway|SMPDB	0.022316377	0.035038618	0.098344171	0.92358676	0.97566251	-5.86425414
homo after UVB irradiation VS homo before UVB irradiation	Alzheimer disease - Homo sapiens (human)|KEGG	0.243378246	-0.03100793	1.020364896	0.33312555	0.48987159	-5.83876344
hetero after UVB irradiation VS hetero before UVB irradiation	Alzheimer disease - Homo sapiens (human)|KEGG	0.033473023	0.001859614	0.158970585	0.87743662	0.9555609	-6.33285267
WT after UVB irradiation VS WT before UVB irradiation	Alzheimer disease - Homo sapiens (human)|KEGG	0.179849673	-0.03537274	0.671588909	0.51693737	0.82895075	-5.64601109
homo after UVB irradiation VS homo before UVB irradiation	Alzheimers Disease|Wikipathways	0.575744524	-0.02585351	3.639964055	0.00503342	0.04061675	-2.00377514
hetero after UVB irradiation VS hetero before UVB irradiation	Alzheimers Disease|Wikipathways	0.517404527	0.000460036	3.017325981	0.01566456	0.11352943	-3.04465069
WT after UVB irradiation VS WT before UVB irradiation	Alzheimers Disease|Wikipathways	0.494605633	-0.01044725	2.402221532	0.03698656	0.39022568	-3.60408821
homo after UVB irradiation VS homo before UVB irradiation	amb2 Integrin signaling|PID	0.892199638	0.019127462	5.692873423	0.00025364	0.01666204	0.89599342
hetero after UVB irradiation VS hetero before UVB irradiation	amb2 Integrin signaling|PID	0.810574535	0.06604779	4.000309363	0.00355243	0.06529021	-1.59195984
WT after UVB irradiation VS WT before UVB irradiation	amb2 Integrin signaling|PID	0.634133632	0.03949638	2.18391832	0.05367362	0.41493406	-3.92421289
homo after UVB irradiation VS homo before UVB irradiation	Amiloride Action Pathway|SMPDB	0.234726031	0.017120546	1.156747303	0.27595732	0.43614438	-5.69767199
hetero after UVB irradiation VS hetero before UVB irradiation	Amiloride Action Pathway|SMPDB	-0.03255037	0.028925632	-0.18154472	0.86023155	0.94503144	-6.32869738
WT after UVB irradiation VS WT before UVB irradiation	Amiloride Action Pathway|SMPDB	-0.15908359	0.010252305	-0.80382265	0.44003996	0.79685269	-5.55266936
homo after UVB irradiation VS homo before UVB irradiation	Amine compound SLC transporters|Reactome	-0.24785423	0.06457219	-1.57163449	0.14908702	0.29497601	-5.19450242
hetero after UVB irradiation VS hetero before UVB irradiation	Amine compound SLC transporters|Reactome	-0.37453116	0.017769645	-2.41492723	0.0406965	0.17712536	-3.96477139
WT after UVB irradiation VS WT before UVB irradiation	Amine compound SLC transporters|Reactome	-0.39446005	0.066962107	-1.79776659	0.10217874	0.50394202	-4.46565811
homo after UVB irradiation VS homo before UVB irradiation	Amine ligand-binding receptors|Reactome	0.298420549	-0.01686916	1.540890466	0.15633921	0.30417351	-5.23507292
hetero after UVB irradiation VS hetero before UVB irradiation	Amine ligand-binding receptors|Reactome	-0.04299286	0.017350508	-0.22589154	0.82666915	0.93322694	-6.31894535
WT after UVB irradiation VS WT before UVB irradiation	Amine ligand-binding receptors|Reactome	0.075651002	0.039217189	0.290400751	0.77739516	0.93502208	-5.82662022
homo after UVB irradiation VS homo before UVB irradiation	Amine Oxidase reactions|Reactome	0.68502852	-0.02954598	3.332973755	0.00825953	0.05379888	-2.48685917
hetero after UVB irradiation VS hetero before UVB irradiation	Amine Oxidase reactions|Reactome	0.659074951	0.015882826	3.558504332	0.00681793	0.08550202	-2.23127391
WT after UVB irradiation VS WT before UVB irradiation	Amine Oxidase reactions|Reactome	0.826955982	-0.06344812	4.181541034	0.00184989	0.16246967	-1.00053758
homo after UVB irradiation VS homo before UVB irradiation	Amine-derived hormones|Reactome	0.151674179	0.000591883	0.763202671	0.46408614	0.6088463	-6.06507124
hetero after UVB irradiation VS hetero before UVB irradiation	Amine-derived hormones|Reactome	-0.14430111	0.033741171	-0.78999658	0.4511877	0.67639412	-6.02028336
WT after UVB irradiation VS WT before UVB irradiation	Amine-derived hormones|Reactome	0.001573742	0.062060874	0.00765682	0.99404019	0.99775419	-5.86912149
homo after UVB irradiation VS homo before UVB irradiation	Amino acid and oligopeptide SLC transporters|Reactome	-0.00254993	0.013132582	-0.01487197	0.98844556	0.99205816	-6.36832943
hetero after UVB irradiation VS hetero before UVB irradiation	Amino acid and oligopeptide SLC transporters|Reactome	-0.18166613	0.014625033	-1.03856528	0.32788905	0.55663982	-5.79696653

WT after UVB irradiation VS WT before UVB irradiation	Amino acid and oligopeptide SLC transporters|Reactome	-0.19597951	-0.00983101	-0.96355075	0.35781229	0.74302468	-5.42081345
homo after UVB irradiation VS homo before UVB irradiation	Amino acid conjugation of benzoic acid|Wikipathways	-0.40295717	0.096729331	-1.1830477	0.26587917	0.42933282	-5.66893513
hetero after UVB irradiation VS hetero before UVB irradiation	Amino acid conjugation of benzoic acid|Wikipathways	-0.9757063	-0.0223746	-4.8152865	0.00114837	0.04138456	-0.4899364
WT after UVB irradiation VS WT before UVB irradiation	Amino acid conjugation of benzoic acid|Wikipathways	-0.87940935	-0.01831533	-3.82655006	0.00328835	0.20197958	-1.49491675
homo after UVB irradiation VS homo before UVB irradiation	Amino Acid conjugation|Reactome	0.08093173	0.023459488	0.224217303	0.82739153	0.89374727	-6.34151017
hetero after UVB irradiation VS hetero before UVB irradiation	Amino Acid conjugation|Reactome	-0.17694323	-0.00969811	-1.0654327	0.31625519	0.54576835	-5.76996718
WT after UVB irradiation VS WT before UVB irradiation	Amino Acid conjugation|Reactome	0.05439466	0.064488051	0.170020456	0.86835729	0.96280353	-5.85452964
homo after UVB irradiation VS homo before UVB irradiation	Amino Acid metabolism|Wikipathways	-0.20154549	0.025608643	-0.96012109	0.36106526	0.5161628	-5.89662986
hetero after UVB irradiation VS hetero before UVB irradiation	Amino Acid metabolism|Wikipathways	-0.34638712	-0.01522302	-1.81102635	0.1058313	0.29415277	-4.85199592
WT after UVB irradiation VS WT before UVB irradiation	Amino Acid metabolism|Wikipathways	-0.13622393	-0.04516441	-0.61467727	0.55238656	0.84516674	-5.6815175
homo after UVB irradiation VS homo before UVB irradiation	Amino acid synthesis and interconversion (transamination)|Reactome	0.011078517	-0.00325626	0.047994171	0.96272633	0.97728066	-6.36721077
hetero after UVB irradiation VS hetero before UVB irradiation	Amino acid synthesis and interconversion (transamination)|Reactome	-0.07207246	0.007147772	-0.30795203	0.76559435	0.89270377	-6.29538999
WT after UVB irradiation VS WT before UVB irradiation	Amino acid synthesis and interconversion (transamination)|Reactome	0.099429187	-0.01052643	0.493224949	0.63241955	0.87311368	-5.74748882
homo after UVB irradiation VS homo before UVB irradiation	Amino acid transport across the plasma membrane|Reactome	-0.15314499	0.020532379	-0.92471018	0.37826953	0.53111275	-5.92930259
hetero after UVB irradiation VS hetero before UVB irradiation	Amino acid transport across the plasma membrane|Reactome	-0.16674003	0.035140564	-1.03427681	0.32977614	0.55823827	-5.80122733
WT after UVB irradiation VS WT before UVB irradiation	Amino acid transport across the plasma membrane|Reactome	-0.29393597	-0.03677714	-1.42281977	0.18498458	0.59839062	-4.94160324
homo after UVB irradiation VS homo before UVB irradiation	Amino sugar and nucleotide sugar metabolism - Homo sapiens (human)|KEGG	0.643896029	-0.03221836	3.812502355	0.00382872	0.03517492	-1.73646605
hetero after UVB irradiation VS hetero before UVB irradiation	Amino sugar and nucleotide sugar metabolism - Homo sapiens (human)|KEGG	0.463780799	-0.00880491	2.463291961	0.03767429	0.16931722	-3.89131115
WT after UVB irradiation VS WT before UVB irradiation	Amino sugar and nucleotide sugar metabolism - Homo sapiens (human)|KEGG	0.440396386	-0.01053451	1.969463593	0.07697866	0.46222215	-4.22986202
homo after UVB irradiation VS homo before UVB irradiation	Amino Sugar Metabolism|SMPDB	0.79075295	-0.00257633	4.921856093	0.00072618	0.01666204	-0.11590737
hetero after UVB irradiation VS hetero before UVB irradiation	Amino Sugar Metabolism|SMPDB	0.602895035	0.025817322	2.347921889	0.04528827	0.18731753	-4.06619426
WT after UVB irradiation VS WT before UVB irradiation	Amino Sugar Metabolism|SMPDB	0.514071552	-0.00966104	2.208926071	0.05144363	0.41047076	-3.88793019
homo after UVB irradiation VS homo before UVB irradiation	Aminoacyl-tRNA biosynthesis - Homo sapiens (human)|KEGG	-0.71855989	-0.03108704	-3.7654752	0.00412361	0.03698535	-1.80898356
hetero after UVB irradiation VS hetero before UVB irradiation	Aminoacyl-tRNA biosynthesis - Homo sapiens (human)|KEGG	-0.40697066	-0.08180813	-1.93761317	0.08683349	0.26658789	-4.67260428
WT after UVB irradiation VS WT before UVB irradiation	Aminoacyl-tRNA biosynthesis - Homo sapiens (human)|KEGG	-0.27502391	-0.03268388	-0.93571592	0.37129012	0.74850253	-5.44522499
homo after UVB irradiation VS homo before UVB irradiation	Aminocaproic Acid Action Pathway|SMPDB	0.538210712	0.026444726	3.007777411	0.01409598	0.07305822	-3.00571236
hetero after UVB irradiation VS hetero before UVB irradiation	Aminocaproic Acid Action Pathway|SMPDB	0.558085555	0.09913938	2.984394118	0.01649409	0.11352943	-3.09486545
WT after UVB irradiation VS WT before UVB irradiation	Aminocaproic Acid Action Pathway|SMPDB	0.440879848	0.077377741	1.569633667	0.14732454	0.55622203	-4.76265269
homo after UVB irradiation VS homo before UVB irradiation	Aminosugars metabolism|EHMN	-0.06094857	0.006067166	-0.29547049	0.77406473	0.85410231	-6.32176287
hetero after UVB irradiation VS hetero before UVB irradiation	Aminosugars metabolism|EHMN	0.246443773	-0.01287118	1.346347158	0.2132856	0.43535861	-5.45865585
WT after UVB irradiation VS WT before UVB irradiation	Aminosugars metabolism|EHMN	0.158082053	-0.0821131	0.70090852	0.49921668	0.82066817	-5.62660616
homo after UVB irradiation VS homo before UVB irradiation	Aminosugars metabolism|INOH	0.560774635	-0.0493896	3.437687676	0.00696773	0.04844193	-2.32114478
hetero after UVB irradiation VS hetero before UVB irradiation	Aminosugars metabolism|INOH	0.383017182	-0.02406192	1.675165425	0.13051835	0.32597404	-5.03876647
WT after UVB irradiation VS WT before UVB irradiation	Aminosugars metabolism|INOH	0.270791789	-0.04878911	0.989828927	0.34541762	0.72932143	-5.39723186
homo after UVB irradiation VS homo before UVB irradiation	Amiodarone Action Pathway|SMPDB	0.396415397	-0.03677376	2.343069613	0.04268583	0.13402181	-4.06364635
hetero after UVB irradiation VS hetero before UVB irradiation	Amiodarone Action Pathway|SMPDB	-0.01555055	-0.01775524	-0.07410913	0.94265284	0.97054702	-6.34356282
WT after UVB irradiation VS WT before UVB irradiation	Amiodarone Action Pathway|SMPDB	0.056685205	-0.03978418	0.223404833	0.82768199	0.94525798	-5.84393438
homo after UVB irradiation VS homo before UVB irradiation	Amlodipine Action Pathway|SMPDB	0.396415397	-0.03677376	2.343069613	0.04268583	0.13402181	-4.06364635
hetero after UVB irradiation VS hetero before UVB irradiation	Amlodipine Action Pathway|SMPDB	-0.01555055	-0.01775524	-0.07410913	0.94265284	0.97054702	-6.34356282
WT after UVB irradiation VS WT before UVB irradiation	Amlodipine Action Pathway|SMPDB	0.056685205	-0.03978418	0.223404833	0.82768199	0.94525798	-5.84393438
homo after UVB irradiation VS homo before UVB irradiation	Ammonia Recycling|SMPDB	-0.00675794	0.021449889	-0.02482865	0.98071135	0.98676245	-6.36811705
hetero after UVB irradiation VS hetero before UVB irradiation	Ammonia Recycling|SMPDB	-0.20818199	-0.04485117	-0.77803618	0.45783596	0.68223742	-6.02974494
WT after UVB irradiation VS WT before UVB irradiation	Ammonia Recycling|SMPDB	0.018163909	-0.01602958	0.088945794	0.93086704	0.9788786	-5.86514503
homo after UVB irradiation VS homo before UVB irradiation	Amodiaquine Pathway_ Pharmacokinetics|PharmGKB	-0.20268907	0.043541165	-0.80192458	0.44241895	0.59091566	-6.03456021
hetero after UVB irradiation VS hetero before UVB irradiation	Amodiaquine Pathway_ Pharmacokinetics|PharmGKB	-0.51493933	-0.12456	-1.47777239	0.17584362	0.3928898	-5.29703576
WT after UVB irradiation VS WT before UVB irradiation	Amodiaquine Pathway_ Pharmacokinetics|PharmGKB	-0.6946626	0.001001	-2.30599853	0.04360526	0.39022568	-3.7460715
homo after UVB irradiation VS homo before UVB irradiation	Amoebiasis - Homo sapiens (human)|KEGG	0.587092729	-0.04327028	3.722493703	0.00441402	0.0379263	-1.87549019
hetero after UVB irradiation VS hetero before UVB irradiation	Amoebiasis - Homo sapiens (human)|KEGG	0.49910881	0.039935142	2.587886251	0.03088765	0.15336156	-3.70134436
WT after UVB irradiation VS WT before UVB irradiation	Amoebiasis - Homo sapiens (human)|KEGG	0.278755155	-0.00501155	1.127334811	0.28571521	0.67295584	-5.26569903
homo after UVB irradiation VS homo before UVB irradiation	AMP-activated Protein Kinase (AMPK) Signaling|Wikipathways	0.076108713	-0.02797302	0.4457819	0.66585703	0.77165056	-6.26282022
hetero after UVB irradiation VS hetero before UVB irradiation	AMP-activated Protein Kinase (AMPK) Signaling|Wikipathways	-0.00730538	-0.03140087	-0.04379612	0.9660871	0.98439183	-6.34550086
WT after UVB irradiation VS WT before UVB irradiation	AMP-activated Protein Kinase (AMPK) Signaling|Wikipathways	-0.00041006	-0.02283484	-0.00202543	0.99842346	0.99940779	-5.86914911
homo after UVB irradiation VS homo before UVB irradiation	Amphetamine addiction - Homo sapiens (human)|KEGG	0.193851971	0.019497418	1.254478098	0.23999645	0.40198965	-5.58853574
hetero after UVB irradiation VS hetero before UVB irradiation	Amphetamine addiction - Homo sapiens (human)|KEGG	0.04914899	0.035206116	0.263481227	0.79850901	0.91506577	-6.30903888
WT after UVB irradiation VS WT before UVB irradiation	Amphetamine addiction - Homo sapiens (human)|KEGG	0.062857635	-0.04904759	0.258750414	0.80103373	0.94525798	-5.83535458
homo after UVB irradiation VS homo before UVB irradiation	AMPK inhibits chREBP transcriptional activation activity|Reactome	0.525561893	-0.04606785	2.787881961	0.02031564	0.09100843	-3.3580125
hetero after UVB irradiation VS hetero before UVB irradiation	AMPK inhibits chREBP transcriptional activation activity|Reactome	-0.24600605	-0.01725242	-0.90608789	0.39006329	0.61711505	-5.92215118
WT after UVB irradiation VS WT before UVB irradiation	AMPK inhibits chREBP transcriptional activation activity|Reactome	-0.20842998	-0.08812217	-0.7318089	0.48094934	0.81552033	-5.60534977
homo after UVB irradiation VS homo before UVB irradiation	AMPK signaling pathway - Homo sapiens (human)|KEGG	0.095066322	-0.00710453	0.576713213	0.57769601	0.70060027	-6.19292251
hetero after UVB irradiation VS hetero before UVB irradiation	AMPK signaling pathway - Homo sapiens (human)|KEGG	-0.01123606	-0.01393084	-0.06197958	0.9520242	0.97534868	-6.34445781
WT after UVB irradiation VS WT before UVB irradiation	AMPK signaling pathway - Homo sapiens (human)|KEGG	-0.06385915	-0.02472975	-0.3230808	0.75323503	0.92020735	-5.81656521
homo after UVB irradiation VS homo before UVB irradiation	Amplification of signal from unattached kinetochores via a MAD2 inhibitory signal|Reactome	-0.60534719	-0.01674196	-3.15812329	0.01099777	0.063619	-2.76522639
hetero after UVB irradiation VS hetero before UVB irradiation	Amplification of signal from unattached kinetochores via a MAD2 inhibitory signal|Reactome	0.130878667	-0.02329636	0.442954624	0.66895089	0.82698079	-6.24135591
WT after UVB irradiation VS WT before UVB irradiation	Amplification of signal from unattached kinetochores via a MAD2 inhibitory signal|Reactome	0.066854189	0.027238738	0.228139618	0.82409811	0.94525798	-5.84285717
homo after UVB irradiation VS homo before UVB irradiation	Amplification and Expansion of Oncogenic Pathways as Metastatic Traits|Wikipathways	0.569911941	-0.04224039	2.356344053	0.04175249	0.13402181	-4.04289114
hetero after UVB irradiation VS hetero before UVB irradiation	Amplification and Expansion of Oncogenic Pathways as Metastatic Traits|Wikipathways	0.696732622	0.043516875	4.286069128	0.00236461	0.05498933	-1.1934924
WT after UVB irradiation VS WT before UVB irradiation	Amplification and Expansion of Oncogenic Pathways as Metastatic Traits|Wikipathways	0.462552168	0.059768118	2.208283069	0.05149984	0.41047076	-3.88886454
homo after UVB irradiation VS homo before UVB irradiation	Amplification of signal from the kinetochores|Reactome	-0.60534719	-0.01674196	-3.15812329	0.01099777	0.063619	-2.76522639
hetero after UVB irradiation VS hetero before UVB irradiation	Amplification of signal from the kinetochores|Reactome	0.130878667	-0.02329636	0.442954624	0.66895089	0.82698079	-6.24135591
WT after UVB irradiation VS WT before UVB irradiation	Amplification of signal from the kinetochores|Reactome	0.066854189	0.027238738	0.228139618	0.82409811	0.94525798	-5.84285717
homo after UVB irradiation VS homo before UVB irradiation	Amyloid fiber formation|Reactome	-0.19117812	0.01684609	-0.88539978	0.39804866	0.55064838	-5.96437806
hetero after UVB irradiation VS hetero before UVB irradiation	Amyloid fiber formation|Reactome	0.022617169	-0.00677628	0.107513952	0.9168974	0.97054702	-6.34027488
WT after UVB irradiation VS WT before UVB irradiation	Amyloid fiber formation|Reactome	-0.23202284	-0.026277	-1.09502228	0.29897556	0.68477511	-5.29780039
homo after UVB irradiation VS homo before UVB irradiation	Amyotrophic lateral sclerosis (ALS) - Homo sapiens (human)|KEGG	0.262135349	0.004066979	1.566705216	0.15022931	0.29604965	-5.20103812
hetero after UVB irradiation VS hetero before UVB irradiation	Amyotrophic lateral sclerosis (ALS) - Homo sapiens (human)|KEGG	0.232842279	0.044468051	1.492019483	0.17215349	0.389224	-5.27898516
WT after UVB irradiation VS WT before UVB irradiation	Amyotrophic lateral sclerosis (ALS) - Homo sapiens (human)|KEGG	0.159082677	-0.01205419	0.688178443	0.50686469	0.82465997	-5.63512344
homo after UVB irradiation VS homo before UVB irradiation	Amyotrophic lateral sclerosis (ALS)|Wikipathways	0.483368641	-0.00044636	2.819554844	0.01927111	0.08847577	-3.30729487
hetero after UVB irradiation VS hetero before UVB irradiation	Amyotrophic lateral sclerosis (ALS)|Wikipathways	0.421995789	0.028168218	2.743609401	0.02411545	0.13715811	-3.46309515
WT after UVB irradiation VS WT before UVB irradiation	Amyotrophic lateral sclerosis (ALS)|Wikipathways	0.315219181	-0.02252018	1.445841535	0.17858027	0.59314626	-4.91425358
homo after UVB irradiation VS homo before UVB irradiation	anandamide degradation|HumanCyc	0.113112989	0.036675779	0.278813689	0.78643162	0.86380726	-6.32685654
hetero after UVB irradiation VS hetero before UVB irradiation	anandamide degradation|HumanCyc	-0.23021344	0.038610438	-0.54445974	0.6002338	0.7885114	-6.18857302
WT after UVB irradiation VS WT before UVB irradiation	anandamide degradation|HumanCyc	0.258962078	0.027243889	0.611614059	0.55433311	0.84585342	-5.68334672
homo after UVB irradiation VS homo before UVB irradiation	Anchoring fibril formation|Reactome	1.023987622	-0.0091455	6.016745757	0.00016712	0.01666204	1.292434976
hetero after UVB irradiation VS hetero before UVB irradiation	Anchoring fibril formation|Reactome	0.713447849	-0.03699574	2.939733757	0.01769227	0.11721131	-3.16304187
WT after UVB irradiation VS WT before UVB irradiation	Anchoring fibril formation|Reactome	0.705631635	0.031677132	2.99418253	0.01336089	0.28007727	-2.71680031
homo after UVB irradiation VS homo before UVB irradiation	Anchoring of the basal body to the plasma membrane|Reactome	-0.42792983	0.00753746	-2.37738242	0.04031423	0.13348423	-4.00994345
hetero after UVB irradiation VS hetero before UVB irradiation	Anchoring of the basal body to the plasma membrane|Reactome	-0.08941839	-0.01225846	-0.38201313	0.71190668	0.85775063	-6.26807081
WT after UVB irradiation VS WT before UVB irradiation	Anchoring of the basal body to the plasma membrane|Reactome	-0.19582223	-0.04303976	-0.81494077	0.43393437	0.79685269	-5.54415144
homo after UVB irradiation VS homo before UVB irradiation	Androgen and estrogen biosynthesis and metabolism|EHMN	0.147368964	0.07059208	0.951273886	0.36530927	0.51898012	-5.90488731
hetero after UVB irradiation VS hetero before UVB irradiation	Androgen and estrogen biosynthesis and metabolism|EHMN	0.154008126	0.035582085	0.892067419	0.39711579	0.62480282	-5.93459484
WT after UVB irradiation VS WT before UVB irradiation	Androgen and estrogen biosynthesis and metabolism|EHMN	-0.01200933	0.033486324	-0.05470819	0.95743987	0.98742794	-5.8676352
homo after UVB irradiation VS homo before UVB irradiation	Androgen and Estrogen Metabolism|SMPDB	0.247630977	-0.00081892	1.200495598	0.25935762	0.42308928	-5.64961013
hetero after UVB irradiation VS hetero before UVB irradiation	Androgen and Estrogen Metabolism|SMPDB	0.017963989	0.013614493	0.092630718	0.92836166	0.97054702	-6.34188888
WT after UVB irradiation VS WT before UVB irradiation	Androgen and Estrogen Metabolism|SMPDB	-0.31598762	0.004101129	-1.31107214	0.21890754	0.6260768	-5.07027776
homo after UVB irradiation VS homo before UVB irradiation	androgen biosynthesis|HumanCyc	0.304929005	-0.0352052	1.701024308	0.12175091	0.26055457	-5.01895383
hetero after UVB irradiation VS hetero before UVB irradiation	androgen biosynthesis|HumanCyc	-0.28652602	0.067189119	-1.07073345	0.31399824	0.54335762	-5.76457882
WT after UVB irradiation VS WT before UVB irradiation	androgen biosynthesis|HumanCyc	-0.53706882	0.131448635	-1.86944807	0.09084889	0.48188954	-4.36833708
homo after UVB irradiation VS homo before UVB irradiation	Androgen biosynthesis|Reactome	0.138258318	-0.03590792	0.786042151	0.45122358	0.59996706	-6.0472343
hetero after UVB irradiation VS hetero before UVB irradiation	Androgen biosynthesis|Reactome	-0.36212168	-0.03050032	-1.66146808	0.13328223	0.33088258	-5.05722038
WT after UVB irradiation VS WT before UVB irradiation	Androgen biosynthesis|Reactome	-0.18783298	0.024690146	-0.6817137	0.51077569	0.82661679	-5.63939482
homo after UVB irradiation VS homo before UVB irradiation	Androgen Receptor Network in Prostate Cancer|Wikipathways	0.060578792	-0.01299577	0.35085201	0.73344318	0.82411885	-6.30274956
hetero after UVB irradiation VS hetero before UVB irradiation	Androgen Receptor Network in Prostate Cancer|Wikipathways	0.18421535	0.002503423	1.211504034	0.25858071	0.48448405	-5.61438303
WT after UVB irradiation VS WT before UVB irradiation	Androgen Receptor Network in Prostate Cancer|Wikipathways	-0.12136398	0.005699774	-0.60009782	0.56168574	0.85017866	-5.69014823
homo after UVB irradiation VS homo before UVB irradiation	Androgen receptor signaling pathway|Wikipathways	-0.13246277	0.024965478	-0.65920074	0.5256027	0.66255701	-6.14035677
hetero after UVB irradiation VS hetero before UVB irradiation	Androgen receptor signaling pathway|Wikipathways	0.047953505	-0.02848482	0.265716962	0.79684342	0.91440391	-6.30840248
WT after UVB irradiation VS WT before UVB irradiation	Androgen receptor signaling pathway|Wikipathways	-0.10957743	-0.02911406	-0.47578741	0.64436717	0.87766327	-5.75583882
homo after UVB irradiation VS homo before UVB irradiation	AndrogenReceptor|NetPath	-0.06817367	0.001014583	-0.34575115	0.73715067	0.82682223	-6.3046337
hetero after UVB irradiation VS hetero before UVB irradiation	AndrogenReceptor|NetPath	0.074354009	-0.01977494	0.444758954	0.66769748	0.82651398	-6.24050742
WT after UVB irradiation VS WT before UVB irradiation	AndrogenReceptor|NetPath	0.06819961	-0.01219963	0.309396745	0.76331938	0.92696825	-5.82090327
homo after UVB irradiation VS homo before UVB irradiation	Angiogenesis overview|Wikipathways	0.4727086	-0.03034826	2.651060708	0.0255271	0.10578915	-3.57669149
hetero after UVB irradiation VS hetero before UVB irradiation	Angiogenesis overview|Wikipathways	0.419856407	0.03607223	2.600343803	0.03028098	0.1515046	-3.68230793
WT after UVB irradiation VS WT before UVB irradiation	Angiogenesis overview|Wikipathways	0.408643567	-0.00025245	2.053165835	0.06692424	0.43925282	-4.11184713
homo after UVB irradiation VS homo before UVB irradiation	Angiogenesis|Wikipathways	0.345428601	-0.02432889	1.831767392	0.09883838	0.22792729	-4.8344896
hetero after UVB irradiation VS hetero before UVB irradiation	Angiogenesis|Wikipathways	0.41522684	0.027314422	2.67342082	0.02695783	0.14262729	-3.57054002
WT after UVB irradiation VS WT before UVB irradiation	Angiogenesis|Wikipathways	0.266295029	-0.030354	1.320109636	0.21598347	0.62581393	-5.06013253
homo after UVB irradiation VS homo before UVB irradiation	Angiopoietin Like Protein 8 Regulatory Pathway|Wikipathways	0.082771487	-0.00404338	0.501419065	0.62762205	0.74068811	-6.23518627
hetero after UVB irradiation VS hetero before UVB irradiation	Angiopoietin Like Protein 8 Regulatory Pathway|Wikipathways	-0.02063247	-0.01559128	-0.12613326	0.90258447	0.97054702	-6.33791879
WT after UVB irradiation VS WT before UVB irradiation	Angiopoietin Like Protein 8 Regulatory Pathway|Wikipathways	-0.00094844	-0.00436698	-0.00483078	0.99623986	0.99863683	-5.86913937
homo after UVB irradiation VS homo before UVB irradiation	Angiopoietin receptor Tie2-mediated signaling|PID	0.54449026	-0.0199234	3.072989739	0.01265475	0.06944903	-2.90131329
hetero after UVB irradiation VS hetero before UVB irradiation	Angiopoietin receptor Tie2-mediated signaling|PID	0.571336078	0.033545298	2.85380563	0.02025696	0.12579184	-3.29441261
WT after UVB irradiation VS WT before UVB irradiation	Angiopoietin receptor Tie2-mediated signaling|PID	0.427789837	-0.03520038	1.791181582	0.10328218	0.50425697	-4.47451119
homo after UVB irradiation VS homo before UVB irradiation	angiotensin ii mediated activation of jnk pathway via pyk2 dependent signaling|BioCarta	0.303898834	-0.01869014	1.538967933	0.15680291	0.30427628	-5.23759453
hetero after UVB irradiation VS hetero before UVB irradiation	angiotensin ii mediated activation of jnk pathway via pyk2 dependent signaling|BioCarta	0.397652244	0.020961911	2.427817748	0.03986802	0.17507375	-3.9452111
WT after UVB irradiation VS WT before UVB irradiation	angiotensin ii mediated activation of jnk pathway via pyk2 dependent signaling|BioCarta	0.142286696	0.042860741	0.702258529	0.49840978	0.82026576	-5.62569467
homo after UVB irradiation VS homo before UVB irradiation	Angiotensin Metabolism|SMPDB	0.636038586	-0.11800708	2.436891056	0.03650532	0.12504267	-3.91641947
hetero after UVB irradiation VS hetero before UVB irradiation	Angiotensin Metabolism|SMPDB	-0.02777369	0.003323189	-0.09172332	0.92906122	0.97054702	-6.34197953
WT after UVB irradiation VS WT before UVB irradiation	Angiotensin Metabolism|SMPDB	0.270438532	-0.10210744	0.707878218	0.49505953	0.81710765	-5.62188349
homo after UVB irradiation VS homo before UVB irradiation	Anileridine Action Pathway|SMPDB	0.191132361	0.029768717	1.061313976	0.31508276	0.46923284	-5.79783739
hetero after UVB irradiation VS hetero before UVB irradiation	Anileridine Action Pathway|SMPDB	-0.07904315	0.055005373	-0.44437398	0.66796482	0.82651398	-6.24068873
WT after UVB irradiation VS WT before UVB irradiation	Anileridine Action Pathway|SMPDB	0.022316377	0.035038618	0.098344171	0.92358676	0.97566251	-5.86425414
homo after UVB irradiation VS homo before UVB irradiation	Anistreplase Action Pathway|SMPDB	0.538210712	0.026444726	3.007777411	0.01409598	0.07305822	-3.00571236
hetero after UVB irradiation VS hetero before UVB irradiation	Anistreplase Action Pathway|SMPDB	0.558085555	0.09913938	2.984394118	0.01649409	0.11352943	-3.09486545
WT after UVB irradiation VS WT before UVB irradiation	Anistreplase Action Pathway|SMPDB	0.440879848	0.077377741	1.569633667	0.14732454	0.55622203	-4.76265269
homo after UVB irradiation VS homo before UVB irradiation	Antagonism of Activin by Follistatin|Reactome	0.317875416	-0.04543619	0.929446144	0.37593495	0.52913655	-5.92499143
hetero after UVB irradiation VS hetero before UVB irradiation	Antagonism of Activin by Follistatin|Reactome	0.21205152	0.046599364	0.611846819	0.55678453	0.76066987	-6.14796821
WT after UVB irradiation VS WT before UVB irradiation	Antagonism of Activin by Follistatin|Reactome	0.130670081	0.033985245	0.635769764	0.53908898	0.83903241	-5.66869375
homo after UVB irradiation VS homo before UVB irradiation	anthrax toxin mechanism of action|BioCarta	0.400717301	0.056008434	1.328488111	0.21539948	0.37334177	-5.50176835
hetero after UVB irradiation VS hetero before UVB irradiation	anthrax toxin mechanism of action|BioCarta	0.853320816	-0.01733944	4.372105947	0.00209677	0.05083544	-1.07601916
WT after UVB irradiation VS WT before UVB irradiation	anthrax toxin mechanism of action|BioCarta	0.792223988	-0.03701631	2.552464286	0.02857346	0.35176669	-3.38036856
homo after UVB irradiation VS homo before UVB irradiation	Antiarrhythmic Pathway_ Pharmacodynamics|PharmGKB	0.513242783	-0.04790767	3.095527859	0.01219256	0.06780904	-2.86525973
hetero after UVB irradiation VS hetero before UVB irradiation	Antiarrhythmic Pathway_ Pharmacodynamics|PharmGKB	0.135621887	0.010797898	0.652569624	0.53143043	0.74554014	-6.121339
WT after UVB irradiation VS WT before UVB irradiation	Antiarrhythmic Pathway_ Pharmacodynamics|PharmGKB	0.155700748	0.00391551	0.603874908	0.55926824	0.84911968	-5.68793064
WT after UVB irradiation VS WT before UVB irradiation	Anti-diabetic Drug Nateglinide Pathway_ Pharmacokinetics|PharmGKB	-0.4214302	0.046401692	-1.39621666	0.19262818	0.60496032	-4.97285911
homo after UVB irradiation VS homo before UVB irradiation	Anti-diabetic Drug Potassium Channel Inhibitors Pathway_ Pharmacodynamics|PharmGKB	0.524211823	0.021679317	3.030095453	0.01358471	0.07305822	-2.96997155
hetero after UVB irradiation VS hetero before UVB irradiation	Anti-diabetic Drug Potassium Channel Inhibitors Pathway_ Pharmacodynamics|PharmGKB	0.240631596	0.050617244	1.156619997	0.27916467	0.5098009	-5.67452899
WT after UVB irradiation VS WT before UVB irradiation	Anti-diabetic Drug Potassium Channel Inhibitors Pathway_ Pharmacodynamics|PharmGKB	0.142148532	0.018756412	0.546493472	0.59661261	0.86530281	-5.72022295
homo after UVB irradiation VS homo before UVB irradiation	Anti-diabetic Drug Repaglinide Pathway_ Pharmacokinetics|PharmGKB	0.415217031	0.065958416	1.47224244	0.17366456	0.32484736	-5.32395015
homo after UVB irradiation VS homo before UVB irradiation	Antigen activates B Cell Receptor (BCR) leading to generation of second messengers|Reactome	0.701048807	-0.01637759	3.899698113	0.0033391	0.03256113	-1.60272284

hetero after UVB irradiation VS hetero before UVB irradiation	Antigen activates B Cell Receptor (BCR) leading to generation of second messengers|Reactome	0.619406514	0.019416807	3.226369689	0.01131634	0.10504986	-2.72743474
WT after UVB irradiation VS WT before UVB irradiation	Antigen activates B Cell Receptor (BCR) leading to generation of second messengers|Reactome	0.579406649	-0.00553714	2.380891722	0.0383633	0.39022568	-3.63566364
homo after UVB irradiation VS homo before UVB irradiation	Antigen Presentation: Folding_ assembly and peptide loading of class I MHC|Reactome	0.693891226	-0.01623432	3.461478919	0.00670468	0.04735641	-2.28361845
hetero after UVB irradiation VS hetero before UVB irradiation	Antigen Presentation: Folding_ assembly and peptide loading of class I MHC|Reactome	0.494423429	0.012778855	1.9760242	0.0817425	0.25784242	-4.61729101
WT after UVB irradiation VS WT before UVB irradiation	Antigen Presentation: Folding_ assembly and peptide loading of class I MHC|Reactome	0.504912957	-0.04584988	2.137809897	0.05803091	0.42472304	-3.990794
homo after UVB irradiation VS homo before UVB irradiation	Antigen processing and presentation - Homo sapiens (human)|KEGG	0.203355574	-0.03771948	0.820357027	0.43234535	0.58335959	-6.01957586
hetero after UVB irradiation VS hetero before UVB irradiation	Antigen processing and presentation - Homo sapiens (human)|KEGG	0.138202765	0.008712183	0.685274444	0.51157953	0.73027858	-6.0988362
WT after UVB irradiation VS WT before UVB irradiation	Antigen processing and presentation - Homo sapiens (human)|KEGG	0.348522259	-0.00181789	1.540472794	0.15422133	0.55854784	-4.79902658
homo after UVB irradiation VS homo before UVB irradiation	antigen processing and presentation|BioCarta	0.394060582	-0.09824762	1.670244162	0.12780798	0.26806213	-5.06138575
hetero after UVB irradiation VS hetero before UVB irradiation	antigen processing and presentation|BioCarta	0.11450795	0.059999553	0.337619733	0.74390739	0.87687018	-6.28513076
WT after UVB irradiation VS WT before UVB irradiation	antigen processing and presentation|BioCarta	0.141001007	-0.01015592	0.446441481	0.66471422	0.87935805	-5.76924358
homo after UVB irradiation VS homo before UVB irradiation	Antigen processing: Ubiquitination & Proteasome degradation|Reactome	-0.11339067	-0.00494276	-0.65686372	0.52703934	0.66330255	-6.14193439
hetero after UVB irradiation VS hetero before UVB irradiation	Antigen processing: Ubiquitination & Proteasome degradation|Reactome	0.005979844	-0.01626659	0.03870097	0.97003011	0.98614433	-6.34572884
WT after UVB irradiation VS WT before UVB irradiation	Antigen processing: Ubiquitination & Proteasome degradation|Reactome	-0.1840293	-0.00999085	-0.89076779	0.39381385	0.76452505	-5.48338879
homo after UVB irradiation VS homo before UVB irradiation	Antigen processing-Cross presentation|Reactome	0.944274364	-0.02516461	5.883953663	0.00019796	0.01666204	1.131903556
hetero after UVB irradiation VS hetero before UVB irradiation	Antigen processing-Cross presentation|Reactome	0.715432569	0.053266179	3.358825772	0.0092314	0.09725903	-2.52822416
WT after UVB irradiation VS WT before UVB irradiation	Antigen processing-Cross presentation|Reactome	0.653438678	0.008141662	2.49495642	0.0315437	0.36549871	-3.46624064
homo after UVB irradiation VS homo before UVB irradiation	Antimetabolite Pathway - Folate Cycle_ Pharmacodynamics|PharmGKB	-0.46594591	-0.0028833	-2.05154708	0.069149	0.18442425	-4.51122389
hetero after UVB irradiation VS hetero before UVB irradiation	Antimetabolite Pathway - Folate Cycle_ Pharmacodynamics|PharmGKB	-0.07709368	-0.02903186	-0.35425085	0.73185326	0.86896773	-6.27897858
WT after UVB irradiation VS WT before UVB irradiation	Antimetabolite Pathway - Folate Cycle_ Pharmacodynamics|PharmGKB	0.001217055	-0.02068426	0.003602533	0.99719589	0.99863683	-5.86914462
homo after UVB irradiation VS homo before UVB irradiation	Antimicrobial peptides|Reactome	0.460669491	0.048046789	2.788351818	0.02029973	0.09100843	-3.3572603
hetero after UVB irradiation VS hetero before UVB irradiation	Antimicrobial peptides|Reactome	0.666475099	0.043412929	3.923875666	0.00396889	0.06957772	-1.7006284
WT after UVB irradiation VS WT before UVB irradiation	Antimicrobial peptides|Reactome	0.507297934	0.07208888	2.36757666	0.03924801	0.39022568	-3.65534662
homo after UVB irradiation VS homo before UVB irradiation	Antipyrine Action Pathway|SMPDB	0.40423582	0.045705158	1.979500697	0.07780079	0.19626432	-4.61879689
hetero after UVB irradiation VS hetero before UVB irradiation	Antipyrine Action Pathway|SMPDB	0.482496143	0.064876536	3.04499191	0.01500113	0.1130168	-3.00250826
WT after UVB irradiation VS WT before UVB irradiation	Antipyrine Action Pathway|SMPDB	0.275725339	0.086595857	1.261970114	0.23537138	0.62952955	-5.12455679
homo after UVB irradiation VS homo before UVB irradiation	antisense pathway|BioCarta	-0.50831127	-0.02401801	-1.77068095	0.10899819	0.24253692	-4.92149422
hetero after UVB irradiation VS hetero before UVB irradiation	antisense pathway|BioCarta	0.155952601	-0.01726007	0.418097293	0.6863286	0.8402594	-6.25270955
WT after UVB irradiation VS WT before UVB irradiation	antisense pathway|BioCarta	-0.01997113	-0.00582358	-0.07395708	0.9424913	0.98295421	-5.8663811
homo after UVB irradiation VS homo before UVB irradiation	Antiviral mechanism by IFN-stimulated genes|Reactome	0.347607886	-0.02335997	1.540015468	0.1565501	0.30417351	-5.2362208
hetero after UVB irradiation VS hetero before UVB irradiation	Antiviral mechanism by IFN-stimulated genes|Reactome	0.620930005	0.036520012	3.181568961	0.01212854	0.10610726	-2.7951576
WT after UVB irradiation VS WT before UVB irradiation	Antiviral mechanism by IFN-stimulated genes|Reactome	0.325530443	-0.03890634	1.479128812	0.16965727	0.57650651	-4.87422676
homo after UVB irradiation VS homo before UVB irradiation	Antrafenine Action Pathway|SMPDB	0.40423582	0.045705158	1.979500697	0.07780079	0.19626432	-4.61879689
hetero after UVB irradiation VS hetero before UVB irradiation	Antrafenine Action Pathway|SMPDB	0.482496143	0.064876536	3.04499191	0.01500113	0.1130168	-3.00250826
WT after UVB irradiation VS WT before UVB irradiation	Antrafenine Action Pathway|SMPDB	0.275725339	0.086595857	1.261970114	0.23537138	0.62952955	-5.12455679
homo after UVB irradiation VS homo before UVB irradiation	AOP CYP2E1 Activation Leading To Neurodegeneration|Wikipathways	0.170095981	-0.00946448	0.594338862	0.56632937	0.69195165	-6.18223422
hetero after UVB irradiation VS hetero before UVB irradiation	AOP CYP2E1 Activation Leading To Neurodegeneration|Wikipathways	-0.17662474	-0.0625179	-0.59977066	0.56443603	0.76437184	-6.15556455
WT after UVB irradiation VS WT before UVB irradiation	AOP CYP2E1 Activation Leading To Neurodegeneration|Wikipathways	-0.0865199	-0.12864847	-0.25571751	0.80331043	0.94525798	-5.83613948
homo after UVB irradiation VS homo before UVB irradiation	AP-1 transcription factor network|PID	0.067971973	0.015987023	0.410732703	0.69048425	0.79254678	-6.2786318
hetero after UVB irradiation VS hetero before UVB irradiation	AP-1 transcription factor network|PID	0.070354047	0.056426654	0.405688268	0.69507878	0.84665128	-6.25814238
WT after UVB irradiation VS WT before UVB irradiation	AP-1 transcription factor network|PID	0.039834194	-0.02349931	0.178968876	0.86150751	0.95916804	-5.85295273
homo after UVB irradiation VS homo before UVB irradiation	APC/C:Cdc20 mediated degradation of Cyclin B|Reactome	-0.54219941	0.042746614	-2.62672637	0.02658614	0.10800029	-3.61548345
hetero after UVB irradiation VS hetero before UVB irradiation	APC/C:Cdc20 mediated degradation of Cyclin B|Reactome	0.161033863	-0.01700247	0.822486553	0.43345716	0.65966535	-5.99395958
WT after UVB irradiation VS WT before UVB irradiation	APC/C:Cdc20 mediated degradation of Cyclin B|Reactome	-0.16760928	-0.08461017	-0.68895793	0.50639435	0.82465997	-5.63460595
homo after UVB irradiation VS homo before UVB irradiation	APC/C:Cdc20 mediated degradation of mitotic proteins|Reactome	-0.57883214	0.061699837	-3.02080775	0.01379513	0.07305822	-2.9848439
hetero after UVB irradiation VS hetero before UVB irradiation	APC/C:Cdc20 mediated degradation of mitotic proteins|Reactome	0.2559365	0.012842332	1.19151846	0.26592788	0.49392798	-5.63651167
WT after UVB irradiation VS WT before UVB irradiation	APC/C:Cdc20 mediated degradation of mitotic proteins|Reactome	-0.05969582	-0.0607564	-0.22823471	0.82402618	0.94525798	-5.84283531
homo after UVB irradiation VS homo before UVB irradiation	APC/C:Cdc20 mediated degradation of Securin|Reactome	-0.4761211	0.035010848	-2.33527641	0.0432433	0.13470334	-4.07581892
hetero after UVB irradiation VS hetero before UVB irradiation	APC/C:Cdc20 mediated degradation of Securin|Reactome	0.170577794	-0.01141657	0.934660083	0.37597187	0.60404776	-5.89630412
WT after UVB irradiation VS WT before UVB irradiation	APC/C:Cdc20 mediated degradation of Securin|Reactome	-0.13143027	-0.07279877	-0.56320205	0.58560372	0.86119304	-5.71113003
homo after UVB irradiation VS homo before UVB irradiation	APC/C:Cdh1 mediated degradation of Cdc20 and other APC/C:Cdh1 targeted proteins in late mitosis/early G1|Reactome	-0.4644629	0.026691773	-2.21309136	0.05297426	0.15396594	-4.26532845
hetero after UVB irradiation VS hetero before UVB irradiation	APC/C:Cdh1 mediated degradation of Cdc20 and other APC/C:Cdh1 targeted proteins in late mitosis/early G1|Reactome	0.309093999	-0.00990795	1.563440644	0.15467268	0.36486693	-5.18705287
WT after UVB irradiation VS WT before UVB irradiation	APC/C:Cdh1 mediated degradation of Cdc20 and other APC/C:Cdh1 targeted proteins in late mitosis/early G1|Reactome	-0.03856291	-0.0659021	-0.15560795	0.87941347	0.96754549	-5.85690042
homo after UVB irradiation VS homo before UVB irradiation	APC/C-mediated degradation of cell cycle proteins|Reactome	-0.58568333	0.049850163	-3.25100941	0.00944274	0.0581856	-2.617124
hetero after UVB irradiation VS hetero before UVB irradiation	APC/C-mediated degradation of cell cycle proteins|Reactome	0.217623595	0.003438561	0.957035644	0.36519933	0.59333249	-5.87561187
WT after UVB irradiation VS WT before UVB irradiation	APC/C-mediated degradation of cell cycle proteins|Reactome	-0.09434864	-0.05460128	-0.34701733	0.73571261	0.91453443	-5.80853609
homo after UVB irradiation VS homo before UVB irradiation	APC:Cdc20 mediated degradation of cell cycle proteins prior to satisfation of the cell cycle checkpoint|Reactome	-0.56455307	0.068296773	-2.99861953	0.01431144	0.07367082	-3.02038093
hetero after UVB irradiation VS hetero before UVB irradiation	APC:Cdc20 mediated degradation of cell cycle proteins prior to satisfation of the cell cycle checkpoint|Reactome	0.234264859	0.005523284	1.151479983	0.28115846	0.51114365	-5.68006023
WT after UVB irradiation VS WT before UVB irradiation	APC:Cdc20 mediated degradation of cell cycle proteins prior to satisfation of the cell cycle checkpoint|Reactome	-0.08668212	-0.05740992	-0.34025502	0.74064732	0.91734632	-5.81086113
homo after UVB irradiation VS homo before UVB irradiation	APC-Cdc20 mediated degradation of Nek2A|Reactome	-0.58494532	0.048323465	-2.95451193	0.0153973	0.07691241	-3.09104738
hetero after UVB irradiation VS hetero before UVB irradiation	APC-Cdc20 mediated degradation of Nek2A|Reactome	0.170995022	-0.01597034	0.884760945	0.40082702	0.62933846	-5.94101639
WT after UVB irradiation VS WT before UVB irradiation	APC-Cdc20 mediated degradation of Nek2A|Reactome	-0.14334472	-0.07154078	-0.56217552	0.58627692	0.86138389	-5.71169606
homo after UVB irradiation VS homo before UVB irradiation	Apelin signaling pathway - Homo sapiens (human)|KEGG	0.405900444	-0.03575575	2.543026366	0.03057637	0.11634307	-3.74858192
hetero after UVB irradiation VS hetero before UVB irradiation	Apelin signaling pathway - Homo sapiens (human)|KEGG	0.243837575	0.003910899	1.29334943	0.23021884	0.45170905	-5.52115534
WT after UVB irradiation VS WT before UVB irradiation	Apelin signaling pathway - Homo sapiens (human)|KEGG	0.229546208	-0.01241257	1.072429667	0.30852533	0.69398681	-5.31981844
homo after UVB irradiation VS homo before UVB irradiation	APEX1-Independent Resolution of AP Sites via the Single Nucleotide Replacement Pathway|Reactome	-0.50642807	-0.05012937	-1.83038773	0.09905782	0.22820282	-4.83646965
hetero after UVB irradiation VS hetero before UVB irradiation	APEX1-Independent Resolution of AP Sites via the Single Nucleotide Replacement Pathway|Reactome	-0.41082947	-0.10413045	-1.17749185	0.27118546	0.50063318	-5.65188804
WT after UVB irradiation VS WT before UVB irradiation	APEX1-Independent Resolution of AP Sites via the Single Nucleotide Replacement Pathway|Reactome	-0.18857867	0.018651054	-0.72675703	0.48390688	0.81670462	-5.60888099
homo after UVB irradiation VS homo before UVB irradiation	APOBEC3G mediated resistance to HIV-1 infection|Reactome	-0.50498562	-0.06419899	-1.60309184	0.14197759	0.28595927	-5.15252116
hetero after UVB irradiation VS hetero before UVB irradiation	APOBEC3G mediated resistance to HIV-1 infection|Reactome	-0.15841461	-0.07959004	-0.49831085	0.63101082	0.81063904	-6.21383741
WT after UVB irradiation VS WT before UVB irradiation	APOBEC3G mediated resistance to HIV-1 infection|Reactome	0.046888648	-0.04340228	0.124869045	0.90308189	0.97566251	-5.86125879
homo after UVB irradiation VS homo before UVB irradiation	ApoE and miR-146 in inflammation and atherosclerosis|Wikipathways	0.674474849	-0.0471286	3.476429777	0.00654469	0.04701822	-2.26006147
hetero after UVB irradiation VS hetero before UVB irradiation	ApoE and miR-146 in inflammation and atherosclerosis|Wikipathways	0.539283191	0.029357896	2.945205291	0.01754077	0.11671652	-3.15468492
WT after UVB irradiation VS WT before UVB irradiation	ApoE and miR-146 in inflammation and atherosclerosis|Wikipathways	0.525294792	-0.06304729	1.919644342	0.08361989	0.470709	-4.29920764
homo after UVB irradiation VS homo before UVB irradiation	Apoptosis - Homo sapiens (human)|KEGG	0.406796563	-0.03742578	2.141962884	0.05958743	0.16702037	-4.3743281
hetero after UVB irradiation VS hetero before UVB irradiation	Apoptosis - Homo sapiens (human)|KEGG	0.477201384	-0.0089949	2.988796476	0.01638061	0.11352943	-3.08814969
WT after UVB irradiation VS WT before UVB irradiation	Apoptosis - Homo sapiens (human)|KEGG	0.382966722	-0.02477982	1.882597706	0.08890101	0.48106209	-4.35030254
homo after UVB irradiation VS homo before UVB irradiation	Apoptosis - multiple species - Homo sapiens (human)|KEGG	0.191414218	-0.03621467	0.987579825	0.34812342	0.50413306	-5.87060636
hetero after UVB irradiation VS hetero before UVB irradiation	Apoptosis - multiple species - Homo sapiens (human)|KEGG	0.295014565	-0.03013905	1.705285234	0.12462497	0.31649308	-4.9979335
WT after UVB irradiation VS WT before UVB irradiation	Apoptosis - multiple species - Homo sapiens (human)|KEGG	0.444542687	-0.04750966	1.942844457	0.08046314	0.46574799	-4.26700231
homo after UVB irradiation VS homo before UVB irradiation	Apoptosis induced DNA fragmentation|Reactome	-0.20152655	-0.03668943	-0.92803906	0.37662748	0.52945846	-5.9262742
hetero after UVB irradiation VS hetero before UVB irradiation	Apoptosis induced DNA fragmentation|Reactome	0.114619369	-0.02256632	0.569157729	0.58409845	0.77618681	-6.17419784
WT after UVB irradiation VS WT before UVB irradiation	Apoptosis induced DNA fragmentation|Reactome	0.043030214	-0.04456098	0.172899872	0.8661519	0.96152545	-5.85403099
homo after UVB irradiation VS homo before UVB irradiation	Apoptosis Modulation and Signaling|Wikipathways	0.461139623	-0.03589413	2.647924892	0.02566116	0.10578915	-3.58169248
hetero after UVB irradiation VS hetero before UVB irradiation	Apoptosis Modulation and Signaling|Wikipathways	0.432900722	-0.00902359	2.374073033	0.04343768	0.18293959	-4.02666378
WT after UVB irradiation VS WT before UVB irradiation	Apoptosis Modulation and Signaling|Wikipathways	0.438856687	-0.02411382	2.059956323	0.06616548	0.43803373	-4.10219605
homo after UVB irradiation VS homo before UVB irradiation	Apoptosis Modulation by HSP70|Wikipathways	0.471032546	-0.00395247	2.685731835	0.02409097	0.10226192	-3.52135977
hetero after UVB irradiation VS hetero before UVB irradiation	Apoptosis Modulation by HSP70|Wikipathways	0.405910137	-0.03360994	1.912242879	0.09036062	0.27214648	-4.70892593
WT after UVB irradiation VS WT before UVB irradiation	Apoptosis Modulation by HSP70|Wikipathways	0.605793923	-0.02797068	2.832530402	0.01764026	0.3051632	-2.95982363
homo after UVB irradiation VS homo before UVB irradiation	Apoptosis|Reactome	0.241191875	-0.04282777	1.219979624	0.25222829	0.4151674	-5.62778811
hetero after UVB irradiation VS hetero before UVB irradiation	Apoptosis|Reactome	0.28752115	-0.00534934	1.91557178	0.08989016	0.27169717	-4.70417003
WT after UVB irradiation VS WT before UVB irradiation	Apoptosis|Reactome	0.225286696	-0.02318676	1.167709851	0.2697964	0.66316477	-5.22460063
homo after UVB irradiation VS homo before UVB irradiation	Apoptosis|Wikipathways	0.344063687	-0.01223187	1.784154908	0.10667767	0.23957183	-4.90242266
hetero after UVB irradiation VS hetero before UVB irradiation	Apoptosis|Wikipathways	0.429960987	-0.04053313	2.628624368	0.02894819	0.14775569	-3.63907194
WT after UVB irradiation VS WT before UVB irradiation	Apoptosis|Wikipathways	0.375333241	-0.0261748	1.777104451	0.10567776	0.50446894	-4.49338562
homo after UVB irradiation VS homo before UVB irradiation	Apoptosis-related network due to altered Notch3 in ovarian cancer|Wikipathways	0.015674909	-0.00672202	0.071092881	0.94481517	0.96640369	-6.36573336
hetero after UVB irradiation VS hetero before UVB irradiation	Apoptosis-related network due to altered Notch3 in ovarian cancer|Wikipathways	0.124500068	-0.01103188	0.807256344	0.44170858	0.66732109	-6.00641182
WT after UVB irradiation VS WT before UVB irradiation	Apoptosis-related network due to altered Notch3 in ovarian cancer|Wikipathways	0.124850133	-0.0415295	0.564321593	0.58486998	0.8608125	-5.71051162
homo after UVB irradiation VS homo before UVB irradiation	Apoptotic cleavage of cell adhesion proteins|Reactome	0.031656059	0.037767235	0.114762022	0.91105061	0.94787581	-6.36137687
hetero after UVB irradiation VS hetero before UVB irradiation	Apoptotic cleavage of cell adhesion proteins|Reactome	0.121273477	-0.00275826	0.360404183	0.72741298	0.86703671	-6.27662963
WT after UVB irradiation VS WT before UVB irradiation	Apoptotic cleavage of cell adhesion proteins|Reactome	0.049698396	0.102881183	0.139685912	0.89165921	0.97397725	-5.85927671
homo after UVB irradiation VS homo before UVB irradiation	Apoptotic cleavage of cellular proteins|Reactome	0.256070634	-0.03815665	1.166622407	0.27213811	0.4359939	-5.68693795
hetero after UVB irradiation VS hetero before UVB irradiation	Apoptotic cleavage of cellular proteins|Reactome	0.091966867	-0.00435248	0.489200123	0.63718039	0.81534761	-6.21857683
WT after UVB irradiation VS WT before UVB irradiation	Apoptotic cleavage of cellular proteins|Reactome	0.160697437	0.009048035	0.699256309	0.50020529	0.82085432	-5.62771954
homo after UVB irradiation VS homo before UVB irradiation	apoptotic dna-fragmentation and tissue homeostasis|BioCarta	0.283888231	-0.00332297	1.442632949	0.18163993	0.33536915	-5.36152294
hetero after UVB irradiation VS hetero before UVB irradiation	apoptotic dna-fragmentation and tissue homeostasis|BioCarta	0.304856552	-0.01938603	1.709561301	0.12380847	0.31597198	-4.99210881
WT after UVB irradiation VS WT before UVB irradiation	apoptotic dna-fragmentation and tissue homeostasis|BioCarta	0.263555451	-0.03215081	1.184863316	0.26324901	0.65841086	-5.20681516
homo after UVB irradiation VS homo before UVB irradiation	Apoptotic execution phase|Reactome	0.110942103	-0.03217077	0.539157119	0.60232689	0.72184372	-6.21469565
hetero after UVB irradiation VS hetero before UVB irradiation	Apoptotic execution phase|Reactome	0.064504516	-0.03307544	0.365768204	0.72355111	0.86482318	-6.27455003
WT after UVB irradiation VS WT before UVB irradiation	Apoptotic execution phase|Reactome	0.115445939	0.005187911	0.518267115	0.61545418	0.87257466	-5.73499924
homo after UVB irradiation VS homo before UVB irradiation	Apoptotic factor-mediated response|Reactome	0.070385393	-0.03899006	0.199378598	0.84621774	0.90528768	-6.34713573
hetero after UVB irradiation VS hetero before UVB irradiation	Apoptotic factor-mediated response|Reactome	0.338734224	0.044128543	1.494641658	0.17148184	0.38828327	-5.27565224
WT after UVB irradiation VS WT before UVB irradiation	Apoptotic factor-mediated response|Reactome	0.3794619	-0.11213634	1.424359755	0.18455014	0.59839062	-4.93978241
homo after UVB irradiation VS homo before UVB irradiation	apoptotic signaling in response to dna damage|BioCarta	0.468642057	-0.04412894	2.292670669	0.04641961	0.14027264	-4.14219586
hetero after UVB irradiation VS hetero before UVB irradiation	apoptotic signaling in response to dna damage|BioCarta	0.56213671	-0.02170167	3.661372862	0.00584392	0.08047311	-2.08010037
WT after UVB irradiation VS WT before UVB irradiation	apoptotic signaling in response to dna damage|BioCarta	0.608837232	-0.00974577	2.900641009	0.01568976	0.2924023	-2.85736921
homo after UVB irradiation VS homo before UVB irradiation	Apparent mineralocorticoid excess syndrome|SMPDB	0.235351843	-0.00442303	1.337763903	0.2124708	0.36957778	-5.49065548
hetero after UVB irradiation VS hetero before UVB irradiation	Apparent mineralocorticoid excess syndrome|SMPDB	0.307187072	0.103522065	1.723908534	0.12110487	0.3132781	-4.97251635
WT after UVB irradiation VS WT before UVB irradiation	Apparent mineralocorticoid excess syndrome|SMPDB	0.03938021	0.054409473	0.163687683	0.87321181	0.96283151	-5.85559705
homo after UVB irradiation VS homo before UVB irradiation	Aprotinin Action Pathway|SMPDB	0.538210712	0.026444726	3.007777411	0.01409598	0.07305822	-3.00571236
hetero after UVB irradiation VS hetero before UVB irradiation	Aprotinin Action Pathway|SMPDB	0.558085555	0.09913938	2.984394118	0.01649409	0.11352943	-3.09486545
WT after UVB irradiation VS WT before UVB irradiation	Aprotinin Action Pathway|SMPDB	0.440879848	0.077377741	1.569633667	0.14732454	0.55622203	-4.76265269
homo after UVB irradiation VS homo before UVB irradiation	Aquaporin-mediated transport|Reactome	0.581646428	-0.02174103	3.690400327	0.00464482	0.03884292	-1.92528696
hetero after UVB irradiation VS hetero before UVB irradiation	Aquaporin-mediated transport|Reactome	0.479490875	0.03720917	2.914357613	0.01841281	0.11936114	-3.20181444
WT after UVB irradiation VS WT before UVB irradiation	Aquaporin-mediated transport|Reactome	0.256755867	-0.00044647	1.056757618	0.31528543	0.69898474	-5.33488212
homo after UVB irradiation VS homo before UVB irradiation	Arachidonate Epoxygenase - Epoxide Hydrolase|Wikipathways	0.262762162	0.012765835	0.898869264	0.39119081	0.54440025	-5.95250301
hetero after UVB irradiation VS hetero before UVB irradiation	Arachidonate Epoxygenase - Epoxide Hydrolase|Wikipathways	0.18704926	-0.03140141	0.612451682	0.55640286	0.76066987	-6.14758409
WT after UVB irradiation VS WT before UVB irradiation	Arachidonate Epoxygenase - Epoxide Hydrolase|Wikipathways	0.123442077	0.060707204	0.401955897	0.69610535	0.89774324	-5.78800085
homo after UVB irradiation VS homo before UVB irradiation	Arachidonate production from DAG|Reactome	0.32534735	-0.09022249	1.273839963	0.23334746	0.39505544	-5.56616854
hetero after UVB irradiation VS hetero before UVB irradiation	Arachidonate production from DAG|Reactome	-0.09264142	0.033150933	-0.47093939	0.64963601	0.82411382	-6.2278274
WT after UVB irradiation VS WT before UVB irradiation	Arachidonate production from DAG|Reactome	0.177918342	-0.04745238	0.781125476	0.45267986	0.80754423	-5.56974155
homo after UVB irradiation VS homo before UVB irradiation	Arachidonic acid metabolism - Homo sapiens (human)|KEGG	0.408455253	0.048691316	2.1370649	0.06007107	0.16751767	-4.38179364
hetero after UVB irradiation VS hetero before UVB irradiation	Arachidonic acid metabolism - Homo sapiens (human)|KEGG	0.419154161	0.053920377	2.66649449	0.02725623	0.14296189	-3.58113925
WT after UVB irradiation VS WT before UVB irradiation	Arachidonic acid metabolism - Homo sapiens (human)|KEGG	0.265068185	0.06018228	1.239103349	0.24337709	0.63440287	-5.14933828
homo after UVB irradiation VS homo before UVB irradiation	Arachidonic acid metabolism|EHMN	0.096549828	0.027822792	0.549156825	0.59571484	0.71693041	-6.20903217
hetero after UVB irradiation VS hetero before UVB irradiation	Arachidonic acid metabolism|EHMN	0.094999373	0.019774584	0.616415689	0.55390536	0.75862399	-6.14505819
WT after UVB irradiation VS WT before UVB irradiation	Arachidonic acid metabolism|EHMN	0.085934174	-0.01597272	0.446432822	0.66472026	0.87935805	-5.76924742
homo after UVB irradiation VS homo before UVB irradiation	Arachidonic acid metabolism|Reactome	0.548224322	0.025146087	3.215365787	0.01001072	0.05951688	-2.67390173
hetero after UVB irradiation VS hetero before UVB irradiation	Arachidonic acid metabolism|Reactome	0.354357841	0.058146757	1.874332689	0.09588603	0.27962159	-4.76286773
WT after UVB irradiation VS WT before UVB irradiation	Arachidonic acid metabolism|Reactome	0.236182743	0.065728593	1.092400602	0.30007193	0.68493452	-5.30037356
homo after UVB irradiation VS homo before UVB irradiation	Arachidonic Acid Metabolism|SMPDB	0.40423582	0.045705158	1.979500697	0.07780079	0.19626432	-4.61879689
hetero after UVB irradiation VS hetero before UVB irradiation	Arachidonic Acid Metabolism|SMPDB	0.482496143	0.064876536	3.04499191	0.01500113	0.1130168	-3.00250826
WT after UVB irradiation VS WT before UVB irradiation	Arachidonic Acid Metabolism|SMPDB	0.275725339	0.086595857	1.261970114	0.23537138	0.62952955	-5.12455679
homo after UVB irradiation VS homo before UVB irradiation	Arbutamine Action Pathway|SMPDB	0.396415397	-0.03677376	2.343069613	0.04268583	0.13402181	-4.06364635
hetero after UVB irradiation VS hetero before UVB irradiation	Arbutamine Action Pathway|SMPDB	-0.01555055	-0.01775524	-0.07410913	0.94265284	0.97054702	-6.34356282
WT after UVB irradiation VS WT before UVB irradiation	Arbutamine Action Pathway|SMPDB	0.056685205	-0.03978418	0.223404833	0.82768199	0.94525798	-5.84393438
homo after UVB irradiation VS homo before UVB irradiation	Ardeparin Action Pathway|SMPDB	0.542957634	0.032474294	3.216401628	0.00999373	0.05951688	-2.67225071
hetero after UVB irradiation VS hetero before UVB irradiation	Ardeparin Action Pathway|SMPDB	0.507034173	0.109310797	2.703272449	0.02570936	0.14017854	-3.52484947

WT after UVB irradiation VS WT before UVB irradiation	Ardeparin Action Pathway|SMPDB	0.380045687	0.058400764	1.387851186	0.19508646	0.60625027	-4.98260907
homo after UVB irradiation VS homo before UVB irradiation	Arf1 pathway|PID	0.22217518	-0.03838935	0.885094078	0.39820528	0.55064838	-5.96464583
hetero after UVB irradiation VS hetero before UVB irradiation	Arf1 pathway|PID	0.598928855	0.003324756	3.09019369	0.01397924	0.1130168	-2.93374787
WT after UVB irradiation VS WT before UVB irradiation	Arf1 pathway|PID	0.43286417	-0.03076193	2.165687485	0.05535757	0.41769317	-3.95058872
homo after UVB irradiation VS homo before UVB irradiation	Arf6 downstream pathway|PID	0.238971721	-0.01652305	0.805269352	0.44057942	0.59091566	-6.03186303
hetero after UVB irradiation VS hetero before UVB irradiation	Arf6 downstream pathway|PID	0.617460883	-0.00017518	3.531488814	0.00710122	0.08637	-2.27118833
WT after UVB irradiation VS WT before UVB irradiation	Arf6 downstream pathway|PID	0.322037141	-0.00261198	1.291257376	0.22543336	0.62952955	-5.09235413
homo after UVB irradiation VS homo before UVB irradiation	Arf6 signaling events|PID	0.58391828	-0.06796065	3.724480584	0.00440014	0.0379263	-1.87241113
hetero after UVB irradiation VS hetero before UVB irradiation	Arf6 signaling events|PID	0.485028276	0.051149632	2.41390693	0.04076281	0.17712536	-3.96631899
WT after UVB irradiation VS WT before UVB irradiation	Arf6 signaling events|PID	0.292530038	0.014957604	1.294719717	0.22428163	0.62952955	-5.08851328
homo after UVB irradiation VS homo before UVB irradiation	Arf6 trafficking events|PID	0.542214425	-0.02792466	2.90654531	0.016674	0.08099305	-3.16791475
hetero after UVB irradiation VS hetero before UVB irradiation	Arf6 trafficking events|PID	0.682895973	0.001394127	4.564448716	0.00160892	0.04856602	-0.81770747
WT after UVB irradiation VS WT before UVB irradiation	Arf6 trafficking events|PID	0.480684915	-0.02289455	2.328495036	0.04196138	0.39022568	-3.71298703
homo after UVB irradiation VS homo before UVB irradiation	Argatroban Action Pathway|SMPDB	0.538210712	0.026444726	3.007777411	0.01409598	0.07305822	-3.00571236
hetero after UVB irradiation VS hetero before UVB irradiation	Argatroban Action Pathway|SMPDB	0.558085555	0.09913938	2.984394118	0.01649409	0.11352943	-3.09486545
WT after UVB irradiation VS WT before UVB irradiation	Argatroban Action Pathway|SMPDB	0.440879848	0.077377741	1.569633667	0.14732454	0.55622203	-4.76265269
homo after UVB irradiation VS homo before UVB irradiation	Arginine and proline metabolism - Homo sapiens (human)|KEGG	0.185999533	-0.02113582	0.977399542	0.35288103	0.50783175	-5.88032387
hetero after UVB irradiation VS hetero before UVB irradiation	Arginine and proline metabolism - Homo sapiens (human)|KEGG	0.027980044	-0.0143717	0.165724927	0.87228108	0.95207307	-6.33166674
WT after UVB irradiation VS WT before UVB irradiation	Arginine and proline metabolism - Homo sapiens (human)|KEGG	0.082238643	-0.04834859	0.336526264	0.74337363	0.9185045	-5.81212407
homo after UVB irradiation VS homo before UVB irradiation	Arginine and Proline Metabolism|SMPDB	-0.34739547	0.00502943	-1.63262028	0.13558154	0.277359	-5.11269582
hetero after UVB irradiation VS hetero before UVB irradiation	Arginine and Proline Metabolism|SMPDB	-0.56351352	-0.04220085	-3.24793085	0.01094591	0.10415756	-2.6949004
WT after UVB irradiation VS WT before UVB irradiation	Arginine and Proline Metabolism|SMPDB	-0.47021304	-0.04055952	-2.31093774	0.04323906	0.39022568	-3.73881388
homo after UVB irradiation VS homo before UVB irradiation	Arginine biosynthesis - Homo sapiens (human)|KEGG	-0.04221929	0.023063438	-0.25303581	0.80569382	0.87946402	-6.3341662
hetero after UVB irradiation VS hetero before UVB irradiation	Arginine biosynthesis - Homo sapiens (human)|KEGG	-0.05302324	-0.02067413	-0.2453493	0.81205652	0.92298761	-6.31400465
WT after UVB irradiation VS WT before UVB irradiation	Arginine biosynthesis - Homo sapiens (human)|KEGG	-0.11587446	0.03118693	-0.52193406	0.61298939	0.87257466	-5.73312133
homo after UVB irradiation VS homo before UVB irradiation	Arginine Proline metabolism|INOH	-0.08174062	-0.00770343	-0.43016735	0.67677894	0.78113565	-6.27001784
hetero after UVB irradiation VS hetero before UVB irradiation	Arginine Proline metabolism|INOH	-0.1665199	-0.02956163	-0.92403036	0.38117031	0.60875062	-5.90599599
WT after UVB irradiation VS WT before UVB irradiation	Arginine Proline metabolism|INOH	-0.07591836	-0.03501487	-0.32319019	0.75315461	0.92020735	-5.81652979
homo after UVB irradiation VS homo before UVB irradiation	Arginine: Glycine Amidinotransferase Deficiency (AGAT Deficiency)|SMPDB	-0.34739547	0.00502943	-1.63262028	0.13558154	0.277359	-5.11269582
hetero after UVB irradiation VS hetero before UVB irradiation	Arginine: Glycine Amidinotransferase Deficiency (AGAT Deficiency)|SMPDB	-0.56351352	-0.04220085	-3.24793085	0.01094591	0.10415756	-2.6949004
WT after UVB irradiation VS WT before UVB irradiation	Arginine: Glycine Amidinotransferase Deficiency (AGAT Deficiency)|SMPDB	-0.47021304	-0.04055952	-2.31093774	0.04323906	0.39022568	-3.73881388
homo after UVB irradiation VS homo before UVB irradiation	Argininemia|SMPDB	-0.37671284	0.065264039	-1.86404069	0.09383246	0.21982504	-4.78798165
hetero after UVB irradiation VS hetero before UVB irradiation	Argininemia|SMPDB	-0.31260554	-0.01518237	-1.48265131	0.17457213	0.39158454	-5.29086545
WT after UVB irradiation VS WT before UVB irradiation	Argininemia|SMPDB	-0.12033773	0.030196148	-0.57832296	0.57573553	0.8562298	-5.70268083
homo after UVB irradiation VS homo before UVB irradiation	Argininosuccinic Aciduria|SMPDB	-0.37671284	0.065264039	-1.86404069	0.09383246	0.21982504	-4.78798165
hetero after UVB irradiation VS hetero before UVB irradiation	Argininosuccinic Aciduria|SMPDB	-0.31260554	-0.01518237	-1.48265131	0.17457213	0.39158454	-5.29086545
WT after UVB irradiation VS WT before UVB irradiation	Argininosuccinic Aciduria|SMPDB	-0.12033773	0.030196148	-0.57832296	0.57573553	0.8562298	-5.70268083
homo after UVB irradiation VS homo before UVB irradiation	ARL13B-mediated ciliary trafficking of INPP5E|Reactome	0.630661104	0.003891047	2.721213788	0.02270539	0.09808212	-3.46466918
hetero after UVB irradiation VS hetero before UVB irradiation	ARL13B-mediated ciliary trafficking of INPP5E|Reactome	-0.24950026	0.014689825	-0.65078219	0.5325286	0.74685088	-6.12254037
WT after UVB irradiation VS WT before UVB irradiation	ARL13B-mediated ciliary trafficking of INPP5E|Reactome	-0.35609606	-0.15391635	-1.14900633	0.27708158	0.66671843	-5.2437738
homo after UVB irradiation VS homo before UVB irradiation	ARMS-mediated activation|Reactome	0.417707633	0.042082674	2.053937313	0.0688783	0.18380965	-4.50763099
hetero after UVB irradiation VS hetero before UVB irradiation	ARMS-mediated activation|Reactome	0.093995979	-0.03817283	0.408933106	0.69278597	0.84544001	-6.25673691
WT after UVB irradiation VS WT before UVB irradiation	ARMS-mediated activation|Reactome	-0.04998633	-0.00678458	-0.17047524	0.86800889	0.96280353	-5.85445143
homo after UVB irradiation VS homo before UVB irradiation	Aromatase deficiency|SMPDB	0.247630977	-0.00081892	1.200495598	0.25935762	0.42308928	-5.64961013
hetero after UVB irradiation VS hetero before UVB irradiation	Aromatase deficiency|SMPDB	0.017963989	0.013614493	0.092630718	0.92836166	0.97054702	-6.34188888
WT after UVB irradiation VS WT before UVB irradiation	Aromatase deficiency|SMPDB	-0.31598762	0.004101129	-1.31107214	0.21890754	0.6260768	-5.07027776
homo after UVB irradiation VS homo before UVB irradiation	Aromatase Inhibitor Pathway (Breast Cell)_ Pharmacodynamics|PharmGKB	-0.2246365	0.00456032	-0.59535895	0.56567536	0.69153245	-6.18160651
hetero after UVB irradiation VS hetero before UVB irradiation	Aromatase Inhibitor Pathway (Breast Cell)_ Pharmacodynamics|PharmGKB	-0.34250239	-0.03806438	-0.95696274	0.36523405	0.59333249	-5.87567993
WT after UVB irradiation VS WT before UVB irradiation	Aromatase Inhibitor Pathway (Breast Cell)_ Pharmacodynamics|PharmGKB	-0.206806	-0.04301359	-0.68998021	0.50577792	0.82465997	-5.63392649
homo after UVB irradiation VS homo before UVB irradiation	Aromatase Inhibitor Pathway (Multiple Tissues)_ Pharmacodynamics|PharmGKB	-0.08068625	0.081019975	-0.19901287	0.84649574	0.90528768	-6.34721368
hetero after UVB irradiation VS hetero before UVB irradiation	Aromatase Inhibitor Pathway (Multiple Tissues)_ Pharmacodynamics|PharmGKB	-0.2191217	0.048253418	-0.83979187	0.42421056	0.6512468	-5.97957206
WT after UVB irradiation VS WT before UVB irradiation	Aromatase Inhibitor Pathway (Multiple Tissues)_ Pharmacodynamics|PharmGKB	-0.18061104	0.050857668	-0.46627155	0.65093245	0.87935805	-5.76027477
homo after UVB irradiation VS homo before UVB irradiation	Aromatic L-Aminoacid Decarboxylase Deficiency|SMPDB	0.30723314	0.017078188	1.126215861	0.28803498	0.44703185	-5.73042792
hetero after UVB irradiation VS hetero before UVB irradiation	Aromatic L-Aminoacid Decarboxylase Deficiency|SMPDB	-0.33561907	0.132015706	-1.53743203	0.16084568	0.37252774	-5.22080329
WT after UVB irradiation VS WT before UVB irradiation	Aromatic L-Aminoacid Decarboxylase Deficiency|SMPDB	0.513543589	0.096381175	1.948116725	0.07976147	0.46479598	-4.25966199
homo after UVB irradiation VS homo before UVB irradiation	-arrestins in gpcr desensitization|BioCarta	0.396875357	0.044211486	2.113303432	0.06247153	0.1717309	-4.41793305
hetero after UVB irradiation VS hetero before UVB irradiation	-arrestins in gpcr desensitization|BioCarta	0.309278566	0.011367309	1.884438414	0.09438259	0.27748818	-4.74852854
WT after UVB irradiation VS WT before UVB irradiation	-arrestins in gpcr desensitization|BioCarta	0.296456862	-0.02772042	1.168471169	0.26950309	0.66280056	-5.22381533
homo after UVB irradiation VS homo before UVB irradiation	Arrhythmogenic right ventricular cardiomyopathy (ARVC) - Homo sapiens (human)|KEGG	0.427399878	-0.01790142	2.421064102	0.03748217	0.12690988	-3.94133821
hetero after UVB irradiation VS hetero before UVB irradiation	Arrhythmogenic right ventricular cardiomyopathy (ARVC) - Homo sapiens (human)|KEGG	0.207042473	-0.00256392	1.154917213	0.27982391	0.51023746	-5.67636334
WT after UVB irradiation VS WT before UVB irradiation	Arrhythmogenic right ventricular cardiomyopathy (ARVC) - Homo sapiens (human)|KEGG	0.208117083	-0.00476853	0.894413447	0.39195202	0.76452505	-5.48035201
homo after UVB irradiation VS homo before UVB irradiation	Arrhythmogenic Right Ventricular Cardiomyopathy|Wikipathways	0.435689866	-0.01617626	2.424968215	0.03723883	0.12651226	-3.9351943
hetero after UVB irradiation VS hetero before UVB irradiation	Arrhythmogenic Right Ventricular Cardiomyopathy|Wikipathways	0.220728546	-0.0040536	1.233592279	0.25065537	0.4748492	-5.58963048
WT after UVB irradiation VS WT before UVB irradiation	Arrhythmogenic Right Ventricular Cardiomyopathy|Wikipathways	0.22520334	-4.88E-05	0.979026553	0.35047413	0.73579208	-5.40698809
homo after UVB irradiation VS homo before UVB irradiation	arsenate detoxification I (glutaredoxin)|HumanCyc	0.708206652	0.035101469	3.041024712	0.0133413	0.07226143	-2.95247316
hetero after UVB irradiation VS hetero before UVB irradiation	arsenate detoxification I (glutaredoxin)|HumanCyc	0.825058381	0.079856598	3.61965611	0.00621988	0.08279755	-2.14124851
WT after UVB irradiation VS WT before UVB irradiation	arsenate detoxification I (glutaredoxin)|HumanCyc	0.907313406	-0.02152257	3.483414682	0.00581621	0.21421457	-1.98996114
homo after UVB irradiation VS homo before UVB irradiation	Artemether Metabolism Pathway|SMPDB	-0.68524673	0.034641094	-3.04133457	0.01333447	0.07226143	-2.9519771
hetero after UVB irradiation VS hetero before UVB irradiation	Artemether Metabolism Pathway|SMPDB	-0.59598783	-0.03181997	-1.89244512	0.09320731	0.27569039	-4.73714678
WT after UVB irradiation VS WT before UVB irradiation	Artemether Metabolism Pathway|SMPDB	-0.85511058	0.148941207	-4.37574902	0.0013599	0.15645713	-0.73874013
homo after UVB irradiation VS homo before UVB irradiation	Artemisinin and Derivatives Pathway_ Pharmacokinetics|PharmGKB	-0.71493715	0.077995328	-3.01175277	0.01400349	0.07305822	-2.99934535
hetero after UVB irradiation VS hetero before UVB irradiation	Artemisinin and Derivatives Pathway_ Pharmacokinetics|PharmGKB	-0.71525286	0.104032006	-2.78896201	0.02244352	0.13389735	-3.39365844
WT after UVB irradiation VS WT before UVB irradiation	Artemisinin and Derivatives Pathway_ Pharmacokinetics|PharmGKB	-0.83070864	0.102135034	-3.93166108	0.00276876	0.18595459	-1.34650428
homo after UVB irradiation VS homo before UVB irradiation	Aryl Hydrocarbon Receptor Pathway|Wikipathways	-0.21669859	0.026412104	-1.14040428	0.28237143	0.44245297	-5.71528731
hetero after UVB irradiation VS hetero before UVB irradiation	Aryl Hydrocarbon Receptor Pathway|Wikipathways	-0.03860192	0.008205449	-0.22022357	0.83093948	0.93531602	-6.32030879
WT after UVB irradiation VS WT before UVB irradiation	Aryl Hydrocarbon Receptor Pathway|Wikipathways	-0.19986624	-0.01941071	-0.95769438	0.36061814	0.74374432	-5.42599835
homo after UVB irradiation VS homo before UVB irradiation	Aryl hydrocarbon receptor signalling|Reactome	-0.37400494	-0.02907802	-1.67787648	0.1262811	0.26608191	-5.05090178
hetero after UVB irradiation VS hetero before UVB irradiation	Aryl hydrocarbon receptor signalling|Reactome	-0.49338145	0.061776982	-1.97423197	0.08197354	0.25808464	-4.61988021
WT after UVB irradiation VS WT before UVB irradiation	Aryl hydrocarbon receptor signalling|Reactome	-0.33825479	-0.07108252	-1.71828046	0.11624642	0.51734642	-4.5714743
homo after UVB irradiation VS homo before UVB irradiation	Aryl Hydrocarbon Receptor|Wikipathways	0.074529148	-0.01395776	0.369908891	0.71965673	0.81577771	-6.29547307
hetero after UVB irradiation VS hetero before UVB irradiation	Aryl Hydrocarbon Receptor|Wikipathways	0.149994444	0.004326173	0.977489488	0.35555343	0.58358392	-5.85635594
WT after UVB irradiation VS WT before UVB irradiation	Aryl Hydrocarbon Receptor|Wikipathways	0.003235013	-0.00998125	0.016687133	0.98701182	0.99664845	-5.86901013
homo after UVB irradiation VS homo before UVB irradiation	Arylamine metabolism|Wikipathways	0.400117662	0.106943799	1.949989072	0.08163323	0.20262084	-4.66243491
hetero after UVB irradiation VS hetero before UVB irradiation	Arylamine metabolism|Wikipathways	0.182676687	-0.07832912	0.581355412	0.57621871	0.77169106	-6.16688104
WT after UVB irradiation VS WT before UVB irradiation	Arylamine metabolism|Wikipathways	0.457164195	0.137321358	1.472127315	0.17150148	0.57997722	-4.88269239
homo after UVB irradiation VS homo before UVB irradiation	Ascorbate and aldarate metabolism - Homo sapiens (human)|KEGG	-0.42660649	0.110654493	-2.14130733	0.05965194	0.16705151	-4.3753276
hetero after UVB irradiation VS hetero before UVB irradiation	Ascorbate and aldarate metabolism - Homo sapiens (human)|KEGG	-0.18168688	0.00558377	-0.96842237	0.35980571	0.5882333	-5.86493188
WT after UVB irradiation VS WT before UVB irradiation	Ascorbate and aldarate metabolism - Homo sapiens (human)|KEGG	-0.01684359	0.068925891	-0.0772525	0.93993427	0.98118396	-5.86612882
homo after UVB irradiation VS homo before UVB irradiation	ascorbate recycling (cytosolic)|HumanCyc	1.115149632	-0.01649581	5.177146648	0.00050783	0.01666204	0.229857463
hetero after UVB irradiation VS hetero before UVB irradiation	ascorbate recycling (cytosolic)|HumanCyc	1.060186335	0.048850932	5.202307852	0.00069484	0.03228824	-0.00463267
WT after UVB irradiation VS WT before UVB irradiation	ascorbate recycling (cytosolic)|HumanCyc	1.032761052	0.03429543	4.480928248	0.00115364	0.15645713	-0.59966352
homo after UVB irradiation VS homo before UVB irradiation	asparagine biosynthesis|HumanCyc	-0.00821114	0.020093972	-0.02120187	0.98352838	0.98879492	-6.36820674
hetero after UVB irradiation VS hetero before UVB irradiation	asparagine biosynthesis|HumanCyc	0.146286699	-0.01838061	0.40591006	0.69492195	0.84665128	-6.25804665
WT after UVB irradiation VS WT before UVB irradiation	asparagine biosynthesis|HumanCyc	0.320107825	0.08348891	1.052814108	0.31700402	0.70177284	-5.33864522
homo after UVB irradiation VS homo before UVB irradiation	asparagine degradation|HumanCyc	0.20490324	-0.08397123	0.544885715	0.59853428	0.71899698	-6.21146311
hetero after UVB irradiation VS hetero before UVB irradiation	asparagine degradation|HumanCyc	-0.61374202	-0.02912507	-1.83123255	0.102556	0.28762124	-4.82368225
WT after UVB irradiation VS WT before UVB irradiation	asparagine degradation|HumanCyc	-0.65192907	-0.18579658	-2.45381486	0.03385362	0.37872086	-3.52750227
homo after UVB irradiation VS homo before UVB irradiation	Asparagine N-linked glycosylation|Reactome	0.432932177	-0.03036324	2.653564154	0.02542058	0.10578915	-3.57269856
hetero after UVB irradiation VS hetero before UVB irradiation	Asparagine N-linked glycosylation|Reactome	0.444115285	0.009642333	2.700788848	0.02581094	0.14017854	-3.52865132
WT after UVB irradiation VS WT before UVB irradiation	Asparagine N-linked glycosylation|Reactome	0.368293111	-0.02221695	1.804925556	0.1009914	0.50352369	-4.45601625
homo after UVB irradiation VS homo before UVB irradiation	aspartate biosynthesis|HumanCyc	-0.12102386	-0.0063784	-0.30971863	0.76353884	0.84687345	-6.31717583
hetero after UVB irradiation VS hetero before UVB irradiation	aspartate biosynthesis|HumanCyc	-0.86851748	-0.02945987	-2.84664866	0.02048715	0.12653175	-3.30536327
WT after UVB irradiation VS WT before UVB irradiation	aspartate biosynthesis|HumanCyc	-0.59252348	-0.00692501	-1.692006	0.12126958	0.52647129	-4.60592903
homo after UVB irradiation VS homo before UVB irradiation	Aspartate Metabolism|SMPDB	-0.45356441	0.050143346	-2.44562708	0.03597696	0.12504267	-3.90265183
hetero after UVB irradiation VS hetero before UVB irradiation	Aspartate Metabolism|SMPDB	-0.36442322	-0.0375773	-1.84647526	0.1001486	0.28471271	-4.80223908
WT after UVB irradiation VS WT before UVB irradiation	Aspartate Metabolism|SMPDB	-0.06304373	-0.04233882	-0.24741384	0.80955351	0.94525798	-5.83824172
homo after UVB irradiation VS homo before UVB irradiation	aspirin blocks signaling pathway involved in platelet activation|BioCarta	0.590131096	0.008971303	3.369870234	0.00777811	0.05184649	-2.42837189
hetero after UVB irradiation VS hetero before UVB irradiation	aspirin blocks signaling pathway involved in platelet activation|BioCarta	0.523602437	0.036107449	3.005345407	0.01596122	0.11352943	-3.06291263
WT after UVB irradiation VS WT before UVB irradiation	aspirin blocks signaling pathway involved in platelet activation|BioCarta	0.488265571	0.025855514	2.425691466	0.03552759	0.38724228	-3.56928438
homo after UVB irradiation VS homo before UVB irradiation	aspirin triggered resolvin D biosynthesis|HumanCyc	1.006710653	0.064890502	6.119288351	0.00014687	0.01666204	1.414502442
hetero after UVB irradiation VS hetero before UVB irradiation	aspirin triggered resolvin D biosynthesis|HumanCyc	1.137325436	0.020416142	6.57433205	0.00013792	0.02920766	1.521872713
WT after UVB irradiation VS WT before UVB irradiation	aspirin triggered resolvin D biosynthesis|HumanCyc	0.51845401	-0.01837107	1.489591671	0.1669333	0.57064699	-4.86153038
homo after UVB irradiation VS homo before UVB irradiation	aspirin triggered resolvin E biosynthesis|HumanCyc	1.006710653	0.064890502	6.119288351	0.00014687	0.01666204	1.414502442
hetero after UVB irradiation VS hetero before UVB irradiation	aspirin triggered resolvin E biosynthesis|HumanCyc	1.137325436	0.020416142	6.57433205	0.00013792	0.02920766	1.521872713
WT after UVB irradiation VS WT before UVB irradiation	aspirin triggered resolvin E biosynthesis|HumanCyc	0.51845401	-0.01837107	1.489591671	0.1669333	0.57064699	-4.86153038
homo after UVB irradiation VS homo before UVB irradiation	aspirin-triggered lipoxin biosynthesis|HumanCyc	1.006710653	0.064890502	6.119288351	0.00014687	0.01666204	1.414502442
hetero after UVB irradiation VS hetero before UVB irradiation	aspirin-triggered lipoxin biosynthesis|HumanCyc	1.137325436	0.020416142	6.57433205	0.00013792	0.02920766	1.521872713
WT after UVB irradiation VS WT before UVB irradiation	aspirin-triggered lipoxin biosynthesis|HumanCyc	0.51845401	-0.01837107	1.489591671	0.1669333	0.57064699	-4.86153038
homo after UVB irradiation VS homo before UVB irradiation	Assembly of active LPL and LIPC lipase complexes|Reactome	0.284786707	-0.01815394	1.427454331	0.18584978	0.34107318	-5.38059908
hetero after UVB irradiation VS hetero before UVB irradiation	Assembly of active LPL and LIPC lipase complexes|Reactome	0.091247173	-0.00911724	0.335223089	0.74565071	0.87775722	-6.28599362
WT after UVB irradiation VS WT before UVB irradiation	Assembly of active LPL and LIPC lipase complexes|Reactome	0.190055765	0.005991892	0.919436407	0.37933946	0.75122805	-5.45922812
homo after UVB irradiation VS homo before UVB irradiation	Assembly of collagen fibrils and other multimeric structures|Reactome	0.829976321	-0.00433749	5.211951043	0.00048401	0.01666204	0.276173996
hetero after UVB irradiation VS hetero before UVB irradiation	Assembly of collagen fibrils and other multimeric structures|Reactome	0.575691648	0.029023291	2.725161222	0.02483151	0.13853371	-3.49133995
WT after UVB irradiation VS WT before UVB irradiation	Assembly of collagen fibrils and other multimeric structures|Reactome	0.57001662	0.017327227	2.542629211	0.02906106	0.3548797	-3.39507234
homo after UVB irradiation VS homo before UVB irradiation	Assembly Of The HIV Virion|Reactome	0.456375976	-0.02050258	1.848510741	0.09621109	0.22367608	-4.81040652
hetero after UVB irradiation VS hetero before UVB irradiation	Assembly Of The HIV Virion|Reactome	0.527191594	0.025350167	1.983472508	0.08078897	0.25607584	-4.60652207
WT after UVB irradiation VS WT before UVB irradiation	Assembly Of The HIV Virion|Reactome	0.183890412	-0.04778508	0.781525992	0.45245478	0.80748768	-5.569444
homo after UVB irradiation VS homo before UVB irradiation	Assembly of the ORC complex at the origin of replication|Reactome	-0.22601519	-0.02396238	-0.70039573	0.50066605	0.64052709	-6.11171649
hetero after UVB irradiation VS hetero before UVB irradiation	Assembly of the ORC complex at the origin of replication|Reactome	-0.27208365	0.004037543	-1.03873533	0.3278144	0.55663982	-5.7967973
WT after UVB irradiation VS WT before UVB irradiation	Assembly of the ORC complex at the origin of replication|Reactome	-0.35965769	-0.03818096	-1.12345912	0.28728114	0.67386388	-5.26958687
homo after UVB irradiation VS homo before UVB irradiation	Assembly of the pre-replicative complex|Reactome	-0.40500686	-0.02188146	-1.38612879	0.1977391	0.35391572	-5.4318844
hetero after UVB irradiation VS hetero before UVB irradiation	Assembly of the pre-replicative complex|Reactome	0.202995299	0.034179837	0.710078735	0.49683418	0.71538528	-6.08111653
WT after UVB irradiation VS WT before UVB irradiation	Assembly of the pre-replicative complex|Reactome	-0.00663955	-0.04014589	-0.02368034	0.98156972	0.99419581	-5.86886712
homo after UVB irradiation VS homo before UVB irradiation	Assembly of Viral Components at the Budding Site|Reactome	0.080196881	-0.02834002	0.22576152	0.82622478	0.89312082	-6.34113888
hetero after UVB irradiation VS hetero before UVB irradiation	Assembly of Viral Components at the Budding Site|Reactome	0.652664311	-0.09629075	1.685932647	0.12838275	0.3229899	-5.02420914
WT after UVB irradiation VS WT before UVB irradiation	Assembly of Viral Components at the Budding Site|Reactome	0.35174681	-0.01930461	0.890629702	0.39388449	0.76452505	-5.48350361
homo after UVB irradiation VS homo before UVB irradiation	Association Between Physico-Chemical Features and Toxicity Associated Pathways|Wikipathways	0.131560169	0.016450638	0.621183953	0.54926045	0.67913558	-6.1653845
hetero after UVB irradiation VS hetero before UVB irradiation	Association Between Physico-Chemical Features and Toxicity Associated Pathways|Wikipathways	0.081102101	-0.00328406	0.474048782	0.64750673	0.82239471	-6.22627575
WT after UVB irradiation VS WT before UVB irradiation	Association Between Physico-Chemical Features and Toxicity Associated Pathways|Wikipathways	0.086121719	0.021151774	0.431799966	0.67497502	0.88200598	-5.77562596
homo after UVB irradiation VS homo before UVB irradiation	Association of TriC/CCT with target proteins during biosynthesis|Reactome	-0.06329023	-0.00690782	-0.36491428	0.72325999	0.81823714	-6.29741631
hetero after UVB irradiation VS hetero before UVB irradiation	Association of TriC/CCT with target proteins during biosynthesis|Reactome	-0.26928549	-0.02481305	-1.64350883	0.1369873	0.33641601	-5.08130397
WT after UVB irradiation VS WT before UVB irradiation	Association of TriC/CCT with target proteins during biosynthesis|Reactome	0.24972545	0.021927445	1.078189661	0.30606873	0.69096189	-5.31423869
homo after UVB irradiation VS homo before UVB irradiation	Asthma - Homo sapiens (human)|KEGG	-0.03835989	-0.0163822	-0.14836118	0.88519531	0.93057836	-6.35663583
hetero after UVB irradiation VS hetero before UVB irradiation	Asthma - Homo sapiens (human)|KEGG	-0.16990459	0.03186312	-0.60968863	0.55814755	0.76153239	-6.14933593
WT after UVB irradiation VS WT before UVB irradiation	Asthma - Homo sapiens (human)|KEGG	0.161219302	0.010531139	0.464654432	0.65205128	0.87935805	-5.76102011
homo after UVB irradiation VS homo before UVB irradiation	Astrocytic Glutamate-Glutamine Uptake And Metabolism|Reactome	-0.29809726	-0.02650975	-1.12964363	0.2866586	0.44641309	-5.72678318
hetero after UVB irradiation VS hetero before UVB irradiation	Astrocytic Glutamate-Glutamine Uptake And Metabolism|Reactome	-0.27005399	0.0474308	-0.85815227	0.41455074	0.64192892	-5.96403258
WT after UVB irradiation VS WT before UVB irradiation	Astrocytic Glutamate-Glutamine Uptake And Metabolism|Reactome	-0.13026708	-0.02061759	-0.38944581	0.70504583	0.90126444	-5.79293369

homo after UVB irradiation VS homo before UVB irradiation	Asymmetric localization of PCP proteins|Reactome	-0.04095008	0.022913674	-0.17730355	0.86303609	0.91685035	-6.35158623
hetero after UVB irradiation VS hetero before UVB irradiation	Asymmetric localization of PCP proteins|Reactome	-0.00404702	-0.00384653	-0.01543541	0.98804407	0.99369649	-6.34641205
WT after UVB irradiation VS WT before UVB irradiation	Asymmetric localization of PCP proteins|Reactome	-0.35858477	0.045206528	-1.30352711	0.22137377	0.62795901	-5.07871115
homo after UVB irradiation VS homo before UVB irradiation	Atenolol Action Pathway|SMPDB	0.396415397	-0.03677376	2.343069613	0.04268583	0.13402181	-4.06364635
hetero after UVB irradiation VS hetero before UVB irradiation	Atenolol Action Pathway|SMPDB	-0.01555055	-0.01775524	-0.07410913	0.94265284	0.97054702	-6.34356282
WT after UVB irradiation VS WT before UVB irradiation	Atenolol Action Pathway|SMPDB	0.056685205	-0.03978418	0.223404833	0.82768199	0.94525798	-5.84393438
homo after UVB irradiation VS homo before UVB irradiation	ATF-2 transcription factor network|PID	-0.18300427	-0.00033991	-1.08644813	0.3043844	0.46544514	-5.77209663
hetero after UVB irradiation VS hetero before UVB irradiation	ATF-2 transcription factor network|PID	0.074878294	0.015859057	0.472100284	0.64884065	0.82363337	-6.22724922
WT after UVB irradiation VS WT before UVB irradiation	ATF-2 transcription factor network|PID	-0.18587661	-0.009375	-0.88054728	0.39906634	0.76730004	-5.49184656
homo after UVB irradiation VS homo before UVB irradiation	ATF4 activates genes|Wikipathways	0.580048693	0.001896382	2.459860724	0.03513229	0.12483583	-3.88020067
hetero after UVB irradiation VS hetero before UVB irradiation	ATF4 activates genes|Wikipathways	0.627991144	0.196564297	2.644819456	0.02821193	0.14497177	-3.61430152
WT after UVB irradiation VS WT before UVB irradiation	ATF4 activates genes|Wikipathways	0.326941246	0.018722118	1.006600376	0.33767374	0.72206121	-5.38191365
homo after UVB irradiation VS homo before UVB irradiation	ATF6 (ATF6-alpha) activates chaperone genes|Wikipathways	0.234916138	-0.03461275	0.708586294	0.4957963	0.63607921	-6.10583606
hetero after UVB irradiation VS hetero before UVB irradiation	ATF6 (ATF6-alpha) activates chaperone genes|Wikipathways	0.647631453	-0.10188809	1.807102177	0.10647867	0.29485406	-4.85747951
WT after UVB irradiation VS WT before UVB irradiation	ATF6 (ATF6-alpha) activates chaperone genes|Wikipathways	0.603890414	-0.12860741	1.681119429	0.12340764	0.52904005	-4.62012519
homo after UVB irradiation VS homo before UVB irradiation	ATF6 (ATF6-alpha) activates chaperones|Reactome	-1.15802914	-0.01763894	-7.13328767	4.40E-05	0.01666204	2.53617285
hetero after UVB irradiation VS hetero before UVB irradiation	ATF6 (ATF6-alpha) activates chaperones|Reactome	-0.44687584	-0.05914394	-1.58422713	0.14989316	0.35767419	-5.15986245
WT after UVB irradiation VS WT before UVB irradiation	ATF6 (ATF6-alpha) activates chaperones|Reactome	-0.75359889	-0.02809191	-2.44226719	0.03453137	0.38092943	-3.54466815
homo after UVB irradiation VS homo before UVB irradiation	ATM pathway|PID	-0.53406473	-0.03500713	-2.87149986	0.01767456	0.08397714	-3.22407519
hetero after UVB irradiation VS hetero before UVB irradiation	ATM pathway|PID	-0.11323969	-0.09268244	-0.52011481	0.61636966	0.80108722	-6.20216152
WT after UVB irradiation VS WT before UVB irradiation	ATM pathway|PID	-0.07810555	0.007460695	-0.2767228	0.78758319	0.93972043	-5.83051639
homo after UVB irradiation VS homo before UVB irradiation	ATM Signaling Network in Development and Disease|Wikipathways	-0.42663048	-0.05390164	-2.47882041	0.03403763	0.12324808	-3.85025886
hetero after UVB irradiation VS hetero before UVB irradiation	ATM Signaling Network in Development and Disease|Wikipathways	-0.17571807	-0.05671839	-0.7761241	0.45890482	0.68333492	-6.03124603
WT after UVB irradiation VS WT before UVB irradiation	ATM Signaling Network in Development and Disease|Wikipathways	-0.17419855	-0.02492783	-0.68207175	0.51055861	0.82655837	-5.6391592
homo after UVB irradiation VS homo before UVB irradiation	atm signaling pathway|BioCarta	-0.33938568	-0.03829697	-1.7190422	0.11832758	0.25703318	-4.99393138
hetero after UVB irradiation VS hetero before UVB irradiation	atm signaling pathway|BioCarta	0.251334604	-0.06039276	1.427438033	0.18944468	0.40788326	-5.36000112
WT after UVB irradiation VS WT before UVB irradiation	atm signaling pathway|BioCarta	0.016992848	-0.04436619	0.081039055	0.93699705	0.98056728	-5.86582538
homo after UVB irradiation VS homo before UVB irradiation	ATM Signaling Pathway|Wikipathways	-0.46042517	-0.05454166	-2.11909538	0.06187804	0.17056013	-4.409136
hetero after UVB irradiation VS hetero before UVB irradiation	ATM Signaling Pathway|Wikipathways	0.239232713	-0.03910773	0.971599519	0.35831141	0.58663196	-5.86193404
WT after UVB irradiation VS WT before UVB irradiation	ATM Signaling Pathway|Wikipathways	-0.09408303	-0.03739773	-0.32926999	0.74868975	0.9185045	-5.81454284
homo after UVB irradiation VS homo before UVB irradiation	Atorvastatin Action Pathway|SMPDB	-0.19230642	-0.01987665	-0.61925406	0.55047761	0.67913558	-6.16661869
hetero after UVB irradiation VS hetero before UVB irradiation	Atorvastatin Action Pathway|SMPDB	0.185820603	0.008098609	0.631890782	0.54421801	0.75147406	-6.13505553
WT after UVB irradiation VS WT before UVB irradiation	Atorvastatin Action Pathway|SMPDB	-0.14894747	0.045069977	-0.50755372	0.62268399	0.87257466	-5.74041409
homo after UVB irradiation VS homo before UVB irradiation	Atorvastatin/Lovastatin/Simvastatin Pathway_ Pharmacokinetics|PharmGKB	0.112045761	0.132465596	0.592698126	0.56738217	0.69195165	-6.18324176
hetero after UVB irradiation VS hetero before UVB irradiation	Atorvastatin/Lovastatin/Simvastatin Pathway_ Pharmacokinetics|PharmGKB	-0.37920945	0.08617089	-2.06067205	0.071516	0.23970209	-4.49413675
WT after UVB irradiation VS WT before UVB irradiation	Atorvastatin/Lovastatin/Simvastatin Pathway_ Pharmacokinetics|PharmGKB	-0.2357514	0.031500193	-0.77306218	0.45722678	0.80891799	-5.57570344
homo after UVB irradiation VS homo before UVB irradiation	ATP sensitive Potassium channels|Reactome	0.640249798	-0.04340881	2.101266377	0.06372251	0.17433295	-4.43619003
hetero after UVB irradiation VS hetero before UVB irradiation	ATP sensitive Potassium channels|Reactome	0.168650769	-0.01543856	0.427816231	0.67950991	0.8342843	-6.24834501
WT after UVB irradiation VS WT before UVB irradiation	ATP sensitive Potassium channels|Reactome	0.236793411	0.009278204	0.632730073	0.54099386	0.83916339	-5.67056632
homo after UVB irradiation VS homo before UVB irradiation	ATR signaling pathway|PID	-0.54664381	-0.0247458	-2.75744629	0.02137336	0.09383729	-3.40672322
hetero after UVB irradiation VS hetero before UVB irradiation	ATR signaling pathway|PID	0.163342591	-0.02443652	0.588873752	0.57139161	0.7680339	-6.16230003
WT after UVB irradiation VS WT before UVB irradiation	ATR signaling pathway|PID	-0.01234432	0.0062694	-0.04165174	0.96758964	0.98924463	-5.8682724
homo after UVB irradiation VS homo before UVB irradiation	ATR Signaling|Wikipathways	-0.8208821	-0.03820394	-4.93141344	0.0007164	0.01666204	-0.10277206
hetero after UVB irradiation VS hetero before UVB irradiation	ATR Signaling|Wikipathways	-0.08783406	-0.03144628	-0.33021861	0.74929595	0.88068359	-6.2877761
WT after UVB irradiation VS WT before UVB irradiation	ATR Signaling|Wikipathways	-0.23635866	-0.0450341	-0.7147177	0.49100087	0.81710765	-5.61720829
homo after UVB irradiation VS homo before UVB irradiation	Attachment of GPI anchor to uPAR|Reactome	0.460579638	-0.0574864	2.541461559	0.0306564	0.11645352	-3.75106471
hetero after UVB irradiation VS hetero before UVB irradiation	Attachment of GPI anchor to uPAR|Reactome	0.374164405	0.051170706	1.797310812	0.10811017	0.29786212	-4.87114008
WT after UVB irradiation VS WT before UVB irradiation	Attachment of GPI anchor to uPAR|Reactome	0.608153764	0.036008492	2.353388659	0.04021266	0.39022568	-3.67629541
homo after UVB irradiation VS homo before UVB irradiation	attenuation of gpcr signaling|BioCarta	0.493788392	-0.0062565	2.592118658	0.02816861	0.11225652	-3.67058262
hetero after UVB irradiation VS hetero before UVB irradiation	attenuation of gpcr signaling|BioCarta	0.548251963	0.04042176	2.916237942	0.01835839	0.11936114	-3.19894069
WT after UVB irradiation VS WT before UVB irradiation	attenuation of gpcr signaling|BioCarta	0.333473042	-0.03718406	1.278199651	0.22982081	0.62952955	-5.10677524
homo after UVB irradiation VS homo before UVB irradiation	Attenuation phase|Reactome	-0.45718018	0.007664859	-2.57570839	0.02895161	0.11412829	-3.69667801
hetero after UVB irradiation VS hetero before UVB irradiation	Attenuation phase|Reactome	-0.39361842	-0.00652436	-2.55750276	0.03241944	0.15685114	-3.74774651
WT after UVB irradiation VS WT before UVB irradiation	Attenuation phase|Reactome	-0.43142176	-0.01067953	-1.80022708	0.10176922	0.50394202	-4.46234628
homo after UVB irradiation VS homo before UVB irradiation	Atypical NF-kappaB pathway|PID	0.489578163	-0.05665988	2.266971975	0.04844472	0.14396371	-4.18208539
hetero after UVB irradiation VS hetero before UVB irradiation	Atypical NF-kappaB pathway|PID	0.655232049	0.077873011	2.970180305	0.01686605	0.11437413	-3.11655445
WT after UVB irradiation VS WT before UVB irradiation	Atypical NF-kappaB pathway|PID	0.54091508	-0.04008144	2.052012884	0.06705388	0.43936166	-4.11348468
homo after UVB irradiation VS homo before UVB irradiation	AUF1 (hnRNP D0) binds and destabilizes mRNA|Reactome	-0.7330956	0.036062318	-3.62850622	0.00512635	0.04120936	-2.02164385
hetero after UVB irradiation VS hetero before UVB irradiation	AUF1 (hnRNP D0) binds and destabilizes mRNA|Reactome	-0.31931312	-0.0254599	-1.21142022	0.25861117	0.48448405	-5.61447636
WT after UVB irradiation VS WT before UVB irradiation	AUF1 (hnRNP D0) binds and destabilizes mRNA|Reactome	-0.40358305	-0.02909044	-1.338299	0.21019668	0.62538648	-5.03957082
homo after UVB irradiation VS homo before UVB irradiation	AURKA Activation by TPX2|Reactome	-0.4951894	-0.00185274	-2.76493297	0.02110812	0.09285134	-3.39474387
hetero after UVB irradiation VS hetero before UVB irradiation	AURKA Activation by TPX2|Reactome	0.020672989	0.001955051	0.079118894	0.93878494	0.97054702	-6.34314668
WT after UVB irradiation VS WT before UVB irradiation	AURKA Activation by TPX2|Reactome	-0.11657744	-0.03029886	-0.46110635	0.65450926	0.87935805	-5.76264678
homo after UVB irradiation VS homo before UVB irradiation	Aurora A signaling|PID	-0.16467305	-0.0470922	-0.68764085	0.50830816	0.64758232	-6.12075153
hetero after UVB irradiation VS hetero before UVB irradiation	Aurora A signaling|PID	0.559243967	-0.01570221	3.352576435	0.0093201	0.09765748	-2.53758605
WT after UVB irradiation VS WT before UVB irradiation	Aurora A signaling|PID	0.379211391	-0.04356956	1.630363723	0.13382931	0.54709793	-4.68567226
homo after UVB irradiation VS homo before UVB irradiation	Aurora B signaling|PID	-0.44966397	-0.00385742	-1.91915579	0.0858272	0.20862332	-4.70774538
hetero after UVB irradiation VS hetero before UVB irradiation	Aurora B signaling|PID	0.068705513	-0.03649143	0.239854419	0.81617554	0.92648152	-6.31544068
WT after UVB irradiation VS WT before UVB irradiation	Aurora B signaling|PID	-0.09810209	-0.02287818	-0.35805806	0.72768287	0.91040977	-5.80464425
homo after UVB irradiation VS homo before UVB irradiation	Aurora C signaling|PID	-0.45798677	0.02077694	-1.34460703	0.21033173	0.36838308	-5.48242392
hetero after UVB irradiation VS hetero before UVB irradiation	Aurora C signaling|PID	0.024852621	0.047775276	0.07756242	0.93998648	0.97054702	-6.34327888
WT after UVB irradiation VS WT before UVB irradiation	Aurora C signaling|PID	-0.02244363	0.06207891	-0.08587626	0.93324628	0.98009095	-5.86541666
homo after UVB irradiation VS homo before UVB irradiation	Autodegradation of Cdh1 by Cdh1:APC/C|Reactome	-0.40245999	0.016427842	-1.98290644	0.07736964	0.19626432	-4.61374444
hetero after UVB irradiation VS hetero before UVB irradiation	Autodegradation of Cdh1 by Cdh1:APC/C|Reactome	0.141254198	-0.00361531	0.831396089	0.42867943	0.65540741	-5.98658381
WT after UVB irradiation VS WT before UVB irradiation	Autodegradation of Cdh1 by Cdh1:APC/C|Reactome	-0.15314622	-0.0875125	-0.69139324	0.50492661	0.82448724	-5.6329858
homo after UVB irradiation VS homo before UVB irradiation	Autodegradation of the E3 ubiquitin ligase COP1|Reactome	-0.54780349	0.054744224	-2.54242895	0.0306069	0.11636227	-3.74952983
hetero after UVB irradiation VS hetero before UVB irradiation	Autodegradation of the E3 ubiquitin ligase COP1|Reactome	0.229204513	0.06228664	0.996862737	0.34659404	0.57530067	-5.83782144
WT after UVB irradiation VS WT before UVB irradiation	Autodegradation of the E3 ubiquitin ligase COP1|Reactome	-0.76458327	0.006379364	-3.57036153	0.00502767	0.20968398	-1.86311548
homo after UVB irradiation VS homo before UVB irradiation	Autoimmune thyroid disease - Homo sapiens (human)|KEGG	0.233157372	-0.07485072	0.835079211	0.42441153	0.57588797	-6.00739702
hetero after UVB irradiation VS hetero before UVB irradiation	Autoimmune thyroid disease - Homo sapiens (human)|KEGG	-0.19933489	-0.02559477	-0.68139532	0.51390988	0.73158211	-6.10155669
WT after UVB irradiation VS WT before UVB irradiation	Autoimmune thyroid disease - Homo sapiens (human)|KEGG	0.119447496	-0.05084758	0.354539499	0.73023818	0.91086739	-5.80589742
homo after UVB irradiation VS homo before UVB irradiation	Autointegration results in viral DNA circles|Reactome	-0.72016939	-0.04289266	-2.76617486	0.02106445	0.09274857	-3.39275657
hetero after UVB irradiation VS hetero before UVB irradiation	Autointegration results in viral DNA circles|Reactome	-0.34909744	-0.05229416	-1.08743415	0.30696955	0.53592103	-5.74747096
WT after UVB irradiation VS WT before UVB irradiation	Autointegration results in viral DNA circles|Reactome	-0.14480261	-0.04989433	-0.37290872	0.71693662	0.90536666	-5.79922254
homo after UVB irradiation VS homo before UVB irradiation	Autophagy - animal - Homo sapiens (human)|KEGG	0.140150281	-0.02317515	0.70614684	0.49724361	0.63711097	-6.10759388
hetero after UVB irradiation VS hetero before UVB irradiation	Autophagy - animal - Homo sapiens (human)|KEGG	0.144862607	-0.00852994	0.935052836	0.3757808	0.60404776	-5.8959443
WT after UVB irradiation VS WT before UVB irradiation	Autophagy - animal - Homo sapiens (human)|KEGG	-0.01889015	-0.01475832	-0.09248972	0.92812098	0.97756654	-5.86481958
homo after UVB irradiation VS homo before UVB irradiation	Autophagy - other - Homo sapiens (human)|KEGG	-0.2851171	0.020146535	-1.4291807	0.18536676	0.34032353	-5.37843582
hetero after UVB irradiation VS hetero before UVB irradiation	Autophagy - other - Homo sapiens (human)|KEGG	-0.21364868	-0.05520729	-1.10632614	0.29916819	0.53033405	-5.72788108
WT after UVB irradiation VS WT before UVB irradiation	Autophagy - other - Homo sapiens (human)|KEGG	-0.24229686	-0.01849736	-0.92507103	0.37653952	0.75077508	-5.45440448
homo after UVB irradiation VS homo before UVB irradiation	Axon guidance - Homo sapiens (human)|KEGG	0.079630191	-0.01218671	0.455403883	0.65916747	0.76623184	-6.25826259
hetero after UVB irradiation VS hetero before UVB irradiation	Axon guidance - Homo sapiens (human)|KEGG	0.073321767	0.007601308	0.449035743	0.66473092	0.82651398	-6.23848315
WT after UVB irradiation VS WT before UVB irradiation	Axon guidance - Homo sapiens (human)|KEGG	0.004732729	-0.00841503	0.023826281	0.98145616	0.99419581	-5.86886361
homo after UVB irradiation VS homo before UVB irradiation	Axon guidance|Reactome	0.270830445	-0.02040793	1.515632394	0.16252874	0.31207158	-5.26805427
hetero after UVB irradiation VS hetero before UVB irradiation	Axon guidance|Reactome	0.219303866	0.010752811	1.406175498	0.19546103	0.4161797	-5.38620975
WT after UVB irradiation VS WT before UVB irradiation	Axon guidance|Reactome	0.110241514	-0.00528699	0.574173738	0.57843435	0.85909258	-5.70502008
homo after UVB irradiation VS homo before UVB irradiation	Axonal growth inhibition (RHOA activation)|Reactome	0.231346832	-0.07271496	0.992219936	0.34597077	0.50149286	-5.86615026
hetero after UVB irradiation VS hetero before UVB irradiation	Axonal growth inhibition (RHOA activation)|Reactome	0.224197067	0.00813038	1.103867068	0.30017471	0.53033405	-5.73044514
WT after UVB irradiation VS WT before UVB irradiation	Axonal growth inhibition (RHOA activation)|Reactome	0.251792339	0.096013302	1.292858078	0.22490029	0.62952955	-5.09057932
homo after UVB irradiation VS homo before UVB irradiation	Axonal growth stimulation|Reactome	0.563179516	0.008159155	2.50542975	0.03255821	0.12029246	-3.80817045
hetero after UVB irradiation VS hetero before UVB irradiation	Axonal growth stimulation|Reactome	0.305116673	0.02524732	1.253005648	0.24385666	0.4668201	-5.56762366
WT after UVB irradiation VS WT before UVB irradiation	Axonal growth stimulation|Reactome	0.558282762	0.180532933	1.6557063	0.12853112	0.53952355	-4.6530776
homo after UVB irradiation VS homo before UVB irradiation	Azathioprine Action Pathway|SMPDB	0.1869187	-0.04175754	1.06533095	0.31335381	0.46923284	-5.79375484
hetero after UVB irradiation VS hetero before UVB irradiation	Azathioprine Action Pathway|SMPDB	0.029059741	-0.03702953	0.148746722	0.88525209	0.9597952	-6.33455446
WT after UVB irradiation VS WT before UVB irradiation	Azathioprine Action Pathway|SMPDB	0.208528988	-0.04951637	0.893857571	0.39223551	0.76452505	-5.48081572
homo after UVB irradiation VS homo before UVB irradiation	B cell receptor signaling pathway - Homo sapiens (human)|KEGG	0.581678731	-0.03230738	3.499048515	0.00631015	0.04624743	-2.22446082
hetero after UVB irradiation VS hetero before UVB irradiation	B cell receptor signaling pathway - Homo sapiens (human)|KEGG	0.623352884	0.02738791	3.861589163	0.00434677	0.07183716	-1.78980801
WT after UVB irradiation VS WT before UVB irradiation	B cell receptor signaling pathway - Homo sapiens (human)|KEGG	0.520248118	0.01547555	2.37407862	0.03881353	0.39022568	-3.64573785
homo after UVB irradiation VS homo before UVB irradiation	B Cell Receptor Signaling Pathway|Wikipathways	0.343258736	-0.01835556	1.856056681	0.09504839	0.22192484	-4.79952072
hetero after UVB irradiation VS hetero before UVB irradiation	B Cell Receptor Signaling Pathway|Wikipathways	0.387641244	0.028753129	2.3375447	0.04604418	0.18856188	-4.08186013
WT after UVB irradiation VS WT before UVB irradiation	B Cell Receptor Signaling Pathway|Wikipathways	0.29672615	-0.01513199	1.36782256	0.20107999	0.61591705	-5.00579701
homo after UVB irradiation VS homo before UVB irradiation	B cell receptor signaling|INOH	0.002855387	-0.01467173	0.011395182	0.99114662	0.99334524	-6.3683785
hetero after UVB irradiation VS hetero before UVB irradiation	B cell receptor signaling|INOH	0.232729085	0.000127839	1.07619038	0.31168792	0.54022992	-5.75901067
WT after UVB irradiation VS WT before UVB irradiation	B cell receptor signaling|INOH	0.149296576	-0.01519512	0.617057131	0.55087693	0.84516674	-5.68009052
homo after UVB irradiation VS homo before UVB irradiation	b cell survival pathway|BioCarta	0.303344528	-0.04632257	1.208454701	0.25642589	0.41995717	-5.6407266
hetero after UVB irradiation VS hetero before UVB irradiation	b cell survival pathway|BioCarta	0.628166907	-0.01869064	3.511605964	0.00731765	0.0874386	-2.30061905
WT after UVB irradiation VS WT before UVB irradiation	b cell survival pathway|BioCarta	0.512202795	-0.02889305	2.396273509	0.03736554	0.39022568	-3.61289871
homo after UVB irradiation VS homo before UVB irradiation	Bacterial invasion of epithelial cells - Homo sapiens (human)|KEGG	0.516678948	-0.03960153	2.449265349	0.03575916	0.12504267	-3.89691535
hetero after UVB irradiation VS hetero before UVB irradiation	Bacterial invasion of epithelial cells - Homo sapiens (human)|KEGG	0.568476276	0.018846159	3.290814524	0.01024637	0.10264385	-2.63031148
WT after UVB irradiation VS WT before UVB irradiation	Bacterial invasion of epithelial cells - Homo sapiens (human)|KEGG	0.577026892	-0.00142247	2.97823157	0.01373169	0.28307166	-2.74075285
homo after UVB irradiation VS homo before UVB irradiation	BARD1 signaling events|PID	-0.61170743	-0.02656674	-2.73225317	0.0222909	0.09702597	-3.44701959
hetero after UVB irradiation VS hetero before UVB irradiation	BARD1 signaling events|PID	-0.12797165	-0.09054089	-0.43080065	0.67742227	0.83248604	-6.24698553
WT after UVB irradiation VS WT before UVB irradiation	BARD1 signaling events|PID	-0.07914974	0.021136288	-0.26245609	0.79825466	0.94477083	-5.83438319
homo after UVB irradiation VS homo before UVB irradiation	Basal cell carcinoma - Homo sapiens (human)|KEGG	-0.42278804	0.026766609	-2.60911498	0.02737999	0.11018326	-3.64353349
hetero after UVB irradiation VS hetero before UVB irradiation	Basal cell carcinoma - Homo sapiens (human)|KEGG	-0.34009619	0.009487616	-1.69455095	0.12669659	0.31975806	-5.01252524
WT after UVB irradiation VS WT before UVB irradiation	Basal cell carcinoma - Homo sapiens (human)|KEGG	-0.33125582	0.051107552	-1.33072133	0.21259156	0.62581393	-5.04815987
homo after UVB irradiation VS homo before UVB irradiation	Basal transcription factors - Homo sapiens (human)|KEGG	-0.69815991	0.039511682	-3.54611445	0.00584978	0.04451679	-2.15053377
hetero after UVB irradiation VS hetero before UVB irradiation	Basal transcription factors - Homo sapiens (human)|KEGG	-0.38417114	-0.07453977	-1.65724456	0.13414522	0.33284372	-5.06289571
WT after UVB irradiation VS WT before UVB irradiation	Basal transcription factors - Homo sapiens (human)|KEGG	-0.30422901	-0.01892333	-1.06407282	0.31211616	0.69567326	-5.32787242
homo after UVB irradiation VS homo before UVB irradiation	Base excision repair - Homo sapiens (human)|KEGG	-0.36258949	-0.01731397	-1.88708459	0.09040394	0.21571189	-4.75455598
hetero after UVB irradiation VS hetero before UVB irradiation	Base excision repair - Homo sapiens (human)|KEGG	-0.00029599	-0.04288223	-0.00135144	0.99895316	0.99964322	-6.34654031
WT after UVB irradiation VS WT before UVB irradiation	Base excision repair - Homo sapiens (human)|KEGG	0.145726933	0.02349963	0.562590129	0.58600497	0.86137261	-5.71146756
homo after UVB irradiation VS homo before UVB irradiation	Base Excision Repair|Reactome	-0.45223265	-0.01747997	-2.17447565	0.05647101	0.16165213	-4.32463734
hetero after UVB irradiation VS hetero before UVB irradiation	Base Excision Repair|Reactome	0.050472591	-0.02334625	0.221855754	0.82970915	0.93424267	-6.31991968
WT after UVB irradiation VS WT before UVB irradiation	Base Excision Repair|Reactome	0.132031072	0.050326638	0.486666908	0.63690017	0.87636259	-5.75066267
homo after UVB irradiation VS homo before UVB irradiation	Base-Excision Repair_ AP Site Formation|Reactome	-0.62930802	-0.05369783	-3.54140783	0.00589419	0.04470579	-2.15791703
hetero after UVB irradiation VS hetero before UVB irradiation	Base-Excision Repair_ AP Site Formation|Reactome	-0.35308914	-0.09357971	-1.43234657	0.18807895	0.40570935	-5.35391736
WT after UVB irradiation VS WT before UVB irradiation	Base-Excision Repair_ AP Site Formation|Reactome	0.107767851	0.017748211	0.446744605	0.66450254	0.87935805	-5.76910929
homo after UVB irradiation VS homo before UVB irradiation	basic mechanism of action of ppara pparb(d) and pparg and effects on gene expression|BioCarta	0.351070135	0.035116289	1.283203132	0.23018707	0.39086432	-5.55526654
hetero after UVB irradiation VS hetero before UVB irradiation	basic mechanism of action of ppara pparb(d) and pparg and effects on gene expression|BioCarta	-0.00489816	0.006013782	-0.0192654	0.98507781	0.99251462	-6.34633995
WT after UVB irradiation VS WT before UVB irradiation	basic mechanism of action of ppara pparb(d) and pparg and effects on gene expression|BioCarta	0.059804367	-0.01095853	0.200205853	0.84530027	0.95361858	-5.84888902
homo after UVB irradiation VS homo before UVB irradiation	basic mechanisms of sumoylation|BioCarta	-0.45932633	-0.00521354	-2.0788767	0.06611367	0.17862426	-4.47005592
hetero after UVB irradiation VS hetero before UVB irradiation	basic mechanisms of sumoylation|BioCarta	0.072911857	0.00337061	0.243187448	0.81367634	0.92395036	-6.31457345
WT after UVB irradiation VS WT before UVB irradiation	basic mechanisms of sumoylation|BioCarta	0.035079043	0.02128541	0.128342896	0.90040163	0.97566251	-5.86081394
homo after UVB irradiation VS homo before UVB irradiation	Basigin interactions|Reactome	0.23258023	-0.01099019	1.156080445	0.27621675	0.4362524	-5.69839442
hetero after UVB irradiation VS hetero before UVB irradiation	Basigin interactions|Reactome	0.228955033	-0.00208646	1.359675951	0.20919803	0.43126397	-5.44268589
WT after UVB irradiation VS WT before UVB irradiation	Basigin interactions|Reactome	0.320739803	-0.01626287	1.540752294	0.15415391	0.55854784	-4.79867982
homo after UVB irradiation VS homo before UVB irradiation	BBSome-mediated cargo-targeting to cilium|Reactome	-0.59945307	-0.03309909	-3.6099176	0.00528096	0.04193541	-2.05066253

hetero after UVB irradiation VS hetero before UVB irradiation	BBSome-mediated cargo-targeting to cilium|Reactome	-0.63101779	-0.06252163	-3.24573091	0.01098312	0.10415769	-2.69821817
WT after UVB irradiation VS WT before UVB irradiation	BBSome-mediated cargo-targeting to cilium|Reactome	-0.45742727	-0.08046257	-2.04055679	0.06835507	0.44205977	-4.12973848
homo after UVB irradiation VS homo before UVB irradiation	bcr signaling pathway|BioCarta	0.242946319	-0.00208094	1.412175898	0.19017189	0.34647455	-5.39967185
hetero after UVB irradiation VS hetero before UVB irradiation	bcr signaling pathway|BioCarta	0.414834245	0.020226754	2.63968794	0.02844313	0.14599527	-3.62215096
WT after UVB irradiation VS WT before UVB irradiation	bcr signaling pathway|BioCarta	0.245349196	0.017733473	0.97771192	0.35109318	0.73619953	-5.40816949
homo after UVB irradiation VS homo before UVB irradiation	BCR signaling pathway|PID	0.421406628	-0.04343543	2.467996518	0.03465835	0.12421508	-3.86735729
hetero after UVB irradiation VS hetero before UVB irradiation	BCR signaling pathway|PID	0.579125333	7.77E-05	3.789690297	0.00483116	0.07419102	-1.89342932
WT after UVB irradiation VS WT before UVB irradiation	BCR signaling pathway|PID	0.297585086	-0.0135902	1.245269237	0.24119693	0.63358831	-5.14268771
homo after UVB irradiation VS homo before UVB irradiation	BCR|NetPath	0.36587403	-0.02054985	1.916577704	0.08618692	0.20921289	-4.71152053
hetero after UVB irradiation VS hetero before UVB irradiation	BCR|NetPath	0.503932184	0.007889216	3.275490086	0.01049082	0.10391574	-2.65337354
WT after UVB irradiation VS WT before UVB irradiation	BCR|NetPath	0.370023402	-0.01526756	1.776460317	0.10578858	0.50446894	-4.49424758
homo after UVB irradiation VS homo before UVB irradiation	BDNF activates NTRK2 (TRKB) signaling|Reactome	0.581869796	-0.11300673	1.567928556	0.1499451	0.29561714	-5.19941719
hetero after UVB irradiation VS hetero before UVB irradiation	BDNF activates NTRK2 (TRKB) signaling|Reactome	0.073813415	-0.02969325	0.167133545	0.87120669	0.95135604	-6.33141324
WT after UVB irradiation VS WT before UVB irradiation	BDNF activates NTRK2 (TRKB) signaling|Reactome	0.41270393	-0.07381485	1.040166859	0.32256354	0.70648859	-5.3506392
homo after UVB irradiation VS homo before UVB irradiation	BDNF|NetPath	-0.03447516	-0.03402204	-0.20160625	0.84452493	0.90528595	-6.34665789
hetero after UVB irradiation VS hetero before UVB irradiation	BDNF|NetPath	0.069488629	-0.02845599	0.453360052	0.66173772	0.82651398	-6.23641765
WT after UVB irradiation VS WT before UVB irradiation	BDNF|NetPath	0.145993114	-0.02504169	0.745398031	0.4730504	0.81341158	-5.5957431
homo after UVB irradiation VS homo before UVB irradiation	BDNF-TrkB Signaling|Wikipathways	0.204060528	-0.02229359	0.931969644	0.37469524	0.52771698	-5.92268685
hetero after UVB irradiation VS hetero before UVB irradiation	BDNF-TrkB Signaling|Wikipathways	0.196560173	-0.01552204	1.105040478	0.29969409	0.53033405	-5.72922216
WT after UVB irradiation VS WT before UVB irradiation	BDNF-TrkB Signaling|Wikipathways	0.066435094	-0.04081143	0.326141199	0.75098625	0.9192587	-5.81556989
homo after UVB irradiation VS homo before UVB irradiation	Benazepril Action Pathway|SMPDB	0.636038586	-0.11800708	2.436891056	0.03650532	0.12504267	-3.91641947
hetero after UVB irradiation VS hetero before UVB irradiation	Benazepril Action Pathway|SMPDB	-0.02777369	0.003323189	-0.09172332	0.92906122	0.97054702	-6.34197953
WT after UVB irradiation VS WT before UVB irradiation	Benazepril Action Pathway|SMPDB	0.270438532	-0.10210744	0.707878218	0.49505953	0.81710765	-5.62188349
homo after UVB irradiation VS homo before UVB irradiation	Benazepril Metabolism Pathway|SMPDB	1.076747846	-0.04193589	4.959558579	0.00068841	0.01666204	-0.06417629
hetero after UVB irradiation VS hetero before UVB irradiation	Benazepril Metabolism Pathway|SMPDB	0.553630209	0.003105397	1.56066683	0.15532075	0.36486693	-5.19066683
WT after UVB irradiation VS WT before UVB irradiation	Benazepril Metabolism Pathway|SMPDB	0.585930211	-0.05239369	1.72512433	0.11496929	0.51527453	-4.56245573
homo after UVB irradiation VS homo before UVB irradiation	Bendroflumethiazide Action Pathway|SMPDB	0.234726031	0.017120546	1.156747303	0.27595732	0.43614438	-5.69767199
hetero after UVB irradiation VS hetero before UVB irradiation	Bendroflumethiazide Action Pathway|SMPDB	-0.03255037	0.028925632	-0.18154472	0.86023155	0.94503144	-6.32869738
WT after UVB irradiation VS WT before UVB irradiation	Bendroflumethiazide Action Pathway|SMPDB	-0.15908359	0.010252305	-0.80382265	0.44003996	0.79685269	-5.55266936
homo after UVB irradiation VS homo before UVB irradiation	Benzene metabolism|Wikipathways	0.946928621	-0.02579847	5.317729719	0.00041871	0.01666204	0.415728093
hetero after UVB irradiation VS hetero before UVB irradiation	Benzene metabolism|Wikipathways	0.296577829	0.008974905	0.836080494	0.42618204	0.65304481	-5.98267894
WT after UVB irradiation VS WT before UVB irradiation	Benzene metabolism|Wikipathways	0.352474401	0.048310647	0.902083595	0.38805505	0.76130447	-5.47392889
homo after UVB irradiation VS homo before UVB irradiation	Benzo(a)pyrene metabolism|Wikipathways	0.389951317	0.023361356	1.674482613	0.12695801	0.26689238	-5.05556678
hetero after UVB irradiation VS hetero before UVB irradiation	Benzo(a)pyrene metabolism|Wikipathways	0.07376203	-0.01818583	0.274810572	0.79008022	0.91030982	-6.30575954
WT after UVB irradiation VS WT before UVB irradiation	Benzo(a)pyrene metabolism|Wikipathways	-0.08959918	-0.009864	-0.36237392	0.72455332	0.90882588	-5.80309068
homo after UVB irradiation VS homo before UVB irradiation	Benzocaine Action Pathway|SMPDB	0.191132361	0.029768717	1.061313976	0.31508276	0.46923284	-5.79783739
hetero after UVB irradiation VS hetero before UVB irradiation	Benzocaine Action Pathway|SMPDB	-0.07904315	0.055005373	-0.44437398	0.66796482	0.82651398	-6.24068873
WT after UVB irradiation VS WT before UVB irradiation	Benzocaine Action Pathway|SMPDB	0.022316377	0.035038618	0.098344171	0.92358676	0.97566251	-5.86425414
homo after UVB irradiation VS homo before UVB irradiation	Benzodiazepine Pathway_ Pharmacodynamics|PharmGKB	0.16221027	0.021053194	0.82373932	0.4305138	0.58181948	-6.01679437
hetero after UVB irradiation VS hetero before UVB irradiation	Benzodiazepine Pathway_ Pharmacodynamics|PharmGKB	-0.37403612	-0.06927437	-2.15001907	0.06206849	0.22346619	-4.36249237
WT after UVB irradiation VS WT before UVB irradiation	Benzodiazepine Pathway_ Pharmacodynamics|PharmGKB	0.011526347	-0.01498706	0.047032451	0.96340596	0.98829178	-5.86803071
homo after UVB irradiation VS homo before UVB irradiation	Benzodiazepine Pathway_ Pharmacokinetics|PharmGKB	-0.24478251	0.137197239	-1.09102684	0.30246605	0.46312664	-5.76735739
hetero after UVB irradiation VS hetero before UVB irradiation	Benzodiazepine Pathway_ Pharmacokinetics|PharmGKB	-0.52936872	0.071493976	-1.76280029	0.11404884	0.3086904	-4.91903429
WT after UVB irradiation VS WT before UVB irradiation	Benzodiazepine Pathway_ Pharmacokinetics|PharmGKB	-0.20480312	0.227906882	-0.98005506	0.34999037	0.73549869	-5.40606292
homo after UVB irradiation VS homo before UVB irradiation	Beta defensins|Reactome	0.77653781	-0.09396854	3.523386369	0.00606755	0.04556651	-2.18620691
hetero after UVB irradiation VS hetero before UVB irradiation	Beta defensins|Reactome	0.57892449	0.01851615	2.645665772	0.02817398	0.14497177	-3.61300689
WT after UVB irradiation VS WT before UVB irradiation	Beta defensins|Reactome	0.49563273	-0.0246282	1.392097406	0.19383537	0.60625027	-4.97766484
homo after UVB irradiation VS homo before UVB irradiation	Beta oxidation of butanoyl-CoA to acetyl-CoA|Reactome	-0.12817104	-0.01188752	-0.38779858	0.70681067	0.80621971	-6.28830268
hetero after UVB irradiation VS hetero before UVB irradiation	Beta oxidation of butanoyl-CoA to acetyl-CoA|Reactome	-0.67381888	-0.03140591	-2.38242127	0.04286294	0.18194424	-4.01402959
WT after UVB irradiation VS WT before UVB irradiation	Beta oxidation of butanoyl-CoA to acetyl-CoA|Reactome	-0.4553965	-0.07846779	-1.31857321	0.21647827	0.62581393	-5.06186061
homo after UVB irradiation VS homo before UVB irradiation	Beta oxidation of decanoyl-CoA to octanoyl-CoA-CoA|Reactome	-0.52918292	-0.04100155	-2.13557661	0.06021878	0.16751767	-4.38406103
hetero after UVB irradiation VS hetero before UVB irradiation	Beta oxidation of decanoyl-CoA to octanoyl-CoA-CoA|Reactome	-0.79151598	0.026908227	-3.29895912	0.01011889	0.10249308	-2.61806319
WT after UVB irradiation VS WT before UVB irradiation	Beta oxidation of decanoyl-CoA to octanoyl-CoA-CoA|Reactome	-0.47816577	0.023569746	-1.59782737	0.1409164	0.55622203	-4.72711704
homo after UVB irradiation VS homo before UVB irradiation	Beta oxidation of hexanoyl-CoA to butanoyl-CoA|Reactome	-0.46434233	0.076664883	-1.74901184	0.1128277	0.24815504	-4.95201912
hetero after UVB irradiation VS hetero before UVB irradiation	Beta oxidation of hexanoyl-CoA to butanoyl-CoA|Reactome	-0.66307208	0.078360656	-1.90552267	0.09131751	0.27391014	-4.71851757
WT after UVB irradiation VS WT before UVB irradiation	Beta oxidation of hexanoyl-CoA to butanoyl-CoA|Reactome	-0.5211099	-0.01799117	-1.70473639	0.1188118	0.52274903	-4.58926888
homo after UVB irradiation VS homo before UVB irradiation	Beta oxidation of lauroyl-CoA to decanoyl-CoA-CoA|Reactome	-0.58301184	-0.00644843	-1.97501459	0.07837219	0.1973786	-4.62544693
hetero after UVB irradiation VS hetero before UVB irradiation	Beta oxidation of lauroyl-CoA to decanoyl-CoA-CoA|Reactome	-0.70930974	0.039779874	-2.4869074	0.0362815	0.16681566	-3.85537694
WT after UVB irradiation VS WT before UVB irradiation	Beta oxidation of lauroyl-CoA to decanoyl-CoA-CoA|Reactome	-0.66029511	-0.02284399	-2.28660114	0.0450728	0.39434384	-3.77453883
homo after UVB irradiation VS homo before UVB irradiation	Beta oxidation of myristoyl-CoA to lauroyl-CoA|Reactome	-0.40342567	-0.13235757	-1.00920379	0.33817596	0.49364176	-5.84969709
hetero after UVB irradiation VS hetero before UVB irradiation	Beta oxidation of myristoyl-CoA to lauroyl-CoA|Reactome	-0.47906575	-0.05019494	-1.18806656	0.26721398	0.4961146	-5.64030763
WT after UVB irradiation VS WT before UVB irradiation	Beta oxidation of myristoyl-CoA to lauroyl-CoA|Reactome	-0.94725627	0.045675828	-3.5371585	0.00531484	0.20968398	-1.91145105
homo after UVB irradiation VS homo before UVB irradiation	Beta oxidation of octanoyl-CoA to hexanoyl-CoA|Reactome	-0.5681347	0.004678284	-1.76850588	0.10937712	0.24265581	-4.92456641
hetero after UVB irradiation VS hetero before UVB irradiation	Beta oxidation of octanoyl-CoA to hexanoyl-CoA|Reactome	-1.0132623	0.041777021	-4.01169947	0.00349448	0.06529021	-1.57583967
WT after UVB irradiation VS WT before UVB irradiation	Beta oxidation of octanoyl-CoA to hexanoyl-CoA|Reactome	-0.62464462	0.021305178	-2.11811332	0.05999375	0.42812412	-4.01910398
homo after UVB irradiation VS homo before UVB irradiation	Beta oxidation of palmitoyl-CoA to myristoyl-CoA|Reactome	-0.55897942	-0.0557875	-1.52324779	0.16064025	0.3092257	-5.25814405
hetero after UVB irradiation VS hetero before UVB irradiation	Beta oxidation of palmitoyl-CoA to myristoyl-CoA|Reactome	-0.9466564	-0.05473077	-3.89278823	0.00415292	0.07036798	-1.74506907
WT after UVB irradiation VS WT before UVB irradiation	Beta oxidation of palmitoyl-CoA to myristoyl-CoA|Reactome	-0.88740053	0.096429967	-3.23611697	0.00883608	0.24856491	-2.35505249
homo after UVB irradiation VS homo before UVB irradiation	Beta Oxidation of Very Long Chain Fatty Acids|SMPDB	0.161860886	-0.05103371	0.630831613	0.54319908	0.67708436	-6.15916189
hetero after UVB irradiation VS hetero before UVB irradiation	Beta Oxidation of Very Long Chain Fatty Acids|SMPDB	-0.12884308	-0.07596147	-0.45167658	0.66290222	0.82651398	-6.237224
WT after UVB irradiation VS WT before UVB irradiation	Beta Oxidation of Very Long Chain Fatty Acids|SMPDB	-0.13599861	-0.0440626	-0.59210605	0.56682	0.85125516	-5.69479783
homo after UVB irradiation VS homo before UVB irradiation	Beta Ureidopropionase Deficiency|SMPDB	-0.15230025	-0.02916717	-0.66373722	0.52282067	0.65944729	-6.13727985
hetero after UVB irradiation VS hetero before UVB irradiation	Beta Ureidopropionase Deficiency|SMPDB	-0.07293889	-0.06090355	-0.36429729	0.72460928	0.86482318	-6.27512326
WT after UVB irradiation VS WT before UVB irradiation	Beta Ureidopropionase Deficiency|SMPDB	0.197121499	-0.04726474	0.926444646	0.37585917	0.75077508	-5.45322485
homo after UVB irradiation VS homo before UVB irradiation	Beta1 integrin cell surface interactions|PID	0.724963336	-0.00943811	4.510141107	0.00131875	0.02074181	-0.6956614
hetero after UVB irradiation VS hetero before UVB irradiation	Beta1 integrin cell surface interactions|PID	0.557227525	0.054654186	2.574189965	0.03156887	0.15507377	-3.72226654
WT after UVB irradiation VS WT before UVB irradiation	Beta1 integrin cell surface interactions|PID	0.425760577	0.015696517	1.577084854	0.14560642	0.55622203	-4.75329586
homo after UVB irradiation VS homo before UVB irradiation	Beta2 integrin cell surface interactions|PID	0.826584489	-0.00624685	4.821324002	0.00083817	0.01759873	-0.25497028
hetero after UVB irradiation VS hetero before UVB irradiation	Beta2 integrin cell surface interactions|PID	0.620258344	0.058687828	3.340693883	0.00949123	0.0985871	-2.55539744
WT after UVB irradiation VS WT before UVB irradiation	Beta2 integrin cell surface interactions|PID	0.562592493	0.02822454	1.935430009	0.08145968	0.46795768	-4.27731165
homo after UVB irradiation VS homo before UVB irradiation	Beta3 integrin cell surface interactions|PID	0.582775952	-0.03327408	3.521865993	0.00608242	0.04556964	-2.18859499
hetero after UVB irradiation VS hetero before UVB irradiation	Beta3 integrin cell surface interactions|PID	0.520149318	0.0546329	2.559426092	0.03232023	0.15685114	-3.74481039
WT after UVB irradiation VS WT before UVB irradiation	Beta3 integrin cell surface interactions|PID	0.420197875	0.012977783	1.529855513	0.15680183	0.56072663	-4.8121716
homo after UVB irradiation VS homo before UVB irradiation	Beta5 beta6 beta7 and beta8 integrin cell surface interactions|PID	0.823088985	-0.00677416	4.967642267	0.00068059	0.01666204	-0.05311479
hetero after UVB irradiation VS hetero before UVB irradiation	Beta5 beta6 beta7 and beta8 integrin cell surface interactions|PID	0.75013956	0.018174028	3.684456118	0.00564631	0.08008296	-2.04636022
WT after UVB irradiation VS WT before UVB irradiation	Beta5 beta6 beta7 and beta8 integrin cell surface interactions|PID	0.625030022	-0.00225321	2.353475551	0.04020668	0.39022568	-3.67616719
homo after UVB irradiation VS homo before UVB irradiation	Beta-agonist/Beta-blocker Pathway_ Pharmacodynamics|PharmGKB	0.442799955	-0.04324943	2.821154848	0.01921981	0.08846538	-3.3047322
hetero after UVB irradiation VS hetero before UVB irradiation	Beta-agonist/Beta-blocker Pathway_ Pharmacodynamics|PharmGKB	0.361674229	0.012854966	1.8965392	0.09261173	0.27457285	-4.73131987
WT after UVB irradiation VS WT before UVB irradiation	Beta-agonist/Beta-blocker Pathway_ Pharmacodynamics|PharmGKB	0.297690934	-0.00973248	1.235903805	0.24451468	0.63629547	-5.15278006
homo after UVB irradiation VS homo before UVB irradiation	beta-Alanine metabolism - Homo sapiens (human)|KEGG	-0.0060918	-0.00514952	-0.03178209	0.97531121	0.98523695	-6.36790556
hetero after UVB irradiation VS hetero before UVB irradiation	beta-Alanine metabolism - Homo sapiens (human)|KEGG	-0.32110746	0.007576427	-1.37119798	0.20571888	0.42698348	-5.42880132
WT after UVB irradiation VS WT before UVB irradiation	beta-Alanine metabolism - Homo sapiens (human)|KEGG	0.019278623	-0.03573241	0.08272686	0.93568813	0.98056728	-5.86568545
homo after UVB irradiation VS homo before UVB irradiation	Beta-Alanine Metabolism|SMPDB	0.066798026	0.046789851	0.254634663	0.80449493	0.87857543	-6.33373313
hetero after UVB irradiation VS hetero before UVB irradiation	Beta-Alanine Metabolism|SMPDB	-0.52991759	0.031774511	-1.70647522	0.12439725	0.31623126	-4.99631323
WT after UVB irradiation VS WT before UVB irradiation	Beta-Alanine Metabolism|SMPDB	-0.14708226	-0.03671277	-0.48165088	0.64033764	0.87636259	-5.75306298
homo after UVB irradiation VS homo before UVB irradiation	Beta-catenin independent WNT signaling|Reactome	0.283390244	-0.03415459	1.653083026	0.13130235	0.27325731	-5.08486675
hetero after UVB irradiation VS hetero before UVB irradiation	Beta-catenin independent WNT signaling|Reactome	0.325217104	0.008632683	1.904186176	0.09150896	0.27391014	-4.72042364
WT after UVB irradiation VS WT before UVB irradiation	Beta-catenin independent WNT signaling|Reactome	0.060793311	0.019527229	0.299496488	0.77064444	0.93158633	-5.82392713
homo after UVB irradiation VS homo before UVB irradiation	Beta-catenin independent WNT signaling|Wikipathways	-1.09397778	-0.02307877	-5.72618128	0.00024282	0.01666204	0.937535869
hetero after UVB irradiation VS hetero before UVB irradiation	Beta-catenin independent WNT signaling|Wikipathways	-0.9841652	-0.08492327	-5.1330679	0.00075899	0.03391648	-0.08963554
WT after UVB irradiation VS WT before UVB irradiation	Beta-catenin independent WNT signaling|Wikipathways	-1.16511645	0.014665793	-5.72236788	0.00018642	0.15645713	0.891016857
homo after UVB irradiation VS homo before UVB irradiation	Beta-catenin phosphorylation cascade|Reactome	-0.72001235	0.010588513	-4.47521143	0.00138879	0.02143814	-0.74607857
hetero after UVB irradiation VS hetero before UVB irradiation	Beta-catenin phosphorylation cascade|Reactome	-0.38980588	-0.05943667	-1.86457134	0.09735975	0.28193504	-4.77668992
WT after UVB irradiation VS WT before UVB irradiation	Beta-catenin phosphorylation cascade|Reactome	-0.48994974	0.005349867	-1.95292358	0.07912674	0.46479598	-4.25296278
homo after UVB irradiation VS homo before UVB irradiation	Betaine Metabolism|SMPDB	0.251696718	-0.04735892	1.217900214	0.2529815	0.41625712	-5.6301291
hetero after UVB irradiation VS hetero before UVB irradiation	Betaine Metabolism|SMPDB	0.311495112	0.047243939	1.330263665	0.21830868	0.44009331	-5.47779341
WT after UVB irradiation VS WT before UVB irradiation	Betaine Metabolism|SMPDB	0.641271091	0.041511638	2.443458387	0.03446085	0.38092943	-3.54289803
homo after UVB irradiation VS homo before UVB irradiation	Beta-Ketothiolase Deficiency|SMPDB	-0.23657683	-0.05522798	-0.8011182	0.4428632	0.59091566	-6.03520901
hetero after UVB irradiation VS hetero before UVB irradiation	Beta-Ketothiolase Deficiency|SMPDB	-0.90022729	-0.04719335	-5.31014337	0.00060637	0.03004176	0.126180045
WT after UVB irradiation VS WT before UVB irradiation	Beta-Ketothiolase Deficiency|SMPDB	-0.36319435	-0.13086657	-1.31407353	0.21793282	0.62581393	-5.06691375
homo after UVB irradiation VS homo before UVB irradiation	betaKlotho-mediated ligand binding|Reactome	0.285041309	-0.01134563	0.893016539	0.39416034	0.54703225	-5.9576814
hetero after UVB irradiation VS hetero before UVB irradiation	betaKlotho-mediated ligand binding|Reactome	0.008485178	0.007162283	0.021562687	0.98329872	0.99178481	-6.34628907
WT after UVB irradiation VS WT before UVB irradiation	betaKlotho-mediated ligand binding|Reactome	0.219974701	-0.07936794	0.614976815	0.55219642	0.84516674	-5.68133817
homo after UVB irradiation VS homo before UVB irradiation	Beta-mercaptolactate-cysteine disulfiduria|SMPDB	-0.57249069	0.017193233	-3.29055479	0.00885139	0.05619844	-2.55422007
hetero after UVB irradiation VS hetero before UVB irradiation	Beta-mercaptolactate-cysteine disulfiduria|SMPDB	-0.70250163	-0.01628473	-2.9739972	0.01676532	0.1140542	-3.11072933
WT after UVB irradiation VS WT before UVB irradiation	Beta-mercaptolactate-cysteine disulfiduria|SMPDB	-0.64815523	-0.01177362	-2.71723565	0.02151468	0.31542987	-3.13323441
homo after UVB irradiation VS homo before UVB irradiation	Beta-oxidation of pristanoyl-CoA|Reactome	-0.40535372	-0.08591487	-1.63556545	0.134958	0.277359	-5.10870191
hetero after UVB irradiation VS hetero before UVB irradiation	Beta-oxidation of pristanoyl-CoA|Reactome	-0.65272211	0.010159925	-2.95480085	0.01727829	0.11547575	-3.14003194
WT after UVB irradiation VS WT before UVB irradiation	Beta-oxidation of pristanoyl-CoA|Reactome	-0.76393722	-0.06358844	-3.64064423	0.00447165	0.20968398	-1.76124498
homo after UVB irradiation VS homo before UVB irradiation	Beta-oxidation of very long chain fatty acids|Reactome	0.206667089	-0.03624635	0.918294048	0.38144892	0.53484506	-5.9351141
hetero after UVB irradiation VS hetero before UVB irradiation	Beta-oxidation of very long chain fatty acids|Reactome	-0.09442748	-0.06423797	-0.34379171	0.73942507	0.87358721	-6.28288115
WT after UVB irradiation VS WT before UVB irradiation	Beta-oxidation of very long chain fatty acids|Reactome	0.112368932	-0.04956129	0.46505739	0.65177241	0.87935805	-5.76083462
homo after UVB irradiation VS homo before UVB irradiation	Betaxolol Action Pathway|SMPDB	0.396415397	-0.03677376	2.343069613	0.04268583	0.13402181	-4.06364635
hetero after UVB irradiation VS hetero before UVB irradiation	Betaxolol Action Pathway|SMPDB	-0.01555055	-0.01775524	-0.07410913	0.94265284	0.97054702	-6.34356282
WT after UVB irradiation VS WT before UVB irradiation	Betaxolol Action Pathway|SMPDB	0.056685205	-0.03978418	0.223404833	0.82768199	0.94525798	-5.84393438
homo after UVB irradiation VS homo before UVB irradiation	Betazole Action Pathway|SMPDB	0.21780303	0.051698704	1.19150037	0.26270351	0.42561191	-5.65959884
hetero after UVB irradiation VS hetero before UVB irradiation	Betazole Action Pathway|SMPDB	0.331566302	0.063470812	1.441200644	0.18563717	0.40321767	-5.34291209
WT after UVB irradiation VS WT before UVB irradiation	Betazole Action Pathway|SMPDB	-0.11560861	0.135982321	-0.44330845	0.66690385	0.87935805	-5.77062647
homo after UVB irradiation VS homo before UVB irradiation	Bevacizumab Action Pathway|SMPDB	1.057487254	-0.08050205	4.907864281	0.00074076	0.01666498	-0.13516395
hetero after UVB irradiation VS hetero before UVB irradiation	Bevacizumab Action Pathway|SMPDB	1.095078369	0.025726236	5.353206107	0.00057454	0.03004176	0.177885194
WT after UVB irradiation VS WT before UVB irradiation	Bevacizumab Action Pathway|SMPDB	0.761002659	0.062697285	2.18514203	0.05356237	0.41493406	-3.92244018
homo after UVB irradiation VS homo before UVB irradiation	Bevantolol Action Pathway|SMPDB	0.396415397	-0.03677376	2.343069613	0.04268583	0.13402181	-4.06364635
hetero after UVB irradiation VS hetero before UVB irradiation	Bevantolol Action Pathway|SMPDB	-0.01555055	-0.01775524	-0.07410913	0.94265284	0.97054702	-6.34356282
WT after UVB irradiation VS WT before UVB irradiation	Bevantolol Action Pathway|SMPDB	0.056685205	-0.03978418	0.223404833	0.82768199	0.94525798	-5.84393438
homo after UVB irradiation VS homo before UVB irradiation	BH3-only proteins associate with and inactivate anti-apoptotic BCL-2 members|Reactome	0.499962301	-0.0536437	2.568854258	0.02928505	0.11431817	-3.70757092
hetero after UVB irradiation VS hetero before UVB irradiation	BH3-only proteins associate with and inactivate anti-apoptotic BCL-2 members|Reactome	0.229033391	0.003306651	0.721224612	0.49029655	0.71035417	-6.07297284
WT after UVB irradiation VS WT before UVB irradiation	BH3-only proteins associate with and inactivate anti-apoptotic BCL-2 members|Reactome	0.361153585	-0.0372991	1.536708616	0.15513192	0.5591533	-4.80369301
homo after UVB irradiation VS homo before UVB irradiation	Bicarbonate transporters|Reactome	-0.00951551	-0.03458631	-0.04574172	0.96447426	0.97798567	-6.36732418
hetero after UVB irradiation VS hetero before UVB irradiation	Bicarbonate transporters|Reactome	-0.3652299	-0.02329297	-2.0862172	0.06868076	0.23513821	-4.4566598
WT after UVB irradiation VS WT before UVB irradiation	Bicarbonate transporters|Reactome	-0.4381316	-0.01408034	-1.90081203	0.08626689	0.47656734	-4.32523323
homo after UVB irradiation VS homo before UVB irradiation	Bile acid and bile salt metabolism|Reactome	0.121360656	0.028618387	0.685504166	0.50959531	0.64886006	-6.12225045
hetero after UVB irradiation VS hetero before UVB irradiation	Bile acid and bile salt metabolism|Reactome	-0.14008769	0.035218874	-0.72285369	0.48934562	0.70942663	-6.0717732
WT after UVB irradiation VS WT before UVB irradiation	Bile acid and bile salt metabolism|Reactome	-0.01253209	0.029820695	-0.0574614	0.95530054	0.98742794	-5.8674788
homo after UVB irradiation VS homo before UVB irradiation	bile acid biosynthesis_ neutral pathway|HumanCyc	0.047325796	-0.00664343	0.238778389	0.81640784	0.8875301	-6.33790897
hetero after UVB irradiation VS hetero before UVB irradiation	bile acid biosynthesis_ neutral pathway|HumanCyc	-0.18720343	0.035470648	-0.77284819	0.46073992	0.68466055	-6.03381038
WT after UVB irradiation VS WT before UVB irradiation	bile acid biosynthesis_ neutral pathway|HumanCyc	-0.06683918	0.029374029	-0.23656338	0.81773248	0.94525798	-5.84088547
homo after UVB irradiation VS homo before UVB irradiation	Bile acid biosynthesis|EHMN	0.118684693	-0.01512128	0.718055895	0.49020299	0.63156514	-6.09896109
hetero after UVB irradiation VS hetero before UVB irradiation	Bile acid biosynthesis|EHMN	0.004202871	0.0054653	0.022710916	0.98240953	0.99118272	-6.3462615

WT after UVB irradiation VS WT before UVB irradiation	Bile acid biosynthesis|EHMN	-0.00774893	0.012809543	-0.03901005	0.96964403	0.98979979	-5.86838033
homo after UVB irradiation VS homo before UVB irradiation	Bile Acid Biosynthesis|SMPDB	0.143425895	0.01576019	0.783036509	0.45290274	0.6002769	-6.04960791
hetero after UVB irradiation VS hetero before UVB irradiation	Bile Acid Biosynthesis|SMPDB	-0.01773938	0.040584313	-0.07540586	0.94165152	0.97054702	-6.34345771
WT after UVB irradiation VS WT before UVB irradiation	Bile Acid Biosynthesis|SMPDB	0.061518516	0.026229795	0.23751225	0.8170163	0.94525798	-5.84065895
homo after UVB irradiation VS homo before UVB irradiation	Bile Acids synthesis and enterohepatic circulation|Wikipathways	0.070180468	0.112921009	0.265827842	0.79611701	0.87255648	-6.33062606
hetero after UVB irradiation VS hetero before UVB irradiation	Bile Acids synthesis and enterohepatic circulation|Wikipathways	-0.21723918	-0.02470882	-0.79124069	0.45049989	0.67567572	-6.01929206
WT after UVB irradiation VS WT before UVB irradiation	Bile Acids synthesis and enterohepatic circulation|Wikipathways	-0.07967471	0.237567487	-0.22314657	0.82787759	0.94525798	-5.8439925
homo after UVB irradiation VS homo before UVB irradiation	Bile salt and organic anion SLC transporters|Reactome	0.378802552	-0.03114747	2.074816624	0.06655639	0.17939578	-4.47618378
hetero after UVB irradiation VS hetero before UVB irradiation	Bile salt and organic anion SLC transporters|Reactome	0.115221152	-0.02466067	0.611662072	0.55690113	0.76066987	-6.14808546
WT after UVB irradiation VS WT before UVB irradiation	Bile salt and organic anion SLC transporters|Reactome	0.034177992	0.00844226	0.133988808	0.89604833	0.97566251	-5.860065
homo after UVB irradiation VS homo before UVB irradiation	Bile secretion - Homo sapiens (human)|KEGG	0.596843616	0.03408346	3.729585258	0.00436467	0.0379263	-1.86450252
hetero after UVB irradiation VS hetero before UVB irradiation	Bile secretion - Homo sapiens (human)|KEGG	0.236476614	0.042124664	1.269460288	0.23821491	0.4612216	-5.54878959
WT after UVB irradiation VS WT before UVB irradiation	Bile secretion - Homo sapiens (human)|KEGG	0.332666518	0.031305456	1.499012189	0.1645132	0.56790008	-4.85005254
homo after UVB irradiation VS homo before UVB irradiation	Binding and entry of HIV virion|Reactome	0.498072507	-0.12103648	1.648632038	0.13222264	0.27454688	-5.09093584
hetero after UVB irradiation VS hetero before UVB irradiation	Binding and entry of HIV virion|Reactome	0.999695419	0.107272845	4.862842982	0.00107836	0.04104387	-0.42896617
WT after UVB irradiation VS WT before UVB irradiation	Binding and entry of HIV virion|Reactome	0.906622223	-0.17234771	3.171453146	0.00986508	0.25745039	-2.45138981
homo after UVB irradiation VS homo before UVB irradiation	Binding and Uptake of Ligands by Scavenger Receptors|Reactome	0.444830236	-0.01902704	2.181244271	0.05584234	0.16036233	-4.31426387
hetero after UVB irradiation VS hetero before UVB irradiation	Binding and Uptake of Ligands by Scavenger Receptors|Reactome	0.412729928	0.057864718	2.043869519	0.07344238	0.24222169	-4.51871209
WT after UVB irradiation VS WT before UVB irradiation	Binding and Uptake of Ligands by Scavenger Receptors|Reactome	0.399076172	0.016955176	1.43566069	0.18138863	0.598227	-4.92638241
homo after UVB irradiation VS homo before UVB irradiation	Binding of TCF/LEF:CTNNB1 to target gene promoters|Reactome	-0.14400123	0.007919855	-0.66546389	0.52176409	0.65938966	-6.13610367
hetero after UVB irradiation VS hetero before UVB irradiation	Binding of TCF/LEF:CTNNB1 to target gene promoters|Reactome	-0.16270892	-0.0089775	-0.68565008	0.51135422	0.73018563	-6.09857204
WT after UVB irradiation VS WT before UVB irradiation	Binding of TCF/LEF:CTNNB1 to target gene promoters|Reactome	-0.37535436	0.117689052	-1.59072139	0.14250784	0.55622203	-4.736107
homo after UVB irradiation VS homo before UVB irradiation	bioactive peptide induced signaling pathway|BioCarta	0.291229971	-0.01169848	1.390166492	0.19654949	0.35304681	-5.42691628
hetero after UVB irradiation VS hetero before UVB irradiation	bioactive peptide induced signaling pathway|BioCarta	0.229144478	0.017116338	1.224981693	0.25372071	0.4792619	-5.59931625
WT after UVB irradiation VS WT before UVB irradiation	bioactive peptide induced signaling pathway|BioCarta	0.174663648	-0.00695229	0.768977145	0.4595416	0.80891799	-5.57870311
homo after UVB irradiation VS homo before UVB irradiation	Biogenic Amine Synthesis|Wikipathways	0.545893362	-0.03157358	2.289241481	0.04668498	0.14070205	-4.14752525
hetero after UVB irradiation VS hetero before UVB irradiation	Biogenic Amine Synthesis|Wikipathways	-0.00034554	0.035997314	-0.00130999	0.99898527	0.99964322	-6.34654037
WT after UVB irradiation VS WT before UVB irradiation	Biogenic Amine Synthesis|Wikipathways	0.39738996	0.054629876	1.348598641	0.2069777	0.62198427	-5.02784413
homo after UVB irradiation VS homo before UVB irradiation	Biogenic amines are oxidatively deaminated to aldehydes by MAOA and MAOB|Reactome	0.449346563	-0.07747094	1.271628085	0.23409926	0.39574129	-5.56873584
hetero after UVB irradiation VS hetero before UVB irradiation	Biogenic amines are oxidatively deaminated to aldehydes by MAOA and MAOB|Reactome	0.198326803	0.060811856	0.432570694	0.67618549	0.8318605	-6.24617496
WT after UVB irradiation VS WT before UVB irradiation	Biogenic amines are oxidatively deaminated to aldehydes by MAOA and MAOB|Reactome	0.500063884	0.035509258	1.345120182	0.20806019	0.62329858	-5.03181126
homo after UVB irradiation VS homo before UVB irradiation	Biological oxidations|Reactome	0.175433169	0.000613578	0.988724115	0.34759164	0.50368246	-5.86950901
hetero after UVB irradiation VS hetero before UVB irradiation	Biological oxidations|Reactome	-0.00505987	0.006900362	-0.02684869	0.97920546	0.98989252	-6.34615026
WT after UVB irradiation VS WT before UVB irradiation	Biological oxidations|Reactome	0.086015136	0.001997783	0.380221795	0.71166823	0.90420468	-5.79647409
homo after UVB irradiation VS homo before UVB irradiation	Biopterin metabolism|EHMN	0.099427901	-0.01879056	0.532596566	0.60668576	0.72573414	-6.21835827
hetero after UVB irradiation VS hetero before UVB irradiation	Biopterin metabolism|EHMN	-0.00319641	0.01259682	-0.01938296	0.98498676	0.99251462	-6.34633749
WT after UVB irradiation VS WT before UVB irradiation	Biopterin metabolism|EHMN	-0.23219453	-0.07678334	-1.03045024	0.32688443	0.71049278	-5.35977614
homo after UVB irradiation VS homo before UVB irradiation	Biosynthesis of A2E_ implicated in retinal degradation|Reactome	0.151963221	-0.00337558	0.370120082	0.71950453	0.81577771	-6.29539034
hetero after UVB irradiation VS hetero before UVB irradiation	Biosynthesis of A2E_ implicated in retinal degradation|Reactome	-0.2697141	0.01289775	-0.64818052	0.53412945	0.74771561	-6.12428369
WT after UVB irradiation VS WT before UVB irradiation	Biosynthesis of A2E_ implicated in retinal degradation|Reactome	0.317720968	-0.00400158	0.731626644	0.48105584	0.81552033	-5.60547755
homo after UVB irradiation VS homo before UVB irradiation	Biosynthesis of aspirin-triggered D-series resolvins|Reactome	1.165232396	0.051525676	6.179117376	0.00013629	0.01666204	1.484966671
hetero after UVB irradiation VS hetero before UVB irradiation	Biosynthesis of aspirin-triggered D-series resolvins|Reactome	0.777727107	0.060207402	2.7114099	0.02537937	0.14017854	-3.51239236
WT after UVB irradiation VS WT before UVB irradiation	Biosynthesis of aspirin-triggered D-series resolvins|Reactome	0.625002892	0.045011267	1.986098056	0.07487362	0.45723857	-4.20655371
homo after UVB irradiation VS homo before UVB irradiation	Biosynthesis of DHA-derived SPMs|Reactome	0.648991561	0.029463986	3.254520087	0.00938862	0.05808098	-2.61153573
hetero after UVB irradiation VS hetero before UVB irradiation	Biosynthesis of DHA-derived SPMs|Reactome	0.279783072	0.047416413	1.236133944	0.24975642	0.47452679	-5.58676257
WT after UVB irradiation VS WT before UVB irradiation	Biosynthesis of DHA-derived SPMs|Reactome	0.096449629	0.067828331	0.375934014	0.71475527	0.90473595	-5.79809183
homo after UVB irradiation VS homo before UVB irradiation	Biosynthesis of DHA-derived sulfido conjugates|Reactome	0.351714302	-0.00870156	1.028201303	0.32961362	0.48580239	-5.8310297
hetero after UVB irradiation VS hetero before UVB irradiation	Biosynthesis of DHA-derived sulfido conjugates|Reactome	0.789529254	0.033448651	2.981614808	0.01656614	0.11354659	-3.09910571
WT after UVB irradiation VS WT before UVB irradiation	Biosynthesis of DHA-derived sulfido conjugates|Reactome	0.211396264	-0.03512927	0.456573166	0.65765598	0.87935805	-5.76470775
homo after UVB irradiation VS homo before UVB irradiation	Biosynthesis of DPA-derived SPMs|Reactome	0.556321783	0.051204546	2.378545336	0.04023616	0.13332244	-4.00812037
hetero after UVB irradiation VS hetero before UVB irradiation	Biosynthesis of DPA-derived SPMs|Reactome	0.716867654	0.106821083	3.198902877	0.0118073	0.10542662	-2.76893591
WT after UVB irradiation VS WT before UVB irradiation	Biosynthesis of DPA-derived SPMs|Reactome	0.481614224	0.018685209	2.168440378	0.05510008	0.41769317	-3.94661005
homo after UVB irradiation VS homo before UVB irradiation	Biosynthesis of DPAn-3 SPMs|Reactome	0.556321783	0.051204546	2.378545336	0.04023616	0.13332244	-4.00812037
hetero after UVB irradiation VS hetero before UVB irradiation	Biosynthesis of DPAn-3 SPMs|Reactome	0.716867654	0.106821083	3.198902877	0.0118073	0.10542662	-2.76893591
WT after UVB irradiation VS WT before UVB irradiation	Biosynthesis of DPAn-3 SPMs|Reactome	0.481614224	0.018685209	2.168440378	0.05510008	0.41769317	-3.94661005
homo after UVB irradiation VS homo before UVB irradiation	Biosynthesis of DPAn-3-derived 13-series resolvins|Reactome	1.124627237	-0.01546968	5.320526807	0.00041712	0.01666204	0.419393541
hetero after UVB irradiation VS hetero before UVB irradiation	Biosynthesis of DPAn-3-derived 13-series resolvins|Reactome	1.060306813	0.059313086	4.314997529	0.00227066	0.05380538	-1.15386252
WT after UVB irradiation VS WT before UVB irradiation	Biosynthesis of DPAn-3-derived 13-series resolvins|Reactome	0.785677262	0.000746408	2.304088826	0.04374766	0.39022568	-3.74887665
homo after UVB irradiation VS homo before UVB irradiation	Biosynthesis of DPAn-3-derived maresins|Reactome	0.113494996	0.01307249	0.291944979	0.77667682	0.85494367	-6.32286518
hetero after UVB irradiation VS hetero before UVB irradiation	Biosynthesis of DPAn-3-derived maresins|Reactome	0.558805295	0.016555579	2.533179537	0.03370089	0.16051059	-3.78486177
WT after UVB irradiation VS WT before UVB irradiation	Biosynthesis of DPAn-3-derived maresins|Reactome	0.294276733	0.038851735	1.395367526	0.19287651	0.60499108	-4.97385051
homo after UVB irradiation VS homo before UVB irradiation	Biosynthesis of DPAn-3-derived protectins and resolvins|Reactome	1.130148334	-0.04157466	5.613300672	0.00028163	0.01666204	0.796026052
hetero after UVB irradiation VS hetero before UVB irradiation	Biosynthesis of DPAn-3-derived protectins and resolvins|Reactome	1.055188628	0.013837018	5.670874875	0.00038902	0.03004176	0.549993297
WT after UVB irradiation VS WT before UVB irradiation	Biosynthesis of DPAn-3-derived protectins and resolvins|Reactome	0.863170772	-0.01933524	2.75415854	0.02018891	0.31542987	-3.07771909
homo after UVB irradiation VS homo before UVB irradiation	Biosynthesis of DPAn-6 SPMs|Reactome	-0.02834682	-0.03886316	-0.0751249	0.94169171	0.96450524	-6.36541678
hetero after UVB irradiation VS hetero before UVB irradiation	Biosynthesis of DPAn-6 SPMs|Reactome	0.177918029	0.085480754	0.439162305	0.67158887	0.82819347	-6.24312852
WT after UVB irradiation VS WT before UVB irradiation	Biosynthesis of DPAn-6 SPMs|Reactome	0.473703595	0.004540544	1.895104627	0.08708437	0.47748624	-4.33309957
homo after UVB irradiation VS homo before UVB irradiation	Biosynthesis of D-series resolvins|Reactome	1.000278175	0.030155671	5.318628026	0.0004182	0.01666204	0.416905419
hetero after UVB irradiation VS hetero before UVB irradiation	Biosynthesis of D-series resolvins|Reactome	0.481840157	0.07558701	1.452674495	0.18251424	0.40149609	-5.32859118
WT after UVB irradiation VS WT before UVB irradiation	Biosynthesis of D-series resolvins|Reactome	0.41232653	0.06078428	1.243435099	0.24184379	0.63358831	-5.14466847
homo after UVB irradiation VS homo before UVB irradiation	Biosynthesis of electrophilic ?-3 PUFA oxo-derivatives|Reactome	1.006710653	0.064890502	6.119288351	0.00014687	0.01666204	1.414502442
hetero after UVB irradiation VS hetero before UVB irradiation	Biosynthesis of electrophilic ?-3 PUFA oxo-derivatives|Reactome	1.137325436	0.020416142	6.57433205	0.00013792	0.02920766	1.521872713
WT after UVB irradiation VS WT before UVB irradiation	Biosynthesis of electrophilic ?-3 PUFA oxo-derivatives|Reactome	0.51845401	-0.01837107	1.489591671	0.1669333	0.57064699	-4.86153038
homo after UVB irradiation VS homo before UVB irradiation	Biosynthesis of EPA-derived SPMs|Reactome	1.063360084	0.003397857	6.46441366	9.60E-05	0.01666204	1.813414465
hetero after UVB irradiation VS hetero before UVB irradiation	Biosynthesis of EPA-derived SPMs|Reactome	0.679162102	0.073588091	3.305445214	0.01001855	0.10192984	-2.60831341
WT after UVB irradiation VS WT before UVB irradiation	Biosynthesis of EPA-derived SPMs|Reactome	0.479645766	0.053197785	1.510482001	0.16160783	0.56340685	-4.83601926
homo after UVB irradiation VS homo before UVB irradiation	Biosynthesis of E-series 18(R)-resolvins|Reactome	1.194839714	-0.01604137	7.218304907	4.00E-05	0.01666204	2.623480731
hetero after UVB irradiation VS hetero before UVB irradiation	Biosynthesis of E-series 18(R)-resolvins|Reactome	0.75406778	0.016545171	3.490449193	0.0075556	0.08807784	-2.33198533
WT after UVB irradiation VS WT before UVB irradiation	Biosynthesis of E-series 18(R)-resolvins|Reactome	0.67119187	0.020681897	2.099715599	0.06188427	0.43347466	-4.04547193
homo after UVB irradiation VS homo before UVB irradiation	Biosynthesis of E-series 18(S)-resolvins|Reactome	1.05612034	-0.02391956	6.028907056	0.00016456	0.01666204	1.30699787
hetero after UVB irradiation VS hetero before UVB irradiation	Biosynthesis of E-series 18(S)-resolvins|Reactome	0.495789899	0.04757365	1.922700258	0.08889054	0.26992877	-4.69397559
WT after UVB irradiation VS WT before UVB irradiation	Biosynthesis of E-series 18(S)-resolvins|Reactome	0.475284861	0.040992623	1.388122959	0.19500618	0.60625027	-4.98229291
homo after UVB irradiation VS homo before UVB irradiation	Biosynthesis of maresin conjugates in tissue regeneration (MCTR)|Reactome	0.351714302	-0.00870156	1.028201303	0.32961362	0.48580239	-5.8310297
hetero after UVB irradiation VS hetero before UVB irradiation	Biosynthesis of maresin conjugates in tissue regeneration (MCTR)|Reactome	0.789529254	0.033448651	2.981614808	0.01656614	0.11354659	-3.09910571
WT after UVB irradiation VS WT before UVB irradiation	Biosynthesis of maresin conjugates in tissue regeneration (MCTR)|Reactome	0.211396264	-0.03512927	0.456573166	0.65765598	0.87935805	-5.76470775
homo after UVB irradiation VS homo before UVB irradiation	Biosynthesis of maresin-like SPMs|Reactome	0.724884405	0.178503475	2.270197395	0.04818589	0.14342685	-4.17708524
hetero after UVB irradiation VS hetero before UVB irradiation	Biosynthesis of maresin-like SPMs|Reactome	-0.14760988	0.040949837	-0.3635581	0.72514129	0.86482318	-6.27541048
WT after UVB irradiation VS WT before UVB irradiation	Biosynthesis of maresin-like SPMs|Reactome	-0.53107171	0.216013909	-1.6785765	0.12391192	0.52904005	-4.62343435
homo after UVB irradiation VS homo before UVB irradiation	Biosynthesis of maresins|Reactome	0.862083168	0.090352507	3.150637589	0.01113407	0.06427076	-2.77718069
hetero after UVB irradiation VS hetero before UVB irradiation	Biosynthesis of maresins|Reactome	0.159555208	0.070852992	0.451287507	0.6631715	0.82651398	-6.23740995
WT after UVB irradiation VS WT before UVB irradiation	Biosynthesis of maresins|Reactome	0.220339142	0.167552208	0.750948601	0.46984785	0.81218589	-5.59177409
homo after UVB irradiation VS homo before UVB irradiation	Biosynthesis of protectin and resolvin conjugates in tissue regeneration (PCTR and RCTR)|Reactome	0.833457588	0.014889828	2.883879817	0.01731431	0.08295606	-3.20423701
hetero after UVB irradiation VS hetero before UVB irradiation	Biosynthesis of protectin and resolvin conjugates in tissue regeneration (PCTR and RCTR)|Reactome	1.008840031	0.039914705	4.569156203	0.00159864	0.04856602	-0.81146089
WT after UVB irradiation VS WT before UVB irradiation	Biosynthesis of protectin and resolvin conjugates in tissue regeneration (PCTR and RCTR)|Reactome	0.918496403	-0.04534428	2.907980507	0.01549302	0.29238268	-2.84633231
homo after UVB irradiation VS homo before UVB irradiation	Biosynthesis of protectins|Reactome	0.452306583	-0.06469458	1.890187601	0.08995132	0.21498584	-4.75004149
hetero after UVB irradiation VS hetero before UVB irradiation	Biosynthesis of protectins|Reactome	0.218586579	0.02134245	0.701634982	0.5018234	0.72041293	-6.08721128
WT after UVB irradiation VS WT before UVB irradiation	Biosynthesis of protectins|Reactome	0.298889243	-0.0398823	0.952082542	0.36332175	0.74528402	-5.43094243
homo after UVB irradiation VS homo before UVB irradiation	Biosynthesis of specialized proresolving mediators (SPMs)|Reactome	0.717903868	0.022217403	3.844529487	0.00364064	0.03413771	-1.68723217
hetero after UVB irradiation VS hetero before UVB irradiation	Biosynthesis of specialized proresolving mediators (SPMs)|Reactome	0.370788879	0.064853154	1.698869799	0.1258593	0.31888088	-5.00665957
WT after UVB irradiation VS WT before UVB irradiation	Biosynthesis of specialized proresolving mediators (SPMs)|Reactome	0.170481117	0.092851132	0.680707603	0.51138599	0.8267256	-5.64005628
homo after UVB irradiation VS homo before UVB irradiation	Biosynthesis of the N-glycan precursor (dolichol lipid-linked oligosaccharide_ LLO) and transfer to a nascent protein|Reactome	0.416101911	-0.02777012	2.286851323	0.04687082	0.14089015	-4.15123868
hetero after UVB irradiation VS hetero before UVB irradiation	Biosynthesis of the N-glycan precursor (dolichol lipid-linked oligosaccharide_ LLO) and transfer to a nascent protein|Reactome	0.302581111	-0.0077365	1.602920185	0.14570929	0.349733	-5.1352503
WT after UVB irradiation VS WT before UVB irradiation	Biosynthesis of the N-glycan precursor (dolichol lipid-linked oligosaccharide_ LLO) and transfer to a nascent protein|Reactome	0.346231982	-0.02133042	1.639309434	0.13193722	0.54612111	-4.67419747
homo after UVB irradiation VS homo before UVB irradiation	Biosynthesis of unsaturated fatty acids - Homo sapiens (human)|KEGG	-0.2423486	-0.0355059	-1.16466595	0.27289141	0.43614438	-5.68906994
hetero after UVB irradiation VS hetero before UVB irradiation	Biosynthesis of unsaturated fatty acids - Homo sapiens (human)|KEGG	-0.35060045	-0.07083833	-1.58854697	0.14891676	0.35555924	-5.15418808
WT after UVB irradiation VS WT before UVB irradiation	Biosynthesis of unsaturated fatty acids - Homo sapiens (human)|KEGG	-0.2676223	-0.05687901	-1.16905419	0.26927865	0.66280056	-5.22321368
homo after UVB irradiation VS homo before UVB irradiation	Biotin metabolism - Homo sapiens (human)|KEGG	-0.09110349	-0.07644896	-0.29365951	0.77540614	0.85468276	-6.32233073
hetero after UVB irradiation VS hetero before UVB irradiation	Biotin metabolism - Homo sapiens (human)|KEGG	-0.15357683	-0.08581325	-0.55516836	0.59320841	0.78464854	-6.18241277
WT after UVB irradiation VS WT before UVB irradiation	Biotin metabolism - Homo sapiens (human)|KEGG	0.121018938	-0.11916833	0.376537314	0.71432059	0.90443641	-5.79786529
homo after UVB irradiation VS homo before UVB irradiation	Biotin Metabolism|SMPDB	0.526884498	-0.07424403	2.530982847	0.03119777	0.11752935	-3.76768491
hetero after UVB irradiation VS hetero before UVB irradiation	Biotin Metabolism|SMPDB	0.118669319	-0.00962251	0.574549436	0.58060805	0.77397513	-6.17098126
WT after UVB irradiation VS WT before UVB irradiation	Biotin Metabolism|SMPDB	0.295241913	-0.10880856	0.874075054	0.40241759	0.77020249	-5.49715984
homo after UVB irradiation VS homo before UVB irradiation	Biotin transport and metabolism|Reactome	-0.32756684	-0.03250099	-0.98829974	0.34778879	0.50380825	-5.86991609
hetero after UVB irradiation VS hetero before UVB irradiation	Biotin transport and metabolism|Reactome	-0.83511276	-0.04363873	-4.54573279	0.00165053	0.04894587	-0.84257836
WT after UVB irradiation VS WT before UVB irradiation	Biotin transport and metabolism|Reactome	-0.02470101	-0.06480623	-0.11164644	0.9132952	0.97566251	-5.86284072
homo after UVB irradiation VS homo before UVB irradiation	biotin-carboxyl carrier protein assembly|HumanCyc	-0.52542718	-0.02991937	-1.35389951	0.20745612	0.36559037	-5.47120133
hetero after UVB irradiation VS hetero before UVB irradiation	biotin-carboxyl carrier protein assembly|HumanCyc	-0.63714538	-0.01469711	-2.91905654	0.01827714	0.11936114	-3.19463319
WT after UVB irradiation VS WT before UVB irradiation	biotin-carboxyl carrier protein assembly|HumanCyc	-0.51981735	-0.09276198	-1.77961584	0.10524669	0.50446894	-4.49002354
homo after UVB irradiation VS homo before UVB irradiation	Biotinidase Deficiency|SMPDB	0.526884498	-0.07424403	2.530982847	0.03119777	0.11752935	-3.76768491
hetero after UVB irradiation VS hetero before UVB irradiation	Biotinidase Deficiency|SMPDB	0.118669319	-0.00962251	0.574549436	0.58060805	0.77397513	-6.17098126
WT after UVB irradiation VS WT before UVB irradiation	Biotinidase Deficiency|SMPDB	0.295241913	-0.10880856	0.874075054	0.40241759	0.77020249	-5.49715984
homo after UVB irradiation VS homo before UVB irradiation	Bisoprolol Action Pathway|SMPDB	0.396415397	-0.03677376	2.343069613	0.04268583	0.13402181	-4.06364635
hetero after UVB irradiation VS hetero before UVB irradiation	Bisoprolol Action Pathway|SMPDB	-0.01555055	-0.01775524	-0.07410913	0.94265284	0.97054702	-6.34356282
WT after UVB irradiation VS WT before UVB irradiation	Bisoprolol Action Pathway|SMPDB	0.056685205	-0.03978418	0.223404833	0.82768199	0.94525798	-5.84393438
homo after UVB irradiation VS homo before UVB irradiation	Bisphosphonate Pathway_ Pharmacodynamics|PharmGKB	0.260174928	-0.03841774	1.016948189	0.33466556	0.4915994	-5.84212068
hetero after UVB irradiation VS hetero before UVB irradiation	Bisphosphonate Pathway_ Pharmacodynamics|PharmGKB	0.631123725	-0.02634281	2.913565793	0.01843577	0.11936114	-3.20302463
WT after UVB irradiation VS WT before UVB irradiation	Bisphosphonate Pathway_ Pharmacodynamics|PharmGKB	0.302749919	-0.03481471	1.234987881	0.24484113	0.63648666	-5.15376416
homo after UVB irradiation VS homo before UVB irradiation	Bivalirudin Action Pathway|SMPDB	0.538210712	0.026444726	3.007777411	0.01409598	0.07305822	-3.00571236
hetero after UVB irradiation VS hetero before UVB irradiation	Bivalirudin Action Pathway|SMPDB	0.558085555	0.09913938	2.984394118	0.01649409	0.11352943	-3.09486545
WT after UVB irradiation VS WT before UVB irradiation	Bivalirudin Action Pathway|SMPDB	0.440879848	0.077377741	1.569633667	0.14732454	0.55622203	-4.76265269
homo after UVB irradiation VS homo before UVB irradiation	Bladder cancer - Homo sapiens (human)|KEGG	0.1128921	0.01406245	0.527820872	0.60986908	0.72758029	-6.22099793
hetero after UVB irradiation VS hetero before UVB irradiation	Bladder cancer - Homo sapiens (human)|KEGG	0.259432478	0.048577631	1.405379673	0.1956894	0.4161797	-5.3871861
WT after UVB irradiation VS WT before UVB irradiation	Bladder cancer - Homo sapiens (human)|KEGG	0.05941413	0.013955337	0.275656843	0.78837897	0.93991114	-5.83081226
homo after UVB irradiation VS homo before UVB irradiation	Bladder Cancer|Wikipathways	0.220231875	-0.00843501	1.065135026	0.31343797	0.46923284	-5.79395424
hetero after UVB irradiation VS hetero before UVB irradiation	Bladder Cancer|Wikipathways	0.297558245	0.035127385	1.723102015	0.12125539	0.31331055	-4.97361972
WT after UVB irradiation VS WT before UVB irradiation	Bladder Cancer|Wikipathways	0.125037408	0.000665787	0.602691836	0.56002483	0.84914787	-5.68862663
homo after UVB irradiation VS homo before UVB irradiation	Blood Clotting Cascade|Wikipathways	0.246525321	0.007832087	1.495691742	0.16756633	0.31760392	-5.29386361
hetero after UVB irradiation VS hetero before UVB irradiation	Blood Clotting Cascade|Wikipathways	0.517586906	0.056917805	2.613814981	0.02963852	0.14964797	-3.66171619
WT after UVB irradiation VS WT before UVB irradiation	Blood Clotting Cascade|Wikipathways	0.402963828	-0.00738056	1.6201727	0.13601423	0.54838491	-4.69870286
homo after UVB irradiation VS homo before UVB irradiation	Blue diaper syndrome|SMPDB	0.234726031	0.017120546	1.156747303	0.27595732	0.43614438	-5.69767199
hetero after UVB irradiation VS hetero before UVB irradiation	Blue diaper syndrome|SMPDB	-0.03255037	0.028925632	-0.18154472	0.86023155	0.94503144	-6.32869738
WT after UVB irradiation VS WT before UVB irradiation	Blue diaper syndrome|SMPDB	-0.15908359	0.010252305	-0.80382265	0.44003996	0.79685269	-5.55266936
homo after UVB irradiation VS homo before UVB irradiation	BMAL1:CLOCK_NPAS2 activates circadian gene expression|Reactome	0.218136063	0.067398715	1.302007458	0.22394711	0.38369325	-5.53320556
hetero after UVB irradiation VS hetero before UVB irradiation	BMAL1:CLOCK_NPAS2 activates circadian gene expression|Reactome	0.092994074	0.005304851	0.396419732	0.70164614	0.84920422	-6.26209767
WT after UVB irradiation VS WT before UVB irradiation	BMAL1:CLOCK_NPAS2 activates circadian gene expression|Reactome	-0.16987497	-0.04582904	-0.66279887	0.52232264	0.83116638	-5.65168226
homo after UVB irradiation VS homo before UVB irradiation	BMAL1-CLOCK_NPAS2 activates circadian gene expression|Wikipathways	-0.15481901	0.072792144	-0.75731123	0.46744235	0.61261245	-6.0695973
hetero after UVB irradiation VS hetero before UVB irradiation	BMAL1-CLOCK_NPAS2 activates circadian gene expression|Wikipathways	-0.26523936	-0.00494648	-1.25687484	0.24252013	0.46543442	-5.56320982
WT after UVB irradiation VS WT before UVB irradiation	BMAL1-CLOCK_NPAS2 activates circadian gene expression|Wikipathways	-0.352757	-0.02882198	-1.40091546	0.19125894	0.60496032	-4.96736606

homo after UVB irradiation VS homo before UVB irradiation	BMP receptor signaling|PID	-0.16919168	-0.02708495	-0.73206462	0.48200156	0.62470029	-6.0886415
hetero after UVB irradiation VS hetero before UVB irradiation	BMP receptor signaling|PID	-0.49252737	-0.01778878	-3.03499467	0.01523744	0.11352943	-3.0177319
WT after UVB irradiation VS WT before UVB irradiation	BMP receptor signaling|PID	-0.35503356	-0.00820699	-1.51940567	0.1593784	0.56072663	-4.8250571
homo after UVB irradiation VS homo before UVB irradiation	BMP signaling Dro|INOH	-0.40187854	-0.01832861	-1.93869468	0.08314657	0.20455133	-4.67906644
hetero after UVB irradiation VS hetero before UVB irradiation	BMP signaling Dro|INOH	-0.54374576	-0.0083636	-3.04556788	0.01498764	0.1130168	-3.00163135
WT after UVB irradiation VS WT before UVB irradiation	BMP signaling Dro|INOH	-0.56415249	-0.05486618	-2.64204216	0.02448992	0.33026211	-3.24617007
homo after UVB irradiation VS homo before UVB irradiation	BMP Signaling Pathway in Eyelid Development|Wikipathways	-0.35296869	-0.00068605	-1.94951498	0.08169623	0.20262084	-4.66313381
hetero after UVB irradiation VS hetero before UVB irradiation	BMP Signaling Pathway in Eyelid Development|Wikipathways	-0.43167292	0.016832146	-2.22692885	0.05492183	0.20875475	-4.24802083
WT after UVB irradiation VS WT before UVB irradiation	BMP Signaling Pathway in Eyelid Development|Wikipathways	-0.46710746	0.066303195	-1.90018487	0.08635637	0.47656734	-4.32609812
homo after UVB irradiation VS homo before UVB irradiation	BMP Signalling Pathway|HumanCyc	-0.25265584	0.004951722	-1.08893589	0.30334094	0.46400493	-5.76952355
hetero after UVB irradiation VS hetero before UVB irradiation	BMP Signalling Pathway|HumanCyc	-0.43063981	-0.05133099	-2.64709637	0.02810995	0.14497177	-3.61081843
WT after UVB irradiation VS WT before UVB irradiation	BMP Signalling Pathway|HumanCyc	-0.52024255	-0.016197	-2.32363808	0.04231111	0.39022568	-3.720136
homo after UVB irradiation VS homo before UVB irradiation	BMP2 signaling TAK1|INOH	-0.42002976	-0.05669143	-2.18806927	0.05521529	0.15896155	-4.30379427
hetero after UVB irradiation VS hetero before UVB irradiation	BMP2 signaling TAK1|INOH	-0.34377693	-0.05831692	-1.74947802	0.11642169	0.30887663	-4.93741451
WT after UVB irradiation VS WT before UVB irradiation	BMP2 signaling TAK1|INOH	-0.62545589	-0.0423655	-2.68306764	0.02281903	0.31772718	-3.18457689
homo after UVB irradiation VS homo before UVB irradiation	BMP2 signaling TGF-beta MV|INOH	-0.1827047	-0.01642068	-0.85662948	0.41297811	0.56389296	-5.9892356
hetero after UVB irradiation VS hetero before UVB irradiation	BMP2 signaling TGF-beta MV|INOH	-0.25084289	0.014727181	-1.56901643	0.15337733	0.36392185	-5.17977793
WT after UVB irradiation VS WT before UVB irradiation	BMP2 signaling TGF-beta MV|INOH	-0.3669908	-0.01788965	-1.87111138	0.09060034	0.48188205	-4.36605888
homo after UVB irradiation VS homo before UVB irradiation	BMP2-WNT4-FOXO1 Pathway in Human Primary Endometrial Stromal Cell Differentiation|Wikipathways	-0.81086731	0.00913802	-4.73563772	0.00094826	0.01805472	-0.37478191
hetero after UVB irradiation VS hetero before UVB irradiation	BMP2-WNT4-FOXO1 Pathway in Human Primary Endometrial Stromal Cell Differentiation|Wikipathways	-0.63332342	-0.03675995	-3.28829491	0.01028615	0.10281636	-2.63410184
WT after UVB irradiation VS WT before UVB irradiation	BMP2-WNT4-FOXO1 Pathway in Human Primary Endometrial Stromal Cell Differentiation|Wikipathways	-0.6126925	-0.01596087	-2.70472398	0.02198342	0.31542987	-3.15203903
homo after UVB irradiation VS homo before UVB irradiation	Bone Morphogenic Protein (BMP) Signalling and Regulation|Wikipathways	-0.68054244	0.023787723	-2.9988158	0.01430678	0.07367082	-3.02006653
hetero after UVB irradiation VS hetero before UVB irradiation	Bone Morphogenic Protein (BMP) Signalling and Regulation|Wikipathways	-0.5667169	-0.07225829	-2.99537177	0.01621263	0.11352943	-3.07812082
WT after UVB irradiation VS WT before UVB irradiation	Bone Morphogenic Protein (BMP) Signalling and Regulation|Wikipathways	-0.48084734	-0.00108001	-2.17455161	0.05453257	0.41769317	-3.9377724
homo after UVB irradiation VS homo before UVB irradiation	bone remodeling|BioCarta	0.355098008	-0.01905529	1.702636398	0.12144098	0.26032841	-5.01672049
hetero after UVB irradiation VS hetero before UVB irradiation	bone remodeling|BioCarta	0.522953655	0.034669811	2.757181497	0.02360216	0.13628011	-3.44231525
WT after UVB irradiation VS WT before UVB irradiation	bone remodeling|BioCarta	0.22087568	-0.03805309	0.774884477	0.45619659	0.80891799	-5.57436081
homo after UVB irradiation VS homo before UVB irradiation	Bopindolol Action Pathway|SMPDB	0.396415397	-0.03677376	2.343069613	0.04268583	0.13402181	-4.06364635
hetero after UVB irradiation VS hetero before UVB irradiation	Bopindolol Action Pathway|SMPDB	-0.01555055	-0.01775524	-0.07410913	0.94265284	0.97054702	-6.34356282
WT after UVB irradiation VS WT before UVB irradiation	Bopindolol Action Pathway|SMPDB	0.056685205	-0.03978418	0.223404833	0.82768199	0.94525798	-5.84393438
homo after UVB irradiation VS homo before UVB irradiation	Brain-Derived Neurotrophic Factor (BDNF) signaling pathway|Wikipathways	0.196106932	-0.01095569	1.152316652	0.27768467	0.43781361	-5.70246601
hetero after UVB irradiation VS hetero before UVB irradiation	Brain-Derived Neurotrophic Factor (BDNF) signaling pathway|Wikipathways	0.151967918	-0.01020112	0.999675809	0.34530737	0.5746298	-5.83510647
WT after UVB irradiation VS WT before UVB irradiation	Brain-Derived Neurotrophic Factor (BDNF) signaling pathway|Wikipathways	0.098288348	-0.01053019	0.505257223	0.62423927	0.87257466	-5.74156088
homo after UVB irradiation VS homo before UVB irradiation	Branched-chain amino acid catabolism|Reactome	-0.38885034	-0.00957928	-1.21200158	0.25512806	0.41874827	-5.63675411
hetero after UVB irradiation VS hetero before UVB irradiation	Branched-chain amino acid catabolism|Reactome	-0.92089984	-0.07353438	-5.63182447	0.00040782	0.03004176	0.505128424
WT after UVB irradiation VS WT before UVB irradiation	Branched-chain amino acid catabolism|Reactome	-0.37880546	-0.09334663	-1.33305062	0.21185299	0.62581393	-5.04552319
homo after UVB irradiation VS homo before UVB irradiation	brca1 dependent ub ligase activity|BioCarta	-0.26589528	-0.02082021	-1.01217746	0.3368248	0.49261436	-5.84679338
hetero after UVB irradiation VS hetero before UVB irradiation	brca1 dependent ub ligase activity|BioCarta	0.065607801	-0.04498259	0.26311241	0.79878388	0.91506577	-6.30914336
WT after UVB irradiation VS WT before UVB irradiation	brca1 dependent ub ligase activity|BioCarta	0.237735673	0.002450464	0.793758685	0.44561548	0.802811	-5.56029186
homo after UVB irradiation VS homo before UVB irradiation	Breakdown of the nuclear lamina|Reactome	0.332481598	0.009303738	1.425810026	0.18631084	0.34164469	-5.38265798
hetero after UVB irradiation VS hetero before UVB irradiation	Breakdown of the nuclear lamina|Reactome	0.352095622	0.028382348	1.635125871	0.13874874	0.33884849	-5.09250129
WT after UVB irradiation VS WT before UVB irradiation	Breakdown of the nuclear lamina|Reactome	0.400699752	0.010901207	1.675612575	0.12450203	0.52904005	-4.62728806
homo after UVB irradiation VS homo before UVB irradiation	Breast cancer - Homo sapiens (human)|KEGG	-0.15645768	-0.00202697	-0.97340938	0.3547588	0.50970065	-5.88411042
hetero after UVB irradiation VS hetero before UVB irradiation	Breast cancer - Homo sapiens (human)|KEGG	-0.08309081	0.013101062	-0.5040275	0.62715511	0.80760501	-6.21082159
WT after UVB irradiation VS WT before UVB irradiation	Breast cancer - Homo sapiens (human)|KEGG	-0.13276703	0.023704823	-0.65424985	0.52759205	0.83278813	-5.6571327
homo after UVB irradiation VS homo before UVB irradiation	Breast cancer pathway|Wikipathways	-0.2230377	-0.00634365	-1.37172053	0.20203427	0.35894742	-5.44953611
hetero after UVB irradiation VS hetero before UVB irradiation	Breast cancer pathway|Wikipathways	-0.09553933	-0.00024218	-0.59603873	0.56681271	0.76437184	-6.15788404
WT after UVB irradiation VS WT before UVB irradiation	Breast cancer pathway|Wikipathways	-0.14683249	0.014528156	-0.71150585	0.49290426	0.81710765	-5.6194088
homo after UVB irradiation VS homo before UVB irradiation	Bromfenac Action Pathway|SMPDB	0.40423582	0.045705158	1.979500697	0.07780079	0.19626432	-4.61879689
hetero after UVB irradiation VS hetero before UVB irradiation	Bromfenac Action Pathway|SMPDB	0.482496143	0.064876536	3.04499191	0.01500113	0.1130168	-3.00250826
WT after UVB irradiation VS WT before UVB irradiation	Bromfenac Action Pathway|SMPDB	0.275725339	0.086595857	1.261970114	0.23537138	0.62952955	-5.12455679
homo after UVB irradiation VS homo before UVB irradiation	btg family proteins and cell cycle regulation|BioCarta	-0.51806416	-0.06562449	-1.8598274	0.09447231	0.22098427	-4.79407373
hetero after UVB irradiation VS hetero before UVB irradiation	btg family proteins and cell cycle regulation|BioCarta	-0.01230794	-0.01858575	-0.04403663	0.965901	0.98439183	-6.3454894
WT after UVB irradiation VS WT before UVB irradiation	btg family proteins and cell cycle regulation|BioCarta	-0.04657585	-0.03133294	-0.18869393	0.85407686	0.95632621	-5.8511479
homo after UVB irradiation VS homo before UVB irradiation	Budding and maturation of HIV virion|Reactome	0.439579394	-0.04372666	1.923548735	0.08521752	0.20754519	-4.70130787
hetero after UVB irradiation VS hetero before UVB irradiation	Budding and maturation of HIV virion|Reactome	0.333148349	0.000696515	1.524807161	0.16392088	0.37677831	-5.237075
WT after UVB irradiation VS WT before UVB irradiation	Budding and maturation of HIV virion|Reactome	0.123373956	-0.03420527	0.483349797	0.63917237	0.87636259	-5.75225265
homo after UVB irradiation VS homo before UVB irradiation	Bumetanide Action Pathway|SMPDB	0.234726031	0.017120546	1.156747303	0.27595732	0.43614438	-5.69767199
hetero after UVB irradiation VS hetero before UVB irradiation	Bumetanide Action Pathway|SMPDB	-0.03255037	0.028925632	-0.18154472	0.86023155	0.94503144	-6.32869738
WT after UVB irradiation VS WT before UVB irradiation	Bumetanide Action Pathway|SMPDB	-0.15908359	0.010252305	-0.80382265	0.44003996	0.79685269	-5.55266936
homo after UVB irradiation VS homo before UVB irradiation	Bupivacaine Action Pathway|SMPDB	0.191132361	0.029768717	1.061313976	0.31508276	0.46923284	-5.79783739
hetero after UVB irradiation VS hetero before UVB irradiation	Bupivacaine Action Pathway|SMPDB	-0.07904315	0.055005373	-0.44437398	0.66796482	0.82651398	-6.24068873
WT after UVB irradiation VS WT before UVB irradiation	Bupivacaine Action Pathway|SMPDB	0.022316377	0.035038618	0.098344171	0.92358676	0.97566251	-5.86425414
homo after UVB irradiation VS homo before UVB irradiation	Bupranolol Action Pathway|SMPDB	0.396415397	-0.03677376	2.343069613	0.04268583	0.13402181	-4.06364635
hetero after UVB irradiation VS hetero before UVB irradiation	Bupranolol Action Pathway|SMPDB	-0.01555055	-0.01775524	-0.07410913	0.94265284	0.97054702	-6.34356282
WT after UVB irradiation VS WT before UVB irradiation	Bupranolol Action Pathway|SMPDB	0.056685205	-0.03978418	0.223404833	0.82768199	0.94525798	-5.84393438
homo after UVB irradiation VS homo before UVB irradiation	Buprenorphine Action Pathway|SMPDB	0.191132361	0.029768717	1.061313976	0.31508276	0.46923284	-5.79783739
hetero after UVB irradiation VS hetero before UVB irradiation	Buprenorphine Action Pathway|SMPDB	-0.07904315	0.055005373	-0.44437398	0.66796482	0.82651398	-6.24068873
WT after UVB irradiation VS WT before UVB irradiation	Buprenorphine Action Pathway|SMPDB	0.022316377	0.035038618	0.098344171	0.92358676	0.97566251	-5.86425414
homo after UVB irradiation VS homo before UVB irradiation	bupropion degradation|HumanCyc	-0.37664642	0.062954483	-1.42538314	0.1864307	0.34172725	-5.38319227
hetero after UVB irradiation VS hetero before UVB irradiation	bupropion degradation|HumanCyc	0.008312124	-0.00825173	0.029177609	0.97740224	0.98924321	-6.34607948
WT after UVB irradiation VS WT before UVB irradiation	bupropion degradation|HumanCyc	-0.07204357	-0.0547575	-0.26283161	0.79797319	0.94477083	-5.83428399
homo after UVB irradiation VS homo before UVB irradiation	Busulfan Pathway_ Pharmacodynamics|PharmGKB	0.15926006	-0.02371832	0.951019589	0.36543179	0.51899271	-5.90512373
hetero after UVB irradiation VS hetero before UVB irradiation	Busulfan Pathway_ Pharmacodynamics|PharmGKB	0.0402454	-0.01907704	0.208905381	0.83948436	0.93932613	-6.32292892
WT after UVB irradiation VS WT before UVB irradiation	Busulfan Pathway_ Pharmacodynamics|PharmGKB	0.31757829	-0.01539125	1.502028195	0.16374487	0.56782293	-4.84636868
homo after UVB irradiation VS homo before UVB irradiation	Butanoate metabolism - Homo sapiens (human)|KEGG	-0.27742728	0.004629639	-1.2995977	0.22473881	0.38418473	-5.53604496
hetero after UVB irradiation VS hetero before UVB irradiation	Butanoate metabolism - Homo sapiens (human)|KEGG	-0.38394351	-0.02626773	-2.20418263	0.05694682	0.2131064	-4.28197605
WT after UVB irradiation VS WT before UVB irradiation	Butanoate metabolism - Homo sapiens (human)|KEGG	-0.23858306	0.028492576	-0.85525067	0.41227504	0.77856786	-5.51242414
homo after UVB irradiation VS homo before UVB irradiation	Butanoate metabolism|EHMN	0.132143097	0.006037593	0.801158252	0.44284113	0.59091566	-6.0351768
hetero after UVB irradiation VS hetero before UVB irradiation	Butanoate metabolism|EHMN	-0.0903663	-0.01117568	-0.50705018	0.62512127	0.80602623	-6.2092139
WT after UVB irradiation VS WT before UVB irradiation	Butanoate metabolism|EHMN	0.014642276	0.029141208	0.065845232	0.94878841	0.98569533	-5.86695531
homo after UVB irradiation VS homo before UVB irradiation	Butanoate metabolism|INOH	-0.31047736	-0.0182432	-1.29270767	0.22701531	0.38662884	-5.54414352
hetero after UVB irradiation VS hetero before UVB irradiation	Butanoate metabolism|INOH	-0.53550554	-0.05521028	-3.02560856	0.01546281	0.11352943	-3.03202988
WT after UVB irradiation VS WT before UVB irradiation	Butanoate metabolism|INOH	-0.34432137	0.005247263	-1.20028785	0.25747015	0.65181051	-5.19066
homo after UVB irradiation VS homo before UVB irradiation	Butyrate Metabolism|SMPDB	0.035174059	0.00457248	0.161459408	0.87515265	0.92358867	-6.35446111
hetero after UVB irradiation VS hetero before UVB irradiation	Butyrate Metabolism|SMPDB	-0.37089838	0.030104937	-1.24983272	0.24495726	0.46814053	-5.57123639
WT after UVB irradiation VS WT before UVB irradiation	Butyrate Metabolism|SMPDB	-0.37679555	-0.06234159	-1.21011064	0.2538432	0.64837914	-5.18029269
homo after UVB irradiation VS homo before UVB irradiation	Butyrate Response Factor 1 (BRF1) binds and destabilizes mRNA|Reactome	-0.68271679	0.021674265	-4.15230411	0.00225918	0.02672385	-1.22089477
hetero after UVB irradiation VS hetero before UVB irradiation	Butyrate Response Factor 1 (BRF1) binds and destabilizes mRNA|Reactome	-0.40245755	-0.05145648	-1.64174815	0.13735555	0.33695727	-5.0836581
WT after UVB irradiation VS WT before UVB irradiation	Butyrate Response Factor 1 (BRF1) binds and destabilizes mRNA|Reactome	-0.11401873	0.055446131	-0.37104747	0.71827997	0.90615488	-5.79991378
homo after UVB irradiation VS homo before UVB irradiation	Butyrophilin (BTN) family interactions|Reactome	-0.38911515	-0.03688402	-1.43237624	0.18447554	0.33895989	-5.37442722
hetero after UVB irradiation VS hetero before UVB irradiation	Butyrophilin (BTN) family interactions|Reactome	-0.28623201	-0.04896986	-1.03172716	0.33090204	0.55930944	-5.80375413
WT after UVB irradiation VS WT before UVB irradiation	Butyrophilin (BTN) family interactions|Reactome	-0.54791447	0.019157914	-2.73711129	0.02079053	0.31542987	-3.10335397
homo after UVB irradiation VS homo before UVB irradiation	B-WICH complex positively regulates rRNA expression|Reactome	-0.75709179	-0.00076791	-4.61976665	0.00112235	0.01929633	-0.53866354
hetero after UVB irradiation VS hetero before UVB irradiation	B-WICH complex positively regulates rRNA expression|Reactome	-0.4680743	-0.04858386	-1.98024788	0.08120047	0.25702203	-4.611186
WT after UVB irradiation VS WT before UVB irradiation	B-WICH complex positively regulates rRNA expression|Reactome	-0.42972856	-0.01951686	-1.65196497	0.1293013	0.54076834	-4.65790647
homo after UVB irradiation VS homo before UVB irradiation	C20 prostanoid biosynthesis|HumanCyc	0.246831812	0.052778397	1.484940451	0.17033871	0.32096638	-5.3076938
hetero after UVB irradiation VS hetero before UVB irradiation	C20 prostanoid biosynthesis|HumanCyc	0.048227485	0.098506369	0.22354076	0.82843953	0.93350491	-6.31951499
WT after UVB irradiation VS WT before UVB irradiation	C20 prostanoid biosynthesis|HumanCyc	0.12487832	0.085186204	0.53486963	0.60433503	0.87002106	-5.7263972
homo after UVB irradiation VS homo before UVB irradiation	C21-steroid hormone biosynthesis and metabolism|EHMN	0.307563951	0.070325742	1.783530432	0.10678421	0.23959543	-4.90330809
hetero after UVB irradiation VS hetero before UVB irradiation	C21-steroid hormone biosynthesis and metabolism|EHMN	0.296391748	0.048225108	1.447596073	0.18389074	0.40321767	-5.33493796
WT after UVB irradiation VS WT before UVB irradiation	C21-steroid hormone biosynthesis and metabolism|EHMN	0.079310723	0.033515114	0.379305704	0.71232733	0.90425832	-5.79682122
homo after UVB irradiation VS homo before UVB irradiation	C6 deamination of adenosine|Reactome	0.544034782	-0.07960566	1.637930322	0.1344592	0.277359	-5.10549214
hetero after UVB irradiation VS hetero before UVB irradiation	C6 deamination of adenosine|Reactome	0.660054131	0.017061716	2.04785425	0.07298105	0.24158558	-4.51288957
WT after UVB irradiation VS WT before UVB irradiation	C6 deamination of adenosine|Reactome	0.295358316	-0.12669247	1.110293303	0.29265056	0.67947895	-5.28271793
homo after UVB irradiation VS homo before UVB irradiation	Ca2+ activated K+ channels|Reactome	0.175673495	-0.01978625	0.977756114	0.35271358	0.50783175	-5.87998488
hetero after UVB irradiation VS hetero before UVB irradiation	Ca2+ activated K+ channels|Reactome	-0.076435	-0.03082797	-0.40428208	0.69607342	0.84695747	-6.25874811
WT after UVB irradiation VS WT before UVB irradiation	Ca2+ activated K+ channels|Reactome	-0.14641165	0.007851747	-0.51563326	0.61722765	0.87257466	-5.73634037
homo after UVB irradiation VS homo before UVB irradiation	Ca2+ pathway|Reactome	0.234983484	-0.01239987	1.293239078	0.22683906	0.38647281	-5.54351996
hetero after UVB irradiation VS hetero before UVB irradiation	Ca2+ pathway|Reactome	0.191375131	0.007854712	1.03769642	0.32827071	0.55664357	-5.79783087
WT after UVB irradiation VS WT before UVB irradiation	Ca2+ pathway|Reactome	-0.01592497	-0.01093389	-0.0784936	0.93897145	0.98062137	-5.86603096
homo after UVB irradiation VS homo before UVB irradiation	ca-calmodulin-dependent protein kinase activation|BioCarta	0.308913873	-0.06065922	1.152444512	0.2776347	0.43781361	-5.70232786
hetero after UVB irradiation VS hetero before UVB irradiation	ca-calmodulin-dependent protein kinase activation|BioCarta	-0.28371582	-0.09593775	-0.89034966	0.39798609	0.62595603	-5.93610845
WT after UVB irradiation VS WT before UVB irradiation	ca-calmodulin-dependent protein kinase activation|BioCarta	0.165374107	-0.0442668	0.623913392	0.5465409	0.84264773	-5.67595111
homo after UVB irradiation VS homo before UVB irradiation	Ca-dependent events|Reactome	0.430314379	-0.03859654	2.306747073	0.04534571	0.13845639	-4.12029854
hetero after UVB irradiation VS hetero before UVB irradiation	Ca-dependent events|Reactome	0.322697412	0.00085511	1.669625218	0.13162987	0.32821059	-5.0462394
WT after UVB irradiation VS WT before UVB irradiation	Ca-dependent events|Reactome	0.310874333	-0.02251432	1.245281195	0.24119271	0.63358831	-5.14267479
homo after UVB irradiation VS homo before UVB irradiation	cadmium induces dna synthesis and proliferation in macrophages|BioCarta	0.313674588	-0.00801712	1.579747686	0.14722371	0.29239819	-5.18371982
hetero after UVB irradiation VS hetero before UVB irradiation	cadmium induces dna synthesis and proliferation in macrophages|BioCarta	0.404479649	-0.00191798	2.282788313	0.05024528	0.19880033	-4.16431277
WT after UVB irradiation VS WT before UVB irradiation	cadmium induces dna synthesis and proliferation in macrophages|BioCarta	0.01861383	0.021485817	0.078223966	0.93918062	0.98062137	-5.86605235
homo after UVB irradiation VS homo before UVB irradiation	Caffeine and Theobromine metabolism|Wikipathways	0.788853105	0.037468873	2.62589787	0.02662297	0.10805374	-3.61680351
hetero after UVB irradiation VS hetero before UVB irradiation	Caffeine and Theobromine metabolism|Wikipathways	0.006052937	-0.07365223	0.016701691	0.98706333	0.99338368	-6.34638997
WT after UVB irradiation VS WT before UVB irradiation	Caffeine and Theobromine metabolism|Wikipathways	0.689768566	0.01532372	3.141399333	0.01038432	0.26101395	-2.4962633
homo after UVB irradiation VS homo before UVB irradiation	Caffeine metabolism - Homo sapiens (human)|KEGG	0.86422878	0.027254229	2.825506454	0.01908099	0.08811647	-3.29776212
hetero after UVB irradiation VS hetero before UVB irradiation	Caffeine metabolism - Homo sapiens (human)|KEGG	0.317295214	-0.03396336	1.013788202	0.33890703	0.5677486	-5.82139632
WT after UVB irradiation VS WT before UVB irradiation	Caffeine metabolism - Homo sapiens (human)|KEGG	0.78175896	0.021880189	3.629044165	0.00455882	0.20968398	-1.77801593
homo after UVB irradiation VS homo before UVB irradiation	Caffeine Metabolism|SMPDB	0.785793749	0.149978125	2.449850503	0.03572425	0.12504267	-3.89599259
hetero after UVB irradiation VS hetero before UVB irradiation	Caffeine Metabolism|SMPDB	0.118525547	-0.00745944	0.315784941	0.75984637	0.88865506	-6.29277069
WT after UVB irradiation VS WT before UVB irradiation	Caffeine Metabolism|SMPDB	-0.30041957	0.152668064	-1.23914347	0.24336285	0.63440287	-5.14929508
homo after UVB irradiation VS homo before UVB irradiation	Caffeine Pathway_ Pharmacokinetics|PharmGKB	0.799946348	0.064191119	2.781924998	0.02051841	0.09131291	-3.36754851
hetero after UVB irradiation VS hetero before UVB irradiation	Caffeine Pathway_ Pharmacokinetics|PharmGKB	-0.1741616	-0.10658117	-0.56449938	0.58712341	0.7797519	-6.17695435
WT after UVB irradiation VS WT before UVB irradiation	Caffeine Pathway_ Pharmacokinetics|PharmGKB	0.053152555	0.16023432	0.241927849	0.81368586	0.94525798	-5.83959303
homo after UVB irradiation VS homo before UVB irradiation	Calcineurin activates NFAT|Reactome	0.439308362	-0.09128675	1.649550341	0.1320323	0.27427638	-5.08968441
hetero after UVB irradiation VS hetero before UVB irradiation	Calcineurin activates NFAT|Reactome	0.085096064	-0.03747866	0.249112601	0.80923905	0.92095935	-6.31300265
WT after UVB irradiation VS WT before UVB irradiation	Calcineurin activates NFAT|Reactome	0.367968754	-0.05996386	1.165305169	0.27072447	0.66327566	-5.22707858
homo after UVB irradiation VS homo before UVB irradiation	Calcineurin-regulated NFAT-dependent transcription in lymphocytes|PID	0.118972129	-0.03393868	0.713024553	0.49316987	0.63423973	-6.10262402
hetero after UVB irradiation VS hetero before UVB irradiation	Calcineurin-regulated NFAT-dependent transcription in lymphocytes|PID	0.182971183	-0.00179037	1.003543659	0.34354417	0.57260541	-5.83136375
WT after UVB irradiation VS WT before UVB irradiation	Calcineurin-regulated NFAT-dependent transcription in lymphocytes|PID	0.093854154	-0.04626008	0.438245779	0.67044902	0.87984445	-5.77284133
homo after UVB irradiation VS homo before UVB irradiation	Calcitonin-like ligand receptors|Reactome	0.978853525	-0.03215316	5.856697076	0.00020502	0.01666204	1.098608191
hetero after UVB irradiation VS hetero before UVB irradiation	Calcitonin-like ligand receptors|Reactome	0.428538435	0.050023042	1.681680729	0.12922222	0.32389948	-5.02996308
WT after UVB irradiation VS WT before UVB irradiation	Calcitonin-like ligand receptors|Reactome	0.64123356	0.002308577	2.219290952	0.05054577	0.41004423	-3.87285875
homo after UVB irradiation VS homo before UVB irradiation	Calcium Regulation in the Cardiac Cell|Wikipathways	0.427153755	-0.02619245	2.628791038	0.0264946	0.10782028	-3.61219362
hetero after UVB irradiation VS hetero before UVB irradiation	Calcium Regulation in the Cardiac Cell|Wikipathways	0.266291582	0.004418363	1.388759868	0.20051182	0.42000591	-5.4074988
WT after UVB irradiation VS WT before UVB irradiation	Calcium Regulation in the Cardiac Cell|Wikipathways	0.225285233	-0.01487174	0.935192475	0.37154702	0.74850253	-5.44567841
homo after UVB irradiation VS homo before UVB irradiation	calcium signaling by hbx of hepatitis b virus|BioCarta	0.264537166	-0.00385871	1.132767969	0.28540858	0.44583495	-5.72345381
hetero after UVB irradiation VS hetero before UVB irradiation	calcium signaling by hbx of hepatitis b virus|BioCarta	0.732238537	0.034812905	4.121957857	0.00298297	0.06148411	-1.42079938
WT after UVB irradiation VS WT before UVB irradiation	calcium signaling by hbx of hepatitis b virus|BioCarta	0.469690203	0.023575696	2.153129029	0.05654693	0.41923674	-3.96872003
homo after UVB irradiation VS homo before UVB irradiation	Calcium signaling in the CD4+ TCR pathway|PID	0.195117999	-0.05752833	1.246020193	0.24294942	0.40456129	-5.59823091

hetero after UVB irradiation VS hetero before UVB irradiation	Calcium signaling in the CD4+ TCR pathway|PID	0.082425101	0.010075177	0.359567261	0.72801628	0.86752894	-6.27695142
WT after UVB irradiation VS WT before UVB irradiation	Calcium signaling in the CD4+ TCR pathway|PID	0.156292715	-0.04139793	0.653971047	0.52776442	0.83278813	-5.65730936
homo after UVB irradiation VS homo before UVB irradiation	Calcium signaling pathway - Homo sapiens (human)|KEGG	0.360298005	-0.02017473	2.120525477	0.06173234	0.17041708	-4.40696271
hetero after UVB irradiation VS hetero before UVB irradiation	Calcium signaling pathway - Homo sapiens (human)|KEGG	0.112716753	0.002432709	0.535392116	0.60621736	0.79332148	-6.19370198
WT after UVB irradiation VS WT before UVB irradiation	Calcium signaling pathway - Homo sapiens (human)|KEGG	0.168639616	-0.00082114	0.673414804	0.5158229	0.82895075	-5.64482455
homo after UVB irradiation VS homo before UVB irradiation	Calmodulin induced events|Reactome	0.473516245	-0.0388142	2.641222205	0.02595008	0.10655402	-3.59237986
hetero after UVB irradiation VS hetero before UVB irradiation	Calmodulin induced events|Reactome	0.353178057	0.014248241	1.653514828	0.13491156	0.3336554	-5.06790164
WT after UVB irradiation VS WT before UVB irradiation	Calmodulin induced events|Reactome	0.367302447	-0.02877446	1.419377483	0.18595884	0.59934652	-4.94566878
homo after UVB irradiation VS homo before UVB irradiation	Calnexin/calreticulin cycle|Reactome	0.340311447	-0.01848518	1.609254855	0.14062074	0.28372705	-5.14424202
hetero after UVB irradiation VS hetero before UVB irradiation	Calnexin/calreticulin cycle|Reactome	0.548435653	0.026049587	3.099108879	0.01378638	0.1130168	-2.92020097
WT after UVB irradiation VS WT before UVB irradiation	Calnexin/calreticulin cycle|Reactome	0.299634801	0.012175574	1.27158855	0.23206875	0.62952955	-5.11403773
homo after UVB irradiation VS homo before UVB irradiation	Caloric restriction and aging|Wikipathways	-0.10407335	0.035222546	-0.46582161	0.65196039	0.75959458	-6.25322326
hetero after UVB irradiation VS hetero before UVB irradiation	Caloric restriction and aging|Wikipathways	-0.31456596	-0.0259009	-1.32096846	0.22125737	0.44238028	-5.48878652
WT after UVB irradiation VS WT before UVB irradiation	Caloric restriction and aging|Wikipathways	-0.19885422	-0.02973919	-0.87380852	0.40255602	0.77020249	-5.49737794
homo after UVB irradiation VS homo before UVB irradiation	CaM pathway|Reactome	0.473516245	-0.0388142	2.641222205	0.02595008	0.10655402	-3.59237986
hetero after UVB irradiation VS hetero before UVB irradiation	CaM pathway|Reactome	0.353178057	0.014248241	1.653514828	0.13491156	0.3336554	-5.06790164
WT after UVB irradiation VS WT before UVB irradiation	CaM pathway|Reactome	0.367302447	-0.02877446	1.419377483	0.18595884	0.59934652	-4.94566878
homo after UVB irradiation VS homo before UVB irradiation	CaMK IV-mediated phosphorylation of CREB|Reactome	0.323979766	0.021456418	0.899507612	0.39086789	0.5441167	-5.95193649
hetero after UVB irradiation VS hetero before UVB irradiation	CaMK IV-mediated phosphorylation of CREB|Reactome	0.164379997	0.041773073	0.47720164	0.64535116	0.82080804	-6.22469254
WT after UVB irradiation VS WT before UVB irradiation	CaMK IV-mediated phosphorylation of CREB|Reactome	0.469560674	-0.12603594	1.435076842	0.18155083	0.598227	-4.92707634
homo after UVB irradiation VS homo before UVB irradiation	cAMP signaling pathway - Homo sapiens (human)|KEGG	0.376003385	-0.01319983	2.401717746	0.03871139	0.12940262	-3.97175461
hetero after UVB irradiation VS hetero before UVB irradiation	cAMP signaling pathway - Homo sapiens (human)|KEGG	0.11506429	0.013619821	0.6402036	0.53905569	0.75123046	-6.12958953
WT after UVB irradiation VS WT before UVB irradiation	cAMP signaling pathway - Homo sapiens (human)|KEGG	0.161951574	-0.01851655	0.714793478	0.49095602	0.81710765	-5.61715627
homo after UVB irradiation VS homo before UVB irradiation	Cam-PDE 1 activation|Reactome	0.60875932	0.0124572	1.92552831	0.08494412	0.20724091	-4.69840498
hetero after UVB irradiation VS hetero before UVB irradiation	Cam-PDE 1 activation|Reactome	0.256229743	-0.06020762	0.684539131	0.51202076	0.7303506	-6.09935295
WT after UVB irradiation VS WT before UVB irradiation	Cam-PDE 1 activation|Reactome	0.372864168	-0.09464626	1.079516419	0.30550501	0.69096189	-5.31295016
homo after UVB irradiation VS homo before UVB irradiation	Canavan Disease|SMPDB	-0.45356441	0.050143346	-2.44562708	0.03597696	0.12504267	-3.90265183
hetero after UVB irradiation VS hetero before UVB irradiation	Canavan Disease|SMPDB	-0.36442322	-0.0375773	-1.84647526	0.1001486	0.28471271	-4.80223908
WT after UVB irradiation VS WT before UVB irradiation	Canavan Disease|SMPDB	-0.06304373	-0.04233882	-0.24741384	0.80955351	0.94525798	-5.83824172
homo after UVB irradiation VS homo before UVB irradiation	Candesartan Action Pathway|SMPDB	0.636038586	-0.11800708	2.436891056	0.03650532	0.12504267	-3.91641947
hetero after UVB irradiation VS hetero before UVB irradiation	Candesartan Action Pathway|SMPDB	-0.02777369	0.003323189	-0.09172332	0.92906122	0.97054702	-6.34197953
WT after UVB irradiation VS WT before UVB irradiation	Candesartan Action Pathway|SMPDB	0.270438532	-0.10210744	0.707878218	0.49505953	0.81710765	-5.62188349
homo after UVB irradiation VS homo before UVB irradiation	Cannabinoid receptor signaling|Wikipathways	0.538784355	-0.03833128	2.79790847	0.01997892	0.09043427	-3.34195972
hetero after UVB irradiation VS hetero before UVB irradiation	Cannabinoid receptor signaling|Wikipathways	0.092090996	0.014565898	0.334279171	0.74633774	0.8781124	-6.28633182
WT after UVB irradiation VS WT before UVB irradiation	Cannabinoid receptor signaling|Wikipathways	0.279007142	-0.00681288	1.047091607	0.3195105	0.70304529	-5.34408627
homo after UVB irradiation VS homo before UVB irradiation	Canonical and Non-canonical Notch signaling|Wikipathways	0.192159228	0.044619359	0.891354131	0.3950067	0.54804029	-5.9591471
hetero after UVB irradiation VS hetero before UVB irradiation	Canonical and Non-canonical Notch signaling|Wikipathways	0.361896965	0.03651278	2.185010238	0.0587105	0.21639222	-4.31053277
WT after UVB irradiation VS WT before UVB irradiation	Canonical and Non-canonical Notch signaling|Wikipathways	0.097049952	-0.0377105	0.489993324	0.63462554	0.8751011	-5.74905785
homo after UVB irradiation VS homo before UVB irradiation	Canonical and Non-Canonical TGF-B signaling|Wikipathways	0.05711954	0.012632601	0.231727543	0.82172133	0.89057194	-6.33968072
hetero after UVB irradiation VS hetero before UVB irradiation	Canonical and Non-Canonical TGF-B signaling|Wikipathways	-0.15726952	-0.08932911	-0.6644006	0.52419616	0.73931303	-6.11331249
WT after UVB irradiation VS WT before UVB irradiation	Canonical and Non-Canonical TGF-B signaling|Wikipathways	-0.08864226	-0.04087999	-0.33058925	0.74772219	0.9185045	-5.81410692
homo after UVB irradiation VS homo before UVB irradiation	Canonical NF-kappaB pathway|PID	0.129320152	-0.02608508	0.569418277	0.58243691	0.70466533	-6.19725872
hetero after UVB irradiation VS hetero before UVB irradiation	Canonical NF-kappaB pathway|PID	0.263184831	-0.05263002	1.143098389	0.28443421	0.5136743	-5.68904175
WT after UVB irradiation VS WT before UVB irradiation	Canonical NF-kappaB pathway|PID	0.019727839	-0.04690208	0.084327673	0.93444687	0.98056728	-5.86555008
homo after UVB irradiation VS homo before UVB irradiation	Canonical Wnt signaling pathway|PID	-0.57161851	0.006781719	-3.02704927	0.01365336	0.07305822	-2.97484918
hetero after UVB irradiation VS hetero before UVB irradiation	Canonical Wnt signaling pathway|PID	-0.42876463	-0.0614068	-2.05591818	0.07205601	0.24008135	-4.50109593
WT after UVB irradiation VS WT before UVB irradiation	Canonical Wnt signaling pathway|PID	-0.47993859	-0.01291453	-1.83880241	0.09554285	0.49254317	-4.41015289
homo after UVB irradiation VS homo before UVB irradiation	Cap-dependent Translation Initiation|Reactome	-0.9616441	0.025393751	-5.42439674	0.00036236	0.01666204	0.554607925
hetero after UVB irradiation VS hetero before UVB irradiation	Cap-dependent Translation Initiation|Reactome	-0.27370492	-0.01616192	-0.82660804	0.43124249	0.6573924	-5.99055596
WT after UVB irradiation VS WT before UVB irradiation	Cap-dependent Translation Initiation|Reactome	-0.59454382	-0.01466313	-1.98364867	0.07518015	0.45723857	-4.20999049
homo after UVB irradiation VS homo before UVB irradiation	Capecitabine Action Pathway|SMPDB	0.02464428	0.063631408	0.082398189	0.93606003	0.96219047	-6.3648016
hetero after UVB irradiation VS hetero before UVB irradiation	Capecitabine Action Pathway|SMPDB	-0.1998933	0.018696354	-0.79525143	0.44828734	0.67324339	-6.01608723
WT after UVB irradiation VS WT before UVB irradiation	Capecitabine Action Pathway|SMPDB	0.040092122	-0.00711836	0.152627333	0.88170346	0.96830103	-5.85736466
homo after UVB irradiation VS homo before UVB irradiation	Capecitabine Metabolism Pathway|SMPDB	0.02464428	0.063631408	0.082398189	0.93606003	0.96219047	-6.3648016
hetero after UVB irradiation VS hetero before UVB irradiation	Capecitabine Metabolism Pathway|SMPDB	-0.1998933	0.018696354	-0.79525143	0.44828734	0.67324339	-6.01608723
WT after UVB irradiation VS WT before UVB irradiation	Capecitabine Metabolism Pathway|SMPDB	0.040092122	-0.00711836	0.152627333	0.88170346	0.96830103	-5.85736466
homo after UVB irradiation VS homo before UVB irradiation	Captopril Action Pathway|SMPDB	0.636038586	-0.11800708	2.436891056	0.03650532	0.12504267	-3.91641947
hetero after UVB irradiation VS hetero before UVB irradiation	Captopril Action Pathway|SMPDB	-0.02777369	0.003323189	-0.09172332	0.92906122	0.97054702	-6.34197953
WT after UVB irradiation VS WT before UVB irradiation	Captopril Action Pathway|SMPDB	0.270438532	-0.10210744	0.707878218	0.49505953	0.81710765	-5.62188349
homo after UVB irradiation VS homo before UVB irradiation	Carbamazepine Metabolism Pathway|SMPDB	0.230052768	0.046947394	0.771765282	0.45923619	0.6060325	-6.05843812
hetero after UVB irradiation VS hetero before UVB irradiation	Carbamazepine Metabolism Pathway|SMPDB	-0.10905224	0.071810837	-0.43893254	0.67174885	0.82819347	-6.24323544
WT after UVB irradiation VS WT before UVB irradiation	Carbamazepine Metabolism Pathway|SMPDB	-0.22789838	0.120064937	-0.75861895	0.46544506	0.80910175	-5.58624643
homo after UVB irradiation VS homo before UVB irradiation	Carbamazepine Pathway_ Pharmacokinetics|PharmGKB	0.564541573	0.080435472	2.433590163	0.03670695	0.12535822	-3.92161913
hetero after UVB irradiation VS hetero before UVB irradiation	Carbamazepine Pathway_ Pharmacokinetics|PharmGKB	0.00924971	0.022393857	0.048011105	0.96282597	0.98288035	-6.34529099
WT after UVB irradiation VS WT before UVB irradiation	Carbamazepine Pathway_ Pharmacokinetics|PharmGKB	0.058617307	0.11211179	0.217901506	0.8318528	0.94823415	-5.84515835
homo after UVB irradiation VS homo before UVB irradiation	Carbamoyl Phosphate Synthetase Deficiency|SMPDB	-0.37671284	0.065264039	-1.86404069	0.09383246	0.21982504	-4.78798165
hetero after UVB irradiation VS hetero before UVB irradiation	Carbamoyl Phosphate Synthetase Deficiency|SMPDB	-0.31260554	-0.01518237	-1.48265131	0.17457213	0.39158454	-5.29086545
WT after UVB irradiation VS WT before UVB irradiation	Carbamoyl Phosphate Synthetase Deficiency|SMPDB	-0.12033773	0.030196148	-0.57832296	0.57573553	0.8562298	-5.70268083
homo after UVB irradiation VS homo before UVB irradiation	Carbohydrate digestion and absorption - Homo sapiens (human)|KEGG	0.567150584	0.005791648	3.006048668	0.0141364	0.07314252	-3.00848125
hetero after UVB irradiation VS hetero before UVB irradiation	Carbohydrate digestion and absorption - Homo sapiens (human)|KEGG	0.452263741	0.063933071	2.19243014	0.05802167	0.21518532	-4.29948812
WT after UVB irradiation VS WT before UVB irradiation	Carbohydrate digestion and absorption - Homo sapiens (human)|KEGG	0.412145709	0.086455441	1.722210168	0.11551154	0.51627893	-4.56629809
homo after UVB irradiation VS homo before UVB irradiation	Carboxyterminal post-translational modifications of tubulin|Reactome	-0.15996951	0.015775642	-0.96838697	0.35713279	0.51182307	-5.88885868
hetero after UVB irradiation VS hetero before UVB irradiation	Carboxyterminal post-translational modifications of tubulin|Reactome	-0.00190207	-0.0130546	-0.0121403	0.99059623	0.9943047	-6.34646134
WT after UVB irradiation VS WT before UVB irradiation	Carboxyterminal post-translational modifications of tubulin|Reactome	0.034600921	-0.00653811	0.175660696	0.86403846	0.96034647	-5.85354506
homo after UVB irradiation VS homo before UVB irradiation	Cardiac conduction|Reactome	0.254717259	-0.01468286	1.4978801	0.16700688	0.31707003	-5.2910412
hetero after UVB irradiation VS hetero before UVB irradiation	Cardiac conduction|Reactome	-0.13135147	-0.00016921	-0.68755963	0.5102098	0.72923683	-6.09722713
WT after UVB irradiation VS WT before UVB irradiation	Cardiac conduction|Reactome	0.047746897	-0.02334751	0.195609423	0.84880196	0.95387225	-5.84980685
homo after UVB irradiation VS homo before UVB irradiation	Cardiac conduction|Wikipathways	0.422589463	0.057716203	1.496568049	0.16734211	0.31731065	-5.2927337
hetero after UVB irradiation VS hetero before UVB irradiation	Cardiac conduction|Wikipathways	-0.4159576	0.047761009	-1.43840589	0.1864049	0.40374874	-5.3463902
WT after UVB irradiation VS WT before UVB irradiation	Cardiac Hypertrophic Response|Wikipathways	0.395596872	-0.02485496	2.302908982	0.04563608	0.13900891	-4.12627237
hetero after UVB irradiation VS hetero before UVB irradiation	Cardiac Hypertrophic Response|Wikipathways	0.193107328	-0.02243835	1.104843909	0.29977456	0.53033405	-5.7294271
WT after UVB irradiation VS WT before UVB irradiation	Cardiac Hypertrophic Response|Wikipathways	0.323605845	-0.00818113	1.583545822	0.14413097	0.55622203	-4.7451622
homo after UVB irradiation VS homo before UVB irradiation	Cardiac muscle contraction - Homo sapiens (human)|KEGG	0.222625107	-0.0103425	1.002821088	0.34108979	0.49715253	-5.85590658
hetero after UVB irradiation VS hetero before UVB irradiation	Cardiac muscle contraction - Homo sapiens (human)|KEGG	0.000299328	0.047471965	0.001612071	0.99875127	0.99964322	-6.34653989
WT after UVB irradiation VS WT before UVB irradiation	Cardiac muscle contraction - Homo sapiens (human)|KEGG	0.110585707	-0.02540773	0.415165295	0.68671777	0.89123048	-5.78262885
homo after UVB irradiation VS homo before UVB irradiation	Cardiac Progenitor Differentiation|Wikipathways	0.368835526	0.035274558	2.249750299	0.04984983	0.14684796	-4.20875118
hetero after UVB irradiation VS hetero before UVB irradiation	Cardiac Progenitor Differentiation|Wikipathways	0.170483679	0.05138858	1.123311816	0.29228867	0.52324352	-5.71005583
WT after UVB irradiation VS WT before UVB irradiation	Cardiac Progenitor Differentiation|Wikipathways	0.044168766	0.007333114	0.197094793	0.84766998	0.95372763	-5.84951256
homo after UVB irradiation VS homo before UVB irradiation	cardiac protection against ros|BioCarta	-0.04974232	-0.03407834	-0.1691831	0.8692416	0.91927589	-6.35309305
hetero after UVB irradiation VS hetero before UVB irradiation	cardiac protection against ros|BioCarta	-0.30964436	-0.02403827	-1.06957483	0.31449048	0.54338429	-5.7657583
WT after UVB irradiation VS WT before UVB irradiation	cardiac protection against ros|BioCarta	-0.13383303	-0.0952983	-0.39531306	0.70084676	0.90028732	-5.79063896
homo after UVB irradiation VS homo before UVB irradiation	cardiolipin biosynthesis|HumanCyc	0.186810947	-0.09384366	0.588263044	0.57023346	0.69442834	-6.18595232
hetero after UVB irradiation VS hetero before UVB irradiation	cardiolipin biosynthesis|HumanCyc	0.057815185	-0.03279941	0.153180782	0.88186086	0.95839814	-6.3338302
WT after UVB irradiation VS WT before UVB irradiation	cardiolipin biosynthesis|HumanCyc	0.451010679	-0.02117117	1.410397776	0.18852092	0.60208046	-4.95624479
homo after UVB irradiation VS homo before UVB irradiation	Carfentanil Action Pathway|SMPDB	0.191132361	0.029768717	1.061313976	0.31508276	0.46923284	-5.79783739
hetero after UVB irradiation VS hetero before UVB irradiation	Carfentanil Action Pathway|SMPDB	-0.07904315	0.055005373	-0.44437398	0.66796482	0.82651398	-6.24068873
WT after UVB irradiation VS WT before UVB irradiation	Carfentanil Action Pathway|SMPDB	0.022316377	0.035038618	0.098344171	0.92358676	0.97566251	-5.86425414
homo after UVB irradiation VS homo before UVB irradiation	Cargo concentration in the ER|Reactome	0.461255838	0.007140268	2.236665083	0.05094393	0.14949227	-4.22897557
hetero after UVB irradiation VS hetero before UVB irradiation	Cargo concentration in the ER|Reactome	0.353274143	0.021250241	1.63455284	0.1388699	0.33884849	-5.09326566
WT after UVB irradiation VS WT before UVB irradiation	Cargo concentration in the ER|Reactome	0.291581277	-0.01375777	1.261469973	0.23554416	0.62952955	-5.12510223
homo after UVB irradiation VS homo before UVB irradiation	Cargo recognition for clathrin-mediated endocytosis|Reactome	0.067449302	-0.02903371	0.374380119	0.71643721	0.81354197	-6.29371172
hetero after UVB irradiation VS hetero before UVB irradiation	Cargo recognition for clathrin-mediated endocytosis|Reactome	0.352036004	-0.01278476	2.28748168	0.04987077	0.19835162	-4.15726008
WT after UVB irradiation VS WT before UVB irradiation	Cargo recognition for clathrin-mediated endocytosis|Reactome	0.151446868	-0.00115435	0.746310913	0.47252273	0.81337567	-5.59509212
homo after UVB irradiation VS homo before UVB irradiation	Cargo trafficking to the periciliary membrane|Reactome	-0.16534572	-0.03118877	-0.99574942	0.34433999	0.49944612	-5.86274949
hetero after UVB irradiation VS hetero before UVB irradiation	Cargo trafficking to the periciliary membrane|Reactome	-0.33113551	-0.0428111	-2.05896884	0.07170902	0.23970209	-4.49663062
WT after UVB irradiation VS WT before UVB irradiation	Cargo trafficking to the periciliary membrane|Reactome	-0.32755548	-0.05419337	-1.58889527	0.14291937	0.55622203	-4.73841365
homo after UVB irradiation VS homo before UVB irradiation	carm1 and regulation of the estrogen receptor|BioCarta	-0.17506933	0.020961437	-0.86776086	0.40715641	0.55844883	-5.97970051
hetero after UVB irradiation VS hetero before UVB irradiation	carm1 and regulation of the estrogen receptor|BioCarta	-0.13448525	0.020054695	-0.5495221	0.59690707	0.78655752	-6.18567473
WT after UVB irradiation VS WT before UVB irradiation	carm1 and regulation of the estrogen receptor|BioCarta	-0.02531277	-0.014463	-0.10606465	0.91761174	0.97566251	-5.86345556
homo after UVB irradiation VS homo before UVB irradiation	Carnitine palmitoyl transferase deficiency (I)|SMPDB	-0.19843953	0.039801734	-0.74439404	0.47485571	0.61754234	-6.07941248
hetero after UVB irradiation VS hetero before UVB irradiation	Carnitine palmitoyl transferase deficiency (I)|SMPDB	-0.48316271	0.016700705	-1.75538636	0.11536374	0.30887493	-4.92927061
WT after UVB irradiation VS WT before UVB irradiation	Carnitine palmitoyl transferase deficiency (I)|SMPDB	-0.40763842	0.012220754	-1.51649601	0.16010237	0.56072663	-4.82863564
homo after UVB irradiation VS homo before UVB irradiation	Carnitine palmitoyl transferase deficiency (II)|SMPDB	-0.19843953	0.039801734	-0.74439404	0.47485571	0.61754234	-6.07941248
hetero after UVB irradiation VS hetero before UVB irradiation	Carnitine palmitoyl transferase deficiency (II)|SMPDB	-0.48316271	0.016700705	-1.75538636	0.11536374	0.30887493	-4.92927061
WT after UVB irradiation VS WT before UVB irradiation	Carnitine palmitoyl transferase deficiency (II)|SMPDB	-0.40763842	0.012220754	-1.51649601	0.16010237	0.56072663	-4.82863564
homo after UVB irradiation VS homo before UVB irradiation	Carnitine synthesis|Reactome	-0.36314535	0.036123294	-1.23467762	0.24695618	0.40914439	-5.61115985
hetero after UVB irradiation VS hetero before UVB irradiation	Carnitine synthesis|Reactome	-0.45742016	-0.00749115	-1.72602608	0.1207105	0.3132781	-4.96961829
WT after UVB irradiation VS WT before UVB irradiation	Carnitine synthesis|Reactome	-0.28515237	0.014344301	-0.77926883	0.45372421	0.80754423	-5.57111914
homo after UVB irradiation VS homo before UVB irradiation	Carnitine Synthesis|SMPDB	-0.45530958	0.048270802	-1.51403672	0.1629269	0.31259728	-5.27012706
hetero after UVB irradiation VS hetero before UVB irradiation	Carnitine Synthesis|SMPDB	-0.75283883	-0.12217234	-3.58613628	0.00654049	0.08469194	-2.19053878
WT after UVB irradiation VS WT before UVB irradiation	Carnitine Synthesis|SMPDB	-0.42893722	-0.00110127	-1.26941408	0.23281205	0.62952955	-5.1164207
homo after UVB irradiation VS homo before UVB irradiation	Carnitine-acylcarnitine translocase deficiency|SMPDB	0.161860886	-0.05103371	0.630831613	0.54319908	0.67708436	-6.15916189
hetero after UVB irradiation VS hetero before UVB irradiation	Carnitine-acylcarnitine translocase deficiency|SMPDB	-0.12884308	-0.07596147	-0.45167658	0.66290222	0.82651398	-6.237224
WT after UVB irradiation VS WT before UVB irradiation	Carnitine-acylcarnitine translocase deficiency|SMPDB	-0.13599861	-0.0440626	-0.59210605	0.56682	0.85125516	-5.69479783
homo after UVB irradiation VS homo before UVB irradiation	carnosine biosynthesis|HumanCyc	0.572978794	-0.00791087	1.537965645	0.15704513	0.30448751	-5.23890841
hetero after UVB irradiation VS hetero before UVB irradiation	carnosine biosynthesis|HumanCyc	-0.72860899	-0.00173902	-2.13490347	0.06357655	0.22631041	-4.38487138
WT after UVB irradiation VS WT before UVB irradiation	carnosine biosynthesis|HumanCyc	-0.38261699	-0.06203479	-0.92180557	0.37816038	0.75122805	-5.45720293
homo after UVB irradiation VS homo before UVB irradiation	Carnosinuria_ carnosinemia|SMPDB	0.066798026	0.046789851	0.254634663	0.80449493	0.87857543	-6.33373313
hetero after UVB irradiation VS hetero before UVB irradiation	Carnosinuria_ carnosinemia|SMPDB	-0.52991759	0.031774511	-1.70647522	0.12439725	0.31623126	-4.99631323
WT after UVB irradiation VS WT before UVB irradiation	Carnosinuria_ carnosinemia|SMPDB	-0.14708226	-0.03671277	-0.48165088	0.64033764	0.87636259	-5.75306298
homo after UVB irradiation VS homo before UVB irradiation	Carprofen Action Pathway|SMPDB	0.40423582	0.045705158	1.979500697	0.07780079	0.19626432	-4.61879689
hetero after UVB irradiation VS hetero before UVB irradiation	Carprofen Action Pathway|SMPDB	0.482496143	0.064876536	3.04499191	0.01500113	0.1130168	-3.00250826
WT after UVB irradiation VS WT before UVB irradiation	Carprofen Action Pathway|SMPDB	0.275725339	0.086595857	1.261970114	0.23537138	0.62952955	-5.12455679
homo after UVB irradiation VS homo before UVB irradiation	Carteolol Action Pathway|SMPDB	0.396415397	-0.03677376	2.343069613	0.04268583	0.13402181	-4.06364635
hetero after UVB irradiation VS hetero before UVB irradiation	Carteolol Action Pathway|SMPDB	-0.01555055	-0.01775524	-0.07410913	0.94265284	0.97054702	-6.34356282
WT after UVB irradiation VS WT before UVB irradiation	Carteolol Action Pathway|SMPDB	0.056685205	-0.03978418	0.223404833	0.82768199	0.94525798	-5.84393438
homo after UVB irradiation VS homo before UVB irradiation	Carvedilol Action Pathway|SMPDB	0.363732495	-0.0288084	2.171554099	0.05674447	0.16193454	-4.32911185
hetero after UVB irradiation VS hetero before UVB irradiation	Carvedilol Action Pathway|SMPDB	-0.04095736	-0.02227496	-0.19326116	0.85133209	0.94503144	-6.32632526
WT after UVB irradiation VS WT before UVB irradiation	Carvedilol Action Pathway|SMPDB	0.033066635	-0.04439883	0.13025274	0.89892864	0.97566251	-5.86056419
homo after UVB irradiation VS homo before UVB irradiation	CASP8 activity is inhibited|Reactome	0.849614473	-0.03215571	4.542786992	0.00125669	0.02014127	-0.64871182
hetero after UVB irradiation VS hetero before UVB irradiation	CASP8 activity is inhibited|Reactome	0.683693345	-0.01188812	2.738727154	0.02430288	0.13715811	-3.47057017
WT after UVB irradiation VS WT before UVB irradiation	CASP8 activity is inhibited|Reactome	0.733182815	-0.06921519	3.457990495	0.00607019	0.21490368	-2.02721426
homo after UVB irradiation VS homo before UVB irradiation	Caspase activation via extrinsic apoptotic signalling pathway|Reactome	0.608137156	-0.01411789	3.288598084	0.00887973	0.05619844	-2.55733023
hetero after UVB irradiation VS hetero before UVB irradiation	Caspase activation via extrinsic apoptotic signalling pathway|Reactome	0.695994634	0.015555475	4.495965921	0.00176691	0.04958933	-0.90898983
WT after UVB irradiation VS WT before UVB irradiation	Caspase activation via extrinsic apoptotic signalling pathway|Reactome	0.530442968	-0.02474672	2.688514765	0.02260591	0.31689014	-3.17639419
homo after UVB irradiation VS homo before UVB irradiation	caspase cascade in apoptosis|BioCarta	0.310351968	-0.04423313	1.860977614	0.09429723	0.22080058	-4.79241121
hetero after UVB irradiation VS hetero before UVB irradiation	caspase cascade in apoptosis|BioCarta	0.395332212	-0.01906996	2.288532848	0.04978727	0.19835162	-4.15568011
WT after UVB irradiation VS WT before UVB irradiation	caspase cascade in apoptosis|BioCarta	0.588939323	0.002941202	2.988250544	0.01349758	0.28019754	-2.72570697

homo after UVB irradiation VS homo before UVB irradiation	Caspase Cascade in Apoptosis|PID	0.382751326	-0.01723256	2.274266389	0.0478613	0.14273983	-4.17077473
hetero after UVB irradiation VS hetero before UVB irradiation	Caspase Cascade in Apoptosis|PID	0.286536496	-0.00323332	1.902114036	0.09180655	0.27403683	-4.72337787
WT after UVB irradiation VS WT before UVB irradiation	Caspase Cascade in Apoptosis|PID	0.413163486	0.001927669	2.104224986	0.06141571	0.4321811	-4.03901584
homo after UVB irradiation VS homo before UVB irradiation	Caspase-mediated cleavage of cytoskeletal proteins|Reactome	0.937927178	-0.00552061	5.355534045	0.00039773	0.01666204	0.465161028
hetero after UVB irradiation VS hetero before UVB irradiation	Caspase-mediated cleavage of cytoskeletal proteins|Reactome	0.476172523	0.03510171	2.384377437	0.04272937	0.18194424	-4.01106814
WT after UVB irradiation VS WT before UVB irradiation	Caspase-mediated cleavage of cytoskeletal proteins|Reactome	0.58674747	0.049447889	2.704193077	0.02200354	0.31542987	-3.15283686
homo after UVB irradiation VS homo before UVB irradiation	Catabolism of glucuronate to xylulose-5-phosphate|Reactome	0.369140023	0.048600975	1.132293658	0.28559807	0.44597838	-5.72395969
hetero after UVB irradiation VS hetero before UVB irradiation	Catabolism of glucuronate to xylulose-5-phosphate|Reactome	0.163037266	0.019507977	0.607728869	0.55938693	0.76201005	-6.15057409
WT after UVB irradiation VS WT before UVB irradiation	Catabolism of glucuronate to xylulose-5-phosphate|Reactome	0.281042655	0.027348863	0.891393735	0.39349374	0.76452505	-5.48286813
homo after UVB irradiation VS homo before UVB irradiation	Catalytic cycle of mammalian Flavin-containing MonoOxygenases (FMOs)|Wikipathways	-0.1080259	-0.00592193	-0.29524871	0.77422896	0.85410231	-6.3218326
hetero after UVB irradiation VS hetero before UVB irradiation	Catalytic cycle of mammalian Flavin-containing MonoOxygenases (FMOs)|Wikipathways	-0.86247158	-0.0412728	-3.7887471	0.00483788	0.07419102	-1.89479342
WT after UVB irradiation VS WT before UVB irradiation	Catalytic cycle of mammalian Flavin-containing MonoOxygenases (FMOs)|Wikipathways	-0.08707261	-0.12934091	-0.26307142	0.79779348	0.94477083	-5.83422057
homo after UVB irradiation VS homo before UVB irradiation	catecholamine biosynthesis|HumanCyc	0.30723314	0.017078188	1.126215861	0.28803498	0.44703185	-5.73042792
hetero after UVB irradiation VS hetero before UVB irradiation	catecholamine biosynthesis|HumanCyc	-0.33561907	0.132015706	-1.53743203	0.16084568	0.37252774	-5.22080329
WT after UVB irradiation VS WT before UVB irradiation	catecholamine biosynthesis|HumanCyc	0.513543589	0.096381175	1.948116725	0.07976147	0.46479598	-4.25966199
homo after UVB irradiation VS homo before UVB irradiation	Catecholamine biosynthesis|Reactome	0.30723314	0.017078188	1.126215861	0.28803498	0.44703185	-5.73042792
hetero after UVB irradiation VS hetero before UVB irradiation	Catecholamine biosynthesis|Reactome	-0.33561907	0.132015706	-1.53743203	0.16084568	0.37252774	-5.22080329
WT after UVB irradiation VS WT before UVB irradiation	Catecholamine biosynthesis|Reactome	0.513543589	0.096381175	1.948116725	0.07976147	0.46479598	-4.25966199
homo after UVB irradiation VS homo before UVB irradiation	Catecholamine Biosynthesis|SMPDB	0.30723314	0.017078188	1.126215861	0.28803498	0.44703185	-5.73042792
hetero after UVB irradiation VS hetero before UVB irradiation	Catecholamine Biosynthesis|SMPDB	-0.33561907	0.132015706	-1.53743203	0.16084568	0.37252774	-5.22080329
WT after UVB irradiation VS WT before UVB irradiation	Catecholamine Biosynthesis|SMPDB	0.513543589	0.096381175	1.948116725	0.07976147	0.46479598	-4.25966199
homo after UVB irradiation VS homo before UVB irradiation	Cation-coupled Chloride cotransporters|Reactome	0.608466321	0.022405497	2.087851723	0.065145	0.17716026	-4.45649535
hetero after UVB irradiation VS hetero before UVB irradiation	Cation-coupled Chloride cotransporters|Reactome	-0.43250336	0.016177793	-2.38690082	0.04255768	0.18179748	-4.00724742
WT after UVB irradiation VS WT before UVB irradiation	Cation-coupled Chloride cotransporters|Reactome	-0.07985206	-0.06540511	-0.27568008	0.78836162	0.93991114	-5.83080583
homo after UVB irradiation VS homo before UVB irradiation	cbl mediated ligand-induced downregulation of egf receptors pathway|BioCarta	0.226724451	-0.0288128	1.153863079	0.2770808	0.43716342	-5.70079431
hetero after UVB irradiation VS hetero before UVB irradiation	cbl mediated ligand-induced downregulation of egf receptors pathway|BioCarta	0.373223488	0.058081189	1.964058173	0.08329701	0.26094005	-4.63456289
WT after UVB irradiation VS WT before UVB irradiation	cbl mediated ligand-induced downregulation of egf receptors pathway|BioCarta	0.384710111	0.037976542	1.735002101	0.11314843	0.51512295	-4.54940773
homo after UVB irradiation VS homo before UVB irradiation	ccr3 signaling in eosinophils|BioCarta	0.438839379	-0.03156213	2.499101909	0.03290413	0.12089941	-3.81818602
hetero after UVB irradiation VS hetero before UVB irradiation	ccr3 signaling in eosinophils|BioCarta	0.40230366	0.032934252	2.144152249	0.06264962	0.22414204	-4.37118325
WT after UVB irradiation VS WT before UVB irradiation	ccr3 signaling in eosinophils|BioCarta	0.398071186	0.033739996	1.929556462	0.08225729	0.46860292	-4.28546721
homo after UVB irradiation VS homo before UVB irradiation	CD209 (DC-SIGN) signaling|Reactome	0.31019289	-0.04255513	1.51368534	0.1630147	0.31259728	-5.27058332
hetero after UVB irradiation VS hetero before UVB irradiation	CD209 (DC-SIGN) signaling|Reactome	0.40009767	0.014399152	2.174321297	0.05971695	0.21845092	-4.32642737
WT after UVB irradiation VS WT before UVB irradiation	CD209 (DC-SIGN) signaling|Reactome	0.395281048	-0.05693732	1.929531232	0.08226073	0.46860292	-4.28550222
homo after UVB irradiation VS homo before UVB irradiation	CD22 mediated BCR regulation|Reactome	0.982224005	-0.05396096	4.916829021	0.00073138	0.01666498	-0.12282236
hetero after UVB irradiation VS hetero before UVB irradiation	CD22 mediated BCR regulation|Reactome	0.99454533	0.068600656	4.367700617	0.00210966	0.05087735	-1.08200542
WT after UVB irradiation VS WT before UVB irradiation	CD22 mediated BCR regulation|Reactome	0.735376834	-0.02361125	2.596410201	0.02649195	0.3446972	-3.31458951
homo after UVB irradiation VS homo before UVB irradiation	CD28 co-stimulation|Reactome	0.440256613	-0.02488904	2.566922252	0.02937973	0.11431817	-3.71064066
hetero after UVB irradiation VS hetero before UVB irradiation	CD28 co-stimulation|Reactome	0.453994487	0.047610365	2.685749034	0.02643491	0.14142055	-3.55167224
WT after UVB irradiation VS WT before UVB irradiation	CD28 co-stimulation|Reactome	0.194249963	-0.00614007	1.012152939	0.33513851	0.71891855	-5.37679672
homo after UVB irradiation VS homo before UVB irradiation	CD28 dependent PI3K/Akt signaling|Reactome	0.30927431	-0.03372957	1.943395282	0.08251355	0.20394538	-4.67214931
hetero after UVB irradiation VS hetero before UVB irradiation	CD28 dependent PI3K/Akt signaling|Reactome	0.353881059	0.035305272	2.150821665	0.06198941	0.22335787	-4.36130295
WT after UVB irradiation VS WT before UVB irradiation	CD28 dependent PI3K/Akt signaling|Reactome	-0.01310436	-0.02187411	-0.0646449	0.94972054	0.98599084	-5.86703462
homo after UVB irradiation VS homo before UVB irradiation	CD28 dependent Vav1 pathway|Reactome	0.511385715	-0.03444442	2.650905047	0.02553374	0.10578915	-3.57693975
hetero after UVB irradiation VS hetero before UVB irradiation	CD28 dependent Vav1 pathway|Reactome	0.554405773	0.081957504	3.036865988	0.01519291	0.11352943	-3.01488187
WT after UVB irradiation VS WT before UVB irradiation	CD28 dependent Vav1 pathway|Reactome	0.489169367	-0.05686045	2.285379883	0.04516678	0.39441053	-3.77632923
homo after UVB irradiation VS homo before UVB irradiation	CD4 T cell receptor signaling|INOH	-0.05840746	-0.01664572	-0.25983504	0.8005992	0.87562289	-6.33230596
hetero after UVB irradiation VS hetero before UVB irradiation	CD4 T cell receptor signaling|INOH	0.146717858	-0.00139806	0.807692305	0.44147091	0.66718316	-6.00605813
WT after UVB irradiation VS WT before UVB irradiation	CD4 T cell receptor signaling|INOH	0.0966573	-0.02077912	0.470144917	0.64825628	0.87935805	-5.75847946
homo after UVB irradiation VS homo before UVB irradiation	CD4 T cell receptor signaling-ERK cascade|INOH	-0.10576935	-0.02709757	-0.55405185	0.59249225	0.7145588	-6.20622429
hetero after UVB irradiation VS hetero before UVB irradiation	CD4 T cell receptor signaling-ERK cascade|INOH	-0.05790322	-0.00297059	-0.30989862	0.76416445	0.89233049	-6.29474506
WT after UVB irradiation VS WT before UVB irradiation	CD4 T cell receptor signaling-ERK cascade|INOH	0.130277155	0.010481029	0.559912508	0.5877625	0.86161188	-5.71294047
homo after UVB irradiation VS homo before UVB irradiation	CD4 T cell receptor signaling-JNK cascade|INOH	0.019984888	-0.02614578	0.112367004	0.91289822	0.94906495	-6.36166874
hetero after UVB irradiation VS hetero before UVB irradiation	CD4 T cell receptor signaling-JNK cascade|INOH	-0.01853337	0.00250627	-0.08894148	0.93120626	0.97054702	-6.34225191
WT after UVB irradiation VS WT before UVB irradiation	CD4 T cell receptor signaling-JNK cascade|INOH	0.144838431	-0.04655208	0.510417711	0.62074709	0.87257466	-5.73897702
homo after UVB irradiation VS homo before UVB irradiation	CD4 T cell receptor signaling-NFkB cascade|INOH	-0.08151173	-0.0266853	-0.33239482	0.74689216	0.83524604	-6.30943979
hetero after UVB irradiation VS hetero before UVB irradiation	CD4 T cell receptor signaling-NFkB cascade|INOH	0.166359372	0.010990433	0.789837556	0.45127567	0.67639412	-6.02040997
WT after UVB irradiation VS WT before UVB irradiation	CD4 T cell receptor signaling-NFkB cascade|INOH	0.111414665	-0.02863389	0.50977919	0.62117866	0.87257466	-5.73929807
homo after UVB irradiation VS homo before UVB irradiation	CD40/CD40L signaling|PID	0.22063891	-0.03319195	1.14475925	0.28065082	0.44111933	-5.71061159
hetero after UVB irradiation VS hetero before UVB irradiation	CD40/CD40L signaling|PID	0.241424964	-0.02063845	1.237610386	0.24923545	0.47404445	-5.58509477
WT after UVB irradiation VS WT before UVB irradiation	CD40/CD40L signaling|PID	0.139768288	-0.08525698	0.567618644	0.582712	0.85958498	-5.70868373
homo after UVB irradiation VS homo before UVB irradiation	cd40l signaling pathway|BioCarta	0.025593187	-0.02766853	0.12040047	0.90670313	0.94520696	-6.3606655
hetero after UVB irradiation VS hetero before UVB irradiation	cd40l signaling pathway|BioCarta	0.202603252	-0.12132859	0.799905145	0.44572927	0.67049863	-6.01235124
WT after UVB irradiation VS WT before UVB irradiation	cd40l signaling pathway|BioCarta	-0.18821211	-0.0871551	-0.77738773	0.45478391	0.80866776	-5.57251193
homo after UVB irradiation VS homo before UVB irradiation	Cdc20:Phospho-APC/C mediated degradation of Cyclin A|Reactome	-0.54307527	0.064248872	-2.82662558	0.01904546	0.08811647	-3.29596953
hetero after UVB irradiation VS hetero before UVB irradiation	Cdc20:Phospho-APC/C mediated degradation of Cyclin A|Reactome	0.251622161	0.003917455	1.296057705	0.22932676	0.45170905	-5.51800106
WT after UVB irradiation VS WT before UVB irradiation	Cdc20:Phospho-APC/C mediated degradation of Cyclin A|Reactome	-0.06687246	-0.06273188	-0.26791639	0.79416508	0.94244265	-5.83292705
homo after UVB irradiation VS homo before UVB irradiation	cdc25 and chk1 regulatory pathway in response to dna damage|BioCarta	-0.29652249	-0.01672051	-1.72523994	0.11717049	0.25512659	-4.98529351
hetero after UVB irradiation VS hetero before UVB irradiation	cdc25 and chk1 regulatory pathway in response to dna damage|BioCarta	0.041446275	-0.0322492	0.180077392	0.86134761	0.9455738	-6.32898409
WT after UVB irradiation VS WT before UVB irradiation	cdc25 and chk1 regulatory pathway in response to dna damage|BioCarta	-0.11362297	-0.02039522	-0.43020513	0.67609695	0.88217037	-5.77630882
homo after UVB irradiation VS homo before UVB irradiation	CDC42 signaling events|PID	0.28979349	-0.03122612	1.324508503	0.21666633	0.37430892	-5.50652019
hetero after UVB irradiation VS hetero before UVB irradiation	CDC42 signaling events|PID	0.431662684	0.00109407	2.554548156	0.03257243	0.15685114	-3.75225658
WT after UVB irradiation VS WT before UVB irradiation	CDC42 signaling events|PID	0.262863582	0.013158368	1.288913402	0.22621583	0.62952955	-5.09495032
homo after UVB irradiation VS homo before UVB irradiation	CDC6 association with the ORC:origin complex|Reactome	-0.32061565	-0.0184276	-0.96992209	0.35640593	0.51094176	-5.88740947
hetero after UVB irradiation VS hetero before UVB irradiation	CDC6 association with the ORC:origin complex|Reactome	-0.19291284	0.012994414	-0.66079814	0.5263926	0.74166846	-6.11577024
WT after UVB irradiation VS WT before UVB irradiation	CDC6 association with the ORC:origin complex|Reactome	-0.3523546	-0.02136243	-1.12109491	0.28823965	0.67436922	-5.2719535
homo after UVB irradiation VS homo before UVB irradiation	cdk regulation of dna replication|BioCarta	-0.47354878	-0.02271305	-1.48184804	0.17114352	0.32186978	-5.31166062
hetero after UVB irradiation VS hetero before UVB irradiation	cdk regulation of dna replication|BioCarta	0.145192248	-0.00714212	0.48822544	0.6378422	0.81573646	-6.21907898
WT after UVB irradiation VS WT before UVB irradiation	cdk regulation of dna replication|BioCarta	0.113270136	-0.04078944	0.369076785	0.71970342	0.90672477	-5.800642
homo after UVB irradiation VS homo before UVB irradiation	CDK-mediated phosphorylation and removal of Cdc6|Reactome	-0.38057594	0.036106436	-1.95691047	0.08071871	0.20118642	-4.6522237
hetero after UVB irradiation VS hetero before UVB irradiation	CDK-mediated phosphorylation and removal of Cdc6|Reactome	0.218547408	0.002237498	1.095799417	0.30349563	0.53328183	-5.73882755
WT after UVB irradiation VS WT before UVB irradiation	CDK-mediated phosphorylation and removal of Cdc6|Reactome	-0.034813	-0.04724589	-0.14478675	0.88773271	0.9710839	-5.85854321
homo after UVB irradiation VS homo before UVB irradiation	CDO in myogenesis|Reactome	0.26030841	-0.02417554	1.438865076	0.18267721	0.33687347	-5.36627012
hetero after UVB irradiation VS hetero before UVB irradiation	CDO in myogenesis|Reactome	-0.01372665	-0.04502388	-0.05127785	0.960299	0.98139974	-6.34511508
WT after UVB irradiation VS WT before UVB irradiation	CDO in myogenesis|Reactome	-0.13249739	-0.0314992	-0.54975231	0.59445691	0.86485362	-5.7184696
homo after UVB irradiation VS homo before UVB irradiation	CDP-diacylglycerol biosynthesis|HumanCyc	-0.18730079	-0.05414068	-0.90565151	0.38776952	0.54134863	-5.94646682
hetero after UVB irradiation VS hetero before UVB irradiation	CDP-diacylglycerol biosynthesis|HumanCyc	-0.23871754	-0.01906289	-1.1840082	0.26873252	0.49783052	-5.64476059
WT after UVB irradiation VS WT before UVB irradiation	CDP-diacylglycerol biosynthesis|HumanCyc	-0.10469024	0.026247657	-0.45709218	0.65729535	0.87935805	-5.76447277
homo after UVB irradiation VS homo before UVB irradiation	CDT1 association with the CDC6:ORC:origin complex|Reactome	-0.3059165	-0.02703172	-1.13399614	0.28491837	0.44528503	-5.72214315
hetero after UVB irradiation VS hetero before UVB irradiation	CDT1 association with the CDC6:ORC:origin complex|Reactome	0.153232051	0.031061196	0.550478712	0.59627956	0.78595784	-6.18512425
WT after UVB irradiation VS WT before UVB irradiation	CDT1 association with the CDC6:ORC:origin complex|Reactome	-0.28229256	-0.02733807	-1.10206996	0.29604354	0.68285796	-5.29085962
homo after UVB irradiation VS homo before UVB irradiation	Celecoxib Action Pathway|SMPDB	0.37170376	0.058296263	1.799898794	0.10402399	0.23618774	-4.88005154
hetero after UVB irradiation VS hetero before UVB irradiation	Celecoxib Action Pathway|SMPDB	0.482496143	0.064876536	3.04499191	0.01500113	0.1130168	-3.00250826
WT after UVB irradiation VS WT before UVB irradiation	Celecoxib Action Pathway|SMPDB	0.242923394	0.09712304	1.12533769	0.28652129	0.6736679	-5.26770368
homo after UVB irradiation VS homo before UVB irradiation	Celecoxib Metabolism Pathway|SMPDB	0.294927933	0.242235457	1.506727672	0.16476168	0.31411349	-5.27960493
hetero after UVB irradiation VS hetero before UVB irradiation	Celecoxib Metabolism Pathway|SMPDB	0.616784032	0.1238265	2.383798016	0.04276889	0.18194424	-4.01194537
WT after UVB irradiation VS WT before UVB irradiation	Celecoxib Metabolism Pathway|SMPDB	-0.08015769	0.202333443	-0.29459325	0.7742811	0.9344243	-5.82538902
homo after UVB irradiation VS homo before UVB irradiation	Celecoxib Pathway_ Pharmacodynamics|PharmGKB	0.438253193	0.003697368	2.595628416	0.02800392	0.11196663	-3.66499872
hetero after UVB irradiation VS hetero before UVB irradiation	Celecoxib Pathway_ Pharmacodynamics|PharmGKB	0.263499872	0.029403096	1.40173392	0.19673856	0.417313	-5.39165453
WT after UVB irradiation VS WT before UVB irradiation	Celecoxib Pathway_ Pharmacodynamics|PharmGKB	0.19589261	-0.01035322	0.838604585	0.42112845	0.78758953	-5.52568553
WT after UVB irradiation VS WT before UVB irradiation	Celecoxib Pathway_ Pharmacokinetics|PharmGKB	-0.4214302	0.046401692	-1.39621666	0.19262818	0.60496032	-4.97285911
homo after UVB irradiation VS homo before UVB irradiation	Cell adhesion molecules (CAMs) - Homo sapiens (human)|KEGG	0.189529602	-0.0385403	1.141269239	0.28202903	0.44245297	-5.71435971
hetero after UVB irradiation VS hetero before UVB irradiation	Cell adhesion molecules (CAMs) - Homo sapiens (human)|KEGG	0.130232235	0.013042706	0.647279888	0.53468429	0.74826251	-6.12488571
WT after UVB irradiation VS WT before UVB irradiation	Cell adhesion molecules (CAMs) - Homo sapiens (human)|KEGG	0.084192005	-0.04681436	0.328628806	0.74916016	0.9185045	-5.8147541
homo after UVB irradiation VS homo before UVB irradiation	Cell cycle - Homo sapiens (human)|KEGG	-0.47638762	-0.00301015	-2.5514646	0.03014835	0.11538756	-3.73518963
hetero after UVB irradiation VS hetero before UVB irradiation	Cell cycle - Homo sapiens (human)|KEGG	0.081653387	-0.0030472	0.369175035	0.72110265	0.86426134	-6.27321378
WT after UVB irradiation VS WT before UVB irradiation	Cell cycle - Homo sapiens (human)|KEGG	-0.03125119	-0.01456316	-0.12309162	0.90445376	0.97566251	-5.86148169
homo after UVB irradiation VS homo before UVB irradiation	Cell Cycle Checkpoints|Reactome	-0.53709631	-0.01470921	-2.82140582	0.01921178	0.08846538	-3.30433023
hetero after UVB irradiation VS hetero before UVB irradiation	Cell Cycle Checkpoints|Reactome	0.126780034	-0.00469321	0.487448027	0.63837031	0.81595398	-6.21947882
WT after UVB irradiation VS WT before UVB irradiation	Cell Cycle Checkpoints|Reactome	-0.00708354	0.006132959	-0.02592849	0.97982041	0.99308473	-5.86881063
homo after UVB irradiation VS homo before UVB irradiation	Cell Cycle Checkpoints|Wikipathways	-0.5643224	-0.01497266	-1.48649716	0.16993482	0.32049666	-5.30569503
hetero after UVB irradiation VS hetero before UVB irradiation	Cell Cycle Checkpoints|Wikipathways	-0.20197503	-0.03132311	-0.48348632	0.64106496	0.81779292	-6.22150708
WT after UVB irradiation VS WT before UVB irradiation	Cell Cycle Checkpoints|Wikipathways	-1.094234	-0.03588978	-4.37290589	0.00136599	0.15645713	-0.74252629
homo after UVB irradiation VS homo before UVB irradiation	cell cycle: g1/s check point|BioCarta	-0.40617023	0.044772021	-2.16631373	0.0572382	0.162632	-4.33713319
hetero after UVB irradiation VS hetero before UVB irradiation	cell cycle: g1/s check point|BioCarta	0.137297927	0.006267748	0.659938593	0.52691749	0.74191436	-6.11635488
WT after UVB irradiation VS WT before UVB irradiation	cell cycle: g1/s check point|BioCarta	-0.06106139	-0.02160802	-0.22009132	0.83019254	0.94716196	-5.84467495
homo after UVB irradiation VS homo before UVB irradiation	cell cycle: g2/m checkpoint|BioCarta	-0.43597271	-0.04364347	-2.67262522	0.02462406	0.10366003	-3.54228459
hetero after UVB irradiation VS hetero before UVB irradiation	cell cycle: g2/m checkpoint|BioCarta	0.042883701	0.010549093	0.168062763	0.87049811	0.95129447	-6.33124484
WT after UVB irradiation VS WT before UVB irradiation	cell cycle: g2/m checkpoint|BioCarta	-0.1774452	-0.02070941	-0.66128251	0.52325501	0.83120196	-5.65265372
homo after UVB irradiation VS homo before UVB irradiation	Cell Cycle_ Mitotic|Reactome	-0.50107932	-0.01722034	-2.6935897	0.02377695	0.10155807	-3.50881029
hetero after UVB irradiation VS hetero before UVB irradiation	Cell Cycle_ Mitotic|Reactome	0.112040992	-0.00086491	0.475806963	0.64630425	0.82171681	-6.2253941
WT after UVB irradiation VS WT before UVB irradiation	Cell Cycle_ Mitotic|Reactome	-0.00614518	0.005004274	-0.02332671	0.98184489	0.99425402	-5.86887554
homo after UVB irradiation VS homo before UVB irradiation	Cell Cycle|Reactome	-0.49475553	-0.01572709	-2.68164401	0.02425598	0.10270243	-3.52788701
hetero after UVB irradiation VS hetero before UVB irradiation	Cell Cycle|Reactome	0.100599231	-0.00880105	0.432298174	0.67637584	0.8318605	-6.24629996
WT after UVB irradiation VS WT before UVB irradiation	Cell Cycle|Reactome	-0.00940127	0.008276937	-0.03592388	0.97204438	0.99045333	-5.86849746
homo after UVB irradiation VS homo before UVB irradiation	Cell Cycle|Wikipathways	-0.46193915	-0.00582821	-2.47412565	0.03430549	0.12359784	-3.85767674
hetero after UVB irradiation VS hetero before UVB irradiation	Cell Cycle|Wikipathways	0.095210815	0.001886004	0.437705161	0.67260376	0.82879911	-6.24380575
WT after UVB irradiation VS WT before UVB irradiation	Cell Cycle|Wikipathways	-0.02545197	-0.01510924	-0.1016757	0.9210078	0.97566251	-5.86391692
homo after UVB irradiation VS homo before UVB irradiation	Cell death signalling via NRAGE_ NRIF and NADE|Reactome	0.313810412	-0.05110566	1.843593995	0.09697578	0.22511003	-4.8174888
hetero after UVB irradiation VS hetero before UVB irradiation	Cell death signalling via NRAGE_ NRIF and NADE|Reactome	0.338981908	0.021774161	1.958311003	0.08405366	0.26213234	-4.64284557
WT after UVB irradiation VS WT before UVB irradiation	Cell death signalling via NRAGE_ NRIF and NADE|Reactome	0.202282731	-0.00961189	1.044086897	0.32083256	0.70512141	-5.34693385
homo after UVB irradiation VS homo before UVB irradiation	Cell Differentiation - Index expanded|Wikipathways	-0.42168124	0.037061421	-2.41723414	0.03772241	0.12739684	-3.94736355
hetero after UVB irradiation VS hetero before UVB irradiation	Cell Differentiation - Index expanded|Wikipathways	-0.2516352	-0.03213649	-1.04040873	0.32708042	0.55613609	-5.79513082
WT after UVB irradiation VS WT before UVB irradiation	Cell Differentiation - Index expanded|Wikipathways	-0.28185019	0.005225989	-1.147193	0.27779605	0.66789028	-5.24562038
homo after UVB irradiation VS homo before UVB irradiation	Cell Differentiation - Index|Wikipathways	-0.21539492	0.079601671	-1.13113189	0.28606262	0.44628615	-5.72519811
hetero after UVB irradiation VS hetero before UVB irradiation	Cell Differentiation - Index|Wikipathways	-0.36877001	-0.02890189	-1.48843931	0.17307428	0.3907244	-5.28353039
WT after UVB irradiation VS WT before UVB irradiation	Cell Differentiation - Index|Wikipathways	-0.19740656	0.023341575	-0.7825221	0.4518953	0.80680448	-5.56870339
homo after UVB irradiation VS homo before UVB irradiation	Cell junction organization|Reactome	0.221130661	0.026244347	1.225070347	0.250392	0.41288908	-5.62204494
hetero after UVB irradiation VS hetero before UVB irradiation	Cell junction organization|Reactome	0.234768171	0.045228781	1.391201936	0.19979684	0.41977191	-5.40452336
WT after UVB irradiation VS WT before UVB irradiation	Cell junction organization|Reactome	-0.04128457	-0.00624298	-0.19858902	0.84653162	0.9536885	-5.84921428
homo after UVB irradiation VS homo before UVB irradiation	Cell surface interactions at the vascular wall|Reactome	0.441708792	-0.02380566	2.429924656	0.03693215	0.12584491	-3.92739157
hetero after UVB irradiation VS hetero before UVB irradiation	Cell surface interactions at the vascular wall|Reactome	0.524537329	0.023410385	3.133024217	0.01307763	0.11038491	-2.86871343
WT after UVB irradiation VS WT before UVB irradiation	Cell surface interactions at the vascular wall|Reactome	0.339933228	-0.00834121	1.485111392	0.16809505	0.57375943	-4.86697373
homo after UVB irradiation VS homo before UVB irradiation	cell to cell adhesion signaling|BioCarta	0.560785487	-0.07342456	2.475082164	0.03425075	0.12352996	-3.85616561
hetero after UVB irradiation VS hetero before UVB irradiation	cell to cell adhesion signaling|BioCarta	0.371807998	0.030911273	1.988609522	0.08013757	0.25471898	-4.59908693
WT after UVB irradiation VS WT before UVB irradiation	cell to cell adhesion signaling|BioCarta	0.508761025	0.029022574	2.2192594	0.05054848	0.41004423	-3.87290466
homo after UVB irradiation VS homo before UVB irradiation	Cell-Cell communication|Reactome	0.278277971	-0.00053485	1.435095378	0.1837201	0.33797985	-5.37101182
hetero after UVB irradiation VS hetero before UVB irradiation	Cell-Cell communication|Reactome	0.313290529	0.043492316	1.831752051	0.10247306	0.28762124	-4.82295261
WT after UVB irradiation VS WT before UVB irradiation	Cell-Cell communication|Reactome	0.133794666	-0.00043906	0.663498941	0.52189252	0.83116638	-5.65123308

homo after UVB irradiation VS homo before UVB irradiation	Cell-cell junction organization|Reactome	-0.16820383	0.048109451	-1.00174516	0.34158282	0.49735552	-5.8569502
hetero after UVB irradiation VS hetero before UVB irradiation	Cell-cell junction organization|Reactome	-0.13382327	0.02414086	-0.78785494	0.45237337	0.67714871	-6.02198668
WT after UVB irradiation VS WT before UVB irradiation	Cell-cell junction organization|Reactome	-0.41015818	0.025140905	-1.9104472	0.084903	0.47344569	-4.31193102
homo after UVB irradiation VS homo before UVB irradiation	Cell-extracellular matrix interactions|Reactome	0.96798409	-0.02646879	6.032019305	0.00016392	0.01666204	1.310720993
hetero after UVB irradiation VS hetero before UVB irradiation	Cell-extracellular matrix interactions|Reactome	0.862960572	0.071218883	3.605052932	0.00635746	0.0835081	-2.1627052
WT after UVB irradiation VS WT before UVB irradiation	Cell-extracellular matrix interactions|Reactome	0.494811547	-0.06530295	1.634447894	0.13296249	0.54653478	-4.68043764
homo after UVB irradiation VS homo before UVB irradiation	Cell-type Dependent Selectivity of CCK2R Signaling|Wikipathways	0.111232848	-0.03960734	0.451320071	0.66200285	0.76815883	-6.26020833
hetero after UVB irradiation VS hetero before UVB irradiation	Cell-type Dependent Selectivity of CCK2R Signaling|Wikipathways	-0.0589466	-0.01708592	-0.20584675	0.84179744	0.94031753	-6.32361352
WT after UVB irradiation VS WT before UVB irradiation	Cell-type Dependent Selectivity of CCK2R Signaling|Wikipathways	0.063986969	0.01750714	0.195592692	0.84881472	0.95387225	-5.84981015
homo after UVB irradiation VS homo before UVB irradiation	Cellular hexose transport|Reactome	0.579136287	0.001571347	3.022076143	0.0137662	0.07305822	-2.98281271
hetero after UVB irradiation VS hetero before UVB irradiation	Cellular hexose transport|Reactome	0.315036234	0.03223831	1.772898031	0.11228042	0.30517241	-4.90506206
WT after UVB irradiation VS WT before UVB irradiation	Cellular hexose transport|Reactome	0.410398675	0.081004737	1.596477062	0.1412176	0.55622203	-4.72882707
homo after UVB irradiation VS homo before UVB irradiation	Cellular response to heat stress|Reactome	-0.60981701	0.007062672	-3.70111403	0.0045664	0.03846279	-1.90865047
hetero after UVB irradiation VS hetero before UVB irradiation	Cellular response to heat stress|Reactome	-0.27726362	-0.04217591	-1.40461361	0.19590945	0.4161797	-5.38812561
WT after UVB irradiation VS WT before UVB irradiation	Cellular response to heat stress|Reactome	-0.25432775	-0.01587648	-1.02720204	0.32833851	0.71204479	-5.36281541
homo after UVB irradiation VS homo before UVB irradiation	Cellular response to heat stress|Wikipathways	-0.11578956	0.0009306	-0.60534134	0.55929783	0.68575561	-6.17541137
hetero after UVB irradiation VS hetero before UVB irradiation	Cellular response to heat stress|Wikipathways	-0.12534302	0.003982008	-0.6754048	0.51752146	0.73507723	-6.10573083
WT after UVB irradiation VS WT before UVB irradiation	Cellular response to heat stress|Wikipathways	-0.19522376	0.017850451	-0.83334118	0.42395449	0.79068324	-5.52983218
homo after UVB irradiation VS homo before UVB irradiation	Cellular response to hypoxia|Reactome	0.248076222	-0.01164082	1.172640443	0.26983138	0.43325305	-5.68036342
hetero after UVB irradiation VS hetero before UVB irradiation	Cellular response to hypoxia|Reactome	0.340237115	0.014109785	1.78160339	0.1107764	0.30216567	-4.89298866
WT after UVB irradiation VS WT before UVB irradiation	Cellular response to hypoxia|Reactome	-0.0815399	0.0026454	-0.374079	0.71609248	0.90536666	-5.7987862
homo after UVB irradiation VS homo before UVB irradiation	Cellular responses to external stimuli|Reactome	-0.22019394	0.003857826	-1.34646818	0.20975311	0.36751063	-5.48018031
hetero after UVB irradiation VS hetero before UVB irradiation	Cellular responses to external stimuli|Reactome	-0.03774102	-0.01832005	-0.22833907	0.82482702	0.93322694	-6.31834602
WT after UVB irradiation VS WT before UVB irradiation	Cellular responses to external stimuli|Reactome	-0.11885648	-0.02495003	-0.57854865	0.57558893	0.8562298	-5.70255315
homo after UVB irradiation VS homo before UVB irradiation	Cellular responses to stress|Reactome	-0.29263269	0.005015362	-1.80752626	0.10276042	0.23382702	-4.8691803
hetero after UVB irradiation VS hetero before UVB irradiation	Cellular responses to stress|Reactome	-0.03271793	-0.01723076	-0.19352137	0.85113469	0.94503144	-6.32627091
WT after UVB irradiation VS WT before UVB irradiation	Cellular responses to stress|Reactome	-0.11700681	-0.0267296	-0.56554648	0.58406777	0.86058971	-5.7098337
homo after UVB irradiation VS homo before UVB irradiation	Cellular roles of Anthrax toxin|PID	0.583468057	0.034944881	2.895540833	0.01698181	0.0820518	-3.18555004
hetero after UVB irradiation VS hetero before UVB irradiation	Cellular roles of Anthrax toxin|PID	0.652402757	-0.0024451	4.144578568	0.0028883	0.06144665	-1.38921954
WT after UVB irradiation VS WT before UVB irradiation	Cellular roles of Anthrax toxin|PID	0.457606226	0.076248523	2.168706369	0.05507526	0.41769317	-3.94622554
homo after UVB irradiation VS homo before UVB irradiation	Cellular senescence - Homo sapiens (human)|KEGG	-0.01838839	-0.01613792	-0.114657	0.91113161	0.94787581	-6.36138979
hetero after UVB irradiation VS hetero before UVB irradiation	Cellular senescence - Homo sapiens (human)|KEGG	0.132912859	-0.01388248	0.862959963	0.41204696	0.63968325	-5.95991719
WT after UVB irradiation VS WT before UVB irradiation	Cellular senescence - Homo sapiens (human)|KEGG	0.010947311	-0.0359189	0.055052085	0.95717263	0.98742794	-5.86761608
homo after UVB irradiation VS homo before UVB irradiation	Cellular Senescence|Reactome	-0.29343234	0.024161642	-1.6518749	0.13155157	0.27352662	-5.08651493
hetero after UVB irradiation VS hetero before UVB irradiation	Cellular Senescence|Reactome	0.012060844	0.00235317	0.064581882	0.95001293	0.9739614	-6.34427922
WT after UVB irradiation VS WT before UVB irradiation	Cellular Senescence|Reactome	-0.17626266	-0.01451364	-0.86646191	0.40638447	0.7725109	-5.50336721
homo after UVB irradiation VS homo before UVB irradiation	Central carbon metabolism in cancer - Homo sapiens (human)|KEGG	0.299824835	-0.03496482	1.690543475	0.12378346	0.26290983	-5.03344715
hetero after UVB irradiation VS hetero before UVB irradiation	Central carbon metabolism in cancer - Homo sapiens (human)|KEGG	0.243488331	0.000240293	1.589062283	0.14880067	0.35546827	-5.15351065
WT after UVB irradiation VS WT before UVB irradiation	Central carbon metabolism in cancer - Homo sapiens (human)|KEGG	0.192675372	0.01161402	0.958950209	0.36001512	0.74374432	-5.42488868
homo after UVB irradiation VS homo before UVB irradiation	Centrosome maturation|Reactome	-0.46559019	-0.00279485	-2.63553562	0.02619776	0.10699372	-3.60144484
hetero after UVB irradiation VS hetero before UVB irradiation	Centrosome maturation|Reactome	-0.02486017	-0.01446198	-0.10147379	0.92154771	0.97054702	-6.34095877
WT after UVB irradiation VS WT before UVB irradiation	Centrosome maturation|Reactome	-0.07093494	-0.02355644	-0.285332	0.78116559	0.93661609	-5.8280856
homo after UVB irradiation VS homo before UVB irradiation	ceramide <i>de novo</i> biosynthesis|HumanCyc	1.037714721	-0.00015627	6.383815606	0.00010591	0.01666204	1.721881995
hetero after UVB irradiation VS hetero before UVB irradiation	ceramide <i>de novo</i> biosynthesis|HumanCyc	0.519353597	0.080843993	1.76858676	0.1130323	0.30666948	-4.91103182
WT after UVB irradiation VS WT before UVB irradiation	ceramide <i>de novo</i> biosynthesis|HumanCyc	0.38664312	0.012522462	1.358196626	0.20401525	0.61829669	-5.01686244
homo after UVB irradiation VS homo before UVB irradiation	ceramide signaling pathway|BioCarta	0.351615152	-0.03408946	1.966858877	0.07942117	0.19914171	-4.63752158
hetero after UVB irradiation VS hetero before UVB irradiation	ceramide signaling pathway|BioCarta	0.347050747	-0.02465922	1.727057029	0.12051893	0.3132781	-4.96820674
WT after UVB irradiation VS WT before UVB irradiation	ceramide signaling pathway|BioCarta	0.444823164	-0.02292627	2.304328974	0.04372973	0.39022568	-3.74852393
homo after UVB irradiation VS homo before UVB irradiation	Ceramide signaling pathway|PID	0.39037659	-0.02200812	2.504682909	0.03259885	0.12029246	-3.80935275
hetero after UVB irradiation VS hetero before UVB irradiation	Ceramide signaling pathway|PID	0.354850019	-0.00725665	2.033488308	0.07465755	0.2448123	-4.53386472
WT after UVB irradiation VS WT before UVB irradiation	Ceramide signaling pathway|PID	0.511089998	-0.02294097	2.576564536	0.02741263	0.35054739	-3.34430996
homo after UVB irradiation VS homo before UVB irradiation	Ceramide signalling|Reactome	-0.39715391	-0.03976774	-1.74217623	0.11406112	0.25026577	-4.96161022
hetero after UVB irradiation VS hetero before UVB irradiation	Ceramide signalling|Reactome	-0.60452978	-0.04076311	-2.668847	0.0271545	0.14275688	-3.57753936
WT after UVB irradiation VS WT before UVB irradiation	Ceramide signalling|Reactome	-0.08687221	-0.00666988	-0.30814158	0.76424672	0.92719694	-5.82129197
homo after UVB irradiation VS homo before UVB irradiation	Cerebrotendinous Xanthomatosis (CTX)|SMPDB	0.143425895	0.01576019	0.783036509	0.45290274	0.6002769	-6.04960791
hetero after UVB irradiation VS hetero before UVB irradiation	Cerebrotendinous Xanthomatosis (CTX)|SMPDB	-0.01773938	0.040584313	-0.07540586	0.94165152	0.97054702	-6.34345771
WT after UVB irradiation VS WT before UVB irradiation	Cerebrotendinous Xanthomatosis (CTX)|SMPDB	0.061518516	0.026229795	0.23751225	0.8170163	0.94525798	-5.84065895
homo after UVB irradiation VS homo before UVB irradiation	Cerivastatin Action Pathway|SMPDB	-0.19230642	-0.01987665	-0.61925406	0.55047761	0.67913558	-6.16661869
hetero after UVB irradiation VS hetero before UVB irradiation	Cerivastatin Action Pathway|SMPDB	0.185820603	0.008098609	0.631890782	0.54421801	0.75147406	-6.13505553
WT after UVB irradiation VS WT before UVB irradiation	Cerivastatin Action Pathway|SMPDB	-0.14894747	0.045069977	-0.50755372	0.62268399	0.87257466	-5.74041409
homo after UVB irradiation VS homo before UVB irradiation	Cetuximab Action Pathway|SMPDB	-1.02021206	0.004763088	-4.21042005	0.00206751	0.02537706	-1.13430282
hetero after UVB irradiation VS hetero before UVB irradiation	Cetuximab Action Pathway|SMPDB	-0.7208662	-0.04088773	-2.69458506	0.02606648	0.14043856	-3.53814761
WT after UVB irradiation VS WT before UVB irradiation	Cetuximab Action Pathway|SMPDB	-0.88905476	-0.02311791	-2.71673052	0.02153341	0.31542987	-3.1339937
homo after UVB irradiation VS homo before UVB irradiation	cGMP effects|Reactome	0.355822641	-0.03629742	1.732025317	0.11591555	0.25360346	-4.97581881
hetero after UVB irradiation VS hetero before UVB irradiation	cGMP effects|Reactome	0.247628311	-0.02665604	0.99993195	0.3451904	0.5746298	-5.83485896
WT after UVB irradiation VS WT before UVB irradiation	cGMP effects|Reactome	0.168661338	0.019314525	0.546479399	0.59662193	0.86530281	-5.7202305
homo after UVB irradiation VS homo before UVB irradiation	cGMP-PKG signaling pathway - Homo sapiens (human)|KEGG	0.404938169	-0.03756502	2.482345738	0.03383786	0.1228169	-3.84468712
hetero after UVB irradiation VS hetero before UVB irradiation	cGMP-PKG signaling pathway - Homo sapiens (human)|KEGG	0.138177203	-0.0041822	0.671386827	0.51995254	0.73623731	-6.10851205
WT after UVB irradiation VS WT before UVB irradiation	cGMP-PKG signaling pathway - Homo sapiens (human)|KEGG	0.167404671	-0.03327183	0.664855059	0.52105993	0.83116638	-5.65036173
homo after UVB irradiation VS homo before UVB irradiation	Chagas disease (American trypanosomiasis) - Homo sapiens (human)|KEGG	0.31598894	-0.02564911	1.830947042	0.0989688	0.22811285	-4.83566702
hetero after UVB irradiation VS hetero before UVB irradiation	Chagas disease (American trypanosomiasis) - Homo sapiens (human)|KEGG	0.376096093	0.032266945	2.078640841	0.06951008	0.23555906	-4.46778909
WT after UVB irradiation VS WT before UVB irradiation	Chagas disease (American trypanosomiasis) - Homo sapiens (human)|KEGG	0.233911757	-0.02880992	0.965342152	0.35695719	0.74236042	-5.41922229
homo after UVB irradiation VS homo before UVB irradiation	chaperones modulate interferon signaling pathway|BioCarta	-0.0689755	0.043865107	-0.30432817	0.76751522	0.84977558	-6.31893613
hetero after UVB irradiation VS hetero before UVB irradiation	chaperones modulate interferon signaling pathway|BioCarta	-0.24141867	-0.04542734	-0.98648506	0.35137196	0.58048328	-5.84778538
WT after UVB irradiation VS WT before UVB irradiation	chaperones modulate interferon signaling pathway|BioCarta	-0.30815305	-0.0719472	-1.37243129	0.19968727	0.61247263	-5.00048086
homo after UVB irradiation VS homo before UVB irradiation	Chaperonin-mediated protein folding|Reactome	-0.01201822	-0.01841125	-0.06577437	0.94893675	0.9693166	-6.36612428
hetero after UVB irradiation VS hetero before UVB irradiation	Chaperonin-mediated protein folding|Reactome	0.022122947	-0.01357569	0.129129611	0.90028449	0.96973277	-6.33750468
WT after UVB irradiation VS WT before UVB irradiation	Chaperonin-mediated protein folding|Reactome	0.138014478	-0.02560666	0.66772686	0.51929938	0.82992425	-5.64851118
homo after UVB irradiation VS homo before UVB irradiation	Chemical carcinogenesis - Homo sapiens (human)|KEGG	0.05274248	0.043299483	0.256192414	0.80332738	0.87814049	-6.33330861
hetero after UVB irradiation VS hetero before UVB irradiation	Chemical carcinogenesis - Homo sapiens (human)|KEGG	0.106974414	0.00481233	0.595234579	0.56732557	0.76437184	-6.1583821
WT after UVB irradiation VS WT before UVB irradiation	Chemical carcinogenesis - Homo sapiens (human)|KEGG	0.013973635	0.033205447	0.06251433	0.95137524	0.98681143	-5.86717181
homo after UVB irradiation VS homo before UVB irradiation	Chemical Compounds to monitor Proteins|Wikipathways	-0.15808432	0.024039227	-0.53731724	0.60354766	0.72235875	-6.21572706
hetero after UVB irradiation VS hetero before UVB irradiation	Chemical Compounds to monitor Proteins|Wikipathways	0.016842177	0.059721201	0.064561027	0.95002904	0.9739614	-6.34428068
WT after UVB irradiation VS WT before UVB irradiation	Chemical Compounds to monitor Proteins|Wikipathways	-0.14149054	-0.03245668	-0.51462947	0.61790422	0.87257466	-5.73684979
homo after UVB irradiation VS homo before UVB irradiation	Chemokine receptors bind chemokines|Reactome	0.332763808	0.01829622	1.542056265	0.15605861	0.30417351	-5.23354295
hetero after UVB irradiation VS hetero before UVB irradiation	Chemokine receptors bind chemokines|Reactome	0.23215519	0.034109523	1.125651163	0.29135112	0.52220936	-5.70758528
WT after UVB irradiation VS WT before UVB irradiation	Chemokine receptors bind chemokines|Reactome	0.116090517	0.005953506	0.437663692	0.67085717	0.87984445	-5.77309442
homo after UVB irradiation VS homo before UVB irradiation	Chemokine signaling pathway - Homo sapiens (human)|KEGG	0.544554085	-0.02149279	3.171728824	0.01075442	0.06263355	-2.74350555
hetero after UVB irradiation VS hetero before UVB irradiation	Chemokine signaling pathway - Homo sapiens (human)|KEGG	0.551017192	0.032668999	3.338338485	0.00952554	0.0985871	-2.5589297
WT after UVB irradiation VS WT before UVB irradiation	Chemokine signaling pathway - Homo sapiens (human)|KEGG	0.417278032	-0.00372186	1.871007637	0.09061582	0.48188205	-4.36620099
homo after UVB irradiation VS homo before UVB irradiation	Chemokine signaling pathway|Wikipathways	0.51203921	-0.0234356	2.976828365	0.01483776	0.07510999	-3.05529024
hetero after UVB irradiation VS hetero before UVB irradiation	Chemokine signaling pathway|Wikipathways	0.514167718	0.026346886	3.156696865	0.0126055	0.10799973	-2.83282267
WT after UVB irradiation VS WT before UVB irradiation	Chemokine signaling pathway|Wikipathways	0.373263453	-0.00993949	1.669058632	0.12581587	0.53232197	-4.6357969
homo after UVB irradiation VS homo before UVB irradiation	CHILD Syndrome|SMPDB	-0.19230642	-0.01987665	-0.61925406	0.55047761	0.67913558	-6.16661869
hetero after UVB irradiation VS hetero before UVB irradiation	CHILD Syndrome|SMPDB	0.185820603	0.008098609	0.631890782	0.54421801	0.75147406	-6.13505553
WT after UVB irradiation VS WT before UVB irradiation	CHILD Syndrome|SMPDB	-0.14894747	0.045069977	-0.50755372	0.62268399	0.87257466	-5.74041409
homo after UVB irradiation VS homo before UVB irradiation	Chk1/Chk2(Cds1) mediated inactivation of Cyclin B:Cdk1 complex|Reactome	-0.18992774	-0.02276883	-0.90356033	0.38882214	0.54176438	-5.948332
hetero after UVB irradiation VS hetero before UVB irradiation	Chk1/Chk2(Cds1) mediated inactivation of Cyclin B:Cdk1 complex|Reactome	0.466766808	-0.01669272	1.912759906	0.0902874	0.27214648	-4.70818747
WT after UVB irradiation VS WT before UVB irradiation	Chk1/Chk2(Cds1) mediated inactivation of Cyclin B:Cdk1 complex|Reactome	0.234231997	0.026068257	0.919155268	0.37947955	0.75122805	-5.45946816
homo after UVB irradiation VS homo before UVB irradiation	CHL1 interactions|Reactome	0.48277948	0.066314435	2.742318766	0.02191963	0.0959588	-3.43092254
hetero after UVB irradiation VS hetero before UVB irradiation	CHL1 interactions|Reactome	0.745505286	0.041713529	3.81922338	0.00462558	0.07411472	-1.85077917
WT after UVB irradiation VS WT before UVB irradiation	CHL1 interactions|Reactome	0.249226982	-0.00737993	0.672484705	0.51639043	0.82895075	-5.64542933
homo after UVB irradiation VS homo before UVB irradiation	Chloroprocaine Action Pathway|SMPDB	0.191132361	0.029768717	1.061313976	0.31508276	0.46923284	-5.79783739
hetero after UVB irradiation VS hetero before UVB irradiation	Chloroprocaine Action Pathway|SMPDB	-0.07904315	0.055005373	-0.44437398	0.66796482	0.82651398	-6.24068873
WT after UVB irradiation VS WT before UVB irradiation	Chloroprocaine Action Pathway|SMPDB	0.022316377	0.035038618	0.098344171	0.92358676	0.97566251	-5.86425414
homo after UVB irradiation VS homo before UVB irradiation	Chlorothiazide Action Pathway|SMPDB	0.234726031	0.017120546	1.156747303	0.27595732	0.43614438	-5.69767199
hetero after UVB irradiation VS hetero before UVB irradiation	Chlorothiazide Action Pathway|SMPDB	-0.03255037	0.028925632	-0.18154472	0.86023155	0.94503144	-6.32869738
WT after UVB irradiation VS WT before UVB irradiation	Chlorothiazide Action Pathway|SMPDB	-0.15908359	0.010252305	-0.80382265	0.44003996	0.79685269	-5.55266936
homo after UVB irradiation VS homo before UVB irradiation	Chlorthalidone Action Pathway|SMPDB	0.234726031	0.017120546	1.156747303	0.27595732	0.43614438	-5.69767199
hetero after UVB irradiation VS hetero before UVB irradiation	Chlorthalidone Action Pathway|SMPDB	-0.03255037	0.028925632	-0.18154472	0.86023155	0.94503144	-6.32869738
WT after UVB irradiation VS WT before UVB irradiation	Chlorthalidone Action Pathway|SMPDB	-0.15908359	0.010252305	-0.80382265	0.44003996	0.79685269	-5.55266936
homo after UVB irradiation VS homo before UVB irradiation	cholesterol biosynthesis I|HumanCyc	-0.15495558	-0.00067938	-0.46361493	0.65348386	0.76059325	-6.25429977
hetero after UVB irradiation VS hetero before UVB irradiation	cholesterol biosynthesis I|HumanCyc	0.36230699	0.004745716	1.466693197	0.17876134	0.39630067	-5.3110041
WT after UVB irradiation VS WT before UVB irradiation	cholesterol biosynthesis I|HumanCyc	-0.15025749	0.115182744	-0.47495628	0.64493933	0.87766327	-5.75622967
homo after UVB irradiation VS homo before UVB irradiation	cholesterol biosynthesis II (via 24_25-dihydrolanosterol)|HumanCyc	-0.15495558	-0.00067938	-0.46361493	0.65348386	0.76059325	-6.25429977
hetero after UVB irradiation VS hetero before UVB irradiation	cholesterol biosynthesis II (via 24_25-dihydrolanosterol)|HumanCyc	0.36230699	0.004745716	1.466693197	0.17876134	0.39630067	-5.3110041
WT after UVB irradiation VS WT before UVB irradiation	cholesterol biosynthesis II (via 24_25-dihydrolanosterol)|HumanCyc	-0.15025749	0.115182744	-0.47495628	0.64493933	0.87766327	-5.75622967
homo after UVB irradiation VS homo before UVB irradiation	cholesterol biosynthesis III (via desmosterol)|HumanCyc	-0.15495558	-0.00067938	-0.46361493	0.65348386	0.76059325	-6.25429977
hetero after UVB irradiation VS hetero before UVB irradiation	cholesterol biosynthesis III (via desmosterol)|HumanCyc	0.36230699	0.004745716	1.466693197	0.17876134	0.39630067	-5.3110041
WT after UVB irradiation VS WT before UVB irradiation	cholesterol biosynthesis III (via desmosterol)|HumanCyc	-0.15025749	0.115182744	-0.47495628	0.64493933	0.87766327	-5.75622967
homo after UVB irradiation VS homo before UVB irradiation	Cholesterol Biosynthesis Pathway|Wikipathways	-0.18247059	-0.02619339	-0.53335938	0.6061781	0.72531688	-6.21793457
hetero after UVB irradiation VS hetero before UVB irradiation	Cholesterol Biosynthesis Pathway|Wikipathways	0.236148682	-0.00822509	0.659446377	0.52721822	0.74191436	-6.11668937
WT after UVB irradiation VS WT before UVB irradiation	Cholesterol Biosynthesis Pathway|Wikipathways	-0.05121719	0.075031608	-0.15405492	0.88060651	0.96769248	-5.85714343
homo after UVB irradiation VS homo before UVB irradiation	Cholesterol biosynthesis via desmosterol|Reactome	0.522955234	-0.06104275	1.509477454	0.16406929	0.31331676	-5.27604241
hetero after UVB irradiation VS hetero before UVB irradiation	Cholesterol biosynthesis via desmosterol|Reactome	0.711082265	-0.01071815	3.410825001	0.00852686	0.09297946	-2.45047886
WT after UVB irradiation VS WT before UVB irradiation	Cholesterol biosynthesis via desmosterol|Reactome	0.328654812	0.05951777	0.891899996	0.39323498	0.76452505	-5.48244679
homo after UVB irradiation VS homo before UVB irradiation	Cholesterol biosynthesis via lathosterol|Reactome	0.522955234	-0.06104275	1.509477454	0.16406929	0.31331676	-5.27604241
hetero after UVB irradiation VS hetero before UVB irradiation	Cholesterol biosynthesis via lathosterol|Reactome	0.711082265	-0.01071815	3.410825001	0.00852686	0.09297946	-2.45047886
WT after UVB irradiation VS WT before UVB irradiation	Cholesterol biosynthesis via lathosterol|Reactome	0.328654812	0.05951777	0.891899996	0.39323498	0.76452505	-5.48244679
homo after UVB irradiation VS homo before UVB irradiation	Cholesterol biosynthesis_ regulation and transport|Wikipathways	-0.28370759	-0.01212924	-0.85265194	0.41507225	0.56607524	-5.99261735
hetero after UVB irradiation VS hetero before UVB irradiation	Cholesterol biosynthesis_ regulation and transport|Wikipathways	0.293441755	-0.03198396	0.832723363	0.4279708	0.65525392	-5.98547929
WT after UVB irradiation VS WT before UVB irradiation	Cholesterol biosynthesis_ regulation and transport|Wikipathways	0.052947134	0.087493795	0.138292265	0.89273254	0.97398699	-5.85947257
homo after UVB irradiation VS homo before UVB irradiation	Cholesterol biosynthesis|Reactome	-0.22684134	-0.01562823	-0.78370785	0.45252732	0.6002769	-6.04907842
hetero after UVB irradiation VS hetero before UVB irradiation	Cholesterol biosynthesis|Reactome	0.190264549	0.022998441	0.658131657	0.52802196	0.74212892	-6.11758169
WT after UVB irradiation VS WT before UVB irradiation	Cholesterol biosynthesis|Reactome	-0.0767223	0.058210669	-0.25168705	0.80633893	0.94525798	-5.83716842
homo after UVB irradiation VS homo before UVB irradiation	Cholesterol metabolism - Homo sapiens (human)|KEGG	0.414665831	0.030028593	2.515815711	0.03199828	0.11936615	-3.79172298
hetero after UVB irradiation VS hetero before UVB irradiation	Cholesterol metabolism - Homo sapiens (human)|KEGG	0.20134944	0.014141534	1.030273022	0.33154549	0.55949068	-5.80519309
WT after UVB irradiation VS WT before UVB irradiation	Cholesterol metabolism - Homo sapiens (human)|KEGG	0.167322367	-0.03650492	0.691701492	0.50474101	0.82448724	-5.63278037
homo after UVB irradiation VS homo before UVB irradiation	Cholesteryl ester storage disease|SMPDB	-0.19230642	-0.01987665	-0.61925406	0.55047761	0.67913558	-6.16661869
hetero after UVB irradiation VS hetero before UVB irradiation	Cholesteryl ester storage disease|SMPDB	0.185820603	0.008098609	0.631890782	0.54421801	0.75147406	-6.13505553
WT after UVB irradiation VS WT before UVB irradiation	Cholesteryl ester storage disease|SMPDB	-0.14894747	0.045069977	-0.50755372	0.62268399	0.87257466	-5.74041409
homo after UVB irradiation VS homo before UVB irradiation	Choline catabolism|Reactome	0.024138572	0.026069071	0.103755378	0.91954601	0.95153785	-6.36266747
hetero after UVB irradiation VS hetero before UVB irradiation	Choline catabolism|Reactome	0.181741147	0.10773466	0.889430011	0.39845259	0.62647357	-5.93691783
WT after UVB irradiation VS WT before UVB irradiation	Choline catabolism|Reactome	0.449660724	0.075141496	1.604841345	0.13936104	0.55478524	-4.7182216
homo after UVB irradiation VS homo before UVB irradiation	choline degradation|HumanCyc	1.189912781	-0.03086465	7.282751079	3.72E-05	0.01666204	2.689001592
hetero after UVB irradiation VS hetero before UVB irradiation	choline degradation|HumanCyc	0.997225992	0.159399746	3.508930567	0.0073473	0.0874386	-2.30458267
WT after UVB irradiation VS WT before UVB irradiation	choline degradation|HumanCyc	0.817388886	-0.00561412	2.705620748	0.02194949	0.31542987	-3.15069136
homo after UVB irradiation VS homo before UVB irradiation	Choline metabolism in cancer - Homo sapiens (human)|KEGG	0.332455575	-0.0047112	1.923275309	0.08525535	0.20754519	-4.70170874

hetero after UVB irradiation VS hetero before UVB irradiation	Choline metabolism in cancer - Homo sapiens (human)|KEGG	0.288217314	0.001210392	1.82569529	0.10344395	0.28926228	-4.8314541
WT after UVB irradiation VS WT before UVB irradiation	Choline metabolism in cancer - Homo sapiens (human)|KEGG	0.065869528	0.020696379	0.333059273	0.74591189	0.9185045	-5.81328616
homo after UVB irradiation VS homo before UVB irradiation	Cholinergic synapse - Homo sapiens (human)|KEGG	0.436650637	-0.04908996	2.732488503	0.02228215	0.09702597	-3.44664328
hetero after UVB irradiation VS hetero before UVB irradiation	Cholinergic synapse - Homo sapiens (human)|KEGG	0.227166897	0.027040457	1.175218453	0.2720455	0.5012059	-5.65436809
WT after UVB irradiation VS WT before UVB irradiation	Cholinergic synapse - Homo sapiens (human)|KEGG	0.157609616	-0.0070225	0.656186126	0.52639584	0.83205707	-5.65590387
homo after UVB irradiation VS homo before UVB irradiation	Chondrodysplasia Punctata II_ X Linked Dominant (CDPX2)|SMPDB	-0.19230642	-0.01987665	-0.61925406	0.55047761	0.67913558	-6.16661869
hetero after UVB irradiation VS hetero before UVB irradiation	Chondrodysplasia Punctata II_ X Linked Dominant (CDPX2)|SMPDB	0.185820603	0.008098609	0.631890782	0.54421801	0.75147406	-6.13505553
WT after UVB irradiation VS WT before UVB irradiation	Chondrodysplasia Punctata II_ X Linked Dominant (CDPX2)|SMPDB	-0.14894747	0.045069977	-0.50755372	0.62268399	0.87257466	-5.74041409
homo after UVB irradiation VS homo before UVB irradiation	chondroitin and dermatan biosynthesis|HumanCyc	0.186498749	-0.00985029	0.871057754	0.40544313	0.5575582	-5.97685638
hetero after UVB irradiation VS hetero before UVB irradiation	chondroitin and dermatan biosynthesis|HumanCyc	0.14904321	0.100032672	0.670585782	0.52043805	0.73623731	-6.10906476
WT after UVB irradiation VS WT before UVB irradiation	chondroitin and dermatan biosynthesis|HumanCyc	0.017850561	-0.08082086	0.050754667	0.96051249	0.98815178	-5.86784636
homo after UVB irradiation VS homo before UVB irradiation	chondroitin sulfate biosynthesis (late stages)|HumanCyc	0.493260663	-0.00742143	2.431129354	0.03685798	0.125686	-3.9254946
hetero after UVB irradiation VS hetero before UVB irradiation	chondroitin sulfate biosynthesis (late stages)|HumanCyc	0.268936074	0.082742847	1.078790933	0.31059161	0.53909998	-5.75634965
WT after UVB irradiation VS WT before UVB irradiation	chondroitin sulfate biosynthesis (late stages)|HumanCyc	0.197056845	-0.03519467	0.588144506	0.56937467	0.85284819	-5.69708122
homo after UVB irradiation VS homo before UVB irradiation	chondroitin sulfate biosynthesis|HumanCyc	0.48402951	-0.01938541	2.833330209	0.01883397	0.08765675	-3.28522974
hetero after UVB irradiation VS hetero before UVB irradiation	chondroitin sulfate biosynthesis|HumanCyc	0.345576709	0.059211049	1.470278647	0.17781247	0.39515808	-5.30649029
WT after UVB irradiation VS WT before UVB irradiation	chondroitin sulfate biosynthesis|HumanCyc	0.355630781	-0.04246174	1.208317684	0.25450215	0.64897339	-5.18218961
homo after UVB irradiation VS homo before UVB irradiation	Chondroitin sulfate biosynthesis|Reactome	0.46363438	0.000660317	2.532822992	0.03110202	0.11752935	-3.76476701
hetero after UVB irradiation VS hetero before UVB irradiation	Chondroitin sulfate biosynthesis|Reactome	0.179590807	0.068373613	0.846259349	0.42079018	0.64743517	-5.97413037
WT after UVB irradiation VS WT before UVB irradiation	Chondroitin sulfate biosynthesis|Reactome	0.158456602	-0.00957792	0.585400524	0.57114789	0.8541036	-5.69865449
homo after UVB irradiation VS homo before UVB irradiation	chondroitin sulfate degradation (metazoa)|HumanCyc	0.186814689	0.04856387	1.040521535	0.3241489	0.48028464	-5.81877641
hetero after UVB irradiation VS hetero before UVB irradiation	chondroitin sulfate degradation (metazoa)|HumanCyc	0.342722053	-0.04983869	1.831608409	0.10249598	0.28762124	-4.82315436
WT after UVB irradiation VS WT before UVB irradiation	chondroitin sulfate degradation (metazoa)|HumanCyc	0.309612573	0.015250983	1.315069742	0.21761009	0.62581393	-5.06579602
homo after UVB irradiation VS homo before UVB irradiation	Chondroitin sulfate/dermatan sulfate metabolism|Reactome	0.539742583	-0.01289494	3.278693102	0.00902463	0.05691501	-2.57307785
hetero after UVB irradiation VS hetero before UVB irradiation	Chondroitin sulfate/dermatan sulfate metabolism|Reactome	0.313132428	-0.00367516	1.360205446	0.20903705	0.43119765	-5.44204943
WT after UVB irradiation VS WT before UVB irradiation	Chondroitin sulfate/dermatan sulfate metabolism|Reactome	0.33242278	-0.01175292	1.313823123	0.218014	0.62581393	-5.06719461
homo after UVB irradiation VS homo before UVB irradiation	ChREBP activates metabolic gene expression|Reactome	0.171152522	-0.000273	0.833974421	0.42500344	0.57609992	-6.00831741
hetero after UVB irradiation VS hetero before UVB irradiation	ChREBP activates metabolic gene expression|Reactome	-0.49492834	0.02276423	-1.27817702	0.23527072	0.45788382	-5.5387459
WT after UVB irradiation VS WT before UVB irradiation	ChREBP activates metabolic gene expression|Reactome	0.290494251	-0.08090861	0.991405691	0.34468405	0.7291211	-5.39580055
homo after UVB irradiation VS homo before UVB irradiation	chrebp regulation by carbohydrates and camp|BioCarta	0.480175986	0.008611225	3.035645213	0.01346055	0.07249235	-2.9610857
hetero after UVB irradiation VS hetero before UVB irradiation	chrebp regulation by carbohydrates and camp|BioCarta	0.261968645	0.036552443	1.407808215	0.19499323	0.4161797	-5.38420564
WT after UVB irradiation VS WT before UVB irradiation	chrebp regulation by carbohydrates and camp|BioCarta	0.136703711	-0.02682785	0.494393072	0.63162308	0.87303764	-5.74691926
homo after UVB irradiation VS homo before UVB irradiation	Chromatin modifying enzymes|Reactome	-0.47808898	-0.00563022	-2.55147435	0.03014786	0.11538756	-3.73517414
hetero after UVB irradiation VS hetero before UVB irradiation	Chromatin modifying enzymes|Reactome	-0.25543579	-0.06797148	-1.32134235	0.22113811	0.44238028	-5.48834528
WT after UVB irradiation VS WT before UVB irradiation	Chromatin modifying enzymes|Reactome	-0.25050874	-0.01990048	-0.95470473	0.36205664	0.74374432	-5.42863522
homo after UVB irradiation VS homo before UVB irradiation	Chromatin organization|Reactome	-0.47808898	-0.00563022	-2.55147435	0.03014786	0.11538756	-3.73517414
hetero after UVB irradiation VS hetero before UVB irradiation	Chromatin organization|Reactome	-0.25543579	-0.06797148	-1.32134235	0.22113811	0.44238028	-5.48834528
WT after UVB irradiation VS WT before UVB irradiation	Chromatin organization|Reactome	-0.25050874	-0.01990048	-0.95470473	0.36205664	0.74374432	-5.42863522
homo after UVB irradiation VS homo before UVB irradiation	chromatin remodeling by hswi/snf atp-dependent complexes|BioCarta	-0.35454462	0.054286792	-1.46023569	0.1768612	0.32961153	-5.33924286
hetero after UVB irradiation VS hetero before UVB irradiation	chromatin remodeling by hswi/snf atp-dependent complexes|BioCarta	0.134600468	0.043836321	0.493437327	0.6343073	0.81332653	-6.21638288
WT after UVB irradiation VS WT before UVB irradiation	chromatin remodeling by hswi/snf atp-dependent complexes|BioCarta	-0.03321537	0.020177954	-0.11081893	0.91393495	0.97566251	-5.86293385
homo after UVB irradiation VS homo before UVB irradiation	Chromosomal and microsatellite instability in colorectal cancer|Wikipathways	0.073895831	-0.02532091	0.38982574	0.70536105	0.80552596	-6.28746949
hetero after UVB irradiation VS hetero before UVB irradiation	Chromosomal and microsatellite instability in colorectal cancer|Wikipathways	0.224379319	-0.01949598	1.323536091	0.22043949	0.44184836	-5.48575482
WT after UVB irradiation VS WT before UVB irradiation	Chromosomal and microsatellite instability in colorectal cancer|Wikipathways	0.102787533	0.008956128	0.533608547	0.60517595	0.8702632	-5.72705955
homo after UVB irradiation VS homo before UVB irradiation	Chromosome Maintenance|Reactome	-0.49261753	-0.01931791	-1.98800237	0.07672874	0.19626432	-4.60617834
hetero after UVB irradiation VS hetero before UVB irradiation	Chromosome Maintenance|Reactome	0.142727462	-0.02052124	0.518333568	0.6175591	0.80199705	-6.20313294
WT after UVB irradiation VS WT before UVB irradiation	Chromosome Maintenance|Reactome	0.164160985	0.058472485	0.588555827	0.56910913	0.85280226	-5.69684481
homo after UVB irradiation VS homo before UVB irradiation	Chronic myeloid leukemia - Homo sapiens (human)|KEGG	0.134172832	-0.03386522	0.666362314	0.52121484	0.65906036	-6.13549059
hetero after UVB irradiation VS hetero before UVB irradiation	Chronic myeloid leukemia - Homo sapiens (human)|KEGG	0.320738083	-0.01338027	1.974721788	0.08191034	0.25808464	-4.61917266
WT after UVB irradiation VS WT before UVB irradiation	Chronic myeloid leukemia - Homo sapiens (human)|KEGG	0.15691149	-0.02629117	0.730589057	0.48166244	0.81552033	-5.60620443
homo after UVB irradiation VS homo before UVB irradiation	Chylomicron assembly|Reactome	0.602121789	0.060841859	2.484992055	0.03368866	0.12247008	-3.84050377
hetero after UVB irradiation VS hetero before UVB irradiation	Chylomicron assembly|Reactome	0.497282372	0.012964731	2.758526301	0.02355191	0.13628011	-3.44025625
WT after UVB irradiation VS WT before UVB irradiation	Chylomicron assembly|Reactome	0.288715586	0.065273409	1.139573424	0.28081403	0.66935161	-5.25335573
homo after UVB irradiation VS homo before UVB irradiation	Chylomicron clearance|Reactome	-0.15617856	0.061920791	-0.48126125	0.64134866	0.75106386	-6.24555502
hetero after UVB irradiation VS hetero before UVB irradiation	Chylomicron clearance|Reactome	0.197284315	0.019136897	0.672144596	0.51949352	0.73623731	-6.10798867
WT after UVB irradiation VS WT before UVB irradiation	Chylomicron clearance|Reactome	0.318522991	0.095269492	1.178878804	0.26551877	0.66118679	-5.21304187
homo after UVB irradiation VS homo before UVB irradiation	Chylomicron remodeling|Reactome	0.795110916	0.074728535	3.830397171	0.00372241	0.03447567	-1.70894164
hetero after UVB irradiation VS hetero before UVB irradiation	Chylomicron remodeling|Reactome	0.541032033	0.059622218	2.45582798	0.03812557	0.16974934	-3.90266021
WT after UVB irradiation VS WT before UVB irradiation	Chylomicron remodeling|Reactome	0.591768258	0.109746683	2.289625	0.04484091	0.39382393	-3.77010479
homo after UVB irradiation VS homo before UVB irradiation	Cilazapril Action Pathway|SMPDB	0.636038586	-0.11800708	2.436891056	0.03650532	0.12504267	-3.91641947
hetero after UVB irradiation VS hetero before UVB irradiation	Cilazapril Action Pathway|SMPDB	-0.02777369	0.003323189	-0.09172332	0.92906122	0.97054702	-6.34197953
WT after UVB irradiation VS WT before UVB irradiation	Cilazapril Action Pathway|SMPDB	0.270438532	-0.10210744	0.707878218	0.49505953	0.81710765	-5.62188349
homo after UVB irradiation VS homo before UVB irradiation	Cilazapril Metabolism Pathway|SMPDB	1.076747846	-0.04193589	4.959558579	0.00068841	0.01666204	-0.06417629
hetero after UVB irradiation VS hetero before UVB irradiation	Cilazapril Metabolism Pathway|SMPDB	0.553630209	0.003105397	1.56066683	0.15532075	0.36486693	-5.19066683
WT after UVB irradiation VS WT before UVB irradiation	Cilazapril Metabolism Pathway|SMPDB	0.585930211	-0.05239369	1.72512433	0.11496929	0.51527453	-4.56245573
homo after UVB irradiation VS homo before UVB irradiation	Ciliary landscape|Wikipathways	-0.30800421	-0.01109233	-1.94214394	0.08268162	0.20395693	-4.67399138
hetero after UVB irradiation VS hetero before UVB irradiation	Ciliary landscape|Wikipathways	-0.13840913	-0.02566396	-0.77555342	0.45922415	0.68347523	-6.03169342
WT after UVB irradiation VS WT before UVB irradiation	Ciliary landscape|Wikipathways	-0.12255486	-0.01893633	-0.57501889	0.57788407	0.85855451	-5.70454487
homo after UVB irradiation VS homo before UVB irradiation	Cilium Assembly|Reactome	-0.33562017	-0.00940249	-1.92421384	0.08512557	0.20751915	-4.70033269
hetero after UVB irradiation VS hetero before UVB irradiation	Cilium Assembly|Reactome	-0.20243167	-0.03780732	-1.03103203	0.33120951	0.55930944	-5.8044422
WT after UVB irradiation VS WT before UVB irradiation	Cilium Assembly|Reactome	-0.26737207	-0.04972335	-1.26028395	0.23595429	0.62952955	-5.12639507
homo after UVB irradiation VS homo before UVB irradiation	Cimetidine Action Pathway|SMPDB	0.21780303	0.051698704	1.19150037	0.26270351	0.42561191	-5.65959884
hetero after UVB irradiation VS hetero before UVB irradiation	Cimetidine Action Pathway|SMPDB	0.331566302	0.063470812	1.441200644	0.18563717	0.40321767	-5.34291209
WT after UVB irradiation VS WT before UVB irradiation	Cimetidine Action Pathway|SMPDB	-0.11560861	0.135982321	-0.44330845	0.66690385	0.87935805	-5.77062647
homo after UVB irradiation VS homo before UVB irradiation	Cimetidine Metabolism Pathway|SMPDB	1.089753148	-0.04475232	4.967623568	0.00068061	0.01666204	-0.05314037
hetero after UVB irradiation VS hetero before UVB irradiation	Cimetidine Metabolism Pathway|SMPDB	1.104362048	0.029998137	5.039706695	0.00085589	0.03680415	-0.2055051
WT after UVB irradiation VS WT before UVB irradiation	Cimetidine Metabolism Pathway|SMPDB	1.059857768	0.071821028	4.446387427	0.00121746	0.15645713	-0.64512292
homo after UVB irradiation VS homo before UVB irradiation	Circadian Clock|Reactome	-0.29609834	0.028856092	-1.68313181	0.12523937	0.26466492	-5.0436684
hetero after UVB irradiation VS hetero before UVB irradiation	Circadian Clock|Reactome	-0.37309699	-0.04929234	-2.5000979	0.03552621	0.16574051	-3.83528914
WT after UVB irradiation VS WT before UVB irradiation	Circadian Clock|Reactome	-0.53408544	-0.0236432	-2.68814947	0.02262014	0.31689014	-3.17694296
homo after UVB irradiation VS homo before UVB irradiation	Circadian Clock|Wikipathways	0.223745833	-0.01012648	1.099346261	0.29900447	0.45968162	-5.75870719
hetero after UVB irradiation VS hetero before UVB irradiation	Circadian Clock|Wikipathways	-0.05581033	-0.03480062	-0.21659613	0.83367556	0.93635724	-6.32116341
WT after UVB irradiation VS WT before UVB irradiation	Circadian Clock|Wikipathways	-0.27160279	-0.06115393	-1.02366195	0.32992877	0.71290873	-5.36611909
homo after UVB irradiation VS homo before UVB irradiation	Circadian entrainment - Homo sapiens (human)|KEGG	0.364409431	-0.02607956	2.159639345	0.05787305	0.16382415	-4.34734098
hetero after UVB irradiation VS hetero before UVB irradiation	Circadian entrainment - Homo sapiens (human)|KEGG	0.160785514	0.022252369	0.766848633	0.46411335	0.68805201	-6.03848247
WT after UVB irradiation VS WT before UVB irradiation	Circadian entrainment - Homo sapiens (human)|KEGG	0.073092534	-0.02414783	0.276617975	0.78766144	0.93972043	-5.83054553
homo after UVB irradiation VS homo before UVB irradiation	Circadian rhythm - Homo sapiens (human)|KEGG	-0.1448732	-0.00654427	-0.80614598	0.44009815	0.59091566	-6.03115452
hetero after UVB irradiation VS hetero before UVB irradiation	Circadian rhythm - Homo sapiens (human)|KEGG	-0.23545752	-0.04752992	-1.09154936	0.3052567	0.53493274	-5.74322508
WT after UVB irradiation VS WT before UVB irradiation	Circadian rhythm - Homo sapiens (human)|KEGG	-0.33923044	-0.02102149	-1.69185213	0.12129956	0.52647129	-4.60613
homo after UVB irradiation VS homo before UVB irradiation	Circadian rhythm pathway|PID	-0.20251699	0.01798085	-1.18234493	0.26614458	0.42955679	-5.66970919
hetero after UVB irradiation VS hetero before UVB irradiation	Circadian rhythm pathway|PID	0.008757444	-0.04468912	0.049318826	0.96181433	0.98206759	-6.34522196
WT after UVB irradiation VS WT before UVB irradiation	Circadian rhythm pathway|PID	-0.0250244	-0.07114131	-0.12829217	0.90044076	0.97566251	-5.86082052
homo after UVB irradiation VS homo before UVB irradiation	Circadian rythm related genes|Wikipathways	1.102427104	-0.02946902	4.702515614	0.00099487	0.01831679	-0.42141013
hetero after UVB irradiation VS hetero before UVB irradiation	Circadian rythm related genes|Wikipathways	0.85340455	0.017969898	2.797170872	0.02215383	0.13269008	-3.38109144
WT after UVB irradiation VS WT before UVB irradiation	Circadian rythm related genes|Wikipathways	0.349692107	-0.05183388	1.000122977	0.34064918	0.72554886	-5.38785433
homo after UVB irradiation VS homo before UVB irradiation	Citalopram Action Pathway|SMPDB	0.34533219	0.042904911	2.158975925	0.05793653	0.16387075	-4.34835509
hetero after UVB irradiation VS hetero before UVB irradiation	Citalopram Action Pathway|SMPDB	-0.00631207	0.054089602	-0.03194276	0.97526144	0.988327	-6.3459878
WT after UVB irradiation VS WT before UVB irradiation	Citalopram Action Pathway|SMPDB	0.107367163	0.050117311	0.441687175	0.66803823	0.87935805	-5.77133841
homo after UVB irradiation VS homo before UVB irradiation	Citalopram Metabolism Pathway|SMPDB	0.579225579	-0.08589532	2.217150875	0.05261911	0.15312865	-4.25907601
hetero after UVB irradiation VS hetero before UVB irradiation	Citalopram Metabolism Pathway|SMPDB	0.22050413	0.090588581	0.604875318	0.56119438	0.76343066	-6.15237042
WT after UVB irradiation VS WT before UVB irradiation	Citalopram Metabolism Pathway|SMPDB	0.208983649	0.014071995	0.596319572	0.56410982	0.85109394	-5.69235361
homo after UVB irradiation VS homo before UVB irradiation	Citalopram Pathway_ Pharmacokinetics|PharmGKB	-0.37684247	-0.08003597	-0.87628748	0.40273576	0.55488578	-5.97232617
hetero after UVB irradiation VS hetero before UVB irradiation	Citalopram Pathway_ Pharmacokinetics|PharmGKB	-0.13710652	-0.12477915	-0.400264	0.69891897	0.84778456	-6.26046782
WT after UVB irradiation VS WT before UVB irradiation	Citalopram Pathway_ Pharmacokinetics|PharmGKB	-0.24368965	-0.11004384	-0.60684261	0.55737287	0.84822456	-5.68617924
homo after UVB irradiation VS homo before UVB irradiation	Citrate cycle (TCA cycle) - Homo sapiens (human)|KEGG	-0.15215414	0.009359864	-0.55413039	0.59244063	0.7145588	-6.20617905
hetero after UVB irradiation VS hetero before UVB irradiation	Citrate cycle (TCA cycle) - Homo sapiens (human)|KEGG	-0.39544841	-0.0400202	-1.5401974	0.16017901	0.37252774	-5.21722933
WT after UVB irradiation VS WT before UVB irradiation	Citrate cycle (TCA cycle) - Homo sapiens (human)|KEGG	-0.1396974	-0.09674315	-0.51948826	0.61463281	0.87257466	-5.73437526
homo after UVB irradiation VS homo before UVB irradiation	Citrate cycle|INOH	-0.08387534	0.018953192	-0.30885125	0.76417819	0.84731365	-6.31746113
hetero after UVB irradiation VS hetero before UVB irradiation	Citrate cycle|INOH	-0.21301081	-0.00059191	-0.88078908	0.40285482	0.63121769	-5.94448894
WT after UVB irradiation VS WT before UVB irradiation	Citrate cycle|INOH	0.04848638	-0.06625684	0.179115931	0.86139504	0.95916804	-5.85292614
homo after UVB irradiation VS homo before UVB irradiation	Citric acid cycle (TCA cycle)|Reactome	-0.31564463	0.01346484	-1.06539163	0.31332775	0.46923284	-5.79369308
hetero after UVB irradiation VS hetero before UVB irradiation	Citric acid cycle (TCA cycle)|Reactome	-0.49306075	-0.00638096	-1.92544653	0.08850827	0.2694861	-4.69004446
WT after UVB irradiation VS WT before UVB irradiation	Citric acid cycle (TCA cycle)|Reactome	-0.20802424	-0.12657503	-0.75125158	0.46967343	0.81218589	-5.59155669
homo after UVB irradiation VS homo before UVB irradiation	Citric Acid Cycle|SMPDB	-0.23678174	0.014544733	-0.76379894	0.46374733	0.6088463	-6.06461144
hetero after UVB irradiation VS hetero before UVB irradiation	Citric Acid Cycle|SMPDB	-0.47814985	0.004803127	-1.74993278	0.11633994	0.30887493	-4.93678811
WT after UVB irradiation VS WT before UVB irradiation	Citric Acid Cycle|SMPDB	-0.1568339	-0.12513755	-0.50250429	0.62610623	0.87257466	-5.74292911
homo after UVB irradiation VS homo before UVB irradiation	Citrullinemia Type I|SMPDB	-0.37671284	0.065264039	-1.86404069	0.09383246	0.21982504	-4.78798165
hetero after UVB irradiation VS hetero before UVB irradiation	Citrullinemia Type I|SMPDB	-0.31260554	-0.01518237	-1.48265131	0.17457213	0.39158454	-5.29086545
WT after UVB irradiation VS WT before UVB irradiation	Citrullinemia Type I|SMPDB	-0.12033773	0.030196148	-0.57832296	0.57573553	0.8562298	-5.70268083
homo after UVB irradiation VS homo before UVB irradiation	citrulline-nitric oxide cycle|HumanCyc	-0.02191995	0.021188827	-0.12575185	0.90258	0.94250012	-6.35995891
hetero after UVB irradiation VS hetero before UVB irradiation	citrulline-nitric oxide cycle|HumanCyc	0.35501783	0.042763238	1.432876823	0.18793193	0.40558415	-5.3532594
WT after UVB irradiation VS WT before UVB irradiation	citrulline-nitric oxide cycle|HumanCyc	0.005043496	0.079318399	0.019000329	0.9852116	0.99548728	-5.86896831
homo after UVB irradiation VS homo before UVB irradiation	Class A/1 (Rhodopsin-like receptors)|Reactome	0.321043947	0.018617766	1.875034639	0.09218188	0.21763312	-4.7720569
hetero after UVB irradiation VS hetero before UVB irradiation	Class A/1 (Rhodopsin-like receptors)|Reactome	0.197889966	0.031397618	1.240839581	0.24809916	0.47275751	-5.58144232
WT after UVB irradiation VS WT before UVB irradiation	Class A/1 (Rhodopsin-like receptors)|Reactome	0.188147309	0.044226668	0.813592136	0.43467196	0.79685269	-5.54519007
homo after UVB irradiation VS homo before UVB irradiation	Class B/2 (Secretin family receptors)|Reactome	0.102969082	-0.00410424	0.610517583	0.55600693	0.68429316	-6.17216154
hetero after UVB irradiation VS hetero before UVB irradiation	Class B/2 (Secretin family receptors)|Reactome	0.014543153	0.014056965	0.075736601	0.94139614	0.97054702	-6.34343061
WT after UVB irradiation VS WT before UVB irradiation	Class B/2 (Secretin family receptors)|Reactome	-0.09537079	-0.00362734	-0.48211916	0.64001634	0.87636259	-5.7528399
homo after UVB irradiation VS homo before UVB irradiation	Class C/3 (Metabotropic glutamate/pheromone receptors)|Reactome	0.102983782	-0.00400375	0.593172997	0.56707735	0.69195165	-6.18295042
hetero after UVB irradiation VS hetero before UVB irradiation	Class C/3 (Metabotropic glutamate/pheromone receptors)|Reactome	-0.19510875	0.051913101	-1.08867626	0.30645176	0.535791	-5.74619068
WT after UVB irradiation VS WT before UVB irradiation	Class C/3 (Metabotropic glutamate/pheromone receptors)|Reactome	-0.26850383	0.114745388	-1.34402146	0.20840309	0.62329858	-5.03306292
homo after UVB irradiation VS homo before UVB irradiation	Class I MHC mediated antigen processing & presentation|Reactome	0.074326739	-0.00923973	0.426802014	0.67914344	0.78327076	-6.27153685
hetero after UVB irradiation VS hetero before UVB irradiation	Class I MHC mediated antigen processing & presentation|Reactome	0.146014132	-0.00412299	0.954824817	0.36625345	0.5945097	-5.87767381
WT after UVB irradiation VS WT before UVB irradiation	Class I MHC mediated antigen processing & presentation|Reactome	-0.00592555	-0.01876448	-0.03002721	0.97663144	0.99265119	-5.86869445
homo after UVB irradiation VS homo before UVB irradiation	Class I PI3K signaling events mediated by Akt|PID	0.053188881	-0.03830327	0.238564308	0.81656903	0.8875301	-6.33796354
hetero after UVB irradiation VS hetero before UVB irradiation	Class I PI3K signaling events mediated by Akt|PID	0.151009386	0.009990271	0.964465855	0.36167306	0.58991729	-5.86865421
WT after UVB irradiation VS WT before UVB irradiation	Class I PI3K signaling events mediated by Akt|PID	0.000903093	-0.06153978	0.004004387	0.9968831	0.99863683	-5.86914307
homo after UVB irradiation VS homo before UVB irradiation	Class I PI3K signaling events|PID	0.393042106	-0.05113868	1.8209409	0.10057255	0.23064503	-4.85000921
hetero after UVB irradiation VS hetero before UVB irradiation	Class I PI3K signaling events|PID	0.620488943	0.017467544	4.007320314	0.00351664	0.06529021	-1.58203512
WT after UVB irradiation VS WT before UVB irradiation	Class I PI3K signaling events|PID	0.308860913	0.003411798	1.519367815	0.1593878	0.56072663	-4.82510368
homo after UVB irradiation VS homo before UVB irradiation	Class IB PI3K non-lipid kinase events|PID	0.517800217	-0.02637293	1.882900919	0.09101756	0.21600114	-4.76063767
hetero after UVB irradiation VS hetero before UVB irradiation	Class IB PI3K non-lipid kinase events|PID	0.583009559	0.039360543	2.455658596	0.03813588	0.16974934	-3.90291772
WT after UVB irradiation VS WT before UVB irradiation	Class IB PI3K non-lipid kinase events|PID	0.615632861	-0.01028393	2.479301756	0.03240374	0.36974198	-3.48956949
homo after UVB irradiation VS homo before UVB irradiation	Classical antibody-mediated complement activation|Reactome	0.780883354	-0.03333382	2.441251212	0.03624066	0.12504267	-3.90954919
hetero after UVB irradiation VS hetero before UVB irradiation	Classical antibody-mediated complement activation|Reactome	0.699924503	0.022570947	2.091150608	0.06814591	0.23495391	-4.4494066
WT after UVB irradiation VS WT before UVB irradiation	Classical antibody-mediated complement activation|Reactome	0.727129468	-0.02755661	2.117660543	0.0600396	0.42812412	-4.01975378
homo after UVB irradiation VS homo before UVB irradiation	classical complement pathway|BioCarta	0.834604518	-0.07979989	3.03921501	0.01338129	0.07239217	-2.9553704
hetero after UVB irradiation VS hetero before UVB irradiation	classical complement pathway|BioCarta	0.356674555	0.109796774	1.2918413	0.23071688	0.45210986	-5.52290996

WT after UVB irradiation VS WT before UVB irradiation	classical complement pathway|BioCarta	0.47300956	-0.00942378	1.497491284	0.16490185	0.56840025	-4.85190855
homo after UVB irradiation VS homo before UVB irradiation	Classical Kir channels|Reactome	0.904432746	-0.01888768	3.800229261	0.00390348	0.03564656	-1.75536604
hetero after UVB irradiation VS hetero before UVB irradiation	Classical Kir channels|Reactome	-0.00644115	-0.04406676	-0.01922249	0.98511104	0.99251462	-6.34634085
WT after UVB irradiation VS WT before UVB irradiation	Classical Kir channels|Reactome	0.72000203	0.013708661	2.034316411	0.06907387	0.44306234	-4.13857877
homo after UVB irradiation VS homo before UVB irradiation	Clathrin derived vesicle budding|Reactome	0.424388856	-0.04130465	2.346725185	0.04242678	0.13402181	-4.05793335
hetero after UVB irradiation VS hetero before UVB irradiation	Clathrin derived vesicle budding|Reactome	0.513699754	0.010539723	3.460578813	0.00790555	0.089711	-2.37635598
WT after UVB irradiation VS WT before UVB irradiation	Clathrin derived vesicle budding|Reactome	0.367398802	0.015230747	1.789012264	0.10364808	0.50425697	-4.47742435
homo after UVB irradiation VS homo before UVB irradiation	Clathrin-mediated endocytosis|Reactome	0.227007466	-0.02354031	1.252613067	0.24064505	0.40233809	-5.59067757
hetero after UVB irradiation VS hetero before UVB irradiation	Clathrin-mediated endocytosis|Reactome	0.458591816	-0.00073673	3.011521016	0.01580758	0.11352943	-3.05349827
WT after UVB irradiation VS WT before UVB irradiation	Clathrin-mediated endocytosis|Reactome	0.314270724	-0.00707205	1.577568783	0.14549545	0.55622203	-4.7526873
homo after UVB irradiation VS homo before UVB irradiation	Clearance of Nuclear Envelope Membranes from Chromatin|Reactome	-0.34782683	-0.03078361	-1.47973268	0.17169595	0.3224861	-5.31437121
hetero after UVB irradiation VS hetero before UVB irradiation	Clearance of Nuclear Envelope Membranes from Chromatin|Reactome	-0.29315374	-0.08640644	-1.36223974	0.20841954	0.43043782	-5.43960275
WT after UVB irradiation VS WT before UVB irradiation	Clearance of Nuclear Envelope Membranes from Chromatin|Reactome	0.073450293	-0.01640136	0.327328912	0.75011418	0.91881722	-5.81518114
homo after UVB irradiation VS homo before UVB irradiation	Cleavage of Growing Transcript in the Termination Region|Reactome	-0.80741953	-0.00051288	-4.68778227	0.00101638	0.01843909	-0.44220742
hetero after UVB irradiation VS hetero before UVB irradiation	Cleavage of Growing Transcript in the Termination Region|Reactome	-0.3146229	-0.05596631	-1.10404532	0.30010166	0.53033405	-5.73025941
WT after UVB irradiation VS WT before UVB irradiation	Cleavage of Growing Transcript in the Termination Region|Reactome	-0.40160517	-0.05380581	-1.32341012	0.21492372	0.62581393	-5.0564157
homo after UVB irradiation VS homo before UVB irradiation	Cleavage of the damaged purine|Reactome	-0.20582373	0.015435386	-0.82269915	0.4310765	0.58181948	-6.01765082
hetero after UVB irradiation VS hetero before UVB irradiation	Cleavage of the damaged purine|Reactome	0.171991434	-0.05342382	0.515799017	0.61925362	0.80231894	-6.20450981
WT after UVB irradiation VS WT before UVB irradiation	Cleavage of the damaged purine|Reactome	0.62990354	0.108869935	1.999454535	0.07322271	0.45403763	-4.18778491
homo after UVB irradiation VS homo before UVB irradiation	Cleavage of the damaged pyrimidine|Reactome	-0.78356809	-0.06735188	-4.03947359	0.00268707	0.02974154	-1.39037379
hetero after UVB irradiation VS hetero before UVB irradiation	Cleavage of the damaged pyrimidine|Reactome	-0.52063106	-0.10015914	-1.97323455	0.0821024	0.25808464	-4.62132082
WT after UVB irradiation VS WT before UVB irradiation	Cleavage of the damaged pyrimidine|Reactome	-0.03096487	-0.00387426	-0.10858657	0.91566112	0.97566251	-5.86318166
homo after UVB irradiation VS homo before UVB irradiation	CLEC7A (Dectin-1) induces NFAT activation|Reactome	0.581784254	-0.06263016	2.557527434	0.02984452	0.11509489	-3.72556343
hetero after UVB irradiation VS hetero before UVB irradiation	CLEC7A (Dectin-1) induces NFAT activation|Reactome	0.050130406	-0.03875646	0.158791973	0.87757304	0.9555609	-6.33288336
WT after UVB irradiation VS WT before UVB irradiation	CLEC7A (Dectin-1) induces NFAT activation|Reactome	0.192250608	-0.09515075	0.589779143	0.56831978	0.85238635	-5.69614076
homo after UVB irradiation VS homo before UVB irradiation	CLEC7A (Dectin-1) signaling|Reactome	0.566522617	-0.0534412	3.16147041	0.01093738	0.06361795	-2.75988202
hetero after UVB irradiation VS hetero before UVB irradiation	CLEC7A (Dectin-1) signaling|Reactome	0.517183915	0.039779545	2.736876705	0.0243743	0.13715811	-3.4734033
WT after UVB irradiation VS WT before UVB irradiation	CLEC7A (Dectin-1) signaling|Reactome	0.425088527	-0.03667627	2.037403285	0.06871742	0.44205977	-4.13420702
homo after UVB irradiation VS homo before UVB irradiation	CLEC7A/inflammasome pathway|Reactome	0.487821886	-0.02620869	2.557517342	0.02984502	0.11509489	-3.72557946
hetero after UVB irradiation VS hetero before UVB irradiation	CLEC7A/inflammasome pathway|Reactome	0.582257682	0.008139908	2.884002742	0.01931463	0.12176495	-3.2482217
WT after UVB irradiation VS WT before UVB irradiation	CLEC7A/inflammasome pathway|Reactome	0.447964773	-0.05121632	1.729989261	0.11406919	0.51527453	-4.55603404
homo after UVB irradiation VS homo before UVB irradiation	Clopidogrel Action Pathway|SMPDB	-0.2010916	0.054447148	-0.74675099	0.47349743	0.61754234	-6.07763267
hetero after UVB irradiation VS hetero before UVB irradiation	Clopidogrel Action Pathway|SMPDB	0.039836216	0.06004002	0.152366223	0.88248366	0.95842588	-6.33396484
WT after UVB irradiation VS WT before UVB irradiation	Clopidogrel Action Pathway|SMPDB	-0.22991261	0.1204075	-0.83262345	0.42434084	0.79068324	-5.53039588
homo after UVB irradiation VS homo before UVB irradiation	Clopidogrel Metabolism Pathway|SMPDB	-0.2010916	0.054447148	-0.74675099	0.47349743	0.61754234	-6.07763267
hetero after UVB irradiation VS hetero before UVB irradiation	Clopidogrel Metabolism Pathway|SMPDB	0.039836216	0.06004002	0.152366223	0.88248366	0.95842588	-6.33396484
WT after UVB irradiation VS WT before UVB irradiation	Clopidogrel Metabolism Pathway|SMPDB	-0.22991261	0.1204075	-0.83262345	0.42434084	0.79068324	-5.53039588
homo after UVB irradiation VS homo before UVB irradiation	Clopidogrel Pathway_ Pharmacokinetics|PharmGKB	-0.29621268	0.009842643	-1.0621984	0.31470147	0.46923284	-5.79693956
hetero after UVB irradiation VS hetero before UVB irradiation	Clopidogrel Pathway_ Pharmacokinetics|PharmGKB	-0.12157677	0.053537113	-0.5086646	0.62403638	0.80561526	-6.20835154
WT after UVB irradiation VS WT before UVB irradiation	Clopidogrel Pathway_ Pharmacokinetics|PharmGKB	-0.35793099	0.061020438	-1.32850361	0.21329674	0.62581393	-5.05066737
homo after UVB irradiation VS homo before UVB irradiation	CMP phosphorylation|HumanCyc	0.071789458	-0.03331797	0.24789293	0.80955377	0.88262237	-6.33554097
hetero after UVB irradiation VS hetero before UVB irradiation	CMP phosphorylation|HumanCyc	0.034611737	-0.01640602	0.141740042	0.89061596	0.96325071	-6.33565579
WT after UVB irradiation VS WT before UVB irradiation	CMP phosphorylation|HumanCyc	0.09453227	-0.07563305	0.409038296	0.69106525	0.89400112	-5.78514142
homo after UVB irradiation VS homo before UVB irradiation	CMP-<i>N</i>-acetylneuraminate biosynthesis I (eukaryotes)|HumanCyc	-0.28086903	-0.0167205	-0.94779827	0.36698643	0.52028198	-5.90811412
hetero after UVB irradiation VS hetero before UVB irradiation	CMP-<i>N</i>-acetylneuraminate biosynthesis I (eukaryotes)|HumanCyc	0.184319732	-0.02055078	0.570621935	0.58314943	0.77583045	-6.17332709
WT after UVB irradiation VS WT before UVB irradiation	CMP-<i>N</i>-acetylneuraminate biosynthesis I (eukaryotes)|HumanCyc	0.1954903	0.044632766	0.766973304	0.46067987	0.80891799	-5.58016943
homo after UVB irradiation VS homo before UVB irradiation	C-MYB transcription factor network|PID	-0.00682434	-0.00634194	-0.04267038	0.96685799	0.97951488	-6.36747006
hetero after UVB irradiation VS hetero before UVB irradiation	C-MYB transcription factor network|PID	0.261285065	0.02425245	1.695756229	0.12646241	0.31934829	-5.01088899
WT after UVB irradiation VS WT before UVB irradiation	C-MYB transcription factor network|PID	0.067206496	0.024189773	0.329451354	0.7485567	0.9185045	-5.81448302
homo after UVB irradiation VS homo before UVB irradiation	C-MYC pathway|PID	-0.47437143	0.019575692	-2.54761636	0.0303428	0.1157437	-3.74129794
hetero after UVB irradiation VS hetero before UVB irradiation	C-MYC pathway|PID	-0.10977197	-0.0567266	-0.59779324	0.56569464	0.76437184	-6.15679522
WT after UVB irradiation VS WT before UVB irradiation	C-MYC pathway|PID	-0.13210678	-0.00672273	-0.48714785	0.63657105	0.87636259	-5.75043128
homo after UVB irradiation VS homo before UVB irradiation	Coagulation|SMPDB	0.538210712	0.026444726	3.007777411	0.01409598	0.07305822	-3.00571236
hetero after UVB irradiation VS hetero before UVB irradiation	Coagulation|SMPDB	0.558085555	0.09913938	2.984394118	0.01649409	0.11352943	-3.09486545
WT after UVB irradiation VS WT before UVB irradiation	Coagulation|SMPDB	0.440879848	0.077377741	1.569633667	0.14732454	0.55622203	-4.76265269
homo after UVB irradiation VS homo before UVB irradiation	Cobalamin (Cbl_ vitamin B12) transport and metabolism|Reactome	0.220330614	-0.08043135	1.256751536	0.23920775	0.40081562	-5.58592186
hetero after UVB irradiation VS hetero before UVB irradiation	Cobalamin (Cbl_ vitamin B12) transport and metabolism|Reactome	0.067908995	-0.01965025	0.353292147	0.73254604	0.86896773	-6.27934102
WT after UVB irradiation VS WT before UVB irradiation	Cobalamin (Cbl_ vitamin B12) transport and metabolism|Reactome	0.138446158	-0.01286147	0.597853939	0.56312469	0.85017866	-5.69145954
homo after UVB irradiation VS homo before UVB irradiation	Cocaine Action Pathway|SMPDB	0.191132361	0.029768717	1.061313976	0.31508276	0.46923284	-5.79783739
hetero after UVB irradiation VS hetero before UVB irradiation	Cocaine Action Pathway|SMPDB	-0.07904315	0.055005373	-0.44437398	0.66796482	0.82651398	-6.24068873
WT after UVB irradiation VS WT before UVB irradiation	Cocaine Action Pathway|SMPDB	0.022316377	0.035038618	0.098344171	0.92358676	0.97566251	-5.86425414
homo after UVB irradiation VS homo before UVB irradiation	Cocaine addiction - Homo sapiens (human)|KEGG	0.21281215	0.02684787	1.267173983	0.23561924	0.39728119	-5.57389623
hetero after UVB irradiation VS hetero before UVB irradiation	Cocaine addiction - Homo sapiens (human)|KEGG	0.186706467	0.053434438	0.883283664	0.40158038	0.63008722	-5.94230946
WT after UVB irradiation VS WT before UVB irradiation	Cocaine addiction - Homo sapiens (human)|KEGG	0.210465466	-0.00936445	0.888887328	0.39477661	0.76452505	-5.4849511
homo after UVB irradiation VS homo before UVB irradiation	Cocaine metabolism|Wikipathways	-0.65987409	-0.01925944	-1.99673628	0.07564201	0.19626432	-4.5931935
hetero after UVB irradiation VS hetero before UVB irradiation	Cocaine metabolism|Wikipathways	-1.03960417	-0.06506842	-4.80103515	0.00117029	0.04167338	-0.50828041
WT after UVB irradiation VS WT before UVB irradiation	Cocaine metabolism|Wikipathways	-0.63038295	-0.03443843	-1.77979103	0.10521668	0.50446894	-4.48978893
homo after UVB irradiation VS homo before UVB irradiation	Codeine Action Pathway|SMPDB	0.152034158	0.049146715	0.84848008	0.41727655	0.5678941	-5.99614992
hetero after UVB irradiation VS hetero before UVB irradiation	Codeine Action Pathway|SMPDB	-0.07904315	0.055005373	-0.44437398	0.66796482	0.82651398	-6.24068873
WT after UVB irradiation VS WT before UVB irradiation	Codeine Action Pathway|SMPDB	0.006841267	0.042478642	0.029703217	0.97688351	0.99265119	-5.86870425
homo after UVB irradiation VS homo before UVB irradiation	Codeine and Morphine Metabolism|Wikipathways	-0.01662376	0.201859381	-0.09607313	0.92548193	0.95561974	-6.36349141
hetero after UVB irradiation VS hetero before UVB irradiation	Codeine and Morphine Metabolism|Wikipathways	-0.00133037	-0.04724438	-0.00685071	0.9946934	0.99666135	-6.34651584
WT after UVB irradiation VS WT before UVB irradiation	Codeine and Morphine Metabolism|Wikipathways	0.303749922	-0.03665159	0.949076671	0.36477589	0.74545864	-5.43358083
homo after UVB irradiation VS homo before UVB irradiation	Codeine and Morphine Pathway_ Pharmacokinetics|PharmGKB	0.242829912	0.036916619	0.899991384	0.39062329	0.54394203	-5.95150694
hetero after UVB irradiation VS hetero before UVB irradiation	Codeine and Morphine Pathway_ Pharmacokinetics|PharmGKB	0.008141616	-0.00468027	0.028513005	0.97791681	0.98924321	-6.34610028
WT after UVB irradiation VS WT before UVB irradiation	Codeine and Morphine Pathway_ Pharmacokinetics|PharmGKB	0.278073104	-0.01870545	0.720494393	0.48758901	0.81710765	-5.61322822
homo after UVB irradiation VS homo before UVB irradiation	Codeine Metabolism Pathway|SMPDB	-0.31908549	0.043737575	-1.07015448	0.31128734	0.46923284	-5.78883674
hetero after UVB irradiation VS hetero before UVB irradiation	Codeine Metabolism Pathway|SMPDB	0.335010248	0.048982465	1.094308446	0.30411252	0.53415987	-5.7403717
WT after UVB irradiation VS WT before UVB irradiation	Codeine Metabolism Pathway|SMPDB	-0.59882161	0.136940491	-1.98783623	0.07465681	0.45723857	-4.20411387
homo after UVB irradiation VS homo before UVB irradiation	coenzyme A biosynthesis|HumanCyc	0.268614681	-0.04654425	0.82851512	0.42793661	0.57912228	-6.01285013
hetero after UVB irradiation VS hetero before UVB irradiation	coenzyme A biosynthesis|HumanCyc	-0.19520978	0.001078478	-0.70383896	0.50051809	0.71899195	-6.08562666
WT after UVB irradiation VS WT before UVB irradiation	coenzyme A biosynthesis|HumanCyc	0.101147607	-0.06654026	0.450988415	0.66154229	0.87935805	-5.76722003
homo after UVB irradiation VS homo before UVB irradiation	Coenzyme A biosynthesis|Reactome	0.12468797	-0.05034156	0.416709702	0.68625635	0.78967906	-6.27602343
hetero after UVB irradiation VS hetero before UVB irradiation	Coenzyme A biosynthesis|Reactome	-0.32383056	-0.03184527	-1.38751946	0.20087583	0.42057511	-5.40900889
WT after UVB irradiation VS WT before UVB irradiation	Coenzyme A biosynthesis|Reactome	-0.09252386	-0.05619623	-0.38727181	0.70660436	0.90152666	-5.79377553
homo after UVB irradiation VS homo before UVB irradiation	Cohesin Loading onto Chromatin|Reactome	-1.02460944	-0.00980873	-5.52627932	0.00031611	0.01666204	0.685531432
hetero after UVB irradiation VS hetero before UVB irradiation	Cohesin Loading onto Chromatin|Reactome	-0.74061243	-0.06351916	-3.63998251	0.00603359	0.0822456	-2.11142681
WT after UVB irradiation VS WT before UVB irradiation	Cohesin Loading onto Chromatin|Reactome	-0.63442606	-0.04207458	-2.14325529	0.05749919	0.42266366	-3.982953
homo after UVB irradiation VS homo before UVB irradiation	Collagen biosynthesis and modifying enzymes|Reactome	0.684930395	-0.01637908	4.266386454	0.00189917	0.02423495	-1.05137616
hetero after UVB irradiation VS hetero before UVB irradiation	Collagen biosynthesis and modifying enzymes|Reactome	0.259797694	0.013992919	1.146993807	0.28290799	0.51297702	-5.68487339
WT after UVB irradiation VS WT before UVB irradiation	Collagen biosynthesis and modifying enzymes|Reactome	0.262158448	-0.00263125	1.00617541	0.33786836	0.72206121	-5.38230435
homo after UVB irradiation VS homo before UVB irradiation	Collagen chain trimerization|Reactome	0.534929028	-0.01452838	3.276892286	0.00905124	0.05693716	-2.57594158
hetero after UVB irradiation VS hetero before UVB irradiation	Collagen chain trimerization|Reactome	0.123502895	0.033608353	0.609604223	0.5582009	0.76153239	-6.14938934
WT after UVB irradiation VS WT before UVB irradiation	Collagen chain trimerization|Reactome	0.188195998	-0.0081798	0.769738902	0.45910937	0.80891799	-5.57814481
homo after UVB irradiation VS homo before UVB irradiation	Collagen degradation|Reactome	0.652092198	0.006093782	3.86859208	0.00350574	0.03355809	-1.65032518
hetero after UVB irradiation VS hetero before UVB irradiation	Collagen degradation|Reactome	0.42402673	0.076003324	2.362064725	0.0442779	0.18566576	-4.04482467
WT after UVB irradiation VS WT before UVB irradiation	Collagen degradation|Reactome	0.490941811	-0.01519229	2.316474845	0.04283208	0.39022568	-3.73067351
homo after UVB irradiation VS homo before UVB irradiation	Collagen formation|Reactome	0.765258056	-0.01244137	4.849969017	0.00080449	0.01724556	-0.2151803
hetero after UVB irradiation VS hetero before UVB irradiation	Collagen formation|Reactome	0.421231873	0.034662266	1.87809079	0.0953243	0.27959342	-4.75753876
WT after UVB irradiation VS WT before UVB irradiation	Collagen formation|Reactome	0.388924918	0.016545197	1.575362633	0.14600196	0.55622203	-4.75546078
homo after UVB irradiation VS homo before UVB irradiation	Collecting duct acid secretion - Homo sapiens (human)|KEGG	0.142879245	-0.0262662	0.550155854	0.59505638	0.71693041	-6.208461
hetero after UVB irradiation VS hetero before UVB irradiation	Collecting duct acid secretion - Homo sapiens (human)|KEGG	0.286208378	0.020425823	1.436048741	0.18705457	0.40464865	-5.34932058
WT after UVB irradiation VS WT before UVB irradiation	Collecting duct acid secretion - Homo sapiens (human)|KEGG	0.195627166	0.03443946	0.800551791	0.44184696	0.79860542	-5.5551559
homo after UVB irradiation VS homo before UVB irradiation	Colorectal cancer - Homo sapiens (human)|KEGG	0.009215545	-0.01637647	0.04852926	0.96231112	0.97710919	-6.36718302
hetero after UVB irradiation VS hetero before UVB irradiation	Colorectal cancer - Homo sapiens (human)|KEGG	0.161369976	-0.0052991	0.961975423	0.36285216	0.59130501	-5.87099103
WT after UVB irradiation VS WT before UVB irradiation	Colorectal cancer - Homo sapiens (human)|KEGG	0.045706839	0.025504482	0.236724215	0.81761107	0.94525798	-5.84084714
homo after UVB irradiation VS homo before UVB irradiation	Common Pathway of Fibrin Clot Formation|Reactome	0.632639067	0.055187647	3.655326291	0.00491157	0.03990434	-1.97983941
hetero after UVB irradiation VS hetero before UVB irradiation	Common Pathway of Fibrin Clot Formation|Reactome	0.301645265	0.083314672	1.481137309	0.17496582	0.39186333	-5.29278143
WT after UVB irradiation VS WT before UVB irradiation	Common Pathway of Fibrin Clot Formation|Reactome	0.158941047	0.000267819	0.555241897	0.5908349	0.86254944	-5.71549396
homo after UVB irradiation VS homo before UVB irradiation	Common Pathways Underlying Drug Addiction|Wikipathways	0.248180616	-0.01905103	1.553337988	0.15336616	0.30028727	-5.21870248
hetero after UVB irradiation VS hetero before UVB irradiation	Common Pathways Underlying Drug Addiction|Wikipathways	0.209352614	0.049932056	1.36970682	0.20616633	0.42713914	-5.43060235
WT after UVB irradiation VS WT before UVB irradiation	Common Pathways Underlying Drug Addiction|Wikipathways	0.264669117	-0.00335256	1.158951548	0.27318877	0.66470598	-5.23360755
homo after UVB irradiation VS homo before UVB irradiation	Competing endogenous RNAs (ceRNAs) regulate PTEN translation|Reactome	-0.06600531	-0.0126454	-0.22091229	0.82989015	0.89474926	-6.34229637
hetero after UVB irradiation VS hetero before UVB irradiation	Competing endogenous RNAs (ceRNAs) regulate PTEN translation|Reactome	-0.2611407	-0.00927373	-1.29662538	0.22914014	0.45170905	-5.51733934
WT after UVB irradiation VS WT before UVB irradiation	Competing endogenous RNAs (ceRNAs) regulate PTEN translation|Reactome	-0.22650327	0.042886887	-0.78954021	0.44796632	0.80421615	-5.56346203
homo after UVB irradiation VS homo before UVB irradiation	Complement Activation|Wikipathways	0.724012198	-0.07191063	2.54493795	0.03047887	0.11616573	-3.74554861
hetero after UVB irradiation VS hetero before UVB irradiation	Complement Activation|Wikipathways	0.22160523	0.056292816	0.74859722	0.47447565	0.69660117	-6.05250131
WT after UVB irradiation VS WT before UVB irradiation	Complement Activation|Wikipathways	0.372414981	-0.03265322	1.10846302	0.29340312	0.67984377	-5.28453404
homo after UVB irradiation VS homo before UVB irradiation	Complement and coagulation cascades - Homo sapiens (human)|KEGG	0.566334711	0.007890326	3.036150384	0.0134493	0.07249235	-2.96027689
hetero after UVB irradiation VS hetero before UVB irradiation	Complement and coagulation cascades - Homo sapiens (human)|KEGG	0.483834819	0.06561333	2.556144581	0.03248968	0.15685114	-3.74981976
WT after UVB irradiation VS WT before UVB irradiation	Complement and coagulation cascades - Homo sapiens (human)|KEGG	0.332764266	-0.01557168	1.170908854	0.26856566	0.66238094	-5.22129829
homo after UVB irradiation VS homo before UVB irradiation	Complement and Coagulation Cascades|Wikipathways	0.607326046	0.029730199	3.314082541	0.00851788	0.05493312	-2.51684301
hetero after UVB irradiation VS hetero before UVB irradiation	Complement and Coagulation Cascades|Wikipathways	0.504560769	0.059956182	2.655424965	0.02774012	0.14438349	-3.59807669
WT after UVB irradiation VS WT before UVB irradiation	Complement and Coagulation Cascades|Wikipathways	0.373910262	-0.00263573	1.355706783	0.20478031	0.61875813	-5.0197162
homo after UVB irradiation VS homo before UVB irradiation	Complement cascade|Reactome	0.686601789	-0.02056051	3.35320946	0.00799179	0.05250432	-2.45476964
hetero after UVB irradiation VS hetero before UVB irradiation	Complement cascade|Reactome	0.418748597	0.08976799	1.938734704	0.08668063	0.2664129	-4.67099463
WT after UVB irradiation VS WT before UVB irradiation	Complement cascade|Reactome	0.422008204	0.008761602	1.518670598	0.15956103	0.56072663	-4.82596153
homo after UVB irradiation VS homo before UVB irradiation	Complex I biogenesis|Reactome	-0.10288664	0.000389582	-0.32966193	0.7488913	0.83645736	-6.31040042
hetero after UVB irradiation VS hetero before UVB irradiation	Complex I biogenesis|Reactome	-0.49191825	-0.07591654	-2.21753801	0.05574912	0.2098759	-4.26204901
WT after UVB irradiation VS WT before UVB irradiation	Complex I biogenesis|Reactome	-0.09705995	-0.0739475	-0.31567852	0.75868418	0.92423014	-5.81893466
homo after UVB irradiation VS homo before UVB irradiation	Composition of Lipid Particles|Wikipathways	0.659425851	0.034665899	2.527787363	0.03136475	0.11777258	-3.7727512
hetero after UVB irradiation VS hetero before UVB irradiation	Composition of Lipid Particles|Wikipathways	0.495296646	0.064535141	1.807959387	0.10633694	0.29469241	-4.85628208
WT after UVB irradiation VS WT before UVB irradiation	Composition of Lipid Particles|Wikipathways	0.337582349	-0.06355704	0.836181416	0.42242792	0.78872784	-5.52759735
homo after UVB irradiation VS homo before UVB irradiation	Condensation of Prometaphase Chromosomes|Reactome	-0.5823794	0.065932776	-2.24777674	0.05001337	0.14723471	-4.21180354
hetero after UVB irradiation VS hetero before UVB irradiation	Condensation of Prometaphase Chromosomes|Reactome	0.542524673	0.034840654	1.701500341	0.12535183	0.31812563	-5.00308345
WT after UVB irradiation VS WT before UVB irradiation	Condensation of Prometaphase Chromosomes|Reactome	0.058607242	-0.00590147	0.178144406	0.86213812	0.95916804	-5.85310138
homo after UVB irradiation VS homo before UVB irradiation	Condensation of Prophase Chromosomes|Reactome	-0.8095229	0.037066216	-4.42047863	0.00150661	0.02211705	-0.82545749
hetero after UVB irradiation VS hetero before UVB irradiation	Condensation of Prophase Chromosomes|Reactome	-0.24686497	0.037707368	-0.79403474	0.44895776	0.67380622	-6.01706091
WT after UVB irradiation VS WT before UVB irradiation	Condensation of Prophase Chromosomes|Reactome	-0.32729816	-0.04100737	-1.20397152	0.25610516	0.65122698	-5.18677932
homo after UVB irradiation VS homo before UVB irradiation	Congenital Bile Acid Synthesis Defect Type II|SMPDB	0.143425895	0.01576019	0.783036509	0.45290274	0.6002769	-6.04960791
hetero after UVB irradiation VS hetero before UVB irradiation	Congenital Bile Acid Synthesis Defect Type II|SMPDB	-0.01773938	0.040584313	-0.07540586	0.94165152	0.97054702	-6.34345771
WT after UVB irradiation VS WT before UVB irradiation	Congenital Bile Acid Synthesis Defect Type II|SMPDB	0.061518516	0.026229795	0.23751225	0.8170163	0.94525798	-5.84065895
homo after UVB irradiation VS homo before UVB irradiation	Congenital Bile Acid Synthesis Defect Type III|SMPDB	0.143425895	0.01576019	0.783036509	0.45290274	0.6002769	-6.04960791
hetero after UVB irradiation VS hetero before UVB irradiation	Congenital Bile Acid Synthesis Defect Type III|SMPDB	-0.01773938	0.040584313	-0.07540586	0.94165152	0.97054702	-6.34345771
WT after UVB irradiation VS WT before UVB irradiation	Congenital Bile Acid Synthesis Defect Type III|SMPDB	0.061518516	0.026229795	0.23751225	0.8170163	0.94525798	-5.84065895
homo after UVB irradiation VS homo before UVB irradiation	Congenital disorder of glycosylation CDG-IId|SMPDB	0.737643047	0.024031742	3.897326755	0.00335151	0.03256113	-1.60634747
hetero after UVB irradiation VS hetero before UVB irradiation	Congenital disorder of glycosylation CDG-IId|SMPDB	0.744733789	0.085967304	4.116781712	0.0030051	0.06148411	-1.42803661
WT after UVB irradiation VS WT before UVB irradiation	Congenital disorder of glycosylation CDG-IId|SMPDB	0.584123909	0.04155907	2.501560947	0.03118765	0.36428127	-3.45639206

homo after UVB irradiation VS homo before UVB irradiation	Congenital Erythropoietic Porphyria (CEP) or Gunther Disease|SMPDB	0.388154593	-0.02664649	1.768839848	0.10931886	0.24265581	-4.92409481
hetero after UVB irradiation VS hetero before UVB irradiation	Congenital Erythropoietic Porphyria (CEP) or Gunther Disease|SMPDB	0.451496919	0.003377552	2.70023395	0.02583369	0.14017854	-3.52950073
WT after UVB irradiation VS WT before UVB irradiation	Congenital Erythropoietic Porphyria (CEP) or Gunther Disease|SMPDB	0.381835405	-0.00693641	1.754704281	0.10959456	0.5076251	-4.52327279
homo after UVB irradiation VS homo before UVB irradiation	Congenital lactic acidosis|SMPDB	-0.23678174	0.014544733	-0.76379894	0.46374733	0.6088463	-6.06461144
hetero after UVB irradiation VS hetero before UVB irradiation	Congenital lactic acidosis|SMPDB	-0.47814985	0.004803127	-1.74993278	0.11633994	0.30887493	-4.93678811
WT after UVB irradiation VS WT before UVB irradiation	Congenital lactic acidosis|SMPDB	-0.1568339	-0.12513755	-0.50250429	0.62610623	0.87257466	-5.74292911
homo after UVB irradiation VS homo before UVB irradiation	Congenital Lipoid Adrenal Hyperplasia (CLAH) or Lipoid CAH|SMPDB	0.235351843	-0.00442303	1.337763903	0.2124708	0.36957778	-5.49065548
hetero after UVB irradiation VS hetero before UVB irradiation	Congenital Lipoid Adrenal Hyperplasia (CLAH) or Lipoid CAH|SMPDB	0.307187072	0.103522065	1.723908534	0.12110487	0.3132781	-4.97251635
WT after UVB irradiation VS WT before UVB irradiation	Congenital Lipoid Adrenal Hyperplasia (CLAH) or Lipoid CAH|SMPDB	0.03938021	0.054409473	0.163687683	0.87321181	0.96283151	-5.85559705
homo after UVB irradiation VS homo before UVB irradiation	Conjugation of benzoate with glycine|Reactome	0.4251612	-0.10872382	0.978431641	0.35239651	0.50783175	-5.87934239
hetero after UVB irradiation VS hetero before UVB irradiation	Conjugation of benzoate with glycine|Reactome	0.246426612	0.031985469	1.476377946	0.17620852	0.39293465	-5.29879716
WT after UVB irradiation VS WT before UVB irradiation	Conjugation of benzoate with glycine|Reactome	0.245382705	0.116787662	0.671552832	0.51695941	0.82895075	-5.64603451
homo after UVB irradiation VS homo before UVB irradiation	Conjugation of carboxylic acids|Reactome	0.08093173	0.023459488	0.224217303	0.82739153	0.89374727	-6.34151017
hetero after UVB irradiation VS hetero before UVB irradiation	Conjugation of carboxylic acids|Reactome	-0.17694323	-0.00969811	-1.0654327	0.31625519	0.54576835	-5.76996718
WT after UVB irradiation VS WT before UVB irradiation	Conjugation of carboxylic acids|Reactome	0.05439466	0.064488051	0.170020456	0.86835729	0.96280353	-5.85452964
homo after UVB irradiation VS homo before UVB irradiation	Conjugation of phenylacetate with glutamine|Reactome	0.670559973	-0.01279821	1.868760791	0.09312045	0.21939524	-4.78114958
hetero after UVB irradiation VS hetero before UVB irradiation	Conjugation of phenylacetate with glutamine|Reactome	1.076993147	0.041570917	4.704973834	0.00133044	0.04394311	-0.63280464
WT after UVB irradiation VS WT before UVB irradiation	Conjugation of phenylacetate with glutamine|Reactome	0.730774813	0.08210487	2.225703565	0.04999786	0.40887857	-3.86352471
homo after UVB irradiation VS homo before UVB irradiation	Conjugation of salicylate with glycine|Reactome	-0.15689144	0.099652834	-0.43215558	0.67538376	0.77972243	-6.26911503
hetero after UVB irradiation VS hetero before UVB irradiation	Conjugation of salicylate with glycine|Reactome	-0.49386274	-0.02264761	-2.32800786	0.04674994	0.19042558	-4.09624665
WT after UVB irradiation VS WT before UVB irradiation	Conjugation of salicylate with glycine|Reactome	-0.19000836	-0.06892079	-0.6145364	0.55247599	0.84516674	-5.6816018
homo after UVB irradiation VS homo before UVB irradiation	Constitutive Androstane Receptor Pathway|Wikipathways	-0.3137815	0.001776442	-1.68971361	0.12394571	0.26290983	-5.03459276
hetero after UVB irradiation VS hetero before UVB irradiation	Constitutive Androstane Receptor Pathway|Wikipathways	-0.26240763	-0.00869702	-1.70844732	0.1240207	0.31597895	-4.99362688
WT after UVB irradiation VS WT before UVB irradiation	Constitutive Androstane Receptor Pathway|Wikipathways	-0.26441701	0.014761746	-1.27202024	0.23192142	0.62952955	-5.11356431
homo after UVB irradiation VS homo before UVB irradiation	Constitutive Signaling by Aberrant PI3K in Cancer|Reactome	0.013786004	-0.00010347	0.081129974	0.93704174	0.96254952	-6.36491295
hetero after UVB irradiation VS hetero before UVB irradiation	Constitutive Signaling by Aberrant PI3K in Cancer|Reactome	-0.05170699	0.029801439	-0.2905422	0.77842462	0.90221039	-6.30098135
WT after UVB irradiation VS WT before UVB irradiation	Constitutive Signaling by Aberrant PI3K in Cancer|Reactome	-0.13933793	0.008490323	-0.6616196	0.52304766	0.83120196	-5.65243794
homo after UVB irradiation VS homo before UVB irradiation	Constitutive Signaling by AKT1 E17K in Cancer|Reactome	-0.1599693	-0.00379947	-0.73830406	0.47837682	0.62106015	-6.08398814
hetero after UVB irradiation VS hetero before UVB irradiation	Constitutive Signaling by AKT1 E17K in Cancer|Reactome	-0.00410703	-0.04379221	-0.02444739	0.98106485	0.99041257	-6.34621707
WT after UVB irradiation VS WT before UVB irradiation	Constitutive Signaling by AKT1 E17K in Cancer|Reactome	-0.18694785	-0.02771117	-0.85388272	0.41299777	0.77895025	-5.51352235
homo after UVB irradiation VS homo before UVB irradiation	Constitutive Signaling by EGFRvIII|Reactome	-0.1508093	0.012438294	-0.54497549	0.59847495	0.71899698	-6.2114122
hetero after UVB irradiation VS hetero before UVB irradiation	Constitutive Signaling by EGFRvIII|Reactome	0.123883972	0.028147153	0.544370106	0.60029279	0.7885114	-6.18862411
WT after UVB irradiation VS WT before UVB irradiation	Constitutive Signaling by EGFRvIII|Reactome	-0.15552704	0.012092346	-0.59367495	0.56581003	0.85125516	-5.69388961
homo after UVB irradiation VS homo before UVB irradiation	Constitutive Signaling by Ligand-Responsive EGFR Cancer Variants|Reactome	-0.09549357	0.004161018	-0.36590867	0.72254203	0.81762749	-6.29703148
hetero after UVB irradiation VS hetero before UVB irradiation	Constitutive Signaling by Ligand-Responsive EGFR Cancer Variants|Reactome	0.05338722	0.025662055	0.245035157	0.81229184	0.92298761	-6.31408761
WT after UVB irradiation VS WT before UVB irradiation	Constitutive Signaling by Ligand-Responsive EGFR Cancer Variants|Reactome	-0.10752269	0.01800905	-0.45003185	0.66220901	0.87935805	-5.76764737
homo after UVB irradiation VS homo before UVB irradiation	Constitutive Signaling by NOTCH1 HD Domain Mutants|Reactome	0.065072391	0.016481871	0.30273082	0.76869491	0.85025701	-6.31945194
hetero after UVB irradiation VS hetero before UVB irradiation	Constitutive Signaling by NOTCH1 HD Domain Mutants|Reactome	0.177856081	0.021991238	0.97072226	0.35872354	0.58688511	-5.86276257
WT after UVB irradiation VS WT before UVB irradiation	Constitutive Signaling by NOTCH1 HD Domain Mutants|Reactome	-0.39144991	0.025306019	-1.6526366	0.12916274	0.54068397	-4.65704005
homo after UVB irradiation VS homo before UVB irradiation	Constitutive Signaling by NOTCH1 HD+PEST Domain Mutants|Reactome	0.005065895	0.00041336	0.02691679	0.97908955	0.98606224	-6.36805899
hetero after UVB irradiation VS hetero before UVB irradiation	Constitutive Signaling by NOTCH1 HD+PEST Domain Mutants|Reactome	0.107491188	-0.0016007	0.596752392	0.56635777	0.76437184	-6.1574415
WT after UVB irradiation VS WT before UVB irradiation	Constitutive Signaling by NOTCH1 HD+PEST Domain Mutants|Reactome	-0.11781569	0.018354062	-0.49804015	0.62913956	0.87289644	-5.74513277
homo after UVB irradiation VS homo before UVB irradiation	Constitutive Signaling by NOTCH1 PEST Domain Mutants|Reactome	0.005065895	0.00041336	0.02691679	0.97908955	0.98606224	-6.36805899
hetero after UVB irradiation VS hetero before UVB irradiation	Constitutive Signaling by NOTCH1 PEST Domain Mutants|Reactome	0.107491188	-0.0016007	0.596752392	0.56635777	0.76437184	-6.1574415
WT after UVB irradiation VS WT before UVB irradiation	Constitutive Signaling by NOTCH1 PEST Domain Mutants|Reactome	-0.11781569	0.018354062	-0.49804015	0.62913956	0.87289644	-5.74513277
homo after UVB irradiation VS homo before UVB irradiation	Constitutive Signaling by NOTCH1 t(7;9)(NOTCH1:M1580_K2555) Translocation Mutant|Reactome	0.256226502	-0.00369095	0.882983866	0.39928759	0.55113275	-5.96649206
hetero after UVB irradiation VS hetero before UVB irradiation	Constitutive Signaling by NOTCH1 t(7;9)(NOTCH1:M1580_K2555) Translocation Mutant|Reactome	0.460194318	0.038419756	2.041996388	0.07366022	0.24223901	-4.52144791
WT after UVB irradiation VS WT before UVB irradiation	Constitutive Signaling by NOTCH1 t(7;9)(NOTCH1:M1580_K2555) Translocation Mutant|Reactome	0.279255534	0.013743116	1.089118438	0.30144887	0.6862996	-5.3035883
homo after UVB irradiation VS homo before UVB irradiation	Control of immune tolerance by vasoactive intestinalpeptide|Wikipathways	0.752433065	-0.05624376	3.78596971	0.00399227	0.03616803	-1.77734785
hetero after UVB irradiation VS hetero before UVB irradiation	Control of immune tolerance by vasoactive intestinalpeptide|Wikipathways	0.498206678	0.047901014	1.82714271	0.10321114	0.28878846	-4.82942349
WT after UVB irradiation VS WT before UVB irradiation	Control of immune tolerance by vasoactive intestinalpeptide|Wikipathways	0.270322278	-0.01782221	0.771843991	0.45791628	0.80891799	-5.57659943
homo after UVB irradiation VS homo before UVB irradiation	control of skeletal myogenesis by hdac and calcium/calmodulin-dependent kinase (camk)|BioCarta	0.39132904	-0.0737921	2.297164509	0.0460741	0.13996961	-4.13520883
hetero after UVB irradiation VS hetero before UVB irradiation	control of skeletal myogenesis by hdac and calcium/calmodulin-dependent kinase (camk)|BioCarta	0.256380619	-0.00660508	0.818291326	0.43571945	0.66222383	-5.99740931
WT after UVB irradiation VS WT before UVB irradiation	control of skeletal myogenesis by hdac and calcium/calmodulin-dependent kinase (camk)|BioCarta	-0.04892269	-0.02636913	-0.19591815	0.84856666	0.95387225	-5.84974586
homo after UVB irradiation VS homo before UVB irradiation	Conversion from APC/C:Cdc20 to APC/C:Cdh1 in late anaphase|Reactome	-0.51969491	0.006801458	-2.69385452	0.02376644	0.10155807	-3.5083873
hetero after UVB irradiation VS hetero before UVB irradiation	Conversion from APC/C:Cdc20 to APC/C:Cdh1 in late anaphase|Reactome	0.090248418	-0.0265603	0.55394864	0.59400635	0.78483643	-6.18312005
WT after UVB irradiation VS WT before UVB irradiation	Conversion from APC/C:Cdc20 to APC/C:Cdh1 in late anaphase|Reactome	-0.02295186	-0.07465128	-0.09632408	0.92515099	0.97601399	-5.86445316
homo after UVB irradiation VS homo before UVB irradiation	Cooperation of PDCL (PhLP1) and TRiC/CCT in G-protein beta folding|Reactome	0.072878707	-0.00905778	0.362604793	0.72492855	0.81931281	-6.29830619
hetero after UVB irradiation VS hetero before UVB irradiation	Cooperation of PDCL (PhLP1) and TRiC/CCT in G-protein beta folding|Reactome	0.113188082	0.012154406	0.639727492	0.53935057	0.75133249	-6.12990433
WT after UVB irradiation VS WT before UVB irradiation	Cooperation of PDCL (PhLP1) and TRiC/CCT in G-protein beta folding|Reactome	0.082506738	-0.00867242	0.424866388	0.6798587	0.88509541	-5.77857704
homo after UVB irradiation VS homo before UVB irradiation	Cooperation of Prefoldin and TriC/CCT in actin and tubulin folding|Reactome	-0.54878301	0.02952787	-2.40266709	0.03865015	0.12940262	-3.97026319
hetero after UVB irradiation VS hetero before UVB irradiation	Cooperation of Prefoldin and TriC/CCT in actin and tubulin folding|Reactome	-0.35791943	-0.04755759	-1.4340662	0.18760252	0.40525701	-5.35178306
WT after UVB irradiation VS WT before UVB irradiation	Cooperation of Prefoldin and TriC/CCT in actin and tubulin folding|Reactome	-0.24563159	-0.01875318	-0.86228633	0.40857159	0.77553883	-5.50675216
homo after UVB irradiation VS homo before UVB irradiation	COPI-dependent Golgi-to-ER retrograde traffic|Reactome	0.201710769	-0.02695523	1.014989231	0.33555092	0.49208395	-5.8440415
hetero after UVB irradiation VS hetero before UVB irradiation	COPI-dependent Golgi-to-ER retrograde traffic|Reactome	0.370968353	0.001488008	2.390379573	0.04232212	0.18131011	-4.00197914
WT after UVB irradiation VS WT before UVB irradiation	COPI-dependent Golgi-to-ER retrograde traffic|Reactome	0.325226803	-0.03689938	1.551487794	0.15158357	0.55727585	-4.78533325
homo after UVB irradiation VS homo before UVB irradiation	COPII-mediated vesicle transport|Reactome	0.324729618	-0.02956672	1.737126331	0.11498024	0.25191929	-4.96868388
hetero after UVB irradiation VS hetero before UVB irradiation	COPII-mediated vesicle transport|Reactome	0.35503648	0.015116828	2.146532949	0.06241317	0.22396997	-4.36765731
WT after UVB irradiation VS WT before UVB irradiation	COPII-mediated vesicle transport|Reactome	0.323059523	-0.03052549	1.596881469	0.14112733	0.55622203	-4.72831501
homo after UVB irradiation VS homo before UVB irradiation	COPI-independent Golgi-to-ER retrograde traffic|Reactome	0.256097825	-0.02693394	1.184851879	0.26519877	0.42863631	-5.66694643
hetero after UVB irradiation VS hetero before UVB irradiation	COPI-independent Golgi-to-ER retrograde traffic|Reactome	0.345188024	0.015736956	1.716330901	0.12252593	0.31410189	-4.98287371
WT after UVB irradiation VS WT before UVB irradiation	COPI-independent Golgi-to-ER retrograde traffic|Reactome	0.059487195	0.039817408	0.236651519	0.81766594	0.94525798	-5.84086447
homo after UVB irradiation VS homo before UVB irradiation	COPI-mediated anterograde transport|Reactome	0.577281312	-0.05174494	3.241053454	0.00959795	0.05838531	-2.63297578
hetero after UVB irradiation VS hetero before UVB irradiation	COPI-mediated anterograde transport|Reactome	0.521497069	0.006566696	2.821154972	0.0213292	0.12945204	-3.34437813
WT after UVB irradiation VS WT before UVB irradiation	COPI-mediated anterograde transport|Reactome	0.359587187	-0.01968368	1.620027617	0.13604557	0.54838491	-4.69888804
homo after UVB irradiation VS homo before UVB irradiation	Copper homeostasis|Wikipathways	0.057719253	-0.00863613	0.313703507	0.76060398	0.84515876	-6.31585509
hetero after UVB irradiation VS hetero before UVB irradiation	Copper homeostasis|Wikipathways	0.393394708	0.010371351	2.129288926	0.06414576	0.22717668	-4.39317319
WT after UVB irradiation VS WT before UVB irradiation	Copper homeostasis|Wikipathways	0.119427788	-0.0021211	0.534295256	0.60471796	0.8701156	-5.72669905
homo after UVB irradiation VS homo before UVB irradiation	Coregulation of Androgen receptor activity|PID	-0.00902452	0.002858414	-0.04732538	0.9632453	0.97759014	-6.36724501
hetero after UVB irradiation VS hetero before UVB irradiation	Coregulation of Androgen receptor activity|PID	-0.01218429	-0.03803375	-0.06517114	0.94955755	0.9739614	-6.34423777
WT after UVB irradiation VS WT before UVB irradiation	Coregulation of Androgen receptor activity|PID	0.024338418	0.0087524	0.118846369	0.90773171	0.97566251	-5.8620012
homo after UVB irradiation VS homo before UVB irradiation	Cori Cycle|Wikipathways	0.705763043	-0.01052395	4.031896558	0.00271872	0.02978124	-1.40181808
hetero after UVB irradiation VS hetero before UVB irradiation	Cori Cycle|Wikipathways	0.740595264	0.062923934	4.566149799	0.0016052	0.04856602	-0.8154498
WT after UVB irradiation VS WT before UVB irradiation	Cori Cycle|Wikipathways	0.604306345	-0.01424611	2.849633852	0.01712858	0.30353778	-2.93409244
homo after UVB irradiation VS homo before UVB irradiation	corticosteroids and cardioprotection|BioCarta	0.392929107	0.000905869	1.876769255	0.09192394	0.21724881	-4.76954059
hetero after UVB irradiation VS hetero before UVB irradiation	corticosteroids and cardioprotection|BioCarta	0.266373142	0.037672696	1.096123916	0.3033615	0.53328183	-5.73849127
WT after UVB irradiation VS WT before UVB irradiation	corticosteroids and cardioprotection|BioCarta	0.081478229	-0.00277599	0.304608863	0.76685886	0.92873095	-5.82237768
homo after UVB irradiation VS homo before UVB irradiation	Corticosterone methyl oxidase I deficiency (CMO I)|SMPDB	0.235351843	-0.00442303	1.337763903	0.2124708	0.36957778	-5.49065548
hetero after UVB irradiation VS hetero before UVB irradiation	Corticosterone methyl oxidase I deficiency (CMO I)|SMPDB	0.307187072	0.103522065	1.723908534	0.12110487	0.3132781	-4.97251635
WT after UVB irradiation VS WT before UVB irradiation	Corticosterone methyl oxidase I deficiency (CMO I)|SMPDB	0.03938021	0.054409473	0.163687683	0.87321181	0.96283151	-5.85559705
homo after UVB irradiation VS homo before UVB irradiation	Corticosterone methyl oxidase II deficiency - CMO II|SMPDB	0.235351843	-0.00442303	1.337763903	0.2124708	0.36957778	-5.49065548
hetero after UVB irradiation VS hetero before UVB irradiation	Corticosterone methyl oxidase II deficiency - CMO II|SMPDB	0.307187072	0.103522065	1.723908534	0.12110487	0.3132781	-4.97251635
WT after UVB irradiation VS WT before UVB irradiation	Corticosterone methyl oxidase II deficiency - CMO II|SMPDB	0.03938021	0.054409473	0.163687683	0.87321181	0.96283151	-5.85559705
homo after UVB irradiation VS homo before UVB irradiation	Corticotropin Activation of Cortisol Production|SMPDB	0.752701153	-0.0399557	3.684476433	0.00468878	0.03913891	-1.93449121
hetero after UVB irradiation VS hetero before UVB irradiation	Corticotropin Activation of Cortisol Production|SMPDB	0.873449964	0.06624564	4.142120594	0.00289843	0.06144665	-1.39264721
WT after UVB irradiation VS WT before UVB irradiation	Corticotropin Activation of Cortisol Production|SMPDB	0.809414734	-0.11834976	2.938697819	0.01469633	0.28439881	-2.80015227
homo after UVB irradiation VS homo before UVB irradiation	Corticotropin-releasing hormone signaling pathway|Wikipathways	0.230403863	-0.00174906	1.348445142	0.20913995	0.36695387	-5.47779483
hetero after UVB irradiation VS hetero before UVB irradiation	Corticotropin-releasing hormone signaling pathway|Wikipathways	0.364594404	0.032899691	2.342205066	0.04570316	0.18854754	-4.07482611
WT after UVB irradiation VS WT before UVB irradiation	Corticotropin-releasing hormone signaling pathway|Wikipathways	0.218199102	0.011962413	1.023161165	0.33015419	0.71290873	-5.3665857
homo after UVB irradiation VS homo before UVB irradiation	Cortisol synthesis and secretion - Homo sapiens (human)|KEGG	0.62237967	-0.02683102	3.893422527	0.00337204	0.03262019	-1.61231665
hetero after UVB irradiation VS hetero before UVB irradiation	Cortisol synthesis and secretion - Homo sapiens (human)|KEGG	0.287872599	0.033291865	1.29558566	0.22948203	0.45170905	-5.51855115
WT after UVB irradiation VS WT before UVB irradiation	Cortisol synthesis and secretion - Homo sapiens (human)|KEGG	0.291648703	0.024926859	1.160495109	0.27258847	0.66431878	-5.23202383
homo after UVB irradiation VS homo before UVB irradiation	Costimulation by the CD28 family|Reactome	0.071450218	-0.02646629	0.388330914	0.70642987	0.80598671	-6.28808429
hetero after UVB irradiation VS hetero before UVB irradiation	Costimulation by the CD28 family|Reactome	0.237018723	0.04280918	1.335343837	0.2167113	0.43793611	-5.47176441
WT after UVB irradiation VS WT before UVB irradiation	Costimulation by the CD28 family|Reactome	0.106086859	-0.00760677	0.506614285	0.62331998	0.87257466	-5.74088381
homo after UVB irradiation VS homo before UVB irradiation	COX reactions|Reactome	0.047548045	0.023442677	0.117189471	0.90917855	0.94692273	-6.36107478
hetero after UVB irradiation VS hetero before UVB irradiation	COX reactions|Reactome	0.015568391	0.042254104	0.037085411	0.97128054	0.98664958	-6.34579525
WT after UVB irradiation VS WT before UVB irradiation	COX reactions|Reactome	-0.2011984	0.015529431	-0.49851764	0.62881476	0.87289644	-5.74489795
homo after UVB irradiation VS homo before UVB irradiation	creatine biosynthesis|HumanCyc	1.129356626	-0.06410878	5.569864437	0.0002983	0.01666204	0.741026302
hetero after UVB irradiation VS hetero before UVB irradiation	creatine biosynthesis|HumanCyc	0.621082673	0.11995899	1.705211398	0.12463911	0.31649308	-4.99803402
WT after UVB irradiation VS WT before UVB irradiation	creatine biosynthesis|HumanCyc	0.544268889	-0.04967032	1.496035105	0.16527469	0.56923795	-4.8536845
homo after UVB irradiation VS homo before UVB irradiation	Creatine deficiency_ guanidinoacetate methyltransferase deficiency|SMPDB	-0.34739547	0.00502943	-1.63262028	0.13558154	0.277359	-5.11269582
hetero after UVB irradiation VS hetero before UVB irradiation	Creatine deficiency_ guanidinoacetate methyltransferase deficiency|SMPDB	-0.56351352	-0.04220085	-3.24793085	0.01094591	0.10415756	-2.6949004
WT after UVB irradiation VS WT before UVB irradiation	Creatine deficiency_ guanidinoacetate methyltransferase deficiency|SMPDB	-0.47021304	-0.04055952	-2.31093774	0.04323906	0.39022568	-3.73881388
homo after UVB irradiation VS homo before UVB irradiation	Creatine metabolism|Reactome	0.563819718	-0.03052616	2.479336142	0.03400833	0.12323972	-3.84944383
hetero after UVB irradiation VS hetero before UVB irradiation	Creatine metabolism|Reactome	0.167373058	0.000266403	0.565377286	0.58655268	0.77922095	-6.17643646
WT after UVB irradiation VS WT before UVB irradiation	Creatine metabolism|Reactome	0.247623189	-0.00223414	0.76485197	0.46188685	0.80891799	-5.58171806
homo after UVB irradiation VS homo before UVB irradiation	creatine-phosphate biosynthesis|HumanCyc	0.455837034	-0.02129898	1.628139709	0.1365351	0.27843648	-5.11876435
hetero after UVB irradiation VS hetero before UVB irradiation	creatine-phosphate biosynthesis|HumanCyc	0.151089019	-0.01839007	0.457328335	0.65899658	0.82651398	-6.23450564
WT after UVB irradiation VS WT before UVB irradiation	creatine-phosphate biosynthesis|HumanCyc	0.092660008	0.102146326	0.255104978	0.80377047	0.94525798	-5.83629689
homo after UVB irradiation VS homo before UVB irradiation	Creation of C4 and C2 activators|Reactome	0.820428026	-0.02983877	3.488969863	0.00641356	0.04648301	-2.24031834
hetero after UVB irradiation VS hetero before UVB irradiation	Creation of C4 and C2 activators|Reactome	0.578751404	0.025243504	2.772845476	0.02302363	0.13611114	-3.41833274
WT after UVB irradiation VS WT before UVB irradiation	Creation of C4 and C2 activators|Reactome	0.187366643	-0.04063026	0.65568004	0.52670834	0.83205707	-5.65622537
homo after UVB irradiation VS homo before UVB irradiation	CREB phosphorylation through the activation of Adenylate Cyclase|Reactome	0.248183921	-0.05081429	0.866642318	0.40773882	0.559078	-5.98066336
hetero after UVB irradiation VS hetero before UVB irradiation	CREB phosphorylation through the activation of Adenylate Cyclase|Reactome	0.247405717	0.065066313	0.997599813	0.34625656	0.57530067	-5.83711065
WT after UVB irradiation VS WT before UVB irradiation	CREB phosphorylation through the activation of Adenylate Cyclase|Reactome	0.29114323	0.002537491	1.267132943	0.23359391	0.62952955	-5.1189175
homo after UVB irradiation VS homo before UVB irradiation	CREB phosphorylation through the activation of CaMKII|Reactome	0.175496097	0.055249774	0.720787146	0.48859713	0.63056374	-6.09696305
hetero after UVB irradiation VS hetero before UVB irradiation	CREB phosphorylation through the activation of CaMKII|Reactome	-0.0191629	0.035072687	-0.07004323	0.94579321	0.97180465	-6.34388058
WT after UVB irradiation VS WT before UVB irradiation	CREB phosphorylation through the activation of CaMKII|Reactome	0.138075905	-0.06380485	0.488199858	0.63585143	0.87626237	-5.74992439
homo after UVB irradiation VS homo before UVB irradiation	CREB phosphorylation through the activation of CaMKK|Reactome	0.236269686	-0.05252368	0.674525947	0.51623994	0.65421914	-6.1298854
hetero after UVB irradiation VS hetero before UVB irradiation	CREB phosphorylation through the activation of CaMKK|Reactome	-0.21901028	-0.09194847	-0.75094095	0.4731366	0.69499085	-6.05071753
WT after UVB irradiation VS WT before UVB irradiation	CREB phosphorylation through the activation of CaMKK|Reactome	0.2184358	-0.11801993	0.880834871	0.39891788	0.76730004	-5.49160969
homo after UVB irradiation VS homo before UVB irradiation	CREB phosphorylation through the activation of Ras|Reactome	0.023033334	0.011146564	0.139675862	0.89186651	0.93572208	-6.35797695
hetero after UVB irradiation VS hetero before UVB irradiation	CREB phosphorylation through the activation of Ras|Reactome	-0.15902114	-0.01480915	-0.93448434	0.3760574	0.60404776	-5.89646508
WT after UVB irradiation VS WT before UVB irradiation	CREB phosphorylation through the activation of Ras|Reactome	-0.00646818	-0.04670696	-0.02783394	0.97833784	0.99268361	-5.86875873
homo after UVB irradiation VS homo before UVB irradiation	CREB phosphorylation|Reactome	-0.23133812	-0.02203944	-1.20946223	0.25605668	0.41965356	-5.63959902
hetero after UVB irradiation VS hetero before UVB irradiation	CREB phosphorylation|Reactome	0.408704329	0.002690485	1.46146728	0.18015234	0.39822229	-5.31757171
WT after UVB irradiation VS WT before UVB irradiation	CREB phosphorylation|Reactome	-0.00496857	-0.17385613	-0.01526276	0.98812036	0.99664845	-5.86903318
homo after UVB irradiation VS homo before UVB irradiation	CREB3 factors activate genes|Reactome	0.453789933	0.026506288	1.802870188	0.10353005	0.23537222	-4.87581903
hetero after UVB irradiation VS hetero before UVB irradiation	CREB3 factors activate genes|Reactome	-0.03626555	-0.04685979	-0.13800768	0.89347566	0.96526714	-6.33622086
WT after UVB irradiation VS WT before UVB irradiation	CREB3 factors activate genes|Reactome	-0.01383061	0.051536147	-0.05037098	0.96081072	0.98815178	-5.86786601
homo after UVB irradiation VS homo before UVB irradiation	CRH|NetPath	0.449669465	-0.04902667	2.60891876	0.02738896	0.11018326	-3.64384588
hetero after UVB irradiation VS hetero before UVB irradiation	CRH|NetPath	0.627569462	0.033588846	3.642600743	0.00601003	0.0822456	-2.10758926
WT after UVB irradiation VS WT before UVB irradiation	CRH|NetPath	0.480656801	-0.02450613	2.157477844	0.05613232	0.41769317	-3.96244497
homo after UVB irradiation VS homo before UVB irradiation	Cristae formation|Reactome	-0.12379019	-0.00788214	-0.35693959	0.729028	0.8217964	-6.3004658
hetero after UVB irradiation VS hetero before UVB irradiation	Cristae formation|Reactome	-0.30692402	-0.03008504	-1.22217205	0.2547276	0.47937588	-5.60246665
WT after UVB irradiation VS WT before UVB irradiation	Cristae formation|Reactome	-0.15226012	-0.01538689	-0.38351393	0.70930171	0.90329752	-5.79521992
homo after UVB irradiation VS homo before UVB irradiation	CRMPs in Sema3A signaling|Reactome	0.286370069	-0.0460518	1.668135519	0.12823277	0.26846864	-5.06427783

hetero after UVB irradiation VS hetero before UVB irradiation	CRMPs in Sema3A signaling|Reactome	0.158527912	0.036908237	0.998706889	0.34575014	0.57509341	-5.83604227
WT after UVB irradiation VS WT before UVB irradiation	CRMPs in Sema3A signaling|Reactome	0.303419887	0.030510045	1.512899694	0.16100115	0.56258013	-4.83305307
homo after UVB irradiation VS homo before UVB irradiation	Crosslinking of collagen fibrils|Reactome	0.908317705	0.03538708	5.095745984	0.00056859	0.01666204	0.120760862
hetero after UVB irradiation VS hetero before UVB irradiation	Crosslinking of collagen fibrils|Reactome	0.52030869	0.02526684	1.81230085	0.10562184	0.29415277	-4.85021388
WT after UVB irradiation VS WT before UVB irradiation	Crosslinking of collagen fibrils|Reactome	0.316884025	-0.07500447	1.078503477	0.30593532	0.69096189	-5.31393402
homo after UVB irradiation VS homo before UVB irradiation	Cross-presentation of particulate exogenous antigens (phagosomes)|Reactome	1.178669164	-0.01955694	7.364703975	3.40E-05	0.01666204	2.771505252
hetero after UVB irradiation VS hetero before UVB irradiation	Cross-presentation of particulate exogenous antigens (phagosomes)|Reactome	0.954312014	0.103723684	3.965611337	0.00373539	0.06783236	-1.64118533
WT after UVB irradiation VS WT before UVB irradiation	Cross-presentation of particulate exogenous antigens (phagosomes)|Reactome	0.747286583	0.06490767	2.365529557	0.03938579	0.39022568	-3.65837078
homo after UVB irradiation VS homo before UVB irradiation	Cross-presentation of soluble exogenous antigens (endosomes)|Reactome	0.342540172	-0.01619262	1.295641808	0.22604353	0.3856931	-5.54069834
hetero after UVB irradiation VS hetero before UVB irradiation	Cross-presentation of soluble exogenous antigens (endosomes)|Reactome	0.429333684	0.071341996	1.529770807	0.16270561	0.37607109	-5.23068633
WT after UVB irradiation VS WT before UVB irradiation	Cross-presentation of soluble exogenous antigens (endosomes)|Reactome	0.338899147	0.035473487	0.955059102	0.36188592	0.74374432	-5.42832302
homo after UVB irradiation VS homo before UVB irradiation	CS/DS degradation|Reactome	0.3728852	0.045010563	1.841534139	0.09729783	0.22539923	-4.82045339
hetero after UVB irradiation VS hetero before UVB irradiation	CS/DS degradation|Reactome	0.091479021	-0.13202668	0.354845777	0.73142349	0.86896773	-6.27875319
WT after UVB irradiation VS WT before UVB irradiation	CS/DS degradation|Reactome	-0.02664348	-0.14434679	-0.08991265	0.93011776	0.9788786	-5.8650575
homo after UVB irradiation VS homo before UVB irradiation	c-src mediated regulation of Cx43 function and closure of gap junctions|Reactome	-0.33369828	0.085068035	-1.01068862	0.33750078	0.49312914	-5.84824804
hetero after UVB irradiation VS hetero before UVB irradiation	c-src mediated regulation of Cx43 function and closure of gap junctions|Reactome	0.118464661	0.084729439	0.370066031	0.72046286	0.86372165	-6.27286234
WT after UVB irradiation VS WT before UVB irradiation	c-src mediated regulation of Cx43 function and closure of gap junctions|Reactome	-0.0581366	0.088252379	-0.15423083	0.88047136	0.96769248	-5.85711602
homo after UVB irradiation VS homo before UVB irradiation	ctcf: first multivalent nuclear factor|BioCarta	-0.26568301	0.002518557	-1.42309582	0.18707406	0.34276892	-5.38605327
hetero after UVB irradiation VS hetero before UVB irradiation	ctcf: first multivalent nuclear factor|BioCarta	-0.13746314	-0.02464575	-0.80076051	0.44526017	0.67012749	-6.01166253
WT after UVB irradiation VS WT before UVB irradiation	ctcf: first multivalent nuclear factor|BioCarta	-0.23747216	-0.06087755	-1.07889832	0.30576753	0.69096189	-5.3135506
homo after UVB irradiation VS homo before UVB irradiation	CTLA4 inhibitory signaling|Reactome	-0.02515545	-0.03660599	-0.13291063	0.89706912	0.93988472	-6.35896579
hetero after UVB irradiation VS hetero before UVB irradiation	CTLA4 inhibitory signaling|Reactome	0.201393102	0.042605564	1.222255747	0.25469756	0.47937588	-5.60237287
WT after UVB irradiation VS WT before UVB irradiation	CTLA4 inhibitory signaling|Reactome	-0.06730831	0.007289874	-0.32977926	0.74831619	0.9185045	-5.81437477
homo after UVB irradiation VS homo before UVB irradiation	C-type lectin receptor signaling pathway - Homo sapiens (human)|KEGG	0.53594958	-0.0249814	3.037473083	0.01341991	0.07249235	-2.95815921
hetero after UVB irradiation VS hetero before UVB irradiation	C-type lectin receptor signaling pathway - Homo sapiens (human)|KEGG	0.528233322	0.038760239	3.102211377	0.01371992	0.1130168	-2.91548783
WT after UVB irradiation VS WT before UVB irradiation	C-type lectin receptor signaling pathway - Homo sapiens (human)|KEGG	0.378398424	0.006393655	1.644045923	0.13094512	0.54366034	-4.66810823
homo after UVB irradiation VS homo before UVB irradiation	C-type lectin receptors (CLRs)|Reactome	0.515099128	-0.04164741	2.986315735	0.01460622	0.07468309	-3.04009065
hetero after UVB irradiation VS hetero before UVB irradiation	C-type lectin receptors (CLRs)|Reactome	0.472998485	0.031831125	3.000989063	0.01607053	0.11352943	-3.06955476
WT after UVB irradiation VS WT before UVB irradiation	C-type lectin receptors (CLRs)|Reactome	0.378428225	-0.01635491	1.899337279	0.08647743	0.47656734	-4.32726679
homo after UVB irradiation VS homo before UVB irradiation	C-type lectin receptors (CLRs)|Wikipathways	-0.06294648	0.066343372	-0.22156786	0.82939437	0.89464085	-6.34214134
hetero after UVB irradiation VS hetero before UVB irradiation	C-type lectin receptors (CLRs)|Wikipathways	-0.11376091	0.027538808	-0.55403123	0.5939523	0.78483643	-6.1830722
WT after UVB irradiation VS WT before UVB irradiation	C-type lectin receptors (CLRs)|Wikipathways	-0.23445433	-0.11016077	-0.76048381	0.46437864	0.80891799	-5.58489501
homo after UVB irradiation VS homo before UVB irradiation	Cushing syndrome - Homo sapiens (human)|KEGG	0.162481034	-0.00820953	0.972926372	0.3549866	0.50986751	-5.88456793
hetero after UVB irradiation VS hetero before UVB irradiation	Cushing syndrome - Homo sapiens (human)|KEGG	0.003076389	0.003749235	0.017478458	0.98646174	0.99321683	-6.34637557
WT after UVB irradiation VS WT before UVB irradiation	Cushing syndrome - Homo sapiens (human)|KEGG	0.089829155	0.014161	0.440872647	0.66860847	0.87948693	-5.77169515
homo after UVB irradiation VS homo before UVB irradiation	CXCR3-mediated signaling events|PID	0.241813983	-0.03574582	1.234311838	0.24708629	0.40921146	-5.61157539
hetero after UVB irradiation VS hetero before UVB irradiation	CXCR3-mediated signaling events|PID	0.468501211	5.59E-05	2.957677162	0.01720041	0.11529333	-3.13564036
WT after UVB irradiation VS WT before UVB irradiation	CXCR3-mediated signaling events|PID	0.34212926	0.013596823	1.686641163	0.122319	0.52731779	-4.61293073
homo after UVB irradiation VS homo before UVB irradiation	cxcr4 signaling pathway|BioCarta	0.432389511	0.017113945	1.744513986	0.11363791	0.24957705	-4.95833217
hetero after UVB irradiation VS hetero before UVB irradiation	cxcr4 signaling pathway|BioCarta	0.612953035	0.049031946	3.245123411	0.01099342	0.10415769	-2.69913442
WT after UVB irradiation VS WT before UVB irradiation	cxcr4 signaling pathway|BioCarta	0.34610234	0.034559975	1.690852999	0.12149443	0.52647129	-4.60743479
homo after UVB irradiation VS homo before UVB irradiation	CXCR4-mediated signaling events|PID	0.479305584	-0.03725791	2.490103292	0.03340234	0.12201208	-3.83242169
hetero after UVB irradiation VS hetero before UVB irradiation	CXCR4-mediated signaling events|PID	0.645601971	0.042199835	3.738314605	0.00521228	0.07738625	-1.96790646
WT after UVB irradiation VS WT before UVB irradiation	CXCR4-mediated signaling events|PID	0.451291025	-0.01686538	2.081463373	0.06381586	0.43544914	-4.07155718
homo after UVB irradiation VS homo before UVB irradiation	Cyclin A/B1/B2 associated events during G2/M transition|Reactome	-0.57532391	0.023054331	-3.35889107	0.00791824	0.05229475	-2.4457652
hetero after UVB irradiation VS hetero before UVB irradiation	Cyclin A/B1/B2 associated events during G2/M transition|Reactome	0.086171821	-0.01263109	0.357287808	0.72966043	0.86896773	-6.27782416
WT after UVB irradiation VS WT before UVB irradiation	Cyclin A/B1/B2 associated events during G2/M transition|Reactome	-0.18773889	0.026560714	-0.65031205	0.53002971	0.83380183	-5.65962154
homo after UVB irradiation VS homo before UVB irradiation	Cyclin A:Cdk2-associated events at S phase entry|Reactome	-0.20306119	0.015358612	-1.03252166	0.32768942	0.48374714	-5.82674593
hetero after UVB irradiation VS hetero before UVB irradiation	Cyclin A:Cdk2-associated events at S phase entry|Reactome	0.257112304	0.024951815	1.4258981	0.18987493	0.40842375	-5.36190718
WT after UVB irradiation VS WT before UVB irradiation	Cyclin A:Cdk2-associated events at S phase entry|Reactome	-0.05143049	-0.0198123	-0.21540988	0.83374293	0.94931538	-5.84570257
homo after UVB irradiation VS homo before UVB irradiation	Cyclin D associated events in G1|Reactome	-0.20984066	0.004713549	-1.14068093	0.28226188	0.44245297	-5.71499069
hetero after UVB irradiation VS hetero before UVB irradiation	Cyclin D associated events in G1|Reactome	0.137165512	0.00881504	0.753784499	0.47151529	0.69305602	-6.04854683
WT after UVB irradiation VS WT before UVB irradiation	Cyclin D associated events in G1|Reactome	-0.12232611	-0.02428181	-0.49379233	0.63203262	0.87303764	-5.74721233
homo after UVB irradiation VS homo before UVB irradiation	Cyclin E associated events during G1/S transition|Reactome	-0.33202552	0.013920726	-1.8168986	0.10122724	0.23145299	-4.85579301
hetero after UVB irradiation VS hetero before UVB irradiation	Cyclin E associated events during G1/S transition|Reactome	0.162698547	-0.00413496	0.932058042	0.37723958	0.60405274	-5.89868486
WT after UVB irradiation VS WT before UVB irradiation	Cyclin E associated events during G1/S transition|Reactome	-0.15398981	-0.04219539	-0.67071497	0.51747131	0.82895075	-5.64657798
homo after UVB irradiation VS homo before UVB irradiation	cyclin e destruction pathway|BioCarta	-0.295238	-0.04785265	-1.1145234	0.29276902	0.45292679	-5.74279751
hetero after UVB irradiation VS hetero before UVB irradiation	cyclin e destruction pathway|BioCarta	-0.18218799	-0.08072169	-0.72469119	0.48827445	0.70846522	-6.07041724
WT after UVB irradiation VS WT before UVB irradiation	cyclin e destruction pathway|BioCarta	-0.05071356	0.012828448	-0.19320362	0.85063615	0.95502804	-5.85027881
homo after UVB irradiation VS homo before UVB irradiation	cycling of ran in nucleocytoplasmic transport|BioCarta	-0.9102309	0.008524181	-4.40922741	0.00153213	0.02224473	-0.84183196
hetero after UVB irradiation VS hetero before UVB irradiation	cycling of ran in nucleocytoplasmic transport|BioCarta	-0.16717158	0.034273974	-0.48373827	0.64089342	0.81779292	-6.22137855
WT after UVB irradiation VS WT before UVB irradiation	cycling of ran in nucleocytoplasmic transport|BioCarta	-0.17277104	-0.03647662	-0.50428642	0.62489733	0.87257466	-5.74204419
homo after UVB irradiation VS homo before UVB irradiation	cyclins and cell cycle regulation|BioCarta	-0.12994016	0.046342913	-0.60386592	0.56023788	0.6867238	-6.17633302
hetero after UVB irradiation VS hetero before UVB irradiation	cyclins and cell cycle regulation|BioCarta	0.231601485	-0.01149382	1.101427869	0.30117572	0.53143706	-5.7329843
WT after UVB irradiation VS WT before UVB irradiation	cyclins and cell cycle regulation|BioCarta	0.232426475	-0.04820309	0.924244605	0.37694926	0.75077508	-5.45511349
homo after UVB irradiation VS homo before UVB irradiation	Cyclophosphamide Action Pathway|SMPDB	-0.09653661	0.011767812	-0.35608695	0.72964577	0.8217964	-6.30078797
hetero after UVB irradiation VS hetero before UVB irradiation	Cyclophosphamide Action Pathway|SMPDB	-0.18112932	-0.07520002	-0.51328958	0.62093373	0.80306263	-6.2058668
WT after UVB irradiation VS WT before UVB irradiation	Cyclophosphamide Action Pathway|SMPDB	-0.50215858	-0.00146229	-1.83246814	0.09654057	0.49428338	-4.41875771
homo after UVB irradiation VS homo before UVB irradiation	Cyclophosphamide Metabolism Pathway|SMPDB	-0.09653661	0.011767812	-0.35608695	0.72964577	0.8217964	-6.30078797
hetero after UVB irradiation VS hetero before UVB irradiation	Cyclophosphamide Metabolism Pathway|SMPDB	-0.18112932	-0.07520002	-0.51328958	0.62093373	0.80306263	-6.2058668
WT after UVB irradiation VS WT before UVB irradiation	Cyclophosphamide Metabolism Pathway|SMPDB	-0.50215858	-0.00146229	-1.83246814	0.09654057	0.49428338	-4.41875771
homo after UVB irradiation VS homo before UVB irradiation	Cyclophosphamide Pathway_ Pharmacodynamics|PharmGKB	0.430703919	-0.03810173	1.802679287	0.10356172	0.23537222	-4.87609105
hetero after UVB irradiation VS hetero before UVB irradiation	Cyclophosphamide Pathway_ Pharmacodynamics|PharmGKB	0.269824564	-0.00538242	1.131890273	0.28886238	0.51871614	-5.70097793
WT after UVB irradiation VS WT before UVB irradiation	Cyclophosphamide Pathway_ Pharmacodynamics|PharmGKB	0.342425443	-0.00914909	1.424793405	0.18442796	0.59839062	-4.93926944
homo after UVB irradiation VS homo before UVB irradiation	Cyclophosphamide Pathway_ Pharmacokinetics|PharmGKB	-0.45007548	0.127299644	-2.09520619	0.0643614	0.17578232	-4.44536855
hetero after UVB irradiation VS hetero before UVB irradiation	Cyclophosphamide Pathway_ Pharmacokinetics|PharmGKB	-0.71525286	0.104032006	-2.78896201	0.02244352	0.13389735	-3.39365844
WT after UVB irradiation VS WT before UVB irradiation	Cyclophosphamide Pathway_ Pharmacokinetics|PharmGKB	-0.75959428	0.071672811	-3.06200261	0.01189451	0.27435478	-2.61507041
homo after UVB irradiation VS homo before UVB irradiation	Cyclothiazide Action Pathway|SMPDB	0.234726031	0.017120546	1.156747303	0.27595732	0.43614438	-5.69767199
hetero after UVB irradiation VS hetero before UVB irradiation	Cyclothiazide Action Pathway|SMPDB	-0.03255037	0.028925632	-0.18154472	0.86023155	0.94503144	-6.32869738
WT after UVB irradiation VS WT before UVB irradiation	Cyclothiazide Action Pathway|SMPDB	-0.15908359	0.010252305	-0.80382265	0.44003996	0.79685269	-5.55266936
homo after UVB irradiation VS homo before UVB irradiation	CYP2E1 reactions|Reactome	-0.43307855	0.104368729	-1.92536931	0.08496605	0.20724091	-4.6986382
hetero after UVB irradiation VS hetero before UVB irradiation	CYP2E1 reactions|Reactome	-0.48900187	0.015784848	-1.77361374	0.11215605	0.30501627	-4.90407041
WT after UVB irradiation VS WT before UVB irradiation	CYP2E1 reactions|Reactome	-0.77305748	0.049232884	-3.5206792	0.00546363	0.20968398	-1.93548916
homo after UVB irradiation VS homo before UVB irradiation	Cystathionine Beta-Synthase Deficiency|SMPDB	-0.09396972	-0.02692441	-0.3795694	0.71270801	0.81002335	-6.29164178
hetero after UVB irradiation VS hetero before UVB irradiation	Cystathionine Beta-Synthase Deficiency|SMPDB	0.075091809	-0.02022482	0.352613458	0.73303662	0.86896773	-6.27959702
WT after UVB irradiation VS WT before UVB irradiation	Cystathionine Beta-Synthase Deficiency|SMPDB	0.176060787	0.019583595	0.866300336	0.40646895	0.7725109	-5.50349844
homo after UVB irradiation VS homo before UVB irradiation	Cysteine and methionine metabolism - Homo sapiens (human)|KEGG	-0.1775448	0.002261423	-1.02421103	0.33139838	0.48780303	-5.83497356
hetero after UVB irradiation VS hetero before UVB irradiation	Cysteine and methionine metabolism - Homo sapiens (human)|KEGG	-0.32489011	-0.00415236	-2.04618044	0.07317449	0.24186319	-4.51533578
WT after UVB irradiation VS WT before UVB irradiation	Cysteine and methionine metabolism - Homo sapiens (human)|KEGG	-0.10747984	-0.01152401	-0.55453503	0.59130063	0.86254944	-5.71587867
homo after UVB irradiation VS homo before UVB irradiation	cysteine biosynthesis/homocysteine degradation (trans-sulfuration)|HumanCyc	-1.03096392	0.009185618	-5.50480244	0.0003253	0.01666204	0.658072793
hetero after UVB irradiation VS hetero before UVB irradiation	cysteine biosynthesis/homocysteine degradation (trans-sulfuration)|HumanCyc	-0.67635827	-0.13184633	-2.08448047	0.06887001	0.23513821	-4.45921201
WT after UVB irradiation VS WT before UVB irradiation	cysteine biosynthesis/homocysteine degradation (trans-sulfuration)|HumanCyc	-1.00471126	-0.08504096	-3.60367118	0.00475571	0.20968398	-1.81475856
homo after UVB irradiation VS homo before UVB irradiation	cysteine biosynthesis|HumanCyc	-0.81516568	-0.00155771	-4.59307257	0.00116712	0.0196644	-0.57671938
hetero after UVB irradiation VS hetero before UVB irradiation	cysteine biosynthesis|HumanCyc	-0.84370331	-0.02299902	-4.3420663	0.00218638	0.05245011	-1.11690069
WT after UVB irradiation VS WT before UVB irradiation	cysteine biosynthesis|HumanCyc	-0.69648265	0.00173465	-3.39749886	0.00672164	0.22490313	-2.11612763
homo after UVB irradiation VS homo before UVB irradiation	Cysteine formation from homocysteine|Reactome	-1.03096392	0.009185618	-5.50480244	0.0003253	0.01666204	0.658072793
hetero after UVB irradiation VS hetero before UVB irradiation	Cysteine formation from homocysteine|Reactome	-0.67635827	-0.13184633	-2.08448047	0.06887001	0.23513821	-4.45921201
WT after UVB irradiation VS WT before UVB irradiation	Cysteine formation from homocysteine|Reactome	-1.00471126	-0.08504096	-3.60367118	0.00475571	0.20968398	-1.81475856
homo after UVB irradiation VS homo before UVB irradiation	Cysteine Metabolism|SMPDB	-0.57249069	0.017193233	-3.29055479	0.00885139	0.05619844	-2.55422007
hetero after UVB irradiation VS hetero before UVB irradiation	Cysteine Metabolism|SMPDB	-0.70250163	-0.01628473	-2.9739972	0.01676532	0.1140542	-3.11072933
WT after UVB irradiation VS WT before UVB irradiation	Cysteine Metabolism|SMPDB	-0.64815523	-0.01177362	-2.71723565	0.02151468	0.31542987	-3.13323441
homo after UVB irradiation VS homo before UVB irradiation	cystic fibrosis transmembrane conductance regulator (cftr) and beta 2 adrenergic receptor (b2ar) pathway|BioCarta	0.171606104	-0.01494334	0.867787172	0.40714272	0.55844883	-5.97967784
hetero after UVB irradiation VS hetero before UVB irradiation	cystic fibrosis transmembrane conductance regulator (cftr) and beta 2 adrenergic receptor (b2ar) pathway|BioCarta	0.049132521	-0.00360413	0.285637624	0.78205225	0.90426031	-6.30249899
WT after UVB irradiation VS WT before UVB irradiation	cystic fibrosis transmembrane conductance regulator (cftr) and beta 2 adrenergic receptor (b2ar) pathway|BioCarta	-0.00785272	-0.07267988	-0.03278494	0.97448606	0.99163945	-5.86860671
homo after UVB irradiation VS homo before UVB irradiation	Cystinosis_ ocular nonnephropathic|SMPDB	-0.57249069	0.017193233	-3.29055479	0.00885139	0.05619844	-2.55422007
hetero after UVB irradiation VS hetero before UVB irradiation	Cystinosis_ ocular nonnephropathic|SMPDB	-0.70250163	-0.01628473	-2.9739972	0.01676532	0.1140542	-3.11072933
WT after UVB irradiation VS WT before UVB irradiation	Cystinosis_ ocular nonnephropathic|SMPDB	-0.64815523	-0.01177362	-2.71723565	0.02151468	0.31542987	-3.13323441
homo after UVB irradiation VS homo before UVB irradiation	Cystinuria|SMPDB	0.234726031	0.017120546	1.156747303	0.27595732	0.43614438	-5.69767199
hetero after UVB irradiation VS hetero before UVB irradiation	Cystinuria|SMPDB	-0.03255037	0.028925632	-0.18154472	0.86023155	0.94503144	-6.32869738
WT after UVB irradiation VS WT before UVB irradiation	Cystinuria|SMPDB	-0.15908359	0.010252305	-0.80382265	0.44003996	0.79685269	-5.55266936
homo after UVB irradiation VS homo before UVB irradiation	Cytochrome c-mediated apoptotic response|Reactome	0.559708311	0.022472492	1.657754002	0.13034279	0.27177943	-5.07848833
hetero after UVB irradiation VS hetero before UVB irradiation	Cytochrome c-mediated apoptotic response|Reactome	0.655477124	0.073652291	3.066446317	0.01450676	0.1130168	-2.96985691
WT after UVB irradiation VS WT before UVB irradiation	Cytochrome c-mediated apoptotic response|Reactome	0.796091303	-0.05990195	3.524175982	0.0054317	0.20968398	-1.93038582
homo after UVB irradiation VS homo before UVB irradiation	Cytochrome P450 - arranged by substrate type|Reactome	0.167004437	0.039766349	0.828958215	0.42769804	0.57897102	-6.0124832
hetero after UVB irradiation VS hetero before UVB irradiation	Cytochrome P450 - arranged by substrate type|Reactome	0.061460848	0.038493602	0.315348882	0.76016595	0.88865506	-6.29291819
WT after UVB irradiation VS WT before UVB irradiation	Cytochrome P450 - arranged by substrate type|Reactome	-0.04065435	0.022866618	-0.17027203	0.86816455	0.96280353	-5.8544864
homo after UVB irradiation VS homo before UVB irradiation	Cytokine Signaling in Immune system|Reactome	0.496188815	-0.0165698	2.929459737	0.01605115	0.07889081	-3.13119292
hetero after UVB irradiation VS hetero before UVB irradiation	Cytokine Signaling in Immune system|Reactome	0.453668895	0.033721942	2.577210222	0.03141735	0.15507377	-3.71765354
WT after UVB irradiation VS WT before UVB irradiation	Cytokine Signaling in Immune system|Reactome	0.350932662	0.00558832	1.551358365	0.15161433	0.55727585	-4.78549447
homo after UVB irradiation VS homo before UVB irradiation	Cytokine-cytokine receptor interaction - Homo sapiens (human)|KEGG	0.458746727	-0.01048677	2.771201339	0.02088861	0.09232406	-3.38471253
hetero after UVB irradiation VS hetero before UVB irradiation	Cytokine-cytokine receptor interaction - Homo sapiens (human)|KEGG	0.261435577	0.02062159	1.375352702	0.20447661	0.42537853	-5.4237768
WT after UVB irradiation VS WT before UVB irradiation	Cytokine-cytokine receptor interaction - Homo sapiens (human)|KEGG	0.187954951	-0.0071975	0.747367803	0.4719123	0.81329188	-5.59433757
homo after UVB irradiation VS homo before UVB irradiation	Cytokines and Inflammatory Response|Wikipathways	0.415784388	0.047609921	2.565977969	0.02942612	0.11434865	-3.7121409
hetero after UVB irradiation VS hetero before UVB irradiation	Cytokines and Inflammatory Response|Wikipathways	0.321631596	0.071064386	1.73135892	0.11972257	0.31314253	-4.96231259
WT after UVB irradiation VS WT before UVB irradiation	Cytokines and Inflammatory Response|Wikipathways	0.423408005	-0.01799061	1.339438378	0.20983854	0.62472791	-5.03827654
homo after UVB irradiation VS homo before UVB irradiation	Cytoplasmic Ribosomal Proteins|Wikipathways	-0.91000531	0.023117128	-4.76818379	0.00090472	0.01796071	-0.32913539
hetero after UVB irradiation VS hetero before UVB irradiation	Cytoplasmic Ribosomal Proteins|Wikipathways	-0.11949412	0.003759383	-0.35109059	0.73413789	0.8693688	-6.28016972
WT after UVB irradiation VS WT before UVB irradiation	Cytoplasmic Ribosomal Proteins|Wikipathways	-0.58623945	0.001111437	-2.03650665	0.06882078	0.44205977	-4.13547711
homo after UVB irradiation VS homo before UVB irradiation	Cytosolic DNA-sensing pathway - Homo sapiens (human)|KEGG	0.342104781	-0.00885539	1.701924525	0.12157775	0.26037712	-5.01770683
hetero after UVB irradiation VS hetero before UVB irradiation	Cytosolic DNA-sensing pathway - Homo sapiens (human)|KEGG	0.550203664	0.059659557	3.531051252	0.00710591	0.08637	-2.27183551
WT after UVB irradiation VS WT before UVB irradiation	Cytosolic DNA-sensing pathway - Homo sapiens (human)|KEGG	0.514453935	-0.00952648	2.497345952	0.03141442	0.3650627	-3.46267783
homo after UVB irradiation VS homo before UVB irradiation	Cytosolic iron-sulfur cluster assembly|Reactome	-1.19892168	-0.07313707	-5.88645355	0.00019732	0.01666204	1.134951401
hetero after UVB irradiation VS hetero before UVB irradiation	Cytosolic iron-sulfur cluster assembly|Reactome	-0.91831788	-0.01998971	-4.59426389	0.00154496	0.04803647	-0.77820562
WT after UVB irradiation VS WT before UVB irradiation	Cytosolic iron-sulfur cluster assembly|Reactome	-0.59274802	-0.05340312	-1.55772388	0.15010784	0.55622203	-4.77755583
homo after UVB irradiation VS homo before UVB irradiation	Cytosolic sensors of pathogen-associated DNA|Reactome	0.28582844	-0.02610313	1.657928818	0.130307	0.27177943	-5.07824943
hetero after UVB irradiation VS hetero before UVB irradiation	Cytosolic sensors of pathogen-associated DNA|Reactome	0.416010742	0.011554552	2.449078987	0.0385383	0.17060506	-3.91291847
WT after UVB irradiation VS WT before UVB irradiation	Cytosolic sensors of pathogen-associated DNA|Reactome	0.346088639	-0.02351176	1.666820769	0.12626732	0.53296014	-4.63869826
homo after UVB irradiation VS homo before UVB irradiation	Cytosolic sulfonation of small molecules|Reactome	0.603017319	0.015332114	3.75480375	0.00419379	0.03725456	-1.82547582
hetero after UVB irradiation VS hetero before UVB irradiation	Cytosolic sulfonation of small molecules|Reactome	0.208865317	-0.00066652	1.116374541	0.29508306	0.52649833	-5.71736008
WT after UVB irradiation VS WT before UVB irradiation	Cytosolic sulfonation of small molecules|Reactome	0.099615692	-0.01748957	0.503598842	0.6253636	0.87257466	-5.74238596
homo after UVB irradiation VS homo before UVB irradiation	Cytosolic tRNA aminoacylation|Reactome	-0.89745224	8.68E-05	-4.94928617	0.00069849	0.01666204	-0.07824808
hetero after UVB irradiation VS hetero before UVB irradiation	Cytosolic tRNA aminoacylation|Reactome	-0.38403273	-0.06837171	-1.46713726	0.17864358	0.39630067	-5.31044541
WT after UVB irradiation VS WT before UVB irradiation	Cytosolic tRNA aminoacylation|Reactome	-0.27810024	0.001792717	-0.78016298	0.45322106	0.80754423	-5.57045606
homo after UVB irradiation VS homo before UVB irradiation	D-<i>myo</i>-inositol (1_3_4)-trisphosphate biosynthesis|HumanCyc	0.765856812	-0.00992861	4.199456295	0.00210231	0.02552962	-1.15060146
hetero after UVB irradiation VS hetero before UVB irradiation	D-<i>myo</i>-inositol (1_3_4)-trisphosphate biosynthesis|HumanCyc	0.305343061	0.08262842	1.040411718	0.32707911	0.55613609	-5.79512784
WT after UVB irradiation VS WT before UVB irradiation	D-<i>myo</i>-inositol (1_3_4)-trisphosphate biosynthesis|HumanCyc	0.369095506	-0.01816162	1.306587915	0.22037053	0.62628016	-5.07529398
homo after UVB irradiation VS homo before UVB irradiation	D-<i>myo</i>-inositol (1_4_5)-trisphosphate biosynthesis|HumanCyc	0.234000103	-0.03753081	1.224390667	0.25063654	0.41314311	-5.62281272
hetero after UVB irradiation VS hetero before UVB irradiation	D-<i>myo</i>-inositol (1_4_5)-trisphosphate biosynthesis|HumanCyc	0.224525319	-0.0135308	1.298915934	0.22838844	0.45170905	-5.51466744

WT after UVB irradiation VS WT before UVB irradiation	D-<i>myo</i>-inositol (1_4_5)-trisphosphate biosynthesis|HumanCyc	0.16143149	0.091695355	0.813291937	0.43483626	0.79685269	-5.54542106
homo after UVB irradiation VS homo before UVB irradiation	D-<i>myo</i>-inositol (1_4_5)-trisphosphate degradation|HumanCyc	0.703770954	-0.07306266	3.608831588	0.00529014	0.04193541	-2.052359
hetero after UVB irradiation VS hetero before UVB irradiation	D-<i>myo</i>-inositol (1_4_5)-trisphosphate degradation|HumanCyc	0.323826192	0.036126009	1.282586145	0.23379313	0.4557866	-5.5336482
WT after UVB irradiation VS WT before UVB irradiation	D-<i>myo</i>-inositol (1_4_5)-trisphosphate degradation|HumanCyc	0.444746912	-0.01946127	1.649744153	0.12976042	0.54169637	-4.66077009
homo after UVB irradiation VS homo before UVB irradiation	D-<i>myo</i>-inositol (1_4_5_6)-tetrakisphosphate biosynthesis|HumanCyc	0.227558486	0.009764834	0.755622966	0.46840701	0.61352449	-6.0708886
hetero after UVB irradiation VS hetero before UVB irradiation	D-<i>myo</i>-inositol (1_4_5_6)-tetrakisphosphate biosynthesis|HumanCyc	-0.18563786	0.075567234	-0.71804012	0.4921588	0.71214597	-6.07531098
WT after UVB irradiation VS WT before UVB irradiation	D-<i>myo</i>-inositol (1_4_5_6)-tetrakisphosphate biosynthesis|HumanCyc	0.132676051	0.085257842	0.566868824	0.58320239	0.8600211	-5.7091003
homo after UVB irradiation VS homo before UVB irradiation	D-<i>myo</i>-inositol (3_4_5_6)-tetrakisphosphate biosynthesis|HumanCyc	0.748332656	-0.04579846	2.304721078	0.04549876	0.13868313	-4.12345222
hetero after UVB irradiation VS hetero before UVB irradiation	D-<i>myo</i>-inositol (3_4_5_6)-tetrakisphosphate biosynthesis|HumanCyc	0.199164456	0.023756292	0.543368556	0.60095218	0.7891501	-6.18919447
WT after UVB irradiation VS WT before UVB irradiation	D-<i>myo</i>-inositol (3_4_5_6)-tetrakisphosphate biosynthesis|HumanCyc	0.489937514	0.036411411	1.502370873	0.16365777	0.56782293	-4.84594984
homo after UVB irradiation VS homo before UVB irradiation	D-<i>myo</i>-inositol-5-phosphate metabolism|HumanCyc	0.298698266	-0.00799748	1.582792577	0.14652977	0.2920362	-5.179665
hetero after UVB irradiation VS hetero before UVB irradiation	D-<i>myo</i>-inositol-5-phosphate metabolism|HumanCyc	0.030809314	-0.01134088	0.162987162	0.87437004	0.95379399	-6.33215334
WT after UVB irradiation VS WT before UVB irradiation	D-<i>myo</i>-inositol-5-phosphate metabolism|HumanCyc	0.091612217	0.081133408	0.451206431	0.66139038	0.87935805	-5.76712251
homo after UVB irradiation VS homo before UVB irradiation	d4gdi signaling pathway|BioCarta	0.784721224	-0.12825536	2.936065319	0.01587605	0.07819852	-3.12060729
hetero after UVB irradiation VS hetero before UVB irradiation	d4gdi signaling pathway|BioCarta	0.812519657	-0.04328569	4.54431752	0.00165372	0.04894587	-0.84446139
WT after UVB irradiation VS WT before UVB irradiation	d4gdi signaling pathway|BioCarta	0.506860728	0.04098296	2.185757858	0.05350647	0.41493406	-3.92154796
homo after UVB irradiation VS homo before UVB irradiation	DAG and IP3 signaling|Reactome	0.471646286	-0.04553234	2.606268684	0.02751049	0.11028351	-3.64806477
hetero after UVB irradiation VS hetero before UVB irradiation	DAG and IP3 signaling|Reactome	0.306411207	0.024071282	1.581085683	0.15060684	0.35828077	-5.16398385
WT after UVB irradiation VS WT before UVB irradiation	DAG and IP3 signaling|Reactome	0.302933916	-0.04370135	1.308134989	0.21986488	0.62621406	-5.07356469
homo after UVB irradiation VS homo before UVB irradiation	DAP12 interactions|Reactome	0.522947675	-0.04889033	3.126229492	0.0115907	0.0658248	-2.81617603
hetero after UVB irradiation VS hetero before UVB irradiation	DAP12 interactions|Reactome	0.671590817	0.015929257	4.08847715	0.00312925	0.06228434	-1.46768405
WT after UVB irradiation VS WT before UVB irradiation	DAP12 interactions|Reactome	0.546127803	0.028581306	2.644748212	0.02437604	0.33026211	-3.24210949
homo after UVB irradiation VS homo before UVB irradiation	DAP12 signaling|Reactome	0.469974025	-0.0518493	2.686121869	0.02407529	0.10226192	-3.52073694
hetero after UVB irradiation VS hetero before UVB irradiation	DAP12 signaling|Reactome	0.636521922	-0.00930731	3.899370601	0.00411321	0.07021718	-1.73564776
WT after UVB irradiation VS WT before UVB irradiation	DAP12 signaling|Reactome	0.457216664	0.014460835	2.253045737	0.04772536	0.401167	-3.82364663
homo after UVB irradiation VS homo before UVB irradiation	D-Arginine and D-ornithine metabolism - Homo sapiens (human)|KEGG	-0.19155898	-0.01406147	-0.52765185	0.6099819	0.72758029	-6.22109094
hetero after UVB irradiation VS hetero before UVB irradiation	D-Arginine and D-ornithine metabolism - Homo sapiens (human)|KEGG	-1.01476099	-0.06204584	-5.42226339	0.00052722	0.03004176	0.260168995
WT after UVB irradiation VS WT before UVB irradiation	D-Arginine and D-ornithine metabolism - Homo sapiens (human)|KEGG	0.05631233	-0.0091435	0.14597812	0.88681608	0.97031361	-5.85836811
homo after UVB irradiation VS homo before UVB irradiation	D-Arginine and D-Ornithine Metabolism|SMPDB	-0.19155898	-0.01406147	-0.52765185	0.6099819	0.72758029	-6.22109094
hetero after UVB irradiation VS hetero before UVB irradiation	D-Arginine and D-Ornithine Metabolism|SMPDB	-1.01476099	-0.06204584	-5.42226339	0.00052722	0.03004176	0.260168995
WT after UVB irradiation VS WT before UVB irradiation	D-Arginine and D-Ornithine Metabolism|SMPDB	0.05631233	-0.0091435	0.14597812	0.88681608	0.97031361	-5.85836811
homo after UVB irradiation VS homo before UVB irradiation	DARPP-32 events|Reactome	0.045788609	-0.02650094	0.249004514	0.80871901	0.88203765	-6.33524618
hetero after UVB irradiation VS hetero before UVB irradiation	DARPP-32 events|Reactome	0.005425037	-0.04382886	0.031685161	0.97546087	0.988327	-6.34599669
WT after UVB irradiation VS WT before UVB irradiation	DARPP-32 events|Reactome	0.153070343	-0.05160256	0.675014176	0.51484788	0.8287185	-5.64378282
homo after UVB irradiation VS homo before UVB irradiation	DCC mediated attractive signaling|Reactome	0.083782148	-0.02755814	0.447773342	0.66446993	0.77043416	-6.26188458
hetero after UVB irradiation VS hetero before UVB irradiation	DCC mediated attractive signaling|Reactome	0.054899933	0.00882615	0.297712506	0.77313148	0.89850415	-6.29871707
WT after UVB irradiation VS WT before UVB irradiation	DCC mediated attractive signaling|Reactome	-0.2133789	-0.10002935	-1.07039282	0.30939762	0.69398681	-5.321786
homo after UVB irradiation VS homo before UVB irradiation	DDX1 as a regulatory component of the Drosha microprocessor|Wikipathways	-0.60186774	-0.05375333	-2.0505953	0.06925707	0.18460466	-4.51265413
hetero after UVB irradiation VS hetero before UVB irradiation	DDX1 as a regulatory component of the Drosha microprocessor|Wikipathways	-0.5562194	-0.07950672	-1.73160149	0.11967782	0.31314253	-4.96198004
WT after UVB irradiation VS WT before UVB irradiation	DDX1 as a regulatory component of the Drosha microprocessor|Wikipathways	-0.34224284	-0.00816394	-0.90468673	0.38673869	0.76061898	-5.47173852
homo after UVB irradiation VS homo before UVB irradiation	DDX58/IFIH1-mediated induction of interferon-alpha/beta|Reactome	0.391031233	-0.04621895	2.14110661	0.05967171	0.16705151	-4.37563362
hetero after UVB irradiation VS hetero before UVB irradiation	DDX58/IFIH1-mediated induction of interferon-alpha/beta|Reactome	0.504487567	-0.00452226	2.78733529	0.02250139	0.1340671	-3.39614886
WT after UVB irradiation VS WT before UVB irradiation	DDX58/IFIH1-mediated induction of interferon-alpha/beta|Reactome	0.467564593	-0.0080243	2.385864882	0.03803789	0.39022568	-3.62830649
hetero after UVB irradiation VS hetero before UVB irradiation	DDX58-IFIH1-mediated induction of interferon-alpha-beta|Wikipathways	0.333105604	0.055731528	1.092739339	0.30476281	0.53447821	-5.74199509
homo after UVB irradiation VS homo before UVB irradiation	De novo fatty acid biosynthesis|EHMN	-0.50940406	0.01993228	-2.88567224	0.01726277	0.08288309	-3.20136467
hetero after UVB irradiation VS hetero before UVB irradiation	De novo fatty acid biosynthesis|EHMN	-0.11440268	-0.05545654	-0.54654876	0.5988598	0.78821916	-6.18738004
WT after UVB irradiation VS WT before UVB irradiation	De novo fatty acid biosynthesis|EHMN	-0.02336445	0.001152566	-0.08552041	0.93352215	0.98009095	-5.86544753
homo after UVB irradiation VS homo before UVB irradiation	Deactivation of the beta-catenin transactivating complex|Reactome	0.096397349	0.014841693	0.460324525	0.65575866	0.76285199	-6.25589591
hetero after UVB irradiation VS hetero before UVB irradiation	Deactivation of the beta-catenin transactivating complex|Reactome	0.273675057	-0.02767594	1.339572212	0.21538935	0.43741933	-5.46673512
WT after UVB irradiation VS WT before UVB irradiation	Deactivation of the beta-catenin transactivating complex|Reactome	-0.05103237	0.023422121	-0.20969	0.83808619	0.94951669	-5.84692844
homo after UVB irradiation VS homo before UVB irradiation	Deadenylation of mRNA|Reactome	-0.51430347	-0.05174679	-2.22856604	0.05163279	0.15118527	-4.24147716
hetero after UVB irradiation VS hetero before UVB irradiation	Deadenylation of mRNA|Reactome	0.193508457	-0.04387058	0.713114016	0.49504838	0.71428009	-6.0789099
WT after UVB irradiation VS WT before UVB irradiation	Deadenylation of mRNA|Reactome	-0.42359874	0.037455341	-1.39733793	0.19230069	0.60496032	-4.9715494
homo after UVB irradiation VS homo before UVB irradiation	Deadenylation-dependent mRNA decay|Reactome	-0.61023237	-0.00598757	-3.69716043	0.00459517	0.03856904	-1.91478823
hetero after UVB irradiation VS hetero before UVB irradiation	Deadenylation-dependent mRNA decay|Reactome	-0.0759955	-0.04616798	-0.31235036	0.76236484	0.89053278	-6.29392711
WT after UVB irradiation VS WT before UVB irradiation	Deadenylation-dependent mRNA decay|Reactome	-0.29766004	0.014322975	-0.99043359	0.34513617	0.72932143	-5.3966832
homo after UVB irradiation VS homo before UVB irradiation	Death Receptor Signalling|Reactome	0.345376286	-0.03918118	1.886026398	0.09055878	0.21571189	-4.75609479
hetero after UVB irradiation VS hetero before UVB irradiation	Death Receptor Signalling|Reactome	0.386726732	0.013176329	2.372100353	0.04357461	0.18322239	-4.0296482
WT after UVB irradiation VS WT before UVB irradiation	Death Receptor Signalling|Reactome	0.278631012	-0.01199127	1.443204293	0.17930415	0.59499502	-4.91740058
homo after UVB irradiation VS homo before UVB irradiation	Dectin-1 mediated noncanonical NF-kB signaling|Reactome	0.045441022	-0.03299775	0.183770222	0.8581014	0.9134577	-6.35033611
hetero after UVB irradiation VS hetero before UVB irradiation	Dectin-1 mediated noncanonical NF-kB signaling|Reactome	0.076072626	-0.05009513	0.377439054	0.7151775	0.85921866	-6.26992267
WT after UVB irradiation VS WT before UVB irradiation	Dectin-1 mediated noncanonical NF-kB signaling|Reactome	0.141549645	-0.05582595	0.608181946	0.55651867	0.84793065	-5.68538623
homo after UVB irradiation VS homo before UVB irradiation	Dectin-2 family|Reactome	0.777809222	0.022434237	4.608555086	0.00114093	0.01943833	-0.55463339
hetero after UVB irradiation VS hetero before UVB irradiation	Dectin-2 family|Reactome	0.597665336	0.052424946	3.843487327	0.00446365	0.07266178	-1.81582875
WT after UVB irradiation VS WT before UVB irradiation	Dectin-2 family|Reactome	0.206779254	0.024870483	0.730612025	0.481649	0.81552033	-5.60618835
homo after UVB irradiation VS homo before UVB irradiation	Defective ABCC8 can cause hypoglycemias and hyperglycemias|Reactome	0.17582008	-0.04081759	0.468512098	0.65010519	0.75801335	-6.25190414
hetero after UVB irradiation VS hetero before UVB irradiation	Defective ABCC8 can cause hypoglycemias and hyperglycemias|Reactome	0.310498313	0.01267002	0.805010464	0.44293433	0.66850818	-6.0082313
WT after UVB irradiation VS WT before UVB irradiation	Defective ABCC8 can cause hypoglycemias and hyperglycemias|Reactome	0.137214654	-0.02935871	0.308858756	0.76371681	0.92696825	-5.82107007
homo after UVB irradiation VS homo before UVB irradiation	Defective CFTR causes cystic fibrosis|Reactome	0.088930617	-0.0401129	0.38598535	0.70810835	0.80749818	-6.28904438
hetero after UVB irradiation VS hetero before UVB irradiation	Defective CFTR causes cystic fibrosis|Reactome	0.265190142	0.045632544	1.44637891	0.184222	0.40321767	-5.33645717
WT after UVB irradiation VS WT before UVB irradiation	Defective CFTR causes cystic fibrosis|Reactome	-0.06089499	0.015709269	-0.25667145	0.80259413	0.94525798	-5.83589359
homo after UVB irradiation VS homo before UVB irradiation	Defensins|Reactome	0.645277161	-0.10257234	2.531759538	0.03115732	0.11752935	-3.76645336
hetero after UVB irradiation VS hetero before UVB irradiation	Defensins|Reactome	0.508336396	0.085646124	2.133021052	0.06376684	0.22647381	-4.38765542
WT after UVB irradiation VS WT before UVB irradiation	Defensins|Reactome	0.351038922	-0.04708261	1.086183777	0.30268414	0.68749071	-5.30645636
homo after UVB irradiation VS homo before UVB irradiation	Degradation of AXIN|Reactome	-0.66983432	0.054411729	-3.2926702	0.00882086	0.05619844	-2.55085795
hetero after UVB irradiation VS hetero before UVB irradiation	Degradation of AXIN|Reactome	-0.39877038	-0.01114961	-1.48564825	0.17379514	0.39118926	-5.28706949
WT after UVB irradiation VS WT before UVB irradiation	Degradation of AXIN|Reactome	-0.82710503	-0.08919975	-2.95059203	0.01439909	0.28345104	-2.7822766
homo after UVB irradiation VS homo before UVB irradiation	Degradation of beta catenin|PID	-0.65222916	0.015143187	-2.99248468	0.01445765	0.07420453	-3.03020818
hetero after UVB irradiation VS hetero before UVB irradiation	Degradation of beta catenin|PID	-0.53816399	-0.09730808	-2.15703944	0.06138003	0.22186375	-4.35208459
WT after UVB irradiation VS WT before UVB irradiation	Degradation of beta catenin|PID	-0.54730563	-0.01692734	-1.98529131	0.07497445	0.45723857	-4.20768584
homo after UVB irradiation VS homo before UVB irradiation	Degradation of beta-catenin by the destruction complex|Reactome	-0.46391495	0.00822727	-2.58716538	0.02840269	0.11277104	-3.6784615
hetero after UVB irradiation VS hetero before UVB irradiation	Degradation of beta-catenin by the destruction complex|Reactome	-0.28152429	-0.02796434	-1.52302172	0.16436	0.37722849	-5.23937023
WT after UVB irradiation VS WT before UVB irradiation	Degradation of beta-catenin by the destruction complex|Reactome	-0.39501751	0.033269777	-1.63621679	0.13258862	0.54650924	-4.67816829
homo after UVB irradiation VS homo before UVB irradiation	Degradation of beta-catenin by the destruction complex|Wikipathways	-1.18022417	0.004930147	-7.05553586	4.80E-05	0.01666204	2.455448144
hetero after UVB irradiation VS hetero before UVB irradiation	Degradation of beta-catenin by the destruction complex|Wikipathways	-1.14520033	-0.02867975	-6.79659259	0.00010851	0.02920766	1.742391011
WT after UVB irradiation VS WT before UVB irradiation	Degradation of beta-catenin by the destruction complex|Wikipathways	-1.00683689	-0.06659497	-4.4978627	0.00112365	0.15645713	-0.57745268
homo after UVB irradiation VS homo before UVB irradiation	Degradation of cysteine and homocysteine|Reactome	-0.00611104	-0.04357796	-0.0268369	0.9791516	0.98606224	-6.3680613
hetero after UVB irradiation VS hetero before UVB irradiation	Degradation of cysteine and homocysteine|Reactome	-0.0882096	0.036102459	-0.3160801	0.75963008	0.88865506	-6.29267074
WT after UVB irradiation VS WT before UVB irradiation	Degradation of cysteine and homocysteine|Reactome	0.016730393	0.027276246	0.07045726	0.94520768	0.98295421	-5.866637
homo after UVB irradiation VS homo before UVB irradiation	Degradation of DVL|Reactome	-0.01992247	0.032641621	-0.08290884	0.93566477	0.96219047	-6.36475628
hetero after UVB irradiation VS hetero before UVB irradiation	Degradation of DVL|Reactome	-0.19715649	0.007683112	-1.0365704	0.32876584	0.55727583	-5.79895023
WT after UVB irradiation VS WT before UVB irradiation	Degradation of DVL|Reactome	-0.46497642	0.071237019	-1.91819613	0.08382073	0.47086009	-4.30121277
homo after UVB irradiation VS homo before UVB irradiation	Degradation of GABA|Reactome	-0.96078212	-0.06336452	-4.40813768	0.00153462	0.02224473	-0.84341892
hetero after UVB irradiation VS hetero before UVB irradiation	Degradation of GABA|Reactome	-0.24947082	0.110968118	-0.57540571	0.5800548	0.77397513	-6.17046785
WT after UVB irradiation VS WT before UVB irradiation	Degradation of GABA|Reactome	-0.22371324	0.065559803	-0.68008487	0.51176395	0.82675132	-5.64046526
homo after UVB irradiation VS homo before UVB irradiation	Degradation of GLI1 by the proteasome|Reactome	-0.09806228	0.013298546	-0.5214304	0.61414225	0.73044374	-6.22449512
hetero after UVB irradiation VS hetero before UVB irradiation	Degradation of GLI1 by the proteasome|Reactome	0.361410931	0.067943892	2.147784468	0.06228921	0.22396997	-4.36580335
WT after UVB irradiation VS WT before UVB irradiation	Degradation of GLI1 by the proteasome|Reactome	-0.156621	0.028436735	-0.67665828	0.51384674	0.82865045	-5.64270961
homo after UVB irradiation VS homo before UVB irradiation	Degradation of GLI2 by the proteasome|Reactome	-0.18041993	0.013503395	-0.95326672	0.36435015	0.51814121	-5.90303277
hetero after UVB irradiation VS hetero before UVB irradiation	Degradation of GLI2 by the proteasome|Reactome	0.309012761	0.039076328	1.424698879	0.19021057	0.40895273	-5.36339068
WT after UVB irradiation VS WT before UVB irradiation	Degradation of GLI2 by the proteasome|Reactome	-0.15590175	0.020545904	-0.66148388	0.52313114	0.83120196	-5.65252483
homo after UVB irradiation VS homo before UVB irradiation	Degradation of Superoxides|SMPDB	0.419047585	0.011690562	1.965309364	0.07962196	0.19933837	-4.63981344
hetero after UVB irradiation VS hetero before UVB irradiation	Degradation of Superoxides|SMPDB	0.258402514	0.043473436	0.97564999	0.35641307	0.58457386	-5.8581009
WT after UVB irradiation VS WT before UVB irradiation	Degradation of Superoxides|SMPDB	0.128235654	0.090733486	0.461067633	0.65453611	0.87935805	-5.76266447
homo after UVB irradiation VS homo before UVB irradiation	Degradation of the extracellular matrix|Reactome	0.64050682	-0.00624209	3.949055647	0.0030916	0.03143932	-1.52744408
hetero after UVB irradiation VS hetero before UVB irradiation	Degradation of the extracellular matrix|Reactome	0.497543316	0.042347376	2.842558685	0.02061991	0.12673224	-3.31162169
WT after UVB irradiation VS WT before UVB irradiation	Degradation of the extracellular matrix|Reactome	0.457385459	0.023674518	2.191951402	0.05294735	0.41470276	-3.9125707
homo after UVB irradiation VS homo before UVB irradiation	degradation of the rar and rxr by the proteasome|BioCarta	0.966638794	0.024902657	4.654879557	0.00106625	0.01874686	-0.48877678
hetero after UVB irradiation VS hetero before UVB irradiation	degradation of the rar and rxr by the proteasome|BioCarta	0.289570288	0.031434636	0.756162423	0.47016227	0.69247644	-6.0467261
WT after UVB irradiation VS WT before UVB irradiation	degradation of the rar and rxr by the proteasome|BioCarta	0.63355427	-0.03479439	2.542615517	0.02906175	0.3548797	-3.39509281
homo after UVB irradiation VS homo before UVB irradiation	Degradation pathway of sphingolipids_ including diseases|Wikipathways	0.970628657	-0.07984763	5.238742744	0.0004665	0.01666204	0.311692913
hetero after UVB irradiation VS hetero before UVB irradiation	Degradation pathway of sphingolipids_ including diseases|Wikipathways	1.093344399	0.069342924	6.4803522	0.00015291	0.02920766	1.426497013
WT after UVB irradiation VS WT before UVB irradiation	Degradation pathway of sphingolipids_ including diseases|Wikipathways	0.468643664	-0.10764129	1.308111557	0.21987253	0.62621406	-5.0735909
homo after UVB irradiation VS homo before UVB irradiation	Demo complete|Wikipathways	-0.38764358	-0.04242154	-1.58790144	0.145372	0.29099892	-5.17285175
hetero after UVB irradiation VS hetero before UVB irradiation	Demo complete|Wikipathways	-0.00312767	-0.03923389	-0.00901445	0.9930174	0.99537155	-6.34649722
WT after UVB irradiation VS WT before UVB irradiation	Demo complete|Wikipathways	-0.55109617	0.070343314	-2.1625178	0.05565547	0.41769317	-3.95516792
homo after UVB irradiation VS homo before UVB irradiation	Demo|Wikipathways	-1.00103545	0.027522366	-5.06337633	0.00059489	0.01666204	0.077077567
hetero after UVB irradiation VS hetero before UVB irradiation	Demo|Wikipathways	-1.06067946	-0.03610542	-4.37610498	0.00208514	0.05083544	-1.0705877
WT after UVB irradiation VS WT before UVB irradiation	Demo|Wikipathways	-1.12363418	-0.03951815	-4.56284025	0.00101602	0.15645713	-0.49270043
homo after UVB irradiation VS homo before UVB irradiation	Dengue-2 Interactions with Blood Clotting Cascade|Wikipathways	0.259935224	-0.00404067	1.584365452	0.14617245	0.29145127	-5.1775687
hetero after UVB irradiation VS hetero before UVB irradiation	Dengue-2 Interactions with Blood Clotting Cascade|Wikipathways	0.487660751	0.044890144	2.544842704	0.03308016	0.15838168	-3.7670685
WT after UVB irradiation VS WT before UVB irradiation	Dengue-2 Interactions with Blood Clotting Cascade|Wikipathways	0.332710338	-0.0211273	1.320691783	0.21579623	0.62581393	-5.0594774
homo after UVB irradiation VS homo before UVB irradiation	Dengue-2 Interactions with Complement and Coagulation Cascades|Wikipathways	0.608747817	0.033462111	3.316394177	0.00848582	0.05480378	-2.51317268
hetero after UVB irradiation VS hetero before UVB irradiation	Dengue-2 Interactions with Complement and Coagulation Cascades|Wikipathways	0.494758222	0.064050581	2.611767237	0.02973528	0.14964797	-3.66484673
WT after UVB irradiation VS WT before UVB irradiation	Dengue-2 Interactions with Complement and Coagulation Cascades|Wikipathways	0.36246777	-0.00492069	1.304356401	0.22110158	0.62764193	-5.07778585
homo after UVB irradiation VS homo before UVB irradiation	Depolymerisation of the Nuclear Lamina|Reactome	-0.27745659	0.035480952	-1.53445922	0.15789514	0.30587578	-5.24350099
hetero after UVB irradiation VS hetero before UVB irradiation	Depolymerisation of the Nuclear Lamina|Reactome	0.228242702	-0.03711873	1.115719521	0.295348	0.52649833	-5.71804804
WT after UVB irradiation VS WT before UVB irradiation	Depolymerisation of the Nuclear Lamina|Reactome	-0.03401542	-0.11403241	-0.13858074	0.89251036	0.97397725	-5.85943219
homo after UVB irradiation VS homo before UVB irradiation	Deposition of new CENPA-containing nucleosomes at the centromere|Reactome	-0.50190868	0.033606094	-1.86522951	0.09365265	0.21982504	-4.78626162
hetero after UVB irradiation VS hetero before UVB irradiation	Deposition of new CENPA-containing nucleosomes at the centromere|Reactome	0.183280885	-0.03869092	0.608379259	0.55897544	0.76190086	-6.15016359
WT after UVB irradiation VS WT before UVB irradiation	Deposition of new CENPA-containing nucleosomes at the centromere|Reactome	0.271286949	0.065901858	0.954443616	0.36218248	0.74374432	-5.4288652
homo after UVB irradiation VS homo before UVB irradiation	Depurination|Reactome	-0.20582373	0.015435386	-0.82269915	0.4310765	0.58181948	-6.01765082
hetero after UVB irradiation VS hetero before UVB irradiation	Depurination|Reactome	0.171991434	-0.05342382	0.515799017	0.61925362	0.80231894	-6.20450981
WT after UVB irradiation VS WT before UVB irradiation	Depurination|Reactome	0.62990354	0.108869935	1.999454535	0.07322271	0.45403763	-4.18778491
homo after UVB irradiation VS homo before UVB irradiation	Depyrimidination|Reactome	-0.78356809	-0.06735188	-4.03947359	0.00268707	0.02974154	-1.39037379
hetero after UVB irradiation VS hetero before UVB irradiation	Depyrimidination|Reactome	-0.52063106	-0.10015914	-1.97323455	0.0821024	0.25808464	-4.62132082
WT after UVB irradiation VS WT before UVB irradiation	Depyrimidination|Reactome	-0.03096487	-0.00387426	-0.10858657	0.91566112	0.97566251	-5.86318166
homo after UVB irradiation VS homo before UVB irradiation	Deregulated CDK5 triggers multiple neurodegenerative pathways in Alzheimer_s disease models|Reactome	0.257711588	-0.01036428	1.211473663	0.25532089	0.41874827	-5.6373459
hetero after UVB irradiation VS hetero before UVB irradiation	Deregulated CDK5 triggers multiple neurodegenerative pathways in Alzheimer_s disease models|Reactome	0.47642371	0.036407496	2.675724658	0.02685931	0.14252149	-3.5670143
WT after UVB irradiation VS WT before UVB irradiation	Deregulated CDK5 triggers multiple neurodegenerative pathways in Alzheimer_s disease models|Reactome	0.226971887	0.050305015	0.798911108	0.44275521	0.79860542	-5.55639984
homo after UVB irradiation VS homo before UVB irradiation	Deregulated CDK5 triggers multiple neurodegenerative pathways in Alzheimer_s disease models|Wikipathways	0.720910224	-0.08713437	2.95375419	0.01541667	0.07691241	-3.09226158
hetero after UVB irradiation VS hetero before UVB irradiation	Deregulated CDK5 triggers multiple neurodegenerative pathways in Alzheimer_s disease models|Wikipathways	0.524210362	0.048967647	1.945470272	0.08576805	0.2650378	-4.66132071
WT after UVB irradiation VS WT before UVB irradiation	Deregulated CDK5 triggers multiple neurodegenerative pathways in Alzheimer_s disease models|Wikipathways	0.550557121	-0.06331779	2.225039592	0.05005432	0.40887857	-3.86449151
homo after UVB irradiation VS homo before UVB irradiation	deregulation of cdk5 in alzheimers disease|BioCarta	-0.0492863	-0.03351113	-0.22336306	0.82803715	0.89402167	-6.34171449
hetero after UVB irradiation VS hetero before UVB irradiation	deregulation of cdk5 in alzheimers disease|BioCarta	0.273186157	0.055843359	1.195296939	0.26452589	0.49192534	-5.63234773
WT after UVB irradiation VS WT before UVB irradiation	deregulation of cdk5 in alzheimers disease|BioCarta	0.391696428	-0.0227838	1.632482183	0.13337906	0.54656243	-4.68295795
homo after UVB irradiation VS homo before UVB irradiation	Deregulation of Rab and Rab Effector Genes in Bladder Cancer|Wikipathways	-0.37257384	-0.02527818	-1.6288502	0.1363835	0.27825158	-5.11780266
hetero after UVB irradiation VS hetero before UVB irradiation	Deregulation of Rab and Rab Effector Genes in Bladder Cancer|Wikipathways	-0.16100403	0.0081103	-1.03118028	0.33114392	0.55930944	-5.80429549

WT after UVB irradiation VS WT before UVB irradiation	Deregulation of Rab and Rab Effector Genes in Bladder Cancer|Wikipathways	-0.15232936	0.041232141	-0.69837671	0.50073209	0.82142366	-5.62831132
homo after UVB irradiation VS homo before UVB irradiation	dermatan sulfate biosynthesis (late stages)|HumanCyc	0.745832655	-0.03560788	2.801581576	0.01985698	0.09021589	-3.33607831
hetero after UVB irradiation VS hetero before UVB irradiation	dermatan sulfate biosynthesis (late stages)|HumanCyc	0.607250225	0.062980779	1.783686945	0.11041923	0.30216567	-4.89009522
WT after UVB irradiation VS WT before UVB irradiation	dermatan sulfate biosynthesis (late stages)|HumanCyc	0.689888661	0.054745138	2.426317605	0.03548945	0.38724228	-3.56835504
homo after UVB irradiation VS homo before UVB irradiation	dermatan sulfate biosynthesis|HumanCyc	0.404959845	-0.00492938	2.332274499	0.04345994	0.13517581	-4.08050523
hetero after UVB irradiation VS hetero before UVB irradiation	dermatan sulfate biosynthesis|HumanCyc	0.408305428	0.050407223	1.804061025	0.10698292	0.29559041	-4.86172576
WT after UVB irradiation VS WT before UVB irradiation	dermatan sulfate biosynthesis|HumanCyc	0.40299573	-0.02883776	1.476619954	0.17031613	0.5778854	-4.87726309
homo after UVB irradiation VS homo before UVB irradiation	Dermatan sulfate biosynthesis|Reactome	0.545596454	0.031250757	2.876859895	0.01751766	0.08340524	-3.21548617
hetero after UVB irradiation VS hetero before UVB irradiation	Dermatan sulfate biosynthesis|Reactome	0.143518272	0.016073267	0.528839397	0.610561	0.7972333	-6.19735844
WT after UVB irradiation VS WT before UVB irradiation	Dermatan sulfate biosynthesis|Reactome	0.308093599	-0.01226969	1.399592669	0.19164357	0.60496032	-4.96891365
homo after UVB irradiation VS homo before UVB irradiation	dermatan sulfate degradation (metazoa)|HumanCyc	0.30055058	0.04165786	1.815417316	0.10146814	0.23176764	-4.85791097
hetero after UVB irradiation VS hetero before UVB irradiation	dermatan sulfate degradation (metazoa)|HumanCyc	0.147032162	-0.12333371	0.577991374	0.57838594	0.7737843	-6.16891324
WT after UVB irradiation VS WT before UVB irradiation	dermatan sulfate degradation (metazoa)|HumanCyc	0.09175477	-0.04621468	0.331081227	0.74736149	0.9185045	-5.81394392
homo after UVB irradiation VS homo before UVB irradiation	Desipramine Action Pathway|SMPDB	0.191132361	0.029768717	1.061313976	0.31508276	0.46923284	-5.79783739
hetero after UVB irradiation VS hetero before UVB irradiation	Desipramine Action Pathway|SMPDB	-0.07904315	0.055005373	-0.44437398	0.66796482	0.82651398	-6.24068873
WT after UVB irradiation VS WT before UVB irradiation	Desipramine Action Pathway|SMPDB	0.022316377	0.035038618	0.098344171	0.92358676	0.97566251	-5.86425414
homo after UVB irradiation VS homo before UVB irradiation	Desipramine Metabolism Pathway|SMPDB	0.197169582	-0.08331033	0.736198587	0.47959803	0.62211494	-6.08556231
hetero after UVB irradiation VS hetero before UVB irradiation	Desipramine Metabolism Pathway|SMPDB	-0.1211451	-0.08865763	-0.39070534	0.70570842	0.85253617	-6.26449241
WT after UVB irradiation VS WT before UVB irradiation	Desipramine Metabolism Pathway|SMPDB	-0.33173712	-0.01126547	-0.94436865	0.3670619	0.74581032	-5.43769949
homo after UVB irradiation VS homo before UVB irradiation	Desmosterolosis|SMPDB	-0.19230642	-0.01987665	-0.61925406	0.55047761	0.67913558	-6.16661869
hetero after UVB irradiation VS hetero before UVB irradiation	Desmosterolosis|SMPDB	0.185820603	0.008098609	0.631890782	0.54421801	0.75147406	-6.13505553
WT after UVB irradiation VS WT before UVB irradiation	Desmosterolosis|SMPDB	-0.14894747	0.045069977	-0.50755372	0.62268399	0.87257466	-5.74041409
homo after UVB irradiation VS homo before UVB irradiation	Detoxification of Reactive Oxygen Species|Reactome	0.581942358	-0.03455714	2.712725289	0.02302938	0.09929382	-3.47823689
hetero after UVB irradiation VS hetero before UVB irradiation	Detoxification of Reactive Oxygen Species|Reactome	0.564300689	0.020703721	2.992701903	0.01628062	0.11352943	-3.08219275
WT after UVB irradiation VS WT before UVB irradiation	Detoxification of Reactive Oxygen Species|Reactome	0.500777808	-0.02340507	1.943755292	0.08034151	0.46563411	-4.26573476
homo after UVB irradiation VS homo before UVB irradiation	Deubiquitination|Reactome	-0.2351763	-0.00803425	-1.29239277	0.22711982	0.3866626	-5.54451296
hetero after UVB irradiation VS hetero before UVB irradiation	Deubiquitination|Reactome	-0.04050189	-0.0401922	-0.21710081	0.83329475	0.93635724	-6.32104535
WT after UVB irradiation VS WT before UVB irradiation	Deubiquitination|Reactome	-0.11277338	-0.01864905	-0.47225308	0.64680191	0.87935805	-5.75749637
homo after UVB irradiation VS homo before UVB irradiation	Development and heterogeneity of the ILC family|Wikipathways	0.251134449	0.026823212	1.554404766	0.15311371	0.29992158	-5.21729596
hetero after UVB irradiation VS hetero before UVB irradiation	Development and heterogeneity of the ILC family|Wikipathways	0.154350274	0.034243987	0.868134303	0.40936413	0.63634069	-5.95546659
WT after UVB irradiation VS WT before UVB irradiation	Development and heterogeneity of the ILC family|Wikipathways	0.025020562	-0.01327661	0.094186286	0.92680672	0.97640745	-5.86465928
homo after UVB irradiation VS homo before UVB irradiation	Development of pulmonary dendritic cells and macrophage subsets|Wikipathways	0.225855008	-0.02743876	1.122345434	0.28959534	0.44899569	-5.7345333
hetero after UVB irradiation VS hetero before UVB irradiation	Development of pulmonary dendritic cells and macrophage subsets|Wikipathways	0.259839268	-0.01561478	1.046372498	0.32447492	0.55421891	-5.78917506
WT after UVB irradiation VS WT before UVB irradiation	Development of pulmonary dendritic cells and macrophage subsets|Wikipathways	0.243524957	-0.04330863	0.727017958	0.48375384	0.81670462	-5.60869914
homo after UVB irradiation VS homo before UVB irradiation	Developmental Biology|Reactome	0.151603355	-0.0034908	0.875718333	0.40302979	0.55512338	-5.97282029
hetero after UVB irradiation VS hetero before UVB irradiation	Developmental Biology|Reactome	0.112955695	0.020140108	0.744651441	0.47673557	0.69847661	-6.05549341
WT after UVB irradiation VS WT before UVB irradiation	Developmental Biology|Reactome	-0.02077624	0.016070653	-0.10635478	0.9173873	0.97566251	-5.86342438
homo after UVB irradiation VS homo before UVB irradiation	DEx/H-box helicases activate type I IFN and inflammatory cytokines production|Reactome	0.503142914	-0.04253816	2.806860315	0.01968306	0.08963384	-3.32762538
hetero after UVB irradiation VS hetero before UVB irradiation	DEx/H-box helicases activate type I IFN and inflammatory cytokines production|Reactome	0.569514753	0.049257292	3.243882753	0.01101448	0.10415769	-2.70100571
WT after UVB irradiation VS WT before UVB irradiation	DEx/H-box helicases activate type I IFN and inflammatory cytokines production|Reactome	0.428467425	0.009380194	1.710617213	0.11769169	0.52190644	-4.58155123
homo after UVB irradiation VS homo before UVB irradiation	Dezocine Action Pathway|SMPDB	0.191132361	0.029768717	1.061313976	0.31508276	0.46923284	-5.79783739
hetero after UVB irradiation VS hetero before UVB irradiation	Dezocine Action Pathway|SMPDB	-0.07904315	0.055005373	-0.44437398	0.66796482	0.82651398	-6.24068873
WT after UVB irradiation VS WT before UVB irradiation	Dezocine Action Pathway|SMPDB	0.022316377	0.035038618	0.098344171	0.92358676	0.97566251	-5.86425414
homo after UVB irradiation VS homo before UVB irradiation	D-galactose degradation V (Leloir pathway)|HumanCyc	0.59540819	0.006200978	3.007758298	0.01409643	0.07305822	-3.00574298
hetero after UVB irradiation VS hetero before UVB irradiation	D-galactose degradation V (Leloir pathway)|HumanCyc	0.565867757	0.044485449	2.491570859	0.03601264	0.16681566	-3.84827627
WT after UVB irradiation VS WT before UVB irradiation	D-galactose degradation V (Leloir pathway)|HumanCyc	0.442529323	0.037928395	1.758819534	0.1088652	0.5076251	-4.51779575
homo after UVB irradiation VS homo before UVB irradiation	D-glucuronate degradation|HumanCyc	-0.24765061	-0.07649967	-0.68445963	0.51022527	0.64930004	-6.12298167
hetero after UVB irradiation VS hetero before UVB irradiation	D-glucuronate degradation|HumanCyc	-0.36272443	0.011403622	-0.96580458	0.36104043	0.58927951	-5.86739609
WT after UVB irradiation VS WT before UVB irradiation	D-glucuronate degradation|HumanCyc	-0.03500099	0.10252401	-0.1016409	0.92103473	0.97566251	-5.8639205
homo after UVB irradiation VS homo before UVB irradiation	D-Glutamine and D-glutamate metabolism - Homo sapiens (human)|KEGG	0.702909317	-0.13339635	3.624014004	0.00516328	0.04133911	-2.02865333
hetero after UVB irradiation VS hetero before UVB irradiation	D-Glutamine and D-glutamate metabolism - Homo sapiens (human)|KEGG	0.562607842	0.003737026	2.152472218	0.06182707	0.22312571	-4.35885654
WT after UVB irradiation VS WT before UVB irradiation	D-Glutamine and D-glutamate metabolism - Homo sapiens (human)|KEGG	0.608497955	-0.01616951	2.060652029	0.06608821	0.43803373	-4.10120664
homo after UVB irradiation VS homo before UVB irradiation	D-glyceric acidura|SMPDB	0.512939135	0.00160955	2.795906579	0.02004569	0.09043427	-3.34516503
hetero after UVB irradiation VS hetero before UVB irradiation	D-glyceric acidura|SMPDB	0.12253787	0.061964979	0.508050343	0.62444904	0.80561526	-6.20867996
WT after UVB irradiation VS WT before UVB irradiation	D-glyceric acidura|SMPDB	0.284092323	-0.00634299	1.317391439	0.21685951	0.62581393	-5.06318888
homo after UVB irradiation VS homo before UVB irradiation	Dibucaine Action Pathway|SMPDB	0.191132361	0.029768717	1.061313976	0.31508276	0.46923284	-5.79783739
hetero after UVB irradiation VS hetero before UVB irradiation	Dibucaine Action Pathway|SMPDB	-0.07904315	0.055005373	-0.44437398	0.66796482	0.82651398	-6.24068873
WT after UVB irradiation VS WT before UVB irradiation	Dibucaine Action Pathway|SMPDB	0.022316377	0.035038618	0.098344171	0.92358676	0.97566251	-5.86425414
homo after UVB irradiation VS homo before UVB irradiation	dicer pathway|BioCarta	-1.00439033	0.011928429	-4.48310979	0.00137261	0.02131751	-0.73466165
hetero after UVB irradiation VS hetero before UVB irradiation	dicer pathway|BioCarta	-0.12914312	0.013042668	-0.29047407	0.77847497	0.90221039	-6.3010026
WT after UVB irradiation VS WT before UVB irradiation	dicer pathway|BioCarta	-0.39762808	0.065808298	-1.02894125	0.32755934	0.71134736	-5.36118901
homo after UVB irradiation VS homo before UVB irradiation	Diclofenac Action Pathway|SMPDB	0.40423582	0.045705158	1.979500697	0.07780079	0.19626432	-4.61879689
hetero after UVB irradiation VS hetero before UVB irradiation	Diclofenac Action Pathway|SMPDB	0.482496143	0.064876536	3.04499191	0.01500113	0.1130168	-3.00250826
WT after UVB irradiation VS WT before UVB irradiation	Diclofenac Action Pathway|SMPDB	0.275725339	0.086595857	1.261970114	0.23537138	0.62952955	-5.12455679
homo after UVB irradiation VS homo before UVB irradiation	Dicoumarol Action Pathway|SMPDB	0.538210712	0.026444726	3.007777411	0.01409598	0.07305822	-3.00571236
hetero after UVB irradiation VS hetero before UVB irradiation	Dicoumarol Action Pathway|SMPDB	0.558085555	0.09913938	2.984394118	0.01649409	0.11352943	-3.09486545
WT after UVB irradiation VS WT before UVB irradiation	Dicoumarol Action Pathway|SMPDB	0.440879848	0.077377741	1.569633667	0.14732454	0.55622203	-4.76265269
homo after UVB irradiation VS homo before UVB irradiation	Dicumarol Action Pathway|SMPDB	0.538210712	0.026444726	3.007777411	0.01409598	0.07305822	-3.00571236
hetero after UVB irradiation VS hetero before UVB irradiation	Dicumarol Action Pathway|SMPDB	0.558085555	0.09913938	2.984394118	0.01649409	0.11352943	-3.09486545
WT after UVB irradiation VS WT before UVB irradiation	Dicumarol Action Pathway|SMPDB	0.440879848	0.077377741	1.569633667	0.14732454	0.55622203	-4.76265269
homo after UVB irradiation VS homo before UVB irradiation	Differentiation of white and brown adipocyte|Wikipathways	0.17688408	-0.0021533	0.962451907	0.35995318	0.51521825	-5.89444403
hetero after UVB irradiation VS hetero before UVB irradiation	Differentiation of white and brown adipocyte|Wikipathways	0.02838861	-0.0364157	0.113529958	0.91226904	0.97054702	-6.33955454
WT after UVB irradiation VS WT before UVB irradiation	Differentiation of white and brown adipocyte|Wikipathways	-0.16080394	-0.0209445	-0.72039686	0.4876465	0.81710765	-5.61329566
homo after UVB irradiation VS homo before UVB irradiation	Differentiation Pathway|Wikipathways	-0.00729331	0.056995316	-0.04157069	0.96771157	0.9797275	-6.36751982
hetero after UVB irradiation VS hetero before UVB irradiation	Differentiation Pathway|Wikipathways	-0.07769057	0.034950426	-0.42979443	0.67812581	0.833126	-6.2474449
WT after UVB irradiation VS WT before UVB irradiation	Differentiation Pathway|Wikipathways	0.014668902	0.034264025	0.065466059	0.94908285	0.98577698	-5.86698052
homo after UVB irradiation VS homo before UVB irradiation	Diflunisal Action Pathway|SMPDB	0.40423582	0.045705158	1.979500697	0.07780079	0.19626432	-4.61879689
hetero after UVB irradiation VS hetero before UVB irradiation	Diflunisal Action Pathway|SMPDB	0.482496143	0.064876536	3.04499191	0.01500113	0.1130168	-3.00250826
WT after UVB irradiation VS WT before UVB irradiation	Diflunisal Action Pathway|SMPDB	0.275725339	0.086595857	1.261970114	0.23537138	0.62952955	-5.12455679
homo after UVB irradiation VS homo before UVB irradiation	Digestion and absorption|Reactome	0.289089672	0.085940071	1.594434958	0.14390325	0.28881857	-5.16412063
hetero after UVB irradiation VS hetero before UVB irradiation	Digestion and absorption|Reactome	0.102115105	0.079948956	0.539559342	0.60346355	0.79129581	-6.19135481
WT after UVB irradiation VS WT before UVB irradiation	Digestion and absorption|Reactome	0.036109052	0.164743886	0.175723293	0.86399056	0.96034647	-5.85353396
homo after UVB irradiation VS homo before UVB irradiation	Digestion of dietary carbohydrate|Reactome	-0.17854922	0.180439671	-0.68315716	0.51101145	0.65006375	-6.12389206
hetero after UVB irradiation VS hetero before UVB irradiation	Digestion of dietary carbohydrate|Reactome	0.127087869	0.085192517	0.448181056	0.66532327	0.82651398	-6.23888916
WT after UVB irradiation VS WT before UVB irradiation	Digestion of dietary carbohydrate|Reactome	-0.03603862	0.14383499	-0.13289964	0.89688786	0.97566251	-5.86021198
homo after UVB irradiation VS homo before UVB irradiation	Digestion of dietary lipid|Reactome	0.454826817	0.286500555	1.749027649	0.11282486	0.24815504	-4.95199691
hetero after UVB irradiation VS hetero before UVB irradiation	Digestion of dietary lipid|Reactome	-0.34621523	0.01633338	-1.05008918	0.32285926	0.55364654	-5.78545025
WT after UVB irradiation VS WT before UVB irradiation	Digestion of dietary lipid|Reactome	0.124563162	0.218775228	0.4717631	0.6471398	0.87935805	-5.75772523
homo after UVB irradiation VS homo before UVB irradiation	Digestion|Reactome	0.052347643	0.177945113	0.268548707	0.79408458	0.87074692	-6.32985089
hetero after UVB irradiation VS hetero before UVB irradiation	Digestion|Reactome	-0.05359644	0.104308075	-0.27257146	0.7917438	0.91160777	-6.30641841
WT after UVB irradiation VS WT before UVB irradiation	Digestion|Reactome	-0.02199137	0.194595409	-0.10656153	0.91722737	0.97566251	-5.8634021
homo after UVB irradiation VS homo before UVB irradiation	Dihydromorphine Action Pathway|SMPDB	0.191132361	0.029768717	1.061313976	0.31508276	0.46923284	-5.79783739
hetero after UVB irradiation VS hetero before UVB irradiation	Dihydromorphine Action Pathway|SMPDB	-0.07904315	0.055005373	-0.44437398	0.66796482	0.82651398	-6.24068873
WT after UVB irradiation VS WT before UVB irradiation	Dihydromorphine Action Pathway|SMPDB	0.022316377	0.035038618	0.098344171	0.92358676	0.97566251	-5.86425414
homo after UVB irradiation VS homo before UVB irradiation	Dihydropyrimidinase Deficiency|SMPDB	-0.15230025	-0.02916717	-0.66373722	0.52282067	0.65944729	-6.13727985
hetero after UVB irradiation VS hetero before UVB irradiation	Dihydropyrimidinase Deficiency|SMPDB	-0.07293889	-0.06090355	-0.36429729	0.72460928	0.86482318	-6.27512326
WT after UVB irradiation VS WT before UVB irradiation	Dihydropyrimidinase Deficiency|SMPDB	0.197121499	-0.04726474	0.926444646	0.37585917	0.75077508	-5.45322485
homo after UVB irradiation VS homo before UVB irradiation	Dihydropyrimidine Dehydrogenase Deficiency (DHPD)|SMPDB	-0.11801782	0.021953564	-0.50783827	0.62328187	0.73728109	-6.23179983
hetero after UVB irradiation VS hetero before UVB irradiation	Dihydropyrimidine Dehydrogenase Deficiency (DHPD)|SMPDB	-0.03882851	0.032366748	-0.2244119	0.82778335	0.93322694	-6.31930458
WT after UVB irradiation VS WT before UVB irradiation	Dihydropyrimidine Dehydrogenase Deficiency (DHPD)|SMPDB	0.143497804	-0.01134274	0.603206807	0.55969543	0.84911968	-5.68832383
homo after UVB irradiation VS homo before UVB irradiation	Dilated cardiomyopathy (DCM) - Homo sapiens (human)|KEGG	0.563886477	-0.01178476	3.478002242	0.00652809	0.04701822	-2.257585
hetero after UVB irradiation VS hetero before UVB irradiation	Dilated cardiomyopathy (DCM) - Homo sapiens (human)|KEGG	0.311953527	0.046472468	1.456270324	0.18154507	0.39994316	-5.32408943
WT after UVB irradiation VS WT before UVB irradiation	Dilated cardiomyopathy (DCM) - Homo sapiens (human)|KEGG	0.339360502	0.003131811	1.282919365	0.22822694	0.62952955	-5.10157446
homo after UVB irradiation VS homo before UVB irradiation	Diltiazem Action Pathway|SMPDB	0.396415397	-0.03677376	2.343069613	0.04268583	0.13402181	-4.06364635
hetero after UVB irradiation VS hetero before UVB irradiation	Diltiazem Action Pathway|SMPDB	-0.01555055	-0.01775524	-0.07410913	0.94265284	0.97054702	-6.34356282
WT after UVB irradiation VS WT before UVB irradiation	Diltiazem Action Pathway|SMPDB	0.056685205	-0.03978418	0.223404833	0.82768199	0.94525798	-5.84393438
homo after UVB irradiation VS homo before UVB irradiation	Dimerization of procaspase-8|Reactome	0.849614473	-0.03215571	4.542786992	0.00125669	0.02014127	-0.64871182
hetero after UVB irradiation VS hetero before UVB irradiation	Dimerization of procaspase-8|Reactome	0.683693345	-0.01188812	2.738727154	0.02430288	0.13715811	-3.47057017
WT after UVB irradiation VS WT before UVB irradiation	Dimerization of procaspase-8|Reactome	0.733182815	-0.06921519	3.457990495	0.00607019	0.21490368	-2.02721426
homo after UVB irradiation VS homo before UVB irradiation	Dimethyl-branched-chain fatty acid mitochondrial beta-oxidation|EHMN	-0.30135347	-0.05206463	-0.9219175	0.37965105	0.53288862	-5.93183623
hetero after UVB irradiation VS hetero before UVB irradiation	Dimethyl-branched-chain fatty acid mitochondrial beta-oxidation|EHMN	-0.68618797	0.041027845	-2.73733373	0.02435664	0.13715811	-3.47270358
WT after UVB irradiation VS WT before UVB irradiation	Dimethyl-branched-chain fatty acid mitochondrial beta-oxidation|EHMN	-0.58548956	-0.00760239	-2.1626325	0.05564467	0.41769317	-3.95500225
homo after UVB irradiation VS homo before UVB irradiation	Dimethylglycine Dehydrogenase Deficiency|SMPDB	-0.11801782	0.021953564	-0.50783827	0.62328187	0.73728109	-6.23179983
hetero after UVB irradiation VS hetero before UVB irradiation	Dimethylglycine Dehydrogenase Deficiency|SMPDB	-0.11801782	0.021953564	-0.50783827	0.62328187	0.73728109	-6.23179983
WT after UVB irradiation VS WT before UVB irradiation	Dimethylglycine Dehydrogenase Deficiency|SMPDB	-0.03882851	0.032366748	-0.2244119	0.82778335	0.93322694	-6.31930458
homo after UVB irradiation VS homo before UVB irradiation	Dimethylglycine Dehydrogenase Deficiency|SMPDB	-0.03882851	0.032366748	-0.2244119	0.82778335	0.93322694	-6.31930458
hetero after UVB irradiation VS hetero before UVB irradiation	Dimethylglycine Dehydrogenase Deficiency|SMPDB	0.143497804	-0.01134274	0.603206807	0.55969543	0.84911968	-5.68832383
WT after UVB irradiation VS WT before UVB irradiation	Dimethylglycine Dehydrogenase Deficiency|SMPDB	0.143497804	-0.01134274	0.603206807	0.55969543	0.84911968	-5.68832383
homo after UVB irradiation VS homo before UVB irradiation	Dimethylthiambutene Action Pathway|SMPDB	0.191132361	0.029768717	1.061313976	0.31508276	0.46923284	-5.79783739
hetero after UVB irradiation VS hetero before UVB irradiation	Dimethylthiambutene Action Pathway|SMPDB	-0.07904315	0.055005373	-0.44437398	0.66796482	0.82651398	-6.24068873
WT after UVB irradiation VS WT before UVB irradiation	Dimethylthiambutene Action Pathway|SMPDB	0.022316377	0.035038618	0.098344171	0.92358676	0.97566251	-5.86425414
homo after UVB irradiation VS homo before UVB irradiation	Diphenoxylate Action Pathway|SMPDB	0.191132361	0.029768717	1.061313976	0.31508276	0.46923284	-5.79783739
hetero after UVB irradiation VS hetero before UVB irradiation	Diphenoxylate Action Pathway|SMPDB	-0.07904315	0.055005373	-0.44437398	0.66796482	0.82651398	-6.24068873
WT after UVB irradiation VS WT before UVB irradiation	Diphenoxylate Action Pathway|SMPDB	0.022316377	0.035038618	0.098344171	0.92358676	0.97566251	-5.86425414
homo after UVB irradiation VS homo before UVB irradiation	diphthamide biosynthesis|HumanCyc	-0.87383805	0.096244433	-3.46350425	0.00668277	0.04730781	-2.28042613
hetero after UVB irradiation VS hetero before UVB irradiation	diphthamide biosynthesis|HumanCyc	-0.46930883	-0.03612039	-1.86623148	0.09710761	0.28138365	-4.77434113
WT after UVB irradiation VS WT before UVB irradiation	diphthamide biosynthesis|HumanCyc	-0.18397457	0.078771816	-0.43288452	0.67421253	0.88126177	-5.7751602
homo after UVB irradiation VS homo before UVB irradiation	Direct p53 effectors|PID	-0.00805417	0.001655981	-0.04222177	0.96720619	0.97959824	-6.36749051
hetero after UVB irradiation VS hetero before UVB irradiation	Direct p53 effectors|PID	0.073334824	0.008542298	0.444286353	0.66802567	0.82651398	-6.24072998
WT after UVB irradiation VS WT before UVB irradiation	Direct p53 effectors|PID	-0.09012753	0.016784557	-0.44957272	0.66252913	0.87935805	-5.76785217
homo after UVB irradiation VS homo before UVB irradiation	Disassembly of the destruction complex and recruitment of AXIN to the membrane|Reactome	-0.43799524	0.023546535	-2.42637327	0.03715163	0.126457	-3.93298269
hetero after UVB irradiation VS hetero before UVB irradiation	Disassembly of the destruction complex and recruitment of AXIN to the membrane|Reactome	-0.33579892	-0.02697037	-1.69737722	0.12614809	0.31934829	-5.00868753
WT after UVB irradiation VS WT before UVB irradiation	Disassembly of the destruction complex and recruitment of AXIN to the membrane|Reactome	-0.36078656	0.062207601	-1.37465474	0.19901827	0.61083093	-4.99791192
homo after UVB irradiation VS homo before UVB irradiation	Disease|Reactome	-0.08702767	-0.00031872	-0.49098173	0.63471113	0.7464505	-6.24060538
hetero after UVB irradiation VS hetero before UVB irradiation	Disease|Reactome	0.096607943	-0.01062925	0.595286003	0.56729277	0.76437184	-6.15835027
WT after UVB irradiation VS WT before UVB irradiation	Disease|Reactome	0.021631997	-0.00448291	0.098443048	0.92351021	0.97566251	-5.8642443
homo after UVB irradiation VS homo before UVB irradiation	Diseases associated with visual transduction|Reactome	0.151963221	-0.00337558	0.370120082	0.71950453	0.81577771	-6.29539034
hetero after UVB irradiation VS hetero before UVB irradiation	Diseases associated with visual transduction|Reactome	-0.2697141	0.01289775	-0.64818052	0.53412945	0.74771561	-6.12428369
WT after UVB irradiation VS WT before UVB irradiation	Diseases associated with visual transduction|Reactome	0.317720968	-0.00400158	0.731626644	0.48105584	0.81552033	-5.60547755
homo after UVB irradiation VS homo before UVB irradiation	Diseases of metabolism|Reactome	0.75853214	-0.0102717	2.294747761	0.0462596	0.14003669	-4.13896681
hetero after UVB irradiation VS hetero before UVB irradiation	Diseases of metabolism|Reactome	0.084135561	0.009875163	0.197001018	0.84849606	0.94443102	-6.32553714
WT after UVB irradiation VS WT before UVB irradiation	Diseases of metabolism|Reactome	0.005390724	0.12172669	0.013005468	0.98987719	0.99676022	-5.86906551
homo after UVB irradiation VS homo before UVB irradiation	Diseases of signal transduction|Reactome	0.101626771	-0.00927896	0.577173734	0.57739744	0.7004242	-6.19264705
hetero after UVB irradiation VS hetero before UVB irradiation	Diseases of signal transduction|Reactome	0.136057979	0.004408882	0.920375177	0.38296991	0.61076867	-5.90930794
WT after UVB irradiation VS WT before UVB irradiation	Diseases of signal transduction|Reactome	0.024191704	-0.01132026	0.124187388	0.90360798	0.97566251	-5.86134465
homo after UVB irradiation VS homo before UVB irradiation	Disinhibition of SNARE formation|Reactome	0.359301981	0.006543339	1.435349425	0.18364966	0.33797985	-5.37069251
hetero after UVB irradiation VS hetero before UVB irradiation	Disinhibition of SNARE formation|Reactome	0.009147408	-0.0312679	0.038007551	0.9705668	0.98614433	-6.34575769
WT after UVB irradiation VS WT before UVB irradiation	Disinhibition of SNARE formation|Reactome	-0.42273967	-0.06121131	-1.54121421	0.15404253	0.55854784	-4.79810666

homo after UVB irradiation VS homo before UVB irradiation	Disopyramide Action Pathway|SMPDB	0.396415397	-0.03677376	2.343069613	0.04268583	0.13402181	-4.06364635
hetero after UVB irradiation VS hetero before UVB irradiation	Disopyramide Action Pathway|SMPDB	-0.01555055	-0.01775524	-0.07410913	0.94265284	0.97054702	-6.34356282
WT after UVB irradiation VS WT before UVB irradiation	Disopyramide Action Pathway|SMPDB	0.056685205	-0.03978418	0.223404833	0.82768199	0.94525798	-5.84393438
homo after UVB irradiation VS homo before UVB irradiation	Disorders of Folate Metabolism and Transport|Wikipathways	-0.12133122	0.038389352	-0.31150189	0.76222497	0.84648814	-6.31658683
hetero after UVB irradiation VS hetero before UVB irradiation	Disorders of Folate Metabolism and Transport|Wikipathways	-0.07274637	-0.08544769	-0.23702765	0.81829689	0.92781025	-6.31616693
WT after UVB irradiation VS WT before UVB irradiation	Disorders of Folate Metabolism and Transport|Wikipathways	0.631735338	-0.19253053	2.058883105	0.06628486	0.43803373	-4.1037221
homo after UVB irradiation VS homo before UVB irradiation	Disorders of the Krebs cycle|Wikipathways	-0.58296057	-0.03696571	-1.5375332	0.15714975	0.30456101	-5.23947514
hetero after UVB irradiation VS hetero before UVB irradiation	Disorders of the Krebs cycle|Wikipathways	-0.95737325	-0.03823779	-3.49147815	0.00754384	0.08807784	-2.33045866
WT after UVB irradiation VS WT before UVB irradiation	Disorders of the Krebs cycle|Wikipathways	0.194605129	-0.07542867	0.520380278	0.61403317	0.87257466	-5.73391858
homo after UVB irradiation VS homo before UVB irradiation	Disorders of transmembrane transporters|Reactome	0.097181515	-0.04093626	0.424768631	0.68057389	0.7845242	-6.2724491
hetero after UVB irradiation VS hetero before UVB irradiation	Disorders of transmembrane transporters|Reactome	0.280255404	0.045519023	1.501738893	0.16967558	0.38622363	-5.26661473
WT after UVB irradiation VS WT before UVB irradiation	Disorders of transmembrane transporters|Reactome	-0.01049528	0.005990406	-0.04801053	0.9626456	0.98815178	-5.86798363
homo after UVB irradiation VS homo before UVB irradiation	Displacement of DNA glycosylase by APEX1|Reactome	-0.70694566	-0.02915433	-3.42679931	0.00709168	0.04876599	-2.33833526
hetero after UVB irradiation VS hetero before UVB irradiation	Displacement of DNA glycosylase by APEX1|Reactome	-0.27210228	-0.04978846	-1.15799164	0.27863454	0.5094329	-5.67304995
WT after UVB irradiation VS WT before UVB irradiation	Displacement of DNA glycosylase by APEX1|Reactome	0.048099904	0.014094176	0.192279088	0.85134127	0.95502804	-5.85045864
homo after UVB irradiation VS homo before UVB irradiation	Dissolution of Fibrin Clot|Reactome	0.278954004	0.023617904	1.419889896	0.187979	0.34387505	-5.39005838
hetero after UVB irradiation VS hetero before UVB irradiation	Dissolution of Fibrin Clot|Reactome	0.561610115	0.060080911	1.934580659	0.08724807	0.26707636	-4.6769549
WT after UVB irradiation VS WT before UVB irradiation	Dissolution of Fibrin Clot|Reactome	0.326698299	-0.03162967	1.200786781	0.25728493	0.65181051	-5.19013489
homo after UVB irradiation VS homo before UVB irradiation	Disulfiram Action Pathway|SMPDB	0.078405823	0.020830426	0.301680776	0.76947074	0.850703	-6.31978956
hetero after UVB irradiation VS hetero before UVB irradiation	Disulfiram Action Pathway|SMPDB	-0.4346031	0.015861715	-1.85104646	0.09943708	0.28344855	-4.79579451
WT after UVB irradiation VS WT before UVB irradiation	Disulfiram Action Pathway|SMPDB	0.031467304	0.002575672	0.119274312	0.90740119	0.97566251	-5.86194965
homo after UVB irradiation VS homo before UVB irradiation	Di-unsaturated fatty acid beta-oxidation|EHMN	-0.37771187	-0.00031125	-1.38167967	0.19905703	0.35545342	-5.43734787
hetero after UVB irradiation VS hetero before UVB irradiation	Di-unsaturated fatty acid beta-oxidation|EHMN	-0.5738755	-0.04751084	-2.75933678	0.02352168	0.13628011	-3.43901535
WT after UVB irradiation VS WT before UVB irradiation	Di-unsaturated fatty acid beta-oxidation|EHMN	-0.35836996	-0.04269642	-1.41575533	0.18698872	0.59994616	-4.94993999
homo after UVB irradiation VS homo before UVB irradiation	Diuretics Pathway_ Pharmacodynamics|PharmGKB	-0.01457018	0.01671334	-0.07219767	0.94395923	0.96574453	-6.36564835
hetero after UVB irradiation VS hetero before UVB irradiation	Diuretics Pathway_ Pharmacodynamics|PharmGKB	-0.13711756	0.007422061	-0.7543496	0.47119353	0.69305602	-6.0481146
WT after UVB irradiation VS WT before UVB irradiation	Diuretics Pathway_ Pharmacodynamics|PharmGKB	-0.2289082	0.004395844	-1.16397548	0.27123874	0.66327566	-5.22844716
homo after UVB irradiation VS homo before UVB irradiation	D-mannose degradation|HumanCyc	-0.69553512	-0.01633946	-1.91132667	0.08692396	0.21040556	-4.71920319
hetero after UVB irradiation VS hetero before UVB irradiation	D-mannose degradation|HumanCyc	0.068484359	-0.00921268	0.157085461	0.87887664	0.95629499	-6.3331749
WT after UVB irradiation VS WT before UVB irradiation	D-mannose degradation|HumanCyc	1.114179677	0.014140283	4.4819479	0.00115181	0.15645713	-0.59832473
homo after UVB irradiation VS homo before UVB irradiation	DNA Damage Bypass|Reactome	-0.26969985	0.00804939	-1.41158256	0.19034147	0.34647455	-5.40040992
hetero after UVB irradiation VS hetero before UVB irradiation	DNA Damage Bypass|Reactome	0.195161322	-0.02258667	0.996190033	0.34690226	0.5755723	-5.8384698
WT after UVB irradiation VS WT before UVB irradiation	DNA Damage Bypass|Reactome	0.071035086	0.012634206	0.293470589	0.77511457	0.93442888	-5.82572041
homo after UVB irradiation VS homo before UVB irradiation	DNA Damage Recognition in GG-NER|Reactome	-0.51366596	-0.00066258	-2.95324715	0.01542965	0.07691241	-3.09307406
hetero after UVB irradiation VS hetero before UVB irradiation	DNA Damage Recognition in GG-NER|Reactome	-0.11114841	-0.02637008	-0.60396923	0.561769	0.76386225	-6.15293919
WT after UVB irradiation VS WT before UVB irradiation	DNA Damage Recognition in GG-NER|Reactome	-0.09584603	0.023051519	-0.35909327	0.72693173	0.91030908	-5.80427326
homo after UVB irradiation VS homo before UVB irradiation	DNA Damage Response (only ATM dependent)|Wikipathways	0.009019869	-0.00856981	0.050778682	0.96056579	0.9766073	-6.36706303
hetero after UVB irradiation VS hetero before UVB irradiation	DNA Damage Response (only ATM dependent)|Wikipathways	0.004063996	-0.01027944	0.02588377	0.97995261	0.99027303	-6.34617786
WT after UVB irradiation VS WT before UVB irradiation	DNA Damage Response (only ATM dependent)|Wikipathways	0.000158119	0.034774552	0.000813005	0.99936718	0.99958605	-5.86915086
homo after UVB irradiation VS homo before UVB irradiation	DNA Damage Response|Wikipathways	-0.41274707	-0.00938618	-2.29551885	0.04620033	0.14003669	-4.13776789
hetero after UVB irradiation VS hetero before UVB irradiation	DNA Damage Response|Wikipathways	0.092852129	-0.03601582	0.414707109	0.6887143	0.84227523	-6.2542094
WT after UVB irradiation VS WT before UVB irradiation	DNA Damage Response|Wikipathways	-0.03732476	-0.04055935	-0.15154532	0.88253505	0.96830103	-5.85753098
homo after UVB irradiation VS homo before UVB irradiation	DNA Damage Reversal|Reactome	0.087420945	-0.0592878	0.487583226	0.63702791	0.74869226	-6.24234657
hetero after UVB irradiation VS hetero before UVB irradiation	DNA Damage Reversal|Reactome	0.420197489	0.049990409	1.95069414	0.0850666	0.26412369	-4.65380979
WT after UVB irradiation VS WT before UVB irradiation	DNA Damage Reversal|Reactome	0.354584029	0.001136126	1.590255645	0.1426127	0.55622203	-4.73669544
homo after UVB irradiation VS homo before UVB irradiation	DNA Damage/Telomere Stress Induced Senescence|Reactome	-0.78432504	-0.02277471	-4.08294026	0.00251287	0.02882851	-1.32487407
hetero after UVB irradiation VS hetero before UVB irradiation	DNA Damage/Telomere Stress Induced Senescence|Reactome	-0.27003767	-0.04013029	-0.99321436	0.34826815	0.57744861	-5.84133368
WT after UVB irradiation VS WT before UVB irradiation	DNA Damage/Telomere Stress Induced Senescence|Reactome	-0.35408184	-0.01187625	-1.14099942	0.28024727	0.66921967	-5.25191101
homo after UVB irradiation VS homo before UVB irradiation	DNA Damage-Telomere Stress Induced Senescence|Wikipathways	-0.5643224	-0.01497266	-1.48649716	0.16993482	0.32049666	-5.30569503
hetero after UVB irradiation VS hetero before UVB irradiation	DNA Damage-Telomere Stress Induced Senescence|Wikipathways	-0.20197503	-0.03132311	-0.48348632	0.64106496	0.81779292	-6.22150708
WT after UVB irradiation VS WT before UVB irradiation	DNA Damage-Telomere Stress Induced Senescence|Wikipathways	-1.094234	-0.03588978	-4.37290589	0.00136599	0.15645713	-0.74252629
homo after UVB irradiation VS homo before UVB irradiation	DNA Double Strand Break Response|Reactome	-0.55730122	0.030039204	-3.18141342	0.01058459	0.0621477	-2.72804971
hetero after UVB irradiation VS hetero before UVB irradiation	DNA Double Strand Break Response|Reactome	-0.13823568	-0.03374409	-0.63160134	0.54439828	0.75147406	-6.13524468
WT after UVB irradiation VS WT before UVB irradiation	DNA Double Strand Break Response|Reactome	-0.27902294	-0.03159594	-1.18046971	0.26491387	0.66112659	-5.21138885
homo after UVB irradiation VS homo before UVB irradiation	DNA Double-Strand Break Repair|Reactome	-0.51778018	-0.01279611	-2.82568796	0.01907523	0.08811647	-3.29747139
hetero after UVB irradiation VS hetero before UVB irradiation	DNA Double-Strand Break Repair|Reactome	0.002725171	-0.02083699	0.011189159	0.99133294	0.9947197	-6.34647338
WT after UVB irradiation VS WT before UVB irradiation	DNA Double-Strand Break Repair|Reactome	-0.07431623	-0.00074541	-0.28351495	0.78251867	0.93661609	-5.82860473
homo after UVB irradiation VS homo before UVB irradiation	DNA IR-damage and cellular response via ATR|Wikipathways	-0.77456097	0.010665176	-2.3726533	0.04063322	0.13402181	-4.01735517
hetero after UVB irradiation VS hetero before UVB irradiation	DNA IR-damage and cellular response via ATR|Wikipathways	-0.40407428	-0.01896363	-1.02855253	0.33230803	0.56036256	-5.80689361
WT after UVB irradiation VS WT before UVB irradiation	DNA IR-damage and cellular response via ATR|Wikipathways	-0.40318467	0.056934337	-1.00995197	0.33614175	0.71971842	-5.37882771
homo after UVB irradiation VS homo before UVB irradiation	DNA IR-Double Strand Breaks (DSBs) and cellular response via ATM|Wikipathways	-0.39253139	-0.00993026	-1.20203354	0.25878902	0.42261469	-5.64789687
hetero after UVB irradiation VS hetero before UVB irradiation	DNA IR-Double Strand Breaks (DSBs) and cellular response via ATM|Wikipathways	0.211866073	-0.11098467	0.480236347	0.64327971	0.81947147	-6.22315928
WT after UVB irradiation VS WT before UVB irradiation	DNA IR-Double Strand Breaks (DSBs) and cellular response via ATM|Wikipathways	-0.28904799	-0.0700875	-0.78410381	0.45100785	0.80616896	-5.5675257
homo after UVB irradiation VS homo before UVB irradiation	DNA methylation|Reactome	-0.36370871	0.082734082	-1.36279058	0.2047359	0.36219455	-5.46041567
hetero after UVB irradiation VS hetero before UVB irradiation	DNA methylation|Reactome	-0.00277902	0.043119951	-0.01250428	0.99031431	0.9943047	-6.34645648
WT after UVB irradiation VS WT before UVB irradiation	DNA methylation|Reactome	0.006692456	0.03328064	0.0302117	0.97648791	0.99265119	-5.86868882
homo after UVB irradiation VS homo before UVB irradiation	DNA Mismatch Repair|Wikipathways	-0.71710764	-0.056326	-2.55027977	0.03020808	0.11551936	-3.73707046
hetero after UVB irradiation VS hetero before UVB irradiation	DNA Mismatch Repair|Wikipathways	0.289907242	0.036042172	0.950647168	0.3682515	0.59623032	-5.88155972
WT after UVB irradiation VS WT before UVB irradiation	DNA Mismatch Repair|Wikipathways	0.134709075	0.096303697	0.403405335	0.6950726	0.8972291	-5.78741958
homo after UVB irradiation VS homo before UVB irradiation	DNA Repair|Reactome	-0.51988006	-0.01134733	-3.0594268	0.01294152	0.070683	-2.92301721
hetero after UVB irradiation VS hetero before UVB irradiation	DNA Repair|Reactome	-0.06301245	-0.03669266	-0.29140533	0.7777868	0.90207436	-6.30071165
WT after UVB irradiation VS WT before UVB irradiation	DNA Repair|Reactome	-0.06905921	0.007422789	-0.26526908	0.79614702	0.94345353	-5.83363672
homo after UVB irradiation VS homo before UVB irradiation	DNA replication - Homo sapiens (human)|KEGG	-0.47726317	-0.0359358	-1.68022776	0.12581405	0.26534271	-5.04766695
hetero after UVB irradiation VS hetero before UVB irradiation	DNA replication - Homo sapiens (human)|KEGG	0.250482248	0.012574456	0.980677669	0.3540672	0.5819117	-5.85332547
WT after UVB irradiation VS WT before UVB irradiation	DNA replication - Homo sapiens (human)|KEGG	0.208010619	0.043640857	0.765456439	0.46154272	0.80891799	-5.58127716
homo after UVB irradiation VS homo before UVB irradiation	DNA replication initiation|Reactome	-0.40904328	-0.05035554	-1.38407172	0.19834753	0.35446372	-5.43441188
hetero after UVB irradiation VS hetero before UVB irradiation	DNA replication initiation|Reactome	0.089272505	0.009809254	0.337052249	0.74432004	0.87687018	-6.28533561
WT after UVB irradiation VS WT before UVB irradiation	DNA replication initiation|Reactome	0.251641815	-0.04278636	1.049767987	0.31833638	0.70267871	-5.34154442
homo after UVB irradiation VS homo before UVB irradiation	DNA Replication Pre-Initiation|Reactome	-0.45162445	-0.03568692	-1.59964727	0.14274103	0.28699056	-5.15714076
hetero after UVB irradiation VS hetero before UVB irradiation	DNA Replication Pre-Initiation|Reactome	0.163426575	0.017239772	0.556558774	0.59229952	0.78411304	-6.18160476
WT after UVB irradiation VS WT before UVB irradiation	DNA Replication Pre-Initiation|Reactome	0.042039874	-0.02506154	0.151945732	0.88222729	0.96830103	-5.85746957
homo after UVB irradiation VS homo before UVB irradiation	DNA Replication|Reactome	-0.441128	-0.03137175	-1.81992081	0.10073739	0.23090709	-4.85146933
hetero after UVB irradiation VS hetero before UVB irradiation	DNA Replication|Reactome	0.1957314	0.023228958	0.76998949	0.46234528	0.68643967	-6.03604048
WT after UVB irradiation VS WT before UVB irradiation	DNA Replication|Reactome	0.063939812	-0.00605097	0.240356473	0.81487062	0.94525798	-5.83997458
homo after UVB irradiation VS homo before UVB irradiation	DNA Replication|Wikipathways	-0.41957738	-0.02308691	-1.50544396	0.16508579	0.31460005	-5.28126674
hetero after UVB irradiation VS hetero before UVB irradiation	DNA Replication|Wikipathways	0.303743705	0.018716876	1.124657545	0.29174904	0.52251165	-5.70863508
WT after UVB irradiation VS WT before UVB irradiation	DNA Replication|Wikipathways	0.088820492	0.002156651	0.304009215	0.76730256	0.92902194	-5.82256075
homo after UVB irradiation VS homo before UVB irradiation	DNA strand elongation|Reactome	-0.55223084	-0.06251916	-1.93462407	0.08369845	0.20546621	-4.68505103
hetero after UVB irradiation VS hetero before UVB irradiation	DNA strand elongation|Reactome	0.263620731	-0.00290279	0.913013387	0.38661314	0.61421495	-5.91594608
WT after UVB irradiation VS WT before UVB irradiation	DNA strand elongation|Reactome	0.173770063	0.057381206	0.581373592	0.57375566	0.8562298	-5.70095095
homo after UVB irradiation VS homo before UVB irradiation	DNA-PK pathway in nonhomologous end joining|PID	-0.30368711	-0.05247248	-1.49126834	0.16870216	0.31922671	-5.29956106
hetero after UVB irradiation VS hetero before UVB irradiation	DNA-PK pathway in nonhomologous end joining|PID	-0.31035681	-0.01261161	-1.37305395	0.20516314	0.42641751	-5.42655796
WT after UVB irradiation VS WT before UVB irradiation	DNA-PK pathway in nonhomologous end joining|PID	0.193389527	0.076952385	0.867995315	0.40558332	0.7725109	-5.50212065
homo after UVB irradiation VS homo before UVB irradiation	Dobutamine Action Pathway|SMPDB	0.396415397	-0.03677376	2.343069613	0.04268583	0.13402181	-4.06364635
hetero after UVB irradiation VS hetero before UVB irradiation	Dobutamine Action Pathway|SMPDB	-0.01555055	-0.01775524	-0.07410913	0.94265284	0.97054702	-6.34356282
WT after UVB irradiation VS WT before UVB irradiation	Dobutamine Action Pathway|SMPDB	0.056685205	-0.03978418	0.223404833	0.82768199	0.94525798	-5.84393438
homo after UVB irradiation VS homo before UVB irradiation	Docetaxel Action Pathway|SMPDB	0.194768865	0.003265929	0.860816473	0.4107816	0.56240743	-5.9856613
hetero after UVB irradiation VS hetero before UVB irradiation	Docetaxel Action Pathway|SMPDB	0.237367392	0.050865229	0.815830007	0.43705048	0.66334222	-5.99942629
WT after UVB irradiation VS WT before UVB irradiation	Docetaxel Action Pathway|SMPDB	0.585929693	0.017820683	2.275475662	0.04593603	0.39565089	-3.79084056
homo after UVB irradiation VS homo before UVB irradiation	dolichol and dolichyl phosphate biosynthesis|HumanCyc	0.310438241	-0.04304443	0.855465741	0.41359005	0.56439097	-5.99022641
hetero after UVB irradiation VS hetero before UVB irradiation	dolichol and dolichyl phosphate biosynthesis|HumanCyc	0.139074825	0.037123171	0.422666833	0.68311884	0.83700422	-6.25066944
WT after UVB irradiation VS WT before UVB irradiation	dolichol and dolichyl phosphate biosynthesis|HumanCyc	1.114567112	-0.03650163	5.349844982	0.00031478	0.15645713	0.473135895
homo after UVB irradiation VS homo before UVB irradiation	dolichyl-diphosphooligosaccharide biosynthesis|HumanCyc	-0.01691291	-0.07780642	-0.05817302	0.95483013	0.97250823	-6.3666303
hetero after UVB irradiation VS hetero before UVB irradiation	dolichyl-diphosphooligosaccharide biosynthesis|HumanCyc	0.062329936	-0.04507634	0.22796337	0.82510972	0.93322694	-6.31843843
WT after UVB irradiation VS WT before UVB irradiation	dolichyl-diphosphooligosaccharide biosynthesis|HumanCyc	0.425899908	0.012828581	1.776883334	0.10571579	0.50446894	-4.49368153
homo after UVB irradiation VS homo before UVB irradiation	Dopamine Activation of Neurological Reward System|SMPDB	0.875523332	-0.0376863	5.13313463	0.00053977	0.01666204	0.171004849
hetero after UVB irradiation VS hetero before UVB irradiation	Dopamine Activation of Neurological Reward System|SMPDB	0.807417865	0.051809149	3.815575525	0.00465046	0.07411472	-1.85604071
WT after UVB irradiation VS WT before UVB irradiation	Dopamine Activation of Neurological Reward System|SMPDB	0.691041733	-0.10957519	2.693126759	0.02242702	0.31689014	-3.16946526
homo after UVB irradiation VS homo before UVB irradiation	Dopamine beta-hydroxylase deficiency|SMPDB	0.031995945	0.027270645	0.129408983	0.89976401	0.9399608	-6.35945841
hetero after UVB irradiation VS hetero before UVB irradiation	Dopamine beta-hydroxylase deficiency|SMPDB	-0.38264835	0.006355022	-1.73471603	0.11910451	0.31235808	-4.9577083
WT after UVB irradiation VS WT before UVB irradiation	Dopamine beta-hydroxylase deficiency|SMPDB	0.126407683	0.040850924	0.512443468	0.61937892	0.87257466	-5.73795593
homo after UVB irradiation VS homo before UVB irradiation	Dopamine clearance from the synaptic cleft|Reactome	0.126908256	0.108086881	0.7409866	0.47682378	0.61974875	-6.08197673
hetero after UVB irradiation VS hetero before UVB irradiation	Dopamine clearance from the synaptic cleft|Reactome	0.379494136	0.056083545	1.900804602	0.09199507	0.27424037	-4.72524409
WT after UVB irradiation VS WT before UVB irradiation	Dopamine clearance from the synaptic cleft|Reactome	0.939994619	0.044259213	3.873128286	0.00304654	0.19478336	-1.42894863
homo after UVB irradiation VS homo before UVB irradiation	dopamine degradation|HumanCyc	0.038911625	-0.04648509	0.14005297	0.89157667	0.93563297	-6.3579204
hetero after UVB irradiation VS hetero before UVB irradiation	dopamine degradation|HumanCyc	-0.12833774	-0.03593163	-0.35594312	0.73063104	0.86896773	-6.27833649
WT after UVB irradiation VS WT before UVB irradiation	dopamine degradation|HumanCyc	0.272121148	-0.0098318	1.047166382	0.31947766	0.70304529	-5.34401532
homo after UVB irradiation VS homo before UVB irradiation	Dopamine metabolism|Wikipathways	0.373496392	-0.02878331	1.764546756	0.11006996	0.24349779	-4.93015382
hetero after UVB irradiation VS hetero before UVB irradiation	Dopamine metabolism|Wikipathways	0.134937749	0.075210885	0.538510314	0.60415614	0.79138772	-6.19194727
WT after UVB irradiation VS WT before UVB irradiation	Dopamine metabolism|Wikipathways	0.376533753	0.043387448	1.422677802	0.18502468	0.59839062	-4.94177104
homo after UVB irradiation VS homo before UVB irradiation	Dopamine Neurotransmitter Release Cycle|Reactome	0.428021211	-0.00679658	2.581875067	0.02865485	0.11336662	-3.68687435
hetero after UVB irradiation VS hetero before UVB irradiation	Dopamine Neurotransmitter Release Cycle|Reactome	0.013520216	-0.01401518	0.070351985	0.9455547	0.97177865	-6.34385708
WT after UVB irradiation VS WT before UVB irradiation	Dopamine Neurotransmitter Release Cycle|Reactome	0.077763748	0.023804041	0.333113623	0.74587207	0.9185045	-5.81326804
homo after UVB irradiation VS homo before UVB irradiation	Dopamine receptors|Reactome	0.121141521	0.025677757	0.340302272	0.741119	0.82980612	-6.3066167
hetero after UVB irradiation VS hetero before UVB irradiation	Dopamine receptors|Reactome	-0.09490266	0.094675648	-0.3444852	0.73892208	0.87321931	-6.2826259
WT after UVB irradiation VS WT before UVB irradiation	Dopamine receptors|Reactome	0.011217432	0.019409966	0.028999054	0.97743135	0.99265119	-5.86872519
homo after UVB irradiation VS homo before UVB irradiation	Dopaminergic Neurogenesis|Wikipathways	-0.00298026	0.098845656	-0.01610424	0.98748827	0.99183269	-6.36830892
hetero after UVB irradiation VS hetero before UVB irradiation	Dopaminergic Neurogenesis|Wikipathways	-0.07712141	0.097122774	-0.40180777	0.6978251	0.84778456	-6.25980905
WT after UVB irradiation VS WT before UVB irradiation	Dopaminergic Neurogenesis|Wikipathways	-0.13411941	0.111487171	-0.63421476	0.54006297	0.83916339	-5.66965272
homo after UVB irradiation VS homo before UVB irradiation	Dopaminergic synapse - Homo sapiens (human)|KEGG	0.246955613	-0.01070912	1.498240443	0.16691492	0.31702725	-5.29057621
hetero after UVB irradiation VS hetero before UVB irradiation	Dopaminergic synapse - Homo sapiens (human)|KEGG	0.241339393	0.04678027	1.396573322	0.19823199	0.41868045	-5.39796757
WT after UVB irradiation VS WT before UVB irradiation	Dopaminergic synapse - Homo sapiens (human)|KEGG	0.157117051	-0.02340144	0.661796332	0.52293896	0.83120196	-5.65232477
homo after UVB irradiation VS homo before UVB irradiation	Dopa-responsive dystonia|SMPDB	0.35847852	0.013300746	1.711755208	0.11970137	0.25817499	-5.00406733
hetero after UVB irradiation VS hetero before UVB irradiation	Dopa-responsive dystonia|SMPDB	0.498149914	0.002599842	2.269426991	0.05132677	0.20064099	-4.18437481
WT after UVB irradiation VS WT before UVB irradiation	Dopa-responsive dystonia|SMPDB	0.467985189	-0.04591589	2.072035951	0.06483587	0.43544914	-4.08500072
homo after UVB irradiation VS homo before UVB irradiation	double stranded rna induced gene expression|BioCarta	0.003604191	0.004431484	0.015016281	0.98833345	0.99205816	-6.36832711
hetero after UVB irradiation VS hetero before UVB irradiation	double stranded rna induced gene expression|BioCarta	0.355511825	0.032326847	1.403977142	0.19609244	0.41629685	-5.38890595
WT after UVB irradiation VS WT before UVB irradiation	double stranded rna induced gene expression|BioCarta	-0.04368359	-0.07366416	-0.16364854	0.87324183	0.96283151	-5.85560352
homo after UVB irradiation VS homo before UVB irradiation	downregulated of mta-3 in er-negative breast tumors|BioCarta	0.135033472	-0.02781679	0.551999381	0.59384234	0.7158089	-6.20740446
hetero after UVB irradiation VS hetero before UVB irradiation	downregulated of mta-3 in er-negative breast tumors|BioCarta	0.23917308	0.012296866	1.476967678	0.17605411	0.39293465	-5.29805235
WT after UVB irradiation VS WT before UVB irradiation	downregulated of mta-3 in er-negative breast tumors|BioCarta	-0.13525027	-0.00725144	-0.51242549	0.61939106	0.87257466	-5.73796501
homo after UVB irradiation VS homo before UVB irradiation	Downregulation of ERBB2 signaling|Reactome	-0.0551742	0.015192671	-0.2970892	0.77286641	0.85383693	-6.32125241
hetero after UVB irradiation VS hetero before UVB irradiation	Downregulation of ERBB2 signaling|Reactome	-0.02768357	0.023422938	-0.15928979	0.87719283	0.9555609	-6.33279772
WT after UVB irradiation VS WT before UVB irradiation	Downregulation of ERBB2 signaling|Reactome	-0.18218471	-0.01088536	-0.88319186	0.3977026	0.7657284	-5.48966599
homo after UVB irradiation VS homo before UVB irradiation	Downregulation of ERBB2:ERBB3 signaling|Reactome	-0.06822965	0.034946588	-0.30193264	0.76928462	0.850703	-6.31970869
hetero after UVB irradiation VS hetero before UVB irradiation	Downregulation of ERBB2:ERBB3 signaling|Reactome	0.311150776	0.05312822	1.435009491	0.18734163	0.40490864	-5.35061167
WT after UVB irradiation VS WT before UVB irradiation	Downregulation of ERBB2:ERBB3 signaling|Reactome	-0.30355851	-0.05116115	-1.28489566	0.22756224	0.62952955	-5.09939278
homo after UVB irradiation VS homo before UVB irradiation	Downregulation of ERBB4 signaling|Reactome	0.040211935	0.097817249	0.219700567	0.83080673	0.89531827	-6.34258172

hetero after UVB irradiation VS hetero before UVB irradiation	Downregulation of ERBB4 signaling|Reactome	0.549962389	0.059507945	2.672608249	0.02699266	0.14262729	-3.57178353
WT after UVB irradiation VS WT before UVB irradiation	Downregulation of ERBB4 signaling|Reactome	-0.67211766	-0.02562556	-3.1353013	0.01049304	0.26101395	-2.50537536
homo after UVB irradiation VS homo before UVB irradiation	Downregulation of SMAD2/3:SMAD4 transcriptional activity|Reactome	-0.07442962	-0.01224218	-0.33056744	0.74822869	0.83620396	-6.31008298
hetero after UVB irradiation VS hetero before UVB irradiation	Downregulation of SMAD2/3:SMAD4 transcriptional activity|Reactome	0.062970068	-0.04463243	0.332017727	0.74798471	0.87941394	-6.28713831
WT after UVB irradiation VS WT before UVB irradiation	Downregulation of SMAD2/3:SMAD4 transcriptional activity|Reactome	-0.34223229	-0.00820907	-1.57154375	0.14688241	0.55622203	-4.76025651
homo after UVB irradiation VS homo before UVB irradiation	Downregulation of TGF-beta receptor signaling|Reactome	-0.3513617	0.021085646	-1.71077964	0.11988639	0.25845196	-5.00542266
hetero after UVB irradiation VS hetero before UVB irradiation	Downregulation of TGF-beta receptor signaling|Reactome	-0.14617089	0.004724047	-0.65903914	0.5274671	0.7420355	-6.11696594
WT after UVB irradiation VS WT before UVB irradiation	Downregulation of TGF-beta receptor signaling|Reactome	-0.52606206	-0.0249634	-2.29849207	0.04416758	0.39111425	-3.75709456
homo after UVB irradiation VS homo before UVB irradiation	Downstream signal transduction|Reactome	0.483766521	-0.03977914	2.030373676	0.07159166	0.18906162	-4.54298515
hetero after UVB irradiation VS hetero before UVB irradiation	Downstream signal transduction|Reactome	0.585967184	0.050542723	3.315437809	0.00986601	0.10128218	-2.59330038
WT after UVB irradiation VS WT before UVB irradiation	Downstream signal transduction|Reactome	0.491043017	-0.00949872	2.336735699	0.04137444	0.39022568	-3.7008501
homo after UVB irradiation VS homo before UVB irradiation	Downstream signaling events of B Cell Receptor (BCR)|Reactome	0.197108561	-0.04718254	0.957080945	0.36251954	0.51727006	-5.89947438
hetero after UVB irradiation VS hetero before UVB irradiation	Downstream signaling events of B Cell Receptor (BCR)|Reactome	0.278349141	-0.03778632	1.471833557	0.17740235	0.39482418	-5.3045308
WT after UVB irradiation VS WT before UVB irradiation	Downstream signaling events of B Cell Receptor (BCR)|Reactome	0.06951185	-0.07360954	0.313485124	0.76030152	0.92494383	-5.81962644
homo after UVB irradiation VS homo before UVB irradiation	Downstream signaling in naïve CD8+ T cells|PID	0.171192694	-0.02216905	0.919350107	0.3809243	0.53434727	-5.93415987
hetero after UVB irradiation VS hetero before UVB irradiation	Downstream signaling in naïve CD8+ T cells|PID	0.271530404	0.024294465	1.344114192	0.21397703	0.43637221	-5.46132157
WT after UVB irradiation VS WT before UVB irradiation	Downstream signaling in naïve CD8+ T cells|PID	0.277002636	-0.00396649	1.155140618	0.27467534	0.66570326	-5.2375109
homo after UVB irradiation VS homo before UVB irradiation	Downstream signaling of activated FGFR1|Reactome	-0.11468093	0.026705435	-0.59273764	0.5673568	0.69195165	-6.18321753
hetero after UVB irradiation VS hetero before UVB irradiation	Downstream signaling of activated FGFR1|Reactome	-0.04879465	0.003225975	-0.26986028	0.79375962	0.91224316	-6.30720911
WT after UVB irradiation VS WT before UVB irradiation	Downstream signaling of activated FGFR1|Reactome	-0.30559222	0.010165925	-1.38002315	0.19741078	0.60852749	-4.99169812
homo after UVB irradiation VS homo before UVB irradiation	Downstream signaling of activated FGFR2|Reactome	-0.23960913	0.014757862	-1.34177928	0.21121344	0.36936061	-5.4858288
hetero after UVB irradiation VS hetero before UVB irradiation	Downstream signaling of activated FGFR2|Reactome	-0.10025386	0.019028006	-0.59533317	0.56726268	0.76437184	-6.15832107
WT after UVB irradiation VS WT before UVB irradiation	Downstream signaling of activated FGFR2|Reactome	-0.34444408	0.012542072	-1.5881471	0.14308828	0.55622203	-4.73935827
homo after UVB irradiation VS homo before UVB irradiation	Downstream signaling of activated FGFR3|Reactome	-0.18125996	0.032407726	-0.83459293	0.42467199	0.57589911	-6.00780227
hetero after UVB irradiation VS hetero before UVB irradiation	Downstream signaling of activated FGFR3|Reactome	-0.07388076	0.012827365	-0.42327335	0.68269332	0.8369328	-6.25039706
WT after UVB irradiation VS WT before UVB irradiation	Downstream signaling of activated FGFR3|Reactome	-0.33389352	0.007516085	-1.48186373	0.16894156	0.57535875	-4.87091327
homo after UVB irradiation VS homo before UVB irradiation	Downstream signaling of activated FGFR4|Reactome	-0.05525159	0.007655333	-0.30497031	0.76704115	0.84945668	-6.31872802
hetero after UVB irradiation VS hetero before UVB irradiation	Downstream signaling of activated FGFR4|Reactome	-0.05282028	0.002018937	-0.32011242	0.75667747	0.88730021	-6.29129606
WT after UVB irradiation VS WT before UVB irradiation	Downstream signaling of activated FGFR4|Reactome	-0.30573613	0.007899169	-1.40177211	0.19101021	0.60496032	-4.96636333
homo after UVB irradiation VS homo before UVB irradiation	Downstream TCR signaling|Reactome	0.053920686	-0.04936218	0.245901509	0.81104987	0.88340976	-6.33606582
hetero after UVB irradiation VS hetero before UVB irradiation	Downstream TCR signaling|Reactome	0.175218182	0.002501591	0.915747316	0.38525724	0.6127015	-5.91348595
WT after UVB irradiation VS WT before UVB irradiation	Downstream TCR signaling|Reactome	0.056812166	-0.06147808	0.237440243	0.81707064	0.94525798	-5.84067617
WT after UVB irradiation VS WT before UVB irradiation	Doxepin Metabolism Pathway|SMPDB	-0.4214302	0.046401692	-1.39621666	0.19262818	0.60496032	-4.97285911
WT after UVB irradiation VS WT before UVB irradiation	Doxepin Pathway_ Pharmacokinetics|PharmGKB	-0.4214302	0.046401692	-1.39621666	0.19262818	0.60496032	-4.97285911
homo after UVB irradiation VS homo before UVB irradiation	Doxorubicin Metabolism Pathway|SMPDB	0.779545685	-0.04061384	3.837955357	0.00367844	0.03413771	-1.69732801
hetero after UVB irradiation VS hetero before UVB irradiation	Doxorubicin Metabolism Pathway|SMPDB	0.540534947	0.057670909	2.382296759	0.04287146	0.18194424	-4.01421808
WT after UVB irradiation VS WT before UVB irradiation	Doxorubicin Metabolism Pathway|SMPDB	0.64448625	0.030926285	2.354134074	0.04016141	0.39022568	-3.67519544
homo after UVB irradiation VS homo before UVB irradiation	Doxorubicin Pathway (Cancer Cell)_ Pharmacodynamics|PharmGKB	0.505672767	0.016917423	2.447344368	0.035874	0.12504267	-3.89994437
hetero after UVB irradiation VS hetero before UVB irradiation	Doxorubicin Pathway (Cancer Cell)_ Pharmacodynamics|PharmGKB	0.610506876	0.055973754	3.81721612	0.00463926	0.07411472	-1.85367414
WT after UVB irradiation VS WT before UVB irradiation	Doxorubicin Pathway (Cancer Cell)_ Pharmacodynamics|PharmGKB	0.69644144	-0.04556506	3.224456368	0.00901316	0.24856491	-2.37240184
homo after UVB irradiation VS homo before UVB irradiation	Doxorubicin Pathway (Cardiomyocyte Cell)_ Pharmacodynamics|PharmGKB	0.22200662	-0.06133847	1.046067561	0.32171147	0.47732908	-5.81322303
hetero after UVB irradiation VS hetero before UVB irradiation	Doxorubicin Pathway (Cardiomyocyte Cell)_ Pharmacodynamics|PharmGKB	0.236596212	-0.01266236	1.338198483	0.21581807	0.43741933	-5.46837016
WT after UVB irradiation VS WT before UVB irradiation	Doxorubicin Pathway (Cardiomyocyte Cell)_ Pharmacodynamics|PharmGKB	0.234128965	-0.01318295	0.899472308	0.3893787	0.76242672	-5.47612079
homo after UVB irradiation VS homo before UVB irradiation	Doxorubicin Pathway_ Pharmacokinetics|PharmGKB	0.848406692	-0.01327361	4.29986442	0.00180548	0.02356086	-1.00199115
hetero after UVB irradiation VS hetero before UVB irradiation	Doxorubicin Pathway_ Pharmacokinetics|PharmGKB	0.541063434	0.065929466	2.255163465	0.05250668	0.20350804	-4.20576477
WT after UVB irradiation VS WT before UVB irradiation	Doxorubicin Pathway_ Pharmacokinetics|PharmGKB	0.563332346	0.050842072	2.016410075	0.07117633	0.44835368	-4.16389151
homo after UVB irradiation VS homo before UVB irradiation	DroToll-like|INOH	0.057871353	0.000589795	0.203148587	0.84335339	0.90478185	-6.34632396
hetero after UVB irradiation VS hetero before UVB irradiation	DroToll-like|INOH	0.227146858	0.015935733	0.884915541	0.40074824	0.62933846	-5.94088097
WT after UVB irradiation VS WT before UVB irradiation	DroToll-like|INOH	0.243191763	-0.00359816	0.90205358	0.38807025	0.76130447	-5.47395411
homo after UVB irradiation VS homo before UVB irradiation	Drug Induction of Bile Acid Pathway|Wikipathways	0.334534902	0.058126133	1.668471636	0.12816497	0.26846864	-5.06381696
hetero after UVB irradiation VS hetero before UVB irradiation	Drug Induction of Bile Acid Pathway|Wikipathways	-0.20926117	-0.02445652	-1.02120035	0.33558175	0.56462961	-5.81413576
WT after UVB irradiation VS WT before UVB irradiation	Drug Induction of Bile Acid Pathway|Wikipathways	0.497151538	0.071204911	1.934627566	0.08156822	0.46799255	-4.27842645
homo after UVB irradiation VS homo before UVB irradiation	Drug metabolism - cytochrome P450 - Homo sapiens (human)|KEGG	0.081638139	0.031539856	0.327917616	0.75016832	0.8371496	-6.3110095
hetero after UVB irradiation VS hetero before UVB irradiation	Drug metabolism - cytochrome P450 - Homo sapiens (human)|KEGG	-0.09047527	0.013621811	-0.42467976	0.68170707	0.83594856	-6.24976401
WT after UVB irradiation VS WT before UVB irradiation	Drug metabolism - cytochrome P450 - Homo sapiens (human)|KEGG	0.02815017	0.000555725	0.110999863	0.91379507	0.97566251	-5.86291355
homo after UVB irradiation VS homo before UVB irradiation	Drug metabolism - other enzymes - Homo sapiens (human)|KEGG	0.047502123	-0.01595965	0.234888574	0.81933799	0.88883281	-6.3388929
hetero after UVB irradiation VS hetero before UVB irradiation	Drug metabolism - other enzymes - Homo sapiens (human)|KEGG	0.121529382	-0.01102331	0.788207597	0.45217798	0.6770786	-6.02170647
WT after UVB irradiation VS WT before UVB irradiation	Drug metabolism - other enzymes - Homo sapiens (human)|KEGG	0.270956375	-0.02009402	1.381787721	0.19688481	0.6083714	-4.98965222
homo after UVB irradiation VS homo before UVB irradiation	DSCAM interactions|Reactome	0.249009267	0.031941695	1.358831307	0.20594349	0.36362644	-5.46522438
hetero after UVB irradiation VS hetero before UVB irradiation	DSCAM interactions|Reactome	-0.06274333	0.031937429	-0.30307668	0.76917978	0.8963654	-6.29698784
WT after UVB irradiation VS WT before UVB irradiation	DSCAM interactions|Reactome	-0.07839331	0.13195028	-0.37733838	0.71374358	0.90428334	-5.79756394
homo after UVB irradiation VS homo before UVB irradiation	dTMP <i>de novo</i> biosynthesis (mitochondrial)|HumanCyc	-0.88888145	-0.02689494	-4.06213086	0.00259472	0.02932936	-1.35619921
hetero after UVB irradiation VS hetero before UVB irradiation	dTMP <i>de novo</i> biosynthesis (mitochondrial)|HumanCyc	0.004569943	-0.0568755	0.013034109	0.98990393	0.9943047	-6.34644914
WT after UVB irradiation VS WT before UVB irradiation	dTMP <i>de novo</i> biosynthesis (mitochondrial)|HumanCyc	0.10093811	0.102259309	0.278240399	0.7864507	0.93901185	-5.83009321
homo after UVB irradiation VS homo before UVB irradiation	Dual hijack model of Vif in HIV infection|Wikipathways	0.210613616	-0.0356296	0.925396353	0.37793064	0.53080015	-5.92867909
hetero after UVB irradiation VS hetero before UVB irradiation	Dual hijack model of Vif in HIV infection|Wikipathways	0.256452158	0.016862726	0.962378092	0.36266132	0.5912054	-5.87061352
WT after UVB irradiation VS WT before UVB irradiation	Dual hijack model of Vif in HIV infection|Wikipathways	0.245958958	0.065415616	0.872094622	0.40344692	0.77093812	-5.49877902
homo after UVB irradiation VS homo before UVB irradiation	Dual Incision in GG-NER|Reactome	-0.17390124	-0.00939339	-0.80244677	0.44213142	0.59091566	-6.03413976
hetero after UVB irradiation VS hetero before UVB irradiation	Dual Incision in GG-NER|Reactome	0.289563807	0.020761882	1.391028557	0.19984753	0.41977191	-5.40473472
WT after UVB irradiation VS WT before UVB irradiation	Dual Incision in GG-NER|Reactome	0.124131268	0.034057352	0.462271495	0.65370161	0.87935805	-5.76211392
homo after UVB irradiation VS homo before UVB irradiation	Dual incision in TC-NER|Reactome	-0.44885196	-0.00361687	-2.39602567	0.03908057	0.13034468	-3.98069431
hetero after UVB irradiation VS hetero before UVB irradiation	Dual incision in TC-NER|Reactome	-0.03444203	-0.01514062	-0.16387946	0.87368909	0.95338158	-6.33199563
WT after UVB irradiation VS WT before UVB irradiation	Dual incision in TC-NER|Reactome	-0.11035173	0.008891821	-0.38714868	0.70669267	0.90152666	-5.79382307
homo after UVB irradiation VS homo before UVB irradiation	E2F mediated regulation of DNA replication|Reactome	-0.57387338	-0.03390333	-2.4658661	0.03478184	0.12436481	-3.87072116
hetero after UVB irradiation VS hetero before UVB irradiation	E2F mediated regulation of DNA replication|Reactome	-0.12359365	0.024526106	-0.40987077	0.69212403	0.84508474	-6.25632877
WT after UVB irradiation VS WT before UVB irradiation	E2F mediated regulation of DNA replication|Reactome	-0.23967037	-0.05838657	-0.89251894	0.39291877	0.76452505	-5.4819314
homo after UVB irradiation VS homo before UVB irradiation	E2F transcription factor network|PID	-0.45470917	-0.03603083	-2.40924894	0.03822821	0.12862933	-3.95991986
hetero after UVB irradiation VS hetero before UVB irradiation	E2F transcription factor network|PID	0.008866966	-0.01554987	0.039620221	0.96931866	0.98614433	-6.34568979
WT after UVB irradiation VS WT before UVB irradiation	E2F transcription factor network|PID	-0.1170331	-0.03077435	-0.4635655	0.65280519	0.87935805	-5.76152061
homo after UVB irradiation VS homo before UVB irradiation	e2f1 destruction pathway|BioCarta	-0.37045432	-0.02007625	-1.49039527	0.16892714	0.31952001	-5.30068439
hetero after UVB irradiation VS hetero before UVB irradiation	e2f1 destruction pathway|BioCarta	-0.04883118	-0.02925591	-0.20088759	0.84555123	0.9427648	-6.32470226
WT after UVB irradiation VS WT before UVB irradiation	e2f1 destruction pathway|BioCarta	-2.43E-05	0.017115189	-8.77E-05	0.99993177	0.99993177	-5.86915119
homo after UVB irradiation VS homo before UVB irradiation	E2F-enabled inhibition of pre-replication complex formation|Reactome	-0.47865315	-0.01754698	-1.61734884	0.13885641	0.28141074	-5.13334232
hetero after UVB irradiation VS hetero before UVB irradiation	E2F-enabled inhibition of pre-replication complex formation|Reactome	-0.16232936	0.014102294	-0.52189405	0.61518273	0.80022914	-6.20118807
WT after UVB irradiation VS WT before UVB irradiation	E2F-enabled inhibition of pre-replication complex formation|Reactome	-0.23098434	-0.0827034	-0.69683729	0.50165489	0.82205162	-5.62934538
homo after UVB irradiation VS homo before UVB irradiation	E3 ubiquitin ligases ubiquitinate target proteins|Reactome	-0.05406917	-0.00306268	-0.25977957	0.80064073	0.87562289	-6.33232133
hetero after UVB irradiation VS hetero before UVB irradiation	E3 ubiquitin ligases ubiquitinate target proteins|Reactome	0.044164423	0.009536722	0.263244991	0.79868507	0.91506577	-6.30910582
WT after UVB irradiation VS WT before UVB irradiation	E3 ubiquitin ligases ubiquitinate target proteins|Reactome	-0.09658816	-0.00902541	-0.43500066	0.67272591	0.8808312	-5.77424819
homo after UVB irradiation VS homo before UVB irradiation	Early Phase of HIV Life Cycle|Reactome	-0.26991323	-0.06059669	-1.05755344	0.31670795	0.47119209	-5.80164842
hetero after UVB irradiation VS hetero before UVB irradiation	Early Phase of HIV Life Cycle|Reactome	0.08581729	-0.01962392	0.337312658	0.74413067	0.87687018	-6.28524165
WT after UVB irradiation VS WT before UVB irradiation	Early Phase of HIV Life Cycle|Reactome	0.205141711	0.011518167	0.744636204	0.47349104	0.81355664	-5.59628582
homo after UVB irradiation VS homo before UVB irradiation	Ebola Virus Pathway on Host|Wikipathways	0.363270597	-0.0148268	1.638122773	0.13441868	0.277359	-5.10523082
hetero after UVB irradiation VS hetero before UVB irradiation	Ebola Virus Pathway on Host|Wikipathways	0.390576022	0.048735124	2.014961357	0.07687459	0.24866063	-4.56084671
WT after UVB irradiation VS WT before UVB irradiation	Ebola Virus Pathway on Host|Wikipathways	0.388600103	0.009651547	1.770929189	0.10674458	0.50570798	-4.50164304
homo after UVB irradiation VS homo before UVB irradiation	EBV LMP1 signaling|Wikipathways	0.263992057	-0.03041024	1.072487539	0.31029159	0.46923284	-5.78645176
hetero after UVB irradiation VS hetero before UVB irradiation	EBV LMP1 signaling|Wikipathways	0.279914664	-0.02103204	1.123066094	0.29238729	0.52324352	-5.71031512
WT after UVB irradiation VS WT before UVB irradiation	EBV LMP1 signaling|Wikipathways	0.127101288	-0.06948826	0.490787986	0.63408273	0.87461669	-5.74867293
homo after UVB irradiation VS homo before UVB irradiation	E-cadherin signaling in keratinocytes|PID	0.426927232	-0.0344691	1.432776362	0.18436421	0.3388917	-5.3739249
hetero after UVB irradiation VS hetero before UVB irradiation	E-cadherin signaling in keratinocytes|PID	0.613141052	-0.00334822	2.763479028	0.0233678	0.13628011	-3.43267327
WT after UVB irradiation VS WT before UVB irradiation	E-cadherin signaling in keratinocytes|PID	0.279147361	0.003354207	1.283776423	0.22793848	0.62952955	-5.10062862
homo after UVB irradiation VS homo before UVB irradiation	E-cadherin signaling in the nascent adherens junction|PID	0.361910785	-0.02830785	1.513075277	0.16316722	0.31264018	-5.27137534
hetero after UVB irradiation VS hetero before UVB irradiation	E-cadherin signaling in the nascent adherens junction|PID	0.591275822	0.004560536	3.471018948	0.00778133	0.08956216	-2.36083649
WT after UVB irradiation VS WT before UVB irradiation	E-cadherin signaling in the nascent adherens junction|PID	0.371003416	0.023864662	1.809348448	0.10026418	0.50131283	-4.45005054
homo after UVB irradiation VS homo before UVB irradiation	ECM proteoglycans|Reactome	0.556013562	0.038362854	3.46769946	0.00663763	0.0470948	-2.2738148
hetero after UVB irradiation VS hetero before UVB irradiation	ECM proteoglycans|Reactome	0.241231982	0.042306325	1.119707148	0.29373802	0.52483649	-5.71385532
WT after UVB irradiation VS WT before UVB irradiation	ECM proteoglycans|Reactome	0.256193488	0.009484007	0.985175118	0.34758942	0.73221443	-5.4014456
homo after UVB irradiation VS homo before UVB irradiation	ECM-receptor interaction - Homo sapiens (human)|KEGG	0.514458586	-0.01920161	3.160251696	0.01095933	0.063619	-2.76182789
hetero after UVB irradiation VS hetero before UVB irradiation	ECM-receptor interaction - Homo sapiens (human)|KEGG	0.375319992	0.00533235	1.846191736	0.10019289	0.28471271	-4.80263858
WT after UVB irradiation VS WT before UVB irradiation	ECM-receptor interaction - Homo sapiens (human)|KEGG	0.314098807	0.006842726	1.307804469	0.21997283	0.62621406	-5.07393426
homo after UVB irradiation VS homo before UVB irradiation	Ectoderm Differentiation|Wikipathways	-0.01342988	-0.00936675	-0.08281495	0.93573744	0.96219047	-6.36476463
hetero after UVB irradiation VS hetero before UVB irradiation	Ectoderm Differentiation|Wikipathways	-0.1081305	-0.00713692	-0.6470099	0.53485069	0.74826564	-6.12506604
WT after UVB irradiation VS WT before UVB irradiation	Ectoderm Differentiation|Wikipathways	-0.14963349	-0.00345892	-0.75583118	0.46704217	0.809788	-5.58826118
homo after UVB irradiation VS homo before UVB irradiation	EDA Signalling in Hair Follicle Development|Wikipathways	-0.09871991	-0.02010378	-0.54263721	0.6000214	0.72035794	-6.21273572
hetero after UVB irradiation VS hetero before UVB irradiation	EDA Signalling in Hair Follicle Development|Wikipathways	-0.03910767	0.016916078	-0.22777936	0.82524818	0.93322694	-6.31848364
WT after UVB irradiation VS WT before UVB irradiation	EDA Signalling in Hair Follicle Development|Wikipathways	-0.13630851	0.055046771	-0.59243563	0.56660775	0.85125516	-5.69460722
homo after UVB irradiation VS homo before UVB irradiation	Effects of Botulinum toxin|PID	0.55028489	-0.01668415	3.510902792	0.00619075	0.04588471	-2.20582153
hetero after UVB irradiation VS hetero before UVB irradiation	Effects of Botulinum toxin|PID	0.202114197	0.003482555	0.875866748	0.40537796	0.63319136	-5.94877454
WT after UVB irradiation VS WT before UVB irradiation	Effects of Botulinum toxin|PID	0.29748443	0.051882167	1.344334858	0.20830524	0.62329858	-5.03270596
homo after UVB irradiation VS homo before UVB irradiation	effects of calcineurin in keratinocyte differentiation|BioCarta	0.010753376	-0.03890481	0.046841364	0.9636209	0.977754	-6.36726949
hetero after UVB irradiation VS hetero before UVB irradiation	effects of calcineurin in keratinocyte differentiation|BioCarta	0.118085793	-0.02846769	0.739828388	0.47950743	0.70051118	-6.059132
WT after UVB irradiation VS WT before UVB irradiation	effects of calcineurin in keratinocyte differentiation|BioCarta	-0.23733317	-0.09568225	-0.93935824	0.36950604	0.747689	-5.44206414
homo after UVB irradiation VS homo before UVB irradiation	Effects of Nitric Oxide|Wikipathways	0.620940356	-0.01556512	2.864880812	0.01787029	0.08446763	-3.23468131
hetero after UVB irradiation VS hetero before UVB irradiation	Effects of Nitric Oxide|Wikipathways	0.61551761	0.080534617	2.49444146	0.03584814	0.16681566	-3.84390472
WT after UVB irradiation VS WT before UVB irradiation	Effects of Nitric Oxide|Wikipathways	0.404897559	0.023886067	1.224971621	0.24843411	0.64146154	-5.16449192
homo after UVB irradiation VS homo before UVB irradiation	Effects of PIP2 hydrolysis|Reactome	0.378325141	-0.03754229	2.145626589	0.05922812	0.16661003	-4.36874032
hetero after UVB irradiation VS hetero before UVB irradiation	Effects of PIP2 hydrolysis|Reactome	0.19897596	0.020932909	0.850563225	0.4185247	0.64556195	-5.97048974
WT after UVB irradiation VS WT before UVB irradiation	Effects of PIP2 hydrolysis|Reactome	-0.04950517	-0.02681452	-0.23814159	0.81654139	0.94525798	-5.84050821
homo after UVB irradiation VS homo before UVB irradiation	egf signaling pathway|BioCarta	0.225774167	-0.02063624	1.101339135	0.29817986	0.45887739	-5.75662762
hetero after UVB irradiation VS hetero before UVB irradiation	egf signaling pathway|BioCarta	0.364950439	0.002545824	1.977380591	0.08156805	0.25776716	-4.61533093
WT after UVB irradiation VS WT before UVB irradiation	egf signaling pathway|BioCarta	0.129955398	0.012107431	0.635613039	0.5391871	0.83903241	-5.6687905
homo after UVB irradiation VS homo before UVB irradiation	EGF|INOH	0.19360161	-0.02948569	0.904104521	0.38854802	0.54154769	-5.94784697
hetero after UVB irradiation VS hetero before UVB irradiation	EGF|INOH	0.334188158	0.009703297	1.994537709	0.0793921	0.25322957	-4.59049872
WT after UVB irradiation VS WT before UVB irradiation	EGF|INOH	0.277541381	0.037796535	1.224493355	0.24860673	0.64146154	-5.16500259
homo after UVB irradiation VS homo before UVB irradiation	EGF-Core|Signalink	0.018811377	0.008051792	0.106624833	0.91733017	0.95153785	-6.36234351
hetero after UVB irradiation VS hetero before UVB irradiation	EGF-Core|Signalink	0.212623509	-0.0254581	1.338704512	0.21566006	0.43741933	-5.467768
WT after UVB irradiation VS WT before UVB irradiation	EGF-Core|Signalink	-0.01368955	-0.01585408	-0.07023561	0.94537974	0.98295421	-5.86665279
homo after UVB irradiation VS homo before UVB irradiation	EGF-EGFR Signaling Pathway|Wikipathways	0.265694302	-0.01751408	1.484454098	0.17046506	0.32096638	-5.30831801
hetero after UVB irradiation VS hetero before UVB irradiation	EGF-EGFR Signaling Pathway|Wikipathways	0.379284988	0.006668945	2.444743141	0.03880581	0.17155858	-3.91950694
WT after UVB irradiation VS WT before UVB irradiation	EGF-EGFR Signaling Pathway|Wikipathways	0.186504754	-0.01842215	0.949744707	0.36445235	0.74545864	-5.43299505
homo after UVB irradiation VS homo before UVB irradiation	EGF-Ncore|Signalink	-0.27069307	0.003905709	-1.34883822	0.20901822	0.36695387	-5.47732024
hetero after UVB irradiation VS hetero before UVB irradiation	EGF-Ncore|Signalink	-0.1073646	-0.03373373	-0.60158719	0.56328124	0.764281	-6.15443073
WT after UVB irradiation VS WT before UVB irradiation	EGF-Ncore|Signalink	-0.15271872	-0.04714916	-0.71956642	0.4881361	0.81710765	-5.61386951
homo after UVB irradiation VS homo before UVB irradiation	EGFR downregulation|Reactome	0.02886532	-0.04141317	0.133889619	0.89631593	0.93952675	-6.35882572
hetero after UVB irradiation VS hetero before UVB irradiation	EGFR downregulation|Reactome	0.297403116	0.02097243	1.921004023	0.08912744	0.270288	-4.69640263
WT after UVB irradiation VS WT before UVB irradiation	EGFR downregulation|Reactome	-0.13820923	-0.02903028	-0.62950072	0.54302184	0.83985745	-5.6725467
homo after UVB irradiation VS homo before UVB irradiation	EGFR Inhibitor Pathway_ Pharmacodynamics|PharmGKB	0.307190925	0.001899916	1.62022605	0.13823404	0.28077252	-5.12946052
hetero after UVB irradiation VS hetero before UVB irradiation	EGFR Inhibitor Pathway_ Pharmacodynamics|PharmGKB	0.436353937	0.034430958	2.75428279	0.02371084	0.13628011	-3.44675339
WT after UVB irradiation VS WT before UVB irradiation	EGFR Inhibitor Pathway_ Pharmacodynamics|PharmGKB	0.169689342	0.007201431	0.787756052	0.44896303	0.80503107	-5.56479838

homo after UVB irradiation VS homo before UVB irradiation	EGFR interacts with phospholipase C-gamma|Reactome	0.078479888	0.041250564	0.349087619	0.73472479	0.82507461	-6.30340433
hetero after UVB irradiation VS hetero before UVB irradiation	EGFR interacts with phospholipase C-gamma|Reactome	-0.31896472	0.057567434	-1.7307823	0.11982903	0.31317702	-4.96310302
WT after UVB irradiation VS WT before UVB irradiation	EGFR interacts with phospholipase C-gamma|Reactome	-0.40059841	0.044651624	-1.42212944	0.18517962	0.59839062	-4.94241906
homo after UVB irradiation VS homo before UVB irradiation	EGFR Transactivation by Gastrin|Reactome	-0.02355974	-0.00309007	-0.08383189	0.93495035	0.9620059	-6.36467364
hetero after UVB irradiation VS hetero before UVB irradiation	EGFR Transactivation by Gastrin|Reactome	0.29064976	0.061083412	1.160593758	0.27763108	0.50861835	-5.67024067
WT after UVB irradiation VS WT before UVB irradiation	EGFR Transactivation by Gastrin|Reactome	-0.06758612	0.033682526	-0.2460971	0.8105448	0.94525798	-5.83856879
homo after UVB irradiation VS homo before UVB irradiation	EGFR1|NetPath	0.151043052	-0.03114946	0.758686398	0.46665755	0.61175951	-6.0685436
hetero after UVB irradiation VS hetero before UVB irradiation	EGFR1|NetPath	0.275337919	0.001688091	1.8456492	0.10027769	0.28477614	-4.80340299
WT after UVB irradiation VS WT before UVB irradiation	EGFR1|NetPath	0.139874007	-0.02241963	0.710061146	0.4937619	0.81710765	-5.6203957
homo after UVB irradiation VS homo before UVB irradiation	EGFR-dependent Endothelin signaling events|PID	-0.12578311	-0.01813364	-0.5703505	0.58182989	0.70411749	-6.19670745
hetero after UVB irradiation VS hetero before UVB irradiation	EGFR-dependent Endothelin signaling events|PID	-0.26743368	-0.00827589	-1.40626553	0.19543521	0.4161797	-5.38609929
WT after UVB irradiation VS WT before UVB irradiation	EGFR-dependent Endothelin signaling events|PID	-0.00222161	0.021738862	-0.00958045	0.99254295	0.99712795	-5.86910469
homo after UVB irradiation VS homo before UVB irradiation	Eicosanoid ligand-binding receptors|Reactome	0.568347838	-0.02233343	3.083225986	0.01244265	0.06880589	-2.88493661
hetero after UVB irradiation VS hetero before UVB irradiation	Eicosanoid ligand-binding receptors|Reactome	0.329653974	0.059620273	1.408176207	0.19488792	0.4161797	-5.38375374
WT after UVB irradiation VS WT before UVB irradiation	Eicosanoid ligand-binding receptors|Reactome	0.434712895	0.000917046	1.790864938	0.10333552	0.50425697	-4.47493651
homo after UVB irradiation VS homo before UVB irradiation	eicosanoid metabolism|BioCarta	0.770774216	-0.01495025	4.313052557	0.00176993	0.02356086	-0.98258231
hetero after UVB irradiation VS hetero before UVB irradiation	eicosanoid metabolism|BioCarta	0.39324124	0.066870571	1.554702983	0.15672239	0.36651649	-5.1984254
WT after UVB irradiation VS WT before UVB irradiation	eicosanoid metabolism|BioCarta	0.363507513	0.033142253	1.194037303	0.2597996	0.65444279	-5.19722502
homo after UVB irradiation VS homo before UVB irradiation	Eicosanoid Synthesis|Wikipathways	0.113722684	0.004714763	0.543422825	0.59950158	0.71996955	-6.21229164
hetero after UVB irradiation VS hetero before UVB irradiation	Eicosanoid Synthesis|Wikipathways	0.154679127	0.012896538	0.977075291	0.35574686	0.58369121	-5.85674908
WT after UVB irradiation VS WT before UVB irradiation	Eicosanoid Synthesis|Wikipathways	0.085914576	0.012758547	0.437928908	0.67067119	0.87984445	-5.77297914
homo after UVB irradiation VS homo before UVB irradiation	Eicosanoids|Reactome	0.614134643	0.071824355	3.406312429	0.00733115	0.04981254	-2.37070652
hetero after UVB irradiation VS hetero before UVB irradiation	Eicosanoids|Reactome	0.403370343	0.092874444	1.803934244	0.10700399	0.29559041	-4.86190271
WT after UVB irradiation VS WT before UVB irradiation	Eicosanoids|Reactome	0.296033369	0.020847078	0.980318547	0.34986652	0.73549869	-5.40582579
homo after UVB irradiation VS homo before UVB irradiation	eicosapentaenoate biosynthesis|HumanCyc	-0.47201084	-0.035129	-2.97796502	0.01480982	0.07510999	-3.05346915
hetero after UVB irradiation VS hetero before UVB irradiation	eicosapentaenoate biosynthesis|HumanCyc	-0.32050803	0.029765098	-1.86320404	0.09756788	0.28213748	-4.77862379
WT after UVB irradiation VS WT before UVB irradiation	eicosapentaenoate biosynthesis|HumanCyc	-0.3804015	0.012337585	-1.75998036	0.10866027	0.5076251	-4.51624968
homo after UVB irradiation VS homo before UVB irradiation	eIF5A regulation in response to inhibition of the nuclear export system|Wikipathways	-0.3387572	0.003963684	-1.31712288	0.21903398	0.37739969	-5.51531344
hetero after UVB irradiation VS hetero before UVB irradiation	eIF5A regulation in response to inhibition of the nuclear export system|Wikipathways	0.004730436	0.112084114	0.010963612	0.99150764	0.9947197	-6.34647609
WT after UVB irradiation VS WT before UVB irradiation	eIF5A regulation in response to inhibition of the nuclear export system|Wikipathways	-0.22287006	0.025617307	-0.59966536	0.56196291	0.85017866	-5.69040131
homo after UVB irradiation VS homo before UVB irradiation	Elastic fibre formation|Reactome	0.56831	-0.02280157	3.065438579	0.0128136	0.07023635	-2.91339626
hetero after UVB irradiation VS hetero before UVB irradiation	Elastic fibre formation|Reactome	0.452025712	0.032748424	1.911985764	0.09039705	0.27214648	-4.70929314
WT after UVB irradiation VS WT before UVB irradiation	Elastic fibre formation|Reactome	0.426215796	-0.02311187	1.885698731	0.08844733	0.48106209	-4.34604166
homo after UVB irradiation VS homo before UVB irradiation	Electric Transmission Across Gap Junctions|Reactome	0.078969267	-0.10837512	0.261114452	0.79964164	0.87515909	-6.33195048
hetero after UVB irradiation VS hetero before UVB irradiation	Electric Transmission Across Gap Junctions|Reactome	0.374514444	-0.08153992	1.044571376	0.32526011	0.55421891	-5.7909765
WT after UVB irradiation VS WT before UVB irradiation	Electric Transmission Across Gap Junctions|Reactome	-0.22834221	0.041621614	-0.80708273	0.43824379	0.79685269	-5.55018224
homo after UVB irradiation VS homo before UVB irradiation	Electron Transport Chain (OXPHOS system in mitochondria)|Wikipathways	-0.00675903	-0.01902214	-0.02241295	0.98258767	0.98843254	-6.36817836
hetero after UVB irradiation VS hetero before UVB irradiation	Electron Transport Chain (OXPHOS system in mitochondria)|Wikipathways	-0.26176918	-0.04831133	-1.03532509	0.32931409	0.55799763	-5.80018707
WT after UVB irradiation VS WT before UVB irradiation	Electron Transport Chain (OXPHOS system in mitochondria)|Wikipathways	-0.02888325	-0.07803748	-0.08900538	0.93082085	0.9788786	-5.86513966
homo after UVB irradiation VS homo before UVB irradiation	Electron transport from NADPH to Ferredoxin|Reactome	0.304373394	0.00114655	1.644678534	0.13304495	0.27587795	-5.09631926
hetero after UVB irradiation VS hetero before UVB irradiation	Electron transport from NADPH to Ferredoxin|Reactome	0.028721585	0.01388405	0.07101209	0.9450448	0.97147366	-6.34380649
WT after UVB irradiation VS WT before UVB irradiation	Electron transport from NADPH to Ferredoxin|Reactome	0.186971648	0.074717217	0.572099823	0.57978588	0.85958498	-5.70618344
homo after UVB irradiation VS homo before UVB irradiation	Elevation of cytosolic Ca2+ levels|Reactome	0.447262316	-0.03568101	2.335196793	0.04324904	0.13470334	-4.07594323
hetero after UVB irradiation VS hetero before UVB irradiation	Elevation of cytosolic Ca2+ levels|Reactome	0.231600118	0.012245125	0.896304054	0.39497513	0.62250836	-5.93085145
WT after UVB irradiation VS WT before UVB irradiation	Elevation of cytosolic Ca2+ levels|Reactome	0.018826587	-0.08491384	0.080817512	0.93716887	0.98056728	-5.86584353
homo after UVB irradiation VS homo before UVB irradiation	Enalapril Action Pathway|SMPDB	0.636038586	-0.11800708	2.436891056	0.03650532	0.12504267	-3.91641947
hetero after UVB irradiation VS hetero before UVB irradiation	Enalapril Action Pathway|SMPDB	-0.02777369	0.003323189	-0.09172332	0.92906122	0.97054702	-6.34197953
WT after UVB irradiation VS WT before UVB irradiation	Enalapril Action Pathway|SMPDB	0.270438532	-0.10210744	0.707878218	0.49505953	0.81710765	-5.62188349
homo after UVB irradiation VS homo before UVB irradiation	Enalapril Metabolism Pathway|SMPDB	1.076747846	-0.04193589	4.959558579	0.00068841	0.01666204	-0.06417629
hetero after UVB irradiation VS hetero before UVB irradiation	Enalapril Metabolism Pathway|SMPDB	0.553630209	0.003105397	1.56066683	0.15532075	0.36486693	-5.19066683
WT after UVB irradiation VS WT before UVB irradiation	Enalapril Metabolism Pathway|SMPDB	0.585930211	-0.05239369	1.72512433	0.11496929	0.51527453	-4.56245573
homo after UVB irradiation VS homo before UVB irradiation	Endochondral Ossification|Wikipathways	0.317624944	-0.0317594	1.851425743	0.09576037	0.22296882	-4.80620367
hetero after UVB irradiation VS hetero before UVB irradiation	Endochondral Ossification|Wikipathways	0.078960881	-0.0153143	0.458448651	0.65822369	0.82651398	-6.23396298
WT after UVB irradiation VS WT before UVB irradiation	Endochondral Ossification|Wikipathways	-0.10398706	-0.02011994	-0.49557949	0.63081465	0.87303764	-5.74633947
homo after UVB irradiation VS homo before UVB irradiation	Endocrine and other factor-regulated calcium reabsorption - Homo sapiens (human)|KEGG	0.670993459	-0.03961467	4.181649546	0.00216015	0.02581999	-1.17710983
hetero after UVB irradiation VS hetero before UVB irradiation	Endocrine and other factor-regulated calcium reabsorption - Homo sapiens (human)|KEGG	0.540047091	0.040262881	2.997549912	0.01615738	0.11352943	-3.0747991
WT after UVB irradiation VS WT before UVB irradiation	Endocrine and other factor-regulated calcium reabsorption - Homo sapiens (human)|KEGG	0.508673376	0.011573714	2.260781209	0.04710077	0.401167	-3.81234188
homo after UVB irradiation VS homo before UVB irradiation	Endocytosis - Homo sapiens (human)|KEGG	0.413352152	-0.0212316	2.166803681	0.05719186	0.16260153	-4.33638348
hetero after UVB irradiation VS hetero before UVB irradiation	Endocytosis - Homo sapiens (human)|KEGG	0.548438653	0.00150175	3.668257458	0.00578423	0.08047311	-2.07003019
WT after UVB irradiation VS WT before UVB irradiation	Endocytosis - Homo sapiens (human)|KEGG	0.356504526	-0.00459478	1.783401804	0.10459989	0.50446894	-4.4849509
homo after UVB irradiation VS homo before UVB irradiation	endocytotic role of ndk phosphins and dynamin|BioCarta	0.422336314	-0.0056839	2.370244025	0.04079668	0.13402181	-4.02112988
hetero after UVB irradiation VS hetero before UVB irradiation	endocytotic role of ndk phosphins and dynamin|BioCarta	0.296589014	-0.00020587	1.524865764	0.16390649	0.37677831	-5.23699964
WT after UVB irradiation VS WT before UVB irradiation	endocytotic role of ndk phosphins and dynamin|BioCarta	0.10091865	-0.07595621	0.402427824	0.69576902	0.89762065	-5.78781181
homo after UVB irradiation VS homo before UVB irradiation	Endoderm Differentiation|Wikipathways	-0.20794688	0.053344275	-1.13409618	0.28487847	0.44528503	-5.72203633
hetero after UVB irradiation VS hetero before UVB irradiation	Endoderm Differentiation|Wikipathways	-0.01410667	0.04612436	-0.07277024	0.94368685	0.97091178	-6.34366944
WT after UVB irradiation VS WT before UVB irradiation	Endoderm Differentiation|Wikipathways	-0.09444383	0.032957466	-0.42243169	0.68157729	0.88657462	-5.77960239
homo after UVB irradiation VS homo before UVB irradiation	Endogenous sterols|Reactome	0.030255492	0.003037074	0.146633466	0.88652163	0.93161283	-6.35690902
hetero after UVB irradiation VS hetero before UVB irradiation	Endogenous sterols|Reactome	-0.18064238	0.000403946	-0.83935789	0.42444077	0.65138081	-5.97993596
WT after UVB irradiation VS WT before UVB irradiation	Endogenous sterols|Reactome	-0.17133814	-0.01333217	-0.69999642	0.49976229	0.82071714	-5.62722109
homo after UVB irradiation VS homo before UVB irradiation	Endogenous TLR signaling|PID	0.81008974	0.007516399	4.992367174	0.00065726	0.01666204	-0.01934775
hetero after UVB irradiation VS hetero before UVB irradiation	Endogenous TLR signaling|PID	0.786364777	0.030292802	4.587247456	0.00155976	0.04803647	-0.78748852
WT after UVB irradiation VS WT before UVB irradiation	Endogenous TLR signaling|PID	0.565622057	-0.01914664	2.353128375	0.04023057	0.39022568	-3.67667947
homo after UVB irradiation VS homo before UVB irradiation	Endohydrolysis of 1_4-alpha-D-glucosidic linkages in polysaccharides by alpha-amylase|EHMN	-0.66367967	0.106519783	-1.90383566	0.08798566	0.21222531	-4.73014785
hetero after UVB irradiation VS hetero before UVB irradiation	Endohydrolysis of 1_4-alpha-D-glucosidic linkages in polysaccharides by alpha-amylase|EHMN	-0.42028883	0.127463748	-1.1024505	0.30075573	0.53112925	-5.73192026
WT after UVB irradiation VS WT before UVB irradiation	Endohydrolysis of 1_4-alpha-D-glucosidic linkages in polysaccharides by alpha-amylase|EHMN	-0.3178039	0.039207149	-0.84106795	0.41981021	0.78544581	-5.52373713
homo after UVB irradiation VS homo before UVB irradiation	Endometrial cancer - Homo sapiens (human)|KEGG	0.120777459	-0.015845	0.603080732	0.56073852	0.68696862	-6.17682265
hetero after UVB irradiation VS hetero before UVB irradiation	Endometrial cancer - Homo sapiens (human)|KEGG	0.136011979	-0.00351514	0.763101627	0.46622848	0.68896154	-6.04138443
WT after UVB irradiation VS WT before UVB irradiation	Endometrial cancer - Homo sapiens (human)|KEGG	0.060200517	0.026589865	0.295217318	0.77381792	0.93418621	-5.82520427
homo after UVB irradiation VS homo before UVB irradiation	Endometrial cancer|Wikipathways	0.149310522	-0.02269928	0.76310381	0.46414233	0.6088463	-6.06514745
hetero after UVB irradiation VS hetero before UVB irradiation	Endometrial cancer|Wikipathways	0.048670595	0.003467685	0.278799371	0.78711949	0.90873624	-6.3045727
WT after UVB irradiation VS WT before UVB irradiation	Endometrial cancer|Wikipathways	-0.00209186	0.009998507	-0.01052537	0.99180749	0.99712795	-5.86909507
homo after UVB irradiation VS homo before UVB irradiation	Endosomal Sorting Complex Required For Transport (ESCRT)|Reactome	0.398889941	-0.02401298	1.705752817	0.12084388	0.26005561	-5.01240005
hetero after UVB irradiation VS hetero before UVB irradiation	Endosomal Sorting Complex Required For Transport (ESCRT)|Reactome	0.294930041	0.007364842	1.238102561	0.24906199	0.47399772	-5.5845385
WT after UVB irradiation VS WT before UVB irradiation	Endosomal Sorting Complex Required For Transport (ESCRT)|Reactome	0.164567713	0.0104356	0.621405777	0.5481245	0.84342472	-5.67746994
homo after UVB irradiation VS homo before UVB irradiation	Endosomal/Vacuolar pathway|Reactome	0.940585216	-0.00641833	5.17409997	0.00050997	0.01666204	0.22579364
hetero after UVB irradiation VS hetero before UVB irradiation	Endosomal/Vacuolar pathway|Reactome	0.708094875	0.045846521	2.60583476	0.03001742	0.15035098	-3.67391534
WT after UVB irradiation VS WT before UVB irradiation	Endosomal/Vacuolar pathway|Reactome	0.664410539	0.017502323	2.287819609	0.04497922	0.39428043	-3.7727523
homo after UVB irradiation VS homo before UVB irradiation	Endothelin Pathways|Wikipathways	0.186243437	-0.03236224	1.17257073	0.26985801	0.43325305	-5.68043972
hetero after UVB irradiation VS hetero before UVB irradiation	Endothelin Pathways|Wikipathways	0.170855952	-0.02784725	0.990511835	0.34951217	0.57899653	-5.84392885
WT after UVB irradiation VS WT before UVB irradiation	Endothelin Pathways|Wikipathways	0.199120359	-0.01167157	0.907923105	0.38510649	0.7587495	-5.46900797
homo after UVB irradiation VS homo before UVB irradiation	Endothelins|PID	0.414899907	-0.03934685	2.592888514	0.0281324	0.11225652	-3.66935789
hetero after UVB irradiation VS hetero before UVB irradiation	Endothelins|PID	0.467748639	0.019926235	2.757403824	0.02359385	0.13628011	-3.44197485
WT after UVB irradiation VS WT before UVB irradiation	Endothelins|PID	0.353467126	-0.00226559	1.69975755	0.11976763	0.52405201	-4.59579224
homo after UVB irradiation VS homo before UVB irradiation	Energy dependent regulation of mTOR by LKB1-AMPK|Reactome	0.302591428	-0.04488401	1.877908484	0.0917549	0.21706551	-4.76788743
hetero after UVB irradiation VS hetero before UVB irradiation	Energy dependent regulation of mTOR by LKB1-AMPK|Reactome	0.112224206	-0.02658259	0.569984941	0.58356219	0.77592663	-6.17370616
WT after UVB irradiation VS WT before UVB irradiation	Energy dependent regulation of mTOR by LKB1-AMPK|Reactome	0.099860828	-0.01769456	0.512297458	0.61947748	0.87257466	-5.73802966
homo after UVB irradiation VS homo before UVB irradiation	Energy Metabolism|Wikipathways	-0.29334825	0.010105338	-1.53268932	0.15832573	0.30644989	-5.24581681
hetero after UVB irradiation VS hetero before UVB irradiation	Energy Metabolism|Wikipathways	-0.41394424	-0.02280215	-2.5734883	0.03160418	0.15507377	-3.72333818
WT after UVB irradiation VS WT before UVB irradiation	Energy Metabolism|Wikipathways	-0.38265997	-0.02349927	-1.76477951	0.10781675	0.5076251	-4.50985257
homo after UVB irradiation VS homo before UVB irradiation	eNOS activation and regulation|Reactome	0.697013033	-0.05019342	4.394538113	0.00156613	0.02229782	-0.86323905
hetero after UVB irradiation VS hetero before UVB irradiation	eNOS activation and regulation|Reactome	0.59814461	-0.02679821	2.895645733	0.01896346	0.1205973	-3.23041836
WT after UVB irradiation VS WT before UVB irradiation	eNOS activation and regulation|Reactome	0.513201481	0.019218643	2.383541888	0.03818956	0.39022568	-3.63174344
homo after UVB irradiation VS homo before UVB irradiation	eNOS activation|Reactome	0.502140706	-0.02934335	2.741604844	0.02194576	0.09598115	-3.43206439
hetero after UVB irradiation VS hetero before UVB irradiation	eNOS activation|Reactome	0.342313736	0.009453022	1.293060652	0.23031414	0.45170905	-5.52149142
WT after UVB irradiation VS WT before UVB irradiation	eNOS activation|Reactome	0.44466412	0.005213089	1.856411772	0.09281895	0.48552482	-4.38616211
homo after UVB irradiation VS homo before UVB irradiation	Enoxaparin Action Pathway|SMPDB	0.542957634	0.032474294	3.216401628	0.00999373	0.05951688	-2.67225071
hetero after UVB irradiation VS hetero before UVB irradiation	Enoxaparin Action Pathway|SMPDB	0.507034173	0.109310797	2.703272449	0.02570936	0.14017854	-3.52484947
WT after UVB irradiation VS WT before UVB irradiation	Enoxaparin Action Pathway|SMPDB	0.380045687	0.058400764	1.387851186	0.19508646	0.60625027	-4.98260907
homo after UVB irradiation VS homo before UVB irradiation	Entry of Influenza Virion into Host Cell via Endocytosis|Reactome	1.094770633	-0.03078596	5.614113191	0.00028133	0.01666204	0.797051976
hetero after UVB irradiation VS hetero before UVB irradiation	Entry of Influenza Virion into Host Cell via Endocytosis|Reactome	1.068902692	0.032712215	5.86245514	0.00030952	0.03004176	0.766584983
WT after UVB irradiation VS WT before UVB irradiation	Entry of Influenza Virion into Host Cell via Endocytosis|Reactome	1.0627229	0.028811064	4.401952072	0.00130511	0.15645713	-0.70391183
homo after UVB irradiation VS homo before UVB irradiation	Enzymatic degradation of dopamine by COMT|Reactome	0.425703357	0.005899757	1.877612174	0.09179884	0.21706551	-4.76831745
hetero after UVB irradiation VS hetero before UVB irradiation	Enzymatic degradation of dopamine by COMT|Reactome	0.237891918	-0.01410789	1.203385584	0.26154493	0.48777487	-5.62340296
WT after UVB irradiation VS WT before UVB irradiation	Enzymatic degradation of dopamine by COMT|Reactome	0.98667826	0.013760519	3.611368751	0.00469506	0.20968398	-1.80360317
homo after UVB irradiation VS homo before UVB irradiation	Enzymatic degradation of Dopamine by monoamine oxidase|Reactome	1.116780111	-0.03793857	5.186414373	0.00050137	0.01666204	0.242209945
hetero after UVB irradiation VS hetero before UVB irradiation	Enzymatic degradation of Dopamine by monoamine oxidase|Reactome	0.585546456	0.073106689	1.581220934	0.15057605	0.35828077	-5.1638065
WT after UVB irradiation VS WT before UVB irradiation	Enzymatic degradation of Dopamine by monoamine oxidase|Reactome	0.786779097	0.019042696	2.31747774	0.04275877	0.39022568	-3.72919864
homo after UVB irradiation VS homo before UVB irradiation	EPHA forward signaling|PID	0.282421127	-0.00124341	1.407801716	0.19142508	0.34719862	-5.40510829
hetero after UVB irradiation VS hetero before UVB irradiation	EPHA forward signaling|PID	0.476614323	0.044957438	2.824649026	0.02121172	0.12891069	-3.33903029
WT after UVB irradiation VS WT before UVB irradiation	EPHA forward signaling|PID	0.171394406	-0.03479072	0.808030544	0.4377225	0.79685269	-5.54945751
homo after UVB irradiation VS homo before UVB irradiation	EPHA2 forward signaling|PID	0.380798446	-0.05140177	1.700592115	0.12183412	0.26055457	-5.01955239
hetero after UVB irradiation VS hetero before UVB irradiation	EPHA2 forward signaling|PID	0.756556905	0.021976764	4.943894637	0.00096947	0.03910488	-0.32591677
WT after UVB irradiation VS WT before UVB irradiation	EPHA2 forward signaling|PID	0.519290389	0.049691357	2.45273335	0.03391654	0.37872086	-3.52911053
homo after UVB irradiation VS homo before UVB irradiation	EPHA-mediated growth cone collapse|Reactome	0.025684081	0.024960395	0.119859469	0.90712012	0.94542581	-6.36073523
hetero after UVB irradiation VS hetero before UVB irradiation	EPHA-mediated growth cone collapse|Reactome	0.054753497	0.009945335	0.272917437	0.79148668	0.91160777	-6.30631695
WT after UVB irradiation VS WT before UVB irradiation	EPHA-mediated growth cone collapse|Reactome	-0.23642879	-0.02179037	-1.12913818	0.28498885	0.6726326	-5.26388656
homo after UVB irradiation VS homo before UVB irradiation	EPHB forward signaling|PID	0.381190859	-0.04185729	1.499430578	0.1666115	0.31658265	-5.28903998
hetero after UVB irradiation VS hetero before UVB irradiation	EPHB forward signaling|PID	0.572296662	-0.03021455	3.445069956	0.00809394	0.09122567	-2.39943233
WT after UVB irradiation VS WT before UVB irradiation	EPHB forward signaling|PID	0.277830866	-0.01759419	1.312348289	0.21849266	0.6260768	-5.06884807
homo after UVB irradiation VS homo before UVB irradiation	EPHB-mediated forward signaling|Reactome	0.482146077	-0.06574234	1.950144934	0.08161253	0.20262084	-4.66220512
hetero after UVB irradiation VS hetero before UVB irradiation	EPHB-mediated forward signaling|Reactome	0.630656046	0.019243108	3.66556106	0.00580753	0.08047311	-2.07397352
WT after UVB irradiation VS WT before UVB irradiation	EPHB-mediated forward signaling|Reactome	0.407252993	-0.01392196	1.922223088	0.08326338	0.470709	-4.29563569
homo after UVB irradiation VS homo before UVB irradiation	EPH-ephrin mediated repulsion of cells|Reactome	0.106877896	-0.04372226	0.413629386	0.68843383	0.79059076	-6.27737221
hetero after UVB irradiation VS hetero before UVB irradiation	EPH-ephrin mediated repulsion of cells|Reactome	0.711353772	0.017401142	3.875086256	0.00426175	0.07141563	-1.77043646
WT after UVB irradiation VS WT before UVB irradiation	EPH-ephrin mediated repulsion of cells|Reactome	0.21929906	0.036699896	0.9737483	0.35296447	0.73775227	-5.41172358
homo after UVB irradiation VS homo before UVB irradiation	EPH-Ephrin signaling|Reactome	0.246987613	-0.01194772	1.040435374	0.32418687	0.48028464	-5.8188625
hetero after UVB irradiation VS hetero before UVB irradiation	EPH-Ephrin signaling|Reactome	0.33539997	-0.0011271	1.574847729	0.15203307	0.36129653	-5.17215494
WT after UVB irradiation VS WT before UVB irradiation	EPH-Ephrin signaling|Reactome	0.252735205	0.0022392	1.141348214	0.28010878	0.66921967	-5.25155743
homo after UVB irradiation VS homo before UVB irradiation	Ephrin A reverse signaling|PID	-0.34660956	0.053255758	-1.22012724	0.25217489	0.4151674	-5.62762182
hetero after UVB irradiation VS hetero before UVB irradiation	Ephrin A reverse signaling|PID	-0.34349936	-0.05815382	-1.09187218	0.30512266	0.53490349	-5.74289151
WT after UVB irradiation VS WT before UVB irradiation	Ephrin A reverse signaling|PID	-0.15726018	-0.08562577	-0.58848123	0.56915728	0.85280226	-5.69688769
homo after UVB irradiation VS homo before UVB irradiation	Ephrin B reverse signaling|PID	0.30024578	-0.0578766	1.419429866	0.18810917	0.34397535	-5.39063262
hetero after UVB irradiation VS hetero before UVB irradiation	Ephrin B reverse signaling|PID	0.431541048	0.005661733	2.226175495	0.05498775	0.20875475	-4.24914669
WT after UVB irradiation VS WT before UVB irradiation	Ephrin B reverse signaling|PID	0.097470998	0.010925449	0.501667831	0.62667404	0.87283328	-5.74334344
homo after UVB irradiation VS homo before UVB irradiation	Ephrin signaling|Reactome	0.113027506	-0.00427031	0.452453053	0.66121566	0.76783079	-6.2596702
hetero after UVB irradiation VS hetero before UVB irradiation	Ephrin signaling|Reactome	0.24699204	-0.00963229	1.08654878	0.30733905	0.53610845	-5.74838289
WT after UVB irradiation VS WT before UVB irradiation	Ephrin signaling|Reactome	0.371298229	0.015229894	1.612050486	0.13777829	0.55341552	-4.70905618
homo after UVB irradiation VS homo before UVB irradiation	Epigenetic regulation of gene expression|Reactome	-0.64501474	0.006611729	-3.72979552	0.00436321	0.0379263	-1.86417684

hetero after UVB irradiation VS hetero before UVB irradiation	Epigenetic regulation of gene expression|Reactome	-0.37640573	-0.04565609	-1.59266174	0.14799206	0.35372199	-5.14877561
WT after UVB irradiation VS WT before UVB irradiation	Epigenetic regulation of gene expression|Reactome	-0.29723897	-0.02189783	-1.10791329	0.29362945	0.68002318	-5.28507907
homo after UVB irradiation VS homo before UVB irradiation	Epinephrine Action Pathway|SMPDB	0.396415397	-0.03677376	2.343069613	0.04268583	0.13402181	-4.06364635
hetero after UVB irradiation VS hetero before UVB irradiation	Epinephrine Action Pathway|SMPDB	-0.01555055	-0.01775524	-0.07410913	0.94265284	0.97054702	-6.34356282
WT after UVB irradiation VS WT before UVB irradiation	Epinephrine Action Pathway|SMPDB	0.056685205	-0.03978418	0.223404833	0.82768199	0.94525798	-5.84393438
homo after UVB irradiation VS homo before UVB irradiation	Epithelial cell signaling in Helicobacter pylori infection - Homo sapiens (human)|KEGG	0.40215348	-0.03945428	1.85560517	0.09511759	0.22192484	-4.80017263
hetero after UVB irradiation VS hetero before UVB irradiation	Epithelial cell signaling in Helicobacter pylori infection - Homo sapiens (human)|KEGG	0.56726042	0.016836396	3.739106652	0.00520617	0.07738625	-1.96675557
WT after UVB irradiation VS WT before UVB irradiation	Epithelial cell signaling in Helicobacter pylori infection - Homo sapiens (human)|KEGG	0.43092	0.006717724	2.194291456	0.05273757	0.41454814	-3.90917703
homo after UVB irradiation VS homo before UVB irradiation	Epithelial to mesenchymal transition in colorectal cancer|Wikipathways	-0.07282099	-0.00514113	-0.42423566	0.68094905	0.78475854	-6.27268752
hetero after UVB irradiation VS hetero before UVB irradiation	Epithelial to mesenchymal transition in colorectal cancer|Wikipathways	0.039410179	0.008969559	0.264104922	0.79804426	0.91506577	-6.30886188
WT after UVB irradiation VS WT before UVB irradiation	Epithelial to mesenchymal transition in colorectal cancer|Wikipathways	-0.17932026	0.015865111	-0.91729931	0.38040528	0.75273436	-5.46105122
homo after UVB irradiation VS homo before UVB irradiation	Eplerenone Action Pathway|SMPDB	0.234726031	0.017120546	1.156747303	0.27595732	0.43614438	-5.69767199
hetero after UVB irradiation VS hetero before UVB irradiation	Eplerenone Action Pathway|SMPDB	-0.03255037	0.028925632	-0.18154472	0.86023155	0.94503144	-6.32869738
WT after UVB irradiation VS WT before UVB irradiation	Eplerenone Action Pathway|SMPDB	-0.15908359	0.010252305	-0.80382265	0.44003996	0.79685269	-5.55266936
homo after UVB irradiation VS homo before UVB irradiation	EPO Receptor Signaling|Wikipathways	0.582601879	-0.04171284	3.521303291	0.00608793	0.04556964	-2.1894789
hetero after UVB irradiation VS hetero before UVB irradiation	EPO Receptor Signaling|Wikipathways	0.568562517	0.019544288	3.702147847	0.00549966	0.07940727	-2.02054734
WT after UVB irradiation VS WT before UVB irradiation	EPO Receptor Signaling|Wikipathways	0.452622735	-0.00619479	2.124314727	0.05936901	0.42631804	-4.01019951
homo after UVB irradiation VS homo before UVB irradiation	epo signaling pathway|BioCarta	0.656107054	-0.03166266	3.508682483	0.00621293	0.04588471	-2.20931168
hetero after UVB irradiation VS hetero before UVB irradiation	epo signaling pathway|BioCarta	0.677098102	0.060480214	3.692342864	0.00558043	0.08000318	-2.03484814
WT after UVB irradiation VS WT before UVB irradiation	epo signaling pathway|BioCarta	0.505679796	0.024736794	2.562655873	0.02807674	0.35160213	-3.36512469
homo after UVB irradiation VS homo before UVB irradiation	EPO signaling pathway|PID	0.410683125	-0.03413909	1.989777569	0.07650667	0.19626432	-4.60354088
hetero after UVB irradiation VS hetero before UVB irradiation	EPO signaling pathway|PID	0.446375729	0.03304707	2.767239976	0.02322897	0.13628011	-3.42691503
WT after UVB irradiation VS WT before UVB irradiation	EPO signaling pathway|PID	0.505732142	-0.01356316	2.357022839	0.03996339	0.39022568	-3.67093196
homo after UVB irradiation VS homo before UVB irradiation	EPO signaling|INOH	0.097713673	-0.01671212	0.570767252	0.58155863	0.70403417	-6.19646074
hetero after UVB irradiation VS hetero before UVB irradiation	EPO signaling|INOH	0.207616695	-0.00066684	1.342413456	0.21450494	0.43637221	-5.46335003
WT after UVB irradiation VS WT before UVB irradiation	EPO signaling|INOH	0.084433601	-0.03586431	0.420357661	0.6830428	0.88772239	-5.78047138
homo after UVB irradiation VS homo before UVB irradiation	epoxysqualene biosynthesis|HumanCyc	-0.3672054	0.028192118	-0.92739299	0.37694577	0.52974281	-5.92686266
hetero after UVB irradiation VS hetero before UVB irradiation	epoxysqualene biosynthesis|HumanCyc	0.508706317	0.006216512	1.380545531	0.20293302	0.42313299	-5.41748361
WT after UVB irradiation VS WT before UVB irradiation	epoxysqualene biosynthesis|HumanCyc	0.308133163	0.034350935	0.773966971	0.45671509	0.80891799	-5.57503715
homo after UVB irradiation VS homo before UVB irradiation	Eprosartan Action Pathway|SMPDB	0.636038586	-0.11800708	2.436891056	0.03650532	0.12504267	-3.91641947
hetero after UVB irradiation VS hetero before UVB irradiation	Eprosartan Action Pathway|SMPDB	-0.02777369	0.003323189	-0.09172332	0.92906122	0.97054702	-6.34197953
WT after UVB irradiation VS WT before UVB irradiation	Eprosartan Action Pathway|SMPDB	0.270438532	-0.10210744	0.707878218	0.49505953	0.81710765	-5.62188349
homo after UVB irradiation VS homo before UVB irradiation	Epstein-Barr virus infection - Homo sapiens (human)|KEGG	0.26425655	-0.03555367	1.372541317	0.20178747	0.35894742	-5.44853379
hetero after UVB irradiation VS hetero before UVB irradiation	Epstein-Barr virus infection - Homo sapiens (human)|KEGG	0.383408054	0.022808696	2.452955659	0.03830068	0.1700514	-3.90702648
WT after UVB irradiation VS WT before UVB irradiation	Epstein-Barr virus infection - Homo sapiens (human)|KEGG	0.373549141	0.025738111	1.841220168	0.09516454	0.49164755	-4.40686497
homo after UVB irradiation VS homo before UVB irradiation	Eptifibatide Action Pathway|SMPDB	0.250990463	0.059108895	0.688272445	0.50792806	0.64727869	-6.12030765
hetero after UVB irradiation VS hetero before UVB irradiation	Eptifibatide Action Pathway|SMPDB	0.140468489	-0.09168173	0.363509274	0.72517643	0.86482318	-6.27542943
WT after UVB irradiation VS WT before UVB irradiation	Eptifibatide Action Pathway|SMPDB	0.031786681	-0.05176445	0.082324754	0.93599995	0.98056728	-5.86571905
homo after UVB irradiation VS homo before UVB irradiation	er associated degradation (erad) pathway|BioCarta	0.128030874	-0.01942824	0.755094235	0.46870938	0.61374449	-6.07129249
hetero after UVB irradiation VS hetero before UVB irradiation	er associated degradation (erad) pathway|BioCarta	0.150511308	-0.02401982	0.843170332	0.42242143	0.64915606	-5.97673382
WT after UVB irradiation VS WT before UVB irradiation	er associated degradation (erad) pathway|BioCarta	0.515141541	0.042620356	2.24416915	0.04845195	0.40306021	-3.83660685
homo after UVB irradiation VS homo before UVB irradiation	ER Quality Control Compartment (ERQC)|Reactome	0.390898857	-0.03872707	2.100240486	0.06383023	0.17452027	-4.43774442
hetero after UVB irradiation VS hetero before UVB irradiation	ER Quality Control Compartment (ERQC)|Reactome	0.381312191	0.030120934	2.116620169	0.06544847	0.22964907	-4.41188387
WT after UVB irradiation VS WT before UVB irradiation	ER Quality Control Compartment (ERQC)|Reactome	0.196674393	0.015859536	0.951875378	0.36342183	0.74528402	-5.43112449
homo after UVB irradiation VS homo before UVB irradiation	ER to Golgi Anterograde Transport|Reactome	0.485103816	-0.04121761	2.673277052	0.02459727	0.10366003	-3.54124416
hetero after UVB irradiation VS hetero before UVB irradiation	ER to Golgi Anterograde Transport|Reactome	0.478595512	0.010600681	2.847418396	0.02046227	0.12653175	-3.30418548
WT after UVB irradiation VS WT before UVB irradiation	ER to Golgi Anterograde Transport|Reactome	0.364795545	-0.02800941	1.802960514	0.10131604	0.50394202	-4.45866458
homo after UVB irradiation VS homo before UVB irradiation	ErbB receptor signaling network|PID	-0.42771264	0.061972037	-2.30650823	0.04536372	0.13845639	-4.12067035
hetero after UVB irradiation VS hetero before UVB irradiation	ErbB receptor signaling network|PID	-0.54413475	-0.04917546	-2.94327671	0.01759402	0.11690018	-3.1576304
WT after UVB irradiation VS WT before UVB irradiation	ErbB receptor signaling network|PID	-0.41934823	-0.01075041	-1.99882614	0.07329961	0.45403763	-4.188669
homo after UVB irradiation VS homo before UVB irradiation	ErbB signaling pathway - Homo sapiens (human)|KEGG	0.072697161	0.01383653	0.350069061	0.73401179	0.82447671	-6.30304051
hetero after UVB irradiation VS hetero before UVB irradiation	ErbB signaling pathway - Homo sapiens (human)|KEGG	0.173805689	0.002856036	1.162378228	0.27694461	0.50756476	-5.66831153
WT after UVB irradiation VS WT before UVB irradiation	ErbB signaling pathway - Homo sapiens (human)|KEGG	0.04962488	0.005979534	0.255627474	0.80337804	0.94525798	-5.83616264
homo after UVB irradiation VS homo before UVB irradiation	ErbB Signaling Pathway|Wikipathways	0.066651282	0.010819058	0.316264691	0.75871979	0.84413123	-6.31499749
hetero after UVB irradiation VS hetero before UVB irradiation	ErbB Signaling Pathway|Wikipathways	0.202145565	-0.00112391	1.333261346	0.2173649	0.43877289	-5.47423762
WT after UVB irradiation VS WT before UVB irradiation	ErbB Signaling Pathway|Wikipathways	0.050701252	-0.00077517	0.262567797	0.79817093	0.94477083	-5.8343537
homo after UVB irradiation VS homo before UVB irradiation	ErbB1 downstream signaling|PID	0.235684399	-0.04243801	1.071702866	0.31062622	0.46923284	-5.78725434
hetero after UVB irradiation VS hetero before UVB irradiation	ErbB1 downstream signaling|PID	0.351903198	0.012789993	2.303479377	0.04861487	0.19626802	-4.13319922
WT after UVB irradiation VS WT before UVB irradiation	ErbB1 downstream signaling|PID	0.207063931	0.026794226	1.026887008	0.32847979	0.71204479	-5.36310977
homo after UVB irradiation VS homo before UVB irradiation	ERBB2 Activates PTK6 Signaling|Reactome	-0.17561726	0.055798477	-0.85833057	0.41208474	0.56379199	-5.98778522
hetero after UVB irradiation VS hetero before UVB irradiation	ERBB2 Activates PTK6 Signaling|Reactome	-0.40869534	-0.04646406	-2.40274183	0.0414955	0.17944638	-3.98324819
WT after UVB irradiation VS WT before UVB irradiation	ERBB2 Activates PTK6 Signaling|Reactome	-0.26397699	0.030384354	-1.03897535	0.32309107	0.70648859	-5.35176328
homo after UVB irradiation VS homo before UVB irradiation	ERBB2 Regulates Cell Motility|Reactome	-0.11194386	0.077433323	-0.56171006	0.58746933	0.70943797	-6.2017847
hetero after UVB irradiation VS hetero before UVB irradiation	ERBB2 Regulates Cell Motility|Reactome	-0.21797374	-0.04628645	-1.38409678	0.2018832	0.42210258	-5.41317142
WT after UVB irradiation VS WT before UVB irradiation	ERBB2 Regulates Cell Motility|Reactome	-0.17927628	0.047472798	-0.75963042	0.46486645	0.80891799	-5.5855138
homo after UVB irradiation VS homo before UVB irradiation	ErbB2/ErbB3 signaling events|PID	0.195769431	-0.01176549	0.945631753	0.36803471	0.52090715	-5.91012065
hetero after UVB irradiation VS hetero before UVB irradiation	ErbB2/ErbB3 signaling events|PID	0.362449074	-0.01180956	2.373646987	0.04346722	0.18293959	-4.02730837
WT after UVB irradiation VS WT before UVB irradiation	ErbB2/ErbB3 signaling events|PID	0.280418539	0.042404069	1.467334033	0.17277399	0.58147297	-4.88847382
homo after UVB irradiation VS homo before UVB irradiation	ErbB4 signaling events|PID	0.009300754	0.01618198	0.04901282	0.96193591	0.97710919	-6.36715769
hetero after UVB irradiation VS hetero before UVB irradiation	ErbB4 signaling events|PID	0.16237248	-0.02256417	0.956891728	0.36526788	0.59333249	-5.87574621
WT after UVB irradiation VS WT before UVB irradiation	ErbB4 signaling events|PID	-0.11510217	-0.00497325	-0.52110371	0.61354708	0.87257466	-5.73354766
homo after UVB irradiation VS homo before UVB irradiation	ERCC6 (CSB) and EHMT2 (G9a) positively regulate rRNA expression|Reactome	-0.53777921	0.050650381	-2.59198794	0.02817476	0.11225652	-3.67079056
hetero after UVB irradiation VS hetero before UVB irradiation	ERCC6 (CSB) and EHMT2 (G9a) positively regulate rRNA expression|Reactome	-0.24042238	0.022762554	-0.82441176	0.43242168	0.65858414	-5.99237149
WT after UVB irradiation VS WT before UVB irradiation	ERCC6 (CSB) and EHMT2 (G9a) positively regulate rRNA expression|Reactome	-0.24065053	0.071154759	-0.88398511	0.39729417	0.7654467	-5.48901084
homo after UVB irradiation VS homo before UVB irradiation	erk and pi-3 kinase are necessary for collagen binding in corneal epithelia|BioCarta	0.623100362	-0.02295611	2.859195401	0.01804017	0.08518242	-3.24379107
hetero after UVB irradiation VS hetero before UVB irradiation	erk and pi-3 kinase are necessary for collagen binding in corneal epithelia|BioCarta	0.509042901	-0.01197525	2.967559782	0.01693556	0.11437413	-3.12055411
WT after UVB irradiation VS WT before UVB irradiation	erk and pi-3 kinase are necessary for collagen binding in corneal epithelia|BioCarta	0.496016716	0.017983463	2.557134732	0.02834476	0.35176669	-3.37338371
homo after UVB irradiation VS homo before UVB irradiation	ERK Pathway in Huntington_s Disease|Wikipathways	0.120571096	-0.0542544	0.586508792	0.57136346	0.69550668	-6.18701925
hetero after UVB irradiation VS hetero before UVB irradiation	ERK Pathway in Huntington_s Disease|Wikipathways	0.150438642	-0.01166439	0.917599205	0.38434076	0.61213953	-5.91181613
WT after UVB irradiation VS WT before UVB irradiation	ERK Pathway in Huntington_s Disease|Wikipathways	0.211901645	-0.00649509	0.831807578	0.42478031	0.7908939	-5.53103615
homo after UVB irradiation VS homo before UVB irradiation	ERK/MAPK targets|Reactome	0.0512339	-0.01598405	0.32668262	0.75107295	0.83786931	-6.31143884
hetero after UVB irradiation VS hetero before UVB irradiation	ERK/MAPK targets|Reactome	0.358957862	0.008047412	1.916417029	0.08977107	0.27151728	-4.70296196
WT after UVB irradiation VS WT before UVB irradiation	ERK/MAPK targets|Reactome	0.17990451	-0.00433042	0.845495301	0.41744801	0.78391656	-5.520223
homo after UVB irradiation VS homo before UVB irradiation	erk1/erk2 mapk signaling pathway|BioCarta	-0.05558928	-0.03020182	-0.32607458	0.75151848	0.83816154	-6.31164963
hetero after UVB irradiation VS hetero before UVB irradiation	erk1/erk2 mapk signaling pathway|BioCarta	0.249480947	0.00217571	1.379015186	0.20338687	0.42369167	-5.41933976
WT after UVB irradiation VS WT before UVB irradiation	erk1/erk2 mapk signaling pathway|BioCarta	0.233901078	0.031230002	1.185459767	0.26302364	0.65841086	-5.2061933
homo after UVB irradiation VS homo before UVB irradiation	ERKs are inactivated|Reactome	0.002464236	-0.02053779	0.012003126	0.99067431	0.99328478	-6.36837085
hetero after UVB irradiation VS hetero before UVB irradiation	ERKs are inactivated|Reactome	0.37322307	0.000261975	2.341352461	0.04576536	0.18854754	-4.07611316
WT after UVB irradiation VS WT before UVB irradiation	ERKs are inactivated|Reactome	0.092693458	0.041423789	0.368325741	0.7202462	0.90672477	-5.80091854
homo after UVB irradiation VS homo before UVB irradiation	Erlotinib Action Pathway|SMPDB	-0.00954399	0.027600782	-0.02798687	0.97825849	0.98606224	-6.36802743
hetero after UVB irradiation VS hetero before UVB irradiation	Erlotinib Action Pathway|SMPDB	0.450480736	0.077000723	1.834989604	0.10195761	0.28686591	-4.81840356
WT after UVB irradiation VS WT before UVB irradiation	Erlotinib Action Pathway|SMPDB	0.041335014	-0.03262342	0.127514023	0.90104104	0.97566251	-5.86092118
homo after UVB irradiation VS homo before UVB irradiation	Erlotinib Pathway_ Pharmacokinetics|PharmGKB	-0.29415127	5.83E-05	-1.22588561	0.25009894	0.41255482	-5.62112361
hetero after UVB irradiation VS hetero before UVB irradiation	Erlotinib Pathway_ Pharmacokinetics|PharmGKB	-0.25095117	-0.0019153	-1.11398975	0.29604856	0.52748019	-5.71986337
WT after UVB irradiation VS WT before UVB irradiation	Erlotinib Pathway_ Pharmacokinetics|PharmGKB	-0.46654151	-0.04358841	-2.12797084	0.05900361	0.42631804	-4.00494595
homo after UVB irradiation VS homo before UVB irradiation	ER-Phagosome pathway|Reactome	0.863158209	-0.032726	4.722674954	0.00096621	0.01815527	-0.39300962
hetero after UVB irradiation VS hetero before UVB irradiation	ER-Phagosome pathway|Reactome	0.664984633	0.02169389	3.413400904	0.00849346	0.09297946	-2.44663481
WT after UVB irradiation VS WT before UVB irradiation	ER-Phagosome pathway|Reactome	0.553768102	-0.02470829	2.156269061	0.05624727	0.41769317	-3.96418955
homo after UVB irradiation VS homo before UVB irradiation	Erythrocytes take up carbon dioxide and release oxygen|Reactome	-0.11472985	-0.05123043	-0.48638821	0.63784355	0.74907244	-6.2429561
hetero after UVB irradiation VS hetero before UVB irradiation	Erythrocytes take up carbon dioxide and release oxygen|Reactome	-0.06140183	-0.10427143	-0.30837226	0.76528559	0.89270377	-6.2952511
WT after UVB irradiation VS WT before UVB irradiation	Erythrocytes take up carbon dioxide and release oxygen|Reactome	-0.14373732	0.004995015	-0.63136589	0.54185001	0.83916339	-5.67140403
homo after UVB irradiation VS homo before UVB irradiation	Erythrocytes take up oxygen and release carbon dioxide|Reactome	-0.06656013	-0.05321002	-0.26016266	0.80035397	0.87562289	-6.33221509
hetero after UVB irradiation VS hetero before UVB irradiation	Erythrocytes take up oxygen and release carbon dioxide|Reactome	0.262851506	-0.04529543	1.049085535	0.32329493	0.55397681	-5.78645708
WT after UVB irradiation VS WT before UVB irradiation	Erythrocytes take up oxygen and release carbon dioxide|Reactome	0.1063142	0.028344716	0.351957009	0.73211587	0.91205892	-5.80680953
homo after UVB irradiation VS homo before UVB irradiation	erythropoietin mediated neuroprotection through nf-kb|BioCarta	-0.01493522	-0.03759577	-0.0637761	0.95048571	0.97003079	-6.36626332
hetero after UVB irradiation VS hetero before UVB irradiation	erythropoietin mediated neuroprotection through nf-kb|BioCarta	0.325168914	-0.04812554	1.496787384	0.17093396	0.38742764	-5.27292245
WT after UVB irradiation VS WT before UVB irradiation	erythropoietin mediated neuroprotection through nf-kb|BioCarta	-0.1845396	-0.05567817	-0.76586568	0.46130982	0.80891799	-5.58097849
homo after UVB irradiation VS homo before UVB irradiation	ESC Pluripotency Pathways|Wikipathways	-0.15074982	0.000391088	-0.93468185	0.37336612	0.5263321	-5.92020419
hetero after UVB irradiation VS hetero before UVB irradiation	ESC Pluripotency Pathways|Wikipathways	-0.21682598	0.005919274	-1.25695875	0.24249121	0.46543442	-5.56311399
WT after UVB irradiation VS WT before UVB irradiation	ESC Pluripotency Pathways|Wikipathways	-0.15315229	0.028324419	-0.77626037	0.45541977	0.80867112	-5.57334524
homo after UVB irradiation VS homo before UVB irradiation	Escitalopram Action Pathway|SMPDB	0.191132361	0.029768717	1.061313976	0.31508276	0.46923284	-5.79783739
hetero after UVB irradiation VS hetero before UVB irradiation	Escitalopram Action Pathway|SMPDB	-0.07904315	0.055005373	-0.44437398	0.66796482	0.82651398	-6.24068873
WT after UVB irradiation VS WT before UVB irradiation	Escitalopram Action Pathway|SMPDB	0.022316377	0.035038618	0.098344171	0.92358676	0.97566251	-5.86425414
homo after UVB irradiation VS homo before UVB irradiation	Esmolol Action Pathway|SMPDB	0.396415397	-0.03677376	2.343069613	0.04268583	0.13402181	-4.06364635
hetero after UVB irradiation VS hetero before UVB irradiation	Esmolol Action Pathway|SMPDB	-0.01555055	-0.01775524	-0.07410913	0.94265284	0.97054702	-6.34356282
WT after UVB irradiation VS WT before UVB irradiation	Esmolol Action Pathway|SMPDB	0.056685205	-0.03978418	0.223404833	0.82768199	0.94525798	-5.84393438
homo after UVB irradiation VS homo before UVB irradiation	Esomeprazole Action Pathway|SMPDB	0.21780303	0.051698704	1.19150037	0.26270351	0.42561191	-5.65959884
hetero after UVB irradiation VS hetero before UVB irradiation	Esomeprazole Action Pathway|SMPDB	0.331566302	0.063470812	1.441200644	0.18563717	0.40321767	-5.34291209
WT after UVB irradiation VS WT before UVB irradiation	Esomeprazole Action Pathway|SMPDB	-0.11560861	0.135982321	-0.44330845	0.66690385	0.87935805	-5.77062647
WT after UVB irradiation VS WT before UVB irradiation	Esomeprazole Metabolism Pathway|SMPDB	-0.06647614	0.033610835	-0.18292054	0.85874942	0.9134577	-6.35050291
WT after UVB irradiation VS WT before UVB irradiation	Esomeprazole Metabolism Pathway|SMPDB	-0.4370633	0.058240551	-1.4704397	0.17194858	0.57997722	-4.88472923
homo after UVB irradiation VS homo before UVB irradiation	ESR-mediated signaling|Reactome	-0.54531184	0.041840831	-3.22432265	0.00986475	0.05934449	-2.65962734
hetero after UVB irradiation VS hetero before UVB irradiation	ESR-mediated signaling|Reactome	-0.41643377	-0.03634885	-2.35444947	0.04481912	0.18704365	-4.05633387
WT after UVB irradiation VS WT before UVB irradiation	ESR-mediated signaling|Reactome	-0.38630048	0.004749177	-1.67635506	0.12435396	0.52904005	-4.62632302
homo after UVB irradiation VS homo before UVB irradiation	ESR-mediated signaling|Wikipathways	-0.57270553	0.031340399	-3.56691327	0.00565763	0.04348946	-2.11793261
hetero after UVB irradiation VS hetero before UVB irradiation	ESR-mediated signaling|Wikipathways	-0.42971247	-0.04787245	-2.22017068	0.05551596	0.20938837	-4.25811764
WT after UVB irradiation VS WT before UVB irradiation	ESR-mediated signaling|Wikipathways	-0.42459334	0.030873634	-1.76327598	0.10808037	0.5076251	-4.51185763
homo after UVB irradiation VS homo before UVB irradiation	Establishment of Sister Chromatid Cohesion|Reactome	-1.05429593	-0.03986669	-5.6441127	0.00027041	0.01666204	0.834856212
hetero after UVB irradiation VS hetero before UVB irradiation	Establishment of Sister Chromatid Cohesion|Reactome	-0.42706655	-0.08241502	-1.83828983	0.10143469	0.28640434	-4.81376311
WT after UVB irradiation VS WT before UVB irradiation	Establishment of Sister Chromatid Cohesion|Reactome	-0.42350167	-0.05257751	-1.30120724	0.22213666	0.62934127	-5.08129746
homo after UVB irradiation VS homo before UVB irradiation	estradiol biosynthesis I|HumanCyc	-0.1542137	-0.09206323	-0.57787576	0.57694245	0.70016088	-6.19222674
hetero after UVB irradiation VS hetero before UVB irradiation	estradiol biosynthesis I|HumanCyc	-0.06080769	-0.04668839	-0.18593255	0.85689614	0.94503144	-6.32782624
WT after UVB irradiation VS WT before UVB irradiation	estradiol biosynthesis I|HumanCyc	-0.49041556	-0.1412853	-1.52140841	0.15888175	0.56072663	-4.8225916
homo after UVB irradiation VS homo before UVB irradiation	estradiol biosynthesis II|HumanCyc	0.184849238	-0.01037525	0.48216169	0.64073239	0.75095947	-6.24510047
hetero after UVB irradiation VS hetero before UVB irradiation	estradiol biosynthesis II|HumanCyc	0.443823365	0.003188208	1.158997237	0.2782464	0.50892741	-5.67196484
WT after UVB irradiation VS WT before UVB irradiation	estradiol biosynthesis II|HumanCyc	0.015508698	-0.06466795	0.034801648	0.97291729	0.99045333	-5.86853767
homo after UVB irradiation VS homo before UVB irradiation	Estrogen biosynthesis|Reactome	0.405433292	-0.01133685	1.630321698	0.13606998	0.27798457	-5.11581017
hetero after UVB irradiation VS hetero before UVB irradiation	Estrogen biosynthesis|Reactome	0.339808859	0.054719111	1.888165169	0.09383381	0.27672034	-4.74323313
WT after UVB irradiation VS WT before UVB irradiation	Estrogen biosynthesis|Reactome	0.077636097	0.044518	0.260104055	0.80001823	0.94525798	-5.83500132
homo after UVB irradiation VS homo before UVB irradiation	Estrogen Metabolism Pathway|PharmGKB	-0.20919665	0.096434788	-0.79436477	0.44659547	0.59438796	-6.04062041
hetero after UVB irradiation VS hetero before UVB irradiation	Estrogen Metabolism Pathway|PharmGKB	-0.31959042	0.012873586	-1.36323673	0.20811748	0.43001491	-5.43840281
WT after UVB irradiation VS WT before UVB irradiation	Estrogen Metabolism Pathway|PharmGKB	-0.31708502	-0.03242459	-1.18342622	0.26379265	0.6590487	-5.20831253
homo after UVB irradiation VS homo before UVB irradiation	Estrogen metabolism|Wikipathways	-0.07897383	0.081808545	-0.42013788	0.68383654	0.78720899	-6.274511
hetero after UVB irradiation VS hetero before UVB irradiation	Estrogen metabolism|Wikipathways	-0.02156587	-0.01489181	-0.09680494	0.92514444	0.97054702	-6.34146037
WT after UVB irradiation VS WT before UVB irradiation	Estrogen metabolism|Wikipathways	0.166256013	0.006952877	0.628007804	0.54396084	0.8402851	-5.67345908
homo after UVB irradiation VS homo before UVB irradiation	Estrogen Receptor Pathway|Wikipathways	-0.11693237	0.028098125	-0.51279486	0.61994099	0.73561606	-6.22915714
hetero after UVB irradiation VS hetero before UVB irradiation	Estrogen Receptor Pathway|Wikipathways	-0.31804441	-0.05014336	-1.28796152	0.23200228	0.45326463	-5.52741765
WT after UVB irradiation VS WT before UVB irradiation	Estrogen Receptor Pathway|Wikipathways	-0.49722133	-0.01926827	-2.42720967	0.03543519	0.38724228	-3.56703094

homo after UVB irradiation VS homo before UVB irradiation	estrogen responsive protein efp controls cell cycle and breast tumors growth|BioCarta	-0.0790587	0.060792603	-0.42699616	0.67900693	0.78327076	-6.27144953
hetero after UVB irradiation VS hetero before UVB irradiation	estrogen responsive protein efp controls cell cycle and breast tumors growth|BioCarta	0.48427544	-0.00085665	2.354470049	0.04481764	0.18704365	-4.05630277
WT after UVB irradiation VS WT before UVB irradiation	estrogen responsive protein efp controls cell cycle and breast tumors growth|BioCarta	0.359282028	0.059193896	1.438851285	0.18050447	0.59650067	-4.92258712
homo after UVB irradiation VS homo before UVB irradiation	Estrogen signaling pathway - Homo sapiens (human)|KEGG	0.151316997	-0.00648942	0.887776629	0.39683241	0.54957136	-5.96229351
hetero after UVB irradiation VS hetero before UVB irradiation	Estrogen signaling pathway - Homo sapiens (human)|KEGG	0.050745073	0.039483888	0.324657956	0.75335412	0.88469054	-6.28972606
WT after UVB irradiation VS WT before UVB irradiation	Estrogen signaling pathway - Homo sapiens (human)|KEGG	-0.04129689	0.026226981	-0.21610899	0.83321248	0.94894798	-5.8455505
homo after UVB irradiation VS homo before UVB irradiation	Estrogen signaling pathway|Wikipathways	0.232471308	0.004370153	1.274008353	0.23329031	0.39505544	-5.56597297
hetero after UVB irradiation VS hetero before UVB irradiation	Estrogen signaling pathway|Wikipathways	0.450367777	0.027646482	2.145510341	0.06251462	0.22396997	-4.36917197
WT after UVB irradiation VS WT before UVB irradiation	Estrogen signaling pathway|Wikipathways	0.394116123	-0.0062567	1.701711957	0.1193916	0.5237862	-4.5932327
homo after UVB irradiation VS homo before UVB irradiation	Estrogen-dependent gene expression|Reactome	-0.50547498	0.043212382	-2.95884587	0.01528697	0.07678802	-3.0841028
hetero after UVB irradiation VS hetero before UVB irradiation	Estrogen-dependent gene expression|Reactome	-0.35560476	-0.0293436	-1.93601874	0.08705123	0.26690451	-4.67489203
WT after UVB irradiation VS WT before UVB irradiation	Estrogen-dependent gene expression|Reactome	-0.36075506	0.009454413	-1.56738221	0.14784719	0.55622203	-4.765475
homo after UVB irradiation VS homo before UVB irradiation	Ethacrynic Acid Action Pathway|SMPDB	0.234726031	0.017120546	1.156747303	0.27595732	0.43614438	-5.69767199
hetero after UVB irradiation VS hetero before UVB irradiation	Ethacrynic Acid Action Pathway|SMPDB	-0.03255037	0.028925632	-0.18154472	0.86023155	0.94503144	-6.32869738
WT after UVB irradiation VS WT before UVB irradiation	Ethacrynic Acid Action Pathway|SMPDB	-0.15908359	0.010252305	-0.80382265	0.44003996	0.79685269	-5.55266936
homo after UVB irradiation VS homo before UVB irradiation	ethanol degradation II|HumanCyc	-0.00926507	0.039663404	-0.03597304	0.97205709	0.98255916	-6.36775301
hetero after UVB irradiation VS hetero before UVB irradiation	ethanol degradation II|HumanCyc	-0.3648989	-0.02625408	-1.43121937	0.18839181	0.406192	-5.35531553
WT after UVB irradiation VS WT before UVB irradiation	ethanol degradation II|HumanCyc	-0.14484348	-0.07982719	-0.51478817	0.61779723	0.87257466	-5.73676931
homo after UVB irradiation VS homo before UVB irradiation	ethanol degradation IV|HumanCyc	-0.00347701	0.032686971	-0.01259994	0.99021065	0.99325611	-6.36836296
hetero after UVB irradiation VS hetero before UVB irradiation	ethanol degradation IV|HumanCyc	-0.42113981	-0.07120545	-1.44508553	0.18457458	0.40321767	-5.3380707
WT after UVB irradiation VS WT before UVB irradiation	ethanol degradation IV|HumanCyc	-0.24808381	-0.04139715	-0.84155672	0.41954899	0.78544581	-5.52334996
homo after UVB irradiation VS homo before UVB irradiation	Ethanol Degradation|SMPDB	0.055518024	0.030118806	0.184158252	0.8578055	0.9134577	-6.35025969
hetero after UVB irradiation VS hetero before UVB irradiation	Ethanol Degradation|SMPDB	-0.57271164	-0.00379079	-1.94684292	0.0855832	0.26482567	-4.6593478
WT after UVB irradiation VS WT before UVB irradiation	Ethanol Degradation|SMPDB	-0.21798857	0.030810225	-0.7308411	0.48151504	0.81552033	-5.60602794
homo after UVB irradiation VS homo before UVB irradiation	Ethanol effects on histone modifications|Wikipathways	0.00910377	0.031317812	0.04848764	0.96234341	0.97710919	-6.36718519
hetero after UVB irradiation VS hetero before UVB irradiation	Ethanol effects on histone modifications|Wikipathways	-0.01807083	0.001720084	-0.10034231	0.92241919	0.97054702	-6.34108249
WT after UVB irradiation VS WT before UVB irradiation	Ethanol effects on histone modifications|Wikipathways	0.265913301	0.010420458	1.304286355	0.22112456	0.62764193	-5.07786402
homo after UVB irradiation VS homo before UVB irradiation	Ethanol metabolism resulting in production of ROS by CYP2E1|Wikipathways	0.121774833	0.056018798	0.542188413	0.60031845	0.72038214	-6.21298914
hetero after UVB irradiation VS hetero before UVB irradiation	Ethanol metabolism resulting in production of ROS by CYP2E1|Wikipathways	0.028781883	0.068432581	0.096849482	0.92511012	0.97054702	-6.3414557
WT after UVB irradiation VS WT before UVB irradiation	Ethanol metabolism resulting in production of ROS by CYP2E1|Wikipathways	-0.19086143	-0.02144877	-0.73305013	0.48022442	0.81552033	-5.60447883
homo after UVB irradiation VS homo before UVB irradiation	Ethanol oxidation|Reactome	-0.16945264	0.031218514	-0.59122485	0.56832845	0.69273564	-6.18414427
hetero after UVB irradiation VS hetero before UVB irradiation	Ethanol oxidation|Reactome	-0.43527265	-0.03866724	-1.8551091	0.09880873	0.28271827	-4.7900616
WT after UVB irradiation VS WT before UVB irradiation	Ethanol oxidation|Reactome	-0.28447506	-0.05879048	-1.20848586	0.25444029	0.64897339	-5.18201177
homo after UVB irradiation VS homo before UVB irradiation	Ether lipid metabolism - Homo sapiens (human)|KEGG	0.146351286	0.027440113	0.873245717	0.40430891	0.55654943	-5.97496386
hetero after UVB irradiation VS hetero before UVB irradiation	Ether lipid metabolism - Homo sapiens (human)|KEGG	0.321489839	-0.00686286	2.058786406	0.07172973	0.23970209	-4.49689772
WT after UVB irradiation VS WT before UVB irradiation	Ether lipid metabolism - Homo sapiens (human)|KEGG	0.220717281	0.008852353	1.088312592	0.30178769	0.68672863	-5.30437645
homo after UVB irradiation VS homo before UVB irradiation	Ethylmalonic Encephalopathy|SMPDB	-0.19843953	0.039801734	-0.74439404	0.47485571	0.61754234	-6.07941248
hetero after UVB irradiation VS hetero before UVB irradiation	Ethylmalonic Encephalopathy|SMPDB	-0.48316271	0.016700705	-1.75538636	0.11536374	0.30887493	-4.92927061
WT after UVB irradiation VS WT before UVB irradiation	Ethylmalonic Encephalopathy|SMPDB	-0.40763842	0.012220754	-1.51649601	0.16010237	0.56072663	-4.82863564
homo after UVB irradiation VS homo before UVB irradiation	Ethylmorphine Action Pathway|SMPDB	0.191132361	0.029768717	1.061313976	0.31508276	0.46923284	-5.79783739
hetero after UVB irradiation VS hetero before UVB irradiation	Ethylmorphine Action Pathway|SMPDB	-0.07904315	0.055005373	-0.44437398	0.66796482	0.82651398	-6.24068873
WT after UVB irradiation VS WT before UVB irradiation	Ethylmorphine Action Pathway|SMPDB	0.022316377	0.035038618	0.098344171	0.92358676	0.97566251	-5.86425414
homo after UVB irradiation VS homo before UVB irradiation	Etodolac Action Pathway|SMPDB	0.40423582	0.045705158	1.979500697	0.07780079	0.19626432	-4.61879689
hetero after UVB irradiation VS hetero before UVB irradiation	Etodolac Action Pathway|SMPDB	0.482496143	0.064876536	3.04499191	0.01500113	0.1130168	-3.00250826
WT after UVB irradiation VS WT before UVB irradiation	Etodolac Action Pathway|SMPDB	0.275725339	0.086595857	1.261970114	0.23537138	0.62952955	-5.12455679
homo after UVB irradiation VS homo before UVB irradiation	Etoposide Action Pathway|SMPDB	-0.109809	0.007481266	-0.49301193	0.63332911	0.74607346	-6.23955976
hetero after UVB irradiation VS hetero before UVB irradiation	Etoposide Action Pathway|SMPDB	0.233839477	0.023463399	1.095907136	0.3034511	0.53328183	-5.73871593
WT after UVB irradiation VS WT before UVB irradiation	Etoposide Action Pathway|SMPDB	0.223448145	0.084789547	1.039170948	0.32300443	0.70648859	-5.35157882
homo after UVB irradiation VS homo before UVB irradiation	Etoposide Metabolism Pathway|SMPDB	-0.109809	0.007481266	-0.49301193	0.63332911	0.74607346	-6.23955976
hetero after UVB irradiation VS hetero before UVB irradiation	Etoposide Metabolism Pathway|SMPDB	0.233839477	0.023463399	1.095907136	0.3034511	0.53328183	-5.73871593
WT after UVB irradiation VS WT before UVB irradiation	Etoposide Metabolism Pathway|SMPDB	0.223448145	0.084789547	1.039170948	0.32300443	0.70648859	-5.35157882
homo after UVB irradiation VS homo before UVB irradiation	Etoposide Pathway_ Pharmacokinetics/Pharmacodynamics|PharmGKB	-0.10620078	0.014703745	-0.48996271	0.63540536	0.74697757	-6.24112867
hetero after UVB irradiation VS hetero before UVB irradiation	Etoposide Pathway_ Pharmacokinetics/Pharmacodynamics|PharmGKB	-0.02149445	0.047082313	-0.09530544	0.9263	0.97054702	-6.34161647
WT after UVB irradiation VS WT before UVB irradiation	Etoposide Pathway_ Pharmacokinetics/Pharmacodynamics|PharmGKB	-0.07870247	0.112156958	-0.3818076	0.71052789	0.90414624	-5.79587127
homo after UVB irradiation VS homo before UVB irradiation	Etoricoxib Action Pathway|SMPDB	0.40423582	0.045705158	1.979500697	0.07780079	0.19626432	-4.61879689
hetero after UVB irradiation VS hetero before UVB irradiation	Etoricoxib Action Pathway|SMPDB	0.482496143	0.064876536	3.04499191	0.01500113	0.1130168	-3.00250826
WT after UVB irradiation VS WT before UVB irradiation	Etoricoxib Action Pathway|SMPDB	0.275725339	0.086595857	1.261970114	0.23537138	0.62952955	-5.12455679
homo after UVB irradiation VS homo before UVB irradiation	eukaryotic protein translation|BioCarta	-0.76354588	-0.00518897	-4.2874645	0.00183959	0.02379486	-1.02026355
hetero after UVB irradiation VS hetero before UVB irradiation	eukaryotic protein translation|BioCarta	-0.09543698	0.014273775	-0.29494745	0.77517118	0.90044332	-6.29959664
WT after UVB irradiation VS WT before UVB irradiation	eukaryotic protein translation|BioCarta	-0.45164917	-0.01325463	-1.67876076	0.12387532	0.52904005	-4.62319465
homo after UVB irradiation VS homo before UVB irradiation	Eukaryotic Transcription Initiation|Wikipathways	-0.77005961	0.018324834	-4.42212299	0.00150292	0.02211705	-0.82306598
hetero after UVB irradiation VS hetero before UVB irradiation	Eukaryotic Transcription Initiation|Wikipathways	-0.41365004	-0.06893217	-2.20188033	0.05715583	0.21310989	-4.28540839
WT after UVB irradiation VS WT before UVB irradiation	Eukaryotic Transcription Initiation|Wikipathways	-0.33209314	-0.03988232	-1.11382255	0.29120365	0.6781882	-5.27920956
homo after UVB irradiation VS homo before UVB irradiation	Eukaryotic Translation Elongation|Reactome	-0.93498455	0.025394	-4.94519734	0.00070255	0.01666204	-0.08385399
hetero after UVB irradiation VS hetero before UVB irradiation	Eukaryotic Translation Elongation|Reactome	-0.13098217	0.006667166	-0.39074321	0.70568146	0.85253617	-6.26447665
WT after UVB irradiation VS WT before UVB irradiation	Eukaryotic Translation Elongation|Reactome	-0.60217445	0.014138582	-2.03665752	0.06880338	0.44205977	-4.13526342
homo after UVB irradiation VS homo before UVB irradiation	Eukaryotic Translation Initiation|Reactome	-0.9616441	0.025393751	-5.42439674	0.00036236	0.01666204	0.554607925
hetero after UVB irradiation VS hetero before UVB irradiation	Eukaryotic Translation Initiation|Reactome	-0.27370492	-0.01616192	-0.82660804	0.43124249	0.6573924	-5.99055596
WT after UVB irradiation VS WT before UVB irradiation	Eukaryotic Translation Initiation|Reactome	-0.59454382	-0.01466313	-1.98364867	0.07518015	0.45723857	-4.20999049
homo after UVB irradiation VS homo before UVB irradiation	Eukaryotic Translation Termination|Reactome	-0.91284328	0.028798885	-4.8351799	0.0008217	0.01753207	-0.23570694
hetero after UVB irradiation VS hetero before UVB irradiation	Eukaryotic Translation Termination|Reactome	-0.16461	-0.01599913	-0.50056675	0.62948784	0.80936688	-6.21265117
WT after UVB irradiation VS WT before UVB irradiation	Eukaryotic Translation Termination|Reactome	-0.57411506	0.002536661	-1.96523328	0.07752279	0.46277213	-4.23577754
homo after UVB irradiation VS homo before UVB irradiation	eumelanin biosynthesis|HumanCyc	-0.87601117	0.062534169	-3.32049111	0.00842931	0.05459326	-2.50666863
hetero after UVB irradiation VS hetero before UVB irradiation	eumelanin biosynthesis|HumanCyc	-0.73923434	0.002918901	-2.91446197	0.01840978	0.11936114	-3.20165495
WT after UVB irradiation VS WT before UVB irradiation	eumelanin biosynthesis|HumanCyc	-0.94261533	0.100275536	-3.8082717	0.00338865	0.20197958	-1.52089001
homo after UVB irradiation VS homo before UVB irradiation	EV release from cardiac cells and their functional effects|Wikipathways	0.295396387	0.043365689	1.35652505	0.20664968	0.36459135	-5.46802117
hetero after UVB irradiation VS hetero before UVB irradiation	EV release from cardiac cells and their functional effects|Wikipathways	0.285516681	0.031797679	1.049699849	0.32302821	0.55372794	-5.78584091
WT after UVB irradiation VS WT before UVB irradiation	EV release from cardiac cells and their functional effects|Wikipathways	0.172942632	0.04001087	0.688270138	0.50680935	0.82465997	-5.63506259
homo after UVB irradiation VS homo before UVB irradiation	Evolocumab Mechanism|Wikipathways	0.614474587	0.045253128	1.77845028	0.10765453	0.24079424	-4.91050563
hetero after UVB irradiation VS hetero before UVB irradiation	Evolocumab Mechanism|Wikipathways	0.776358909	0.037286384	2.618640193	0.02941178	0.14913595	-3.65433897
WT after UVB irradiation VS WT before UVB irradiation	Evolocumab Mechanism|Wikipathways	0.709600779	-0.03032083	2.05914282	0.06625595	0.43803373	-4.10335283
homo after UVB irradiation VS homo before UVB irradiation	Excitatory Neural Signalling Through 5-HTR 4 and Serotonin|SMPDB	0.503170469	0.000741612	2.649105727	0.0256106	0.10578915	-3.57980936
hetero after UVB irradiation VS hetero before UVB irradiation	Excitatory Neural Signalling Through 5-HTR 4 and Serotonin|SMPDB	0.848800921	0.040164885	4.4371921	0.00191587	0.04958933	-0.98793924
WT after UVB irradiation VS WT before UVB irradiation	Excitatory Neural Signalling Through 5-HTR 4 and Serotonin|SMPDB	0.726046823	-0.12679237	3.1948682	0.009479	0.25023459	-2.41647074
homo after UVB irradiation VS homo before UVB irradiation	Excitatory Neural Signalling Through 5-HTR 6 and Serotonin|SMPDB	0.505700533	-0.01378513	3.098956308	0.01212379	0.06750878	-2.85977684
hetero after UVB irradiation VS hetero before UVB irradiation	Excitatory Neural Signalling Through 5-HTR 6 and Serotonin|SMPDB	0.760775338	0.031846196	3.739085891	0.00520633	0.07738625	-1.96678574
WT after UVB irradiation VS WT before UVB irradiation	Excitatory Neural Signalling Through 5-HTR 6 and Serotonin|SMPDB	0.705577346	-0.11605788	2.926468017	0.01500843	0.2892131	-2.81853595
homo after UVB irradiation VS homo before UVB irradiation	Excitatory Neural Signalling Through 5-HTR 7 and Serotonin|SMPDB	0.598594045	0.01709927	3.124586729	0.01162212	0.06584328	-2.81880146
hetero after UVB irradiation VS hetero before UVB irradiation	Excitatory Neural Signalling Through 5-HTR 7 and Serotonin|SMPDB	0.895699992	0.042823275	4.862810172	0.00107841	0.04104387	-0.4290081
WT after UVB irradiation VS WT before UVB irradiation	Excitatory Neural Signalling Through 5-HTR 7 and Serotonin|SMPDB	0.737065396	-0.11118505	3.221028207	0.00906591	0.24856491	-2.37750444
homo after UVB irradiation VS homo before UVB irradiation	Exercise-induced Circadian Regulation|Wikipathways	-0.33404073	-0.01297614	-1.97307287	0.07862073	0.19761678	-4.62832344
hetero after UVB irradiation VS hetero before UVB irradiation	Exercise-induced Circadian Regulation|Wikipathways	0.027562107	-0.01743814	0.161933148	0.87517454	0.95431712	-6.33233853
WT after UVB irradiation VS WT before UVB irradiation	Exercise-induced Circadian Regulation|Wikipathways	-0.30824114	-0.07026443	-1.4213494	0.1854002	0.59839062	-4.9433406
homo after UVB irradiation VS homo before UVB irradiation	Export of Viral Ribonucleoproteins from Nucleus|Reactome	-0.78876262	-0.01316435	-4.29819648	0.00181003	0.02356086	-1.00444769
hetero after UVB irradiation VS hetero before UVB irradiation	Export of Viral Ribonucleoproteins from Nucleus|Reactome	-0.25633234	-0.0697783	-0.90733471	0.38944051	0.61655778	-5.9210369
WT after UVB irradiation VS WT before UVB irradiation	Export of Viral Ribonucleoproteins from Nucleus|Reactome	-0.15981245	-0.01571445	-0.47921627	0.64200929	0.87745357	-5.75421949
homo after UVB irradiation VS homo before UVB irradiation	Expression and Processing of Neurotrophins|Reactome	0.393747226	-0.0787386	1.795293373	0.10479387	0.2370387	-4.88660513
hetero after UVB irradiation VS hetero before UVB irradiation	Expression and Processing of Neurotrophins|Reactome	0.480751157	0.022217775	1.628535985	0.14014788	0.34069016	-5.10128348
WT after UVB irradiation VS WT before UVB irradiation	Expression and Processing of Neurotrophins|Reactome	0.595352451	0.036212764	2.855413395	0.01695907	0.30353778	-2.92539776
homo after UVB irradiation VS homo before UVB irradiation	exRNA mechanism of action and biogenesis|Wikipathways	-0.83595603	-0.0475636	-3.72897466	0.00436889	0.0379263	-1.86544835
hetero after UVB irradiation VS hetero before UVB irradiation	exRNA mechanism of action and biogenesis|Wikipathways	-0.59322706	-0.11791372	-2.30017315	0.04887183	0.19678251	-4.13817451
WT after UVB irradiation VS WT before UVB irradiation	exRNA mechanism of action and biogenesis|Wikipathways	-0.54180026	-0.07196111	-1.49747108	0.16490701	0.56840025	-4.8519332
homo after UVB irradiation VS homo before UVB irradiation	Extension of Telomeres|Reactome	-0.56951259	-0.05312075	-2.14049366	0.05973211	0.16711814	-4.37656807
hetero after UVB irradiation VS hetero before UVB irradiation	Extension of Telomeres|Reactome	0.078897302	-0.02443281	0.293232315	0.77643732	0.90142672	-6.30013818
WT after UVB irradiation VS WT before UVB irradiation	Extension of Telomeres|Reactome	0.091447707	0.054175077	0.31475591	0.75936434	0.92455796	-5.81922622
homo after UVB irradiation VS homo before UVB irradiation	Extracellular matrix organization|Reactome	0.678568856	-0.0016409	4.302851674	0.00179736	0.02356086	-0.99759257
hetero after UVB irradiation VS hetero before UVB irradiation	Extracellular matrix organization|Reactome	0.440961496	0.039663409	2.088650036	0.0684165	0.23513821	-4.45308361
WT after UVB irradiation VS WT before UVB irradiation	Extracellular matrix organization|Reactome	0.396800793	0.025547803	1.660488226	0.12755272	0.53719353	-4.64689722
homo after UVB irradiation VS homo before UVB irradiation	Extracellular vesicle-mediated signaling in recipient cells|Wikipathways	-0.07553102	0.027223454	-0.3328225	0.74657948	0.83510091	-6.30928876
hetero after UVB irradiation VS hetero before UVB irradiation	Extracellular vesicle-mediated signaling in recipient cells|Wikipathways	-0.14371175	-0.04109783	-0.89456906	0.39585078	0.62309352	-5.93238621
WT after UVB irradiation VS WT before UVB irradiation	Extracellular vesicle-mediated signaling in recipient cells|Wikipathways	-0.17380461	0.033866936	-0.74605034	0.47267331	0.81337567	-5.59527801
homo after UVB irradiation VS homo before UVB irradiation	Extracellular vesicles in the crosstalk of cardiac cells|Wikipathways	0.621485082	-0.04194022	3.364398338	0.00784763	0.05200618	-2.43703939
hetero after UVB irradiation VS hetero before UVB irradiation	Extracellular vesicles in the crosstalk of cardiac cells|Wikipathways	0.445833649	0.078741651	2.376491729	0.04327038	0.18278703	-4.02300406
WT after UVB irradiation VS WT before UVB irradiation	Extracellular vesicles in the crosstalk of cardiac cells|Wikipathways	0.308980674	-0.04312659	1.007853208	0.33710048	0.72143293	-5.38076108
homo after UVB irradiation VS homo before UVB irradiation	Extrinsic Pathway of Fibrin Clot Formation|Reactome	0.975823622	0.021025973	4.970102091	0.00067823	0.01666204	-0.04975094
hetero after UVB irradiation VS hetero before UVB irradiation	Extrinsic Pathway of Fibrin Clot Formation|Reactome	0.728328025	0.034541093	2.517757358	0.03453976	0.16285121	-3.8083783
WT after UVB irradiation VS WT before UVB irradiation	Extrinsic Pathway of Fibrin Clot Formation|Reactome	0.997247414	0.088624122	3.717908112	0.00393352	0.20870715	-1.64998615
homo after UVB irradiation VS homo before UVB irradiation	extrinsic prothrombin activation pathway|BioCarta	0.60760229	0.077864943	3.642114123	0.00501617	0.04060967	-2.00042362
hetero after UVB irradiation VS hetero before UVB irradiation	extrinsic prothrombin activation pathway|BioCarta	0.334105788	0.026670223	1.493044274	0.17189072	0.38882278	-5.27768299
WT after UVB irradiation VS WT before UVB irradiation	extrinsic prothrombin activation pathway|BioCarta	0.442942767	0.028867831	1.542215901	0.15380125	0.55854784	-4.79686341
homo after UVB irradiation VS homo before UVB irradiation	FABP4 in ovarian cancer|Wikipathways	-0.47647449	-0.00579854	-1.29336077	0.22679871	0.38647281	-5.54337713
hetero after UVB irradiation VS hetero before UVB irradiation	FABP4 in ovarian cancer|Wikipathways	-1.03703704	-0.06370205	-4.33827658	0.00219798	0.0524522	-1.12206845
WT after UVB irradiation VS WT before UVB irradiation	FABP4 in ovarian cancer|Wikipathways	-0.41027555	-0.08900914	-1.15337798	0.27536507	0.66577703	-5.23931305
homo after UVB irradiation VS homo before UVB irradiation	Fabry disease|SMPDB	0.410096132	-0.05671339	1.580382344	0.14707883	0.29223756	-5.18287502
hetero after UVB irradiation VS hetero before UVB irradiation	Fabry disease|SMPDB	0.381719767	-0.02021245	2.484597089	0.03641544	0.16681566	-3.85889413
WT after UVB irradiation VS WT before UVB irradiation	Fabry disease|SMPDB	0.327957029	0.011268274	1.427782053	0.1835878	0.59839062	-4.93573151
homo after UVB irradiation VS homo before UVB irradiation	Factors and pathways affecting insulin-like growth factor (IGF1)-Akt signaling|Wikipathways	0.117598611	-0.03158478	0.596247534	0.56510602	0.69102145	-6.18105891
hetero after UVB irradiation VS hetero before UVB irradiation	Factors and pathways affecting insulin-like growth factor (IGF1)-Akt signaling|Wikipathways	-0.03777039	-0.04482478	-0.18407146	0.85831049	0.94503144	-6.32819825
WT after UVB irradiation VS WT before UVB irradiation	Factors and pathways affecting insulin-like growth factor (IGF1)-Akt signaling|Wikipathways	-0.1043995	-0.0852836	-0.45453855	0.6590706	0.87935805	-5.76562644
homo after UVB irradiation VS homo before UVB irradiation	Factors involved in megakaryocyte development and platelet production|Reactome	-0.06059172	-0.01528737	-0.35790618	0.72832791	0.82152798	-6.30009967
hetero after UVB irradiation VS hetero before UVB irradiation	Factors involved in megakaryocyte development and platelet production|Reactome	0.117204998	-0.01457329	0.686771216	0.51068211	0.72945442	-6.09778282
WT after UVB irradiation VS WT before UVB irradiation	Factors involved in megakaryocyte development and platelet production|Reactome	0.005892209	-0.00669326	0.02899246	0.97743648	0.99265119	-5.86872539
hetero after UVB irradiation VS hetero before UVB irradiation	Factors involved in megakaryocyte development and platelet production|Wikipathways	0.333105604	0.055731528	1.092739339	0.30476281	0.53447821	-5.74199509
homo after UVB irradiation VS homo before UVB irradiation	Familial Hypercholanemia (FHCA)|SMPDB	0.143425895	0.01576019	0.783036509	0.45290274	0.6002769	-6.04960791
hetero after UVB irradiation VS hetero before UVB irradiation	Familial Hypercholanemia (FHCA)|SMPDB	-0.01773938	0.040584313	-0.07540586	0.94165152	0.97054702	-6.34345771
WT after UVB irradiation VS WT before UVB irradiation	Familial Hypercholanemia (FHCA)|SMPDB	0.061518516	0.026229795	0.23751225	0.8170163	0.94525798	-5.84065895
homo after UVB irradiation VS homo before UVB irradiation	Familial lipoprotein lipase deficiency|SMPDB	0.512939135	0.00160955	2.795906579	0.02004569	0.09043427	-3.34516503
hetero after UVB irradiation VS hetero before UVB irradiation	Familial lipoprotein lipase deficiency|SMPDB	0.12253787	0.061964979	0.508050343	0.62444904	0.80561526	-6.20867996
WT after UVB irradiation VS WT before UVB irradiation	Familial lipoprotein lipase deficiency|SMPDB	0.284092323	-0.00634299	1.317391439	0.21685951	0.62581393	-5.06318888
homo after UVB irradiation VS homo before UVB irradiation	Famotidine Action Pathway|SMPDB	0.21780303	0.051698704	1.19150037	0.26270351	0.42561191	-5.65959884
hetero after UVB irradiation VS hetero before UVB irradiation	Famotidine Action Pathway|SMPDB	0.331566302	0.063470812	1.441200644	0.18563717	0.40321767	-5.34291209
WT after UVB irradiation VS WT before UVB irradiation	Famotidine Action Pathway|SMPDB	-0.11560861	0.135982321	-0.44330845	0.66690385	0.87935805	-5.77062647
homo after UVB irradiation VS homo before UVB irradiation	Fanconi anemia pathway - Homo sapiens (human)|KEGG	-0.48639104	-0.03211821	-2.2809073	0.04733611	0.14163609	-4.16046925
hetero after UVB irradiation VS hetero before UVB irradiation	Fanconi anemia pathway - Homo sapiens (human)|KEGG	0.085735003	-0.0469325	0.324217932	0.75367559	0.88469054	-6.28987898
WT after UVB irradiation VS WT before UVB irradiation	Fanconi anemia pathway - Homo sapiens (human)|KEGG	0.099051299	0.043907448	0.356447823	0.72885185	0.9105948	-5.80521925
homo after UVB irradiation VS homo before UVB irradiation	Fanconi anemia pathway|PID	-0.55772742	-0.01393021	-2.57466156	0.02900229	0.11412829	-3.69834193
hetero after UVB irradiation VS hetero before UVB irradiation	Fanconi anemia pathway|PID	0.14764689	-0.03706788	0.53262647	0.60804864	0.79548958	-6.19525035
WT after UVB irradiation VS WT before UVB irradiation	Fanconi anemia pathway|PID	0.112037603	0.046619748	0.387455316	0.70647274	0.90152666	-5.79370464

homo after UVB irradiation VS homo before UVB irradiation	Fanconi Anemia Pathway|Reactome	-0.38198131	-0.00258349	-1.99927199	0.07532924	0.19626432	-4.58941958
hetero after UVB irradiation VS hetero before UVB irradiation	Fanconi Anemia Pathway|Reactome	0.235275764	-0.01540258	1.000395032	0.34497899	0.57450283	-5.83441137
WT after UVB irradiation VS WT before UVB irradiation	Fanconi Anemia Pathway|Reactome	0.09799128	0.025391236	0.353601833	0.73091973	0.91105633	-5.80622935
homo after UVB irradiation VS homo before UVB irradiation	Fanconi-bickel syndrome|SMPDB	0.441748628	-0.03359755	1.766880832	0.10966101	0.24271071	-4.92686053
hetero after UVB irradiation VS hetero before UVB irradiation	Fanconi-bickel syndrome|SMPDB	0.197724182	0.033762374	0.765455745	0.46489888	0.68805201	-6.03956266
WT after UVB irradiation VS WT before UVB irradiation	Fanconi-bickel syndrome|SMPDB	0.075540742	-0.03429086	0.238329189	0.81639984	0.94525798	-5.8404632
homo after UVB irradiation VS homo before UVB irradiation	Farnesoid X Receptor Pathway|Wikipathways	0.005092625	0.074016274	0.028963876	0.97749973	0.98606224	-6.36799754
hetero after UVB irradiation VS hetero before UVB irradiation	Farnesoid X Receptor Pathway|Wikipathways	-0.48634527	0.018042588	-2.18156984	0.05903261	0.21664304	-4.31565078
WT after UVB irradiation VS WT before UVB irradiation	Farnesoid X Receptor Pathway|Wikipathways	0.053474744	0.004211694	0.192871271	0.85088961	0.95502804	-5.85034356
homo after UVB irradiation VS homo before UVB irradiation	FAS (CD95) signaling pathway|PID	0.179808322	-0.01144706	0.998212821	0.34320518	0.49859207	-5.86037018
hetero after UVB irradiation VS hetero before UVB irradiation	FAS (CD95) signaling pathway|PID	0.399361855	0.007462711	2.240083677	0.05378325	0.20588996	-4.22834769
WT after UVB irradiation VS WT before UVB irradiation	FAS (CD95) signaling pathway|PID	0.263420114	-0.04569694	1.262008884	0.23535799	0.62952955	-5.1245145
homo after UVB irradiation VS homo before UVB irradiation	Fas Ligand (FasL) pathway and Stress induction of Heat Shock Proteins (HSP) regulation|Wikipathways	0.206176701	-0.0457015	1.080748402	0.30678556	0.46864358	-5.77797475
hetero after UVB irradiation VS hetero before UVB irradiation	Fas Ligand (FasL) pathway and Stress induction of Heat Shock Proteins (HSP) regulation|Wikipathways	0.255043071	0.009410499	1.389991638	0.20015091	0.4197851	-5.4059984
WT after UVB irradiation VS WT before UVB irradiation	Fas Ligand (FasL) pathway and Stress induction of Heat Shock Proteins (HSP) regulation|Wikipathways	0.25337741	-0.00189555	1.308103703	0.2198751	0.62621406	-5.07359968
homo after UVB irradiation VS homo before UVB irradiation	fas signaling pathway (cd95)|BioCarta	0.09669087	-0.00947669	0.504929994	0.62524635	0.73909372	-6.23333913
hetero after UVB irradiation VS hetero before UVB irradiation	fas signaling pathway (cd95)|BioCarta	0.19775512	0.004549743	0.835968935	0.4262414	0.65304481	-5.98277215
WT after UVB irradiation VS WT before UVB irradiation	fas signaling pathway (cd95)|BioCarta	0.127862794	0.017531155	0.56041157	0.58743471	0.86161188	-5.71266644
homo after UVB irradiation VS homo before UVB irradiation	Fas|INOH	0.466331054	-0.02715779	2.037364973	0.07077615	0.18768759	-4.53251096
hetero after UVB irradiation VS hetero before UVB irradiation	Fas|INOH	0.562930933	0.02731457	3.208962651	0.01162495	0.10542662	-2.75372895
WT after UVB irradiation VS WT before UVB irradiation	Fas|INOH	0.444677518	0.010135221	2.142881046	0.05753558	0.42266366	-3.98349208
homo after UVB irradiation VS homo before UVB irradiation	FasL/ CD95L signaling|Reactome	0.984992242	-0.05012315	5.308630999	0.00042394	0.01666204	0.403795838
hetero after UVB irradiation VS hetero before UVB irradiation	FasL/ CD95L signaling|Reactome	0.629746787	-0.03983808	1.874960522	0.09579197	0.27962159	-4.76197775
WT after UVB irradiation VS WT before UVB irradiation	FasL/ CD95L signaling|Reactome	0.706617033	-0.1283164	3.084856087	0.01143818	0.26821873	-2.58083773
homo after UVB irradiation VS homo before UVB irradiation	Fat digestion and absorption - Homo sapiens (human)|KEGG	0.271557912	0.082285576	1.692533398	0.1233952	0.26258979	-5.03069895
hetero after UVB irradiation VS hetero before UVB irradiation	Fat digestion and absorption - Homo sapiens (human)|KEGG	0.118664936	0.02932414	0.731005718	0.48460484	0.70547073	-6.06573443
WT after UVB irradiation VS WT before UVB irradiation	Fat digestion and absorption - Homo sapiens (human)|KEGG	0.141544157	0.07644931	0.621746035	0.54790946	0.84342472	-5.67726418
homo after UVB irradiation VS homo before UVB irradiation	fatty acid α-oxidation III|HumanCyc	-0.16056223	-0.02482858	-0.65570045	0.52775531	0.66365485	-6.14271777
hetero after UVB irradiation VS hetero before UVB irradiation	fatty acid α-oxidation III|HumanCyc	-0.65216642	-0.14789402	-2.55777366	0.03240544	0.15685114	-3.74733296
WT after UVB irradiation VS WT before UVB irradiation	fatty acid α-oxidation III|HumanCyc	-0.47895295	-0.05957997	-1.96902291	0.07703518	0.46222215	-4.23047848
homo after UVB irradiation VS homo before UVB irradiation	fatty acid α-oxidation|HumanCyc	0.478670501	0.040803201	2.566647099	0.02939324	0.11431817	-3.71107782
hetero after UVB irradiation VS hetero before UVB irradiation	fatty acid α-oxidation|HumanCyc	0.408658427	-0.0450395	1.420715486	0.19132918	0.41074913	-5.36831298
WT after UVB irradiation VS WT before UVB irradiation	fatty acid α-oxidation|HumanCyc	0.209205316	-0.04860556	0.718903945	0.48852689	0.81710765	-5.61432688
homo after UVB irradiation VS homo before UVB irradiation	fatty acid β-oxidation (peroxisome)|HumanCyc	-0.08280317	-0.0514691	-0.31419574	0.76024173	0.84500091	-6.3156908
hetero after UVB irradiation VS hetero before UVB irradiation	fatty acid β-oxidation (peroxisome)|HumanCyc	-0.31318264	-0.03013638	-1.26303031	0.24040643	0.46293726	-5.556169
WT after UVB irradiation VS WT before UVB irradiation	fatty acid β-oxidation (peroxisome)|HumanCyc	-0.24289566	-0.02387432	-0.88485435	0.39684695	0.76537164	-5.48829237
homo after UVB irradiation VS homo before UVB irradiation	fatty acid β-oxidation (unsaturated_ odd number)|HumanCyc	-0.2864037	0.0110298	-0.89317746	0.39407848	0.54703225	-5.9575394
hetero after UVB irradiation VS hetero before UVB irradiation	fatty acid β-oxidation (unsaturated_ odd number)|HumanCyc	-0.34314979	-0.11230318	-1.1394518	0.28586892	0.51562743	-5.69293457
WT after UVB irradiation VS WT before UVB irradiation	fatty acid β-oxidation (unsaturated_ odd number)|HumanCyc	-0.20601622	0.032667338	-0.6140566	0.55278066	0.84516674	-5.68188882
homo after UVB irradiation VS homo before UVB irradiation	fatty acid β-oxidation|HumanCyc	-0.17086209	-0.02678679	-0.63987199	0.53755466	0.67264307	-6.15325127
hetero after UVB irradiation VS hetero before UVB irradiation	fatty acid β-oxidation|HumanCyc	-0.3154814	-0.05661286	-1.27272236	0.23710951	0.45989156	-5.54503629
WT after UVB irradiation VS WT before UVB irradiation	fatty acid β-oxidation|HumanCyc	-0.21553432	-0.03773401	-0.74869141	0.47114852	0.81275627	-5.59339127
homo after UVB irradiation VS homo before UVB irradiation	fatty acid activation|HumanCyc	-0.01315098	0.015191753	-0.04859126	0.96226301	0.97710919	-6.36717979
hetero after UVB irradiation VS hetero before UVB irradiation	fatty acid activation|HumanCyc	0.023664277	-0.05428518	0.091582546	0.92916975	0.97054702	-6.34199352
WT after UVB irradiation VS WT before UVB irradiation	fatty acid activation|HumanCyc	-0.19347113	-0.04507564	-0.69126346	0.50500476	0.82448724	-5.63307228
homo after UVB irradiation VS homo before UVB irradiation	Fatty Acid Beta Oxidation|Wikipathways	-0.25194105	0.003775971	-1.07607904	0.30876353	0.46923284	-5.78277246
hetero after UVB irradiation VS hetero before UVB irradiation	Fatty Acid Beta Oxidation|Wikipathways	-0.51147002	-0.03329645	-2.62605431	0.02906681	0.14819519	-3.64300217
WT after UVB irradiation VS WT before UVB irradiation	Fatty Acid Beta Oxidation|Wikipathways	-0.41071015	-0.06370347	-1.81844585	0.09878348	0.49761536	-4.43775867
homo after UVB irradiation VS homo before UVB irradiation	Fatty acid biosynthesis - Homo sapiens (human)|KEGG	-0.14022621	0.018046307	-0.50477363	0.62535206	0.73909372	-6.23342166
hetero after UVB irradiation VS hetero before UVB irradiation	Fatty acid biosynthesis - Homo sapiens (human)|KEGG	0.120159739	-0.00720994	0.570195892	0.58342548	0.77592663	-6.17358067
WT after UVB irradiation VS WT before UVB irradiation	Fatty acid biosynthesis - Homo sapiens (human)|KEGG	0.230027021	0.018516704	0.935314921	0.37148691	0.74850253	-5.44557236
homo after UVB irradiation VS homo before UVB irradiation	fatty acid biosynthesis initiation|HumanCyc	-0.36931918	-0.02988018	-0.94389792	0.3688752	0.52161169	-5.91172374
hetero after UVB irradiation VS hetero before UVB irradiation	fatty acid biosynthesis initiation|HumanCyc	-0.19068684	-0.11702266	-0.91727521	0.38450098	0.61213953	-5.91210847
WT after UVB irradiation VS WT before UVB irradiation	fatty acid biosynthesis initiation|HumanCyc	0.516716503	0.087695988	2.058068736	0.06637558	0.43803373	-4.10487991
homo after UVB irradiation VS homo before UVB irradiation	Fatty Acid Biosynthesis|SMPDB	-0.40678415	-0.02683895	-1.13034444	0.28637784	0.44641309	-5.72603698
hetero after UVB irradiation VS hetero before UVB irradiation	Fatty Acid Biosynthesis|SMPDB	0.038041246	-0.09845606	0.111647447	0.91371696	0.97054702	-6.33978415
WT after UVB irradiation VS WT before UVB irradiation	Fatty Acid Biosynthesis|SMPDB	0.847574424	0.007431558	2.765985928	0.0197818	0.31542987	-3.05993072
homo after UVB irradiation VS homo before UVB irradiation	Fatty Acid Biosynthesis|Wikipathways	-0.26095123	-0.01697779	-0.93173234	0.37481169	0.52771822	-5.92290379
hetero after UVB irradiation VS hetero before UVB irradiation	Fatty Acid Biosynthesis|Wikipathways	-0.548368	-0.04512636	-2.88876508	0.01917018	0.12135791	-3.24093913
WT after UVB irradiation VS WT before UVB irradiation	Fatty Acid Biosynthesis|Wikipathways	-0.15224467	-0.09019997	-0.60059185	0.56136921	0.85017866	-5.68985891
homo after UVB irradiation VS homo before UVB irradiation	Fatty acid degradation - Homo sapiens (human)|KEGG	-0.03158651	-0.01645479	-0.13132563	0.89828877	0.9399608	-6.35919039
hetero after UVB irradiation VS hetero before UVB irradiation	Fatty acid degradation - Homo sapiens (human)|KEGG	-0.2014564	-0.03227416	-1.01386224	0.33887369	0.5677486	-5.821324
WT after UVB irradiation VS WT before UVB irradiation	Fatty acid degradation - Homo sapiens (human)|KEGG	-0.12657183	-0.02624442	-0.49515027	0.63110706	0.87303764	-5.74654938
homo after UVB irradiation VS homo before UVB irradiation	Fatty acid elongation - Homo sapiens (human)|KEGG	-0.31497582	-0.02518302	-1.54869344	0.15446959	0.30167159	-5.2248197
hetero after UVB irradiation VS hetero before UVB irradiation	Fatty acid elongation - Homo sapiens (human)|KEGG	-0.4335497	-0.03641328	-2.65358651	0.02782133	0.14438349	-3.60088945
WT after UVB irradiation VS WT before UVB irradiation	Fatty acid elongation - Homo sapiens (human)|KEGG	-0.42840174	0.000845194	-1.97822058	0.07586366	0.46034175	-4.21760101
homo after UVB irradiation VS homo before UVB irradiation	fatty acid elongation -- saturated|HumanCyc	-0.02199304	-0.00035205	-0.0526088	0.95914596	0.97559823	-6.36696139
hetero after UVB irradiation VS hetero before UVB irradiation	fatty acid elongation -- saturated|HumanCyc	-0.018798	-0.00204956	-0.04551337	0.96475838	0.9837433	-6.34541768
WT after UVB irradiation VS WT before UVB irradiation	fatty acid elongation -- saturated|HumanCyc	0.80562294	-0.02602061	2.309903838	0.04331546	0.39022568	-3.74033338
homo after UVB irradiation VS homo before UVB irradiation	Fatty Acid Elongation In Mitochondria|SMPDB	-0.83801153	0.022760834	-3.4725845	0.00658545	0.04701822	-2.2661183
hetero after UVB irradiation VS hetero before UVB irradiation	Fatty Acid Elongation In Mitochondria|SMPDB	-0.7961592	0.030443028	-3.1957153	0.0118657	0.10542662	-2.77375612
WT after UVB irradiation VS WT before UVB irradiation	Fatty Acid Elongation In Mitochondria|SMPDB	-0.48656077	-0.0330546	-2.25189377	0.04781905	0.401167	-3.8253293
homo after UVB irradiation VS homo before UVB irradiation	Fatty acid metabolism|Reactome	0.069990724	-0.02206891	0.353146072	0.73177813	0.8234219	-6.30189343
hetero after UVB irradiation VS hetero before UVB irradiation	Fatty acid metabolism|Reactome	-0.08059848	-0.00905736	-0.38361181	0.71076498	0.85660147	-6.26741849
WT after UVB irradiation VS WT before UVB irradiation	Fatty acid metabolism|Reactome	-0.06989686	0.014813168	-0.29895943	0.77104248	0.93182086	-5.82408841
homo after UVB irradiation VS homo before UVB irradiation	Fatty acid Metabolism|SMPDB	-0.19843953	0.039801734	-0.74439404	0.47485571	0.61754234	-6.07941248
hetero after UVB irradiation VS hetero before UVB irradiation	Fatty acid Metabolism|SMPDB	-0.48316271	0.016700705	-1.75538636	0.11536374	0.30887493	-4.92927061
WT after UVB irradiation VS WT before UVB irradiation	Fatty acid Metabolism|SMPDB	-0.40763842	0.012220754	-1.51649601	0.16010237	0.56072663	-4.82863564
homo after UVB irradiation VS homo before UVB irradiation	Fatty Acid Omega Oxidation|Wikipathways	0.011226688	-0.01705205	0.037222672	0.97108689	0.98205598	-6.36770387
hetero after UVB irradiation VS hetero before UVB irradiation	Fatty Acid Omega Oxidation|Wikipathways	-0.35323905	-0.04524522	-1.34697325	0.21309208	0.43535861	-5.45790792
WT after UVB irradiation VS WT before UVB irradiation	Fatty Acid Omega Oxidation|Wikipathways	-0.17521302	-0.04091875	-0.62450056	0.54617047	0.84264773	-5.67559465
homo after UVB irradiation VS homo before UVB irradiation	Fatty Acids bound to GPR40 (FFAR1) regulate insulin secretion|Reactome	0.353476268	-0.02054789	1.95622048	0.08080944	0.20118642	-4.65324229
hetero after UVB irradiation VS hetero before UVB irradiation	Fatty Acids bound to GPR40 (FFAR1) regulate insulin secretion|Reactome	0.552605435	0.002323181	2.529800623	0.0338829	0.16053874	-3.79001525
WT after UVB irradiation VS WT before UVB irradiation	Fatty Acids bound to GPR40 (FFAR1) regulate insulin secretion|Reactome	0.547455038	0.046868101	2.605922804	0.02606159	0.34104092	-3.30033552
homo after UVB irradiation VS homo before UVB irradiation	Fatty acids|Reactome	0.474347446	0.121231809	2.796809402	0.02001555	0.09043427	-3.34371949
hetero after UVB irradiation VS hetero before UVB irradiation	Fatty acids|Reactome	0.376664562	0.084984642	1.680880588	0.12938076	0.32392126	-5.0310451
WT after UVB irradiation VS WT before UVB irradiation	Fatty acids|Reactome	0.110175821	0.036222057	0.3803292	0.71159097	0.90420468	-5.79643334
homo after UVB irradiation VS homo before UVB irradiation	Fatty acyl-CoA biosynthesis|Reactome	-0.00204783	-0.03449934	-0.00765498	0.99405246	0.99523987	-6.36841678
hetero after UVB irradiation VS hetero before UVB irradiation	Fatty acyl-CoA biosynthesis|Reactome	-0.0215517	-0.00408653	-0.09805044	0.92418476	0.97054702	-6.34132886
WT after UVB irradiation VS WT before UVB irradiation	Fatty acyl-CoA biosynthesis|Reactome	0.061763988	-0.02069441	0.240505301	0.81475839	0.94525798	-5.83993855
homo after UVB irradiation VS homo before UVB irradiation	FBXL7 down-regulates AURKA during mitotic entry and in early mitosis|Reactome	-0.4789036	0.01395849	-2.48680653	0.03358674	0.1222943	-3.83763497
hetero after UVB irradiation VS hetero before UVB irradiation	FBXL7 down-regulates AURKA during mitotic entry and in early mitosis|Reactome	0.122391404	0.018484784	0.542414284	0.60158079	0.78974805	-6.189737
WT after UVB irradiation VS WT before UVB irradiation	FBXL7 down-regulates AURKA during mitotic entry and in early mitosis|Reactome	-0.32016211	0.001546593	-1.18906353	0.26166523	0.65669403	-5.20243113
homo after UVB irradiation VS homo before UVB irradiation	Fc epsilon receptor (FCERI) signaling|Reactome	0.368048642	-0.0361971	2.00040259	0.07519018	0.19618193	-4.5877363
hetero after UVB irradiation VS hetero before UVB irradiation	Fc epsilon receptor (FCERI) signaling|Reactome	0.370544637	0.011863675	2.186046556	0.05861382	0.21639222	-4.30899073
WT after UVB irradiation VS WT before UVB irradiation	Fc epsilon receptor (FCERI) signaling|Reactome	0.261894349	-0.00800876	1.272088333	0.23189819	0.62952955	-5.11348962
homo after UVB irradiation VS homo before UVB irradiation	fc epsilon receptor i signaling in mast cells|BioCarta	0.356259004	-0.01290615	1.961951109	0.08005879	0.20008125	-4.64477814
hetero after UVB irradiation VS hetero before UVB irradiation	fc epsilon receptor i signaling in mast cells|BioCarta	0.621895882	0.002162816	4.017620829	0.00346475	0.06529021	-1.56746691
WT after UVB irradiation VS WT before UVB irradiation	fc epsilon receptor i signaling in mast cells|BioCarta	0.396473714	0.022757351	1.970940773	0.07678951	0.46222215	-4.22779523
homo after UVB irradiation VS homo before UVB irradiation	Fc Epsilon Receptor I Signaling in Mast Cells|SMPDB	0.314755479	-0.02634815	1.835764981	0.0982051	0.22704025	-4.82874856
hetero after UVB irradiation VS hetero before UVB irradiation	Fc Epsilon Receptor I Signaling in Mast Cells|SMPDB	0.496316647	0.000182617	3.054346878	0.01478345	0.1130168	-2.98826772
WT after UVB irradiation VS WT before UVB irradiation	Fc Epsilon Receptor I Signaling in Mast Cells|SMPDB	0.278599448	0.013337371	1.376294576	0.19852608	0.61055124	-4.99601553
homo after UVB irradiation VS homo before UVB irradiation	Fc epsilon RI signaling pathway - Homo sapiens (human)|KEGG	0.359575345	-0.00343512	2.079428156	0.06605375	0.17862118	-4.46922328
hetero after UVB irradiation VS hetero before UVB irradiation	Fc epsilon RI signaling pathway - Homo sapiens (human)|KEGG	0.502279298	0.003829784	3.146648829	0.01280369	0.10928694	-2.84805188
WT after UVB irradiation VS WT before UVB irradiation	Fc epsilon RI signaling pathway - Homo sapiens (human)|KEGG	0.408077875	0.034949552	2.111344128	0.06068284	0.43072383	-4.02881428
homo after UVB irradiation VS homo before UVB irradiation	Fc gamma R-mediated phagocytosis - Homo sapiens (human)|KEGG	0.593796518	-0.03240678	3.264990985	0.00922913	0.05749002	-2.59487269
hetero after UVB irradiation VS hetero before UVB irradiation	Fc gamma R-mediated phagocytosis - Homo sapiens (human)|KEGG	0.64320203	0.009993255	4.096382511	0.00309403	0.06222118	-1.45659838
WT after UVB irradiation VS WT before UVB irradiation	Fc gamma R-mediated phagocytosis - Homo sapiens (human)|KEGG	0.572438233	-0.00355112	2.967783817	0.0139802	0.28307166	-2.75644612
homo after UVB irradiation VS homo before UVB irradiation	Fc-epsilon receptor I signaling in mast cells|PID	0.513115105	-0.03220986	2.872665715	0.01764031	0.08390172	-3.22220702
hetero after UVB irradiation VS hetero before UVB irradiation	Fc-epsilon receptor I signaling in mast cells|PID	0.61622818	0.004736594	4.079916015	0.00316787	0.06277895	-1.4797
WT after UVB irradiation VS WT before UVB irradiation	Fc-epsilon receptor I signaling in mast cells|PID	0.480984841	-0.01118166	2.291369033	0.04470769	0.39341048	-3.76754679
homo after UVB irradiation VS homo before UVB irradiation	FCERI mediated Ca+2 mobilization|Reactome	0.567698959	-0.05228395	3.231339371	0.00975194	0.05874321	-2.64844801
hetero after UVB irradiation VS hetero before UVB irradiation	FCERI mediated Ca+2 mobilization|Reactome	0.373027478	0.043096405	1.648047759	0.13604211	0.33582478	-5.07522936
WT after UVB irradiation VS WT before UVB irradiation	FCERI mediated Ca+2 mobilization|Reactome	0.504341827	0.017530175	2.160763013	0.05582106	0.41769317	-3.95770218
homo after UVB irradiation VS homo before UVB irradiation	FCERI mediated MAPK activation|Reactome	0.27855565	-0.04838412	1.325111894	0.21647385	0.37419996	-5.50580033
hetero after UVB irradiation VS hetero before UVB irradiation	FCERI mediated MAPK activation|Reactome	0.523318428	0.004709193	2.964978077	0.01700434	0.11465353	-3.1244948
WT after UVB irradiation VS WT before UVB irradiation	FCERI mediated MAPK activation|Reactome	0.391237785	0.069980208	1.997500864	0.07346203	0.45403763	-4.19053319
homo after UVB irradiation VS homo before UVB irradiation	FCERI mediated NF-kB activation|Reactome	0.419053032	-0.04416925	2.228326399	0.05165331	0.15118527	-4.24184688
hetero after UVB irradiation VS hetero before UVB irradiation	FCERI mediated NF-kB activation|Reactome	0.442476265	-0.01913942	2.380803343	0.04297373	0.18220863	-4.0164787
WT after UVB irradiation VS WT before UVB irradiation	FCERI mediated NF-kB activation|Reactome	0.139205764	-0.09311726	0.616932355	0.55095602	0.84516674	-5.68016547
homo after UVB irradiation VS homo before UVB irradiation	Fcgamma receptor (FCGR) dependent phagocytosis|Reactome	0.498909045	-0.01782913	2.639623712	0.02601947	0.10674293	-3.59492823
hetero after UVB irradiation VS hetero before UVB irradiation	Fcgamma receptor (FCGR) dependent phagocytosis|Reactome	0.561291109	0.022646943	3.536521706	0.00704752	0.08628369	-2.2637459
WT after UVB irradiation VS WT before UVB irradiation	Fcgamma receptor (FCGR) dependent phagocytosis|Reactome	0.55480281	-0.00327458	2.852267076	0.01705114	0.30353778	-2.93013103
homo after UVB irradiation VS homo before UVB irradiation	FCGR activation|Reactome	0.457502857	-0.05928417	1.50429085	0.1653774	0.31489292	-5.28275876
hetero after UVB irradiation VS hetero before UVB irradiation	FCGR activation|Reactome	0.54366314	0.076918587	2.563342182	0.03211919	0.15640947	-3.73883165
WT after UVB irradiation VS WT before UVB irradiation	FCGR activation|Reactome	0.535828209	0.087263699	1.38768219	0.19513639	0.60625027	-4.98280564
homo after UVB irradiation VS homo before UVB irradiation	Felbamate Metabolism Pathway|SMPDB	-0.21057582	-0.04257838	-0.54085587	0.60120092	0.7210621	-6.21374042
hetero after UVB irradiation VS hetero before UVB irradiation	Felbamate Metabolism Pathway|SMPDB	-0.65787371	-0.02552071	-2.18704977	0.05852037	0.21639222	-4.30749779
WT after UVB irradiation VS WT before UVB irradiation	Felbamate Metabolism Pathway|SMPDB	-0.59281316	-0.00736485	-1.99503617	0.073765	0.45463532	-4.19399891
homo after UVB irradiation VS homo before UVB irradiation	Felbamate Metabolism|Wikipathways	0.388460901	0.020895461	1.066764261	0.31273867	0.46923284	-5.79229523
hetero after UVB irradiation VS hetero before UVB irradiation	Felbamate Metabolism|Wikipathways	-0.14760988	0.040949837	-0.3635581	0.72514129	0.86482318	-6.27541048
WT after UVB irradiation VS WT before UVB irradiation	Felbamate Metabolism|Wikipathways	-0.06962327	0.086915055	-0.17823013	0.86207255	0.95916804	-5.85308595
homo after UVB irradiation VS homo before UVB irradiation	Felodipine Action Pathway|SMPDB	0.396415397	-0.03677376	2.343069613	0.04268583	0.13402181	-4.06364635
hetero after UVB irradiation VS hetero before UVB irradiation	Felodipine Action Pathway|SMPDB	-0.01555055	-0.01775524	-0.07410913	0.94265284	0.97054702	-6.34356282
WT after UVB irradiation VS WT before UVB irradiation	Felodipine Action Pathway|SMPDB	0.056685205	-0.03978418	0.223404833	0.82768199	0.94525798	-5.84393438
homo after UVB irradiation VS homo before UVB irradiation	Felodipine Metabolism Pathway|SMPDB	0.24667064	0.02426039	1.045925472	0.32177374	0.47732908	-5.8133656
hetero after UVB irradiation VS hetero before UVB irradiation	Felodipine Metabolism Pathway|SMPDB	-0.59737788	-0.16095249	-1.99033008	0.07992052	0.25428326	-4.59659523
WT after UVB irradiation VS WT before UVB irradiation	Felodipine Metabolism Pathway|SMPDB	-0.22615208	-0.160842	-0.72070179	0.48746679	0.81710765	-5.61308479
homo after UVB irradiation VS homo before UVB irradiation	Fenoprofen Action Pathway|SMPDB	0.40423582	0.045705158	1.979500697	0.07780079	0.19626432	-4.61879689
hetero after UVB irradiation VS hetero before UVB irradiation	Fenoprofen Action Pathway|SMPDB	0.482496143	0.064876536	3.04499191	0.01500113	0.1130168	-3.00250826
WT after UVB irradiation VS WT before UVB irradiation	Fenoprofen Action Pathway|SMPDB	0.275725339	0.086595857	1.261970114	0.23537138	0.62952955	-5.12455679
homo after UVB irradiation VS homo before UVB irradiation	Fentanyl Action Pathway|SMPDB	0.191132361	0.029768717	1.061313976	0.31508276	0.46923284	-5.79783739
hetero after UVB irradiation VS hetero before UVB irradiation	Fentanyl Action Pathway|SMPDB	-0.07904315	0.055005373	-0.44437398	0.66796482	0.82651398	-6.24068873
WT after UVB irradiation VS WT before UVB irradiation	Fentanyl Action Pathway|SMPDB	0.022316377	0.035038618	0.098344171	0.92358676	0.97566251	-5.86425414
homo after UVB irradiation VS homo before UVB irradiation	Ferroptosis - Homo sapiens (human)|KEGG	0.086258912	-0.03304476	0.43545147	0.67307381	0.77795793	-6.26760964
hetero after UVB irradiation VS hetero before UVB irradiation	Ferroptosis - Homo sapiens (human)|KEGG	0.202538372	0.018536162	1.351500064	0.21169732	0.43347545	-5.45249363
WT after UVB irradiation VS WT before UVB irradiation	Ferroptosis - Homo sapiens (human)|KEGG	0.14225229	-0.05471985	0.65275278	0.52851802	0.83278813	-5.65808052
homo after UVB irradiation VS homo before UVB irradiation	Ferroptosis|Wikipathways	0.086258912	-0.03304476	0.43545147	0.67307381	0.77795793	-6.26760964

hetero after UVB irradiation VS hetero before UVB irradiation	Ferroptosis|Wikipathways	0.202538372	0.018536162	1.351500064	0.21169732	0.43347545	-5.45249363
WT after UVB irradiation VS WT before UVB irradiation	Ferroptosis|Wikipathways	0.14225229	-0.05471985	0.65275278	0.52851802	0.83278813	-5.65808052
homo after UVB irradiation VS homo before UVB irradiation	Fertilization|Reactome	-0.05451861	0.039015424	-0.31349976	0.76075394	0.84515876	-6.31592302
hetero after UVB irradiation VS hetero before UVB irradiation	Fertilization|Reactome	-0.21244072	0.027318139	-1.15979632	0.27793828	0.50863663	-5.67110208
WT after UVB irradiation VS WT before UVB irradiation	Fertilization|Reactome	-0.00339381	0.011251775	-0.014586	0.98864707	0.99672211	-5.86904341
homo after UVB irradiation VS homo before UVB irradiation	FGF signaling pathway|PID	0.386700659	-0.02672569	2.054213519	0.06884708	0.18380965	-4.50721572
hetero after UVB irradiation VS hetero before UVB irradiation	FGF signaling pathway|PID	0.419526597	0.027655076	2.448844784	0.0385527	0.17060506	-3.91327439
WT after UVB irradiation VS WT before UVB irradiation	FGF signaling pathway|PID	0.19656295	0.009377763	1.000744892	0.34036266	0.72554886	-5.38728528
homo after UVB irradiation VS homo before UVB irradiation	FGF|INOH	0.092973115	-0.02056578	0.539660698	0.60199299	0.72163298	-6.21441278
hetero after UVB irradiation VS hetero before UVB irradiation	FGF|INOH	0.226302621	0.022672804	1.478879546	0.17555437	0.3924359	-5.29563657
WT after UVB irradiation VS WT before UVB irradiation	FGF|INOH	0.124580415	0.002921482	0.596067161	0.56427197	0.85109394	-5.69250048
homo after UVB irradiation VS homo before UVB irradiation	FGFR1 ligand binding and activation|Reactome	-0.05818685	0.020460424	-0.25436309	0.80469853	0.87858764	-6.33380688
hetero after UVB irradiation VS hetero before UVB irradiation	FGFR1 ligand binding and activation|Reactome	-0.24460504	0.01651682	-1.25716883	0.24241883	0.46543442	-5.56287407
WT after UVB irradiation VS WT before UVB irradiation	FGFR1 ligand binding and activation|Reactome	-0.39388814	0.066186788	-1.56200071	0.14910307	0.55622203	-4.77221156
homo after UVB irradiation VS homo before UVB irradiation	FGFR1 mutant receptor activation|Reactome	0.218454001	0.023804757	1.140538221	0.28231839	0.44245297	-5.71514371
hetero after UVB irradiation VS hetero before UVB irradiation	FGFR1 mutant receptor activation|Reactome	0.181533332	0.021082049	0.958652109	0.36443002	0.59328506	-5.87410186
WT after UVB irradiation VS WT before UVB irradiation	FGFR1 mutant receptor activation|Reactome	-0.14043143	-0.05014154	-0.6134864	0.55314285	0.84516674	-5.68222964
homo after UVB irradiation VS homo before UVB irradiation	FGFR1b ligand binding and activation|Reactome	0.059820538	-0.07851557	0.207130207	0.84033087	0.90259956	-6.34545026
hetero after UVB irradiation VS hetero before UVB irradiation	FGFR1b ligand binding and activation|Reactome	-0.55508525	-0.01446773	-3.05739201	0.0147133	0.1130168	-2.98363336
WT after UVB irradiation VS WT before UVB irradiation	FGFR1b ligand binding and activation|Reactome	-0.33532997	-0.11362617	-1.3398934	0.20969566	0.62472791	-5.03775945
homo after UVB irradiation VS homo before UVB irradiation	FGFR1c and Klotho ligand binding and activation|Reactome	1.09579422	-0.05103473	5.736512805	0.00023957	0.01666204	0.950385468
hetero after UVB irradiation VS hetero before UVB irradiation	FGFR1c and Klotho ligand binding and activation|Reactome	0.717029238	0.122694048	2.753830485	0.02372785	0.13628011	-3.44744591
WT after UVB irradiation VS WT before UVB irradiation	FGFR1c and Klotho ligand binding and activation|Reactome	0.513220947	-0.00905719	1.564306847	0.14856374	0.55622203	-4.76932636
homo after UVB irradiation VS homo before UVB irradiation	FGFR1c ligand binding and activation|Reactome	0.112662175	0.03253101	0.528670135	0.60930236	0.72733975	-6.22053015
hetero after UVB irradiation VS hetero before UVB irradiation	FGFR1c ligand binding and activation|Reactome	-0.23913244	0.027601515	-1.17641724	0.27159172	0.50077469	-5.65306075
WT after UVB irradiation VS WT before UVB irradiation	FGFR1c ligand binding and activation|Reactome	-0.38240016	0.054426905	-1.31016674	0.21920228	0.6260768	-5.07129153
homo after UVB irradiation VS homo before UVB irradiation	FGFR2 alternative splicing|Reactome	-0.79084817	-0.01178494	-4.50852193	0.00132192	0.02074181	-0.69799433
hetero after UVB irradiation VS hetero before UVB irradiation	FGFR2 alternative splicing|Reactome	-0.26481958	-0.0594142	-1.14306329	0.28444799	0.5136743	-5.68907926
WT after UVB irradiation VS WT before UVB irradiation	FGFR2 alternative splicing|Reactome	-0.33006462	-0.02559485	-1.03096701	0.32665354	0.71049278	-5.35929192
homo after UVB irradiation VS homo before UVB irradiation	FGFR2 ligand binding and activation|Reactome	-0.4180541	0.072736329	-1.94969512	0.08167228	0.20262084	-4.66286827
hetero after UVB irradiation VS hetero before UVB irradiation	FGFR2 ligand binding and activation|Reactome	-0.27306916	0.011893204	-1.42670304	0.18964993	0.40813238	-5.36091102
WT after UVB irradiation VS WT before UVB irradiation	FGFR2 ligand binding and activation|Reactome	-0.50755672	0.047829128	-2.37672356	0.03863814	0.39022568	-3.64182758
homo after UVB irradiation VS homo before UVB irradiation	FGFR2 mutant receptor activation|Reactome	-0.31990956	0.058499883	-1.32496308	0.21652131	0.37419996	-5.50597789
hetero after UVB irradiation VS hetero before UVB irradiation	FGFR2 mutant receptor activation|Reactome	-0.35814887	0.041228686	-1.74985853	0.11635328	0.30887493	-4.93689038
WT after UVB irradiation VS WT before UVB irradiation	FGFR2 mutant receptor activation|Reactome	-0.5602751	0.043099089	-2.25524081	0.04754731	0.401167	-3.82043972
homo after UVB irradiation VS homo before UVB irradiation	FGFR2b ligand binding and activation|Reactome	-0.44380093	-0.02343028	-2.17409353	0.0565067	0.16165213	-4.32522269
hetero after UVB irradiation VS hetero before UVB irradiation	FGFR2b ligand binding and activation|Reactome	-0.28515555	-0.03350847	-1.4468878	0.18408344	0.40321767	-5.33582209
WT after UVB irradiation VS WT before UVB irradiation	FGFR2b ligand binding and activation|Reactome	-0.26132707	0.029369751	-1.11908457	0.28905665	0.67456398	-5.27396292
homo after UVB irradiation VS homo before UVB irradiation	FGFR2c ligand binding and activation|Reactome	-0.2575522	0.054879897	-1.07978616	0.30719237	0.46888942	-5.77896475
hetero after UVB irradiation VS hetero before UVB irradiation	FGFR2c ligand binding and activation|Reactome	-0.42886982	0.028376359	-2.09193603	0.06806114	0.23495391	-4.4482514
WT after UVB irradiation VS WT before UVB irradiation	FGFR2c ligand binding and activation|Reactome	-0.64709933	0.039705315	-2.4775946	0.03249891	0.36974198	-3.4921122
homo after UVB irradiation VS homo before UVB irradiation	FGFR3 ligand binding and activation|Reactome	-0.22859914	0.053273276	-1.1277698	0.28741037	0.44697403	-5.72877666
hetero after UVB irradiation VS hetero before UVB irradiation	FGFR3 ligand binding and activation|Reactome	-0.25636576	-0.00600285	-1.28979349	0.23139458	0.45275431	-5.5252903
WT after UVB irradiation VS WT before UVB irradiation	FGFR3 ligand binding and activation|Reactome	-0.49049323	0.054218054	-1.79624005	0.10243358	0.50410801	-4.46771179
homo after UVB irradiation VS homo before UVB irradiation	FGFR3 mutant receptor activation|Reactome	-0.26599503	0.061152615	-1.23364561	0.2473234	0.40930723	-5.61233202
hetero after UVB irradiation VS hetero before UVB irradiation	FGFR3 mutant receptor activation|Reactome	-0.36764465	0.018934928	-1.85348374	0.09905967	0.28290349	-4.79235578
WT after UVB irradiation VS WT before UVB irradiation	FGFR3 mutant receptor activation|Reactome	-0.57433163	0.049260151	-2.21583632	0.05084334	0.41025002	-3.87788422
homo after UVB irradiation VS homo before UVB irradiation	FGFR3b ligand binding and activation|Reactome	-0.37453929	0.031243808	-1.88626445	0.09052392	0.21571189	-4.75574865
hetero after UVB irradiation VS hetero before UVB irradiation	FGFR3b ligand binding and activation|Reactome	-0.43902286	-0.00791168	-2.18346317	0.05885514	0.21664304	-4.31283446
WT after UVB irradiation VS WT before UVB irradiation	FGFR3b ligand binding and activation|Reactome	-0.74020698	0.00996642	-3.25964867	0.00848951	0.24856491	-2.32007305
homo after UVB irradiation VS homo before UVB irradiation	FGFR3c ligand binding and activation|Reactome	-0.22859914	0.053273276	-1.1277698	0.28741037	0.44697403	-5.72877666
hetero after UVB irradiation VS hetero before UVB irradiation	FGFR3c ligand binding and activation|Reactome	-0.25636576	-0.00600285	-1.28979349	0.23139458	0.45275431	-5.5252903
WT after UVB irradiation VS WT before UVB irradiation	FGFR3c ligand binding and activation|Reactome	-0.49049323	0.054218054	-1.79624005	0.10243358	0.50410801	-4.46771179
homo after UVB irradiation VS homo before UVB irradiation	FGFR4 ligand binding and activation|Reactome	-0.03354143	0.041369947	-0.14064118	0.8911246	0.93558863	-6.35783187
hetero after UVB irradiation VS hetero before UVB irradiation	FGFR4 ligand binding and activation|Reactome	-0.29269118	0.029365553	-1.2619819	0.24076535	0.46332993	-5.55736983
WT after UVB irradiation VS WT before UVB irradiation	FGFR4 ligand binding and activation|Reactome	-0.3969532	0.013568094	-1.38308796	0.196498	0.60799889	-4.9881436
homo after UVB irradiation VS homo before UVB irradiation	FGFR4 mutant receptor activation|Reactome	-0.44288436	-0.02951044	-1.21264138	0.25489452	0.41874827	-5.63603664
hetero after UVB irradiation VS hetero before UVB irradiation	FGFR4 mutant receptor activation|Reactome	-1.03591909	-0.05275035	-4.59213692	0.00154943	0.04803647	-0.78101878
WT after UVB irradiation VS WT before UVB irradiation	FGFR4 mutant receptor activation|Reactome	-0.77742531	-0.02461073	-2.51529562	0.03045986	0.36321198	-3.43589941
homo after UVB irradiation VS homo before UVB irradiation	FGFRL1 modulation of FGFR1 signaling|Reactome	-0.40068394	0.05787883	-1.94793229	0.08190687	0.20292282	-4.66546651
hetero after UVB irradiation VS hetero before UVB irradiation	FGFRL1 modulation of FGFR1 signaling|Reactome	-0.35926537	0.05550849	-2.16534218	0.06057545	0.22017775	-4.33976451
WT after UVB irradiation VS WT before UVB irradiation	FGFRL1 modulation of FGFR1 signaling|Reactome	-0.5234149	0.073538624	-2.07445796	0.06457235	0.43544914	-4.08154889
homo after UVB irradiation VS homo before UVB irradiation	Fibrin Complement Receptor 3 Signaling Pathway|Wikipathways	0.795493996	-0.00418378	4.965813437	0.00068235	0.01666204	-0.05561639
hetero after UVB irradiation VS hetero before UVB irradiation	Fibrin Complement Receptor 3 Signaling Pathway|Wikipathways	0.894260324	0.036218846	5.96210052	0.00027535	0.02988205	0.87695522
WT after UVB irradiation VS WT before UVB irradiation	Fibrin Complement Receptor 3 Signaling Pathway|Wikipathways	0.78608512	-0.00431835	3.614953452	0.00466709	0.20968398	-1.79841072
homo after UVB irradiation VS homo before UVB irradiation	fibrinolysis pathway|BioCarta	0.178183307	0.010787048	0.950394954	0.36573287	0.51909738	-5.90570423
hetero after UVB irradiation VS hetero before UVB irradiation	fibrinolysis pathway|BioCarta	0.674391463	0.01316896	3.857485693	0.00437298	0.07183716	-1.79570256
WT after UVB irradiation VS WT before UVB irradiation	fibrinolysis pathway|BioCarta	0.440509961	-0.03637227	1.936224485	0.08135235	0.4679297	-4.27620773
homo after UVB irradiation VS homo before UVB irradiation	Fibroblast growth factor-1|NetPath	0.348736533	-0.02510716	1.788041784	0.10601678	0.23881234	-4.89690826
hetero after UVB irradiation VS hetero before UVB irradiation	Fibroblast growth factor-1|NetPath	0.478372368	-0.00953036	3.077002953	0.01426975	0.1130168	-2.95380076
WT after UVB irradiation VS WT before UVB irradiation	Fibroblast growth factor-1|NetPath	0.385069964	-0.01611752	1.947135844	0.07989158	0.46479598	-4.26102822
homo after UVB irradiation VS homo before UVB irradiation	Fibronectin matrix formation|Reactome	1.038285448	-0.00874721	5.180039308	0.0005058	0.01666204	0.233714447
hetero after UVB irradiation VS hetero before UVB irradiation	Fibronectin matrix formation|Reactome	0.682629933	0.057775333	2.159094863	0.06117988	0.22149158	-4.34903575
WT after UVB irradiation VS WT before UVB irradiation	Fibronectin matrix formation|Reactome	0.581127969	-0.00281893	1.798669118	0.10202835	0.50394202	-4.46444355
homo after UVB irradiation VS homo before UVB irradiation	Ficolins bind to repetitive carbohydrate structures on the target cell surface|Reactome	0.309658286	0.018597469	0.896884769	0.39219591	0.54530041	-5.95426203
hetero after UVB irradiation VS hetero before UVB irradiation	Ficolins bind to repetitive carbohydrate structures on the target cell surface|Reactome	-0.34980194	0.055133847	-1.05122143	0.3223683	0.55349684	-5.78431353
WT after UVB irradiation VS WT before UVB irradiation	Ficolins bind to repetitive carbohydrate structures on the target cell surface|Reactome	-0.58414502	0.001639567	-1.64272901	0.13122029	0.54430796	-4.6698022
homo after UVB irradiation VS homo before UVB irradiation	fig-met-1-last-solution|Wikipathways	0.202061811	0.000340692	0.883819889	0.39885856	0.55104029	-5.96576107
hetero after UVB irradiation VS hetero before UVB irradiation	fig-met-1-last-solution|Wikipathways	-0.03222129	0.009584823	-0.12822018	0.90098245	0.96992868	-6.33763139
WT after UVB irradiation VS WT before UVB irradiation	fig-met-1-last-solution|Wikipathways	0.191188621	-0.03636994	0.706963096	0.49560414	0.81771102	-5.62250597
homo after UVB irradiation VS homo before UVB irradiation	Flavan-3-ols|Wikipathways	1.011969848	-0.00971256	4.023922695	0.00275245	0.02978124	-1.41387018
hetero after UVB irradiation VS hetero before UVB irradiation	Flavan-3-ols|Wikipathways	0.393640147	-0.00020703	1.04238256	0.3262163	0.5554329	-5.79316252
WT after UVB irradiation VS WT before UVB irradiation	Flavan-3-ols|Wikipathways	0.599448487	-0.05276689	1.685103404	0.1226213	0.52731779	-4.61493556
homo after UVB irradiation VS homo before UVB irradiation	flavin biosynthesis|HumanCyc	-0.96168363	-0.04561348	-4.30366528	0.00179515	0.02356086	-0.99639481
hetero after UVB irradiation VS hetero before UVB irradiation	flavin biosynthesis|HumanCyc	-0.17711767	-0.08664513	-0.74598263	0.47597236	0.69780702	-6.05448551
WT after UVB irradiation VS WT before UVB irradiation	flavin biosynthesis|HumanCyc	-0.53652949	-0.04045939	-1.88146561	0.08906718	0.48106209	-4.35185732
homo after UVB irradiation VS homo before UVB irradiation	Flecainide Action Pathway|SMPDB	0.396415397	-0.03677376	2.343069613	0.04268583	0.13402181	-4.06364635
hetero after UVB irradiation VS hetero before UVB irradiation	Flecainide Action Pathway|SMPDB	-0.01555055	-0.01775524	-0.07410913	0.94265284	0.97054702	-6.34356282
WT after UVB irradiation VS WT before UVB irradiation	Flecainide Action Pathway|SMPDB	0.056685205	-0.03978418	0.223404833	0.82768199	0.94525798	-5.84393438
homo after UVB irradiation VS homo before UVB irradiation	Fluid shear stress and atherosclerosis - Homo sapiens (human)|KEGG	0.479429704	-0.02314027	2.776781674	0.02069513	0.09183086	-3.37578121
hetero after UVB irradiation VS hetero before UVB irradiation	Fluid shear stress and atherosclerosis - Homo sapiens (human)|KEGG	0.539087646	0.021693847	3.40205071	0.00864166	0.09312575	-2.46357803
WT after UVB irradiation VS WT before UVB irradiation	Fluid shear stress and atherosclerosis - Homo sapiens (human)|KEGG	0.402552939	-0.00052829	1.851381065	0.09358971	0.48767729	-4.39302619
homo after UVB irradiation VS homo before UVB irradiation	Fluoropyrimidine Activity|Wikipathways	-0.0569028	-0.03968534	-0.27367365	0.79026077	0.86725925	-6.32836973
hetero after UVB irradiation VS hetero before UVB irradiation	Fluoropyrimidine Activity|Wikipathways	0.2508241	0.005737891	1.518926401	0.16537117	0.3789652	-5.24462926
WT after UVB irradiation VS WT before UVB irradiation	Fluoropyrimidine Activity|Wikipathways	0.30209007	0.017518121	1.560163804	0.1495339	0.55622203	-4.77450797
homo after UVB irradiation VS homo before UVB irradiation	Fluoropyrimidine Pathway_ Pharmacodynamics|PharmGKB	-0.50341677	0.015574961	-2.56328078	0.02955902	0.11457257	-3.71642569
hetero after UVB irradiation VS hetero before UVB irradiation	Fluoropyrimidine Pathway_ Pharmacodynamics|PharmGKB	0.165243357	0.009192764	0.780138809	0.45666249	0.68110852	-6.0280906
WT after UVB irradiation VS WT before UVB irradiation	Fluoropyrimidine Pathway_ Pharmacodynamics|PharmGKB	0.168016896	0.018179338	0.663167358	0.52209622	0.83116638	-5.65144589
homo after UVB irradiation VS homo before UVB irradiation	Fluoropyrimidine Pathway_ Pharmacokinetics|PharmGKB	0.091096342	-0.04421161	0.40903355	0.69168823	0.79350142	-6.27936668
hetero after UVB irradiation VS hetero before UVB irradiation	Fluoropyrimidine Pathway_ Pharmacokinetics|PharmGKB	0.295222295	-0.00640942	1.610747251	0.14398908	0.34739964	-5.12490064
WT after UVB irradiation VS WT before UVB irradiation	Fluoropyrimidine Pathway_ Pharmacokinetics|PharmGKB	0.335445106	0.010509716	1.734482341	0.11324358	0.51512295	-4.55009522
homo after UVB irradiation VS homo before UVB irradiation	Fluorouracil Action Pathway|SMPDB	-0.66037111	-0.01689861	-1.87938723	0.09153593	0.21666824	-4.76574094
hetero after UVB irradiation VS hetero before UVB irradiation	Fluorouracil Action Pathway|SMPDB	0.185619941	-0.0180113	0.439651165	0.67124854	0.82819347	-6.24290083
WT after UVB irradiation VS WT before UVB irradiation	Fluorouracil Action Pathway|SMPDB	0.878356244	0.039870623	2.991871386	0.01341398	0.28007727	-2.72027026
homo after UVB irradiation VS homo before UVB irradiation	Fluorouracil Metabolism Pathway|SMPDB	-0.66037111	-0.01689861	-1.87938723	0.09153593	0.21666824	-4.76574094
hetero after UVB irradiation VS hetero before UVB irradiation	Fluorouracil Metabolism Pathway|SMPDB	0.185619941	-0.0180113	0.439651165	0.67124854	0.82819347	-6.24290083
WT after UVB irradiation VS WT before UVB irradiation	Fluorouracil Metabolism Pathway|SMPDB	0.878356244	0.039870623	2.991871386	0.01341398	0.28007727	-2.72027026
homo after UVB irradiation VS homo before UVB irradiation	Fluoxetine Action Pathway|SMPDB	0.141737442	0.028564559	0.782554201	0.45317257	0.60045287	-6.04998805
hetero after UVB irradiation VS hetero before UVB irradiation	Fluoxetine Action Pathway|SMPDB	-0.11378967	0.064388575	-0.67136433	0.51996617	0.73623731	-6.10852758
WT after UVB irradiation VS WT before UVB irradiation	Fluoxetine Action Pathway|SMPDB	-0.0018052	0.043506696	-0.00823637	0.9935891	0.99773998	-5.86911683
homo after UVB irradiation VS homo before UVB irradiation	Fluoxetine Metabolism Pathway|SMPDB	-0.86041213	0.056640538	-3.43746095	0.00697029	0.04844193	-2.32150263
hetero after UVB irradiation VS hetero before UVB irradiation	Fluoxetine Metabolism Pathway|SMPDB	-0.92164071	0.060219939	-4.63202355	0.00146787	0.04691448	-0.7283874
WT after UVB irradiation VS WT before UVB irradiation	Fluoxetine Metabolism Pathway|SMPDB	-0.8461784	0.090761032	-3.74534468	0.00375904	0.20870715	-1.61066986
homo after UVB irradiation VS homo before UVB irradiation	Fluoxetine Pathway_ Pharmacokinetics|PharmGKB	-0.86041213	0.056640538	-3.43746095	0.00697029	0.04844193	-2.32150263
hetero after UVB irradiation VS hetero before UVB irradiation	Fluoxetine Pathway_ Pharmacokinetics|PharmGKB	-0.92164071	0.060219939	-4.63202355	0.00146787	0.04691448	-0.7283874
WT after UVB irradiation VS WT before UVB irradiation	Fluoxetine Pathway_ Pharmacokinetics|PharmGKB	-0.8461784	0.090761032	-3.74534468	0.00375904	0.20870715	-1.61066986
homo after UVB irradiation VS homo before UVB irradiation	Flurbiprofen Action Pathway|SMPDB	0.40423582	0.045705158	1.979500697	0.07780079	0.19626432	-4.61879689
hetero after UVB irradiation VS hetero before UVB irradiation	Flurbiprofen Action Pathway|SMPDB	0.482496143	0.064876536	3.04499191	0.01500113	0.1130168	-3.00250826
WT after UVB irradiation VS WT before UVB irradiation	Flurbiprofen Action Pathway|SMPDB	0.275725339	0.086595857	1.261970114	0.23537138	0.62952955	-5.12455679
homo after UVB irradiation VS homo before UVB irradiation	Fluvastatin Action Pathway|SMPDB	-0.19230642	-0.01987665	-0.61925406	0.55047761	0.67913558	-6.16661869
hetero after UVB irradiation VS hetero before UVB irradiation	Fluvastatin Action Pathway|SMPDB	0.185820603	0.008098609	0.631890782	0.54421801	0.75147406	-6.13505553
WT after UVB irradiation VS WT before UVB irradiation	Fluvastatin Action Pathway|SMPDB	-0.14894747	0.045069977	-0.50755372	0.62268399	0.87257466	-5.74041409
homo after UVB irradiation VS homo before UVB irradiation	Fluvastatin Pathway_ Pharmacokinetics|PharmGKB	-0.31595753	0.173633641	-1.5856279	0.14588622	0.2913279	-5.1758853
hetero after UVB irradiation VS hetero before UVB irradiation	Fluvastatin Pathway_ Pharmacokinetics|PharmGKB	-0.59639325	0.086962376	-3.29234244	0.01022233	0.10264385	-2.62801325
WT after UVB irradiation VS WT before UVB irradiation	Fluvastatin Pathway_ Pharmacokinetics|PharmGKB	-0.63586609	0.121833717	-2.77524165	0.01946896	0.31542987	-3.04600879
homo after UVB irradiation VS homo before UVB irradiation	fmlp induced chemokine gene expression in hmc-1 cells|BioCarta	0.304231099	-0.01510726	1.894744771	0.08929044	0.21424076	-4.74340563
hetero after UVB irradiation VS hetero before UVB irradiation	fmlp induced chemokine gene expression in hmc-1 cells|BioCarta	0.326056223	-0.00748511	1.909306319	0.09077758	0.27275162	-4.71311878
WT after UVB irradiation VS WT before UVB irradiation	fmlp induced chemokine gene expression in hmc-1 cells|BioCarta	0.285555318	0.033919174	1.297204227	0.22345817	0.62952955	-5.08575279
homo after UVB irradiation VS homo before UVB irradiation	FMO oxidises nucleophiles|Reactome	0.189396904	0.010904488	0.498580468	0.62954611	0.74238315	-6.23667077
hetero after UVB irradiation VS hetero before UVB irradiation	FMO oxidises nucleophiles|Reactome	-0.59092709	0.011399668	-1.90267743	0.09172555	0.27397446	-4.72257476
WT after UVB irradiation VS WT before UVB irradiation	FMO oxidises nucleophiles|Reactome	0.263281425	-0.09332455	0.706003592	0.49617557	0.81835818	-5.62315786
homo after UVB irradiation VS homo before UVB irradiation	Focal adhesion - Homo sapiens (human)|KEGG	0.476896207	-0.01786141	2.95979952	0.0152628	0.07675106	-3.08257472
hetero after UVB irradiation VS hetero before UVB irradiation	Focal adhesion - Homo sapiens (human)|KEGG	0.397371036	0.010391309	2.201799471	0.05716318	0.21310989	-4.28552892
WT after UVB irradiation VS WT before UVB irradiation	Focal adhesion - Homo sapiens (human)|KEGG	0.352707385	0.004064549	1.597609265	0.14096501	0.55622203	-4.7273933
homo after UVB irradiation VS homo before UVB irradiation	Focal Adhesion|Wikipathways	0.503375863	-0.02200549	3.122131139	0.01166924	0.06594278	-2.82272613
hetero after UVB irradiation VS hetero before UVB irradiation	Focal Adhesion|Wikipathways	0.431173715	0.016288811	2.452710529	0.03831566	0.1700514	-3.90739908
WT after UVB irradiation VS WT before UVB irradiation	Focal Adhesion|Wikipathways	0.369043611	0.003684436	1.693688286	0.12094218	0.52647129	-4.6037311
homo after UVB irradiation VS homo before UVB irradiation	Focal Adhesion-PI3K-Akt-mTOR-signaling pathway|Wikipathways	0.392561494	-0.01233714	2.462285041	0.0349904	0.12452545	-3.87637437
hetero after UVB irradiation VS hetero before UVB irradiation	Focal Adhesion-PI3K-Akt-mTOR-signaling pathway|Wikipathways	0.314011823	0.005600541	1.774664781	0.11197364	0.30470201	-4.90261384
WT after UVB irradiation VS WT before UVB irradiation	Focal Adhesion-PI3K-Akt-mTOR-signaling pathway|Wikipathways	0.163813223	-0.02539229	0.762221335	0.46338644	0.80891799	-5.58363323
homo after UVB irradiation VS homo before UVB irradiation	Folate biosynthesis - Homo sapiens (human)|KEGG	0.457807149	-0.03651917	2.491345782	0.03333311	0.12185666	-3.8304566
hetero after UVB irradiation VS hetero before UVB irradiation	Folate biosynthesis - Homo sapiens (human)|KEGG	0.269857604	-0.00360631	1.372093245	0.20545065	0.42682044	-5.42771943
WT after UVB irradiation VS WT before UVB irradiation	Folate biosynthesis - Homo sapiens (human)|KEGG	0.341751565	0.018486042	1.524174308	0.15819807	0.56072663	-4.81918345
homo after UVB irradiation VS homo before UVB irradiation	Folate malabsorption_ hereditary|SMPDB	0.210604765	-0.01290295	0.884608242	0.39845428	0.55064838	-5.96507122
hetero after UVB irradiation VS hetero before UVB irradiation	Folate malabsorption_ hereditary|SMPDB	0.256530919	-0.05900519	1.406879866	0.1952591	0.4161797	-5.38534533
WT after UVB irradiation VS WT before UVB irradiation	Folate malabsorption_ hereditary|SMPDB	0.359853909	-0.03380361	1.522274352	0.15866743	0.56072663	-4.82152497
homo after UVB irradiation VS homo before UVB irradiation	Folate metabolism|INOH	-0.08185766	-0.00202004	-0.3988933	0.69889222	0.80002307	-6.28369123
hetero after UVB irradiation VS hetero before UVB irradiation	Folate metabolism|INOH	0.019860982	-0.03570696	0.099941336	0.92272805	0.97054702	-6.34112601

WT after UVB irradiation VS WT before UVB irradiation	Folate metabolism|INOH	0.244673192	-0.07682809	1.070616354	0.3093018	0.69398681	-5.32157021
homo after UVB irradiation VS homo before UVB irradiation	Folate Metabolism|SMPDB	0.210604765	-0.01290295	0.884608242	0.39845428	0.55064838	-5.96507122
hetero after UVB irradiation VS hetero before UVB irradiation	Folate Metabolism|SMPDB	0.256530919	-0.05900519	1.406879866	0.1952591	0.4161797	-5.38534533
WT after UVB irradiation VS WT before UVB irradiation	Folate Metabolism|SMPDB	0.359853909	-0.03380361	1.522274352	0.15866743	0.56072663	-4.82152497
homo after UVB irradiation VS homo before UVB irradiation	Folate Metabolism|Wikipathways	0.279295302	0.006813156	1.675210724	0.12681251	0.26670931	-5.05456638
hetero after UVB irradiation VS hetero before UVB irradiation	Folate Metabolism|Wikipathways	0.328940835	0.012230288	1.763540724	0.11391829	0.30852025	-4.91801094
WT after UVB irradiation VS WT before UVB irradiation	Folate Metabolism|Wikipathways	0.371429805	-0.02270324	1.577281819	0.14556125	0.55622203	-4.75304818
homo after UVB irradiation VS homo before UVB irradiation	folate polyglutamylation|HumanCyc	-0.51968405	-0.03965505	-1.62324891	0.13758285	0.27994034	-5.12537816
hetero after UVB irradiation VS hetero before UVB irradiation	folate polyglutamylation|HumanCyc	-0.46538255	-0.10901921	-1.4730495	0.17708222	0.39430568	-5.30299765
WT after UVB irradiation VS WT before UVB irradiation	folate polyglutamylation|HumanCyc	0.241196555	0.145676037	0.686238993	0.5080361	0.82540052	-5.63640869
homo after UVB irradiation VS homo before UVB irradiation	folate transformations|HumanCyc	-0.10295743	-0.05407377	-0.40872287	0.69190848	0.79350142	-6.27950073
hetero after UVB irradiation VS hetero before UVB irradiation	folate transformations|HumanCyc	0.248714753	-0.04737485	1.117536742	0.29461344	0.52598827	-5.71613871
WT after UVB irradiation VS WT before UVB irradiation	folate transformations|HumanCyc	0.179393226	-0.10197985	0.640269993	0.53627596	0.83767333	-5.66590634
homo after UVB irradiation VS homo before UVB irradiation	Folate-Alcohol and Cancer Pathway Hypotheses|Wikipathways	0.229195697	-0.01651596	0.967234744	0.35767906	0.51244512	-5.8899452
hetero after UVB irradiation VS hetero before UVB irradiation	Folate-Alcohol and Cancer Pathway Hypotheses|Wikipathways	0.307109127	0.002705269	1.014295955	0.33867844	0.5677486	-5.82090026
WT after UVB irradiation VS WT before UVB irradiation	Folate-Alcohol and Cancer Pathway Hypotheses|Wikipathways	-0.07206682	-0.07010164	-0.26621531	0.79543844	0.94308602	-5.83338386
homo after UVB irradiation VS homo before UVB irradiation	Folding of actin by CCT/TriC|Reactome	-0.69950875	0.018830223	-3.47980942	0.00650907	0.04701822	-2.25473917
hetero after UVB irradiation VS hetero before UVB irradiation	Folding of actin by CCT/TriC|Reactome	-0.57256288	-0.01554237	-2.22543017	0.05505304	0.20875475	-4.25026047
WT after UVB irradiation VS WT before UVB irradiation	Folding of actin by CCT/TriC|Reactome	-0.12313173	0.01861823	-0.36913515	0.71966124	0.90672477	-5.80062049
homo after UVB irradiation VS homo before UVB irradiation	Follicle Stimulating Hormone (FSH) signaling pathway|Wikipathways	-0.01780486	-0.02474732	-0.08894093	0.93099719	0.95961548	-6.36419975
hetero after UVB irradiation VS hetero before UVB irradiation	Follicle Stimulating Hormone (FSH) signaling pathway|Wikipathways	0.116577864	-0.01917922	0.583343898	0.57493979	0.7705309	-6.1656747
WT after UVB irradiation VS WT before UVB irradiation	Follicle Stimulating Hormone (FSH) signaling pathway|Wikipathways	0.068337715	-0.01155043	0.318196829	0.75682877	0.92319364	-5.81813459
homo after UVB irradiation VS homo before UVB irradiation	Fondaparinux Action Pathway|SMPDB	0.542957634	0.032474294	3.216401628	0.00999373	0.05951688	-2.67225071
hetero after UVB irradiation VS hetero before UVB irradiation	Fondaparinux Action Pathway|SMPDB	0.507034173	0.109310797	2.703272449	0.02570936	0.14017854	-3.52484947
WT after UVB irradiation VS WT before UVB irradiation	Fondaparinux Action Pathway|SMPDB	0.380045687	0.058400764	1.387851186	0.19508646	0.60625027	-4.98260907
homo after UVB irradiation VS homo before UVB irradiation	Forasartan Action Pathway|SMPDB	0.636038586	-0.11800708	2.436891056	0.03650532	0.12504267	-3.91641947
hetero after UVB irradiation VS hetero before UVB irradiation	Forasartan Action Pathway|SMPDB	-0.02777369	0.003323189	-0.09172332	0.92906122	0.97054702	-6.34197953
WT after UVB irradiation VS WT before UVB irradiation	Forasartan Action Pathway|SMPDB	0.270438532	-0.10210744	0.707878218	0.49505953	0.81710765	-5.62188349
homo after UVB irradiation VS homo before UVB irradiation	formaldehyde oxidation|HumanCyc	1.09798122	-0.00192582	5.528264585	0.00031527	0.01666204	0.688065859
hetero after UVB irradiation VS hetero before UVB irradiation	formaldehyde oxidation|HumanCyc	0.860308677	0.022369659	2.898660109	0.01887362	0.1205973	-3.2258097
WT after UVB irradiation VS WT before UVB irradiation	formaldehyde oxidation|HumanCyc	0.6595994	-0.01032608	1.919132909	0.08369076	0.470709	-4.29991581
homo after UVB irradiation VS homo before UVB irradiation	Formation of a pool of free 40S subunits|Reactome	-0.94578158	0.022245045	-4.96886538	0.00067942	0.01666204	-0.05144204
hetero after UVB irradiation VS hetero before UVB irradiation	Formation of a pool of free 40S subunits|Reactome	-0.21071304	-0.01229519	-0.62272347	0.54994464	0.75605031	-6.14100824
WT after UVB irradiation VS WT before UVB irradiation	Formation of a pool of free 40S subunits|Reactome	-0.59866906	-0.00705469	-1.99565971	0.07368824	0.45463532	-4.19312226
homo after UVB irradiation VS homo before UVB irradiation	Formation of annular gap junctions|Reactome	0.893341927	-0.07039528	5.163454312	0.00051754	0.01666204	0.211582002
hetero after UVB irradiation VS hetero before UVB irradiation	Formation of annular gap junctions|Reactome	0.906777985	0.041598967	5.971205894	0.00027244	0.02988205	0.886963444
WT after UVB irradiation VS WT before UVB irradiation	Formation of annular gap junctions|Reactome	0.887400869	0.054693126	4.335119529	0.00144971	0.15689229	-0.79297839
homo after UVB irradiation VS homo before UVB irradiation	Formation of apoptosome|Reactome	0.337614377	0.03095562	0.913227542	0.38397301	0.5369742	-5.93967943
hetero after UVB irradiation VS hetero before UVB irradiation	Formation of apoptosome|Reactome	0.403779825	0.087211422	1.465302386	0.17913061	0.39692626	-5.31275331
WT after UVB irradiation VS WT before UVB irradiation	Formation of apoptosome|Reactome	0.484709491	-0.11093405	1.932913873	0.08180048	0.46860292	-4.28080659
homo after UVB irradiation VS homo before UVB irradiation	Formation of ATP by chemiosmotic coupling|Reactome	-0.12379019	-0.00788214	-0.35693959	0.729028	0.8217964	-6.3004658
hetero after UVB irradiation VS hetero before UVB irradiation	Formation of ATP by chemiosmotic coupling|Reactome	-0.30692402	-0.03008504	-1.22217205	0.2547276	0.47937588	-5.60246665
WT after UVB irradiation VS WT before UVB irradiation	Formation of ATP by chemiosmotic coupling|Reactome	-0.15226012	-0.01538689	-0.38351393	0.70930171	0.90329752	-5.79521992
homo after UVB irradiation VS homo before UVB irradiation	Formation of editosomes by ADAR proteins|Reactome	0.544034782	-0.07960566	1.637930322	0.1344592	0.277359	-5.10549214
hetero after UVB irradiation VS hetero before UVB irradiation	Formation of editosomes by ADAR proteins|Reactome	0.660054131	0.017061716	2.04785425	0.07298105	0.24158558	-4.51288957
WT after UVB irradiation VS WT before UVB irradiation	Formation of editosomes by ADAR proteins|Reactome	0.295358316	-0.12669247	1.110293303	0.29265056	0.67947895	-5.28271793
homo after UVB irradiation VS homo before UVB irradiation	Formation of Fibrin Clot (Clotting Cascade)|Reactome	0.533039308	0.025950532	3.016117658	0.01390265	0.07305822	-2.99235482
hetero after UVB irradiation VS hetero before UVB irradiation	Formation of Fibrin Clot (Clotting Cascade)|Reactome	0.302735215	0.047500393	1.721144366	0.12162147	0.31336951	-4.97629694
WT after UVB irradiation VS WT before UVB irradiation	Formation of Fibrin Clot (Clotting Cascade)|Reactome	0.308003775	-0.01421613	1.254457015	0.23797773	0.63195092	-5.13273431
homo after UVB irradiation VS homo before UVB irradiation	Formation of HIV elongation complex in the absence of HIV Tat|Reactome	-0.64643842	0.019124449	-3.57824384	0.00555574	0.04299579	-2.10019048
hetero after UVB irradiation VS hetero before UVB irradiation	Formation of HIV elongation complex in the absence of HIV Tat|Reactome	-0.30449327	-0.05148675	-1.34649544	0.21323976	0.43535861	-5.45847873
WT after UVB irradiation VS WT before UVB irradiation	Formation of HIV elongation complex in the absence of HIV Tat|Reactome	-0.22606579	-0.01012777	-0.7249748	0.48495297	0.81710765	-5.61012155
homo after UVB irradiation VS homo before UVB irradiation	Formation of HIV-1 elongation complex containing HIV-1 Tat|Reactome	-0.66651563	0.024864965	-3.59445591	0.00541328	0.04239626	-2.07482703
hetero after UVB irradiation VS hetero before UVB irradiation	Formation of HIV-1 elongation complex containing HIV-1 Tat|Reactome	-0.30434514	-0.05206909	-1.35335126	0.2111292	0.43289558	-5.45027621
WT after UVB irradiation VS WT before UVB irradiation	Formation of HIV-1 elongation complex containing HIV-1 Tat|Reactome	-0.21592557	-0.00811919	-0.68432856	0.50919158	0.82580894	-5.63767152
homo after UVB irradiation VS homo before UVB irradiation	Formation of Incision Complex in GG-NER|Reactome	-0.27200138	0.009400507	-1.37341702	0.20152445	0.35887701	-5.44746398
hetero after UVB irradiation VS hetero before UVB irradiation	Formation of Incision Complex in GG-NER|Reactome	0.092478061	-0.01238113	0.492402472	0.63500839	0.8134818	-6.21692035
WT after UVB irradiation VS WT before UVB irradiation	Formation of Incision Complex in GG-NER|Reactome	-0.05471041	0.004709163	-0.19362175	0.8503173	0.95502804	-5.8501972
homo after UVB irradiation VS homo before UVB irradiation	Formation of RNA Pol II elongation complex|Reactome	-0.62341397	0.005455488	-3.4144186	0.00723541	0.04931566	-2.35789387
hetero after UVB irradiation VS hetero before UVB irradiation	Formation of RNA Pol II elongation complex|Reactome	-0.27680582	-0.05308823	-1.18198544	0.26949201	0.49831423	-5.64697602
WT after UVB irradiation VS WT before UVB irradiation	Formation of RNA Pol II elongation complex|Reactome	-0.29210507	-0.01506203	-0.9861841	0.34711771	0.73189593	-5.40053339
homo after UVB irradiation VS homo before UVB irradiation	Formation of Senescence-Associated Heterochromatin Foci (SAHF)|Reactome	-0.81849976	-0.04004444	-4.68002452	0.00102791	0.01844436	-0.45317199
hetero after UVB irradiation VS hetero before UVB irradiation	Formation of Senescence-Associated Heterochromatin Foci (SAHF)|Reactome	-0.38960781	-0.02567655	-1.2845737	0.23312961	0.45507698	-5.53134645
WT after UVB irradiation VS WT before UVB irradiation	Formation of Senescence-Associated Heterochromatin Foci (SAHF)|Reactome	-0.41216568	-0.03333541	-1.32272758	0.21514252	0.62581393	-5.05718485
homo after UVB irradiation VS homo before UVB irradiation	Formation of TC-NER Pre-Incision Complex|Reactome	-0.70957816	0.003513396	-4.34464848	0.00168774	0.02286718	-0.93618856
hetero after UVB irradiation VS hetero before UVB irradiation	Formation of TC-NER Pre-Incision Complex|Reactome	-0.3605037	-0.06380802	-1.81887137	0.10454817	0.29199176	-4.84101856
WT after UVB irradiation VS WT before UVB irradiation	Formation of TC-NER Pre-Incision Complex|Reactome	-0.29594564	-0.00585425	-1.02073908	0.33124609	0.71318926	-5.36883994
homo after UVB irradiation VS homo before UVB irradiation	Formation of the active cofactor_ UDP-glucuronate|Reactome	0.489805034	-0.06084003	2.135674392	0.06020906	0.16751767	-4.38391207
hetero after UVB irradiation VS hetero before UVB irradiation	Formation of the active cofactor_ UDP-glucuronate|Reactome	0.374145417	0.031141119	1.194144438	0.26495288	0.49251844	-5.63361879
WT after UVB irradiation VS WT before UVB irradiation	Formation of the active cofactor_ UDP-glucuronate|Reactome	0.215372424	-0.11793637	0.596039088	0.56429	0.85109394	-5.69251681
homo after UVB irradiation VS homo before UVB irradiation	Formation of the beta-catenin:TCF transactivating complex|Reactome	-0.34993514	0.040442658	-1.78002599	0.10738388	0.24059004	-4.90827422
hetero after UVB irradiation VS hetero before UVB irradiation	Formation of the beta-catenin:TCF transactivating complex|Reactome	-0.18634146	-0.00899751	-0.97236753	0.35795088	0.58625229	-5.86120821
WT after UVB irradiation VS WT before UVB irradiation	Formation of the beta-catenin:TCF transactivating complex|Reactome	-0.28106488	0.059098502	-1.14088343	0.28029334	0.66921967	-5.25202858
homo after UVB irradiation VS homo before UVB irradiation	Formation of the cornified envelope|Reactome	0.11134121	-0.00281214	0.47768125	0.64380173	0.75335692	-6.24735425
hetero after UVB irradiation VS hetero before UVB irradiation	Formation of the cornified envelope|Reactome	0.404600342	0.035315512	1.857014535	0.09851532	0.28258275	-4.78737106
WT after UVB irradiation VS WT before UVB irradiation	Formation of the cornified envelope|Reactome	0.108481608	0.106743351	0.32264045	0.7535588	0.92020735	-5.81670766
homo after UVB irradiation VS homo before UVB irradiation	Formation of the Early Elongation Complex|Reactome	-0.73718014	0.009736168	-4.02830289	0.00273386	0.02978124	-1.40724866
hetero after UVB irradiation VS hetero before UVB irradiation	Formation of the Early Elongation Complex|Reactome	-0.27312086	-0.06650294	-1.22112068	0.25510522	0.47969043	-5.60364425
WT after UVB irradiation VS WT before UVB irradiation	Formation of the Early Elongation Complex|Reactome	-0.21177086	0.010317359	-0.62797549	0.54398118	0.8402851	-5.6734788
homo after UVB irradiation VS homo before UVB irradiation	Formation of the Editosome|Reactome	0.733981786	0.027234617	3.66137419	0.00486445	0.03966267	-1.97042322
hetero after UVB irradiation VS hetero before UVB irradiation	Formation of the Editosome|Reactome	0.518011488	0.091761844	2.20108417	0.05722828	0.21310989	-4.28659513
WT after UVB irradiation VS WT before UVB irradiation	Formation of the Editosome|Reactome	0.748592347	0.097083739	2.838560665	0.01745812	0.30353778	-2.95075132
homo after UVB irradiation VS homo before UVB irradiation	Formation of the HIV-1 Early Elongation Complex|Reactome	-0.73718014	0.009736168	-4.02830289	0.00273386	0.02978124	-1.40724866
hetero after UVB irradiation VS hetero before UVB irradiation	Formation of the HIV-1 Early Elongation Complex|Reactome	-0.27312086	-0.06650294	-1.22112068	0.25510522	0.47969043	-5.60364425
WT after UVB irradiation VS WT before UVB irradiation	Formation of the HIV-1 Early Elongation Complex|Reactome	-0.21177086	0.010317359	-0.62797549	0.54398118	0.8402851	-5.6734788
homo after UVB irradiation VS homo before UVB irradiation	Formation of the ternary complex_ and subsequently_ the 43S complex|Reactome	-0.95087676	0.021164165	-4.97179336	0.00067661	0.01666204	-0.04743867
hetero after UVB irradiation VS hetero before UVB irradiation	Formation of the ternary complex_ and subsequently_ the 43S complex|Reactome	-0.27388296	-0.01426016	-0.81510906	0.43744088	0.66348076	-6.00001611
WT after UVB irradiation VS WT before UVB irradiation	Formation of the ternary complex_ and subsequently_ the 43S complex|Reactome	-0.55247416	-0.02378402	-1.76990311	0.10692279	0.50602735	-4.50301375
homo after UVB irradiation VS homo before UVB irradiation	Formation of tubulin folding intermediates by CCT/TriC|Reactome	-0.64128766	0.02363482	-3.12660566	0.01158352	0.0658248	-2.81557487
hetero after UVB irradiation VS hetero before UVB irradiation	Formation of tubulin folding intermediates by CCT/TriC|Reactome	-0.36909044	-0.04206747	-1.36608061	0.20725792	0.42901071	-5.43497701
WT after UVB irradiation VS WT before UVB irradiation	Formation of tubulin folding intermediates by CCT/TriC|Reactome	-0.27558378	-0.0055919	-0.94796725	0.36531365	0.74545864	-5.43455289
homo after UVB irradiation VS homo before UVB irradiation	Formyl peptide receptors bind formyl peptides and many other ligands|Reactome	0.919491191	0.019840762	5.814563807	0.00021648	0.01666204	1.046907746
hetero after UVB irradiation VS hetero before UVB irradiation	Formyl peptide receptors bind formyl peptides and many other ligands|Reactome	0.75852237	0.033333141	3.342851549	0.00945991	0.0985871	-2.55216217
WT after UVB irradiation VS WT before UVB irradiation	Formyl peptide receptors bind formyl peptides and many other ligands|Reactome	0.568344829	0.044574893	2.181688446	0.05387692	0.41493406	-3.92744244
homo after UVB irradiation VS homo before UVB irradiation	fosb gene expression and drug abuse|BioCarta	-0.50241079	0.010607184	-2.07792379	0.06621732	0.17879851	-4.4714945
hetero after UVB irradiation VS hetero before UVB irradiation	fosb gene expression and drug abuse|BioCarta	-0.20141119	0.034818879	-0.6407512	0.53871665	0.75113812	-6.12922719
WT after UVB irradiation VS WT before UVB irradiation	fosb gene expression and drug abuse|BioCarta	-0.16399507	0.081613083	-0.47735793	0.64328668	0.87745357	-5.75509849
homo after UVB irradiation VS homo before UVB irradiation	Fosinopril Action Pathway|SMPDB	0.636038586	-0.11800708	2.436891056	0.03650532	0.12504267	-3.91641947
hetero after UVB irradiation VS hetero before UVB irradiation	Fosinopril Action Pathway|SMPDB	-0.02777369	0.003323189	-0.09172332	0.92906122	0.97054702	-6.34197953
WT after UVB irradiation VS WT before UVB irradiation	Fosinopril Action Pathway|SMPDB	0.270438532	-0.10210744	0.707878218	0.49505953	0.81710765	-5.62188349
homo after UVB irradiation VS homo before UVB irradiation	Fosinopril Metabolism Pathway|SMPDB	1.076747846	-0.04193589	4.959558579	0.00068841	0.01666204	-0.06417629
hetero after UVB irradiation VS hetero before UVB irradiation	Fosinopril Metabolism Pathway|SMPDB	0.553630209	0.003105397	1.56066683	0.15532075	0.36486693	-5.19066683
WT after UVB irradiation VS WT before UVB irradiation	Fosinopril Metabolism Pathway|SMPDB	0.585930211	-0.05239369	1.72512433	0.11496929	0.51527453	-4.56245573
homo after UVB irradiation VS homo before UVB irradiation	Fosphenytoin (Antiarrhythmic) Action Pathway|SMPDB	0.396415397	-0.03677376	2.343069613	0.04268583	0.13402181	-4.06364635
hetero after UVB irradiation VS hetero before UVB irradiation	Fosphenytoin (Antiarrhythmic) Action Pathway|SMPDB	-0.01555055	-0.01775524	-0.07410913	0.94265284	0.97054702	-6.34356282
WT after UVB irradiation VS WT before UVB irradiation	Fosphenytoin (Antiarrhythmic) Action Pathway|SMPDB	0.056685205	-0.03978418	0.223404833	0.82768199	0.94525798	-5.84393438
homo after UVB irradiation VS homo before UVB irradiation	Fosphenytoin (Antiarrhythmic) Metabolism Pathway|SMPDB	0.042410873	-0.01164074	0.180877879	0.86030775	0.91480326	-6.35090075
hetero after UVB irradiation VS hetero before UVB irradiation	Fosphenytoin (Antiarrhythmic) Metabolism Pathway|SMPDB	-0.67835384	-0.07423511	-3.02386253	0.01550512	0.11352943	-3.03469014
WT after UVB irradiation VS WT before UVB irradiation	Fosphenytoin (Antiarrhythmic) Metabolism Pathway|SMPDB	-0.50045171	-0.07491755	-2.09830348	0.0620317	0.43384195	-4.04749272
homo after UVB irradiation VS homo before UVB irradiation	FOXA1 transcription factor network|PID	-0.00633215	0.071446293	-0.03575031	0.97223002	0.98255916	-6.36776159
hetero after UVB irradiation VS hetero before UVB irradiation	FOXA1 transcription factor network|PID	-0.02813339	0.02333513	-0.17834111	0.86266866	0.94644961	-6.32932037
WT after UVB irradiation VS WT before UVB irradiation	FOXA1 transcription factor network|PID	-0.09137073	0.006628581	-0.45647885	0.65772153	0.87935805	-5.76475043
homo after UVB irradiation VS homo before UVB irradiation	FOXA2 and FOXA3 transcription factor networks|PID	0.084713394	0.066309135	0.441038741	0.66916615	0.7743063	-6.26503261
hetero after UVB irradiation VS hetero before UVB irradiation	FOXA2 and FOXA3 transcription factor networks|PID	-0.09733836	0.03057398	-0.58058556	0.57671427	0.77200107	-6.16734707
WT after UVB irradiation VS WT before UVB irradiation	FOXA2 and FOXA3 transcription factor networks|PID	-0.11685511	0.012545295	-0.5457172	0.59712672	0.86531555	-5.72063917
homo after UVB irradiation VS homo before UVB irradiation	FOXM1 transcription factor network|PID	-0.46008897	0.017974104	-2.22637182	0.05182098	0.1514818	-4.24486198
hetero after UVB irradiation VS hetero before UVB irradiation	FOXM1 transcription factor network|PID	0.11773709	-0.00368315	0.513836375	0.62056744	0.80306263	-6.20557165
WT after UVB irradiation VS WT before UVB irradiation	FOXM1 transcription factor network|PID	0.041527075	-0.0106338	0.165116168	0.87211628	0.96283151	-5.85535979
homo after UVB irradiation VS homo before UVB irradiation	FoxO family signaling|PID	-0.24423122	0.040503237	-1.04643494	0.32155051	0.47730807	-5.81285434
hetero after UVB irradiation VS hetero before UVB irradiation	FoxO family signaling|PID	-0.10342006	-0.03264752	-0.50548116	0.62617658	0.80713969	-6.21004955
WT after UVB irradiation VS WT before UVB irradiation	FoxO family signaling|PID	-0.23577148	-0.02229442	-1.0710741	0.30910565	0.69398681	-5.32112821
homo after UVB irradiation VS homo before UVB irradiation	FoxO signaling pathway - Homo sapiens (human)|KEGG	-0.08673527	-0.00546286	-0.47420155	0.64619042	0.75537774	-6.24909082
hetero after UVB irradiation VS hetero before UVB irradiation	FoxO signaling pathway - Homo sapiens (human)|KEGG	-0.03447726	-0.01069949	-0.20487231	0.8425347	0.94055184	-6.32382952
WT after UVB irradiation VS WT before UVB irradiation	FoxO signaling pathway - Homo sapiens (human)|KEGG	-0.18831831	-0.03638825	-0.96092093	0.3590703	0.74336993	-5.42314494
homo after UVB irradiation VS homo before UVB irradiation	Free fatty acid receptors|Reactome	-0.35977375	0.020882004	-1.59140928	0.14458177	0.28992551	-5.16816652
hetero after UVB irradiation VS hetero before UVB irradiation	Free fatty acid receptors|Reactome	-0.11733666	-0.0077056	-0.28376977	0.78343528	0.90510815	-6.30307031
WT after UVB irradiation VS WT before UVB irradiation	Free fatty acid receptors|Reactome	0.078183477	0.019864074	0.262246261	0.79841194	0.94477083	-5.83443856
homo after UVB irradiation VS homo before UVB irradiation	Free fatty acids regulate insulin secretion|Reactome	0.353476268	-0.02054789	1.95622048	0.08080944	0.20118642	-4.65324229
hetero after UVB irradiation VS hetero before UVB irradiation	Free fatty acids regulate insulin secretion|Reactome	0.552605435	0.002323181	2.529800623	0.0338829	0.16053874	-3.79001525
WT after UVB irradiation VS WT before UVB irradiation	Free fatty acids regulate insulin secretion|Reactome	0.547455038	0.046868101	2.605922804	0.02606159	0.34104092	-3.30033552
homo after UVB irradiation VS homo before UVB irradiation	Frs2-mediated activation|Reactome	0.305466396	0.008879376	1.52405937	0.16044013	0.30897074	-5.25708618
hetero after UVB irradiation VS hetero before UVB irradiation	Frs2-mediated activation|Reactome	0.193047954	-0.00644007	1.105004848	0.29970867	0.53033405	-5.72925931
WT after UVB irradiation VS WT before UVB irradiation	Frs2-mediated activation|Reactome	0.189132842	0.05673257	0.729146174	0.48250677	0.81552033	-5.60721372
homo after UVB irradiation VS homo before UVB irradiation	FRS-mediated FGFR1 signaling|Reactome	-0.18080789	0.026607653	-1.03173612	0.32803865	0.48410617	-5.82752587
hetero after UVB irradiation VS hetero before UVB irradiation	FRS-mediated FGFR1 signaling|Reactome	-0.17478858	0.003420625	-0.93438611	0.37610521	0.60404776	-5.89655504
WT after UVB irradiation VS WT before UVB irradiation	FRS-mediated FGFR1 signaling|Reactome	-0.28940128	0.02005921	-1.20380609	0.25616634	0.65122698	-5.18695378
homo after UVB irradiation VS homo before UVB irradiation	FRS-mediated FGFR2 signaling|Reactome	-0.37278686	0.032211445	-2.10336283	0.06350291	0.17425138	-4.43301276
hetero after UVB irradiation VS hetero before UVB irradiation	FRS-mediated FGFR2 signaling|Reactome	-0.24754212	0.012639405	-1.31544828	0.22302448	0.44410029	-5.49529152
WT after UVB irradiation VS WT before UVB irradiation	FRS-mediated FGFR2 signaling|Reactome	-0.43377487	0.011694809	-1.82931626	0.0970406	0.49496258	-4.42303442
homo after UVB irradiation VS homo before UVB irradiation	FRS-mediated FGFR3 signaling|Reactome	-0.2830487	0.043151705	-1.37867452	0.19995145	0.35677152	-5.44103172
hetero after UVB irradiation VS hetero before UVB irradiation	FRS-mediated FGFR3 signaling|Reactome	-0.24711475	-0.00882633	-1.30165985	0.2274907	0.45082721	-5.51146263
WT after UVB irradiation VS WT before UVB irradiation	FRS-mediated FGFR3 signaling|Reactome	-0.42138389	0.011704443	-1.67879833	0.12386786	0.52904005	-4.62314578
homo after UVB irradiation VS homo before UVB irradiation	FRS-mediated FGFR4 signaling|Reactome	-0.16688375	0.017914954	-0.96078428	0.36074858	0.51603322	-5.89600836
hetero after UVB irradiation VS hetero before UVB irradiation	FRS-mediated FGFR4 signaling|Reactome	-0.22232674	-0.02439698	-1.23448195	0.2503404	0.47484459	-5.58862707
WT after UVB irradiation VS WT before UVB irradiation	FRS-mediated FGFR4 signaling|Reactome	-0.38805652	0.017938388	-1.63392353	0.1330735	0.54653478	-4.68111011
homo after UVB irradiation VS homo before UVB irradiation	fructose 2_6-bisphosphate synthesis|HumanCyc	0.41537031	-0.0294785	2.317407409	0.04454864	0.13725311	-4.10369347
hetero after UVB irradiation VS hetero before UVB irradiation	fructose 2_6-bisphosphate synthesis|HumanCyc	0.325735849	-0.06226928	1.395542884	0.19853137	0.41868045	-5.39922642
WT after UVB irradiation VS WT before UVB irradiation	fructose 2_6-bisphosphate synthesis|HumanCyc	-0.00863127	-0.03900647	-0.02835017	0.9779362	0.99268361	-5.86874404

homo after UVB irradiation VS homo before UVB irradiation	Fructose and Mannose Degradation|SMPDB	0.646134756	-0.02493519	2.787404746	0.02033181	0.09100843	-3.35877647
hetero after UVB irradiation VS hetero before UVB irradiation	Fructose and Mannose Degradation|SMPDB	0.437523013	0.017432895	2.391772938	0.04222813	0.18123901	-3.99986867
WT after UVB irradiation VS WT before UVB irradiation	Fructose and Mannose Degradation|SMPDB	0.510604418	0.046596932	2.250154608	0.04796085	0.401167	-3.82786925
homo after UVB irradiation VS homo before UVB irradiation	Fructose and mannose metabolism - Homo sapiens (human)|KEGG	0.729461895	-0.01481097	4.195904245	0.00211371	0.02555896	-1.15588564
hetero after UVB irradiation VS hetero before UVB irradiation	Fructose and mannose metabolism - Homo sapiens (human)|KEGG	0.603196871	0.019739021	3.577765251	0.00662326	0.08470408	-2.20286966
WT after UVB irradiation VS WT before UVB irradiation	Fructose and mannose metabolism - Homo sapiens (human)|KEGG	0.52514147	0.053428333	2.468307644	0.03302151	0.37053866	-3.50593984
homo after UVB irradiation VS homo before UVB irradiation	Fructose and mannose metabolism|EHMN	0.572738021	0.013143527	3.123798893	0.01163722	0.06584328	-2.8200606
hetero after UVB irradiation VS hetero before UVB irradiation	Fructose and mannose metabolism|EHMN	0.298991666	0.035790561	1.306292588	0.22598174	0.44842175	-5.5060417
WT after UVB irradiation VS WT before UVB irradiation	Fructose and mannose metabolism|EHMN	0.543179908	0.037004419	2.653940954	0.02399312	0.32905879	-3.22831291
homo after UVB irradiation VS homo before UVB irradiation	Fructose biosynthesis|Reactome	0.959788062	-0.01081132	4.811389114	0.00085021	0.01759873	-0.26880141
hetero after UVB irradiation VS hetero before UVB irradiation	Fructose biosynthesis|Reactome	1.237562278	-0.01714596	6.96855692	9.05E-05	0.02920766	1.908249269
WT after UVB irradiation VS WT before UVB irradiation	Fructose biosynthesis|Reactome	0.744293402	0.068371073	2.076722965	0.06432683	0.43544914	-4.07831958
homo after UVB irradiation VS homo before UVB irradiation	Fructose catabolism|Reactome	0.242628502	-0.04590004	1.071023204	0.31091629	0.46923284	-5.78794915
hetero after UVB irradiation VS hetero before UVB irradiation	Fructose catabolism|Reactome	-0.42557908	0.018566901	-1.70912413	0.12389172	0.31597198	-4.99270462
WT after UVB irradiation VS WT before UVB irradiation	Fructose catabolism|Reactome	0.19090454	0.094371458	0.858981477	0.41030835	0.77688258	-5.50942139
homo after UVB irradiation VS homo before UVB irradiation	Fructose intolerance_ hereditary|SMPDB	0.646134756	-0.02493519	2.787404746	0.02033181	0.09100843	-3.35877647
hetero after UVB irradiation VS hetero before UVB irradiation	Fructose intolerance_ hereditary|SMPDB	0.437523013	0.017432895	2.391772938	0.04222813	0.18123901	-3.99986867
WT after UVB irradiation VS WT before UVB irradiation	Fructose intolerance_ hereditary|SMPDB	0.510604418	0.046596932	2.250154608	0.04796085	0.401167	-3.82786925
homo after UVB irradiation VS homo before UVB irradiation	Fructose Mannose metabolism|INOH	0.65817974	-0.03652356	3.624915833	0.00515584	0.04133911	-2.02724598
hetero after UVB irradiation VS hetero before UVB irradiation	Fructose Mannose metabolism|INOH	0.503757436	0.001422167	2.768653814	0.023177	0.13628011	-3.42475036
WT after UVB irradiation VS WT before UVB irradiation	Fructose Mannose metabolism|INOH	0.345163721	0.018677876	1.521966322	0.15874364	0.56072663	-4.82190443
homo after UVB irradiation VS homo before UVB irradiation	Fructose metabolism|Reactome	0.4819091	-0.0266655	2.439233075	0.03636292	0.12504267	-3.91272945
hetero after UVB irradiation VS hetero before UVB irradiation	Fructose metabolism|Reactome	0.1755673	-0.00910558	0.794644393	0.44862174	0.67352369	-6.01657318
WT after UVB irradiation VS WT before UVB irradiation	Fructose metabolism|Reactome	0.352628426	0.012149478	1.551108574	0.15167372	0.55727585	-4.78580561
homo after UVB irradiation VS homo before UVB irradiation	Fructose-1_6-diphosphatase deficiency|SMPDB	0.252684369	-0.00151752	1.090705456	0.3026004	0.46312664	-5.76769054
hetero after UVB irradiation VS hetero before UVB irradiation	Fructose-1_6-diphosphatase deficiency|SMPDB	-0.06167553	0.005820603	-0.24893092	0.809375	0.92095935	-6.31305137
WT after UVB irradiation VS WT before UVB irradiation	Fructose-1_6-diphosphatase deficiency|SMPDB	-0.01454403	-0.00173466	-0.04854099	0.96223323	0.98815178	-5.86795769
homo after UVB irradiation VS homo before UVB irradiation	Fructosuria|SMPDB	0.646134756	-0.02493519	2.787404746	0.02033181	0.09100843	-3.35877647
hetero after UVB irradiation VS hetero before UVB irradiation	Fructosuria|SMPDB	0.437523013	0.017432895	2.391772938	0.04222813	0.18123901	-3.99986867
WT after UVB irradiation VS WT before UVB irradiation	Fructosuria|SMPDB	0.510604418	0.046596932	2.250154608	0.04796085	0.401167	-3.82786925
homo after UVB irradiation VS homo before UVB irradiation	FSH|NetPath	0.018389258	0.006740268	0.066738959	0.94818911	0.9693166	-6.36605564
hetero after UVB irradiation VS hetero before UVB irradiation	FSH|NetPath	0.259621916	-0.0706472	1.106795315	0.29897646	0.53033405	-5.7273914
WT after UVB irradiation VS WT before UVB irradiation	FSH|NetPath	0.148870452	0.06082095	0.560583165	0.58732203	0.86161188	-5.71257217
homo after UVB irradiation VS homo before UVB irradiation	FTO Obesity Variant Mechanism|Wikipathways	0.135124941	-0.04121264	0.581950464	0.57430549	0.69741459	-6.18977779
hetero after UVB irradiation VS hetero before UVB irradiation	FTO Obesity Variant Mechanism|Wikipathways	0.117693699	-0.00605575	0.576343974	0.57944891	0.77397513	-6.16990447
WT after UVB irradiation VS WT before UVB irradiation	FTO Obesity Variant Mechanism|Wikipathways	-0.08914274	0.025316089	-0.32729077	0.75014218	0.91881722	-5.81519365
homo after UVB irradiation VS homo before UVB irradiation	Fumarase deficiency|SMPDB	-0.23678174	0.014544733	-0.76379894	0.46374733	0.6088463	-6.06461144
hetero after UVB irradiation VS hetero before UVB irradiation	Fumarase deficiency|SMPDB	-0.47814985	0.004803127	-1.74993278	0.11633994	0.30887493	-4.93678811
WT after UVB irradiation VS WT before UVB irradiation	Fumarase deficiency|SMPDB	-0.1568339	-0.12513755	-0.50250429	0.62610623	0.87257466	-5.74292911
homo after UVB irradiation VS homo before UVB irradiation	Furosemide Action Pathway|SMPDB	0.234726031	0.017120546	1.156747303	0.27595732	0.43614438	-5.69767199
hetero after UVB irradiation VS hetero before UVB irradiation	Furosemide Action Pathway|SMPDB	-0.03255037	0.028925632	-0.18154472	0.86023155	0.94503144	-6.32869738
WT after UVB irradiation VS WT before UVB irradiation	Furosemide Action Pathway|SMPDB	-0.15908359	0.010252305	-0.80382265	0.44003996	0.79685269	-5.55266936
homo after UVB irradiation VS homo before UVB irradiation	G alpha (12/13) signalling events|Reactome	0.331845469	-0.05474779	2.021456888	0.07264478	0.19106915	-4.55632495
hetero after UVB irradiation VS hetero before UVB irradiation	G alpha (12/13) signalling events|Reactome	0.338710572	0.015363071	2.016054071	0.07674209	0.24857939	-4.55925751
WT after UVB irradiation VS WT before UVB irradiation	G alpha (12/13) signalling events|Reactome	0.112508635	-0.02822312	0.546457518	0.59663642	0.86530281	-5.72024224
homo after UVB irradiation VS homo before UVB irradiation	G alpha (i) signalling events|Reactome	0.356721831	0.000103169	2.216370778	0.05268718	0.15322908	-4.26027776
hetero after UVB irradiation VS hetero before UVB irradiation	G alpha (i) signalling events|Reactome	0.297796082	0.032076695	1.859064616	0.09820055	0.28257457	-4.78447507
WT after UVB irradiation VS WT before UVB irradiation	G alpha (i) signalling events|Reactome	0.20843435	0.031842798	0.975415715	0.35217637	0.73722538	-5.41022988
homo after UVB irradiation VS homo before UVB irradiation	G alpha (q) signalling events|Reactome	0.340437559	-0.0081836	2.168465136	0.057035	0.16240116	-4.33384079
hetero after UVB irradiation VS hetero before UVB irradiation	G alpha (q) signalling events|Reactome	0.294579171	0.03815495	1.871898479	0.09625155	0.280149	-4.76631723
WT after UVB irradiation VS WT before UVB irradiation	G alpha (q) signalling events|Reactome	0.144548153	0.027865888	0.671566459	0.51695108	0.82895075	-5.64602566
homo after UVB irradiation VS homo before UVB irradiation	G alpha (s) signalling events|Reactome	0.401957821	0.026142463	2.513952406	0.03209803	0.11947408	-3.79467455
hetero after UVB irradiation VS hetero before UVB irradiation	G alpha (s) signalling events|Reactome	0.291504991	0.072861708	1.839723687	0.10120828	0.28605405	-4.81174591
WT after UVB irradiation VS WT before UVB irradiation	G alpha (s) signalling events|Reactome	0.155289524	0.03200334	0.652674562	0.52856643	0.83278813	-5.65812998
homo after UVB irradiation VS homo before UVB irradiation	G alpha (z) signalling events|Reactome	0.465315716	-0.03466305	2.724074783	0.02259723	0.09770733	-3.46009556
hetero after UVB irradiation VS hetero before UVB irradiation	G alpha (z) signalling events|Reactome	0.407581185	0.020871405	2.416629287	0.04058613	0.17702544	-3.96218949
WT after UVB irradiation VS WT before UVB irradiation	G alpha (z) signalling events|Reactome	0.156824466	-0.0283829	0.662819157	0.52231018	0.83116638	-5.65166925
homo after UVB irradiation VS homo before UVB irradiation	G beta:gamma signalling through PI3Kgamma|Reactome	0.616332926	-0.03711349	3.704591711	0.00454124	0.0383987	-1.90325301
hetero after UVB irradiation VS hetero before UVB irradiation	G beta:gamma signalling through PI3Kgamma|Reactome	0.632383651	0.029121101	3.475997114	0.00772283	0.08930222	-2.3534406
WT after UVB irradiation VS WT before UVB irradiation	G beta:gamma signalling through PI3Kgamma|Reactome	0.413245265	-0.01038871	1.612151269	0.13775628	0.55341552	-4.70892789
homo after UVB irradiation VS homo before UVB irradiation	G beta:gamma signalling through PLC beta|Reactome	0.508509098	0.016070456	2.211067171	0.05315222	0.15418872	-4.26844487
hetero after UVB irradiation VS hetero before UVB irradiation	G beta:gamma signalling through PLC beta|Reactome	0.691874725	0.032186549	3.462371038	0.00788407	0.089711	-2.37369094
WT after UVB irradiation VS WT before UVB irradiation	G beta:gamma signalling through PLC beta|Reactome	0.300058669	-0.04196881	1.196301661	0.25895378	0.65411611	-5.19484963
homo after UVB irradiation VS homo before UVB irradiation	G protein gated Potassium channels|Reactome	0.227909476	-0.0130962	1.107580443	0.29560878	0.45577992	-5.75009619
hetero after UVB irradiation VS hetero before UVB irradiation	G protein gated Potassium channels|Reactome	0.111975855	0.033657182	0.713384829	0.49488925	0.71428009	-6.07871261
WT after UVB irradiation VS WT before UVB irradiation	G protein gated Potassium channels|Reactome	-0.00386805	-0.05146768	-0.01498999	0.98833265	0.99664845	-5.86903736
homo after UVB irradiation VS homo before UVB irradiation	G Protein Signaling Pathways|Wikipathways	0.315357978	-0.03353082	2.004108744	0.07473606	0.1953319	-4.58221595
hetero after UVB irradiation VS hetero before UVB irradiation	G Protein Signaling Pathways|Wikipathways	0.293890162	0.012432981	1.610528035	0.144037	0.34739964	-5.12519086
WT after UVB irradiation VS WT before UVB irradiation	G Protein Signaling Pathways|Wikipathways	0.121726893	-0.04806795	0.527446187	0.60929385	0.87257466	-5.73027499
homo after UVB irradiation VS homo before UVB irradiation	G(M2)-Gangliosidosis: Variant B_ Tay-sachs disease|SMPDB	0.79075295	-0.00257633	4.921856093	0.00072618	0.01666204	-0.11590737
hetero after UVB irradiation VS hetero before UVB irradiation	G(M2)-Gangliosidosis: Variant B_ Tay-sachs disease|SMPDB	0.602895035	0.025817322	2.347921889	0.04528827	0.18731753	-4.06619426
WT after UVB irradiation VS WT before UVB irradiation	G(M2)-Gangliosidosis: Variant B_ Tay-sachs disease|SMPDB	0.514071552	-0.00966104	2.208926071	0.05144363	0.41047076	-3.88793019
homo after UVB irradiation VS homo before UVB irradiation	G0 and Early G1|Reactome	-0.67412448	0.011762492	-3.0822741	0.01246221	0.06880589	-2.88645937
hetero after UVB irradiation VS hetero before UVB irradiation	G0 and Early G1|Reactome	-0.05261539	0.009649644	-0.18596571	0.85687095	0.94503144	-6.32781958
WT after UVB irradiation VS WT before UVB irradiation	G0 and Early G1|Reactome	-0.10154964	-0.01627756	-0.32261887	0.75357467	0.92020735	-5.81671464
homo after UVB irradiation VS homo before UVB irradiation	G1 Phase|Reactome	-0.20984066	0.004713549	-1.14068093	0.28226188	0.44245297	-5.71499069
hetero after UVB irradiation VS hetero before UVB irradiation	G1 Phase|Reactome	0.137165512	0.00881504	0.753784499	0.47151529	0.69305602	-6.04854683
WT after UVB irradiation VS WT before UVB irradiation	G1 Phase|Reactome	-0.12232611	-0.02428181	-0.49379233	0.63203262	0.87303764	-5.74721233
homo after UVB irradiation VS homo before UVB irradiation	G1 to S cell cycle control|Wikipathways	-0.36213702	-0.03457467	-1.63180455	0.13575469	0.27758886	-5.11380132
hetero after UVB irradiation VS hetero before UVB irradiation	G1 to S cell cycle control|Wikipathways	0.072104762	0.002103152	0.317060674	0.75891169	0.88865506	-6.29233801
WT after UVB irradiation VS WT before UVB irradiation	G1 to S cell cycle control|Wikipathways	0.081225681	-0.02866524	0.328915524	0.74894979	0.9185045	-5.81465968
homo after UVB irradiation VS homo before UVB irradiation	G1/S DNA Damage Checkpoints|Reactome	-0.47440755	0.024229453	-2.52269959	0.03163245	0.11829139	-3.78081562
hetero after UVB irradiation VS hetero before UVB irradiation	G1/S DNA Damage Checkpoints|Reactome	0.220736461	0.033417191	0.951460995	0.36786164	0.59605878	-5.8808038
WT after UVB irradiation VS WT before UVB irradiation	G1/S DNA Damage Checkpoints|Reactome	-0.19034024	-0.03776853	-0.75486506	0.46759648	0.81013396	-5.58895788
homo after UVB irradiation VS homo before UVB irradiation	G1/S Transition|Reactome	-0.48907699	-0.03754963	-2.10200167	0.06364541	0.17433295	-4.43507578
hetero after UVB irradiation VS hetero before UVB irradiation	G1/S Transition|Reactome	0.165612537	0.021196892	0.666010302	0.52321652	0.7387921	-6.11221041
WT after UVB irradiation VS WT before UVB irradiation	G1/S Transition|Reactome	0.019086856	-0.01937771	0.072326718	0.94375661	0.98295421	-5.86650185
homo after UVB irradiation VS homo before UVB irradiation	G1/S-Specific Transcription|Reactome	-0.53435365	-0.03020835	-1.71266811	0.11952847	0.25817499	-5.00279871
hetero after UVB irradiation VS hetero before UVB irradiation	G1/S-Specific Transcription|Reactome	0.395803645	0.055572445	1.337665513	0.2159846	0.43741933	-5.46900423
WT after UVB irradiation VS WT before UVB irradiation	G1/S-Specific Transcription|Reactome	0.300773995	-0.00216916	0.991405511	0.34468414	0.7291211	-5.39580072
homo after UVB irradiation VS homo before UVB irradiation	G13 Signaling Pathway|Wikipathways	0.399564275	-0.0101053	2.231973135	0.0513419	0.15046671	-4.23621954
hetero after UVB irradiation VS hetero before UVB irradiation	G13 Signaling Pathway|Wikipathways	0.360991955	0.027086552	2.313072374	0.04787687	0.19425953	-4.11875584
WT after UVB irradiation VS WT before UVB irradiation	G13 Signaling Pathway|Wikipathways	0.253900392	-0.01660222	1.242676623	0.24211169	0.63358831	-5.14548698
homo after UVB irradiation VS homo before UVB irradiation	G2 Phase|Reactome	-0.32586319	0.035258427	-1.16155279	0.27409349	0.43614438	-5.69245696
hetero after UVB irradiation VS hetero before UVB irradiation	G2 Phase|Reactome	0.382198077	0.040152048	1.477309561	0.17596466	0.39293465	-5.29762049
WT after UVB irradiation VS WT before UVB irradiation	G2 Phase|Reactome	0.290793402	0.033335098	0.981961329	0.34909505	0.73436992	-5.40434611
homo after UVB irradiation VS homo before UVB irradiation	G2/M Checkpoints|Reactome	-0.44779014	-0.01503865	-2.11891717	0.06189622	0.17056013	-4.40940679
hetero after UVB irradiation VS hetero before UVB irradiation	G2/M Checkpoints|Reactome	0.107314918	-0.01228529	0.413280935	0.68971901	0.84327769	-6.25483686
WT after UVB irradiation VS WT before UVB irradiation	G2/M Checkpoints|Reactome	-0.01148729	0.014358402	-0.04279964	0.96669702	0.98922368	-5.8682233
homo after UVB irradiation VS homo before UVB irradiation	G2/M DNA damage checkpoint|Reactome	-0.45386969	-0.00091625	-2.31658207	0.04460986	0.13727975	-4.10497971
hetero after UVB irradiation VS hetero before UVB irradiation	G2/M DNA damage checkpoint|Reactome	0.068537121	-0.01764579	0.276364366	0.78892647	0.90984182	-6.30529921
WT after UVB irradiation VS WT before UVB irradiation	G2/M DNA damage checkpoint|Reactome	-0.05893367	0.011985528	-0.22428797	0.8270132	0.94525798	-5.84373516
homo after UVB irradiation VS homo before UVB irradiation	G2/M DNA replication checkpoint|Reactome	-0.66238287	0.021353131	-2.50594953	0.03252996	0.12029246	-3.80734758
hetero after UVB irradiation VS hetero before UVB irradiation	G2/M DNA replication checkpoint|Reactome	0.2687299	0.028368651	0.925396699	0.38049919	0.6078918	-5.90475522
WT after UVB irradiation VS WT before UVB irradiation	G2/M DNA replication checkpoint|Reactome	-0.09987357	0.027225368	-0.34941083	0.73396899	0.9131126	-5.80770246
homo after UVB irradiation VS homo before UVB irradiation	G2/M Transition|Reactome	-0.55204197	0.016244724	-3.27331654	0.00910431	0.05710202	-2.5816285
hetero after UVB irradiation VS hetero before UVB irradiation	G2/M Transition|Reactome	-0.01047135	-0.01447275	-0.04524467	0.96496628	0.9837433	-6.3454309
WT after UVB irradiation VS WT before UVB irradiation	G2/M Transition|Reactome	-0.16908149	-0.02269117	-0.64182158	0.53530807	0.83767333	-5.66494113
homo after UVB irradiation VS homo before UVB irradiation	GAB1 signalosome|Reactome	0.042521444	0.002736028	0.175217053	0.86462963	0.91750838	-6.35198009
hetero after UVB irradiation VS hetero before UVB irradiation	GAB1 signalosome|Reactome	0.221905055	0.030325702	1.327404065	0.21921224	0.4411344	-5.48118062
WT after UVB irradiation VS WT before UVB irradiation	GAB1 signalosome|Reactome	0.026119807	0.037478813	0.102524377	0.92035098	0.97566251	-5.86382923
homo after UVB irradiation VS homo before UVB irradiation	GABA A (rho) receptor activation|Reactome	-0.43586296	0.163165167	-1.35056419	0.20848441	0.36655365	-5.47523532
hetero after UVB irradiation VS hetero before UVB irradiation	GABA A (rho) receptor activation|Reactome	-0.79315968	0.115273416	-3.72414612	0.00532292	0.07849967	-1.98850816
WT after UVB irradiation VS WT before UVB irradiation	GABA A (rho) receptor activation|Reactome	-0.65965513	0.16036137	-2.25464339	0.04759571	0.401167	-3.8213126
homo after UVB irradiation VS homo before UVB irradiation	GABA A receptor activation|Reactome	-0.27368503	0.100234937	-1.01386899	0.33605802	0.4922959	-5.84513861
hetero after UVB irradiation VS hetero before UVB irradiation	GABA A receptor activation|Reactome	-0.85843901	-0.04295275	-4.18675573	0.00272026	0.05916047	-1.33054836
WT after UVB irradiation VS WT before UVB irradiation	GABA A receptor activation|Reactome	-0.73928167	0.019378314	-3.25778829	0.00851639	0.24856491	-2.32283686
homo after UVB irradiation VS homo before UVB irradiation	GABA B receptor activation|Reactome	0.360763269	-0.02159689	2.006470782	0.07444799	0.19493053	-4.57869567
hetero after UVB irradiation VS hetero before UVB irradiation	GABA B receptor activation|Reactome	0.21007887	0.036901545	1.25563105	0.2429491	0.46586538	-5.56462969
WT after UVB irradiation VS WT before UVB irradiation	GABA B receptor activation|Reactome	0.113131058	-0.03418705	0.462751502	0.65336902	0.87935805	-5.76189402
homo after UVB irradiation VS homo before UVB irradiation	GABA receptor activation|Reactome	0.188233733	0.018868432	1.009746007	0.33792929	0.49359729	-5.84916814
hetero after UVB irradiation VS hetero before UVB irradiation	GABA receptor activation|Reactome	-0.07664886	0.021965801	-0.44795243	0.66548176	0.82651398	-6.23899764
WT after UVB irradiation VS WT before UVB irradiation	GABA receptor activation|Reactome	-0.09173898	-0.01211636	-0.38124385	0.7109332	0.90420468	-5.79608585
homo after UVB irradiation VS homo before UVB irradiation	GABA receptor Signaling|Wikipathways	-0.13689549	0.06172924	-0.65323085	0.52927722	0.66532749	-6.14437668
hetero after UVB irradiation VS hetero before UVB irradiation	GABA receptor Signaling|Wikipathways	-0.30667976	-0.03411467	-1.39472694	0.1987687	0.41868045	-5.40022282
WT after UVB irradiation VS WT before UVB irradiation	GABA receptor Signaling|Wikipathways	-0.29557337	-0.017978	-1.41434247	0.18739175	0.59994616	-4.95160413
homo after UVB irradiation VS homo before UVB irradiation	GABA shunt|HumanCyc	0.318812193	0.006713046	1.556368759	0.1526499	0.29939838	-5.21470504
hetero after UVB irradiation VS hetero before UVB irradiation	GABA shunt|HumanCyc	0.01269222	0.059129488	0.035608626	0.97242363	0.98705712	-6.34585348
WT after UVB irradiation VS WT before UVB irradiation	GABA shunt|HumanCyc	0.032323095	-0.04436434	0.130884191	0.89844172	0.97566251	-5.8604808
homo after UVB irradiation VS homo before UVB irradiation	GABA synthesis_ release_ reuptake and degradation|Reactome	0.109411225	0.03995959	0.650967203	0.53067449	0.66677483	-6.14589223
hetero after UVB irradiation VS hetero before UVB irradiation	GABA synthesis_ release_ reuptake and degradation|Reactome	-0.18113638	0.010704761	-0.98409181	0.3524808	0.58105154	-5.85007159
WT after UVB irradiation VS WT before UVB irradiation	GABA synthesis_ release_ reuptake and degradation|Reactome	0.058789324	-1.77E-06	0.261571876	0.79891751	0.94500291	-5.83461621
homo after UVB irradiation VS homo before UVB irradiation	GABA synthesis|Reactome	0.81879556	0.026901093	2.759181106	0.0213116	0.09365619	-3.40394752
hetero after UVB irradiation VS hetero before UVB irradiation	GABA synthesis|Reactome	1.049748463	0.052957373	4.274162601	0.00240449	0.05507363	-1.20984202
WT after UVB irradiation VS WT before UVB irradiation	GABA synthesis|Reactome	0.518753499	0.021044556	1.611328678	0.13793604	0.55342759	-4.70997488
homo after UVB irradiation VS homo before UVB irradiation	GABAergic synapse - Homo sapiens (human)|KEGG	0.179811838	0.012783702	1.127978073	0.28732674	0.44697403	-5.7285552
hetero after UVB irradiation VS hetero before UVB irradiation	GABAergic synapse - Homo sapiens (human)|KEGG	0.002438733	0.001153822	0.01388989	0.9892411	0.9942582	-6.34643664
WT after UVB irradiation VS WT before UVB irradiation	GABAergic synapse - Homo sapiens (human)|KEGG	-0.01614762	-0.04473056	-0.06499873	0.94944576	0.9859297	-5.86701139
homo after UVB irradiation VS homo before UVB irradiation	GABA-Transaminase Deficiency|SMPDB	0.066798026	0.046789851	0.254634663	0.80449493	0.87857543	-6.33373313
hetero after UVB irradiation VS hetero before UVB irradiation	GABA-Transaminase Deficiency|SMPDB	-0.52991759	0.031774511	-1.70647522	0.12439725	0.31623126	-4.99631323
WT after UVB irradiation VS WT before UVB irradiation	GABA-Transaminase Deficiency|SMPDB	-0.14708226	-0.03671277	-0.48165088	0.64033764	0.87636259	-5.75306298
homo after UVB irradiation VS homo before UVB irradiation	Galactose catabolism|Reactome	0.362351346	0.034779299	1.692426379	0.12341605	0.26258979	-5.03084679
hetero after UVB irradiation VS hetero before UVB irradiation	Galactose catabolism|Reactome	0.294104176	0.036579587	1.150950483	0.28136451	0.51114365	-5.68062903
WT after UVB irradiation VS WT before UVB irradiation	Galactose catabolism|Reactome	0.171419437	0.014158121	0.857152271	0.41127181	0.7774331	-5.51089502
homo after UVB irradiation VS homo before UVB irradiation	Galactose metabolism - Homo sapiens (human)|KEGG	0.793040479	-0.02050973	4.258130012	0.00192306	0.02442117	-1.06358107
hetero after UVB irradiation VS hetero before UVB irradiation	Galactose metabolism - Homo sapiens (human)|KEGG	0.656264462	0.059306528	3.43214388	0.00825456	0.09193881	-2.41868559
WT after UVB irradiation VS WT before UVB irradiation	Galactose metabolism - Homo sapiens (human)|KEGG	0.547738351	0.04591315	2.063453659	0.06577789	0.43803373	-4.09722112
homo after UVB irradiation VS homo before UVB irradiation	Galactose metabolism|EHMN	0.661979381	-0.01767319	4.000699455	0.00285324	0.03029741	-1.44901992

hetero after UVB irradiation VS hetero before UVB irradiation	Galactose metabolism|EHMN	0.68104672	0.04780935	3.76486579	0.00501148	0.07564846	-1.92937185
WT after UVB irradiation VS WT before UVB irradiation	Galactose metabolism|EHMN	0.667162394	0.014434511	3.278474971	0.0082223	0.24856491	-2.29212047
homo after UVB irradiation VS homo before UVB irradiation	Galactose metabolism|INOH	1.034698998	-0.03841528	6.124808872	0.00014585	0.01666204	1.421027568
hetero after UVB irradiation VS hetero before UVB irradiation	Galactose metabolism|INOH	0.884898074	0.093771658	3.945101198	0.00384825	0.06828702	-1.67036575
WT after UVB irradiation VS WT before UVB irradiation	Galactose metabolism|INOH	0.705634913	0.042102865	2.53154505	0.02962047	0.35882409	-3.41163515
homo after UVB irradiation VS homo before UVB irradiation	Galactose Metabolism|SMPDB	0.761269273	-0.01404971	3.826638993	0.00374447	0.03453993	-1.71471893
hetero after UVB irradiation VS hetero before UVB irradiation	Galactose Metabolism|SMPDB	0.811677434	0.083576787	4.866424798	0.00107328	0.04104387	-0.42438924
WT after UVB irradiation VS WT before UVB irradiation	Galactose Metabolism|SMPDB	0.60957604	0.029545218	2.35299562	0.04023971	0.39022568	-3.67687536
homo after UVB irradiation VS homo before UVB irradiation	Galactosemia II (GALK)|SMPDB	0.698574264	-0.05912759	4.348137644	0.00167892	0.02281533	-0.93107444
hetero after UVB irradiation VS hetero before UVB irradiation	Galactosemia II (GALK)|SMPDB	0.346014476	0.023648214	1.782710589	0.11058647	0.30216567	-4.89145127
WT after UVB irradiation VS WT before UVB irradiation	Galactosemia II (GALK)|SMPDB	0.461260912	0.002017987	1.791110278	0.10329419	0.50425697	-4.47460697
homo after UVB irradiation VS homo before UVB irradiation	Galactosemia III|SMPDB	0.698574264	-0.05912759	4.348137644	0.00167892	0.02281533	-0.93107444
hetero after UVB irradiation VS hetero before UVB irradiation	Galactosemia III|SMPDB	0.346014476	0.023648214	1.782710589	0.11058647	0.30216567	-4.89145127
WT after UVB irradiation VS WT before UVB irradiation	Galactosemia III|SMPDB	0.461260912	0.002017987	1.791110278	0.10329419	0.50425697	-4.47460697
homo after UVB irradiation VS homo before UVB irradiation	Galactosemia|SMPDB	0.761269273	-0.01404971	3.826638993	0.00374447	0.03453993	-1.71471893
hetero after UVB irradiation VS hetero before UVB irradiation	Galactosemia|SMPDB	0.811677434	0.083576787	4.866424798	0.00107328	0.04104387	-0.42438924
WT after UVB irradiation VS WT before UVB irradiation	Galactosemia|SMPDB	0.60957604	0.029545218	2.35299562	0.04023971	0.39022568	-3.67687536
homo after UVB irradiation VS homo before UVB irradiation	Gamma carboxylation_ hypusine formation and arylsulfatase activation|Reactome	0.219963618	0.001298746	1.184249416	0.26542581	0.42885148	-5.66761076
hetero after UVB irradiation VS hetero before UVB irradiation	Gamma carboxylation_ hypusine formation and arylsulfatase activation|Reactome	0.256197287	-0.03194191	1.237442779	0.24929455	0.47404445	-5.58528417
WT after UVB irradiation VS WT before UVB irradiation	Gamma carboxylation_ hypusine formation and arylsulfatase activation|Reactome	0.457907161	-0.03889948	2.184326092	0.05363653	0.41493406	-3.92362221
homo after UVB irradiation VS homo before UVB irradiation	gamma-aminobutyric acid receptor life cycle pathway|BioCarta	-0.09167978	0.018023371	-0.56411879	0.58589424	0.70809769	-6.20037656
hetero after UVB irradiation VS hetero before UVB irradiation	gamma-aminobutyric acid receptor life cycle pathway|BioCarta	-0.0258507	-0.02366763	-0.11684454	0.90972049	0.97054702	-6.33914097
WT after UVB irradiation VS WT before UVB irradiation	gamma-aminobutyric acid receptor life cycle pathway|BioCarta	-0.04924971	-0.08399663	-0.21317877	0.83543639	0.94951669	-5.84618462
homo after UVB irradiation VS homo before UVB irradiation	Gamma-carboxylation of protein precursors|Reactome	0.736244844	0.027002639	3.555308963	0.00576401	0.0440108	-2.13611657
hetero after UVB irradiation VS hetero before UVB irradiation	Gamma-carboxylation of protein precursors|Reactome	0.50538135	0.1254432	2.04997908	0.07273619	0.24158558	-4.50978334
WT after UVB irradiation VS WT before UVB irradiation	Gamma-carboxylation of protein precursors|Reactome	0.521311546	0.041570395	1.857860901	0.09259802	0.48552482	-4.38418335
homo after UVB irradiation VS homo before UVB irradiation	Gamma-carboxylation_ transport_ and amino-terminal cleavage of proteins|Reactome	0.776108566	0.035510119	4.152905283	0.0022571	0.02672385	-1.21999655
hetero after UVB irradiation VS hetero before UVB irradiation	Gamma-carboxylation_ transport_ and amino-terminal cleavage of proteins|Reactome	0.541691451	0.108415185	2.315949441	0.04765771	0.19360413	-4.11442189
WT after UVB irradiation VS WT before UVB irradiation	Gamma-carboxylation_ transport_ and amino-terminal cleavage of proteins|Reactome	0.632849483	0.030835835	2.693582455	0.02240943	0.31689014	-3.1687806
homo after UVB irradiation VS homo before UVB irradiation	Gamma-cystathionase deficiency (CTH)|SMPDB	-1.03096392	0.009185618	-5.50480244	0.0003253	0.01666204	0.658072793
hetero after UVB irradiation VS hetero before UVB irradiation	Gamma-cystathionase deficiency (CTH)|SMPDB	-0.67635827	-0.13184633	-2.08448047	0.06887001	0.23513821	-4.45921201
WT after UVB irradiation VS WT before UVB irradiation	Gamma-cystathionase deficiency (CTH)|SMPDB	-1.00471126	-0.08504096	-3.60367118	0.00475571	0.20968398	-1.81475856
homo after UVB irradiation VS homo before UVB irradiation	Gamma-Glutamyltransferase Deficiency|SMPDB	-0.03868231	-0.05039994	-0.170337	0.86835923	0.9186887	-6.35288321
hetero after UVB irradiation VS hetero before UVB irradiation	Gamma-Glutamyltransferase Deficiency|SMPDB	0.017134286	0.027343289	0.102228327	0.92096661	0.97054702	-6.34087549
WT after UVB irradiation VS WT before UVB irradiation	Gamma-Glutamyltransferase Deficiency|SMPDB	0.144724093	-0.01736596	0.615162034	0.55207887	0.84516674	-5.68122724
homo after UVB irradiation VS homo before UVB irradiation	Gamma-glutamyl-transpeptidase deficiency|SMPDB	-0.03868231	-0.05039994	-0.170337	0.86835923	0.9186887	-6.35288321
hetero after UVB irradiation VS hetero before UVB irradiation	Gamma-glutamyl-transpeptidase deficiency|SMPDB	0.017134286	0.027343289	0.102228327	0.92096661	0.97054702	-6.34087549
WT after UVB irradiation VS WT before UVB irradiation	Gamma-glutamyl-transpeptidase deficiency|SMPDB	0.144724093	-0.01736596	0.615162034	0.55207887	0.84516674	-5.68122724
homo after UVB irradiation VS homo before UVB irradiation	Ganglio Sphingolipid Metabolism|Wikipathways	0.143471486	-0.04932185	0.777774798	0.45585218	0.60277262	-6.05374403
hetero after UVB irradiation VS hetero before UVB irradiation	Ganglio Sphingolipid Metabolism|Wikipathways	0.007423698	-0.05998537	0.035448026	0.97254794	0.98705712	-6.34585967
WT after UVB irradiation VS WT before UVB irradiation	Ganglio Sphingolipid Metabolism|Wikipathways	0.064757091	-0.01368916	0.31994145	0.75554434	0.92187309	-5.81757667
homo after UVB irradiation VS homo before UVB irradiation	Gap junction - Homo sapiens (human)|KEGG	0.386651064	-0.03690843	2.349019844	0.04226496	0.13402181	-4.05434617
hetero after UVB irradiation VS hetero before UVB irradiation	Gap junction - Homo sapiens (human)|KEGG	0.407092713	0.003586354	2.485563654	0.03635934	0.16681566	-3.85742269
WT after UVB irradiation VS WT before UVB irradiation	Gap junction - Homo sapiens (human)|KEGG	0.312672182	-0.01803727	1.527572952	0.1573615	0.56072663	-4.81499063
homo after UVB irradiation VS homo before UVB irradiation	Gap junction assembly|Reactome	-0.60044815	0.017203699	-1.72632664	0.11696868	0.2549303	-4.98377736
hetero after UVB irradiation VS hetero before UVB irradiation	Gap junction assembly|Reactome	-0.18654347	-0.03778519	-0.50338777	0.62758599	0.80760501	-6.21116069
WT after UVB irradiation VS WT before UVB irradiation	Gap junction assembly|Reactome	-0.43070416	0.030729037	-1.1431942	0.27937671	0.66906841	-5.24968476
homo after UVB irradiation VS homo before UVB irradiation	Gap junction degradation|Reactome	0.67484106	-0.05593903	3.556281445	0.00575501	0.0440108	-2.13459219
hetero after UVB irradiation VS hetero before UVB irradiation	Gap junction degradation|Reactome	0.734410782	0.02738485	4.902526801	0.00102348	0.04092112	-0.37837596
WT after UVB irradiation VS WT before UVB irradiation	Gap junction degradation|Reactome	0.663366826	0.066660018	3.010417424	0.01299393	0.27886745	-2.69243077
homo after UVB irradiation VS homo before UVB irradiation	Gap junction trafficking and regulation|Reactome	0.420111246	-0.04471669	1.855769192	0.09509244	0.22192484	-4.79993582
hetero after UVB irradiation VS hetero before UVB irradiation	Gap junction trafficking and regulation|Reactome	0.668251924	0.021851301	4.006806381	0.00351925	0.06529021	-1.5827624
WT after UVB irradiation VS WT before UVB irradiation	Gap junction trafficking and regulation|Reactome	0.440081427	0.080291961	1.850998518	0.09364856	0.48767729	-4.39354781
homo after UVB irradiation VS homo before UVB irradiation	Gap junction trafficking|Reactome	0.531829657	-0.07029674	2.566908228	0.02938042	0.11431817	-3.71066294
hetero after UVB irradiation VS hetero before UVB irradiation	Gap junction trafficking|Reactome	0.670193237	0.013811124	4.396433558	0.00202709	0.0502626	-1.04301724
WT after UVB irradiation VS WT before UVB irradiation	Gap junction trafficking|Reactome	0.503829683	0.082821294	2.100972165	0.06175336	0.43347466	-4.04367335
homo after UVB irradiation VS homo before UVB irradiation	Gap-filling DNA repair synthesis and ligation in GG-NER|Reactome	-0.22675877	0.011690452	-0.91692668	0.38212896	0.53514858	-5.93634828
hetero after UVB irradiation VS hetero before UVB irradiation	Gap-filling DNA repair synthesis and ligation in GG-NER|Reactome	0.397075113	0.032048632	1.939794213	0.08653647	0.26632899	-4.66947371
WT after UVB irradiation VS WT before UVB irradiation	Gap-filling DNA repair synthesis and ligation in GG-NER|Reactome	0.056031856	0.039096476	0.211180329	0.83695399	0.94951669	-5.84661218
homo after UVB irradiation VS homo before UVB irradiation	Gap-filling DNA repair synthesis and ligation in TC-NER|Reactome	-0.52852012	0.001700423	-2.95564297	0.01536843	0.07691241	-3.08923503
hetero after UVB irradiation VS hetero before UVB irradiation	Gap-filling DNA repair synthesis and ligation in TC-NER|Reactome	-0.07750306	-0.01718791	-0.37893852	0.71410458	0.85921866	-6.26931797
WT after UVB irradiation VS WT before UVB irradiation	Gap-filling DNA repair synthesis and ligation in TC-NER|Reactome	-0.14750643	0.011997455	-0.51539969	0.61738505	0.87257466	-5.73645899
homo after UVB irradiation VS homo before UVB irradiation	Gastric Acid Production|SMPDB	0.21780303	0.051698704	1.19150037	0.26270351	0.42561191	-5.65959884
hetero after UVB irradiation VS hetero before UVB irradiation	Gastric Acid Production|SMPDB	0.331566302	0.063470812	1.441200644	0.18563717	0.40321767	-5.34291209
WT after UVB irradiation VS WT before UVB irradiation	Gastric Acid Production|SMPDB	-0.11560861	0.135982321	-0.44330845	0.66690385	0.87935805	-5.77062647
homo after UVB irradiation VS homo before UVB irradiation	Gastric acid production|Wikipathways	-0.06438905	-0.05826928	-0.22218275	0.82892943	0.89456199	-6.34199552
hetero after UVB irradiation VS hetero before UVB irradiation	Gastric acid production|Wikipathways	0.39716303	-0.021843	1.442651997	0.18523957	0.40321767	-5.34110429
WT after UVB irradiation VS WT before UVB irradiation	Gastric acid production|Wikipathways	-0.04230719	-0.02700761	-0.09559273	0.92571738	0.97616053	-5.86452419
homo after UVB irradiation VS homo before UVB irradiation	Gastric acid secretion - Homo sapiens (human)|KEGG	0.421524587	-0.02346373	2.432738577	0.03675915	0.12544265	-3.92296036
hetero after UVB irradiation VS hetero before UVB irradiation	Gastric acid secretion - Homo sapiens (human)|KEGG	0.192074598	0.025953721	0.984552856	0.35226699	0.58090916	-5.8496315
WT after UVB irradiation VS WT before UVB irradiation	Gastric acid secretion - Homo sapiens (human)|KEGG	0.120317455	-0.00099544	0.533570019	0.60520165	0.8702632	-5.72707976
homo after UVB irradiation VS homo before UVB irradiation	Gastric cancer - Homo sapiens (human)|KEGG	-0.20087391	0.00827503	-1.26971393	0.23475148	0.39655023	-5.57095508
hetero after UVB irradiation VS hetero before UVB irradiation	Gastric cancer - Homo sapiens (human)|KEGG	-0.21031474	0.003423801	-1.22316909	0.25436991	0.47937588	-5.60134926
WT after UVB irradiation VS WT before UVB irradiation	Gastric cancer - Homo sapiens (human)|KEGG	-0.19249456	0.037836097	-0.95456062	0.36212609	0.74374432	-5.42876216
homo after UVB irradiation VS homo before UVB irradiation	Gastric Cancer Network 1|Wikipathways	-0.56308457	-0.03807168	-2.52409487	0.03155881	0.11811274	-3.77860426
hetero after UVB irradiation VS hetero before UVB irradiation	Gastric Cancer Network 1|Wikipathways	-0.04846272	-0.01200364	-0.16752955	0.8709047	0.95129447	-6.33134159
WT after UVB irradiation VS WT before UVB irradiation	Gastric Cancer Network 1|Wikipathways	0.106040715	0.049243106	0.392098222	0.70314625	0.90059561	-5.79190042
homo after UVB irradiation VS homo before UVB irradiation	Gastric Cancer Network 2|Wikipathways	-0.60602845	-0.01861444	-3.35595181	0.0079562	0.05234584	-2.45042316
hetero after UVB irradiation VS hetero before UVB irradiation	Gastric Cancer Network 2|Wikipathways	-0.1856718	-0.05576026	-0.75402393	0.47137894	0.69305602	-6.04836372
WT after UVB irradiation VS WT before UVB irradiation	Gastric Cancer Network 2|Wikipathways	-0.1119473	-0.00477581	-0.39875954	0.6983851	0.89871083	-5.78927554
homo after UVB irradiation VS homo before UVB irradiation	Gastric ulcer formation|Wikipathways	0.397252343	0.064013718	1.704038238	0.12117206	0.26019636	-5.01477755
hetero after UVB irradiation VS hetero before UVB irradiation	Gastric ulcer formation|Wikipathways	0.021061235	-0.00610113	0.072547114	0.94385917	0.97091178	-6.34368701
WT after UVB irradiation VS WT before UVB irradiation	Gastric ulcer formation|Wikipathways	0.401940244	0.016815684	1.442716472	0.17943832	0.59499502	-4.9179823
homo after UVB irradiation VS homo before UVB irradiation	Gastrin|NetPath	0.299599727	-0.02890623	1.439221648	0.18257883	0.33687347	-5.36582121
hetero after UVB irradiation VS hetero before UVB irradiation	Gastrin|NetPath	0.405662384	0.002122095	2.585609995	0.03099983	0.15358392	-3.70482207
WT after UVB irradiation VS WT before UVB irradiation	Gastrin|NetPath	0.245206593	-0.01779657	1.219277174	0.25049569	0.64403178	-5.1705629
homo after UVB irradiation VS homo before UVB irradiation	Gastrin-CREB signalling pathway via PKC and MAPK|Reactome	0.042946311	-0.01985886	0.184869999	0.8572628	0.9134577	-6.35011908
hetero after UVB irradiation VS hetero before UVB irradiation	Gastrin-CREB signalling pathway via PKC and MAPK|Reactome	0.329260886	0.016698636	1.571168495	0.15287999	0.36293072	-5.17696638
WT after UVB irradiation VS WT before UVB irradiation	Gastrin-CREB signalling pathway via PKC and MAPK|Reactome	0.047759464	0.044838672	0.185693153	0.85636812	0.95782364	-5.85171491
homo after UVB irradiation VS homo before UVB irradiation	gata3 participate in activating the th2 cytokine genes expression|BioCarta	0.080574182	-0.01727489	0.376029341	0.71525116	0.81259935	-6.29305686
hetero after UVB irradiation VS hetero before UVB irradiation	gata3 participate in activating the th2 cytokine genes expression|BioCarta	-0.00574184	0.020585541	-0.02909428	0.97746676	0.98924321	-6.34608211
WT after UVB irradiation VS WT before UVB irradiation	gata3 participate in activating the th2 cytokine genes expression|BioCarta	0.008966748	-0.0594093	0.037925256	0.97048772	0.99043964	-5.8684226
homo after UVB irradiation VS homo before UVB irradiation	Gaucher Disease|SMPDB	0.410096132	-0.05671339	1.580382344	0.14707883	0.29223756	-5.18287502
hetero after UVB irradiation VS hetero before UVB irradiation	Gaucher Disease|SMPDB	0.381719767	-0.02021245	2.484597089	0.03641544	0.16681566	-3.85889413
WT after UVB irradiation VS WT before UVB irradiation	Gaucher Disease|SMPDB	0.327957029	0.011268274	1.427782053	0.1835878	0.59839062	-4.93573151
homo after UVB irradiation VS homo before UVB irradiation	GDP-fucose biosynthesis|Reactome	0.688604044	-0.02812351	2.852797093	0.01823331	0.08582814	-3.25404266
hetero after UVB irradiation VS hetero before UVB irradiation	GDP-fucose biosynthesis|Reactome	0.398276888	-0.05583999	1.667445476	0.13206956	0.32858792	-5.04917628
WT after UVB irradiation VS WT before UVB irradiation	GDP-fucose biosynthesis|Reactome	0.54453529	-0.10368614	1.987048667	0.07475497	0.45723857	-4.20521945
homo after UVB irradiation VS homo before UVB irradiation	GDP-glucose biosynthesis II|HumanCyc	0.600791855	-0.02901424	2.305514524	0.04543876	0.13859277	-4.12221722
hetero after UVB irradiation VS hetero before UVB irradiation	GDP-glucose biosynthesis II|HumanCyc	0.522034571	0.090347619	1.960541648	0.0837592	0.2618393	-4.63963182
WT after UVB irradiation VS WT before UVB irradiation	GDP-glucose biosynthesis II|HumanCyc	0.242325952	0.038515346	0.811695893	0.43571045	0.79685269	-5.54664792
homo after UVB irradiation VS homo before UVB irradiation	GDP-L-fucose biosynthesis I (from GDP-D-mannose)|HumanCyc	0.783734796	0.033062661	2.319421447	0.04439961	0.13688629	-4.10055423
hetero after UVB irradiation VS hetero before UVB irradiation	GDP-L-fucose biosynthesis I (from GDP-D-mannose)|HumanCyc	0.126740917	-0.01719851	0.400699977	0.69860998	0.84778456	-6.26028202
WT after UVB irradiation VS WT before UVB irradiation	GDP-L-fucose biosynthesis I (from GDP-D-mannose)|HumanCyc	1.127373892	-0.07046202	4.650359738	0.00088799	0.15645713	-0.3797351
homo after UVB irradiation VS homo before UVB irradiation	GDP-L-fucose biosynthesis II (from L-fucose)|HumanCyc	0.531643472	-0.02087475	1.40973516	0.19087028	0.34680211	-5.40270664
hetero after UVB irradiation VS hetero before UVB irradiation	GDP-L-fucose biosynthesis II (from L-fucose)|HumanCyc	0.633749457	0.008425978	1.641032824	0.13750542	0.33714348	-5.08461419
WT after UVB irradiation VS WT before UVB irradiation	GDP-L-fucose biosynthesis II (from L-fucose)|HumanCyc	0.058219817	0.014658622	0.125090963	0.90291063	0.97566251	-5.86123073
homo after UVB irradiation VS homo before UVB irradiation	GDP-mannose biosynthesis|HumanCyc	0.831249048	-0.03962629	4.038729164	0.00269016	0.02974154	-1.39149781
hetero after UVB irradiation VS hetero before UVB irradiation	GDP-mannose biosynthesis|HumanCyc	0.893542094	-0.00492767	5.210763607	0.00068742	0.03228824	0.005693923
WT after UVB irradiation VS WT before UVB irradiation	GDP-mannose biosynthesis|HumanCyc	0.899068427	-0.07873031	4.048974294	0.00228889	0.17027242	-1.18284382
homo after UVB irradiation VS homo before UVB irradiation	Gefitinib Action Pathway|SMPDB	-1.02021206	0.004763088	-4.21042005	0.00206751	0.02537706	-1.13430282
hetero after UVB irradiation VS hetero before UVB irradiation	Gefitinib Action Pathway|SMPDB	-0.7208662	-0.04088773	-2.69458506	0.02606648	0.14043856	-3.53814761
WT after UVB irradiation VS WT before UVB irradiation	Gefitinib Action Pathway|SMPDB	-0.88905476	-0.02311791	-2.71673052	0.02153341	0.31542987	-3.1339937
homo after UVB irradiation VS homo before UVB irradiation	Gefitinib Pathway_ Pharmacokinetics|PharmGKB	-0.27952451	0.005825621	-1.06630869	0.31293409	0.46923284	-5.79275933
hetero after UVB irradiation VS hetero before UVB irradiation	Gefitinib Pathway_ Pharmacokinetics|PharmGKB	0.15696476	0.058357124	0.563345826	0.58787382	0.78049383	-6.17763371
WT after UVB irradiation VS WT before UVB irradiation	Gefitinib Pathway_ Pharmacokinetics|PharmGKB	-0.18123963	0.049805897	-0.77364553	0.45689683	0.80891799	-5.57527394
homo after UVB irradiation VS homo before UVB irradiation	Gemcitabine Action Pathway|SMPDB	-0.25120335	-0.00907249	-1.2680424	0.23532226	0.39694717	-5.57289111
hetero after UVB irradiation VS hetero before UVB irradiation	Gemcitabine Action Pathway|SMPDB	0.215673563	0.065390025	0.933567855	0.37650362	0.60404911	-5.8973041
WT after UVB irradiation VS WT before UVB irradiation	Gemcitabine Action Pathway|SMPDB	0.041788611	0.004979925	0.151127147	0.88285647	0.96830103	-5.85759495
homo after UVB irradiation VS homo before UVB irradiation	Gemcitabine Metabolism Pathway|SMPDB	-0.25120335	-0.00907249	-1.2680424	0.23532226	0.39694717	-5.57289111
hetero after UVB irradiation VS hetero before UVB irradiation	Gemcitabine Metabolism Pathway|SMPDB	0.215673563	0.065390025	0.933567855	0.37650362	0.60404911	-5.8973041
WT after UVB irradiation VS WT before UVB irradiation	Gemcitabine Metabolism Pathway|SMPDB	0.041788611	0.004979925	0.151127147	0.88285647	0.96830103	-5.85759495
homo after UVB irradiation VS homo before UVB irradiation	Gemcitabine Pathway_ Pharmacodynamics|PharmGKB	0.282211984	-0.0297041	1.287655616	0.22869669	0.38862266	-5.55006291
hetero after UVB irradiation VS hetero before UVB irradiation	Gemcitabine Pathway_ Pharmacodynamics|PharmGKB	0.514940767	0.070014802	2.679031786	0.02671854	0.14252149	-3.56195301
WT after UVB irradiation VS WT before UVB irradiation	Gemcitabine Pathway_ Pharmacodynamics|PharmGKB	0.266277353	-0.05565419	1.123832184	0.28713012	0.67385573	-5.26921307
homo after UVB irradiation VS homo before UVB irradiation	Gene and protein expression by JAK-STAT signaling after Interleukin-12 stimulation|Wikipathways	0.042902417	-0.02680229	0.20266242	0.84372263	0.90483546	-6.34642949
hetero after UVB irradiation VS hetero before UVB irradiation	Gene and protein expression by JAK-STAT signaling after Interleukin-12 stimulation|Wikipathways	0.295396199	0.000922012	1.509745516	0.16765817	0.38349848	-5.25639039
WT after UVB irradiation VS WT before UVB irradiation	Gene and protein expression by JAK-STAT signaling after Interleukin-12 stimulation|Wikipathways	0.273809456	-0.00373526	1.038828206	0.32315626	0.70648859	-5.35190202
homo after UVB irradiation VS homo before UVB irradiation	Gene expression (Transcription)|Reactome	-0.39666726	-0.00097798	-2.33181848	0.04349294	0.13518637	-4.08121701
hetero after UVB irradiation VS hetero before UVB irradiation	Gene expression (Transcription)|Reactome	-0.12772372	-0.03774694	-0.68719891	0.51042586	0.72931695	-6.09748144
WT after UVB irradiation VS WT before UVB irradiation	Gene expression (Transcription)|Reactome	-0.20918007	-0.01102635	-0.85865387	0.41048079	0.77688258	-5.50968551
homo after UVB irradiation VS homo before UVB irradiation	Gene regulatory network modelling somitogenesis|Wikipathways	0.09747674	0.027162962	0.341138734	0.74050929	0.8293358	-6.30631428
hetero after UVB irradiation VS hetero before UVB irradiation	Gene regulatory network modelling somitogenesis|Wikipathways	0.15072218	0.000757395	0.645259124	0.53593046	0.74924265	-6.12623373
WT after UVB irradiation VS WT before UVB irradiation	Gene regulatory network modelling somitogenesis|Wikipathways	-0.25086834	-0.01097898	-0.82652281	0.42763441	0.79358242	-5.53517042
homo after UVB irradiation VS homo before UVB irradiation	Gene Silencing by RNA|Reactome	-0.60840332	0.012531989	-3.6667285	0.00482314	0.03957638	-1.96209021
hetero after UVB irradiation VS hetero before UVB irradiation	Gene Silencing by RNA|Reactome	-0.21714029	-0.04210306	-0.96569522	0.36109207	0.58927951	-5.86749891
WT after UVB irradiation VS WT before UVB irradiation	Gene Silencing by RNA|Reactome	-0.17527111	0.004566039	-0.62755757	0.54424421	0.8402851	-5.67373384
homo after UVB irradiation VS homo before UVB irradiation	generation of amyloid b-peptide by ps1|BioCarta	0.38427753	-0.08424891	1.334581933	0.21347166	0.3711773	-5.49447348
hetero after UVB irradiation VS hetero before UVB irradiation	generation of amyloid b-peptide by ps1|BioCarta	0.872626099	0.015646616	3.664311597	0.00581836	0.08047311	-2.07580111
WT after UVB irradiation VS WT before UVB irradiation	generation of amyloid b-peptide by ps1|BioCarta	0.764430317	0.033379507	2.392128915	0.03763186	0.39022568	-3.61903545
homo after UVB irradiation VS homo before UVB irradiation	Generation of second messenger molecules|Reactome	0.082343898	-0.04972551	0.363855207	0.72402496	0.81889967	-6.29782507
hetero after UVB irradiation VS hetero before UVB irradiation	Generation of second messenger molecules|Reactome	0.159399274	0.032543745	0.706633392	0.49886616	0.71729714	-6.08361119
WT after UVB irradiation VS WT before UVB irradiation	Generation of second messenger molecules|Reactome	0.347017625	-0.01354042	1.202221357	0.25675296	0.65122698	-5.18862417
homo after UVB irradiation VS homo before UVB irradiation	Generic Transcription Pathway|Reactome	-0.30372636	-0.00324681	-1.77825763	0.10768766	0.24079424	-4.91077838
hetero after UVB irradiation VS hetero before UVB irradiation	Generic Transcription Pathway|Reactome	-0.09416521	-0.02230587	-0.55252804	0.59493644	0.7848684	-6.18394199
WT after UVB irradiation VS WT before UVB irradiation	Generic Transcription Pathway|Reactome	-0.17479661	-0.00196793	-0.75767358	0.46598627	0.80910175	-5.58693039
homo after UVB irradiation VS homo before UVB irradiation	Genes targeted by miRNAs in adipocytes|Wikipathways	0.294661126	-0.05317651	1.356803289	0.20656437	0.36458172	-5.46768392
hetero after UVB irradiation VS hetero before UVB irradiation	Genes targeted by miRNAs in adipocytes|Wikipathways	0.102622616	0.001461479	0.411844632	0.69073151	0.84428915	-6.25546667

WT after UVB irradiation VS WT before UVB irradiation	Genes targeted by miRNAs in adipocytes|Wikipathways	-0.02722404	-0.08384309	-0.10964522	0.91484246	0.97566251	-5.86306477
homo after UVB irradiation VS homo before UVB irradiation	Genotoxicity pathway|Wikipathways	-0.25043095	-0.03784544	-1.46476711	0.17564882	0.32828997	-5.33348034
hetero after UVB irradiation VS hetero before UVB irradiation	Genotoxicity pathway|Wikipathways	-0.00240334	-0.05339992	-0.01357078	0.98948826	0.99428737	-6.34644139
WT after UVB irradiation VS WT before UVB irradiation	Genotoxicity pathway|Wikipathways	-0.18015182	-0.00338355	-0.90956197	0.38428182	0.75745148	-5.46762213
homo after UVB irradiation VS homo before UVB irradiation	geranylgeranyldiphosphate biosynthesis|HumanCyc	-0.9936216	0.020087806	-3.8580019	0.00356445	0.03390684	-1.66655941
hetero after UVB irradiation VS hetero before UVB irradiation	geranylgeranyldiphosphate biosynthesis|HumanCyc	-0.67854999	-0.02921144	-2.15581954	0.06149912	0.22211807	-4.35389373
WT after UVB irradiation VS WT before UVB irradiation	geranylgeranyldiphosphate biosynthesis|HumanCyc	-1.05786735	-0.05909063	-3.52307728	0.00544171	0.20968398	-1.93198915
homo after UVB irradiation VS homo before UVB irradiation	Ghrelin|NetPath	0.407132349	-0.04425704	2.459192112	0.03517153	0.12483995	-3.88125583
hetero after UVB irradiation VS hetero before UVB irradiation	Ghrelin|NetPath	0.389771311	0.014207681	2.252282452	0.05274824	0.20392406	-4.21008177
WT after UVB irradiation VS WT before UVB irradiation	Ghrelin|NetPath	0.314758181	-0.0172788	1.463909524	0.17368811	0.58411898	-4.89259719
homo after UVB irradiation VS homo before UVB irradiation	GLI proteins bind promoters of Hh responsive genes to promote transcription|Reactome	-0.75824381	0.039908252	-4.3565391	0.00165788	0.02273231	-0.9187678
hetero after UVB irradiation VS hetero before UVB irradiation	GLI proteins bind promoters of Hh responsive genes to promote transcription|Reactome	-0.43955864	0.035086414	-1.55991139	0.15549767	0.36496311	-5.19165048
WT after UVB irradiation VS WT before UVB irradiation	GLI proteins bind promoters of Hh responsive genes to promote transcription|Reactome	-0.53256958	0.044436321	-1.77229005	0.10650864	0.50548239	-4.49982451
homo after UVB irradiation VS homo before UVB irradiation	GLI3 is processed to GLI3R by the proteasome|Reactome	-0.29883354	-0.02371043	-1.73950121	0.11454715	0.25121148	-4.96535852
hetero after UVB irradiation VS hetero before UVB irradiation	GLI3 is processed to GLI3R by the proteasome|Reactome	0.228814657	0.055988203	1.097362177	0.30285009	0.53328183	-5.73720738
WT after UVB irradiation VS WT before UVB irradiation	GLI3 is processed to GLI3R by the proteasome|Reactome	-0.21030988	0.010539049	-0.92442161	0.37686147	0.75077508	-5.45496167
homo after UVB irradiation VS homo before UVB irradiation	Glial Cell Differentiation|Wikipathways	0.576258297	-0.09797404	2.596528966	0.02796182	0.11189627	-3.66356583
hetero after UVB irradiation VS hetero before UVB irradiation	Glial Cell Differentiation|Wikipathways	0.258680075	0.053295215	0.812164555	0.43903782	0.66482869	-6.00242045
WT after UVB irradiation VS WT before UVB irradiation	Glial Cell Differentiation|Wikipathways	0.244701256	-0.00084862	0.752819054	0.46877176	0.81124692	-5.59043072
homo after UVB irradiation VS homo before UVB irradiation	Glibenclamide Action Pathway|SMPDB	-0.17556465	0.032733531	-0.6220452	0.54871777	0.67913558	-6.16483259
hetero after UVB irradiation VS hetero before UVB irradiation	Glibenclamide Action Pathway|SMPDB	-0.35455332	-0.0590526	-1.26742043	0.23890834	0.4612216	-5.55113336
WT after UVB irradiation VS WT before UVB irradiation	Glibenclamide Action Pathway|SMPDB	-0.23688751	-0.13206249	-0.65900303	0.52465845	0.83140705	-5.65411026
homo after UVB irradiation VS homo before UVB irradiation	Gliclazide Action Pathway|SMPDB	-0.17556465	0.032733531	-0.6220452	0.54871777	0.67913558	-6.16483259
hetero after UVB irradiation VS hetero before UVB irradiation	Gliclazide Action Pathway|SMPDB	-0.35455332	-0.0590526	-1.26742043	0.23890834	0.4612216	-5.55113336
WT after UVB irradiation VS WT before UVB irradiation	Gliclazide Action Pathway|SMPDB	-0.23688751	-0.13206249	-0.65900303	0.52465845	0.83140705	-5.65411026
homo after UVB irradiation VS homo before UVB irradiation	Glioma - Homo sapiens (human)|KEGG	0.314207277	-0.00871569	1.796320635	0.10462169	0.2370387	-4.885144
hetero after UVB irradiation VS hetero before UVB irradiation	Glioma - Homo sapiens (human)|KEGG	0.365493635	0.008297441	2.389759168	0.04236403	0.18131011	-4.00291878
WT after UVB irradiation VS WT before UVB irradiation	Glioma - Homo sapiens (human)|KEGG	0.24885011	0.011087374	1.242881381	0.24203935	0.63358831	-5.14526605
homo after UVB irradiation VS homo before UVB irradiation	Global Genome Nucleotide Excision Repair (GG-NER)|Reactome	-0.3622261	-0.00946296	-1.95280467	0.08126003	0.20208786	-4.65828278
hetero after UVB irradiation VS hetero before UVB irradiation	Global Genome Nucleotide Excision Repair (GG-NER)|Reactome	0.061851175	-0.02750872	0.321582831	0.75560183	0.88626689	-6.29079055
WT after UVB irradiation VS WT before UVB irradiation	Global Genome Nucleotide Excision Repair (GG-NER)|Reactome	-0.0135244	0.009916929	-0.05088926	0.96040787	0.98815178	-5.86783943
homo after UVB irradiation VS homo before UVB irradiation	Globo Sphingolipid Metabolism|Wikipathways	-0.04608034	0.023719244	-0.24453972	0.81207342	0.88427872	-6.33642233
hetero after UVB irradiation VS hetero before UVB irradiation	Globo Sphingolipid Metabolism|Wikipathways	-0.01987587	-0.01771595	-0.12260269	0.90529575	0.97054702	-6.3383943
WT after UVB irradiation VS WT before UVB irradiation	Globo Sphingolipid Metabolism|Wikipathways	-0.12399716	-0.01881757	-0.5410459	0.6002253	0.86775212	-5.72313206
homo after UVB irradiation VS homo before UVB irradiation	Globoid Cell Leukodystrophy|SMPDB	0.410096132	-0.05671339	1.580382344	0.14707883	0.29223756	-5.18287502
hetero after UVB irradiation VS hetero before UVB irradiation	Globoid Cell Leukodystrophy|SMPDB	0.381719767	-0.02021245	2.484597089	0.03641544	0.16681566	-3.85889413
WT after UVB irradiation VS WT before UVB irradiation	Globoid Cell Leukodystrophy|SMPDB	0.327957029	0.011268274	1.427782053	0.1835878	0.59839062	-4.93573151
homo after UVB irradiation VS homo before UVB irradiation	Glucagon signaling in metabolic regulation|Reactome	0.474303153	-0.0525269	2.877530821	0.01749812	0.08340524	-3.21441105
hetero after UVB irradiation VS hetero before UVB irradiation	Glucagon signaling in metabolic regulation|Reactome	0.438506311	0.03980299	2.386259025	0.04260128	0.18181334	-4.00821924
WT after UVB irradiation VS WT before UVB irradiation	Glucagon signaling in metabolic regulation|Reactome	0.298714435	-0.04920619	1.124485226	0.28686591	0.6736679	-5.26855853
homo after UVB irradiation VS homo before UVB irradiation	Glucagon signaling pathway - Homo sapiens (human)|KEGG	0.292630567	-0.00637208	1.704610445	0.12106245	0.26012759	-5.01398424
hetero after UVB irradiation VS hetero before UVB irradiation	Glucagon signaling pathway - Homo sapiens (human)|KEGG	0.008537692	-0.006979	0.038769221	0.96997728	0.98614433	-6.34572597
WT after UVB irradiation VS WT before UVB irradiation	Glucagon signaling pathway - Homo sapiens (human)|KEGG	0.094323714	-0.0453403	0.378256478	0.71308251	0.90425832	-5.79721779
homo after UVB irradiation VS homo before UVB irradiation	Glucagon-like Peptide-1 (GLP1) regulates insulin secretion|Reactome	0.371904667	-0.02890877	2.225614478	0.05188608	0.15157508	-4.24603004
hetero after UVB irradiation VS hetero before UVB irradiation	Glucagon-like Peptide-1 (GLP1) regulates insulin secretion|Reactome	0.452323903	0.017532503	2.47527004	0.03696126	0.16802342	-3.87308976
WT after UVB irradiation VS WT before UVB irradiation	Glucagon-like Peptide-1 (GLP1) regulates insulin secretion|Reactome	0.173707764	-0.06463256	0.693749085	0.50350922	0.82373662	-5.63141362
homo after UVB irradiation VS homo before UVB irradiation	Glucagon-type ligand receptors|Reactome	0.444497171	-0.00255871	2.093905276	0.06449935	0.17603349	-4.44733771
hetero after UVB irradiation VS hetero before UVB irradiation	Glucagon-type ligand receptors|Reactome	0.545817635	0.025943264	2.994262278	0.01624072	0.11352943	-3.07980537
WT after UVB irradiation VS WT before UVB irradiation	Glucagon-type ligand receptors|Reactome	0.104121611	-0.06117762	0.388014583	0.7060717	0.90148993	-5.79348841
homo after UVB irradiation VS homo before UVB irradiation	Glucocorticoid and Mineralcorticoid Metabolism|Wikipathways	0.034641976	-0.01315163	0.176583636	0.86358585	0.91700767	-6.35172265
hetero after UVB irradiation VS hetero before UVB irradiation	Glucocorticoid and Mineralcorticoid Metabolism|Wikipathways	0.184714317	0.080053708	1.030792803	0.33131538	0.55930944	-5.80467892
WT after UVB irradiation VS WT before UVB irradiation	Glucocorticoid and Mineralcorticoid Metabolism|Wikipathways	-0.14445002	0.0005639	-0.57895166	0.5753272	0.8562298	-5.70232502
homo after UVB irradiation VS homo before UVB irradiation	glucocorticoid biosynthesis|HumanCyc	0.241685466	0.017581266	1.170035694	0.27082786	0.43450457	-5.68321209
hetero after UVB irradiation VS hetero before UVB irradiation	glucocorticoid biosynthesis|HumanCyc	-0.15472168	0.104588128	-0.53841546	0.60421879	0.79138772	-6.19200079
WT after UVB irradiation VS WT before UVB irradiation	glucocorticoid biosynthesis|HumanCyc	-0.20337291	0.200208825	-0.54699505	0.59628056	0.86530281	-5.71995372
homo after UVB irradiation VS homo before UVB irradiation	Glucocorticoid biosynthesis|Reactome	0.009006886	-0.02626041	0.045151138	0.9649326	0.97821542	-6.36735302
hetero after UVB irradiation VS hetero before UVB irradiation	Glucocorticoid biosynthesis|Reactome	-0.00804074	0.073536344	-0.03816403	0.97044569	0.98614433	-6.34575123
WT after UVB irradiation VS WT before UVB irradiation	Glucocorticoid biosynthesis|Reactome	0.028255572	0.033997106	0.108509172	0.91572098	0.97566251	-5.86319016
homo after UVB irradiation VS homo before UVB irradiation	Glucocorticoid Pathway (HPA Axis)_ Pharmacodynamics|PharmGKB	0.24028133	0.021355993	1.184867593	0.26519285	0.42863631	-5.6669291
hetero after UVB irradiation VS hetero before UVB irradiation	Glucocorticoid Pathway (HPA Axis)_ Pharmacodynamics|PharmGKB	-0.08831487	0.03557549	-0.30374599	0.76868721	0.89608089	-6.29676995
WT after UVB irradiation VS WT before UVB irradiation	Glucocorticoid Pathway (HPA Axis)_ Pharmacodynamics|PharmGKB	0.32832993	0.06814264	1.111013387	0.29235489	0.67947895	-5.28200279
homo after UVB irradiation VS homo before UVB irradiation	Glucocorticoid Pathway (Peripheral Tissue)_ Pharmacodynamics|PharmGKB	-0.51596059	0.038953508	-3.14706647	0.01119971	0.06448659	-2.78288446
hetero after UVB irradiation VS hetero before UVB irradiation	Glucocorticoid Pathway (Peripheral Tissue)_ Pharmacodynamics|PharmGKB	-0.36691925	0.03712752	-1.66451058	0.13266371	0.32988609	-5.05312768
WT after UVB irradiation VS WT before UVB irradiation	Glucocorticoid Pathway (Peripheral Tissue)_ Pharmacodynamics|PharmGKB	-0.52512411	-0.01440639	-1.93845323	0.08105196	0.46678978	-4.27310995
homo after UVB irradiation VS homo before UVB irradiation	Glucocorticoid Receptor Pathway|Wikipathways	0.122770175	0.025636587	0.597707529	0.56417125	0.6905322	-6.18015753
hetero after UVB irradiation VS hetero before UVB irradiation	Glucocorticoid Receptor Pathway|Wikipathways	0.106351008	0.007179935	0.67272037	0.5191449	0.73623731	-6.10759063
WT after UVB irradiation VS WT before UVB irradiation	Glucocorticoid Receptor Pathway|Wikipathways	-0.16591062	-0.01835399	-0.78410036	0.45100978	0.80616896	-5.56752826
homo after UVB irradiation VS homo before UVB irradiation	Glucocorticoid receptor regulatory network|PID	0.143273027	0.009460247	0.778615925	0.45537985	0.60268533	-6.05308448
hetero after UVB irradiation VS hetero before UVB irradiation	Glucocorticoid receptor regulatory network|PID	0.220828965	0.023979196	1.417201613	0.1923207	0.41232255	-5.37264824
WT after UVB irradiation VS WT before UVB irradiation	Glucocorticoid receptor regulatory network|PID	0.174046427	0.040250682	0.896588155	0.39084434	0.76436977	-5.47853555
homo after UVB irradiation VS homo before UVB irradiation	gluconeogenesis|HumanCyc	0.332343142	0.011952775	1.642276259	0.13354686	0.27639803	-5.09958701
hetero after UVB irradiation VS hetero before UVB irradiation	gluconeogenesis|HumanCyc	-0.00615591	0.039102041	-0.03116936	0.97586019	0.988327	-6.34601428
WT after UVB irradiation VS WT before UVB irradiation	gluconeogenesis|HumanCyc	0.162252836	0.052900882	0.60884862	0.55609375	0.84793065	-5.68499089
homo after UVB irradiation VS homo before UVB irradiation	Gluconeogenesis|Reactome	0.363372757	-0.00063227	1.642029887	0.13359843	0.27639803	-5.099922
hetero after UVB irradiation VS hetero before UVB irradiation	Gluconeogenesis|Reactome	0.029975453	0.042612043	0.141970899	0.89043913	0.96325071	-6.33562034
WT after UVB irradiation VS WT before UVB irradiation	Gluconeogenesis|Reactome	0.238897448	0.044051918	0.914571157	0.38176896	0.75477872	-5.4633734
homo after UVB irradiation VS homo before UVB irradiation	Gluconeogenesis|SMPDB	0.252684369	-0.00151752	1.090705456	0.3026004	0.46312664	-5.76769054
hetero after UVB irradiation VS hetero before UVB irradiation	Gluconeogenesis|SMPDB	-0.06167553	0.005820603	-0.24893092	0.809375	0.92095935	-6.31305137
WT after UVB irradiation VS WT before UVB irradiation	Gluconeogenesis|SMPDB	-0.01454403	-0.00173466	-0.04854099	0.96223323	0.98815178	-5.86795769
homo after UVB irradiation VS homo before UVB irradiation	Glucose metabolism|Reactome	0.021863608	0.00131305	0.105231255	0.91840622	0.95153785	-6.36250194
hetero after UVB irradiation VS hetero before UVB irradiation	Glucose metabolism|Reactome	0.050913407	-0.01804989	0.292780253	0.77677115	0.90158465	-6.30028041
WT after UVB irradiation VS WT before UVB irradiation	Glucose metabolism|Reactome	0.229387698	0.019280055	1.140818627	0.28031908	0.66921967	-5.25209425
homo after UVB irradiation VS homo before UVB irradiation	Glucose Transporter Defect (SGLT2)|SMPDB	0.234726031	0.017120546	1.156747303	0.27595732	0.43614438	-5.69767199
homo after UVB irradiation VS homo before UVB irradiation	Glucose Transporter Defect (SGLT2)|SMPDB	0.234726031	0.017120546	1.156747303	0.27595732	0.43614438	-5.69767199
hetero after UVB irradiation VS hetero before UVB irradiation	Glucose Transporter Defect (SGLT2)|SMPDB	-0.03255037	0.028925632	-0.18154472	0.86023155	0.94503144	-6.32869738
hetero after UVB irradiation VS hetero before UVB irradiation	Glucose Transporter Defect (SGLT2)|SMPDB	-0.03255037	0.028925632	-0.18154472	0.86023155	0.94503144	-6.32869738
WT after UVB irradiation VS WT before UVB irradiation	Glucose Transporter Defect (SGLT2)|SMPDB	-0.15908359	0.010252305	-0.80382265	0.44003996	0.79685269	-5.55266936
WT after UVB irradiation VS WT before UVB irradiation	Glucose Transporter Defect (SGLT2)|SMPDB	-0.15908359	0.010252305	-0.80382265	0.44003996	0.79685269	-5.55266936
homo after UVB irradiation VS homo before UVB irradiation	Glucose-6-phosphate dehydrogenase deficiency|SMPDB	-0.14766847	-0.01149858	-0.59272914	0.56736226	0.69195165	-6.18322274
hetero after UVB irradiation VS hetero before UVB irradiation	Glucose-6-phosphate dehydrogenase deficiency|SMPDB	-0.05432787	-0.01134205	-0.2708573	0.79301813	0.91162084	-6.30691924
WT after UVB irradiation VS WT before UVB irradiation	Glucose-6-phosphate dehydrogenase deficiency|SMPDB	0.010991275	0.045530165	0.039987875	0.96888357	0.98924463	-5.8683412
homo after UVB irradiation VS homo before UVB irradiation	Glucose-Alanine Cycle|SMPDB	0.223493996	-0.07690742	0.740734324	0.47696969	0.61976199	-6.08216617
hetero after UVB irradiation VS hetero before UVB irradiation	Glucose-Alanine Cycle|SMPDB	0.013990524	-0.03649432	0.045780437	0.96455175	0.9837433	-6.34540445
WT after UVB irradiation VS WT before UVB irradiation	Glucose-Alanine Cycle|SMPDB	0.125602783	0.014157332	0.425008313	0.67975858	0.88509541	-5.77851709
homo after UVB irradiation VS homo before UVB irradiation	Glucuronidation|Reactome	-0.48958571	0.139769759	-2.47610015	0.03419258	0.1235153	-3.85455726
hetero after UVB irradiation VS hetero before UVB irradiation	Glucuronidation|Reactome	-0.01301218	-0.03498133	-0.07648629	0.9408173	0.97054702	-6.34336875
WT after UVB irradiation VS WT before UVB irradiation	Glucuronidation|Reactome	-0.24249694	0.004373531	-0.96846802	0.35546865	0.74095177	-5.41644004
homo after UVB irradiation VS homo before UVB irradiation	Glucuronidation|Wikipathways	-0.15375691	0.116904064	-0.69678839	0.50282017	0.64253487	-6.11428692
hetero after UVB irradiation VS hetero before UVB irradiation	Glucuronidation|Wikipathways	-0.08114667	-0.02952389	-0.43205139	0.67654824	0.8318605	-6.2464131
WT after UVB irradiation VS WT before UVB irradiation	Glucuronidation|Wikipathways	-0.19374786	-0.02349149	-0.74125838	0.47544789	0.81430085	-5.59868621
homo after UVB irradiation VS homo before UVB irradiation	GLUT-1 deficiency syndrome|SMPDB	0.737643047	0.024031742	3.897326755	0.00335151	0.03256113	-1.60634747
hetero after UVB irradiation VS hetero before UVB irradiation	GLUT-1 deficiency syndrome|SMPDB	0.744733789	0.085967304	4.116781712	0.0030051	0.06148411	-1.42803661
WT after UVB irradiation VS WT before UVB irradiation	GLUT-1 deficiency syndrome|SMPDB	0.584123909	0.04155907	2.501560947	0.03118765	0.36428127	-3.45639206
homo after UVB irradiation VS homo before UVB irradiation	Glutamate binding_ activation of AMPA receptors and synaptic plasticity|Reactome	-0.04041758	0.00810068	-0.2472849	0.81001048	0.88269877	-6.33570166
hetero after UVB irradiation VS hetero before UVB irradiation	Glutamate binding_ activation of AMPA receptors and synaptic plasticity|Reactome	-0.09762573	0.0025896	-0.60225434	0.56285746	0.76403561	-6.15401353
WT after UVB irradiation VS WT before UVB irradiation	Glutamate binding_ activation of AMPA receptors and synaptic plasticity|Reactome	-0.28153268	-0.03498425	-1.32484296	0.21446499	0.62581393	-5.05480016
homo after UVB irradiation VS homo before UVB irradiation	glutamate biosynthesis/degradation|HumanCyc	0.714670311	-0.00378976	2.101256514	0.06372354	0.17433295	-4.43620497
hetero after UVB irradiation VS hetero before UVB irradiation	glutamate biosynthesis/degradation|HumanCyc	0.398029108	0.031343809	1.00690705	0.34201647	0.57144834	-5.82810002
WT after UVB irradiation VS WT before UVB irradiation	glutamate biosynthesis/degradation|HumanCyc	0.152350148	-0.03742406	0.392534627	0.70283392	0.90059561	-5.79172976
homo after UVB irradiation VS homo before UVB irradiation	glutamate dependent acid resistance|HumanCyc	0.792621544	0.055284126	2.271765693	0.04806053	0.14314702	-4.17465335
hetero after UVB irradiation VS hetero before UVB irradiation	glutamate dependent acid resistance|HumanCyc	0.326919358	0.067520323	0.670841312	0.52028318	0.73623731	-6.10888851
WT after UVB irradiation VS WT before UVB irradiation	glutamate dependent acid resistance|HumanCyc	0.382378394	0.070503933	0.970545927	0.35448166	0.73956955	-5.41458651
homo after UVB irradiation VS homo before UVB irradiation	Glutamate Glutamine metabolism|INOH	-0.38651877	0.00528329	-2.45777246	0.03525497	0.12483995	-3.88349605
hetero after UVB irradiation VS hetero before UVB irradiation	Glutamate Glutamine metabolism|INOH	-0.48587861	-0.03033347	-2.86910425	0.01977378	0.12380339	-3.27100836
WT after UVB irradiation VS WT before UVB irradiation	Glutamate Glutamine metabolism|INOH	-0.2451053	-0.01221416	-1.22242742	0.2493535	0.64238639	-5.16720686
homo after UVB irradiation VS homo before UVB irradiation	Glutamate Metabolism|SMPDB	-0.24547682	-0.00810915	-1.40068944	0.19347783	0.34903982	-5.41392471
hetero after UVB irradiation VS hetero before UVB irradiation	Glutamate Metabolism|SMPDB	-0.17636771	-0.04343396	-1.04504584	0.32505313	0.55421891	-5.79050219
WT after UVB irradiation VS WT before UVB irradiation	Glutamate Metabolism|SMPDB	-0.06328445	0.007258138	-0.28249238	0.78328047	0.93661609	-5.82889545
homo after UVB irradiation VS homo before UVB irradiation	Glutamate Neurotransmitter Release Cycle|Reactome	0.289870168	0.003042494	1.507772574	0.16449827	0.31390031	-5.27825165
hetero after UVB irradiation VS hetero before UVB irradiation	Glutamate Neurotransmitter Release Cycle|Reactome	0.058421402	-0.00590206	0.258705382	0.80207058	0.916937	-6.31038062
WT after UVB irradiation VS WT before UVB irradiation	Glutamate Neurotransmitter Release Cycle|Reactome	-0.02529498	-0.00707919	-0.10456658	0.91877072	0.97566251	-5.86361522
homo after UVB irradiation VS homo before UVB irradiation	glutamate removal from folates|HumanCyc	0.98786448	-0.03431494	3.674141802	0.00476653	0.03957638	-1.95055792
hetero after UVB irradiation VS hetero before UVB irradiation	glutamate removal from folates|HumanCyc	1.062956752	0.043910316	4.395923094	0.00202853	0.0502626	-1.04370874
WT after UVB irradiation VS WT before UVB irradiation	glutamate removal from folates|HumanCyc	0.449337563	0.044888142	1.121045251	0.28825981	0.67436922	-5.27200317
homo after UVB irradiation VS homo before UVB irradiation	Glutamatergic synapse - Homo sapiens (human)|KEGG	0.355214263	-0.0159474	2.293192777	0.04637934	0.14024375	-4.14138426
hetero after UVB irradiation VS hetero before UVB irradiation	Glutamatergic synapse - Homo sapiens (human)|KEGG	0.300645281	0.032646423	1.739742423	0.11818464	0.31191986	-4.95080702
WT after UVB irradiation VS WT before UVB irradiation	Glutamatergic synapse - Homo sapiens (human)|KEGG	0.10180603	0.006039573	0.436623278	0.67158698	0.87984445	-5.77354599
homo after UVB irradiation VS homo before UVB irradiation	glutamine biosynthesis|HumanCyc	0.519715043	-0.00128396	1.352597863	0.20785691	0.36615535	-5.47277643
hetero after UVB irradiation VS hetero before UVB irradiation	glutamine biosynthesis|HumanCyc	-0.54323748	0.055628015	-1.52551163	0.16374792	0.37677831	-5.23616898
WT after UVB irradiation VS WT before UVB irradiation	glutamine biosynthesis|HumanCyc	-0.04905559	-0.06211773	-0.11256172	0.91258767	0.97566251	-5.86273689
homo after UVB irradiation VS homo before UVB irradiation	glutamine degradation/glutamate biosynthesis|HumanCyc	0.004222984	0.043350989	0.015396127	0.98803836	0.99205816	-6.3683209
hetero after UVB irradiation VS hetero before UVB irradiation	glutamine degradation/glutamate biosynthesis|HumanCyc	0.577623521	-0.06679585	2.284345933	0.05012068	0.19880033	-4.16197247
WT after UVB irradiation VS WT before UVB irradiation	glutamine degradation/glutamate biosynthesis|HumanCyc	0.729033379	0.013764943	2.912474208	0.01537379	0.29238268	-2.83957531
homo after UVB irradiation VS homo before UVB irradiation	Glutaminolysis and Cancer|SMPDB	-0.09039029	-0.01110332	-0.37492371	0.71604619	0.81330023	-6.29349618
hetero after UVB irradiation VS hetero before UVB irradiation	Glutaminolysis and Cancer|SMPDB	-0.20601296	-0.03783083	-0.76285956	0.46636533	0.68896154	-6.04157147
WT after UVB irradiation VS WT before UVB irradiation	Glutaminolysis and Cancer|SMPDB	-0.04837248	-0.06733736	-0.19254203	0.85114072	0.95502804	-5.85040759
homo after UVB irradiation VS homo before UVB irradiation	Glutaric Aciduria Type I|SMPDB	-0.19843953	0.039801734	-0.74439404	0.47485571	0.61754234	-6.07941248
homo after UVB irradiation VS homo before UVB irradiation	Glutaric Aciduria Type I|SMPDB	-0.02100802	-0.06308448	-0.07524201	0.941601	0.96450524	-6.36540732
hetero after UVB irradiation VS hetero before UVB irradiation	Glutaric Aciduria Type I|SMPDB	-0.48316271	0.016700705	-1.75538636	0.11536374	0.30887493	-4.92927061
hetero after UVB irradiation VS hetero before UVB irradiation	Glutaric Aciduria Type I|SMPDB	-0.10000854	-0.05054657	-0.39990823	0.69917117	0.84778456	-6.26061929
WT after UVB irradiation VS WT before UVB irradiation	Glutaric Aciduria Type I|SMPDB	-0.40763842	0.012220754	-1.51649601	0.16010237	0.56072663	-4.82863564
WT after UVB irradiation VS WT before UVB irradiation	Glutaric Aciduria Type I|SMPDB	-0.11826398	-0.1134336	-0.44264387	0.66736875	0.87935805	-5.77091861
homo after UVB irradiation VS homo before UVB irradiation	glutaryl-CoA degradation|HumanCyc	-0.31887575	-0.07456184	-0.91585014	0.38266497	0.53563711	-5.93731888
hetero after UVB irradiation VS hetero before UVB irradiation	glutaryl-CoA degradation|HumanCyc	-0.53335074	-0.10671321	-1.50420721	0.16905136	0.38617647	-5.26346598
WT after UVB irradiation VS WT before UVB irradiation	glutaryl-CoA degradation|HumanCyc	-0.30041948	-0.0232709	-0.7364577	0.47823784	0.81552033	-5.60208105
homo after UVB irradiation VS homo before UVB irradiation	glutathione biosynthesis|HumanCyc	-0.61757849	0.030654024	-1.79871798	0.10422088	0.23651718	-4.8817326
hetero after UVB irradiation VS hetero before UVB irradiation	glutathione biosynthesis|HumanCyc	-0.39061679	-0.03140954	-1.07797813	0.31093394	0.53928344	-5.75718187
WT after UVB irradiation VS WT before UVB irradiation	glutathione biosynthesis|HumanCyc	-0.59026526	-0.05840894	-1.55835315	0.14995963	0.55622203	-4.77677004

homo after UVB irradiation VS homo before UVB irradiation	Glutathione conjugation|Reactome	0.269891546	-0.03272262	1.123914128	0.28896212	0.44816611	-5.73287066
hetero after UVB irradiation VS hetero before UVB irradiation	Glutathione conjugation|Reactome	0.318433254	0.038726948	1.613893669	0.14330277	0.34688172	-5.1207329
WT after UVB irradiation VS WT before UVB irradiation	Glutathione conjugation|Reactome	0.196075161	0.043155941	0.882540522	0.39803818	0.76605156	-5.49020356
homo after UVB irradiation VS homo before UVB irradiation	Glutathione metabolism - Homo sapiens (human)|KEGG	0.133423507	-0.03126251	0.603155027	0.56069113	0.68696862	-6.17677635
hetero after UVB irradiation VS hetero before UVB irradiation	Glutathione metabolism - Homo sapiens (human)|KEGG	0.2247129	0.013208309	1.342231444	0.21456151	0.43637221	-5.46356701
WT after UVB irradiation VS WT before UVB irradiation	Glutathione metabolism - Homo sapiens (human)|KEGG	0.162030855	0.017705981	0.815445054	0.43365878	0.79685269	-5.54376268
homo after UVB irradiation VS homo before UVB irradiation	Glutathione Metabolism|SMPDB	-0.03868231	-0.05039994	-0.170337	0.86835923	0.9186887	-6.35288321
hetero after UVB irradiation VS hetero before UVB irradiation	Glutathione Metabolism|SMPDB	0.017134286	0.027343289	0.102228327	0.92096661	0.97054702	-6.34087549
WT after UVB irradiation VS WT before UVB irradiation	Glutathione Metabolism|SMPDB	0.144724093	-0.01736596	0.615162034	0.55207887	0.84516674	-5.68122724
homo after UVB irradiation VS homo before UVB irradiation	Glutathione metabolism|Wikipathways	0.409559859	-0.03307211	1.99319385	0.07608101	0.19626432	-4.5984627
hetero after UVB irradiation VS hetero before UVB irradiation	Glutathione metabolism|Wikipathways	0.343008199	0.018549682	1.654802667	0.1346465	0.33336163	-5.06617377
WT after UVB irradiation VS WT before UVB irradiation	Glutathione metabolism|Wikipathways	0.188488245	0.01633731	0.854012674	0.41292908	0.77895025	-5.51341808
homo after UVB irradiation VS homo before UVB irradiation	glutathione redox reactions I|HumanCyc	0.85265837	0.005082664	4.491461074	0.00135573	0.02112713	-0.7226005
hetero after UVB irradiation VS hetero before UVB irradiation	glutathione redox reactions I|HumanCyc	0.283128063	0.031906587	0.985436688	0.35185737	0.58065383	-5.8487874
WT after UVB irradiation VS WT before UVB irradiation	glutathione redox reactions I|HumanCyc	0.452608309	-0.00269244	1.458585483	0.17511754	0.58633563	-4.89899594
homo after UVB irradiation VS homo before UVB irradiation	glutathione redox reactions II|HumanCyc	0.121563556	-0.0213619	0.472246828	0.64753413	0.75613204	-6.25006104
hetero after UVB irradiation VS hetero before UVB irradiation	glutathione redox reactions II|HumanCyc	0.018920659	-0.00348228	0.04661434	0.96390657	0.9835429	-6.34536267
WT after UVB irradiation VS WT before UVB irradiation	glutathione redox reactions II|HumanCyc	0.36385449	-0.02269799	1.429523164	0.18309985	0.59839062	-4.93366823
homo after UVB irradiation VS homo before UVB irradiation	Glutathione synthesis and recycling|Reactome	-0.08889524	0.007275507	-0.40857813	0.69201109	0.79350142	-6.27956315
hetero after UVB irradiation VS hetero before UVB irradiation	Glutathione synthesis and recycling|Reactome	0.224479704	0.074681564	0.985823005	0.35167844	0.58056875	-5.84841826
WT after UVB irradiation VS WT before UVB irradiation	Glutathione synthesis and recycling|Reactome	-0.01753519	0.029483963	-0.07112421	0.94468997	0.98295421	-5.86658919
homo after UVB irradiation VS homo before UVB irradiation	Glutathione Synthetase Deficiency|SMPDB	-0.03868231	-0.05039994	-0.170337	0.86835923	0.9186887	-6.35288321
hetero after UVB irradiation VS hetero before UVB irradiation	Glutathione Synthetase Deficiency|SMPDB	0.017134286	0.027343289	0.102228327	0.92096661	0.97054702	-6.34087549
WT after UVB irradiation VS WT before UVB irradiation	Glutathione Synthetase Deficiency|SMPDB	0.144724093	-0.01736596	0.615162034	0.55207887	0.84516674	-5.68122724
homo after UVB irradiation VS homo before UVB irradiation	glutathione-mediated detoxification|HumanCyc	0.377143243	-0.04849945	1.226042968	0.2500424	0.41255482	-5.62094572
hetero after UVB irradiation VS hetero before UVB irradiation	glutathione-mediated detoxification|HumanCyc	0.18326864	0.03649934	0.741419146	0.47859206	0.69976736	-6.05793419
WT after UVB irradiation VS WT before UVB irradiation	glutathione-mediated detoxification|HumanCyc	0.229815553	-0.02137007	0.757396656	0.46614488	0.80910175	-5.5871306
homo after UVB irradiation VS homo before UVB irradiation	glycerol degradation|HumanCyc	-0.1885937	0.020429412	-0.57982152	0.57568241	0.6989008	-6.19105931
hetero after UVB irradiation VS hetero before UVB irradiation	glycerol degradation|HumanCyc	-0.30185929	0.004587255	-0.90080734	0.39270883	0.61972322	-5.92685651
WT after UVB irradiation VS WT before UVB irradiation	glycerol degradation|HumanCyc	-0.23844962	0.008564631	-1.15453921	0.27491052	0.66570326	-5.23812602
homo after UVB irradiation VS homo before UVB irradiation	Glycerol Kinase Deficiency|SMPDB	0.512939135	0.00160955	2.795906579	0.02004569	0.09043427	-3.34516503
hetero after UVB irradiation VS hetero before UVB irradiation	Glycerol Kinase Deficiency|SMPDB	0.12253787	0.061964979	0.508050343	0.62444904	0.80561526	-6.20867996
WT after UVB irradiation VS WT before UVB irradiation	Glycerol Kinase Deficiency|SMPDB	0.284092323	-0.00634299	1.317391439	0.21685951	0.62581393	-5.06318888
homo after UVB irradiation VS homo before UVB irradiation	Glycerol Phosphate Shuttle|SMPDB	1.082696587	0.013930166	6.569074027	8.47E-05	0.01666204	1.930817354
hetero after UVB irradiation VS hetero before UVB irradiation	Glycerol Phosphate Shuttle|SMPDB	0.552312551	0.051137721	1.842495731	0.10077193	0.28564273	-4.80784431
WT after UVB irradiation VS WT before UVB irradiation	Glycerol Phosphate Shuttle|SMPDB	0.731634896	-0.02653285	2.398101596	0.03724866	0.39022568	-3.6101913
homo after UVB irradiation VS homo before UVB irradiation	glycerol-3-phosphate shuttle|HumanCyc	1.072881289	0.033283076	5.371500165	0.00038921	0.01666204	0.485968411
hetero after UVB irradiation VS hetero before UVB irradiation	glycerol-3-phosphate shuttle|HumanCyc	0.350143929	-0.0188597	1.078856362	0.31056406	0.53909998	-5.75628264
WT after UVB irradiation VS WT before UVB irradiation	glycerol-3-phosphate shuttle|HumanCyc	0.8502737	-0.03472049	2.956567497	0.01425207	0.28307166	-2.77329756
homo after UVB irradiation VS homo before UVB irradiation	Glycerolipid metabolism - Homo sapiens (human)|KEGG	0.213650391	0.002953187	1.303244074	0.22354174	0.38315206	-5.53174706
hetero after UVB irradiation VS hetero before UVB irradiation	Glycerolipid metabolism - Homo sapiens (human)|KEGG	0.093084391	0.023999776	0.517151765	0.61834892	0.80231894	-6.20377573
WT after UVB irradiation VS WT before UVB irradiation	Glycerolipid metabolism - Homo sapiens (human)|KEGG	0.11705338	0.037912757	0.568815355	0.58192979	0.85958498	-5.70801781
homo after UVB irradiation VS homo before UVB irradiation	Glycerolipid Metabolism|SMPDB	0.512939135	0.00160955	2.795906579	0.02004569	0.09043427	-3.34516503
hetero after UVB irradiation VS hetero before UVB irradiation	Glycerolipid Metabolism|SMPDB	0.12253787	0.061964979	0.508050343	0.62444904	0.80561526	-6.20867996
WT after UVB irradiation VS WT before UVB irradiation	Glycerolipid Metabolism|SMPDB	0.284092323	-0.00634299	1.317391439	0.21685951	0.62581393	-5.06318888
homo after UVB irradiation VS homo before UVB irradiation	Glycerophospholipid biosynthesis|Reactome	-0.03010777	-0.01534854	-0.15902136	0.87702023	0.92482087	-6.35487976
hetero after UVB irradiation VS hetero before UVB irradiation	Glycerophospholipid biosynthesis|Reactome	-0.09917793	-0.02818391	-0.60476037	0.56126726	0.76343066	-6.15244262
WT after UVB irradiation VS WT before UVB irradiation	Glycerophospholipid biosynthesis|Reactome	-0.06992559	-0.00864141	-0.32922273	0.74872442	0.9185045	-5.81455843
homo after UVB irradiation VS homo before UVB irradiation	Glycerophospholipid catabolism|Reactome	-0.07490463	-0.05245809	-0.36040031	0.72652267	0.82013506	-6.29915048
hetero after UVB irradiation VS hetero before UVB irradiation	Glycerophospholipid catabolism|Reactome	0.095094842	0.001921212	0.301210045	0.7705541	0.89710998	-6.297593
WT after UVB irradiation VS WT before UVB irradiation	Glycerophospholipid catabolism|Reactome	-0.112791	0.036525852	-0.43381106	0.67356144	0.88104551	-5.77476141
homo after UVB irradiation VS homo before UVB irradiation	Glycerophospholipid metabolism - Homo sapiens (human)|KEGG	0.251977833	-0.01284552	1.526237024	0.15990426	0.3083289	-5.25424603
hetero after UVB irradiation VS hetero before UVB irradiation	Glycerophospholipid metabolism - Homo sapiens (human)|KEGG	0.124266831	-0.01156512	0.718318875	0.49199561	0.71213591	-6.07510668
WT after UVB irradiation VS WT before UVB irradiation	Glycerophospholipid metabolism - Homo sapiens (human)|KEGG	0.15004291	0.002203979	0.745037672	0.4732588	0.81346365	-5.59599988
homo after UVB irradiation VS homo before UVB irradiation	Glycerophospholipid metabolism|EHMN	0.11903048	0.002005126	0.718850797	0.48973528	0.63135722	-6.09838028
hetero after UVB irradiation VS hetero before UVB irradiation	Glycerophospholipid metabolism|EHMN	0.142365188	0.008988308	0.882905681	0.4017733	0.63017299	-5.94264002
WT after UVB irradiation VS WT before UVB irradiation	Glycerophospholipid metabolism|EHMN	0.103419165	-0.03428796	0.50890026	0.62177296	0.87257466	-5.73973939
homo after UVB irradiation VS homo before UVB irradiation	Glycine and Serine Metabolism|SMPDB	-0.11801782	0.021953564	-0.50783827	0.62328187	0.73728109	-6.23179983
hetero after UVB irradiation VS hetero before UVB irradiation	Glycine and Serine Metabolism|SMPDB	-0.03882851	0.032366748	-0.2244119	0.82778335	0.93322694	-6.31930458
WT after UVB irradiation VS WT before UVB irradiation	Glycine and Serine Metabolism|SMPDB	0.143497804	-0.01134274	0.603206807	0.55969543	0.84911968	-5.68832383
homo after UVB irradiation VS homo before UVB irradiation	glycine betaine degradation|HumanCyc	-0.64831026	0.085094502	-2.92954788	0.0160488	0.07889081	-3.13105167
hetero after UVB irradiation VS hetero before UVB irradiation	glycine betaine degradation|HumanCyc	-0.25777388	0.006471741	-1.56042809	0.15537664	0.36486693	-5.19097772
WT after UVB irradiation VS WT before UVB irradiation	glycine betaine degradation|HumanCyc	0.20736269	0.074706346	0.536259088	0.6034092	0.86948343	-5.72566572
homo after UVB irradiation VS homo before UVB irradiation	glycine biosynthesis|HumanCyc	-0.3314782	0.176544289	-1.11234189	0.29365898	0.45360179	-5.74509453
hetero after UVB irradiation VS hetero before UVB irradiation	glycine biosynthesis|HumanCyc	-0.47492279	0.018600109	-1.4892971	0.17285327	0.39041882	-5.28244193
WT after UVB irradiation VS WT before UVB irradiation	glycine biosynthesis|HumanCyc	-0.12993107	-0.00925101	-0.39202022	0.70320209	0.90059561	-5.7919309
homo after UVB irradiation VS homo before UVB irradiation	glycine cleavage|HumanCyc	-0.62375475	-0.07107438	-1.7022337	0.12151833	0.26037199	-5.01727848
hetero after UVB irradiation VS hetero before UVB irradiation	glycine cleavage|HumanCyc	-0.55232133	-0.04183779	-1.68230349	0.12909895	0.32389948	-5.02912076
WT after UVB irradiation VS WT before UVB irradiation	glycine cleavage|HumanCyc	-0.73250703	-0.04594409	-2.92099231	0.01515034	0.28974126	-2.82676805
homo after UVB irradiation VS homo before UVB irradiation	Glycine degradation|Reactome	-0.46178105	-0.07765309	-1.21270946	0.25486968	0.41874827	-5.63596028
hetero after UVB irradiation VS hetero before UVB irradiation	Glycine degradation|Reactome	-0.50248783	-0.06424131	-1.44693504	0.18407058	0.40321767	-5.33576312
WT after UVB irradiation VS WT before UVB irradiation	Glycine degradation|Reactome	-0.61934898	-0.04636164	-2.18183683	0.05386337	0.41493406	-3.92722757
homo after UVB irradiation VS homo before UVB irradiation	Glycine Metabolism|Wikipathways	-0.70186435	0.019175671	-2.45612297	0.03535218	0.12483995	-3.88609865
hetero after UVB irradiation VS hetero before UVB irradiation	Glycine Metabolism|Wikipathways	0.28647897	-0.10716899	1.027879823	0.33260655	0.56065852	-5.80755791
WT after UVB irradiation VS WT before UVB irradiation	Glycine Metabolism|Wikipathways	-0.25499521	-0.01348219	-0.70933135	0.49419549	0.81710765	-5.62089356
homo after UVB irradiation VS homo before UVB irradiation	Glycine N-methyltransferase Deficiency|SMPDB	-0.09396972	-0.02692441	-0.3795694	0.71270801	0.81002335	-6.29164178
hetero after UVB irradiation VS hetero before UVB irradiation	Glycine N-methyltransferase Deficiency|SMPDB	0.075091809	-0.02022482	0.352613458	0.73303662	0.86896773	-6.27959702
WT after UVB irradiation VS WT before UVB irradiation	Glycine N-methyltransferase Deficiency|SMPDB	0.176060787	0.019583595	0.866300336	0.40646895	0.7725109	-5.50349844
homo after UVB irradiation VS homo before UVB irradiation	Glycine Serine metabolism|INOH	-0.3494365	0.060344203	-1.6974415	0.12244228	0.26137048	-5.02391346
hetero after UVB irradiation VS hetero before UVB irradiation	Glycine Serine metabolism|INOH	-0.26018435	0.001773949	-1.60709049	0.14479045	0.34862908	-5.12973916
WT after UVB irradiation VS WT before UVB irradiation	Glycine Serine metabolism|INOH	-0.04484929	0.000783949	-0.20953754	0.83820204	0.94951669	-5.84696067
homo after UVB irradiation VS homo before UVB irradiation	glycine/serine biosynthesis|HumanCyc	-0.97331195	-0.0515519	-3.89896388	0.00334294	0.03256113	-1.60384504
hetero after UVB irradiation VS hetero before UVB irradiation	glycine/serine biosynthesis|HumanCyc	-0.40818441	-0.05925432	-1.40052508	0.19708751	0.41782552	-5.3931346
WT after UVB irradiation VS WT before UVB irradiation	glycine/serine biosynthesis|HumanCyc	-0.18547238	0.010496557	-0.45107018	0.66148532	0.87935805	-5.76718346
homo after UVB irradiation VS homo before UVB irradiation	Glycine_ serine and threonine metabolism - Homo sapiens (human)|KEGG	-0.03492755	0.006411354	-0.16690518	0.87098405	0.91994291	-6.35350313
hetero after UVB irradiation VS hetero before UVB irradiation	Glycine_ serine and threonine metabolism - Homo sapiens (human)|KEGG	-0.1062744	0.034701794	-0.66684406	0.52270955	0.73830496	-6.11163864
WT after UVB irradiation VS WT before UVB irradiation	Glycine_ serine and threonine metabolism - Homo sapiens (human)|KEGG	0.016291871	-0.01876222	0.076956552	0.94016389	0.98119937	-5.86615192
homo after UVB irradiation VS homo before UVB irradiation	Glycine_ serine_ alanine and threonine metabolism|EHMN	-0.23351741	0.016615554	-1.46329724	0.17604129	0.32873067	-5.33535075
hetero after UVB irradiation VS hetero before UVB irradiation	Glycine_ serine_ alanine and threonine metabolism|EHMN	-0.13844082	0.001996949	-0.81039324	0.4400004	0.6656229	-6.00386326
WT after UVB irradiation VS WT before UVB irradiation	Glycine_ serine_ alanine and threonine metabolism|EHMN	-0.05360155	-0.01602837	-0.25298892	0.80536034	0.94525798	-5.83683783
homo after UVB irradiation VS homo before UVB irradiation	glycoaminoglycan-protein linkage region biosynthesis|HumanCyc	0.143992853	-0.01877242	0.729731047	0.48336169	0.62610765	-6.09037285
hetero after UVB irradiation VS hetero before UVB irradiation	glycoaminoglycan-protein linkage region biosynthesis|HumanCyc	0.519788889	0.014241314	2.05513031	0.07214588	0.24020521	-4.50224882
WT after UVB irradiation VS WT before UVB irradiation	glycoaminoglycan-protein linkage region biosynthesis|HumanCyc	0.576545196	-0.02540524	2.577607369	0.02736347	0.35054739	-3.34274884
homo after UVB irradiation VS homo before UVB irradiation	glycogen biosynthesis|HumanCyc	0.593374124	-0.03785016	2.320441033	0.04432434	0.13674659	-4.09896478
hetero after UVB irradiation VS hetero before UVB irradiation	glycogen biosynthesis|HumanCyc	0.303944652	0.002964702	1.01567701	0.33805729	0.56712002	-5.81955002
WT after UVB irradiation VS WT before UVB irradiation	glycogen biosynthesis|HumanCyc	0.147127917	-0.09079839	0.437407571	0.6710368	0.87984445	-5.77320568
homo after UVB irradiation VS homo before UVB irradiation	Glycogen breakdown (glycogenolysis)|Reactome	0.196894207	-0.05540101	0.768567998	0.46104328	0.60754106	-6.06092254
hetero after UVB irradiation VS hetero before UVB irradiation	Glycogen breakdown (glycogenolysis)|Reactome	-0.29688322	-0.04248519	-1.1596989	0.27797583	0.50863663	-5.67120729
WT after UVB irradiation VS WT before UVB irradiation	Glycogen breakdown (glycogenolysis)|Reactome	-0.00034637	-0.0490527	-0.00122781	0.9990443	0.999482	-5.86915043
homo after UVB irradiation VS homo before UVB irradiation	Glycogen metabolism|Reactome	0.293519811	-0.075522	1.191341443	0.26276294	0.42561191	-5.65977483
hetero after UVB irradiation VS hetero before UVB irradiation	Glycogen metabolism|Reactome	-0.16223799	-0.04968736	-0.59959778	0.56454601	0.76437184	-6.15567229
WT after UVB irradiation VS WT before UVB irradiation	Glycogen metabolism|Reactome	0.044673849	-0.05240121	0.14685416	0.88614217	0.97031361	-5.85823845
homo after UVB irradiation VS homo before UVB irradiation	Glycogen Storage Disease Type 1A (GSD1A) or Von Gierke Disease|SMPDB	0.252684369	-0.00151752	1.090705456	0.3026004	0.46312664	-5.76769054
hetero after UVB irradiation VS hetero before UVB irradiation	Glycogen Storage Disease Type 1A (GSD1A) or Von Gierke Disease|SMPDB	-0.06167553	0.005820603	-0.24893092	0.809375	0.92095935	-6.31305137
WT after UVB irradiation VS WT before UVB irradiation	Glycogen Storage Disease Type 1A (GSD1A) or Von Gierke Disease|SMPDB	-0.01454403	-0.00173466	-0.04854099	0.96223323	0.98815178	-5.86795769
homo after UVB irradiation VS homo before UVB irradiation	Glycogen Synthesis and Degradation|Wikipathways	-0.00913022	-0.04129764	-0.04602527	0.96425421	0.97798567	-6.36731021
hetero after UVB irradiation VS hetero before UVB irradiation	Glycogen Synthesis and Degradation|Wikipathways	-0.13097632	-0.01873766	-0.595609	0.56708674	0.76437184	-6.15815027
WT after UVB irradiation VS WT before UVB irradiation	Glycogen Synthesis and Degradation|Wikipathways	0.05945788	-0.01328947	0.248391828	0.80881748	0.94525798	-5.83799768
homo after UVB irradiation VS homo before UVB irradiation	Glycogen synthesis|Reactome	0.367315421	-0.02071672	1.942806694	0.08259257	0.20394538	-4.67301582
hetero after UVB irradiation VS hetero before UVB irradiation	Glycogen synthesis|Reactome	-0.06213606	-0.04784033	-0.20116141	0.84534386	0.9427648	-6.32464283
WT after UVB irradiation VS WT before UVB irradiation	Glycogen synthesis|Reactome	-0.08126035	-0.10416297	-0.23429717	0.81944365	0.94525798	-5.84142286
homo after UVB irradiation VS homo before UVB irradiation	Glycogen synthetase deficiency|SMPDB	0.768442354	-0.02313676	4.373424779	0.00161642	0.02229782	-0.89406544
hetero after UVB irradiation VS hetero before UVB irradiation	Glycogen synthetase deficiency|SMPDB	0.409967917	0.043436941	1.396269407	0.19832025	0.41868045	-5.39833891
WT after UVB irradiation VS WT before UVB irradiation	Glycogen synthetase deficiency|SMPDB	0.202660553	-0.06041924	0.567608804	0.58271843	0.85958498	-5.7086892
homo after UVB irradiation VS homo before UVB irradiation	glycogenolysis|HumanCyc	0.172716001	0.040761959	0.807747553	0.43921979	0.59071504	-6.02985837
hetero after UVB irradiation VS hetero before UVB irradiation	glycogenolysis|HumanCyc	-0.21275977	0.043139253	-0.93193205	0.37730104	0.60405274	-5.8988
WT after UVB irradiation VS WT before UVB irradiation	glycogenolysis|HumanCyc	0.01954411	-0.06536892	0.062582478	0.95132231	0.98681143	-5.86716749
homo after UVB irradiation VS homo before UVB irradiation	Glycogenosis_ Type IA. Von gierke disease|SMPDB	0.252684369	-0.00151752	1.090705456	0.3026004	0.46312664	-5.76769054
hetero after UVB irradiation VS hetero before UVB irradiation	Glycogenosis_ Type IA. Von gierke disease|SMPDB	-0.06167553	0.005820603	-0.24893092	0.809375	0.92095935	-6.31305137
WT after UVB irradiation VS WT before UVB irradiation	Glycogenosis_ Type IA. Von gierke disease|SMPDB	-0.01454403	-0.00173466	-0.04854099	0.96223323	0.98815178	-5.86795769
homo after UVB irradiation VS homo before UVB irradiation	Glycogenosis_ Type IB|SMPDB	0.252684369	-0.00151752	1.090705456	0.3026004	0.46312664	-5.76769054
hetero after UVB irradiation VS hetero before UVB irradiation	Glycogenosis_ Type IB|SMPDB	-0.06167553	0.005820603	-0.24893092	0.809375	0.92095935	-6.31305137
WT after UVB irradiation VS WT before UVB irradiation	Glycogenosis_ Type IB|SMPDB	-0.01454403	-0.00173466	-0.04854099	0.96223323	0.98815178	-5.86795769
homo after UVB irradiation VS homo before UVB irradiation	Glycogenosis_ Type IC|SMPDB	0.252684369	-0.00151752	1.090705456	0.3026004	0.46312664	-5.76769054
hetero after UVB irradiation VS hetero before UVB irradiation	Glycogenosis_ Type IC|SMPDB	-0.06167553	0.005820603	-0.24893092	0.809375	0.92095935	-6.31305137
WT after UVB irradiation VS WT before UVB irradiation	Glycogenosis_ Type IC|SMPDB	-0.01454403	-0.00173466	-0.04854099	0.96223323	0.98815178	-5.86795769
homo after UVB irradiation VS homo before UVB irradiation	Glycogenosis_ Type III. Cori disease_ Debrancher glycogenosis|SMPDB	0.768442354	-0.02313676	4.373424779	0.00161642	0.02229782	-0.89406544
hetero after UVB irradiation VS hetero before UVB irradiation	Glycogenosis_ Type III. Cori disease_ Debrancher glycogenosis|SMPDB	0.409967917	0.043436941	1.396269407	0.19832025	0.41868045	-5.39833891
WT after UVB irradiation VS WT before UVB irradiation	Glycogenosis_ Type III. Cori disease_ Debrancher glycogenosis|SMPDB	0.202660553	-0.06041924	0.567608804	0.58271843	0.85958498	-5.7086892
homo after UVB irradiation VS homo before UVB irradiation	Glycogenosis_ Type IV. Amylopectinosis_ Anderson disease|SMPDB	0.768442354	-0.02313676	4.373424779	0.00161642	0.02229782	-0.89406544
hetero after UVB irradiation VS hetero before UVB irradiation	Glycogenosis_ Type IV. Amylopectinosis_ Anderson disease|SMPDB	0.409967917	0.043436941	1.396269407	0.19832025	0.41868045	-5.39833891
WT after UVB irradiation VS WT before UVB irradiation	Glycogenosis_ Type IV. Amylopectinosis_ Anderson disease|SMPDB	0.202660553	-0.06041924	0.567608804	0.58271843	0.85958498	-5.7086892
homo after UVB irradiation VS homo before UVB irradiation	Glycogenosis_ Type VI. Hers disease|SMPDB	0.768442354	-0.02313676	4.373424779	0.00161642	0.02229782	-0.89406544
hetero after UVB irradiation VS hetero before UVB irradiation	Glycogenosis_ Type VI. Hers disease|SMPDB	0.409967917	0.043436941	1.396269407	0.19832025	0.41868045	-5.39833891
WT after UVB irradiation VS WT before UVB irradiation	Glycogenosis_ Type VI. Hers disease|SMPDB	0.202660553	-0.06041924	0.567608804	0.58271843	0.85958498	-5.7086892
homo after UVB irradiation VS homo before UVB irradiation	Glycogenosis_ Type VII. Tarui disease|SMPDB	0.441748628	-0.03359755	1.766880832	0.10966101	0.24271071	-4.92686053
hetero after UVB irradiation VS hetero before UVB irradiation	Glycogenosis_ Type VII. Tarui disease|SMPDB	0.197724182	0.033762374	0.765455745	0.46489888	0.68805201	-6.03956266
WT after UVB irradiation VS WT before UVB irradiation	Glycogenosis_ Type VII. Tarui disease|SMPDB	0.075540742	-0.03429086	0.238329189	0.81639984	0.94525798	-5.8404632
homo after UVB irradiation VS homo before UVB irradiation	Glycolysis / Gluconeogenesis - Homo sapiens (human)|KEGG	0.351205215	-0.0022428	1.780001287	0.10738812	0.24059004	-4.9083092
hetero after UVB irradiation VS hetero before UVB irradiation	Glycolysis / Gluconeogenesis - Homo sapiens (human)|KEGG	0.130898896	0.016291039	0.644741706	0.53624982	0.74930309	-6.12657828
WT after UVB irradiation VS WT before UVB irradiation	Glycolysis / Gluconeogenesis - Homo sapiens (human)|KEGG	0.178929106	0.026166771	0.738519438	0.47703838	0.81525267	-5.60062547
homo after UVB irradiation VS homo before UVB irradiation	Glycolysis and Gluconeogenesis|EHMN	-0.13490137	0.013505722	-0.66997466	0.51900994	0.65681801	-6.13301797
hetero after UVB irradiation VS hetero before UVB irradiation	Glycolysis and Gluconeogenesis|EHMN	-0.06945641	-0.01526679	-0.34160167	0.74101437	0.87478464	-6.28368392
WT after UVB irradiation VS WT before UVB irradiation	Glycolysis and Gluconeogenesis|EHMN	0.122074796	-0.05051441	0.611803276	0.55421275	0.84585342	-5.68323398
homo after UVB irradiation VS homo before UVB irradiation	Glycolysis and Gluconeogenesis|Wikipathways	0.406131351	-0.02822195	2.006418019	0.07445441	0.19493053	-4.57877432
hetero after UVB irradiation VS hetero before UVB irradiation	Glycolysis and Gluconeogenesis|Wikipathways	0.139556866	0.010144508	0.555149938	0.59322046	0.78464854	-6.18242346
WT after UVB irradiation VS WT before UVB irradiation	Glycolysis and Gluconeogenesis|Wikipathways	0.206685321	0.022132354	0.759814995	0.46476092	0.80891799	-5.58538002
homo after UVB irradiation VS homo before UVB irradiation	Glycolysis Gluconeogenesis|INOH	0.466920274	-0.00838287	2.347354296	0.04238235	0.13402181	-4.05694995

hetero after UVB irradiation VS hetero before UVB irradiation	Glycolysis Gluconeogenesis|INOH	0.178000711	0.03998101	0.852556797	0.41747821	0.6443839	-5.96879814
WT after UVB irradiation VS WT before UVB irradiation	Glycolysis Gluconeogenesis|INOH	0.231525647	0.012794328	0.919700481	0.37920791	0.75122805	-5.45900261
homo after UVB irradiation VS homo before UVB irradiation	Glycolysis Pathway D (2)|Wikipathways	0.687993859	-0.03373394	3.416637097	0.00720943	0.04931566	-2.35438824
hetero after UVB irradiation VS hetero before UVB irradiation	Glycolysis Pathway D (2)|Wikipathways	0.405495388	0.03797864	1.805332018	0.10677191	0.29530725	-4.85995148
WT after UVB irradiation VS WT before UVB irradiation	Glycolysis Pathway D (2)|Wikipathways	0.427049797	0.025796362	1.7500876	0.11041808	0.50951142	-4.52940976
homo after UVB irradiation VS homo before UVB irradiation	glycolysis|HumanCyc	0.766231786	-0.02810268	3.949212311	0.00309085	0.03143932	-1.52720564
hetero after UVB irradiation VS hetero before UVB irradiation	glycolysis|HumanCyc	0.51318637	0.034771041	2.342009807	0.0457174	0.18854754	-4.07512087
WT after UVB irradiation VS WT before UVB irradiation	glycolysis|HumanCyc	0.471270407	0.042641702	1.89840581	0.08661066	0.47656734	-4.32855086
homo after UVB irradiation VS homo before UVB irradiation	Glycolysis|Reactome	-0.02219845	-0.01941336	-0.10519001	0.91843807	0.95153785	-6.3625066
hetero after UVB irradiation VS hetero before UVB irradiation	Glycolysis|Reactome	0.137688339	-0.02813294	0.72691085	0.4869825	0.70735061	-6.06877522
WT after UVB irradiation VS WT before UVB irradiation	Glycolysis|Reactome	0.242189128	0.002265432	1.079384335	0.30556109	0.69096189	-5.31307849
homo after UVB irradiation VS homo before UVB irradiation	Glycolysis|SMPDB	0.441748628	-0.03359755	1.766880832	0.10966101	0.24271071	-4.92686053
hetero after UVB irradiation VS hetero before UVB irradiation	Glycolysis|SMPDB	0.197724182	0.033762374	0.765455745	0.46489888	0.68805201	-6.03956266
WT after UVB irradiation VS WT before UVB irradiation	Glycolysis|SMPDB	0.075540742	-0.03429086	0.238329189	0.81639984	0.94525798	-5.8404632
homo after UVB irradiation VS homo before UVB irradiation	Glycoprotein hormones|Reactome	0.353606191	-0.01534229	1.394518276	0.19527419	0.35117052	-5.42155125
hetero after UVB irradiation VS hetero before UVB irradiation	Glycoprotein hormones|Reactome	0.428422585	0.099273929	1.662509102	0.1330703	0.33071671	-5.05582043
WT after UVB irradiation VS WT before UVB irradiation	Glycoprotein hormones|Reactome	0.660857349	0.101054631	2.858948038	0.01685624	0.30353778	-2.9200804
homo after UVB irradiation VS homo before UVB irradiation	Glycosaminoglycan biosynthesis - chondroitin sulfate / dermatan sulfate - Homo sapiens (human)|KEGG	0.561978443	-0.0038003	2.910582317	0.01656251	0.08070907	-3.16144516
hetero after UVB irradiation VS hetero before UVB irradiation	Glycosaminoglycan biosynthesis - chondroitin sulfate / dermatan sulfate - Homo sapiens (human)|KEGG	0.384966214	0.063306314	1.460472816	0.18041812	0.39861647	-5.31881996
WT after UVB irradiation VS WT before UVB irradiation	Glycosaminoglycan biosynthesis - chondroitin sulfate / dermatan sulfate - Homo sapiens (human)|KEGG	0.420651579	-0.04598176	1.431545013	0.18253462	0.59839062	-4.93127028
homo after UVB irradiation VS homo before UVB irradiation	Glycosaminoglycan biosynthesis - heparan sulfate / heparin - Homo sapiens (human)|KEGG	0.352503041	-0.05022303	2.021929949	0.07258854	0.19103127	-4.55561778
hetero after UVB irradiation VS hetero before UVB irradiation	Glycosaminoglycan biosynthesis - heparan sulfate / heparin - Homo sapiens (human)|KEGG	0.282966461	0.011220451	1.454989977	0.18188963	0.40050867	-5.32569309
WT after UVB irradiation VS WT before UVB irradiation	Glycosaminoglycan biosynthesis - heparan sulfate / heparin - Homo sapiens (human)|KEGG	0.315673586	0.01841962	1.586207808	0.14352693	0.55622203	-4.7418056
homo after UVB irradiation VS homo before UVB irradiation	Glycosaminoglycan biosynthesis - keratan sulfate - Homo sapiens (human)|KEGG	0.498316582	0.004080261	2.307178939	0.04531315	0.13845639	-4.1196262
hetero after UVB irradiation VS hetero before UVB irradiation	Glycosaminoglycan biosynthesis - keratan sulfate - Homo sapiens (human)|KEGG	0.312228692	0.044026479	1.640477548	0.13762187	0.33724756	-5.08535621
WT after UVB irradiation VS WT before UVB irradiation	Glycosaminoglycan biosynthesis - keratan sulfate - Homo sapiens (human)|KEGG	0.465754848	-0.07537008	1.790057372	0.10347165	0.50425697	-4.47602109
homo after UVB irradiation VS homo before UVB irradiation	Glycosaminoglycan degradation - Homo sapiens (human)|KEGG	0.632195228	-0.01119082	3.575344711	0.00558163	0.04312303	-2.1047289
hetero after UVB irradiation VS hetero before UVB irradiation	Glycosaminoglycan degradation - Homo sapiens (human)|KEGG	0.518963914	-0.00229067	2.111017791	0.06603275	0.23081079	-4.42014837
WT after UVB irradiation VS WT before UVB irradiation	Glycosaminoglycan degradation - Homo sapiens (human)|KEGG	0.530234154	0.013156973	2.191035787	0.05302965	0.41470276	-3.9138983
homo after UVB irradiation VS homo before UVB irradiation	Glycosaminoglycan metabolism|Reactome	0.54737607	-0.01202081	3.392082966	0.00750241	0.05059971	-2.39321048
hetero after UVB irradiation VS hetero before UVB irradiation	Glycosaminoglycan metabolism|Reactome	0.36205729	0.024689784	1.836676701	0.10168997	0.28675832	-4.81603176
WT after UVB irradiation VS WT before UVB irradiation	Glycosaminoglycan metabolism|Reactome	0.402482253	-0.00642204	1.816570898	0.099087	0.49761536	-4.4402943
homo after UVB irradiation VS homo before UVB irradiation	Glycosphingolipid biosynthesis - ganglio series - Homo sapiens (human)|KEGG	0.440162746	-0.03663099	2.168652425	0.05701735	0.16240116	-4.33355412
hetero after UVB irradiation VS hetero before UVB irradiation	Glycosphingolipid biosynthesis - ganglio series - Homo sapiens (human)|KEGG	0.338926781	0.0018874	1.531310559	0.16233027	0.37558445	-5.2287022
WT after UVB irradiation VS WT before UVB irradiation	Glycosphingolipid biosynthesis - ganglio series - Homo sapiens (human)|KEGG	0.314130019	-0.06032621	1.290979459	0.22552602	0.62952955	-5.09266212
homo after UVB irradiation VS homo before UVB irradiation	Glycosphingolipid biosynthesis - ganglioseries|EHMN	0.65747885	0.001501635	4.06033358	0.00260193	0.02933429	-1.35890753
hetero after UVB irradiation VS hetero before UVB irradiation	Glycosphingolipid biosynthesis - ganglioseries|EHMN	0.672683183	0.00882567	3.560115913	0.00680141	0.08550202	-2.2288956
WT after UVB irradiation VS WT before UVB irradiation	Glycosphingolipid biosynthesis - ganglioseries|EHMN	0.56301312	-0.04584595	2.707484106	0.02187914	0.31542987	-3.14789102
homo after UVB irradiation VS homo before UVB irradiation	Glycosphingolipid biosynthesis - globo and isoglobo series - Homo sapiens (human)|KEGG	0.104528834	-0.02908031	0.518678376	0.6159872	0.732066	-6.22598881
hetero after UVB irradiation VS hetero before UVB irradiation	Glycosphingolipid biosynthesis - globo and isoglobo series - Homo sapiens (human)|KEGG	0.295756156	-0.02331645	1.718246152	0.1221653	0.31406061	-4.98025786
WT after UVB irradiation VS WT before UVB irradiation	Glycosphingolipid biosynthesis - globo and isoglobo series - Homo sapiens (human)|KEGG	-0.01366585	-0.01919332	-0.05721662	0.95549072	0.98742794	-5.86749302
homo after UVB irradiation VS homo before UVB irradiation	Glycosphingolipid biosynthesis - globoseries|EHMN	0.372468884	-0.05450274	2.273844896	0.04789482	0.14274657	-4.17142855
hetero after UVB irradiation VS hetero before UVB irradiation	Glycosphingolipid biosynthesis - globoseries|EHMN	0.562186695	-0.02176725	3.417201978	0.00844443	0.0927463	-2.4409637
WT after UVB irradiation VS WT before UVB irradiation	Glycosphingolipid biosynthesis - globoseries|EHMN	0.32077264	-0.09554997	1.555695213	0.15058652	0.55641476	-4.78008788
homo after UVB irradiation VS homo before UVB irradiation	Glycosphingolipid biosynthesis - lacto and neolacto series - Homo sapiens (human)|KEGG	0.12994307	0.006056867	0.727123166	0.48488457	0.62736836	-6.09230191
hetero after UVB irradiation VS hetero before UVB irradiation	Glycosphingolipid biosynthesis - lacto and neolacto series - Homo sapiens (human)|KEGG	0.079817571	0.023190806	0.427662222	0.67961773	0.8342843	-6.24841492
WT after UVB irradiation VS WT before UVB irradiation	Glycosphingolipid biosynthesis - lacto and neolacto series - Homo sapiens (human)|KEGG	0.081389015	-0.03921422	0.328722719	0.74909125	0.9185045	-5.81472318
homo after UVB irradiation VS homo before UVB irradiation	Glycosphingolipid biosynthesis - lactoseries|EHMN	-0.80995151	0.090644457	-4.38198568	0.00159583	0.02229782	-0.88155801
hetero after UVB irradiation VS hetero before UVB irradiation	Glycosphingolipid biosynthesis - lactoseries|EHMN	-1.04081979	-0.01800749	-5.65116333	0.00039839	0.03004176	0.527377283
WT after UVB irradiation VS WT before UVB irradiation	Glycosphingolipid biosynthesis - lactoseries|EHMN	-0.37498765	0.020525558	-1.26544091	0.23417523	0.62952955	-5.12076746
homo after UVB irradiation VS homo before UVB irradiation	Glycosphingolipid biosynthesis - neolactoseries|EHMN	0.320921819	0.016480487	1.897201836	0.08893601	0.21361485	-4.739825
hetero after UVB irradiation VS hetero before UVB irradiation	Glycosphingolipid biosynthesis - neolactoseries|EHMN	0.480751296	-0.00802478	2.543080464	0.0331732	0.1586605	-3.76975743
WT after UVB irradiation VS WT before UVB irradiation	Glycosphingolipid biosynthesis - neolactoseries|EHMN	0.362746162	0.0133035	1.731738486	0.11374712	0.51527453	-4.55372288
homo after UVB irradiation VS homo before UVB irradiation	Glycosphingolipid metabolism|EHMN	0.002393442	-0.00515258	0.012063562	0.99062736	0.99328478	-6.36837007
hetero after UVB irradiation VS hetero before UVB irradiation	Glycosphingolipid metabolism|EHMN	0.174653164	-0.02239875	0.911817487	0.38720733	0.61473991	-5.91702033
WT after UVB irradiation VS WT before UVB irradiation	Glycosphingolipid metabolism|EHMN	0.156454263	-0.0200634	0.681035423	0.51118709	0.8267256	-5.63984085
homo after UVB irradiation VS homo before UVB irradiation	Glycosphingolipid metabolism|Reactome	0.472219305	-0.07014878	1.915069065	0.08639807	0.20961401	-4.71372868
hetero after UVB irradiation VS hetero before UVB irradiation	Glycosphingolipid metabolism|Reactome	0.602425014	-0.0306976	2.886193211	0.01924805	0.12168185	-3.24487195
WT after UVB irradiation VS WT before UVB irradiation	Glycosphingolipid metabolism|Reactome	0.578154398	-0.03323189	2.863133096	0.01673531	0.30353778	-2.9137847
homo after UVB irradiation VS homo before UVB irradiation	Glycosylphosphatidylinositol (GPI)-anchor biosynthesis - Homo sapiens (human)|KEGG	-0.0335886	-0.04090418	-0.19882659	0.84663734	0.90528768	-6.34725333
hetero after UVB irradiation VS hetero before UVB irradiation	Glycosylphosphatidylinositol (GPI)-anchor biosynthesis - Homo sapiens (human)|KEGG	0.090855668	-0.01882791	0.471913863	0.64896834	0.82363337	-6.22734216
WT after UVB irradiation VS WT before UVB irradiation	Glycosylphosphatidylinositol (GPI)-anchor biosynthesis - Homo sapiens (human)|KEGG	0.177584427	0.040069795	0.765993652	0.46123701	0.80891799	-5.58088507
homo after UVB irradiation VS homo before UVB irradiation	Glyoxylate and dicarboxylate metabolism - Homo sapiens (human)|KEGG	-0.1467434	-0.0191011	-0.62057362	0.54964522	0.67913558	-6.1657752
hetero after UVB irradiation VS hetero before UVB irradiation	Glyoxylate and dicarboxylate metabolism - Homo sapiens (human)|KEGG	-0.47256001	-0.00653716	-2.25073793	0.05287819	0.20425321	-4.21239564
WT after UVB irradiation VS WT before UVB irradiation	Glyoxylate and dicarboxylate metabolism - Homo sapiens (human)|KEGG	-0.00479319	-0.0531665	-0.01896063	0.9852425	0.99548728	-5.86896907
homo after UVB irradiation VS homo before UVB irradiation	Glyoxylate metabolism and glycine degradation|Reactome	-0.41949092	-0.01249216	-1.76230785	0.11046355	0.24413193	-4.93331085
hetero after UVB irradiation VS hetero before UVB irradiation	Glyoxylate metabolism and glycine degradation|Reactome	-0.69555183	-0.01712965	-3.80965304	0.00469116	0.07419102	-1.864587
WT after UVB irradiation VS WT before UVB irradiation	Glyoxylate metabolism and glycine degradation|Reactome	-0.40836444	-0.03552117	-1.87521274	0.08999014	0.48188205	-4.3604376
homo after UVB irradiation VS homo before UVB irradiation	Glypican 1 network|PID	0.5954628	-0.06026926	3.392926751	0.00749214	0.05059971	-2.39187558
hetero after UVB irradiation VS hetero before UVB irradiation	Glypican 1 network|PID	0.563198629	0.056258425	3.367758917	0.00910614	0.09677373	-2.51484848
WT after UVB irradiation VS WT before UVB irradiation	Glypican 1 network|PID	0.254844797	-0.04656326	1.030168742	0.32701025	0.71049278	-5.36003984
homo after UVB irradiation VS homo before UVB irradiation	Glypican 2 network|PID	1.099905152	0.036480483	4.552032413	0.00123969	0.02014127	-0.63544572
hetero after UVB irradiation VS hetero before UVB irradiation	Glypican 2 network|PID	-0.07174834	-0.04065977	-0.16962101	0.86931013	0.95085052	-6.33096038
WT after UVB irradiation VS WT before UVB irradiation	Glypican 2 network|PID	0.29674163	-0.01285071	0.700647004	0.49937308	0.82066817	-5.62678255
homo after UVB irradiation VS homo before UVB irradiation	Glypican 3 network|PID	-0.12260178	0.032030242	-0.4914773	0.63437364	0.74634116	-6.24035053
hetero after UVB irradiation VS hetero before UVB irradiation	Glypican 3 network|PID	-0.02062799	0.045930769	-0.09569706	0.92599818	0.97054702	-6.34157594
WT after UVB irradiation VS WT before UVB irradiation	Glypican 3 network|PID	0.086695826	0.076079338	0.392235816	0.70304777	0.90059561	-5.79184663
homo after UVB irradiation VS homo before UVB irradiation	GMCSF-mediated signaling events|PID	0.517331492	-0.03592918	2.978990042	0.01478468	0.07509103	-3.05182694
hetero after UVB irradiation VS hetero before UVB irradiation	GMCSF-mediated signaling events|PID	0.693685605	0.023984175	4.500567795	0.00175579	0.04958933	-0.90283192
WT after UVB irradiation VS WT before UVB irradiation	GMCSF-mediated signaling events|PID	0.572877706	-0.01677033	2.739222999	0.02071504	0.31542987	-3.10017878
homo after UVB irradiation VS homo before UVB irradiation	GnRH signaling pathway - Homo sapiens (human)|KEGG	0.475819774	-0.03164828	2.907318634	0.01665258	0.08097517	-3.16667544
hetero after UVB irradiation VS hetero before UVB irradiation	GnRH signaling pathway - Homo sapiens (human)|KEGG	0.366507498	0.016144829	2.217401441	0.05576124	0.2098759	-4.26225292
WT after UVB irradiation VS WT before UVB irradiation	GnRH signaling pathway - Homo sapiens (human)|KEGG	0.260802694	0.011132539	1.226064984	0.24803984	0.64108543	-5.16332393
homo after UVB irradiation VS homo before UVB irradiation	Golgi Associated Vesicle Biogenesis|Reactome	0.363724568	-0.03821844	1.973656411	0.07854596	0.19759827	-4.62745909
hetero after UVB irradiation VS hetero before UVB irradiation	Golgi Associated Vesicle Biogenesis|Reactome	0.492296466	-0.00688254	3.122085797	0.01330198	0.11120965	-2.88531078
WT after UVB irradiation VS WT before UVB irradiation	Golgi Associated Vesicle Biogenesis|Reactome	0.315712696	0.013928925	1.4774314	0.17010279	0.57759066	-4.87628139
homo after UVB irradiation VS homo before UVB irradiation	Golgi Cisternae Pericentriolar Stack Reorganization|Reactome	-0.11426999	-0.00515543	-0.5586921	0.58944603	0.71144874	-6.20354106
hetero after UVB irradiation VS hetero before UVB irradiation	Golgi Cisternae Pericentriolar Stack Reorganization|Reactome	0.511317096	0.018695177	2.862812083	0.01997105	0.12483305	-3.28063365
WT after UVB irradiation VS WT before UVB irradiation	Golgi Cisternae Pericentriolar Stack Reorganization|Reactome	0.277771582	-0.00605564	1.133174169	0.28336846	0.67189698	-5.25982226
homo after UVB irradiation VS homo before UVB irradiation	Golgi-to-ER retrograde transport|Reactome	0.216177396	-0.02501133	1.127801442	0.28739766	0.44697403	-5.72874301
hetero after UVB irradiation VS hetero before UVB irradiation	Golgi-to-ER retrograde transport|Reactome	0.37447865	0.011695201	2.349059012	0.04520619	0.18731753	-4.06447688
WT after UVB irradiation VS WT before UVB irradiation	Golgi-to-ER retrograde transport|Reactome	0.267147992	-0.01049376	1.229769487	0.24670776	0.63911291	-5.15936104
homo after UVB irradiation VS homo before UVB irradiation	Gout or Kelley-Seegmiller Syndrome|SMPDB	0.019015587	-0.03943216	0.101997834	0.92090359	0.95153785	-6.36286155
hetero after UVB irradiation VS hetero before UVB irradiation	Gout or Kelley-Seegmiller Syndrome|SMPDB	-0.02085195	-0.0438046	-0.1098564	0.91509485	0.97054702	-6.33999905
WT after UVB irradiation VS WT before UVB irradiation	Gout or Kelley-Seegmiller Syndrome|SMPDB	0.165884344	-0.0622813	0.759490799	0.46494629	0.80891799	-5.58561498
homo after UVB irradiation VS homo before UVB irradiation	GP1b-IX-V activation signalling|Reactome	0.538682888	0.02435595	1.834892812	0.09834294	0.22724387	-4.83000159
hetero after UVB irradiation VS hetero before UVB irradiation	GP1b-IX-V activation signalling|Reactome	0.503979405	0.007919503	2.292378919	0.04948293	0.19801197	-4.14989799
WT after UVB irradiation VS WT before UVB irradiation	GP1b-IX-V activation signalling|Reactome	0.629177248	-0.04426831	2.29725486	0.04426094	0.39111425	-3.75891057
homo after UVB irradiation VS homo before UVB irradiation	GPCR Adenosine A2A receptor|INOH	0.450130456	-0.02102555	2.41136276	0.03809367	0.12846062	-3.95659679
hetero after UVB irradiation VS hetero before UVB irradiation	GPCR Adenosine A2A receptor|INOH	0.523460034	0.021641719	2.755524	0.02366424	0.13628011	-3.44485301
WT after UVB irradiation VS WT before UVB irradiation	GPCR Adenosine A2A receptor|INOH	0.341945094	-0.03306561	1.53858276	0.15467796	0.5588223	-4.80137048
homo after UVB irradiation VS homo before UVB irradiation	GPCR Dopamine D1like receptor|INOH	0.283162546	-0.02060657	1.371953412	0.20196422	0.35894742	-5.44925176
hetero after UVB irradiation VS hetero before UVB irradiation	GPCR Dopamine D1like receptor|INOH	0.094116189	0.025260094	0.460141448	0.65705668	0.82651398	-6.23314063
WT after UVB irradiation VS WT before UVB irradiation	GPCR Dopamine D1like receptor|INOH	0.258193282	-0.00468871	1.017405578	0.33275327	0.71581921	-5.37193546
homo after UVB irradiation VS homo before UVB irradiation	GPCR downstream signalling|Reactome	0.388843549	0.000646522	2.477748528	0.03409861	0.12327989	-3.85195268
hetero after UVB irradiation VS hetero before UVB irradiation	GPCR downstream signalling|Reactome	0.295937544	0.045203364	1.861925971	0.09776281	0.28215302	-4.78043095
WT after UVB irradiation VS WT before UVB irradiation	GPCR downstream signalling|Reactome	0.193231697	0.02615929	0.878843908	0.39994645	0.76778286	-5.49324813
homo after UVB irradiation VS homo before UVB irradiation	GPCR GroupI metabotropic glutamate receptor|INOH	0.299558994	-0.03888285	1.72380378	0.11743769	0.2553431	-4.98729649
hetero after UVB irradiation VS hetero before UVB irradiation	GPCR GroupI metabotropic glutamate receptor|INOH	0.348698819	0.022544648	2.003752692	0.07824655	0.25169216	-4.57713217
WT after UVB irradiation VS WT before UVB irradiation	GPCR GroupI metabotropic glutamate receptor|INOH	0.066120416	0.006792657	0.332085425	0.74662544	0.9185045	-5.81361047
homo after UVB irradiation VS homo before UVB irradiation	GPCR ligand binding|Reactome	0.262382701	0.013278851	1.555782925	0.15278812	0.29954081	-5.21547807
hetero after UVB irradiation VS hetero before UVB irradiation	GPCR ligand binding|Reactome	0.147582806	0.031771384	0.916110129	0.38507756	0.61262951	-5.91315902
WT after UVB irradiation VS WT before UVB irradiation	GPCR ligand binding|Reactome	0.099833236	0.034124795	0.456620413	0.65762315	0.87935805	-5.76468637
homo after UVB irradiation VS homo before UVB irradiation	GPCR signaling-cholera toxin|INOH	0.248361748	0.009931397	1.529557723	0.15909014	0.30753835	-5.24991051
hetero after UVB irradiation VS hetero before UVB irradiation	GPCR signaling-cholera toxin|INOH	0.112570835	0.036157356	0.655432684	0.52967427	0.74422173	-6.11940851
WT after UVB irradiation VS WT before UVB irradiation	GPCR signaling-cholera toxin|INOH	0.078879412	0.025934465	0.356443679	0.72885486	0.9105948	-5.80522073
homo after UVB irradiation VS homo before UVB irradiation	GPCR signaling-G alpha i|INOH	0.229514332	0.011544546	1.408994167	0.19108274	0.34691205	-5.40362732
hetero after UVB irradiation VS hetero before UVB irradiation	GPCR signaling-G alpha i|INOH	0.088919218	0.039576212	0.522713179	0.6146367	0.79997547	-6.20073887
WT after UVB irradiation VS WT before UVB irradiation	GPCR signaling-G alpha i|INOH	0.058582971	0.027783422	0.266491023	0.79523201	0.94308602	-5.83331001
homo after UVB irradiation VS homo before UVB irradiation	GPCR signaling-G alpha q|INOH	0.21144431	0.00664571	1.328462577	0.21540759	0.37334177	-5.50179887
hetero after UVB irradiation VS hetero before UVB irradiation	GPCR signaling-G alpha q|INOH	0.094870577	0.04000813	0.574351386	0.58073606	0.77397513	-6.1710999
WT after UVB irradiation VS WT before UVB irradiation	GPCR signaling-G alpha q|INOH	0.057720589	0.029115768	0.269783459	0.79276821	0.94187628	-5.83242237
homo after UVB irradiation VS homo before UVB irradiation	GPCR signaling-G alpha s Epac and ERK|INOH	0.240872184	0.010098348	1.482176357	0.17105791	0.32186978	-5.3112397
hetero after UVB irradiation VS hetero before UVB irradiation	GPCR signaling-G alpha s Epac and ERK|INOH	0.113150406	0.039015457	0.669893032	0.52085814	0.73660298	-6.10954227
WT after UVB irradiation VS WT before UVB irradiation	GPCR signaling-G alpha s Epac and ERK|INOH	0.067567662	0.027640605	0.308773739	0.76377962	0.92696825	-5.8210964
homo after UVB irradiation VS homo before UVB irradiation	GPCR signaling-G alpha s PKA and ERK|INOH	0.259296246	-0.00765366	1.598773449	0.14293529	0.28725463	-5.15831179
hetero after UVB irradiation VS hetero before UVB irradiation	GPCR signaling-G alpha s PKA and ERK|INOH	0.149578808	0.034278039	0.87812924	0.40421684	0.63291665	-5.94680718
WT after UVB irradiation VS WT before UVB irradiation	GPCR signaling-G alpha s PKA and ERK|INOH	0.094380798	0.028210316	0.432928361	0.67418171	0.88126177	-5.77514135
homo after UVB irradiation VS homo before UVB irradiation	GPCR signaling-pertussis toxin|INOH	0.229514332	0.011544546	1.408994167	0.19108274	0.34691205	-5.40362732
hetero after UVB irradiation VS hetero before UVB irradiation	GPCR signaling-pertussis toxin|INOH	0.088919218	0.039576212	0.522713179	0.6146367	0.79997547	-6.20073887
WT after UVB irradiation VS WT before UVB irradiation	GPCR signaling-pertussis toxin|INOH	0.058582971	0.027783422	0.266491023	0.79523201	0.94308602	-5.83331001
homo after UVB irradiation VS homo before UVB irradiation	GPCRs_ Class A Rhodopsin-like|Wikipathways	0.198399626	0.008507443	1.172711562	0.26980421	0.43325305	-5.68028558
hetero after UVB irradiation VS hetero before UVB irradiation	GPCRs_ Class A Rhodopsin-like|Wikipathways	0.125597507	0.033188338	0.802751041	0.4441698	0.66948609	-6.01005739
WT after UVB irradiation VS WT before UVB irradiation	GPCRs_ Class A Rhodopsin-like|Wikipathways	0.160593133	0.020255833	0.674916105	0.51490763	0.8287185	-5.64384676
homo after UVB irradiation VS homo before UVB irradiation	GPCRs_ Class B Secretin-like|Wikipathways	0.311464363	-0.01489475	1.993993109	0.07598175	0.19626432	-4.59727416
hetero after UVB irradiation VS hetero before UVB irradiation	GPCRs_ Class B Secretin-like|Wikipathways	0.163615662	0.003934295	0.766066478	0.46455434	0.68805201	-6.03908924
WT after UVB irradiation VS WT before UVB irradiation	GPCRs_ Class B Secretin-like|Wikipathways	-0.03601415	-0.07593377	-0.16314054	0.87363149	0.96283151	-5.85568738
homo after UVB irradiation VS homo before UVB irradiation	GPCRs_ Class C Metabotropic glutamate_ pheromone|Wikipathways	0.131047062	-0.04295023	0.673593821	0.51680652	0.6545738	-6.13052853
hetero after UVB irradiation VS hetero before UVB irradiation	GPCRs_ Class C Metabotropic glutamate_ pheromone|Wikipathways	-0.18239451	0.045125052	-1.01150987	0.33993417	0.56880321	-5.82361982
WT after UVB irradiation VS WT before UVB irradiation	GPCRs_ Class C Metabotropic glutamate_ pheromone|Wikipathways	-0.02663103	0.032533213	-0.10964476	0.91484282	0.97566251	-5.86306482
homo after UVB irradiation VS homo before UVB irradiation	GPCRs_ Other|Wikipathways	0.463573609	-0.00466264	2.956952364	0.01533507	0.07691241	-3.08713688
hetero after UVB irradiation VS hetero before UVB irradiation	GPCRs_ Other|Wikipathways	0.38807844	0.056500069	2.367949015	0.04386417	0.18410028	-4.03592736
WT after UVB irradiation VS WT before UVB irradiation	GPCRs_ Other|Wikipathways	0.276871999	0.016811679	1.141827901	0.27991841	0.66921967	-5.25107103
homo after UVB irradiation VS homo before UVB irradiation	GPR40 Pathway|Wikipathways	0.258447839	-0.04066314	1.159825317	0.27476234	0.43614438	-5.6943335
hetero after UVB irradiation VS hetero before UVB irradiation	GPR40 Pathway|Wikipathways	0.012214586	0.030470559	0.068163487	0.9472454	0.972639	-6.34402144
WT after UVB irradiation VS WT before UVB irradiation	GPR40 Pathway|Wikipathways	0.122071342	0.056381961	0.561715912	0.58657848	0.86138389	-5.71194917
homo after UVB irradiation VS homo before UVB irradiation	G-protein activation|Reactome	0.443810977	-0.00358487	1.890648646	0.08988426	0.21498584	-4.74937045
hetero after UVB irradiation VS hetero before UVB irradiation	G-protein activation|Reactome	0.52566071	0.039913934	3.012497125	0.01578344	0.11352943	-3.05201042

WT after UVB irradiation VS WT before UVB irradiation	G-protein activation|Reactome	0.207230148	-0.01499004	0.890180144	0.39411454	0.76452505	-5.48387731
homo after UVB irradiation VS homo before UVB irradiation	G-protein beta:gamma signalling|Reactome	0.649422771	-0.03359174	3.927416421	0.00319764	0.03208887	-1.56040883
hetero after UVB irradiation VS hetero before UVB irradiation	G-protein beta:gamma signalling|Reactome	0.667919868	0.028786762	3.665093994	0.00581158	0.08047311	-2.07465667
WT after UVB irradiation VS WT before UVB irradiation	G-protein beta:gamma signalling|Reactome	0.439763047	-0.00619582	1.73958163	0.11231315	0.51412987	-4.54334591
homo after UVB irradiation VS homo before UVB irradiation	G-protein mediated events|Reactome	0.525659294	-0.04204515	3.111449389	0.01187653	0.06653773	-2.83980106
hetero after UVB irradiation VS hetero before UVB irradiation	G-protein mediated events|Reactome	0.356553398	0.016546496	1.798779852	0.1078639	0.29778539	-4.86909252
WT after UVB irradiation VS WT before UVB irradiation	G-protein mediated events|Reactome	0.283354308	-0.01308677	1.165028566	0.27083139	0.66327566	-5.22736337
homo after UVB irradiation VS homo before UVB irradiation	g-protein signaling through tubby proteins|BioCarta	0.409938012	0.03333114	1.586142316	0.14576973	0.2913279	-5.17519913
hetero after UVB irradiation VS hetero before UVB irradiation	g-protein signaling through tubby proteins|BioCarta	0.23817319	0.072550496	1.05115755	0.32239599	0.55349684	-5.78437769
WT after UVB irradiation VS WT before UVB irradiation	g-protein signaling through tubby proteins|BioCarta	0.310906307	-0.02103394	1.199832099	0.25763943	0.65187771	-5.19113953
homo after UVB irradiation VS homo before UVB irradiation	GPVI-mediated activation cascade|Reactome	0.384400632	-0.02335404	1.673576104	0.12713937	0.26715064	-5.05681197
hetero after UVB irradiation VS hetero before UVB irradiation	GPVI-mediated activation cascade|Reactome	0.597642816	0.0148709	3.894015545	0.00414548	0.07036798	-1.74331196
WT after UVB irradiation VS WT before UVB irradiation	GPVI-mediated activation cascade|Reactome	0.454127047	0.012511713	2.128697194	0.05893127	0.42631804	-4.00390189
homo after UVB irradiation VS homo before UVB irradiation	Graft-versus-host disease - Homo sapiens (human)|KEGG	0.286753578	-0.02025483	0.994577251	0.34488095	0.5000719	-5.86387999
hetero after UVB irradiation VS hetero before UVB irradiation	Graft-versus-host disease - Homo sapiens (human)|KEGG	0.144290622	0.027423014	0.451669366	0.66290722	0.82651398	-6.23722745
WT after UVB irradiation VS WT before UVB irradiation	Graft-versus-host disease - Homo sapiens (human)|KEGG	0.281770492	-0.03008767	0.831449254	0.42497342	0.7908939	-5.53131719
homo after UVB irradiation VS homo before UVB irradiation	granzyme a mediated apoptosis pathway|BioCarta	-0.48241369	0.031798024	-2.43383466	0.03669198	0.12535822	-3.92123404
hetero after UVB irradiation VS hetero before UVB irradiation	granzyme a mediated apoptosis pathway|BioCarta	-0.39182107	-0.058447	-1.56281518	0.1548186	0.36486693	-5.18786808
WT after UVB irradiation VS WT before UVB irradiation	granzyme a mediated apoptosis pathway|BioCarta	-0.15637721	-0.029126	-0.59012779	0.56809492	0.85232901	-5.69593986
homo after UVB irradiation VS homo before UVB irradiation	GRB2 events in EGFR signaling|Reactome	-0.23951422	0.001387778	-1.01544178	0.33534623	0.49203423	-5.84359802
hetero after UVB irradiation VS hetero before UVB irradiation	GRB2 events in EGFR signaling|Reactome	-0.13473056	0.041229194	-0.52836747	0.61087446	0.7972333	-6.19762016
WT after UVB irradiation VS WT before UVB irradiation	GRB2 events in EGFR signaling|Reactome	-0.24653479	0.034980061	-0.99550751	0.34278112	0.72738377	-5.39206853
homo after UVB irradiation VS homo before UVB irradiation	GRB2 events in ERBB2 signaling|Reactome	-0.17151975	0.066272659	-0.95521933	0.36341217	0.51763025	-5.90121258
hetero after UVB irradiation VS hetero before UVB irradiation	GRB2 events in ERBB2 signaling|Reactome	-0.31131295	-0.01994648	-1.93041531	0.08782061	0.26825812	-4.68292678
WT after UVB irradiation VS WT before UVB irradiation	GRB2 events in ERBB2 signaling|Reactome	-0.2282877	0.020826947	-1.00540862	0.33821973	0.7221059	-5.38300896
homo after UVB irradiation VS homo before UVB irradiation	GRB2:SOS provides linkage to MAPK signaling for Integrins|Reactome	0.673090165	-0.01858712	4.013231616	0.00279837	0.02999383	-1.43004273
hetero after UVB irradiation VS hetero before UVB irradiation	GRB2:SOS provides linkage to MAPK signaling for Integrins|Reactome	0.525877174	0.02217441	2.512669744	0.03482109	0.1634547	-3.81613314
WT after UVB irradiation VS WT before UVB irradiation	GRB2:SOS provides linkage to MAPK signaling for Integrins|Reactome	0.645529281	0.005702746	2.942676038	0.01459623	0.28372475	-2.79417304
homo after UVB irradiation VS homo before UVB irradiation	GRB7 events in ERBB2 signaling|Reactome	-0.89235939	0.070898562	-4.02118857	0.00276412	0.02978529	-1.41800465
hetero after UVB irradiation VS hetero before UVB irradiation	GRB7 events in ERBB2 signaling|Reactome	-0.67678706	-0.1046421	-1.99524338	0.07930381	0.25322957	-4.58947584
WT after UVB irradiation VS WT before UVB irradiation	GRB7 events in ERBB2 signaling|Reactome	-0.69362919	-0.03647907	-2.13387704	0.05841786	0.42483181	-3.99645322
homo after UVB irradiation VS homo before UVB irradiation	Growth hormone receptor signaling|Reactome	0.64122501	-0.04543321	3.745616581	0.00425521	0.03746619	-1.83968484
hetero after UVB irradiation VS hetero before UVB irradiation	Growth hormone receptor signaling|Reactome	0.39065949	0.003948716	1.751715997	0.1160199	0.30887493	-4.93433117
WT after UVB irradiation VS WT before UVB irradiation	Growth hormone receptor signaling|Reactome	0.232007672	-0.08075489	0.75964363	0.4648589	0.80891799	-5.58550423
homo after UVB irradiation VS homo before UVB irradiation	growth hormone signaling pathway|BioCarta	0.464916272	-0.00409484	2.652858636	0.02545056	0.10578915	-3.57382388
hetero after UVB irradiation VS hetero before UVB irradiation	growth hormone signaling pathway|BioCarta	0.274480791	0.00922309	1.517306723	0.16577261	0.37969425	-5.24670702
WT after UVB irradiation VS WT before UVB irradiation	growth hormone signaling pathway|BioCarta	0.100353168	-0.03525787	0.430988775	0.67554557	0.88217037	-5.77597359
homo after UVB irradiation VS homo before UVB irradiation	Growth hormone signaling|INOH	0.661858201	-0.06168834	3.945248818	0.00310999	0.03154271	-1.5332389
hetero after UVB irradiation VS hetero before UVB irradiation	Growth hormone signaling|INOH	0.274836994	0.001118279	1.164889727	0.27598079	0.5064092	-5.66559282
WT after UVB irradiation VS WT before UVB irradiation	Growth hormone signaling|INOH	0.154678504	-0.09185528	0.459391323	0.65569892	0.87935805	-5.76342879
homo after UVB irradiation VS homo before UVB irradiation	g-secretase mediated erbb4 signaling pathway|BioCarta	0.131313436	0.03488933	0.630151827	0.54362489	0.67708436	-6.15960322
hetero after UVB irradiation VS hetero before UVB irradiation	g-secretase mediated erbb4 signaling pathway|BioCarta	-0.09858469	-7.43E-05	-0.48083306	0.64287279	0.81928142	-6.22285672
WT after UVB irradiation VS WT before UVB irradiation	g-secretase mediated erbb4 signaling pathway|BioCarta	0.117659919	0.031403856	0.501755795	0.62661431	0.87283328	-5.7432999
homo after UVB irradiation VS homo before UVB irradiation	GTP hydrolysis and joining of the 60S ribosomal subunit|Reactome	-0.97379771	0.022815229	-5.43151105	0.00035891	0.01666204	0.563804855
hetero after UVB irradiation VS hetero before UVB irradiation	GTP hydrolysis and joining of the 60S ribosomal subunit|Reactome	-0.25758477	-0.01219528	-0.7777637	0.45798818	0.68223742	-6.02995905
WT after UVB irradiation VS WT before UVB irradiation	GTP hydrolysis and joining of the 60S ribosomal subunit|Reactome	-0.60845188	-0.01253128	-2.05638531	0.06656348	0.43803373	-4.10727278
homo after UVB irradiation VS homo before UVB irradiation	Guanidinoacetate Methyltransferase Deficiency (GAMT Deficiency)|SMPDB	-0.34739547	0.00502943	-1.63262028	0.13558154	0.277359	-5.11269582
hetero after UVB irradiation VS hetero before UVB irradiation	Guanidinoacetate Methyltransferase Deficiency (GAMT Deficiency)|SMPDB	-0.56351352	-0.04220085	-3.24793085	0.01094591	0.10415756	-2.6949004
WT after UVB irradiation VS WT before UVB irradiation	Guanidinoacetate Methyltransferase Deficiency (GAMT Deficiency)|SMPDB	-0.47021304	-0.04055952	-2.31093774	0.04323906	0.39022568	-3.73881388
homo after UVB irradiation VS homo before UVB irradiation	guanine and guanosine salvage|HumanCyc	-0.32715535	-0.06179471	-1.02422536	0.33139196	0.48780303	-5.83495942
hetero after UVB irradiation VS hetero before UVB irradiation	guanine and guanosine salvage|HumanCyc	-0.27347597	0.141230739	-0.62157821	0.55066253	0.75605031	-6.14174635
WT after UVB irradiation VS WT before UVB irradiation	guanine and guanosine salvage|HumanCyc	0.088657767	-0.04403722	0.234828713	0.81904221	0.94525798	-5.84129727
homo after UVB irradiation VS homo before UVB irradiation	guanosine deoxyribonucleotides <i>de novo</i> biosynthesis|HumanCyc	-0.26766388	-0.07651359	-1.13478893	0.2846023	0.44528503	-5.72129654
hetero after UVB irradiation VS hetero before UVB irradiation	guanosine deoxyribonucleotides <i>de novo</i> biosynthesis|HumanCyc	-0.15783943	-0.04297759	-0.59914586	0.56483354	0.76437184	-6.1559538
WT after UVB irradiation VS WT before UVB irradiation	guanosine deoxyribonucleotides <i>de novo</i> biosynthesis|HumanCyc	-0.05360404	-0.09347628	-0.22827277	0.82399739	0.94525798	-5.84282656
homo after UVB irradiation VS homo before UVB irradiation	guanosine nucleotides <i>de novo</i> biosynthesis|HumanCyc	-0.28219211	-0.04061941	-1.43647987	0.18333648	0.33754612	-5.36927125
hetero after UVB irradiation VS hetero before UVB irradiation	guanosine nucleotides <i>de novo</i> biosynthesis|HumanCyc	-0.15793573	-0.05175316	-0.73404172	0.48284683	0.70381063	-6.06347027
WT after UVB irradiation VS WT before UVB irradiation	guanosine nucleotides <i>de novo</i> biosynthesis|HumanCyc	0.040164574	-0.06117979	0.174393889	0.86500808	0.96095643	-5.85376898
homo after UVB irradiation VS homo before UVB irradiation	guanosine nucleotides degradation|HumanCyc	0.629120756	0.0110337	2.316480181	0.04461742	0.13727975	-4.1051385
hetero after UVB irradiation VS hetero before UVB irradiation	guanosine nucleotides degradation|HumanCyc	0.64216519	0.118444381	3.196235886	0.01185614	0.10542662	-2.77296884
WT after UVB irradiation VS WT before UVB irradiation	guanosine nucleotides degradation|HumanCyc	0.495946205	-0.0189831	1.491784463	0.16636724	0.57064699	-4.85886261
homo after UVB irradiation VS homo before UVB irradiation	guanosine ribonucleotides <i>de novo</i> biosynthesis|HumanCyc	-0.11645502	-0.03426375	-0.55265915	0.59340817	0.71547444	-6.20702554
hetero after UVB irradiation VS hetero before UVB irradiation	guanosine ribonucleotides <i>de novo</i> biosynthesis|HumanCyc	-0.26875415	-0.08137188	-1.28576524	0.23273259	0.45449663	-5.52996542
WT after UVB irradiation VS WT before UVB irradiation	guanosine ribonucleotides <i>de novo</i> biosynthesis|HumanCyc	-0.00977844	-0.05934031	-0.04284826	0.96665921	0.98922368	-5.86822119
homo after UVB irradiation VS homo before UVB irradiation	Gut-Liver Indole Metabolism|Wikipathways	0.388460901	0.020895461	1.066764261	0.31273867	0.46923284	-5.79229523
hetero after UVB irradiation VS hetero before UVB irradiation	Gut-Liver Indole Metabolism|Wikipathways	-0.14760988	0.040949837	-0.3635581	0.72514129	0.86482318	-6.27541048
WT after UVB irradiation VS WT before UVB irradiation	Gut-Liver Indole Metabolism|Wikipathways	-0.06962327	0.086915055	-0.17823013	0.86207255	0.95916804	-5.85308595
homo after UVB irradiation VS homo before UVB irradiation	H19 action Rb-E2F1 signaling and CDK-Beta-catenin activity|Wikipathways	-0.39788283	0.009212078	-1.62917948	0.13631328	0.27825158	-5.11735688
hetero after UVB irradiation VS hetero before UVB irradiation	H19 action Rb-E2F1 signaling and CDK-Beta-catenin activity|Wikipathways	-0.02357233	0.001557777	-0.09511124	0.92644967	0.97054702	-6.34163651
WT after UVB irradiation VS WT before UVB irradiation	H19 action Rb-E2F1 signaling and CDK-Beta-catenin activity|Wikipathways	-0.00200521	-0.00146655	-0.00723869	0.99436564	0.99786155	-5.86912465
homo after UVB irradiation VS homo before UVB irradiation	Hair Follicle Development- Induction (Part 1 of 3)|Wikipathways	-0.38218726	-0.00536068	-2.19883197	0.05424031	0.15674764	-4.28726462
hetero after UVB irradiation VS hetero before UVB irradiation	Hair Follicle Development- Induction (Part 1 of 3)|Wikipathways	-0.37118676	-0.02681599	-2.21475321	0.05599679	0.2105886	-4.26620639
WT after UVB irradiation VS WT before UVB irradiation	Hair Follicle Development- Induction (Part 1 of 3)|Wikipathways	-0.30689812	0.023866788	-1.53903907	0.15456761	0.5588223	-4.80080474
homo after UVB irradiation VS homo before UVB irradiation	Hartnup Disorder|SMPDB	0.234726031	0.017120546	1.156747303	0.27595732	0.43614438	-5.69767199
hetero after UVB irradiation VS hetero before UVB irradiation	Hartnup Disorder|SMPDB	-0.03255037	0.028925632	-0.18154472	0.86023155	0.94503144	-6.32869738
WT after UVB irradiation VS WT before UVB irradiation	Hartnup Disorder|SMPDB	-0.15908359	0.010252305	-0.80382265	0.44003996	0.79685269	-5.55266936
homo after UVB irradiation VS homo before UVB irradiation	HATs acetylate histones|Reactome	-0.49949265	-0.0103065	-2.80467908	0.01975474	0.08984077	-3.33111831
hetero after UVB irradiation VS hetero before UVB irradiation	HATs acetylate histones|Reactome	-0.22880907	-0.06702845	-1.22435469	0.25394512	0.47932892	-5.60001973
WT after UVB irradiation VS WT before UVB irradiation	HATs acetylate histones|Reactome	-0.23944158	-0.0359664	-0.90622178	0.38596391	0.75978327	-5.47044441
homo after UVB irradiation VS homo before UVB irradiation	Hawkinsinuria|SMPDB	0.031995945	0.027270645	0.129408983	0.89976401	0.9399608	-6.35945841
hetero after UVB irradiation VS hetero before UVB irradiation	Hawkinsinuria|SMPDB	-0.38264835	0.006355022	-1.73471603	0.11910451	0.31235808	-4.9577083
WT after UVB irradiation VS WT before UVB irradiation	Hawkinsinuria|SMPDB	0.126407683	0.040850924	0.512443468	0.61937892	0.87257466	-5.73795593
homo after UVB irradiation VS homo before UVB irradiation	HCN channels|Reactome	0.249498234	0.022470036	0.715523692	0.49169478	0.63295132	-6.10080747
hetero after UVB irradiation VS hetero before UVB irradiation	HCN channels|Reactome	0.047662796	0.081053733	0.14980995	0.88443869	0.95959808	-6.33438272
WT after UVB irradiation VS WT before UVB irradiation	HCN channels|Reactome	0.346387259	-0.04328278	1.279138245	0.22950312	0.62952955	-5.10574204
homo after UVB irradiation VS homo before UVB irradiation	HDAC6 interactions|Wikipathways	0.173653713	0.00785789	0.787714725	0.45029095	0.59890139	-6.04591
hetero after UVB irradiation VS hetero before UVB irradiation	HDAC6 interactions|Wikipathways	0.075722522	0.042587684	0.474398736	0.6472673	0.82239471	-6.22610051
WT after UVB irradiation VS WT before UVB irradiation	HDAC6 interactions|Wikipathways	0.125139231	-0.06335104	0.552906869	0.59237411	0.86350864	-5.71676305
homo after UVB irradiation VS homo before UVB irradiation	HDACs deacetylate histones|Reactome	-0.33413382	0.042057386	-1.6896777	0.12395273	0.26290983	-5.03464232
hetero after UVB irradiation VS hetero before UVB irradiation	HDACs deacetylate histones|Reactome	-0.15697199	-0.0015968	-0.6465086	0.53515972	0.74846824	-6.12540067
WT after UVB irradiation VS WT before UVB irradiation	HDACs deacetylate histones|Reactome	-0.17381593	0.036038986	-0.58236593	0.57311244	0.85592037	-5.70038641
homo after UVB irradiation VS homo before UVB irradiation	HDL assembly|Reactome	0.719363459	-0.01699996	3.874265848	0.00347471	0.03340106	-1.64163336
hetero after UVB irradiation VS hetero before UVB irradiation	HDL assembly|Reactome	0.619422387	0.050338164	1.888053694	0.09385018	0.27672034	-4.74339158
WT after UVB irradiation VS WT before UVB irradiation	HDL assembly|Reactome	0.574696413	-0.00428131	1.798462289	0.1020628	0.50394202	-4.46472191
homo after UVB irradiation VS homo before UVB irradiation	HDL clearance|Reactome	0.898088832	-0.05517017	4.470281639	0.00139899	0.02143814	-0.75320937
hetero after UVB irradiation VS hetero before UVB irradiation	HDL clearance|Reactome	0.515391686	0.096774308	1.997687619	0.07899872	0.25304019	-4.58593198
WT after UVB irradiation VS WT before UVB irradiation	HDL clearance|Reactome	0.667024589	0.036980856	2.1943858	0.05272913	0.41454814	-3.90904019
homo after UVB irradiation VS homo before UVB irradiation	HDL remodeling|Reactome	0.555462163	-0.0276645	2.924137559	0.01619367	0.07948172	-3.13972198
hetero after UVB irradiation VS hetero before UVB irradiation	HDL remodeling|Reactome	0.350207951	-0.01737475	2.016260486	0.07671708	0.24857939	-4.55895727
WT after UVB irradiation VS WT before UVB irradiation	HDL remodeling|Reactome	0.547062735	-0.04059963	2.418529732	0.0359667	0.38924151	-3.57991106
homo after UVB irradiation VS homo before UVB irradiation	HDMs demethylate histones|Reactome	-0.61035719	-0.01773741	-2.62153626	0.02681766	0.10874728	-3.6237521
hetero after UVB irradiation VS hetero before UVB irradiation	HDMs demethylate histones|Reactome	-0.34185765	-0.05860543	-1.54922269	0.15802038	0.36879513	-5.20554082
WT after UVB irradiation VS WT before UVB irradiation	HDMs demethylate histones|Reactome	-0.38511373	0.003931488	-1.52090047	0.15900759	0.56072663	-4.82321709
homo after UVB irradiation VS homo before UVB irradiation	HDR through Homologous Recombination (HR) or Single Strand Annealing (SSA)|Reactome	-0.50683361	-0.00373381	-2.66355577	0.02499988	0.10482042	-3.55675849
hetero after UVB irradiation VS hetero before UVB irradiation	HDR through Homologous Recombination (HR) or Single Strand Annealing (SSA)|Reactome	0.095150132	-0.00563522	0.378024756	0.71475833	0.85921866	-6.26968674
WT after UVB irradiation VS WT before UVB irradiation	HDR through Homologous Recombination (HR) or Single Strand Annealing (SSA)|Reactome	-0.01922531	0.004977173	-0.07206353	0.94396089	0.98295421	-5.86652109
homo after UVB irradiation VS homo before UVB irradiation	HDR through Homologous Recombination (HRR)|Reactome	-0.51105582	-0.02862355	-2.38804113	0.03960429	0.13161078	-3.99322702
hetero after UVB irradiation VS hetero before UVB irradiation	HDR through Homologous Recombination (HRR)|Reactome	0.067386843	-0.03350522	0.2585629	0.80217691	0.916937	-6.31042028
WT after UVB irradiation VS WT before UVB irradiation	HDR through Homologous Recombination (HRR)|Reactome	-0.03266985	0.009819124	-0.11578494	0.9100967	0.97566251	-5.86236455
homo after UVB irradiation VS homo before UVB irradiation	HDR through MMEJ (alt-NHEJ)|Reactome	-0.65766764	-0.01591049	-2.94702315	0.01558985	0.07717742	-3.10304755
hetero after UVB irradiation VS hetero before UVB irradiation	HDR through MMEJ (alt-NHEJ)|Reactome	-0.3060559	-0.07360107	-1.22232705	0.25467197	0.47937588	-5.60229299
WT after UVB irradiation VS WT before UVB irradiation	HDR through MMEJ (alt-NHEJ)|Reactome	-0.07830057	0.028242128	-0.24250594	0.81325013	0.94525798	-5.83945205
homo after UVB irradiation VS homo before UVB irradiation	HDR through Single Strand Annealing (SSA)|Reactome	-0.52161254	-0.04756458	-2.33784166	0.04305902	0.13447844	-4.07181318
hetero after UVB irradiation VS hetero before UVB irradiation	HDR through Single Strand Annealing (SSA)|Reactome	0.09209119	-0.05641051	0.351974704	0.73349846	0.86906316	-6.27983753
WT after UVB irradiation VS WT before UVB irradiation	HDR through Single Strand Annealing (SSA)|Reactome	-0.09199877	0.013732633	-0.30141229	0.76922509	0.93036308	-5.82334951
homo after UVB irradiation VS homo before UVB irradiation	Heart Development|Wikipathways	0.015273702	0.032108629	0.088894658	0.93103298	0.95961548	-6.36420417
hetero after UVB irradiation VS hetero before UVB irradiation	Heart Development|Wikipathways	-0.18316	0.018572944	-1.05711861	0.31982056	0.55029901	-5.77837802
WT after UVB irradiation VS WT before UVB irradiation	Heart Development|Wikipathways	-0.10434473	0.036660672	-0.45155858	0.66114504	0.87935805	-5.7669649
homo after UVB irradiation VS homo before UVB irradiation	Hedgehog _off_ state|Reactome	-0.08690097	-0.00335123	-0.471692	0.64791578	0.75613204	-6.25033573
hetero after UVB irradiation VS hetero before UVB irradiation	Hedgehog _off_ state|Reactome	0.029332536	0.024868779	0.176307092	0.86421681	0.94735456	-6.32971021
WT after UVB irradiation VS WT before UVB irradiation	Hedgehog _off_ state|Reactome	-0.22495755	-0.00123152	-1.12008905	0.28864821	0.67456398	-5.27295924
homo after UVB irradiation VS homo before UVB irradiation	Hedgehog _off_ state|Wikipathways	-0.67512922	0.075833611	-3.46014774	0.00671913	0.04735641	-2.28571685
hetero after UVB irradiation VS hetero before UVB irradiation	Hedgehog _off_ state|Wikipathways	-0.4644228	0.0261044	-1.52011145	0.165078	0.37848367	-5.24310829
WT after UVB irradiation VS WT before UVB irradiation	Hedgehog _off_ state|Wikipathways	-0.64890551	-0.00542435	-2.03806381	0.06864137	0.44205977	-4.13327125
homo after UVB irradiation VS homo before UVB irradiation	Hedgehog _on_ state|Reactome	-0.20067963	0.015266074	-1.14861767	0.27913334	0.43949063	-5.70645788
hetero after UVB irradiation VS hetero before UVB irradiation	Hedgehog _on_ state|Reactome	-0.08484485	0.01127521	-0.44192092	0.66966947	0.82741166	-6.24184052
WT after UVB irradiation VS WT before UVB irradiation	Hedgehog _on_ state|Reactome	-0.31523948	0.020079938	-1.40014138	0.19148394	0.60496032	-4.9682718
homo after UVB irradiation VS homo before UVB irradiation	Hedgehog _on_ state|Wikipathways	-0.84081282	0.050063158	-3.85049933	0.00360667	0.03395476	-1.67806895
hetero after UVB irradiation VS hetero before UVB irradiation	Hedgehog _on_ state|Wikipathways	-0.59858285	0.007485459	-2.11875213	0.06522746	0.22947406	-4.40873727
WT after UVB irradiation VS WT before UVB irradiation	Hedgehog _on_ state|Wikipathways	-0.54868398	0.081440599	-1.69566019	0.12055943	0.52608786	-4.60115335
homo after UVB irradiation VS homo before UVB irradiation	Hedgehog ligand biogenesis|Reactome	0.433157195	-0.00975857	2.25250962	0.04962206	0.14627134	-4.20448233
hetero after UVB irradiation VS hetero before UVB irradiation	Hedgehog ligand biogenesis|Reactome	0.416472179	0.035098703	2.28887061	0.04976047	0.19835162	-4.1551724
WT after UVB irradiation VS WT before UVB irradiation	Hedgehog ligand biogenesis|Reactome	0.241186419	0.077291693	1.173197575	0.26768787	0.66208473	-5.21893154
homo after UVB irradiation VS homo before UVB irradiation	Hedgehog signaling events mediated by Gli proteins|PID	-0.36616543	0.026786225	-2.08116296	0.06586561	0.1785881	-4.46660344
hetero after UVB irradiation VS hetero before UVB irradiation	Hedgehog signaling events mediated by Gli proteins|PID	-0.03244141	0.020708265	-0.15554481	0.88005386	0.95689063	-6.33343542
WT after UVB irradiation VS WT before UVB irradiation	Hedgehog signaling events mediated by Gli proteins|PID	-0.11322814	-0.00106237	-0.47551779	0.64455276	0.87766327	-5.75596569
homo after UVB irradiation VS homo before UVB irradiation	Hedgehog signaling pathway - Homo sapiens (human)|KEGG	-0.21126204	0.01081887	-1.12125503	0.29003613	0.44952646	-5.73568797
hetero after UVB irradiation VS hetero before UVB irradiation	Hedgehog signaling pathway - Homo sapiens (human)|KEGG	-0.01269987	-0.00494311	-0.0731458	0.94339679	0.97087437	-6.34363973
WT after UVB irradiation VS WT before UVB irradiation	Hedgehog signaling pathway - Homo sapiens (human)|KEGG	-0.16214085	0.02389886	-0.73831752	0.47715577	0.81525267	-5.60076819
homo after UVB irradiation VS homo before UVB irradiation	Hedgehog Signaling Pathway|Wikipathways	-0.38515636	0.050177847	-2.05482984	0.06877748	0.18375539	-4.50628901
homo after UVB irradiation VS homo before UVB irradiation	Hedgehog Signaling Pathway|Wikipathways	-0.17001476	0.007262644	-0.88921355	0.39609839	0.54888228	-5.96103101
hetero after UVB irradiation VS hetero before UVB irradiation	Hedgehog Signaling Pathway|Wikipathways	-0.26237294	-0.0462893	-1.20813772	0.25980646	0.48618872	-5.61812825
hetero after UVB irradiation VS hetero before UVB irradiation	Hedgehog Signaling Pathway|Wikipathways	0.013444829	-0.00180249	0.079167536	0.93874739	0.97054702	-6.34314251
WT after UVB irradiation VS WT before UVB irradiation	Hedgehog Signaling Pathway|Wikipathways	-0.3038758	0.008914666	-1.07712826	0.30652028	0.69163939	-5.31526861
WT after UVB irradiation VS WT before UVB irradiation	Hedgehog Signaling Pathway|Wikipathways	-0.13583532	0.016547601	-0.64394793	0.5339833	0.83689146	-5.66361489
homo after UVB irradiation VS homo before UVB irradiation	Hedgehog|INOH	-0.23043213	0.013920412	-0.99845764	0.34309256	0.49859207	-5.86013347
hetero after UVB irradiation VS hetero before UVB irradiation	Hedgehog|INOH	-0.07006087	0.023575041	-0.2712831	0.79270152	0.91162084	-6.30679512
WT after UVB irradiation VS WT before UVB irradiation	Hedgehog|INOH	-0.02245321	-0.01461789	-0.08250481	0.93586032	0.98056728	-5.86570403

homo after UVB irradiation VS homo before UVB irradiation	Hedgehog|NetPath	-0.27816877	0.038434008	-1.34864556	0.20907788	0.36695387	-5.47755287
hetero after UVB irradiation VS hetero before UVB irradiation	Hedgehog|NetPath	-0.16197297	-0.01214826	-0.69585607	0.5052561	0.72374522	-6.0913453
WT after UVB irradiation VS WT before UVB irradiation	Hedgehog|NetPath	-0.21230927	-0.0162391	-0.80889971	0.43724482	0.79685269	-5.54879227
homo after UVB irradiation VS homo before UVB irradiation	Hematopoietic cell lineage - Homo sapiens (human)|KEGG	0.238137101	-0.01917687	1.264913132	0.23639388	0.39783533	-5.57651075
hetero after UVB irradiation VS hetero before UVB irradiation	Hematopoietic cell lineage - Homo sapiens (human)|KEGG	0.082874255	-0.00611859	0.396133586	0.70184932	0.84922463	-6.26221838
WT after UVB irradiation VS WT before UVB irradiation	Hematopoietic cell lineage - Homo sapiens (human)|KEGG	0.229382461	-0.03035865	0.767922158	0.46014065	0.80891799	-5.57947552
homo after UVB irradiation VS homo before UVB irradiation	Hematopoietic Stem Cell Differentiation|Wikipathways	0.553320422	-0.01138397	3.431290941	0.00704028	0.0487059	-2.3312427
hetero after UVB irradiation VS hetero before UVB irradiation	Hematopoietic Stem Cell Differentiation|Wikipathways	0.599895162	0.021947869	3.611563199	0.00629573	0.08320066	-2.1531363
WT after UVB irradiation VS WT before UVB irradiation	Hematopoietic Stem Cell Differentiation|Wikipathways	0.548853636	0.062647886	2.423267549	0.03567562	0.38792988	-3.57288165
homo after UVB irradiation VS homo before UVB irradiation	Hematopoietic Stem Cell Gene Regulation by GABP alpha-beta Complex|Wikipathways	-0.48920821	0.010898093	-2.15536149	0.05828352	0.16457796	-4.35387842
hetero after UVB irradiation VS hetero before UVB irradiation	Hematopoietic Stem Cell Gene Regulation by GABP alpha-beta Complex|Wikipathways	-0.2036532	-0.09346718	-1.1019173	0.30097466	0.53131003	-5.73247515
WT after UVB irradiation VS WT before UVB irradiation	Hematopoietic Stem Cell Gene Regulation by GABP alpha-beta Complex|Wikipathways	-0.11715245	-0.00396854	-0.44603453	0.66499844	0.87935805	-5.76942373
homo after UVB irradiation VS homo before UVB irradiation	heme biosynthesis from uroporphyrinogen-III I|HumanCyc	1.000766319	-0.03828262	5.637335055	0.00027284	0.01666204	0.82632797
hetero after UVB irradiation VS hetero before UVB irradiation	heme biosynthesis from uroporphyrinogen-III I|HumanCyc	0.634558384	0.194201084	2.208504567	0.05655649	0.21216828	-4.27553051
WT after UVB irradiation VS WT before UVB irradiation	heme biosynthesis from uroporphyrinogen-III I|HumanCyc	0.693532404	-0.0247585	2.331556276	0.0417424	0.39022568	-3.7084795
homo after UVB irradiation VS homo before UVB irradiation	heme biosynthesis|HumanCyc	0.284190576	-0.0380209	1.264216523	0.23663298	0.39783533	-5.57731567
hetero after UVB irradiation VS hetero before UVB irradiation	heme biosynthesis|HumanCyc	0.084145905	0.004688598	0.347665803	0.7366169	0.87139887	-6.28144886
WT after UVB irradiation VS WT before UVB irradiation	heme biosynthesis|HumanCyc	0.145466888	-0.02245161	0.594610096	0.5652085	0.85125516	-5.69334719
homo after UVB irradiation VS homo before UVB irradiation	Heme biosynthesis|Reactome	0.081703037	-0.03600764	0.353101656	0.73181036	0.8234219	-6.30191006
hetero after UVB irradiation VS hetero before UVB irradiation	Heme biosynthesis|Reactome	0.197315223	-0.00439226	1.154828741	0.27985819	0.51023746	-5.67645859
WT after UVB irradiation VS WT before UVB irradiation	Heme biosynthesis|Reactome	0.147776553	0.004562412	0.578290081	0.57575689	0.8562298	-5.70269943
homo after UVB irradiation VS homo before UVB irradiation	Heme Biosynthesis|Wikipathways	0.284190576	-0.0380209	1.264216523	0.23663298	0.39783533	-5.57731567
hetero after UVB irradiation VS hetero before UVB irradiation	Heme Biosynthesis|Wikipathways	0.084145905	0.004688598	0.347665803	0.7366169	0.87139887	-6.28144886
WT after UVB irradiation VS WT before UVB irradiation	Heme Biosynthesis|Wikipathways	0.145466888	-0.02245161	0.594610096	0.5652085	0.85125516	-5.69334719
homo after UVB irradiation VS homo before UVB irradiation	heme degradation|HumanCyc	0.984438889	-0.06275736	4.760227748	0.00091516	0.01797515	-0.34027825
hetero after UVB irradiation VS hetero before UVB irradiation	heme degradation|HumanCyc	1.149420624	0.008198538	6.093193141	0.00023653	0.02988205	1.019810421
WT after UVB irradiation VS WT before UVB irradiation	heme degradation|HumanCyc	1.093623163	-0.05145194	5.152822133	0.0004186	0.15645713	0.241950761
homo after UVB irradiation VS homo before UVB irradiation	Heme degradation|Reactome	0.219682757	0.079545719	0.928274197	0.37651169	0.52945846	-5.92605995
hetero after UVB irradiation VS hetero before UVB irradiation	Heme degradation|Reactome	0.553415165	-0.00114883	2.87661354	0.01954096	0.1227581	-3.25952248
WT after UVB irradiation VS WT before UVB irradiation	Heme degradation|Reactome	0.51993955	-0.07049614	2.249037089	0.04805217	0.40119609	-3.82950106
homo after UVB irradiation VS homo before UVB irradiation	hemoglobins chaperone|BioCarta	-0.02597696	-0.02982262	-0.09088053	0.92949692	0.95889808	-6.36401251
hetero after UVB irradiation VS hetero before UVB irradiation	hemoglobins chaperone|BioCarta	0.045040627	0.003387509	0.201094938	0.8453942	0.9427648	-6.32465726
WT after UVB irradiation VS WT before UVB irradiation	hemoglobins chaperone|BioCarta	0.286539578	-0.04559624	1.183799998	0.26365117	0.6590487	-5.2079232
homo after UVB irradiation VS homo before UVB irradiation	Hemostasis|Reactome	0.382530761	-0.01435488	2.353454554	0.04195393	0.13402181	-4.04741126
hetero after UVB irradiation VS hetero before UVB irradiation	Hemostasis|Reactome	0.407241808	0.011630544	2.552461714	0.03268091	0.15685114	-3.75544119
WT after UVB irradiation VS WT before UVB irradiation	Hemostasis|Reactome	0.25999464	-0.01622014	1.252537568	0.23864735	0.63196069	-5.13481798
homo after UVB irradiation VS homo before UVB irradiation	heparan sulfate biosynthesis (late stages)|HumanCyc	0.375828743	-0.02434009	1.978160439	0.07797109	0.1965853	-4.62078425
hetero after UVB irradiation VS hetero before UVB irradiation	heparan sulfate biosynthesis (late stages)|HumanCyc	0.174482539	0.005346157	0.888661154	0.39884289	0.62665491	-5.93759396
WT after UVB irradiation VS WT before UVB irradiation	heparan sulfate biosynthesis (late stages)|HumanCyc	0.193281297	0.039085288	0.896337888	0.3909717	0.76436977	-5.47874478
homo after UVB irradiation VS homo before UVB irradiation	heparan sulfate biosynthesis|HumanCyc	0.324423008	-0.01653551	1.887321685	0.09036928	0.21571189	-4.75421115
hetero after UVB irradiation VS hetero before UVB irradiation	heparan sulfate biosynthesis|HumanCyc	0.265785818	0.020427515	1.464132004	0.17944187	0.39742277	-5.31422455
WT after UVB irradiation VS WT before UVB irradiation	heparan sulfate biosynthesis|HumanCyc	0.300546561	0.023665945	1.46241648	0.17408795	0.58503287	-4.89439308
homo after UVB irradiation VS homo before UVB irradiation	Heparan sulfate/heparin (HS-GAG) metabolism|Reactome	0.483117541	-0.0140621	2.896550486	0.01695333	0.08200096	-3.18393203
hetero after UVB irradiation VS hetero before UVB irradiation	Heparan sulfate/heparin (HS-GAG) metabolism|Reactome	0.351399545	0.015030191	1.718300877	0.12215502	0.31406061	-4.9801831
WT after UVB irradiation VS WT before UVB irradiation	Heparan sulfate/heparin (HS-GAG) metabolism|Reactome	0.3680089	0.018322625	1.634152301	0.13302506	0.54653478	-4.68081674
homo after UVB irradiation VS homo before UVB irradiation	Heparin Action Pathway|SMPDB	0.542957634	0.032474294	3.216401628	0.00999373	0.05951688	-2.67225071
hetero after UVB irradiation VS hetero before UVB irradiation	Heparin Action Pathway|SMPDB	0.507034173	0.109310797	2.703272449	0.02570936	0.14017854	-3.52484947
WT after UVB irradiation VS WT before UVB irradiation	Heparin Action Pathway|SMPDB	0.380045687	0.058400764	1.387851186	0.19508646	0.60625027	-4.98260907
homo after UVB irradiation VS homo before UVB irradiation	Hepatitis B - Homo sapiens (human)|KEGG	0.398776646	-0.02145582	2.298933807	0.04593875	0.13974439	-4.13245699
hetero after UVB irradiation VS hetero before UVB irradiation	Hepatitis B - Homo sapiens (human)|KEGG	0.54720442	0.013564255	3.520630507	0.00721857	0.08681326	-2.28725519
WT after UVB irradiation VS WT before UVB irradiation	Hepatitis B - Homo sapiens (human)|KEGG	0.446423557	-0.01847514	2.274635242	0.04600189	0.39565089	-3.79207121
homo after UVB irradiation VS homo before UVB irradiation	Hepatitis C - Homo sapiens (human)|KEGG	0.327756631	-0.01727174	1.819196869	0.10085453	0.23103646	-4.85250532
hetero after UVB irradiation VS hetero before UVB irradiation	Hepatitis C - Homo sapiens (human)|KEGG	0.4258791	0.015869672	2.753510407	0.02373989	0.13628011	-3.44793597
WT after UVB irradiation VS WT before UVB irradiation	Hepatitis C - Homo sapiens (human)|KEGG	0.324679341	0.004027022	1.637850019	0.13224426	0.54612111	-4.6760718
homo after UVB irradiation VS homo before UVB irradiation	Hepatitis C and Hepatocellular Carcinoma|Wikipathways	0.275407595	-0.008809	1.343984196	0.21052567	0.36858136	-5.48317428
hetero after UVB irradiation VS hetero before UVB irradiation	Hepatitis C and Hepatocellular Carcinoma|Wikipathways	0.495412824	0.023834711	2.91497151	0.01839502	0.11936114	-3.2008762
WT after UVB irradiation VS WT before UVB irradiation	Hepatitis C and Hepatocellular Carcinoma|Wikipathways	0.370218583	0.003592817	1.814412826	0.0994374	0.49849683	-4.44321134
homo after UVB irradiation VS homo before UVB irradiation	Hepatocellular carcinoma - Homo sapiens (human)|KEGG	-0.03006487	-0.01486335	-0.18279878	0.8588423	0.9134577	-6.35052675
hetero after UVB irradiation VS hetero before UVB irradiation	Hepatocellular carcinoma - Homo sapiens (human)|KEGG	0.02783139	0.00239514	0.167969369	0.87056932	0.95129447	-6.33126181
WT after UVB irradiation VS WT before UVB irradiation	Hepatocellular carcinoma - Homo sapiens (human)|KEGG	0.007244955	0.047528676	0.035617514	0.97228267	0.99045333	-5.86850856
homo after UVB irradiation VS homo before UVB irradiation	Hereditary Coproporphyria (HCP)|SMPDB	0.388154593	-0.02664649	1.768839848	0.10931886	0.24265581	-4.92409481
hetero after UVB irradiation VS hetero before UVB irradiation	Hereditary Coproporphyria (HCP)|SMPDB	0.451496919	0.003377552	2.70023395	0.02583369	0.14017854	-3.52950073
WT after UVB irradiation VS WT before UVB irradiation	Hereditary Coproporphyria (HCP)|SMPDB	0.381835405	-0.00693641	1.754704281	0.10959456	0.5076251	-4.52327279
homo after UVB irradiation VS homo before UVB irradiation	Hereditary leiomyomatosis and renal cell carcinoma pathway|Wikipathways	0.465722106	-0.03715022	2.175563918	0.05636947	0.16165213	-4.32297013
hetero after UVB irradiation VS hetero before UVB irradiation	Hereditary leiomyomatosis and renal cell carcinoma pathway|Wikipathways	0.51121528	0.007550266	2.418040694	0.04049483	0.1767964	-3.96004829
WT after UVB irradiation VS WT before UVB irradiation	Hereditary leiomyomatosis and renal cell carcinoma pathway|Wikipathways	0.315169971	-0.02050045	1.503910194	0.163267	0.56749503	-4.84406768
homo after UVB irradiation VS homo before UVB irradiation	Heroin Action Pathway|SMPDB	0.142319268	0.02212538	0.89862111	0.39131639	0.5444091	-5.95272314
hetero after UVB irradiation VS hetero before UVB irradiation	Heroin Action Pathway|SMPDB	-0.17860343	0.042804681	-1.01191792	0.33975003	0.56880321	-5.82322188
WT after UVB irradiation VS WT before UVB irradiation	Heroin Action Pathway|SMPDB	-0.06741597	0.040611317	-0.29018869	0.77755278	0.93502208	-5.82668203
homo after UVB irradiation VS homo before UVB irradiation	Heroin Metabolism Pathway|SMPDB	-0.3836974	-0.00584462	-1.09942613	0.29897139	0.45968162	-5.7586239
hetero after UVB irradiation VS hetero before UVB irradiation	Heroin Metabolism Pathway|SMPDB	-0.58800065	0.102774769	-2.81255124	0.02162133	0.13052983	-3.35754736
WT after UVB irradiation VS WT before UVB irradiation	Heroin Metabolism Pathway|SMPDB	-1.10619862	0.164449001	-5.52026004	0.00024711	0.15645713	0.667403205
homo after UVB irradiation VS homo before UVB irradiation	Heroin metabolism|Wikipathways	-0.3836974	-0.00584462	-1.09942613	0.29897139	0.45968162	-5.7586239
hetero after UVB irradiation VS hetero before UVB irradiation	Heroin metabolism|Wikipathways	-0.84120552	0.012857729	-3.62989129	0.00612532	0.0822456	-2.12622559
WT after UVB irradiation VS WT before UVB irradiation	Heroin metabolism|Wikipathways	-0.97471547	0.059619429	-3.65302777	0.00438049	0.20968398	-1.74336031
homo after UVB irradiation VS homo before UVB irradiation	Herpes simplex infection - Homo sapiens (human)|KEGG	0.06016616	-0.01962779	0.352279906	0.73240663	0.82388979	-6.30221732
hetero after UVB irradiation VS hetero before UVB irradiation	Herpes simplex infection - Homo sapiens (human)|KEGG	0.244096834	0.00938074	1.537994461	0.16070989	0.37252774	-5.22007669
WT after UVB irradiation VS WT before UVB irradiation	Herpes simplex infection - Homo sapiens (human)|KEGG	0.206295706	-0.00809342	1.040672686	0.32233979	0.70648859	-5.3501617
homo after UVB irradiation VS homo before UVB irradiation	Hfe effect on hepcidin production|Wikipathways	-0.05929626	-0.0325373	-0.22581728	0.82618266	0.89312082	-6.34112542
hetero after UVB irradiation VS hetero before UVB irradiation	Hfe effect on hepcidin production|Wikipathways	-0.25703047	-0.033755	-0.84047698	0.42384733	0.65090839	-5.97899728
WT after UVB irradiation VS WT before UVB irradiation	Hfe effect on hepcidin production|Wikipathways	-0.73393722	0.013360238	-3.54700591	0.00522794	0.20968398	-1.89710194
homo after UVB irradiation VS homo before UVB irradiation	HGF|INOH	0.331477206	-0.03253471	1.847911315	0.09630401	0.22377818	-4.81127041
hetero after UVB irradiation VS hetero before UVB irradiation	HGF|INOH	0.541972751	0.017964103	3.567044712	0.00673088	0.08522036	-2.21867384
WT after UVB irradiation VS WT before UVB irradiation	HGF|INOH	0.458191956	0.039073659	2.169573547	0.05499442	0.41769317	-3.94497189
homo after UVB irradiation VS homo before UVB irradiation	Hh mutants abrogate ligand secretion|Reactome	0.353144435	-0.05913315	1.411194223	0.19045252	0.34647455	-5.40089286
hetero after UVB irradiation VS hetero before UVB irradiation	Hh mutants abrogate ligand secretion|Reactome	0.626706694	0.079331994	3.718404706	0.00536846	0.07849967	-1.99686405
WT after UVB irradiation VS WT before UVB irradiation	Hh mutants abrogate ligand secretion|Reactome	0.014138139	0.06383666	0.055646796	0.9567105	0.98742794	-5.86758274
homo after UVB irradiation VS homo before UVB irradiation	Hh mutants that don_t undergo autocatalytic processing are degraded by ERAD|Reactome	0.353144435	-0.05913315	1.411194223	0.19045252	0.34647455	-5.40089286
hetero after UVB irradiation VS hetero before UVB irradiation	Hh mutants that don_t undergo autocatalytic processing are degraded by ERAD|Reactome	0.626706694	0.079331994	3.718404706	0.00536846	0.07849967	-1.99686405
WT after UVB irradiation VS WT before UVB irradiation	Hh mutants that don_t undergo autocatalytic processing are degraded by ERAD|Reactome	0.014138139	0.06383666	0.055646796	0.9567105	0.98742794	-5.86758274
homo after UVB irradiation VS homo before UVB irradiation	HH-Core|Signalink	-0.23366376	-0.01664554	-1.17616375	0.26848815	0.43225104	-5.67650275
hetero after UVB irradiation VS hetero before UVB irradiation	HH-Core|Signalink	-0.08311326	0.042303005	-0.51464503	0.62002594	0.80281275	-6.20513461
WT after UVB irradiation VS WT before UVB irradiation	HH-Core|Signalink	-0.1865836	0.025728068	-0.96606356	0.35661325	0.74232121	-5.41858083
homo after UVB irradiation VS homo before UVB irradiation	HH-Ncore|Signalink	-0.17818096	0.00107138	-1.1311463	0.28605686	0.44628615	-5.72518275
hetero after UVB irradiation VS hetero before UVB irradiation	HH-Ncore|Signalink	-0.33777539	-0.00502908	-1.68602088	0.12836539	0.3229899	-5.02408967
WT after UVB irradiation VS WT before UVB irradiation	HH-Ncore|Signalink	0.134293208	0.010583113	0.671026488	0.51728095	0.82895075	-5.64637599
homo after UVB irradiation VS homo before UVB irradiation	HIF-1 signaling pathway - Homo sapiens (human)|KEGG	0.424835883	-0.00996651	2.409567939	0.03820788	0.12862933	-3.95941841
hetero after UVB irradiation VS hetero before UVB irradiation	HIF-1 signaling pathway - Homo sapiens (human)|KEGG	0.285923148	0.022331995	1.55645321	0.15630987	0.36611531	-5.19615012
WT after UVB irradiation VS WT before UVB irradiation	HIF-1 signaling pathway - Homo sapiens (human)|KEGG	0.181538967	-0.02252305	0.75759927	0.46602883	0.80910175	-5.58698412
homo after UVB irradiation VS homo before UVB irradiation	HIF1A and PPARG regulation of glycolysis|Wikipathways	1.005520695	0.032811448	5.162235737	0.00051842	0.01666204	0.209954062
hetero after UVB irradiation VS hetero before UVB irradiation	HIF1A and PPARG regulation of glycolysis|Wikipathways	0.797598595	0.051442875	3.878467753	0.00424073	0.07132558	-1.76558725
WT after UVB irradiation VS WT before UVB irradiation	HIF1A and PPARG regulation of glycolysis|Wikipathways	0.693526766	0.063332259	2.241220869	0.04869564	0.40361703	-3.84090856
homo after UVB irradiation VS homo before UVB irradiation	HIF-1-alpha transcription factor network|PID	0.378194767	-0.00276373	1.98653399	0.0769129	0.19626432	-4.60835925
hetero after UVB irradiation VS hetero before UVB irradiation	HIF-1-alpha transcription factor network|PID	0.359675274	0.014096264	1.797628332	0.1080569	0.29786212	-4.87069758
WT after UVB irradiation VS WT before UVB irradiation	HIF-1-alpha transcription factor network|PID	0.202787574	0.005405678	0.860172501	0.40968186	0.7766779	-5.50846044
homo after UVB irradiation VS homo before UVB irradiation	HIF-2-alpha transcription factor network|PID	0.344621999	-0.0376504	1.988544818	0.07666082	0.19626432	-4.6053725
hetero after UVB irradiation VS hetero before UVB irradiation	HIF-2-alpha transcription factor network|PID	0.377433063	0.081695753	1.950208459	0.08513159	0.26414552	-4.65450841
WT after UVB irradiation VS WT before UVB irradiation	HIF-2-alpha transcription factor network|PID	0.225054882	0.004675114	0.952100016	0.36331331	0.74528402	-5.43092707
homo after UVB irradiation VS homo before UVB irradiation	Highly calcium permeable nicotinic acetylcholine receptors|Reactome	0.05719494	-0.02255186	0.187313433	0.85540031	0.91256024	-6.3496323
hetero after UVB irradiation VS hetero before UVB irradiation	Highly calcium permeable nicotinic acetylcholine receptors|Reactome	-0.55926556	0.054110339	-3.52498002	0.00717132	0.08667895	-2.28081764
WT after UVB irradiation VS WT before UVB irradiation	Highly calcium permeable nicotinic acetylcholine receptors|Reactome	-0.88414615	-0.01965496	-4.40974578	0.00128927	0.15645713	-0.69357569
homo after UVB irradiation VS homo before UVB irradiation	Highly calcium permeable postsynaptic nicotinic acetylcholine receptors|Reactome	-0.07984634	-0.01796203	-0.30381561	0.76789369	0.8497825	-6.31910193
hetero after UVB irradiation VS hetero before UVB irradiation	Highly calcium permeable postsynaptic nicotinic acetylcholine receptors|Reactome	-0.53948236	0.019065514	-3.33596683	0.00956022	0.0985871	-2.5624869
WT after UVB irradiation VS WT before UVB irradiation	Highly calcium permeable postsynaptic nicotinic acetylcholine receptors|Reactome	-0.748664	-0.00456409	-3.7429375	0.00377402	0.20870715	-1.61411519
homo after UVB irradiation VS homo before UVB irradiation	Highly sodium permeable acetylcholine nicotinic receptors|Reactome	0.133050256	0.029431798	0.7023091	0.4995258	0.63924743	-6.11034827
hetero after UVB irradiation VS hetero before UVB irradiation	Highly sodium permeable acetylcholine nicotinic receptors|Reactome	-0.5712227	0.062243011	-3.19593053	0.01186175	0.10542662	-2.77343062
WT after UVB irradiation VS WT before UVB irradiation	Highly sodium permeable acetylcholine nicotinic receptors|Reactome	-0.77217324	-0.0021988	-3.3724485	0.00701215	0.22552445	-2.15305713
homo after UVB irradiation VS homo before UVB irradiation	Hippo signaling pathway - Homo sapiens (human)|KEGG	-0.2180489	0.008372445	-1.21058909	0.25564426	0.41912808	-5.6383371
hetero after UVB irradiation VS hetero before UVB irradiation	Hippo signaling pathway - Homo sapiens (human)|KEGG	-0.20193138	-0.01169945	-1.20569164	0.26070012	0.48679686	-5.62084512
WT after UVB irradiation VS WT before UVB irradiation	Hippo signaling pathway - Homo sapiens (human)|KEGG	-0.31124845	0.005622271	-1.41394101	0.1875064	0.59994616	-4.9520768
homo after UVB irradiation VS homo before UVB irradiation	Hippo signaling pathway - multiple species - Homo sapiens (human)|KEGG	-0.11717965	0.005224639	-0.53783699	0.60320267	0.72232452	-6.21543603
hetero after UVB irradiation VS hetero before UVB irradiation	Hippo signaling pathway - multiple species - Homo sapiens (human)|KEGG	-0.01190758	-0.01912961	-0.07183396	0.94440998	0.97125918	-6.34374284
WT after UVB irradiation VS WT before UVB irradiation	Hippo signaling pathway - multiple species - Homo sapiens (human)|KEGG	-0.18585595	-0.00650557	-0.94508542	0.36671321	0.74581032	-5.43707353
homo after UVB irradiation VS homo before UVB irradiation	histamine biosynthesis|HumanCyc	1.073641484	-0.01457919	4.80934	0.00085271	0.01759873	-0.27165612
hetero after UVB irradiation VS hetero before UVB irradiation	histamine biosynthesis|HumanCyc	1.053433534	0.031488727	5.063306436	0.00083019	0.03638465	-0.17607954
WT after UVB irradiation VS WT before UVB irradiation	histamine biosynthesis|HumanCyc	0.641454666	-0.00792022	1.846636165	0.0943221	0.49001597	-4.39949278
homo after UVB irradiation VS homo before UVB irradiation	histamine degradation|HumanCyc	0.014260273	0.004956421	0.037425195	0.97092966	0.98205598	-6.36769575
hetero after UVB irradiation VS hetero before UVB irradiation	histamine degradation|HumanCyc	-0.05880966	-0.02001537	-0.20741349	0.84061239	0.93932613	-6.32326409
WT after UVB irradiation VS WT before UVB irradiation	histamine degradation|HumanCyc	-0.0373131	-0.12043038	-0.11910226	0.90753407	0.97566251	-5.8619704
homo after UVB irradiation VS homo before UVB irradiation	Histamine receptors|Reactome	0.232486557	0.032989747	0.784857263	0.45188505	0.6002769	-6.04817097
hetero after UVB irradiation VS hetero before UVB irradiation	Histamine receptors|Reactome	0.086363003	-0.09772409	0.258438728	0.80226958	0.916937	-6.31045482
WT after UVB irradiation VS WT before UVB irradiation	Histamine receptors|Reactome	-0.0444743	-0.04571872	-0.13202277	0.89756385	0.97566251	-5.86032944
homo after UVB irradiation VS homo before UVB irradiation	Histidine catabolism|Reactome	0.707352498	-0.00135708	3.666377681	0.00482583	0.03957638	-1.9626361
hetero after UVB irradiation VS hetero before UVB irradiation	Histidine catabolism|Reactome	-0.00182558	-0.03885418	-0.00936828	0.99274333	0.99536386	-6.34649369
WT after UVB irradiation VS WT before UVB irradiation	Histidine catabolism|Reactome	0.063896695	-0.08149334	0.204618242	0.8419421	0.95176969	-5.84798807
homo after UVB irradiation VS homo before UVB irradiation	histidine degradation|HumanCyc	0.038667577	-0.01085948	0.154478174	0.88050249	0.92763599	-6.35564298
hetero after UVB irradiation VS hetero before UVB irradiation	histidine degradation|HumanCyc	-0.33447181	-0.02095959	-1.60551537	0.14513688	0.34927871	-5.13182158
WT after UVB irradiation VS WT before UVB irradiation	histidine degradation|HumanCyc	0.133265113	-0.11006881	0.538622419	0.60183616	0.86843152	-5.72441745
homo after UVB irradiation VS homo before UVB irradiation	Histidine degradation|INOH	0.261276476	-0.00109115	1.368041479	0.20314363	0.36035501	-5.45402403
hetero after UVB irradiation VS hetero before UVB irradiation	Histidine degradation|INOH	0.019347878	0.033749327	0.093978582	0.92732266	0.97054702	-6.34175257
WT after UVB irradiation VS WT before UVB irradiation	Histidine degradation|INOH	0.169230982	-0.02643511	0.772119627	0.45776021	0.80891799	-5.5763968
homo after UVB irradiation VS homo before UVB irradiation	Histidine metabolism - Homo sapiens (human)|KEGG	0.400561977	-0.00161442	2.000453248	0.07518396	0.19618193	-4.58766088
hetero after UVB irradiation VS hetero before UVB irradiation	Histidine metabolism - Homo sapiens (human)|KEGG	-0.10537612	0.014509668	-0.53949263	0.60350758	0.79129581	-6.19139252
WT after UVB irradiation VS WT before UVB irradiation	Histidine metabolism - Homo sapiens (human)|KEGG	0.114763164	-0.05385371	0.461703872	0.65409502	0.87935805	-5.76237367
homo after UVB irradiation VS homo before UVB irradiation	Histidine metabolism|EHMN	0.003289319	-0.00373383	0.018452555	0.98566402	0.99049643	-6.36826532
hetero after UVB irradiation VS hetero before UVB irradiation	Histidine metabolism|EHMN	-0.1925764	0.005599011	-0.7886449	0.45193578	0.67696625	-6.02135885
WT after UVB irradiation VS WT before UVB irradiation	Histidine metabolism|EHMN	-0.19867606	-0.03559102	-0.92757528	0.37529984	0.75077508	-5.4522528
homo after UVB irradiation VS homo before UVB irradiation	Histidine Metabolism|SMPDB	0.497960703	-0.02512164	2.567478868	0.02935242	0.11431817	-3.70975629

hetero after UVB irradiation VS hetero before UVB irradiation	Histidine Metabolism|SMPDB	-0.13494541	0.02003813	-0.59758015	0.56583037	0.76437184	-6.15692762
WT after UVB irradiation VS WT before UVB irradiation	Histidine Metabolism|SMPDB	0.06579821	-0.06804772	0.271218891	0.79169479	0.9410906	-5.83203202
homo after UVB irradiation VS homo before UVB irradiation	Histidine_ lysine_ phenylalanine_ tyrosine_ proline and tryptophan catabolism|Reactome	0.167129724	-0.00716703	0.809125701	0.4384649	0.59004737	-6.02874125
hetero after UVB irradiation VS hetero before UVB irradiation	Histidine_ lysine_ phenylalanine_ tyrosine_ proline and tryptophan catabolism|Reactome	-0.24890634	0.012337026	-1.48185281	0.17477967	0.39185722	-5.29187609
WT after UVB irradiation VS WT before UVB irradiation	Histidine_ lysine_ phenylalanine_ tyrosine_ proline and tryptophan catabolism|Reactome	0.008899802	-0.0446405	0.03456215	0.97310358	0.99045333	-5.86854608
homo after UVB irradiation VS homo before UVB irradiation	Histidinemia|SMPDB	0.497960703	-0.02512164	2.567478868	0.02935242	0.11431817	-3.70975629
hetero after UVB irradiation VS hetero before UVB irradiation	Histidinemia|SMPDB	-0.13494541	0.02003813	-0.59758015	0.56583037	0.76437184	-6.15692762
WT after UVB irradiation VS WT before UVB irradiation	Histidinemia|SMPDB	0.06579821	-0.06804772	0.271218891	0.79169479	0.9410906	-5.83203202
homo after UVB irradiation VS homo before UVB irradiation	Histone Modifications|Wikipathways	-0.53991594	0.022573069	-2.28841939	0.04674882	0.14080151	-4.1488026
hetero after UVB irradiation VS hetero before UVB irradiation	Histone Modifications|Wikipathways	-0.46857411	-0.07401013	-2.24233946	0.05359036	0.20578337	-4.2249716
WT after UVB irradiation VS WT before UVB irradiation	Histone Modifications|Wikipathways	-0.39612969	-0.00742112	-1.46786943	0.17263145	0.58142171	-4.88782862
homo after UVB irradiation VS homo before UVB irradiation	HIV elongation arrest and recovery|Reactome	-0.59337696	0.013820482	-2.91653162	0.01639958	0.08000053	-3.15191098
hetero after UVB irradiation VS hetero before UVB irradiation	HIV elongation arrest and recovery|Reactome	-0.30796546	-0.0389977	-1.31604362	0.22283332	0.44410029	-5.49459081
WT after UVB irradiation VS WT before UVB irradiation	HIV elongation arrest and recovery|Reactome	-0.19369301	0.012329594	-0.59982097	0.56186317	0.85017866	-5.69031027
homo after UVB irradiation VS homo before UVB irradiation	HIV Infection|Reactome	-0.35016569	0.013917863	-1.88179858	0.09117989	0.21616167	-4.76223915
hetero after UVB irradiation VS hetero before UVB irradiation	HIV Infection|Reactome	-0.02070996	-0.03030967	-0.10308644	0.92030581	0.97054702	-6.34078004
WT after UVB irradiation VS WT before UVB irradiation	HIV Infection|Reactome	0.016603637	0.001587911	0.061662678	0.95203674	0.98717655	-5.86722536
homo after UVB irradiation VS homo before UVB irradiation	HIV Life Cycle|Reactome	-0.4737693	0.017251019	-2.53765618	0.03085191	0.11700151	-3.75710156
hetero after UVB irradiation VS hetero before UVB irradiation	HIV Life Cycle|Reactome	-0.13884267	-0.0365762	-0.62164892	0.55061819	0.75605031	-6.14170082
WT after UVB irradiation VS WT before UVB irradiation	HIV Life Cycle|Reactome	-0.108481	-0.00479606	-0.37519837	0.71528545	0.90515618	-5.7983676
homo after UVB irradiation VS homo before UVB irradiation	HIV Transcription Elongation|Reactome	-0.66651563	0.024864965	-3.59445591	0.00541328	0.04239626	-2.07482703
hetero after UVB irradiation VS hetero before UVB irradiation	HIV Transcription Elongation|Reactome	-0.30434514	-0.05206909	-1.35335126	0.2111292	0.43289558	-5.45027621
WT after UVB irradiation VS WT before UVB irradiation	HIV Transcription Elongation|Reactome	-0.21592557	-0.00811919	-0.68432856	0.50919158	0.82580894	-5.63767152
homo after UVB irradiation VS homo before UVB irradiation	HIV Transcription Initiation|Reactome	-0.70093034	0.029785556	-3.66498681	0.00483654	0.03957638	-1.96480049
hetero after UVB irradiation VS hetero before UVB irradiation	HIV Transcription Initiation|Reactome	-0.40314601	-0.06740223	-1.89602939	0.09268569	0.27457285	-4.73204572
WT after UVB irradiation VS WT before UVB irradiation	HIV Transcription Initiation|Reactome	-0.27178022	-0.03117844	-0.94159333	0.36841429	0.74581032	-5.44011948
homo after UVB irradiation VS homo before UVB irradiation	hiv-1 defeats host-mediated resistance by cem15|BioCarta	0.792061249	-0.1367704	3.596708485	0.00539379	0.04239626	-2.07130504
hetero after UVB irradiation VS hetero before UVB irradiation	hiv-1 defeats host-mediated resistance by cem15|BioCarta	1.136242504	0.027972413	7.484961918	5.34E-05	0.02920766	2.382415912
WT after UVB irradiation VS WT before UVB irradiation	hiv-1 defeats host-mediated resistance by cem15|BioCarta	0.583562165	-0.06987919	1.826738156	0.09745137	0.49506164	-4.42653013
homo after UVB irradiation VS homo before UVB irradiation	hiv-1 nef: negative effector of fas and tnf|BioCarta	0.201329642	-0.01183104	1.107938919	0.29546163	0.45577992	-5.7497202
hetero after UVB irradiation VS hetero before UVB irradiation	hiv-1 nef: negative effector of fas and tnf|BioCarta	0.248996969	-0.0363796	1.314258976	0.22340676	0.44447315	-5.4966907
WT after UVB irradiation VS WT before UVB irradiation	hiv-1 nef: negative effector of fas and tnf|BioCarta	0.237145523	-0.04550376	1.173003227	0.26776232	0.66208473	-5.21913264
homo after UVB irradiation VS homo before UVB irradiation	HIV-1 Nef: Negative effector of Fas and TNF-alpha|PID	0.399708396	-0.02645165	2.177933458	0.05614899	0.16114161	-4.31933917
hetero after UVB irradiation VS hetero before UVB irradiation	HIV-1 Nef: Negative effector of Fas and TNF-alpha|PID	0.375088262	-0.03322214	1.861258072	0.09786482	0.28215302	-4.78137515
WT after UVB irradiation VS WT before UVB irradiation	HIV-1 Nef: Negative effector of Fas and TNF-alpha|PID	0.360007744	-0.05575871	1.695412765	0.1206074	0.52608786	-4.60147689
homo after UVB irradiation VS homo before UVB irradiation	homocarnosine biosynthesis|HumanCyc	0.572978794	-0.00791087	1.537965645	0.15704513	0.30448751	-5.23890841
hetero after UVB irradiation VS hetero before UVB irradiation	homocarnosine biosynthesis|HumanCyc	-0.72860899	-0.00173902	-2.13490347	0.06357655	0.22631041	-4.38487138
WT after UVB irradiation VS WT before UVB irradiation	homocarnosine biosynthesis|HumanCyc	-0.38261699	-0.06203479	-0.92180557	0.37816038	0.75122805	-5.45720293
homo after UVB irradiation VS homo before UVB irradiation	Homocarnosinosis|SMPDB	-0.24547682	-0.00810915	-1.40068944	0.19347783	0.34903982	-5.41392471
hetero after UVB irradiation VS hetero before UVB irradiation	Homocarnosinosis|SMPDB	-0.17636771	-0.04343396	-1.04504584	0.32505313	0.55421891	-5.79050219
WT after UVB irradiation VS WT before UVB irradiation	Homocarnosinosis|SMPDB	-0.06328445	0.007258138	-0.28249238	0.78328047	0.93661609	-5.82889545
homo after UVB irradiation VS homo before UVB irradiation	Homocysteine Degradation|SMPDB	-1.03096392	0.009185618	-5.50480244	0.0003253	0.01666204	0.658072793
hetero after UVB irradiation VS hetero before UVB irradiation	Homocysteine Degradation|SMPDB	-0.67635827	-0.13184633	-2.08448047	0.06887001	0.23513821	-4.45921201
WT after UVB irradiation VS WT before UVB irradiation	Homocysteine Degradation|SMPDB	-1.00471126	-0.08504096	-3.60367118	0.00475571	0.20968398	-1.81475856
homo after UVB irradiation VS homo before UVB irradiation	Homocystinuria_ cystathionine beta-synthase deficiency|SMPDB	-1.03096392	0.009185618	-5.50480244	0.0003253	0.01666204	0.658072793
hetero after UVB irradiation VS hetero before UVB irradiation	Homocystinuria_ cystathionine beta-synthase deficiency|SMPDB	-0.67635827	-0.13184633	-2.08448047	0.06887001	0.23513821	-4.45921201
WT after UVB irradiation VS WT before UVB irradiation	Homocystinuria_ cystathionine beta-synthase deficiency|SMPDB	-1.00471126	-0.08504096	-3.60367118	0.00475571	0.20968398	-1.81475856
homo after UVB irradiation VS homo before UVB irradiation	Homocystinuria-megaloblastic anemia due to defect in cobalamin metabolism_ cblG complementation type|SMPDB	-0.09396972	-0.02692441	-0.3795694	0.71270801	0.81002335	-6.29164178
hetero after UVB irradiation VS hetero before UVB irradiation	Homocystinuria-megaloblastic anemia due to defect in cobalamin metabolism_ cblG complementation type|SMPDB	0.075091809	-0.02022482	0.352613458	0.73303662	0.86896773	-6.27959702
WT after UVB irradiation VS WT before UVB irradiation	Homocystinuria-megaloblastic anemia due to defect in cobalamin metabolism_ cblG complementation type|SMPDB	0.176060787	0.019583595	0.866300336	0.40646895	0.7725109	-5.50349844
homo after UVB irradiation VS homo before UVB irradiation	Homologous DNA Pairing and Strand Exchange|Reactome	-0.63342609	-0.04144269	-2.69524296	0.02371141	0.10146796	-3.50616954
hetero after UVB irradiation VS hetero before UVB irradiation	Homologous DNA Pairing and Strand Exchange|Reactome	-0.1026194	-0.06603928	-0.37463007	0.71718923	0.86115609	-6.27104923
WT after UVB irradiation VS WT before UVB irradiation	Homologous DNA Pairing and Strand Exchange|Reactome	-0.11484835	0.006236711	-0.39905028	0.6981776	0.89869704	-5.78916
homo after UVB irradiation VS homo before UVB irradiation	Homologous recombination - Homo sapiens (human)|KEGG	-0.49330916	-0.0293085	-2.18384438	0.05560264	0.15977449	-4.31027643
hetero after UVB irradiation VS hetero before UVB irradiation	Homologous recombination - Homo sapiens (human)|KEGG	-0.04111259	-0.04021254	-0.1565174	0.87931066	0.9565389	-6.33327126
WT after UVB irradiation VS WT before UVB irradiation	Homologous recombination - Homo sapiens (human)|KEGG	0.094169977	-0.00884426	0.386479652	0.7071726	0.9016352	-5.79408114
homo after UVB irradiation VS homo before UVB irradiation	Homologous recombination|Wikipathways	-0.87037803	0.017075686	-4.71072126	0.0009831	0.0182528	-0.40984219
hetero after UVB irradiation VS hetero before UVB irradiation	Homologous recombination|Wikipathways	0.164372956	-0.03749069	0.522824301	0.61456265	0.79997547	-6.20067788
WT after UVB irradiation VS WT before UVB irradiation	Homologous recombination|Wikipathways	-0.08513406	0.024563407	-0.26931272	0.79312033	0.94204956	-5.83254994
homo after UVB irradiation VS homo before UVB irradiation	Homology Directed Repair|Reactome	-0.52382545	-0.00343953	-2.81866878	0.01929958	0.08847577	-3.30871403
hetero after UVB irradiation VS hetero before UVB irradiation	Homology Directed Repair|Reactome	0.079787341	-0.00898055	0.318262731	0.75803136	0.88797403	-6.29192876
WT after UVB irradiation VS WT before UVB irradiation	Homology Directed Repair|Reactome	-0.02629617	0.008718464	-0.09786034	0.92396138	0.97566251	-5.86430218
homo after UVB irradiation VS homo before UVB irradiation	hop pathway in cardiac development|BioCarta	-0.52765188	0.136423411	-1.84177535	0.09726007	0.22539923	-4.82010631
hetero after UVB irradiation VS hetero before UVB irradiation	hop pathway in cardiac development|BioCarta	-0.61787978	-0.04537881	-2.22305743	0.05526141	0.20875475	-4.25380563
WT after UVB irradiation VS WT before UVB irradiation	hop pathway in cardiac development|BioCarta	-0.5172326	0.048785167	-1.42311781	0.18490044	0.59839062	-4.94125094
homo after UVB irradiation VS homo before UVB irradiation	Hormonal control of Pubertal Growth Spurt|Wikipathways	1.038311796	-0.01600812	3.844157415	0.00364277	0.03413771	-1.68780341
hetero after UVB irradiation VS hetero before UVB irradiation	Hormonal control of Pubertal Growth Spurt|Wikipathways	-0.05647682	0.015216446	-0.13716973	0.89411792	0.9657321	-6.33634567
WT after UVB irradiation VS WT before UVB irradiation	Hormonal control of Pubertal Growth Spurt|Wikipathways	-0.33459808	0.020153014	-0.81321356	0.43487916	0.79685269	-5.54548136
homo after UVB irradiation VS homo before UVB irradiation	Hormone ligand-binding receptors|Reactome	0.334512248	0.071616326	1.707821219	0.12044906	0.25942	-5.0095303
hetero after UVB irradiation VS hetero before UVB irradiation	Hormone ligand-binding receptors|Reactome	-0.80301196	-0.14313976	-3.13926826	0.01295134	0.10972528	-2.8592428
WT after UVB irradiation VS WT before UVB irradiation	Hormone ligand-binding receptors|Reactome	-0.26070897	0.116648986	-0.90171674	0.38824082	0.76131207	-5.47423715
homo after UVB irradiation VS homo before UVB irradiation	Host Interactions of HIV factors|Reactome	-0.24833201	0.007178648	-1.25795039	0.2387927	0.40041406	-5.58454214
hetero after UVB irradiation VS hetero before UVB irradiation	Host Interactions of HIV factors|Reactome	0.170610353	-0.02014228	0.911534196	0.38734818	0.61473991	-5.91727464
WT after UVB irradiation VS WT before UVB irradiation	Host Interactions of HIV factors|Reactome	0.178614059	-0.00386143	0.701390352	0.4989286	0.82062552	-5.62628102
homo after UVB irradiation VS homo before UVB irradiation	Host Interactions with Influenza Factors|Reactome	-0.39833539	-0.04945337	-1.58590233	0.14582406	0.2913279	-5.17551926
hetero after UVB irradiation VS hetero before UVB irradiation	Host Interactions with Influenza Factors|Reactome	0.521627804	0.03463471	1.839587624	0.10122975	0.28605405	-4.81193736
WT after UVB irradiation VS WT before UVB irradiation	Host Interactions with Influenza Factors|Reactome	0.064994202	-0.14672171	0.210788385	0.83725172	0.94951669	-5.84669557
homo after UVB irradiation VS homo before UVB irradiation	how does salmonella hijack a cell|BioCarta	0.849780383	-0.05538058	3.995053085	0.00287833	0.03044128	-1.457577
hetero after UVB irradiation VS hetero before UVB irradiation	how does salmonella hijack a cell|BioCarta	0.870968537	0.041932235	5.330326199	0.00059122	0.03004176	0.150451245
WT after UVB irradiation VS WT before UVB irradiation	how does salmonella hijack a cell|BioCarta	0.880703342	0.064374666	4.161676559	0.00190958	0.1645483	-1.02767267
homo after UVB irradiation VS homo before UVB irradiation	how progesterone initiates the oocyte maturation|BioCarta	-0.00278445	-0.01053094	-0.01681577	0.98693552	0.99149561	-6.36829633
hetero after UVB irradiation VS hetero before UVB irradiation	how progesterone initiates the oocyte maturation|BioCarta	0.170859367	-0.02215931	1.085409036	0.30781521	0.5363233	-5.749556
WT after UVB irradiation VS WT before UVB irradiation	how progesterone initiates the oocyte maturation|BioCarta	0.223952165	0.032992854	1.070783988	0.30922995	0.69398681	-5.32140836
homo after UVB irradiation VS homo before UVB irradiation	HSF1 activation|Reactome	-0.4415165	0.015240696	-2.64715264	0.02569428	0.10578915	-3.58292397
hetero after UVB irradiation VS hetero before UVB irradiation	HSF1 activation|Reactome	-0.26529697	-0.0385032	-1.64784864	0.13608345	0.33582478	-5.07549603
WT after UVB irradiation VS WT before UVB irradiation	HSF1 activation|Reactome	-0.272183	-0.01812898	-1.0941491	0.29934037	0.68477511	-5.29865794
homo after UVB irradiation VS homo before UVB irradiation	HSF1-dependent transactivation|Reactome	-0.39687024	0.000173821	-2.28409561	0.04708597	0.14135079	-4.15551883
hetero after UVB irradiation VS hetero before UVB irradiation	HSF1-dependent transactivation|Reactome	-0.4812941	-0.01419238	-3.13975853	0.01294148	0.10972528	-2.85849929
WT after UVB irradiation VS WT before UVB irradiation	HSF1-dependent transactivation|Reactome	-0.45461462	-0.00399641	-2.12676717	0.05912367	0.42631804	-4.00667583
homo after UVB irradiation VS homo before UVB irradiation	HS-GAG biosynthesis|Reactome	0.480293073	-0.03349346	2.843643874	0.01851327	0.08696668	-3.26870735
hetero after UVB irradiation VS hetero before UVB irradiation	HS-GAG biosynthesis|Reactome	0.237531919	0.030755778	1.084129676	0.3083504	0.53651817	-5.75087171
WT after UVB irradiation VS WT before UVB irradiation	HS-GAG biosynthesis|Reactome	0.237225401	0.040654872	0.999858128	0.34077125	0.72554886	-5.38809657
homo after UVB irradiation VS homo before UVB irradiation	HS-GAG degradation|Reactome	0.722192548	-0.0457108	4.37809883	0.00160514	0.02229782	-0.88723531
hetero after UVB irradiation VS hetero before UVB irradiation	HS-GAG degradation|Reactome	0.474628392	-0.02242465	1.714543299	0.1228634	0.31461313	-4.985314
WT after UVB irradiation VS WT before UVB irradiation	HS-GAG degradation|Reactome	0.5311921	0.014892392	1.717793344	0.11633781	0.51734642	-4.57211552
homo after UVB irradiation VS homo before UVB irradiation	HSP90 chaperone cycle for steroid hormone receptors (SHR)|Reactome	-0.52259528	-0.00307225	-2.74514804	0.0218164	0.09569038	-3.42639723
hetero after UVB irradiation VS hetero before UVB irradiation	HSP90 chaperone cycle for steroid hormone receptors (SHR)|Reactome	-0.65050583	-0.03493342	-3.22376153	0.01136203	0.10504986	-2.73137292
WT after UVB irradiation VS WT before UVB irradiation	HSP90 chaperone cycle for steroid hormone receptors (SHR)|Reactome	-0.82441411	-0.01365681	-4.03918882	0.00232536	0.17027242	-1.196413
homo after UVB irradiation VS homo before UVB irradiation	Human Complement System|Wikipathways	0.669723647	-0.04591739	3.586733763	0.00548065	0.0427042	-2.08690485
hetero after UVB irradiation VS hetero before UVB irradiation	Human Complement System|Wikipathways	0.637100877	0.027728733	3.524328247	0.00717838	0.08667895	-2.28178216
WT after UVB irradiation VS WT before UVB irradiation	Human Complement System|Wikipathways	0.485386084	-0.00059625	1.927018154	0.08260424	0.46863796	-4.28898862
homo after UVB irradiation VS homo before UVB irradiation	human cytomegalovirus and map kinase pathways|BioCarta	-0.00275475	-0.01162762	-0.01469656	0.98858183	0.99205816	-6.36833221
hetero after UVB irradiation VS hetero before UVB irradiation	human cytomegalovirus and map kinase pathways|BioCarta	0.235258226	8.78E-05	1.392928348	0.19929272	0.41937961	-5.40241796
WT after UVB irradiation VS WT before UVB irradiation	human cytomegalovirus and map kinase pathways|BioCarta	-0.04261363	-0.03114052	-0.20202395	0.84391616	0.95302431	-5.84852015
homo after UVB irradiation VS homo before UVB irradiation	Human cytomegalovirus infection - Homo sapiens (human)|KEGG	0.510314269	-0.03206545	3.054084226	0.01305631	0.07097034	-2.93156804
hetero after UVB irradiation VS hetero before UVB irradiation	Human cytomegalovirus infection - Homo sapiens (human)|KEGG	0.519348929	0.032633124	3.21386717	0.01153712	0.10542662	-2.74631793
WT after UVB irradiation VS WT before UVB irradiation	Human cytomegalovirus infection - Homo sapiens (human)|KEGG	0.372562505	-0.03676426	1.781249351	0.10496717	0.50446894	-4.4878355
homo after UVB irradiation VS homo before UVB irradiation	Human immunodeficiency virus 1 infection - Homo sapiens (human)|KEGG	0.410418597	-0.02548697	2.386395599	0.03971309	0.13178049	-3.99580879
hetero after UVB irradiation VS hetero before UVB irradiation	Human immunodeficiency virus 1 infection - Homo sapiens (human)|KEGG	0.532804755	0.024222237	3.55487913	0.00685524	0.08550202	-2.236625
WT after UVB irradiation VS WT before UVB irradiation	Human immunodeficiency virus 1 infection - Homo sapiens (human)|KEGG	0.408025807	-0.00432003	2.108021243	0.06102387	0.43075115	-4.03357727
homo after UVB irradiation VS homo before UVB irradiation	Human papillomavirus infection - Homo sapiens (human)|KEGG	0.238228552	-0.01922682	1.388128596	0.19714915	0.35356756	-5.42942495
hetero after UVB irradiation VS hetero before UVB irradiation	Human papillomavirus infection - Homo sapiens (human)|KEGG	0.276597868	0.002971252	1.848337612	0.09985814	0.28447089	-4.79961426
WT after UVB irradiation VS WT before UVB irradiation	Human papillomavirus infection - Homo sapiens (human)|KEGG	0.245070029	0.00589242	1.253637799	0.23826333	0.63195092	-5.1336239
homo after UVB irradiation VS homo before UVB irradiation	Human T-cell leukemia virus 1 infection - Homo sapiens (human)|KEGG	0.011839762	-0.02033968	0.074415672	0.94224105	0.96485145	-6.36547373
hetero after UVB irradiation VS hetero before UVB irradiation	Human T-cell leukemia virus 1 infection - Homo sapiens (human)|KEGG	0.17571883	-0.0044874	1.150583293	0.28150746	0.511152	-5.68102336
WT after UVB irradiation VS WT before UVB irradiation	Human T-cell leukemia virus 1 infection - Homo sapiens (human)|KEGG	0.216183772	-0.02497954	1.043680732	0.32101158	0.70517551	-5.34731828
homo after UVB irradiation VS homo before UVB irradiation	Human Thyroid Stimulating Hormone (TSH) signaling pathway|Wikipathways	0.331062979	-0.02777868	1.88198598	0.09115227	0.21616167	-4.76196692
hetero after UVB irradiation VS hetero before UVB irradiation	Human Thyroid Stimulating Hormone (TSH) signaling pathway|Wikipathways	0.417153456	-0.00603661	2.657353962	0.02765517	0.14438349	-3.59512533
WT after UVB irradiation VS WT before UVB irradiation	Human Thyroid Stimulating Hormone (TSH) signaling pathway|Wikipathways	0.317345534	-0.01925731	1.60527696	0.13926494	0.55478524	-4.71766842
homo after UVB irradiation VS homo before UVB irradiation	Huntington disease - Homo sapiens (human)|KEGG	-0.03029922	-0.01730684	-0.14282524	0.88944641	0.93425634	-6.35749995
hetero after UVB irradiation VS hetero before UVB irradiation	Huntington disease - Homo sapiens (human)|KEGG	-0.13315812	-0.02159282	-0.68443791	0.51208152	0.7303506	-6.09942404
WT after UVB irradiation VS WT before UVB irradiation	Huntington disease - Homo sapiens (human)|KEGG	-0.02613877	-0.0251658	-0.10753161	0.91647702	0.97566251	-5.86329702
homo after UVB irradiation VS homo before UVB irradiation	HuR (ELAVL1) binds and stabilizes mRNA|Reactome	-0.2471456	-0.00600462	-1.24887957	0.24194779	0.40353094	-5.59495843
hetero after UVB irradiation VS hetero before UVB irradiation	HuR (ELAVL1) binds and stabilizes mRNA|Reactome	0.109127575	0.030827925	0.445143642	0.66743039	0.82651398	-6.2403261
WT after UVB irradiation VS WT before UVB irradiation	HuR (ELAVL1) binds and stabilizes mRNA|Reactome	0.047528359	-0.0584601	0.192709866	0.85101271	0.95502804	-5.85037496
homo after UVB irradiation VS homo before UVB irradiation	Hyaluronan biosynthesis and export|Reactome	0.71518891	-0.03014287	1.885890791	0.09057864	0.21571189	-4.75629197
hetero after UVB irradiation VS hetero before UVB irradiation	Hyaluronan biosynthesis and export|Reactome	0.864628986	0.106510464	2.804147029	0.02191065	0.13157939	-3.3704121
WT after UVB irradiation VS WT before UVB irradiation	Hyaluronan biosynthesis and export|Reactome	1.03114389	-0.02104068	5.181423649	0.00040149	0.15645713	0.275951506
homo after UVB irradiation VS homo before UVB irradiation	Hyaluronan metabolism|Reactome	0.544091684	0.00070696	2.581415956	0.02867684	0.11336662	-3.68760434
hetero after UVB irradiation VS hetero before UVB irradiation	Hyaluronan metabolism|Reactome	0.663749544	-0.00100752	3.426758932	0.00832247	0.09229635	-2.42671172
WT after UVB irradiation VS WT before UVB irradiation	Hyaluronan metabolism|Reactome	0.834347413	-0.10652891	4.089892675	0.00214276	0.17027242	-1.12626909
homo after UVB irradiation VS homo before UVB irradiation	Hyaluronan uptake and degradation|Reactome	0.52232843	0.015975274	2.574306846	0.02901948	0.11412829	-3.69890572
hetero after UVB irradiation VS hetero before UVB irradiation	Hyaluronan uptake and degradation|Reactome	0.598919445	-0.01062247	3.02719006	0.0154246	0.11352943	-3.02962043
WT after UVB irradiation VS WT before UVB irradiation	Hyaluronan uptake and degradation|Reactome	0.792085127	-0.12581698	3.843396228	0.00319866	0.20011336	-1.47102125
homo after UVB irradiation VS homo before UVB irradiation	Hydrochlorothiazide Action Pathway|SMPDB	0.234726031	0.017120546	1.156747303	0.27595732	0.43614438	-5.69767199
hetero after UVB irradiation VS hetero before UVB irradiation	Hydrochlorothiazide Action Pathway|SMPDB	-0.03255037	0.028925632	-0.18154472	0.86023155	0.94503144	-6.32869738
WT after UVB irradiation VS WT before UVB irradiation	Hydrochlorothiazide Action Pathway|SMPDB	-0.15908359	0.010252305	-0.80382265	0.44003996	0.79685269	-5.55266936
homo after UVB irradiation VS homo before UVB irradiation	Hydrocodone Action Pathway|SMPDB	0.191132361	0.029768717	1.061313976	0.31508276	0.46923284	-5.79783739
hetero after UVB irradiation VS hetero before UVB irradiation	Hydrocodone Action Pathway|SMPDB	-0.07904315	0.055005373	-0.44437398	0.66796482	0.82651398	-6.24068873
WT after UVB irradiation VS WT before UVB irradiation	Hydrocodone Action Pathway|SMPDB	0.022316377	0.035038618	0.098344171	0.92358676	0.97566251	-5.86425414
homo after UVB irradiation VS homo before UVB irradiation	Hydroflumethiazide Action Pathway|SMPDB	0.234726031	0.017120546	1.156747303	0.27595732	0.43614438	-5.69767199
hetero after UVB irradiation VS hetero before UVB irradiation	Hydroflumethiazide Action Pathway|SMPDB	-0.03255037	0.028925632	-0.18154472	0.86023155	0.94503144	-6.32869738
WT after UVB irradiation VS WT before UVB irradiation	Hydroflumethiazide Action Pathway|SMPDB	-0.15908359	0.010252305	-0.80382265	0.44003996	0.79685269	-5.55266936
homo after UVB irradiation VS homo before UVB irradiation	hydrogen sulfide biosynthesis (trans-sulfuration)|HumanCyc	-1.03096392	0.009185618	-5.50480244	0.0003253	0.01666204	0.658072793
hetero after UVB irradiation VS hetero before UVB irradiation	hydrogen sulfide biosynthesis (trans-sulfuration)|HumanCyc	-0.67635827	-0.13184633	-2.08448047	0.06887001	0.23513821	-4.45921201
WT after UVB irradiation VS WT before UVB irradiation	hydrogen sulfide biosynthesis (trans-sulfuration)|HumanCyc	-1.00471126	-0.08504096	-3.60367118	0.00475571	0.20968398	-1.81475856
homo after UVB irradiation VS homo before UVB irradiation	Hydrolysis of LPC|Reactome	0.282601314	0.034627763	1.561412018	0.15146458	0.2978412	-5.20804329
hetero after UVB irradiation VS hetero before UVB irradiation	Hydrolysis of LPC|Reactome	0.401233018	0.098429185	1.53811455	0.16068091	0.37252774	-5.21992153

WT after UVB irradiation VS WT before UVB irradiation	Hydrolysis of LPC|Reactome	0.26000947	0.004265092	1.051132777	0.31773891	0.70237831	-5.34024627
homo after UVB irradiation VS homo before UVB irradiation	Hydrolysis of LPE|Reactome	-0.33967944	-0.00895178	-0.81395168	0.43582831	0.58719151	-6.02481636
hetero after UVB irradiation VS hetero before UVB irradiation	Hydrolysis of LPE|Reactome	-0.41114007	-0.01457489	-1.03247346	0.33057218	0.55929769	-5.80301502
WT after UVB irradiation VS WT before UVB irradiation	Hydrolysis of LPE|Reactome	-0.74900702	-0.11311836	-3.54960292	0.00520527	0.20968398	-1.89331963
homo after UVB irradiation VS homo before UVB irradiation	Hydromorphone Action Pathway|SMPDB	0.191132361	0.029768717	1.061313976	0.31508276	0.46923284	-5.79783739
hetero after UVB irradiation VS hetero before UVB irradiation	Hydromorphone Action Pathway|SMPDB	-0.07904315	0.055005373	-0.44437398	0.66796482	0.82651398	-6.24068873
WT after UVB irradiation VS WT before UVB irradiation	Hydromorphone Action Pathway|SMPDB	0.022316377	0.035038618	0.098344171	0.92358676	0.97566251	-5.86425414
homo after UVB irradiation VS homo before UVB irradiation	Hydroxycarboxylic acid-binding receptors|Reactome	1.176101886	0.056751126	6.762806223	6.73E-05	0.01666204	2.143855016
hetero after UVB irradiation VS hetero before UVB irradiation	Hydroxycarboxylic acid-binding receptors|Reactome	1.187900757	0.136103588	6.373089441	0.00017223	0.02920766	1.316063283
WT after UVB irradiation VS WT before UVB irradiation	Hydroxycarboxylic acid-binding receptors|Reactome	0.949873805	0.009024984	3.380937264	0.00691228	0.22490313	-2.14053597
homo after UVB irradiation VS homo before UVB irradiation	Hypercholesterolemia|SMPDB	-0.19230642	-0.01987665	-0.61925406	0.55047761	0.67913558	-6.16661869
hetero after UVB irradiation VS hetero before UVB irradiation	Hypercholesterolemia|SMPDB	0.185820603	0.008098609	0.631890782	0.54421801	0.75147406	-6.13505553
WT after UVB irradiation VS WT before UVB irradiation	Hypercholesterolemia|SMPDB	-0.14894747	0.045069977	-0.50755372	0.62268399	0.87257466	-5.74041409
homo after UVB irradiation VS homo before UVB irradiation	Hyperglycinemia_ non-ketotic|SMPDB	-0.11801782	0.021953564	-0.50783827	0.62328187	0.73728109	-6.23179983
hetero after UVB irradiation VS hetero before UVB irradiation	Hyperglycinemia_ non-ketotic|SMPDB	-0.03882851	0.032366748	-0.2244119	0.82778335	0.93322694	-6.31930458
WT after UVB irradiation VS WT before UVB irradiation	Hyperglycinemia_ non-ketotic|SMPDB	0.143497804	-0.01134274	0.603206807	0.55969543	0.84911968	-5.68832383
homo after UVB irradiation VS homo before UVB irradiation	Hyper-IgD syndrome|SMPDB	-0.19230642	-0.01987665	-0.61925406	0.55047761	0.67913558	-6.16661869
hetero after UVB irradiation VS hetero before UVB irradiation	Hyper-IgD syndrome|SMPDB	0.185820603	0.008098609	0.631890782	0.54421801	0.75147406	-6.13505553
WT after UVB irradiation VS WT before UVB irradiation	Hyper-IgD syndrome|SMPDB	-0.14894747	0.045069977	-0.50755372	0.62268399	0.87257466	-5.74041409
homo after UVB irradiation VS homo before UVB irradiation	Hyperinsulinism-Hyperammonemia Syndrome|SMPDB	-0.24547682	-0.00810915	-1.40068944	0.19347783	0.34903982	-5.41392471
hetero after UVB irradiation VS hetero before UVB irradiation	Hyperinsulinism-Hyperammonemia Syndrome|SMPDB	-0.17636771	-0.04343396	-1.04504584	0.32505313	0.55421891	-5.79050219
WT after UVB irradiation VS WT before UVB irradiation	Hyperinsulinism-Hyperammonemia Syndrome|SMPDB	-0.06328445	0.007258138	-0.28249238	0.78328047	0.93661609	-5.82889545
homo after UVB irradiation VS homo before UVB irradiation	Hyperlysinemia I_ Familial|SMPDB	-0.02100802	-0.06308448	-0.07524201	0.941601	0.96450524	-6.36540732
hetero after UVB irradiation VS hetero before UVB irradiation	Hyperlysinemia I_ Familial|SMPDB	-0.10000854	-0.05054657	-0.39990823	0.69917117	0.84778456	-6.26061929
WT after UVB irradiation VS WT before UVB irradiation	Hyperlysinemia I_ Familial|SMPDB	-0.11826398	-0.1134336	-0.44264387	0.66736875	0.87935805	-5.77091861
homo after UVB irradiation VS homo before UVB irradiation	Hyperlysinemia II or Saccharopinuria|SMPDB	-0.02100802	-0.06308448	-0.07524201	0.941601	0.96450524	-6.36540732
hetero after UVB irradiation VS hetero before UVB irradiation	Hyperlysinemia II or Saccharopinuria|SMPDB	-0.10000854	-0.05054657	-0.39990823	0.69917117	0.84778456	-6.26061929
WT after UVB irradiation VS WT before UVB irradiation	Hyperlysinemia II or Saccharopinuria|SMPDB	-0.11826398	-0.1134336	-0.44264387	0.66736875	0.87935805	-5.77091861
homo after UVB irradiation VS homo before UVB irradiation	Hypermethioninemia|SMPDB	-0.09396972	-0.02692441	-0.3795694	0.71270801	0.81002335	-6.29164178
hetero after UVB irradiation VS hetero before UVB irradiation	Hypermethioninemia|SMPDB	0.075091809	-0.02022482	0.352613458	0.73303662	0.86896773	-6.27959702
WT after UVB irradiation VS WT before UVB irradiation	Hypermethioninemia|SMPDB	0.176060787	0.019583595	0.866300336	0.40646895	0.7725109	-5.50349844
homo after UVB irradiation VS homo before UVB irradiation	Hyperornithinemia with gyrate atrophy (HOGA)|SMPDB	-0.34739547	0.00502943	-1.63262028	0.13558154	0.277359	-5.11269582
hetero after UVB irradiation VS hetero before UVB irradiation	Hyperornithinemia with gyrate atrophy (HOGA)|SMPDB	-0.56351352	-0.04220085	-3.24793085	0.01094591	0.10415756	-2.6949004
WT after UVB irradiation VS WT before UVB irradiation	Hyperornithinemia with gyrate atrophy (HOGA)|SMPDB	-0.47021304	-0.04055952	-2.31093774	0.04323906	0.39022568	-3.73881388
homo after UVB irradiation VS homo before UVB irradiation	Hyperornithinemia-hyperammonemia-homocitrullinuria [HHH-syndrome]|SMPDB	-0.34739547	0.00502943	-1.63262028	0.13558154	0.277359	-5.11269582
hetero after UVB irradiation VS hetero before UVB irradiation	Hyperornithinemia-hyperammonemia-homocitrullinuria [HHH-syndrome]|SMPDB	-0.56351352	-0.04220085	-3.24793085	0.01094591	0.10415756	-2.6949004
WT after UVB irradiation VS WT before UVB irradiation	Hyperornithinemia-hyperammonemia-homocitrullinuria [HHH-syndrome]|SMPDB	-0.47021304	-0.04055952	-2.31093774	0.04323906	0.39022568	-3.73881388
homo after UVB irradiation VS homo before UVB irradiation	Hyperphenylalaniemia due to guanosine triphosphate cyclohydrolase deficiency|SMPDB	0.35847852	0.013300746	1.711755208	0.11970137	0.25817499	-5.00406733
hetero after UVB irradiation VS hetero before UVB irradiation	Hyperphenylalaniemia due to guanosine triphosphate cyclohydrolase deficiency|SMPDB	0.498149914	0.002599842	2.269426991	0.05132677	0.20064099	-4.18437481
WT after UVB irradiation VS WT before UVB irradiation	Hyperphenylalaniemia due to guanosine triphosphate cyclohydrolase deficiency|SMPDB	0.467985189	-0.04591589	2.072035951	0.06483587	0.43544914	-4.08500072
homo after UVB irradiation VS homo before UVB irradiation	Hyperphenylalaninemia due to 6-pyruvoyltetrahydropterin synthase deficiency (ptps)|SMPDB	0.35847852	0.013300746	1.711755208	0.11970137	0.25817499	-5.00406733
hetero after UVB irradiation VS hetero before UVB irradiation	Hyperphenylalaninemia due to 6-pyruvoyltetrahydropterin synthase deficiency (ptps)|SMPDB	0.498149914	0.002599842	2.269426991	0.05132677	0.20064099	-4.18437481
WT after UVB irradiation VS WT before UVB irradiation	Hyperphenylalaninemia due to 6-pyruvoyltetrahydropterin synthase deficiency (ptps)|SMPDB	0.467985189	-0.04591589	2.072035951	0.06483587	0.43544914	-4.08500072
homo after UVB irradiation VS homo before UVB irradiation	Hyperphenylalaninemia due to dhpr-deficiency|SMPDB	0.35847852	0.013300746	1.711755208	0.11970137	0.25817499	-5.00406733
hetero after UVB irradiation VS hetero before UVB irradiation	Hyperphenylalaninemia due to dhpr-deficiency|SMPDB	0.498149914	0.002599842	2.269426991	0.05132677	0.20064099	-4.18437481
WT after UVB irradiation VS WT before UVB irradiation	Hyperphenylalaninemia due to dhpr-deficiency|SMPDB	0.467985189	-0.04591589	2.072035951	0.06483587	0.43544914	-4.08500072
homo after UVB irradiation VS homo before UVB irradiation	Hyperprolinemia Type I|SMPDB	-0.34739547	0.00502943	-1.63262028	0.13558154	0.277359	-5.11269582
hetero after UVB irradiation VS hetero before UVB irradiation	Hyperprolinemia Type I|SMPDB	-0.56351352	-0.04220085	-3.24793085	0.01094591	0.10415756	-2.6949004
WT after UVB irradiation VS WT before UVB irradiation	Hyperprolinemia Type I|SMPDB	-0.47021304	-0.04055952	-2.31093774	0.04323906	0.39022568	-3.73881388
homo after UVB irradiation VS homo before UVB irradiation	Hyperprolinemia Type II|SMPDB	-0.34739547	0.00502943	-1.63262028	0.13558154	0.277359	-5.11269582
hetero after UVB irradiation VS hetero before UVB irradiation	Hyperprolinemia Type II|SMPDB	-0.56351352	-0.04220085	-3.24793085	0.01094591	0.10415756	-2.6949004
WT after UVB irradiation VS WT before UVB irradiation	Hyperprolinemia Type II|SMPDB	-0.47021304	-0.04055952	-2.31093774	0.04323906	0.39022568	-3.73881388
homo after UVB irradiation VS homo before UVB irradiation	Hypertrophic cardiomyopathy (HCM) - Homo sapiens (human)|KEGG	0.552102479	-0.00806193	3.414332317	0.00723642	0.04931566	-2.35803022
hetero after UVB irradiation VS hetero before UVB irradiation	Hypertrophic cardiomyopathy (HCM) - Homo sapiens (human)|KEGG	0.278342963	0.040973112	1.338917475	0.21559359	0.43741933	-5.46751454
WT after UVB irradiation VS WT before UVB irradiation	Hypertrophic cardiomyopathy (HCM) - Homo sapiens (human)|KEGG	0.277642784	-0.02518309	1.024765735	0.32943231	0.71256659	-5.36508999
homo after UVB irradiation VS homo before UVB irradiation	Hypertrophy Model|Wikipathways	0.250692552	0.041397043	0.946784419	0.36747671	0.52061441	-5.90905358
hetero after UVB irradiation VS hetero before UVB irradiation	Hypertrophy Model|Wikipathways	0.27687572	0.029551882	1.192123506	0.26570297	0.49371143	-5.63584553
WT after UVB irradiation VS WT before UVB irradiation	Hypertrophy Model|Wikipathways	0.091870509	0.013943873	0.355992826	0.7291823	0.9105948	-5.80538127
homo after UVB irradiation VS homo before UVB irradiation	Hypoacetylaspartia|SMPDB	-0.45356441	0.050143346	-2.44562708	0.03597696	0.12504267	-3.90265183
hetero after UVB irradiation VS hetero before UVB irradiation	Hypoacetylaspartia|SMPDB	-0.36442322	-0.0375773	-1.84647526	0.1001486	0.28471271	-4.80223908
WT after UVB irradiation VS WT before UVB irradiation	Hypoacetylaspartia|SMPDB	-0.06304373	-0.04233882	-0.24741384	0.80955351	0.94525798	-5.83824172
homo after UVB irradiation VS homo before UVB irradiation	Hypophosphatasia|SMPDB	0.659234177	-0.08426311	3.104885328	0.01200579	0.06709723	-2.85029594
hetero after UVB irradiation VS hetero before UVB irradiation	Hypophosphatasia|SMPDB	0.368557199	-0.00112402	1.358565524	0.20953599	0.43157028	-5.44402013
WT after UVB irradiation VS WT before UVB irradiation	Hypophosphatasia|SMPDB	0.713384492	0.082523133	2.765196844	0.0198087	0.31542987	-3.06111756
homo after UVB irradiation VS homo before UVB irradiation	Hypothesized Pathways in Pathogenesis of Cardiovascular Disease|Wikipathways	0.328440592	-0.02840153	1.615965203	0.13915661	0.281894	-5.13520772
hetero after UVB irradiation VS hetero before UVB irradiation	Hypothesized Pathways in Pathogenesis of Cardiovascular Disease|Wikipathways	0.317774936	0.022139151	1.296692864	0.22911796	0.45170905	-5.51726067
WT after UVB irradiation VS WT before UVB irradiation	Hypothesized Pathways in Pathogenesis of Cardiovascular Disease|Wikipathways	0.318235301	-0.00087427	1.351196613	0.20617229	0.62110082	-5.02487673
homo after UVB irradiation VS homo before UVB irradiation	Hypothetical Craniofacial Development Pathway|Wikipathways	-0.16261676	0.001584672	-0.50065138	0.62814211	0.74111031	-6.23558854
hetero after UVB irradiation VS hetero before UVB irradiation	Hypothetical Craniofacial Development Pathway|Wikipathways	0.141319191	-0.00774329	0.672178661	0.51947289	0.73623731	-6.10796513
WT after UVB irradiation VS WT before UVB irradiation	Hypothetical Craniofacial Development Pathway|Wikipathways	-0.18905485	-0.00021949	-0.78487422	0.450576	0.80602452	-5.56695131
homo after UVB irradiation VS homo before UVB irradiation	hypoxia and p53 in the cardiovascular system|BioCarta	-0.3078166	0.014137438	-1.59973994	0.14272045	0.28699056	-5.15701656
hetero after UVB irradiation VS hetero before UVB irradiation	hypoxia and p53 in the cardiovascular system|BioCarta	0.098386958	0.000733926	0.404979057	0.69558035	0.84689449	-6.25844813
WT after UVB irradiation VS WT before UVB irradiation	hypoxia and p53 in the cardiovascular system|BioCarta	-0.14046426	-0.03940625	-0.6014086	0.56084612	0.85011093	-5.68938011
homo after UVB irradiation VS homo before UVB irradiation	hypoxia-inducible factor in the cardivascular system|BioCarta	-0.06596626	-0.02762057	-0.32852329	0.74972481	0.8371496	-6.31079837
hetero after UVB irradiation VS hetero before UVB irradiation	hypoxia-inducible factor in the cardivascular system|BioCarta	0.183331001	-0.021546	0.809808166	0.44031865	0.66588335	-6.00433924
WT after UVB irradiation VS WT before UVB irradiation	hypoxia-inducible factor in the cardivascular system|BioCarta	-0.06806166	-0.02177341	-0.2767725	0.7875461	0.93972043	-5.83050257
homo after UVB irradiation VS homo before UVB irradiation	Hypoxia-mediated EMT and Stemness|Wikipathways	0.003691793	-0.06255254	0.009964089	0.99225845	0.99416331	-6.36839492
hetero after UVB irradiation VS hetero before UVB irradiation	Hypoxia-mediated EMT and Stemness|Wikipathways	0.261453398	-0.02755566	0.927251232	0.37958965	0.60686413	-5.90306875
WT after UVB irradiation VS WT before UVB irradiation	Hypoxia-mediated EMT and Stemness|Wikipathways	-0.16499919	0.040143788	-0.62662679	0.5448303	0.8409057	-5.6743013
homo after UVB irradiation VS homo before UVB irradiation	Hypoxic and oxygen homeostasis regulation of HIF-1-alpha|PID	-0.01886259	-0.01301717	-0.10272324	0.92034323	0.95153785	-6.36278185
hetero after UVB irradiation VS hetero before UVB irradiation	Hypoxic and oxygen homeostasis regulation of HIF-1-alpha|PID	0.13767117	0.039704305	0.804457171	0.44323666	0.66874303	-6.00867888
WT after UVB irradiation VS WT before UVB irradiation	Hypoxic and oxygen homeostasis regulation of HIF-1-alpha|PID	-0.13011812	-0.00332384	-0.5728462	0.57929928	0.8595386	-5.70576521
homo after UVB irradiation VS homo before UVB irradiation	hypusine biosynthesis|HumanCyc	-1.02481705	0.088239527	-4.55316689	0.00123762	0.02014127	-0.6338188
hetero after UVB irradiation VS hetero before UVB irradiation	hypusine biosynthesis|HumanCyc	-0.8698397	-0.11680737	-3.01686553	0.01567586	0.11352943	-3.04535242
WT after UVB irradiation VS WT before UVB irradiation	hypusine biosynthesis|HumanCyc	-0.7273789	-0.0734353	-2.23907325	0.0488739	0.40436065	-3.84404112
homo after UVB irradiation VS homo before UVB irradiation	Hypusine synthesis from eIF5A-lysine|Reactome	-0.94274025	0.031743442	-4.80897863	0.00085315	0.01759873	-0.27215963
hetero after UVB irradiation VS hetero before UVB irradiation	Hypusine synthesis from eIF5A-lysine|Reactome	-0.79090099	-0.07296174	-3.57969321	0.0066041	0.08470408	-2.20002895
WT after UVB irradiation VS WT before UVB irradiation	Hypusine synthesis from eIF5A-lysine|Reactome	-0.55163944	-0.09166622	-1.69022263	0.12161752	0.52647129	-4.60825779
homo after UVB irradiation VS homo before UVB irradiation	Ibandronate Action Pathway|SMPDB	-0.19230642	-0.01987665	-0.61925406	0.55047761	0.67913558	-6.16661869
hetero after UVB irradiation VS hetero before UVB irradiation	Ibandronate Action Pathway|SMPDB	0.185820603	0.008098609	0.631890782	0.54421801	0.75147406	-6.13505553
WT after UVB irradiation VS WT before UVB irradiation	Ibandronate Action Pathway|SMPDB	-0.14894747	0.045069977	-0.50755372	0.62268399	0.87257466	-5.74041409
homo after UVB irradiation VS homo before UVB irradiation	Ibuprofen Action Pathway|SMPDB	0.176062113	0.05418829	0.834669082	0.42463119	0.57589911	-6.00773882
hetero after UVB irradiation VS hetero before UVB irradiation	Ibuprofen Action Pathway|SMPDB	0.419536954	0.04582289	2.69817797	0.02591817	0.14030289	-3.53264791
WT after UVB irradiation VS WT before UVB irradiation	Ibuprofen Action Pathway|SMPDB	0.30015271	0.076839824	1.539450201	0.15446824	0.5588223	-4.80029494
homo after UVB irradiation VS homo before UVB irradiation	Ibuprofen Metabolism Pathway|SMPDB	-0.15782922	0.102409268	-0.84340494	0.41996894	0.57053799	-6.0004274
hetero after UVB irradiation VS hetero before UVB irradiation	Ibuprofen Metabolism Pathway|SMPDB	0.197884104	-0.00705169	0.838895438	0.42468617	0.65153805	-5.98032355
WT after UVB irradiation VS WT before UVB irradiation	Ibuprofen Metabolism Pathway|SMPDB	0.235372144	0.108698034	0.936188835	0.37105813	0.74850253	-5.44481516
homo after UVB irradiation VS homo before UVB irradiation	Ibuprofen Pathway_ Pharmacokinetics|PharmGKB	0.001808813	0.211270932	0.011365456	0.99116972	0.99334524	-6.36837886
hetero after UVB irradiation VS hetero before UVB irradiation	Ibuprofen Pathway_ Pharmacokinetics|PharmGKB	-0.51961102	0.166042629	-2.51671247	0.03459735	0.16285121	-3.80997112
WT after UVB irradiation VS WT before UVB irradiation	Ibuprofen Pathway_ Pharmacokinetics|PharmGKB	-0.34123983	0.073304422	-1.13059737	0.28440218	0.67194245	-5.2624184
homo after UVB irradiation VS homo before UVB irradiation	Ibutilide Action Pathway|SMPDB	0.396415397	-0.03677376	2.343069613	0.04268583	0.13402181	-4.06364635
hetero after UVB irradiation VS hetero before UVB irradiation	Ibutilide Action Pathway|SMPDB	-0.01555055	-0.01775524	-0.07410913	0.94265284	0.97054702	-6.34356282
WT after UVB irradiation VS WT before UVB irradiation	Ibutilide Action Pathway|SMPDB	0.056685205	-0.03978418	0.223404833	0.82768199	0.94525798	-5.84393438
homo after UVB irradiation VS homo before UVB irradiation	ID signaling pathway|Wikipathways	-0.34111246	-0.02482839	-1.64384995	0.13321788	0.27598585	-5.09744664
hetero after UVB irradiation VS hetero before UVB irradiation	ID signaling pathway|Wikipathways	-0.17928158	-0.05771819	-0.9052266	0.39049391	0.61758197	-5.92292018
WT after UVB irradiation VS WT before UVB irradiation	ID signaling pathway|Wikipathways	-0.03238681	0.038584287	-0.14083158	0.89077703	0.97348138	-5.85911425
homo after UVB irradiation VS homo before UVB irradiation	ID|NetPath	-0.0828466	0.004064195	-0.3900277	0.7052167	0.80552596	-6.28738626
hetero after UVB irradiation VS hetero before UVB irradiation	ID|NetPath	0.078801515	-0.02542689	0.387307906	0.70812834	0.85500635	-6.2659003
WT after UVB irradiation VS WT before UVB irradiation	ID|NetPath	-0.01905909	0.067516477	-0.08678076	0.93254511	0.97986866	-5.8653376
homo after UVB irradiation VS homo before UVB irradiation	ifn alpha signaling pathway|BioCarta	0.81315054	-0.05908201	4.183616215	0.00215368	0.02581999	-1.17417987
hetero after UVB irradiation VS hetero before UVB irradiation	ifn alpha signaling pathway|BioCarta	0.7648532	0.062525096	3.867470863	0.0043095	0.07183716	-1.7813632
WT after UVB irradiation VS WT before UVB irradiation	ifn alpha signaling pathway|BioCarta	0.899764043	-0.00027162	3.602750575	0.00476302	0.20968398	-1.8160932
homo after UVB irradiation VS homo before UVB irradiation	IFN alpha signaling|INOH	1.142202974	-0.01147806	7.170636245	4.22E-05	0.01666204	2.574650621
hetero after UVB irradiation VS hetero before UVB irradiation	IFN alpha signaling|INOH	1.047365937	0.07089424	5.83972943	0.00031795	0.03004176	0.741195611
WT after UVB irradiation VS WT before UVB irradiation	IFN alpha signaling|INOH	1.03503586	0.025944315	4.255987951	0.00164306	0.15689229	-0.89942641
homo after UVB irradiation VS homo before UVB irradiation	ifn gamma signaling pathway|BioCarta	0.831640774	0.021718793	3.578480426	0.00555363	0.04299579	-2.09982015
hetero after UVB irradiation VS hetero before UVB irradiation	ifn gamma signaling pathway|BioCarta	0.689814696	0.077918263	2.356216641	0.04469294	0.18702113	-4.05366363
WT after UVB irradiation VS WT before UVB irradiation	ifn gamma signaling pathway|BioCarta	0.71145201	-0.05253753	2.31518417	0.04292661	0.39022568	-3.73257139
homo after UVB irradiation VS homo before UVB irradiation	IFN gamma signaling|INOH	0.624614332	0.020295084	2.257367563	0.04922351	0.14535514	-4.19696337
hetero after UVB irradiation VS hetero before UVB irradiation	IFN gamma signaling|INOH	0.60298834	0.041967044	2.059119501	0.07169193	0.23970209	-4.49641005
WT after UVB irradiation VS WT before UVB irradiation	IFN gamma signaling|INOH	0.579087356	-0.10456762	1.821088448	0.09835716	0.49761536	-4.43418287
homo after UVB irradiation VS homo before UVB irradiation	IFN-gamma pathway|PID	0.351260098	-0.01226481	1.688180905	0.12424588	0.26312925	-5.03670784
hetero after UVB irradiation VS hetero before UVB irradiation	IFN-gamma pathway|PID	0.397245979	0.030913301	2.102798494	0.06689914	0.23308674	-4.43226229
WT after UVB irradiation VS WT before UVB irradiation	IFN-gamma pathway|PID	0.231739484	-0.01443534	1.074516389	0.30763362	0.69353727	-5.3177997
homo after UVB irradiation VS homo before UVB irradiation	Ifosfamide Action Pathway|SMPDB	-0.6187322	0.070665967	-2.69792037	0.02360566	0.10120509	-3.50189262
hetero after UVB irradiation VS hetero before UVB irradiation	Ifosfamide Action Pathway|SMPDB	-0.85220121	0.052222958	-3.574791	0.00665293	0.08470408	-2.20725291
WT after UVB irradiation VS WT before UVB irradiation	Ifosfamide Action Pathway|SMPDB	-0.96778609	0.047360415	-4.42962202	0.00124978	0.15645713	-0.6672634
homo after UVB irradiation VS homo before UVB irradiation	Ifosfamide Metabolism Pathway|SMPDB	-0.6187322	0.070665967	-2.69792037	0.02360566	0.10120509	-3.50189262
hetero after UVB irradiation VS hetero before UVB irradiation	Ifosfamide Metabolism Pathway|SMPDB	-0.85220121	0.052222958	-3.574791	0.00665293	0.08470408	-2.20725291
WT after UVB irradiation VS WT before UVB irradiation	Ifosfamide Metabolism Pathway|SMPDB	-0.96778609	0.047360415	-4.42962202	0.00124978	0.15645713	-0.6672634
homo after UVB irradiation VS homo before UVB irradiation	Ifosfamide Pathway_ Pharmacodynamics|PharmGKB	0.267657503	-0.01307344	1.013525619	0.33621356	0.4922959	-5.8454747
hetero after UVB irradiation VS hetero before UVB irradiation	Ifosfamide Pathway_ Pharmacodynamics|PharmGKB	0.033705849	-0.00142063	0.11246664	0.91308684	0.97054702	-6.3396847
WT after UVB irradiation VS WT before UVB irradiation	Ifosfamide Pathway_ Pharmacodynamics|PharmGKB	0.050948379	-0.06508409	0.179208492	0.86132425	0.95916804	-5.8529094
homo after UVB irradiation VS homo before UVB irradiation	Ifosfamide Pathway_ Pharmacokinetics|PharmGKB	-0.49381778	0.091344807	-2.1513828	0.05866781	0.16535631	-4.35995507
hetero after UVB irradiation VS hetero before UVB irradiation	Ifosfamide Pathway_ Pharmacokinetics|PharmGKB	-0.80283968	0.118460248	-2.82594238	0.0211684	0.12881919	-3.33705079
WT after UVB irradiation VS WT before UVB irradiation	Ifosfamide Pathway_ Pharmacokinetics|PharmGKB	-0.91697448	0.104616538	-4.24374143	0.00167534	0.15689229	-0.91599539
homo after UVB irradiation VS homo before UVB irradiation	IGF signaling|INOH	0.330883053	-0.05368627	2.082281424	0.06574458	0.17836587	-4.46491399
hetero after UVB irradiation VS hetero before UVB irradiation	IGF signaling|INOH	0.229054128	-0.00354358	1.28975637	0.23140688	0.45275431	-5.52533343
WT after UVB irradiation VS WT before UVB irradiation	IGF signaling|INOH	0.241155816	0.025351834	1.163025524	0.27160662	0.6636851	-5.22942419
homo after UVB irradiation VS homo before UVB irradiation	IGF1 pathway|PID	0.133834915	-0.03695632	0.606490953	0.55856599	0.68504225	-6.17469181
hetero after UVB irradiation VS hetero before UVB irradiation	IGF1 pathway|PID	0.301042657	-0.02677102	1.977722007	0.0815242	0.25776716	-4.61483748
WT after UVB irradiation VS WT before UVB irradiation	IGF1 pathway|PID	0.125090113	-0.09683911	0.63374455	0.54035769	0.83916339	-5.66994228
homo after UVB irradiation VS homo before UVB irradiation	igf-1 signaling pathway|BioCarta	0.034088004	-0.0311041	0.154161486	0.88074533	0.92767778	-6.35569536
hetero after UVB irradiation VS hetero before UVB irradiation	igf-1 signaling pathway|BioCarta	0.386884471	-0.00041648	2.283017594	0.05022692	0.19880033	-4.1639683
WT after UVB irradiation VS WT before UVB irradiation	igf-1 signaling pathway|BioCarta	0.196822522	0.005250942	0.947570855	0.36550593	0.74545864	-5.43489997
homo after UVB irradiation VS homo before UVB irradiation	IGF1R signaling cascade|Reactome	-0.06177599	-0.01697084	-0.37036269	0.71932971	0.81577771	-6.29529524
hetero after UVB irradiation VS hetero before UVB irradiation	IGF1R signaling cascade|Reactome	-0.15305674	0.010077338	-0.95177499	0.3677113	0.59602707	-5.880512
WT after UVB irradiation VS WT before UVB irradiation	IGF1R signaling cascade|Reactome	-0.3174807	-0.01264154	-1.62454017	0.135074	0.54785342	-4.6931239

homo after UVB irradiation VS homo before UVB irradiation	IGF-Core|Signalink	0.168207885	-0.0407514	0.825812539	0.42939373	0.58092201	-6.01508455
hetero after UVB irradiation VS hetero before UVB irradiation	IGF-Core|Signalink	-0.00015367	-0.04420906	-0.00054504	0.9995778	0.99979715	-6.34654114
WT after UVB irradiation VS WT before UVB irradiation	IGF-Core|Signalink	-0.18232543	-0.1174942	-0.73793774	0.4773766	0.81532496	-5.60103652
homo after UVB irradiation VS homo before UVB irradiation	IGF-Ncore|Signalink	-0.04012484	0.034538807	-0.1318902	0.89785431	0.9399608	-6.3591107
hetero after UVB irradiation VS hetero before UVB irradiation	IGF-Ncore|Signalink	0.008495953	-0.02072164	0.034815552	0.97303753	0.98711441	-6.34588378
WT after UVB irradiation VS WT before UVB irradiation	IGF-Ncore|Signalink	-0.17876403	-0.06188378	-0.78919983	0.44815636	0.80421615	-5.56371719
homo after UVB irradiation VS homo before UVB irradiation	IKK complex recruitment mediated by RIP1|Reactome	0.432241115	-0.02183033	2.458308795	0.03522342	0.12483995	-3.88264973
hetero after UVB irradiation VS hetero before UVB irradiation	IKK complex recruitment mediated by RIP1|Reactome	0.582794546	0.00870973	3.58928246	0.00650967	0.08456271	-2.18590652
WT after UVB irradiation VS WT before UVB irradiation	IKK complex recruitment mediated by RIP1|Reactome	0.153145245	-0.00582043	0.724114313	0.48545854	0.81710765	-5.61071954
homo after UVB irradiation VS homo before UVB irradiation	il 2 signaling pathway|BioCarta	0.470082322	-0.07925413	2.727853998	0.02245515	0.09736962	-3.45405352
hetero after UVB irradiation VS hetero before UVB irradiation	il 2 signaling pathway|BioCarta	0.488280707	0.028026639	2.685916892	0.02642786	0.14142055	-3.55141532
WT after UVB irradiation VS WT before UVB irradiation	il 2 signaling pathway|BioCarta	0.439808315	-0.04608288	1.662791537	0.12708383	0.53591123	-4.64391695
homo after UVB irradiation VS homo before UVB irradiation	il 3 signaling pathway|BioCarta	0.60081632	-0.03476383	3.728772391	0.0043703	0.0379263	-1.86576169
hetero after UVB irradiation VS hetero before UVB irradiation	il 3 signaling pathway|BioCarta	0.611353066	0.050744106	3.402550427	0.00863508	0.09312575	-2.46283179
WT after UVB irradiation VS WT before UVB irradiation	il 3 signaling pathway|BioCarta	0.477302965	0.026871935	2.031734751	0.06937333	0.44435906	-4.1422332
homo after UVB irradiation VS homo before UVB irradiation	il 4 signaling pathway|BioCarta	0.601393281	-0.05611769	3.5390226	0.00591684	0.04480312	-2.16165955
hetero after UVB irradiation VS hetero before UVB irradiation	il 4 signaling pathway|BioCarta	0.54009056	0.002355399	2.41388121	0.04076448	0.17712536	-3.966358
WT after UVB irradiation VS WT before UVB irradiation	il 4 signaling pathway|BioCarta	0.560717906	0.023271874	2.267037533	0.04660142	0.39955937	-3.80319168
homo after UVB irradiation VS homo before UVB irradiation	il 6 signaling pathway|BioCarta	0.17164413	-0.04313008	1.032762699	0.32758232	0.48374714	-5.82650651
hetero after UVB irradiation VS hetero before UVB irradiation	il 6 signaling pathway|BioCarta	0.15553682	0.013905164	0.731478302	0.48433092	0.70529722	-6.06538253
WT after UVB irradiation VS WT before UVB irradiation	il 6 signaling pathway|BioCarta	0.108397456	-0.06188651	0.442577364	0.66741527	0.87935805	-5.77094782
homo after UVB irradiation VS homo before UVB irradiation	IL1 and megakaryocytes in obesity|Wikipathways	0.847420627	-0.04261599	4.960037635	0.00068795	0.01666204	-0.06352047
hetero after UVB irradiation VS hetero before UVB irradiation	IL1 and megakaryocytes in obesity|Wikipathways	0.780964729	0.063386932	3.882733038	0.00421437	0.07114476	-1.75947296
WT after UVB irradiation VS WT before UVB irradiation	IL1 and megakaryocytes in obesity|Wikipathways	0.615421432	0.014344668	2.066887721	0.06539941	0.43794589	-4.0923334
homo after UVB irradiation VS homo before UVB irradiation	IL-1 JNK|INOH	-0.05729543	0.013224047	-0.212418	0.83632106	0.89977426	-6.34426398
hetero after UVB irradiation VS hetero before UVB irradiation	IL-1 JNK|INOH	0.147356572	-0.01477109	0.614676135	0.55500056	0.75966743	-6.14616848
WT after UVB irradiation VS WT before UVB irradiation	IL-1 JNK|INOH	0.090367716	-0.00687978	0.322941665	0.75333733	0.92020735	-5.81661024
homo after UVB irradiation VS homo before UVB irradiation	IL-1 NFkB|INOH	-0.02233742	-0.00151212	-0.07537142	0.94150077	0.96450524	-6.36539686
hetero after UVB irradiation VS hetero before UVB irradiation	IL-1 NFkB|INOH	0.207072573	-0.00106128	0.90062669	0.39279956	0.61972322	-5.92701708
WT after UVB irradiation VS WT before UVB irradiation	IL-1 NFkB|INOH	0.083294572	-0.0088823	0.292141916	0.77610137	0.9344727	-5.826111
homo after UVB irradiation VS homo before UVB irradiation	IL-1 p38|INOH	-0.11387559	-0.00622451	-0.45410787	0.66006667	0.76688662	-6.25888189
hetero after UVB irradiation VS hetero before UVB irradiation	IL-1 p38|INOH	0.144868519	-0.02004177	0.638006107	0.54041752	0.75147406	-6.13104075
WT after UVB irradiation VS WT before UVB irradiation	IL-1 p38|INOH	0.093868704	0.001787217	0.343594654	0.73820871	0.91673557	-5.80971846
homo after UVB irradiation VS homo before UVB irradiation	IL-1 signaling pathway|Wikipathways	0.316578878	-0.03115384	1.52424896	0.16039341	0.30897074	-5.256839
hetero after UVB irradiation VS hetero before UVB irradiation	IL-1 signaling pathway|Wikipathways	0.375252924	-0.02183502	2.194212379	0.0578574	0.21475083	-4.29683388
WT after UVB irradiation VS WT before UVB irradiation	IL-1 signaling pathway|Wikipathways	0.148479226	-0.03338228	0.76006612	0.46461735	0.80891799	-5.58519795
homo after UVB irradiation VS homo before UVB irradiation	IL1|NetPath	0.381435667	-0.02875776	1.794806108	0.10487563	0.2370387	-4.88729805
hetero after UVB irradiation VS hetero before UVB irradiation	IL1|NetPath	0.328533316	-0.00821387	1.91386111	0.09013163	0.27214648	-4.7066144
WT after UVB irradiation VS WT before UVB irradiation	IL1|NetPath	0.152107622	-0.04282022	0.748523121	0.47124559	0.81275627	-5.59351167
homo after UVB irradiation VS homo before UVB irradiation	il-10 anti-inflammatory signaling pathway|BioCarta	0.823249798	-3.10E-05	4.470198597	0.00139916	0.02143814	-0.75332952
hetero after UVB irradiation VS hetero before UVB irradiation	il-10 anti-inflammatory signaling pathway|BioCarta	0.808358453	0.076899528	4.429856334	0.00193539	0.04958933	-0.99783246
WT after UVB irradiation VS WT before UVB irradiation	il-10 anti-inflammatory signaling pathway|BioCarta	0.668657945	0.016071114	3.041389795	0.012322	0.27611764	-2.64596857
homo after UVB irradiation VS homo before UVB irradiation	IL-10 signaling|INOH	1.183467929	-0.00713548	7.261858488	3.81E-05	0.01666204	2.667822759
hetero after UVB irradiation VS hetero before UVB irradiation	IL-10 signaling|INOH	1.04852112	0.084434914	6.25804291	0.00019599	0.02988205	1.195720991
WT after UVB irradiation VS WT before UVB irradiation	IL-10 signaling|INOH	0.772002996	-0.02839374	2.703431154	0.02203244	0.31542987	-3.15398186
homo after UVB irradiation VS homo before UVB irradiation	IL11|NetPath	0.434634841	-0.03405995	1.936018357	0.08350903	0.20511149	-4.68300173
hetero after UVB irradiation VS hetero before UVB irradiation	IL11|NetPath	0.558487833	0.004104246	3.441827757	0.00813391	0.09131619	-2.40425986
WT after UVB irradiation VS WT before UVB irradiation	IL11|NetPath	0.567363014	-0.01295627	2.837838229	0.01747984	0.30353778	-2.95183819
homo after UVB irradiation VS homo before UVB irradiation	il12 and stat4 dependent signaling pathway in th1 development|BioCarta	0.266926909	-0.04486767	1.700294782	0.1218914	0.26055457	-5.01996413
hetero after UVB irradiation VS hetero before UVB irradiation	il12 and stat4 dependent signaling pathway in th1 development|BioCarta	0.341601986	0.036747015	1.912215583	0.09036449	0.27214648	-4.70896492
WT after UVB irradiation VS WT before UVB irradiation	il12 and stat4 dependent signaling pathway in th1 development|BioCarta	0.214569117	-0.09601245	0.829555934	0.42599476	0.79182665	-5.53280039
homo after UVB irradiation VS homo before UVB irradiation	IL12 signaling mediated by STAT4|PID	0.153819041	-0.00372983	0.782329138	0.45329852	0.60045287	-6.05016537
hetero after UVB irradiation VS hetero before UVB irradiation	IL12 signaling mediated by STAT4|PID	0.227135548	0.012533238	1.104624257	0.2998645	0.53033405	-5.72965607
WT after UVB irradiation VS WT before UVB irradiation	IL12 signaling mediated by STAT4|PID	0.161457008	-0.06080142	0.511925474	0.61972862	0.87257466	-5.73821739
homo after UVB irradiation VS homo before UVB irradiation	IL-12 signaling|INOH	0.368990096	-0.05632654	2.04748534	0.06961133	0.18533254	-4.51732588
hetero after UVB irradiation VS hetero before UVB irradiation	IL-12 signaling|INOH	0.371567005	0.025592901	1.440380079	0.18586229	0.40321767	-5.3439337
WT after UVB irradiation VS WT before UVB irradiation	IL-12 signaling|INOH	0.270838289	-0.09803556	0.830068319	0.4257182	0.79167634	-5.53239928
homo after UVB irradiation VS homo before UVB irradiation	IL12-mediated signaling events|PID	0.46967248	-0.02044292	2.412442528	0.03802512	0.12832425	-3.9548991
hetero after UVB irradiation VS hetero before UVB irradiation	IL12-mediated signaling events|PID	0.344826772	0.019420864	1.559074736	0.15569382	0.36523542	-5.19273959
WT after UVB irradiation VS WT before UVB irradiation	IL12-mediated signaling events|PID	0.378839568	-0.04860122	1.342396802	0.208911	0.62359252	-5.03491246
homo after UVB irradiation VS homo before UVB irradiation	IL-13 signaling|INOH	0.448633503	-0.01284943	2.559612083	0.02974075	0.11488687	-3.72225282
hetero after UVB irradiation VS hetero before UVB irradiation	IL-13 signaling|INOH	0.202220883	-0.01917341	0.803368805	0.44383177	0.66919789	-6.00955854
WT after UVB irradiation VS WT before UVB irradiation	IL-13 signaling|INOH	0.362077339	-0.02213891	1.24244807	0.24219247	0.63358831	-5.14573355
homo after UVB irradiation VS homo before UVB irradiation	IL-17 signaling pathway - Homo sapiens (human)|KEGG	0.247946305	0.026406367	1.305847358	0.22269036	0.38216685	-5.5286736
hetero after UVB irradiation VS hetero before UVB irradiation	IL-17 signaling pathway - Homo sapiens (human)|KEGG	0.363463601	0.02227646	1.955609987	0.08441153	0.26244731	-4.6467353
WT after UVB irradiation VS WT before UVB irradiation	IL-17 signaling pathway - Homo sapiens (human)|KEGG	0.256111195	0.016527924	1.236675437	0.24423994	0.63594286	-5.15195059
homo after UVB irradiation VS homo before UVB irradiation	IL17 signaling pathway|Wikipathways	0.351662003	-0.03940091	1.606145316	0.14130388	0.28472794	-5.14842144
hetero after UVB irradiation VS hetero before UVB irradiation	IL17 signaling pathway|Wikipathways	0.298112748	-0.03145246	1.564551291	0.15441388	0.36486693	-5.18560487
WT after UVB irradiation VS WT before UVB irradiation	IL17 signaling pathway|Wikipathways	0.259714242	0.006033731	1.302008856	0.2218728	0.62898392	-5.08040414
homo after UVB irradiation VS homo before UVB irradiation	IL1-mediated signaling events|PID	0.411471542	-0.05403211	1.623062124	0.13762301	0.27994034	-5.12563053
hetero after UVB irradiation VS hetero before UVB irradiation	IL1-mediated signaling events|PID	0.527718684	0.028007832	2.835475379	0.02085191	0.12723297	-3.32246119
WT after UVB irradiation VS WT before UVB irradiation	IL1-mediated signaling events|PID	0.254082038	-0.05596763	1.228241427	0.24725653	0.63942274	-5.16099672
homo after UVB irradiation VS homo before UVB irradiation	il-2 receptor beta chain in t cell activation|BioCarta	0.284094775	-0.02854106	1.782716727	0.10692317	0.23978939	-4.9044616
hetero after UVB irradiation VS hetero before UVB irradiation	il-2 receptor beta chain in t cell activation|BioCarta	0.30962321	-0.03909324	1.901627218	0.09187659	0.27406643	-4.72407175
WT after UVB irradiation VS WT before UVB irradiation	il-2 receptor beta chain in t cell activation|BioCarta	0.377772769	-0.02931817	1.685401682	0.12256261	0.52731779	-4.61454676
homo after UVB irradiation VS homo before UVB irradiation	IL2 signaling events mediated by PI3K|PID	0.032592914	-0.03206797	0.190835041	0.85271765	0.9103364	-6.34891957
hetero after UVB irradiation VS hetero before UVB irradiation	IL2 signaling events mediated by PI3K|PID	-0.01002188	-0.02979154	-0.05063849	0.96079353	0.9814915	-6.34515042
WT after UVB irradiation VS WT before UVB irradiation	IL2 signaling events mediated by PI3K|PID	0.11123452	-0.03017389	0.492578916	0.63286024	0.87345806	-5.74780326
homo after UVB irradiation VS homo before UVB irradiation	IL2 signaling events mediated by STAT5|PID	0.038992352	-0.05837675	0.195199108	0.84939599	0.90742679	-6.34801806
hetero after UVB irradiation VS hetero before UVB irradiation	IL2 signaling events mediated by STAT5|PID	0.108334693	0.002435003	0.507368018	0.6249076	0.80597874	-6.20904433
WT after UVB irradiation VS WT before UVB irradiation	IL2 signaling events mediated by STAT5|PID	0.213473748	-0.07591038	0.841946952	0.4193405	0.78544581	-5.5230407
homo after UVB irradiation VS homo before UVB irradiation	IL-2 Signaling Pathway|Wikipathways	0.172777808	-0.03084991	0.973504151	0.35471411	0.50970065	-5.88402063
hetero after UVB irradiation VS hetero before UVB irradiation	IL-2 Signaling Pathway|Wikipathways	0.29100161	-0.01030334	1.903664319	0.09158382	0.27391014	-4.72116776
WT after UVB irradiation VS WT before UVB irradiation	IL-2 Signaling Pathway|Wikipathways	0.211289006	-0.00296316	1.013927146	0.33433142	0.71819925	-5.37515696
homo after UVB irradiation VS homo before UVB irradiation	IL-2 signaling|INOH	0.805127434	-0.09052011	3.567677255	0.0056507	0.04348946	-2.11673591
hetero after UVB irradiation VS hetero before UVB irradiation	IL-2 signaling|INOH	0.579962347	0.017065167	1.868285121	0.09679656	0.2808394	-4.77143448
WT after UVB irradiation VS WT before UVB irradiation	IL-2 signaling|INOH	0.540637494	-0.01719231	1.586004228	0.14357304	0.55622203	-4.74206241
homo after UVB irradiation VS homo before UVB irradiation	IL2|NetPath	0.303765364	-0.02359018	1.759494204	0.11096001	0.24463709	-4.93727556
hetero after UVB irradiation VS hetero before UVB irradiation	IL2|NetPath	0.454581735	-0.01076567	2.896677987	0.01893264	0.1205973	-3.22884013
WT after UVB irradiation VS WT before UVB irradiation	IL2|NetPath	0.285698098	-0.03568976	1.325814285	0.21415449	0.62581393	-5.0537043
homo after UVB irradiation VS homo before UVB irradiation	il22 soluble receptor signaling pathway|BioCarta	0.891716167	-0.01277577	5.561237038	0.00030174	0.01666204	0.730065841
hetero after UVB irradiation VS hetero before UVB irradiation	il22 soluble receptor signaling pathway|BioCarta	0.746977917	0.035003924	3.601620584	0.00639027	0.08365054	-2.16775223
WT after UVB irradiation VS WT before UVB irradiation	il22 soluble receptor signaling pathway|BioCarta	0.67087768	-0.03824877	2.655097126	0.02394539	0.32905879	-3.22657745
homo after UVB irradiation VS homo before UVB irradiation	IL-23 signaling|INOH	0.616809786	-0.04170539	2.421915475	0.03742897	0.12687503	-3.93999857
hetero after UVB irradiation VS hetero before UVB irradiation	IL-23 signaling|INOH	0.625721047	0.001285137	2.288999195	0.04975027	0.19835162	-4.15497911
WT after UVB irradiation VS WT before UVB irradiation	IL-23 signaling|INOH	0.524688028	-0.09448808	1.710541392	0.11770607	0.52190644	-4.58165082
homo after UVB irradiation VS homo before UVB irradiation	IL23-mediated signaling events|PID	0.632861177	0.007890986	3.254479257	0.00938925	0.05808098	-2.61160072
hetero after UVB irradiation VS hetero before UVB irradiation	IL23-mediated signaling events|PID	0.626736105	0.045108583	2.950423897	0.01739751	0.11610228	-3.14671535
WT after UVB irradiation VS WT before UVB irradiation	IL23-mediated signaling events|PID	0.514140718	0.017796465	1.779281811	0.10530394	0.50446894	-4.49047085
homo after UVB irradiation VS homo before UVB irradiation	IL27-mediated signaling events|PID	0.779442162	0.010357287	4.622841654	0.00111732	0.01929633	-0.53428693
hetero after UVB irradiation VS hetero before UVB irradiation	IL27-mediated signaling events|PID	0.584753018	0.037772338	2.969303192	0.01688928	0.11437413	-3.11789314
WT after UVB irradiation VS WT before UVB irradiation	IL27-mediated signaling events|PID	0.529436809	-6.67E-05	1.927812944	0.08249546	0.46860292	-4.2878862
homo after UVB irradiation VS homo before UVB irradiation	IL2-mediated signaling events|PID	0.165945831	-0.01502846	0.985684928	0.34900536	0.50461003	-5.87242128
hetero after UVB irradiation VS hetero before UVB irradiation	IL2-mediated signaling events|PID	0.388107868	-0.00479834	2.461277068	0.03779558	0.16931722	-3.89437523
WT after UVB irradiation VS WT before UVB irradiation	IL2-mediated signaling events|PID	0.128206868	-0.03241369	0.585403848	0.57114574	0.8541036	-5.69865258
homo after UVB irradiation VS homo before UVB irradiation	IL-3 Signaling Pathway|Wikipathways	0.63875013	-0.04262315	3.745108063	0.00425864	0.03746619	-1.8404716
hetero after UVB irradiation VS hetero before UVB irradiation	IL-3 Signaling Pathway|Wikipathways	0.567566366	0.040426059	2.889811866	0.01913858	0.12132638	-3.23933847
WT after UVB irradiation VS WT before UVB irradiation	IL-3 Signaling Pathway|Wikipathways	0.636248305	0.003482965	3.07717105	0.01158961	0.27004966	-2.59234633
homo after UVB irradiation VS homo before UVB irradiation	IL-3 signaling|INOH	1.019585649	-0.04113964	5.798555462	0.00022101	0.01666204	1.027190253
hetero after UVB irradiation VS hetero before UVB irradiation	IL-3 signaling|INOH	0.679248875	0.041893007	2.092136196	0.06803955	0.23495391	-4.44795698
WT after UVB irradiation VS WT before UVB irradiation	IL-3 signaling|INOH	0.522004037	-0.04916892	1.57157082	0.14687616	0.55622203	-4.76022253
homo after UVB irradiation VS homo before UVB irradiation	IL3|NetPath	0.512009109	-0.03209496	2.493573998	0.0332093	0.12150134	-3.8269321
hetero after UVB irradiation VS hetero before UVB irradiation	IL3|NetPath	0.582547035	0.01229116	3.764761802	0.00501225	0.07564846	-1.92952259
WT after UVB irradiation VS WT before UVB irradiation	IL3|NetPath	0.471566777	-0.01701927	2.339427959	0.04118443	0.39022568	-3.6968829
homo after UVB irradiation VS homo before UVB irradiation	IL3-mediated signaling events|PID	0.439990153	-0.05102819	2.162748927	0.05757643	0.16318684	-4.3425864
hetero after UVB irradiation VS hetero before UVB irradiation	IL3-mediated signaling events|PID	0.690559285	0.021101265	4.043790927	0.00333656	0.06477425	-1.53052522
WT after UVB irradiation VS WT before UVB irradiation	IL3-mediated signaling events|PID	0.390891572	-0.05913559	1.561808226	0.14914817	0.55622203	-4.77245227
homo after UVB irradiation VS homo before UVB irradiation	IL-4 Signaling Pathway|Wikipathways	0.482215223	-0.03641578	2.768044372	0.02099887	0.0926385	-3.38976482
hetero after UVB irradiation VS hetero before UVB irradiation	IL-4 Signaling Pathway|Wikipathways	0.548882698	0.03873644	3.004083472	0.01599281	0.11352943	-3.06483661
WT after UVB irradiation VS WT before UVB irradiation	IL-4 Signaling Pathway|Wikipathways	0.510773109	-0.04629188	2.265561198	0.04671879	0.39955937	-3.80535147
homo after UVB irradiation VS homo before UVB irradiation	IL-4 signaling|INOH	1.068289035	-0.0661077	6.295806241	0.00011797	0.01666204	1.620807377
hetero after UVB irradiation VS hetero before UVB irradiation	IL-4 signaling|INOH	0.850124825	0.094343804	3.19959145	0.01179472	0.10542662	-2.76789476
WT after UVB irradiation VS WT before UVB irradiation	IL-4 signaling|INOH	1.005131564	0.037608752	4.06116318	0.0022443	0.17027242	-1.16596319
homo after UVB irradiation VS homo before UVB irradiation	IL4|NetPath	0.484746036	-0.03725597	2.561015634	0.0296711	0.11488687	-3.72002364
hetero after UVB irradiation VS hetero before UVB irradiation	IL4|NetPath	0.610370526	0.026261346	3.292422678	0.01022107	0.10264385	-2.62789257
WT after UVB irradiation VS WT before UVB irradiation	IL4|NetPath	0.551022922	-0.05881666	2.483082703	0.03219393	0.36849539	-3.48393706
homo after UVB irradiation VS homo before UVB irradiation	IL4-mediated signaling events|PID	0.375730435	-0.02683325	2.197820123	0.05433126	0.15685522	-4.28881965
hetero after UVB irradiation VS hetero before UVB irradiation	IL4-mediated signaling events|PID	0.384658666	0.011609407	1.983029711	0.08084536	0.25607584	-4.60716266
WT after UVB irradiation VS WT before UVB irradiation	IL4-mediated signaling events|PID	0.289207445	-0.00471893	1.230268183	0.24652888	0.63911291	-5.15882691
homo after UVB irradiation VS homo before UVB irradiation	IL-5 Signaling Pathway|Wikipathways	0.338760396	-0.04720209	1.774931434	0.10826119	0.24160342	-4.91548538
hetero after UVB irradiation VS hetero before UVB irradiation	IL-5 Signaling Pathway|Wikipathways	0.506727776	-0.00536327	3.404815294	0.00860531	0.09312575	-2.45944991
WT after UVB irradiation VS WT before UVB irradiation	IL-5 Signaling Pathway|Wikipathways	0.401792778	0.03381177	1.959645447	0.07824707	0.46387039	-4.24358379
homo after UVB irradiation VS homo before UVB irradiation	IL-5 signaling|INOH	0.615717213	-0.05063597	3.377732695	0.00767934	0.05141328	-2.41592171
hetero after UVB irradiation VS hetero before UVB irradiation	IL-5 signaling|INOH	0.415456156	0.029017839	1.404486635	0.19594595	0.4161797	-5.38828131
WT after UVB irradiation VS WT before UVB irradiation	IL-5 signaling|INOH	0.473916497	0.01040058	1.598122922	0.14085055	0.55622203	-4.72674264
homo after UVB irradiation VS homo before UVB irradiation	IL5|NetPath	0.45157582	-0.04773898	2.435997677	0.03655978	0.12504267	-3.91782688
hetero after UVB irradiation VS hetero before UVB irradiation	IL5|NetPath	0.675157114	-0.01655907	4.496455382	0.00176573	0.04958933	-0.90833471
WT after UVB irradiation VS WT before UVB irradiation	IL5|NetPath	0.490733787	-0.02103595	2.412862772	0.03631793	0.39022568	-3.58831579
homo after UVB irradiation VS homo before UVB irradiation	IL5-mediated signaling events|PID	0.210195429	-0.04817896	1.049749569	0.32010099	0.47593002	-5.80952335
hetero after UVB irradiation VS hetero before UVB irradiation	IL5-mediated signaling events|PID	0.382763257	0.032376665	1.851668454	0.09934064	0.28335083	-4.79491711
WT after UVB irradiation VS WT before UVB irradiation	IL5-mediated signaling events|PID	0.085548079	-0.08084232	0.273051323	0.79032515	0.94093571	-5.83153074
homo after UVB irradiation VS homo before UVB irradiation	IL-6 signaling pathway|Wikipathways	0.402082509	-0.03175354	2.025597172	0.07215397	0.19019614	-4.55013363
hetero after UVB irradiation VS hetero before UVB irradiation	IL-6 signaling pathway|Wikipathways	0.369480713	0.023705505	2.46261311	0.03771511	0.16931722	-3.89234352
WT after UVB irradiation VS WT before UVB irradiation	IL-6 signaling pathway|Wikipathways	0.369258268	0.017588865	1.872502527	0.09039293	0.48188205	-4.36415278
homo after UVB irradiation VS homo before UVB irradiation	IL-6 signaling|INOH	0.718581382	-0.00533673	3.798192288	0.00391603	0.03568983	-1.75850464
hetero after UVB irradiation VS hetero before UVB irradiation	IL-6 signaling|INOH	0.221985565	0.001371876	0.737813928	0.48066825	0.70175717	-6.06064563
WT after UVB irradiation VS WT before UVB irradiation	IL-6 signaling|INOH	0.211078549	-0.11078906	0.571702298	0.58004513	0.85958498	-5.70640598
homo after UVB irradiation VS homo before UVB irradiation	IL6|NetPath	0.289976988	-0.02769995	1.4222617	0.18730914	0.34306199	-5.38709587

hetero after UVB irradiation VS hetero before UVB irradiation	IL6|NetPath	0.313027903	0.010558285	1.959076824	0.08395246	0.26209266	-4.64174238
WT after UVB irradiation VS WT before UVB irradiation	IL6|NetPath	0.197941158	0.005050444	1.03373636	0.32541829	0.70939634	-5.35669367
homo after UVB irradiation VS homo before UVB irradiation	IL6-mediated signaling events|PID	0.318221082	-0.0223951	1.613760782	0.13963608	0.28261451	-5.13817786
hetero after UVB irradiation VS hetero before UVB irradiation	IL6-mediated signaling events|PID	0.37769196	0.0166129	2.301832632	0.04874269	0.19660989	-4.13567745
WT after UVB irradiation VS WT before UVB irradiation	IL6-mediated signaling events|PID	0.206539616	-0.02344956	0.863932659	0.40770829	0.77432416	-5.50541922
homo after UVB irradiation VS homo before UVB irradiation	IL-6-type cytokine receptor ligand interactions|Reactome	0.379206857	-0.05152959	1.883318454	0.09095615	0.21596764	-4.76003097
hetero after UVB irradiation VS hetero before UVB irradiation	IL-6-type cytokine receptor ligand interactions|Reactome	0.200809834	0.015943153	1.09003723	0.30588521	0.53552381	-5.74478661
WT after UVB irradiation VS WT before UVB irradiation	IL-6-type cytokine receptor ligand interactions|Reactome	0.397401301	-0.03200432	1.510799377	0.16152808	0.56340685	-4.83563004
homo after UVB irradiation VS homo before UVB irradiation	il-7 signal transduction|BioCarta	0.10911205	-0.06318411	0.64094397	0.53688765	0.67222756	-6.15254531
hetero after UVB irradiation VS hetero before UVB irradiation	il-7 signal transduction|BioCarta	0.269473837	0.031883043	1.429038501	0.18899843	0.40711476	-5.35801882
WT after UVB irradiation VS WT before UVB irradiation	il-7 signal transduction|BioCarta	0.134365097	-0.10633429	0.46008967	0.65521438	0.87935805	-5.7631107
homo after UVB irradiation VS homo before UVB irradiation	IL-7 Signaling Pathway|Wikipathways	0.38177174	-0.04109292	2.137439534	0.06003394	0.16751767	-4.38122281
hetero after UVB irradiation VS hetero before UVB irradiation	IL-7 Signaling Pathway|Wikipathways	0.330169916	0.006742297	1.817665087	0.10474452	0.29236099	-4.84270777
WT after UVB irradiation VS WT before UVB irradiation	IL-7 Signaling Pathway|Wikipathways	0.509749625	-0.06039593	2.489854339	0.0318215	0.36606743	-3.47384623
homo after UVB irradiation VS homo before UVB irradiation	IL-7 signaling|INOH	0.086742366	-0.0194572	0.507953858	0.62320385	0.73728109	-6.23173848
hetero after UVB irradiation VS hetero before UVB irradiation	IL-7 signaling|INOH	0.179494079	-0.0054218	1.151723508	0.28106374	0.51114365	-5.67979857
WT after UVB irradiation VS WT before UVB irradiation	IL-7 signaling|INOH	0.063543247	-0.0405061	0.313149579	0.76054905	0.92494383	-5.81973185
homo after UVB irradiation VS homo before UVB irradiation	IL-7|NetPath	0.23083844	-0.02789916	1.107436186	0.29566801	0.45577992	-5.75024747
hetero after UVB irradiation VS hetero before UVB irradiation	IL-7|NetPath	0.189992028	-0.01376724	1.091026256	0.30547401	0.53510782	-5.74376546
WT after UVB irradiation VS WT before UVB irradiation	IL-7|NetPath	0.248204589	-0.06381264	1.14456995	0.27883211	0.66906841	-5.24828765
homo after UVB irradiation VS homo before UVB irradiation	IL8- and CXCR1-mediated signaling events|PID	0.732857928	-0.04670583	4.462339493	0.00141559	0.02159829	-0.76470533
hetero after UVB irradiation VS hetero before UVB irradiation	IL8- and CXCR1-mediated signaling events|PID	0.869317947	0.02109158	5.037412098	0.00085844	0.03680415	-0.20837106
WT after UVB irradiation VS WT before UVB irradiation	IL8- and CXCR1-mediated signaling events|PID	0.635247706	0.018158032	2.87107392	0.01650823	0.30278353	-2.90183954
homo after UVB irradiation VS homo before UVB irradiation	IL8- and CXCR2-mediated signaling events|PID	0.831267181	-0.04608453	4.875016776	0.00077622	0.01695809	-0.18049563
hetero after UVB irradiation VS hetero before UVB irradiation	IL8- and CXCR2-mediated signaling events|PID	0.943525766	0.034045925	5.556120317	0.00044715	0.03004176	0.417455068
WT after UVB irradiation VS WT before UVB irradiation	IL8- and CXCR2-mediated signaling events|PID	0.776835645	0.018643388	3.805274669	0.00340539	0.20197958	-1.52515331
homo after UVB irradiation VS homo before UVB irradiation	IL-9 Signaling Pathway|Wikipathways	0.849092555	-0.01897541	4.785823101	0.00088203	0.01776424	-0.30446682
hetero after UVB irradiation VS hetero before UVB irradiation	IL-9 Signaling Pathway|Wikipathways	0.747264366	0.046856898	3.773575267	0.00494742	0.07516773	-1.91675218
WT after UVB irradiation VS WT before UVB irradiation	IL-9 Signaling Pathway|Wikipathways	0.794532617	-0.02939538	3.890213384	0.00296257	0.19328637	-1.40483111
homo after UVB irradiation VS homo before UVB irradiation	IL9|NetPath	0.738750665	-0.02290667	3.930448833	0.00318256	0.03200781	-1.55578565
hetero after UVB irradiation VS hetero before UVB irradiation	IL9|NetPath	0.622461194	0.020229401	2.629535409	0.02890625	0.14770707	-3.6376787
WT after UVB irradiation VS WT before UVB irradiation	IL9|NetPath	0.554380148	-0.00807444	2.358186922	0.03988386	0.39022568	-3.6692136
homo after UVB irradiation VS homo before UVB irradiation	Imatinib and Chronic Myeloid Leukemia|Wikipathways	-0.23105088	-0.05186097	-1.18203412	0.26626203	0.4295945	-5.67005142
hetero after UVB irradiation VS hetero before UVB irradiation	Imatinib and Chronic Myeloid Leukemia|Wikipathways	0.013902167	-0.04602111	0.078504499	0.93925921	0.97054702	-6.34319918
WT after UVB irradiation VS WT before UVB irradiation	Imatinib and Chronic Myeloid Leukemia|Wikipathways	-0.07618158	-0.08282467	-0.29955161	0.77060359	0.93158633	-5.82391056
homo after UVB irradiation VS homo before UVB irradiation	Imatinib Pathway_ Pharmacokinetics/Pharmacodynamics|PharmGKB	-0.40689832	-0.00651969	-1.82456999	0.09998812	0.22976637	-4.84481164
hetero after UVB irradiation VS hetero before UVB irradiation	Imatinib Pathway_ Pharmacokinetics/Pharmacodynamics|PharmGKB	0.065824118	0.004070832	0.431117563	0.67720076	0.83243826	-6.24684064
WT after UVB irradiation VS WT before UVB irradiation	Imatinib Pathway_ Pharmacokinetics/Pharmacodynamics|PharmGKB	-0.37394161	0.002305049	-1.31931721	0.21623855	0.62581393	-5.06102398
homo after UVB irradiation VS homo before UVB irradiation	Iminoglycinuria|SMPDB	0.234726031	0.017120546	1.156747303	0.27595732	0.43614438	-5.69767199
hetero after UVB irradiation VS hetero before UVB irradiation	Iminoglycinuria|SMPDB	-0.03255037	0.028925632	-0.18154472	0.86023155	0.94503144	-6.32869738
WT after UVB irradiation VS WT before UVB irradiation	Iminoglycinuria|SMPDB	-0.15908359	0.010252305	-0.80382265	0.44003996	0.79685269	-5.55266936
homo after UVB irradiation VS homo before UVB irradiation	Imipramine Action Pathway|SMPDB	0.191132361	0.029768717	1.061313976	0.31508276	0.46923284	-5.79783739
hetero after UVB irradiation VS hetero before UVB irradiation	Imipramine Action Pathway|SMPDB	-0.07904315	0.055005373	-0.44437398	0.66796482	0.82651398	-6.24068873
WT after UVB irradiation VS WT before UVB irradiation	Imipramine Action Pathway|SMPDB	0.022316377	0.035038618	0.098344171	0.92358676	0.97566251	-5.86425414
homo after UVB irradiation VS homo before UVB irradiation	Imipramine Metabolism Pathway|SMPDB	0.197169582	-0.08331033	0.736198587	0.47959803	0.62211494	-6.08556231
hetero after UVB irradiation VS hetero before UVB irradiation	Imipramine Metabolism Pathway|SMPDB	-0.1211451	-0.08865763	-0.39070534	0.70570842	0.85253617	-6.26449241
WT after UVB irradiation VS WT before UVB irradiation	Imipramine Metabolism Pathway|SMPDB	-0.33173712	-0.01126547	-0.94436865	0.3670619	0.74581032	-5.43769949
homo after UVB irradiation VS homo before UVB irradiation	Immune System|Reactome	0.484974854	-0.02447446	2.887699087	0.01720468	0.0828656	-3.19811664
hetero after UVB irradiation VS hetero before UVB irradiation	Immune System|Reactome	0.500217616	0.020444101	3.167419422	0.01239752	0.10716984	-2.81657928
WT after UVB irradiation VS WT before UVB irradiation	Immune System|Reactome	0.395131639	-0.01209677	1.92243163	0.08323461	0.470709	-4.29534674
homo after UVB irradiation VS homo before UVB irradiation	Immunoregulatory interactions between a Lymphoid and a non-Lymphoid cell|Reactome	0.77260163	-0.01440939	4.456213683	0.00142853	0.02159829	-0.77357885
hetero after UVB irradiation VS hetero before UVB irradiation	Immunoregulatory interactions between a Lymphoid and a non-Lymphoid cell|Reactome	0.572880584	0.048312623	2.568303927	0.03186628	0.1555519	-3.73125547
WT after UVB irradiation VS WT before UVB irradiation	Immunoregulatory interactions between a Lymphoid and a non-Lymphoid cell|Reactome	0.448181514	-0.00341569	1.433833959	0.18189651	0.59839062	-4.92855297
homo after UVB irradiation VS homo before UVB irradiation	Import of palmitoyl-CoA into the mitochondrial matrix|Reactome	0.114307406	-0.00495408	0.525291794	0.61155838	0.72907978	-6.22238677
hetero after UVB irradiation VS hetero before UVB irradiation	Import of palmitoyl-CoA into the mitochondrial matrix|Reactome	-0.15768876	-0.04325938	-0.75607946	0.47020943	0.69247644	-6.0467897
WT after UVB irradiation VS WT before UVB irradiation	Import of palmitoyl-CoA into the mitochondrial matrix|Reactome	-0.32726509	-0.02394386	-1.67222675	0.12517922	0.53064492	-4.63168598
homo after UVB irradiation VS homo before UVB irradiation	Inactivation of APC/C via direct inhibition of the APC/C complex|Reactome	-0.63084404	0.017603632	-3.36685914	0.00781628	0.05187376	-2.43314119
hetero after UVB irradiation VS hetero before UVB irradiation	Inactivation of APC/C via direct inhibition of the APC/C complex|Reactome	0.144484838	-0.03873269	0.813367947	0.43838468	0.66406028	-6.00143871
WT after UVB irradiation VS WT before UVB irradiation	Inactivation of APC/C via direct inhibition of the APC/C complex|Reactome	0.014443841	-0.08495071	0.05539309	0.95690764	0.98742794	-5.86759701
homo after UVB irradiation VS homo before UVB irradiation	Inactivation of CDC42 and RAC1|Reactome	-0.26669874	-0.00715072	-1.24842695	0.24210612	0.40353094	-5.5954768
hetero after UVB irradiation VS hetero before UVB irradiation	Inactivation of CDC42 and RAC1|Reactome	-0.31470868	-0.0273155	-1.73687067	0.11870938	0.31235808	-4.95475106
WT after UVB irradiation VS WT before UVB irradiation	Inactivation of CDC42 and RAC1|Reactome	-0.4966805	-0.01889391	-2.32103329	0.04249983	0.39022568	-3.72396864
homo after UVB irradiation VS homo before UVB irradiation	inactivation of gsk3 by akt causes accumulation of b-catenin in alveolar macrophages|BioCarta	0.033987718	0.010634078	0.205627256	0.84147146	0.90318728	-6.34578203
hetero after UVB irradiation VS hetero before UVB irradiation	inactivation of gsk3 by akt causes accumulation of b-catenin in alveolar macrophages|BioCarta	0.17246268	-0.00690781	1.111896674	0.29689802	0.52861765	-5.7220572
WT after UVB irradiation VS WT before UVB irradiation	inactivation of gsk3 by akt causes accumulation of b-catenin in alveolar macrophages|BioCarta	-0.05835565	0.006151597	-0.2970443	0.77246247	0.93294159	-5.82466121
homo after UVB irradiation VS homo before UVB irradiation	Inactivation_ recovery and regulation of the phototransduction cascade|Reactome	-0.05737574	0.03311874	-0.28787974	0.77969246	0.85764292	-6.32412012
hetero after UVB irradiation VS hetero before UVB irradiation	Inactivation_ recovery and regulation of the phototransduction cascade|Reactome	0.128348411	0.010775346	0.622325531	0.55019402	0.75605031	-6.14126485
WT after UVB irradiation VS WT before UVB irradiation	Inactivation_ recovery and regulation of the phototransduction cascade|Reactome	-0.35132424	-0.01824564	-1.76119579	0.10844607	0.5076251	-4.51463034
homo after UVB irradiation VS homo before UVB irradiation	Incretin synthesis_ secretion_ and inactivation|Reactome	-0.43721953	-0.04508373	-1.91340564	0.08663145	0.2099571	-4.71616255
hetero after UVB irradiation VS hetero before UVB irradiation	Incretin synthesis_ secretion_ and inactivation|Reactome	-0.05698059	-0.01514542	-0.18420698	0.85820748	0.94503144	-6.32817129
WT after UVB irradiation VS WT before UVB irradiation	Incretin synthesis_ secretion_ and inactivation|Reactome	0.075397122	-0.03746968	0.251456374	0.80651237	0.94525798	-5.83722682
homo after UVB irradiation VS homo before UVB irradiation	Indapamide Action Pathway|SMPDB	0.234726031	0.017120546	1.156747303	0.27595732	0.43614438	-5.69767199
hetero after UVB irradiation VS hetero before UVB irradiation	Indapamide Action Pathway|SMPDB	-0.03255037	0.028925632	-0.18154472	0.86023155	0.94503144	-6.32869738
WT after UVB irradiation VS WT before UVB irradiation	Indapamide Action Pathway|SMPDB	-0.15908359	0.010252305	-0.80382265	0.44003996	0.79685269	-5.55266936
homo after UVB irradiation VS homo before UVB irradiation	Indomethacin Action Pathway|SMPDB	0.40423582	0.045705158	1.979500697	0.07780079	0.19626432	-4.61879689
hetero after UVB irradiation VS hetero before UVB irradiation	Indomethacin Action Pathway|SMPDB	0.482496143	0.064876536	3.04499191	0.01500113	0.1130168	-3.00250826
WT after UVB irradiation VS WT before UVB irradiation	Indomethacin Action Pathway|SMPDB	0.275725339	0.086595857	1.261970114	0.23537138	0.62952955	-5.12455679
homo after UVB irradiation VS homo before UVB irradiation	induction of apoptosis through dr3 and dr4/5 death receptors|BioCarta	0.883380446	-0.04406487	5.383193623	0.0003831	0.01666204	0.501181243
hetero after UVB irradiation VS hetero before UVB irradiation	induction of apoptosis through dr3 and dr4/5 death receptors|BioCarta	0.593457313	-0.00886203	3.142436682	0.01288774	0.10959386	-2.85443814
WT after UVB irradiation VS WT before UVB irradiation	induction of apoptosis through dr3 and dr4/5 death receptors|BioCarta	0.657674608	0.027813008	3.2374522	0.00881603	0.24856491	-2.35306652
homo after UVB irradiation VS homo before UVB irradiation	Infectious disease|Reactome	-0.28103458	0.011332158	-1.54625126	0.15505261	0.30268073	-5.22803199
hetero after UVB irradiation VS hetero before UVB irradiation	Infectious disease|Reactome	0.020305615	-0.02171467	0.105843759	0.91818295	0.97054702	-6.34046793
WT after UVB irradiation VS WT before UVB irradiation	Infectious disease|Reactome	-0.009296	-0.00746287	-0.03644857	0.97163626	0.99045333	-5.86847823
homo after UVB irradiation VS homo before UVB irradiation	Inflammasomes|Reactome	0.527827244	-0.02444797	2.530010355	0.0312485	0.11752935	-3.76922686
hetero after UVB irradiation VS hetero before UVB irradiation	Inflammasomes|Reactome	0.573600765	0.046861167	3.853711336	0.00439723	0.07183716	-1.80112644
WT after UVB irradiation VS WT before UVB irradiation	Inflammasomes|Reactome	0.373868511	-0.03605416	1.573149614	0.14651161	0.55622203	-4.75824067
homo after UVB irradiation VS homo before UVB irradiation	Inflammatory bowel disease (IBD) - Homo sapiens (human)|KEGG	0.266755653	-0.02169022	1.169127532	0.27117598	0.43491027	-5.6842042
hetero after UVB irradiation VS hetero before UVB irradiation	Inflammatory bowel disease (IBD) - Homo sapiens (human)|KEGG	0.234930017	0.025205271	1.086999383	0.30715095	0.53598546	-5.74791884
WT after UVB irradiation VS WT before UVB irradiation	Inflammatory bowel disease (IBD) - Homo sapiens (human)|KEGG	0.298396789	-0.01043713	1.0204545	0.33137456	0.71318926	-5.36910452
homo after UVB irradiation VS homo before UVB irradiation	Inflammatory mediator regulation of TRP channels - Homo sapiens (human)|KEGG	0.323582412	-0.0100571	1.960078897	0.0803033	0.20058254	-4.64754448
hetero after UVB irradiation VS hetero before UVB irradiation	Inflammatory mediator regulation of TRP channels - Homo sapiens (human)|KEGG	0.33670685	0.054821233	2.231557146	0.05451856	0.20829471	-4.2411021
WT after UVB irradiation VS WT before UVB irradiation	Inflammatory mediator regulation of TRP channels - Homo sapiens (human)|KEGG	0.114778119	0.01525335	0.573628684	0.57878939	0.85923416	-5.70532621
homo after UVB irradiation VS homo before UVB irradiation	Inflammatory Response Pathway|Wikipathways	0.553836778	-0.08389296	2.510732101	0.03227114	0.11979678	-3.79977486
hetero after UVB irradiation VS hetero before UVB irradiation	Inflammatory Response Pathway|Wikipathways	0.35142801	0.055550048	1.321442591	0.22110615	0.44238028	-5.48822698
WT after UVB irradiation VS WT before UVB irradiation	Inflammatory Response Pathway|Wikipathways	0.340050042	-0.01685181	1.032310331	0.32605392	0.71010408	-5.35803228
homo after UVB irradiation VS homo before UVB irradiation	influence of ras and rho proteins on g1 to s transition|BioCarta	0.226359049	-0.02166761	1.150378421	0.27844301	0.43870628	-5.7045589
hetero after UVB irradiation VS hetero before UVB irradiation	influence of ras and rho proteins on g1 to s transition|BioCarta	0.507875823	-0.02569446	2.962453607	0.01707187	0.11493885	-3.1283484
WT after UVB irradiation VS WT before UVB irradiation	influence of ras and rho proteins on g1 to s transition|BioCarta	0.284135524	0.02742381	1.249070678	0.23986071	0.63283875	-5.13857578
homo after UVB irradiation VS homo before UVB irradiation	Influenza A - Homo sapiens (human)|KEGG	0.324548313	-0.01528354	1.768239345	0.10942364	0.24265581	-4.92494275
hetero after UVB irradiation VS hetero before UVB irradiation	Influenza A - Homo sapiens (human)|KEGG	0.476634996	0.035000698	3.122155848	0.01330053	0.11120965	-2.88520447
WT after UVB irradiation VS WT before UVB irradiation	Influenza A - Homo sapiens (human)|KEGG	0.369786643	0.00148144	1.766982681	0.10743151	0.5076251	-4.50691302
homo after UVB irradiation VS homo before UVB irradiation	Influenza A virus infection|Wikipathways	-0.50824221	-0.01638088	-1.40764986	0.19146871	0.34719862	-5.40529683
hetero after UVB irradiation VS hetero before UVB irradiation	Influenza A virus infection|Wikipathways	-0.97828292	-0.05386829	-3.79311673	0.00480682	0.07419102	-1.8884749
WT after UVB irradiation VS WT before UVB irradiation	Influenza A virus infection|Wikipathways	-0.76228981	-0.05108748	-2.30042349	0.04402223	0.39111425	-3.7542591
homo after UVB irradiation VS homo before UVB irradiation	Influenza Infection|Reactome	-0.64965875	-0.00352653	-3.59062888	0.00544656	0.04254196	-2.08081194
hetero after UVB irradiation VS hetero before UVB irradiation	Influenza Infection|Reactome	-0.12751915	-0.05316873	-0.52988151	0.60986911	0.79700362	-6.19677975
WT after UVB irradiation VS WT before UVB irradiation	Influenza Infection|Reactome	-0.09932422	-0.02187141	-0.32209713	0.75395834	0.92042977	-5.81688317
homo after UVB irradiation VS homo before UVB irradiation	Influenza Life Cycle|Reactome	-0.64939937	-0.00080534	-3.48924108	0.00641075	0.04648301	-2.23989149
hetero after UVB irradiation VS hetero before UVB irradiation	Influenza Life Cycle|Reactome	-0.19457329	-0.05836188	-0.79720008	0.447215	0.67229748	-6.01452513
WT after UVB irradiation VS WT before UVB irradiation	Influenza Life Cycle|Reactome	-0.09142177	-0.0090752	-0.29384489	0.77483665	0.93442888	-5.82561006
homo after UVB irradiation VS homo before UVB irradiation	Influenza Viral RNA Transcription and Replication|Reactome	-0.76458269	-0.00727764	-4.19521944	0.00211592	0.02555896	-1.15690459
hetero after UVB irradiation VS hetero before UVB irradiation	Influenza Viral RNA Transcription and Replication|Reactome	-0.28944784	-0.05725261	-1.16841602	0.27463211	0.50474725	-5.66176855
WT after UVB irradiation VS WT before UVB irradiation	Influenza Viral RNA Transcription and Replication|Reactome	-0.14122226	0.016990699	-0.43451174	0.67306925	0.88083472	-5.77445928
homo after UVB irradiation VS homo before UVB irradiation	Inhibition of voltage gated Ca2+ channels via Gbeta/gamma subunits|Reactome	0.227909476	-0.0130962	1.107580443	0.29560878	0.45577992	-5.75009619
hetero after UVB irradiation VS hetero before UVB irradiation	Inhibition of voltage gated Ca2+ channels via Gbeta/gamma subunits|Reactome	0.111975855	0.033657182	0.713384829	0.49488925	0.71428009	-6.07871261
WT after UVB irradiation VS WT before UVB irradiation	Inhibition of voltage gated Ca2+ channels via Gbeta/gamma subunits|Reactome	-0.00386805	-0.05146768	-0.01498999	0.98833265	0.99664845	-5.86903736
homo after UVB irradiation VS homo before UVB irradiation	Inhibition of adenylate cyclase pathway|Reactome	0.491541476	-0.06690044	2.289704283	0.04664908	0.14068672	-4.14680612
hetero after UVB irradiation VS hetero before UVB irradiation	Inhibition of adenylate cyclase pathway|Reactome	0.314368875	0.055650203	1.346396421	0.21327037	0.43535861	-5.45859701
WT after UVB irradiation VS WT before UVB irradiation	Inhibition of adenylate cyclase pathway|Reactome	0.347682046	-0.02244335	1.322676585	0.21515887	0.62581393	-5.05724231
homo after UVB irradiation VS homo before UVB irradiation	inhibition of cellular proliferation by gleevec|BioCarta	0.261444353	-0.01240093	1.420169499	0.18789993	0.34387505	-5.38970931
hetero after UVB irradiation VS hetero before UVB irradiation	inhibition of cellular proliferation by gleevec|BioCarta	0.309107372	0.013769252	2.020320612	0.07622683	0.24781874	-4.55304975
WT after UVB irradiation VS WT before UVB irradiation	inhibition of cellular proliferation by gleevec|BioCarta	0.07291885	0.003211748	0.342737831	0.73883408	0.91673557	-5.81001266
homo after UVB irradiation VS homo before UVB irradiation	Inhibition of exosome biogenesis and secretion by Manumycin A in CRPC cells|Wikipathways	0.302165492	-0.0373766	1.811982741	0.10202874	0.23269891	-4.86281872
hetero after UVB irradiation VS hetero before UVB irradiation	Inhibition of exosome biogenesis and secretion by Manumycin A in CRPC cells|Wikipathways	0.345230081	0.011462423	2.071244939	0.07032902	0.23727584	-4.47864189
WT after UVB irradiation VS WT before UVB irradiation	Inhibition of exosome biogenesis and secretion by Manumycin A in CRPC cells|Wikipathways	0.158833325	-0.04562328	0.74864269	0.47117662	0.81275627	-5.59342613
homo after UVB irradiation VS homo before UVB irradiation	Inhibition of Host mRNA Processing and RNA Silencing|Reactome	-0.71023469	0.06663706	-2.45564455	0.03538042	0.12484313	-3.88685346
hetero after UVB irradiation VS hetero before UVB irradiation	Inhibition of Host mRNA Processing and RNA Silencing|Reactome	-0.05529814	0.046926315	-0.13856886	0.89304558	0.96526008	-6.33613686
WT after UVB irradiation VS WT before UVB irradiation	Inhibition of Host mRNA Processing and RNA Silencing|Reactome	-0.26974164	0.047708322	-0.61616546	0.55144227	0.84516674	-5.68062577
homo after UVB irradiation VS homo before UVB irradiation	inhibition of huntingtons disease neurodegeneration by histone deacetylase inhibitors|BioCarta	-0.61698901	-0.03677846	-2.04135711	0.07031446	0.18687767	-4.5265242
hetero after UVB irradiation VS hetero before UVB irradiation	inhibition of huntingtons disease neurodegeneration by histone deacetylase inhibitors|BioCarta	-0.46176818	-0.0368403	-1.64625178	0.13641539	0.33602293	-5.07763397
WT after UVB irradiation VS WT before UVB irradiation	inhibition of huntingtons disease neurodegeneration by histone deacetylase inhibitors|BioCarta	-0.33759513	-0.09219787	-0.97725661	0.35130777	0.73631143	-5.40857835
homo after UVB irradiation VS homo before UVB irradiation	inhibition of matrix metalloproteinases|BioCarta	0.510882485	-0.05013729	2.554843642	0.02997863	0.115124	-3.72982499
hetero after UVB irradiation VS hetero before UVB irradiation	inhibition of matrix metalloproteinases|BioCarta	-0.09864052	-0.05432493	-0.32839919	0.75062287	0.88201573	-6.28841767
WT after UVB irradiation VS WT before UVB irradiation	inhibition of matrix metalloproteinases|BioCarta	-0.10239336	-0.08765099	-0.33633403	0.74351428	0.9185045	-5.81218881
homo after UVB irradiation VS homo before UVB irradiation	Inhibition of PKR|Reactome	1.07554672	-0.04829357	4.809790967	0.00085216	0.01759873	-0.2710278
hetero after UVB irradiation VS hetero before UVB irradiation	Inhibition of PKR|Reactome	1.076372068	0.039728381	4.408823331	0.00199256	0.05017737	-1.02624631
WT after UVB irradiation VS WT before UVB irradiation	Inhibition of PKR|Reactome	1.072463768	0.025834007	4.636807644	0.00090664	0.15645713	-0.39713762
homo after UVB irradiation VS homo before UVB irradiation	Inhibition of replication initiation of damaged DNA by RB1/E2F1|Reactome	-0.67654521	-0.04780822	-3.2767676	0.00905308	0.05693716	-2.57613987
hetero after UVB irradiation VS hetero before UVB irradiation	Inhibition of replication initiation of damaged DNA by RB1/E2F1|Reactome	-0.10180784	0.004088262	-0.34283412	0.74011982	0.87395496	-6.28323277
WT after UVB irradiation VS WT before UVB irradiation	Inhibition of replication initiation of damaged DNA by RB1/E2F1|Reactome	-0.19884583	-0.01647131	-0.76964589	0.45916214	0.80891799	-5.578213
homo after UVB irradiation VS homo before UVB irradiation	Inhibition of Signaling by Overexpressed EGFR|Reactome	-0.51530931	-0.03364261	-2.35238494	0.04202874	0.13402181	-4.04908417
hetero after UVB irradiation VS hetero before UVB irradiation	Inhibition of Signaling by Overexpressed EGFR|Reactome	-0.88562402	0.006290704	-4.4253647	0.00194745	0.04958933	-1.00389429
WT after UVB irradiation VS WT before UVB irradiation	Inhibition of Signaling by Overexpressed EGFR|Reactome	-0.58256044	0.040464599	-1.96237306	0.07789274	0.46277213	-4.23977438
homo after UVB irradiation VS homo before UVB irradiation	Inhibition of the proteolytic activity of APC/C required for the onset of anaphase by mitotic spindle checkpoint components|Reactome	-0.63084404	0.017603632	-3.36685914	0.00781628	0.05187376	-2.43314119
hetero after UVB irradiation VS hetero before UVB irradiation	Inhibition of the proteolytic activity of APC/C required for the onset of anaphase by mitotic spindle checkpoint components|Reactome	0.144484838	-0.03873269	0.813367947	0.43838468	0.66406028	-6.00143871
WT after UVB irradiation VS WT before UVB irradiation	Inhibition of the proteolytic activity of APC/C required for the onset of anaphase by mitotic spindle checkpoint components|Reactome	0.014443841	-0.08495071	0.05539309	0.95690764	0.98742794	-5.86759701
homo after UVB irradiation VS homo before UVB irradiation	Inhibition of TSC complex formation by PKB|Reactome	0.514498664	-0.05721763	2.567074693	0.02937225	0.11431817	-3.71039846
hetero after UVB irradiation VS hetero before UVB irradiation	Inhibition of TSC complex formation by PKB|Reactome	0.016909134	0.033044754	0.051797173	0.95989731	0.98139974	-6.34508605

WT after UVB irradiation VS WT before UVB irradiation	Inhibition of TSC complex formation by PKB|Reactome	-0.25909948	-0.1541775	-0.85121518	0.4144096	0.77949285	-5.51565956
homo after UVB irradiation VS homo before UVB irradiation	Initial triggering of complement|Reactome	0.693627289	-0.03873186	2.986597417	0.0145994	0.07468309	-3.03963939
hetero after UVB irradiation VS hetero before UVB irradiation	Initial triggering of complement|Reactome	0.335383245	0.032629945	1.213981803	0.25768153	0.48333845	-5.61162175
WT after UVB irradiation VS WT before UVB irradiation	Initial triggering of complement|Reactome	0.33068646	-0.02642444	1.057783699	0.31483942	0.69891341	-5.33390117
homo after UVB irradiation VS homo before UVB irradiation	Initiation of Nuclear Envelope Reformation|Reactome	-0.27069257	-0.01304465	-1.48450677	0.17045138	0.32096638	-5.30825042
hetero after UVB irradiation VS hetero before UVB irradiation	Initiation of Nuclear Envelope Reformation|Reactome	-0.07586628	-0.05159094	-0.44428114	0.66802929	0.82651398	-6.24073243
WT after UVB irradiation VS WT before UVB irradiation	Initiation of Nuclear Envelope Reformation|Reactome	0.007404765	-0.02239639	0.031612407	0.97539821	0.99212553	-5.86864496
homo after UVB irradiation VS homo before UVB irradiation	Initiation of transcription and translation elongation at the HIV-1 LTR|Wikipathways	-0.01125323	-0.03142966	-0.05731322	0.95549693	0.97297033	-6.36668363
hetero after UVB irradiation VS hetero before UVB irradiation	Initiation of transcription and translation elongation at the HIV-1 LTR|Wikipathways	0.123021223	-0.00898431	0.742832427	0.47777976	0.69910758	-6.05686813
WT after UVB irradiation VS WT before UVB irradiation	Initiation of transcription and translation elongation at the HIV-1 LTR|Wikipathways	0.072530921	-0.05423477	0.330410467	0.74785328	0.9185045	-5.81416609
homo after UVB irradiation VS homo before UVB irradiation	InlA-mediated entry of Listeria monocytogenes into host cells|Reactome	-0.18086443	0.095969344	-0.58251006	0.57394386	0.69732889	-6.18944022
hetero after UVB irradiation VS hetero before UVB irradiation	InlA-mediated entry of Listeria monocytogenes into host cells|Reactome	0.151178497	0.039890055	0.502211642	0.62837855	0.80830062	-6.21178305
WT after UVB irradiation VS WT before UVB irradiation	InlA-mediated entry of Listeria monocytogenes into host cells|Reactome	-0.48139691	0.055372498	-1.45923824	0.17494174	0.58617675	-4.89821219
homo after UVB irradiation VS homo before UVB irradiation	InlB-mediated entry of Listeria monocytogenes into host cell|Reactome	0.035626112	0.004714867	0.173547523	0.86590518	0.9179761	-6.35229191
hetero after UVB irradiation VS hetero before UVB irradiation	InlB-mediated entry of Listeria monocytogenes into host cell|Reactome	0.565849041	0.034341471	3.4782254	0.00769679	0.08930222	-2.350131
WT after UVB irradiation VS WT before UVB irradiation	InlB-mediated entry of Listeria monocytogenes into host cell|Reactome	-0.15676283	-0.02426266	-0.70010755	0.4996958	0.82071714	-5.62714621
homo after UVB irradiation VS homo before UVB irradiation	Innate Immune System|Reactome	0.585297202	-0.02340157	3.528956002	0.00601341	0.04530898	-2.17746046
hetero after UVB irradiation VS hetero before UVB irradiation	Innate Immune System|Reactome	0.618345337	0.024068142	4.030536123	0.00340084	0.06516742	-1.5492229
WT after UVB irradiation VS WT before UVB irradiation	Innate Immune System|Reactome	0.52077734	-0.00493275	2.621487886	0.02537236	0.33782961	-3.27700173
homo after UVB irradiation VS homo before UVB irradiation	inosine-5_-phosphate biosynthesis|HumanCyc	-0.5310017	-0.02652553	-1.68511606	0.12484808	0.26428203	-5.04093425
hetero after UVB irradiation VS hetero before UVB irradiation	inosine-5_-phosphate biosynthesis|HumanCyc	-0.67589913	-0.08753566	-2.23251986	0.05443504	0.2081501	-4.23966256
WT after UVB irradiation VS WT before UVB irradiation	inosine-5_-phosphate biosynthesis|HumanCyc	-0.10940288	0.001458012	-0.27893599	0.78593179	0.93869978	-5.82989849
homo after UVB irradiation VS homo before UVB irradiation	Inositol Metabolism|SMPDB	-0.14676596	-0.00836473	-0.69151306	0.5059806	0.64569799	-6.11802441
hetero after UVB irradiation VS hetero before UVB irradiation	Inositol Metabolism|SMPDB	-0.14251468	-0.02832938	-0.84164422	0.42322896	0.65017783	-5.97801709
WT after UVB irradiation VS WT before UVB irradiation	Inositol Metabolism|SMPDB	0.184375143	-0.02926978	0.843398326	0.41856572	0.78472481	-5.52188941
homo after UVB irradiation VS homo before UVB irradiation	Inositol phosphate metabolism - Homo sapiens (human)|KEGG	0.278654169	-0.03932041	1.635682319	0.13493331	0.277359	-5.10854336
hetero after UVB irradiation VS hetero before UVB irradiation	Inositol phosphate metabolism - Homo sapiens (human)|KEGG	0.146741091	0.007718287	0.948428674	0.3693158	0.59714134	-5.88361775
WT after UVB irradiation VS WT before UVB irradiation	Inositol phosphate metabolism - Homo sapiens (human)|KEGG	0.097949194	0.006658084	0.485184837	0.63791489	0.87636259	-5.75137434
homo after UVB irradiation VS homo before UVB irradiation	Inositol phosphate metabolism|INOH	0.437939613	-0.04481119	2.496490506	0.03304794	0.12110506	-3.82231809
hetero after UVB irradiation VS hetero before UVB irradiation	Inositol phosphate metabolism|INOH	0.361622828	0.023234365	2.026581097	0.0754768	0.24625858	-4.54393327
WT after UVB irradiation VS WT before UVB irradiation	Inositol phosphate metabolism|INOH	0.307249783	0.01326401	1.490118451	0.16679716	0.57064699	-4.86088971
homo after UVB irradiation VS homo before UVB irradiation	Inositol phosphate metabolism|Reactome	0.444425948	-0.02729318	2.514889871	0.03204781	0.11945329	-3.7931896
hetero after UVB irradiation VS hetero before UVB irradiation	Inositol phosphate metabolism|Reactome	0.242122238	0.016529325	1.500339301	0.17003044	0.38653303	-5.26839886
WT after UVB irradiation VS WT before UVB irradiation	Inositol phosphate metabolism|Reactome	0.285009333	0.036302419	1.425095575	0.18434287	0.59839062	-4.93891195
homo after UVB irradiation VS homo before UVB irradiation	Inositol Phosphate Metabolism|SMPDB	0.333555205	-0.01636124	1.578412639	0.14752889	0.29262246	-5.18549628
hetero after UVB irradiation VS hetero before UVB irradiation	Inositol Phosphate Metabolism|SMPDB	0.237897296	-0.00274914	1.39469923	0.19877676	0.41868045	-5.40025666
WT after UVB irradiation VS WT before UVB irradiation	Inositol Phosphate Metabolism|SMPDB	0.339711769	-0.03994697	1.70757404	0.11827012	0.52237876	-4.58554659
homo after UVB irradiation VS homo before UVB irradiation	inositol pyrophosphates biosynthesis|HumanCyc	-0.25692665	-0.01386037	-0.98582145	0.34894176	0.50461003	-5.87229061
hetero after UVB irradiation VS hetero before UVB irradiation	inositol pyrophosphates biosynthesis|HumanCyc	-0.54742432	-0.06138556	-2.40134739	0.04158793	0.17950546	-3.98536172
WT after UVB irradiation VS WT before UVB irradiation	inositol pyrophosphates biosynthesis|HumanCyc	-0.29632212	0.028237205	-1.18607978	0.26278954	0.65834329	-5.20554663
homo after UVB irradiation VS homo before UVB irradiation	Inositol transporters|Reactome	0.123005574	0.046734208	0.379432403	0.71280636	0.81002335	-6.29169678
hetero after UVB irradiation VS hetero before UVB irradiation	Inositol transporters|Reactome	0.160093968	0.103518386	0.377977748	0.71479197	0.85921866	-6.26970569
WT after UVB irradiation VS WT before UVB irradiation	Inositol transporters|Reactome	0.458808713	0.038219326	1.333333044	0.21176358	0.62581393	-5.04520328
homo after UVB irradiation VS homo before UVB irradiation	Insulin effects increased synthesis of Xylulose-5-Phosphate|Reactome	-1.00911256	0.013264989	-4.95132534	0.00069648	0.01666204	-0.07545335
hetero after UVB irradiation VS hetero before UVB irradiation	Insulin effects increased synthesis of Xylulose-5-Phosphate|Reactome	-0.64882726	-0.13013885	-1.75753498	0.11498122	0.30887493	-4.926306
WT after UVB irradiation VS WT before UVB irradiation	Insulin effects increased synthesis of Xylulose-5-Phosphate|Reactome	-0.41227088	-0.02093182	-1.01020055	0.33602833	0.71971842	-5.3785985
homo after UVB irradiation VS homo before UVB irradiation	insulin Mam|INOH	0.054871842	-0.02456878	0.295521099	0.77402726	0.85410231	-6.32174695
hetero after UVB irradiation VS hetero before UVB irradiation	insulin Mam|INOH	0.090213745	-0.01056604	0.552685264	0.59483347	0.7848684	-6.18385111
WT after UVB irradiation VS WT before UVB irradiation	insulin Mam|INOH	0.015276548	-0.00514564	0.078844299	0.9386994	0.98056728	-5.86600302
homo after UVB irradiation VS homo before UVB irradiation	Insulin Pathway|PID	0.352173679	-0.0675407	1.845344854	0.09670283	0.22459059	-4.81496776
hetero after UVB irradiation VS hetero before UVB irradiation	Insulin Pathway|PID	0.216505905	-0.0388735	1.235218188	0.25008	0.47455878	-5.58779634
WT after UVB irradiation VS WT before UVB irradiation	Insulin Pathway|PID	0.095382118	-0.05015635	0.463559696	0.65280921	0.87935805	-5.76152328
homo after UVB irradiation VS homo before UVB irradiation	Insulin processing|Reactome	-0.02365763	0.056727203	-0.08923456	0.93077004	0.95961548	-6.36417166
hetero after UVB irradiation VS hetero before UVB irradiation	Insulin processing|Reactome	0.118552728	0.026508997	0.563114232	0.58802453	0.78049383	-6.17776995
WT after UVB irradiation VS WT before UVB irradiation	Insulin processing|Reactome	-0.08549031	0.075951421	-0.36583039	0.72205076	0.90793111	-5.80183341
homo after UVB irradiation VS homo before UVB irradiation	Insulin receptor recycling|Reactome	0.307082925	-0.04391065	1.140912319	0.28217028	0.44245297	-5.71474255
hetero after UVB irradiation VS hetero before UVB irradiation	Insulin receptor recycling|Reactome	0.309568562	-0.00916404	1.711212513	0.1234945	0.31587425	-4.98985778
WT after UVB irradiation VS WT before UVB irradiation	Insulin receptor recycling|Reactome	0.322182679	-0.0195849	1.380125571	0.19738022	0.60852749	-4.99157942
homo after UVB irradiation VS homo before UVB irradiation	Insulin receptor signalling cascade|Reactome	-0.01347875	-0.02530155	-0.0795196	0.93828848	0.9636134	-6.36505185
hetero after UVB irradiation VS hetero before UVB irradiation	Insulin receptor signalling cascade|Reactome	-0.16929576	0.00584075	-1.07013875	0.31425082	0.54338429	-5.76518435
WT after UVB irradiation VS WT before UVB irradiation	Insulin receptor signalling cascade|Reactome	-0.30152656	0.002773409	-1.54425468	0.15331118	0.55854784	-4.7943315
homo after UVB irradiation VS homo before UVB irradiation	Insulin resistance - Homo sapiens (human)|KEGG	0.268489221	-0.02014705	1.716113363	0.11887801	0.2578608	-4.9980079
hetero after UVB irradiation VS hetero before UVB irradiation	Insulin resistance - Homo sapiens (human)|KEGG	0.012064331	-0.02699966	0.068845899	0.94671818	0.97253162	-6.34397074
WT after UVB irradiation VS WT before UVB irradiation	Insulin resistance - Homo sapiens (human)|KEGG	0.091816171	-0.04550797	0.408846336	0.69120165	0.89400112	-5.78521955
homo after UVB irradiation VS homo before UVB irradiation	Insulin secretion - Homo sapiens (human)|KEGG	0.514200083	-0.03301629	3.262662054	0.00926436	0.05763086	-2.5985783
hetero after UVB irradiation VS hetero before UVB irradiation	Insulin secretion - Homo sapiens (human)|KEGG	0.136608445	0.012749156	0.648383488	0.53400445	0.74771561	-6.12414791
WT after UVB irradiation VS WT before UVB irradiation	Insulin secretion - Homo sapiens (human)|KEGG	0.156050988	-0.02483091	0.617606151	0.550529	0.84516674	-5.67976061
homo after UVB irradiation VS homo before UVB irradiation	Insulin signaling pathway - Homo sapiens (human)|KEGG	0.204813831	-0.03268776	1.317454668	0.21892716	0.37735802	-5.51491914
hetero after UVB irradiation VS hetero before UVB irradiation	Insulin signaling pathway - Homo sapiens (human)|KEGG	0.020202238	-0.01395511	0.121682842	0.90600236	0.97054702	-6.33851598
WT after UVB irradiation VS WT before UVB irradiation	Insulin signaling pathway - Homo sapiens (human)|KEGG	0.095179187	-0.04306504	0.443272282	0.66692915	0.87935805	-5.77064238
homo after UVB irradiation VS homo before UVB irradiation	insulin signaling pathway|BioCarta	0.03720625	-0.04634958	0.198423785	0.84694356	0.90528768	-6.34733894
hetero after UVB irradiation VS hetero before UVB irradiation	insulin signaling pathway|BioCarta	-0.13783717	-0.011554	-0.65049917	0.53270261	0.74686511	-6.12273032
WT after UVB irradiation VS WT before UVB irradiation	insulin signaling pathway|BioCarta	-0.16244953	-0.03499954	-0.7153091	0.4906509	0.81710765	-5.61680214
homo after UVB irradiation VS homo before UVB irradiation	Insulin Signaling|Wikipathways	0.208221056	-0.01902353	1.272396568	0.23383784	0.39544576	-5.56784423
hetero after UVB irradiation VS hetero before UVB irradiation	Insulin Signaling|Wikipathways	0.097994947	-0.01877311	0.600390508	0.56404184	0.76437184	-6.15517801
WT after UVB irradiation VS WT before UVB irradiation	Insulin Signaling|Wikipathways	0.080535195	-0.01920284	0.401742868	0.69625719	0.89774324	-5.78808611
homo after UVB irradiation VS homo before UVB irradiation	Insulin Signalling|SMPDB	0.32186835	-0.05320673	1.899378356	0.08862315	0.21308757	-4.73665155
hetero after UVB irradiation VS hetero before UVB irradiation	Insulin Signalling|SMPDB	0.247286329	-0.02309791	1.281935787	0.23401058	0.45601549	-5.53440086
WT after UVB irradiation VS WT before UVB irradiation	Insulin Signalling|SMPDB	0.316414607	-0.00502542	1.501127883	0.1639739	0.56790008	-4.84746882
homo after UVB irradiation VS homo before UVB irradiation	insulin|INOH	0.09311992	-0.02986817	0.491800236	0.63415377	0.74627477	-6.24018432
hetero after UVB irradiation VS hetero before UVB irradiation	insulin|INOH	0.104266556	-0.01026232	0.625839129	0.54799441	0.75415414	-6.13899398
WT after UVB irradiation VS WT before UVB irradiation	insulin|INOH	0.02533335	-0.0094275	0.130974189	0.89837232	0.97566251	-5.86046889
homo after UVB irradiation VS homo before UVB irradiation	Insulin-like Growth Factor-2 mRNA Binding Proteins (IGF2BPs/IMPs/VICKZs) bind RNA|Reactome	0.364772251	0.01156027	1.350794278	0.20841334	0.36655365	-5.47495725
hetero after UVB irradiation VS hetero before UVB irradiation	Insulin-like Growth Factor-2 mRNA Binding Proteins (IGF2BPs/IMPs/VICKZs) bind RNA|Reactome	0.348152669	-0.03624358	1.342958397	0.21433567	0.43637221	-5.46270025
WT after UVB irradiation VS WT before UVB irradiation	Insulin-like Growth Factor-2 mRNA Binding Proteins (IGF2BPs/IMPs/VICKZs) bind RNA|Reactome	0.138870031	0.038597639	0.361379146	0.72527419	0.90898113	-5.80345037
homo after UVB irradiation VS homo before UVB irradiation	Insulin-mediated glucose transport|PID	0.461298815	-0.06894237	2.900198284	0.01685083	0.08176505	-3.17808625
hetero after UVB irradiation VS hetero before UVB irradiation	Insulin-mediated glucose transport|PID	0.244332159	0.000464242	1.261840916	0.24081365	0.46332993	-5.55753126
WT after UVB irradiation VS WT before UVB irradiation	Insulin-mediated glucose transport|PID	0.203131113	-0.06920976	0.943955692	0.36726291	0.74581032	-5.43805994
homo after UVB irradiation VS homo before UVB irradiation	Integrated Breast Cancer Pathway|Wikipathways	-0.20964986	0.003991624	-1.14677307	0.27985799	0.44047969	-5.70844495
hetero after UVB irradiation VS hetero before UVB irradiation	Integrated Breast Cancer Pathway|Wikipathways	0.118241318	-0.03629431	0.626209413	0.5477629	0.75406321	-6.13875398
WT after UVB irradiation VS WT before UVB irradiation	Integrated Breast Cancer Pathway|Wikipathways	-0.10467228	-0.05113692	-0.5083805	0.62212453	0.87257466	-5.74000002
homo after UVB irradiation VS homo before UVB irradiation	Integrated Cancer Pathway|Wikipathways	-0.32299323	-0.00791481	-1.90163887	0.08829931	0.21253275	-4.73335401
hetero after UVB irradiation VS hetero before UVB irradiation	Integrated Cancer Pathway|Wikipathways	0.044908832	-0.03505803	0.218870833	0.83195953	0.93531759	-6.32062914
WT after UVB irradiation VS WT before UVB irradiation	Integrated Cancer Pathway|Wikipathways	-0.45043769	-0.03503742	-1.83873388	0.09555359	0.49254317	-4.41024605
homo after UVB irradiation VS homo before UVB irradiation	Integrated Lung Cancer Pathway|Wikipathways	0.443905959	-0.01253332	2.169065836	0.05697839	0.16240116	-4.33292133
hetero after UVB irradiation VS hetero before UVB irradiation	Integrated Lung Cancer Pathway|Wikipathways	0.68148316	0.011965838	4.373809582	0.0020918	0.05083544	-1.07370498
WT after UVB irradiation VS WT before UVB irradiation	Integrated Lung Cancer Pathway|Wikipathways	0.388299278	0.003742997	1.914336362	0.08435819	0.4721371	-4.30655379
homo after UVB irradiation VS homo before UVB irradiation	Integration of energy metabolism|Reactome	0.334371589	-0.05280907	2.037363381	0.07077634	0.18768759	-4.53251335
hetero after UVB irradiation VS hetero before UVB irradiation	Integration of energy metabolism|Reactome	0.217519122	0.034616508	1.124901291	0.29165139	0.52251165	-5.70837762
WT after UVB irradiation VS WT before UVB irradiation	Integration of energy metabolism|Reactome	0.128470678	-0.02552845	0.551106196	0.59356253	0.8647222	-5.71773829
homo after UVB irradiation VS homo before UVB irradiation	Integration of provirus|Reactome	-0.46378481	-0.08372948	-2.19765356	0.05434625	0.15685522	-4.28907561
hetero after UVB irradiation VS hetero before UVB irradiation	Integration of provirus|Reactome	-0.35567104	-0.09946127	-1.36506221	0.20756538	0.42931834	-5.43620431
WT after UVB irradiation VS WT before UVB irradiation	Integration of provirus|Reactome	0.021156441	-0.00710736	0.070759425	0.94497313	0.98295421	-5.8666154
homo after UVB irradiation VS homo before UVB irradiation	Integration of viral DNA into host genomic DNA|Reactome	-0.72016939	-0.04289266	-2.76617486	0.02106445	0.09274857	-3.39275657
hetero after UVB irradiation VS hetero before UVB irradiation	Integration of viral DNA into host genomic DNA|Reactome	-0.34909744	-0.05229416	-1.08743415	0.30696955	0.53592103	-5.74747096
WT after UVB irradiation VS WT before UVB irradiation	Integration of viral DNA into host genomic DNA|Reactome	-0.14480261	-0.04989433	-0.37290872	0.71693662	0.90536666	-5.79922254
homo after UVB irradiation VS homo before UVB irradiation	Integrin alphaIIb beta3 signaling|Reactome	0.70180328	-0.02226647	4.33452503	0.00171363	0.02308086	-0.95103702
hetero after UVB irradiation VS hetero before UVB irradiation	Integrin alphaIIb beta3 signaling|Reactome	0.657494755	-0.00196073	3.378851385	0.00895309	0.09578437	-2.49825077
WT after UVB irradiation VS WT before UVB irradiation	Integrin alphaIIb beta3 signaling|Reactome	0.525640116	-0.01752301	2.399277414	0.03717367	0.39022568	-3.60844969
homo after UVB irradiation VS homo before UVB irradiation	Integrin cell surface interactions|Reactome	0.729091996	0.001154445	4.459489173	0.0014216	0.02159829	-0.76883343
hetero after UVB irradiation VS hetero before UVB irradiation	Integrin cell surface interactions|Reactome	0.657019412	0.038949828	3.635488287	0.00607427	0.0822456	-2.11801601
WT after UVB irradiation VS WT before UVB irradiation	Integrin cell surface interactions|Reactome	0.43993107	0.017403408	1.737615137	0.11267114	0.51456909	-4.54594988
homo after UVB irradiation VS homo before UVB irradiation	integrin signaling pathway|BioCarta	0.459208874	-0.03817766	2.451259766	0.03564032	0.12504267	-3.89377007
hetero after UVB irradiation VS hetero before UVB irradiation	integrin signaling pathway|BioCarta	0.360627016	0.020064268	2.355777946	0.04472423	0.18702113	-4.05432654
WT after UVB irradiation VS WT before UVB irradiation	integrin signaling pathway|BioCarta	0.365329502	-0.03344182	1.755719725	0.10941418	0.5076251	-4.5219219
homo after UVB irradiation VS homo before UVB irradiation	Integrin signaling|Reactome	0.70180328	-0.02226647	4.33452503	0.00171363	0.02308086	-0.95103702
hetero after UVB irradiation VS hetero before UVB irradiation	Integrin signaling|Reactome	0.657494755	-0.00196073	3.378851385	0.00895309	0.09578437	-2.49825077
WT after UVB irradiation VS WT before UVB irradiation	Integrin signaling|Reactome	0.525640116	-0.01752301	2.399277414	0.03717367	0.39022568	-3.60844969
homo after UVB irradiation VS homo before UVB irradiation	Integrin|INOH	0.543501742	-0.03953134	3.304891731	0.00864659	0.05544988	-2.53143945
hetero after UVB irradiation VS hetero before UVB irradiation	Integrin|INOH	0.447123061	0.032451892	2.588273931	0.03086859	0.15336156	-3.70075204
WT after UVB irradiation VS WT before UVB irradiation	Integrin|INOH	0.401719603	0.022565255	1.846007412	0.09441954	0.49001597	-4.40034912
homo after UVB irradiation VS homo before UVB irradiation	Integrin-linked kinase signaling|PID	0.163258354	-0.01121557	0.822960633	0.430935	0.58181948	-6.01743561
hetero after UVB irradiation VS hetero before UVB irradiation	Integrin-linked kinase signaling|PID	0.433578613	-0.00996277	2.77608451	0.02290582	0.13559055	-3.41337368
WT after UVB irradiation VS WT before UVB irradiation	Integrin-linked kinase signaling|PID	0.310935462	-0.07165502	1.365028824	0.20192821	0.61620632	-5.00901384
homo after UVB irradiation VS homo before UVB irradiation	Integrin-mediated Cell Adhesion|Wikipathways	0.486170936	-0.00568416	3.115701535	0.01179357	0.06636109	-2.83300342
hetero after UVB irradiation VS hetero before UVB irradiation	Integrin-mediated Cell Adhesion|Wikipathways	0.440905045	0.000229564	2.490857668	0.03605363	0.16681566	-3.84936228
WT after UVB irradiation VS WT before UVB irradiation	Integrin-mediated Cell Adhesion|Wikipathways	0.412852693	0.007915193	1.953918167	0.078996	0.46479598	-4.25157584
homo after UVB irradiation VS homo before UVB irradiation	Integrins in angiogenesis|PID	0.502940385	-0.0244265	2.967811891	0.01506129	0.07607282	-3.06973649
hetero after UVB irradiation VS hetero before UVB irradiation	Integrins in angiogenesis|PID	0.378714771	0.003929188	2.04290267	0.07355474	0.24223901	-4.52012433
WT after UVB irradiation VS WT before UVB irradiation	Integrins in angiogenesis|PID	0.369201876	-0.0072647	1.686873983	0.12227329	0.52731779	-4.61262711
homo after UVB irradiation VS homo before UVB irradiation	Interaction between L1 and Ankyrins|Reactome	0.595605577	0.007919962	3.390825329	0.00751775	0.05062836	-2.39520022
hetero after UVB irradiation VS hetero before UVB irradiation	Interaction between L1 and Ankyrins|Reactome	-0.06696018	0.024992893	-0.3327088	0.74748127	0.87923087	-6.28689242
WT after UVB irradiation VS WT before UVB irradiation	Interaction between L1 and Ankyrins|Reactome	0.034607359	-0.00041925	0.14025623	0.89122004	0.97373252	-5.859196
homo after UVB irradiation VS homo before UVB irradiation	Interaction between PHLDA1 and AURKA|Reactome	-0.46014907	0.028155767	-1.07601759	0.30878963	0.46923284	-5.7828355
hetero after UVB irradiation VS hetero before UVB irradiation	Interaction between PHLDA1 and AURKA|Reactome	0.836270635	0.03583414	2.758298349	0.02356042	0.13628011	-3.44060526
WT after UVB irradiation VS WT before UVB irradiation	Interaction between PHLDA1 and AURKA|Reactome	-0.0652964	-0.15682679	-0.16489918	0.87228267	0.96283151	-5.85539596
homo after UVB irradiation VS homo before UVB irradiation	Interaction With Cumulus Cells|Reactome	-0.64206428	0.024685222	-2.1533541	0.05847711	0.16492061	-4.35694474
WT after UVB irradiation VS WT before UVB irradiation	Interaction With Cumulus Cells|Reactome	0.361344364	0.044680807	1.174006972	0.26737798	0.66185108	-5.21809373
homo after UVB irradiation VS homo before UVB irradiation	Interaction With The Zona Pellucida|Reactome	0.02277563	0.05360429	0.093427622	0.92752721	0.95708231	-6.36376051
hetero after UVB irradiation VS hetero before UVB irradiation	Interaction With The Zona Pellucida|Reactome	-0.1400868	0.050829919	-0.4426843	0.66913877	0.82698875	-6.24148274
WT after UVB irradiation VS WT before UVB irradiation	Interaction With The Zona Pellucida|Reactome	-0.15329025	0.101197816	-0.7523711	0.46902933	0.8113852	-5.59075271
homo after UVB irradiation VS homo before UVB irradiation	Interactions of Rev with host cellular proteins|Reactome	-0.73476088	-0.00974656	-3.74252976	0.00427606	0.03747505	-1.84446117
hetero after UVB irradiation VS hetero before UVB irradiation	Interactions of Rev with host cellular proteins|Reactome	-0.09143061	-0.06266232	-0.33815138	0.74352088	0.87687018	-6.28493854
WT after UVB irradiation VS WT before UVB irradiation	Interactions of Rev with host cellular proteins|Reactome	-0.0402056	0.005188007	-0.12130099	0.90583616	0.97566251	-5.86170303
homo after UVB irradiation VS homo before UVB irradiation	Interactions of Tat with host cellular proteins|Reactome	0.624562494	0.006099203	1.733781453	0.11559277	0.25313985	-4.97336363
hetero after UVB irradiation VS hetero before UVB irradiation	Interactions of Tat with host cellular proteins|Reactome	0.200919431	0.02902075	0.528324546	0.61090298	0.7972333	-6.19764395
WT after UVB irradiation VS WT before UVB irradiation	Interactions of Tat with host cellular proteins|Reactome	0.19013453	-0.10240108	0.702523832	0.49825131	0.82026576	-5.62551536
homo after UVB irradiation VS homo before UVB irradiation	Interactions of Vpr with host cellular proteins|Reactome	-0.69207819	-0.00842544	-3.50481489	0.00625177	0.04604127	-2.21539233

hetero after UVB irradiation VS hetero before UVB irradiation	Interactions of Vpr with host cellular proteins|Reactome	-0.21586454	-0.06501532	-0.8319186	0.42840037	0.65540741	-5.98614917
WT after UVB irradiation VS WT before UVB irradiation	Interactions of Vpr with host cellular proteins|Reactome	-0.10025104	-0.02155164	-0.31380166	0.76006804	0.9249216	-5.8195269
homo after UVB irradiation VS homo before UVB irradiation	Interactome of polycomb repressive complex 2 (PRC2)|Wikipathways	-0.51710775	-0.01079385	-2.53625279	0.03092433	0.11717882	-3.75932756
hetero after UVB irradiation VS hetero before UVB irradiation	Interactome of polycomb repressive complex 2 (PRC2)|Wikipathways	-0.19576042	-0.04152845	-0.76288699	0.46634983	0.68896154	-6.04155029
WT after UVB irradiation VS WT before UVB irradiation	Interactome of polycomb repressive complex 2 (PRC2)|Wikipathways	-0.45510167	-0.04386428	-1.69960808	0.11979643	0.52405201	-4.59598792
homo after UVB irradiation VS homo before UVB irradiation	Interconversion of 2-oxoglutarate and 2-hydroxyglutarate|Reactome	-0.52283006	0.071624	-1.37184295	0.20199744	0.35894742	-5.44938664
hetero after UVB irradiation VS hetero before UVB irradiation	Interconversion of 2-oxoglutarate and 2-hydroxyglutarate|Reactome	-0.77133557	0.017107852	-3.17237579	0.0123026	0.10701384	-2.80907387
WT after UVB irradiation VS WT before UVB irradiation	Interconversion of 2-oxoglutarate and 2-hydroxyglutarate|Reactome	-0.14441075	-0.13499512	-0.36178441	0.72498048	0.90898113	-5.80330396
homo after UVB irradiation VS homo before UVB irradiation	Interconversion of nucleotide di- and triphosphates|Reactome	-0.01337178	-0.03057112	-0.06295159	0.95112491	0.97016767	-6.36631943
hetero after UVB irradiation VS hetero before UVB irradiation	Interconversion of nucleotide di- and triphosphates|Reactome	0.010242255	-0.01938096	0.043585154	0.96625034	0.98439183	-6.34551086
WT after UVB irradiation VS WT before UVB irradiation	Interconversion of nucleotide di- and triphosphates|Reactome	0.086857147	-0.03069248	0.378945806	0.71258633	0.90425832	-5.79695737
homo after UVB irradiation VS homo before UVB irradiation	Interconversion of polyamines|Reactome	0.514188452	-0.03761697	1.372291593	0.20186253	0.35894742	-5.44883879
hetero after UVB irradiation VS hetero before UVB irradiation	Interconversion of polyamines|Reactome	0.777178307	0.032244171	2.568268112	0.0318681	0.1555519	-3.73131017
WT after UVB irradiation VS WT before UVB irradiation	Interconversion of polyamines|Reactome	0.570714754	-0.13448014	1.827399885	0.09734579	0.49506164	-4.42563309
homo after UVB irradiation VS homo before UVB irradiation	Interferon alpha/beta signaling|Reactome	0.92257226	-0.03123351	5.145758185	0.00053039	0.01666204	0.187917301
hetero after UVB irradiation VS hetero before UVB irradiation	Interferon alpha/beta signaling|Reactome	0.756902689	0.027423254	3.79364375	0.00480309	0.07419102	-1.887713
WT after UVB irradiation VS WT before UVB irradiation	Interferon alpha/beta signaling|Reactome	0.720221701	0.009986644	3.047725233	0.01218897	0.27611764	-2.63646978
homo after UVB irradiation VS homo before UVB irradiation	Interferon alpha-beta signaling|Wikipathways	0.917070449	-0.01319683	4.522178699	0.0012955	0.02061012	-0.67833028
hetero after UVB irradiation VS hetero before UVB irradiation	Interferon alpha-beta signaling|Wikipathways	0.709355836	0.014094489	2.913753608	0.01843032	0.11936114	-3.20273758
WT after UVB irradiation VS WT before UVB irradiation	Interferon alpha-beta signaling|Wikipathways	0.749849997	0.00947627	2.998557702	0.01326097	0.28007727	-2.71023196
homo after UVB irradiation VS homo before UVB irradiation	Interferon gamma signaling|Reactome	0.564941188	-0.01823039	2.947230285	0.01558449	0.07717742	-3.10271563
hetero after UVB irradiation VS hetero before UVB irradiation	Interferon gamma signaling|Reactome	0.41187701	0.0435866	1.77584377	0.11176936	0.30432781	-4.9009795
WT after UVB irradiation VS WT before UVB irradiation	Interferon gamma signaling|Reactome	0.451001501	0.03091847	1.756491434	0.10927727	0.5076251	-4.52089501
homo after UVB irradiation VS homo before UVB irradiation	Interferon gamma signaling|Wikipathways	0.964053331	-0.04699592	4.776652394	0.00089375	0.01782281	-0.31728582
hetero after UVB irradiation VS hetero before UVB irradiation	Interferon gamma signaling|Wikipathways	0.729579347	0.088188261	2.126081606	0.06447316	0.22815889	-4.39791299
WT after UVB irradiation VS WT before UVB irradiation	Interferon gamma signaling|Wikipathways	0.772477385	0.075934147	2.592782872	0.0266579	0.34587112	-3.32002347
homo after UVB irradiation VS homo before UVB irradiation	Interferon Signaling|Reactome	0.599825387	-0.01836955	3.387608353	0.00755712	0.05066933	-2.40029043
hetero after UVB irradiation VS hetero before UVB irradiation	Interferon Signaling|Reactome	0.50749727	0.030712629	2.476097004	0.03691253	0.16802342	-3.87183137
WT after UVB irradiation VS WT before UVB irradiation	Interferon Signaling|Reactome	0.477543178	0.012271077	2.0132436	0.07155435	0.44888558	-4.16835927
homo after UVB irradiation VS homo before UVB irradiation	Interferon type I signaling pathways|Wikipathways	0.17808637	-0.03327453	1.138826952	0.28299667	0.44328055	-5.71697753
hetero after UVB irradiation VS hetero before UVB irradiation	Interferon type I signaling pathways|Wikipathways	0.3240719	0.004071543	2.134638927	0.06360326	0.22631041	-4.38526266
WT after UVB irradiation VS WT before UVB irradiation	Interferon type I signaling pathways|Wikipathways	0.145386077	-0.04913167	0.688079779	0.50692425	0.82465997	-5.6351889
homo after UVB irradiation VS homo before UVB irradiation	Interleukin receptor SHC signaling|Reactome	0.612390213	-0.04764033	3.387844959	0.00755422	0.05066933	-2.39991602
hetero after UVB irradiation VS hetero before UVB irradiation	Interleukin receptor SHC signaling|Reactome	0.53378668	0.055488069	2.427993436	0.03985685	0.17507375	-3.94494441
WT after UVB irradiation VS WT before UVB irradiation	Interleukin receptor SHC signaling|Reactome	0.488979745	0.009701284	1.870163088	0.09074197	0.48188205	-4.36735784
homo after UVB irradiation VS homo before UVB irradiation	Interleukin-1 family signaling|Reactome	0.503289207	-0.03938318	2.450447543	0.03568867	0.12504267	-3.89505104
hetero after UVB irradiation VS hetero before UVB irradiation	Interleukin-1 family signaling|Reactome	0.513888689	0.025989102	3.157826187	0.01258342	0.10799973	-2.83111149
WT after UVB irradiation VS WT before UVB irradiation	Interleukin-1 family signaling|Reactome	0.244493085	0.007788576	1.253196745	0.23841722	0.63195092	-5.13410266
homo after UVB irradiation VS homo before UVB irradiation	Interleukin-1 family signaling|Wikipathways	0.080966585	-0.00275874	0.356277764	0.7295075	0.8217964	-6.30071594
hetero after UVB irradiation VS hetero before UVB irradiation	Interleukin-1 family signaling|Wikipathways	0.141992671	0.018013206	0.756623021	0.46990049	0.69247644	-6.04637285
WT after UVB irradiation VS WT before UVB irradiation	Interleukin-1 family signaling|Wikipathways	-0.04120671	-0.0068508	-0.19658358	0.84805953	0.95372763	-5.84961409
homo after UVB irradiation VS homo before UVB irradiation	Interleukin-1 Induced Activation of NF-kappa-B|Wikipathways	0.388163695	-0.07208342	2.036238561	0.07090694	0.18768759	-4.53419941
hetero after UVB irradiation VS hetero before UVB irradiation	Interleukin-1 Induced Activation of NF-kappa-B|Wikipathways	0.484019426	-0.01357037	2.537760501	0.03345569	0.15967648	-3.77787393
WT after UVB irradiation VS WT before UVB irradiation	Interleukin-1 Induced Activation of NF-kappa-B|Wikipathways	0.286367479	-0.07347677	1.243871877	0.24168962	0.63358831	-5.14419696
homo after UVB irradiation VS homo before UVB irradiation	Interleukin-1 processing|Reactome	0.990473706	-0.02784224	4.132237822	0.00232966	0.02721394	-1.25090533
hetero after UVB irradiation VS hetero before UVB irradiation	Interleukin-1 processing|Reactome	0.987240049	0.052393841	5.724579478	0.00036469	0.03004176	0.611296484
WT after UVB irradiation VS WT before UVB irradiation	Interleukin-1 processing|Reactome	0.912658684	0.02839318	3.992503212	0.00250796	0.17621298	-1.26135806
homo after UVB irradiation VS homo before UVB irradiation	Interleukin-1 signaling|Reactome	0.368019913	-0.03696928	1.662122251	0.12945116	0.27051441	-5.07251467
hetero after UVB irradiation VS hetero before UVB irradiation	Interleukin-1 signaling|Reactome	0.465152819	-0.00329459	2.748714503	0.02392105	0.13697507	-3.45527887
WT after UVB irradiation VS WT before UVB irradiation	Interleukin-1 signaling|Reactome	0.13723984	-0.01911483	0.704278639	0.49720387	0.81975814	-5.62432781
homo after UVB irradiation VS homo before UVB irradiation	Interleukin-10 signaling|Reactome	1.183467929	-0.00713548	7.261858488	3.81E-05	0.01666204	2.667822759
hetero after UVB irradiation VS hetero before UVB irradiation	Interleukin-10 signaling|Reactome	1.04852112	0.084434914	6.25804291	0.00019599	0.02988205	1.195720991
WT after UVB irradiation VS WT before UVB irradiation	Interleukin-10 signaling|Reactome	0.772002996	-0.02839374	2.703431154	0.02203244	0.31542987	-3.15398186
homo after UVB irradiation VS homo before UVB irradiation	Interleukin-10 signaling|Wikipathways	0.693137971	0.024968967	3.61107959	0.00527116	0.04193048	-2.04884748
hetero after UVB irradiation VS hetero before UVB irradiation	Interleukin-10 signaling|Wikipathways	0.569807528	0.051259787	3.034731597	0.01524371	0.11352943	-3.01813258
WT after UVB irradiation VS WT before UVB irradiation	Interleukin-10 signaling|Wikipathways	0.3384556	-0.01484255	1.16437637	0.27108362	0.66327566	-5.22803467
homo after UVB irradiation VS homo before UVB irradiation	Interleukin-11 Signaling Pathway|Wikipathways	0.243728227	-0.03943404	1.229697607	0.24873234	0.41104302	-5.6168098
hetero after UVB irradiation VS hetero before UVB irradiation	Interleukin-11 Signaling Pathway|Wikipathways	0.494111771	-0.00760241	3.17393287	0.01227294	0.10695993	-2.80671637
WT after UVB irradiation VS WT before UVB irradiation	Interleukin-11 Signaling Pathway|Wikipathways	0.452403597	-0.02343598	2.118810427	0.05992321	0.42812412	-4.01810342
homo after UVB irradiation VS homo before UVB irradiation	Interleukin-12 family signaling|Reactome	0.76143199	0.00456822	4.246352042	0.0019577	0.02455727	-1.08100895
hetero after UVB irradiation VS hetero before UVB irradiation	Interleukin-12 family signaling|Reactome	0.571656697	0.013783218	2.070378113	0.07042561	0.23742598	-4.47991314
WT after UVB irradiation VS WT before UVB irradiation	Interleukin-12 family signaling|Reactome	0.541255952	-0.01662798	1.870779632	0.09064986	0.48188205	-4.36651333
homo after UVB irradiation VS homo before UVB irradiation	Interleukin-12 family signaling|Wikipathways	1.045193416	0.002355563	6.04477287	0.0001613	0.01666204	1.325961976
hetero after UVB irradiation VS hetero before UVB irradiation	Interleukin-12 family signaling|Wikipathways	1.068489094	0.095486783	4.467247621	0.00183808	0.04958933	-0.94749642
WT after UVB irradiation VS WT before UVB irradiation	Interleukin-12 family signaling|Wikipathways	0.340722466	-0.08179519	0.955377005	0.36173281	0.74374432	-5.42804286
homo after UVB irradiation VS homo before UVB irradiation	Interleukin-12 signaling|Reactome	0.580571981	-0.03928249	3.234141696	0.00970726	0.0585513	-2.64398401
hetero after UVB irradiation VS hetero before UVB irradiation	Interleukin-12 signaling|Reactome	0.516374218	0.007720722	1.967817305	0.08280563	0.2599606	-4.62914079
WT after UVB irradiation VS WT before UVB irradiation	Interleukin-12 signaling|Reactome	0.429002319	-0.0689478	1.405858278	0.18982752	0.60496032	-4.96157492
homo after UVB irradiation VS homo before UVB irradiation	Interleukin-15 signaling|Reactome	0.91337441	-0.04942813	4.937132995	0.00071062	0.01666204	-0.09491837
hetero after UVB irradiation VS hetero before UVB irradiation	Interleukin-15 signaling|Reactome	0.699538666	0.019857798	2.395345984	0.04198807	0.18071921	-3.9944559
WT after UVB irradiation VS WT before UVB irradiation	Interleukin-15 signaling|Reactome	0.698354485	0.018014102	2.39388938	0.03751851	0.39022568	-3.61642904
homo after UVB irradiation VS homo before UVB irradiation	Interleukin-17 signaling|Reactome	0.154328249	-0.01313828	0.856985189	0.41279119	0.56380634	-5.98893251
hetero after UVB irradiation VS hetero before UVB irradiation	Interleukin-17 signaling|Reactome	0.368629581	0.009636885	2.376489101	0.04327056	0.18278703	-4.02300803
WT after UVB irradiation VS WT before UVB irradiation	Interleukin-17 signaling|Reactome	0.023449524	-0.00470322	0.118596826	0.90792445	0.97566251	-5.86203117
homo after UVB irradiation VS homo before UVB irradiation	Interleukin-18 signaling|Reactome	1.106258238	-0.0361061	6.362399003	0.00010872	0.01666204	1.697394575
hetero after UVB irradiation VS hetero before UVB irradiation	Interleukin-18 signaling|Reactome	0.899995668	0.032024655	3.689264577	0.00560605	0.08008296	-2.03934048
WT after UVB irradiation VS WT before UVB irradiation	Interleukin-18 signaling|Reactome	0.903222586	0.12168661	3.382506484	0.00689398	0.22490313	-2.1382221
homo after UVB irradiation VS homo before UVB irradiation	Interleukin-2 family signaling|Reactome	0.578510908	-0.05430409	3.255833806	0.00936846	0.05808098	-2.60944476
hetero after UVB irradiation VS hetero before UVB irradiation	Interleukin-2 family signaling|Reactome	0.546684583	0.065564106	2.470074021	0.03726889	0.16852339	-3.88099537
WT after UVB irradiation VS WT before UVB irradiation	Interleukin-2 family signaling|Reactome	0.441686531	-0.02266988	1.658586383	0.12794104	0.53754069	-4.64935638
homo after UVB irradiation VS homo before UVB irradiation	Interleukin-2 signaling|Reactome	0.70082252	-0.08044401	3.90185074	0.00332789	0.03256113	-1.59943317
hetero after UVB irradiation VS hetero before UVB irradiation	Interleukin-2 signaling|Reactome	0.74946404	0.059145072	3.618494217	0.00623071	0.08279755	-2.14295473
WT after UVB irradiation VS WT before UVB irradiation	Interleukin-2 signaling|Reactome	0.642231605	-0.04902796	2.242694656	0.04857368	0.40333815	-3.8387584
homo after UVB irradiation VS homo before UVB irradiation	Interleukin-20 family signaling|Reactome	0.495815418	0.003091182	2.685544357	0.02409852	0.10226192	-3.52165915
hetero after UVB irradiation VS hetero before UVB irradiation	Interleukin-20 family signaling|Reactome	0.328769311	0.005329785	1.320267999	0.22148094	0.44257349	-5.48961291
WT after UVB irradiation VS WT before UVB irradiation	Interleukin-20 family signaling|Reactome	0.280981695	0.039994397	1.403425953	0.19053077	0.60496032	-4.96442634
homo after UVB irradiation VS homo before UVB irradiation	Interleukin-20 family signaling|Wikipathways	0.549536117	-0.02306991	1.389530092	0.19673659	0.35310505	-5.42769995
hetero after UVB irradiation VS hetero before UVB irradiation	Interleukin-20 family signaling|Wikipathways	0.425190982	0.010682566	1.07280757	0.31311854	0.54224708	-5.76246493
WT after UVB irradiation VS WT before UVB irradiation	Interleukin-20 family signaling|Wikipathways	0.194729531	-0.06450208	0.591763237	0.56704082	0.85130684	-5.69499599
homo after UVB irradiation VS homo before UVB irradiation	Interleukin-23 signaling|Reactome	0.599784119	-0.01241319	2.733668422	0.02223832	0.096982	-3.44475654
hetero after UVB irradiation VS hetero before UVB irradiation	Interleukin-23 signaling|Reactome	0.639761429	-0.01456431	2.175132509	0.05963998	0.21834459	-4.32522176
WT after UVB irradiation VS WT before UVB irradiation	Interleukin-23 signaling|Reactome	0.623831511	-0.03067793	2.076205747	0.06438282	0.43544914	-4.0790571
homo after UVB irradiation VS homo before UVB irradiation	Interleukin-27 signaling|Reactome	1.085454555	0.050342962	6.752114325	6.82E-05	0.01666204	2.132241035
hetero after UVB irradiation VS hetero before UVB irradiation	Interleukin-27 signaling|Reactome	0.840011387	0.060097597	3.673442048	0.0057397	0.08047311	-2.06245063
WT after UVB irradiation VS WT before UVB irradiation	Interleukin-27 signaling|Reactome	0.697173017	-0.02715771	2.483200682	0.0321874	0.36849539	-3.48376128
homo after UVB irradiation VS homo before UVB irradiation	Interleukin-3_ 5 and GM-CSF signaling|Reactome	0.508513169	-0.02628398	2.816789452	0.0193601	0.08858881	-3.31172399
hetero after UVB irradiation VS hetero before UVB irradiation	Interleukin-3_ 5 and GM-CSF signaling|Reactome	0.522337018	0.043669552	2.645121225	0.02819839	0.14497177	-3.6138399
WT after UVB irradiation VS WT before UVB irradiation	Interleukin-3_ 5 and GM-CSF signaling|Reactome	0.440784533	-0.00259701	1.971997515	0.07665445	0.46222215	-4.22631632
homo after UVB irradiation VS homo before UVB irradiation	Interleukin-33 signaling|Reactome	1.093643618	0.004197229	6.105043141	0.00014951	0.01666204	1.397643027
hetero after UVB irradiation VS hetero before UVB irradiation	Interleukin-33 signaling|Reactome	0.910236467	0.06339618	3.474616882	0.007739	0.08930222	-2.35549088
WT after UVB irradiation VS WT before UVB irradiation	Interleukin-33 signaling|Reactome	0.377036053	0.06185004	1.179696794	0.26520762	0.66118679	-5.21219215
homo after UVB irradiation VS homo before UVB irradiation	Interleukin-36 pathway|Reactome	0.706469171	-0.00955803	3.987670592	0.00291148	0.03063096	-1.46877161
hetero after UVB irradiation VS hetero before UVB irradiation	Interleukin-36 pathway|Reactome	0.585616448	0.075873827	2.481556084	0.0365925	0.16733232	-3.86352315
WT after UVB irradiation VS WT before UVB irradiation	Interleukin-36 pathway|Reactome	0.292497381	0.127236388	1.35224893	0.2058468	0.62078923	-5.02367369
homo after UVB irradiation VS homo before UVB irradiation	Interleukin-37 signaling|Reactome	0.867314509	-0.0599284	3.713390589	0.00447824	0.03821988	-1.88960298
hetero after UVB irradiation VS hetero before UVB irradiation	Interleukin-37 signaling|Reactome	0.491822384	0.040493424	1.434970459	0.18735242	0.40490864	-5.35066015
WT after UVB irradiation VS WT before UVB irradiation	Interleukin-37 signaling|Reactome	0.371266039	-0.06262167	0.967380878	0.35598584	0.74135309	-5.41740851
homo after UVB irradiation VS homo before UVB irradiation	Interleukin-38 signaling|Reactome	-0.1997647	0.078703118	-0.64173051	0.53639855	0.671933	-6.15202664
hetero after UVB irradiation VS hetero before UVB irradiation	Interleukin-38 signaling|Reactome	-0.47641771	0.045524089	-2.59697239	0.03044396	0.15198638	-3.68746039
WT after UVB irradiation VS WT before UVB irradiation	Interleukin-38 signaling|Reactome	-0.52924335	0.004795192	-2.07678782	0.06431982	0.43544914	-4.0782271
homo after UVB irradiation VS homo before UVB irradiation	Interleukin-4 and 13 signaling|Reactome	0.850793729	-0.03198746	5.220957114	0.00047805	0.01666204	0.288126798
hetero after UVB irradiation VS hetero before UVB irradiation	Interleukin-4 and 13 signaling|Reactome	0.733447957	0.074612213	3.505043472	0.0073906	0.08749704	-2.3103429
WT after UVB irradiation VS WT before UVB irradiation	Interleukin-4 and 13 signaling|Reactome	0.743199896	0.005839319	2.82617018	0.01783445	0.30735819	-2.96939243
homo after UVB irradiation VS homo before UVB irradiation	Interleukin-4 and Interleukin-13 signaling|Wikipathways	0.45152934	0.001161356	2.709507205	0.02315343	0.09964047	-3.48337971
hetero after UVB irradiation VS hetero before UVB irradiation	Interleukin-4 and Interleukin-13 signaling|Wikipathways	0.374997088	0.044381293	2.112463144	0.06588153	0.23045894	-4.4180168
WT after UVB irradiation VS WT before UVB irradiation	Interleukin-4 and Interleukin-13 signaling|Wikipathways	0.121326275	-0.04469382	0.449572066	0.66252959	0.87935805	-5.76785246
homo after UVB irradiation VS homo before UVB irradiation	Interleukin-6 family signaling|Reactome	0.368327443	-0.03309402	2.059933844	0.06820363	0.18264973	-4.49861078
hetero after UVB irradiation VS hetero before UVB irradiation	Interleukin-6 family signaling|Reactome	0.196853627	0.018291601	1.138411135	0.28627942	0.5159595	-5.69404385
WT after UVB irradiation VS WT before UVB irradiation	Interleukin-6 family signaling|Reactome	0.339392949	-0.05113917	1.324065512	0.21471379	0.62581393	-5.05567688
homo after UVB irradiation VS homo before UVB irradiation	Interleukin-6 signaling|Reactome	0.61975036	-0.00047697	3.725732006	0.00439141	0.0379263	-1.87047204
hetero after UVB irradiation VS hetero before UVB irradiation	Interleukin-6 signaling|Reactome	0.458130228	0.055606698	2.65376046	0.02781363	0.14438349	-3.60062331
WT after UVB irradiation VS WT before UVB irradiation	Interleukin-6 signaling|Reactome	0.415768861	-0.06749239	1.53813398	0.15478656	0.5588223	-4.80192678
homo after UVB irradiation VS homo before UVB irradiation	Interleukin-7 signaling|Reactome	0.478804109	-0.04256952	2.343701251	0.04264096	0.13402181	-4.06265936
hetero after UVB irradiation VS hetero before UVB irradiation	Interleukin-7 signaling|Reactome	0.240862329	0.051204765	1.164560848	0.27610685	0.50643663	-5.66594907
WT after UVB irradiation VS WT before UVB irradiation	Interleukin-7 signaling|Reactome	0.280371475	-0.07109304	1.055663217	0.31576166	0.69936155	-5.33592756
homo after UVB irradiation VS homo before UVB irradiation	Interleukin-7 signaling|Wikipathways	-0.23991764	0.01916179	-0.79815002	0.44450099	0.59189021	-6.03759226
hetero after UVB irradiation VS hetero before UVB irradiation	Interleukin-7 signaling|Wikipathways	-0.18420503	-0.0834472	-0.65958193	0.52713539	0.74191436	-6.11659727
WT after UVB irradiation VS WT before UVB irradiation	Interleukin-7 signaling|Wikipathways	-0.49305632	0.006686783	-1.55788847	0.15006906	0.55622203	-4.77735032
homo after UVB irradiation VS homo before UVB irradiation	Interleukin-9 signaling|Reactome	0.929915458	-0.09849263	4.913444989	0.00073491	0.01666498	-0.12747956
hetero after UVB irradiation VS hetero before UVB irradiation	Interleukin-9 signaling|Reactome	0.695937055	-0.00414847	2.492733343	0.03594593	0.16681566	-3.84650602
WT after UVB irradiation VS WT before UVB irradiation	Interleukin-9 signaling|Reactome	0.388467258	-0.12958919	0.948591337	0.36501107	0.74545864	-5.43400618
homo after UVB irradiation VS homo before UVB irradiation	internal ribosome entry pathway|BioCarta	0.039649569	-0.02282505	0.152279223	0.88218892	0.928984	-6.35600449
hetero after UVB irradiation VS hetero before UVB irradiation	internal ribosome entry pathway|BioCarta	0.45313439	0.024822781	1.715028132	0.12277178	0.31455525	-4.98465226
WT after UVB irradiation VS WT before UVB irradiation	internal ribosome entry pathway|BioCarta	0.355880542	0.026010613	1.641575364	0.13146177	0.54481479	-4.67128556
homo after UVB irradiation VS homo before UVB irradiation	Internalization of ErbB1|PID	0.458624583	-0.03888577	2.356912537	0.04171297	0.13402181	-4.0420017
hetero after UVB irradiation VS hetero before UVB irradiation	Internalization of ErbB1|PID	0.510388019	-0.00018195	3.33851607	0.00952295	0.0985871	-2.55866337
WT after UVB irradiation VS WT before UVB irradiation	Internalization of ErbB1|PID	0.319472657	-0.01612258	1.624608616	0.13505931	0.54785342	-4.69303641
homo after UVB irradiation VS homo before UVB irradiation	Intestinal absorption|Reactome	0.693744892	-0.02531972	3.670508294	0.00479419	0.03957638	-1.95620953
hetero after UVB irradiation VS hetero before UVB irradiation	Intestinal absorption|Reactome	0.223901891	0.064260579	0.774183314	0.45999142	0.68405903	-6.03276638
WT after UVB irradiation VS WT before UVB irradiation	Intestinal absorption|Reactome	0.026480438	0.017077899	0.085591143	0.93346731	0.98009095	-5.8654414
homo after UVB irradiation VS homo before UVB irradiation	Intestinal hexose absorption|Reactome	0.550056927	-0.03327539	2.867968772	0.0177787	0.08412182	-3.22973333
hetero after UVB irradiation VS hetero before UVB irradiation	Intestinal hexose absorption|Reactome	-0.0677389	0.037434757	-0.21077043	0.83807472	0.93902276	-6.32250657
WT after UVB irradiation VS WT before UVB irradiation	Intestinal hexose absorption|Reactome	-0.21872473	0.0004111	-0.68071471	0.51138168	0.8267256	-5.64005161
homo after UVB irradiation VS homo before UVB irradiation	Intestinal immune network for IgA production - Homo sapiens (human)|KEGG	0.407047586	-0.07424107	1.812257328	0.10198382	0.23269891	-4.86242652
hetero after UVB irradiation VS hetero before UVB irradiation	Intestinal immune network for IgA production - Homo sapiens (human)|KEGG	0.087479852	0.023893981	0.377545269	0.71510148	0.85921866	-6.26987991
WT after UVB irradiation VS WT before UVB irradiation	Intestinal immune network for IgA production - Homo sapiens (human)|KEGG	0.16695269	-0.00375923	0.550785006	0.59377465	0.8647222	-5.71791194
homo after UVB irradiation VS homo before UVB irradiation	Intestinal infectious diseases|Reactome	-1.02385686	0.020129225	-5.30929929	0.00042355	0.01666204	0.404672706
hetero after UVB irradiation VS hetero before UVB irradiation	Intestinal infectious diseases|Reactome	-0.73142455	-0.03792725	-2.72709106	0.0247556	0.13846576	-3.48838538

WT after UVB irradiation VS WT before UVB irradiation	Intestinal infectious diseases|Reactome	-0.8078207	-0.00709088	-2.79964363	0.01866781	0.31542987	-3.00930048
homo after UVB irradiation VS homo before UVB irradiation	Intestinal lipid absorption|Reactome	1.091782638	-0.03255467	5.312872826	0.00042149	0.01666204	0.409360339
hetero after UVB irradiation VS hetero before UVB irradiation	Intestinal lipid absorption|Reactome	1.100345734	0.043351345	4.783132359	0.00119848	0.04202057	-0.53137211
WT after UVB irradiation VS WT before UVB irradiation	Intestinal lipid absorption|Reactome	0.607202836	0.040886567	1.627487699	0.13444273	0.54709793	-4.68935415
homo after UVB irradiation VS homo before UVB irradiation	Intracellular oxygen transport|Reactome	0.345816528	-0.00027419	1.237332813	0.24601343	0.40847175	-5.60814082
hetero after UVB irradiation VS hetero before UVB irradiation	Intracellular oxygen transport|Reactome	0.382534179	0.065729771	1.058706782	0.31913709	0.5497456	-5.77677521
WT after UVB irradiation VS WT before UVB irradiation	Intracellular oxygen transport|Reactome	0.436825588	0.081129384	1.150945662	0.27631907	0.66649013	-5.2417965
homo after UVB irradiation VS homo before UVB irradiation	Intracellular signaling by second messengers|Reactome	0.075823832	-0.01348844	0.44198807	0.66850324	0.77373531	-6.26459162
hetero after UVB irradiation VS hetero before UVB irradiation	Intracellular signaling by second messengers|Reactome	0.085193388	0.001387213	0.558307979	0.59115716	0.783511	-6.18058558
WT after UVB irradiation VS WT before UVB irradiation	Intracellular signaling by second messengers|Reactome	-0.01834442	0.004899811	-0.09447374	0.92658406	0.97639811	-5.86463183
homo after UVB irradiation VS homo before UVB irradiation	Intracellular Signalling Through Adenosine Receptor A2a and Adenosine|SMPDB	0.247034828	-0.02307922	1.306346461	0.22252744	0.38212121	-5.52808388
hetero after UVB irradiation VS hetero before UVB irradiation	Intracellular Signalling Through Adenosine Receptor A2a and Adenosine|SMPDB	0.523109388	-0.02432114	3.270097443	0.01057828	0.10415756	-2.66149405
WT after UVB irradiation VS WT before UVB irradiation	Intracellular Signalling Through Adenosine Receptor A2a and Adenosine|SMPDB	0.242546059	-0.05083936	1.177488801	0.26604818	0.66118679	-5.21448481
homo after UVB irradiation VS homo before UVB irradiation	Intracellular Signalling Through Adenosine Receptor A2b and Adenosine|SMPDB	0.238518696	-0.01600443	1.260136083	0.23803753	0.39958801	-5.58202433
hetero after UVB irradiation VS hetero before UVB irradiation	Intracellular Signalling Through Adenosine Receptor A2b and Adenosine|SMPDB	0.503194999	-0.03289433	3.121131801	0.01332174	0.11120965	-2.88675871
WT after UVB irradiation VS WT before UVB irradiation	Intracellular Signalling Through Adenosine Receptor A2b and Adenosine|SMPDB	0.258112511	-0.04473559	1.24605976	0.24091856	0.63358831	-5.14183335
homo after UVB irradiation VS homo before UVB irradiation	Intracellular Signalling Through FSH Receptor and Follicle Stimulating Hormone|SMPDB	0.503170469	0.000741612	2.649105727	0.0256106	0.10578915	-3.57980936
hetero after UVB irradiation VS hetero before UVB irradiation	Intracellular Signalling Through FSH Receptor and Follicle Stimulating Hormone|SMPDB	0.848800921	0.040164885	4.4371921	0.00191587	0.04958933	-0.98793924
WT after UVB irradiation VS WT before UVB irradiation	Intracellular Signalling Through FSH Receptor and Follicle Stimulating Hormone|SMPDB	0.726046823	-0.12679237	3.1948682	0.009479	0.25023459	-2.41647074
homo after UVB irradiation VS homo before UVB irradiation	Intracellular Signalling Through Histamine H2 Receptor and Histamine|SMPDB	0.633678894	-0.00810128	3.761854159	0.00414728	0.03698535	-1.81457823
hetero after UVB irradiation VS hetero before UVB irradiation	Intracellular Signalling Through Histamine H2 Receptor and Histamine|SMPDB	0.85519289	0.042919731	4.42941263	0.00193658	0.04958933	-0.99843113
WT after UVB irradiation VS WT before UVB irradiation	Intracellular Signalling Through Histamine H2 Receptor and Histamine|SMPDB	0.749860264	-0.08565304	3.39998949	0.00669344	0.22490313	-2.11245933
homo after UVB irradiation VS homo before UVB irradiation	Intracellular Signalling Through LHCGR Receptor and Luteinizing Hormone/Choriogonadotropin|SMPDB	0.382078	0.055534907	1.865801767	0.09356622	0.21982504	-4.78543348
hetero after UVB irradiation VS hetero before UVB irradiation	Intracellular Signalling Through LHCGR Receptor and Luteinizing Hormone/Choriogonadotropin|SMPDB	0.848800921	0.040164885	4.4371921	0.00191587	0.04958933	-0.98793924
WT after UVB irradiation VS WT before UVB irradiation	Intracellular Signalling Through LHCGR Receptor and Luteinizing Hormone/Choriogonadotropin|SMPDB	0.6088905	-0.06862424	2.627279466	0.02512056	0.33545493	-3.26831634
homo after UVB irradiation VS homo before UVB irradiation	Intracellular Signalling Through PGD2 receptor and Prostaglandin D2|SMPDB	0.486693979	-0.04141625	2.221440108	0.05224637	0.15243255	-4.25246624
hetero after UVB irradiation VS hetero before UVB irradiation	Intracellular Signalling Through PGD2 receptor and Prostaglandin D2|SMPDB	0.791847058	0.071677726	3.523405762	0.00718838	0.08667895	-2.28314738
WT after UVB irradiation VS WT before UVB irradiation	Intracellular Signalling Through PGD2 receptor and Prostaglandin D2|SMPDB	0.622032954	-0.10961871	2.715609703	0.02157502	0.31542987	-3.13567839
homo after UVB irradiation VS homo before UVB irradiation	Intracellular Signalling Through Prostacyclin Receptor and Prostacyclin|SMPDB	0.531808118	-0.08700021	2.356385274	0.04174962	0.13402181	-4.04282664
hetero after UVB irradiation VS hetero before UVB irradiation	Intracellular Signalling Through Prostacyclin Receptor and Prostacyclin|SMPDB	0.800628978	0.048651515	3.576007765	0.00664078	0.08470408	-2.20545959
WT after UVB irradiation VS WT before UVB irradiation	Intracellular Signalling Through Prostacyclin Receptor and Prostacyclin|SMPDB	0.668057748	-0.06040623	2.571925641	0.02763238	0.35054739	-3.35125359
homo after UVB irradiation VS homo before UVB irradiation	Intraflagellar transport|Reactome	-0.24642002	-0.0252156	-1.20728754	0.25685413	0.42035697	-5.64203198
hetero after UVB irradiation VS hetero before UVB irradiation	Intraflagellar transport|Reactome	-0.27725551	-0.03898077	-1.39332712	0.19917644	0.4193285	-5.40193142
WT after UVB irradiation VS WT before UVB irradiation	Intraflagellar transport|Reactome	-0.36959119	-0.03309769	-1.81774502	0.09889683	0.49761536	-4.43870659
homo after UVB irradiation VS homo before UVB irradiation	Intra-Golgi and retrograde Golgi-to-ER traffic|Reactome	0.257435324	-0.02734523	1.437904111	0.18294257	0.33695675	-5.36747961
hetero after UVB irradiation VS hetero before UVB irradiation	Intra-Golgi and retrograde Golgi-to-ER traffic|Reactome	0.324213227	0.003222878	2.002528107	0.07839787	0.25179907	-4.57890962
WT after UVB irradiation VS WT before UVB irradiation	Intra-Golgi and retrograde Golgi-to-ER traffic|Reactome	0.186665687	-0.00761208	0.885774029	0.39637417	0.76478278	-5.48753156
homo after UVB irradiation VS homo before UVB irradiation	Intra-Golgi traffic|Reactome	0.312597565	-0.03667375	1.457484264	0.17760089	0.33035788	-5.34273642
hetero after UVB irradiation VS hetero before UVB irradiation	Intra-Golgi traffic|Reactome	0.27633185	-0.01504335	1.684098295	0.1287443	0.32349311	-5.02669234
WT after UVB irradiation VS WT before UVB irradiation	Intra-Golgi traffic|Reactome	0.079378154	-0.01467203	0.395112559	0.70099008	0.90028732	-5.79071792
homo after UVB irradiation VS homo before UVB irradiation	Intrinsic Pathway for Apoptosis|Reactome	0.280180831	-0.08460486	1.32545985	0.21636292	0.37419996	-5.50538511
hetero after UVB irradiation VS hetero before UVB irradiation	Intrinsic Pathway for Apoptosis|Reactome	0.447024996	-0.01464251	2.993164278	0.01626882	0.11352943	-3.08148754
WT after UVB irradiation VS WT before UVB irradiation	Intrinsic Pathway for Apoptosis|Reactome	0.331839587	-0.02376827	1.691223348	0.12142217	0.52647129	-4.60695119
homo after UVB irradiation VS homo before UVB irradiation	Intrinsic Pathway for Apoptosis|Wikipathways	-0.22490455	-0.10348359	-0.72723895	0.4848169	0.62736836	-6.0922164
hetero after UVB irradiation VS hetero before UVB irradiation	Intrinsic Pathway for Apoptosis|Wikipathways	-0.37393108	0.062255763	-1.10383198	0.30018909	0.53033405	-5.73048169
WT after UVB irradiation VS WT before UVB irradiation	Intrinsic Pathway for Apoptosis|Wikipathways	-0.13221801	-0.13777803	-0.51780219	0.61576704	0.87257466	-5.73523644
homo after UVB irradiation VS homo before UVB irradiation	Intrinsic Pathway of Fibrin Clot Formation|Reactome	0.374235278	0.028297785	1.990143896	0.07646092	0.19626432	-4.60299651
hetero after UVB irradiation VS hetero before UVB irradiation	Intrinsic Pathway of Fibrin Clot Formation|Reactome	0.0365705	0.057299867	0.205422371	0.84211851	0.94031753	-6.32370771
WT after UVB irradiation VS WT before UVB irradiation	Intrinsic Pathway of Fibrin Clot Formation|Reactome	0.106702965	-0.02002642	0.49717603	0.62972755	0.87292043	-5.74555718
homo after UVB irradiation VS homo before UVB irradiation	intrinsic prothrombin activation pathway|BioCarta	0.350981638	0.04377211	1.965243029	0.07963057	0.19933837	-4.63991154
hetero after UVB irradiation VS hetero before UVB irradiation	intrinsic prothrombin activation pathway|BioCarta	0.298230459	0.020991363	1.836420127	0.10173063	0.28675832	-4.81639252
WT after UVB irradiation VS WT before UVB irradiation	intrinsic prothrombin activation pathway|BioCarta	0.119459463	0.036506238	0.443416256	0.66682845	0.87935805	-5.77057904
homo after UVB irradiation VS homo before UVB irradiation	Invadopodia formation|Reactome	1.118845478	-0.02899028	6.663595875	7.57E-05	0.01666204	2.035447659
hetero after UVB irradiation VS hetero before UVB irradiation	Invadopodia formation|Reactome	0.898319688	0.052009059	3.377381045	0.00897322	0.09578437	-2.50045013
WT after UVB irradiation VS WT before UVB irradiation	Invadopodia formation|Reactome	0.69628632	0.089557825	2.093599094	0.06252528	0.43544914	-4.05422171
homo after UVB irradiation VS homo before UVB irradiation	Inwardly rectifying K+ channels|Reactome	0.386771533	0.013032774	1.94835467	0.0818506	0.20289352	-4.66484405
hetero after UVB irradiation VS hetero before UVB irradiation	Inwardly rectifying K+ channels|Reactome	0.168610673	0.04293926	0.925819528	0.38029168	0.6077733	-5.90437095
WT after UVB irradiation VS WT before UVB irradiation	Inwardly rectifying K+ channels|Reactome	0.06806541	-0.03484455	0.251417405	0.80654167	0.94525798	-5.83723668
homo after UVB irradiation VS homo before UVB irradiation	Ion channel transport|Reactome	0.16527277	-0.01035523	0.963904945	0.35926118	0.51438901	-5.89307919
hetero after UVB irradiation VS hetero before UVB irradiation	Ion channel transport|Reactome	0.034823717	0.012233155	0.219774568	0.83127801	0.93531602	-6.32041534
WT after UVB irradiation VS WT before UVB irradiation	Ion channel transport|Reactome	-0.03043934	0.002793669	-0.15633318	0.87885646	0.96716565	-5.85678611
homo after UVB irradiation VS homo before UVB irradiation	ion channels and their functional role in vascular endothelium|BioCarta	0.407605722	-0.01786192	2.513716313	0.03211069	0.11947408	-3.79504851
hetero after UVB irradiation VS hetero before UVB irradiation	ion channels and their functional role in vascular endothelium|BioCarta	0.115645999	0.047657988	0.575776638	0.57981523	0.77397513	-6.17024523
WT after UVB irradiation VS WT before UVB irradiation	ion channels and their functional role in vascular endothelium|BioCarta	0.130596567	0.008930188	0.448883346	0.66300991	0.87935805	-5.7681593
homo after UVB irradiation VS homo before UVB irradiation	Ion homeostasis|Reactome	0.324080104	-0.0502757	1.807410902	0.10277942	0.23382702	-4.86934488
hetero after UVB irradiation VS hetero before UVB irradiation	Ion homeostasis|Reactome	-0.02285274	-0.00575477	-0.10002724	0.92266188	0.97054702	-6.3411167
WT after UVB irradiation VS WT before UVB irradiation	Ion homeostasis|Reactome	0.137649873	-0.03358937	0.524251068	0.61143459	0.87257466	-5.73192831
homo after UVB irradiation VS homo before UVB irradiation	Ion influx/efflux at host-pathogen interface|Reactome	-0.01620924	-0.00150336	-0.08467053	0.93430132	0.96168165	-6.36459777
hetero after UVB irradiation VS hetero before UVB irradiation	Ion influx/efflux at host-pathogen interface|Reactome	0.294518936	0.06344122	1.018936433	0.33659475	0.56570754	-5.81635771
WT after UVB irradiation VS WT before UVB irradiation	Ion influx/efflux at host-pathogen interface|Reactome	0.32022362	0.061477663	1.245745566	0.24102916	0.63358831	-5.14217296
homo after UVB irradiation VS homo before UVB irradiation	Ion transport by P-type ATPases|Reactome	0.249510379	-0.00301512	1.339974797	0.21177771	0.36957778	-5.48799907
hetero after UVB irradiation VS hetero before UVB irradiation	Ion transport by P-type ATPases|Reactome	0.103775469	0.030592773	0.612827189	0.556166	0.76057744	-6.14734545
WT after UVB irradiation VS WT before UVB irradiation	Ion transport by P-type ATPases|Reactome	0.149739165	0.00572932	0.776891466	0.45506375	0.80866776	-5.57287889
homo after UVB irradiation VS homo before UVB irradiation	ionomycin and phorbal ester signaling pathway|BioCarta	-0.05801062	0.077812083	-0.35140741	0.73303993	0.82411885	-6.30254279
hetero after UVB irradiation VS hetero before UVB irradiation	ionomycin and phorbal ester signaling pathway|BioCarta	0.24143869	0.034451114	0.928580537	0.37893868	0.60603597	-5.90185823
WT after UVB irradiation VS WT before UVB irradiation	ionomycin and phorbal ester signaling pathway|BioCarta	-0.10610494	-0.02460116	-0.50492265	0.62446603	0.87257466	-5.74172755
homo after UVB irradiation VS homo before UVB irradiation	Ionotropic activity of kainate receptors|Reactome	-0.4255247	-0.05440509	-1.68269443	0.12532577	0.26466492	-5.04427086
hetero after UVB irradiation VS hetero before UVB irradiation	Ionotropic activity of kainate receptors|Reactome	-0.32439968	-0.04214232	-1.61046316	0.14405119	0.34739964	-5.12527674
WT after UVB irradiation VS WT before UVB irradiation	Ionotropic activity of kainate receptors|Reactome	-0.13972547	0.069563579	-0.63121751	0.54194318	0.83916339	-5.67149504
homo after UVB irradiation VS homo before UVB irradiation	IRAK1 recruits IKK complex upon TLR7/8 or 9 stimulation|Reactome	0.299763468	-0.06065958	1.449058506	0.17988275	0.33360654	-5.35340946
hetero after UVB irradiation VS hetero before UVB irradiation	IRAK1 recruits IKK complex upon TLR7/8 or 9 stimulation|Reactome	0.200111763	-0.03696362	0.938014118	0.37434241	0.60270317	-5.89322745
WT after UVB irradiation VS WT before UVB irradiation	IRAK1 recruits IKK complex upon TLR7/8 or 9 stimulation|Reactome	0.044379884	-0.00890479	0.200000168	0.84545689	0.95361858	-5.84893055
homo after UVB irradiation VS homo before UVB irradiation	IRAK1 recruits IKK complex|Reactome	0.299763468	-0.06065958	1.449058506	0.17988275	0.33360654	-5.35340946
hetero after UVB irradiation VS hetero before UVB irradiation	IRAK1 recruits IKK complex|Reactome	0.200111763	-0.03696362	0.938014118	0.37434241	0.60270317	-5.89322745
WT after UVB irradiation VS WT before UVB irradiation	IRAK1 recruits IKK complex|Reactome	0.044379884	-0.00890479	0.200000168	0.84545689	0.95361858	-5.84893055
homo after UVB irradiation VS homo before UVB irradiation	IRAK2 mediated activation of TAK1 complex upon TLR7/8 or 9 stimulation|Reactome	0.305805203	-0.0893148	1.343329819	0.21072959	0.36865567	-5.48396239
hetero after UVB irradiation VS hetero before UVB irradiation	IRAK2 mediated activation of TAK1 complex upon TLR7/8 or 9 stimulation|Reactome	0.470494954	0.119816978	1.253942438	0.24353251	0.46639545	-5.56655585
WT after UVB irradiation VS WT before UVB irradiation	IRAK2 mediated activation of TAK1 complex upon TLR7/8 or 9 stimulation|Reactome	0.269062545	0.063738132	0.8258475	0.42800004	0.79361599	-5.53569708
homo after UVB irradiation VS homo before UVB irradiation	IRAK2 mediated activation of TAK1 complex|Reactome	0.305805203	-0.0893148	1.343329819	0.21072959	0.36865567	-5.48396239
hetero after UVB irradiation VS hetero before UVB irradiation	IRAK2 mediated activation of TAK1 complex|Reactome	0.470494954	0.119816978	1.253942438	0.24353251	0.46639545	-5.56655585
WT after UVB irradiation VS WT before UVB irradiation	IRAK2 mediated activation of TAK1 complex|Reactome	0.269062545	0.063738132	0.8258475	0.42800004	0.79361599	-5.53569708
homo after UVB irradiation VS homo before UVB irradiation	Irbesartan Action Pathway|SMPDB	0.636038586	-0.11800708	2.436891056	0.03650532	0.12504267	-3.91641947
hetero after UVB irradiation VS hetero before UVB irradiation	Irbesartan Action Pathway|SMPDB	-0.02777369	0.003323189	-0.09172332	0.92906122	0.97054702	-6.34197953
WT after UVB irradiation VS WT before UVB irradiation	Irbesartan Action Pathway|SMPDB	0.270438532	-0.10210744	0.707878218	0.49505953	0.81710765	-5.62188349
homo after UVB irradiation VS homo before UVB irradiation	IRE1alpha activates chaperones|Reactome	0.204028727	-0.03514026	0.665486475	0.52175028	0.65938966	-6.13608827
hetero after UVB irradiation VS hetero before UVB irradiation	IRE1alpha activates chaperones|Reactome	0.285290241	-0.02945646	0.872600663	0.4070583	0.63366521	-5.95160718
WT after UVB irradiation VS WT before UVB irradiation	IRE1alpha activates chaperones|Reactome	0.106771939	-0.09329895	0.363053627	0.72406093	0.90882588	-5.80284436
homo after UVB irradiation VS homo before UVB irradiation	IRF3 mediated activation of type 1 IFN|Reactome	1.117392098	-0.04909295	5.795323358	0.00022194	0.01666204	1.023204307
hetero after UVB irradiation VS hetero before UVB irradiation	IRF3 mediated activation of type 1 IFN|Reactome	0.804492936	0.160005258	2.743142824	0.0241333	0.13715811	-3.46380951
WT after UVB irradiation VS WT before UVB irradiation	IRF3 mediated activation of type 1 IFN|Reactome	0.768052522	-0.07875203	3.415221145	0.00652363	0.22490313	-2.09003924
homo after UVB irradiation VS homo before UVB irradiation	IRF3-mediated induction of type I IFN|Reactome	0.417100116	-0.01602026	2.143537975	0.0594327	0.16679145	-4.37192618
hetero after UVB irradiation VS hetero before UVB irradiation	IRF3-mediated induction of type I IFN|Reactome	0.369637063	-0.01682238	1.708825354	0.12394864	0.31597198	-4.99311177
WT after UVB irradiation VS WT before UVB irradiation	IRF3-mediated induction of type I IFN|Reactome	0.562554284	-0.02425355	2.338013789	0.04128413	0.39022568	-3.69896688
homo after UVB irradiation VS homo before UVB irradiation	Irinotecan Action Pathway|SMPDB	-0.17727385	0.019282749	-1.01296387	0.33646815	0.4922959	-5.84602433
hetero after UVB irradiation VS hetero before UVB irradiation	Irinotecan Action Pathway|SMPDB	0.10519638	0.025664886	0.591922207	0.56944089	0.76631579	-6.16042716
WT after UVB irradiation VS WT before UVB irradiation	Irinotecan Action Pathway|SMPDB	0.218610486	0.047350117	0.978466496	0.35073776	0.73579208	-5.40749154
homo after UVB irradiation VS homo before UVB irradiation	Irinotecan Metabolism Pathway|SMPDB	-0.17727385	0.019282749	-1.01296387	0.33646815	0.4922959	-5.84602433
hetero after UVB irradiation VS hetero before UVB irradiation	Irinotecan Metabolism Pathway|SMPDB	0.10519638	0.025664886	0.591922207	0.56944089	0.76631579	-6.16042716
WT after UVB irradiation VS WT before UVB irradiation	Irinotecan Metabolism Pathway|SMPDB	0.218610486	0.047350117	0.978466496	0.35073776	0.73579208	-5.40749154
homo after UVB irradiation VS homo before UVB irradiation	Irinotecan Pathway_ Pharmacodynamics|PharmGKB	-0.35517973	0.071072046	-1.90172168	0.08828746	0.21253275	-4.73323318
hetero after UVB irradiation VS hetero before UVB irradiation	Irinotecan Pathway_ Pharmacodynamics|PharmGKB	-0.30714137	0.017950658	-1.91805622	0.08954056	0.27117998	-4.70061861
WT after UVB irradiation VS WT before UVB irradiation	Irinotecan Pathway_ Pharmacodynamics|PharmGKB	-0.26567981	-0.03733039	-1.05976011	0.31398168	0.6974486	-5.3320096
homo after UVB irradiation VS homo before UVB irradiation	Irinotecan Pathway_ Pharmacokinetics|PharmGKB	-0.02875097	0.057336705	-0.13838099	0.89286189	0.9365512	-6.35817
hetero after UVB irradiation VS hetero before UVB irradiation	Irinotecan Pathway_ Pharmacokinetics|PharmGKB	-0.29769299	0.037114321	-1.4530804	0.18240461	0.40144867	-5.32808333
WT after UVB irradiation VS WT before UVB irradiation	Irinotecan Pathway_ Pharmacokinetics|PharmGKB	-0.00737625	0.038700207	-0.02434627	0.98105154	0.99411191	-5.86885092
homo after UVB irradiation VS homo before UVB irradiation	Irinotecan Pathway|Wikipathways	-0.11704086	0.052093593	-0.58812007	0.57032551	0.69442834	-6.18603939
hetero after UVB irradiation VS hetero before UVB irradiation	Irinotecan Pathway|Wikipathways	-0.52393656	0.033986308	-2.9287016	0.01800188	0.11857306	-3.17989526
WT after UVB irradiation VS WT before UVB irradiation	Irinotecan Pathway|Wikipathways	-0.22290049	0.078850202	-0.87623101	0.4012991	0.76940932	-5.49539362
homo after UVB irradiation VS homo before UVB irradiation	Iron metabolism in placenta|Wikipathways	0.271962948	0.007675895	1.589620368	0.14498429	0.29047751	-5.17055659
hetero after UVB irradiation VS hetero before UVB irradiation	Iron metabolism in placenta|Wikipathways	-0.00938813	-0.01851804	-0.05189508	0.95982158	0.98139974	-6.34508054
WT after UVB irradiation VS WT before UVB irradiation	Iron metabolism in placenta|Wikipathways	0.308642356	-0.06746071	1.345851935	0.20783208	0.6232233	-5.03097728
homo after UVB irradiation VS homo before UVB irradiation	Iron uptake and transport|Reactome	0.280607967	-0.02079745	1.272791792	0.23370348	0.39536938	-5.56738553
hetero after UVB irradiation VS hetero before UVB irradiation	Iron uptake and transport|Reactome	0.328303653	0.019296952	1.859893417	0.09807357	0.28242841	-4.78330393
WT after UVB irradiation VS WT before UVB irradiation	Iron uptake and transport|Reactome	0.331718279	-0.00678952	1.533895237	0.15581557	0.5603226	-4.80717638
homo after UVB irradiation VS homo before UVB irradiation	iron-sulfur cluster biosynthesis|HumanCyc	-0.66206925	-0.01998426	-1.94354128	0.08249396	0.20394538	-4.67193437
hetero after UVB irradiation VS hetero before UVB irradiation	iron-sulfur cluster biosynthesis|HumanCyc	0.104631182	-0.0187566	0.249247268	0.80913828	0.92095935	-6.31296652
WT after UVB irradiation VS WT before UVB irradiation	iron-sulfur cluster biosynthesis|HumanCyc	0.017582053	0.011465655	0.04119885	0.96794183	0.98924463	-5.8682914
homo after UVB irradiation VS homo before UVB irradiation	IRS activation|Reactome	0.195548323	-0.08487732	0.980086226	0.35162081	0.50726717	-5.87776722
hetero after UVB irradiation VS hetero before UVB irradiation	IRS activation|Reactome	-0.5356645	-0.04194724	-1.41933636	0.1917178	0.4112234	-5.37001525
WT after UVB irradiation VS WT before UVB irradiation	IRS activation|Reactome	-0.3412968	-0.21119763	-0.97430247	0.3527024	0.73755198	-5.41122737
homo after UVB irradiation VS homo before UVB irradiation	IRS-mediated signalling|Reactome	-0.12613679	0.001107786	-0.75144788	0.47079811	0.61612616	-6.07407107
hetero after UVB irradiation VS hetero before UVB irradiation	IRS-mediated signalling|Reactome	-0.2180791	0.017380816	-1.363227	0.20812042	0.43001491	-5.43841452
WT after UVB irradiation VS WT before UVB irradiation	IRS-mediated signalling|Reactome	-0.37813024	0.009366734	-1.91936054	0.08365921	0.470709	-4.29960062
homo after UVB irradiation VS homo before UVB irradiation	IRS-related events triggered by IGF1R|Reactome	-0.08905738	-0.00687544	-0.52446519	0.61211103	0.72935777	-6.22283933
hetero after UVB irradiation VS hetero before UVB irradiation	IRS-related events triggered by IGF1R|Reactome	-0.17877106	0.012105192	-1.11921435	0.2939366	0.52498552	-5.71437405
WT after UVB irradiation VS WT before UVB irradiation	IRS-related events triggered by IGF1R|Reactome	-0.34751472	-0.00916097	-1.77184194	0.10658628	0.50548239	-4.5004234
homo after UVB irradiation VS homo before UVB irradiation	ISG15 antiviral mechanism|Reactome	0.347607886	-0.02335997	1.540015468	0.1565501	0.30417351	-5.2362208
hetero after UVB irradiation VS hetero before UVB irradiation	ISG15 antiviral mechanism|Reactome	0.620930005	0.036520012	3.181568961	0.01212854	0.10610726	-2.7951576
WT after UVB irradiation VS WT before UVB irradiation	ISG15 antiviral mechanism|Reactome	0.325530443	-0.03890634	1.479128812	0.16965727	0.57650651	-4.87422676
homo after UVB irradiation VS homo before UVB irradiation	Isobutyryl-coa dehydrogenase deficiency|SMPDB	-0.23657683	-0.05522798	-0.8011182	0.4428632	0.59091566	-6.03520901
hetero after UVB irradiation VS hetero before UVB irradiation	Isobutyryl-coa dehydrogenase deficiency|SMPDB	-0.90022729	-0.04719335	-5.31014337	0.00060637	0.03004176	0.126180045
WT after UVB irradiation VS WT before UVB irradiation	Isobutyryl-coa dehydrogenase deficiency|SMPDB	-0.36319435	-0.13086657	-1.31407353	0.21793282	0.62581393	-5.06691375
homo after UVB irradiation VS homo before UVB irradiation	isoleucine degradation|HumanCyc	-0.39923482	0.004104881	-1.23853671	0.24558694	0.40791196	-5.60677045
hetero after UVB irradiation VS hetero before UVB irradiation	isoleucine degradation|HumanCyc	-0.92827016	-0.07904725	-5.50201297	0.0004778	0.03004176	0.354226893
WT after UVB irradiation VS WT before UVB irradiation	isoleucine degradation|HumanCyc	-0.29953034	-0.09438873	-1.16124728	0.27229632	0.66431878	-5.23125152
homo after UVB irradiation VS homo before UVB irradiation	Isoprenaline Action Pathway|SMPDB	0.396415397	-0.03677376	2.343069613	0.04268583	0.13402181	-4.06364635
hetero after UVB irradiation VS hetero before UVB irradiation	Isoprenaline Action Pathway|SMPDB	-0.01555055	-0.01775524	-0.07410913	0.94265284	0.97054702	-6.34356282
WT after UVB irradiation VS WT before UVB irradiation	Isoprenaline Action Pathway|SMPDB	0.056685205	-0.03978418	0.223404833	0.82768199	0.94525798	-5.84393438

homo after UVB irradiation VS homo before UVB irradiation	Isovaleric acidemia|SMPDB	-0.23657683	-0.05522798	-0.8011182	0.4428632	0.59091566	-6.03520901
hetero after UVB irradiation VS hetero before UVB irradiation	Isovaleric acidemia|SMPDB	-0.90022729	-0.04719335	-5.31014337	0.00060637	0.03004176	0.126180045
WT after UVB irradiation VS WT before UVB irradiation	Isovaleric acidemia|SMPDB	-0.36319435	-0.13086657	-1.31407353	0.21793282	0.62581393	-5.06691375
homo after UVB irradiation VS homo before UVB irradiation	Isovaleric Aciduria|SMPDB	-0.23657683	-0.05522798	-0.8011182	0.4428632	0.59091566	-6.03520901
hetero after UVB irradiation VS hetero before UVB irradiation	Isovaleric Aciduria|SMPDB	-0.90022729	-0.04719335	-5.31014337	0.00060637	0.03004176	0.126180045
WT after UVB irradiation VS WT before UVB irradiation	Isovaleric Aciduria|SMPDB	-0.36319435	-0.13086657	-1.31407353	0.21793282	0.62581393	-5.06691375
homo after UVB irradiation VS homo before UVB irradiation	Isradipine Action Pathway|SMPDB	0.396415397	-0.03677376	2.343069613	0.04268583	0.13402181	-4.06364635
hetero after UVB irradiation VS hetero before UVB irradiation	Isradipine Action Pathway|SMPDB	-0.01555055	-0.01775524	-0.07410913	0.94265284	0.97054702	-6.34356282
WT after UVB irradiation VS WT before UVB irradiation	Isradipine Action Pathway|SMPDB	0.056685205	-0.03978418	0.223404833	0.82768199	0.94525798	-5.84393438
homo after UVB irradiation VS homo before UVB irradiation	JAK STAT MolecularVariation 1|INOH	0.631351196	-0.01102862	3.853523712	0.00358959	0.03395341	-1.67342847
hetero after UVB irradiation VS hetero before UVB irradiation	JAK STAT MolecularVariation 1|INOH	0.425033608	0.03317374	1.87434895	0.0958836	0.27962159	-4.76284468
WT after UVB irradiation VS WT before UVB irradiation	JAK STAT MolecularVariation 1|INOH	0.345055013	-0.03139097	1.140006924	0.28064164	0.66929002	-5.25291668
homo after UVB irradiation VS homo before UVB irradiation	JAK STAT MolecularVariation 2|INOH	0.688393998	-0.02700287	3.972851682	0.00297926	0.03091661	-1.49126449
hetero after UVB irradiation VS hetero before UVB irradiation	JAK STAT MolecularVariation 2|INOH	0.468340379	0.041625962	1.872546577	0.0961541	0.280149	-4.76539898
WT after UVB irradiation VS WT before UVB irradiation	JAK STAT MolecularVariation 2|INOH	0.367388053	-0.06270112	1.203543893	0.25626332	0.65122698	-5.18723026
homo after UVB irradiation VS homo before UVB irradiation	JAK STAT pathway and regulation|INOH	0.118070036	-0.01398316	0.646301177	0.53356154	0.6696652	-6.1490011
hetero after UVB irradiation VS hetero before UVB irradiation	JAK STAT pathway and regulation|INOH	0.194315442	-0.00900129	1.289647149	0.23144308	0.45275431	-5.52546031
WT after UVB irradiation VS WT before UVB irradiation	JAK STAT pathway and regulation|INOH	0.158418474	-0.03222096	0.745497502	0.47299288	0.81341158	-5.5956722
homo after UVB irradiation VS homo before UVB irradiation	JAK-STAT signaling pathway - Homo sapiens (human)|KEGG	0.355962392	-0.01715597	2.205292107	0.05366314	0.1552911	-4.27733161
hetero after UVB irradiation VS hetero before UVB irradiation	JAK-STAT signaling pathway - Homo sapiens (human)|KEGG	0.200589751	0.014739913	1.082136556	0.30918563	0.53747829	-5.75291916
WT after UVB irradiation VS WT before UVB irradiation	JAK-STAT signaling pathway - Homo sapiens (human)|KEGG	0.204413214	-0.02613045	0.826558134	0.42761529	0.79358242	-5.53514287
homo after UVB irradiation VS homo before UVB irradiation	JAK-STAT|Wikipathways	0.308098984	-0.02348398	1.758777425	0.11108681	0.24479845	-4.93828509
hetero after UVB irradiation VS hetero before UVB irradiation	JAK-STAT|Wikipathways	0.32035886	-0.01094281	2.117882235	0.06531755	0.22954309	-4.41002127
WT after UVB irradiation VS WT before UVB irradiation	JAK-STAT|Wikipathways	0.240248868	-0.04314324	1.14998946	0.27669483	0.66649013	-5.24277174
homo after UVB irradiation VS homo before UVB irradiation	JAK-STAT-Core|Signalink	0.606006583	-0.02066594	3.775808231	0.00405683	0.03653548	-1.79302709
hetero after UVB irradiation VS hetero before UVB irradiation	JAK-STAT-Core|Signalink	0.374428644	0.025182493	1.751376116	0.11608083	0.30887493	-4.93479955
WT after UVB irradiation VS WT before UVB irradiation	JAK-STAT-Core|Signalink	0.327277958	-0.01709802	1.19213794	0.26051078	0.65515019	-5.19921499
homo after UVB irradiation VS homo before UVB irradiation	JAK-STAT-Ncore|Signalink	-0.17528248	-0.01591474	-0.72250198	0.48759057	0.62962063	-6.09570511
hetero after UVB irradiation VS hetero before UVB irradiation	JAK-STAT-Ncore|Signalink	-0.04207512	-0.03260782	-0.21881939	0.83199832	0.93531759	-6.32064128
WT after UVB irradiation VS WT before UVB irradiation	JAK-STAT-Ncore|Signalink	-0.24802491	-0.06571713	-1.11691753	0.28993935	0.67593313	-5.27612591
homo after UVB irradiation VS homo before UVB irradiation	JNK (c-Jun kinases) phosphorylation and activation mediated by activated human TAK1|Reactome	0.523177367	-0.01260504	2.91829111	0.01635171	0.07993781	-3.14909128
hetero after UVB irradiation VS hetero before UVB irradiation	JNK (c-Jun kinases) phosphorylation and activation mediated by activated human TAK1|Reactome	0.37892165	0.036153327	1.695747179	0.12646417	0.31934829	-5.01090128
WT after UVB irradiation VS WT before UVB irradiation	JNK (c-Jun kinases) phosphorylation and activation mediated by activated human TAK1|Reactome	0.292852412	0.010094128	1.318204677	0.2165971	0.62581393	-5.06227492
homo after UVB irradiation VS homo before UVB irradiation	JNK signaling in the CD4+ TCR pathway|PID	0.32538203	-0.04823822	1.775790141	0.10811286	0.241504	-4.9142706
hetero after UVB irradiation VS hetero before UVB irradiation	JNK signaling in the CD4+ TCR pathway|PID	0.113426581	-0.01047729	0.671693058	0.51976701	0.73623731	-6.10830061
WT after UVB irradiation VS WT before UVB irradiation	JNK signaling in the CD4+ TCR pathway|PID	-0.03043993	-0.05108856	-0.12445946	0.90339799	0.97566251	-5.86131043
homo after UVB irradiation VS homo before UVB irradiation	Josephin domain DUBs|Reactome	-0.37605775	0.006074513	-1.59545056	0.14367614	0.28848955	-5.16276162
hetero after UVB irradiation VS hetero before UVB irradiation	Josephin domain DUBs|Reactome	0.085093472	0.004903333	0.516264795	0.61894204	0.80231894	-6.20425725
WT after UVB irradiation VS WT before UVB irradiation	Josephin domain DUBs|Reactome	-0.39710143	-0.03170627	-1.54290497	0.15363546	0.55854784	-4.7960079
homo after UVB irradiation VS homo before UVB irradiation	Joubert syndrome|SMPDB	0.204887351	-0.03452263	1.001726165	0.34159152	0.49735552	-5.85696862
hetero after UVB irradiation VS hetero before UVB irradiation	Joubert syndrome|SMPDB	0.194527838	-0.01339579	0.989499008	0.34997925	0.57902193	-5.84490002
WT after UVB irradiation VS WT before UVB irradiation	Joubert syndrome|SMPDB	0.153809442	-0.05379699	0.682327223	0.51040375	0.82655837	-5.63899101
homo after UVB irradiation VS homo before UVB irradiation	Kaposi sarcoma-associated herpesvirus infection - Homo sapiens (human)|KEGG	0.514214768	-0.02154753	3.157600759	0.01100723	0.063619	-2.76606076
hetero after UVB irradiation VS hetero before UVB irradiation	Kaposi sarcoma-associated herpesvirus infection - Homo sapiens (human)|KEGG	0.532395975	0.021866807	3.46002411	0.0079122	0.089711	-2.37718089
WT after UVB irradiation VS WT before UVB irradiation	Kaposi sarcoma-associated herpesvirus infection - Homo sapiens (human)|KEGG	0.449903715	-0.00595466	2.181910853	0.05385661	0.41493406	-3.92712037
homo after UVB irradiation VS homo before UVB irradiation	Kennedy pathway from Sphingolipids|Wikipathways	0.581251658	-0.0470329	3.713996659	0.00447393	0.03821988	-1.88866308
hetero after UVB irradiation VS hetero before UVB irradiation	Kennedy pathway from Sphingolipids|Wikipathways	0.353517896	-0.03161296	1.636458326	0.13846739	0.33859139	-5.0907234
WT after UVB irradiation VS WT before UVB irradiation	Kennedy pathway from Sphingolipids|Wikipathways	0.32545787	-0.01320937	1.516739575	0.16004166	0.56072663	-4.82833624
homo after UVB irradiation VS homo before UVB irradiation	Keratan sulfate biosynthesis|Reactome	0.422807508	-0.02974707	2.456717379	0.03531712	0.12483995	-3.88516081
hetero after UVB irradiation VS hetero before UVB irradiation	Keratan sulfate biosynthesis|Reactome	0.181360276	0.041192306	0.783514633	0.45478266	0.67919377	-6.02542647
WT after UVB irradiation VS WT before UVB irradiation	Keratan sulfate biosynthesis|Reactome	0.302199315	-0.01636118	1.166624148	0.27021511	0.66327566	-5.22571988
homo after UVB irradiation VS homo before UVB irradiation	Keratan sulfate degradation|Reactome	0.496784311	-0.02785272	2.31132358	0.04500183	0.13809028	-4.11317221
hetero after UVB irradiation VS hetero before UVB irradiation	Keratan sulfate degradation|Reactome	0.870877844	-0.05740855	4.791534558	0.00118516	0.04187568	-0.52052803
WT after UVB irradiation VS WT before UVB irradiation	Keratan sulfate degradation|Reactome	0.40031488	-0.12876638	1.067055528	0.3108309	0.6940288	-5.32500345
homo after UVB irradiation VS homo before UVB irradiation	Keratan sulfate/keratin metabolism|Reactome	0.454988224	-0.01020051	2.585065182	0.02850253	0.11306912	-3.68180157
hetero after UVB irradiation VS hetero before UVB irradiation	Keratan sulfate/keratin metabolism|Reactome	0.306026428	0.032755542	1.385236729	0.2015472	0.42159346	-5.41178576
WT after UVB irradiation VS WT before UVB irradiation	Keratan sulfate/keratin metabolism|Reactome	0.30939565	-0.03824709	1.141273674	0.28013837	0.66921967	-5.251633
homo after UVB irradiation VS homo before UVB irradiation	Keratinization|Reactome	-0.20405732	0.03243579	-0.95577524	0.36314544	0.51751626	-5.90069382
hetero after UVB irradiation VS hetero before UVB irradiation	Keratinization|Reactome	-0.05984117	0.004045838	-0.28862532	0.77984174	0.90262028	-6.30157751
WT after UVB irradiation VS WT before UVB irradiation	Keratinization|Reactome	-0.18761524	0.096637346	-0.63247979	0.54115088	0.83916339	-5.67072014
homo after UVB irradiation VS homo before UVB irradiation	keratinocyte differentiation|BioCarta	0.096337395	-0.01942687	0.523207818	0.61295218	0.72940831	-6.22352646
hetero after UVB irradiation VS hetero before UVB irradiation	keratinocyte differentiation|BioCarta	0.26686244	-0.00993203	1.685129194	0.128541	0.32316043	-5.02529695
WT after UVB irradiation VS WT before UVB irradiation	keratinocyte differentiation|BioCarta	-0.00793124	-0.01810548	-0.04105685	0.96805225	0.98924463	-5.86829732
homo after UVB irradiation VS homo before UVB irradiation	Ketobemidone Action Pathway|SMPDB	0.191132361	0.029768717	1.061313976	0.31508276	0.46923284	-5.79783739
hetero after UVB irradiation VS hetero before UVB irradiation	Ketobemidone Action Pathway|SMPDB	-0.07904315	0.055005373	-0.44437398	0.66796482	0.82651398	-6.24068873
WT after UVB irradiation VS WT before UVB irradiation	Ketobemidone Action Pathway|SMPDB	0.022316377	0.035038618	0.098344171	0.92358676	0.97566251	-5.86425414
homo after UVB irradiation VS homo before UVB irradiation	ketogenesis|HumanCyc	-0.24325692	-0.01247016	-1.06824202	0.31210541	0.46923284	-5.79078876
hetero after UVB irradiation VS hetero before UVB irradiation	ketogenesis|HumanCyc	-0.16206036	-0.03457093	-0.76855236	0.46315373	0.68741605	-6.03715891
WT after UVB irradiation VS WT before UVB irradiation	ketogenesis|HumanCyc	-0.31509183	-0.00666963	-1.2186296	0.25073101	0.64403178	-5.171252
homo after UVB irradiation VS homo before UVB irradiation	ketolysis|HumanCyc	-0.44975479	-0.0622915	-1.44917823	0.17985015	0.33360654	-5.35325807
hetero after UVB irradiation VS hetero before UVB irradiation	ketolysis|HumanCyc	-0.51691501	-0.05012507	-2.11290262	0.06583561	0.23045894	-4.41736859
WT after UVB irradiation VS WT before UVB irradiation	ketolysis|HumanCyc	-0.58167694	-0.01201008	-2.17185442	0.05478234	0.41769317	-3.94167379
homo after UVB irradiation VS homo before UVB irradiation	Ketone body metabolism|Reactome	-0.17412775	-0.00210666	-0.74999018	0.47163479	0.61686751	-6.07517855
hetero after UVB irradiation VS hetero before UVB irradiation	Ketone body metabolism|Reactome	-0.37772771	-0.07512384	-2.25420083	0.05258727	0.20364722	-4.20720734
WT after UVB irradiation VS WT before UVB irradiation	Ketone body metabolism|Reactome	-0.41370261	-0.04152806	-1.73511255	0.11312822	0.51512295	-4.54926162
homo after UVB irradiation VS homo before UVB irradiation	Ketone Body Metabolism|SMPDB	-0.58381137	-0.00610195	-3.51265809	0.00617327	0.04588471	-2.20306267
hetero after UVB irradiation VS hetero before UVB irradiation	Ketone Body Metabolism|SMPDB	-0.44524322	-0.05678469	-2.22372135	0.05520303	0.20875475	-4.25281375
WT after UVB irradiation VS WT before UVB irradiation	Ketone Body Metabolism|SMPDB	-0.66271453	-0.08062143	-3.00987236	0.01300608	0.27886745	-2.69324878
homo after UVB irradiation VS homo before UVB irradiation	Ketoprofen Action Pathway|SMPDB	0.40423582	0.045705158	1.979500697	0.07780079	0.19626432	-4.61879689
hetero after UVB irradiation VS hetero before UVB irradiation	Ketoprofen Action Pathway|SMPDB	0.482496143	0.064876536	3.04499191	0.01500113	0.1130168	-3.00250826
WT after UVB irradiation VS WT before UVB irradiation	Ketoprofen Action Pathway|SMPDB	0.275725339	0.086595857	1.261970114	0.23537138	0.62952955	-5.12455679
homo after UVB irradiation VS homo before UVB irradiation	Ketorolac Action Pathway|SMPDB	0.40423582	0.045705158	1.979500697	0.07780079	0.19626432	-4.61879689
hetero after UVB irradiation VS hetero before UVB irradiation	Ketorolac Action Pathway|SMPDB	0.482496143	0.064876536	3.04499191	0.01500113	0.1130168	-3.00250826
WT after UVB irradiation VS WT before UVB irradiation	Ketorolac Action Pathway|SMPDB	0.275725339	0.086595857	1.261970114	0.23537138	0.62952955	-5.12455679
homo after UVB irradiation VS homo before UVB irradiation	Kidney Function|SMPDB	0.234726031	0.017120546	1.156747303	0.27595732	0.43614438	-5.69767199
hetero after UVB irradiation VS hetero before UVB irradiation	Kidney Function|SMPDB	-0.03255037	0.028925632	-0.18154472	0.86023155	0.94503144	-6.32869738
WT after UVB irradiation VS WT before UVB irradiation	Kidney Function|SMPDB	-0.15908359	0.010252305	-0.80382265	0.44003996	0.79685269	-5.55266936
homo after UVB irradiation VS homo before UVB irradiation	Kinesins|Reactome	-0.25686191	0.003024535	-1.40506675	0.19221223	0.34818728	-5.40850195
hetero after UVB irradiation VS hetero before UVB irradiation	Kinesins|Reactome	0.066535605	-0.02764154	0.272486765	0.79180675	0.91160777	-6.30644323
WT after UVB irradiation VS WT before UVB irradiation	Kinesins|Reactome	-0.05747749	0.00507959	-0.20998047	0.8378655	0.94951669	-5.84686698
homo after UVB irradiation VS homo before UVB irradiation	Kit receptor signaling pathway|Wikipathways	0.3128627	-0.02410967	1.69354352	0.12319853	0.26247468	-5.02930327
hetero after UVB irradiation VS hetero before UVB irradiation	Kit receptor signaling pathway|Wikipathways	0.496674066	0.012844617	3.198551258	0.01181373	0.10542662	-2.76946758
WT after UVB irradiation VS WT before UVB irradiation	Kit receptor signaling pathway|Wikipathways	0.306544703	-0.01481425	1.469029106	0.17232306	0.58081137	-4.88643062
homo after UVB irradiation VS homo before UVB irradiation	KitReceptor|NetPath	0.245074836	-0.01962253	1.25112928	0.2411621	0.40276012	-5.59237997
hetero after UVB irradiation VS hetero before UVB irradiation	KitReceptor|NetPath	0.509141044	0.008436209	3.072470955	0.014371	0.1130168	-2.96069289
WT after UVB irradiation VS WT before UVB irradiation	KitReceptor|NetPath	0.124676151	-0.04871796	0.557947574	0.58905402	0.86161188	-5.71401717
homo after UVB irradiation VS homo before UVB irradiation	Krabbe disease|SMPDB	0.410096132	-0.05671339	1.580382344	0.14707883	0.29223756	-5.18287502
hetero after UVB irradiation VS hetero before UVB irradiation	Krabbe disease|SMPDB	0.381719767	-0.02021245	2.484597089	0.03641544	0.16681566	-3.85889413
WT after UVB irradiation VS WT before UVB irradiation	Krabbe disease|SMPDB	0.327957029	0.011268274	1.427782053	0.1835878	0.59839062	-4.93573151
homo after UVB irradiation VS homo before UVB irradiation	KSRP (KHSRP) binds and destabilizes mRNA|Reactome	-0.79868203	0.014939966	-4.88891833	0.000761	0.01684857	-0.16128929
hetero after UVB irradiation VS hetero before UVB irradiation	KSRP (KHSRP) binds and destabilizes mRNA|Reactome	-0.3412549	-0.01986008	-1.47100495	0.1776208	0.39500859	-5.30557516
WT after UVB irradiation VS WT before UVB irradiation	KSRP (KHSRP) binds and destabilizes mRNA|Reactome	-0.10757152	0.043921848	-0.33414883	0.74511385	0.9185045	-5.81292222
homo after UVB irradiation VS homo before UVB irradiation	L13a-mediated translational silencing of Ceruloplasmin expression|Reactome	-0.96897149	0.024283161	-5.36838469	0.00039086	0.01666204	0.481911518
hetero after UVB irradiation VS hetero before UVB irradiation	L13a-mediated translational silencing of Ceruloplasmin expression|Reactome	-0.24945123	-0.01457705	-0.75276966	0.47209351	0.69368221	-6.04932236
WT after UVB irradiation VS WT before UVB irradiation	L13a-mediated translational silencing of Ceruloplasmin expression|Reactome	-0.60759156	-0.0138022	-2.05055774	0.06721785	0.43980503	-4.115551
homo after UVB irradiation VS homo before UVB irradiation	L1CAM interactions|Reactome	0.458881101	-0.00250063	2.818802294	0.01929529	0.08847577	-3.30850019
hetero after UVB irradiation VS hetero before UVB irradiation	L1CAM interactions|Reactome	0.403125185	0.028293051	2.646281905	0.02814639	0.14497177	-3.61206436
WT after UVB irradiation VS WT before UVB irradiation	L1CAM interactions|Reactome	0.228022528	0.002101037	1.153888233	0.27516526	0.66577703	-5.23879157
homo after UVB irradiation VS homo before UVB irradiation	Labetalol Action Pathway|SMPDB	0.363732495	-0.0288084	2.171554099	0.05674447	0.16193454	-4.32911185
hetero after UVB irradiation VS hetero before UVB irradiation	Labetalol Action Pathway|SMPDB	-0.04095736	-0.02227496	-0.19326116	0.85133209	0.94503144	-6.32632526
WT after UVB irradiation VS WT before UVB irradiation	Labetalol Action Pathway|SMPDB	0.033066635	-0.04439883	0.13025274	0.89892864	0.97566251	-5.86056419
homo after UVB irradiation VS homo before UVB irradiation	lactate fermentation (reoxidation of cytosolic NADH)|HumanCyc	0.140914157	0.016824384	0.415436158	0.68715625	0.78999012	-6.27658225
hetero after UVB irradiation VS hetero before UVB irradiation	lactate fermentation (reoxidation of cytosolic NADH)|HumanCyc	0.127123705	0.041773184	0.479775432	0.64359411	0.81964291	-6.22339274
WT after UVB irradiation VS WT before UVB irradiation	lactate fermentation (reoxidation of cytosolic NADH)|HumanCyc	-0.01899267	-0.05384596	-0.06085057	0.95266756	0.98725499	-5.86727574
homo after UVB irradiation VS homo before UVB irradiation	Lactic Acidemia|SMPDB	-0.39210009	0.022178168	-1.25326746	0.24041731	0.40210456	-5.58992632
hetero after UVB irradiation VS hetero before UVB irradiation	Lactic Acidemia|SMPDB	-0.35251865	0.016423987	-1.29321274	0.23026395	0.45170905	-5.52131443
WT after UVB irradiation VS WT before UVB irradiation	Lactic Acidemia|SMPDB	0.415408645	-0.09593973	1.608715996	0.13850837	0.55342759	-4.71329834
homo after UVB irradiation VS homo before UVB irradiation	lactose degradation III|HumanCyc	0.304837368	0.016280645	0.996401874	0.34403915	0.49934395	-5.86211977
hetero after UVB irradiation VS hetero before UVB irradiation	lactose degradation III|HumanCyc	0.498611779	-0.03435129	1.382605189	0.20232357	0.42263558	-5.41498345
WT after UVB irradiation VS WT before UVB irradiation	lactose degradation III|HumanCyc	-0.33415556	-0.14700649	-1.25799317	0.2367481	0.63082179	-5.12888973
homo after UVB irradiation VS homo before UVB irradiation	Lactose Degradation|SMPDB	0.555862112	0.013045501	2.471862393	0.03443537	0.12370725	-3.86125189
hetero after UVB irradiation VS hetero before UVB irradiation	Lactose Degradation|SMPDB	0.464005584	0.000509078	2.46424583	0.037617	0.16931722	-3.88986048
WT after UVB irradiation VS WT before UVB irradiation	Lactose Degradation|SMPDB	0.597296689	0.032545014	2.724746167	0.02123812	0.31542987	-3.12194441
homo after UVB irradiation VS homo before UVB irradiation	Lactose Intolerance|SMPDB	0.555862112	0.013045501	2.471862393	0.03443537	0.12370725	-3.86125189
hetero after UVB irradiation VS hetero before UVB irradiation	Lactose Intolerance|SMPDB	0.464005584	0.000509078	2.46424583	0.037617	0.16931722	-3.88986048
WT after UVB irradiation VS WT before UVB irradiation	Lactose Intolerance|SMPDB	0.597296689	0.032545014	2.724746167	0.02123812	0.31542987	-3.12194441
homo after UVB irradiation VS homo before UVB irradiation	Lactose synthesis|Reactome	1.116214533	-0.03312021	5.751080314	0.00023507	0.01666204	0.968474416
hetero after UVB irradiation VS hetero before UVB irradiation	Lactose synthesis|Reactome	1.132337341	0.037273676	6.107602619	0.00023264	0.02988205	1.03535158
WT after UVB irradiation VS WT before UVB irradiation	Lactose synthesis|Reactome	0.940152191	0.065995479	3.466854261	0.00598037	0.21490368	-2.01421848
homo after UVB irradiation VS homo before UVB irradiation	Lactose Synthesis|SMPDB	0.737643047	0.024031742	3.897326755	0.00335151	0.03256113	-1.60634747
hetero after UVB irradiation VS hetero before UVB irradiation	Lactose Synthesis|SMPDB	0.744733789	0.085967304	4.116781712	0.0030051	0.06148411	-1.42803661
WT after UVB irradiation VS WT before UVB irradiation	Lactose Synthesis|SMPDB	0.584123909	0.04155907	2.501560947	0.03118765	0.36428127	-3.45639206
homo after UVB irradiation VS homo before UVB irradiation	Lagging Strand Synthesis|Reactome	-0.48747526	-0.02238768	-1.67148278	0.12755906	0.26766751	-5.05968605
hetero after UVB irradiation VS hetero before UVB irradiation	Lagging Strand Synthesis|Reactome	0.306332805	0.028967131	1.131677503	0.28894697	0.51871614	-5.70120369
WT after UVB irradiation VS WT before UVB irradiation	Lagging Strand Synthesis|Reactome	0.120368872	0.050546615	0.406610864	0.69279094	0.89511744	-5.78612689
homo after UVB irradiation VS homo before UVB irradiation	Lamin A-processing pathway|Wikipathways	0.343667303	-0.14730056	1.075532735	0.30899559	0.46923284	-5.78333274
hetero after UVB irradiation VS hetero before UVB irradiation	Lamin A-processing pathway|Wikipathways	0.616667427	-0.07981067	1.757197707	0.11504119	0.30887493	-4.92677146
WT after UVB irradiation VS WT before UVB irradiation	Lamin A-processing pathway|Wikipathways	0.200371161	-0.01035361	0.852138107	0.41392076	0.77942111	-5.51492076
homo after UVB irradiation VS homo before UVB irradiation	Laminin interactions|Reactome	0.785438133	-0.04687502	3.633194865	0.00508811	0.04097406	-2.01433014
hetero after UVB irradiation VS hetero before UVB irradiation	Laminin interactions|Reactome	0.693753529	0.052933093	3.41752186	0.00844032	0.0927463	-2.44048651
WT after UVB irradiation VS WT before UVB irradiation	Laminin interactions|Reactome	0.642112883	-0.02999831	2.738820188	0.02072942	0.31542987	-3.10078446
homo after UVB irradiation VS homo before UVB irradiation	Lamivudine Metabolism Pathway|SMPDB	0.562691305	0.005546746	3.124326494	0.0116271	0.06584328	-2.81921737
hetero after UVB irradiation VS hetero before UVB irradiation	Lamivudine Metabolism Pathway|SMPDB	0.328974429	0.022150431	1.996317162	0.07916964	0.25322957	-4.58791916
WT after UVB irradiation VS WT before UVB irradiation	Lamivudine Metabolism Pathway|SMPDB	0.511251289	-0.00744425	2.476756937	0.03254571	0.36974198	-3.49335975
homo after UVB irradiation VS homo before UVB irradiation	Lamivudine Pathway_ Pharmacokinetics/Pharmacodynamics|PharmGKB	0.594000797	-0.00397972	3.446406179	0.00687011	0.04810019	-2.30738728
hetero after UVB irradiation VS hetero before UVB irradiation	Lamivudine Pathway_ Pharmacokinetics/Pharmacodynamics|PharmGKB	0.400953906	0.020525333	2.418272402	0.04047986	0.1767964	-3.95969676
WT after UVB irradiation VS WT before UVB irradiation	Lamivudine Pathway_ Pharmacokinetics/Pharmacodynamics|PharmGKB	0.528528052	-0.01207285	2.520754673	0.03017527	0.36196871	-3.42775004
homo after UVB irradiation VS homo before UVB irradiation	lanosterol biosynthesis|HumanCyc	-0.56001491	0.004328197	-1.6382472	0.13439249	0.277359	-5.10506187

hetero after UVB irradiation VS hetero before UVB irradiation	lanosterol biosynthesis|HumanCyc	-0.30109931	0.011945428	-0.72122364	0.49029711	0.71035417	-6.07297355
WT after UVB irradiation VS WT before UVB irradiation	lanosterol biosynthesis|HumanCyc	-0.8586179	-0.03020879	-2.72675854	0.02116462	0.31542987	-3.11891913
homo after UVB irradiation VS homo before UVB irradiation	Lansoprazole Action Pathway|SMPDB	0.21780303	0.051698704	1.19150037	0.26270351	0.42561191	-5.65959884
hetero after UVB irradiation VS hetero before UVB irradiation	Lansoprazole Action Pathway|SMPDB	0.331566302	0.063470812	1.441200644	0.18563717	0.40321767	-5.34291209
WT after UVB irradiation VS WT before UVB irradiation	Lansoprazole Action Pathway|SMPDB	-0.11560861	0.135982321	-0.44330845	0.66690385	0.87935805	-5.77062647
homo after UVB irradiation VS homo before UVB irradiation	Lansoprazole Metabolism Pathway|SMPDB	-0.06647614	0.033610835	-0.18292054	0.85874942	0.9134577	-6.35050291
WT after UVB irradiation VS WT before UVB irradiation	Lansoprazole Metabolism Pathway|SMPDB	-0.4370633	0.058240551	-1.4704397	0.17194858	0.57997722	-4.88472923
homo after UVB irradiation VS homo before UVB irradiation	L-arginine:glycine amidinotransferase deficiency|SMPDB	-0.34739547	0.00502943	-1.63262028	0.13558154	0.277359	-5.11269582
hetero after UVB irradiation VS hetero before UVB irradiation	L-arginine:glycine amidinotransferase deficiency|SMPDB	-0.56351352	-0.04220085	-3.24793085	0.01094591	0.10415756	-2.6949004
WT after UVB irradiation VS WT before UVB irradiation	L-arginine:glycine amidinotransferase deficiency|SMPDB	-0.47021304	-0.04055952	-2.31093774	0.04323906	0.39022568	-3.73881388
homo after UVB irradiation VS homo before UVB irradiation	Late Phase of HIV Life Cycle|Reactome	-0.48765367	0.023813889	-2.63422853	0.02625503	0.10713177	-3.60352816
hetero after UVB irradiation VS hetero before UVB irradiation	Late Phase of HIV Life Cycle|Reactome	-0.15666679	-0.03921959	-0.70252953	0.50129335	0.71987873	-6.08656865
WT after UVB irradiation VS WT before UVB irradiation	Late Phase of HIV Life Cycle|Reactome	-0.1292001	-0.00974333	-0.44182866	0.6679392	0.87935805	-5.77127638
homo after UVB irradiation VS homo before UVB irradiation	Latent infection of Homo sapiens with Mycobacterium tuberculosis|Reactome	1.019010934	-0.02342197	4.307824036	0.00178393	0.02356086	-0.99027397
hetero after UVB irradiation VS hetero before UVB irradiation	Latent infection of Homo sapiens with Mycobacterium tuberculosis|Reactome	1.044821233	0.05989276	4.854999212	0.00108958	0.04104387	-0.43899655
WT after UVB irradiation VS WT before UVB irradiation	Latent infection of Homo sapiens with Mycobacterium tuberculosis|Reactome	0.459621405	0.016400241	1.150166408	0.27662526	0.66649013	-5.24259131
homo after UVB irradiation VS homo before UVB irradiation	L-carnitine biosynthesis|HumanCyc	0.132102434	-0.08045837	0.335909317	0.74432418	0.83278222	-6.30819305
hetero after UVB irradiation VS hetero before UVB irradiation	L-carnitine biosynthesis|HumanCyc	-0.02247639	-0.01784464	-0.04933295	0.96180341	0.98206759	-6.3452212
WT after UVB irradiation VS WT before UVB irradiation	L-carnitine biosynthesis|HumanCyc	-0.03024013	-0.026498	-0.0789146	0.93864487	0.98056728	-5.86599741
homo after UVB irradiation VS homo before UVB irradiation	lck and fyn tyrosine kinases in initiation of tcr activation|BioCarta	-0.47161094	0.063252633	-1.6829838	0.1252686	0.26466492	-5.04387228
hetero after UVB irradiation VS hetero before UVB irradiation	lck and fyn tyrosine kinases in initiation of tcr activation|BioCarta	-0.25525413	0.018006437	-1.05765222	0.31959079	0.55014439	-5.77783969
WT after UVB irradiation VS WT before UVB irradiation	lck and fyn tyrosine kinases in initiation of tcr activation|BioCarta	-0.01118376	-0.11120812	-0.02997267	0.97667387	0.99265119	-5.86869611
homo after UVB irradiation VS homo before UVB irradiation	L-cysteine degradation I|HumanCyc	0.361935335	-0.02581491	0.889928733	0.39573341	0.54874665	-5.96040201
hetero after UVB irradiation VS hetero before UVB irradiation	L-cysteine degradation I|HumanCyc	-0.42865185	-0.04905235	-1.08587139	0.30762198	0.53619691	-5.74908022
WT after UVB irradiation VS WT before UVB irradiation	L-cysteine degradation I|HumanCyc	-0.20922449	-0.02891785	-0.54199935	0.59959217	0.86711129	-5.72262487
homo after UVB irradiation VS homo before UVB irradiation	L-cysteine degradation II|HumanCyc	-0.35038928	-0.11171897	-0.88577304	0.39785749	0.55064838	-5.96405102
hetero after UVB irradiation VS hetero before UVB irradiation	L-cysteine degradation II|HumanCyc	-0.66275074	0.081606344	-1.64695207	0.13626973	0.33602293	-5.07669652
WT after UVB irradiation VS WT before UVB irradiation	L-cysteine degradation II|HumanCyc	-0.42483092	-0.00156969	-1.08767295	0.30205683	0.68699877	-5.30500173
homo after UVB irradiation VS homo before UVB irradiation	LDL clearance|Reactome	0.419721925	-0.01353126	2.144875754	0.05930158	0.16661003	-4.36988572
hetero after UVB irradiation VS hetero before UVB irradiation	LDL clearance|Reactome	0.650957504	0.031676396	3.546539472	0.0069419	0.08598141	-2.24894091
WT after UVB irradiation VS WT before UVB irradiation	LDL clearance|Reactome	0.443634152	-0.01007842	1.882182508	0.08896192	0.48106209	-4.35087281
homo after UVB irradiation VS homo before UVB irradiation	LDL remodeling|Reactome	-0.27718274	0.049810823	-0.69923595	0.50135799	0.64118759	-6.1125442
hetero after UVB irradiation VS hetero before UVB irradiation	LDL remodeling|Reactome	-0.67465789	0.009543921	-2.0786898	0.06950469	0.23555906	-4.46771722
WT after UVB irradiation VS WT before UVB irradiation	LDL remodeling|Reactome	-0.7045116	-0.01586095	-2.42080285	0.03582676	0.38864795	-3.57653879
homo after UVB irradiation VS homo before UVB irradiation	L-dopa degradation|HumanCyc	1.011969848	-0.00971256	4.023922695	0.00275245	0.02978124	-1.41387018
hetero after UVB irradiation VS hetero before UVB irradiation	L-dopa degradation|HumanCyc	0.393640147	-0.00020703	1.04238256	0.3262163	0.5554329	-5.79316252
WT after UVB irradiation VS WT before UVB irradiation	L-dopa degradation|HumanCyc	0.599448487	-0.05276689	1.685103404	0.1226213	0.52731779	-4.61493556
homo after UVB irradiation VS homo before UVB irradiation	L-dopachrome biosynthesis|HumanCyc	-1.0234841	0.007517396	-5.10903186	0.00055817	0.01666204	0.138640915
hetero after UVB irradiation VS hetero before UVB irradiation	L-dopachrome biosynthesis|HumanCyc	-0.58609196	-0.02703766	-1.86848388	0.0967665	0.2808394	-4.77115309
WT after UVB irradiation VS WT before UVB irradiation	L-dopachrome biosynthesis|HumanCyc	-0.849039	0.021479961	-2.95394328	0.01431645	0.28307166	-2.77724071
homo after UVB irradiation VS homo before UVB irradiation	Leading Strand Synthesis|Reactome	-0.44537839	-0.0092232	-1.56961564	0.14955393	0.29522837	-5.19718062
hetero after UVB irradiation VS hetero before UVB irradiation	Leading Strand Synthesis|Reactome	0.368900781	-0.00233809	1.325603217	0.2197829	0.44126832	-5.48331134
WT after UVB irradiation VS WT before UVB irradiation	Leading Strand Synthesis|Reactome	0.040265502	0.012547595	0.134706475	0.89549522	0.97566251	-5.85996749
homo after UVB irradiation VS homo before UVB irradiation	lectin induced complement pathway|BioCarta	0.693003092	-0.06580477	2.373352066	0.04058593	0.13402181	-4.01626022
hetero after UVB irradiation VS hetero before UVB irradiation	lectin induced complement pathway|BioCarta	0.067896132	0.083496603	0.259005854	0.80184636	0.916937	-6.31029692
WT after UVB irradiation VS WT before UVB irradiation	lectin induced complement pathway|BioCarta	0.269494352	-0.04382327	0.852791791	0.41357476	0.77942111	-5.51439708
homo after UVB irradiation VS homo before UVB irradiation	Lectin pathway of complement activation|Reactome	0.557100127	-0.01111135	1.801971911	0.10367914	0.23552187	-4.8770989
hetero after UVB irradiation VS hetero before UVB irradiation	Lectin pathway of complement activation|Reactome	0.466287289	0.049200169	2.398317658	0.04178946	0.18020468	-3.98995322
WT after UVB irradiation VS WT before UVB irradiation	Lectin pathway of complement activation|Reactome	-0.12535511	-0.02241908	-0.45974259	0.65545518	0.87935805	-5.76326885
homo after UVB irradiation VS homo before UVB irradiation	Legionellosis - Homo sapiens (human)|KEGG	0.52633986	-0.017624	2.774820424	0.02076292	0.09195295	-3.3789203
hetero after UVB irradiation VS hetero before UVB irradiation	Legionellosis - Homo sapiens (human)|KEGG	0.554350723	-0.00355836	3.185336408	0.01205795	0.10610726	-2.78945645
WT after UVB irradiation VS WT before UVB irradiation	Legionellosis - Homo sapiens (human)|KEGG	0.376477477	0.002077785	1.55146292	0.15158948	0.55727585	-4.78536423
homo after UVB irradiation VS homo before UVB irradiation	Leigh Syndrome|SMPDB	-0.1559239	-0.03195163	-0.60679304	0.55837377	0.6849905	-6.17450252
hetero after UVB irradiation VS hetero before UVB irradiation	Leigh Syndrome|SMPDB	-0.2311653	-0.04505886	-1.14490839	0.28372423	0.51297702	-5.68710621
WT after UVB irradiation VS WT before UVB irradiation	Leigh Syndrome|SMPDB	-0.05726125	-0.08194106	-0.22775541	0.82438877	0.94525798	-5.84294542
homo after UVB irradiation VS homo before UVB irradiation	Leishmaniasis - Homo sapiens (human)|KEGG	0.496146124	-0.03428374	2.477716723	0.03410042	0.12327989	-3.85200293
hetero after UVB irradiation VS hetero before UVB irradiation	Leishmaniasis - Homo sapiens (human)|KEGG	0.55300852	0.054805116	2.610853375	0.02977857	0.14964797	-3.66624377
WT after UVB irradiation VS WT before UVB irradiation	Leishmaniasis - Homo sapiens (human)|KEGG	0.508677663	-0.0022938	1.958379222	0.07841207	0.46387039	-4.24535151
homo after UVB irradiation VS homo before UVB irradiation	Lepirudin Action Pathway|SMPDB	0.538210712	0.026444726	3.007777411	0.01409598	0.07305822	-3.00571236
hetero after UVB irradiation VS hetero before UVB irradiation	Lepirudin Action Pathway|SMPDB	0.558085555	0.09913938	2.984394118	0.01649409	0.11352943	-3.09486545
WT after UVB irradiation VS WT before UVB irradiation	Lepirudin Action Pathway|SMPDB	0.440879848	0.077377741	1.569633667	0.14732454	0.55622203	-4.76265269
homo after UVB irradiation VS homo before UVB irradiation	Leptin and adiponectin|Wikipathways	-0.09068389	-0.01624578	-0.42771349	0.67850267	0.78292726	-6.27112657
hetero after UVB irradiation VS hetero before UVB irradiation	Leptin and adiponectin|Wikipathways	-0.02460694	-0.10254694	-0.11850287	0.90844583	0.97054702	-6.3389296
WT after UVB irradiation VS WT before UVB irradiation	Leptin and adiponectin|Wikipathways	-0.08464538	-0.06025743	-0.31739759	0.75741745	0.92353903	-5.81838918
homo after UVB irradiation VS homo before UVB irradiation	Leptin Insulin Overlap|Wikipathways	0.188385276	-0.0172742	1.127317369	0.28759212	0.44703185	-5.7292576
hetero after UVB irradiation VS hetero before UVB irradiation	Leptin Insulin Overlap|Wikipathways	0.016322294	-0.07960563	0.068575022	0.94692745	0.97253162	-6.34399093
WT after UVB irradiation VS WT before UVB irradiation	Leptin Insulin Overlap|Wikipathways	-0.10112427	-0.05824895	-0.39033283	0.70441033	0.90113315	-5.7925889
homo after UVB irradiation VS homo before UVB irradiation	Leptin signaling pathway|Wikipathways	0.140552255	-0.03914299	0.718618263	0.48987207	0.63135722	-6.09855024
hetero after UVB irradiation VS hetero before UVB irradiation	Leptin signaling pathway|Wikipathways	0.189311028	-0.02718439	1.206739966	0.26031681	0.48628034	-5.61968121
WT after UVB irradiation VS WT before UVB irradiation	Leptin signaling pathway|Wikipathways	0.095090385	-0.02957997	0.449507806	0.66257439	0.87935805	-5.76788111
homo after UVB irradiation VS homo before UVB irradiation	Leptin|NetPath	0.118791639	-0.04895446	0.589097351	0.56969648	0.69403258	-6.18544386
hetero after UVB irradiation VS hetero before UVB irradiation	Leptin|NetPath	0.130763522	0.00523357	0.845331809	0.42127953	0.6476196	-5.97491295
WT after UVB irradiation VS WT before UVB irradiation	Leptin|NetPath	0.045348684	-0.02670141	0.219433276	0.83069136	0.94737089	-5.84482071
homo after UVB irradiation VS homo before UVB irradiation	Lesch-Nyhan Syndrome (LNS)|SMPDB	0.019015587	-0.03943216	0.101997834	0.92090359	0.95153785	-6.36286155
hetero after UVB irradiation VS hetero before UVB irradiation	Lesch-Nyhan Syndrome (LNS)|SMPDB	-0.02085195	-0.0438046	-0.1098564	0.91509485	0.97054702	-6.33999905
WT after UVB irradiation VS WT before UVB irradiation	Lesch-Nyhan Syndrome (LNS)|SMPDB	0.165884344	-0.0622813	0.759490799	0.46494629	0.80891799	-5.58561498
homo after UVB irradiation VS homo before UVB irradiation	let-7 inhibition of ES cell reprogramming|Wikipathways	-0.40336455	0.095253775	-1.46088014	0.17668834	0.32942382	-5.338424
hetero after UVB irradiation VS hetero before UVB irradiation	let-7 inhibition of ES cell reprogramming|Wikipathways	-0.55628242	0.063997489	-2.96747527	0.01693781	0.11437413	-3.12068311
WT after UVB irradiation VS WT before UVB irradiation	let-7 inhibition of ES cell reprogramming|Wikipathways	-0.75531446	0.023605933	-3.27196325	0.00831374	0.24856491	-2.30178551
homo after UVB irradiation VS homo before UVB irradiation	leucine degradation|HumanCyc	-0.28728688	0.001645716	-0.87889644	0.40138986	0.5535325	-5.97005761
hetero after UVB irradiation VS hetero before UVB irradiation	leucine degradation|HumanCyc	-0.72521925	-0.05447932	-3.94054651	0.00387381	0.06843726	-1.67685417
WT after UVB irradiation VS WT before UVB irradiation	leucine degradation|HumanCyc	-0.12716717	-0.11841949	-0.44442409	0.66612377	0.87935805	-5.77013511
homo after UVB irradiation VS homo before UVB irradiation	Leucine Stimulation on Insulin Signaling|SMPDB	0.049540294	0.006031658	0.275263656	0.78907564	0.86629463	-6.3279046
hetero after UVB irradiation VS hetero before UVB irradiation	Leucine Stimulation on Insulin Signaling|SMPDB	-0.22260323	-0.01344131	-0.84582835	0.42101752	0.64743517	-5.9744941
WT after UVB irradiation VS WT before UVB irradiation	Leucine Stimulation on Insulin Signaling|SMPDB	-0.27795559	-0.04222703	-0.958471	0.36024514	0.74374432	-5.42531225
homo after UVB irradiation VS homo before UVB irradiation	Leukocyte transendothelial migration - Homo sapiens (human)|KEGG	0.438119039	-0.01105909	2.405601571	0.03846146	0.12903383	-3.96565238
hetero after UVB irradiation VS hetero before UVB irradiation	Leukocyte transendothelial migration - Homo sapiens (human)|KEGG	0.508080645	0.033816907	3.171016204	0.01232856	0.1070354	-2.81113251
WT after UVB irradiation VS WT before UVB irradiation	Leukocyte transendothelial migration - Homo sapiens (human)|KEGG	0.373236468	-0.00532682	1.779787285	0.10521733	0.50446894	-4.48979395
homo after UVB irradiation VS homo before UVB irradiation	leukotriene biosynthesis|HumanCyc	0.876482013	0.019346811	4.364739592	0.0016376	0.02252198	-0.90676578
hetero after UVB irradiation VS hetero before UVB irradiation	leukotriene biosynthesis|HumanCyc	0.879757644	0.000745226	3.430911189	0.00827006	0.09193881	-2.42052261
WT after UVB irradiation VS WT before UVB irradiation	leukotriene biosynthesis|HumanCyc	0.666592038	-0.0287964	2.375809915	0.03869864	0.39022568	-3.64317841
homo after UVB irradiation VS homo before UVB irradiation	Leukotriene C4 Synthesis Deficiency|SMPDB	0.40423582	0.045705158	1.979500697	0.07780079	0.19626432	-4.61879689
hetero after UVB irradiation VS hetero before UVB irradiation	Leukotriene C4 Synthesis Deficiency|SMPDB	0.482496143	0.064876536	3.04499191	0.01500113	0.1130168	-3.00250826
WT after UVB irradiation VS WT before UVB irradiation	Leukotriene C4 Synthesis Deficiency|SMPDB	0.275725339	0.086595857	1.261970114	0.23537138	0.62952955	-5.12455679
homo after UVB irradiation VS homo before UVB irradiation	Leukotriene metabolism|EHMN	-0.28939936	0.009932353	-1.60954034	0.14055817	0.28372617	-5.14385809
hetero after UVB irradiation VS hetero before UVB irradiation	Leukotriene metabolism|EHMN	-0.28369602	-0.01606237	-1.89886752	0.09227463	0.27457285	-4.72800397
WT after UVB irradiation VS WT before UVB irradiation	Leukotriene metabolism|EHMN	-0.20282435	-0.02784993	-1.03235113	0.32603572	0.71010408	-5.357994
homo after UVB irradiation VS homo before UVB irradiation	Leukotriene modifiers pathway_ Pharmacodynamics|PharmGKB	0.780936885	0.02267472	3.898054311	0.0033477	0.03256113	-1.60523532
hetero after UVB irradiation VS hetero before UVB irradiation	Leukotriene modifiers pathway_ Pharmacodynamics|PharmGKB	0.900693406	-0.04406233	4.994013974	0.00090815	0.03797547	-0.26273965
WT after UVB irradiation VS WT before UVB irradiation	Leukotriene modifiers pathway_ Pharmacodynamics|PharmGKB	0.629127405	0.023176138	2.948069147	0.01446162	0.28346023	-2.7860679
homo after UVB irradiation VS homo before UVB irradiation	Leukotriene receptors|Reactome	1.055482628	-0.01959678	5.697969714	0.00025195	0.01666204	0.90236119
hetero after UVB irradiation VS hetero before UVB irradiation	Leukotriene receptors|Reactome	0.604103363	-0.05940246	2.13190995	0.06387942	0.22658552	-4.3892984
WT after UVB irradiation VS WT before UVB irradiation	Leukotriene receptors|Reactome	0.727237673	0.015767362	3.216036298	0.00914329	0.24856491	-2.38493618
homo after UVB irradiation VS homo before UVB irradiation	Levallorphan Action Pathway|SMPDB	0.191132361	0.029768717	1.061313976	0.31508276	0.46923284	-5.79783739
hetero after UVB irradiation VS hetero before UVB irradiation	Levallorphan Action Pathway|SMPDB	-0.07904315	0.055005373	-0.44437398	0.66796482	0.82651398	-6.24068873
WT after UVB irradiation VS WT before UVB irradiation	Levallorphan Action Pathway|SMPDB	0.022316377	0.035038618	0.098344171	0.92358676	0.97566251	-5.86425414
homo after UVB irradiation VS homo before UVB irradiation	Levobunolol Action Pathway|SMPDB	0.396415397	-0.03677376	2.343069613	0.04268583	0.13402181	-4.06364635
hetero after UVB irradiation VS hetero before UVB irradiation	Levobunolol Action Pathway|SMPDB	-0.01555055	-0.01775524	-0.07410913	0.94265284	0.97054702	-6.34356282
WT after UVB irradiation VS WT before UVB irradiation	Levobunolol Action Pathway|SMPDB	0.056685205	-0.03978418	0.223404833	0.82768199	0.94525798	-5.84393438
homo after UVB irradiation VS homo before UVB irradiation	Levobupivacaine Action Pathway|SMPDB	0.191132361	0.029768717	1.061313976	0.31508276	0.46923284	-5.79783739
hetero after UVB irradiation VS hetero before UVB irradiation	Levobupivacaine Action Pathway|SMPDB	-0.07904315	0.055005373	-0.44437398	0.66796482	0.82651398	-6.24068873
WT after UVB irradiation VS WT before UVB irradiation	Levobupivacaine Action Pathway|SMPDB	0.022316377	0.035038618	0.098344171	0.92358676	0.97566251	-5.86425414
homo after UVB irradiation VS homo before UVB irradiation	Levomethadyl Acetate Action Action Pathway|SMPDB	0.191132361	0.029768717	1.061313976	0.31508276	0.46923284	-5.79783739
hetero after UVB irradiation VS hetero before UVB irradiation	Levomethadyl Acetate Action Action Pathway|SMPDB	-0.07904315	0.055005373	-0.44437398	0.66796482	0.82651398	-6.24068873
WT after UVB irradiation VS WT before UVB irradiation	Levomethadyl Acetate Action Action Pathway|SMPDB	0.022316377	0.035038618	0.098344171	0.92358676	0.97566251	-5.86425414
homo after UVB irradiation VS homo before UVB irradiation	Levomethadyl Acetate Metabolism Pathway|SMPDB	0.873508946	-0.00608847	2.782632297	0.02049422	0.09129427	-3.3664163
hetero after UVB irradiation VS hetero before UVB irradiation	Levomethadyl Acetate Metabolism Pathway|SMPDB	0.894105956	0.043579074	2.862115742	0.01999301	0.12483305	-3.28169891
WT after UVB irradiation VS WT before UVB irradiation	Levomethadyl Acetate Metabolism Pathway|SMPDB	0.436109556	0.06978914	1.078504427	0.30593492	0.69096189	-5.3139331
homo after UVB irradiation VS homo before UVB irradiation	Levorphanol Action Pathway|SMPDB	0.191132361	0.029768717	1.061313976	0.31508276	0.46923284	-5.79783739
hetero after UVB irradiation VS hetero before UVB irradiation	Levorphanol Action Pathway|SMPDB	-0.07904315	0.055005373	-0.44437398	0.66796482	0.82651398	-6.24068873
WT after UVB irradiation VS WT before UVB irradiation	Levorphanol Action Pathway|SMPDB	0.022316377	0.035038618	0.098344171	0.92358676	0.97566251	-5.86425414
homo after UVB irradiation VS homo before UVB irradiation	LGI-ADAM interactions|Reactome	0.235264282	0.022540316	1.091976407	0.30206939	0.46312664	-5.76637262
hetero after UVB irradiation VS hetero before UVB irradiation	LGI-ADAM interactions|Reactome	-0.13218152	-0.00377883	-0.66526459	0.52367021	0.73920373	-6.11272126
WT after UVB irradiation VS WT before UVB irradiation	LGI-ADAM interactions|Reactome	-0.05934552	-0.0656299	-0.2304917	0.82231934	0.94525798	-5.84231375
homo after UVB irradiation VS homo before UVB irradiation	L-glutamine tRNA biosynthesis|HumanCyc	-0.80165757	0.042601591	-2.49763596	0.03298478	0.12106794	-3.82050571
hetero after UVB irradiation VS hetero before UVB irradiation	L-glutamine tRNA biosynthesis|HumanCyc	-1.03192547	-0.03875776	-4.55926667	0.00162033	0.04858852	-0.82458796
WT after UVB irradiation VS WT before UVB irradiation	L-glutamine tRNA biosynthesis|HumanCyc	-0.41794146	-0.09133584	-0.92440392	0.37687024	0.75077508	-5.45497685
homo after UVB irradiation VS homo before UVB irradiation	Lidocaine (Antiarrhythmic) Action Pathway|SMPDB	0.396415397	-0.03677376	2.343069613	0.04268583	0.13402181	-4.06364635
hetero after UVB irradiation VS hetero before UVB irradiation	Lidocaine (Antiarrhythmic) Action Pathway|SMPDB	-0.01555055	-0.01775524	-0.07410913	0.94265284	0.97054702	-6.34356282
WT after UVB irradiation VS WT before UVB irradiation	Lidocaine (Antiarrhythmic) Action Pathway|SMPDB	0.056685205	-0.03978418	0.223404833	0.82768199	0.94525798	-5.84393438
homo after UVB irradiation VS homo before UVB irradiation	Lidocaine (Local Anaesthetic) Action Pathway|SMPDB	0.191132361	0.029768717	1.061313976	0.31508276	0.46923284	-5.79783739
hetero after UVB irradiation VS hetero before UVB irradiation	Lidocaine (Local Anaesthetic) Action Pathway|SMPDB	-0.07904315	0.055005373	-0.44437398	0.66796482	0.82651398	-6.24068873
WT after UVB irradiation VS WT before UVB irradiation	Lidocaine (Local Anaesthetic) Action Pathway|SMPDB	0.022316377	0.035038618	0.098344171	0.92358676	0.97566251	-5.86425414
homo after UVB irradiation VS homo before UVB irradiation	Lidocaine (Local Anaesthetic) Metabolism Pathway|SMPDB	1.090788602	-0.02000497	5.599662037	0.00028675	0.01666204	0.77878938
hetero after UVB irradiation VS hetero before UVB irradiation	Lidocaine (Local Anaesthetic) Metabolism Pathway|SMPDB	0.508084384	0.023683829	1.319045548	0.22187157	0.44296567	-5.49105448
WT after UVB irradiation VS WT before UVB irradiation	Lidocaine (Local Anaesthetic) Metabolism Pathway|SMPDB	0.546785263	-0.05289129	1.455310782	0.17600177	0.58755921	-4.90292451
homo after UVB irradiation VS homo before UVB irradiation	LIF signaling|INOH	0.276736272	-0.01854974	1.063627643	0.31408604	0.46923284	-5.79548742
hetero after UVB irradiation VS hetero before UVB irradiation	LIF signaling|INOH	-0.0224387	0.009362238	-0.12345648	0.90463996	0.97054702	-6.33828054
WT after UVB irradiation VS WT before UVB irradiation	LIF signaling|INOH	0.050773592	-0.06545538	0.234404203	0.81936281	0.94525798	-5.84139759
homo after UVB irradiation VS homo before UVB irradiation	Ligand-dependent caspase activation|Reactome	0.816073954	-0.00782356	5.108257265	0.00055877	0.01666204	0.137599265
hetero after UVB irradiation VS hetero before UVB irradiation	Ligand-dependent caspase activation|Reactome	0.719755752	0.019233961	3.499457606	0.0074533	0.08801074	-2.31862354
WT after UVB irradiation VS WT before UVB irradiation	Ligand-dependent caspase activation|Reactome	0.650561448	-0.04452083	2.764674823	0.01982652	0.31542987	-3.06190272
homo after UVB irradiation VS homo before UVB irradiation	Ligand-independent caspase activation via DCC|Reactome	0.298858467	0.015976287	1.051154031	0.3194883	0.47517381	-5.80810947
hetero after UVB irradiation VS hetero before UVB irradiation	Ligand-independent caspase activation via DCC|Reactome	0.458198692	-0.0091592	2.50676768	0.03515035	0.16432337	-3.82512746
WT after UVB irradiation VS WT before UVB irradiation	Ligand-independent caspase activation via DCC|Reactome	0.264576855	-0.01105964	1.08269079	0.30415947	0.69006274	-5.30986228
homo after UVB irradiation VS homo before UVB irradiation	Ligand-receptor interactions|Reactome	-0.00675384	0.016761929	-0.03089455	0.97600042	0.98571509	-6.36793545
hetero after UVB irradiation VS hetero before UVB irradiation	Ligand-receptor interactions|Reactome	-0.12549303	0.016644034	-0.54597909	0.59923432	0.78848442	-6.18770578
WT after UVB irradiation VS WT before UVB irradiation	Ligand-receptor interactions|Reactome	-0.01491757	0.029033401	-0.05739185	0.95535458	0.98742794	-5.86748285

homo after UVB irradiation VS homo before UVB irradiation	links between pyk2 and map kinases|BioCarta	0.347736168	-0.02261342	1.688851991	0.12411437	0.26297272	-5.03578189
hetero after UVB irradiation VS hetero before UVB irradiation	links between pyk2 and map kinases|BioCarta	0.523636885	-0.00899478	3.013960412	0.01574732	0.11352943	-3.04978008
WT after UVB irradiation VS WT before UVB irradiation	links between pyk2 and map kinases|BioCarta	0.325247227	0.045900386	1.435087741	0.1815478	0.598227	-4.92706339
homo after UVB irradiation VS homo before UVB irradiation	Linoleate metabolism|EHMN	0.004736628	0.10157615	0.029526395	0.97706289	0.98606224	-6.36797986
hetero after UVB irradiation VS hetero before UVB irradiation	Linoleate metabolism|EHMN	0.030371373	0.032997919	0.18623802	0.85666405	0.94503144	-6.32776482
WT after UVB irradiation VS WT before UVB irradiation	Linoleate metabolism|EHMN	-0.13504715	0.03289052	-0.65009374	0.53016504	0.83380183	-5.65975911
homo after UVB irradiation VS homo before UVB irradiation	Linoleic acid (LA) metabolism|Reactome	-0.25117464	-0.01635272	-1.0477665	0.3209676	0.47690793	-5.81151721
hetero after UVB irradiation VS hetero before UVB irradiation	Linoleic acid (LA) metabolism|Reactome	0.273085054	-0.02536629	1.310246501	0.22470059	0.44685222	-5.50140518
WT after UVB irradiation VS WT before UVB irradiation	Linoleic acid (LA) metabolism|Reactome	0.075496541	-0.05923774	0.327180386	0.75022322	0.91881722	-5.81522983
homo after UVB irradiation VS homo before UVB irradiation	Linoleic acid metabolism - Homo sapiens (human)|KEGG	0.226432582	0.076410527	1.045650348	0.32189435	0.4773529	-5.8136416
hetero after UVB irradiation VS hetero before UVB irradiation	Linoleic acid metabolism - Homo sapiens (human)|KEGG	0.181906397	0.034020289	1.120132863	0.29356655	0.52473582	-5.71340706
WT after UVB irradiation VS WT before UVB irradiation	Linoleic acid metabolism - Homo sapiens (human)|KEGG	0.07418154	0.056858459	0.305604286	0.76612252	0.92808529	-5.822073
homo after UVB irradiation VS homo before UVB irradiation	Lipid Metabolism Pathway|Wikipathways	0.253380782	-0.06513807	1.446266194	0.18064454	0.3341193	-5.35693804
hetero after UVB irradiation VS hetero before UVB irradiation	Lipid Metabolism Pathway|Wikipathways	0.038647512	-0.04398407	0.166668478	0.87156138	0.95151539	-6.33149717
WT after UVB irradiation VS WT before UVB irradiation	Lipid Metabolism Pathway|Wikipathways	0.092188396	-0.08977262	0.392745413	0.70268308	0.90059561	-5.79164727
homo after UVB irradiation VS homo before UVB irradiation	Lipid particle organization|Reactome	0.1144066	-0.0258679	0.408745544	0.6918924	0.79350142	-6.27949095
hetero after UVB irradiation VS hetero before UVB irradiation	Lipid particle organization|Reactome	-0.54634618	-0.12246673	-1.83285676	0.1022969	0.28746564	-4.82140076
WT after UVB irradiation VS WT before UVB irradiation	Lipid particle organization|Reactome	-0.67855496	-0.14023002	-2.56237597	0.02809026	0.35160213	-3.36554345
homo after UVB irradiation VS homo before UVB irradiation	Lipid storage and perilipins in skeletal muscle|Wikipathways	-0.58924752	0.023014888	-1.69929894	0.12208341	0.26072631	-5.0213429
hetero after UVB irradiation VS hetero before UVB irradiation	Lipid storage and perilipins in skeletal muscle|Wikipathways	-1.01013381	-0.00614572	-5.20346205	0.00069382	0.03228824	-0.00322242
WT after UVB irradiation VS WT before UVB irradiation	Lipid storage and perilipins in skeletal muscle|Wikipathways	-0.70962308	-0.01630652	-2.94255066	0.01459937	0.28372475	-2.79436147
homo after UVB irradiation VS homo before UVB irradiation	lipoate biosynthesis and incorporation|HumanCyc	-0.67367861	0.044323608	-2.26229834	0.04882218	0.14466065	-4.18932729
hetero after UVB irradiation VS hetero before UVB irradiation	lipoate biosynthesis and incorporation|HumanCyc	-1.01695675	0.009923473	-5.0276352	0.00086937	0.03680415	-0.22059221
WT after UVB irradiation VS WT before UVB irradiation	lipoate biosynthesis and incorporation|HumanCyc	-0.4667147	-0.02946974	-1.50090839	0.16402977	0.56790008	-4.84773696
homo after UVB irradiation VS homo before UVB irradiation	Lipoate metabolism|EHMN	-0.27487532	0.030111867	-1.45740055	0.17762343	0.33035788	-5.34284265
hetero after UVB irradiation VS hetero before UVB irradiation	Lipoate metabolism|EHMN	-0.40593942	0.018498053	-2.11680008	0.06542979	0.22964907	-4.41161837
WT after UVB irradiation VS WT before UVB irradiation	Lipoate metabolism|EHMN	-0.33887417	-0.0355969	-1.50337888	0.16340179	0.56749503	-4.84471746
homo after UVB irradiation VS homo before UVB irradiation	lipoate salvage|HumanCyc	-0.53248053	-0.02216861	-1.61177265	0.14006977	0.28327069	-5.14085469
hetero after UVB irradiation VS hetero before UVB irradiation	lipoate salvage|HumanCyc	-0.99666938	0.05995092	-4.16227718	0.00281647	0.06026979	-1.36456614
WT after UVB irradiation VS WT before UVB irradiation	lipoate salvage|HumanCyc	-0.38382473	0.00482188	-1.39798645	0.19211149	0.60496032	-4.97079157
homo after UVB irradiation VS homo before UVB irradiation	Lipoic acid metabolism - Homo sapiens (human)|KEGG	-0.60674936	0.062201897	-1.77549539	0.10816375	0.241504	-4.91468761
hetero after UVB irradiation VS hetero before UVB irradiation	Lipoic acid metabolism - Homo sapiens (human)|KEGG	-0.96389419	-0.07411847	-4.03339847	0.00338685	0.06516742	-1.54518296
WT after UVB irradiation VS WT before UVB irradiation	Lipoic acid metabolism - Homo sapiens (human)|KEGG	-0.27115987	-0.01638421	-0.72441565	0.48528146	0.81710765	-5.6105102
homo after UVB irradiation VS homo before UVB irradiation	lipoxin biosynthesis|HumanCyc	0.37230333	-0.0264397	1.165384818	0.27261443	0.43614438	-5.68828688
hetero after UVB irradiation VS hetero before UVB irradiation	lipoxin biosynthesis|HumanCyc	0.627921815	0.0558953	2.676755891	0.02681533	0.14252149	-3.5654361
WT after UVB irradiation VS WT before UVB irradiation	lipoxin biosynthesis|HumanCyc	0.564516512	0.010862666	2.709988452	0.02178496	0.31542987	-3.14412723
homo after UVB irradiation VS homo before UVB irradiation	Lisinopril Action Pathway|SMPDB	0.636038586	-0.11800708	2.436891056	0.03650532	0.12504267	-3.91641947
hetero after UVB irradiation VS hetero before UVB irradiation	Lisinopril Action Pathway|SMPDB	-0.02777369	0.003323189	-0.09172332	0.92906122	0.97054702	-6.34197953
WT after UVB irradiation VS WT before UVB irradiation	Lisinopril Action Pathway|SMPDB	0.270438532	-0.10210744	0.707878218	0.49505953	0.81710765	-5.62188349
homo after UVB irradiation VS homo before UVB irradiation	lissencephaly gene (lis1) in neuronal migration and development|BioCarta	-0.07514171	0.022595401	-0.34459574	0.73799145	0.82743722	-6.30505675
hetero after UVB irradiation VS hetero before UVB irradiation	lissencephaly gene (lis1) in neuronal migration and development|BioCarta	0.125355749	-0.0113793	0.645078083	0.53604219	0.74924265	-6.12635432
WT after UVB irradiation VS WT before UVB irradiation	lissencephaly gene (lis1) in neuronal migration and development|BioCarta	0.011817186	0.050691238	0.051966767	0.95957039	0.98815178	-5.8677833
homo after UVB irradiation VS homo before UVB irradiation	Lissencephaly gene (LIS1) in neuronal migration and development|PID	0.200134573	-0.02322728	1.235150421	0.24678809	0.40910289	-5.6106226
hetero after UVB irradiation VS hetero before UVB irradiation	Lissencephaly gene (LIS1) in neuronal migration and development|PID	0.363702295	0.022714812	2.051377662	0.07257546	0.24145909	-4.50773825
WT after UVB irradiation VS WT before UVB irradiation	Lissencephaly gene (LIS1) in neuronal migration and development|PID	0.324713812	0.017444873	1.597100885	0.14107838	0.55622203	-4.72803716
homo after UVB irradiation VS homo before UVB irradiation	Listeria monocytogenes entry into host cells|Reactome	-0.02773992	-0.00345176	-0.11525534	0.91067011	0.94787581	-6.36131598
hetero after UVB irradiation VS hetero before UVB irradiation	Listeria monocytogenes entry into host cells|Reactome	0.489813115	0.03209572	2.770616175	0.02310507	0.13623921	-3.42174589
WT after UVB irradiation VS WT before UVB irradiation	Listeria monocytogenes entry into host cells|Reactome	-0.13625162	-0.00987782	-0.53007182	0.60753755	0.87257466	-5.72890927
homo after UVB irradiation VS homo before UVB irradiation	Liver steatosis AOP|Wikipathways	-0.13166307	-0.00887686	-0.69441419	0.50424103	0.64383796	-6.11597217
hetero after UVB irradiation VS hetero before UVB irradiation	Liver steatosis AOP|Wikipathways	-0.31940889	-0.02963325	-1.78934978	0.10945391	0.30108082	-4.88222388
WT after UVB irradiation VS WT before UVB irradiation	Liver steatosis AOP|Wikipathways	-0.23801719	-0.03179957	-1.13690508	0.28187697	0.67048548	-5.25605546
homo after UVB irradiation VS homo before UVB irradiation	Liver X Receptor Pathway|Wikipathways	-0.12327306	0.058333055	-0.44992126	0.66297534	0.76909182	-6.26087094
hetero after UVB irradiation VS hetero before UVB irradiation	Liver X Receptor Pathway|Wikipathways	-0.40104836	-0.00229338	-1.75751229	0.11498526	0.30887493	-4.92633731
WT after UVB irradiation VS WT before UVB irradiation	Liver X Receptor Pathway|Wikipathways	0.49245989	0.029105766	2.111377267	0.06067945	0.43072383	-4.02876677
homo after UVB irradiation VS homo before UVB irradiation	LKB1 signaling events|PID	-0.01042532	0.00657373	-0.05047491	0.96080147	0.97662946	-6.36707955
hetero after UVB irradiation VS hetero before UVB irradiation	LKB1 signaling events|PID	-0.05708435	-0.04179723	-0.37122561	0.71963055	0.86340512	-6.27240372
WT after UVB irradiation VS WT before UVB irradiation	LKB1 signaling events|PID	-0.07879667	-0.02513674	-0.34834652	0.73474413	0.913828	-5.80807384
homo after UVB irradiation VS homo before UVB irradiation	L-kynurenine degradation|HumanCyc	-0.06834647	-0.00719782	-0.34445047	0.73809719	0.82743722	-6.30510984
hetero after UVB irradiation VS hetero before UVB irradiation	L-kynurenine degradation|HumanCyc	-0.18820231	-0.01314906	-0.93222096	0.37716011	0.60405274	-5.89853595
WT after UVB irradiation VS WT before UVB irradiation	L-kynurenine degradation|HumanCyc	-0.33280881	-0.02234533	-1.26164952	0.23548212	0.62952955	-5.12490644
homo after UVB irradiation VS homo before UVB irradiation	LncRNA involvement in canonical Wnt signaling and colorectal cancer|Wikipathways	-0.42029765	0.029617386	-2.64213068	0.02591073	0.10655402	-3.59093148
hetero after UVB irradiation VS hetero before UVB irradiation	LncRNA involvement in canonical Wnt signaling and colorectal cancer|Wikipathways	-0.32157865	-0.01905397	-1.62945279	0.13995246	0.34069016	-5.10006273
WT after UVB irradiation VS WT before UVB irradiation	LncRNA involvement in canonical Wnt signaling and colorectal cancer|Wikipathways	-0.31629039	0.021418527	-1.29582849	0.22391383	0.62952955	-5.08728179
homo after UVB irradiation VS homo before UVB irradiation	LncRNA-mediated mechanisms of therapeutic resistance|Wikipathways	-0.26525705	0.046814183	-0.91816425	0.38151344	0.53484506	-5.93523132
hetero after UVB irradiation VS hetero before UVB irradiation	LncRNA-mediated mechanisms of therapeutic resistance|Wikipathways	0.152444301	-0.0279673	0.643933866	0.53674866	0.74954056	-6.12711573
WT after UVB irradiation VS WT before UVB irradiation	LncRNA-mediated mechanisms of therapeutic resistance|Wikipathways	-0.12424788	-0.05485781	-0.56360927	0.58533678	0.86119304	-5.71090522
homo after UVB irradiation VS homo before UVB irradiation	Localization of the PINCH-ILK-PARVIN complex to focal adhesions|Reactome	0.920367205	-0.0516453	3.534001265	0.00596481	0.04509157	-2.16954001
hetero after UVB irradiation VS hetero before UVB irradiation	Localization of the PINCH-ILK-PARVIN complex to focal adhesions|Reactome	0.826847467	0.068904904	3.004026565	0.01599423	0.11352943	-3.06492338
WT after UVB irradiation VS WT before UVB irradiation	Localization of the PINCH-ILK-PARVIN complex to focal adhesions|Reactome	0.476054667	-0.06225055	1.273769151	0.23132531	0.62952955	-5.11164518
homo after UVB irradiation VS homo before UVB irradiation	Long chain acyl-CoA dehydrogenase deficiency (LCAD)|SMPDB	-0.19843953	0.039801734	-0.74439404	0.47485571	0.61754234	-6.07941248
hetero after UVB irradiation VS hetero before UVB irradiation	Long chain acyl-CoA dehydrogenase deficiency (LCAD)|SMPDB	-0.48316271	0.016700705	-1.75538636	0.11536374	0.30887493	-4.92927061
WT after UVB irradiation VS WT before UVB irradiation	Long chain acyl-CoA dehydrogenase deficiency (LCAD)|SMPDB	-0.40763842	0.012220754	-1.51649601	0.16010237	0.56072663	-4.82863564
homo after UVB irradiation VS homo before UVB irradiation	Long-chain-3-hydroxyacyl-coa dehydrogenase deficiency (LCHAD)|SMPDB	-0.83801153	0.022760834	-3.4725845	0.00658545	0.04701822	-2.2661183
hetero after UVB irradiation VS hetero before UVB irradiation	Long-chain-3-hydroxyacyl-coa dehydrogenase deficiency (LCHAD)|SMPDB	-0.7961592	0.030443028	-3.1957153	0.0118657	0.10542662	-2.77375612
WT after UVB irradiation VS WT before UVB irradiation	Long-chain-3-hydroxyacyl-coa dehydrogenase deficiency (LCHAD)|SMPDB	-0.48656077	-0.0330546	-2.25189377	0.04781905	0.401167	-3.8253293
homo after UVB irradiation VS homo before UVB irradiation	Longevity regulating pathway - Homo sapiens (human)|KEGG	0.220556642	-0.0410121	1.24903306	0.24189412	0.40353094	-5.59478261
hetero after UVB irradiation VS hetero before UVB irradiation	Longevity regulating pathway - Homo sapiens (human)|KEGG	0.051346378	-0.00102548	0.324436833	0.75351566	0.88469054	-6.28980293
WT after UVB irradiation VS WT before UVB irradiation	Longevity regulating pathway - Homo sapiens (human)|KEGG	-0.088111	-0.04565632	-0.45090903	0.66159761	0.87935805	-5.76725552
homo after UVB irradiation VS homo before UVB irradiation	Longevity regulating pathway - multiple species - Homo sapiens (human)|KEGG	0.060880442	-0.00956475	0.339271872	0.74187033	0.83044372	-6.30698823
hetero after UVB irradiation VS hetero before UVB irradiation	Longevity regulating pathway - multiple species - Homo sapiens (human)|KEGG	-0.01887476	0.012523006	-0.11632824	0.9101174	0.97054702	-6.33920617
WT after UVB irradiation VS WT before UVB irradiation	Longevity regulating pathway - multiple species - Homo sapiens (human)|KEGG	-0.09542693	-0.02291338	-0.49358602	0.6321733	0.87303764	-5.7473129
homo after UVB irradiation VS homo before UVB irradiation	Long-term depression - Homo sapiens (human)|KEGG	0.32202058	-0.02039122	2.029319644	0.07171539	0.18927888	-4.54456313
hetero after UVB irradiation VS hetero before UVB irradiation	Long-term depression - Homo sapiens (human)|KEGG	0.375736152	0.02487337	2.466886468	0.03745887	0.16904708	-3.88584418
WT after UVB irradiation VS WT before UVB irradiation	Long-term depression - Homo sapiens (human)|KEGG	0.157158885	0.006109764	0.777999352	0.45443918	0.80850166	-5.5720594
homo after UVB irradiation VS homo before UVB irradiation	Long-term potentiation - Homo sapiens (human)|KEGG	0.274649531	-0.03856293	1.723182902	0.11755338	0.25547298	-4.98816216
hetero after UVB irradiation VS hetero before UVB irradiation	Long-term potentiation - Homo sapiens (human)|KEGG	0.169618752	0.02402614	1.00344651	0.34358837	0.57260541	-5.83145789
WT after UVB irradiation VS WT before UVB irradiation	Long-term potentiation - Homo sapiens (human)|KEGG	0.063618452	-0.0194854	0.30899298	0.76361765	0.92696825	-5.82102848
homo after UVB irradiation VS homo before UVB irradiation	Lornoxicam Action Pathway|SMPDB	0.40423582	0.045705158	1.979500697	0.07780079	0.19626432	-4.61879689
hetero after UVB irradiation VS hetero before UVB irradiation	Lornoxicam Action Pathway|SMPDB	0.482496143	0.064876536	3.04499191	0.01500113	0.1130168	-3.00250826
WT after UVB irradiation VS WT before UVB irradiation	Lornoxicam Action Pathway|SMPDB	0.275725339	0.086595857	1.261970114	0.23537138	0.62952955	-5.12455679
homo after UVB irradiation VS homo before UVB irradiation	Losartan Action Pathway|SMPDB	0.636038586	-0.11800708	2.436891056	0.03650532	0.12504267	-3.91641947
hetero after UVB irradiation VS hetero before UVB irradiation	Losartan Action Pathway|SMPDB	-0.02777369	0.003323189	-0.09172332	0.92906122	0.97054702	-6.34197953
WT after UVB irradiation VS WT before UVB irradiation	Losartan Action Pathway|SMPDB	0.270438532	-0.10210744	0.707878218	0.49505953	0.81710765	-5.62188349
homo after UVB irradiation VS homo before UVB irradiation	Losartan Pathway_ Pharmacokinetics|PharmGKB	-0.00989697	0.003766021	-0.02545663	0.9802236	0.98670656	-6.36810008
hetero after UVB irradiation VS hetero before UVB irradiation	Losartan Pathway_ Pharmacokinetics|PharmGKB	-0.05923523	-0.00889464	-0.15758483	0.87849513	0.95610812	-6.33308992
WT after UVB irradiation VS WT before UVB irradiation	Losartan Pathway_ Pharmacokinetics|PharmGKB	-0.42357612	0.015480758	-1.44654077	0.17838877	0.59314626	-4.91341858
homo after UVB irradiation VS homo before UVB irradiation	Loss of Function of SMAD2/3 in Cancer|Reactome	-0.21250582	0.002665563	-0.67944745	0.5132547	0.65152098	-6.12647645
hetero after UVB irradiation VS hetero before UVB irradiation	Loss of Function of SMAD2/3 in Cancer|Reactome	-0.41047651	-0.03660938	-1.88598158	0.09415498	0.27705513	-4.74633631
WT after UVB irradiation VS WT before UVB irradiation	Loss of Function of SMAD2/3 in Cancer|Reactome	-0.44904362	-0.05506262	-1.96758166	0.07722029	0.46222215	-4.23249425
homo after UVB irradiation VS homo before UVB irradiation	Loss of Function of TGFBR1 in Cancer|Reactome	0.063839792	-0.05333939	0.226646754	0.82555614	0.89312082	-6.34092489
hetero after UVB irradiation VS hetero before UVB irradiation	Loss of Function of TGFBR1 in Cancer|Reactome	0.150449946	-0.08314986	0.873939959	0.40636867	0.63319136	-5.95044668
WT after UVB irradiation VS WT before UVB irradiation	Loss of Function of TGFBR1 in Cancer|Reactome	0.00330265	-0.13359433	0.009794744	0.99237615	0.99712795	-5.86910259
homo after UVB irradiation VS homo before UVB irradiation	Loss of Function of TGFBR2 in Cancer|Reactome	0.063839792	-0.05333939	0.226646754	0.82555614	0.89312082	-6.34092489
hetero after UVB irradiation VS hetero before UVB irradiation	Loss of Function of TGFBR2 in Cancer|Reactome	0.150449946	-0.08314986	0.873939959	0.40636867	0.63319136	-5.95044668
WT after UVB irradiation VS WT before UVB irradiation	Loss of Function of TGFBR2 in Cancer|Reactome	0.00330265	-0.13359433	0.009794744	0.99237615	0.99712795	-5.86910259
homo after UVB irradiation VS homo before UVB irradiation	Loss of Nlp from mitotic centrosomes|Reactome	-0.46795907	0.002237457	-2.61514175	0.02710569	0.10950729	-3.63393697
hetero after UVB irradiation VS hetero before UVB irradiation	Loss of Nlp from mitotic centrosomes|Reactome	-0.01493484	-0.00580824	-0.05799005	0.95510832	0.97784899	-6.34471734
WT after UVB irradiation VS WT before UVB irradiation	Loss of Nlp from mitotic centrosomes|Reactome	-0.14115336	-0.02980665	-0.56461597	0.58467713	0.86080608	-5.71034882
homo after UVB irradiation VS homo before UVB irradiation	Loss of proteins required for interphase microtubule organization from the centrosome|Reactome	-0.46795907	0.002237457	-2.61514175	0.02710569	0.10950729	-3.63393697
hetero after UVB irradiation VS hetero before UVB irradiation	Loss of proteins required for interphase microtubule organization from the centrosome|Reactome	-0.01493484	-0.00580824	-0.05799005	0.95510832	0.97784899	-6.34471734
WT after UVB irradiation VS WT before UVB irradiation	Loss of proteins required for interphase microtubule organization from the centrosome|Reactome	-0.14115336	-0.02980665	-0.56461597	0.58467713	0.86080608	-5.71034882
homo after UVB irradiation VS homo before UVB irradiation	Lovastatin Action Pathway|SMPDB	-0.19230642	-0.01987665	-0.61925406	0.55047761	0.67913558	-6.16661869
hetero after UVB irradiation VS hetero before UVB irradiation	Lovastatin Action Pathway|SMPDB	0.185820603	0.008098609	0.631890782	0.54421801	0.75147406	-6.13505553
WT after UVB irradiation VS WT before UVB irradiation	Lovastatin Action Pathway|SMPDB	-0.14894747	0.045069977	-0.50755372	0.62268399	0.87257466	-5.74041409
homo after UVB irradiation VS homo before UVB irradiation	LPA receptor mediated events|PID	0.4557146	-0.03029603	2.401749892	0.03870931	0.12940262	-3.97170411
hetero after UVB irradiation VS hetero before UVB irradiation	LPA receptor mediated events|PID	0.444708568	0.043725352	2.505692429	0.03521067	0.16443671	-3.82676584
WT after UVB irradiation VS WT before UVB irradiation	LPA receptor mediated events|PID	0.399916365	-0.00767418	1.950062591	0.07950396	0.46479598	-4.25695086
homo after UVB irradiation VS homo before UVB irradiation	LPA4-mediated signaling events|PID	0.389211191	-0.07114889	1.588549646	0.14522568	0.29083354	-5.17198641
hetero after UVB irradiation VS hetero before UVB irradiation	LPA4-mediated signaling events|PID	0.231526332	0.043859313	0.982686717	0.35313304	0.58149581	-5.8514118
WT after UVB irradiation VS WT before UVB irradiation	LPA4-mediated signaling events|PID	0.164047105	-0.07563144	0.614029612	0.5527978	0.84516674	-5.68190495
homo after UVB irradiation VS homo before UVB irradiation	LRR FLII-interacting protein 1 (LRRFIP1) activates type I IFN production|Reactome	-0.22820946	0.085874122	-0.69905972	0.50146318	0.64118759	-6.11266986
hetero after UVB irradiation VS hetero before UVB irradiation	LRR FLII-interacting protein 1 (LRRFIP1) activates type I IFN production|Reactome	0.163021075	0.009877166	0.489221171	0.6371661	0.81534761	-6.21856598
WT after UVB irradiation VS WT before UVB irradiation	LRR FLII-interacting protein 1 (LRRFIP1) activates type I IFN production|Reactome	-0.18852739	0.038716916	-0.53718643	0.60279171	0.86926104	-5.72517653
homo after UVB irradiation VS homo before UVB irradiation	L-serine degradation|HumanCyc	0.463924785	-0.03129142	1.197716716	0.26038758	0.42446614	-5.65270176
hetero after UVB irradiation VS hetero before UVB irradiation	L-serine degradation|HumanCyc	-0.07875287	0.030205163	-0.1765344	0.86404377	0.94735456	-6.32966686
WT after UVB irradiation VS WT before UVB irradiation	L-serine degradation|HumanCyc	-0.31506707	-0.08735046	-0.82013565	0.43110102	0.79682615	-5.54013674
homo after UVB irradiation VS homo before UVB irradiation	LTF danger signal response pathway|Wikipathways	0.737251691	-0.00898854	4.02828327	0.00273395	0.02978124	-1.40727832
hetero after UVB irradiation VS hetero before UVB irradiation	LTF danger signal response pathway|Wikipathways	0.643528035	0.005894303	2.849312901	0.02040115	0.12634297	-3.30128668
WT after UVB irradiation VS WT before UVB irradiation	LTF danger signal response pathway|Wikipathways	0.63935116	-0.01047871	2.758153522	0.02005047	0.31542987	-3.07171088
homo after UVB irradiation VS homo before UVB irradiation	Lumiracoxib Action Pathway|SMPDB	0.40423582	0.045705158	1.979500697	0.07780079	0.19626432	-4.61879689
hetero after UVB irradiation VS hetero before UVB irradiation	Lumiracoxib Action Pathway|SMPDB	0.482496143	0.064876536	3.04499191	0.01500113	0.1130168	-3.00250826
WT after UVB irradiation VS WT before UVB irradiation	Lumiracoxib Action Pathway|SMPDB	0.275725339	0.086595857	1.261970114	0.23537138	0.62952955	-5.12455679
homo after UVB irradiation VS homo before UVB irradiation	Lung fibrosis|Wikipathways	0.458137592	0.001557669	2.835788609	0.01875702	0.0875711	-3.28129156
hetero after UVB irradiation VS hetero before UVB irradiation	Lung fibrosis|Wikipathways	0.361763447	-0.00315313	2.098083733	0.06740111	0.23397886	-4.4392051
WT after UVB irradiation VS WT before UVB irradiation	Lung fibrosis|Wikipathways	0.221809785	-0.02495948	0.86382994	0.40776212	0.77432416	-5.50550245
homo after UVB irradiation VS homo before UVB irradiation	Lysine catabolism|Reactome	0.175203577	-0.01711934	0.745616339	0.474151	0.61754234	-6.0784901
hetero after UVB irradiation VS hetero before UVB irradiation	Lysine catabolism|Reactome	0.078419272	0.018449832	0.44435308	0.66797933	0.82651398	-6.24069857
WT after UVB irradiation VS WT before UVB irradiation	Lysine catabolism|Reactome	0.096248564	-0.03354017	0.396320421	0.70012687	0.8996847	-5.79024163
homo after UVB irradiation VS homo before UVB irradiation	Lysine degradation - Homo sapiens (human)|KEGG	-0.22629046	-0.02839556	-1.32832156	0.21545238	0.37334177	-5.50196742
hetero after UVB irradiation VS hetero before UVB irradiation	Lysine degradation - Homo sapiens (human)|KEGG	-0.32630898	-0.05851427	-2.0568643	0.07194822	0.23989758	-4.4997113
WT after UVB irradiation VS WT before UVB irradiation	Lysine degradation - Homo sapiens (human)|KEGG	-0.15076687	-0.04805564	-0.72808501	0.48312833	0.81599375	-5.60795486
homo after UVB irradiation VS homo before UVB irradiation	lysine degradation I (saccharopine pathway)|HumanCyc	0.54513221	-0.04453816	2.25761719	0.04920311	0.14535514	-4.19657689
hetero after UVB irradiation VS hetero before UVB irradiation	lysine degradation I (saccharopine pathway)|HumanCyc	0.48200601	0.072336809	2.103590888	0.06681514	0.23300949	-4.431095
WT after UVB irradiation VS WT before UVB irradiation	lysine degradation I (saccharopine pathway)|HumanCyc	0.269001949	-0.10987143	0.936021856	0.37114003	0.74850253	-5.44495988
homo after UVB irradiation VS homo before UVB irradiation	lysine degradation II (pipecolate pathway)|HumanCyc	0.193248513	0.000386795	0.749714622	0.47179306	0.6168978	-6.07538768
hetero after UVB irradiation VS hetero before UVB irradiation	lysine degradation II (pipecolate pathway)|HumanCyc	0.040589738	0.050447911	0.209061757	0.83936614	0.93932613	-6.32289365
WT after UVB irradiation VS WT before UVB irradiation	lysine degradation II (pipecolate pathway)|HumanCyc	-0.27190984	-0.04011475	-0.96253724	0.35829674	0.74302468	-5.4217126
homo after UVB irradiation VS homo before UVB irradiation	Lysine degradation|INOH	-0.26679253	-0.00484559	-1.22809096	0.24930758	0.41169562	-5.61862912

hetero after UVB irradiation VS hetero before UVB irradiation	Lysine degradation|INOH	-0.32976571	0.023312165	-1.84210381	0.10083352	0.28564273	-4.80839608
WT after UVB irradiation VS WT before UVB irradiation	Lysine degradation|INOH	0.034902979	0.024092252	0.137857521	0.89306741	0.97411963	-5.85953326
homo after UVB irradiation VS homo before UVB irradiation	Lysine Degradation|SMPDB	-0.02100802	-0.06308448	-0.07524201	0.941601	0.96450524	-6.36540732
hetero after UVB irradiation VS hetero before UVB irradiation	Lysine Degradation|SMPDB	-0.10000854	-0.05054657	-0.39990823	0.69917117	0.84778456	-6.26061929
WT after UVB irradiation VS WT before UVB irradiation	Lysine Degradation|SMPDB	-0.11826398	-0.1134336	-0.44264387	0.66736875	0.87935805	-5.77091861
homo after UVB irradiation VS homo before UVB irradiation	Lysine metabolism|EHMN	-0.53749701	-0.02799211	-2.9486638	0.01554746	0.07716271	-3.1004185
hetero after UVB irradiation VS hetero before UVB irradiation	Lysine metabolism|EHMN	-0.17227714	-0.04197153	-0.77512629	0.45946326	0.68349659	-6.03202809
WT after UVB irradiation VS WT before UVB irradiation	Lysine metabolism|EHMN	-0.12948666	-0.02086813	-0.48324489	0.63924429	0.87636259	-5.75230277
homo after UVB irradiation VS homo before UVB irradiation	Lysinuric protein intolerance (LPI)|SMPDB	0.234726031	0.017120546	1.156747303	0.27595732	0.43614438	-5.69767199
hetero after UVB irradiation VS hetero before UVB irradiation	Lysinuric protein intolerance (LPI)|SMPDB	-0.03255037	0.028925632	-0.18154472	0.86023155	0.94503144	-6.32869738
WT after UVB irradiation VS WT before UVB irradiation	Lysinuric protein intolerance (LPI)|SMPDB	-0.15908359	0.010252305	-0.80382265	0.44003996	0.79685269	-5.55266936
homo after UVB irradiation VS homo before UVB irradiation	Lysinuric Protein Intolerance|SMPDB	0.234726031	0.017120546	1.156747303	0.27595732	0.43614438	-5.69767199
hetero after UVB irradiation VS hetero before UVB irradiation	Lysinuric Protein Intolerance|SMPDB	-0.03255037	0.028925632	-0.18154472	0.86023155	0.94503144	-6.32869738
WT after UVB irradiation VS WT before UVB irradiation	Lysinuric Protein Intolerance|SMPDB	-0.15908359	0.010252305	-0.80382265	0.44003996	0.79685269	-5.55266936
homo after UVB irradiation VS homo before UVB irradiation	Lysosomal Acid Lipase Deficiency (Wolman Disease)|SMPDB	-0.19230642	-0.01987665	-0.61925406	0.55047761	0.67913558	-6.16661869
hetero after UVB irradiation VS hetero before UVB irradiation	Lysosomal Acid Lipase Deficiency (Wolman Disease)|SMPDB	0.185820603	0.008098609	0.631890782	0.54421801	0.75147406	-6.13505553
WT after UVB irradiation VS WT before UVB irradiation	Lysosomal Acid Lipase Deficiency (Wolman Disease)|SMPDB	-0.14894747	0.045069977	-0.50755372	0.62268399	0.87257466	-5.74041409
homo after UVB irradiation VS homo before UVB irradiation	Lysosomal oligosaccharide catabolism|Reactome	1.008864511	-0.03336622	5.448135677	0.00035097	0.01666204	0.585264064
hetero after UVB irradiation VS hetero before UVB irradiation	Lysosomal oligosaccharide catabolism|Reactome	0.354946817	-0.00420497	1.03789725	0.32818246	0.55664357	-5.79763114
WT after UVB irradiation VS WT before UVB irradiation	Lysosomal oligosaccharide catabolism|Reactome	1.024566898	0.095935773	3.549743216	0.00520404	0.20968398	-1.89311532
homo after UVB irradiation VS homo before UVB irradiation	Lysosome - Homo sapiens (human)|KEGG	0.572867515	-0.03062455	3.333176091	0.00825681	0.05379888	-2.48653817
hetero after UVB irradiation VS hetero before UVB irradiation	Lysosome - Homo sapiens (human)|KEGG	0.592783764	-0.00607847	3.855195033	0.00438768	0.07183716	-1.79899408
WT after UVB irradiation VS WT before UVB irradiation	Lysosome - Homo sapiens (human)|KEGG	0.580741599	-0.02245451	2.896047169	0.01581418	0.2924023	-2.86427774
homo after UVB irradiation VS homo before UVB irradiation	Lysosome Vesicle Biogenesis|Reactome	0.433267989	-0.04719814	2.23920896	0.05072941	0.14895852	-4.22504629
hetero after UVB irradiation VS hetero before UVB irradiation	Lysosome Vesicle Biogenesis|Reactome	0.498197397	0.009322737	3.232353986	0.01121223	0.10504986	-2.71840093
WT after UVB irradiation VS WT before UVB irradiation	Lysosome Vesicle Biogenesis|Reactome	0.29437503	0.028457394	1.317795766	0.21672901	0.62581393	-5.06273452
homo after UVB irradiation VS homo before UVB irradiation	Lysosphingolipid and LPA receptors|Reactome	0.43870608	0.013586897	2.567241444	0.02936407	0.11431817	-3.71013352
hetero after UVB irradiation VS hetero before UVB irradiation	Lysosphingolipid and LPA receptors|Reactome	0.432011977	-0.01524497	2.170936519	0.06003916	0.21945348	-4.33145657
WT after UVB irradiation VS WT before UVB irradiation	Lysosphingolipid and LPA receptors|Reactome	0.273902398	0.066513315	1.060003236	0.31387629	0.6974486	-5.33177672
homo after UVB irradiation VS homo before UVB irradiation	M Phase|Reactome	-0.52977971	-0.00667602	-2.99237736	0.01446022	0.07420453	-3.0303801
hetero after UVB irradiation VS hetero before UVB irradiation	M Phase|Reactome	0.071667883	-0.00156545	0.297927709	0.7729728	0.89850415	-6.29864828
WT after UVB irradiation VS WT before UVB irradiation	M Phase|Reactome	-0.06098584	-0.01012297	-0.23339795	0.82012291	0.94525798	-5.84163467
homo after UVB irradiation VS homo before UVB irradiation	M/G1 Transition|Reactome	-0.45162445	-0.03568692	-1.59964727	0.14274103	0.28699056	-5.15714076
hetero after UVB irradiation VS hetero before UVB irradiation	M/G1 Transition|Reactome	0.163426575	0.017239772	0.556558774	0.59229952	0.78411304	-6.18160476
WT after UVB irradiation VS WT before UVB irradiation	M/G1 Transition|Reactome	0.042039874	-0.02506154	0.151945732	0.88222729	0.96830103	-5.85746957
homo after UVB irradiation VS homo before UVB irradiation	Macroautophagy|Reactome	0.071209973	-0.03332978	0.382845	0.71035816	0.80920668	-6.29032097
hetero after UVB irradiation VS hetero before UVB irradiation	Macroautophagy|Reactome	-0.06535716	-0.05185678	-0.39129382	0.70528961	0.85253617	-6.26424734
WT after UVB irradiation VS WT before UVB irradiation	Macroautophagy|Reactome	-0.1032318	-0.02297722	-0.48478349	0.63818982	0.87636259	-5.75156671
homo after UVB irradiation VS homo before UVB irradiation	Macrophage markers|Wikipathways	0.580105955	-0.02711239	2.530043282	0.03124678	0.11752935	-3.76917465
hetero after UVB irradiation VS hetero before UVB irradiation	Macrophage markers|Wikipathways	0.5169191	0.075619775	2.165545736	0.06055586	0.22017775	-4.33946231
WT after UVB irradiation VS WT before UVB irradiation	Macrophage markers|Wikipathways	0.576396467	0.067321996	1.774859001	0.10606455	0.50446894	-4.49638977
homo after UVB irradiation VS homo before UVB irradiation	Magnesium salicylate Action Pathway|SMPDB	0.40423582	0.045705158	1.979500697	0.07780079	0.19626432	-4.61879689
hetero after UVB irradiation VS hetero before UVB irradiation	Magnesium salicylate Action Pathway|SMPDB	0.482496143	0.064876536	3.04499191	0.01500113	0.1130168	-3.00250826
WT after UVB irradiation VS WT before UVB irradiation	Magnesium salicylate Action Pathway|SMPDB	0.275725339	0.086595857	1.261970114	0.23537138	0.62952955	-5.12455679
homo after UVB irradiation VS homo before UVB irradiation	Malaria - Homo sapiens (human)|KEGG	0.78418812	-0.0118839	4.765087162	0.00090877	0.01796298	-0.33347118
hetero after UVB irradiation VS hetero before UVB irradiation	Malaria - Homo sapiens (human)|KEGG	0.665342587	0.06851213	3.214232538	0.0115306	0.10542662	-2.74576591
WT after UVB irradiation VS WT before UVB irradiation	Malaria - Homo sapiens (human)|KEGG	0.441754701	-0.01287819	1.395853892	0.19273424	0.60496032	-4.9732827
homo after UVB irradiation VS homo before UVB irradiation	malate-aspartate shuttle|HumanCyc	-0.49604186	0.025762864	-1.89369521	0.08944225	0.21433488	-4.74493453
hetero after UVB irradiation VS hetero before UVB irradiation	malate-aspartate shuttle|HumanCyc	-0.68793524	0.057770118	-2.3342945	0.04628351	0.18935052	-4.08676429
WT after UVB irradiation VS WT before UVB irradiation	malate-aspartate shuttle|HumanCyc	-0.78817205	-0.12198058	-2.50901434	0.03079059	0.36428127	-3.44527324
homo after UVB irradiation VS homo before UVB irradiation	Malate-Aspartate Shuttle|SMPDB	-0.21202955	-0.00770396	-0.74887705	0.47227435	0.61720095	-6.07602295
hetero after UVB irradiation VS hetero before UVB irradiation	Malate-Aspartate Shuttle|SMPDB	-0.19468432	0.131578037	-0.49699216	0.63190193	0.81132648	-6.21452849
WT after UVB irradiation VS WT before UVB irradiation	Malate-Aspartate Shuttle|SMPDB	-0.01726	-0.06226296	-0.04478503	0.96515327	0.98922368	-5.86813522
homo after UVB irradiation VS homo before UVB irradiation	Malate-aspartate shuttle|Wikipathways	-0.39102056	-0.07167522	-1.39760221	0.19437471	0.34996646	-5.41774272
hetero after UVB irradiation VS hetero before UVB irradiation	Malate-aspartate shuttle|Wikipathways	-0.28810929	0.065186481	-0.97526052	0.35659527	0.58466232	-5.85847002
WT after UVB irradiation VS WT before UVB irradiation	Malate-aspartate shuttle|Wikipathways	-0.45340371	-0.13321451	-1.56860804	0.14756243	0.55622203	-4.76393865
homo after UVB irradiation VS homo before UVB irradiation	Malonic Aciduria|SMPDB	-0.25733603	-0.09162056	-0.7770169	0.45627806	0.60277262	-6.05433779
hetero after UVB irradiation VS hetero before UVB irradiation	Malonic Aciduria|SMPDB	-0.83573623	-0.01284435	-4.81326218	0.00115145	0.04138456	-0.49254
WT after UVB irradiation VS WT before UVB irradiation	Malonic Aciduria|SMPDB	-0.18571921	-0.14542889	-0.63906971	0.5370254	0.83767333	-5.66665155
homo after UVB irradiation VS homo before UVB irradiation	Malonyl-coa decarboxylase deficiency|SMPDB	-0.25733603	-0.09162056	-0.7770169	0.45627806	0.60277262	-6.05433779
hetero after UVB irradiation VS hetero before UVB irradiation	Malonyl-coa decarboxylase deficiency|SMPDB	-0.83573623	-0.01284435	-4.81326218	0.00115145	0.04138456	-0.49254
WT after UVB irradiation VS WT before UVB irradiation	Malonyl-coa decarboxylase deficiency|SMPDB	-0.18571921	-0.14542889	-0.63906971	0.5370254	0.83767333	-5.66665155
homo after UVB irradiation VS homo before UVB irradiation	Mammary gland development pathway - Embryonic development (Stage 1 of 4)|Wikipathways	0.153117269	0.098518089	0.675463028	0.51567072	0.65367921	-6.12923805
hetero after UVB irradiation VS hetero before UVB irradiation	Mammary gland development pathway - Embryonic development (Stage 1 of 4)|Wikipathways	0.173748899	-0.02464406	0.932690715	0.37693106	0.60405274	-5.89810648
WT after UVB irradiation VS WT before UVB irradiation	Mammary gland development pathway - Embryonic development (Stage 1 of 4)|Wikipathways	0.276925017	0.041779338	1.365329159	0.20183688	0.61620632	-5.00866823
homo after UVB irradiation VS homo before UVB irradiation	Mammary gland development pathway - Involution (Stage 4 of 4)|Wikipathways	0.68545717	-0.00540721	3.476151331	0.00654763	0.04701822	-2.26050001
hetero after UVB irradiation VS hetero before UVB irradiation	Mammary gland development pathway - Involution (Stage 4 of 4)|Wikipathways	0.727429825	-0.00066991	4.453312984	0.00187371	0.04958933	-0.96622873
WT after UVB irradiation VS WT before UVB irradiation	Mammary gland development pathway - Involution (Stage 4 of 4)|Wikipathways	0.530594301	0.0382995	2.224078144	0.05013619	0.40887857	-3.86589132
homo after UVB irradiation VS homo before UVB irradiation	Mammary gland development pathway - Pregnancy and lactation (Stage 3 of 4)|Wikipathways	-0.28269605	0.000482929	-1.66782323	0.12829579	0.26846864	-5.06470599
hetero after UVB irradiation VS hetero before UVB irradiation	Mammary gland development pathway - Pregnancy and lactation (Stage 3 of 4)|Wikipathways	-0.11773387	-0.0311396	-0.74799995	0.47481728	0.6966202	-6.05295511
WT after UVB irradiation VS WT before UVB irradiation	Mammary gland development pathway - Pregnancy and lactation (Stage 3 of 4)|Wikipathways	-0.23935525	0.038100716	-1.06780427	0.31050889	0.6940288	-5.32428227
homo after UVB irradiation VS homo before UVB irradiation	Mammary gland development pathway - Puberty (Stage 2 of 4)|Wikipathways	-0.12423146	-0.01897939	-0.59028807	0.56893059	0.69328452	-6.18471704
hetero after UVB irradiation VS hetero before UVB irradiation	Mammary gland development pathway - Puberty (Stage 2 of 4)|Wikipathways	-0.15852967	-0.08428617	-0.5869893	0.57259936	0.76943039	-6.16345333
WT after UVB irradiation VS WT before UVB irradiation	Mammary gland development pathway - Puberty (Stage 2 of 4)|Wikipathways	-0.20428588	-0.03477662	-0.71641752	0.48999538	0.81710765	-5.61604011
homo after UVB irradiation VS homo before UVB irradiation	Mannose type O-glycan biosynthesis - Homo sapiens (human)|KEGG	-0.20804614	0.017790865	-1.01543228	0.33535053	0.49203423	-5.84360733
hetero after UVB irradiation VS hetero before UVB irradiation	Mannose type O-glycan biosynthesis - Homo sapiens (human)|KEGG	-0.01975575	-0.04938616	-0.09861489	0.92374989	0.97054702	-6.34126871
WT after UVB irradiation VS WT before UVB irradiation	Mannose type O-glycan biosynthesis - Homo sapiens (human)|KEGG	-0.11588858	-0.08093918	-0.4548041	0.65888589	0.87935805	-5.76550676
homo after UVB irradiation VS homo before UVB irradiation	MAP kinase activation|Reactome	0.154328249	-0.01313828	0.856985189	0.41279119	0.56380634	-5.98893251
hetero after UVB irradiation VS hetero before UVB irradiation	MAP kinase activation|Reactome	0.368629581	0.009636885	2.376489101	0.04327056	0.18278703	-4.02300803
WT after UVB irradiation VS WT before UVB irradiation	MAP kinase activation|Reactome	0.023449524	-0.00470322	0.118596826	0.90792445	0.97566251	-5.86203117
homo after UVB irradiation VS homo before UVB irradiation	MAP kinase cascade|HumanCyc	0.244095794	-0.04505327	0.869383586	0.40631251	0.55796178	-5.97830177
hetero after UVB irradiation VS hetero before UVB irradiation	MAP kinase cascade|HumanCyc	0.511919368	-0.00850371	2.456775054	0.03806801	0.16974934	-3.90122041
WT after UVB irradiation VS WT before UVB irradiation	MAP kinase cascade|HumanCyc	0.30542454	0.071258934	1.022446744	0.33047598	0.71290873	-5.36725106
homo after UVB irradiation VS homo before UVB irradiation	map kinase inactivation of smrt corepressor|BioCarta	0.177089881	-0.03137522	1.014335208	0.33584691	0.49228795	-5.84468214
hetero after UVB irradiation VS hetero before UVB irradiation	map kinase inactivation of smrt corepressor|BioCarta	0.245469926	0.008563162	1.391260986	0.19977958	0.41977191	-5.40445138
WT after UVB irradiation VS WT before UVB irradiation	map kinase inactivation of smrt corepressor|BioCarta	0.247534232	0.052916508	1.21295812	0.25279951	0.64679852	-5.17727592
homo after UVB irradiation VS homo before UVB irradiation	MAP2K and MAPK activation|Reactome	0.397788488	-0.0212198	2.346474467	0.0424445	0.13402181	-4.05832524
hetero after UVB irradiation VS hetero before UVB irradiation	MAP2K and MAPK activation|Reactome	0.381461043	-0.0112011	2.262419988	0.05190309	0.20220025	-4.19488621
WT after UVB irradiation VS WT before UVB irradiation	MAP2K and MAPK activation|Reactome	0.498089659	0.040073265	2.411120989	0.03642655	0.39022568	-3.59089833
homo after UVB irradiation VS homo before UVB irradiation	MAP3K8 (TPL2)-dependent MAPK1/3 activation|Reactome	0.185079982	-0.01857185	0.824299073	0.4302112	0.58181948	-6.0163331
hetero after UVB irradiation VS hetero before UVB irradiation	MAP3K8 (TPL2)-dependent MAPK1/3 activation|Reactome	0.385904934	0.024584307	2.270739456	0.05121952	0.20064099	-4.18240521
WT after UVB irradiation VS WT before UVB irradiation	MAP3K8 (TPL2)-dependent MAPK1/3 activation|Reactome	-0.10967099	-0.01333075	-0.54489215	0.59767338	0.86570705	-5.72108092
homo after UVB irradiation VS homo before UVB irradiation	MAPK and NFkB Signalling Pathways Inhibited by Yersinia YopJ|Wikipathways	0.573880837	-0.07081112	2.797927308	0.01997829	0.09043427	-3.34192956
hetero after UVB irradiation VS hetero before UVB irradiation	MAPK and NFkB Signalling Pathways Inhibited by Yersinia YopJ|Wikipathways	0.653108035	-0.0149071	2.90323152	0.01873821	0.12046367	-3.21882103
WT after UVB irradiation VS WT before UVB irradiation	MAPK and NFkB Signalling Pathways Inhibited by Yersinia YopJ|Wikipathways	0.697197786	0.017510581	3.143828318	0.01034133	0.26101395	-2.49263442
homo after UVB irradiation VS homo before UVB irradiation	MAPK Cascade|Wikipathways	0.05921047	-0.03099521	0.360608576	0.72637201	0.82013506	-6.29907093
hetero after UVB irradiation VS hetero before UVB irradiation	MAPK Cascade|Wikipathways	0.186901349	-0.01975134	1.081731744	0.30935548	0.53756854	-5.75333467
WT after UVB irradiation VS WT before UVB irradiation	MAPK Cascade|Wikipathways	0.060606791	0.035883054	0.294400271	0.77442435	0.9344243	-5.82544607
homo after UVB irradiation VS homo before UVB irradiation	MAPK family signaling cascades|Reactome	0.16155048	-0.01045464	1.028975812	0.32926805	0.48567999	-5.8302628
hetero after UVB irradiation VS hetero before UVB irradiation	MAPK family signaling cascades|Reactome	0.146288382	0.013582432	0.901595749	0.39231302	0.61959901	-5.92615541
WT after UVB irradiation VS WT before UVB irradiation	MAPK family signaling cascades|Reactome	0.089190584	-0.0054147	0.448016591	0.66361464	0.87935805	-5.76854482
homo after UVB irradiation VS homo before UVB irradiation	MAPK signaling pathway - Homo sapiens (human)|KEGG	0.218990837	0.001944287	1.362370593	0.20486371	0.36228029	-5.4609262
hetero after UVB irradiation VS hetero before UVB irradiation	MAPK signaling pathway - Homo sapiens (human)|KEGG	0.176832815	-0.0020147	1.135490465	0.28743402	0.51701826	-5.69715317
WT after UVB irradiation VS WT before UVB irradiation	MAPK signaling pathway - Homo sapiens (human)|KEGG	0.066394409	-0.0326073	0.314348464	0.75966478	0.92467725	-5.81935471
homo after UVB irradiation VS homo before UVB irradiation	MAPK Signaling Pathway|Wikipathways	0.15803755	-0.00029247	0.984901037	0.34937069	0.50497833	-5.87317126
hetero after UVB irradiation VS hetero before UVB irradiation	MAPK Signaling Pathway|Wikipathways	0.115964572	0.00179442	0.755622402	0.47046932	0.69263538	-6.04714002
WT after UVB irradiation VS WT before UVB irradiation	MAPK Signaling Pathway|Wikipathways	0.018175588	-0.0284942	0.08662383	0.93266676	0.97986866	-5.86535138
homo after UVB irradiation VS homo before UVB irradiation	MAPK targets/ Nuclear events mediated by MAP kinases|Reactome	0.026491687	0.014567668	0.158432955	0.87747107	0.92499926	-6.35497985
hetero after UVB irradiation VS hetero before UVB irradiation	MAPK targets/ Nuclear events mediated by MAP kinases|Reactome	0.3576138	-0.00140346	2.063747774	0.07116871	0.23939999	-4.48963163
WT after UVB irradiation VS WT before UVB irradiation	MAPK targets/ Nuclear events mediated by MAP kinases|Reactome	0.064554693	-0.02082707	0.302803995	0.76819459	0.92971761	-5.82292764
homo after UVB irradiation VS homo before UVB irradiation	MAPK1 (ERK2) activation|Reactome	0.199651383	-0.01832859	0.870109146	0.40593558	0.55777967	-5.97767565
hetero after UVB irradiation VS hetero before UVB irradiation	MAPK1 (ERK2) activation|Reactome	0.017539831	-0.00069706	0.080026268	0.93808456	0.97054702	-6.3430684
WT after UVB irradiation VS WT before UVB irradiation	MAPK1 (ERK2) activation|Reactome	0.20852868	-0.01330314	0.866504936	0.40636197	0.7725109	-5.50333225
homo after UVB irradiation VS homo before UVB irradiation	MAPK1/MAPK3 signaling|Reactome	0.146435489	-0.01060797	0.944690611	0.36849076	0.5212295	-5.91099112
hetero after UVB irradiation VS hetero before UVB irradiation	MAPK1/MAPK3 signaling|Reactome	0.155139361	0.015651625	0.939494315	0.37362494	0.60239918	-5.89186685
WT after UVB irradiation VS WT before UVB irradiation	MAPK1/MAPK3 signaling|Reactome	0.088187843	-0.00585386	0.43952464	0.66955268	0.87984445	-5.77228414
homo after UVB irradiation VS homo before UVB irradiation	MAPK3 (ERK1) activation|Reactome	0.450692085	0.021382016	2.795373931	0.0200635	0.09043427	-3.34601785
hetero after UVB irradiation VS hetero before UVB irradiation	MAPK3 (ERK1) activation|Reactome	0.500671793	0.047259875	2.974880897	0.01674208	0.1140542	-3.10938078
WT after UVB irradiation VS WT before UVB irradiation	MAPK3 (ERK1) activation|Reactome	0.528672497	-0.06431203	2.3338134	0.04158166	0.39022568	-3.70515516
homo after UVB irradiation VS homo before UVB irradiation	MAPK6/MAPK4 signaling|Reactome	0.077187431	0.017097318	0.370962702	0.7188974	0.81577771	-6.29505979
hetero after UVB irradiation VS hetero before UVB irradiation	MAPK6/MAPK4 signaling|Reactome	0.151847434	-0.00847836	0.810946318	0.43969968	0.66550548	-6.00341305
WT after UVB irradiation VS WT before UVB irradiation	MAPK6/MAPK4 signaling|Reactome	-0.01780032	0.004896526	-0.08159808	0.93656349	0.98056728	-5.86577935
homo after UVB irradiation VS homo before UVB irradiation	MAPK6-MAPK4 signaling|Wikipathways	0.434817495	0.044909893	2.581541803	0.02867081	0.11336662	-3.68740424
hetero after UVB irradiation VS hetero before UVB irradiation	MAPK6-MAPK4 signaling|Wikipathways	0.267512168	0.00270408	0.781806416	0.45573324	0.68016768	-6.0267758
WT after UVB irradiation VS WT before UVB irradiation	MAPK6-MAPK4 signaling|Wikipathways	0.106140323	0.020730021	0.367705859	0.72069431	0.90672477	-5.80114637
homo after UVB irradiation VS homo before UVB irradiation	mapkinase signaling pathway|BioCarta	0.05984851	0.002792204	0.367114744	0.72167162	0.81745289	-6.29656334
hetero after UVB irradiation VS hetero before UVB irradiation	mapkinase signaling pathway|BioCarta	0.14629796	-0.02457305	0.869237748	0.4087936	0.63571519	-5.95451462
WT after UVB irradiation VS WT before UVB irradiation	mapkinase signaling pathway|BioCarta	0.057734369	0.0039942	0.290386236	0.77740595	0.93502208	-5.82662445
homo after UVB irradiation VS homo before UVB irradiation	Maple Syrup Urine Disease|SMPDB	-0.23657683	-0.05522798	-0.8011182	0.4428632	0.59091566	-6.03520901
hetero after UVB irradiation VS hetero before UVB irradiation	Maple Syrup Urine Disease|SMPDB	-0.90022729	-0.04719335	-5.31014337	0.00060637	0.03004176	0.126180045
WT after UVB irradiation VS WT before UVB irradiation	Maple Syrup Urine Disease|SMPDB	-0.36319435	-0.13086657	-1.31407353	0.21793282	0.62581393	-5.06691375
homo after UVB irradiation VS homo before UVB irradiation	MASTL Facilitates Mitotic Progression|Reactome	-0.21849375	0.007241124	-0.99613933	0.34416018	0.49934395	-5.8623732
hetero after UVB irradiation VS hetero before UVB irradiation	MASTL Facilitates Mitotic Progression|Reactome	0.20553783	0.023382278	0.8770946	0.40474753	0.63319136	-5.94770738
WT after UVB irradiation VS WT before UVB irradiation	MASTL Facilitates Mitotic Progression|Reactome	-0.1273788	0.00553362	-0.5100612	0.62098803	0.87257466	-5.73915632
homo after UVB irradiation VS homo before UVB irradiation	Matrix Metalloproteinases|Wikipathways	0.823538131	-0.01121534	5.201467932	0.00049106	0.01666204	0.262244214
hetero after UVB irradiation VS hetero before UVB irradiation	Matrix Metalloproteinases|Wikipathways	0.517077204	0.067749849	2.0617289	0.07139648	0.23970209	-4.49258897
WT after UVB irradiation VS WT before UVB irradiation	Matrix Metalloproteinases|Wikipathways	0.429640039	0.022412407	1.674826982	0.12465886	0.52910502	-4.6283089
homo after UVB irradiation VS homo before UVB irradiation	Maturity onset diabetes of the young - Homo sapiens (human)|KEGG	0.512044776	0.114414861	2.920386964	0.01629488	0.07974534	-3.14573254
hetero after UVB irradiation VS hetero before UVB irradiation	Maturity onset diabetes of the young - Homo sapiens (human)|KEGG	0.41438771	0.125076513	2.338976919	0.04593911	0.18854754	-4.0796987
WT after UVB irradiation VS WT before UVB irradiation	Maturity onset diabetes of the young - Homo sapiens (human)|KEGG	0.236064926	0.112414079	1.025793148	0.32897072	0.71204479	-5.3641313
homo after UVB irradiation VS homo before UVB irradiation	mcalpain and friends in cell motility|BioCarta	0.346991938	-0.03901925	1.509602546	0.16403785	0.31331676	-5.27588025
hetero after UVB irradiation VS hetero before UVB irradiation	mcalpain and friends in cell motility|BioCarta	0.327908818	-0.00113308	2.081335189	0.06921404	0.23555906	-4.46383257
WT after UVB irradiation VS WT before UVB irradiation	mcalpain and friends in cell motility|BioCarta	0.326783507	0.039527678	1.556673411	0.15035554	0.55622203	-4.7788672
homo after UVB irradiation VS homo before UVB irradiation	Measles - Homo sapiens (human)|KEGG	0.445867454	-0.01034657	2.648021997	0.025657	0.10578915	-3.58153763
hetero after UVB irradiation VS hetero before UVB irradiation	Measles - Homo sapiens (human)|KEGG	0.51400607	0.020001893	3.175034547	0.01225199	0.10695993	-2.80504849
WT after UVB irradiation VS WT before UVB irradiation	Measles - Homo sapiens (human)|KEGG	0.373274938	-0.03072616	1.763922917	0.10796687	0.5076251	-4.510995
homo after UVB irradiation VS homo before UVB irradiation	mechanism of acetaminophen activity and toxicity|BioCarta	0.535035386	-0.03154901	1.933474429	0.08385494	0.20555644	-4.6867403
hetero after UVB irradiation VS hetero before UVB irradiation	mechanism of acetaminophen activity and toxicity|BioCarta	-0.00093964	-0.0106219	-0.00428629	0.9966798	0.9984322	-6.34653133

WT after UVB irradiation VS WT before UVB irradiation	mechanism of acetaminophen activity and toxicity|BioCarta	-0.13918378	0.015608039	-0.38825189	0.70590156	0.90148993	-5.79339658
homo after UVB irradiation VS homo before UVB irradiation	mechanism of gene regulation by peroxisome proliferators via ppara|BioCarta	-0.0374131	0.014582895	-0.22261181	0.82860504	0.89442332	-6.34189353
hetero after UVB irradiation VS hetero before UVB irradiation	mechanism of gene regulation by peroxisome proliferators via ppara|BioCarta	-0.14555862	-0.01157973	-0.87641003	0.40509894	0.63319136	-5.94830252
WT after UVB irradiation VS WT before UVB irradiation	mechanism of gene regulation by peroxisome proliferators via ppara|BioCarta	-0.23931254	-0.01958056	-1.22480506	0.24849421	0.64146154	-5.16466978
homo after UVB irradiation VS homo before UVB irradiation	mechanism of protein import into the nucleus|BioCarta	-0.89256545	-0.0008136	-4.22396912	0.00202536	0.02512983	-1.11418493
hetero after UVB irradiation VS hetero before UVB irradiation	mechanism of protein import into the nucleus|BioCarta	-0.01047493	0.041874577	-0.03125778	0.97579174	0.988327	-6.34601128
WT after UVB irradiation VS WT before UVB irradiation	mechanism of protein import into the nucleus|BioCarta	-0.16207661	-0.03780586	-0.48299723	0.6394141	0.87636259	-5.75242104
homo after UVB irradiation VS homo before UVB irradiation	MECP2 and Associated Rett Syndrome|Wikipathways	-0.07432995	0.003020487	-0.41999492	0.68393737	0.78720899	-6.27457431
hetero after UVB irradiation VS hetero before UVB irradiation	MECP2 and Associated Rett Syndrome|Wikipathways	-0.20767733	-0.00378612	-1.24114547	0.24799174	0.47275047	-5.581096
WT after UVB irradiation VS WT before UVB irradiation	MECP2 and Associated Rett Syndrome|Wikipathways	-0.17372556	-0.00182341	-0.8838015	0.39738868	0.7654467	-5.48916253
homo after UVB irradiation VS homo before UVB irradiation	Medium chain acyl-coa dehydrogenase deficiency (MCAD)|SMPDB	-0.19843953	0.039801734	-0.74439404	0.47485571	0.61754234	-6.07941248
hetero after UVB irradiation VS hetero before UVB irradiation	Medium chain acyl-coa dehydrogenase deficiency (MCAD)|SMPDB	-0.48316271	0.016700705	-1.75538636	0.11536374	0.30887493	-4.92927061
WT after UVB irradiation VS WT before UVB irradiation	Medium chain acyl-coa dehydrogenase deficiency (MCAD)|SMPDB	-0.40763842	0.012220754	-1.51649601	0.16010237	0.56072663	-4.82863564
homo after UVB irradiation VS homo before UVB irradiation	Mefenamic Acid Action Pathway|SMPDB	0.40423582	0.045705158	1.979500697	0.07780079	0.19626432	-4.61879689
hetero after UVB irradiation VS hetero before UVB irradiation	Mefenamic Acid Action Pathway|SMPDB	0.482496143	0.064876536	3.04499191	0.01500113	0.1130168	-3.00250826
WT after UVB irradiation VS WT before UVB irradiation	Mefenamic Acid Action Pathway|SMPDB	0.275725339	0.086595857	1.261970114	0.23537138	0.62952955	-5.12455679
homo after UVB irradiation VS homo before UVB irradiation	Meiosis|Reactome	-0.51875783	0.044063874	-2.62784332	0.02653658	0.10789495	-3.61370374
hetero after UVB irradiation VS hetero before UVB irradiation	Meiosis|Reactome	-0.4903907	0.02536824	-2.33908538	0.04593116	0.18854754	-4.07953501
WT after UVB irradiation VS WT before UVB irradiation	Meiosis|Reactome	-0.49289476	0.069696314	-2.16213776	0.05569129	0.41769317	-3.95571682
homo after UVB irradiation VS homo before UVB irradiation	Meiotic recombination|Reactome	-0.37088019	0.059904501	-1.23905654	0.24540298	0.40775473	-5.60617844
hetero after UVB irradiation VS hetero before UVB irradiation	Meiotic recombination|Reactome	-0.25884063	0.029563326	-0.99154026	0.34903837	0.57851523	-5.84294193
WT after UVB irradiation VS WT before UVB irradiation	Meiotic recombination|Reactome	-0.33097103	0.095249917	-1.31854121	0.21648859	0.62581393	-5.06189659
homo after UVB irradiation VS homo before UVB irradiation	Meiotic synapsis|Reactome	-0.52728141	0.058051741	-2.9646385	0.01514077	0.07630548	-3.07482113
hetero after UVB irradiation VS hetero before UVB irradiation	Meiotic synapsis|Reactome	-0.42194091	-0.00021156	-1.95588598	0.0843749	0.26244731	-4.64633793
WT after UVB irradiation VS WT before UVB irradiation	Meiotic synapsis|Reactome	-0.54863154	0.049384678	-2.33437733	0.04154159	0.39022568	-3.70432448
homo after UVB irradiation VS homo before UVB irradiation	Melanin biosynthesis|Reactome	-1.01837352	0.017389593	-5.1945859	0.00049574	0.01666204	0.253089739
hetero after UVB irradiation VS hetero before UVB irradiation	Melanin biosynthesis|Reactome	-0.66256652	-0.05605599	-2.03714041	0.07422785	0.24392975	-4.52853677
WT after UVB irradiation VS WT before UVB irradiation	Melanin biosynthesis|Reactome	-0.86523523	0.01276715	-3.0410592	0.01232899	0.27611764	-2.64646429
homo after UVB irradiation VS homo before UVB irradiation	melanocyte development and pigmentation pathway|BioCarta	-0.29973851	-0.04965511	-1.77176021	0.10881062	0.24229081	-4.91996914
hetero after UVB irradiation VS hetero before UVB irradiation	melanocyte development and pigmentation pathway|BioCarta	0.030998234	-0.02248357	0.132620136	0.89760644	0.96851647	-6.33701007
WT after UVB irradiation VS WT before UVB irradiation	melanocyte development and pigmentation pathway|BioCarta	-0.09665693	0.017234013	-0.36774931	0.72066289	0.90672477	-5.80113041
homo after UVB irradiation VS homo before UVB irradiation	Melanogenesis - Homo sapiens (human)|KEGG	0.129152155	-0.01434812	0.800017152	0.44347027	0.59103481	-6.03609397
hetero after UVB irradiation VS hetero before UVB irradiation	Melanogenesis - Homo sapiens (human)|KEGG	0.001906806	0.011489413	0.010761231	0.99166439	0.9947197	-6.34647848
WT after UVB irradiation VS WT before UVB irradiation	Melanogenesis - Homo sapiens (human)|KEGG	0.005369956	0.02518972	0.026801339	0.97914126	0.99283707	-5.86878731
homo after UVB irradiation VS homo before UVB irradiation	Melanoma - Homo sapiens (human)|KEGG	0.220717078	0.003802629	1.3468254	0.2096422	0.36745732	-5.47974944
hetero after UVB irradiation VS hetero before UVB irradiation	Melanoma - Homo sapiens (human)|KEGG	0.333657292	0.007558085	2.209064453	0.05650611	0.21215392	-4.27469531
WT after UVB irradiation VS WT before UVB irradiation	Melanoma - Homo sapiens (human)|KEGG	0.133971692	0.005464566	0.639256301	0.53690886	0.83767333	-5.66653579
homo after UVB irradiation VS homo before UVB irradiation	melatonin degradation I|HumanCyc	0.084543743	0.048881115	0.2947063	0.77463068	0.85413274	-6.32200291
hetero after UVB irradiation VS hetero before UVB irradiation	melatonin degradation I|HumanCyc	0.20637087	-0.07551983	0.767735768	0.46361351	0.68765063	-6.0377936
WT after UVB irradiation VS WT before UVB irradiation	melatonin degradation I|HumanCyc	0.255936984	-0.07313008	0.990060249	0.34530993	0.72932143	-5.39702199
homo after UVB irradiation VS homo before UVB irradiation	melatonin degradation II|HumanCyc	1.082132207	0.003499834	4.680333577	0.00102745	0.01844436	-0.45273499
hetero after UVB irradiation VS hetero before UVB irradiation	melatonin degradation II|HumanCyc	0.99828168	0.035960499	3.863913338	0.004332	0.07183716	-1.78647043
WT after UVB irradiation VS WT before UVB irradiation	melatonin degradation II|HumanCyc	0.778669321	-0.00682134	2.157812295	0.05610056	0.41769317	-3.96196222
homo after UVB irradiation VS homo before UVB irradiation	Melatonin metabolism and effects|Wikipathways	-0.39773723	-0.00972149	-2.34187965	0.0427705	0.13403575	-4.06550563
hetero after UVB irradiation VS hetero before UVB irradiation	Melatonin metabolism and effects|Wikipathways	-0.43050589	-0.04301429	-2.91714575	0.01833218	0.11936114	-3.19755331
WT after UVB irradiation VS WT before UVB irradiation	Melatonin metabolism and effects|Wikipathways	-0.47976036	-0.0282424	-2.50158397	0.03118641	0.36428127	-3.45635772
homo after UVB irradiation VS homo before UVB irradiation	Meloxicam Action Pathway|SMPDB	0.40423582	0.045705158	1.979500697	0.07780079	0.19626432	-4.61879689
hetero after UVB irradiation VS hetero before UVB irradiation	Meloxicam Action Pathway|SMPDB	0.482496143	0.064876536	3.04499191	0.01500113	0.1130168	-3.00250826
WT after UVB irradiation VS WT before UVB irradiation	Meloxicam Action Pathway|SMPDB	0.275725339	0.086595857	1.261970114	0.23537138	0.62952955	-5.12455679
homo after UVB irradiation VS homo before UVB irradiation	Membrane binding and targetting of GAG proteins|Reactome	0.435813323	-0.01188239	1.783899575	0.10672122	0.23957183	-4.90278471
hetero after UVB irradiation VS hetero before UVB irradiation	Membrane binding and targetting of GAG proteins|Reactome	0.48723579	0.00367111	1.785379393	0.11012989	0.30202891	-4.88774383
WT after UVB irradiation VS WT before UVB irradiation	Membrane binding and targetting of GAG proteins|Reactome	0.022125954	-0.02862519	0.089561473	0.9303899	0.9788786	-5.8650894
homo after UVB irradiation VS homo before UVB irradiation	Membrane Trafficking|Reactome	0.273388834	-0.02667227	1.555311602	0.1528994	0.29963032	-5.21609989
hetero after UVB irradiation VS hetero before UVB irradiation	Membrane Trafficking|Reactome	0.343638021	0.004912873	2.276893623	0.0507196	0.19980978	-4.17316659
WT after UVB irradiation VS WT before UVB irradiation	Membrane Trafficking|Reactome	0.219624968	-0.00681296	1.118489703	0.28929875	0.67478416	-5.27455699
homo after UVB irradiation VS homo before UVB irradiation	Mepivacaine Action Pathway|SMPDB	0.191132361	0.029768717	1.061313976	0.31508276	0.46923284	-5.79783739
hetero after UVB irradiation VS hetero before UVB irradiation	Mepivacaine Action Pathway|SMPDB	-0.07904315	0.055005373	-0.44437398	0.66796482	0.82651398	-6.24068873
WT after UVB irradiation VS WT before UVB irradiation	Mepivacaine Action Pathway|SMPDB	0.022316377	0.035038618	0.098344171	0.92358676	0.97566251	-5.86425414
homo after UVB irradiation VS homo before UVB irradiation	Mercaptopurine Action Pathway|SMPDB	0.1869187	-0.04175754	1.06533095	0.31335381	0.46923284	-5.79375484
hetero after UVB irradiation VS hetero before UVB irradiation	Mercaptopurine Action Pathway|SMPDB	0.029059741	-0.03702953	0.148746722	0.88525209	0.9597952	-6.33455446
WT after UVB irradiation VS WT before UVB irradiation	Mercaptopurine Action Pathway|SMPDB	0.208528988	-0.04951637	0.893857571	0.39223551	0.76452505	-5.48081572
homo after UVB irradiation VS homo before UVB irradiation	Mercaptopurine Metabolism Pathway|SMPDB	0.331004877	-0.05155217	1.902192892	0.08822011	0.21253275	-4.73254557
hetero after UVB irradiation VS hetero before UVB irradiation	Mercaptopurine Metabolism Pathway|SMPDB	0.25110504	0.015340223	1.017317166	0.33732073	0.56671871	-5.81794463
WT after UVB irradiation VS WT before UVB irradiation	Mercaptopurine Metabolism Pathway|SMPDB	0.311661416	-0.00956702	1.129230824	0.28495158	0.6726326	-5.26379339
homo after UVB irradiation VS homo before UVB irradiation	Mesodermal Commitment Pathway|Wikipathways	-0.15670093	-7.68E-05	-0.95905731	0.36157364	0.51659178	-5.89762602
hetero after UVB irradiation VS hetero before UVB irradiation	Mesodermal Commitment Pathway|Wikipathways	-0.09944062	-0.00491628	-0.63656636	0.54131086	0.75147406	-6.13198911
WT after UVB irradiation VS WT before UVB irradiation	Mesodermal Commitment Pathway|Wikipathways	-0.21663775	0.006872754	-1.09385427	0.29946363	0.68477511	-5.29894737
homo after UVB irradiation VS homo before UVB irradiation	MET activates PI3K/AKT signaling|Reactome	0.024459198	-0.0419043	0.095658668	0.92580232	0.95573443	-6.36353406
hetero after UVB irradiation VS hetero before UVB irradiation	MET activates PI3K/AKT signaling|Reactome	0.349330302	0.039771439	1.696155472	0.12638493	0.31934829	-5.01034687
WT after UVB irradiation VS WT before UVB irradiation	MET activates PI3K/AKT signaling|Reactome	-0.01567974	-0.04707285	-0.05769028	0.95512271	0.98742794	-5.86746546
homo after UVB irradiation VS homo before UVB irradiation	MET activates PTK2 signaling|Reactome	0.585317923	-0.0465094	2.472482639	0.03439973	0.12370725	-3.86027217
hetero after UVB irradiation VS hetero before UVB irradiation	MET activates PTK2 signaling|Reactome	0.42122308	0.003382241	2.048935285	0.07285638	0.24158558	-4.51130936
WT after UVB irradiation VS WT before UVB irradiation	MET activates PTK2 signaling|Reactome	0.364104077	-0.09785024	1.54107377	0.15407639	0.55854784	-4.79828094
homo after UVB irradiation VS homo before UVB irradiation	MET activates PTPN11|Reactome	-0.27758351	-0.07967404	-0.98215011	0.35065498	0.50621571	-5.87579937
hetero after UVB irradiation VS hetero before UVB irradiation	MET activates PTPN11|Reactome	-0.12277455	-0.00347812	-0.67106346	0.5201485	0.73623731	-6.10873524
WT after UVB irradiation VS WT before UVB irradiation	MET activates PTPN11|Reactome	-0.25143448	-0.01335504	-0.70243012	0.49830728	0.82026576	-5.62557871
homo after UVB irradiation VS homo before UVB irradiation	MET activates RAP1 and RAC1|Reactome	0.346226784	-0.0366199	1.395369528	0.19502555	0.35086157	-5.42050053
hetero after UVB irradiation VS hetero before UVB irradiation	MET activates RAP1 and RAC1|Reactome	0.399344553	-0.03272784	1.655710958	0.13445984	0.33336163	-5.06495473
WT after UVB irradiation VS WT before UVB irradiation	MET activates RAP1 and RAC1|Reactome	0.147656034	-0.09155229	0.446741328	0.66450483	0.87935805	-5.76911075
homo after UVB irradiation VS homo before UVB irradiation	MET activates RAS signaling|Reactome	-0.02650762	-0.01968473	-0.11284139	0.91253221	0.9489005	-6.36161141
hetero after UVB irradiation VS hetero before UVB irradiation	MET activates RAS signaling|Reactome	0.222242209	0.008079467	1.099203889	0.30209071	0.53245533	-5.7352958
WT after UVB irradiation VS WT before UVB irradiation	MET activates RAS signaling|Reactome	-0.10279007	-0.00496135	-0.39141891	0.70363255	0.90089427	-5.7921657
homo after UVB irradiation VS homo before UVB irradiation	MET activates STAT3|Reactome	0.40530911	-0.01016712	2.333179351	0.04339453	0.13506436	-4.07909281
hetero after UVB irradiation VS hetero before UVB irradiation	MET activates STAT3|Reactome	0.520607352	-0.0371338	1.907811273	0.09099056	0.27321145	-4.7152525
WT after UVB irradiation VS WT before UVB irradiation	MET activates STAT3|Reactome	0.050033522	-0.13191791	0.14438374	0.88804282	0.97119051	-5.85860211
homo after UVB irradiation VS homo before UVB irradiation	MET in type 1 papillary renal cell carcinoma|Wikipathways	0.219592605	-0.01069558	0.94676561	0.36748582	0.52061441	-5.909071
hetero after UVB irradiation VS hetero before UVB irradiation	MET in type 1 papillary renal cell carcinoma|Wikipathways	0.516622615	0.010308586	3.202348983	0.0117445	0.10542662	-2.76372566
WT after UVB irradiation VS WT before UVB irradiation	MET in type 1 papillary renal cell carcinoma|Wikipathways	0.318581002	0.020170395	1.543062787	0.15359752	0.55854784	-4.79581192
homo after UVB irradiation VS homo before UVB irradiation	MET interacts with TNS proteins|Reactome	0.395901708	-0.02420525	1.584769603	0.14608076	0.2913957	-5.17702987
hetero after UVB irradiation VS hetero before UVB irradiation	MET interacts with TNS proteins|Reactome	0.291797401	-0.01619962	0.99697643	0.34654197	0.57530067	-5.83771183
WT after UVB irradiation VS WT before UVB irradiation	MET interacts with TNS proteins|Reactome	-0.11217343	-0.08690795	-0.46337998	0.65293368	0.87935805	-5.76160577
homo after UVB irradiation VS homo before UVB irradiation	MET promotes cell motility|Reactome	0.543528586	-0.01216	2.420417286	0.03752263	0.12690988	-3.94235592
hetero after UVB irradiation VS hetero before UVB irradiation	MET promotes cell motility|Reactome	0.434506004	-0.00338799	1.984725227	0.08062965	0.25607584	-4.60470952
WT after UVB irradiation VS WT before UVB irradiation	MET promotes cell motility|Reactome	0.303192866	-0.08823809	1.392919798	0.19359385	0.60625027	-4.97670613
homo after UVB irradiation VS homo before UVB irradiation	MET Receptor Activation|Reactome	0.346405946	-0.07538546	1.657904155	0.13031205	0.27177943	-5.07828313
hetero after UVB irradiation VS hetero before UVB irradiation	MET Receptor Activation|Reactome	0.081579381	0.036389184	0.456630567	0.65947818	0.82651398	-6.23484299
WT after UVB irradiation VS WT before UVB irradiation	MET Receptor Activation|Reactome	-0.03629696	-0.00258377	-0.15229206	0.88196112	0.96830103	-5.85741633
homo after UVB irradiation VS homo before UVB irradiation	Metabolic reprogramming in colon cancer|Wikipathways	0.144046157	0.017538858	0.610139823	0.55624672	0.68440381	-6.17239958
hetero after UVB irradiation VS hetero before UVB irradiation	Metabolic reprogramming in colon cancer|Wikipathways	0.162731629	-0.00183361	0.64825875	0.53408127	0.74771561	-6.12423136
WT after UVB irradiation VS WT before UVB irradiation	Metabolic reprogramming in colon cancer|Wikipathways	0.383058903	-0.03244367	1.782818932	0.10469923	0.50446894	-4.4857322
homo after UVB irradiation VS homo before UVB irradiation	Metabolism of amino acids and derivatives|Reactome	-0.37359324	-0.00681655	-2.03630862	0.0708988	0.18768759	-4.5340944
hetero after UVB irradiation VS hetero before UVB irradiation	Metabolism of amino acids and derivatives|Reactome	-0.26579496	-0.01867378	-1.32536126	0.21985966	0.44126832	-5.48359747
WT after UVB irradiation VS WT before UVB irradiation	Metabolism of amino acids and derivatives|Reactome	-0.31058065	-0.00960039	-1.58795295	0.14313214	0.55622203	-4.73960335
homo after UVB irradiation VS homo before UVB irradiation	metabolism of anandamide an endogenous cannabinoid|BioCarta	0.574182839	0.054696517	2.879665621	0.01743609	0.08336461	-3.21099014
hetero after UVB irradiation VS hetero before UVB irradiation	metabolism of anandamide an endogenous cannabinoid|BioCarta	0.2918494	0.022014523	1.461705432	0.18008874	0.39822229	-5.31727271
WT after UVB irradiation VS WT before UVB irradiation	metabolism of anandamide an endogenous cannabinoid|BioCarta	0.917751066	-0.01525957	4.075268169	0.00219383	0.17027242	-1.1464585
homo after UVB irradiation VS homo before UVB irradiation	Metabolism of Angiotensinogen to Angiotensins|Reactome	0.508453677	-0.03001323	2.422204954	0.0374109	0.12687503	-3.93954305
hetero after UVB irradiation VS hetero before UVB irradiation	Metabolism of Angiotensinogen to Angiotensins|Reactome	0.252329162	0.032328953	1.341966664	0.21464381	0.43637221	-5.46388264
WT after UVB irradiation VS WT before UVB irradiation	Metabolism of Angiotensinogen to Angiotensins|Reactome	0.135822158	-0.03007125	0.576901959	0.57665903	0.85729225	-5.70348373
homo after UVB irradiation VS homo before UVB irradiation	Metabolism of carbohydrates|Reactome	0.34034125	-0.00857394	1.965700679	0.07957121	0.19933837	-4.63923472
hetero after UVB irradiation VS hetero before UVB irradiation	Metabolism of carbohydrates|Reactome	0.228760554	0.007473497	1.316915851	0.22255352	0.44410029	-5.49356385
WT after UVB irradiation VS WT before UVB irradiation	Metabolism of carbohydrates|Reactome	0.341782305	0.000872872	1.693908249	0.12089943	0.52647129	-4.60344363
homo after UVB irradiation VS homo before UVB irradiation	Metabolism of cofactors|Reactome	-0.27820375	-0.04279065	-1.23636957	0.2463551	0.40873472	-5.60923659
hetero after UVB irradiation VS hetero before UVB irradiation	Metabolism of cofactors|Reactome	-0.24343572	-0.03939124	-1.3916128	0.19967677	0.41977191	-5.40402245
WT after UVB irradiation VS WT before UVB irradiation	Metabolism of cofactors|Reactome	-0.18552458	-0.08433157	-0.81338819	0.43478357	0.79685269	-5.54534701
homo after UVB irradiation VS homo before UVB irradiation	Metabolism of Dichloroethylene by CYP450|Wikipathways	0.388460901	0.020895461	1.066764261	0.31273867	0.46923284	-5.79229523
hetero after UVB irradiation VS hetero before UVB irradiation	Metabolism of Dichloroethylene by CYP450|Wikipathways	-0.14760988	0.040949837	-0.3635581	0.72514129	0.86482318	-6.27541048
WT after UVB irradiation VS WT before UVB irradiation	Metabolism of Dichloroethylene by CYP450|Wikipathways	-0.06962327	0.086915055	-0.17823013	0.86207255	0.95916804	-5.85308595
homo after UVB irradiation VS homo before UVB irradiation	Metabolism of fat-soluble vitamins|Reactome	0.416882936	0.014810491	2.575996679	0.02893767	0.11412829	-3.69621975
hetero after UVB irradiation VS hetero before UVB irradiation	Metabolism of fat-soluble vitamins|Reactome	0.27026495	0.041499759	1.486387624	0.17360392	0.39118926	-5.28613232
WT after UVB irradiation VS WT before UVB irradiation	Metabolism of fat-soluble vitamins|Reactome	0.303586611	0.048401484	1.28614322	0.22714346	0.62952955	-5.09801437
homo after UVB irradiation VS homo before UVB irradiation	Metabolism of folate and pterines|Reactome	-0.22816294	-0.02142621	-1.01289531	0.33649923	0.4922959	-5.8460914
hetero after UVB irradiation VS hetero before UVB irradiation	Metabolism of folate and pterines|Reactome	0.02503377	-0.07133745	0.109985213	0.91499574	0.97054702	-6.33998371
WT after UVB irradiation VS WT before UVB irradiation	Metabolism of folate and pterines|Reactome	0.110948708	-0.04281233	0.406433823	0.69291687	0.89511744	-5.78619854
homo after UVB irradiation VS homo before UVB irradiation	Metabolism of ingested H2SeO4 and H2SeO3 into H2Se|Reactome	-0.72800598	0.017706821	-2.82628456	0.01905628	0.08811647	-3.29651578
hetero after UVB irradiation VS hetero before UVB irradiation	Metabolism of ingested H2SeO4 and H2SeO3 into H2Se|Reactome	-0.84930588	-0.03937964	-3.47603418	0.0077224	0.08930222	-2.35338554
WT after UVB irradiation VS WT before UVB irradiation	Metabolism of ingested H2SeO4 and H2SeO3 into H2Se|Reactome	-0.4109139	-0.09174524	-1.57166374	0.14685468	0.55622203	-4.76010592
homo after UVB irradiation VS homo before UVB irradiation	Metabolism of ingested MeSeO2H into MeSeH|Reactome	-1.03748343	0.037007124	-4.96394684	0.00068416	0.01666204	-0.0581702
hetero after UVB irradiation VS hetero before UVB irradiation	Metabolism of ingested MeSeO2H into MeSeH|Reactome	-0.93720887	-0.03635385	-3.82973184	0.00455469	0.07388006	-1.83563245
WT after UVB irradiation VS WT before UVB irradiation	Metabolism of ingested MeSeO2H into MeSeH|Reactome	-0.91401796	-0.04177811	-2.76723533	0.01973928	0.31542987	-3.0580515
homo after UVB irradiation VS homo before UVB irradiation	Metabolism of ingested SeMet_ Sec_ MeSec into H2Se|Reactome	-0.08855571	0.030191765	-0.33754804	0.74312793	0.83164758	-6.30760735
hetero after UVB irradiation VS hetero before UVB irradiation	Metabolism of ingested SeMet_ Sec_ MeSec into H2Se|Reactome	0.080734395	-0.03925213	0.319065257	0.75744384	0.8877421	-6.29165469
WT after UVB irradiation VS WT before UVB irradiation	Metabolism of ingested SeMet_ Sec_ MeSec into H2Se|Reactome	-0.06802642	-0.03339948	-0.28422385	0.78199069	0.93661609	-5.82840259
homo after UVB irradiation VS homo before UVB irradiation	Metabolism of lipids|Reactome	0.093031735	-0.01888309	0.541253472	0.60093754	0.72093558	-6.21351643
hetero after UVB irradiation VS hetero before UVB irradiation	Metabolism of lipids|Reactome	0.031031639	-0.00682115	0.184801311	0.85775577	0.94503144	-6.3280528
WT after UVB irradiation VS WT before UVB irradiation	Metabolism of lipids|Reactome	0.018484769	0.008626002	0.088981196	0.9308396	0.9788786	-5.86514184
homo after UVB irradiation VS homo before UVB irradiation	Metabolism of nitric oxide|Reactome	0.697013033	-0.05019342	4.394538113	0.00156613	0.02229782	-0.86323905
hetero after UVB irradiation VS hetero before UVB irradiation	Metabolism of nitric oxide|Reactome	0.59814461	-0.02679821	2.895645733	0.01896346	0.1205973	-3.23041836
WT after UVB irradiation VS WT before UVB irradiation	Metabolism of nitric oxide|Reactome	0.513201481	0.019218643	2.383541888	0.03818956	0.39022568	-3.63174344
homo after UVB irradiation VS homo before UVB irradiation	Metabolism of non-coding RNA|Reactome	-0.85617848	-0.01442054	-4.88630847	0.00076383	0.01684857	-0.16489268
hetero after UVB irradiation VS hetero before UVB irradiation	Metabolism of non-coding RNA|Reactome	-0.27099951	-0.08049357	-1.00423945	0.34322771	0.57242294	-5.83068927
WT after UVB irradiation VS WT before UVB irradiation	Metabolism of non-coding RNA|Reactome	-0.21352358	-0.01442229	-0.63963086	0.53667496	0.83767333	-5.66630332
homo after UVB irradiation VS homo before UVB irradiation	Metabolism of nucleotides|Reactome	-0.01457757	-0.02572759	-0.08121867	0.93697308	0.96254952	-6.36490522
hetero after UVB irradiation VS hetero before UVB irradiation	Metabolism of nucleotides|Reactome	0.019514107	-0.03445669	0.101217854	0.92174482	0.97054702	-6.34098687
WT after UVB irradiation VS WT before UVB irradiation	Metabolism of nucleotides|Reactome	0.180490698	-0.03187491	0.841284332	0.41969455	0.78544581	-5.52356575
homo after UVB irradiation VS homo before UVB irradiation	Metabolism of polyamines|Reactome	0.118201839	-0.02077046	0.562855335	0.58672014	0.70890822	-6.20111587
hetero after UVB irradiation VS hetero before UVB irradiation	Metabolism of polyamines|Reactome	0.114096862	-0.03039754	0.618825036	0.55239055	0.75745973	-6.14351569
WT after UVB irradiation VS WT before UVB irradiation	Metabolism of polyamines|Reactome	0.117384477	-0.00220063	0.571065309	0.58046069	0.85958498	-5.70676228

homo after UVB irradiation VS homo before UVB irradiation	Metabolism of porphyrins|Reactome	0.125269773	0.010103436	0.576420219	0.57788602	0.7006446	-6.19309766
hetero after UVB irradiation VS hetero before UVB irradiation	Metabolism of porphyrins|Reactome	0.355410986	0.017024633	2.205896759	0.05679169	0.21270052	-4.27942003
WT after UVB irradiation VS WT before UVB irradiation	Metabolism of porphyrins|Reactome	0.311720927	-0.02049329	1.490341404	0.16673957	0.57064699	-4.86061851
homo after UVB irradiation VS homo before UVB irradiation	Metabolism of proteins|Reactome	-0.07657985	-0.01269615	-0.47260465	0.64728806	0.75613204	-6.24988372
hetero after UVB irradiation VS hetero before UVB irradiation	Metabolism of proteins|Reactome	0.044539202	-0.01770914	0.293609131	0.77615909	0.90133328	-6.30001947
WT after UVB irradiation VS WT before UVB irradiation	Metabolism of proteins|Reactome	0.022594554	-0.0197213	0.112001029	0.91302109	0.97566251	-5.86280059
homo after UVB irradiation VS homo before UVB irradiation	Metabolism of RNA|Reactome	-0.79963352	0.00543958	-4.92674932	0.00072116	0.01666204	-0.10918043
hetero after UVB irradiation VS hetero before UVB irradiation	Metabolism of RNA|Reactome	-0.30250394	-0.06938686	-1.29196933	0.23067456	0.45210986	-5.52276106
WT after UVB irradiation VS WT before UVB irradiation	Metabolism of RNA|Reactome	-0.34697303	-0.02992106	-1.1872968	0.26233049	0.65791507	-5.20427657
homo after UVB irradiation VS homo before UVB irradiation	Metabolism of serotonin|Reactome	0.790387281	-0.01373253	2.775057385	0.02075472	0.09195295	-3.37854103
hetero after UVB irradiation VS hetero before UVB irradiation	Metabolism of serotonin|Reactome	0.271791101	0.073049071	0.611769209	0.55683351	0.76066987	-6.14801747
WT after UVB irradiation VS WT before UVB irradiation	Metabolism of serotonin|Reactome	0.684447473	0.004370207	1.872639942	0.09037247	0.48188205	-4.36396447
homo after UVB irradiation VS homo before UVB irradiation	Metabolism of Spingolipids in ER and Golgi apparatus|Wikipathways	0.849703153	-0.04649656	4.070706257	0.00256065	0.02922724	-1.34328311
hetero after UVB irradiation VS hetero before UVB irradiation	Metabolism of Spingolipids in ER and Golgi apparatus|Wikipathways	0.385021764	-0.00561073	1.064524011	0.31664336	0.54586249	-5.77088887
WT after UVB irradiation VS WT before UVB irradiation	Metabolism of Spingolipids in ER and Golgi apparatus|Wikipathways	0.946165199	-0.01298136	4.682093997	0.0008459	0.15645713	-0.33911369
homo after UVB irradiation VS homo before UVB irradiation	Metabolism of steroid hormones|Reactome	0.435205744	0.006894832	2.347733914	0.04235557	0.13402181	-4.05635652
hetero after UVB irradiation VS hetero before UVB irradiation	Metabolism of steroid hormones|Reactome	0.337901437	0.032290678	1.721633806	0.12152985	0.31331055	-4.97562773
WT after UVB irradiation VS WT before UVB irradiation	Metabolism of steroid hormones|Reactome	0.159055049	0.031758176	0.697974408	0.50097315	0.82152401	-5.62858175
homo after UVB irradiation VS homo before UVB irradiation	Metabolism of steroids|Reactome	-0.03708431	0.013338394	-0.21891568	0.83140058	0.89574683	-6.34276572
hetero after UVB irradiation VS hetero before UVB irradiation	Metabolism of steroids|Reactome	0.009277831	0.011758171	0.053140925	0.95885803	0.98102691	-6.34500958
WT after UVB irradiation VS WT before UVB irradiation	Metabolism of steroids|Reactome	0.010631768	0.025942978	0.049782508	0.96126814	0.98815178	-5.86789586
WT after UVB irradiation VS WT before UVB irradiation	Metabolism of Tetrahydrocannabinol (THC)|Wikipathways	-0.4214302	0.046401692	-1.39621666	0.19262818	0.60496032	-4.97285911
homo after UVB irradiation VS homo before UVB irradiation	Metabolism of vitamin K|Reactome	0.005005613	-0.13760669	0.014040177	0.98909175	0.99235178	-6.36834235
hetero after UVB irradiation VS hetero before UVB irradiation	Metabolism of vitamin K|Reactome	0.222505465	-0.02104355	0.652815787	0.5312793	0.74554014	-6.12117332
WT after UVB irradiation VS WT before UVB irradiation	Metabolism of vitamin K|Reactome	0.433017145	0.036374642	1.787529838	0.1038988	0.50425697	-4.47941414
homo after UVB irradiation VS homo before UVB irradiation	Metabolism of vitamins and cofactors|Reactome	0.061330482	-0.00778985	0.367970043	0.72105462	0.81695667	-6.29623046
hetero after UVB irradiation VS hetero before UVB irradiation	Metabolism of vitamins and cofactors|Reactome	-0.02874295	-0.01711576	-0.1676543	0.87080957	0.95129447	-6.33131898
WT after UVB irradiation VS WT before UVB irradiation	Metabolism of vitamins and cofactors|Reactome	0.104284744	-0.01526348	0.518581286	0.61524281	0.87257466	-5.73483884
homo after UVB irradiation VS homo before UVB irradiation	Metabolism of water-soluble vitamins and cofactors|Reactome	-0.03337246	-0.02575017	-0.18503558	0.85713655	0.9134577	-6.3500863
hetero after UVB irradiation VS hetero before UVB irradiation	Metabolism of water-soluble vitamins and cofactors|Reactome	-0.11247623	-0.03417929	-0.61819934	0.55278371	0.75777088	-6.1439168
WT after UVB irradiation VS WT before UVB irradiation	Metabolism of water-soluble vitamins and cofactors|Reactome	0.036558851	-0.0357706	0.182907287	0.85849653	0.95916804	-5.85223324
homo after UVB irradiation VS homo before UVB irradiation	Metabolism of xenobiotics by cytochrome P450 - Homo sapiens (human)|KEGG	0.011592891	0.046302381	0.055278829	0.9570748	0.97414256	-6.36680667
hetero after UVB irradiation VS hetero before UVB irradiation	Metabolism of xenobiotics by cytochrome P450 - Homo sapiens (human)|KEGG	0.088957955	0.009513207	0.451225828	0.66321419	0.82651398	-6.23743941
WT after UVB irradiation VS WT before UVB irradiation	Metabolism of xenobiotics by cytochrome P450 - Homo sapiens (human)|KEGG	0.010151626	0.014979515	0.042953097	0.96657769	0.98922368	-5.86821663
homo after UVB irradiation VS homo before UVB irradiation	Metabolism|Reactome	0.046509709	-0.01455233	0.261214996	0.7995664	0.87515909	-6.33192247
hetero after UVB irradiation VS hetero before UVB irradiation	Metabolism|Reactome	-0.03048905	-0.01198579	-0.19220438	0.85213388	0.94503144	-6.32654525
WT after UVB irradiation VS WT before UVB irradiation	Metabolism|Reactome	0.032858212	-0.01402021	0.163112353	0.87365311	0.96283151	-5.85569202
homo after UVB irradiation VS homo before UVB irradiation	Metachromatic Leukodystrophy (MLD)|SMPDB	0.410096132	-0.05671339	1.580382344	0.14707883	0.29223756	-5.18287502
hetero after UVB irradiation VS hetero before UVB irradiation	Metachromatic Leukodystrophy (MLD)|SMPDB	0.381719767	-0.02021245	2.484597089	0.03641544	0.16681566	-3.85889413
WT after UVB irradiation VS WT before UVB irradiation	Metachromatic Leukodystrophy (MLD)|SMPDB	0.327957029	0.011268274	1.427782053	0.1835878	0.59839062	-4.93573151
homo after UVB irradiation VS homo before UVB irradiation	Metal ion SLC transporters|Reactome	0.059983478	-0.01106912	0.266471645	0.79563596	0.8722386	-6.33044334
hetero after UVB irradiation VS hetero before UVB irradiation	Metal ion SLC transporters|Reactome	0.077122062	0.064682336	0.515397961	0.61952198	0.80243853	-6.2047271
WT after UVB irradiation VS WT before UVB irradiation	Metal ion SLC transporters|Reactome	0.237080384	0.043035058	1.180622062	0.264856	0.66112659	-5.21123046
homo after UVB irradiation VS homo before UVB irradiation	Metal sequestration by antimicrobial proteins|Reactome	0.984899685	0.030716998	4.654076549	0.0010675	0.01874686	-0.48991548
hetero after UVB irradiation VS hetero before UVB irradiation	Metal sequestration by antimicrobial proteins|Reactome	1.059883215	0.040998882	5.770190537	0.00034534	0.03004176	0.66300068
WT after UVB irradiation VS WT before UVB irradiation	Metal sequestration by antimicrobial proteins|Reactome	0.700945834	0.040763946	2.383363781	0.03820121	0.39022568	-3.63200692
homo after UVB irradiation VS homo before UVB irradiation	Metalloprotease DUBs|Reactome	-0.29486098	0.012805312	-1.44714624	0.18040415	0.3341193	-5.35582641
hetero after UVB irradiation VS hetero before UVB irradiation	Metalloprotease DUBs|Reactome	0.063056952	0.037529412	0.230380587	0.82329135	0.93254522	-6.31784122
WT after UVB irradiation VS WT before UVB irradiation	Metalloprotease DUBs|Reactome	-0.15995618	-0.04434604	-0.63137712	0.54184296	0.83916339	-5.67139714
homo after UVB irradiation VS homo before UVB irradiation	Metapathway biotransformation Phase I and II|Wikipathways	0.242851464	0.041018703	1.38589441	0.19780835	0.35391572	-5.4321725
hetero after UVB irradiation VS hetero before UVB irradiation	Metapathway biotransformation Phase I and II|Wikipathways	0.129917357	0.019844425	0.734351272	0.48266781	0.70381063	-6.06323896
WT after UVB irradiation VS WT before UVB irradiation	Metapathway biotransformation Phase I and II|Wikipathways	0.132671301	0.018380596	0.58874473	0.5689872	0.85280226	-5.69673618
homo after UVB irradiation VS homo before UVB irradiation	Metastatic brain tumor|Wikipathways	-0.4219918	0.040514758	-1.93973026	0.08300672	0.20442756	-4.67754312
hetero after UVB irradiation VS hetero before UVB irradiation	Metastatic brain tumor|Wikipathways	-0.17960865	-0.01447195	-0.88887824	0.39873266	0.62665491	-5.9374031
WT after UVB irradiation VS WT before UVB irradiation	Metastatic brain tumor|Wikipathways	-0.23601003	0.018475139	-0.81359671	0.43466946	0.79685269	-5.54518656
homo after UVB irradiation VS homo before UVB irradiation	Metformin Pathway_ Pharmacodynamic|PharmGKB	-0.15737977	0.002096717	-0.72381167	0.4868227	0.62916288	-6.09474257
hetero after UVB irradiation VS hetero before UVB irradiation	Metformin Pathway_ Pharmacodynamic|PharmGKB	-0.44890215	-0.06905625	-1.96012708	0.08381385	0.2618393	-4.6402292
WT after UVB irradiation VS WT before UVB irradiation	Metformin Pathway_ Pharmacodynamic|PharmGKB	-0.19653494	-0.09485201	-0.7182328	0.488923	0.81710765	-5.61478985
homo after UVB irradiation VS homo before UVB irradiation	Metformin Pathway_ Pharmacokinetics|PharmGKB	0.505018033	0.003508503	2.133567681	0.06041871	0.16790738	-4.3871208
hetero after UVB irradiation VS hetero before UVB irradiation	Metformin Pathway_ Pharmacokinetics|PharmGKB	-0.15876708	-0.07887302	-0.49739985	0.63162636	0.81120117	-6.21431502
WT after UVB irradiation VS WT before UVB irradiation	Metformin Pathway_ Pharmacokinetics|PharmGKB	0.033884011	-0.02004574	0.118561444	0.90795178	0.97566251	-5.86203541
homo after UVB irradiation VS homo before UVB irradiation	Methadone Action Pathway|SMPDB	0.206009113	0.046776634	1.134559502	0.28469374	0.44528503	-5.72154159
hetero after UVB irradiation VS hetero before UVB irradiation	Methadone Action Pathway|SMPDB	-0.05374216	0.042666571	-0.29221213	0.77719077	0.90184204	-6.30045884
WT after UVB irradiation VS WT before UVB irradiation	Methadone Action Pathway|SMPDB	0.01874832	0.032649421	0.083079126	0.93541497	0.98056728	-5.86565589
homo after UVB irradiation VS homo before UVB irradiation	Methadone Metabolism Pathway|SMPDB	0.26457845	0.050523009	1.188493006	0.26382986	0.42717984	-5.66292615
hetero after UVB irradiation VS hetero before UVB irradiation	Methadone Metabolism Pathway|SMPDB	0.083721034	0.19827456	0.385686163	0.70928471	0.85571569	-6.26656817
WT after UVB irradiation VS WT before UVB irradiation	Methadone Metabolism Pathway|SMPDB	0.004115212	0.058013302	0.01665566	0.98703632	0.99664845	-5.86901066
homo after UVB irradiation VS homo before UVB irradiation	Methadyl Acetate Action Pathway|SMPDB	0.191132361	0.029768717	1.061313976	0.31508276	0.46923284	-5.79783739
hetero after UVB irradiation VS hetero before UVB irradiation	Methadyl Acetate Action Pathway|SMPDB	-0.07904315	0.055005373	-0.44437398	0.66796482	0.82651398	-6.24068873
WT after UVB irradiation VS WT before UVB irradiation	Methadyl Acetate Action Pathway|SMPDB	0.022316377	0.035038618	0.098344171	0.92358676	0.97566251	-5.86425414
homo after UVB irradiation VS homo before UVB irradiation	Methionine Adenosyltransferase Deficiency|SMPDB	-0.09396972	-0.02692441	-0.3795694	0.71270801	0.81002335	-6.29164178
hetero after UVB irradiation VS hetero before UVB irradiation	Methionine Adenosyltransferase Deficiency|SMPDB	0.075091809	-0.02022482	0.352613458	0.73303662	0.86896773	-6.27959702
WT after UVB irradiation VS WT before UVB irradiation	Methionine Adenosyltransferase Deficiency|SMPDB	0.176060787	0.019583595	0.866300336	0.40646895	0.7725109	-5.50349844
homo after UVB irradiation VS homo before UVB irradiation	Methionine and cysteine metabolism|EHMN	-0.09771879	0.011047588	-0.59734472	0.56440346	0.6905322	-6.18038171
hetero after UVB irradiation VS hetero before UVB irradiation	Methionine and cysteine metabolism|EHMN	-0.01389422	-0.02397566	-0.08505672	0.93420273	0.97054702	-6.34261827
WT after UVB irradiation VS WT before UVB irradiation	Methionine and cysteine metabolism|EHMN	0.076112867	-0.04758526	0.360735624	0.72574067	0.90931623	-5.80368255
homo after UVB irradiation VS homo before UVB irradiation	Methionine Cysteine metabolism|INOH	-0.32368256	0.049944688	-1.6224723	0.13774989	0.27994034	-5.12642737
hetero after UVB irradiation VS hetero before UVB irradiation	Methionine Cysteine metabolism|INOH	-0.34171914	0.020541355	-1.89808072	0.09238842	0.27457285	-4.72912468
WT after UVB irradiation VS WT before UVB irradiation	Methionine Cysteine metabolism|INOH	-0.18857863	0.022444894	-0.89642713	0.39092629	0.76436977	-5.47867018
homo after UVB irradiation VS homo before UVB irradiation	Methionine De Novo and Salvage Pathway|Wikipathways	0.270420772	-0.0210206	1.221206201	0.25178485	0.41473651	-5.6264059
hetero after UVB irradiation VS hetero before UVB irradiation	Methionine De Novo and Salvage Pathway|Wikipathways	0.062296372	-0.03142958	0.283574637	0.78357981	0.90510815	-6.30312978
WT after UVB irradiation VS WT before UVB irradiation	Methionine De Novo and Salvage Pathway|Wikipathways	0.420754621	-0.02784761	2.037578734	0.06869721	0.44205977	-4.13395847
homo after UVB irradiation VS homo before UVB irradiation	methionine degradation|HumanCyc	-0.57277639	-0.05503133	-2.33105808	0.04354803	0.13524454	-4.0824038
hetero after UVB irradiation VS hetero before UVB irradiation	methionine degradation|HumanCyc	-0.84724864	-0.02916516	-4.30891092	0.00229009	0.05380538	-1.16218972
WT after UVB irradiation VS WT before UVB irradiation	methionine degradation|HumanCyc	-0.36021951	-0.07450183	-1.42560505	0.18419947	0.59839062	-4.93830909
homo after UVB irradiation VS homo before UVB irradiation	Methionine Metabolism|SMPDB	-0.09396972	-0.02692441	-0.3795694	0.71270801	0.81002335	-6.29164178
hetero after UVB irradiation VS hetero before UVB irradiation	Methionine Metabolism|SMPDB	0.075091809	-0.02022482	0.352613458	0.73303662	0.86896773	-6.27959702
WT after UVB irradiation VS WT before UVB irradiation	Methionine Metabolism|SMPDB	0.176060787	0.019583595	0.866300336	0.40646895	0.7725109	-5.50349844
homo after UVB irradiation VS homo before UVB irradiation	Methionine salvage pathway|Reactome	0.335989112	-0.04936044	1.522661378	0.16078498	0.30937388	-5.25890821
hetero after UVB irradiation VS hetero before UVB irradiation	Methionine salvage pathway|Reactome	-0.19108307	0.032470717	-0.74295225	0.47771093	0.69910758	-6.05677767
WT after UVB irradiation VS WT before UVB irradiation	Methionine salvage pathway|Reactome	0.069034224	-0.06755191	0.27418173	0.78948061	0.94041679	-5.83121985
homo after UVB irradiation VS homo before UVB irradiation	methionine salvage|HumanCyc	0.0756302	-0.0634475	0.277833681	0.78716124	0.86440072	-6.32714718
hetero after UVB irradiation VS hetero before UVB irradiation	methionine salvage|HumanCyc	0.825840675	0.090330735	4.122696615	0.00297983	0.06148411	-1.41976679
WT after UVB irradiation VS WT before UVB irradiation	methionine salvage|HumanCyc	0.747837627	-0.04759047	2.228022217	0.04980116	0.40833376	-3.86014796
homo after UVB irradiation VS homo before UVB irradiation	Methotrexate Action Pathway|SMPDB	0.210604765	-0.01290295	0.884608242	0.39845428	0.55064838	-5.96507122
hetero after UVB irradiation VS hetero before UVB irradiation	Methotrexate Action Pathway|SMPDB	0.256530919	-0.05900519	1.406879866	0.1952591	0.4161797	-5.38534533
WT after UVB irradiation VS WT before UVB irradiation	Methotrexate Action Pathway|SMPDB	0.359853909	-0.03380361	1.522274352	0.15866743	0.56072663	-4.82152497
homo after UVB irradiation VS homo before UVB irradiation	Methotrexate Pathway (Brain Cell)_ Pharmacokinetics|PharmGKB	0.570536615	0.016080596	2.071910071	0.06687507	0.18004219	-4.4805681
hetero after UVB irradiation VS hetero before UVB irradiation	Methotrexate Pathway (Brain Cell)_ Pharmacokinetics|PharmGKB	0.709365593	0.048590163	2.653111775	0.02784234	0.14438349	-3.60161576
WT after UVB irradiation VS WT before UVB irradiation	Methotrexate Pathway (Brain Cell)_ Pharmacokinetics|PharmGKB	0.593998592	-0.02118296	2.141232524	0.05769616	0.42295081	-3.98586636
homo after UVB irradiation VS homo before UVB irradiation	Methotrexate Pathway (Cancer Cell)_ Pharmacodynamics|PharmGKB	-0.32133252	0.000830918	-1.23027016	0.2485276	0.41085338	-5.61616105
hetero after UVB irradiation VS hetero before UVB irradiation	Methotrexate Pathway (Cancer Cell)_ Pharmacodynamics|PharmGKB	-0.00774321	-0.02400534	-0.03648475	0.97174546	0.98668207	-6.34581922
WT after UVB irradiation VS WT before UVB irradiation	Methotrexate Pathway (Cancer Cell)_ Pharmacodynamics|PharmGKB	0.077296601	-0.01052597	0.250747814	0.80704517	0.94525798	-5.83740588
homo after UVB irradiation VS homo before UVB irradiation	Methotrexate Pathway_ Pharmacokinetics|PharmGKB	0.460342788	0.096970328	1.725337527	0.11715236	0.25512659	-4.98515737
hetero after UVB irradiation VS hetero before UVB irradiation	Methotrexate Pathway_ Pharmacokinetics|PharmGKB	0.289718794	0.026867106	1.268303762	0.23860785	0.4612216	-5.55011873
WT after UVB irradiation VS WT before UVB irradiation	Methotrexate Pathway_ Pharmacokinetics|PharmGKB	0.697683382	0.067774656	3.062736423	0.01187958	0.27435478	-2.6139708
homo after UVB irradiation VS homo before UVB irradiation	Methyclothiazide Action Pathway|SMPDB	0.234726031	0.017120546	1.156747303	0.27595732	0.43614438	-5.69767199
hetero after UVB irradiation VS hetero before UVB irradiation	Methyclothiazide Action Pathway|SMPDB	-0.03255037	0.028925632	-0.18154472	0.86023155	0.94503144	-6.32869738
WT after UVB irradiation VS WT before UVB irradiation	Methyclothiazide Action Pathway|SMPDB	-0.15908359	0.010252305	-0.80382265	0.44003996	0.79685269	-5.55266936
homo after UVB irradiation VS homo before UVB irradiation	Methylation of MeSeH for excretion|Reactome	-0.81336978	-0.004556	-2.52836131	0.0313347	0.11775656	-3.77184131
hetero after UVB irradiation VS hetero before UVB irradiation	Methylation of MeSeH for excretion|Reactome	-0.65714345	-0.06417821	-1.75236404	0.11590379	0.30887493	-4.93343801
WT after UVB irradiation VS WT before UVB irradiation	Methylation of MeSeH for excretion|Reactome	-0.85816176	-0.09524994	-2.63007636	0.02499985	0.33482204	-3.26412137
homo after UVB irradiation VS homo before UVB irradiation	Methylation Pathways|Wikipathways	0.426486226	-0.0648221	1.811462295	0.10211394	0.23277696	-4.86356203
hetero after UVB irradiation VS hetero before UVB irradiation	Methylation Pathways|Wikipathways	-0.28473813	-0.01898267	-1.12862269	0.29016367	0.52049037	-5.70444169
WT after UVB irradiation VS WT before UVB irradiation	Methylation Pathways|Wikipathways	0.020766444	-0.150108	0.063053098	0.95095678	0.98681143	-5.86713755
homo after UVB irradiation VS homo before UVB irradiation	Methylation|Reactome	0.350129404	-0.03793011	1.886912751	0.09042907	0.21571189	-4.75480589
hetero after UVB irradiation VS hetero before UVB irradiation	Methylation|Reactome	-0.10280433	0.001701828	-0.48756045	0.63829393	0.81595398	-6.21942104
WT after UVB irradiation VS WT before UVB irradiation	Methylation|Reactome	0.393447886	-0.0795517	1.388365127	0.19493467	0.60625027	-4.98201116
homo after UVB irradiation VS homo before UVB irradiation	Methylene Blue Pathway_ Pharmacodynamics|PharmGKB	0.374339549	-0.0129161	1.461409862	0.17654636	0.32929357	-5.33775075
hetero after UVB irradiation VS hetero before UVB irradiation	Methylene Blue Pathway_ Pharmacodynamics|PharmGKB	0.402281931	0.061505698	2.091388237	0.06812025	0.23495391	-4.4490571
WT after UVB irradiation VS WT before UVB irradiation	Methylene Blue Pathway_ Pharmacodynamics|PharmGKB	0.237268472	-0.05808792	0.860570128	0.40947285	0.77660403	-5.50813937
homo after UVB irradiation VS homo before UVB irradiation	Methylenetetrahydrofolate Reductase Deficiency (MTHFRD)|SMPDB	0.210604765	-0.01290295	0.884608242	0.39845428	0.55064838	-5.96507122
homo after UVB irradiation VS homo before UVB irradiation	Methylenetetrahydrofolate Reductase Deficiency (MTHFRD)|SMPDB	-0.09396972	-0.02692441	-0.3795694	0.71270801	0.81002335	-6.29164178
hetero after UVB irradiation VS hetero before UVB irradiation	Methylenetetrahydrofolate Reductase Deficiency (MTHFRD)|SMPDB	0.256530919	-0.05900519	1.406879866	0.1952591	0.4161797	-5.38534533
hetero after UVB irradiation VS hetero before UVB irradiation	Methylenetetrahydrofolate Reductase Deficiency (MTHFRD)|SMPDB	0.075091809	-0.02022482	0.352613458	0.73303662	0.86896773	-6.27959702
WT after UVB irradiation VS WT before UVB irradiation	Methylenetetrahydrofolate Reductase Deficiency (MTHFRD)|SMPDB	0.359853909	-0.03380361	1.522274352	0.15866743	0.56072663	-4.82152497
WT after UVB irradiation VS WT before UVB irradiation	Methylenetetrahydrofolate Reductase Deficiency (MTHFRD)|SMPDB	0.176060787	0.019583595	0.866300336	0.40646895	0.7725109	-5.50349844
homo after UVB irradiation VS homo before UVB irradiation	methylglyoxal degradation I|HumanCyc	1.094689818	0.010272288	4.542527002	0.00125718	0.02014127	-0.64908507
hetero after UVB irradiation VS hetero before UVB irradiation	methylglyoxal degradation I|HumanCyc	1.014841638	-0.00299866	5.639355378	0.00040412	0.03004176	0.513799665
WT after UVB irradiation VS WT before UVB irradiation	methylglyoxal degradation I|HumanCyc	0.64205161	0.065849611	1.889300813	0.08792305	0.48023726	-4.34108857
homo after UVB irradiation VS homo before UVB irradiation	methylglyoxal degradation III|HumanCyc	0.883295176	-0.04964869	4.062045791	0.00259506	0.02932936	-1.35632739
hetero after UVB irradiation VS hetero before UVB irradiation	methylglyoxal degradation III|HumanCyc	0.502120331	0.125890725	1.361541672	0.20863126	0.43067993	-5.44044258
WT after UVB irradiation VS WT before UVB irradiation	methylglyoxal degradation III|HumanCyc	0.581480539	0.124899674	1.855131318	0.09301457	0.48552482	-4.38790998
homo after UVB irradiation VS homo before UVB irradiation	methylglyoxal degradation VI|HumanCyc	0.888336647	-0.04841783	2.98333231	0.01467863	0.07488562	-3.04487022
hetero after UVB irradiation VS hetero before UVB irradiation	methylglyoxal degradation VI|HumanCyc	0.49280583	-0.00320891	1.320874982	0.2212872	0.44238028	-5.48889682
WT after UVB irradiation VS WT before UVB irradiation	methylglyoxal degradation VI|HumanCyc	0.510252742	-0.01185959	1.269790806	0.23268314	0.62952955	-5.11600806
homo after UVB irradiation VS homo before UVB irradiation	Methylmalonate Semialdehyde Dehydrogenase Deficiency|SMPDB	-0.23657683	-0.05522798	-0.8011182	0.4428632	0.59091566	-6.03520901
hetero after UVB irradiation VS hetero before UVB irradiation	Methylmalonate Semialdehyde Dehydrogenase Deficiency|SMPDB	-0.90022729	-0.04719335	-5.31014337	0.00060637	0.03004176	0.126180045
WT after UVB irradiation VS WT before UVB irradiation	Methylmalonate Semialdehyde Dehydrogenase Deficiency|SMPDB	-0.36319435	-0.13086657	-1.31407353	0.21793282	0.62581393	-5.06691375
homo after UVB irradiation VS homo before UVB irradiation	Methylmalonic Aciduria Due to Cobalamin-Related Disorders|SMPDB	-0.25733603	-0.09162056	-0.7770169	0.45627806	0.60277262	-6.05433779
hetero after UVB irradiation VS hetero before UVB irradiation	Methylmalonic Aciduria Due to Cobalamin-Related Disorders|SMPDB	-0.83573623	-0.01284435	-4.81326218	0.00115145	0.04138456	-0.49254
WT after UVB irradiation VS WT before UVB irradiation	Methylmalonic Aciduria Due to Cobalamin-Related Disorders|SMPDB	-0.18571921	-0.14542889	-0.63906971	0.5370254	0.83767333	-5.66665155
homo after UVB irradiation VS homo before UVB irradiation	Methylmalonic Aciduria|SMPDB	-0.23657683	-0.05522798	-0.8011182	0.4428632	0.59091566	-6.03520901
hetero after UVB irradiation VS hetero before UVB irradiation	Methylmalonic Aciduria|SMPDB	-0.90022729	-0.04719335	-5.31014337	0.00060637	0.03004176	0.126180045
WT after UVB irradiation VS WT before UVB irradiation	Methylmalonic Aciduria|SMPDB	-0.36319435	-0.13086657	-1.31407353	0.21793282	0.62581393	-5.06691375
homo after UVB irradiation VS homo before UVB irradiation	Metiamide Action Pathway|SMPDB	0.21780303	0.051698704	1.19150037	0.26270351	0.42561191	-5.65959884
hetero after UVB irradiation VS hetero before UVB irradiation	Metiamide Action Pathway|SMPDB	0.331566302	0.063470812	1.441200644	0.18563717	0.40321767	-5.34291209
WT after UVB irradiation VS WT before UVB irradiation	Metiamide Action Pathway|SMPDB	-0.11560861	0.135982321	-0.44330845	0.66690385	0.87935805	-5.77062647

homo after UVB irradiation VS homo before UVB irradiation	Metipranolol Action Pathway|SMPDB	0.396415397	-0.03677376	2.343069613	0.04268583	0.13402181	-4.06364635
hetero after UVB irradiation VS hetero before UVB irradiation	Metipranolol Action Pathway|SMPDB	-0.01555055	-0.01775524	-0.07410913	0.94265284	0.97054702	-6.34356282
WT after UVB irradiation VS WT before UVB irradiation	Metipranolol Action Pathway|SMPDB	0.056685205	-0.03978418	0.223404833	0.82768199	0.94525798	-5.84393438
homo after UVB irradiation VS homo before UVB irradiation	Metolazone Action Pathway|SMPDB	0.234726031	0.017120546	1.156747303	0.27595732	0.43614438	-5.69767199
hetero after UVB irradiation VS hetero before UVB irradiation	Metolazone Action Pathway|SMPDB	-0.03255037	0.028925632	-0.18154472	0.86023155	0.94503144	-6.32869738
WT after UVB irradiation VS WT before UVB irradiation	Metolazone Action Pathway|SMPDB	-0.15908359	0.010252305	-0.80382265	0.44003996	0.79685269	-5.55266936
homo after UVB irradiation VS homo before UVB irradiation	Metoprolol Action Pathway|SMPDB	0.396415397	-0.03677376	2.343069613	0.04268583	0.13402181	-4.06364635
hetero after UVB irradiation VS hetero before UVB irradiation	Metoprolol Action Pathway|SMPDB	-0.01555055	-0.01775524	-0.07410913	0.94265284	0.97054702	-6.34356282
WT after UVB irradiation VS WT before UVB irradiation	Metoprolol Action Pathway|SMPDB	0.056685205	-0.03978418	0.223404833	0.82768199	0.94525798	-5.84393438
homo after UVB irradiation VS homo before UVB irradiation	mets affect on macrophage differentiation|BioCarta	-0.08314919	-0.00622871	-0.50901081	0.62249073	0.73728109	-6.23117686
hetero after UVB irradiation VS hetero before UVB irradiation	mets affect on macrophage differentiation|BioCarta	0.195226251	-0.02761867	0.949875271	0.36862155	0.59623032	-5.88227623
WT after UVB irradiation VS WT before UVB irradiation	mets affect on macrophage differentiation|BioCarta	-0.00142966	-0.0238959	-0.00682646	0.9946865	0.99796425	-5.86912758
homo after UVB irradiation VS homo before UVB irradiation	mevalonate pathway|HumanCyc	-0.08324566	0.00209274	-0.31995736	0.75600614	0.84131709	-6.31374902
hetero after UVB irradiation VS hetero before UVB irradiation	mevalonate pathway|HumanCyc	-0.18021886	0.010342625	-0.49274254	0.63477796	0.81341522	-6.21674385
WT after UVB irradiation VS WT before UVB irradiation	mevalonate pathway|HumanCyc	-0.07995649	0.079294782	-0.24023597	0.8149615	0.94525798	-5.84000374
homo after UVB irradiation VS homo before UVB irradiation	Mevalonate pathway|Wikipathways	-0.16999309	-0.03592324	-0.49251552	0.6336669	0.74618219	-6.23981581
hetero after UVB irradiation VS hetero before UVB irradiation	Mevalonate pathway|Wikipathways	0.125315847	-0.03529715	0.298742235	0.77237235	0.89807989	-6.29838746
WT after UVB irradiation VS WT before UVB irradiation	Mevalonate pathway|Wikipathways	0.167898722	0.059264249	0.448859853	0.6630263	0.87935805	-5.76816976
homo after UVB irradiation VS homo before UVB irradiation	Mevalonic aciduria|SMPDB	-0.19230642	-0.01987665	-0.61925406	0.55047761	0.67913558	-6.16661869
hetero after UVB irradiation VS hetero before UVB irradiation	Mevalonic aciduria|SMPDB	0.185820603	0.008098609	0.631890782	0.54421801	0.75147406	-6.13505553
WT after UVB irradiation VS WT before UVB irradiation	Mevalonic aciduria|SMPDB	-0.14894747	0.045069977	-0.50755372	0.62268399	0.87257466	-5.74041409
homo after UVB irradiation VS homo before UVB irradiation	Mexiletine Action Pathway|SMPDB	0.396415397	-0.03677376	2.343069613	0.04268583	0.13402181	-4.06364635
hetero after UVB irradiation VS hetero before UVB irradiation	Mexiletine Action Pathway|SMPDB	-0.01555055	-0.01775524	-0.07410913	0.94265284	0.97054702	-6.34356282
WT after UVB irradiation VS WT before UVB irradiation	Mexiletine Action Pathway|SMPDB	0.056685205	-0.03978418	0.223404833	0.82768199	0.94525798	-5.84393438
homo after UVB irradiation VS homo before UVB irradiation	MFAP5-mediated ovarian cancer cell motility and invasiveness|Wikipathways	0.260609746	-0.03294025	1.347637623	0.20939022	0.36715659	-5.47876949
hetero after UVB irradiation VS hetero before UVB irradiation	MFAP5-mediated ovarian cancer cell motility and invasiveness|Wikipathways	0.380703619	-0.02191558	2.184834752	0.05872689	0.21639222	-4.31079387
WT after UVB irradiation VS WT before UVB irradiation	MFAP5-mediated ovarian cancer cell motility and invasiveness|Wikipathways	0.194722043	-0.02820311	0.92046132	0.37882907	0.75122805	-5.45835255
homo after UVB irradiation VS homo before UVB irradiation	MGMT-mediated DNA damage reversal|Reactome	0.991633532	-0.03893307	4.703142224	0.00099396	0.01831679	-0.42052638
hetero after UVB irradiation VS hetero before UVB irradiation	MGMT-mediated DNA damage reversal|Reactome	1.044779827	0.059789247	3.902928244	0.00409191	0.07021718	-1.73055831
WT after UVB irradiation VS WT before UVB irradiation	MGMT-mediated DNA damage reversal|Reactome	1.103729966	0.042254981	4.228047506	0.00171767	0.15689229	-0.93726541
homo after UVB irradiation VS homo before UVB irradiation	MHC class II antigen presentation|Reactome	0.476366328	-0.03449482	2.281377114	0.04729917	0.14161836	-4.15973988
hetero after UVB irradiation VS hetero before UVB irradiation	MHC class II antigen presentation|Reactome	0.428533751	0.023139504	2.062577708	0.07130063	0.23966688	-4.4913457
WT after UVB irradiation VS WT before UVB irradiation	MHC class II antigen presentation|Reactome	0.480025252	0.006761671	2.310920636	0.04324032	0.39022568	-3.73883902
homo after UVB irradiation VS homo before UVB irradiation	Microglia Pathogen Phagocytosis Pathway|Wikipathways	0.859223103	-0.01606299	5.228165225	0.00047333	0.01666204	0.297683826
hetero after UVB irradiation VS hetero before UVB irradiation	Microglia Pathogen Phagocytosis Pathway|Wikipathways	0.971554807	0.003785411	6.234993807	0.00020117	0.02988205	1.171372712
WT after UVB irradiation VS WT before UVB irradiation	Microglia Pathogen Phagocytosis Pathway|Wikipathways	0.902023398	0.047607427	4.255033104	0.00164555	0.15689229	-0.90071737
homo after UVB irradiation VS homo before UVB irradiation	MicroRNA (miRNA) biogenesis|Reactome	-0.45809918	-0.06762288	-1.94311635	0.08255099	0.20394538	-4.67255997
hetero after UVB irradiation VS hetero before UVB irradiation	MicroRNA (miRNA) biogenesis|Reactome	-0.18029529	-0.09783047	-0.80348927	0.44376587	0.66919789	-6.00946122
WT after UVB irradiation VS WT before UVB irradiation	MicroRNA (miRNA) biogenesis|Reactome	0.015739143	-0.01104333	0.055571498	0.95676901	0.98742794	-5.86758699
homo after UVB irradiation VS homo before UVB irradiation	MicroRNA for Targeting Cancer Growth and Vascularization in Glioblastoma|Wikipathways	0.938483464	-0.03252652	4.72935977	0.00095691	0.01805472	-0.38360636
hetero after UVB irradiation VS hetero before UVB irradiation	MicroRNA for Targeting Cancer Growth and Vascularization in Glioblastoma|Wikipathways	0.762763006	0.057144404	3.70147007	0.0055052	0.07940727	-2.02153549
WT after UVB irradiation VS WT before UVB irradiation	MicroRNA for Targeting Cancer Growth and Vascularization in Glioblastoma|Wikipathways	0.463272954	-0.00045814	1.455758148	0.17588075	0.58755921	-4.90238814
homo after UVB irradiation VS homo before UVB irradiation	MicroRNA network associated with chronic lymphocytic leukemia|Wikipathways	-0.38084758	0.021119642	-1.27278414	0.23370608	0.39536938	-5.56739441
hetero after UVB irradiation VS hetero before UVB irradiation	MicroRNA network associated with chronic lymphocytic leukemia|Wikipathways	-0.16982405	-0.19030364	-0.55297921	0.59464097	0.7848684	-6.18368116
WT after UVB irradiation VS WT before UVB irradiation	MicroRNA network associated with chronic lymphocytic leukemia|Wikipathways	-0.30550471	-0.08532385	-1.34897472	0.20686094	0.62198427	-5.02741481
homo after UVB irradiation VS homo before UVB irradiation	MicroRNAs in cancer - Homo sapiens (human)|KEGG	-0.02068593	-0.00672321	-0.10287752	0.92022406	0.95153785	-6.36276482
hetero after UVB irradiation VS hetero before UVB irradiation	MicroRNAs in cancer - Homo sapiens (human)|KEGG	0.110168125	-0.00742135	0.688094704	0.5098894	0.7290075	-6.09684968
WT after UVB irradiation VS WT before UVB irradiation	MicroRNAs in cancer - Homo sapiens (human)|KEGG	-0.03862687	0.002435368	-0.18919552	0.85369401	0.95632621	-5.85105224
homo after UVB irradiation VS homo before UVB irradiation	MicroRNAs in cardiomyocyte hypertrophy|Wikipathways	0.251641403	-0.00453559	1.41527477	0.18928834	0.34516395	-5.39581393
hetero after UVB irradiation VS hetero before UVB irradiation	MicroRNAs in cardiomyocyte hypertrophy|Wikipathways	0.014062302	-0.03207368	0.0875376	0.93228899	0.97054702	-6.34238619
WT after UVB irradiation VS WT before UVB irradiation	MicroRNAs in cardiomyocyte hypertrophy|Wikipathways	0.163853115	-0.01518009	0.822186409	0.42998593	0.79574324	-5.53854581
homo after UVB irradiation VS homo before UVB irradiation	Microtubule-dependent trafficking of connexons from Golgi to the plasma membrane|Reactome	-0.47340954	-0.02427104	-1.22907819	0.24895398	0.41126045	-5.61751141
hetero after UVB irradiation VS hetero before UVB irradiation	Microtubule-dependent trafficking of connexons from Golgi to the plasma membrane|Reactome	-0.32349958	-0.00720452	-0.81487917	0.43756541	0.66348076	-6.00020409
WT after UVB irradiation VS WT before UVB irradiation	Microtubule-dependent trafficking of connexons from Golgi to the plasma membrane|Reactome	-0.47015405	0.038294873	-1.2644186	0.23452704	0.62952955	-5.12188435
homo after UVB irradiation VS homo before UVB irradiation	Mineral absorption - Homo sapiens (human)|KEGG	0.116090143	-0.00292711	0.583307287	0.5734289	0.69713225	-6.18895878
hetero after UVB irradiation VS hetero before UVB irradiation	Mineral absorption - Homo sapiens (human)|KEGG	0.08649995	0.047313033	0.498560737	0.63084202	0.81063904	-6.21370626
WT after UVB irradiation VS WT before UVB irradiation	Mineral absorption - Homo sapiens (human)|KEGG	0.19847165	0.02553249	0.955347084	0.36174722	0.74374432	-5.42806923
homo after UVB irradiation VS homo before UVB irradiation	mineralocorticoid biosynthesis|HumanCyc	0.901963221	-0.00122184	3.742869583	0.00427376	0.03747505	-1.84393529
hetero after UVB irradiation VS hetero before UVB irradiation	mineralocorticoid biosynthesis|HumanCyc	0.670020496	0.052895265	2.151829174	0.06189026	0.22317708	-4.3598097
WT after UVB irradiation VS WT before UVB irradiation	mineralocorticoid biosynthesis|HumanCyc	0.589123178	0.096950094	1.647766516	0.13017049	0.54238366	-4.66331842
homo after UVB irradiation VS homo before UVB irradiation	Mineralocorticoid biosynthesis|Reactome	0.141473704	0.083744287	0.618276096	0.55109499	0.6797136	-6.16724276
hetero after UVB irradiation VS hetero before UVB irradiation	Mineralocorticoid biosynthesis|Reactome	-0.1847301	0.127100886	-0.64136303	0.538338	0.75106967	-6.12882202
WT after UVB irradiation VS WT before UVB irradiation	Mineralocorticoid biosynthesis|Reactome	-0.38933239	0.218620788	-1.33955376	0.2098023	0.62472791	-5.03814543
homo after UVB irradiation VS homo before UVB irradiation	Minus-strand DNA synthesis|Reactome	-0.08403744	-0.00320991	-0.2001813	0.84560766	0.90528595	-6.34696415
hetero after UVB irradiation VS hetero before UVB irradiation	Minus-strand DNA synthesis|Reactome	0.471357887	-0.00478231	1.150987769	0.28134999	0.51114365	-5.68058898
WT after UVB irradiation VS WT before UVB irradiation	Minus-strand DNA synthesis|Reactome	0.926001949	0.004188178	3.205753029	0.00930482	0.24856491	-2.40025129
homo after UVB irradiation VS homo before UVB irradiation	mir-124 predicted interactions with cell cycle and differentiation|Wikipathways	0.305343151	-0.01931246	1.61113811	0.14020845	0.28327069	-5.14170864
hetero after UVB irradiation VS hetero before UVB irradiation	mir-124 predicted interactions with cell cycle and differentiation|Wikipathways	0.701406443	0.000199871	3.804500245	0.00472688	0.07419102	-1.87202656
WT after UVB irradiation VS WT before UVB irradiation	mir-124 predicted interactions with cell cycle and differentiation|Wikipathways	0.294421444	-0.09939383	1.109371241	0.2930295	0.67966352	-5.28363314
homo after UVB irradiation VS homo before UVB irradiation	miR-222 in Exercise-Induced Cardiac Growth|Wikipathways	-0.97252643	0.073226491	-3.85498146	0.00358139	0.03395341	-1.67119217
hetero after UVB irradiation VS hetero before UVB irradiation	miR-222 in Exercise-Induced Cardiac Growth|Wikipathways	-0.67340433	0.059887052	-2.74616911	0.02401778	0.13715811	-3.45917606
WT after UVB irradiation VS WT before UVB irradiation	miR-222 in Exercise-Induced Cardiac Growth|Wikipathways	-0.93959287	-0.13775776	-3.70027884	0.00405006	0.20968398	-1.67530249
homo after UVB irradiation VS homo before UVB irradiation	mir34a and TGIF2 in osteoclastogenesis|Wikipathways	-0.13725978	-0.0364894	-0.36035678	0.72655416	0.82013506	-6.2991671
hetero after UVB irradiation VS hetero before UVB irradiation	mir34a and TGIF2 in osteoclastogenesis|Wikipathways	0.405357845	0.018197627	1.004333937	0.34318475	0.57242294	-5.83059765
WT after UVB irradiation VS WT before UVB irradiation	mir34a and TGIF2 in osteoclastogenesis|Wikipathways	0.459165267	0.037444797	1.096111704	0.29852087	0.68477511	-5.29672973
homo after UVB irradiation VS homo before UVB irradiation	miR-509-3p alteration of YAP1-ECM axis|Wikipathways	0.341811484	-0.0413656	1.576173295	0.14804205	0.29344734	-5.18847413
hetero after UVB irradiation VS hetero before UVB irradiation	miR-509-3p alteration of YAP1-ECM axis|Wikipathways	0.034407288	0.005557528	0.129511279	0.89999159	0.96973277	-6.33745124
WT after UVB irradiation VS WT before UVB irradiation	miR-509-3p alteration of YAP1-ECM axis|Wikipathways	0.196950861	-0.04655471	0.663559758	0.52185517	0.83116638	-5.65119404
homo after UVB irradiation VS homo before UVB irradiation	miR-517 relationship with ARCN1 and USP1|Wikipathways	-0.48609879	0.038932029	-2.21905717	0.05245313	0.15274299	-4.25613883
hetero after UVB irradiation VS hetero before UVB irradiation	miR-517 relationship with ARCN1 and USP1|Wikipathways	-0.16405874	-0.14970302	-0.65854585	0.52776867	0.74212892	-6.11730075
WT after UVB irradiation VS WT before UVB irradiation	miR-517 relationship with ARCN1 and USP1|Wikipathways	-0.58793323	-0.06821224	-2.56066366	0.02817316	0.35160213	-3.36810506
homo after UVB irradiation VS homo before UVB irradiation	miRNA Biogenesis|Wikipathways	-0.85812771	-0.04469479	-3.95680383	0.00305454	0.03140179	-1.51565553
hetero after UVB irradiation VS hetero before UVB irradiation	miRNA Biogenesis|Wikipathways	-0.34695058	-0.16135852	-1.08736967	0.30699645	0.53592103	-5.7475374
WT after UVB irradiation VS WT before UVB irradiation	miRNA Biogenesis|Wikipathways	-0.43655474	-0.0667691	-1.22194593	0.2495278	0.64238639	-5.1677202
homo after UVB irradiation VS homo before UVB irradiation	miRNA degrading enzymes|Wikipathways	-1.05757066	0.017236567	-5.89620031	0.00019487	0.01666204	1.146825099
hetero after UVB irradiation VS hetero before UVB irradiation	miRNA degrading enzymes|Wikipathways	-0.29001798	-0.02914514	-0.66476625	0.52397353	0.73931303	-6.11306236
WT after UVB irradiation VS WT before UVB irradiation	miRNA degrading enzymes|Wikipathways	-0.67310594	0.014979403	-2.05718469	0.06647419	0.43803373	-4.1061366
homo after UVB irradiation VS homo before UVB irradiation	miRNA Regulation of DNA Damage Response|Wikipathways	-0.41108691	-0.00452727	-2.32881909	0.04371063	0.13549404	-4.08589776
hetero after UVB irradiation VS hetero before UVB irradiation	miRNA Regulation of DNA Damage Response|Wikipathways	0.079532611	-0.03863368	0.355825717	0.73071581	0.86896773	-6.27838113
WT after UVB irradiation VS WT before UVB irradiation	miRNA Regulation of DNA Damage Response|Wikipathways	-0.04855039	-0.0379529	-0.19890043	0.84629442	0.9536885	-5.84915184
homo after UVB irradiation VS homo before UVB irradiation	miRNA regulation of p53 pathway in prostate cancer|Wikipathways	0.391309897	-0.03285573	1.885661564	0.09061222	0.21571189	-4.75662525
hetero after UVB irradiation VS hetero before UVB irradiation	miRNA regulation of p53 pathway in prostate cancer|Wikipathways	0.57688503	-0.04204505	3.195774491	0.01186461	0.10542662	-2.7736666
WT after UVB irradiation VS WT before UVB irradiation	miRNA regulation of p53 pathway in prostate cancer|Wikipathways	0.559114514	-0.00199416	2.664689753	0.02355298	0.3249742	-3.21217651
homo after UVB irradiation VS homo before UVB irradiation	miRNA regulation of prostate cancer signaling pathways|Wikipathways	0.15080639	-0.00687325	0.649247288	0.53173759	0.66792678	-6.14704055
hetero after UVB irradiation VS hetero before UVB irradiation	miRNA regulation of prostate cancer signaling pathways|Wikipathways	0.274603665	-0.04633095	1.681849543	0.1291888	0.32389948	-5.02973476
WT after UVB irradiation VS WT before UVB irradiation	miRNA regulation of prostate cancer signaling pathways|Wikipathways	0.088022764	-0.02759077	0.440786839	0.66866856	0.87948693	-5.77173269
homo after UVB irradiation VS homo before UVB irradiation	miRNA targets in ECM and membrane receptors|Wikipathways	0.933022041	-0.00915703	5.103302916	0.00056264	0.01666204	0.130934468
hetero after UVB irradiation VS hetero before UVB irradiation	miRNA targets in ECM and membrane receptors|Wikipathways	0.659080363	0.088090076	2.194773214	0.0578058	0.21473417	-4.29599854
WT after UVB irradiation VS WT before UVB irradiation	miRNA targets in ECM and membrane receptors|Wikipathways	0.606825057	0.057495505	2.030483351	0.06951893	0.444668	-4.14400401
homo after UVB irradiation VS homo before UVB irradiation	miRNAs involved in DNA damage response|Wikipathways	-0.30196945	-0.0460181	-1.43177267	0.18464359	0.33913219	-5.3751848
hetero after UVB irradiation VS hetero before UVB irradiation	miRNAs involved in DNA damage response|Wikipathways	0.190740243	-0.03894489	0.952144294	0.36753454	0.59595249	-5.88016871
WT after UVB irradiation VS WT before UVB irradiation	miRNAs involved in DNA damage response|Wikipathways	-0.01086557	-0.05906205	-0.04312318	0.96644544	0.98922368	-5.86820922
homo after UVB irradiation VS homo before UVB irradiation	miRNAs involvement in the immune response in sepsis|Wikipathways	0.688837477	-0.03446239	4.276516487	0.00187029	0.023988	-1.03641536
hetero after UVB irradiation VS hetero before UVB irradiation	miRNAs involvement in the immune response in sepsis|Wikipathways	0.645081674	0.014823256	3.928168345	0.00394417	0.06941134	-1.69450278
WT after UVB irradiation VS WT before UVB irradiation	miRNAs involvement in the immune response in sepsis|Wikipathways	0.540979227	-0.03349881	2.22927378	0.0496953	0.40833376	-3.85832487
homo after UVB irradiation VS homo before UVB irradiation	miRs in Muscle Cell Differentiation|Wikipathways	-0.115102	-0.01447807	-0.71167922	0.49396507	0.63444291	-6.10359955
hetero after UVB irradiation VS hetero before UVB irradiation	miRs in Muscle Cell Differentiation|Wikipathways	-0.1014418	-0.04274399	-0.54836984	0.5976634	0.78732653	-6.1863366
WT after UVB irradiation VS WT before UVB irradiation	miRs in Muscle Cell Differentiation|Wikipathways	-0.18463701	-0.0640055	-0.74050424	0.47588548	0.81430085	-5.5992208
homo after UVB irradiation VS homo before UVB irradiation	miR-targeted genes in adipocytes - TarBase|Wikipathways	0.189947239	-0.01833746	0.870890024	0.40553018	0.5575582	-5.9770013
hetero after UVB irradiation VS hetero before UVB irradiation	miR-targeted genes in adipocytes - TarBase|Wikipathways	0.59922433	-0.01585911	3.959244668	0.00377004	0.06818992	-1.65023681
WT after UVB irradiation VS WT before UVB irradiation	miR-targeted genes in adipocytes - TarBase|Wikipathways	0.171949758	-0.05905122	0.661526983	0.52310462	0.83120196	-5.65249723
homo after UVB irradiation VS homo before UVB irradiation	miR-targeted genes in epithelium - TarBase|Wikipathways	0.050321189	-0.02875552	0.293274987	0.77569105	0.85468276	-6.32245086
hetero after UVB irradiation VS hetero before UVB irradiation	miR-targeted genes in epithelium - TarBase|Wikipathways	0.218054273	-0.00947288	1.444320663	0.18478337	0.40321767	-5.33902449
WT after UVB irradiation VS WT before UVB irradiation	miR-targeted genes in epithelium - TarBase|Wikipathways	0.048552826	-0.02567475	0.233320234	0.82018163	0.94525798	-5.84165294
homo after UVB irradiation VS homo before UVB irradiation	miR-targeted genes in leukocytes - TarBase|Wikipathways	0.235406921	-0.04087015	1.149198645	0.27890541	0.43928323	-5.70583153
hetero after UVB irradiation VS hetero before UVB irradiation	miR-targeted genes in leukocytes - TarBase|Wikipathways	0.372585768	-0.00288144	2.526178238	0.03407914	0.16130084	-3.79553938
WT after UVB irradiation VS WT before UVB irradiation	miR-targeted genes in leukocytes - TarBase|Wikipathways	0.282651658	-0.03685761	1.277108227	0.23019069	0.62952955	-5.10797601
homo after UVB irradiation VS homo before UVB irradiation	miR-targeted genes in lymphocytes - TarBase|Wikipathways	-0.03351603	-0.01659558	-0.19668214	0.8482679	0.90643371	-6.3477071
hetero after UVB irradiation VS hetero before UVB irradiation	miR-targeted genes in lymphocytes - TarBase|Wikipathways	0.149710902	-0.02189345	0.99707838	0.34649528	0.57530067	-5.83761353
WT after UVB irradiation VS WT before UVB irradiation	miR-targeted genes in lymphocytes - TarBase|Wikipathways	0.049845959	-0.02435532	0.234851257	0.81902518	0.94525798	-5.84129194
homo after UVB irradiation VS homo before UVB irradiation	miR-targeted genes in muscle cell - TarBase|Wikipathways	0.031996325	-0.0229496	0.175769173	0.86420789	0.91745484	-6.35187632
hetero after UVB irradiation VS hetero before UVB irradiation	miR-targeted genes in muscle cell - TarBase|Wikipathways	0.163713229	-0.01718521	1.074102382	0.31257035	0.54150348	-5.76114373
WT after UVB irradiation VS WT before UVB irradiation	miR-targeted genes in muscle cell - TarBase|Wikipathways	0.013285647	-0.02215987	0.063944096	0.95026479	0.98633166	-5.86708025
homo after UVB irradiation VS homo before UVB irradiation	miR-targeted genes in squamous cell - TarBase|Wikipathways	0.172982841	-0.01470806	0.872274206	0.40481226	0.55707438	-5.97580468
hetero after UVB irradiation VS hetero before UVB irradiation	miR-targeted genes in squamous cell - TarBase|Wikipathways	0.212175982	-0.00499388	1.411408089	0.19396523	0.41545748	-5.37978192
WT after UVB irradiation VS WT before UVB irradiation	miR-targeted genes in squamous cell - TarBase|Wikipathways	0.092096632	-0.03269428	0.428548492	0.67726325	0.88322709	-5.77701557
homo after UVB irradiation VS homo before UVB irradiation	Miscellaneous substrates|Reactome	0.708251752	0.093472685	3.91625955	0.00325381	0.03242281	-1.57742872
hetero after UVB irradiation VS hetero before UVB irradiation	Miscellaneous substrates|Reactome	0.537335053	0.00733066	2.900979349	0.01880479	0.12055168	-3.22226402
WT after UVB irradiation VS WT before UVB irradiation	Miscellaneous substrates|Reactome	0.384085991	0.083759347	1.379830419	0.1974683	0.60852749	-4.99192148
homo after UVB irradiation VS homo before UVB irradiation	Miscellaneous transport and binding events|Reactome	0.009570381	0.000338438	0.034783025	0.97298104	0.98310056	-6.36779824
hetero after UVB irradiation VS hetero before UVB irradiation	Miscellaneous transport and binding events|Reactome	0.048547659	-0.01906258	0.285868548	0.78188132	0.90426031	-6.3024281
WT after UVB irradiation VS WT before UVB irradiation	Miscellaneous transport and binding events|Reactome	-0.20125006	-0.00601844	-0.93718285	0.37057085	0.74850253	-5.44395319
homo after UVB irradiation VS homo before UVB irradiation	Mismatch repair - Homo sapiens (human)|KEGG	-0.46140506	-0.0623727	-1.57603716	0.1480733	0.29344734	-5.18865508
hetero after UVB irradiation VS hetero before UVB irradiation	Mismatch repair - Homo sapiens (human)|KEGG	0.268515779	0.00013215	1.126607326	0.29096861	0.52172892	-5.70657441
WT after UVB irradiation VS WT before UVB irradiation	Mismatch repair - Homo sapiens (human)|KEGG	0.062022084	0.038794761	0.220885336	0.82959075	0.94671188	-5.84449848
homo after UVB irradiation VS homo before UVB irradiation	Mismatch repair (MMR) directed by MSH2:MSH3 (MutSbeta)|Reactome	-0.64077392	-0.07113775	-2.33725411	0.04310116	0.13451804	-4.07273074
hetero after UVB irradiation VS hetero before UVB irradiation	Mismatch repair (MMR) directed by MSH2:MSH3 (MutSbeta)|Reactome	0.163428063	-0.01298963	0.546991786	0.59856863	0.78806349	-6.18712649
WT after UVB irradiation VS WT before UVB irradiation	Mismatch repair (MMR) directed by MSH2:MSH3 (MutSbeta)|Reactome	-0.01892531	0.065336053	-0.05960184	0.95363761	0.98742794	-5.86735191
homo after UVB irradiation VS homo before UVB irradiation	Mismatch repair (MMR) directed by MSH2:MSH6 (MutSalpha)|Reactome	-0.61092847	-0.05997601	-2.22053978	0.0523244	0.15256271	-4.25385397
hetero after UVB irradiation VS hetero before UVB irradiation	Mismatch repair (MMR) directed by MSH2:MSH6 (MutSalpha)|Reactome	0.154370641	-0.03430206	0.539310494	0.60362781	0.79129581	-6.19149545
WT after UVB irradiation VS WT before UVB irradiation	Mismatch repair (MMR) directed by MSH2:MSH6 (MutSalpha)|Reactome	0.100829377	0.081627822	0.326648172	0.75061397	0.91904933	-5.81540412
homo after UVB irradiation VS homo before UVB irradiation	Mismatch Repair|Reactome	-0.6632171	-0.07408082	-2.49466757	0.03314871	0.1213769	-3.82520215
hetero after UVB irradiation VS hetero before UVB irradiation	Mismatch Repair|Reactome	0.151508805	-0.02993862	0.530542604	0.60943041	0.79683988	-6.19641208
WT after UVB irradiation VS WT before UVB irradiation	Mismatch Repair|Reactome	0.027904637	0.066954854	0.090624211	0.92956637	0.97886318	-5.86499247
homo after UVB irradiation VS homo before UVB irradiation	Mitochondrial ABC transporters|Reactome	-0.62514297	-0.09370095	-1.95643643	0.08078103	0.20118642	-4.65292351
hetero after UVB irradiation VS hetero before UVB irradiation	Mitochondrial ABC transporters|Reactome	-0.60679739	-0.03692488	-1.91130347	0.09049381	0.27225794	-4.71026749
WT after UVB irradiation VS WT before UVB irradiation	Mitochondrial ABC transporters|Reactome	-0.82797446	0.036995543	-3.24159589	0.00875411	0.24856491	-2.34690425
homo after UVB irradiation VS homo before UVB irradiation	Mitochondrial Beta-Oxidation of Long Chain Saturated Fatty Acids|SMPDB	-0.45035081	0.007571908	-1.45109906	0.17932781	0.33298527	-5.35082818

hetero after UVB irradiation VS hetero before UVB irradiation	Mitochondrial Beta-Oxidation of Long Chain Saturated Fatty Acids|SMPDB	-0.40158231	0.039403838	-1.40481867	0.19585053	0.4161797	-5.38787416
WT after UVB irradiation VS WT before UVB irradiation	Mitochondrial Beta-Oxidation of Long Chain Saturated Fatty Acids|SMPDB	-0.42515526	-0.00483749	-1.60590896	0.13912563	0.55478524	-4.71686571
homo after UVB irradiation VS homo before UVB irradiation	Mitochondrial Beta-Oxidation of Medium Chain Saturated Fatty Acids|SMPDB	-0.43995595	0.006690227	-1.40849456	0.19122611	0.34703435	-5.40424791
hetero after UVB irradiation VS hetero before UVB irradiation	Mitochondrial Beta-Oxidation of Medium Chain Saturated Fatty Acids|SMPDB	-0.74180988	0.056450044	-2.72624239	0.02478896	0.13846576	-3.48968468
WT after UVB irradiation VS WT before UVB irradiation	Mitochondrial Beta-Oxidation of Medium Chain Saturated Fatty Acids|SMPDB	-0.51590914	0.024650687	-1.79558241	0.10254354	0.50410801	-4.46859626
homo after UVB irradiation VS homo before UVB irradiation	Mitochondrial Beta-Oxidation of Short Chain Saturated Fatty Acids|SMPDB	-0.26160094	-0.00501681	-0.93530424	0.37306159	0.52622775	-5.91963363
hetero after UVB irradiation VS hetero before UVB irradiation	Mitochondrial Beta-Oxidation of Short Chain Saturated Fatty Acids|SMPDB	-0.73349142	0.030699923	-2.99472961	0.01622895	0.11352943	-3.07910018
WT after UVB irradiation VS WT before UVB irradiation	Mitochondrial Beta-Oxidation of Short Chain Saturated Fatty Acids|SMPDB	-0.63617819	-0.14866565	-2.12479626	0.05932076	0.42631804	-4.00950774
homo after UVB irradiation VS homo before UVB irradiation	Mitochondrial biogenesis|Reactome	-0.16554935	-0.01854022	-0.91063016	0.38527162	0.53862529	-5.94201174
hetero after UVB irradiation VS hetero before UVB irradiation	Mitochondrial biogenesis|Reactome	-0.33569633	-0.03820732	-2.03481078	0.07450167	0.24447705	-4.53193573
WT after UVB irradiation VS WT before UVB irradiation	Mitochondrial biogenesis|Reactome	-0.16710832	-0.03604816	-0.77446165	0.45643549	0.80891799	-5.57467258
homo after UVB irradiation VS homo before UVB irradiation	Mitochondrial biogenesis|Wikipathways	-0.51573981	-0.00950793	-3.01503127	0.01392768	0.07305822	-2.99409467
hetero after UVB irradiation VS hetero before UVB irradiation	Mitochondrial biogenesis|Wikipathways	-0.55283914	-0.0211139	-2.40897083	0.04108512	0.17817886	-3.97380479
WT after UVB irradiation VS WT before UVB irradiation	Mitochondrial biogenesis|Wikipathways	-0.37249177	-0.01349186	-1.73820459	0.11256373	0.51456909	-4.5451695
homo after UVB irradiation VS homo before UVB irradiation	Mitochondrial calcium ion transport|Reactome	-0.29050572	0.021041269	-1.26800743	0.23533421	0.39694717	-5.57293159
hetero after UVB irradiation VS hetero before UVB irradiation	Mitochondrial calcium ion transport|Reactome	-0.36923167	-0.03907184	-1.9164447	0.08976718	0.27151728	-4.70292241
WT after UVB irradiation VS WT before UVB irradiation	Mitochondrial calcium ion transport|Reactome	0.120511917	-0.0461435	0.593158168	0.56614259	0.85125516	-5.69418901
homo after UVB irradiation VS homo before UVB irradiation	Mitochondrial complex II deficiency|SMPDB	-0.23678174	0.014544733	-0.76379894	0.46374733	0.6088463	-6.06461144
hetero after UVB irradiation VS hetero before UVB irradiation	Mitochondrial complex II deficiency|SMPDB	-0.47814985	0.004803127	-1.74993278	0.11633994	0.30887493	-4.93678811
WT after UVB irradiation VS WT before UVB irradiation	Mitochondrial complex II deficiency|SMPDB	-0.1568339	-0.12513755	-0.50250429	0.62610623	0.87257466	-5.74292911
homo after UVB irradiation VS homo before UVB irradiation	Mitochondrial DNA depletion syndrome|SMPDB	0.019015587	-0.03943216	0.101997834	0.92090359	0.95153785	-6.36286155
hetero after UVB irradiation VS hetero before UVB irradiation	Mitochondrial DNA depletion syndrome|SMPDB	-0.02085195	-0.0438046	-0.1098564	0.91509485	0.97054702	-6.33999905
WT after UVB irradiation VS WT before UVB irradiation	Mitochondrial DNA depletion syndrome|SMPDB	0.165884344	-0.0622813	0.759490799	0.46494629	0.80891799	-5.58561498
homo after UVB irradiation VS homo before UVB irradiation	Mitochondrial Electron Transport Chain|SMPDB	0.243947809	-0.01055054	0.798161419	0.4444947	0.59189021	-6.03758312
hetero after UVB irradiation VS hetero before UVB irradiation	Mitochondrial Electron Transport Chain|SMPDB	-0.31074783	-0.00170871	-1.2522244	0.24412727	0.46714194	-5.56851377
WT after UVB irradiation VS WT before UVB irradiation	Mitochondrial Electron Transport Chain|SMPDB	0.117626688	-0.08063487	0.357157112	0.72833684	0.9105948	-5.80496628
homo after UVB irradiation VS homo before UVB irradiation	mitochondrial fatty acid beta-oxidation of saturated fatty acids|Reactome	-0.25747959	0.011315255	-1.07816639	0.30787811	0.46923284	-5.78062971
hetero after UVB irradiation VS hetero before UVB irradiation	mitochondrial fatty acid beta-oxidation of saturated fatty acids|Reactome	-0.66042936	-0.00704291	-2.71395564	0.02527702	0.14017854	-3.50849511
WT after UVB irradiation VS WT before UVB irradiation	mitochondrial fatty acid beta-oxidation of saturated fatty acids|Reactome	-0.49502443	0.02846604	-1.70037641	0.11964845	0.52405201	-4.59498193
homo after UVB irradiation VS homo before UVB irradiation	mitochondrial fatty acid beta-oxidation of unsaturated fatty acids|Reactome	-0.17432754	-0.08050127	-0.48593536	0.63815277	0.74924287	-6.24318671
hetero after UVB irradiation VS hetero before UVB irradiation	mitochondrial fatty acid beta-oxidation of unsaturated fatty acids|Reactome	-0.82808433	-0.04677735	-2.98363153	0.01651382	0.11352943	-3.09602886
WT after UVB irradiation VS WT before UVB irradiation	mitochondrial fatty acid beta-oxidation of unsaturated fatty acids|Reactome	-0.29590392	-0.04668436	-0.75429979	0.467921	0.81015326	-5.58936515
homo after UVB irradiation VS homo before UVB irradiation	Mitochondrial Fatty Acid Beta-Oxidation|Reactome	-0.41910893	0.004711961	-1.7614627	0.11061246	0.24418567	-4.93450208
hetero after UVB irradiation VS hetero before UVB irradiation	Mitochondrial Fatty Acid Beta-Oxidation|Reactome	-0.63155791	-0.0179167	-3.50964506	0.00733937	0.0874386	-2.30352407
WT after UVB irradiation VS WT before UVB irradiation	Mitochondrial Fatty Acid Beta-Oxidation|Reactome	-0.41844766	0.027304438	-1.74929723	0.11055963	0.50951142	-4.5304596
homo after UVB irradiation VS homo before UVB irradiation	Mitochondrial fatty acid synthesis and respiration|Wikipathways	-0.07009244	-0.0532236	-0.3827961	0.71039322	0.80920668	-6.29034077
hetero after UVB irradiation VS hetero before UVB irradiation	Mitochondrial fatty acid synthesis and respiration|Wikipathways	0.03186192	-0.04236044	0.182430195	0.85955822	0.94503144	-6.32852324
WT after UVB irradiation VS WT before UVB irradiation	Mitochondrial fatty acid synthesis and respiration|Wikipathways	-0.28733243	-0.07718706	-1.24225644	0.24226022	0.63358831	-5.14594026
homo after UVB irradiation VS homo before UVB irradiation	Mitochondrial fatty acid synthesis pathway|Wikipathways	-0.4809512	-0.08440141	-1.2956612	0.22603712	0.3856931	-5.54067555
hetero after UVB irradiation VS hetero before UVB irradiation	Mitochondrial fatty acid synthesis pathway|Wikipathways	0.006894341	-0.05564981	0.033414972	0.97412173	0.98779265	-6.34593561
WT after UVB irradiation VS WT before UVB irradiation	Mitochondrial fatty acid synthesis pathway|Wikipathways	0.109010348	0.033612985	0.429973759	0.67625979	0.88217037	-5.77640769
homo after UVB irradiation VS homo before UVB irradiation	Mitochondrial Gene Expression|Wikipathways	-0.55153537	-0.00978387	-2.46834181	0.03463837	0.12421508	-3.86681203
hetero after UVB irradiation VS hetero before UVB irradiation	Mitochondrial Gene Expression|Wikipathways	-0.28926727	-0.08545935	-1.20429399	0.26121187	0.48735313	-5.62239577
WT after UVB irradiation VS WT before UVB irradiation	Mitochondrial Gene Expression|Wikipathways	-0.23033735	-0.06768553	-0.90414247	0.38701365	0.76061898	-5.47219692
homo after UVB irradiation VS homo before UVB irradiation	Mitochondrial iron-sulfur cluster biogenesis|Reactome	0.088734755	0.028460748	0.351248676	0.73315517	0.82411885	-6.30260192
hetero after UVB irradiation VS hetero before UVB irradiation	Mitochondrial iron-sulfur cluster biogenesis|Reactome	0.053557077	-0.02656648	0.190389799	0.85351107	0.94503144	-6.3269202
WT after UVB irradiation VS WT before UVB irradiation	Mitochondrial iron-sulfur cluster biogenesis|Reactome	0.15143557	-0.04615418	0.516292444	0.61678356	0.87257466	-5.73600533
homo after UVB irradiation VS homo before UVB irradiation	mitochondrial L-carnitine shuttle|HumanCyc	0.220536452	-0.05583005	0.819165346	0.43299191	0.5840594	-6.02055351
hetero after UVB irradiation VS hetero before UVB irradiation	mitochondrial L-carnitine shuttle|HumanCyc	0.317801301	-0.05598517	1.462029606	0.18000221	0.39822229	-5.31686567
WT after UVB irradiation VS WT before UVB irradiation	mitochondrial L-carnitine shuttle|HumanCyc	-0.20212857	-0.08325025	-0.84376862	0.4183682	0.78467662	-5.5215954
homo after UVB irradiation VS homo before UVB irradiation	Mitochondrial LC-Fatty Acid Beta-Oxidation|Wikipathways	-0.11572842	-0.02186799	-0.41534776	0.68721874	0.78999012	-6.27662097
hetero after UVB irradiation VS hetero before UVB irradiation	Mitochondrial LC-Fatty Acid Beta-Oxidation|Wikipathways	-0.45746677	-0.04532469	-1.7681979	0.11310035	0.30666948	-4.91156996
WT after UVB irradiation VS WT before UVB irradiation	Mitochondrial LC-Fatty Acid Beta-Oxidation|Wikipathways	-0.33826585	0.035187142	-1.24803022	0.24022584	0.63307062	-5.13970209
homo after UVB irradiation VS homo before UVB irradiation	Mitochondrial protein import|Reactome	-0.27316767	-0.01181139	-1.15540185	0.27648096	0.43651869	-5.69912924
hetero after UVB irradiation VS hetero before UVB irradiation	Mitochondrial protein import|Reactome	-0.2400615	-0.04918854	-1.24626644	0.24619922	0.46992296	-5.57528958
WT after UVB irradiation VS WT before UVB irradiation	Mitochondrial protein import|Reactome	-0.12091557	-0.06423997	-0.51898612	0.61497049	0.87257466	-5.73463201
homo after UVB irradiation VS homo before UVB irradiation	Mitochondrial transcription initiation|Reactome	-0.92775745	-0.00345869	-3.66905897	0.00480527	0.03957638	-1.95846424
hetero after UVB irradiation VS hetero before UVB irradiation	Mitochondrial transcription initiation|Reactome	-1.03893763	0.056707151	-4.06847805	0.00322027	0.06341888	-1.49577104
WT after UVB irradiation VS WT before UVB irradiation	Mitochondrial transcription initiation|Reactome	-0.72068758	-0.00498417	-2.55085333	0.02865277	0.35176669	-3.38277746
homo after UVB irradiation VS homo before UVB irradiation	Mitochondrial transcription termination|Reactome	0.214504639	0.015428264	0.511555542	0.62077547	0.73641486	-6.22982018
hetero after UVB irradiation VS hetero before UVB irradiation	Mitochondrial transcription termination|Reactome	0.734695568	-0.02643728	2.197432806	0.05756172	0.21403988	-4.2920365
WT after UVB irradiation VS WT before UVB irradiation	Mitochondrial transcription termination|Reactome	0.466504945	0.062304327	1.214576405	0.25220789	0.64637118	-5.17555914
homo after UVB irradiation VS homo before UVB irradiation	Mitochondrial translation elongation|Reactome	-0.53812492	0.027144687	-2.41038752	0.03815568	0.12857479	-3.95813002
hetero after UVB irradiation VS hetero before UVB irradiation	Mitochondrial translation elongation|Reactome	-0.20306889	-0.06368437	-1.10443702	0.29994118	0.53033405	-5.72985123
WT after UVB irradiation VS WT before UVB irradiation	Mitochondrial translation elongation|Reactome	-0.05404178	-0.04655836	-0.21210034	0.83625525	0.94951669	-5.84641584
homo after UVB irradiation VS homo before UVB irradiation	Mitochondrial translation initiation|Reactome	-0.50760525	0.011812303	-2.34344471	0.04265918	0.13402181	-4.06306025
hetero after UVB irradiation VS hetero before UVB irradiation	Mitochondrial translation initiation|Reactome	-0.23232504	-0.06578236	-1.25789059	0.24217027	0.46543442	-5.56204956
WT after UVB irradiation VS WT before UVB irradiation	Mitochondrial translation initiation|Reactome	-0.06003678	-0.04967432	-0.23840597	0.81634191	0.94525798	-5.84044477
homo after UVB irradiation VS homo before UVB irradiation	Mitochondrial translation termination|Reactome	-0.53117136	0.023766474	-2.43770003	0.03645607	0.12504267	-3.91514495
hetero after UVB irradiation VS hetero before UVB irradiation	Mitochondrial translation termination|Reactome	-0.24558885	-0.06790065	-1.29876205	0.22843887	0.45170905	-5.51484704
WT after UVB irradiation VS WT before UVB irradiation	Mitochondrial translation termination|Reactome	-0.0723666	-0.04721859	-0.28932412	0.77819551	0.93502208	-5.82693359
homo after UVB irradiation VS homo before UVB irradiation	Mitochondrial translation|Reactome	-0.54469524	0.018269806	-2.60697829	0.0274779	0.11028351	-3.64693514
hetero after UVB irradiation VS hetero before UVB irradiation	Mitochondrial translation|Reactome	-0.23566189	-0.06533132	-1.29998835	0.22803721	0.45170905	-5.51341542
WT after UVB irradiation VS WT before UVB irradiation	Mitochondrial translation|Reactome	-0.09670129	-0.04415746	-0.38321476	0.70951664	0.90329752	-5.79533433
homo after UVB irradiation VS homo before UVB irradiation	Mitochondrial tRNA aminoacylation|Reactome	-0.6775513	-0.0595058	-2.7301235	0.02237026	0.09709374	-3.45042487
hetero after UVB irradiation VS hetero before UVB irradiation	Mitochondrial tRNA aminoacylation|Reactome	-0.35940033	-0.08806906	-1.8087592	0.10620486	0.29463284	-4.85516462
WT after UVB irradiation VS WT before UVB irradiation	Mitochondrial tRNA aminoacylation|Reactome	-0.14246179	-0.02486705	-0.49949388	0.62815097	0.87289644	-5.7444172
homo after UVB irradiation VS homo before UVB irradiation	Mitochondrial Uncoupling Proteins|Reactome	-0.39693893	0.063733281	-2.06477594	0.06766347	0.18152256	-4.49132037
hetero after UVB irradiation VS hetero before UVB irradiation	Mitochondrial Uncoupling Proteins|Reactome	-0.30411846	0.102455619	-0.89576362	0.39524774	0.62250836	-5.93132977
WT after UVB irradiation VS WT before UVB irradiation	Mitochondrial Uncoupling Proteins|Reactome	-0.18097992	0.114353525	-0.7619752	0.46352692	0.80891799	-5.58381213
homo after UVB irradiation VS homo before UVB irradiation	Mitophagy - animal - Homo sapiens (human)|KEGG	-0.02922196	-0.03459203	-0.151278	0.882957	0.92936414	-6.35616738
hetero after UVB irradiation VS hetero before UVB irradiation	Mitophagy - animal - Homo sapiens (human)|KEGG	0.010395618	-0.02332146	0.066764349	0.94832643	0.97331049	-6.34412379
WT after UVB irradiation VS WT before UVB irradiation	Mitophagy - animal - Homo sapiens (human)|KEGG	-0.18151428	-0.0196717	-0.92631128	0.37592519	0.75077508	-5.45333944
homo after UVB irradiation VS homo before UVB irradiation	Mitophagy|Reactome	-0.12263971	0.032102622	-0.66510345	0.52198454	0.65944729	-6.13634943
hetero after UVB irradiation VS hetero before UVB irradiation	Mitophagy|Reactome	0.092363282	-0.01174224	0.52461656	0.61336888	0.79900982	-6.19969253
WT after UVB irradiation VS WT before UVB irradiation	Mitophagy|Reactome	-0.33332054	-0.02603906	-1.44021396	0.18012798	0.59611921	-4.92096457
homo after UVB irradiation VS homo before UVB irradiation	Mitotic Anaphase|Reactome	-0.59617304	-0.01660224	-3.24497086	0.00953657	0.05835911	-2.62673782
hetero after UVB irradiation VS hetero before UVB irradiation	Mitotic Anaphase|Reactome	0.116884312	-0.01670073	0.455505512	0.66025504	0.82651398	-6.23538588
WT after UVB irradiation VS WT before UVB irradiation	Mitotic Anaphase|Reactome	-0.01328012	0.006629793	-0.04732319	0.96317993	0.9882819	-5.86801681
homo after UVB irradiation VS homo before UVB irradiation	Mitotic G1-G1/S phases|Reactome	-0.41570522	-0.02083999	-1.9275012	0.08467246	0.20674569	-4.69551068
hetero after UVB irradiation VS hetero before UVB irradiation	Mitotic G1-G1/S phases|Reactome	0.165314082	0.014712924	0.724257679	0.48852703	0.70846522	-6.07073742
WT after UVB irradiation VS WT before UVB irradiation	Mitotic G1-G1/S phases|Reactome	0.031895582	-0.00194206	0.120295613	0.90661248	0.97566251	-5.86182589
homo after UVB irradiation VS homo before UVB irradiation	Mitotic G1-G1-S phases|Wikipathways	-0.57309306	-0.03224272	-1.83386704	0.09850529	0.22736591	-4.83147495
hetero after UVB irradiation VS hetero before UVB irradiation	Mitotic G1-G1-S phases|Wikipathways	0.328217991	0.062556461	1.052917329	0.32163402	0.55300184	-5.78260919
WT after UVB irradiation VS WT before UVB irradiation	Mitotic G1-G1-S phases|Wikipathways	0.341294581	0.045522872	1.072429261	0.3085255	0.69398681	-5.31981883
homo after UVB irradiation VS homo before UVB irradiation	Mitotic G2-G2/M phases|Reactome	-0.54383473	0.012929742	-3.21134641	0.01007695	0.05975501	-2.68030878
hetero after UVB irradiation VS hetero before UVB irradiation	Mitotic G2-G2/M phases|Reactome	-0.00946046	-0.01234537	-0.04118634	0.96810663	0.98540197	-6.34562115
WT after UVB irradiation VS WT before UVB irradiation	Mitotic G2-G2/M phases|Reactome	-0.15960122	-0.02020201	-0.60761345	0.55688116	0.84804143	-5.68572303
homo after UVB irradiation VS homo before UVB irradiation	Mitotic G2-G2-M phases|Wikipathways	-0.88625867	-0.00809681	-3.7229601	0.00441076	0.0379263	-1.87476737
hetero after UVB irradiation VS hetero before UVB irradiation	Mitotic G2-G2-M phases|Wikipathways	0.146041163	0.008076682	0.407066543	0.69410448	0.84591664	-6.25754671
WT after UVB irradiation VS WT before UVB irradiation	Mitotic G2-G2-M phases|Wikipathways	-0.2339185	-0.03831763	-0.62146761	0.54808541	0.84342472	-5.67743256
homo after UVB irradiation VS homo before UVB irradiation	Mitotic Metaphase and Anaphase|Reactome	-0.60122915	-0.01708208	-3.26926119	0.00916489	0.05732449	-2.58807917
hetero after UVB irradiation VS hetero before UVB irradiation	Mitotic Metaphase and Anaphase|Reactome	0.120122603	-0.017062	0.4674093	0.65205752	0.82580666	-6.22957727
WT after UVB irradiation VS WT before UVB irradiation	Mitotic Metaphase and Anaphase|Reactome	-0.01510195	0.007298927	-0.05366819	0.95824807	0.98785634	-5.86769228
homo after UVB irradiation VS homo before UVB irradiation	Mitotic Metaphase/Anaphase Transition|Reactome	-0.81013138	-0.0108853	-3.08277078	0.012452	0.06880589	-2.88566482
hetero after UVB irradiation VS hetero before UVB irradiation	Mitotic Metaphase/Anaphase Transition|Reactome	0.638324798	0.056816528	1.734991655	0.11905389	0.31235808	-4.95733009
WT after UVB irradiation VS WT before UVB irradiation	Mitotic Metaphase/Anaphase Transition|Reactome	-0.05719196	0.068190593	-0.13699165	0.89373444	0.97461442	-5.85965358
homo after UVB irradiation VS homo before UVB irradiation	Mitotic Prometaphase|Reactome	-0.56592146	-0.01712766	-3.02328079	0.01373878	0.07305822	-2.98088365
hetero after UVB irradiation VS hetero before UVB irradiation	Mitotic Prometaphase|Reactome	0.060178154	0.003410094	0.224042229	0.82806179	0.93330999	-6.31939397
WT after UVB irradiation VS WT before UVB irradiation	Mitotic Prometaphase|Reactome	-0.0059881	0.005704758	-0.02195081	0.98291555	0.99445621	-5.8689071
homo after UVB irradiation VS homo before UVB irradiation	Mitotic Prophase|Reactome	-0.44274877	0.008319776	-2.55632103	0.02990473	0.115124	-3.72747915
hetero after UVB irradiation VS hetero before UVB irradiation	Mitotic Prophase|Reactome	-0.05773643	-0.03588588	-0.26305654	0.79882552	0.91506577	-6.30915917
WT after UVB irradiation VS WT before UVB irradiation	Mitotic Prophase|Reactome	-0.09826777	-0.02599485	-0.39276875	0.70266638	0.90059561	-5.79163814
homo after UVB irradiation VS homo before UVB irradiation	Mitotic Spindle Checkpoint|Reactome	-0.59278516	-0.01406767	-3.23932813	0.00962512	0.05841986	-2.63572341
hetero after UVB irradiation VS hetero before UVB irradiation	Mitotic Spindle Checkpoint|Reactome	0.145518908	-0.01714008	0.528926126	0.61050341	0.7972333	-6.19731032
WT after UVB irradiation VS WT before UVB irradiation	Mitotic Spindle Checkpoint|Reactome	0.033049873	0.01562636	0.116152023	0.90981308	0.97566251	-5.86232148
homo after UVB irradiation VS homo before UVB irradiation	Mitotic Telophase/Cytokinesis|Reactome	-0.97241276	-0.01036617	-5.18108158	0.00050507	0.01666204	0.235103845
hetero after UVB irradiation VS hetero before UVB irradiation	Mitotic Telophase/Cytokinesis|Reactome	-0.5078528	-0.08113135	-1.96732149	0.08287028	0.2599606	-4.62985615
WT after UVB irradiation VS WT before UVB irradiation	Mitotic Telophase/Cytokinesis|Reactome	-0.46294748	-0.05393565	-1.36186385	0.20289278	0.61688026	-5.0126529
homo after UVB irradiation VS homo before UVB irradiation	MNGIE (Mitochondrial Neurogastrointestinal Encephalopathy)|SMPDB	-0.15230025	-0.02916717	-0.66373722	0.52282067	0.65944729	-6.13727985
hetero after UVB irradiation VS hetero before UVB irradiation	MNGIE (Mitochondrial Neurogastrointestinal Encephalopathy)|SMPDB	-0.07293889	-0.06090355	-0.36429729	0.72460928	0.86482318	-6.27512326
WT after UVB irradiation VS WT before UVB irradiation	MNGIE (Mitochondrial Neurogastrointestinal Encephalopathy)|SMPDB	0.197121499	-0.04726474	0.926444646	0.37585917	0.75077508	-5.45322485
homo after UVB irradiation VS homo before UVB irradiation	Model for regulation of MSMP expression in cancer cells and its proangiogenic role in ovarian tumors|Wikipathways	0.311472396	-0.00650332	1.31915753	0.21837957	0.37669857	-5.51289433
hetero after UVB irradiation VS hetero before UVB irradiation	Model for regulation of MSMP expression in cancer cells and its proangiogenic role in ovarian tumors|Wikipathways	0.459092032	0.080992174	1.401679714	0.1967542	0.417313	-5.39172092
WT after UVB irradiation VS WT before UVB irradiation	Model for regulation of MSMP expression in cancer cells and its proangiogenic role in ovarian tumors|Wikipathways	0.392197258	0.089853462	1.551927979	0.15147899	0.55727585	-4.78478485
homo after UVB irradiation VS homo before UVB irradiation	Moexipril Action Pathway|SMPDB	0.636038586	-0.11800708	2.436891056	0.03650532	0.12504267	-3.91641947
hetero after UVB irradiation VS hetero before UVB irradiation	Moexipril Action Pathway|SMPDB	-0.02777369	0.003323189	-0.09172332	0.92906122	0.97054702	-6.34197953
WT after UVB irradiation VS WT before UVB irradiation	Moexipril Action Pathway|SMPDB	0.270438532	-0.10210744	0.707878218	0.49505953	0.81710765	-5.62188349
homo after UVB irradiation VS homo before UVB irradiation	Moexipril Metabolism Pathway|SMPDB	1.076747846	-0.04193589	4.959558579	0.00068841	0.01666204	-0.06417629
hetero after UVB irradiation VS hetero before UVB irradiation	Moexipril Metabolism Pathway|SMPDB	0.553630209	0.003105397	1.56066683	0.15532075	0.36486693	-5.19066683
WT after UVB irradiation VS WT before UVB irradiation	Moexipril Metabolism Pathway|SMPDB	0.585930211	-0.05239369	1.72512433	0.11496929	0.51527453	-4.56245573
homo after UVB irradiation VS homo before UVB irradiation	Molecules associated with elastic fibres|Reactome	0.485802308	-0.02429537	2.555139753	0.0299638	0.115124	-3.72935483
hetero after UVB irradiation VS hetero before UVB irradiation	Molecules associated with elastic fibres|Reactome	0.359551009	0.036914661	1.497746931	0.17068945	0.3872586	-5.271701
WT after UVB irradiation VS WT before UVB irradiation	Molecules associated with elastic fibres|Reactome	0.307518653	-0.03024879	1.310557291	0.2190751	0.6260768	-5.07085429
homo after UVB irradiation VS homo before UVB irradiation	molybdenum cofactor biosynthesis|HumanCyc	-0.29464483	-0.05189167	-1.00546034	0.33988266	0.49581604	-5.85334277
hetero after UVB irradiation VS hetero before UVB irradiation	molybdenum cofactor biosynthesis|HumanCyc	-0.14041155	-0.11345486	-0.32163476	0.75556385	0.88626689	-6.29077266
WT after UVB irradiation VS WT before UVB irradiation	molybdenum cofactor biosynthesis|HumanCyc	-0.37473077	-0.02168993	-1.22935411	0.24685684	0.63911291	-5.15980582
homo after UVB irradiation VS homo before UVB irradiation	Molybdenum cofactor biosynthesis|Reactome	-0.44478902	0.029991503	-1.98055922	0.07766654	0.19626432	-4.61722692
hetero after UVB irradiation VS hetero before UVB irradiation	Molybdenum cofactor biosynthesis|Reactome	0.049543236	-0.08826711	0.146769391	0.88676519	0.9605218	-6.33487061
WT after UVB irradiation VS WT before UVB irradiation	Molybdenum cofactor biosynthesis|Reactome	-0.32558212	-0.0210821	-1.23952618	0.24322708	0.63440287	-5.14888296
homo after UVB irradiation VS homo before UVB irradiation	Molybdenum Cofactor Deficiency|SMPDB	0.019015587	-0.03943216	0.101997834	0.92090359	0.95153785	-6.36286155
hetero after UVB irradiation VS hetero before UVB irradiation	Molybdenum Cofactor Deficiency|SMPDB	-0.02085195	-0.0438046	-0.1098564	0.91509485	0.97054702	-6.33999905
WT after UVB irradiation VS WT before UVB irradiation	Molybdenum Cofactor Deficiency|SMPDB	0.165884344	-0.0622813	0.759490799	0.46494629	0.80891799	-5.58561498
homo after UVB irradiation VS homo before UVB irradiation	Monoamine GPCRs|Wikipathways	0.336676684	-0.03577079	1.795639702	0.10473579	0.2370387	-4.88611257
hetero after UVB irradiation VS hetero before UVB irradiation	Monoamine GPCRs|Wikipathways	-0.03189003	0.011285896	-0.1588661	0.87751642	0.9555609	-6.33287063
WT after UVB irradiation VS WT before UVB irradiation	Monoamine GPCRs|Wikipathways	0.202653169	0.031037599	0.736535407	0.47819259	0.81552033	-5.60202625
homo after UVB irradiation VS homo before UVB irradiation	Monoamine oxidase-a deficiency (MAO-A)|SMPDB	0.031995945	0.027270645	0.129408983	0.89976401	0.9399608	-6.35945841
hetero after UVB irradiation VS hetero before UVB irradiation	Monoamine oxidase-a deficiency (MAO-A)|SMPDB	-0.38264835	0.006355022	-1.73471603	0.11910451	0.31235808	-4.9577083

WT after UVB irradiation VS WT before UVB irradiation	Monoamine oxidase-a deficiency (MAO-A)|SMPDB	0.126407683	0.040850924	0.512443468	0.61937892	0.87257466	-5.73795593
homo after UVB irradiation VS homo before UVB irradiation	Monoamine Transport|Wikipathways	0.215381481	0.054620445	1.275084131	0.23292546	0.39463363	-5.56472309
hetero after UVB irradiation VS hetero before UVB irradiation	Monoamine Transport|Wikipathways	0.143077731	0.028929304	0.783954193	0.45453827	0.67919377	-6.02507886
WT after UVB irradiation VS WT before UVB irradiation	Monoamine Transport|Wikipathways	0.152054663	-0.00163801	0.63335392	0.5406026	0.83916339	-5.67018268
homo after UVB irradiation VS homo before UVB irradiation	Mono-unsaturated fatty acid beta-oxidation|EHMN	-0.27818304	-0.01682094	-0.98099677	0.35119446	0.50681223	-5.87689946
hetero after UVB irradiation VS hetero before UVB irradiation	Mono-unsaturated fatty acid beta-oxidation|EHMN	-0.49038911	-0.03466577	-2.37376955	0.04345872	0.18293959	-4.02712294
WT after UVB irradiation VS WT before UVB irradiation	Mono-unsaturated fatty acid beta-oxidation|EHMN	-0.36877304	-0.03402597	-1.47216126	0.17149249	0.57997722	-4.8826514
homo after UVB irradiation VS homo before UVB irradiation	Morphine Action Pathway|SMPDB	-0.00479399	0.053934027	-0.02653378	0.97938702	0.98608184	-6.36806999
hetero after UVB irradiation VS hetero before UVB irradiation	Morphine Action Pathway|SMPDB	-0.00659826	0.064042146	-0.04266653	0.96696118	0.98467584	-6.34555383
WT after UVB irradiation VS WT before UVB irradiation	Morphine Action Pathway|SMPDB	0.081145447	0.018536671	0.380563507	0.71142245	0.90420468	-5.7963444
homo after UVB irradiation VS homo before UVB irradiation	Morphine addiction - Homo sapiens (human)|KEGG	0.387236943	0.000153243	2.335647502	0.0432166	0.13470334	-4.0752395
hetero after UVB irradiation VS hetero before UVB irradiation	Morphine addiction - Homo sapiens (human)|KEGG	0.256176559	0.036979678	1.377010808	0.20398263	0.42473862	-5.42176894
WT after UVB irradiation VS WT before UVB irradiation	Morphine addiction - Homo sapiens (human)|KEGG	0.05333354	-0.02910457	0.204240286	0.84222962	0.95185911	-5.84806601
homo after UVB irradiation VS homo before UVB irradiation	Morphine Metabolism Pathway|SMPDB	-0.36112121	0.048770879	-1.54118819	0.15626751	0.30417351	-5.23468226
hetero after UVB irradiation VS hetero before UVB irradiation	Morphine Metabolism Pathway|SMPDB	0.176433322	0.010159222	0.693553294	0.50662804	0.72525459	-6.09298416
WT after UVB irradiation VS WT before UVB irradiation	Morphine Metabolism Pathway|SMPDB	0.250390302	0.036429895	0.807630948	0.43794222	0.79685269	-5.54976314
homo after UVB irradiation VS homo before UVB irradiation	mRNA 3_-end processing|Reactome	-0.8520644	0.001777339	-4.79759609	0.00086722	0.01759873	-0.28803007
hetero after UVB irradiation VS hetero before UVB irradiation	mRNA 3_-end processing|Reactome	-0.3435226	-0.05403506	-1.13581007	0.28730749	0.51699469	-5.69681321
WT after UVB irradiation VS WT before UVB irradiation	mRNA 3_-end processing|Reactome	-0.48211891	-0.06897623	-1.55897868	0.14981243	0.55622203	-4.77598872
homo after UVB irradiation VS homo before UVB irradiation	mRNA capping|HumanCyc	-0.56132334	-0.04779869	-2.87740354	0.01750182	0.08340524	-3.21461501
hetero after UVB irradiation VS hetero before UVB irradiation	mRNA capping|HumanCyc	-0.05932733	0.022997855	-0.16283582	0.87448554	0.95379399	-6.33218001
WT after UVB irradiation VS WT before UVB irradiation	mRNA capping|HumanCyc	-0.38046819	0.027401412	-0.91972492	0.37919574	0.75122805	-5.45898173
homo after UVB irradiation VS homo before UVB irradiation	mRNA Capping|Reactome	-0.75324357	0.026390518	-4.10319207	0.00243582	0.02808576	-1.29444598
hetero after UVB irradiation VS hetero before UVB irradiation	mRNA Capping|Reactome	-0.36856365	-0.07433623	-1.75664996	0.11513864	0.30887493	-4.92752732
WT after UVB irradiation VS WT before UVB irradiation	mRNA Capping|Reactome	-0.25342483	0.012187229	-0.78255412	0.45187733	0.80680448	-5.56867957
homo after UVB irradiation VS homo before UVB irradiation	mRNA decay by 3_ to 5_ exoribonuclease|Reactome	-0.81835499	0.01090385	-4.79821634	0.00086645	0.01759873	-0.28716473
hetero after UVB irradiation VS hetero before UVB irradiation	mRNA decay by 3_ to 5_ exoribonuclease|Reactome	-0.36421632	-0.04526558	-1.27542963	0.23619537	0.4587041	-5.54191646
WT after UVB irradiation VS WT before UVB irradiation	mRNA decay by 3_ to 5_ exoribonuclease|Reactome	-0.13384271	0.075008871	-0.38886548	0.70546172	0.90126444	-5.79315885
homo after UVB irradiation VS homo before UVB irradiation	mRNA decay by 5_ to 3_ exoribonuclease|Reactome	-0.41761116	-0.02634165	-2.61936705	0.02691502	0.10904525	-3.62720743
hetero after UVB irradiation VS hetero before UVB irradiation	mRNA decay by 5_ to 3_ exoribonuclease|Reactome	-0.08576589	-0.02222706	-0.34585154	0.73793147	0.87250107	-6.28212155
WT after UVB irradiation VS WT before UVB irradiation	mRNA decay by 5_ to 3_ exoribonuclease|Reactome	-0.10850441	0.01126046	-0.38074099	0.7112948	0.90420468	-5.79627699
homo after UVB irradiation VS homo before UVB irradiation	mRNA Editing: A to I Conversion|Reactome	0.544034782	-0.07960566	1.637930322	0.1344592	0.277359	-5.10549214
hetero after UVB irradiation VS hetero before UVB irradiation	mRNA Editing: A to I Conversion|Reactome	0.660054131	0.017061716	2.04785425	0.07298105	0.24158558	-4.51288957
WT after UVB irradiation VS WT before UVB irradiation	mRNA Editing: A to I Conversion|Reactome	0.295358316	-0.12669247	1.110293303	0.29265056	0.67947895	-5.28271793
homo after UVB irradiation VS homo before UVB irradiation	mRNA Editing: C to U Conversion|Reactome	0.733981786	0.027234617	3.66137419	0.00486445	0.03966267	-1.97042322
hetero after UVB irradiation VS hetero before UVB irradiation	mRNA Editing: C to U Conversion|Reactome	0.518011488	0.091761844	2.20108417	0.05722828	0.21310989	-4.28659513
WT after UVB irradiation VS WT before UVB irradiation	mRNA Editing: C to U Conversion|Reactome	0.748592347	0.097083739	2.838560665	0.01745812	0.30353778	-2.95075132
homo after UVB irradiation VS homo before UVB irradiation	mRNA Editing|Reactome	0.635155158	-0.00033981	3.838154668	0.00367729	0.03413771	-1.69702185
hetero after UVB irradiation VS hetero before UVB irradiation	mRNA Editing|Reactome	0.516722916	0.054655796	2.835629852	0.02084683	0.12723297	-3.32222479
WT after UVB irradiation VS WT before UVB irradiation	mRNA Editing|Reactome	0.531575705	-0.0086643	2.37494969	0.03875568	0.39022568	-3.64445016
homo after UVB irradiation VS homo before UVB irradiation	mRNA Processing|Wikipathways	-0.81348513	0.000397601	-4.77655631	0.00089387	0.01782281	-0.3174202
hetero after UVB irradiation VS hetero before UVB irradiation	mRNA Processing|Wikipathways	-0.28870679	-0.08412771	-1.09985938	0.30182081	0.53218539	-5.73461488
WT after UVB irradiation VS WT before UVB irradiation	mRNA Processing|Wikipathways	-0.35868152	-0.02795556	-1.16121631	0.27230834	0.66431878	-5.23128332
homo after UVB irradiation VS homo before UVB irradiation	mRNA Splicing - Major Pathway|Reactome	-0.83668039	0.003855495	-5.01590017	0.00063585	0.01666204	0.012699237
hetero after UVB irradiation VS hetero before UVB irradiation	mRNA Splicing - Major Pathway|Reactome	-0.37299186	-0.08148403	-1.52611079	0.16360093	0.37677831	-5.23539822
WT after UVB irradiation VS WT before UVB irradiation	mRNA Splicing - Major Pathway|Reactome	-0.32875729	-0.02926736	-1.08173874	0.30456254	0.69063413	-5.31078912
homo after UVB irradiation VS homo before UVB irradiation	mRNA Splicing - Minor Pathway|Reactome	-0.70668116	-0.00499197	-3.88897846	0.00339557	0.03277835	-1.61911355
hetero after UVB irradiation VS hetero before UVB irradiation	mRNA Splicing - Minor Pathway|Reactome	-0.3678271	-0.07338054	-1.63584974	0.13859583	0.33872374	-5.09153552
WT after UVB irradiation VS WT before UVB irradiation	mRNA Splicing - Minor Pathway|Reactome	-0.24511496	0.013183142	-0.737573	0.47758875	0.81538237	-5.6012941
homo after UVB irradiation VS homo before UVB irradiation	mRNA Splicing|Reactome	-0.82153214	0.000915972	-4.90772103	0.00074091	0.01666498	-0.13536126
hetero after UVB irradiation VS hetero before UVB irradiation	mRNA Splicing|Reactome	-0.38065074	-0.0843089	-1.58312102	0.1501441	0.35774479	-5.16131409
WT after UVB irradiation VS WT before UVB irradiation	mRNA Splicing|Reactome	-0.33617323	-0.02699083	-1.10678222	0.29409555	0.68075741	-5.28619981
homo after UVB irradiation VS homo before UVB irradiation	mRNA surveillance pathway - Homo sapiens (human)|KEGG	-0.57174961	0.006587219	-3.49197377	0.00638255	0.04648301	-2.23559109
hetero after UVB irradiation VS hetero before UVB irradiation	mRNA surveillance pathway - Homo sapiens (human)|KEGG	-0.24017619	-0.05435901	-1.11574344	0.29533832	0.52649833	-5.71802292
WT after UVB irradiation VS WT before UVB irradiation	mRNA surveillance pathway - Homo sapiens (human)|KEGG	-0.38450892	-0.06332529	-1.42518182	0.18431859	0.59839062	-4.93880991
homo after UVB irradiation VS homo before UVB irradiation	mRNA_ protein_ and metabolite inducation pathway by cyclosporin A|Wikipathways	-0.46112801	-0.00251436	-2.61797109	0.02697787	0.10920296	-3.62943089
hetero after UVB irradiation VS hetero before UVB irradiation	mRNA_ protein_ and metabolite inducation pathway by cyclosporin A|Wikipathways	-0.30173623	-0.06799137	-1.71997146	0.12184129	0.31375853	-4.97790029
WT after UVB irradiation VS WT before UVB irradiation	mRNA_ protein_ and metabolite inducation pathway by cyclosporin A|Wikipathways	-0.24506383	0.020556154	-1.03418634	0.32521791	0.70929809	-5.35627096
homo after UVB irradiation VS homo before UVB irradiation	Mtb iron assimilation by chelation|Reactome	1.019010934	-0.02342197	4.307824036	0.00178393	0.02356086	-0.99027397
hetero after UVB irradiation VS hetero before UVB irradiation	Mtb iron assimilation by chelation|Reactome	1.044821233	0.05989276	4.854999212	0.00108958	0.04104387	-0.43899655
WT after UVB irradiation VS WT before UVB irradiation	Mtb iron assimilation by chelation|Reactome	0.459621405	0.016400241	1.150166408	0.27662526	0.66649013	-5.24259131
homo after UVB irradiation VS homo before UVB irradiation	MTF1 activates gene expression|Reactome	0.05251671	0.062497611	0.174913978	0.86486116	0.91750838	-6.35203692
hetero after UVB irradiation VS hetero before UVB irradiation	MTF1 activates gene expression|Reactome	0.476492888	0.05632829	1.696368358	0.12634363	0.31934829	-5.01005777
WT after UVB irradiation VS WT before UVB irradiation	MTF1 activates gene expression|Reactome	-0.39494132	0.090456807	-1.7395119	0.11232583	0.51412987	-4.54343826
homo after UVB irradiation VS homo before UVB irradiation	MTF1 activates gene expression|Wikipathways	0.564264832	0.006933079	1.890006716	0.08997765	0.21498584	-4.75030474
hetero after UVB irradiation VS hetero before UVB irradiation	MTF1 activates gene expression|Wikipathways	0.294039404	0.020849211	0.857002372	0.41515117	0.64192892	-5.96501405
WT after UVB irradiation VS WT before UVB irradiation	MTF1 activates gene expression|Wikipathways	-0.2773607	0.124940384	-0.8178766	0.43233161	0.79685269	-5.54188529
homo after UVB irradiation VS homo before UVB irradiation	MTHFR deficiency|Wikipathways	0.454017156	-0.05853586	1.610307403	0.14039018	0.2835124	-5.14282632
hetero after UVB irradiation VS hetero before UVB irradiation	MTHFR deficiency|Wikipathways	0.658352365	0.035533617	2.909573617	0.018552	0.1199433	-3.20912645
WT after UVB irradiation VS WT before UVB irradiation	MTHFR deficiency|Wikipathways	0.579561157	-0.06861055	2.649795556	0.02416505	0.32984203	-3.23453482
homo after UVB irradiation VS homo before UVB irradiation	mTOR signaling pathway - Homo sapiens (human)|KEGG	-0.05095055	-0.01258499	-0.29560596	0.77396442	0.85410231	-6.32172026
hetero after UVB irradiation VS hetero before UVB irradiation	mTOR signaling pathway - Homo sapiens (human)|KEGG	-0.02227251	-0.01324759	-0.14156235	0.89075207	0.96325071	-6.33568303
WT after UVB irradiation VS WT before UVB irradiation	mTOR signaling pathway - Homo sapiens (human)|KEGG	-0.04315871	0.01771767	-0.20178856	0.84409533	0.95302431	-5.84856809
homo after UVB irradiation VS homo before UVB irradiation	mtor signaling pathway|BioCarta	-0.17694122	0.00771204	-0.95809082	0.36203598	0.51690316	-5.89853031
hetero after UVB irradiation VS hetero before UVB irradiation	mtor signaling pathway|BioCarta	-0.10708647	-0.0395208	-0.62087189	0.55110555	0.75638033	-6.14220095
WT after UVB irradiation VS WT before UVB irradiation	mtor signaling pathway|BioCarta	-0.23422843	-0.05476643	-1.12074298	0.28838255	0.67436922	-5.27230547
homo after UVB irradiation VS homo before UVB irradiation	mTOR signaling pathway|PID	0.050833659	-0.01153166	0.265258846	0.79654225	0.87260362	-6.33078718
hetero after UVB irradiation VS hetero before UVB irradiation	mTOR signaling pathway|PID	0.177696797	-0.01788296	0.995954467	0.34701024	0.5755723	-5.83869676
WT after UVB irradiation VS WT before UVB irradiation	mTOR signaling pathway|PID	-0.01390902	0.009568802	-0.06113602	0.95244583	0.98724923	-5.86725811
homo after UVB irradiation VS homo before UVB irradiation	mTOR signalling|Reactome	0.213690379	-0.04583087	1.29040633	0.22777995	0.38735877	-5.54684194
hetero after UVB irradiation VS hetero before UVB irradiation	mTOR signalling|Reactome	0.173138813	-0.0136429	0.978927831	0.35488234	0.58269226	-5.85498971
WT after UVB irradiation VS WT before UVB irradiation	mTOR signalling|Reactome	0.074127879	-0.01751418	0.389063212	0.70532	0.90126444	-5.79308217
homo after UVB irradiation VS homo before UVB irradiation	mTORC1-mediated signalling|Reactome	0.145628647	-0.03066571	0.78206935	0.45344393	0.60047129	-6.05037
hetero after UVB irradiation VS hetero before UVB irradiation	mTORC1-mediated signalling|Reactome	0.194169303	-0.00436169	0.943332218	0.37176937	0.60046946	-5.88833099
WT after UVB irradiation VS WT before UVB irradiation	mTORC1-mediated signalling|Reactome	0.13887042	0.015004376	0.649765131	0.5303688	0.83380183	-5.65996613
homo after UVB irradiation VS homo before UVB irradiation	mucin core 1 and core 2 <i>O</i>-glycosylation|HumanCyc	0.181133764	-0.01219929	1.047469553	0.32109752	0.47694577	-5.81181551
hetero after UVB irradiation VS hetero before UVB irradiation	mucin core 1 and core 2 <i>O</i>-glycosylation|HumanCyc	0.258366403	-0.00656805	1.32256969	0.22074702	0.44227029	-5.48689633
WT after UVB irradiation VS WT before UVB irradiation	mucin core 1 and core 2 <i>O</i>-glycosylation|HumanCyc	0.358673603	-0.0195673	1.630923761	0.13371015	0.54709793	-4.68495489
homo after UVB irradiation VS homo before UVB irradiation	Mucin type O-glycan biosynthesis - Homo sapiens (human)|KEGG	0.130059508	-0.01105163	0.724115225	0.48664484	0.62911107	-6.09451925
hetero after UVB irradiation VS hetero before UVB irradiation	Mucin type O-glycan biosynthesis - Homo sapiens (human)|KEGG	0.139895026	-0.02056457	0.688921028	0.50939486	0.72852895	-6.09626626
WT after UVB irradiation VS WT before UVB irradiation	Mucin type O-glycan biosynthesis - Homo sapiens (human)|KEGG	0.232270249	-0.00878581	1.040233082	0.32253424	0.70648859	-5.3505767
homo after UVB irradiation VS homo before UVB irradiation	Mucopolysaccharidosis VI. Sly syndrome|SMPDB	0.768442354	-0.02313676	4.373424779	0.00161642	0.02229782	-0.89406544
hetero after UVB irradiation VS hetero before UVB irradiation	Mucopolysaccharidosis VI. Sly syndrome|SMPDB	0.409967917	0.043436941	1.396269407	0.19832025	0.41868045	-5.39833891
WT after UVB irradiation VS WT before UVB irradiation	Mucopolysaccharidosis VI. Sly syndrome|SMPDB	0.202660553	-0.06041924	0.567608804	0.58271843	0.85958498	-5.7086892
homo after UVB irradiation VS homo before UVB irradiation	multi-drug resistance factors|BioCarta	0.215690567	0.033773855	0.784012068	0.45235727	0.6002769	-6.04883836
hetero after UVB irradiation VS hetero before UVB irradiation	multi-drug resistance factors|BioCarta	0.270383803	0.020137154	1.155343113	0.2796589	0.51023746	-5.67590471
WT after UVB irradiation VS WT before UVB irradiation	multi-drug resistance factors|BioCarta	0.602702426	0.041582689	1.749690182	0.11048923	0.50951142	-4.52993768
homo after UVB irradiation VS homo before UVB irradiation	Multifunctional anion exchangers|Reactome	0.265741281	-0.02172226	1.401236498	0.19331927	0.34903982	-5.41324758
hetero after UVB irradiation VS hetero before UVB irradiation	Multifunctional anion exchangers|Reactome	-0.00023064	-0.04497835	-0.00089775	0.99930459	0.99974327	-6.34654086
WT after UVB irradiation VS WT before UVB irradiation	Multifunctional anion exchangers|Reactome	-0.16769728	-0.03670329	-0.6673576	0.51952556	0.82992425	-5.64874953
homo after UVB irradiation VS homo before UVB irradiation	multiple antiapoptotic pathways from igf-1r signaling lead to bad phosphorylation|BioCarta	0.476465956	-0.08707326	2.647152274	0.0256943	0.10578915	-3.58292456
hetero after UVB irradiation VS hetero before UVB irradiation	multiple antiapoptotic pathways from igf-1r signaling lead to bad phosphorylation|BioCarta	0.57950331	0.005349386	3.268742827	0.01060037	0.10415756	-2.6635343
WT after UVB irradiation VS WT before UVB irradiation	multiple antiapoptotic pathways from igf-1r signaling lead to bad phosphorylation|BioCarta	0.478869029	-0.04322975	2.404926273	0.03681547	0.39022568	-3.60008047
homo after UVB irradiation VS homo before UVB irradiation	Multiple carboxylase deficiency_ neonatal or early onset form|SMPDB	0.526884498	-0.07424403	2.530982847	0.03119777	0.11752935	-3.76768491
hetero after UVB irradiation VS hetero before UVB irradiation	Multiple carboxylase deficiency_ neonatal or early onset form|SMPDB	0.118669319	-0.00962251	0.574549436	0.58060805	0.77397513	-6.17098126
WT after UVB irradiation VS WT before UVB irradiation	Multiple carboxylase deficiency_ neonatal or early onset form|SMPDB	0.295241913	-0.10880856	0.874075054	0.40241759	0.77020249	-5.49715984
homo after UVB irradiation VS homo before UVB irradiation	multi-step regulation of transcription by pitx2|BioCarta	-0.61348575	0.030108551	-3.94409376	0.00311559	0.03154271	-1.53499752
hetero after UVB irradiation VS hetero before UVB irradiation	multi-step regulation of transcription by pitx2|BioCarta	-0.41989912	0.007142634	-2.12510728	0.06457294	0.22833471	-4.39935248
WT after UVB irradiation VS WT before UVB irradiation	multi-step regulation of transcription by pitx2|BioCarta	-0.43598745	0.01342362	-1.75613984	0.10933963	0.5076251	-4.52136289
homo after UVB irradiation VS homo before UVB irradiation	Muscarinic acetylcholine receptors|Reactome	-0.28867376	0.012696759	-1.28038247	0.23113539	0.3923287	-5.5585566
hetero after UVB irradiation VS hetero before UVB irradiation	Muscarinic acetylcholine receptors|Reactome	-0.39221907	0.002118027	-1.60907179	0.14435575	0.34776612	-5.12711826
WT after UVB irradiation VS WT before UVB irradiation	Muscarinic acetylcholine receptors|Reactome	-0.47987569	0.021476962	-1.6762528	0.12437435	0.52904005	-4.62645594
homo after UVB irradiation VS homo before UVB irradiation	Muscle contraction|Reactome	0.265580306	-0.01244037	1.473960637	0.17321125	0.32479777	-5.32175545
hetero after UVB irradiation VS hetero before UVB irradiation	Muscle contraction|Reactome	-0.09311933	0.008167853	-0.46324557	0.65491927	0.82651398	-6.23162519
WT after UVB irradiation VS WT before UVB irradiation	Muscle contraction|Reactome	0.104516823	-0.0125922	0.414531295	0.68716708	0.89156024	-5.78289051
homo after UVB irradiation VS homo before UVB irradiation	Muscle/Heart Contraction|SMPDB	0.396415397	-0.03677376	2.343069613	0.04268583	0.13402181	-4.06364635
hetero after UVB irradiation VS hetero before UVB irradiation	Muscle/Heart Contraction|SMPDB	-0.01555055	-0.01775524	-0.07410913	0.94265284	0.97054702	-6.34356282
WT after UVB irradiation VS WT before UVB irradiation	Muscle/Heart Contraction|SMPDB	0.056685205	-0.03978418	0.223404833	0.82768199	0.94525798	-5.84393438
homo after UVB irradiation VS homo before UVB irradiation	Mycophenolic Acid Metabolism Pathway|SMPDB	0.071992463	0.136287106	0.406875293	0.69321884	0.79464283	-6.28029589
hetero after UVB irradiation VS hetero before UVB irradiation	Mycophenolic Acid Metabolism Pathway|SMPDB	-0.11179642	0.061085948	-0.62771437	0.54682257	0.75322371	-6.13777724
WT after UVB irradiation VS WT before UVB irradiation	Mycophenolic Acid Metabolism Pathway|SMPDB	0.019836415	0.091086724	0.070434079	0.94522568	0.98295421	-5.86663866
homo after UVB irradiation VS homo before UVB irradiation	Mycophenolic acid Pathway_ Pharmacokinetics/Pharmacodynamics|PharmGKB	-0.02990765	0.130416684	-0.14024326	0.89143042	0.93563297	-6.3578918
hetero after UVB irradiation VS hetero before UVB irradiation	Mycophenolic acid Pathway_ Pharmacokinetics/Pharmacodynamics|PharmGKB	-0.65187727	0.035875471	-3.63086864	0.00611638	0.0822456	-2.12479174
WT after UVB irradiation VS WT before UVB irradiation	Mycophenolic acid Pathway_ Pharmacokinetics/Pharmacodynamics|PharmGKB	-0.20759471	0.133324886	-0.87229945	0.40334038	0.77093812	-5.4986117
homo after UVB irradiation VS homo before UVB irradiation	Mycophenolic acid Pathway_ Pharmacokinetics|PharmGKB	0.052607217	0.141328368	0.292666242	0.77614219	0.8547673	-6.32264072
hetero after UVB irradiation VS hetero before UVB irradiation	Mycophenolic acid Pathway_ Pharmacokinetics|PharmGKB	-0.11179642	0.061085948	-0.62771437	0.54682257	0.75322371	-6.13777724
WT after UVB irradiation VS WT before UVB irradiation	Mycophenolic acid Pathway_ Pharmacokinetics|PharmGKB	0.019836415	0.091086724	0.070434079	0.94522568	0.98295421	-5.86663866
homo after UVB irradiation VS homo before UVB irradiation	MyD88 cascade initiated on plasma membrane|Reactome	0.124432945	-0.01092111	0.706090574	0.49727702	0.63711097	-6.10763436
hetero after UVB irradiation VS hetero before UVB irradiation	MyD88 cascade initiated on plasma membrane|Reactome	0.319792807	-0.00723436	1.994764204	0.07936376	0.25322957	-4.59017043
WT after UVB irradiation VS WT before UVB irradiation	MyD88 cascade initiated on plasma membrane|Reactome	-0.00861276	-0.00116419	-0.04467189	0.96524124	0.98922368	-5.86814035
homo after UVB irradiation VS homo before UVB irradiation	MyD88 dependent cascade initiated on endosome|Reactome	0.150388964	-0.0212724	0.857597139	0.41246975	0.56380634	-5.98841085
hetero after UVB irradiation VS hetero before UVB irradiation	MyD88 dependent cascade initiated on endosome|Reactome	0.356267365	0.000893068	2.224567353	0.05512872	0.20875475	-4.25154973
WT after UVB irradiation VS WT before UVB irradiation	MyD88 dependent cascade initiated on endosome|Reactome	0.018925204	0.002605536	0.097553812	0.92419873	0.97566251	-5.8643325
homo after UVB irradiation VS homo before UVB irradiation	MyD88:Mal cascade initiated on plasma membrane|Reactome	0.245026221	-0.0159036	1.415841049	0.18912726	0.34500802	-5.39510836
hetero after UVB irradiation VS hetero before UVB irradiation	MyD88:Mal cascade initiated on plasma membrane|Reactome	0.426793791	0.004836032	2.701355627	0.02578772	0.14017854	-3.52778371
WT after UVB irradiation VS WT before UVB irradiation	MyD88:Mal cascade initiated on plasma membrane|Reactome	0.09202684	-0.01046908	0.464896089	0.65188403	0.87935805	-5.76090889
homo after UVB irradiation VS homo before UVB irradiation	MyD88-independent TLR4 cascade|Reactome	0.227850331	-0.01635389	1.264615506	0.23649601	0.39783533	-5.57685469
hetero after UVB irradiation VS hetero before UVB irradiation	MyD88-independent TLR4 cascade|Reactome	0.438982416	0.001289394	2.755449486	0.02366704	0.13628011	-3.44496709
WT after UVB irradiation VS WT before UVB irradiation	MyD88-independent TLR4 cascade|Reactome	0.136806375	-0.00208081	0.685595715	0.50842499	0.82544506	-5.63683426
homo after UVB irradiation VS homo before UVB irradiation	Myoadenylate deaminase deficiency|SMPDB	0.019015587	-0.03943216	0.101997834	0.92090359	0.95153785	-6.36286155
hetero after UVB irradiation VS hetero before UVB irradiation	Myoadenylate deaminase deficiency|SMPDB	-0.02085195	-0.0438046	-0.1098564	0.91509485	0.97054702	-6.33999905
WT after UVB irradiation VS WT before UVB irradiation	Myoadenylate deaminase deficiency|SMPDB	0.165884344	-0.0622813	0.759490799	0.46494629	0.80891799	-5.58561498
homo after UVB irradiation VS homo before UVB irradiation	Myogenesis|Reactome	0.26030841	-0.02417554	1.438865076	0.18267721	0.33687347	-5.36627012
hetero after UVB irradiation VS hetero before UVB irradiation	Myogenesis|Reactome	-0.01372665	-0.04502388	-0.05127785	0.960299	0.98139974	-6.34511508
WT after UVB irradiation VS WT before UVB irradiation	Myogenesis|Reactome	-0.13249739	-0.0314992	-0.54975231	0.59445691	0.86485362	-5.7184696
homo after UVB irradiation VS homo before UVB irradiation	Myometrial Relaxation and Contraction Pathways|Wikipathways	0.395125254	-0.0249403	2.442288768	0.03617797	0.12504267	-3.90791397
hetero after UVB irradiation VS hetero before UVB irradiation	Myometrial Relaxation and Contraction Pathways|Wikipathways	0.323899656	0.034829526	1.966042994	0.08303722	0.26030511	-4.63170047
WT after UVB irradiation VS WT before UVB irradiation	Myometrial Relaxation and Contraction Pathways|Wikipathways	0.187053918	-0.01496475	0.807335421	0.43810477	0.79685269	-5.5499891

homo after UVB irradiation VS homo before UVB irradiation	Na+/Cl- dependent neurotransmitter transporters|Reactome	-0.00776375	0.076186045	-0.04024543	0.96874028	0.98055157	-6.36757807
hetero after UVB irradiation VS hetero before UVB irradiation	Na+/Cl- dependent neurotransmitter transporters|Reactome	-0.29653975	0.01937291	-1.72342165	0.12119572	0.31331055	-4.97318247
WT after UVB irradiation VS WT before UVB irradiation	Na+/Cl- dependent neurotransmitter transporters|Reactome	-0.25119503	0.054258087	-1.23154241	0.2460723	0.63911291	-5.15746143
homo after UVB irradiation VS homo before UVB irradiation	Nabumetone Action Pathway|SMPDB	0.40423582	0.045705158	1.979500697	0.07780079	0.19626432	-4.61879689
hetero after UVB irradiation VS hetero before UVB irradiation	Nabumetone Action Pathway|SMPDB	0.482496143	0.064876536	3.04499191	0.01500113	0.1130168	-3.00250826
WT after UVB irradiation VS WT before UVB irradiation	Nabumetone Action Pathway|SMPDB	0.275725339	0.086595857	1.261970114	0.23537138	0.62952955	-5.12455679
homo after UVB irradiation VS homo before UVB irradiation	NAD <i>de novo</i> biosynthesis|HumanCyc	0.417940991	-0.00335212	2.350959352	0.04212865	0.13402181	-4.05131357
hetero after UVB irradiation VS hetero before UVB irradiation	NAD <i>de novo</i> biosynthesis|HumanCyc	-0.04948657	-0.01237402	-0.23956285	0.81639428	0.92648152	-6.31551598
WT after UVB irradiation VS WT before UVB irradiation	NAD <i>de novo</i> biosynthesis|HumanCyc	0.215100387	-0.03663982	0.687946794	0.50700452	0.82465997	-5.63527712
homo after UVB irradiation VS homo before UVB irradiation	NAD biosynthesis from 2-amino-3-carboxymuconate semialdehyde|HumanCyc	0.607265138	0.009929983	2.673768095	0.02457711	0.10366003	-3.54046036
hetero after UVB irradiation VS hetero before UVB irradiation	NAD biosynthesis from 2-amino-3-carboxymuconate semialdehyde|HumanCyc	-0.43241225	-0.02573235	-1.69898361	0.12583731	0.31888088	-5.0065049
WT after UVB irradiation VS WT before UVB irradiation	NAD biosynthesis from 2-amino-3-carboxymuconate semialdehyde|HumanCyc	0.200665514	0.007568843	0.734501938	0.4793774	0.81552033	-5.60345845
homo after UVB irradiation VS homo before UVB irradiation	NAD Biosynthesis II (from tryptophan)|Wikipathways	0.449291327	0.020921581	2.257073191	0.04924757	0.14535514	-4.19741911
hetero after UVB irradiation VS hetero before UVB irradiation	NAD Biosynthesis II (from tryptophan)|Wikipathways	0.10651019	-0.0132857	0.422671316	0.6831157	0.83700422	-6.25066742
WT after UVB irradiation VS WT before UVB irradiation	NAD Biosynthesis II (from tryptophan)|Wikipathways	0.386393122	-0.0312376	1.291786577	0.22525701	0.62952955	-5.09176753
homo after UVB irradiation VS homo before UVB irradiation	NAD metabolism_ sirtuins and aging|Wikipathways	-0.19183919	-0.01681648	-1.09164374	0.30220831	0.46312664	-5.7667177
hetero after UVB irradiation VS hetero before UVB irradiation	NAD metabolism_ sirtuins and aging|Wikipathways	-0.5127317	-0.02860371	-2.69364298	0.02610551	0.14048279	-3.53958964
WT after UVB irradiation VS WT before UVB irradiation	NAD metabolism_ sirtuins and aging|Wikipathways	-0.19712877	0.00620538	-0.94702288	0.36577186	0.74545864	-5.43537959
homo after UVB irradiation VS homo before UVB irradiation	NAD phosphorylation and dephosphorylation|HumanCyc	-0.66890325	-0.00252651	-1.94165115	0.0827479	0.20401021	-4.67471666
hetero after UVB irradiation VS hetero before UVB irradiation	NAD phosphorylation and dephosphorylation|HumanCyc	0.077593524	-0.0257748	0.192179248	0.85215296	0.94503144	-6.32655047
WT after UVB irradiation VS WT before UVB irradiation	NAD phosphorylation and dephosphorylation|HumanCyc	-0.1230329	0.001368415	-0.31726333	0.75751636	0.92353903	-5.81843189
homo after UVB irradiation VS homo before UVB irradiation	NAD salvage|HumanCyc	-0.33799165	-0.02922053	-1.57913162	0.14736447	0.2925505	-5.18453969
hetero after UVB irradiation VS hetero before UVB irradiation	NAD salvage|HumanCyc	-0.70356874	-0.05005834	-2.51290906	0.0348078	0.1634547	-3.8157684
WT after UVB irradiation VS WT before UVB irradiation	NAD salvage|HumanCyc	-0.40813437	-0.05191947	-1.07019923	0.30948062	0.69398681	-5.32197285
homo after UVB irradiation VS homo before UVB irradiation	NAD+ biosynthetic pathways|Wikipathways	0.408078145	-0.08377392	2.342767079	0.04270734	0.13402181	-4.06411907
hetero after UVB irradiation VS hetero before UVB irradiation	NAD+ biosynthetic pathways|Wikipathways	0.004670237	-0.03024624	0.028180251	0.97817445	0.98924321	-6.34611051
WT after UVB irradiation VS WT before UVB irradiation	NAD+ biosynthetic pathways|Wikipathways	0.133524197	-0.01076368	0.630890356	0.54214864	0.83916339	-5.67169565
homo after UVB irradiation VS homo before UVB irradiation	NAD+ metabolism|Wikipathways	-0.04857186	-0.03256015	-0.30094842	0.770012	0.85109533	-6.32002436
hetero after UVB irradiation VS hetero before UVB irradiation	NAD+ metabolism|Wikipathways	-0.12167588	-0.04089208	-0.66009476	0.52682211	0.74191436	-6.11624871
WT after UVB irradiation VS WT before UVB irradiation	NAD+ metabolism|Wikipathways	-0.10042701	-0.07258837	-0.44777053	0.66378636	0.87935805	-5.76865414
homo after UVB irradiation VS homo before UVB irradiation	NADE modulates death signalling|Reactome	-0.17195279	0.083619183	-0.63027839	0.5435456	0.67708436	-6.15952109
hetero after UVB irradiation VS hetero before UVB irradiation	NADE modulates death signalling|Reactome	-0.05147256	-0.03046604	-0.15761126	0.87847493	0.95610812	-6.33308541
WT after UVB irradiation VS WT before UVB irradiation	NADE modulates death signalling|Reactome	0.377785839	0.095367838	1.261204184	0.23563602	0.62952955	-5.12539203
homo after UVB irradiation VS homo before UVB irradiation	NADH repair|HumanCyc	0.547778477	0.035443957	1.506747644	0.16475664	0.31411349	-5.27957907
hetero after UVB irradiation VS hetero before UVB irradiation	NADH repair|HumanCyc	0.459022031	0.126511147	1.337333619	0.21608836	0.43741933	-5.469399
WT after UVB irradiation VS WT before UVB irradiation	NADH repair|HumanCyc	0.318256027	-0.01898711	0.915233668	0.38143748	0.75444997	-5.46281
homo after UVB irradiation VS homo before UVB irradiation	Nadolol Action Pathway|SMPDB	0.396415397	-0.03677376	2.343069613	0.04268583	0.13402181	-4.06364635
hetero after UVB irradiation VS hetero before UVB irradiation	Nadolol Action Pathway|SMPDB	-0.01555055	-0.01775524	-0.07410913	0.94265284	0.97054702	-6.34356282
WT after UVB irradiation VS WT before UVB irradiation	Nadolol Action Pathway|SMPDB	0.056685205	-0.03978418	0.223404833	0.82768199	0.94525798	-5.84393438
homo after UVB irradiation VS homo before UVB irradiation	NADPH regeneration|Reactome	0.75853214	-0.0102717	2.294747761	0.0462596	0.14003669	-4.13896681
hetero after UVB irradiation VS hetero before UVB irradiation	NADPH regeneration|Reactome	0.084135561	0.009875163	0.197001018	0.84849606	0.94443102	-6.32553714
WT after UVB irradiation VS WT before UVB irradiation	NADPH regeneration|Reactome	0.005390724	0.12172669	0.013005468	0.98987719	0.99676022	-5.86906551
homo after UVB irradiation VS homo before UVB irradiation	Nalbuphine Action Pathway|SMPDB	0.161806559	0.043920545	0.896537684	0.39237189	0.54537901	-5.95456934
hetero after UVB irradiation VS hetero before UVB irradiation	Nalbuphine Action Pathway|SMPDB	-0.10403569	0.069661108	-0.60607049	0.56043694	0.76275652	-6.15161899
WT after UVB irradiation VS WT before UVB irradiation	Nalbuphine Action Pathway|SMPDB	0.048288971	0.049090135	0.211171304	0.83696085	0.94951669	-5.84661411
homo after UVB irradiation VS homo before UVB irradiation	Naloxone Action Pathway|SMPDB	0.191132361	0.029768717	1.061313976	0.31508276	0.46923284	-5.79783739
hetero after UVB irradiation VS hetero before UVB irradiation	Naloxone Action Pathway|SMPDB	-0.07904315	0.055005373	-0.44437398	0.66796482	0.82651398	-6.24068873
WT after UVB irradiation VS WT before UVB irradiation	Naloxone Action Pathway|SMPDB	0.022316377	0.035038618	0.098344171	0.92358676	0.97566251	-5.86425414
homo after UVB irradiation VS homo before UVB irradiation	Naltrexone Action Pathway|SMPDB	0.191132361	0.029768717	1.061313976	0.31508276	0.46923284	-5.79783739
hetero after UVB irradiation VS hetero before UVB irradiation	Naltrexone Action Pathway|SMPDB	-0.07904315	0.055005373	-0.44437398	0.66796482	0.82651398	-6.24068873
WT after UVB irradiation VS WT before UVB irradiation	Naltrexone Action Pathway|SMPDB	0.022316377	0.035038618	0.098344171	0.92358676	0.97566251	-5.86425414
homo after UVB irradiation VS homo before UVB irradiation	Nanomaterial induced apoptosis|Wikipathways	0.525788972	-0.04815905	2.826552204	0.01904779	0.08811647	-3.29608707
hetero after UVB irradiation VS hetero before UVB irradiation	Nanomaterial induced apoptosis|Wikipathways	0.473181574	0.014835701	2.760132863	0.02349203	0.13628011	-3.43779649
WT after UVB irradiation VS WT before UVB irradiation	Nanomaterial induced apoptosis|Wikipathways	0.554520359	-0.04310192	2.108236649	0.0610017	0.43075115	-4.03326859
homo after UVB irradiation VS homo before UVB irradiation	Nanomaterial induced inflammasome activation|Wikipathways	1.096421326	-0.03345959	6.111580788	0.00014829	0.01666204	1.405384351
hetero after UVB irradiation VS hetero before UVB irradiation	Nanomaterial induced inflammasome activation|Wikipathways	1.10169956	-0.00367669	5.964628687	0.00027454	0.02988205	0.87973536
WT after UVB irradiation VS WT before UVB irradiation	Nanomaterial induced inflammasome activation|Wikipathways	0.819353055	0.025207891	2.789871106	0.0189846	0.31542987	-3.02400205
homo after UVB irradiation VS homo before UVB irradiation	Nanoparticle triggered autophagic cell death|Wikipathways	-0.070432	0.013079709	-0.30970423	0.76354945	0.84687345	-6.31718058
hetero after UVB irradiation VS hetero before UVB irradiation	Nanoparticle triggered autophagic cell death|Wikipathways	-0.22552848	-0.06364834	-1.05760762	0.31960999	0.55014439	-5.7778847
WT after UVB irradiation VS WT before UVB irradiation	Nanoparticle triggered autophagic cell death|Wikipathways	-0.2896581	-0.03896058	-1.40050188	0.19137913	0.60496032	-4.96785003
homo after UVB irradiation VS homo before UVB irradiation	Nanoparticle triggered regulated necrosis|Wikipathways	0.569418274	-0.05780438	3.449519245	0.0068356	0.0479437	-2.30247655
hetero after UVB irradiation VS hetero before UVB irradiation	Nanoparticle triggered regulated necrosis|Wikipathways	0.609276173	0.024595482	3.363612979	0.00916405	0.09691354	-2.52105524
WT after UVB irradiation VS WT before UVB irradiation	Nanoparticle triggered regulated necrosis|Wikipathways	0.629671106	-0.07556421	2.704616723	0.02198748	0.31542987	-3.15220022
homo after UVB irradiation VS homo before UVB irradiation	Naproxen Action Pathway|SMPDB	0.40423582	0.045705158	1.979500697	0.07780079	0.19626432	-4.61879689
hetero after UVB irradiation VS hetero before UVB irradiation	Naproxen Action Pathway|SMPDB	0.482496143	0.064876536	3.04499191	0.01500113	0.1130168	-3.00250826
WT after UVB irradiation VS WT before UVB irradiation	Naproxen Action Pathway|SMPDB	0.275725339	0.086595857	1.261970114	0.23537138	0.62952955	-5.12455679
homo after UVB irradiation VS homo before UVB irradiation	Nateglinide Action Pathway|SMPDB	-0.17556465	0.032733531	-0.6220452	0.54871777	0.67913558	-6.16483259
hetero after UVB irradiation VS hetero before UVB irradiation	Nateglinide Action Pathway|SMPDB	-0.35455332	-0.0590526	-1.26742043	0.23890834	0.4612216	-5.55113336
WT after UVB irradiation VS WT before UVB irradiation	Nateglinide Action Pathway|SMPDB	-0.23688751	-0.13206249	-0.65900303	0.52465845	0.83140705	-5.65411026
homo after UVB irradiation VS homo before UVB irradiation	Natural killer cell mediated cytotoxicity - Homo sapiens (human)|KEGG	0.528570121	-0.01577738	3.265863965	0.00921596	0.05748641	-2.59348376
hetero after UVB irradiation VS hetero before UVB irradiation	Natural killer cell mediated cytotoxicity - Homo sapiens (human)|KEGG	0.541428496	0.041829655	3.01857878	0.01563387	0.11352943	-3.04274147
WT after UVB irradiation VS WT before UVB irradiation	Natural killer cell mediated cytotoxicity - Homo sapiens (human)|KEGG	0.545077623	0.010973313	2.329118075	0.04191672	0.39022568	-3.71206974
homo after UVB irradiation VS homo before UVB irradiation	N-cadherin signaling events|PID	0.217716121	-0.03265348	1.048392892	0.32069367	0.47665602	-5.81088772
hetero after UVB irradiation VS hetero before UVB irradiation	N-cadherin signaling events|PID	0.134838616	0.008659106	0.846754383	0.42052917	0.64733939	-5.97371241
WT after UVB irradiation VS WT before UVB irradiation	N-cadherin signaling events|PID	0.131300766	-0.02516587	0.670101176	0.5178465	0.82924438	-5.64697572
homo after UVB irradiation VS homo before UVB irradiation	NCAM signaling for neurite out-growth|Reactome	0.500829091	-0.02781876	3.015689108	0.01391252	0.07305822	-2.99304114
hetero after UVB irradiation VS hetero before UVB irradiation	NCAM signaling for neurite out-growth|Reactome	0.218104389	-0.01238601	1.257595384	0.24227191	0.46543442	-5.56238683
WT after UVB irradiation VS WT before UVB irradiation	NCAM signaling for neurite out-growth|Reactome	0.197381744	-0.00414884	0.993785655	0.34357899	0.72813236	-5.39363667
homo after UVB irradiation VS homo before UVB irradiation	NCAM1 interactions|Reactome	0.600914404	-0.02427255	3.59487803	0.00540962	0.04239626	-2.07416699
hetero after UVB irradiation VS hetero before UVB irradiation	NCAM1 interactions|Reactome	0.211160397	0.00226063	1.027223339	0.33289806	0.56094247	-5.80820587
WT after UVB irradiation VS WT before UVB irradiation	NCAM1 interactions|Reactome	0.27464038	0.02021592	1.164528854	0.27102463	0.66327566	-5.22787774
homo after UVB irradiation VS homo before UVB irradiation	ncRNAs involved in STAT3 signaling in hepatocellular carcinoma|Wikipathways	0.577586351	-0.02773549	2.889253542	0.01716027	0.08273895	-3.19562561
hetero after UVB irradiation VS hetero before UVB irradiation	ncRNAs involved in STAT3 signaling in hepatocellular carcinoma|Wikipathways	0.221330896	-0.0033355	0.858777861	0.41422434	0.64192892	-5.96349816
WT after UVB irradiation VS WT before UVB irradiation	ncRNAs involved in STAT3 signaling in hepatocellular carcinoma|Wikipathways	0.16373987	-0.12790611	0.520590771	0.61389172	0.87257466	-5.7338107
homo after UVB irradiation VS homo before UVB irradiation	ncRNAs involved in Wnt signaling in hepatocellular carcinoma|Wikipathways	-0.40600362	0.027832566	-2.55157873	0.0301426	0.11538756	-3.73500844
hetero after UVB irradiation VS hetero before UVB irradiation	ncRNAs involved in Wnt signaling in hepatocellular carcinoma|Wikipathways	-0.29995125	-0.02494104	-1.55714959	0.15614601	0.36594993	-5.19524444
WT after UVB irradiation VS WT before UVB irradiation	ncRNAs involved in Wnt signaling in hepatocellular carcinoma|Wikipathways	-0.29894939	0.018184008	-1.25060656	0.23932256	0.63251512	-5.13691193
homo after UVB irradiation VS homo before UVB irradiation	Nebivolol Action Pathway|SMPDB	0.396415397	-0.03677376	2.343069613	0.04268583	0.13402181	-4.06364635
hetero after UVB irradiation VS hetero before UVB irradiation	Nebivolol Action Pathway|SMPDB	-0.01555055	-0.01775524	-0.07410913	0.94265284	0.97054702	-6.34356282
WT after UVB irradiation VS WT before UVB irradiation	Nebivolol Action Pathway|SMPDB	0.056685205	-0.03978418	0.223404833	0.82768199	0.94525798	-5.84393438
homo after UVB irradiation VS homo before UVB irradiation	Necroptosis - Homo sapiens (human)|KEGG	0.556241743	-0.01509174	3.373750558	0.0077292	0.05159579	-2.42222682
hetero after UVB irradiation VS hetero before UVB irradiation	Necroptosis - Homo sapiens (human)|KEGG	0.456327946	0.014044074	2.572256264	0.03166627	0.15519875	-3.72521979
WT after UVB irradiation VS WT before UVB irradiation	Necroptosis - Homo sapiens (human)|KEGG	0.503285389	-0.00903165	2.372308808	0.03893133	0.39022568	-3.64835385
homo after UVB irradiation VS homo before UVB irradiation	Nectin adhesion pathway|PID	0.464906072	-0.04904428	2.058611161	0.0683519	0.1829395	-4.50060121
hetero after UVB irradiation VS hetero before UVB irradiation	Nectin adhesion pathway|PID	0.598970996	-0.00149903	3.589048102	0.00651196	0.08456271	-2.18625154
WT after UVB irradiation VS WT before UVB irradiation	Nectin adhesion pathway|PID	0.437606689	-0.02619856	2.143567879	0.05746881	0.42266366	-3.98250271
homo after UVB irradiation VS homo before UVB irradiation	Nectin/Necl trans heterodimerization|Reactome	-1.0488582	-0.00602442	-5.67789031	0.00025867	0.01666204	0.877247986
hetero after UVB irradiation VS hetero before UVB irradiation	Nectin/Necl trans heterodimerization|Reactome	-0.62695727	-0.03775823	-3.05281296	0.01481892	0.1130168	-2.99060238
WT after UVB irradiation VS WT before UVB irradiation	Nectin/Necl trans heterodimerization|Reactome	-0.8724624	-0.06833262	-3.80857619	0.00338695	0.20197958	-1.52045694
homo after UVB irradiation VS homo before UVB irradiation	Neddylation|Reactome	-0.11252176	-0.01907175	-0.66969935	0.51917778	0.65684837	-6.13320684
hetero after UVB irradiation VS hetero before UVB irradiation	Neddylation|Reactome	-0.01422742	-0.02749388	-0.08601922	0.93346021	0.97054702	-6.34252902
WT after UVB irradiation VS WT before UVB irradiation	Neddylation|Reactome	-0.07445438	-0.01255212	-0.33607207	0.74370598	0.9185045	-5.81227698
homo after UVB irradiation VS homo before UVB irradiation	Nef and signal transduction|Reactome	-2.47E-05	-0.03639213	-0.00010878	0.99991548	0.99991548	-6.36844826
hetero after UVB irradiation VS hetero before UVB irradiation	Nef and signal transduction|Reactome	0.391435137	0.042188685	2.279909305	0.05047639	0.19936861	-4.16863761
WT after UVB irradiation VS WT before UVB irradiation	Nef and signal transduction|Reactome	0.409381281	-0.05152599	1.378061715	0.19799683	0.60974478	-4.99397027
homo after UVB irradiation VS homo before UVB irradiation	Nef Mediated CD4 Down-regulation|Reactome	0.134786995	-0.07657226	0.523870794	0.61250859	0.72940831	-6.22316435
hetero after UVB irradiation VS hetero before UVB irradiation	Nef Mediated CD4 Down-regulation|Reactome	0.536163631	0.041799717	2.726373603	0.0247838	0.13846576	-3.4894838
WT after UVB irradiation VS WT before UVB irradiation	Nef Mediated CD4 Down-regulation|Reactome	0.599286734	-0.04500629	2.256446068	0.04744982	0.401167	-3.81867854
homo after UVB irradiation VS homo before UVB irradiation	Nef Mediated CD8 Down-regulation|Reactome	0.296980579	-0.04805588	1.196855201	0.26070755	0.42483608	-5.65365917
hetero after UVB irradiation VS hetero before UVB irradiation	Nef Mediated CD8 Down-regulation|Reactome	0.591123086	0.013275434	3.63406378	0.00608722	0.0822456	-2.12010508
WT after UVB irradiation VS WT before UVB irradiation	Nef Mediated CD8 Down-regulation|Reactome	0.697361811	0.03446683	2.560575264	0.02817744	0.35160213	-3.36823729
homo after UVB irradiation VS homo before UVB irradiation	Nef mediated downregulation of CD28 cell surface expression|Reactome	1.114054322	-0.05552566	5.989958445	0.00017289	0.01666204	1.260275826
hetero after UVB irradiation VS hetero before UVB irradiation	Nef mediated downregulation of CD28 cell surface expression|Reactome	1.125017681	0.049292957	6.483838066	0.00015232	0.02920766	1.430057602
WT after UVB irradiation VS WT before UVB irradiation	Nef mediated downregulation of CD28 cell surface expression|Reactome	0.887487886	0.064819249	4.118871429	0.00204521	0.16821036	-1.08636529
homo after UVB irradiation VS homo before UVB irradiation	Nef mediated downregulation of MHC class I complex cell surface expression|Reactome	0.698539386	-0.04236379	3.508127971	0.00621849	0.04588471	-2.21018341
hetero after UVB irradiation VS hetero before UVB irradiation	Nef mediated downregulation of MHC class I complex cell surface expression|Reactome	0.690452007	-0.02206557	4.489840719	0.00178184	0.04958933	-0.9171915
WT after UVB irradiation VS WT before UVB irradiation	Nef mediated downregulation of MHC class I complex cell surface expression|Reactome	0.519007208	0.023945925	2.504378518	0.03103696	0.36428127	-3.45218941
homo after UVB irradiation VS homo before UVB irradiation	Nef-mediates down modulation of cell surface receptors by recruiting them to clathrin adapters|Reactome	0.503909098	-0.04155013	2.540403612	0.03071063	0.11656255	-3.75274317
hetero after UVB irradiation VS hetero before UVB irradiation	Nef-mediates down modulation of cell surface receptors by recruiting them to clathrin adapters|Reactome	0.719094506	0.008587261	4.742380246	0.00126542	0.04336692	-0.58413342
WT after UVB irradiation VS WT before UVB irradiation	Nef-mediates down modulation of cell surface receptors by recruiting them to clathrin adapters|Reactome	0.605629761	0.002623725	2.999171898	0.01324701	0.28007727	-2.70930993
homo after UVB irradiation VS homo before UVB irradiation	Negative epigenetic regulation of rRNA expression|Reactome	-0.67459543	0.005696476	-3.90626893	0.00330498	0.03256113	-1.59268309
hetero after UVB irradiation VS hetero before UVB irradiation	Negative epigenetic regulation of rRNA expression|Reactome	-0.41447389	-0.0478678	-1.79224014	0.10896425	0.29991489	-4.87820218
WT after UVB irradiation VS WT before UVB irradiation	Negative epigenetic regulation of rRNA expression|Reactome	-0.27749981	-0.0088028	-0.98440094	0.34795168	0.73263961	-5.40214501
homo after UVB irradiation VS homo before UVB irradiation	Negative feedback regulation of MAPK pathway|Reactome	0.246829255	-0.01959747	1.156960279	0.2758745	0.43614438	-5.69744121
hetero after UVB irradiation VS hetero before UVB irradiation	Negative feedback regulation of MAPK pathway|Reactome	0.398830047	0.014648767	1.7797246	0.1110994	0.30270882	-4.89559651
WT after UVB irradiation VS WT before UVB irradiation	Negative feedback regulation of MAPK pathway|Reactome	0.383162464	0.071708325	1.417766833	0.18641619	0.59955123	-4.9475689
homo after UVB irradiation VS homo before UVB irradiation	Negative regulation of activity of TFAP2 (AP-2) family transcription factors|Reactome	-0.45676156	-0.06194026	-2.11166751	0.06264015	0.17209081	-4.42041632
hetero after UVB irradiation VS hetero before UVB irradiation	Negative regulation of activity of TFAP2 (AP-2) family transcription factors|Reactome	-0.05155784	-0.05033616	-0.17821011	0.86276835	0.94644961	-6.32934561
WT after UVB irradiation VS WT before UVB irradiation	Negative regulation of activity of TFAP2 (AP-2) family transcription factors|Reactome	-0.17218863	0.073831039	-0.53936749	0.60134068	0.86826522	-5.72402285
homo after UVB irradiation VS homo before UVB irradiation	Negative regulation of FGFR1 signaling|Reactome	-0.16301498	0.023678256	-0.86819029	0.40693296	0.55844883	-5.97933057
hetero after UVB irradiation VS hetero before UVB irradiation	Negative regulation of FGFR1 signaling|Reactome	-0.08341891	0.04377799	-0.47397216	0.64755915	0.82239471	-6.2263141
WT after UVB irradiation VS WT before UVB irradiation	Negative regulation of FGFR1 signaling|Reactome	-0.3040581	0.03749573	-1.32137238	0.2155775	0.62581393	-5.05871124
homo after UVB irradiation VS homo before UVB irradiation	Negative regulation of FGFR2 signaling|Reactome	-0.30515877	0.029339124	-1.70310165	0.12135167	0.26025915	-5.01607575
hetero after UVB irradiation VS hetero before UVB irradiation	Negative regulation of FGFR2 signaling|Reactome	-0.16375332	0.03311388	-0.9732901	0.35751816	0.58575405	-5.8603357
WT after UVB irradiation VS WT before UVB irradiation	Negative regulation of FGFR2 signaling|Reactome	-0.39242685	0.040098303	-1.75039967	0.11036224	0.50951142	-4.52899517
homo after UVB irradiation VS homo before UVB irradiation	Negative regulation of FGFR3 signaling|Reactome	-0.23986157	0.032788105	-1.1963769	0.26088534	0.42497412	-5.6541905
hetero after UVB irradiation VS hetero before UVB irradiation	Negative regulation of FGFR3 signaling|Reactome	-0.14206287	0.024535667	-0.82593533	0.43160344	0.65772266	-5.99111248
WT after UVB irradiation VS WT before UVB irradiation	Negative regulation of FGFR3 signaling|Reactome	-0.38523238	0.044703393	-1.67548386	0.12452771	0.52904005	-4.62745534
homo after UVB irradiation VS homo before UVB irradiation	Negative regulation of FGFR4 signaling|Reactome	-0.17359218	0.015395808	-0.95590077	0.36308523	0.51751626	-5.90057664
hetero after UVB irradiation VS hetero before UVB irradiation	Negative regulation of FGFR4 signaling|Reactome	-0.14186615	0.036203241	-0.93959532	0.37357602	0.60239918	-5.89177394
WT after UVB irradiation VS WT before UVB irradiation	Negative regulation of FGFR4 signaling|Reactome	-0.33747161	0.044539013	-1.56283963	0.14890668	0.55622203	-4.77116227
homo after UVB irradiation VS homo before UVB irradiation	Negative regulation of MAPK pathway|Reactome	-0.27232496	0.006963637	-1.2309704	0.2482774	0.41058841	-5.61536733
hetero after UVB irradiation VS hetero before UVB irradiation	Negative regulation of MAPK pathway|Reactome	0.042749446	-0.00401171	0.213687178	0.83587144	0.93724527	-6.32183862
WT after UVB irradiation VS WT before UVB irradiation	Negative regulation of MAPK pathway|Reactome	-0.25482881	0.018982922	-1.03455383	0.32505433	0.70928005	-5.35592564
homo after UVB irradiation VS homo before UVB irradiation	Negative regulation of MET activity|Reactome	0.091654968	-0.01687251	0.398847113	0.6989251	0.80002307	-6.28371069
hetero after UVB irradiation VS hetero before UVB irradiation	Negative regulation of MET activity|Reactome	0.557907262	-0.01009985	3.556789865	0.00683555	0.08550202	-2.23380441
WT after UVB irradiation VS WT before UVB irradiation	Negative regulation of MET activity|Reactome	0.026713905	-0.02563547	0.112568463	0.91258246	0.97566251	-5.86273613
homo after UVB irradiation VS homo before UVB irradiation	Negative regulation of TCF-dependent signaling by DVL-interacting proteins|Reactome	-0.37498407	-0.03181562	-1.31066906	0.22112061	0.37999124	-5.52297002

hetero after UVB irradiation VS hetero before UVB irradiation	Negative regulation of TCF-dependent signaling by DVL-interacting proteins|Reactome	-0.31270894	-0.14257612	-1.26559348	0.23953082	0.46222755	-5.55323036
WT after UVB irradiation VS WT before UVB irradiation	Negative regulation of TCF-dependent signaling by DVL-interacting proteins|Reactome	-0.69818028	-0.07854516	-3.03233369	0.01251473	0.27745036	-2.6595496
homo after UVB irradiation VS homo before UVB irradiation	Negative regulation of TCF-dependent signaling by WNT ligand antagonists|Reactome	-0.41721558	-0.00626443	-2.52580631	0.03146872	0.11796894	-3.77589159
hetero after UVB irradiation VS hetero before UVB irradiation	Negative regulation of TCF-dependent signaling by WNT ligand antagonists|Reactome	-0.33143684	-0.04982297	-1.50253546	0.16947391	0.38622363	-5.2655989
WT after UVB irradiation VS WT before UVB irradiation	Negative regulation of TCF-dependent signaling by WNT ligand antagonists|Reactome	-0.35478097	0.063467213	-1.30788749	0.21994571	0.62621406	-5.07384144
homo after UVB irradiation VS homo before UVB irradiation	Negative regulation of the PI3K/AKT network|Reactome	0.179835775	-0.00251639	1.12008059	0.29051148	0.45011042	-5.73693067
hetero after UVB irradiation VS hetero before UVB irradiation	Negative regulation of the PI3K/AKT network|Reactome	0.089841154	0.016364036	0.526867949	0.61187103	0.79797086	-6.1984503
WT after UVB irradiation VS WT before UVB irradiation	Negative regulation of the PI3K/AKT network|Reactome	-0.03861099	0.021587789	-0.18832856	0.85435577	0.95633402	-5.85121742
homo after UVB irradiation VS homo before UVB irradiation	Negative regulators of DDX58/IFIH1 signaling|Reactome	0.622460564	-0.02822423	3.397277009	0.00743942	0.05032354	-2.3849942
hetero after UVB irradiation VS hetero before UVB irradiation	Negative regulators of DDX58/IFIH1 signaling|Reactome	0.630748049	-0.00928128	3.493708089	0.00751842	0.08807784	-2.32715049
WT after UVB irradiation VS WT before UVB irradiation	Negative regulators of DDX58/IFIH1 signaling|Reactome	0.625067317	-0.03293838	3.161477553	0.01003442	0.25758249	-2.46627787
homo after UVB irradiation VS homo before UVB irradiation	Neomycin_ kanamycin and gentamicin biosynthesis - Homo sapiens (human)|KEGG	0.976007578	-0.00337657	4.315618685	0.0017631	0.02356086	-0.97880878
hetero after UVB irradiation VS hetero before UVB irradiation	Neomycin_ kanamycin and gentamicin biosynthesis - Homo sapiens (human)|KEGG	0.637077056	-0.00911584	2.096594387	0.06756043	0.23417525	-4.44139736
WT after UVB irradiation VS WT before UVB irradiation	Neomycin_ kanamycin and gentamicin biosynthesis - Homo sapiens (human)|KEGG	0.701713427	0.021065685	2.523084398	0.03005462	0.36196871	-3.42427148
homo after UVB irradiation VS homo before UVB irradiation	Neovascularisation processes|Wikipathways	0.153811151	-0.02396039	0.679648962	0.51313269	0.65152098	-6.12633639
hetero after UVB irradiation VS hetero before UVB irradiation	Neovascularisation processes|Wikipathways	0.281022221	0.003701711	1.86289254	0.09761536	0.28213748	-4.77906429
WT after UVB irradiation VS WT before UVB irradiation	Neovascularisation processes|Wikipathways	0.049824574	0.007557995	0.244454006	0.81178227	0.94525798	-5.8389745
homo after UVB irradiation VS homo before UVB irradiation	NEP/NS2 Interacts with the Cellular Export Machinery|Reactome	-0.76937683	-0.01851125	-4.06914422	0.00256683	0.02922724	-1.34563506
hetero after UVB irradiation VS hetero before UVB irradiation	NEP/NS2 Interacts with the Cellular Export Machinery|Reactome	-0.21700666	-0.0761736	-0.75991261	0.46803362	0.6901641	-6.04384453
WT after UVB irradiation VS WT before UVB irradiation	NEP/NS2 Interacts with the Cellular Export Machinery|Reactome	-0.12307951	-0.00460315	-0.36237887	0.72454974	0.90882588	-5.80308889
homo after UVB irradiation VS homo before UVB irradiation	Nepafenac Action Pathway|SMPDB	0.40423582	0.045705158	1.979500697	0.07780079	0.19626432	-4.61879689
hetero after UVB irradiation VS hetero before UVB irradiation	Nepafenac Action Pathway|SMPDB	0.482496143	0.064876536	3.04499191	0.01500113	0.1130168	-3.00250826
WT after UVB irradiation VS WT before UVB irradiation	Nepafenac Action Pathway|SMPDB	0.275725339	0.086595857	1.261970114	0.23537138	0.62952955	-5.12455679
homo after UVB irradiation VS homo before UVB irradiation	Nephrin family interactions|Reactome	0.191466407	-0.01973962	0.727473867	0.4846796	0.62736836	-6.09204285
hetero after UVB irradiation VS hetero before UVB irradiation	Nephrin family interactions|Reactome	0.285694557	0.015799967	1.35032401	0.21205892	0.4340209	-5.45390135
WT after UVB irradiation VS WT before UVB irradiation	Nephrin family interactions|Reactome	0.198073745	-0.04318608	0.884118998	0.39722527	0.7654467	-5.48890022
homo after UVB irradiation VS homo before UVB irradiation	Nephrin/Neph1 signaling in the kidney podocyte|PID	0.289708663	-0.03428115	1.321193707	0.21772631	0.3758557	-5.51047087
hetero after UVB irradiation VS hetero before UVB irradiation	Nephrin/Neph1 signaling in the kidney podocyte|PID	0.443742262	0.009888489	2.978286579	0.01665286	0.11396958	-3.1041839
WT after UVB irradiation VS WT before UVB irradiation	Nephrin/Neph1 signaling in the kidney podocyte|PID	0.229566591	0.013505405	1.11932907	0.28895719	0.67456398	-5.27371868
homo after UVB irradiation VS homo before UVB irradiation	nerve growth factor pathway (ngf)|BioCarta	0.197399798	-0.01112956	0.848039456	0.41750983	0.56804228	-5.99652216
hetero after UVB irradiation VS hetero before UVB irradiation	nerve growth factor pathway (ngf)|BioCarta	0.487164879	0.01999644	2.531776991	0.03377632	0.16053874	-3.78700099
WT after UVB irradiation VS WT before UVB irradiation	nerve growth factor pathway (ngf)|BioCarta	0.332437856	0.04640574	1.563651747	0.14871678	0.55622203	-4.7701462
homo after UVB irradiation VS homo before UVB irradiation	Netrin mediated repulsion signals|Reactome	-0.28737191	0.04636374	-1.17676406	0.26825982	0.43205444	-5.67584411
hetero after UVB irradiation VS hetero before UVB irradiation	Netrin mediated repulsion signals|Reactome	-0.3474159	-0.02076156	-1.56644037	0.15397458	0.3647693	-5.18314073
WT after UVB irradiation VS WT before UVB irradiation	Netrin mediated repulsion signals|Reactome	-0.40314439	-0.0401859	-1.72540178	0.11491778	0.51527453	-4.56208974
homo after UVB irradiation VS homo before UVB irradiation	Netrin-1 signaling|Reactome	-0.00176526	-0.00725889	-0.00947403	0.99263919	0.99416331	-6.36840004
hetero after UVB irradiation VS hetero before UVB irradiation	Netrin-1 signaling|Reactome	-0.20628023	0.000777075	-1.29405525	0.22998607	0.45170905	-5.52033371
WT after UVB irradiation VS WT before UVB irradiation	Netrin-1 signaling|Reactome	-0.20569642	-0.0050426	-1.02455959	0.32952499	0.71256659	-5.36528225
homo after UVB irradiation VS homo before UVB irradiation	Netrin-mediated signaling events|PID	0.46718395	-0.01305273	2.496946768	0.03302277	0.12110506	-3.82159619
hetero after UVB irradiation VS hetero before UVB irradiation	Netrin-mediated signaling events|PID	0.419288759	0.010658477	2.480128605	0.03667592	0.16750384	-3.86569585
WT after UVB irradiation VS WT before UVB irradiation	Netrin-mediated signaling events|PID	0.237038739	-0.01485414	1.202062389	0.25681187	0.65122698	-5.18879164
homo after UVB irradiation VS homo before UVB irradiation	Neural Crest Differentiation|Wikipathways	-0.16211258	0.03124074	-0.92601426	0.37762565	0.53053499	-5.9281173
hetero after UVB irradiation VS hetero before UVB irradiation	Neural Crest Differentiation|Wikipathways	-0.11854977	-0.0026529	-0.74738523	0.47516906	0.69685347	-6.05342183
WT after UVB irradiation VS WT before UVB irradiation	Neural Crest Differentiation|Wikipathways	-0.16234062	0.055338539	-0.80301532	0.44048552	0.79734338	-5.55328392
homo after UVB irradiation VS homo before UVB irradiation	Neurexins and neuroligins|Reactome	0.140306297	0.004635368	0.810981573	0.43744971	0.58886842	-6.02723429
hetero after UVB irradiation VS hetero before UVB irradiation	Neurexins and neuroligins|Reactome	0.120140471	0.013552029	0.766122022	0.46452302	0.68805201	-6.03904617
WT after UVB irradiation VS WT before UVB irradiation	Neurexins and neuroligins|Reactome	-0.10288521	-0.01428171	-0.47826273	0.64266458	0.87745357	-5.75467092
homo after UVB irradiation VS homo before UVB irradiation	Neuroactive ligand-receptor interaction - Homo sapiens (human)|KEGG	0.123919033	0.014575915	0.718608644	0.48987773	0.63135722	-6.09855727
hetero after UVB irradiation VS hetero before UVB irradiation	Neuroactive ligand-receptor interaction - Homo sapiens (human)|KEGG	-0.09635815	0.011687017	-0.59066883	0.57024247	0.76694163	-6.16119827
WT after UVB irradiation VS WT before UVB irradiation	Neuroactive ligand-receptor interaction - Homo sapiens (human)|KEGG	-0.05783162	0.022451956	-0.25346934	0.8049993	0.94525798	-5.83671541
homo after UVB irradiation VS homo before UVB irradiation	Neurodegenerative Diseases|Reactome	0.257711588	-0.01036428	1.211473663	0.25532089	0.41874827	-5.6373459
hetero after UVB irradiation VS hetero before UVB irradiation	Neurodegenerative Diseases|Reactome	0.47642371	0.036407496	2.675724658	0.02685931	0.14252149	-3.5670143
WT after UVB irradiation VS WT before UVB irradiation	Neurodegenerative Diseases|Reactome	0.226971887	0.050305015	0.798911108	0.44275521	0.79860542	-5.55639984
homo after UVB irradiation VS homo before UVB irradiation	Neurofascin interactions|Reactome	0.147516924	-0.03185633	0.584474121	0.57267564	0.69654687	-6.18825303
hetero after UVB irradiation VS hetero before UVB irradiation	Neurofascin interactions|Reactome	-0.19696145	0.069441438	-0.80596977	0.44241049	0.66793872	-6.00745466
WT after UVB irradiation VS WT before UVB irradiation	Neurofascin interactions|Reactome	-0.19779861	0.071887199	-0.72508081	0.48489071	0.81710765	-5.61004784
homo after UVB irradiation VS homo before UVB irradiation	Neuronal System|Reactome	0.131350862	0.010473357	0.799727793	0.4436299	0.59107503	-6.03632637
hetero after UVB irradiation VS hetero before UVB irradiation	Neuronal System|Reactome	-0.03909132	0.007180889	-0.24467007	0.81256535	0.92298761	-6.31418389
WT after UVB irradiation VS WT before UVB irradiation	Neuronal System|Reactome	-0.07692929	-0.00866071	-0.3551485	0.72979565	0.91067215	-5.80568138
homo after UVB irradiation VS homo before UVB irradiation	Neurophilin interactions with VEGF and VEGFR|Reactome	0.63958658	-0.01608204	3.113244257	0.01184144	0.06650432	-2.83693163
hetero after UVB irradiation VS hetero before UVB irradiation	Neurophilin interactions with VEGF and VEGFR|Reactome	0.656685129	-0.00659862	2.873324515	0.01964258	0.12315116	-3.26455306
WT after UVB irradiation VS WT before UVB irradiation	Neurophilin interactions with VEGF and VEGFR|Reactome	0.498227894	0.013404189	1.760994248	0.10848156	0.5076251	-4.5148989
homo after UVB irradiation VS homo before UVB irradiation	neuroregulin receptor degredation protein-1 controls erbb3 receptor recycling|BioCarta	-0.18053398	0.084102521	-0.87768245	0.40201573	0.55406092	-5.97111391
hetero after UVB irradiation VS hetero before UVB irradiation	neuroregulin receptor degredation protein-1 controls erbb3 receptor recycling|BioCarta	-0.36067162	-0.02589388	-1.55496321	0.15666099	0.36651649	-5.19808719
WT after UVB irradiation VS WT before UVB irradiation	neuroregulin receptor degredation protein-1 controls erbb3 receptor recycling|BioCarta	-0.38875551	0.014815845	-1.68654494	0.12233789	0.52731779	-4.61305621
homo after UVB irradiation VS homo before UVB irradiation	Neurotoxicity of clostridium toxins|Reactome	-0.03437653	0.031245511	-0.19322146	0.85090087	0.90882185	-6.3484291
hetero after UVB irradiation VS hetero before UVB irradiation	Neurotoxicity of clostridium toxins|Reactome	-0.60581429	-0.03618362	-3.02138826	0.01556527	0.11352943	-3.03846023
WT after UVB irradiation VS WT before UVB irradiation	Neurotoxicity of clostridium toxins|Reactome	-0.42622821	0.015509806	-1.52399557	0.15824217	0.56072663	-4.8194038
homo after UVB irradiation VS homo before UVB irradiation	Neurotransmitter clearance|Reactome	0.08748317	0.04931082	0.416302371	0.68654412	0.78981115	-6.27620234
hetero after UVB irradiation VS hetero before UVB irradiation	Neurotransmitter clearance|Reactome	-0.16660393	0.009701622	-0.66341605	0.5247959	0.73987618	-6.11398539
WT after UVB irradiation VS WT before UVB irradiation	Neurotransmitter clearance|Reactome	0.306112346	-0.03983481	1.000424153	0.34051041	0.72554886	-5.38757879
homo after UVB irradiation VS homo before UVB irradiation	Neurotransmitter Disorders|Wikipathways	0.714097106	0.072637621	3.471475838	0.00659726	0.04701822	-2.26786484
hetero after UVB irradiation VS hetero before UVB irradiation	Neurotransmitter Disorders|Wikipathways	0.422654714	0.155833997	1.202705377	0.26179455	0.48804071	-5.62415678
WT after UVB irradiation VS WT before UVB irradiation	Neurotransmitter Disorders|Wikipathways	0.515933502	-0.01045493	1.628802902	0.1341619	0.54709793	-4.68767086
homo after UVB irradiation VS homo before UVB irradiation	Neurotransmitter receptors and postsynaptic signal transmission|Reactome	0.070743265	0.014171282	0.415969999	0.68677897	0.78988231	-6.2763482
hetero after UVB irradiation VS hetero before UVB irradiation	Neurotransmitter receptors and postsynaptic signal transmission|Reactome	-0.09670043	0.008858206	-0.59316878	0.56864429	0.76592218	-6.15965876
WT after UVB irradiation VS WT before UVB irradiation	Neurotransmitter receptors and postsynaptic signal transmission|Reactome	-0.09778037	-0.02339128	-0.45461788	0.65901542	0.87935805	-5.7655907
homo after UVB irradiation VS homo before UVB irradiation	Neurotransmitter release cycle|Reactome	0.302713448	0.02889475	1.86695921	0.09339162	0.21980728	-4.78375815
hetero after UVB irradiation VS hetero before UVB irradiation	Neurotransmitter release cycle|Reactome	0.03687209	-0.00315154	0.215280371	0.83466859	0.93635724	-6.32146994
WT after UVB irradiation VS WT before UVB irradiation	Neurotransmitter release cycle|Reactome	0.017024122	-0.00563483	0.075677258	0.94115647	0.98201088	-5.86625078
homo after UVB irradiation VS homo before UVB irradiation	Neurotransmitter uptake and metabolism In glial cells|Reactome	-0.29809726	-0.02650975	-1.12964363	0.2866586	0.44641309	-5.72678318
hetero after UVB irradiation VS hetero before UVB irradiation	Neurotransmitter uptake and metabolism In glial cells|Reactome	-0.27005399	0.0474308	-0.85815227	0.41455074	0.64192892	-5.96403258
WT after UVB irradiation VS WT before UVB irradiation	Neurotransmitter uptake and metabolism In glial cells|Reactome	-0.13026708	-0.02061759	-0.38944581	0.70504583	0.90126444	-5.79293369
homo after UVB irradiation VS homo before UVB irradiation	Neurotrophic factor-mediated Trk receptor signaling|PID	0.258133258	-0.01665732	1.397000516	0.19454993	0.35014386	-5.41848622
hetero after UVB irradiation VS hetero before UVB irradiation	Neurotrophic factor-mediated Trk receptor signaling|PID	0.296973144	-0.00431305	1.823733031	0.10376035	0.28996913	-4.83420593
WT after UVB irradiation VS WT before UVB irradiation	Neurotrophic factor-mediated Trk receptor signaling|PID	0.207049946	-0.01566028	1.016345322	0.33323372	0.71651524	-5.37291834
homo after UVB irradiation VS homo before UVB irradiation	Neurotrophin signaling pathway - Homo sapiens (human)|KEGG	0.27837832	-0.03172034	1.668000469	0.12826002	0.26846864	-5.06446299
hetero after UVB irradiation VS hetero before UVB irradiation	Neurotrophin signaling pathway - Homo sapiens (human)|KEGG	0.298862954	0.002722064	1.948422294	0.08537098	0.26434846	-4.65707714
WT after UVB irradiation VS WT before UVB irradiation	Neurotrophin signaling pathway - Homo sapiens (human)|KEGG	0.185494692	0.006172455	0.955178156	0.36182857	0.74374432	-5.42821811
homo after UVB irradiation VS homo before UVB irradiation	Neutrophil degranulation|Reactome	0.658269031	-0.03224256	3.922393399	0.0032228	0.03227042	-1.5680695
hetero after UVB irradiation VS hetero before UVB irradiation	Neutrophil degranulation|Reactome	0.68684199	0.02016149	4.465553767	0.00184237	0.04958933	-0.94977179
WT after UVB irradiation VS WT before UVB irradiation	Neutrophil degranulation|Reactome	0.579337798	0.003061767	2.902944223	0.01562775	0.2924023	-2.85390561
homo after UVB irradiation VS homo before UVB irradiation	Nevirapine Metabolism Pathway|SMPDB	-0.55087761	0.058884314	-1.99612938	0.07571705	0.19626432	-4.5940965
hetero after UVB irradiation VS hetero before UVB irradiation	Nevirapine Metabolism Pathway|SMPDB	-0.5789025	0.118018592	-3.0855485	0.01408084	0.1130168	-2.94080837
WT after UVB irradiation VS WT before UVB irradiation	Nevirapine Metabolism Pathway|SMPDB	-0.5790886	0.078999664	-2.01572796	0.0712576	0.44835368	-4.16485416
homo after UVB irradiation VS homo before UVB irradiation	Nevirapine Pathway_ Pharmacokinetics|PharmGKB	-0.44466088	0.029484401	-1.69691413	0.12254435	0.26146613	-5.02464303
hetero after UVB irradiation VS hetero before UVB irradiation	Nevirapine Pathway_ Pharmacokinetics|PharmGKB	-0.40059345	0.121055986	-1.9374588	0.08685454	0.26658789	-4.6728258
WT after UVB irradiation VS WT before UVB irradiation	Nevirapine Pathway_ Pharmacokinetics|PharmGKB	-0.37656296	0.057663335	-1.17919253	0.2653994	0.66118679	-5.21271603
homo after UVB irradiation VS homo before UVB irradiation	nfat and hypertrophy of the heart|BioCarta	0.233197262	-0.02900042	1.412584628	0.19005515	0.34642388	-5.39916331
hetero after UVB irradiation VS hetero before UVB irradiation	nfat and hypertrophy of the heart|BioCarta	0.041964477	0.003038886	0.227632915	0.82535839	0.93322694	-6.31851959
WT after UVB irradiation VS WT before UVB irradiation	nfat and hypertrophy of the heart|BioCarta	0.124407472	-0.00929538	0.534862105	0.60434004	0.87002106	-5.72640115
homo after UVB irradiation VS homo before UVB irradiation	NFG and proNGF binds to p75NTR|Reactome	-0.10375451	-0.00994191	-0.40668398	0.69335458	0.79464283	-6.28037803
hetero after UVB irradiation VS hetero before UVB irradiation	NFG and proNGF binds to p75NTR|Reactome	-0.44216296	-0.07199849	-1.68072222	0.12941216	0.32392126	-5.03125923
WT after UVB irradiation VS WT before UVB irradiation	NFG and proNGF binds to p75NTR|Reactome	0.425235864	0.116469514	1.122084449	0.28783817	0.67413174	-5.27096341
homo after UVB irradiation VS homo before UVB irradiation	NF-kappa B signaling pathway - Homo sapiens (human)|KEGG	0.383103055	-0.01721582	2.226789703	0.05178508	0.15147386	-4.24421742
hetero after UVB irradiation VS hetero before UVB irradiation	NF-kappa B signaling pathway - Homo sapiens (human)|KEGG	0.421020803	-0.01322784	2.555158654	0.03254076	0.15685114	-3.75132471
WT after UVB irradiation VS WT before UVB irradiation	NF-kappa B signaling pathway - Homo sapiens (human)|KEGG	0.329912365	-0.03208377	1.404302725	0.19027702	0.60496032	-4.96339886
homo after UVB irradiation VS homo before UVB irradiation	nfkb activation by nontypeable hemophilus influenzae|BioCarta	0.11438714	0.029147231	0.60282979	0.56089857	0.68698038	-6.17697901
hetero after UVB irradiation VS hetero before UVB irradiation	nfkb activation by nontypeable hemophilus influenzae|BioCarta	0.191845555	0.039416296	1.040324468	0.32711735	0.55613609	-5.79521478
WT after UVB irradiation VS WT before UVB irradiation	nfkb activation by nontypeable hemophilus influenzae|BioCarta	-0.04037169	-0.0516265	-0.16270143	0.87396834	0.96294654	-5.85575966
homo after UVB irradiation VS homo before UVB irradiation	NF-kB activation through FADD/RIP-1 pathway mediated by caspase-8 and -10|Reactome	0.85107651	-0.04010864	4.745079657	0.0009354	0.01805472	-0.36152196
hetero after UVB irradiation VS hetero before UVB irradiation	NF-kB activation through FADD/RIP-1 pathway mediated by caspase-8 and -10|Reactome	0.887568423	0.044657449	4.111653301	0.0030272	0.06159804	-1.43521114
WT after UVB irradiation VS WT before UVB irradiation	NF-kB activation through FADD/RIP-1 pathway mediated by caspase-8 and -10|Reactome	0.984380925	-0.03243078	5.024370393	0.00050559	0.15645713	0.087404886
homo after UVB irradiation VS homo before UVB irradiation	NF-kB is activated and signals survival|Reactome	0.349886103	-0.04556418	1.774161002	0.10839444	0.24166455	-4.91657504
hetero after UVB irradiation VS hetero before UVB irradiation	NF-kB is activated and signals survival|Reactome	0.332989346	-0.03610483	1.379193115	0.20333406	0.42369167	-5.41912402
WT after UVB irradiation VS WT before UVB irradiation	NF-kB is activated and signals survival|Reactome	-0.05800012	0.005742776	-0.2431227	0.81278532	0.94525798	-5.83930126
homo after UVB irradiation VS homo before UVB irradiation	nf-kb signaling pathway|BioCarta	0.503711666	-0.03763026	2.309242156	0.04515791	0.13838323	-4.11641374
hetero after UVB irradiation VS hetero before UVB irradiation	nf-kb signaling pathway|BioCarta	0.518068593	-0.03579128	2.19732128	0.05757194	0.21403988	-4.29220266
WT after UVB irradiation VS WT before UVB irradiation	nf-kb signaling pathway|BioCarta	0.571972106	-0.06196059	2.616039199	0.02561154	0.33903743	-3.28517136
homo after UVB irradiation VS homo before UVB irradiation	NGF processing|Reactome	0.393747226	-0.0787386	1.795293373	0.10479387	0.2370387	-4.88660513
hetero after UVB irradiation VS hetero before UVB irradiation	NGF processing|Reactome	0.480751157	0.022217775	1.628535985	0.14014788	0.34069016	-5.10128348
WT after UVB irradiation VS WT before UVB irradiation	NGF processing|Reactome	0.595352451	0.036212764	2.855413395	0.01695907	0.30353778	-2.92539776
homo after UVB irradiation VS homo before UVB irradiation	NGF|INOH	0.203427726	-0.00651171	1.141290385	0.28202067	0.44245297	-5.71433703
hetero after UVB irradiation VS hetero before UVB irradiation	NGF|INOH	0.240225149	0.001538913	1.330341991	0.21828398	0.44009331	-5.47770057
WT after UVB irradiation VS WT before UVB irradiation	NGF|INOH	0.447092219	0.066021818	2.02347927	0.07033918	0.44678587	-4.15390799
homo after UVB irradiation VS homo before UVB irradiation	NGF-independant TRKA activation|Reactome	0.638437128	0.081556699	2.724502461	0.0225811	0.09770733	-3.45941184
hetero after UVB irradiation VS hetero before UVB irradiation	NGF-independant TRKA activation|Reactome	0.207466087	0.004160524	0.980496319	0.35415162	0.5819117	-5.85349805
WT after UVB irradiation VS WT before UVB irradiation	NGF-independant TRKA activation|Reactome	0.21851385	0.004735553	0.749802973	0.47050771	0.81271132	-5.59259542
homo after UVB irradiation VS homo before UVB irradiation	N-glycan antennae elongation in the medial/trans-Golgi|Reactome	0.263884744	0.032345566	1.364783926	0.2041302	0.36140306	-5.45799118
hetero after UVB irradiation VS hetero before UVB irradiation	N-glycan antennae elongation in the medial/trans-Golgi|Reactome	0.134134552	0.015363208	0.631153798	0.54467709	0.75163129	-6.135537
WT after UVB irradiation VS WT before UVB irradiation	N-glycan antennae elongation in the medial/trans-Golgi|Reactome	0.003826679	-0.01633617	0.014960277	0.98835578	0.99664845	-5.86903781
homo after UVB irradiation VS homo before UVB irradiation	N-Glycan antennae elongation|Reactome	0.157029772	-0.00519485	0.612496343	0.5547518	0.68330098	-6.17091247
hetero after UVB irradiation VS hetero before UVB irradiation	N-Glycan antennae elongation|Reactome	0.043596737	0.017054682	0.215372952	0.83459871	0.93635724	-6.32144843
WT after UVB irradiation VS WT before UVB irradiation	N-Glycan antennae elongation|Reactome	-0.17782872	-0.03070439	-0.6867727	0.50771358	0.82517008	-5.63605533
homo after UVB irradiation VS homo before UVB irradiation	N-Glycan biosynthesis - Homo sapiens (human)|KEGG	0.35507409	-0.03300393	2.256434145	0.04929984	0.14541543	-4.19840842
hetero after UVB irradiation VS hetero before UVB irradiation	N-Glycan biosynthesis - Homo sapiens (human)|KEGG	0.338142885	-0.01445071	1.613539813	0.14337981	0.34688172	-5.12120182
WT after UVB irradiation VS WT before UVB irradiation	N-Glycan biosynthesis - Homo sapiens (human)|KEGG	0.403438177	-0.00944595	1.808154324	0.10046004	0.50142188	-4.45166187
homo after UVB irradiation VS homo before UVB irradiation	N-Glycan biosynthesis|EHMN	0.558210809	-0.03416899	3.501752505	0.00628271	0.04612031	-2.22020801
hetero after UVB irradiation VS hetero before UVB irradiation	N-Glycan biosynthesis|EHMN	0.455989196	0.002078977	2.067276204	0.07077232	0.23824241	-4.48446094
WT after UVB irradiation VS WT before UVB irradiation	N-Glycan biosynthesis|EHMN	0.570057127	-0.01636318	2.671209107	0.02328996	0.32329864	-3.20238727
homo after UVB irradiation VS homo before UVB irradiation	N-glycan trimming and elongation in the cis-Golgi|Reactome	0.732670984	-0.05757297	3.22282718	0.00988897	0.0594119	-2.66201033
hetero after UVB irradiation VS hetero before UVB irradiation	N-glycan trimming and elongation in the cis-Golgi|Reactome	0.725402387	0.017642528	4.043154999	0.00333961	0.06477425	-1.53142168
WT after UVB irradiation VS WT before UVB irradiation	N-glycan trimming and elongation in the cis-Golgi|Reactome	0.353515809	-0.04962942	1.15513314	0.27467827	0.66570326	-5.23751855
homo after UVB irradiation VS homo before UVB irradiation	N-glycan trimming in the ER and Calnexin/Calreticulin cycle|Reactome	0.061476202	-0.0176866	0.279270997	0.78609124	0.86364114	-6.32672057
hetero after UVB irradiation VS hetero before UVB irradiation	N-glycan trimming in the ER and Calnexin/Calreticulin cycle|Reactome	0.27562512	-0.00545234	1.450362695	0.18313972	0.40248354	-5.33148198
WT after UVB irradiation VS WT before UVB irradiation	N-glycan trimming in the ER and Calnexin/Calreticulin cycle|Reactome	0.127377862	0.003009153	0.495769225	0.6306854	0.87303764	-5.74624662
homo after UVB irradiation VS homo before UVB irradiation	NHR|Signalink	0.011827518	0.026412477	0.062897678	0.9511667	0.97016767	-6.36632308
hetero after UVB irradiation VS hetero before UVB irradiation	NHR|Signalink	-0.38432268	0.013955373	-2.22874983	0.05476281	0.20875475	-4.24529906

WT after UVB irradiation VS WT before UVB irradiation	NHR|Signalink	-0.26096172	0.051070374	-1.11064984	0.29250413	0.67947895	-5.28236389
homo after UVB irradiation VS homo before UVB irradiation	NICD traffics to nucleus|Reactome	-0.34877969	0.012416797	-1.50298274	0.16570876	0.31526092	-5.2844505
hetero after UVB irradiation VS hetero before UVB irradiation	NICD traffics to nucleus|Reactome	-0.13364681	-0.03963393	-0.5835319	0.57481896	0.7705309	-6.16556045
WT after UVB irradiation VS WT before UVB irradiation	NICD traffics to nucleus|Reactome	-0.17777877	-0.04280642	-0.73036313	0.48179458	0.81552033	-5.60636259
homo after UVB irradiation VS homo before UVB irradiation	Nicotinamide salvaging|Reactome	0.184421351	-0.00870299	0.960487497	0.36089027	0.51607422	-5.89628652
hetero after UVB irradiation VS hetero before UVB irradiation	Nicotinamide salvaging|Reactome	-0.10624529	0.000234199	-0.50523982	0.62633899	0.80713969	-6.21017787
WT after UVB irradiation VS WT before UVB irradiation	Nicotinamide salvaging|Reactome	0.11256103	-0.02284094	0.489383933	0.63504195	0.87541098	-5.74935262
homo after UVB irradiation VS homo before UVB irradiation	Nicotinate and nicotinamide metabolism - Homo sapiens (human)|KEGG	0.508792225	-0.01989758	3.147487543	0.01119195	0.06448659	-2.7822119
hetero after UVB irradiation VS hetero before UVB irradiation	Nicotinate and nicotinamide metabolism - Homo sapiens (human)|KEGG	0.277395392	0.018702285	1.329867393	0.2184337	0.44015067	-5.47826307
WT after UVB irradiation VS WT before UVB irradiation	Nicotinate and nicotinamide metabolism - Homo sapiens (human)|KEGG	0.155226065	-0.03626259	0.640740042	0.53598264	0.83767333	-5.66561416
homo after UVB irradiation VS homo before UVB irradiation	Nicotinate and Nicotinamide Metabolism|SMPDB	0.486057684	0.023217182	2.953564584	0.01542152	0.07691241	-3.0925654
hetero after UVB irradiation VS hetero before UVB irradiation	Nicotinate and Nicotinamide Metabolism|SMPDB	0.212937917	0.035142437	1.025289043	0.33375814	0.56197621	-5.8101132
WT after UVB irradiation VS WT before UVB irradiation	Nicotinate and Nicotinamide Metabolism|SMPDB	0.21003956	-0.0224995	0.748969002	0.47098844	0.81275627	-5.59319262
homo after UVB irradiation VS homo before UVB irradiation	Nicotinate metabolism|Reactome	0.321583353	-0.00563566	1.986222245	0.07695205	0.19626432	-4.6088222
hetero after UVB irradiation VS hetero before UVB irradiation	Nicotinate metabolism|Reactome	0.113861954	-0.01089736	0.598457068	0.56527194	0.76437184	-6.15638249
WT after UVB irradiation VS WT before UVB irradiation	Nicotinate metabolism|Reactome	0.128786947	-0.04520856	0.536677318	0.60313067	0.86947531	-5.72544519
homo after UVB irradiation VS homo before UVB irradiation	Nicotinate Nicotinamide metabolism|INOH	0.211277339	-0.00073265	1.159647596	0.27483123	0.43614438	-5.69452644
hetero after UVB irradiation VS hetero before UVB irradiation	Nicotinate Nicotinamide metabolism|INOH	0.091904948	0.034126921	0.447491841	0.66580112	0.82651398	-6.23921603
WT after UVB irradiation VS WT before UVB irradiation	Nicotinate Nicotinamide metabolism|INOH	0.071772286	-0.05815477	0.306788051	0.76524718	0.92751695	-5.8217094
homo after UVB irradiation VS homo before UVB irradiation	Nicotine Action Pathway|SMPDB	0.064990694	0.078371873	0.395993558	0.70095816	0.80134576	-6.28490862
hetero after UVB irradiation VS hetero before UVB irradiation	Nicotine Action Pathway|SMPDB	-0.17583902	0.045404657	-0.98185245	0.35352073	0.58157426	-5.85220683
WT after UVB irradiation VS WT before UVB irradiation	Nicotine Action Pathway|SMPDB	-0.06423548	0.032234998	-0.28980224	0.77784005	0.93502208	-5.82679457
homo after UVB irradiation VS homo before UVB irradiation	Nicotine Activity on Chromaffin Cells|Wikipathways	0.21416965	0.05671292	0.658299015	0.52615675	0.66255701	-6.14096609
hetero after UVB irradiation VS hetero before UVB irradiation	Nicotine Activity on Chromaffin Cells|Wikipathways	-0.2262246	-0.04226961	-0.87585717	0.40538289	0.63319136	-5.94878287
WT after UVB irradiation VS WT before UVB irradiation	Nicotine Activity on Chromaffin Cells|Wikipathways	-0.30347486	0.009493876	-1.10101244	0.29648208	0.68285796	-5.29190327
homo after UVB irradiation VS homo before UVB irradiation	Nicotine Activity on Dopaminergic Neurons|Wikipathways	0.089803392	0.040960183	0.467772263	0.65061508	0.75841421	-6.2522676
hetero after UVB irradiation VS hetero before UVB irradiation	Nicotine Activity on Dopaminergic Neurons|Wikipathways	0.184431788	0.040396336	0.83044559	0.4291874	0.65540741	-5.98737388
WT after UVB irradiation VS WT before UVB irradiation	Nicotine Activity on Dopaminergic Neurons|Wikipathways	0.259813092	-0.02588066	1.21307907	0.25275525	0.64679852	-5.17714767
homo after UVB irradiation VS homo before UVB irradiation	Nicotine addiction - Homo sapiens (human)|KEGG	-0.21916981	0.066283977	-1.03270807	0.32760659	0.48374714	-5.82656078
hetero after UVB irradiation VS hetero before UVB irradiation	Nicotine addiction - Homo sapiens (human)|KEGG	-0.51072454	0.002797565	-2.65299377	0.02784756	0.14438349	-3.6017963
WT after UVB irradiation VS WT before UVB irradiation	Nicotine addiction - Homo sapiens (human)|KEGG	-0.56301901	0.016361519	-2.56904918	0.02776951	0.35131125	-3.35555848
homo after UVB irradiation VS homo before UVB irradiation	nicotine degradation III|HumanCyc	-0.08656644	0.165812374	-0.40077357	0.69755399	0.79905457	-6.28289726
hetero after UVB irradiation VS hetero before UVB irradiation	nicotine degradation III|HumanCyc	0.126967217	-0.05778351	0.47013301	0.65018877	0.82458554	-6.22822822
WT after UVB irradiation VS WT before UVB irradiation	nicotine degradation III|HumanCyc	0.048977275	-0.01637268	0.223113334	0.82790277	0.94525798	-5.84399997
homo after UVB irradiation VS homo before UVB irradiation	nicotine degradation IV|HumanCyc	-0.27312169	0.063664167	-1.22172119	0.25159886	0.41457971	-5.62582527
hetero after UVB irradiation VS hetero before UVB irradiation	nicotine degradation IV|HumanCyc	-0.40571298	-0.0180075	-1.87841357	0.0952762	0.27959342	-4.75708086
WT after UVB irradiation VS WT before UVB irradiation	nicotine degradation IV|HumanCyc	-0.11471614	-0.04865961	-0.44154896	0.66813498	0.87935805	-5.77139899
homo after UVB irradiation VS homo before UVB irradiation	Nicotine Metabolism Pathway|SMPDB	-0.44449162	0.140963625	-1.94251111	0.08263227	0.20394538	-4.67345092
hetero after UVB irradiation VS hetero before UVB irradiation	Nicotine Metabolism Pathway|SMPDB	-0.69332103	0.083239541	-3.42124825	0.00839256	0.0927463	-2.43492844
WT after UVB irradiation VS WT before UVB irradiation	Nicotine Metabolism Pathway|SMPDB	-0.79754583	0.054234214	-3.52357155	0.00543721	0.20968398	-1.93126784
homo after UVB irradiation VS homo before UVB irradiation	Nicotine Metabolism|Wikipathways	-0.3994521	0.173745036	-1.58200117	0.14670985	0.29223756	-5.18071932
hetero after UVB irradiation VS hetero before UVB irradiation	Nicotine Metabolism|Wikipathways	-0.3040901	0.055118112	-1.06957475	0.31449051	0.54338429	-5.76575839
WT after UVB irradiation VS WT before UVB irradiation	Nicotine Metabolism|Wikipathways	-0.54729751	0.05387175	-2.20043543	0.05219057	0.41452927	-3.90026188
homo after UVB irradiation VS homo before UVB irradiation	Nicotine Pathway (Chromaffin Cell)_ Pharmacodynamics|PharmGKB	0.21416965	0.05671292	0.658299015	0.52615675	0.66255701	-6.14096609
hetero after UVB irradiation VS hetero before UVB irradiation	Nicotine Pathway (Chromaffin Cell)_ Pharmacodynamics|PharmGKB	-0.2262246	-0.04226961	-0.87585717	0.40538289	0.63319136	-5.94878287
WT after UVB irradiation VS WT before UVB irradiation	Nicotine Pathway (Chromaffin Cell)_ Pharmacodynamics|PharmGKB	-0.30347486	0.009493876	-1.10101244	0.29648208	0.68285796	-5.29190327
homo after UVB irradiation VS homo before UVB irradiation	Nicotine Pathway (Dopaminergic Neuron)_ Pharmacodynamics|PharmGKB	0.440546407	-0.00832703	2.577830525	0.02884914	0.11394911	-3.69330462
hetero after UVB irradiation VS hetero before UVB irradiation	Nicotine Pathway (Dopaminergic Neuron)_ Pharmacodynamics|PharmGKB	0.232474698	0.012459034	1.498086852	0.17060291	0.3872586	-5.27126819
WT after UVB irradiation VS WT before UVB irradiation	Nicotine Pathway (Dopaminergic Neuron)_ Pharmacodynamics|PharmGKB	0.312684145	0.006818857	1.441697434	0.17971888	0.59519662	-4.91919709
homo after UVB irradiation VS homo before UVB irradiation	Nicotine Pathway_ Pharmacokinetics|PharmGKB	-0.46889476	0.203612236	-1.89306786	0.0895331	0.21437238	-4.74584824
hetero after UVB irradiation VS hetero before UVB irradiation	Nicotine Pathway_ Pharmacokinetics|PharmGKB	-0.3040901	0.055118112	-1.06957475	0.31449051	0.54338429	-5.76575839
WT after UVB irradiation VS WT before UVB irradiation	Nicotine Pathway_ Pharmacokinetics|PharmGKB	-0.55574417	0.072374273	-2.2748759	0.04598302	0.39565089	-3.79171882
homo after UVB irradiation VS homo before UVB irradiation	Nifedipine Action Pathway|SMPDB	0.396415397	-0.03677376	2.343069613	0.04268583	0.13402181	-4.06364635
hetero after UVB irradiation VS hetero before UVB irradiation	Nifedipine Action Pathway|SMPDB	-0.01555055	-0.01775524	-0.07410913	0.94265284	0.97054702	-6.34356282
WT after UVB irradiation VS WT before UVB irradiation	Nifedipine Action Pathway|SMPDB	0.056685205	-0.03978418	0.223404833	0.82768199	0.94525798	-5.84393438
homo after UVB irradiation VS homo before UVB irradiation	Nifedipine Activity|Wikipathways	0.173022396	0.001694688	0.933640737	0.37387591	0.52688809	-5.92115789
hetero after UVB irradiation VS hetero before UVB irradiation	Nifedipine Activity|Wikipathways	0.436672358	0.033358961	2.132710974	0.06379824	0.22647381	-4.38811395
WT after UVB irradiation VS WT before UVB irradiation	Nifedipine Activity|Wikipathways	0.512281986	0.016476088	2.042569521	0.06812474	0.44205977	-4.12688514
homo after UVB irradiation VS homo before UVB irradiation	NIK-->noncanonical NF-kB signaling|Reactome	0.045441022	-0.03299775	0.183770222	0.8581014	0.9134577	-6.35033611
hetero after UVB irradiation VS hetero before UVB irradiation	NIK-->noncanonical NF-kB signaling|Reactome	0.076072626	-0.05009513	0.377439054	0.7151775	0.85921866	-6.26992267
WT after UVB irradiation VS WT before UVB irradiation	NIK-->noncanonical NF-kB signaling|Reactome	0.141549645	-0.05582595	0.608181946	0.55651867	0.84793065	-5.68538623
homo after UVB irradiation VS homo before UVB irradiation	Nimodipine Action Pathway|SMPDB	0.396415397	-0.03677376	2.343069613	0.04268583	0.13402181	-4.06364635
hetero after UVB irradiation VS hetero before UVB irradiation	Nimodipine Action Pathway|SMPDB	-0.01555055	-0.01775524	-0.07410913	0.94265284	0.97054702	-6.34356282
WT after UVB irradiation VS WT before UVB irradiation	Nimodipine Action Pathway|SMPDB	0.056685205	-0.03978418	0.223404833	0.82768199	0.94525798	-5.84393438
homo after UVB irradiation VS homo before UVB irradiation	Nisoldipine Action Pathway|SMPDB	0.396415397	-0.03677376	2.343069613	0.04268583	0.13402181	-4.06364635
hetero after UVB irradiation VS hetero before UVB irradiation	Nisoldipine Action Pathway|SMPDB	-0.01555055	-0.01775524	-0.07410913	0.94265284	0.97054702	-6.34356282
WT after UVB irradiation VS WT before UVB irradiation	Nisoldipine Action Pathway|SMPDB	0.056685205	-0.03978418	0.223404833	0.82768199	0.94525798	-5.84393438
homo after UVB irradiation VS homo before UVB irradiation	Nitrendipine Action Pathway|SMPDB	0.396415397	-0.03677376	2.343069613	0.04268583	0.13402181	-4.06364635
hetero after UVB irradiation VS hetero before UVB irradiation	Nitrendipine Action Pathway|SMPDB	-0.01555055	-0.01775524	-0.07410913	0.94265284	0.97054702	-6.34356282
WT after UVB irradiation VS WT before UVB irradiation	Nitrendipine Action Pathway|SMPDB	0.056685205	-0.03978418	0.223404833	0.82768199	0.94525798	-5.84393438
homo after UVB irradiation VS homo before UVB irradiation	nitric oxide signaling pathway|BioCarta	0.136949037	-0.00724407	0.764917664	0.46311209	0.6088463	-6.06374791
hetero after UVB irradiation VS hetero before UVB irradiation	nitric oxide signaling pathway|BioCarta	-0.03294154	-0.04369406	-0.14616119	0.8872307	0.9607977	-6.33496701
WT after UVB irradiation VS WT before UVB irradiation	nitric oxide signaling pathway|BioCarta	0.031116086	-0.07253781	0.104040319	0.9191779	0.97566251	-5.86367077
homo after UVB irradiation VS homo before UVB irradiation	Nitric oxide stimulates guanylate cyclase|Reactome	0.530416742	-0.0101349	2.829175205	0.01896475	0.08809059	-3.29188552
hetero after UVB irradiation VS hetero before UVB irradiation	Nitric oxide stimulates guanylate cyclase|Reactome	0.313781241	0.00062169	1.337285719	0.21610334	0.43741933	-5.46945597
WT after UVB irradiation VS WT before UVB irradiation	Nitric oxide stimulates guanylate cyclase|Reactome	0.206514686	-0.00387029	0.647141099	0.53199745	0.83483909	-5.66161573
homo after UVB irradiation VS homo before UVB irradiation	Nitrogen metabolism - Homo sapiens (human)|KEGG	0.283501829	0.018252844	1.511842825	0.16347574	0.31309993	-5.27297481
hetero after UVB irradiation VS hetero before UVB irradiation	Nitrogen metabolism - Homo sapiens (human)|KEGG	-0.1128441	-0.02031334	-0.71348272	0.49483173	0.71428009	-6.07864129
WT after UVB irradiation VS WT before UVB irradiation	Nitrogen metabolism - Homo sapiens (human)|KEGG	-0.02128442	0.076202325	-0.10330958	0.91974334	0.97566251	-5.86374744
homo after UVB irradiation VS homo before UVB irradiation	Nizatidine Action Pathway|SMPDB	0.21780303	0.051698704	1.19150037	0.26270351	0.42561191	-5.65959884
hetero after UVB irradiation VS hetero before UVB irradiation	Nizatidine Action Pathway|SMPDB	0.331566302	0.063470812	1.441200644	0.18563717	0.40321767	-5.34291209
WT after UVB irradiation VS WT before UVB irradiation	Nizatidine Action Pathway|SMPDB	-0.11560861	0.135982321	-0.44330845	0.66690385	0.87935805	-5.77062647
homo after UVB irradiation VS homo before UVB irradiation	NLR Proteins|Wikipathways	0.200387273	-0.05258909	0.738973602	0.47798889	0.62090961	-6.08348671
hetero after UVB irradiation VS hetero before UVB irradiation	NLR Proteins|Wikipathways	0.340771906	0.006708223	1.357812935	0.2097653	0.43184744	-5.44492402
WT after UVB irradiation VS WT before UVB irradiation	NLR Proteins|Wikipathways	0.114801174	-0.0247616	0.462355225	0.65364359	0.87935805	-5.76207558
homo after UVB irradiation VS homo before UVB irradiation	no2-dependent il-12 pathway in nk cells|BioCarta	0.407799773	-0.02476672	2.48001838	0.03396961	0.12319718	-3.84836563
hetero after UVB irradiation VS hetero before UVB irradiation	no2-dependent il-12 pathway in nk cells|BioCarta	0.460581474	0.00852593	1.834142503	0.10209224	0.2870675	-4.81959413
WT after UVB irradiation VS WT before UVB irradiation	no2-dependent il-12 pathway in nk cells|BioCarta	0.310741642	-0.09032786	0.963101014	0.35802721	0.74302468	-5.42121254
homo after UVB irradiation VS homo before UVB irradiation	NO-cGMP-PKG mediated Neuroprotection|Wikipathways	0.427516289	0.030911072	2.460195123	0.03511269	0.12483583	-3.87967293
hetero after UVB irradiation VS hetero before UVB irradiation	NO-cGMP-PKG mediated Neuroprotection|Wikipathways	0.184635237	0.02092059	0.837682447	0.42533031	0.6520873	-5.98133933
WT after UVB irradiation VS WT before UVB irradiation	NO-cGMP-PKG mediated Neuroprotection|Wikipathways	0.237946398	-0.04939111	0.832920368	0.42418098	0.79068324	-5.53016273
homo after UVB irradiation VS homo before UVB irradiation	NOD1/2 Signaling Pathway|Reactome	0.380801738	-0.05119874	2.060282082	0.06816465	0.18264973	-4.49808666
hetero after UVB irradiation VS hetero before UVB irradiation	NOD1/2 Signaling Pathway|Reactome	0.517554329	0.046546735	2.937188061	0.01776322	0.11751009	-3.16693043
WT after UVB irradiation VS WT before UVB irradiation	NOD1/2 Signaling Pathway|Reactome	0.35641328	0.025612637	1.708967446	0.11800495	0.52194851	-4.58371765
homo after UVB irradiation VS homo before UVB irradiation	NOD-like receptor signaling pathway - Homo sapiens (human)|KEGG	0.558629465	-0.02125599	3.357680598	0.00793385	0.05229475	-2.44768341
hetero after UVB irradiation VS hetero before UVB irradiation	NOD-like receptor signaling pathway - Homo sapiens (human)|KEGG	0.576419594	0.023238936	3.559660616	0.00680607	0.08550202	-2.22956748
WT after UVB irradiation VS WT before UVB irradiation	NOD-like receptor signaling pathway - Homo sapiens (human)|KEGG	0.455127238	-0.01400604	2.077998437	0.06418897	0.43544914	-4.07650057
homo after UVB irradiation VS homo before UVB irradiation	Non Ketotic Hyperglycinemia|SMPDB	-0.11801782	0.021953564	-0.50783827	0.62328187	0.73728109	-6.23179983
hetero after UVB irradiation VS hetero before UVB irradiation	Non Ketotic Hyperglycinemia|SMPDB	-0.03882851	0.032366748	-0.2244119	0.82778335	0.93322694	-6.31930458
WT after UVB irradiation VS WT before UVB irradiation	Non Ketotic Hyperglycinemia|SMPDB	0.143497804	-0.01134274	0.603206807	0.55969543	0.84911968	-5.68832383
homo after UVB irradiation VS homo before UVB irradiation	Non-alcoholic fatty liver disease (NAFLD) - Homo sapiens (human)|KEGG	0.205295196	-0.02799875	0.928882517	0.37621224	0.52936367	-5.92550545
hetero after UVB irradiation VS hetero before UVB irradiation	Non-alcoholic fatty liver disease (NAFLD) - Homo sapiens (human)|KEGG	-0.0971102	-0.00272873	-0.4771164	0.6454094	0.82080804	-6.22473547
WT after UVB irradiation VS WT before UVB irradiation	Non-alcoholic fatty liver disease (NAFLD) - Homo sapiens (human)|KEGG	0.120041428	-0.04344741	0.438677222	0.67014656	0.87984445	-5.77265353
homo after UVB irradiation VS homo before UVB irradiation	Nonalcoholic fatty liver disease|Wikipathways	0.223713862	-0.02869284	1.039211635	0.32472663	0.48061646	-5.82008465
hetero after UVB irradiation VS hetero before UVB irradiation	Nonalcoholic fatty liver disease|Wikipathways	-0.06241575	-0.00119546	-0.30287259	0.76933	0.8963654	-6.29705418
WT after UVB irradiation VS WT before UVB irradiation	Nonalcoholic fatty liver disease|Wikipathways	0.13379085	-0.04085084	0.49524733	0.63104093	0.87303764	-5.74650192
homo after UVB irradiation VS homo before UVB irradiation	Noncanonical activation of NOTCH3|Reactome	0.893762608	-0.03346979	3.722173465	0.00441627	0.0379263	-1.8759865
hetero after UVB irradiation VS hetero before UVB irradiation	Noncanonical activation of NOTCH3|Reactome	1.130398424	0.118532546	5.515207016	0.00047012	0.03004176	0.369688696
WT after UVB irradiation VS WT before UVB irradiation	Noncanonical activation of NOTCH3|Reactome	0.972348499	-0.03175993	4.546100615	0.00104266	0.15645713	-0.51446282
homo after UVB irradiation VS homo before UVB irradiation	Noncanonical Wnt signaling pathway|PID	0.077809526	0.010476075	0.356175597	0.72958153	0.8217964	-6.30075451
hetero after UVB irradiation VS hetero before UVB irradiation	Noncanonical Wnt signaling pathway|PID	0.100916809	-0.03730442	0.554619651	0.59356731	0.78483643	-6.18273113
WT after UVB irradiation VS WT before UVB irradiation	Noncanonical Wnt signaling pathway|PID	-0.06608074	0.021014926	-0.32132589	0.75452563	0.92087615	-5.81713181
homo after UVB irradiation VS homo before UVB irradiation	Non-genomic actions of 1_25 dihydroxyvitamin D3|Wikipathways	0.368142018	-0.04081491	2.2660209	0.0485213	0.14404959	-4.18355942
hetero after UVB irradiation VS hetero before UVB irradiation	Non-genomic actions of 1_25 dihydroxyvitamin D3|Wikipathways	0.477864963	0.02081536	3.10872821	0.01358139	0.11267501	-2.90558987
WT after UVB irradiation VS WT before UVB irradiation	Non-genomic actions of 1_25 dihydroxyvitamin D3|Wikipathways	0.35164231	0.021827555	1.678560626	0.12391507	0.52904005	-4.623455
homo after UVB irradiation VS homo before UVB irradiation	Nongenotropic Androgen signaling|PID	0.485408565	-0.03297031	2.730526694	0.02235522	0.09709374	-3.44978018
hetero after UVB irradiation VS hetero before UVB irradiation	Nongenotropic Androgen signaling|PID	0.5866875	0.005045217	3.546674071	0.00694049	0.08598141	-2.24874207
WT after UVB irradiation VS WT before UVB irradiation	Nongenotropic Androgen signaling|PID	0.516159968	0.051163565	2.705045065	0.02197127	0.31542987	-3.1515565
homo after UVB irradiation VS homo before UVB irradiation	Non-homologous end joining|Wikipathways	-0.5744929	-0.07794549	-2.13803989	0.05997449	0.1674884	-4.38030799
hetero after UVB irradiation VS hetero before UVB irradiation	Non-homologous end joining|Wikipathways	-0.49123006	-0.119238	-1.5823183	0.15032645	0.35798743	-5.16236724
WT after UVB irradiation VS WT before UVB irradiation	Non-homologous end joining|Wikipathways	-0.36034888	0.029017653	-1.50742703	0.16237728	0.56565755	-4.83976326
homo after UVB irradiation VS homo before UVB irradiation	Non-homologous end-joining - Homo sapiens (human)|KEGG	-0.34175833	-0.01871602	-1.23500007	0.24684154	0.40910289	-5.61079347
hetero after UVB irradiation VS hetero before UVB irradiation	Non-homologous end-joining - Homo sapiens (human)|KEGG	-0.09634763	-0.02562554	-0.3870137	0.70833806	0.85503307	-6.26602166
WT after UVB irradiation VS WT before UVB irradiation	Non-homologous end-joining - Homo sapiens (human)|KEGG	0.096458292	0.034193486	0.393840959	0.70189933	0.90059561	-5.79121783
homo after UVB irradiation VS homo before UVB irradiation	Nonhomologous End-Joining (NHEJ)|Reactome	-0.56807395	-0.0012577	-3.31816266	0.00846138	0.05472333	-2.51036501
hetero after UVB irradiation VS hetero before UVB irradiation	Nonhomologous End-Joining (NHEJ)|Reactome	-0.15792414	-0.03302121	-0.60308627	0.56232928	0.76403561	-6.1534927
WT after UVB irradiation VS WT before UVB irradiation	Nonhomologous End-Joining (NHEJ)|Reactome	-0.26010693	-0.02114795	-1.09503801	0.29896899	0.68477511	-5.29778493
homo after UVB irradiation VS homo before UVB irradiation	Non-integrin membrane-ECM interactions|Reactome	0.724115931	-0.02652488	3.858453703	0.00356193	0.03390684	-1.66586653
hetero after UVB irradiation VS hetero before UVB irradiation	Non-integrin membrane-ECM interactions|Reactome	0.445698992	0.045003207	2.023902618	0.07579681	0.24694916	-4.54783475
WT after UVB irradiation VS WT before UVB irradiation	Non-integrin membrane-ECM interactions|Reactome	0.438203825	0.00147075	1.977929724	0.07590045	0.46034175	-4.21800858
homo after UVB irradiation VS homo before UVB irradiation	Nonsense Mediated Decay (NMD) enhanced by the Exon Junction Complex (EJC)|Reactome	-0.94424035	0.028961175	-5.26579089	0.0004495	0.01666204	0.347433047
hetero after UVB irradiation VS hetero before UVB irradiation	Nonsense Mediated Decay (NMD) enhanced by the Exon Junction Complex (EJC)|Reactome	-0.2265561	-0.03142519	-0.71886244	0.49167749	0.71190152	-6.07470809
WT after UVB irradiation VS WT before UVB irradiation	Nonsense Mediated Decay (NMD) enhanced by the Exon Junction Complex (EJC)|Reactome	-0.57476546	-0.00219345	-1.92827924	0.0824317	0.46860292	-4.28723935
homo after UVB irradiation VS homo before UVB irradiation	Nonsense Mediated Decay (NMD) independent of the Exon Junction Complex (EJC)|Reactome	-0.92144047	0.023367648	-4.82483618	0.00083396	0.01759873	-0.2500845
hetero after UVB irradiation VS hetero before UVB irradiation	Nonsense Mediated Decay (NMD) independent of the Exon Junction Complex (EJC)|Reactome	-0.12396889	0.005745984	-0.37740946	0.71519868	0.85921866	-6.26993458
WT after UVB irradiation VS WT before UVB irradiation	Nonsense Mediated Decay (NMD) independent of the Exon Junction Complex (EJC)|Reactome	-0.56797846	0.004236837	-1.87454628	0.09008904	0.48188205	-4.3613514
homo after UVB irradiation VS homo before UVB irradiation	Nonsense-Mediated Decay (NMD)|Reactome	-0.94424035	0.028961175	-5.26579089	0.0004495	0.01666204	0.347433047
hetero after UVB irradiation VS hetero before UVB irradiation	Nonsense-Mediated Decay (NMD)|Reactome	-0.2265561	-0.03142519	-0.71886244	0.49167749	0.71190152	-6.07470809
WT after UVB irradiation VS WT before UVB irradiation	Nonsense-Mediated Decay (NMD)|Reactome	-0.57476546	-0.00219345	-1.92827924	0.0824317	0.46860292	-4.28723935
homo after UVB irradiation VS homo before UVB irradiation	Non-small cell lung cancer - Homo sapiens (human)|KEGG	0.17757161	-0.01247264	0.955938984	0.36306691	0.51751626	-5.90054097
hetero after UVB irradiation VS hetero before UVB irradiation	Non-small cell lung cancer - Homo sapiens (human)|KEGG	0.340886536	0.004380278	2.078834974	0.06948871	0.23555906	-4.46750406
WT after UVB irradiation VS WT before UVB irradiation	Non-small cell lung cancer - Homo sapiens (human)|KEGG	0.113297814	0.019588036	0.566101638	0.58370438	0.8604835	-5.70952599
homo after UVB irradiation VS homo before UVB irradiation	Non-small cell lung cancer|Wikipathways	0.185744373	-0.0128808	1.023711528	0.33162231	0.48797534	-5.83546639
hetero after UVB irradiation VS hetero before UVB irradiation	Non-small cell lung cancer|Wikipathways	0.345815866	0.007297652	2.060928494	0.07148698	0.23970209	-4.4937612
WT after UVB irradiation VS WT before UVB irradiation	Non-small cell lung cancer|Wikipathways	0.12561711	0.015490701	0.632339181	0.5412391	0.83916339	-5.67080653
homo after UVB irradiation VS homo before UVB irradiation	noradrenaline and adrenaline degradation|HumanCyc	0.463246829	0.032337004	1.896354616	0.08905807	0.21379556	-4.74105986
hetero after UVB irradiation VS hetero before UVB irradiation	noradrenaline and adrenaline degradation|HumanCyc	-0.38981588	0.011578228	-1.88145323	0.09482436	0.27848545	-4.75276735
WT after UVB irradiation VS WT before UVB irradiation	noradrenaline and adrenaline degradation|HumanCyc	0.285989319	0.036165002	1.133129645	0.2833863	0.67189698	-5.25986716

homo after UVB irradiation VS homo before UVB irradiation	NoRC negatively regulates rRNA expression|Reactome	-0.6407904	0.009415556	-3.7049514	0.00453865	0.0383987	-1.90269484
hetero after UVB irradiation VS hetero before UVB irradiation	NoRC negatively regulates rRNA expression|Reactome	-0.39951652	-0.04732262	-1.72872606	0.12020938	0.3132781	-4.96592075
WT after UVB irradiation VS WT before UVB irradiation	NoRC negatively regulates rRNA expression|Reactome	-0.2696335	-0.00654249	-0.95987144	0.35957324	0.74374432	-5.42407392
homo after UVB irradiation VS homo before UVB irradiation	Norepinephrine Neurotransmitter Release Cycle|Reactome	0.355327812	0.011508254	1.998089961	0.07547488	0.19626432	-4.59117903
hetero after UVB irradiation VS hetero before UVB irradiation	Norepinephrine Neurotransmitter Release Cycle|Reactome	-0.0871296	-0.03850951	-0.49022042	0.63648797	0.81491914	-6.21805017
WT after UVB irradiation VS WT before UVB irradiation	Norepinephrine Neurotransmitter Release Cycle|Reactome	0.047389146	-0.01435154	0.179108891	0.86140042	0.95916804	-5.85292741
homo after UVB irradiation VS homo before UVB irradiation	NOSIP mediated eNOS trafficking|Reactome	1.132720144	-0.03714561	7.273537778	3.76E-05	0.01666204	2.679669395
hetero after UVB irradiation VS hetero before UVB irradiation	NOSIP mediated eNOS trafficking|Reactome	1.182060943	-0.00664124	7.467176846	5.44E-05	0.02920766	2.366662596
WT after UVB irradiation VS WT before UVB irradiation	NOSIP mediated eNOS trafficking|Reactome	0.703759308	0.003464451	3.090982289	0.01131891	0.2678418	-2.57166577
homo after UVB irradiation VS homo before UVB irradiation	NOSTRIN mediated eNOS trafficking|Reactome	1.062021001	-0.08588616	6.171731066	0.00013755	0.01666204	1.476297403
hetero after UVB irradiation VS hetero before UVB irradiation	NOSTRIN mediated eNOS trafficking|Reactome	0.85750578	0.035148032	4.09359417	0.0031064	0.06222118	-1.46050738
WT after UVB irradiation VS WT before UVB irradiation	NOSTRIN mediated eNOS trafficking|Reactome	0.970805429	-0.03627256	4.690721378	0.00083482	0.15645713	-0.32810158
homo after UVB irradiation VS homo before UVB irradiation	Notch signaling pathway - Homo sapiens (human)|KEGG	0.09664171	-0.0245329	0.459538735	0.65630247	0.76329013	-6.25627548
hetero after UVB irradiation VS hetero before UVB irradiation	Notch signaling pathway - Homo sapiens (human)|KEGG	0.246776843	0.013323219	1.333622212	0.21725152	0.43873833	-5.47380923
WT after UVB irradiation VS WT before UVB irradiation	Notch signaling pathway - Homo sapiens (human)|KEGG	0.113492753	0.030440629	0.485604853	0.63762724	0.87636259	-5.75117287
homo after UVB irradiation VS homo before UVB irradiation	Notch signaling pathway|PID	0.035483233	-0.00914105	0.19122524	0.85242053	0.91023202	-6.34883979
hetero after UVB irradiation VS hetero before UVB irradiation	Notch signaling pathway|PID	0.232570624	0.015568494	1.485693978	0.17378331	0.39118926	-5.28701154
WT after UVB irradiation VS WT before UVB irradiation	Notch signaling pathway|PID	0.095002164	-0.01063172	0.431019156	0.6755242	0.88217037	-5.77596058
homo after UVB irradiation VS homo before UVB irradiation	Notch Signaling Pathway|Wikipathways	0.024421604	-0.03013559	0.125225226	0.90298562	0.94250012	-6.3600298
hetero after UVB irradiation VS hetero before UVB irradiation	Notch Signaling Pathway|Wikipathways	0.241297362	0.004792198	1.527880684	0.16316743	0.37656564	-5.23312043
WT after UVB irradiation VS WT before UVB irradiation	Notch Signaling Pathway|Wikipathways	0.105935819	0.025474952	0.471674552	0.64720087	0.87935805	-5.75776657
homo after UVB irradiation VS homo before UVB irradiation	Notch Signaling|Wikipathways	0.215091053	-0.03523734	1.01737342	0.3344736	0.4915994	-5.84170334
hetero after UVB irradiation VS hetero before UVB irradiation	Notch Signaling|Wikipathways	0.311728625	1.76E-06	1.67673789	0.13020444	0.32572549	-5.03664328
WT after UVB irradiation VS WT before UVB irradiation	Notch Signaling|Wikipathways	0.163503662	0.028134378	0.721094139	0.48723563	0.81710765	-5.61281336
homo after UVB irradiation VS homo before UVB irradiation	Notch|INOH	-0.12744646	0.00818687	-0.51297885	0.61981715	0.73561606	-6.22905858
hetero after UVB irradiation VS hetero before UVB irradiation	Notch|INOH	0.124160658	0.003160504	0.521334885	0.61555562	0.80048574	-6.20149433
WT after UVB irradiation VS WT before UVB irradiation	Notch|INOH	0.04367192	-0.0064225	0.156780448	0.87851297	0.96716565	-5.85671535
homo after UVB irradiation VS homo before UVB irradiation	Notch|NetPath	0.00479616	-0.0232343	0.021674289	0.98316142	0.98879186	-6.36819586
hetero after UVB irradiation VS hetero before UVB irradiation	Notch|NetPath	0.206968903	0.013858344	1.132169118	0.28875154	0.51871614	-5.70068201
WT after UVB irradiation VS WT before UVB irradiation	Notch|NetPath	0.106028635	0.025282678	0.444050318	0.66638507	0.87935805	-5.77029986
homo after UVB irradiation VS homo before UVB irradiation	NOTCH1 Intracellular Domain Regulates Transcription|Reactome	-0.26885831	0.037462114	-1.38685738	0.19752399	0.35391572	-5.43098861
hetero after UVB irradiation VS hetero before UVB irradiation	NOTCH1 Intracellular Domain Regulates Transcription|Reactome	-0.15200876	-0.0023866	-0.70057818	0.50245005	0.72085846	-6.08796955
WT after UVB irradiation VS WT before UVB irradiation	NOTCH1 Intracellular Domain Regulates Transcription|Reactome	-0.22435521	0.022188284	-0.92514355	0.37650357	0.75077508	-5.45434224
homo after UVB irradiation VS homo before UVB irradiation	NOTCH1 regulation of human endothelial cell calcification|Wikipathways	0.28987091	-0.02023217	1.198723112	0.26001419	0.42400885	-5.6515827
hetero after UVB irradiation VS hetero before UVB irradiation	NOTCH1 regulation of human endothelial cell calcification|Wikipathways	0.278015848	0.007833604	1.407195423	0.19516869	0.4161797	-5.38495799
WT after UVB irradiation VS WT before UVB irradiation	NOTCH1 regulation of human endothelial cell calcification|Wikipathways	0.113345548	-0.03664942	0.477287138	0.64333536	0.87745357	-5.75513191
homo after UVB irradiation VS homo before UVB irradiation	NOTCH2 Activation and Transmission of Signal to the Nucleus|Reactome	0.288412011	-0.03428073	1.476452135	0.17255575	0.32370154	-5.31857021
hetero after UVB irradiation VS hetero before UVB irradiation	NOTCH2 Activation and Transmission of Signal to the Nucleus|Reactome	0.41930961	0.011164276	2.339528598	0.0458987	0.18854754	-4.07886608
WT after UVB irradiation VS WT before UVB irradiation	NOTCH2 Activation and Transmission of Signal to the Nucleus|Reactome	-0.08385894	0.014811413	-0.35985718	0.72637764	0.90986469	-5.80399883
homo after UVB irradiation VS homo before UVB irradiation	NOTCH2 intracellular domain regulates transcription|Reactome	-0.29403145	0.010227035	-1.14072933	0.28224272	0.44245297	-5.71493878
hetero after UVB irradiation VS hetero before UVB irradiation	NOTCH2 intracellular domain regulates transcription|Reactome	0.023196052	0.02044451	0.111457815	0.91386283	0.97054702	-6.33980707
WT after UVB irradiation VS WT before UVB irradiation	NOTCH2 intracellular domain regulates transcription|Reactome	-0.18918946	-0.04914412	-0.66971762	0.51808104	0.82932917	-5.6472241
homo after UVB irradiation VS homo before UVB irradiation	NOTCH3 Activation and Transmission of Signal to the Nucleus|Reactome	0.279109909	-0.06415329	1.459216449	0.1771349	0.32987917	-5.34053749
hetero after UVB irradiation VS hetero before UVB irradiation	NOTCH3 Activation and Transmission of Signal to the Nucleus|Reactome	0.465695993	0.029824517	2.573573812	0.03159987	0.15507377	-3.72320758
WT after UVB irradiation VS WT before UVB irradiation	NOTCH3 Activation and Transmission of Signal to the Nucleus|Reactome	0.028506598	0.014517101	0.121791923	0.90545712	0.97566251	-5.86164267
homo after UVB irradiation VS homo before UVB irradiation	NOTCH3 Intracellular Domain Regulates Transcription|Reactome	-0.19607483	-0.00397511	-0.84675177	0.41819209	0.56863166	-5.99760905
hetero after UVB irradiation VS hetero before UVB irradiation	NOTCH3 Intracellular Domain Regulates Transcription|Reactome	0.010902061	0.025934083	0.051975893	0.95975908	0.98139974	-6.34507599
WT after UVB irradiation VS WT before UVB irradiation	NOTCH3 Intracellular Domain Regulates Transcription|Reactome	0.023861318	-0.02455424	0.102153586	0.92063794	0.97566251	-5.86386763
homo after UVB irradiation VS homo before UVB irradiation	NOTCH-Core|Signalink	0.35854227	0.00408013	1.761259447	0.1106483	0.24418567	-4.93478852
hetero after UVB irradiation VS hetero before UVB irradiation	NOTCH-Core|Signalink	0.458405761	-0.00551098	2.559464417	0.03231826	0.15685114	-3.74475188
WT after UVB irradiation VS WT before UVB irradiation	NOTCH-Core|Signalink	0.246688994	0.031070204	1.140278748	0.28053359	0.66929002	-5.25264132
homo after UVB irradiation VS homo before UVB irradiation	Notch-HLH transcription pathway|Reactome	-0.34877969	0.012416797	-1.50298274	0.16570876	0.31526092	-5.2844505
hetero after UVB irradiation VS hetero before UVB irradiation	Notch-HLH transcription pathway|Reactome	-0.13364681	-0.03963393	-0.5835319	0.57481896	0.7705309	-6.16556045
WT after UVB irradiation VS WT before UVB irradiation	Notch-HLH transcription pathway|Reactome	-0.17777877	-0.04280642	-0.73036313	0.48179458	0.81552033	-5.60636259
homo after UVB irradiation VS homo before UVB irradiation	Notch-mediated HES/HEY network|PID	0.085368535	-0.01251179	0.471815207	0.64783102	0.75613204	-6.25027476
hetero after UVB irradiation VS hetero before UVB irradiation	Notch-mediated HES/HEY network|PID	0.156306509	-0.00871407	0.933777408	0.37640156	0.60404911	-5.89711232
WT after UVB irradiation VS WT before UVB irradiation	Notch-mediated HES/HEY network|PID	0.074234193	0.000561068	0.370804213	0.71845562	0.90615488	-5.80000387
homo after UVB irradiation VS homo before UVB irradiation	NOTCH-Ncore|Signalink	0.416871888	-0.06640182	2.353712629	0.0419359	0.13402181	-4.04700759
hetero after UVB irradiation VS hetero before UVB irradiation	NOTCH-Ncore|Signalink	0.238295442	0.011441935	1.248421586	0.24544806	0.46888192	-5.57284112
WT after UVB irradiation VS WT before UVB irradiation	NOTCH-Ncore|Signalink	0.337822912	-0.01992734	1.500280571	0.1641897	0.56790008	-4.84850383
homo after UVB irradiation VS homo before UVB irradiation	NR1D1 (REV-ERBA) represses gene expression|Reactome	-0.09230373	0.074271574	-0.39649904	0.70059784	0.80113442	-6.28469703
hetero after UVB irradiation VS hetero before UVB irradiation	NR1D1 (REV-ERBA) represses gene expression|Reactome	-0.23899898	-0.03996208	-0.72441702	0.48843418	0.70846522	-6.07061975
WT after UVB irradiation VS WT before UVB irradiation	NR1D1 (REV-ERBA) represses gene expression|Reactome	-0.4730344	0.064016548	-1.51939338	0.15938146	0.56072663	-4.82507222
homo after UVB irradiation VS homo before UVB irradiation	NR1D1 (REV-ERBA) represses gene expression|Wikipathways	-0.91434725	0.011348575	-3.07992522	0.01251064	0.06898983	-2.89021705
hetero after UVB irradiation VS hetero before UVB irradiation	NR1D1 (REV-ERBA) represses gene expression|Wikipathways	-1.05645612	-0.0805333	-5.45650782	0.00050535	0.03004176	0.300683978
WT after UVB irradiation VS WT before UVB irradiation	NR1D1 (REV-ERBA) represses gene expression|Wikipathways	-0.98820261	-0.06941594	-4.39534443	0.0013187	0.15645713	-0.71268328
homo after UVB irradiation VS homo before UVB irradiation	NRAGE signals death through JNK|Reactome	0.292170358	-0.0639396	1.63120762	0.13588153	0.27772385	-5.11461011
hetero after UVB irradiation VS hetero before UVB irradiation	NRAGE signals death through JNK|Reactome	0.269963065	0.009291807	1.468885548	0.17818062	0.39578326	-5.30824485
WT after UVB irradiation VS WT before UVB irradiation	NRAGE signals death through JNK|Reactome	0.195484455	-0.02684568	0.958375795	0.36029085	0.74374432	-5.42539639
homo after UVB irradiation VS homo before UVB irradiation	NrCAM interactions|Reactome	0.014258953	-0.02702736	0.059421452	0.953862	0.97176299	-6.36655145
hetero after UVB irradiation VS hetero before UVB irradiation	NrCAM interactions|Reactome	-0.23393957	0.035049124	-1.10490072	0.2997513	0.53033405	-5.72936787
WT after UVB irradiation VS WT before UVB irradiation	NrCAM interactions|Reactome	-0.3680651	0.067081075	-1.83040122	0.09686821	0.49485134	-4.42156264
homo after UVB irradiation VS homo before UVB irradiation	NRF2 pathway|Wikipathways	0.235349197	0.010419484	1.459162843	0.1771493	0.32987917	-5.34060557
hetero after UVB irradiation VS hetero before UVB irradiation	NRF2 pathway|Wikipathways	0.127876822	0.017620726	0.727767472	0.48648449	0.7068525	-6.06814035
WT after UVB irradiation VS WT before UVB irradiation	NRF2 pathway|Wikipathways	0.147627609	0.026679766	0.719682518	0.48806763	0.81710765	-5.61378932
homo after UVB irradiation VS homo before UVB irradiation	NRF2-ARE regulation|Wikipathways	0.020017278	-0.02180951	0.087846322	0.93184399	0.96023463	-6.36430363
hetero after UVB irradiation VS hetero before UVB irradiation	NRF2-ARE regulation|Wikipathways	0.370239776	-0.01530384	1.954915185	0.08450382	0.26255517	-4.64773558
WT after UVB irradiation VS WT before UVB irradiation	NRF2-ARE regulation|Wikipathways	0.062587096	0.021698974	0.267767226	0.79427672	0.94244265	-5.83296722
homo after UVB irradiation VS homo before UVB irradiation	NRIF signals cell death from the nucleus|Reactome	0.368826911	-0.02169308	1.829592174	0.09918456	0.22827633	-4.8376111
hetero after UVB irradiation VS hetero before UVB irradiation	NRIF signals cell death from the nucleus|Reactome	0.424845583	0.013589948	2.047824379	0.0729845	0.24158558	-4.51293323
WT after UVB irradiation VS WT before UVB irradiation	NRIF signals cell death from the nucleus|Reactome	0.128885459	0.028597498	0.607046721	0.55724265	0.84822456	-5.68605849
homo after UVB irradiation VS homo before UVB irradiation	NS1 Mediated Effects on Host Pathways|Reactome	-0.39833539	-0.04945337	-1.58590233	0.14582406	0.2913279	-5.17551926
hetero after UVB irradiation VS hetero before UVB irradiation	NS1 Mediated Effects on Host Pathways|Reactome	0.521627804	0.03463471	1.839587624	0.10122975	0.28605405	-4.81193736
WT after UVB irradiation VS WT before UVB irradiation	NS1 Mediated Effects on Host Pathways|Reactome	0.064994202	-0.14672171	0.210788385	0.83725172	0.94951669	-5.84669557
homo after UVB irradiation VS homo before UVB irradiation	NTF3 activates NTRK2 (TRKB) signaling|Reactome	1.088634858	-0.00931909	4.618680071	0.00112414	0.01929633	-0.5402104
hetero after UVB irradiation VS hetero before UVB irradiation	NTF3 activates NTRK2 (TRKB) signaling|Reactome	0.377988945	0.033993748	0.960637702	0.36348668	0.59212733	-5.87224425
WT after UVB irradiation VS WT before UVB irradiation	NTF3 activates NTRK2 (TRKB) signaling|Reactome	0.739192635	-0.00698721	2.058756007	0.06629901	0.43803373	-4.10390281
homo after UVB irradiation VS homo before UVB irradiation	NTF4 activates NTRK2 (TRKB) signaling|Reactome	0.970697688	0.026576128	4.433791337	0.00147701	0.02189616	-0.8061078
hetero after UVB irradiation VS hetero before UVB irradiation	NTF4 activates NTRK2 (TRKB) signaling|Reactome	0.589433768	0.024730157	1.600304551	0.14628829	0.35056889	-5.13870316
WT after UVB irradiation VS WT before UVB irradiation	NTF4 activates NTRK2 (TRKB) signaling|Reactome	0.814998732	0.024180793	3.244831162	0.00870608	0.24856491	-2.34209387
homo after UVB irradiation VS homo before UVB irradiation	Nuclear Envelope Breakdown|Reactome	-0.49992965	-0.00681129	-2.69722222	0.02363319	0.10122807	-3.50300789
hetero after UVB irradiation VS hetero before UVB irradiation	Nuclear Envelope Breakdown|Reactome	-0.03604329	-0.07611371	-0.15180717	0.88291115	0.95842588	-6.33405683
WT after UVB irradiation VS WT before UVB irradiation	Nuclear Envelope Breakdown|Reactome	-0.05825188	-0.03044938	-0.19840535	0.84667153	0.9536885	-5.84925107
homo after UVB irradiation VS homo before UVB irradiation	Nuclear Envelope Reassembly|Reactome	-0.27069257	-0.01304465	-1.48450677	0.17045138	0.32096638	-5.30825042
hetero after UVB irradiation VS hetero before UVB irradiation	Nuclear Envelope Reassembly|Reactome	-0.07586628	-0.05159094	-0.44428114	0.66802929	0.82651398	-6.24073243
WT after UVB irradiation VS WT before UVB irradiation	Nuclear Envelope Reassembly|Reactome	0.007404765	-0.02239639	0.031612407	0.97539821	0.99212553	-5.86864496
homo after UVB irradiation VS homo before UVB irradiation	Nuclear Events (kinase and transcription factor activation)|Reactome	-0.00699873	-0.01451325	-0.04305115	0.96656244	0.97943278	-6.36745252
hetero after UVB irradiation VS hetero before UVB irradiation	Nuclear Events (kinase and transcription factor activation)|Reactome	0.383231743	-0.00990475	2.00542813	0.07803999	0.25138252	-4.57469974
WT after UVB irradiation VS WT before UVB irradiation	Nuclear Events (kinase and transcription factor activation)|Reactome	0.156928875	-0.0414009	0.6875913	0.50721915	0.82465997	-5.63551288
homo after UVB irradiation VS homo before UVB irradiation	Nuclear import of Rev protein|Reactome	-0.71811115	-0.01207996	-3.51645985	0.00613559	0.04585122	-2.19708829
hetero after UVB irradiation VS hetero before UVB irradiation	Nuclear import of Rev protein|Reactome	-0.05464033	-0.07357518	-0.20770193	0.84039427	0.93932613	-6.32319947
WT after UVB irradiation VS WT before UVB irradiation	Nuclear import of Rev protein|Reactome	-0.01657574	0.009622078	-0.05007931	0.96103744	0.98815178	-5.86788085
homo after UVB irradiation VS homo before UVB irradiation	Nuclear Pore Complex (NPC) Disassembly|Reactome	-0.63256136	-0.02128754	-3.25986663	0.00930683	0.0578163	-2.60302659
hetero after UVB irradiation VS hetero before UVB irradiation	Nuclear Pore Complex (NPC) Disassembly|Reactome	-0.07916222	-0.07198167	-0.28913952	0.77946152	0.90262028	-6.30141797
WT after UVB irradiation VS WT before UVB irradiation	Nuclear Pore Complex (NPC) Disassembly|Reactome	-0.06990008	-0.00210239	-0.20837944	0.83908215	0.94994749	-5.84720471
homo after UVB irradiation VS homo before UVB irradiation	Nuclear Receptor transcription pathway|Reactome	-0.03766861	0.022736631	-0.21267902	0.83612325	0.89977345	-6.34420465
hetero after UVB irradiation VS hetero before UVB irradiation	Nuclear Receptor transcription pathway|Reactome	-0.37197778	0.009395354	-2.18173312	0.05901729	0.21664304	-4.31540793
WT after UVB irradiation VS WT before UVB irradiation	Nuclear Receptor transcription pathway|Reactome	-0.22973905	0.037308597	-0.9973048	0.34194977	0.72704124	-5.39042936
homo after UVB irradiation VS homo before UVB irradiation	receptors coordinate the activities of chromatin remodeling complexes and coactivators to facilitate initiation of transcription in carcinoma cells|	0.064997515	-0.01394008	0.297820069	0.77232556	0.85344591	-6.32102103
hetero after UVB irradiation VS hetero before UVB irradiation	receptors coordinate the activities of chromatin remodeling complexes and coactivators to facilitate initiation of transcription in carcinoma cells|	-0.22107884	-0.00575883	-1.16673768	0.27527335	0.50543332	-5.66358974
WT after UVB irradiation VS WT before UVB irradiation	receptors coordinate the activities of chromatin remodeling complexes and coactivators to facilitate initiation of transcription in carcinoma cells|	-0.08417146	-0.01680475	-0.35194654	0.73212349	0.91205892	-5.80681322
homo after UVB irradiation VS homo before UVB irradiation	Nuclear Receptors in Lipid Metabolism and Toxicity|Wikipathways	0.178705594	0.030684047	1.028864186	0.32931784	0.48567999	-5.83037335
hetero after UVB irradiation VS hetero before UVB irradiation	Nuclear Receptors in Lipid Metabolism and Toxicity|Wikipathways	-0.18757376	0.013461505	-0.95804666	0.36471802	0.59328506	-5.87466768
WT after UVB irradiation VS WT before UVB irradiation	Nuclear Receptors in Lipid Metabolism and Toxicity|Wikipathways	0.127428725	0.016034712	0.555736111	0.5905094	0.86244209	-5.71522471
homo after UVB irradiation VS homo before UVB irradiation	Nuclear Receptors Meta-Pathway|Wikipathways	0.096836872	0.015591203	0.594155031	0.56644727	0.69195165	-6.18234724
hetero after UVB irradiation VS hetero before UVB irradiation	Nuclear Receptors Meta-Pathway|Wikipathways	0.018239803	-0.00093847	0.109826705	0.9151177	0.97054702	-6.34000258
WT after UVB irradiation VS WT before UVB irradiation	Nuclear Receptors Meta-Pathway|Wikipathways	-0.0213064	0.001411176	-0.10475032	0.91862855	0.97566251	-5.86359576
homo after UVB irradiation VS homo before UVB irradiation	Nuclear Receptors|Wikipathways	0.02266992	0.009361816	0.109917493	0.91478843	0.95059717	-6.3619609
hetero after UVB irradiation VS hetero before UVB irradiation	Nuclear Receptors|Wikipathways	-0.37573812	-0.01090306	-2.18284077	0.05891342	0.21664304	-4.31376034
WT after UVB irradiation VS WT before UVB irradiation	Nuclear Receptors|Wikipathways	-0.26750538	0.038795498	-1.05753567	0.31494719	0.69891341	-5.33413836
homo after UVB irradiation VS homo before UVB irradiation	Nuclear signaling by ERBB4|Reactome	0.341766015	0.003824472	1.930112197	0.08431419	0.20609132	-4.69167836
hetero after UVB irradiation VS hetero before UVB irradiation	Nuclear signaling by ERBB4|Reactome	0.225277703	-0.00608069	1.283807005	0.23338537	0.45519048	-5.53223462
WT after UVB irradiation VS WT before UVB irradiation	Nuclear signaling by ERBB4|Reactome	0.20282308	0.023430986	0.889270353	0.39458038	0.76452505	-5.4846331
homo after UVB irradiation VS homo before UVB irradiation	Nucleobase biosynthesis|Reactome	-0.55361098	-0.025195	-2.4562902	0.03534231	0.12483995	-3.8858348
hetero after UVB irradiation VS hetero before UVB irradiation	Nucleobase biosynthesis|Reactome	-0.5896711	-0.10962632	-2.11908873	0.06519263	0.22947406	-4.40824041
WT after UVB irradiation VS WT before UVB irradiation	Nucleobase biosynthesis|Reactome	-0.00049865	-0.00449797	-0.00172729	0.99865552	0.99940779	-5.86914968
homo after UVB irradiation VS homo before UVB irradiation	Nucleobase catabolism|Reactome	-0.04364314	0.005155333	-0.23607228	0.81844601	0.88825957	-6.33859517
hetero after UVB irradiation VS hetero before UVB irradiation	Nucleobase catabolism|Reactome	0.033011458	0.000442644	0.170782346	0.86842498	0.95036761	-6.33074667
WT after UVB irradiation VS WT before UVB irradiation	Nucleobase catabolism|Reactome	0.071406936	-0.03124483	0.341785012	0.73952974	0.91673557	-5.81033899
homo after UVB irradiation VS homo before UVB irradiation	Nucleosome assembly|Reactome	-0.50190868	0.033606094	-1.86522951	0.09365265	0.21982504	-4.78626162
hetero after UVB irradiation VS hetero before UVB irradiation	Nucleosome assembly|Reactome	0.183280885	-0.03869092	0.608379259	0.55897544	0.76190086	-6.15016359
WT after UVB irradiation VS WT before UVB irradiation	Nucleosome assembly|Reactome	0.271286949	0.065901858	0.954443616	0.36218248	0.74374432	-5.4288652
homo after UVB irradiation VS homo before UVB irradiation	Nucleotide excision repair - Homo sapiens (human)|KEGG	-0.29076978	-0.0316323	-1.37703061	0.20044217	0.3573678	-5.44304466
hetero after UVB irradiation VS hetero before UVB irradiation	Nucleotide excision repair - Homo sapiens (human)|KEGG	0.146402337	-0.03242381	0.754550902	0.47107894	0.69305602	-6.04796055
WT after UVB irradiation VS WT before UVB irradiation	Nucleotide excision repair - Homo sapiens (human)|KEGG	0.029141436	0.02789369	0.105404658	0.91812231	0.97566251	-5.86352618
homo after UVB irradiation VS homo before UVB irradiation	Nucleotide Excision Repair|Reactome	-0.48207007	-0.00231752	-2.78048522	0.02056772	0.09140661	-3.36985318
hetero after UVB irradiation VS hetero before UVB irradiation	Nucleotide Excision Repair|Reactome	-0.09337553	-0.0336402	-0.48071869	0.64295077	0.81928142	-6.22291474
WT after UVB irradiation VS WT before UVB irradiation	Nucleotide Excision Repair|Reactome	-0.10922802	0.003931452	-0.39410296	0.70171195	0.90059561	-5.79111496
homo after UVB irradiation VS homo before UVB irradiation	Nucleotide Excision Repair|SMPDB	-0.24033186	-0.05846785	-0.85628346	0.41316	0.56397266	-5.98953031
hetero after UVB irradiation VS hetero before UVB irradiation	Nucleotide Excision Repair|SMPDB	0.372465392	-0.0208913	1.549865031	0.15786774	0.36862765	-5.20470753
WT after UVB irradiation VS WT before UVB irradiation	Nucleotide Excision Repair|SMPDB	0.06013004	0.035902153	0.181855861	0.85930013	0.95916804	-5.85242684
homo after UVB irradiation VS homo before UVB irradiation	Nucleotide GPCRs|Wikipathways	0.317074763	-0.01660144	1.377697988	0.20024283	0.35715186	-5.44222766
hetero after UVB irradiation VS hetero before UVB irradiation	Nucleotide GPCRs|Wikipathways	0.626511029	-0.02897869	3.609438992	0.0063158	0.08320066	-2.15625792
WT after UVB irradiation VS WT before UVB irradiation	Nucleotide GPCRs|Wikipathways	0.476458211	0.009052825	2.110814659	0.06073706	0.43072383	-4.02957338
homo after UVB irradiation VS homo before UVB irradiation	Nucleotide Metabolism|Wikipathways	-0.39739823	-0.04288399	-1.93912592	0.08308831	0.20451818	-4.67843215
hetero after UVB irradiation VS hetero before UVB irradiation	Nucleotide Metabolism|Wikipathways	0.344328138	-0.05552002	1.827191758	0.10320326	0.28878846	-4.82935467
WT after UVB irradiation VS WT before UVB irradiation	Nucleotide Metabolism|Wikipathways	0.378038673	-0.10014488	1.600064614	0.14041862	0.55622203	-4.72428202
homo after UVB irradiation VS homo before UVB irradiation	Nucleotide salvage|Reactome	0.392939589	-0.03556793	2.21188209	0.05308051	0.15407858	-4.26719032

hetero after UVB irradiation VS hetero before UVB irradiation	Nucleotide salvage|Reactome	0.526823482	-0.04348353	2.989778636	0.0163554	0.11352943	-3.08665154
WT after UVB irradiation VS WT before UVB irradiation	Nucleotide salvage|Reactome	0.427558134	-0.04050493	1.895056375	0.08709132	0.47748624	-4.33316603
homo after UVB irradiation VS homo before UVB irradiation	Nucleotide Sugars Metabolism|SMPDB	0.698574264	-0.05912759	4.348137644	0.00167892	0.02281533	-0.93107444
hetero after UVB irradiation VS hetero before UVB irradiation	Nucleotide Sugars Metabolism|SMPDB	0.346014476	0.023648214	1.782710589	0.11058647	0.30216567	-4.89145127
WT after UVB irradiation VS WT before UVB irradiation	Nucleotide Sugars Metabolism|SMPDB	0.461260912	0.002017987	1.791110278	0.10329419	0.50425697	-4.47460697
homo after UVB irradiation VS homo before UVB irradiation	Nucleotide-binding domain_ leucine rich repeat containing receptor (NLR) signaling pathways|Reactome	0.450614594	-0.03979317	2.436118013	0.03655244	0.12504267	-3.91763731
hetero after UVB irradiation VS hetero before UVB irradiation	Nucleotide-binding domain_ leucine rich repeat containing receptor (NLR) signaling pathways|Reactome	0.539531102	0.056622731	3.282924266	0.01037148	0.1029917	-2.64218305
WT after UVB irradiation VS WT before UVB irradiation	Nucleotide-binding domain_ leucine rich repeat containing receptor (NLR) signaling pathways|Reactome	0.353929383	-0.01163892	1.719043128	0.11610347	0.51731174	-4.57047018
homo after UVB irradiation VS homo before UVB irradiation	Nucleotide-binding domain_ leucine rich repeat containing receptor (NLR) signaling pathways|Wikipathways	0.94694334	0.009753976	4.130359432	0.00233637	0.02721394	-1.25371751
hetero after UVB irradiation VS hetero before UVB irradiation	Nucleotide-binding domain_ leucine rich repeat containing receptor (NLR) signaling pathways|Wikipathways	1.043537668	0.036519471	5.518045082	0.00046848	0.03004176	0.373010877
WT after UVB irradiation VS WT before UVB irradiation	Nucleotide-binding domain_ leucine rich repeat containing receptor (NLR) signaling pathways|Wikipathways	0.941303311	0.02614501	3.015535463	0.01288038	0.27886745	-2.68475038
homo after UVB irradiation VS homo before UVB irradiation	Nucleotide-binding Oligomerization Domain (NOD) pathway|Wikipathways	0.731359729	-0.03321785	4.26604372	0.00190016	0.02423495	-1.0518826
hetero after UVB irradiation VS hetero before UVB irradiation	Nucleotide-binding Oligomerization Domain (NOD) pathway|Wikipathways	0.663466256	0.013414207	4.241754135	0.00251672	0.05623149	-1.25445743
WT after UVB irradiation VS WT before UVB irradiation	Nucleotide-binding Oligomerization Domain (NOD) pathway|Wikipathways	0.547990155	0.018054415	2.705387969	0.02195829	0.31542987	-3.15104119
homo after UVB irradiation VS homo before UVB irradiation	Nucleotide-like (purinergic) receptors|Reactome	0.254359272	-0.01308986	1.125972004	0.28813309	0.44703218	-5.7306869
hetero after UVB irradiation VS hetero before UVB irradiation	Nucleotide-like (purinergic) receptors|Reactome	0.546744575	-0.00392607	3.311162104	0.00993098	0.10144594	-2.59972312
WT after UVB irradiation VS WT before UVB irradiation	Nucleotide-like (purinergic) receptors|Reactome	0.466656014	0.012933607	1.761261476	0.10843451	0.5076251	-4.51454281
homo after UVB irradiation VS homo before UVB irradiation	O2/CO2 exchange in erythrocytes|Reactome	-0.11472985	-0.05123043	-0.48638821	0.63784355	0.74907244	-6.2429561
hetero after UVB irradiation VS hetero before UVB irradiation	O2/CO2 exchange in erythrocytes|Reactome	-0.06140183	-0.10427143	-0.30837226	0.76528559	0.89270377	-6.2952511
WT after UVB irradiation VS WT before UVB irradiation	O2/CO2 exchange in erythrocytes|Reactome	-0.14373732	0.004995015	-0.63136589	0.54185001	0.83916339	-5.67140403
homo after UVB irradiation VS homo before UVB irradiation	O-Glycan biosynthesis|EHMN	0.460070739	-0.00222989	2.884021041	0.01731024	0.08295606	-3.2040107
hetero after UVB irradiation VS hetero before UVB irradiation	O-Glycan biosynthesis|EHMN	0.483345624	0.017477247	2.460324479	0.03785306	0.16931722	-3.89582374
WT after UVB irradiation VS WT before UVB irradiation	O-Glycan biosynthesis|EHMN	0.514740631	-0.0075248	2.55449296	0.0284739	0.35176669	-3.37733478
homo after UVB irradiation VS homo before UVB irradiation	O-glycosylation of TSR domain-containing proteins|Reactome	0.358955555	-0.02724476	2.089405122	0.06497873	0.17691883	-4.45414627
hetero after UVB irradiation VS hetero before UVB irradiation	O-glycosylation of TSR domain-containing proteins|Reactome	0.04743058	-0.01058597	0.199466372	0.84662778	0.94350353	-6.32500943
WT after UVB irradiation VS WT before UVB irradiation	O-glycosylation of TSR domain-containing proteins|Reactome	0.069538228	-0.02240852	0.271790471	0.79126749	0.9410906	-5.83187601
homo after UVB irradiation VS homo before UVB irradiation	oleate biosynthesis|HumanCyc	-1.02752541	-0.04287861	-5.12574717	0.00054534	0.01666204	0.161095424
hetero after UVB irradiation VS hetero before UVB irradiation	oleate biosynthesis|HumanCyc	-0.58892225	-0.00542262	-2.59817463	0.03038574	0.15186205	-3.68562308
WT after UVB irradiation VS WT before UVB irradiation	oleate biosynthesis|HumanCyc	-0.4457811	-0.04037904	-1.70214167	0.11930907	0.5237862	-4.59266973
homo after UVB irradiation VS homo before UVB irradiation	Olfactory bulb development and olfactory learning|Wikipathways	0.547110694	0.020717888	3.02650562	0.01366565	0.07305822	-2.9757197
hetero after UVB irradiation VS hetero before UVB irradiation	Olfactory bulb development and olfactory learning|Wikipathways	0.16480945	0.028981305	0.673682788	0.51856251	0.73586797	-6.10692462
WT after UVB irradiation VS WT before UVB irradiation	Olfactory bulb development and olfactory learning|Wikipathways	0.292399384	0.059745451	1.060349334	0.3137263	0.6974486	-5.33144513
homo after UVB irradiation VS homo before UVB irradiation	Olfactory receptor activity|Wikipathways	0.137394533	0.061819498	0.677121861	0.51466403	0.65258427	-6.12809009
hetero after UVB irradiation VS hetero before UVB irradiation	Olfactory receptor activity|Wikipathways	0.081276926	0.138939787	0.455480228	0.66027251	0.82651398	-6.23539807
WT after UVB irradiation VS WT before UVB irradiation	Olfactory receptor activity|Wikipathways	0.073601162	0.077436084	0.330633825	0.74768951	0.9185045	-5.81409216
homo after UVB irradiation VS homo before UVB irradiation	Olfactory Signaling Pathway|Reactome	0.139268178	0.05775634	0.681492906	0.51201708	0.65085467	-6.12505304
hetero after UVB irradiation VS hetero before UVB irradiation	Olfactory Signaling Pathway|Reactome	0.189191635	0.121592683	1.096722208	0.30311431	0.53328183	-5.73787106
WT after UVB irradiation VS WT before UVB irradiation	Olfactory Signaling Pathway|Reactome	0.097813009	0.070244151	0.446726537	0.66451516	0.87935805	-5.7691173
homo after UVB irradiation VS homo before UVB irradiation	Olfactory transduction - Homo sapiens (human)|KEGG	0.208993423	0.049699265	1.24520555	0.24323541	0.40488985	-5.59916228
hetero after UVB irradiation VS hetero before UVB irradiation	Olfactory transduction - Homo sapiens (human)|KEGG	0.17907515	0.109460376	1.160097287	0.27782231	0.50863663	-5.67077702
WT after UVB irradiation VS WT before UVB irradiation	Olfactory transduction - Homo sapiens (human)|KEGG	0.081439543	0.049176706	0.342738173	0.73883383	0.91673557	-5.81001255
homo after UVB irradiation VS homo before UVB irradiation	Oligomerization of connexins into connexons|Reactome	-0.60044815	0.017203699	-1.72632664	0.11696868	0.2549303	-4.98377736
hetero after UVB irradiation VS hetero before UVB irradiation	Oligomerization of connexins into connexons|Reactome	-0.18654347	-0.03778519	-0.50338777	0.62758599	0.80760501	-6.21116069
WT after UVB irradiation VS WT before UVB irradiation	Oligomerization of connexins into connexons|Reactome	-0.43070416	0.030729037	-1.1431942	0.27937671	0.66906841	-5.24968476
homo after UVB irradiation VS homo before UVB irradiation	O-linked glycosylation of mucins|Reactome	0.224798273	0.010148962	1.368783164	0.20291957	0.36009746	-5.45311992
hetero after UVB irradiation VS hetero before UVB irradiation	O-linked glycosylation of mucins|Reactome	0.169652307	-0.00326553	1.111221207	0.29717257	0.52889986	-5.72276453
WT after UVB irradiation VS WT before UVB irradiation	O-linked glycosylation of mucins|Reactome	0.076800718	-0.03272914	0.332795518	0.74610512	0.9185045	-5.81337409
homo after UVB irradiation VS homo before UVB irradiation	O-linked glycosylation|Reactome	0.233817793	-0.00847626	1.365871288	0.20380044	0.36109926	-5.45666765
hetero after UVB irradiation VS hetero before UVB irradiation	O-linked glycosylation|Reactome	0.076781304	0.002577973	0.40212933	0.69759735	0.84778456	-6.25967153
WT after UVB irradiation VS WT before UVB irradiation	O-linked glycosylation|Reactome	0.057973849	-0.03789649	0.243020351	0.81286245	0.94525798	-5.83932631
homo after UVB irradiation VS homo before UVB irradiation	Olmesartan Action Pathway|SMPDB	0.636038586	-0.11800708	2.436891056	0.03650532	0.12504267	-3.91641947
hetero after UVB irradiation VS hetero before UVB irradiation	Olmesartan Action Pathway|SMPDB	-0.02777369	0.003323189	-0.09172332	0.92906122	0.97054702	-6.34197953
WT after UVB irradiation VS WT before UVB irradiation	Olmesartan Action Pathway|SMPDB	0.270438532	-0.10210744	0.707878218	0.49505953	0.81710765	-5.62188349
homo after UVB irradiation VS homo before UVB irradiation	Omega-3 fatty acid metabolism|EHMN	-0.23947473	-0.02415116	-1.23612937	0.24644036	0.40873472	-5.60950974
hetero after UVB irradiation VS hetero before UVB irradiation	Omega-3 fatty acid metabolism|EHMN	-0.16856217	-0.02063523	-0.6992222	0.50325482	0.72148515	-6.08894098
WT after UVB irradiation VS WT before UVB irradiation	Omega-3 fatty acid metabolism|EHMN	-0.23686752	-0.04977157	-1.12216444	0.28780573	0.67413174	-5.27088334
homo after UVB irradiation VS homo before UVB irradiation	Omega-6 fatty acid metabolism|EHMN	-0.43185252	-0.00456602	-1.93354134	0.08384583	0.20555644	-4.68664199
hetero after UVB irradiation VS hetero before UVB irradiation	Omega-6 fatty acid metabolism|EHMN	-0.54508802	-0.0356536	-2.63803945	0.02851781	0.14611598	-3.62467243
WT after UVB irradiation VS WT before UVB irradiation	Omega-6 fatty acid metabolism|EHMN	-0.32701529	-0.03473919	-1.40641249	0.18966759	0.60489657	-4.96092477
homo after UVB irradiation VS homo before UVB irradiation	Omeprazole Action Pathway|SMPDB	0.21780303	0.051698704	1.19150037	0.26270351	0.42561191	-5.65959884
hetero after UVB irradiation VS hetero before UVB irradiation	Omeprazole Action Pathway|SMPDB	0.331566302	0.063470812	1.441200644	0.18563717	0.40321767	-5.34291209
WT after UVB irradiation VS WT before UVB irradiation	Omeprazole Action Pathway|SMPDB	-0.11560861	0.135982321	-0.44330845	0.66690385	0.87935805	-5.77062647
homo after UVB irradiation VS homo before UVB irradiation	Omeprazole Metabolism Pathway|SMPDB	-0.06647614	0.033610835	-0.18292054	0.85874942	0.9134577	-6.35050291
WT after UVB irradiation VS WT before UVB irradiation	Omeprazole Metabolism Pathway|SMPDB	-0.4370633	0.058240551	-1.4704397	0.17194858	0.57997722	-4.88472923
homo after UVB irradiation VS homo before UVB irradiation	Oncogene Induced Senescence|Reactome	-0.04098022	0.02276281	-0.20973031	0.83835856	0.90132922	-6.34487065
hetero after UVB irradiation VS hetero before UVB irradiation	Oncogene Induced Senescence|Reactome	0.291091781	0.03232554	1.603027877	0.1456855	0.349733	-5.13510808
WT after UVB irradiation VS WT before UVB irradiation	Oncogene Induced Senescence|Reactome	-0.13342064	-0.00701889	-0.64012755	0.53636487	0.83767333	-5.66599485
homo after UVB irradiation VS homo before UVB irradiation	Oncogene Induced Senescence|Wikipathways	0.344155292	-0.05401807	0.889496979	0.39595372	0.54885388	-5.96078179
hetero after UVB irradiation VS hetero before UVB irradiation	Oncogene Induced Senescence|Wikipathways	0.432239285	0.031765015	1.335194225	0.2167582	0.43793611	-5.47194217
WT after UVB irradiation VS WT before UVB irradiation	Oncogene Induced Senescence|Wikipathways	-0.03834524	0.03784967	-0.10195644	0.92079051	0.97566251	-5.86388799
homo after UVB irradiation VS homo before UVB irradiation	Oncogenic MAPK signaling|Reactome	0.340791272	-0.01725633	2.056003862	0.06864508	0.18350903	-4.50452346
hetero after UVB irradiation VS hetero before UVB irradiation	Oncogenic MAPK signaling|Reactome	0.338442952	0.0030045	2.075078899	0.06990333	0.23618929	-4.47301733
WT after UVB irradiation VS WT before UVB irradiation	Oncogenic MAPK signaling|Reactome	0.374521058	0.011910226	1.885390129	0.08849239	0.48106209	-4.34646582
homo after UVB irradiation VS homo before UVB irradiation	Oncostatin M Signaling Pathway|Wikipathways	0.441022596	-0.03715009	2.306797883	0.04534187	0.13845639	-4.12021944
hetero after UVB irradiation VS hetero before UVB irradiation	Oncostatin M Signaling Pathway|Wikipathways	0.522060397	0.012388399	3.237838875	0.01111769	0.10448339	-2.71012364
WT after UVB irradiation VS WT before UVB irradiation	Oncostatin M Signaling Pathway|Wikipathways	0.407806166	-0.00485411	2.007063536	0.0722976	0.45254499	-4.17707165
homo after UVB irradiation VS homo before UVB irradiation	Oncostatin_M|NetPath	0.350963401	-0.03155589	1.578747822	0.14745222	0.29259749	-5.18505035
hetero after UVB irradiation VS hetero before UVB irradiation	Oncostatin_M|NetPath	0.379543688	0.020031763	2.556118779	0.03249101	0.15685114	-3.74985914
WT after UVB irradiation VS WT before UVB irradiation	Oncostatin_M|NetPath	0.3177991	0.011610491	1.583357737	0.14417374	0.55622203	-4.74539925
homo after UVB irradiation VS homo before UVB irradiation	One carbon metabolism and related pathways|Wikipathways	0.255540987	0.007946179	1.618852411	0.13853086	0.28112529	-5.13131425
hetero after UVB irradiation VS hetero before UVB irradiation	One carbon metabolism and related pathways|Wikipathways	0.153021007	0.01960248	0.846087258	0.42088094	0.64743517	-5.97427562
WT after UVB irradiation VS WT before UVB irradiation	One carbon metabolism and related pathways|Wikipathways	0.218252103	-0.00782066	1.031888186	0.32624227	0.71017561	-5.35842827
homo after UVB irradiation VS homo before UVB irradiation	One Carbon Metabolism|Wikipathways	-0.12158525	-0.03516096	-0.53985646	0.60186322	0.72163298	-6.21430274
hetero after UVB irradiation VS hetero before UVB irradiation	One Carbon Metabolism|Wikipathways	0.011818556	-0.06117377	0.062874031	0.95133285	0.97485952	-6.34439725
WT after UVB irradiation VS WT before UVB irradiation	One Carbon Metabolism|Wikipathways	0.279010384	-0.07302911	1.152049448	0.27588582	0.66629855	-5.24067
homo after UVB irradiation VS homo before UVB irradiation	One carbon pool by folate - Homo sapiens (human)|KEGG	-0.23830334	-0.03657712	-1.01295949	0.33647014	0.4922959	-5.84602862
hetero after UVB irradiation VS hetero before UVB irradiation	One carbon pool by folate - Homo sapiens (human)|KEGG	-0.03337995	-0.05839532	-0.15181224	0.88290727	0.95842588	-6.334056
WT after UVB irradiation VS WT before UVB irradiation	One carbon pool by folate - Homo sapiens (human)|KEGG	-0.02485518	-0.08003842	-0.08144679	0.93668083	0.98056728	-5.86579184
homo after UVB irradiation VS homo before UVB irradiation	Oocyte meiosis - Homo sapiens (human)|KEGG	-0.10168938	-0.00097188	-0.62506132	0.54681973	0.67913558	-6.16289423
hetero after UVB irradiation VS hetero before UVB irradiation	Oocyte meiosis - Homo sapiens (human)|KEGG	0.15745623	0.005067291	0.988396076	0.35048841	0.57944367	-5.84595669
WT after UVB irradiation VS WT before UVB irradiation	Oocyte meiosis - Homo sapiens (human)|KEGG	-0.01136495	-0.00405373	-0.0569881	0.95566828	0.98742794	-5.86750624
homo after UVB irradiation VS homo before UVB irradiation	Opioid Signalling|Reactome	0.411947988	-0.01949904	2.633267898	0.02629719	0.10720802	-3.60505922
hetero after UVB irradiation VS hetero before UVB irradiation	Opioid Signalling|Reactome	0.366117138	0.020240208	2.109580325	0.06618348	0.23116038	-4.42226791
WT after UVB irradiation VS WT before UVB irradiation	Opioid Signalling|Reactome	0.247461734	-0.00692957	1.092335004	0.3000994	0.68493452	-5.30043789
homo after UVB irradiation VS homo before UVB irradiation	opposing roles of aif in apoptosis and cell survival|BioCarta	-0.78711639	0.085165608	-3.49651726	0.00633596	0.04636215	-2.22844257
hetero after UVB irradiation VS hetero before UVB irradiation	opposing roles of aif in apoptosis and cell survival|BioCarta	-0.73877505	-0.1348571	-2.84222332	0.02063083	0.12673224	-3.31213487
WT after UVB irradiation VS WT before UVB irradiation	opposing roles of aif in apoptosis and cell survival|BioCarta	0.617866476	-0.13158644	2.446078528	0.03430622	0.38092943	-3.53900399
homo after UVB irradiation VS homo before UVB irradiation	Opsins|Reactome	0.473887517	0.128105504	1.689864555	0.12391618	0.26290983	-5.0343844
hetero after UVB irradiation VS hetero before UVB irradiation	Opsins|Reactome	0.291002951	0.113602637	1.202325516	0.26193403	0.48810111	-5.62457763
WT after UVB irradiation VS WT before UVB irradiation	Opsins|Reactome	0.56004282	0.134792672	1.755830718	0.10939448	0.5076251	-4.52177422
homo after UVB irradiation VS homo before UVB irradiation	Optineurin and Myosin Phosphatase Negatively Regulate PLK1|Reactome	-0.53618062	-0.00640484	-2.34520863	0.04253406	0.13402181	-4.06030369
hetero after UVB irradiation VS hetero before UVB irradiation	Optineurin and Myosin Phosphatase Negatively Regulate PLK1|Reactome	0.328643982	-0.03546571	1.250345037	0.24477927	0.46814053	-5.57065348
WT after UVB irradiation VS WT before UVB irradiation	Optineurin and Myosin Phosphatase Negatively Regulate PLK1|Reactome	-0.3931529	-0.00102579	-0.9952352	0.34290722	0.72738377	-5.39231667
homo after UVB irradiation VS homo before UVB irradiation	Orc1 removal from chromatin|Reactome	-0.52211333	-0.02072219	-2.0050005	0.07462718	0.19515904	-4.5808871
hetero after UVB irradiation VS hetero before UVB irradiation	Orc1 removal from chromatin|Reactome	0.12766375	0.062765997	0.461227657	0.65630837	0.82651398	-6.23261144
WT after UVB irradiation VS WT before UVB irradiation	Orc1 removal from chromatin|Reactome	-0.0648935	-0.00973175	-0.23732035	0.81716112	0.94525798	-5.84070483
homo after UVB irradiation VS homo before UVB irradiation	Orexin and neuropeptides FF and QRFP bind to their respective receptors|Reactome	0.152506956	0.057484199	0.677539481	0.51441077	0.65244433	-6.12780069
hetero after UVB irradiation VS hetero before UVB irradiation	Orexin and neuropeptides FF and QRFP bind to their respective receptors|Reactome	-0.22091395	0.064793552	-0.71066775	0.49648731	0.71538528	-6.08068897
WT after UVB irradiation VS WT before UVB irradiation	Orexin and neuropeptides FF and QRFP bind to their respective receptors|Reactome	0.279269943	0.08213471	1.346220866	0.20771715	0.6232233	-5.03055669
homo after UVB irradiation VS homo before UVB irradiation	Organelle biogenesis and maintenance|Reactome	-0.31313372	-0.00988736	-1.81360407	0.10176374	0.23232663	-4.86050249
hetero after UVB irradiation VS hetero before UVB irradiation	Organelle biogenesis and maintenance|Reactome	-0.22241866	-0.04704204	-1.21001216	0.25912335	0.48524413	-5.61604372
WT after UVB irradiation VS WT before UVB irradiation	Organelle biogenesis and maintenance|Reactome	-0.25786056	-0.04171332	-1.28752725	0.22667961	0.62952955	-5.09648409
homo after UVB irradiation VS homo before UVB irradiation	Organic anion transport|Reactome	0.440117119	0.116881765	1.58969384	0.14496774	0.29047751	-5.17045846
hetero after UVB irradiation VS hetero before UVB irradiation	Organic anion transport|Reactome	-0.81454662	0.022948845	-3.01724572	0.01566653	0.11352943	-3.044773
WT after UVB irradiation VS WT before UVB irradiation	Organic anion transport|Reactome	-0.37551238	0.088756615	-1.21517489	0.25198938	0.64617378	-5.17492381
homo after UVB irradiation VS homo before UVB irradiation	Organic anion transporters|Reactome	0.175567879	-0.01609272	0.95646993	0.36281234	0.51751626	-5.9000452
hetero after UVB irradiation VS hetero before UVB irradiation	Organic anion transporters|Reactome	-0.39944501	-0.09723686	-1.40706544	0.19520592	0.4161797	-5.38511755
WT after UVB irradiation VS WT before UVB irradiation	Organic anion transporters|Reactome	-0.12046075	-0.02872803	-0.50305447	0.62573289	0.87257466	-5.74265623
homo after UVB irradiation VS homo before UVB irradiation	Organic cation transport|Reactome	0.497496533	0.044664668	2.715511076	0.02292254	0.09892658	-3.47378454
hetero after UVB irradiation VS hetero before UVB irradiation	Organic cation transport|Reactome	0.003157527	0.020724939	0.016206002	0.98744724	0.99353248	-6.34639882
WT after UVB irradiation VS WT before UVB irradiation	Organic cation transport|Reactome	0.227834447	0.029005702	1.138740545	0.28114547	0.66979206	-5.25419892
homo after UVB irradiation VS homo before UVB irradiation	Organic cation/anion/zwitterion transport|Reactome	0.47619226	0.053439748	2.58143947	0.02867571	0.11336662	-3.68756695
hetero after UVB irradiation VS hetero before UVB irradiation	Organic cation/anion/zwitterion transport|Reactome	-0.14363451	0.029876623	-0.70997892	0.49689297	0.71538528	-6.08118895
WT after UVB irradiation VS WT before UVB irradiation	Organic cation/anion/zwitterion transport|Reactome	0.133892498	0.035614333	0.646641017	0.53230817	0.83483909	-5.66192942
homo after UVB irradiation VS homo before UVB irradiation	ornithine <i>de novo </i> biosynthesis|HumanCyc	0.388574968	0.046899247	1.03703386	0.32568886	0.48157233	-5.82225681
hetero after UVB irradiation VS hetero before UVB irradiation	ornithine <i>de novo </i> biosynthesis|HumanCyc	0.089602165	-0.05984413	0.257837235	0.80271853	0.91722012	-6.31062193
WT after UVB irradiation VS WT before UVB irradiation	ornithine <i>de novo </i> biosynthesis|HumanCyc	0.210812284	-0.12579696	0.678535229	0.51270524	0.82768641	-5.64148152
homo after UVB irradiation VS homo before UVB irradiation	Ornithine Aminotransferase Deficiency (OAT Deficiency)|SMPDB	-0.34739547	0.00502943	-1.63262028	0.13558154	0.277359	-5.11269582
hetero after UVB irradiation VS hetero before UVB irradiation	Ornithine Aminotransferase Deficiency (OAT Deficiency)|SMPDB	-0.56351352	-0.04220085	-3.24793085	0.01094591	0.10415756	-2.6949004
WT after UVB irradiation VS WT before UVB irradiation	Ornithine Aminotransferase Deficiency (OAT Deficiency)|SMPDB	-0.47021304	-0.04055952	-2.31093774	0.04323906	0.39022568	-3.73881388
homo after UVB irradiation VS homo before UVB irradiation	Ornithine Transcarbamylase Deficiency (OTC Deficiency)|SMPDB	-0.37671284	0.065264039	-1.86404069	0.09383246	0.21982504	-4.78798165
hetero after UVB irradiation VS hetero before UVB irradiation	Ornithine Transcarbamylase Deficiency (OTC Deficiency)|SMPDB	-0.31260554	-0.01518237	-1.48265131	0.17457213	0.39158454	-5.29086545
WT after UVB irradiation VS WT before UVB irradiation	Ornithine Transcarbamylase Deficiency (OTC Deficiency)|SMPDB	-0.12033773	0.030196148	-0.57832296	0.57573553	0.8562298	-5.70268083
homo after UVB irradiation VS homo before UVB irradiation	Osteoblast Signaling|Wikipathways	0.666910739	0.000497422	3.267847649	0.0091861	0.05737857	-2.59032786
hetero after UVB irradiation VS hetero before UVB irradiation	Osteoblast Signaling|Wikipathways	0.599850651	0.065394299	2.329855316	0.04661239	0.19011835	-4.09346054
WT after UVB irradiation VS WT before UVB irradiation	Osteoblast Signaling|Wikipathways	0.685945169	0.103315289	2.375223062	0.03873754	0.39022568	-3.64404602
homo after UVB irradiation VS homo before UVB irradiation	Osteoclast differentiation - Homo sapiens (human)|KEGG	0.570599274	-0.01646598	3.457190694	0.00675132	0.04749851	-2.29037873
hetero after UVB irradiation VS hetero before UVB irradiation	Osteoclast differentiation - Homo sapiens (human)|KEGG	0.552400977	0.036878409	3.15006715	0.0127359	0.10891229	-2.84287012
WT after UVB irradiation VS WT before UVB irradiation	Osteoclast differentiation - Homo sapiens (human)|KEGG	0.477188876	0.003189234	1.920150996	0.08354973	0.470709	-4.298506
homo after UVB irradiation VS homo before UVB irradiation	Osteoclast Signaling|Wikipathways	0.531108902	-0.03894472	3.357438129	0.00793698	0.05229475	-2.44806766
hetero after UVB irradiation VS hetero before UVB irradiation	Osteoclast Signaling|Wikipathways	0.365200116	0.021834042	1.782381229	0.11064293	0.30216567	-4.89190864
WT after UVB irradiation VS WT before UVB irradiation	Osteoclast Signaling|Wikipathways	0.568618027	-0.00535495	2.366770514	0.03930221	0.39022568	-3.65653759
homo after UVB irradiation VS homo before UVB irradiation	Osteopontin Signaling|Wikipathways	0.715725581	-0.05222738	3.189038973	0.01045282	0.06166351	-2.71588317
hetero after UVB irradiation VS hetero before UVB irradiation	Osteopontin Signaling|Wikipathways	0.662727689	-0.03634805	3.206255343	0.01167373	0.10542662	-2.75782068
WT after UVB irradiation VS WT before UVB irradiation	Osteopontin Signaling|Wikipathways	0.708346021	0.009896169	3.161189858	0.01003934	0.25758249	-2.46670734
homo after UVB irradiation VS homo before UVB irradiation	Osteopontin-mediated events|PID	0.48791059	-0.06340212	2.309821146	0.04511444	0.13834286	-4.11551211
hetero after UVB irradiation VS hetero before UVB irradiation	Osteopontin-mediated events|PID	0.509364909	-0.01699382	2.99078924	0.01632951	0.11352943	-3.08511004
WT after UVB irradiation VS WT before UVB irradiation	Osteopontin-mediated events|PID	0.369310063	0.007182862	1.708686242	0.11805842	0.52194851	-4.58408681

homo after UVB irradiation VS homo before UVB irradiation	Other glycan degradation - Homo sapiens (human)|KEGG	0.489906497	-0.0750369	2.085711044	0.0653748	0.17757366	-4.45973156
hetero after UVB irradiation VS hetero before UVB irradiation	Other glycan degradation - Homo sapiens (human)|KEGG	0.561825459	-0.04098192	2.70481947	0.02564629	0.14017854	-3.52248129
WT after UVB irradiation VS WT before UVB irradiation	Other glycan degradation - Homo sapiens (human)|KEGG	0.598080996	-0.00102431	2.752695057	0.02023987	0.31542987	-3.07992002
homo after UVB irradiation VS homo before UVB irradiation	Other interleukin signaling|Reactome	0.827004799	-0.02155322	5.11675731	0.0005522	0.01666204	0.14902456
hetero after UVB irradiation VS hetero before UVB irradiation	Other interleukin signaling|Reactome	0.568121508	0.050939904	2.929827819	0.01797002	0.1185345	-3.17817457
WT after UVB irradiation VS WT before UVB irradiation	Other interleukin signaling|Reactome	0.724968517	0.064555765	3.22057172	0.00907296	0.24856491	-2.37818396
homo after UVB irradiation VS homo before UVB irradiation	Other semaphorin interactions|Reactome	0.220850835	-0.07322841	1.248290337	0.24215392	0.40353094	-5.59563322
hetero after UVB irradiation VS hetero before UVB irradiation	Other semaphorin interactions|Reactome	0.389419523	0.003551026	2.24991732	0.05294736	0.20434723	-4.21362488
WT after UVB irradiation VS WT before UVB irradiation	Other semaphorin interactions|Reactome	0.220516097	-0.04543028	0.888434192	0.39500886	0.76452505	-5.48532716
homo after UVB irradiation VS homo before UVB irradiation	Other types of O-glycan biosynthesis - Homo sapiens (human)|KEGG	0.410118393	-0.01191721	2.139690881	0.0598113	0.16723724	-4.37779177
hetero after UVB irradiation VS hetero before UVB irradiation	Other types of O-glycan biosynthesis - Homo sapiens (human)|KEGG	0.360187475	0.013960629	1.39653819	0.19824219	0.41868045	-5.3980105
WT after UVB irradiation VS WT before UVB irradiation	Other types of O-glycan biosynthesis - Homo sapiens (human)|KEGG	0.343422339	-0.09136777	1.396242296	0.19262069	0.60496032	-4.97282917
homo after UVB irradiation VS homo before UVB irradiation	Ovarian Infertility Genes|Wikipathways	-0.24839111	0.009360455	-1.16866537	0.27135328	0.4350418	-5.68470886
hetero after UVB irradiation VS hetero before UVB irradiation	Ovarian Infertility Genes|Wikipathways	-0.38194941	-0.03746489	-2.14652054	0.0624144	0.22396997	-4.36767569
WT after UVB irradiation VS WT before UVB irradiation	Ovarian Infertility Genes|Wikipathways	-0.42562727	-0.0352769	-2.19824402	0.05238504	0.41454814	-3.9034425
homo after UVB irradiation VS homo before UVB irradiation	Ovarian steroidogenesis - Homo sapiens (human)|KEGG	0.339734147	-0.02002401	2.037743273	0.07073228	0.18768759	-4.53194383
hetero after UVB irradiation VS hetero before UVB irradiation	Ovarian steroidogenesis - Homo sapiens (human)|KEGG	0.316654512	0.017153765	1.895868835	0.092709	0.27457285	-4.7322743
WT after UVB irradiation VS WT before UVB irradiation	Ovarian steroidogenesis - Homo sapiens (human)|KEGG	0.002676095	-0.00999078	0.012294045	0.99043089	0.99676022	-5.86907462
homo after UVB irradiation VS homo before UVB irradiation	Ovarian tumor domain proteases|Reactome	0.318941209	-0.02294249	1.681869174	0.12548894	0.26477934	-5.04540735
hetero after UVB irradiation VS hetero before UVB irradiation	Ovarian tumor domain proteases|Reactome	0.358750497	0.017713402	2.219900689	0.05553983	0.20938837	-4.25852087
WT after UVB irradiation VS WT before UVB irradiation	Ovarian tumor domain proteases|Reactome	0.118884003	0.017642756	0.598275859	0.56285396	0.85017866	-5.69121332
homo after UVB irradiation VS homo before UVB irradiation	Overview of nanoparticle effects|Wikipathways	0.71196931	-0.03490283	4.389624031	0.00157769	0.02229782	-0.87040779
hetero after UVB irradiation VS hetero before UVB irradiation	Overview of nanoparticle effects|Wikipathways	0.623222667	0.080214467	3.901626997	0.00409969	0.07021718	-1.73241962
WT after UVB irradiation VS WT before UVB irradiation	Overview of nanoparticle effects|Wikipathways	0.49251633	-0.00242005	2.19923006	0.05229745	0.41454814	-3.90201146
homo after UVB irradiation VS homo before UVB irradiation	overview of telomerase protein component gene htert transcriptional regulation|BioCarta	-0.11465623	0.052165415	-0.53168123	0.60729522	0.72589267	-6.21886593
hetero after UVB irradiation VS hetero before UVB irradiation	overview of telomerase protein component gene htert transcriptional regulation|BioCarta	-0.0214468	0.03174493	-0.13683469	0.89437473	0.96578063	-6.33639536
WT after UVB irradiation VS WT before UVB irradiation	overview of telomerase protein component gene htert transcriptional regulation|BioCarta	-0.01953341	0.100400611	-0.08052755	0.93739377	0.98056728	-5.86586722
homo after UVB irradiation VS homo before UVB irradiation	overview of telomerase rna component gene hterc transcriptional regulation|BioCarta	-0.3825657	-0.01399975	-1.46815328	0.17474756	0.3267393	-5.32916705
hetero after UVB irradiation VS hetero before UVB irradiation	overview of telomerase rna component gene hterc transcriptional regulation|BioCarta	-0.16541789	-0.02186375	-0.63570437	0.54184613	0.75147406	-6.13255598
WT after UVB irradiation VS WT before UVB irradiation	overview of telomerase rna component gene hterc transcriptional regulation|BioCarta	-0.20170617	-0.01493886	-0.54857624	0.5952344	0.8649398	-5.71910349
homo after UVB irradiation VS homo before UVB irradiation	Oxaprozin Action Pathway|SMPDB	0.40423582	0.045705158	1.979500697	0.07780079	0.19626432	-4.61879689
hetero after UVB irradiation VS hetero before UVB irradiation	Oxaprozin Action Pathway|SMPDB	0.482496143	0.064876536	3.04499191	0.01500113	0.1130168	-3.00250826
WT after UVB irradiation VS WT before UVB irradiation	Oxaprozin Action Pathway|SMPDB	0.275725339	0.086595857	1.261970114	0.23537138	0.62952955	-5.12455679
homo after UVB irradiation VS homo before UVB irradiation	Oxidation by Cytochrome P450|Wikipathways	0.138050477	0.034365992	0.754704267	0.46893248	0.61386058	-6.07159022
hetero after UVB irradiation VS hetero before UVB irradiation	Oxidation by Cytochrome P450|Wikipathways	0.105285427	0.024348569	0.511963387	0.62182259	0.80359154	-6.20658145
WT after UVB irradiation VS WT before UVB irradiation	Oxidation by Cytochrome P450|Wikipathways	-0.02262872	0.012025914	-0.0958545	0.92551465	0.97616053	-5.86449883
homo after UVB irradiation VS homo before UVB irradiation	Oxidation of Branched Chain Fatty Acids|SMPDB	0.300836817	-0.05951636	1.179047299	0.26739282	0.43096209	-5.67333677
hetero after UVB irradiation VS hetero before UVB irradiation	Oxidation of Branched Chain Fatty Acids|SMPDB	0.105258864	-0.05756042	0.331965471	0.74802279	0.87941394	-6.28715688
WT after UVB irradiation VS WT before UVB irradiation	Oxidation of Branched Chain Fatty Acids|SMPDB	0.134666912	-0.05063532	0.531589903	0.60652329	0.87177925	-5.72811672
homo after UVB irradiation VS homo before UVB irradiation	Oxidative Damage|Wikipathways	0.202552032	-0.01101645	1.251427626	0.24105807	0.40273367	-5.59203778
hetero after UVB irradiation VS hetero before UVB irradiation	Oxidative Damage|Wikipathways	0.270265892	0.008970054	1.43336464	0.18779676	0.40548444	-5.35265397
WT after UVB irradiation VS WT before UVB irradiation	Oxidative Damage|Wikipathways	0.355453488	-0.06356295	1.723765993	0.11522175	0.51589975	-4.56424712
homo after UVB irradiation VS homo before UVB irradiation	oxidative ethanol degradation III|HumanCyc	-0.10399776	0.022377233	-0.3590846	0.72747475	0.82097126	-6.299652
hetero after UVB irradiation VS hetero before UVB irradiation	oxidative ethanol degradation III|HumanCyc	-0.61161637	-0.01685085	-2.38998237	0.04234895	0.18131011	-4.00258073
WT after UVB irradiation VS WT before UVB irradiation	oxidative ethanol degradation III|HumanCyc	-0.33336175	-0.0181582	-1.20221685	0.25675463	0.65122698	-5.18862891
homo after UVB irradiation VS homo before UVB irradiation	Oxidative phosphorylation - Homo sapiens (human)|KEGG	0.00825982	-0.025271	0.029647134	0.97696912	0.98606224	-6.36797602
hetero after UVB irradiation VS hetero before UVB irradiation	Oxidative phosphorylation - Homo sapiens (human)|KEGG	-0.15844892	-0.04534866	-0.66433805	0.52423425	0.73931303	-6.11335527
WT after UVB irradiation VS WT before UVB irradiation	Oxidative phosphorylation - Homo sapiens (human)|KEGG	-0.00681375	-0.04862601	-0.02270869	0.9823258	0.99430008	-5.86888996
homo after UVB irradiation VS homo before UVB irradiation	Oxidative phosphorylation|Wikipathways	0.017639277	-0.02294901	0.05212098	0.95952441	0.9757658	-6.36698884
hetero after UVB irradiation VS hetero before UVB irradiation	Oxidative phosphorylation|Wikipathways	-0.23027636	-0.03176878	-0.87695521	0.40481906	0.63319136	-5.94782859
WT after UVB irradiation VS WT before UVB irradiation	Oxidative phosphorylation|Wikipathways	0.003508976	-0.06777615	0.010098874	0.99213944	0.99712795	-5.86909953
homo after UVB irradiation VS homo before UVB irradiation	oxidative stress induced gene expression via nrf2|BioCarta	0.338070207	-0.0238873	1.570593969	0.1493275	0.29522837	-5.19588302
hetero after UVB irradiation VS hetero before UVB irradiation	oxidative stress induced gene expression via nrf2|BioCarta	0.455960296	0.00621052	2.691400734	0.02619865	0.14081773	-3.54302175
WT after UVB irradiation VS WT before UVB irradiation	oxidative stress induced gene expression via nrf2|BioCarta	0.289596662	-0.03998489	1.238730103	0.24350957	0.63440287	-5.14974011
homo after UVB irradiation VS homo before UVB irradiation	Oxidative Stress Induced Senescence|Reactome	-0.23439892	0.032081781	-1.29427246	0.22649662	0.38632185	-5.54230685
hetero after UVB irradiation VS hetero before UVB irradiation	Oxidative Stress Induced Senescence|Reactome	-0.00928822	0.013213204	-0.04697671	0.96362622	0.983477	-6.34534427
WT after UVB irradiation VS WT before UVB irradiation	Oxidative Stress Induced Senescence|Reactome	-0.25574687	0.009324369	-1.29060819	0.22564985	0.62952955	-5.0930735
homo after UVB irradiation VS homo before UVB irradiation	Oxidative Stress Induced Senescence|Wikipathways	-0.01240154	0.043509903	-0.03978022	0.96910141	0.9806997	-6.36759807
hetero after UVB irradiation VS hetero before UVB irradiation	Oxidative Stress Induced Senescence|Wikipathways	0.298717684	-0.01339524	1.029064597	0.33208094	0.56018686	-5.80638772
WT after UVB irradiation VS WT before UVB irradiation	Oxidative Stress Induced Senescence|Wikipathways	-0.12356385	0.012585004	-0.55820494	0.58888477	0.86161188	-5.71387634
homo after UVB irradiation VS homo before UVB irradiation	Oxidative Stress Pathway (Erythrocyte)|PharmGKB	0.573258087	-0.07746011	2.281910034	0.0472573	0.14158585	-4.15891251
homo after UVB irradiation VS homo before UVB irradiation	Oxidative Stress Pathway (Erythrocyte)|PharmGKB	0.573258087	-0.07746011	2.281910034	0.0472573	0.14158585	-4.15891251
hetero after UVB irradiation VS hetero before UVB irradiation	Oxidative Stress Pathway (Erythrocyte)|PharmGKB	0.210856326	0.091368828	0.58336559	0.57492585	0.7705309	-6.16566152
hetero after UVB irradiation VS hetero before UVB irradiation	Oxidative Stress Pathway (Erythrocyte)|PharmGKB	0.210856326	0.091368828	0.58336559	0.57492585	0.7705309	-6.16566152
WT after UVB irradiation VS WT before UVB irradiation	Oxidative Stress Pathway (Erythrocyte)|PharmGKB	0.199509683	-0.11826803	0.598119045	0.56295457	0.85017866	-5.69130485
WT after UVB irradiation VS WT before UVB irradiation	Oxidative Stress Pathway (Erythrocyte)|PharmGKB	0.199509683	-0.11826803	0.598119045	0.56295457	0.85017866	-5.69130485
homo after UVB irradiation VS homo before UVB irradiation	Oxidative Stress Regulatory Pathway (Erythrocyte)|PharmGKB	0.273703965	-0.01537068	1.142901199	0.2813839	0.44211938	-5.71260812
hetero after UVB irradiation VS hetero before UVB irradiation	Oxidative Stress Regulatory Pathway (Erythrocyte)|PharmGKB	0.16009197	0.062721292	0.599897776	0.56435518	0.76437184	-6.15548531
WT after UVB irradiation VS WT before UVB irradiation	Oxidative Stress Regulatory Pathway (Erythrocyte)|PharmGKB	-0.10536315	-0.0971178	-0.45276308	0.66030619	0.87935805	-5.76642491
homo after UVB irradiation VS homo before UVB irradiation	Oxidative Stress|Wikipathways	0.297911058	-0.00435717	1.622411283	0.13776302	0.27994034	-5.12650979
hetero after UVB irradiation VS hetero before UVB irradiation	Oxidative Stress|Wikipathways	0.422434868	0.035594861	2.291849543	0.04952471	0.19801197	-4.15069396
WT after UVB irradiation VS WT before UVB irradiation	Oxidative Stress|Wikipathways	0.301295358	-0.01187195	1.19730298	0.25858046	0.65353456	-5.19379816
homo after UVB irradiation VS homo before UVB irradiation	oxidized GTP and dGTP detoxification|HumanCyc	0.835031478	0.000144964	2.548471317	0.03029949	0.1156968	-3.73994098
hetero after UVB irradiation VS hetero before UVB irradiation	oxidized GTP and dGTP detoxification|HumanCyc	0.474711716	0.005175662	1.204663105	0.26107664	0.4873003	-5.62198638
WT after UVB irradiation VS WT before UVB irradiation	oxidized GTP and dGTP detoxification|HumanCyc	0.226244532	0.066409571	0.584280906	0.57187228	0.85490694	-5.69929445
homo after UVB irradiation VS homo before UVB irradiation	Oxprenolol Action Pathway|SMPDB	0.396415397	-0.03677376	2.343069613	0.04268583	0.13402181	-4.06364635
hetero after UVB irradiation VS hetero before UVB irradiation	Oxprenolol Action Pathway|SMPDB	-0.01555055	-0.01775524	-0.07410913	0.94265284	0.97054702	-6.34356282
WT after UVB irradiation VS WT before UVB irradiation	Oxprenolol Action Pathway|SMPDB	0.056685205	-0.03978418	0.223404833	0.82768199	0.94525798	-5.84393438
homo after UVB irradiation VS homo before UVB irradiation	Oxybuprocaine Action Pathway|SMPDB	0.191132361	0.029768717	1.061313976	0.31508276	0.46923284	-5.79783739
hetero after UVB irradiation VS hetero before UVB irradiation	Oxybuprocaine Action Pathway|SMPDB	-0.07904315	0.055005373	-0.44437398	0.66796482	0.82651398	-6.24068873
WT after UVB irradiation VS WT before UVB irradiation	Oxybuprocaine Action Pathway|SMPDB	0.022316377	0.035038618	0.098344171	0.92358676	0.97566251	-5.86425414
homo after UVB irradiation VS homo before UVB irradiation	Oxycodone Action Pathway|SMPDB	0.191132361	0.029768717	1.061313976	0.31508276	0.46923284	-5.79783739
hetero after UVB irradiation VS hetero before UVB irradiation	Oxycodone Action Pathway|SMPDB	-0.07904315	0.055005373	-0.44437398	0.66796482	0.82651398	-6.24068873
WT after UVB irradiation VS WT before UVB irradiation	Oxycodone Action Pathway|SMPDB	0.022316377	0.035038618	0.098344171	0.92358676	0.97566251	-5.86425414
homo after UVB irradiation VS homo before UVB irradiation	Oxygen-dependent asparagine hydroxylation of Hypoxia-inducible Factor Alpha|Reactome	0.892548738	-0.10644221	3.979532801	0.0029485	0.03080689	-1.48111994
hetero after UVB irradiation VS hetero before UVB irradiation	Oxygen-dependent asparagine hydroxylation of Hypoxia-inducible Factor Alpha|Reactome	0.77863908	0.049761778	3.948019595	0.00383198	0.06828702	-1.66620991
WT after UVB irradiation VS WT before UVB irradiation	Oxygen-dependent asparagine hydroxylation of Hypoxia-inducible Factor Alpha|Reactome	0.326909834	0.009244512	1.249475925	0.23971863	0.63283875	-5.1381369
homo after UVB irradiation VS homo before UVB irradiation	Oxygen-dependent proline hydroxylation of Hypoxia-inducible Factor Alpha|Reactome	0.333977389	-0.00735387	1.571564993	0.14910307	0.29497601	-5.19459465
hetero after UVB irradiation VS hetero before UVB irradiation	Oxygen-dependent proline hydroxylation of Hypoxia-inducible Factor Alpha|Reactome	0.37988903	0.016907325	1.920111385	0.08925234	0.27048683	-4.69767954
WT after UVB irradiation VS WT before UVB irradiation	Oxygen-dependent proline hydroxylation of Hypoxia-inducible Factor Alpha|Reactome	-0.00780693	0.030067282	-0.03570102	0.97221772	0.99045333	-5.86850555
homo after UVB irradiation VS homo before UVB irradiation	Oxymorphone Action Pathway|SMPDB	0.191132361	0.029768717	1.061313976	0.31508276	0.46923284	-5.79783739
hetero after UVB irradiation VS hetero before UVB irradiation	Oxymorphone Action Pathway|SMPDB	-0.07904315	0.055005373	-0.44437398	0.66796482	0.82651398	-6.24068873
WT after UVB irradiation VS WT before UVB irradiation	Oxymorphone Action Pathway|SMPDB	0.022316377	0.035038618	0.098344171	0.92358676	0.97566251	-5.86425414
homo after UVB irradiation VS homo before UVB irradiation	Oxytocin signaling pathway - Homo sapiens (human)|KEGG	0.437117054	-0.0270364	2.78621831	0.02037206	0.09100843	-3.3606758
hetero after UVB irradiation VS hetero before UVB irradiation	Oxytocin signaling pathway - Homo sapiens (human)|KEGG	0.248014395	0.034734878	1.335317591	0.21671952	0.43793611	-5.47179559
WT after UVB irradiation VS WT before UVB irradiation	Oxytocin signaling pathway - Homo sapiens (human)|KEGG	0.186559988	-0.01426472	0.81171632	0.43569926	0.79685269	-5.54663223
homo after UVB irradiation VS homo before UVB irradiation	p130Cas linkage to MAPK signaling for integrins|Reactome	0.850149694	-0.01730845	5.250479328	0.00045904	0.01666204	0.327215734
hetero after UVB irradiation VS hetero before UVB irradiation	p130Cas linkage to MAPK signaling for integrins|Reactome	0.614650734	0.011493737	2.48566526	0.03635345	0.16681566	-3.857268
WT after UVB irradiation VS WT before UVB irradiation	p130Cas linkage to MAPK signaling for integrins|Reactome	0.6906734	-0.02231323	2.908782272	0.01547168	0.29238268	-2.8451267
homo after UVB irradiation VS homo before UVB irradiation	P2Y receptors|Reactome	0.22639601	-0.020936	0.961818091	0.36025534	0.51548915	-5.89503884
hetero after UVB irradiation VS hetero before UVB irradiation	P2Y receptors|Reactome	0.562563114	-0.0161658	3.239645205	0.01108674	0.10440777	-2.70739825
WT after UVB irradiation VS WT before UVB irradiation	P2Y receptors|Reactome	0.511580953	0.030075901	1.98974953	0.07441884	0.45723857	-4.20142727
homo after UVB irradiation VS homo before UVB irradiation	p38 mapk signaling pathway|BioCarta	-0.25706059	0.028595541	-1.60720144	0.14107153	0.28438525	-5.14700245
hetero after UVB irradiation VS hetero before UVB irradiation	p38 mapk signaling pathway|BioCarta	-0.07584204	-0.04703851	-0.46782771	0.65177027	0.82567229	-6.22937051
WT after UVB irradiation VS WT before UVB irradiation	p38 mapk signaling pathway|BioCarta	-0.25099031	-0.02129686	-1.26277942	0.23509203	0.62952955	-5.12367387
homo after UVB irradiation VS homo before UVB irradiation	p38 MAPK signaling pathway|PID	0.277628274	-0.00113031	1.675588909	0.12673699	0.26670931	-5.05404667
hetero after UVB irradiation VS hetero before UVB irradiation	p38 MAPK signaling pathway|PID	0.218537108	-0.0609012	1.076122228	0.3117167	0.54022992	-5.75908034
WT after UVB irradiation VS WT before UVB irradiation	p38 MAPK signaling pathway|PID	0.04728531	-0.04826495	0.240587642	0.8146963	0.94525798	-5.83991861
homo after UVB irradiation VS homo before UVB irradiation	p38 MAPK Signaling Pathway|Wikipathways	0.009727304	-0.03281085	0.053453237	0.95849089	0.97536648	-6.36691329
hetero after UVB irradiation VS hetero before UVB irradiation	p38 MAPK Signaling Pathway|Wikipathways	0.256875603	-0.02370426	1.6137716	0.14332934	0.34688172	-5.12089466
WT after UVB irradiation VS WT before UVB irradiation	p38 MAPK Signaling Pathway|Wikipathways	-0.03406724	-0.02353499	-0.16582703	0.87157122	0.96283151	-5.85524096
homo after UVB irradiation VS homo before UVB irradiation	p38 signaling mediated by MAPKAP kinases|PID	0.377477823	-0.06336254	1.693299668	0.12324598	0.26247468	-5.02964024
hetero after UVB irradiation VS hetero before UVB irradiation	p38 signaling mediated by MAPKAP kinases|PID	0.579284162	-0.00919166	3.856637015	0.00437842	0.07183716	-1.79692197
WT after UVB irradiation VS WT before UVB irradiation	p38 signaling mediated by MAPKAP kinases|PID	0.524131686	-0.02702766	2.185046461	0.05357105	0.41493406	-3.92257864
homo after UVB irradiation VS homo before UVB irradiation	p38MAPK events|Reactome	0.016804289	0.015436055	0.062780866	0.95125726	0.97016767	-6.36633096
hetero after UVB irradiation VS hetero before UVB irradiation	p38MAPK events|Reactome	0.667561591	0.053113215	3.496145707	0.00749074	0.08807784	-2.32353487
WT after UVB irradiation VS WT before UVB irradiation	p38MAPK events|Reactome	0.254290371	0.046146479	0.899278597	0.38947702	0.76242672	-5.47628318
homo after UVB irradiation VS homo before UVB irradiation	p53 pathway|PID	-0.60637216	0.020174226	-3.59515606	0.00540721	0.04239626	-2.07373226
hetero after UVB irradiation VS hetero before UVB irradiation	p53 pathway|PID	-0.1193818	-0.00306028	-0.52228847	0.61491978	0.80011543	-6.20097185
WT after UVB irradiation VS WT before UVB irradiation	p53 pathway|PID	-0.36339394	-0.06920782	-1.48275981	0.16870762	0.57499084	-4.86982681
homo after UVB irradiation VS homo before UVB irradiation	p53 signaling pathway - Homo sapiens (human)|KEGG	0.115745134	0.008229391	0.644704728	0.53455145	0.67053899	-6.15006009
hetero after UVB irradiation VS hetero before UVB irradiation	p53 signaling pathway - Homo sapiens (human)|KEGG	0.348494129	-0.01499557	1.967632337	0.08282975	0.2599606	-4.62940767
WT after UVB irradiation VS WT before UVB irradiation	p53 signaling pathway - Homo sapiens (human)|KEGG	0.20076982	-0.02462705	1.035448727	0.32465626	0.70875006	-5.35508433
homo after UVB irradiation VS homo before UVB irradiation	p53 signaling pathway|BioCarta	-0.4663114	-0.03491383	-2.13025599	0.06074969	0.16831499	-4.39216278
hetero after UVB irradiation VS hetero before UVB irradiation	p53 signaling pathway|BioCarta	-0.13741582	-0.04229348	-0.58421681	0.57437888	0.7705309	-6.16514394
WT after UVB irradiation VS WT before UVB irradiation	p53 signaling pathway|BioCarta	-0.13635142	-0.08377665	-0.48175156	0.64026855	0.87636259	-5.75301504
homo after UVB irradiation VS homo before UVB irradiation	p53-Dependent G1 DNA Damage Response|Reactome	-0.41738713	0.018048059	-2.27638284	0.04769331	0.14242487	-4.16749122
hetero after UVB irradiation VS hetero before UVB irradiation	p53-Dependent G1 DNA Damage Response|Reactome	0.26410713	0.023570726	1.153824236	0.28024772	0.51053921	-5.67753974
WT after UVB irradiation VS WT before UVB irradiation	p53-Dependent G1 DNA Damage Response|Reactome	-0.22049348	-0.04885037	-0.93295619	0.37264599	0.7494118	-5.44761316
homo after UVB irradiation VS homo before UVB irradiation	p53-Dependent G1/S DNA damage checkpoint|Reactome	-0.41738713	0.018048059	-2.27638284	0.04769331	0.14242487	-4.16749122
hetero after UVB irradiation VS hetero before UVB irradiation	p53-Dependent G1/S DNA damage checkpoint|Reactome	0.26410713	0.023570726	1.153824236	0.28024772	0.51053921	-5.67753974
WT after UVB irradiation VS WT before UVB irradiation	p53-Dependent G1/S DNA damage checkpoint|Reactome	-0.22049348	-0.04885037	-0.93295619	0.37264599	0.7494118	-5.44761316
homo after UVB irradiation VS homo before UVB irradiation	p53-Independent DNA Damage Response|Reactome	-0.49558194	0.042913081	-2.01507448	0.0734076	0.19274245	-4.56585995
hetero after UVB irradiation VS hetero before UVB irradiation	p53-Independent DNA Damage Response|Reactome	0.065090439	0.068477923	0.190850087	0.85316168	0.94503144	-6.32682542
WT after UVB irradiation VS WT before UVB irradiation	p53-Independent DNA Damage Response|Reactome	-0.4239123	-0.00307825	-1.55750263	0.15015998	0.55622203	-4.77783208
homo after UVB irradiation VS homo before UVB irradiation	p53-Independent G1/S DNA damage checkpoint|Reactome	-0.49558194	0.042913081	-2.01507448	0.0734076	0.19274245	-4.56585995
hetero after UVB irradiation VS hetero before UVB irradiation	p53-Independent G1/S DNA damage checkpoint|Reactome	0.065090439	0.068477923	0.190850087	0.85316168	0.94503144	-6.32682542
WT after UVB irradiation VS WT before UVB irradiation	p53-Independent G1/S DNA damage checkpoint|Reactome	-0.4239123	-0.00307825	-1.55750263	0.15015998	0.55622203	-4.77783208
homo after UVB irradiation VS homo before UVB irradiation	p73 transcription factor network|PID	-0.10167635	-0.00719095	-0.60922845	0.55682549	0.68493135	-6.17297329
hetero after UVB irradiation VS hetero before UVB irradiation	p73 transcription factor network|PID	0.313853605	-0.03250874	1.807846208	0.10635564	0.29469241	-4.85644019
WT after UVB irradiation VS WT before UVB irradiation	p73 transcription factor network|PID	0.084942505	-0.01600653	0.399397354	0.69792993	0.89864566	-5.78902197
homo after UVB irradiation VS homo before UVB irradiation	p75 NTR receptor-mediated signalling|Reactome	0.320223947	-0.0533483	1.755268211	0.11170953	0.24593333	-4.94322463
hetero after UVB irradiation VS hetero before UVB irradiation	p75 NTR receptor-mediated signalling|Reactome	0.382551793	0.010402157	2.320364643	0.04732331	0.19241719	-4.107769
WT after UVB irradiation VS WT before UVB irradiation	p75 NTR receptor-mediated signalling|Reactome	0.203551213	-0.00121086	1.0514908	0.31758231	0.70237831	-5.33990551
homo after UVB irradiation VS homo before UVB irradiation	p75(NTR)-mediated signaling|PID	0.376742665	-0.03990594	2.16792638	0.05708582	0.16240116	-4.33466537
hetero after UVB irradiation VS hetero before UVB irradiation	p75(NTR)-mediated signaling|PID	0.408799592	0.013064437	2.619643242	0.02936487	0.14913595	-3.65280533
WT after UVB irradiation VS WT before UVB irradiation	p75(NTR)-mediated signaling|PID	0.403942953	-0.00540952	2.095061149	0.06237149	0.43544914	-4.05213097
homo after UVB irradiation VS homo before UVB irradiation	p75NTR negatively regulates cell cycle via SC1|Reactome	-0.10171847	0.014135903	-0.45726179	0.65787942	0.76492931	-6.25737185

hetero after UVB irradiation VS hetero before UVB irradiation	p75NTR negatively regulates cell cycle via SC1|Reactome	-0.04713309	-0.03649792	-0.15010759	0.88421102	0.95957948	-6.33433443
WT after UVB irradiation VS WT before UVB irradiation	p75NTR negatively regulates cell cycle via SC1|Reactome	0.071923665	0.063536012	0.216149284	0.83318191	0.94894798	-5.84554172
homo after UVB irradiation VS homo before UVB irradiation	p75NTR recruits signalling complexes|Reactome	0.412736936	-0.0086391	2.186748786	0.05533608	0.1592089	-4.30582066
hetero after UVB irradiation VS hetero before UVB irradiation	p75NTR recruits signalling complexes|Reactome	0.235836089	-0.0345088	0.984860205	0.3521245	0.58088436	-5.84933803
WT after UVB irradiation VS WT before UVB irradiation	p75NTR recruits signalling complexes|Reactome	0.254510619	0.049277837	1.125696356	0.2863764	0.6736679	-5.26734386
homo after UVB irradiation VS homo before UVB irradiation	p75NTR regulates axonogenesis|Reactome	0.303594364	-0.0499456	1.517023684	0.16218227	0.31153734	-5.26624592
hetero after UVB irradiation VS hetero before UVB irradiation	p75NTR regulates axonogenesis|Reactome	0.24417279	0.010587011	1.175695641	0.27186479	0.50107551	-5.65384781
WT after UVB irradiation VS WT before UVB irradiation	p75NTR regulates axonogenesis|Reactome	0.341682663	0.099339976	1.763307871	0.10807477	0.5076251	-4.51181511
homo after UVB irradiation VS homo before UVB irradiation	p75NTR signals via NF-kB|Reactome	0.365195639	-0.03316197	1.607286699	0.14105278	0.28438525	-5.14688787
hetero after UVB irradiation VS hetero before UVB irradiation	p75NTR signals via NF-kB|Reactome	0.414022319	-0.02345077	1.89598465	0.09269219	0.27457285	-4.73210941
WT after UVB irradiation VS WT before UVB irradiation	p75NTR signals via NF-kB|Reactome	0.008104915	0.025865035	0.036446005	0.97163825	0.99045333	-5.86847832
homo after UVB irradiation VS homo before UVB irradiation	Packaging Of Telomere Ends|Reactome	-0.26954596	0.033483331	-1.18055364	0.26682205	0.43034598	-5.67168063
hetero after UVB irradiation VS hetero before UVB irradiation	Packaging Of Telomere Ends|Reactome	0.128002401	-0.01596526	0.468352355	0.65141019	0.82567229	-6.22911102
WT after UVB irradiation VS WT before UVB irradiation	Packaging Of Telomere Ends|Reactome	-0.13535556	0.071487863	-0.56066443	0.58726867	0.86161188	-5.71252751
homo after UVB irradiation VS homo before UVB irradiation	Paclitaxel Action Pathway|SMPDB	0.194768865	0.003265929	0.860816473	0.4107816	0.56240743	-5.9856613
hetero after UVB irradiation VS hetero before UVB irradiation	Paclitaxel Action Pathway|SMPDB	0.237367392	0.050865229	0.815830007	0.43705048	0.66334222	-5.99942629
WT after UVB irradiation VS WT before UVB irradiation	Paclitaxel Action Pathway|SMPDB	0.585929693	0.017820683	2.275475662	0.04593603	0.39565089	-3.79084056
homo after UVB irradiation VS homo before UVB irradiation	palmitate biosynthesis|HumanCyc	-0.70958522	-0.03595114	-4.05745032	0.00261352	0.02939245	-1.36325322
hetero after UVB irradiation VS hetero before UVB irradiation	palmitate biosynthesis|HumanCyc	-0.39871056	-0.04248003	-2.42778974	0.0398698	0.17507375	-3.94525361
WT after UVB irradiation VS WT before UVB irradiation	palmitate biosynthesis|HumanCyc	-0.20587279	0.001027657	-0.69961067	0.49999316	0.82080113	-5.62748095
homo after UVB irradiation VS homo before UVB irradiation	Pamidronate Action Pathway|SMPDB	-0.19230642	-0.01987665	-0.61925406	0.55047761	0.67913558	-6.16661869
hetero after UVB irradiation VS hetero before UVB irradiation	Pamidronate Action Pathway|SMPDB	0.185820603	0.008098609	0.631890782	0.54421801	0.75147406	-6.13505553
WT after UVB irradiation VS WT before UVB irradiation	Pamidronate Action Pathway|SMPDB	-0.14894747	0.045069977	-0.50755372	0.62268399	0.87257466	-5.74041409
homo after UVB irradiation VS homo before UVB irradiation	Pancreas Function|SMPDB	-0.17556465	0.032733531	-0.6220452	0.54871777	0.67913558	-6.16483259
hetero after UVB irradiation VS hetero before UVB irradiation	Pancreas Function|SMPDB	-0.35455332	-0.0590526	-1.26742043	0.23890834	0.4612216	-5.55113336
WT after UVB irradiation VS WT before UVB irradiation	Pancreas Function|SMPDB	-0.23688751	-0.13206249	-0.65900303	0.52465845	0.83140705	-5.65411026
homo after UVB irradiation VS homo before UVB irradiation	Pancreatic adenocarcinoma pathway|Wikipathways	0.213132078	-0.01467763	1.068282636	0.31208802	0.46923284	-5.79074733
hetero after UVB irradiation VS hetero before UVB irradiation	Pancreatic adenocarcinoma pathway|Wikipathways	0.384883514	0.000965834	2.268817124	0.05137667	0.20066399	-4.18528996
WT after UVB irradiation VS WT before UVB irradiation	Pancreatic adenocarcinoma pathway|Wikipathways	0.287150065	0.011430046	1.432131057	0.18237106	0.59839062	-4.93057482
homo after UVB irradiation VS homo before UVB irradiation	Pancreatic cancer - Homo sapiens (human)|KEGG	0.262998604	-0.02179075	1.405827413	0.19199302	0.34801117	-5.40755851
hetero after UVB irradiation VS hetero before UVB irradiation	Pancreatic cancer - Homo sapiens (human)|KEGG	0.386617346	-0.00552436	2.282820164	0.05024273	0.19880033	-4.16426492
WT after UVB irradiation VS WT before UVB irradiation	Pancreatic cancer - Homo sapiens (human)|KEGG	0.307625546	-0.00251575	1.54922425	0.15212237	0.55762063	-4.7881518
homo after UVB irradiation VS homo before UVB irradiation	Pancreatic secretion - Homo sapiens (human)|KEGG	0.486140958	-0.0056902	2.980367274	0.01475096	0.0750032	-3.04962045
hetero after UVB irradiation VS hetero before UVB irradiation	Pancreatic secretion - Homo sapiens (human)|KEGG	0.278538544	0.035930181	1.595271676	0.14740824	0.3525114	-5.14533878
WT after UVB irradiation VS WT before UVB irradiation	Pancreatic secretion - Homo sapiens (human)|KEGG	0.153810274	-0.01644507	0.691663598	0.50476382	0.82448724	-5.63280563
homo after UVB irradiation VS homo before UVB irradiation	Panitumumab Action Pathway|SMPDB	-1.02021206	0.004763088	-4.21042005	0.00206751	0.02537706	-1.13430282
hetero after UVB irradiation VS hetero before UVB irradiation	Panitumumab Action Pathway|SMPDB	-0.7208662	-0.04088773	-2.69458506	0.02606648	0.14043856	-3.53814761
WT after UVB irradiation VS WT before UVB irradiation	Panitumumab Action Pathway|SMPDB	-0.88905476	-0.02311791	-2.71673052	0.02153341	0.31542987	-3.1339937
homo after UVB irradiation VS homo before UVB irradiation	Pantoprazole Action Pathway|SMPDB	0.21780303	0.051698704	1.19150037	0.26270351	0.42561191	-5.65959884
hetero after UVB irradiation VS hetero before UVB irradiation	Pantoprazole Action Pathway|SMPDB	0.331566302	0.063470812	1.441200644	0.18563717	0.40321767	-5.34291209
WT after UVB irradiation VS WT before UVB irradiation	Pantoprazole Action Pathway|SMPDB	-0.11560861	0.135982321	-0.44330845	0.66690385	0.87935805	-5.77062647
homo after UVB irradiation VS homo before UVB irradiation	Pantoprazole Metabolism Pathway|SMPDB	-0.06647614	0.033610835	-0.18292054	0.85874942	0.9134577	-6.35050291
WT after UVB irradiation VS WT before UVB irradiation	Pantoprazole Metabolism Pathway|SMPDB	-0.4370633	0.058240551	-1.4704397	0.17194858	0.57997722	-4.88472923
homo after UVB irradiation VS homo before UVB irradiation	Pantothenate and CoA biosynthesis - Homo sapiens (human)|KEGG	0.078827615	-0.00143523	0.315261086	0.75945791	0.84454088	-6.31533436
hetero after UVB irradiation VS hetero before UVB irradiation	Pantothenate and CoA biosynthesis - Homo sapiens (human)|KEGG	-0.2936132	-0.01085298	-1.55709446	0.15615898	0.36594993	-5.19531616
WT after UVB irradiation VS WT before UVB irradiation	Pantothenate and CoA biosynthesis - Homo sapiens (human)|KEGG	0.031164432	-0.00116936	0.129594694	0.89943613	0.97566251	-5.86065065
homo after UVB irradiation VS homo before UVB irradiation	Pantothenate and CoA Biosynthesis|SMPDB	0.155053813	0.028391202	0.658442482	0.52606857	0.66255701	-6.1408692
hetero after UVB irradiation VS hetero before UVB irradiation	Pantothenate and CoA Biosynthesis|SMPDB	-0.20525427	-0.05850953	-0.7434293	0.47743696	0.69910758	-6.05641737
WT after UVB irradiation VS WT before UVB irradiation	Pantothenate and CoA Biosynthesis|SMPDB	0.048570937	-0.02548106	0.164861273	0.87231174	0.96283151	-5.85540227
homo after UVB irradiation VS homo before UVB irradiation	PAOs oxidise polyamines to amines|Reactome	0.492795948	-0.0472877	1.259825889	0.23814459	0.39962079	-5.58238185
hetero after UVB irradiation VS hetero before UVB irradiation	PAOs oxidise polyamines to amines|Reactome	0.677825891	0.004088929	2.027156193	0.07540826	0.24621119	-4.54309537
WT after UVB irradiation VS WT before UVB irradiation	PAOs oxidise polyamines to amines|Reactome	0.780939001	-0.06156973	2.556143482	0.02839315	0.35176669	-3.37486629
homo after UVB irradiation VS homo before UVB irradiation	PAR1-mediated thrombin signaling events|PID	0.657903149	-0.04186897	3.95460475	0.00306501	0.03140179	-1.51900054
hetero after UVB irradiation VS hetero before UVB irradiation	PAR1-mediated thrombin signaling events|PID	0.632410028	0.064793683	2.837032368	0.02080069	0.12723297	-3.32007847
WT after UVB irradiation VS WT before UVB irradiation	PAR1-mediated thrombin signaling events|PID	0.494436365	-0.05612381	2.019711104	0.07078425	0.44774469	-4.15923119
homo after UVB irradiation VS homo before UVB irradiation	PAR4-mediated thrombin signaling events|PID	0.597532809	-0.01598534	3.243449151	0.00956036	0.05835911	-2.62916084
hetero after UVB irradiation VS hetero before UVB irradiation	PAR4-mediated thrombin signaling events|PID	0.436555785	0.071005033	2.014161995	0.07697166	0.24866063	-4.56200911
WT after UVB irradiation VS WT before UVB irradiation	PAR4-mediated thrombin signaling events|PID	0.39372973	-0.08454872	1.536240287	0.15524554	0.5591533	-4.80427313
homo after UVB irradiation VS homo before UVB irradiation	Paradoxical activation of RAF signaling by kinase inactive BRAF|Reactome	0.44624235	-0.03179323	2.689989071	0.02392033	0.10188454	-3.5145611
hetero after UVB irradiation VS hetero before UVB irradiation	Paradoxical activation of RAF signaling by kinase inactive BRAF|Reactome	0.514012692	-0.00838014	3.13003322	0.01313858	0.11049016	-2.87325098
WT after UVB irradiation VS WT before UVB irradiation	Paradoxical activation of RAF signaling by kinase inactive BRAF|Reactome	0.533138453	0.03058589	2.572376948	0.02761092	0.35054739	-3.35057812
homo after UVB irradiation VS homo before UVB irradiation	Parathyroid hormone synthesis_ secretion and action - Homo sapiens (human)|KEGG	0.396718996	-0.02287348	2.363779287	0.04123851	0.13402181	-4.03125433
hetero after UVB irradiation VS hetero before UVB irradiation	Parathyroid hormone synthesis_ secretion and action - Homo sapiens (human)|KEGG	0.336648733	0.044882521	1.962843793	0.08345635	0.26125963	-4.63631372
WT after UVB irradiation VS WT before UVB irradiation	Parathyroid hormone synthesis_ secretion and action - Homo sapiens (human)|KEGG	0.201323713	0.017250774	0.924465683	0.37683961	0.75077508	-5.45492387
homo after UVB irradiation VS homo before UVB irradiation	Parkinson disease - Homo sapiens (human)|KEGG	0.116734069	-0.01656263	0.415084074	0.68740513	0.7900055	-6.27673645
hetero after UVB irradiation VS hetero before UVB irradiation	Parkinson disease - Homo sapiens (human)|KEGG	-0.07417722	-0.00256752	-0.31373661	0.76134799	0.88957296	-6.29346185
WT after UVB irradiation VS WT before UVB irradiation	Parkinson disease - Homo sapiens (human)|KEGG	0.084655978	-0.04797658	0.293470917	0.77511432	0.93442888	-5.82572031
homo after UVB irradiation VS homo before UVB irradiation	Parkinsons Disease Pathway|Wikipathways	0.260634384	-0.02091196	1.464497698	0.1757207	0.32828997	-5.33382326
hetero after UVB irradiation VS hetero before UVB irradiation	Parkinsons Disease Pathway|Wikipathways	0.399756033	0.016442092	2.473696426	0.03705415	0.16802342	-3.87548417
WT after UVB irradiation VS WT before UVB irradiation	Parkinsons Disease Pathway|Wikipathways	0.415206373	-0.01120355	2.041302079	0.0682697	0.44205977	-4.12868204
homo after UVB irradiation VS homo before UVB irradiation	Parkin-Ubiquitin Proteasomal System pathway|Wikipathways	-0.42351878	0.008697787	-1.70551475	0.1208894	0.26005561	-5.01273024
hetero after UVB irradiation VS hetero before UVB irradiation	Parkin-Ubiquitin Proteasomal System pathway|Wikipathways	-0.12978575	-0.00632691	-0.55150583	0.59560619	0.78552459	-6.18453221
WT after UVB irradiation VS WT before UVB irradiation	Parkin-Ubiquitin Proteasomal System pathway|Wikipathways	-0.17058312	-0.01266413	-0.61005885	0.55532286	0.84708066	-5.6842722
homo after UVB irradiation VS homo before UVB irradiation	Passive transport by Aquaporins|Reactome	0.754055961	0.045020738	4.458233469	0.00142425	0.02159829	-0.77065246
hetero after UVB irradiation VS hetero before UVB irradiation	Passive transport by Aquaporins|Reactome	0.387624025	0.039535402	2.015891512	0.07676179	0.24857939	-4.55949394
WT after UVB irradiation VS WT before UVB irradiation	Passive transport by Aquaporins|Reactome	0.182527672	0.123189533	0.740676678	0.4757854	0.81430085	-5.59909861
homo after UVB irradiation VS homo before UVB irradiation	Pathogenic Escherichia coli infection - Homo sapiens (human)|KEGG	0.158677903	-0.0093537	0.631354867	0.54287144	0.67708436	-6.15882189
hetero after UVB irradiation VS hetero before UVB irradiation	Pathogenic Escherichia coli infection - Homo sapiens (human)|KEGG	0.405458066	0.002171471	2.461998512	0.03775211	0.16931722	-3.89327815
WT after UVB irradiation VS WT before UVB irradiation	Pathogenic Escherichia coli infection - Homo sapiens (human)|KEGG	0.166045206	-0.06040853	0.730096726	0.48195043	0.81552033	-5.60654901
homo after UVB irradiation VS homo before UVB irradiation	Pathogenic Escherichia coli infection|Wikipathways	0.158677903	-0.0093537	0.631354867	0.54287144	0.67708436	-6.15882189
hetero after UVB irradiation VS hetero before UVB irradiation	Pathogenic Escherichia coli infection|Wikipathways	0.405458066	0.002171471	2.461998512	0.03775211	0.16931722	-3.89327815
WT after UVB irradiation VS WT before UVB irradiation	Pathogenic Escherichia coli infection|Wikipathways	0.166045206	-0.06040853	0.730096726	0.48195043	0.81552033	-5.60654901
homo after UVB irradiation VS homo before UVB irradiation	Pathway_PA165959425|PharmGKB	0.372374143	-0.04757537	1.700011792	0.12194593	0.26055457	-5.02035598
hetero after UVB irradiation VS hetero before UVB irradiation	Pathway_PA165959425|PharmGKB	0.460366052	0.022163409	2.941535808	0.01764223	0.11704988	-3.16028938
WT after UVB irradiation VS WT before UVB irradiation	Pathway_PA165959425|PharmGKB	0.433467682	0.043171041	2.092434286	0.06264807	0.43544914	-4.05588704
homo after UVB irradiation VS homo before UVB irradiation	Pathway_PA165964473|PharmGKB	0.015683832	-0.00199626	0.065962204	0.94879115	0.9693166	-6.36611099
hetero after UVB irradiation VS hetero before UVB irradiation	Pathway_PA165964473|PharmGKB	0.019467979	-0.01932862	0.101196715	0.9217611	0.97054702	-6.34098919
WT after UVB irradiation VS WT before UVB irradiation	Pathway_PA165964473|PharmGKB	0.044367292	-0.04143481	0.210194523	0.83770287	0.94951669	-5.84682163
homo after UVB irradiation VS homo before UVB irradiation	Pathway_PA165980337|PharmGKB	0.573258087	-0.07746011	2.281910034	0.0472573	0.14158585	-4.15891251
hetero after UVB irradiation VS hetero before UVB irradiation	Pathway_PA165980337|PharmGKB	0.210856326	0.091368828	0.58336559	0.57492585	0.7705309	-6.16566152
WT after UVB irradiation VS WT before UVB irradiation	Pathway_PA165980337|PharmGKB	0.199509683	-0.11826803	0.598119045	0.56295457	0.85017866	-5.69130485
homo after UVB irradiation VS homo before UVB irradiation	Pathway_PA165986194 -need delete|PharmGKB	0.254595207	0.043149964	1.540078891	0.1565348	0.30417351	-5.23613761
hetero after UVB irradiation VS hetero before UVB irradiation	Pathway_PA165986194 -need delete|PharmGKB	0.003198058	-0.00161008	0.012308593	0.99046588	0.9943047	-6.34645911
WT after UVB irradiation VS WT before UVB irradiation	Pathway_PA165986194 -need delete|PharmGKB	0.177058396	-0.02271735	0.729080397	0.48254529	0.81552033	-5.60725969
homo after UVB irradiation VS homo before UVB irradiation	Pathways Affected in Adenoid Cystic Carcinoma|Wikipathways	-0.58719117	-0.006534	-2.92367214	0.01620619	0.07948172	-3.14046784
hetero after UVB irradiation VS hetero before UVB irradiation	Pathways Affected in Adenoid Cystic Carcinoma|Wikipathways	-0.47821322	-0.03616737	-2.61183018	0.0297323	0.14964797	-3.66475051
WT after UVB irradiation VS WT before UVB irradiation	Pathways Affected in Adenoid Cystic Carcinoma|Wikipathways	-0.48719586	-0.00238108	-2.12094604	0.0597076	0.42807631	-4.01503756
homo after UVB irradiation VS homo before UVB irradiation	Pathways in cancer - Homo sapiens (human)|KEGG	0.235611572	-0.01585525	1.489492948	0.16915993	0.31964135	-5.30184494
hetero after UVB irradiation VS hetero before UVB irradiation	Pathways in cancer - Homo sapiens (human)|KEGG	0.283340915	0.003403891	1.843635779	0.10059299	0.28531601	-4.80623903
WT after UVB irradiation VS WT before UVB irradiation	Pathways in cancer - Homo sapiens (human)|KEGG	0.174873417	-0.00782605	0.890041759	0.39418537	0.76452505	-5.48399231
homo after UVB irradiation VS homo before UVB irradiation	Pathways in clear cell renal cell carcinoma|Wikipathways	0.285428709	-0.02332889	1.784866546	0.10655639	0.23957183	-4.90141347
hetero after UVB irradiation VS hetero before UVB irradiation	Pathways in clear cell renal cell carcinoma|Wikipathways	0.189810797	-0.00917988	0.917338414	0.38446972	0.61213953	-5.91205145
WT after UVB irradiation VS WT before UVB irradiation	Pathways in clear cell renal cell carcinoma|Wikipathways	0.112089608	-0.00866474	0.521416265	0.61333713	0.87257466	-5.73338726
homo after UVB irradiation VS homo before UVB irradiation	Pausing and recovery of HIV elongation|Reactome	-0.59337696	0.013820482	-2.91653162	0.01639958	0.08000053	-3.15191098
hetero after UVB irradiation VS hetero before UVB irradiation	Pausing and recovery of HIV elongation|Reactome	-0.30796546	-0.0389977	-1.31604362	0.22283332	0.44410029	-5.49459081
WT after UVB irradiation VS WT before UVB irradiation	Pausing and recovery of HIV elongation|Reactome	-0.19369301	0.012329594	-0.59982097	0.56186317	0.85017866	-5.69031027
homo after UVB irradiation VS homo before UVB irradiation	Pausing and recovery of Tat-mediated HIV elongation|Reactome	-0.61583101	0.026175894	-2.83281055	0.01885027	0.08765675	-3.28606218
hetero after UVB irradiation VS hetero before UVB irradiation	Pausing and recovery of Tat-mediated HIV elongation|Reactome	-0.29583947	-0.03522075	-1.27197184	0.23736346	0.45999261	-5.5459004
WT after UVB irradiation VS WT before UVB irradiation	Pausing and recovery of Tat-mediated HIV elongation|Reactome	-0.17905057	0.013402429	-0.54950196	0.59462237	0.86485362	-5.71860464
homo after UVB irradiation VS homo before UVB irradiation	PCNA-Dependent Long Patch Base Excision Repair|Reactome	-0.36282774	-0.02272213	-1.36494503	0.20408132	0.36140306	-5.45779513
hetero after UVB irradiation VS hetero before UVB irradiation	PCNA-Dependent Long Patch Base Excision Repair|Reactome	0.314040878	0.024484244	1.35210937	0.21151018	0.43347545	-5.45176399
WT after UVB irradiation VS WT before UVB irradiation	PCNA-Dependent Long Patch Base Excision Repair|Reactome	0.138201097	0.047246906	0.478719369	0.64235073	0.87745357	-5.75445484
homo after UVB irradiation VS homo before UVB irradiation	PCP/CE pathway|Reactome	0.180436514	-0.02982228	0.913315469	0.3839291	0.5369742	-5.93960038
hetero after UVB irradiation VS hetero before UVB irradiation	PCP/CE pathway|Reactome	0.301929797	0.000475277	1.72209015	0.12144448	0.31331055	-4.97500369
WT after UVB irradiation VS WT before UVB irradiation	PCP/CE pathway|Reactome	0.036028227	0.031011374	0.147839141	0.88538457	0.97031361	-5.85809174
homo after UVB irradiation VS homo before UVB irradiation	PD-1 signaling|Reactome	-0.28520857	0.015702802	-1.00491895	0.34013001	0.49601841	-5.85386912
hetero after UVB irradiation VS hetero before UVB irradiation	PD-1 signaling|Reactome	-0.06550058	0.036289335	-0.25519714	0.80468996	0.91901198	-6.31135085
WT after UVB irradiation VS WT before UVB irradiation	PD-1 signaling|Reactome	0.101122483	-0.08326279	0.323523832	0.75290934	0.92020735	-5.81642169
homo after UVB irradiation VS homo before UVB irradiation	PDE3B signalling|Reactome	1.034153104	0.064792843	4.442289607	0.00145843	0.0217621	-0.79376996
hetero after UVB irradiation VS hetero before UVB irradiation	PDE3B signalling|Reactome	0.544751551	0.058897515	1.619313171	0.14212762	0.34495084	-5.11354453
WT after UVB irradiation VS WT before UVB irradiation	PDE3B signalling|Reactome	0.334156357	-0.04402326	1.079631058	0.30545633	0.69096189	-5.31283877
homo after UVB irradiation VS homo before UVB irradiation	PDGF Pathway|Wikipathways	0.508363915	-0.06139745	1.968192901	0.07924869	0.1988184	-4.63554787
hetero after UVB irradiation VS hetero before UVB irradiation	PDGF Pathway|Wikipathways	0.523794843	-0.04139501	2.157978678	0.06128849	0.22170869	-4.35069149
WT after UVB irradiation VS WT before UVB irradiation	PDGF Pathway|Wikipathways	0.361045947	0.052839093	1.290168134	0.22579669	0.62952955	-5.09356098
homo after UVB irradiation VS homo before UVB irradiation	PDGF receptor signaling network|PID	1.165338504	0.019118357	7.396300884	3.28E-05	0.01666204	2.803072455
hetero after UVB irradiation VS hetero before UVB irradiation	PDGF receptor signaling network|PID	0.873047905	0.137985963	3.223743425	0.01136235	0.10504986	-2.73140026
WT after UVB irradiation VS WT before UVB irradiation	PDGF receptor signaling network|PID	0.873479598	0.081377993	3.120828671	0.01075574	0.26333964	-2.52701031
homo after UVB irradiation VS homo before UVB irradiation	pdgf signaling pathway|BioCarta	0.354736753	-0.03403191	1.679703655	0.12591802	0.26543937	-5.04838821
hetero after UVB irradiation VS hetero before UVB irradiation	pdgf signaling pathway|BioCarta	0.624582879	0.025073196	3.537429405	0.00703788	0.08628369	-2.26240395
WT after UVB irradiation VS WT before UVB irradiation	pdgf signaling pathway|BioCarta	0.371762687	0.026737282	1.62795257	0.13434341	0.54709793	-4.68875926
homo after UVB irradiation VS homo before UVB irradiation	PDGF|INOH	0.514609141	-0.03726415	2.680821148	0.02428933	0.10270243	-3.52920081
hetero after UVB irradiation VS hetero before UVB irradiation	PDGF|INOH	0.59375062	0.01456975	3.910777509	0.00404534	0.07021718	-1.7193358
WT after UVB irradiation VS WT before UVB irradiation	PDGF|INOH	0.458825268	0.007177929	2.305163543	0.04366747	0.39022568	-3.74729807
homo after UVB irradiation VS homo before UVB irradiation	PDGFR-alpha signaling pathway|PID	0.358319177	-0.03623342	1.809867414	0.10237544	0.23314027	-4.86583924
hetero after UVB irradiation VS hetero before UVB irradiation	PDGFR-alpha signaling pathway|PID	0.500312482	0.090347498	2.573437346	0.03160674	0.15507377	-3.723416
WT after UVB irradiation VS WT before UVB irradiation	PDGFR-alpha signaling pathway|PID	0.361309531	0.055340733	1.6472329	0.13028134	0.54238366	-4.66400574
homo after UVB irradiation VS homo before UVB irradiation	PDGFR-beta pathway|Wikipathways	0.522020613	-0.03720871	2.705580784	0.02330569	0.10020128	-3.48965388
hetero after UVB irradiation VS hetero before UVB irradiation	PDGFR-beta pathway|Wikipathways	0.617536884	0.023970481	3.71014775	0.00543467	0.07914133	-2.0088886
WT after UVB irradiation VS WT before UVB irradiation	PDGFR-beta pathway|Wikipathways	0.4264041	0.021305648	1.877266545	0.08968601	0.48188205	-4.35762069
homo after UVB irradiation VS homo before UVB irradiation	PDGFR-beta signaling pathway|PID	0.470419386	-0.04593491	2.340022178	0.04290298	0.13426663	-4.0684074
hetero after UVB irradiation VS hetero before UVB irradiation	PDGFR-beta signaling pathway|PID	0.587040812	0.004933542	3.786177413	0.00485625	0.07419102	-1.89851041
WT after UVB irradiation VS WT before UVB irradiation	PDGFR-beta signaling pathway|PID	0.47331223	-0.0062515	2.425840894	0.03551849	0.38724228	-3.5690626
homo after UVB irradiation VS homo before UVB irradiation	PECAM1 interactions|Reactome	0.450848188	-0.06545658	2.119154496	0.06187201	0.17056013	-4.40904618
hetero after UVB irradiation VS hetero before UVB irradiation	PECAM1 interactions|Reactome	0.707844915	0.012945499	4.251368671	0.00248285	0.05610569	-1.24120424
WT after UVB irradiation VS WT before UVB irradiation	PECAM1 interactions|Reactome	0.385436966	-0.02171733	1.897062404	0.08680315	0.47705171	-4.33040234
homo after UVB irradiation VS homo before UVB irradiation	pelp1 modulation of estrogen receptor activity|BioCarta	-0.47246063	0.077241895	-2.40789681	0.03831452	0.12873001	-3.96204519
hetero after UVB irradiation VS hetero before UVB irradiation	pelp1 modulation of estrogen receptor activity|BioCarta	-0.28874059	0.034305232	-1.03416937	0.32982353	0.55823827	-5.80133391
WT after UVB irradiation VS WT before UVB irradiation	pelp1 modulation of estrogen receptor activity|BioCarta	-0.16654528	0.018528335	-0.68890257	0.50642775	0.82465997	-5.63464272

homo after UVB irradiation VS homo before UVB irradiation	Penbutolol Action Pathway|SMPDB	0.396415397	-0.03677376	2.343069613	0.04268583	0.13402181	-4.06364635
hetero after UVB irradiation VS hetero before UVB irradiation	Penbutolol Action Pathway|SMPDB	-0.01555055	-0.01775524	-0.07410913	0.94265284	0.97054702	-6.34356282
WT after UVB irradiation VS WT before UVB irradiation	Penbutolol Action Pathway|SMPDB	0.056685205	-0.03978418	0.223404833	0.82768199	0.94525798	-5.84393438
homo after UVB irradiation VS homo before UVB irradiation	Pentazocine Action Pathway|SMPDB	0.191132361	0.029768717	1.061313976	0.31508276	0.46923284	-5.79783739
hetero after UVB irradiation VS hetero before UVB irradiation	Pentazocine Action Pathway|SMPDB	-0.07904315	0.055005373	-0.44437398	0.66796482	0.82651398	-6.24068873
WT after UVB irradiation VS WT before UVB irradiation	Pentazocine Action Pathway|SMPDB	0.022316377	0.035038618	0.098344171	0.92358676	0.97566251	-5.86425414
homo after UVB irradiation VS homo before UVB irradiation	Pentose and glucuronate interconversions - Homo sapiens (human)|KEGG	-0.00176483	0.098105547	-0.00692575	0.99461903	0.99546557	-6.36842249
hetero after UVB irradiation VS hetero before UVB irradiation	Pentose and glucuronate interconversions - Homo sapiens (human)|KEGG	0.338688334	-0.01697982	2.098626144	0.06734317	0.23395593	-4.43840659
WT after UVB irradiation VS WT before UVB irradiation	Pentose and glucuronate interconversions - Homo sapiens (human)|KEGG	0.175295107	-0.01225129	0.707946803	0.49501873	0.81710765	-5.6218368
homo after UVB irradiation VS homo before UVB irradiation	Pentose phosphate cycle|INOH	0.119645274	0.000911596	0.664367662	0.52243474	0.65944729	-6.13685072
hetero after UVB irradiation VS hetero before UVB irradiation	Pentose phosphate cycle|INOH	0.101047089	-0.04078066	0.464661035	0.65394574	0.82651398	-6.23093095
WT after UVB irradiation VS WT before UVB irradiation	Pentose phosphate cycle|INOH	0.204081781	0.046364501	0.938060723	0.37014088	0.74850253	-5.4431913
homo after UVB irradiation VS homo before UVB irradiation	Pentose Phosphate Metabolism|Wikipathways	-0.72547504	-0.00366272	-3.23874686	0.00963429	0.05841986	-2.63664914
hetero after UVB irradiation VS hetero before UVB irradiation	Pentose Phosphate Metabolism|Wikipathways	-0.06534804	-0.0234737	-0.22992334	0.82363523	0.93270295	-6.31795467
WT after UVB irradiation VS WT before UVB irradiation	Pentose Phosphate Metabolism|Wikipathways	-0.07199461	0.068576036	-0.18257175	0.85875296	0.95916804	-5.85229514
homo after UVB irradiation VS homo before UVB irradiation	Pentose phosphate pathway - Homo sapiens (human)|KEGG	0.123621202	-0.00950097	0.629767441	0.54386576	0.67719963	-6.15985258
hetero after UVB irradiation VS hetero before UVB irradiation	Pentose phosphate pathway - Homo sapiens (human)|KEGG	0.09242027	0.009897836	0.545386638	0.59962395	0.7885114	-6.18804421
WT after UVB irradiation VS WT before UVB irradiation	Pentose phosphate pathway - Homo sapiens (human)|KEGG	0.161626785	0.040377801	0.729026865	0.48257663	0.81552033	-5.60729709
homo after UVB irradiation VS homo before UVB irradiation	Pentose Phosphate Pathway (Erythrocyte)|PharmGKB	0.381672698	0.000471925	1.329945443	0.21493712	0.37301091	-5.50002583
hetero after UVB irradiation VS hetero before UVB irradiation	Pentose Phosphate Pathway (Erythrocyte)|PharmGKB	0.876690697	0.022468924	3.490652784	0.00755327	0.08807784	-2.33168325
WT after UVB irradiation VS WT before UVB irradiation	Pentose Phosphate Pathway (Erythrocyte)|PharmGKB	0.736228249	0.04592652	2.755445886	0.0201442	0.31542987	-3.07578303
homo after UVB irradiation VS homo before UVB irradiation	Pentose phosphate pathway (hexose monophosphate shunt)|Reactome	-0.5736787	-0.01640065	-3.08383521	0.01243014	0.06880589	-2.88396203
hetero after UVB irradiation VS hetero before UVB irradiation	Pentose phosphate pathway (hexose monophosphate shunt)|Reactome	-0.13144135	-0.01602223	-0.57372453	0.58114132	0.77428885	-6.17147519
WT after UVB irradiation VS WT before UVB irradiation	Pentose phosphate pathway (hexose monophosphate shunt)|Reactome	-0.14575985	0.018110775	-0.43507291	0.67267517	0.8808312	-5.77421698
homo after UVB irradiation VS homo before UVB irradiation	pentose phosphate pathway (non-oxidative branch)|HumanCyc	-0.77442233	-0.06958917	-3.97652236	0.00296232	0.03081082	-1.48569023
hetero after UVB irradiation VS hetero before UVB irradiation	pentose phosphate pathway (non-oxidative branch)|HumanCyc	-0.51052318	-0.09793793	-2.65377148	0.02781315	0.14438349	-3.60060646
WT after UVB irradiation VS WT before UVB irradiation	pentose phosphate pathway (non-oxidative branch)|HumanCyc	-0.398725	-0.03389945	-1.17597736	0.26662479	0.66142066	-5.21605239
homo after UVB irradiation VS homo before UVB irradiation	pentose phosphate pathway (oxidative branch)|HumanCyc	0.234974843	0.026930731	0.747679002	0.47296331	0.61754234	-6.07693054
hetero after UVB irradiation VS hetero before UVB irradiation	pentose phosphate pathway (oxidative branch)|HumanCyc	0.535070343	0.081669143	1.983118418	0.08083406	0.25607584	-4.60703433
WT after UVB irradiation VS WT before UVB irradiation	pentose phosphate pathway (oxidative branch)|HumanCyc	0.477452547	0.008547051	1.391638794	0.19397016	0.60625027	-4.97819932
homo after UVB irradiation VS homo before UVB irradiation	Pentose phosphate pathway|EHMN	-0.42122427	0.005754367	-2.22336322	0.05208009	0.15204455	-4.24950153
hetero after UVB irradiation VS hetero before UVB irradiation	Pentose phosphate pathway|EHMN	-0.02804934	-0.04098421	-0.11341416	0.91235809	0.97054702	-6.33956878
WT after UVB irradiation VS WT before UVB irradiation	Pentose phosphate pathway|EHMN	0.000523354	-0.01712647	0.001604257	0.99875129	0.99940779	-5.86914989
homo after UVB irradiation VS homo before UVB irradiation	pentose phosphate pathway|HumanCyc	-0.44791795	-0.01779991	-2.20863762	0.05336659	0.15471227	-4.2721843
hetero after UVB irradiation VS hetero before UVB irradiation	pentose phosphate pathway|HumanCyc	-0.17123539	-0.05940803	-0.76361499	0.46593832	0.68896154	-6.04098757
WT after UVB irradiation VS WT before UVB irradiation	pentose phosphate pathway|HumanCyc	-0.03146232	0.006531648	-0.09460963	0.92647881	0.97639811	-5.86461883
homo after UVB irradiation VS homo before UVB irradiation	Pentose Phosphate Pathway|SMPDB	-0.14766847	-0.01149858	-0.59272914	0.56736226	0.69195165	-6.18322274
hetero after UVB irradiation VS hetero before UVB irradiation	Pentose Phosphate Pathway|SMPDB	-0.05432787	-0.01134205	-0.2708573	0.79301813	0.91162084	-6.30691924
WT after UVB irradiation VS WT before UVB irradiation	Pentose Phosphate Pathway|SMPDB	0.010991275	0.045530165	0.039987875	0.96888357	0.98924463	-5.8683412
homo after UVB irradiation VS homo before UVB irradiation	Peptide chain elongation|Reactome	-0.94673073	0.023343124	-4.99739795	0.00065262	0.01666204	-0.01248933
hetero after UVB irradiation VS hetero before UVB irradiation	Peptide chain elongation|Reactome	-0.08608619	0.015643926	-0.25309705	0.80625921	0.92057351	-6.31192541
WT after UVB irradiation VS WT before UVB irradiation	Peptide chain elongation|Reactome	-0.61234719	0.012460069	-2.06258417	0.06587405	0.43803373	-4.09845823
homo after UVB irradiation VS homo before UVB irradiation	Peptide GPCRs|Wikipathways	0.246676612	0.008640599	1.386798209	0.19754145	0.35391572	-5.43106137
hetero after UVB irradiation VS hetero before UVB irradiation	Peptide GPCRs|Wikipathways	0.187909774	0.05911997	1.110531021	0.2974533	0.52919287	-5.72348695
WT after UVB irradiation VS WT before UVB irradiation	Peptide GPCRs|Wikipathways	0.200750499	0.04284507	0.879683158	0.39951265	0.76730004	-5.49255786
homo after UVB irradiation VS homo before UVB irradiation	Peptide hormone biosynthesis|Reactome	0.264584991	0.01592808	1.037559522	0.3254564	0.4813845	-5.82173283
hetero after UVB irradiation VS hetero before UVB irradiation	Peptide hormone biosynthesis|Reactome	0.278387069	0.075253357	1.211984328	0.25840621	0.48448405	-5.61384808
WT after UVB irradiation VS WT before UVB irradiation	Peptide hormone biosynthesis|Reactome	0.532933698	0.132745627	2.698796879	0.02220903	0.31597707	-3.16094573
homo after UVB irradiation VS homo before UVB irradiation	Peptide hormone metabolism|Reactome	0.143617149	0.007290291	0.748690784	0.47238142	0.61720095	-6.07616414
hetero after UVB irradiation VS hetero before UVB irradiation	Peptide hormone metabolism|Reactome	0.153841962	0.047854763	0.861900476	0.41259781	0.64032033	-5.96082576
WT after UVB irradiation VS WT before UVB irradiation	Peptide hormone metabolism|Reactome	0.146520298	-0.01265032	0.660912268	0.52348281	0.83127468	-5.65289061
homo after UVB irradiation VS homo before UVB irradiation	Peptide ligand-binding receptors|Reactome	0.201864593	0.038893255	1.135726752	0.28422875	0.44505778	-5.72029448
hetero after UVB irradiation VS hetero before UVB irradiation	Peptide ligand-binding receptors|Reactome	0.124007764	0.046446391	0.783997843	0.45451401	0.67919377	-6.02504433
WT after UVB irradiation VS WT before UVB irradiation	Peptide ligand-binding receptors|Reactome	0.079911051	0.046107623	0.364542187	0.72298305	0.90882588	-5.80230335
homo after UVB irradiation VS homo before UVB irradiation	Perindopril Action Pathway|SMPDB	0.636038586	-0.11800708	2.436891056	0.03650532	0.12504267	-3.91641947
hetero after UVB irradiation VS hetero before UVB irradiation	Perindopril Action Pathway|SMPDB	-0.02777369	0.003323189	-0.09172332	0.92906122	0.97054702	-6.34197953
WT after UVB irradiation VS WT before UVB irradiation	Perindopril Action Pathway|SMPDB	0.270438532	-0.10210744	0.707878218	0.49505953	0.81710765	-5.62188349
homo after UVB irradiation VS homo before UVB irradiation	PERK regulates gene expression|Reactome	-0.42405008	0.006471056	-1.64422495	0.13313959	0.27594887	-5.09693645
hetero after UVB irradiation VS hetero before UVB irradiation	PERK regulates gene expression|Reactome	-0.25302291	-0.04882797	-0.77874047	0.45744268	0.68204898	-6.02919124
WT after UVB irradiation VS WT before UVB irradiation	PERK regulates gene expression|Reactome	0.031573939	-0.07333188	0.097030184	0.92460419	0.97566251	-5.86438406
homo after UVB irradiation VS homo before UVB irradiation	Peroxisomal lipid metabolism|Reactome	0.054980312	-0.04451037	0.235847944	0.81861504	0.88825957	-6.33865171
hetero after UVB irradiation VS hetero before UVB irradiation	Peroxisomal lipid metabolism|Reactome	-0.22782826	-0.03459168	-0.87346141	0.40661499	0.63319136	-5.95086151
WT after UVB irradiation VS WT before UVB irradiation	Peroxisomal lipid metabolism|Reactome	-0.1987818	-0.04984102	-0.75763869	0.46600625	0.80910175	-5.58695562
homo after UVB irradiation VS homo before UVB irradiation	Peroxisomal protein import|Reactome	0.119071372	-0.02399992	0.656614022	0.52719298	0.66331307	-6.14210266
hetero after UVB irradiation VS hetero before UVB irradiation	Peroxisomal protein import|Reactome	-0.18318075	-0.0232196	-0.99015386	0.3496772	0.57899653	-5.84427219
WT after UVB irradiation VS WT before UVB irradiation	Peroxisomal protein import|Reactome	-0.0456823	-0.02955077	-0.19161899	0.85184479	0.95516208	-5.85058652
homo after UVB irradiation VS homo before UVB irradiation	Peroxisome - Homo sapiens (human)|KEGG	0.164541822	-0.05252408	0.800585164	0.44315703	0.59096232	-6.03563757
hetero after UVB irradiation VS hetero before UVB irradiation	Peroxisome - Homo sapiens (human)|KEGG	-0.19403765	-0.0525439	-0.98352445	0.35274406	0.58106521	-5.85061293
WT after UVB irradiation VS WT before UVB irradiation	Peroxisome - Homo sapiens (human)|KEGG	0.0416542	-0.03620736	0.16592458	0.87149643	0.96283151	-5.85522461
homo after UVB irradiation VS homo before UVB irradiation	Pertussis - Homo sapiens (human)|KEGG	0.774665867	-0.0156388	4.734414092	0.00094994	0.01805472	-0.37650139
hetero after UVB irradiation VS hetero before UVB irradiation	Pertussis - Homo sapiens (human)|KEGG	0.706780636	0.047130622	4.182466251	0.00273686	0.05916047	-1.33650273
WT after UVB irradiation VS WT before UVB irradiation	Pertussis - Homo sapiens (human)|KEGG	0.615242046	0.026860669	2.567255234	0.02785538	0.3514241	-3.358243
homo after UVB irradiation VS homo before UVB irradiation	pertussis toxin-insensitive ccr5 signaling in macrophage|BioCarta	0.562757898	0.038493364	1.760264832	0.11082383	0.24445489	-4.93618998
hetero after UVB irradiation VS hetero before UVB irradiation	pertussis toxin-insensitive ccr5 signaling in macrophage|BioCarta	0.944409737	0.075951879	3.784699271	0.00486685	0.07419102	-1.90064892
WT after UVB irradiation VS WT before UVB irradiation	pertussis toxin-insensitive ccr5 signaling in macrophage|BioCarta	0.32482666	0.058012756	1.165472223	0.27065992	0.66327566	-5.22690656
homo after UVB irradiation VS homo before UVB irradiation	Phagosome - Homo sapiens (human)|KEGG	0.464960446	-0.03044839	2.46393367	0.03489423	0.12437708	-3.87377196
hetero after UVB irradiation VS hetero before UVB irradiation	Phagosome - Homo sapiens (human)|KEGG	0.517719899	0.011124174	3.251049795	0.01089339	0.10415756	-2.6901974
WT after UVB irradiation VS WT before UVB irradiation	Phagosome - Homo sapiens (human)|KEGG	0.418116522	-0.01683193	1.843145615	0.09486425	0.49111758	-4.40424518
homo after UVB irradiation VS homo before UVB irradiation	Phase 0 - rapid depolarisation|Reactome	0.126088625	0.01363096	0.554792436	0.59200551	0.71434914	-6.20579745
hetero after UVB irradiation VS hetero before UVB irradiation	Phase 0 - rapid depolarisation|Reactome	-0.38814377	-0.05302408	-1.85849369	0.09828811	0.28258275	-4.78528169
WT after UVB irradiation VS WT before UVB irradiation	Phase 0 - rapid depolarisation|Reactome	-0.12999258	-0.06214282	-0.49207303	0.63320545	0.87367048	-5.74804923
homo after UVB irradiation VS homo before UVB irradiation	Phase 1 - inactivation of fast Na+ channels|Reactome	0.297460113	0.030916999	0.971049283	0.35587292	0.51072928	-5.88634417
hetero after UVB irradiation VS hetero before UVB irradiation	Phase 1 - inactivation of fast Na+ channels|Reactome	-0.28318614	0.009054803	-1.20688283	0.26026461	0.48628034	-5.61952255
WT after UVB irradiation VS WT before UVB irradiation	Phase 1 - inactivation of fast Na+ channels|Reactome	-0.04267425	0.008815676	-0.19204984	0.85151613	0.95502804	-5.8505031
homo after UVB irradiation VS homo before UVB irradiation	Phase 2 - plateau phase|Reactome	0.2151451	-0.01295343	1.017065128	0.33461276	0.4915994	-5.84200592
hetero after UVB irradiation VS hetero before UVB irradiation	Phase 2 - plateau phase|Reactome	-0.22986643	0.020635729	-1.23316099	0.25080818	0.47494128	-5.59011672
WT after UVB irradiation VS WT before UVB irradiation	Phase 2 - plateau phase|Reactome	-0.08188155	0.019110332	-0.34995623	0.73357189	0.9131126	-5.80751173
homo after UVB irradiation VS homo before UVB irradiation	Phase 3 - rapid repolarisation|Reactome	0.174501504	0.013752995	0.630325213	0.54351627	0.67708436	-6.1594907
hetero after UVB irradiation VS hetero before UVB irradiation	Phase 3 - rapid repolarisation|Reactome	-0.34334881	0.039598611	-1.7091792	0.12388123	0.31597198	-4.99262957
WT after UVB irradiation VS WT before UVB irradiation	Phase 3 - rapid repolarisation|Reactome	-0.19833797	0.094274944	-0.82212653	0.43001846	0.79574324	-5.53859231
homo after UVB irradiation VS homo before UVB irradiation	Phase 4 - resting membrane potential|Reactome	0.348526423	-0.03562494	1.974049014	0.07849569	0.19758066	-4.6268775
hetero after UVB irradiation VS hetero before UVB irradiation	Phase 4 - resting membrane potential|Reactome	0.400483508	0.004887982	2.11370656	0.0657517	0.23035839	-4.41618272
WT after UVB irradiation VS WT before UVB irradiation	Phase 4 - resting membrane potential|Reactome	0.327168427	0.041628917	1.658721486	0.12791342	0.53754069	-4.64918173
homo after UVB irradiation VS homo before UVB irradiation	Phase I - Functionalization of compounds|Reactome	0.134029064	0.005455622	0.695085148	0.50383924	0.64350489	-6.11549644
hetero after UVB irradiation VS hetero before UVB irradiation	Phase I - Functionalization of compounds|Reactome	-0.08097766	0.00363288	-0.41617076	0.68768386	0.84146659	-6.2535633
WT after UVB irradiation VS WT before UVB irradiation	Phase I - Functionalization of compounds|Reactome	-0.03049249	-0.00286088	-0.12809448	0.90059326	0.97566251	-5.86084615
homo after UVB irradiation VS homo before UVB irradiation	Phase I biotransformations_ non P450|Wikipathways	0.770120727	-0.01495285	4.03668878	0.00269865	0.02976339	-1.39457903
hetero after UVB irradiation VS hetero before UVB irradiation	Phase I biotransformations_ non P450|Wikipathways	-0.02052314	0.030408956	-0.09432702	0.92705409	0.97054702	-6.34171702
WT after UVB irradiation VS WT before UVB irradiation	Phase I biotransformations_ non P450|Wikipathways	-0.02131344	0.018960627	-0.08126009	0.93682562	0.98056728	-5.86580722
homo after UVB irradiation VS homo before UVB irradiation	Phase II - Conjugation of compounds|Reactome	0.241850785	-0.00122398	1.301267244	0.22419006	0.38376668	-5.53407813
hetero after UVB irradiation VS hetero before UVB irradiation	Phase II - Conjugation of compounds|Reactome	0.146716446	-0.0108999	0.831363227	0.42869699	0.65540741	-5.98661114
WT after UVB irradiation VS WT before UVB irradiation	Phase II - Conjugation of compounds|Reactome	0.191713875	0.000358848	0.899309339	0.38946141	0.76242672	-5.47625741
homo after UVB irradiation VS homo before UVB irradiation	Phenindione Action Pathway|SMPDB	0.538210712	0.026444726	3.007777411	0.01409598	0.07305822	-3.00571236
hetero after UVB irradiation VS hetero before UVB irradiation	Phenindione Action Pathway|SMPDB	0.558085555	0.09913938	2.984394118	0.01649409	0.11352943	-3.09486545
WT after UVB irradiation VS WT before UVB irradiation	Phenindione Action Pathway|SMPDB	0.440879848	0.077377741	1.569633667	0.14732454	0.55622203	-4.76265269
homo after UVB irradiation VS homo before UVB irradiation	Phenprocoumon Action Pathway|SMPDB	0.538210712	0.026444726	3.007777411	0.01409598	0.07305822	-3.00571236
hetero after UVB irradiation VS hetero before UVB irradiation	Phenprocoumon Action Pathway|SMPDB	0.558085555	0.09913938	2.984394118	0.01649409	0.11352943	-3.09486545
WT after UVB irradiation VS WT before UVB irradiation	Phenprocoumon Action Pathway|SMPDB	0.440879848	0.077377741	1.569633667	0.14732454	0.55622203	-4.76265269
homo after UVB irradiation VS homo before UVB irradiation	Phenylacetate Metabolism|SMPDB	0.4251612	-0.10872382	0.978431641	0.35239651	0.50783175	-5.87934239
hetero after UVB irradiation VS hetero before UVB irradiation	Phenylacetate Metabolism|SMPDB	0.246426612	0.031985469	1.476377946	0.17620852	0.39293465	-5.29879716
WT after UVB irradiation VS WT before UVB irradiation	Phenylacetate Metabolism|SMPDB	0.245382705	0.116787662	0.671552832	0.51695941	0.82895075	-5.64603451
homo after UVB irradiation VS homo before UVB irradiation	Phenylalanine and tyrosine catabolism|Reactome	0.300728191	0.025846004	1.185380161	0.2649998	0.42861817	-5.6663637
hetero after UVB irradiation VS hetero before UVB irradiation	Phenylalanine and tyrosine catabolism|Reactome	-0.26399491	-0.03443263	-1.00637764	0.34225659	0.57164	-5.8286143
WT after UVB irradiation VS WT before UVB irradiation	Phenylalanine and tyrosine catabolism|Reactome	0.444110942	0.017766062	1.915500712	0.08419572	0.47180597	-4.30494307
homo after UVB irradiation VS homo before UVB irradiation	Phenylalanine and Tyrosine Metabolism|SMPDB	-0.04803592	0.079291694	-0.16825705	0.86994987	0.91927589	-6.35326043
hetero after UVB irradiation VS hetero before UVB irradiation	Phenylalanine and Tyrosine Metabolism|SMPDB	-0.45282658	-0.01182911	-2.07966958	0.0693969	0.23555906	-4.46627862
WT after UVB irradiation VS WT before UVB irradiation	Phenylalanine and Tyrosine Metabolism|SMPDB	0.43481789	0.042180167	2.082624193	0.06369133	0.43544914	-4.06990044
homo after UVB irradiation VS homo before UVB irradiation	phenylalanine degradation/tyrosine biosynthesis|HumanCyc	1.043568984	-0.06633146	5.14213264	0.00053307	0.01666204	0.183062624
hetero after UVB irradiation VS hetero before UVB irradiation	phenylalanine degradation/tyrosine biosynthesis|HumanCyc	-0.16394819	0.018434941	-0.49304236	0.63457483	0.8133836	-6.21658814
WT after UVB irradiation VS WT before UVB irradiation	phenylalanine degradation/tyrosine biosynthesis|HumanCyc	0.401444214	-0.02143887	1.011425443	0.33546987	0.7192915	-5.37746842
homo after UVB irradiation VS homo before UVB irradiation	Phenylalanine degradation|INOH	-0.11360018	-0.02957893	-0.47095708	0.64842147	0.75643649	-6.25069911
hetero after UVB irradiation VS hetero before UVB irradiation	Phenylalanine degradation|INOH	-0.34611937	0.047196042	-1.34219809	0.21457187	0.43637221	-5.46360678
WT after UVB irradiation VS WT before UVB irradiation	Phenylalanine degradation|INOH	-0.10853421	-0.01861049	-0.44928635	0.66272883	0.87935805	-5.76797981
homo after UVB irradiation VS homo before UVB irradiation	Phenylalanine metabolism - Homo sapiens (human)|KEGG	0.037240557	-0.01157797	0.18817544	0.85474348	0.91207261	-6.34945906
hetero after UVB irradiation VS hetero before UVB irradiation	Phenylalanine metabolism - Homo sapiens (human)|KEGG	-0.12490333	0.002893258	-0.44923061	0.6645959	0.82651398	-6.23839048
WT after UVB irradiation VS WT before UVB irradiation	Phenylalanine metabolism - Homo sapiens (human)|KEGG	0.257163325	0.031384277	1.064108487	0.31210077	0.69567326	-5.32783816
homo after UVB irradiation VS homo before UVB irradiation	Phenylalanine_ tyrosine and tryptophan biosynthesis - Homo sapiens (human)|KEGG	-0.2548948	0.115448844	-0.72324635	0.48715406	0.62941297	-6.09515824
hetero after UVB irradiation VS hetero before UVB irradiation	Phenylalanine_ tyrosine and tryptophan biosynthesis - Homo sapiens (human)|KEGG	-0.3348161	0.037257077	-0.91159877	0.38731607	0.61473991	-5.91721668
WT after UVB irradiation VS WT before UVB irradiation	Phenylalanine_ tyrosine and tryptophan biosynthesis - Homo sapiens (human)|KEGG	-0.27443935	0.078379774	-0.82918195	0.4261967	0.79187971	-5.53309302
homo after UVB irradiation VS homo before UVB irradiation	Phenylbutazone Action Pathway|SMPDB	0.40423582	0.045705158	1.979500697	0.07780079	0.19626432	-4.61879689
hetero after UVB irradiation VS hetero before UVB irradiation	Phenylbutazone Action Pathway|SMPDB	0.482496143	0.064876536	3.04499191	0.01500113	0.1130168	-3.00250826
WT after UVB irradiation VS WT before UVB irradiation	Phenylbutazone Action Pathway|SMPDB	0.275725339	0.086595857	1.261970114	0.23537138	0.62952955	-5.12455679
homo after UVB irradiation VS homo before UVB irradiation	phenylethylamine degradation I|HumanCyc	-0.52855526	-0.01406606	-2.31604742	0.04464956	0.13728612	-4.10581289
hetero after UVB irradiation VS hetero before UVB irradiation	phenylethylamine degradation I|HumanCyc	-0.84930714	0.089760551	-2.87622438	0.01955296	0.1227581	-3.26011769
WT after UVB irradiation VS WT before UVB irradiation	phenylethylamine degradation I|HumanCyc	-0.40796694	-0.07895894	-1.91573317	0.08416332	0.47180597	-4.30462145
homo after UVB irradiation VS homo before UVB irradiation	Phenylketonuria|SMPDB	-0.04803592	0.079291694	-0.16825705	0.86994987	0.91927589	-6.35326043
hetero after UVB irradiation VS hetero before UVB irradiation	Phenylketonuria|SMPDB	-0.45282658	-0.01182911	-2.07966958	0.0693969	0.23555906	-4.46627862
WT after UVB irradiation VS WT before UVB irradiation	Phenylketonuria|SMPDB	0.43481789	0.042180167	2.082624193	0.06369133	0.43544914	-4.06990044
homo after UVB irradiation VS homo before UVB irradiation	Phenytoin (Antiarrhythmic) Action Pathway|SMPDB	0.308109388	-0.01579811	1.817663553	0.10110305	0.23139675	-4.85469895
hetero after UVB irradiation VS hetero before UVB irradiation	Phenytoin (Antiarrhythmic) Action Pathway|SMPDB	0.054516648	-0.02856672	0.288178422	0.78017226	0.90277358	-6.30171594
WT after UVB irradiation VS WT before UVB irradiation	Phenytoin (Antiarrhythmic) Action Pathway|SMPDB	0.101972311	-0.0083023	0.437096267	0.67125516	0.87984445	-5.77334083
homo after UVB irradiation VS homo before UVB irradiation	Phenytoin Pathway_ Pharmacokinetics|PharmGKB	0.142952043	0.105215112	0.756021996	0.46817889	0.61340167	-6.07058362
hetero after UVB irradiation VS hetero before UVB irradiation	Phenytoin Pathway_ Pharmacokinetics|PharmGKB	0.056087242	0.011236341	0.288665673	0.7798119	0.90262028	-6.301565
WT after UVB irradiation VS WT before UVB irradiation	Phenytoin Pathway_ Pharmacokinetics|PharmGKB	-0.03525003	0.065244843	-0.16404516	0.87293763	0.96283151	-5.85553787
homo after UVB irradiation VS homo before UVB irradiation	Phosphate bond hydrolysis by NTPDase proteins|Reactome	-0.11370035	-0.07210327	-0.53058492	0.6080256	0.72657548	-6.21947288
hetero after UVB irradiation VS hetero before UVB irradiation	Phosphate bond hydrolysis by NTPDase proteins|Reactome	-0.31509049	-0.02911845	-1.0533226	0.32145874	0.55290904	-5.78220159
WT after UVB irradiation VS WT before UVB irradiation	Phosphate bond hydrolysis by NTPDase proteins|Reactome	-0.21974908	-0.04529515	-0.82008775	0.43112709	0.79682615	-5.54017386
homo after UVB irradiation VS homo before UVB irradiation	Phosphate bond hydrolysis by NUDT proteins|Reactome	-0.19423518	-0.04022895	-0.76282297	0.46430197	0.6088463	-6.06536388

hetero after UVB irradiation VS hetero before UVB irradiation	Phosphate bond hydrolysis by NUDT proteins|Reactome	0.158872818	-0.0206239	0.585070604	0.57383054	0.7705309	-6.1646241
WT after UVB irradiation VS WT before UVB irradiation	Phosphate bond hydrolysis by NUDT proteins|Reactome	0.050804925	-0.0065159	0.233128288	0.82032665	0.94525798	-5.84169804
homo after UVB irradiation VS homo before UVB irradiation	phosphatidylcholine biosynthesis pathway|BioCarta	0.369019229	-0.05481113	1.438602087	0.1827498	0.33687347	-5.36660117
hetero after UVB irradiation VS hetero before UVB irradiation	phosphatidylcholine biosynthesis pathway|BioCarta	0.361977388	0.058094626	0.93451161	0.37604413	0.60404776	-5.89644011
WT after UVB irradiation VS WT before UVB irradiation	phosphatidylcholine biosynthesis pathway|BioCarta	0.161591656	-0.0593974	0.453113803	0.66006203	0.87935805	-5.76626742
homo after UVB irradiation VS homo before UVB irradiation	phosphatidylcholine biosynthesis|HumanCyc	0.738006489	-0.01948916	4.230167419	0.00200638	0.02496215	-1.10499048
hetero after UVB irradiation VS hetero before UVB irradiation	phosphatidylcholine biosynthesis|HumanCyc	0.338160874	-0.02752049	1.348677276	0.21256613	0.43486374	-5.45587117
WT after UVB irradiation VS WT before UVB irradiation	phosphatidylcholine biosynthesis|HumanCyc	0.023585	-0.06178517	0.073917241	0.94252222	0.98295421	-5.86638408
homo after UVB irradiation VS homo before UVB irradiation	phosphatidylethanolamine biosynthesis II|HumanCyc	0.384065184	-0.00902747	1.87629889	0.09199382	0.21730148	-4.77022303
hetero after UVB irradiation VS hetero before UVB irradiation	phosphatidylethanolamine biosynthesis II|HumanCyc	0.211535144	-0.04081264	0.937367025	0.37465637	0.60299568	-5.89382172
WT after UVB irradiation VS WT before UVB irradiation	phosphatidylethanolamine biosynthesis II|HumanCyc	0.138452684	-0.00752727	0.503055378	0.62573227	0.87257466	-5.74265578
homo after UVB irradiation VS homo before UVB irradiation	Phosphatidylinositol phosphate metabolism|EHMN	0.049257527	-0.01535593	0.296150649	0.77356114	0.85410231	-6.32154872
hetero after UVB irradiation VS hetero before UVB irradiation	Phosphatidylinositol phosphate metabolism|EHMN	0.146071004	0.00042276	0.920826146	0.38274755	0.61076867	-5.9088999
WT after UVB irradiation VS WT before UVB irradiation	Phosphatidylinositol phosphate metabolism|EHMN	0.186091415	-0.01226239	0.935584591	0.37135456	0.74850253	-5.44533877
homo after UVB irradiation VS homo before UVB irradiation	Phosphatidylinositol Phosphate Metabolism|SMPDB	0.204887351	-0.03452263	1.001726165	0.34159152	0.49735552	-5.85696862
hetero after UVB irradiation VS hetero before UVB irradiation	Phosphatidylinositol Phosphate Metabolism|SMPDB	0.194527838	-0.01339579	0.989499008	0.34997925	0.57902193	-5.84490002
WT after UVB irradiation VS WT before UVB irradiation	Phosphatidylinositol Phosphate Metabolism|SMPDB	0.153809442	-0.05379699	0.682327223	0.51040375	0.82655837	-5.63899101
homo after UVB irradiation VS homo before UVB irradiation	Phosphatidylinositol signaling system - Homo sapiens (human)|KEGG	0.343323047	-0.04123779	2.02498357	0.07222651	0.19019614	-4.5510515
hetero after UVB irradiation VS hetero before UVB irradiation	Phosphatidylinositol signaling system - Homo sapiens (human)|KEGG	0.170765612	0.008829854	0.974097515	0.35713978	0.58534452	-5.85957157
WT after UVB irradiation VS WT before UVB irradiation	Phosphatidylinositol signaling system - Homo sapiens (human)|KEGG	0.075860352	0.012282766	0.37852298	0.71289066	0.90425832	-5.79711717
homo after UVB irradiation VS homo before UVB irradiation	phosphatidylserine biosynthesis I|HumanCyc	1.038974486	-0.02122681	4.18226282	0.00215813	0.02581999	-1.17619611
hetero after UVB irradiation VS hetero before UVB irradiation	phosphatidylserine biosynthesis I|HumanCyc	0.652133408	0.004699501	1.645276317	0.13661853	0.33605356	-5.07893946
WT after UVB irradiation VS WT before UVB irradiation	phosphatidylserine biosynthesis I|HumanCyc	0.648172337	0.057597811	1.729558874	0.11414857	0.51527453	-4.55660252
homo after UVB irradiation VS homo before UVB irradiation	phosphatidylserine biosynthesis II|HumanCyc	-0.56001491	0.011286448	-1.51366279	0.16302033	0.31259728	-5.2706126
hetero after UVB irradiation VS hetero before UVB irradiation	phosphatidylserine biosynthesis II|HumanCyc	-0.93911351	-0.06674534	-3.53525908	0.00706095	0.08628369	-2.26561274
WT after UVB irradiation VS WT before UVB irradiation	phosphatidylserine biosynthesis II|HumanCyc	-0.51634841	0.040492629	-1.34254399	0.20886495	0.62359252	-5.03474496
homo after UVB irradiation VS homo before UVB irradiation	Phosphodiesterases in neuronal function|Wikipathways	0.349806089	-0.01189772	1.717069308	0.1186981	0.25759292	-4.99667775
hetero after UVB irradiation VS hetero before UVB irradiation	Phosphodiesterases in neuronal function|Wikipathways	0.198923284	0.023526951	0.950030516	0.3685471	0.59623032	-5.88213216
WT after UVB irradiation VS WT before UVB irradiation	Phosphodiesterases in neuronal function|Wikipathways	0.109857964	-0.01492749	0.399684841	0.69772481	0.89864566	-5.78890755
homo after UVB irradiation VS homo before UVB irradiation	Phosphoenolpyruvate carboxykinase deficiency 1 (PEPCK1)|SMPDB	0.252684369	-0.00151752	1.090705456	0.3026004	0.46312664	-5.76769054
hetero after UVB irradiation VS hetero before UVB irradiation	Phosphoenolpyruvate carboxykinase deficiency 1 (PEPCK1)|SMPDB	-0.06167553	0.005820603	-0.24893092	0.809375	0.92095935	-6.31305137
WT after UVB irradiation VS WT before UVB irradiation	Phosphoenolpyruvate carboxykinase deficiency 1 (PEPCK1)|SMPDB	-0.01454403	-0.00173466	-0.04854099	0.96223323	0.98815178	-5.86795769
homo after UVB irradiation VS homo before UVB irradiation	phosphoinositides and their downstream targets|BioCarta	0.311168893	-0.01931016	1.339453229	0.21194104	0.36957778	-5.488626
hetero after UVB irradiation VS hetero before UVB irradiation	phosphoinositides and their downstream targets|BioCarta	0.459175741	-0.00526782	3.029006751	0.01538082	0.11352943	-3.02685283
WT after UVB irradiation VS WT before UVB irradiation	phosphoinositides and their downstream targets|BioCarta	0.408776461	0.038827544	2.043012501	0.06807415	0.44205977	-4.12625702
homo after UVB irradiation VS homo before UVB irradiation	phospholipase c delta in phospholipid associated cell signaling|BioCarta	-0.38390059	-0.00297148	-1.13389425	0.28495902	0.44528503	-5.72225192
hetero after UVB irradiation VS hetero before UVB irradiation	phospholipase c delta in phospholipid associated cell signaling|BioCarta	-0.39582806	-0.034375	-1.61821248	0.14236558	0.34534449	-5.11500546
WT after UVB irradiation VS WT before UVB irradiation	phospholipase c delta in phospholipid associated cell signaling|BioCarta	-0.38543669	0.03031459	-1.26353156	0.23483264	0.62952955	-5.12285295
homo after UVB irradiation VS homo before UVB irradiation	phospholipase c signaling pathway|BioCarta	0.729657117	-0.04634742	3.21433313	0.0100277	0.05954026	-2.67554775
hetero after UVB irradiation VS hetero before UVB irradiation	phospholipase c signaling pathway|BioCarta	0.882286568	0.030734078	4.138139242	0.00291492	0.06148411	-1.39820122
WT after UVB irradiation VS WT before UVB irradiation	phospholipase c signaling pathway|BioCarta	-0.01381894	0.00765122	-0.06979798	0.94571947	0.98295421	-5.86668382
homo after UVB irradiation VS homo before UVB irradiation	phospholipase c-epsilon pathway|BioCarta	0.459139352	-0.02838737	2.842235424	0.01855674	0.08707783	-3.27096377
hetero after UVB irradiation VS hetero before UVB irradiation	phospholipase c-epsilon pathway|BioCarta	0.28596683	-0.00406238	1.293580175	0.23014272	0.45170905	-5.52088677
WT after UVB irradiation VS WT before UVB irradiation	phospholipase c-epsilon pathway|BioCarta	0.352144361	-0.10599663	1.132565448	0.28361239	0.67189698	-5.26043595
homo after UVB irradiation VS homo before UVB irradiation	Phospholipase C-mediated cascade: FGFR1|Reactome	0.060600238	0.046856736	0.29346343	0.77555142	0.85468276	-6.322392
hetero after UVB irradiation VS hetero before UVB irradiation	Phospholipase C-mediated cascade: FGFR1|Reactome	-0.1479498	0.031323916	-0.78366688	0.45469801	0.67919377	-6.02530609
WT after UVB irradiation VS WT before UVB irradiation	Phospholipase C-mediated cascade: FGFR1|Reactome	-0.35958111	0.066845599	-1.59618578	0.14128264	0.55622203	-4.72919585
homo after UVB irradiation VS homo before UVB irradiation	Phospholipase C-mediated cascade; FGFR2|Reactome	-0.22363761	0.054332256	-0.9709442	0.35592259	0.51072928	-5.88644353
hetero after UVB irradiation VS hetero before UVB irradiation	Phospholipase C-mediated cascade; FGFR2|Reactome	-0.29446276	0.038190254	-1.49785359	0.17066229	0.3872586	-5.2715652
WT after UVB irradiation VS WT before UVB irradiation	Phospholipase C-mediated cascade; FGFR2|Reactome	-0.52036304	0.048224956	-2.30924131	0.0433645	0.39022568	-3.74130701
homo after UVB irradiation VS homo before UVB irradiation	Phospholipase C-mediated cascade; FGFR3|Reactome	-0.14736321	0.082695741	-0.67093485	0.5184248	0.6562594	-6.13235869
hetero after UVB irradiation VS hetero before UVB irradiation	Phospholipase C-mediated cascade; FGFR3|Reactome	-0.28334983	0.020601963	-1.48087229	0.17503481	0.39186333	-5.2931167
WT after UVB irradiation VS WT before UVB irradiation	Phospholipase C-mediated cascade; FGFR3|Reactome	-0.53390223	0.04746663	-2.30783847	0.04346849	0.39022568	-3.74336833
homo after UVB irradiation VS homo before UVB irradiation	Phospholipase C-mediated cascade; FGFR4|Reactome	0.047506709	0.045161287	0.210368331	0.83787477	0.9012335	-6.34472732
hetero after UVB irradiation VS hetero before UVB irradiation	Phospholipase C-mediated cascade; FGFR4|Reactome	-0.21732175	0.031726266	-1.01047624	0.34040094	0.56937522	-5.8246273
WT after UVB irradiation VS WT before UVB irradiation	Phospholipase C-mediated cascade; FGFR4|Reactome	-0.35086536	0.0196506	-1.36395895	0.20225384	0.61620632	-5.0102446
homo after UVB irradiation VS homo before UVB irradiation	Phospholipase D signaling pathway - Homo sapiens (human)|KEGG	0.485091641	-0.01142664	2.951563921	0.01547281	0.07695951	-3.09577128
hetero after UVB irradiation VS hetero before UVB irradiation	Phospholipase D signaling pathway - Homo sapiens (human)|KEGG	0.428646386	0.017657737	2.672670351	0.02699	0.14262729	-3.57168849
WT after UVB irradiation VS WT before UVB irradiation	Phospholipase D signaling pathway - Homo sapiens (human)|KEGG	0.32173403	0.012218828	1.620144584	0.13602031	0.54838491	-4.69873874
homo after UVB irradiation VS homo before UVB irradiation	phospholipases|HumanCyc	0.249974422	0.029514228	1.296510869	0.22575636	0.38559559	-5.53967688
hetero after UVB irradiation VS hetero before UVB irradiation	phospholipases|HumanCyc	0.286300216	0.033018932	1.923879176	0.08872625	0.26960949	-4.69228828
WT after UVB irradiation VS WT before UVB irradiation	phospholipases|HumanCyc	0.090566212	0.057834181	0.413705413	0.68775257	0.89181317	-5.78323079
homo after UVB irradiation VS homo before UVB irradiation	Phospholipid Biosynthesis|SMPDB	0.287284825	-0.02596263	1.528065056	0.15945564	0.30798411	-5.25186003
hetero after UVB irradiation VS hetero before UVB irradiation	Phospholipid Biosynthesis|SMPDB	0.031703385	-0.00301222	0.12901171	0.90037497	0.96973277	-6.33752116
WT after UVB irradiation VS WT before UVB irradiation	Phospholipid Biosynthesis|SMPDB	0.274715595	-0.02009338	1.104619116	0.29498852	0.68163568	-5.28834073
homo after UVB irradiation VS homo before UVB irradiation	Phospholipid metabolism|Reactome	0.085385786	-0.02543768	0.492217185	0.63386994	0.74618219	-6.23996957
hetero after UVB irradiation VS hetero before UVB irradiation	Phospholipid metabolism|Reactome	0.039001685	-0.01715125	0.244589111	0.812626	0.92298761	-6.31420522
WT after UVB irradiation VS WT before UVB irradiation	Phospholipid metabolism|Reactome	0.025422692	-0.00676721	0.126131409	0.90210776	0.97566251	-5.86109854
homo after UVB irradiation VS homo before UVB irradiation	phospholipids as signalling intermediaries|BioCarta	0.476247384	-0.03592619	2.780141861	0.0205795	0.09140661	-3.37040279
hetero after UVB irradiation VS hetero before UVB irradiation	phospholipids as signalling intermediaries|BioCarta	0.504914022	0.022355486	3.253289089	0.01085584	0.10415756	-2.68682131
WT after UVB irradiation VS WT before UVB irradiation	phospholipids as signalling intermediaries|BioCarta	0.439406401	0.031549472	2.22842963	0.04976668	0.40833376	-3.85955453
homo after UVB irradiation VS homo before UVB irradiation	Phosphonate and phosphinate metabolism - Homo sapiens (human)|KEGG	0.247018824	0.067025247	1.371715867	0.20203567	0.35894742	-5.44954181
hetero after UVB irradiation VS hetero before UVB irradiation	Phosphonate and phosphinate metabolism - Homo sapiens (human)|KEGG	0.02725903	-0.00359519	0.115423699	0.91081283	0.97054702	-6.3393197
WT after UVB irradiation VS WT before UVB irradiation	Phosphonate and phosphinate metabolism - Homo sapiens (human)|KEGG	-0.20036995	-0.00519922	-0.99474517	0.34313421	0.72752735	-5.39276308
homo after UVB irradiation VS homo before UVB irradiation	phospho-PLA2 pathway|Reactome	-0.12091931	0.077513983	-0.34611767	0.73688403	0.82682223	-6.30449922
hetero after UVB irradiation VS hetero before UVB irradiation	phospho-PLA2 pathway|Reactome	-0.20265408	-0.01991795	-0.53879527	0.60396797	0.79138772	-6.19178644
WT after UVB irradiation VS WT before UVB irradiation	phospho-PLA2 pathway|Reactome	-0.10773595	0.035662781	-0.28943624	0.77811215	0.93502208	-5.82690101
homo after UVB irradiation VS homo before UVB irradiation	Phosphorylation of CD3 and TCR zeta chains|Reactome	-0.30811032	0.020246383	-1.11308761	0.29335452	0.45328485	-5.74430971
hetero after UVB irradiation VS hetero before UVB irradiation	Phosphorylation of CD3 and TCR zeta chains|Reactome	-0.12419889	0.018387214	-0.50968256	0.6233528	0.80534072	-6.20780646
WT after UVB irradiation VS WT before UVB irradiation	Phosphorylation of CD3 and TCR zeta chains|Reactome	0.113217078	-0.0616886	0.344059036	0.73786986	0.91671698	-5.80955871
homo after UVB irradiation VS homo before UVB irradiation	Phosphorylation of Emi1|Reactome	-0.70255509	-0.0656873	-2.68568143	0.024093	0.10226192	-3.52144027
hetero after UVB irradiation VS hetero before UVB irradiation	Phosphorylation of Emi1|Reactome	0.612282496	0.056267678	1.811370066	0.10577478	0.29415277	-4.85151538
WT after UVB irradiation VS WT before UVB irradiation	Phosphorylation of Emi1|Reactome	0.14326503	0.05564399	0.388811505	0.70550041	0.90126444	-5.79317978
homo after UVB irradiation VS homo before UVB irradiation	phosphorylation of mek1 by cdk5/p35 down regulates the map kinase pathway|BioCarta	0.345616937	-0.04321672	1.79439882	0.10494402	0.2370387	-4.88787718
hetero after UVB irradiation VS hetero before UVB irradiation	phosphorylation of mek1 by cdk5/p35 down regulates the map kinase pathway|BioCarta	0.426124185	-0.00059058	2.440470189	0.03907127	0.17223099	-3.92599837
WT after UVB irradiation VS WT before UVB irradiation	phosphorylation of mek1 by cdk5/p35 down regulates the map kinase pathway|BioCarta	0.516398311	0.117703983	2.134393396	0.05836692	0.42483181	-3.99571039
homo after UVB irradiation VS homo before UVB irradiation	Phosphorylation of proteins involved in G1/S transition by active Cyclin E:Cdk2 complexes|Reactome	-0.54712336	-0.01481392	-1.63397426	0.13529456	0.277359	-5.11086019
hetero after UVB irradiation VS hetero before UVB irradiation	Phosphorylation of proteins involved in G1/S transition by active Cyclin E:Cdk2 complexes|Reactome	-0.20237588	-0.03862404	-0.55994091	0.59009182	0.78232651	-6.17963147
WT after UVB irradiation VS WT before UVB irradiation	Phosphorylation of proteins involved in G1/S transition by active Cyclin E:Cdk2 complexes|Reactome	0.043290542	0.110933892	0.103883628	0.91929914	0.97566251	-5.86368726
homo after UVB irradiation VS homo before UVB irradiation	Phosphorylation of proteins involved in the G2/M transition by Cyclin A:Cdc2 complexes|Reactome	-0.56673931	0.082497975	-1.69023514	0.12384372	0.26290983	-5.03387283
hetero after UVB irradiation VS hetero before UVB irradiation	Phosphorylation of proteins involved in the G2/M transition by Cyclin A:Cdc2 complexes|Reactome	0.716880008	0.097508964	2.338593307	0.04596723	0.18854754	-4.08027765
WT after UVB irradiation VS WT before UVB irradiation	Phosphorylation of proteins involved in the G2/M transition by Cyclin A:Cdc2 complexes|Reactome	0.411981719	0.026921064	1.098109371	0.29768851	0.68387495	-5.29476433
homo after UVB irradiation VS homo before UVB irradiation	Phosphorylation of the APC/C|Reactome	-0.58738089	0.02130056	-3.11614299	0.01178499	0.06636109	-2.83229773
hetero after UVB irradiation VS hetero before UVB irradiation	Phosphorylation of the APC/C|Reactome	0.130631582	-0.03917338	0.734147301	0.48278576	0.70381063	-6.06339139
WT after UVB irradiation VS WT before UVB irradiation	Phosphorylation of the APC/C|Reactome	-0.01765717	-0.08871931	-0.06989923	0.94564086	0.98295421	-5.86667666
homo after UVB irradiation VS homo before UVB irradiation	Photodynamic therapy-induced AP-1 survival signaling.|Wikipathways	0.140118259	-0.02648432	0.794289621	0.44663712	0.59438796	-6.0406804
hetero after UVB irradiation VS hetero before UVB irradiation	Photodynamic therapy-induced AP-1 survival signaling.|Wikipathways	0.381922526	0.003266401	2.425349703	0.04002533	0.17558752	-3.94895726
WT after UVB irradiation VS WT before UVB irradiation	Photodynamic therapy-induced AP-1 survival signaling.|Wikipathways	0.098701481	-0.05559544	0.450347453	0.661989	0.87935805	-5.76750647
homo after UVB irradiation VS homo before UVB irradiation	Photodynamic therapy-induced HIF-1 survival signaling|Wikipathways	0.602194535	-0.02733095	3.021805875	0.01377236	0.07305822	-2.98324551
hetero after UVB irradiation VS hetero before UVB irradiation	Photodynamic therapy-induced HIF-1 survival signaling|Wikipathways	0.719082709	0.026523589	3.944731512	0.00385032	0.06828702	-1.67089228
WT after UVB irradiation VS WT before UVB irradiation	Photodynamic therapy-induced HIF-1 survival signaling|Wikipathways	0.522138184	0.003075841	2.295930167	0.04436111	0.39111425	-3.76085473
homo after UVB irradiation VS homo before UVB irradiation	Photodynamic therapy-induced NFE2L2 (NRF2) survival signaling|Wikipathways	0.346241746	-0.06030166	1.893500075	0.0894705	0.21433488	-4.74521875
hetero after UVB irradiation VS hetero before UVB irradiation	Photodynamic therapy-induced NFE2L2 (NRF2) survival signaling|Wikipathways	0.209372717	0.014524371	1.171715693	0.27337494	0.5028422	-5.6581826
WT after UVB irradiation VS WT before UVB irradiation	Photodynamic therapy-induced NFE2L2 (NRF2) survival signaling|Wikipathways	0.214066149	-0.000427	0.985708867	0.34733983	0.7320263	-5.40096314
homo after UVB irradiation VS homo before UVB irradiation	Photodynamic therapy-induced NF-kB survival signaling|Wikipathways	0.577302854	0.023671253	3.469977467	0.00661324	0.04703437	-2.27022548
hetero after UVB irradiation VS hetero before UVB irradiation	Photodynamic therapy-induced NF-kB survival signaling|Wikipathways	0.524411702	-0.02645825	2.329580752	0.0466328	0.19011835	-4.09387463
WT after UVB irradiation VS WT before UVB irradiation	Photodynamic therapy-induced NF-kB survival signaling|Wikipathways	0.432971578	-0.04706506	1.544751246	0.15319203	0.55854784	-4.79371454
homo after UVB irradiation VS homo before UVB irradiation	Photodynamic therapy-induced unfolded protein response|Wikipathways	0.08916665	0.026483189	0.345701975	0.73718644	0.82682223	-6.30465174
hetero after UVB irradiation VS hetero before UVB irradiation	Photodynamic therapy-induced unfolded protein response|Wikipathways	0.391667732	-0.03328986	1.565759018	0.1541329	0.36486693	-5.18402967
WT after UVB irradiation VS WT before UVB irradiation	Photodynamic therapy-induced unfolded protein response|Wikipathways	0.203270615	-0.04050857	0.802656136	0.44068384	0.79738633	-5.55355716
homo after UVB irradiation VS homo before UVB irradiation	Phototransduction - Homo sapiens (human)|KEGG	0.400999494	0.045258368	2.136187661	0.06015809	0.16751767	-4.38313016
hetero after UVB irradiation VS hetero before UVB irradiation	Phototransduction - Homo sapiens (human)|KEGG	0.100284778	0.069213682	0.621519704	0.55069922	0.75605031	-6.14178402
WT after UVB irradiation VS WT before UVB irradiation	Phototransduction - Homo sapiens (human)|KEGG	-0.30061587	0.02911812	-1.564307	0.14856371	0.55622203	-4.76932617
homo after UVB irradiation VS homo before UVB irradiation	Physiological and Pathological Hypertrophy of the Heart|Wikipathways	0.12496658	-0.0019125	0.640878865	0.53692814	0.67222756	-6.15258821
hetero after UVB irradiation VS hetero before UVB irradiation	Physiological and Pathological Hypertrophy of the Heart|Wikipathways	0.121582525	-0.02885863	0.606309125	0.56028578	0.76275652	-6.1514688
WT after UVB irradiation VS WT before UVB irradiation	Physiological and Pathological Hypertrophy of the Heart|Wikipathways	0.086159211	-0.02381552	0.351094413	0.73274347	0.91258234	-5.80711275
homo after UVB irradiation VS homo before UVB irradiation	Physiological factors|Reactome	0.343318164	0.068094735	1.533314918	0.15817342	0.30628491	-5.24499843
hetero after UVB irradiation VS hetero before UVB irradiation	Physiological factors|Reactome	-0.05105361	0.0192473	-0.1748854	0.86529928	0.94783573	-6.32998005
WT after UVB irradiation VS WT before UVB irradiation	Physiological factors|Reactome	-0.20808657	-0.07390045	-0.63566404	0.53915517	0.83903241	-5.66875902
homo after UVB irradiation VS homo before UVB irradiation	Phytanic acid peroxisomal oxidation|EHMN	0.160997567	-0.04240669	0.656224567	0.52743266	0.66343182	-6.14236498
hetero after UVB irradiation VS hetero before UVB irradiation	Phytanic acid peroxisomal oxidation|EHMN	-0.03307691	-0.03265716	-0.10604945	0.91802462	0.97054702	-6.34044432
WT after UVB irradiation VS WT before UVB irradiation	Phytanic acid peroxisomal oxidation|EHMN	-0.17087714	-0.09840363	-0.60301155	0.55982031	0.84911968	-5.68843867
homo after UVB irradiation VS homo before UVB irradiation	Phytanic Acid Peroxisomal Oxidation|SMPDB	0.309508869	0.005460472	1.563469103	0.15098345	0.29702303	-5.20532249
hetero after UVB irradiation VS hetero before UVB irradiation	Phytanic Acid Peroxisomal Oxidation|SMPDB	0.084800099	-0.0542149	0.263185751	0.79872922	0.91506577	-6.3091226
WT after UVB irradiation VS WT before UVB irradiation	Phytanic Acid Peroxisomal Oxidation|SMPDB	-0.07150324	-0.07402423	-0.29688223	0.77258268	0.93294159	-5.82470951
homo after UVB irradiation VS homo before UVB irradiation	Phytochemical activity on NRF2 transcriptional activation|Wikipathways	0.060760779	-0.02339381	0.23354733	0.82034902	0.8897182	-6.33922846
hetero after UVB irradiation VS hetero before UVB irradiation	Phytochemical activity on NRF2 transcriptional activation|Wikipathways	0.607501751	-0.03931855	3.611127526	0.00629984	0.08320066	-2.1537765
WT after UVB irradiation VS WT before UVB irradiation	Phytochemical activity on NRF2 transcriptional activation|Wikipathways	0.294725886	-0.01149159	1.27320108	0.2315188	0.62952955	-5.11226874
homo after UVB irradiation VS homo before UVB irradiation	phytol degradation|HumanCyc	0.272578161	0.025040479	1.426386737	0.18614902	0.3414851	-5.38193603
hetero after UVB irradiation VS hetero before UVB irradiation	phytol degradation|HumanCyc	-1.10302397	-0.10845791	-5.28876255	0.00062289	0.0302034	0.100395325
WT after UVB irradiation VS WT before UVB irradiation	phytol degradation|HumanCyc	-0.06457593	0.034109613	-0.21179319	0.83648851	0.94951669	-5.84648149
homo after UVB irradiation VS homo before UVB irradiation	PI and PC transport between ER and Golgi membranes|Reactome	-1.02617628	0.021661696	-4.54783088	0.00124739	0.02014127	-0.64147278
hetero after UVB irradiation VS hetero before UVB irradiation	PI and PC transport between ER and Golgi membranes|Reactome	-0.44589363	-0.01068257	-1.08924436	0.30621517	0.53578839	-5.74560475
WT after UVB irradiation VS WT before UVB irradiation	PI and PC transport between ER and Golgi membranes|Reactome	-0.28060791	0.058302751	-0.72580419	0.48446598	0.81710765	-5.60954458
homo after UVB irradiation VS homo before UVB irradiation	PI Metabolism|Reactome	0.226115605	-0.03513212	1.389186105	0.19683778	0.35314786	-5.42812344
hetero after UVB irradiation VS hetero before UVB irradiation	PI Metabolism|Reactome	0.210379564	0.000130158	1.328269147	0.21893856	0.44077826	-5.48015642
WT after UVB irradiation VS WT before UVB irradiation	PI Metabolism|Reactome	0.186879608	-0.00190388	0.930616874	0.37379808	0.75071937	-5.44963294
homo after UVB irradiation VS homo before UVB irradiation	PI-3K cascade:FGFR1|Reactome	-0.1180305	-0.0108489	-0.56706358	0.58397172	0.70596105	-6.19864741
hetero after UVB irradiation VS hetero before UVB irradiation	PI-3K cascade:FGFR1|Reactome	-0.14611545	0.040647109	-0.86008299	0.41354397	0.64157026	-5.96238218
WT after UVB irradiation VS WT before UVB irradiation	PI-3K cascade:FGFR1|Reactome	-0.33532588	0.048981518	-1.46113748	0.1744311	0.58532465	-4.8959306
homo after UVB irradiation VS homo before UVB irradiation	PI-3K cascade:FGFR2|Reactome	-0.31691225	-0.00475905	-1.52644628	0.15985285	0.3083289	-5.25397299
hetero after UVB irradiation VS hetero before UVB irradiation	PI-3K cascade:FGFR2|Reactome	-0.21420872	0.039306823	-1.18675998	0.26770211	0.4964458	-5.64174242
WT after UVB irradiation VS WT before UVB irradiation	PI-3K cascade:FGFR2|Reactome	-0.47203233	0.033184866	-2.04211127	0.06817712	0.44205977	-4.12753487
homo after UVB irradiation VS homo before UVB irradiation	PI-3K cascade:FGFR3|Reactome	-0.27286562	0.001104304	-1.31395165	0.22005723	0.3784487	-5.51907886
hetero after UVB irradiation VS hetero before UVB irradiation	PI-3K cascade:FGFR3|Reactome	-0.22975159	0.023567437	-1.29445976	0.22985276	0.45170905	-5.51986269
WT after UVB irradiation VS WT before UVB irradiation	PI-3K cascade:FGFR3|Reactome	-0.48102388	0.029132592	-1.9986298	0.07332365	0.45403763	-4.18894521
homo after UVB irradiation VS homo before UVB irradiation	PI-3K cascade:FGFR4|Reactome	-0.0951055	-0.00308823	-0.48552662	0.63843193	0.74937794	-6.24339468
hetero after UVB irradiation VS hetero before UVB irradiation	PI-3K cascade:FGFR4|Reactome	-0.20906968	0.013185378	-1.04674369	0.32431328	0.55421891	-5.78880351

WT after UVB irradiation VS WT before UVB irradiation	PI-3K cascade:FGFR4|Reactome	-0.38919445	0.029624666	-1.53761545	0.15491212	0.55883384	-4.80256942
homo after UVB irradiation VS homo before UVB irradiation	PI3K Cascade|Reactome	-0.1054812	-0.00255374	-0.57774524	0.57702702	0.70016088	-6.19230492
hetero after UVB irradiation VS hetero before UVB irradiation	PI3K Cascade|Reactome	-0.23280837	0.024030328	-1.33165847	0.21786911	0.43959602	-5.47613955
WT after UVB irradiation VS WT before UVB irradiation	PI3K Cascade|Reactome	-0.37911149	0.01989721	-1.93956321	0.08090274	0.46678978	-4.27156663
homo after UVB irradiation VS homo before UVB irradiation	PI3K events in ERBB2 signaling|Reactome	-0.17878768	0.048797136	-0.89361438	0.39385628	0.54703225	-5.95715374
hetero after UVB irradiation VS hetero before UVB irradiation	PI3K events in ERBB2 signaling|Reactome	-0.28664063	-0.02328725	-1.68961374	0.12766004	0.32201132	-5.01922211
WT after UVB irradiation VS WT before UVB irradiation	PI3K events in ERBB2 signaling|Reactome	-0.19481827	0.030140568	-0.96554862	0.35685873	0.74236042	-5.41903874
homo after UVB irradiation VS homo before UVB irradiation	PI3K events in ERBB4 signaling|Reactome	0.121231454	0.064376503	0.718565125	0.48990334	0.63135722	-6.09858908
hetero after UVB irradiation VS hetero before UVB irradiation	PI3K events in ERBB4 signaling|Reactome	-0.00065263	-0.00488241	-0.00266883	0.99793269	0.99946764	-6.34653743
WT after UVB irradiation VS WT before UVB irradiation	PI3K events in ERBB4 signaling|Reactome	-0.05692479	-0.02961853	-0.2474033	0.80956145	0.94525798	-5.83824434
homo after UVB irradiation VS homo before UVB irradiation	PI3K/AKT activation|Reactome	0.160267428	0.024260097	0.978230562	0.35249087	0.50783175	-5.87953368
hetero after UVB irradiation VS hetero before UVB irradiation	PI3K/AKT activation|Reactome	-0.00706571	-0.03311966	-0.03961935	0.96931933	0.98614433	-6.34568983
WT after UVB irradiation VS WT before UVB irradiation	PI3K/AKT activation|Reactome	-0.09844636	0.027922357	-0.4809541	0.64081584	0.87675433	-5.75339454
homo after UVB irradiation VS homo before UVB irradiation	PI3K/AKT Signaling in Cancer|Reactome	-0.05722447	0.010404779	-0.34723957	0.73606809	0.82617672	-6.30408669
hetero after UVB irradiation VS hetero before UVB irradiation	PI3K/AKT Signaling in Cancer|Reactome	-0.06229687	0.00259051	-0.40108056	0.69834029	0.84778456	-6.26011967
WT after UVB irradiation VS WT before UVB irradiation	PI3K/AKT Signaling in Cancer|Reactome	-0.16432654	0.00079239	-0.84472361	0.4178591	0.784366	-5.52083666
homo after UVB irradiation VS homo before UVB irradiation	PI3K-Akt signaling pathway - Homo sapiens (human)|KEGG	0.319762603	-0.00853782	1.99265942	0.07614745	0.19626432	-4.59925733
hetero after UVB irradiation VS hetero before UVB irradiation	PI3K-Akt signaling pathway - Homo sapiens (human)|KEGG	0.275952039	-0.00110738	1.62875513	0.14010115	0.34069016	-5.10099171
WT after UVB irradiation VS WT before UVB irradiation	PI3K-Akt signaling pathway - Homo sapiens (human)|KEGG	0.132255935	-0.02453024	0.623799704	0.54661264	0.84264773	-5.67602009
homo after UVB irradiation VS homo before UVB irradiation	PI3K-Akt Signaling Pathway|Wikipathways	0.332329503	-0.01163866	2.116532757	0.06213996	0.17102534	-4.41302919
hetero after UVB irradiation VS hetero before UVB irradiation	PI3K-Akt Signaling Pathway|Wikipathways	0.284157695	-0.00049541	1.642592927	0.13717875	0.33670476	-5.08252874
WT after UVB irradiation VS WT before UVB irradiation	PI3K-Akt Signaling Pathway|Wikipathways	0.145320279	-0.02505746	0.665624531	0.52058786	0.8310118	-5.6498666
homo after UVB irradiation VS homo before UVB irradiation	PI3K-AKT-mTOR - VitD3 Signalling|Wikipathways	0.324707649	-0.02943701	1.911831119	0.0868529	0.21038213	-4.71846552
hetero after UVB irradiation VS hetero before UVB irradiation	PI3K-AKT-mTOR - VitD3 Signalling|Wikipathways	0.235815671	-0.00710387	1.050850466	0.32252909	0.55349684	-5.78468606
WT after UVB irradiation VS WT before UVB irradiation	PI3K-AKT-mTOR - VitD3 Signalling|Wikipathways	-0.03170059	-0.03974837	-0.12963686	0.89940361	0.97566251	-5.86064513
homo after UVB irradiation VS homo before UVB irradiation	PI3K-AKT-mTOR signaling pathway and therapeutic opportunities|Wikipathways	-0.01531803	-0.04350456	-0.0773865	0.93994018	0.96450524	-6.36523157
hetero after UVB irradiation VS hetero before UVB irradiation	PI3K-AKT-mTOR signaling pathway and therapeutic opportunities|Wikipathways	-0.06344769	-0.00136974	-0.34819973	0.7362302	0.87139373	-6.28125024
WT after UVB irradiation VS WT before UVB irradiation	PI3K-AKT-mTOR signaling pathway and therapeutic opportunities|Wikipathways	-0.19618205	-0.00056199	-0.91289486	0.38260858	0.75519583	-5.46479738
homo after UVB irradiation VS homo before UVB irradiation	PI5P Regulates TP53 Acetylation|Reactome	0.157574966	-0.05332845	0.801693388	0.44254629	0.59091566	-6.03474628
hetero after UVB irradiation VS hetero before UVB irradiation	PI5P Regulates TP53 Acetylation|Reactome	0.0632699	-0.00624233	0.318332964	0.75797994	0.88797403	-6.2919048
WT after UVB irradiation VS WT before UVB irradiation	PI5P Regulates TP53 Acetylation|Reactome	0.443284695	0.014730417	2.156686398	0.05620756	0.41769317	-3.96358726
homo after UVB irradiation VS homo before UVB irradiation	PI5P_ PP2A and IER3 Regulate PI3K/AKT Signaling|Reactome	0.157756344	-0.00041398	0.974721287	0.3541406	0.50913287	-5.88286684
hetero after UVB irradiation VS hetero before UVB irradiation	PI5P_ PP2A and IER3 Regulate PI3K/AKT Signaling|Reactome	0.073759422	0.018790041	0.433269221	0.67569769	0.83171215	-6.24585421
WT after UVB irradiation VS WT before UVB irradiation	PI5P_ PP2A and IER3 Regulate PI3K/AKT Signaling|Reactome	-0.03043636	0.022500528	-0.14702821	0.88600829	0.97031361	-5.8582126
homo after UVB irradiation VS homo before UVB irradiation	Pilocytic astrocytoma|Wikipathways	-0.46689231	-0.05185262	-1.52682769	0.15975918	0.30830956	-5.25347526
hetero after UVB irradiation VS hetero before UVB irradiation	Pilocytic astrocytoma|Wikipathways	-0.23398272	0.094926664	-0.70946255	0.49719721	0.71538528	-6.08156348
WT after UVB irradiation VS WT before UVB irradiation	Pilocytic astrocytoma|Wikipathways	-0.29963397	0.023168084	-0.85950168	0.41003464	0.77688258	-5.50900181
homo after UVB irradiation VS homo before UVB irradiation	Pindolol Action Pathway|SMPDB	0.396415397	-0.03677376	2.343069613	0.04268583	0.13402181	-4.06364635
hetero after UVB irradiation VS hetero before UVB irradiation	Pindolol Action Pathway|SMPDB	-0.01555055	-0.01775524	-0.07410913	0.94265284	0.97054702	-6.34356282
WT after UVB irradiation VS WT before UVB irradiation	Pindolol Action Pathway|SMPDB	0.056685205	-0.03978418	0.223404833	0.82768199	0.94525798	-5.84393438
homo after UVB irradiation VS homo before UVB irradiation	Pink/Parkin Mediated Mitophagy|Reactome	-0.27736542	0.036361371	-1.48156956	0.17121615	0.32186978	-5.3120176
hetero after UVB irradiation VS hetero before UVB irradiation	Pink/Parkin Mediated Mitophagy|Reactome	-0.09643819	-0.02875088	-0.53229377	0.60826913	0.7955497	-6.19543611
WT after UVB irradiation VS WT before UVB irradiation	Pink/Parkin Mediated Mitophagy|Reactome	-0.50593906	-0.02442891	-2.37858583	0.03851511	0.39022568	-3.63907389
homo after UVB irradiation VS homo before UVB irradiation	PIP3 activates AKT signaling|Reactome	-0.0049817	-0.00878573	-0.02901851	0.9774573	0.98606224	-6.36799583
hetero after UVB irradiation VS hetero before UVB irradiation	PIP3 activates AKT signaling|Reactome	0.046873332	-0.00578035	0.307988427	0.76556761	0.89270377	-6.29537797
WT after UVB irradiation VS WT before UVB irradiation	PIP3 activates AKT signaling|Reactome	-0.06741148	0.009111053	-0.33726734	0.74283149	0.9185045	-5.81187415
homo after UVB irradiation VS homo before UVB irradiation	Pirenzepine Action Pathway|SMPDB	0.21780303	0.051698704	1.19150037	0.26270351	0.42561191	-5.65959884
hetero after UVB irradiation VS hetero before UVB irradiation	Pirenzepine Action Pathway|SMPDB	0.331566302	0.063470812	1.441200644	0.18563717	0.40321767	-5.34291209
WT after UVB irradiation VS WT before UVB irradiation	Pirenzepine Action Pathway|SMPDB	-0.11560861	0.135982321	-0.44330845	0.66690385	0.87935805	-5.77062647
homo after UVB irradiation VS homo before UVB irradiation	Piroxicam Action Pathway|SMPDB	0.40423582	0.045705158	1.979500697	0.07780079	0.19626432	-4.61879689
hetero after UVB irradiation VS hetero before UVB irradiation	Piroxicam Action Pathway|SMPDB	0.482496143	0.064876536	3.04499191	0.01500113	0.1130168	-3.00250826
WT after UVB irradiation VS WT before UVB irradiation	Piroxicam Action Pathway|SMPDB	0.275725339	0.086595857	1.261970114	0.23537138	0.62952955	-5.12455679
homo after UVB irradiation VS homo before UVB irradiation	PIWI-interacting RNA (piRNA) biogenesis|Reactome	-0.38090182	0.045105384	-1.52455114	0.16031898	0.30897074	-5.25644501
hetero after UVB irradiation VS hetero before UVB irradiation	PIWI-interacting RNA (piRNA) biogenesis|Reactome	-0.36149482	0.033235497	-1.80627484	0.10661563	0.29505406	-4.85863499
WT after UVB irradiation VS WT before UVB irradiation	PIWI-interacting RNA (piRNA) biogenesis|Reactome	-0.21233548	0.11884427	-0.79859315	0.44293136	0.79860542	-5.55664065
homo after UVB irradiation VS homo before UVB irradiation	PKA activation in glucagon signalling|Reactome	0.538863001	-0.05992784	2.946587529	0.01560113	0.07717742	-3.10374561
hetero after UVB irradiation VS hetero before UVB irradiation	PKA activation in glucagon signalling|Reactome	0.297107042	0.023517093	1.32940737	0.21857892	0.44024865	-5.47880819
WT after UVB irradiation VS WT before UVB irradiation	PKA activation in glucagon signalling|Reactome	0.325643977	-0.03324765	1.103828347	0.29531548	0.68185329	-5.28912258
homo after UVB irradiation VS homo before UVB irradiation	PKA activation|Reactome	0.498621053	-0.06635032	2.555821475	0.0299297	0.115124	-3.72827237
hetero after UVB irradiation VS hetero before UVB irradiation	PKA activation|Reactome	0.272221208	0.02920123	1.308565248	0.2252446	0.44754354	-5.5033778
WT after UVB irradiation VS WT before UVB irradiation	PKA activation|Reactome	0.331955566	-0.02815923	1.154820915	0.27480034	0.66570326	-5.23783792
homo after UVB irradiation VS homo before UVB irradiation	PKA-mediated phosphorylation of CREB|Reactome	0.419004632	-0.04364919	1.997973013	0.07548931	0.19626432	-4.59135309
hetero after UVB irradiation VS hetero before UVB irradiation	PKA-mediated phosphorylation of CREB|Reactome	0.272898414	0.032202078	1.309315205	0.22500179	0.44725607	-5.50249808
WT after UVB irradiation VS WT before UVB irradiation	PKA-mediated phosphorylation of CREB|Reactome	0.376753683	-0.05618843	1.432068944	0.18238839	0.59839062	-4.93064854
homo after UVB irradiation VS homo before UVB irradiation	PKA-mediated phosphorylation of key metabolic factors|Reactome	0.940895389	-0.04098315	4.630707592	0.00110454	0.01917617	-0.52309826
hetero after UVB irradiation VS hetero before UVB irradiation	PKA-mediated phosphorylation of key metabolic factors|Reactome	0.231725916	0.003371824	0.640111845	0.53911251	0.75123046	-6.12965021
WT after UVB irradiation VS WT before UVB irradiation	PKA-mediated phosphorylation of key metabolic factors|Reactome	0.586321395	0.010929589	1.717004044	0.11648603	0.51750165	-4.57315433
homo after UVB irradiation VS homo before UVB irradiation	PKB-mediated events|Reactome	1.034153104	0.064792843	4.442289607	0.00145843	0.0217621	-0.79376996
hetero after UVB irradiation VS hetero before UVB irradiation	PKB-mediated events|Reactome	0.544751551	0.058897515	1.619313171	0.14212762	0.34495084	-5.11354453
WT after UVB irradiation VS WT before UVB irradiation	PKB-mediated events|Reactome	0.334156357	-0.04402326	1.079631058	0.30545633	0.69096189	-5.31283877
homo after UVB irradiation VS homo before UVB irradiation	pkc-catalyzed phosphorylation of inhibitory phosphoprotein of myosin phosphatase|BioCarta	0.62333592	-0.02494567	3.471155144	0.00660067	0.04701822	-2.26837007
hetero after UVB irradiation VS hetero before UVB irradiation	pkc-catalyzed phosphorylation of inhibitory phosphoprotein of myosin phosphatase|BioCarta	0.43991357	0.04820373	2.485906671	0.03633946	0.16681566	-3.85690048
WT after UVB irradiation VS WT before UVB irradiation	pkc-catalyzed phosphorylation of inhibitory phosphoprotein of myosin phosphatase|BioCarta	0.17293105	-0.05893425	0.728868325	0.48266947	0.81552033	-5.60740787
homo after UVB irradiation VS homo before UVB irradiation	PKMTs methylate histone lysines|Reactome	-0.43770375	-0.02137212	-1.89964229	0.08858528	0.21308757	-4.73626663
hetero after UVB irradiation VS hetero before UVB irradiation	PKMTs methylate histone lysines|Reactome	-0.23321041	-0.06497991	-1.08988998	0.30594647	0.53552381	-5.74493859
WT after UVB irradiation VS WT before UVB irradiation	PKMTs methylate histone lysines|Reactome	-0.190469	-0.02318273	-0.77339332	0.45703946	0.80891799	-5.57545967
homo after UVB irradiation VS homo before UVB irradiation	Plasma lipoprotein assembly_ remodeling_ and clearance|Reactome	0.590839386	-0.0222557	3.708431779	0.00451363	0.03837847	-1.89729469
hetero after UVB irradiation VS hetero before UVB irradiation	Plasma lipoprotein assembly_ remodeling_ and clearance|Reactome	0.39942917	0.038314664	1.810941527	0.10584526	0.29415277	-4.8521145
WT after UVB irradiation VS WT before UVB irradiation	Plasma lipoprotein assembly_ remodeling_ and clearance|Reactome	0.435128881	-0.00624422	1.835284072	0.09609585	0.49311206	-4.41493403
homo after UVB irradiation VS homo before UVB irradiation	Plasma lipoprotein assembly_ remodeling_ and clearance|Wikipathways	0.73652224	0.060536804	2.466609523	0.0347387	0.1243079	-3.86954737
hetero after UVB irradiation VS hetero before UVB irradiation	Plasma lipoprotein assembly_ remodeling_ and clearance|Wikipathways	0.017238805	0.053033008	0.088950287	0.93119946	0.97054702	-6.34225106
WT after UVB irradiation VS WT before UVB irradiation	Plasma lipoprotein assembly_ remodeling_ and clearance|Wikipathways	0.502919894	0.065259593	1.705598443	0.11864701	0.52273774	-4.58813841
homo after UVB irradiation VS homo before UVB irradiation	Plasma lipoprotein assembly|Reactome	0.674088502	0.018531444	3.900315162	0.00333589	0.03256113	-1.6017798
hetero after UVB irradiation VS hetero before UVB irradiation	Plasma lipoprotein assembly|Reactome	0.529102402	0.029600965	2.341606514	0.04574682	0.18854754	-4.07572966
WT after UVB irradiation VS WT before UVB irradiation	Plasma lipoprotein assembly|Reactome	0.521012652	0.039388701	1.920838176	0.08345467	0.470709	-4.29755424
homo after UVB irradiation VS homo before UVB irradiation	Plasma lipoprotein clearance|Reactome	0.645279404	-0.01951988	3.749616465	0.00422836	0.03741603	-1.83349735
hetero after UVB irradiation VS hetero before UVB irradiation	Plasma lipoprotein clearance|Reactome	0.53228385	0.062100139	2.756356086	0.02363306	0.13628011	-3.44357902
WT after UVB irradiation VS WT before UVB irradiation	Plasma lipoprotein clearance|Reactome	0.622640396	0.027260934	2.8477369	0.01718458	0.30353778	-2.93694624
homo after UVB irradiation VS homo before UVB irradiation	Plasma lipoprotein remodeling|Reactome	0.329112786	-0.01442558	1.767705795	0.10951681	0.24271071	-4.92569603
hetero after UVB irradiation VS hetero before UVB irradiation	Plasma lipoprotein remodeling|Reactome	0.116719847	0.021846013	0.529826906	0.60990536	0.79700362	-6.19681009
WT after UVB irradiation VS WT before UVB irradiation	Plasma lipoprotein remodeling|Reactome	0.245978669	-0.00718907	1.091157298	0.30059295	0.68537595	-5.3015922
homo after UVB irradiation VS homo before UVB irradiation	Plasma membrane estrogen receptor signaling|PID	0.400762974	-0.04373535	2.400929067	0.03876233	0.12947243	-3.97299353
hetero after UVB irradiation VS hetero before UVB irradiation	Plasma membrane estrogen receptor signaling|PID	0.521150543	0.042673171	2.534607417	0.03362427	0.16031318	-3.78268379
WT after UVB irradiation VS WT before UVB irradiation	Plasma membrane estrogen receptor signaling|PID	0.490279844	-0.01526997	2.390126277	0.0377612	0.39022568	-3.62199995
homo after UVB irradiation VS homo before UVB irradiation	Plasmalogen biosynthesis|Reactome	-0.87623029	0.031902035	-3.41799912	0.00719353	0.04931566	-2.3522362
hetero after UVB irradiation VS hetero before UVB irradiation	Plasmalogen biosynthesis|Reactome	-0.43644009	0.028640986	-1.44081151	0.1857439	0.40321767	-5.34339661
WT after UVB irradiation VS WT before UVB irradiation	Plasmalogen biosynthesis|Reactome	-0.41898085	0.068134468	-1.25631747	0.23733014	0.63195092	-5.13071254
homo after UVB irradiation VS homo before UVB irradiation	Plasmalogen Synthesis|SMPDB	-0.34088506	-0.01706757	-1.38547601	0.19793201	0.35399825	-5.43268672
hetero after UVB irradiation VS hetero before UVB irradiation	Plasmalogen Synthesis|SMPDB	0.052360829	-0.03251061	0.156193791	0.87955793	0.95657959	-6.33332599
WT after UVB irradiation VS WT before UVB irradiation	Plasmalogen Synthesis|SMPDB	0.151987418	0.009711208	0.52813952	0.60882982	0.87257466	-5.72991497
homo after UVB irradiation VS homo before UVB irradiation	Platelet activation - Homo sapiens (human)|KEGG	0.522981376	-0.03450144	3.076928653	0.0125727	0.06924841	-2.89501114
hetero after UVB irradiation VS hetero before UVB irradiation	Platelet activation - Homo sapiens (human)|KEGG	0.486357481	0.045227119	2.576128947	0.03147151	0.15507377	-3.71930507
WT after UVB irradiation VS WT before UVB irradiation	Platelet activation - Homo sapiens (human)|KEGG	0.405561016	0.009875165	1.823208826	0.09801631	0.49731716	-4.431312
homo after UVB irradiation VS homo before UVB irradiation	Platelet activation_ signaling and aggregation|Reactome	0.542135851	-0.01448051	3.335442046	0.00822638	0.05373627	-2.4829434
hetero after UVB irradiation VS hetero before UVB irradiation	Platelet activation_ signaling and aggregation|Reactome	0.480497429	0.034647053	2.685806379	0.0264325	0.14142055	-3.55158447
WT after UVB irradiation VS WT before UVB irradiation	Platelet activation_ signaling and aggregation|Reactome	0.353471328	0.002556221	1.660142819	0.12762316	0.53719353	-4.64734395
homo after UVB irradiation VS homo before UVB irradiation	Platelet Adhesion to exposed collagen|Reactome	0.321018522	-0.00880326	1.3512366	0.20827676	0.36647078	-5.47442259
hetero after UVB irradiation VS hetero before UVB irradiation	Platelet Adhesion to exposed collagen|Reactome	0.513764782	-0.00997589	2.516624925	0.03460218	0.16285121	-3.81010456
WT after UVB irradiation VS WT before UVB irradiation	Platelet Adhesion to exposed collagen|Reactome	0.381552803	-0.00587161	1.413888664	0.18752136	0.59994616	-4.95213842
homo after UVB irradiation VS homo before UVB irradiation	Platelet Aggregation (Plug Formation)|Reactome	0.611572992	-0.02174756	3.652164424	0.0049364	0.04003478	-1.98476381
hetero after UVB irradiation VS hetero before UVB irradiation	Platelet Aggregation (Plug Formation)|Reactome	0.434948099	0.015340293	2.286499096	0.04994894	0.19848935	-4.15873684
WT after UVB irradiation VS WT before UVB irradiation	Platelet Aggregation (Plug Formation)|Reactome	0.350010023	0.003491077	1.602217035	0.13994119	0.55526622	-4.7215524
homo after UVB irradiation VS homo before UVB irradiation	Platelet Aggregation Inhibitor Pathway_ Pharmacodynamics|PharmGKB	0.557938215	-0.02925656	2.943693872	0.01567625	0.07746508	-3.10838259
hetero after UVB irradiation VS hetero before UVB irradiation	Platelet Aggregation Inhibitor Pathway_ Pharmacodynamics|PharmGKB	0.547060662	0.042631169	2.413999572	0.04075678	0.17712536	-3.96617847
WT after UVB irradiation VS WT before UVB irradiation	Platelet Aggregation Inhibitor Pathway_ Pharmacodynamics|PharmGKB	0.395515188	0.039581021	1.515305935	0.1603993	0.56104412	-4.83009812
homo after UVB irradiation VS homo before UVB irradiation	platelet amyloid precursor protein pathway|BioCarta	0.562932475	-0.00948482	3.55977416	0.00572283	0.04391671	-2.12911803
hetero after UVB irradiation VS hetero before UVB irradiation	platelet amyloid precursor protein pathway|BioCarta	1.023475078	0.035865443	6.08208336	0.00023958	0.02988205	1.0078064
WT after UVB irradiation VS WT before UVB irradiation	platelet amyloid precursor protein pathway|BioCarta	0.806998902	-0.0005284	3.237926248	0.00880893	0.24856491	-2.35236147
homo after UVB irradiation VS homo before UVB irradiation	Platelet calcium homeostasis|Reactome	0.328151274	-0.04824981	1.713363313	0.11939696	0.25817499	-5.00183238
hetero after UVB irradiation VS hetero before UVB irradiation	Platelet calcium homeostasis|Reactome	0.167537706	0.020000838	0.730295954	0.48501642	0.70584446	-6.06626256
WT after UVB irradiation VS WT before UVB irradiation	Platelet calcium homeostasis|Reactome	0.144786514	-0.07388374	0.627574699	0.54423343	0.8402851	-5.67372339
homo after UVB irradiation VS homo before UVB irradiation	Platelet degranulation|Reactome	0.554098278	0.001196632	3.432651325	0.00702478	0.04867246	-2.3290949
hetero after UVB irradiation VS hetero before UVB irradiation	Platelet degranulation|Reactome	0.399743983	0.028325588	1.942827904	0.08612495	0.2656005	-4.66511721
WT after UVB irradiation VS WT before UVB irradiation	Platelet degranulation|Reactome	0.363479562	-0.00149053	1.588742751	0.14295379	0.55622203	-4.73860624
homo after UVB irradiation VS homo before UVB irradiation	Platelet homeostasis|Reactome	0.330685104	-0.01672148	2.079307691	0.06606683	0.17862118	-4.46940517
hetero after UVB irradiation VS hetero before UVB irradiation	Platelet homeostasis|Reactome	0.333911989	0.009518929	1.78849159	0.10959969	0.3013	-4.88341745
WT after UVB irradiation VS WT before UVB irradiation	Platelet homeostasis|Reactome	0.166952801	-0.04524802	0.69744043	0.50129321	0.82175381	-5.62894048
homo after UVB irradiation VS homo before UVB irradiation	Platelet sensitization by LDL|Reactome	-0.13787232	0.005332682	-0.72664217	0.48516578	0.62737665	-6.09265703
hetero after UVB irradiation VS hetero before UVB irradiation	Platelet sensitization by LDL|Reactome	0.175411196	0.016690411	1.026675552	0.33314146	0.56114515	-5.8087463
WT after UVB irradiation VS WT before UVB irradiation	Platelet sensitization by LDL|Reactome	0.056001042	0.051433418	0.271644375	0.7913767	0.9410906	-5.83191592
homo after UVB irradiation VS homo before UVB irradiation	Platelet-mediated interactions with vascular and circulating cells|Wikipathways	0.833022193	-0.02790618	4.447602741	0.00144695	0.0217621	-0.78606194
hetero after UVB irradiation VS hetero before UVB irradiation	Platelet-mediated interactions with vascular and circulating cells|Wikipathways	0.765988989	0.04009359	3.788777399	0.00483767	0.07419102	-1.89474959
WT after UVB irradiation VS WT before UVB irradiation	Platelet-mediated interactions with vascular and circulating cells|Wikipathways	0.425326203	-0.02542769	1.171134464	0.26847903	0.66238094	-5.22106514
homo after UVB irradiation VS homo before UVB irradiation	Platinum Pathway_ Pharmacokinetics/Pharmacodynamics|PharmGKB	0.479628152	0.009599649	2.606437086	0.02750276	0.11028351	-3.64779669
hetero after UVB irradiation VS hetero before UVB irradiation	Platinum Pathway_ Pharmacokinetics/Pharmacodynamics|PharmGKB	0.202014277	0.007591721	1.085864074	0.30762504	0.53619691	-5.74908775
WT after UVB irradiation VS WT before UVB irradiation	Platinum Pathway_ Pharmacokinetics/Pharmacodynamics|PharmGKB	0.324369914	0.008664904	1.429523114	0.18309987	0.59839062	-4.93366829
homo after UVB irradiation VS homo before UVB irradiation	PLC beta mediated events|Reactome	0.528333248	-0.04235665	3.103726103	0.01202877	0.0671005	-2.85214952
hetero after UVB irradiation VS hetero before UVB irradiation	PLC beta mediated events|Reactome	0.350909987	0.014149498	1.754582148	0.11550721	0.30887493	-4.93037983
WT after UVB irradiation VS WT before UVB irradiation	PLC beta mediated events|Reactome	0.286256758	-0.01140742	1.194461164	0.25964111	0.65440449	-5.19678063
homo after UVB irradiation VS homo before UVB irradiation	PLCG1 events in ERBB2 signaling|Reactome	-0.19759745	0.062700711	-0.88252076	0.39952539	0.55129433	-5.96689674
hetero after UVB irradiation VS hetero before UVB irradiation	PLCG1 events in ERBB2 signaling|Reactome	-0.57824604	0.035856098	-2.75160661	0.02381164	0.13652004	-3.45085084
WT after UVB irradiation VS WT before UVB irradiation	PLCG1 events in ERBB2 signaling|Reactome	-0.48749159	0.062965883	-1.89897886	0.08652868	0.47656734	-4.32776092
homo after UVB irradiation VS homo before UVB irradiation	PLC-gamma1 signalling|Reactome	0.619794756	0.112458966	2.771130862	0.02089106	0.09232406	-3.38482532
hetero after UVB irradiation VS hetero before UVB irradiation	PLC-gamma1 signalling|Reactome	0.183348027	-0.02667191	0.934493809	0.37605279	0.60404776	-5.89645641
WT after UVB irradiation VS WT before UVB irradiation	PLC-gamma1 signalling|Reactome	0.389392889	0.156202611	1.095344466	0.29884103	0.68477511	-5.29748384
homo after UVB irradiation VS homo before UVB irradiation	Plexin-D1 Signaling|PID	0.510868673	-0.0269756	3.234700317	0.00969838	0.0585513	-2.64309421
hetero after UVB irradiation VS hetero before UVB irradiation	Plexin-D1 Signaling|PID	0.582941138	-0.00584267	3.408963769	0.00855107	0.09302099	-2.45325682
WT after UVB irradiation VS WT before UVB irradiation	Plexin-D1 Signaling|PID	0.36459278	0.019111892	1.455517769	0.17594577	0.58755921	-4.90267635

homo after UVB irradiation VS homo before UVB irradiation	PLK1 signaling events|PID	-0.60665122	0.015296543	-3.24412898	0.00954972	0.05835911	-2.62807833
hetero after UVB irradiation VS hetero before UVB irradiation	PLK1 signaling events|PID	0.132337138	0.003474215	0.439109023	0.67162597	0.82819347	-6.24315332
WT after UVB irradiation VS WT before UVB irradiation	PLK1 signaling events|PID	-0.01538157	0.003406824	-0.05342237	0.95843911	0.98785634	-5.86770561
homo after UVB irradiation VS homo before UVB irradiation	PLK3 signaling events|PID	-0.54564566	-0.09155762	-1.75659792	0.1114732	0.24553141	-4.94135351
hetero after UVB irradiation VS hetero before UVB irradiation	PLK3 signaling events|PID	0.194587842	0.043814755	0.571834103	0.58236442	0.77501227	-6.17260467
WT after UVB irradiation VS WT before UVB irradiation	PLK3 signaling events|PID	0.125668816	0.069369626	0.386838735	0.70691499	0.90155844	-5.79394268
homo after UVB irradiation VS homo before UVB irradiation	Plus-strand DNA synthesis|Reactome	-0.08403744	-0.00320991	-0.2001813	0.84560766	0.90528595	-6.34696415
hetero after UVB irradiation VS hetero before UVB irradiation	Plus-strand DNA synthesis|Reactome	0.471357887	-0.00478231	1.150987769	0.28134999	0.51114365	-5.68058898
WT after UVB irradiation VS WT before UVB irradiation	Plus-strand DNA synthesis|Reactome	0.926001949	0.004188178	3.205753029	0.00930482	0.24856491	-2.40025129
homo after UVB irradiation VS homo before UVB irradiation	POLB-Dependent Long Patch Base Excision Repair|Reactome	-0.47433157	0.025594269	-1.8844712	0.09078679	0.21591872	-4.75835567
hetero after UVB irradiation VS hetero before UVB irradiation	POLB-Dependent Long Patch Base Excision Repair|Reactome	-0.10739926	-0.05097799	-0.37059945	0.72007994	0.86371694	-6.27265154
WT after UVB irradiation VS WT before UVB irradiation	POLB-Dependent Long Patch Base Excision Repair|Reactome	0.162107327	0.091081173	0.442682323	0.66734184	0.87935805	-5.77090171
homo after UVB irradiation VS homo before UVB irradiation	Polo-like kinase mediated events|Reactome	-0.84465054	0.00882411	-4.59953441	0.00115611	0.01955108	-0.5674969
hetero after UVB irradiation VS hetero before UVB irradiation	Polo-like kinase mediated events|Reactome	-0.09619793	-0.03483264	-0.3454047	0.73825538	0.87265768	-6.28228671
WT after UVB irradiation VS WT before UVB irradiation	Polo-like kinase mediated events|Reactome	-0.30640482	0.004316155	-0.93293841	0.37265474	0.7494118	-5.44762852
homo after UVB irradiation VS homo before UVB irradiation	polyadenylation of mrna|BioCarta	-0.96935093	-0.00363657	-5.23817673	0.00046686	0.01666204	0.31094374
hetero after UVB irradiation VS hetero before UVB irradiation	polyadenylation of mrna|BioCarta	-0.39567985	-0.05808539	-1.10962985	0.29782017	0.52943227	-5.72442971
WT after UVB irradiation VS WT before UVB irradiation	polyadenylation of mrna|BioCarta	-0.46514708	-0.04744544	-1.27645854	0.23041109	0.62952955	-5.10869045
homo after UVB irradiation VS homo before UVB irradiation	Polycystic Kidney Disease Pathway|Wikipathways	0.141345489	-0.0207031	0.702344583	0.49950467	0.63924743	-6.11032287
hetero after UVB irradiation VS hetero before UVB irradiation	Polycystic Kidney Disease Pathway|Wikipathways	-0.0396183	0.001943979	-0.18616397	0.85672031	0.94503144	-6.32777972
WT after UVB irradiation VS WT before UVB irradiation	Polycystic Kidney Disease Pathway|Wikipathways	-0.073623	-0.01023876	-0.38521112	0.70808297	0.90229211	-5.79456927
homo after UVB irradiation VS homo before UVB irradiation	Polymerase switching on the C-strand of the telomere|Reactome	-0.44537839	-0.0092232	-1.56961564	0.14955393	0.29522837	-5.19718062
hetero after UVB irradiation VS hetero before UVB irradiation	Polymerase switching on the C-strand of the telomere|Reactome	0.368900781	-0.00233809	1.325603217	0.2197829	0.44126832	-5.48331134
WT after UVB irradiation VS WT before UVB irradiation	Polymerase switching on the C-strand of the telomere|Reactome	0.040265502	0.012547595	0.134706475	0.89549522	0.97566251	-5.85996749
homo after UVB irradiation VS homo before UVB irradiation	Polymerase switching|Reactome	-0.44537839	-0.0092232	-1.56961564	0.14955393	0.29522837	-5.19718062
hetero after UVB irradiation VS hetero before UVB irradiation	Polymerase switching|Reactome	0.368900781	-0.00233809	1.325603217	0.2197829	0.44126832	-5.48331134
WT after UVB irradiation VS WT before UVB irradiation	Polymerase switching|Reactome	0.040265502	0.012547595	0.134706475	0.89549522	0.97566251	-5.85996749
homo after UVB irradiation VS homo before UVB irradiation	Polyol Pathway|Wikipathways	0.939493048	-0.00640464	5.434008937	0.0003577	0.01666204	0.567032019
hetero after UVB irradiation VS hetero before UVB irradiation	Polyol Pathway|Wikipathways	0.641804824	-0.05318533	2.057842	0.071837	0.23970209	-4.49828023
WT after UVB irradiation VS WT before UVB irradiation	Polyol Pathway|Wikipathways	0.529935956	0.08908897	1.679640518	0.12370069	0.52904005	-4.62205004
homo after UVB irradiation VS homo before UVB irradiation	Polythiazide Action Pathway|SMPDB	0.234726031	0.017120546	1.156747303	0.27595732	0.43614438	-5.69767199
hetero after UVB irradiation VS hetero before UVB irradiation	Polythiazide Action Pathway|SMPDB	-0.03255037	0.028925632	-0.18154472	0.86023155	0.94503144	-6.32869738
WT after UVB irradiation VS WT before UVB irradiation	Polythiazide Action Pathway|SMPDB	-0.15908359	0.010252305	-0.80382265	0.44003996	0.79685269	-5.55266936
homo after UVB irradiation VS homo before UVB irradiation	Porphyria Variegata (PV)|SMPDB	0.388154593	-0.02664649	1.768839848	0.10931886	0.24265581	-4.92409481
hetero after UVB irradiation VS hetero before UVB irradiation	Porphyria Variegata (PV)|SMPDB	0.451496919	0.003377552	2.70023395	0.02583369	0.14017854	-3.52950073
WT after UVB irradiation VS WT before UVB irradiation	Porphyria Variegata (PV)|SMPDB	0.381835405	-0.00693641	1.754704281	0.10959456	0.5076251	-4.52327279
homo after UVB irradiation VS homo before UVB irradiation	Porphyrin and chlorophyll metabolism - Homo sapiens (human)|KEGG	-0.04634592	0.039305712	-0.20803639	0.83964335	0.9020733	-6.34524907
hetero after UVB irradiation VS hetero before UVB irradiation	Porphyrin and chlorophyll metabolism - Homo sapiens (human)|KEGG	0.145633775	0.015018632	0.941256008	0.37277235	0.60145004	-5.89024524
WT after UVB irradiation VS WT before UVB irradiation	Porphyrin and chlorophyll metabolism - Homo sapiens (human)|KEGG	0.224674421	-0.00352974	1.067973632	0.31043609	0.6940288	-5.32411909
homo after UVB irradiation VS homo before UVB irradiation	Porphyrin metabolism|EHMN	0.130466171	0.062928215	0.706054943	0.49729818	0.63711097	-6.10765999
hetero after UVB irradiation VS hetero before UVB irradiation	Porphyrin metabolism|EHMN	0.224466525	0.069912911	1.323552594	0.22043424	0.44184836	-5.48573532
WT after UVB irradiation VS WT before UVB irradiation	Porphyrin metabolism|EHMN	0.414489172	0.040101063	1.899285737	0.0864848	0.47656734	-4.32733785
homo after UVB irradiation VS homo before UVB irradiation	Porphyrin metabolism|INOH	0.220017501	-0.01540177	0.953403171	0.36428454	0.51814121	-5.90290567
hetero after UVB irradiation VS hetero before UVB irradiation	Porphyrin metabolism|INOH	-0.05837879	0.02386681	-0.20345964	0.84360381	0.94128426	-6.32414086
WT after UVB irradiation VS WT before UVB irradiation	Porphyrin metabolism|INOH	0.108743318	-0.02292216	0.385275802	0.70803654	0.90229211	-5.79454442
homo after UVB irradiation VS homo before UVB irradiation	Porphyrin Metabolism|SMPDB	0.388154593	-0.02664649	1.768839848	0.10931886	0.24265581	-4.92409481
hetero after UVB irradiation VS hetero before UVB irradiation	Porphyrin Metabolism|SMPDB	0.451496919	0.003377552	2.70023395	0.02583369	0.14017854	-3.52950073
WT after UVB irradiation VS WT before UVB irradiation	Porphyrin Metabolism|SMPDB	0.381835405	-0.00693641	1.754704281	0.10959456	0.5076251	-4.52327279
homo after UVB irradiation VS homo before UVB irradiation	Positive epigenetic regulation of rRNA expression|Reactome	-0.70425583	0.01175774	-4.20204823	0.00209403	0.02552962	-1.14674674
hetero after UVB irradiation VS hetero before UVB irradiation	Positive epigenetic regulation of rRNA expression|Reactome	-0.41004044	-0.04272323	-1.68583334	0.1284023	0.3229899	-5.02434361
WT after UVB irradiation VS WT before UVB irradiation	Positive epigenetic regulation of rRNA expression|Reactome	-0.35316178	-0.00588924	-1.31108685	0.21890275	0.6260768	-5.07026128
homo after UVB irradiation VS homo before UVB irradiation	Post NMDA receptor activation events|Reactome	0.133188896	-0.01429676	0.745929856	0.47397035	0.61754234	-6.0782533
hetero after UVB irradiation VS hetero before UVB irradiation	Post NMDA receptor activation events|Reactome	-0.07828702	-0.00786036	-0.4092893	0.69253449	0.84535945	-6.25658198
WT after UVB irradiation VS WT before UVB irradiation	Post NMDA receptor activation events|Reactome	0.056959408	-0.04059294	0.246208699	0.81046077	0.94525798	-5.83854113
homo after UVB irradiation VS homo before UVB irradiation	Post-chaperonin tubulin folding pathway|Reactome	-0.24914617	0.039648772	-1.03456202	0.32678365	0.48303468	-5.82471792
hetero after UVB irradiation VS hetero before UVB irradiation	Post-chaperonin tubulin folding pathway|Reactome	-0.05012874	-0.01177839	-0.18922693	0.85439391	0.94503144	-6.32715863
WT after UVB irradiation VS WT before UVB irradiation	Post-chaperonin tubulin folding pathway|Reactome	-0.15520959	-0.04283389	-0.5831407	0.57261051	0.8554505	-5.69994502
homo after UVB irradiation VS homo before UVB irradiation	Postsynaptic nicotinic acetylcholine receptors|Reactome	0.106608129	0.018460278	0.48363748	0.63972296	0.75031467	-6.24435373
hetero after UVB irradiation VS hetero before UVB irradiation	Postsynaptic nicotinic acetylcholine receptors|Reactome	-0.39673811	-0.00107584	-2.11855793	0.06524756	0.22947406	-4.40902394
WT after UVB irradiation VS WT before UVB irradiation	Postsynaptic nicotinic acetylcholine receptors|Reactome	-0.66670677	-0.00479849	-3.09744745	0.01119442	0.26766971	-2.56198852
homo after UVB irradiation VS homo before UVB irradiation	Post-transcriptional silencing by small RNAs|Reactome	-0.19572451	0.002241391	-0.63885633	0.53818708	0.67306551	-6.15391915
hetero after UVB irradiation VS hetero before UVB irradiation	Post-transcriptional silencing by small RNAs|Reactome	-0.48970935	-0.027321	-2.59501086	0.03053919	0.15229502	-3.69045796
WT after UVB irradiation VS WT before UVB irradiation	Post-transcriptional silencing by small RNAs|Reactome	-0.36531002	0.017268104	-1.13792646	0.28146973	0.66986569	-5.25502263
homo after UVB irradiation VS homo before UVB irradiation	Post-translational modification: synthesis of GPI-anchored proteins|Reactome	0.061420418	-0.00897083	0.369481178	0.71996501	0.81592461	-6.29564048
hetero after UVB irradiation VS hetero before UVB irradiation	Post-translational modification: synthesis of GPI-anchored proteins|Reactome	-0.04295062	0.013874603	-0.25184501	0.80719521	0.92095935	-6.31226573
WT after UVB irradiation VS WT before UVB irradiation	Post-translational modification: synthesis of GPI-anchored proteins|Reactome	0.0260703	0.046605475	0.129653254	0.89939096	0.97566251	-5.86064298
homo after UVB irradiation VS homo before UVB irradiation	Post-translational protein modification|Reactome	0.08604161	-0.01666556	0.538137387	0.60300333	0.72227524	-6.21526771
hetero after UVB irradiation VS hetero before UVB irradiation	Post-translational protein modification|Reactome	0.132659403	-0.00808945	0.894486101	0.39589268	0.62309352	-5.93245953
WT after UVB irradiation VS WT before UVB irradiation	Post-translational protein modification|Reactome	0.101434214	-0.00952775	0.514436187	0.61803454	0.87257466	-5.73694777
homo after UVB irradiation VS homo before UVB irradiation	Post-translational protein phosphorylation|Reactome	0.621082888	0.03141203	3.53013736	0.00600199	0.0452977	-2.17560565
hetero after UVB irradiation VS hetero before UVB irradiation	Post-translational protein phosphorylation|Reactome	0.341373414	0.043364907	1.743857043	0.11743655	0.31030895	-4.94515094
WT after UVB irradiation VS WT before UVB irradiation	Post-translational protein phosphorylation|Reactome	0.349219488	0.030433312	1.506136061	0.16270339	0.56636157	-4.84134403
homo after UVB irradiation VS homo before UVB irradiation	Posttranslational regulation of adherens junction stability and dissassembly|PID	0.318552887	-0.0076382	1.730645698	0.11616971	0.25403779	-4.97774673
hetero after UVB irradiation VS hetero before UVB irradiation	Posttranslational regulation of adherens junction stability and dissassembly|PID	0.28920002	-7.86E-06	1.754103036	0.11559277	0.30887493	-4.93104054
WT after UVB irradiation VS WT before UVB irradiation	Posttranslational regulation of adherens junction stability and dissassembly|PID	0.0884775	0.001584083	0.419184535	0.68387234	0.88829493	-5.78096109
homo after UVB irradiation VS homo before UVB irradiation	Potassium Channels|Reactome	0.230760734	0.002262123	1.236382132	0.24635064	0.40873472	-5.6092223
hetero after UVB irradiation VS hetero before UVB irradiation	Potassium Channels|Reactome	0.032573444	-0.00083268	0.19445107	0.8504295	0.94503144	-6.32607613
WT after UVB irradiation VS WT before UVB irradiation	Potassium Channels|Reactome	-0.04754344	0.010144546	-0.19692627	0.8477984	0.95372763	-5.84954606
homo after UVB irradiation VS homo before UVB irradiation	Potassium transport channels|Reactome	-0.12369628	0.13311311	-0.43779373	0.6714344	0.77653736	-6.26653315
hetero after UVB irradiation VS hetero before UVB irradiation	Potassium transport channels|Reactome	-0.42658286	-0.15955126	-1.74744119	0.11678849	0.3094895	-4.94021918
WT after UVB irradiation VS WT before UVB irradiation	Potassium transport channels|Reactome	-0.51316979	-0.018556	-1.76051341	0.10856628	0.5076251	-4.51553955
homo after UVB irradiation VS homo before UVB irradiation	POU5F1 (OCT4)_ SOX2_ NANOG activate genes related to proliferation|Reactome	0.219444788	0.038673367	1.187768406	0.26410183	0.42746861	-5.66372692
hetero after UVB irradiation VS hetero before UVB irradiation	POU5F1 (OCT4)_ SOX2_ NANOG activate genes related to proliferation|Reactome	0.209291262	0.103491785	1.10117462	0.30127981	0.53143706	-5.73324769
WT after UVB irradiation VS WT before UVB irradiation	POU5F1 (OCT4)_ SOX2_ NANOG activate genes related to proliferation|Reactome	-0.28272773	-0.1062228	-1.22069683	0.24998044	0.64318912	-5.16905128
homo after UVB irradiation VS homo before UVB irradiation	POU5F1 (OCT4)_ SOX2_ NANOG activate genes related to proliferation|Wikipathways	0.291497853	-0.02100329	1.299885013	0.2246443	0.38416699	-5.53570661
hetero after UVB irradiation VS hetero before UVB irradiation	POU5F1 (OCT4)_ SOX2_ NANOG activate genes related to proliferation|Wikipathways	0.224920317	0.079546673	0.919483325	0.38340994	0.61104284	-5.91011443
WT after UVB irradiation VS WT before UVB irradiation	POU5F1 (OCT4)_ SOX2_ NANOG activate genes related to proliferation|Wikipathways	-0.22189457	-0.08989763	-0.95687703	0.36101101	0.74374432	-5.42671992
homo after UVB irradiation VS homo before UVB irradiation	POU5F1 (OCT4)_ SOX2_ NANOG repress genes related to differentiation|Reactome	0.19729829	0.097148151	0.852021879	0.41540464	0.56635939	-5.99315181
hetero after UVB irradiation VS hetero before UVB irradiation	POU5F1 (OCT4)_ SOX2_ NANOG repress genes related to differentiation|Reactome	0.256000189	0.080000883	1.380922802	0.20282127	0.42313299	-5.41702583
WT after UVB irradiation VS WT before UVB irradiation	POU5F1 (OCT4)_ SOX2_ NANOG repress genes related to differentiation|Reactome	-0.03049409	-0.04908596	-0.11363697	0.91175659	0.97566251	-5.86261385
homo after UVB irradiation VS homo before UVB irradiation	POU5F1 (OCT4)_ SOX2_ NANOG repress genes related to differentiation|Wikipathways	0.246839392	0.094199989	0.948147677	0.36681757	0.52028198	-5.90779017
hetero after UVB irradiation VS hetero before UVB irradiation	POU5F1 (OCT4)_ SOX2_ NANOG repress genes related to differentiation|Wikipathways	0.237558349	0.079578406	0.950157239	0.36848634	0.59623032	-5.88201455
WT after UVB irradiation VS WT before UVB irradiation	POU5F1 (OCT4)_ SOX2_ NANOG repress genes related to differentiation|Wikipathways	0.117787844	-0.00915505	0.406254088	0.69304473	0.89511744	-5.78627126
homo after UVB irradiation VS homo before UVB irradiation	PP2A-mediated dephosphorylation of key metabolic factors|Reactome	-0.08855586	0.015491661	-0.35155055	0.73293602	0.82411885	-6.30248945
hetero after UVB irradiation VS hetero before UVB irradiation	PP2A-mediated dephosphorylation of key metabolic factors|Reactome	-0.33451039	0.024521935	-1.95811042	0.08408019	0.26213234	-4.6431345
WT after UVB irradiation VS WT before UVB irradiation	PP2A-mediated dephosphorylation of key metabolic factors|Reactome	-0.22647563	0.008122753	-0.99921523	0.34106771	0.72584166	-5.38868439
homo after UVB irradiation VS homo before UVB irradiation	PPAR Alpha Pathway|Wikipathways	0.141014391	0.061761976	0.726657401	0.48515688	0.62737665	-6.09264579
hetero after UVB irradiation VS hetero before UVB irradiation	PPAR Alpha Pathway|Wikipathways	0.065310462	0.004260818	0.352132008	0.73338471	0.86906316	-6.27977834
WT after UVB irradiation VS WT before UVB irradiation	PPAR Alpha Pathway|Wikipathways	0.181800172	0.01463223	0.823123255	0.42947717	0.79538615	-5.53781788
homo after UVB irradiation VS homo before UVB irradiation	PPAR signaling pathway - Homo sapiens (human)|KEGG	0.161299455	0.015690982	0.801617178	0.44258827	0.59091566	-6.03480761
hetero after UVB irradiation VS hetero before UVB irradiation	PPAR signaling pathway - Homo sapiens (human)|KEGG	-0.05579962	-0.01771858	-0.25902456	0.8018324	0.916937	-6.3102917
WT after UVB irradiation VS WT before UVB irradiation	PPAR signaling pathway - Homo sapiens (human)|KEGG	-0.1460364	-0.01053625	-0.62168298	0.54794931	0.84342472	-5.67730232
homo after UVB irradiation VS homo before UVB irradiation	PPAR signaling pathway|Wikipathways	0.16678023	0.016660386	0.839062214	0.42228222	0.57351	-6.00407016
hetero after UVB irradiation VS hetero before UVB irradiation	PPAR signaling pathway|Wikipathways	-0.05949868	-0.0276319	-0.27488555	0.79002453	0.91030982	-6.30573738
WT after UVB irradiation VS WT before UVB irradiation	PPAR signaling pathway|Wikipathways	-0.10049478	-0.00861437	-0.41195918	0.68899122	0.89316574	-5.78394811
homo after UVB irradiation VS homo before UVB irradiation	Practolol Action Pathway|SMPDB	0.396415397	-0.03677376	2.343069613	0.04268583	0.13402181	-4.06364635
hetero after UVB irradiation VS hetero before UVB irradiation	Practolol Action Pathway|SMPDB	-0.01555055	-0.01775524	-0.07410913	0.94265284	0.97054702	-6.34356282
WT after UVB irradiation VS WT before UVB irradiation	Practolol Action Pathway|SMPDB	0.056685205	-0.03978418	0.223404833	0.82768199	0.94525798	-5.84393438
homo after UVB irradiation VS homo before UVB irradiation	Prader-Willi and Angelman Syndrome|Wikipathways	-0.05335871	0.070215696	-0.24886432	0.80882428	0.88203765	-6.33528343
hetero after UVB irradiation VS hetero before UVB irradiation	Prader-Willi and Angelman Syndrome|Wikipathways	-0.14313277	0.026600337	-0.77386391	0.46017042	0.68410201	-6.03301628
WT after UVB irradiation VS WT before UVB irradiation	Prader-Willi and Angelman Syndrome|Wikipathways	-0.06153892	0.009892241	-0.28682616	0.78005352	0.93661609	-5.82765627
homo after UVB irradiation VS homo before UVB irradiation	Pravastatin Action Pathway|SMPDB	-0.19230642	-0.01987665	-0.61925406	0.55047761	0.67913558	-6.16661869
hetero after UVB irradiation VS hetero before UVB irradiation	Pravastatin Action Pathway|SMPDB	0.185820603	0.008098609	0.631890782	0.54421801	0.75147406	-6.13505553
WT after UVB irradiation VS WT before UVB irradiation	Pravastatin Action Pathway|SMPDB	-0.14894747	0.045069977	-0.50755372	0.62268399	0.87257466	-5.74041409
homo after UVB irradiation VS homo before UVB irradiation	Pravastatin Pathway_ Pharmacokinetics|PharmGKB	0.638880758	-0.00894597	2.563991739	0.02952393	0.11453378	-3.7152963
hetero after UVB irradiation VS hetero before UVB irradiation	Pravastatin Pathway_ Pharmacokinetics|PharmGKB	0.091701183	0.0524632	0.415777937	0.68796035	0.84157898	-6.25373692
WT after UVB irradiation VS WT before UVB irradiation	Pravastatin Pathway_ Pharmacokinetics|PharmGKB	0.444829502	0.066448422	1.231046572	0.24624989	0.63911291	-5.1579929
homo after UVB irradiation VS homo before UVB irradiation	PRC2 methylates histones and DNA|Reactome	-0.572987	0.034012587	-2.56800879	0.02932645	0.11431817	-3.70891431
hetero after UVB irradiation VS hetero before UVB irradiation	PRC2 methylates histones and DNA|Reactome	-0.09129729	0.006993753	-0.36867753	0.72145999	0.86446232	-6.27340967
WT after UVB irradiation VS WT before UVB irradiation	PRC2 methylates histones and DNA|Reactome	-0.14852649	0.026344987	-0.64760783	0.53170756	0.83483909	-5.66132277
homo after UVB irradiation VS homo before UVB irradiation	Prednisolone Action Pathway|SMPDB	-0.34731654	0.07340037	-1.55760719	0.15235807	0.29908295	-5.21307031
hetero after UVB irradiation VS hetero before UVB irradiation	Prednisolone Action Pathway|SMPDB	-0.74673605	0.080571121	-3.11655194	0.013417	0.11159616	-2.89371067
WT after UVB irradiation VS WT before UVB irradiation	Prednisolone Action Pathway|SMPDB	-0.75860849	0.00885957	-2.47231496	0.032795	0.36981426	-3.4999742
homo after UVB irradiation VS homo before UVB irradiation	Prednisolone Metabolism Pathway|SMPDB	-0.34731654	0.07340037	-1.55760719	0.15235807	0.29908295	-5.21307031
hetero after UVB irradiation VS hetero before UVB irradiation	Prednisolone Metabolism Pathway|SMPDB	-0.74673605	0.080571121	-3.11655194	0.013417	0.11159616	-2.89371067
WT after UVB irradiation VS WT before UVB irradiation	Prednisolone Metabolism Pathway|SMPDB	-0.75860849	0.00885957	-2.47231496	0.032795	0.36981426	-3.4999742
homo after UVB irradiation VS homo before UVB irradiation	Prefoldin mediated transfer of substrate to CCT/TriC|Reactome	-0.59885631	0.031958891	-2.59816909	0.0278853	0.11168796	-3.66095602
hetero after UVB irradiation VS hetero before UVB irradiation	Prefoldin mediated transfer of substrate to CCT/TriC|Reactome	-0.39637588	-0.04297847	-1.60440775	0.14538093	0.34931275	-5.1332853
WT after UVB irradiation VS WT before UVB irradiation	Prefoldin mediated transfer of substrate to CCT/TriC|Reactome	-0.24573313	-0.02467641	-0.82484122	0.42854526	0.79398224	-5.53648116
homo after UVB irradiation VS homo before UVB irradiation	Pregnane X Receptor pathway|Wikipathways	-0.34892995	0.027961282	-1.8907101	0.08987532	0.21498584	-4.74928101
hetero after UVB irradiation VS hetero before UVB irradiation	Pregnane X Receptor pathway|Wikipathways	-0.27671765	0.007421743	-1.74433693	0.11734959	0.31030895	-4.94449088
WT after UVB irradiation VS WT before UVB irradiation	Pregnane X Receptor pathway|Wikipathways	-0.26721896	0.023164375	-1.26017183	0.23599309	0.62952955	-5.12651723
homo after UVB irradiation VS homo before UVB irradiation	pregnenolone biosynthesis|HumanCyc	0.971255799	-0.01147283	4.253634872	0.0019362	0.02442184	-1.07023013
hetero after UVB irradiation VS hetero before UVB irradiation	pregnenolone biosynthesis|HumanCyc	0.670765791	0.016955469	1.835128343	0.10193557	0.28686591	-4.81820855
WT after UVB irradiation VS WT before UVB irradiation	pregnenolone biosynthesis|HumanCyc	0.430967635	-0.04130124	1.174169475	0.2673158	0.66185108	-5.21792547
homo after UVB irradiation VS homo before UVB irradiation	Pregnenolone biosynthesis|Reactome	0.632376986	-0.03868954	3.024610175	0.01370858	0.07305822	-2.97875487
hetero after UVB irradiation VS hetero before UVB irradiation	Pregnenolone biosynthesis|Reactome	0.559299592	0.018621515	2.443296202	0.0388955	0.17178846	-3.92170528
WT after UVB irradiation VS WT before UVB irradiation	Pregnenolone biosynthesis|Reactome	0.401944973	0.02848179	1.588618789	0.14298177	0.55622203	-4.73876276
homo after UVB irradiation VS homo before UVB irradiation	Preimplantation Embryo|Wikipathways	0.022485414	0.036832998	0.124563571	0.90349528	0.94272383	-6.36011845
hetero after UVB irradiation VS hetero before UVB irradiation	Preimplantation Embryo|Wikipathways	0.072362831	0.059635869	0.409878596	0.69211851	0.84508474	-6.25632536
WT after UVB irradiation VS WT before UVB irradiation	Preimplantation Embryo|Wikipathways	-0.02193198	0.025644808	-0.11254349	0.91260177	0.97566251	-5.86273897
homo after UVB irradiation VS homo before UVB irradiation	Pre-NOTCH Expression and Processing|Reactome	-0.15927814	-0.01789657	-0.76333365	0.4640117	0.6088463	-6.06497026
hetero after UVB irradiation VS hetero before UVB irradiation	Pre-NOTCH Expression and Processing|Reactome	0.018468808	-0.00716657	0.123731388	0.90442883	0.97054702	-6.33824374
WT after UVB irradiation VS WT before UVB irradiation	Pre-NOTCH Expression and Processing|Reactome	0.063350329	-0.01661733	0.284333815	0.7819088	0.93661609	-5.82837118
homo after UVB irradiation VS homo before UVB irradiation	Pre-NOTCH Expression and Processing|Wikipathways	-0.41867164	0.046067181	-1.24677937	0.24268315	0.40426533	-5.59736257
hetero after UVB irradiation VS hetero before UVB irradiation	Pre-NOTCH Expression and Processing|Wikipathways	-0.01141629	-0.01956071	-0.03847722	0.97020329	0.98614433	-6.34573821
WT after UVB irradiation VS WT before UVB irradiation	Pre-NOTCH Expression and Processing|Wikipathways	-0.12549439	0.086545313	-0.3403624	0.74056887	0.91734632	-5.81082456
homo after UVB irradiation VS homo before UVB irradiation	Pre-NOTCH Processing in Golgi|Reactome	0.44849019	-0.05857057	2.130612806	0.06071395	0.16831499	-4.39161966

hetero after UVB irradiation VS hetero before UVB irradiation	Pre-NOTCH Processing in Golgi|Reactome	0.538291007	0.043782065	2.802513032	0.02196736	0.13174638	-3.37291343
WT after UVB irradiation VS WT before UVB irradiation	Pre-NOTCH Processing in Golgi|Reactome	0.578833106	-0.02658791	2.96578724	0.0140282	0.28307166	-2.75944551
homo after UVB irradiation VS homo before UVB irradiation	Pre-NOTCH Processing in the Endoplasmic Reticulum|Reactome	-0.03986578	0.0166398	-0.14129301	0.89062369	0.93527778	-6.35773335
hetero after UVB irradiation VS hetero before UVB irradiation	Pre-NOTCH Processing in the Endoplasmic Reticulum|Reactome	-0.05256995	-0.00551327	-0.27990657	0.78629831	0.90801817	-6.30424028
WT after UVB irradiation VS WT before UVB irradiation	Pre-NOTCH Processing in the Endoplasmic Reticulum|Reactome	0.038446691	-0.00300993	0.138647076	0.89245926	0.97397725	-5.85942289
homo after UVB irradiation VS homo before UVB irradiation	Pre-NOTCH Transcription and Translation|Reactome	-0.40210404	0.008597681	-1.70476801	0.12103228	0.26012759	-5.01376577
hetero after UVB irradiation VS hetero before UVB irradiation	Pre-NOTCH Transcription and Translation|Reactome	-0.12440882	-0.00862319	-0.69916139	0.50329093	0.72148515	-6.08898451
WT after UVB irradiation VS WT before UVB irradiation	Pre-NOTCH Transcription and Translation|Reactome	-0.1690708	-0.0228188	-0.68281726	0.51010678	0.82655837	-5.63866825
homo after UVB irradiation VS homo before UVB irradiation	Presenilin action in Notch and Wnt signaling|PID	-0.29199371	0.004256139	-1.70384852	0.12120842	0.26019636	-5.01504056
hetero after UVB irradiation VS hetero before UVB irradiation	Presenilin action in Notch and Wnt signaling|PID	0.041748746	-0.01750002	0.2571311	0.80324567	0.91759242	-6.31081761
WT after UVB irradiation VS WT before UVB irradiation	Presenilin action in Notch and Wnt signaling|PID	-0.08867448	0.044683752	-0.36351727	0.72372513	0.90882588	-5.80267608
homo after UVB irradiation VS homo before UVB irradiation	Presynaptic depolarization and calcium channel opening|Reactome	-0.18282701	0.037881629	-0.86083304	0.41077292	0.56240743	-5.98564712
hetero after UVB irradiation VS hetero before UVB irradiation	Presynaptic depolarization and calcium channel opening|Reactome	-0.41367645	0.008577873	-2.25533434	0.05249239	0.20350804	-4.20550869
WT after UVB irradiation VS WT before UVB irradiation	Presynaptic depolarization and calcium channel opening|Reactome	-0.37185139	-0.00559903	-1.67599855	0.12442504	0.52904005	-4.62678642
homo after UVB irradiation VS homo before UVB irradiation	Presynaptic function of Kainate receptors|Reactome	0.43728693	0.007259109	1.945670345	0.0822088	0.20345008	-4.66879904
hetero after UVB irradiation VS hetero before UVB irradiation	Presynaptic function of Kainate receptors|Reactome	0.637381978	0.0250606	3.212510923	0.01156134	0.10542662	-2.7483671
WT after UVB irradiation VS WT before UVB irradiation	Presynaptic function of Kainate receptors|Reactome	0.232663706	-0.03703901	0.947601437	0.36549109	0.74545864	-5.4348732
homo after UVB irradiation VS homo before UVB irradiation	Presynaptic nicotinic acetylcholine receptors|Reactome	0.260127974	0.018283828	1.079139525	0.30746599	0.4690577	-5.77962966
hetero after UVB irradiation VS hetero before UVB irradiation	Presynaptic nicotinic acetylcholine receptors|Reactome	-0.40334949	0.049113463	-2.25838621	0.05223777	0.20305209	-4.20093432
WT after UVB irradiation VS WT before UVB irradiation	Presynaptic nicotinic acetylcholine receptors|Reactome	-0.76434682	0.010812885	-3.62725119	0.00457245	0.20968398	-1.78060968
homo after UVB irradiation VS homo before UVB irradiation	Presynaptic phase of homologous DNA pairing and strand exchange|Reactome	-0.59707488	-0.04485326	-2.52122177	0.03171064	0.11848672	-3.78315758
hetero after UVB irradiation VS hetero before UVB irradiation	Presynaptic phase of homologous DNA pairing and strand exchange|Reactome	-0.08198022	-0.07381242	-0.30178761	0.77012877	0.89684388	-6.29740615
WT after UVB irradiation VS WT before UVB irradiation	Presynaptic phase of homologous DNA pairing and strand exchange|Reactome	-0.21153953	-0.01074846	-0.71309987	0.49195905	0.81710765	-5.61831781
homo after UVB irradiation VS homo before UVB irradiation	Prilocaine Action Pathway|SMPDB	0.191132361	0.029768717	1.061313976	0.31508276	0.46923284	-5.79783739
hetero after UVB irradiation VS hetero before UVB irradiation	Prilocaine Action Pathway|SMPDB	-0.07904315	0.055005373	-0.44437398	0.66796482	0.82651398	-6.24068873
WT after UVB irradiation VS WT before UVB irradiation	Prilocaine Action Pathway|SMPDB	0.022316377	0.035038618	0.098344171	0.92358676	0.97566251	-5.86425414
homo after UVB irradiation VS homo before UVB irradiation	Primary bile acid biosynthesis - Homo sapiens (human)|KEGG	0.101083748	0.023137642	0.570693164	0.58160685	0.70403417	-6.19650461
hetero after UVB irradiation VS hetero before UVB irradiation	Primary bile acid biosynthesis - Homo sapiens (human)|KEGG	-0.06217904	0.042014047	-0.2670517	0.79584959	0.91372353	-6.30802003
WT after UVB irradiation VS WT before UVB irradiation	Primary bile acid biosynthesis - Homo sapiens (human)|KEGG	0.061272538	0.03778392	0.243777477	0.81229195	0.94525798	-5.83914077
homo after UVB irradiation VS homo before UVB irradiation	Primary Focal Segmental Glomerulosclerosis FSGS|Wikipathways	0.24697685	-0.01295841	1.157597019	0.27562702	0.43614438	-5.69675103
hetero after UVB irradiation VS hetero before UVB irradiation	Primary Focal Segmental Glomerulosclerosis FSGS|Wikipathways	0.28449351	-0.01093233	1.727860862	0.12036975	0.3132781	-4.9671059
WT after UVB irradiation VS WT before UVB irradiation	Primary Focal Segmental Glomerulosclerosis FSGS|Wikipathways	0.214582083	-0.01249148	1.051208601	0.31770574	0.70237831	-5.34017411
homo after UVB irradiation VS homo before UVB irradiation	Primary hyperoxaluria II_ PH2|SMPDB	-0.1559239	-0.03195163	-0.60679304	0.55837377	0.6849905	-6.17450252
hetero after UVB irradiation VS hetero before UVB irradiation	Primary hyperoxaluria II_ PH2|SMPDB	-0.2311653	-0.04505886	-1.14490839	0.28372423	0.51297702	-5.68710621
WT after UVB irradiation VS WT before UVB irradiation	Primary hyperoxaluria II_ PH2|SMPDB	-0.05726125	-0.08194106	-0.22775541	0.82438877	0.94525798	-5.84294542
homo after UVB irradiation VS homo before UVB irradiation	Primary Hyperoxaluria Type I|SMPDB	-0.39210009	0.022178168	-1.25326746	0.24041731	0.40210456	-5.58992632
hetero after UVB irradiation VS hetero before UVB irradiation	Primary Hyperoxaluria Type I|SMPDB	-0.35251865	0.016423987	-1.29321274	0.23026395	0.45170905	-5.52131443
WT after UVB irradiation VS WT before UVB irradiation	Primary Hyperoxaluria Type I|SMPDB	0.415408645	-0.09593973	1.608715996	0.13850837	0.55342759	-4.71329834
homo after UVB irradiation VS homo before UVB irradiation	Primary immunodeficiency - Homo sapiens (human)|KEGG	0.487628639	-0.0084884	2.400461022	0.0387926	0.1294788	-3.97327873
hetero after UVB irradiation VS hetero before UVB irradiation	Primary immunodeficiency - Homo sapiens (human)|KEGG	0.378675808	0.017304288	2.005599942	0.07801884	0.25138252	-4.57445027
WT after UVB irradiation VS WT before UVB irradiation	Primary immunodeficiency - Homo sapiens (human)|KEGG	0.411506142	-0.00758292	1.80895652	0.10032843	0.50131283	-4.45057945
homo after UVB irradiation VS homo before UVB irradiation	Prion disease pathway|Wikipathways	0.080263449	0.007667457	0.464384847	0.65295213	0.76055597	-6.25392473
hetero after UVB irradiation VS hetero before UVB irradiation	Prion disease pathway|Wikipathways	0.120847605	0.001498202	0.763904801	0.46577456	0.68896154	-6.04076343
WT after UVB irradiation VS WT before UVB irradiation	Prion disease pathway|Wikipathways	0.16632853	0.012668712	0.856012482	0.41187293	0.77824728	-5.5118119
homo after UVB irradiation VS homo before UVB irradiation	Prion diseases - Homo sapiens (human)|KEGG	0.366116461	-0.01477341	2.075954455	0.06643203	0.17916635	-4.47446687
hetero after UVB irradiation VS hetero before UVB irradiation	Prion diseases - Homo sapiens (human)|KEGG	0.179387424	0.055805878	0.824344695	0.43245773	0.65858414	-5.99242686
WT after UVB irradiation VS WT before UVB irradiation	Prion diseases - Homo sapiens (human)|KEGG	0.241650196	0.002468916	1.066842699	0.31092247	0.6940288	-5.32520837
homo after UVB irradiation VS homo before UVB irradiation	prion pathway|BioCarta	0.39665838	-0.0251638	1.883497416	0.09092984	0.21596764	-4.75977091
hetero after UVB irradiation VS hetero before UVB irradiation	prion pathway|BioCarta	0.269368957	0.001915889	1.598743397	0.14663486	0.35121476	-5.14076263
WT after UVB irradiation VS WT before UVB irradiation	prion pathway|BioCarta	0.142612926	-0.05921358	0.558600258	0.58862486	0.86161188	-5.71365992
homo after UVB irradiation VS homo before UVB irradiation	Procainamide (Antiarrhythmic) Action Pathway|SMPDB	0.396415397	-0.03677376	2.343069613	0.04268583	0.13402181	-4.06364635
hetero after UVB irradiation VS hetero before UVB irradiation	Procainamide (Antiarrhythmic) Action Pathway|SMPDB	-0.01555055	-0.01775524	-0.07410913	0.94265284	0.97054702	-6.34356282
WT after UVB irradiation VS WT before UVB irradiation	Procainamide (Antiarrhythmic) Action Pathway|SMPDB	0.056685205	-0.03978418	0.223404833	0.82768199	0.94525798	-5.84393438
homo after UVB irradiation VS homo before UVB irradiation	Procaine Action Pathway|SMPDB	0.191132361	0.029768717	1.061313976	0.31508276	0.46923284	-5.79783739
hetero after UVB irradiation VS hetero before UVB irradiation	Procaine Action Pathway|SMPDB	-0.07904315	0.055005373	-0.44437398	0.66796482	0.82651398	-6.24068873
WT after UVB irradiation VS WT before UVB irradiation	Procaine Action Pathway|SMPDB	0.022316377	0.035038618	0.098344171	0.92358676	0.97566251	-5.86425414
homo after UVB irradiation VS homo before UVB irradiation	Processing and activation of SUMO|Reactome	-0.50593644	-0.00436083	-2.40832461	0.03828719	0.12873001	-3.96137279
hetero after UVB irradiation VS hetero before UVB irradiation	Processing and activation of SUMO|Reactome	0.06266741	-0.04085411	0.208377731	0.83988328	0.93932613	-6.32304773
WT after UVB irradiation VS WT before UVB irradiation	Processing and activation of SUMO|Reactome	-0.20800355	-0.00225319	-0.69414647	0.50327037	0.82373662	-5.63114794
homo after UVB irradiation VS homo before UVB irradiation	Processing of Capped Intron-Containing Pre-mRNA|Reactome	-0.80918817	-0.00331274	-4.80110116	0.00086286	0.01759873	-0.28314081
hetero after UVB irradiation VS hetero before UVB irradiation	Processing of Capped Intron-Containing Pre-mRNA|Reactome	-0.33347975	-0.07716193	-1.38974817	0.2002222	0.4197851	-5.40629504
WT after UVB irradiation VS WT before UVB irradiation	Processing of Capped Intron-Containing Pre-mRNA|Reactome	-0.32509868	-0.03247135	-1.07316559	0.30821063	0.69398681	-5.31910684
homo after UVB irradiation VS homo before UVB irradiation	Processing of Capped Intronless Pre-mRNA|Reactome	-0.77243258	-0.00313559	-4.54445521	0.00125361	0.02014127	-0.64631713
hetero after UVB irradiation VS hetero before UVB irradiation	Processing of Capped Intronless Pre-mRNA|Reactome	-0.1374196	-0.0517134	-0.51756916	0.61806991	0.80231894	-6.20354886
WT after UVB irradiation VS WT before UVB irradiation	Processing of Capped Intronless Pre-mRNA|Reactome	-0.2260891	-0.00459746	-0.69106039	0.50512706	0.82448724	-5.63320755
homo after UVB irradiation VS homo before UVB irradiation	Processing of DNA double-strand break ends|Reactome	-0.54616612	0.007135502	-2.8381947	0.01868201	0.08739967	-3.27743708
hetero after UVB irradiation VS hetero before UVB irradiation	Processing of DNA double-strand break ends|Reactome	0.022184538	-0.01229933	0.085717387	0.93369305	0.97054702	-6.34255711
WT after UVB irradiation VS WT before UVB irradiation	Processing of DNA double-strand break ends|Reactome	-0.1371083	-0.00802415	-0.51099159	0.62035935	0.87257466	-5.73868815
homo after UVB irradiation VS homo before UVB irradiation	Processing of Intronless Pre-mRNAs|Reactome	-0.82780817	-0.0111917	-4.30697003	0.00178623	0.02356086	-0.99153069
hetero after UVB irradiation VS hetero before UVB irradiation	Processing of Intronless Pre-mRNAs|Reactome	-0.21227918	-0.06166457	-0.72568732	0.48769438	0.70815896	-6.06968088
WT after UVB irradiation VS WT before UVB irradiation	Processing of Intronless Pre-mRNAs|Reactome	-0.37954199	-0.02873234	-1.06817631	0.31034899	0.6940288	-5.32392379
homo after UVB irradiation VS homo before UVB irradiation	Processing of SMDT1|Reactome	-0.50108123	0.012167897	-2.09513615	0.06436882	0.17578232	-4.44547458
hetero after UVB irradiation VS hetero before UVB irradiation	Processing of SMDT1|Reactome	-0.5968422	-0.0224086	-3.57715129	0.00662937	0.08470408	-2.20377439
WT after UVB irradiation VS WT before UVB irradiation	Processing of SMDT1|Reactome	0.125637676	-0.09714886	0.623373683	0.54688151	0.84264773	-5.67627848
homo after UVB irradiation VS homo before UVB irradiation	Processive synthesis on the C-strand of the telomere|Reactome	-0.47991929	-0.01019566	-1.54999516	0.15415963	0.30132401	-5.22310631
hetero after UVB irradiation VS hetero before UVB irradiation	Processive synthesis on the C-strand of the telomere|Reactome	0.307905292	0.012310049	1.080817839	0.30973921	0.53803024	-5.75427228
WT after UVB irradiation VS WT before UVB irradiation	Processive synthesis on the C-strand of the telomere|Reactome	0.191133613	0.0858589	0.576259674	0.57707671	0.85763402	-5.70384603
homo after UVB irradiation VS homo before UVB irradiation	Processive synthesis on the lagging strand|Reactome	-0.5523956	-0.03174811	-1.82561192	0.09982092	0.22949764	-4.84331852
hetero after UVB irradiation VS hetero before UVB irradiation	Processive synthesis on the lagging strand|Reactome	0.182074395	0.014850053	0.581208312	0.57631338	0.77169106	-6.16697013
WT after UVB irradiation VS WT before UVB irradiation	Processive synthesis on the lagging strand|Reactome	0.123587233	0.057108561	0.405468701	0.69360355	0.89558592	-5.78658862
homo after UVB irradiation VS homo before UVB irradiation	progesterone biosynthesis|HumanCyc	-0.4662442	-0.01886597	-1.28392345	0.22994542	0.39059924	-5.55442555
hetero after UVB irradiation VS hetero before UVB irradiation	progesterone biosynthesis|HumanCyc	-1.00395421	-0.03995611	-4.44202763	0.00190312	0.04958933	-0.98142266
WT after UVB irradiation VS WT before UVB irradiation	progesterone biosynthesis|HumanCyc	-0.74394616	-0.01811287	-2.64908418	0.02419468	0.32984203	-3.23560247
homo after UVB irradiation VS homo before UVB irradiation	Progesterone-mediated oocyte maturation - Homo sapiens (human)|KEGG	-0.02158885	-0.00200341	-0.12510987	0.90307447	0.94250012	-6.36004529
hetero after UVB irradiation VS hetero before UVB irradiation	Progesterone-mediated oocyte maturation - Homo sapiens (human)|KEGG	0.357853064	0.024440113	2.258416984	0.0522352	0.20305209	-4.20088819
WT after UVB irradiation VS WT before UVB irradiation	Progesterone-mediated oocyte maturation - Homo sapiens (human)|KEGG	0.265995071	0.009648674	1.28727445	0.22676427	0.62952955	-5.09676368
homo after UVB irradiation VS homo before UVB irradiation	Programmed Cell Death|Reactome	0.258952307	-0.04237228	1.32337151	0.21702942	0.37479438	-5.50787605
hetero after UVB irradiation VS hetero before UVB irradiation	Programmed Cell Death|Reactome	0.317208989	-0.00208142	2.123874647	0.06469938	0.22842741	-4.40117333
WT after UVB irradiation VS WT before UVB irradiation	Programmed Cell Death|Reactome	0.242371005	-0.01958489	1.261582631	0.23550523	0.62952955	-5.12497938
homo after UVB irradiation VS homo before UVB irradiation	Progressive trimming of alpha-1_2-linked mannose residues from Man9/8/7GlcNAc2 to produce Man5GlcNAc2|Reactome	0.603182393	-0.05391969	2.167928375	0.05708563	0.16240116	-4.33466231
hetero after UVB irradiation VS hetero before UVB irradiation	Progressive trimming of alpha-1_2-linked mannose residues from Man9/8/7GlcNAc2 to produce Man5GlcNAc2|Reactome	0.320813326	-0.03405726	1.276123147	0.23596168	0.45864108	-5.54111655
WT after UVB irradiation VS WT before UVB irradiation	Progressive trimming of alpha-1_2-linked mannose residues from Man9/8/7GlcNAc2 to produce Man5GlcNAc2|Reactome	0.280325205	-0.01675155	0.950731753	0.3639747	0.74541365	-5.43212893
homo after UVB irradiation VS homo before UVB irradiation	Prolactin receptor signaling|Reactome	-0.09037851	-0.08904308	-0.39507405	0.70161381	0.80189454	-6.28529287
hetero after UVB irradiation VS hetero before UVB irradiation	Prolactin receptor signaling|Reactome	-0.29782948	-0.04120915	-1.2783843	0.23520108	0.45788382	-5.53850651
WT after UVB irradiation VS WT before UVB irradiation	Prolactin receptor signaling|Reactome	-0.22457268	0.004585721	-1.0983996	0.29756773	0.68387495	-5.29447857
homo after UVB irradiation VS homo before UVB irradiation	Prolactin signaling pathway - Homo sapiens (human)|KEGG	0.268949551	-0.00273264	1.71828161	0.1184703	0.25722082	-4.99499035
hetero after UVB irradiation VS hetero before UVB irradiation	Prolactin signaling pathway - Homo sapiens (human)|KEGG	0.325531216	0.028626142	2.160886933	0.0610059	0.22121311	-4.34637695
WT after UVB irradiation VS WT before UVB irradiation	Prolactin signaling pathway - Homo sapiens (human)|KEGG	0.222263277	0.02861138	1.09357663	0.29957974	0.68477511	-5.29921988
homo after UVB irradiation VS homo before UVB irradiation	Prolactin Signaling Pathway|Wikipathways	0.434621559	-0.02742701	2.240172765	0.05064837	0.14881624	-4.22355728
hetero after UVB irradiation VS hetero before UVB irradiation	Prolactin Signaling Pathway|Wikipathways	0.544605803	0.002170938	3.553869176	0.00686567	0.08550202	-2.23811605
WT after UVB irradiation VS WT before UVB irradiation	Prolactin Signaling Pathway|Wikipathways	0.406177628	-0.00626086	1.963576714	0.07773685	0.46277213	-4.23809268
homo after UVB irradiation VS homo before UVB irradiation	Prolactin|NetPath	0.364691855	-0.01704227	1.946018288	0.08216229	0.20344524	-4.66828651
hetero after UVB irradiation VS hetero before UVB irradiation	Prolactin|NetPath	0.509011133	0.010557264	3.301819814	0.01007451	0.10227082	-2.61376256
WT after UVB irradiation VS WT before UVB irradiation	Prolactin|NetPath	0.326055534	0.00294053	1.620049959	0.13604074	0.54838491	-4.69885953
homo after UVB irradiation VS homo before UVB irradiation	Prolidase Deficiency (PD)|SMPDB	-0.34739547	0.00502943	-1.63262028	0.13558154	0.277359	-5.11269582
hetero after UVB irradiation VS hetero before UVB irradiation	Prolidase Deficiency (PD)|SMPDB	-0.56351352	-0.04220085	-3.24793085	0.01094591	0.10415756	-2.6949004
WT after UVB irradiation VS WT before UVB irradiation	Prolidase Deficiency (PD)|SMPDB	-0.47021304	-0.04055952	-2.31093774	0.04323906	0.39022568	-3.73881388
homo after UVB irradiation VS homo before UVB irradiation	proline biosynthesis|HumanCyc	-0.17583958	-0.00538891	-0.51125675	0.62097675	0.7364623	-6.22997981
hetero after UVB irradiation VS hetero before UVB irradiation	proline biosynthesis|HumanCyc	-0.1035519	0.011621214	-0.4185248	0.68602802	0.84011707	-6.25251959
WT after UVB irradiation VS WT before UVB irradiation	proline biosynthesis|HumanCyc	0.503321937	0.052310823	2.255396846	0.04753468	0.401167	-3.82021172
homo after UVB irradiation VS homo before UVB irradiation	Proline catabolism|Reactome	-0.66835784	-0.04119624	-2.21204766	0.05306595	0.15407858	-4.26693541
hetero after UVB irradiation VS hetero before UVB irradiation	Proline catabolism|Reactome	-1.17848479	-0.01294269	-5.99927998	0.00026368	0.02988205	0.917740287
WT after UVB irradiation VS WT before UVB irradiation	Proline catabolism|Reactome	-0.79330624	-0.14211761	-3.2575665	0.00851961	0.24856491	-2.32316637
homo after UVB irradiation VS homo before UVB irradiation	proline degradation|HumanCyc	-0.83079631	-0.00448379	-2.67017663	0.02472496	0.10395413	-3.54619275
hetero after UVB irradiation VS hetero before UVB irradiation	proline degradation|HumanCyc	-1.22621155	-0.03200115	-6.92865914	9.43E-05	0.02920766	1.87013196
WT after UVB irradiation VS WT before UVB irradiation	proline degradation|HumanCyc	-0.98404348	-0.07243146	-4.42617835	0.00125653	0.15645713	-0.67181722
homo after UVB irradiation VS homo before UVB irradiation	Prolinemia Type II|SMPDB	-0.34739547	0.00502943	-1.63262028	0.13558154	0.277359	-5.11269582
hetero after UVB irradiation VS hetero before UVB irradiation	Prolinemia Type II|SMPDB	-0.56351352	-0.04220085	-3.24793085	0.01094591	0.10415756	-2.6949004
WT after UVB irradiation VS WT before UVB irradiation	Prolinemia Type II|SMPDB	-0.47021304	-0.04055952	-2.31093774	0.04323906	0.39022568	-3.73881388
homo after UVB irradiation VS homo before UVB irradiation	Prolonged ERK activation events|Reactome	0.307514048	-0.01944207	1.366358851	0.20365272	0.36105098	-5.45607396
hetero after UVB irradiation VS hetero before UVB irradiation	Prolonged ERK activation events|Reactome	0.149063417	-0.0369206	0.741246705	0.47869123	0.69976736	-6.05806415
WT after UVB irradiation VS WT before UVB irradiation	Prolonged ERK activation events|Reactome	0.026446902	0.002461557	0.104121598	0.91911501	0.97566251	-5.86366221
homo after UVB irradiation VS homo before UVB irradiation	Propanoate metabolism - Homo sapiens (human)|KEGG	-0.32304827	-0.01900493	-1.11476637	0.29267003	0.45292679	-5.74254147
hetero after UVB irradiation VS hetero before UVB irradiation	Propanoate metabolism - Homo sapiens (human)|KEGG	-0.69148124	-0.02995892	-3.90094254	0.00410378	0.07021718	-1.73339877
WT after UVB irradiation VS WT before UVB irradiation	Propanoate metabolism - Homo sapiens (human)|KEGG	-0.31289774	-0.10872019	-1.21625217	0.25159644	0.64552861	-5.17377964
homo after UVB irradiation VS homo before UVB irradiation	Propanoate metabolism|EHMN	-0.09711537	-0.06746116	-0.35075627	0.7335127	0.82411885	-6.30278537
hetero after UVB irradiation VS hetero before UVB irradiation	Propanoate metabolism|EHMN	-0.51555802	-0.05581177	-2.38830899	0.04246217	0.18155964	-4.00511501
WT after UVB irradiation VS WT before UVB irradiation	Propanoate metabolism|EHMN	-0.03504644	-0.055532	-0.12671763	0.90165546	0.97566251	-5.86102358
homo after UVB irradiation VS homo before UVB irradiation	Propanoate metabolism|INOH	-0.0863892	0.002302455	-0.39751436	0.69987433	0.80070814	-6.28427122
hetero after UVB irradiation VS hetero before UVB irradiation	Propanoate metabolism|INOH	-0.40911174	-0.01531961	-1.85581865	0.09869937	0.28258275	-4.78905981
WT after UVB irradiation VS WT before UVB irradiation	Propanoate metabolism|INOH	-0.13908157	-0.03749331	-0.54299098	0.59893407	0.8664725	-5.72209649
homo after UVB irradiation VS homo before UVB irradiation	Propanoate Metabolism|SMPDB	-0.25733603	-0.09162056	-0.7770169	0.45627806	0.60277262	-6.05433779
hetero after UVB irradiation VS hetero before UVB irradiation	Propanoate Metabolism|SMPDB	-0.83573623	-0.01284435	-4.81326218	0.00115145	0.04138456	-0.49254
WT after UVB irradiation VS WT before UVB irradiation	Propanoate Metabolism|SMPDB	-0.18571921	-0.14542889	-0.63906971	0.5370254	0.83767333	-5.66665155
homo after UVB irradiation VS homo before UVB irradiation	Proparacaine Action Pathway|SMPDB	0.191132361	0.029768717	1.061313976	0.31508276	0.46923284	-5.79783739
hetero after UVB irradiation VS hetero before UVB irradiation	Proparacaine Action Pathway|SMPDB	-0.07904315	0.055005373	-0.44437398	0.66796482	0.82651398	-6.24068873
WT after UVB irradiation VS WT before UVB irradiation	Proparacaine Action Pathway|SMPDB	0.022316377	0.035038618	0.098344171	0.92358676	0.97566251	-5.86425414
homo after UVB irradiation VS homo before UVB irradiation	Propionic Acidemia|SMPDB	-0.23657683	-0.05522798	-0.8011182	0.4428632	0.59091566	-6.03520901
hetero after UVB irradiation VS hetero before UVB irradiation	Propionic Acidemia|SMPDB	-0.90022729	-0.04719335	-5.31014337	0.00060637	0.03004176	0.126180045
WT after UVB irradiation VS WT before UVB irradiation	Propionic Acidemia|SMPDB	-0.36319435	-0.13086657	-1.31407353	0.21793282	0.62581393	-5.06691375
homo after UVB irradiation VS homo before UVB irradiation	Propionyl-CoA catabolism|Reactome	0.094194353	-0.10950846	0.23683434	0.8178719	0.88813008	-6.33840271
hetero after UVB irradiation VS hetero before UVB irradiation	Propionyl-CoA catabolism|Reactome	-1.18381972	-0.05688687	-6.05117461	0.00024828	0.02988205	0.974310121
WT after UVB irradiation VS WT before UVB irradiation	Propionyl-CoA catabolism|Reactome	0.077922218	-0.10787548	0.198273703	0.84677181	0.9536885	-5.84927741
homo after UVB irradiation VS homo before UVB irradiation	propionyl-CoA degradation|HumanCyc	0.090007565	-0.13727663	0.223620067	0.82784289	0.89402167	-6.3416531
hetero after UVB irradiation VS hetero before UVB irradiation	propionyl-CoA degradation|HumanCyc	-1.22685762	-0.05512391	-6.56347815	0.00013956	0.02920766	1.510923014
WT after UVB irradiation VS WT before UVB irradiation	propionyl-CoA degradation|HumanCyc	0.222893672	-0.04567589	0.569155395	0.58170764	0.85958498	-5.70782836
homo after UVB irradiation VS homo before UVB irradiation	Propoxyphene Action Pathway|SMPDB	0.191132361	0.029768717	1.061313976	0.31508276	0.46923284	-5.79783739
hetero after UVB irradiation VS hetero before UVB irradiation	Propoxyphene Action Pathway|SMPDB	-0.07904315	0.055005373	-0.44437398	0.66796482	0.82651398	-6.24068873

WT after UVB irradiation VS WT before UVB irradiation	Propoxyphene Action Pathway|SMPDB	0.022316377	0.035038618	0.098344171	0.92358676	0.97566251	-5.86425414
homo after UVB irradiation VS homo before UVB irradiation	Propranolol Action Pathway|SMPDB	0.396415397	-0.03677376	2.343069613	0.04268583	0.13402181	-4.06364635
hetero after UVB irradiation VS hetero before UVB irradiation	Propranolol Action Pathway|SMPDB	-0.01555055	-0.01775524	-0.07410913	0.94265284	0.97054702	-6.34356282
WT after UVB irradiation VS WT before UVB irradiation	Propranolol Action Pathway|SMPDB	0.056685205	-0.03978418	0.223404833	0.82768199	0.94525798	-5.84393438
homo after UVB irradiation VS homo before UVB irradiation	Proprotein convertase subtilisin-kexin type 9 (PCSK9) mediated LDL receptor degradation|Wikipathways	0.614474587	0.045253128	1.77845028	0.10765453	0.24079424	-4.91050563
hetero after UVB irradiation VS hetero before UVB irradiation	Proprotein convertase subtilisin-kexin type 9 (PCSK9) mediated LDL receptor degradation|Wikipathways	0.776358909	0.037286384	2.618640193	0.02941178	0.14913595	-3.65433897
WT after UVB irradiation VS WT before UVB irradiation	Proprotein convertase subtilisin-kexin type 9 (PCSK9) mediated LDL receptor degradation|Wikipathways	0.709600779	-0.03032083	2.05914282	0.06625595	0.43803373	-4.10335283
homo after UVB irradiation VS homo before UVB irradiation	Prostacyclin signalling through prostacyclin receptor|Reactome	0.466375544	0.02274067	2.091179762	0.06478928	0.17650826	-4.4514619
hetero after UVB irradiation VS hetero before UVB irradiation	Prostacyclin signalling through prostacyclin receptor|Reactome	0.652256786	0.035790446	3.628832082	0.00613504	0.0822456	-2.12777966
WT after UVB irradiation VS WT before UVB irradiation	Prostacyclin signalling through prostacyclin receptor|Reactome	0.241890582	-0.0611006	0.885892919	0.39631308	0.76478278	-5.48743315
homo after UVB irradiation VS homo before UVB irradiation	Prostaglandin formation from arachidonate|EHMN	0.412466056	0.002876499	2.548359254	0.03030516	0.1156968	-3.74011885
hetero after UVB irradiation VS hetero before UVB irradiation	Prostaglandin formation from arachidonate|EHMN	0.229228156	0.001670693	1.307758254	0.22550611	0.44786792	-5.50432407
WT after UVB irradiation VS WT before UVB irradiation	Prostaglandin formation from arachidonate|EHMN	0.107950555	0.022931762	0.531418567	0.60663772	0.87177925	-5.72820628
homo after UVB irradiation VS homo before UVB irradiation	Prostaglandin formation from dihomo gama-linoleic acid|EHMN	-0.40187222	-0.00758898	-1.93239682	0.08400188	0.20565821	-4.68832334
hetero after UVB irradiation VS hetero before UVB irradiation	Prostaglandin formation from dihomo gama-linoleic acid|EHMN	-0.02598808	0.010496847	-0.11231795	0.91320121	0.97054702	-6.33970281
WT after UVB irradiation VS WT before UVB irradiation	Prostaglandin formation from dihomo gama-linoleic acid|EHMN	-0.02298075	0.004088762	-0.0794745	0.93821055	0.98056728	-5.86595251
homo after UVB irradiation VS homo before UVB irradiation	Prostaglandin Leukotriene metabolism|INOH	0.473774468	0.037798411	2.612662876	0.02721817	0.10968948	-3.63788443
hetero after UVB irradiation VS hetero before UVB irradiation	Prostaglandin Leukotriene metabolism|INOH	0.478449326	0.074589489	3.206065213	0.01167717	0.10542662	-2.75810806
WT after UVB irradiation VS WT before UVB irradiation	Prostaglandin Leukotriene metabolism|INOH	0.262754879	0.077550715	1.335790781	0.21098688	0.62581393	-5.04241743
homo after UVB irradiation VS homo before UVB irradiation	Prostaglandin Synthesis and Regulation|Wikipathways	0.356380956	0.033679192	2.161381428	0.0577067	0.16345457	-4.34467758
hetero after UVB irradiation VS hetero before UVB irradiation	Prostaglandin Synthesis and Regulation|Wikipathways	0.208682799	0.024787307	0.988600659	0.35039393	0.57944367	-5.84576076
WT after UVB irradiation VS WT before UVB irradiation	Prostaglandin Synthesis and Regulation|Wikipathways	0.113590489	-0.00511786	0.486370106	0.63710331	0.87636259	-5.75080535
homo after UVB irradiation VS homo before UVB irradiation	Prostanoid ligand receptors|Reactome	0.281356704	-0.00840188	1.446695101	0.18052734	0.3341193	-5.35639631
hetero after UVB irradiation VS hetero before UVB irradiation	Prostanoid ligand receptors|Reactome	0.17597143	0.070447033	0.765381444	0.4649408	0.68805201	-6.03962023
WT after UVB irradiation VS WT before UVB irradiation	Prostanoid ligand receptors|Reactome	0.243205243	-0.03998786	0.922622938	0.3777542	0.75122805	-5.45650323
homo after UVB irradiation VS homo before UVB irradiation	Prostate cancer - Homo sapiens (human)|KEGG	0.151591102	-0.00253597	0.801515437	0.44264432	0.59091566	-6.03488947
hetero after UVB irradiation VS hetero before UVB irradiation	Prostate cancer - Homo sapiens (human)|KEGG	0.191337013	-0.01520587	1.269136001	0.23832504	0.4612216	-5.54916236
WT after UVB irradiation VS WT before UVB irradiation	Prostate cancer - Homo sapiens (human)|KEGG	0.130983195	-0.00700031	0.673094229	0.51601847	0.82895075	-5.64503309
homo after UVB irradiation VS homo before UVB irradiation	Proteasome - Homo sapiens (human)|KEGG	-0.31323374	0.049183283	-1.07119936	0.31084108	0.46923284	-5.7877691
hetero after UVB irradiation VS hetero before UVB irradiation	Proteasome - Homo sapiens (human)|KEGG	-0.11636285	-0.01430853	-0.38614826	0.70895514	0.85555137	-6.26637814
WT after UVB irradiation VS WT before UVB irradiation	Proteasome - Homo sapiens (human)|KEGG	-0.01904893	-0.04979547	-0.05783913	0.95500706	0.98742794	-5.86745675
homo after UVB irradiation VS homo before UVB irradiation	proteasome complex|BioCarta	-0.44871789	0.050671791	-1.53932371	0.156717	0.30423888	-5.23712803
hetero after UVB irradiation VS hetero before UVB irradiation	proteasome complex|BioCarta	-0.22663218	-0.02542178	-0.67844816	0.51568473	0.73273731	-6.10361436
WT after UVB irradiation VS WT before UVB irradiation	proteasome complex|BioCarta	-0.10710967	-0.00064103	-0.28000591	0.78513386	0.93817538	-5.82959805
homo after UVB irradiation VS homo before UVB irradiation	Proteasome Degradation|Wikipathways	-0.21707697	-8.03E-05	-0.80611728	0.4401139	0.59091566	-6.03117773
hetero after UVB irradiation VS hetero before UVB irradiation	Proteasome Degradation|Wikipathways	0.103676759	-0.00065953	0.404503658	0.69591666	0.84695747	-6.2586528
WT after UVB irradiation VS WT before UVB irradiation	Proteasome Degradation|Wikipathways	0.141682597	-0.05797966	0.484918019	0.63809765	0.87636259	-5.75150225
homo after UVB irradiation VS homo before UVB irradiation	protein <i>O</i>-[<i>N</i>-acetyl]-glucosylation|HumanCyc	0.656146455	-0.03255467	1.908523133	0.08731989	0.21117722	-4.72330137
hetero after UVB irradiation VS hetero before UVB irradiation	protein <i>O</i>-[<i>N</i>-acetyl]-glucosylation|HumanCyc	0.588079415	0.021986212	1.55317788	0.15708264	0.3671706	-5.2004069
WT after UVB irradiation VS WT before UVB irradiation	protein <i>O</i>-[<i>N</i>-acetyl]-glucosylation|HumanCyc	-0.02525347	0.014389086	-0.06150456	0.95215956	0.98717655	-5.86723522
homo after UVB irradiation VS homo before UVB irradiation	Protein alkylation leading to liver fibrosis|Wikipathways	0.36223107	-0.04678034	2.150982248	0.05870664	0.16536366	-4.36056664
hetero after UVB irradiation VS hetero before UVB irradiation	Protein alkylation leading to liver fibrosis|Wikipathways	0.224285104	0.022725617	1.113755727	0.29614344	0.52748019	-5.7201088
WT after UVB irradiation VS WT before UVB irradiation	Protein alkylation leading to liver fibrosis|Wikipathways	0.222551822	-0.01517576	0.969526479	0.35496564	0.74024114	-5.41549628
homo after UVB irradiation VS homo before UVB irradiation	protein citrullination|HumanCyc	-0.99028596	0.072161549	-4.72970518	0.00095643	0.01805472	-0.38312068
hetero after UVB irradiation VS hetero before UVB irradiation	protein citrullination|HumanCyc	-0.55231866	-0.02273723	-2.24890884	0.05303249	0.20450262	-4.2151354
WT after UVB irradiation VS WT before UVB irradiation	protein citrullination|HumanCyc	-0.54881637	0.054846995	-2.53363598	0.02951413	0.35848683	-3.40851142
homo after UVB irradiation VS homo before UVB irradiation	Protein digestion and absorption - Homo sapiens (human)|KEGG	0.2076683	-0.00175468	1.175135452	0.26887963	0.43255711	-5.67763039
hetero after UVB irradiation VS hetero before UVB irradiation	Protein digestion and absorption - Homo sapiens (human)|KEGG	0.114143872	0.05537619	0.683780279	0.51247636	0.73064349	-6.09988571
WT after UVB irradiation VS WT before UVB irradiation	Protein digestion and absorption - Homo sapiens (human)|KEGG	0.012817505	-0.01386526	0.058814714	0.95424911	0.98742794	-5.86739911
homo after UVB irradiation VS homo before UVB irradiation	Protein export - Homo sapiens (human)|KEGG	0.105011472	-0.03967549	0.38918993	0.70581558	0.80552596	-6.28773127
hetero after UVB irradiation VS hetero before UVB irradiation	Protein export - Homo sapiens (human)|KEGG	0.108906138	-0.00583481	0.466097525	0.65295845	0.82651398	-6.23022434
WT after UVB irradiation VS WT before UVB irradiation	Protein export - Homo sapiens (human)|KEGG	0.260182685	-0.0822644	0.92592395	0.37611697	0.75077508	-5.45367218
homo after UVB irradiation VS homo before UVB irradiation	Protein folding|Reactome	0.007089865	-0.01502019	0.038545575	0.97005987	0.98145211	-6.36765002
hetero after UVB irradiation VS hetero before UVB irradiation	Protein folding|Reactome	0.019756375	-0.01426789	0.112752302	0.91286713	0.97054702	-6.33964986
WT after UVB irradiation VS WT before UVB irradiation	Protein folding|Reactome	0.13622664	-0.0330113	0.646856162	0.53217448	0.83483909	-5.66179449
homo after UVB irradiation VS homo before UVB irradiation	protein kinase a at the centrosome|BioCarta	0.246880762	0.006537566	1.35935103	0.20578463	0.3634865	-5.46459368
hetero after UVB irradiation VS hetero before UVB irradiation	protein kinase a at the centrosome|BioCarta	0.535534162	-0.03709045	3.353591146	0.00930563	0.09765748	-2.53606569
WT after UVB irradiation VS WT before UVB irradiation	protein kinase a at the centrosome|BioCarta	0.618221995	-0.10622501	2.384557754	0.03812316	0.39022568	-3.63024051
homo after UVB irradiation VS homo before UVB irradiation	Protein methylation|Reactome	-0.47094771	-0.02294607	-2.86817669	0.01777255	0.08412182	-3.22940017
hetero after UVB irradiation VS hetero before UVB irradiation	Protein methylation|Reactome	-0.1878171	-0.05168707	-0.8285518	0.43020072	0.65624327	-5.98894576
WT after UVB irradiation VS WT before UVB irradiation	Protein methylation|Reactome	-0.39098877	-0.04086814	-1.81780943	0.09888641	0.49761536	-4.43861948
homo after UVB irradiation VS homo before UVB irradiation	Protein processing in endoplasmic reticulum - Homo sapiens (human)|KEGG	0.126449018	-0.01813114	0.60828403	0.5574256	0.6849905	-6.17356697
hetero after UVB irradiation VS hetero before UVB irradiation	Protein processing in endoplasmic reticulum - Homo sapiens (human)|KEGG	0.132164817	-0.04669528	0.668243418	0.52185933	0.73763142	-6.11067755
WT after UVB irradiation VS WT before UVB irradiation	Protein processing in endoplasmic reticulum - Homo sapiens (human)|KEGG	0.146354376	-0.00914492	0.637947528	0.53772663	0.83844231	-5.66734711
homo after UVB irradiation VS homo before UVB irradiation	Protein repair|Reactome	0.996359243	0.05257518	4.087686705	0.00249458	0.02869082	-1.31773747
hetero after UVB irradiation VS hetero before UVB irradiation	Protein repair|Reactome	0.703624996	0.003055865	2.738060096	0.0243286	0.13715811	-3.47159147
WT after UVB irradiation VS WT before UVB irradiation	Protein repair|Reactome	0.554107359	0.007572616	1.828297907	0.09720266	0.49496258	-4.42441549
homo after UVB irradiation VS homo before UVB irradiation	Protein ubiquitination|Reactome	-0.04666165	-0.00075041	-0.226506	0.82566245	0.89312082	-6.34095897
hetero after UVB irradiation VS hetero before UVB irradiation	Protein ubiquitination|Reactome	0.21515334	0.006986696	1.232655198	0.25098748	0.47497442	-5.59068682
WT after UVB irradiation VS WT before UVB irradiation	Protein ubiquitination|Reactome	-0.10568993	-0.02238627	-0.4527733	0.66029907	0.87935805	-5.76642032
homo after UVB irradiation VS homo before UVB irradiation	Protein-protein interactions at synapses|Reactome	0.052637732	0.01210375	0.315779729	0.75907643	0.84432229	-6.3151604
hetero after UVB irradiation VS hetero before UVB irradiation	Protein-protein interactions at synapses|Reactome	0.042312849	0.023360187	0.271206475	0.79275849	0.91162084	-6.30681747
WT after UVB irradiation VS WT before UVB irradiation	Protein-protein interactions at synapses|Reactome	-0.07324535	-0.00584363	-0.35002291	0.73352335	0.9131126	-5.80748839
homo after UVB irradiation VS homo before UVB irradiation	Proteoglycan biosynthesis|EHMN	0.689835963	-0.01847045	4.203845048	0.00208831	0.02552962	-1.14407508
hetero after UVB irradiation VS hetero before UVB irradiation	Proteoglycan biosynthesis|EHMN	0.558918463	0.035891385	2.45872195	0.03794996	0.16941813	-3.8982604
WT after UVB irradiation VS WT before UVB irradiation	Proteoglycan biosynthesis|EHMN	0.572461001	-0.03000302	2.381848711	0.03830047	0.39022568	-3.63424813
homo after UVB irradiation VS homo before UVB irradiation	Proteoglycan syndecan-mediated signaling events|PID	0.611708844	-0.03913939	3.389145546	0.00753828	0.05066933	-2.39785803
hetero after UVB irradiation VS hetero before UVB irradiation	Proteoglycan syndecan-mediated signaling events|PID	0.398948675	0.013392826	1.380587842	0.20292049	0.42313299	-5.41743227
WT after UVB irradiation VS WT before UVB irradiation	Proteoglycan syndecan-mediated signaling events|PID	0.264056974	-0.01568447	0.793816194	0.44558349	0.802811	-5.56024854
homo after UVB irradiation VS homo before UVB irradiation	Proteoglycans in cancer - Homo sapiens (human)|KEGG	0.264942044	-0.01376824	1.602490734	0.14211055	0.28610087	-5.15332771
hetero after UVB irradiation VS hetero before UVB irradiation	Proteoglycans in cancer - Homo sapiens (human)|KEGG	0.235911807	0.009406339	1.493988489	0.17164893	0.38846862	-5.27648276
WT after UVB irradiation VS WT before UVB irradiation	Proteoglycans in cancer - Homo sapiens (human)|KEGG	0.144240091	0.003853515	0.738667831	0.47695212	0.81525267	-5.60052057
homo after UVB irradiation VS homo before UVB irradiation	proteolysis and signaling pathway of notch|BioCarta	0.377794668	0.081966087	1.567981744	0.14993275	0.29561714	-5.1993467
hetero after UVB irradiation VS hetero before UVB irradiation	proteolysis and signaling pathway of notch|BioCarta	0.550706894	0.013538738	2.7778743	0.02284099	0.1353826	-3.41063348
WT after UVB irradiation VS WT before UVB irradiation	proteolysis and signaling pathway of notch|BioCarta	0.437255738	0.041493982	1.846306212	0.09437322	0.49001597	-4.39994218
homo after UVB irradiation VS homo before UVB irradiation	Proton Pump Inhibitor Pathway_ Pharmacodynamics|PharmGKB	0.242908489	-0.01422019	1.4201347	0.18790977	0.34387505	-5.38975276
hetero after UVB irradiation VS hetero before UVB irradiation	Proton Pump Inhibitor Pathway_ Pharmacodynamics|PharmGKB	0.161634799	0.035612651	0.998238163	0.34596449	0.57509341	-5.83649473
WT after UVB irradiation VS WT before UVB irradiation	Proton Pump Inhibitor Pathway_ Pharmacodynamics|PharmGKB	0.064206796	0.030984344	0.284524345	0.78176692	0.93661609	-5.82831675
homo after UVB irradiation VS homo before UVB irradiation	Proton Pump Inhibitor Pathway_ Pharmacokinetics|PharmGKB	0.287856196	0.010644467	0.709584915	0.49520459	0.63549864	-6.1051149
hetero after UVB irradiation VS hetero before UVB irradiation	Proton Pump Inhibitor Pathway_ Pharmacokinetics|PharmGKB	0.537854792	0.010206405	1.458066707	0.18106259	0.39948891	-5.32183804
WT after UVB irradiation VS WT before UVB irradiation	Proton Pump Inhibitor Pathway_ Pharmacokinetics|PharmGKB	0.455764964	0.018826066	1.134786012	0.28272335	0.67144957	-5.25819606
homo after UVB irradiation VS homo before UVB irradiation	Proton/oligopeptide cotransporters|Reactome	0.478263641	0.017589213	1.257386252	0.23898793	0.40059431	-5.5851915
hetero after UVB irradiation VS hetero before UVB irradiation	Proton/oligopeptide cotransporters|Reactome	0.439907631	0.017968596	1.354731309	0.21070652	0.43289558	-5.4486219
WT after UVB irradiation VS WT before UVB irradiation	Proton/oligopeptide cotransporters|Reactome	0.462750105	0.052527949	1.938880511	0.08099449	0.46678978	-4.2725159
homo after UVB irradiation VS homo before UVB irradiation	Proton-coupled monocarboxylate transport|Reactome	0.550707411	-0.03864562	2.965444074	0.01512055	0.07628779	-3.07353037
hetero after UVB irradiation VS hetero before UVB irradiation	Proton-coupled monocarboxylate transport|Reactome	0.583820239	0.041063801	2.675718394	0.02685958	0.14252149	-3.56702388
WT after UVB irradiation VS WT before UVB irradiation	Proton-coupled monocarboxylate transport|Reactome	0.439313407	0.022579677	1.636667273	0.13249356	0.54650924	-4.67759014
homo after UVB irradiation VS homo before UVB irradiation	Proton-coupled neutral amino acid transporters|Reactome	0.079162177	-0.03319309	0.20978311	0.83831852	0.90132922	-6.3448588
hetero after UVB irradiation VS hetero before UVB irradiation	Proton-coupled neutral amino acid transporters|Reactome	0.197477618	0.042469786	0.471755846	0.64907658	0.82363337	-6.22742091
WT after UVB irradiation VS WT before UVB irradiation	Proton-coupled neutral amino acid transporters|Reactome	0.983341087	-0.1297646	4.032973926	0.00234884	0.17027242	-1.20503885
homo after UVB irradiation VS homo before UVB irradiation	Proximal tubule bicarbonate reclamation - Homo sapiens (human)|KEGG	0.336958411	-0.03450247	1.849917192	0.09599338	0.22339743	-4.80837906
hetero after UVB irradiation VS hetero before UVB irradiation	Proximal tubule bicarbonate reclamation - Homo sapiens (human)|KEGG	-0.0043421	-0.04236543	-0.02444196	0.98106906	0.99041257	-6.34621722
WT after UVB irradiation VS WT before UVB irradiation	Proximal tubule bicarbonate reclamation - Homo sapiens (human)|KEGG	0.119274639	-0.0020389	0.557459629	0.58937498	0.86161188	-5.71428399
homo after UVB irradiation VS homo before UVB irradiation	PRPP biosynthesis|HumanCyc	-0.1437614	0.004937334	-0.52325245	0.61292232	0.72940831	-6.22350209
hetero after UVB irradiation VS hetero before UVB irradiation	PRPP biosynthesis|HumanCyc	-0.14064702	-0.04077113	-0.52504472	0.61308388	0.79886687	-6.19945667
WT after UVB irradiation VS WT before UVB irradiation	PRPP biosynthesis|HumanCyc	-0.11784593	0.007349038	-0.36310157	0.7240262	0.90882588	-5.80282697
homo after UVB irradiation VS homo before UVB irradiation	pten dependent cell cycle arrest and apoptosis|BioCarta	0.529024802	-0.01837366	2.793615939	0.02012237	0.09061022	-3.34883253
hetero after UVB irradiation VS hetero before UVB irradiation	pten dependent cell cycle arrest and apoptosis|BioCarta	0.214265477	0.06042822	0.966257593	0.36082653	0.58926812	-5.86697003
WT after UVB irradiation VS WT before UVB irradiation	pten dependent cell cycle arrest and apoptosis|BioCarta	0.225386817	0.00357762	0.942876168	0.36778873	0.74581032	-5.43900161
homo after UVB irradiation VS homo before UVB irradiation	PTEN Regulation|Reactome	-0.04158742	-0.02239738	-0.22065763	0.83008276	0.89474926	-6.34235647
hetero after UVB irradiation VS hetero before UVB irradiation	PTEN Regulation|Reactome	0.081751639	-0.01785548	0.455265982	0.6604205	0.82651398	-6.2355013
WT after UVB irradiation VS WT before UVB irradiation	PTEN Regulation|Reactome	-0.01261109	0.006403276	-0.05584054	0.95655996	0.98742794	-5.86757181
homo after UVB irradiation VS homo before UVB irradiation	PTEN Regulation|Wikipathways	0.708996024	-0.00393473	2.046339142	0.06974233	0.18552288	-4.51904705
hetero after UVB irradiation VS hetero before UVB irradiation	PTEN Regulation|Wikipathways	0.376663975	0.050473056	0.883788634	0.40132275	0.62989983	-5.94186766
WT after UVB irradiation VS WT before UVB irradiation	PTEN Regulation|Wikipathways	0.301963467	-0.05807468	0.818189648	0.43216094	0.79685269	-5.54164323
homo after UVB irradiation VS homo before UVB irradiation	Pterine Biosynthesis|SMPDB	0.35847852	0.013300746	1.711755208	0.11970137	0.25817499	-5.00406733
hetero after UVB irradiation VS hetero before UVB irradiation	Pterine Biosynthesis|SMPDB	0.498149914	0.002599842	2.269426991	0.05132677	0.20064099	-4.18437481
WT after UVB irradiation VS WT before UVB irradiation	Pterine Biosynthesis|SMPDB	0.467985189	-0.04591589	2.072035951	0.06483587	0.43544914	-4.08500072
homo after UVB irradiation VS homo before UVB irradiation	PTF1A related regulatory pathway|Wikipathways	-0.21938977	0.056423377	-1.13106751	0.28608839	0.44628615	-5.72526672
hetero after UVB irradiation VS hetero before UVB irradiation	PTF1A related regulatory pathway|Wikipathways	-0.17778252	0.049060142	-0.74544066	0.47628299	0.6980379	-6.05489605
WT after UVB irradiation VS WT before UVB irradiation	PTF1A related regulatory pathway|Wikipathways	-0.05429185	0.182801474	-0.24573591	0.81081678	0.94525798	-5.8386582
homo after UVB irradiation VS homo before UVB irradiation	PTK6 Activates STAT3|Reactome	1.034018795	-0.05185131	6.026649628	0.00016504	0.01666204	1.304296402
hetero after UVB irradiation VS hetero before UVB irradiation	PTK6 Activates STAT3|Reactome	0.769953801	-1.54E-06	3.626498204	0.0061565	0.08229128	-2.13120442
WT after UVB irradiation VS WT before UVB irradiation	PTK6 Activates STAT3|Reactome	0.683829232	0.035401308	2.802174541	0.01858663	0.31542987	-3.00549294
homo after UVB irradiation VS homo before UVB irradiation	PTK6 Down-Regulation|Reactome	0.143855177	-0.10769099	0.47426326	0.64614802	0.75537774	-6.24906013
hetero after UVB irradiation VS hetero before UVB irradiation	PTK6 Down-Regulation|Reactome	0.358110013	0.005745955	1.105274695	0.29959823	0.53033405	-5.72897793
WT after UVB irradiation VS WT before UVB irradiation	PTK6 Down-Regulation|Reactome	-0.01100698	0.0729507	-0.03511666	0.97267225	0.99045333	-5.86852651
homo after UVB irradiation VS homo before UVB irradiation	PTK6 Expression|Reactome	0.574884197	-0.04886615	2.651830278	0.02549431	0.10578915	-3.57546409
hetero after UVB irradiation VS hetero before UVB irradiation	PTK6 Expression|Reactome	0.577124781	0.080189112	2.267320876	0.05149933	0.20097082	-4.18753496
WT after UVB irradiation VS WT before UVB irradiation	PTK6 Expression|Reactome	0.483748588	-0.02712589	1.644788231	0.13079025	0.54351142	-4.66715306
homo after UVB irradiation VS homo before UVB irradiation	PTK6 promotes HIF1A stabilization|Reactome	0.349369953	-0.04854267	1.481460318	0.17124465	0.32186978	-5.31215761
hetero after UVB irradiation VS hetero before UVB irradiation	PTK6 promotes HIF1A stabilization|Reactome	0.363882525	-0.01705604	1.646038969	0.13645968	0.33602293	-5.07791882
WT after UVB irradiation VS WT before UVB irradiation	PTK6 promotes HIF1A stabilization|Reactome	0.135527418	0.052068591	0.539746349	0.60108882	0.86824073	-5.723822
homo after UVB irradiation VS homo before UVB irradiation	PTK6 Regulates Cell Cycle|Reactome	-0.372624	-0.03167547	-1.58494292	0.14604146	0.2913957	-5.17679877
hetero after UVB irradiation VS hetero before UVB irradiation	PTK6 Regulates Cell Cycle|Reactome	0.124860884	-0.07809281	0.520913006	0.61583704	0.80062328	-6.2017252
WT after UVB irradiation VS WT before UVB irradiation	PTK6 Regulates Cell Cycle|Reactome	-0.00902207	0.085383631	-0.02733468	0.97872629	0.99283707	-5.86877269
homo after UVB irradiation VS homo before UVB irradiation	PTK6 Regulates Proteins Involved in RNA Processing|Reactome	-0.21266076	-0.00743914	-0.80435075	0.44108412	0.59091566	-6.03260475
hetero after UVB irradiation VS hetero before UVB irradiation	PTK6 Regulates Proteins Involved in RNA Processing|Reactome	0.245635299	0.05855487	0.756087814	0.47020469	0.69247644	-6.0467833
WT after UVB irradiation VS WT before UVB irradiation	PTK6 Regulates Proteins Involved in RNA Processing|Reactome	0.20724398	0.024222001	0.828848768	0.42637666	0.79189192	-5.53335363
homo after UVB irradiation VS homo before UVB irradiation	PTK6 Regulates RHO GTPases_ RAS GTPase and MAP kinases|Reactome	0.348359695	-0.06795589	1.49196746	0.16852219	0.31901838	-5.29866125
hetero after UVB irradiation VS hetero before UVB irradiation	PTK6 Regulates RHO GTPases_ RAS GTPase and MAP kinases|Reactome	0.701354474	-0.03298108	4.436138059	0.00191867	0.04958933	-0.98936021
WT after UVB irradiation VS WT before UVB irradiation	PTK6 Regulates RHO GTPases_ RAS GTPase and MAP kinases|Reactome	0.283841297	0.014955768	1.153330741	0.27538357	0.66577703	-5.23936132
homo after UVB irradiation VS homo before UVB irradiation	PTK6 Regulates RTKs and Their Effectors AKT1 and DOK1|Reactome	0.475087824	-0.01769406	1.963388787	0.07987151	0.19976593	-4.64265314
hetero after UVB irradiation VS hetero before UVB irradiation	PTK6 Regulates RTKs and Their Effectors AKT1 and DOK1|Reactome	0.825251366	0.049234166	4.184480659	0.00272905	0.05916047	-1.33370611
WT after UVB irradiation VS WT before UVB irradiation	PTK6 Regulates RTKs and Their Effectors AKT1 and DOK1|Reactome	0.174750409	0.014822087	0.789797419	0.44782275	0.80421615	-5.56326916
homo after UVB irradiation VS homo before UVB irradiation	Purine catabolism|Reactome	0.008453293	-0.00611885	0.049829343	0.96130236	0.97692111	-6.36711433
hetero after UVB irradiation VS hetero before UVB irradiation	Purine catabolism|Reactome	0.184601598	-0.00424722	0.867872487	0.40949958	0.63634069	-5.95569231
WT after UVB irradiation VS WT before UVB irradiation	Purine catabolism|Reactome	0.039119212	-0.04820511	0.156405246	0.87880112	0.96716565	-5.85677472

homo after UVB irradiation VS homo before UVB irradiation	purine deoxyribonucleosides degradation|HumanCyc	0.279091339	0.049351677	0.762925083	0.46424392	0.6088463	-6.06528519
hetero after UVB irradiation VS hetero before UVB irradiation	purine deoxyribonucleosides degradation|HumanCyc	0.42449252	0.172044722	1.301755502	0.22745945	0.45082721	-5.51135082
WT after UVB irradiation VS WT before UVB irradiation	purine deoxyribonucleosides degradation|HumanCyc	0.470391088	-0.03565739	1.634974045	0.13285119	0.54653478	-4.67976277
homo after UVB irradiation VS homo before UVB irradiation	purine deoxyribonucleosides salvage|HumanCyc	0.224800853	-0.01534101	0.824112044	0.43031229	0.58181948	-6.01648725
hetero after UVB irradiation VS hetero before UVB irradiation	purine deoxyribonucleosides salvage|HumanCyc	0.053464517	-0.06165083	0.212631007	0.83666908	0.9377877	-6.32208154
WT after UVB irradiation VS WT before UVB irradiation	purine deoxyribonucleosides salvage|HumanCyc	0.079798032	-0.0652427	0.373649574	0.71640219	0.90536666	-5.79894647
homo after UVB irradiation VS homo before UVB irradiation	Purine metabolism - Homo sapiens (human)|KEGG	-0.04711439	-0.02789616	-0.27132323	0.79201374	0.86868477	-6.32905245
hetero after UVB irradiation VS hetero before UVB irradiation	Purine metabolism - Homo sapiens (human)|KEGG	0.034951042	-0.02958341	0.207677858	0.84041247	0.93932613	-6.32320487
WT after UVB irradiation VS WT before UVB irradiation	Purine metabolism - Homo sapiens (human)|KEGG	0.117376662	-0.04823972	0.559266324	0.58818706	0.86161188	-5.71329494
homo after UVB irradiation VS homo before UVB irradiation	Purine metabolism|EHMN	-0.04729468	-0.00567081	-0.28502105	0.78181538	0.85915019	-6.32499225
hetero after UVB irradiation VS hetero before UVB irradiation	Purine metabolism|EHMN	0.051160552	0.004465582	0.302612798	0.76952124	0.8963654	-6.29713857
WT after UVB irradiation VS WT before UVB irradiation	Purine metabolism|EHMN	0.07730822	-0.00988668	0.379834131	0.71194711	0.90420468	-5.79662108
homo after UVB irradiation VS homo before UVB irradiation	Purine Metabolism|SMPDB	0.019015587	-0.03943216	0.101997834	0.92090359	0.95153785	-6.36286155
hetero after UVB irradiation VS hetero before UVB irradiation	Purine Metabolism|SMPDB	-0.02085195	-0.0438046	-0.1098564	0.91509485	0.97054702	-6.33999905
WT after UVB irradiation VS WT before UVB irradiation	Purine Metabolism|SMPDB	0.165884344	-0.0622813	0.759490799	0.46494629	0.80891799	-5.58561498
homo after UVB irradiation VS homo before UVB irradiation	Purine metabolism|Wikipathways	0.302236963	-0.02170312	1.326692096	0.21597045	0.37381389	-5.50391407
hetero after UVB irradiation VS hetero before UVB irradiation	Purine metabolism|Wikipathways	0.305637176	-0.04454762	1.207458292	0.26005443	0.48618872	-5.61888328
WT after UVB irradiation VS WT before UVB irradiation	Purine metabolism|Wikipathways	0.568457936	-0.02725107	2.744000312	0.02054527	0.31542987	-3.09299523
homo after UVB irradiation VS homo before UVB irradiation	Purine Nucleoside Phosphorylase Deficiency|SMPDB	0.019015587	-0.03943216	0.101997834	0.92090359	0.95153785	-6.36286155
hetero after UVB irradiation VS hetero before UVB irradiation	Purine Nucleoside Phosphorylase Deficiency|SMPDB	-0.02085195	-0.0438046	-0.1098564	0.91509485	0.97054702	-6.33999905
WT after UVB irradiation VS WT before UVB irradiation	Purine Nucleoside Phosphorylase Deficiency|SMPDB	0.165884344	-0.0622813	0.759490799	0.46494629	0.80891799	-5.58561498
homo after UVB irradiation VS homo before UVB irradiation	purine nucleotides <i>de novo</i> biosynthesis|HumanCyc	-0.01189734	-0.02689678	-0.0646064	0.94984208	0.96980745	-6.36620607
hetero after UVB irradiation VS hetero before UVB irradiation	purine nucleotides <i>de novo</i> biosynthesis|HumanCyc	-0.09580287	-0.03274715	-0.55359363	0.59423871	0.78485658	-6.18332563
WT after UVB irradiation VS WT before UVB irradiation	purine nucleotides <i>de novo</i> biosynthesis|HumanCyc	0.165225006	-0.02350113	0.716622759	0.48987406	0.81710765	-5.6158989
homo after UVB irradiation VS homo before UVB irradiation	purine nucleotides degradation|HumanCyc	0.402707666	-0.02740461	2.121987678	0.0615837	0.17010961	-4.40474013
hetero after UVB irradiation VS hetero before UVB irradiation	purine nucleotides degradation|HumanCyc	0.16665118	0.04815935	0.877845367	0.4043624	0.63292713	-5.94705425
WT after UVB irradiation VS WT before UVB irradiation	purine nucleotides degradation|HumanCyc	0.124561649	0.004183766	0.494932969	0.63125513	0.87303764	-5.74665558
homo after UVB irradiation VS homo before UVB irradiation	Purine nucleotides nucleosides metabolism|INOH	0.282199769	-0.03443359	1.622477119	0.13774885	0.27994034	-5.12642085
hetero after UVB irradiation VS hetero before UVB irradiation	Purine nucleotides nucleosides metabolism|INOH	0.099346889	-0.03383192	0.561165807	0.58929338	0.78149527	-6.17891408
WT after UVB irradiation VS WT before UVB irradiation	Purine nucleotides nucleosides metabolism|INOH	0.196397768	-0.05193891	0.909846307	0.38413887	0.75745148	-5.46738148
homo after UVB irradiation VS homo before UVB irradiation	Purine ribonucleoside monophosphate biosynthesis|Reactome	-0.46171959	-0.00454307	-1.9057606	0.08771168	0.21178822	-4.72733715
hetero after UVB irradiation VS hetero before UVB irradiation	Purine ribonucleoside monophosphate biosynthesis|Reactome	-0.59093007	-0.10538654	-2.22461184	0.05512482	0.20875475	-4.25148326
WT after UVB irradiation VS WT before UVB irradiation	Purine ribonucleoside monophosphate biosynthesis|Reactome	-0.07717872	-0.04455922	-0.27886629	0.78598379	0.93869978	-5.82991803
homo after UVB irradiation VS homo before UVB irradiation	purine ribonucleosides degradation to ribose-1-phosphate|HumanCyc	-0.09392514	-0.03761276	-0.24637983	0.81069045	0.88322896	-6.33594014
hetero after UVB irradiation VS hetero before UVB irradiation	purine ribonucleosides degradation to ribose-1-phosphate|HumanCyc	0.109267697	0.114965336	0.327387965	0.75136074	0.88265522	-6.28877276
WT after UVB irradiation VS WT before UVB irradiation	purine ribonucleosides degradation to ribose-1-phosphate|HumanCyc	-0.1832281	-0.00480301	-0.56179422	0.58652709	0.86138389	-5.71190606
homo after UVB irradiation VS homo before UVB irradiation	Purine salvage|Reactome	0.366774149	-0.04999813	1.818946762	0.10089503	0.23103646	-4.8528632
hetero after UVB irradiation VS hetero before UVB irradiation	Purine salvage|Reactome	0.387351335	-0.00992177	2.002142901	0.07844552	0.25179907	-4.57946867
WT after UVB irradiation VS WT before UVB irradiation	Purine salvage|Reactome	0.406151767	-0.01188175	1.812628326	0.09972801	0.49940551	-4.44562222
homo after UVB irradiation VS homo before UVB irradiation	Putative anti-Inflammatory metabolites formation from EPA|EHMN	-0.17379388	0.003504202	-0.94624341	0.36773854	0.52081084	-5.90955455
hetero after UVB irradiation VS hetero before UVB irradiation	Putative anti-Inflammatory metabolites formation from EPA|EHMN	0.049711415	0.012115228	0.260990284	0.80036601	0.9161397	-6.30974171
WT after UVB irradiation VS WT before UVB irradiation	Putative anti-Inflammatory metabolites formation from EPA|EHMN	0.030144611	-0.04328346	0.125834856	0.90233659	0.97566251	-5.86113633
homo after UVB irradiation VS homo before UVB irradiation	putrescine biosynthesis I|HumanCyc	-1.03690358	-0.00320991	-4.78493479	0.00088315	0.01776424	-0.30570793
hetero after UVB irradiation VS hetero before UVB irradiation	putrescine biosynthesis I|HumanCyc	-1.07363932	-0.04184005	-4.74927376	0.00125382	0.04329482	-0.57518917
WT after UVB irradiation VS WT before UVB irradiation	putrescine biosynthesis I|HumanCyc	-0.30237814	-0.05085941	-0.73532799	0.47889587	0.81552033	-5.60287708
homo after UVB irradiation VS homo before UVB irradiation	putrescine biosynthesis II|HumanCyc	-0.67304202	0.030984416	-2.12218039	0.06156414	0.17010961	-4.40444717
hetero after UVB irradiation VS hetero before UVB irradiation	putrescine biosynthesis II|HumanCyc	-0.13897854	-0.02933163	-0.35621542	0.73043445	0.86896773	-6.27823289
WT after UVB irradiation VS WT before UVB irradiation	putrescine biosynthesis II|HumanCyc	0.198578175	0.042767103	0.740995598	0.47560034	0.81430085	-5.59887255
homo after UVB irradiation VS homo before UVB irradiation	putrescine degradation III|HumanCyc	0.097305154	0.028717927	0.538779022	0.60257766	0.72195476	-6.21490788
hetero after UVB irradiation VS hetero before UVB irradiation	putrescine degradation III|HumanCyc	-0.13119437	0.031657224	-0.64301723	0.53731502	0.74987197	-6.12772482
WT after UVB irradiation VS WT before UVB irradiation	putrescine degradation III|HumanCyc	0.107758044	-0.01563923	0.430325655	0.67601214	0.88217037	-5.7762573
homo after UVB irradiation VS homo before UVB irradiation	pyridoxal 5_-phosphate salvage|HumanCyc	0.106915991	-0.0211306	0.372211231	0.71799819	0.81511182	-6.29456867
hetero after UVB irradiation VS hetero before UVB irradiation	pyridoxal 5_-phosphate salvage|HumanCyc	0.436232573	0.001619417	2.135184153	0.06354822	0.22631041	-4.3844562
WT after UVB irradiation VS WT before UVB irradiation	pyridoxal 5_-phosphate salvage|HumanCyc	0.682156802	0.047066662	2.599929451	0.02633192	0.34359389	-3.30931674
homo after UVB irradiation VS homo before UVB irradiation	Pyridoxine dependency with seizures|SMPDB	-0.02100802	-0.06308448	-0.07524201	0.941601	0.96450524	-6.36540732
hetero after UVB irradiation VS hetero before UVB irradiation	Pyridoxine dependency with seizures|SMPDB	-0.10000854	-0.05054657	-0.39990823	0.69917117	0.84778456	-6.26061929
WT after UVB irradiation VS WT before UVB irradiation	Pyridoxine dependency with seizures|SMPDB	-0.11826398	-0.1134336	-0.44264387	0.66736875	0.87935805	-5.77091861
homo after UVB irradiation VS homo before UVB irradiation	Pyrimidine biosynthesis|Reactome	-0.84202168	-0.09722435	-2.97749022	0.01482149	0.07510999	-3.05422985
hetero after UVB irradiation VS hetero before UVB irradiation	Pyrimidine biosynthesis|Reactome	-0.57905062	-0.01768283	-1.74466925	0.1172894	0.31030895	-4.94403374
WT after UVB irradiation VS WT before UVB irradiation	Pyrimidine biosynthesis|Reactome	0.319661237	0.135101974	0.909784347	0.38417002	0.75745148	-5.46743393
homo after UVB irradiation VS homo before UVB irradiation	Pyrimidine catabolism|Reactome	-0.02629634	0.052633339	-0.12983136	0.89943887	0.9399608	-6.35939968
hetero after UVB irradiation VS hetero before UVB irradiation	Pyrimidine catabolism|Reactome	0.180283135	0.018259598	1.084631954	0.3081402	0.53651817	-5.7503553
WT after UVB irradiation VS WT before UVB irradiation	Pyrimidine catabolism|Reactome	0.077411032	-0.01281603	0.330394588	0.74786493	0.9185045	-5.81417135
homo after UVB irradiation VS homo before UVB irradiation	pyrimidine deoxyribonucleosides degradation|HumanCyc	0.332394643	-0.00210254	1.544084485	0.15557151	0.30343422	-5.23087957
hetero after UVB irradiation VS hetero before UVB irradiation	pyrimidine deoxyribonucleosides degradation|HumanCyc	0.521931621	0.000114525	1.840793601	0.10103966	0.28604891	-4.81024031
WT after UVB irradiation VS WT before UVB irradiation	pyrimidine deoxyribonucleosides degradation|HumanCyc	0.258878518	-0.04564568	0.788172405	0.4487303	0.80492981	-5.56448677
homo after UVB irradiation VS homo before UVB irradiation	pyrimidine deoxyribonucleosides salvage|HumanCyc	0.266832093	0.010744973	1.027941072	0.3297298	0.4858168	-5.83128727
hetero after UVB irradiation VS hetero before UVB irradiation	pyrimidine deoxyribonucleosides salvage|HumanCyc	0.950188008	0.078366518	4.244595599	0.00250666	0.05623149	-1.25053909
WT after UVB irradiation VS WT before UVB irradiation	pyrimidine deoxyribonucleosides salvage|HumanCyc	0.939101867	-0.0556094	3.948195749	0.00269519	0.18515985	-1.32330848
homo after UVB irradiation VS homo before UVB irradiation	pyrimidine deoxyribonucleotide phosphorylation|HumanCyc	0.065377213	-0.03329399	0.224721384	0.82701062	0.89374727	-6.34138925
hetero after UVB irradiation VS hetero before UVB irradiation	pyrimidine deoxyribonucleotide phosphorylation|HumanCyc	-0.08522028	-0.02769564	-0.35109444	0.73413511	0.8693688	-6.28016827
WT after UVB irradiation VS WT before UVB irradiation	pyrimidine deoxyribonucleotide phosphorylation|HumanCyc	0.151099051	-0.04108998	0.65257199	0.52862991	0.83278813	-5.65819484
homo after UVB irradiation VS homo before UVB irradiation	pyrimidine deoxyribonucleotides <i>de novo</i> biosynthesis|HumanCyc	-0.25833768	-0.05061722	-1.10454349	0.29685767	0.45739605	-5.75327782
hetero after UVB irradiation VS hetero before UVB irradiation	pyrimidine deoxyribonucleotides <i>de novo</i> biosynthesis|HumanCyc	-0.1543867	-0.03309873	-0.5512067	0.59580225	0.78555587	-6.18470474
WT after UVB irradiation VS WT before UVB irradiation	pyrimidine deoxyribonucleotides <i>de novo</i> biosynthesis|HumanCyc	0.186586466	-0.08505704	0.774836609	0.45622364	0.80891799	-5.57439611
homo after UVB irradiation VS homo before UVB irradiation	pyrimidine deoxyribonucleotides biosynthesis from CTP|HumanCyc	-0.274202	-0.05281	-1.1873683	0.26425209	0.42756026	-5.66416893
hetero after UVB irradiation VS hetero before UVB irradiation	pyrimidine deoxyribonucleotides biosynthesis from CTP|HumanCyc	-0.16587835	-0.03772951	-0.62909068	0.54596344	0.75249512	-6.13688213
WT after UVB irradiation VS WT before UVB irradiation	pyrimidine deoxyribonucleotides biosynthesis from CTP|HumanCyc	0.1456139	-0.07386421	0.580361345	0.5744122	0.8562298	-5.70152589
homo after UVB irradiation VS homo before UVB irradiation	Pyrimidine metabolism - Homo sapiens (human)|KEGG	-0.40985268	-0.02573719	-2.44575339	0.03596938	0.12504267	-3.90245271
hetero after UVB irradiation VS hetero before UVB irradiation	Pyrimidine metabolism - Homo sapiens (human)|KEGG	0.010523681	-0.04702828	0.050604977	0.96081945	0.9814915	-6.34515226
WT after UVB irradiation VS WT before UVB irradiation	Pyrimidine metabolism - Homo sapiens (human)|KEGG	0.062673394	-0.02049789	0.256940278	0.8023923	0.94525798	-5.83582413
homo after UVB irradiation VS homo before UVB irradiation	Pyrimidine metabolism|EHMN	-0.29428058	-0.00982854	-1.63420675	0.13524534	0.277359	-5.11054491
hetero after UVB irradiation VS hetero before UVB irradiation	Pyrimidine metabolism|EHMN	0.019124884	-0.02413388	0.098985968	0.92346401	0.97054702	-6.34122898
WT after UVB irradiation VS WT before UVB irradiation	Pyrimidine metabolism|EHMN	0.05872863	-0.02814796	0.243082055	0.81281595	0.94525798	-5.83931121
homo after UVB irradiation VS homo before UVB irradiation	Pyrimidine Metabolism|SMPDB	-0.15230025	-0.02916717	-0.66373722	0.52282067	0.65944729	-6.13727985
hetero after UVB irradiation VS hetero before UVB irradiation	Pyrimidine Metabolism|SMPDB	-0.07293889	-0.06090355	-0.36429729	0.72460928	0.86482318	-6.27512326
WT after UVB irradiation VS WT before UVB irradiation	Pyrimidine Metabolism|SMPDB	0.197121499	-0.04726474	0.926444646	0.37585917	0.75077508	-5.45322485
homo after UVB irradiation VS homo before UVB irradiation	Pyrimidine metabolism|Wikipathways	-0.43710646	-0.02118841	-2.59115815	0.02821385	0.11231425	-3.67211059
hetero after UVB irradiation VS hetero before UVB irradiation	Pyrimidine metabolism|Wikipathways	0.02489175	-0.03326065	0.114201856	0.91175234	0.97054702	-6.33947166
WT after UVB irradiation VS WT before UVB irradiation	Pyrimidine metabolism|Wikipathways	0.09421766	0.010311958	0.358573062	0.72730915	0.91040977	-5.80445982
homo after UVB irradiation VS homo before UVB irradiation	Pyrimidine nucleotides nucleosides metabolism|INOH	-0.19788051	-0.06087336	-1.08050928	0.30688662	0.46864358	-5.77822083
hetero after UVB irradiation VS hetero before UVB irradiation	Pyrimidine nucleotides nucleosides metabolism|INOH	-0.02559371	-0.04039148	-0.1304515	0.89927013	0.96973277	-6.33731891
WT after UVB irradiation VS WT before UVB irradiation	Pyrimidine nucleotides nucleosides metabolism|INOH	0.134104398	-0.05057063	0.578944162	0.57533207	0.8562298	-5.70232926
homo after UVB irradiation VS homo before UVB irradiation	pyrimidine ribonucleosides degradation|HumanCyc	-0.12220256	0.011869548	-0.6429085	0.53566653	0.67156874	-6.15124875
homo after UVB irradiation VS homo before UVB irradiation	pyrimidine ribonucleosides degradation|HumanCyc	0.057218022	-0.05212171	0.199781447	0.84591155	0.90528768	-6.34704971
hetero after UVB irradiation VS hetero before UVB irradiation	pyrimidine ribonucleosides degradation|HumanCyc	0.689678695	0.004091313	1.861888297	0.09776856	0.28215302	-4.78048422
hetero after UVB irradiation VS hetero before UVB irradiation	pyrimidine ribonucleosides degradation|HumanCyc	0.073153984	0.001646221	0.266033121	0.79660798	0.91436393	-6.30831206
WT after UVB irradiation VS WT before UVB irradiation	pyrimidine ribonucleosides degradation|HumanCyc	0.102545002	-0.02532254	0.307274123	0.76488785	0.92732753	-5.82155971
WT after UVB irradiation VS WT before UVB irradiation	pyrimidine ribonucleosides degradation|HumanCyc	-0.04568143	0.001776317	-0.20516528	0.84152599	0.95175499	-5.84787502
homo after UVB irradiation VS homo before UVB irradiation	pyrimidine ribonucleosides salvage I|HumanCyc	0.78205661	-0.09215243	3.430337132	0.00705116	0.0487074	-2.33274868
hetero after UVB irradiation VS hetero before UVB irradiation	pyrimidine ribonucleosides salvage I|HumanCyc	0.75938165	-0.13750753	3.001320152	0.0160622	0.11352943	-3.06904992
WT after UVB irradiation VS WT before UVB irradiation	pyrimidine ribonucleosides salvage I|HumanCyc	0.451450423	-0.05926676	1.495377477	0.16544332	0.56938932	-4.8544862
homo after UVB irradiation VS homo before UVB irradiation	Pyrimidine salvage|Reactome	0.303020326	-0.05775286	1.330637432	0.21471787	0.37277634	-5.49919798
hetero after UVB irradiation VS hetero before UVB irradiation	Pyrimidine salvage|Reactome	0.812362869	-0.02766323	3.90462604	0.00408179	0.07021718	-1.72813013
WT after UVB irradiation VS WT before UVB irradiation	Pyrimidine salvage|Reactome	0.56090597	-0.06949003	2.13192053	0.05861128	0.42556076	-3.99926736
homo after UVB irradiation VS homo before UVB irradiation	Pyrophosphate hydrolysis|Reactome	-1.03093213	0.02298293	-5.44514758	0.00035238	0.01666204	0.581410316
hetero after UVB irradiation VS hetero before UVB irradiation	Pyrophosphate hydrolysis|Reactome	-0.71315064	-0.0353279	-2.61142568	0.02975145	0.14964797	-3.66536888
WT after UVB irradiation VS WT before UVB irradiation	Pyrophosphate hydrolysis|Reactome	-0.85424908	-0.08332947	-4.04850039	0.00229064	0.17027242	-1.18350062
homo after UVB irradiation VS homo before UVB irradiation	Pyruvaldehyde Degradation|SMPDB	1.094689818	0.010272288	4.542527002	0.00125718	0.02014127	-0.64908507
hetero after UVB irradiation VS hetero before UVB irradiation	Pyruvaldehyde Degradation|SMPDB	1.014841638	-0.00299866	5.639355378	0.00040412	0.03004176	0.513799665
WT after UVB irradiation VS WT before UVB irradiation	Pyruvaldehyde Degradation|SMPDB	0.64205161	0.065849611	1.889300813	0.08792305	0.48023726	-4.34108857
homo after UVB irradiation VS homo before UVB irradiation	Pyruvate Carboxylase Deficiency|SMPDB	-0.39210009	0.022178168	-1.25326746	0.24041731	0.40210456	-5.58992632
hetero after UVB irradiation VS hetero before UVB irradiation	Pyruvate Carboxylase Deficiency|SMPDB	-0.35251865	0.016423987	-1.29321274	0.23026395	0.45170905	-5.52131443
WT after UVB irradiation VS WT before UVB irradiation	Pyruvate Carboxylase Deficiency|SMPDB	0.415408645	-0.09593973	1.608715996	0.13850837	0.55342759	-4.71329834
homo after UVB irradiation VS homo before UVB irradiation	Pyruvate Decarboxylase E1 Component Deficiency (PDHE1 Deficiency)|SMPDB	-0.1559239	-0.03195163	-0.60679304	0.55837377	0.6849905	-6.17450252
hetero after UVB irradiation VS hetero before UVB irradiation	Pyruvate Decarboxylase E1 Component Deficiency (PDHE1 Deficiency)|SMPDB	-0.2311653	-0.04505886	-1.14490839	0.28372423	0.51297702	-5.68710621
WT after UVB irradiation VS WT before UVB irradiation	Pyruvate Decarboxylase E1 Component Deficiency (PDHE1 Deficiency)|SMPDB	-0.05726125	-0.08194106	-0.22775541	0.82438877	0.94525798	-5.84294542
homo after UVB irradiation VS homo before UVB irradiation	pyruvate decarboxylation to acetyl CoA|HumanCyc	-0.41475087	-0.11558559	-1.22712947	0.24965234	0.41211591	-5.61971706
hetero after UVB irradiation VS hetero before UVB irradiation	pyruvate decarboxylation to acetyl CoA|HumanCyc	-0.65601836	-0.06975517	-2.44082418	0.03904921	0.17223099	-3.92546065
WT after UVB irradiation VS WT before UVB irradiation	pyruvate decarboxylation to acetyl CoA|HumanCyc	-0.40592299	-0.16110582	-1.15959446	0.27293861	0.66445131	-5.2329481
homo after UVB irradiation VS homo before UVB irradiation	Pyruvate Dehydrogenase Complex Deficiency|SMPDB	-0.1559239	-0.03195163	-0.60679304	0.55837377	0.6849905	-6.17450252
hetero after UVB irradiation VS hetero before UVB irradiation	Pyruvate Dehydrogenase Complex Deficiency|SMPDB	-0.2311653	-0.04505886	-1.14490839	0.28372423	0.51297702	-5.68710621
WT after UVB irradiation VS WT before UVB irradiation	Pyruvate Dehydrogenase Complex Deficiency|SMPDB	-0.05726125	-0.08194106	-0.22775541	0.82438877	0.94525798	-5.84294542
homo after UVB irradiation VS homo before UVB irradiation	Pyruvate dehydrogenase deficiency (E2)|SMPDB	-0.23678174	0.014544733	-0.76379894	0.46374733	0.6088463	-6.06461144
hetero after UVB irradiation VS hetero before UVB irradiation	Pyruvate dehydrogenase deficiency (E2)|SMPDB	-0.47814985	0.004803127	-1.74993278	0.11633994	0.30887493	-4.93678811
WT after UVB irradiation VS WT before UVB irradiation	Pyruvate dehydrogenase deficiency (E2)|SMPDB	-0.1568339	-0.12513755	-0.50250429	0.62610623	0.87257466	-5.74292911
homo after UVB irradiation VS homo before UVB irradiation	Pyruvate dehydrogenase deficiency (E3)|SMPDB	-0.23678174	0.014544733	-0.76379894	0.46374733	0.6088463	-6.06461144
hetero after UVB irradiation VS hetero before UVB irradiation	Pyruvate dehydrogenase deficiency (E3)|SMPDB	-0.47814985	0.004803127	-1.74993278	0.11633994	0.30887493	-4.93678811
WT after UVB irradiation VS WT before UVB irradiation	Pyruvate dehydrogenase deficiency (E3)|SMPDB	-0.1568339	-0.12513755	-0.50250429	0.62610623	0.87257466	-5.74292911
homo after UVB irradiation VS homo before UVB irradiation	Pyruvate kinase deficiency|SMPDB	-0.1559239	-0.03195163	-0.60679304	0.55837377	0.6849905	-6.17450252
hetero after UVB irradiation VS hetero before UVB irradiation	Pyruvate kinase deficiency|SMPDB	-0.2311653	-0.04505886	-1.14490839	0.28372423	0.51297702	-5.68710621
WT after UVB irradiation VS WT before UVB irradiation	Pyruvate kinase deficiency|SMPDB	-0.05726125	-0.08194106	-0.22775541	0.82438877	0.94525798	-5.84294542
homo after UVB irradiation VS homo before UVB irradiation	Pyruvate metabolism - Homo sapiens (human)|KEGG	-0.15236647	0.015156207	-0.69658962	0.50293903	0.64253487	-6.11442822
hetero after UVB irradiation VS hetero before UVB irradiation	Pyruvate metabolism - Homo sapiens (human)|KEGG	-0.15619682	-0.0323661	-0.76019032	0.46787624	0.6901553	-6.04363065
WT after UVB irradiation VS WT before UVB irradiation	Pyruvate metabolism - Homo sapiens (human)|KEGG	-0.04609411	-0.04740282	-0.18048961	0.8603446	0.95916804	-5.85267675
homo after UVB irradiation VS homo before UVB irradiation	Pyruvate metabolism and Citric Acid (TCA) cycle|Reactome	-0.10343581	-0.01210301	-0.45186943	0.6616211	0.76791102	-6.25994757
hetero after UVB irradiation VS hetero before UVB irradiation	Pyruvate metabolism and Citric Acid (TCA) cycle|Reactome	-0.33020052	-0.01453947	-1.50141361	0.16975799	0.38622363	-5.26702946
WT after UVB irradiation VS WT before UVB irradiation	Pyruvate metabolism and Citric Acid (TCA) cycle|Reactome	-0.11552222	-0.079431	-0.45031895	0.66200888	0.87935805	-5.7675192
homo after UVB irradiation VS homo before UVB irradiation	Pyruvate metabolism|INOH	-0.07362354	-0.01341467	-0.31137106	0.76232134	0.84648814	-6.31663016
hetero after UVB irradiation VS hetero before UVB irradiation	Pyruvate metabolism|INOH	-0.13570415	-0.04231617	-0.68171604	0.51371696	0.73158211	-6.10133228
WT after UVB irradiation VS WT before UVB irradiation	Pyruvate metabolism|INOH	-0.02976047	-0.05240263	-0.1191201	0.90752029	0.97566251	-5.86196825
homo after UVB irradiation VS homo before UVB irradiation	Pyruvate metabolism|Reactome	0.128777768	0.008422691	0.745812471	0.47403798	0.61754234	-6.07834197
hetero after UVB irradiation VS hetero before UVB irradiation	Pyruvate metabolism|Reactome	-0.16453688	-0.02889464	-0.77265811	0.46084655	0.68466055	-6.03395888
WT after UVB irradiation VS WT before UVB irradiation	Pyruvate metabolism|Reactome	-0.02044398	-0.03365559	-0.08232081	0.93600301	0.98056728	-5.86571938
homo after UVB irradiation VS homo before UVB irradiation	Pyruvate Metabolism|SMPDB	-0.1559239	-0.03195163	-0.60679304	0.55837377	0.6849905	-6.17450252
hetero after UVB irradiation VS hetero before UVB irradiation	Pyruvate Metabolism|SMPDB	-0.2311653	-0.04505886	-1.14490839	0.28372423	0.51297702	-5.68710621
WT after UVB irradiation VS WT before UVB irradiation	Pyruvate Metabolism|SMPDB	-0.05726125	-0.08194106	-0.22775541	0.82438877	0.94525798	-5.84294542
homo after UVB irradiation VS homo before UVB irradiation	Quercetin and Nf-kB- AP-1 Induced Cell Apoptosis|Wikipathways	0.496809145	-0.02475197	1.917561897	0.08604943	0.20899026	-4.7100796

hetero after UVB irradiation VS hetero before UVB irradiation	Quercetin and Nf-kB- AP-1 Induced Cell Apoptosis|Wikipathways	0.511446087	0.07693866	1.942194634	0.0862107	0.26568518	-4.66602681
WT after UVB irradiation VS WT before UVB irradiation	Quercetin and Nf-kB- AP-1 Induced Cell Apoptosis|Wikipathways	0.181196549	-0.00785883	0.641702879	0.53538208	0.83767333	-5.66501505
homo after UVB irradiation VS homo before UVB irradiation	Quinapril Action Pathway|SMPDB	0.636038586	-0.11800708	2.436891056	0.03650532	0.12504267	-3.91641947
hetero after UVB irradiation VS hetero before UVB irradiation	Quinapril Action Pathway|SMPDB	-0.02777369	0.003323189	-0.09172332	0.92906122	0.97054702	-6.34197953
WT after UVB irradiation VS WT before UVB irradiation	Quinapril Action Pathway|SMPDB	0.270438532	-0.10210744	0.707878218	0.49505953	0.81710765	-5.62188349
homo after UVB irradiation VS homo before UVB irradiation	Quinapril Metabolism Pathway|SMPDB	1.076747846	-0.04193589	4.959558579	0.00068841	0.01666204	-0.06417629
hetero after UVB irradiation VS hetero before UVB irradiation	Quinapril Metabolism Pathway|SMPDB	0.553630209	0.003105397	1.56066683	0.15532075	0.36486693	-5.19066683
WT after UVB irradiation VS WT before UVB irradiation	Quinapril Metabolism Pathway|SMPDB	0.585930211	-0.05239369	1.72512433	0.11496929	0.51527453	-4.56245573
homo after UVB irradiation VS homo before UVB irradiation	Quinethazone Action Pathway|SMPDB	0.234726031	0.017120546	1.156747303	0.27595732	0.43614438	-5.69767199
hetero after UVB irradiation VS hetero before UVB irradiation	Quinethazone Action Pathway|SMPDB	-0.03255037	0.028925632	-0.18154472	0.86023155	0.94503144	-6.32869738
WT after UVB irradiation VS WT before UVB irradiation	Quinethazone Action Pathway|SMPDB	-0.15908359	0.010252305	-0.80382265	0.44003996	0.79685269	-5.55266936
homo after UVB irradiation VS homo before UVB irradiation	Quinidine Action Pathway|SMPDB	0.396415397	-0.03677376	2.343069613	0.04268583	0.13402181	-4.06364635
hetero after UVB irradiation VS hetero before UVB irradiation	Quinidine Action Pathway|SMPDB	-0.01555055	-0.01775524	-0.07410913	0.94265284	0.97054702	-6.34356282
WT after UVB irradiation VS WT before UVB irradiation	Quinidine Action Pathway|SMPDB	0.056685205	-0.03978418	0.223404833	0.82768199	0.94525798	-5.84393438
homo after UVB irradiation VS homo before UVB irradiation	RA biosynthesis pathway|Reactome	0.577174062	0.017670788	3.746625594	0.00424842	0.03746619	-1.8381238
hetero after UVB irradiation VS hetero before UVB irradiation	RA biosynthesis pathway|Reactome	0.18562825	0.012132805	0.857830858	0.41471851	0.64192892	-5.96430702
WT after UVB irradiation VS WT before UVB irradiation	RA biosynthesis pathway|Reactome	0.130202759	0.018619821	0.496547718	0.63015526	0.87303764	-5.74586533
homo after UVB irradiation VS homo before UVB irradiation	RAB GEFs exchange GTP for GDP on RABs|Reactome	0.166577474	-0.02627513	0.916938961	0.38212285	0.53514858	-5.9363372
hetero after UVB irradiation VS hetero before UVB irradiation	RAB GEFs exchange GTP for GDP on RABs|Reactome	0.273550192	0.003183091	1.710570192	0.12361654	0.31597198	-4.99073355
WT after UVB irradiation VS WT before UVB irradiation	RAB GEFs exchange GTP for GDP on RABs|Reactome	0.22546932	-0.01568815	1.160108528	0.27273872	0.66431878	-5.23242061
homo after UVB irradiation VS homo before UVB irradiation	RAB geranylgeranylation|Reactome	0.3243795	-0.02189833	1.98198221	0.07748642	0.19626432	-4.61511588
hetero after UVB irradiation VS hetero before UVB irradiation	RAB geranylgeranylation|Reactome	0.345635051	0.022734505	1.912729804	0.09029166	0.27214648	-4.70823047
WT after UVB irradiation VS WT before UVB irradiation	RAB geranylgeranylation|Reactome	0.130346618	0.010436534	0.63089862	0.54214344	0.83916339	-5.67169059
homo after UVB irradiation VS homo before UVB irradiation	Rab regulation of trafficking|Reactome	0.145285875	-0.02647916	0.810963019	0.43745985	0.58886842	-6.02724937
hetero after UVB irradiation VS hetero before UVB irradiation	Rab regulation of trafficking|Reactome	0.224968262	-0.00674519	1.480701736	0.17507923	0.39186333	-5.29333244
WT after UVB irradiation VS WT before UVB irradiation	Rab regulation of trafficking|Reactome	0.144214663	-0.01751579	0.734710416	0.47925584	0.81552033	-5.60331178
homo after UVB irradiation VS homo before UVB irradiation	Rabeprazole Action Pathway|SMPDB	0.21780303	0.051698704	1.19150037	0.26270351	0.42561191	-5.65959884
hetero after UVB irradiation VS hetero before UVB irradiation	Rabeprazole Action Pathway|SMPDB	0.331566302	0.063470812	1.441200644	0.18563717	0.40321767	-5.34291209
WT after UVB irradiation VS WT before UVB irradiation	Rabeprazole Action Pathway|SMPDB	-0.11560861	0.135982321	-0.44330845	0.66690385	0.87935805	-5.77062647
homo after UVB irradiation VS homo before UVB irradiation	Rabeprazole Metabolism Pathway|SMPDB	-0.06647614	0.033610835	-0.18292054	0.85874942	0.9134577	-6.35050291
WT after UVB irradiation VS WT before UVB irradiation	Rabeprazole Metabolism Pathway|SMPDB	-0.4370633	0.058240551	-1.4704397	0.17194858	0.57997722	-4.88472923
homo after UVB irradiation VS homo before UVB irradiation	rac1 cell motility signaling pathway|BioCarta	0.404272905	-0.04105547	2.127467321	0.06102976	0.1689884	-4.39640653
hetero after UVB irradiation VS hetero before UVB irradiation	rac1 cell motility signaling pathway|BioCarta	0.374663451	0.011071669	2.074047008	0.07001765	0.23640034	-4.47453145
WT after UVB irradiation VS WT before UVB irradiation	rac1 cell motility signaling pathway|BioCarta	0.342272606	-0.04487097	1.686232323	0.1223993	0.52731779	-4.61346384
homo after UVB irradiation VS homo before UVB irradiation	RAC1 signaling pathway|PID	0.439387285	-0.02332515	2.146148321	0.05917712	0.16658615	-4.36794435
hetero after UVB irradiation VS hetero before UVB irradiation	RAC1 signaling pathway|PID	0.545464269	0.005431284	3.285192565	0.01033535	0.10285702	-2.63876962
WT after UVB irradiation VS WT before UVB irradiation	RAC1 signaling pathway|PID	0.415846593	-0.00858309	2.099848891	0.06187038	0.43347466	-4.04528116
homo after UVB irradiation VS homo before UVB irradiation	RAC1-PAK1-p38-MMP2 Pathway|Wikipathways	0.261397273	-0.02155377	1.348269895	0.20919425	0.36695387	-5.47800638
hetero after UVB irradiation VS hetero before UVB irradiation	RAC1-PAK1-p38-MMP2 Pathway|Wikipathways	0.417357005	0.00880886	2.586597469	0.03095111	0.15350943	-3.70331341
WT after UVB irradiation VS WT before UVB irradiation	RAC1-PAK1-p38-MMP2 Pathway|Wikipathways	0.285947562	-0.02148461	1.395854565	0.19273404	0.60496032	-4.97328192
homo after UVB irradiation VS homo before UVB irradiation	RAF activation|Reactome	0.032154967	-0.02817511	0.14504936	0.88773803	0.93267645	-6.35715671
hetero after UVB irradiation VS hetero before UVB irradiation	RAF activation|Reactome	0.301017216	0.001567678	1.711337686	0.12347072	0.31587425	-4.9896871
WT after UVB irradiation VS WT before UVB irradiation	RAF activation|Reactome	0.108582736	-0.00838744	0.497158711	0.62973934	0.87292043	-5.74556568
homo after UVB irradiation VS homo before UVB irradiation	RAF/MAP kinase cascade|Reactome	0.150839242	-0.01065445	0.970708478	0.35603401	0.51072928	-5.88666637
hetero after UVB irradiation VS hetero before UVB irradiation	RAF/MAP kinase cascade|Reactome	0.150695802	0.014269587	0.906746615	0.38973417	0.61680845	-5.92156263
WT after UVB irradiation VS WT before UVB irradiation	RAF/MAP kinase cascade|Reactome	0.082827147	-0.00131741	0.41401421	0.68753363	0.89178247	-5.78310364
homo after UVB irradiation VS homo before UVB irradiation	RAF-independent MAPK1/3 activation|Reactome	-0.1535089	-0.00879755	-0.77024141	0.4600969	0.60681758	-6.05962336
hetero after UVB irradiation VS hetero before UVB irradiation	RAF-independent MAPK1/3 activation|Reactome	0.156651323	-0.01043175	0.850686298	0.41846004	0.64556195	-5.9703854
WT after UVB irradiation VS WT before UVB irradiation	RAF-independent MAPK1/3 activation|Reactome	-0.05205642	-0.00920323	-0.21276026	0.83575415	0.94951669	-5.84627449
homo after UVB irradiation VS homo before UVB irradiation	RAGE|NetPath	0.310058754	-0.04314085	1.541851849	0.15610778	0.30417351	-5.23381127
hetero after UVB irradiation VS hetero before UVB irradiation	RAGE|NetPath	0.475461674	-0.00387971	2.734438368	0.02446875	0.13718149	-3.4771365
WT after UVB irradiation VS WT before UVB irradiation	RAGE|NetPath	0.414231758	-0.03040521	2.067311213	0.06535288	0.43794589	-4.09173045
homo after UVB irradiation VS homo before UVB irradiation	RalA downstream regulated genes|Wikipathways	0.208722218	-0.05860741	0.871646642	0.40513764	0.55735416	-5.9763474
hetero after UVB irradiation VS hetero before UVB irradiation	RalA downstream regulated genes|Wikipathways	0.554290409	-0.02265916	3.242002628	0.01104648	0.10424401	-2.70384178
WT after UVB irradiation VS WT before UVB irradiation	RalA downstream regulated genes|Wikipathways	0.123661237	0.011673281	0.390797341	0.70407764	0.90113315	-5.79240804
homo after UVB irradiation VS homo before UVB irradiation	Ramipril Action Pathway|SMPDB	0.636038586	-0.11800708	2.436891056	0.03650532	0.12504267	-3.91641947
hetero after UVB irradiation VS hetero before UVB irradiation	Ramipril Action Pathway|SMPDB	-0.02777369	0.003323189	-0.09172332	0.92906122	0.97054702	-6.34197953
WT after UVB irradiation VS WT before UVB irradiation	Ramipril Action Pathway|SMPDB	0.270438532	-0.10210744	0.707878218	0.49505953	0.81710765	-5.62188349
homo after UVB irradiation VS homo before UVB irradiation	Ramipril Metabolism Pathway|SMPDB	1.076747846	-0.04193589	4.959558579	0.00068841	0.01666204	-0.06417629
hetero after UVB irradiation VS hetero before UVB irradiation	Ramipril Metabolism Pathway|SMPDB	0.553630209	0.003105397	1.56066683	0.15532075	0.36486693	-5.19066683
WT after UVB irradiation VS WT before UVB irradiation	Ramipril Metabolism Pathway|SMPDB	0.585930211	-0.05239369	1.72512433	0.11496929	0.51527453	-4.56245573
homo after UVB irradiation VS homo before UVB irradiation	Ranitidine Action Pathway|SMPDB	0.21780303	0.051698704	1.19150037	0.26270351	0.42561191	-5.65959884
hetero after UVB irradiation VS hetero before UVB irradiation	Ranitidine Action Pathway|SMPDB	0.331566302	0.063470812	1.441200644	0.18563717	0.40321767	-5.34291209
WT after UVB irradiation VS WT before UVB irradiation	Ranitidine Action Pathway|SMPDB	-0.11560861	0.135982321	-0.44330845	0.66690385	0.87935805	-5.77062647
homo after UVB irradiation VS homo before UVB irradiation	RANKL|NetPath	0.426433251	-0.01611948	2.294480018	0.04628019	0.14003669	-4.13938309
hetero after UVB irradiation VS hetero before UVB irradiation	RANKL|NetPath	0.503037065	-0.00159008	2.89540475	0.01897066	0.1205973	-3.23078681
WT after UVB irradiation VS WT before UVB irradiation	RANKL|NetPath	0.526099338	-0.04996055	2.52033453	0.03019708	0.36196871	-3.42837733
homo after UVB irradiation VS homo before UVB irradiation	RANKL-RANK (Receptor activator of NFKB (ligand)) Signaling Pathway|Wikipathways	0.611113104	-0.03071694	3.402170661	0.00738058	0.04999956	-2.37725507
hetero after UVB irradiation VS hetero before UVB irradiation	RANKL-RANK (Receptor activator of NFKB (ligand)) Signaling Pathway|Wikipathways	0.556925313	0.023336555	3.366849576	0.00911881	0.09677373	-2.51620968
WT after UVB irradiation VS WT before UVB irradiation	RANKL-RANK (Receptor activator of NFKB (ligand)) Signaling Pathway|Wikipathways	0.500315251	-0.00760561	2.448906143	0.03414011	0.38028752	-3.53480083
homo after UVB irradiation VS homo before UVB irradiation	Rap1 signaling pathway - Homo sapiens (human)|KEGG	0.409390224	-0.02104513	2.509569069	0.03233389	0.11993222	-3.80161662
hetero after UVB irradiation VS hetero before UVB irradiation	Rap1 signaling pathway - Homo sapiens (human)|KEGG	0.387716264	0.019001038	2.245761061	0.05329908	0.20528072	-4.21984928
WT after UVB irradiation VS WT before UVB irradiation	Rap1 signaling pathway - Homo sapiens (human)|KEGG	0.276091095	-0.0027296	1.322260372	0.2152924	0.62581393	-5.0577112
homo after UVB irradiation VS homo before UVB irradiation	Rap1 signalling|Reactome	0.672140777	0.006803649	4.152630016	0.00225805	0.02672385	-1.22040782
hetero after UVB irradiation VS hetero before UVB irradiation	Rap1 signalling|Reactome	0.464776664	-0.00835863	1.928436074	0.08809392	0.26876095	-4.68576279
WT after UVB irradiation VS WT before UVB irradiation	Rap1 signalling|Reactome	0.458763917	-0.02971015	2.350759186	0.04039397	0.39022568	-3.680175
homo after UVB irradiation VS homo before UVB irradiation	Rapid glucocorticoid signaling|PID	0.296490062	-0.0699155	1.671495259	0.12755656	0.26766751	-5.05966892
hetero after UVB irradiation VS hetero before UVB irradiation	Rapid glucocorticoid signaling|PID	0.692046317	0.044130231	3.309998913	0.00994874	0.10144594	-2.60147071
WT after UVB irradiation VS WT before UVB irradiation	Rapid glucocorticoid signaling|PID	0.619913388	-0.11963147	2.630880399	0.02496526	0.33482204	-3.26291535
homo after UVB irradiation VS homo before UVB irradiation	Rapoport-Luebering glycolytic shunt|HumanCyc	-0.1655489	0.042392343	-0.65719925	0.52683294	0.66322558	-6.1417082
hetero after UVB irradiation VS hetero before UVB irradiation	Rapoport-Luebering glycolytic shunt|HumanCyc	-0.23827618	0.107369068	-1.21941626	0.25571839	0.48044699	-5.60555183
WT after UVB irradiation VS WT before UVB irradiation	Rapoport-Luebering glycolytic shunt|HumanCyc	-0.26107219	0.080967691	-1.09423139	0.29930598	0.68477511	-5.29857715
homo after UVB irradiation VS homo before UVB irradiation	Ras activation upon Ca2+ influx through NMDA receptor|Reactome	0.049124805	0.021849364	0.294913279	0.77447738	0.85413274	-6.32193796
hetero after UVB irradiation VS hetero before UVB irradiation	Ras activation upon Ca2+ influx through NMDA receptor|Reactome	-0.23079282	-0.0015357	-0.95378112	0.36675187	0.59508238	-5.8786459
WT after UVB irradiation VS WT before UVB irradiation	Ras activation upon Ca2+ influx through NMDA receptor|Reactome	-0.03056831	-0.05393405	-0.10016879	0.92217419	0.97566251	-5.86407084
homo after UVB irradiation VS homo before UVB irradiation	Ras signaling in the CD4+ TCR pathway|PID	0.059082718	0.001912237	0.249082627	0.80866036	0.88203765	-6.33522542
hetero after UVB irradiation VS hetero before UVB irradiation	Ras signaling in the CD4+ TCR pathway|PID	0.39418249	-0.00033209	1.81481368	0.10521001	0.29330106	-4.84669885
WT after UVB irradiation VS WT before UVB irradiation	Ras signaling in the CD4+ TCR pathway|PID	0.176948609	0.055396547	0.852095488	0.41394332	0.77942111	-5.51495489
homo after UVB irradiation VS homo before UVB irradiation	Ras signaling pathway - Homo sapiens (human)|KEGG	0.393895864	-0.00348844	2.401691317	0.03871309	0.12940262	-3.97179613
hetero after UVB irradiation VS hetero before UVB irradiation	Ras signaling pathway - Homo sapiens (human)|KEGG	0.389021587	0.019246262	2.472558841	0.03712145	0.16802342	-3.87721502
WT after UVB irradiation VS WT before UVB irradiation	Ras signaling pathway - Homo sapiens (human)|KEGG	0.268241684	-0.00011126	1.29825301	0.22311131	0.62952955	-5.08458641
homo after UVB irradiation VS homo before UVB irradiation	ras signaling pathway|BioCarta	0.457262889	-0.06892565	1.690883692	0.123717	0.26290983	-5.03297741
hetero after UVB irradiation VS hetero before UVB irradiation	ras signaling pathway|BioCarta	0.599931309	0.016872874	3.744959226	0.00516124	0.07738465	-1.95825404
WT after UVB irradiation VS WT before UVB irradiation	ras signaling pathway|BioCarta	0.493108032	-0.02657683	2.581050001	0.0272018	0.35054739	-3.33759471
homo after UVB irradiation VS homo before UVB irradiation	Ras Signaling|Wikipathways	0.355782957	-0.01742822	2.086053025	0.06533803	0.17757366	-4.45921464
hetero after UVB irradiation VS hetero before UVB irradiation	Ras Signaling|Wikipathways	0.385031264	0.015610491	2.472647182	0.03711622	0.16802342	-3.87708061
WT after UVB irradiation VS WT before UVB irradiation	Ras Signaling|Wikipathways	0.277523293	-0.01118302	1.374663618	0.1990156	0.61083093	-4.99790166
homo after UVB irradiation VS homo before UVB irradiation	ras-independent pathway in nk cell-mediated cytotoxicity|BioCarta	0.845251729	-0.02852261	4.039295847	0.0026878	0.02974154	-1.39064216
hetero after UVB irradiation VS hetero before UVB irradiation	ras-independent pathway in nk cell-mediated cytotoxicity|BioCarta	0.728237181	0.056843051	2.88527188	0.01927602	0.12168991	-3.24628087
WT after UVB irradiation VS WT before UVB irradiation	ras-independent pathway in nk cell-mediated cytotoxicity|BioCarta	0.688858889	0.058721102	2.371038873	0.03901607	0.39022568	-3.65023072
homo after UVB irradiation VS homo before UVB irradiation	rb tumor suppressor/checkpoint signaling in response to dna damage|BioCarta	-0.46165334	-0.02624928	-2.8416422	0.01857508	0.08707783	-3.27191415
hetero after UVB irradiation VS hetero before UVB irradiation	rb tumor suppressor/checkpoint signaling in response to dna damage|BioCarta	-0.0297212	-0.02347937	-0.15177238	0.88293775	0.95842588	-6.33406254
WT after UVB irradiation VS WT before UVB irradiation	rb tumor suppressor/checkpoint signaling in response to dna damage|BioCarta	-0.11882026	-0.00678721	-0.4510059	0.66153011	0.87935805	-5.76721221
homo after UVB irradiation VS homo before UVB irradiation	Reactions specific to the complex N-glycan synthesis pathway|Reactome	0.331978741	0.02162906	1.500661103	0.16629829	0.31611907	-5.28745085
hetero after UVB irradiation VS hetero before UVB irradiation	Reactions specific to the complex N-glycan synthesis pathway|Reactome	0.319127328	0.003078641	0.919797208	0.38325503	0.6110096	-5.90983066
WT after UVB irradiation VS WT before UVB irradiation	Reactions specific to the complex N-glycan synthesis pathway|Reactome	0.236985178	0.006040562	0.711077974	0.49315817	0.81710765	-5.61970128
homo after UVB irradiation VS homo before UVB irradiation	Reactions specific to the hybrid N-glycan synthesis pathway|Reactome	1.021247515	-0.01758201	3.794190073	0.00394083	0.03577298	-1.76467278
hetero after UVB irradiation VS hetero before UVB irradiation	Reactions specific to the hybrid N-glycan synthesis pathway|Reactome	0.461586237	0.000600377	1.232229974	0.2511383	0.47497442	-5.59116598
WT after UVB irradiation VS WT before UVB irradiation	Reactions specific to the hybrid N-glycan synthesis pathway|Reactome	0.632207501	-0.0286745	1.623766402	0.13524015	0.54804063	-4.6941129
homo after UVB irradiation VS homo before UVB irradiation	Receptor Mediated Mitophagy|Reactome	0.219990758	-0.00381592	0.857375855	0.41258597	0.56380634	-5.98859952
hetero after UVB irradiation VS hetero before UVB irradiation	Receptor Mediated Mitophagy|Reactome	0.420260149	0.034371303	1.675905002	0.13037062	0.3257836	-5.03776799
WT after UVB irradiation VS WT before UVB irradiation	Receptor Mediated Mitophagy|Reactome	0.16941372	0.022651034	0.598753042	0.56254787	0.85017866	-5.69093466
homo after UVB irradiation VS homo before UVB irradiation	Receptor-ligand binding initiates the second proteolytic cleavage of Notch receptor|Reactome	0.037408765	0.030802323	0.15143377	0.88283749	0.92936414	-6.35614211
hetero after UVB irradiation VS hetero before UVB irradiation	Receptor-ligand binding initiates the second proteolytic cleavage of Notch receptor|Reactome	0.407579793	0.024776787	1.948439716	0.08536864	0.26434846	-4.65705209
WT after UVB irradiation VS WT before UVB irradiation	Receptor-ligand binding initiates the second proteolytic cleavage of Notch receptor|Reactome	-0.31238382	-0.0316089	-1.38693618	0.19535695	0.60652289	-4.9836732
homo after UVB irradiation VS homo before UVB irradiation	Receptor-type tyrosine-protein phosphatases|Reactome	0.046899782	0.016610203	0.208548581	0.83925482	0.9020733	-6.34513496
hetero after UVB irradiation VS hetero before UVB irradiation	Receptor-type tyrosine-protein phosphatases|Reactome	-0.02341196	0.043642878	-0.13980621	0.89209743	0.96446397	-6.33595044
WT after UVB irradiation VS WT before UVB irradiation	Receptor-type tyrosine-protein phosphatases|Reactome	-0.12231733	-0.00948078	-0.53893541	0.60162799	0.86840551	-5.72425175
homo after UVB irradiation VS homo before UVB irradiation	Recognition and association of DNA glycosylase with site containing an affected purine|Reactome	-0.20582373	0.015435386	-0.82269915	0.4310765	0.58181948	-6.01765082
hetero after UVB irradiation VS hetero before UVB irradiation	Recognition and association of DNA glycosylase with site containing an affected purine|Reactome	0.171991434	-0.05342382	0.515799017	0.61925362	0.80231894	-6.20450981
WT after UVB irradiation VS WT before UVB irradiation	Recognition and association of DNA glycosylase with site containing an affected purine|Reactome	0.62990354	0.108869935	1.999454535	0.07322271	0.45403763	-4.18778491
homo after UVB irradiation VS homo before UVB irradiation	Recognition and association of DNA glycosylase with site containing an affected pyrimidine|Reactome	-0.78356809	-0.06735188	-4.03947359	0.00268707	0.02974154	-1.39037379
hetero after UVB irradiation VS hetero before UVB irradiation	Recognition and association of DNA glycosylase with site containing an affected pyrimidine|Reactome	-0.52063106	-0.10015914	-1.97323455	0.0821024	0.25808464	-4.62132082
WT after UVB irradiation VS WT before UVB irradiation	Recognition and association of DNA glycosylase with site containing an affected pyrimidine|Reactome	-0.03096487	-0.00387426	-0.10858657	0.91566112	0.97566251	-5.86318166
homo after UVB irradiation VS homo before UVB irradiation	Recognition of DNA damage by PCNA-containing replication complex|Reactome	-0.43845369	0.006807347	-2.09303294	0.06459201	0.17618107	-4.4486579
hetero after UVB irradiation VS hetero before UVB irradiation	Recognition of DNA damage by PCNA-containing replication complex|Reactome	0.220625612	0.011070568	0.949925267	0.36859758	0.59623032	-5.88222983
WT after UVB irradiation VS WT before UVB irradiation	Recognition of DNA damage by PCNA-containing replication complex|Reactome	-0.07901991	0.014565389	-0.28932836	0.77819237	0.93502208	-5.82693236
homo after UVB irradiation VS homo before UVB irradiation	Recruitment and ATM-mediated phosphorylation of repair and signaling proteins at DNA double strand breaks|Reactome	-0.53637831	0.031317291	-3.03552065	0.01346332	0.07249235	-2.96128513
hetero after UVB irradiation VS hetero before UVB irradiation	Recruitment and ATM-mediated phosphorylation of repair and signaling proteins at DNA double strand breaks|Reactome	-0.15128537	-0.03703086	-0.70915317	0.49737954	0.71538528	-6.08178775
WT after UVB irradiation VS WT before UVB irradiation	Recruitment and ATM-mediated phosphorylation of repair and signaling proteins at DNA double strand breaks|Reactome	-0.28444182	-0.02746429	-1.19463025	0.2595779	0.65440449	-5.19660332
homo after UVB irradiation VS homo before UVB irradiation	Recruitment of mitotic centrosome proteins and complexes|Reactome	-0.46559019	-0.00279485	-2.63553562	0.02619776	0.10699372	-3.60144484
hetero after UVB irradiation VS hetero before UVB irradiation	Recruitment of mitotic centrosome proteins and complexes|Reactome	-0.02486017	-0.01446198	-0.10147379	0.92154771	0.97054702	-6.34095877
WT after UVB irradiation VS WT before UVB irradiation	Recruitment of mitotic centrosome proteins and complexes|Reactome	-0.07093494	-0.02355644	-0.285332	0.78116559	0.93661609	-5.8280856
homo after UVB irradiation VS homo before UVB irradiation	Recruitment of NuMA to mitotic centrosomes|Reactome	-0.46729952	-0.00272787	-2.66850498	0.02479409	0.10405312	-3.54886065
hetero after UVB irradiation VS hetero before UVB irradiation	Recruitment of NuMA to mitotic centrosomes|Reactome	-0.02022784	-0.01460416	-0.08211221	0.93647469	0.97054702	-6.34288507
WT after UVB irradiation VS WT before UVB irradiation	Recruitment of NuMA to mitotic centrosomes|Reactome	-0.08019237	-0.02558508	-0.3239412	0.75260257	0.92020735	-5.81628631
homo after UVB irradiation VS homo before UVB irradiation	Recycling of bile acids and salts|Reactome	0.106046328	0.003357995	0.583255611	0.57346227	0.69713225	-6.18899
hetero after UVB irradiation VS hetero before UVB irradiation	Recycling of bile acids and salts|Reactome	-0.10439297	0.000774814	-0.47909299	0.64405977	0.81977783	-6.22373803
WT after UVB irradiation VS WT before UVB irradiation	Recycling of bile acids and salts|Reactome	0.214906035	0.039430452	0.927665163	0.3752554	0.75077508	-5.45217548
homo after UVB irradiation VS homo before UVB irradiation	Recycling of eIF2:GDP|Reactome	-1.01474566	0.024045013	-6.15863755	0.00013982	0.01666204	1.46090885
hetero after UVB irradiation VS hetero before UVB irradiation	Recycling of eIF2:GDP|Reactome	-0.77808198	-0.1415746	-2.38561446	0.04264512	0.18183017	-4.00919521
WT after UVB irradiation VS WT before UVB irradiation	Recycling of eIF2:GDP|Reactome	-0.52118727	-0.05624602	-1.48329118	0.16856904	0.57494757	-4.86918236
homo after UVB irradiation VS homo before UVB irradiation	Recycling pathway of L1|Reactome	0.395875388	-0.02830859	2.33785584	0.043058	0.13447844	-4.07179102
hetero after UVB irradiation VS hetero before UVB irradiation	Recycling pathway of L1|Reactome	0.531153978	-0.00109326	3.166532696	0.01241458	0.10716984	-2.81792224
WT after UVB irradiation VS WT before UVB irradiation	Recycling pathway of L1|Reactome	0.348249407	-0.00969686	1.671853654	0.12525404	0.53064492	-4.63217032
homo after UVB irradiation VS homo before UVB irradiation	Reduction of cytosolic Ca++ levels|Reactome	0.18219691	-0.0830276	0.941343032	0.37011623	0.52279924	-5.91408155
hetero after UVB irradiation VS hetero before UVB irradiation	Reduction of cytosolic Ca++ levels|Reactome	0.037882162	0.009292023	0.169382304	0.8694921	0.95085052	-6.33100412
WT after UVB irradiation VS WT before UVB irradiation	Reduction of cytosolic Ca++ levels|Reactome	0.234070004	-0.03546796	0.989136106	0.34574031	0.72966542	-5.39786018

homo after UVB irradiation VS homo before UVB irradiation	Reelin signaling pathway|PID	0.036213794	-0.03139675	0.20826329	0.83947122	0.9020733	-6.34519855
hetero after UVB irradiation VS hetero before UVB irradiation	Reelin signaling pathway|PID	0.066423536	-0.00235635	0.377862063	0.71487476	0.85921866	-6.26975231
WT after UVB irradiation VS WT before UVB irradiation	Reelin signaling pathway|PID	0.042679909	-0.01944524	0.196600503	0.84804664	0.95372763	-5.84961073
homo after UVB irradiation VS homo before UVB irradiation	Reelin signalling pathway|Reactome	0.221452029	-0.02571272	0.78004909	0.45457579	0.60179561	-6.05195925
hetero after UVB irradiation VS hetero before UVB irradiation	Reelin signalling pathway|Reactome	-0.37162716	0.004311152	-1.32572142	0.2197454	0.44126832	-5.48317154
WT after UVB irradiation VS WT before UVB irradiation	Reelin signalling pathway|Reactome	-0.13238005	-0.06964961	-0.46348343	0.65286203	0.87935805	-5.76155829
homo after UVB irradiation VS homo before UVB irradiation	Refsum Disease|SMPDB	0.309508869	0.005460472	1.563469103	0.15098345	0.29702303	-5.20532249
hetero after UVB irradiation VS hetero before UVB irradiation	Refsum Disease|SMPDB	0.084800099	-0.0542149	0.263185751	0.79872922	0.91506577	-6.3091226
WT after UVB irradiation VS WT before UVB irradiation	Refsum Disease|SMPDB	-0.07150324	-0.07402423	-0.29688223	0.77258268	0.93294159	-5.82470951
homo after UVB irradiation VS homo before UVB irradiation	Regulated Necrosis|Reactome	0.574668591	-0.02068998	2.981907535	0.01471334	0.07489533	-3.04715282
hetero after UVB irradiation VS hetero before UVB irradiation	Regulated Necrosis|Reactome	0.553467011	-0.00013774	2.145130163	0.06255239	0.22396997	-4.36973503
WT after UVB irradiation VS WT before UVB irradiation	Regulated Necrosis|Reactome	0.551958599	-0.07601754	2.310719951	0.04325514	0.39022568	-3.73913397
homo after UVB irradiation VS homo before UVB irradiation	Regulated proteolysis of p75NTR|Reactome	0.759157081	-0.03701793	3.954322207	0.00306636	0.03140179	-1.51943036
hetero after UVB irradiation VS hetero before UVB irradiation	Regulated proteolysis of p75NTR|Reactome	0.874047132	-0.01522776	5.47281865	0.00049528	0.03004176	0.319914495
WT after UVB irradiation VS WT before UVB irradiation	Regulated proteolysis of p75NTR|Reactome	0.717554005	-0.0206829	3.208205041	0.00926604	0.24856491	-2.39659874
homo after UVB irradiation VS homo before UVB irradiation	Regulation by c-FLIP|Reactome	0.849614473	-0.03215571	4.542786992	0.00125669	0.02014127	-0.64871182
hetero after UVB irradiation VS hetero before UVB irradiation	Regulation by c-FLIP|Reactome	0.683693345	-0.01188812	2.738727154	0.02430288	0.13715811	-3.47057017
WT after UVB irradiation VS WT before UVB irradiation	Regulation by c-FLIP|Reactome	0.733182815	-0.06921519	3.457990495	0.00607019	0.21490368	-2.02721426
homo after UVB irradiation VS homo before UVB irradiation	Regulation by TREX1|Reactome	1.100811796	-0.04245361	5.214671399	0.0004822	0.01666204	0.27978583
hetero after UVB irradiation VS hetero before UVB irradiation	Regulation by TREX1|Reactome	1.111111111	0.045421609	4.675002854	0.00138514	0.04542068	-0.67196788
WT after UVB irradiation VS WT before UVB irradiation	Regulation by TREX1|Reactome	1.00379424	-0.02123116	3.714938453	0.0039529	0.20870715	-1.65424777
homo after UVB irradiation VS homo before UVB irradiation	Regulation of actin cytoskeleton - Homo sapiens (human)|KEGG	0.389132888	-0.00814157	2.188870596	0.05514212	0.15895134	-4.30256439
hetero after UVB irradiation VS hetero before UVB irradiation	Regulation of actin cytoskeleton - Homo sapiens (human)|KEGG	0.373015755	0.003007147	2.433667795	0.03949763	0.1739422	-3.93632945
WT after UVB irradiation VS WT before UVB irradiation	Regulation of actin cytoskeleton - Homo sapiens (human)|KEGG	0.273371273	0.000552699	1.384278994	0.19614424	0.60772778	-4.98676086
homo after UVB irradiation VS homo before UVB irradiation	Regulation of Actin Cytoskeleton|Wikipathways	0.310805005	-0.00786691	1.897358556	0.08891345	0.21361485	-4.73959654
hetero after UVB irradiation VS hetero before UVB irradiation	Regulation of Actin Cytoskeleton|Wikipathways	0.280671345	0.011760375	1.838007152	0.10147938	0.28640434	-4.81416072
WT after UVB irradiation VS WT before UVB irradiation	Regulation of Actin Cytoskeleton|Wikipathways	0.20907081	0.001076785	1.070069979	0.30953605	0.69398681	-5.32209758
homo after UVB irradiation VS homo before UVB irradiation	Regulation of actin dynamics for phagocytic cup formation|Reactome	0.443954046	-0.0191904	2.312075367	0.04494558	0.13809028	-4.11200124
hetero after UVB irradiation VS hetero before UVB irradiation	Regulation of actin dynamics for phagocytic cup formation|Reactome	0.578703481	0.014457321	3.673222379	0.00574158	0.08047311	-2.06277171
WT after UVB irradiation VS WT before UVB irradiation	Regulation of actin dynamics for phagocytic cup formation|Reactome	0.557596011	-0.00339208	2.766525576	0.01976342	0.31542987	-3.05911904
homo after UVB irradiation VS homo before UVB irradiation	Regulation of Androgen receptor activity|PID	-0.37888089	0.017484678	-1.83684284	0.098035	0.22676181	-4.82719966
hetero after UVB irradiation VS hetero before UVB irradiation	Regulation of Androgen receptor activity|PID	-0.30760475	-0.03438975	-1.6708299	0.13138744	0.32796493	-5.04461548
WT after UVB irradiation VS WT before UVB irradiation	Regulation of Androgen receptor activity|PID	-0.44971356	-0.01773702	-2.00674709	0.07233585	0.45254499	-4.17751749
homo after UVB irradiation VS homo before UVB irradiation	Regulation of APC/C activators between G1/S and early anaphase|Reactome	-0.55791733	0.048233755	-3.09306768	0.01224216	0.06800204	-2.86919438
hetero after UVB irradiation VS hetero before UVB irradiation	Regulation of APC/C activators between G1/S and early anaphase|Reactome	0.215014507	-0.00575687	1.048376471	0.32360301	0.55421891	-5.78716796
WT after UVB irradiation VS WT before UVB irradiation	Regulation of APC/C activators between G1/S and early anaphase|Reactome	-0.09304123	-0.05043891	-0.36920594	0.7196101	0.90672477	-5.80059439
homo after UVB irradiation VS homo before UVB irradiation	Regulation of Apoptosis by Parathyroid Hormone-related Protein|Wikipathways	0.313078974	-0.0729825	1.932849605	0.08394011	0.20561724	-4.68765823
hetero after UVB irradiation VS hetero before UVB irradiation	Regulation of Apoptosis by Parathyroid Hormone-related Protein|Wikipathways	0.17705775	-0.03129422	1.034598133	0.32963446	0.55823827	-5.80090856
WT after UVB irradiation VS WT before UVB irradiation	Regulation of Apoptosis by Parathyroid Hormone-related Protein|Wikipathways	0.327330955	0.073683119	1.58072157	0.1447743	0.55622203	-4.74871994
homo after UVB irradiation VS homo before UVB irradiation	Regulation of Apoptosis|Reactome	-0.41834802	-0.0444504	-1.78518473	0.1065022	0.23957183	-4.90096218
hetero after UVB irradiation VS hetero before UVB irradiation	Regulation of Apoptosis|Reactome	-0.18825426	-0.03562391	-0.60139788	0.56340153	0.764281	-6.15454904
WT after UVB irradiation VS WT before UVB irradiation	Regulation of Apoptosis|Reactome	-0.22169468	-0.13421491	-0.87698885	0.40090646	0.76901426	-5.49477191
homo after UVB irradiation VS homo before UVB irradiation	regulation of bad phosphorylation|BioCarta	0.38276383	-0.07893979	2.13896874	0.05988263	0.1673342	-4.37889244
hetero after UVB irradiation VS hetero before UVB irradiation	regulation of bad phosphorylation|BioCarta	0.399165057	-0.01433537	1.876113853	0.09561941	0.27962159	-4.76034256
WT after UVB irradiation VS WT before UVB irradiation	regulation of bad phosphorylation|BioCarta	0.186363556	-0.06321812	0.651965178	0.52900555	0.83291538	-5.65857837
homo after UVB irradiation VS homo before UVB irradiation	Regulation of beta-cell development|Reactome	0.634629129	-0.03415062	3.13262261	0.01146927	0.06546084	-2.80595973
hetero after UVB irradiation VS hetero before UVB irradiation	Regulation of beta-cell development|Reactome	0.42794189	0.054973673	1.53918473	0.16042285	0.37252774	-5.21853851
WT after UVB irradiation VS WT before UVB irradiation	Regulation of beta-cell development|Reactome	0.316370395	-0.03720487	1.253541651	0.23829687	0.63195092	-5.13372828
homo after UVB irradiation VS homo before UVB irradiation	Regulation of beta-cell development|Wikipathways	0.346285917	0.111115628	1.748710907	0.11288174	0.24815504	-4.95244175
hetero after UVB irradiation VS hetero before UVB irradiation	Regulation of beta-cell development|Wikipathways	0.406507046	0.136972789	2.309048184	0.04818509	0.19487813	-4.1248161
WT after UVB irradiation VS WT before UVB irradiation	Regulation of beta-cell development|Wikipathways	0.146456057	0.074204531	0.524071568	0.61155497	0.87257466	-5.73202091
homo after UVB irradiation VS homo before UVB irradiation	Regulation of CDC42 activity|PID	0.295487284	-0.05138899	1.645569146	0.13285931	0.27561817	-5.09510713
hetero after UVB irradiation VS hetero before UVB irradiation	Regulation of CDC42 activity|PID	0.35171145	0.024613263	2.178697691	0.05930285	0.21739382	-4.31992193
WT after UVB irradiation VS WT before UVB irradiation	Regulation of CDC42 activity|PID	0.255724632	0.000426792	1.251601684	0.23897441	0.63196069	-5.13583312
homo after UVB irradiation VS homo before UVB irradiation	regulation of cell cycle progression by plk3|BioCarta	0.154892422	-0.03955522	0.776618715	0.45650191	0.60277262	-6.05464954
hetero after UVB irradiation VS hetero before UVB irradiation	regulation of cell cycle progression by plk3|BioCarta	0.469750217	0.023371259	2.307807243	0.04828054	0.19509104	-4.12668451
WT after UVB irradiation VS WT before UVB irradiation	regulation of cell cycle progression by plk3|BioCarta	0.476280392	-0.01919627	2.29673145	0.04430049	0.39111425	-3.75967877
homo after UVB irradiation VS homo before UVB irradiation	Regulation of cholesterol biosynthesis by SREBP (SREBF)|Reactome	-0.38313841	-0.05290047	-1.58548246	0.14591917	0.2913279	-5.17607928
hetero after UVB irradiation VS hetero before UVB irradiation	Regulation of cholesterol biosynthesis by SREBP (SREBF)|Reactome	-0.06819845	0.008296165	-0.29998021	0.77146004	0.89765169	-6.29798973
WT after UVB irradiation VS WT before UVB irradiation	Regulation of cholesterol biosynthesis by SREBP (SREBF)|Reactome	-0.04436737	0.014473167	-0.17444653	0.86496778	0.96095643	-5.85375971
homo after UVB irradiation VS homo before UVB irradiation	regulation of ck1/cdk5 by type 1 glutamate receptors|BioCarta	0.495335538	-0.03579162	2.877377607	0.01750258	0.08340524	-3.21465657
hetero after UVB irradiation VS hetero before UVB irradiation	regulation of ck1/cdk5 by type 1 glutamate receptors|BioCarta	0.452479209	0.033191986	2.130605836	0.06401181	0.22687855	-4.3912265
WT after UVB irradiation VS WT before UVB irradiation	regulation of ck1/cdk5 by type 1 glutamate receptors|BioCarta	0.492781677	0.009703981	2.017660709	0.07102754	0.44835368	-4.16212622
homo after UVB irradiation VS homo before UVB irradiation	Regulation of commissural axon pathfinding by SLIT and ROBO|Reactome	0.174574831	0.054884081	0.88305949	0.39924877	0.55113275	-5.96642596
hetero after UVB irradiation VS hetero before UVB irradiation	Regulation of commissural axon pathfinding by SLIT and ROBO|Reactome	0.25945962	0.017021044	0.954872265	0.3662308	0.5945097	-5.8776296
WT after UVB irradiation VS WT before UVB irradiation	Regulation of commissural axon pathfinding by SLIT and ROBO|Reactome	0.152877777	0.077193633	0.657149527	0.52580126	0.83177497	-5.65529123
homo after UVB irradiation VS homo before UVB irradiation	Regulation of Complement cascade|Reactome	0.674203994	-0.02827224	3.052949695	0.01308081	0.07101902	-2.93338397
hetero after UVB irradiation VS hetero before UVB irradiation	Regulation of Complement cascade|Reactome	0.466067809	0.111803752	2.097380759	0.06747626	0.2340615	-4.44023991
WT after UVB irradiation VS WT before UVB irradiation	Regulation of Complement cascade|Reactome	0.507370983	0.007118169	1.816166253	0.09915261	0.49761536	-4.44084137
homo after UVB irradiation VS homo before UVB irradiation	Regulation of cortical dendrite branching|Reactome	0.213314851	0.002837864	1.101850903	0.29796838	0.45881213	-5.75609312
hetero after UVB irradiation VS hetero before UVB irradiation	Regulation of cortical dendrite branching|Reactome	0.234501256	0.006323685	0.729418291	0.48552568	0.70613467	-6.06691501
WT after UVB irradiation VS WT before UVB irradiation	Regulation of cortical dendrite branching|Reactome	0.073658437	0.021460345	0.224607675	0.82677113	0.94525798	-5.84366284
homo after UVB irradiation VS homo before UVB irradiation	Regulation of cytoplasmic and nuclear SMAD2/3 signaling|PID	-0.43862324	0.017186726	-2.48840175	0.03349739	0.1221638	-3.83511254
hetero after UVB irradiation VS hetero before UVB irradiation	Regulation of cytoplasmic and nuclear SMAD2/3 signaling|PID	-0.16688576	-0.04508909	-0.96633596	0.36078954	0.58926812	-5.86689631
WT after UVB irradiation VS WT before UVB irradiation	Regulation of cytoplasmic and nuclear SMAD2/3 signaling|PID	-0.09906978	-0.00199005	-0.41070528	0.68988125	0.89330527	-5.78446139
homo after UVB irradiation VS homo before UVB irradiation	Regulation of cytoskeletal remodeling and cell spreading by IPP complex components|Reactome	0.995302193	-0.04414568	5.478090004	0.00033714	0.01666204	0.623816044
hetero after UVB irradiation VS hetero before UVB irradiation	Regulation of cytoskeletal remodeling and cell spreading by IPP complex components|Reactome	0.974769854	0.077527721	4.277531789	0.00239313	0.05507363	-1.20521329
WT after UVB irradiation VS WT before UVB irradiation	Regulation of cytoskeletal remodeling and cell spreading by IPP complex components|Reactome	0.593413694	-0.03698797	1.958567195	0.07838756	0.46387039	-4.24508912
homo after UVB irradiation VS homo before UVB irradiation	regulation of eif2|BioCarta	-0.68227517	-0.03851392	-3.97807264	0.00295519	0.03080689	-1.48333653
hetero after UVB irradiation VS hetero before UVB irradiation	regulation of eif2|BioCarta	-0.4443269	-0.06610406	-1.74837188	0.11662075	0.30922478	-4.93893782
WT after UVB irradiation VS WT before UVB irradiation	regulation of eif2|BioCarta	-0.35381795	-0.05244736	-1.17714379	0.26617971	0.66118679	-5.21484276
homo after UVB irradiation VS homo before UVB irradiation	regulation of eif-4e and p70s6 kinase|BioCarta	-0.02170436	0.013239948	-0.11536859	0.91058277	0.94787581	-6.36130197
hetero after UVB irradiation VS hetero before UVB irradiation	regulation of eif-4e and p70s6 kinase|BioCarta	0.014851646	-0.03087951	0.086144332	0.9333637	0.97054702	-6.34251734
WT after UVB irradiation VS WT before UVB irradiation	regulation of eif-4e and p70s6 kinase|BioCarta	-0.16631159	-0.02179972	-0.74692064	0.47217051	0.81337567	-5.59465694
homo after UVB irradiation VS homo before UVB irradiation	Regulation of expression of SLITs and ROBOs|Reactome	0.026613135	0.0540921	0.16133896	0.87524489	0.92358867	-6.35448194
hetero after UVB irradiation VS hetero before UVB irradiation	Regulation of expression of SLITs and ROBOs|Reactome	0.234389574	0.091074259	1.045500195	0.32485501	0.55421891	-5.79004782
WT after UVB irradiation VS WT before UVB irradiation	Regulation of expression of SLITs and ROBOs|Reactome	-0.32248562	0.075904695	-1.36453438	0.20207864	0.61620632	-5.00958271
homo after UVB irradiation VS homo before UVB irradiation	Regulation of FZD by ubiquitination|Reactome	-0.46707513	-0.03803554	-2.80704551	0.01967699	0.08963384	-3.32732882
hetero after UVB irradiation VS hetero before UVB irradiation	Regulation of FZD by ubiquitination|Reactome	-0.28789161	-0.02439134	-1.38158322	0.20262577	0.42307296	-5.41622428
WT after UVB irradiation VS WT before UVB irradiation	Regulation of FZD by ubiquitination|Reactome	-0.56140765	0.033653889	-2.50284849	0.0311187	0.36428127	-3.45447166
homo after UVB irradiation VS homo before UVB irradiation	Regulation of gap junction activity|Reactome	-0.33369828	0.085068035	-1.01068862	0.33750078	0.49312914	-5.84824804
hetero after UVB irradiation VS hetero before UVB irradiation	Regulation of gap junction activity|Reactome	0.118464661	0.084729439	0.370066031	0.72046286	0.86372165	-6.27286234
WT after UVB irradiation VS WT before UVB irradiation	Regulation of gap junction activity|Reactome	-0.0581366	0.088252379	-0.15423083	0.88047136	0.96769248	-5.85711602
homo after UVB irradiation VS homo before UVB irradiation	Regulation of gene expression by Hypoxia-inducible Factor|Reactome	0.231069598	-0.01762341	0.765130392	0.46299136	0.6088463	-6.06358358
hetero after UVB irradiation VS hetero before UVB irradiation	Regulation of gene expression by Hypoxia-inducible Factor|Reactome	0.165904354	-0.03650431	0.461759892	0.65594185	0.82651398	-6.23235171
WT after UVB irradiation VS WT before UVB irradiation	Regulation of gene expression by Hypoxia-inducible Factor|Reactome	-0.09513236	-0.10901454	-0.24431604	0.8118862	0.94525798	-5.83900845
homo after UVB irradiation VS homo before UVB irradiation	Regulation of gene expression in beta cells|Reactome	0.634629129	-0.03415062	3.13262261	0.01146927	0.06546084	-2.80595973
hetero after UVB irradiation VS hetero before UVB irradiation	Regulation of gene expression in beta cells|Reactome	0.42794189	0.054973673	1.53918473	0.16042285	0.37252774	-5.21853851
WT after UVB irradiation VS WT before UVB irradiation	Regulation of gene expression in beta cells|Reactome	0.316370395	-0.03720487	1.253541651	0.23829687	0.63195092	-5.13372828
homo after UVB irradiation VS homo before UVB irradiation	Regulation of Glucokinase by Glucokinase Regulatory Protein|Reactome	-0.66117361	-0.00530641	-3.14556831	0.01122736	0.06453768	-2.78527749
hetero after UVB irradiation VS hetero before UVB irradiation	Regulation of Glucokinase by Glucokinase Regulatory Protein|Reactome	-0.2002794	-0.0684295	-0.72521382	0.48797005	0.7083336	-6.07003101
WT after UVB irradiation VS WT before UVB irradiation	Regulation of Glucokinase by Glucokinase Regulatory Protein|Reactome	-0.06314027	0.000744669	-0.19356392	0.8503614	0.95502804	-5.8502085
homo after UVB irradiation VS homo before UVB irradiation	Regulation of HSF1-mediated heat shock response|Reactome	-0.62907602	0.000773095	-3.76373711	0.00413496	0.03698535	-1.81166881
hetero after UVB irradiation VS hetero before UVB irradiation	Regulation of HSF1-mediated heat shock response|Reactome	-0.23946146	-0.04883156	-1.17319618	0.27281239	0.50241571	-5.65657133
WT after UVB irradiation VS WT before UVB irradiation	Regulation of HSF1-mediated heat shock response|Reactome	-0.22720839	-0.01580966	-0.8869616	0.39576425	0.76476626	-5.48654814
homo after UVB irradiation VS homo before UVB irradiation	Regulation of Hypoxia-inducible Factor (HIF) by oxygen|Reactome	0.248076222	-0.01164082	1.172640443	0.26983138	0.43325305	-5.68036342
hetero after UVB irradiation VS hetero before UVB irradiation	Regulation of Hypoxia-inducible Factor (HIF) by oxygen|Reactome	0.340237115	0.014109785	1.78160339	0.1107764	0.30216567	-4.89298866
WT after UVB irradiation VS WT before UVB irradiation	Regulation of Hypoxia-inducible Factor (HIF) by oxygen|Reactome	-0.0815399	0.0026454	-0.374079	0.71609248	0.90536666	-5.7987862
homo after UVB irradiation VS homo before UVB irradiation	Regulation of Hypoxia-inducible Factor (HIF) by oxygen|Wikipathways	0.896933756	-0.00701872	3.639786838	0.00503484	0.04061675	-2.00405141
hetero after UVB irradiation VS hetero before UVB irradiation	Regulation of Hypoxia-inducible Factor (HIF) by oxygen|Wikipathways	1.090191912	0.009576177	5.195023462	0.0007013	0.03228824	-0.01353818
WT after UVB irradiation VS WT before UVB irradiation	Regulation of Hypoxia-inducible Factor (HIF) by oxygen|Wikipathways	0.870009342	0.051159506	3.304091165	0.00787254	0.24795776	-2.25413459
homo after UVB irradiation VS homo before UVB irradiation	Regulation of IFNA signaling|Reactome	0.744471788	-0.04429435	3.374498292	0.00771981	0.05159579	-2.4210428
hetero after UVB irradiation VS hetero before UVB irradiation	Regulation of IFNA signaling|Reactome	0.866958376	0.02855265	5.145568694	0.00074695	0.03370881	-0.07423034
WT after UVB irradiation VS WT before UVB irradiation	Regulation of IFNA signaling|Reactome	0.808942527	-0.00010616	3.494783467	0.00570621	0.21421457	-1.97332601
homo after UVB irradiation VS homo before UVB irradiation	Regulation of IFNG signaling|Reactome	0.124676199	-0.04447126	0.492155865	0.63391168	0.74618219	-6.24000116
hetero after UVB irradiation VS hetero before UVB irradiation	Regulation of IFNG signaling|Reactome	0.425497859	-0.00647671	2.553821918	0.03261015	0.15685114	-3.75336508
WT after UVB irradiation VS WT before UVB irradiation	Regulation of IFNG signaling|Reactome	0.140028074	-0.09244527	0.505988523	0.6237438	0.87257466	-5.74119623
homo after UVB irradiation VS homo before UVB irradiation	Regulation of innate immune responses to cytosolic DNA|Reactome	0.509548471	-0.00993204	2.649734099	0.02558373	0.10578915	-3.57880723
hetero after UVB irradiation VS hetero before UVB irradiation	Regulation of innate immune responses to cytosolic DNA|Reactome	0.589797859	0.02773857	2.90479158	0.01869222	0.12044273	-3.21643619
WT after UVB irradiation VS WT before UVB irradiation	Regulation of innate immune responses to cytosolic DNA|Reactome	0.542484653	-0.0580426	2.468822296	0.03299233	0.37053866	-3.50517377
homo after UVB irradiation VS homo before UVB irradiation	Regulation of insulin secretion|Reactome	0.369808537	-0.04829695	2.18475795	0.05551866	0.15963363	-4.30887507
hetero after UVB irradiation VS hetero before UVB irradiation	Regulation of insulin secretion|Reactome	0.333390949	0.032563964	1.716781752	0.12244095	0.31407031	-4.98225806
WT after UVB irradiation VS WT before UVB irradiation	Regulation of insulin secretion|Reactome	0.203368082	-0.02765087	0.844277967	0.41809661	0.78448942	-5.52119081
homo after UVB irradiation VS homo before UVB irradiation	Regulation of Insulin-like Growth Factor (IGF) transport and uptake by Insulin-like Growth Factor Binding Proteins (IGFBPs)|Reactome	0.625670196	0.024043141	3.666584668	0.00482424	0.03957638	-1.96231402
hetero after UVB irradiation VS hetero before UVB irradiation	Regulation of Insulin-like Growth Factor (IGF) transport and uptake by Insulin-like Growth Factor Binding Proteins (IGFBPs)|Reactome	0.336807169	0.041589325	1.717680593	0.12227169	0.31407031	-4.98103044
WT after UVB irradiation VS WT before UVB irradiation	Regulation of Insulin-like Growth Factor (IGF) transport and uptake by Insulin-like Growth Factor Binding Proteins (IGFBPs)|Reactome	0.351251535	0.034515479	1.524799332	0.15804393	0.56072663	-4.81841278
homo after UVB irradiation VS homo before UVB irradiation	Regulation of KIT signaling|Reactome	-0.09010038	-0.07215077	-0.47219331	0.64757094	0.75613204	-6.25008755
hetero after UVB irradiation VS hetero before UVB irradiation	Regulation of KIT signaling|Reactome	0.511508515	0.030461804	2.635073627	0.02865268	0.14657566	-3.62920866
WT after UVB irradiation VS WT before UVB irradiation	Regulation of KIT signaling|Reactome	0.006938363	-0.06339741	0.028121341	0.97811423	0.99268361	-5.86875059
homo after UVB irradiation VS homo before UVB irradiation	Regulation of lipid metabolism by Peroxisome proliferator-activated receptor alpha (PPARalpha)|Reactome	-0.18195971	0.033584713	-0.89329744	0.39401745	0.54703225	-5.95743351
hetero after UVB irradiation VS hetero before UVB irradiation	Regulation of lipid metabolism by Peroxisome proliferator-activated receptor alpha (PPARalpha)|Reactome	-0.36010966	-0.02277475	-2.16396597	0.0607081	0.22048406	-4.34180744
WT after UVB irradiation VS WT before UVB irradiation	Regulation of lipid metabolism by Peroxisome proliferator-activated receptor alpha (PPARalpha)|Reactome	-0.34986734	-0.00983824	-1.42170118	0.18530069	0.59839062	-4.94292505
homo after UVB irradiation VS homo before UVB irradiation	Regulation of lipid metabolism by Peroxisome proliferator-activated receptor alpha (PPARalpha)|Wikipathways	0.138918185	0.025578974	0.768437301	0.46111725	0.60754106	-6.0610239
hetero after UVB irradiation VS hetero before UVB irradiation	Regulation of lipid metabolism by Peroxisome proliferator-activated receptor alpha (PPARalpha)|Wikipathways	-0.00753297	-0.00107186	-0.03867668	0.97004891	0.98614433	-6.34572986
WT after UVB irradiation VS WT before UVB irradiation	Regulation of lipid metabolism by Peroxisome proliferator-activated receptor alpha (PPARalpha)|Wikipathways	-0.05945236	-0.00339971	-0.2870892	0.7798578	0.93661609	-5.82758046
homo after UVB irradiation VS homo before UVB irradiation	Regulation of lipolysis in adipocytes - Homo sapiens (human)|KEGG	0.394430312	-0.06701448	2.132907872	0.06048451	0.16798801	-4.38812555
hetero after UVB irradiation VS hetero before UVB irradiation	Regulation of lipolysis in adipocytes - Homo sapiens (human)|KEGG	0.122947866	0.01237144	0.545248334	0.59971492	0.7885114	-6.18812317
WT after UVB irradiation VS WT before UVB irradiation	Regulation of lipolysis in adipocytes - Homo sapiens (human)|KEGG	0.245099487	-0.03949765	0.876953684	0.40092467	0.76901426	-5.49480077
homo after UVB irradiation VS homo before UVB irradiation	regulation of map kinase pathways through dual specificity phosphatases|BioCarta	-0.47469801	0.024427219	-2.46704349	0.03471354	0.1243079	-3.86886216
hetero after UVB irradiation VS hetero before UVB irradiation	regulation of map kinase pathways through dual specificity phosphatases|BioCarta	0.110243299	0.002574242	0.622016092	0.55038799	0.75605031	-6.14146428
WT after UVB irradiation VS WT before UVB irradiation	regulation of map kinase pathways through dual specificity phosphatases|BioCarta	-0.21567239	0.006894022	-0.98278392	0.34870922	0.73389632	-5.40360444
homo after UVB irradiation VS homo before UVB irradiation	Regulation of Microtubule Cytoskeleton|Wikipathways	0.268186129	-0.0391594	1.513438602	0.16307637	0.31259728	-5.27090368
hetero after UVB irradiation VS hetero before UVB irradiation	Regulation of Microtubule Cytoskeleton|Wikipathways	0.481081605	0.015520585	3.209028549	0.01162377	0.10542662	-2.75362936
WT after UVB irradiation VS WT before UVB irradiation	Regulation of Microtubule Cytoskeleton|Wikipathways	0.164663114	-0.00039779	0.770629952	0.45860411	0.80891799	-5.57749112
homo after UVB irradiation VS homo before UVB irradiation	Regulation of mitotic cell cycle|Reactome	-0.58568333	0.049850163	-3.25100941	0.00944274	0.0581856	-2.617124
hetero after UVB irradiation VS hetero before UVB irradiation	Regulation of mitotic cell cycle|Reactome	0.217623595	0.003438561	0.957035644	0.36519933	0.59333249	-5.87561187
WT after UVB irradiation VS WT before UVB irradiation	Regulation of mitotic cell cycle|Reactome	-0.09434864	-0.05460128	-0.34701733	0.73571261	0.91453443	-5.80853609
homo after UVB irradiation VS homo before UVB irradiation	Regulation of mRNA stability by proteins that bind AU-rich elements|Reactome	-0.60015404	0.012459924	-3.58266557	0.0055165	0.04291029	-2.09327014

hetero after UVB irradiation VS hetero before UVB irradiation	Regulation of mRNA stability by proteins that bind AU-rich elements|Reactome	-0.17194259	-0.03429724	-0.73882487	0.48008548	0.70113092	-6.05988648
WT after UVB irradiation VS WT before UVB irradiation	Regulation of mRNA stability by proteins that bind AU-rich elements|Reactome	-0.07736746	0.015994031	-0.28125202	0.78420484	0.93731053	-5.8292467
homo after UVB irradiation VS homo before UVB irradiation	Regulation of necroptotic cell death|Reactome	0.849614473	-0.03215571	4.542786992	0.00125669	0.02014127	-0.64871182
hetero after UVB irradiation VS hetero before UVB irradiation	Regulation of necroptotic cell death|Reactome	0.683693345	-0.01188812	2.738727154	0.02430288	0.13715811	-3.47057017
WT after UVB irradiation VS WT before UVB irradiation	Regulation of necroptotic cell death|Reactome	0.733182815	-0.06921519	3.457990495	0.00607019	0.21490368	-2.02721426
homo after UVB irradiation VS homo before UVB irradiation	Regulation of nuclear beta catenin signaling and target gene transcription|PID	0.071218841	0.024727352	0.390227811	0.70507368	0.80552596	-6.28730374
hetero after UVB irradiation VS hetero before UVB irradiation	Regulation of nuclear beta catenin signaling and target gene transcription|PID	0.044384325	-0.01238612	0.291850272	0.77745807	0.9019226	-6.30057231
WT after UVB irradiation VS WT before UVB irradiation	Regulation of nuclear beta catenin signaling and target gene transcription|PID	-0.04075029	0.057292518	-0.18861201	0.8541394	0.95632621	-5.8511635
homo after UVB irradiation VS homo before UVB irradiation	Regulation of nuclear SMAD2/3 signaling|PID	-0.21566398	0.018085952	-1.11014393	0.29455778	0.45483627	-5.7474054
hetero after UVB irradiation VS hetero before UVB irradiation	Regulation of nuclear SMAD2/3 signaling|PID	-0.05177943	0.005420557	-0.30944125	0.76450033	0.89234123	-6.29489695
WT after UVB irradiation VS WT before UVB irradiation	Regulation of nuclear SMAD2/3 signaling|PID	-0.23174989	0.022522288	-1.10441719	0.29507198	0.68163568	-5.28854042
homo after UVB irradiation VS homo before UVB irradiation	Regulation of ornithine decarboxylase (ODC)|Reactome	0.259665374	-0.02420759	0.870227203	0.40587427	0.55777967	-5.97757373
hetero after UVB irradiation VS hetero before UVB irradiation	Regulation of ornithine decarboxylase (ODC)|Reactome	0.056187159	-0.01889691	0.21603295	0.83410057	0.93635724	-6.32129484
WT after UVB irradiation VS WT before UVB irradiation	Regulation of ornithine decarboxylase (ODC)|Reactome	0.159012682	-0.08334163	0.505449354	0.62410908	0.87257466	-5.74146513
homo after UVB irradiation VS homo before UVB irradiation	regulation of p27 phosphorylation during cell cycle progression|BioCarta	-0.39514386	-0.03991655	-1.72865468	0.1165374	0.25447622	-4.98052769
hetero after UVB irradiation VS hetero before UVB irradiation	regulation of p27 phosphorylation during cell cycle progression|BioCarta	0.000594917	-0.00255501	0.002155288	0.99833049	0.99964322	-6.34653878
WT after UVB irradiation VS WT before UVB irradiation	regulation of p27 phosphorylation during cell cycle progression|BioCarta	0.086170815	0.011756637	0.312953236	0.7606939	0.92494383	-5.81979348
homo after UVB irradiation VS homo before UVB irradiation	Regulation of p38-alpha and p38-beta|PID	0.201486394	-0.02172705	0.9777077	0.35273631	0.50783175	-5.88003091
hetero after UVB irradiation VS hetero before UVB irradiation	Regulation of p38-alpha and p38-beta|PID	0.370595052	0.024889258	2.312022074	0.04795713	0.19430096	-4.12033773
WT after UVB irradiation VS WT before UVB irradiation	Regulation of p38-alpha and p38-beta|PID	0.154736311	-0.03230054	0.76044213	0.46440245	0.80891799	-5.58492524
homo after UVB irradiation VS homo before UVB irradiation	Regulation of PAK-2p34 activity by PS-GAP/RHG10|Reactome	-0.68054162	-0.05051209	-1.82200344	0.10040111	0.23044084	-4.84848794
hetero after UVB irradiation VS hetero before UVB irradiation	Regulation of PAK-2p34 activity by PS-GAP/RHG10|Reactome	-0.52266829	0.012199334	-1.44381367	0.18492187	0.40321767	-5.33965655
WT after UVB irradiation VS WT before UVB irradiation	Regulation of PAK-2p34 activity by PS-GAP/RHG10|Reactome	-0.53781344	-0.14539715	-1.66872533	0.12588301	0.53232197	-4.63622915
homo after UVB irradiation VS homo before UVB irradiation	regulation of pgc-1a|BioCarta	-0.02633273	-0.00903574	-0.11308113	0.91234725	0.9489005	-6.36158235
hetero after UVB irradiation VS hetero before UVB irradiation	regulation of pgc-1a|BioCarta	-0.44811104	-0.10058876	-1.65499311	0.13460734	0.33336163	-5.0659182
WT after UVB irradiation VS WT before UVB irradiation	regulation of pgc-1a|BioCarta	-0.28242069	-0.02966249	-0.99540715	0.34282759	0.72738377	-5.39215999
homo after UVB irradiation VS homo before UVB irradiation	Regulation of PLK1 Activity at G2/M Transition|Reactome	-0.50004536	0.012707652	-2.84853482	0.01836314	0.08635025	-3.26087153
hetero after UVB irradiation VS hetero before UVB irradiation	Regulation of PLK1 Activity at G2/M Transition|Reactome	-0.00217298	-0.01087597	-0.00879451	0.99318776	0.99537155	-6.34649934
WT after UVB irradiation VS WT before UVB irradiation	Regulation of PLK1 Activity at G2/M Transition|Reactome	-0.2158063	-0.04139872	-0.84960119	0.41526542	0.78045975	-5.5169499
homo after UVB irradiation VS homo before UVB irradiation	Regulation of PTEN gene transcription|Reactome	-0.08729396	-0.02308892	-0.43318811	0.67465971	0.77908352	-6.2686446
hetero after UVB irradiation VS hetero before UVB irradiation	Regulation of PTEN gene transcription|Reactome	0.048261244	-0.0229579	0.23718568	0.81817825	0.92781025	-6.31612655
WT after UVB irradiation VS WT before UVB irradiation	Regulation of PTEN gene transcription|Reactome	0.046290055	0.011714934	0.202435125	0.84360321	0.95293986	-5.84843627
homo after UVB irradiation VS homo before UVB irradiation	Regulation of PTEN localization|Reactome	-0.21129372	0.041180786	-1.13020884	0.28643215	0.44641309	-5.72618139
hetero after UVB irradiation VS hetero before UVB irradiation	Regulation of PTEN localization|Reactome	0.211260001	0.018064686	0.982094781	0.35340809	0.58157426	-5.85197596
WT after UVB irradiation VS WT before UVB irradiation	Regulation of PTEN localization|Reactome	-0.45358606	-0.01737185	-2.13531354	0.05827624	0.42483181	-3.99438653
homo after UVB irradiation VS homo before UVB irradiation	Regulation of PTEN mRNA translation|Reactome	-0.06600531	-0.0126454	-0.22091229	0.82989015	0.89474926	-6.34229637
hetero after UVB irradiation VS hetero before UVB irradiation	Regulation of PTEN mRNA translation|Reactome	-0.2611407	-0.00927373	-1.29662538	0.22914014	0.45170905	-5.51733934
WT after UVB irradiation VS WT before UVB irradiation	Regulation of PTEN mRNA translation|Reactome	-0.22650327	0.042886887	-0.78954021	0.44796632	0.80421615	-5.56346203
homo after UVB irradiation VS homo before UVB irradiation	Regulation of PTEN stability and activity|Reactome	0.174538751	0.002385684	0.965641958	0.35843522	0.51336738	-5.89144542
hetero after UVB irradiation VS hetero before UVB irradiation	Regulation of PTEN stability and activity|Reactome	0.36620337	-0.00047314	2.146259683	0.06244026	0.22396997	-4.36806209
WT after UVB irradiation VS WT before UVB irradiation	Regulation of PTEN stability and activity|Reactome	-0.06275767	-0.01602514	-0.31172258	0.76160204	0.92580157	-5.82017891
homo after UVB irradiation VS homo before UVB irradiation	Regulation of pyruvate dehydrogenase (PDH) complex|Reactome	-0.15426354	-0.00845761	-0.72808038	0.48432525	0.62717785	-6.09159457
hetero after UVB irradiation VS hetero before UVB irradiation	Regulation of pyruvate dehydrogenase (PDH) complex|Reactome	-0.50696853	-0.04109334	-2.55349401	0.0326272	0.15685114	-3.75386559
WT after UVB irradiation VS WT before UVB irradiation	Regulation of pyruvate dehydrogenase (PDH) complex|Reactome	-0.3684993	-0.07876434	-1.45210443	0.17687127	0.58918386	-4.90676576
homo after UVB irradiation VS homo before UVB irradiation	Regulation of RAC1 activity|PID	0.399580414	-0.04031282	2.498951595	0.03291239	0.12089941	-3.81842389
hetero after UVB irradiation VS hetero before UVB irradiation	Regulation of RAC1 activity|PID	0.402396854	0.027883051	2.325869684	0.04690964	0.19090548	-4.0994707
WT after UVB irradiation VS WT before UVB irradiation	Regulation of RAC1 activity|PID	0.446268915	-0.03876295	2.218167827	0.05064232	0.41007712	-3.8744928
homo after UVB irradiation VS homo before UVB irradiation	Regulation of RAS by GAPs|Reactome	0.038653716	0.015502476	0.167656104	0.87040956	0.91954884	-6.35336856
hetero after UVB irradiation VS hetero before UVB irradiation	Regulation of RAS by GAPs|Reactome	0.312218515	0.004043532	1.356218338	0.21025188	0.43265377	-5.44683816
WT after UVB irradiation VS WT before UVB irradiation	Regulation of RAS by GAPs|Reactome	-0.0494552	0.017842627	-0.22161128	0.82904066	0.94632059	-5.8443366
homo after UVB irradiation VS homo before UVB irradiation	Regulation of Ras family activation|PID	0.133831283	-0.04658327	0.732977197	0.48147032	0.62418895	-6.08796309
hetero after UVB irradiation VS hetero before UVB irradiation	Regulation of Ras family activation|PID	0.090153371	-0.01320752	0.455231517	0.66044431	0.82651398	-6.23551791
WT after UVB irradiation VS WT before UVB irradiation	Regulation of Ras family activation|PID	-0.02173852	-0.07021811	-0.10834723	0.91584622	0.97566251	-5.86320793
homo after UVB irradiation VS homo before UVB irradiation	Regulation of retinoblastoma protein|PID	-0.28859502	0.005183135	-1.73209542	0.11590265	0.25360346	-4.97572083
hetero after UVB irradiation VS hetero before UVB irradiation	Regulation of retinoblastoma protein|PID	0.07210563	-0.01083977	0.45208273	0.66262119	0.82651398	-6.23702972
WT after UVB irradiation VS WT before UVB irradiation	Regulation of retinoblastoma protein|PID	-0.12034871	-0.01873783	-0.51070203	0.62055497	0.87257466	-5.73883394
homo after UVB irradiation VS homo before UVB irradiation	Regulation of RhoA activity|PID	0.105905173	-0.0283754	0.573315538	0.57990149	0.70290157	-6.19494852
hetero after UVB irradiation VS hetero before UVB irradiation	Regulation of RhoA activity|PID	0.086534068	0.009166505	0.481552366	0.64238243	0.8192443	-6.22249152
WT after UVB irradiation VS WT before UVB irradiation	Regulation of RhoA activity|PID	0.067994413	-0.02882949	0.328564054	0.74920767	0.9185045	-5.81477541
homo after UVB irradiation VS homo before UVB irradiation	Regulation of RUNX1 Expression and Activity|Reactome	0.125834507	-0.03335927	0.504302158	0.62567085	0.73909372	-6.23367034
hetero after UVB irradiation VS hetero before UVB irradiation	Regulation of RUNX1 Expression and Activity|Reactome	0.166066357	0.035221156	0.853825471	0.41681319	0.64357538	-5.96771992
WT after UVB irradiation VS WT before UVB irradiation	Regulation of RUNX1 Expression and Activity|Reactome	0.203463849	0.031698349	0.804975597	0.43940418	0.79685269	-5.55179077
homo after UVB irradiation VS homo before UVB irradiation	Regulation of RUNX2 expression and activity|Reactome	-0.08125649	0.047744655	-0.51637394	0.61753427	0.73352276	-6.22723384
hetero after UVB irradiation VS hetero before UVB irradiation	Regulation of RUNX2 expression and activity|Reactome	-0.01162629	0.062233298	-0.07128882	0.94483105	0.97147303	-6.34378514
WT after UVB irradiation VS WT before UVB irradiation	Regulation of RUNX2 expression and activity|Reactome	-0.16200263	0.008847617	-0.76967887	0.45914343	0.80891799	-5.57818882
homo after UVB irradiation VS homo before UVB irradiation	Regulation of RUNX2 expression and activity|Wikipathways	0.31076522	-0.15547248	0.83676438	0.42350975	0.57483517	-6.00599112
hetero after UVB irradiation VS hetero before UVB irradiation	Regulation of RUNX2 expression and activity|Wikipathways	0.639058115	-0.03295477	1.890381921	0.09350882	0.27640286	-4.74008143
WT after UVB irradiation VS WT before UVB irradiation	Regulation of RUNX2 expression and activity|Wikipathways	0.520775276	-0.02447451	1.491570608	0.16642237	0.57064699	-4.85912289
homo after UVB irradiation VS homo before UVB irradiation	Regulation of RUNX3 expression and activity|Reactome	-0.00064322	0.009135867	-0.00288043	0.99776202	0.99819926	-6.36844381
hetero after UVB irradiation VS hetero before UVB irradiation	Regulation of RUNX3 expression and activity|Reactome	0.563452116	0.042509534	2.766775586	0.02324607	0.13628011	-3.42762603
WT after UVB irradiation VS WT before UVB irradiation	Regulation of RUNX3 expression and activity|Reactome	-0.18740485	-0.02383661	-0.66485837	0.5210579	0.83116638	-5.6503596
homo after UVB irradiation VS homo before UVB irradiation	Regulation of signaling by CBL|Reactome	0.329683032	-0.01709095	1.44530067	0.1809086	0.33428922	-5.35815716
hetero after UVB irradiation VS hetero before UVB irradiation	Regulation of signaling by CBL|Reactome	0.505397534	0.002686382	2.916851737	0.01834067	0.11936114	-3.19800264
WT after UVB irradiation VS WT before UVB irradiation	Regulation of signaling by CBL|Reactome	0.194418413	0.027737238	0.858156408	0.41074273	0.77688258	-5.51008641
homo after UVB irradiation VS homo before UVB irradiation	Regulation of signaling by NODAL|Reactome	0.194149241	0.015027298	0.950546866	0.36565963	0.51909738	-5.90556308
hetero after UVB irradiation VS hetero before UVB irradiation	Regulation of signaling by NODAL|Reactome	-0.06215966	0.012853143	-0.35360157	0.73232241	0.86896773	-6.27922414
WT after UVB irradiation VS WT before UVB irradiation	Regulation of signaling by NODAL|Reactome	0.114773751	0.042316956	0.515830088	0.61709503	0.87257466	-5.73624037
homo after UVB irradiation VS homo before UVB irradiation	Regulation of sister chromatid separation at the metaphase-anaphase transition|Wikipathways	-0.49131437	0.005524551	-1.97021505	0.0789879	0.19835132	-4.63255508
hetero after UVB irradiation VS hetero before UVB irradiation	Regulation of sister chromatid separation at the metaphase-anaphase transition|Wikipathways	0.337880255	-0.01221854	1.315637567	0.22296368	0.44410029	-5.49506876
WT after UVB irradiation VS WT before UVB irradiation	Regulation of sister chromatid separation at the metaphase-anaphase transition|Wikipathways	0.079064583	0.041224756	0.253571762	0.80492233	0.94525798	-5.83668928
homo after UVB irradiation VS homo before UVB irradiation	regulation of spermatogenesis by crem|BioCarta	0.447764849	0.000560672	2.296262672	0.04614323	0.14003669	-4.13661128
hetero after UVB irradiation VS hetero before UVB irradiation	regulation of spermatogenesis by crem|BioCarta	0.382560486	0.036043969	1.516063208	0.16608141	0.38021047	-5.24830139
WT after UVB irradiation VS WT before UVB irradiation	regulation of spermatogenesis by crem|BioCarta	0.007283868	-0.12387343	0.02683211	0.97911732	0.99283707	-5.86878648
homo after UVB irradiation VS homo before UVB irradiation	regulation of splicing through sam68|BioCarta	0.020838725	0.003479911	0.087475202	0.93213111	0.96031377	-6.36433857
hetero after UVB irradiation VS hetero before UVB irradiation	regulation of splicing through sam68|BioCarta	0.454106667	0.020630831	1.725861003	0.1207412	0.3132781	-4.96984427
WT after UVB irradiation VS WT before UVB irradiation	regulation of splicing through sam68|BioCarta	0.296826916	0.118119494	0.937848069	0.370245	0.74850253	-5.44337591
homo after UVB irradiation VS homo before UVB irradiation	Regulation of Telomerase|PID	-0.35041918	-0.01023781	-1.93066319	0.08423877	0.20601726	-4.69086937
hetero after UVB irradiation VS hetero before UVB irradiation	Regulation of Telomerase|PID	-0.114999	-0.02486735	-0.61662386	0.55377438	0.75862399	-6.14492513
WT after UVB irradiation VS WT before UVB irradiation	Regulation of Telomerase|PID	-0.10107931	0.002305082	-0.42528658	0.6795623	0.88509541	-5.7783995
homo after UVB irradiation VS homo before UVB irradiation	Regulation of thyroid hormone activity|Reactome	-0.70260239	-0.00206258	-3.83861	0.00367466	0.03413771	-1.69632245
hetero after UVB irradiation VS hetero before UVB irradiation	Regulation of thyroid hormone activity|Reactome	0.196635659	0.159834482	0.761007771	0.46741318	0.68969547	-6.0430007
WT after UVB irradiation VS WT before UVB irradiation	Regulation of thyroid hormone activity|Reactome	-0.48613714	-0.00993653	-1.55643176	0.15041257	0.55622203	-4.77916879
homo after UVB irradiation VS homo before UVB irradiation	Regulation of TLR by endogenous ligand|Reactome	0.843698029	0.038051984	4.997913966	0.00065214	0.01666204	-0.01178608
hetero after UVB irradiation VS hetero before UVB irradiation	Regulation of TLR by endogenous ligand|Reactome	0.763299176	0.058322834	4.638423678	0.00145522	0.04691448	-0.7199666
WT after UVB irradiation VS WT before UVB irradiation	Regulation of TLR by endogenous ligand|Reactome	0.402934896	0.02961314	1.360812863	0.20321393	0.61688026	-5.01386007
homo after UVB irradiation VS homo before UVB irradiation	Regulation of TNFR1 signaling|Reactome	0.432683161	-0.02801201	1.950665088	0.08154348	0.20262084	-4.66143821
hetero after UVB irradiation VS hetero before UVB irradiation	Regulation of TNFR1 signaling|Reactome	0.441065851	0.009690888	2.018670094	0.07642576	0.24828839	-4.55545173
WT after UVB irradiation VS WT before UVB irradiation	Regulation of TNFR1 signaling|Reactome	0.545621403	-0.00732757	2.686296363	0.02269247	0.31693117	-3.1797268
homo after UVB irradiation VS homo before UVB irradiation	Regulation of toll-like receptor signaling pathway|Wikipathways	0.602860435	-0.00173783	3.70920625	0.00450808	0.03837847	-1.89609321
hetero after UVB irradiation VS hetero before UVB irradiation	Regulation of toll-like receptor signaling pathway|Wikipathways	0.613631118	0.050652353	4.063814175	0.0032419	0.06341888	-1.50232975
WT after UVB irradiation VS WT before UVB irradiation	Regulation of toll-like receptor signaling pathway|Wikipathways	0.445315147	0.010144643	2.053015831	0.06694109	0.43925282	-4.1120602
homo after UVB irradiation VS homo before UVB irradiation	Regulation of TP53 Activity through Acetylation|Reactome	-0.17814621	-0.04888693	-1.01680288	0.33473117	0.4915994	-5.84226326
hetero after UVB irradiation VS hetero before UVB irradiation	Regulation of TP53 Activity through Acetylation|Reactome	-0.02744142	-0.03810212	-0.14304128	0.88961935	0.96315558	-6.33545525
WT after UVB irradiation VS WT before UVB irradiation	Regulation of TP53 Activity through Acetylation|Reactome	0.124483696	0.02428604	0.474996386	0.64491172	0.87766327	-5.75621082
homo after UVB irradiation VS homo before UVB irradiation	Regulation of TP53 Activity through Association with Co-factors|Reactome	0.280872842	-0.00489883	1.688876886	0.12410949	0.26297272	-5.03574753
hetero after UVB irradiation VS hetero before UVB irradiation	Regulation of TP53 Activity through Association with Co-factors|Reactome	0.248519646	-0.01187253	1.088735137	0.30642723	0.535791	-5.74612996
WT after UVB irradiation VS WT before UVB irradiation	Regulation of TP53 Activity through Association with Co-factors|Reactome	0.014508814	0.064029753	0.056337701	0.95617364	0.98742794	-5.86754356
homo after UVB irradiation VS homo before UVB irradiation	Regulation of TP53 Activity through Association with Co-factors|Wikipathways	0.358018555	-0.01520046	0.95451421	0.36375068	0.51773242	-5.90187023
hetero after UVB irradiation VS hetero before UVB irradiation	Regulation of TP53 Activity through Association with Co-factors|Wikipathways	0.765169865	0.024077179	2.243971594	0.05345122	0.20542215	-4.22252842
WT after UVB irradiation VS WT before UVB irradiation	Regulation of TP53 Activity through Association with Co-factors|Wikipathways	0.3238581	0.040451162	0.761644887	0.46371547	0.80891799	-5.58405213
homo after UVB irradiation VS homo before UVB irradiation	Regulation of TP53 Activity through Methylation|Reactome	-0.56367274	0.054198341	-3.17845042	0.01063626	0.06218329	-2.73277795
hetero after UVB irradiation VS hetero before UVB irradiation	Regulation of TP53 Activity through Methylation|Reactome	-0.20954189	0.02287833	-0.84885401	0.41942338	0.646292	-5.97193741
WT after UVB irradiation VS WT before UVB irradiation	Regulation of TP53 Activity through Methylation|Reactome	-0.52520473	-0.01663964	-2.0416154	0.06823384	0.44205977	-4.12823786
homo after UVB irradiation VS homo before UVB irradiation	Regulation of TP53 Activity through Phosphorylation|Reactome	-0.49347496	-0.00856841	-2.77066278	0.02090738	0.09232406	-3.38557444
hetero after UVB irradiation VS hetero before UVB irradiation	Regulation of TP53 Activity through Phosphorylation|Reactome	-0.03033703	-0.01827307	-0.12979316	0.89977529	0.96973277	-6.33741167
WT after UVB irradiation VS WT before UVB irradiation	Regulation of TP53 Activity through Phosphorylation|Reactome	-0.24377121	-0.01632718	-0.93117963	0.3735207	0.75049232	-5.44914744
homo after UVB irradiation VS homo before UVB irradiation	Regulation of TP53 Activity|Reactome	-0.40982501	-0.00587199	-2.46455141	0.03485826	0.124366	-3.87279675
hetero after UVB irradiation VS hetero before UVB irradiation	Regulation of TP53 Activity|Reactome	-0.06195814	-0.01789798	-0.31570181	0.7599073	0.88865506	-6.29279883
WT after UVB irradiation VS WT before UVB irradiation	Regulation of TP53 Activity|Reactome	-0.23552087	-0.00739723	-0.90379328	0.38719013	0.76061898	-5.4724909
homo after UVB irradiation VS homo before UVB irradiation	Regulation of TP53 Degradation|Reactome	-0.19031409	0.00289427	-1.04136145	0.32377886	0.47999165	-5.81793686
hetero after UVB irradiation VS hetero before UVB irradiation	Regulation of TP53 Degradation|Reactome	0.224438376	0.024738269	1.178418724	0.27083546	0.50018963	-5.65087594
WT after UVB irradiation VS WT before UVB irradiation	Regulation of TP53 Degradation|Reactome	-0.26790759	-0.01224251	-1.1490474	0.27706542	0.66671843	-5.24373196
homo after UVB irradiation VS homo before UVB irradiation	Regulation of TP53 Expression and Degradation|Reactome	-0.2268531	0.006840746	-1.26862122	0.23512448	0.39694717	-5.5722209
hetero after UVB irradiation VS hetero before UVB irradiation	Regulation of TP53 Expression and Degradation|Reactome	0.193052062	0.025278633	1.015732445	0.33803237	0.56712002	-5.8194958
WT after UVB irradiation VS WT before UVB irradiation	Regulation of TP53 Expression and Degradation|Reactome	-0.28207027	-0.014806	-1.21349923	0.25260157	0.64679852	-5.17670206
homo after UVB irradiation VS homo before UVB irradiation	Regulation of TP53 Expression and Degradation|Wikipathways	-0.16296972	-0.04783602	-0.73082748	0.48272232	0.6254569	-6.08955998
hetero after UVB irradiation VS hetero before UVB irradiation	Regulation of TP53 Expression and Degradation|Wikipathways	0.023536715	-0.05705291	0.125987916	0.90269606	0.97054702	-6.33793863
WT after UVB irradiation VS WT before UVB irradiation	Regulation of TP53 Expression and Degradation|Wikipathways	-0.36700605	0.010666741	-1.74387582	0.11153502	0.51348834	-4.53765464
homo after UVB irradiation VS homo before UVB irradiation	Regulation of TP53 Expression|Reactome	-0.73721775	-0.04197118	-2.4991781	0.03289994	0.12089941	-3.81806546
hetero after UVB irradiation VS hetero before UVB irradiation	Regulation of TP53 Expression|Reactome	-0.38657138	-0.05439626	-0.95353939	0.36686738	0.59508238	-5.87887092
WT after UVB irradiation VS WT before UVB irradiation	Regulation of TP53 Expression|Reactome	-0.19687826	0.085388083	-0.53967897	0.60113361	0.86824073	-5.72385773
homo after UVB irradiation VS homo before UVB irradiation	regulation of transcriptional activity by pml|BioCarta	-0.33445604	-0.00407294	-1.46164484	0.17648341	0.32929357	-5.33745205
hetero after UVB irradiation VS hetero before UVB irradiation	regulation of transcriptional activity by pml|BioCarta	0.11039594	-0.02323481	0.438047499	0.67236526	0.82872927	-6.24364683
WT after UVB irradiation VS WT before UVB irradiation	regulation of transcriptional activity by pml|BioCarta	-0.35424102	0.020709503	-1.30669119	0.22033675	0.62628016	-5.07517858
homo after UVB irradiation VS homo before UVB irradiation	Regulation of Wnt-B-catenin Signaling by Small Molecule Compounds|Wikipathways	-0.24589062	0.02299213	-1.04709493	0.32126149	0.47703413	-5.81219175
hetero after UVB irradiation VS hetero before UVB irradiation	Regulation of Wnt-B-catenin Signaling by Small Molecule Compounds|Wikipathways	-0.29008778	0.002254516	-1.72197931	0.12146521	0.31331055	-4.97515527
WT after UVB irradiation VS WT before UVB irradiation	Regulation of Wnt-B-catenin Signaling by Small Molecule Compounds|Wikipathways	-0.31491603	0.064900228	-1.22868089	0.2470986	0.63937638	-5.16052645
homo after UVB irradiation VS homo before UVB irradiation	regulators of bone mineralization|BioCarta	0.001277021	0.012093001	0.006677988	0.99481152	0.99546557	-6.3684243
hetero after UVB irradiation VS hetero before UVB irradiation	regulators of bone mineralization|BioCarta	0.1463088	-0.00703931	0.644413534	0.53645243	0.74935648	-6.12679669
WT after UVB irradiation VS WT before UVB irradiation	regulators of bone mineralization|BioCarta	0.187794626	0.081463108	0.919848355	0.37913426	0.75122805	-5.4588763
homo after UVB irradiation VS homo before UVB irradiation	Relationship between inflammation_ COX-2 and EGFR|Wikipathways	0.343240706	-0.00610577	1.664850014	0.12889717	0.26948008	-5.06878018
hetero after UVB irradiation VS hetero before UVB irradiation	Relationship between inflammation_ COX-2 and EGFR|Wikipathways	0.482604934	0.051953116	3.034531982	0.01524847	0.11352943	-3.01843661
WT after UVB irradiation VS WT before UVB irradiation	Relationship between inflammation_ COX-2 and EGFR|Wikipathways	0.211964114	0.040378048	0.920513485	0.3788031	0.75122805	-5.45830796
homo after UVB irradiation VS homo before UVB irradiation	Relaxin receptors|Reactome	0.849455292	0.03286151	4.932041011	0.00071577	0.01666204	-0.10191006
hetero after UVB irradiation VS hetero before UVB irradiation	Relaxin receptors|Reactome	-0.48885774	-0.03679139	-2.33949523	0.04590115	0.18854754	-4.07891644
WT after UVB irradiation VS WT before UVB irradiation	Relaxin receptors|Reactome	-0.40252179	-0.11155154	-1.52619523	0.15770016	0.56072663	-4.81669095
homo after UVB irradiation VS homo before UVB irradiation	Relaxin signaling pathway - Homo sapiens (human)|KEGG	0.392835588	-0.02956508	2.503781793	0.03264795	0.1203152	-3.8107792
hetero after UVB irradiation VS hetero before UVB irradiation	Relaxin signaling pathway - Homo sapiens (human)|KEGG	0.382710837	0.021136064	2.296393052	0.04916727	0.1971015	-4.1438612

WT after UVB irradiation VS WT before UVB irradiation	Relaxin signaling pathway - Homo sapiens (human)|KEGG	0.258353584	0.005114276	1.177164759	0.26617172	0.66118679	-5.21482101
homo after UVB irradiation VS homo before UVB irradiation	Release of apoptotic factors from the mitochondria|Reactome	0.244704679	-0.0305756	0.584918078	0.57238918	0.69638396	-6.18798416
hetero after UVB irradiation VS hetero before UVB irradiation	Release of apoptotic factors from the mitochondria|Reactome	0.209805529	0.080436231	0.602701961	0.56257324	0.76403561	-6.15373338
WT after UVB irradiation VS WT before UVB irradiation	Release of apoptotic factors from the mitochondria|Reactome	0.130913964	-0.02321729	0.363679146	0.7236079	0.90882588	-5.80261728
homo after UVB irradiation VS homo before UVB irradiation	Release of Hh-Np from the secreting cell|Reactome	0.156017127	0.024985754	0.800834059	0.44301981	0.59095193	-6.03543749
hetero after UVB irradiation VS hetero before UVB irradiation	Release of Hh-Np from the secreting cell|Reactome	-0.01533827	0.018937548	-0.06423525	0.95028081	0.97400043	-6.34430343
WT after UVB irradiation VS WT before UVB irradiation	Release of Hh-Np from the secreting cell|Reactome	-0.07068582	0.080728379	-0.30161845	0.76907241	0.93036308	-5.82328713
homo after UVB irradiation VS homo before UVB irradiation	Remifentanil Action Pathway|SMPDB	0.191132361	0.029768717	1.061313976	0.31508276	0.46923284	-5.79783739
hetero after UVB irradiation VS hetero before UVB irradiation	Remifentanil Action Pathway|SMPDB	-0.07904315	0.055005373	-0.44437398	0.66796482	0.82651398	-6.24068873
WT after UVB irradiation VS WT before UVB irradiation	Remifentanil Action Pathway|SMPDB	0.022316377	0.035038618	0.098344171	0.92358676	0.97566251	-5.86425414
homo after UVB irradiation VS homo before UVB irradiation	Removal of aminoterminal propeptides from gamma-carboxylated proteins|Reactome	0.69771706	0.052552116	3.700054377	0.00457409	0.03846279	-1.91029536
hetero after UVB irradiation VS hetero before UVB irradiation	Removal of aminoterminal propeptides from gamma-carboxylated proteins|Reactome	0.542028211	0.102886331	2.300869977	0.04881756	0.1967378	-4.13712602
WT after UVB irradiation VS WT before UVB irradiation	Removal of aminoterminal propeptides from gamma-carboxylated proteins|Reactome	0.695300925	0.010041019	3.139094964	0.01042526	0.26101395	-2.49970636
homo after UVB irradiation VS homo before UVB irradiation	Removal of the Flap Intermediate from the C-strand|Reactome	-0.49721994	-0.0212222	-1.61115482	0.14020479	0.28327069	-5.14168615
hetero after UVB irradiation VS hetero before UVB irradiation	Removal of the Flap Intermediate from the C-strand|Reactome	0.236185137	0.011928106	0.815585262	0.43718298	0.66334222	-5.99962657
WT after UVB irradiation VS WT before UVB irradiation	Removal of the Flap Intermediate from the C-strand|Reactome	0.138396799	0.067221887	0.407771817	0.69196538	0.89473552	-5.78565628
homo after UVB irradiation VS homo before UVB irradiation	Removal of the Flap Intermediate|Reactome	-0.57055454	-0.03528796	-1.90475773	0.08785432	0.21202051	-4.72880164
hetero after UVB irradiation VS hetero before UVB irradiation	Removal of the Flap Intermediate|Reactome	0.118072204	0.009463711	0.378143604	0.71467329	0.85921866	-6.26963883
WT after UVB irradiation VS WT before UVB irradiation	Removal of the Flap Intermediate|Reactome	0.073220569	0.040674736	0.236053577	0.81811733	0.94525798	-5.84100681
homo after UVB irradiation VS homo before UVB irradiation	Renal cell carcinoma - Homo sapiens (human)|KEGG	0.255379453	-0.01988959	1.17085218	0.27051519	0.43415548	-5.68231965
hetero after UVB irradiation VS hetero before UVB irradiation	Renal cell carcinoma - Homo sapiens (human)|KEGG	0.425048471	0.027952117	2.817337897	0.0214583	0.12985515	-3.35022058
WT after UVB irradiation VS WT before UVB irradiation	Renal cell carcinoma - Homo sapiens (human)|KEGG	0.271887308	0.025159303	1.384498503	0.1960791	0.60772778	-4.98650593
homo after UVB irradiation VS homo before UVB irradiation	Renin secretion - Homo sapiens (human)|KEGG	0.690915556	-0.02125877	4.131148912	0.00233355	0.02721394	-1.2525355
hetero after UVB irradiation VS hetero before UVB irradiation	Renin secretion - Homo sapiens (human)|KEGG	0.308729154	0.00564471	1.22304809	0.2544133	0.47937588	-5.6014849
WT after UVB irradiation VS WT before UVB irradiation	Renin secretion - Homo sapiens (human)|KEGG	0.33679759	-0.05100135	1.155768272	0.27443007	0.66570326	-5.23686868
homo after UVB irradiation VS homo before UVB irradiation	Renin-angiotensin system - Homo sapiens (human)|KEGG	0.324403166	-0.03985808	1.993963699	0.0759854	0.19626432	-4.59731789
hetero after UVB irradiation VS hetero before UVB irradiation	Renin-angiotensin system - Homo sapiens (human)|KEGG	0.346364625	-0.02693103	2.102417559	0.06693957	0.23308674	-4.4328234
WT after UVB irradiation VS WT before UVB irradiation	Renin-angiotensin system - Homo sapiens (human)|KEGG	0.341536273	-0.07170297	1.709732364	0.11785961	0.52194851	-4.58271331
homo after UVB irradiation VS homo before UVB irradiation	Repaglinide Action Pathway|SMPDB	-0.17556465	0.032733531	-0.6220452	0.54871777	0.67913558	-6.16483259
hetero after UVB irradiation VS hetero before UVB irradiation	Repaglinide Action Pathway|SMPDB	-0.35455332	-0.0590526	-1.26742043	0.23890834	0.4612216	-5.55113336
WT after UVB irradiation VS WT before UVB irradiation	Repaglinide Action Pathway|SMPDB	-0.23688751	-0.13206249	-0.65900303	0.52465845	0.83140705	-5.65411026
homo after UVB irradiation VS homo before UVB irradiation	repression of pain sensation by the transcriptional regulator dream|BioCarta	0.026447039	-0.00362347	0.129338941	0.89981793	0.9399608	-6.35946813
hetero after UVB irradiation VS hetero before UVB irradiation	repression of pain sensation by the transcriptional regulator dream|BioCarta	0.236233088	0.009467313	1.274610363	0.2364717	0.45904514	-5.54286104
WT after UVB irradiation VS WT before UVB irradiation	repression of pain sensation by the transcriptional regulator dream|BioCarta	0.323275099	-0.01943697	1.54866414	0.15225595	0.55762063	-4.78884887
homo after UVB irradiation VS homo before UVB irradiation	Repression of WNT target genes|Reactome	-0.23984998	-0.00977809	-1.085626	0.30472985	0.46581737	-5.77294596
hetero after UVB irradiation VS hetero before UVB irradiation	Repression of WNT target genes|Reactome	-0.11091629	-0.0511192	-0.57498622	0.5803258	0.77397513	-6.17071945
WT after UVB irradiation VS WT before UVB irradiation	Repression of WNT target genes|Reactome	-0.23045053	0.058070544	-0.89594619	0.3911711	0.76443235	-5.47907215
homo after UVB irradiation VS homo before UVB irradiation	Reproduction|Reactome	-0.27582495	0.046892036	-1.65515034	0.13087688	0.27262036	-5.08204492
hetero after UVB irradiation VS hetero before UVB irradiation	Reproduction|Reactome	-0.34232783	0.005498584	-1.84443992	0.10046695	0.28513596	-4.80510649
WT after UVB irradiation VS WT before UVB irradiation	Reproduction|Reactome	-0.24970252	0.03144768	-1.16708735	0.27003641	0.66327566	-5.22524247
homo after UVB irradiation VS homo before UVB irradiation	Rescinnamine Action Pathway|SMPDB	0.636038586	-0.11800708	2.436891056	0.03650532	0.12504267	-3.91641947
hetero after UVB irradiation VS hetero before UVB irradiation	Rescinnamine Action Pathway|SMPDB	-0.02777369	0.003323189	-0.09172332	0.92906122	0.97054702	-6.34197953
WT after UVB irradiation VS WT before UVB irradiation	Rescinnamine Action Pathway|SMPDB	0.270438532	-0.10210744	0.707878218	0.49505953	0.81710765	-5.62188349
homo after UVB irradiation VS homo before UVB irradiation	Resistin as a regulator of inflammation|Wikipathways	0.438587173	-0.04311975	2.606530103	0.02749848	0.11028351	-3.64764861
hetero after UVB irradiation VS hetero before UVB irradiation	Resistin as a regulator of inflammation|Wikipathways	0.509152543	0.046869081	2.850291551	0.02036965	0.12631953	-3.29978926
WT after UVB irradiation VS WT before UVB irradiation	Resistin as a regulator of inflammation|Wikipathways	0.354034193	0.021236053	1.60347635	0.13966253	0.55478524	-4.71995444
homo after UVB irradiation VS homo before UVB irradiation	Resolution of Abasic Sites (AP sites)|Reactome	-0.45223265	-0.01747997	-2.17447565	0.05647101	0.16165213	-4.32463734
hetero after UVB irradiation VS hetero before UVB irradiation	Resolution of Abasic Sites (AP sites)|Reactome	0.050472591	-0.02334625	0.221855754	0.82970915	0.93424267	-6.31991968
WT after UVB irradiation VS WT before UVB irradiation	Resolution of Abasic Sites (AP sites)|Reactome	0.132031072	0.050326638	0.486666908	0.63690017	0.87636259	-5.75066267
homo after UVB irradiation VS homo before UVB irradiation	Resolution of AP sites via the multiple-nucleotide patch replacement pathway|Reactome	-0.33424013	-0.00178112	-1.3153982	0.21958998	0.37807235	-5.51736204
hetero after UVB irradiation VS hetero before UVB irradiation	Resolution of AP sites via the multiple-nucleotide patch replacement pathway|Reactome	0.245478321	0.007714514	1.038203911	0.32804775	0.55664357	-5.79732609
WT after UVB irradiation VS WT before UVB irradiation	Resolution of AP sites via the multiple-nucleotide patch replacement pathway|Reactome	0.186463589	0.056438093	0.647089978	0.53202921	0.83483909	-5.66164781
homo after UVB irradiation VS homo before UVB irradiation	Resolution of AP sites via the single-nucleotide replacement pathway|Reactome	-0.51917482	0.027725709	-1.65769863	0.13035413	0.27177943	-5.07856399
hetero after UVB irradiation VS hetero before UVB irradiation	Resolution of AP sites via the single-nucleotide replacement pathway|Reactome	-0.44099994	-0.09377456	-1.48583557	0.17374668	0.39118926	-5.28683209
WT after UVB irradiation VS WT before UVB irradiation	Resolution of AP sites via the single-nucleotide replacement pathway|Reactome	-0.4302013	-0.01692667	-1.13145802	0.28405659	0.67189698	-5.26155179
homo after UVB irradiation VS homo before UVB irradiation	Resolution of D-loop Structures through Holliday Junction Intermediates|Reactome	-0.61256419	-0.04007772	-2.48622187	0.03361955	0.12231622	-3.8385594
hetero after UVB irradiation VS hetero before UVB irradiation	Resolution of D-loop Structures through Holliday Junction Intermediates|Reactome	-0.00974234	-0.0883648	-0.03498083	0.97290959	0.98711441	-6.34587752
WT after UVB irradiation VS WT before UVB irradiation	Resolution of D-loop Structures through Holliday Junction Intermediates|Reactome	-0.03583028	0.017944169	-0.11994552	0.90688283	0.97566251	-5.86186844
homo after UVB irradiation VS homo before UVB irradiation	Resolution of D-loop Structures through Synthesis-Dependent Strand Annealing (SDSA)|Reactome	-0.64775567	-0.04253713	-2.42523992	0.03722195	0.12651226	-3.93476664
hetero after UVB irradiation VS hetero before UVB irradiation	Resolution of D-loop Structures through Synthesis-Dependent Strand Annealing (SDSA)|Reactome	-0.1483892	-0.08974254	-0.51631071	0.61891133	0.80231894	-6.20423235
WT after UVB irradiation VS WT before UVB irradiation	Resolution of D-loop Structures through Synthesis-Dependent Strand Annealing (SDSA)|Reactome	-0.08571498	0.000487179	-0.2927248	0.77566841	0.9344727	-5.82593986
homo after UVB irradiation VS homo before UVB irradiation	Resolution of D-Loop Structures|Reactome	-0.58137672	-0.04355717	-2.34229706	0.04274078	0.13403462	-4.06485345
hetero after UVB irradiation VS hetero before UVB irradiation	Resolution of D-Loop Structures|Reactome	-0.00871758	-0.07718042	-0.03103077	0.97596749	0.988327	-6.34601896
WT after UVB irradiation VS WT before UVB irradiation	Resolution of D-Loop Structures|Reactome	-0.00340873	0.016874849	-0.01149578	0.99105219	0.99712795	-5.86908424
homo after UVB irradiation VS homo before UVB irradiation	Resolution of Sister Chromatid Cohesion|Reactome	-0.65896981	-0.01853762	-3.47236482	0.00658779	0.04701822	-2.26646436
hetero after UVB irradiation VS hetero before UVB irradiation	Resolution of Sister Chromatid Cohesion|Reactome	0.091208892	-0.01967424	0.314686224	0.7606517	0.88898729	-6.29314197
WT after UVB irradiation VS WT before UVB irradiation	Resolution of Sister Chromatid Cohesion|Reactome	0.024601557	0.017332335	0.083355355	0.93520078	0.98056728	-5.86563261
homo after UVB irradiation VS homo before UVB irradiation	resolvin D biosynthesis|HumanCyc	1.130148334	-0.04157466	5.613300672	0.00028163	0.01666204	0.796026052
hetero after UVB irradiation VS hetero before UVB irradiation	resolvin D biosynthesis|HumanCyc	1.055188628	0.013837018	5.670874875	0.00038902	0.03004176	0.549993297
WT after UVB irradiation VS WT before UVB irradiation	resolvin D biosynthesis|HumanCyc	0.863170772	-0.01933524	2.75415854	0.02018891	0.31542987	-3.07771909
homo after UVB irradiation VS homo before UVB irradiation	Respiratory electron transport_ ATP synthesis by chemiosmotic coupling_ and heat production by uncoupling proteins.|Reactome	-0.07126521	0.005035181	-0.24428076	0.8122681	0.88427872	-6.3364899
hetero after UVB irradiation VS hetero before UVB irradiation	Respiratory electron transport_ ATP synthesis by chemiosmotic coupling_ and heat production by uncoupling proteins.|Reactome	-0.43674101	-0.05225825	-2.03595179	0.07436744	0.24421238	-4.53027114
WT after UVB irradiation VS WT before UVB irradiation	Respiratory electron transport_ ATP synthesis by chemiosmotic coupling_ and heat production by uncoupling proteins.|Reactome	-0.08818571	-0.06049744	-0.29245523	0.77586864	0.9344727	-5.82601905
homo after UVB irradiation VS homo before UVB irradiation	Respiratory electron transport|Reactome	-0.0709908	0.010251659	-0.23911849	0.81615179	0.8875301	-6.33782219
hetero after UVB irradiation VS hetero before UVB irradiation	Respiratory electron transport|Reactome	-0.44270291	-0.05366808	-2.10393337	0.06677886	0.23300949	-4.43059045
WT after UVB irradiation VS WT before UVB irradiation	Respiratory electron transport|Reactome	-0.07082757	-0.0680355	-0.23598951	0.8181657	0.94525798	-5.84102204
homo after UVB irradiation VS homo before UVB irradiation	Response of Mtb to phagocytosis|Reactome	1.019010934	-0.02342197	4.307824036	0.00178393	0.02356086	-0.99027397
hetero after UVB irradiation VS hetero before UVB irradiation	Response of Mtb to phagocytosis|Reactome	1.044821233	0.05989276	4.854999212	0.00108958	0.04104387	-0.43899655
WT after UVB irradiation VS WT before UVB irradiation	Response of Mtb to phagocytosis|Reactome	0.459621405	0.016400241	1.150166408	0.27662526	0.66649013	-5.24259131
homo after UVB irradiation VS homo before UVB irradiation	Response to elevated platelet cytosolic Ca2+|Reactome	0.536863147	0.001245013	3.306306801	0.00862664	0.05539977	-2.52919172
hetero after UVB irradiation VS hetero before UVB irradiation	Response to elevated platelet cytosolic Ca2+|Reactome	0.386986786	0.029002109	1.874604674	0.09584527	0.27962159	-4.76248219
WT after UVB irradiation VS WT before UVB irradiation	Response to elevated platelet cytosolic Ca2+|Reactome	0.335382686	0.004080183	1.455552135	0.17593647	0.58755921	-4.90263515
homo after UVB irradiation VS homo before UVB irradiation	Response to metal ions|Reactome	0.05251671	0.062497611	0.174913978	0.86486116	0.91750838	-6.35203692
hetero after UVB irradiation VS hetero before UVB irradiation	Response to metal ions|Reactome	0.476492888	0.05632829	1.696368358	0.12634363	0.31934829	-5.01005777
WT after UVB irradiation VS WT before UVB irradiation	Response to metal ions|Reactome	-0.39494132	0.090456807	-1.7395119	0.11232583	0.51412987	-4.54343826
homo after UVB irradiation VS homo before UVB irradiation	RET signaling|Reactome	0.214702398	-0.01468766	1.164838646	0.27282485	0.43614438	-5.68888186
hetero after UVB irradiation VS hetero before UVB irradiation	RET signaling|Reactome	0.194183633	-0.00990435	1.294965518	0.22968616	0.45170905	-5.51927363
WT after UVB irradiation VS WT before UVB irradiation	RET signaling|Reactome	0.239131421	0.024748309	1.195172387	0.25937533	0.65440449	-5.19603469
homo after UVB irradiation VS homo before UVB irradiation	Reteplase Action Pathway|SMPDB	0.538210712	0.026444726	3.007777411	0.01409598	0.07305822	-3.00571236
hetero after UVB irradiation VS hetero before UVB irradiation	Reteplase Action Pathway|SMPDB	0.558085555	0.09913938	2.984394118	0.01649409	0.11352943	-3.09486545
WT after UVB irradiation VS WT before UVB irradiation	Reteplase Action Pathway|SMPDB	0.440879848	0.077377741	1.569633667	0.14732454	0.55622203	-4.76265269
homo after UVB irradiation VS homo before UVB irradiation	retinoate biosynthesis I|HumanCyc	0.690201847	0.005353282	3.507930542	0.00622046	0.04588471	-2.21049378
hetero after UVB irradiation VS hetero before UVB irradiation	retinoate biosynthesis I|HumanCyc	0.344032223	0.042084692	1.439130843	0.18620548	0.40374874	-5.34548836
WT after UVB irradiation VS WT before UVB irradiation	retinoate biosynthesis I|HumanCyc	0.359859131	0.044775653	1.143570721	0.27922758	0.66906841	-5.24930252
homo after UVB irradiation VS homo before UVB irradiation	retinoate biosynthesis II|HumanCyc	0.965432434	-0.00196704	5.46280897	0.00034412	0.01666204	0.604167169
hetero after UVB irradiation VS hetero before UVB irradiation	retinoate biosynthesis II|HumanCyc	0.712342053	0.019285642	3.68313086	0.00565746	0.08008296	-2.04829545
WT after UVB irradiation VS WT before UVB irradiation	retinoate biosynthesis II|HumanCyc	0.679617267	0.091449128	2.991155654	0.01343046	0.28007727	-2.7213449
homo after UVB irradiation VS homo before UVB irradiation	Retinoblastoma Gene in Cancer|Wikipathways	-0.53424788	-0.04399223	-2.30795951	0.04525435	0.13845639	-4.11841092
hetero after UVB irradiation VS hetero before UVB irradiation	Retinoblastoma Gene in Cancer|Wikipathways	0.088638529	-0.01056789	0.346100701	0.73775088	0.87250107	-6.28202937
WT after UVB irradiation VS WT before UVB irradiation	Retinoblastoma Gene in Cancer|Wikipathways	0.023569839	0.024712448	0.080196389	0.93765062	0.98056728	-5.86589416
homo after UVB irradiation VS homo before UVB irradiation	Retinoic acid receptors-mediated signaling|PID	-0.12109364	0.006412227	-0.58258023	0.57389853	0.69732889	-6.18939786
hetero after UVB irradiation VS hetero before UVB irradiation	Retinoic acid receptors-mediated signaling|PID	-0.05265073	-0.03272376	-0.28878529	0.77972345	0.90262028	-6.3015279
WT after UVB irradiation VS WT before UVB irradiation	Retinoic acid receptors-mediated signaling|PID	-0.15575253	-0.01058668	-0.67915706	0.5123274	0.8273689	-5.64107397
homo after UVB irradiation VS homo before UVB irradiation	Retinoid cycle disease events|Reactome	0.151963221	-0.00337558	0.370120082	0.71950453	0.81577771	-6.29539034
hetero after UVB irradiation VS hetero before UVB irradiation	Retinoid cycle disease events|Reactome	-0.2697141	0.01289775	-0.64818052	0.53412945	0.74771561	-6.12428369
WT after UVB irradiation VS WT before UVB irradiation	Retinoid cycle disease events|Reactome	0.317720968	-0.00400158	0.731626644	0.48105584	0.81552033	-5.60547755
homo after UVB irradiation VS homo before UVB irradiation	Retinoid metabolism and transport|Reactome	0.469234702	0.019166053	2.702535542	0.02342447	0.10061724	-3.49451948
hetero after UVB irradiation VS hetero before UVB irradiation	Retinoid metabolism and transport|Reactome	0.285592399	0.053888217	1.487646039	0.17327889	0.39099267	-5.28453665
WT after UVB irradiation VS WT before UVB irradiation	Retinoid metabolism and transport|Reactome	0.338619174	0.053343277	1.385453653	0.19579587	0.60747265	-4.98539637
homo after UVB irradiation VS homo before UVB irradiation	retinol biosynthesis|HumanCyc	0.308439405	0.030340562	1.9059108	0.08769033	0.21178822	-4.72711779
hetero after UVB irradiation VS hetero before UVB irradiation	retinol biosynthesis|HumanCyc	-0.13432916	0.051476821	-0.74283878	0.47777611	0.69910758	-6.05686334
WT after UVB irradiation VS WT before UVB irradiation	retinol biosynthesis|HumanCyc	0.025941702	0.081050816	0.105560363	0.91800185	0.97566251	-5.86350956
homo after UVB irradiation VS homo before UVB irradiation	Retinol metabolism - Homo sapiens (human)|KEGG	0.027980594	0.080404662	0.166500987	0.87129331	0.92005672	-6.35357532
hetero after UVB irradiation VS hetero before UVB irradiation	Retinol metabolism - Homo sapiens (human)|KEGG	-0.08079412	0.007326958	-0.41948908	0.68535026	0.83951263	-6.25209042
WT after UVB irradiation VS WT before UVB irradiation	Retinol metabolism - Homo sapiens (human)|KEGG	-0.09692323	0.042775518	-0.41122545	0.68951197	0.89330527	-5.78424864
homo after UVB irradiation VS homo before UVB irradiation	Retinol Metabolism|SMPDB	-0.11250963	0.020293984	-0.63167954	0.54266821	0.67708436	-6.15861079
hetero after UVB irradiation VS hetero before UVB irradiation	Retinol Metabolism|SMPDB	0.025383371	0.031188844	0.134162656	0.8964234	0.96753442	-6.3367873
WT after UVB irradiation VS WT before UVB irradiation	Retinol Metabolism|SMPDB	-0.01537733	0.00324887	-0.07239348	0.94370479	0.98295421	-5.86649696
homo after UVB irradiation VS homo before UVB irradiation	Retrograde endocannabinoid signaling - Homo sapiens (human)|KEGG	0.204985269	-0.0144101	0.999096342	0.34279885	0.49847757	-5.85951567
hetero after UVB irradiation VS hetero before UVB irradiation	Retrograde endocannabinoid signaling - Homo sapiens (human)|KEGG	-0.08224169	0.01775261	-0.38543092	0.70946679	0.85571569	-6.26667304
WT after UVB irradiation VS WT before UVB irradiation	Retrograde endocannabinoid signaling - Homo sapiens (human)|KEGG	0.039271007	-0.02703989	0.158288295	0.87735518	0.96621199	-5.85647531
homo after UVB irradiation VS homo before UVB irradiation	Retrograde neurotrophin signalling|Reactome	0.744183611	-0.01294186	4.40461974	0.00154271	0.02229116	-0.8485433
hetero after UVB irradiation VS hetero before UVB irradiation	Retrograde neurotrophin signalling|Reactome	0.65231193	0.010599348	4.116081041	0.00300811	0.06148411	-1.42901659
WT after UVB irradiation VS WT before UVB irradiation	Retrograde neurotrophin signalling|Reactome	0.694436081	0.013941685	3.338789953	0.00742299	0.23542226	-2.20277266
homo after UVB irradiation VS homo before UVB irradiation	Retrograde transport at the Trans-Golgi-Network|Reactome	-0.02782397	-0.03436623	-0.15790779	0.8778735	0.92507972	-6.35506886
hetero after UVB irradiation VS hetero before UVB irradiation	Retrograde transport at the Trans-Golgi-Network|Reactome	-0.04184002	-0.03131872	-0.22217819	0.82946616	0.93424267	-6.31984247
WT after UVB irradiation VS WT before UVB irradiation	Retrograde transport at the Trans-Golgi-Network|Reactome	-0.19675642	-0.02815928	-0.90040572	0.38890519	0.76228756	-5.47533791
homo after UVB irradiation VS homo before UVB irradiation	Rett syndrome causing genes|Wikipathways	-0.28821513	0.038271983	-1.77778648	0.10776873	0.24085757	-4.91144538
hetero after UVB irradiation VS hetero before UVB irradiation	Rett syndrome causing genes|Wikipathways	-0.2960976	-0.04854406	-2.00139047	0.07853869	0.25192071	-4.58056055
WT after UVB irradiation VS WT before UVB irradiation	Rett syndrome causing genes|Wikipathways	-0.42456849	-0.0159508	-2.08219937	0.06373688	0.43544914	-4.07050679
homo after UVB irradiation VS homo before UVB irradiation	Reuptake of GABA|Reactome	-0.18736179	0.121621395	-0.90057361	0.39032905	0.54369812	-5.95098971
hetero after UVB irradiation VS hetero before UVB irradiation	Reuptake of GABA|Reactome	-0.47165833	-0.00991564	-2.18233214	0.05896109	0.21664304	-4.31451693
WT after UVB irradiation VS WT before UVB irradiation	Reuptake of GABA|Reactome	-0.08629477	-0.03414089	-0.27023445	0.79243091	0.94172052	-5.83229995
homo after UVB irradiation VS homo before UVB irradiation	Reversal of alkylation damage by DNA dioxygenases|Reactome	-0.04671703	-0.07550252	-0.23705565	0.81770519	0.88813008	-6.33834671
hetero after UVB irradiation VS hetero before UVB irradiation	Reversal of alkylation damage by DNA dioxygenases|Reactome	0.286166314	0.041348673	1.283800679	0.23338748	0.45519048	-5.53224195
WT after UVB irradiation VS WT before UVB irradiation	Reversal of alkylation damage by DNA dioxygenases|Reactome	0.169522302	0.021192413	0.713901904	0.49148389	0.81710765	-5.61776805
homo after UVB irradiation VS homo before UVB irradiation	reversal of insulin resistance by leptin|BioCarta	0.065779466	-0.03693039	0.285391862	0.7815399	0.85905421	-6.3248796
hetero after UVB irradiation VS hetero before UVB irradiation	reversal of insulin resistance by leptin|BioCarta	-0.14435436	-0.04760054	-0.66789514	0.52207085	0.73763142	-6.11091692
WT after UVB irradiation VS WT before UVB irradiation	reversal of insulin resistance by leptin|BioCarta	-0.2125441	-0.07681012	-0.74408424	0.47381045	0.81372871	-5.59667872
homo after UVB irradiation VS homo before UVB irradiation	Reverse Transcription of HIV RNA|Reactome	-0.08403744	-0.00320991	-0.2001813	0.84560766	0.90528595	-6.34696415
hetero after UVB irradiation VS hetero before UVB irradiation	Reverse Transcription of HIV RNA|Reactome	0.471357887	-0.00478231	1.150987769	0.28134999	0.51114365	-5.68058898
WT after UVB irradiation VS WT before UVB irradiation	Reverse Transcription of HIV RNA|Reactome	0.926001949	0.004188178	3.205753029	0.00930482	0.24856491	-2.40025129
homo after UVB irradiation VS homo before UVB irradiation	Reversible hydration of carbon dioxide|Reactome	0.354587544	0.007748923	1.771622557	0.10883453	0.24229081	-4.92016368
hetero after UVB irradiation VS hetero before UVB irradiation	Reversible hydration of carbon dioxide|Reactome	0.092316878	0.015583828	0.535932356	0.60585998	0.7930815	-6.19339865
WT after UVB irradiation VS WT before UVB irradiation	Reversible hydration of carbon dioxide|Reactome	0.056743077	0.146253292	0.231154105	0.82181857	0.94525798	-5.84215971
homo after UVB irradiation VS homo before UVB irradiation	Rev-mediated nuclear export of HIV RNA|Reactome	-0.79134147	-0.01411847	-4.24185664	0.00197109	0.0246115	-1.08766617
hetero after UVB irradiation VS hetero before UVB irradiation	Rev-mediated nuclear export of HIV RNA|Reactome	-0.21469581	-0.06426347	-0.72845631	0.48608426	0.70672155	-6.06762935
WT after UVB irradiation VS WT before UVB irradiation	Rev-mediated nuclear export of HIV RNA|Reactome	-0.13363436	-0.00756913	-0.39272518	0.70269756	0.90059561	-5.79165519

homo after UVB irradiation VS homo before UVB irradiation	Rhesus glycoproteins mediate ammonium transport.|Reactome	-0.27113803	0.00869212	-1.41089463	0.19053824	0.34647455	-5.40126539
hetero after UVB irradiation VS hetero before UVB irradiation	Rhesus glycoproteins mediate ammonium transport.|Reactome	0.247291437	0.054884562	0.966709292	0.36061335	0.58926812	-5.86654505
WT after UVB irradiation VS WT before UVB irradiation	Rhesus glycoproteins mediate ammonium transport.|Reactome	0.116334662	0.040375989	0.333023635	0.745938	0.9185045	-5.81329805
homo after UVB irradiation VS homo before UVB irradiation	Rheumatoid arthritis - Homo sapiens (human)|KEGG	0.518337881	-0.00534026	2.783037171	0.02048039	0.09129427	-3.3657682
hetero after UVB irradiation VS hetero before UVB irradiation	Rheumatoid arthritis - Homo sapiens (human)|KEGG	0.447895087	0.052945946	2.466921406	0.03745679	0.16904708	-3.88579104
WT after UVB irradiation VS WT before UVB irradiation	Rheumatoid arthritis - Homo sapiens (human)|KEGG	0.464712331	0.051327736	1.888676547	0.0880137	0.48023726	-4.34194726
homo after UVB irradiation VS homo before UVB irradiation	rho cell motility signaling pathway|BioCarta	0.54713239	-0.03173405	2.832750667	0.01885215	0.08765675	-3.28615811
hetero after UVB irradiation VS hetero before UVB irradiation	rho cell motility signaling pathway|BioCarta	0.441197928	0.025023068	2.453109044	0.03829131	0.1700514	-3.90679334
WT after UVB irradiation VS WT before UVB irradiation	rho cell motility signaling pathway|BioCarta	0.371991213	-0.03852611	1.855078824	0.0930226	0.48552482	-4.38798163
homo after UVB irradiation VS homo before UVB irradiation	Rho GTPase cycle|Reactome	0.286448274	-0.03970281	1.724622575	0.11728529	0.25525482	-4.98615464
hetero after UVB irradiation VS hetero before UVB irradiation	Rho GTPase cycle|Reactome	0.306260912	0.005629519	1.857319526	0.09846843	0.28258275	-4.7869403
WT after UVB irradiation VS WT before UVB irradiation	Rho GTPase cycle|Reactome	0.207775267	-0.03300376	1.067033393	0.31084042	0.6940288	-5.32502476
homo after UVB irradiation VS homo before UVB irradiation	RHO GTPase Effectors|Reactome	-0.1486959	0.006256071	-0.8664097	0.40786001	0.559078	-5.98086347
hetero after UVB irradiation VS hetero before UVB irradiation	RHO GTPase Effectors|Reactome	0.302380973	0.006169306	1.567896339	0.15363676	0.36415827	-5.18124046
WT after UVB irradiation VS WT before UVB irradiation	RHO GTPase Effectors|Reactome	0.214877386	0.012091373	0.94721975	0.3656763	0.74545864	-5.43520731
homo after UVB irradiation VS homo before UVB irradiation	RHO GTPases activate CIT|Reactome	-0.1043678	0.022832683	-0.45353935	0.6604613	0.76714992	-6.25915303
hetero after UVB irradiation VS hetero before UVB irradiation	RHO GTPases activate CIT|Reactome	0.321892109	0.004334006	1.498980309	0.17037563	0.38712469	-5.27013033
WT after UVB irradiation VS WT before UVB irradiation	RHO GTPases activate CIT|Reactome	0.011943219	-0.00961199	0.055630184	0.95672341	0.98742794	-5.86758368
homo after UVB irradiation VS homo before UVB irradiation	RHO GTPases Activate Formins|Reactome	-0.46365042	-0.01330719	-2.52051397	0.03174815	0.11852989	-3.78427918
hetero after UVB irradiation VS hetero before UVB irradiation	RHO GTPases Activate Formins|Reactome	0.251207524	-0.00081017	1.00820194	0.34142969	0.57067713	-5.82684123
WT after UVB irradiation VS WT before UVB irradiation	RHO GTPases Activate Formins|Reactome	0.133303442	0.02636875	0.497991638	0.62917256	0.87289644	-5.74515662
homo after UVB irradiation VS homo before UVB irradiation	RHO GTPases Activate Formins|Wikipathways	1.102634195	-0.01002319	5.059537853	0.00059809	0.01666204	0.071886173
hetero after UVB irradiation VS hetero before UVB irradiation	RHO GTPases Activate Formins|Wikipathways	0.811502391	0.054613585	2.539800757	0.03334707	0.15932488	-3.77476135
WT after UVB irradiation VS WT before UVB irradiation	RHO GTPases Activate Formins|Wikipathways	0.773320064	-0.01675271	2.28415886	0.04526094	0.39447846	-3.77811905
homo after UVB irradiation VS homo before UVB irradiation	RHO GTPases activate IQGAPs|Reactome	0.276295864	0.019654345	0.941860888	0.36986444	0.52279924	-5.91360406
hetero after UVB irradiation VS hetero before UVB irradiation	RHO GTPases activate IQGAPs|Reactome	0.496218392	-0.04130687	1.857484236	0.09844312	0.28258275	-4.78670766
WT after UVB irradiation VS WT before UVB irradiation	RHO GTPases activate IQGAPs|Reactome	0.329057829	0.006372458	1.217126446	0.2512779	0.64507374	-5.17285054
homo after UVB irradiation VS homo before UVB irradiation	RHO GTPases activate KTN1|Reactome	0.281319155	-0.03592493	0.984388976	0.34960948	0.50516357	-5.87366091
hetero after UVB irradiation VS hetero before UVB irradiation	RHO GTPases activate KTN1|Reactome	0.474096545	-0.00572134	2.703624866	0.02569498	0.14017854	-3.52430999
WT after UVB irradiation VS WT before UVB irradiation	RHO GTPases activate KTN1|Reactome	0.341874061	-0.0644689	1.318472602	0.21651071	0.62581393	-5.06197373
homo after UVB irradiation VS homo before UVB irradiation	RHO GTPases Activate NADPH Oxidases|Reactome	0.689446283	0.022844302	3.234640081	0.00969933	0.0585513	-2.64319015
hetero after UVB irradiation VS hetero before UVB irradiation	RHO GTPases Activate NADPH Oxidases|Reactome	0.650222929	0.032713753	3.692199682	0.00558162	0.08000318	-2.03505707
WT after UVB irradiation VS WT before UVB irradiation	RHO GTPases Activate NADPH Oxidases|Reactome	0.881446059	0.026365484	4.376662601	0.00135795	0.15645713	-0.73752383
homo after UVB irradiation VS homo before UVB irradiation	RHO GTPases activate PAKs|Reactome	0.117545168	0.035035488	0.481707565	0.64104316	0.75095947	-6.24532982
hetero after UVB irradiation VS hetero before UVB irradiation	RHO GTPases activate PAKs|Reactome	0.056649862	0.00288563	0.227154255	0.82571863	0.93322694	-6.31863694
WT after UVB irradiation VS WT before UVB irradiation	RHO GTPases activate PAKs|Reactome	0.158170999	0.043740453	0.721298176	0.48711545	0.81710765	-5.61267215
homo after UVB irradiation VS homo before UVB irradiation	RHO GTPases activate PKNs|Reactome	0.066363592	0.00764426	0.306776699	0.76570811	0.84859788	-6.3181403
hetero after UVB irradiation VS hetero before UVB irradiation	RHO GTPases activate PKNs|Reactome	0.25694702	0.010550018	1.565217099	0.15425892	0.36486693	-5.18473656
WT after UVB irradiation VS WT before UVB irradiation	RHO GTPases activate PKNs|Reactome	-0.01014867	0.03526419	-0.05103518	0.96029446	0.98815178	-5.8678319
homo after UVB irradiation VS homo before UVB irradiation	RHO GTPases Activate Rhotekin and Rhophilins|Reactome	0.163462429	0.023450964	0.63353727	0.54150618	0.67666043	-6.157401
hetero after UVB irradiation VS hetero before UVB irradiation	RHO GTPases Activate Rhotekin and Rhophilins|Reactome	0.173786768	-0.01890753	0.661412161	0.52601784	0.7413696	-6.11535218
[truncated: 645,817 more chars]
