# Supplementary material for: UVB-Induced Skin Autoinflammation Due to Nlrp1b Mutation and Its Inhibition by Anti-IL-1β Antibody
Source: Front Immunol. 2022 Jun 17;13:876390. doi: 10.3389/fimmu.2022.876390 (PMC9248282; doi:10.3389/fimmu.2022.876390)
Supplement: Supplementary file 7 [file Table_4.pdf]

Supplemental Table S4 Gene Ontology analysis of lesional skin

condition	GO	logFC	AveExpr	t	P.Value	adj.P.Val	B
homo after UVB irradiation VS homo before UVB irradiation	GO_1_4_ALPHA_OLIGOGLUCAN_PHOSPHORYLASE_ACTIVITY	-0.02838031	-0.04001743	-0.12493954	0.90353437	0.94731486	-6.54229787
hetero after UVB irradiation VS hetero before UVB irradiation	GO_1_4_ALPHA_OLIGOGLUCAN_PHOSPHORYLASE_ACTIVITY1	-0.06059312	-0.12238836	-0.29951855	0.77231877	0.9114711	-6.18311915
WT after UVB irradiation VS WT before UVB irradiation	GO_1_4_ALPHA_OLIGOGLUCAN_PHOSPHORYLASE_ACTIVITY2	0.295035416	0.002737259	1.450908476	0.18441381	0.68726847	-5.01610755
homo after UVB irradiation VS homo before UVB irradiation	GO_1_ACYL_2_LYSOPHOSPHATIDYLSERINE_ACYLHYDROLASE_ACTIVITY	0.123614096	-0.00961262	0.548627064	0.59765593	0.74819842	-6.38863916
hetero after UVB irradiation VS hetero before UVB irradiation	GO_1_ACYL_2_LYSOPHOSPHATIDYLSERINE_ACYLHYDROLASE_ACTIVITY1	-0.40983835	-0.01097396	-1.58371242	0.15262449	0.47093079	-5.05786899
WT after UVB irradiation VS WT before UVB irradiation	GO_1_ACYL_2_LYSOPHOSPHATIDYLSERINE_ACYLHYDROLASE_ACTIVITY2	-0.07419648	-0.04592732	-0.25787049	0.80293949	0.95333317	-5.96113136
homo after UVB irradiation VS homo before UVB irradiation	GO_1_ACYLGLYCEROL_3_PHOSPHATE_O_ACYLTRANSFERASE_ACTIVITY	0.47129144	-0.07386443	3.238206761	0.01128327	0.07115692	-2.86571654
hetero after UVB irradiation VS hetero before UVB irradiation	GO_1_ACYLGLYCEROL_3_PHOSPHATE_O_ACYLTRANSFERASE_ACTIVITY1	0.264707276	0.008913233	1.656838137	0.13685126	0.44981284	-4.96277187
WT after UVB irradiation VS WT before UVB irradiation	GO_1_ACYLGLYCEROL_3_PHOSPHATE_O_ACYLTRANSFERASE_ACTIVITY2	0.5659118	0.032889849	3.439992844	0.00866247	0.31908946	-2.28543985
homo after UVB irradiation VS homo before UVB irradiation	GO_1_ACYLGLYCEROPHOSPHOCHOLINE_O_ACYLTRANSFERASE_ACTIVITY	0.010716553	-0.03038124	0.035302537	0.97266992	0.98603757	-6.55017279
hetero after UVB irradiation VS hetero before UVB irradiation	GO_1_ACYLGLYCEROPHOSPHOCHOLINE_O_ACYLTRANSFERASE_ACTIVITY1	0.003201001	0.042246647	0.01418077	0.98903942	0.99599741	-6.23137506
WT after UVB irradiation VS WT before UVB irradiation	GO_1_ACYLGLYCEROPHOSPHOCHOLINE_O_ACYLTRANSFERASE_ACTIVITY2	0.206057787	0.059428611	0.834666365	0.42784139	0.82753247	-5.64433484
homo after UVB irradiation VS homo before UVB irradiation	GO_1_ALKYL_2_ACETYLGLYCEROPHOSPHOCHOLINE_ESTERASE_ACTIVITY	-0.1458336	0.023361277	-0.73816365	0.48069314	0.65797131	-6.26137413
hetero after UVB irradiation VS hetero before UVB irradiation	GO_1_ALKYL_2_ACETYLGLYCEROPHOSPHOCHOLINE_ESTERASE_ACTIVITY1	0.026948273	-0.04672443	0.122066715	0.90591211	0.96546627	-6.22341151
WT after UVB irradiation VS WT before UVB irradiation	GO_1_ALKYL_2_ACETYLGLYCEROPHOSPHOCHOLINE_ESTERASE_ACTIVITY2	-0.20976278	-0.02877534	-0.95114846	0.36905112	0.79984535	-5.54504251
homo after UVB irradiation VS homo before UVB irradiation	GO_1_PHOSPHATIDYLINOSITOL_3_KINASE_ACTIVITY	-0.4073343	-0.00656843	-2.22903474	0.05509557	0.17439841	-4.42395679
hetero after UVB irradiation VS hetero before UVB irradiation	GO_1_PHOSPHATIDYLINOSITOL_3_KINASE_ACTIVITY1	0.037158222	0.053286947	0.209459824	0.83942104	0.93872655	-6.20775992
WT after UVB irradiation VS WT before UVB irradiation	GO_1_PHOSPHATIDYLINOSITOL_3_KINASE_ACTIVITY2	-0.13853099	0.019125165	-0.71828114	0.49278468	0.85306847	-5.73266145
homo after UVB irradiation VS homo before UVB irradiation	GO_1_PHOSPHATIDYLINOSITOL_3_KINASE_REGULATOR_ACTIVITY	0.354268712	-0.04737541	2.649581741	0.02827563	0.11915509	-3.77732192
hetero after UVB irradiation VS hetero before UVB irradiation	GO_1_PHOSPHATIDYLINOSITOL_3_KINASE_REGULATOR_ACTIVITY1	0.202504516	0.039577189	1.501232264	0.17238989	0.49537636	-5.16255592
WT after UVB irradiation VS WT before UVB irradiation	GO_1_PHOSPHATIDYLINOSITOL_3_KINASE_REGULATOR_ACTIVITY2	0.119249161	0.036178773	0.617229984	0.5540382	0.87677659	-5.79990935
homo after UVB irradiation VS homo before UVB irradiation	GO_1_PHOSPHATIDYLINOSITOL_4_KINASE_ACTIVITY	-0.07888701	0.00089989	-0.2611763	0.80029862	0.88652441	-6.51357548
hetero after UVB irradiation VS hetero before UVB irradiation	GO_1_PHOSPHATIDYLINOSITOL_4_KINASE_ACTIVITY1	0.063071991	-0.09861745	0.399835412	0.69993292	0.88146568	-6.14568988
WT after UVB irradiation VS WT before UVB irradiation	GO_1_PHOSPHATIDYLINOSITOL_4_KINASE_ACTIVITY2	0.696585332	-0.00704646	3.783035881	0.00524671	0.27455937	-1.82936479
homo after UVB irradiation VS homo before UVB irradiation	GO_1_PHOSPHATIDYLINOSITOL_4_PHOSPHATE_3_KINASE_ACTIVITY	-0.07669536	-0.00206507	-0.45954261	0.6576014	0.79172912	-6.43643081
hetero after UVB irradiation VS hetero before UVB irradiation	GO_1_PHOSPHATIDYLINOSITOL_4_PHOSPHATE_3_KINASE_ACTIVITY1	0.374634513	0.029029385	2.236185028	0.05636596	0.31374431	-4.15602592
WT after UVB irradiation VS WT before UVB irradiation	GO_1_PHOSPHATIDYLINOSITOL_4_PHOSPHATE_3_KINASE_ACTIVITY2	0.057963725	-0.03885177	0.251223682	0.80789893	0.95404687	-5.96290182
homo after UVB irradiation VS homo before UVB irradiation	GO_1_PHOSPHATIDYLINOSITOL_4_PHOSPHATE_5_KINASE_ACTIVITY	0.002174354	-0.00152127	0.013912907	0.98922696	0.99468893	-6.55075048
hetero after UVB irradiation VS hetero before UVB irradiation	GO_1_PHOSPHATIDYLINOSITOL_4_PHOSPHATE_5_KINASE_ACTIVITY1	0.02865578	-0.08256403	0.1237368	0.90463216	0.96509505	-6.22318933
WT after UVB irradiation VS WT before UVB irradiation	GO_1_PHOSPHATIDYLINOSITOL_4_PHOSPHATE_5_KINASE_ACTIVITY2	-0.15634252	-0.03338971	-0.77529392	0.46020517	0.83956579	-5.69080432
homo after UVB irradiation VS homo before UVB irradiation	GO_1_PHOSPHATIDYLINOSITOL_BINDING	0.222933397	-0.02357451	0.903687431	0.39154691	0.58088446	-6.1237071
hetero after UVB irradiation VS hetero before UVB irradiation	GO_1_PHOSPHATIDYLINOSITOL_BINDING1	0.181758143	0.058431841	1.228333545	0.25486157	0.58243013	-5.48567811
WT after UVB irradiation VS WT before UVB irradiation	GO_1_PHOSPHATIDYLINOSITOL_BINDING2	0.116549273	-0.01067538	0.744604155	0.47756149	0.84915768	-5.71367864
homo after UVB irradiation VS homo before UVB irradiation	GO_10_FORMYLTETRAHYDROFOLATE_METABOLIC_PROCESS	0.033502225	-0.08236544	0.162552404	0.87474545	0.93129057	-6.53637848
hetero after UVB irradiation VS hetero before UVB irradiation	GO_10_FORMYLTETRAHYDROFOLATE_METABOLIC_PROCESS1	-0.04635491	-0.02031295	-0.17763468	0.86350636	0.94819913	-6.21440766
WT after UVB irradiation VS WT before UVB irradiation	GO_10_FORMYLTETRAHYDROFOLATE_METABOLIC_PROCESS2	0.107244508	-0.11874701	0.369026443	0.72157064	0.93154998	-5.9248447
homo after UVB irradiation VS homo before UVB irradiation	GO_11_CIS_RETINAL_BINDING	0.218003403	0.264957908	1.437079975	0.18717594	0.37200745	-5.54073634
hetero after UVB irradiation VS hetero before UVB irradiation	GO_11_CIS_RETINAL_BINDING1	-0.56086488	0.156972813	-1.56969188	0.1558323	0.47491659	-5.07586437
WT after UVB irradiation VS WT before UVB irradiation	GO_11_CIS_RETINAL_BINDING2	-0.11968258	0.109839458	-0.34487848	0.73897243	0.93478445	-5.93379143
homo after UVB irradiation VS homo before UVB irradiation	GO_14_3_3_PROTEIN_BINDING	0.31177539	-0.03040507	1.92309742	0.08923743	0.23369597	-4.87820841
hetero after UVB irradiation VS hetero before UVB irradiation	GO_14_3_3_PROTEIN_BINDING1	0.08182509	0.020736089	0.438111684	0.67310673	0.86714482	-6.12869166
WT after UVB irradiation VS WT before UVB irradiation	GO_14_3_3_PROTEIN_BINDING2	0.101156734	-0.02873593	0.623018984	0.5504115	0.87546015	-5.79630307
homo after UVB irradiation VS homo before UVB irradiation	GO_2_ACYLGLYCEROL_O_ACYLTRANSFERASE_ACTIVITY	0.238794461	0.042569877	0.956403241	0.36580011	0.55593329	-6.07505895
hetero after UVB irradiation VS hetero before UVB irradiation	GO_2_ACYLGLYCEROL_O_ACYLTRANSFERASE_ACTIVITY1	0.031978918	0.007747694	0.119844843	0.90761541	0.96555872	-6.22370244
WT after UVB irradiation VS WT before UVB irradiation	GO_2_ACYLGLYCEROL_O_ACYLTRANSFERASE_ACTIVITY2	0.328166266	-0.03682686	1.209469914	0.26060623	0.74056733	-5.29028018
homo after UVB irradiation VS homo before UVB irradiation	GO_2_IRON_2_SULFUR_CLUSTER_BINDING	-0.16342108	-0.01690976	-0.76691249	0.46431384	0.64373162	-6.23916954
hetero after UVB irradiation VS hetero before UVB irradiation	GO_2_IRON_2_SULFUR_CLUSTER_BINDING1	-0.42451413	-0.05390262	-1.8276673	0.10571063	0.40296205	-4.73341904
WT after UVB irradiation VS WT before UVB irradiation	GO_2_IRON_2_SULFUR_CLUSTER_BINDING2	-0.20462019	-0.07700887	-0.86675896	0.41102012	0.81998431	-5.61803056
homo after UVB irradiation VS homo before UVB irradiation	GO_2_OXOGLUTARATE_DEPENDENT_DIOXYGENASE_ACTIVITY	0.05180055	-0.04610091	0.375521322	0.71665614	0.83174948	-6.47412484
hetero after UVB irradiation VS hetero before UVB irradiation	GO_2_OXOGLUTARATE_DEPENDENT_DIOXYGENASE_ACTIVITY1	-0.05837256	-0.04115533	-0.41050841	0.69240523	0.87708228	-6.14109926
WT after UVB irradiation VS WT before UVB irradiation	GO_2_OXOGLUTARATE_DEPENDENT_DIOXYGENASE_ACTIVITY2	-0.00392362	-0.03454567	-0.02565283	0.98015517	0.99512555	-5.99571945
homo after UVB irradiation VS homo before UVB irradiation	GO_2_OXOGLUTARATE_METABOLIC_PROCESS	-0.2199131	0.05243468	-0.91117571	0.38781151	0.57719964	-6.11693293
hetero after UVB irradiation VS hetero before UVB irradiation	GO_2_OXOGLUTARATE_METABOLIC_PROCESS1	-0.36698597	-0.01567274	-1.52329829	0.16688759	0.48812553	-5.13483219
WT after UVB irradiation VS WT before UVB irradiation	GO_2_OXOGLUTARATE_METABOLIC_PROCESS2	-0.3442682	-0.03175577	-1.68785835	0.12946097	0.63548424	-4.72094524
homo after UVB irradiation VS homo before UVB irradiation	GO_2FE_2S_CLUSTER_ASSEMBLY	-0.39626093	-0.14221056	-1.2494203	0.24547728	0.43932104	-5.76702415
hetero after UVB irradiation VS hetero before UVB irradiation	GO_2FE_2S_CLUSTER_ASSEMBLY1	0.26979508	-0.05081709	0.850475373	0.4202351	0.72213684	-5.85689938
WT after UVB irradiation VS WT before UVB irradiation	GO_2FE_2S_CLUSTER_ASSEMBLY2	-0.00806063	-0.01753539	-0.02768108	0.97858656	0.9945584	-5.99566238
homo after UVB irradiation VS homo before UVB irradiation	GO_3_5_CYCLIC_AMP_PHOSPHODIESTERASE_ACTIVITY	0.758379736	-0.02642209	4.132674166	0.0030118	0.03623886	-1.535927
hetero after UVB irradiation VS hetero before UVB irradiation	GO_3_5_CYCLIC_AMP_PHOSPHODIESTERASE_ACTIVITY1	0.452966474	0.005520269	2.003932542	0.0806965	0.3643689	-4.48826367
WT after UVB irradiation VS WT before UVB irradiation	GO_3_5_CYCLIC_AMP_PHOSPHODIESTERASE_ACTIVITY2	0.110286831	-0.00938427	0.362062909	0.7265717	0.93275498	-5.9274847
homo after UVB irradiation VS homo before UVB irradiation	GO_3_5_CYCLIC_GMP_PHOSPHODIESTERASE_ACTIVITY	0.203418872	-0.02120094	0.887352577	0.39978505	0.58837167	-6.13832521
hetero after UVB irradiation VS hetero before UVB irradiation	GO_3_5_CYCLIC_GMP_PHOSPHODIESTERASE_ACTIVITY1	0.124599187	-0.03741302	0.461248357	0.65712704	0.85970091	-6.11770141
WT after UVB irradiation VS WT before UVB irradiation	GO_3_5_CYCLIC_GMP_PHOSPHODIESTERASE_ACTIVITY2	0.21399162	-0.01247802	0.699876323	0.50361122	0.85763155	-5.74557968
homo after UVB irradiation VS homo before UVB irradiation	GO_3_5_DNA_HELICASE_ACTIVITY	-0.6135777	0.011531893	-2.96703276	0.01716623	0.08960809	-3.28430968
hetero after UVB irradiation VS hetero before UVB irradiation	GO_3_5_DNA_HELICASE_ACTIVITY1	-0.30272986	-0.08437631	-1.00872562	0.34317418	0.66125335	-5.71385002
WT after UVB irradiation VS WT before UVB irradiation	GO_3_5_DNA_HELICASE_ACTIVITY2	-0.13644439	0.031944932	-0.43297223	0.67633625	0.91685973	-5.89834666
homo after UVB irradiation VS homo before UVB irradiation	GO_3_5_EXODEOXYRIBONUCLEASE_ACTIVITY	0.098944622	0.005477501	0.55163701	0.59568264	0.74685545	-6.38688661
hetero after UVB irradiation VS hetero before UVB irradiation	GO_3_5_EXODEOXYRIBONUCLEASE_ACTIVITY1	0.324169891	0.048999587	1.883431769	0.09708672	0.38897068	-4.65667737
WT after UVB irradiation VS WT before UVB irradiation	GO_3_5_EXODEOXYRIBONUCLEASE_ACTIVITY2	0.341805018	-0.06298245	1.899440714	0.09360585	0.59116964	-4.44125986
homo after UVB irradiation VS homo before UVB irradiation	GO_3_5_EXONUCLEASE_ACTIVITY	-0.48177858	-0.01181377	-3.4094202	0.0086925	0.06193877	-2.60417219
hetero after UVB irradiation VS hetero before UVB irradiation	GO_3_5_EXONUCLEASE_ACTIVITY1	-0.10216622	-0.04853842	-0.46850767	0.65215132	0.85713917	-6.11414291
WT after UVB irradiation VS WT before UVB irradiation	GO_3_5_EXONUCLEASE_ACTIVITY2	-0.22906429	-0.01574751	-0.89296353	0.39763668	0.81477652	-5.5959508
homo after UVB irradiation VS homo before UVB irradiation	GO_3_BETA_HYDROXY_DELTA5_STEROID_DEHYDROGENASE_ACTIVITY	-0.28672673	-0.06089277	-1.4298985	0.18916407	0.37479781	-5.54975553
hetero after UVB irradiation VS hetero before UVB irradiation	GO_3_BETA_HYDROXY_DELTA5_STEROID_DEHYDROGENASE_ACTIVITY1	0.027762518	-0.03800398	0.111424813	0.91407481	0.96745438	-6.22475661
WT after UVB irradiation VS WT before UVB irradiation	GO_3_BETA_HYDROXY_DELTA5_STEROID_DEHYDROGENASE_ACTIVITY2	-0.41494148	0.074084764	-1.48224637	0.17610946	0.68050466	-4.97839402
homo after UVB irradiation VS homo before UVB irradiation	GO_3_CHLOROALLYL_ALDEHYDE_DEHYDROGENASE_ACTIVITY	0.04485379	0.020317153	0.160842428	0.87605035	0.93214183	-6.53668101
hetero after UVB irradiation VS hetero before UVB irradiation	GO_3_CHLOROALLYL_ALDEHYDE_DEHYDROGENASE_ACTIVITY1	-0.1715079	0.06230958	-0.69660095	0.50614557	0.77620911	-5.97643374
WT after UVB irradiation VS WT before UVB irradiation	GO_3_CHLOROALLYL_ALDEHYDE_DEHYDROGENASE_ACTIVITY2	0.168235481	0.074295956	0.93135429	0.37860082	0.80338597	-5.56264805
homo after UVB irradiation VS homo before UVB irradiation	GO_3_HYDROXYACYL_COA_DEHYDRATASE_ACTIVITY	0.544025262	-0.05097892	3.229815844	0.01142943	0.07168046	-2.878598
hetero after UVB irradiation VS hetero before UVB irradiation	GO_3_HYDROXYACYL_COA_DEHYDRATASE_ACTIVITY1	0.34217433	-0.00918102	0.980981061	0.35585479	0.67182617	-5.74030952
WT after UVB irradiation VS WT before UVB irradiation	GO_3_HYDROXYACYL_COA_DEHYDRATASE_ACTIVITY2	0.184364233	-0.04812899	0.543569624	0.60137747	0.89567499	-5.8431081
homo after UVB irradiation VS homo before UVB irradiation	GO_3_HYDROXYACYL_COA_DEHYDROGENASE_ACTIVITY	-0.56252891	0.034652809	-1.8003018	0.10802205	0.26285956	-5.05390797
hetero after UVB irradiation VS hetero before UVB irradiation	GO_3_HYDROXYACYL_COA_DEHYDROGENASE_ACTIVITY1	-0.75260552	-0.04465157	-3.70217074	0.00622447	0.13546947	-2.04476542
WT after UVB irradiation VS WT before UVB irradiation	GO_3_HYDROXYACYL_COA_DEHYDROGENASE_ACTIVITY2	-0.76377788	0.06516393	-3.62398439	0.0066068	0.30328715	-2.03860315
homo after UVB irradiation VS homo before UVB irradiation	GO_3_KETO_STEROL_REDUCTASE_ACTIVITY	0.388463787	-0.12342092	2.233656251	0.0546934	0.17369341	-4.41695611
hetero after UVB irradiation VS hetero before UVB irradiation	GO_3_KETO_STEROL_REDUCTASE_ACTIVITY1	0.38594574	0.048833247	1.012032394	0.34168616	0.66014434	-5.71065884
WT after UVB irradiation VS WT before UVB irradiation	GO_3_KETO_STEROL_REDUCTASE_ACTIVITY2	0.29345516	0.000394135	0.798668801	0.44727166	0.83464692	-5.6728543
homo after UVB irradiation VS homo before UVB irradiation	GO_3_OXO_ARACHIDOYL_COA_SYNTHASE_ACTIVITY	-0.05212036	-0.00407433	-0.15492969	0.88056548	0.93512556	-6.53770258
hetero after UVB irradiation VS hetero before UVB irradiation	GO_3_OXO_ARACHIDOYL_COA_SYNTHASE_ACTIVITY1	-0.13897606	-0.10812771	-0.4541682	0.6619976	0.86164211	-6.12112146
WT after UVB irradiation VS WT before UVB irradiation	GO_3_OXO_ARACHIDOYL_COA_SYNTHASE_ACTIVITY2	-0.3134251	-0.10279086	-0.97697216	0.35686294	0.79119413	-5.52163852
homo after UVB irradiation VS homo before UVB irradiation	GO_3_PHOSPHOADENOSINE_5_PHOSPHOSULFATE_BINDING	0.117297288	0.09146954	0.65089991	0.53265256	0.69971883	-6.32417207
hetero after UVB irradiation VS hetero before UVB irradiation	GO_3_PHOSPHOADENOSINE_5_PHOSPHOSULFATE_BINDING1	0.096089472	-0.038767	0.423730016	0.68313043	0.87267532	-6.13525222
WT after UVB irradiation VS WT before UVB irradiation	GO_3_PHOSPHOADENOSINE_5_PHOSPHOSULFATE_BINDING2	0.249909565	0.033700338	1.304781688	0.22782431	0.71951396	-5.18574183
homo after UVB irradiation VS homo before UVB irradiation	GO_3_PHOSPHOADENOSINE_5_PHOSPHOSULFATE_BIOSYNTHETIC_PROCESS	0.661822077	-0.02035386	4.937869014	0.00101089	0.02161993	-0.4352976
hetero after UVB irradiation VS hetero before UVB irradiation	GO_3_PHOSPHOADENOSINE_5_PHOSPHOSULFATE_BIOSYNTHETIC_PROCESS1	0.285020938	-0.05048748	1.212444939	0.2605552	0.58691513	-5.50322996
WT after UVB irradiation VS WT before UVB irradiation	GO_3_PHOSPHOADENOSINE_5_PHOSPHOSULFATE_BIOSYNTHETIC_PROCESS2	0.173090338	-0.08435851	0.899845204	0.39417448	0.81365684	-5.59006403
homo after UVB irradiation VS homo before UVB irradiation	GO_3_PHOSPHOADENOSINE_5_PHOSPHOSULFATE_METABOLIC_PROCESS	0.745316028	0.034669209	6.110771387	0.00024295	0.01196149	0.986215421
hetero after UVB irradiation VS hetero before UVB irradiation	GO_3_PHOSPHOADENOSINE_5_PHOSPHOSULFATE_METABOLIC_PROCESS1	0.322180721	0.021828339	2.142266683	0.06518409	0.33409111	-4.29136693
WT after UVB irradiation VS WT before UVB irradiation	GO_3_PHOSPHOADENOSINE_5_PHOSPHOSULFATE_METABOLIC_PROCESS2	0.156702709	-0.03105715	1.11466536	0.29698028	0.7587209	-5.38890615
homo after UVB irradiation VS homo before UVB irradiation	GO_3_UTR_MEDIATED_MRNA_DESTABILIZATION	-0.13996491	-0.02792953	-1.10853309	0.29861086	0.49290833	-5.92268582
hetero after UVB irradiation VS hetero before UVB irradiation	GO_3_UTR_MEDIATED_MRNA_DESTABILIZATION1	-0.01104592	0.0245741	-0.08291097	0.93599679	0.97622057	-6.22775785
WT after UVB irradiation VS WT before UVB irradiation	GO_3_UTR_MEDIATED_MRNA_DESTABILIZATION2	-0.26714281	-0.00560773	-1.82170976	0.10552432	0.60971039	-4.54546313
homo after UVB irradiation VS homo before UVB irradiation	GO_3_UTR_MEDIATED_MRNA_STABILIZATION	-0.40716086	0.005538422	-2.52336267	0.03453399	0.13312907	-3.97288602
hetero after UVB irradiation VS hetero before UVB irradiation	GO_3_UTR_MEDIATED_MRNA_STABILIZATION1	0.016275604	-0.01971995	0.065304917	0.94956311	0.98092529	-6.22917196
WT after UVB irradiation VS WT before UVB irradiation	GO_3_UTR_MEDIATED_MRNA_STABILIZATION2	-0.17984411	0.068193209	-0.77794603	0.45872526	0.8390123	-5.68879052
homo after UVB irradiation VS homo before UVB irradiation	GO_3M_COMPLEX	0.642124489	-0.06093341	2.708998132	0.02574164	0.11329612	-3.68506229
hetero after UVB irradiation VS hetero before UVB irradiation	GO_3M_COMPLEX1	0.273460778	-0.05658897	1.097776992	0.30481928	0.62665244	-5.62520434
WT after UVB irradiation VS WT before UVB irradiation	GO_3M_COMPLEX2	0.522567465	-0.02034285	2.241762117	0.05488285	0.5232848	-3.96807164
homo after UVB irradiation VS homo before UVB irradiation	GO_4_GALACTOSYL_N_ACETYLGLUCOSAMINIDE_3_ALPHA_L_FUCOSYLTRANSFERASE_ACTIVITY	-0.18067024	0.032478555	-0.85541171	0.41624938	0.60233301	-6.16627159
hetero after UVB irradiation VS hetero before UVB irradiation	GO_4_GALACTOSYL_N_ACETYLGLUCOSAMINIDE_3_ALPHA_L_FUCOSYLTRANSFERASE_ACTIVITY1	0.29638406	-0.02163551	1.048526392	0.32559253	0.64521291	-5.6749187
WT after UVB irradiation VS WT before UVB irradiation	GO_4_GALACTOSYL_N_ACETYLGLUCOSAMINIDE_3_ALPHA_L_FUCOSYLTRANSFERASE_ACTIVITY2	-0.17449925	0.031645008	-0.68866727	0.51027786	0.85882417	-5.75330286
homo after UVB irradiation VS homo before UVB irradiation	GO_4_IRON_4_SULFUR_CLUSTER_BINDING	-0.25588463	0.005055455	-1.23316962	0.25117844	0.44575145	-5.78564023
hetero after UVB irradiation VS hetero before UVB irradiation	GO_4_IRON_4_SULFUR_CLUSTER_BINDING1	0.059853968	-0.02945185	0.381753549	0.71276689	0.88718833	-6.15320227
WT after UVB irradiation VS WT before UVB irradiation	GO_4_IRON_4_SULFUR_CLUSTER_BINDING2	0.08745707	-0.05821228	0.591361993	0.57041326	0.883893	-5.81565052
homo after UVB irradiation VS homo before UVB irradiation	GO_5_3_DNA_HELICASE_ACTIVITY	-0.68945907	0.007515951	-2.97315543	0.01700304	0.08901955	-3.27481687
hetero after UVB irradiation VS hetero before UVB irradiation	GO_5_3_DNA_HELICASE_ACTIVITY1	-0.22370385	-0.10272186	-0.84723868	0.42193134	0.72389129	-5.85961943
WT after UVB irradiation VS WT before UVB irradiation	GO_5_3_DNA_HELICASE_ACTIVITY2	-0.01038095	0.08715563	-0.02992776	0.97684915	0.99416176	-5.99559409
homo after UVB irradiation VS homo before UVB irradiation	GO_5_3_EXODEOXYRIBONUCLEASE_ACTIVITY	0.381757598	-0.01489347	2.374044966	0.04377038	0.15168242	-4.20288425
hetero after UVB irradiation VS hetero before UVB irradiation	GO_5_3_EXODEOXYRIBONUCLEASE_ACTIVITY1	0.562735966	0.017840674	3.092394392	0.01517695	0.18251925	-2.90559986
WT after UVB irradiation VS WT before UVB irradiation	GO_5_3_EXODEOXYRIBONUCLEASE_ACTIVITY2	0.592176403	0.042227903	2.506918721	0.03620973	0.47615783	-3.59249215
homo after UVB irradiation VS homo before UVB irradiation	GO_5_3_EXONUCLEASE_ACTIVITY	-0.14627531	-0.03393456	-0.86792897	0.40974115	0.59636554	-6.15542102
hetero after UVB irradiation VS hetero before UVB irradiation	GO_5_3_EXONUCLEASE_ACTIVITY1	0.401789997	-0.00188327	1.877083086	0.09803358	0.38992672	-4.66545537
WT after UVB irradiation VS WT before UVB irradiation	GO_5_3_EXONUCLEASE_ACTIVITY2	0.207521472	0.037553209	0.69672413	0.50548044	0.85763155	-5.74776267
homo after UVB irradiation VS homo before UVB irradiation	GO_5_DEOXYRIBOSE_5_PHOSPHATE_LYASE_ACTIVITY	-0.78510425	-0.09791245	-2.55901957	0.03263565	0.12850951	-3.91771815
hetero after UVB irradiation VS hetero before UVB irradiation	GO_5_DEOXYRIBOSE_5_PHOSPHATE_LYASE_ACTIVITY1	-0.80806126	-0.01352266	-3.23991501	0.01218092	0.17097045	-2.69336348
WT after UVB irradiation VS WT before UVB irradiation	GO_5_DEOXYRIBOSE_5_PHOSPHATE_LYASE_ACTIVITY2	0.16056612	0.008116829	0.552985818	0.59520539	0.8931505	-5.83786674
homo after UVB irradiation VS homo before UVB irradiation	GO_5_PHOSPHORIBOSE_1_DIPHOSPHATE_METABOLIC_PROCESS	-0.28920007	-0.04260197	-1.66534153	0.1329126	0.29981524	-5.24118691
hetero after UVB irradiation VS hetero before UVB irradiation	GO_5_PHOSPHORIBOSE_1_DIPHOSPHATE_METABOLIC_PROCESS1	-0.04221287	-0.06973834	-0.21771511	0.83320168	0.93560757	-6.20585897
WT after UVB irradiation VS WT before UVB irradiation	GO_5_PHOSPHORIBOSE_1_DIPHOSPHATE_METABOLIC_PROCESS2	0.026118349	0.043842975	0.108176902	0.91648783	0.97976028	-5.9898967
homo after UVB irradiation VS homo before UVB irradiation	GO_5S_CLASS_RRNA_TRANSCRIPTION_BY_RNA_POLYMERASE_III	-0.58982268	0.008873644	-2.32921901	0.04699899	0.15866897	-4.27150986
hetero after UVB irradiation VS hetero before UVB irradiation	GO_5S_CLASS_RRNA_TRANSCRIPTION_BY_RNA_POLYMERASE_III1	-0.05826038	-0.00869435	-0.21457911	0.83556282	0.93666051	-6.20658965
WT after UVB irradiation VS WT before UVB irradiation	GO_5S_CLASS_RRNA_TRANSCRIPTION_BY_RNA_POLYMERASE_III2	-0.22602471	0.041698641	-0.65222638	0.53232835	0.86897754	-5.77764676
homo after UVB irradiation VS homo before UVB irradiation	GO_5S_RRNA_BINDING	-0.72929197	0.026276869	-5.06421554	0.00085908	0.02007867	-0.27184237
hetero after UVB irradiation VS hetero before UVB irradiation	GO_5S_RRNA_BINDING1	-0.19846383	-0.04134813	-0.83813731	0.42672686	0.72769923	-5.86722208
WT after UVB irradiation VS WT before UVB irradiation	GO_5S_RRNA_BINDING2	-0.4620414	-0.04704989	-1.67904182	0.13120291	0.63700751	-4.73230632
homo after UVB irradiation VS homo before UVB irradiation	GO_7S_RNA_BINDING	-0.15081427	-0.00327651	-0.54646947	0.5990726	0.74919356	-6.38988994
hetero after UVB irradiation VS hetero before UVB irradiation	GO_7S_RNA_BINDING1	0.184401103	-0.04471184	0.893268746	0.39826142	0.70539829	-5.82014028
WT after UVB irradiation VS WT before UVB irradiation	GO_7S_RNA_BINDING2	0.135277886	-0.03671687	0.455204303	0.66091918	0.91168296	-5.88819055
homo after UVB irradiation VS homo before UVB irradiation	GO_7SK_SNRNA_BINDING	0.108803803	0.029832093	0.433850169	0.67541588	0.80438319	-6.44872729
hetero after UVB irradiation VS hetero before UVB irradiation	GO_7SK_SNRNA_BINDING1	-0.31767419	0.008234699	-1.1434705	0.28650275	0.61181397	-5.57761426
WT after UVB irradiation VS WT before UVB irradiation	GO_7SK_SNRNA_BINDING2	-0.14401961	0.08465665	-0.4554985	0.66071629	0.91158787	-5.88805292
homo after UVB irradiation VS homo before UVB irradiation	GO_90S_PRERIBOSOME	-0.91895825	-0.02600095	-5.92218027	0.00030177	0.01276035	0.771872175
hetero after UVB irradiation VS hetero before UVB irradiation	GO_90S_PRERIBOSOME1	-0.44641057	-0.02912223	-1.45895989	0.18338605	0.50787377	-5.21505819
WT after UVB irradiation VS WT before UVB irradiation	GO_90S_PRERIBOSOME2	-0.28518536	-0.03058416	-0.78016341	0.45749038	0.83880233	-5.68710231
homo after UVB irradiation VS homo before UVB irradiation	GO_9PLUS0_NON_MOTILE_CILIUM	0.051933346	0.030007258	0.363965975	0.72494961	0.83729212	-6.47873727
hetero after UVB irradiation VS hetero before UVB irradiation	GO_9PLUS0_NON_MOTILE_CILIUM1	-0.20976392	0.043750554	-1.53471561	0.16410309	0.48556351	-5.12040455
WT after UVB irradiation VS WT before UVB irradiation	GO_9PLUS0_NON_MOTILE_CILIUM2	-0.09087068	0.005198647	-0.52306574	0.61493553	0.90020288	-5.85423247
homo after UVB irradiation VS homo before UVB irradiation	GO_9PLUS2_MOTILE_CILIUM	0.031987817	0.025583672	0.229838781	0.82376119	0.90140825	-6.52195816
hetero after UVB irradiation VS hetero before UVB irradiation	GO_9PLUS2_MOTILE_CILIUM1	-0.20652254	0.019399484	-1.36870199	0.20896306	0.53418041	-5.3243105
WT after UVB irradiation VS WT before UVB irradiation	GO_9PLUS2_MOTILE_CILIUM2	-0.07656805	0.035689483	-0.54788959	0.59854156	0.89476186	-5.84071379

homo after UVB irradiation VS homo before UVB irradiation	GO_A_BAND	0.228010706	-0.01264456	1.026108978	0.33371404	0.5266408	-6.00738631
hetero after UVB irradiation VS hetero before UVB irradiation	GO_A_BAND1	-0.37162285	-0.01813855	-1.62888605	0.14269473	0.45783941	-4.99936167
WT after UVB irradiation VS WT before UVB irradiation	GO_A_BAND2	-0.12053362	-0.01895567	-0.46304389	0.65552296	0.91019499	-5.88449438
homo after UVB irradiation VS homo before UVB irradiation	GO_ABSCISSION	-0.46046585	0.029976333	-1.73708862	0.11908534	0.27943113	-5.14245283
hetero after UVB irradiation VS hetero before UVB irradiation	GO_ABSCISSION1	-0.11657009	-0.10514603	-0.65955057	0.52840676	0.79043843	-6.00211728
WT after UVB irradiation VS WT before UVB irradiation	GO_ABSCISSION2	0.176589027	-0.00181159	0.544151548	0.60099503	0.89567499	-5.84278659
homo after UVB irradiation VS homo before UVB irradiation	GO_ACETATE_ESTER_TRANSPORT	0.165310579	0.083815539	0.73589792	0.48199978	0.65920952	-6.26309263
hetero after UVB irradiation VS hetero before UVB irradiation	GO_ACETATE_ESTER_TRANSPORT1	-0.27257955	0.038288243	-1.39003645	0.20265101	0.52703584	-5.29885164
WT after UVB irradiation VS WT before UVB irradiation	GO_ACETATE_ESTER_TRANSPORT2	-0.46294897	0.081271912	-1.91928856	0.09077468	0.58414903	-4.41441645
homo after UVB irradiation VS homo before UVB irradiation	GO_ACETYL_COA_BIOSYNTHETIC_PROCESS	-0.28719339	-0.01826538	-1.16460855	0.27644643	0.47248323	-5.8623132
homo after UVB irradiation VS homo before UVB irradiation	GO_ACETYL_COA_BIOSYNTHETIC_PROCESS_FROM_PYRUVATE	-0.25283134	-0.01720552	-1.12112256	0.29351523	0.48826562	-5.90932047
hetero after UVB irradiation VS hetero before UVB irradiation	GO_ACETYL_COA_BIOSYNTHETIC_PROCESS_FROM_PYRUVATE1	-0.69017495	-0.03720222	-3.67467487	0.00647285	0.13614289	-2.08254055
WT after UVB irradiation VS WT before UVB irradiation	GO_ACETYL_COA_BIOSYNTHETIC_PROCESS_FROM_PYRUVATE2	-0.4307142	-0.10994469	-1.66470482	0.13408181	0.6413761	-4.75072471
hetero after UVB irradiation VS hetero before UVB irradiation	GO_ACETYL_COA_BIOSYNTHETIC_PROCESS1	-0.56727345	-0.0672949	-2.79580762	0.02377854	0.22123339	-3.33750731
WT after UVB irradiation VS WT before UVB irradiation	GO_ACETYL_COA_BIOSYNTHETIC_PROCESS2	-0.35813267	-0.09488009	-1.50181251	0.17109452	0.67808426	-4.9546262
homo after UVB irradiation VS homo before UVB irradiation	GO_ACETYL_COA_C_ACYLTRANSFERASE_ACTIVITY	-0.47225312	0.003044661	-1.79564048	0.10880383	0.2636467	-5.06048411
hetero after UVB irradiation VS hetero before UVB irradiation	GO_ACETYL_COA_C_ACYLTRANSFERASE_ACTIVITY1	-0.56467395	0.004498125	-2.42024806	0.0423865	0.27740933	-3.88813751
WT after UVB irradiation VS WT before UVB irradiation	GO_ACETYL_COA_C_ACYLTRANSFERASE_ACTIVITY2	-0.36936916	-0.05118576	-1.42738543	0.1908725	0.69285981	-5.04412127
homo after UVB irradiation VS homo before UVB irradiation	GO_ACETYL_COA_HYDROLASE_ACTIVITY	-0.14572993	-8.64E-06	-1.1446019	0.28419732	0.47930284	-5.88410036
hetero after UVB irradiation VS hetero before UVB irradiation	GO_ACETYL_COA_HYDROLASE_ACTIVITY1	0.208938735	0.06308278	0.731885538	0.48550446	0.76360092	-5.95083087
WT after UVB irradiation VS WT before UVB irradiation	GO_ACETYL_COA_HYDROLASE_ACTIVITY2	-0.09076429	-0.04893683	-0.3339105	0.74693011	0.93606982	-5.93766131
homo after UVB irradiation VS homo before UVB irradiation	GO_ACETYL_COA_METABOLIC_PROCESS	-0.13577645	-0.01228048	-0.63539685	0.54222815	0.70722598	-6.3345882
hetero after UVB irradiation VS hetero before UVB irradiation	GO_ACETYL_COA_METABOLIC_PROCESS1	-0.2371883	-0.03522826	-1.05211826	0.32404095	0.64380615	-5.6713499
WT after UVB irradiation VS WT before UVB irradiation	GO_ACETYL_COA_METABOLIC_PROCESS2	-0.16628814	-0.04206375	-0.75085923	0.47398959	0.84774103	-5.70908093
homo after UVB irradiation VS homo before UVB irradiation	GO_ACETYLCHOLINE_BINDING	0.200543881	-0.02606773	0.838934968	0.42492626	0.60986548	-6.18035337
hetero after UVB irradiation VS hetero before UVB irradiation	GO_ACETYLCHOLINE_BINDING1	-0.45926253	-0.02718151	-2.68009893	0.02838928	0.23720962	-3.50714808
WT after UVB irradiation VS WT before UVB irradiation	GO_ACETYLCHOLINE_BINDING2	-0.53917246	0.004495156	-2.75331531	0.02464681	0.42639272	-3.2419993
homo after UVB irradiation VS homo before UVB irradiation	GO_ACETYLCHOLINE_GATED_CATION_SELECTIVE_CHANNEL_ACTIVITY	0.118164072	-0.00756556	0.545459274	0.59973651	0.74952177	-6.39047399
hetero after UVB irradiation VS hetero before UVB irradiation	GO_ACETYLCHOLINE_GATED_CATION_SELECTIVE_CHANNEL_ACTIVITY1	-0.36537392	-0.01854606	-1.81109532	0.10841075	0.40648574	-4.75606188
WT after UVB irradiation VS WT before UVB irradiation	GO_ACETYLCHOLINE_GATED_CATION_SELECTIVE_CHANNEL_ACTIVITY2	-0.53353467	-0.01849974	-2.57693316	0.03245093	0.45650945	-3.49286003
homo after UVB irradiation VS homo before UVB irradiation	GO_ACETYLCHOLINE_GATED_CHANNEL_COMPLEX	0.162991674	-0.01494184	0.740620191	0.47927906	0.65688874	-6.25950568
hetero after UVB irradiation VS hetero before UVB irradiation	GO_ACETYLCHOLINE_GATED_CHANNEL_COMPLEX1	-0.36735819	-0.02736888	-1.9685368	0.08521135	0.37178552	-4.53806879
WT after UVB irradiation VS WT before UVB irradiation	GO_ACETYLCHOLINE_GATED_CHANNEL_COMPLEX2	-0.5894536	0.013551798	-3.50067746	0.00791816	0.3154082	-2.20348546
homo after UVB irradiation VS homo before UVB irradiation	GO_ACETYLCHOLINE_METABOLIC_PROCESS	-0.3768809	0.007691132	-1.17647125	0.27193197	0.46705076	-5.8492676
hetero after UVB irradiation VS hetero before UVB irradiation	GO_ACETYLCHOLINE_METABOLIC_PROCESS1	-0.78220194	0.033167769	-3.84455808	0.00509152	0.12799059	-1.8509465
WT after UVB irradiation VS WT before UVB irradiation	GO_ACETYLCHOLINE_METABOLIC_PROCESS2	-0.58537754	-0.0756509	-2.01954945	0.07768508	0.56130885	-4.27752268
homo after UVB irradiation VS homo before UVB irradiation	GO_ACETYLCHOLINE_RECEPTOR_ACTIVITY	0.02494292	-0.01765941	0.125192862	0.90333992	0.94720457	-6.54226316
hetero after UVB irradiation VS hetero before UVB irradiation	GO_ACETYLCHOLINE_RECEPTOR_ACTIVITY1	-0.50365347	0.025715712	-3.52747167	0.00799552	0.14509294	-2.28660363
WT after UVB irradiation VS WT before UVB irradiation	GO_ACETYLCHOLINE_RECEPTOR_ACTIVITY2	-0.56718619	-0.00550508	-3.41443038	0.00899784	0.31908946	-2.32011512
homo after UVB irradiation VS homo before UVB irradiation	GO_ACETYLCHOLINE_RECEPTOR_BINDING	0.541077302	-0.03318282	3.191440934	0.01212358	0.07408036	-2.93757739
hetero after UVB irradiation VS hetero before UVB irradiation	GO_ACETYLCHOLINE_RECEPTOR_BINDING1	0.243595443	0.0102382	0.955239638	0.36793406	0.68165913	-5.76434754
WT after UVB irradiation VS WT before UVB irradiation	GO_ACETYLCHOLINE_RECEPTOR_BINDING2	0.140109103	0.034018462	0.550299394	0.59696275	0.89380878	-5.83937056
homo after UVB irradiation VS homo before UVB irradiation	GO_ACETYLCHOLINE_RECEPTOR_INHIBITOR_ACTIVITY	0.151557014	0.031535916	0.699247018	0.50345315	0.67712888	-6.29024506
hetero after UVB irradiation VS hetero before UVB irradiation	GO_ACETYLCHOLINE_RECEPTOR_INHIBITOR_ACTIVITY1	0.125363489	0.06741483	0.498336135	0.63190272	0.8484405	-6.09897255
WT after UVB irradiation VS WT before UVB irradiation	GO_ACETYLCHOLINE_RECEPTOR_INHIBITOR_ACTIVITY2	0.274696153	0.073089225	1.207945733	0.26116076	0.74128665	-5.29190936
homo after UVB irradiation VS homo before UVB irradiation	GO_ACETYLESTERASE_ACTIVITY	-0.30536179	-0.01459869	-1.3118069	0.22458501	0.41547601	-5.69404895
hetero after UVB irradiation VS hetero before UVB irradiation	GO_ACETYLESTERASE_ACTIVITY1	-0.39001621	0.043583231	-2.46460578	0.03957562	0.27162031	-3.82322948
WT after UVB irradiation VS WT before UVB irradiation	GO_ACETYLESTERASE_ACTIVITY2	-0.32225559	0.022813058	-1.43327917	0.18923587	0.6913973	-5.0371266
homo after UVB irradiation VS homo before UVB irradiation	GO_ACETYLGALACTOSAMINYLTRANSFERASE_ACTIVITY	0.169517172	-0.01417846	1.131897881	0.28920911	0.48410378	-5.89779344
hetero after UVB irradiation VS hetero before UVB irradiation	GO_ACETYLGALACTOSAMINYLTRANSFERASE_ACTIVITY1	0.191798277	-0.00110236	1.101707557	0.30320776	0.62506123	-5.62116527
WT after UVB irradiation VS WT before UVB irradiation	GO_ACETYLGALACTOSAMINYLTRANSFERASE_ACTIVITY2	0.243009423	-0.01094926	1.375362449	0.20586388	0.70285634	-5.10513865
homo after UVB irradiation VS homo before UVB irradiation	GO_ACETYLGLUCOSAMINYLTRANSFERASE_ACTIVITY	0.346517145	-0.00134086	2.537834744	0.0337504	0.13127429	-3.95050403
hetero after UVB irradiation VS hetero before UVB irradiation	GO_ACETYLGLUCOSAMINYLTRANSFERASE_ACTIVITY1	0.226851648	-0.01861277	1.436189657	0.18956387	0.5145903	-5.24299549
WT after UVB irradiation VS WT before UVB irradiation	GO_ACETYLGLUCOSAMINYLTRANSFERASE_ACTIVITY2	0.203208558	-0.03627889	1.191347315	0.26726273	0.74336868	-5.30956124
homo after UVB irradiation VS homo before UVB irradiation	GO_ACETYLTRANSFERASE_ACTIVITY	-0.35249516	-0.0312316	-2.6980586	0.02619015	0.11423533	-3.70205461
hetero after UVB irradiation VS hetero before UVB irradiation	GO_ACETYLTRANSFERASE_ACTIVITY1	-0.19847749	-0.02996747	-1.21567922	0.25938772	0.58569628	-5.49966949
WT after UVB irradiation VS WT before UVB irradiation	GO_ACETYLTRANSFERASE_ACTIVITY2	-0.20603207	-0.03739079	-1.03964796	0.32854728	0.77673377	-5.46284017
homo after UVB irradiation VS homo before UVB irradiation	GO_ACID_AMINO_ACID_LIGASE_ACTIVITY	0.152646072	-0.01654941	0.956873268	0.3655763	0.55570761	-6.07461524
hetero after UVB irradiation VS hetero before UVB irradiation	GO_ACID_AMINO_ACID_LIGASE_ACTIVITY1	0.108981839	-0.01371554	0.735272806	0.48355201	0.7626384	-5.94831517
WT after UVB irradiation VS WT before UVB irradiation	GO_ACID_AMINO_ACID_LIGASE_ACTIVITY2	0.306157948	0.066347	1.810154476	0.10741432	0.61228673	-4.56081948
homo after UVB irradiation VS homo before UVB irradiation	GO_ACID_PHOSPHATASE_ACTIVITY	-0.5011629	0.017260491	-3.09143938	0.01414737	0.08092872	-3.09174561
hetero after UVB irradiation VS hetero before UVB irradiation	GO_ACID_PHOSPHATASE_ACTIVITY1	-0.44435896	-0.00992108	-2.45112393	0.0404094	0.27277266	-3.84296717
WT after UVB irradiation VS WT before UVB irradiation	GO_ACID_PHOSPHATASE_ACTIVITY2	-0.23683318	0.04248338	-1.02697056	0.33413082	0.77844475	-5.47495682
homo after UVB irradiation VS homo before UVB irradiation	GO_ACID_SECRETION	-0.12425595	0.017795337	-0.82493138	0.43239884	0.61641021	-6.19214027
hetero after UVB irradiation VS hetero before UVB irradiation	GO_ACID_SECRETION1	-0.18899815	-0.00212317	-1.17953535	0.27268367	0.59910746	-5.5390935
WT after UVB irradiation VS WT before UVB irradiation	GO_ACID_SECRETION2	-0.07156137	0.022015621	-0.3832291	0.7114148	0.92897515	-5.91930997
homo after UVB irradiation VS homo before UVB irradiation	GO_ACID_THIOL_LIGASE_ACTIVITY	-0.25994704	0.026228235	-1.1749754	0.27249791	0.46747869	-5.85091778
hetero after UVB irradiation VS hetero before UVB irradiation	GO_ACID_THIOL_LIGASE_ACTIVITY1	-0.31108068	-0.00209444	-2.29905449	0.05113616	0.30062359	-4.06485225
WT after UVB irradiation VS WT before UVB irradiation	GO_ACID_THIOL_LIGASE_ACTIVITY2	-0.23699553	0.028999768	-0.95937341	0.36513594	0.79670383	-5.5376414
homo after UVB irradiation VS homo before UVB irradiation	GO_ACIDIC_AMINO_ACID_TRANSMEMBRANE_TRANSPORTER_ACTIVITY	0.014092782	0.009644398	0.101836352	0.92129559	0.9575686	-6.54516871
hetero after UVB irradiation VS hetero before UVB irradiation	GO_ACIDIC_AMINO_ACID_TRANSMEMBRANE_TRANSPORTER_ACTIVITY1	-0.25561509	0.008399987	-1.1669488	0.2774433	0.60400502	-5.55263128
WT after UVB irradiation VS WT before UVB irradiation	GO_ACIDIC_AMINO_ACID_TRANSMEMBRANE_TRANSPORTER_ACTIVITY2	-0.03962307	-0.02024849	-0.18852635	0.85510319	0.9663792	-5.97735634
homo after UVB irradiation VS homo before UVB irradiation	GO_ACIDIC_AMINO_ACID_TRANSPORT	-0.03087218	0.022039417	-0.23238014	0.82185111	0.90044226	-6.52131767
hetero after UVB irradiation VS hetero before UVB irradiation	GO_ACIDIC_AMINO_ACID_TRANSPORT1	-0.06076701	0.019301788	-0.42610433	0.68147089	0.87177843	-6.1341835
WT after UVB irradiation VS WT before UVB irradiation	GO_ACIDIC_AMINO_ACID_TRANSPORT2	-0.08817541	-0.01390437	-0.52516638	0.61353912	0.89976585	-5.85311103
homo after UVB irradiation VS homo before UVB irradiation	GO_ACROSIN_BINDING	-0.13961355	0.035297328	-0.37640995	0.71601998	0.83114423	-6.47376437
hetero after UVB irradiation VS hetero before UVB irradiation	GO_ACROSIN_BINDING1	-0.89929964	-0.10146554	-3.72922086	0.00599004	0.1340952	-2.00771084
WT after UVB irradiation VS WT before UVB irradiation	GO_ACROSIN_BINDING2	-0.83550448	0.176445783	-5.05965411	0.00094193	0.19353487	-0.30854237
homo after UVB irradiation VS homo before UVB irradiation	GO_ACROSOMAL_MEMBRANE	0.051215397	0.00929968	0.361000357	0.72708434	0.83877296	-6.47989853
hetero after UVB irradiation VS hetero before UVB irradiation	GO_ACROSOMAL_MEMBRANE1	-0.09527299	0.033086932	-0.6338829	0.54417484	0.79834348	-6.01917639
WT after UVB irradiation VS WT before UVB irradiation	GO_ACROSOMAL_MEMBRANE2	-0.08461393	0.022347781	-0.50413902	0.62759186	0.90339257	-5.86414751
homo after UVB irradiation VS homo before UVB irradiation	GO_ACROSOMAL_VESICLE	-0.02174323	0.023462317	-0.17196775	0.86756788	0.92712717	-6.53465581
homo after UVB irradiation VS homo before UVB irradiation	GO_ACROSOMAL_VESICLE_EXOCYTOSIS	0.298224993	0.05569579	1.378128899	0.20404871	0.39356252	-5.61396268
hetero after UVB irradiation VS hetero before UVB irradiation	GO_ACROSOMAL_VESICLE_EXOCYTOSIS1	-0.11435316	-0.06746504	-0.483956	0.64162454	0.85287918	-6.10639593
WT after UVB irradiation VS WT before UVB irradiation	GO_ACROSOMAL_VESICLE_EXOCYTOSIS2	0.185040697	-0.02548738	0.900982059	0.39360462	0.81339076	-5.58908803
hetero after UVB irradiation VS hetero before UVB irradiation	GO_ACROSOMAL_VESICLE1	0.024427023	0.042687207	0.179190649	0.86232503	0.94810134	-6.21410783
WT after UVB irradiation VS WT before UVB irradiation	GO_ACROSOMAL_VESICLE2	-0.07085313	0.053426372	-0.4547102	0.66126	0.91169919	-5.88842151
homo after UVB irradiation VS homo before UVB irradiation	GO_ACROSOME_ASSEMBLY	-0.40833443	-0.00568828	-2.30606797	0.04875824	0.16212827	-4.30685869
hetero after UVB irradiation VS hetero before UVB irradiation	GO_ACROSOME_ASSEMBLY1	-0.09074573	-0.00576589	-0.45815752	0.65925115	0.86028652	-6.11920059
WT after UVB irradiation VS WT before UVB irradiation	GO_ACROSOME_ASSEMBLY2	-0.30220642	0.001199999	-1.92907549	0.08940898	0.58307649	-4.40114697
homo after UVB irradiation VS homo before UVB irradiation	GO_ACROSOME_REACTION	0.299271073	0.050727195	1.873775797	0.09637665	0.24565808	-4.94932738
hetero after UVB irradiation VS hetero before UVB irradiation	GO_ACROSOME_REACTION1	-0.04043836	0.042492839	-0.32086329	0.7566865	0.90565384	-6.17602829
WT after UVB irradiation VS WT before UVB irradiation	GO_ACROSOME_REACTION2	0.025905178	0.053852849	0.147025365	0.88670669	0.97086341	-5.98467685
homo after UVB irradiation VS homo before UVB irradiation	GO_ACTIN_BASED_CELL_PROJECTION	0.15644177	-0.00313649	1.161293919	0.27771863	0.47357164	-5.86594151
hetero after UVB irradiation VS hetero before UVB irradiation	GO_ACTIN_BASED_CELL_PROJECTION1	0.110068879	0.004759873	0.866438265	0.4119399	0.71580135	-5.84335983
WT after UVB irradiation VS WT before UVB irradiation	GO_ACTIN_BASED_CELL_PROJECTION2	0.118366512	0.009349553	0.84863827	0.42045984	0.82383319	-5.63298364
homo after UVB irradiation VS homo before UVB irradiation	GO_ACTIN_BINDING	0.330565604	-0.01598989	2.407453619	0.04150901	0.14686671	-4.15159593
hetero after UVB irradiation VS hetero before UVB irradiation	GO_ACTIN_BINDING1	0.150116943	-0.00116605	1.013822096	0.34088288	0.65976755	-5.7089284
WT after UVB irradiation VS WT before UVB irradiation	GO_ACTIN_BINDING2	0.087584997	0.001076326	0.554288546	0.59435421	0.8931505	-5.83713506
homo after UVB irradiation VS homo before UVB irradiation	GO_ACTIN_CROSSLINK_FORMATION	0.356337013	0.046381872	2.677082963	0.02707257	0.1164073	-3.73462935
hetero after UVB irradiation VS hetero before UVB irradiation	GO_ACTIN_CROSSLINK_FORMATION1	0.020792851	0.045293738	0.102094083	0.9212408	0.96983444	-6.22583541
WT after UVB irradiation VS WT before UVB irradiation	GO_ACTIN_CROSSLINK_FORMATION2	0.355201652	0.033775361	1.675066824	0.13199533	0.63801344	-4.73741994
homo after UVB irradiation VS homo before UVB irradiation	GO_ACTIN_CYTOSKELETON	0.307627047	-0.01103066	2.06734448	0.07114857	0.20472647	-4.66653603
homo after UVB irradiation VS homo before UVB irradiation	GO_ACTIN_CYTOSKELETON_REORGANIZATION	0.320613799	-0.03094064	2.220102644	0.05588114	0.17571661	-4.43747772
hetero after UVB irradiation VS hetero before UVB irradiation	GO_ACTIN_CYTOSKELETON_REORGANIZATION1	0.306778166	0.00096399	2.323223084	0.04925725	0.29581029	-4.02970149
WT after UVB irradiation VS WT before UVB irradiation	GO_ACTIN_CYTOSKELETON_REORGANIZATION2	0.264434598	0.008472438	1.831718197	0.10391275	0.60721285	-4.53213326
hetero after UVB irradiation VS hetero before UVB irradiation	GO_ACTIN_CYTOSKELETON1	0.194651962	0.000471259	1.403174204	0.19884737	0.52312209	-5.28305919
WT after UVB irradiation VS WT before UVB irradiation	GO_ACTIN_CYTOSKELETON2	0.13520123	0.006197881	0.908516203	0.38984313	0.81061225	-5.58259481
homo after UVB irradiation VS homo before UVB irradiation	GO_ACTIN_DEPENDENT_ATPASE_ACTIVITY	0.200803157	0.025161586	1.062107145	0.31800862	0.51164993	-5.97100141
hetero after UVB irradiation VS hetero before UVB irradiation	GO_ACTIN_DEPENDENT_ATPASE_ACTIVITY1	0.221646286	0.025126318	1.088162752	0.30878981	0.62929293	-5.63503996
WT after UVB irradiation VS WT before UVB irradiation	GO_ACTIN_DEPENDENT_ATPASE_ACTIVITY2	0.266865261	0.027950638	1.307202496	0.22703922	0.71906685	-5.1830207
homo after UVB irradiation VS homo before UVB irradiation	GO_ACTIN_FILAMENT	0.37342383	-0.02155339	2.605625019	0.03031268	0.12366244	-3.84550971
homo after UVB irradiation VS homo before UVB irradiation	GO_ACTIN_FILAMENT_BASED_MOVEMENT	0.315957639	-0.00744492	2.409345837	0.04138448	0.14666445	-4.14868768
hetero after UVB irradiation VS hetero before UVB irradiation	GO_ACTIN_FILAMENT_BASED_MOVEMENT1	-0.05055186	-0.01208242	-0.29663278	0.77444096	0.91152166	-6.18404128
WT after UVB irradiation VS WT before UVB irradiation	GO_ACTIN_FILAMENT_BASED_MOVEMENT2	-0.01080398	-0.02767722	-0.05432988	0.95798886	0.98896463	-5.99450948
homo after UVB irradiation VS homo before UVB irradiation	GO_ACTIN_FILAMENT_BASED_PROCESS	0.297131692	-0.02754764	2.19479268	0.05816757	0.18048334	-4.4757209
hetero after UVB irradiation VS hetero before UVB irradiation	GO_ACTIN_FILAMENT_BASED_PROCESS1	0.198292311	-0.00212987	1.411254539	0.19653913	0.52076339	-5.27330327
WT after UVB irradiation VS WT before UVB irradiation	GO_ACTIN_FILAMENT_BASED_PROCESS2	0.138038469	-0.01098929	0.900029064	0.39408228	0.81363974	-5.58990625
homo after UVB irradiation VS homo before UVB irradiation	GO_ACTIN_FILAMENT_BASED_TRANSPORT	0.20379064	0.021989576	0.962294395	0.36300231	0.55356139	-6.06948501
hetero after UVB irradiation VS hetero before UVB irradiation	GO_ACTIN_FILAMENT_BASED_TRANSPORT1	0.197559927	-0.02058787	0.954289807	0.36838557	0.68175554	-5.76522497
WT after UVB irradiation VS WT before UVB irradiation	GO_ACTIN_FILAMENT_BASED_TRANSPORT2	0.168409536	0.018319504	0.831409358	0.429575	0.82802534	-5.64695844
homo after UVB irradiation VS homo before UVB irradiation	GO_ACTIN_FILAMENT_BINDING	0.419671365	-0.00213256	2.898005502	0.0191222	0.09517243	-3.39141585
hetero after UVB irradiation VS hetero before UVB irradiation	GO_ACTIN_FILAMENT_BINDING1	0.232116106	0.006210447	1.504321686	0.17160985	0.49454803	-5.15868725
WT after UVB irradiation VS WT before UVB irradiation	GO_ACTIN_FILAMENT_BINDING2	0.168057451	0.00913653	1.047606644	0.32507906	0.77400637	-5.45517668
homo after UVB irradiation VS homo before UVB irradiation	GO_ACTIN_FILAMENT_BUNDLE	0.476000281	-0.00369387	2.628303755	0.02924372	0.1214948	-3.81033802
homo after UVB irradiation VS homo before UVB irradiation	GO_ACTIN_FILAMENT_BUNDLE_ORGANIZATION	0.279912879	-0.01597704	1.833086325	0.1026708	0.25489787	-5.00745441
hetero after UVB irradiation VS hetero before UVB irradiation	GO_ACTIN_FILAMENT_BUNDLE_ORGANIZATION1	0.25567461	-0.00144516	1.752277958	0.11852958	0.42267339	-4.83577742
WT after UVB irradiation VS WT before UVB irradiation	GO_ACTIN_FILAMENT_BUNDLE_ORGANIZATION2	0.165701937	-0.0262509	1.056875476	0.32107582	0.77346379	-5.44619684
hetero after UVB irradiation VS hetero before UVB irradiation	GO_ACTIN_FILAMENT_BUNDLE1	0.345876582	-0.00329713	1.920979162	0.0916634	0.37876929	-4.60455808
WT after UVB irradiation VS WT before UVB irradiation	GO_ACTIN_FILAMENT_BUNDLE2	0.305035052	-0.01129017	1.633706038	0.14050625	0.65051831	-4.79030294
homo after UVB irradiation VS homo before UVB irradiation	GO_ACTIN_FILAMENT_DEPOLYMERIZATION	0.476810245	-0.02644511	3.713188045	0.0055212	0.04887601	-2.14748012
hetero after UVB irradiation VS hetero before UVB irradiation	GO_ACTIN_FILAMENT_DEPOLYMERIZATION1	0.241135482	0.002525513	1.533262748	0.16445508	0.48576486	-5.12224357
WT after UVB irradiation VS WT before UVB irradiation	GO_ACTIN_FILAMENT_DEPOLYMERIZATION2	0.156086749	0.034978378	0.847325969	0.42114933	0.82392955	-5.63405642
homo after UVB irradiation VS homo before UVB irradiation	GO_ACTIN_FILAMENT_NETWORK_FORMATION	0.277139159	0.027419589	1.64591606	0.13690264	0.30577754	-5.26757388
hetero after UVB irradiation VS hetero before UVB irradiation	GO_ACTIN_FILAMENT_NETWORK_FORMATION1	0.25129007	0.002827894	1.760992254	0.11697624	0.42037002	-4.82403305
WT after UVB irradiation VS WT before UVB irradiation	GO_ACTIN_FILAMENT_NETWORK_FORMATION2	0.181045344	0.164835645	0.896936363	0.39563527	0.81453088	-5.59255679
homo after UVB irradiation VS homo before UVB irradiation	GO_ACTIN_FILAMENT_ORGANIZATION	0.364681072	-0.0205631	2.508654762	0.03534918	0.13468829	-3.99561915
hetero after UVB irradiation VS hetero before UVB irradiation	GO_ACTIN_FILAMENT_ORGANIZATION1	0.28157273	0.000398896	1.976811547	0.08413446	0.37033946	-4.52644895
WT after UVB irradiation VS WT before UVB irradiation	GO_ACTIN_FILAMENT_ORGANIZATION2	0.238068363	-0.01367873	1.568892667	0.15485723	0.65981549	-4.87191302
homo after UVB irradiation VS homo before UVB irradiation	GO_ACTIN_FILAMENT_REORGANIZATION	0.424942188	-0.02097852	1.905235502	0.09176274	0.2380701	-4.90404484
hetero after UVB irradiation VS hetero before UVB irradiation	GO_ACTIN_FILAMENT_REORGANIZATION1	-0.218779	-0.11123433	-0.76326156	0.46761565	0.7530936	-5.92714508
WT after UVB irradiation VS WT before UVB irradiation	GO_ACTIN_FILAMENT_REORGANIZATION2	0.175146433	-0.08346249	0.46812465	0.65203713	0.90930021	-5.88206707
homo after UVB irradiation VS homo before UVB irradiation	GO_ACTIN_FILAMENT_SEVERING	0.720410018	-0.01307331	4.203872739	0.00272421	0.03454156	-1.43459538
hetero after UVB irradiation VS hetero before UVB irradiation	GO_ACTIN_FILAMENT_SEVERING1	0.486724515	-0.00449693	2.287454637	0.05206326	0.30329469	-4.08170423
WT after UVB irradiation VS WT before UVB irradiation	GO_ACTIN_FILAMENT_SEVERING2	0.309498215	0.009963444	1.582306211	0.15178224	0.65936135	-4.8551549
hetero after UVB irradiation VS hetero before UVB irradiation	GO_ACTIN_FILAMENT1	0.230869929	0.024670356	1.466018232	0.18150764	0.50681846	-5.20634867
WT after UVB irradiation VS WT before UVB irradiation	GO_ACTIN_FILAMENT2	0.179782807	0.0305917	1.250814255	0.24593224	0.73489776	-5.24556768
homo after UVB irradiation VS homo before UVB irradiation	GO_ACTIN_MEDIATED_CELL_CONTRACTION	0.308896881	-0.00328508	1.996870407	0.0794942	0.21768342	-4.77059078
hetero after UVB irradiation VS hetero before UVB irradiation	GO_ACTIN_MEDIATED_CELL_CONTRACTION1	-0.13615257	-0.02236359	-0.67502817	0.51903488	0.78616125	-5.99153924
WT after UVB irradiation VS WT before UVB irradiation	GO_ACTIN_MEDIATED_CELL_CONTRACTION2	-0.03585483	-0.04466368	-0.15439739	0.88107527	0.9693507	-5.98350784
homo after UVB irradiation VS homo before UVB irradiation	GO_ACTIN_MODIFICATION	0.668169961	0.039870395	2.849595423	0.02063011	0.09963465	-3.46660143
hetero after UVB irradiation VS hetero before UVB irradiation	GO_ACTIN_MODIFICATION1	0.753793729	0.017553927	3.449493117	0.00895298	0.15043564	-2.39589748

WT after UVB irradiation VS WT before UVB irradiation	GO_ACTIN_MODIFICATION2	0.205980723	-0.05577604	1.134775597	0.28894076	0.75486529	-5.368456
homo after UVB irradiation VS homo before UVB irradiation	GO_ACTIN_MONOMER_BINDING	0.283198805	-0.0373626	1.765846999	0.11392719	0.27155928	-5.10234249
hetero after UVB irradiation VS hetero before UVB irradiation	GO_ACTIN_MONOMER_BINDING1	0.112227903	0.010146003	0.650752698	0.53377986	0.79279875	-6.00803267
WT after UVB irradiation VS WT before UVB irradiation	GO_ACTIN_MONOMER_BINDING2	0.06379639	-0.02920024	0.349292905	0.73577894	0.93474796	-5.93219966
homo after UVB irradiation VS homo before UVB irradiation	GO_ACTIN_NUCLEATION	0.357295941	-0.03686243	1.985177517	0.08096728	0.22000269	-4.78774137
hetero after UVB irradiation VS hetero before UVB irradiation	GO_ACTIN_NUCLEATION1	0.326269215	0.005775575	1.933562711	0.08991183	0.37657277	-4.58701532
WT after UVB irradiation VS WT before UVB irradiation	GO_ACTIN_NUCLEATION2	0.415510316	0.005249461	2.885974125	0.02006726	0.39829087	-3.05404004
homo after UVB irradiation VS homo before UVB irradiation	GO_ACTIN_POLYMERIZATION_DEPENDENT_CELL_MOTILITY	0.587042874	-0.04513762	3.304977449	0.01018773	0.06753831	-2.76341335
hetero after UVB irradiation VS hetero before UVB irradiation	GO_ACTIN_POLYMERIZATION_DEPENDENT_CELL_MOTILITY1	0.733208933	0.011247655	4.195360039	0.00314288	0.11536387	-1.38701286
WT after UVB irradiation VS WT before UVB irradiation	GO_ACTIN_POLYMERIZATION_DEPENDENT_CELL_MOTILITY2	0.87289419	-0.01152312	5.486554842	0.00055898	0.18842845	0.134895671
homo after UVB irradiation VS homo before UVB irradiation	GO_ACTIN_POLYMERIZATION_OR_DEPOLYMERIZATION	0.474639672	-0.02009908	3.414164373	0.00863032	0.06173419	-2.59696327
hetero after UVB irradiation VS hetero before UVB irradiation	GO_ACTIN_POLYMERIZATION_OR_DEPOLYMERIZATION1	0.365583606	0.002544705	2.527920136	0.0358873	0.26025233	-3.73044505
WT after UVB irradiation VS WT before UVB irradiation	GO_ACTIN_POLYMERIZATION_OR_DEPOLYMERIZATION2	0.327485568	0.004641406	2.308900567	0.04939714	0.50822195	-3.87341029
homo after UVB irradiation VS homo before UVB irradiation	GO_ACTININ_BINDING	0.538536965	-0.04645758	3.233559771	0.01136397	0.07148258	-2.87284978
hetero after UVB irradiation VS hetero before UVB irradiation	GO_ACTININ_BINDING1	0.191412715	-0.01675927	0.786861547	0.4544526	0.74559734	-5.90876928
WT after UVB irradiation VS WT before UVB irradiation	GO_ACTININ_BINDING2	0.188738104	-0.02772999	0.67783664	0.51677153	0.86137677	-5.7606607
homo after UVB irradiation VS homo before UVB irradiation	GO_ACTION_POTENTIAL	0.332041514	-0.01479464	2.675084869	0.02715819	0.11660559	-3.73773184
hetero after UVB irradiation VS hetero before UVB irradiation	GO_ACTION_POTENTIAL1	-0.03022981	-9.15E-06	-0.18828946	0.85542443	0.94499137	-6.21230261
WT after UVB irradiation VS WT before UVB irradiation	GO_ACTION_POTENTIAL2	-0.00419849	-0.0114605	-0.0206959	0.98398913	0.99634469	-5.99584067
homo after UVB irradiation VS homo before UVB irradiation	GO_ACTIVATED_T_CELL_PROLIFERATION	0.486148335	-0.03008421	3.691610322	0.00569982	0.05000724	-2.17956787
hetero after UVB irradiation VS hetero before UVB irradiation	GO_ACTIVATED_T_CELL_PROLIFERATION1	0.398480362	0.000726987	2.135197261	0.06590052	0.33426479	-4.30150574
WT after UVB irradiation VS WT before UVB irradiation	GO_ACTIVATED_T_CELL_PROLIFERATION2	0.222875905	-0.04881827	0.996140296	0.34801342	0.78574573	-5.50395274
homo after UVB irradiation VS homo before UVB irradiation	GO_ACTIVATING_TRANSCRIPTION_FACTOR_BINDING	0.069507397	0.011730021	0.520804903	0.61606126	0.76140837	-6.40441561
hetero after UVB irradiation VS hetero before UVB irradiation	GO_ACTIVATING_TRANSCRIPTION_FACTOR_BINDING1	0.071612474	0.012193487	0.521254429	0.61656593	0.84102847	-6.08672182
WT after UVB irradiation VS WT before UVB irradiation	GO_ACTIVATING_TRANSCRIPTION_FACTOR_BINDING2	-0.12317925	-0.03179648	-0.86525631	0.41179716	0.82044248	-5.6192804
homo after UVB irradiation VS homo before UVB irradiation	GO_ACTIVATION_INDUCED_CELL_DEATH_OF_T_CELLS	0.379127045	-0.03016976	1.75659987	0.11556262	0.27390583	-5.11527183
hetero after UVB irradiation VS hetero before UVB irradiation	GO_ACTIVATION_INDUCED_CELL_DEATH_OF_T_CELLS1	0.150210132	-0.03225151	0.441567672	0.67070824	0.86572523	-6.1270841
WT after UVB irradiation VS WT before UVB irradiation	GO_ACTIVATION_INDUCED_CELL_DEATH_OF_T_CELLS2	0.320561598	-0.07908665	1.335796648	0.21793891	0.71478521	-5.15064247
homo after UVB irradiation VS homo before UVB irradiation	GO_ACTIVATION_OF_ADENYLATE_CYCLASE_ACTIVITY	0.582930012	-0.01542409	4.697339175	0.00138681	0.02511063	-0.75347356
hetero after UVB irradiation VS hetero before UVB irradiation	GO_ACTIVATION_OF_ADENYLATE_CYCLASE_ACTIVITY1	0.01475633	0.012822135	0.08038802	0.93793958	0.97722007	-6.22798108
WT after UVB irradiation VS WT before UVB irradiation	GO_ACTIVATION_OF_ADENYLATE_CYCLASE_ACTIVITY2	0.130732832	-0.02812314	0.643963809	0.53740735	0.87025652	-5.78300229
homo after UVB irradiation VS homo before UVB irradiation	GO_ACTIVATION_OF_CYSTEINE_TYPE_ENDOPEPTIDASE_ACTIVITY	0.560107728	-0.02767403	4.347397319	0.00223026	0.03158532	-1.23262894
homo after UVB irradiation VS homo before UVB irradiation	GO_ACTIVATION_OF_CYSTEINE_TYPE_ENDOPEPTIDASE_ACTIVITY_INVOLVED_IN_APOPTOTIC_PROCESS	0.385484129	-0.00744017	2.918703188	0.01851263	0.0934377	-3.35928577
homo after UVB irradiation VS homo before UVB irradiation	GO_ACTIVATION_OF_CYSTEINE_TYPE_ENDOPEPTIDASE_ACTIVITY_INVOLVED_IN_APOPTOTIC_PROCESS_BY_CYTOCHROME_C	0.343551657	-0.00106273	0.861773366	0.41293268	0.5990609	-6.1607734
hetero after UVB irradiation VS hetero before UVB irradiation	GO_ACTIVATION_OF_CYSTEINE_TYPE_ENDOPEPTIDASE_ACTIVITY_INVOLVED_IN_APOPTOTIC_PROCESS_BY_CYTOCHROME_C1	0.657268745	0.079178993	2.950869494	0.01878355	0.19947197	-3.11099264
WT after UVB irradiation VS WT before UVB irradiation	GO_ACTIVATION_OF_CYSTEINE_TYPE_ENDOPEPTIDASE_ACTIVITY_INVOLVED_IN_APOPTOTIC_PROCESS_BY_CYTOCHROME_C2	0.50002756	0.004037565	2.140605586	0.06430648	0.53678963	-4.10979923
hetero after UVB irradiation VS hetero before UVB irradiation	GO_ACTIVATION_OF_CYSTEINE_TYPE_ENDOPEPTIDASE_ACTIVITY_INVOLVED_IN_APOPTOTIC_PROCESS1	0.29778074	0.010992477	2.290525386	0.05181621	0.30233295	-4.07724436
WT after UVB irradiation VS WT before UVB irradiation	GO_ACTIVATION_OF_CYSTEINE_TYPE_ENDOPEPTIDASE_ACTIVITY_INVOLVED_IN_APOPTOTIC_PROCESS2	0.372523265	-0.02980403	2.325213192	0.0481489	0.50693606	-3.85035285
hetero after UVB irradiation VS hetero before UVB irradiation	GO_ACTIVATION_OF_CYSTEINE_TYPE_ENDOPEPTIDASE_ACTIVITY1	0.55418557	0.056437356	4.15005387	0.00334153	0.11632799	-1.44580439
WT after UVB irradiation VS WT before UVB irradiation	GO_ACTIVATION_OF_CYSTEINE_TYPE_ENDOPEPTIDASE_ACTIVITY2	0.517669577	0.014748848	3.457328924	0.00844257	0.31908946	-2.26197482
homo after UVB irradiation VS homo before UVB irradiation	GO_ACTIVATION_OF_GTPASE_ACTIVITY	0.098669486	-0.01515252	0.558029002	0.59150385	0.74379552	-6.38313535
hetero after UVB irradiation VS hetero before UVB irradiation	GO_ACTIVATION_OF_GTPASE_ACTIVITY1	0.080896644	-0.0281307	0.607951437	0.56038677	0.80863165	-6.03579158
WT after UVB irradiation VS WT before UVB irradiation	GO_ACTIVATION_OF_GTPASE_ACTIVITY2	-0.03283613	-0.00531322	-0.21786963	0.8329207	0.96075976	-5.97109814
homo after UVB irradiation VS homo before UVB irradiation	GO_ACTIVATION_OF_IMMUNE_RESPONSE	0.333382191	-0.02035227	2.248975947	0.05338087	0.17114445	-4.39372642
hetero after UVB irradiation VS hetero before UVB irradiation	GO_ACTIVATION_OF_IMMUNE_RESPONSE1	0.383395517	0.016848521	3.01250044	0.01711376	0.1913886	-3.0213612
WT after UVB irradiation VS WT before UVB irradiation	GO_ACTIVATION_OF_IMMUNE_RESPONSE2	0.343224714	0.001831042	2.122758735	0.06612694	0.537335	-4.13466878
homo after UVB irradiation VS homo before UVB irradiation	GO_ACTIVATION_OF_INNATE_IMMUNE_RESPONSE	0.100813188	-0.00606534	0.515212908	0.61979607	0.76367112	-6.40749393
hetero after UVB irradiation VS hetero before UVB irradiation	GO_ACTIVATION_OF_INNATE_IMMUNE_RESPONSE1	0.248509393	0.008336733	1.368638514	0.2089821	0.53418041	-5.3243859
WT after UVB irradiation VS WT before UVB irradiation	GO_ACTIVATION_OF_INNATE_IMMUNE_RESPONSE2	0.244252873	-0.00247627	1.272256166	0.23859781	0.72898299	-5.22199221
homo after UVB irradiation VS homo before UVB irradiation	GO_ACTIVATION_OF_JANUS_KINASE_ACTIVITY	0.31229591	-0.04223799	1.821112121	0.1045957	0.25789787	-5.02446149
hetero after UVB irradiation VS hetero before UVB irradiation	GO_ACTIVATION_OF_JANUS_KINASE_ACTIVITY1	0.240640355	0.005114224	1.023258409	0.33667153	0.65647312	-5.69976625
WT after UVB irradiation VS WT before UVB irradiation	GO_ACTIVATION_OF_JANUS_KINASE_ACTIVITY2	0.216356762	-0.05900098	0.665833335	0.52402773	0.8649007	-5.76869438
homo after UVB irradiation VS homo before UVB irradiation	GO_ACTIVATION_OF_JNKK_ACTIVITY	0.216077083	-0.01248398	1.275958879	0.23639887	0.42867266	-5.73626987
hetero after UVB irradiation VS hetero before UVB irradiation	GO_ACTIVATION_OF_JNKK_ACTIVITY1	0.371021943	-0.01438738	2.464502517	0.03958194	0.27162031	-3.8233807
WT after UVB irradiation VS WT before UVB irradiation	GO_ACTIVATION_OF_JNKK_ACTIVITY2	-0.00662622	0.026981529	-0.03461574	0.97322424	0.99307736	-5.99543446
homo after UVB irradiation VS homo before UVB irradiation	GO_ACTIVATION_OF_JUN_KINASE_ACTIVITY	0.218768653	0.011148737	1.491760743	0.17263086	0.35254824	-5.47120005
hetero after UVB irradiation VS hetero before UVB irradiation	GO_ACTIVATION_OF_JUN_KINASE_ACTIVITY1	0.100311362	0.004815029	0.71662315	0.49436519	0.76861874	-5.96204089
WT after UVB irradiation VS WT before UVB irradiation	GO_ACTIVATION_OF_JUN_KINASE_ACTIVITY2	-0.00424793	0.006579536	-0.02642032	0.9795616	0.99508405	-5.99569837
homo after UVB irradiation VS homo before UVB irradiation	GO_ACTIVATION_OF_MAPK_ACTIVITY	0.200078606	0.008407615	1.415111736	0.19331587	0.37976505	-5.56824105
hetero after UVB irradiation VS hetero before UVB irradiation	GO_ACTIVATION_OF_MAPK_ACTIVITY1	0.24928774	-9.58E-05	1.994785168	0.08184054	0.36628163	-4.50115972
WT after UVB irradiation VS WT before UVB irradiation	GO_ACTIVATION_OF_MAPK_ACTIVITY2	0.128899356	-0.00347351	0.889432048	0.39942186	0.81573919	-5.59895754
homo after UVB irradiation VS homo before UVB irradiation	GO_ACTIVATION_OF_MAPKK_ACTIVITY	0.194759895	0.007467486	1.175318936	0.27236786	0.46741859	-5.85053894
hetero after UVB irradiation VS hetero before UVB irradiation	GO_ACTIVATION_OF_MAPKK_ACTIVITY1	0.112913578	-0.00016269	0.698335269	0.50511818	0.77535877	-5.97520118
WT after UVB irradiation VS WT before UVB irradiation	GO_ACTIVATION_OF_MAPKK_ACTIVITY2	-0.02885833	0.023481955	-0.18299519	0.8593006	0.9674015	-5.97843575
homo after UVB irradiation VS homo before UVB irradiation	GO_ACTIVATION_OF_MAPKKK_ACTIVITY	0.303053226	-0.00726879	1.378265988	0.20400799	0.39353542	-5.61379457
hetero after UVB irradiation VS hetero before UVB irradiation	GO_ACTIVATION_OF_MAPKKK_ACTIVITY1	-0.06394416	-0.02423479	-0.31497427	0.76098789	0.90682777	-6.17803215
WT after UVB irradiation VS WT before UVB irradiation	GO_ACTIVATION_OF_MAPKKK_ACTIVITY2	-0.09731829	-0.07987932	-0.55304017	0.59516987	0.8931505	-5.83783625
homo after UVB irradiation VS homo before UVB irradiation	GO_ACTIVATION_OF_NF_KAPPAB_INDUCING_KINASE_ACTIVITY	0.396199889	-0.08953831	2.429630776	0.04007284	0.14370716	-4.1174895
hetero after UVB irradiation VS hetero before UVB irradiation	GO_ACTIVATION_OF_NF_KAPPAB_INDUCING_KINASE_ACTIVITY1	0.24368143	0.005277999	1.102443856	0.30290663	0.62486932	-5.62040748
WT after UVB irradiation VS WT before UVB irradiation	GO_ACTIVATION_OF_NF_KAPPAB_INDUCING_KINASE_ACTIVITY2	0.282782372	-0.03372253	1.373152729	0.2065227	0.70312257	-5.10770099
homo after UVB irradiation VS homo before UVB irradiation	GO_ACTIVATION_OF_PHOSPHOLIPASE_C_ACTIVITY	0.504571076	0.01419834	3.647360861	0.00608558	0.05167143	-2.24554836
hetero after UVB irradiation VS hetero before UVB irradiation	GO_ACTIVATION_OF_PHOSPHOLIPASE_C_ACTIVITY1	0.320369898	0.003157678	1.35172986	0.21410575	0.54114961	-5.34439559
WT after UVB irradiation VS WT before UVB irradiation	GO_ACTIVATION_OF_PHOSPHOLIPASE_C_ACTIVITY2	0.360573818	-0.01365877	1.402765387	0.19784411	0.69702831	-5.0731618
homo after UVB irradiation VS homo before UVB irradiation	GO_ACTIVATION_OF_PHOSPHOLIPASE_D_ACTIVITY	0.479839561	0.058729811	3.072062753	0.01457865	0.08223895	-3.12168802
hetero after UVB irradiation VS hetero before UVB irradiation	GO_ACTIVATION_OF_PHOSPHOLIPASE_D_ACTIVITY1	0.132546097	0.026353052	0.471438474	0.65014771	0.85672259	-6.11269138
WT after UVB irradiation VS WT before UVB irradiation	GO_ACTIVATION_OF_PHOSPHOLIPASE_D_ACTIVITY2	-0.18936601	-0.09063212	-0.64976687	0.53383717	0.8692322	-5.77924733
homo after UVB irradiation VS homo before UVB irradiation	GO_ACTIVATION_OF_PROTEIN_KINASE_A_ACTIVITY	0.452246832	-0.04048349	2.588852574	0.03112871	0.12512052	-3.87150795
hetero after UVB irradiation VS hetero before UVB irradiation	GO_ACTIVATION_OF_PROTEIN_KINASE_A_ACTIVITY1	0.246602076	0.010575436	1.169483808	0.27647926	0.60333154	-5.54991274
WT after UVB irradiation VS WT before UVB irradiation	GO_ACTIVATION_OF_PROTEIN_KINASE_A_ACTIVITY2	0.388730229	0.007908938	1.44659942	0.18558234	0.68726847	-5.02125843
homo after UVB irradiation VS homo before UVB irradiation	GO_ACTIVATION_OF_PROTEIN_KINASE_ACTIVITY	0.206768079	-0.00816821	1.580468545	0.15116347	0.32517775	-5.35531986
hetero after UVB irradiation VS hetero before UVB irradiation	GO_ACTIVATION_OF_PROTEIN_KINASE_ACTIVITY1	0.261564919	0.006152848	2.059591225	0.07406341	0.35255978	-4.40944474
WT after UVB irradiation VS WT before UVB irradiation	GO_ACTIVATION_OF_PROTEIN_KINASE_ACTIVITY2	0.12093527	-0.01805771	0.781220915	0.45690223	0.83880233	-5.68629574
homo after UVB irradiation VS homo before UVB irradiation	GO_ACTIVATION_OF_PROTEIN_KINASE_B_ACTIVITY	0.299058288	-0.06454993	2.236973838	0.05440649	0.17300297	-4.41192858
hetero after UVB irradiation VS hetero before UVB irradiation	GO_ACTIVATION_OF_PROTEIN_KINASE_B_ACTIVITY1	0.071061584	0.011239861	0.412337993	0.69111844	0.87620446	-6.14030072
WT after UVB irradiation VS WT before UVB irradiation	GO_ACTIVATION_OF_PROTEIN_KINASE_B_ACTIVITY2	0.029450974	0.006880025	0.170836805	0.86854369	0.96790654	-5.98069644
homo after UVB irradiation VS homo before UVB irradiation	GO_ACTIVATION_OF_PROTEIN_KINASE_C_ACTIVITY	-0.72127614	-0.01289726	-3.89216634	0.00425023	0.04225671	-1.88362057
hetero after UVB irradiation VS hetero before UVB irradiation	GO_ACTIVATION_OF_PROTEIN_KINASE_C_ACTIVITY1	-0.2301328	-0.08180037	-0.77924491	0.45867326	0.74772822	-5.91475193
WT after UVB irradiation VS WT before UVB irradiation	GO_ACTIVATION_OF_PROTEIN_KINASE_C_ACTIVITY2	-0.15477642	-0.1073039	-0.58457072	0.57475764	0.88586474	-5.8196814
homo after UVB irradiation VS homo before UVB irradiation	GO_ACTIVATION_OF_STORE_OPERATED_CALCIUM_CHANNEL_ACTIVITY	0.490711255	-0.03119592	2.440466316	0.03938938	0.14260771	-4.10080908
hetero after UVB irradiation VS hetero before UVB irradiation	GO_ACTIVATION_OF_STORE_OPERATED_CALCIUM_CHANNEL_ACTIVITY1	0.463958518	-0.04488868	1.474248569	0.17933894	0.50330437	-5.19616384
WT after UVB irradiation VS WT before UVB irradiation	GO_ACTIVATION_OF_STORE_OPERATED_CALCIUM_CHANNEL_ACTIVITY2	0.207348541	0.000121209	1.003448266	0.34468363	0.78398526	-5.49714048
homo after UVB irradiation VS homo before UVB irradiation	GO_ACTIVATION_OF_TRANSMEMBRANE_RECEPTOR_PROTEIN_TYROSINE_KINASE_ACTIVITY	0.484807081	0.017914738	3.588099648	0.00664615	0.05379154	-2.33427827
hetero after UVB irradiation VS hetero before UVB irradiation	GO_ACTIVATION_OF_TRANSMEMBRANE_RECEPTOR_PROTEIN_TYROSINE_KINASE_ACTIVITY1	0.25660606	0.058410018	1.283882061	0.23576745	0.56422064	-5.42314141
WT after UVB irradiation VS WT before UVB irradiation	GO_ACTIVATION_OF_TRANSMEMBRANE_RECEPTOR_PROTEIN_TYROSINE_KINASE_ACTIVITY2	0.155768498	0.022145537	0.708240928	0.49867226	0.85541458	-5.73974502
homo after UVB irradiation VS homo before UVB irradiation	GO_ACTIVE_ION_TRANSMEMBRANE_TRANSPORTER_ACTIVITY	0.115756586	0.028915048	0.810290728	0.4403075	0.62305524	-6.20428372
hetero after UVB irradiation VS hetero before UVB irradiation	GO_ACTIVE_ION_TRANSMEMBRANE_TRANSPORTER_ACTIVITY1	-0.05290893	0.016030971	-0.42438371	0.68267334	0.87233156	-6.13495855
WT after UVB irradiation VS WT before UVB irradiation	GO_ACTIVE_ION_TRANSMEMBRANE_TRANSPORTER_ACTIVITY2	-0.06053298	0.015764754	-0.42678198	0.68065837	0.91716791	-5.90108841
homo after UVB irradiation VS homo before UVB irradiation	GO_ACTIVE_TRANSMEMBRANE_TRANSPORTER_ACTIVITY	0.117638691	0.01131159	0.893921552	0.39645732	0.58522024	-6.13247293
hetero after UVB irradiation VS hetero before UVB irradiation	GO_ACTIVE_TRANSMEMBRANE_TRANSPORTER_ACTIVITY1	-0.06389301	0.00597749	-0.51321059	0.62192652	0.84401411	-6.09108009
WT after UVB irradiation VS WT before UVB irradiation	GO_ACTIVE_TRANSMEMBRANE_TRANSPORTER_ACTIVITY2	-0.03125766	0.010363233	-0.21228973	0.83712736	0.96188496	-5.972357
homo after UVB irradiation VS homo before UVB irradiation	GO_ACTIVIN_ACTIVATED_RECEPTOR_ACTIVITY	0.174162011	-0.06496244	0.800063269	0.44589039	0.62818078	-6.21265691
hetero after UVB irradiation VS hetero before UVB irradiation	GO_ACTIVIN_ACTIVATED_RECEPTOR_ACTIVITY1	0.273838712	-0.0196577	1.632038823	0.14202433	0.45755083	-4.99524912
WT after UVB irradiation VS WT before UVB irradiation	GO_ACTIVIN_ACTIVATED_RECEPTOR_ACTIVITY2	-0.31736819	0.009895698	-1.75636301	0.1166395	0.62406566	-4.63180936
homo after UVB irradiation VS homo before UVB irradiation	GO_ACTIVIN_BINDING	0.257846722	-0.06652578	1.068809486	0.31514889	0.50894405	-5.96412198
hetero after UVB irradiation VS hetero before UVB irradiation	GO_ACTIVIN_BINDING1	0.281195323	0.00445325	1.664126482	0.13536433	0.44725394	-4.95318441
WT after UVB irradiation VS WT before UVB irradiation	GO_ACTIVIN_BINDING2	-0.12593308	-0.05388971	-0.782362	0.45626816	0.83880233	-5.68542437
homo after UVB irradiation VS homo before UVB irradiation	GO_ACTIVIN_RECEPTOR_ACTIVITY_TYPE_I	0.157476071	-0.05949002	0.786749866	0.45322928	0.6344436	-6.22342025
hetero after UVB irradiation VS hetero before UVB irradiation	GO_ACTIVIN_RECEPTOR_ACTIVITY_TYPE_I1	0.663338119	-0.02853044	2.815682385	0.02306795	0.21672816	-3.3084097
WT after UVB irradiation VS WT before UVB irradiation	GO_ACTIVIN_RECEPTOR_ACTIVITY_TYPE_I2	-0.21322645	-0.10312023	-1.17364645	0.27389831	0.74597065	-5.32820189
homo after UVB irradiation VS homo before UVB irradiation	GO_ACTIVIN_RECEPTOR_BINDING	0.123296735	0.023691935	0.889559876	0.39866465	0.58748694	-6.1363627
hetero after UVB irradiation VS hetero before UVB irradiation	GO_ACTIVIN_RECEPTOR_BINDING1	-0.01225436	0.052735842	-0.06476106	0.94998248	0.98092529	-6.2292103
WT after UVB irradiation VS WT before UVB irradiation	GO_ACTIVIN_RECEPTOR_BINDING2	-0.14060842	0.097695223	-0.71226712	0.49630595	0.85524978	-5.73691493
homo after UVB irradiation VS homo before UVB irradiation	GO_ACTIVIN_RECEPTOR_COMPLEX	0.022979781	-0.0785878	0.086704781	0.93295442	0.96403461	-6.54673274
hetero after UVB irradiation VS hetero before UVB irradiation	GO_ACTIVIN_RECEPTOR_COMPLEX1	0.407955111	-0.01583165	2.128220707	0.06661515	0.33582955	-4.31150405
WT after UVB irradiation VS WT before UVB irradiation	GO_ACTIVIN_RECEPTOR_COMPLEX2	-0.54777381	0.016637765	-2.77993881	0.02364826	0.41944387	-3.204208
homo after UVB irradiation VS homo before UVB irradiation	GO_ACTIVIN_RECEPTOR_SIGNALING_PATHWAY	-0.06460892	-0.00305848	-0.39009408	0.70625351	0.82480081	-6.46810959
hetero after UVB irradiation VS hetero before UVB irradiation	GO_ACTIVIN_RECEPTOR_SIGNALING_PATHWAY1	-0.07488979	0.059483603	-0.46567543	0.65409041	0.8581629	-6.11553751
WT after UVB irradiation VS WT before UVB irradiation	GO_ACTIVIN_RECEPTOR_SIGNALING_PATHWAY2	-0.14827668	0.067439816	-0.92214489	0.38310535	0.80533983	-5.57073906
homo after UVB irradiation VS homo before UVB irradiation	GO_ACTOMYOSIN	0.366135632	-0.00801851	1.850856608	0.09987562	0.25022926	-4.98213144
homo after UVB irradiation VS homo before UVB irradiation	GO_ACTOMYOSIN_CONTRACTILE_RING_ORGANIZATION	-0.64309737	-0.02028738	-2.62900091	0.02921147	0.12139501	-3.80925652
hetero after UVB irradiation VS hetero before UVB irradiation	GO_ACTOMYOSIN_CONTRACTILE_RING_ORGANIZATION1	0.264321078	-0.03532187	1.021997084	0.33723211	0.65709719	-5.70099465
WT after UVB irradiation VS WT before UVB irradiation	GO_ACTOMYOSIN_CONTRACTILE_RING_ORGANIZATION2	0.301748578	-0.01437732	0.983919772	0.35363598	0.78942569	-5.51525883
homo after UVB irradiation VS homo before UVB irradiation	GO_ACTOMYOSIN_STRUCTURE_ORGANIZATION	0.098529512	-0.02517013	0.702596099	0.50146814	0.6753208	-6.28781479
hetero after UVB irradiation VS hetero before UVB irradiation	GO_ACTOMYOSIN_STRUCTURE_ORGANIZATION1	0.040961244	-0.01139492	0.249114547	0.80966406	0.92615526	-6.19796681
WT after UVB irradiation VS WT before UVB irradiation	GO_ACTOMYOSIN_STRUCTURE_ORGANIZATION2	0.021171074	-0.0449989	0.11837031	0.90865795	0.97832631	-5.98868025
hetero after UVB irradiation VS hetero before UVB irradiation	GO_ACTOMYOSIN1	0.298141194	-0.02119754	1.89868089	0.09484805	0.38472677	-4.63555179
WT after UVB irradiation VS WT before UVB irradiation	GO_ACTOMYOSIN2	0.204724196	-0.02605031	1.098662955	0.30350432	0.76462443	-5.40499187
homo after UVB irradiation VS homo before UVB irradiation	GO_ACUTE_INFLAMMATORY_RESPONSE	0.589525942	0.002699092	4.622907169	0.00153197	0.02656559	-0.85378704
homo after UVB irradiation VS homo before UVB irradiation	GO_ACUTE_INFLAMMATORY_RESPONSE_TO_ANTIGENIC_STIMULUS	0.619320713	-0.02431457	3.122220329	0.01348937	0.07868088	-3.04422451
hetero after UVB irradiation VS hetero before UVB irradiation	GO_ACUTE_INFLAMMATORY_RESPONSE_TO_ANTIGENIC_STIMULUS1	0.295182204	0.058394463	1.296568082	0.23158062	0.56028918	-5.40861057
WT after UVB irradiation VS WT before UVB irradiation	GO_ACUTE_INFLAMMATORY_RESPONSE_TO_ANTIGENIC_STIMULUS2	0.440194878	0.016107603	1.972294047	0.08361037	0.5707311	-4.34230048
hetero after UVB irradiation VS hetero before UVB irradiation	GO_ACUTE_INFLAMMATORY_RESPONSE1	0.364723725	0.061975865	1.987052875	0.08281986	0.36857799	-4.51204745
WT after UVB irradiation VS WT before UVB irradiation	GO_ACUTE_INFLAMMATORY_RESPONSE2	0.271654038	0.012809941	1.211259844	0.25995626	0.74028841	-5.28836518
homo after UVB irradiation VS homo before UVB irradiation	GO_ACUTE_PHASE_RESPONSE	0.527903787	0.023895864	4.126716272	0.00303731	0.03639967	-1.54444022
hetero after UVB irradiation VS hetero before UVB irradiation	GO_ACUTE_PHASE_RESPONSE1	0.313032745	0.037030225	1.807706737	0.10897085	0.40749463	-4.76068222
WT after UVB irradiation VS WT before UVB irradiation	GO_ACUTE_PHASE_RESPONSE2	0.239491041	0.004969178	0.950334385	0.36944031	0.79984535	-5.54577232
homo after UVB irradiation VS homo before UVB irradiation	GO_ACYL_CARNITINE_TRANSPORT	-0.20783047	0.025773471	-0.78780736	0.4526434	0.63418122	-6.22257097
hetero after UVB irradiation VS hetero before UVB irradiation	GO_ACYL_CARNITINE_TRANSPORT1	-0.41268201	0.036407261	-1.31115171	0.2268459	0.55603622	-5.39179501
WT after UVB irradiation VS WT before UVB irradiation	GO_ACYL_CARNITINE_TRANSPORT2	-0.48987067	-0.0420417	-2.57036468	0.03278606	0.45936878	-3.50220895
homo after UVB irradiation VS homo before UVB irradiation	GO_ACYL_COA_BINDING	-0.15215642	-0.02295721	-0.83832132	0.42525183	0.6100954	-6.18087338
hetero after UVB irradiation VS hetero before UVB irradiation	GO_ACYL_COA_BINDING1	-0.43446982	-0.03979196	-3.03523116	0.01653771	0.18990694	-2.98837353
WT after UVB irradiation VS WT before UVB irradiation	GO_ACYL_COA_BINDING2	-0.26512904	-0.01132674	-1.29001988	0.23266174	0.72451161	-5.20226617
homo after UVB irradiation VS homo before UVB irradiation	GO_ACYL_COA_DEHYDROGENASE_ACTIVITY	0.244385411	-0.01876437	1.107102069	0.29919449	0.49354047	-5.924198
hetero after UVB irradiation VS hetero before UVB irradiation	GO_ACYL_COA_DEHYDROGENASE_ACTIVITY1	-0.49846705	-0.02689727	-2.16032239	0.06338895	0.33086924	-4.26543887
WT after UVB irradiation VS WT before UVB irradiation	GO_ACYL_COA_DEHYDROGENASE_ACTIVITY2	-0.16870237	0.039135277	-0.51815624	0.61820564	0.90020288	-5.8568371
homo after UVB irradiation VS homo before UVB irradiation	GO_ACYL_COA_LIGASE_ACTIVITY	-0.15901117	0.027422365	-0.74566945	0.47638099	0.65431673	-6.25564819
hetero after UVB irradiation VS hetero before UVB irradiation	GO_ACYL_COA_LIGASE_ACTIVITY1	-0.23343023	-0.00314795	-1.48391643	0.17682096	0.50051267	-5.18416046
WT after UVB irradiation VS WT before UVB irradiation	GO_ACYL_COA_LIGASE_ACTIVITY2	-0.26471807	0.051133926	-1.08608856	0.30871012	0.76668381	-5.41751367
homo after UVB irradiation VS homo before UVB irradiation	GO_ACYL_COA_OXIDASE_ACTIVITY	-0.22253977	-0.05731315	-0.74947804	0.47420258	0.65261022	-6.25272341
hetero after UVB irradiation VS hetero before UVB irradiation	GO_ACYL_COA_OXIDASE_ACTIVITY1	-0.31471314	-0.09904252	-1.01023843	0.34249281	0.66080334	-5.71239108
WT after UVB irradiation VS WT before UVB irradiation	GO_ACYL_COA_OXIDASE_ACTIVITY2	-0.49565754	-0.09267739	-1.85040196	0.10096638	0.60045927	-4.50717818
homo after UVB irradiation VS homo before UVB irradiation	GO_ACYLGLYCEROL_ACYL_CHAIN_REMODELING	-0.04917792	-0.04635129	-0.18109424	0.86062287	0.92341937	-6.53289412

hetero after UVB irradiation VS hetero before UVB irradiation	GO_ACYLGLYCEROL_ACYL_CHAIN_REMODELING1	-0.34033591	-0.06919839	-1.26041948	0.24368003	0.57159915	-5.44977474
WT after UVB irradiation VS WT before UVB irradiation	GO_ACYLGLYCEROL_ACYL_CHAIN_REMODELING2	0.063513575	-0.06970884	0.222305665	0.8295805	0.95961989	-5.97007435
homo after UVB irradiation VS homo before UVB irradiation	GO_ACYLGLYCEROL_HOMEOSTASIS	0.311827425	0.01112237	2.192415472	0.05838699	0.18104996	-4.47930741
hetero after UVB irradiation VS hetero before UVB irradiation	GO_ACYLGLYCEROL_HOMEOSTASIS1	-0.12872567	0.025559008	-0.77060118	0.46349493	0.75147819	-5.92148132
WT after UVB irradiation VS WT before UVB irradiation	GO_ACYLGLYCEROL_HOMEOSTASIS2	0.109677464	0.037109443	0.604850725	0.56184023	0.88048211	-5.80751872
homo after UVB irradiation VS homo before UVB irradiation	GO_ACYLGLYCEROL_LIPASE_ACTIVITY	0.0389655	0.001176999	0.245839266	0.81175645	0.89338519	-6.57180982
hetero after UVB irradiation VS hetero before UVB irradiation	GO_ACYLGLYCEROL_LIPASE_ACTIVITY1	-0.20941606	0.00778204	-0.80358409	0.44527851	0.73977264	-5.89546158
WT after UVB irradiation VS WT before UVB irradiation	GO_ACYLGLYCEROL_LIPASE_ACTIVITY2	-0.06926991	0.055823973	-0.34586837	0.73825585	0.93478445	-5.9334362
homo after UVB irradiation VS homo before UVB irradiation	GO_ACYLGLYCEROL_O_ACYLTRANSFERASE_ACTIVITY	0.459489384	-0.03784671	3.005082485	0.01617776	0.08690758	-3.22533949
hetero after UVB irradiation VS hetero before UVB irradiation	GO_ACYLGLYCEROL_O_ACYLTRANSFERASE_ACTIVITY1	0.247638745	0.004363755	1.576916349	0.15417181	0.47198468	-5.06660166
WT after UVB irradiation VS WT before UVB irradiation	GO_ACYLGLYCEROL_O_ACYLTRANSFERASE_ACTIVITY2	0.535901873	0.034454317	2.950242918	0.01817441	0.38810404	-2.96336007
homo after UVB irradiation VS homo before UVB irradiation	GO_ACYLGLYCEROL_TRANSPORT	0.066408486	0.020576145	0.356832218	0.73008894	0.84072747	-6.4815151
hetero after UVB irradiation VS hetero before UVB irradiation	GO_ACYLGLYCEROL_TRANSPORT1	-0.17599809	-0.03690504	-0.81311993	0.44010416	0.73710622	-5.88776755
WT after UVB irradiation VS WT before UVB irradiation	GO_ACYLGLYCEROL_TRANSPORT2	-0.91024382	-0.07163937	-4.71661165	0.00145971	0.20031801	-0.68843576
homo after UVB irradiation VS homo before UVB irradiation	GO_ADA2_GCN5_ADA3_TRANSCRIPTION_ACTIVATOR_COMPLEX	-0.4218426	-0.06174226	-2.3012605	0.04913168	0.16278316	-4.31419056
hetero after UVB irradiation VS hetero before UVB irradiation	GO_ADA2_GCN5_ADA3_TRANSCRIPTION_ACTIVATOR_COMPLEX1	-0.29047273	-0.09148032	-1.15108036	0.28354042	0.60906208	-5.5695555
WT after UVB irradiation VS WT before UVB irradiation	GO_ADA2_GCN5_ADA3_TRANSCRIPTION_ACTIVATOR_COMPLEX2	-0.20859509	-0.05489712	-0.72251601	0.49031474	0.85206058	-5.72964757
homo after UVB irradiation VS homo before UVB irradiation	GO_ADAPTATION_OF_SIGNALING_PATHWAY	0.754193077	-0.00419188	5.599082422	0.00044226	0.01560266	0.392211819
hetero after UVB irradiation VS hetero before UVB irradiation	GO_ADAPTATION_OF_SIGNALING_PATHWAY1	0.511555567	0.034678889	3.301321792	0.01112383	0.16496673	-2.60567683
WT after UVB irradiation VS WT before UVB irradiation	GO_ADAPTATION_OF_SIGNALING_PATHWAY2	0.545317383	0.044751303	2.639717454	0.0294184	0.44931137	-3.40350433
homo after UVB irradiation VS homo before UVB irradiation	GO_ADAPTIVE_IMMUNE_RESPONSE	0.390515799	-0.01599244	2.412630093	0.04116924	0.14618249	-4.14363913
homo after UVB irradiation VS homo before UVB irradiation	TIVE_IMMUNE_RESPONSE_BASED_ON_SOMATIC_RECOMBINATION_OF_IMMUNE_RECEPTORS_BUILT_FROM_IMMUNOGLOBULIN_SUPERFAMILY_	0.404520323	-0.01185632	2.746983661	0.02424435	0.1093775	-3.62604626
hetero after UVB irradiation VS hetero before UVB irradiation	TIVE_IMMUNE_RESPONSE_BASED_ON_SOMATIC_RECOMBINATION_OF_IMMUNE_RECEPTORS_BUILT_FROM_IMMUNOGLOBULIN_SUPERFAMILY_	0.285690195	0.029442821	1.84744678	0.10257147	0.39756884	-4.70629387
WT after UVB irradiation VS WT before UVB irradiation	TIVE_IMMUNE_RESPONSE_BASED_ON_SOMATIC_RECOMBINATION_OF_IMMUNE_RECEPTORS_BUILT_FROM_IMMUNOGLOBULIN_SUPERFAMILY_	0.294231339	-0.00625741	1.446705267	0.18555356	0.68726847	-5.02113201
hetero after UVB irradiation VS hetero before UVB irradiation	GO_ADAPTIVE_IMMUNE_RESPONSE1	0.299552076	0.029528974	1.824269752	0.10625895	0.40364873	-4.73806751
WT after UVB irradiation VS WT before UVB irradiation	GO_ADAPTIVE_IMMUNE_RESPONSE2	0.280716323	-0.01078803	1.260514527	0.24259108	0.73248003	-5.23493436
homo after UVB irradiation VS homo before UVB irradiation	GO_ADAPTIVE_THERMOGENESIS	0.17818356	-0.02281849	1.471274332	0.17795898	0.35977589	-5.49742822
hetero after UVB irradiation VS hetero before UVB irradiation	GO_ADAPTIVE_THERMOGENESIS1	0.015854937	-0.00930029	0.099912303	0.92291753	0.97050086	-6.2260741
WT after UVB irradiation VS WT before UVB irradiation	GO_ADAPTIVE_THERMOGENESIS2	-0.01391825	-0.01269454	-0.0724669	0.94398833	0.98531933	-5.99329669
homo after UVB irradiation VS homo before UVB irradiation	GO_ADENOHYPOPHYSIS_DEVELOPMENT	-0.22120373	0.089302983	-1.52875874	0.16336633	0.3410379	-5.42331556
hetero after UVB irradiation VS hetero before UVB irradiation	GO_ADENOHYPOPHYSIS_DEVELOPMENT1	0.233861114	0.175475183	1.203518039	0.26380019	0.59036447	-5.51302412
WT after UVB irradiation VS WT before UVB irradiation	GO_ADENOHYPOPHYSIS_DEVELOPMENT2	-0.05506807	0.121701154	-0.26351217	0.79873742	0.95209758	-5.959593
homo after UVB irradiation VS homo before UVB irradiation	GO_ADENOSINE_DEAMINASE_ACTIVITY	-0.09275957	0.014819057	-0.44431898	0.66813012	0.79938351	-6.44379821
hetero after UVB irradiation VS hetero before UVB irradiation	GO_ADENOSINE_DEAMINASE_ACTIVITY1	-0.19237586	0.068085906	-0.73247208	0.48516601	0.76337374	-5.95039597
WT after UVB irradiation VS WT before UVB irradiation	GO_ADENOSINE_DEAMINASE_ACTIVITY2	0.003203206	0.002962594	0.017390865	0.98654567	0.99704551	-5.99590709
homo after UVB irradiation VS homo before UVB irradiation	GO_ADENOSINE_METABOLIC_PROCESS	-0.1198823	-0.02520449	-0.91977454	0.38355403	0.57334956	-6.10909799
hetero after UVB irradiation VS hetero before UVB irradiation	GO_ADENOSINE_METABOLIC_PROCESS1	-0.04440119	0.015183179	-0.27276415	0.79206995	0.91891235	-6.19133336
WT after UVB irradiation VS WT before UVB irradiation	GO_ADENOSINE_METABOLIC_PROCESS2	-0.19363608	-0.1271143	-0.82282705	0.4341664	0.82957545	-5.65383076
homo after UVB irradiation VS homo before UVB irradiation	GO_ADENOSINE_RECEPTOR_BINDING	0.46395991	-0.00220734	2.347709421	0.0456394	0.1555788	-4.24323021
hetero after UVB irradiation VS hetero before UVB irradiation	GO_ADENOSINE_RECEPTOR_BINDING1	0.571490158	0.062421995	1.840154964	0.10371828	0.39980851	-4.71630615
WT after UVB irradiation VS WT before UVB irradiation	GO_ADENOSINE_RECEPTOR_BINDING2	0.027081724	0.169056177	0.062549223	0.95164187	0.98676072	-5.99400281
homo after UVB irradiation VS homo before UVB irradiation	GO_ADENOSINE_TO_INOSINE_EDITING	-0.22126498	0.092970777	-1.07088465	0.31426754	0.5081795	-5.96198538
hetero after UVB irradiation VS hetero before UVB irradiation	GO_ADENOSINE_TO_INOSINE_EDITING1	-0.4433723	0.016459608	-1.97667523	0.08415209	0.37033946	-4.52664049
WT after UVB irradiation VS WT before UVB irradiation	GO_ADENOSINE_TO_INOSINE_EDITING2	-0.17841079	-0.02147229	-1.09571125	0.30472004	0.76513806	-5.40794061
homo after UVB irradiation VS homo before UVB irradiation	GO_ADENYL_NUCLEOTIDE_BINDING	-0.13361719	-0.00783955	-1.09472956	0.30427821	0.49841642	-5.93721198
hetero after UVB irradiation VS hetero before UVB irradiation	GO_ADENYL_NUCLEOTIDE_BINDING1	-0.03427024	-0.01997542	-0.24639131	0.81169768	0.92705695	-6.1986927
WT after UVB irradiation VS WT before UVB irradiation	GO_ADENYL_NUCLEOTIDE_BINDING2	-0.02072392	-0.01422369	-0.13413511	0.89656967	0.97390508	-5.98658423
homo after UVB irradiation VS homo before UVB irradiation	GO_ADENYL_NUCLEOTIDE_EXCHANGE_FACTOR_ACTIVITY	-0.36369524	0.042383159	-2.48865521	0.03648889	0.13689581	-4.02650769
hetero after UVB irradiation VS hetero before UVB irradiation	GO_ADENYL_NUCLEOTIDE_EXCHANGE_FACTOR_ACTIVITY1	-0.17360808	-0.0433548	-0.86807554	0.41109571	0.71492968	-5.84195944
WT after UVB irradiation VS WT before UVB irradiation	GO_ADENYL_NUCLEOTIDE_EXCHANGE_FACTOR_ACTIVITY2	-0.36240786	-0.0268843	-1.56876279	0.15488727	0.65981549	-4.87207493
homo after UVB irradiation VS homo before UVB irradiation	GO_ADENYLATE_CYCLASE_ACTIVATING_ADRENERGIC_RECEPTOR_SIGNALING_PATHWAY_INVOLVED_IN_HEART_PROCESS	0.749373122	0.063123072	3.759187846	0.00515997	0.04674385	-2.07926906
hetero after UVB irradiation VS hetero before UVB irradiation	GO_ADENYLATE_CYCLASE_ACTIVATING_ADRENERGIC_RECEPTOR_SIGNALING_PATHWAY_INVOLVED_IN_HEART_PROCESS1	0.460638689	-0.00031975	1.674073434	0.13335912	0.44392017	-4.94006904
WT after UVB irradiation VS WT before UVB irradiation	GO_ADENYLATE_CYCLASE_ACTIVATING_ADRENERGIC_RECEPTOR_SIGNALING_PATHWAY_INVOLVED_IN_HEART_PROCESS2	0.129303551	0.04342082	0.536208749	0.60622626	0.89704906	-5.84714738
homo after UVB irradiation VS homo before UVB irradiation	GO_ADENYLATE_CYCLASE_ACTIVATING_DOPAMINE_RECEPTOR_SIGNALING_PATHWAY	0.321667049	0.034205815	1.275626976	0.23651065	0.42871731	-5.73665716
hetero after UVB irradiation VS hetero before UVB irradiation	GO_ADENYLATE_CYCLASE_ACTIVATING_DOPAMINE_RECEPTOR_SIGNALING_PATHWAY1	0.247877899	0.06081221	1.765570972	0.11616775	0.41924211	-4.81785281
WT after UVB irradiation VS WT before UVB irradiation	GO_ADENYLATE_CYCLASE_ACTIVATING_DOPAMINE_RECEPTOR_SIGNALING_PATHWAY2	0.418722464	-0.07475418	1.725181044	0.12232157	0.63115364	-4.67256682
homo after UVB irradiation VS homo before UVB irradiation	GO_ADENYLATE_CYCLASE_ACTIVATING_G_PROTEIN_COUPLED_RECEPTOR_SIGNALING_PATHWAY	0.457488866	-0.00487692	3.573667974	0.00679078	0.05427157	-2.35594802
hetero after UVB irradiation VS hetero before UVB irradiation	GO_ADENYLATE_CYCLASE_ACTIVATING_G_PROTEIN_COUPLED_RECEPTOR_SIGNALING_PATHWAY1	0.217092467	0.039924554	1.155206396	0.28194468	0.60762024	-5.56517045
WT after UVB irradiation VS WT before UVB irradiation	GO_ADENYLATE_CYCLASE_ACTIVATING_G_PROTEIN_COUPLED_RECEPTOR_SIGNALING_PATHWAY2	0.125129658	0.00774375	0.619936493	0.55234088	0.87634603	-5.79822711
homo after UVB irradiation VS homo before UVB irradiation	GO_ADENYLATE_CYCLASE_ACTIVATOR_ACTIVITY	1.081307713	-0.03414045	5.844486077	0.00033041	0.01344886	0.682024054
hetero after UVB irradiation VS hetero before UVB irradiation	GO_ADENYLATE_CYCLASE_ACTIVATOR_ACTIVITY1	0.832411212	0.044656824	4.384114832	0.00244261	0.10988755	-1.14577263
WT after UVB irradiation VS WT before UVB irradiation	GO_ADENYLATE_CYCLASE_ACTIVATOR_ACTIVITY2	0.821748582	0.043918425	2.353198443	0.04608041	0.50534441	-3.81075146
homo after UVB irradiation VS homo before UVB irradiation	GO_ADENYLATE_CYCLASE_ACTIVITY	0.323321198	-0.05413826	1.13161573	0.28932122	0.48410448	-5.8980963
hetero after UVB irradiation VS hetero before UVB irradiation	GO_ADENYLATE_CYCLASE_ACTIVITY1	0.078574089	0.055800206	0.395059439	0.70331299	0.88183935	-6.14770656
WT after UVB irradiation VS WT before UVB irradiation	GO_ADENYLATE_CYCLASE_ACTIVITY2	0.198845191	-0.01197142	0.745087931	0.47728461	0.84910837	-5.71332423
homo after UVB irradiation VS homo before UVB irradiation	GO_ADENYLATE_CYCLASE_BINDING	0.602087706	-0.03987776	3.272740132	0.01070203	0.06927216	-2.81276038
hetero after UVB irradiation VS hetero before UVB irradiation	GO_ADENYLATE_CYCLASE_BINDING1	0.129850043	-0.01891992	0.509322538	0.62452629	0.8448012	-6.09316404
WT after UVB irradiation VS WT before UVB irradiation	GO_ADENYLATE_CYCLASE_BINDING2	0.273764433	-0.01419121	1.016988791	0.33857827	0.78064003	-5.48441815
homo after UVB irradiation VS homo before UVB irradiation	GO_ADENYLATE_CYCLASE_INHIBITING_ADRENERGIC_RECEPTOR_SIGNALING_PATHWAY	0.165605228	0.001126645	0.432722405	0.67620293	0.80475159	-6.44925159
hetero after UVB irradiation VS hetero before UVB irradiation	GO_ADENYLATE_CYCLASE_INHIBITING_ADRENERGIC_RECEPTOR_SIGNALING_PATHWAY1	-0.00730587	0.013719778	-0.01916475	0.98518767	0.99448825	-6.23128493
WT after UVB irradiation VS WT before UVB irradiation	GO_ADENYLATE_CYCLASE_INHIBITING_ADRENERGIC_RECEPTOR_SIGNALING_PATHWAY2	0.099668262	-0.11502272	0.26694113	0.79618683	0.95157276	-5.95864202
homo after UVB irradiation VS homo before UVB irradiation	GO_ADENYLATE_CYCLASE_INHIBITING_DOPAMINE_RECEPTOR_SIGNALING_PATHWAY	-0.33408779	0.022001853	-1.81158361	0.10615166	0.2599967	-5.03796189
hetero after UVB irradiation VS hetero before UVB irradiation	GO_ADENYLATE_CYCLASE_INHIBITING_DOPAMINE_RECEPTOR_SIGNALING_PATHWAY1	0.049760971	0.054610751	0.223905562	0.8285461	0.93461067	-6.20438588
WT after UVB irradiation VS WT before UVB irradiation	GO_ADENYLATE_CYCLASE_INHIBITING_DOPAMINE_RECEPTOR_SIGNALING_PATHWAY2	0.101893474	0.000767873	0.394192585	0.70361667	0.92570885	-5.91490006
homo after UVB irradiation VS homo before UVB irradiation	GO_ADENYLATE_CYCLASE_INHIBITING_G_PROTEIN_COUPLED_ACETYLCHOLINE_RECEPTOR_SIGNALING_PATHWAY	0.119147196	-0.00211157	0.796216198	0.44800276	0.62979726	-6.21578299
hetero after UVB irradiation VS hetero before UVB irradiation	GO_ADENYLATE_CYCLASE_INHIBITING_G_PROTEIN_COUPLED_ACETYLCHOLINE_RECEPTOR_SIGNALING_PATHWAY1	-0.12685757	0.041638777	-0.5943508	0.569001	0.81359633	-6.04425449
WT after UVB irradiation VS WT before UVB irradiation	GO_ADENYLATE_CYCLASE_INHIBITING_G_PROTEIN_COUPLED_ACETYLCHOLINE_RECEPTOR_SIGNALING_PATHWAY2	-0.27803026	-0.00840689	-1.39901316	0.19892592	0.69740523	-5.0775621
homo after UVB irradiation VS homo before UVB irradiation	GO_ADENYLATE_CYCLASE_INHIBITING_G_PROTEIN_COUPLED_GLUTAMATE_RECEPTOR_SIGNALING_PATHWAY	-0.33844689	0.033874185	-1.70373837	0.1253384	0.28900294	-5.1885909
hetero after UVB irradiation VS hetero before UVB irradiation	GO_ADENYLATE_CYCLASE_INHIBITING_G_PROTEIN_COUPLED_GLUTAMATE_RECEPTOR_SIGNALING_PATHWAY1	-0.29968163	0.048593364	-1.53896374	0.16307779	0.48457024	-5.11502214
WT after UVB irradiation VS WT before UVB irradiation	GO_ADENYLATE_CYCLASE_INHIBITING_G_PROTEIN_COUPLED_GLUTAMATE_RECEPTOR_SIGNALING_PATHWAY2	-0.27879879	0.17693584	-1.64002836	0.13917346	0.64810012	-4.78225854
homo after UVB irradiation VS homo before UVB irradiation	GO_ADENYLATE_CYCLASE_INHIBITING_G_PROTEIN_COUPLED_RECEPTOR_SIGNALING_PATHWAY	0.172819043	-0.01629418	1.308190644	0.22575366	0.41650725	-5.69834279
hetero after UVB irradiation VS hetero before UVB irradiation	GO_ADENYLATE_CYCLASE_INHIBITING_G_PROTEIN_COUPLED_RECEPTOR_SIGNALING_PATHWAY1	0.046941442	0.039904734	0.375281144	0.71738493	0.88933311	-6.15581007
WT after UVB irradiation VS WT before UVB irradiation	GO_ADENYLATE_CYCLASE_INHIBITING_G_PROTEIN_COUPLED_RECEPTOR_SIGNALING_PATHWAY2	0.06625189	0.038909832	0.342350298	0.7408038	0.93520732	-5.93469422
homo after UVB irradiation VS homo before UVB irradiation	GO_ADENYLATE_CYCLASE_INHIBITING_SEROTONIN_RECEPTOR_SIGNALING_PATHWAY	0.590926747	0.022958537	3.608055633	0.0064515	0.05315736	-2.30435296
hetero after UVB irradiation VS hetero before UVB irradiation	GO_ADENYLATE_CYCLASE_INHIBITING_SEROTONIN_RECEPTOR_SIGNALING_PATHWAY1	0.086481657	0.118171381	0.488408759	0.6386062	0.85182425	-6.10411911
WT after UVB irradiation VS WT before UVB irradiation	GO_ADENYLATE_CYCLASE_INHIBITING_SEROTONIN_RECEPTOR_SIGNALING_PATHWAY2	0.392570272	0.065290746	1.70231323	0.12665127	0.63366527	-4.70226241
homo after UVB irradiation VS homo before UVB irradiation	GO_ADENYLATE_KINASE_ACTIVITY	0.544550933	-0.01137988	3.323217903	0.00990828	0.06646202	-2.73553124
hetero after UVB irradiation VS hetero before UVB irradiation	GO_ADENYLATE_KINASE_ACTIVITY1	0.323803533	0.050917504	2.51252692	0.03675059	0.26299308	-3.75301488
WT after UVB irradiation VS WT before UVB irradiation	GO_ADENYLATE_KINASE_ACTIVITY2	0.279917069	0.035360545	1.525719861	0.16514017	0.66947139	-4.92536132
homo after UVB irradiation VS homo before UVB irradiation	GO_ADENYLYLTRANSFERASE_ACTIVITY	-0.09707235	-0.00608919	-0.56920712	0.58423478	0.73802026	-6.37647915
hetero after UVB irradiation VS hetero before UVB irradiation	GO_ADENYLYLTRANSFERASE_ACTIVITY1	-0.05219945	0.023663075	-0.30848108	0.76574086	0.90794023	-6.18019968
WT after UVB irradiation VS WT before UVB irradiation	GO_ADENYLYLTRANSFERASE_ACTIVITY2	-0.14801037	-0.00962891	-0.78186923	0.45654191	0.83880233	-5.6858008
homo after UVB irradiation VS homo before UVB irradiation	GO_ADHERENS_JUNCTION	0.070453203	0.009757306	0.414792765	0.68877168	0.81391709	-6.45741168
hetero after UVB irradiation VS hetero before UVB irradiation	GO_ADHERENS_JUNCTION_ASSEMBLY	0.055902915	0.021873612	0.259832373	0.80130063	0.88721439	-6.5139566
hetero after UVB irradiation VS hetero before UVB irradiation	GO_ADHERENS_JUNCTION_ASSEMBLY1	0.167436158	-0.00502354	1.005557227	0.34460458	0.66219367	-5.71690019
WT after UVB irradiation VS WT before UVB irradiation	GO_ADHERENS_JUNCTION_ASSEMBLY2	0.206604044	-0.0304552	1.121856277	0.29408524	0.75745298	-5.38162357
homo after UVB irradiation VS homo before UVB irradiation	GO_ADHERENS_JUNCTION_MAINTENANCE	-0.07641139	-0.03769129	-0.24363257	0.81340903	0.89464687	-6.51839829
hetero after UVB irradiation VS hetero before UVB irradiation	GO_ADHERENS_JUNCTION_MAINTENANCE1	0.077216486	-0.01102997	0.351850598	0.73420529	0.89615016	-6.16489053
WT after UVB irradiation VS WT before UVB irradiation	GO_ADHERENS_JUNCTION_MAINTENANCE2	-0.24080521	-0.00240176	-0.61054109	0.55824601	0.87964603	-5.80403828
homo after UVB irradiation VS homo before UVB irradiation	GO_ADHERENS_JUNCTION_ORGANIZATION	0.031322718	0.018442928	0.166578584	0.87167464	0.92946038	-6.53565362
hetero after UVB irradiation VS hetero before UVB irradiation	GO_ADHERENS_JUNCTION_ORGANIZATION1	0.022579295	0.009814019	0.148437519	0.88573835	0.95858053	-6.21955242
WT after UVB irradiation VS WT before UVB irradiation	GO_ADHERENS_JUNCTION_ORGANIZATION2	-0.12814943	0.006697922	-0.73092976	0.48543115	0.85147728	-5.72361394
hetero after UVB irradiation VS hetero before UVB irradiation	GO_ADHERENS_JUNCTION1	0.02981577	-0.01562855	0.20373508	0.84374105	0.94020865	-6.20903552
WT after UVB irradiation VS WT before UVB irradiation	GO_ADHERENS_JUNCTION2	-0.11176565	0.013909795	-0.69245746	0.50801747	0.85795535	-5.75070366
homo after UVB irradiation VS homo before UVB irradiation	GO_ADHESION_OF_SYMBIONT_TO_HOST	0.02352454	0.025570021	0.09210738	0.92878962	0.96136393	-6.54620307
homo after UVB irradiation VS homo before UVB irradiation	GO_ADHESION_OF_SYMBIONT_TO_HOST_CELL	-0.14442299	0.031844588	-0.56314021	0.58817393	0.74102985	-6.38010695
hetero after UVB irradiation VS hetero before UVB irradiation	GO_ADHESION_OF_SYMBIONT_TO_HOST_CELL1	0.202271148	-0.00849364	1.158195581	0.28079319	0.60652504	-5.5619868
WT after UVB irradiation VS WT before UVB irradiation	GO_ADHESION_OF_SYMBIONT_TO_HOST_CELL2	-0.03546791	-0.02827373	-0.19010124	0.85390895	0.9663792	-5.97704318
hetero after UVB irradiation VS hetero before UVB irradiation	GO_ADHESION_OF_SYMBIONT_TO_HOST1	0.269783847	0.016870774	1.698457102	0.12855951	0.43612669	-4.90777181
WT after UVB irradiation VS WT before UVB irradiation	GO_ADHESION_OF_SYMBIONT_TO_HOST2	0.067305504	-0.03410586	0.444974552	0.66799237	0.91409156	-5.89292369
homo after UVB irradiation VS homo before UVB irradiation	GO_ADIPONECTIN_ACTIVATED_SIGNALING_PATHWAY	0.3219161	-0.07141016	1.462242456	0.1803538	0.36304475	-5.50892508
hetero after UVB irradiation VS hetero before UVB irradiation	GO_ADIPONECTIN_ACTIVATED_SIGNALING_PATHWAY1	0.200275211	-0.01327308	1.079481632	0.31241012	0.63316074	-5.64386691
WT after UVB irradiation VS WT before UVB irradiation	GO_ADIPONECTIN_ACTIVATED_SIGNALING_PATHWAY2	0.026690241	-0.00941431	0.093150673	0.92804737	0.98222728	-5.99149082
homo after UVB irradiation VS homo before UVB irradiation	GO_ADIPOSE_TISSUE_DEVELOPMENT	-0.03467532	0.002206343	-0.25310002	0.806326	0.89007116	-6.51583666
hetero after UVB irradiation VS hetero before UVB irradiation	GO_ADIPOSE_TISSUE_DEVELOPMENT1	-0.18254971	0.001166433	-1.30333936	0.22937188	0.55747992	-5.40081768
WT after UVB irradiation VS WT before UVB irradiation	GO_ADIPOSE_TISSUE_DEVELOPMENT2	-0.06535268	-0.03747116	-0.38213012	0.71219849	0.92925846	-5.91974542
homo after UVB irradiation VS homo before UVB irradiation	GO_ADP_BINDING	0.062241013	-0.00704384	0.379267857	0.71397562	0.8299068	-6.47259947
hetero after UVB irradiation VS hetero before UVB irradiation	GO_ADP_BINDING1	0.09402199	0.02545058	0.527429605	0.61246723	0.83896555	-6.08333323
WT after UVB irradiation VS WT before UVB irradiation	GO_ADP_BINDING2	0.110304146	-0.00720201	0.515069042	0.6202666	0.90087565	-5.85846323
homo after UVB irradiation VS homo before UVB irradiation	GO_ADP_BIOSYNTHETIC_PROCESS	0.824473703	-0.01384809	5.584881944	0.0004499	0.01573263	0.37515894
hetero after UVB irradiation VS hetero before UVB irradiation	GO_ADP_BIOSYNTHETIC_PROCESS1	0.354929751	0.066660961	1.439129363	0.18875608	0.51443012	-5.23940255
WT after UVB irradiation VS WT before UVB irradiation	GO_ADP_BIOSYNTHETIC_PROCESS2	0.162278584	-0.06284978	0.683349035	0.51346011	0.85974355	-5.75692871
homo after UVB irradiation VS homo before UVB irradiation	GO_ADP_RIBOSE_DIPHOSPHATASE_ACTIVITY	0.438860968	-0.14160819	1.700933204	0.12587798	0.28974908	-5.19245269
hetero after UVB irradiation VS hetero before UVB irradiation	GO_ADP_RIBOSE_DIPHOSPHATASE_ACTIVITY1	0.635789668	-0.081749	3.298995012	0.01116207	0.16508899	-2.60899177
WT after UVB irradiation VS WT before UVB irradiation	GO_ADP_RIBOSE_DIPHOSPHATASE_ACTIVITY2	0.43617297	-0.06149788	1.872008318	0.09765794	0.59779444	-4.47820779
homo after UVB irradiation VS homo before UVB irradiation	GO_ADP_RIBOSYLATION_FACTOR_BINDING	-0.42677519	-0.03395271	-1.85468435	0.09928301	0.2492302	-4.97666398
hetero after UVB irradiation VS hetero before UVB irradiation	GO_ADP_RIBOSYLATION_FACTOR_BINDING1	-0.41933779	-0.06481186	-2.20677765	0.05899177	0.32049595	-4.19852435
WT after UVB irradiation VS WT before UVB irradiation	GO_ADP_RIBOSYLATION_FACTOR_BINDING2	-0.41934711	-0.07480941	-1.96001513	0.08521998	0.57584638	-4.3590597
homo after UVB irradiation VS homo before UVB irradiation	GO_ADRENAL_GLAND_DEVELOPMENT	0.172490432	0.015574606	1.144299628	0.28431575	0.47933823	-5.88442745
hetero after UVB irradiation VS hetero before UVB irradiation	GO_ADRENAL_GLAND_DEVELOPMENT1	-0.13683732	0.035135667	-0.71345629	0.49621669	0.76978275	-5.96434111
WT after UVB irradiation VS WT before UVB irradiation	GO_ADRENAL_GLAND_DEVELOPMENT2	0.01644887	-0.01938304	0.089653957	0.93074007	0.98303258	-5.99182773
homo after UVB irradiation VS homo before UVB irradiation	GO_ADRENERGIC_RECEPTOR_ACTIVITY	0.292279419	-0.05951494	1.358030327	0.21009476	0.40035748	-5.63849596
hetero after UVB irradiation VS hetero before UVB irradiation	GO_ADRENERGIC_RECEPTOR_ACTIVITY1	-0.35960714	-0.0308612	-1.53797197	0.16331664	0.48473598	-5.11627941
WT after UVB irradiation VS WT before UVB irradiation	GO_ADRENERGIC_RECEPTOR_ACTIVITY2	-0.07959043	-0.07310525	-0.23979226	0.81644987	0.95637428	-5.96584031
homo after UVB irradiation VS homo before UVB irradiation	GO_ADRENERGIC_RECEPTOR_BINDING	0.268916234	-0.01358605	1.76920065	0.11333942	0.27072675	-5.09764601
hetero after UVB irradiation VS hetero before UVB irradiation	GO_ADRENERGIC_RECEPTOR_BINDING1	-0.02759172	-0.03544141	-0.11822634	0.90885649	0.9660078	-6.22391101
WT after UVB irradiation VS WT before UVB irradiation	GO_ADRENERGIC_RECEPTOR_BINDING2	0.004200672	-0.05614534	0.025977912	0.97990375	0.99512555	-5.9957106
homo after UVB irradiation VS homo before UVB irradiation	GO_ADRENERGIC_RECEPTOR_SIGNALING_PATHWAY	0.241086081	-0.01558649	1.71294928	0.12358167	0.2865545	-5.17588955
hetero after UVB irradiation VS hetero before UVB irradiation	GO_ADRENERGIC_RECEPTOR_SIGNALING_PATHWAY1	-0.38838375	0.015165181	-2.25792078	0.05450017	0.30930942	-4.12455088
WT after UVB irradiation VS WT before UVB irradiation	GO_ADRENERGIC_RECEPTOR_SIGNALING_PATHWAY2	-0.19317426	0.00229652	-0.88819879	0.40004663	0.81573919	-5.60000527
homo after UVB irradiation VS homo before UVB irradiation	GO_ADULT_BEHAVIOR	0.004989894	0.018173604	0.034536562	0.97326264	0.98636336	-6.55020215
hetero after UVB irradiation VS hetero before UVB irradiation	GO_ADULT_BEHAVIOR1	-0.05652923	0.027957676	-0.43126819	0.67786796	0.87031213	-6.13183949
WT after UVB irradiation VS WT before UVB irradiation	GO_ADULT_BEHAVIOR2	-0.0179722	0.037632112	-0.1085662	0.91618861	0.97967628	-5.98985225
homo after UVB irradiation VS homo before UVB irradiation	GO_ADULT_FEEDING_BEHAVIOR	-0.14201232	0.093785366	-0.65633161	0.5293218	0.69693093	-6.32046909
hetero after UVB irradiation VS hetero before UVB irradiation	GO_ADULT_FEEDING_BEHAVIOR1	-0.21022218	0.063144348	-0.83576047	0.42798547	0.72883481	-5.86919631
WT after UVB irradiation VS WT before UVB irradiation	GO_ADULT_FEEDING_BEHAVIOR2	-0.2282647	0.079365137	-1.07666941	0.3126556	0.77011405	-5.42682462
homo after UVB irradiation VS homo before UVB irradiation	GO_ADULT_HEART_DEVELOPMENT	-0.26448968	0.084318778	-1.6560645	0.13480464	0.30281716	-5.25380754
hetero after UVB irradiation VS hetero before UVB irradiation	GO_ADULT_HEART_DEVELOPMENT1	-0.42010094	0.067787039	-2.67280361	0.02870918	0.23772875	-3.51785283
WT after UVB irradiation VS WT before UVB irradiation	GO_ADULT_HEART_DEVELOPMENT2	-0.3192598	-0.03495943	-1.19542512	0.26575288	0.74237377	-5.30523996
homo after UVB irradiation VS homo before UVB irradiation	GO_ADULT_LOCOMOTORY_BEHAVIOR	0.050209422	0.017204657	0.316194041	0.75963405	0.8608447	-6.49631957
hetero after UVB irradiation VS hetero before UVB irradiation	GO_ADULT_LOCOMOTORY_BEHAVIOR1	-0.02759838	0.022293449	-0.1963213	0.84934403	0.94258929	-6.2106355
WT after UVB irradiation VS WT before UVB irradiation	GO_ADULT_LOCOMOTORY_BEHAVIOR2	0.131916987	0.044884184	0.682802628	0.51378776	0.85974355	-5.75729983

homo after UVB irradiation VS homo before UVB irradiation	GO_AEROBIC_ELECTRON_TRANSPORT_CHAIN	-0.12806856	-0.00510034	-0.4964031	0.63244423	0.77298157	-6.41761953
hetero after UVB irradiation VS hetero before UVB irradiation	GO_AEROBIC_ELECTRON_TRANSPORT_CHAIN1	0.043884469	0.040304901	0.169869118	0.86940764	0.95080444	-6.21586529
WT after UVB irradiation VS WT before UVB irradiation	GO_AEROBIC_ELECTRON_TRANSPORT_CHAIN2	-0.0664228	-0.05210839	-0.26141944	0.80029533	0.9524928	-5.96016746
homo after UVB irradiation VS homo before UVB irradiation	GO_AEROBIC_RESPIRATION	-0.08579465	0.021100459	-0.39428995	0.70327027	0.82287974	-6.46633674
hetero after UVB irradiation VS hetero before UVB irradiation	GO_AEROBIC_RESPIRATION1	-0.17653971	-0.02752995	-0.96094455	0.36523085	0.67975709	-5.7590631
WT after UVB irradiation VS WT before UVB irradiation	GO_AEROBIC_RESPIRATION2	-0.06967706	-0.06208538	-0.31043963	0.76406667	0.94098943	-5.94553395
homo after UVB irradiation VS homo before UVB irradiation	GO_AGGREPHAGY	0.096973612	-0.00099212	0.465560852	0.65346117	0.78909035	-6.43345331
hetero after UVB irradiation VS hetero before UVB irradiation	GO_AGGREPHAGY1	0.335324133	0.044013789	1.691237384	0.12996363	0.43834133	-4.91735606
WT after UVB irradiation VS WT before UVB irradiation	GO_AGGREPHAGY2	-0.0600544	-0.01577501	-0.27309065	0.79161912	0.94920966	-5.95690631
homo after UVB irradiation VS homo before UVB irradiation	GO_AGGRESOME	-0.05260307	0.025645066	-0.30025888	0.77133746	0.86773177	-6.50164874
hetero after UVB irradiation VS hetero before UVB irradiation	GO_AGGRESOME_ASSEMBLY	0.248986412	-0.05992524	1.479736541	0.17574074	0.35675044	-5.48661948
WT after UVB irradiation VS WT before UVB irradiation	GO_AGGRESOME_ASSEMBLY1	0.090081113	0.01908274	0.473262485	0.64890227	0.8557563	-6.1117837
homo after UVB irradiation VS homo before UVB irradiation	GO_AGGRESOME_ASSEMBLY2	0.288109222	-0.0253153	1.40073469	0.19842894	0.69702831	-5.07554409
hetero after UVB irradiation VS hetero before UVB irradiation	GO_AGGRESOME1	0.123031733	-0.00722071	0.714286953	0.49573062	0.76966121	-5.96373863
WT after UVB irradiation VS WT before UVB irradiation	GO_AGGRESOME2	-0.1311505	-0.04325467	-0.87925131	0.40460045	0.81613006	-5.60757139
homo after UVB irradiation VS homo before UVB irradiation	GO_AGGRESSIVE_BEHAVIOR	-0.28792136	0.066489554	-1.46912861	0.17852537	0.36059388	-5.50016326
hetero after UVB irradiation VS hetero before UVB irradiation	GO_AGGRESSIVE_BEHAVIOR1	-0.38995818	0.057716175	-2.23620177	0.0563645	0.31374431	-4.15600169
WT after UVB irradiation VS WT before UVB irradiation	GO_AGGRESSIVE_BEHAVIOR2	-0.33380653	0.036032908	-1.39628151	0.19971672	0.69758436	-5.08076125
homo after UVB irradiation VS homo before UVB irradiation	GO_AGING	0.139459272	-0.01386632	1.112928938	0.2968237	0.49149769	-5.91803166
hetero after UVB irradiation VS hetero before UVB irradiation	GO_AGING1	0.179489836	-0.00297619	1.331644819	0.22033265	0.54746446	-5.36796795
WT after UVB irradiation VS WT before UVB irradiation	GO_AGING2	0.12163236	-0.0091728	0.857520554	0.41581386	0.8213754	-5.6256865
homo after UVB irradiation VS homo before UVB irradiation	GO_AIM2_INFLAMMASOME_COMPLEX	1.217320412	-0.02816965	8.172390333	2.96E-05	0.00847131	3.013940801
hetero after UVB irradiation VS hetero before UVB irradiation	GO_AIM2_INFLAMMASOME_COMPLEX1	1.097042842	0.014772059	5.95529597	0.00036539	0.07788488	0.628001292
WT after UVB irradiation VS WT before UVB irradiation	GO_AIM2_INFLAMMASOME_COMPLEX2	1.066718614	0.077732075	5.204102162	0.00078723	0.18842845	-0.15490116
homo after UVB irradiation VS homo before UVB irradiation	GO_ALANINE_TRANSMEMBRANE_TRANSPORTER_ACTIVITY	-0.27257921	-0.09687383	-1.36389187	0.20831577	0.39822021	-5.63136435
hetero after UVB irradiation VS hetero before UVB irradiation	GO_ALANINE_TRANSMEMBRANE_TRANSPORTER_ACTIVITY1	-0.48153404	-0.00461491	-2.34802583	0.04740094	0.29034796	-3.99357729
WT after UVB irradiation VS WT before UVB irradiation	GO_ALANINE_TRANSMEMBRANE_TRANSPORTER_ACTIVITY2	-0.03651113	0.027357225	-0.13730907	0.89413927	0.97322079	-5.98613072
homo after UVB irradiation VS homo before UVB irradiation	GO_ALANINE_TRANSPORT	-0.21364573	-0.09553412	-1.11429767	0.29626897	0.49074433	-5.91657973
hetero after UVB irradiation VS hetero before UVB irradiation	GO_ALANINE_TRANSPORT1	-0.37197542	-0.0031741	-2.27355607	0.05319621	0.30612069	-4.1018787
WT after UVB irradiation VS WT before UVB irradiation	GO_ALANINE_TRANSPORT2	-0.12856411	-0.00694318	-0.63356586	0.5438401	0.87202956	-5.78965484
homo after UVB irradiation VS homo before UVB irradiation	GO_ALCOHOL_BINDING	0.49081658	0.00077473	3.923036138	0.00406454	0.04159165	-1.83854029
hetero after UVB irradiation VS hetero before UVB irradiation	GO_ALCOHOL_BINDING1	0.191518145	0.032316003	1.289994474	0.23374218	0.56214284	-5.41615144
WT after UVB irradiation VS WT before UVB irradiation	GO_ALCOHOL_BINDING2	0.237352056	0.011321406	1.538926684	0.16193115	0.66526114	-4.90909194
homo after UVB irradiation VS homo before UVB irradiation	GO_ALCOHOL_BIOSYNTHETIC_PROCESS	0.107486544	-0.01866129	0.87449722	0.40635494	0.593377	-6.14967497
hetero after UVB irradiation VS hetero before UVB irradiation	GO_ALCOHOL_BIOSYNTHETIC_PROCESS1	0.132023796	0.005291915	0.873738653	0.40818527	0.71330228	-5.83709904
WT after UVB irradiation VS WT before UVB irradiation	GO_ALCOHOL_BIOSYNTHETIC_PROCESS2	0.145774972	0.015823373	0.81948338	0.43596434	0.83024038	-5.65649209
homo after UVB irradiation VS homo before UVB irradiation	GO_ALCOHOL_CATABOLIC_PROCESS	0.411318484	0.02219163	2.920096382	0.01847233	0.09338684	-3.35712345
hetero after UVB irradiation VS hetero before UVB irradiation	GO_ALCOHOL_CATABOLIC_PROCESS1	0.221287617	0.07279394	1.445487073	0.18701947	0.51246214	-5.23161797
WT after UVB irradiation VS WT before UVB irradiation	GO_ALCOHOL_CATABOLIC_PROCESS2	0.266999338	0.051491039	1.555578643	0.15796503	0.66180255	-4.88847646
homo after UVB irradiation VS homo before UVB irradiation	GO_ALCOHOL_DEHYDROGENASE_ACTIVITY_ZINC_DEPENDENT	0.272337695	-0.04474928	0.756117982	0.47042029	0.64933048	-6.24759331
hetero after UVB irradiation VS hetero before UVB irradiation	GO_ALCOHOL_DEHYDROGENASE_ACTIVITY_ZINC_DEPENDENT1	-0.06965017	0.019394995	-0.21823514	0.83281031	0.9354456	-6.20573679
WT after UVB irradiation VS WT before UVB irradiation	GO_ALCOHOL_DEHYDROGENASE_ACTIVITY_ZINC_DEPENDENT2	-0.19116175	-0.09175838	-1.0199324	0.33726203	0.78034487	-5.48163528
homo after UVB irradiation VS homo before UVB irradiation	GO_ALCOHOL_DEHYDROGENASE_NAD_P_PLUS_ACTIVITY	0.226652746	-0.0717292	1.082411805	0.30940686	0.50379753	-5.9500603
hetero after UVB irradiation VS hetero before UVB irradiation	GO_ALCOHOL_DEHYDROGENASE_NAD_P_PLUS_ACTIVITY1	0.011351527	-0.01149711	0.058982007	0.95443975	0.98300834	-6.22959792
WT after UVB irradiation VS WT before UVB irradiation	GO_ALCOHOL_DEHYDROGENASE_NAD_P_PLUS_ACTIVITY2	0.104909411	-0.04035424	0.507248539	0.62550336	0.90205599	-5.86254196
homo after UVB irradiation VS homo before UVB irradiation	GO_ALCOHOL_DEHYDROGENASE_NADPPLUS_ACTIVITY	0.418782175	0.022970405	3.075333595	0.0145049	0.0819836	-3.11663219
hetero after UVB irradiation VS hetero before UVB irradiation	GO_ALCOHOL_DEHYDROGENASE_NADPPLUS_ACTIVITY1	0.136607743	0.040478713	0.619369985	0.55321353	0.80394119	-6.02855252
WT after UVB irradiation VS WT before UVB irradiation	GO_ALCOHOL_DEHYDROGENASE_NADPPLUS_ACTIVITY2	0.213800217	0.028762931	0.864795958	0.41203542	0.82044248	-5.61966295
homo after UVB irradiation VS homo before UVB irradiation	GO_ALCOHOL_METABOLIC_PROCESS	0.286741924	-0.00715678	2.381354067	0.04326534	0.15047527	-4.19167329
hetero after UVB irradiation VS hetero before UVB irradiation	GO_ALCOHOL_METABOLIC_PROCESS1	0.143241209	0.011493074	0.909360122	0.39021683	0.69998178	-5.80594023
WT after UVB irradiation VS WT before UVB irradiation	GO_ALCOHOL_METABOLIC_PROCESS2	0.181456557	0.014918747	1.025467233	0.33479775	0.77878473	-5.47638623
homo after UVB irradiation VS homo before UVB irradiation	GO_ALDEHYDE_BIOSYNTHETIC_PROCESS	0.194260145	-0.0603815	1.451315358	0.18328907	0.36690135	-5.52277928
hetero after UVB irradiation VS hetero before UVB irradiation	GO_ALDEHYDE_BIOSYNTHETIC_PROCESS1	-0.02324253	0.018416991	-0.14521764	0.88819713	0.9591099	-6.22006373
WT after UVB irradiation VS WT before UVB irradiation	GO_ALDEHYDE_BIOSYNTHETIC_PROCESS2	0.017633425	0.015327369	0.083731912	0.93530258	0.98322796	-5.99236899
homo after UVB irradiation VS homo before UVB irradiation	GO_ALDEHYDE_CATABOLIC_PROCESS	0.657841002	0.024122107	3.220139808	0.01160044	0.07224597	-2.89345902
hetero after UVB irradiation VS hetero before UVB irradiation	GO_ALDEHYDE_CATABOLIC_PROCESS1	0.350932172	0.090615818	1.501752731	0.17225825	0.49529282	-5.16190447
WT after UVB irradiation VS WT before UVB irradiation	GO_ALDEHYDE_CATABOLIC_PROCESS2	0.440931644	0.018264651	1.947245548	0.08692553	0.5790399	-4.37645516
homo after UVB irradiation VS homo before UVB irradiation	GO_ALDEHYDE_DEHYDROGENASE_NAD_P_PLUS_ACTIVITY	0.270722369	-0.02233112	1.649393321	0.13618044	0.3048566	-5.26286169
hetero after UVB irradiation VS hetero before UVB irradiation	GO_ALDEHYDE_DEHYDROGENASE_NAD_P_PLUS_ACTIVITY1	-0.06307386	0.018643838	-0.34515084	0.73904372	0.89814933	-6.16738293
WT after UVB irradiation VS WT before UVB irradiation	GO_ALDEHYDE_DEHYDROGENASE_NAD_P_PLUS_ACTIVITY2	0.080862086	-0.03979227	0.456481419	0.66003866	0.91122546	-5.8875925
homo after UVB irradiation VS homo before UVB irradiation	GO_ALDEHYDE_LYASE_ACTIVITY	-0.24708358	0.021072914	-1.42343887	0.19096811	0.37692143	-5.55784513
hetero after UVB irradiation VS hetero before UVB irradiation	GO_ALDEHYDE_LYASE_ACTIVITY1	-0.08568823	-0.10245717	-0.4882776	0.638695	0.85182425	-6.10418645
WT after UVB irradiation VS WT before UVB irradiation	GO_ALDEHYDE_LYASE_ACTIVITY2	-0.25745509	0.060026122	-1.28310265	0.23495826	0.72628385	-5.20996829
homo after UVB irradiation VS homo before UVB irradiation	GO_ALDITOL_BIOSYNTHETIC_PROCESS	-0.02106615	-0.02460426	-0.08805144	0.93191609	0.96324759	-6.5466037
hetero after UVB irradiation VS hetero before UVB irradiation	GO_ALDITOL_BIOSYNTHETIC_PROCESS1	-0.09180338	-0.03955929	-0.45247259	0.66316661	0.86213521	-6.1219331
WT after UVB irradiation VS WT before UVB irradiation	GO_ALDITOL_BIOSYNTHETIC_PROCESS2	-0.00123529	-0.00281851	-0.00639196	0.99505466	0.99875662	-5.99604511
homo after UVB irradiation VS homo before UVB irradiation	GO_ALDITOL_CATABOLIC_PROCESS	0.102284793	0.008459042	0.417954424	0.68654777	0.81233046	-6.45599676
hetero after UVB irradiation VS hetero before UVB irradiation	GO_ALDITOL_CATABOLIC_PROCESS1	0.368167297	0.059859397	2.434522699	0.04146056	0.27438818	-3.86726017
WT after UVB irradiation VS WT before UVB irradiation	GO_ALDITOL_CATABOLIC_PROCESS2	0.118501914	0.025982855	0.556307937	0.59303609	0.8931505	-5.83599772
homo after UVB irradiation VS homo before UVB irradiation	GO_ALDITOL_METABOLIC_PROCESS	0.22771006	0.015609425	1.428558148	0.18953717	0.37523478	-5.55143589
hetero after UVB irradiation VS hetero before UVB irradiation	GO_ALDITOL_METABOLIC_PROCESS1	0.065983335	-0.03011228	0.467709327	0.65269762	0.85725021	-6.11453682
WT after UVB irradiation VS WT before UVB irradiation	GO_ALDITOL_METABOLIC_PROCESS2	0.18672146	-0.02002581	0.867975032	0.41039204	0.81906347	-5.61701779
homo after UVB irradiation VS homo before UVB irradiation	GO_ALDITOL_NADPPLUS_1_OXIDOREDUCTASE_ACTIVITY	0.518111227	0.052520172	3.063828239	0.01476605	0.08257891	-3.13441886
hetero after UVB irradiation VS hetero before UVB irradiation	GO_ALDITOL_NADPPLUS_1_OXIDOREDUCTASE_ACTIVITY1	0.405231768	0.067900813	2.107013808	0.06883455	0.3402756	-4.34185038
WT after UVB irradiation VS WT before UVB irradiation	GO_ALDITOL_NADPPLUS_1_OXIDOREDUCTASE_ACTIVITY2	0.305772892	0.01920693	1.277156343	0.23694771	0.72853545	-5.21656819
homo after UVB irradiation VS homo before UVB irradiation	GO_ALDITOL_PHOSPHATE_METABOLIC_PROCESS	-0.02662241	0.009727962	-0.10728051	0.91710564	0.95462566	-6.54454473
hetero after UVB irradiation VS hetero before UVB irradiation	GO_ALDITOL_PHOSPHATE_METABOLIC_PROCESS1	-0.34127208	0.046416437	-1.36102388	0.21127617	0.53727475	-5.33341554
WT after UVB irradiation VS WT before UVB irradiation	GO_ALDITOL_PHOSPHATE_METABOLIC_PROCESS2	-0.07881361	0.004837107	-0.39844447	0.70060231	0.92463338	-5.91315766
homo after UVB irradiation VS homo before UVB irradiation	GO_ALDO_KETO_REDUCTASE_NADP_ACTIVITY	0.464261824	0.019110555	3.569327881	0.00683492	0.05432461	-2.36246948
hetero after UVB irradiation VS hetero before UVB irradiation	GO_ALDO_KETO_REDUCTASE_NADP_ACTIVITY1	0.140682056	0.060406225	0.658333114	0.52914832	0.79049147	-6.00294008
WT after UVB irradiation VS WT before UVB irradiation	GO_ALDO_KETO_REDUCTASE_NADP_ACTIVITY2	0.316307626	0.048557542	1.318311297	0.22346598	0.71863455	-5.17049339
homo after UVB irradiation VS homo before UVB irradiation	GO_ALDOSTERONE_METABOLIC_PROCESS	0.139339249	-0.06949208	0.718172422	0.49230096	0.66815831	-6.27637694
hetero after UVB irradiation VS hetero before UVB irradiation	GO_ALDOSTERONE_METABOLIC_PROCESS1	-0.15713381	0.057974039	-0.7025812	0.50260851	0.77390992	-5.9721723
WT after UVB irradiation VS WT before UVB irradiation	GO_ALDOSTERONE_METABOLIC_PROCESS2	-0.11993201	0.060487263	-0.48968296	0.6373479	0.9057274	-5.87149018
homo after UVB irradiation VS homo before UVB irradiation	GO_ALKALI_METAL_ION_BINDING	-0.12457264	0.057569738	-0.6702778	0.52082782	0.69034726	-6.31083494
hetero after UVB irradiation VS hetero before UVB irradiation	GO_ALKALI_METAL_ION_BINDING1	-0.04577337	-0.00675581	-0.34243743	0.74100685	0.89905419	-6.16837915
WT after UVB irradiation VS WT before UVB irradiation	GO_ALKALI_METAL_ION_BINDING2	-0.02921455	-0.05394149	-0.13308693	0.89737256	0.97409039	-5.98673168
homo after UVB irradiation VS homo before UVB irradiation	GO_ALKALOID_METABOLIC_PROCESS	-0.28920481	-0.05361758	-0.88056403	0.40324492	0.59031859	-6.14433574
hetero after UVB irradiation VS hetero before UVB irradiation	GO_ALKALOID_METABOLIC_PROCESS1	-0.46284547	0.025739	-1.69584742	0.12906542	0.43680947	-4.91123825
WT after UVB irradiation VS WT before UVB irradiation	GO_ALKALOID_METABOLIC_PROCESS2	-0.06834411	-0.0650362	-0.2520849	0.80725582	0.95404687	-5.96267499
homo after UVB irradiation VS homo before UVB irradiation	GO_ALKANE_1_MONOOXYGENASE_ACTIVITY	0.836963996	0.103596404	5.628409468	0.00042694	0.01544198	0.427331225
hetero after UVB irradiation VS hetero before UVB irradiation	GO_ALKANE_1_MONOOXYGENASE_ACTIVITY1	0.812990314	0.094773344	5.126013478	0.00095408	0.08430141	-0.25649279
WT after UVB irradiation VS WT before UVB irradiation	GO_ALKANE_1_MONOOXYGENASE_ACTIVITY2	0.709413416	0.127308637	2.258721845	0.05344247	0.51991848	-3.94419851
homo after UVB irradiation VS homo before UVB irradiation	GO_ALPHA_1_3_FUCOSYLTRANSFERASE_ACTIVITY	0.279524253	0.010299664	1.871323464	0.09674542	0.24603489	-4.95284498
hetero after UVB irradiation VS hetero before UVB irradiation	GO_ALPHA_1_3_FUCOSYLTRANSFERASE_ACTIVITY1	0.669587871	0.046892997	4.658705712	0.00170877	0.09735383	-0.8056374
WT after UVB irradiation VS WT before UVB irradiation	GO_ALPHA_1_3_FUCOSYLTRANSFERASE_ACTIVITY2	0.169822385	-0.08379884	0.674136945	0.51900141	0.86248918	-5.76315045
homo after UVB irradiation VS homo before UVB irradiation	GO_ALPHA_2A_ADRENERGIC_RECEPTOR_BINDING	0.412350198	-0.09944956	1.944937064	0.08623983	0.22889884	-4.84649862
hetero after UVB irradiation VS hetero before UVB irradiation	GO_ALPHA_2A_ADRENERGIC_RECEPTOR_BINDING1	0.116736249	-0.00369735	0.283332734	0.78424769	0.91575615	-6.18817839
WT after UVB irradiation VS WT before UVB irradiation	GO_ALPHA_2A_ADRENERGIC_RECEPTOR_BINDING2	0.109305873	0.06232381	0.33151431	0.74867298	0.93642732	-5.93849061
homo after UVB irradiation VS homo before UVB irradiation	GO_ALPHA_ACTININ_BINDING	0.598666939	-0.04941445	3.429368749	0.00843419	0.06107216	-2.57387469
hetero after UVB irradiation VS hetero before UVB irradiation	GO_ALPHA_ACTININ_BINDING1	0.219890266	-0.02894731	0.752599872	0.47364477	0.75680588	-5.9352896
WT after UVB irradiation VS WT before UVB irradiation	GO_ALPHA_ACTININ_BINDING2	0.219555753	-0.02518719	0.802637061	0.44510068	0.83340629	-5.66976221
homo after UVB irradiation VS homo before UVB irradiation	GO_ALPHA_ADRENERGIC_RECEPTOR_ACTIVITY	0.257373298	-0.09392385	1.005098682	0.34315167	0.53576349	-6.02817547
hetero after UVB irradiation VS hetero before UVB irradiation	GO_ALPHA_ADRENERGIC_RECEPTOR_ACTIVITY1	-0.32267297	-0.05619163	-1.46238818	0.18247155	0.50688348	-5.21083081
WT after UVB irradiation VS WT before UVB irradiation	GO_ALPHA_ADRENERGIC_RECEPTOR_ACTIVITY2	-0.28266322	-0.07399562	-0.96094304	0.36439231	0.79568799	-5.53622333
homo after UVB irradiation VS homo before UVB irradiation	GO_ALPHA_AMINO_ACID_BIOSYNTHETIC_PROCESS	-0.11912402	0.005584021	-0.84707354	0.42062477	0.605654	-6.17342646
hetero after UVB irradiation VS hetero before UVB irradiation	GO_ALPHA_AMINO_ACID_BIOSYNTHETIC_PROCESS1	-0.03917769	-0.00057544	-0.28887239	0.78015793	0.91401375	-6.18647778
WT after UVB irradiation VS WT before UVB irradiation	GO_ALPHA_AMINO_ACID_BIOSYNTHETIC_PROCESS2	0.086375988	-0.00011221	0.49230053	0.63557572	0.90550211	-5.87017549
homo after UVB irradiation VS homo before UVB irradiation	GO_ALPHA_AMINO_ACID_CATABOLIC_PROCESS	0.104600642	-0.00966404	0.556715893	0.59236099	0.74414498	-6.38390924
hetero after UVB irradiation VS hetero before UVB irradiation	GO_ALPHA_AMINO_ACID_CATABOLIC_PROCESS1	-0.26363856	-0.01104292	-1.62318805	0.14391373	0.45992217	-5.0067848
WT after UVB irradiation VS WT before UVB irradiation	GO_ALPHA_AMINO_ACID_CATABOLIC_PROCESS2	-0.00023756	-0.05127228	-0.00102796	0.99920468	0.99997598	-5.99606611
homo after UVB irradiation VS homo before UVB irradiation	GO_ALPHA_AMINO_ACID_METABOLIC_PROCESS	-0.05364962	-0.0004275	-0.34111845	0.74146015	0.8478673	-6.48744574
hetero after UVB irradiation VS hetero before UVB irradiation	GO_ALPHA_AMINO_ACID_METABOLIC_PROCESS1	-0.23132362	-0.0174115	-1.65659617	0.13690089	0.44981284	-4.96308984
WT after UVB irradiation VS WT before UVB irradiation	GO_ALPHA_AMINO_ACID_METABOLIC_PROCESS2	-0.0059268	-0.02768201	-0.03562577	0.97244333	0.99307736	-5.99539703
homo after UVB irradiation VS homo before UVB irradiation	GO_ALPHA_AMYLASE_ACTIVITY_RELEASING_MALTOHEXAOSE	-0.66367967	0.106519783	-1.88273788	0.0950403	0.24357584	-4.93645704
hetero after UVB irradiation VS hetero before UVB irradiation	GO_ALPHA_AMYLASE_ACTIVITY_RELEASING_MALTOHEXAOSE1	-0.42028883	0.127463748	-1.09378224	0.30646409	0.627591	-5.62929868
WT after UVB irradiation VS WT before UVB irradiation	GO_ALPHA_AMYLASE_ACTIVITY_RELEASING_MALTOHEXAOSE2	-0.3178039	0.039207149	-0.81510349	0.43832723	0.83056493	-5.65996445
homo after UVB irradiation VS homo before UVB irradiation	GO_ALPHA_BETA_T_CELL_ACTIVATION	0.404416763	-0.02044197	2.724197033	0.0251315	0.11160566	-3.66145073
hetero after UVB irradiation VS hetero before UVB irradiation	GO_ALPHA_BETA_T_CELL_ACTIVATION_INVOLVED_IN_IMMUNE_RESPONSE	0.281745758	-0.01821448	1.663864674	0.13321217	0.30035324	-5.24319835
WT after UVB irradiation VS WT before UVB irradiation	GO_ALPHA_BETA_T_CELL_ACTIVATION_INVOLVED_IN_IMMUNE_RESPONSE1	0.264966929	0.029522312	1.525893795	0.16625088	0.48771331	-5.13155726
homo after UVB irradiation VS homo before UVB irradiation	GO_ALPHA_BETA_T_CELL_ACTIVATION_INVOLVED_IN_IMMUNE_RESPONSE2	0.253030523	-0.01301267	1.173129441	0.27409412	0.74597065	-5.32874346
hetero after UVB irradiation VS hetero before UVB irradiation	GO_ALPHA_BETA_T_CELL_ACTIVATION1	0.306019519	0.035976767	1.782657567	0.11319689	0.41521928	-4.79473318
WT after UVB irradiation VS WT before UVB irradiation	GO_ALPHA_BETA_T_CELL_ACTIVATION2	0.22863489	-0.01393561	1.02671197	0.33424546	0.77844475	-5.4752028
homo after UVB irradiation VS homo before UVB irradiation	GO_ALPHA_BETA_T_CELL_DIFFERENTIATION	0.294767101	-0.01507794	2.010525255	0.07780661	0.21471242	-4.75052008
hetero after UVB irradiation VS hetero before UVB irradiation	GO_ALPHA_BETA_T_CELL_DIFFERENTIATION1	0.206931076	0.036804575	1.312736855	0.22633628	0.55534056	-5.38996018
WT after UVB irradiation VS WT before UVB irradiation	GO_ALPHA_BETA_T_CELL_DIFFERENTIATION2	0.197084907	-0.02220202	0.982618357	0.35423877	0.78942569	-5.51645653
homo after UVB irradiation VS homo before UVB irradiation	GO_ALPHA_BETA_T_CELL_PROLIFERATION	0.741237737	-0.05292862	4.333367506	0.002274	0.03195943	-1.25223416
hetero after UVB irradiation VS hetero before UVB irradiation	GO_ALPHA_BETA_T_CELL_PROLIFERATION1	0.513702927	0.019850461	2.087143043	0.07097995	0.34427001	-4.37021955
WT after UVB irradiation VS WT before UVB irradiation	GO_ALPHA_BETA_T_CELL_PROLIFERATION2	0.4417131	0.022464372	1.727924476	0.12181149	0.63058669	-4.66899304
homo after UVB irradiation VS homo before UVB irradiation	GO_ALPHA_BETA_T_CELL_RECEPTOR_COMPLEX	-0.43231067	0.007786446	-1.81644355	0.10535536	0.25886782	-5.03107981
hetero after UVB irradiation VS hetero before UVB irradiation	GO_ALPHA_BETA_T_CELL_RECEPTOR_COMPLEX1	-0.28299434	-0.00394208	-1.01188813	0.34175098	0.66014434	-5.71079823
WT after UVB irradiation VS WT before UVB irradiation	GO_ALPHA_BETA_T_CELL_RECEPTOR_COMPLEX2	0.160889901	-0.0398687	0.408239818	0.69367941	0.92180005	-5.9090754
homo after UVB irradiation VS homo before UVB irradiation	GO_ALPHA_CATENIN_BINDING	0.204306497	0.043616797	1.065225737	0.31667549	0.51000547	-5.96780448
hetero after UVB irradiation VS hetero before UVB irradiation	GO_ALPHA_CATENIN_BINDING1	0.351220954	0.000749059	1.272146604	0.23969755	0.5680482	-5.43650227
WT after UVB irradiation VS WT before UVB irradiation	GO_ALPHA_CATENIN_BINDING2	0.099679534	0.139434646	0.331850739	0.74842818	0.93642732	-5.93837452
homo after UVB irradiation VS homo before UVB irradiation	GO_ALPHA_DNA_POLYMERASE_PRIMASE_COMPLEX	-0.68865637	-0.07109434	-2.16131183	0.06133392	0.18686726	-4.52614395
hetero after UVB irradiation VS hetero before UVB irradiation	GO_ALPHA_DNA_POLYMERASE_PRIMASE_COMPLEX1	0.353528361	-0.0394055	0.989076013	0.35211879	0.66862242	-5.73264804
WT after UVB irradiation VS WT before UVB irradiation	GO_ALPHA_DNA_POLYMERASE_PRIMASE_COMPLEX2	0.218783503	-0.02140527	0.790072036	0.45199944	0.83611442	-5.67950849
homo after UVB irradiation VS homo before UVB irradiation	GO_ALPHA_GLUCOSIDASE_ACTIVITY	0.605386148	0.014991196	3.498918076	0.00759511	0.05790843	-2.46856054
hetero after UVB irradiation VS hetero before UVB irradiation	GO_ALPHA_GLUCOSIDASE_ACTIVITY1	0.568283574	0.012966078	1.685059344	0.13117646	0.44005505	-4.92554325
WT after UVB irradiation VS WT before UVB irradiation	GO_ALPHA_GLUCOSIDASE_ACTIVITY2	0.363600577	-0.05759843	2.011815604	0.07862622	0.56242712	-4.28815358
homo after UVB irradiation VS homo before UVB irradiation	GO_ALPHA_LINOLENIC_ACID_METABOLIC_PROCESS	0.200806268	-0.04769281	1.04218132	0.32662969	0.52007707	-5.99125968
hetero after UVB irradiation VS hetero before UVB irradiation	GO_ALPHA_LINOLENIC_ACID_METABOLIC_PROCESS1	0.172604527	-0.0234803	0.700772263	0.50367676	0.77419315	-5.9734647
WT after UVB irradiation VS WT before UVB irradiation	GO_ALPHA_LINOLENIC_ACID_METABOLIC_PROCESS2	0.162942968	-0.07876283	0.840206171	0.42490391	0.82634514	-5.63985282
homo after UVB irradiation VS homo before UVB irradiation	GO_ALPHA_N_ACETYLGALACTOSAMINIDE_ALPHA_2_6_SIALYLTRANSFERASE_ACTIVITY	0.674941418	0.002492041	2.925964445	0.01830357	0.09290211	-3.34801644
hetero after UVB irradiation VS hetero before UVB irradiation	GO_ALPHA_N_ACETYLGALACTOSAMINIDE_ALPHA_2_6_SIALYLTRANSFERASE_ACTIVITY1	0.131839775	0.03667357	0.51296896	0.62208792	0.84401411	-6.09121003
WT after UVB irradiation VS WT before UVB irradiation	GO_ALPHA_N_ACETYLGALACTOSAMINIDE_ALPHA_2_6_SIALYLTRANSFERASE_ACTIVITY2	-0.06551482	-0.05022987	-0.30020936	0.7715801	0.94202287	-5.94879044
homo after UVB irradiation VS homo before UVB irradiation	GO_ALPHA_N_ACETYLNEURAMINATE_ALPHA_2_8_SIALYLTRANSFERASE_ACTIVITY	-0.00553327	-0.04329409	-0.02334977	0.981921	0.99114063	-6.5505575
hetero after UVB irradiation VS hetero before UVB irradiation	GO_ALPHA_N_ACETYLNEURAMINATE_ALPHA_2_8_SIALYLTRANSFERASE_ACTIVITY1	-0.45202009	-0.00076632	-2.26598308	0.05382386	0.30765655	-4.11286318
WT after UVB irradiation VS WT before UVB irradiation	GO_ALPHA_N_ACETYLNEURAMINATE_ALPHA_2_8_SIALYLTRANSFERASE_ACTIVITY2	-0.54890644	0.024132458	-2.98803217	0.01714887	0.38271751	-2.9101844
homo after UVB irradiation VS homo before UVB irradiation	GO_ALPHA_TUBULIN_BINDING	-0.31687948	-0.03188717	-2.37570644	0.04365506	0.15158064	-4.20033633
hetero after UVB irradiation VS hetero before UVB irradiation	GO_ALPHA_TUBULIN_BINDING1	-0.24272111	-0.03081546	-1.98418919	0.08318543	0.36861389	-4.51607661

WT after UVB irradiation VS WT before UVB irradiation	GO_ALPHA_TUBULIN_BINDING2	-0.2080161	0.025877368	-1.39649702	0.19965423	0.69758436	-5.08050899
homo after UVB irradiation VS homo before UVB irradiation	GO_ALTERNATIVE_MRNA_SPLICING_VIA_SPLICEOSOME	-0.69881635	-0.01805445	-4.94367496	0.00100331	0.02151484	-0.42773087
hetero after UVB irradiation VS hetero before UVB irradiation	GO_ALTERNATIVE_MRNA_SPLICING_VIA_SPLICEOSOME1	-0.30307405	-0.06120486	-1.43080747	0.19105072	0.51579406	-5.24956291
WT after UVB irradiation VS WT before UVB irradiation	GO_ALTERNATIVE_MRNA_SPLICING_VIA_SPLICEOSOME2	-0.47661442	-0.0486373	-2.34916638	0.04637287	0.50534441	-3.81646046
homo after UVB irradiation VS homo before UVB irradiation	GO_ALVEOLAR_LAMELLAR_BODY	0.440113506	0.066761002	1.749316114	0.11686619	0.27580271	-5.12543472
hetero after UVB irradiation VS hetero before UVB irradiation	GO_ALVEOLAR_LAMELLAR_BODY1	0.397808673	-0.02298096	1.986208041	0.08292755	0.36857799	-4.5132363
WT after UVB irradiation VS WT before UVB irradiation	GO_ALVEOLAR_LAMELLAR_BODY2	0.323943302	-0.01440178	1.235845333	0.25116355	0.73564768	-5.26187086
homo after UVB irradiation VS homo before UVB irradiation	GO_AMACRINE_CELL_DIFFERENTIATION	-0.66206094	0.11259795	-3.62421706	0.0062983	0.0525376	-2.28015178
hetero after UVB irradiation VS hetero before UVB irradiation	GO_AMACRINE_CELL_DIFFERENTIATION1	-0.82076879	-0.01867237	-3.29589818	0.01121317	0.16546158	-2.61340474
WT after UVB irradiation VS WT before UVB irradiation	GO_AMACRINE_CELL_DIFFERENTIATION2	-0.4620633	0.1815155	-1.7891745	0.11092766	0.61576748	-4.58860616
homo after UVB irradiation VS homo before UVB irradiation	GO_AMEBOIDAL_TYPE_CELL_MIGRATION	0.325888693	-0.03081508	2.614973863	0.0298673	0.12287072	-3.83101332
hetero after UVB irradiation VS hetero before UVB irradiation	GO_AMEBOIDAL_TYPE_CELL_MIGRATION1	0.289071221	0.012168493	1.879594207	0.09765801	0.3897511	-4.6619846
WT after UVB irradiation VS WT before UVB irradiation	GO_AMEBOIDAL_TYPE_CELL_MIGRATION2	0.207457238	-0.01940014	1.276731328	0.23709045	0.72854168	-5.21703917
homo after UVB irradiation VS homo before UVB irradiation	GO_AMELOGENESIS	0.258182304	-0.02086862	1.480773382	0.17547063	0.35627685	-5.48529268
hetero after UVB irradiation VS hetero before UVB irradiation	GO_AMELOGENESIS1	0.397979313	0.043699284	2.508568865	0.03697596	0.26371309	-3.75881719
WT after UVB irradiation VS WT before UVB irradiation	GO_AMELOGENESIS2	0.089232359	0.001466832	0.538137797	0.60495353	0.89591658	-5.84609374
homo after UVB irradiation VS homo before UVB irradiation	GO_AMIDE_BINDING	0.156740323	-0.01005306	1.289566225	0.23185428	0.42308318	-5.72033413
hetero after UVB irradiation VS hetero before UVB irradiation	GO_AMIDE_BINDING1	0.028996683	0.002456765	0.192446413	0.85227615	0.94335585	-6.21144839
WT after UVB irradiation VS WT before UVB irradiation	GO_AMIDE_BINDING2	0.052577409	-0.01364654	0.314767385	0.76089615	0.93919119	-5.94412433
homo after UVB irradiation VS homo before UVB irradiation	GO_AMIDE_BIOSYNTHETIC_PROCESS	-0.50631151	-0.0009077	-4.06062121	0.00333628	0.03804745	-1.63922929
hetero after UVB irradiation VS hetero before UVB irradiation	GO_AMIDE_BIOSYNTHETIC_PROCESS1	-0.21049661	-0.04271901	-1.22201693	0.25711259	0.58440439	-5.4926742
WT after UVB irradiation VS WT before UVB irradiation	GO_AMIDE_BIOSYNTHETIC_PROCESS2	-0.25098826	-0.01871651	-1.37357922	0.2063954	0.70308793	-5.10720663
homo after UVB irradiation VS homo before UVB irradiation	GO_AMIDE_TRANSMEMBRANE_TRANSPORTER_ACTIVITY	0.102365022	-0.0047224	0.550975634	0.59611593	0.74720825	-6.38727246
hetero after UVB irradiation VS hetero before UVB irradiation	GO_AMIDE_TRANSMEMBRANE_TRANSPORTER_ACTIVITY1	0.101982186	-0.02085113	0.593407545	0.56960124	0.8137684	-6.04483496
WT after UVB irradiation VS WT before UVB irradiation	GO_AMIDE_TRANSMEMBRANE_TRANSPORTER_ACTIVITY2	0.104603621	-0.04162935	0.63183998	0.54491225	0.87281776	-5.79074963
homo after UVB irradiation VS homo before UVB irradiation	GO_AMIDINE_LYASE_ACTIVITY	-0.07767046	0.027931352	-0.47760659	0.64521243	0.78320308	-6.42738349
hetero after UVB irradiation VS hetero before UVB irradiation	GO_AMIDINE_LYASE_ACTIVITY1	0.269804717	-0.02304164	1.437213986	0.18928205	0.51444052	-5.24174402
WT after UVB irradiation VS WT before UVB irradiation	GO_AMIDINE_LYASE_ACTIVITY2	0.112274661	0.012534804	0.684061216	0.51303325	0.85974355	-5.7564446
homo after UVB irradiation VS homo before UVB irradiation	GO_AMINE_BINDING	0.590868517	-0.03203713	3.715918471	0.00549902	0.04873819	-2.14342387
hetero after UVB irradiation VS hetero before UVB irradiation	GO_AMINE_BINDING1	0.018468664	0.049712002	0.081384972	0.93717183	0.97688512	-6.2278937
WT after UVB irradiation VS WT before UVB irradiation	GO_AMINE_BINDING2	0.346436771	0.094102397	1.480995161	0.17643456	0.68058088	-4.97990821
homo after UVB irradiation VS homo before UVB irradiation	GO_AMINE_BIOSYNTHETIC_PROCESS	0.161541037	-0.02489112	0.738911795	0.48026219	0.65767982	-6.26080566
hetero after UVB irradiation VS hetero before UVB irradiation	GO_AMINE_BIOSYNTHETIC_PROCESS1	0.102017775	-0.02295847	0.683391949	0.51401362	0.78258272	-5.98573262
WT after UVB irradiation VS WT before UVB irradiation	GO_AMINE_BIOSYNTHETIC_PROCESS2	0.240990439	-0.05734548	1.152066675	0.28216894	0.75292132	-5.35066647
homo after UVB irradiation VS homo before UVB irradiation	GO_AMINE_CATABOLIC_PROCESS	0.387213779	-0.03813994	2.836405421	0.02106183	0.10070646	-3.48709339
hetero after UVB irradiation VS hetero before UVB irradiation	GO_AMINE_CATABOLIC_PROCESS1	0.29139627	0.052578106	1.971204452	0.08486273	0.37147425	-4.53432433
WT after UVB irradiation VS WT before UVB irradiation	GO_AMINE_CATABOLIC_PROCESS2	0.223573962	-0.04353794	1.355972217	0.21170747	0.70907106	-5.12753901
homo after UVB irradiation VS homo before UVB irradiation	GO_AMINE_METABOLIC_PROCESS	-0.06312619	-0.00902581	-0.36529339	0.72399492	0.83689721	-6.47821451
hetero after UVB irradiation VS hetero before UVB irradiation	GO_AMINE_METABOLIC_PROCESS1	-0.04027588	-0.00653623	-0.24001756	0.81646329	0.92946554	-6.20036094
WT after UVB irradiation VS WT before UVB irradiation	GO_AMINE_METABOLIC_PROCESS2	0.169647673	-0.01294719	1.108408129	0.29951788	0.76180374	-5.39521586
homo after UVB irradiation VS homo before UVB irradiation	GO_AMINE_TRANSPORT	0.023206863	0.026888866	0.148254146	0.88566866	0.93810991	-6.53881023
hetero after UVB irradiation VS hetero before UVB irradiation	GO_AMINE_TRANSPORT1	-0.02518073	0.035992238	-0.18162234	0.86047956	0.94732472	-6.21363405
WT after UVB irradiation VS WT before UVB irradiation	GO_AMINE_TRANSPORT2	-0.00837589	0.021587563	-0.04897026	0.96212932	0.990522	-5.99480151
homo after UVB irradiation VS homo before UVB irradiation	GO_AMINO_ACID_ACTIVATION	-0.78927349	-0.03684492	-4.715635	0.00135346	0.02471899	-0.72894967
hetero after UVB irradiation VS hetero before UVB irradiation	GO_AMINO_ACID_ACTIVATION1	-0.49775292	-0.06026442	-2.48518786	0.03833622	0.26840285	-3.79308243
WT after UVB irradiation VS WT before UVB irradiation	GO_AMINO_ACID_ACTIVATION2	-0.3482646	-0.03771328	-1.36148389	0.21003204	0.7066673	-5.12119109
homo after UVB irradiation VS homo before UVB irradiation	GO_AMINO_ACID_BETAINE_BIOSYNTHETIC_PROCESS	0.397928714	-0.09533537	1.646538844	0.13677303	0.30558057	-5.26673028
hetero after UVB irradiation VS hetero before UVB irradiation	GO_AMINO_ACID_BETAINE_BIOSYNTHETIC_PROCESS1	-0.00928317	-0.02520124	-0.04723503	0.96350508	0.98664387	-6.23027432
WT after UVB irradiation VS WT before UVB irradiation	GO_AMINO_ACID_BETAINE_BIOSYNTHETIC_PROCESS2	0.009277291	0.00460237	0.034515966	0.97330138	0.99307736	-5.9954381
homo after UVB irradiation VS homo before UVB irradiation	GO_AMINO_ACID_BETAINE_METABOLIC_PROCESS	-0.00188804	-0.03569661	-0.00844011	0.9934645	0.99636779	-6.55081762
hetero after UVB irradiation VS hetero before UVB irradiation	GO_AMINO_ACID_BETAINE_METABOLIC_PROCESS1	0.030980248	-0.02130044	0.122480573	0.9055949	0.96539217	-6.22335673
WT after UVB irradiation VS WT before UVB irradiation	GO_AMINO_ACID_BETAINE_METABOLIC_PROCESS2	-0.02187289	-0.03038452	-0.08886038	0.93135131	0.98320919	-5.9919024
homo after UVB irradiation VS homo before UVB irradiation	GO_AMINO_ACID_BETAINE_TRANSPORT	-0.17805538	0.047272215	-0.68912828	0.50948042	0.68169395	-6.29752511
hetero after UVB irradiation VS hetero before UVB irradiation	GO_AMINO_ACID_BETAINE_TRANSPORT1	-0.04482508	0.077953189	-0.24526681	0.81253786	0.9274295	-6.19899015
WT after UVB irradiation VS WT before UVB irradiation	GO_AMINO_ACID_BETAINE_TRANSPORT2	-0.65099476	-0.05504658	-2.80352777	0.02279832	0.41742287	-3.17075007
homo after UVB irradiation VS homo before UVB irradiation	GO_AMINO_ACID_BINDING	-0.28031315	0.019762152	-2.09877225	0.06770677	0.19871695	-4.61977797
hetero after UVB irradiation VS hetero before UVB irradiation	GO_AMINO_ACID_BINDING1	-0.30134831	0.019534149	-2.14129794	0.06528181	0.33409111	-4.29275672
WT after UVB irradiation VS WT before UVB irradiation	GO_AMINO_ACID_BINDING2	-0.24165036	0.041499918	-1.42830662	0.19061588	0.69285981	-5.04302908
homo after UVB irradiation VS homo before UVB irradiation	GO_AMINO_ACID_CATION_SYMPORTER_ACTIVITY	-0.13696458	0.034268961	-0.79518416	0.44857058	0.6300139	-6.21661942
hetero after UVB irradiation VS hetero before UVB irradiation	GO_AMINO_ACID_CATION_SYMPORTER_ACTIVITY1	-0.15480407	0.011367712	-1.05916548	0.32101356	0.64072448	-5.66432164
WT after UVB irradiation VS WT before UVB irradiation	GO_AMINO_ACID_CATION_SYMPORTER_ACTIVITY2	-0.13195248	-0.00718094	-0.68274844	0.51382026	0.85974355	-5.75733662
homo after UVB irradiation VS homo before UVB irradiation	GO_AMINO_ACID_HOMEOSTASIS	-0.42903672	0.014314328	-2.51417037	0.03504122	0.13423812	-3.98709568
hetero after UVB irradiation VS hetero before UVB irradiation	GO_AMINO_ACID_HOMEOSTASIS1	0.274723292	-0.01505132	0.947471145	0.37163906	0.68523129	-5.77150381
WT after UVB irradiation VS WT before UVB irradiation	GO_AMINO_ACID_HOMEOSTASIS2	-0.13851059	-0.00458643	-0.45852175	0.65863309	0.91105574	-5.88663374
homo after UVB irradiation VS homo before UVB irradiation	GO_AMINO_ACID_IMPORT	-0.12052869	0.029953956	-0.95972759	0.36421937	0.55450909	-6.07191696
hetero after UVB irradiation VS hetero before UVB irradiation	GO_AMINO_ACID_IMPORT1	-0.28161649	0.022445293	-2.16410167	0.06301943	0.32964008	-4.26000597
WT after UVB irradiation VS WT before UVB irradiation	GO_AMINO_ACID_IMPORT2	-0.14332911	0.00715701	-0.78242884	0.45623104	0.83880233	-5.6853733
homo after UVB irradiation VS homo before UVB irradiation	GO_AMINO_ACID_SALVAGE	0.156167468	-0.01412396	0.663546301	0.52491723	0.69354495	-6.31550781
hetero after UVB irradiation VS hetero before UVB irradiation	GO_AMINO_ACID_SALVAGE1	0.165619002	0.015622431	0.547450567	0.59927885	0.83280104	-6.07209254
WT after UVB irradiation VS WT before UVB irradiation	GO_AMINO_ACID_SALVAGE2	0.420898509	-0.05167486	1.710919532	0.12500535	0.63170411	-4.69110625
homo after UVB irradiation VS homo before UVB irradiation	GO_AMINO_ACID_SODIUM_SYMPORTER_ACTIVITY	-0.24424703	0.022314431	-1.44103934	0.18608767	0.37054321	-5.53575234
hetero after UVB irradiation VS hetero before UVB irradiation	GO_AMINO_ACID_SODIUM_SYMPORTER_ACTIVITY1	-0.2240996	0.014339975	-1.34783124	0.21530241	0.54268132	-5.34898791
WT after UVB irradiation VS WT before UVB irradiation	GO_AMINO_ACID_SODIUM_SYMPORTER_ACTIVITY2	-0.38246537	0.020570453	-2.03655358	0.07565407	0.55851649	-4.25411039
homo after UVB irradiation VS homo before UVB irradiation	GO_AMINO_ACID_TRANSMEMBRANE_TRANSPORT	0.021720746	0.012200091	0.172906349	0.86685306	0.92676596	-6.5344788
hetero after UVB irradiation VS hetero before UVB irradiation	GO_AMINO_ACID_TRANSMEMBRANE_TRANSPORT1	-0.16446501	0.006949167	-1.2592273	0.24408799	0.57161579	-5.45111956
WT after UVB irradiation VS WT before UVB irradiation	GO_AMINO_ACID_TRANSMEMBRANE_TRANSPORT2	-0.1043819	0.009665174	-0.60610609	0.56104615	0.88029724	-5.80675343
homo after UVB irradiation VS homo before UVB irradiation	GO_AMINO_ACID_TRANSMEMBRANE_TRANSPORTER_ACTIVITY	-0.11305324	0.013115741	-0.81945826	0.43534385	0.61838186	-6.19670144
hetero after UVB irradiation VS hetero before UVB irradiation	GO_AMINO_ACID_TRANSMEMBRANE_TRANSPORTER_ACTIVITY1	-0.26566928	0.002591258	-1.90781202	0.0935313	0.38241837	-4.62287439
WT after UVB irradiation VS WT before UVB irradiation	GO_AMINO_ACID_TRANSMEMBRANE_TRANSPORTER_ACTIVITY2	-0.12269013	-0.0030465	-0.75650953	0.47077814	0.84640265	-5.70489927
homo after UVB irradiation VS homo before UVB irradiation	GO_AMINO_ACID_TRANSPORT	0.002879267	0.010174616	0.021249623	0.98354679	0.99194842	-6.55060891
hetero after UVB irradiation VS hetero before UVB irradiation	GO_AMINO_ACID_TRANSPORT1	-0.16720401	0.012712008	-1.33562206	0.21908736	0.54626422	-5.36331727
WT after UVB irradiation VS WT before UVB irradiation	GO_AMINO_ACID_TRANSPORT2	-0.08636693	0.005667415	-0.51996143	0.61700218	0.90020288	-5.85588206
homo after UVB irradiation VS homo before UVB irradiation	GO_AMINO_SUGAR_BIOSYNTHETIC_PROCESS	0.404372951	-0.04346046	2.577009051	0.03171833	0.12619774	-3.88985839
hetero after UVB irradiation VS hetero before UVB irradiation	GO_AMINO_SUGAR_BIOSYNTHETIC_PROCESS1	0.341298226	-0.0196954	1.606614901	0.14751383	0.4653087	-5.02830535
WT after UVB irradiation VS WT before UVB irradiation	GO_AMINO_SUGAR_BIOSYNTHETIC_PROCESS2	0.276660956	-0.0515049	1.061155027	0.31924048	0.77323848	-5.44203094
homo after UVB irradiation VS homo before UVB irradiation	GO_AMINO_SUGAR_CATABOLIC_PROCESS	0.882621539	0.011978167	5.997118803	0.00027671	0.01222573	0.857676173
hetero after UVB irradiation VS hetero before UVB irradiation	GO_AMINO_SUGAR_CATABOLIC_PROCESS1	0.93470326	0.020971911	7.405523357	8.34E-05	0.07788488	1.924345684
WT after UVB irradiation VS WT before UVB irradiation	GO_AMINO_SUGAR_CATABOLIC_PROCESS2	0.78842953	0.021387639	4.61703018	0.00166296	0.2011505	-0.80270602
homo after UVB irradiation VS homo before UVB irradiation	GO_AMINO_SUGAR_METABOLIC_PROCESS	0.485936608	-0.0163283	3.597962399	0.00654918	0.05360305	-2.31948267
hetero after UVB irradiation VS hetero before UVB irradiation	GO_AMINO_SUGAR_METABOLIC_PROCESS1	0.268329937	0.004751355	1.542140195	0.16231492	0.48356934	-5.11099255
WT after UVB irradiation VS WT before UVB irradiation	GO_AMINO_SUGAR_METABOLIC_PROCESS2	0.290435975	-0.02434444	1.287680888	0.23343616	0.7246145	-5.20487351
homo after UVB irradiation VS homo before UVB irradiation	GO_AMINOACYL_TRNA_EDITING_ACTIVITY	-0.40953047	-0.04227773	-1.34263293	0.21483013	0.40524712	-5.6571373
hetero after UVB irradiation VS hetero before UVB irradiation	GO_AMINOACYL_TRNA_EDITING_ACTIVITY1	-0.44268853	-0.14947138	-1.91667946	0.09226938	0.37946684	-4.61054378
WT after UVB irradiation VS WT before UVB irradiation	GO_AMINOACYL_TRNA_EDITING_ACTIVITY2	0.366858262	-0.02934519	1.26484234	0.24111273	0.73118354	-5.23017308
homo after UVB irradiation VS homo before UVB irradiation	GO_AMINOACYL_TRNA_METABOLISM_INVOLVED_IN_TRANSLATIONAL_FIDELITY	-0.40953047	-0.04227773	-1.34263293	0.21483013	0.40524712	-5.6571373
hetero after UVB irradiation VS hetero before UVB irradiation	GO_AMINOACYL_TRNA_METABOLISM_INVOLVED_IN_TRANSLATIONAL_FIDELITY1	-0.44268853	-0.14947138	-1.91667946	0.09226938	0.37946684	-4.61054378
WT after UVB irradiation VS WT before UVB irradiation	GO_AMINOACYL_TRNA_METABOLISM_INVOLVED_IN_TRANSLATIONAL_FIDELITY2	0.366858262	-0.02934519	1.26484234	0.24111273	0.73118354	-5.23017308
homo after UVB irradiation VS homo before UVB irradiation	GO_AMINOACYL_TRNA_SYNTHETASE_MULTIENZYME_COMPLEX	-1.05940102	0.042645292	-6.09425028	0.00024756	0.01196149	0.967648777
hetero after UVB irradiation VS hetero before UVB irradiation	GO_AMINOACYL_TRNA_SYNTHETASE_MULTIENZYME_COMPLEX1	-0.66390791	0.031776397	-1.93929799	0.08912424	0.37595306	-4.5790076
WT after UVB irradiation VS WT before UVB irradiation	GO_AMINOACYL_TRNA_SYNTHETASE_MULTIENZYME_COMPLEX2	-0.80335884	-0.11676856	-2.3827561	0.04399218	0.49758639	-3.76886983
homo after UVB irradiation VS homo before UVB irradiation	GO_AMINOACYLASE_ACTIVITY	0.364552365	-0.07210037	1.188919032	0.26725949	0.46252472	-5.83547804
hetero after UVB irradiation VS hetero before UVB irradiation	GO_AMINOACYLASE_ACTIVITY1	-0.24679392	-0.01439481	-0.77499834	0.46103787	0.7487737	-5.918066
WT after UVB irradiation VS WT before UVB irradiation	GO_AMINOACYLASE_ACTIVITY2	0.314066876	-0.01779994	1.037733891	0.32938565	0.77711776	-5.46467672
homo after UVB irradiation VS homo before UVB irradiation	GO_AMINOGLYCAN_BIOSYNTHETIC_PROCESS	0.501031695	-0.00695215	3.967773781	0.00381066	0.04047966	-1.77344065
hetero after UVB irradiation VS hetero before UVB irradiation	GO_AMINOGLYCAN_BIOSYNTHETIC_PROCESS1	0.309642247	0.033102478	1.613015079	0.14611388	0.46297111	-5.02000708
WT after UVB irradiation VS WT before UVB irradiation	GO_AMINOGLYCAN_BIOSYNTHETIC_PROCESS2	0.38127573	0.003569102	1.877260776	0.09686938	0.59686908	-4.47114753
homo after UVB irradiation VS homo before UVB irradiation	GO_AMINOGLYCAN_CATABOLIC_PROCESS	0.50515639	-0.00830668	3.831789702	0.00464008	0.04431913	-1.97216254
hetero after UVB irradiation VS hetero before UVB irradiation	GO_AMINOGLYCAN_CATABOLIC_PROCESS1	0.313587421	-0.00477854	1.691476363	0.12991693	0.43834133	-4.9170391
WT after UVB irradiation VS WT before UVB irradiation	GO_AMINOGLYCAN_CATABOLIC_PROCESS2	0.401642642	0.0322226	2.371345468	0.04478686	0.50107628	-3.78504431
homo after UVB irradiation VS homo before UVB irradiation	GO_AMINOGLYCAN_METABOLIC_PROCESS	0.497981941	-0.00363597	4.080396177	0.00324365	0.0375267	-1.61080313
hetero after UVB irradiation VS hetero before UVB irradiation	GO_AMINOGLYCAN_METABOLIC_PROCESS1	0.321369673	0.017845567	1.802142048	0.10989658	0.40919352	-4.76826251
WT after UVB irradiation VS WT before UVB irradiation	GO_AMINOGLYCAN_METABOLIC_PROCESS2	0.386383292	0.014917525	2.08113529	0.07057104	0.54753775	-4.19248896
homo after UVB irradiation VS homo before UVB irradiation	GO_AMINOGLYCOSIDE_ANTIBIOTIC_METABOLIC_PROCESS	0.282185038	0.04997342	1.473492102	0.17737525	0.35900789	-5.49459893
hetero after UVB irradiation VS hetero before UVB irradiation	GO_AMINOGLYCOSIDE_ANTIBIOTIC_METABOLIC_PROCESS1	0.294026219	0.052847312	1.768038322	0.11573427	0.41917522	-4.81451976
WT after UVB irradiation VS WT before UVB irradiation	GO_AMINOGLYCOSIDE_ANTIBIOTIC_METABOLIC_PROCESS2	0.318144059	0.032286536	1.305056705	0.227735	0.71951396	-5.18543286
homo after UVB irradiation VS homo before UVB irradiation	GO_AMINOPEPTIDASE_ACTIVITY	0.469027984	-0.05410018	3.188667595	0.01217543	0.07415303	-2.94184397
hetero after UVB irradiation VS hetero before UVB irradiation	GO_AMINOPEPTIDASE_ACTIVITY1	0.419365273	-0.00862409	2.912739347	0.01990091	0.20478228	-3.16657289
WT after UVB irradiation VS WT before UVB irradiation	GO_AMINOPEPTIDASE_ACTIVITY2	0.361471418	-0.02038159	2.188246377	0.05968464	0.52638053	-4.04320327
homo after UVB irradiation VS homo before UVB irradiation	GO_AMMONIA_LYASE_ACTIVITY	0.51624528	-0.03261059	1.883294588	0.09495787	0.24347243	-4.93565676
hetero after UVB irradiation VS hetero before UVB irradiation	GO_AMMONIA_LYASE_ACTIVITY1	-0.07185911	0.016472678	-0.35519606	0.73179399	0.8944957	-6.16362861
WT after UVB irradiation VS WT before UVB irradiation	GO_AMMONIA_LYASE_ACTIVITY2	-0.05767367	-0.11759702	-0.24480432	0.81269745	0.95523506	-5.96456852
homo after UVB irradiation VS homo before UVB irradiation	GO_AMMONIUM_ION_METABOLIC_PROCESS	0.077803234	0.008359562	0.436963922	0.67324504	0.80335101	-6.44727294
hetero after UVB irradiation VS hetero before UVB irradiation	GO_AMMONIUM_ION_METABOLIC_PROCESS1	0.077357137	0.043026989	0.557497457	0.59271927	0.82733693	-6.06630601
WT after UVB irradiation VS WT before UVB irradiation	GO_AMMONIUM_ION_METABOLIC_PROCESS2	0.108337609	0.021577306	0.620113572	0.55222994	0.87634603	-5.79811681
homo after UVB irradiation VS homo before UVB irradiation	GO_AMMONIUM_TRANSMEMBRANE_TRANSPORTER_ACTIVITY	0.049951092	-0.00610122	0.223979035	0.82817009	0.9038759	-6.52340847
hetero after UVB irradiation VS hetero before UVB irradiation	GO_AMMONIUM_TRANSMEMBRANE_TRANSPORTER_ACTIVITY1	-0.137098	0.028898572	-0.81728654	0.4378563	0.73571027	-5.88438182
WT after UVB irradiation VS WT before UVB irradiation	GO_AMMONIUM_TRANSMEMBRANE_TRANSPORTER_ACTIVITY2	-0.05796951	-0.00175268	-0.27823785	0.78780246	0.9469715	-5.95542371
homo after UVB irradiation VS homo before UVB irradiation	GO_AMMONIUM_TRANSPORT	0.049951092	-0.00610122	0.223979035	0.82817009	0.9038759	-6.52340847
hetero after UVB irradiation VS hetero before UVB irradiation	GO_AMMONIUM_TRANSPORT1	-0.137098	0.028898572	-0.81728654	0.4378563	0.73571027	-5.88438182
WT after UVB irradiation VS WT before UVB irradiation	GO_AMMONIUM_TRANSPORT2	-0.05796951	-0.00175268	-0.27823785	0.78780246	0.9469715	-5.95542371
homo after UVB irradiation VS homo before UVB irradiation	GO_AMP_ACTIVATED_PROTEIN_KINASE_ACTIVITY	0.62045216	-0.03953911	3.51228594	0.00744419	0.0572405	-2.44837661
hetero after UVB irradiation VS hetero before UVB irradiation	GO_AMP_ACTIVATED_PROTEIN_KINASE_ACTIVITY1	0.22624494	0.027186106	0.873347023	0.40838606	0.71330228	-5.83743599
WT after UVB irradiation VS WT before UVB irradiation	GO_AMP_ACTIVATED_PROTEIN_KINASE_ACTIVITY2	0.214723	-0.04557293	0.656520972	0.52969996	0.86747113	-5.77483904
homo after UVB irradiation VS homo before UVB irradiation	GO_AMP_BINDING	0.230347291	-0.02552873	1.327996847	0.21941571	0.41022649	-5.67473147
hetero after UVB irradiation VS hetero before UVB irradiation	GO_AMP_BINDING1	0.106563834	0.016978162	0.524902803	0.61414262	0.83965967	-6.08472428
WT after UVB irradiation VS WT before UVB irradiation	GO_AMP_BINDING2	0.118777647	0.003276275	0.543973974	0.60111172	0.89567499	-5.84288473
homo after UVB irradiation VS homo before UVB irradiation	GO_AMP_BIOSYNTHETIC_PROCESS	-0.16843511	0.039508055	-0.57057794	0.58334675	0.73752982	-6.37565447
hetero after UVB irradiation VS hetero before UVB irradiation	GO_AMP_BIOSYNTHETIC_PROCESS1	-0.05650813	-0.10743706	-0.1846091	0.85821408	0.94611931	-6.21304347
WT after UVB irradiation VS WT before UVB irradiation	GO_AMP_BIOSYNTHETIC_PROCESS2	0.083259231	-0.00916094	0.217642799	0.83309159	0.96075976	-5.97114995
homo after UVB irradiation VS homo before UVB irradiation	GO_AMP_METABOLIC_PROCESS	0.200596751	-0.0356096	1.325707498	0.22014048	0.41100873	-5.67747234
hetero after UVB irradiation VS hetero before UVB irradiation	GO_AMP_METABOLIC_PROCESS1	-0.0255309	-0.04838145	-0.090421	0.93021645	0.9744077	-6.22705262
WT after UVB irradiation VS WT before UVB irradiation	GO_AMP_METABOLIC_PROCESS2	0.146131616	-0.05110991	0.683342819	0.51346384	0.85974355	-5.75693294
homo after UVB irradiation VS homo before UVB irradiation	GO_AMPA_GLUTAMATE_RECEPTOR_CLUSTERING	0.486055454	0.018130006	2.682869437	0.02682615	0.11581874	-3.72564403
hetero after UVB irradiation VS hetero before UVB irradiation	GO_AMPA_GLUTAMATE_RECEPTOR_CLUSTERING1	0.143592969	-0.04818913	0.529141786	0.61133335	0.83821176	-6.08238712
WT after UVB irradiation VS WT before UVB irradiation	GO_AMPA_GLUTAMATE_RECEPTOR_CLUSTERING2	0.257771171	-0.07186731	1.162703271	0.2780674	0.74917538	-5.3396299
homo after UVB irradiation VS homo before UVB irradiation	GO_AMPA_GLUTAMATE_RECEPTOR_COMPLEX	0.285847504	0.065312819	1.711431354	0.1238696	0.28687125	-5.17798489
hetero after UVB irradiation VS hetero before UVB irradiation	GO_AMPA_GLUTAMATE_RECEPTOR_COMPLEX1	-0.07104163	0.059237662	-0.39619085	0.70251162	0.88151888	-6.14723092
WT after UVB irradiation VS WT before UVB irradiation	GO_AMPA_GLUTAMATE_RECEPTOR_COMPLEX2	0.049679791	0.059950556	0.251916662	0.80738144	0.95404687	-5.96271936
homo after UVB irradiation VS homo before UVB irradiation	GO_AMYGDALA_DEVELOPMENT	-0.19135011	0.035684831	-0.54411191	0.60062264	0.75024448	-6.39125142
hetero after UVB irradiation VS hetero before UVB irradiation	GO_AMYGDALA_DEVELOPMENT1	0.209420081	0.098483679	0.74305066	0.47908817	0.76048031	-5.94250058
WT after UVB irradiation VS WT before UVB irradiation	GO_AMYGDALA_DEVELOPMENT2	-0.36124222	-0.05419278	-1.13680522	0.28813917	0.75467539	-5.36637768
homo after UVB irradiation VS homo before UVB irradiation	GO_AMYLASE_ACTIVITY	-0.09010061	0.160084742	-0.38770473	0.70795473	0.82561333	-6.46911099

hetero after UVB irradiation VS hetero before UVB irradiation	GO_AMYLASE_ACTIVITY1	0.149301336	0.136929468	0.638035793	0.54160469	0.79722035	-6.01645746
WT after UVB irradiation VS WT before UVB irradiation	GO_AMYLASE_ACTIVITY2	0.205130251	0.154090716	0.543079964	0.60169937	0.89567499	-5.84337838
homo after UVB irradiation VS homo before UVB irradiation	GO_AMYLOID_BETA_BINDING	0.40617057	-0.01877468	3.333952356	0.0097476	0.06588762	-2.71913636
hetero after UVB irradiation VS hetero before UVB irradiation	GO_AMYLOID_BETA_BINDING1	0.209287233	0.023267335	1.095277667	0.30584753	0.6274085	-5.62776724
WT after UVB irradiation VS WT before UVB irradiation	GO_AMYLOID_BETA_BINDING2	0.154225623	-0.01076263	0.683344468	0.51346285	0.85974355	-5.75693182
homo after UVB irradiation VS homo before UVB irradiation	GO_AMYLOID_BETA_CLEARANCE	0.483750042	-0.00142688	3.22308015	0.01154819	0.07214932	-2.88894232
homo after UVB irradiation VS homo before UVB irradiation	GO_AMYLOID_BETA_CLEARANCE_BY_CELLULAR_CATABOLIC_PROCESS	0.47831616	-0.06188194	2.341295635	0.04610651	0.15677275	-4.25304418
hetero after UVB irradiation VS hetero before UVB irradiation	GO_AMYLOID_BETA_CLEARANCE_BY_CELLULAR_CATABOLIC_PROCESS1	0.218266908	0.005647273	1.150209713	0.28387808	0.60942163	-5.5704794
WT after UVB irradiation VS WT before UVB irradiation	GO_AMYLOID_BETA_CLEARANCE_BY_CELLULAR_CATABOLIC_PROCESS2	0.303760638	-0.06981429	1.29678546	0.23043397	0.72256251	-5.19470752
homo after UVB irradiation VS homo before UVB irradiation	GO_AMYLOID_BETA_CLEARANCE_BY_TRANSCYTOSIS	0.816061982	0.030813911	3.083991162	0.01431156	0.08157415	-3.10325277
hetero after UVB irradiation VS hetero before UVB irradiation	GO_AMYLOID_BETA_CLEARANCE_BY_TRANSCYTOSIS1	0.963505656	0.023262243	5.494908522	0.0006155	0.08125048	0.150870617
WT after UVB irradiation VS WT before UVB irradiation	GO_AMYLOID_BETA_CLEARANCE_BY_TRANSCYTOSIS2	0.790592418	-0.02797093	4.423923712	0.0021502	0.21585363	-1.02942488
hetero after UVB irradiation VS hetero before UVB irradiation	GO_AMYLOID_BETA_CLEARANCE1	0.382868118	0.004419734	2.197840587	0.05981356	0.32192177	-4.21141914
WT after UVB irradiation VS WT before UVB irradiation	GO_AMYLOID_BETA_CLEARANCE2	0.254222944	-0.07059922	1.386798508	0.2024833	0.69852129	-5.0918388
homo after UVB irradiation VS homo before UVB irradiation	GO_AMYLOID_BETA_FORMATION	0.260424872	0.000285963	1.652937712	0.13544788	0.3037031	-5.25805347
hetero after UVB irradiation VS hetero before UVB irradiation	GO_AMYLOID_BETA_FORMATION1	0.395366407	0.007880069	3.106363673	0.01486261	0.18054811	-2.88541429
WT after UVB irradiation VS WT before UVB irradiation	GO_AMYLOID_BETA_FORMATION2	0.248214929	-0.03289724	1.576335512	0.15314409	0.65981549	-4.86262305
homo after UVB irradiation VS homo before UVB irradiation	GO_AMYLOID_BETA_METABOLIC_PROCESS	0.30963682	-0.02330647	2.045357503	0.07365685	0.20842839	-4.69912175
hetero after UVB irradiation VS hetero before UVB irradiation	GO_AMYLOID_BETA_METABOLIC_PROCESS1	0.265215003	0.003276614	1.984915351	0.08309259	0.36861389	-4.51505508
WT after UVB irradiation VS WT before UVB irradiation	GO_AMYLOID_BETA_METABOLIC_PROCESS2	0.251275378	-0.03293241	1.53814137	0.16212039	0.66546953	-4.91006138
homo after UVB irradiation VS homo before UVB irradiation	GO_AMYLOID_FIBRIL_FORMATION	0.496536359	-0.01925482	2.663235097	0.02767169	0.11761266	-3.75612918
hetero after UVB irradiation VS hetero before UVB irradiation	GO_AMYLOID_FIBRIL_FORMATION1	0.211374847	-0.00551578	0.877039898	0.40649547	0.71194505	-5.83425384
WT after UVB irradiation VS WT before UVB irradiation	GO_AMYLOID_FIBRIL_FORMATION2	0.131885826	-0.02241717	0.616357607	0.55458593	0.87726708	-5.80045016
homo after UVB irradiation VS homo before UVB irradiation	GO_AMYLOID_PRECURSOR_PROTEIN_BIOSYNTHETIC_PROCESS	-0.22948454	-0.0488423	-1.39606499	0.19878062	0.38763573	-5.59188084
hetero after UVB irradiation VS hetero before UVB irradiation	GO_AMYLOID_PRECURSOR_PROTEIN_BIOSYNTHETIC_PROCESS1	-0.28766922	-0.03610684	-1.98283331	0.08335907	0.36912975	-4.51798373
WT after UVB irradiation VS WT before UVB irradiation	GO_AMYLOID_PRECURSOR_PROTEIN_BIOSYNTHETIC_PROCESS2	-0.05863761	0.00099612	-0.25737762	0.80330691	0.95360379	-5.96126421
homo after UVB irradiation VS homo before UVB irradiation	GO_AMYLOID_PRECURSOR_PROTEIN_CATABOLIC_PROCESS	0.336191651	-0.02686303	1.867324637	0.0973496	0.24687886	-4.95857698
hetero after UVB irradiation VS hetero before UVB irradiation	GO_AMYLOID_PRECURSOR_PROTEIN_CATABOLIC_PROCESS1	0.371220766	-0.00106768	2.45538864	0.04014374	0.27243899	-3.83672442
WT after UVB irradiation VS WT before UVB irradiation	GO_AMYLOID_PRECURSOR_PROTEIN_CATABOLIC_PROCESS2	0.291125906	-0.04703515	1.656707214	0.13571293	0.64396005	-4.76096824
homo after UVB irradiation VS homo before UVB irradiation	GO_AMYLOID_PRECURSOR_PROTEIN_METABOLIC_PROCESS	0.25841775	-0.01203294	1.513855535	0.1670435	0.345537	-5.44268276
hetero after UVB irradiation VS hetero before UVB irradiation	GO_AMYLOID_PRECURSOR_PROTEIN_METABOLIC_PROCESS1	0.207636217	0.01349109	1.361734072	0.2110613	0.53706164	-5.33257466
WT after UVB irradiation VS WT before UVB irradiation	GO_AMYLOID_PRECURSOR_PROTEIN_METABOLIC_PROCESS2	0.207323167	-0.0246671	1.371050045	0.2071513	0.7035516	-5.11013693
homo after UVB irradiation VS homo before UVB irradiation	GO_ANAGEN	-0.07863864	0.070785354	-0.24288475	0.8139693	0.89480687	-6.51859653
hetero after UVB irradiation VS hetero before UVB irradiation	GO_ANAGEN1	0.258642539	-0.03926968	1.042642513	0.3281467	0.64786363	-5.68074517
WT after UVB irradiation VS WT before UVB irradiation	GO_ANAGEN2	-0.28713753	0.038629952	-0.77051964	0.46287731	0.84111321	-5.69441475
homo after UVB irradiation VS homo before UVB irradiation	GO_ANAPHASE_PROMOTING_COMPLEX	-0.42740498	0.008310038	-2.42092953	0.04063029	0.14499783	-4.13087669
homo after UVB irradiation VS homo before UVB irradiation	GO_ANAPHASE_PROMOTING_COMPLEX_BINDING	-0.15731853	0.02654869	-0.65022033	0.53307017	0.69993997	-6.32463341
hetero after UVB irradiation VS hetero before UVB irradiation	GO_ANAPHASE_PROMOTING_COMPLEX_BINDING1	0.705045087	0.091295447	2.562946936	0.03399903	0.25433187	-3.67906763
WT after UVB irradiation VS WT before UVB irradiation	GO_ANAPHASE_PROMOTING_COMPLEX_BINDING2	0.245375419	0.002502353	0.896009334	0.39610163	0.81467202	-5.59334984
homo after UVB irradiation VS homo before UVB irradiation	GO_ANAPHASE_PROMOTING_COMPLEX_DEPENDENT_CATABOLIC_PROCESS	-0.49554774	0.047221572	-2.45511547	0.03848393	0.14081512	-4.07824174
hetero after UVB irradiation VS hetero before UVB irradiation	GO_ANAPHASE_PROMOTING_COMPLEX_DEPENDENT_CATABOLIC_PROCESS1	0.116801885	0.001425246	0.535409064	0.60719245	0.83580295	-6.07889969
WT after UVB irradiation VS WT before UVB irradiation	GO_ANAPHASE_PROMOTING_COMPLEX_DEPENDENT_CATABOLIC_PROCESS2	-0.03694714	-0.03830177	-0.13510695	0.89582538	0.97368307	-5.98644649
hetero after UVB irradiation VS hetero before UVB irradiation	GO_ANAPHASE_PROMOTING_COMPLEX1	0.277440703	-0.0390799	1.588110916	0.15163061	0.46987377	-5.05220726
WT after UVB irradiation VS WT before UVB irradiation	GO_ANAPHASE_PROMOTING_COMPLEX2	0.164723477	-0.08853818	0.843773085	0.42302001	0.82491361	-5.63695397
homo after UVB irradiation VS homo before UVB irradiation	GO_ANATOMICAL_STRUCTURE_ARRANGEMENT	-0.52308208	0.029799795	-3.9415857	0.00395715	0.04117099	-1.81151446
hetero after UVB irradiation VS hetero before UVB irradiation	GO_ANATOMICAL_STRUCTURE_ARRANGEMENT1	-0.49179903	-0.03633423	-2.66241333	0.0291712	0.2392555	-3.53309992
WT after UVB irradiation VS WT before UVB irradiation	GO_ANATOMICAL_STRUCTURE_ARRANGEMENT2	-0.46513085	-0.02546795	-2.26684879	0.05276562	0.51991848	-3.93274888
homo after UVB irradiation VS homo before UVB irradiation	GO_ANATOMICAL_STRUCTURE_FORMATION_INVOLVED_IN_MORPHOGENESIS	0.29251948	-0.01306875	2.339436683	0.04624278	0.15705517	-4.25588773
hetero after UVB irradiation VS hetero before UVB irradiation	GO_ANATOMICAL_STRUCTURE_FORMATION_INVOLVED_IN_MORPHOGENESIS1	0.216360141	0.016234085	1.489418446	0.1754021	0.49840812	-5.17731033
WT after UVB irradiation VS WT before UVB irradiation	GO_ANATOMICAL_STRUCTURE_FORMATION_INVOLVED_IN_MORPHOGENESIS2	0.14988749	0.003208442	0.935940119	0.37637234	0.80338597	-5.55859532
homo after UVB irradiation VS homo before UVB irradiation	GO_ANATOMICAL_STRUCTURE_HOMEOSTASIS	0.012767365	-0.00565302	0.106592361	0.9176351	0.95508892	-6.54462539
hetero after UVB irradiation VS hetero before UVB irradiation	GO_ANATOMICAL_STRUCTURE_HOMEOSTASIS1	0.083694958	-0.01124762	0.656557136	0.53023122	0.79127567	-6.00413792
WT after UVB irradiation VS WT before UVB irradiation	GO_ANATOMICAL_STRUCTURE_HOMEOSTASIS2	0.080171793	-0.00105182	0.56401258	0.58802176	0.89199176	-5.83162341
homo after UVB irradiation VS homo before UVB irradiation	GO_ANATOMICAL_STRUCTURE_MATURATION	0.136515393	-0.00358522	1.123345393	0.29262277	0.48727535	-5.90694916
hetero after UVB irradiation VS hetero before UVB irradiation	GO_ANATOMICAL_STRUCTURE_MATURATION1	0.095848318	0.003184752	0.756754174	0.47128945	0.75516855	-5.9321278
WT after UVB irradiation VS WT before UVB irradiation	GO_ANATOMICAL_STRUCTURE_MATURATION2	0.096854373	0.018881907	0.681126529	0.51479363	0.86004864	-5.75843661
homo after UVB irradiation VS homo before UVB irradiation	GO_ANATOMICAL_STRUCTURE_REGRESSION	0.261645285	0.049788574	1.562864385	0.15522249	0.32991766	-5.37860477
hetero after UVB irradiation VS hetero before UVB irradiation	GO_ANATOMICAL_STRUCTURE_REGRESSION1	0.229694772	0.075305997	1.707583482	0.12680478	0.43343324	-4.89563106
WT after UVB irradiation VS WT before UVB irradiation	GO_ANATOMICAL_STRUCTURE_REGRESSION2	0.080887352	0.074204012	0.491792937	0.63591918	0.9055032	-5.87043094
homo after UVB irradiation VS homo before UVB irradiation	GO_ANCHORED_COMPONENT_OF_EXTERNAL_SIDE_OF_PLASMA_MEMBRANE	0.72672604	-0.01419846	5.008010678	0.00092331	0.02088233	-0.34424202
hetero after UVB irradiation VS hetero before UVB irradiation	GO_ANCHORED_COMPONENT_OF_EXTERNAL_SIDE_OF_PLASMA_MEMBRANE1	0.51705788	0.028265233	2.345202432	0.04760866	0.29093095	-3.99769191
WT after UVB irradiation VS WT before UVB irradiation	GO_ANCHORED_COMPONENT_OF_EXTERNAL_SIDE_OF_PLASMA_MEMBRANE2	0.449826185	0.035443818	2.390141398	0.04348539	0.49624823	-3.75839731
homo after UVB irradiation VS homo before UVB irradiation	GO_ANCHORED_COMPONENT_OF_MEMBRANE	0.288486792	-0.00474952	2.304959641	0.04884408	0.16217762	-4.30854927
hetero after UVB irradiation VS hetero before UVB irradiation	GO_ANCHORED_COMPONENT_OF_MEMBRANE1	0.110556451	0.042842677	0.656813049	0.53007509	0.79119847	-6.00396549
WT after UVB irradiation VS WT before UVB irradiation	GO_ANCHORED_COMPONENT_OF_MEMBRANE2	0.101685629	0.033883364	0.591831726	0.57011346	0.88382859	-5.81537014
homo after UVB irradiation VS homo before UVB irradiation	GO_ANCHORED_COMPONENT_OF_PLASMA_MEMBRANE	0.433372021	0.004038428	3.248871355	0.01110032	0.07061828	-2.8493525
hetero after UVB irradiation VS hetero before UVB irradiation	GO_ANCHORED_COMPONENT_OF_PLASMA_MEMBRANE1	0.161874449	0.031049089	0.946931159	0.37189763	0.68555056	-5.77199953
WT after UVB irradiation VS WT before UVB irradiation	GO_ANCHORED_COMPONENT_OF_PLASMA_MEMBRANE2	0.181043266	0.005126958	0.888479965	0.39990413	0.81573919	-5.5997665
homo after UVB irradiation VS homo before UVB irradiation	GO_ANCHORED_COMPONENT_OF_POSTSYNAPTIC_MEMBRANE	0.165041589	0.004413684	0.883683484	0.40165241	0.58944732	-6.14157853
hetero after UVB irradiation VS hetero before UVB irradiation	GO_ANCHORED_COMPONENT_OF_POSTSYNAPTIC_MEMBRANE1	-0.09708385	-0.06157959	-0.40704673	0.69484282	0.87828513	-6.14260087
WT after UVB irradiation VS WT before UVB irradiation	GO_ANCHORED_COMPONENT_OF_POSTSYNAPTIC_MEMBRANE2	-0.02895486	0.020097609	-0.17554765	0.86495976	0.96790654	-5.97983881
homo after UVB irradiation VS homo before UVB irradiation	GO_ANCHORED_COMPONENT_OF_PRESYNAPTIC_MEMBRANE	-0.14181533	0.050903605	-0.578763	0.57806002	0.73441264	-6.37069232
hetero after UVB irradiation VS hetero before UVB irradiation	GO_ANCHORED_COMPONENT_OF_PRESYNAPTIC_MEMBRANE1	-0.38359634	0.045617431	-2.03664405	0.07673113	0.35706231	-4.44201137
WT after UVB irradiation VS WT before UVB irradiation	GO_ANCHORED_COMPONENT_OF_PRESYNAPTIC_MEMBRANE2	-0.24475787	-0.05467957	-1.13479687	0.28893234	0.75486529	-5.36843423
homo after UVB irradiation VS homo before UVB irradiation	GO_ANCHORED_COMPONENT_OF_SYNAPTIC_MEMBRANE	0.010979334	0.0288487	0.051479611	0.96015659	0.97912218	-6.5494025
hetero after UVB irradiation VS hetero before UVB irradiation	GO_ANCHORED_COMPONENT_OF_SYNAPTIC_MEMBRANE1	-0.13963257	0.015322304	-0.81991099	0.43644451	0.73484426	-5.88224179
WT after UVB irradiation VS WT before UVB irradiation	GO_ANCHORED_COMPONENT_OF_SYNAPTIC_MEMBRANE2	-0.24930711	-0.03302573	-1.52140306	0.16620138	0.67086399	-4.93066339
homo after UVB irradiation VS homo before UVB irradiation	GO_ANCHORED_COMPONENT_OF_SYNAPTIC_VESICLE_MEMBRANE	0.299746537	0.012300939	1.400719726	0.19743287	0.38607469	-5.58612162
hetero after UVB irradiation VS hetero before UVB irradiation	GO_ANCHORED_COMPONENT_OF_SYNAPTIC_VESICLE_MEMBRANE1	0.409693454	0.049259886	1.917627837	0.09213539	0.37946684	-4.6092239
WT after UVB irradiation VS WT before UVB irradiation	GO_ANCHORED_COMPONENT_OF_SYNAPTIC_VESICLE_MEMBRANE2	0.147347716	0.067767297	0.724235472	0.48931415	0.85181184	-5.72841946
homo after UVB irradiation VS homo before UVB irradiation	GO_ANCHORING_JUNCTION	0.139949152	-0.00999797	0.872582401	0.40734006	0.59433629	-6.15135381
hetero after UVB irradiation VS hetero before UVB irradiation	GO_ANCHORING_JUNCTION1	0.193248345	-0.00148234	1.559071197	0.1583029	0.47822063	-5.08944286
WT after UVB irradiation VS WT before UVB irradiation	GO_ANCHORING_JUNCTION2	0.026934892	-0.00862623	0.180371861	0.86129302	0.9676681	-5.97893656
homo after UVB irradiation VS homo before UVB irradiation	GO_ANDROGEN_BIOSYNTHETIC_PROCESS	0.027602511	-0.051273	0.121196066	0.90640866	0.94891264	-6.54280261
hetero after UVB irradiation VS hetero before UVB irradiation	GO_ANDROGEN_BIOSYNTHETIC_PROCESS1	-0.22755137	-0.00551204	-0.87330538	0.40840742	0.71330228	-5.83747181
WT after UVB irradiation VS WT before UVB irradiation	GO_ANDROGEN_BIOSYNTHETIC_PROCESS2	-0.21514988	0.023024843	-1.13946694	0.28709064	0.75424544	-5.36364812
homo after UVB irradiation VS homo before UVB irradiation	GO_ANDROGEN_CATABOLIC_PROCESS	0.877329017	0.055548721	5.129251792	0.00079076	0.01938401	-0.18869194
hetero after UVB irradiation VS hetero before UVB irradiation	GO_ANDROGEN_CATABOLIC_PROCESS1	0.452271698	0.083189535	2.028772629	0.07766763	0.35870535	-4.45316
WT after UVB irradiation VS WT before UVB irradiation	GO_ANDROGEN_CATABOLIC_PROCESS2	0.375797325	-0.05862786	1.245358938	0.24782812	0.73564768	-5.25152417
homo after UVB irradiation VS homo before UVB irradiation	GO_ANDROGEN_METABOLIC_PROCESS	0.025519441	0.003917791	0.134776571	0.89598865	0.94335791	-6.54089861
hetero after UVB irradiation VS hetero before UVB irradiation	GO_ANDROGEN_METABOLIC_PROCESS1	-0.07963126	0.023771566	-0.51451378	0.6210564	0.84367202	-6.09037829
WT after UVB irradiation VS WT before UVB irradiation	GO_ANDROGEN_METABOLIC_PROCESS2	-0.14495765	0.076642683	-0.79694777	0.44821545	0.8349077	-5.67419131
homo after UVB irradiation VS homo before UVB irradiation	GO_ANDROGEN_RECEPTOR_BINDING	-0.14316957	0.004258488	-0.84132988	0.42365731	0.60887329	-6.17832084
hetero after UVB irradiation VS hetero before UVB irradiation	GO_ANDROGEN_RECEPTOR_BINDING1	0.066199612	0.035978508	0.37768082	0.71567131	0.88832822	-6.15484822
WT after UVB irradiation VS WT before UVB irradiation	GO_ANDROGEN_RECEPTOR_BINDING2	-0.15181651	-0.0326055	-0.73291966	0.48428077	0.85146732	-5.72217808
homo after UVB irradiation VS homo before UVB irradiation	GO_ANDROGEN_RECEPTOR_SIGNALING_PATHWAY	-0.30835334	0.032739063	-1.7655551	0.11397848	0.27163767	-5.10275108
hetero after UVB irradiation VS hetero before UVB irradiation	GO_ANDROGEN_RECEPTOR_SIGNALING_PATHWAY1	-0.15911907	0.01476894	-0.84149884	0.42495124	0.72623423	-5.86442201
WT after UVB irradiation VS WT before UVB irradiation	GO_ANDROGEN_RECEPTOR_SIGNALING_PATHWAY2	-0.32645423	0.003868795	-1.62593435	0.14216058	0.65167133	-4.80017166
homo after UVB irradiation VS homo before UVB irradiation	GO_ANDROSTERONE_DEHYDROGENASE_ACTIVITY	0.040448347	0.058098036	0.142713754	0.88990835	0.94024173	-6.53969268
hetero after UVB irradiation VS hetero before UVB irradiation	GO_ANDROSTERONE_DEHYDROGENASE_ACTIVITY1	-0.12686053	0.043572277	-0.59155739	0.57077963	0.81424167	-6.04597111
WT after UVB irradiation VS WT before UVB irradiation	GO_ANDROSTERONE_DEHYDROGENASE_ACTIVITY2	-0.42457135	-0.02227635	-2.02828179	0.07663555	0.55925897	-4.26550601
homo after UVB irradiation VS homo before UVB irradiation	GO_ANGIOGENESIS_INVOLVED_IN_WOUND_HEALING	0.606558822	-0.02360707	4.67313046	0.00143232	0.02549668	-0.78600433
hetero after UVB irradiation VS hetero before UVB irradiation	GO_ANGIOGENESIS_INVOLVED_IN_WOUND_HEALING1	0.458926323	0.057340427	2.384512873	0.04479708	0.28321579	-3.9403512
WT after UVB irradiation VS WT before UVB irradiation	GO_ANGIOGENESIS_INVOLVED_IN_WOUND_HEALING2	0.286620572	-0.03213429	0.97888996	0.35596998	0.79088361	-5.51988099
homo after UVB irradiation VS homo before UVB irradiation	GO_ANGIOTENSIN_ACTIVATED_SIGNALING_PATHWAY	0.246631359	-0.02819395	1.695647607	0.1269005	0.29137556	-5.19972108
hetero after UVB irradiation VS hetero before UVB irradiation	GO_ANGIOTENSIN_ACTIVATED_SIGNALING_PATHWAY1	0.104925931	0.013621433	0.55443299	0.59471586	0.82914089	-6.06808128
WT after UVB irradiation VS WT before UVB irradiation	GO_ANGIOTENSIN_ACTIVATED_SIGNALING_PATHWAY2	0.232805504	-0.00587508	1.162338695	0.27820718	0.74917538	-5.34000935
homo after UVB irradiation VS homo before UVB irradiation	GO_ANGIOTENSIN_RECEPTOR_BINDING	0.700486889	0.001406967	3.800799816	0.00485482	0.0454297	-2.01779683
hetero after UVB irradiation VS hetero before UVB irradiation	GO_ANGIOTENSIN_RECEPTOR_BINDING1	0.726714525	0.000874004	4.626321697	0.00178128	0.09780003	-0.84507977
WT after UVB irradiation VS WT before UVB irradiation	GO_ANGIOTENSIN_RECEPTOR_BINDING2	0.538926696	-0.07028978	1.94985147	0.08657483	0.57723988	-4.37290804
homo after UVB irradiation VS homo before UVB irradiation	GO_ANIMAL_ORGAN_FORMATION	-0.15659105	0.015082153	-1.19416942	0.2653084	0.46049825	-5.82963108
hetero after UVB irradiation VS hetero before UVB irradiation	GO_ANIMAL_ORGAN_FORMATION1	-0.30804885	-0.00986074	-2.0518667	0.07495115	0.35391027	-4.42041812
WT after UVB irradiation VS WT before UVB irradiation	GO_ANIMAL_ORGAN_FORMATION2	-0.26163331	0.016694025	-1.67642807	0.13172346	0.63774381	-4.73566937
homo after UVB irradiation VS homo before UVB irradiation	GO_ANIMAL_ORGAN_MATURATION	0.233505127	-0.00405478	1.409409123	0.19493814	0.382286	-5.5753392
hetero after UVB irradiation VS hetero before UVB irradiation	GO_ANIMAL_ORGAN_MATURATION1	-0.11550609	0.027216957	-0.65432985	0.53159121	0.79183076	-6.00563607
WT after UVB irradiation VS WT before UVB irradiation	GO_ANIMAL_ORGAN_MATURATION2	0.180881492	-0.00471453	1.115797253	0.29652308	0.7587209	-5.38776205
homo after UVB irradiation VS homo before UVB irradiation	GO_ANIMAL_ORGAN_MORPHOGENESIS	0.070292155	0.001230191	0.56928687	0.5841831	0.73801815	-6.37643122
hetero after UVB irradiation VS hetero before UVB irradiation	GO_ANIMAL_ORGAN_MORPHOGENESIS1	0.030338498	0.013261445	0.220516644	0.83109388	0.93540378	-6.20519736
WT after UVB irradiation VS WT before UVB irradiation	GO_ANIMAL_ORGAN_MORPHOGENESIS2	-0.00014409	0.010342972	-0.00099147	0.99923291	0.99997598	-5.99606615
homo after UVB irradiation VS homo before UVB irradiation	GO_ANIMAL_ORGAN_REGENERATION	0.090624481	0.015415535	0.678693298	0.51574289	0.68627754	-6.3049337
hetero after UVB irradiation VS hetero before UVB irradiation	GO_ANIMAL_ORGAN_REGENERATION1	0.24081074	0.041504631	1.465450759	0.18165802	0.50686486	-5.20704975
WT after UVB irradiation VS WT before UVB irradiation	GO_ANIMAL_ORGAN_REGENERATION2	0.122348139	-0.02008564	0.725098944	0.48881218	0.8517724	-5.72780178
homo after UVB irradiation VS homo before UVB irradiation	GO_ANION_CATION_SYMPORTER_ACTIVITY	0.122893361	0.000701121	0.557519318	0.59183647	0.74395022	-6.38343594
hetero after UVB irradiation VS hetero before UVB irradiation	GO_ANION_CATION_SYMPORTER_ACTIVITY1	-0.4264859	-0.01993876	-2.65344696	0.02957603	0.24111818	-3.54625824
WT after UVB irradiation VS WT before UVB irradiation	GO_ANION_CATION_SYMPORTER_ACTIVITY2	-0.22375465	-0.01798147	-1.08994144	0.30710758	0.76592641	-5.41368802
homo after UVB irradiation VS homo before UVB irradiation	GO_ANION_CHANNEL_ACTIVITY	-0.07451329	0.038484297	-0.55105981	0.59606077	0.74720258	-6.38722337
hetero after UVB irradiation VS hetero before UVB irradiation	GO_ANION_CHANNEL_ACTIVITY1	-0.30490716	-0.00808412	-2.13969337	0.06544398	0.33409179	-4.29505838
WT after UVB irradiation VS WT before UVB irradiation	GO_ANION_CHANNEL_ACTIVITY2	-0.36210198	0.017401261	-2.41926379	0.04154348	0.4941814	-3.71707367
homo after UVB irradiation VS homo before UVB irradiation	GO_ANION_HOMEOSTASIS	0.284818758	-0.00226606	1.950587623	0.08548008	0.22790055	-4.83827342
hetero after UVB irradiation VS hetero before UVB irradiation	GO_ANION_HOMEOSTASIS1	-0.09446386	-0.00471566	-0.66953491	0.52234929	0.78779499	-5.9953185
WT after UVB irradiation VS WT before UVB irradiation	GO_ANION_HOMEOSTASIS2	-0.01720151	0.010077226	-0.09940019	0.9232373	0.98103299	-5.99085664
homo after UVB irradiation VS homo before UVB irradiation	GO_ANION_SODIUM_SYMPORTER_ACTIVITY	0.008937792	0.005966406	0.037515161	0.97095785	0.98524548	-6.55008438
hetero after UVB irradiation VS hetero before UVB irradiation	GO_ANION_SODIUM_SYMPORTER_ACTIVITY1	-0.39413889	-0.01464825	-2.37107011	0.04573924	0.28562342	-3.95997195
WT after UVB irradiation VS WT before UVB irradiation	GO_ANION_SODIUM_SYMPORTER_ACTIVITY2	-0.43341773	0.012293925	-2.48382386	0.03754405	0.47615783	-3.62533908
homo after UVB irradiation VS homo before UVB irradiation	GO_ANION_TRANSMEMBRANE_TRANSPORT	0.106234246	0.005961082	0.858054537	0.41486925	0.60104315	-6.1639916
hetero after UVB irradiation VS hetero before UVB irradiation	GO_ANION_TRANSMEMBRANE_TRANSPORT1	-0.14797441	-0.00560573	-1.10988018	0.29987876	0.62289006	-5.61273378
WT after UVB irradiation VS WT before UVB irradiation	GO_ANION_TRANSMEMBRANE_TRANSPORT2	-0.10526245	0.000624336	-0.66423259	0.5250001	0.86563125	-5.7697561
homo after UVB irradiation VS homo before UVB irradiation	GO_ANION_TRANSMEMBRANE_TRANSPORTER_ACTIVITY	0.05614041	0.021824509	0.439241857	0.67165896	0.80210984	-6.4462027
hetero after UVB irradiation VS hetero before UVB irradiation	GO_ANION_TRANSMEMBRANE_TRANSPORTER_ACTIVITY1	-0.15586628	0.003910957	-1.24405835	0.24932914	0.57694983	-5.4681583
WT after UVB irradiation VS WT before UVB irradiation	GO_ANION_TRANSMEMBRANE_TRANSPORTER_ACTIVITY2	-0.11542944	0.010816454	-0.73551754	0.48278155	0.85139062	-5.72029841
homo after UVB irradiation VS homo before UVB irradiation	GO_ANION_TRANSPORT	0.12463922	0.009889183	0.991887364	0.34918903	0.54115858	-6.04107612
hetero after UVB irradiation VS hetero before UVB irradiation	GO_ANION_TRANSPORT1	-0.04402169	0.013013576	-0.33762669	0.74449234	0.90030853	-6.17012665
WT after UVB irradiation VS WT before UVB irradiation	GO_ANION_TRANSPORT2	-0.02089557	0.009247989	-0.13400385	0.89667021	0.97390508	-5.98660276
homo after UVB irradiation VS homo before UVB irradiation	GO_ANKYRIN_BINDING	0.416488226	-0.02160515	2.542151998	0.03352014	0.13075779	-3.94382468
hetero after UVB irradiation VS hetero before UVB irradiation	GO_ANKYRIN_BINDING1	0.229878219	0.024851673	1.461943301	0.18258999	0.50688952	-5.21137969
WT after UVB irradiation VS WT before UVB irradiation	GO_ANKYRIN_BINDING2	0.189298742	0.075417783	0.962987691	0.36342532	0.79550655	-5.53437337
homo after UVB irradiation VS homo before UVB irradiation	GO_ANKYRIN_REPEAT_BINDING	0.649361311	-0.04757174	2.852245391	0.02054448	0.09940161	-3.46248472
hetero after UVB irradiation VS hetero before UVB irradiation	GO_ANKYRIN_REPEAT_BINDING1	0.432052119	0.045541675	1.566615679	0.15654426	0.47542876	-5.07980203
WT after UVB irradiation VS WT before UVB irradiation	GO_ANKYRIN_REPEAT_BINDING2	0.037332884	-0.02641053	0.140796278	0.89147037	0.97267492	-5.9856203
homo after UVB irradiation VS homo before UVB irradiation	GO_ANNEALING_ACTIVITY	-0.37223347	-0.02572315	-1.66916059	0.13214082	0.29857634	-5.2359814
hetero after UVB irradiation VS hetero before UVB irradiation	GO_ANNEALING_ACTIVITY1	-0.11063867	-0.04144757	-0.36982881	0.72128482	0.89103348	-6.15797353
WT after UVB irradiation VS WT before UVB irradiation	GO_ANNEALING_ACTIVITY2	-0.26690288	-0.03374715	-1.30032084	0.22927705	0.72156087	-5.19074779

homo after UVB irradiation VS homo before UVB irradiation	GO_ANNEALING_HELICASE_ACTIVITY	-0.15441401	0.003763005	-0.4836897	0.64106635	0.78011149	-6.42426251
hetero after UVB irradiation VS hetero before UVB irradiation	GO_ANNEALING_HELICASE_ACTIVITY1	-0.06436583	-0.02381242	-0.22794915	0.82550898	0.93364073	-6.20340167
WT after UVB irradiation VS WT before UVB irradiation	GO_ANNEALING_HELICASE_ACTIVITY2	0.19690235	0.019949953	0.69495194	0.50653324	0.85763155	-5.74898616
homo after UVB irradiation VS homo before UVB irradiation	GO_ANNULATE_LAMELLAE	-0.02116114	-0.03488803	-0.1144672	0.91157881	0.95151443	-6.54367143
hetero after UVB irradiation VS hetero before UVB irradiation	GO_ANNULATE_LAMELLAE1	0.550585577	-0.01596183	2.501274285	0.037395	0.26516129	-3.76950944
WT after UVB irradiation VS WT before UVB irradiation	GO_ANNULATE_LAMELLAE2	-0.06930708	0.042642724	-0.20778171	0.84053002	0.96316991	-5.97335047
homo after UVB irradiation VS homo before UVB irradiation	GO_ANOIKIS	0.343741	-0.02184026	1.79857571	0.10831092	0.26310194	-5.05634396
hetero after UVB irradiation VS hetero before UVB irradiation	GO_ANOIKIS1	0.290574952	-0.01530117	1.941061496	0.0888834	0.37595306	-4.57654383
WT after UVB irradiation VS WT before UVB irradiation	GO_ANOIKIS2	0.224836437	0.015487114	1.377959222	0.20509199	0.70190963	-5.10212438
homo after UVB irradiation VS homo before UVB irradiation	GO_ANTERIOR_COMMISSURE_MORPHOGENESIS	-0.65565283	0.001421388	-2.59423398	0.03086449	0.1247153	-3.86316782
hetero after UVB irradiation VS hetero before UVB irradiation	GO_ANTERIOR_COMMISSURE_MORPHOGENESIS1	-0.5145407	0.063572568	-1.80816404	0.1088951	0.40740642	-4.76005888
WT after UVB irradiation VS WT before UVB irradiation	GO_ANTERIOR_COMMISSURE_MORPHOGENESIS2	-0.72082215	-0.01924699	-3.4431248	0.0086223	0.31908946	-2.28119755
homo after UVB irradiation VS homo before UVB irradiation	GO_ANTERIOR_HEAD_DEVELOPMENT	-0.1353648	0.035377965	-0.54385254	0.6007933	0.75026713	-6.39140087
hetero after UVB irradiation VS hetero before UVB irradiation	GO_ANTERIOR_HEAD_DEVELOPMENT1	0.097351071	-0.01810228	0.596055916	0.56791688	0.81261967	-6.04320304
WT after UVB irradiation VS WT before UVB irradiation	GO_ANTERIOR_HEAD_DEVELOPMENT2	0.106065582	0.021724761	0.589643621	0.57151074	0.8839005	-5.81667445
homo after UVB irradiation VS homo before UVB irradiation	GO_ANTERIOR_POSTERIOR_AXIS_SPECIFICATION	-0.20118859	0.065682818	-1.29985036	0.22846862	0.41960316	-5.70821636
hetero after UVB irradiation VS hetero before UVB irradiation	GO_ANTERIOR_POSTERIOR_AXIS_SPECIFICATION1	-0.14199667	0.034332167	-0.74408697	0.47849546	0.75997674	-5.94172187
WT after UVB irradiation VS WT before UVB irradiation	GO_ANTERIOR_POSTERIOR_AXIS_SPECIFICATION2	-0.19803518	0.043057476	-1.27630588	0.23723341	0.72854168	-5.21751054
homo after UVB irradiation VS homo before UVB irradiation	GO_ANTERIOR_POSTERIOR_AXON_GUIDANCE	0.039175317	0.062700988	0.169004536	0.86982545	0.92853112	-6.53520836
hetero after UVB irradiation VS hetero before UVB irradiation	GO_ANTERIOR_POSTERIOR_AXON_GUIDANCE1	-0.02671181	0.02364462	-0.10953234	0.91552757	0.96799239	-6.22498301
WT after UVB irradiation VS WT before UVB irradiation	GO_ANTERIOR_POSTERIOR_AXON_GUIDANCE2	-0.41487666	0.082647682	-2.43882681	0.0402882	0.48863525	-3.68929253
homo after UVB irradiation VS homo before UVB irradiation	GO_ANTERIOR_POSTERIOR_PATTERN_SPECIFICATION	-0.16612813	0.026618675	-1.34462058	0.21421375	0.40486069	-5.65473846
hetero after UVB irradiation VS hetero before UVB irradiation	GO_ANTERIOR_POSTERIOR_PATTERN_SPECIFICATION1	-0.07250563	0.029508428	-0.54650397	0.59989892	0.83297985	-6.07263272
WT after UVB irradiation VS WT before UVB irradiation	GO_ANTERIOR_POSTERIOR_PATTERN_SPECIFICATION2	-0.10020825	0.029174952	-0.60478083	0.56188446	0.88048211	-5.80756129
homo after UVB irradiation VS homo before UVB irradiation	GO_ANTEROGRADE_AXONAL_PROTEIN_TRANSPORT	0.098278998	-0.02861097	0.563696116	0.58781238	0.74089061	-6.37977603
hetero after UVB irradiation VS hetero before UVB irradiation	GO_ANTEROGRADE_AXONAL_PROTEIN_TRANSPORT1	-0.29840872	-0.08624358	-1.36799055	0.20917647	0.53447757	-5.32515545
WT after UVB irradiation VS WT before UVB irradiation	GO_ANTEROGRADE_AXONAL_PROTEIN_TRANSPORT2	-0.06062883	-0.06880344	-0.29499064	0.77542282	0.94304554	-5.95041061
homo after UVB irradiation VS homo before UVB irradiation	GO_ANTEROGRADE_AXONAL_TRANSPORT	0.13798957	-0.03221362	1.006000492	0.34274246	0.5355056	-6.02729001
homo after UVB irradiation VS homo before UVB irradiation	GO_ANTEROGRADE_AXONAL_TRANSPORT_OF_MITOCHONDRION	-0.2886936	-0.04360757	-1.58505503	0.15012182	0.32372589	-5.34923073
hetero after UVB irradiation VS hetero before UVB irradiation	GO_ANTEROGRADE_AXONAL_TRANSPORT_OF_MITOCHONDRION1	-0.77735813	-0.06872204	-4.46353666	0.00220027	0.10610192	-1.04606837
WT after UVB irradiation VS WT before UVB irradiation	GO_ANTEROGRADE_AXONAL_TRANSPORT_OF_MITOCHONDRION2	-0.49786403	-0.12687925	-2.20407999	0.05822225	0.52549586	-4.02100758
hetero after UVB irradiation VS hetero before UVB irradiation	GO_ANTEROGRADE_AXONAL_TRANSPORT1	-0.13431438	-0.0303889	-0.93057324	0.3797938	0.69239404	-5.78691086
WT after UVB irradiation VS WT before UVB irradiation	GO_ANTEROGRADE_AXONAL_TRANSPORT2	0.139579867	-0.03110431	0.994081859	0.34895572	0.78651615	-5.50586466
homo after UVB irradiation VS homo before UVB irradiation	GO_ANTEROGRADE_DENDRITIC_TRANSPORT	0.164102623	0.016347528	0.744318081	0.4771555	0.65498172	-6.25668284
hetero after UVB irradiation VS hetero before UVB irradiation	GO_ANTEROGRADE_DENDRITIC_TRANSPORT1	0.052681217	0.004992217	0.261034092	0.80078135	0.92279029	-6.19469731
WT after UVB irradiation VS WT before UVB irradiation	GO_ANTEROGRADE_DENDRITIC_TRANSPORT2	-0.05504098	-0.09887722	-0.23069375	0.8232745	0.95856757	-5.96808276
homo after UVB irradiation VS homo before UVB irradiation	GO_ANTEROGRADE_NEURONAL_DENSE_CORE_VESICLE_TRANSPORT	-0.26633468	-0.01596259	-1.06553612	0.31654304	0.50995913	-5.96748591
hetero after UVB irradiation VS hetero before UVB irradiation	GO_ANTEROGRADE_NEURONAL_DENSE_CORE_VESICLE_TRANSPORT1	-0.19097283	-0.0735841	-0.63040422	0.54633333	0.7993465	-6.02144163
WT after UVB irradiation VS WT before UVB irradiation	GO_ANTEROGRADE_NEURONAL_DENSE_CORE_VESICLE_TRANSPORT2	-0.3637847	0.00625791	-1.35675894	0.21146762	0.70891132	-5.12663387
homo after UVB irradiation VS homo before UVB irradiation	GO_ANTIBACTERIAL_HUMORAL_RESPONSE	0.766174766	0.019745594	5.23371839	0.00069307	0.01856187	-0.05653046
hetero after UVB irradiation VS hetero before UVB irradiation	GO_ANTIBACTERIAL_HUMORAL_RESPONSE1	0.587494878	0.056102678	3.061339682	0.015901	0.18566181	-2.95053418
WT after UVB irradiation VS WT before UVB irradiation	GO_ANTIBACTERIAL_HUMORAL_RESPONSE2	0.42893563	0.048693497	1.780799508	0.11236011	0.61708154	-4.59966347
homo after UVB irradiation VS homo before UVB irradiation	GO_ANTIFUNGAL_HUMORAL_RESPONSE	1.043727843	-0.02133291	7.283322721	6.94E-05	0.00942429	2.206147332
hetero after UVB irradiation VS hetero before UVB irradiation	GO_ANTIFUNGAL_HUMORAL_RESPONSE1	0.918847234	0.122685533	3.945409121	0.00442397	0.12651442	-1.71555015
WT after UVB irradiation VS WT before UVB irradiation	GO_ANTIFUNGAL_HUMORAL_RESPONSE2	1.042076005	0.012885461	5.010210537	0.00100227	0.19353487	-0.36198887
homo after UVB irradiation VS homo before UVB irradiation	GO_ANTIFUNGAL_INNATE_IMMUNE_RESPONSE	0.668018939	-0.03300703	2.154096219	0.06203814	0.18790943	-4.53698486
hetero after UVB irradiation VS hetero before UVB irradiation	GO_ANTIFUNGAL_INNATE_IMMUNE_RESPONSE1	0.281285344	0.041595075	0.801990008	0.44614754	0.74052375	-5.89674032
WT after UVB irradiation VS WT before UVB irradiation	GO_ANTIFUNGAL_INNATE_IMMUNE_RESPONSE2	0.774947562	0.089608409	2.747471255	0.02487175	0.42827578	-3.25029856
homo after UVB irradiation VS homo before UVB irradiation	GO_ANTIGEN_BINDING	0.337628051	-0.01071887	1.696077469	0.12681706	0.2913167	-5.19913036
hetero after UVB irradiation VS hetero before UVB irradiation	GO_ANTIGEN_BINDING1	0.128394674	-0.00014831	0.655563254	0.53083782	0.79153627	-6.004807
WT after UVB irradiation VS WT before UVB irradiation	GO_ANTIGEN_BINDING2	0.137279722	-0.0224264	0.535301322	0.60682545	0.89713202	-5.84764179
homo after UVB irradiation VS homo before UVB irradiation	GO_ANTIGEN_PROCESSING_AND_PRESENTATION	0.244536903	-0.03789234	1.496289763	0.17147221	0.35105652	-5.46537381
homo after UVB irradiation VS homo before UVB irradiation	GO_ANTIGEN_PROCESSING_AND_PRESENTATION_ENDOGENOUS_LIPID_ANTIGEN_VIA_MHC_CLASS_IB	1.017022863	-0.03439778	3.974981182	0.00377137	0.04021247	-1.76297883
hetero after UVB irradiation VS hetero before UVB irradiation	GO_ANTIGEN_PROCESSING_AND_PRESENTATION_ENDOGENOUS_LIPID_ANTIGEN_VIA_MHC_CLASS_IB1	0.281100553	0.050266029	0.637876879	0.54170291	0.79722035	-6.0165618
WT after UVB irradiation VS WT before UVB irradiation	GO_ANTIGEN_PROCESSING_AND_PRESENTATION_ENDOGENOUS_LIPID_ANTIGEN_VIA_MHC_CLASS_IB2	0.608778586	-0.04872385	1.705449281	0.1260492	0.63290509	-4.69820001
homo after UVB irradiation VS homo before UVB irradiation	GO_ANTIGEN_PROCESSING_AND_PRESENTATION_OF_ENDOGENOUS_ANTIGEN	0.886045402	-0.0400518	5.920667153	0.0003023	0.01276035	0.770131021
hetero after UVB irradiation VS hetero before UVB irradiation	GO_ANTIGEN_PROCESSING_AND_PRESENTATION_OF_ENDOGENOUS_ANTIGEN1	0.542603596	0.063564858	2.025828345	0.07802078	0.3598858	-4.45732709
WT after UVB irradiation VS WT before UVB irradiation	GO_ANTIGEN_PROCESSING_AND_PRESENTATION_OF_ENDOGENOUS_ANTIGEN2	0.660107281	0.051412357	2.226992935	0.05616849	0.5232848	-3.98883759
homo after UVB irradiation VS homo before UVB irradiation	GO_ANTIGEN_PROCESSING_AND_PRESENTATION_OF_ENDOGENOUS_PEPTIDE_ANTIGEN	1.12864677	-0.02155862	8.947786219	1.49E-05	0.00847131	3.646581074
homo after UVB irradiation VS homo before UVB irradiation	GO_ANTIGEN_PROCESSING_AND_PRESENTATION_OF_ENDOGENOUS_PEPTIDE_ANTIGEN_VIA_MHC_CLASS_I_VIA_ER_PATHWAY	1.133843369	0.001162244	8.500142273	2.20E-05	0.00847131	3.289030825
hetero after UVB irradiation VS hetero before UVB irradiation	GO_ANTIGEN_PROCESSING_AND_PRESENTATION_OF_ENDOGENOUS_PEPTIDE_ANTIGEN_VIA_MHC_CLASS_I_VIA_ER_PATHWAY1	0.555997414	0.047377647	1.49875012	0.1730189	0.49554869	-5.16566107
WT after UVB irradiation VS WT before UVB irradiation	GO_ANTIGEN_PROCESSING_AND_PRESENTATION_OF_ENDOGENOUS_PEPTIDE_ANTIGEN_VIA_MHC_CLASS_I_VIA_ER_PATHWAY2	0.694520002	0.038784257	1.951411641	0.08636552	0.57723988	-4.37078367
hetero after UVB irradiation VS hetero before UVB irradiation	GO_ANTIGEN_PROCESSING_AND_PRESENTATION_OF_ENDOGENOUS_PEPTIDE_ANTIGEN1	0.689056868	0.083123035	2.484588447	0.03837175	0.2684222	-3.79396064
WT after UVB irradiation VS WT before UVB irradiation	GO_ANTIGEN_PROCESSING_AND_PRESENTATION_OF_ENDOGENOUS_PEPTIDE_ANTIGEN2	0.72964017	0.045712588	2.491600935	0.03708926	0.47615783	-3.61427948
homo after UVB irradiation VS homo before UVB irradiation	GO_ANTIGEN_PROCESSING_AND_PRESENTATION_OF_EXOGENOUS_PEPTIDE_ANTIGEN_VIA_MHC_CLASS_I	0.132961325	-0.02833337	0.554071881	0.59408894	0.7455246	-6.38546238
homo after UVB irradiation VS homo before UVB irradiation	GO_ANTIGEN_PROCESSING_AND_PRESENTATION_OF_EXOGENOUS_PEPTIDE_ANTIGEN_VIA_MHC_CLASS_I_TAP_INDEPENDENT	0.945462027	-0.04979904	5.714010873	0.00038543	0.01473521	0.529083459
hetero after UVB irradiation VS hetero before UVB irradiation	GO_ANTIGEN_PROCESSING_AND_PRESENTATION_OF_EXOGENOUS_PEPTIDE_ANTIGEN_VIA_MHC_CLASS_I_TAP_INDEPENDENT1	0.51330431	-0.02366747	1.403518527	0.19874853	0.52310096	-5.28264412
WT after UVB irradiation VS WT before UVB irradiation	GO_ANTIGEN_PROCESSING_AND_PRESENTATION_OF_EXOGENOUS_PEPTIDE_ANTIGEN_VIA_MHC_CLASS_I_TAP_INDEPENDENT2	0.446149153	-0.06207075	1.277089174	0.23697026	0.72853545	-5.21664263
hetero after UVB irradiation VS hetero before UVB irradiation	GO_ANTIGEN_PROCESSING_AND_PRESENTATION_OF_EXOGENOUS_PEPTIDE_ANTIGEN_VIA_MHC_CLASS_I1	0.316251132	0.00034445	1.543133859	0.16207694	0.48325062	-5.10973114
WT after UVB irradiation VS WT before UVB irradiation	GO_ANTIGEN_PROCESSING_AND_PRESENTATION_OF_EXOGENOUS_PEPTIDE_ANTIGEN_VIA_MHC_CLASS_I2	0.335333499	-0.04322494	1.462125372	0.18140242	0.68481851	-5.00265949
homo after UVB irradiation VS homo before UVB irradiation	GO_ANTIGEN_PROCESSING_AND_PRESENTATION_OF_LIPID_ANTIGEN_VIA_MHC_CLASS_IB	0.647037717	-0.04081207	2.260704981	0.05239706	0.16934371	-4.3759177
hetero after UVB irradiation VS hetero before UVB irradiation	GO_ANTIGEN_PROCESSING_AND_PRESENTATION_OF_LIPID_ANTIGEN_VIA_MHC_CLASS_IB1	-0.13471366	-0.06589804	-0.44214783	0.670306	0.86556131	-6.12681306
WT after UVB irradiation VS WT before UVB irradiation	GO_ANTIGEN_PROCESSING_AND_PRESENTATION_OF_LIPID_ANTIGEN_VIA_MHC_CLASS_IB2	0.59951802	0.000479612	2.881209562	0.02021548	0.39829087	-3.06077377
homo after UVB irradiation VS homo before UVB irradiation	GO_ANTIGEN_PROCESSING_AND_PRESENTATION_OF_PEPTIDE_ANTIGEN	0.189399323	-0.02838217	1.092671026	0.30513062	0.49953544	-5.93936671
homo after UVB irradiation VS homo before UVB irradiation	GO_ANTIGEN_PROCESSING_AND_PRESENTATION_OF_PEPTIDE_ANTIGEN_VIA_MHC_CLASS_I	0.250044667	-0.02725003	1.142598347	0.28498307	0.47986854	-5.88626724
hetero after UVB irradiation VS hetero before UVB irradiation	GO_ANTIGEN_PROCESSING_AND_PRESENTATION_OF_PEPTIDE_ANTIGEN_VIA_MHC_CLASS_I1	0.365590788	0.013368719	2.051124495	0.075037	0.35391027	-4.42147191
WT after UVB irradiation VS WT before UVB irradiation	GO_ANTIGEN_PROCESSING_AND_PRESENTATION_OF_PEPTIDE_ANTIGEN_VIA_MHC_CLASS_I2	0.385505501	-0.03456963	1.858383984	0.09973194	0.59874718	-4.49648941
homo after UVB irradiation VS homo before UVB irradiation	GO_ANTIGEN_PROCESSING_AND_PRESENTATION_OF_PEPTIDE_ANTIGEN_VIA_MHC_CLASS_IB	1.098177226	-0.03244243	8.067041833	3.26E-05	0.00847131	2.9230233
hetero after UVB irradiation VS hetero before UVB irradiation	GO_ANTIGEN_PROCESSING_AND_PRESENTATION_OF_PEPTIDE_ANTIGEN_VIA_MHC_CLASS_IB1	0.452861901	0.031064261	1.206922776	0.26255862	0.58915982	-5.50929436
WT after UVB irradiation VS WT before UVB irradiation	GO_ANTIGEN_PROCESSING_AND_PRESENTATION_OF_PEPTIDE_ANTIGEN_VIA_MHC_CLASS_IB2	0.70547312	0.056189113	2.094530167	0.06910993	0.54421307	-4.17391071
hetero after UVB irradiation VS hetero before UVB irradiation	GO_ANTIGEN_PROCESSING_AND_PRESENTATION_OF_PEPTIDE_ANTIGEN1	0.364902121	0.010794995	2.500066014	0.03746488	0.26552915	-3.77128034
WT after UVB irradiation VS WT before UVB irradiation	GO_ANTIGEN_PROCESSING_AND_PRESENTATION_OF_PEPTIDE_ANTIGEN2	0.342632437	0.003629734	1.966490819	0.08436742	0.573209	-4.35022506
homo after UVB irradiation VS homo before UVB irradiation	GO_ANTIGEN_PROCESSING_AND_PRESENTATION_OF_PEPTIDE_OR_POLYSACCHARIDE_ANTIGEN_VIA_MHC_CLASS_II	0.179757239	-0.01643124	1.089394152	0.3064914	0.5009851	-5.9427905
hetero after UVB irradiation VS hetero before UVB irradiation	GO_ANTIGEN_PROCESSING_AND_PRESENTATION_OF_PEPTIDE_OR_POLYSACCHARIDE_ANTIGEN_VIA_MHC_CLASS_II1	0.40982225	0.01005123	3.077818081	0.0155124	0.18429049	-2.92668071
WT after UVB irradiation VS WT before UVB irradiation	GO_ANTIGEN_PROCESSING_AND_PRESENTATION_OF_PEPTIDE_OR_POLYSACCHARIDE_ANTIGEN_VIA_MHC_CLASS_II2	0.296217943	0.04057673	1.967086596	0.08428939	0.57320671	-4.34941181
homo after UVB irradiation VS homo before UVB irradiation	GO_ANTIGEN_PROCESSING_AND_PRESENTATION_VIA_MHC_CLASS_IB	0.978891109	-0.02303533	5.944919975	0.00029391	0.01275671	0.797997625
hetero after UVB irradiation VS hetero before UVB irradiation	GO_ANTIGEN_PROCESSING_AND_PRESENTATION_VIA_MHC_CLASS_IB1	0.198552877	0.04861429	0.690238014	0.50992619	0.77957635	-5.9809327
WT after UVB irradiation VS WT before UVB irradiation	GO_ANTIGEN_PROCESSING_AND_PRESENTATION_VIA_MHC_CLASS_IB2	0.645310565	0.041395878	2.239656221	0.05506437	0.5232848	-3.97103398
hetero after UVB irradiation VS hetero before UVB irradiation	GO_ANTIGEN_PROCESSING_AND_PRESENTATION1	0.381557482	0.006532024	2.697522066	0.02764001	0.23528578	-3.48158513
WT after UVB irradiation VS WT before UVB irradiation	GO_ANTIGEN_PROCESSING_AND_PRESENTATION2	0.362360772	0.004501278	2.184104538	0.06007312	0.52638053	-4.04900443
homo after UVB irradiation VS homo before UVB irradiation	GO_ANTIGEN_RECEPTOR_MEDIATED_SIGNALING_PATHWAY	0.137253377	-0.03699275	0.81418045	0.43819676	0.62111897	-6.20107545
hetero after UVB irradiation VS hetero before UVB irradiation	GO_ANTIGEN_RECEPTOR_MEDIATED_SIGNALING_PATHWAY1	0.248413459	-0.00037686	1.765214185	0.11623056	0.41924211	-4.81833462
WT after UVB irradiation VS WT before UVB irradiation	GO_ANTIGEN_RECEPTOR_MEDIATED_SIGNALING_PATHWAY2	0.234729176	-0.00060177	1.473138102	0.17848824	0.68201735	-4.9894008
homo after UVB irradiation VS homo before UVB irradiation	GO_ANTIMICROBIAL_HUMORAL_IMMUNE_RESPONSE_MEDIATED_BY_ANTIMICROBIAL_PEPTIDE	0.755293524	0.012714003	4.695669461	0.0013899	0.0251357	-0.75571429
hetero after UVB irradiation VS hetero before UVB irradiation	GO_ANTIMICROBIAL_HUMORAL_IMMUNE_RESPONSE_MEDIATED_BY_ANTIMICROBIAL_PEPTIDE1	0.714964254	0.053737237	4.905918941	0.00125052	0.09106857	-0.51047564
WT after UVB irradiation VS WT before UVB irradiation	GO_ANTIMICROBIAL_HUMORAL_IMMUNE_RESPONSE_MEDIATED_BY_ANTIMICROBIAL_PEPTIDE2	0.488601584	0.095914115	2.678991576	0.02767003	0.44102721	-3.34763113
homo after UVB irradiation VS homo before UVB irradiation	GO_ANTIMICROBIAL_HUMORAL_RESPONSE	0.6657436	0.029114681	4.728677353	0.00133021	0.02450673	-0.71149988
hetero after UVB irradiation VS hetero before UVB irradiation	GO_ANTIMICROBIAL_HUMORAL_RESPONSE1	0.636684596	0.059219933	4.296779634	0.00274297	0.11244771	-1.25664691
WT after UVB irradiation VS WT before UVB irradiation	GO_ANTIMICROBIAL_HUMORAL_RESPONSE2	0.48744556	0.072165863	2.74834862	0.02483784	0.42819162	-3.24905251
homo after UVB irradiation VS homo before UVB irradiation	GO_ANTIMICROBIAL_PEPTIDE_PRODUCTION	0.997516287	-0.09165048	5.070731077	0.00085195	0.01999553	-0.26348187
hetero after UVB irradiation VS hetero before UVB irradiation	GO_ANTIMICROBIAL_PEPTIDE_PRODUCTION1	0.708680178	0.01574453	3.707603563	0.00617661	0.13522564	-2.03731463
WT after UVB irradiation VS WT before UVB irradiation	GO_ANTIMICROBIAL_PEPTIDE_PRODUCTION2	0.301464093	0.122941727	0.878693205	0.40488572	0.81613006	-5.60804126
homo after UVB irradiation VS homo before UVB irradiation	GO_ANTIOXIDANT_ACTIVITY	0.45577614	-0.00646574	2.846244977	0.0207389	0.09972109	-3.47180646
hetero after UVB irradiation VS hetero before UVB irradiation	GO_ANTIOXIDANT_ACTIVITY1	0.320799587	0.021644913	1.892101635	0.09580779	0.38611944	-4.64467356
WT after UVB irradiation VS WT before UVB irradiation	GO_ANTIOXIDANT_ACTIVITY2	0.321590755	0.023785696	1.481402513	0.17632866	0.68058088	-4.97941532
homo after UVB irradiation VS homo before UVB irradiation	GO_ANTIPORTER_ACTIVITY	0.122184059	-0.01143644	0.850735007	0.4186995	0.60416041	-6.17029182
hetero after UVB irradiation VS hetero before UVB irradiation	GO_ANTIPORTER_ACTIVITY1	-0.05122673	-0.00524916	-0.3654913	0.72439353	0.89176195	-6.15967282
WT after UVB irradiation VS WT before UVB irradiation	GO_ANTIPORTER_ACTIVITY2	0.022619912	0.009342956	0.136439978	0.89480465	0.97322079	-5.98625595
homo after UVB irradiation VS homo before UVB irradiation	GO_ANTRAL_OVARIAN_FOLLICLE_GROWTH	0.503318895	0.03216116	2.732360175	0.02480993	0.1107919	-3.64876797
hetero after UVB irradiation VS hetero before UVB irradiation	GO_ANTRAL_OVARIAN_FOLLICLE_GROWTH1	-0.17508301	0.031537988	-0.78529105	0.45532071	0.7461091	-5.91000691
WT after UVB irradiation VS WT before UVB irradiation	GO_ANTRAL_OVARIAN_FOLLICLE_GROWTH2	-0.34253117	0.051360064	-1.82369571	0.10520267	0.60922187	-4.54282026
homo after UVB irradiation VS homo before UVB irradiation	GO_AORTA_DEVELOPMENT	0.122416278	-0.01170016	0.852926711	0.41755001	0.60346515	-6.16841006
hetero after UVB irradiation VS hetero before UVB irradiation	GO_AORTA_DEVELOPMENT1	0.066386397	-0.00648475	0.437950887	0.67321842	0.86714482	-6.12876617
WT after UVB irradiation VS WT before UVB irradiation	GO_AORTA_DEVELOPMENT2	0.0330103	0.00040322	0.21460342	0.8353824	0.96163722	-5.97183894
homo after UVB irradiation VS homo before UVB irradiation	GO_AORTA_MORPHOGENESIS	0.144687891	-0.00884752	0.945461484	0.37103887	0.56129375	-6.08533896
hetero after UVB irradiation VS hetero before UVB irradiation	GO_AORTA_MORPHOGENESIS1	0.02730873	-0.00599178	0.152374839	0.88273351	0.95748425	-6.21891203
WT after UVB irradiation VS WT before UVB irradiation	GO_AORTA_MORPHOGENESIS2	0.100399933	0.024500081	0.475084696	0.64727667	0.90907607	-5.87870137
homo after UVB irradiation VS homo before UVB irradiation	GO_AORTA_SMOOTH_MUSCLE_TISSUE_MORPHOGENESIS	0.022719456	-0.14074543	0.058965477	0.95436983	0.97606456	-6.54894891
hetero after UVB irradiation VS hetero before UVB irradiation	GO_AORTA_SMOOTH_MUSCLE_TISSUE_MORPHOGENESIS1	-0.52887058	-0.12710128	-1.63216517	0.14199752	0.45755083	-4.99508424
WT after UVB irradiation VS WT before UVB irradiation	GO_AORTA_SMOOTH_MUSCLE_TISSUE_MORPHOGENESIS2	-0.0488312	-0.00368082	-0.11759895	0.90925009	0.97832631	-5.98877612
homo after UVB irradiation VS homo before UVB irradiation	GO_AORTIC_VALVE_DEVELOPMENT	0.239177519	0.019538457	1.763277904	0.11437938	0.27202199	-5.10593761
hetero after UVB irradiation VS hetero before UVB irradiation	GO_AORTIC_VALVE_DEVELOPMENT1	0.209201054	0.015027288	1.491763706	0.1748004	0.49736582	-5.17438628
WT after UVB irradiation VS WT before UVB irradiation	GO_AORTIC_VALVE_DEVELOPMENT2	0.098203324	0.044042629	0.610546902	0.55824234	0.87964603	-5.80403471
homo after UVB irradiation VS homo before UVB irradiation	GO_AP_1_ADAPTOR_COMPLEX	0.422795433	-0.03713709	1.723918748	0.12151927	0.28342973	-5.16072179
homo after UVB irradiation VS homo before UVB irradiation	GO_AP_1_ADAPTOR_COMPLEX_BINDING	0.120283875	-0.07974348	0.418679101	0.68603848	0.81211032	-6.455671
hetero after UVB irradiation VS hetero before UVB irradiation	GO_AP_1_ADAPTOR_COMPLEX_BINDING1	0.722172604	0.004899456	3.759627564	0.00573775	0.13234683	-1.96618774
WT after UVB irradiation VS WT before UVB irradiation	GO_AP_1_ADAPTOR_COMPLEX_BINDING2	0.617259031	0.164895403	2.198816262	0.05870444	0.52638053	-4.02838957
hetero after UVB irradiation VS hetero before UVB irradiation	GO_AP_1_ADAPTOR_COMPLEX1	0.125469746	0.083100794	0.486990588	0.63956674	0.8522738	-6.10484638
WT after UVB irradiation VS WT before UVB irradiation	GO_AP_1_ADAPTOR_COMPLEX2	0.067765546	0.126256266	0.200177678	0.84627758	0.96503502	-5.97497843
homo after UVB irradiation VS homo before UVB irradiation	GO_AP_2_ADAPTOR_COMPLEX_BINDING	0.013704266	-0.07098666	0.080078468	0.93806555	0.96709748	-6.54733875
hetero after UVB irradiation VS hetero before UVB irradiation	GO_AP_2_ADAPTOR_COMPLEX_BINDING1	0.479274299	-0.00539719	2.894724548	0.02045266	0.20617329	-3.19286238
WT after UVB irradiation VS WT before UVB irradiation	GO_AP_2_ADAPTOR_COMPLEX_BINDING2	0.335637096	-0.00475123	1.20958555	0.2605642	0.74056733	-5.29015652
homo after UVB irradiation VS homo before UVB irradiation	GO_AP_3_ADAPTOR_COMPLEX	0.410239269	-0.04143672	2.980317108	0.0168142	0.0885787	-3.26371491
hetero after UVB irradiation VS hetero before UVB irradiation	GO_AP_3_ADAPTOR_COMPLEX1	0.082888586	-0.038172	0.394409087	0.70377381	0.88201854	-6.14797938
WT after UVB irradiation VS WT before UVB irradiation	GO_AP_3_ADAPTOR_COMPLEX2	0.659255393	0.014897552	2.949351424	0.01819936	0.38810404	-2.96461589
homo after UVB irradiation VS homo before UVB irradiation	GO_AP_4_ADAPTOR_COMPLEX	-0.52763464	-0.0037705	-2.0028092	0.07875594	0.21642485	-4.76186715
hetero after UVB irradiation VS hetero before UVB irradiation	GO_AP_4_ADAPTOR_COMPLEX1	-0.36554556	-0.02207147	-1.03208101	0.33277048	0.65240229	-5.6911421
WT after UVB irradiation VS WT before UVB irradiation	GO_AP_4_ADAPTOR_COMPLEX2	-0.47565039	0.03954527	-1.65797213	0.13545374	0.64335078	-4.75934959
homo after UVB irradiation VS homo before UVB irradiation	GO_AP_TYPE_MEMBRANE_COAT_ADAPTOR_COMPLEX	0.250989973	-0.03445116	1.76170031	0.11465788	0.27256655	-5.1081441
hetero after UVB irradiation VS hetero before UVB irradiation	GO_AP_TYPE_MEMBRANE_COAT_ADAPTOR_COMPLEX1	0.259425694	0.026026339	1.341823373	0.21715779	0.54440713	-5.35604901
WT after UVB irradiation VS WT before UVB irradiation	GO_AP_TYPE_MEMBRANE_COAT_ADAPTOR_COMPLEX2	0.265252862	0.04058335	1.346297875	0.21467611	0.71222466	-5.13864356
homo after UVB irradiation VS homo before UVB irradiation	GO_APICAL_CORTEX	-0.24681405	-0.01084409	-1.26404335	0.24043961	0.43328457	-5.75013184
hetero after UVB irradiation VS hetero before UVB irradiation	GO_APICAL_CORTEX1	-0.10576815	0.004911063	-0.33482678	0.74652383	0.90058797	-6.17113267
WT after UVB irradiation VS WT before UVB irradiation	GO_APICAL_CORTEX2	-0.1119398	0.034639108	-0.34680134	0.73758073	0.93478445	-5.93310049
homo after UVB irradiation VS homo before UVB irradiation	GO_APICAL_DENDRITE	0.147344668	-9.86E-05	0.977068801	0.35605581	0.54771135	-6.05538671
hetero after UVB irradiation VS hetero before UVB irradiation	GO_APICAL_DENDRITE1	-0.18456579	0.004911133	-0.91398474	0.38792689	0.69807916	-5.80182155
WT after UVB irradiation VS WT before UVB irradiation	GO_APICAL_DENDRITE2	-0.31819697	0.035135703	-1.78346875	0.11190169	0.61576748	-4.59614152
homo after UVB irradiation VS homo before UVB irradiation	GO_APICAL_JUNCTION_ASSEMBLY	-0.17784297	0.037062992	-0.94581518	0.37086866	0.56115132	-6.08500813
hetero after UVB irradiation VS hetero before UVB irradiation	GO_APICAL_JUNCTION_ASSEMBLY1	-0.04580304	-0.01749623	-0.25772199	0.80324657	0.92371161	-6.19562088
WT after UVB irradiation VS WT before UVB irradiation	GO_APICAL_JUNCTION_ASSEMBLY2	-0.22918891	-0.02221184	-1.2376449	0.25052978	0.73564768	-5.25991772
homo after UVB irradiation VS homo before UVB irradiation	GO_APICAL_JUNCTION_COMPLEX	-0.24338161	0.018317316	-1.53507457	0.16182993	0.33878908	-5.41507638
hetero after UVB irradiation VS hetero before UVB irradiation	GO_APICAL_JUNCTION_COMPLEX1	-0.16992555	-0.0235082	-1.05597098	0.32238312	0.64170619	-5.66751185

WT after UVB irradiation VS WT before UVB irradiation	GO_APICAL_JUNCTION_COMPLEX2	-0.30876895	-0.03102487	-1.6954252	0.12798303	0.63467098	-4.71117376
homo after UVB irradiation VS homo before UVB irradiation	GO_APICAL_PART_OF_CELL	0.068540494	0.004348592	0.496045601	0.63268588	0.77321287	-6.41780855
hetero after UVB irradiation VS hetero before UVB irradiation	GO_APICAL_PART_OF_CELL1	0.003532559	0.001505356	0.027989118	0.97836905	0.99256196	-6.2310593
WT after UVB irradiation VS WT before UVB irradiation	GO_APICAL_PART_OF_CELL2	-0.08034472	0.014936857	-0.57342099	0.58193048	0.88904835	-5.8262068
homo after UVB irradiation VS homo before UVB irradiation	GO_APICAL_PLASMA_MEMBRANE	0.056972607	0.004706988	0.409922996	0.69220334	0.81568671	-6.45957086
hetero after UVB irradiation VS hetero before UVB irradiation	GO_APICAL_PLASMA_MEMBRANE1	-0.02977556	0.002433788	-0.23594427	0.81951316	0.93071163	-6.20140452
WT after UVB irradiation VS WT before UVB irradiation	GO_APICAL_PLASMA_MEMBRANE2	-0.10822983	0.018052827	-0.76935472	0.46353087	0.84167898	-5.6952928
homo after UVB irradiation VS homo before UVB irradiation	GO_APICAL_PROTEIN_LOCALIZATION	-0.18277507	-0.02031404	-0.87863236	0.40423331	0.59127051	-6.14603906
hetero after UVB irradiation VS hetero before UVB irradiation	GO_APICAL_PROTEIN_LOCALIZATION1	-0.21661794	-0.0158003	-1.41059559	0.19672648	0.5210771	-5.27410008
WT after UVB irradiation VS WT before UVB irradiation	GO_APICAL_PROTEIN_LOCALIZATION2	-0.30984254	-0.03295839	-2.03101643	0.07630972	0.55881014	-4.26173999
homo after UVB irradiation VS homo before UVB irradiation	GO_APICOLATERAL_PLASMA_MEMBRANE	-0.5030075	0.002228571	-3.03826796	0.01536387	0.08414192	-3.17395815
hetero after UVB irradiation VS hetero before UVB irradiation	GO_APICOLATERAL_PLASMA_MEMBRANE1	-0.53072116	0.024211597	-2.99848295	0.01747942	0.19363832	-3.04172341
WT after UVB irradiation VS WT before UVB irradiation	GO_APICOLATERAL_PLASMA_MEMBRANE2	-0.62585281	0.015238491	-3.38759373	0.00936475	0.32213413	-2.35661415
homo after UVB irradiation VS homo before UVB irradiation	GO_APOLIPOPROTEIN_A_I_BINDING	0.517088554	0.028073958	2.306168315	0.04875047	0.16212827	-4.30670562
hetero after UVB irradiation VS hetero before UVB irradiation	GO_APOLIPOPROTEIN_A_I_BINDING1	-0.06974438	-0.02175221	-0.5382629	0.60531187	0.83488689	-6.07729907
WT after UVB irradiation VS WT before UVB irradiation	GO_APOLIPOPROTEIN_A_I_BINDING2	0.174493194	0.014164248	0.613379678	0.55645806	0.87872408	-5.80229105
homo after UVB irradiation VS homo before UVB irradiation	GO_APOLIPOPROTEIN_A_I_MEDIATED_SIGNALING_PATHWAY	1.044432593	-0.00593176	6.294650274	0.0001975	0.01103958	1.190170512
hetero after UVB irradiation VS hetero before UVB irradiation	GO_APOLIPOPROTEIN_A_I_MEDIATED_SIGNALING_PATHWAY1	0.862685996	0.034945018	3.897843748	0.00472627	0.1277306	-1.77920998
WT after UVB irradiation VS WT before UVB irradiation	GO_APOLIPOPROTEIN_A_I_MEDIATED_SIGNALING_PATHWAY2	0.851245917	-0.03520427	3.639639457	0.0064576	0.30156568	-2.01783282
homo after UVB irradiation VS homo before UVB irradiation	GO_APOLIPOPROTEIN_BINDING	0.173820074	-0.03454525	0.992255515	0.34901971	0.54101299	-6.04071844
hetero after UVB irradiation VS hetero before UVB irradiation	GO_APOLIPOPROTEIN_BINDING1	0.026852879	-0.02159739	0.160138468	0.87681455	0.95484139	-6.2176005
WT after UVB irradiation VS WT before UVB irradiation	GO_APOLIPOPROTEIN_BINDING2	0.191687351	-0.04737709	0.861155229	0.41392316	0.8207826	-5.62268245
homo after UVB irradiation VS homo before UVB irradiation	GO_APOLIPOPROTEIN_RECEPTOR_BINDING	0.993749482	-0.00631133	6.428648046	0.00017027	0.01060214	1.335728359
hetero after UVB irradiation VS hetero before UVB irradiation	GO_APOLIPOPROTEIN_RECEPTOR_BINDING1	0.745539501	0.073042103	3.989952637	0.00415967	0.12588577	-1.65626012
WT after UVB irradiation VS WT before UVB irradiation	GO_APOLIPOPROTEIN_RECEPTOR_BINDING2	0.577869913	0.058077216	2.582258983	0.03218176	0.45445578	-3.4852797
homo after UVB irradiation VS homo before UVB irradiation	GO_APOPTOTIC_CELL_CLEARANCE	0.693452944	-0.00322295	5.339743721	0.00060721	0.01768499	0.075842312
hetero after UVB irradiation VS hetero before UVB irradiation	GO_APOPTOTIC_CELL_CLEARANCE1	0.553670398	0.030190445	3.44887202	0.00896108	0.15043564	-2.39677118
WT after UVB irradiation VS WT before UVB irradiation	GO_APOPTOTIC_CELL_CLEARANCE2	0.495456881	-0.0190371	2.246699916	0.05445956	0.52185694	-3.96112395
homo after UVB irradiation VS homo before UVB irradiation	GO_APOPTOTIC_CHROMOSOME_CONDENSATION	-0.04742578	0.021742623	-0.25950066	0.80154801	0.88742171	-6.51405037
hetero after UVB irradiation VS hetero before UVB irradiation	GO_APOPTOTIC_CHROMOSOME_CONDENSATION1	0.307468404	-0.04355621	1.329234904	0.22109016	0.54852054	-5.37078177
WT after UVB irradiation VS WT before UVB irradiation	GO_APOPTOTIC_CHROMOSOME_CONDENSATION2	0.321221516	0.050445643	1.448158464	0.18515881	0.68726847	-5.0193958
homo after UVB irradiation VS homo before UVB irradiation	GO_APOPTOTIC_DNA_FRAGMENTATION	-0.08862886	0.003741358	-0.45961422	0.65755206	0.79172912	-6.4363956
hetero after UVB irradiation VS hetero before UVB irradiation	GO_APOPTOTIC_DNA_FRAGMENTATION1	-0.06171657	-0.01567494	-0.41948199	0.68610421	0.87376352	-6.13715014
WT after UVB irradiation VS WT before UVB irradiation	GO_APOPTOTIC_DNA_FRAGMENTATION2	0.148188254	-0.00825936	0.985155498	0.35306434	0.78942569	-5.51412046
homo after UVB irradiation VS homo before UVB irradiation	GO_APOPTOTIC_MITOCHONDRIAL_CHANGES	0.106189503	-0.00975493	0.709715061	0.49726511	0.67191872	-6.28261476
hetero after UVB irradiation VS hetero before UVB irradiation	GO_APOPTOTIC_MITOCHONDRIAL_CHANGES1	0.110396554	0.000211711	0.731175727	0.48591425	0.76391974	-5.95135677
WT after UVB irradiation VS WT before UVB irradiation	GO_APOPTOTIC_MITOCHONDRIAL_CHANGES2	0.072774147	0.020722446	0.497614504	0.63198564	0.9044038	-5.86748629
homo after UVB irradiation VS homo before UVB irradiation	GO_APOPTOTIC_PROCESS	0.094501447	-0.00921829	0.736030055	0.48192351	0.6591663	-6.26299254
homo after UVB irradiation VS homo before UVB irradiation	GO_APOPTOTIC_PROCESS_IN_BONE_MARROW_CELL	-0.74397196	0.059205466	-4.77967791	0.00124337	0.02367539	-0.64352285
hetero after UVB irradiation VS hetero before UVB irradiation	GO_APOPTOTIC_PROCESS_IN_BONE_MARROW_CELL1	-0.643319	-0.06916507	-3.08593695	0.01532461	0.18280516	-2.91493661
WT after UVB irradiation VS WT before UVB irradiation	GO_APOPTOTIC_PROCESS_IN_BONE_MARROW_CELL2	-0.38471733	-0.07207038	-1.69513187	0.12804003	0.63467098	-4.71155291
homo after UVB irradiation VS homo before UVB irradiation	GO_APOPTOTIC_PROCESS_INVOLVED_IN_BLOOD_VESSEL_MORPHOGENESIS	0.606061304	-0.0532227	4.149004702	0.00294307	0.03577508	-1.51261898
hetero after UVB irradiation VS hetero before UVB irradiation	GO_APOPTOTIC_PROCESS_INVOLVED_IN_BLOOD_VESSEL_MORPHOGENESIS1	0.81530306	0.006654379	5.006924073	0.00110355	0.089745	-0.39289352
WT after UVB irradiation VS WT before UVB irradiation	GO_APOPTOTIC_PROCESS_INVOLVED_IN_BLOOD_VESSEL_MORPHOGENESIS2	0.585645957	0.09892395	2.982931487	0.01728371	0.38271751	-2.91735526
homo after UVB irradiation VS homo before UVB irradiation	GO_APOPTOTIC_PROCESS_INVOLVED_IN_DEVELOPMENT	0.239209403	0.017784636	1.612676042	0.14398498	0.31603466	-5.31236664
hetero after UVB irradiation VS hetero before UVB irradiation	GO_APOPTOTIC_PROCESS_INVOLVED_IN_DEVELOPMENT1	0.121221567	0.029864322	0.795049589	0.44994469	0.74207367	-5.90228283
WT after UVB irradiation VS WT before UVB irradiation	GO_APOPTOTIC_PROCESS_INVOLVED_IN_DEVELOPMENT2	0.060114881	0.021214845	0.386409196	0.70914909	0.92765828	-5.91804313
homo after UVB irradiation VS homo before UVB irradiation	GO_APOPTOTIC_PROCESS_INVOLVED_IN_HEART_MORPHOGENESIS	-0.081771	-0.01346151	-0.32360996	0.75420953	0.85680293	-6.49374781
hetero after UVB irradiation VS hetero before UVB irradiation	GO_APOPTOTIC_PROCESS_INVOLVED_IN_HEART_MORPHOGENESIS1	-0.55628119	0.017683059	-2.16420855	0.06300901	0.32964008	-4.2598523
WT after UVB irradiation VS WT before UVB irradiation	GO_APOPTOTIC_PROCESS_INVOLVED_IN_HEART_MORPHOGENESIS2	-0.63660025	-0.06640252	-2.85894338	0.02092332	0.40609596	-3.09226127
homo after UVB irradiation VS homo before UVB irradiation	GO_APOPTOTIC_PROCESS_INVOLVED_IN_MORPHOGENESIS	0.139560283	0.025596676	0.744202303	0.47722189	0.65498172	-6.2567714
hetero after UVB irradiation VS hetero before UVB irradiation	GO_APOPTOTIC_PROCESS_INVOLVED_IN_MORPHOGENESIS1	-0.03671068	0.02158711	-0.23600136	0.81947039	0.93071163	-6.20139001
WT after UVB irradiation VS WT before UVB irradiation	GO_APOPTOTIC_PROCESS_INVOLVED_IN_MORPHOGENESIS2	-0.12575662	0.041729377	-0.79468081	0.44946066	0.83495728	-5.67594871
hetero after UVB irradiation VS hetero before UVB irradiation	GO_APOPTOTIC_PROCESS1	0.129193442	0.003472607	1.030492342	0.33347034	0.65330016	-5.69269915
WT after UVB irradiation VS WT before UVB irradiation	GO_APOPTOTIC_PROCESS2	0.057562494	-0.0136505	0.407112268	0.69447477	0.9219193	-5.90955015
homo after UVB irradiation VS homo before UVB irradiation	GO_APOPTOTIC_SIGNALING_PATHWAY	0.093503157	-0.01817745	0.675303312	0.51778755	0.68781986	-6.30731879
hetero after UVB irradiation VS hetero before UVB irradiation	GO_APOPTOTIC_SIGNALING_PATHWAY1	0.162336011	0.006832208	1.288587825	0.23420694	0.56254359	-5.41776191
WT after UVB irradiation VS WT before UVB irradiation	GO_APOPTOTIC_SIGNALING_PATHWAY2	0.108461534	-0.01395383	0.753668287	0.47239121	0.84713539	-5.70700538
homo after UVB irradiation VS homo before UVB irradiation	GO_APPENDAGE_DEVELOPMENT	-0.09569476	0.007279852	-0.73232765	0.48406339	0.66111277	-6.26579121
hetero after UVB irradiation VS hetero before UVB irradiation	GO_APPENDAGE_DEVELOPMENT1	-0.13576956	0.023434029	-1.0163372	0.33975647	0.65952819	-5.70649265
WT after UVB irradiation VS WT before UVB irradiation	GO_APPENDAGE_DEVELOPMENT2	-0.12826827	0.005358024	-0.88214089	0.40312576	0.81613006	-5.60513472
homo after UVB irradiation VS homo before UVB irradiation	GO_APPENDAGE_MORPHOGENESIS	-0.06506296	0.005741954	-0.50558264	0.62625535	0.76894329	-6.41272223
hetero after UVB irradiation VS hetero before UVB irradiation	GO_APPENDAGE_MORPHOGENESIS1	-0.11132739	0.012188734	-0.82333614	0.43460672	0.7340639	-5.87944024
WT after UVB irradiation VS WT before UVB irradiation	GO_APPENDAGE_MORPHOGENESIS2	-0.13861183	0.002438128	-0.92419847	0.38209751	0.8049904	-5.56894041
homo after UVB irradiation VS homo before UVB irradiation	GO_ARACHIDONATE_COA_LIGASE_ACTIVITY	-0.12554054	0.005807434	-0.59285557	0.56902078	0.72724828	-6.36199647
hetero after UVB irradiation VS hetero before UVB irradiation	GO_ARACHIDONATE_COA_LIGASE_ACTIVITY1	-0.10456327	-0.02279692	-0.54137428	0.60326514	0.83419966	-6.07554502
WT after UVB irradiation VS WT before UVB irradiation	GO_ARACHIDONATE_COA_LIGASE_ACTIVITY2	-0.32053844	0.036305902	-1.33262189	0.21893366	0.71478521	-5.1542587
homo after UVB irradiation VS homo before UVB irradiation	GO_ARACHIDONIC_ACID_EPOXYGENASE_ACTIVITY	-0.00694393	0.125598588	-0.03393269	0.97372995	0.98656593	-6.55022484
hetero after UVB irradiation VS hetero before UVB irradiation	GO_ARACHIDONIC_ACID_EPOXYGENASE_ACTIVITY1	-0.02207527	0.002927271	-0.1079158	0.91676878	0.96807214	-6.22517334
WT after UVB irradiation VS WT before UVB irradiation	GO_ARACHIDONIC_ACID_EPOXYGENASE_ACTIVITY2	-0.12002953	0.045430576	-0.40936803	0.69288398	0.92156983	-5.90859912
homo after UVB irradiation VS homo before UVB irradiation	GO_ARACHIDONIC_ACID_METABOLIC_PROCESS	0.153487281	0.015082952	0.908418389	0.38918394	0.57842556	-6.11943261
hetero after UVB irradiation VS hetero before UVB irradiation	GO_ARACHIDONIC_ACID_METABOLIC_PROCESS1	0.112279317	0.04280606	0.74055076	0.48051995	0.76122341	-5.94437523
WT after UVB irradiation VS WT before UVB irradiation	GO_ARACHIDONIC_ACID_METABOLIC_PROCESS2	-0.01809986	0.038678074	-0.09478469	0.9267894	0.98199735	-5.99132897
homo after UVB irradiation VS homo before UVB irradiation	GO_ARACHIDONIC_ACID_MONOOXYGENASE_ACTIVITY	0.001530399	0.109239345	0.008628859	0.99331836	0.99634161	-6.55081585
hetero after UVB irradiation VS hetero before UVB irradiation	GO_ARACHIDONIC_ACID_MONOOXYGENASE_ACTIVITY1	-0.0547856	0.025191037	-0.32052203	0.75693552	0.90565384	-6.17614539
WT after UVB irradiation VS WT before UVB irradiation	GO_ARACHIDONIC_ACID_MONOOXYGENASE_ACTIVITY2	-0.18272736	0.048201226	-0.63519194	0.54283111	0.87128113	-5.78862091
homo after UVB irradiation VS homo before UVB irradiation	GO_ARACHIDONIC_ACID_SECRETION	0.093556827	0.045381359	0.649120899	0.5337462	0.7003817	-6.32537885
hetero after UVB irradiation VS hetero before UVB irradiation	GO_ARACHIDONIC_ACID_SECRETION1	0.113912046	0.051907266	0.813947948	0.43965682	0.73705703	-5.88709587
WT after UVB irradiation VS WT before UVB irradiation	GO_ARACHIDONIC_ACID_SECRETION2	-0.07408886	-0.00841906	-0.35944386	0.72845626	0.93296251	-5.92846505
homo after UVB irradiation VS homo before UVB irradiation	GO_ARF_GUANYL_NUCLEOTIDE_EXCHANGE_FACTOR_ACTIVITY	0.179371586	-0.00014963	1.267408665	0.23929256	0.43187624	-5.74622559
hetero after UVB irradiation VS hetero before UVB irradiation	GO_ARF_GUANYL_NUCLEOTIDE_EXCHANGE_FACTOR_ACTIVITY1	0.160362308	-0.04744324	1.010922922	0.34218486	0.66045887	-5.71173042
WT after UVB irradiation VS WT before UVB irradiation	GO_ARF_GUANYL_NUCLEOTIDE_EXCHANGE_FACTOR_ACTIVITY2	-0.1544319	-0.05951255	-0.86801548	0.41037116	0.81906347	-5.61698408
homo after UVB irradiation VS homo before UVB irradiation	GO_ARF_PROTEIN_SIGNAL_TRANSDUCTION	0.161542	0.01169152	0.958795569	0.36466203	0.55471751	-6.07279873
hetero after UVB irradiation VS hetero before UVB irradiation	GO_ARF_PROTEIN_SIGNAL_TRANSDUCTION1	0.233868947	-0.02932001	1.57889106	0.15372074	0.47184575	-5.06406615
WT after UVB irradiation VS WT before UVB irradiation	GO_ARF_PROTEIN_SIGNAL_TRANSDUCTION2	-0.04025322	-0.06019112	-0.23114945	0.8229323	0.95856757	-5.96797248
homo after UVB irradiation VS homo before UVB irradiation	GO_ARGININE_BINDING	0.437490353	-0.0359197	2.37422259	0.04375804	0.15168242	-4.20261187
hetero after UVB irradiation VS hetero before UVB irradiation	GO_ARGININE_BINDING1	0.499341549	0.037382239	3.141016971	0.01411196	0.17802516	-2.83541627
WT after UVB irradiation VS WT before UVB irradiation	GO_ARGININE_BINDING2	0.303282719	0.175411719	1.398633705	0.19903561	0.69740523	-5.07800672
homo after UVB irradiation VS homo before UVB irradiation	GO_ARGININE_BIOSYNTHETIC_PROCESS	-0.11276885	0.115650043	-0.4900575	0.63674045	0.77695055	-6.42095553
hetero after UVB irradiation VS hetero before UVB irradiation	GO_ARGININE_BIOSYNTHETIC_PROCESS1	0.282966316	0.003115244	0.915962723	0.38695046	0.69764483	-5.80005486
WT after UVB irradiation VS WT before UVB irradiation	GO_ARGININE_BIOSYNTHETIC_PROCESS2	0.172593148	0.101847179	0.719653325	0.4919835	0.85299376	-5.73168659
homo after UVB irradiation VS homo before UVB irradiation	GO_ARGININE_CATABOLIC_PROCESS	0.640285508	-0.05542024	4.049602661	0.00338911	0.03823813	-1.65509262
hetero after UVB irradiation VS hetero before UVB irradiation	GO_ARGININE_CATABOLIC_PROCESS1	0.58109984	-0.04170747	2.654467561	0.02952966	0.24111818	-3.54476046
WT after UVB irradiation VS WT before UVB irradiation	GO_ARGININE_CATABOLIC_PROCESS2	0.032075143	-0.04031752	0.115017018	0.91123259	0.97857624	-5.98909246
homo after UVB irradiation VS homo before UVB irradiation	GO_ARGININE_METABOLIC_PROCESS	0.248727702	0.003100792	1.653764588	0.1352775	0.30350294	-5.25693102
hetero after UVB irradiation VS hetero before UVB irradiation	GO_ARGININE_METABOLIC_PROCESS1	0.369964933	-0.04784579	2.480942113	0.03858862	0.26884499	-3.79930262
WT after UVB irradiation VS WT before UVB irradiation	GO_ARGININE_METABOLIC_PROCESS2	0.00340862	0.028564694	0.019373806	0.98501178	0.99679464	-5.99586862
homo after UVB irradiation VS homo before UVB irradiation	GO_ARGININE_TRANSMEMBRANE_TRANSPORT	-0.20050971	0.003721696	-0.9968872	0.34689481	0.53882877	-6.03620955
hetero after UVB irradiation VS hetero before UVB irradiation	GO_ARGININE_TRANSMEMBRANE_TRANSPORT1	-0.32274248	-0.01259036	-1.75990554	0.1171689	0.4206677	-4.82549893
WT after UVB irradiation VS WT before UVB irradiation	GO_ARGININE_TRANSMEMBRANE_TRANSPORT2	-0.13784993	-0.03353896	-0.84849865	0.42053316	0.82383319	-5.63309784
homo after UVB irradiation VS homo before UVB irradiation	GO_ARGININE_TRANSMEMBRANE_TRANSPORTER_ACTIVITY	-0.18310465	0.018891732	-0.84004167	0.42433955	0.60931971	-6.17941474
hetero after UVB irradiation VS hetero before UVB irradiation	GO_ARGININE_TRANSMEMBRANE_TRANSPORTER_ACTIVITY1	-0.26366215	-0.01748407	-1.16455911	0.27835459	0.60400502	-5.55519025
WT after UVB irradiation VS WT before UVB irradiation	GO_ARGININE_TRANSMEMBRANE_TRANSPORTER_ACTIVITY2	-0.0529789	-0.02296876	-0.3171283	0.75916856	0.93913078	-5.94334732
homo after UVB irradiation VS homo before UVB irradiation	GO_ARGININE_TRANSPORT	0.037717318	-0.02450013	0.158655266	0.87771998	0.93340449	-6.53706333
hetero after UVB irradiation VS hetero before UVB irradiation	GO_ARGININE_TRANSPORT1	0.021962715	0.018630586	0.117997863	0.90903171	0.96611804	-6.22394022
WT after UVB irradiation VS WT before UVB irradiation	GO_ARGININE_TRANSPORT2	0.002839998	0.002566239	0.017840716	0.98619768	0.99693925	-5.99589872
homo after UVB irradiation VS homo before UVB irradiation	GO_ARMADILLO_REPEAT_DOMAIN_BINDING	-0.58817577	0.059619288	-3.88635638	0.00428618	0.04234489	-1.89211955
hetero after UVB irradiation VS hetero before UVB irradiation	GO_ARMADILLO_REPEAT_DOMAIN_BINDING1	-0.07268152	-0.04346629	-0.2724642	0.79229233	0.91891235	-6.19142118
WT after UVB irradiation VS WT before UVB irradiation	GO_ARMADILLO_REPEAT_DOMAIN_BINDING2	-0.12470468	0.0945037	-0.36610098	0.72366993	0.93154998	-5.92595971
homo after UVB irradiation VS homo before UVB irradiation	GO_AROMATASE_ACTIVITY	0.215722353	0.105220301	1.187388855	0.26783031	0.46298924	-5.83717867
hetero after UVB irradiation VS hetero before UVB irradiation	GO_AROMATASE_ACTIVITY1	0.048642325	0.0761642	0.248320002	0.81025724	0.92633113	-6.19817941
WT after UVB irradiation VS WT before UVB irradiation	GO_AROMATASE_ACTIVITY2	-0.02972691	0.03457218	-0.12111701	0.90654989	0.97761021	-5.98833381
homo after UVB irradiation VS homo before UVB irradiation	GO_AROMATIC_AMINO_ACID_FAMILY_CATABOLIC_PROCESS	0.475494756	-0.01494374	2.795773022	0.0224511	0.10433784	-3.55023006
hetero after UVB irradiation VS hetero before UVB irradiation	GO_AROMATIC_AMINO_ACID_FAMILY_CATABOLIC_PROCESS1	-0.11300663	0.01618789	-0.60454366	0.56253802	0.80978619	-6.03792837
WT after UVB irradiation VS WT before UVB irradiation	GO_AROMATIC_AMINO_ACID_FAMILY_CATABOLIC_PROCESS2	0.193092108	-0.03560958	0.736625785	0.48214292	0.85127391	-5.71949481
homo after UVB irradiation VS homo before UVB irradiation	GO_AROMATIC_AMINO_ACID_FAMILY_METABOLIC_PROCESS	0.270726952	-0.01754161	1.540730245	0.16046511	0.33660037	-5.40768262
hetero after UVB irradiation VS hetero before UVB irradiation	GO_AROMATIC_AMINO_ACID_FAMILY_METABOLIC_PROCESS1	-0.11123268	-0.00346636	-0.72014799	0.49230963	0.76753004	-5.9594702
WT after UVB irradiation VS WT before UVB irradiation	GO_AROMATIC_AMINO_ACID_FAMILY_METABOLIC_PROCESS2	0.047135252	-0.0281421	0.198298472	0.84769951	0.96522688	-5.9753715
homo after UVB irradiation VS homo before UVB irradiation	GO_AROMATIC_AMINO_ACID_TRANSMEMBRANE_TRANSPORTER_ACTIVITY	-0.60700219	-0.01935934	-4.04250467	0.00342362	0.03848613	-1.66532076
hetero after UVB irradiation VS hetero before UVB irradiation	GO_AROMATIC_AMINO_ACID_TRANSMEMBRANE_TRANSPORTER_ACTIVITY1	-0.46466959	-0.02319486	-2.73431511	0.02612387	0.23027706	-3.42762052
WT after UVB irradiation VS WT before UVB irradiation	GO_AROMATIC_AMINO_ACID_TRANSMEMBRANE_TRANSPORTER_ACTIVITY2	-0.33312613	-0.02066626	-1.45116592	0.18434421	0.68726847	-5.01579955
homo after UVB irradiation VS homo before UVB irradiation	GO_AROMATIC_AMINO_ACID_TRANSPORT	-0.71351308	-0.02476081	-4.717974	0.00134925	0.02467279	-0.72581827
hetero after UVB irradiation VS hetero before UVB irradiation	GO_AROMATIC_AMINO_ACID_TRANSPORT1	-0.48302589	-0.04253554	-2.27387754	0.05316973	0.30612069	-4.10141229
WT after UVB irradiation VS WT before UVB irradiation	GO_AROMATIC_AMINO_ACID_TRANSPORT2	-0.43377832	-0.04332444	-1.78838721	0.11106158	0.61576748	-4.58964645
homo after UVB irradiation VS homo before UVB irradiation	GO_ARP2_3_COMPLEX_BINDING	0.30781865	-0.02480926	1.611158738	0.14431609	0.31649168	-5.31440021
hetero after UVB irradiation VS hetero before UVB irradiation	GO_ARP2_3_COMPLEX_BINDING1	-0.05988328	-0.02451616	-0.30672345	0.76702926	0.90866119	-6.18077883
WT after UVB irradiation VS WT before UVB irradiation	GO_ARP2_3_COMPLEX_BINDING2	-0.08804816	-0.03208266	-0.54194479	0.60244599	0.89591106	-5.84400411
homo after UVB irradiation VS homo before UVB irradiation	GO_ARP2_3_COMPLEX_MEDIATED_ACTIN_NUCLEATION	0.443166481	-0.04619308	2.305290072	0.04881847	0.16216553	-4.30804527
hetero after UVB irradiation VS hetero before UVB irradiation	GO_ARP2_3_COMPLEX_MEDIATED_ACTIN_NUCLEATION1	0.472727069	-0.01337765	2.834745154	0.02240702	0.2152311	-3.28051601
WT after UVB irradiation VS WT before UVB irradiation	GO_ARP2_3_COMPLEX_MEDIATED_ACTIN_NUCLEATION2	0.508920737	-0.00834601	3.121864802	0.01397608	0.36090271	-2.72285821
homo after UVB irradiation VS homo before UVB irradiation	GO_ARP2_3_PROTEIN_COMPLEX	0.790619578	-0.07685935	3.126793754	0.01339436	0.07831242	-3.0371687
hetero after UVB irradiation VS hetero before UVB irradiation	GO_ARP2_3_PROTEIN_COMPLEX1	0.95543108	-0.01329262	4.818952905	0.00139428	0.09333375	-0.61311349
WT after UVB irradiation VS WT before UVB irradiation	GO_ARP2_3_PROTEIN_COMPLEX2	0.879888821	-0.00453883	4.874286762	0.00119098	0.19353487	-0.51118264
homo after UVB irradiation VS homo before UVB irradiation	GO_ARRESTIN_FAMILY_PROTEIN_BINDING	0.249111327	0.013290779	1.026746108	0.33343098	0.52641928	-6.0067507
hetero after UVB irradiation VS hetero before UVB irradiation	GO_ARRESTIN_FAMILY_PROTEIN_BINDING1	0.288156728	0.040888573	1.43487425	0.18992631	0.5147346	-5.24460186
WT after UVB irradiation VS WT before UVB irradiation	GO_ARRESTIN_FAMILY_PROTEIN_BINDING2	0.591926283	-0.0245685	3.706691482	0.00585797	0.28530066	-1.9293008
homo after UVB irradiation VS homo before UVB irradiation	GO_ARTERIAL_ENDOTHELIAL_CELL_DIFFERENTIATION	-0.04350718	-0.02943183	-0.15565932	0.88000806	0.93493729	-6.53757858
hetero after UVB irradiation VS hetero before UVB irradiation	GO_ARTERIAL_ENDOTHELIAL_CELL_DIFFERENTIATION1	0.07994522	-0.07004339	0.307998764	0.76609434	0.90806394	-6.18035893
WT after UVB irradiation VS WT before UVB irradiation	GO_ARTERIAL_ENDOTHELIAL_CELL_DIFFERENTIATION2	0.013787397	-0.01870697	0.063074799	0.95123613	0.98676072	-5.99396799
homo after UVB irradiation VS homo before UVB irradiation	GO_ARTERY_DEVELOPMENT	0.097665564	0.004027707	0.684814696	0.51206344	0.6834918	-6.30059987
hetero after UVB irradiation VS hetero before UVB irradiation	GO_ARTERY_DEVELOPMENT1	0.018592916	0.014400536	0.119922219	0.90755609	0.96555872	-6.2236924
WT after UVB irradiation VS WT before UVB irradiation	GO_ARTERY_DEVELOPMENT2	-0.07349361	0.013096969	-0.48393673	0.64124699	0.90671989	-5.87435317
homo after UVB irradiation VS homo before UVB irradiation	GO_ARTERY_MORPHOGENESIS	0.141936738	0.002173809	0.997324268	0.3466948	0.53877161	-6.03578322
hetero after UVB irradiation VS hetero before UVB irradiation	GO_ARTERY_MORPHOGENESIS1	-0.01533391	0.000451987	-0.0853825	0.93409403	0.97579263	-6.22753249
WT after UVB irradiation VS WT before UVB irradiation	GO_ARTERY_MORPHOGENESIS2	-0.05664714	0.017660185	-0.32562574	0.75296252	0.93734883	-5.94050392
homo after UVB irradiation VS homo before UVB irradiation	GO_ARTERY_SMOOTH_MUSCLE_CONTRACTION	0.526122515	-0.05636881	4.263923898	0.00250454	0.03335693	-1.34971439
hetero after UVB irradiation VS hetero before UVB irradiation	GO_ARTERY_SMOOTH_MUSCLE_CONTRACTION1	0.307083373	0.005068794	1.557325361	0.15871241	0.47878838	-5.09167047
WT after UVB irradiation VS WT before UVB irradiation	GO_ARTERY_SMOOTH_MUSCLE_CONTRACTION2	0.172505591	0.085137435	0.853532497	0.41789537	0.82297693	-5.62897059
homo after UVB irradiation VS homo before UVB irradiation	GO_ARYL_HYDROCARBON_RECEPTOR_BINDING	-0.41969681	0.046972284	-2.22433969	0.05550713	0.17498798	-4.43106548
hetero after UVB irradiation VS hetero before UVB irradiation	GO_ARYL_HYDROCARBON_RECEPTOR_BINDING1	-0.47171533	0.028567864	-1.74308436	0.12018933	0.42499731	-4.84814191
WT after UVB irradiation VS WT before UVB irradiation	GO_ARYL_HYDROCARBON_RECEPTOR_BINDING2	-0.49658836	-0.0716427	-2.66096298	0.02845903	0.44441257	-3.37327604
homo after UVB irradiation VS homo before UVB irradiation	GO_ARYL_SULFOTRANSFERASE_ACTIVITY	0.420435719	0.044131047	1.497411871	0.17118621	0.35067329	-5.46392875

WT after UVB irradiation VS WT before UVB irradiation	GO_ARYL_SULFOTRANSFERASE_ACTIVITY1	-0.15898489	0.040326761	-0.39793132	0.70096581	0.92474097	-5.9133689
homo after UVB irradiation VS homo before UVB irradiation	GO_ARYLESTERASE_ACTIVITY	0.590886107	0.022792348	3.679104125	0.00580614	0.05041635	-2.19819179
hetero after UVB irradiation VS hetero before UVB irradiation	GO_ARYLESTERASE_ACTIVITY1	0.044025353	-0.03576145	0.181699377	0.8604211	0.94732472	-6.21361894
WT after UVB irradiation VS WT before UVB irradiation	GO_ARYLESTERASE_ACTIVITY2	0.142112792	0.07864364	0.612198097	0.5572019	0.87951956	-5.80301923
homo after UVB irradiation VS homo before UVB irradiation	GO_ARYLSULFATASE_ACTIVITY	0.583310991	-0.04847032	2.503757456	0.0356249	0.13504151	-4.00318543
hetero after UVB irradiation VS hetero before UVB irradiation	GO_ARYLSULFATASE_ACTIVITY1	0.404230646	-0.02270321	1.348710975	0.21503187	0.54230223	-5.34795234
WT after UVB irradiation VS WT before UVB irradiation	GO_ARYLSULFATASE_ACTIVITY2	0.289748639	-0.04041857	0.905094995	0.39154796	0.81255424	-5.58554873
homo after UVB irradiation VS homo before UVB irradiation	GO_ASCENDING_AORTA_DEVELOPMENT	0.180489036	-0.03752545	0.895671566	0.39557415	0.58450864	-6.13090786
hetero after UVB irradiation VS hetero before UVB irradiation	GO_ASCENDING_AORTA_DEVELOPMENT1	0.603960896	-0.00792807	2.829199183	0.02259726	0.21581359	-3.28862957
WT after UVB irradiation VS WT before UVB irradiation	GO_ASCENDING_AORTA_DEVELOPMENT2	0.056980634	-0.03346607	0.166492636	0.87185153	0.96897064	-5.98146679
homo after UVB irradiation VS homo before UVB irradiation	GO_ASPARAGINE_METABOLIC_PROCESS	0.27355113	-0.05997162	0.850576069	0.41878294	0.60418159	-6.17042812
hetero after UVB irradiation VS hetero before UVB irradiation	GO_ASPARAGINE_METABOLIC_PROCESS1	0.159136995	-0.00431505	0.710062381	0.49820586	0.77140877	-5.96679637
WT after UVB irradiation VS WT before UVB irradiation	GO_ASPARAGINE_METABOLIC_PROCESS2	0.26841741	0.0002332	1.129534496	0.29101899	0.75646944	-5.3738107
homo after UVB irradiation VS homo before UVB irradiation	GO_ASPARTATE_FAMILY_AMINO_ACID_BIOSYNTHETIC_PROCESS	-0.17053395	0.004372682	-1.25634379	0.24308126	0.43662257	-5.75904275
hetero after UVB irradiation VS hetero before UVB irradiation	GO_ASPARTATE_FAMILY_AMINO_ACID_BIOSYNTHETIC_PROCESS1	0.211782047	0.014374082	1.358661727	0.21199225	0.53868738	-5.33621051
WT after UVB irradiation VS WT before UVB irradiation	GO_ASPARTATE_FAMILY_AMINO_ACID_BIOSYNTHETIC_PROCESS2	0.191126077	-0.02766183	1.059091593	0.32012439	0.77325115	-5.44404113
homo after UVB irradiation VS homo before UVB irradiation	GO_ASPARTATE_FAMILY_AMINO_ACID_CATABOLIC_PROCESS	0.032661171	0.006668555	0.120146243	0.90721499	0.94943667	-6.54294142
hetero after UVB irradiation VS hetero before UVB irradiation	GO_ASPARTATE_FAMILY_AMINO_ACID_CATABOLIC_PROCESS1	-0.27995427	0.015346925	-1.65830522	0.13655075	0.44956333	-4.96084353
WT after UVB irradiation VS WT before UVB irradiation	GO_ASPARTATE_FAMILY_AMINO_ACID_CATABOLIC_PROCESS2	-0.10828721	-0.03902992	-0.48152645	0.64288601	0.90762265	-5.87554461
homo after UVB irradiation VS homo before UVB irradiation	GO_ASPARTATE_FAMILY_AMINO_ACID_METABOLIC_PROCESS	-0.1017989	-0.01070906	-0.57854896	0.57819793	0.73447766	-6.37082291
hetero after UVB irradiation VS hetero before UVB irradiation	GO_ASPARTATE_FAMILY_AMINO_ACID_METABOLIC_PROCESS1	-0.14349292	-0.00125306	-0.98861581	0.35233038	0.66877878	-5.7330849
WT after UVB irradiation VS WT before UVB irradiation	GO_ASPARTATE_FAMILY_AMINO_ACID_METABOLIC_PROCESS2	0.016511358	-0.02375698	0.106236077	0.91797976	0.98043563	-5.99011594
homo after UVB irradiation VS homo before UVB irradiation	GO_ASPARTATE_METABOLIC_PROCESS	-0.3694128	0.032030903	-1.25238588	0.24444858	0.43831442	-5.76360908
hetero after UVB irradiation VS hetero before UVB irradiation	GO_ASPARTATE_METABOLIC_PROCESS1	-0.54250577	0.082762523	-2.01347351	0.07951978	0.36294274	-4.47479482
WT after UVB irradiation VS WT before UVB irradiation	GO_ASPARTATE_METABOLIC_PROCESS2	-0.42012233	0.062625642	-1.90176718	0.09326968	0.59021472	-4.43811814
homo after UVB irradiation VS homo before UVB irradiation	GO_ASPARTATE_TRANSMEMBRANE_TRANSPORT	0.148023946	-0.07177029	0.794172174	0.44912784	0.63067652	-6.21743869
hetero after UVB irradiation VS hetero before UVB irradiation	GO_ASPARTATE_TRANSMEMBRANE_TRANSPORT1	-0.07317546	0.008438589	-0.31947855	0.75769712	0.9059386	-6.17650272
WT after UVB irradiation VS WT before UVB irradiation	GO_ASPARTATE_TRANSMEMBRANE_TRANSPORT2	0.094436758	0.046509914	0.472463938	0.64906719	0.9092481	-5.8799742
homo after UVB irradiation VS homo before UVB irradiation	GO_ASPARTIC_ENDOPEPTIDASE_ACTIVITY_INTRAMEMBRANE_CLEAVING	0.347873561	-0.00853824	1.620789634	0.1422261	0.31342826	-5.30147576
hetero after UVB irradiation VS hetero before UVB irradiation	GO_ASPARTIC_ENDOPEPTIDASE_ACTIVITY_INTRAMEMBRANE_CLEAVING1	0.76441437	-0.11167834	2.994033376	0.01759718	0.19405039	-3.04819001
WT after UVB irradiation VS WT before UVB irradiation	GO_ASPARTIC_ENDOPEPTIDASE_ACTIVITY_INTRAMEMBRANE_CLEAVING2	0.537144567	-0.0372503	2.96603964	0.01773818	0.3854098	-2.94111783
homo after UVB irradiation VS homo before UVB irradiation	GO_ASPARTIC_TYPE_ENDOPEPTIDASE_INHIBITOR_ACTIVITY	0.74046501	0.022023247	5.705766287	0.00038923	0.01480375	0.519332207
hetero after UVB irradiation VS hetero before UVB irradiation	GO_ASPARTIC_TYPE_ENDOPEPTIDASE_INHIBITOR_ACTIVITY1	0.119576106	0.086604103	0.421443983	0.68473001	0.87343327	-6.13627583
WT after UVB irradiation VS WT before UVB irradiation	GO_ASPARTIC_TYPE_ENDOPEPTIDASE_INHIBITOR_ACTIVITY2	0.226751564	-0.0524774	0.814529081	0.43863776	0.83056493	-5.66041868
homo after UVB irradiation VS homo before UVB irradiation	GO_ASPARTIC_TYPE_PEPTIDASE_ACTIVITY	0.307108491	0.003249829	1.82749643	0.10356521	0.25651423	-5.0153995
hetero after UVB irradiation VS hetero before UVB irradiation	GO_ASPARTIC_TYPE_PEPTIDASE_ACTIVITY1	0.119804216	0.015539891	0.871424939	0.40937257	0.71386216	-5.83908792
WT after UVB irradiation VS WT before UVB irradiation	GO_ASPARTIC_TYPE_PEPTIDASE_ACTIVITY2	0.283135042	0.034512279	1.878636131	0.0966639	0.59686908	-4.46929768
homo after UVB irradiation VS homo before UVB irradiation	GO_ASSOCIATIVE_LEARNING	0.250329764	0.008373526	1.784993291	0.11060947	0.26653558	-5.07547767
hetero after UVB irradiation VS hetero before UVB irradiation	GO_ASSOCIATIVE_LEARNING1	0.120690639	0.029598971	0.812958738	0.44019128	0.73711558	-5.88789825
WT after UVB irradiation VS WT before UVB irradiation	GO_ASSOCIATIVE_LEARNING2	0.192347006	0.023073066	1.033766571	0.3311286	0.77805919	-5.46847529
homo after UVB irradiation VS homo before UVB irradiation	GO_ASTER	-0.56489523	0.027942618	-3.43466593	0.00836696	0.06088328	-2.56583607
hetero after UVB irradiation VS hetero before UVB irradiation	GO_ASTER1	-0.25894788	-0.03293586	-0.81428744	0.4394735	0.73683342	-5.88682031
WT after UVB irradiation VS WT before UVB irradiation	GO_ASTER2	-0.34919598	-0.03221794	-1.23685232	0.25080874	0.73564768	-5.26077816
homo after UVB irradiation VS homo before UVB irradiation	GO_ASTRAL_MICROTUBULE_ORGANIZATION	-0.14631871	0.004536953	-0.54550162	0.59970868	0.74952177	-6.39044953
hetero after UVB irradiation VS hetero before UVB irradiation	GO_ASTRAL_MICROTUBULE_ORGANIZATION1	0.026702761	-0.01910927	0.134924902	0.89606545	0.96152498	-6.22162339
WT after UVB irradiation VS WT before UVB irradiation	GO_ASTRAL_MICROTUBULE_ORGANIZATION2	-0.27917636	-0.04043193	-1.09627936	0.30448575	0.76513806	-5.40737351
homo after UVB irradiation VS homo before UVB irradiation	GO_ASTROCYTE_ACTIVATION	0.476235909	-0.00986643	2.810006452	0.02195403	0.10309081	-3.52811219
hetero after UVB irradiation VS hetero before UVB irradiation	GO_ASTROCYTE_ACTIVATION1	0.41767478	0.04467935	2.649114722	0.02977369	0.24132636	-3.55261607
WT after UVB irradiation VS WT before UVB irradiation	GO_ASTROCYTE_ACTIVATION2	0.25924851	-0.03620211	1.076228215	0.31284138	0.77011405	-5.4272593
homo after UVB irradiation VS homo before UVB irradiation	GO_ASTROCYTE_CELL_MIGRATION	0.50341809	-0.05057773	2.013051778	0.07749817	0.21428526	-4.74680152
hetero after UVB irradiation VS hetero before UVB irradiation	GO_ASTROCYTE_CELL_MIGRATION1	0.452183552	-0.02922786	2.310584163	0.05023104	0.29827332	-4.04808994
WT after UVB irradiation VS WT before UVB irradiation	GO_ASTROCYTE_CELL_MIGRATION2	0.40430297	-0.0127977	1.953236921	0.08612125	0.57689121	-4.36829767
homo after UVB irradiation VS homo before UVB irradiation	GO_ASTROCYTE_DEVELOPMENT	0.43023706	0.006422439	2.69818068	0.0261851	0.11423533	-3.70186499
hetero after UVB irradiation VS hetero before UVB irradiation	GO_ASTROCYTE_DEVELOPMENT1	0.307125801	0.035547866	1.825573329	0.10604826	0.40361985	-4.73628436
WT after UVB irradiation VS WT before UVB irradiation	GO_ASTROCYTE_DEVELOPMENT2	0.132990457	-0.06745917	0.599541765	0.56520556	0.88183483	-5.81073918
homo after UVB irradiation VS homo before UVB irradiation	GO_ASTROCYTE_DIFFERENTIATION	0.207582441	0.00427404	1.63803182	0.13855313	0.30801875	-5.27823985
hetero after UVB irradiation VS hetero before UVB irradiation	GO_ASTROCYTE_DIFFERENTIATION1	0.188822989	0.03322581	1.165373097	0.27804391	0.60400502	-5.55431901
WT after UVB irradiation VS WT before UVB irradiation	GO_ASTROCYTE_DIFFERENTIATION2	0.056241079	-0.01435933	0.307809846	0.76599557	0.94123239	-5.94638123
homo after UVB irradiation VS homo before UVB irradiation	GO_ASTROCYTE_END_FOOT	0.347340387	0.02551856	2.222381859	0.05567965	0.17530269	-4.43402876
hetero after UVB irradiation VS hetero before UVB irradiation	GO_ASTROCYTE_END_FOOT1	-0.16631409	0.013029145	-0.74208361	0.4796417	0.76097951	-5.94322641
WT after UVB irradiation VS WT before UVB irradiation	GO_ASTROCYTE_END_FOOT2	-0.44755688	-0.00863217	-1.71108003	0.12497485	0.63170411	-4.69089796
homo after UVB irradiation VS homo before UVB irradiation	GO_ASTROCYTE_PROJECTION	0.051894255	0.022727317	0.290725839	0.77836883	0.87239325	-6.504708
hetero after UVB irradiation VS hetero before UVB irradiation	GO_ASTROCYTE_PROJECTION1	0.008886103	0.109022253	0.065515565	0.94940068	0.98092529	-6.22915703
WT after UVB irradiation VS WT before UVB irradiation	GO_ASTROCYTE_PROJECTION2	-0.31128846	0.083290829	-2.01109707	0.07871422	0.56251126	-4.28914071
homo after UVB irradiation VS homo before UVB irradiation	GO_ASYMMETRIC_CELL_DIVISION	-0.0276997	-0.00378951	-0.21162819	0.83748385	0.90944045	-6.52634412
hetero after UVB irradiation VS hetero before UVB irradiation	GO_ASYMMETRIC_CELL_DIVISION1	0.321232948	0.012279642	1.838156965	0.10403464	0.40029539	-4.71904705
WT after UVB irradiation VS WT before UVB irradiation	GO_ASYMMETRIC_CELL_DIVISION2	0.162185202	0.00883827	0.902862694	0.39266325	0.81282718	-5.5874713
homo after UVB irradiation VS homo before UVB irradiation	GO_ASYMMETRIC_GLUTAMATERGIC_EXCITATORY_SYNAPSE	0.10043909	0.073190795	0.341987881	0.74082921	0.84752947	-6.48712439
hetero after UVB irradiation VS hetero before UVB irradiation	GO_ASYMMETRIC_GLUTAMATERGIC_EXCITATORY_SYNAPSE1	-0.85784036	-0.08254537	-4.21690791	0.00305291	0.11447176	-1.35917101
WT after UVB irradiation VS WT before UVB irradiation	GO_ASYMMETRIC_GLUTAMATERGIC_EXCITATORY_SYNAPSE2	-0.22754636	0.06468734	-0.66146445	0.52668421	0.86617234	-5.77158675
homo after UVB irradiation VS homo before UVB irradiation	GO_ASYMMETRIC_NEUROBLAST_DIVISION	0.117725982	-0.03201757	0.5611421	0.58947446	0.74216147	-6.38129388
hetero after UVB irradiation VS hetero before UVB irradiation	GO_ASYMMETRIC_NEUROBLAST_DIVISION1	0.29000244	-0.02776181	1.578676155	0.15376977	0.47184575	-5.06434216
WT after UVB irradiation VS WT before UVB irradiation	GO_ASYMMETRIC_NEUROBLAST_DIVISION2	0.138782578	0.065866066	0.550316634	0.59695147	0.89380878	-5.83936094
homo after UVB irradiation VS homo before UVB irradiation	GO_ASYMMETRIC_STEM_CELL_DIVISION	-0.24164933	-0.04473707	-1.54488626	0.15946877	0.33550924	-5.40223995
hetero after UVB irradiation VS hetero before UVB irradiation	GO_ASYMMETRIC_STEM_CELL_DIVISION1	0.037675277	-0.00553214	0.204800505	0.84293663	0.93980906	-6.20880076
WT after UVB irradiation VS WT before UVB irradiation	GO_ASYMMETRIC_STEM_CELL_DIVISION2	-0.28439812	-0.03267547	-1.7274556	0.12189852	0.63058669	-4.669604
homo after UVB irradiation VS homo before UVB irradiation	GO_ATF6_MEDIATED_UNFOLDED_PROTEIN_RESPONSE	-0.42894612	-0.02300576	-2.34322993	0.04596514	0.1564664	-4.25008495
hetero after UVB irradiation VS hetero before UVB irradiation	GO_ATF6_MEDIATED_UNFOLDED_PROTEIN_RESPONSE1	-0.03063043	-0.0567067	-0.10852947	0.91629756	0.96799239	-6.22510142
WT after UVB irradiation VS WT before UVB irradiation	GO_ATF6_MEDIATED_UNFOLDED_PROTEIN_RESPONSE2	0.010559416	-0.09786719	0.037057328	0.97133658	0.99295046	-5.99534214
homo after UVB irradiation VS homo before UVB irradiation	GO_ATG1_ULK1_KINASE_COMPLEX	-0.23210908	-0.02389628	-1.24939434	0.2454863	0.43932104	-5.76705402
hetero after UVB irradiation VS hetero before UVB irradiation	GO_ATG1_ULK1_KINASE_COMPLEX1	0.026910682	0.00143044	0.11115434	0.91428242	0.96745438	-6.2247892
WT after UVB irradiation VS WT before UVB irradiation	GO_ATG1_ULK1_KINASE_COMPLEX2	-0.32004051	-0.08593616	-1.35750963	0.21123897	0.70891132	-5.1257699
homo after UVB irradiation VS homo before UVB irradiation	GO_ATP_ACTIVATED_INWARD_RECTIFIER_POTASSIUM_CHANNEL_ACTIVITY	0.446412773	0.08339615	1.721236058	0.12202069	0.28408433	-5.16443535
hetero after UVB irradiation VS hetero before UVB irradiation	GO_ATP_ACTIVATED_INWARD_RECTIFIER_POTASSIUM_CHANNEL_ACTIVITY1	0.286645702	-0.0232601	0.995843533	0.34901835	0.66595626	-5.72620577
WT after UVB irradiation VS WT before UVB irradiation	GO_ATP_ACTIVATED_INWARD_RECTIFIER_POTASSIUM_CHANNEL_ACTIVITY2	0.002229298	0.026443038	0.006293254	0.99513102	0.99875662	-5.99604577
homo after UVB irradiation VS homo before UVB irradiation	GO_ATP_BINDING_CASSETTE_ABC_TRANSPORTER_COMPLEX	-0.12946302	0.034221767	-0.47208562	0.64898676	0.78565198	-6.43018372
hetero after UVB irradiation VS hetero before UVB irradiation	GO_ATP_BINDING_CASSETTE_ABC_TRANSPORTER_COMPLEX1	-0.0658447	-0.01586508	-0.28612002	0.78218902	0.91452453	-6.18732675
WT after UVB irradiation VS WT before UVB irradiation	GO_ATP_BINDING_CASSETTE_ABC_TRANSPORTER_COMPLEX2	0.044418707	0.097428152	0.177804331	0.86324408	0.96771169	-5.97941977
homo after UVB irradiation VS homo before UVB irradiation	GO_ATP_BIOSYNTHETIC_PROCESS	0.299327803	-0.07177621	1.440216411	0.1863134	0.37086202	-5.53678889
hetero after UVB irradiation VS hetero before UVB irradiation	GO_ATP_BIOSYNTHETIC_PROCESS1	0.086383609	-0.00329843	0.479086196	0.64493374	0.85438728	-6.10886355
WT after UVB irradiation VS WT before UVB irradiation	GO_ATP_BIOSYNTHETIC_PROCESS2	0.283524867	-0.01994433	1.149903668	0.28300897	0.75292132	-5.35290215
homo after UVB irradiation VS homo before UVB irradiation	GO_ATP_DEPENDENT_CHROMATIN_REMODELING	-0.62337127	-0.01418581	-4.30826146	0.0023546	0.03234184	-1.28739202
hetero after UVB irradiation VS hetero before UVB irradiation	GO_ATP_DEPENDENT_CHROMATIN_REMODELING1	-0.17649773	-0.02992439	-0.76331064	0.46758802	0.7530936	-5.92710736
WT after UVB irradiation VS WT before UVB irradiation	GO_ATP_DEPENDENT_CHROMATIN_REMODELING2	-0.08910606	0.020493964	-0.33850321	0.74359394	0.93561604	-5.93605561
homo after UVB irradiation VS homo before UVB irradiation	GO_ATP_DEPENDENT_MICROTUBULE_MOTOR_ACTIVITY	-0.26502705	0.013677322	-2.01189096	0.07763973	0.21451593	-4.74851022
hetero after UVB irradiation VS hetero before UVB irradiation	GO_ATP_DEPENDENT_MICROTUBULE_MOTOR_ACTIVITY_MINUS_END_DIRECTED	-0.45711121	0.04606898	-3.1358637	0.01320799	0.07774646	-3.02317956
hetero after UVB irradiation VS hetero before UVB irradiation	GO_ATP_DEPENDENT_MICROTUBULE_MOTOR_ACTIVITY_MINUS_END_DIRECTED1	-0.42378325	-0.02515722	-2.16688348	0.06274879	0.32880724	-4.25600571
WT after UVB irradiation VS WT before UVB irradiation	GO_ATP_DEPENDENT_MICROTUBULE_MOTOR_ACTIVITY_MINUS_END_DIRECTED2	-0.47682303	-0.03292508	-2.93061455	0.01873203	0.38933605	-2.99102363
homo after UVB irradiation VS homo before UVB irradiation	GO_ATP_DEPENDENT_MICROTUBULE_MOTOR_ACTIVITY_PLUS_END_DIRECTED	-0.06193227	-0.0113091	-0.33720478	0.74430287	0.84993248	-6.48888247
hetero after UVB irradiation VS hetero before UVB irradiation	GO_ATP_DEPENDENT_MICROTUBULE_MOTOR_ACTIVITY_PLUS_END_DIRECTED1	0.221060513	-0.06585811	1.060868074	0.32028548	0.63987714	-5.66261842
WT after UVB irradiation VS WT before UVB irradiation	GO_ATP_DEPENDENT_MICROTUBULE_MOTOR_ACTIVITY_PLUS_END_DIRECTED2	0.046516232	-0.02524935	0.200965454	0.84568167	0.96472824	-5.97481256
hetero after UVB irradiation VS hetero before UVB irradiation	GO_ATP_DEPENDENT_MICROTUBULE_MOTOR_ACTIVITY1	-0.11301943	-0.04519426	-0.59867272	0.56625541	0.81186016	-6.04158407
WT after UVB irradiation VS WT before UVB irradiation	GO_ATP_DEPENDENT_MICROTUBULE_MOTOR_ACTIVITY2	-0.16800001	-0.03844048	-0.83292984	0.42876509	0.82753247	-5.64573472
homo after UVB irradiation VS homo before UVB irradiation	GO_ATP_DEPENDENT_PEPTIDASE_ACTIVITY	-1.04697406	-0.01713597	-7.15868213	7.87E-05	0.00984522	2.085218698
hetero after UVB irradiation VS hetero before UVB irradiation	GO_ATP_DEPENDENT_PEPTIDASE_ACTIVITY1	-0.7515506	-0.08298787	-3.11972247	0.01456838	0.17960875	-2.86612714
WT after UVB irradiation VS WT before UVB irradiation	GO_ATP_DEPENDENT_PEPTIDASE_ACTIVITY2	-0.5465172	-0.1994297	-2.34280107	0.04683834	0.50583727	-3.82547089
homo after UVB irradiation VS homo before UVB irradiation	GO_ATP_DEPENDENT_PROTEIN_BINDING	-0.23816155	0.055231566	-1.03444035	0.33002716	0.52322771	-5.99905081
hetero after UVB irradiation VS hetero before UVB irradiation	GO_ATP_DEPENDENT_PROTEIN_BINDING1	-0.00389676	-0.04332335	-0.01810239	0.98600866	0.99474198	-6.2313064
WT after UVB irradiation VS WT before UVB irradiation	GO_ATP_DEPENDENT_PROTEIN_BINDING2	-0.1184674	-0.02514001	-0.4338275	0.67574009	0.91685973	-5.89796489
homo after UVB irradiation VS homo before UVB irradiation	GO_ATP_GATED_ION_CHANNEL_ACTIVITY	0.262714122	-0.01029438	1.217343216	0.25683574	0.45226321	-5.80360974
hetero after UVB irradiation VS hetero before UVB irradiation	GO_ATP_GATED_ION_CHANNEL_ACTIVITY1	0.150816324	-0.00956697	0.648087065	0.53541436	0.79370848	-6.00981095
WT after UVB irradiation VS WT before UVB irradiation	GO_ATP_GATED_ION_CHANNEL_ACTIVITY2	-0.25547802	-0.1128816	-1.14182551	0.28616411	0.75424544	-5.36122568
homo after UVB irradiation VS homo before UVB irradiation	GO_ATP_METABOLIC_PROCESS	0.074773599	-0.01667317	0.410334447	0.69191311	0.81554007	-6.45938938
hetero after UVB irradiation VS hetero before UVB irradiation	GO_ATP_METABOLIC_PROCESS1	-0.00861324	-0.00676591	-0.05239754	0.9595203	0.98542968	-6.22999547
WT after UVB irradiation VS WT before UVB irradiation	GO_ATP_METABOLIC_PROCESS2	0.159812382	-0.03285301	0.893751212	0.39723929	0.81477652	-5.59527885
homo after UVB irradiation VS homo before UVB irradiation	GO_ATP_SYNTHESIS_COUPLED_ELECTRON_TRANSPORT	-0.00376214	-0.00239808	-0.01278082	0.9901035	0.99491641	-6.55076707
hetero after UVB irradiation VS hetero before UVB irradiation	GO_ATP_SYNTHESIS_COUPLED_ELECTRON_TRANSPORT1	-0.26674488	-0.03301161	-1.10019056	0.30382892	0.62554758	-5.62272538
WT after UVB irradiation VS WT before UVB irradiation	GO_ATP_SYNTHESIS_COUPLED_ELECTRON_TRANSPORT2	0.014032231	-0.07539596	0.046844044	0.96377223	0.99054347	-5.99490897
homo after UVB irradiation VS homo before UVB irradiation	GO_ATP_SYNTHESIS_COUPLED_PROTON_TRANSPORT	-0.12091726	-0.03342155	-0.39966691	0.69945521	0.82070322	-6.46403816
hetero after UVB irradiation VS hetero before UVB irradiation	GO_ATP_SYNTHESIS_COUPLED_PROTON_TRANSPORT1	-0.14677821	-0.06263061	-0.63284232	0.54481997	0.79870659	-6.01985516
WT after UVB irradiation VS WT before UVB irradiation	GO_ATP_SYNTHESIS_COUPLED_PROTON_TRANSPORT2	0.035721215	-0.03082565	0.113429542	0.91245184	0.97884691	-5.98928349
homo after UVB irradiation VS homo before UVB irradiation	GO_ATP_TRANSMEMBRANE_TRANSPORTER_ACTIVITY	-0.2167709	0.004248393	-1.48920686	0.17328727	0.35353859	-5.47448101
hetero after UVB irradiation VS hetero before UVB irradiation	GO_ATP_TRANSMEMBRANE_TRANSPORTER_ACTIVITY1	-0.29840119	-0.01619573	-2.13440066	0.06598174	0.33426479	-4.30264775
WT after UVB irradiation VS WT before UVB irradiation	GO_ATP_TRANSMEMBRANE_TRANSPORTER_ACTIVITY2	-0.04655026	-0.03444058	-0.28110076	0.78568223	0.94642844	-5.95458734
homo after UVB irradiation VS homo before UVB irradiation	GO_ATP_TRANSPORT	-0.25999097	0.00401521	-1.81449268	0.10567433	0.25921478	-5.03384333
hetero after UVB irradiation VS hetero before UVB irradiation	GO_ATP_TRANSPORT1	-0.32870546	-0.00708322	-2.45004164	0.0404771	0.27281954	-3.84455132
WT after UVB irradiation VS WT before UVB irradiation	GO_ATP_TRANSPORT2	-0.0631169	-0.01398956	-0.38921389	0.70715338	0.92691191	-5.91691749
homo after UVB irradiation VS homo before UVB irradiation	GO_ATPASE_ACTIVATOR_ACTIVITY	0.017806755	-0.00475217	0.115210284	0.91100763	0.95136506	-6.54357791
hetero after UVB irradiation VS hetero before UVB irradiation	GO_ATPASE_ACTIVATOR_ACTIVITY1	-0.01408681	-0.01860747	-0.08743598	0.93251346	0.97506864	-6.22734023
WT after UVB irradiation VS WT before UVB irradiation	GO_ATPASE_ACTIVATOR_ACTIVITY2	0.060871547	0.009216189	0.331870411	0.74841387	0.93642732	-5.93836773
WT after UVB irradiation VS WT before UVB irradiation	GO_ATPASE_ACTIVITY	-0.45216678	0.002399731	-3.64536344	0.00610364	0.0517098	-2.2485323
homo after UVB irradiation VS homo before UVB irradiation	GO_ATPASE_ACTIVITY_COUPLED_TO_TRANSMEMBRANE_MOVEMENT_OF_IONS_ROTATIONAL_MECHANISM	0.241398171	-0.06105126	0.86190907	0.41286213	0.59901744	-6.16065574
hetero after UVB irradiation VS hetero before UVB irradiation	GO_ATPASE_ACTIVITY_COUPLED_TO_TRANSMEMBRANE_MOVEMENT_OF_IONS_ROTATIONAL_MECHANISM1	0.29863206	0.00165608	1.852447576	0.10179196	0.39668105	-4.69941907
WT after UVB irradiation VS WT before UVB irradiation	GO_ATPASE_ACTIVITY_COUPLED_TO_TRANSMEMBRANE_MOVEMENT_OF_IONS_ROTATIONAL_MECHANISM2	0.265061418	-0.04367314	0.997286797	0.34748942	0.78529818	-5.50288653
hetero after UVB irradiation VS hetero before UVB irradiation	GO_ATPASE_ACTIVITY1	-0.18869217	-0.04699086	-1.01250566	0.3414736	0.66014434	-5.71020147
WT after UVB irradiation VS WT before UVB irradiation	GO_ATPASE_ACTIVITY2	-0.10641851	-0.00437123	-0.50964911	0.62389347	0.90175221	-5.86129616
homo after UVB irradiation VS homo before UVB irradiation	GO_ATPASE_BINDING	0.104816007	-0.02477891	0.708791839	0.49780892	0.67223459	-6.28329174
hetero after UVB irradiation VS hetero before UVB irradiation	GO_ATPASE_BINDING1	-0.02564056	0.001438739	-0.19479217	0.85050083	0.9428285	-6.2109582
WT after UVB irradiation VS WT before UVB irradiation	GO_ATPASE_BINDING2	0.015534001	0.020013827	0.097578673	0.92463893	0.98118223	-5.99104572
homo after UVB irradiation VS homo before UVB irradiation	GO_ATPASE_COMPLEX	-0.51599579	-0.00509171	-3.74386626	0.00527742	0.04742925	-2.10195911
hetero after UVB irradiation VS hetero before UVB irradiation	GO_ATPASE_COMPLEX1	-0.08408119	-0.01679966	-0.39485253	0.70345958	0.88183935	-6.1477934
WT after UVB irradiation VS WT before UVB irradiation	GO_ATPASE_COMPLEX2	-0.06480527	0.033168735	-0.24629646	0.81158131	0.95508327	-5.96418489
homo after UVB irradiation VS homo before UVB irradiation	GO_ATPASE_COUPLED_AMIDE_TRANSPORTER_ACTIVITY	0.866948182	-0.08553535	4.608256138	0.00156244	0.02682706	-0.87363522
hetero after UVB irradiation VS hetero before UVB irradiation	GO_ATPASE_COUPLED_AMIDE_TRANSPORTER_ACTIVITY1	0.705313929	0.044756556	2.622285875	0.03102832	0.24624966	-3.59199052
WT after UVB irradiation VS WT before UVB irradiation	GO_ATPASE_COUPLED_AMIDE_TRANSPORTER_ACTIVITY2	0.464373184	0.007796486	1.512566419	0.1683928	0.67379949	-4.94149244
homo after UVB irradiation VS homo before UVB irradiation	GO_ATPASE_COUPLED_INTRAMEMBRANE_LIPID_TRANSPORTER_ACTIVITY	-0.02334481	-0.06986081	-0.11397481	0.91195732	0.9516556	-6.54373306
hetero after UVB irradiation VS hetero before UVB irradiation	GO_ATPASE_COUPLED_INTRAMEMBRANE_LIPID_TRANSPORTER_ACTIVITY1	0.005453094	-0.03140132	0.028789894	0.97775037	0.99241192	-6.23103464
WT after UVB irradiation VS WT before UVB irradiation	GO_ATPASE_COUPLED_INTRAMEMBRANE_LIPID_TRANSPORTER_ACTIVITY2	0.311329182	-0.08692306	1.235032681	0.25145019	0.73564768	-5.26275225
homo after UVB irradiation VS homo before UVB irradiation	GO_ATPASE_COUPLED_ION_TRANSMEMBRANE_TRANSPORTER_ACTIVITY	0.141422539	-0.00689457	0.809450776	0.44076421	0.62338101	-6.2049748
hetero after UVB irradiation VS hetero before UVB irradiation	GO_ATPASE_COUPLED_ION_TRANSMEMBRANE_TRANSPORTER_ACTIVITY1	0.117813638	0.012292421	0.769063425	0.46435626	0.75184081	-5.92267179
WT after UVB irradiation VS WT before UVB irradiation	GO_ATPASE_COUPLED_ION_TRANSMEMBRANE_TRANSPORTER_ACTIVITY2	0.193725834	-0.00580387	1.269174316	0.23964054	0.72963866	-5.22539665
homo after UVB irradiation VS homo before UVB irradiation	GO_ATPASE_COUPLED_LIPID_TRANSMEMBRANE_TRANSPORTER_ACTIVITY	0.611393749	-0.02149444	1.911650058	0.09084812	0.23644544	-4.89477681

hetero after UVB irradiation VS hetero before UVB irradiation	GO_ATPASE_COUPLED_LIPID_TRANSMEMBRANE_TRANSPORTER_ACTIVITY1	0.665147545	0.025632624	3.609813802	0.00710172	0.13963559	-2.17208126
WT after UVB irradiation VS WT before UVB irradiation	GO_ATPASE_COUPLED_LIPID_TRANSMEMBRANE_TRANSPORTER_ACTIVITY2	0.781925189	-0.0037878	3.617527465	0.0066694	0.30328715	-2.04718077
homo after UVB irradiation VS homo before UVB irradiation	GO_ATPASE_COUPLED_XENOBIOTIC_TRANSMEMBRANE_TRANSPORTER_ACTIVITY	0.580491178	0.02551328	2.042656488	0.07397083	0.208945	-4.70311736
hetero after UVB irradiation VS hetero before UVB irradiation	GO_ATPASE_COUPLED_XENOBIOTIC_TRANSMEMBRANE_TRANSPORTER_ACTIVITY1	0.384097981	0.040751101	1.535267877	0.16396947	0.48526545	-5.11970526
WT after UVB irradiation VS WT before UVB irradiation	GO_ATPASE_COUPLED_XENOBIOTIC_TRANSMEMBRANE_TRANSPORTER_ACTIVITY2	0.869850512	0.066104376	3.206701029	0.01228814	0.34717171	-2.60503585
homo after UVB irradiation VS homo before UVB irradiation	GO_ATPASE_DEPENDENT_TRANSMEMBRANE_TRANSPORT_COMPLEX	0.166920851	0.059409266	0.922006594	0.38245447	0.57262444	-6.10705445
hetero after UVB irradiation VS hetero before UVB irradiation	GO_ATPASE_DEPENDENT_TRANSMEMBRANE_TRANSPORT_COMPLEX1	-0.0477231	-0.00465261	-0.31872674	0.75824602	0.90625937	-6.17675947
WT after UVB irradiation VS WT before UVB irradiation	GO_ATPASE_DEPENDENT_TRANSMEMBRANE_TRANSPORT_COMPLEX2	0.112679115	0.047535206	0.613282364	0.5565193	0.87872408	-5.80235107
homo after UVB irradiation VS homo before UVB irradiation	GO_ATPASE_INHIBITOR_ACTIVITY	-0.39029912	0.016456052	-2.184512	0.05912236	0.18251169	-4.49122454
hetero after UVB irradiation VS hetero before UVB irradiation	GO_ATPASE_INHIBITOR_ACTIVITY1	-0.61341144	-0.03651173	-2.44187879	0.04099142	0.27357274	-3.85649746
WT after UVB irradiation VS WT before UVB irradiation	GO_ATPASE_INHIBITOR_ACTIVITY2	-0.61999184	-0.02807013	-3.53984932	0.00747394	0.31411149	-2.15086293
homo after UVB irradiation VS homo before UVB irradiation	GO_ATPASE_REGULATOR_ACTIVITY	-0.11184694	-0.01085457	-0.82906925	0.43018142	0.61430107	-6.18867482
hetero after UVB irradiation VS hetero before UVB irradiation	GO_ATPASE_REGULATOR_ACTIVITY1	-0.13118433	-0.01722283	-0.73748508	0.4822796	0.76203732	-5.9466667
WT after UVB irradiation VS WT before UVB irradiation	GO_ATPASE_REGULATOR_ACTIVITY2	-0.18562587	-0.01432051	-1.13393674	0.28927258	0.75486529	-5.36931421
homo after UVB irradiation VS homo before UVB irradiation	GO_ATRIAL_CARDIAC_MUSCLE_CELL_MEMBRANE_REPOLARIZATION	0.454142781	-0.00472634	1.93144113	0.08808065	0.23198397	-4.86610921
hetero after UVB irradiation VS hetero before UVB irradiation	GO_ATRIAL_CARDIAC_MUSCLE_CELL_MEMBRANE_REPOLARIZATION1	-0.07124975	0.041092577	-0.26365747	0.79883044	0.92219856	-6.19395758
WT after UVB irradiation VS WT before UVB irradiation	GO_ATRIAL_CARDIAC_MUSCLE_CELL_MEMBRANE_REPOLARIZATION2	-0.0079464	0.01836106	-0.03448092	0.97332848	0.99307736	-5.99543937
homo after UVB irradiation VS homo before UVB irradiation	GO_ATRIAL_CARDIAC_MUSCLE_CELL_TO_AV_NODE_CELL_SIGNALING	0.472207259	-0.04452895	3.189925489	0.01215188	0.07410957	-2.93990872
hetero after UVB irradiation VS hetero before UVB irradiation	GO_ATRIAL_CARDIAC_MUSCLE_CELL_TO_AV_NODE_CELL_SIGNALING1	-0.03932975	-0.00335759	-0.18083869	0.86107419	0.94760937	-6.21378742
WT after UVB irradiation VS WT before UVB irradiation	GO_ATRIAL_CARDIAC_MUSCLE_CELL_TO_AV_NODE_CELL_SIGNALING2	-0.02964987	-0.01453501	-0.14397809	0.88903649	0.97167171	-5.98514346
homo after UVB irradiation VS homo before UVB irradiation	GO_ATRIAL_CARDIAC_MUSCLE_TISSUE_DEVELOPMENT	0.051716696	-0.00599778	0.180114253	0.86136801	0.9237759	-6.53308762
hetero after UVB irradiation VS hetero before UVB irradiation	GO_ATRIAL_CARDIAC_MUSCLE_TISSUE_DEVELOPMENT1	0.139589217	0.055709623	0.438842225	0.67259939	0.86681615	-6.12835285
WT after UVB irradiation VS WT before UVB irradiation	GO_ATRIAL_CARDIAC_MUSCLE_TISSUE_DEVELOPMENT2	0.418544405	0.017182293	1.159524697	0.27928798	0.75044661	-5.34293539
homo after UVB irradiation VS homo before UVB irradiation	GO_ATRIAL_SEPTUM_DEVELOPMENT	-0.23297741	-0.0294958	-1.2285901	0.25280473	0.44753352	-5.79085627
hetero after UVB irradiation VS hetero before UVB irradiation	GO_ATRIAL_SEPTUM_DEVELOPMENT1	-0.18801754	-0.04582375	-1.19972777	0.26518805	0.59190967	-5.51716786
WT after UVB irradiation VS WT before UVB irradiation	GO_ATRIAL_SEPTUM_DEVELOPMENT2	-0.47689987	-0.0407988	-3.23067621	0.01185091	0.34347216	-2.57188275
homo after UVB irradiation VS homo before UVB irradiation	GO_ATRIAL_SEPTUM_MORPHOGENESIS	-0.15613937	-0.02565718	-0.76179813	0.46720048	0.64615534	-6.24317357
hetero after UVB irradiation VS hetero before UVB irradiation	GO_ATRIAL_SEPTUM_MORPHOGENESIS1	-0.14471601	-0.0406218	-0.96203173	0.36471739	0.67934483	-5.75805325
WT after UVB irradiation VS WT before UVB irradiation	GO_ATRIAL_SEPTUM_MORPHOGENESIS2	-0.42344205	0.009106845	-2.33274178	0.04758348	0.50650586	-3.83970469
homo after UVB irradiation VS homo before UVB irradiation	GO_ATRIAL_SEPTUM_PRIMUM_MORPHOGENESIS	-0.32134547	-0.02378064	-1.29904908	0.2287309	0.4199194	-5.70916278
hetero after UVB irradiation VS hetero before UVB irradiation	GO_ATRIAL_SEPTUM_PRIMUM_MORPHOGENESIS1	-0.20272481	-0.00586908	-0.81542613	0.438859	0.7363081	-5.88589536
WT after UVB irradiation VS WT before UVB irradiation	GO_ATRIAL_SEPTUM_PRIMUM_MORPHOGENESIS2	-0.74179245	-0.0709293	-3.65458646	0.00631849	0.29981324	-1.99803705
homo after UVB irradiation VS homo before UVB irradiation	GO_ATRIOVENTRICULAR_CANAL_DEVELOPMENT	-0.53331288	0.079127505	-3.48699693	0.00773243	0.05848666	-2.48657621
hetero after UVB irradiation VS hetero before UVB irradiation	GO_ATRIOVENTRICULAR_CANAL_DEVELOPMENT1	0.001473915	0.036807392	0.006003208	0.99535986	0.99792976	-6.23146457
WT after UVB irradiation VS WT before UVB irradiation	GO_ATRIOVENTRICULAR_CANAL_DEVELOPMENT2	0.027350349	0.025564854	0.106418849	0.91783925	0.98042373	-5.99009547
homo after UVB irradiation VS homo before UVB irradiation	GO_ATRIOVENTRICULAR_VALVE_DEVELOPMENT	-0.02117795	0.009862549	-0.11778819	0.90902654	0.95030792	-6.54324881
hetero after UVB irradiation VS hetero before UVB irradiation	GO_ATRIOVENTRICULAR_VALVE_DEVELOPMENT1	-0.08971805	-0.04123771	-0.70721599	0.49987804	0.77248814	-5.96884765
WT after UVB irradiation VS WT before UVB irradiation	GO_ATRIOVENTRICULAR_VALVE_DEVELOPMENT2	-0.25134922	0.044860017	-1.5001087	0.1715261	0.67808426	-4.95670251
homo after UVB irradiation VS homo before UVB irradiation	GO_ATRIOVENTRICULAR_VALVE_FORMATION	0.27283425	0.040401571	1.242256103	0.24797727	0.44238401	-5.77525158
hetero after UVB irradiation VS hetero before UVB irradiation	GO_ATRIOVENTRICULAR_VALVE_FORMATION1	0.234113095	0.010459063	1.125547203	0.29357885	0.61756748	-5.59644599
WT after UVB irradiation VS WT before UVB irradiation	GO_ATRIOVENTRICULAR_VALVE_FORMATION2	0.396672352	0.056631109	2.096882297	0.06885643	0.54354218	-4.17064548
homo after UVB irradiation VS homo before UVB irradiation	GO_ATTACHMENT_OF_GPI_ANCHOR_TO_PROTEIN	0.385371889	-0.10137281	2.400765323	0.04195217	0.14772032	-4.1618727
hetero after UVB irradiation VS hetero before UVB irradiation	GO_ATTACHMENT_OF_GPI_ANCHOR_TO_PROTEIN1	0.230221954	0.051776759	0.989213941	0.35205539	0.66862242	-5.73251708
WT after UVB irradiation VS WT before UVB irradiation	GO_ATTACHMENT_OF_GPI_ANCHOR_TO_PROTEIN2	0.498127593	0.044917806	1.58894958	0.15027988	0.65844472	-4.8468291
homo after UVB irradiation VS homo before UVB irradiation	GO_ATTACHMENT_OF_MITOTIC_SPINDLE_MICROTUBULES_TO_KINETOCHORE	-0.54347392	-0.08202659	-2.6662963	0.0275381	0.11730681	-3.75137687
hetero after UVB irradiation VS hetero before UVB irradiation	GO_ATTACHMENT_OF_MITOTIC_SPINDLE_MICROTUBULES_TO_KINETOCHORE1	0.236150495	-0.01114393	0.7951853	0.44987023	0.74207367	-5.90217484
WT after UVB irradiation VS WT before UVB irradiation	GO_ATTACHMENT_OF_MITOTIC_SPINDLE_MICROTUBULES_TO_KINETOCHORE2	0.048065197	0.02570323	0.16677504	0.87163641	0.96897064	-5.98141731
homo after UVB irradiation VS homo before UVB irradiation	GO_ATTACHMENT_OF_SPINDLE_MICROTUBULES_TO_KINETOCHORE	-0.53980902	-0.04212956	-2.98147356	0.01678391	0.08852043	-3.26192238
hetero after UVB irradiation VS hetero before UVB irradiation	GO_ATTACHMENT_OF_SPINDLE_MICROTUBULES_TO_KINETOCHORE1	0.211680299	0.003490992	0.780948876	0.45772674	0.74723043	-5.91341781
WT after UVB irradiation VS WT before UVB irradiation	GO_ATTACHMENT_OF_SPINDLE_MICROTUBULES_TO_KINETOCHORE2	-0.14128867	0.006920329	-0.46126495	0.65674559	0.91069298	-5.88533834
homo after UVB irradiation VS homo before UVB irradiation	GO_AU_RICH_ELEMENT_BINDING	-0.55528072	-0.04612797	-3.44619128	0.00822265	0.06037312	-2.5483558
hetero after UVB irradiation VS hetero before UVB irradiation	GO_AU_RICH_ELEMENT_BINDING1	-0.20178226	-0.02439723	-1.42075497	0.19385532	0.51887906	-5.26179156
WT after UVB irradiation VS WT before UVB irradiation	GO_AU_RICH_ELEMENT_BINDING2	-0.27106849	-0.00709311	-1.6444536	0.1382475	0.64683525	-4.77661942
homo after UVB irradiation VS homo before UVB irradiation	GO_AUDITORY_RECEPTOR_CELL_DEVELOPMENT	0.152312138	-0.01528653	1.123989172	0.29236469	0.48723809	-5.90626173
hetero after UVB irradiation VS hetero before UVB irradiation	GO_AUDITORY_RECEPTOR_CELL_DEVELOPMENT1	0.222032113	0.021693913	1.257100392	0.24481724	0.572323	-5.45351674
WT after UVB irradiation VS WT before UVB irradiation	GO_AUDITORY_RECEPTOR_CELL_DEVELOPMENT2	-0.07590692	-0.04121487	-0.52633362	0.61276391	0.8996674	-5.85248609
homo after UVB irradiation VS homo before UVB irradiation	GO_AUDITORY_RECEPTOR_CELL_FATE_COMMITMENT	-0.56358918	-0.00092537	-3.03939108	0.01533708	0.08404354	-3.17222011
hetero after UVB irradiation VS hetero before UVB irradiation	GO_AUDITORY_RECEPTOR_CELL_FATE_COMMITMENT1	-0.21155604	-0.04821056	-0.90922382	0.39028447	0.69998178	-5.80606137
WT after UVB irradiation VS WT before UVB irradiation	GO_AUDITORY_RECEPTOR_CELL_FATE_COMMITMENT2	-0.20199669	0.061263805	-0.81927695	0.43607551	0.83024038	-5.65665609
homo after UVB irradiation VS homo before UVB irradiation	GO_AUDITORY_RECEPTOR_CELL_MORPHOGENESIS	0.314338256	-0.02886998	2.26790755	0.05180183	0.16834169	-4.36497181
hetero after UVB irradiation VS hetero before UVB irradiation	GO_AUDITORY_RECEPTOR_CELL_MORPHOGENESIS1	0.26224259	0.040986942	1.236302481	0.25204519	0.57985326	-5.47681788
WT after UVB irradiation VS WT before UVB irradiation	GO_AUDITORY_RECEPTOR_CELL_MORPHOGENESIS2	-0.11620068	0.025964147	-0.77087754	0.46267663	0.84105926	-5.69414476
homo after UVB irradiation VS homo before UVB irradiation	GO_AUTOCRINE_SIGNALING	0.408218637	0.03040206	2.168704957	0.0606205	0.18536608	-4.5150266
hetero after UVB irradiation VS hetero before UVB irradiation	GO_AUTOCRINE_SIGNALING1	0.635781423	0.033220672	3.461757704	0.00879465	0.14994695	-2.37865501
WT after UVB irradiation VS WT before UVB irradiation	GO_AUTOCRINE_SIGNALING2	0.424255916	0.150559559	2.112710364	0.06717405	0.54021113	-4.14865112
homo after UVB irradiation VS homo before UVB irradiation	GO_AUTOLYSOSOME	0.80050391	0.023595917	3.655477167	0.0060128	0.05125992	-2.2334284
hetero after UVB irradiation VS hetero before UVB irradiation	GO_AUTOLYSOSOME1	0.609903297	0.037035488	2.92935011	0.01940587	0.20164937	-3.14234921
WT after UVB irradiation VS WT before UVB irradiation	GO_AUTOLYSOSOME2	0.609740037	-0.03403187	2.495101182	0.03688639	0.47615783	-3.60930136
homo after UVB irradiation VS homo before UVB irradiation	GO_AUTONOMIC_NERVOUS_SYSTEM_DEVELOPMENT	-0.1716805	0.049510797	-1.09816257	0.30286084	0.49729132	-5.93361184
hetero after UVB irradiation VS hetero before UVB irradiation	GO_AUTONOMIC_NERVOUS_SYSTEM_DEVELOPMENT1	-0.10533243	0.000239115	-0.78714235	0.4542975	0.74542576	-5.90854778
WT after UVB irradiation VS WT before UVB irradiation	GO_AUTONOMIC_NERVOUS_SYSTEM_DEVELOPMENT2	-0.08598578	0.041216976	-0.57277472	0.58234776	0.88924937	-5.8265815
homo after UVB irradiation VS homo before UVB irradiation	GO_AUTOPHAGIC_CELL_DEATH	0.367228564	-0.00795401	2.205942169	0.05714923	0.17819836	-4.45888701
hetero after UVB irradiation VS hetero before UVB irradiation	GO_AUTOPHAGIC_CELL_DEATH1	0.425211949	0.035571923	2.139453153	0.06546829	0.33409179	-4.29540292
WT after UVB irradiation VS WT before UVB irradiation	GO_AUTOPHAGIC_CELL_DEATH2	0.270564458	-0.03919839	1.435515818	0.18861801	0.69085152	-5.0344679
homo after UVB irradiation VS homo before UVB irradiation	GO_AUTOPHAGOSOME	0.316250023	-0.01779448	1.608049206	0.14499683	0.31723301	-5.31856472
homo after UVB irradiation VS homo before UVB irradiation	GO_AUTOPHAGOSOME_MATURATION	0.26120553	-0.03953531	1.354615852	0.21113706	0.40151412	-5.64264138
hetero after UVB irradiation VS hetero before UVB irradiation	GO_AUTOPHAGOSOME_MATURATION1	0.214242834	0.006387188	1.521625406	0.16729914	0.48884476	-5.13694144
WT after UVB irradiation VS WT before UVB irradiation	GO_AUTOPHAGOSOME_MATURATION2	0.075935029	-0.05776357	0.437624978	0.67309599	0.91558199	-5.89626115
homo after UVB irradiation VS homo before UVB irradiation	GO_AUTOPHAGOSOME_MEMBRANE	0.371340855	-0.03205502	1.67439663	0.13108937	0.29701917	-5.22883511
hetero after UVB irradiation VS hetero before UVB irradiation	GO_AUTOPHAGOSOME_MEMBRANE1	0.301096277	0.018359062	2.104350405	0.06911838	0.34103104	-4.34565661
WT after UVB irradiation VS WT before UVB irradiation	GO_AUTOPHAGOSOME_MEMBRANE2	0.194172132	-0.07088815	1.191553882	0.26718607	0.74336868	-5.30934258
homo after UVB irradiation VS homo before UVB irradiation	GO_AUTOPHAGOSOME_ORGANIZATION	0.084670734	-0.01744594	0.443069305	0.66899789	0.80022712	-6.44439249
hetero after UVB irradiation VS hetero before UVB irradiation	GO_AUTOPHAGOSOME_ORGANIZATION1	0.087842269	-0.03937745	0.660741656	0.52768189	0.79029868	-6.00131099
WT after UVB irradiation VS WT before UVB irradiation	GO_AUTOPHAGOSOME_ORGANIZATION2	-0.03475193	-0.0314611	-0.22174267	0.83000417	0.95977959	-5.9702054
hetero after UVB irradiation VS hetero before UVB irradiation	GO_AUTOPHAGOSOME1	0.304513086	0.003439877	2.346597293	0.04750592	0.2906291	-3.99565921
WT after UVB irradiation VS WT before UVB irradiation	GO_AUTOPHAGOSOME2	0.308756057	-0.0431993	1.940925536	0.08778173	0.57920555	-4.38505177
homo after UVB irradiation VS homo before UVB irradiation	GO_AUTOPHAGY_OF_MITOCHONDRION	-0.12840547	-0.01861203	-0.85618839	0.41584345	0.60215894	-6.16560214
hetero after UVB irradiation VS hetero before UVB irradiation	GO_AUTOPHAGY_OF_MITOCHONDRION1	-0.19788741	-0.05557211	-1.35394489	0.21342842	0.54045743	-5.34178285
WT after UVB irradiation VS WT before UVB irradiation	GO_AUTOPHAGY_OF_MITOCHONDRION2	-0.04752384	-0.01228953	-0.25557132	0.80465393	0.95377263	-5.96174892
homo after UVB irradiation VS homo before UVB irradiation	GO_AUTOPHAGY_OF_NUCLEUS	-0.28200158	-0.01739592	-1.55077396	0.15806679	0.33387816	-5.39451585
hetero after UVB irradiation VS hetero before UVB irradiation	GO_AUTOPHAGY_OF_NUCLEUS1	-0.06860639	-0.01146267	-0.27550023	0.79004241	0.918108	-6.19052785
WT after UVB irradiation VS WT before UVB irradiation	GO_AUTOPHAGY_OF_NUCLEUS2	-0.16486554	0.025005527	-0.64057635	0.539498	0.87025652	-5.78518024
homo after UVB irradiation VS homo before UVB irradiation	GO_AUTOPHAGY_OF_PEROXISOME	-0.21401167	-0.03698555	-0.58939472	0.57123319	0.72888882	-6.36414979
hetero after UVB irradiation VS hetero before UVB irradiation	GO_AUTOPHAGY_OF_PEROXISOME1	-0.63191806	-0.01354748	-2.56736354	0.03376824	0.25357971	-3.67258774
WT after UVB irradiation VS WT before UVB irradiation	GO_AUTOPHAGY_OF_PEROXISOME2	-0.29800249	0.023019406	-1.06188906	0.3189265	0.77323848	-5.44131515
homo after UVB irradiation VS homo before UVB irradiation	GO_AV_NODE_CELL_TO_BUNDLE_OF_HIS_CELL_COMMUNICATION	0.503261218	-0.03221993	2.893475045	0.01925836	0.09559222	-3.3984501
hetero after UVB irradiation VS hetero before UVB irradiation	GO_AV_NODE_CELL_TO_BUNDLE_OF_HIS_CELL_COMMUNICATION1	-0.15651803	-0.04221559	-0.62738222	0.54821257	0.80025851	-6.02340037
WT after UVB irradiation VS WT before UVB irradiation	GO_AV_NODE_CELL_TO_BUNDLE_OF_HIS_CELL_COMMUNICATION2	-0.03797451	-0.03460023	-0.11860857	0.90847506	0.97832631	-5.98865051
homo after UVB irradiation VS homo before UVB irradiation	GO_AXIAL_MESODERM_DEVELOPMENT	-1.06624738	-0.00103948	-6.57725145	0.00014481	0.01016637	1.494181419
hetero after UVB irradiation VS hetero before UVB irradiation	GO_AXIAL_MESODERM_DEVELOPMENT1	-0.84638176	-0.0104218	-3.46177718	0.0087944	0.14994695	-2.37862765
WT after UVB irradiation VS WT before UVB irradiation	GO_AXIAL_MESODERM_DEVELOPMENT2	-0.10998693	0.109702905	-0.36567449	0.72397618	0.93154998	-5.92612155
homo after UVB irradiation VS homo before UVB irradiation	GO_AXIS_ELONGATION	-0.32205414	-0.00638067	-2.58004775	0.03156598	0.12597827	-3.88515085
homo after UVB irradiation VS homo before UVB irradiation	GO_AXIS_ELONGATION_INVOLVED_IN_SOMITOGENESIS	-0.6822004	-0.02959453	-4.94946435	0.00099581	0.02151484	-0.42019103
hetero after UVB irradiation VS hetero before UVB irradiation	GO_AXIS_ELONGATION_INVOLVED_IN_SOMITOGENESIS1	-0.6479124	-0.11015765	-3.45627267	0.00886509	0.15004408	-2.3863639
WT after UVB irradiation VS WT before UVB irradiation	GO_AXIS_ELONGATION_INVOLVED_IN_SOMITOGENESIS2	-0.58751766	-0.08393973	-2.56671678	0.0329737	0.460248	-3.50740094
hetero after UVB irradiation VS hetero before UVB irradiation	GO_AXIS_ELONGATION1	-0.28927938	-0.00932871	-1.68830909	0.13053719	0.43937198	-4.92123831
WT after UVB irradiation VS WT before UVB irradiation	GO_AXIS_ELONGATION2	-0.37597977	0.006469309	-2.37731191	0.04436955	0.49817448	-3.77658786
homo after UVB irradiation VS homo before UVB irradiation	GO_AXIS_SPECIFICATION	-0.31349035	0.040519892	-2.49908781	0.03588982	0.13545424	-4.01039843
hetero after UVB irradiation VS hetero before UVB irradiation	GO_AXIS_SPECIFICATION1	-0.26892355	0.009840255	-1.56936322	0.15590822	0.47498806	-5.07628525
WT after UVB irradiation VS WT before UVB irradiation	GO_AXIS_SPECIFICATION2	-0.26316376	0.041142631	-1.7567726	0.11656653	0.62406566	-4.63127201
homo after UVB irradiation VS homo before UVB irradiation	GO_AXO_DENDRITIC_PROTEIN_TRANSPORT	-0.16531006	-0.00154863	-0.96588667	0.36130411	0.55226916	-6.06607281
hetero after UVB irradiation VS hetero before UVB irradiation	GO_AXO_DENDRITIC_PROTEIN_TRANSPORT1	-0.19810893	-0.03038798	-1.12274269	0.2946987	0.6181342	-5.59937358
WT after UVB irradiation VS WT before UVB irradiation	GO_AXO_DENDRITIC_PROTEIN_TRANSPORT2	-0.1752772	-0.07670978	-0.87530949	0.40661834	0.81694579	-5.61088485
homo after UVB irradiation VS homo before UVB irradiation	GO_AXO_DENDRITIC_TRANSPORT	-0.00235511	-0.01291713	-0.01701927	0.98682188	0.99310041	-6.55069775
hetero after UVB irradiation VS hetero before UVB irradiation	GO_AXO_DENDRITIC_TRANSPORT1	-0.05369538	-0.02374154	-0.393762	0.70423244	0.88236851	-6.14825039
WT after UVB irradiation VS WT before UVB irradiation	GO_AXO_DENDRITIC_TRANSPORT2	0.0650671	-0.03050949	0.445431071	0.66767596	0.91400778	-5.89271464
homo after UVB irradiation VS homo before UVB irradiation	GO_AXOLEMMA	0.117159722	-0.02498439	0.591550911	0.56985423	0.72795524	-6.36280958
hetero after UVB irradiation VS hetero before UVB irradiation	GO_AXOLEMMA1	0.130291546	0.024320598	0.712982402	0.49649413	0.7699703	-5.96468455
WT after UVB irradiation VS WT before UVB irradiation	GO_AXOLEMMA2	0.097376452	0.013240783	0.37821523	0.7149932	0.93029655	-5.92128685
homo after UVB irradiation VS homo before UVB irradiation	GO_AXON	0.127070763	-0.00231512	1.034255293	0.33010871	0.52324535	-5.99923651
homo after UVB irradiation VS homo before UVB irradiation	GO_AXON_CHOICE_POINT_RECOGNITION	0.157505881	0.079349742	1.01359089	0.33931288	0.53217559	-6.01981278
hetero after UVB irradiation VS hetero before UVB irradiation	GO_AXON_CHOICE_POINT_RECOGNITION1	0.26228715	0.10508377	1.219222562	0.25811366	0.58489002	-5.49576149
WT after UVB irradiation VS WT before UVB irradiation	GO_AXON_CHOICE_POINT_RECOGNITION2	-0.24651114	0.022103521	-1.22681152	0.25436526	0.73589193	-5.27164721
homo after UVB irradiation VS homo before UVB irradiation	GO_AXON_CYTOPLASM	0.007500085	-0.01652246	0.056377445	0.95637014	0.97709207	-6.54911268
hetero after UVB irradiation VS hetero before UVB irradiation	GO_AXON_CYTOPLASM1	-0.06663403	-0.02118355	-0.48867616	0.63842517	0.85182425	-6.10398175
WT after UVB irradiation VS WT before UVB irradiation	GO_AXON_CYTOPLASM2	0.065686033	-0.03018876	0.471559213	0.64968586	0.9092481	-5.88041206
homo after UVB irradiation VS homo before UVB irradiation	GO_AXON_DEVELOPMENT	0.159582058	-0.00845732	1.217288186	0.25685559	0.45226321	-5.80367195
hetero after UVB irradiation VS hetero before UVB irradiation	GO_AXON_DEVELOPMENT1	0.118347492	0.018704636	0.880299127	0.4048321	0.71132442	-5.83143628
WT after UVB irradiation VS WT before UVB irradiation	GO_AXON_DEVELOPMENT2	0.056231173	0.005826216	0.377992059	0.71515265	0.93029655	-5.92137426
homo after UVB irradiation VS homo before UVB irradiation	GO_AXON_ENSHEATHMENT_IN_CENTRAL_NERVOUS_SYSTEM	-0.25082237	0.009113048	-1.60200334	0.14632874	0.31900918	-5.32664991
hetero after UVB irradiation VS hetero before UVB irradiation	GO_AXON_ENSHEATHMENT_IN_CENTRAL_NERVOUS_SYSTEM1	0.148000407	0.015546393	0.799236991	0.44765111	0.74149757	-5.8989437
WT after UVB irradiation VS WT before UVB irradiation	GO_AXON_ENSHEATHMENT_IN_CENTRAL_NERVOUS_SYSTEM2	-0.13289424	0.060867149	-0.72770998	0.48729628	0.85164239	-5.72593007
homo after UVB irradiation VS homo before UVB irradiation	GO_AXON_EXTENSION	0.200456711	-0.02463563	1.474587149	0.17708765	0.35867175	-5.49320103
hetero after UVB irradiation VS hetero before UVB irradiation	GO_AXON_EXTENSION1	0.095285354	0.011116813	0.689721542	0.51023384	0.7798043	-5.98129628
WT after UVB irradiation VS WT before UVB irradiation	GO_AXON_EXTENSION2	0.088501695	0.005332883	0.557334675	0.59236652	0.89307494	-5.83541799
homo after UVB irradiation VS homo before UVB irradiation	GO_AXON_GUIDANCE_RECEPTOR_ACTIVITY	-0.01498289	-0.03306139	-0.09698239	0.92503351	0.95935902	-6.54569771
hetero after UVB irradiation VS hetero before UVB irradiation	GO_AXON_GUIDANCE_RECEPTOR_ACTIVITY1	-0.0732682	0.022087122	-0.50038656	0.63052266	0.84800118	-6.09789749
WT after UVB irradiation VS WT before UVB irradiation	GO_AXON_GUIDANCE_RECEPTOR_ACTIVITY2	-0.14718712	0.049951228	-0.56678616	0.58622241	0.89109011	-5.83003516
homo after UVB irradiation VS homo before UVB irradiation	GO_AXON_HILLOCK	-0.33777372	-0.00137518	-1.33620554	0.21683369	0.40746401	-5.66487889
hetero after UVB irradiation VS hetero before UVB irradiation	GO_AXON_HILLOCK1	-0.06093119	-0.03033787	-0.21825304	0.83279684	0.9354456	-6.20573258
WT after UVB irradiation VS WT before UVB irradiation	GO_AXON_HILLOCK2	0.148379097	-0.0149668	0.814915396	0.4384289	0.83056493	-5.66011322
homo after UVB irradiation VS homo before UVB irradiation	GO_AXON_INITIAL_SEGMENT	0.473213959	-0.03051843	2.217419487	0.05611928	0.1760786	-4.44153688
hetero after UVB irradiation VS hetero before UVB irradiation	GO_AXON_INITIAL_SEGMENT1	0.309597434	0.031752442	1.657652394	0.1366844	0.44980262	-4.9617017
WT after UVB irradiation VS WT before UVB irradiation	GO_AXON_INITIAL_SEGMENT2	0.372604648	0.027161258	1.623744024	0.14263004	0.65167133	-4.80294901
homo after UVB irradiation VS homo before UVB irradiation	GO_AXON_MIDLINE_CHOICE_POINT_RECOGNITION	0.175851098	0.093536455	0.914349681	0.38623603	0.57593354	-6.11404789
hetero after UVB irradiation VS hetero before UVB irradiation	GO_AXON_MIDLINE_CHOICE_POINT_RECOGNITION1	0.659158819	0.099416908	4.204875393	0.0031028	0.11503745	-1.3747086
WT after UVB irradiation VS WT before UVB irradiation	GO_AXON_MIDLINE_CHOICE_POINT_RECOGNITION2	-0.07972913	0.047179453	-0.3207526	0.75651928	0.93854182	-5.94214351
hetero after UVB irradiation VS hetero before UVB irradiation	GO_AXON1	0.032028687	0.003933937	0.247925737	0.81055163	0.92652154	-6.19828466
WT after UVB irradiation VS WT before UVB irradiation	GO_AXON2	0.020162464	-0.00834607	0.136894039	0.89445701	0.97322079	-5.98619062
homo after UVB irradiation VS homo before UVB irradiation	GO_AXONAL_FASCICULATION	0.210168116	-0.01385415	1.3449623	0.21410793	0.40476438	-5.65432582
hetero after UVB irradiation VS hetero before UVB irradiation	GO_AXONAL_FASCICULATION1	0.138736283	0.044343574	0.820784018	0.43597557	0.73478547	-5.88152864
WT after UVB irradiation VS WT before UVB irradiation	GO_AXONAL_FASCICULATION2	0.287328841	-0.00828187	1.450289735	0.1845812	0.68726847	-5.0168477

homo after UVB irradiation VS homo before UVB irradiation	GO_AXONAL_GROWTH_CONE	-0.08463487	0.003973973	-0.44495582	0.66768809	0.79924345	-6.44349475
hetero after UVB irradiation VS hetero before UVB irradiation	GO_AXONAL_GROWTH_CONE1	-0.28507428	-0.03293417	-1.79091223	0.11178744	0.4125528	-4.78353247
WT after UVB irradiation VS WT before UVB irradiation	GO_AXONAL_GROWTH_CONE2	-0.31863467	0.00428727	-1.88091353	0.09632456	0.59686908	-4.46623358
homo after UVB irradiation VS homo before UVB irradiation	GO_AXONAL_TRANSPORT	0.022656043	-0.01603998	0.178063501	0.86292779	0.92457891	-6.53348917
homo after UVB irradiation VS homo before UVB irradiation	GO_AXONAL_TRANSPORT_OF_MITOCHONDRION	-0.18562469	-0.01169618	-1.2850142	0.23336632	0.42466738	-5.72567751
hetero after UVB irradiation VS hetero before UVB irradiation	GO_AXONAL_TRANSPORT_OF_MITOCHONDRION1	-0.25329899	0.002675806	-1.38663573	0.20364588	0.5282121	-5.30292536
WT after UVB irradiation VS WT before UVB irradiation	GO_AXONAL_TRANSPORT_OF_MITOCHONDRION2	-0.25943142	-0.00017529	-1.60452359	0.14681075	0.65371647	-4.82724466
hetero after UVB irradiation VS hetero before UVB irradiation	GO_AXONAL_TRANSPORT1	-0.12613719	-0.03253115	-0.92019785	0.38486587	0.69630711	-5.79626189
WT after UVB irradiation VS WT before UVB irradiation	GO_AXONAL_TRANSPORT2	0.055162611	-0.03283283	0.39727846	0.7014284	0.92477252	-5.91363727
homo after UVB irradiation VS homo before UVB irradiation	GO_AXONEMAL_DYNEIN_COMPLEX	-0.3812026	0.038242619	-2.53417033	0.0339471	0.13183086	-3.9561725
homo after UVB irradiation VS homo before UVB irradiation	GO_AXONEMAL_DYNEIN_COMPLEX_ASSEMBLY	-0.21194205	-0.00920704	-1.43795279	0.18693556	0.37175105	-5.53963834
hetero after UVB irradiation VS hetero before UVB irradiation	GO_AXONEMAL_DYNEIN_COMPLEX_ASSEMBLY1	-0.32425221	0.008039923	-2.24376494	0.05570819	0.31172928	-4.14505552
WT after UVB irradiation VS WT before UVB irradiation	GO_AXONEMAL_DYNEIN_COMPLEX_ASSEMBLY2	-0.23588887	0.030791534	-1.65830215	0.13538619	0.64335078	-4.75892719
hetero after UVB irradiation VS hetero before UVB irradiation	GO_AXONEMAL_DYNEIN_COMPLEX1	-0.48390529	0.018901529	-2.25088607	0.0550972	0.31051949	-4.13474331
WT after UVB irradiation VS WT before UVB irradiation	GO_AXONEMAL_DYNEIN_COMPLEX2	-0.36133734	0.044597659	-2.13104714	0.06527528	0.53678963	-4.12312449
homo after UVB irradiation VS homo before UVB irradiation	GO_AXONEMAL_MICROTUBULE	0.038362664	0.046550011	0.124080114	0.90419412	0.9475315	-6.5424151
hetero after UVB irradiation VS hetero before UVB irradiation	GO_AXONEMAL_MICROTUBULE1	-0.34594753	-0.01689884	-1.70508865	0.12728223	0.43416011	-4.89895272
WT after UVB irradiation VS WT before UVB irradiation	GO_AXONEMAL_MICROTUBULE2	-0.15797087	0.035357239	-0.62949249	0.54637255	0.87364475	-5.79223441
homo after UVB irradiation VS homo before UVB irradiation	GO_AXONEME_ASSEMBLY	-0.05653792	-0.00343247	-0.46000831	0.65728058	0.7915366	-6.43620172
hetero after UVB irradiation VS hetero before UVB irradiation	GO_AXONEME_ASSEMBLY1	-0.24149646	0.011648998	-1.67405617	0.13336257	0.44392017	-4.94009183
WT after UVB irradiation VS WT before UVB irradiation	GO_AXONEME_ASSEMBLY2	-0.22172249	-0.00019295	-1.41521709	0.19429084	0.69334561	-5.05851065
homo after UVB irradiation VS homo before UVB irradiation	GO_AXONOGENESIS_INVOLVED_IN_INNERVATION	0.198239269	0.035252408	0.746868247	0.4756946	0.65390047	-6.25472898
hetero after UVB irradiation VS hetero before UVB irradiation	GO_AXONOGENESIS_INVOLVED_IN_INNERVATION1	-0.17656529	0.010889655	-0.70287062	0.50243772	0.77390992	-5.97196525
WT after UVB irradiation VS WT before UVB irradiation	GO_AXONOGENESIS_INVOLVED_IN_INNERVATION2	0.088311168	0.043524246	0.305351767	0.7678001	0.94123239	-5.94716683
homo after UVB irradiation VS homo before UVB irradiation	GO_AZOLE_TRANSMEMBRANE_TRANSPORT	-0.33017174	0.03290885	-2.18473902	0.05910111	0.18249739	-4.49088238
hetero after UVB irradiation VS hetero before UVB irradiation	GO_AZOLE_TRANSMEMBRANE_TRANSPORT1	-0.47447804	0.070213549	-2.590682	0.03257594	0.25034181	-3.63837146
WT after UVB irradiation VS WT before UVB irradiation	GO_AZOLE_TRANSMEMBRANE_TRANSPORT2	-0.15600616	0.017077394	-0.99633976	0.34792222	0.78574573	-5.50376731
homo after UVB irradiation VS homo before UVB irradiation	GO_AZOLE_TRANSMEMBRANE_TRANSPORTER_ACTIVITY	-0.27063679	0.039629062	-2.02076829	0.07656341	0.21265693	-4.73543488
hetero after UVB irradiation VS hetero before UVB irradiation	GO_AZOLE_TRANSMEMBRANE_TRANSPORTER_ACTIVITY1	-0.44110811	0.087023601	-2.41182555	0.0429426	0.27767067	-3.90045067
WT after UVB irradiation VS WT before UVB irradiation	GO_AZOLE_TRANSMEMBRANE_TRANSPORTER_ACTIVITY2	-0.1303867	0.038137968	-0.93220786	0.3781853	0.80338597	-5.56189491
homo after UVB irradiation VS homo before UVB irradiation	GO_AZUROPHIL_GRANULE	0.665093063	-0.02535033	5.125490699	0.00079454	0.01939486	-0.19348233
homo after UVB irradiation VS homo before UVB irradiation	GO_AZUROPHIL_GRANULE_LUMEN	0.614326482	-0.03044367	4.549786264	0.0016907	0.02787404	-0.95318131
hetero after UVB irradiation VS hetero before UVB irradiation	GO_AZUROPHIL_GRANULE_LUMEN1	0.671133685	0.023297458	4.902520333	0.00125582	0.09106857	-0.51446237
WT after UVB irradiation VS WT before UVB irradiation	GO_AZUROPHIL_GRANULE_LUMEN2	0.629451637	-0.02339294	4.337932457	0.00241513	0.22523179	-1.13255382
homo after UVB irradiation VS homo before UVB irradiation	GO_AZUROPHIL_GRANULE_MEMBRANE	0.649399862	-0.02095918	4.799508055	0.00121128	0.02347563	-0.61720305
hetero after UVB irradiation VS hetero before UVB irradiation	GO_AZUROPHIL_GRANULE_MEMBRANE1	0.649793032	0.004220062	4.738841164	0.00154279	0.09569098	-0.70880997
WT after UVB irradiation VS WT before UVB irradiation	GO_AZUROPHIL_GRANULE_MEMBRANE2	0.649626246	-0.04127171	3.30050343	0.01066819	0.33609774	-2.47571084
hetero after UVB irradiation VS hetero before UVB irradiation	GO_AZUROPHIL_GRANULE1	0.686913673	0.016064269	5.16701831	0.00090786	0.08366654	-0.21008422
WT after UVB irradiation VS WT before UVB irradiation	GO_AZUROPHIL_GRANULE2	0.635629772	-0.02289313	4.005690077	0.00382255	0.26532716	-1.54338314
homo after UVB irradiation VS homo before UVB irradiation	GO_B_1_B_CELL_DIFFERENTIATION	0.667392869	-0.00783233	4.477398154	0.00186548	0.02922381	-1.05240247
hetero after UVB irradiation VS hetero before UVB irradiation	GO_B_1_B_CELL_DIFFERENTIATION1	0.549934178	0.023105621	2.717568847	0.02680303	0.23196771	-3.45217896
WT after UVB irradiation VS WT before UVB irradiation	GO_B_1_B_CELL_DIFFERENTIATION2	0.370584914	-0.11159586	0.970989399	0.35965946	0.79231624	-5.52710415
homo after UVB irradiation VS homo before UVB irradiation	GO_B_CELL_ACTIVATION	0.37849113	-0.01186137	2.964262363	0.0172406	0.08974236	-3.28860545
homo after UVB irradiation VS homo before UVB irradiation	GO_B_CELL_ACTIVATION_INVOLVED_IN_IMMUNE_RESPONSE	0.286006851	-0.00759834	2.019024789	0.07677366	0.21308047	-4.73800434
hetero after UVB irradiation VS hetero before UVB irradiation	GO_B_CELL_ACTIVATION_INVOLVED_IN_IMMUNE_RESPONSE1	0.256187352	0.025864054	1.877249704	0.09800862	0.38992672	-4.66522513
WT after UVB irradiation VS WT before UVB irradiation	GO_B_CELL_ACTIVATION_INVOLVED_IN_IMMUNE_RESPONSE2	0.326518295	-0.02583886	1.919904712	0.09068812	0.58389378	-4.41358169
hetero after UVB irradiation VS hetero before UVB irradiation	GO_B_CELL_ACTIVATION1	0.337808374	0.023984775	2.450657795	0.04043854	0.27281954	-3.84364945
WT after UVB irradiation VS WT before UVB irradiation	GO_B_CELL_ACTIVATION2	0.281757477	-0.00735114	1.532928307	0.16338164	0.66778023	-4.91649026
homo after UVB irradiation VS homo before UVB irradiation	GO_B_CELL_APOPTOTIC_PROCESS	0.369843373	-0.01218707	2.13449682	0.06399119	0.19162296	-4.566383
hetero after UVB irradiation VS hetero before UVB irradiation	GO_B_CELL_APOPTOTIC_PROCESS1	0.453777097	0.022985012	2.663777389	0.02911011	0.2392116	-3.53109819
WT after UVB irradiation VS WT before UVB irradiation	GO_B_CELL_APOPTOTIC_PROCESS2	0.318694538	-0.05541974	1.456890654	0.18280233	0.68604157	-5.00894258
homo after UVB irradiation VS homo before UVB irradiation	GO_B_CELL_CHEMOTAXIS	0.681690936	-0.02694443	4.341472336	0.00224862	0.03175393	-1.24090483
hetero after UVB irradiation VS hetero before UVB irradiation	GO_B_CELL_CHEMOTAXIS1	0.528749132	0.047018059	2.457887288	0.03998892	0.27243899	-3.83306648
WT after UVB irradiation VS WT before UVB irradiation	GO_B_CELL_CHEMOTAXIS2	0.522066855	0.011443912	1.761321979	0.11575891	0.62254163	-4.62530022
homo after UVB irradiation VS homo before UVB irradiation	GO_B_CELL_DIFFERENTIATION	0.471582823	-0.02249952	3.686565548	0.00574245	0.05029016	-2.18707812
hetero after UVB irradiation VS hetero before UVB irradiation	GO_B_CELL_DIFFERENTIATION1	0.41781852	0.021487304	2.861475772	0.02151313	0.21149087	-3.24142982
WT after UVB irradiation VS WT before UVB irradiation	GO_B_CELL_DIFFERENTIATION2	0.267576467	0.002161874	1.276279928	0.23724213	0.72854168	-5.21753928
homo after UVB irradiation VS homo before UVB irradiation	GO_B_CELL_HOMEOSTASIS	0.388459725	-0.04491535	3.157583127	0.01277263	0.07647347	-2.98970179
hetero after UVB irradiation VS hetero before UVB irradiation	GO_B_CELL_HOMEOSTASIS1	0.565137237	0.02940301	4.12758043	0.00344506	0.11632799	-1.47509309
WT after UVB irradiation VS WT before UVB irradiation	GO_B_CELL_HOMEOSTASIS2	0.293936963	-0.02413111	1.23225128	0.2524333	0.73565635	-5.26576601
homo after UVB irradiation VS homo before UVB irradiation	GO_B_CELL_LINEAGE_COMMITMENT	-0.05226434	-0.04731212	-0.30378537	0.76874201	0.86644205	-6.50049258
hetero after UVB irradiation VS hetero before UVB irradiation	GO_B_CELL_LINEAGE_COMMITMENT1	-0.04259198	-0.01950225	-0.21695075	0.83377701	0.93574283	-6.20603802
WT after UVB irradiation VS WT before UVB irradiation	GO_B_CELL_LINEAGE_COMMITMENT2	-0.05571606	0.007682823	-0.32622974	0.75252211	0.93734883	-5.94029902
homo after UVB irradiation VS homo before UVB irradiation	GO_B_CELL_MEDIATED_IMMUNITY	0.271446184	-0.00625023	1.660864324	0.13382266	0.30126941	-5.24728208
hetero after UVB irradiation VS hetero before UVB irradiation	GO_B_CELL_MEDIATED_IMMUNITY1	0.174087002	0.037584421	1.131520142	0.29120525	0.61561958	-5.59019368
WT after UVB irradiation VS WT before UVB irradiation	GO_B_CELL_MEDIATED_IMMUNITY2	0.265090297	-0.01284028	1.265565811	0.24086634	0.73118354	-5.22937611
homo after UVB irradiation VS homo before UVB irradiation	GO_B_CELL_PROLIFERATION	0.393358337	-0.00751056	2.675923708	0.02712221	0.11648499	-3.73642936
homo after UVB irradiation VS homo before UVB irradiation	GO_B_CELL_PROLIFERATION_INVOLVED_IN_IMMUNE_RESPONSE	0.869521229	-0.058861	4.729039005	0.00132957	0.02450673	-0.7110164
hetero after UVB irradiation VS hetero before UVB irradiation	GO_B_CELL_PROLIFERATION_INVOLVED_IN_IMMUNE_RESPONSE1	0.972599447	0.09518399	4.279959062	0.00280529	0.11250952	-1.27814894
WT after UVB irradiation VS WT before UVB irradiation	GO_B_CELL_PROLIFERATION_INVOLVED_IN_IMMUNE_RESPONSE2	0.603423031	-0.03628947	1.654402304	0.1361864	0.64454865	-4.76391627
hetero after UVB irradiation VS hetero before UVB irradiation	GO_B_CELL_PROLIFERATION1	0.312130307	0.016852452	2.190329877	0.06051296	0.32391145	-4.22224812
WT after UVB irradiation VS WT before UVB irradiation	GO_B_CELL_PROLIFERATION2	0.155813046	-0.0127141	0.713061581	0.49583987	0.85500221	-5.73635483
homo after UVB irradiation VS homo before UVB irradiation	GO_B_CELL_RECEPTOR_SIGNALING_PATHWAY	0.540372579	-0.02054028	3.644177641	0.00611438	0.05174208	-2.25030398
hetero after UVB irradiation VS hetero before UVB irradiation	GO_B_CELL_RECEPTOR_SIGNALING_PATHWAY1	0.556547839	0.02924032	3.405074496	0.00955222	0.15519858	-2.45850421
WT after UVB irradiation VS WT before UVB irradiation	GO_B_CELL_RECEPTOR_SIGNALING_PATHWAY2	0.357211522	-0.01385175	1.468795963	0.17963227	0.68255081	-4.99463499
homo after UVB irradiation VS homo before UVB irradiation	GO_BARBED_END_ACTIN_FILAMENT_CAPPING	0.572003201	-0.02451147	3.73938162	0.00531233	0.04768492	-2.10860613
hetero after UVB irradiation VS hetero before UVB irradiation	GO_BARBED_END_ACTIN_FILAMENT_CAPPING1	0.510760035	0.014551489	3.325209758	0.0107392	0.16269771	-2.57167962
WT after UVB irradiation VS WT before UVB irradiation	GO_BARBED_END_ACTIN_FILAMENT_CAPPING2	0.19442562	0.049726965	0.950336275	0.36943941	0.79984535	-5.54577063
homo after UVB irradiation VS homo before UVB irradiation	GO_BARR_BODY	-0.61246258	0.089740363	-2.46814362	0.03769626	0.13923445	-4.05815666
hetero after UVB irradiation VS hetero before UVB irradiation	GO_BARR_BODY1	-0.33243825	-0.0546305	-0.95403803	0.36850533	0.68175554	-5.76545745
WT after UVB irradiation VS WT before UVB irradiation	GO_BARR_BODY2	-0.60583492	-0.03996271	-1.63646331	0.13992356	0.64925417	-4.78679641
homo after UVB irradiation VS homo before UVB irradiation	GO_BASAL_CORTEX	-0.11873694	-0.07163975	-0.59196548	0.56958931	0.72774281	-6.36255138
hetero after UVB irradiation VS hetero before UVB irradiation	GO_BASAL_CORTEX1	0.200832715	0.035931004	0.721597155	0.49146612	0.76741525	-5.95841013
WT after UVB irradiation VS WT before UVB irradiation	GO_BASAL_CORTEX2	-0.68194852	0.077963499	-2.91902654	0.01906954	0.39193477	-3.00736857
homo after UVB irradiation VS homo before UVB irradiation	GO_BASAL_DENDRITE	0.422166764	-0.06713028	1.85275247	0.09958169	0.24983457	-4.979424
hetero after UVB irradiation VS hetero before UVB irradiation	GO_BASAL_DENDRITE1	-0.01016502	0.034928947	-0.0792161	0.93884217	0.97753	-6.22808243
WT after UVB irradiation VS WT before UVB irradiation	GO_BASAL_DENDRITE2	-0.32449102	-0.0294227	-1.70884322	0.12540061	0.63222988	-4.69379994
homo after UVB irradiation VS homo before UVB irradiation	GO_BASAL_PART_OF_CELL	0.185372743	-0.0104665	1.314823087	0.2236142	0.41449341	-5.69046171
hetero after UVB irradiation VS hetero before UVB irradiation	GO_BASAL_PART_OF_CELL1	0.277640811	0.019906143	1.963474164	0.08587677	0.37193478	-4.54517074
WT after UVB irradiation VS WT before UVB irradiation	GO_BASAL_PART_OF_CELL2	-0.00454661	0.003792788	-0.02609603	0.9798124	0.99512555	-5.99570735
homo after UVB irradiation VS homo before UVB irradiation	GO_BASAL_PLASMA_MEMBRANE	0.080933847	-0.00446699	0.517945488	0.61796955	0.76269068	-6.40599357
hetero after UVB irradiation VS hetero before UVB irradiation	GO_BASAL_PLASMA_MEMBRANE1	0.06792325	0.027306455	0.490780609	0.63700134	0.85099142	-6.10289832
WT after UVB irradiation VS WT before UVB irradiation	GO_BASAL_PLASMA_MEMBRANE2	-0.10007811	0.006049303	-0.64835212	0.53470624	0.86957641	-5.78016554
homo after UVB irradiation VS homo before UVB irradiation	GO_BASAL_TRANSCRIPTION_MACHINERY_BINDING	-0.51627269	-0.00178584	-3.22879565	0.01144734	0.07173178	-2.88016454
hetero after UVB irradiation VS hetero before UVB irradiation	GO_BASAL_TRANSCRIPTION_MACHINERY_BINDING1	-0.25873144	-0.08656327	-1.45637107	0.18407932	0.50859587	-5.2182468
WT after UVB irradiation VS WT before UVB irradiation	GO_BASAL_TRANSCRIPTION_MACHINERY_BINDING2	-0.2835274	-0.03633989	-1.06089479	0.31935185	0.77323848	-5.44228462
homo after UVB irradiation VS homo before UVB irradiation	GO_BASE_CONVERSION_OR_SUBSTITUTION_EDITING	0.149570416	0.057563609	1.188603552	0.26737709	0.46261208	-5.83582879
hetero after UVB irradiation VS hetero before UVB irradiation	GO_BASE_CONVERSION_OR_SUBSTITUTION_EDITING1	-0.08447989	0.030108536	-0.49504497	0.63412111	0.84957966	-6.10068952
WT after UVB irradiation VS WT before UVB irradiation	GO_BASE_CONVERSION_OR_SUBSTITUTION_EDITING2	0.21711193	-0.00118308	1.125262209	0.29272193	0.75699426	-5.37816258
homo after UVB irradiation VS homo before UVB irradiation	GO_BASE_EXCISION_REPAIR	-0.48521856	-0.00770194	-3.03653155	0.01540538	0.08429262	-3.17664539
homo after UVB irradiation VS homo before UVB irradiation	GO_BASE_EXCISION_REPAIR_AP_SITE_FORMATION	-0.62930802	-0.05369783	-4.1611809	0.00289292	0.03554278	-1.49526578
hetero after UVB irradiation VS hetero before UVB irradiation	GO_BASE_EXCISION_REPAIR_AP_SITE_FORMATION1	-0.35308914	-0.09357971	-1.48019007	0.17778773	0.50161295	-5.18879206
WT after UVB irradiation VS WT before UVB irradiation	GO_BASE_EXCISION_REPAIR_AP_SITE_FORMATION2	0.107767851	0.017748211	0.49867143	0.63127282	0.90411526	-5.8669482
homo after UVB irradiation VS homo before UVB irradiation	GO_BASE_EXCISION_REPAIR_GAP_FILLING	-0.66295286	0.002867307	-2.95024535	0.01762204	0.09098965	-3.31034431
hetero after UVB irradiation VS hetero before UVB irradiation	GO_BASE_EXCISION_REPAIR_GAP_FILLING1	-0.29408558	0.020636366	-1.13904448	0.28823719	0.61322317	-5.58228412
WT after UVB irradiation VS WT before UVB irradiation	GO_BASE_EXCISION_REPAIR_GAP_FILLING2	0.033297086	-0.00833482	0.096477392	0.92548648	0.98141737	-5.99115835
hetero after UVB irradiation VS hetero before UVB irradiation	GO_BASE_EXCISION_REPAIR1	-0.2318313	-0.04296769	-0.93572393	0.37729424	0.69022487	-5.78223781
WT after UVB irradiation VS WT before UVB irradiation	GO_BASE_EXCISION_REPAIR2	-0.03946915	0.025127768	-0.14905151	0.88515824	0.97059594	-5.98436122
homo after UVB irradiation VS homo before UVB irradiation	GO_BASEMENT_MEMBRANE	0.449756966	-0.01324734	3.590604622	0.00662138	0.05377637	-2.33051937
homo after UVB irradiation VS homo before UVB irradiation	GO_BASEMENT_MEMBRANE_ASSEMBLY	0.141374916	-0.03550252	0.869281879	0.40904205	0.59579869	-6.15424041
hetero after UVB irradiation VS hetero before UVB irradiation	GO_BASEMENT_MEMBRANE_ASSEMBLY1	0.140544298	-0.00878781	0.893617692	0.3980857	0.70539829	-5.81983452
WT after UVB irradiation VS WT before UVB irradiation	GO_BASEMENT_MEMBRANE_ASSEMBLY2	-0.15814258	-0.02472995	-0.89225146	0.39799618	0.81477652	-5.59655785
homo after UVB irradiation VS homo before UVB irradiation	GO_BASEMENT_MEMBRANE_COLLAGEN_TRIMER	0.274771645	-0.01141361	1.544073131	0.15966327	0.33553843	-5.40330543
hetero after UVB irradiation VS hetero before UVB irradiation	GO_BASEMENT_MEMBRANE_COLLAGEN_TRIMER1	0.296194194	-0.02786513	1.342132024	0.21706214	0.54440713	-5.35568672
WT after UVB irradiation VS WT before UVB irradiation	GO_BASEMENT_MEMBRANE_COLLAGEN_TRIMER2	0.182394527	0.004739902	0.766671005	0.46503887	0.84275802	-5.69731131
homo after UVB irradiation VS homo before UVB irradiation	GO_BASEMENT_MEMBRANE_ORGANIZATION	0.600834541	-0.05377482	4.05673748	0.0033548	0.03812829	-1.64481869
hetero after UVB irradiation VS hetero before UVB irradiation	GO_BASEMENT_MEMBRANE_ORGANIZATION1	0.472801245	0.009986414	2.956735173	0.01861755	0.1993618	-3.10245075
WT after UVB irradiation VS WT before UVB irradiation	GO_BASEMENT_MEMBRANE_ORGANIZATION2	0.262068437	0.019928619	1.422945336	0.19211366	0.69334561	-5.04937994
hetero after UVB irradiation VS hetero before UVB irradiation	GO_BASEMENT_MEMBRANE1	0.151431989	0.012865118	0.71856664	0.49323114	0.76797706	-5.96062485
WT after UVB irradiation VS WT before UVB irradiation	GO_BASEMENT_MEMBRANE2	0.19246668	0.013912331	0.885760424	0.40128398	0.81600492	-5.60207335
homo after UVB irradiation VS homo before UVB irradiation	GO_BASIC_AMINO_ACID_TRANSMEMBRANE_TRANSPORT	0.073667622	0.01720984	0.447841001	0.66568725	0.7976742	-6.44211475
hetero after UVB irradiation VS hetero before UVB irradiation	GO_BASIC_AMINO_ACID_TRANSMEMBRANE_TRANSPORT1	-0.15799297	-0.02274341	-0.92287675	0.38355153	0.69523507	-5.79385546
WT after UVB irradiation VS WT before UVB irradiation	GO_BASIC_AMINO_ACID_TRANSMEMBRANE_TRANSPORT2	0.01229891	-0.02759306	0.062586132	0.95161337	0.98676072	-5.99400038
homo after UVB irradiation VS homo before UVB irradiation	GO_BASIC_AMINO_ACID_TRANSMEMBRANE_TRANSPORTER_ACTIVITY	0.06904274	0.033272	0.434420647	0.67501791	0.80407304	-6.44846158
hetero after UVB irradiation VS hetero before UVB irradiation	GO_BASIC_AMINO_ACID_TRANSMEMBRANE_TRANSPORTER_ACTIVITY1	-0.04056504	-0.00815386	-0.23077447	0.82338876	0.93305762	-6.20270367
WT after UVB irradiation VS WT before UVB irradiation	GO_BASIC_AMINO_ACID_TRANSMEMBRANE_TRANSPORTER_ACTIVITY2	0.167707936	0.005884879	1.050552045	0.32380275	0.77346379	-5.45232949
homo after UVB irradiation VS homo before UVB irradiation	GO_BASIC_AMINO_ACID_TRANSPORT	0.130514342	-0.00031215	0.662386786	0.52562361	0.693942	-6.31630846
hetero after UVB irradiation VS hetero before UVB irradiation	GO_BASIC_AMINO_ACID_TRANSPORT1	0.11560086	-0.00038655	0.655578782	0.53082834	0.79153627	-6.00479655
WT after UVB irradiation VS WT before UVB irradiation	GO_BASIC_AMINO_ACID_TRANSPORT2	0.093895782	-0.00205741	0.562349508	0.58910214	0.89215883	-5.8325723
homo after UVB irradiation VS homo before UVB irradiation	GO_BASOLATERAL_PLASMA_MEMBRANE	0.118217653	-0.00422426	0.799962233	0.44594578	0.62818078	-6.21273918
hetero after UVB irradiation VS hetero before UVB irradiation	GO_BASOLATERAL_PLASMA_MEMBRANE1	-0.0630689	-0.0060111	-0.51072226	0.62358971	0.84445518	-6.09241551
WT after UVB irradiation VS WT before UVB irradiation	GO_BASOLATERAL_PLASMA_MEMBRANE2	-0.06891212	0.019268014	-0.47302452	0.64868399	0.90916798	-5.8797025
homo after UVB irradiation VS homo before UVB irradiation	GO_BBSOME	-0.24005816	-0.00906512	-0.78072544	0.45657669	0.63757302	-6.22823979
hetero after UVB irradiation VS hetero before UVB irradiation	GO_BBSOME1	-0.7406959	-0.10576258	-4.28616877	0.00278211	0.11250952	-1.27020542
WT after UVB irradiation VS WT before UVB irradiation	GO_BBSOME2	-0.22118768	-0.0912239	-0.78381423	0.45546206	0.83847974	-5.68431385
homo after UVB irradiation VS homo before UVB irradiation	GO_BCL_2_FAMILY_PROTEIN_COMPLEX	0.397576585	-0.06694799	1.555506884	0.15694783	0.33229255	-5.38829521
hetero after UVB irradiation VS hetero before UVB irradiation	GO_BCL_2_FAMILY_PROTEIN_COMPLEX1	0.340947278	-0.01205479	2.362442635	0.04635439	0.28788102	-3.97255786
WT after UVB irradiation VS WT before UVB irradiation	GO_BCL_2_FAMILY_PROTEIN_COMPLEX2	0.356600935	-0.07748025	1.257941347	0.24347366	0.73311008	-5.23776026
homo after UVB irradiation VS homo before UVB irradiation	GO_BEHAVIOR	0.078052773	0.002487128	0.625751473	0.54823688	0.71155072	-6.34095392
hetero after UVB irradiation VS hetero before UVB irradiation	GO_BEHAVIOR1	-0.00698714	0.010951157	-0.05197353	0.95984754	0.98542968	-6.23001946
WT after UVB irradiation VS WT before UVB irradiation	GO_BEHAVIOR2	-0.0265861	0.002214423	-0.16355268	0.87409166	0.96914919	-5.98197696
homo after UVB irradiation VS homo before UVB irradiation	GO_BEHAVIORAL_RESPONSE_TO_COCAINE	0.140534606	-0.00436648	0.876426065	0.40536432	0.59249239	-6.14798076
hetero after UVB irradiation VS hetero before UVB irradiation	GO_BEHAVIORAL_RESPONSE_TO_COCAINE1	0.358519689	-0.00892154	1.781453703	0.11340384	0.41533559	-4.796365
WT after UVB irradiation VS WT before UVB irradiation	GO_BEHAVIORAL_RESPONSE_TO_COCAINE2	0.253640234	0.036256343	1.142897493	0.2857438	0.75392834	-5.36012351
homo after UVB irradiation VS homo before UVB irradiation	GO_BEHAVIORAL_RESPONSE_TO_ETHANOL	-0.25566359	0.047977114	-1.15658066	0.2795358	0.47480546	-5.87108807
hetero after UVB irradiation VS hetero before UVB irradiation	GO_BEHAVIORAL_RESPONSE_TO_ETHANOL1	0.026836699	0.054962619	0.093615785	0.9277588	0.97320032	-6.22673412
WT after UVB irradiation VS WT before UVB irradiation	GO_BEHAVIORAL_RESPONSE_TO_ETHANOL2	-0.21880037	0.095850319	-1.08845066	0.30772686	0.7665674	-5.41516943
homo after UVB irradiation VS homo before UVB irradiation	GO_BEHAVIORAL_RESPONSE_TO_NICOTINE	-0.42881503	0.00378928	-1.58888292	0.14925743	0.3224963	-5.34414166
hetero after UVB irradiation VS hetero before UVB irradiation	GO_BEHAVIORAL_RESPONSE_TO_NICOTINE1	-1.04178606	0.013318056	-7.49268882	7.69E-05	0.07788488	1.993209158
WT after UVB irradiation VS WT before UVB irradiation	GO_BEHAVIORAL_RESPONSE_TO_NICOTINE2	-1.10882175	-0.01805624	-7.25549926	8.24E-05	0.18842845	1.657091778
homo after UVB irradiation VS homo before UVB irradiation	GO_BEHAVIORAL_RESPONSE_TO_PAIN	0.211298632	-0.03918738	1.098414958	0.30275684	0.49729132	-5.93334683
hetero after UVB irradiation VS hetero before UVB irradiation	GO_BEHAVIORAL_RESPONSE_TO_PAIN1	0.257850677	0.012019	1.518921927	0.16796615	0.48947934	-5.14034755

WT after UVB irradiation VS WT before UVB irradiation	GO_BEHAVIORAL_RESPONSE_TO_PAIN2	0.068057819	0.010704187	0.450985649	0.66383177	0.91232901	-5.89015481
homo after UVB irradiation VS homo before UVB irradiation	GO_BENZENE_CONTAINING_COMPOUND_METABOLIC_PROCESS	0.284988985	-0.03681178	1.399750321	0.1977129	0.38636004	-5.58732202
hetero after UVB irradiation VS hetero before UVB irradiation	GO_BENZENE_CONTAINING_COMPOUND_METABOLIC_PROCESS1	0.153450779	0.023897054	0.75363087	0.47305952	0.75662211	-5.93450631
WT after UVB irradiation VS WT before UVB irradiation	GO_BENZENE_CONTAINING_COMPOUND_METABOLIC_PROCESS2	0.073187532	0.002106884	0.3138583	0.76156175	0.93974135	-5.94442201
homo after UVB irradiation VS homo before UVB irradiation	GO_BENZODIAZEPINE_RECEPTOR_ACTIVITY	-0.10505828	0.093081111	-0.43984995	0.67123585	0.8018902	-6.4459161
hetero after UVB irradiation VS hetero before UVB irradiation	GO_BENZODIAZEPINE_RECEPTOR_ACTIVITY1	-0.69428041	-0.06984285	-2.77227496	0.02464928	0.22452146	-3.37197897
WT after UVB irradiation VS WT before UVB irradiation	GO_BENZODIAZEPINE_RECEPTOR_ACTIVITY2	-0.44860201	0.039136756	-1.92059869	0.09059071	0.58379396	-4.41264137
homo after UVB irradiation VS homo before UVB irradiation	GO_BENZODIAZEPINE_RECEPTOR_BINDING	-0.78417921	-0.041472	-2.84990169	0.0206202	0.09963465	-3.46612564
hetero after UVB irradiation VS hetero before UVB irradiation	GO_BENZODIAZEPINE_RECEPTOR_BINDING1	-0.8221962	-0.09576708	-2.64690062	0.02987524	0.24142332	-3.55586544
WT after UVB irradiation VS WT before UVB irradiation	GO_BENZODIAZEPINE_RECEPTOR_BINDING2	-0.37108187	0.001797517	-1.10711821	0.30004314	0.76208892	-5.39651343
homo after UVB irradiation VS homo before UVB irradiation	GO_BERGMANN_GLIAL_CELL_DIFFERENTIATION	-0.18592808	-0.0179665	-1.05311462	0.32187719	0.51551352	-5.98018006
hetero after UVB irradiation VS hetero before UVB irradiation	GO_BERGMANN_GLIAL_CELL_DIFFERENTIATION1	-0.04622879	0.044198566	-0.2967465	0.77435729	0.91152166	-6.1840051
WT after UVB irradiation VS WT before UVB irradiation	GO_BERGMANN_GLIAL_CELL_DIFFERENTIATION2	-0.11183372	0.048902267	-0.64494892	0.53680028	0.86992784	-5.78236699
homo after UVB irradiation VS homo before UVB irradiation	GO_BETA_1_3_GALACTOSYLTRANSFERASE_ACTIVITY	0.029160212	-0.00370921	0.118875362	0.90819126	0.94991559	-6.54310784
hetero after UVB irradiation VS hetero before UVB irradiation	GO_BETA_1_3_GALACTOSYLTRANSFERASE_ACTIVITY1	0.311933683	0.037676146	1.263081136	0.24277128	0.57107173	-5.44676931
WT after UVB irradiation VS WT before UVB irradiation	GO_BETA_1_3_GALACTOSYLTRANSFERASE_ACTIVITY2	0.341380851	-0.02657668	1.665065697	0.13400864	0.64134459	-4.75026197
homo after UVB irradiation VS homo before UVB irradiation	GO_BETA_1_ADRENERGIC_RECEPTOR_BINDING	0.30675339	-0.08012886	1.669997153	0.13197231	0.29828693	-5.23484037
hetero after UVB irradiation VS hetero before UVB irradiation	GO_BETA_1_ADRENERGIC_RECEPTOR_BINDING1	-0.04274243	0.010668744	-0.14068756	0.89165859	0.9603178	-6.22076423
WT after UVB irradiation VS WT before UVB irradiation	GO_BETA_1_ADRENERGIC_RECEPTOR_BINDING2	-0.23611112	-0.07840683	-1.05715007	0.32095781	0.77346379	-5.44592991
homo after UVB irradiation VS homo before UVB irradiation	GO_BETA_2_ADRENERGIC_RECEPTOR_BINDING	-0.36831164	-0.04092491	-1.78585719	0.11046192	0.26639754	-5.07426255
hetero after UVB irradiation VS hetero before UVB irradiation	GO_BETA_2_ADRENERGIC_RECEPTOR_BINDING1	-0.33335636	-0.1519592	-1.23581303	0.25221742	0.5800182	-5.47736316
WT after UVB irradiation VS WT before UVB irradiation	GO_BETA_2_ADRENERGIC_RECEPTOR_BINDING2	-0.3623545	-0.13055674	-2.18043363	0.06041951	0.52788521	-4.05414425
homo after UVB irradiation VS homo before UVB irradiation	GO_BETA_2_MICROGLOBULIN_BINDING	1.152175657	-0.00800057	7.885428617	3.86E-05	0.00847131	2.76334809
hetero after UVB irradiation VS hetero before UVB irradiation	GO_BETA_2_MICROGLOBULIN_BINDING1	0.636390207	0.123935002	1.978028634	0.08397718	0.37023221	-4.52473862
WT after UVB irradiation VS WT before UVB irradiation	GO_BETA_2_MICROGLOBULIN_BINDING2	0.727330501	0.021952435	2.14389458	0.06397639	0.53678963	-4.10521119
homo after UVB irradiation VS homo before UVB irradiation	GO_BETA_CATENIN_BINDING	-0.15738582	0.003816722	-0.90219039	0.39229678	0.58130929	-6.1250559
hetero after UVB irradiation VS hetero before UVB irradiation	GO_BETA_CATENIN_BINDING1	-0.22542751	-0.00574558	-1.18129727	0.27202278	0.59829603	-5.53719047
WT after UVB irradiation VS WT before UVB irradiation	GO_BETA_CATENIN_BINDING2	-0.18201957	0.043902804	-0.87305136	0.40777754	0.81732515	-5.61277755
homo after UVB irradiation VS homo before UVB irradiation	GO_BETA_CATENIN_DESTRUCTION_COMPLEX	-0.63567484	0.024330531	-4.22270382	0.00265318	0.03401579	-1.40792005
homo after UVB irradiation VS homo before UVB irradiation	GO_BETA_CATENIN_DESTRUCTION_COMPLEX_ASSEMBLY	-0.99478524	0.045056055	-4.9912708	0.00094344	0.02093582	-0.36590221
hetero after UVB irradiation VS hetero before UVB irradiation	GO_BETA_CATENIN_DESTRUCTION_COMPLEX_ASSEMBLY1	-0.65145551	-0.10250423	-1.96209338	0.08605912	0.37207396	-4.54710677
WT after UVB irradiation VS WT before UVB irradiation	GO_BETA_CATENIN_DESTRUCTION_COMPLEX_ASSEMBLY2	-0.68781296	-0.07777436	-1.93307892	0.08885602	0.58148482	-4.39571281
homo after UVB irradiation VS homo before UVB irradiation	GO_BETA_CATENIN_DESTRUCTION_COMPLEX_DISASSEMBLY	-0.44959112	0.033415665	-2.58062515	0.03153712	0.12595216	-3.88425629
hetero after UVB irradiation VS hetero before UVB irradiation	GO_BETA_CATENIN_DESTRUCTION_COMPLEX_DISASSEMBLY1	-0.45482858	-0.02310217	-1.93991544	0.08903984	0.37595306	-4.57814505
WT after UVB irradiation VS WT before UVB irradiation	GO_BETA_CATENIN_DESTRUCTION_COMPLEX_DISASSEMBLY2	-0.38972516	0.077612929	-1.50061279	0.17139831	0.67808426	-4.95608835
hetero after UVB irradiation VS hetero before UVB irradiation	GO_BETA_CATENIN_DESTRUCTION_COMPLEX1	-0.60005793	-0.07923307	-2.39469966	0.04409616	0.28081987	-3.9254749
WT after UVB irradiation VS WT before UVB irradiation	GO_BETA_CATENIN_DESTRUCTION_COMPLEX2	-0.4247121	0.012637628	-1.55617555	0.1578245	0.66180255	-4.88773539
homo after UVB irradiation VS homo before UVB irradiation	GO_BETA_CATENIN_TCF_COMPLEX	0.22237582	0.025586109	0.900146547	0.39332222	0.58234735	-6.1268944
homo after UVB irradiation VS homo before UVB irradiation	GO_BETA_CATENIN_TCF_COMPLEX_ASSEMBLY	-0.36338611	0.000620707	-2.06485837	0.07142798	0.20519033	-4.67022589
hetero after UVB irradiation VS hetero before UVB irradiation	GO_BETA_CATENIN_TCF_COMPLEX_ASSEMBLY1	-0.24769803	-0.01077112	-1.47176092	0.17999199	0.50426464	-5.19924553
WT after UVB irradiation VS WT before UVB irradiation	GO_BETA_CATENIN_TCF_COMPLEX_ASSEMBLY2	-0.21275502	0.079511787	-0.81308131	0.43942112	0.83100258	-5.66156236
hetero after UVB irradiation VS hetero before UVB irradiation	GO_BETA_CATENIN_TCF_COMPLEX1	0.137139534	0.027683801	0.602102278	0.56408215	0.81055567	-6.0394525
WT after UVB irradiation VS WT before UVB irradiation	GO_BETA_CATENIN_TCF_COMPLEX2	-0.13689071	0.108316777	-0.49481527	0.6338755	0.905002	-5.86890625
homo after UVB irradiation VS homo before UVB irradiation	GO_BETA_GALACTOSIDASE_ACTIVITY	0.153036516	-0.01551347	1.039502481	0.32780235	0.52112106	-5.99396091
hetero after UVB irradiation VS hetero before UVB irradiation	GO_BETA_GALACTOSIDASE_ACTIVITY1	0.350950302	0.01422583	0.903628907	0.39306838	0.70256648	-5.8110212
WT after UVB irradiation VS WT before UVB irradiation	GO_BETA_GALACTOSIDASE_ACTIVITY2	0.083033939	-0.08065201	0.277906706	0.78804782	0.9469715	-5.95551991
homo after UVB irradiation VS homo before UVB irradiation	GO_BETA_GLUCOSIDASE_ACTIVITY	-0.16985428	0.056990824	-0.52971083	0.61013747	0.75687615	-6.39944887
hetero after UVB irradiation VS hetero before UVB irradiation	GO_BETA_GLUCOSIDASE_ACTIVITY1	0.206156281	-0.06086262	1.341969114	0.21711262	0.54440713	-5.35587795
WT after UVB irradiation VS WT before UVB irradiation	GO_BETA_GLUCOSIDASE_ACTIVITY2	0.147542513	-0.00115044	0.583572376	0.57539786	0.88606346	-5.82027037
homo after UVB irradiation VS homo before UVB irradiation	GO_BETA_N_ACETYLGLUCOSAMINYLGLYCOPEPTIDE_BETA_1_4_GALACTOSYLTRANSFERASE_ACTIVITY	0.876876989	-0.07059032	5.930494822	0.00029887	0.01276035	0.781433673
hetero after UVB irradiation VS hetero before UVB irradiation	GO_BETA_N_ACETYLGLUCOSAMINYLGLYCOPEPTIDE_BETA_1_4_GALACTOSYLTRANSFERASE_ACTIVITY1	0.842271553	0.067341963	3.154754469	0.0138255	0.17734093	-2.81562659
WT after UVB irradiation VS WT before UVB irradiation	GO_BETA_N_ACETYLGLUCOSAMINYLGLYCOPEPTIDE_BETA_1_4_GALACTOSYLTRANSFERASE_ACTIVITY2	0.601539508	-0.082219	2.386096248	0.04376225	0.49685404	-3.76413379
homo after UVB irradiation VS homo before UVB irradiation	GO_BETA_N_ACETYLHEXOSAMINIDASE_ACTIVITY	0.479127767	-0.04152176	2.086516952	0.0690292	0.20104262	-4.63803598
hetero after UVB irradiation VS hetero before UVB irradiation	GO_BETA_N_ACETYLHEXOSAMINIDASE_ACTIVITY1	0.483151198	-0.00174918	2.191905448	0.06036557	0.323869	-4.21997706
WT after UVB irradiation VS WT before UVB irradiation	GO_BETA_N_ACETYLHEXOSAMINIDASE_ACTIVITY2	0.188324253	-0.05945082	0.677027316	0.51725882	0.86168548	-5.76120638
homo after UVB irradiation VS homo before UVB irradiation	GO_BETA_TUBULIN_BINDING	-0.35093166	-0.02904138	-2.44903177	0.03885739	0.14171516	-4.08761602
hetero after UVB irradiation VS hetero before UVB irradiation	GO_BETA_TUBULIN_BINDING1	-0.24583199	-0.04833734	-1.25737277	0.24472375	0.57219833	-5.4532099
WT after UVB irradiation VS WT before UVB irradiation	GO_BETA_TUBULIN_BINDING2	-0.18105456	-0.0009746	-1.11133688	0.29832799	0.75997207	-5.39226571
homo after UVB irradiation VS homo before UVB irradiation	GO_BH_DOMAIN_BINDING	0.192873119	-0.02831352	1.054139691	0.32143437	0.51513976	-5.97913676
hetero after UVB irradiation VS hetero before UVB irradiation	GO_BH_DOMAIN_BINDING1	0.21391826	-0.05768523	1.432349558	0.19062366	0.51550175	-5.24768266
WT after UVB irradiation VS WT before UVB irradiation	GO_BH_DOMAIN_BINDING2	-0.02273922	-0.07505793	-0.09821914	0.92414606	0.98116648	-5.99097963
homo after UVB irradiation VS homo before UVB irradiation	GO_BH3_DOMAIN_BINDING	-0.03565777	-0.04839898	-0.16531035	0.87264169	0.92999014	-6.53588385
hetero after UVB irradiation VS hetero before UVB irradiation	GO_BH3_DOMAIN_BINDING1	0.052676796	-0.0641126	0.274777285	0.79057797	0.91823508	-6.19074145
WT after UVB irradiation VS WT before UVB irradiation	GO_BH3_DOMAIN_BINDING2	-0.18843777	-0.08597062	-0.80580314	0.44337371	0.83340629	-5.66728592
homo after UVB irradiation VS homo before UVB irradiation	GO_BHLH_TRANSCRIPTION_FACTOR_BINDING	0.256172558	0.039875336	1.644562219	0.13718477	0.3062223	-5.2694072
hetero after UVB irradiation VS hetero before UVB irradiation	GO_BHLH_TRANSCRIPTION_FACTOR_BINDING1	0.289007846	0.055054634	1.979148566	0.0838327	0.36997436	-4.52316454
WT after UVB irradiation VS WT before UVB irradiation	GO_BHLH_TRANSCRIPTION_FACTOR_BINDING2	0.20758666	-0.01021798	1.169813634	0.27535269	0.74698979	-5.33221293
homo after UVB irradiation VS homo before UVB irradiation	GO_BICARBONATE_TRANSMEMBRANE_TRANSPORTER_ACTIVITY	-0.03607162	-0.01562759	-0.23327813	0.82117648	0.89997039	-6.5210897
hetero after UVB irradiation VS hetero before UVB irradiation	GO_BICARBONATE_TRANSMEMBRANE_TRANSPORTER_ACTIVITY1	-0.46155319	-0.03694546	-3.32147381	0.01079841	0.16309248	-2.57699228
WT after UVB irradiation VS WT before UVB irradiation	GO_BICARBONATE_TRANSMEMBRANE_TRANSPORTER_ACTIVITY2	-0.57894413	0.01851701	-2.95522923	0.01803552	0.38628476	-2.95633713
homo after UVB irradiation VS homo before UVB irradiation	GO_BICARBONATE_TRANSPORT	0.057374721	-0.02101668	0.361087458	0.72702161	0.83876615	-6.47986455
hetero after UVB irradiation VS hetero before UVB irradiation	GO_BICARBONATE_TRANSPORT1	-0.18628831	-0.05498202	-1.33743804	0.21852079	0.54565069	-5.36119098
WT after UVB irradiation VS WT before UVB irradiation	GO_BICARBONATE_TRANSPORT2	-0.27222842	0.01635727	-1.51510788	0.1677599	0.67272632	-4.93838134
homo after UVB irradiation VS homo before UVB irradiation	GO_BILE_ACID_AND_BILE_SALT_TRANSPORT	0.313201869	0.046566684	1.763595522	0.11432338	0.27197648	-5.10549327
hetero after UVB irradiation VS hetero before UVB irradiation	GO_BILE_ACID_AND_BILE_SALT_TRANSPORT1	0.03986456	0.048949183	0.262055828	0.80002135	0.92259806	-6.19441007
WT after UVB irradiation VS WT before UVB irradiation	GO_BILE_ACID_AND_BILE_SALT_TRANSPORT2	0.249575162	0.067437579	1.418755983	0.19329121	0.69334561	-5.05433308
homo after UVB irradiation VS homo before UVB irradiation	GO_BILE_ACID_BINDING	0.323814962	0.007553144	1.262545801	0.24095152	0.4338362	-5.75186785
hetero after UVB irradiation VS hetero before UVB irradiation	GO_BILE_ACID_BINDING1	-0.13109185	0.054704397	-0.53504308	0.60743385	0.83594367	-6.07910439
WT after UVB irradiation VS WT before UVB irradiation	GO_BILE_ACID_BINDING2	0.042271926	0.003818249	0.214854361	0.8351932	0.96163722	-5.97178241
homo after UVB irradiation VS homo before UVB irradiation	GO_BILE_ACID_BIOSYNTHETIC_PROCESS	0.042334107	0.013900847	0.263363426	0.79866877	0.88582901	-6.51295112
hetero after UVB irradiation VS hetero before UVB irradiation	GO_BILE_ACID_BIOSYNTHETIC_PROCESS1	-0.27804712	0.01597944	-1.65669505	0.13688061	0.44981284	-4.9629599
WT after UVB irradiation VS WT before UVB irradiation	GO_BILE_ACID_BIOSYNTHETIC_PROCESS2	-0.02095764	0.018751415	-0.10119037	0.92186006	0.98046046	-5.99066741
homo after UVB irradiation VS homo before UVB irradiation	GO_BILE_ACID_METABOLIC_PROCESS	0.019524583	0.005554741	0.127264549	0.90174995	0.94665205	-6.54197667
hetero after UVB irradiation VS hetero before UVB irradiation	GO_BILE_ACID_METABOLIC_PROCESS1	-0.24739118	0.023301695	-1.62193074	0.144184	0.46030459	-5.00842112
WT after UVB irradiation VS WT before UVB irradiation	GO_BILE_ACID_METABOLIC_PROCESS2	-0.04129473	0.026082316	-0.23674668	0.81873248	0.95684131	-5.96660045
homo after UVB irradiation VS homo before UVB irradiation	GO_BILE_ACID_SECRETION	-0.24042712	0.030588308	-1.18007606	0.27057206	0.46569068	-5.84528476
hetero after UVB irradiation VS hetero before UVB irradiation	GO_BILE_ACID_SECRETION1	-0.58342398	-0.03599979	-2.63340548	0.03050188	0.24447892	-3.57567104
WT after UVB irradiation VS WT before UVB irradiation	GO_BILE_ACID_SECRETION2	-0.0499074	0.064573622	-0.24280311	0.8141951	0.95562532	-5.96507942
homo after UVB irradiation VS homo before UVB irradiation	GO_BILE_ACID_SODIUM_SYMPORTER_ACTIVITY	0.388468681	0.167487964	1.408515269	0.1951935	0.3825834	-5.57645022
hetero after UVB irradiation VS hetero before UVB irradiation	GO_BILE_ACID_SODIUM_SYMPORTER_ACTIVITY1	0.154101214	0.082991793	0.607441521	0.56070837	0.80900719	-6.03611201
WT after UVB irradiation VS WT before UVB irradiation	GO_BILE_ACID_SODIUM_SYMPORTER_ACTIVITY2	0.023155556	-0.05560734	0.075112351	0.94194783	0.98463518	-5.99309083
homo after UVB irradiation VS homo before UVB irradiation	GO_BILE_ACID_TRANSMEMBRANE_TRANSPORTER_ACTIVITY	0.339678543	0.083546408	1.972908297	0.08254118	0.22268011	-4.80570052
hetero after UVB irradiation VS hetero before UVB irradiation	GO_BILE_ACID_TRANSMEMBRANE_TRANSPORTER_ACTIVITY1	0.267606605	0.059526598	1.7702022	0.11535535	0.41843252	-4.81159513
WT after UVB irradiation VS WT before UVB irradiation	GO_BILE_ACID_TRANSMEMBRANE_TRANSPORTER_ACTIVITY2	0.168685882	0.067702216	0.656042754	0.52999225	0.86765165	-5.77515251
homo after UVB irradiation VS homo before UVB irradiation	GO_BINDING_OF_SPERM_TO_ZONA_PELLUCIDA	-0.18957343	0.10277497	-1.12260448	0.29292	0.48766025	-5.90773994
hetero after UVB irradiation VS hetero before UVB irradiation	GO_BINDING_OF_SPERM_TO_ZONA_PELLUCIDA1	-0.1216106	0.056135847	-0.5731413	0.58258468	0.82130691	-6.05710329
WT after UVB irradiation VS WT before UVB irradiation	GO_BINDING_OF_SPERM_TO_ZONA_PELLUCIDA2	-0.05503786	0.062472178	-0.27692773	0.78877334	0.9471827	-5.95580365
homo after UVB irradiation VS homo before UVB irradiation	GO_BIOACTIVE_LIPID_RECEPTOR_ACTIVITY	0.299745128	0.001494283	1.442278346	0.18574826	0.37026706	-5.53419102
hetero after UVB irradiation VS hetero before UVB irradiation	GO_BIOACTIVE_LIPID_RECEPTOR_ACTIVITY1	0.36569549	-0.00587234	2.043956982	0.07587095	0.35517061	-4.43164335
WT after UVB irradiation VS WT before UVB irradiation	GO_BIOACTIVE_LIPID_RECEPTOR_ACTIVITY2	0.490580716	-0.00067335	2.964337189	0.01778466	0.3854098	-2.943514
homo after UVB irradiation VS homo before UVB irradiation	GO_BIOLOGICAL_ADHESION	0.323859791	-0.01129272	2.51805784	0.0348258	0.13362566	-3.98108701
hetero after UVB irradiation VS hetero before UVB irradiation	GO_BIOLOGICAL_ADHESION1	0.237591354	0.008917	1.607546747	0.14730924	0.46496154	-5.02709813
WT after UVB irradiation VS WT before UVB irradiation	GO_BIOLOGICAL_ADHESION2	0.138900317	-0.00423475	0.802780914	0.44502211	0.83340629	-5.66964988
homo after UVB irradiation VS homo before UVB irradiation	GO_BIOLOGICAL_PHASE	-0.17306589	0.023653939	-0.73402485	0.4830817	0.66007747	-6.26450982
hetero after UVB irradiation VS hetero before UVB irradiation	GO_BIOLOGICAL_PHASE1	0.067833818	0.039683015	0.448255042	0.66607859	0.86317077	-6.12393941
WT after UVB irradiation VS WT before UVB irradiation	GO_BIOLOGICAL_PHASE2	-0.35317316	0.073601529	-1.57974893	0.15236418	0.65975298	-4.85835524
homo after UVB irradiation VS homo before UVB irradiation	GO_BIOMINERALIZATION	0.208908583	-0.00412357	1.555788343	0.15688151	0.3321998	-5.38792495
hetero after UVB irradiation VS hetero before UVB irradiation	GO_BIOMINERALIZATION1	0.098808343	0.013693453	0.687889272	0.5113262	0.78050372	-5.98258421
WT after UVB irradiation VS WT before UVB irradiation	GO_BIOMINERALIZATION2	-0.03352091	0.019114815	-0.21270732	0.83681235	0.96182256	-5.97226391
homo after UVB irradiation VS homo before UVB irradiation	GO_BIOTIN_BINDING	-0.47945888	-0.03012781	-1.39882226	0.19798131	0.38666095	-5.58847073
hetero after UVB irradiation VS hetero before UVB irradiation	GO_BIOTIN_BINDING1	-1.10053362	-0.03575224	-6.52146741	0.00019954	0.07788488	1.169638464
WT after UVB irradiation VS WT before UVB irradiation	GO_BIOTIN_BINDING2	-0.27849887	-0.10214263	-0.92688351	0.3807827	0.80406414	-5.56658389
homo after UVB irradiation VS homo before UVB irradiation	GO_BIOTIN_METABOLIC_PROCESS	-0.29368105	-0.03504309	-0.92143492	0.38273587	0.57270667	-6.10757822
hetero after UVB irradiation VS hetero before UVB irradiation	GO_BIOTIN_METABOLIC_PROCESS1	-0.92092941	-0.04394388	-5.57601817	0.00056034	0.08125048	0.237411557
WT after UVB irradiation VS WT before UVB irradiation	GO_BIOTIN_METABOLIC_PROCESS2	0.07271309	-0.06079337	0.329933502	0.74982361	0.93708901	-5.93903453
WT after UVB irradiation VS WT before UVB irradiation	GO_BITTER_TASTE_RECEPTOR_ACTIVITY	-0.42810641	0.024693662	-1.42742944	0.19086023	0.69285981	-5.04406911
homo after UVB irradiation VS homo before UVB irradiation	GO_BLASTOCYST_DEVELOPMENT	-0.25159723	0.016971231	-1.82720831	0.10361151	0.25658584	-5.01580875
hetero after UVB irradiation VS hetero before UVB irradiation	GO_BLASTOCYST_DEVELOPMENT1	0.002690554	0.017651298	0.017827467	0.98622112	0.99475994	-6.23131176
WT after UVB irradiation VS WT before UVB irradiation	GO_BLASTOCYST_DEVELOPMENT2	-0.06427445	0.011877255	-0.36950273	0.7212291	0.93154998	-5.92466236
homo after UVB irradiation VS homo before UVB irradiation	GO_BLASTOCYST_FORMATION	-0.14422344	0.023172473	-0.96587393	0.36131012	0.55226916	-6.06608492
hetero after UVB irradiation VS hetero before UVB irradiation	GO_BLASTOCYST_FORMATION1	0.105676855	0.021226495	0.635591347	0.54311663	0.79799154	-6.01805979
WT after UVB irradiation VS WT before UVB irradiation	GO_BLASTOCYST_FORMATION2	-0.06642337	0.012083957	-0.38982214	0.70672089	0.92686457	-5.91667234
homo after UVB irradiation VS homo before UVB irradiation	GO_BLASTOCYST_GROWTH	-0.32547355	-0.04497556	-1.8577351	0.09881308	0.24883069	-4.97230312
hetero after UVB irradiation VS hetero before UVB irradiation	GO_BLASTOCYST_GROWTH1	0.127780251	-0.02217421	0.595562259	0.56823063	0.81279673	-6.04350773
WT after UVB irradiation VS WT before UVB irradiation	GO_BLASTOCYST_GROWTH2	0.025181347	0.004728674	0.112934722	0.91283193	0.97884691	-5.98934249
homo after UVB irradiation VS homo before UVB irradiation	GO_BLASTODERM_SEGMENTATION	-0.23062213	0.040818272	-1.32491073	0.2203932	0.41120918	-5.67842554
hetero after UVB irradiation VS hetero before UVB irradiation	GO_BLASTODERM_SEGMENTATION1	-0.12970878	0.021416645	-0.63482553	0.54359083	0.79809106	-6.01856064
WT after UVB irradiation VS WT before UVB irradiation	GO_BLASTODERM_SEGMENTATION2	-0.11727071	0.013442073	-0.6953959	0.50626937	0.85763155	-5.74867992
homo after UVB irradiation VS homo before UVB irradiation	GO_BLEB	0.173992046	-0.06243618	0.63104077	0.54493699	0.70951431	-6.33747405
homo after UVB irradiation VS homo before UVB irradiation	GO_BLEB_ASSEMBLY	-0.06711395	-0.0232811	-0.26880919	0.79461511	0.88339123	-6.51137426
hetero after UVB irradiation VS hetero before UVB irradiation	GO_BLEB_ASSEMBLY1	-0.32333889	-0.0425783	-1.55234549	0.15988577	0.4803083	-5.09801761
WT after UVB irradiation VS WT before UVB irradiation	GO_BLEB_ASSEMBLY2	-0.18760499	-0.11950136	-0.76468277	0.46615817	0.84342918	-5.69880283
hetero after UVB irradiation VS hetero before UVB irradiation	GO_BLEB1	0.641019645	-0.00896827	3.598277949	0.00722024	0.14037142	-2.18806898
WT after UVB irradiation VS WT before UVB irradiation	GO_BLEB2	0.203125657	-0.17318064	0.696101152	0.50585037	0.85763155	-5.74819308
homo after UVB irradiation VS homo before UVB irradiation	GO_BLOC_1_COMPLEX	0.165291282	-0.07515487	0.908428642	0.38917883	0.57842556	-6.11942332
hetero after UVB irradiation VS hetero before UVB irradiation	GO_BLOC_1_COMPLEX1	0.287907835	0.016551542	1.412875703	0.19607886	0.52060431	-5.27134204
WT after UVB irradiation VS WT before UVB irradiation	GO_BLOC_1_COMPLEX2	0.433730443	-0.00900949	2.407736769	0.04230148	0.495137	-3.73343509
homo after UVB irradiation VS homo before UVB irradiation	GO_BLOC_COMPLEX	0.010634664	-0.06975545	0.056986257	0.95589955	0.97690961	-6.54907481
hetero after UVB irradiation VS hetero before UVB irradiation	GO_BLOC_COMPLEX1	0.259168522	0.019034711	1.173271871	0.27504379	0.60180104	-5.54584284
WT after UVB irradiation VS WT before UVB irradiation	GO_BLOC_COMPLEX2	0.574439162	-0.02646088	3.838318806	0.00484677	0.27412617	-1.75758733
homo after UVB irradiation VS homo before UVB irradiation	GO_BLOOD_COAGULATION_INTRINSIC_PATHWAY	0.367997015	0.056044098	2.112106178	0.06629597	0.19613667	-4.59987822
hetero after UVB irradiation VS hetero before UVB irradiation	GO_BLOOD_COAGULATION_INTRINSIC_PATHWAY1	0.107687081	0.02767023	0.64010082	0.5403294	0.79673932	-6.01509954
WT after UVB irradiation VS WT before UVB irradiation	GO_BLOOD_COAGULATION_INTRINSIC_PATHWAY2	0.091092579	-0.01433441	0.461559185	0.65654329	0.91049801	-5.88519896
homo after UVB irradiation VS homo before UVB irradiation	GO_BLOOD_MICROPARTICLE	0.474868892	0.008016484	2.972839911	0.01701141	0.08902036	-3.27530602
hetero after UVB irradiation VS hetero before UVB irradiation	GO_BLOOD_MICROPARTICLE1	0.285364859	0.057722927	1.436065162	0.18959814	0.5145903	-5.24314756
WT after UVB irradiation VS WT before UVB irradiation	GO_BLOOD_MICROPARTICLE2	0.309833349	0.016214726	1.352330301	0.21282084	0.71002533	-5.13172497
homo after UVB irradiation VS homo before UVB irradiation	GO_BLOOD_VESSEL_ENDOTHELIAL_CELL_DIFFERENTIATION	0.351528007	-0.00581512	1.52228481	0.16495471	0.3430435	-5.43174155
hetero after UVB irradiation VS hetero before UVB irradiation	GO_BLOOD_VESSEL_ENDOTHELIAL_CELL_DIFFERENTIATION1	0.36404614	-0.00952005	1.304438655	0.229015	0.55721087	-5.39955012
WT after UVB irradiation VS WT before UVB irradiation	GO_BLOOD_VESSEL_ENDOTHELIAL_CELL_DIFFERENTIATION2	0.208493955	0.029010267	1.100989384	0.30254885	0.76462443	-5.40266373
homo after UVB irradiation VS homo before UVB irradiation	GO_BLOOD_VESSEL_ENDOTHELIAL_CELL_MIGRATION	0.573100429	-0.02388813	4.282368264	0.00244095	0.0330029	-1.32375231
hetero after UVB irradiation VS hetero before UVB irradiation	GO_BLOOD_VESSEL_ENDOTHELIAL_CELL_MIGRATION1	0.434561409	0.034563729	2.703449738	0.02738976	0.23456351	-3.4728892
WT after UVB irradiation VS WT before UVB irradiation	GO_BLOOD_VESSEL_ENDOTHELIAL_CELL_MIGRATION2	0.28730384	0.00123256	1.571792453	0.15418772	0.65981549	-4.86829617

homo after UVB irradiation VS homo before UVB irradiation	GO_BLOOD_VESSEL_ENDOTHELIAL_CELL_PROLIFERATION_INVOLVED_IN_SPROUTING_ANGIOGENESIS	0.417141986	-0.01447755	2.944774387	0.01777328	0.09145057	-3.31883096
hetero after UVB irradiation VS hetero before UVB irradiation	GO_BLOOD_VESSEL_ENDOTHELIAL_CELL_PROLIFERATION_INVOLVED_IN_SPROUTING_ANGIOGENESIS1	0.424151206	0.02218443	2.438736757	0.04119114	0.27359812	-3.8610949
WT after UVB irradiation VS WT before UVB irradiation	GO_BLOOD_VESSEL_ENDOTHELIAL_CELL_PROLIFERATION_INVOLVED_IN_SPROUTING_ANGIOGENESIS2	0.114335717	-0.07282989	0.452396962	0.66285671	0.91223984	-5.88949961
homo after UVB irradiation VS homo before UVB irradiation	GO_BLOOD_VESSEL_MATURATION	0.812326237	-0.06474068	5.959270848	0.00028907	0.01262071	0.814445401
hetero after UVB irradiation VS hetero before UVB irradiation	GO_BLOOD_VESSEL_MATURATION1	0.552086035	0.045180652	2.005700978	0.08047712	0.36432827	-4.48576856
WT after UVB irradiation VS WT before UVB irradiation	GO_BLOOD_VESSEL_MATURATION2	0.503986189	-0.07164788	1.546131812	0.16020417	0.66392783	-4.90018569
homo after UVB irradiation VS homo before UVB irradiation	GO_BLOOD_VESSEL_MORPHOGENESIS	0.440318288	-0.01868683	3.5119957	0.00744743	0.0572405	-2.44881463
hetero after UVB irradiation VS hetero before UVB irradiation	GO_BLOOD_VESSEL_MORPHOGENESIS1	0.340370266	0.02544683	2.014956204	0.07933842	0.3626016	-4.4727001
WT after UVB irradiation VS WT before UVB irradiation	GO_BLOOD_VESSEL_MORPHOGENESIS2	0.243233381	-0.00622419	1.276201453	0.2372685	0.72854168	-5.21762621
homo after UVB irradiation VS homo before UVB irradiation	GO_BLOOD_VESSEL_REMODELING	0.319591754	-0.00249403	1.996180221	0.07958043	0.21787911	-4.77160405
hetero after UVB irradiation VS hetero before UVB irradiation	GO_BLOOD_VESSEL_REMODELING1	0.307380689	-0.00429121	1.749977557	0.11894285	0.42329407	-4.83887374
WT after UVB irradiation VS WT before UVB irradiation	GO_BLOOD_VESSEL_REMODELING2	0.163007647	0.009610229	0.764931281	0.46601817	0.84342918	-5.69861658
homo after UVB irradiation VS homo before UVB irradiation	GO_BMP_BINDING	0.360912325	-0.05756935	1.749690892	0.11679878	0.27573182	-5.12491226
hetero after UVB irradiation VS hetero before UVB irradiation	GO_BMP_BINDING1	-0.29058333	0.033112396	-1.43163941	0.19082022	0.5156874	-5.24854867
WT after UVB irradiation VS WT before UVB irradiation	GO_BMP_BINDING2	-0.00121715	0.013033978	-0.00535208	0.99585919	0.99875662	-5.99605155
homo after UVB irradiation VS homo before UVB irradiation	GO_BMP_RECEPTOR_ACTIVITY	0.105778055	-0.09568403	0.524269724	0.61375306	0.75995586	-6.40249267
hetero after UVB irradiation VS hetero before UVB irradiation	GO_BMP_RECEPTOR_ACTIVITY1	0.04486058	0.041820686	0.203158672	0.84417634	0.94032189	-6.20916202
WT after UVB irradiation VS WT before UVB irradiation	GO_BMP_RECEPTOR_ACTIVITY2	-0.20389991	0.1134912	-0.79830491	0.4474711	0.83480988	-5.67313719
homo after UVB irradiation VS homo before UVB irradiation	GO_BMP_RECEPTOR_BINDING	-0.19937181	-0.00734957	-1.15662153	0.27952	0.47480546	-5.87104351
hetero after UVB irradiation VS hetero before UVB irradiation	GO_BMP_RECEPTOR_BINDING1	-0.04127325	0.000451595	-0.27114486	0.7932707	0.91941572	-6.19180637
WT after UVB irradiation VS WT before UVB irradiation	GO_BMP_RECEPTOR_BINDING2	-0.15740429	0.020196168	-0.83394895	0.42822283	0.82753247	-5.64491346
homo after UVB irradiation VS homo before UVB irradiation	GO_BMP_SIGNALING_PATHWAY_INVOLVED_IN_HEART_DEVELOPMENT	-0.86416094	0.04791507	-5.55488793	0.00046651	0.01609396	0.339037683
hetero after UVB irradiation VS hetero before UVB irradiation	GO_BMP_SIGNALING_PATHWAY_INVOLVED_IN_HEART_DEVELOPMENT1	-0.54860538	0.015799783	-2.71443339	0.0269322	0.23196771	-3.45677779
WT after UVB irradiation VS WT before UVB irradiation	GO_BMP_SIGNALING_PATHWAY_INVOLVED_IN_HEART_DEVELOPMENT2	-0.69640958	-0.02882301	-3.42444543	0.00886484	0.31908946	-2.30651915
homo after UVB irradiation VS homo before UVB irradiation	GO_BODY_FLUID_SECRETION	0.27048093	0.002656225	1.958833629	0.08438286	0.22591397	-4.82625501
hetero after UVB irradiation VS hetero before UVB irradiation	GO_BODY_FLUID_SECRETION1	0.213618333	0.014105783	1.597027048	0.14963402	0.46792642	-5.04070691
WT after UVB irradiation VS WT before UVB irradiation	GO_BODY_FLUID_SECRETION2	0.222460107	0.023256296	1.499900905	0.1715788	0.67808426	-4.95695565
homo after UVB irradiation VS homo before UVB irradiation	GO_BODY_MORPHOGENESIS	0.025896242	-0.00732426	0.190039086	0.85382849	0.91914308	-6.53107984
hetero after UVB irradiation VS hetero before UVB irradiation	GO_BODY_MORPHOGENESIS1	-0.07986526	0.051694575	-0.48175581	0.64311859	0.85386521	-6.10751371
WT after UVB irradiation VS WT before UVB irradiation	GO_BODY_MORPHOGENESIS2	-0.14990247	0.026792773	-0.86989706	0.40940073	0.81852412	-5.6154147
homo after UVB irradiation VS homo before UVB irradiation	GO_BONE_CELL_DEVELOPMENT	0.504844228	-0.02170875	3.219255685	0.0116162	0.07229873	-2.89481727
hetero after UVB irradiation VS hetero before UVB irradiation	GO_BONE_CELL_DEVELOPMENT1	0.5957889	0.048784063	4.670848497	0.00168242	0.09676143	-0.79089441
WT after UVB irradiation VS WT before UVB irradiation	GO_BONE_CELL_DEVELOPMENT2	0.497912299	0.006558242	3.202886057	0.01235926	0.34717171	-2.61031727
homo after UVB irradiation VS homo before UVB irradiation	GO_BONE_DEVELOPMENT	0.109429572	-0.00471052	0.848736811	0.41974942	0.60526111	-6.17200391
hetero after UVB irradiation VS hetero before UVB irradiation	GO_BONE_DEVELOPMENT1	0.038675343	0.007926478	0.254222998	0.80585344	0.92397674	-6.19658397
WT after UVB irradiation VS WT before UVB irradiation	GO_BONE_DEVELOPMENT2	-0.00521249	0.00137575	-0.03359021	0.97401716	0.99307736	-5.99547136
homo after UVB irradiation VS homo before UVB irradiation	GO_BONE_GROWTH	0.04330131	0.02266601	0.338517656	0.74334879	0.8492373	-6.48840231
hetero after UVB irradiation VS hetero before UVB irradiation	GO_BONE_GROWTH1	-0.14869192	-0.00042249	-0.86556199	0.41239222	0.71608138	-5.84410843
WT after UVB irradiation VS WT before UVB irradiation	GO_BONE_GROWTH2	-0.06149722	-0.01168072	-0.37488217	0.71737616	0.9309626	-5.92258714
homo after UVB irradiation VS homo before UVB irradiation	GO_BONE_MARROW_DEVELOPMENT	0.215340402	-0.03972993	1.180255146	0.27050464	0.46562895	-5.84508667
hetero after UVB irradiation VS hetero before UVB irradiation	GO_BONE_MARROW_DEVELOPMENT1	0.542077079	-0.01156264	2.452577563	0.04031865	0.27269233	-3.84083941
WT after UVB irradiation VS WT before UVB irradiation	GO_BONE_MARROW_DEVELOPMENT2	0.532949517	0.045630183	3.384366746	0.00940992	0.32218592	-2.36100948
homo after UVB irradiation VS homo before UVB irradiation	GO_BONE_MATURATION	0.282774025	0.003388041	1.682199222	0.1295368	0.29485956	-5.21816591
hetero after UVB irradiation VS hetero before UVB irradiation	GO_BONE_MATURATION1	-0.0186602	-0.00682008	-0.09783671	0.92451305	0.97152219	-6.22629641
WT after UVB irradiation VS WT before UVB irradiation	GO_BONE_MATURATION2	0.167253229	-0.04556133	0.828162735	0.43130792	0.82885935	-5.64956518
homo after UVB irradiation VS homo before UVB irradiation	GO_BONE_MINERALIZATION	0.276445686	-0.03010346	2.023652158	0.07621687	0.21209799	-4.73118326
homo after UVB irradiation VS homo before UVB irradiation	GO_BONE_MINERALIZATION_INVOLVED_IN_BONE_MATURATION	0.290072563	0.02624672	1.305242658	0.22671016	0.41751677	-5.70183745
hetero after UVB irradiation VS hetero before UVB irradiation	GO_BONE_MINERALIZATION_INVOLVED_IN_BONE_MATURATION1	-0.17841831	0.014032497	-0.72352032	0.49034816	0.76672432	-5.95700046
WT after UVB irradiation VS WT before UVB irradiation	GO_BONE_MINERALIZATION_INVOLVED_IN_BONE_MATURATION2	0.220558622	-0.03545258	1.099076886	0.30333414	0.76462443	-5.40457789
hetero after UVB irradiation VS hetero before UVB irradiation	GO_BONE_MINERALIZATION1	0.081277335	0.008680724	0.542043165	0.60282563	0.83402069	-6.07516671
WT after UVB irradiation VS WT before UVB irradiation	GO_BONE_MINERALIZATION2	-0.02458517	0.014820282	-0.15159935	0.88321184	0.97008693	-5.98395823
homo after UVB irradiation VS homo before UVB irradiation	GO_BONE_MORPHOGENESIS	-0.00230799	0.011847444	-0.01754768	0.98641278	0.9930995	-6.55068773
hetero after UVB irradiation VS hetero before UVB irradiation	GO_BONE_MORPHOGENESIS1	-0.11020891	0.006107285	-0.63346609	0.5444332	0.79849481	-6.0194484
WT after UVB irradiation VS WT before UVB irradiation	GO_BONE_MORPHOGENESIS2	-0.14544792	1.17E-05	-0.86468411	0.41209332	0.82044248	-5.61975587
homo after UVB irradiation VS homo before UVB irradiation	GO_BONE_REMODELING	0.356148907	-0.00143334	2.539219812	0.03367636	0.13105537	-3.94836127
hetero after UVB irradiation VS hetero before UVB irradiation	GO_BONE_REMODELING1	0.306497784	0.028097646	2.128827561	0.06655268	0.33582955	-4.31063464
WT after UVB irradiation VS WT before UVB irradiation	GO_BONE_REMODELING2	0.242214031	0.013604213	1.412031888	0.19519444	0.69389529	-5.06226559
homo after UVB irradiation VS homo before UVB irradiation	GO_BONE_RESORPTION	0.541918899	-0.01339352	3.681332763	0.00578704	0.05041635	-2.19487155
hetero after UVB irradiation VS hetero before UVB irradiation	GO_BONE_RESORPTION1	0.50357929	0.050136424	3.249169479	0.01201505	0.16987008	-2.68012213
WT after UVB irradiation VS WT before UVB irradiation	GO_BONE_RESORPTION2	0.476354911	0.008093925	2.690365428	0.02718382	0.43942403	-3.33145594
homo after UVB irradiation VS homo before UVB irradiation	GO_BONE_TRABECULA_FORMATION	0.134865017	0.014379671	0.701288585	0.50224252	0.67611684	-6.28876482
hetero after UVB irradiation VS hetero before UVB irradiation	GO_BONE_TRABECULA_FORMATION1	-0.35228898	-0.00080058	-1.71426164	0.12553493	0.43247359	-4.88672942
WT after UVB irradiation VS WT before UVB irradiation	GO_BONE_TRABECULA_FORMATION2	-0.18977361	-0.02345155	-1.05214358	0.32311473	0.77346379	-5.45078855
homo after UVB irradiation VS homo before UVB irradiation	GO_BONE_TRABECULA_MORPHOGENESIS	0.083423745	-0.01479486	0.531436876	0.60899288	0.75621911	-6.39847717
hetero after UVB irradiation VS hetero before UVB irradiation	GO_BONE_TRABECULA_MORPHOGENESIS1	-0.28565804	0.021915212	-1.39580049	0.20097445	0.52553232	-5.29193355
WT after UVB irradiation VS WT before UVB irradiation	GO_BONE_TRABECULA_MORPHOGENESIS2	-0.24180522	-0.02448712	-1.38820954	0.20206954	0.69830181	-5.0901933
homo after UVB irradiation VS homo before UVB irradiation	GO_BORC_COMPLEX	0.623104169	-0.05494578	2.461018424	0.03812502	0.14005747	-4.06914305
hetero after UVB irradiation VS hetero before UVB irradiation	GO_BORC_COMPLEX1	0.098702798	-0.0366953	0.298282294	0.77322766	0.91152166	-6.18351525
WT after UVB irradiation VS WT before UVB irradiation	GO_BORC_COMPLEX2	0.334251511	-0.09789191	1.156620644	0.28040694	0.75145468	-5.3459499
homo after UVB irradiation VS homo before UVB irradiation	GO_BOX_C_D_SNORNP_ASSEMBLY	-0.89448152	0.015966848	-4.27172628	0.00247743	0.03315789	-1.33872559
hetero after UVB irradiation VS hetero before UVB irradiation	GO_BOX_C_D_SNORNP_ASSEMBLY1	-0.7784499	-0.02842778	-3.86741667	0.00493125	0.12780541	-1.82011876
WT after UVB irradiation VS WT before UVB irradiation	GO_BOX_C_D_SNORNP_ASSEMBLY2	-0.30513014	-0.09954371	-1.13057298	0.29060625	0.75624915	-5.3727511
homo after UVB irradiation VS homo before UVB irradiation	GO_BOX_C_D_SNORNP_COMPLEX	-1.02761206	0.007902477	-6.34738677	0.00018625	0.01082079	1.247762605
hetero after UVB irradiation VS hetero before UVB irradiation	GO_BOX_C_D_SNORNP_COMPLEX1	-1.04256534	0.02050613	-4.71145103	0.00159746	0.09569098	-0.74178133
WT after UVB irradiation VS WT before UVB irradiation	GO_BOX_C_D_SNORNP_COMPLEX2	-0.61705033	-0.08924505	-1.69637353	0.12779891	0.63467098	-4.70994779
homo after UVB irradiation VS homo before UVB irradiation	GO_BOX_H_ACA_RNP_COMPLEX	-1.04438507	0.006223852	-5.4757157	0.00051365	0.01654995	0.243021992
hetero after UVB irradiation VS hetero before UVB irradiation	GO_BOX_H_ACA_RNP_COMPLEX1	-0.87144906	0.008501391	-2.74820096	0.02557418	0.22788198	-3.40726199
WT after UVB irradiation VS WT before UVB irradiation	GO_BOX_H_ACA_RNP_COMPLEX2	-0.61339728	-0.07774926	-1.67250151	0.13250907	0.63860701	-4.74071722
homo after UVB irradiation VS homo before UVB irradiation	GO_BOX_H_ACA_SNORNA_BINDING	-1.04438507	0.006223852	-5.4757157	0.00051365	0.01654995	0.243021992
hetero after UVB irradiation VS hetero before UVB irradiation	GO_BOX_H_ACA_SNORNA_BINDING1	-0.87144906	0.008501391	-2.74820096	0.02557418	0.22788198	-3.40726199
WT after UVB irradiation VS WT before UVB irradiation	GO_BOX_H_ACA_SNORNA_BINDING2	-0.61339728	-0.07774926	-1.67250151	0.13250907	0.63860701	-4.74071722
homo after UVB irradiation VS homo before UVB irradiation	GO_BOX_H_ACA_SNORNP_COMPLEX	-1.04438507	0.006223852	-5.4757157	0.00051365	0.01654995	0.243021992
hetero after UVB irradiation VS hetero before UVB irradiation	GO_BOX_H_ACA_SNORNP_COMPLEX1	-0.87144906	0.008501391	-2.74820096	0.02557418	0.22788198	-3.40726199
WT after UVB irradiation VS WT before UVB irradiation	GO_BOX_H_ACA_SNORNP_COMPLEX2	-0.61339728	-0.07774926	-1.67250151	0.13250907	0.63860701	-4.74071722
homo after UVB irradiation VS homo before UVB irradiation	GO_BOX_H_ACA_TELOMERASE_RNP_COMPLEX	-1.04438507	0.006223852	-5.4757157	0.00051365	0.01654995	0.243021992
hetero after UVB irradiation VS hetero before UVB irradiation	GO_BOX_H_ACA_TELOMERASE_RNP_COMPLEX1	-0.87144906	0.008501391	-2.74820096	0.02557418	0.22788198	-3.40726199
WT after UVB irradiation VS WT before UVB irradiation	GO_BOX_H_ACA_TELOMERASE_RNP_COMPLEX2	-0.61339728	-0.07774926	-1.67250151	0.13250907	0.63860701	-4.74071722
homo after UVB irradiation VS homo before UVB irradiation	GO_BRADYKININ_CATABOLIC_PROCESS	0.615974466	-0.09935069	1.832046562	0.10283661	0.25518074	-5.00893301
hetero after UVB irradiation VS hetero before UVB irradiation	GO_BRADYKININ_CATABOLIC_PROCESS1	0.66741527	-0.04632974	3.414283451	0.00942458	0.15459076	-2.44550413
WT after UVB irradiation VS WT before UVB irradiation	GO_BRADYKININ_CATABOLIC_PROCESS2	0.478383759	-0.06171057	1.941247062	0.08773798	0.57920555	-4.38461463
homo after UVB irradiation VS homo before UVB irradiation	GO_BRAIN_DERIVED_NEUROTROPHIC_FACTOR_RECEPTOR_SIGNALING_PATHWAY	0.19053646	-0.07568576	0.644417954	0.53664379	0.70274669	-6.32855472
hetero after UVB irradiation VS hetero before UVB irradiation	GO_BRAIN_DERIVED_NEUROTROPHIC_FACTOR_RECEPTOR_SIGNALING_PATHWAY1	-0.1721495	-0.00795789	-0.549827	0.59772373	0.83191957	-6.07073259
WT after UVB irradiation VS WT before UVB irradiation	GO_BRAIN_DERIVED_NEUROTROPHIC_FACTOR_RECEPTOR_SIGNALING_PATHWAY2	-0.05654987	-0.05604164	-0.15718674	0.87894637	0.9693507	-5.9830507
homo after UVB irradiation VS homo before UVB irradiation	GO_BRAIN_MORPHOGENESIS	-0.06436025	0.019008345	-0.4147006	0.68883656	0.81391709	-6.45745277
hetero after UVB irradiation VS hetero before UVB irradiation	GO_BRAIN_MORPHOGENESIS1	-0.0033294	-0.03547647	-0.01927656	0.98510127	0.99448825	-6.2312826
WT after UVB irradiation VS WT before UVB irradiation	GO_BRAIN_MORPHOGENESIS2	-0.19383847	0.009277697	-1.21568394	0.2583555	0.7393034	-5.28362382
homo after UVB irradiation VS homo before UVB irradiation	GO_BRAINSTEM_DEVELOPMENT	-0.65035438	0.034688286	-2.4181366	0.04081086	0.14536469	-4.13517223
hetero after UVB irradiation VS hetero before UVB irradiation	GO_BRAINSTEM_DEVELOPMENT1	-0.96713189	-0.05258499	-6.11378043	0.00030727	0.07788488	0.784501394
WT after UVB irradiation VS WT before UVB irradiation	GO_BRAINSTEM_DEVELOPMENT2	-0.53833582	0.017257095	-1.94070817	0.08781132	0.57920555	-4.38534728
homo after UVB irradiation VS homo before UVB irradiation	GO_BRANCH_ELONGATION_INVOLVED_IN_URETERIC_BUD_BRANCHING	0.376703686	0.011166775	1.575552496	0.15228723	0.3263582	-5.3618362
hetero after UVB irradiation VS hetero before UVB irradiation	GO_BRANCH_ELONGATION_INVOLVED_IN_URETERIC_BUD_BRANCHING1	-0.16748486	0.051682682	-0.76156078	0.468574	0.75327138	-5.9284509
WT after UVB irradiation VS WT before UVB irradiation	GO_BRANCH_ELONGATION_INVOLVED_IN_URETERIC_BUD_BRANCHING2	-0.46789113	0.029367463	-1.7297241	0.12147796	0.63058669	-4.66664744
homo after UVB irradiation VS homo before UVB irradiation	GO_BRANCH_ELONGATION_OF_AN_EPITHELIUM	-0.05670695	0.009810666	-0.38070702	0.71294707	0.82920554	-6.47200965
hetero after UVB irradiation VS hetero before UVB irradiation	GO_BRANCH_ELONGATION_OF_AN_EPITHELIUM1	-0.09614232	-0.0066403	-0.55280155	0.59578029	0.83012448	-6.06902271
WT after UVB irradiation VS WT before UVB irradiation	GO_BRANCH_ELONGATION_OF_AN_EPITHELIUM2	-0.31099521	0.04358486	-1.86978644	0.09799335	0.59874718	-4.48119236
homo after UVB irradiation VS homo before UVB irradiation	GO_BRANCHED_CHAIN_AMINO_ACID_BIOSYNTHETIC_PROCESS	0.624488606	-0.08465805	2.035001742	0.0748677	0.21017082	-4.714432
hetero after UVB irradiation VS hetero before UVB irradiation	GO_BRANCHED_CHAIN_AMINO_ACID_BIOSYNTHETIC_PROCESS1	0.004166914	0.021359327	0.014519059	0.98877797	0.99586429	-6.23136979
WT after UVB irradiation VS WT before UVB irradiation	GO_BRANCHED_CHAIN_AMINO_ACID_BIOSYNTHETIC_PROCESS2	0.289374712	-0.06333293	1.172223469	0.27443753	0.74597065	-5.32969209
homo after UVB irradiation VS homo before UVB irradiation	GO_BRANCHED_CHAIN_AMINO_ACID_METABOLIC_PROCESS	-0.21708292	-0.0192808	-0.70503993	0.50002279	0.67411258	-6.28603493
hetero after UVB irradiation VS hetero before UVB irradiation	GO_BRANCHED_CHAIN_AMINO_ACID_METABOLIC_PROCESS1	-0.72215308	-0.04318259	-4.43761397	0.00227637	0.10650321	-1.07849301
WT after UVB irradiation VS WT before UVB irradiation	GO_BRANCHED_CHAIN_AMINO_ACID_METABOLIC_PROCESS2	-0.13190173	-0.10360416	-0.4865905	0.6394448	0.90634429	-5.87303491
homo after UVB irradiation VS homo before UVB irradiation	GO_BRANCHED_CHAIN_AMINO_ACID_TRANSMEMBRANE_TRANSPORTER_ACTIVITY	-0.5279295	-0.09446827	-1.7144386	0.12329977	0.28613535	-5.17383286
hetero after UVB irradiation VS hetero before UVB irradiation	GO_BRANCHED_CHAIN_AMINO_ACID_TRANSMEMBRANE_TRANSPORTER_ACTIVITY1	-0.44868805	-0.04456363	-1.83974187	0.10378362	0.39991031	-4.71687294
WT after UVB irradiation VS WT before UVB irradiation	GO_BRANCHED_CHAIN_AMINO_ACID_TRANSMEMBRANE_TRANSPORTER_ACTIVITY2	-0.23373627	-0.03072764	-0.72820969	0.48700651	0.85164239	-5.72557119
homo after UVB irradiation VS homo before UVB irradiation	GO_BRANCHED_CHAIN_AMINO_ACID_TRANSPORT	-0.51720302	-0.05814426	-1.84039788	0.1015119	0.25282889	-4.99704731
hetero after UVB irradiation VS hetero before UVB irradiation	GO_BRANCHED_CHAIN_AMINO_ACID_TRANSPORT1	-0.60550554	0.00255714	-3.36075822	0.01019264	0.15859238	-2.52121
WT after UVB irradiation VS WT before UVB irradiation	GO_BRANCHED_CHAIN_AMINO_ACID_TRANSPORT2	-0.36637325	-0.01134133	-1.42613033	0.19122263	0.69334561	-5.04560871
homo after UVB irradiation VS homo before UVB irradiation	GO_BRANCHING_INVOLVED_IN_BLOOD_VESSEL_MORPHOGENESIS	0.30332479	0.014882994	1.612291287	0.14406888	0.31608276	-5.3128824
hetero after UVB irradiation VS hetero before UVB irradiation	GO_BRANCHING_INVOLVED_IN_BLOOD_VESSEL_MORPHOGENESIS1	0.537341017	0.04997564	3.810696402	0.0053393	0.12893175	-1.89676131
WT after UVB irradiation VS WT before UVB irradiation	GO_BRANCHING_INVOLVED_IN_BLOOD_VESSEL_MORPHOGENESIS2	0.147109945	-0.0235236	0.841292924	0.42432931	0.82612337	-5.63897069
homo after UVB irradiation VS homo before UVB irradiation	GO_BRANCHING_INVOLVED_IN_LABYRINTHINE_LAYER_MORPHOGENESIS	-0.02216872	0.016076861	-0.10979012	0.91517509	0.95335584	-6.54424618
hetero after UVB irradiation VS hetero before UVB irradiation	GO_BRANCHING_INVOLVED_IN_LABYRINTHINE_LAYER_MORPHOGENESIS1	0.177505136	0.031428785	0.845520312	0.42283384	0.7249295	-5.86106003
WT after UVB irradiation VS WT before UVB irradiation	GO_BRANCHING_INVOLVED_IN_LABYRINTHINE_LAYER_MORPHOGENESIS2	-0.20536431	0.025015548	-1.20312195	0.26292216	0.74156847	-5.29705632
homo after UVB irradiation VS homo before UVB irradiation	GO_BRANCHING_INVOLVED_IN_MAMMARY_GLAND_DUCT_MORPHOGENESIS	-0.04202452	-0.03897031	-0.32818808	0.75086794	0.8545857	-6.49213115
hetero after UVB irradiation VS hetero before UVB irradiation	GO_BRANCHING_INVOLVED_IN_MAMMARY_GLAND_DUCT_MORPHOGENESIS1	0.027060856	-0.02408277	0.196219925	0.84942071	0.94258929	-6.21065697
WT after UVB irradiation VS WT before UVB irradiation	GO_BRANCHING_INVOLVED_IN_MAMMARY_GLAND_DUCT_MORPHOGENESIS2	-0.01886053	0.023685025	-0.12570818	0.903028	0.97576564	-5.98773706
homo after UVB irradiation VS homo before UVB irradiation	GO_BRANCHING_INVOLVED_IN_PROSTATE_GLAND_MORPHOGENESIS	-0.71399761	0.01479624	-5.15410006	0.00076623	0.01924501	-0.15709975
hetero after UVB irradiation VS hetero before UVB irradiation	GO_BRANCHING_INVOLVED_IN_PROSTATE_GLAND_MORPHOGENESIS1	-0.65948476	-0.05085497	-2.85737109	0.02164796	0.21196897	-3.24742958
WT after UVB irradiation VS WT before UVB irradiation	GO_BRANCHING_INVOLVED_IN_PROSTATE_GLAND_MORPHOGENESIS2	-0.74541203	0.045613622	-3.41539609	0.00898493	0.31908946	-2.31880353
homo after UVB irradiation VS homo before UVB irradiation	GO_BRANCHING_INVOLVED_IN_SALIVARY_GLAND_MORPHOGENESIS	0.080800401	0.02774949	0.453066176	0.66207084	0.79522867	-6.4395939
hetero after UVB irradiation VS hetero before UVB irradiation	GO_BRANCHING_INVOLVED_IN_SALIVARY_GLAND_MORPHOGENESIS1	0.127826546	0.05852257	0.780752882	0.45783555	0.74732537	-5.91357139
WT after UVB irradiation VS WT before UVB irradiation	GO_BRANCHING_INVOLVED_IN_SALIVARY_GLAND_MORPHOGENESIS2	0.023598972	0.052874734	0.163766249	0.87392889	0.96914919	-5.98194021
homo after UVB irradiation VS homo before UVB irradiation	GO_BRANCHING_MORPHOGENESIS_OF_A_NERVE	0.111894627	-0.02441809	0.657749399	0.52845448	0.69653472	-6.31949798
hetero after UVB irradiation VS hetero before UVB irradiation	GO_BRANCHING_MORPHOGENESIS_OF_A_NERVE1	-0.14728312	0.02548263	-0.49096132	0.63687915	0.85094067	-6.10280508
WT after UVB irradiation VS WT before UVB irradiation	GO_BRANCHING_MORPHOGENESIS_OF_A_NERVE2	-0.16406936	-0.0308636	-0.98707795	0.3521764	0.7885696	-5.51234727
homo after UVB irradiation VS homo before UVB irradiation	GO_BRANCHING_MORPHOGENESIS_OF_AN_EPITHELIAL_TUBE	0.027498509	0.000879908	0.21987858	0.83125914	0.90572006	-6.52440135
hetero after UVB irradiation VS hetero before UVB irradiation	GO_BRANCHING_MORPHOGENESIS_OF_AN_EPITHELIAL_TUBE1	-0.03888206	0.008882035	-0.27190511	0.79270688	0.9192285	-6.19158463
WT after UVB irradiation VS WT before UVB irradiation	GO_BRANCHING_MORPHOGENESIS_OF_AN_EPITHELIAL_TUBE2	-0.09326012	0.001725043	-0.62260342	0.55067138	0.87546015	-5.79656297
homo after UVB irradiation VS homo before UVB irradiation	GO_BRANCHIOMOTOR_NEURON_AXON_GUIDANCE	-0.00844123	-0.00963342	-0.03325033	0.97425799	0.9867622	-6.55024999
hetero after UVB irradiation VS hetero before UVB irradiation	GO_BRANCHIOMOTOR_NEURON_AXON_GUIDANCE1	-0.10656648	0.041111574	-0.37292714	0.7190676	0.89048853	-6.15674787
WT after UVB irradiation VS WT before UVB irradiation	GO_BRANCHIOMOTOR_NEURON_AXON_GUIDANCE2	0.310780576	-0.08416145	1.236591167	0.25090072	0.73564768	-5.2610616
homo after UVB irradiation VS homo before UVB irradiation	GO_BRCA1_A_COMPLEX	-0.0011917	0.012149855	-0.00532592	0.99587591	0.9975659	-6.55084115
hetero after UVB irradiation VS hetero before UVB irradiation	GO_BRCA1_A_COMPLEX1	-0.14435014	-0.0561763	-0.44581283	0.66776757	0.86386453	-6.12509304
WT after UVB irradiation VS WT before UVB irradiation	GO_BRCA1_A_COMPLEX2	0.192050961	-0.08413984	0.860311905	0.4143613	0.82135231	-5.62338039
homo after UVB irradiation VS homo before UVB irradiation	GO_BRISC_COMPLEX	-0.16557605	0.056886295	-0.52739626	0.61167412	0.75814505	-6.40074724
hetero after UVB irradiation VS hetero before UVB irradiation	GO_BRISC_COMPLEX1	-0.01487566	-0.05814501	-0.04783438	0.9630424	0.98664387	-6.23024343
WT after UVB irradiation VS WT before UVB irradiation	GO_BRISC_COMPLEX2	0.526492839	-0.03517035	1.555323519	0.15802513	0.66180255	-4.88879315
homo after UVB irradiation VS homo before UVB irradiation	GO_BRONCHUS_DEVELOPMENT	-0.29351911	0.070577855	-1.50953363	0.1681236	0.34694448	-5.44827946
hetero after UVB irradiation VS hetero before UVB irradiation	GO_BRONCHUS_DEVELOPMENT1	-0.38514767	0.049446539	-2.12610032	0.06683385	0.33629881	-4.31454138
WT after UVB irradiation VS WT before UVB irradiation	GO_BRONCHUS_DEVELOPMENT2	-0.31780376	0.055884929	-1.15301504	0.28180127	0.75255145	-5.34968532
homo after UVB irradiation VS homo before UVB irradiation	GO_BROWN_FAT_CELL_DIFFERENTIATION	0.206167803	-0.02662976	1.365775001	0.20774699	0.39780163	-5.6290691
hetero after UVB irradiation VS hetero before UVB irradiation	GO_BROWN_FAT_CELL_DIFFERENTIATION1	0.087641195	0.006515882	0.531672984	0.60965914	0.83720565	-6.08098322

WT after UVB irradiation VS WT before UVB irradiation	GO_BROWN_FAT_CELL_DIFFERENTIATION2	0.037988835	-0.03815049	0.195193376	0.85005033	0.9656064	-5.97601295
homo after UVB irradiation VS homo before UVB irradiation	GO_BRUSH_BORDER	0.180960175	0.016126397	1.336310915	0.21680072	0.40745392	-5.66475216
homo after UVB irradiation VS homo before UVB irradiation	GO_BRUSH_BORDER_ASSEMBLY	-0.35745791	0.122072335	-1.89602025	0.09309205	0.24041992	-4.91733892
hetero after UVB irradiation VS hetero before UVB irradiation	GO_BRUSH_BORDER_ASSEMBLY1	-0.31419811	0.04300377	-1.15093858	0.28359539	0.60906208	-5.56970599
WT after UVB irradiation VS WT before UVB irradiation	GO_BRUSH_BORDER_ASSEMBLY2	-0.16045072	0.013118147	-0.67520877	0.51835479	0.86195476	-5.7624304
homo after UVB irradiation VS homo before UVB irradiation	GO_BRUSH_BORDER_MEMBRANE	0.087343425	0.037774232	0.61143041	0.55723011	0.71801642	-6.35024212
hetero after UVB irradiation VS hetero before UVB irradiation	GO_BRUSH_BORDER_MEMBRANE1	-0.01252272	-0.01201061	-0.09586392	0.92602988	0.97217839	-6.22650338
WT after UVB irradiation VS WT before UVB irradiation	GO_BRUSH_BORDER_MEMBRANE2	-0.01261185	0.026419715	-0.08872156	0.93145824	0.98320919	-5.9919154
hetero after UVB irradiation VS hetero before UVB irradiation	GO_BRUSH_BORDER1	0.086742351	0.01375494	0.590309075	0.57157549	0.81424167	-6.04673584
WT after UVB irradiation VS WT before UVB irradiation	GO_BRUSH_BORDER2	0.115770152	0.010202209	0.809084781	0.44158854	0.83231427	-5.66471059
homo after UVB irradiation VS homo before UVB irradiation	GO_BUBBLE_DNA_BINDING	-0.11789195	0.031596408	-0.44814908	0.66547377	0.79756326	-6.44196689
hetero after UVB irradiation VS hetero before UVB irradiation	GO_BUBBLE_DNA_BINDING1	0.161122431	-0.0482207	0.790598334	0.45239153	0.74417172	-5.9058161
WT after UVB irradiation VS WT before UVB irradiation	GO_BUBBLE_DNA_BINDING2	-0.17549557	-0.11503945	-0.59538865	0.56784625	0.88290486	-5.81324049
homo after UVB irradiation VS homo before UVB irradiation	GO_BUD_ELONGATION_INVOLVED_IN_LUNG_BRANCHING	-0.33129585	0.033328095	-1.26262603	0.24092407	0.4338362	-5.75177489
hetero after UVB irradiation VS hetero before UVB irradiation	GO_BUD_ELONGATION_INVOLVED_IN_LUNG_BRANCHING1	-0.34831765	0.026962936	-1.27590823	0.23843182	0.56736997	-5.4322282
WT after UVB irradiation VS WT before UVB irradiation	GO_BUD_ELONGATION_INVOLVED_IN_LUNG_BRANCHING2	-0.10490733	-0.02458082	-0.45066549	0.66405306	0.91232901	-5.89030317
homo after UVB irradiation VS homo before UVB irradiation	GO_BUNDLE_OF_HIS_CELL_TO_PURKINJE_MYOCYTE_COMMUNICATION	-0.04775299	0.023599082	-0.30851208	0.76526804	0.86417665	-6.49892224
hetero after UVB irradiation VS hetero before UVB irradiation	GO_BUNDLE_OF_HIS_CELL_TO_PURKINJE_MYOCYTE_COMMUNICATION1	-0.28968396	0.005918122	-1.77408651	0.11467809	0.41761843	-4.80634163
WT after UVB irradiation VS WT before UVB irradiation	GO_BUNDLE_OF_HIS_CELL_TO_PURKINJE_MYOCYTE_COMMUNICATION2	-0.54078816	0.01016559	-3.04069753	0.01581899	0.37470434	-2.83627266
homo after UVB irradiation VS homo before UVB irradiation	GO_BUNDLE_OF_HIS_CELL_TO_PURKINJE_MYOCYTE_SIGNALING	0.147210256	0.040912942	0.908088981	0.38934813	0.57861132	-6.11973082
hetero after UVB irradiation VS hetero before UVB irradiation	GO_BUNDLE_OF_HIS_CELL_TO_PURKINJE_MYOCYTE_SIGNALING1	-0.1345342	0.039768758	-0.49154716	0.63648311	0.85090495	-6.10250259
WT after UVB irradiation VS WT before UVB irradiation	GO_BUNDLE_OF_HIS_CELL_TO_PURKINJE_MYOCYTE_SIGNALING2	-0.26959606	-0.03526624	-1.27070686	0.23912153	0.72928936	-5.22370436
homo after UVB irradiation VS homo before UVB irradiation	GO_BUTYRATE_COA_LIGASE_ACTIVITY	-0.36698342	0.024779109	-1.68854419	0.12828676	0.29332647	-5.20947233
hetero after UVB irradiation VS hetero before UVB irradiation	GO_BUTYRATE_COA_LIGASE_ACTIVITY1	-0.50388119	-0.01944616	-3.41066183	0.00947456	0.1547277	-2.45061543
WT after UVB irradiation VS WT before UVB irradiation	GO_BUTYRATE_COA_LIGASE_ACTIVITY2	-0.27034688	0.048516199	-1.01565634	0.33917537	0.78064003	-5.48567584
homo after UVB irradiation VS homo before UVB irradiation	GO_C_ACETYLTRANSFERASE_ACTIVITY	-0.89423633	-0.03005891	-3.66886115	0.00589479	0.05056585	-2.21345958
hetero after UVB irradiation VS hetero before UVB irradiation	GO_C_ACETYLTRANSFERASE_ACTIVITY1	-0.72763785	-0.13282944	-2.43849125	0.04120679	0.27359812	-3.86145411
WT after UVB irradiation VS WT before UVB irradiation	GO_C_ACETYLTRANSFERASE_ACTIVITY2	-0.5304753	-0.00647832	-2.17762042	0.0606863	0.52811349	-4.05808203
homo after UVB irradiation VS homo before UVB irradiation	GO_C_ACYLTRANSFERASE_ACTIVITY	-0.07843416	-0.02796166	-0.5210659	0.61588723	0.76132081	-6.40427117
hetero after UVB irradiation VS hetero before UVB irradiation	GO_C_ACYLTRANSFERASE_ACTIVITY1	-0.1453301	-0.01398013	-0.91667455	0.38659952	0.69764483	-5.79941833
WT after UVB irradiation VS WT before UVB irradiation	GO_C_ACYLTRANSFERASE_ACTIVITY2	-0.01883843	0.068931677	-0.07301586	0.94356487	0.98531933	-5.99325458
homo after UVB irradiation VS homo before UVB irradiation	GO_C_C_CHEMOKINE_BINDING	0.278738109	-0.02796743	1.180827859	0.27028914	0.46555781	-5.84445304
hetero after UVB irradiation VS hetero before UVB irradiation	GO_C_C_CHEMOKINE_BINDING1	0.141223598	0.018176876	0.617728133	0.55424163	0.80472198	-6.02960091
WT after UVB irradiation VS WT before UVB irradiation	GO_C_C_CHEMOKINE_BINDING2	0.11524993	0.000433415	0.366365678	0.72347988	0.93154998	-5.92585917
homo after UVB irradiation VS homo before UVB irradiation	GO_C_METHYLTRANSFERASE_ACTIVITY	-0.71082821	-0.05683794	-3.06448239	0.01475107	0.08257891	-3.13340739
hetero after UVB irradiation VS hetero before UVB irradiation	GO_C_METHYLTRANSFERASE_ACTIVITY1	-0.81669694	-0.01219455	-3.57486069	0.00746738	0.14177754	-2.22058039
WT after UVB irradiation VS WT before UVB irradiation	GO_C_METHYLTRANSFERASE_ACTIVITY2	-0.17799349	-0.141144	-0.66073889	0.52712618	0.86633267	-5.77206545
homo after UVB irradiation VS homo before UVB irradiation	GO_C_PALMITOYLTRANSFERASE_ACTIVITY	0.837147015	-0.00566987	6.003173736	0.00027479	0.01221056	0.864572459
hetero after UVB irradiation VS hetero before UVB irradiation	GO_C_PALMITOYLTRANSFERASE_ACTIVITY1	0.554887869	0.075164297	2.616243628	0.03131828	0.24668014	-3.60085827
WT after UVB irradiation VS WT before UVB irradiation	GO_C_PALMITOYLTRANSFERASE_ACTIVITY2	0.282324931	0.122777105	1.097055776	0.30416579	0.76505447	-5.40659814
homo after UVB irradiation VS homo before UVB irradiation	GO_C_TERMINAL_PROTEIN_AMINO_ACID_MODIFICATION	0.32536109	-0.00294428	2.233219739	0.05473126	0.17369341	-4.41761748
hetero after UVB irradiation VS hetero before UVB irradiation	GO_C_TERMINAL_PROTEIN_AMINO_ACID_MODIFICATION1	0.075821381	-0.01586157	0.336433092	0.7453581	0.90030853	-6.17055651
WT after UVB irradiation VS WT before UVB irradiation	GO_C_TERMINAL_PROTEIN_AMINO_ACID_MODIFICATION2	0.184703695	0.040601424	0.965120592	0.36241864	0.79519319	-5.5324403
homo after UVB irradiation VS homo before UVB irradiation	GO_C_TERMINAL_PROTEIN_DEGLUTAMYLATION	0.252284476	-0.12783548	0.85924829	0.41424691	0.60055425	-6.16295981
hetero after UVB irradiation VS hetero before UVB irradiation	GO_C_TERMINAL_PROTEIN_DEGLUTAMYLATION1	-0.46643801	-0.13151041	-1.76739205	0.11584766	0.41917522	-4.81539297
WT after UVB irradiation VS WT before UVB irradiation	GO_C_TERMINAL_PROTEIN_DEGLUTAMYLATION2	-0.08351932	0.088238011	-0.18850597	0.85511865	0.9663792	-5.97736038
homo after UVB irradiation VS homo before UVB irradiation	GO_C_TERMINAL_PROTEIN_LIPIDATION	0.299646427	-0.03199641	2.087410669	0.06893192	0.20087447	-4.63670557
hetero after UVB irradiation VS hetero before UVB irradiation	GO_C_TERMINAL_PROTEIN_LIPIDATION1	0.211779239	-0.0063448	0.678945967	0.51667902	0.78479751	-5.98882715
WT after UVB irradiation VS WT before UVB irradiation	GO_C_TERMINAL_PROTEIN_LIPIDATION2	0.131133691	0.024611161	0.471542926	0.649697	0.9092481	-5.88041993
homo after UVB irradiation VS homo before UVB irradiation	GO_C_X_C_CHEMOKINE_BINDING	-0.12203014	0.047130339	-0.53120173	0.60914874	0.75634901	-6.39860972
hetero after UVB irradiation VS hetero before UVB irradiation	GO_C_X_C_CHEMOKINE_BINDING1	0.065049838	0.020483679	0.206533148	0.84162885	0.93968495	-6.20841641
WT after UVB irradiation VS WT before UVB irradiation	GO_C_X_C_CHEMOKINE_BINDING2	-0.09772847	0.053229127	-0.29705341	0.77390314	0.94274743	-5.94977353
homo after UVB irradiation VS homo before UVB irradiation	GO_C_X_C_CHEMOKINE_RECEPTOR_ACTIVITY	0.458544107	-0.05255692	2.397520205	0.04216889	0.14812814	-4.16685734
hetero after UVB irradiation VS hetero before UVB irradiation	GO_C_X_C_CHEMOKINE_RECEPTOR_ACTIVITY1	0.549191926	0.018093855	2.84784434	0.02196426	0.21364842	-3.26135785
WT after UVB irradiation VS WT before UVB irradiation	GO_C_X_C_CHEMOKINE_RECEPTOR_ACTIVITY2	0.209507317	-0.03181288	0.67802712	0.51665688	0.86137677	-5.76053219
homo after UVB irradiation VS homo before UVB irradiation	GO_C_X3_C_CHEMOKINE_BINDING	1.181565948	-0.03534542	9.315803168	1.10E-05	0.00847131	3.925699075
hetero after UVB irradiation VS hetero before UVB irradiation	GO_C_X3_C_CHEMOKINE_BINDING1	1.016461788	0.079393223	4.854623498	0.00133324	0.09333375	-0.57085801
WT after UVB irradiation VS WT before UVB irradiation	GO_C_X3_C_CHEMOKINE_BINDING2	0.993879137	0.089238226	3.472811485	0.00825117	0.31908946	-2.24105402
homo after UVB irradiation VS homo before UVB irradiation	GO_C21_STEROID_HORMONE_BIOSYNTHETIC_PROCESS	0.280092418	0.000161028	1.931997529	0.08800402	0.23190623	-4.8653017
hetero after UVB irradiation VS hetero before UVB irradiation	GO_C21_STEROID_HORMONE_BIOSYNTHETIC_PROCESS1	0.112486036	0.023375634	0.699475529	0.50444342	0.77488727	-5.97438935
WT after UVB irradiation VS WT before UVB irradiation	GO_C21_STEROID_HORMONE_BIOSYNTHETIC_PROCESS2	0.084513833	0.045861028	0.473655517	0.64825279	0.90907607	-5.8793963
homo after UVB irradiation VS homo before UVB irradiation	GO_C21_STEROID_HORMONE_METABOLIC_PROCESS	0.182819261	0.001543503	1.230577696	0.25209782	0.44665717	-5.78859404
hetero after UVB irradiation VS hetero before UVB irradiation	GO_C21_STEROID_HORMONE_METABOLIC_PROCESS1	0.076950974	0.030840879	0.481047308	0.64360008	0.85406424	-6.10787264
WT after UVB irradiation VS WT before UVB irradiation	GO_C21_STEROID_HORMONE_METABOLIC_PROCESS2	-0.02757101	0.048206941	-0.1543226	0.88113237	0.9693507	-5.98351999
homo after UVB irradiation VS homo before UVB irradiation	GO_C2H2_ZINC_FINGER_DOMAIN_BINDING	0.148849413	-0.01125856	0.851628667	0.41823053	0.60383798	-6.16952503
hetero after UVB irradiation VS hetero before UVB irradiation	GO_C2H2_ZINC_FINGER_DOMAIN_BINDING1	-0.09108537	0.030062445	-0.47624961	0.64686521	0.85505449	-6.11029008
WT after UVB irradiation VS WT before UVB irradiation	GO_C2H2_ZINC_FINGER_DOMAIN_BINDING2	0.129945011	0.017432071	0.730525344	0.48566517	0.85147728	-5.72390534
homo after UVB irradiation VS homo before UVB irradiation	GO_C3HC4_TYPE_RING_FINGER_DOMAIN_BINDING	-0.3604377	-0.03901924	-1.39560263	0.19891493	0.38771464	-5.59245228
hetero after UVB irradiation VS hetero before UVB irradiation	GO_C3HC4_TYPE_RING_FINGER_DOMAIN_BINDING1	-0.67142723	-0.01278704	-3.06017603	0.01592883	0.18580692	-2.95221949
WT after UVB irradiation VS WT before UVB irradiation	GO_C3HC4_TYPE_RING_FINGER_DOMAIN_BINDING2	-0.67728614	-0.13410836	-2.58569697	0.03200922	0.45445578	-3.48038635
homo after UVB irradiation VS homo before UVB irradiation	GO_C4_DICARBOXYLATE_TRANSMEMBRANE_TRANSPORTER_ACTIVITY	0.064422593	0.034921778	0.331282482	0.74861245	0.85313198	-6.49102591
hetero after UVB irradiation VS hetero before UVB irradiation	GO_C4_DICARBOXYLATE_TRANSMEMBRANE_TRANSPORTER_ACTIVITY1	-0.28220932	-0.00798761	-1.64774356	0.13872785	0.4522908	-4.97470839
WT after UVB irradiation VS WT before UVB irradiation	GO_C4_DICARBOXYLATE_TRANSMEMBRANE_TRANSPORTER_ACTIVITY2	0.285328522	0.04561125	1.719044138	0.1234698	0.63133281	-4.68055253
homo after UVB irradiation VS homo before UVB irradiation	GO_C4_DICARBOXYLATE_TRANSPORT	0.037556678	0.030502846	0.292850727	0.77679966	0.87127537	-6.50403447
hetero after UVB irradiation VS hetero before UVB irradiation	GO_C4_DICARBOXYLATE_TRANSPORT1	-0.20440311	0.022298632	-1.15806206	0.28084455	0.60652504	-5.56212913
WT after UVB irradiation VS WT before UVB irradiation	GO_C4_DICARBOXYLATE_TRANSPORT2	0.003612358	0.019474001	0.021827443	0.98311388	0.99634469	-5.99581528
homo after UVB irradiation VS homo before UVB irradiation	GO_CADHERIN_BINDING	0.076000711	0.00354077	0.389528664	0.70665593	0.82507261	-6.4683471
homo after UVB irradiation VS homo before UVB irradiation	GO_CADHERIN_BINDING_INVOLVED_IN_CELL_CELL_ADHESION	-0.07337697	-0.02279679	-0.27977276	0.78647427	0.87851039	-6.50810358
hetero after UVB irradiation VS hetero before UVB irradiation	GO_CADHERIN_BINDING_INVOLVED_IN_CELL_CELL_ADHESION1	-0.23223396	-0.06308989	-1.30615303	0.22845938	0.55693814	-5.397572
WT after UVB irradiation VS WT before UVB irradiation	GO_CADHERIN_BINDING_INVOLVED_IN_CELL_CELL_ADHESION2	-0.40944566	-0.04911173	-1.83157027	0.10393639	0.60721285	-4.53233047
hetero after UVB irradiation VS hetero before UVB irradiation	GO_CADHERIN_BINDING1	0.297726711	0.005795555	1.930287654	0.09036458	0.37773929	-4.59158464
WT after UVB irradiation VS WT before UVB irradiation	GO_CADHERIN_BINDING2	0.035882221	0.023376573	0.183621727	0.8588249	0.96716109	-5.97831508
homo after UVB irradiation VS homo before UVB irradiation	GO_CADMIUM_ION_BINDING	0.71799758	-0.01761158	3.054316964	0.01498563	0.08315721	-3.14912803
hetero after UVB irradiation VS hetero before UVB irradiation	GO_CADMIUM_ION_BINDING1	0.539116064	-0.06753455	1.564085387	0.1571321	0.47628027	-5.08303801
WT after UVB irradiation VS WT before UVB irradiation	GO_CADMIUM_ION_BINDING2	0.358012484	0.03050659	1.082668951	0.31013796	0.76742969	-5.42090084
homo after UVB irradiation VS homo before UVB irradiation	GO_CADMIUM_ION_TRANSMEMBRANE_TRANSPORT	-0.17941671	-0.00117121	-0.85480228	0.41656809	0.602676	-6.16679651
hetero after UVB irradiation VS hetero before UVB irradiation	GO_CADMIUM_ION_TRANSMEMBRANE_TRANSPORT1	-0.00823759	0.041542352	-0.03602658	0.97216012	0.99021155	-6.2307803
WT after UVB irradiation VS WT before UVB irradiation	GO_CADMIUM_ION_TRANSMEMBRANE_TRANSPORT2	0.100173184	0.007664683	0.433024914	0.67629952	0.91685973	-5.89832316
homo after UVB irradiation VS homo before UVB irradiation	GO_CAJAL_BODY	-0.7590154	0.001233342	-5.50338536	0.00049661	0.0163216	0.276688915
hetero after UVB irradiation VS hetero before UVB irradiation	GO_CAJAL_BODY1	-0.23946647	-0.05306723	-0.92949153	0.38032029	0.69277918	-5.78788967
WT after UVB irradiation VS WT before UVB irradiation	GO_CAJAL_BODY2	-0.28723753	-0.00973556	-1.02798462	0.33368152	0.77844475	-5.47399173
homo after UVB irradiation VS homo before UVB irradiation	GO_CALCINEURIN_COMPLEX	0.262462506	-0.03920707	1.452553039	0.1829545	0.36646792	-5.5212131
hetero after UVB irradiation VS hetero before UVB irradiation	GO_CALCINEURIN_COMPLEX1	-0.03856215	-0.04225153	-0.13764194	0.89398717	0.96135218	-6.22122276
WT after UVB irradiation VS WT before UVB irradiation	GO_CALCINEURIN_COMPLEX2	0.10922727	-0.2222655	0.269708554	0.79413021	0.950487	-5.95786571
homo after UVB irradiation VS homo before UVB irradiation	GO_CALCINEURIN_MEDIATED_SIGNALING	0.294377454	-0.00392118	2.132928373	0.06415006	0.1919429	-4.56873247
hetero after UVB irradiation VS hetero before UVB irradiation	GO_CALCINEURIN_MEDIATED_SIGNALING1	0.231879552	-0.0137319	1.38205905	0.2049915	0.52974687	-5.30839849
WT after UVB irradiation VS WT before UVB irradiation	GO_CALCINEURIN_MEDIATED_SIGNALING2	0.272189697	0.00440504	1.484450117	0.17553816	0.68033133	-4.97572541
homo after UVB irradiation VS homo before UVB irradiation	GO_CALCITONIN_FAMILY_RECEPTOR_ACTIVITY	0.461788363	-0.04902561	2.083704491	0.06933621	0.20134405	-4.6422216
hetero after UVB irradiation VS hetero before UVB irradiation	GO_CALCITONIN_FAMILY_RECEPTOR_ACTIVITY1	-0.09245315	0.021492766	-0.32523948	0.75349595	0.90402359	-6.1745158
WT after UVB irradiation VS WT before UVB irradiation	GO_CALCITONIN_FAMILY_RECEPTOR_ACTIVITY2	0.171480143	-0.04008187	0.694878658	0.50657681	0.85763155	-5.74903669
homo after UVB irradiation VS homo before UVB irradiation	GO_CALCITONIN_FAMILY_RECEPTOR_COMPLEX	0.756130123	-0.08567091	4.120803474	0.00306284	0.03652486	-1.55289413
hetero after UVB irradiation VS hetero before UVB irradiation	GO_CALCITONIN_FAMILY_RECEPTOR_COMPLEX1	0.171315935	0.018060421	0.535498977	0.60713316	0.83580295	-6.07884938
WT after UVB irradiation VS WT before UVB irradiation	GO_CALCITONIN_FAMILY_RECEPTOR_COMPLEX2	0.221656846	-0.05175302	0.642587559	0.53825615	0.87025652	-5.78388839
homo after UVB irradiation VS homo before UVB irradiation	GO_CALCITONIN_FAMILY_RECEPTOR_SIGNALING_PATHWAY	0.793173322	-0.06426732	4.56266127	0.00166151	0.02776774	-0.93561938
hetero after UVB irradiation VS hetero before UVB irradiation	GO_CALCITONIN_FAMILY_RECEPTOR_SIGNALING_PATHWAY1	0.291500324	0.030704224	0.921900989	0.38402988	0.69552909	-5.79473262
WT after UVB irradiation VS WT before UVB irradiation	GO_CALCITONIN_FAMILY_RECEPTOR_SIGNALING_PATHWAY2	0.371358667	-0.02526895	1.04521852	0.32611676	0.77483578	-5.45748081
homo after UVB irradiation VS homo before UVB irradiation	GO_CALCIUM_ACTIVATED_CATION_CHANNEL_ACTIVITY	0.406460086	0.011087715	3.064534336	0.01474988	0.08257891	-3.13332707
hetero after UVB irradiation VS hetero before UVB irradiation	GO_CALCIUM_ACTIVATED_CATION_CHANNEL_ACTIVITY1	0.045422684	-0.03334969	0.266374319	0.79681162	0.92123735	-6.19318383
WT after UVB irradiation VS WT before UVB irradiation	GO_CALCIUM_ACTIVATED_CATION_CHANNEL_ACTIVITY2	0.02029456	0.045745627	0.102370755	0.9209521	0.98043563	-5.9905408
homo after UVB irradiation VS homo before UVB irradiation	GO_CALCIUM_ACTIVATED_PHOSPHOLIPID_SCRAMBLING	0.350662855	-0.06581648	1.133698186	0.28849461	0.48352752	-5.89585972
hetero after UVB irradiation VS hetero before UVB irradiation	GO_CALCIUM_ACTIVATED_PHOSPHOLIPID_SCRAMBLING1	-0.07132208	-0.01402682	-0.38416475	0.71104973	0.88637166	-6.1522198
WT after UVB irradiation VS WT before UVB irradiation	GO_CALCIUM_ACTIVATED_PHOSPHOLIPID_SCRAMBLING2	0.002879204	0.151784994	0.011188849	0.99134353	0.9985657	-5.99600061
homo after UVB irradiation VS homo before UVB irradiation	GO_CALCIUM_ACTIVATED_POTASSIUM_CHANNEL_ACTIVITY	0.228173246	-0.00849332	1.685386985	0.12890737	0.29415769	-5.21380014
hetero after UVB irradiation VS hetero before UVB irradiation	GO_CALCIUM_ACTIVATED_POTASSIUM_CHANNEL_ACTIVITY1	0.013103608	-0.05981942	0.072918065	0.94369437	0.97923521	-6.22860165
WT after UVB irradiation VS WT before UVB irradiation	GO_CALCIUM_ACTIVATED_POTASSIUM_CHANNEL_ACTIVITY2	-0.17784672	0.050457592	-0.93437426	0.37713218	0.80338597	-5.55998093
homo after UVB irradiation VS homo before UVB irradiation	GO_CALCIUM_AND_CALMODULIN_DEPENDENT_PROTEIN_KINASE_COMPLEX	0.446499355	-0.00850464	2.128175664	0.06463381	0.19270157	-4.57584897
hetero after UVB irradiation VS hetero before UVB irradiation	GO_CALCIUM_AND_CALMODULIN_DEPENDENT_PROTEIN_KINASE_COMPLEX1	-0.0634535	0.06761892	-0.17042071	0.86898818	0.95068131	-6.21576389
WT after UVB irradiation VS WT before UVB irradiation	GO_CALCIUM_AND_CALMODULIN_DEPENDENT_PROTEIN_KINASE_COMPLEX2	-0.19421412	-0.06593393	-0.57298496	0.582212	0.8891784	-5.82645966
homo after UVB irradiation VS homo before UVB irradiation	GO_CALCIUM_CATION_ANTIPORTER_ACTIVITY	-0.34854046	0.030904719	-2.22477354	0.05546898	0.17490506	-4.43040873
hetero after UVB irradiation VS hetero before UVB irradiation	GO_CALCIUM_CATION_ANTIPORTER_ACTIVITY1	-0.09210905	0.01782467	-0.47794266	0.64571205	0.85438728	-6.1094396
WT after UVB irradiation VS WT before UVB irradiation	GO_CALCIUM_CATION_ANTIPORTER_ACTIVITY2	-0.12691139	0.052824955	-0.77568439	0.45998708	0.83947926	-5.69050819
homo after UVB irradiation VS homo before UVB irradiation	GO_CALCIUM_CHANNEL_COMPLEX	0.322335448	-0.01758988	1.953673767	0.08506784	0.2270476	-4.83377751
hetero after UVB irradiation VS hetero before UVB irradiation	GO_CALCIUM_CHANNEL_COMPLEX1	-0.14588612	0.007051522	-0.79891834	0.44782536	0.74157085	-5.89919831
WT after UVB irradiation VS WT before UVB irradiation	GO_CALCIUM_CHANNEL_COMPLEX2	0.090839343	0.008284016	0.430682574	0.67793345	0.91685973	-5.89936516
homo after UVB irradiation VS homo before UVB irradiation	GO_CALCIUM_CHANNEL_INHIBITOR_ACTIVITY	0.797250618	0.033000111	4.469642527	0.00188533	0.02931694	-1.06308131
hetero after UVB irradiation VS hetero before UVB irradiation	GO_CALCIUM_CHANNEL_INHIBITOR_ACTIVITY1	0.424914637	0.034608278	3.12300627	0.014497	0.17916057	-2.86138852
WT after UVB irradiation VS WT before UVB irradiation	GO_CALCIUM_CHANNEL_INHIBITOR_ACTIVITY2	0.539637529	0.027143457	1.935304994	0.08854997	0.5809123	-4.39268966
homo after UVB irradiation VS homo before UVB irradiation	GO_CALCIUM_CHANNEL_REGULATOR_ACTIVITY	0.319602349	0.021559314	1.986864491	0.08075315	0.21966345	-4.78526909
hetero after UVB irradiation VS hetero before UVB irradiation	GO_CALCIUM_CHANNEL_REGULATOR_ACTIVITY1	0.036813228	0.062822361	0.220301298	0.83125585	0.9354456	-6.20524851
WT after UVB irradiation VS WT before UVB irradiation	GO_CALCIUM_CHANNEL_REGULATOR_ACTIVITY2	0.120391578	0.019004455	0.598461251	0.56589191	0.88229844	-5.81139147
homo after UVB irradiation VS homo before UVB irradiation	GO_CALCIUM_DEPENDENT_CELL_CELL_ADHESION_VIA_PLASMA_MEMBRANE_CELL_ADHESION_MOLECULES	0.508522523	0.039417351	3.505440617	0.00752107	0.0575368	-2.45870986
hetero after UVB irradiation VS hetero before UVB irradiation	GO_CALCIUM_DEPENDENT_CELL_CELL_ADHESION_VIA_PLASMA_MEMBRANE_CELL_ADHESION_MOLECULES1	0.309290356	0.02813347	1.989852254	0.08246401	0.36807092	-4.50810711
WT after UVB irradiation VS WT before UVB irradiation	GO_CALCIUM_DEPENDENT_CELL_CELL_ADHESION_VIA_PLASMA_MEMBRANE_CELL_ADHESION_MOLECULES2	0.232776828	0.04104512	1.020330144	0.33708448	0.78034487	-5.48125879
homo after UVB irradiation VS homo before UVB irradiation	GO_CALCIUM_DEPENDENT_CYSTEINE_TYPE_ENDOPEPTIDASE_ACTIVITY	0.220933771	-0.04848881	1.189275422	0.26712668	0.4623491	-5.83508173
hetero after UVB irradiation VS hetero before UVB irradiation	GO_CALCIUM_DEPENDENT_CYSTEINE_TYPE_ENDOPEPTIDASE_ACTIVITY1	-0.01677676	-0.00633291	-0.06197072	0.95213439	0.9818782	-6.22940198
WT after UVB irradiation VS WT before UVB irradiation	GO_CALCIUM_DEPENDENT_CYSTEINE_TYPE_ENDOPEPTIDASE_ACTIVITY2	0.261070255	0.037949602	1.140265602	0.28677663	0.75424544	-5.36282823
homo after UVB irradiation VS homo before UVB irradiation	GO_CALCIUM_DEPENDENT_PHOSPHOLIPASE_A2_ACTIVITY	0.136218828	0.065684522	0.650054005	0.53317241	0.69993997	-6.32474625
hetero after UVB irradiation VS hetero before UVB irradiation	GO_CALCIUM_DEPENDENT_PHOSPHOLIPASE_A2_ACTIVITY1	0.210430924	0.054543391	1.391791726	0.20213917	0.52671122	-5.29674671
WT after UVB irradiation VS WT before UVB irradiation	GO_CALCIUM_DEPENDENT_PHOSPHOLIPASE_A2_ACTIVITY2	0.1273149	0.026314997	0.519397837	0.61737778	0.90020288	-5.85618056
homo after UVB irradiation VS homo before UVB irradiation	GO_CALCIUM_DEPENDENT_PHOSPHOLIPID_BINDING	0.258250129	0.021024718	1.648258345	0.13641578	0.30519825	-5.26440029
hetero after UVB irradiation VS hetero before UVB irradiation	GO_CALCIUM_DEPENDENT_PHOSPHOLIPID_BINDING1	0.222047338	0.008943187	1.51522024	0.16888332	0.49068232	-5.14500615
WT after UVB irradiation VS WT before UVB irradiation	GO_CALCIUM_DEPENDENT_PHOSPHOLIPID_BINDING2	0.160079921	0.024276923	1.037612688	0.32943879	0.77711776	-5.46479293
homo after UVB irradiation VS homo before UVB irradiation	GO_CALCIUM_DEPENDENT_PROTEIN_BINDING	0.415437604	-0.00084888	2.667547141	0.0274837	0.11721876	-3.74943495
hetero after UVB irradiation VS hetero before UVB irradiation	GO_CALCIUM_DEPENDENT_PROTEIN_BINDING1	0.23705172	0.009206233	1.59944787	0.14909608	0.46710442	-5.03757905
WT after UVB irradiation VS WT before UVB irradiation	GO_CALCIUM_DEPENDENT_PROTEIN_BINDING2	0.292309778	0.012780539	1.672855008	0.13243817	0.63860701	-4.740263
homo after UVB irradiation VS homo before UVB irradiation	GO_CALCIUM_DEPENDENT_PROTEIN_KINASE_ACTIVITY	-0.08642854	-0.01981022	-0.5388602	0.60408322	0.75295766	-6.39426454
hetero after UVB irradiation VS hetero before UVB irradiation	GO_CALCIUM_DEPENDENT_PROTEIN_KINASE_ACTIVITY1	-0.03863117	-0.01016739	-0.2712888	0.79316394	0.91941572	-6.19176443
WT after UVB irradiation VS WT before UVB irradiation	GO_CALCIUM_DEPENDENT_PROTEIN_KINASE_ACTIVITY2	-0.22642901	-0.07478157	-0.98224758	0.35441065	0.78955887	-5.51679753
homo after UVB irradiation VS homo before UVB irradiation	GO_CALCIUM_DEPENDENT_PROTEIN_SERINE_THREONINE_PHOSPHATASE_ACTIVITY	-0.15624471	0.00927489	-0.77295397	0.46091915	0.64088257	-6.23440983
hetero after UVB irradiation VS hetero before UVB irradiation	GO_CALCIUM_DEPENDENT_PROTEIN_SERINE_THREONINE_PHOSPHATASE_ACTIVITY1	-0.40275772	-0.03773591	-1.64320129	0.13967397	0.45377126	-4.98065874
WT after UVB irradiation VS WT before UVB irradiation	GO_CALCIUM_DEPENDENT_PROTEIN_SERINE_THREONINE_PHOSPHATASE_ACTIVITY2	-0.1300685	-0.16940585	-0.33683362	0.74480608	0.93574017	-5.9366418
homo after UVB irradiation VS homo before UVB irradiation	GO_CALCIUM_IMPORT_INTO_THE_MITOCHONDRION	-0.23270214	-0.01341533	-1.00749698	0.34206422	0.53512479	-6.02581929

hetero after UVB irradiation VS hetero before UVB irradiation	GO_CALCIUM_IMPORT_INTO_THE_MITOCHONDRION1	-0.41870583	-0.0303722	-2.35913109	0.04659271	0.28821887	-3.97738738
WT after UVB irradiation VS WT before UVB irradiation	GO_CALCIUM_IMPORT_INTO_THE_MITOCHONDRION2	-0.06337055	-0.05910059	-0.29697092	0.77396389	0.94274743	-5.94979909
homo after UVB irradiation VS homo before UVB irradiation	GO_CALCIUM_INDEPENDENT_CELL_CELL_ADHESION_VIA_PLASMA_MEMBRANE_CELL_ADHESION_MOLECULES	-0.61981632	0.056494857	-4.79974094	0.00121091	0.02347563	-0.61689433
hetero after UVB irradiation VS hetero before UVB irradiation	GO_CALCIUM_INDEPENDENT_CELL_CELL_ADHESION_VIA_PLASMA_MEMBRANE_CELL_ADHESION_MOLECULES1	-0.29828367	0.045439193	-2.28246725	0.05246701	0.30468446	-4.08894585
WT after UVB irradiation VS WT before UVB irradiation	GO_CALCIUM_INDEPENDENT_CELL_CELL_ADHESION_VIA_PLASMA_MEMBRANE_CELL_ADHESION_MOLECULES2	-0.75779715	0.005705713	-5.21432678	0.00077738	0.18842845	-0.14416634
homo after UVB irradiation VS homo before UVB irradiation	GO_CALCIUM_INDEPENDENT_PHOSPHOLIPASE_A2_ACTIVITY	0.123536028	-0.00979575	0.714616224	0.49438446	0.66969263	-6.2790078
hetero after UVB irradiation VS hetero before UVB irradiation	GO_CALCIUM_INDEPENDENT_PHOSPHOLIPASE_A2_ACTIVITY1	0.26187541	0.002792618	1.480668744	0.17766328	0.50135768	-5.18819746
WT after UVB irradiation VS WT before UVB irradiation	GO_CALCIUM_INDEPENDENT_PHOSPHOLIPASE_A2_ACTIVITY2	0.03045508	-0.02192084	0.13661115	0.89467359	0.97322079	-5.98623135
homo after UVB irradiation VS homo before UVB irradiation	GO_CALCIUM_ION_BINDING	0.402179731	-0.00049058	3.28999343	0.01042347	0.06834604	-2.78633916
hetero after UVB irradiation VS hetero before UVB irradiation	GO_CALCIUM_ION_BINDING1	0.245850744	0.026582507	1.561898352	0.1576418	0.47686805	-5.08583288
WT after UVB irradiation VS WT before UVB irradiation	GO_CALCIUM_ION_BINDING2	0.185977613	0.018737632	1.036216683	0.33005136	0.77726938	-5.46613068
homo after UVB irradiation VS homo before UVB irradiation	GO_CALCIUM_ION_EXPORT_ACROSS_PLASMA_MEMBRANE	-0.88889016	0.025703914	-6.78505288	0.00011596	0.01007802	1.710624979
hetero after UVB irradiation VS hetero before UVB irradiation	GO_CALCIUM_ION_EXPORT_ACROSS_PLASMA_MEMBRANE1	-0.8101532	-0.02401702	-4.27688	0.00281687	0.11250952	-1.28209012
WT after UVB irradiation VS WT before UVB irradiation	GO_CALCIUM_ION_EXPORT_ACROSS_PLASMA_MEMBRANE2	-0.60338308	-0.01340665	-3.28673414	0.01089118	0.33677148	-2.4946284
homo after UVB irradiation VS homo before UVB irradiation	GO_CALCIUM_ION_IMPORT	0.181026599	-0.02200029	1.166757449	0.27562417	0.4715263	-5.859957
homo after UVB irradiation VS homo before UVB irradiation	GO_CALCIUM_ION_IMPORT_INTO_CYTOSOL	0.092727631	-0.00870182	0.591797019	0.56969696	0.72781733	-6.36265632
hetero after UVB irradiation VS hetero before UVB irradiation	GO_CALCIUM_ION_IMPORT_INTO_CYTOSOL1	-0.14052611	0.02795204	-0.84395883	0.42365511	0.72539508	-5.86236701
WT after UVB irradiation VS WT before UVB irradiation	GO_CALCIUM_ION_IMPORT_INTO_CYTOSOL2	-0.08307752	0.078985096	-0.53097013	0.60968976	0.89837162	-5.84999096
homo after UVB irradiation VS homo before UVB irradiation	GO_CALCIUM_ION_IMPORT_INTO_SARCOPLASMIC_RETICULUM	-0.27372184	-0.01501687	-1.33585749	0.21694265	0.40756494	-5.66529744
hetero after UVB irradiation VS hetero before UVB irradiation	GO_CALCIUM_ION_IMPORT_INTO_SARCOPLASMIC_RETICULUM1	0.369081703	-0.01182944	1.024752479	0.33600843	0.65575193	-5.69830972
WT after UVB irradiation VS WT before UVB irradiation	GO_CALCIUM_ION_IMPORT_INTO_SARCOPLASMIC_RETICULUM2	0.20126278	-0.07396472	0.771545139	0.46230247	0.84099789	-5.69364085
hetero after UVB irradiation VS hetero before UVB irradiation	GO_CALCIUM_ION_IMPORT1	-0.06239251	-0.02025017	-0.39641099	0.70235574	0.88151888	-6.14713822
WT after UVB irradiation VS WT before UVB irradiation	GO_CALCIUM_ION_IMPORT2	0.12084588	0.035195703	0.661881048	0.52643055	0.86617234	-5.77131167
homo after UVB irradiation VS homo before UVB irradiation	GO_CALCIUM_ION_REGULATED_EXOCYTOSIS	0.149691031	-0.0008359	1.061092754	0.31844319	0.5119584	-5.97203975
homo after UVB irradiation VS homo before UVB irradiation	GO_CALCIUM_ION_REGULATED_EXOCYTOSIS_OF_NEUROTRANSMITTER	-0.36038296	0.027733218	-2.53878583	0.03369954	0.13111101	-3.94903267
hetero after UVB irradiation VS hetero before UVB irradiation	GO_CALCIUM_ION_REGULATED_EXOCYTOSIS_OF_NEUROTRANSMITTER1	-0.09208177	-0.01864056	-0.53823155	0.60533251	0.83488689	-6.07731669
WT after UVB irradiation VS WT before UVB irradiation	GO_CALCIUM_ION_REGULATED_EXOCYTOSIS_OF_NEUROTRANSMITTER2	-0.42675338	-0.04899463	-2.76808675	0.02408754	0.42212473	-3.22102804
hetero after UVB irradiation VS hetero before UVB irradiation	GO_CALCIUM_ION_REGULATED_EXOCYTOSIS1	0.056852919	-0.01363878	0.382810321	0.71201408	0.88684729	-6.15277241
WT after UVB irradiation VS WT before UVB irradiation	GO_CALCIUM_ION_REGULATED_EXOCYTOSIS2	-0.03408338	-0.00440058	-0.23185349	0.82240369	0.95836402	-5.96780168
homo after UVB irradiation VS homo before UVB irradiation	GO_CALCIUM_ION_TRANSMEMBRANE_IMPORT_INTO_CYTOSOL	0.364833255	-0.02193454	2.73278127	0.02479346	0.1107919	-3.64811371
hetero after UVB irradiation VS hetero before UVB irradiation	GO_CALCIUM_ION_TRANSMEMBRANE_IMPORT_INTO_CYTOSOL1	0.197862016	0.022756663	1.22311	0.25672187	0.58385284	-5.49146526
WT after UVB irradiation VS WT before UVB irradiation	GO_CALCIUM_ION_TRANSMEMBRANE_IMPORT_INTO_CYTOSOL2	0.186019416	0.004440547	0.846370542	0.42165181	0.82436616	-5.63483661
homo after UVB irradiation VS homo before UVB irradiation	GO_CALCIUM_ION_TRANSMEMBRANE_TRANSPORT	0.182768648	-0.00540276	1.367673051	0.20717506	0.39730765	-5.62675366
homo after UVB irradiation VS homo before UVB irradiation	GO_CALCIUM_ION_TRANSMEMBRANE_TRANSPORT_VIA_HIGH_VOLTAGE_GATED_CALCIUM_CHANNEL	0.583806418	-0.01222443	2.237807363	0.05433464	0.17284247	-4.41066517
hetero after UVB irradiation VS hetero before UVB irradiation	GO_CALCIUM_ION_TRANSMEMBRANE_TRANSPORT_VIA_HIGH_VOLTAGE_GATED_CALCIUM_CHANNEL1	-0.04566396	-0.03324412	-0.14082091	0.89155666	0.96027811	-6.22074392
WT after UVB irradiation VS WT before UVB irradiation	GO_CALCIUM_ION_TRANSMEMBRANE_TRANSPORT_VIA_HIGH_VOLTAGE_GATED_CALCIUM_CHANNEL2	0.174976313	-0.05614988	0.559185906	0.59116032	0.89248172	-5.83437023
hetero after UVB irradiation VS hetero before UVB irradiation	GO_CALCIUM_ION_TRANSMEMBRANE_TRANSPORT1	0.034792597	0.016592247	0.225962331	0.82700088	0.93413454	-6.20388743
WT after UVB irradiation VS WT before UVB irradiation	GO_CALCIUM_ION_TRANSMEMBRANE_TRANSPORT2	0.073051278	0.000137638	0.379231871	0.71426701	0.92987478	-5.92088803
homo after UVB irradiation VS homo before UVB irradiation	GO_CALCIUM_ION_TRANSMEMBRANE_TRANSPORTER_ACTIVITY	0.155116456	0.012541805	1.204589174	0.26147086	0.45695158	-5.81797405
hetero after UVB irradiation VS hetero before UVB irradiation	GO_CALCIUM_ION_TRANSMEMBRANE_TRANSPORTER_ACTIVITY1	-0.02287232	0.020565591	-0.1496362	0.88482334	0.95804067	-6.21935922
WT after UVB irradiation VS WT before UVB irradiation	GO_CALCIUM_ION_TRANSMEMBRANE_TRANSPORTER_ACTIVITY2	0.063767401	0.019026479	0.377584927	0.71544358	0.93029655	-5.92153359
homo after UVB irradiation VS homo before UVB irradiation	GO_CALCIUM_MEDIATED_SIGNALING	0.460164038	-0.01335396	3.446145426	0.00822321	0.06037312	-2.54842532
homo after UVB irradiation VS homo before UVB irradiation	GO_CALCIUM_MEDIATED_SIGNALING_USING_INTRACELLULAR_CALCIUM_SOURCE	0.688632809	-0.00349141	4.996378546	0.00093725	0.02089269	-0.35928844
hetero after UVB irradiation VS hetero before UVB irradiation	GO_CALCIUM_MEDIATED_SIGNALING_USING_INTRACELLULAR_CALCIUM_SOURCE1	0.316237055	0.030637598	1.844516355	0.10303089	0.39866653	-4.71031932
WT after UVB irradiation VS WT before UVB irradiation	GO_CALCIUM_MEDIATED_SIGNALING_USING_INTRACELLULAR_CALCIUM_SOURCE2	0.485077357	0.088847702	2.484465294	0.03750633	0.47615783	-3.62442698
hetero after UVB irradiation VS hetero before UVB irradiation	GO_CALCIUM_MEDIATED_SIGNALING1	0.246570989	0.012474603	1.411809427	0.19638148	0.52060431	-5.27263213
WT after UVB irradiation VS WT before UVB irradiation	GO_CALCIUM_MEDIATED_SIGNALING2	0.274889035	-0.00013855	1.315897694	0.22423823	0.71904338	-5.17322081
homo after UVB irradiation VS homo before UVB irradiation	GO_CALCIUM_POTASSIUM_SODIUM_ANTIPORTER_ACTIVITY	-0.33643998	0.070320451	-1.56485341	0.15475901	0.32926451	-5.37598085
hetero after UVB irradiation VS hetero before UVB irradiation	GO_CALCIUM_POTASSIUM_SODIUM_ANTIPORTER_ACTIVITY1	-0.26962381	0.037965936	-1.56775717	0.15627973	0.4751771	-5.07834133
WT after UVB irradiation VS WT before UVB irradiation	GO_CALCIUM_POTASSIUM_SODIUM_ANTIPORTER_ACTIVITY2	-0.33254507	0.054983957	-1.5628743	0.15625515	0.66180255	-4.87940898
homo after UVB irradiation VS homo before UVB irradiation	GO_CALCIUM_RELEASE_CHANNEL_ACTIVITY	0.698396041	-0.07935671	4.397563734	0.00208111	0.03080331	-1.16277268
hetero after UVB irradiation VS hetero before UVB irradiation	GO_CALCIUM_RELEASE_CHANNEL_ACTIVITY1	0.13137313	-0.03076971	0.579522499	0.57847873	0.8187705	-6.05328242
WT after UVB irradiation VS WT before UVB irradiation	GO_CALCIUM_RELEASE_CHANNEL_ACTIVITY2	0.043522527	0.022753573	0.202060249	0.8448537	0.96467526	-5.97458098
homo after UVB irradiation VS homo before UVB irradiation	GO_CALCIUM_SENSITIVE_GUANYLATE_CYCLASE_ACTIVATOR_ACTIVITY	0.208431756	0.001034683	0.840463201	0.42411622	0.6090583	-6.17905694
hetero after UVB irradiation VS hetero before UVB irradiation	GO_CALCIUM_SENSITIVE_GUANYLATE_CYCLASE_ACTIVATOR_ACTIVITY1	-0.00841162	0.032865194	-0.03011436	0.97672713	0.99212405	-6.23099234
WT after UVB irradiation VS WT before UVB irradiation	GO_CALCIUM_SENSITIVE_GUANYLATE_CYCLASE_ACTIVATOR_ACTIVITY2	0.126480855	0.03369216	0.567191528	0.58595968	0.89098475	-5.82980243
homo after UVB irradiation VS homo before UVB irradiation	GO_CALCIUM_TRANSMEMBRANE_TRANSPORTER_ACTIVITY_PHOSPHORYLATIVE_MECHANISM	-0.2308069	-0.02673219	-1.44204021	0.18581345	0.37029697	-5.53449116
hetero after UVB irradiation VS hetero before UVB irradiation	GO_CALCIUM_TRANSMEMBRANE_TRANSPORTER_ACTIVITY_PHOSPHORYLATIVE_MECHANISM1	-0.2232798	-0.02898668	-1.06808242	0.31721478	0.63719321	-5.65537906
WT after UVB irradiation VS WT before UVB irradiation	GO_CALCIUM_TRANSMEMBRANE_TRANSPORTER_ACTIVITY_PHOSPHORYLATIVE_MECHANISM2	-0.05121866	-0.04667808	-0.21447784	0.83547709	0.96163722	-5.9718672
homo after UVB irradiation VS homo before UVB irradiation	GO_CALMODULIN_BINDING	0.098315443	-0.00542838	0.743087691	0.47786139	0.65567647	-6.25762344
hetero after UVB irradiation VS hetero before UVB irradiation	GO_CALMODULIN_BINDING1	-0.10540095	-0.0041972	-0.76300963	0.46775753	0.7530936	-5.92733867
WT after UVB irradiation VS WT before UVB irradiation	GO_CALMODULIN_BINDING2	-0.02240456	0.007761782	-0.13730787	0.89414019	0.97322079	-5.9861309
homo after UVB irradiation VS homo before UVB irradiation	GO_CALMODULIN_DEPENDENT_KINASE_SIGNALING_PATHWAY	0.602507764	-0.03637388	3.653180309	0.0060333	0.05133852	-2.23685746
hetero after UVB irradiation VS hetero before UVB irradiation	GO_CALMODULIN_DEPENDENT_KINASE_SIGNALING_PATHWAY1	0.624234042	0.047038401	2.644172181	0.03000086	0.24204081	-3.55986967
WT after UVB irradiation VS WT before UVB irradiation	GO_CALMODULIN_DEPENDENT_KINASE_SIGNALING_PATHWAY2	0.735716055	-0.0562128	4.188805873	0.00296194	0.24418663	-1.31454316
homo after UVB irradiation VS homo before UVB irradiation	GO_CALMODULIN_DEPENDENT_PROTEIN_KINASE_ACTIVITY	0.142175043	-0.03566535	0.677481633	0.51647313	0.68693952	-6.30578742
hetero after UVB irradiation VS hetero before UVB irradiation	GO_CALMODULIN_DEPENDENT_PROTEIN_KINASE_ACTIVITY1	-0.21129793	-0.07143536	-1.16210459	0.27929314	0.60499848	-5.55781485
WT after UVB irradiation VS WT before UVB irradiation	GO_CALMODULIN_DEPENDENT_PROTEIN_KINASE_ACTIVITY2	-0.03256354	-0.06067897	-0.13755528	0.89395079	0.97322079	-5.9860951
homo after UVB irradiation VS homo before UVB irradiation	GO_CALMODULIN_DEPENDENT_PROTEIN_PHOSPHATASE_ACTIVITY	-0.28379302	0.016591485	-1.18341156	0.26931868	0.46464949	-5.84159181
hetero after UVB irradiation VS hetero before UVB irradiation	GO_CALMODULIN_DEPENDENT_PROTEIN_PHOSPHATASE_ACTIVITY1	-0.41334191	-0.0315086	-1.70588369	0.1271299	0.43374076	-4.89789441
WT after UVB irradiation VS WT before UVB irradiation	GO_CALMODULIN_DEPENDENT_PROTEIN_PHOSPHATASE_ACTIVITY2	-0.04505718	-0.15051398	-0.1176464	0.90921367	0.97832631	-5.98877024
homo after UVB irradiation VS homo before UVB irradiation	GO_CALYX_OF_HELD	0.426859415	-0.0205472	3.077741776	0.01445085	0.08195662	-3.11291017
hetero after UVB irradiation VS hetero before UVB irradiation	GO_CALYX_OF_HELD1	0.079240614	0.019467308	0.452372853	0.6632354	0.86213521	-6.12198075
WT after UVB irradiation VS WT before UVB irradiation	GO_CALYX_OF_HELD2	0.03482623	-0.03975522	0.155568159	0.88018158	0.9693507	-5.98331696
homo after UVB irradiation VS homo before UVB irradiation	GO_CAMERA_TYPE_EYE_DEVELOPMENT	-0.02814895	0.022251778	-0.22328626	0.82869177	0.90417778	-6.52357749
hetero after UVB irradiation VS hetero before UVB irradiation	GO_CAMERA_TYPE_EYE_DEVELOPMENT1	-0.06250529	0.021283746	-0.47771516	0.64586694	0.85445868	-6.10955405
WT after UVB irradiation VS WT before UVB irradiation	GO_CAMERA_TYPE_EYE_DEVELOPMENT2	-0.09641039	0.024925428	-0.67372899	0.51924766	0.86260697	-5.76342424
homo after UVB irradiation VS homo before UVB irradiation	GO_CAMERA_TYPE_EYE_MORPHOGENESIS	-0.05789759	0.019437335	-0.38348661	0.71096227	0.827472	-6.47086435
hetero after UVB irradiation VS hetero before UVB irradiation	GO_CAMERA_TYPE_EYE_MORPHOGENESIS1	-0.08184487	0.02249279	-0.63902841	0.54099146	0.79694618	-6.01580522
WT after UVB irradiation VS WT before UVB irradiation	GO_CAMERA_TYPE_EYE_MORPHOGENESIS2	-0.08391126	0.013358267	-0.52420718	0.61417654	0.90020288	-5.85362363
homo after UVB irradiation VS homo before UVB irradiation	GO_CAMERA_TYPE_EYE_PHOTORECEPTOR_CELL_DIFFERENTIATION	0.155058332	0.031006438	0.73278095	0.48380107	0.66087682	-6.26544922
hetero after UVB irradiation VS hetero before UVB irradiation	GO_CAMERA_TYPE_EYE_PHOTORECEPTOR_CELL_DIFFERENTIATION1	-0.33415988	0.02908098	-2.37315067	0.04559212	0.28518751	-3.95693601
WT after UVB irradiation VS WT before UVB irradiation	GO_CAMERA_TYPE_EYE_PHOTORECEPTOR_CELL_DIFFERENTIATION2	0.023801901	-0.01902712	0.10568487	0.91840355	0.98043563	-5.99017749
homo after UVB irradiation VS homo before UVB irradiation	GO_CAMP_BINDING	0.283140887	-0.04285107	1.958970138	0.08436481	0.22590663	-4.8260559
hetero after UVB irradiation VS hetero before UVB irradiation	GO_CAMP_BINDING1	0.034331031	-0.01384323	0.158762386	0.87786307	0.95535896	-6.21783768
WT after UVB irradiation VS WT before UVB irradiation	GO_CAMP_BINDING2	0.087785575	-0.05676122	0.318932442	0.75784935	0.93908133	-5.94274974
homo after UVB irradiation VS homo before UVB irradiation	GO_CAMP_BIOSYNTHETIC_PROCESS	0.323321198	-0.05413826	1.13161573	0.28932122	0.48410448	-5.8980963
hetero after UVB irradiation VS hetero before UVB irradiation	GO_CAMP_BIOSYNTHETIC_PROCESS1	0.078574089	0.055800206	0.395059439	0.70331299	0.88183935	-6.14770656
WT after UVB irradiation VS WT before UVB irradiation	GO_CAMP_BIOSYNTHETIC_PROCESS2	0.198845191	-0.01197142	0.745087931	0.47728461	0.84910837	-5.71332423
homo after UVB irradiation VS homo before UVB irradiation	GO_CAMP_CATABOLIC_PROCESS	0.491138668	-0.04724143	2.128975973	0.0645521	0.19256021	-4.57465092
hetero after UVB irradiation VS hetero before UVB irradiation	GO_CAMP_CATABOLIC_PROCESS1	0.183254965	-0.05268216	0.763583236	0.46743454	0.7530936	-5.92689783
WT after UVB irradiation VS WT before UVB irradiation	GO_CAMP_CATABOLIC_PROCESS2	-0.07197714	-0.02071488	-0.25114795	0.8079555	0.95404687	-5.96292173
homo after UVB irradiation VS homo before UVB irradiation	GO_CAMP_DEPENDENT_PROTEIN_KINASE_ACTIVITY	0.521398361	-0.0185579	2.176609404	0.05986674	0.18416791	-4.50312963
hetero after UVB irradiation VS hetero before UVB irradiation	GO_CAMP_DEPENDENT_PROTEIN_KINASE_ACTIVITY1	0.597044869	0.072340749	2.417710815	0.04255325	0.27740933	-3.89184722
WT after UVB irradiation VS WT before UVB irradiation	GO_CAMP_DEPENDENT_PROTEIN_KINASE_ACTIVITY2	0.477207913	-0.12463166	2.457145059	0.03914744	0.48383481	-3.66326532
homo after UVB irradiation VS homo before UVB irradiation	GO_CAMP_DEPENDENT_PROTEIN_KINASE_COMPLEX	0.211959714	0.002341675	1.242079927	0.24803901	0.44241752	-5.7754535
hetero after UVB irradiation VS hetero before UVB irradiation	GO_CAMP_DEPENDENT_PROTEIN_KINASE_COMPLEX1	0.299121993	0.028575368	1.388756873	0.20302485	0.52736467	-5.30038514
WT after UVB irradiation VS WT before UVB irradiation	GO_CAMP_DEPENDENT_PROTEIN_KINASE_COMPLEX2	0.351479208	-0.0408024	1.653048365	0.13646523	0.64462785	-4.76564712
homo after UVB irradiation VS homo before UVB irradiation	GO_CAMP_DEPENDENT_PROTEIN_KINASE_INHIBITOR_ACTIVITY	0.350314853	-0.04738256	2.139792228	0.06345766	0.19052791	-4.55844724
hetero after UVB irradiation VS hetero before UVB irradiation	GO_CAMP_DEPENDENT_PROTEIN_KINASE_INHIBITOR_ACTIVITY1	-0.04615914	-0.01576741	-0.18784335	0.85576246	0.94499137	-6.21239318
WT after UVB irradiation VS WT before UVB irradiation	GO_CAMP_DEPENDENT_PROTEIN_KINASE_INHIBITOR_ACTIVITY2	-0.10272785	-0.07283609	-0.29509818	0.77534357	0.94304554	-5.9503775
homo after UVB irradiation VS homo before UVB irradiation	GO_CAMP_DEPENDENT_PROTEIN_KINASE_REGULATOR_ACTIVITY	0.54568468	-0.04543986	4.250929421	0.00255041	0.03342481	-1.36803601
hetero after UVB irradiation VS hetero before UVB irradiation	GO_CAMP_DEPENDENT_PROTEIN_KINASE_REGULATOR_ACTIVITY1	0.159393416	0.027022976	0.652276428	0.53284691	0.79245001	-6.00701325
WT after UVB irradiation VS WT before UVB irradiation	GO_CAMP_DEPENDENT_PROTEIN_KINASE_REGULATOR_ACTIVITY2	0.170635468	-0.04512158	0.526738826	0.61249492	0.8996674	-5.85226884
homo after UVB irradiation VS homo before UVB irradiation	GO_CAMP_METABOLIC_PROCESS	0.248759947	-0.06295353	1.146393824	0.28349604	0.47855766	-5.88216003
hetero after UVB irradiation VS hetero before UVB irradiation	GO_CAMP_METABOLIC_PROCESS1	0.133200395	-0.01654496	0.668903876	0.52273087	0.78818414	-5.99575089
WT after UVB irradiation VS WT before UVB irradiation	GO_CAMP_METABOLIC_PROCESS2	-0.06365009	-0.00841183	-0.27346633	0.79134035	0.94895251	-5.95679902
homo after UVB irradiation VS homo before UVB irradiation	GO_CAMP_RESPONSE_ELEMENT_BINDING	0.125215382	-0.01072161	0.61275357	0.55639563	0.71743987	-6.34939217
homo after UVB irradiation VS homo before UVB irradiation	GO_CAMP_RESPONSE_ELEMENT_BINDING_PROTEIN_BINDING	0.056925782	-0.04341647	0.199808182	0.84642275	0.91533494	-6.52899937
hetero after UVB irradiation VS hetero before UVB irradiation	GO_CAMP_RESPONSE_ELEMENT_BINDING_PROTEIN_BINDING1	0.160874195	-0.09824931	0.694594133	0.50733603	0.77738842	-5.9778566
WT after UVB irradiation VS WT before UVB irradiation	GO_CAMP_RESPONSE_ELEMENT_BINDING_PROTEIN_BINDING2	-0.11567978	-0.12023727	-0.48946025	0.6374988	0.9057274	-5.87160173
hetero after UVB irradiation VS hetero before UVB irradiation	GO_CAMP_RESPONSE_ELEMENT_BINDING1	0.024922513	-0.02339119	0.150089642	0.88447726	0.95804067	-6.21928574
WT after UVB irradiation VS WT before UVB irradiation	GO_CAMP_RESPONSE_ELEMENT_BINDING2	-0.10731091	-0.02015704	-0.54659604	0.59938997	0.89508174	-5.84143257
homo after UVB irradiation VS homo before UVB irradiation	GO_CANNABINOID_SIGNALING_PATHWAY	-0.10935017	-0.03306317	-0.38417857	0.71046853	0.82736617	-6.47057799
hetero after UVB irradiation VS hetero before UVB irradiation	GO_CANNABINOID_SIGNALING_PATHWAY1	-0.31770065	-0.00286431	-1.09842735	0.30455216	0.62651215	-5.62453675
WT after UVB irradiation VS WT before UVB irradiation	GO_CANNABINOID_SIGNALING_PATHWAY2	-0.01255529	0.006290871	-0.04437843	0.96567762	0.99113261	-5.99502762
homo after UVB irradiation VS homo before UVB irradiation	GO_CANONICAL_WNT_SIGNALING_PATHWAY	-0.17804801	-0.00537674	-1.35776965	0.21017418	0.40040541	-5.63881268
homo after UVB irradiation VS homo before UVB irradiation	GO_CANONICAL_WNT_SIGNALING_PATHWAY_INVOLVED_IN_HEART_DEVELOPMENT	-0.05347337	0.020718703	-0.35471381	0.73161789	0.84150313	-6.48232974
hetero after UVB irradiation VS hetero before UVB irradiation	GO_CANONICAL_WNT_SIGNALING_PATHWAY_INVOLVED_IN_HEART_DEVELOPMENT1	0.217408387	-0.09141893	0.876327729	0.40685958	0.71218831	-5.83486837
WT after UVB irradiation VS WT before UVB irradiation	GO_CANONICAL_WNT_SIGNALING_PATHWAY_INVOLVED_IN_HEART_DEVELOPMENT2	0.303371458	0.082599957	1.315755363	0.22428384	0.71904338	-5.17338155
homo after UVB irradiation VS homo before UVB irradiation	GO_CANONICAL_WNT_SIGNALING_PATHWAY_INVOLVED_IN_OSTEOBLAST_DIFFERENTIATION	-0.19478519	-0.00242954	-0.95438234	0.36676354	0.55705327	-6.07696475
hetero after UVB irradiation VS hetero before UVB irradiation	GO_CANONICAL_WNT_SIGNALING_PATHWAY_INVOLVED_IN_OSTEOBLAST_DIFFERENTIATION1	-0.4762417	0.010750808	-3.25993643	0.01182507	0.16927893	-2.66472829
WT after UVB irradiation VS WT before UVB irradiation	GO_CANONICAL_WNT_SIGNALING_PATHWAY_INVOLVED_IN_OSTEOBLAST_DIFFERENTIATION2	0.12114864	0.014934477	0.588937508	0.57196206	0.88409386	-5.81709442
homo after UVB irradiation VS homo before UVB irradiation	GO_CANONICAL_WNT_SIGNALING_PATHWAY_INVOLVED_IN_REGULATION_OF_CELL_PROLIFERATION	-0.96960932	0.006761569	-6.38279293	0.00017909	0.0106863	1.286205475
hetero after UVB irradiation VS hetero before UVB irradiation	GO_CANONICAL_WNT_SIGNALING_PATHWAY_INVOLVED_IN_REGULATION_OF_CELL_PROLIFERATION1	-0.71569103	-0.12356896	-2.43993774	0.04111469	0.27359812	-3.85933766
WT after UVB irradiation VS WT before UVB irradiation	GO_CANONICAL_WNT_SIGNALING_PATHWAY_INVOLVED_IN_REGULATION_OF_CELL_PROLIFERATION2	-0.66659324	-0.0518858	-2.28134887	0.05157906	0.51572204	-3.91230535
hetero after UVB irradiation VS hetero before UVB irradiation	GO_CANONICAL_WNT_SIGNALING_PATHWAY1	-0.1583105	-0.00150978	-0.98472569	0.35412285	0.67047741	-5.73677144
WT after UVB irradiation VS WT before UVB irradiation	GO_CANONICAL_WNT_SIGNALING_PATHWAY2	-0.1611917	0.011656997	-0.91779954	0.38524432	0.807077	-5.57453445
homo after UVB irradiation VS homo before UVB irradiation	GO_CAP_INDEPENDENT_TRANSLATIONAL_INITIATION	0.004106336	0.067478593	0.013406871	0.98961877	0.99473624	-6.55075807
hetero after UVB irradiation VS hetero before UVB irradiation	GO_CAP_INDEPENDENT_TRANSLATIONAL_INITIATION1	0.208686889	-0.08137094	0.743665741	0.47873632	0.76016713	-5.9420385
WT after UVB irradiation VS WT before UVB irradiation	GO_CAP_INDEPENDENT_TRANSLATIONAL_INITIATION2	-0.06931411	0.027585381	-0.2235618	0.82863527	0.95957725	-5.96978073
homo after UVB irradiation VS homo before UVB irradiation	GO_CARBOHYDRATE_BINDING	0.504304609	-0.00902906	3.965106714	0.00382531	0.04047966	-1.77731383
hetero after UVB irradiation VS hetero before UVB irradiation	GO_CARBOHYDRATE_BINDING1	0.375278301	0.015097817	2.1356482	0.06585459	0.33426479	-4.30085924
WT after UVB irradiation VS WT before UVB irradiation	GO_CARBOHYDRATE_BINDING2	0.315312975	-0.00210704	1.498869876	0.17184051	0.67808426	-4.95821138
homo after UVB irradiation VS homo before UVB irradiation	GO_CARBOHYDRATE_BIOSYNTHETIC_PROCESS	0.330900862	-0.00848611	2.670767647	0.02734414	0.11719938	-3.74443495
hetero after UVB irradiation VS hetero before UVB irradiation	GO_CARBOHYDRATE_BIOSYNTHETIC_PROCESS1	0.142546653	-0.00358339	0.900117292	0.39482304	0.7032845	-5.81412163
WT after UVB irradiation VS WT before UVB irradiation	GO_CARBOHYDRATE_BIOSYNTHETIC_PROCESS2	0.227650431	-0.00420637	1.392330133	0.20086548	0.69762521	-5.08538241
homo after UVB irradiation VS homo before UVB irradiation	GO_CARBOHYDRATE_CATABOLIC_PROCESS	0.166939679	-0.013082	1.238862185	0.24916896	0.44359831	-5.77913796
hetero after UVB irradiation VS hetero before UVB irradiation	GO_CARBOHYDRATE_CATABOLIC_PROCESS1	0.13940186	0.006571288	0.999318941	0.34743424	0.66476179	-5.72288431
WT after UVB irradiation VS WT before UVB irradiation	GO_CARBOHYDRATE_CATABOLIC_PROCESS2	0.242405277	-0.0196087	1.632343102	0.14079509	0.65097221	-4.79203522
homo after UVB irradiation VS homo before UVB irradiation	GO_CARBOHYDRATE_CATION_SYMPORTER_ACTIVITY	0.21673415	0.011765865	1.217670003	0.25671787	0.45220065	-5.80324032
hetero after UVB irradiation VS hetero before UVB irradiation	GO_CARBOHYDRATE_CATION_SYMPORTER_ACTIVITY1	0.077006559	0.065028117	0.332274689	0.74837738	0.90107174	-6.17204257
WT after UVB irradiation VS WT before UVB irradiation	GO_CARBOHYDRATE_CATION_SYMPORTER_ACTIVITY2	0.010213459	0.064096571	0.046900328	0.96372874	0.99054347	-5.99490619
homo after UVB irradiation VS homo before UVB irradiation	GO_CARBOHYDRATE_DERIVATIVE_BIOSYNTHETIC_PROCESS	0.202514484	-0.02158286	1.565306045	0.15465371	0.32926451	-5.37538349
hetero after UVB irradiation VS hetero before UVB irradiation	GO_CARBOHYDRATE_DERIVATIVE_BIOSYNTHETIC_PROCESS1	0.113632311	-0.01452585	0.746992052	0.47683647	0.7595751	-5.9395339
WT after UVB irradiation VS WT before UVB irradiation	GO_CARBOHYDRATE_DERIVATIVE_BIOSYNTHETIC_PROCESS2	0.202093165	-0.0215734	1.301409441	0.2289218	0.72137498	-5.18952716
homo after UVB irradiation VS homo before UVB irradiation	GO_CARBOHYDRATE_DERIVATIVE_CATABOLIC_PROCESS	0.377904998	-0.0241637	2.917160947	0.01855736	0.09355293	-3.36167949
hetero after UVB irradiation VS hetero before UVB irradiation	GO_CARBOHYDRATE_DERIVATIVE_CATABOLIC_PROCESS1	0.291884707	-0.00797238	2.071393857	0.07272679	0.34944857	-4.39265757
WT after UVB irradiation VS WT before UVB irradiation	GO_CARBOHYDRATE_DERIVATIVE_CATABOLIC_PROCESS2	0.350609279	-0.00868148	2.226786317	0.05618668	0.5232848	-3.98912794
homo after UVB irradiation VS homo before UVB irradiation	GO_CARBOHYDRATE_DERIVATIVE_METABOLIC_PROCESS	0.209333675	-0.01880176	1.652882046	0.13545936	0.3037031	-5.25812902
hetero after UVB irradiation VS hetero before UVB irradiation	GO_CARBOHYDRATE_DERIVATIVE_METABOLIC_PROCESS1	0.135059089	-0.01080671	0.929610521	0.38026235	0.69277918	-5.78778204
WT after UVB irradiation VS WT before UVB irradiation	GO_CARBOHYDRATE_DERIVATIVE_METABOLIC_PROCESS2	0.217995948	-0.01665292	1.482287233	0.17609885	0.68050466	-4.97834456

homo after UVB irradiation VS homo before UVB irradiation	GO_CARBOHYDRATE_DERIVATIVE_TRANSMEMBRANE_TRANSPORTER_ACTIVITY	0.054708539	0.005553648	0.435171795	0.67449408	0.80373441	-6.44811121
hetero after UVB irradiation VS hetero before UVB irradiation	GO_CARBOHYDRATE_DERIVATIVE_TRANSMEMBRANE_TRANSPORTER_ACTIVITY1	-0.00470355	0.016884938	-0.02989719	0.97689491	0.99212405	-6.2309994
WT after UVB irradiation VS WT before UVB irradiation	GO_CARBOHYDRATE_DERIVATIVE_TRANSMEMBRANE_TRANSPORTER_ACTIVITY2	0.137304028	-0.0246286	0.795216028	0.44916646	0.83490988	-5.67553418
homo after UVB irradiation VS homo before UVB irradiation	GO_CARBOHYDRATE_DERIVATIVE_TRANSPORT	0.183156014	0.00713523	1.425695062	0.1903363	0.37616082	-5.55502212
hetero after UVB irradiation VS hetero before UVB irradiation	GO_CARBOHYDRATE_DERIVATIVE_TRANSPORT1	0.165146482	0.021966026	1.195924999	0.26658655	0.59300892	-5.52131638
WT after UVB irradiation VS WT before UVB irradiation	GO_CARBOHYDRATE_DERIVATIVE_TRANSPORT2	0.277356543	-0.00496177	1.719407427	0.12340155	0.63133281	-4.68008013
homo after UVB irradiation VS homo before UVB irradiation	GO_CARBOHYDRATE_HOMEOSTASIS	0.135287587	-0.00450637	1.090925835	0.30585474	0.50027693	-5.9411911
hetero after UVB irradiation VS hetero before UVB irradiation	GO_CARBOHYDRATE_HOMEOSTASIS1	-0.06025966	0.011354513	-0.4403682	0.67154024	0.866125	-6.1276434
WT after UVB irradiation VS WT before UVB irradiation	GO_CARBOHYDRATE_HOMEOSTASIS2	0.004675218	-0.01670142	0.029995656	0.97679664	0.99416176	-5.99559195
homo after UVB irradiation VS homo before UVB irradiation	GO_CARBOHYDRATE_IMPORT_ACROSS_PLASMA_MEMBRANE	0.971054616	-0.01005842	5.78530614	0.00035422	0.01391518	0.612975003
hetero after UVB irradiation VS hetero before UVB irradiation	GO_CARBOHYDRATE_IMPORT_ACROSS_PLASMA_MEMBRANE1	0.426209465	0.097456795	1.607968671	0.14721669	0.46496154	-5.02655141
WT after UVB irradiation VS WT before UVB irradiation	GO_CARBOHYDRATE_IMPORT_ACROSS_PLASMA_MEMBRANE2	0.513610135	0.049011906	2.036205467	0.07569513	0.55851649	-4.25459021
homo after UVB irradiation VS homo before UVB irradiation	GO_CARBOHYDRATE_KINASE_ACTIVITY	0.791926552	-0.04669197	6.043097821	0.00026247	0.01214175	0.909907831
hetero after UVB irradiation VS hetero before UVB irradiation	GO_CARBOHYDRATE_KINASE_ACTIVITY1	0.548383844	-0.00671907	3.92505169	0.00455073	0.12742869	-1.74275213
WT after UVB irradiation VS WT before UVB irradiation	GO_CARBOHYDRATE_KINASE_ACTIVITY2	0.527638522	0.062971574	3.069571514	0.01513593	0.36889188	-2.79585502
homo after UVB irradiation VS homo before UVB irradiation	GO_CARBOHYDRATE_MEDIATED_SIGNALING	0.113829058	0.019645678	0.635664214	0.54206214	0.70714384	-6.33441049
hetero after UVB irradiation VS hetero before UVB irradiation	GO_CARBOHYDRATE_MEDIATED_SIGNALING1	0.356853076	0.031679803	2.244753169	0.055623	0.31148881	-4.14362478
WT after UVB irradiation VS WT before UVB irradiation	GO_CARBOHYDRATE_MEDIATED_SIGNALING2	0.112740841	-0.09201506	0.575522077	0.58057499	0.888473	-5.82498594
homo after UVB irradiation VS homo before UVB irradiation	GO_CARBOHYDRATE_METABOLIC_PROCESS	0.216977127	-0.005793	1.767757791	0.11359195	0.27119826	-5.09966708
hetero after UVB irradiation VS hetero before UVB irradiation	GO_CARBOHYDRATE_METABOLIC_PROCESS1	0.155206433	0.003327692	1.068096199	0.31720894	0.63719321	-5.65536519
WT after UVB irradiation VS WT before UVB irradiation	GO_CARBOHYDRATE_METABOLIC_PROCESS2	0.220838774	-0.01181554	1.443850792	0.18633112	0.68862718	-5.02453956
homo after UVB irradiation VS homo before UVB irradiation	GO_CARBOHYDRATE_PHOSPHATASE_ACTIVITY	0.109620378	0.084225003	0.716031085	0.49355485	0.66910107	-6.27796247
hetero after UVB irradiation VS hetero before UVB irradiation	GO_CARBOHYDRATE_PHOSPHATASE_ACTIVITY1	0.030343754	0.086285352	0.1946927	0.85057609	0.94283373	-6.21097911
WT after UVB irradiation VS WT before UVB irradiation	GO_CARBOHYDRATE_PHOSPHATASE_ACTIVITY2	-0.07652478	0.031442758	-0.27725262	0.78853254	0.94699608	-5.9557096
homo after UVB irradiation VS homo before UVB irradiation	GO_CARBOHYDRATE_PHOSPHORYLATION	0.61733794	-0.04842005	4.176095667	0.00283274	0.03518388	-1.47403941
hetero after UVB irradiation VS hetero before UVB irradiation	GO_CARBOHYDRATE_PHOSPHORYLATION1	0.406162018	-0.00709111	3.250542573	0.01199065	0.16980229	-2.67815828
WT after UVB irradiation VS WT before UVB irradiation	GO_CARBOHYDRATE_PHOSPHORYLATION2	0.460034919	0.031544594	3.137606739	0.01364543	0.35726193	-2.70093756
homo after UVB irradiation VS homo before UVB irradiation	GO_CARBOHYDRATE_PROTON_SYMPORTER_ACTIVITY	0.436262503	-0.02485162	2.421361322	0.04060244	0.14499783	-4.13021252
hetero after UVB irradiation VS hetero before UVB irradiation	GO_CARBOHYDRATE_PROTON_SYMPORTER_ACTIVITY1	0.212579023	0.012451472	1.095743326	0.30565575	0.62738297	-5.62729006
WT after UVB irradiation VS WT before UVB irradiation	GO_CARBOHYDRATE_PROTON_SYMPORTER_ACTIVITY2	0.08178204	0.038725853	0.361462862	0.72700329	0.93296251	-5.92770991
homo after UVB irradiation VS homo before UVB irradiation	GO_CARBOHYDRATE_TRANSMEMBRANE_TRANSPORT	0.253180592	0.006532961	2.11830488	0.06564992	0.19471138	-4.59061508
hetero after UVB irradiation VS hetero before UVB irradiation	GO_CARBOHYDRATE_TRANSMEMBRANE_TRANSPORT1	0.024158131	0.019673134	0.146126297	0.88750312	0.9589921	-6.21992057
WT after UVB irradiation VS WT before UVB irradiation	GO_CARBOHYDRATE_TRANSMEMBRANE_TRANSPORT2	0.055584235	-0.00449216	0.324850287	0.75352808	0.93734883	-5.94076643
homo after UVB irradiation VS homo before UVB irradiation	GO_CARBOHYDRATE_TRANSMEMBRANE_TRANSPORTER_ACTIVITY	0.465796665	0.002071088	3.043811688	0.01523211	0.08371643	-3.16537977
hetero after UVB irradiation VS hetero before UVB irradiation	GO_CARBOHYDRATE_TRANSMEMBRANE_TRANSPORTER_ACTIVITY1	0.230850042	0.048456887	1.402538707	0.19902991	0.523293	-5.28382508
WT after UVB irradiation VS WT before UVB irradiation	GO_CARBOHYDRATE_TRANSMEMBRANE_TRANSPORTER_ACTIVITY2	0.274622084	0.049845954	1.330721906	0.21953084	0.71478521	-5.15642036
homo after UVB irradiation VS homo before UVB irradiation	GO_CARBOHYDRATE_TRANSPORT	0.264951809	-0.00212527	2.226073084	0.05535483	0.17476921	-4.42844138
hetero after UVB irradiation VS hetero before UVB irradiation	GO_CARBOHYDRATE_TRANSPORT1	0.047295169	0.012855873	0.312442013	0.7628402	0.90723412	-6.17888269
WT after UVB irradiation VS WT before UVB irradiation	GO_CARBOHYDRATE_TRANSPORT2	0.11670832	-0.00732499	0.69789693	0.50478447	0.85763155	-5.74695148
homo after UVB irradiation VS homo before UVB irradiation	GO_CARBON_CARBON_LYASE_ACTIVITY	-0.01322343	0.003361512	-0.07237826	0.9440088	0.9703774	-6.54798258
hetero after UVB irradiation VS hetero before UVB irradiation	GO_CARBON_CARBON_LYASE_ACTIVITY1	-0.02296089	-0.03239244	-0.13510606	0.89592686	0.96152498	-6.22159693
WT after UVB irradiation VS WT before UVB irradiation	GO_CARBON_CARBON_LYASE_ACTIVITY2	0.198069729	-0.02224407	1.205924143	0.26189775	0.74156847	-5.29406807
homo after UVB irradiation VS homo before UVB irradiation	GO_CARBON_CATABOLITE_REGULATION_OF_TRANSCRIPTION	0.189059459	0.075255389	1.004124307	0.34359422	0.53609853	-6.02913148
hetero after UVB irradiation VS hetero before UVB irradiation	GO_CARBON_CATABOLITE_REGULATION_OF_TRANSCRIPTION1	-0.06560086	-0.0277075	-0.20484566	0.84290254	0.93980906	-6.20879079
WT after UVB irradiation VS WT before UVB irradiation	GO_CARBON_CATABOLITE_REGULATION_OF_TRANSCRIPTION2	0.226737979	-0.04252402	1.179558572	0.2716672	0.7443957	-5.32199715
homo after UVB irradiation VS homo before UVB irradiation	GO_CARBON_DIOXIDE_TRANSPORT	0.489968996	0.048876408	1.844963168	0.10079457	0.25180725	-4.99054065
hetero after UVB irradiation VS hetero before UVB irradiation	GO_CARBON_DIOXIDE_TRANSPORT1	0.640844957	-0.01019249	2.949673113	0.0188176	0.19947197	-3.11273515
WT after UVB irradiation VS WT before UVB irradiation	GO_CARBON_DIOXIDE_TRANSPORT2	0.362254323	0.128111987	1.421096902	0.19263244	0.69334561	-5.0515664
homo after UVB irradiation VS homo before UVB irradiation	GO_CARBON_NITROGEN_LIGASE_ACTIVITY_WITH_GLUTAMINE_AS_AMIDO_N_DONOR	-0.52723374	-0.02479235	-3.46514362	0.00799101	0.05943714	-2.51964058
hetero after UVB irradiation VS hetero before UVB irradiation	GO_CARBON_NITROGEN_LIGASE_ACTIVITY_WITH_GLUTAMINE_AS_AMIDO_N_DONOR1	-0.42128382	-0.05599362	-1.59704533	0.14962995	0.46792642	-5.0406833
WT after UVB irradiation VS WT before UVB irradiation	GO_CARBON_NITROGEN_LIGASE_ACTIVITY_WITH_GLUTAMINE_AS_AMIDO_N_DONOR2	-0.09843642	0.050059663	-0.28132446	0.78551663	0.94642844	-5.95452164
homo after UVB irradiation VS homo before UVB irradiation	GO_CARBON_NITROGEN_LYASE_ACTIVITY	0.340521202	0.040795573	2.239004287	0.05423162	0.17266366	-4.40885077
hetero after UVB irradiation VS hetero before UVB irradiation	GO_CARBON_NITROGEN_LYASE_ACTIVITY1	0.144011267	0.014395436	0.809423257	0.44210513	0.73797548	-5.89075927
WT after UVB irradiation VS WT before UVB irradiation	GO_CARBON_NITROGEN_LYASE_ACTIVITY2	0.200858306	-0.05364863	1.377789267	0.20514243	0.70190963	-5.10232177
homo after UVB irradiation VS homo before UVB irradiation	GO_CARBON_OXYGEN_LYASE_ACTIVITY	-0.07636647	-0.04781783	-0.4002854	0.69901696	0.82050535	-6.46377184
hetero after UVB irradiation VS hetero before UVB irradiation	GO_CARBON_OXYGEN_LYASE_ACTIVITY1	-0.25182023	-0.04613738	-1.7470977	0.11946213	0.42435801	-4.84274765
WT after UVB irradiation VS WT before UVB irradiation	GO_CARBON_OXYGEN_LYASE_ACTIVITY2	-0.04976366	-0.00160151	-0.27756693	0.7882996	0.9469715	-5.9556185
homo after UVB irradiation VS homo before UVB irradiation	GO_CARBON_SULFUR_LYASE_ACTIVITY	0.083327959	0.008475086	0.383979526	0.71061054	0.82744868	-6.47066042
hetero after UVB irradiation VS hetero before UVB irradiation	GO_CARBON_SULFUR_LYASE_ACTIVITY1	0.369031955	-0.00086957	2.082658426	0.07147314	0.34570185	-4.37661313
WT after UVB irradiation VS WT before UVB irradiation	GO_CARBON_SULFUR_LYASE_ACTIVITY2	-0.05836498	0.062147125	-0.24590378	0.81187499	0.95508327	-5.96428607
homo after UVB irradiation VS homo before UVB irradiation	GO_CARBONATE_DEHYDRATASE_ACTIVITY	0.270495618	0.012123769	1.386482108	0.20158041	0.39044271	-5.60370058
hetero after UVB irradiation VS hetero before UVB irradiation	GO_CARBONATE_DEHYDRATASE_ACTIVITY1	0.100283508	-0.00726982	0.570118295	0.58453549	0.82264353	-6.05889984
WT after UVB irradiation VS WT before UVB irradiation	GO_CARBONATE_DEHYDRATASE_ACTIVITY2	0.132069174	0.114708703	0.611471339	0.5576597	0.87953066	-5.80346649
homo after UVB irradiation VS homo before UVB irradiation	GO_CARBONYL_REDUCTASE_NADPH_ACTIVITY	0.455406932	-0.04054627	2.448763301	0.03887396	0.14171516	-4.08802963
hetero after UVB irradiation VS hetero before UVB irradiation	GO_CARBONYL_REDUCTASE_NADPH_ACTIVITY1	0.918207273	0.00234241	3.915850955	0.00460929	0.12761654	-1.75506781
WT after UVB irradiation VS WT before UVB irradiation	GO_CARBONYL_REDUCTASE_NADPH_ACTIVITY2	0.963749653	0.005432705	4.433156413	0.00212368	0.2146515	-1.01843141
homo after UVB irradiation VS homo before UVB irradiation	GO_CARBOXY_LYASE_ACTIVITY	-0.04307833	-0.01559486	-0.21821029	0.83251682	0.90647463	-6.52480011
hetero after UVB irradiation VS hetero before UVB irradiation	GO_CARBOXY_LYASE_ACTIVITY1	-0.1985279	-0.04215498	-1.2881041	0.23436695	0.56254359	-5.41831546
WT after UVB irradiation VS WT before UVB irradiation	GO_CARBOXY_LYASE_ACTIVITY2	0.210784168	-0.01645557	1.18157468	0.27090977	0.74431614	-5.31987636
homo after UVB irradiation VS homo before UVB irradiation	GO_CARBOXY_TERMINAL_DOMAIN_PROTEIN_KINASE_COMPLEX	-0.4738451	0.063212399	-2.36383661	0.04448562	0.15313139	-4.21853256
hetero after UVB irradiation VS hetero before UVB irradiation	GO_CARBOXY_TERMINAL_DOMAIN_PROTEIN_KINASE_COMPLEX1	-0.18730531	-0.05238769	-0.84239684	0.42447777	0.72596129	-5.86367242
WT after UVB irradiation VS WT before UVB irradiation	GO_CARBOXY_TERMINAL_DOMAIN_PROTEIN_KINASE_COMPLEX2	-0.30646464	-0.03434374	-1.17587307	0.27305628	0.74523609	-5.32586758
homo after UVB irradiation VS homo before UVB irradiation	GO_CARBOXYLIC_ESTER_HYDROLASE_ACTIVITY	-0.09361082	0.005705959	-0.61900213	0.55246465	0.71490011	-6.34535572
hetero after UVB irradiation VS hetero before UVB irradiation	GO_CARBOXYLIC_ESTER_HYDROLASE_ACTIVITY1	-0.19922	0.00656183	-1.50460459	0.17153858	0.49447611	-5.15833278
WT after UVB irradiation VS WT before UVB irradiation	GO_CARBOXYLIC_ESTER_HYDROLASE_ACTIVITY2	-0.08522414	0.002202972	-0.55732719	0.5923714	0.89307494	-5.83542221
homo after UVB irradiation VS homo before UVB irradiation	GO_CARBOXYPEPTIDASE_ACTIVITY	0.538779861	-0.01259842	3.464127321	0.00800325	0.05949822	-2.52117946
hetero after UVB irradiation VS hetero before UVB irradiation	GO_CARBOXYPEPTIDASE_ACTIVITY1	0.422278803	0.062361054	2.893157531	0.0205014	0.20617329	-3.19515004
WT after UVB irradiation VS WT before UVB irradiation	GO_CARBOXYPEPTIDASE_ACTIVITY2	0.413697172	0.035547343	1.912048013	0.09179788	0.58643348	-4.42421952
homo after UVB irradiation VS homo before UVB irradiation	GO_CARD_DOMAIN_BINDING	0.955878098	-0.0421437	6.809142206	0.00011305	0.01007802	1.735335483
hetero after UVB irradiation VS hetero before UVB irradiation	GO_CARD_DOMAIN_BINDING1	0.661236484	0.057235104	2.676482679	0.02854739	0.23751402	-3.51245429
WT after UVB irradiation VS WT before UVB irradiation	GO_CARD_DOMAIN_BINDING2	0.637901875	-0.01560779	2.583129316	0.03213799	0.45445578	-3.48404094
homo after UVB irradiation VS homo before UVB irradiation	GO_CARDIAC_ATRIUM_DEVELOPMENT	-0.17351706	-0.02058526	-0.96599234	0.36125424	0.55226916	-6.06597228
hetero after UVB irradiation VS hetero before UVB irradiation	GO_CARDIAC_ATRIUM_DEVELOPMENT1	-0.04561388	-0.02765824	-0.31793879	0.75882146	0.90635732	-6.17702793
WT after UVB irradiation VS WT before UVB irradiation	GO_CARDIAC_ATRIUM_DEVELOPMENT2	-0.21716293	0.001841044	-1.24369521	0.24840874	0.73564768	-5.25333735
homo after UVB irradiation VS homo before UVB irradiation	GO_CARDIAC_ATRIUM_MORPHOGENESIS	-0.15282429	-0.01450843	-0.81790381	0.43618277	0.61921589	-6.19799219
hetero after UVB irradiation VS hetero before UVB irradiation	GO_CARDIAC_ATRIUM_MORPHOGENESIS1	0.006008641	-0.03382062	0.042367156	0.96726343	0.9884024	-6.2305108
WT after UVB irradiation VS WT before UVB irradiation	GO_CARDIAC_ATRIUM_MORPHOGENESIS2	-0.1662114	0.046754683	-0.87858591	0.40494058	0.81613006	-5.60813157
homo after UVB irradiation VS homo before UVB irradiation	GO_CARDIAC_CELL_DEVELOPMENT	0.075414876	0.011808277	0.55752862	0.5918304	0.74395022	-6.38343046
hetero after UVB irradiation VS hetero before UVB irradiation	GO_CARDIAC_CELL_DEVELOPMENT1	-0.15015478	0.013453312	-0.97969306	0.356452	0.67198476	-5.74152407
WT after UVB irradiation VS WT before UVB irradiation	GO_CARDIAC_CELL_DEVELOPMENT2	-0.00425845	-0.02493026	-0.02387318	0.98153159	0.99578204	-5.99576595
homo after UVB irradiation VS homo before UVB irradiation	GO_CARDIAC_CELL_FATE_COMMITMENT	0.295434609	0.021357801	1.542691539	0.15999423	0.33604253	-5.40511512
hetero after UVB irradiation VS hetero before UVB irradiation	GO_CARDIAC_CELL_FATE_COMMITMENT1	0.580742737	0.015076337	3.150845783	0.01390637	0.17751077	-2.82125546
WT after UVB irradiation VS WT before UVB irradiation	GO_CARDIAC_CELL_FATE_COMMITMENT2	0.192631626	0.02855788	1.011038306	0.34125105	0.78168336	-5.49002505
homo after UVB irradiation VS homo before UVB irradiation	GO_CARDIAC_CHAMBER_DEVELOPMENT	0.035414343	0.006578952	0.275017769	0.79000168	0.88051773	-6.50953786
hetero after UVB irradiation VS hetero before UVB irradiation	GO_CARDIAC_CHAMBER_DEVELOPMENT1	-0.00955936	0.012329779	-0.07108935	0.94510375	0.97953855	-6.22874437
WT after UVB irradiation VS WT before UVB irradiation	GO_CARDIAC_CHAMBER_DEVELOPMENT2	-0.09595497	0.013070009	-0.67414249	0.51899807	0.86248918	-5.76314672
homo after UVB irradiation VS homo before UVB irradiation	GO_CARDIAC_CHAMBER_FORMATION	0.262441635	0.009708079	1.098587591	0.30268572	0.49729132	-5.93316554
hetero after UVB irradiation VS hetero before UVB irradiation	GO_CARDIAC_CHAMBER_FORMATION1	0.016369551	0.070365566	0.073958484	0.94289262	0.97870875	-6.22851883
WT after UVB irradiation VS WT before UVB irradiation	GO_CARDIAC_CHAMBER_FORMATION2	-0.17742436	0.016439812	-0.90599958	0.39109667	0.81184605	-5.58476857
homo after UVB irradiation VS homo before UVB irradiation	GO_CARDIAC_CHAMBER_MORPHOGENESIS	0.041906457	0.01391228	0.328253308	0.75082036	0.8545857	-6.49210796
hetero after UVB irradiation VS hetero before UVB irradiation	GO_CARDIAC_CHAMBER_MORPHOGENESIS1	-0.01454775	0.008556485	-0.11007665	0.91510969	0.96772723	-6.22491829
WT after UVB irradiation VS WT before UVB irradiation	GO_CARDIAC_CHAMBER_MORPHOGENESIS2	-0.06886277	0.024486501	-0.45867571	0.65852708	0.91105574	-5.88656123
homo after UVB irradiation VS homo before UVB irradiation	GO_CARDIAC_CONDUCTION	0.326086069	-0.01397971	2.543037857	0.03347309	0.13064332	-3.942454
homo after UVB irradiation VS homo before UVB irradiation	GO_CARDIAC_CONDUCTION_SYSTEM_DEVELOPMENT	0.141641691	-0.06870136	0.617442181	0.5534445	0.71549898	-6.34636691
hetero after UVB irradiation VS hetero before UVB irradiation	GO_CARDIAC_CONDUCTION_SYSTEM_DEVELOPMENT1	-0.10249476	-0.01950623	-0.40554919	0.69589851	0.87879147	-6.1432467
WT after UVB irradiation VS WT before UVB irradiation	GO_CARDIAC_CONDUCTION_SYSTEM_DEVELOPMENT2	-0.36929334	-0.01115545	-2.21846472	0.05692439	0.5233852	-4.00081804
hetero after UVB irradiation VS hetero before UVB irradiation	GO_CARDIAC_CONDUCTION1	-0.01869676	0.00691517	-0.10833592	0.91644618	0.96800858	-6.22512415
WT after UVB irradiation VS WT before UVB irradiation	GO_CARDIAC_CONDUCTION2	0.048038375	0.002195624	0.243235135	0.81387172	0.95548708	-5.96496948
homo after UVB irradiation VS homo before UVB irradiation	GO_CARDIAC_EPITHELIAL_TO_MESENCHYMAL_TRANSITION	-0.10484551	0.015225227	-0.54034246	0.60310542	0.75204606	-6.39341686
hetero after UVB irradiation VS hetero before UVB irradiation	GO_CARDIAC_EPITHELIAL_TO_MESENCHYMAL_TRANSITION1	0.099171346	0.009573846	0.504548462	0.6277262	0.84669911	-6.09570264
WT after UVB irradiation VS WT before UVB irradiation	GO_CARDIAC_EPITHELIAL_TO_MESENCHYMAL_TRANSITION2	-0.00548811	0.018152285	-0.03214139	0.97513741	0.9934792	-5.9955216
homo after UVB irradiation VS homo before UVB irradiation	GO_CARDIAC_LEFT_VENTRICLE_MORPHOGENESIS	0.146621524	0.041471986	1.068571935	0.3152499	0.50894405	-5.96436636
hetero after UVB irradiation VS hetero before UVB irradiation	GO_CARDIAC_LEFT_VENTRICLE_MORPHOGENESIS1	-0.19529579	-0.00082666	-0.89027834	0.39976955	0.70722929	-5.82275666
WT after UVB irradiation VS WT before UVB irradiation	GO_CARDIAC_LEFT_VENTRICLE_MORPHOGENESIS2	0.271577564	0.045102274	1.382609064	0.2037161	0.70036941	-5.09671856
homo after UVB irradiation VS homo before UVB irradiation	GO_CARDIAC_MUSCLE_CELL_ACTION_POTENTIAL	0.254136944	-0.00764751	2.061894361	0.07176249	0.20559096	-4.67462328
homo after UVB irradiation VS homo before UVB irradiation	GO_CARDIAC_MUSCLE_CELL_ACTION_POTENTIAL_INVOLVED_IN_CONTRACTION	0.350668256	-0.01689775	2.752693657	0.0240271	0.10886336	-3.61717368
hetero after UVB irradiation VS hetero before UVB irradiation	GO_CARDIAC_MUSCLE_CELL_ACTION_POTENTIAL_INVOLVED_IN_CONTRACTION1	-0.11859239	-0.01077219	-0.66876398	0.52281548	0.78818414	-5.9958467
WT after UVB irradiation VS WT before UVB irradiation	GO_CARDIAC_MUSCLE_CELL_ACTION_POTENTIAL_INVOLVED_IN_CONTRACTION2	-0.14614226	-0.01120797	-0.73547271	0.4828074	0.85139062	-5.7203309
hetero after UVB irradiation VS hetero before UVB irradiation	GO_CARDIAC_MUSCLE_CELL_ACTION_POTENTIAL1	-0.08289019	0.004028041	-0.47519517	0.64758392	0.85523965	-6.11081833
WT after UVB irradiation VS WT before UVB irradiation	GO_CARDIAC_MUSCLE_CELL_ACTION_POTENTIAL2	-0.15172871	-0.02778399	-0.81106537	0.4405135	0.83181801	-5.66315203
homo after UVB irradiation VS homo before UVB irradiation	GO_CARDIAC_MUSCLE_CELL_CARDIAC_MUSCLE_CELL_ADHESION	-0.16214915	0.001221132	-0.64860288	0.5340649	0.70061301	-6.32572969
hetero after UVB irradiation VS hetero before UVB irradiation	GO_CARDIAC_MUSCLE_CELL_CARDIAC_MUSCLE_CELL_ADHESION1	-0.41676956	-0.02136826	-1.46472587	0.18185029	0.50686486	-5.20794509
WT after UVB irradiation VS WT before UVB irradiation	GO_CARDIAC_MUSCLE_CELL_CARDIAC_MUSCLE_CELL_ADHESION2	-0.58263272	0.020670622	-1.94963741	0.08660358	0.57723988	-4.37319947
homo after UVB irradiation VS homo before UVB irradiation	GO_CARDIAC_MUSCLE_CELL_CONTRACTION	0.321173656	-0.00521355	2.616976117	0.02977277	0.1225979	-3.82790816
hetero after UVB irradiation VS hetero before UVB irradiation	GO_CARDIAC_MUSCLE_CELL_CONTRACTION1	-0.13607302	-0.02253261	-0.72078374	0.49193947	0.76746311	-5.95900538
WT after UVB irradiation VS WT before UVB irradiation	GO_CARDIAC_MUSCLE_CELL_CONTRACTION2	-0.08330003	-0.02086976	-0.41299876	0.69032695	0.92104951	-5.90705784
homo after UVB irradiation VS homo before UVB irradiation	GO_CARDIAC_MUSCLE_CELL_DIFFERENTIATION	0.058060157	-0.00408872	0.449563746	0.66449389	0.79664768	-6.44128669
hetero after UVB irradiation VS hetero before UVB irradiation	GO_CARDIAC_MUSCLE_CELL_DIFFERENTIATION1	-0.12930831	-0.00371877	-0.85477242	0.41799059	0.72088233	-5.85327503
WT after UVB irradiation VS WT before UVB irradiation	GO_CARDIAC_MUSCLE_CELL_DIFFERENTIATION2	-0.04586919	-0.015558	-0.26657831	0.79645658	0.95160795	-5.95874321
homo after UVB irradiation VS homo before UVB irradiation	GO_CARDIAC_MUSCLE_CELL_FATE_COMMITMENT	0.221857743	0.041657897	1.221734631	0.25525554	0.45079057	-5.79863968
hetero after UVB irradiation VS hetero before UVB irradiation	GO_CARDIAC_MUSCLE_CELL_FATE_COMMITMENT1	0.398579948	0.041629892	2.241642448	0.05589161	0.3120771	-4.14812804
WT after UVB irradiation VS WT before UVB irradiation	GO_CARDIAC_MUSCLE_CELL_FATE_COMMITMENT2	0.083819948	0.053860359	0.51615382	0.61954201	0.90083569	-5.85789288
homo after UVB irradiation VS homo before UVB irradiation	GO_CARDIAC_MUSCLE_CELL_MEMBRANE_REPOLARIZATION	0.377537535	-0.02881336	2.589144513	0.03111432	0.12512052	-3.87105553
hetero after UVB irradiation VS hetero before UVB irradiation	GO_CARDIAC_MUSCLE_CELL_MEMBRANE_REPOLARIZATION1	0.042872749	0.037267661	0.22549054	0.82735526	0.93421603	-6.20400217
WT after UVB irradiation VS WT before UVB irradiation	GO_CARDIAC_MUSCLE_CELL_MEMBRANE_REPOLARIZATION2	0.043349993	0.008474918	0.21673432	0.83377615	0.96080232	-5.97135689
homo after UVB irradiation VS homo before UVB irradiation	GO_CARDIAC_MUSCLE_CELL_MYOBLAST_DIFFERENTIATION	-0.04703765	0.033318763	-0.25909744	0.80184875	0.88762149	-6.51416419
hetero after UVB irradiation VS hetero before UVB irradiation	GO_CARDIAC_MUSCLE_CELL_MYOBLAST_DIFFERENTIATION1	-0.03184563	0.015400263	-0.18509157	0.85784825	0.94599872	-6.21294717
WT after UVB irradiation VS WT before UVB irradiation	GO_CARDIAC_MUSCLE_CELL_MYOBLAST_DIFFERENTIATION2	-0.15905196	-0.02880688	-0.68903426	0.51005872	0.85882417	-5.75305174
homo after UVB irradiation VS homo before UVB irradiation	GO_CARDIAC_MUSCLE_CELL_PROLIFERATION	-0.08782874	-0.0170397	-0.54773168	0.59824362	0.7483174	-6.38915878
hetero after UVB irradiation VS hetero before UVB irradiation	GO_CARDIAC_MUSCLE_CELL_PROLIFERATION1	-0.16787822	-0.00608659	-1.12622678	0.29330801	0.61749936	-5.59573581
WT after UVB irradiation VS WT before UVB irradiation	GO_CARDIAC_MUSCLE_CELL_PROLIFERATION2	-0.21165499	-0.01586859	-1.27845199	0.23651302	0.72803976	-5.2151318
homo after UVB irradiation VS homo before UVB irradiation	GO_CARDIAC_MUSCLE_CONTRACTION	0.298145122	-0.00387355	2.189420179	0.05866462	0.18171993	-4.48382507
hetero after UVB irradiation VS hetero before UVB irradiation	GO_CARDIAC_MUSCLE_CONTRACTION1	-0.03591081	-0.00089911	-0.19699293	0.84883606	0.94258929	-6.21049297
WT after UVB irradiation VS WT before UVB irradiation	GO_CARDIAC_MUSCLE_CONTRACTION2	0.002269115	-0.025164	0.010575841	0.99181777	0.9985657	-5.99600765
homo after UVB irradiation VS homo before UVB irradiation	GO_CARDIAC_MUSCLE_FIBER_DEVELOPMENT	0.057052041	-0.00198915	0.237609799	0.81792446	0.89687258	-6.5199778
hetero after UVB irradiation VS hetero before UVB irradiation	GO_CARDIAC_MUSCLE_FIBER_DEVELOPMENT1	-0.23892581	-0.08147745	-0.88946672	0.40017958	0.7076422	-5.82346555
WT after UVB irradiation VS WT before UVB irradiation	GO_CARDIAC_MUSCLE_FIBER_DEVELOPMENT2	-0.05527281	-0.02170904	-0.19363687	0.85122934	0.96597904	-5.97633072
homo after UVB irradiation VS homo before UVB irradiation	GO_CARDIAC_MUSCLE_MYOBLAST_PROLIFERATION	0.41777379	0.066297895	1.281163515	0.23465186	0.42640538	-5.73018788
hetero after UVB irradiation VS hetero before UVB irradiation	GO_CARDIAC_MUSCLE_MYOBLAST_PROLIFERATION1	0.169154713	-0.05854609	0.478139331	0.64557816	0.85438728	-6.10934062
WT after UVB irradiation VS WT before UVB irradiation	GO_CARDIAC_MUSCLE_MYOBLAST_PROLIFERATION2	-0.35367994	-0.12161923	-1.37507599	0.20594918	0.70286588	-5.10547096
homo after UVB irradiation VS homo before UVB irradiation	GO_CARDIAC_MUSCLE_TISSUE_DEVELOPMENT	0.02859858	-0.00710189	0.228888749	0.82447556	0.90163661	-6.52219581
hetero after UVB irradiation VS hetero before UVB irradiation	GO_CARDIAC_MUSCLE_TISSUE_DEVELOPMENT1	-0.07952377	-0.01145398	-0.62338406	0.55070473	0.80124226	-6.02597879

WT after UVB irradiation VS WT before UVB irradiation	GO_CARDIAC_MUSCLE_TISSUE_DEVELOPMENT2	-0.0748919	-0.00725784	-0.50445176	0.62738165	0.90327079	-5.86398645
homo after UVB irradiation VS homo before UVB irradiation	GO_CARDIAC_MUSCLE_TISSUE_MORPHOGENESIS	0.104393458	-0.01329608	0.81656183	0.43690793	0.61976836	-6.19910484
hetero after UVB irradiation VS hetero before UVB irradiation	GO_CARDIAC_MUSCLE_TISSUE_MORPHOGENESIS1	0.004385517	0.001616491	0.03387663	0.97382077	0.99108595	-6.23086179
WT after UVB irradiation VS WT before UVB irradiation	GO_CARDIAC_MUSCLE_TISSUE_MORPHOGENESIS2	0.057243725	0.005689548	0.346961799	0.73746464	0.93478445	-5.93304266
homo after UVB irradiation VS homo before UVB irradiation	GO_CARDIAC_MUSCLE_TISSUE_REGENERATION	-0.65118055	0.086192649	-2.83100202	0.02124136	0.10111454	-3.49548869
hetero after UVB irradiation VS hetero before UVB irradiation	GO_CARDIAC_MUSCLE_TISSUE_REGENERATION1	-0.83188746	-0.04374461	-4.92474524	0.00122158	0.09106857	-0.48842732
WT after UVB irradiation VS WT before UVB irradiation	GO_CARDIAC_MUSCLE_TISSUE_REGENERATION2	-0.94099144	-0.08092773	-4.36494772	0.00232829	0.21952805	-1.10001108
homo after UVB irradiation VS homo before UVB irradiation	GO_CARDIAC_MYOFIBRIL	0.809538986	-0.03976458	3.588208376	0.00664507	0.05379154	-2.3341151
homo after UVB irradiation VS homo before UVB irradiation	GO_CARDIAC_MYOFIBRIL_ASSEMBLY	0.315742765	-0.03261946	1.514623644	0.16685219	0.3453349	-5.44168715
hetero after UVB irradiation VS hetero before UVB irradiation	GO_CARDIAC_MYOFIBRIL_ASSEMBLY1	-0.37029103	-0.009051	-1.65896412	0.13641598	0.44931177	-4.95997722
WT after UVB irradiation VS WT before UVB irradiation	GO_CARDIAC_MYOFIBRIL_ASSEMBLY2	-0.00424884	-0.04217638	-0.01518313	0.98825351	0.9977901	-5.99594503
hetero after UVB irradiation VS hetero before UVB irradiation	GO_CARDIAC_MYOFIBRIL1	0.174430103	0.081101505	1.00051322	0.34689115	0.66382127	-5.72174089
WT after UVB irradiation VS WT before UVB irradiation	GO_CARDIAC_MYOFIBRIL2	0.242910476	-0.06574758	0.757525067	0.47020246	0.84598607	-5.70414484
homo after UVB irradiation VS homo before UVB irradiation	GO_CARDIAC_NEURAL_CREST_CELL_DEVELOPMENT_INVOLVED_IN_OUTFLOW_TRACT_MORPHOGENESIS	0.142967956	0.029298661	0.83525295	0.42688233	0.61160219	-6.18346875
hetero after UVB irradiation VS hetero before UVB irradiation	GO_CARDIAC_NEURAL_CREST_CELL_DEVELOPMENT_INVOLVED_IN_OUTFLOW_TRACT_MORPHOGENESIS1	-0.21378922	0.009508057	-0.98578723	0.35363303	0.6701712	-5.73576656
WT after UVB irradiation VS WT before UVB irradiation	GO_CARDIAC_NEURAL_CREST_CELL_DEVELOPMENT_INVOLVED_IN_OUTFLOW_TRACT_MORPHOGENESIS2	-0.30810932	0.046794655	-1.17285489	0.27419815	0.74597065	-5.329031
homo after UVB irradiation VS homo before UVB irradiation	GO_CARDIAC_NEURAL_CREST_CELL_DIFFERENTIATION_INVOLVED_IN_HEART_DEVELOPMENT	0.133221258	0.012223096	0.675098737	0.5179111	0.68791011	-6.30746238
hetero after UVB irradiation VS hetero before UVB irradiation	GO_CARDIAC_NEURAL_CREST_CELL_DIFFERENTIATION_INVOLVED_IN_HEART_DEVELOPMENT1	-0.19949085	0.034786112	-0.91146814	0.38917179	0.69940265	-5.80406491
WT after UVB irradiation VS WT before UVB irradiation	GO_CARDIAC_NEURAL_CREST_CELL_DIFFERENTIATION_INVOLVED_IN_HEART_DEVELOPMENT2	-0.20316018	0.099806951	-0.77618194	0.45970929	0.83928368	-5.69013068
homo after UVB irradiation VS homo before UVB irradiation	GO_CARDIAC_NEURAL_CREST_CELL_MIGRATION_INVOLVED_IN_OUTFLOW_TRACT_MORPHOGENESIS	0.003162069	0.000653341	0.017376003	0.98654569	0.9930995	-6.55069102
hetero after UVB irradiation VS hetero before UVB irradiation	GO_CARDIAC_NEURAL_CREST_CELL_MIGRATION_INVOLVED_IN_OUTFLOW_TRACT_MORPHOGENESIS1	-0.59200907	-0.00797303	-2.6607408	0.02924628	0.23948838	-3.53555435
WT after UVB irradiation VS WT before UVB irradiation	GO_CARDIAC_NEURAL_CREST_CELL_MIGRATION_INVOLVED_IN_OUTFLOW_TRACT_MORPHOGENESIS2	-0.48469072	0.006366293	-1.90687994	0.09253495	0.58905174	-4.43120929
homo after UVB irradiation VS homo before UVB irradiation	GO_CARDIAC_PACEMAKER_CELL_DIFFERENTIATION	0.557305276	-0.05516101	1.881320237	0.09525051	0.24374747	-4.93849448
hetero after UVB irradiation VS hetero before UVB irradiation	GO_CARDIAC_PACEMAKER_CELL_DIFFERENTIATION1	0.657281678	0.109714431	1.996904498	0.08157409	0.3656462	-4.49817338
WT after UVB irradiation VS WT before UVB irradiation	GO_CARDIAC_PACEMAKER_CELL_DIFFERENTIATION2	0.168259812	-0.07371271	0.641581433	0.53887718	0.87025652	-5.78453511
homo after UVB irradiation VS homo before UVB irradiation	GO_CARDIAC_RIGHT_VENTRICLE_MORPHOGENESIS	-0.11291246	0.030077564	-0.77882248	0.45763747	0.63808027	-6.22975553
hetero after UVB irradiation VS hetero before UVB irradiation	GO_CARDIAC_RIGHT_VENTRICLE_MORPHOGENESIS1	-0.33660616	0.04774592	-2.02012585	0.07870921	0.36152876	-4.46539317
WT after UVB irradiation VS WT before UVB irradiation	GO_CARDIAC_RIGHT_VENTRICLE_MORPHOGENESIS2	-0.40771592	0.059889802	-2.44289551	0.04003196	0.48704381	-3.68351265
homo after UVB irradiation VS homo before UVB irradiation	GO_CARDIAC_SEPTUM_DEVELOPMENT	-0.1274019	0.008790703	-0.92163711	0.38263633	0.57262444	-6.10739301
hetero after UVB irradiation VS hetero before UVB irradiation	GO_CARDIAC_SEPTUM_DEVELOPMENT1	-0.10221043	0.00343902	-0.80916462	0.44224536	0.73804258	-5.89096817
WT after UVB irradiation VS WT before UVB irradiation	GO_CARDIAC_SEPTUM_DEVELOPMENT2	-0.22577494	0.010513937	-1.50851902	0.16940512	0.67524339	-4.94644133
homo after UVB irradiation VS homo before UVB irradiation	GO_CARDIAC_SEPTUM_MORPHOGENESIS	-0.14097424	0.012765227	-0.96123013	0.36350657	0.55404528	-6.07049399
hetero after UVB irradiation VS hetero before UVB irradiation	GO_CARDIAC_SEPTUM_MORPHOGENESIS1	-0.14747167	0.000427832	-1.17000992	0.27627952	0.60324846	-5.54934802
WT after UVB irradiation VS WT before UVB irradiation	GO_CARDIAC_SEPTUM_MORPHOGENESIS2	-0.26828496	0.029674522	-1.78519458	0.11160623	0.61576748	-4.59386327
homo after UVB irradiation VS homo before UVB irradiation	GO_CARDIAC_VASCULAR_SMOOTH_MUSCLE_CELL_DIFFERENTIATION	0.038759147	0.002225157	0.145949947	0.88743144	0.93923016	-6.5391813
hetero after UVB irradiation VS hetero before UVB irradiation	GO_CARDIAC_VASCULAR_SMOOTH_MUSCLE_CELL_DIFFERENTIATION1	0.088247369	0.12423141	0.350475821	0.7351971	0.89626925	-6.16540575
WT after UVB irradiation VS WT before UVB irradiation	GO_CARDIAC_VASCULAR_SMOOTH_MUSCLE_CELL_DIFFERENTIATION2	0.089077523	0.06896813	0.307326312	0.76635042	0.94123239	-5.94653625
homo after UVB irradiation VS homo before UVB irradiation	GO_CARDIAC_VENTRICLE_DEVELOPMENT	0.016907728	0.002042232	0.125964429	0.90274771	0.94702836	-6.54215701
hetero after UVB irradiation VS hetero before UVB irradiation	GO_CARDIAC_VENTRICLE_DEVELOPMENT1	-0.023059	0.006117427	-0.17265734	0.86728776	0.94973642	-6.21534937
WT after UVB irradiation VS WT before UVB irradiation	GO_CARDIAC_VENTRICLE_DEVELOPMENT2	-0.115415	0.019866725	-0.80214594	0.44536897	0.83340629	-5.67014559
homo after UVB irradiation VS homo before UVB irradiation	GO_CARDIAC_VENTRICLE_FORMATION	0.536417776	0.014261186	2.571266475	0.03200828	0.1268384	-3.89875353
hetero after UVB irradiation VS hetero before UVB irradiation	GO_CARDIAC_VENTRICLE_FORMATION1	0.066472016	0.047303217	0.259923481	0.80160772	0.92337675	-6.19500829
WT after UVB irradiation VS WT before UVB irradiation	GO_CARDIAC_VENTRICLE_FORMATION2	-0.13136596	0.04402619	-0.64504428	0.53674153	0.86992784	-5.78230544
homo after UVB irradiation VS homo before UVB irradiation	GO_CARDIAC_VENTRICLE_MORPHOGENESIS	0.136837323	0.003194028	1.105062998	0.30002766	0.49458313	-5.92635023
hetero after UVB irradiation VS hetero before UVB irradiation	GO_CARDIAC_VENTRICLE_MORPHOGENESIS1	0.03014498	0.018117213	0.227009979	0.82621411	0.93407556	-6.20363181
WT after UVB irradiation VS WT before UVB irradiation	GO_CARDIAC_VENTRICLE_MORPHOGENESIS2	0.074770517	0.012473992	0.498023991	0.63170942	0.90435932	-5.86727794
homo after UVB irradiation VS homo before UVB irradiation	GO_CARDIOBLAST_DIFFERENTIATION	-0.07146629	0.015745369	-0.38681327	0.7085899	0.82602584	-6.46948309
hetero after UVB irradiation VS hetero before UVB irradiation	GO_CARDIOBLAST_DIFFERENTIATION1	-0.339048	-0.00622827	-2.00191815	0.0809471	0.36443021	-4.49110503
WT after UVB irradiation VS WT before UVB irradiation	GO_CARDIOBLAST_DIFFERENTIATION2	-0.39699544	0.006457069	-2.03496254	0.0758419	0.55880099	-4.25630324
homo after UVB irradiation VS homo before UVB irradiation	GO_CARDIOBLAST_PROLIFERATION	-0.09610402	0.029918271	-0.67032455	0.52079948	0.69034726	-6.31080233
hetero after UVB irradiation VS hetero before UVB irradiation	GO_CARDIOBLAST_PROLIFERATION1	-0.39335559	-0.03403777	-2.23065044	0.05685112	0.31461576	-4.16403203
WT after UVB irradiation VS WT before UVB irradiation	GO_CARDIOBLAST_PROLIFERATION2	-0.54885193	0.001419314	-3.35276778	0.00986459	0.32934772	-2.40412155
homo after UVB irradiation VS homo before UVB irradiation	GO_CARDIOCYTE_DIFFERENTIATION	0.049141902	-0.00815206	0.390671232	0.70584284	0.82455072	-6.46786682
hetero after UVB irradiation VS hetero before UVB irradiation	GO_CARDIOCYTE_DIFFERENTIATION1	-0.09021059	0.00190293	-0.63213626	0.54525797	0.79883108	-6.02031516
WT after UVB irradiation VS WT before UVB irradiation	GO_CARDIOCYTE_DIFFERENTIATION2	-0.09143995	-0.00848037	-0.56166354	0.58954808	0.89215883	-5.83296294
homo after UVB irradiation VS homo before UVB irradiation	GO_CARDIOLIPIN_ACYL_CHAIN_REMODELING	-0.09671647	-0.07288067	-0.44157948	0.67003312	0.80094603	-6.44509889
hetero after UVB irradiation VS hetero before UVB irradiation	GO_CARDIOLIPIN_ACYL_CHAIN_REMODELING1	-0.27675187	-0.03604709	-1.16510132	0.27814761	0.60400502	-5.55460995
WT after UVB irradiation VS WT before UVB irradiation	GO_CARDIOLIPIN_ACYL_CHAIN_REMODELING2	-0.31488741	0.016782002	-1.5399641	0.16168147	0.66526114	-4.90781091
homo after UVB irradiation VS homo before UVB irradiation	GO_CARDIOLIPIN_BINDING	0.297685934	-0.03800269	1.350170563	0.21250067	0.4026544	-5.64802842
hetero after UVB irradiation VS hetero before UVB irradiation	GO_CARDIOLIPIN_BINDING1	0.310621639	0.011819904	1.379787562	0.20566223	0.53033299	-5.31111093
WT after UVB irradiation VS WT before UVB irradiation	GO_CARDIOLIPIN_BINDING2	0.524165023	-0.04301335	2.793267481	0.02316408	0.41742287	-3.1852998
homo after UVB irradiation VS homo before UVB irradiation	GO_CARDIOLIPIN_BIOSYNTHETIC_PROCESS	0.377585026	-0.02251795	1.896826417	0.09297503	0.24024384	-4.9161769
hetero after UVB irradiation VS hetero before UVB irradiation	GO_CARDIOLIPIN_BIOSYNTHETIC_PROCESS1	0.128316742	-0.00573218	0.427745094	0.68032514	0.87128338	-6.13344163
WT after UVB irradiation VS WT before UVB irradiation	GO_CARDIOLIPIN_BIOSYNTHETIC_PROCESS2	0.404618517	-0.00531126	2.156664997	0.06271045	0.53553824	-4.08738301
homo after UVB irradiation VS homo before UVB irradiation	GO_CARDIOLIPIN_METABOLIC_PROCESS	-0.01212427	-0.08607086	-0.07106158	0.94502544	0.97096509	-6.54808617
hetero after UVB irradiation VS hetero before UVB irradiation	GO_CARDIOLIPIN_METABOLIC_PROCESS1	-0.24376891	-0.02086104	-1.22901905	0.25461827	0.58227336	-5.48491742
WT after UVB irradiation VS WT before UVB irradiation	GO_CARDIOLIPIN_METABOLIC_PROCESS2	0.043458468	-0.03041982	0.248083835	0.81024493	0.95472843	-5.96372234
homo after UVB irradiation VS homo before UVB irradiation	GO_CARGO_ADAPTOR_ACTIVITY	0.370391321	-0.0161802	2.032085849	0.0752121	0.21067969	-4.71873852
hetero after UVB irradiation VS hetero before UVB irradiation	GO_CARGO_ADAPTOR_ACTIVITY1	0.797712359	0.018152476	5.540535034	0.00058378	0.08125048	0.199684941
WT after UVB irradiation VS WT before UVB irradiation	GO_CARGO_ADAPTOR_ACTIVITY2	0.62637992	0.047649681	3.581011761	0.0070355	0.31015346	-2.09580841
homo after UVB irradiation VS homo before UVB irradiation	GO_CARGO_RECEPTOR_ACTIVITY	0.483637858	-0.00335356	3.407103825	0.00872303	0.06209634	-2.6076928
hetero after UVB irradiation VS hetero before UVB irradiation	GO_CARGO_RECEPTOR_ACTIVITY1	0.285183481	0.013394365	1.872666876	0.09869742	0.39071492	-4.67155538
WT after UVB irradiation VS WT before UVB irradiation	GO_CARGO_RECEPTOR_ACTIVITY2	0.335345285	-0.01281694	1.516839977	0.16732978	0.67227452	-4.93625944
homo after UVB irradiation VS homo before UVB irradiation	GO_CARNITINE_BIOSYNTHETIC_PROCESS	0.023495385	-0.09178089	0.079092687	0.93882618	0.96734101	-6.5474248
hetero after UVB irradiation VS hetero before UVB irradiation	GO_CARNITINE_BIOSYNTHETIC_PROCESS1	-0.75013854	-0.07648152	-2.89968031	0.02029931	0.20610175	-3.18562846
WT after UVB irradiation VS WT before UVB irradiation	GO_CARNITINE_BIOSYNTHETIC_PROCESS2	-0.43250196	0.047502198	-1.3244128	0.22152386	0.71673483	-5.16358477
homo after UVB irradiation VS homo before UVB irradiation	GO_CARNITINE_METABOLIC_PROCESS	-0.04873059	-0.08551132	-0.19159741	0.85264611	0.91863172	-6.5307549
hetero after UVB irradiation VS hetero before UVB irradiation	GO_CARNITINE_METABOLIC_PROCESS1	-0.24136801	-0.06627194	-0.88931748	0.40025501	0.70764252	-5.82359584
WT after UVB irradiation VS WT before UVB irradiation	GO_CARNITINE_METABOLIC_PROCESS2	-0.22621659	-0.01111771	-0.94218501	0.37335321	0.8017852	-5.55305108
homo after UVB irradiation VS homo before UVB irradiation	GO_CARNITINE_O_ACYLTRANSFERASE_ACTIVITY	-0.34040868	-0.06027261	-1.45315882	0.18279094	0.36640124	-5.52044625
hetero after UVB irradiation VS hetero before UVB irradiation	GO_CARNITINE_O_ACYLTRANSFERASE_ACTIVITY1	0.034893546	-0.05321274	0.17854555	0.86281476	0.94819913	-6.21423245
WT after UVB irradiation VS WT before UVB irradiation	GO_CARNITINE_O_ACYLTRANSFERASE_ACTIVITY2	-0.36490811	-0.06293515	-2.51709986	0.03563684	0.47351907	-3.57800825
homo after UVB irradiation VS homo before UVB irradiation	GO_CARNITINE_SHUTTLE	-0.1815167	-0.00902821	-0.6693874	0.52136762	0.69064829	-6.31145546
hetero after UVB irradiation VS hetero before UVB irradiation	GO_CARNITINE_SHUTTLE1	-0.33866922	-0.074355	-1.42503387	0.19265713	0.5183896	-5.25659237
WT after UVB irradiation VS WT before UVB irradiation	GO_CARNITINE_SHUTTLE2	-0.45063804	-0.04035191	-2.97352308	0.01753532	0.38336116	-2.93058771
homo after UVB irradiation VS homo before UVB irradiation	GO_CARTILAGE_DEVELOPMENT	0.090581043	0.001429931	0.749816083	0.47400954	0.6524784	-6.25246318
homo after UVB irradiation VS homo before UVB irradiation	GO_CARTILAGE_DEVELOPMENT_INVOLVED_IN_ENDOCHONDRAL_BONE_MORPHOGENESIS	-0.02170921	0.006950002	-0.14875874	0.88528271	0.93786828	-6.5387282
hetero after UVB irradiation VS hetero before UVB irradiation	GO_CARTILAGE_DEVELOPMENT_INVOLVED_IN_ENDOCHONDRAL_BONE_MORPHOGENESIS1	-0.07126939	-0.00172024	-0.40345112	0.69737873	0.88005213	-6.14414768
WT after UVB irradiation VS WT before UVB irradiation	GO_CARTILAGE_DEVELOPMENT_INVOLVED_IN_ENDOCHONDRAL_BONE_MORPHOGENESIS2	-0.037741	-0.00517449	-0.23512506	0.81994861	0.95742852	-5.96700128
hetero after UVB irradiation VS hetero before UVB irradiation	GO_CARTILAGE_DEVELOPMENT1	-0.10117332	-0.00252884	-0.64487864	0.53738569	0.79495371	-6.01194266
WT after UVB irradiation VS WT before UVB irradiation	GO_CARTILAGE_DEVELOPMENT2	-0.06191217	-0.02147499	-0.38506266	0.71010808	0.92809006	-5.91858078
homo after UVB irradiation VS homo before UVB irradiation	GO_CARTILAGE_MORPHOGENESIS	-0.01988033	0.045172384	-0.11233472	0.91321825	0.95237327	-6.54393645
hetero after UVB irradiation VS hetero before UVB irradiation	GO_CARTILAGE_MORPHOGENESIS1	-0.19446709	0.07352084	-1.23306757	0.25318532	0.58110396	-5.48041917
WT after UVB irradiation VS WT before UVB irradiation	GO_CARTILAGE_MORPHOGENESIS2	-0.12749538	0.076055793	-0.77134447	0.46241491	0.84099789	-5.69379235
homo after UVB irradiation VS homo before UVB irradiation	GO_CASPASE_BINDING	1.070151212	-0.02593066	7.697697293	4.62E-05	0.00885602	2.59428867
hetero after UVB irradiation VS hetero before UVB irradiation	GO_CASPASE_BINDING1	0.73822787	0.074683935	2.569812516	0.03364095	0.25343238	-3.66899456
WT after UVB irradiation VS WT before UVB irradiation	GO_CASPASE_BINDING2	0.687440247	-0.12131671	2.609905421	0.03082058	0.45345895	-3.44593055
homo after UVB irradiation VS homo before UVB irradiation	GO_CATALYTIC_ACTIVITY_ACTING_ON_A_GLYCOPROTEIN	0.313460891	0.010742971	1.628318364	0.14061153	0.3110951	-5.29134514
hetero after UVB irradiation VS hetero before UVB irradiation	GO_CATALYTIC_ACTIVITY_ACTING_ON_A_GLYCOPROTEIN1	0.293441275	-0.05237419	1.837349933	0.10416268	0.40029539	-4.72015385
WT after UVB irradiation VS WT before UVB irradiation	GO_CATALYTIC_ACTIVITY_ACTING_ON_A_GLYCOPROTEIN2	0.311694861	0.013121953	2.034737746	0.07586847	0.55880099	-4.25661302
homo after UVB irradiation VS homo before UVB irradiation	GO_CATALYTIC_ACTIVITY_ACTING_ON_A_RRNA	-0.71208697	-0.02642332	-5.59015765	0.00044705	0.01566998	0.381497988
hetero after UVB irradiation VS hetero before UVB irradiation	GO_CATALYTIC_ACTIVITY_ACTING_ON_A_RRNA1	-0.68583994	-0.06740803	-4.17536191	0.00322894	0.11632799	-1.41292144
WT after UVB irradiation VS WT before UVB irradiation	GO_CATALYTIC_ACTIVITY_ACTING_ON_A_RRNA2	-0.20103173	-0.04381278	-0.71023828	0.49749746	0.85541458	-5.73834279
homo after UVB irradiation VS homo before UVB irradiation	GO_CATALYTIC_ACTIVITY_ACTING_ON_A_TRNA	-0.65663417	-0.03198227	-4.34611546	0.00223422	0.03161105	-1.23441895
hetero after UVB irradiation VS hetero before UVB irradiation	GO_CATALYTIC_ACTIVITY_ACTING_ON_A_TRNA1	-0.43799465	-0.07630648	-2.46732737	0.03940943	0.27162031	-3.81924408
WT after UVB irradiation VS WT before UVB irradiation	GO_CATALYTIC_ACTIVITY_ACTING_ON_A_TRNA2	-0.31078352	-0.04390468	-1.20752009	0.26131579	0.74144146	-5.29236407
homo after UVB irradiation VS homo before UVB irradiation	GO_CATALYTIC_ACTIVITY_ACTING_ON_DNA	-0.41335932	-0.02606033	-2.43835051	0.03952191	0.14284223	-4.104067
hetero after UVB irradiation VS hetero before UVB irradiation	GO_CATALYTIC_ACTIVITY_ACTING_ON_DNA1	-0.09042602	-0.04193375	-0.41690111	0.68791378	0.87482399	-6.1382943
WT after UVB irradiation VS WT before UVB irradiation	GO_CATALYTIC_ACTIVITY_ACTING_ON_DNA2	-0.02952081	0.001995287	-0.12212141	0.90577922	0.97708947	-5.98820515
homo after UVB irradiation VS homo before UVB irradiation	GO_CATALYTIC_ACTIVITY_ACTING_ON_RNA	-0.57364776	-0.01890555	-4.5588832	0.00167002	0.02782236	-0.94077011
hetero after UVB irradiation VS hetero before UVB irradiation	GO_CATALYTIC_ACTIVITY_ACTING_ON_RNA1	-0.21695158	-0.07474851	-1.16113114	0.27966609	0.6054215	-5.5588547
WT after UVB irradiation VS WT before UVB irradiation	GO_CATALYTIC_ACTIVITY_ACTING_ON_RNA2	-0.180924	-0.00927641	-0.72744246	0.48745146	0.85164239	-5.72612211
homo after UVB irradiation VS homo before UVB irradiation	GO_CATALYTIC_COMPLEX	-0.33762397	-0.00523127	-2.59527113	0.03081383	0.12461488	-3.86156027
hetero after UVB irradiation VS hetero before UVB irradiation	GO_CATALYTIC_COMPLEX1	-0.11688969	-0.03804179	-0.80651858	0.44368177	0.73893547	-5.893102
WT after UVB irradiation VS WT before UVB irradiation	GO_CATALYTIC_COMPLEX2	-0.10926897	-0.01683472	-0.5842359	0.57497231	0.88587036	-5.81987903
homo after UVB irradiation VS homo before UVB irradiation	GO_CATALYTIC_STEP_1_SPLICEOSOME	-0.97639027	-0.02377854	-7.36739326	6.38E-05	0.00942429	2.286605398
hetero after UVB irradiation VS hetero before UVB irradiation	GO_CATALYTIC_STEP_1_SPLICEOSOME1	-0.33007828	-0.08246412	-1.21406489	0.2599699	0.58638274	-5.50144743
WT after UVB irradiation VS WT before UVB irradiation	GO_CATALYTIC_STEP_1_SPLICEOSOME2	-0.30197731	-0.02425134	-0.90990665	0.3891518	0.81008789	-5.58139172
homo after UVB irradiation VS homo before UVB irradiation	GO_CATALYTIC_STEP_2_SPLICEOSOME	-0.84858204	-0.00254783	-6.21353076	0.00021628	0.01131787	1.100800105
hetero after UVB irradiation VS hetero before UVB irradiation	GO_CATALYTIC_STEP_2_SPLICEOSOME1	-0.36596102	-0.05478863	-1.46094858	0.18285507	0.50719609	-5.21260662
WT after UVB irradiation VS WT before UVB irradiation	GO_CATALYTIC_STEP_2_SPLICEOSOME2	-0.31334532	-0.02974366	-1.01379694	0.34000996	0.78105961	-5.48742881
homo after UVB irradiation VS homo before UVB irradiation	GO_CATECHOLAMINE_BINDING	0.317283521	0.051610518	1.36519363	0.20792244	0.39796518	-5.62977791
hetero after UVB irradiation VS hetero before UVB irradiation	GO_CATECHOLAMINE_BINDING1	0.01554836	0.067344861	0.119586147	0.90781376	0.96555872	-6.22373596
WT after UVB irradiation VS WT before UVB irradiation	GO_CATECHOLAMINE_BINDING2	0.102770874	0.022490553	0.360379785	0.72778258	0.93296251	-5.92811551
homo after UVB irradiation VS homo before UVB irradiation	GO_CATECHOLAMINE_BIOSYNTHETIC_PROCESS	0.259470255	-0.01113084	1.298555655	0.22889254	0.42006805	-5.70974538
hetero after UVB irradiation VS hetero before UVB irradiation	GO_CATECHOLAMINE_BIOSYNTHETIC_PROCESS1	-0.12627729	0.064942701	-0.66453554	0.52537703	0.78916946	-5.99873415
WT after UVB irradiation VS WT before UVB irradiation	GO_CATECHOLAMINE_BIOSYNTHETIC_PROCESS2	0.144244364	0.0325385	0.713932855	0.49532904	0.85462068	-5.73573995
homo after UVB irradiation VS homo before UVB irradiation	GO_CATECHOLAMINE_CATABOLIC_PROCESS	0.052419797	0.086077477	0.248449161	0.8098032	0.89215283	-6.51710707
hetero after UVB irradiation VS hetero before UVB irradiation	GO_CATECHOLAMINE_CATABOLIC_PROCESS1	0.231091473	0.085611386	0.843754254	0.42376279	0.72544775	-5.8625381
WT after UVB irradiation VS WT before UVB irradiation	GO_CATECHOLAMINE_CATABOLIC_PROCESS2	0.596114898	0.037115098	2.136178063	0.06475347	0.53678963	-4.11597314
homo after UVB irradiation VS homo before UVB irradiation	GO_CATECHOLAMINE_METABOLIC_PROCESS	0.214441984	-0.00976566	1.219293615	0.25613292	0.45158744	-5.80140387
hetero after UVB irradiation VS hetero before UVB irradiation	GO_CATECHOLAMINE_METABOLIC_PROCESS1	0.088379075	0.063379124	0.63405942	0.54406545	0.79833369	-6.01906115
WT after UVB irradiation VS WT before UVB irradiation	GO_CATECHOLAMINE_METABOLIC_PROCESS2	0.18849452	0.019952873	0.880711709	0.40385466	0.81613006	-5.6063407
homo after UVB irradiation VS homo before UVB irradiation	GO_CATECHOLAMINE_SECRETION	0.122708387	0.009887712	0.796517961	0.44783682	0.62970252	-6.21553825
hetero after UVB irradiation VS hetero before UVB irradiation	GO_CATECHOLAMINE_SECRETION1	0.030973725	0.031660386	0.191967604	0.85263863	0.94342649	-6.21154772
WT after UVB irradiation VS WT before UVB irradiation	GO_CATECHOLAMINE_SECRETION2	-0.03102703	0.03069332	-0.17543536	0.86504515	0.96790654	-5.97985952
homo after UVB irradiation VS homo before UVB irradiation	GO_CATECHOLAMINE_UPTAKE	0.284501057	-0.02372843	1.389290454	0.20075639	0.3894009	-5.60024188
homo after UVB irradiation VS homo before UVB irradiation	GO_CATECHOLAMINE_UPTAKE_INVOLVED_IN_SYNAPTIC_TRANSMISSION	0.217476391	-0.03703741	0.953573968	0.36714945	0.55740992	-6.07772618
hetero after UVB irradiation VS hetero before UVB irradiation	GO_CATECHOLAMINE_UPTAKE_INVOLVED_IN_SYNAPTIC_TRANSMISSION1	0.081345799	0.052117569	0.49698259	0.63281459	0.84871371	-6.09967997
WT after UVB irradiation VS WT before UVB irradiation	GO_CATECHOLAMINE_UPTAKE_INVOLVED_IN_SYNAPTIC_TRANSMISSION2	0.228294589	0.080536382	0.868773834	0.40997984	0.81879449	-5.61635189
hetero after UVB irradiation VS hetero before UVB irradiation	GO_CATECHOLAMINE_UPTAKE1	0.080885117	0.051919269	0.50260869	0.62902877	0.84727965	-6.09672773
WT after UVB irradiation VS WT before UVB irradiation	GO_CATECHOLAMINE_UPTAKE2	0.200585636	0.021126049	0.785407526	0.45457876	0.83800359	-5.68309344
homo after UVB irradiation VS homo before UVB irradiation	GO_CATENIN_COMPLEX	0.20617313	0.011937427	1.302890534	0.22747579	0.41856112	-5.70462206
hetero after UVB irradiation VS hetero before UVB irradiation	GO_CATENIN_COMPLEX1	-0.08396005	0.047181087	-0.50026811	0.63060234	0.84800118	-6.0979597
WT after UVB irradiation VS WT before UVB irradiation	GO_CATENIN_COMPLEX2	-0.20085843	0.055620913	-0.99421199	0.34889609	0.78651615	-5.50574388
homo after UVB irradiation VS homo before UVB irradiation	GO_CATION_CATION_ANTIPORTER_ACTIVITY	-0.06636714	0.010530776	-0.35752648	0.72958814	0.84061989	-6.4812471
hetero after UVB irradiation VS hetero before UVB irradiation	GO_CATION_CATION_ANTIPORTER_ACTIVITY1	-0.02457647	0.02052122	-0.15350266	0.88187317	0.9571304	-6.21872552
WT after UVB irradiation VS WT before UVB irradiation	GO_CATION_CATION_ANTIPORTER_ACTIVITY2	0.107989486	0.010542339	0.606034142	0.56109164	0.88029724	-5.80679733
homo after UVB irradiation VS homo before UVB irradiation	GO_CATION_CHANNEL_ACTIVITY	0.147127074	0.01750411	1.049941605	0.32325089	0.51653743	-5.98340461
hetero after UVB irradiation VS hetero before UVB irradiation	GO_CATION_CHANNEL_ACTIVITY1	-0.11023484	0.015921375	-0.75046138	0.47486023	0.75784786	-5.93691134
WT after UVB irradiation VS WT before UVB irradiation	GO_CATION_CHANNEL_ACTIVITY2	-0.09210528	0.006722564	-0.51712468	0.61889389	0.9005818	-5.85738147
homo after UVB irradiation VS homo before UVB irradiation	GO_CATION_CHANNEL_COMPLEX	0.16322159	0.019129586	1.132608115	0.28892707	0.48390611	-5.89703084

hetero after UVB irradiation VS hetero before UVB irradiation	GO_CATION_CHANNEL_COMPLEX1	-0.18079107	0.001622084	-1.08135426	0.31162634	0.63234711	-5.64196719
WT after UVB irradiation VS WT before UVB irradiation	GO_CATION_CHANNEL_COMPLEX2	-0.02181719	0.005810607	-0.10357228	0.92002802	0.98043563	-5.99041042
homo after UVB irradiation VS homo before UVB irradiation	GO_CATION_CHLORIDE_SYMPORTER_ACTIVITY	0.32401003	0.037638664	1.370331699	0.20637622	0.39639385	-5.623507
hetero after UVB irradiation VS hetero before UVB irradiation	GO_CATION_CHLORIDE_SYMPORTER_ACTIVITY1	-0.30655045	0.008201144	-1.99030574	0.0824065	0.36807092	-4.50746864
WT after UVB irradiation VS WT before UVB irradiation	GO_CATION_CHLORIDE_SYMPORTER_ACTIVITY2	-0.06813143	-0.01564332	-0.32152365	0.7559561	0.93854182	-5.94188569
homo after UVB irradiation VS homo before UVB irradiation	GO_CATION_TRANSMEMBRANE_TRANSPORT	0.135042373	0.007159343	1.017356832	0.33762104	0.53072543	-6.01608681
hetero after UVB irradiation VS hetero before UVB irradiation	GO_CATION_TRANSMEMBRANE_TRANSPORT1	-0.05802445	0.007490375	-0.41929683	0.68623396	0.87376352	-6.13723245
WT after UVB irradiation VS WT before UVB irradiation	GO_CATION_TRANSMEMBRANE_TRANSPORT2	-0.04283959	0.002443517	-0.253299	0.80634945	0.95404687	-5.96235391
homo after UVB irradiation VS homo before UVB irradiation	GO_CATION_TRANSMEMBRANE_TRANSPORTER_ACTIVITY	0.101349744	0.016092423	0.783387651	0.45509543	0.63609047	-6.22611395
hetero after UVB irradiation VS hetero before UVB irradiation	GO_CATION_TRANSMEMBRANE_TRANSPORTER_ACTIVITY1	-0.0896548	0.013511965	-0.69898499	0.50473363	0.77501035	-5.97473875
WT after UVB irradiation VS WT before UVB irradiation	GO_CATION_TRANSMEMBRANE_TRANSPORTER_ACTIVITY2	-0.08256734	0.012151179	-0.52109437	0.61624752	0.90020288	-5.85528109
homo after UVB irradiation VS homo before UVB irradiation	GO_CATION_TRANSPORT	0.155642173	0.005364566	1.189597341	0.26700676	0.46224998	-5.83472368
hetero after UVB irradiation VS hetero before UVB irradiation	GO_CATION_TRANSPORT1	-0.0282031	0.004497941	-0.20578342	0.84219468	0.93977074	-6.20858312
WT after UVB irradiation VS WT before UVB irradiation	GO_CATION_TRANSPORT2	-0.01732504	-0.00016812	-0.10157077	0.92156744	0.98043563	-5.99062677
homo after UVB irradiation VS homo before UVB irradiation	GO_CATION_TRANSPORTING_ATPASE_COMPLEX	0.367116782	0.039796992	2.021100539	0.07652341	0.21262586	-4.73494516
hetero after UVB irradiation VS hetero before UVB irradiation	GO_CATION_TRANSPORTING_ATPASE_COMPLEX1	0.063985054	0.006519587	0.390933969	0.70623837	0.8835375	-6.14942984
WT after UVB irradiation VS WT before UVB irradiation	GO_CATION_TRANSPORTING_ATPASE_COMPLEX2	0.146810293	0.027568966	0.808129626	0.44210762	0.83246998	-5.66546108
homo after UVB irradiation VS homo before UVB irradiation	GO_CATSPER_COMPLEX	0.010213423	-0.02619575	0.062727347	0.95146287	0.97417176	-6.54869778
hetero after UVB irradiation VS hetero before UVB irradiation	GO_CATSPER_COMPLEX1	-0.42625447	0.046532966	-1.6534811	0.13754121	0.45089754	-4.96718143
WT after UVB irradiation VS WT before UVB irradiation	GO_CATSPER_COMPLEX2	0.040518487	-0.0751282	0.156310252	0.87961521	0.9693507	-5.98319522
homo after UVB irradiation VS homo before UVB irradiation	GO_CAVEOLA	0.318270905	-0.03786442	2.480387903	0.03697078	0.1377543	-4.03926781
hetero after UVB irradiation VS hetero before UVB irradiation	GO_CAVEOLA1	0.049912575	-0.0085173	0.292391578	0.7775636	0.91343573	-6.18538069
WT after UVB irradiation VS WT before UVB irradiation	GO_CAVEOLA2	0.132005042	-0.00877302	0.774264367	0.46078053	0.84000187	-5.6915845
homo after UVB irradiation VS homo before UVB irradiation	GO_CAVEOLIN_MEDIATED_ENDOCYTOSIS	0.438879722	-0.02872441	2.756785051	0.02387267	0.10849676	-3.61081606
hetero after UVB irradiation VS hetero before UVB irradiation	GO_CAVEOLIN_MEDIATED_ENDOCYTOSIS1	0.136788132	0.057870657	0.902584197	0.3935898	0.70256648	-5.8119446
WT after UVB irradiation VS WT before UVB irradiation	GO_CAVEOLIN_MEDIATED_ENDOCYTOSIS2	0.274045225	0.016769273	1.477959728	0.17722546	0.6808946	-4.98357875
homo after UVB irradiation VS homo before UVB irradiation	GO_CCR_CHEMOKINE_RECEPTOR_BINDING	0.196054761	0.013891671	0.92333786	0.38179975	0.57206583	-6.10583371
hetero after UVB irradiation VS hetero before UVB irradiation	GO_CCR_CHEMOKINE_RECEPTOR_BINDING1	0.243634915	0.008502348	1.681402565	0.13189927	0.44121703	-4.930383
WT after UVB irradiation VS WT before UVB irradiation	GO_CCR_CHEMOKINE_RECEPTOR_BINDING2	0.099185637	-0.00742654	0.37290423	0.71879183	0.93096927	-5.92335353
homo after UVB irradiation VS homo before UVB irradiation	GO_CCR1_CHEMOKINE_RECEPTOR_BINDING	0.881419576	0.053695187	3.805837203	0.00481921	0.04526889	-2.01037044
hetero after UVB irradiation VS hetero before UVB irradiation	GO_CCR1_CHEMOKINE_RECEPTOR_BINDING1	1.062710092	0.039807329	5.26283034	0.00080916	0.08366654	-0.10275183
WT after UVB irradiation VS WT before UVB irradiation	GO_CCR1_CHEMOKINE_RECEPTOR_BINDING2	0.890389518	0.04786043	3.001627144	0.0167948	0.37993508	-2.89108221
homo after UVB irradiation VS homo before UVB irradiation	GO_CCR2_CHEMOKINE_RECEPTOR_BINDING	0.93520314	-0.04818443	4.381536076	0.00212756	0.03092226	-1.18504912
hetero after UVB irradiation VS hetero before UVB irradiation	GO_CCR2_CHEMOKINE_RECEPTOR_BINDING1	0.768422733	-0.00021236	3.702883482	0.00621817	0.13546947	-2.0437877
WT after UVB irradiation VS WT before UVB irradiation	GO_CCR2_CHEMOKINE_RECEPTOR_BINDING2	0.714300489	0.069382406	2.922458639	0.01896892	0.39157258	-3.00252658
homo after UVB irradiation VS homo before UVB irradiation	GO_CCR3_CHEMOKINE_RECEPTOR_BINDING	-0.31386224	-0.08935415	-1.03981226	0.32766658	0.52110957	-5.99364882
hetero after UVB irradiation VS hetero before UVB irradiation	GO_CCR3_CHEMOKINE_RECEPTOR_BINDING1	-0.31813458	0.004706285	-1.2791441	0.23734753	0.56648526	-5.42854504
WT after UVB irradiation VS WT before UVB irradiation	GO_CCR3_CHEMOKINE_RECEPTOR_BINDING2	-0.11654602	-0.03932369	-0.42450332	0.68225254	0.91738041	-5.90208818
homo after UVB irradiation VS homo before UVB irradiation	GO_CCR4_NOT_COMPLEX	-0.03645331	-0.01822694	-0.13464995	0.89608571	0.94336694	-6.54091729
hetero after UVB irradiation VS hetero before UVB irradiation	GO_CCR4_NOT_COMPLEX1	0.533247886	0.028682886	2.761513527	0.02505838	0.22633644	-3.38774887
WT after UVB irradiation VS WT before UVB irradiation	GO_CCR4_NOT_COMPLEX2	-0.23258058	0.10498236	-0.92999024	0.37926555	0.80338597	-5.56385049
homo after UVB irradiation VS homo before UVB irradiation	GO_CCR4_NOT_CORE_COMPLEX	-0.10938053	-0.05689479	-0.2943751	0.77567463	0.87053239	-6.50354833
hetero after UVB irradiation VS hetero before UVB irradiation	GO_CCR4_NOT_CORE_COMPLEX1	1.022669235	0.056419657	3.447699444	0.00897639	0.15043564	-2.39842078
WT after UVB irradiation VS WT before UVB irradiation	GO_CCR4_NOT_CORE_COMPLEX2	-0.04795248	0.067073956	-0.14575176	0.88768028	0.97111575	-5.98487305
homo after UVB irradiation VS homo before UVB irradiation	GO_CCR5_CHEMOKINE_RECEPTOR_BINDING	0.815795442	-0.06951107	2.807602664	0.02203718	0.10327171	-3.53184746
hetero after UVB irradiation VS hetero before UVB irradiation	GO_CCR5_CHEMOKINE_RECEPTOR_BINDING1	1.065055255	0.042370445	7.336424317	8.90E-05	0.07788488	1.869078513
WT after UVB irradiation VS WT before UVB irradiation	GO_CCR5_CHEMOKINE_RECEPTOR_BINDING2	0.912436854	-0.02294399	4.505269376	0.00192832	0.20495092	-0.93309625
homo after UVB irradiation VS homo before UVB irradiation	GO_CCR6_CHEMOKINE_RECEPTOR_BINDING	0.052886495	0.184763978	0.14319275	0.88954166	0.94014925	-6.53961771
hetero after UVB irradiation VS hetero before UVB irradiation	GO_CCR6_CHEMOKINE_RECEPTOR_BINDING1	-0.43744695	-0.02049562	-1.26506639	0.24209533	0.57027474	-5.44452497
WT after UVB irradiation VS WT before UVB irradiation	GO_CCR6_CHEMOKINE_RECEPTOR_BINDING2	-0.42765027	0.042213514	-1.12187441	0.29407797	0.75745298	-5.38160517
homo after UVB irradiation VS homo before UVB irradiation	GO_CD4_POSITIVE_ALPHA_BETA_T_CELL_ACTIVATION	0.365486216	-0.01786582	2.46944077	0.03761873	0.13909788	-4.05615614
hetero after UVB irradiation VS hetero before UVB irradiation	GO_CD4_POSITIVE_ALPHA_BETA_T_CELL_ACTIVATION1	0.299481921	0.033681179	1.851852283	0.10188445	0.396782	-4.7002378
WT after UVB irradiation VS WT before UVB irradiation	GO_CD4_POSITIVE_ALPHA_BETA_T_CELL_ACTIVATION2	0.243974148	-0.00816181	1.181000193	0.27112542	0.74431614	-5.32048093
homo after UVB irradiation VS homo before UVB irradiation	GO_CD4_POSITIVE_ALPHA_BETA_T_CELL_CYTOKINE_PRODUCTION	0.300557832	-0.03172076	1.637235305	0.13872088	0.30821545	-5.27931597
hetero after UVB irradiation VS hetero before UVB irradiation	GO_CD4_POSITIVE_ALPHA_BETA_T_CELL_CYTOKINE_PRODUCTION1	-0.00751841	0.031567567	-0.05350789	0.95866342	0.98503151	-6.22993173
WT after UVB irradiation VS WT before UVB irradiation	GO_CD4_POSITIVE_ALPHA_BETA_T_CELL_CYTOKINE_PRODUCTION2	0.085365637	-0.0400749	0.334591616	0.74643498	0.93606982	-5.93742452
homo after UVB irradiation VS homo before UVB irradiation	GO_CD4_POSITIVE_ALPHA_BETA_T_CELL_DIFFERENTIATION	0.343327929	-0.01560489	2.252375165	0.05309389	0.17073578	-4.38856731
hetero after UVB irradiation VS hetero before UVB irradiation	GO_CD4_POSITIVE_ALPHA_BETA_T_CELL_DIFFERENTIATION1	0.264279735	0.041386602	1.635031585	0.14139063	0.45653394	-4.99134185
WT after UVB irradiation VS WT before UVB irradiation	GO_CD4_POSITIVE_ALPHA_BETA_T_CELL_DIFFERENTIATION2	0.279948442	-0.01620159	1.392819205	0.20072298	0.69758436	-5.08481085
homo after UVB irradiation VS homo before UVB irradiation	GO_CD4_POSITIVE_ALPHA_BETA_T_CELL_PROLIFERATION	0.558531499	-0.04886454	3.344008213	0.00959955	0.06534161	-2.70378727
hetero after UVB irradiation VS hetero before UVB irradiation	GO_CD4_POSITIVE_ALPHA_BETA_T_CELL_PROLIFERATION1	0.542503842	0.030468709	2.291508505	0.05173737	0.30223143	-4.07581631
WT after UVB irradiation VS WT before UVB irradiation	GO_CD4_POSITIVE_ALPHA_BETA_T_CELL_PROLIFERATION2	0.227610627	-0.00735953	0.863732931	0.41258598	0.820663	-5.62054567
homo after UVB irradiation VS homo before UVB irradiation	GO_CD4_POSITIVE_CD25_POSITIVE_ALPHA_BETA_REGULATORY_T_CELL_DIFFERENTIATION	0.283256322	0.002877114	1.121630111	0.29331126	0.48809137	-5.90877932
hetero after UVB irradiation VS hetero before UVB irradiation	GO_CD4_POSITIVE_CD25_POSITIVE_ALPHA_BETA_REGULATORY_T_CELL_DIFFERENTIATION1	0.600443678	0.009707138	3.15859165	0.01374658	0.17682818	-2.81010211
WT after UVB irradiation VS WT before UVB irradiation	GO_CD4_POSITIVE_CD25_POSITIVE_ALPHA_BETA_REGULATORY_T_CELL_DIFFERENTIATION2	0.274111925	-0.03314537	1.060371811	0.31957576	0.77323848	-5.44279429
homo after UVB irradiation VS homo before UVB irradiation	GO_CD4_POSITIVE_OR_CD8_POSITIVE_ALPHA_BETA_T_CELL_LINEAGE_COMMITMENT	0.443086505	-0.06485496	2.560531756	0.03255751	0.12837022	-3.91537691
hetero after UVB irradiation VS hetero before UVB irradiation	GO_CD4_POSITIVE_OR_CD8_POSITIVE_ALPHA_BETA_T_CELL_LINEAGE_COMMITMENT1	0.202084334	0.021338141	1.025818264	0.33553603	0.65513148	-5.69726972
WT after UVB irradiation VS WT before UVB irradiation	GO_CD4_POSITIVE_OR_CD8_POSITIVE_ALPHA_BETA_T_CELL_LINEAGE_COMMITMENT2	0.404966549	0.030251012	1.667470138	0.13352204	0.6402309	-4.74717767
homo after UVB irradiation VS homo before UVB irradiation	GO_CD4_RECEPTOR_BINDING	0.158732712	-0.10130594	0.655678398	0.52972169	0.69713619	-6.32091587
hetero after UVB irradiation VS hetero before UVB irradiation	GO_CD4_RECEPTOR_BINDING1	0.486906614	0.043462238	2.98678092	0.01779092	0.19518423	-3.05873313
WT after UVB irradiation VS WT before UVB irradiation	GO_CD4_RECEPTOR_BINDING2	0.251403785	0.015379783	0.736872002	0.48200111	0.85127391	-5.71931613
homo after UVB irradiation VS homo before UVB irradiation	GO_CD40_RECEPTOR_COMPLEX	0.199819374	-0.0082675	1.321109705	0.22160221	0.41264149	-5.68296774
hetero after UVB irradiation VS hetero before UVB irradiation	GO_CD40_RECEPTOR_COMPLEX1	0.092510653	-0.07816552	0.536684629	0.60635151	0.83528483	-6.07818524
WT after UVB irradiation VS WT before UVB irradiation	GO_CD40_RECEPTOR_COMPLEX2	0.33335051	-0.08766873	1.343471093	0.21555025	0.7129431	-5.14187913
homo after UVB irradiation VS homo before UVB irradiation	GO_CD40_SIGNALING_PATHWAY	0.551638581	-0.05588838	2.971743351	0.01704054	0.08914115	-3.27700608
hetero after UVB irradiation VS hetero before UVB irradiation	GO_CD40_SIGNALING_PATHWAY1	0.435845161	0.012585562	2.378796102	0.04519534	0.2842629	-3.94869675
WT after UVB irradiation VS WT before UVB irradiation	GO_CD40_SIGNALING_PATHWAY2	0.519056495	-0.0357836	2.077250871	0.07100036	0.54770112	-4.1978712
homo after UVB irradiation VS homo before UVB irradiation	GO_CD8_POSITIVE_ALPHA_BETA_T_CELL_ACTIVATION	0.517688877	-0.03675981	3.115870511	0.01362245	0.07898711	-3.05402305
hetero after UVB irradiation VS hetero before UVB irradiation	GO_CD8_POSITIVE_ALPHA_BETA_T_CELL_ACTIVATION1	0.313463178	0.027997434	1.271638087	0.23986909	0.5680482	-5.43707944
WT after UVB irradiation VS WT before UVB irradiation	GO_CD8_POSITIVE_ALPHA_BETA_T_CELL_ACTIVATION2	0.200132077	-0.11461223	0.700548477	0.5032132	0.85763155	-5.74511307
homo after UVB irradiation VS homo before UVB irradiation	GO_CD8_POSITIVE_ALPHA_BETA_T_CELL_DIFFERENTIATION	0.492295244	-0.05199196	2.994810748	0.01643864	0.0877977	-3.24125313
hetero after UVB irradiation VS hetero before UVB irradiation	GO_CD8_POSITIVE_ALPHA_BETA_T_CELL_DIFFERENTIATION1	0.358913975	0.057520596	1.503744673	0.17175529	0.49471425	-5.15941013
WT after UVB irradiation VS WT before UVB irradiation	GO_CD8_POSITIVE_ALPHA_BETA_T_CELL_DIFFERENTIATION2	0.263908149	-0.12788242	0.922189945	0.38308322	0.80533983	-5.57069963
homo after UVB irradiation VS homo before UVB irradiation	GO_CD8_POSITIVE_ALPHA_BETA_T_CELL_PROLIFERATION	0.35323353	-0.07621751	1.718925108	0.12245415	0.28475511	-5.16763218
hetero after UVB irradiation VS hetero before UVB irradiation	GO_CD8_POSITIVE_ALPHA_BETA_T_CELL_PROLIFERATION1	0.114023448	0.044422434	0.371035585	0.72042089	0.89079562	-6.15749732
WT after UVB irradiation VS WT before UVB irradiation	GO_CD8_POSITIVE_ALPHA_BETA_T_CELL_PROLIFERATION2	-0.0460481	-0.05621645	-0.15308251	0.88207918	0.96981343	-5.98372051
homo after UVB irradiation VS homo before UVB irradiation	GO_CD95_DEATH_INDUCING_SIGNALING_COMPLEX	0.181726594	-0.03036822	1.040106771	0.32753754	0.52096049	-5.99335203
hetero after UVB irradiation VS hetero before UVB irradiation	GO_CD95_DEATH_INDUCING_SIGNALING_COMPLEX1	0.403682647	0.002495916	2.037174922	0.07666837	0.35706231	-4.44125906
WT after UVB irradiation VS WT before UVB irradiation	GO_CD95_DEATH_INDUCING_SIGNALING_COMPLEX2	0.451367477	-0.07095725	2.311153436	0.04922285	0.50780915	-3.87022715
homo after UVB irradiation VS homo before UVB irradiation	GO_CDC42_PROTEIN_SIGNAL_TRANSDUCTION	0.362749621	-0.01254406	2.020787042	0.07656115	0.21265693	-4.73540725
hetero after UVB irradiation VS hetero before UVB irradiation	GO_CDC42_PROTEIN_SIGNAL_TRANSDUCTION1	0.419933055	0.005875247	1.983538547	0.08326871	0.36887213	-4.51699183
WT after UVB irradiation VS WT before UVB irradiation	GO_CDC42_PROTEIN_SIGNAL_TRANSDUCTION2	0.102113218	0.097728238	0.487804303	0.63862135	0.90634429	-5.87242969
homo after UVB irradiation VS homo before UVB irradiation	GO_CDC73_PAF1_COMPLEX	-0.35435805	-0.03653225	-1.39336597	0.19956575	0.38831642	-5.59521493
hetero after UVB irradiation VS hetero before UVB irradiation	GO_CDC73_PAF1_COMPLEX1	-0.17785179	-0.05987785	-0.7035213	0.50205391	0.77390992	-5.97149949
WT after UVB irradiation VS WT before UVB irradiation	GO_CDC73_PAF1_COMPLEX2	-0.25339562	0.010945363	-0.83939756	0.4253318	0.82664851	-5.64050857
homo after UVB irradiation VS homo before UVB irradiation	GO_CDP_ALCOHOL_PHOSPHATIDYLTRANSFERASE_ACTIVITY	0.106886876	-0.00421291	0.54249188	0.60168901	0.7508352	-6.39218379
hetero after UVB irradiation VS hetero before UVB irradiation	GO_CDP_ALCOHOL_PHOSPHATIDYLTRANSFERASE_ACTIVITY1	0.176234849	-0.04845328	0.791778605	0.45174186	0.74387936	-5.90488087
WT after UVB irradiation VS WT before UVB irradiation	GO_CDP_ALCOHOL_PHOSPHATIDYLTRANSFERASE_ACTIVITY2	0.037252363	0.116376689	0.165487118	0.87261756	0.96914919	-5.9816423
homo after UVB irradiation VS homo before UVB irradiation	GO_CDP_CHOLINE_PATHWAY	0.503278884	-0.0120969	2.784366328	0.02285778	0.10552788	-3.56795581
hetero after UVB irradiation VS hetero before UVB irradiation	GO_CDP_CHOLINE_PATHWAY1	0.19430167	-0.0163781	0.737883412	0.48205072	0.76203732	-5.94636942
WT after UVB irradiation VS WT before UVB irradiation	GO_CDP_CHOLINE_PATHWAY2	-0.15848976	-0.04389683	-0.68220309	0.51414742	0.85974355	-5.75770674
homo after UVB irradiation VS homo before UVB irradiation	GO_CDP_DIACYLGLYCEROL_METABOLIC_PROCESS	0.305464873	-0.1181106	1.776670056	0.11204049	0.26862412	-5.08717179
hetero after UVB irradiation VS hetero before UVB irradiation	GO_CDP_DIACYLGLYCEROL_METABOLIC_PROCESS1	-0.0758395	-0.03975338	-0.2905	0.7789577	0.91391427	-6.185972
WT after UVB irradiation VS WT before UVB irradiation	GO_CDP_DIACYLGLYCEROL_METABOLIC_PROCESS2	0.307673839	0.030270551	1.603392228	0.14706028	0.65371647	-4.82867046
homo after UVB irradiation VS homo before UVB irradiation	GO_CELL_ACTIVATION	0.502928177	-0.02603891	3.768627345	0.00508901	0.04644702	-2.06530481
homo after UVB irradiation VS homo before UVB irradiation	GO_CELL_ACTIVATION_INVOLVED_IN_IMMUNE_RESPONSE	0.59895753	-0.02501426	4.466106101	0.00189446	0.02942787	-1.06795376
hetero after UVB irradiation VS hetero before UVB irradiation	GO_CELL_ACTIVATION_INVOLVED_IN_IMMUNE_RESPONSE1	0.610364935	0.021651505	4.446151343	0.002251	0.10650321	-1.06780163
WT after UVB irradiation VS WT before UVB irradiation	GO_CELL_ACTIVATION_INVOLVED_IN_IMMUNE_RESPONSE2	0.515280433	0.008810346	3.254561991	0.01143154	0.34079835	-2.53891957
hetero after UVB irradiation VS hetero before UVB irradiation	GO_CELL_ACTIVATION1	0.489916439	0.021408441	3.461147099	0.00880246	0.14994695	-2.37951299
WT after UVB irradiation VS WT before UVB irradiation	GO_CELL_ACTIVATION2	0.388868168	0.001762157	2.308599049	0.04942051	0.50822195	-3.87383629
homo after UVB irradiation VS homo before UVB irradiation	GO_CELL_ADHESION_INVOLVED_IN_HEART_MORPHOGENESIS	-0.06528614	-0.08026725	-0.35022413	0.73486247	0.84395832	-6.48404069
hetero after UVB irradiation VS hetero before UVB irradiation	GO_CELL_ADHESION_INVOLVED_IN_HEART_MORPHOGENESIS1	-0.11362012	-0.00901516	-0.36065353	0.72786724	0.89257949	-6.16154526
WT after UVB irradiation VS WT before UVB irradiation	GO_CELL_ADHESION_INVOLVED_IN_HEART_MORPHOGENESIS2	-0.84122229	-0.01403578	-3.11736222	0.01407219	0.36090271	-2.72913273
homo after UVB irradiation VS homo before UVB irradiation	GO_CELL_ADHESION_MEDIATED_BY_INTEGRIN	0.654253039	-0.02101043	4.619208839	0.0015396	0.02666655	-0.8587941
hetero after UVB irradiation VS hetero before UVB irradiation	GO_CELL_ADHESION_MEDIATED_BY_INTEGRIN1	0.492271476	0.034626903	2.412053681	0.04292744	0.27767067	-3.90011721
WT after UVB irradiation VS WT before UVB irradiation	GO_CELL_ADHESION_MEDIATED_BY_INTEGRIN2	0.424025175	0.021746277	2.190698401	0.05945582	0.52638053	-4.03976794
homo after UVB irradiation VS homo before UVB irradiation	GO_CELL_ADHESION_MEDIATOR_ACTIVITY	0.171961502	-0.01634133	0.936597713	0.37532311	0.56532137	-6.09359707
hetero after UVB irradiation VS hetero before UVB irradiation	GO_CELL_ADHESION_MEDIATOR_ACTIVITY1	0.124194748	-0.00302771	0.988439799	0.35241133	0.66877878	-5.73325194
WT after UVB irradiation VS WT before UVB irradiation	GO_CELL_ADHESION_MEDIATOR_ACTIVITY2	-0.01710615	-0.00276315	-0.11528139	0.91102957	0.97857624	-5.98906039
homo after UVB irradiation VS homo before UVB irradiation	GO_CELL_ADHESION_MOLECULE_BINDING	0.236437954	-0.01578976	1.400013424	0.19763686	0.38629499	-5.58699627
hetero after UVB irradiation VS hetero before UVB irradiation	GO_CELL_ADHESION_MOLECULE_BINDING1	0.330191075	0.005851837	2.674240801	0.02864587	0.23751402	-3.51574393
WT after UVB irradiation VS WT before UVB irradiation	GO_CELL_ADHESION_MOLECULE_BINDING2	0.163266007	0.00368229	1.093847434	0.30548967	0.76523073	-5.40979959
homo after UVB irradiation VS homo before UVB irradiation	GO_CELL_ADHESION_MOLECULE_PRODUCTION	0.872907055	-0.04860753	5.002577335	0.00092979	0.02088233	-0.35126749
hetero after UVB irradiation VS hetero before UVB irradiation	GO_CELL_ADHESION_MOLECULE_PRODUCTION1	0.763157224	0.072755099	4.227973013	0.0030078	0.11447176	-1.34490397
WT after UVB irradiation VS WT before UVB irradiation	GO_CELL_ADHESION_MOLECULE_PRODUCTION2	0.536098842	0.058311778	2.381897467	0.04405148	0.49769531	-3.77008719
homo after UVB irradiation VS homo before UVB irradiation	GO_CELL_ADHESIVE_PROTEIN_BINDING_INVOLVED_IN_BUNDLE_OF_HIS_CELL_PURKINJE_MYOCYTE_COMMUNICATION	-0.24870323	-0.00035809	-0.76873386	0.46328868	0.64273301	-6.23773799
hetero after UVB irradiation VS hetero before UVB irradiation	GO_CELL_ADHESIVE_PROTEIN_BINDING_INVOLVED_IN_BUNDLE_OF_HIS_CELL_PURKINJE_MYOCYTE_COMMUNICATION1	-0.15417564	-0.02994617	-0.4750006	0.64771659	0.85523965	-6.11091569
WT after UVB irradiation VS WT before UVB irradiation	GO_CELL_ADHESIVE_PROTEIN_BINDING_INVOLVED_IN_BUNDLE_OF_HIS_CELL_PURKINJE_MYOCYTE_COMMUNICATION2	-0.49288229	0.033719856	-1.28731935	0.23355605	0.72468179	-5.20527626
homo after UVB irradiation VS homo before UVB irradiation	GO_CELL_AGGREGATION	0.169230653	0.03082834	1.015202056	0.33858828	0.53154757	-6.01822002
hetero after UVB irradiation VS hetero before UVB irradiation	GO_CELL_AGGREGATION1	-0.1944536	0.047857363	-1.08109295	0.31173562	0.63238532	-5.64223243
WT after UVB irradiation VS WT before UVB irradiation	GO_CELL_AGGREGATION2	-0.16981775	0.026480906	-0.95719581	0.36616948	0.79769162	-5.53960575
homo after UVB irradiation VS homo before UVB irradiation	GO_CELL_AGING	0.021786092	-0.01396386	0.157675088	0.87846843	0.93377215	-6.53723298
hetero after UVB irradiation VS hetero before UVB irradiation	GO_CELL_AGING1	0.217554221	-0.0112851	1.496442824	0.17360544	0.49630531	-5.16854502
WT after UVB irradiation VS WT before UVB irradiation	GO_CELL_AGING2	0.105831206	-0.0333757	0.683529737	0.51335178	0.85974355	-5.75680592
homo after UVB irradiation VS homo before UVB irradiation	GO_CELL_BODY	0.045564254	-0.00599889	0.370457185	0.72028595	0.83419084	-6.4761634
homo after UVB irradiation VS homo before UVB irradiation	GO_CELL_BODY_FIBER	0.794229103	-0.03969898	4.681630726	0.00141616	0.02530056	-0.77457146
hetero after UVB irradiation VS hetero before UVB irradiation	GO_CELL_BODY_FIBER1	0.252674831	0.004737537	1.627791571	0.14292814	0.45818529	-5.00078847
WT after UVB irradiation VS WT before UVB irradiation	GO_CELL_BODY_FIBER2	0.232488908	0.023119045	1.204813701	0.2623033	0.74156847	-5.2952528
homo after UVB irradiation VS homo before UVB irradiation	GO_CELL_BODY_MEMBRANE	0.20579289	0.007653951	1.577159411	0.15191908	0.32599532	-5.35970737
hetero after UVB irradiation VS hetero before UVB irradiation	GO_CELL_BODY_MEMBRANE1	0.208341719	0.038281436	1.266786892	0.24151081	0.5699238	-5.4425781
WT after UVB irradiation VS WT before UVB irradiation	GO_CELL_BODY_MEMBRANE2	0.171682894	0.047260741	0.924091377	0.38215002	0.8049904	-5.56903429
hetero after UVB irradiation VS hetero before UVB irradiation	GO_CELL_BODY1	-0.09891108	-0.00306355	-0.75956426	0.46970065	0.75417736	-5.9299806
WT after UVB irradiation VS WT before UVB irradiation	GO_CELL_BODY2	-0.0335582	-0.00434712	-0.22139937	0.83026258	0.95977959	-5.97028516
homo after UVB irradiation VS homo before UVB irradiation	GO_CELL_CELL_ADHESION	0.321763635	-0.01555727	2.477835473	0.03712084	0.13815329	-4.04320631
homo after UVB irradiation VS homo before UVB irradiation	GO_CELL_CELL_ADHESION_INVOLVED_IN_GASTRULATION	0.745489538	0.013159471	4.870071098	0.00110418	0.02245522	-0.52405306
hetero after UVB irradiation VS hetero before UVB irradiation	GO_CELL_CELL_ADHESION_INVOLVED_IN_GASTRULATION1	0.506471505	0.030353356	2.535475052	0.03547118	0.25868552	-3.71936566
WT after UVB irradiation VS WT before UVB irradiation	GO_CELL_CELL_ADHESION_INVOLVED_IN_GASTRULATION2	0.558449364	0.063037191	2.261778903	0.05318686	0.51991848	-3.93989231
homo after UVB irradiation VS homo before UVB irradiation	GO_CELL_CELL_ADHESION_MEDIATED_BY_CADHERIN	0.210230973	0.006924622	1.2157973	0.25739393	0.45283289	-5.80535642
hetero after UVB irradiation VS hetero before UVB irradiation	GO_CELL_CELL_ADHESION_MEDIATED_BY_CADHERIN1	0.350981839	-0.05161037	2.442095609	0.04097767	0.27357274	-3.85618019
WT after UVB irradiation VS WT before UVB irradiation	GO_CELL_CELL_ADHESION_MEDIATED_BY_CADHERIN2	0.091025653	0.000956071	0.58938834	0.57167388	0.88391702	-5.81682634
homo after UVB irradiation VS homo before UVB irradiation	GO_CELL_CELL_ADHESION_MEDIATED_BY_INTEGRIN	0.457445132	-0.02678904	1.847502606	0.10039762	0.25117856	-4.98691851
hetero after UVB irradiation VS hetero before UVB irradiation	GO_CELL_CELL_ADHESION_MEDIATED_BY_INTEGRIN1	0.331529766	-0.02584206	1.292113534	0.23304352	0.56147329	-5.41372322

WT after UVB irradiation VS WT before UVB irradiation	GO_CELL_CELL_ADHESION_MEDIATED_BY_INTEGRIN2	0.288226714	-0.04825439	0.914165531	0.38703982	0.8091913	-5.57769754
homo after UVB irradiation VS homo before UVB irradiation	GO_CELL_CELL_ADHESION_VIA_PLASMA_MEMBRANE_ADHESION_MOLECULES	0.334044101	-0.01521487	2.753063019	0.02401312	0.10884752	-3.61659973
hetero after UVB irradiation VS hetero before UVB irradiation	GO_CELL_CELL_ADHESION_VIA_PLASMA_MEMBRANE_ADHESION_MOLECULES1	0.246497503	0.025523687	1.71414583	0.12555685	0.43247359	-4.88688392
WT after UVB irradiation VS WT before UVB irradiation	GO_CELL_CELL_ADHESION_VIA_PLASMA_MEMBRANE_ADHESION_MOLECULES2	0.140449856	0.007677261	0.740554325	0.47988347	0.85038327	-5.71663771
hetero after UVB irradiation VS hetero before UVB irradiation	GO_CELL_CELL_ADHESION1	0.247759711	0.007179522	1.736405478	0.12140873	0.42661488	-4.85710749
WT after UVB irradiation VS WT before UVB irradiation	GO_CELL_CELL_ADHESION2	0.137319897	-0.00347704	0.797208088	0.4480726	0.8349077	-5.67398923
homo after UVB irradiation VS homo before UVB irradiation	GO_CELL_CELL_CONTACT_ZONE	0.367254867	-0.01234985	2.53149804	0.03409127	0.13214731	-3.96030573
hetero after UVB irradiation VS hetero before UVB irradiation	GO_CELL_CELL_CONTACT_ZONE1	0.104953943	-0.02483598	0.70892283	0.49887489	0.77207763	-5.96761847
WT after UVB irradiation VS WT before UVB irradiation	GO_CELL_CELL_CONTACT_ZONE2	0.079302598	-0.01055808	0.439558661	0.67175146	0.91542141	-5.89538818
homo after UVB irradiation VS homo before UVB irradiation	GO_CELL_CELL_JUNCTION	0.099461714	-0.00443356	0.667660914	0.52241526	0.69152247	-6.31265655
homo after UVB irradiation VS homo before UVB irradiation	GO_CELL_CELL_JUNCTION_ASSEMBLY	-0.05327584	0.02796178	-0.32555169	0.75279158	0.85611478	-6.49306483
hetero after UVB irradiation VS hetero before UVB irradiation	GO_CELL_CELL_JUNCTION_ASSEMBLY1	0.042265661	0.004321894	0.280474477	0.78636067	0.91684087	-6.18904323
WT after UVB irradiation VS WT before UVB irradiation	GO_CELL_CELL_JUNCTION_ASSEMBLY2	-0.11226786	-0.00480633	-0.72096091	0.49122081	0.85289953	-5.7307561
homo after UVB irradiation VS homo before UVB irradiation	GO_CELL_CELL_JUNCTION_MAINTENANCE	0.418968407	-0.04043346	2.349537558	0.04550713	0.15527136	-4.24043204
hetero after UVB irradiation VS hetero before UVB irradiation	GO_CELL_CELL_JUNCTION_MAINTENANCE1	0.412922684	0.029382001	1.986294304	0.08291655	0.36857799	-4.51311492
WT after UVB irradiation VS WT before UVB irradiation	GO_CELL_CELL_JUNCTION_MAINTENANCE2	0.08425097	-0.03558953	0.404556889	0.6962788	0.92251627	-5.91062142
homo after UVB irradiation VS homo before UVB irradiation	GO_CELL_CELL_JUNCTION_ORGANIZATION	0.001942161	0.013240167	0.011201674	0.99132621	0.99546635	-6.55078785
hetero after UVB irradiation VS hetero before UVB irradiation	GO_CELL_CELL_JUNCTION_ORGANIZATION1	0.039338018	0.006504867	0.290019111	0.77931225	0.91401375	-6.18612172
WT after UVB irradiation VS WT before UVB irradiation	GO_CELL_CELL_JUNCTION_ORGANIZATION2	-0.11841946	0.001510657	-0.77181781	0.4621497	0.8408841	-5.69343493
hetero after UVB irradiation VS hetero before UVB irradiation	GO_CELL_CELL_JUNCTION1	0.061068759	-0.00444837	0.487910756	0.63894342	0.85207854	-6.10437472
WT after UVB irradiation VS WT before UVB irradiation	GO_CELL_CELL_JUNCTION2	-0.08040745	-0.00037378	-0.53321118	0.60820681	0.89753595	-5.84877766
homo after UVB irradiation VS homo before UVB irradiation	GO_CELL_CELL_RECOGNITION	-0.1493191	0.042993808	-0.92052524	0.38318397	0.57302856	-6.10841114
hetero after UVB irradiation VS hetero before UVB irradiation	GO_CELL_CELL_RECOGNITION1	-0.15616471	0.037838682	-1.15305783	0.28277473	0.60825421	-5.56745527
WT after UVB irradiation VS WT before UVB irradiation	GO_CELL_CELL_RECOGNITION2	-0.01496049	0.018439857	-0.09234735	0.9286659	0.98233894	-5.99156936
homo after UVB irradiation VS homo before UVB irradiation	GO_CELL_CELL_SIGNALING	0.135874158	-0.00249342	1.128576107	0.29053117	0.48532584	-5.90135552
homo after UVB irradiation VS homo before UVB irradiation	GO_CELL_CELL_SIGNALING_BY_WNT	-0.05317251	-0.01538192	-0.39330883	0.70396735	0.82336897	-6.46675292
hetero after UVB irradiation VS hetero before UVB irradiation	GO_CELL_CELL_SIGNALING_BY_WNT1	-0.00807037	0.005032138	-0.05485456	0.95762424	0.9847303	-6.22985262
WT after UVB irradiation VS WT before UVB irradiation	GO_CELL_CELL_SIGNALING_BY_WNT2	-0.05956393	0.019598706	-0.35733913	0.72997216	0.93327298	-5.9292479
homo after UVB irradiation VS homo before UVB irradiation	GO_CELL_CELL_SIGNALING_INVOLVED_IN_CARDIAC_CONDUCTION	0.329590091	-0.03020917	2.443067568	0.03922705	0.14232962	-4.09680313
hetero after UVB irradiation VS hetero before UVB irradiation	GO_CELL_CELL_SIGNALING_INVOLVED_IN_CARDIAC_CONDUCTION1	-0.03940641	0.027683238	-0.18292321	0.85949266	0.94673845	-6.213378
WT after UVB irradiation VS WT before UVB irradiation	GO_CELL_CELL_SIGNALING_INVOLVED_IN_CARDIAC_CONDUCTION2	-0.01179751	-0.00225519	-0.0558157	0.95684126	0.98841297	-5.99442316
hetero after UVB irradiation VS hetero before UVB irradiation	GO_CELL_CELL_SIGNALING1	0.043213948	0.008654207	0.320987145	0.75659613	0.90565384	-6.17598575
WT after UVB irradiation VS WT before UVB irradiation	GO_CELL_CELL_SIGNALING2	0.000951112	0.011253039	0.006372233	0.99506992	0.99875662	-5.99604524
homo after UVB irradiation VS homo before UVB irradiation	GO_CELL_CHEMOTAXIS	0.521957675	-0.01905456	3.761101987	0.0051455	0.04674385	-2.07643647
hetero after UVB irradiation VS hetero before UVB irradiation	GO_CELL_CHEMOTAXIS1	0.43321809	0.019010084	2.785897413	0.02414129	0.22250398	-3.35202188
WT after UVB irradiation VS WT before UVB irradiation	GO_CELL_CHEMOTAXIS2	0.330180412	-0.00331287	1.539393021	0.16181887	0.66526114	-4.90851615
homo after UVB irradiation VS homo before UVB irradiation	GO_CELL_COMMUNICATION_BY_ELECTRICAL_COUPLING	0.527286097	-0.03082906	3.685695322	0.00574984	0.05029665	-2.18837395
homo after UVB irradiation VS homo before UVB irradiation	GO_CELL_COMMUNICATION_BY_ELECTRICAL_COUPLING_INVOLVED_IN_CARDIAC_CONDUCTION	0.650247552	-0.04655346	4.662069411	0.00145365	0.02572123	-0.80089852
hetero after UVB irradiation VS hetero before UVB irradiation	GO_CELL_COMMUNICATION_BY_ELECTRICAL_COUPLING_INVOLVED_IN_CARDIAC_CONDUCTION1	0.238147243	0.029065718	1.182076269	0.27173099	0.59792163	-5.53634846
WT after UVB irradiation VS WT before UVB irradiation	GO_CELL_COMMUNICATION_BY_ELECTRICAL_COUPLING_INVOLVED_IN_CARDIAC_CONDUCTION2	0.300870779	0.005742553	1.273772675	0.2380861	0.72863458	-5.22031501
hetero after UVB irradiation VS hetero before UVB irradiation	GO_CELL_COMMUNICATION_BY_ELECTRICAL_COUPLING1	0.246415006	0.000806292	1.525400937	0.16637161	0.48782273	-5.13217935
WT after UVB irradiation VS WT before UVB irradiation	GO_CELL_COMMUNICATION_BY_ELECTRICAL_COUPLING2	0.03211386	0.023737637	0.176203509	0.86446105	0.96790654	-5.97971757
homo after UVB irradiation VS homo before UVB irradiation	GO_CELL_COMMUNICATION_INVOLVED_IN_CARDIAC_CONDUCTION	0.431887213	-0.02517913	3.410965032	0.0086722	0.06188377	-2.60182453
hetero after UVB irradiation VS hetero before UVB irradiation	GO_CELL_COMMUNICATION_INVOLVED_IN_CARDIAC_CONDUCTION1	0.065507297	0.003661057	0.361483929	0.7272705	0.89236683	-6.16122557
WT after UVB irradiation VS WT before UVB irradiation	GO_CELL_COMMUNICATION_INVOLVED_IN_CARDIAC_CONDUCTION2	0.052956836	0.012181445	0.263327078	0.79887517	0.9521362	-5.95964399
homo after UVB irradiation VS homo before UVB irradiation	GO_CELL_CORTEX	0.231838118	-0.00446094	1.59211419	0.14853127	0.32159312	-5.33984078
homo after UVB irradiation VS homo before UVB irradiation	GO_CELL_CORTEX_REGION	0.017345608	0.03772854	0.10552396	0.91845723	0.95556166	-6.54474959
hetero after UVB irradiation VS hetero before UVB irradiation	GO_CELL_CORTEX_REGION1	0.323177263	0.042785572	1.929653764	0.09045246	0.37789255	-4.59246875
WT after UVB irradiation VS WT before UVB irradiation	GO_CELL_CORTEX_REGION2	0.103748594	0.0226235	0.514222815	0.62083215	0.90087565	-5.85890739
hetero after UVB irradiation VS hetero before UVB irradiation	GO_CELL_CORTEX1	0.213804312	-0.00226926	1.665213438	0.13514386	0.44685353	-4.95175293
WT after UVB irradiation VS WT before UVB irradiation	GO_CELL_CORTEX2	0.1732691	0.007530854	1.201375921	0.26356212	0.74156847	-5.29891589
homo after UVB irradiation VS homo before UVB irradiation	GO_CELL_CYCLE	-0.26098219	-0.00229058	-2.03227781	0.07518938	0.21066447	-4.71845507
homo after UVB irradiation VS homo before UVB irradiation	GO_CELL_CYCLE_ARREST	-0.03346226	-0.01301346	-0.2235581	0.82848705	0.90411521	-6.52351123
hetero after UVB irradiation VS hetero before UVB irradiation	GO_CELL_CYCLE_ARREST1	0.144837549	-0.01315188	1.106454602	0.30127055	0.6237902	-5.61627331
WT after UVB irradiation VS WT before UVB irradiation	GO_CELL_CYCLE_ARREST2	-0.01658378	-0.00745377	-0.11112012	0.91422601	0.97932969	-5.98955667
homo after UVB irradiation VS homo before UVB irradiation	GO_CELL_CYCLE_CHECKPOINT	-0.45550918	-0.01008141	-3.20733171	0.01183094	0.07311103	-2.91314126
hetero after UVB irradiation VS hetero before UVB irradiation	GO_CELL_CYCLE_CHECKPOINT1	-0.01228661	-0.02207245	-0.0603209	0.95340693	0.98257247	-6.22951134
WT after UVB irradiation VS WT before UVB irradiation	GO_CELL_CYCLE_CHECKPOINT2	-0.07908226	0.000618312	-0.34434066	0.73936187	0.93486103	-5.93398402
homo after UVB irradiation VS homo before UVB irradiation	GO_CELL_CYCLE_DNA_REPLICATION	-0.4490435	-0.04770361	-2.03483056	0.07488788	0.21017082	-4.71468488
homo after UVB irradiation VS homo before UVB irradiation	GO_CELL_CYCLE_DNA_REPLICATION_INITIATION	-0.64946993	-0.11793242	-1.91373519	0.09055268	0.23605121	-4.89176161
hetero after UVB irradiation VS hetero before UVB irradiation	GO_CELL_CYCLE_DNA_REPLICATION_INITIATION1	0.734590524	0.071240056	2.55142535	0.03460871	0.25582508	-3.6959702
WT after UVB irradiation VS WT before UVB irradiation	GO_CELL_CYCLE_DNA_REPLICATION_INITIATION2	0.479067021	0.006082834	2.17694061	0.06075095	0.52823908	-4.05903345
hetero after UVB irradiation VS hetero before UVB irradiation	GO_CELL_CYCLE_DNA_REPLICATION1	0.190395566	0.017880283	0.776265927	0.46033118	0.74847936	-5.91707837
WT after UVB irradiation VS WT before UVB irradiation	GO_CELL_CYCLE_DNA_REPLICATION2	0.103670806	0.039732634	0.395893143	0.7024104	0.92532318	-5.91420533
homo after UVB irradiation VS homo before UVB irradiation	GO_CELL_CYCLE_G1_S_PHASE_TRANSITION	-0.29567596	-0.00231944	-2.12290749	0.0651742	0.19375059	-4.58373222
hetero after UVB irradiation VS hetero before UVB irradiation	GO_CELL_CYCLE_G1_S_PHASE_TRANSITION1	0.064683982	-0.01824411	0.410753952	0.69223247	0.87693873	-6.14099229
WT after UVB irradiation VS WT before UVB irradiation	GO_CELL_CYCLE_G1_S_PHASE_TRANSITION2	-0.03760666	-0.00187344	-0.19430117	0.85072611	0.96583938	-5.97619541
homo after UVB irradiation VS homo before UVB irradiation	GO_CELL_CYCLE_G2_M_PHASE_TRANSITION	-0.40584294	-0.00342695	-2.88409469	0.01954349	0.09638803	-3.41301605
hetero after UVB irradiation VS hetero before UVB irradiation	GO_CELL_CYCLE_G2_M_PHASE_TRANSITION1	-0.00252832	-0.0203648	-0.01205049	0.99068585	0.99624062	-6.23140536
WT after UVB irradiation VS WT before UVB irradiation	GO_CELL_CYCLE_G2_M_PHASE_TRANSITION2	-0.06321914	-0.00549813	-0.27786181	0.78808109	0.9469715	-5.95553295
homo after UVB irradiation VS homo before UVB irradiation	GO_CELL_CYCLE_PHASE_TRANSITION	-0.35670393	-0.00691743	-2.64883335	0.02830912	0.11915509	-3.77848342
hetero after UVB irradiation VS hetero before UVB irradiation	GO_CELL_CYCLE_PHASE_TRANSITION1	0.050874221	-0.00970996	0.279063151	0.7874047	0.91722367	-6.1894671
WT after UVB irradiation VS WT before UVB irradiation	GO_CELL_CYCLE_PHASE_TRANSITION2	-0.03036935	0.001537163	-0.14599615	0.88749344	0.97106404	-5.98483553
homo after UVB irradiation VS homo before UVB irradiation	GO_CELL_CYCLE_PROCESS	-0.26931183	-0.00658447	-2.0295288	0.07551538	0.21101695	-4.72251345
hetero after UVB irradiation VS hetero before UVB irradiation	GO_CELL_CYCLE_PROCESS1	0.079482952	-0.00922913	0.488666187	0.63843192	0.85182425	-6.10398688
WT after UVB irradiation VS WT before UVB irradiation	GO_CELL_CYCLE_PROCESS2	-0.0250896	-0.00142204	-0.13459127	0.8962203	0.97377562	-5.9865197
hetero after UVB irradiation VS hetero before UVB irradiation	GO_CELL_CYCLE1	0.048281902	-0.01010486	0.302884047	0.76984638	0.91003052	-6.18203271
WT after UVB irradiation VS WT before UVB irradiation	GO_CELL_CYCLE2	-0.05948476	-0.00692968	-0.32634637	0.75243708	0.93734883	-5.94025942
homo after UVB irradiation VS homo before UVB irradiation	GO_CELL_DEATH_IN_RESPONSE_TO_HYDROGEN_PEROXIDE	0.326157869	-0.02922219	2.608180721	0.03019026	0.12341211	-3.84154717
hetero after UVB irradiation VS hetero before UVB irradiation	GO_CELL_DEATH_IN_RESPONSE_TO_HYDROGEN_PEROXIDE1	0.277372613	0.028260243	1.550310866	0.16036742	0.48106967	-5.1006079
WT after UVB irradiation VS WT before UVB irradiation	GO_CELL_DEATH_IN_RESPONSE_TO_HYDROGEN_PEROXIDE2	0.179676955	0.007241842	0.85521996	0.41701373	0.82238909	-5.62758253
homo after UVB irradiation VS homo before UVB irradiation	GO_CELL_DEATH_IN_RESPONSE_TO_OXIDATIVE_STRESS	0.174872706	-0.02783765	1.280301843	0.23494034	0.42665698	-5.73119594
hetero after UVB irradiation VS hetero before UVB irradiation	GO_CELL_DEATH_IN_RESPONSE_TO_OXIDATIVE_STRESS1	0.170564063	0.004160418	1.332980092	0.21991389	0.54722587	-5.36640754
WT after UVB irradiation VS WT before UVB irradiation	GO_CELL_DEATH_IN_RESPONSE_TO_OXIDATIVE_STRESS2	0.087448869	0.007520427	0.568771206	0.58493649	0.89034634	-5.82889406
homo after UVB irradiation VS homo before UVB irradiation	GO_CELL_DIFFERENTIATION_IN_HINDBRAIN	-0.27646595	0.015971255	-1.60159733	0.14641858	0.31911076	-5.32719232
hetero after UVB irradiation VS hetero before UVB irradiation	GO_CELL_DIFFERENTIATION_IN_HINDBRAIN1	-0.1530224	0.008793112	-0.87699755	0.40651712	0.71194505	-5.83429039
WT after UVB irradiation VS WT before UVB irradiation	GO_CELL_DIFFERENTIATION_IN_HINDBRAIN2	-0.14925654	0.038257044	-0.6637909	0.5252686	0.86563125	-5.77004866
homo after UVB irradiation VS homo before UVB irradiation	GO_CELL_DIFFERENTIATION_IN_SPINAL_CORD	-0.00261867	0.030343572	-0.01925668	0.98508967	0.99235226	-6.55065321
hetero after UVB irradiation VS hetero before UVB irradiation	GO_CELL_DIFFERENTIATION_IN_SPINAL_CORD1	-0.08804206	0.049042976	-0.64602121	0.53668316	0.79463142	-6.01118461
WT after UVB irradiation VS WT before UVB irradiation	GO_CELL_DIFFERENTIATION_IN_SPINAL_CORD2	-0.01250632	0.039598221	-0.07580078	0.9414169	0.98458464	-5.99303605
homo after UVB irradiation VS homo before UVB irradiation	GO_CELL_DIFFERENTIATION_INVOLVED_IN_EMBRYONIC_PLACENTA_DEVELOPMENT	-0.15290337	0.031714438	-0.9055448	0.39061798	0.57991444	-6.12203112
hetero after UVB irradiation VS hetero before UVB irradiation	GO_CELL_DIFFERENTIATION_INVOLVED_IN_EMBRYONIC_PLACENTA_DEVELOPMENT1	0.180183947	0.020332844	0.900999923	0.39438148	0.70309208	-5.81334327
WT after UVB irradiation VS WT before UVB irradiation	GO_CELL_DIFFERENTIATION_INVOLVED_IN_EMBRYONIC_PLACENTA_DEVELOPMENT2	0.080177943	0.033088311	0.435276002	0.67473096	0.9165137	-5.89731669
homo after UVB irradiation VS homo before UVB irradiation	GO_CELL_DIFFERENTIATION_INVOLVED_IN_KIDNEY_DEVELOPMENT	0.039933706	0.030007675	0.287378083	0.78084323	0.87393718	-6.5057594
hetero after UVB irradiation VS hetero before UVB irradiation	GO_CELL_DIFFERENTIATION_INVOLVED_IN_KIDNEY_DEVELOPMENT1	-0.01566901	0.043420556	-0.09276549	0.92841283	0.97347117	-6.22681996
WT after UVB irradiation VS WT before UVB irradiation	GO_CELL_DIFFERENTIATION_INVOLVED_IN_KIDNEY_DEVELOPMENT2	-0.02362758	0.028175721	-0.15993967	0.87684628	0.9693507	-5.98259156
homo after UVB irradiation VS homo before UVB irradiation	GO_CELL_DIFFERENTIATION_INVOLVED_IN_METANEPHROS_DEVELOPMENT	0.079230855	0.046207701	0.480527668	0.64321985	0.78168454	-6.42588947
hetero after UVB irradiation VS hetero before UVB irradiation	GO_CELL_DIFFERENTIATION_INVOLVED_IN_METANEPHROS_DEVELOPMENT1	-0.12226231	0.036572964	-0.67966869	0.51624516	0.78459792	-5.98832534
WT after UVB irradiation VS WT before UVB irradiation	GO_CELL_DIFFERENTIATION_INVOLVED_IN_METANEPHROS_DEVELOPMENT2	0.069730352	0.023428988	0.348216888	0.73655685	0.93474796	-5.93258946
homo after UVB irradiation VS homo before UVB irradiation	GO_CELL_DIFFERENTIATION_INVOLVED_IN_PHENOTYPIC_SWITCHING	-0.46560117	-0.00037442	-1.77484351	0.11235683	0.26907657	-5.08973491
hetero after UVB irradiation VS hetero before UVB irradiation	GO_CELL_DIFFERENTIATION_INVOLVED_IN_PHENOTYPIC_SWITCHING1	0.132115415	-0.06181043	0.397693386	0.701448	0.88146568	-6.14659724
WT after UVB irradiation VS WT before UVB irradiation	GO_CELL_DIFFERENTIATION_INVOLVED_IN_PHENOTYPIC_SWITCHING2	0.687336657	-0.10772164	1.896958647	0.09396578	0.59186437	-4.44461031
homo after UVB irradiation VS homo before UVB irradiation	GO_CELL_DIVISION	-0.23488688	-0.00169439	-1.71243097	0.12367991	0.2866569	-5.17660512
homo after UVB irradiation VS homo before UVB irradiation	GO_CELL_DIVISION_SITE	0.079942502	-0.03272932	0.585224032	0.57390583	0.73110569	-6.3667294
hetero after UVB irradiation VS hetero before UVB irradiation	GO_CELL_DIVISION_SITE1	0.29426065	-0.01140156	2.332710027	0.04853876	0.29346252	-4.01589
WT after UVB irradiation VS WT before UVB irradiation	GO_CELL_DIVISION_SITE2	0.392225588	-0.01754568	2.540360313	0.03436221	0.4670931	-3.54491096
hetero after UVB irradiation VS hetero before UVB irradiation	GO_CELL_DIVISION1	0.197219063	-0.00324014	1.143091671	0.28665087	0.61195316	-5.57801446
WT after UVB irradiation VS WT before UVB irradiation	GO_CELL_DIVISION2	0.041994709	0.012158324	0.206797455	0.84127341	0.96319898	-5.97356457
homo after UVB irradiation VS homo before UVB irradiation	GO_CELL_FATE_COMMITMENT	0.101949475	0.034297467	0.831638382	0.42880862	0.6131759	-6.18651582
homo after UVB irradiation VS homo before UVB irradiation	GO_CELL_FATE_COMMITMENT_INVOLVED_IN_FORMATION_OF_PRIMARY_GERM_LAYER	-0.05980925	0.069989333	-0.39079163	0.70575718	0.82455072	-6.46781613
hetero after UVB irradiation VS hetero before UVB irradiation	GO_CELL_FATE_COMMITMENT_INVOLVED_IN_FORMATION_OF_PRIMARY_GERM_LAYER1	-0.03410543	0.042538993	-0.21483256	0.83537193	0.93666051	-6.20653098
WT after UVB irradiation VS WT before UVB irradiation	GO_CELL_FATE_COMMITMENT_INVOLVED_IN_FORMATION_OF_PRIMARY_GERM_LAYER2	-0.12377313	0.018719448	-0.70269101	0.50194582	0.85706548	-5.74362311
hetero after UVB irradiation VS hetero before UVB irradiation	GO_CELL_FATE_COMMITMENT1	0.000354023	0.021808167	0.002513843	0.99805693	0.99918479	-6.23148069
WT after UVB irradiation VS WT before UVB irradiation	GO_CELL_FATE_COMMITMENT2	-0.01930426	0.031897489	-0.11783478	0.90906905	0.97832631	-5.98874688
homo after UVB irradiation VS homo before UVB irradiation	GO_CELL_FATE_DETERMINATION	0.131866864	0.060618861	0.987520274	0.35120225	0.5430883	-6.04531111
hetero after UVB irradiation VS hetero before UVB irradiation	GO_CELL_FATE_DETERMINATION1	0.103431751	0.054717007	0.602391899	0.56389884	0.81055567	-6.03927198
WT after UVB irradiation VS WT before UVB irradiation	GO_CELL_FATE_DETERMINATION2	-0.06589545	0.037935921	-0.33187821	0.74840819	0.93642732	-5.93836504
homo after UVB irradiation VS homo before UVB irradiation	GO_CELL_FATE_SPECIFICATION	0.083540731	0.074804684	0.606449644	0.56037782	0.72044574	-6.35342652
homo after UVB irradiation VS homo before UVB irradiation	GO_CELL_FATE_SPECIFICATION_INVOLVED_IN_PATTERN_SPECIFICATION	-0.33138673	0.125786499	-1.40172849	0.19714184	0.3856339	-5.58487197
hetero after UVB irradiation VS hetero before UVB irradiation	GO_CELL_FATE_SPECIFICATION_INVOLVED_IN_PATTERN_SPECIFICATION1	0.21401899	0.160665337	0.951949141	0.36950001	0.68290858	-5.76738431
WT after UVB irradiation VS WT before UVB irradiation	GO_CELL_FATE_SPECIFICATION_INVOLVED_IN_PATTERN_SPECIFICATION2	0.151427637	0.060870845	0.801947664	0.44547732	0.83340629	-5.67030032
hetero after UVB irradiation VS hetero before UVB irradiation	GO_CELL_FATE_SPECIFICATION1	0.03413832	0.054318558	0.25322569	0.80659694	0.92458203	-6.1968561
WT after UVB irradiation VS WT before UVB irradiation	GO_CELL_FATE_SPECIFICATION2	-0.02581921	0.018522823	-0.16437614	0.87346409	0.96914919	-5.98183498
homo after UVB irradiation VS homo before UVB irradiation	GO_CELL_GROWTH	0.021809954	0.001881178	0.175554489	0.86483698	0.92575071	-6.53397424
hetero after UVB irradiation VS hetero before UVB irradiation	GO_CELL_GROWTH1	-0.07745257	0.004648724	-0.61686487	0.55478264	0.80503221	-6.03015113
WT after UVB irradiation VS WT before UVB irradiation	GO_CELL_GROWTH2	-0.05148098	-0.00996749	-0.36732136	0.72279389	0.93154998	-5.92549561
homo after UVB irradiation VS homo before UVB irradiation	GO_CELL_JUNCTION_ASSEMBLY	0.196964472	-0.01032843	1.518126603	0.16598219	0.34435524	-5.43714313
hetero after UVB irradiation VS hetero before UVB irradiation	GO_CELL_JUNCTION_ASSEMBLY1	0.158486271	0.005467883	1.233502988	0.25303161	0.5810538	-5.4799348
WT after UVB irradiation VS WT before UVB irradiation	GO_CELL_JUNCTION_ASSEMBLY2	0.050970184	-0.00081966	0.348087854	0.73665016	0.93474796	-5.93263613
homo after UVB irradiation VS homo before UVB irradiation	GO_CELL_JUNCTION_DISASSEMBLY	0.493035039	0.017026174	2.943603132	0.01780583	0.09158612	-3.32064796
hetero after UVB irradiation VS hetero before UVB irradiation	GO_CELL_JUNCTION_DISASSEMBLY1	0.808455967	-0.02092131	5.408194955	0.00068116	0.08306714	0.057153843
WT after UVB irradiation VS WT before UVB irradiation	GO_CELL_JUNCTION_DISASSEMBLY2	0.599526371	0.034783316	2.980001871	0.01736165	0.38271751	-2.92147485
homo after UVB irradiation VS homo before UVB irradiation	GO_CELL_JUNCTION_MAINTENANCE	-0.02522884	0.015054629	-0.16804411	0.87055744	0.92916193	-6.5353854
hetero after UVB irradiation VS hetero before UVB irradiation	GO_CELL_JUNCTION_MAINTENANCE1	0.225402137	0.009728067	1.451793209	0.18531094	0.51022981	-5.22387758
WT after UVB irradiation VS WT before UVB irradiation	GO_CELL_JUNCTION_MAINTENANCE2	-0.11671139	0.00879283	-0.69778838	0.50484886	0.85763155	-5.74702662
homo after UVB irradiation VS homo before UVB irradiation	GO_CELL_JUNCTION_ORGANIZATION	0.153748	-0.01174075	1.208773904	0.25994251	0.45536225	-5.81327255
hetero after UVB irradiation VS hetero before UVB irradiation	GO_CELL_JUNCTION_ORGANIZATION1	0.103692358	0.001827616	0.790932396	0.45220759	0.74416559	-5.90555152
WT after UVB irradiation VS WT before UVB irradiation	GO_CELL_JUNCTION_ORGANIZATION2	0.037101216	-0.0024147	0.249803614	0.80895971	0.95404687	-5.96327418
homo after UVB irradiation VS homo before UVB irradiation	GO_CELL_KILLING	0.563690995	-0.01912509	3.796730282	0.0048838	0.04555642	-2.02379877
hetero after UVB irradiation VS hetero before UVB irradiation	GO_CELL_KILLING1	0.498548569	0.043368661	3.090120068	0.01522879	0.1825855	-2.90888787
WT after UVB irradiation VS WT before UVB irradiation	GO_CELL_KILLING2	0.310242098	-0.01799189	1.460340687	0.18187863	0.68503903	-5.004803
homo after UVB irradiation VS homo before UVB irradiation	GO_CELL_LEADING_EDGE	0.310038068	-0.02694413	2.178362593	0.05970081	0.18373406	-4.50048943
hetero after UVB irradiation VS hetero before UVB irradiation	GO_CELL_LEADING_EDGE1	0.318729294	-0.00112761	2.402033368	0.0435984	0.2791054	-3.91476108
WT after UVB irradiation VS WT before UVB irradiation	GO_CELL_LEADING_EDGE2	0.162800847	-0.01163337	1.166104158	0.27676625	0.74802923	-5.33608627
homo after UVB irradiation VS homo before UVB irradiation	GO_CELL_MATRIX_ADHESION	0.429745456	-0.02305948	3.191185133	0.01212836	0.07408036	-2.9379709
homo after UVB irradiation VS homo before UVB irradiation	GO_CELL_MATRIX_ADHESION_MEDIATOR_ACTIVITY	0.728521082	-0.02515626	3.690009922	0.00571331	0.05006606	-2.18195008
hetero after UVB irradiation VS hetero before UVB irradiation	GO_CELL_MATRIX_ADHESION_MEDIATOR_ACTIVITY1	0.966514667	-0.00228471	6.686049397	0.00016855	0.07788488	1.318240029
WT after UVB irradiation VS WT before UVB irradiation	GO_CELL_MATRIX_ADHESION_MEDIATOR_ACTIVITY2	0.491165898	-0.02085625	1.537967736	0.16216226	0.66546953	-4.91027569
hetero after UVB irradiation VS hetero before UVB irradiation	GO_CELL_MATRIX_ADHESION1	0.328169962	0.011526064	1.938416738	0.08924482	0.37595306	-4.58023851
WT after UVB irradiation VS WT before UVB irradiation	GO_CELL_MATRIX_ADHESION2	0.250445756	-0.00022336	1.44710491	0.18544493	0.68726847	-5.02065463
homo after UVB irradiation VS homo before UVB irradiation	GO_CELL_MATURATION	0.067356397	-0.00313952	0.567574608	0.58529331	0.73897082	-6.37745887
hetero after UVB irradiation VS hetero before UVB irradiation	GO_CELL_MATURATION1	0.037711999	-0.00242	0.278659748	0.7877032	0.91723238	-6.18958787

WT after UVB irradiation VS WT before UVB irradiation	GO_CELL_MATURATION2	0.021919273	0.008346881	0.156114248	0.8797648	0.9693507	-5.98322743
homo after UVB irradiation VS homo before UVB irradiation	GO_CELL_MIGRATION_IN_HINDBRAIN	0.193053581	0.031725461	1.379980709	0.20349928	0.39291363	-5.611691
hetero after UVB irradiation VS hetero before UVB irradiation	GO_CELL_MIGRATION_IN_HINDBRAIN1	-0.10547939	-0.00265475	-0.5912056	0.57100385	0.81424167	-6.04618676
WT after UVB irradiation VS WT before UVB irradiation	GO_CELL_MIGRATION_IN_HINDBRAIN2	-0.05410569	-0.03179606	-0.23019274	0.82365077	0.95856757	-5.96820376
homo after UVB irradiation VS homo before UVB irradiation	GO_CELL_MIGRATION_INVOLVED_IN_GASTRULATION	0.001194075	0.052166783	0.006183026	0.99521222	0.99719147	-6.55083574
hetero after UVB irradiation VS hetero before UVB irradiation	GO_CELL_MIGRATION_INVOLVED_IN_GASTRULATION1	0.081267786	0.091697899	0.472651036	0.64931964	0.85593716	-6.11208834
WT after UVB irradiation VS WT before UVB irradiation	GO_CELL_MIGRATION_INVOLVED_IN_GASTRULATION2	0.026254745	0.155037688	0.1166956	0.90994364	0.97842738	-5.98888759
homo after UVB irradiation VS homo before UVB irradiation	GO_CELL_MIGRATION_INVOLVED_IN_HEART_DEVELOPMENT	0.326862649	-0.03409484	2.016905773	0.07702994	0.21351066	-4.74112624
hetero after UVB irradiation VS hetero before UVB irradiation	GO_CELL_MIGRATION_INVOLVED_IN_HEART_DEVELOPMENT1	-0.12115157	-0.02379103	-0.58630708	0.57413121	0.81578047	-6.04917759
WT after UVB irradiation VS WT before UVB irradiation	GO_CELL_MIGRATION_INVOLVED_IN_HEART_DEVELOPMENT2	-0.00654813	0.029756978	-0.02785104	0.97845513	0.99449427	-5.9956574
homo after UVB irradiation VS homo before UVB irradiation	GO_CELL_MIGRATION_INVOLVED_IN_KIDNEY_DEVELOPMENT	0.740003025	-0.02927648	3.979812584	0.00374527	0.0401449	-1.75596993
hetero after UVB irradiation VS hetero before UVB irradiation	GO_CELL_MIGRATION_INVOLVED_IN_KIDNEY_DEVELOPMENT1	0.324493212	0.067676459	1.265155228	0.24206512	0.57027474	-5.44442449
WT after UVB irradiation VS WT before UVB irradiation	GO_CELL_MIGRATION_INVOLVED_IN_KIDNEY_DEVELOPMENT2	0.555978511	0.125993376	1.959387981	0.08530299	0.57584638	-4.35991484
homo after UVB irradiation VS homo before UVB irradiation	GO_CELL_MIGRATION_INVOLVED_IN_SPROUTING_ANGIOGENESIS	0.575406906	-0.01342184	4.408025093	0.00205138	0.03076451	-1.14825398
hetero after UVB irradiation VS hetero before UVB irradiation	GO_CELL_MIGRATION_INVOLVED_IN_SPROUTING_ANGIOGENESIS1	0.342057875	0.033961654	1.919645555	0.09185094	0.37905759	-4.60641509
WT after UVB irradiation VS WT before UVB irradiation	GO_CELL_MIGRATION_INVOLVED_IN_SPROUTING_ANGIOGENESIS2	0.238487774	0.006725897	1.292421945	0.23186871	0.72404374	-5.1995854
homo after UVB irradiation VS homo before UVB irradiation	GO_CELL_MIGRATION_INVOLVED_IN_VASCULOGENESIS	0.832086321	-0.02240671	3.437095838	0.00833632	0.06072015	-2.56214956
hetero after UVB irradiation VS hetero before UVB irradiation	GO_CELL_MIGRATION_INVOLVED_IN_VASCULOGENESIS1	-0.75634581	-0.24350979	-2.86324978	0.02145514	0.21125909	-3.23883704
WT after UVB irradiation VS WT before UVB irradiation	GO_CELL_MIGRATION_INVOLVED_IN_VASCULOGENESIS2	0.087032202	-0.04058325	0.320889444	0.75641932	0.93854182	-5.94209779
homo after UVB irradiation VS homo before UVB irradiation	GO_CELL_MORPHOGENESIS	0.165447049	-0.0076974	1.264374719	0.24032646	0.43313356	-5.74974751
homo after UVB irradiation VS homo before UVB irradiation	GO_CELL_MORPHOGENESIS_INVOLVED_IN_DIFFERENTIATION	0.166011278	-0.0070523	1.286519262	0.23286547	0.42431216	-5.72391218
hetero after UVB irradiation VS hetero before UVB irradiation	GO_CELL_MORPHOGENESIS_INVOLVED_IN_DIFFERENTIATION1	0.108014095	0.012744532	0.814339352	0.43944547	0.73683342	-5.88677817
WT after UVB irradiation VS WT before UVB irradiation	GO_CELL_MORPHOGENESIS_INVOLVED_IN_DIFFERENTIATION2	0.011279992	-0.0049864	0.07809058	0.93965119	0.9842223	-5.99285026
homo after UVB irradiation VS homo before UVB irradiation	GO_CELL_MORPHOGENESIS_INVOLVED_IN_NEURON_DIFFERENTIATION	0.116981085	-0.00588824	0.898971675	0.39391256	0.58281207	-6.12794969
hetero after UVB irradiation VS hetero before UVB irradiation	GO_CELL_MORPHOGENESIS_INVOLVED_IN_NEURON_DIFFERENTIATION1	0.05401812	0.017079771	0.406073919	0.69552852	0.87852904	-6.14302066
WT after UVB irradiation VS WT before UVB irradiation	GO_CELL_MORPHOGENESIS_INVOLVED_IN_NEURON_DIFFERENTIATION2	-0.04785014	-0.00479856	-0.32811689	0.75114671	0.93734883	-5.93965647
hetero after UVB irradiation VS hetero before UVB irradiation	GO_CELL_MORPHOGENESIS1	0.127699971	0.007498316	1.003025633	0.34575077	0.66286618	-5.71933207
WT after UVB irradiation VS WT before UVB irradiation	GO_CELL_MORPHOGENESIS2	0.027324963	-0.00489707	0.192151432	0.85235489	0.96605575	-5.97663163
homo after UVB irradiation VS homo before UVB irradiation	GO_CELL_MOTILITY	0.324271648	-0.01253018	2.494513357	0.03615127	0.13624547	-4.01746292
homo after UVB irradiation VS homo before UVB irradiation	GO_CELL_MOTILITY_INVOLVED_IN_CEREBRAL_CORTEX_RADIAL_GLIA_GUIDED_MIGRATION	-0.43107125	-0.00868888	-1.53487109	0.16187922	0.33884421	-5.41534212
hetero after UVB irradiation VS hetero before UVB irradiation	GO_CELL_MOTILITY_INVOLVED_IN_CEREBRAL_CORTEX_RADIAL_GLIA_GUIDED_MIGRATION1	-0.07043976	0.018402648	-0.47297569	0.64909802	0.8557563	-6.11192663
WT after UVB irradiation VS WT before UVB irradiation	GO_CELL_MOTILITY_INVOLVED_IN_CEREBRAL_CORTEX_RADIAL_GLIA_GUIDED_MIGRATION2	-0.18854083	-0.03249214	-0.7111117	0.49698429	0.85541458	-5.73772853
hetero after UVB irradiation VS hetero before UVB irradiation	GO_CELL_MOTILITY1	0.274563346	0.012168654	1.96406414	0.08579896	0.37193478	-4.5443434
WT after UVB irradiation VS WT before UVB irradiation	GO_CELL_MOTILITY2	0.204372638	-0.00176465	1.264864678	0.24110512	0.73118354	-5.23014848
homo after UVB irradiation VS homo before UVB irradiation	GO_CELL_PART_MORPHOGENESIS	0.105778367	-0.00936943	0.802113963	0.44476714	0.62705921	-6.21098528
hetero after UVB irradiation VS hetero before UVB irradiation	GO_CELL_PART_MORPHOGENESIS1	0.046170148	0.009414394	0.358423815	0.72947053	0.89320945	-6.16240015
WT after UVB irradiation VS WT before UVB irradiation	GO_CELL_PART_MORPHOGENESIS2	-0.03273685	-0.00589578	-0.22621471	0.82664007	0.95915492	-5.96915527
homo after UVB irradiation VS homo before UVB irradiation	GO_CELL_PROJECTION_ASSEMBLY	-0.05779089	-0.01040537	-0.4653916	0.65357743	0.7891378	-6.43353755
hetero after UVB irradiation VS hetero before UVB irradiation	GO_CELL_PROJECTION_ASSEMBLY1	-0.02533204	-0.01407576	-0.18781753	0.85578203	0.94499137	-6.21239842
WT after UVB irradiation VS WT before UVB irradiation	GO_CELL_PROJECTION_ASSEMBLY2	-0.07110534	-0.01901199	-0.50885678	0.62442459	0.90175221	-5.86170796
homo after UVB irradiation VS homo before UVB irradiation	GO_CELL_PROJECTION_MEMBRANE	0.148174461	-0.00987844	1.161263729	0.27773024	0.47357164	-5.86597453
hetero after UVB irradiation VS hetero before UVB irradiation	GO_CELL_PROJECTION_MEMBRANE1	0.036632035	-0.01354287	0.287601846	0.78109529	0.91401375	-6.18687067
WT after UVB irradiation VS WT before UVB irradiation	GO_CELL_PROJECTION_MEMBRANE2	0.029113951	0.011505335	0.197556956	0.84826076	0.96522688	-5.9755256
homo after UVB irradiation VS homo before UVB irradiation	GO_CELL_PROJECTION_ORGANIZATION	0.037649501	-0.00452171	0.299939912	0.77157237	0.86790864	-6.50175266
hetero after UVB irradiation VS hetero before UVB irradiation	GO_CELL_PROJECTION_ORGANIZATION1	0.032404743	-0.00214528	0.256621069	0.80406651	0.92374885	-6.1959253
WT after UVB irradiation VS WT before UVB irradiation	GO_CELL_PROJECTION_ORGANIZATION2	-0.02535606	-0.01917219	-0.18035267	0.8613076	0.9676681	-5.9789402
homo after UVB irradiation VS homo before UVB irradiation	GO_CELL_PROLIFERATION_IN_BONE_MARROW	0.341373439	-0.0347294	2.038580395	0.0744471	0.20963883	-4.70914398
hetero after UVB irradiation VS hetero before UVB irradiation	GO_CELL_PROLIFERATION_IN_BONE_MARROW1	-0.22242123	-0.04385325	-0.94360544	0.37349304	0.68676179	-5.77504774
WT after UVB irradiation VS WT before UVB irradiation	GO_CELL_PROLIFERATION_IN_BONE_MARROW2	-0.17803751	-0.12575451	-0.62017925	0.55218879	0.87634603	-5.7980759
homo after UVB irradiation VS homo before UVB irradiation	GO_CELL_PROLIFERATION_IN_FOREBRAIN	0.04900645	0.032415319	0.339811654	0.74240889	0.84861374	-6.48792727
hetero after UVB irradiation VS hetero before UVB irradiation	GO_CELL_PROLIFERATION_IN_FOREBRAIN1	0.083155881	0.047584692	0.5271366	0.61266138	0.83897962	-6.08349485
WT after UVB irradiation VS WT before UVB irradiation	GO_CELL_PROLIFERATION_IN_FOREBRAIN2	0.047720215	0.012101606	0.240932549	0.81559571	0.95612852	-5.96555324
homo after UVB irradiation VS homo before UVB irradiation	GO_CELL_PROLIFERATION_IN_HINDBRAIN	-0.18167993	0.031086632	-1.25898058	0.24217388	0.43513454	-5.75599525
hetero after UVB irradiation VS hetero before UVB irradiation	GO_CELL_PROLIFERATION_IN_HINDBRAIN1	-0.08553868	0.026643617	-0.45904845	0.65863853	0.86007701	-6.11876942
WT after UVB irradiation VS WT before UVB irradiation	GO_CELL_PROLIFERATION_IN_HINDBRAIN2	-0.26391146	0.036806414	-1.24604459	0.24758917	0.73564768	-5.25077647
homo after UVB irradiation VS homo before UVB irradiation	GO_CELL_PROLIFERATION_IN_MIDBRAIN	-0.80053203	0.027405306	-5.11335123	0.0008069	0.01952026	-0.20895929
hetero after UVB irradiation VS hetero before UVB irradiation	GO_CELL_PROLIFERATION_IN_MIDBRAIN1	-0.67733895	-0.02137086	-5.24417308	0.00082742	0.08366654	-0.1235313
WT after UVB irradiation VS WT before UVB irradiation	GO_CELL_PROLIFERATION_IN_MIDBRAIN2	-0.86227483	0.01349744	-4.6378752	0.001618	0.2011505	-0.77863722
homo after UVB irradiation VS homo before UVB irradiation	GO_CELL_PROLIFERATION_INVOLVED_IN_HEART_MORPHOGENESIS	-0.09712279	0.047720071	-0.68033875	0.51475224	0.6858251	-6.30377216
hetero after UVB irradiation VS hetero before UVB irradiation	GO_CELL_PROLIFERATION_INVOLVED_IN_HEART_MORPHOGENESIS1	-0.35254066	-0.00999915	-2.1917537	0.06037975	0.323869	-4.22019581
WT after UVB irradiation VS WT before UVB irradiation	GO_CELL_PROLIFERATION_INVOLVED_IN_HEART_MORPHOGENESIS2	-0.41528719	0.036736873	-2.46393436	0.03873296	0.48068087	-3.65361584
homo after UVB irradiation VS homo before UVB irradiation	GO_CELL_PROLIFERATION_INVOLVED_IN_KIDNEY_DEVELOPMENT	-0.01432927	0.033870857	-0.08357916	0.93536494	0.96583588	-6.54702458
hetero after UVB irradiation VS hetero before UVB irradiation	GO_CELL_PROLIFERATION_INVOLVED_IN_KIDNEY_DEVELOPMENT1	-0.31700311	0.00707192	-1.65053129	0.13815012	0.45140459	-4.97105272
WT after UVB irradiation VS WT before UVB irradiation	GO_CELL_PROLIFERATION_INVOLVED_IN_KIDNEY_DEVELOPMENT2	-0.05881596	0.011248502	-0.30991843	0.76444882	0.94098943	-5.94570244
homo after UVB irradiation VS homo before UVB irradiation	GO_CELL_PROLIFERATION_INVOLVED_IN_METANEPHROS_DEVELOPMENT	0.013071075	0.029084964	0.078325977	0.93941783	0.96747777	-6.54749099
hetero after UVB irradiation VS hetero before UVB irradiation	GO_CELL_PROLIFERATION_INVOLVED_IN_METANEPHROS_DEVELOPMENT1	-0.36775858	0.016146886	-1.72988418	0.12261052	0.42875973	-4.8658477
WT after UVB irradiation VS WT before UVB irradiation	GO_CELL_PROLIFERATION_INVOLVED_IN_METANEPHROS_DEVELOPMENT2	0.093524595	0.117965175	0.467583651	0.65240787	0.90930021	-5.88232672
homo after UVB irradiation VS homo before UVB irradiation	GO_CELL_RECOGNITION	0.165542312	0.02247837	1.252623727	0.24436623	0.43822001	-5.76333494
hetero after UVB irradiation VS hetero before UVB irradiation	GO_CELL_RECOGNITION1	0.029328126	0.029718411	0.204102312	0.84346376	0.93997064	-6.20895474
WT after UVB irradiation VS WT before UVB irradiation	GO_CELL_RECOGNITION2	0.034441688	-0.00536588	0.193430794	0.85138547	0.96600764	-5.9763726
homo after UVB irradiation VS homo before UVB irradiation	GO_CELL_REDOX_HOMEOSTASIS	0.382766521	-0.03505583	2.598933253	0.03063562	0.12430297	-3.85588379
hetero after UVB irradiation VS hetero before UVB irradiation	GO_CELL_REDOX_HOMEOSTASIS1	0.455811357	-0.01720296	2.859823238	0.02156731	0.21174131	-3.24384521
WT after UVB irradiation VS WT before UVB irradiation	GO_CELL_REDOX_HOMEOSTASIS2	0.327309017	-0.04854632	1.600930174	0.14760465	0.65441042	-4.83177159
homo after UVB irradiation VS homo before UVB irradiation	GO_CELL_SUBSTRATE_ADHESION	0.361910272	-0.01245081	2.666448148	0.02753149	0.11730681	-3.75114113
hetero after UVB irradiation VS hetero before UVB irradiation	GO_CELL_SUBSTRATE_ADHESION1	0.289376241	0.004143872	1.837562651	0.10412892	0.40029539	-4.71986214
WT after UVB irradiation VS WT before UVB irradiation	GO_CELL_SUBSTRATE_ADHESION2	0.215592077	-0.00998376	1.288879243	0.23303912	0.72459413	-5.20353805
homo after UVB irradiation VS homo before UVB irradiation	GO_CELL_SUBSTRATE_JUNCTION	0.237987269	-0.02539589	1.354268831	0.21124324	0.40157646	-5.64306232
homo after UVB irradiation VS homo before UVB irradiation	GO_CELL_SUBSTRATE_JUNCTION_ORGANIZATION	0.499113866	-0.0334566	3.18993831	0.01215164	0.07410957	-2.939889
hetero after UVB irradiation VS hetero before UVB irradiation	GO_CELL_SUBSTRATE_JUNCTION_ORGANIZATION1	0.451794804	0.012769925	2.609337164	0.03165311	0.24789178	-3.61099424
WT after UVB irradiation VS WT before UVB irradiation	GO_CELL_SUBSTRATE_JUNCTION_ORGANIZATION2	0.380948128	-0.0075404	2.534480769	0.03467996	0.4673719	-3.55327768
hetero after UVB irradiation VS hetero before UVB irradiation	GO_CELL_SUBSTRATE_JUNCTION1	0.407148462	0.006868212	3.278372773	0.01150708	0.16744133	-2.63839861
WT after UVB irradiation VS WT before UVB irradiation	GO_CELL_SUBSTRATE_JUNCTION2	0.208856477	-0.01882154	1.376690256	0.20546888	0.70248163	-5.10359779
homo after UVB irradiation VS homo before UVB irradiation	GO_CELL_SURFACE	0.401013553	-0.02068368	3.121717187	0.01349986	0.07869231	-3.04500083
homo after UVB irradiation VS homo before UVB irradiation	GO_CELL_SURFACE_RECEPTOR_SIGNALING_PATHWAY_INVOLVED_IN_CELL_CELL_SIGNALING	-0.01962301	-0.01798751	-0.15312932	0.88194122	0.93624176	-6.53800609
hetero after UVB irradiation VS hetero before UVB irradiation	GO_CELL_SURFACE_RECEPTOR_SIGNALING_PATHWAY_INVOLVED_IN_CELL_CELL_SIGNALING1	-0.0384201	0.003010807	-0.27082403	0.79350868	0.91941572	-6.19189976
WT after UVB irradiation VS WT before UVB irradiation	GO_CELL_SURFACE_RECEPTOR_SIGNALING_PATHWAY_INVOLVED_IN_CELL_CELL_SIGNALING2	-0.06980739	0.012895722	-0.46068018	0.65714773	0.91099034	-5.8856151
homo after UVB irradiation VS homo before UVB irradiation	GO_CELL_SURFACE_RECEPTOR_SIGNALING_PATHWAY_INVOLVED_IN_HEART_DEVELOPMENT	-0.23463637	0.000209994	-1.63565884	0.13905344	0.30876964	-5.28144506
hetero after UVB irradiation VS hetero before UVB irradiation	GO_CELL_SURFACE_RECEPTOR_SIGNALING_PATHWAY_INVOLVED_IN_HEART_DEVELOPMENT1	0.079364304	-0.0072205	0.542925335	0.60224623	0.83361105	-6.07466711
WT after UVB irradiation VS WT before UVB irradiation	GO_CELL_SURFACE_RECEPTOR_SIGNALING_PATHWAY_INVOLVED_IN_HEART_DEVELOPMENT2	-0.13351777	0.01596518	-0.83331907	0.42855793	0.82753247	-5.64542116
hetero after UVB irradiation VS hetero before UVB irradiation	GO_CELL_SURFACE1	0.215561148	0.012779514	1.356368765	0.21268936	0.53925157	-5.33892081
WT after UVB irradiation VS WT before UVB irradiation	GO_CELL_SURFACE2	0.191480473	-0.00462111	0.972502519	0.35895063	0.79181584	-5.52572429
homo after UVB irradiation VS homo before UVB irradiation	GO_CELL_TIP	-0.65699016	-0.02911984	-3.76560791	0.00511159	0.04651635	-2.06977035
hetero after UVB irradiation VS hetero before UVB irradiation	GO_CELL_TIP1	-0.17318232	0.028227031	-0.7535385	0.47311193	0.75662211	-5.93457653
WT after UVB irradiation VS WT before UVB irradiation	GO_CELL_TIP2	-0.32256567	0.02881731	-1.37924773	0.20470991	0.70139406	-5.10062746
homo after UVB irradiation VS homo before UVB irradiation	GO_CELL_TRAILING_EDGE	0.746698338	-0.02645585	3.881651627	0.00431553	0.04248446	-1.89900515
hetero after UVB irradiation VS hetero before UVB irradiation	GO_CELL_TRAILING_EDGE1	0.704649796	0.039145177	4.313751892	0.0026816	0.11212131	-1.23499927
WT after UVB irradiation VS WT before UVB irradiation	GO_CELL_TRAILING_EDGE2	0.72700634	-0.01723155	3.921967779	0.00430236	0.26635361	-1.64994157
homo after UVB irradiation VS homo before UVB irradiation	GO_CELL_VOLUME_HOMEOSTASIS	0.52792709	-0.043596	3.513392281	0.00743184	0.05721006	-2.44670702
hetero after UVB irradiation VS hetero before UVB irradiation	GO_CELL_VOLUME_HOMEOSTASIS1	0.21872509	-0.0248699	1.443567556	0.18754229	0.51295164	-5.23397032
WT after UVB irradiation VS WT before UVB irradiation	GO_CELL_VOLUME_HOMEOSTASIS2	0.100742858	0.000457864	0.474092802	0.64795405	0.90907607	-5.87918389
homo after UVB irradiation VS homo before UVB irradiation	GO_CELLULAR_ALDEHYDE_METABOLIC_PROCESS	0.357194299	-0.02749719	2.696787652	0.02624277	0.11430477	-3.70402863
hetero after UVB irradiation VS hetero before UVB irradiation	GO_CELLULAR_ALDEHYDE_METABOLIC_PROCESS1	0.097458288	-0.00162331	0.632796751	0.54484824	0.79870659	-6.01988487
WT after UVB irradiation VS WT before UVB irradiation	GO_CELLULAR_ALDEHYDE_METABOLIC_PROCESS2	0.135427986	0.02681018	0.728497206	0.48683984	0.85164239	-5.7253646
homo after UVB irradiation VS homo before UVB irradiation	GO_CELLULAR_AMIDE_METABOLIC_PROCESS	-0.35056954	-0.01099761	-2.76697677	0.02349238	0.10716445	-3.59497878
hetero after UVB irradiation VS hetero before UVB irradiation	GO_CELLULAR_AMIDE_METABOLIC_PROCESS1	-0.14114434	-0.03221593	-0.94673693	0.37199066	0.68562256	-5.77217778
WT after UVB irradiation VS WT before UVB irradiation	GO_CELLULAR_AMIDE_METABOLIC_PROCESS2	-0.15999966	-0.01452435	-0.99655619	0.34782327	0.78574573	-5.50356608
homo after UVB irradiation VS homo before UVB irradiation	GO_CELLULAR_AMINO_ACID_BIOSYNTHETIC_PROCESS	-0.09839364	0.004959438	-0.59873444	0.56527378	0.72429844	-6.35831215
hetero after UVB irradiation VS hetero before UVB irradiation	GO_CELLULAR_AMINO_ACID_BIOSYNTHETIC_PROCESS1	-0.08670552	0.003435453	-0.60552838	0.5619159	0.80941342	-6.03731204
WT after UVB irradiation VS WT before UVB irradiation	GO_CELLULAR_AMINO_ACID_BIOSYNTHETIC_PROCESS2	0.058880667	-0.00244798	0.37909463	0.71436502	0.92987478	-5.92094193
homo after UVB irradiation VS homo before UVB irradiation	GO_CELLULAR_AMINO_ACID_CATABOLIC_PROCESS	0.061613114	-0.01547311	0.321298724	0.7558986	0.85796154	-6.49455556
hetero after UVB irradiation VS hetero before UVB irradiation	GO_CELLULAR_AMINO_ACID_CATABOLIC_PROCESS1	-0.29488613	-0.01144139	-1.81939139	0.10705098	0.40472566	-4.74473637
WT after UVB irradiation VS WT before UVB irradiation	GO_CELLULAR_AMINO_ACID_CATABOLIC_PROCESS2	0.00866435	-0.04680868	0.040956239	0.96832262	0.99198451	-5.99518168
homo after UVB irradiation VS homo before UVB irradiation	GO_CELLULAR_AMINO_ACID_METABOLIC_PROCESS	-0.20800106	-0.01993831	-1.38992601	0.20057031	0.38924462	-5.59945855
hetero after UVB irradiation VS hetero before UVB irradiation	GO_CELLULAR_AMINO_ACID_METABOLIC_PROCESS1	-0.25346767	-0.03339447	-1.83446112	0.10462224	0.40053825	-4.72411423
WT after UVB irradiation VS WT before UVB irradiation	GO_CELLULAR_AMINO_ACID_METABOLIC_PROCESS2	-0.00080409	-0.0210955	-0.0057414	0.99555798	0.99875662	-5.99604927
homo after UVB irradiation VS homo before UVB irradiation	GO_CELLULAR_ANION_HOMEOSTASIS	0.325541172	0.04635941	1.530095068	0.16304017	0.34069438	-5.42157384
hetero after UVB irradiation VS hetero before UVB irradiation	GO_CELLULAR_ANION_HOMEOSTASIS1	-0.04347125	0.003010558	-0.28896606	0.78008884	0.91401375	-6.18644874
WT after UVB irradiation VS WT before UVB irradiation	GO_CELLULAR_ANION_HOMEOSTASIS2	0.155469025	0.038666564	0.763875208	0.46661331	0.84342918	-5.69940769
homo after UVB irradiation VS homo before UVB irradiation	GO_CELLULAR_BIOGENIC_AMINE_METABOLIC_PROCESS	0.154533584	-0.00733769	0.943564249	0.37195284	0.56221531	-6.08711181
hetero after UVB irradiation VS hetero before UVB irradiation	GO_CELLULAR_BIOGENIC_AMINE_METABOLIC_PROCESS1	0.04945773	-0.01131267	0.35385969	0.73275682	0.89537592	-6.16413408
WT after UVB irradiation VS WT before UVB irradiation	GO_CELLULAR_BIOGENIC_AMINE_METABOLIC_PROCESS2	0.131403266	-0.03031959	0.691747773	0.50844023	0.85808011	-5.7511913
homo after UVB irradiation VS homo before UVB irradiation	GO_CELLULAR_CARBOHYDRATE_BIOSYNTHETIC_PROCESS	0.305314387	-0.01038053	2.490938559	0.03635692	0.13669276	-4.02298257
hetero after UVB irradiation VS hetero before UVB irradiation	GO_CELLULAR_CARBOHYDRATE_BIOSYNTHETIC_PROCESS1	0.120773686	-0.01351567	0.729566028	0.4868444	0.76448611	-5.95254775
WT after UVB irradiation VS WT before UVB irradiation	GO_CELLULAR_CARBOHYDRATE_BIOSYNTHETIC_PROCESS2	0.144327074	-0.03483604	0.793398189	0.45016624	0.83530628	-5.67694116
homo after UVB irradiation VS homo before UVB irradiation	GO_CELLULAR_CARBOHYDRATE_CATABOLIC_PROCESS	-0.02502937	0.009223664	-0.18084937	0.86080905	0.92344861	-6.53294257
hetero after UVB irradiation VS hetero before UVB irradiation	GO_CELLULAR_CARBOHYDRATE_CATABOLIC_PROCESS1	-0.11280981	0.051507998	-0.87240378	0.40886997	0.71337032	-5.83824703
WT after UVB irradiation VS WT before UVB irradiation	GO_CELLULAR_CARBOHYDRATE_CATABOLIC_PROCESS2	0.100066121	0.005597031	0.58655646	0.57348543	0.88535896	-5.81850718
homo after UVB irradiation VS homo before UVB irradiation	GO_CELLULAR_CARBOHYDRATE_METABOLIC_PROCESS	0.247857713	-0.02173462	2.065985463	0.07130118	0.20502562	-4.66855324
hetero after UVB irradiation VS hetero before UVB irradiation	GO_CELLULAR_CARBOHYDRATE_METABOLIC_PROCESS1	0.090719284	0.004872769	0.649184064	0.53474134	0.79344322	-6.00907992
WT after UVB irradiation VS WT before UVB irradiation	GO_CELLULAR_CARBOHYDRATE_METABOLIC_PROCESS2	0.119533651	-0.00897617	0.754068895	0.47216355	0.84703593	-5.70670884
homo after UVB irradiation VS homo before UVB irradiation	GO_CELLULAR_CHLORIDE_ION_HOMEOSTASIS	0.422033314	-0.10330967	1.490049983	0.17307032	0.35325018	-5.47339821
hetero after UVB irradiation VS hetero before UVB irradiation	GO_CELLULAR_CHLORIDE_ION_HOMEOSTASIS1	-0.8697667	-0.06897891	-3.88451563	0.00481491	0.12780541	-1.79711166
WT after UVB irradiation VS WT before UVB irradiation	GO_CELLULAR_CHLORIDE_ION_HOMEOSTASIS2	-0.02884497	-0.07989765	-0.07286178	0.94368372	0.98531933	-5.99326643
homo after UVB irradiation VS homo before UVB irradiation	GO_CELLULAR_COMPONENT_ASSEMBLY_INVOLVED_IN_MORPHOGENESIS	0.058563685	-0.00733366	0.371203065	0.71975085	0.83384689	-6.47586483
hetero after UVB irradiation VS hetero before UVB irradiation	GO_CELLULAR_COMPONENT_ASSEMBLY_INVOLVED_IN_MORPHOGENESIS1	-0.1433389	-0.00273334	-0.77839389	0.45914648	0.74772822	-5.91541731
WT after UVB irradiation VS WT before UVB irradiation	GO_CELLULAR_COMPONENT_ASSEMBLY_INVOLVED_IN_MORPHOGENESIS2	-0.09229858	-0.04046284	-0.43472065	0.67511778	0.91661726	-5.89756545
homo after UVB irradiation VS homo before UVB irradiation	GO_CELLULAR_COMPONENT_DISASSEMBLY	0.022459078	-0.00744141	0.165996677	0.87211833	0.92961443	-6.53575948
homo after UVB irradiation VS homo before UVB irradiation	GO_CELLULAR_COMPONENT_DISASSEMBLY_INVOLVED_IN_EXECUTION_PHASE_OF_APOPTOSIS	0.058997667	0.001645111	0.331317079	0.74858725	0.85313198	-6.4910135
hetero after UVB irradiation VS hetero before UVB irradiation	GO_CELLULAR_COMPONENT_DISASSEMBLY_INVOLVED_IN_EXECUTION_PHASE_OF_APOPTOSIS1	0.212906792	0.004658095	1.556616977	0.15887884	0.47894099	-5.09257397
WT after UVB irradiation VS WT before UVB irradiation	GO_CELLULAR_COMPONENT_DISASSEMBLY_INVOLVED_IN_EXECUTION_PHASE_OF_APOPTOSIS2	0.307932781	0.03798679	2.104868091	0.06800253	0.54068603	-4.15955331
hetero after UVB irradiation VS hetero before UVB irradiation	GO_CELLULAR_COMPONENT_DISASSEMBLY1	0.118512819	-0.01173716	0.945616795	0.37252755	0.68644062	-5.77320523
WT after UVB irradiation VS WT before UVB irradiation	GO_CELLULAR_COMPONENT_DISASSEMBLY2	0.118531155	-0.0090947	0.755966592	0.4710861	0.84681445	-5.70530226
homo after UVB irradiation VS homo before UVB irradiation	GO_CELLULAR_COMPONENT_MAINTENANCE	-0.05221911	0.003453011	-0.34504333	0.7386135	0.84640219	-6.48598873
hetero after UVB irradiation VS hetero before UVB irradiation	GO_CELLULAR_COMPONENT_MAINTENANCE1	0.075603377	-0.00878316	0.529331305	0.61120791	0.83821176	-6.08228222
WT after UVB irradiation VS WT before UVB irradiation	GO_CELLULAR_COMPONENT_MAINTENANCE2	-0.22143917	-0.00386888	-1.43474231	0.18883148	0.69085152	-5.03538763
homo after UVB irradiation VS homo before UVB irradiation	GO_CELLULAR_COMPONENT_MORPHOGENESIS	0.106056084	-0.00930275	0.855795182	0.41604892	0.60219559	-6.16594113
hetero after UVB irradiation VS hetero before UVB irradiation	GO_CELLULAR_COMPONENT_MORPHOGENESIS1	0.01761851	0.005916693	0.133184411	0.89739722	0.96178068	-6.22187586
WT after UVB irradiation VS WT before UVB irradiation	GO_CELLULAR_COMPONENT_MORPHOGENESIS2	-0.05296563	-0.00505147	-0.35266941	0.73333997	0.93423996	-5.93096891
homo after UVB irradiation VS homo before UVB irradiation	GO_CELLULAR_COPPER_ION_HOMEOSTASIS	0.104606584	-0.04448345	0.490793899	0.63624113	0.77661681	-6.42057047
hetero after UVB irradiation VS hetero before UVB irradiation	GO_CELLULAR_COPPER_ION_HOMEOSTASIS1	0.540565108	0.008349052	3.686263727	0.0063669	0.13584504	-2.0666058
WT after UVB irradiation VS WT before UVB irradiation	GO_CELLULAR_COPPER_ION_HOMEOSTASIS2	0.208962739	0.043034564	0.798102136	0.44758226	0.83480988	-5.67329479
homo after UVB irradiation VS homo before UVB irradiation	GO_CELLULAR_DEFENSE_RESPONSE	0.628015722	-0.00665733	4.69372391	0.00139351	0.0251701	-0.75832574

hetero after UVB irradiation VS hetero before UVB irradiation	GO_CELLULAR_DEFENSE_RESPONSE1	0.611345029	0.042709661	2.759385789	0.02514009	0.22675257	-3.3908673
WT after UVB irradiation VS WT before UVB irradiation	GO_CELLULAR_DEFENSE_RESPONSE2	0.406341279	-0.01075762	1.498479955	0.17193958	0.67808426	-4.95868617
homo after UVB irradiation VS homo before UVB irradiation	GO_CELLULAR_DETOXIFICATION_OF_ALDEHYDE	0.779419988	-0.03058585	4.811498339	0.00119232	0.02341948	-0.60131904
hetero after UVB irradiation VS hetero before UVB irradiation	GO_CELLULAR_DETOXIFICATION_OF_ALDEHYDE1	0.399204654	0.002557163	1.491662543	0.17482632	0.49736582	-5.17451246
WT after UVB irradiation VS WT before UVB irradiation	GO_CELLULAR_DETOXIFICATION_OF_ALDEHYDE2	0.475785108	-0.00846122	2.091567841	0.0694305	0.54475458	-4.17802178
homo after UVB irradiation VS homo before UVB irradiation	GO_CELLULAR_DIVALENT_INORGANIC_ANION_HOMEOSTASIS	0.283242247	0.096460493	1.203008764	0.26204997	0.45753329	-5.81974667
hetero after UVB irradiation VS hetero before UVB irradiation	GO_CELLULAR_DIVALENT_INORGANIC_ANION_HOMEOSTASIS1	0.404822206	0.096425412	1.742407573	0.12031237	0.42502498	-4.84905106
WT after UVB irradiation VS WT before UVB irradiation	GO_CELLULAR_DIVALENT_INORGANIC_ANION_HOMEOSTASIS2	0.426830125	0.147868103	1.773432763	0.11363444	0.61901197	-4.60937284
homo after UVB irradiation VS homo before UVB irradiation	GO_CELLULAR_EXTRAVASATION	0.710197657	-0.01134364	5.266540245	0.00066515	0.01818244	-0.01536314
hetero after UVB irradiation VS hetero before UVB irradiation	GO_CELLULAR_EXTRAVASATION1	0.748373281	0.014472053	5.327009527	0.00074965	0.08366654	-0.03171797
WT after UVB irradiation VS WT before UVB irradiation	GO_CELLULAR_EXTRAVASATION2	0.426048852	-0.01156375	1.730514851	0.12133168	0.63058669	-4.66561647
homo after UVB irradiation VS homo before UVB irradiation	GO_CELLULAR_GLUCAN_METABOLIC_PROCESS	0.178222992	-0.02712156	1.300565706	0.22823468	0.41933007	-5.70737112
hetero after UVB irradiation VS hetero before UVB irradiation	GO_CELLULAR_GLUCAN_METABOLIC_PROCESS1	-0.17401317	-0.02222206	-1.13056143	0.29158519	0.61588295	-5.59119881
WT after UVB irradiation VS WT before UVB irradiation	GO_CELLULAR_GLUCAN_METABOLIC_PROCESS2	-0.05643098	-0.06557038	-0.28567069	0.78230174	0.94527105	-5.95323491
homo after UVB irradiation VS homo before UVB irradiation	GO_CELLULAR_GLUCOSE_HOMEOSTASIS	0.130742543	0.0064477	0.965830099	0.36133081	0.55226916	-6.06612662
hetero after UVB irradiation VS hetero before UVB irradiation	GO_CELLULAR_GLUCOSE_HOMEOSTASIS1	0.033220522	0.009316555	0.247922602	0.81055397	0.92652154	-6.19828549
WT after UVB irradiation VS WT before UVB irradiation	GO_CELLULAR_GLUCOSE_HOMEOSTASIS2	0.0510571	-0.02617296	0.334270074	0.74666871	0.93606982	-5.93753636
homo after UVB irradiation VS homo before UVB irradiation	GO_CELLULAR_GLUCURONIDATION	-0.90267077	0.04588548	-5.37436795	0.00058174	0.01740349	0.118687941
hetero after UVB irradiation VS hetero before UVB irradiation	GO_CELLULAR_GLUCURONIDATION1	-0.68615502	0.025624635	-3.65450424	0.00666179	0.13729246	-2.11032164
WT after UVB irradiation VS WT before UVB irradiation	GO_CELLULAR_GLUCURONIDATION2	-0.5802663	0.10738412	-3.07749071	0.01495401	0.36596413	-2.7847834
homo after UVB irradiation VS homo before UVB irradiation	GO_CELLULAR_HOMEOSTASIS	0.229082271	-0.01165853	1.800113385	0.10805354	0.26285956	-5.05417392
hetero after UVB irradiation VS hetero before UVB irradiation	GO_CELLULAR_HOMEOSTASIS1	0.150863532	0.006812963	1.165100581	0.27814789	0.60400502	-5.55461074
WT after UVB irradiation VS WT before UVB irradiation	GO_CELLULAR_HOMEOSTASIS2	0.140832554	-0.00622201	0.853303271	0.41801523	0.82297693	-5.62915897
homo after UVB irradiation VS homo before UVB irradiation	GO_CELLULAR_HORMONE_METABOLIC_PROCESS	0.222186452	0.014540299	1.609852747	0.14460164	0.31681841	-5.31614979
hetero after UVB irradiation VS hetero before UVB irradiation	GO_CELLULAR_HORMONE_METABOLIC_PROCESS1	0.025028299	0.03220572	0.151337391	0.88352507	0.95768873	-6.21908238
WT after UVB irradiation VS WT before UVB irradiation	GO_CELLULAR_HORMONE_METABOLIC_PROCESS2	0.027723867	0.047405995	0.146548585	0.88707113	0.9709658	-5.9847505
homo after UVB irradiation VS homo before UVB irradiation	GO_CELLULAR_HYPEROSMOTIC_RESPONSE	-0.24486586	0.07412975	-1.10281002	0.30095037	0.49571656	-5.92872484
hetero after UVB irradiation VS hetero before UVB irradiation	GO_CELLULAR_HYPEROSMOTIC_RESPONSE1	-0.2653523	-0.03989896	-1.42396921	0.19295465	0.51844079	-5.25788684
WT after UVB irradiation VS WT before UVB irradiation	GO_CELLULAR_HYPEROSMOTIC_RESPONSE2	0.018586798	-0.0525051	0.087141588	0.93267535	0.98322796	-5.99206186
homo after UVB irradiation VS homo before UVB irradiation	GO_CELLULAR_HYPEROSMOTIC_SALINITY_RESPONSE	-0.34618385	0.048778949	-1.08528107	0.30820618	0.50275536	-5.94707714
hetero after UVB irradiation VS hetero before UVB irradiation	GO_CELLULAR_HYPEROSMOTIC_SALINITY_RESPONSE1	-0.33432365	-0.02101407	-1.9251392	0.09108074	0.37831177	-4.59876266
WT after UVB irradiation VS WT before UVB irradiation	GO_CELLULAR_HYPEROSMOTIC_SALINITY_RESPONSE2	-0.01140208	-0.04930279	-0.04543238	0.96486311	0.99087638	-5.99497769
homo after UVB irradiation VS homo before UVB irradiation	GO_CELLULAR_HYPOTONIC_RESPONSE	-0.06163524	-0.01984323	-0.2686074	0.7947652	0.88349153	-6.51143326
hetero after UVB irradiation VS hetero before UVB irradiation	GO_CELLULAR_HYPOTONIC_RESPONSE1	-0.225283	-0.05256509	-1.11498874	0.29781275	0.62148563	-5.60744072
WT after UVB irradiation VS WT before UVB irradiation	GO_CELLULAR_HYPOTONIC_RESPONSE2	0.139649757	-0.00302284	0.6387504	0.54062695	0.87025652	-5.78634996
homo after UVB irradiation VS homo before UVB irradiation	GO_CELLULAR_ION_HOMEOSTASIS	0.24235884	-0.01275197	1.795523368	0.10882353	0.2636467	-5.06064923
hetero after UVB irradiation VS hetero before UVB irradiation	GO_CELLULAR_ION_HOMEOSTASIS1	0.120139055	0.015263333	0.920795679	0.38457227	0.69593538	-5.79572534
WT after UVB irradiation VS WT before UVB irradiation	GO_CELLULAR_ION_HOMEOSTASIS2	0.11818974	-0.0050376	0.656398477	0.52977482	0.86747209	-5.77491936
homo after UVB irradiation VS homo before UVB irradiation	GO_CELLULAR_IRON_ION_HOMEOSTASIS	0.098832603	0.003644398	0.662891512	0.52531605	0.69382342	-6.3159601
hetero after UVB irradiation VS hetero before UVB irradiation	GO_CELLULAR_IRON_ION_HOMEOSTASIS1	0.089574225	0.027907832	0.66041376	0.52788138	0.79031582	-6.00153309
WT after UVB irradiation VS WT before UVB irradiation	GO_CELLULAR_IRON_ION_HOMEOSTASIS2	0.179007987	-0.00350556	1.0781063	0.31205117	0.76958855	-5.42540807
homo after UVB irradiation VS homo before UVB irradiation	GO_CELLULAR_KETONE_METABOLIC_PROCESS	0.018077606	-0.02057146	0.135219842	0.89564888	0.94320206	-6.54083307
hetero after UVB irradiation VS hetero before UVB irradiation	GO_CELLULAR_KETONE_METABOLIC_PROCESS1	-0.126148	-0.01600743	-0.97485264	0.35870309	0.67363834	-5.7460774
WT after UVB irradiation VS WT before UVB irradiation	GO_CELLULAR_KETONE_METABOLIC_PROCESS2	-0.00251803	0.000275939	-0.01678211	0.98701658	0.99740162	-5.99591806
homo after UVB irradiation VS homo before UVB irradiation	GO_CELLULAR_LIPID_CATABOLIC_PROCESS	0.092658909	-0.0202066	0.561889188	0.58898801	0.74167557	-6.38085055
hetero after UVB irradiation VS hetero before UVB irradiation	GO_CELLULAR_LIPID_CATABOLIC_PROCESS1	-0.18297096	-0.00635437	-1.22379788	0.25647625	0.58385284	-5.4907041
WT after UVB irradiation VS WT before UVB irradiation	GO_CELLULAR_LIPID_CATABOLIC_PROCESS2	-0.03238683	-0.00722879	-0.17120695	0.86826197	0.96790654	-5.98062989
homo after UVB irradiation VS homo before UVB irradiation	GO_CELLULAR_LIPID_METABOLIC_PROCESS	0.170297526	-0.01643294	1.320909516	0.22166604	0.41270827	-5.68320673
hetero after UVB irradiation VS hetero before UVB irradiation	GO_CELLULAR_LIPID_METABOLIC_PROCESS1	0.032754447	0.0005646	0.221222453	0.83056308	0.93512809	-6.20502936
WT after UVB irradiation VS WT before UVB irradiation	GO_CELLULAR_LIPID_METABOLIC_PROCESS2	0.080119457	0.005892933	0.475318569	0.64711701	0.90907607	-5.87858746
homo after UVB irradiation VS homo before UVB irradiation	GO_CELLULAR_MACROMOLECULE_CATABOLIC_PROCESS	-0.21442326	-0.00600372	-1.51985198	0.16555517	0.34380771	-5.43490282
hetero after UVB irradiation VS hetero before UVB irradiation	GO_CELLULAR_MACROMOLECULE_CATABOLIC_PROCESS1	0.030429943	-0.0123927	0.205412493	0.84247465	0.93977074	-6.20866537
WT after UVB irradiation VS WT before UVB irradiation	GO_CELLULAR_MACROMOLECULE_CATABOLIC_PROCESS2	-0.09180363	-0.01559786	-0.49909396	0.63098797	0.90411526	-5.86673278
homo after UVB irradiation VS homo before UVB irradiation	GO_CELLULAR_MACROMOLECULE_LOCALIZATION	-0.09360982	-0.01489442	-0.7346947	0.4826946	0.6599763	-6.26400337
hetero after UVB irradiation VS hetero before UVB irradiation	GO_CELLULAR_MACROMOLECULE_LOCALIZATION1	0.016071297	-0.01494077	0.125469163	0.90330479	0.96434419	-6.22295569
WT after UVB irradiation VS WT before UVB irradiation	GO_CELLULAR_MACROMOLECULE_LOCALIZATION2	-0.02556664	-0.01849063	-0.16873493	0.87014381	0.96830648	-5.98107162
homo after UVB irradiation VS homo before UVB irradiation	GO_CELLULAR_MAGNESIUM_ION_HOMEOSTASIS	-1.0000058	0.069858354	-6.68302571	0.00012925	0.01007802	1.605094164
hetero after UVB irradiation VS hetero before UVB irradiation	GO_CELLULAR_MAGNESIUM_ION_HOMEOSTASIS1	-0.70019049	-0.1419336	-2.43324977	0.0415423	0.27464073	-3.86912232
WT after UVB irradiation VS WT before UVB irradiation	GO_CELLULAR_MAGNESIUM_ION_HOMEOSTASIS2	-1.06884094	-0.05088514	-6.77430579	0.00013375	0.18842845	1.289252476
homo after UVB irradiation VS homo before UVB irradiation	GO_CELLULAR_METABOLIC_COMPOUND_SALVAGE	0.359175194	-0.05336918	2.579959677	0.03157039	0.12597827	-3.88528729
hetero after UVB irradiation VS hetero before UVB irradiation	GO_CELLULAR_METABOLIC_COMPOUND_SALVAGE1	0.453641268	-0.02493404	3.538068297	0.00787406	0.14436002	-2.27181372
WT after UVB irradiation VS WT before UVB irradiation	GO_CELLULAR_METABOLIC_COMPOUND_SALVAGE2	0.462572143	-0.02941101	2.987210808	0.01717051	0.38271751	-2.91133898
homo after UVB irradiation VS homo before UVB irradiation	GO_CELLULAR_MODIFIED_AMINO_ACID_BIOSYNTHETIC_PROCESS	0.236686493	-0.02598934	1.835290791	0.10232008	0.25428383	-5.0043184
hetero after UVB irradiation VS hetero before UVB irradiation	GO_CELLULAR_MODIFIED_AMINO_ACID_BIOSYNTHETIC_PROCESS1	0.266469091	-0.00268136	1.707880932	0.12674796	0.43339219	-4.8952349
WT after UVB irradiation VS WT before UVB irradiation	GO_CELLULAR_MODIFIED_AMINO_ACID_BIOSYNTHETIC_PROCESS2	0.248569627	0.03865276	1.51085177	0.168821	0.67478101	-4.94358985
homo after UVB irradiation VS homo before UVB irradiation	GO_CELLULAR_MODIFIED_AMINO_ACID_CATABOLIC_PROCESS	0.056011857	-0.00918323	0.305334554	0.7676028	0.86568701	-6.49998051
hetero after UVB irradiation VS hetero before UVB irradiation	GO_CELLULAR_MODIFIED_AMINO_ACID_CATABOLIC_PROCESS1	0.154294388	0.021239032	1.246264197	0.24856116	0.57624014	-5.46568893
WT after UVB irradiation VS WT before UVB irradiation	GO_CELLULAR_MODIFIED_AMINO_ACID_CATABOLIC_PROCESS2	0.085893725	-0.00055838	0.478027851	0.6452688	0.90816985	-5.87726405
homo after UVB irradiation VS homo before UVB irradiation	GO_CELLULAR_MODIFIED_AMINO_ACID_METABOLIC_PROCESS	0.070594401	-0.00815636	0.51778619	0.61807595	0.76269068	-6.40608124
hetero after UVB irradiation VS hetero before UVB irradiation	GO_CELLULAR_MODIFIED_AMINO_ACID_METABOLIC_PROCESS1	0.055437689	-0.00914381	0.419214546	0.68629163	0.87376352	-6.13726901
WT after UVB irradiation VS WT before UVB irradiation	GO_CELLULAR_MODIFIED_AMINO_ACID_METABOLIC_PROCESS2	-0.00322687	0.020630475	-0.02169922	0.98321306	0.99634469	-5.99581823
homo after UVB irradiation VS homo before UVB irradiation	GO_CELLULAR_MONOVALENT_INORGANIC_CATION_HOMEOSTASIS	0.169869709	-0.01493982	1.075907001	0.31214246	0.50612969	-5.95680142
hetero after UVB irradiation VS hetero before UVB irradiation	GO_CELLULAR_MONOVALENT_INORGANIC_CATION_HOMEOSTASIS1	0.05262043	-0.01506978	0.365683204	0.72425588	0.89176195	-6.15959805
WT after UVB irradiation VS WT before UVB irradiation	GO_CELLULAR_MONOVALENT_INORGANIC_CATION_HOMEOSTASIS2	0.12280473	-0.00403775	0.744516047	0.47761193	0.84915768	-5.71374317
homo after UVB irradiation VS homo before UVB irradiation	GO_CELLULAR_NITROGEN_COMPOUND_CATABOLIC_PROCESS	-0.35594249	0.001485389	-2.64791781	0.02835015	0.11925973	-3.77990429
hetero after UVB irradiation VS hetero before UVB irradiation	GO_CELLULAR_NITROGEN_COMPOUND_CATABOLIC_PROCESS1	-0.07244226	-0.01956986	-0.41139804	0.6917794	0.87666335	-6.1407114
WT after UVB irradiation VS WT before UVB irradiation	GO_CELLULAR_NITROGEN_COMPOUND_CATABOLIC_PROCESS2	-0.18959442	-0.01347376	-0.97247161	0.3589651	0.79181584	-5.52575249
homo after UVB irradiation VS homo before UVB irradiation	GO_CELLULAR_OXIDANT_DETOXIFICATION	0.428990795	-0.01042101	2.84810316	0.02067849	0.09967563	-3.46891969
hetero after UVB irradiation VS hetero before UVB irradiation	GO_CELLULAR_OXIDANT_DETOXIFICATION1	0.322258397	0.018801939	1.955679967	0.08691104	0.37259133	-4.55609365
WT after UVB irradiation VS WT before UVB irradiation	GO_CELLULAR_OXIDANT_DETOXIFICATION2	0.305367402	0.017740433	1.519309146	0.16671834	0.67150908	-4.93323241
homo after UVB irradiation VS homo before UVB irradiation	GO_CELLULAR_PIGMENTATION	0.098524986	-0.0159979	0.74259951	0.47814165	0.65587807	-6.25799626
hetero after UVB irradiation VS hetero before UVB irradiation	GO_CELLULAR_PIGMENTATION1	0.010813406	-0.00832857	0.069425213	0.94638647	0.97985397	-6.22887109
WT after UVB irradiation VS WT before UVB irradiation	GO_CELLULAR_PIGMENTATION2	0.198787917	0.013581721	1.276335057	0.2372236	0.72854168	-5.21747821
homo after UVB irradiation VS homo before UVB irradiation	GO_CELLULAR_POLYSACCHARIDE_CATABOLIC_PROCESS	0.040463359	-0.00496654	0.217825511	0.83280697	0.90672366	-6.52489166
hetero after UVB irradiation VS hetero before UVB irradiation	GO_CELLULAR_POLYSACCHARIDE_CATABOLIC_PROCESS1	-0.20857297	0.045329509	-1.48431792	0.17671708	0.50051267	-5.18366106
WT after UVB irradiation VS WT before UVB irradiation	GO_CELLULAR_POLYSACCHARIDE_CATABOLIC_PROCESS2	-0.0016022	-0.00713069	-0.0077256	0.99402286	0.9985657	-5.99603517
homo after UVB irradiation VS homo before UVB irradiation	GO_CELLULAR_POTASSIUM_ION_HOMEOSTASIS	0.350766189	0.016627543	2.496992384	0.03600935	0.13583587	-4.01363466
hetero after UVB irradiation VS hetero before UVB irradiation	GO_CELLULAR_POTASSIUM_ION_HOMEOSTASIS1	-0.09863805	-0.00068087	-0.4944307	0.63453559	0.84988719	-6.10100879
WT after UVB irradiation VS WT before UVB irradiation	GO_CELLULAR_POTASSIUM_ION_HOMEOSTASIS2	0.173424881	0.006088377	0.961560379	0.36410014	0.79568799	-5.53566509
homo after UVB irradiation VS homo before UVB irradiation	GO_CELLULAR_PROCESS_INVOLVED_IN_REPRODUCTION_IN_MULTICELLULAR_ORGANISM	-0.14262079	0.010778396	-1.18546814	0.26854823	0.46383032	-5.83931116
hetero after UVB irradiation VS hetero before UVB irradiation	GO_CELLULAR_PROCESS_INVOLVED_IN_REPRODUCTION_IN_MULTICELLULAR_ORGANISM1	-0.05188589	0.0004041	-0.36944302	0.72156109	0.89103348	-6.15812545
WT after UVB irradiation VS WT before UVB irradiation	GO_CELLULAR_PROCESS_INVOLVED_IN_REPRODUCTION_IN_MULTICELLULAR_ORGANISM2	-0.08071878	0.016476315	-0.57433941	0.58133776	0.88889863	-5.82567365
homo after UVB irradiation VS homo before UVB irradiation	GO_CELLULAR_PROTEIN_CATABOLIC_PROCESS	-0.07468764	-0.0129483	-0.51258324	0.62155643	0.76525182	-6.40893077
hetero after UVB irradiation VS hetero before UVB irradiation	GO_CELLULAR_PROTEIN_CATABOLIC_PROCESS1	0.081002707	-0.01268164	0.623764892	0.55046706	0.80124226	-6.02573384
WT after UVB irradiation VS WT before UVB irradiation	GO_CELLULAR_PROTEIN_CATABOLIC_PROCESS2	-0.00665686	-0.01353293	-0.03877844	0.97000605	0.99229361	-5.99527328
homo after UVB irradiation VS homo before UVB irradiation	GO_CELLULAR_PROTEIN_COMPLEX_DISASSEMBLY	-0.20505357	0.005104606	-1.36439118	0.20816483	0.39822021	-5.63075596
hetero after UVB irradiation VS hetero before UVB irradiation	GO_CELLULAR_PROTEIN_COMPLEX_DISASSEMBLY1	-0.0338784	-0.02751307	-0.25041491	0.80869355	0.92568169	-6.19761742
WT after UVB irradiation VS WT before UVB irradiation	GO_CELLULAR_PROTEIN_COMPLEX_DISASSEMBLY2	-0.0215941	-0.02303934	-0.12790153	0.90134628	0.97530188	-5.98744417
homo after UVB irradiation VS homo before UVB irradiation	GO_CELLULAR_PROTEIN_CONTAINING_COMPLEX_ASSEMBLY	-0.2282648	-0.00636394	-1.8692773	0.09705413	0.2464088	-4.9557786
hetero after UVB irradiation VS hetero before UVB irradiation	GO_CELLULAR_PROTEIN_CONTAINING_COMPLEX_ASSEMBLY1	-0.05441008	-0.02874684	-0.3993747	0.70025867	0.88146568	-6.14588543
WT after UVB irradiation VS WT before UVB irradiation	GO_CELLULAR_PROTEIN_CONTAINING_COMPLEX_ASSEMBLY2	-0.02723018	-0.0122871	-0.16984203	0.86930091	0.96830456	-5.98087458
homo after UVB irradiation VS homo before UVB irradiation	GO_CELLULAR_PROTEIN_CONTAINING_COMPLEX_LOCALIZATION	0.071370477	-0.01326072	0.510527538	0.62293436	0.76618123	-6.41004919
hetero after UVB irradiation VS hetero before UVB irradiation	GO_CELLULAR_PROTEIN_CONTAINING_COMPLEX_LOCALIZATION1	0.156914019	0.016737683	1.174734068	0.27449134	0.60112706	-5.54426943
WT after UVB irradiation VS WT before UVB irradiation	GO_CELLULAR_PROTEIN_CONTAINING_COMPLEX_LOCALIZATION2	0.233397401	-0.01396107	1.514090411	0.16801302	0.67337539	-4.93962719
homo after UVB irradiation VS homo before UVB irradiation	GO_CELLULAR_RESPIRATION	-0.0212055	0.017807522	-0.08593088	0.9335512	0.96446479	-6.54680599
hetero after UVB irradiation VS hetero before UVB irradiation	GO_CELLULAR_RESPIRATION1	-0.25083669	-0.02703365	-1.2913059	0.23330959	0.56170931	-5.41464899
WT after UVB irradiation VS WT before UVB irradiation	GO_CELLULAR_RESPIRATION2	0.021704003	-0.05540693	0.086493771	0.93317444	0.98322796	-5.99212115
homo after UVB irradiation VS homo before UVB irradiation	GO_CELLULAR_RESPONSE_TO_ABIOTIC_STIMULUS	0.059836069	-0.00426633	0.453407033	0.66183525	0.79501041	-6.43942849
hetero after UVB irradiation VS hetero before UVB irradiation	GO_CELLULAR_RESPONSE_TO_ABIOTIC_STIMULUS1	0.110745232	-0.00098369	0.877860557	0.40607618	0.71185533	-5.83354519
WT after UVB irradiation VS WT before UVB irradiation	GO_CELLULAR_RESPONSE_TO_ABIOTIC_STIMULUS2	0.054399563	-0.03056045	0.378436667	0.714835	0.93029655	-5.92120007
homo after UVB irradiation VS homo before UVB irradiation	GO_CELLULAR_RESPONSE_TO_ACID_CHEMICAL	0.410092969	-0.03098798	3.190642113	0.01213849	0.07410957	-2.93880626
hetero after UVB irradiation VS hetero before UVB irradiation	GO_CELLULAR_RESPONSE_TO_ACID_CHEMICAL1	0.105504004	-0.01266457	0.665342577	0.52488755	0.78912283	-5.99818431
WT after UVB irradiation VS WT before UVB irradiation	GO_CELLULAR_RESPONSE_TO_ACID_CHEMICAL2	0.066077221	-0.02868128	0.396075481	0.70228111	0.92532318	-5.91413067
homo after UVB irradiation VS homo before UVB irradiation	GO_CELLULAR_RESPONSE_TO_ACIDIC_PH	0.067616244	0.008612832	0.271935972	0.7922906	0.88180348	-6.51045456
hetero after UVB irradiation VS hetero before UVB irradiation	GO_CELLULAR_RESPONSE_TO_ACIDIC_PH1	-0.0414014	-0.03449622	-0.23928388	0.81701239	0.9298037	-6.20055021
WT after UVB irradiation VS WT before UVB irradiation	GO_CELLULAR_RESPONSE_TO_ACIDIC_PH2	-0.04201755	-0.02952968	-0.21362129	0.83612299	0.96182256	-5.97205953
homo after UVB irradiation VS homo before UVB irradiation	GO_CELLULAR_RESPONSE_TO_ALCOHOL	0.062510451	-0.02110908	0.45921689	0.65782584	0.79180554	-6.43659091
hetero after UVB irradiation VS hetero before UVB irradiation	GO_CELLULAR_RESPONSE_TO_ALCOHOL1	-0.02181362	-0.01616161	-0.17233735	0.86753099	0.94990587	-6.215409
WT after UVB irradiation VS WT before UVB irradiation	GO_CELLULAR_RESPONSE_TO_ALCOHOL2	0.060675922	0.026480412	0.417760309	0.6869799	0.91952231	-5.9050168
homo after UVB irradiation VS homo before UVB irradiation	GO_CELLULAR_RESPONSE_TO_ALDEHYDE	0.602038966	-0.0101397	2.863101036	0.0201975	0.09810877	-3.44562165
hetero after UVB irradiation VS hetero before UVB irradiation	GO_CELLULAR_RESPONSE_TO_ALDEHYDE1	0.300503221	-0.00545182	1.099006982	0.30431425	0.62611003	-5.62394152
WT after UVB irradiation VS WT before UVB irradiation	GO_CELLULAR_RESPONSE_TO_ALDEHYDE2	0.521633492	-0.0226303	2.089377644	0.06966844	0.54504008	-4.18106042
homo after UVB irradiation VS homo before UVB irradiation	GO_CELLULAR_RESPONSE_TO_ALKALOID	0.121759179	0.03402635	0.852153722	0.41795517	0.60367666	-6.1690742
hetero after UVB irradiation VS hetero before UVB irradiation	GO_CELLULAR_RESPONSE_TO_ALKALOID1	0.199123872	0.020897511	1.526229851	0.1661686	0.48756887	-5.13113302
WT after UVB irradiation VS WT before UVB irradiation	GO_CELLULAR_RESPONSE_TO_ALKALOID2	0.189277328	-0.02143053	1.081013129	0.31083121	0.76781656	-5.42253816
homo after UVB irradiation VS homo before UVB irradiation	GO_CELLULAR_RESPONSE_TO_AMYLOID_BETA	0.544654057	-0.03994203	4.242214137	0.00258167	0.03371672	-1.38033837
hetero after UVB irradiation VS hetero before UVB irradiation	GO_CELLULAR_RESPONSE_TO_AMYLOID_BETA1	0.485933593	0.025609422	2.901327456	0.0202486	0.20610175	-3.18322443
WT after UVB irradiation VS WT before UVB irradiation	GO_CELLULAR_RESPONSE_TO_AMYLOID_BETA2	0.446022091	-0.00381865	2.27860777	0.05180131	0.51685731	-3.91617146
homo after UVB irradiation VS homo before UVB irradiation	GO_CELLULAR_RESPONSE_TO_ANTIBIOTIC	-0.17544253	0.018249823	-1.0798629	0.31047656	0.50464837	-5.95270543
hetero after UVB irradiation VS hetero before UVB irradiation	GO_CELLULAR_RESPONSE_TO_ANTIBIOTIC1	0.174011441	-0.02197777	0.807017116	0.4434109	0.73891789	-5.89270042
WT after UVB irradiation VS WT before UVB irradiation	GO_CELLULAR_RESPONSE_TO_ANTIBIOTIC2	-0.13139114	-0.04364359	-0.68434509	0.51286317	0.85974355	-5.75625151
homo after UVB irradiation VS homo before UVB irradiation	GO_CELLULAR_RESPONSE_TO_ARSENIC_CONTAINING_SUBSTANCE	-0.26056457	0.036909394	-0.94929454	0.36919739	0.55965545	-6.08174849
hetero after UVB irradiation VS hetero before UVB irradiation	GO_CELLULAR_RESPONSE_TO_ARSENIC_CONTAINING_SUBSTANCE1	-0.05990624	0.029110817	-0.29174977	0.77803652	0.91343573	-6.18558174
WT after UVB irradiation VS WT before UVB irradiation	GO_CELLULAR_RESPONSE_TO_ARSENIC_CONTAINING_SUBSTANCE2	-0.60103846	-0.01561354	-3.67206424	0.00615986	0.29609459	-1.97493335
homo after UVB irradiation VS homo before UVB irradiation	GO_CELLULAR_RESPONSE_TO_ATP	0.356843552	0.007391699	2.059349341	0.07205092	0.20600399	-4.67839753
hetero after UVB irradiation VS hetero before UVB irradiation	GO_CELLULAR_RESPONSE_TO_ATP1	0.355699848	0.014331086	2.211176018	0.05859144	0.31951981	-4.19217456
WT after UVB irradiation VS WT before UVB irradiation	GO_CELLULAR_RESPONSE_TO_ATP2	0.053483651	-0.00396236	0.347252808	0.73725412	0.93478445	-5.93293772
homo after UVB irradiation VS homo before UVB irradiation	GO_CELLULAR_RESPONSE_TO_BACTERIAL_LIPOPROTEIN	1.046514454	0.030301198	7.857320012	3.97E-05	0.00847131	2.738297058
hetero after UVB irradiation VS hetero before UVB irradiation	GO_CELLULAR_RESPONSE_TO_BACTERIAL_LIPOPROTEIN1	0.94363491	0.085680462	5.455767308	0.00064423	0.08125048	0.108721968
WT after UVB irradiation VS WT before UVB irradiation	GO_CELLULAR_RESPONSE_TO_BACTERIAL_LIPOPROTEIN2	0.392460103	-0.01827302	1.313704757	0.22494185	0.71906685	-5.17569617
homo after UVB irradiation VS homo before UVB irradiation	GO_CELLULAR_RESPONSE_TO_BIOTIC_STIMULUS	0.535542554	-0.01376237	3.887564801	0.00427867	0.04234489	-1.89035146
hetero after UVB irradiation VS hetero before UVB irradiation	GO_CELLULAR_RESPONSE_TO_BIOTIC_STIMULUS1	0.553535377	0.02628662	3.91807953	0.00459503	0.12761654	-1.75208352
WT after UVB irradiation VS WT before UVB irradiation	GO_CELLULAR_RESPONSE_TO_BIOTIC_STIMULUS2	0.368919158	-0.02263026	1.856195303	0.10006899	0.59874718	-4.4994219
homo after UVB irradiation VS homo before UVB irradiation	GO_CELLULAR_RESPONSE_TO_BRAIN_DERIVED_NEUROTROPHIC_FACTOR_STIMULUS	-0.10731449	2.09E-05	-0.56178854	0.58905353	0.74169479	-6.38091031
hetero after UVB irradiation VS hetero before UVB irradiation	GO_CELLULAR_RESPONSE_TO_BRAIN_DERIVED_NEUROTROPHIC_FACTOR_STIMULUS1	-0.24360274	-0.03046668	-1.59477874	0.15013521	0.46837106	-5.04360981
WT after UVB irradiation VS WT before UVB irradiation	GO_CELLULAR_RESPONSE_TO_BRAIN_DERIVED_NEUROTROPHIC_FACTOR_STIMULUS2	-0.3512462	0.000758691	-1.80794261	0.10777972	0.61228673	-4.56375475
homo after UVB irradiation VS homo before UVB irradiation	GO_CELLULAR_RESPONSE_TO_CADMIUM_ION	0.119017014	-0.02309888	0.921847935	0.38253255	0.57262444	-6.10719984
hetero after UVB irradiation VS hetero before UVB irradiation	GO_CELLULAR_RESPONSE_TO_CADMIUM_ION1	0.292523233	0.006902426	1.969358713	0.08510379	0.37178552	-4.53691527
WT after UVB irradiation VS WT before UVB irradiation	GO_CELLULAR_RESPONSE_TO_CADMIUM_ION2	-0.06616025	0.039143289	-0.3184979	0.75816701	0.93908133	-5.94289397
homo after UVB irradiation VS homo before UVB irradiation	GO_CELLULAR_RESPONSE_TO_CAFFEINE	0.272218174	0.005064146	1.10128489	0.30157627	0.49599973	-5.93033026
hetero after UVB irradiation VS hetero before UVB irradiation	GO_CELLULAR_RESPONSE_TO_CAFFEINE1	-0.19845306	-0.06589975	-0.79224352	0.45148613	0.74354122	-5.90451216
WT after UVB irradiation VS WT before UVB irradiation	GO_CELLULAR_RESPONSE_TO_CAFFEINE2	-0.04606349	0.037983468	-0.17830142	0.86286627	0.9676681	-5.97932675

homo after UVB irradiation VS homo before UVB irradiation	GO_CELLULAR_RESPONSE_TO_CALCIUM_ION	0.228547654	0.011507441	1.857272579	0.09888419	0.24883069	-4.97296445
hetero after UVB irradiation VS hetero before UVB irradiation	GO_CELLULAR_RESPONSE_TO_CALCIUM_ION1	0.101287507	0.032948318	0.624626586	0.54992951	0.80098311	-6.02517908
WT after UVB irradiation VS WT before UVB irradiation	GO_CELLULAR_RESPONSE_TO_CALCIUM_ION2	-0.13712625	-0.02110069	-0.85985799	0.41459727	0.8213754	-5.62375582
homo after UVB irradiation VS homo before UVB irradiation	GO_CELLULAR_RESPONSE_TO_CAMP	0.235227332	0.012504556	1.954322103	0.08498148	0.22689918	-4.8328327
hetero after UVB irradiation VS hetero before UVB irradiation	GO_CELLULAR_RESPONSE_TO_CAMP1	-0.01890972	0.002217888	-0.09716868	0.92502664	0.97164672	-6.22636696
WT after UVB irradiation VS WT before UVB irradiation	GO_CELLULAR_RESPONSE_TO_CAMP2	0.017911272	0.004770066	0.099648422	0.92304631	0.98103299	-5.9908306
homo after UVB irradiation VS homo before UVB irradiation	GO_CELLULAR_RESPONSE_TO_CARBOHYDRATE_STIMULUS	0.177255534	-0.00554731	1.286497146	0.23287282	0.42431216	-5.72393813
hetero after UVB irradiation VS hetero before UVB irradiation	GO_CELLULAR_RESPONSE_TO_CARBOHYDRATE_STIMULUS1	0.0780829	-7.41E-05	0.574049839	0.58199908	0.82095894	-6.05656165
WT after UVB irradiation VS WT before UVB irradiation	GO_CELLULAR_RESPONSE_TO_CARBOHYDRATE_STIMULUS2	0.080205201	-0.03937544	0.470994408	0.65007224	0.9092481	-5.880685
homo after UVB irradiation VS homo before UVB irradiation	GO_CELLULAR_RESPONSE_TO_CHEMICAL_STRESS	0.177325867	-0.01236137	1.459248844	0.18115381	0.36415839	-5.51272663
hetero after UVB irradiation VS hetero before UVB irradiation	GO_CELLULAR_RESPONSE_TO_CHEMICAL_STRESS1	0.200864312	0.010003429	1.49179097	0.17479342	0.49736582	-5.17435227
WT after UVB irradiation VS WT before UVB irradiation	GO_CELLULAR_RESPONSE_TO_CHEMICAL_STRESS2	0.131509788	-0.02312954	0.802737308	0.44504593	0.83340629	-5.66968393
homo after UVB irradiation VS homo before UVB irradiation	GO_CELLULAR_RESPONSE_TO_CHOLESTEROL	-0.09616362	-0.00463364	-0.71055759	0.49676916	0.6716898	-6.28199627
hetero after UVB irradiation VS hetero before UVB irradiation	GO_CELLULAR_RESPONSE_TO_CHOLESTEROL1	-0.38054061	-0.01524833	-2.44545764	0.04076511	0.27317438	-3.85126027
WT after UVB irradiation VS WT before UVB irradiation	GO_CELLULAR_RESPONSE_TO_CHOLESTEROL2	-0.0030178	0.110302106	-0.01323251	0.9897625	0.99845632	-5.99597428
homo after UVB irradiation VS homo before UVB irradiation	GO_CELLULAR_RESPONSE_TO_CISPLATIN	-0.13360562	0.043518172	-0.53902067	0.60397732	0.75290533	-6.39417288
hetero after UVB irradiation VS hetero before UVB irradiation	GO_CELLULAR_RESPONSE_TO_CISPLATIN1	0.415260245	0.053145156	1.371170898	0.20822397	0.53315163	-5.32137626
WT after UVB irradiation VS WT before UVB irradiation	GO_CELLULAR_RESPONSE_TO_CISPLATIN2	0.45572751	0.021991702	1.630616855	0.14116171	0.65097221	-4.7942283
homo after UVB irradiation VS homo before UVB irradiation	GO_CELLULAR_RESPONSE_TO_COLD	0.164249751	0.004211063	0.616705612	0.55390751	0.71588834	-6.34684357
hetero after UVB irradiation VS hetero before UVB irradiation	GO_CELLULAR_RESPONSE_TO_COLD1	0.130883371	0.043221064	0.661256758	0.52736859	0.79011584	-6.0009619
WT after UVB irradiation VS WT before UVB irradiation	GO_CELLULAR_RESPONSE_TO_COLD2	-0.03726971	-0.0403501	-0.23430945	0.82056048	0.95768831	-5.96720186
homo after UVB irradiation VS homo before UVB irradiation	GO_CELLULAR_RESPONSE_TO_COPPER_ION	0.084769813	-0.02272523	0.452068116	0.66276089	0.79546698	-6.44007755
hetero after UVB irradiation VS hetero before UVB irradiation	GO_CELLULAR_RESPONSE_TO_COPPER_ION1	0.08034726	0.018352993	0.394771684	0.70351687	0.88183935	-6.14782732
WT after UVB irradiation VS WT before UVB irradiation	GO_CELLULAR_RESPONSE_TO_COPPER_ION2	0.003224245	-0.02336225	0.022345986	0.9827128	0.996184	-5.9958032
homo after UVB irradiation VS homo before UVB irradiation	GO_CELLULAR_RESPONSE_TO_CORTICOSTEROID_STIMULUS	-0.10074914	0.00415667	-0.7417074	0.47865408	0.6562462	-6.25867702
hetero after UVB irradiation VS hetero before UVB irradiation	GO_CELLULAR_RESPONSE_TO_CORTICOSTEROID_STIMULUS1	-0.13510171	-0.03045496	-0.9322501	0.37897871	0.69201952	-5.78539174
WT after UVB irradiation VS WT before UVB irradiation	GO_CELLULAR_RESPONSE_TO_CORTICOSTEROID_STIMULUS2	-0.27709572	-0.02301005	-1.62722683	0.14188423	0.6516606	-4.79853195
homo after UVB irradiation VS homo before UVB irradiation	GO_CELLULAR_RESPONSE_TO_DEXAMETHASONE_STIMULUS	-0.02284843	0.033013522	-0.18398208	0.85842794	0.92216795	-6.53231786
hetero after UVB irradiation VS hetero before UVB irradiation	GO_CELLULAR_RESPONSE_TO_DEXAMETHASONE_STIMULUS1	-0.0200725	-0.01483349	-0.13591526	0.89530782	0.96152498	-6.22147828
WT after UVB irradiation VS WT before UVB irradiation	GO_CELLULAR_RESPONSE_TO_DEXAMETHASONE_STIMULUS2	-0.23784752	-0.00636648	-1.52061957	0.16639464	0.67126456	-4.93162487
homo after UVB irradiation VS homo before UVB irradiation	GO_CELLULAR_RESPONSE_TO_DNA_DAMAGE_STIMULUS	-0.34785406	-0.01032787	-2.55161118	0.03302118	0.12942723	-3.92918632
hetero after UVB irradiation VS hetero before UVB irradiation	GO_CELLULAR_RESPONSE_TO_DNA_DAMAGE_STIMULUS1	-0.01133889	-0.02887261	-0.06578895	0.94918988	0.98092529	-6.22913757
WT after UVB irradiation VS WT before UVB irradiation	GO_CELLULAR_RESPONSE_TO_DNA_DAMAGE_STIMULUS2	-0.03554697	0.005235598	-0.17158565	0.86797376	0.96790654	-5.98056165
homo after UVB irradiation VS homo before UVB irradiation	GO_CELLULAR_RESPONSE_TO_DRUG	0.100676536	-0.00118177	0.784735121	0.45434692	0.63528496	-6.22503559
hetero after UVB irradiation VS hetero before UVB irradiation	GO_CELLULAR_RESPONSE_TO_DRUG1	0.033416511	-0.01002181	0.234015521	0.82095845	0.93142111	-6.20189252
WT after UVB irradiation VS WT before UVB irradiation	GO_CELLULAR_RESPONSE_TO_DRUG2	0.073567578	-0.03015021	0.431865212	0.67710826	0.91685973	-5.89883973
homo after UVB irradiation VS homo before UVB irradiation	GO_CELLULAR_RESPONSE_TO_DSRNA	0.624751829	-0.02867285	3.946210301	0.00393085	0.0410237	-1.80478403
hetero after UVB irradiation VS hetero before UVB irradiation	GO_CELLULAR_RESPONSE_TO_DSRNA1	0.510990544	0.056780472	3.394630315	0.00969921	0.15539797	-2.47326073
WT after UVB irradiation VS WT before UVB irradiation	GO_CELLULAR_RESPONSE_TO_DSRNA2	0.564440549	-0.00689303	3.590037291	0.00694307	0.30954327	-2.08377051
homo after UVB irradiation VS homo before UVB irradiation	GO_CELLULAR_RESPONSE_TO_ELECTRICAL_STIMULUS	0.284625447	0.118029043	1.328113437	0.21937886	0.41020955	-5.6745918
hetero after UVB irradiation VS hetero before UVB irradiation	GO_CELLULAR_RESPONSE_TO_ELECTRICAL_STIMULUS1	0.059211404	0.020969636	0.255292615	0.80505626	0.92374885	-6.19629093
WT after UVB irradiation VS WT before UVB irradiation	GO_CELLULAR_RESPONSE_TO_ELECTRICAL_STIMULUS2	-0.29297092	-0.14260492	-1.61846863	0.14376655	0.65167133	-4.80963105
homo after UVB irradiation VS homo before UVB irradiation	GO_CELLULAR_RESPONSE_TO_ENDOGENOUS_STIMULUS	0.173683731	-0.00774915	1.401419281	0.19723101	0.38575718	-5.58525507
hetero after UVB irradiation VS hetero before UVB irradiation	GO_CELLULAR_RESPONSE_TO_ENDOGENOUS_STIMULUS1	0.098899832	0.001869355	0.747123907	0.47676127	0.7595751	-5.93943442
WT after UVB irradiation VS WT before UVB irradiation	GO_CELLULAR_RESPONSE_TO_ENDOGENOUS_STIMULUS2	0.026452557	-0.0026965	0.184914135	0.85784383	0.9668679	-5.97806488
homo after UVB irradiation VS homo before UVB irradiation	GO_CELLULAR_RESPONSE_TO_EPINEPHRINE_STIMULUS	0.486728959	-0.03216246	2.4905012	0.03638216	0.13669365	-4.02365781
hetero after UVB irradiation VS hetero before UVB irradiation	GO_CELLULAR_RESPONSE_TO_EPINEPHRINE_STIMULUS1	-0.19515971	-0.03499373	-0.94229877	0.37412127	0.6871463	-5.77624307
WT after UVB irradiation VS WT before UVB irradiation	GO_CELLULAR_RESPONSE_TO_EPINEPHRINE_STIMULUS2	-0.12677655	-0.05880322	-0.51444583	0.62068308	0.90087565	-5.8587904
homo after UVB irradiation VS homo before UVB irradiation	GO_CELLULAR_RESPONSE_TO_ESTRADIOL_STIMULUS	-0.34413158	0.007421233	-2.58333388	0.03140206	0.12561676	-3.88005948
hetero after UVB irradiation VS hetero before UVB irradiation	GO_CELLULAR_RESPONSE_TO_ESTRADIOL_STIMULUS1	-0.43732094	-0.00751383	-3.2331479	0.01230374	0.17108413	-2.70305167
WT after UVB irradiation VS WT before UVB irradiation	GO_CELLULAR_RESPONSE_TO_ESTRADIOL_STIMULUS2	-0.32472625	0.010413752	-2.15049558	0.06331893	0.53639346	-4.09599857
homo after UVB irradiation VS homo before UVB irradiation	GO_CELLULAR_RESPONSE_TO_ESTROGEN_STIMULUS	0.230031443	-0.00609869	1.646347196	0.1368129	0.30562337	-5.2669899
hetero after UVB irradiation VS hetero before UVB irradiation	GO_CELLULAR_RESPONSE_TO_ESTROGEN_STIMULUS1	0.168485604	-0.02097956	1.003201565	0.34567102	0.66286618	-5.71916322
WT after UVB irradiation VS WT before UVB irradiation	GO_CELLULAR_RESPONSE_TO_ESTROGEN_STIMULUS2	0.068572546	-0.01124664	0.324616939	0.7536983	0.93734883	-5.94084531
homo after UVB irradiation VS homo before UVB irradiation	GO_CELLULAR_RESPONSE_TO_ETHANOL	0.421512746	0.010407526	1.994283035	0.07981792	0.21816505	-4.77438873
hetero after UVB irradiation VS hetero before UVB irradiation	GO_CELLULAR_RESPONSE_TO_ETHANOL1	0.25094801	0.014397609	1.539478359	0.16295397	0.48449503	-5.11436959
WT after UVB irradiation VS WT before UVB irradiation	GO_CELLULAR_RESPONSE_TO_ETHANOL2	0.178126953	-0.03181122	0.78727864	0.45354291	0.8371398	-5.68165754
homo after UVB irradiation VS homo before UVB irradiation	GO_CELLULAR_RESPONSE_TO_ETHER	0.054088052	0.001703698	0.421178932	0.68428297	0.81049552	-6.45454312
hetero after UVB irradiation VS hetero before UVB irradiation	GO_CELLULAR_RESPONSE_TO_ETHER1	-0.02730937	-0.04920242	-0.09934116	0.92335653	0.97079081	-6.22613574
WT after UVB irradiation VS WT before UVB irradiation	GO_CELLULAR_RESPONSE_TO_ETHER2	0.054422973	-0.08895083	0.198739857	0.84736548	0.96522688	-5.97527951
homo after UVB irradiation VS homo before UVB irradiation	GO_CELLULAR_RESPONSE_TO_EXOGENOUS_DSRNA	0.633154853	-0.0316121	3.668766049	0.00589562	0.05056585	-2.2136014
hetero after UVB irradiation VS hetero before UVB irradiation	GO_CELLULAR_RESPONSE_TO_EXOGENOUS_DSRNA1	0.377807974	0.07845442	2.581178935	0.03305657	0.25182379	-3.65231651
WT after UVB irradiation VS WT before UVB irradiation	GO_CELLULAR_RESPONSE_TO_EXOGENOUS_DSRNA2	0.621106955	0.02269316	3.77491468	0.00530838	0.27482886	-1.83995106
homo after UVB irradiation VS homo before UVB irradiation	GO_CELLULAR_RESPONSE_TO_EXTERNAL_STIMULUS	0.172560215	-0.01366853	1.262263261	0.2410482	0.43393445	-5.75219523
hetero after UVB irradiation VS hetero before UVB irradiation	GO_CELLULAR_RESPONSE_TO_EXTERNAL_STIMULUS1	0.148498794	0.003086161	1.188446357	0.26935461	0.59632699	-5.52944887
WT after UVB irradiation VS WT before UVB irradiation	GO_CELLULAR_RESPONSE_TO_EXTERNAL_STIMULUS2	0.065359337	-0.01686187	0.432057519	0.67697412	0.91685973	-5.89875417
homo after UVB irradiation VS homo before UVB irradiation	GO_CELLULAR_RESPONSE_TO_EXTRACELLULAR_STIMULUS	0.086613204	-0.00567992	0.653206175	0.5312368	0.69808206	-6.32260319
hetero after UVB irradiation VS hetero before UVB irradiation	GO_CELLULAR_RESPONSE_TO_EXTRACELLULAR_STIMULUS1	0.024481275	-0.00124054	0.195919005	0.84964833	0.94258929	-6.21072064
WT after UVB irradiation VS WT before UVB irradiation	GO_CELLULAR_RESPONSE_TO_EXTRACELLULAR_STIMULUS2	-0.01188813	-0.01385173	-0.08235078	0.93636702	0.9832831	-5.99248991
homo after UVB irradiation VS homo before UVB irradiation	GO_CELLULAR_RESPONSE_TO_FATTY_ACID	0.09080468	0.019051257	0.716315361	0.49338828	0.6690802	-6.27775222
hetero after UVB irradiation VS hetero before UVB irradiation	GO_CELLULAR_RESPONSE_TO_FATTY_ACID1	0.220316723	0.004766141	1.650634412	0.13812879	0.45140459	-4.97091744
WT after UVB irradiation VS WT before UVB irradiation	GO_CELLULAR_RESPONSE_TO_FATTY_ACID2	0.159941529	-0.01591212	1.04610796	0.32572998	0.77462252	-5.45662311
homo after UVB irradiation VS homo before UVB irradiation	GO_CELLULAR_RESPONSE_TO_FLUID_SHEAR_STRESS	0.378427271	0.013247319	1.603313676	0.14603913	0.31877143	-5.32489893
hetero after UVB irradiation VS hetero before UVB irradiation	GO_CELLULAR_RESPONSE_TO_FLUID_SHEAR_STRESS1	0.660941103	0.028577596	4.128384583	0.0034413	0.11632799	-1.47404363
WT after UVB irradiation VS WT before UVB irradiation	GO_CELLULAR_RESPONSE_TO_FLUID_SHEAR_STRESS2	0.428366675	0.025746067	2.695633171	0.02696161	0.43942403	-3.32396554
homo after UVB irradiation VS homo before UVB irradiation	GO_CELLULAR_RESPONSE_TO_FOLLICLE_STIMULATING_HORMONE_STIMULUS	-0.43263727	0.059020909	-1.89840991	0.09274559	0.23986097	-4.9138939
hetero after UVB irradiation VS hetero before UVB irradiation	GO_CELLULAR_RESPONSE_TO_FOLLICLE_STIMULATING_HORMONE_STIMULUS1	-0.44248653	0.011723802	-1.84585125	0.10282137	0.39829707	-4.7084859
WT after UVB irradiation VS WT before UVB irradiation	GO_CELLULAR_RESPONSE_TO_FOLLICLE_STIMULATING_HORMONE_STIMULUS2	-0.49484235	-0.0138661	-1.89384799	0.09441873	0.59290945	-4.4488072
homo after UVB irradiation VS homo before UVB irradiation	GO_CELLULAR_RESPONSE_TO_FRUCTOSE_STIMULUS	0.228876602	0.021739084	0.84219084	0.42320177	0.60845563	-6.17758896
hetero after UVB irradiation VS hetero before UVB irradiation	GO_CELLULAR_RESPONSE_TO_FRUCTOSE_STIMULUS1	-0.13991334	-0.0951631	-0.41292918	0.69070288	0.87609212	-6.14004196
WT after UVB irradiation VS WT before UVB irradiation	GO_CELLULAR_RESPONSE_TO_FRUCTOSE_STIMULUS2	0.044475804	-0.08745775	0.187899271	0.85557882	0.9663792	-5.97748032
homo after UVB irradiation VS homo before UVB irradiation	GO_CELLULAR_RESPONSE_TO_GAMMA_RADIATION	-0.46778738	-0.01024791	-3.46541865	0.0079877	0.05943714	-2.51922413
hetero after UVB irradiation VS hetero before UVB irradiation	GO_CELLULAR_RESPONSE_TO_GAMMA_RADIATION1	-0.09467937	-0.02524037	-0.53359966	0.60838641	0.83666184	-6.07991045
WT after UVB irradiation VS WT before UVB irradiation	GO_CELLULAR_RESPONSE_TO_GAMMA_RADIATION2	-0.13766997	-0.03764414	-0.86411249	0.41238934	0.82049743	-5.6202306
homo after UVB irradiation VS homo before UVB irradiation	GO_CELLULAR_RESPONSE_TO_GLUCAGON_STIMULUS	0.252924013	-0.04526098	1.936904676	0.08733095	0.23076456	-4.85817621
hetero after UVB irradiation VS hetero before UVB irradiation	GO_CELLULAR_RESPONSE_TO_GLUCAGON_STIMULUS1	0.232831458	0.002514778	1.632891769	0.14184345	0.45744377	-4.99413588
WT after UVB irradiation VS WT before UVB irradiation	GO_CELLULAR_RESPONSE_TO_GLUCAGON_STIMULUS2	0.118865711	0.001048919	0.702644867	0.5019731	0.85706548	-5.74365524
homo after UVB irradiation VS homo before UVB irradiation	GO_CELLULAR_RESPONSE_TO_GLUCOSE_STARVATION	0.04118101	-0.02408846	0.260990452	0.80043716	0.88652441	-6.5136283
hetero after UVB irradiation VS hetero before UVB irradiation	GO_CELLULAR_RESPONSE_TO_GLUCOSE_STARVATION1	-0.10790061	-0.00348086	-0.71251448	0.49676818	0.77015247	-5.96502348
WT after UVB irradiation VS WT before UVB irradiation	GO_CELLULAR_RESPONSE_TO_GLUCOSE_STARVATION2	-0.1824864	-0.03082548	-1.20164439	0.26346364	0.74156847	-5.29863009
homo after UVB irradiation VS homo before UVB irradiation	GO_CELLULAR_RESPONSE_TO_GONADOTROPIN_STIMULUS	-0.26000004	0.05399637	-1.11253833	0.29698216	0.49159459	-5.91844577
hetero after UVB irradiation VS hetero before UVB irradiation	GO_CELLULAR_RESPONSE_TO_GONADOTROPIN_STIMULUS1	-0.45315223	0.03155761	-2.0747759	0.07234816	0.3485081	-4.38784273
WT after UVB irradiation VS WT before UVB irradiation	GO_CELLULAR_RESPONSE_TO_GONADOTROPIN_STIMULUS2	-0.35137807	0.014108324	-1.43144402	0.18974415	0.69190481	-5.03930631
homo after UVB irradiation VS homo before UVB irradiation	GO_CELLULAR_RESPONSE_TO_GROWTH_HORMONE_STIMULUS	0.764379476	-0.04045189	5.797362445	0.00034922	0.01377928	0.627084926
hetero after UVB irradiation VS hetero before UVB irradiation	GO_CELLULAR_RESPONSE_TO_GROWTH_HORMONE_STIMULUS1	0.533765989	0.031243523	3.076781475	0.01553655	0.18429049	-2.92818058
WT after UVB irradiation VS WT before UVB irradiation	GO_CELLULAR_RESPONSE_TO_GROWTH_HORMONE_STIMULUS2	0.199797371	-0.13024429	0.660389767	0.52733893	0.8664124	-5.77229563
homo after UVB irradiation VS homo before UVB irradiation	GO_CELLULAR_RESPONSE_TO_HEAT	-0.46163647	0.002557623	-3.3539213	0.00945593	0.06469221	-2.68866505
hetero after UVB irradiation VS hetero before UVB irradiation	GO_CELLULAR_RESPONSE_TO_HEAT1	-0.16715196	-0.02685522	-0.93800572	0.37619082	0.68961911	-5.78016111
WT after UVB irradiation VS WT before UVB irradiation	GO_CELLULAR_RESPONSE_TO_HEAT2	-0.18599275	-0.0138182	-0.8188558	0.43630238	0.83024038	-5.65699059
homo after UVB irradiation VS homo before UVB irradiation	GO_CELLULAR_RESPONSE_TO_HEPARIN	-0.18091891	0.068290302	-0.77763604	0.45829967	0.63863345	-6.23069894
hetero after UVB irradiation VS hetero before UVB irradiation	GO_CELLULAR_RESPONSE_TO_HEPARIN1	-0.11326901	0.032121407	-0.36337005	0.72591584	0.89215613	-6.16049681
WT after UVB irradiation VS WT before UVB irradiation	GO_CELLULAR_RESPONSE_TO_HEPARIN2	-0.24166081	0.068493534	-1.36412615	0.20923291	0.7053907	-5.11814246
homo after UVB irradiation VS homo before UVB irradiation	GO_CELLULAR_RESPONSE_TO_HISTAMINE	0.182116468	0.117426997	0.668601611	0.52184428	0.69109361	-6.31200247
hetero after UVB irradiation VS hetero before UVB irradiation	GO_CELLULAR_RESPONSE_TO_HISTAMINE1	-0.37205652	-0.01942009	-1.53255937	0.16462573	0.48576486	-5.12313359
WT after UVB irradiation VS WT before UVB irradiation	GO_CELLULAR_RESPONSE_TO_HISTAMINE2	-0.14152021	0.022226249	-0.54857478	0.59809241	0.89476186	-5.84033242
homo after UVB irradiation VS homo before UVB irradiation	GO_CELLULAR_RESPONSE_TO_HORMONE_STIMULUS	0.166583243	-0.00827102	1.324841921	0.22041503	0.41120918	-5.67850784
hetero after UVB irradiation VS hetero before UVB irradiation	GO_CELLULAR_RESPONSE_TO_HORMONE_STIMULUS1	0.059276933	0.004907113	0.458021248	0.65934487	0.86028652	-6.11926646
WT after UVB irradiation VS WT before UVB irradiation	GO_CELLULAR_RESPONSE_TO_HORMONE_STIMULUS2	0.019666712	0.000907776	0.133723064	0.89688527	0.97390508	-5.98664233
homo after UVB irradiation VS homo before UVB irradiation	GO_CELLULAR_RESPONSE_TO_HYDROGEN_PEROXIDE	0.120191449	0.000658597	0.83879006	0.42500313	0.60989528	-6.1804762
hetero after UVB irradiation VS hetero before UVB irradiation	GO_CELLULAR_RESPONSE_TO_HYDROGEN_PEROXIDE1	0.24954235	0.020649134	1.721677306	0.12413875	0.43059023	-4.87682734
WT after UVB irradiation VS WT before UVB irradiation	GO_CELLULAR_RESPONSE_TO_HYDROGEN_PEROXIDE2	0.033697626	-0.00955347	0.2029379	0.8441901	0.96450382	-5.97439443
homo after UVB irradiation VS homo before UVB irradiation	GO_CELLULAR_RESPONSE_TO_HYDROPEROXIDE	0.016289449	-0.02153488	0.084242154	0.93485357	0.96540477	-6.54696357
hetero after UVB irradiation VS hetero before UVB irradiation	GO_CELLULAR_RESPONSE_TO_HYDROPEROXIDE1	-0.27514029	-0.00199225	-1.29220355	0.23301388	0.56147329	-5.41362002
WT after UVB irradiation VS WT before UVB irradiation	GO_CELLULAR_RESPONSE_TO_HYDROPEROXIDE2	-0.02365972	-0.05541071	-0.06405723	0.95047776	0.98657947	-5.99390212
homo after UVB irradiation VS homo before UVB irradiation	GO_CELLULAR_RESPONSE_TO_HYPEROXIA	0.370630088	0.011710277	1.912485294	0.09072966	0.23638209	-4.89356917
hetero after UVB irradiation VS hetero before UVB irradiation	GO_CELLULAR_RESPONSE_TO_HYPEROXIA1	0.191021472	0.016311801	0.664130531	0.52562279	0.78916946	-5.99900986
WT after UVB irradiation VS WT before UVB irradiation	GO_CELLULAR_RESPONSE_TO_HYPEROXIA2	0.355069174	-0.02680245	1.515451798	0.16767442	0.67272632	-4.93796012
homo after UVB irradiation VS homo before UVB irradiation	GO_CELLULAR_RESPONSE_TO_INCREASED_OXYGEN_LEVELS	0.129834165	0.037273049	0.674348763	0.51836418	0.68827606	-6.30798846
hetero after UVB irradiation VS hetero before UVB irradiation	GO_CELLULAR_RESPONSE_TO_INCREASED_OXYGEN_LEVELS1	0.17701435	-0.02346431	0.807752401	0.44301159	0.73856954	-5.89210774
WT after UVB irradiation VS WT before UVB irradiation	GO_CELLULAR_RESPONSE_TO_INCREASED_OXYGEN_LEVELS2	0.396446407	0.007023635	1.619296424	0.14358767	0.65167133	-4.8085832
homo after UVB irradiation VS homo before UVB irradiation	GO_CELLULAR_RESPONSE_TO_INORGANIC_SUBSTANCE	0.135439409	0.0011946	1.109957605	0.29803078	0.49254997	-5.92117908
hetero after UVB irradiation VS hetero before UVB irradiation	GO_CELLULAR_RESPONSE_TO_INORGANIC_SUBSTANCE1	0.165947059	0.023053365	1.307937119	0.22788239	0.55626675	-5.39551171
WT after UVB irradiation VS WT before UVB irradiation	GO_CELLULAR_RESPONSE_TO_INORGANIC_SUBSTANCE2	0.033182251	-0.01346959	0.210102279	0.838778	0.96241201	-5.9728417
homo after UVB irradiation VS homo before UVB irradiation	GO_CELLULAR_RESPONSE_TO_INSULIN_LIKE_GROWTH_FACTOR_STIMULUS	0.342816998	0.059989481	1.75423539	0.1159843	0.27459704	-5.11857301
hetero after UVB irradiation VS hetero before UVB irradiation	GO_CELLULAR_RESPONSE_TO_INSULIN_LIKE_GROWTH_FACTOR_STIMULUS1	0.442656345	0.036544152	2.506106121	0.0371169	0.26433251	-3.76242722
WT after UVB irradiation VS WT before UVB irradiation	GO_CELLULAR_RESPONSE_TO_INSULIN_LIKE_GROWTH_FACTOR_STIMULUS2	0.064233658	-0.13662888	0.205980264	0.84189075	0.96348476	-5.97374157
homo after UVB irradiation VS homo before UVB irradiation	GO_CELLULAR_RESPONSE_TO_INSULIN_STIMULUS	0.187405585	-0.01502163	1.31571266	0.22332856	0.41418368	-5.6894027
hetero after UVB irradiation VS hetero before UVB irradiation	GO_CELLULAR_RESPONSE_TO_INSULIN_STIMULUS1	0.051812075	-0.00106277	0.391710087	0.70568762	0.88314193	-6.14910697
WT after UVB irradiation VS WT before UVB irradiation	GO_CELLULAR_RESPONSE_TO_INSULIN_STIMULUS2	0.025239018	-0.01699199	0.174618613	0.86566629	0.96790654	-5.98000977
homo after UVB irradiation VS homo before UVB irradiation	GO_CELLULAR_RESPONSE_TO_INTERFERON_ALPHA	0.441812693	0.016637462	2.808502133	0.02200603	0.10322407	-3.53044976
hetero after UVB irradiation VS hetero before UVB irradiation	GO_CELLULAR_RESPONSE_TO_INTERFERON_ALPHA1	0.430714191	0.083364268	2.728124892	0.02637283	0.23118466	-3.43669776
WT after UVB irradiation VS WT before UVB irradiation	GO_CELLULAR_RESPONSE_TO_INTERFERON_ALPHA2	0.220973233	0.017662463	1.180420721	0.27134309	0.74431614	-5.32109054
homo after UVB irradiation VS homo before UVB irradiation	GO_CELLULAR_RESPONSE_TO_INTERFERON_BETA	0.785124135	-0.07515237	4.375520721	0.00214528	0.03093391	-1.19341989
hetero after UVB irradiation VS hetero before UVB irradiation	GO_CELLULAR_RESPONSE_TO_INTERFERON_BETA1	0.751259358	0.03640454	4.613104679	0.00181184	0.09780003	-0.86122926
WT after UVB irradiation VS WT before UVB irradiation	GO_CELLULAR_RESPONSE_TO_INTERFERON_BETA2	0.54489061	0.000716857	1.744394551	0.11879065	0.62626976	-4.64748856
homo after UVB irradiation VS homo before UVB irradiation	GO_CELLULAR_RESPONSE_TO_IONIZING_RADIATION	-0.38134894	-0.01333974	-2.72299902	0.02517906	0.11174957	-3.66331197
hetero after UVB irradiation VS hetero before UVB irradiation	GO_CELLULAR_RESPONSE_TO_IONIZING_RADIATION1	0.029185651	-0.01017976	0.159744214	0.87711493	0.95496996	-6.21766866
WT after UVB irradiation VS WT before UVB irradiation	GO_CELLULAR_RESPONSE_TO_IONIZING_RADIATION2	-0.05350579	-0.01332993	-0.29373025	0.77635188	0.94340554	-5.95079774
homo after UVB irradiation VS homo before UVB irradiation	GO_CELLULAR_RESPONSE_TO_IRON_ION	-0.1084181	-0.0328012	-0.70344729	0.50096442	0.67507369	-6.28719548
hetero after UVB irradiation VS hetero before UVB irradiation	GO_CELLULAR_RESPONSE_TO_IRON_ION1	0.241397326	-0.00601568	1.500022442	0.17269622	0.49543221	-5.16406974
WT after UVB irradiation VS WT before UVB irradiation	GO_CELLULAR_RESPONSE_TO_IRON_ION2	0.215924886	-0.03466138	1.15959378	0.27926141	0.75044661	-5.34286362
homo after UVB irradiation VS homo before UVB irradiation	GO_CELLULAR_RESPONSE_TO_ISOQUINOLINE_ALKALOID	0.540188203	0.024467624	2.257811417	0.05263809	0.16975093	-4.38031299
hetero after UVB irradiation VS hetero before UVB irradiation	GO_CELLULAR_RESPONSE_TO_ISOQUINOLINE_ALKALOID1	0.461104991	0.119815135	1.848819193	0.10235698	0.39705035	-4.70440782
WT after UVB irradiation VS WT before UVB irradiation	GO_CELLULAR_RESPONSE_TO_ISOQUINOLINE_ALKALOID2	0.39347844	0.085025134	1.837061493	0.10306192	0.60538498	-4.52500579
homo after UVB irradiation VS homo before UVB irradiation	GO_CELLULAR_RESPONSE_TO_KETONE	0.020398725	-0.00444478	0.155026663	0.88049139	0.93512556	-6.53768613
hetero after UVB irradiation VS hetero before UVB irradiation	GO_CELLULAR_RESPONSE_TO_KETONE1	0.002418028	-0.01042603	0.017169653	0.98672949	0.99488762	-6.23132424
WT after UVB irradiation VS WT before UVB irradiation	GO_CELLULAR_RESPONSE_TO_KETONE2	-0.04680078	-0.02170371	-0.31637757	0.75971775	0.93913078	-5.94359501
homo after UVB irradiation VS homo before UVB irradiation	GO_CELLULAR_RESPONSE_TO_L_GLUTAMATE	-0.38937268	0.017460183	-2.27109473	0.05154058	0.16782531	-4.36012583
hetero after UVB irradiation VS hetero before UVB irradiation	GO_CELLULAR_RESPONSE_TO_L_GLUTAMATE1	-0.77839881	0.076436692	-3.80030523	0.0054179	0.12957299	-1.91085574
WT after UVB irradiation VS WT before UVB irradiation	GO_CELLULAR_RESPONSE_TO_L_GLUTAMATE2	-0.97860323	-0.00442125	-4.62155987	0.00165308	0.2011505	-0.79746908
homo after UVB irradiation VS homo before UVB irradiation	GO_CELLULAR_RESPONSE_TO_LAMINAR_FLUID_SHEAR_STRESS	0.161492795	0.0292946	0.579872279	0.5773456	0.73377737	-6.37001482
hetero after UVB irradiation VS hetero before UVB irradiation	GO_CELLULAR_RESPONSE_TO_LAMINAR_FLUID_SHEAR_STRESS1	0.36494772	-0.03563567	1.619521561	0.14470317	0.46094742	-5.01155485

WT after UVB irradiation VS WT before UVB irradiation	GO_CELLULAR_RESPONSE_TO_LAMINAR_FLUID_SHEAR_STRESS2	0.367014015	0.027783321	1.700316049	0.12703609	0.63383032	-4.70484786
homo after UVB irradiation VS homo before UVB irradiation	GO_CELLULAR_RESPONSE_TO_LEPTIN_STIMULUS	0.26263699	-0.00077943	1.512037935	0.16749699	0.34608696	-5.44503757
hetero after UVB irradiation VS hetero before UVB irradiation	GO_CELLULAR_RESPONSE_TO_LEPTIN_STIMULUS1	0.18671275	-0.02303182	1.11688273	0.29704968	0.62085908	-5.60547391
WT after UVB irradiation VS WT before UVB irradiation	GO_CELLULAR_RESPONSE_TO_LEPTIN_STIMULUS2	0.093922441	-0.04173636	0.416396825	0.68793759	0.91993413	-5.90560354
homo after UVB irradiation VS homo before UVB irradiation	GO_CELLULAR_RESPONSE_TO_LEUCINE	-0.41793716	-0.04954358	-1.8946037	0.09329801	0.24082538	-4.91938029
homo after UVB irradiation VS homo before UVB irradiation	GO_CELLULAR_RESPONSE_TO_LEUCINE_STARVATION	-0.23208752	-0.05311776	-0.91761889	0.38461814	0.5742422	-6.11106776
hetero after UVB irradiation VS hetero before UVB irradiation	GO_CELLULAR_RESPONSE_TO_LEUCINE_STARVATION1	-0.62943211	-0.04397435	-3.25994214	0.01182497	0.16927893	-2.66472014
WT after UVB irradiation VS WT before UVB irradiation	GO_CELLULAR_RESPONSE_TO_LEUCINE_STARVATION2	-0.70466283	-0.05089795	-3.448771	0.00855037	0.31908946	-2.27355308
hetero after UVB irradiation VS hetero before UVB irradiation	GO_CELLULAR_RESPONSE_TO_LEUCINE1	-0.71659953	-0.03898116	-4.83888669	0.0013598	0.09333375	-0.58947298
WT after UVB irradiation VS WT before UVB irradiation	GO_CELLULAR_RESPONSE_TO_LEUCINE2	-0.35188279	-0.01947431	-1.28889696	0.23303326	0.72459413	-5.2035183
homo after UVB irradiation VS homo before UVB irradiation	GO_CELLULAR_RESPONSE_TO_LIGHT_STIMULUS	-0.1619223	0.01350776	-1.26662843	0.23955809	0.432171	-5.74713186
hetero after UVB irradiation VS hetero before UVB irradiation	GO_CELLULAR_RESPONSE_TO_LIGHT_STIMULUS1	-0.05629706	-0.01457762	-0.41679086	0.68799113	0.87482399	-6.13834302
WT after UVB irradiation VS WT before UVB irradiation	GO_CELLULAR_RESPONSE_TO_LIGHT_STIMULUS2	-0.14789871	-0.03084187	-0.95322396	0.36806022	0.79942934	-5.54317962
homo after UVB irradiation VS homo before UVB irradiation	GO_CELLULAR_RESPONSE_TO_LIPID	0.133796733	-0.0001403	1.09715106	0.30327791	0.49777246	-5.93467346
hetero after UVB irradiation VS hetero before UVB irradiation	GO_CELLULAR_RESPONSE_TO_LIPID1	0.092318046	0.009421568	0.702055954	0.50291854	0.77390992	-5.97254786
WT after UVB irradiation VS WT before UVB irradiation	GO_CELLULAR_RESPONSE_TO_LIPID2	0.055358276	0.006377254	0.376950632	0.71589693	0.93033952	-5.9217815
homo after UVB irradiation VS homo before UVB irradiation	GO_CELLULAR_RESPONSE_TO_LIPOPROTEIN_PARTICLE_STIMULUS	0.597837122	-0.03215328	4.805396965	0.00120193	0.02347563	-0.60939893
hetero after UVB irradiation VS hetero before UVB irradiation	GO_CELLULAR_RESPONSE_TO_LIPOPROTEIN_PARTICLE_STIMULUS1	0.418272529	0.042210521	2.136417109	0.06577635	0.3342272	-4.29975679
WT after UVB irradiation VS WT before UVB irradiation	GO_CELLULAR_RESPONSE_TO_LIPOPROTEIN_PARTICLE_STIMULUS2	0.387956439	0.009971449	2.283615161	0.05139602	0.51559998	-3.90910843
homo after UVB irradiation VS homo before UVB irradiation	GO_CELLULAR_RESPONSE_TO_LITHIUM_ION	0.058146502	0.007705705	0.343721341	0.73957185	0.84713855	-6.48648129
hetero after UVB irradiation VS hetero before UVB irradiation	GO_CELLULAR_RESPONSE_TO_LITHIUM_ION1	-0.08673086	0.019658548	-0.3934594	0.70444696	0.88248944	-6.14837699
WT after UVB irradiation VS WT before UVB irradiation	GO_CELLULAR_RESPONSE_TO_LITHIUM_ION2	-0.18093524	-0.04637952	-1.09341945	0.30566662	0.76523073	-5.41022615
homo after UVB irradiation VS homo before UVB irradiation	GO_CELLULAR_RESPONSE_TO_LOW_DENSITY_LIPOPROTEIN_PARTICLE_STIMULUS	0.498972492	-0.03780657	3.62663982	0.00627567	0.05244054	-2.27652643
hetero after UVB irradiation VS hetero before UVB irradiation	GO_CELLULAR_RESPONSE_TO_LOW_DENSITY_LIPOPROTEIN_PARTICLE_STIMULUS1	0.307837774	0.024243741	1.494777387	0.17402992	0.49666362	-5.17062522
WT after UVB irradiation VS WT before UVB irradiation	GO_CELLULAR_RESPONSE_TO_LOW_DENSITY_LIPOPROTEIN_PARTICLE_STIMULUS2	0.285828769	0.010304484	1.475602507	0.17784182	0.68141335	-4.98642636
homo after UVB irradiation VS homo before UVB irradiation	GO_CELLULAR_RESPONSE_TO_MAGNESIUM_ION	-0.34188402	0.132340829	-1.79702467	0.10857112	0.26334386	-5.05853206
hetero after UVB irradiation VS hetero before UVB irradiation	GO_CELLULAR_RESPONSE_TO_MAGNESIUM_ION1	-0.24078311	0.10953045	-1.38769189	0.20333645	0.52750223	-5.30166082
WT after UVB irradiation VS WT before UVB irradiation	GO_CELLULAR_RESPONSE_TO_MAGNESIUM_ION2	-0.66964237	0.16534439	-2.35088454	0.04624802	0.50534441	-3.81402786
homo after UVB irradiation VS homo before UVB irradiation	GO_CELLULAR_RESPONSE_TO_MANGANESE_ION	0.159303515	-0.04546801	0.620938253	0.55124992	0.71418536	-6.34409746
hetero after UVB irradiation VS hetero before UVB irradiation	GO_CELLULAR_RESPONSE_TO_MANGANESE_ION1	0.057838684	0.069494882	0.270398857	0.79382409	0.9195249	-6.19202335
WT after UVB irradiation VS WT before UVB irradiation	GO_CELLULAR_RESPONSE_TO_MANGANESE_ION2	0.359320501	0.036948752	1.705794333	0.12598312	0.63290509	-4.69775284
homo after UVB irradiation VS homo before UVB irradiation	GO_CELLULAR_RESPONSE_TO_MECHANICAL_STIMULUS	0.46176396	-0.05003174	2.933767589	0.01808162	0.09228787	-3.33590779
hetero after UVB irradiation VS hetero before UVB irradiation	GO_CELLULAR_RESPONSE_TO_MECHANICAL_STIMULUS1	0.493574469	0.000941943	3.695141295	0.00628699	0.13548627	-2.05441232
WT after UVB irradiation VS WT before UVB irradiation	GO_CELLULAR_RESPONSE_TO_MECHANICAL_STIMULUS2	0.303350654	-0.01669902	1.561696166	0.15653013	0.66180255	-4.88087468
homo after UVB irradiation VS homo before UVB irradiation	GO_CELLULAR_RESPONSE_TO_MINERALOCORTICOID_STIMULUS	-0.54448439	-0.04224245	-2.91886877	0.01850784	0.0934377	-3.35902878
hetero after UVB irradiation VS hetero before UVB irradiation	GO_CELLULAR_RESPONSE_TO_MINERALOCORTICOID_STIMULUS1	-0.41745071	-0.03526855	-1.88556365	0.09677073	0.3882406	-4.65372744
WT after UVB irradiation VS WT before UVB irradiation	GO_CELLULAR_RESPONSE_TO_MINERALOCORTICOID_STIMULUS2	-0.14576469	-0.00472873	-0.53018756	0.61020805	0.89866462	-5.85041353
homo after UVB irradiation VS homo before UVB irradiation	GO_CELLULAR_RESPONSE_TO_MOLECULE_OF_BACTERIAL_ORIGIN	0.567313269	-0.01300677	4.098619625	0.00316073	0.03716566	-1.5846572
hetero after UVB irradiation VS hetero before UVB irradiation	GO_CELLULAR_RESPONSE_TO_MOLECULE_OF_BACTERIAL_ORIGIN1	0.588479157	0.029217891	4.11762645	0.00349202	0.11684418	-1.4880923
WT after UVB irradiation VS WT before UVB irradiation	GO_CELLULAR_RESPONSE_TO_MOLECULE_OF_BACTERIAL_ORIGIN2	0.373728911	-0.00782706	1.899683459	0.09357072	0.59116964	-4.44093211
homo after UVB irradiation VS homo before UVB irradiation	GO_CELLULAR_RESPONSE_TO_MURAMYL_DIPEPTIDE	1.055959516	-0.00096876	7.379539014	6.31E-05	0.00942429	2.298156191
hetero after UVB irradiation VS hetero before UVB irradiation	GO_CELLULAR_RESPONSE_TO_MURAMYL_DIPEPTIDE1	0.697272682	0.053268177	3.670090164	0.00651529	0.13621896	-2.08884993
WT after UVB irradiation VS WT before UVB irradiation	GO_CELLULAR_RESPONSE_TO_MURAMYL_DIPEPTIDE2	0.493340277	0.107888622	1.864297638	0.09882663	0.59874718	-4.48855997
homo after UVB irradiation VS homo before UVB irradiation	GO_CELLULAR_RESPONSE_TO_NICOTINE	0.541121875	0.013241685	2.986366332	0.01665638	0.08835006	-3.254339
hetero after UVB irradiation VS hetero before UVB irradiation	GO_CELLULAR_RESPONSE_TO_NICOTINE1	0.426918317	0.026165424	1.973946505	0.08450582	0.37098454	-4.53047385
WT after UVB irradiation VS WT before UVB irradiation	GO_CELLULAR_RESPONSE_TO_NICOTINE2	0.359645482	0.030069928	1.193161759	0.26659004	0.74323208	-5.3076397
homo after UVB irradiation VS homo before UVB irradiation	GO_CELLULAR_RESPONSE_TO_NITRIC_OXIDE	-0.09215359	0.003391445	-0.49005139	0.63674459	0.77695055	-6.42095872
hetero after UVB irradiation VS hetero before UVB irradiation	GO_CELLULAR_RESPONSE_TO_NITRIC_OXIDE1	0.371212461	-0.05026423	2.050644388	0.07509258	0.35391027	-4.42215353
WT after UVB irradiation VS WT before UVB irradiation	GO_CELLULAR_RESPONSE_TO_NITRIC_OXIDE2	0.303944669	-0.05620027	1.621685466	0.14307255	0.65167133	-4.80555769
homo after UVB irradiation VS homo before UVB irradiation	GO_CELLULAR_RESPONSE_TO_NITROGEN_COMPOUND	0.335417507	-0.00897984	2.668823989	0.02742828	0.11721876	-3.74745261
hetero after UVB irradiation VS hetero before UVB irradiation	GO_CELLULAR_RESPONSE_TO_NITROGEN_COMPOUND1	0.219607412	0.010694842	1.642711335	0.13977638	0.454004	-4.98130012
WT after UVB irradiation VS WT before UVB irradiation	GO_CELLULAR_RESPONSE_TO_NITROGEN_COMPOUND2	0.186011545	-0.00738609	1.206688961	0.26161873	0.74156847	-5.29325166
homo after UVB irradiation VS homo before UVB irradiation	GO_CELLULAR_RESPONSE_TO_NITROGEN_STARVATION	-0.08202788	-0.07200742	-0.29338569	0.77640478	0.87108685	-6.50386415
hetero after UVB irradiation VS hetero before UVB irradiation	GO_CELLULAR_RESPONSE_TO_NITROGEN_STARVATION1	-0.21265303	-0.0546913	-0.99350388	0.35008787	0.66699762	-5.7284368
WT after UVB irradiation VS WT before UVB irradiation	GO_CELLULAR_RESPONSE_TO_NITROGEN_STARVATION2	-0.07070838	-0.0799669	-0.2854447	0.78246879	0.94527105	-5.95330229
homo after UVB irradiation VS homo before UVB irradiation	GO_CELLULAR_RESPONSE_TO_NITROSATIVE_STRESS	-0.26247524	0.021383938	-1.58228649	0.15074981	0.3244771	-5.35290743
hetero after UVB irradiation VS hetero before UVB irradiation	GO_CELLULAR_RESPONSE_TO_NITROSATIVE_STRESS1	-0.30729635	-0.02222861	-1.39667155	0.20072214	0.52537574	-5.29088664
WT after UVB irradiation VS WT before UVB irradiation	GO_CELLULAR_RESPONSE_TO_NITROSATIVE_STRESS2	-0.09414418	-0.05144316	-0.33439196	0.74658011	0.93606982	-5.93749398
homo after UVB irradiation VS homo before UVB irradiation	GO_CELLULAR_RESPONSE_TO_NUTRIENT	0.242897082	0.012978238	1.99170144	0.08014218	0.21853978	-4.77817656
hetero after UVB irradiation VS hetero before UVB irradiation	GO_CELLULAR_RESPONSE_TO_NUTRIENT1	0.048428089	0.016082526	0.282066107	0.78518382	0.91626436	-6.1885627
WT after UVB irradiation VS WT before UVB irradiation	GO_CELLULAR_RESPONSE_TO_NUTRIENT2	0.120323403	-0.00781265	0.648016208	0.53491271	0.86957641	-5.7803833
homo after UVB irradiation VS homo before UVB irradiation	GO_CELLULAR_RESPONSE_TO_ORGANIC_CYCLIC_COMPOUND	0.110264183	0.005231222	0.886885214	0.40002257	0.58837167	-6.13874023
hetero after UVB irradiation VS hetero before UVB irradiation	GO_CELLULAR_RESPONSE_TO_ORGANIC_CYCLIC_COMPOUND1	0.041017971	-0.00097323	0.32455896	0.75399176	0.90417736	-6.17475231
WT after UVB irradiation VS WT before UVB irradiation	GO_CELLULAR_RESPONSE_TO_ORGANIC_CYCLIC_COMPOUND2	0.031051521	0.012769437	0.21969899	0.83154279	0.96010372	-5.97067841
homo after UVB irradiation VS homo before UVB irradiation	GO_CELLULAR_RESPONSE_TO_OSMOTIC_STRESS	0.149543718	0.013082121	1.124998206	0.29196057	0.48675786	-5.90518372
hetero after UVB irradiation VS hetero before UVB irradiation	GO_CELLULAR_RESPONSE_TO_OSMOTIC_STRESS1	0.058262945	-0.01213548	0.363238213	0.72601049	0.89215613	-6.16054787
WT after UVB irradiation VS WT before UVB irradiation	GO_CELLULAR_RESPONSE_TO_OSMOTIC_STRESS2	0.17409514	-0.04328296	0.76205047	0.4676428	0.84405637	-5.7007724
homo after UVB irradiation VS homo before UVB irradiation	GO_CELLULAR_RESPONSE_TO_OXIDISED_LOW_DENSITY_LIPOPROTEIN_PARTICLE_STIMULUS	0.572215528	-0.02366703	3.068170607	0.01466691	0.08251607	-3.12770496
hetero after UVB irradiation VS hetero before UVB irradiation	GO_CELLULAR_RESPONSE_TO_OXIDISED_LOW_DENSITY_LIPOPROTEIN_PARTICLE_STIMULUS1	0.449078297	0.042306273	1.946709875	0.08811625	0.3748179	-4.56864785
WT after UVB irradiation VS WT before UVB irradiation	GO_CELLULAR_RESPONSE_TO_OXIDISED_LOW_DENSITY_LIPOPROTEIN_PARTICLE_STIMULUS2	0.209682652	0.025537836	0.845775809	0.42196481	0.82449435	-5.63532189
homo after UVB irradiation VS homo before UVB irradiation	GO_CELLULAR_RESPONSE_TO_OXYGEN_CONTAINING_COMPOUND	0.297913694	-0.01143135	2.458813265	0.0382587	0.14023625	-4.07254237
hetero after UVB irradiation VS hetero before UVB irradiation	GO_CELLULAR_RESPONSE_TO_OXYGEN_CONTAINING_COMPOUND1	0.221267362	0.011756983	1.653147632	0.13760992	0.45089754	-4.96761923
WT after UVB irradiation VS WT before UVB irradiation	GO_CELLULAR_RESPONSE_TO_OXYGEN_CONTAINING_COMPOUND2	0.183573535	-0.00555287	1.149818976	0.28304191	0.75292132	-5.35298963
homo after UVB irradiation VS homo before UVB irradiation	GO_CELLULAR_RESPONSE_TO_OXYGEN_GLUCOSE_DEPRIVATION	-0.29328163	0.002591935	-1.63454815	0.13928818	0.3090476	-5.28294448
hetero after UVB irradiation VS hetero before UVB irradiation	GO_CELLULAR_RESPONSE_TO_OXYGEN_GLUCOSE_DEPRIVATION1	-0.05885211	0.019382853	-0.18201904	0.86017857	0.94722351	-6.21355616
WT after UVB irradiation VS WT before UVB irradiation	GO_CELLULAR_RESPONSE_TO_OXYGEN_GLUCOSE_DEPRIVATION2	0.074374234	-0.08864893	0.287207433	0.78116607	0.9447997	-5.95277532
homo after UVB irradiation VS homo before UVB irradiation	GO_CELLULAR_RESPONSE_TO_OXYGEN_LEVELS	-0.04025763	-0.01559029	-0.24464465	0.81265097	0.89411596	-6.51812904
hetero after UVB irradiation VS hetero before UVB irradiation	GO_CELLULAR_RESPONSE_TO_OXYGEN_LEVELS1	0.064015922	-0.00598682	0.459199199	0.65853491	0.86007701	-6.11869639
WT after UVB irradiation VS WT before UVB irradiation	GO_CELLULAR_RESPONSE_TO_OXYGEN_LEVELS2	-0.0115887	-0.00919114	-0.07168715	0.94458986	0.98531933	-5.99335596
homo after UVB irradiation VS homo before UVB irradiation	GO_CELLULAR_RESPONSE_TO_OXYGEN_RADICAL	0.373515626	-0.01825756	2.508487036	0.03535858	0.13468829	-3.99587831
hetero after UVB irradiation VS hetero before UVB irradiation	GO_CELLULAR_RESPONSE_TO_OXYGEN_RADICAL1	0.288009662	0.022687234	1.766908137	0.11593264	0.41917522	-4.81604672
WT after UVB irradiation VS WT before UVB irradiation	GO_CELLULAR_RESPONSE_TO_OXYGEN_RADICAL2	0.320900387	0.050688475	1.39476329	0.20015743	0.69758436	-5.08253773
homo after UVB irradiation VS homo before UVB irradiation	GO_CELLULAR_RESPONSE_TO_PARATHYROID_HORMONE_STIMULUS	0.201405459	0.051140068	1.001748381	0.34467516	0.53687686	-6.0314596
hetero after UVB irradiation VS hetero before UVB irradiation	GO_CELLULAR_RESPONSE_TO_PARATHYROID_HORMONE_STIMULUS1	-0.13880536	0.01811593	-0.61535332	0.55573067	0.80533165	-6.03111288
WT after UVB irradiation VS WT before UVB irradiation	GO_CELLULAR_RESPONSE_TO_PARATHYROID_HORMONE_STIMULUS2	-0.24199641	0.09443437	-1.48738521	0.17477983	0.68001901	-4.97216786
homo after UVB irradiation VS homo before UVB irradiation	GO_CELLULAR_RESPONSE_TO_PEPTIDE	0.327808419	-0.01410972	2.550555655	0.03307648	0.1295064	-3.93082003
homo after UVB irradiation VS homo before UVB irradiation	GO_CELLULAR_RESPONSE_TO_PEPTIDE_HORMONE_STIMULUS	0.245707911	-0.01531022	1.860448815	0.09839684	0.24838216	-4.96842164
hetero after UVB irradiation VS hetero before UVB irradiation	GO_CELLULAR_RESPONSE_TO_PEPTIDE_HORMONE_STIMULUS1	0.151279232	0.009383001	1.133999567	0.2902245	0.6144265	-5.5875914
WT after UVB irradiation VS WT before UVB irradiation	GO_CELLULAR_RESPONSE_TO_PEPTIDE_HORMONE_STIMULUS2	0.09840609	-0.01453847	0.643004467	0.53799894	0.87025652	-5.78362014
hetero after UVB irradiation VS hetero before UVB irradiation	GO_CELLULAR_RESPONSE_TO_PEPTIDE1	0.238030101	0.010121362	1.750492476	0.11885023	0.42318117	-4.83818081
WT after UVB irradiation VS WT before UVB irradiation	GO_CELLULAR_RESPONSE_TO_PEPTIDE2	0.159437405	-0.01405819	1.023743828	0.33556357	0.77905119	-5.47802295
homo after UVB irradiation VS homo before UVB irradiation	GO_CELLULAR_RESPONSE_TO_PEPTIDOGLYCAN	0.971631636	-0.01436983	4.396372062	0.00208453	0.03080331	-1.1644276
hetero after UVB irradiation VS hetero before UVB irradiation	GO_CELLULAR_RESPONSE_TO_PEPTIDOGLYCAN1	0.82292672	0.061993663	4.155115314	0.00331868	0.11632799	-1.43921953
WT after UVB irradiation VS WT before UVB irradiation	GO_CELLULAR_RESPONSE_TO_PEPTIDOGLYCAN2	0.473843145	-0.04182215	1.530435343	0.1639879	0.66886938	-4.91956067
homo after UVB irradiation VS homo before UVB irradiation	GO_CELLULAR_RESPONSE_TO_PH	0.396050494	0.007090838	2.115867271	0.06590324	0.19520958	-4.59425867
hetero after UVB irradiation VS hetero before UVB irradiation	GO_CELLULAR_RESPONSE_TO_PH1	-0.00739793	-0.06230289	-0.03718057	0.97126882	0.98980532	-6.2307345
WT after UVB irradiation VS WT before UVB irradiation	GO_CELLULAR_RESPONSE_TO_PH2	0.090242099	0.023255863	0.544576024	0.60071615	0.89567499	-5.84255187
homo after UVB irradiation VS homo before UVB irradiation	GO_CELLULAR_RESPONSE_TO_POTASSIUM_ION	0.098784798	0.015419514	0.51095614	0.62264694	0.76589155	-6.40981635
hetero after UVB irradiation VS hetero before UVB irradiation	GO_CELLULAR_RESPONSE_TO_POTASSIUM_ION1	-0.18061602	-0.02605874	-0.96705119	0.3623538	0.67684951	-5.75337934
WT after UVB irradiation VS WT before UVB irradiation	GO_CELLULAR_RESPONSE_TO_POTASSIUM_ION2	-0.33469907	0.012188521	-1.22723146	0.25421568	0.73589193	-5.2711938
homo after UVB irradiation VS homo before UVB irradiation	GO_CELLULAR_RESPONSE_TO_PROSTAGLANDIN_E_STIMULUS	0.301012992	-0.04044876	1.870796381	0.09682485	0.24614028	-4.95360079
hetero after UVB irradiation VS hetero before UVB irradiation	GO_CELLULAR_RESPONSE_TO_PROSTAGLANDIN_E_STIMULUS1	0.450558153	0.000284623	3.009155117	0.01720029	0.19155282	-3.02621939
WT after UVB irradiation VS WT before UVB irradiation	GO_CELLULAR_RESPONSE_TO_PROSTAGLANDIN_E_STIMULUS2	0.174672818	-0.08220887	0.820395804	0.43547321	0.83017793	-5.65576676
homo after UVB irradiation VS homo before UVB irradiation	GO_CELLULAR_RESPONSE_TO_PROSTAGLANDIN_STIMULUS	0.334379862	-0.00662376	2.503376025	0.03564646	0.13508856	-4.00377467
hetero after UVB irradiation VS hetero before UVB irradiation	GO_CELLULAR_RESPONSE_TO_PROSTAGLANDIN_STIMULUS1	0.418106199	0.019293073	2.63120375	0.03060538	0.24470723	-3.57890236
WT after UVB irradiation VS WT before UVB irradiation	GO_CELLULAR_RESPONSE_TO_PROSTAGLANDIN_STIMULUS2	0.101740384	-0.03068965	0.581197546	0.5769224	0.8871242	-5.82166769
homo after UVB irradiation VS homo before UVB irradiation	GO_CELLULAR_RESPONSE_TO_PURINE_CONTAINING_COMPOUND	0.484987608	-0.01702135	2.664234377	0.02762801	0.11750248	-3.7545779
hetero after UVB irradiation VS hetero before UVB irradiation	GO_CELLULAR_RESPONSE_TO_PURINE_CONTAINING_COMPOUND1	0.171422797	-0.01772577	0.663528566	0.52598819	0.78916946	-5.99941938
WT after UVB irradiation VS WT before UVB irradiation	GO_CELLULAR_RESPONSE_TO_PURINE_CONTAINING_COMPOUND2	0.132648629	0.051357179	0.558294106	0.59174122	0.89269323	-5.83487537
homo after UVB irradiation VS homo before UVB irradiation	GO_CELLULAR_RESPONSE_TO_RADIATION	-0.21703411	0.005543887	-1.68465291	0.12905207	0.29428163	-5.21480584
hetero after UVB irradiation VS hetero before UVB irradiation	GO_CELLULAR_RESPONSE_TO_RADIATION1	-0.00812234	-0.0099394	-0.05413474	0.95817968	0.98494862	-6.22989515
WT after UVB irradiation VS WT before UVB irradiation	GO_CELLULAR_RESPONSE_TO_RADIATION2	-0.07746486	-0.01653076	-0.4850272	0.64050614	0.90662539	-5.8738123
homo after UVB irradiation VS homo before UVB irradiation	GO_CELLULAR_RESPONSE_TO_REACTIVE_NITROGEN_SPECIES	-0.13383041	-0.00371131	-0.76646759	0.46456448	0.64388656	-6.23951877
hetero after UVB irradiation VS hetero before UVB irradiation	GO_CELLULAR_RESPONSE_TO_REACTIVE_NITROGEN_SPECIES1	0.237836231	-0.06988337	1.159939754	0.28012307	0.60581798	-5.56012651
WT after UVB irradiation VS WT before UVB irradiation	GO_CELLULAR_RESPONSE_TO_REACTIVE_NITROGEN_SPECIES2	0.169948405	-0.05684143	0.851451797	0.41898426	0.82348328	-5.630679
homo after UVB irradiation VS homo before UVB irradiation	GO_CELLULAR_RESPONSE_TO_REACTIVE_OXYGEN_SPECIES	0.203747893	-0.00698712	1.593608904	0.14819645	0.32119759	-5.33784974
hetero after UVB irradiation VS hetero before UVB irradiation	GO_CELLULAR_RESPONSE_TO_REACTIVE_OXYGEN_SPECIES1	0.305014338	0.027252497	2.228486213	0.05704196	0.31489381	-4.16716176
WT after UVB irradiation VS WT before UVB irradiation	GO_CELLULAR_RESPONSE_TO_REACTIVE_OXYGEN_SPECIES2	0.15508196	0.000707082	0.889230824	0.39952376	0.81573919	-5.59912857
homo after UVB irradiation VS homo before UVB irradiation	GO_CELLULAR_RESPONSE_TO_RETINOIC_ACID	-0.08994342	0.017949128	-0.62041682	0.55157692	0.71418536	-6.34443668
hetero after UVB irradiation VS hetero before UVB irradiation	GO_CELLULAR_RESPONSE_TO_RETINOIC_ACID1	-0.21767924	0.026929598	-1.51972619	0.16776747	0.48938545	-5.13933459
WT after UVB irradiation VS WT before UVB irradiation	GO_CELLULAR_RESPONSE_TO_RETINOIC_ACID2	-0.16060617	0.033869811	-0.89391485	0.39715677	0.81477652	-5.5951392
homo after UVB irradiation VS homo before UVB irradiation	GO_CELLULAR_RESPONSE_TO_SALT	-0.51091286	0.023674532	-1.37353564	0.20541707	0.39506578	-5.61958923
homo after UVB irradiation VS homo before UVB irradiation	GO_CELLULAR_RESPONSE_TO_SALT_STRESS	-0.14666324	0.039454302	-0.59109302	0.57014691	0.72826607	-6.36309456
hetero after UVB irradiation VS hetero before UVB irradiation	GO_CELLULAR_RESPONSE_TO_SALT_STRESS1	-0.253192	0.00143243	-1.4174085	0.19479698	0.51930753	-5.26585155
WT after UVB irradiation VS WT before UVB irradiation	GO_CELLULAR_RESPONSE_TO_SALT_STRESS2	0.125488864	-0.0609975	0.537573091	0.60532596	0.89632703	-5.84640255
hetero after UVB irradiation VS hetero before UVB irradiation	GO_CELLULAR_RESPONSE_TO_SALT1	0.165889199	0.042678706	0.744469582	0.47827674	0.759973	-5.94143412
WT after UVB irradiation VS WT before UVB irradiation	GO_CELLULAR_RESPONSE_TO_SALT2	-0.71411731	-0.11575995	-3.74360129	0.00555347	0.27780541	-1.88086878
homo after UVB irradiation VS homo before UVB irradiation	GO_CELLULAR_RESPONSE_TO_SODIUM_ARSENITE	-0.45968466	-0.00726404	-1.15318592	0.28085056	0.47599766	-5.8747856
hetero after UVB irradiation VS hetero before UVB irradiation	GO_CELLULAR_RESPONSE_TO_SODIUM_ARSENITE1	0.173147635	0.053080674	0.940799552	0.37484303	0.68795818	-5.77761293
WT after UVB irradiation VS WT before UVB irradiation	GO_CELLULAR_RESPONSE_TO_SODIUM_ARSENITE2	-0.59381819	-0.09042282	-3.06860522	0.01515828	0.36889188	-2.79720639
homo after UVB irradiation VS homo before UVB irradiation	GO_CELLULAR_RESPONSE_TO_STARVATION	-0.01079359	-0.01546232	-0.07408227	0.94269327	0.96970058	-6.5478457
hetero after UVB irradiation VS hetero before UVB irradiation	GO_CELLULAR_RESPONSE_TO_STARVATION1	-0.06346887	-0.00648766	-0.48707353	0.63951054	0.8522738	-6.1048039
WT after UVB irradiation VS WT before UVB irradiation	GO_CELLULAR_RESPONSE_TO_STARVATION2	-0.12712772	-0.01299667	-0.84971504	0.41989469	0.82348328	-5.63210236
homo after UVB irradiation VS homo before UVB irradiation	GO_CELLULAR_RESPONSE_TO_STEROID_HORMONE_STIMULUS	-0.10385325	0.015480287	-0.80398083	0.44374626	0.62621818	-6.20946033
hetero after UVB irradiation VS hetero before UVB irradiation	GO_CELLULAR_RESPONSE_TO_STEROID_HORMONE_STIMULUS1	-0.11752252	-0.00690282	-0.94792688	0.37142095	0.68504001	-5.77108526
WT after UVB irradiation VS WT before UVB irradiation	GO_CELLULAR_RESPONSE_TO_STEROID_HORMONE_STIMULUS2	-0.16298536	0.009535887	-1.13648647	0.28826494	0.75467539	-5.36670425
homo after UVB irradiation VS homo before UVB irradiation	GO_CELLULAR_RESPONSE_TO_STEROL	-0.22309143	-0.00433621	-1.68740891	0.12850961	0.29356289	-5.21102898
homo after UVB irradiation VS homo before UVB irradiation	GO_CELLULAR_RESPONSE_TO_STEROL_DEPLETION	0.050533571	-0.02586881	0.335550142	0.74550598	0.85104292	-6.48948505
hetero after UVB irradiation VS hetero before UVB irradiation	GO_CELLULAR_RESPONSE_TO_STEROL_DEPLETION1	-0.10762789	-0.03745585	-0.72065335	0.49201537	0.76746311	-5.95910074
WT after UVB irradiation VS WT before UVB irradiation	GO_CELLULAR_RESPONSE_TO_STEROL_DEPLETION2	0.053359051	0.05697731	0.178706271	0.86255858	0.9676681	-5.97925081
hetero after UVB irradiation VS hetero before UVB irradiation	GO_CELLULAR_RESPONSE_TO_STEROL1	-0.48000003	-0.0222297	-3.12480686	0.01445801	0.17916057	-2.85879064
WT after UVB irradiation VS WT before UVB irradiation	GO_CELLULAR_RESPONSE_TO_STEROL2	-0.16472283	0.072244186	-0.71939923	0.4921318	0.85299376	-5.73186723
homo after UVB irradiation VS homo before UVB irradiation	GO_CELLULAR_RESPONSE_TO_TEMPERATURE_STIMULUS	0.510709183	-0.0721375	1.674428331	0.13108303	0.29701917	-5.22879181
hetero after UVB irradiation VS hetero before UVB irradiation	GO_CELLULAR_RESPONSE_TO_TEMPERATURE_STIMULUS1	0.389414148	-0.01697947	1.427447567	0.19198412	0.51714451	-5.25365561
WT after UVB irradiation VS WT before UVB irradiation	GO_CELLULAR_RESPONSE_TO_TEMPERATURE_STIMULUS2	0.329569477	0.049176779	1.42802529	0.19069422	0.69285981	-5.04336268
homo after UVB irradiation VS homo before UVB irradiation	GO_CELLULAR_RESPONSE_TO_TESTOSTERONE_STIMULUS	-0.09070397	0.02334038	-0.49912605	0.63060518	0.77207671	-6.41617564
hetero after UVB irradiation VS hetero before UVB irradiation	GO_CELLULAR_RESPONSE_TO_TESTOSTERONE_STIMULUS1	-0.11004984	-0.00402639	-0.61830827	0.55387823	0.80466567	-6.02923076
WT after UVB irradiation VS WT before UVB irradiation	GO_CELLULAR_RESPONSE_TO_TESTOSTERONE_STIMULUS2	-0.00886693	-0.03018537	-0.05145429	0.96021018	0.99000127	-5.99466993
homo after UVB irradiation VS homo before UVB irradiation	GO_CELLULAR_RESPONSE_TO_THYROID_HORMONE_STIMULUS	0.045803989	-0.02621874	0.345273298	0.73844684	0.84632339	-6.48590285
hetero after UVB irradiation VS hetero before UVB irradiation	GO_CELLULAR_RESPONSE_TO_THYROID_HORMONE_STIMULUS1	-0.07439415	-0.01127415	-0.51242179	0.6224535	0.84401411	-6.09150407
WT after UVB irradiation VS WT before UVB irradiation	GO_CELLULAR_RESPONSE_TO_THYROID_HORMONE_STIMULUS2	-0.27004954	-0.06973874	-1.29861048	0.22983613	0.72256251	-5.1926643
homo after UVB irradiation VS homo before UVB irradiation	GO_CELLULAR_RESPONSE_TO_TOPOLOGICALLY_INCORRECT_PROTEIN	0.117996549	-0.03206614	0.770822965	0.46211467	0.64163891	-6.2360924

hetero after UVB irradiation VS hetero before UVB irradiation	GO_CELLULAR_RESPONSE_TO_TOPOLOGICALLY_INCORRECT_PROTEIN1	0.174377814	-0.00992857	1.069200906	0.31674079	0.63701027	-5.65425345
WT after UVB irradiation VS WT before UVB irradiation	GO_CELLULAR_RESPONSE_TO_TOPOLOGICALLY_INCORRECT_PROTEIN2	0.173742518	-0.02848883	0.992393322	0.34973013	0.78722502	-5.50743075
homo after UVB irradiation VS homo before UVB irradiation	GO_CELLULAR_RESPONSE_TO_TOXIC_SUBSTANCE	0.437197414	-0.01492491	3.075867423	0.0144929	0.0819836	-3.1158071
hetero after UVB irradiation VS hetero before UVB irradiation	GO_CELLULAR_RESPONSE_TO_TOXIC_SUBSTANCE1	0.286838773	0.018329481	1.757601596	0.11757834	0.42121535	-4.8286055
WT after UVB irradiation VS WT before UVB irradiation	GO_CELLULAR_RESPONSE_TO_TOXIC_SUBSTANCE2	0.318995799	0.018400104	1.525006935	0.16531501	0.66947139	-4.9262375
homo after UVB irradiation VS homo before UVB irradiation	GO_CELLULAR_RESPONSE_TO_UV	-0.28721881	-0.00596595	-1.92732834	0.08864905	0.23275397	-4.87207555
homo after UVB irradiation VS homo before UVB irradiation	GO_CELLULAR_RESPONSE_TO_UV_B	0.117785406	0.022841935	0.536446155	0.60567751	0.75404361	-6.39564045
hetero after UVB irradiation VS hetero before UVB irradiation	GO_CELLULAR_RESPONSE_TO_UV_B1	-0.10463154	-0.03903388	-0.40171812	0.69860243	0.88069863	-6.14488851
WT after UVB irradiation VS WT before UVB irradiation	GO_CELLULAR_RESPONSE_TO_UV_B2	-0.53968629	-0.10561316	-2.6518152	0.02886813	0.44654821	-3.38629071
homo after UVB irradiation VS homo before UVB irradiation	GO_CELLULAR_RESPONSE_TO_UV_C	-0.62174863	0.056274331	-2.201342	0.05756724	0.1792198	-4.46583501
hetero after UVB irradiation VS hetero before UVB irradiation	GO_CELLULAR_RESPONSE_TO_UV_C1	0.147467374	-0.10535486	0.451349186	0.66394166	0.86224091	-6.12246925
WT after UVB irradiation VS WT before UVB irradiation	GO_CELLULAR_RESPONSE_TO_UV_C2	-0.34134419	0.005409996	-1.44690519	0.18549921	0.68726847	-5.0208932
hetero after UVB irradiation VS hetero before UVB irradiation	GO_CELLULAR_RESPONSE_TO_UV1	-0.06794702	-0.03116163	-0.43611455	0.67449459	0.86773527	-6.12961516
WT after UVB irradiation VS WT before UVB irradiation	GO_CELLULAR_RESPONSE_TO_UV2	-0.24222272	-0.01849092	-1.23403974	0.25180078	0.73564768	-5.26382866
homo after UVB irradiation VS homo before UVB irradiation	GO_CELLULAR_RESPONSE_TO_VASCULAR_ENDOTHELIAL_GROWTH_FACTOR_STIMULUS	0.393597069	-0.03176179	2.828252127	0.02133334	0.10135738	-3.49976134
hetero after UVB irradiation VS hetero before UVB irradiation	GO_CELLULAR_RESPONSE_TO_VASCULAR_ENDOTHELIAL_GROWTH_FACTOR_STIMULUS1	0.175298055	-0.00829599	0.803526414	0.44530993	0.73977264	-5.89550788
WT after UVB irradiation VS WT before UVB irradiation	GO_CELLULAR_RESPONSE_TO_VASCULAR_ENDOTHELIAL_GROWTH_FACTOR_STIMULUS2	0.165022956	-0.03718754	0.730434619	0.48571767	0.85147728	-5.72397069
homo after UVB irradiation VS homo before UVB irradiation	GO_CELLULAR_RESPONSE_TO_VIRUS	0.090543697	-0.01781114	0.62751541	0.54713509	0.71068026	-6.33979637
hetero after UVB irradiation VS hetero before UVB irradiation	GO_CELLULAR_RESPONSE_TO_VIRUS1	0.30877042	0.024761445	2.324315922	0.04917394	0.29568488	-4.02811088
WT after UVB irradiation VS WT before UVB irradiation	GO_CELLULAR_RESPONSE_TO_VIRUS2	0.23112128	-0.03536747	1.381093005	0.20416382	0.70085159	-5.09848229
homo after UVB irradiation VS homo before UVB irradiation	GO_CELLULAR_RESPONSE_TO_VITAMIN	0.06470457	0.016843944	0.46120049	0.65645961	0.79117114	-6.43561425
homo after UVB irradiation VS homo before UVB irradiation	GO_CELLULAR_RESPONSE_TO_VITAMIN_D	-0.00508261	-0.01060266	-0.04225049	0.96729433	0.98301511	-6.54987712
hetero after UVB irradiation VS hetero before UVB irradiation	GO_CELLULAR_RESPONSE_TO_VITAMIN_D1	-0.11535133	-0.00879524	-0.78372506	0.45618745	0.74624629	-5.91123889
WT after UVB irradiation VS WT before UVB irradiation	GO_CELLULAR_RESPONSE_TO_VITAMIN_D2	-0.08741727	0.042878719	-0.53012545	0.6102492	0.89866462	-5.85044704
hetero after UVB irradiation VS hetero before UVB irradiation	GO_CELLULAR_RESPONSE_TO_VITAMIN1	0.046579194	0.013399943	0.270283747	0.79390948	0.9195249	-6.19205678
WT after UVB irradiation VS WT before UVB irradiation	GO_CELLULAR_RESPONSE_TO_VITAMIN2	0.008620693	0.014513106	0.043768971	0.96614863	0.99138923	-5.99505596
homo after UVB irradiation VS homo before UVB irradiation	GO_CELLULAR_RESPONSE_TO_X_RAY	-0.5800644	-0.05214044	-2.90949171	0.0187814	0.09420375	-3.37358388
hetero after UVB irradiation VS hetero before UVB irradiation	GO_CELLULAR_RESPONSE_TO_X_RAY1	-0.31213404	-0.0674881	-1.17101472	0.27589839	0.60304498	-5.54826902
WT after UVB irradiation VS WT before UVB irradiation	GO_CELLULAR_RESPONSE_TO_X_RAY2	-0.09543046	0.069589737	-0.30653167	0.76693372	0.94123239	-5.9467905
homo after UVB irradiation VS homo before UVB irradiation	GO_CELLULAR_RESPONSE_TO_ZINC_ION	0.335417906	-0.02880564	2.390404729	0.04264802	0.14920741	-4.1777833
hetero after UVB irradiation VS hetero before UVB irradiation	GO_CELLULAR_RESPONSE_TO_ZINC_ION1	0.789067687	0.030173278	6.093185936	0.00031421	0.07788488	0.764383579
WT after UVB irradiation VS WT before UVB irradiation	GO_CELLULAR_RESPONSE_TO_ZINC_ION2	0.780900489	-0.12341854	3.908197504	0.00438728	0.26635361	-1.66758168
homo after UVB irradiation VS homo before UVB irradiation	GO_CELLULAR_SENESCENCE	0.121256543	-0.01725148	0.85205981	0.41800441	0.6036887	-6.16915485
hetero after UVB irradiation VS hetero before UVB irradiation	GO_CELLULAR_SENESCENCE1	0.218856303	-0.00597175	1.382416378	0.20488617	0.529684	-5.30797156
WT after UVB irradiation VS WT before UVB irradiation	GO_CELLULAR_SENESCENCE2	0.083377219	-0.02093992	0.54048736	0.60340529	0.89591658	-5.84480569
homo after UVB irradiation VS homo before UVB irradiation	GO_CELLULAR_SODIUM_ION_HOMEOSTASIS	0.454357523	0.021718837	2.608823582	0.03015955	0.12333258	-3.84055039
hetero after UVB irradiation VS hetero before UVB irradiation	GO_CELLULAR_SODIUM_ION_HOMEOSTASIS1	-0.10457077	0.055438864	-0.65746824	0.52967551	0.79092206	-6.00352377
WT after UVB irradiation VS WT before UVB irradiation	GO_CELLULAR_SODIUM_ION_HOMEOSTASIS2	0.131108705	0.076204347	0.754628155	0.47184586	0.84699384	-5.70629463
homo after UVB irradiation VS homo before UVB irradiation	GO_CELLULAR_SPHINGOLIPID_HOMEOSTASIS	-0.12330242	-0.02044832	-0.82895843	0.4302407	0.61432628	-6.18876782
hetero after UVB irradiation VS hetero before UVB irradiation	GO_CELLULAR_SPHINGOLIPID_HOMEOSTASIS1	-0.39132714	-0.07571206	-1.24978946	0.24733794	0.57452256	-5.46173659
WT after UVB irradiation VS WT before UVB irradiation	GO_CELLULAR_SPHINGOLIPID_HOMEOSTASIS2	0.222075535	0.02365989	0.794425996	0.44960078	0.83496965	-5.67614599
homo after UVB irradiation VS homo before UVB irradiation	GO_CELLULAR_TRANSITION_METAL_ION_HOMEOSTASIS	0.121979807	0.000438455	0.797101613	0.44751599	0.62949133	-6.21506465
hetero after UVB irradiation VS hetero before UVB irradiation	GO_CELLULAR_TRANSITION_METAL_ION_HOMEOSTASIS1	0.189672328	0.046221593	1.329395513	0.22103961	0.54852054	-5.37059434
WT after UVB irradiation VS WT before UVB irradiation	GO_CELLULAR_TRANSITION_METAL_ION_HOMEOSTASIS2	0.197603154	0.015316564	1.167697755	0.27615826	0.74766532	-5.33442332
homo after UVB irradiation VS homo before UVB irradiation	GO_CELLULAR_TRIGLYCERIDE_HOMEOSTASIS	0.142310101	0.009199408	0.776463848	0.45895455	0.63926949	-6.2316298
hetero after UVB irradiation VS hetero before UVB irradiation	GO_CELLULAR_TRIGLYCERIDE_HOMEOSTASIS1	-0.41895473	0.022671373	-2.08162324	0.07158746	0.34611552	-4.37808848
WT after UVB irradiation VS WT before UVB irradiation	GO_CELLULAR_TRIGLYCERIDE_HOMEOSTASIS2	-0.17379075	0.039993416	-0.70780816	0.49892704	0.85541458	-5.74004838
homo after UVB irradiation VS homo before UVB irradiation	GO_CELLULAR_WATER_HOMEOSTASIS	0.644849685	-0.02116278	4.01993024	0.0035359	0.03892545	-1.69789801
hetero after UVB irradiation VS hetero before UVB irradiation	GO_CELLULAR_WATER_HOMEOSTASIS1	0.290067984	0.075609036	1.03683931	0.3306811	0.65066323	-5.68646776
WT after UVB irradiation VS WT before UVB irradiation	GO_CELLULAR_WATER_HOMEOSTASIS2	0.344877229	0.069589375	1.111154701	0.2984019	0.76001325	-5.39244939
homo after UVB irradiation VS homo before UVB irradiation	GO_CENTRAL_ELEMENT	-0.03771545	0.045894054	-0.15903876	0.87742718	0.93333763	-6.53699667
hetero after UVB irradiation VS hetero before UVB irradiation	GO_CENTRAL_ELEMENT1	0.443575378	0.091927532	2.213008721	0.05842544	0.31883348	-4.18952805
WT after UVB irradiation VS WT before UVB irradiation	GO_CENTRAL_ELEMENT2	0.258944449	0.094428537	1.307111726	0.22706861	0.71906685	-5.18312279
homo after UVB irradiation VS homo before UVB irradiation	GO_CENTRAL_NERVOUS_SYSTEM_DEVELOPMENT	-0.00144547	0.008980765	-0.01213274	0.9906053	0.99505564	-6.55077593
hetero after UVB irradiation VS hetero before UVB irradiation	GO_CENTRAL_NERVOUS_SYSTEM_DEVELOPMENT1	-0.00658074	0.007654588	-0.05206343	0.95977816	0.98542968	-6.23001439
WT after UVB irradiation VS WT before UVB irradiation	GO_CENTRAL_NERVOUS_SYSTEM_DEVELOPMENT2	-0.04394518	-0.00234278	-0.30645389	0.76699082	0.94123239	-5.94681535
homo after UVB irradiation VS homo before UVB irradiation	GO_CENTRAL_NERVOUS_SYSTEM_MATURATION	0.730003258	-0.12363188	3.533075212	0.00721574	0.05610258	-2.41702564
hetero after UVB irradiation VS hetero before UVB irradiation	GO_CENTRAL_NERVOUS_SYSTEM_MATURATION1	0.841232624	0.044052261	3.073019927	0.01562452	0.18450952	-2.93362397
WT after UVB irradiation VS WT before UVB irradiation	GO_CENTRAL_NERVOUS_SYSTEM_MATURATION2	0.656939843	0.011070131	2.35480605	0.04596432	0.50505995	-3.80847496
homo after UVB irradiation VS homo before UVB irradiation	GO_CENTRAL_NERVOUS_SYSTEM_MYELIN_MAINTENANCE	-0.54845049	0.044722908	-2.12151317	0.06531796	0.1939821	-4.58581775
hetero after UVB irradiation VS hetero before UVB irradiation	GO_CENTRAL_NERVOUS_SYSTEM_MYELIN_MAINTENANCE1	-0.34111627	0.074704107	-1.22852084	0.25479507	0.58243013	-5.4854703
WT after UVB irradiation VS WT before UVB irradiation	GO_CENTRAL_NERVOUS_SYSTEM_MYELIN_MAINTENANCE2	-0.62434301	-0.05437117	-3.56805436	0.00717049	0.31015346	-2.11311178
homo after UVB irradiation VS homo before UVB irradiation	GO_CENTRAL_NERVOUS_SYSTEM_NEURON_AXONOGENESIS	0.036571134	-0.01924987	0.198367557	0.84751386	0.91597789	-6.52931266
hetero after UVB irradiation VS hetero before UVB irradiation	GO_CENTRAL_NERVOUS_SYSTEM_NEURON_AXONOGENESIS1	0.123551463	-0.01080028	0.7721144	0.46264838	0.75077966	-5.92030785
WT after UVB irradiation VS WT before UVB irradiation	GO_CENTRAL_NERVOUS_SYSTEM_NEURON_AXONOGENESIS2	-0.09063441	-0.02818712	-0.60350213	0.562694	0.88065281	-5.80833923
homo after UVB irradiation VS homo before UVB irradiation	GO_CENTRAL_NERVOUS_SYSTEM_NEURON_DEVELOPMENT	0.018197556	-0.00012128	0.12404229	0.90422315	0.9475315	-6.54242024
hetero after UVB irradiation VS hetero before UVB irradiation	GO_CENTRAL_NERVOUS_SYSTEM_NEURON_DEVELOPMENT1	0.108278791	0.006993362	0.74039016	0.48061203	0.76122341	-5.94449547
WT after UVB irradiation VS WT before UVB irradiation	GO_CENTRAL_NERVOUS_SYSTEM_NEURON_DEVELOPMENT2	-0.07353333	-0.00786601	-0.48643347	0.63955137	0.90634429	-5.8731131
homo after UVB irradiation VS homo before UVB irradiation	GO_CENTRAL_NERVOUS_SYSTEM_NEURON_DIFFERENTIATION	-0.0821971	0.020716463	-0.66860617	0.52184151	0.69109361	-6.31199929
hetero after UVB irradiation VS hetero before UVB irradiation	GO_CENTRAL_NERVOUS_SYSTEM_NEURON_DIFFERENTIATION1	-0.02793784	0.020461012	-0.21846388	0.83263818	0.9354456	-6.20568296
WT after UVB irradiation VS WT before UVB irradiation	GO_CENTRAL_NERVOUS_SYSTEM_NEURON_DIFFERENTIATION2	-0.09624234	0.010715472	-0.64039356	0.53961095	0.87025652	-5.78529747
homo after UVB irradiation VS homo before UVB irradiation	GO_CENTRAL_NERVOUS_SYSTEM_PROJECTION_NEURON_AXONOGENESIS	0.193206772	-0.02590368	1.072462717	0.31359861	0.50759841	-5.96035849
hetero after UVB irradiation VS hetero before UVB irradiation	GO_CENTRAL_NERVOUS_SYSTEM_PROJECTION_NEURON_AXONOGENESIS1	0.188412359	0.015749868	1.148947884	0.28436802	0.60954889	-5.57181754
WT after UVB irradiation VS WT before UVB irradiation	GO_CENTRAL_NERVOUS_SYSTEM_PROJECTION_NEURON_AXONOGENESIS2	-0.00235157	-0.00025683	-0.01379709	0.98932574	0.99845632	-5.99596622
homo after UVB irradiation VS homo before UVB irradiation	GO_CENTRAL_REGION_OF_GROWTH_CONE	-0.08674104	-0.06505766	-0.3712277	0.71973318	0.83384689	-6.47585495
hetero after UVB irradiation VS hetero before UVB irradiation	GO_CENTRAL_REGION_OF_GROWTH_CONE1	0.081625222	-0.06069226	0.238060801	0.817928	0.93027174	-6.20086446
WT after UVB irradiation VS WT before UVB irradiation	GO_CENTRAL_REGION_OF_GROWTH_CONE2	0.155708425	0.004147196	0.53865121	0.60461504	0.89591658	-5.84581273
homo after UVB irradiation VS homo before UVB irradiation	GO_CENTRIOLAR_SATELLITE	-0.03778208	-0.03015083	-0.27676288	0.7887065	0.87990277	-6.50901427
hetero after UVB irradiation VS hetero before UVB irradiation	GO_CENTRIOLAR_SATELLITE1	0.099976155	-0.0154757	0.682830466	0.51434976	0.78287352	-5.98612441
WT after UVB irradiation VS WT before UVB irradiation	GO_CENTRIOLAR_SATELLITE2	-0.08719199	0.001208453	-0.54495404	0.60046786	0.89567499	-5.8423427
homo after UVB irradiation VS homo before UVB irradiation	GO_CENTRIOLAR_SUBDISTAL_APPENDAGE	-0.08614789	-0.00568535	-0.44821429	0.66542858	0.79756326	-6.44193558
hetero after UVB irradiation VS hetero before UVB irradiation	GO_CENTRIOLAR_SUBDISTAL_APPENDAGE1	-0.28691273	-0.07524716	-1.22683865	0.25539281	0.58262782	-5.48733597
WT after UVB irradiation VS WT before UVB irradiation	GO_CENTRIOLAR_SUBDISTAL_APPENDAGE2	-0.32120739	-0.0524323	-1.2181239	0.25747615	0.73921703	-5.28100392
homo after UVB irradiation VS homo before UVB irradiation	GO_CENTRIOLE	-0.37890408	-0.02485594	-2.28268336	0.05060162	0.1660631	-4.34249379
homo after UVB irradiation VS homo before UVB irradiation	GO_CENTRIOLE_ASSEMBLY	-0.59976545	-0.00881976	-4.23508817	0.00260755	0.03384751	-1.39040573
hetero after UVB irradiation VS hetero before UVB irradiation	GO_CENTRIOLE_ASSEMBLY1	-0.09310512	0.004624052	-0.36218358	0.72676787	0.89222406	-6.16095566
WT after UVB irradiation VS WT before UVB irradiation	GO_CENTRIOLE_ASSEMBLY2	-0.28630039	-0.03487369	-1.1192292	0.29514027	0.7584258	-5.38428802
homo after UVB irradiation VS homo before UVB irradiation	GO_CENTRIOLE_CENTRIOLE_COHESION	-0.22187516	-0.01362426	-0.95975958	0.36420418	0.55450909	-6.07188668
hetero after UVB irradiation VS hetero before UVB irradiation	GO_CENTRIOLE_CENTRIOLE_COHESION1	0.120505448	0.015722431	0.412348718	0.6911109	0.87620446	-6.14029603
WT after UVB irradiation VS WT before UVB irradiation	GO_CENTRIOLE_CENTRIOLE_COHESION2	0.26257893	-0.0007952	1.056891656	0.32106887	0.77346379	-5.44618112
homo after UVB irradiation VS homo before UVB irradiation	GO_CENTRIOLE_ELONGATION	-0.08268025	-0.05530153	-0.41821055	0.68636775	0.81224771	-6.45588169
hetero after UVB irradiation VS hetero before UVB irradiation	GO_CENTRIOLE_ELONGATION1	-0.18260964	-0.04922072	-0.56739	0.58629926	0.82371055	-6.06051376
WT after UVB irradiation VS WT before UVB irradiation	GO_CENTRIOLE_ELONGATION2	-0.49029491	-0.00401369	-1.8829993	0.09601478	0.59686908	-4.46342618
hetero after UVB irradiation VS hetero before UVB irradiation	GO_CENTRIOLE1	-0.07989117	-0.00805346	-0.37545595	0.71726005	0.88933296	-6.15574021
WT after UVB irradiation VS WT before UVB irradiation	GO_CENTRIOLE2	-0.12199483	-0.05108401	-0.62464259	0.54939685	0.87530826	-5.79528619
homo after UVB irradiation VS homo before UVB irradiation	GO_CENTROMERE_COMPLEX_ASSEMBLY	-0.57034443	-0.02814123	-2.58480826	0.0313288	0.12542568	-3.87777499
hetero after UVB irradiation VS hetero before UVB irradiation	GO_CENTROMERE_COMPLEX_ASSEMBLY1	-0.01207253	-0.03673084	-0.04052654	0.96868475	0.98878751	-6.23059352
WT after UVB irradiation VS WT before UVB irradiation	GO_CENTROMERE_COMPLEX_ASSEMBLY2	0.039135034	0.044011266	0.131644761	0.89847743	0.97448415	-5.98693267
homo after UVB irradiation VS homo before UVB irradiation	GO_CENTROMERIC_DNA_BINDING	-0.36590065	0.021394847	-1.20962967	0.25963087	0.45500303	-5.81230971
hetero after UVB irradiation VS hetero before UVB irradiation	GO_CENTROMERIC_DNA_BINDING1	0.173094096	0.001866103	0.814670693	0.4392666	0.73665394	-5.88650912
WT after UVB irradiation VS WT before UVB irradiation	GO_CENTROMERIC_DNA_BINDING2	0.138347674	0.028526612	0.535004013	0.60702184	0.89713202	-5.84780362
homo after UVB irradiation VS homo before UVB irradiation	GO_CENTROMERIC_SISTER_CHROMATID_COHESION	-0.26673638	-0.03050315	-1.15246689	0.28112967	0.47619726	-5.87556776
hetero after UVB irradiation VS hetero before UVB irradiation	GO_CENTROMERIC_SISTER_CHROMATID_COHESION1	0.411894379	0.032126651	1.801504917	0.11000304	0.40948315	-4.76912984
WT after UVB irradiation VS WT before UVB irradiation	GO_CENTROMERIC_SISTER_CHROMATID_COHESION2	-0.05048505	0.052938104	-0.16753144	0.87106029	0.96883047	-5.98128437
homo after UVB irradiation VS homo before UVB irradiation	GO_CENTROSOME	-0.29972877	-0.01002259	-2.29135901	0.04990979	0.16429151	-4.32928176
homo after UVB irradiation VS homo before UVB irradiation	GO_CENTROSOME_DUPLICATION	-0.41255484	0.003086962	-3.04333683	0.01524335	0.08371643	-3.16611451
hetero after UVB irradiation VS hetero before UVB irradiation	GO_CENTROSOME_DUPLICATION1	-0.05119964	-0.0174831	-0.22601013	0.82696498	0.93413454	-6.20387579
WT after UVB irradiation VS WT before UVB irradiation	GO_CENTROSOME_DUPLICATION2	-0.18678355	-0.04507272	-0.82137652	0.43494574	0.83012084	-5.65498639
homo after UVB irradiation VS homo before UVB irradiation	GO_CENTROSOME_SEPARATION	-0.76207315	-0.04309876	-4.19829569	0.00274564	0.03457111	-1.44250572
hetero after UVB irradiation VS hetero before UVB irradiation	GO_CENTROSOME_SEPARATION1	-0.23876027	-0.06793283	-0.79652189	0.44913735	0.74180859	-5.90111047
WT after UVB irradiation VS WT before UVB irradiation	GO_CENTROSOME_SEPARATION2	-0.13595615	-0.0129222	-0.48336947	0.64163255	0.90690671	-5.87463408
hetero after UVB irradiation VS hetero before UVB irradiation	GO_CENTROSOME1	-0.03981629	-0.02393122	-0.23473641	0.82041817	0.93112347	-6.20171059
WT after UVB irradiation VS WT before UVB irradiation	GO_CENTROSOME2	-0.10654754	-0.03323505	-0.57445333	0.58126426	0.88889863	-5.82560746
homo after UVB irradiation VS homo before UVB irradiation	GO_CERAMIDASE_ACTIVITY	-0.04424494	-0.05116551	-0.13580506	0.89520035	0.9429989	-6.54074622
hetero after UVB irradiation VS hetero before UVB irradiation	GO_CERAMIDASE_ACTIVITY1	-0.1893789	-0.02355918	-1.48299892	0.17705856	0.50051267	-5.18530144
WT after UVB irradiation VS WT before UVB irradiation	GO_CERAMIDASE_ACTIVITY2	-0.07754941	-0.01007426	-0.42528847	0.68170305	0.91716791	-5.90174427
homo after UVB irradiation VS homo before UVB irradiation	GO_CERAMIDE_1_PHOSPHATE_BINDING	0.681899482	-0.03752714	3.276051352	0.01064797	0.0691907	-2.80768776
hetero after UVB irradiation VS hetero before UVB irradiation	GO_CERAMIDE_1_PHOSPHATE_BINDING1	0.80042065	0.051814619	4.59768705	0.0018482	0.09881161	-0.8801055
WT after UVB irradiation VS WT before UVB irradiation	GO_CERAMIDE_1_PHOSPHATE_BINDING2	0.292054495	0.072487875	1.280173547	0.23593648	0.72774029	-5.2132218
homo after UVB irradiation VS homo before UVB irradiation	GO_CERAMIDE_1_PHOSPHATE_TRANSPORT	0.741323471	-0.03908715	3.932790902	0.00400768	0.04132163	-1.82432216
hetero after UVB irradiation VS hetero before UVB irradiation	GO_CERAMIDE_1_PHOSPHATE_TRANSPORT1	0.789724739	0.082816225	4.303432643	0.00271874	0.11212131	-1.2481554
WT after UVB irradiation VS WT before UVB irradiation	GO_CERAMIDE_1_PHOSPHATE_TRANSPORT2	0.10941133	0.030532995	0.568031843	0.58541527	0.89079945	-5.82931951
homo after UVB irradiation VS homo before UVB irradiation	GO_CERAMIDE_BINDING	0.595510524	-0.0695111	3.313842393	0.01005089	0.06701003	-2.7498589
hetero after UVB irradiation VS hetero before UVB irradiation	GO_CERAMIDE_BINDING1	0.591494048	0.064423913	3.472954919	0.0086527	0.14984918	-2.36293007
WT after UVB irradiation VS WT before UVB irradiation	GO_CERAMIDE_BINDING2	0.376341205	-0.00842593	2.36449847	0.04527059	0.50230217	-3.7947462
homo after UVB irradiation VS homo before UVB irradiation	GO_CERAMIDE_BIOSYNTHETIC_PROCESS	0.290671988	-0.01528573	1.803115826	0.10755264	0.26238292	-5.04993447
hetero after UVB irradiation VS hetero before UVB irradiation	GO_CERAMIDE_BIOSYNTHETIC_PROCESS1	0.165201066	0.022457109	0.932479859	0.37886712	0.69198704	-5.78518343
WT after UVB irradiation VS WT before UVB irradiation	GO_CERAMIDE_BIOSYNTHETIC_PROCESS2	0.155490504	-0.00466337	1.072273578	0.31451045	0.77104985	-5.43114964
homo after UVB irradiation VS homo before UVB irradiation	GO_CERAMIDE_CATABOLIC_PROCESS	0.26174177	-0.07609336	0.981571243	0.35395879	0.54614908	-6.05105655
hetero after UVB irradiation VS hetero before UVB irradiation	GO_CERAMIDE_CATABOLIC_PROCESS1	0.47271795	-0.05069294	3.266936637	0.01170325	0.1686457	-2.65472663
WT after UVB irradiation VS WT before UVB irradiation	GO_CERAMIDE_CATABOLIC_PROCESS2	0.324126552	-0.02417239	1.920397164	0.09061899	0.58379396	-4.41291445
homo after UVB irradiation VS homo before UVB irradiation	GO_CERAMIDE_METABOLIC_PROCESS	0.32300505	-0.03167196	1.863671256	0.09790472	0.24760581	-4.9638096
hetero after UVB irradiation VS hetero before UVB irradiation	GO_CERAMIDE_METABOLIC_PROCESS1	0.26093401	0.012954402	1.592225586	0.15070621	0.46844437	-5.04690391
WT after UVB irradiation VS WT before UVB irradiation	GO_CERAMIDE_METABOLIC_PROCESS2	0.25181275	-0.00605862	1.714486567	0.12432901	0.63137109	-4.68647534
homo after UVB irradiation VS homo before UVB irradiation	GO_CERAMIDE_TRANSPORT	0.660350505	0.022806599	4.405004238	0.00205992	0.03080331	-1.15244472
hetero after UVB irradiation VS hetero before UVB irradiation	GO_CERAMIDE_TRANSPORT1	0.853584126	0.076328418	5.704997775	0.0004835	0.07788488	0.372821941
WT after UVB irradiation VS WT before UVB irradiation	GO_CERAMIDE_TRANSPORT2	0.340655014	-0.02668958	1.698094819	0.12746534	0.63467098	-4.70772181
homo after UVB irradiation VS homo before UVB irradiation	GO_CEREBELLAR_CORTEX_DEVELOPMENT	-0.17569501	0.024190393	-1.39459229	0.19920869	0.38797027	-5.59370055
hetero after UVB irradiation VS hetero before UVB irradiation	GO_CEREBELLAR_CORTEX_DEVELOPMENT1	-0.06388016	-0.00178598	-0.46984814	0.65123455	0.85686396	-6.11348008
WT after UVB irradiation VS WT before UVB irradiation	GO_CEREBELLAR_CORTEX_DEVELOPMENT2	-0.23550494	0.018044464	-1.600638	0.14766938	0.65441042	-4.83213945
homo after UVB irradiation VS homo before UVB irradiation	GO_CEREBELLAR_CORTEX_FORMATION	-0.26151513	0.025193397	-1.84309058	0.10108822	0.25216339	-4.99321033
hetero after UVB irradiation VS hetero before UVB irradiation	GO_CEREBELLAR_CORTEX_FORMATION1	-0.1190529	-0.00634979	-0.7608907	0.46895194	0.75346851	-5.92896469
WT after UVB irradiation VS WT before UVB irradiation	GO_CEREBELLAR_CORTEX_FORMATION2	-0.24762155	0.03525073	-1.42484378	0.19158212	0.69334561	-5.04713265
homo after UVB irradiation VS homo before UVB irradiation	GO_CEREBELLAR_CORTEX_MORPHOGENESIS	-0.25983825	0.007842694	-1.83825399	0.10185043	0.25350089	-5.00010061
hetero after UVB irradiation VS hetero before UVB irradiation	GO_CEREBELLAR_CORTEX_MORPHOGENESIS1	-0.10191794	-0.00395453	-0.71525896	0.49516222	0.76903628	-5.96303285
WT after UVB irradiation VS WT before UVB irradiation	GO_CEREBELLAR_CORTEX_MORPHOGENESIS2	-0.27725277	0.006961899	-1.58513976	0.15113979	0.6585056	-4.85160585
homo after UVB irradiation VS homo before UVB irradiation	GO_CEREBELLAR_GRANULAR_LAYER_DEVELOPMENT	-0.1990793	0.001096072	-1.50461091	0.16936142	0.34864281	-5.45464329
hetero after UVB irradiation VS hetero before UVB irradiation	GO_CEREBELLAR_GRANULAR_LAYER_DEVELOPMENT1	-0.09164043	-0.03779918	-0.49072032	0.63704211	0.85099142	-6.10292942
WT after UVB irradiation VS WT before UVB irradiation	GO_CEREBELLAR_GRANULAR_LAYER_DEVELOPMENT2	0.008803221	0.024477972	0.041836845	0.96764196	0.99191629	-5.99514321
homo after UVB irradiation VS homo before UVB irradiation	GO_CEREBELLAR_GRANULAR_LAYER_FORMATION	-0.12933597	0.02341189	-0.6905441	0.50863439	0.6812414	-6.29651216
hetero after UVB irradiation VS hetero before UVB irradiation	GO_CEREBELLAR_GRANULAR_LAYER_FORMATION1	-0.25692624	0.040943974	-1.07447292	0.31451412	0.63522726	-5.64893627
WT after UVB irradiation VS WT before UVB irradiation	GO_CEREBELLAR_GRANULAR_LAYER_FORMATION2	0.024803432	0.077130242	0.111385701	0.91402196	0.97932969	-5.98952554

homo after UVB irradiation VS homo before UVB irradiation	GO_CEREBELLAR_GRANULAR_LAYER_MORPHOGENESIS	-0.08925886	0.020815111	-0.41524694	0.68845202	0.81391709	-6.45720906
hetero after UVB irradiation VS hetero before UVB irradiation	GO_CEREBELLAR_GRANULAR_LAYER_MORPHOGENESIS1	-0.08839066	0.019557837	-0.44822858	0.66609688	0.86317077	-6.12395195
WT after UVB irradiation VS WT before UVB irradiation	GO_CEREBELLAR_GRANULAR_LAYER_MORPHOGENESIS2	0.107816693	0.018924276	0.531244908	0.60950783	0.89828294	-5.84984246
homo after UVB irradiation VS homo before UVB irradiation	GO_CEREBELLAR_MOSSY_FIBER	0.206039511	0.025248617	0.667527724	0.52249614	0.69152247	-6.3127491
hetero after UVB irradiation VS hetero before UVB irradiation	GO_CEREBELLAR_MOSSY_FIBER1	0.104687998	-0.04490635	0.278424216	0.7878775	0.91723238	-6.18965831
WT after UVB irradiation VS WT before UVB irradiation	GO_CEREBELLAR_MOSSY_FIBER2	-0.00753998	-0.10589078	-0.02910068	0.97748874	0.99433096	-5.99561985
homo after UVB irradiation VS homo before UVB irradiation	ELLAR_PURKINJE_CELL_GRANULE_CELL_PRECURSOR_CELL_SIGNALING_INVOLVED_IN_REGULATION_OF_GRANULE_CELL_PRECURSOR_CELL_PROL	-0.24431574	0.049781298	-0.99809729	0.34634127	0.53844902	-6.03502883
hetero after UVB irradiation VS hetero before UVB irradiation	ELLAR_PURKINJE_CELL_GRANULE_CELL_PRECURSOR_CELL_SIGNALING_INVOLVED_IN_REGULATION_OF_GRANULE_CELL_PRECURSOR_CELL_PROLI	-0.17425083	0.04055584	-0.53132406	0.60988978	0.83720565	-6.08117711
WT after UVB irradiation VS WT before UVB irradiation	ELLAR_PURKINJE_CELL_GRANULE_CELL_PRECURSOR_CELL_SIGNALING_INVOLVED_IN_REGULATION_OF_GRANULE_CELL_PRECURSOR_CELL_PROLI	-0.46399312	0.074581279	-2.04657908	0.07448085	0.55789253	-4.24028252
homo after UVB irradiation VS homo before UVB irradiation	GO_CEREBELLAR_PURKINJE_CELL_LAYER_DEVELOPMENT	-0.13201906	0.028579931	-0.86762665	0.40989749	0.59653425	-6.15568464
hetero after UVB irradiation VS hetero before UVB irradiation	GO_CEREBELLAR_PURKINJE_CELL_LAYER_DEVELOPMENT1	0.092774242	-0.0115672	0.663855784	0.52578954	0.78916946	-5.99919681
WT after UVB irradiation VS WT before UVB irradiation	GO_CEREBELLAR_PURKINJE_CELL_LAYER_DEVELOPMENT2	-0.28303421	-0.01155383	-1.69703878	0.1276699	0.63467098	-4.70908761
homo after UVB irradiation VS homo before UVB irradiation	GO_CEREBELLAR_PURKINJE_CELL_LAYER_FORMATION	-0.40369657	0.033418994	-2.47008972	0.03758	0.13905493	-4.05515524
hetero after UVB irradiation VS hetero before UVB irradiation	GO_CEREBELLAR_PURKINJE_CELL_LAYER_FORMATION1	-0.10832362	-0.03479537	-0.81360263	0.43984334	0.73710622	-5.88737606
WT after UVB irradiation VS WT before UVB irradiation	GO_CEREBELLAR_PURKINJE_CELL_LAYER_FORMATION2	-0.45756279	-0.01541741	-2.15630095	0.0627462	0.53553824	-4.08789154
homo after UVB irradiation VS homo before UVB irradiation	GO_CEREBELLAR_PURKINJE_CELL_LAYER_MORPHOGENESIS	-0.29143076	0.010281037	-1.60326879	0.14604904	0.31877143	-5.32495891
hetero after UVB irradiation VS hetero before UVB irradiation	GO_CEREBELLAR_PURKINJE_CELL_LAYER_MORPHOGENESIS1	0.130214081	-0.00226693	1.014553131	0.34055519	0.65956586	-5.7082209
WT after UVB irradiation VS WT before UVB irradiation	GO_CEREBELLAR_PURKINJE_CELL_LAYER_MORPHOGENESIS2	-0.3948353	-0.03442777	-1.9717841	0.08367663	0.5707311	-4.34299712
homo after UVB irradiation VS homo before UVB irradiation	GO_CEREBRAL_CORTEX_CELL_MIGRATION	0.248719803	-0.00314478	1.7513717	0.11649692	0.27520407	-5.12256851
hetero after UVB irradiation VS hetero before UVB irradiation	GO_CEREBRAL_CORTEX_CELL_MIGRATION1	0.215701331	0.033561709	1.189083986	0.26911769	0.59622699	-5.52875685
WT after UVB irradiation VS WT before UVB irradiation	GO_CEREBRAL_CORTEX_CELL_MIGRATION2	0.174670923	0.026894374	1.16353713	0.27774792	0.74917538	-5.3387617
homo after UVB irradiation VS homo before UVB irradiation	GO_CEREBRAL_CORTEX_DEVELOPMENT	0.130205713	0.016208201	1.00622486	0.3426407	0.53548037	-6.02706961
hetero after UVB irradiation VS hetero before UVB irradiation	GO_CEREBRAL_CORTEX_DEVELOPMENT1	0.193034373	0.011278966	1.376729315	0.20656829	0.53105475	-5.3147587
WT after UVB irradiation VS WT before UVB irradiation	GO_CEREBRAL_CORTEX_DEVELOPMENT2	0.132557684	0.01333344	0.945088423	0.37195565	0.8012534	-5.55046351
homo after UVB irradiation VS homo before UVB irradiation	GO_CEREBRAL_CORTEX_GABAERGIC_INTERNEURON_DEVELOPMENT	0.309608404	-0.01405544	1.336939199	0.21660419	0.40724017	-5.66399642
hetero after UVB irradiation VS hetero before UVB irradiation	GO_CEREBRAL_CORTEX_GABAERGIC_INTERNEURON_DEVELOPMENT1	0.294235575	0.086510358	1.70706191	0.12690446	0.43357309	-4.89632566
WT after UVB irradiation VS WT before UVB irradiation	GO_CEREBRAL_CORTEX_GABAERGIC_INTERNEURON_DEVELOPMENT2	0.628838574	0.127755495	3.365472891	0.00967905	0.32630845	-2.38677159
homo after UVB irradiation VS homo before UVB irradiation	GO_CEREBRAL_CORTEX_GABAERGIC_INTERNEURON_DIFFERENTIATION	0.128616988	0.020821598	0.586812607	0.57288701	0.73018601	-6.36574884
hetero after UVB irradiation VS hetero before UVB irradiation	GO_CEREBRAL_CORTEX_GABAERGIC_INTERNEURON_DIFFERENTIATION1	0.104733873	0.111102313	0.627884329	0.54790007	0.80023888	-6.02307551
WT after UVB irradiation VS WT before UVB irradiation	GO_CEREBRAL_CORTEX_GABAERGIC_INTERNEURON_DIFFERENTIATION2	0.371652375	0.13130139	2.212936538	0.05741975	0.52369794	-4.0085798
homo after UVB irradiation VS homo before UVB irradiation	GO_CEREBRAL_CORTEX_NEURON_DIFFERENTIATION	-0.19763159	0.040968526	-1.31555744	0.22337838	0.41422402	-5.68958752
hetero after UVB irradiation VS hetero before UVB irradiation	GO_CEREBRAL_CORTEX_NEURON_DIFFERENTIATION1	-0.10580808	0.067937967	-0.55774503	0.59255813	0.82730511	-6.0661622
WT after UVB irradiation VS WT before UVB irradiation	GO_CEREBRAL_CORTEX_NEURON_DIFFERENTIATION2	0.024771968	0.069174206	0.136437546	0.89480651	0.97322079	-5.9862563
homo after UVB irradiation VS homo before UVB irradiation	GO_CEREBRAL_CORTEX_RADIALLY_ORIENTED_CELL_MIGRATION	0.130869708	0.013714634	0.828669196	0.43039546	0.61442835	-6.18901051
hetero after UVB irradiation VS hetero before UVB irradiation	GO_CEREBRAL_CORTEX_RADIALLY_ORIENTED_CELL_MIGRATION1	0.231456896	0.025875677	1.319760492	0.22408998	0.55258552	-5.3818136
WT after UVB irradiation VS WT before UVB irradiation	GO_CEREBRAL_CORTEX_RADIALLY_ORIENTED_CELL_MIGRATION2	0.122284971	0.03037796	0.789375547	0.45238395	0.83625615	-5.68004492
homo after UVB irradiation VS homo before UVB irradiation	GO_CEREBRAL_CORTEX_REGIONALIZATION	0.094154861	0.022929531	0.440165501	0.67101634	0.80173169	-6.44576723
hetero after UVB irradiation VS hetero before UVB irradiation	GO_CEREBRAL_CORTEX_REGIONALIZATION1	0.525139836	0.006889489	2.158749242	0.0635434	0.33099757	-4.26769977
WT after UVB irradiation VS WT before UVB irradiation	GO_CEREBRAL_CORTEX_REGIONALIZATION2	0.033304162	-0.00798574	0.165441847	0.87265205	0.96914919	-5.98165017
homo after UVB irradiation VS homo before UVB irradiation	GO_CEREBRAL_CORTEX_TANGENTIAL_MIGRATION	0.115701597	-0.03136842	0.711356191	0.49629935	0.67123908	-6.28140942
hetero after UVB irradiation VS hetero before UVB irradiation	GO_CEREBRAL_CORTEX_TANGENTIAL_MIGRATION1	-0.26427844	0.058596368	-1.33013548	0.22080682	0.54824591	-5.36973062
WT after UVB irradiation VS WT before UVB irradiation	GO_CEREBRAL_CORTEX_TANGENTIAL_MIGRATION2	-0.04381964	0.092635252	-0.15476689	0.8807932	0.9693507	-5.98344775
homo after UVB irradiation VS homo before UVB irradiation	GO_CEREBROSPINAL_FLUID_CIRCULATION	-0.50017547	0.036902957	-2.68047808	0.02692771	0.11612278	-3.72935746
hetero after UVB irradiation VS hetero before UVB irradiation	GO_CEREBROSPINAL_FLUID_CIRCULATION1	-0.75772614	-0.09421972	-4.06684901	0.00374258	0.12164211	-1.55465711
WT after UVB irradiation VS WT before UVB irradiation	GO_CEREBROSPINAL_FLUID_CIRCULATION2	-0.83208354	0.085293243	-4.45983763	0.00204901	0.21016409	-0.98674841
homo after UVB irradiation VS homo before UVB irradiation	GO_CGMP_BINDING	0.004646444	0.062302766	0.029906887	0.97684554	0.98812908	-6.55036587
hetero after UVB irradiation VS hetero before UVB irradiation	GO_CGMP_BINDING1	-0.23027854	-0.06135299	-1.40497121	0.19833199	0.52299779	-5.28089235
WT after UVB irradiation VS WT before UVB irradiation	GO_CGMP_BINDING2	0.09888893	-0.03518284	0.32617808	0.75255978	0.93734883	-5.94031656
homo after UVB irradiation VS homo before UVB irradiation	GO_CGMP_BIOSYNTHETIC_PROCESS	0.463024812	0.025829828	3.246996365	0.01113226	0.07068793	-2.85222888
hetero after UVB irradiation VS hetero before UVB irradiation	GO_CGMP_BIOSYNTHETIC_PROCESS1	-0.16500364	0.113410997	-0.80043448	0.44699667	0.74085129	-5.89798607
WT after UVB irradiation VS WT before UVB irradiation	GO_CGMP_BIOSYNTHETIC_PROCESS2	-0.17655007	-0.01984654	-0.69496303	0.50652665	0.85763155	-5.74897851
homo after UVB irradiation VS homo before UVB irradiation	GO_CGMP_CATABOLIC_PROCESS	0.322507786	-0.09365175	1.42833405	0.18959961	0.37525771	-5.55171675
hetero after UVB irradiation VS hetero before UVB irradiation	GO_CGMP_CATABOLIC_PROCESS1	0.241330962	0.00422324	0.730684379	0.48619805	0.76412168	-5.95172055
WT after UVB irradiation VS WT before UVB irradiation	GO_CGMP_CATABOLIC_PROCESS2	0.26944581	-0.00018621	0.918868847	0.38471715	0.80652904	-5.57360181
homo after UVB irradiation VS homo before UVB irradiation	GO_CGMP_MEDIATED_SIGNALING	0.633461488	-0.00765968	4.446990727	0.00194462	0.02989252	-1.09432428
hetero after UVB irradiation VS hetero before UVB irradiation	GO_CGMP_MEDIATED_SIGNALING1	0.326168198	0.010448464	1.629071619	0.14265519	0.45781209	-4.99911972
WT after UVB irradiation VS WT before UVB irradiation	GO_CGMP_MEDIATED_SIGNALING2	0.409617539	0.009133915	1.963364505	0.08477799	0.57485421	-4.35449131
homo after UVB irradiation VS homo before UVB irradiation	GO_CGMP_METABOLIC_PROCESS	0.420402058	-0.00428418	2.881306641	0.01962907	0.09663029	-3.41734572
hetero after UVB irradiation VS hetero before UVB irradiation	GO_CGMP_METABOLIC_PROCESS1	-0.05364827	0.032529615	-0.22265117	0.8294889	0.93468067	-6.20468767
WT after UVB irradiation VS WT before UVB irradiation	GO_CGMP_METABOLIC_PROCESS2	-0.02765342	-0.01160953	-0.11114631	0.91420589	0.97932969	-5.9895536
homo after UVB irradiation VS homo before UVB irradiation	GO_CHANGES_TO_DNA_METHYLATION_INVOLVED_IN_EMBRYO_DEVELOPMENT	-0.05110467	-0.04904351	-0.21741359	0.83311761	0.90692805	-6.52498949
hetero after UVB irradiation VS hetero before UVB irradiation	GO_CHANGES_TO_DNA_METHYLATION_INVOLVED_IN_EMBRYO_DEVELOPMENT1	-0.10482504	0.007965521	-0.41820403	0.68699997	0.8742128	-6.13771752
WT after UVB irradiation VS WT before UVB irradiation	GO_CHANGES_TO_DNA_METHYLATION_INVOLVED_IN_EMBRYO_DEVELOPMENT2	-0.03022327	-0.04099906	-0.13279499	0.89759619	0.97409039	-5.98677254
homo after UVB irradiation VS homo before UVB irradiation	GO_CHANNEL_INHIBITOR_ACTIVITY	0.444312668	-0.0560766	2.964291067	0.01723983	0.08974236	-3.28856094
hetero after UVB irradiation VS hetero before UVB irradiation	GO_CHANNEL_INHIBITOR_ACTIVITY1	0.168303568	-0.00560365	1.213742603	0.26008626	0.58645458	-5.50180218
WT after UVB irradiation VS WT before UVB irradiation	GO_CHANNEL_INHIBITOR_ACTIVITY2	0.159084063	-0.02314332	0.742396694	0.47882623	0.84989641	-5.71529328
homo after UVB irradiation VS homo before UVB irradiation	GO_CHANNEL_REGULATOR_ACTIVITY	0.153851361	0.004514144	1.122039941	0.29314664	0.48792748	-5.90834223
hetero after UVB irradiation VS hetero before UVB irradiation	GO_CHANNEL_REGULATOR_ACTIVITY1	-0.09299718	0.019257452	-0.61694063	0.55473514	0.80503221	-6.03010287
WT after UVB irradiation VS WT before UVB irradiation	GO_CHANNEL_REGULATOR_ACTIVITY2	-0.08342607	-0.00199554	-0.46382444	0.65498685	0.91006679	-5.8841231
homo after UVB irradiation VS homo before UVB irradiation	GO_CHAPERONE_BINDING	-0.17019938	-0.01562204	-1.31587605	0.22327613	0.41417456	-5.68920814
hetero after UVB irradiation VS hetero before UVB irradiation	GO_CHAPERONE_BINDING1	-0.0312899	-0.0282758	-0.19112925	0.8532734	0.943838	-6.21172105
WT after UVB irradiation VS WT before UVB irradiation	GO_CHAPERONE_BINDING2	-0.03194493	-0.0056172	-0.17213347	0.86755688	0.96790654	-5.98046268
homo after UVB irradiation VS homo before UVB irradiation	GO_CHAPERONE_COFACTOR_DEPENDENT_PROTEIN_REFOLDING	-0.44741064	0.00962891	-2.88430179	0.01953715	0.09638803	-3.41269443
hetero after UVB irradiation VS hetero before UVB irradiation	GO_CHAPERONE_COFACTOR_DEPENDENT_PROTEIN_REFOLDING1	-0.31421195	-0.00628537	-1.7081635	0.12669401	0.43339219	-4.89485852
WT after UVB irradiation VS WT before UVB irradiation	GO_CHAPERONE_COFACTOR_DEPENDENT_PROTEIN_REFOLDING2	-0.31169289	-0.01484762	-1.42035094	0.19284215	0.69334561	-5.05244832
homo after UVB irradiation VS homo before UVB irradiation	GO_CHAPERONE_COMPLEX	-0.64821853	0.032423666	-4.09261496	0.0031878	0.03724783	-1.59326702
hetero after UVB irradiation VS hetero before UVB irradiation	GO_CHAPERONE_COMPLEX1	-0.37712434	-0.01862064	-1.6811675	0.13194586	0.44121703	-4.93069395
WT after UVB irradiation VS WT before UVB irradiation	GO_CHAPERONE_COMPLEX2	-0.26822689	-0.007665	-1.0226927	0.33603132	0.77905119	-5.47902019
homo after UVB irradiation VS homo before UVB irradiation	GO_CHAPERONE_MEDIATED_AUTOPHAGY	0.065539951	0.001493752	0.307113821	0.76629513	0.86489651	-6.49938925
hetero after UVB irradiation VS hetero before UVB irradiation	GO_CHAPERONE_MEDIATED_AUTOPHAGY1	0.204868169	0.046559029	1.337300246	0.21856373	0.54565069	-5.36135238
WT after UVB irradiation VS WT before UVB irradiation	GO_CHAPERONE_MEDIATED_AUTOPHAGY2	0.030672694	-0.01872417	0.155966281	0.87987772	0.9693507	-5.98325172
homo after UVB irradiation VS homo before UVB irradiation	GO_CHAPERONE_MEDIATED_PROTEIN_COMPLEX_ASSEMBLY	-0.64276681	0.017484535	-3.53536981	0.00719098	0.0559986	-2.4135682
hetero after UVB irradiation VS hetero before UVB irradiation	GO_CHAPERONE_MEDIATED_PROTEIN_COMPLEX_ASSEMBLY1	-0.29154732	-0.07061745	-2.09604189	0.07001117	0.34312588	-4.35752282
WT after UVB irradiation VS WT before UVB irradiation	GO_CHAPERONE_MEDIATED_PROTEIN_COMPLEX_ASSEMBLY2	-0.20791702	-0.01165098	-0.7187383	0.49251766	0.85303943	-5.73233684
homo after UVB irradiation VS homo before UVB irradiation	GO_CHAPERONE_MEDIATED_PROTEIN_FOLDING	-0.32124334	0.015404614	-2.09455142	0.06815942	0.1993866	-4.62606969
hetero after UVB irradiation VS hetero before UVB irradiation	GO_CHAPERONE_MEDIATED_PROTEIN_FOLDING1	-0.27113321	-0.04040743	-1.77213374	0.1150181	0.41818194	-4.80898331
WT after UVB irradiation VS WT before UVB irradiation	GO_CHAPERONE_MEDIATED_PROTEIN_FOLDING2	-0.14602051	-0.04128989	-0.73165001	0.48501456	0.85146975	-5.72309461
homo after UVB irradiation VS homo before UVB irradiation	GO_CHAPERONE_MEDIATED_PROTEIN_TRANSPORT	-0.21995286	0.019930627	-1.41149796	0.19434254	0.38132068	-5.57274119
hetero after UVB irradiation VS hetero before UVB irradiation	GO_CHAPERONE_MEDIATED_PROTEIN_TRANSPORT1	0.117562691	-0.02912272	0.53765054	0.60571513	0.83497629	-6.07764319
WT after UVB irradiation VS WT before UVB irradiation	GO_CHAPERONE_MEDIATED_PROTEIN_TRANSPORT2	0.210715288	-0.04485926	0.927337159	0.38056089	0.80376943	-5.5661852
homo after UVB irradiation VS homo before UVB irradiation	GO_CHAPERONIN_CONTAINING_T_COMPLEX	-0.6699911	0.019323667	-4.14968695	0.00294024	0.03577009	-1.51164609
hetero after UVB irradiation VS hetero before UVB irradiation	GO_CHAPERONIN_CONTAINING_T_COMPLEX1	-0.63649047	-0.02915581	-2.41614234	0.04265667	0.27740933	-3.8941403
WT after UVB irradiation VS WT before UVB irradiation	GO_CHAPERONIN_CONTAINING_T_COMPLEX2	-0.3548386	-0.03385609	-1.02814649	0.33360984	0.77844475	-5.4738376
homo after UVB irradiation VS homo before UVB irradiation	GO_CHECKPOINT_CLAMP_COMPLEX	-0.32911958	0.066201889	-1.75950752	0.11504603	0.27316723	-5.11120959
hetero after UVB irradiation VS hetero before UVB irradiation	GO_CHECKPOINT_CLAMP_COMPLEX1	-0.20924653	-0.03096498	-0.73417644	0.4841834	0.76290553	-5.94913053
WT after UVB irradiation VS WT before UVB irradiation	GO_CHECKPOINT_CLAMP_COMPLEX2	-0.08791408	-0.03536275	-0.34596175	0.73818827	0.93478445	-5.93340264
homo after UVB irradiation VS homo before UVB irradiation	GO_CHEMICAL_HOMEOSTASIS	0.234992803	-0.00891188	1.857206358	0.09889438	0.24883069	-4.97305912
hetero after UVB irradiation VS hetero before UVB irradiation	GO_CHEMICAL_HOMEOSTASIS_WITHIN_A_TISSUE	0.613364187	-0.03373479	3.864411416	0.00442492	0.04298781	-1.92426238
WT after UVB irradiation VS WT before UVB irradiation	GO_CHEMICAL_HOMEOSTASIS_WITHIN_A_TISSUE1	0.740585395	0.039480103	4.426574286	0.00230964	0.10650321	-1.09233645
homo after UVB irradiation VS homo before UVB irradiation	GO_CHEMICAL_HOMEOSTASIS_WITHIN_A_TISSUE2	0.262810388	-0.0185955	1.208701345	0.26088573	0.74093355	-5.29110186
hetero after UVB irradiation VS hetero before UVB irradiation	GO_CHEMICAL_HOMEOSTASIS1	0.112701936	0.012422147	0.845075056	0.42306791	0.7249295	-5.86143292
WT after UVB irradiation VS WT before UVB irradiation	GO_CHEMICAL_HOMEOSTASIS2	0.094426663	-0.00276724	0.558694979	0.59148006	0.89248172	-5.8346484
homo after UVB irradiation VS homo before UVB irradiation	GO_CHEMICAL_SYNAPTIC_TRANSMISSION_POSTSYNAPTIC	0.242447931	-0.02541532	1.924549158	0.08903513	0.23337324	-4.87610463
hetero after UVB irradiation VS hetero before UVB irradiation	GO_CHEMICAL_SYNAPTIC_TRANSMISSION_POSTSYNAPTIC1	-0.06633626	0.025699587	-0.44406288	0.66897905	0.86455201	-6.125916
WT after UVB irradiation VS WT before UVB irradiation	GO_CHEMICAL_SYNAPTIC_TRANSMISSION_POSTSYNAPTIC2	-0.03845228	0.000217656	-0.19931449	0.84693065	0.96518307	-5.97515944
homo after UVB irradiation VS homo before UVB irradiation	GO_CHEMOATTRACTANT_ACTIVITY	0.314608556	0.017450948	2.070237159	0.07082481	0.20425282	-4.66224107
hetero after UVB irradiation VS hetero before UVB irradiation	GO_CHEMOATTRACTANT_ACTIVITY1	0.281085081	0.003907826	1.351635106	0.21413476	0.54114961	-5.3445073
WT after UVB irradiation VS WT before UVB irradiation	GO_CHEMOATTRACTANT_ACTIVITY2	0.100600108	0.016286091	0.586040983	0.57381553	0.88547483	-5.81881235
homo after UVB irradiation VS homo before UVB irradiation	GO_CHEMOKINE_ACTIVITY	0.309052814	0.007242927	1.474397581	0.1771374	0.35867408	-5.49344306
hetero after UVB irradiation VS hetero before UVB irradiation	GO_CHEMOKINE_ACTIVITY1	0.276467198	0.019995096	1.82084134	0.10681499	0.40428853	-4.74275495
WT after UVB irradiation VS WT before UVB irradiation	GO_CHEMOKINE_ACTIVITY2	0.165195234	-0.00823046	0.576075325	0.58021837	0.8883701	-5.82466379
homo after UVB irradiation VS homo before UVB irradiation	GO_CHEMOKINE_BINDING	0.528281352	-0.04868971	2.890982319	0.01933371	0.09580645	-3.40232065
hetero after UVB irradiation VS hetero before UVB irradiation	GO_CHEMOKINE_BINDING1	0.283257355	0.010206854	1.230679712	0.25402967	0.58163375	-5.48307347
WT after UVB irradiation VS WT before UVB irradiation	GO_CHEMOKINE_BINDING2	0.242107189	-0.00084221	0.836013802	0.42712562	0.82735309	-5.64324695
homo after UVB irradiation VS homo before UVB irradiation	GO_CHEMOKINE_BIOSYNTHETIC_PROCESS	0.286227333	-0.01129792	1.427422137	0.1898539	0.37555951	-5.55285935
hetero after UVB irradiation VS hetero before UVB irradiation	GO_CHEMOKINE_BIOSYNTHETIC_PROCESS1	0.427519073	0.055816069	2.155730661	0.06384079	0.33123583	-4.27203702
WT after UVB irradiation VS WT before UVB irradiation	GO_CHEMOKINE_BIOSYNTHETIC_PROCESS2	0.273617012	0.031532254	1.130530334	0.2906232	0.75624915	-5.37279463
homo after UVB irradiation VS homo before UVB irradiation	GO_CHEMOKINE_C_C_MOTIF_LIGAND_5_PRODUCTION	0.200778077	-0.08238601	0.827408162	0.43107063	0.61521366	-6.19006774
hetero after UVB irradiation VS hetero before UVB irradiation	GO_CHEMOKINE_C_C_MOTIF_LIGAND_5_PRODUCTION1	0.414471162	-0.06564941	2.289711209	0.0518816	0.30247526	-4.07842693
WT after UVB irradiation VS WT before UVB irradiation	GO_CHEMOKINE_C_C_MOTIF_LIGAND_5_PRODUCTION2	-0.00690412	-0.02460051	-0.02385707	0.98154405	0.99578204	-5.99576636
homo after UVB irradiation VS homo before UVB irradiation	GO_CHEMOKINE_C_X_C_MOTIF_LIGAND_1_PRODUCTION	0.029774234	0.021278898	0.14208587	0.89038907	0.94024173	-6.53979058
hetero after UVB irradiation VS hetero before UVB irradiation	GO_CHEMOKINE_C_X_C_MOTIF_LIGAND_1_PRODUCTION1	0.986457787	0.035268535	4.738447965	0.00154356	0.09569098	-0.70928238
WT after UVB irradiation VS WT before UVB irradiation	GO_CHEMOKINE_C_X_C_MOTIF_LIGAND_1_PRODUCTION2	0.4848599	-0.01180232	1.541450168	0.16132441	0.66517583	-4.90597509
homo after UVB irradiation VS homo before UVB irradiation	GO_CHEMOKINE_C_X_C_MOTIF_LIGAND_2_PRODUCTION	0.294372946	-0.00150921	1.860112625	0.09844831	0.24842711	-4.96890262
hetero after UVB irradiation VS hetero before UVB irradiation	GO_CHEMOKINE_C_X_C_MOTIF_LIGAND_2_PRODUCTION1	0.341950154	0.011022799	1.709300275	0.12647719	0.43339219	-4.89334409
WT after UVB irradiation VS WT before UVB irradiation	GO_CHEMOKINE_C_X_C_MOTIF_LIGAND_2_PRODUCTION2	0.339483695	0.118454805	1.409103958	0.19602828	0.69404735	-5.06571293
homo after UVB irradiation VS homo before UVB irradiation	GO_CHEMOKINE_PRODUCTION	0.596086567	-0.01988108	4.201682056	0.0027326	0.03454156	-1.43770204
hetero after UVB irradiation VS hetero before UVB irradiation	GO_CHEMOKINE_PRODUCTION1	0.523441518	0.051012947	3.153664877	0.01384799	0.17734093	-2.81719556
WT after UVB irradiation VS WT before UVB irradiation	GO_CHEMOKINE_PRODUCTION2	0.399433179	0.041475625	1.845359163	0.10175372	0.60239077	-4.51392259
homo after UVB irradiation VS homo before UVB irradiation	GO_CHEMOKINE_RECEPTOR_BINDING	0.427228879	-0.00758218	2.126376873	0.06481783	0.19300497	-4.57854126
hetero after UVB irradiation VS hetero before UVB irradiation	GO_CHEMOKINE_RECEPTOR_BINDING1	0.441550199	0.022112529	3.273483073	0.01159053	0.16817096	-2.64537815
WT after UVB irradiation VS WT before UVB irradiation	GO_CHEMOKINE_RECEPTOR_BINDING2	0.281300881	0.00348386	1.105096306	0.30086794	0.76273978	-5.39854514
homo after UVB irradiation VS homo before UVB irradiation	GO_CHEMOKINE_SECRETION	0.831917376	-0.06990299	5.330102687	0.00061451	0.01780829	0.063878562
hetero after UVB irradiation VS hetero before UVB irradiation	GO_CHEMOKINE_SECRETION1	0.786188242	0.038246007	5.225950098	0.00084568	0.08366654	-0.14388356
WT after UVB irradiation VS WT before UVB irradiation	GO_CHEMOKINE_SECRETION2	0.554154675	0.048377118	3.181189803	0.01277202	0.35163556	-2.64038435
homo after UVB irradiation VS homo before UVB irradiation	GO_CHEMOREPELLENT_ACTIVITY	-0.16844887	-0.05137977	-1.19915644	0.26346599	0.45859491	-5.82406072
hetero after UVB irradiation VS hetero before UVB irradiation	GO_CHEMOREPELLENT_ACTIVITY1	-0.28961934	-0.03634517	-2.12161199	0.06729912	0.33776777	-4.32096839
WT after UVB irradiation VS WT before UVB irradiation	GO_CHEMOREPELLENT_ACTIVITY2	-0.09101908	0.014506803	-0.50388166	0.62776488	0.90344513	-5.86427998
homo after UVB irradiation VS homo before UVB irradiation	GO_CHEMOREPULSION_OF_AXON	-0.41861325	0.020830315	-1.7218351	0.12190856	0.2839648	-5.16360635
hetero after UVB irradiation VS hetero before UVB irradiation	GO_CHEMOREPULSION_OF_AXON1	-0.95000574	-0.08257488	-5.55086406	0.00057685	0.08125048	0.210688272
WT after UVB irradiation VS WT before UVB irradiation	GO_CHEMOREPULSION_OF_AXON2	-0.84214718	-0.07758361	-4.65814462	0.00157554	0.2011505	-0.75530871
homo after UVB irradiation VS homo before UVB irradiation	GO_CHEMOSENSORY_BEHAVIOR	0.367163526	-0.02197972	2.220935483	0.05580743	0.17552222	-4.43621754
hetero after UVB irradiation VS hetero before UVB irradiation	GO_CHEMOSENSORY_BEHAVIOR1	0.224295426	0.040506518	1.346901737	0.21558856	0.54296379	-5.35008162
WT after UVB irradiation VS WT before UVB irradiation	GO_CHEMOSENSORY_BEHAVIOR2	0.065233491	0.083615006	0.399359393	0.69995442	0.92425542	-5.91278039
homo after UVB irradiation VS homo before UVB irradiation	GO_CHIASMA_ASSEMBLY	-0.87842387	-0.01260769	-4.86714943	0.00110841	0.02245522	-0.52789427
hetero after UVB irradiation VS hetero before UVB irradiation	GO_CHIASMA_ASSEMBLY1	-0.26421098	0.040641871	-0.91144404	0.38918373	0.69940265	-5.80408637
WT after UVB irradiation VS WT before UVB irradiation	GO_CHIASMA_ASSEMBLY2	-0.24681577	0.005318054	-1.46073168	0.18177421	0.6850334	-5.00433351
homo after UVB irradiation VS homo before UVB irradiation	GO_CHITIN_BINDING	0.395471308	-0.01947587	2.653011537	0.02812267	0.11867493	-3.7719987
hetero after UVB irradiation VS hetero before UVB irradiation	GO_CHITIN_BINDING1	0.537859275	-0.00146993	3.20988386	0.01273601	0.17315837	-2.73639443
WT after UVB irradiation VS WT before UVB irradiation	GO_CHITIN_BINDING2	0.238338181	-0.0285002	1.080120075	0.31120561	0.76835557	-5.42342047
homo after UVB irradiation VS homo before UVB irradiation	GO_CHITIN_METABOLIC_PROCESS	0.646300518	-0.0004568	3.805765039	0.00481972	0.04526889	-2.01047681
hetero after UVB irradiation VS hetero before UVB irradiation	GO_CHITIN_METABOLIC_PROCESS1	0.590655605	0.074845391	3.870039974	0.00491321	0.12780541	-1.81658608

WT after UVB irradiation VS WT before UVB irradiation	GO_CHITIN_METABOLIC_PROCESS2	0.305694531	-0.02218559	1.610894222	0.14541282	0.65365874	-4.81920708
homo after UVB irradiation VS homo before UVB irradiation	GO_CHLORIDE_CHANNEL_COMPLEX	-0.1419741	0.062008085	-0.96929194	0.35969978	0.55111472	-6.06282891
hetero after UVB irradiation VS hetero before UVB irradiation	GO_CHLORIDE_CHANNEL_COMPLEX1	-0.41405004	0.02256901	-2.90188781	0.02023138	0.20610175	-3.18240661
WT after UVB irradiation VS WT before UVB irradiation	GO_CHLORIDE_CHANNEL_COMPLEX2	-0.36286844	0.001194685	-2.19448472	0.05910419	0.52638053	-4.03446182
homo after UVB irradiation VS homo before UVB irradiation	GO_CHLORIDE_CHANNEL_INHIBITOR_ACTIVITY	0.206603154	-0.01778165	0.849949362	0.41911209	0.6045784	-6.17096538
hetero after UVB irradiation VS hetero before UVB irradiation	GO_CHLORIDE_CHANNEL_INHIBITOR_ACTIVITY1	0.276300407	-0.04619727	1.480691674	0.17765732	0.50135768	-5.18816897
WT after UVB irradiation VS WT before UVB irradiation	GO_CHLORIDE_CHANNEL_INHIBITOR_ACTIVITY2	0.241544268	-0.04085152	1.409422768	0.19593734	0.69389529	-5.06533776
homo after UVB irradiation VS homo before UVB irradiation	GO_CHLORIDE_CHANNEL_REGULATOR_ACTIVITY	-0.18635974	0.007746598	-0.9210216	0.38293942	0.572837	-6.10795675
hetero after UVB irradiation VS hetero before UVB irradiation	GO_CHLORIDE_CHANNEL_REGULATOR_ACTIVITY1	-0.12260198	-0.02506157	-0.57151757	0.58363205	0.82191128	-6.05806934
WT after UVB irradiation VS WT before UVB irradiation	GO_CHLORIDE_CHANNEL_REGULATOR_ACTIVITY2	-0.24809968	-0.00019075	-1.41826139	0.19343064	0.69334561	-5.05491729
homo after UVB irradiation VS homo before UVB irradiation	GO_CHLORIDE_ION_BINDING	0.304830559	-0.01119705	1.954463287	0.08496269	0.22689005	-4.83262694
hetero after UVB irradiation VS hetero before UVB irradiation	GO_CHLORIDE_ION_BINDING1	0.276680682	0.002929238	1.452030711	0.18524686	0.51022981	-5.2235857
WT after UVB irradiation VS WT before UVB irradiation	GO_CHLORIDE_ION_BINDING2	-0.06987018	-0.07029209	-0.31505044	0.76068895	0.93919119	-5.94403147
homo after UVB irradiation VS homo before UVB irradiation	GO_CHLORIDE_ION_HOMEOSTASIS	0.667044936	-0.00219309	3.823039244	0.00469967	0.04462871	-1.98503529
hetero after UVB irradiation VS hetero before UVB irradiation	GO_CHLORIDE_ION_HOMEOSTASIS1	-0.25891566	0.001897782	-1.26292099	0.24282588	0.57110912	-5.44695026
WT after UVB irradiation VS WT before UVB irradiation	GO_CHLORIDE_ION_HOMEOSTASIS2	0.026502253	-0.01684993	0.114719363	0.91146118	0.97857624	-5.98912848
homo after UVB irradiation VS homo before UVB irradiation	GO_CHLORIDE_TRANSMEMBRANE_TRANSPORTER_ACTIVITY	0.001389296	0.032369346	0.009601566	0.99256518	0.99607971	-6.55080612
hetero after UVB irradiation VS hetero before UVB irradiation	GO_CHLORIDE_TRANSMEMBRANE_TRANSPORTER_ACTIVITY1	-0.29514303	-0.00899441	-2.19718689	0.05987411	0.32209347	-4.21236193
WT after UVB irradiation VS WT before UVB irradiation	GO_CHLORIDE_TRANSMEMBRANE_TRANSPORTER_ACTIVITY2	-0.36005671	0.031071004	-2.43603651	0.04046488	0.48921067	-3.69325595
homo after UVB irradiation VS homo before UVB irradiation	GO_CHLORIDE_TRANSPORT	-0.00099672	0.019955616	-0.00741704	0.99425669	0.99675584	-6.55082653
hetero after UVB irradiation VS hetero before UVB irradiation	GO_CHLORIDE_TRANSPORT1	-0.26730977	-0.0201965	-1.98977343	0.08247401	0.36807092	-4.50821808
WT after UVB irradiation VS WT before UVB irradiation	GO_CHLORIDE_TRANSPORT2	-0.29675913	0.031742891	-2.03216832	0.07617287	0.55880099	-4.26015327
homo after UVB irradiation VS homo before UVB irradiation	GO_CHOLESTEROL_BINDING	0.464226649	0.002905604	3.411765604	0.0086617	0.06184457	-2.600608
hetero after UVB irradiation VS hetero before UVB irradiation	GO_CHOLESTEROL_BINDING1	0.211939567	0.033383409	1.370730584	0.20835561	0.53337752	-5.3218998
WT after UVB irradiation VS WT before UVB irradiation	GO_CHOLESTEROL_BINDING2	0.309964643	0.025340102	1.956749946	0.085653	0.57584638	-4.363511
homo after UVB irradiation VS homo before UVB irradiation	GO_CHOLESTEROL_CATABOLIC_PROCESS	0.284484532	0.053426525	1.102013095	0.30127729	0.49594615	-5.92956392
hetero after UVB irradiation VS hetero before UVB irradiation	GO_CHOLESTEROL_CATABOLIC_PROCESS1	0.105953078	0.041602649	0.373231388	0.71885003	0.89040133	-6.15662698
WT after UVB irradiation VS WT before UVB irradiation	GO_CHOLESTEROL_CATABOLIC_PROCESS2	0.288375083	0.04517106	1.105858584	0.30055677	0.76247487	-5.39777948
homo after UVB irradiation VS homo before UVB irradiation	GO_CHOLESTEROL_EFFLUX	0.326243893	0.003476798	2.227525644	0.05522753	0.17458128	-4.42624206
hetero after UVB irradiation VS hetero before UVB irradiation	GO_CHOLESTEROL_EFFLUX1	0.100353693	0.017097256	0.596185758	0.56783437	0.81261967	-6.04312286
WT after UVB irradiation VS WT before UVB irradiation	GO_CHOLESTEROL_EFFLUX2	0.150181664	0.040701361	0.763334875	0.466918	0.84378018	-5.6998121
homo after UVB irradiation VS homo before UVB irradiation	GO_CHOLESTEROL_STORAGE	0.509041327	-0.01838278	4.0112517	0.00358011	0.03908552	-1.71044131
hetero after UVB irradiation VS hetero before UVB irradiation	GO_CHOLESTEROL_STORAGE1	0.299067845	0.025616713	1.997549169	0.08149321	0.36539464	-4.4972648
WT after UVB irradiation VS WT before UVB irradiation	GO_CHOLESTEROL_STORAGE2	0.236646815	-0.0358104	0.997272604	0.34749591	0.78529818	-5.50289974
homo after UVB irradiation VS homo before UVB irradiation	GO_CHOLINE_CATABOLIC_PROCESS	0.024138572	0.026069071	0.109847174	0.91513121	0.95335584	-6.54423931
hetero after UVB irradiation VS hetero before UVB irradiation	GO_CHOLINE_CATABOLIC_PROCESS1	0.181741147	0.10773466	0.951293526	0.36981261	0.6832296	-5.76798839
WT after UVB irradiation VS WT before UVB irradiation	GO_CHOLINE_CATABOLIC_PROCESS2	0.449660724	0.075141496	1.675821275	0.13184459	0.63791166	-4.73644979
homo after UVB irradiation VS homo before UVB irradiation	GO_CHOLINE_METABOLIC_PROCESS	-0.07778453	-0.02261772	-0.30357096	0.76889973	0.86648746	-6.50056325
hetero after UVB irradiation VS hetero before UVB irradiation	GO_CHOLINE_METABOLIC_PROCESS1	0.004750717	0.046410175	0.028803586	0.9777398	0.99241192	-6.23103421
WT after UVB irradiation VS WT before UVB irradiation	GO_CHOLINE_METABOLIC_PROCESS2	-0.00887927	-0.02172497	-0.05674481	0.9561237	0.98841297	-5.994368
homo after UVB irradiation VS homo before UVB irradiation	GO_CHOLINE_TRANSMEMBRANE_TRANSPORTER_ACTIVITY	-0.59903576	0.069523489	-2.26990122	0.05163826	0.16799509	-4.36194068
hetero after UVB irradiation VS hetero before UVB irradiation	GO_CHOLINE_TRANSMEMBRANE_TRANSPORTER_ACTIVITY1	-0.84771982	-0.03445076	-3.92360468	0.00455988	0.12742869	-1.74468814
WT after UVB irradiation VS WT before UVB irradiation	GO_CHOLINE_TRANSMEMBRANE_TRANSPORTER_ACTIVITY2	-0.86741942	0.053021031	-5.50269741	0.00054832	0.18842845	0.151038784
homo after UVB irradiation VS homo before UVB irradiation	GO_CHOLINE_TRANSPORT	-0.33534202	0.050803573	-1.2044249	0.261531	0.45695158	-5.81815838
hetero after UVB irradiation VS hetero before UVB irradiation	GO_CHOLINE_TRANSPORT1	-0.45757712	-0.02407291	-2.72348809	0.02656091	0.23166634	-3.44349767
WT after UVB irradiation VS WT before UVB irradiation	GO_CHOLINE_TRANSPORT2	-0.43196143	0.046940237	-2.52716655	0.03507942	0.47026746	-3.56368534
homo after UVB irradiation VS homo before UVB irradiation	GO_CHOLINERGIC_SYNAPSE	0.226196905	0.038036481	1.052142583	0.32229753	0.51585083	-5.98116867
hetero after UVB irradiation VS hetero before UVB irradiation	GO_CHOLINERGIC_SYNAPSE1	0.016610786	-0.04779646	0.085041121	0.93435682	0.9758206	-6.22756402
WT after UVB irradiation VS WT before UVB irradiation	GO_CHOLINERGIC_SYNAPSE2	-0.11933391	-0.03210566	-0.72554829	0.48855108	0.8517724	-5.72748008
homo after UVB irradiation VS homo before UVB irradiation	GO_CHONDROBLAST_DIFFERENTIATION	0.17678448	0.093757709	0.592802908	0.5690544	0.72724828	-6.36202931
hetero after UVB irradiation VS hetero before UVB irradiation	GO_CHONDROBLAST_DIFFERENTIATION1	-0.06818055	-0.05068179	-0.22551928	0.82733367	0.93421603	-6.20399518
WT after UVB irradiation VS WT before UVB irradiation	GO_CHONDROBLAST_DIFFERENTIATION2	-0.03853727	-0.08829168	-0.10332028	0.92022182	0.98043563	-5.99043789
homo after UVB irradiation VS homo before UVB irradiation	GO_CHONDROCYTE_DEVELOPMENT	0.28972053	-0.01284315	2.08343283	0.06936594	0.20134405	-4.64262581
homo after UVB irradiation VS homo before UVB irradiation	GO_CHONDROCYTE_DEVELOPMENT_INVOLVED_IN_ENDOCHONDRAL_BONE_MORPHOGENESIS	0.453017487	0.037360588	2.477470841	0.03714233	0.13816722	-4.04376891
hetero after UVB irradiation VS hetero before UVB irradiation	GO_CHONDROCYTE_DEVELOPMENT_INVOLVED_IN_ENDOCHONDRAL_BONE_MORPHOGENESIS1	0.463232465	-0.02156793	2.567655876	0.03375302	0.25357971	-3.67215882
WT after UVB irradiation VS WT before UVB irradiation	GO_CHONDROCYTE_DEVELOPMENT_INVOLVED_IN_ENDOCHONDRAL_BONE_MORPHOGENESIS2	0.46517363	-0.03711834	2.404946146	0.04248706	0.495137	-3.73739513
hetero after UVB irradiation VS hetero before UVB irradiation	GO_CHONDROCYTE_DEVELOPMENT1	0.197343569	-0.02599379	1.476397999	0.17877638	0.5027681	-5.19349885
WT after UVB irradiation VS WT before UVB irradiation	GO_CHONDROCYTE_DEVELOPMENT2	0.032487369	-0.01323869	0.22549823	0.82717879	0.95924444	-5.96932491
homo after UVB irradiation VS homo before UVB irradiation	GO_CHONDROCYTE_DIFFERENTIATION	0.225305599	-0.01379078	1.609790084	0.14461536	0.31681841	-5.31623372
homo after UVB irradiation VS homo before UVB irradiation	GO_CHONDROCYTE_DIFFERENTIATION_INVOLVED_IN_ENDOCHONDRAL_BONE_MORPHOGENESIS	0.163611387	0.039494754	0.933155621	0.37699658	0.56682002	-6.09678708
hetero after UVB irradiation VS hetero before UVB irradiation	GO_CHONDROCYTE_DIFFERENTIATION_INVOLVED_IN_ENDOCHONDRAL_BONE_MORPHOGENESIS1	0.187182074	-0.03126888	1.128147554	0.29254358	0.61668187	-5.5937269
WT after UVB irradiation VS WT before UVB irradiation	GO_CHONDROCYTE_DIFFERENTIATION_INVOLVED_IN_ENDOCHONDRAL_BONE_MORPHOGENESIS2	0.06880426	-0.00778719	0.416427121	0.6879163	0.91993413	-5.90559052
hetero after UVB irradiation VS hetero before UVB irradiation	GO_CHONDROCYTE_DIFFERENTIATION1	0.007505072	-0.0094145	0.045642946	0.96473418	0.98717061	-6.23035449
WT after UVB irradiation VS WT before UVB irradiation	GO_CHONDROCYTE_DIFFERENTIATION2	0.01108354	-0.03942119	0.071289921	0.94489631	0.98550369	-5.9933859
homo after UVB irradiation VS homo before UVB irradiation	GO_CHONDROCYTE_HYPERTROPHY	0.006208267	0.085330236	0.022514077	0.98256792	0.99139864	-6.55057854
hetero after UVB irradiation VS hetero before UVB irradiation	GO_CHONDROCYTE_HYPERTROPHY1	-0.18454013	0.048379684	-0.67279599	0.52038012	0.78682287	-5.99307825
WT after UVB irradiation VS WT before UVB irradiation	GO_CHONDROCYTE_HYPERTROPHY2	-0.63937197	-0.00612718	-3.35599012	0.00981718	0.3285081	-2.39971917
homo after UVB irradiation VS homo before UVB irradiation	GO_CHONDROCYTE_PROLIFERATION	0.146946676	-0.00656552	0.757578207	0.46959117	0.64868945	-6.24645984
hetero after UVB irradiation VS hetero before UVB irradiation	GO_CHONDROCYTE_PROLIFERATION1	-0.36311435	-0.01007542	-2.5292145	0.03581566	0.25995862	-3.72854694
WT after UVB irradiation VS WT before UVB irradiation	GO_CHONDROCYTE_PROLIFERATION2	-0.12468201	-0.04382694	-0.67108709	0.52084408	0.86304554	-5.76519379
homo after UVB irradiation VS homo before UVB irradiation	GO_CHONDROITIN_SULFATE_BINDING	0.470614031	0.007670107	2.31392435	0.04815401	0.16094253	-4.29487051
hetero after UVB irradiation VS hetero before UVB irradiation	GO_CHONDROITIN_SULFATE_BINDING1	0.065705182	0.01379082	0.195668421	0.84983789	0.94258929	-6.21077359
WT after UVB irradiation VS WT before UVB irradiation	GO_CHONDROITIN_SULFATE_BINDING2	0.244360704	-0.05968623	0.680154693	0.51537741	0.86034214	-5.7590946
homo after UVB irradiation VS homo before UVB irradiation	GO_CHONDROITIN_SULFATE_BIOSYNTHETIC_PROCESS	0.449305712	0.003348363	2.643710304	0.02853946	0.11965125	-3.78643385
hetero after UVB irradiation VS hetero before UVB irradiation	GO_CHONDROITIN_SULFATE_BIOSYNTHETIC_PROCESS1	0.248376798	0.06297901	1.076036131	0.31385627	0.63480958	-5.64735599
WT after UVB irradiation VS WT before UVB irradiation	GO_CHONDROITIN_SULFATE_BIOSYNTHETIC_PROCESS2	0.326802208	-0.04478581	1.31086271	0.22585654	0.71906685	-5.17890041
homo after UVB irradiation VS homo before UVB irradiation	GO_CHONDROITIN_SULFATE_CATABOLIC_PROCESS	0.285971323	0.004634642	1.91173606	0.09083591	0.23644544	-4.89465247
hetero after UVB irradiation VS hetero before UVB irradiation	GO_CHONDROITIN_SULFATE_CATABOLIC_PROCESS1	0.026563752	-0.04453411	0.122703902	0.90542374	0.96537265	-6.2233271
WT after UVB irradiation VS WT before UVB irradiation	GO_CHONDROITIN_SULFATE_CATABOLIC_PROCESS2	0.065183276	-0.00556222	0.351293387	0.73433353	0.93441182	-5.93147186
homo after UVB irradiation VS homo before UVB irradiation	GO_CHONDROITIN_SULFATE_PROTEOGLYCAN_BIOSYNTHETIC_PROCESS	0.358939029	-0.00387807	2.364199041	0.04446003	0.15313139	-4.21797719
hetero after UVB irradiation VS hetero before UVB irradiation	GO_CHONDROITIN_SULFATE_PROTEOGLYCAN_BIOSYNTHETIC_PROCESS1	0.235182667	0.051385792	1.188976469	0.26915763	0.59622699	-5.52887356
WT after UVB irradiation VS WT before UVB irradiation	GO_CHONDROITIN_SULFATE_PROTEOGLYCAN_BIOSYNTHETIC_PROCESS2	0.272035987	-0.03736458	1.092141068	0.30619564	0.76570091	-5.41149953
homo after UVB irradiation VS homo before UVB irradiation	GO_CHONDROITIN_SULFATE_PROTEOGLYCAN_METABOLIC_PROCESS	0.329724727	-0.01192932	2.249676681	0.05332159	0.1710737	-4.39266303
hetero after UVB irradiation VS hetero before UVB irradiation	GO_CHONDROITIN_SULFATE_PROTEOGLYCAN_METABOLIC_PROCESS1	0.169006528	0.009800892	0.903502164	0.39313161	0.70256648	-5.81113327
WT after UVB irradiation VS WT before UVB irradiation	GO_CHONDROITIN_SULFATE_PROTEOGLYCAN_METABOLIC_PROCESS2	0.182838431	-0.02726427	0.77716158	0.45916266	0.8390123	-5.68938677
homo after UVB irradiation VS homo before UVB irradiation	GO_CHONDROITIN_SULFOTRANSFERASE_ACTIVITY	0.565735281	-0.07407892	2.022943593	0.07630187	0.21221009	-4.73222807
hetero after UVB irradiation VS hetero before UVB irradiation	GO_CHONDROITIN_SULFOTRANSFERASE_ACTIVITY1	0.357863273	0.03800364	1.151405897	0.28341425	0.60891973	-5.56920992
WT after UVB irradiation VS WT before UVB irradiation	GO_CHONDROITIN_SULFOTRANSFERASE_ACTIVITY2	0.306554782	0.021818555	0.739296122	0.48060636	0.8504347	-5.71755419
homo after UVB irradiation VS homo before UVB irradiation	GO_CHORIO_ALLANTOIC_FUSION	-0.48780305	0.035999991	-3.58963946	0.00663091	0.05379154	-2.33196758
hetero after UVB irradiation VS hetero before UVB irradiation	GO_CHORIO_ALLANTOIC_FUSION1	-0.24022154	-0.05128602	-1.46557231	0.1816258	0.50686486	-5.20689959
WT after UVB irradiation VS WT before UVB irradiation	GO_CHORIO_ALLANTOIC_FUSION2	-0.37958789	0.007994072	-1.69916695	0.12725798	0.63444123	-4.70633483
homo after UVB irradiation VS homo before UVB irradiation	GO_CHORION_DEVELOPMENT	-0.05009781	0.017325551	-0.1875984	0.85568115	0.92022203	-6.53158348
hetero after UVB irradiation VS hetero before UVB irradiation	GO_CHORION_DEVELOPMENT1	0.508059416	-0.00962703	2.908995874	0.02001427	0.20537595	-3.1720343
WT after UVB irradiation VS WT before UVB irradiation	GO_CHORION_DEVELOPMENT2	-0.07791503	0.00563325	-0.38551991	0.70978237	0.92793255	-5.91839841
homo after UVB irradiation VS homo before UVB irradiation	GO_CHORIONIC_TROPHOBLAST_CELL_DIFFERENTIATION	-0.1351229	0.046739562	-0.49440787	0.63379349	0.77443824	-6.41867282
hetero after UVB irradiation VS hetero before UVB irradiation	GO_CHORIONIC_TROPHOBLAST_CELL_DIFFERENTIATION1	0.424905484	-0.02825251	2.411559301	0.0429603	0.27767067	-3.90083984
WT after UVB irradiation VS WT before UVB irradiation	GO_CHORIONIC_TROPHOBLAST_CELL_DIFFERENTIATION2	-0.08801542	0.039396815	-0.39026048	0.70640928	0.92678537	-5.91649544
homo after UVB irradiation VS homo before UVB irradiation	GO_CHORIONIC_TROPHOBLAST_CELL_PROLIFERATION	-0.22819655	0.08855067	-1.00426456	0.34353049	0.53605578	-6.02899392
hetero after UVB irradiation VS hetero before UVB irradiation	GO_CHORIONIC_TROPHOBLAST_CELL_PROLIFERATION1	0.037913352	0.153477263	0.146535066	0.88719095	0.95898643	-6.21985588
WT after UVB irradiation VS WT before UVB irradiation	GO_CHORIONIC_TROPHOBLAST_CELL_PROLIFERATION2	-0.29166001	0.184693621	-0.97526447	0.3576595	0.79138181	-5.52320127
homo after UVB irradiation VS homo before UVB irradiation	GO_CHROMAFFIN_GRANULE	0.217515005	-0.0358149	0.745579204	0.47643268	0.65432453	-6.25571733
homo after UVB irradiation VS homo before UVB irradiation	GO_CHROMAFFIN_GRANULE_MEMBRANE	0.224879401	-0.05831472	0.734954208	0.48254469	0.65983246	-6.26380705
hetero after UVB irradiation VS hetero before UVB irradiation	GO_CHROMAFFIN_GRANULE_MEMBRANE1	0.146665216	0.053923517	0.852587353	0.41913087	0.72166655	-5.85511991
WT after UVB irradiation VS WT before UVB irradiation	GO_CHROMAFFIN_GRANULE_MEMBRANE2	0.470011635	0.026585589	1.691784889	0.12869209	0.6349412	-4.71587707
hetero after UVB irradiation VS hetero before UVB irradiation	GO_CHROMAFFIN_GRANULE1	0.239604459	0.041239851	1.49370251	0.17430438	0.49691564	-5.17196713
WT after UVB irradiation VS WT before UVB irradiation	GO_CHROMAFFIN_GRANULE2	0.489098409	0.044353462	2.187087288	0.0597931	0.52638053	-4.04482692
homo after UVB irradiation VS homo before UVB irradiation	GO_CHROMATIN	-0.1773069	0.017809573	-1.24638702	0.24653319	0.44044676	-5.77051152
homo after UVB irradiation VS homo before UVB irradiation	GO_CHROMATIN_ASSEMBLY_OR_DISASSEMBLY	-0.41565787	-0.02190651	-2.7317217	0.02483493	0.11085573	-3.64975997
hetero after UVB irradiation VS hetero before UVB irradiation	GO_CHROMATIN_ASSEMBLY_OR_DISASSEMBLY1	-0.05333681	-0.02050025	-0.25782815	0.80316752	0.92371161	-6.19559146
WT after UVB irradiation VS WT before UVB irradiation	GO_CHROMATIN_ASSEMBLY_OR_DISASSEMBLY2	-0.089388	-0.00619437	-0.37532341	0.71706051	0.93082881	-5.92241564
homo after UVB irradiation VS homo before UVB irradiation	GO_CHROMATIN_BINDING	-0.30824426	-0.00139284	-2.08679482	0.06899894	0.20103008	-4.63762236
hetero after UVB irradiation VS hetero before UVB irradiation	GO_CHROMATIN_BINDING1	-0.10967689	-0.03089982	-0.74918264	0.47558802	0.75843259	-5.93787992
WT after UVB irradiation VS WT before UVB irradiation	GO_CHROMATIN_BINDING2	-0.16742273	0.000724267	-0.88456836	0.40188989	0.81601572	-5.60308272
homo after UVB irradiation VS homo before UVB irradiation	GO_CHROMATIN_DISASSEMBLY	-0.28491803	-0.0261119	-1.12062836	0.29371395	0.48843101	-5.90984722
hetero after UVB irradiation VS hetero before UVB irradiation	GO_CHROMATIN_DISASSEMBLY1	-0.03681979	-0.02121901	-0.19339706	0.85155657	0.94321939	-6.21125044
WT after UVB irradiation VS WT before UVB irradiation	GO_CHROMATIN_DISASSEMBLY2	-0.04720733	0.053647294	-0.17128468	0.86820282	0.96790654	-5.9806159
homo after UVB irradiation VS homo before UVB irradiation	GO_CHROMATIN_DNA_BINDING	-0.34287794	0.037996935	-2.09919139	0.06766198	0.19866451	-4.61915298
hetero after UVB irradiation VS hetero before UVB irradiation	GO_CHROMATIN_DNA_BINDING1	-0.36159241	-0.05457052	-2.23131318	0.05679281	0.31461576	-4.16307352
WT after UVB irradiation VS WT before UVB irradiation	GO_CHROMATIN_DNA_BINDING2	-0.42421421	-0.03240033	-2.35121644	0.04622394	0.50534441	-3.81355792
homo after UVB irradiation VS homo before UVB irradiation	GO_CHROMATIN_INSULATOR_SEQUENCE_BINDING	-0.1857698	-0.00757875	-0.81838489	0.43592302	0.61897896	-6.19759294
hetero after UVB irradiation VS hetero before UVB irradiation	GO_CHROMATIN_INSULATOR_SEQUENCE_BINDING1	-0.02491764	-0.08443416	-0.11123328	0.91422183	0.96745438	-6.2247797
WT after UVB irradiation VS WT before UVB irradiation	GO_CHROMATIN_INSULATOR_SEQUENCE_BINDING2	-0.43762917	-0.04919747	-1.81520793	0.10658387	0.61130963	-4.55410819
homo after UVB irradiation VS homo before UVB irradiation	GO_CHROMATIN_MAINTENANCE	-1.13052807	0.061658673	-7.97631259	3.55E-05	0.00847131	2.843723449
hetero after UVB irradiation VS hetero before UVB irradiation	GO_CHROMATIN_MAINTENANCE1	-0.34990185	-0.05285925	-1.1159948	0.29740723	0.62116646	-5.60639628
WT after UVB irradiation VS WT before UVB irradiation	GO_CHROMATIN_MAINTENANCE2	-0.82429462	-0.0815009	-3.55116909	0.00735055	0.31288444	-2.13569785
homo after UVB irradiation VS homo before UVB irradiation	GO_CHROMATIN_MEDIATED_MAINTENANCE_OF_TRANSCRIPTION	-0.24714218	0.008896917	-1.23367994	0.25099775	0.44552138	-5.78505816
hetero after UVB irradiation VS hetero before UVB irradiation	GO_CHROMATIN_MEDIATED_MAINTENANCE_OF_TRANSCRIPTION1	-0.01232185	-0.01149149	-0.05623242	0.95656107	0.9843874	-6.22976965
WT after UVB irradiation VS WT before UVB irradiation	GO_CHROMATIN_MEDIATED_MAINTENANCE_OF_TRANSCRIPTION2	-0.03640181	0.014095273	-0.13863647	0.89312319	0.97322079	-5.98593793
homo after UVB irradiation VS homo before UVB irradiation	GO_CHROMATIN_ORGANIZATION	-0.36415495	-0.00711181	-2.51635827	0.03491981	0.13384719	-3.98371407
homo after UVB irradiation VS homo before UVB irradiation	GO_CHROMATIN_ORGANIZATION_INVOLVED_IN_REGULATION_OF_TRANSCRIPTION	-0.35589064	-0.00078584	-1.97383382	0.08242144	0.22247908	-4.80434711
hetero after UVB irradiation VS hetero before UVB irradiation	GO_CHROMATIN_ORGANIZATION_INVOLVED_IN_REGULATION_OF_TRANSCRIPTION1	-0.11292503	-0.02010212	-0.57165613	0.58354263	0.82191128	-6.057987
WT after UVB irradiation VS WT before UVB irradiation	GO_CHROMATIN_ORGANIZATION_INVOLVED_IN_REGULATION_OF_TRANSCRIPTION2	-0.17258497	-0.0402162	-0.77983577	0.4576727	0.83880233	-5.68735202
hetero after UVB irradiation VS hetero before UVB irradiation	GO_CHROMATIN_ORGANIZATION1	-0.10872766	-0.03650564	-0.66779012	0.52340476	0.7882475	-5.99651316
WT after UVB irradiation VS WT before UVB irradiation	GO_CHROMATIN_ORGANIZATION2	-0.17823472	-0.00706388	-0.86140586	0.41379301	0.820663	-5.62247492
homo after UVB irradiation VS homo before UVB irradiation	GO_CHROMATIN_REMODELING	-0.45751302	-0.00662942	-3.32994092	0.00980732	0.06612115	-2.72526188
homo after UVB irradiation VS homo before UVB irradiation	GO_CHROMATIN_REMODELING_AT_CENTROMERE	-0.53227102	0.004021529	-2.0402499	0.07425167	0.20940535	-4.70667602
hetero after UVB irradiation VS hetero before UVB irradiation	GO_CHROMATIN_REMODELING_AT_CENTROMERE1	0.003964772	-0.04030033	0.012882746	0.99004262	0.99615986	-6.23139411
WT after UVB irradiation VS WT before UVB irradiation	GO_CHROMATIN_REMODELING_AT_CENTROMERE2	0.126224099	0.052790493	0.425314415	0.6816849	0.91716791	-5.9017329
hetero after UVB irradiation VS hetero before UVB irradiation	GO_CHROMATIN_REMODELING1	-0.14698814	-0.02984636	-0.74179735	0.47980563	0.76097951	-5.94344111
WT after UVB irradiation VS WT before UVB irradiation	GO_CHROMATIN_REMODELING2	-0.20955913	-0.02074431	-0.91464034	0.38680488	0.80885357	-5.57728483
homo after UVB irradiation VS homo before UVB irradiation	GO_CHROMATIN_SILENCING	-0.60868063	-0.02488381	-4.03722605	0.00344953	0.03854651	-1.67293185
homo after UVB irradiation VS homo before UVB irradiation	GO_CHROMATIN_SILENCING_AT_TELOMERE	-0.51923022	-0.01861745	-4.09065708	0.00319668	0.03726176	-1.59607547
hetero after UVB irradiation VS hetero before UVB irradiation	GO_CHROMATIN_SILENCING_AT_TELOMERE1	-0.33727352	-0.02766606	-1.38872814	0.20303325	0.52736467	-5.30041956
WT after UVB irradiation VS WT before UVB irradiation	GO_CHROMATIN_SILENCING_AT_TELOMERE2	-0.37504216	-0.03441328	-1.50890966	0.16930718	0.67524339	-4.94596399
homo after UVB irradiation VS homo before UVB irradiation	GO_CHROMATIN_SILENCING_COMPLEX	-0.81999571	-0.04600268	-5.25138083	0.00067789	0.01832165	-0.03435596
hetero after UVB irradiation VS hetero before UVB irradiation	GO_CHROMATIN_SILENCING_COMPLEX1	-0.48455772	-0.11077527	-2.14779068	0.06462962	0.33252442	-4.28343943
WT after UVB irradiation VS WT before UVB irradiation	GO_CHROMATIN_SILENCING_COMPLEX2	-0.49425643	-0.03783689	-1.59894598	0.1480447	0.65449241	-4.83426916
hetero after UVB irradiation VS hetero before UVB irradiation	GO_CHROMATIN_SILENCING1	-0.40438624	-0.01307725	-2.136148	0.06580372	0.33425119	-4.30014264
WT after UVB irradiation VS WT before UVB irradiation	GO_CHROMATIN_SILENCING2	-0.34166049	-0.05095952	-1.63865841	0.13946127	0.64878333	-4.78400285
hetero after UVB irradiation VS hetero before UVB irradiation	GO_CHROMATIN1	-0.03975141	0.006873292	-0.30551852	0.76791297	0.90934305	-6.181174
WT after UVB irradiation VS WT before UVB irradiation	GO_CHROMATIN2	-0.08492236	0.014450072	-0.54788582	0.59854403	0.89476186	-5.84071589
homo after UVB irradiation VS homo before UVB irradiation	GO_CHROMATOID_BODY	-0.26299241	-0.00206396	-1.3514556	0.21210571	0.40216357	-5.64647229

hetero after UVB irradiation VS hetero before UVB irradiation	GO_CHROMATOID_BODY1	0.131526186	-0.013303	0.623403332	0.5506927	0.80124226	-6.0259664
WT after UVB irradiation VS WT before UVB irradiation	GO_CHROMATOID_BODY2	-0.30077199	-0.05600643	-1.48315879	0.17587273	0.68033133	-4.97728939
homo after UVB irradiation VS homo before UVB irradiation	GO_CHROMO_SHADOW_DOMAIN_BINDING	-0.75544309	0.004281515	-3.28704499	0.01047053	0.06852046	-2.79085256
hetero after UVB irradiation VS hetero before UVB irradiation	GO_CHROMO_SHADOW_DOMAIN_BINDING1	-0.43037124	-0.03248837	-1.12378381	0.29428258	0.61784413	-5.59828738
WT after UVB irradiation VS WT before UVB irradiation	GO_CHROMO_SHADOW_DOMAIN_BINDING2	-0.34093981	-0.02035882	-1.04016014	0.32832322	0.77673377	-5.4623483
homo after UVB irradiation VS homo before UVB irradiation	GO_CHROMOCENTER	-0.59968231	0.016552721	-3.37630317	0.00913989	0.06371153	-2.65455529
hetero after UVB irradiation VS hetero before UVB irradiation	GO_CHROMOCENTER1	-0.11858934	0.031462054	-0.40062042	0.69937803	0.8813015	-6.14535619
WT after UVB irradiation VS WT before UVB irradiation	GO_CHROMOCENTER2	-0.13653704	-0.01473442	-0.46511696	0.65409957	0.90967367	-5.883507
homo after UVB irradiation VS homo before UVB irradiation	GO_CHROMOSOMAL_REGION	-0.54907819	-0.01965448	-3.68178471	0.00578318	0.05041635	-2.19419831
hetero after UVB irradiation VS hetero before UVB irradiation	GO_CHROMOSOMAL_REGION1	0.025276869	-0.00841524	0.105673759	0.91849066	0.96916444	-6.22543265
WT after UVB irradiation VS WT before UVB irradiation	GO_CHROMOSOMAL_REGION2	-0.08959159	0.010441835	-0.34392721	0.73966131	0.93499931	-5.93413188
homo after UVB irradiation VS homo before UVB irradiation	GO_CHROMOSOME_CENTROMERIC_REGION	-0.51842802	-0.02175729	-3.33313351	0.00975976	0.0659152	-2.72038664
hetero after UVB irradiation VS hetero before UVB irradiation	GO_CHROMOSOME_CENTROMERIC_REGION1	0.108351197	-0.00353838	0.436819037	0.67400487	0.86749613	-6.12928986
WT after UVB irradiation VS WT before UVB irradiation	GO_CHROMOSOME_CENTROMERIC_REGION2	-0.05025487	0.007215536	-0.18772537	0.85571073	0.9663792	-5.97751463
homo after UVB irradiation VS homo before UVB irradiation	GO_CHROMOSOME_CONDENSATION	-0.57550121	0.017715164	-3.59933857	0.00653577	0.05360305	-2.3174191
hetero after UVB irradiation VS hetero before UVB irradiation	GO_CHROMOSOME_CONDENSATION1	0.085573525	-0.03088832	0.292577279	0.77742678	0.91343573	-6.18532244
WT after UVB irradiation VS WT before UVB irradiation	GO_CHROMOSOME_CONDENSATION2	0.080888183	0.030949523	0.323880343	0.75423572	0.93752161	-5.94109393
homo after UVB irradiation VS homo before UVB irradiation	GO_CHROMOSOME_LOCALIZATION	-0.40455682	-0.00400686	-2.49940469	0.03587178	0.13542779	-4.00990901
homo after UVB irradiation VS homo before UVB irradiation	GO_CHROMOSOME_LOCALIZATION_TO_NUCLEAR_ENVELOPE_INVOLVED_IN_HOMOLOGOUS_CHROMOSOME_SEGREGATION	-0.63040749	0.075735816	-3.87347103	0.00436707	0.04273529	-1.91098489
hetero after UVB irradiation VS hetero before UVB irradiation	GO_CHROMOSOME_LOCALIZATION_TO_NUCLEAR_ENVELOPE_INVOLVED_IN_HOMOLOGOUS_CHROMOSOME_SEGREGATION1	-0.28200634	0.045616478	-1.10254151	0.30286671	0.62486932	-5.62030696
WT after UVB irradiation VS WT before UVB irradiation	GO_CHROMOSOME_LOCALIZATION_TO_NUCLEAR_ENVELOPE_INVOLVED_IN_HOMOLOGOUS_CHROMOSOME_SEGREGATION2	-0.38129719	0.110453163	-1.81814256	0.1061044	0.61036582	-4.55020763
hetero after UVB irradiation VS hetero before UVB irradiation	GO_CHROMOSOME_LOCALIZATION1	0.115933386	0.001369105	0.538868852	0.60491297	0.83482123	-6.07695819
WT after UVB irradiation VS WT before UVB irradiation	GO_CHROMOSOME_LOCALIZATION2	-0.00474373	0.044677748	-0.01874739	0.98549634	0.99685342	-5.99588122
homo after UVB irradiation VS homo before UVB irradiation	GO_CHROMOSOME_MOVEMENT_TOWARDS_SPINDLE_POLE	-0.25308904	-0.01940793	-1.10913196	0.29836688	0.49278121	-5.92205255
hetero after UVB irradiation VS hetero before UVB irradiation	GO_CHROMOSOME_MOVEMENT_TOWARDS_SPINDLE_POLE1	0.417197937	-0.0338751	2.046938099	0.075523	0.35445844	-4.42741398
WT after UVB irradiation VS WT before UVB irradiation	GO_CHROMOSOME_MOVEMENT_TOWARDS_SPINDLE_POLE2	0.677935265	-0.0198838	2.789391293	0.02330382	0.41786039	-3.19079777
homo after UVB irradiation VS homo before UVB irradiation	GO_CHROMOSOME_ORGANIZATION	-0.44410784	-0.01007821	-3.26624322	0.01080893	0.06966632	-2.82271593
homo after UVB irradiation VS homo before UVB irradiation	GO_CHROMOSOME_ORGANIZATION_INVOLVED_IN_MEIOTIC_CELL_CYCLE	-0.34337639	0.0085086	-1.61842819	0.14273599	0.31424063	-5.3046484
hetero after UVB irradiation VS hetero before UVB irradiation	GO_CHROMOSOME_ORGANIZATION_INVOLVED_IN_MEIOTIC_CELL_CYCLE1	0.096743626	0.020062721	0.492759719	0.63566379	0.85031773	-6.10187543
WT after UVB irradiation VS WT before UVB irradiation	GO_CHROMOSOME_ORGANIZATION_INVOLVED_IN_MEIOTIC_CELL_CYCLE2	-0.04217331	0.023640694	-0.22035866	0.83104606	0.96005533	-5.9705262
hetero after UVB irradiation VS hetero before UVB irradiation	GO_CHROMOSOME_ORGANIZATION1	-0.08175346	-0.03850509	-0.44192003	0.67046393	0.86556131	-6.12691953
WT after UVB irradiation VS WT before UVB irradiation	GO_CHROMOSOME_ORGANIZATION2	-0.14339952	0.001211028	-0.63560798	0.54257313	0.87115131	-5.78835598
homo after UVB irradiation VS homo before UVB irradiation	GO_CHROMOSOME_PASSENGER_COMPLEX	-0.30194267	0.027393798	-0.9179587	0.38445026	0.57416582	-6.1107575
hetero after UVB irradiation VS hetero before UVB irradiation	GO_CHROMOSOME_PASSENGER_COMPLEX1	0.473427651	0.043977029	1.48348137	0.17693359	0.50051267	-5.18470153
WT after UVB irradiation VS WT before UVB irradiation	GO_CHROMOSOME_PASSENGER_COMPLEX2	0.211544188	0.027928422	0.562201184	0.58919855	0.89215883	-5.83265681
homo after UVB irradiation VS homo before UVB irradiation	GO_CHROMOSOME_SEGREGATION	-0.46759107	-0.0163559	-3.0780721	0.01444345	0.08194616	-3.11239966
hetero after UVB irradiation VS hetero before UVB irradiation	GO_CHROMOSOME_SEGREGATION1	0.130272897	0.001509699	0.595912974	0.56800772	0.81261967	-6.04329129
WT after UVB irradiation VS WT before UVB irradiation	GO_CHROMOSOME_SEGREGATION2	0.00503673	0.029916435	0.02086438	0.9838588	0.99634469	-5.99583697
homo after UVB irradiation VS homo before UVB irradiation	GO_CHROMOSOME_SEPARATION	-0.50503234	-0.01609474	-3.1411673	0.01310027	0.07727178	-3.01500191
hetero after UVB irradiation VS hetero before UVB irradiation	GO_CHROMOSOME_SEPARATION1	0.22161592	0.011976155	0.991270101	0.35111131	0.6677405	-5.7305631
WT after UVB irradiation VS WT before UVB irradiation	GO_CHROMOSOME_SEPARATION2	0.053440217	0.046595134	0.216794335	0.83373092	0.96080232	-5.97134325
homo after UVB irradiation VS homo before UVB irradiation	GO_CHROMOSOME_TELOMERIC_REGION	-0.61716604	-0.02243071	-4.09733139	0.00316652	0.03717446	-1.5865039
hetero after UVB irradiation VS hetero before UVB irradiation	GO_CHROMOSOME_TELOMERIC_REGION1	-0.13531951	-0.04684172	-0.5851653	0.57486156	0.81620465	-6.04987145
WT after UVB irradiation VS WT before UVB irradiation	GO_CHROMOSOME_TELOMERIC_REGION2	-0.18425692	-0.00349989	-0.72833559	0.48693352	0.85164239	-5.72548073
homo after UVB irradiation VS homo before UVB irradiation	GO_CHRONIC_INFLAMMATORY_RESPONSE	0.607042158	-0.03265528	3.729461452	0.00539043	0.04803534	-2.12331857
hetero after UVB irradiation VS hetero before UVB irradiation	GO_CHRONIC_INFLAMMATORY_RESPONSE1	0.349940403	-0.01471497	2.17718884	0.06175619	0.32651774	-4.24117733
WT after UVB irradiation VS WT before UVB irradiation	GO_CHRONIC_INFLAMMATORY_RESPONSE2	0.412649662	0.044648653	1.777426587	0.1129419	0.61801327	-4.60411094
homo after UVB irradiation VS homo before UVB irradiation	GO_CHYLOMICRON	0.387126229	0.040524665	1.869861847	0.09696584	0.24628656	-4.95494065
homo after UVB irradiation VS homo before UVB irradiation	GO_CHYLOMICRON_ASSEMBLY	0.398611396	0.069838035	1.684929427	0.12899755	0.29426755	-5.21442702
hetero after UVB irradiation VS hetero before UVB irradiation	GO_CHYLOMICRON_ASSEMBLY1	0.329387163	0.056019413	2.195904751	0.05999305	0.32249818	-4.21421093
WT after UVB irradiation VS WT before UVB irradiation	GO_CHYLOMICRON_ASSEMBLY2	0.027708561	0.123245495	0.156023028	0.87983441	0.9693507	-5.98324241
homo after UVB irradiation VS homo before UVB irradiation	GO_CHYLOMICRON_REMODELING	0.795110916	0.074728535	4.19947385	0.00274109	0.03457019	-1.44083427
hetero after UVB irradiation VS hetero before UVB irradiation	GO_CHYLOMICRON_REMODELING1	0.453155336	0.079486893	2.102363339	0.06933088	0.34169887	-4.34849555
WT after UVB irradiation VS WT before UVB irradiation	GO_CHYLOMICRON_REMODELING2	0.486448653	0.132961915	1.865750121	0.09860547	0.59874718	-4.48661104
hetero after UVB irradiation VS hetero before UVB irradiation	GO_CHYLOMICRON1	0.191583121	0.077106868	0.767221841	0.46538919	0.75235709	-5.92409481
WT after UVB irradiation VS WT before UVB irradiation	GO_CHYLOMICRON2	0.19956245	0.081796938	0.860216336	0.41441098	0.82135231	-5.62345945
homo after UVB irradiation VS homo before UVB irradiation	GO_CIA_COMPLEX	0.002352899	-0.13312245	0.008138074	0.99369838	0.99652787	-6.55082037
hetero after UVB irradiation VS hetero before UVB irradiation	GO_CIA_COMPLEX1	0.834298132	-0.03159263	4.056015203	0.00379851	0.12193847	-1.56891381
WT after UVB irradiation VS WT before UVB irradiation	GO_CIA_COMPLEX2	0.45292258	0.020077039	1.452823703	0.18389653	0.68726847	-5.01381543
homo after UVB irradiation VS homo before UVB irradiation	GO_CILIARY_BASAL_BODY	-0.30528371	-0.0008003	-1.84480604	0.10081918	0.25181224	-4.99076469
homo after UVB irradiation VS homo before UVB irradiation	GO_CILIARY_BASAL_BODY_PLASMA_MEMBRANE_DOCKING	-0.43204173	0.003462709	-2.86591949	0.0201084	0.09774032	-3.44124379
hetero after UVB irradiation VS hetero before UVB irradiation	GO_CILIARY_BASAL_BODY_PLASMA_MEMBRANE_DOCKING1	-0.09276229	-0.01324263	-0.40817877	0.69404526	0.87777097	-6.14211115
WT after UVB irradiation VS WT before UVB irradiation	GO_CILIARY_BASAL_BODY_PLASMA_MEMBRANE_DOCKING2	-0.18151574	-0.04175961	-0.8533649	0.417983	0.82297693	-5.62910833
hetero after UVB irradiation VS hetero before UVB irradiation	GO_CILIARY_BASAL_BODY1	-0.19676082	-0.02874908	-1.15274941	0.28289404	0.60842216	-5.567783
WT after UVB irradiation VS WT before UVB irradiation	GO_CILIARY_BASAL_BODY2	-0.25235898	-0.02403386	-1.64314494	0.13852074	0.64754712	-4.77828778
homo after UVB irradiation VS homo before UVB irradiation	GO_CILIARY_BASE	-0.24545765	-0.00162264	-1.18586284	0.26840058	0.46373772	-5.83887315
hetero after UVB irradiation VS hetero before UVB irradiation	GO_CILIARY_BASE1	-0.10906024	0.030090225	-0.53778866	0.60562416	0.83496123	-6.0775656
WT after UVB irradiation VS WT before UVB irradiation	GO_CILIARY_BASE2	-0.17769811	-0.06635851	-0.94232057	0.37328787	0.8017852	-5.55293041
homo after UVB irradiation VS homo before UVB irradiation	GO_CILIARY_MEMBRANE	-0.10636503	0.010144521	-0.79596034	0.44814348	0.62979726	-6.21599044
hetero after UVB irradiation VS hetero before UVB irradiation	GO_CILIARY_MEMBRANE1	-0.32174683	-0.03315186	-2.52539479	0.0360275	0.26057629	-3.73414819
WT after UVB irradiation VS WT before UVB irradiation	GO_CILIARY_MEMBRANE2	-0.21056608	0.002308651	-1.22896161	0.25360018	0.73589193	-5.2693247
homo after UVB irradiation VS homo before UVB irradiation	GO_CILIARY_NEUROTROPHIC_FACTOR_MEDIATED_SIGNALING_PATHWAY	0.124444966	-0.06064898	0.561021832	0.58955279	0.74219677	-6.3813652
hetero after UVB irradiation VS hetero before UVB irradiation	GO_CILIARY_NEUROTROPHIC_FACTOR_MEDIATED_SIGNALING_PATHWAY1	-0.09576618	-0.04192421	-0.36041213	0.72804075	0.89271808	-6.16163806
WT after UVB irradiation VS WT before UVB irradiation	GO_CILIARY_NEUROTROPHIC_FACTOR_MEDIATED_SIGNALING_PATHWAY2	0.144917002	-0.03848901	0.504718753	0.62720222	0.90327079	-5.86384887
homo after UVB irradiation VS homo before UVB irradiation	GO_CILIARY_NEUROTROPHIC_FACTOR_RECEPTOR_ACTIVITY	0.431084093	-0.05980165	2.445427256	0.03908038	0.14213097	-4.09316869
hetero after UVB irradiation VS hetero before UVB irradiation	GO_CILIARY_NEUROTROPHIC_FACTOR_RECEPTOR_ACTIVITY1	0.24382211	-0.01808972	0.978235718	0.35712862	0.67273203	-5.74289681
WT after UVB irradiation VS WT before UVB irradiation	GO_CILIARY_NEUROTROPHIC_FACTOR_RECEPTOR_ACTIVITY2	0.301910089	-0.04395922	0.946363778	0.37134298	0.80092199	-5.54932491
homo after UVB irradiation VS homo before UVB irradiation	GO_CILIARY_NEUROTROPHIC_FACTOR_RECEPTOR_BINDING	0.711395353	-0.04175584	3.906394355	0.00416354	0.04196818	-1.86282675
hetero after UVB irradiation VS hetero before UVB irradiation	GO_CILIARY_NEUROTROPHIC_FACTOR_RECEPTOR_BINDING1	0.412866163	0.031021186	1.336906308	0.21868655	0.54565069	-5.36181376
WT after UVB irradiation VS WT before UVB irradiation	GO_CILIARY_NEUROTROPHIC_FACTOR_RECEPTOR_BINDING2	0.55516516	0.024025534	1.613780157	0.14478355	0.65365874	-4.81556102
homo after UVB irradiation VS homo before UVB irradiation	GO_CILIARY_PLASM	-0.14746516	0.006715189	-1.15018479	0.28201702	0.47704002	-5.8780479
hetero after UVB irradiation VS hetero before UVB irradiation	GO_CILIARY_PLASM1	-0.18328173	0.004643953	-1.12508328	0.29376386	0.61756748	-5.59693064
WT after UVB irradiation VS WT before UVB irradiation	GO_CILIARY_PLASM2	-0.19715753	-0.01722663	-1.30530284	0.2276551	0.71951396	-5.1851563
homo after UVB irradiation VS homo before UVB irradiation	GO_CILIARY_ROOTLET	0.63646458	-0.05959407	4.141069835	0.00297626	0.03597152	-1.52393927
hetero after UVB irradiation VS hetero before UVB irradiation	GO_CILIARY_ROOTLET1	0.163341051	-0.00179337	0.939193993	0.37561714	0.68886484	-5.77907804
WT after UVB irradiation VS WT before UVB irradiation	GO_CILIARY_ROOTLET2	0.37146405	-0.03778043	2.206188803	0.05803017	0.52490436	-4.01804923
homo after UVB irradiation VS homo before UVB irradiation	GO_CILIARY_TIP	-0.34512456	-0.00879553	-1.75622874	0.11562871	0.27397766	-5.11579011
hetero after UVB irradiation VS hetero before UVB irradiation	GO_CILIARY_TIP1	-0.3996806	-0.01185855	-1.86463874	0.09991522	0.39253167	-4.68263155
WT after UVB irradiation VS WT before UVB irradiation	GO_CILIARY_TIP2	-0.44633374	0.0057446	-2.65276051	0.02882558	0.44654821	-3.38494574
homo after UVB irradiation VS homo before UVB irradiation	GO_CILIARY_TRANSITION_FIBER	-0.78545467	-0.02886927	-4.01089069	0.00358196	0.03908552	-1.71096331
hetero after UVB irradiation VS hetero before UVB irradiation	GO_CILIARY_TRANSITION_FIBER1	-0.65317039	-0.09321305	-2.09261367	0.07038284	0.343536	-4.3624157
WT after UVB irradiation VS WT before UVB irradiation	GO_CILIARY_TRANSITION_FIBER2	-0.55735669	-0.0807232	-2.10930211	0.06753288	0.54059322	-4.15339033
homo after UVB irradiation VS homo before UVB irradiation	GO_CILIARY_TRANSITION_ZONE	-0.23000975	-0.00993978	-1.44005069	0.18635889	0.37088309	-5.53699759
hetero after UVB irradiation VS hetero before UVB irradiation	GO_CILIARY_TRANSITION_ZONE1	-0.33051532	-0.02391054	-1.72234052	0.12401459	0.43057867	-4.87594087
WT after UVB irradiation VS WT before UVB irradiation	GO_CILIARY_TRANSITION_ZONE2	-0.36174238	-0.03750552	-2.38884707	0.04357379	0.49624823	-3.7602329
homo after UVB irradiation VS homo before UVB irradiation	GO_CILIUM	-0.08044601	0.003164163	-0.63390918	0.54315236	0.70806532	-6.33557578
homo after UVB irradiation VS homo before UVB irradiation	GO_CILIUM_MOVEMENT	-0.08263853	0.019156832	-0.60538028	0.56105497	0.72092905	-6.35410711
hetero after UVB irradiation VS hetero before UVB irradiation	GO_CILIUM_MOVEMENT1	-0.23330945	0.028709258	-1.43706572	0.18932282	0.51444052	-5.2419252
WT after UVB irradiation VS WT before UVB irradiation	GO_CILIUM_MOVEMENT2	-0.17704131	0.018497144	-1.19752344	0.26497869	0.74156847	-5.30301245
homo after UVB irradiation VS homo before UVB irradiation	GO_CILIUM_OR_FLAGELLUM_DEPENDENT_CELL_MOTILITY	-0.03669008	0.016452638	-0.26806597	0.79516795	0.88387267	-6.51159134
hetero after UVB irradiation VS hetero before UVB irradiation	GO_CILIUM_OR_FLAGELLUM_DEPENDENT_CELL_MOTILITY1	-0.17892763	0.023529151	-1.10056399	0.30367592	0.62541508	-5.62234148
WT after UVB irradiation VS WT before UVB irradiation	GO_CILIUM_OR_FLAGELLUM_DEPENDENT_CELL_MOTILITY2	-0.12881168	0.021523262	-0.8432679	0.42328647	0.82493904	-5.63736515
homo after UVB irradiation VS homo before UVB irradiation	GO_CILIUM_ORGANIZATION	-0.21196725	-0.00506723	-1.61721055	0.14299955	0.31468005	-5.3062834
hetero after UVB irradiation VS hetero before UVB irradiation	GO_CILIUM_ORGANIZATION1	-0.14816592	-0.01368475	-0.94855212	0.37112186	0.68479107	-5.77051077
WT after UVB irradiation VS WT before UVB irradiation	GO_CILIUM_ORGANIZATION2	-0.16566187	-0.03485382	-1.13067077	0.29056741	0.75624915	-5.37265128
hetero after UVB irradiation VS hetero before UVB irradiation	GO_CILIUM1	-0.1689806	0.010493119	-1.16484066	0.2782471	0.60400502	-5.55488894
WT after UVB irradiation VS WT before UVB irradiation	GO_CILIUM2	-0.13222666	-0.00436103	-0.90458593	0.39180209	0.81270918	-5.58598749
homo after UVB irradiation VS homo before UVB irradiation	GO_CIRCADIAN_REGULATION_OF_GENE_EXPRESSION	-0.40495858	0.014394744	-3.15950565	0.01273482	0.07642444	-2.98673995
hetero after UVB irradiation VS hetero before UVB irradiation	GO_CIRCADIAN_REGULATION_OF_GENE_EXPRESSION1	-0.21188384	-0.0058434	-1.59212975	0.15072768	0.46844437	-5.04702752
WT after UVB irradiation VS WT before UVB irradiation	GO_CIRCADIAN_REGULATION_OF_GENE_EXPRESSION2	-0.4654532	0.00383177	-3.26245135	0.01129647	0.34005582	-2.52804682
homo after UVB irradiation VS homo before UVB irradiation	GO_CIRCADIAN_RHYTHM	-0.28690599	0.019999264	-2.28188555	0.05066571	0.16614787	-4.34370825
hetero after UVB irradiation VS hetero before UVB irradiation	GO_CIRCADIAN_RHYTHM1	-0.24771158	-0.01233272	-1.81597015	0.10760977	0.40587247	-4.7494093
WT after UVB irradiation VS WT before UVB irradiation	GO_CIRCADIAN_RHYTHM2	-0.25481029	0.021634095	-1.71628087	0.12399008	0.63137109	-4.68414434
homo after UVB irradiation VS homo before UVB irradiation	GO_CIRCADIAN_SLEEP_WAKE_CYCLE	-0.00684914	-0.02446514	-0.0365242	0.97172461	0.98531737	-6.55012464
homo after UVB irradiation VS homo before UVB irradiation	GO_CIRCADIAN_SLEEP_WAKE_CYCLE_NON_REM_SLEEP	-0.22640114	-0.09063726	-1.076788	0.31177086	0.50574942	-5.95589021
hetero after UVB irradiation VS hetero before UVB irradiation	GO_CIRCADIAN_SLEEP_WAKE_CYCLE_NON_REM_SLEEP1	-0.38266418	-0.04974927	-1.80091358	0.11010194	0.40948315	-4.76993474
WT after UVB irradiation VS WT before UVB irradiation	GO_CIRCADIAN_SLEEP_WAKE_CYCLE_NON_REM_SLEEP2	-0.28906176	-0.00787697	-1.24663108	0.24738493	0.73564768	-5.25013669
homo after UVB irradiation VS homo before UVB irradiation	GO_CIRCADIAN_SLEEP_WAKE_CYCLE_REM_SLEEP	0.275224329	-0.06202971	0.953901982	0.36699282	0.55722946	-6.07741727
hetero after UVB irradiation VS hetero before UVB irradiation	GO_CIRCADIAN_SLEEP_WAKE_CYCLE_REM_SLEEP1	-0.29257417	-0.05806991	-1.19410071	0.2672596	0.59348121	-5.52330336
WT after UVB irradiation VS WT before UVB irradiation	GO_CIRCADIAN_SLEEP_WAKE_CYCLE_REM_SLEEP2	-0.53241697	-0.10676478	-2.69824688	0.02685204	0.43942403	-3.32024927
homo after UVB irradiation VS homo before UVB irradiation	GO_CIRCADIAN_SLEEP_WAKE_CYCLE_SLEEP	-0.0667487	-0.04698635	-0.29722585	0.77357218	0.86903202	-6.50263255
hetero after UVB irradiation VS hetero before UVB irradiation	GO_CIRCADIAN_SLEEP_WAKE_CYCLE_SLEEP1	-0.28033043	0.013231684	-1.6696104	0.1342554	0.44563334	-4.94595803
WT after UVB irradiation VS WT before UVB irradiation	GO_CIRCADIAN_SLEEP_WAKE_CYCLE_SLEEP2	-0.18345267	0.055763728	-1.00879272	0.34226388	0.78172749	-5.49213447
hetero after UVB irradiation VS hetero before UVB irradiation	GO_CIRCADIAN_SLEEP_WAKE_CYCLE1	-0.17047553	0.030028275	-1.045642	0.32684269	0.64640644	-5.677778
WT after UVB irradiation VS WT before UVB irradiation	GO_CIRCADIAN_SLEEP_WAKE_CYCLE2	-0.14655087	0.050509648	-0.77765381	0.45888816	0.8390123	-5.68901269
homo after UVB irradiation VS homo before UVB irradiation	GO_CIRCULATORY_SYSTEM_DEVELOPMENT	0.276630666	-0.02022625	2.250674793	0.05323725	0.17101048	-4.39114824
hetero after UVB irradiation VS hetero before UVB irradiation	GO_CIRCULATORY_SYSTEM_DEVELOPMENT1	0.176552559	0.00798669	1.185790327	0.27034337	0.5968607	-5.53232876
WT after UVB irradiation VS WT before UVB irradiation	GO_CIRCULATORY_SYSTEM_DEVELOPMENT2	0.145160165	-0.01582707	0.857358234	0.41589843	0.8213754	-5.62582042
homo after UVB irradiation VS homo before UVB irradiation	GO_CIRCULATORY_SYSTEM_PROCESS	0.340006407	-0.0055842	2.707676225	0.02579542	0.1134393	-3.68711571
hetero after UVB irradiation VS hetero before UVB irradiation	GO_CIRCULATORY_SYSTEM_PROCESS1	0.087597171	0.013536908	0.536382236	0.60655081	0.83530227	-6.07835476
WT after UVB irradiation VS WT before UVB irradiation	GO_CIRCULATORY_SYSTEM_PROCESS2	0.1003549	-0.01246986	0.490655101	0.63668944	0.90563438	-5.87100268
homo after UVB irradiation VS homo before UVB irradiation	GO_CIS_GOLGI_NETWORK	0.208046816	-0.03505989	1.413691552	0.19371878	0.38029905	-5.5700104
homo after UVB irradiation VS homo before UVB irradiation	GO_CIS_GOLGI_NETWORK_MEMBRANE	0.43281713	-0.01638798	3.054286252	0.01498634	0.08315721	-3.14917554
hetero after UVB irradiation VS hetero before UVB irradiation	GO_CIS_GOLGI_NETWORK_MEMBRANE1	0.45685227	0.050535605	1.706661156	0.1269811	0.43363412	-4.8968593
WT after UVB irradiation VS WT before UVB irradiation	GO_CIS_GOLGI_NETWORK_MEMBRANE2	0.066343049	0.007555365	0.283743473	0.78372675	0.94581744	-5.95380785
hetero after UVB irradiation VS hetero before UVB irradiation	GO_CIS_GOLGI_NETWORK1	0.206819567	-0.00868861	1.216637917	0.25904249	0.58528213	-5.49861287
WT after UVB irradiation VS WT before UVB irradiation	GO_CIS_GOLGI_NETWORK2	0.105695731	-0.02991844	0.723558883	0.48970772	0.85191122	-5.72890302
homo after UVB irradiation VS homo before UVB irradiation	GO_CIS_REGULATORY_REGION_SEQUENCE_SPECIFIC_DNA_BINDING	-0.12263429	0.027281482	-0.7973236	0.44739401	0.62937974	-6.21488445
hetero after UVB irradiation VS hetero before UVB irradiation	GO_CIS_REGULATORY_REGION_SEQUENCE_SPECIFIC_DNA_BINDING1	-0.05855152	0.002124773	-0.45334112	0.6625677	0.86198891	-6.12151772
WT after UVB irradiation VS WT before UVB irradiation	GO_CIS_REGULATORY_REGION_SEQUENCE_SPECIFIC_DNA_BINDING2	-0.11867365	0.012995017	-0.79697656	0.44819965	0.8349077	-5.67416896
homo after UVB irradiation VS homo before UVB irradiation	GO_CIS_TRANS_ISOMERASE_ACTIVITY	0.102946575	-0.03284954	0.650868198	0.53267204	0.69971883	-6.3241936
hetero after UVB irradiation VS hetero before UVB irradiation	GO_CIS_TRANS_ISOMERASE_ACTIVITY1	-0.09538029	-0.01439576	-0.63360839	0.54434499	0.79844479	-6.01935555
WT after UVB irradiation VS WT before UVB irradiation	GO_CIS_TRANS_ISOMERASE_ACTIVITY2	0.263542739	-0.01938794	1.734074202	0.12067526	0.6303734	-4.6609734
homo after UVB irradiation VS homo before UVB irradiation	GO_CITRATE_METABOLIC_PROCESS	-0.29986301	-0.04076859	-0.999627	0.34564247	0.5378159	-6.03353466
hetero after UVB irradiation VS hetero before UVB irradiation	GO_CITRATE_METABOLIC_PROCESS1	-0.14803955	0.015492549	-0.40832046	0.69394546	0.87777097	-6.14204977
WT after UVB irradiation VS WT before UVB irradiation	GO_CITRATE_METABOLIC_PROCESS2	0.211954066	0.037577827	1.04555468	0.32597053	0.77473783	-5.45715671
homo after UVB irradiation VS homo before UVB irradiation	GO_CITRULLINE_BIOSYNTHETIC_PROCESS	-0.26160644	-0.00173502	-0.93265267	0.37724156	0.56713057	-6.09725241
hetero after UVB irradiation VS hetero before UVB irradiation	GO_CITRULLINE_BIOSYNTHETIC_PROCESS1	-0.21546764	-0.04740632	-0.76279624	0.46787772	0.7530936	-5.92750259
WT after UVB irradiation VS WT before UVB irradiation	GO_CITRULLINE_BIOSYNTHETIC_PROCESS2	-0.05265935	0.012811278	-0.16539368	0.87268875	0.96914919	-5.98165855
homo after UVB irradiation VS homo before UVB irradiation	GO_CITRULLINE_METABOLIC_PROCESS	0.022171202	0.028346992	0.092457018	0.92852016	0.96135354	-6.54616769
hetero after UVB irradiation VS hetero before UVB irradiation	GO_CITRULLINE_METABOLIC_PROCESS1	0.201905995	-0.05578737	1.288494233	0.2342379	0.56254359	-5.41786902
WT after UVB irradiation VS WT before UVB irradiation	GO_CITRULLINE_METABOLIC_PROCESS2	0.041060152	0.043113021	0.187194755	0.85611325	0.96638003	-5.97761911

homo after UVB irradiation VS homo before UVB irradiation	GO_CLASS_I_DNA_APURINIC_OR_APYRIMIDINIC_SITE_ENDONUCLEASE_ACTIVITY	-0.53959273	-0.04189626	-2.52073678	0.03467812	0.13335261	-3.97694574
hetero after UVB irradiation VS hetero before UVB irradiation	GO_CLASS_I_DNA_APURINIC_OR_APYRIMIDINIC_SITE_ENDONUCLEASE_ACTIVITY1	-0.59548387	-0.12100039	-1.87029301	0.09905603	0.3913075	-4.67483227
WT after UVB irradiation VS WT before UVB irradiation	GO_CLASS_I_DNA_APURINIC_OR_APYRIMIDINIC_SITE_ENDONUCLEASE_ACTIVITY2	-0.24167697	0.014722515	-0.9496752	0.36975568	0.79984535	-5.54636292
homo after UVB irradiation VS homo before UVB irradiation	GO_CLATHRIN_ADAPTOR_COMPLEX	0.292776502	-0.0626835	1.90793903	0.09137619	0.23742773	-4.90014009
hetero after UVB irradiation VS hetero before UVB irradiation	GO_CLATHRIN_ADAPTOR_COMPLEX1	0.182903678	0.025572025	0.903894707	0.3929358	0.70255187	-5.81078612
WT after UVB irradiation VS WT before UVB irradiation	GO_CLATHRIN_ADAPTOR_COMPLEX2	0.099179959	0.098012611	0.491591434	0.63605555	0.9055032	-5.87053229
homo after UVB irradiation VS homo before UVB irradiation	GO_CLATHRIN_BINDING	0.27583282	-0.0119201	1.899861074	0.0925358	0.23944428	-4.91180104
hetero after UVB irradiation VS hetero before UVB irradiation	GO_CLATHRIN_BINDING1	0.433388921	-0.00542898	3.333058597	0.01061591	0.16189805	-2.56052355
WT after UVB irradiation VS WT before UVB irradiation	GO_CLATHRIN_BINDING2	0.270175201	0.007288266	1.786156084	0.11144194	0.61576748	-4.59259362
homo after UVB irradiation VS homo before UVB irradiation	GO_CLATHRIN_COAT	0.337454914	-0.01083143	2.312260301	0.04828137	0.16122643	-4.29741037
homo after UVB irradiation VS homo before UVB irradiation	GO_CLATHRIN_COAT_ASSEMBLY	0.310886686	-0.02345656	2.227356716	0.05524232	0.17458128	-4.42649785
hetero after UVB irradiation VS hetero before UVB irradiation	GO_CLATHRIN_COAT_ASSEMBLY1	0.403374475	0.006107144	2.722513878	0.02660061	0.23166634	-3.44492641
WT after UVB irradiation VS WT before UVB irradiation	GO_CLATHRIN_COAT_ASSEMBLY2	0.245745933	-0.00571241	1.422652268	0.19219583	0.69334561	-5.04972671
homo after UVB irradiation VS homo before UVB irradiation	GO_CLATHRIN_COAT_OF_COATED_PIT	0.74987023	-0.0098954	5.872114952	0.0003199	0.01314969	0.714079114
hetero after UVB irradiation VS hetero before UVB irradiation	GO_CLATHRIN_COAT_OF_COATED_PIT1	0.690681408	0.05097231	4.698094952	0.00162488	0.09668022	-0.75790568
WT after UVB irradiation VS WT before UVB irradiation	GO_CLATHRIN_COAT_OF_COATED_PIT2	0.538303094	0.070972168	3.610212106	0.00674109	0.30328715	-2.05690643
homo after UVB irradiation VS homo before UVB irradiation	GO_CLATHRIN_COAT_OF_ENDOCYTIC_VESICLE	0.35972669	-0.02279614	2.419632772	0.04071403	0.14507545	-4.1328712
hetero after UVB irradiation VS hetero before UVB irradiation	GO_CLATHRIN_COAT_OF_ENDOCYTIC_VESICLE1	0.204170414	0.038904757	1.009852607	0.34266649	0.66085538	-5.71276332
WT after UVB irradiation VS WT before UVB irradiation	GO_CLATHRIN_COAT_OF_ENDOCYTIC_VESICLE2	0.019358769	0.064081962	0.098902202	0.92362046	0.98103299	-5.99090868
hetero after UVB irradiation VS hetero before UVB irradiation	GO_CLATHRIN_COAT1	0.339656556	0.02716755	2.152546093	0.06415603	0.33204634	-4.27661138
WT after UVB irradiation VS WT before UVB irradiation	GO_CLATHRIN_COAT2	0.214538992	0.060602254	1.252423184	0.24537541	0.73450731	-5.2438077
homo after UVB irradiation VS homo before UVB irradiation	GO_CLATHRIN_COATED_ENDOCYTIC_VESICLE	0.029501773	-0.006508	0.145858204	0.88750164	0.93923016	-6.53919595
homo after UVB irradiation VS homo before UVB irradiation	GO_CLATHRIN_COATED_ENDOCYTIC_VESICLE_MEMBRANE	-0.11521491	-0.03890794	-0.62923447	0.54606259	0.71016013	-6.33866542
hetero after UVB irradiation VS hetero before UVB irradiation	GO_CLATHRIN_COATED_ENDOCYTIC_VESICLE_MEMBRANE1	-0.16063909	-0.01758772	-0.88038239	0.40478967	0.71132442	-5.83136419
WT after UVB irradiation VS WT before UVB irradiation	GO_CLATHRIN_COATED_ENDOCYTIC_VESICLE_MEMBRANE2	-0.16500272	0.076385684	-0.99156598	0.35011004	0.78758749	-5.50819734
hetero after UVB irradiation VS hetero before UVB irradiation	GO_CLATHRIN_COATED_ENDOCYTIC_VESICLE1	0.044530002	0.000861093	0.297032327	0.77414701	0.91152166	-6.18391413
WT after UVB irradiation VS WT before UVB irradiation	GO_CLATHRIN_COATED_ENDOCYTIC_VESICLE2	-0.06466133	0.065327276	-0.44168763	0.6702726	0.9152598	-5.89442282
homo after UVB irradiation VS homo before UVB irradiation	GO_CLATHRIN_COATED_PIT	0.384298898	-0.0078188	2.734195914	0.02473819	0.11075896	-3.64591575
hetero after UVB irradiation VS hetero before UVB irradiation	GO_CLATHRIN_COATED_PIT1	0.324361992	0.016377111	2.554603366	0.03443944	0.25549943	-3.69130817
WT after UVB irradiation VS WT before UVB irradiation	GO_CLATHRIN_COATED_PIT2	0.323418153	0.003053655	2.197856363	0.05879279	0.52638053	-4.02973541
homo after UVB irradiation VS homo before UVB irradiation	GO_CLATHRIN_COATED_VESICLE	0.031879394	-0.01250848	0.189924316	0.85391559	0.91914308	-6.53110367
homo after UVB irradiation VS homo before UVB irradiation	GO_CLATHRIN_COATED_VESICLE_MEMBRANE	0.033881593	-0.0145239	0.193979598	0.85083939	0.91780487	-6.53025308
hetero after UVB irradiation VS hetero before UVB irradiation	GO_CLATHRIN_COATED_VESICLE_MEMBRANE1	0.158820059	0.002227771	1.203113428	0.26394805	0.59036447	-5.51346689
WT after UVB irradiation VS WT before UVB irradiation	GO_CLATHRIN_COATED_VESICLE_MEMBRANE2	0.050693302	0.028998928	0.356523323	0.73056008	0.93332829	-5.92955014
hetero after UVB irradiation VS hetero before UVB irradiation	GO_CLATHRIN_COATED_VESICLE1	0.168520849	-0.00010585	1.227625332	0.25511313	0.58262782	-5.48646369
WT after UVB irradiation VS WT before UVB irradiation	GO_CLATHRIN_COATED_VESICLE2	0.067898619	0.023572575	0.463996766	0.65486852	0.91006679	-5.88404105
homo after UVB irradiation VS homo before UVB irradiation	GO_CLATHRIN_COMPLEX	0.020486688	-0.00512579	0.077705482	0.93989667	0.96765556	-6.54754408
hetero after UVB irradiation VS hetero before UVB irradiation	GO_CLATHRIN_COMPLEX1	0.325063615	0.083382563	1.896243424	0.09520253	0.38540008	-4.63893245
WT after UVB irradiation VS WT before UVB irradiation	GO_CLATHRIN_COMPLEX2	0.219421646	-0.00198722	1.311415302	0.22567845	0.71906685	-5.17827774
homo after UVB irradiation VS homo before UVB irradiation	GO_CLATHRIN_DEPENDENT_ENDOCYTOSIS	0.440208675	-0.02013571	3.017657589	0.0158642	0.08597431	-3.20586349
hetero after UVB irradiation VS hetero before UVB irradiation	GO_CLATHRIN_DEPENDENT_ENDOCYTOSIS1	0.530266597	0.012246865	4.045543143	0.00385343	0.12254574	-1.58271262
WT after UVB irradiation VS WT before UVB irradiation	GO_CLATHRIN_DEPENDENT_ENDOCYTOSIS2	0.347658981	0.000663694	2.310649646	0.04926177	0.50780915	-3.870939
homo after UVB irradiation VS homo before UVB irradiation	GO_CLATHRIN_HEAVY_CHAIN_BINDING	0.713306933	-0.01339542	4.180369671	0.00281574	0.03512419	-1.46796279
hetero after UVB irradiation VS hetero before UVB irradiation	GO_CLATHRIN_HEAVY_CHAIN_BINDING1	0.765372587	0.009772635	3.716135277	0.00610225	0.13479905	-2.02562265
WT after UVB irradiation VS WT before UVB irradiation	GO_CLATHRIN_HEAVY_CHAIN_BINDING2	0.69746934	-0.07011947	3.230236375	0.01185879	0.34347216	-2.57249036
homo after UVB irradiation VS homo before UVB irradiation	GO_CLATHRIN_LIGHT_CHAIN_BINDING	0.519101431	-0.05417974	4.041882869	0.00342666	0.03848613	-1.66621711
hetero after UVB irradiation VS hetero before UVB irradiation	GO_CLATHRIN_LIGHT_CHAIN_BINDING1	0.613073884	0.025213959	2.77811331	0.02443023	0.22374282	-3.36342494
WT after UVB irradiation VS WT before UVB irradiation	GO_CLATHRIN_LIGHT_CHAIN_BINDING2	0.581243026	-0.0020661	2.277040147	0.05192885	0.51716411	-3.91838216
homo after UVB irradiation VS homo before UVB irradiation	GO_CLATHRIN_SCULPTED_GAMMA_AMINOBUTYRIC_ACID_TRANSPORT_VESICLE_MEMBRANE	0.168322693	0.029050289	0.974535398	0.35723983	0.54874447	-6.05781626
hetero after UVB irradiation VS hetero before UVB irradiation	GO_CLATHRIN_SCULPTED_GAMMA_AMINOBUTYRIC_ACID_TRANSPORT_VESICLE_MEMBRANE1	0.223561039	0.052087793	1.29037873	0.23361536	0.56207863	-5.41571131
WT after UVB irradiation VS WT before UVB irradiation	GO_CLATHRIN_SCULPTED_GAMMA_AMINOBUTYRIC_ACID_TRANSPORT_VESICLE_MEMBRANE2	-0.10196569	0.031906385	-0.40199851	0.698087	0.92325416	-5.91168748
homo after UVB irradiation VS homo before UVB irradiation	GO_CLATHRIN_SCULPTED_MONOAMINE_TRANSPORT_VESICLE	-0.35241351	-0.0382562	-1.18376854	0.26918481	0.46464398	-5.84119613
hetero after UVB irradiation VS hetero before UVB irradiation	GO_CLATHRIN_SCULPTED_MONOAMINE_TRANSPORT_VESICLE1	-0.4607233	-0.00775758	-2.60884824	0.03167695	0.24789178	-3.61171179
WT after UVB irradiation VS WT before UVB irradiation	GO_CLATHRIN_SCULPTED_MONOAMINE_TRANSPORT_VESICLE2	-0.59501615	0.026833852	-2.90766795	0.01940648	0.39553294	-3.02339906
homo after UVB irradiation VS homo before UVB irradiation	GO_CLATHRIN_SCULPTED_VESICLE	0.095540978	0.049258772	0.548670136	0.59762767	0.74819842	-6.38861414
hetero after UVB irradiation VS hetero before UVB irradiation	GO_CLATHRIN_SCULPTED_VESICLE1	0.124085834	0.040638273	0.950293254	0.37028992	0.68385458	-5.76890941
WT after UVB irradiation VS WT before UVB irradiation	GO_CLATHRIN_SCULPTED_VESICLE2	-0.15591127	0.023825915	-0.71679958	0.49365066	0.85370213	-5.7337122
homo after UVB irradiation VS homo before UVB irradiation	GO_CLATHRIN_VESICLE_COAT	0.500467614	0.01589023	2.813201953	0.021844	0.1028573	-3.52314672
hetero after UVB irradiation VS hetero before UVB irradiation	GO_CLATHRIN_VESICLE_COAT1	0.460752819	0.027570032	2.80150401	0.02357259	0.21999243	-3.32916597
WT after UVB irradiation VS WT before UVB irradiation	GO_CLATHRIN_VESICLE_COAT2	0.347320842	0.072679976	1.988550575	0.08152435	0.56609447	-4.32006516
homo after UVB irradiation VS homo before UVB irradiation	GO_CLEAVAGE_FURROW	0.055997166	-0.02785947	0.388119132	0.70765956	0.82561333	-6.46893774
homo after UVB irradiation VS homo before UVB irradiation	GO_CLEAVAGE_FURROW_FORMATION	-0.16816985	0.020268024	-0.65690925	0.52896833	0.69678636	-6.32007366
hetero after UVB irradiation VS hetero before UVB irradiation	GO_CLEAVAGE_FURROW_FORMATION1	0.487477601	-0.02256715	2.108307501	0.0686971	0.33988857	-4.34000117
WT after UVB irradiation VS WT before UVB irradiation	GO_CLEAVAGE_FURROW_FORMATION2	0.116431142	0.080430676	0.367891564	0.72238472	0.93154998	-5.92527826
hetero after UVB irradiation VS hetero before UVB irradiation	GO_CLEAVAGE_FURROW1	0.273687594	-0.01168233	2.110085972	0.06850859	0.33988857	-4.33745859
WT after UVB irradiation VS WT before UVB irradiation	GO_CLEAVAGE_FURROW2	0.230659969	0.001071022	1.525137506	0.16528298	0.66947139	-4.92607705
homo after UVB irradiation VS homo before UVB irradiation	GO_CLEAVAGE_INVOLVED_IN_RRNA_PROCESSING	-0.89120211	0.020644034	-5.98469019	0.0002807	0.01236517	0.843503478
hetero after UVB irradiation VS hetero before UVB irradiation	GO_CLEAVAGE_INVOLVED_IN_RRNA_PROCESSING1	-0.57174363	-0.08521786	-2.59520839	0.03234953	0.24945329	-3.63172902
WT after UVB irradiation VS WT before UVB irradiation	GO_CLEAVAGE_INVOLVED_IN_RRNA_PROCESSING2	-0.31200719	0.005201493	-0.91996895	0.38417535	0.80622298	-5.5726414
homo after UVB irradiation VS homo before UVB irradiation	GO_CLUSTER_OF_ACTIN_BASED_CELL_PROJECTIONS	0.1456613	0.021695333	1.137494277	0.28699265	0.48208885	-5.89177499
hetero after UVB irradiation VS hetero before UVB irradiation	GO_CLUSTER_OF_ACTIN_BASED_CELL_PROJECTIONS1	0.00238508	0.022176588	0.018348014	0.98581884	0.99470555	-6.23130155
WT after UVB irradiation VS WT before UVB irradiation	GO_CLUSTER_OF_ACTIN_BASED_CELL_PROJECTIONS2	0.007149874	0.025121885	0.050989681	0.96056911	0.99008999	-5.99469504
homo after UVB irradiation VS homo before UVB irradiation	GO_CLUSTERING_OF_VOLTAGE_GATED_SODIUM_CHANNELS	-0.06674425	-0.04594034	-0.22951568	0.82400412	0.90144838	-6.5220391
hetero after UVB irradiation VS hetero before UVB irradiation	GO_CLUSTERING_OF_VOLTAGE_GATED_SODIUM_CHANNELS1	-0.06948643	0.078232654	-0.31216444	0.76304335	0.90723412	-6.17897552
WT after UVB irradiation VS WT before UVB irradiation	GO_CLUSTERING_OF_VOLTAGE_GATED_SODIUM_CHANNELS2	-0.04757705	0.086393063	-0.24870977	0.80977708	0.95453953	-5.96355958
homo after UVB irradiation VS homo before UVB irradiation	GO_CO_RECEPTOR_BINDING	-0.13768048	0.014362613	-0.5996116	0.56471592	0.72386723	-6.35775958
hetero after UVB irradiation VS hetero before UVB irradiation	GO_CO_RECEPTOR_BINDING1	-0.18262973	0.022192514	-0.70556459	0.50084984	0.77339265	-5.97003443
WT after UVB irradiation VS WT before UVB irradiation	GO_CO_RECEPTOR_BINDING2	-0.01329916	-0.0013902	-0.05033881	0.96107196	0.99026245	-5.99472982
homo after UVB irradiation VS homo before UVB irradiation	GO_CO_SMAD_BINDING	-0.14552882	0.030305165	-0.58771949	0.57230585	0.72982349	-6.36518796
hetero after UVB irradiation VS hetero before UVB irradiation	GO_CO_SMAD_BINDING1	0.045080124	0.072404263	0.252819096	0.80690012	0.92477404	-6.19696675
WT after UVB irradiation VS WT before UVB irradiation	GO_CO_SMAD_BINDING2	-0.12160238	0.077281018	-0.61566754	0.55501943	0.87771516	-5.80087746
homo after UVB irradiation VS homo before UVB irradiation	GO_COA_HYDROLASE_ACTIVITY	-0.49043287	0.027806493	-2.66106531	0.02776678	0.11789461	-3.75949748
hetero after UVB irradiation VS hetero before UVB irradiation	GO_COA_HYDROLASE_ACTIVITY1	-0.34286546	-0.01824011	-1.87823126	0.09786169	0.38986151	-4.66386861
WT after UVB irradiation VS WT before UVB irradiation	GO_COA_HYDROLASE_ACTIVITY2	-0.45260585	0.028273871	-2.90895648	0.01936795	0.39534855	-3.02158008
homo after UVB irradiation VS homo before UVB irradiation	GO_COA_TRANSFERASE_ACTIVITY	-0.19355603	-0.03764071	-0.76628488	0.46466744	0.64391937	-6.23966214
hetero after UVB irradiation VS hetero before UVB irradiation	GO_COA_TRANSFERASE_ACTIVITY1	-0.65780537	0.018179132	-3.8198247	0.00527125	0.1288742	-1.88439343
WT after UVB irradiation VS WT before UVB irradiation	GO_COA_TRANSFERASE_ACTIVITY2	-0.29631877	0.020259434	-0.95372273	0.36782239	0.79928656	-5.54273146
homo after UVB irradiation VS homo before UVB irradiation	GO_COAGULATION	0.459066576	-0.01979573	3.683280794	0.0057704	0.05035773	-2.19196987
hetero after UVB irradiation VS hetero before UVB irradiation	GO_COAGULATION1	0.383295732	0.023071107	2.530591143	0.03573962	0.25953438	-3.72652814
WT after UVB irradiation VS WT before UVB irradiation	GO_COAGULATION2	0.249322468	0.009885597	1.356022742	0.21169206	0.70907106	-5.12748089
homo after UVB irradiation VS homo before UVB irradiation	GO_COATED_VESICLE	0.240296925	-0.01773478	1.515791574	0.16656167	0.34496525	-5.44017277
homo after UVB irradiation VS homo before UVB irradiation	GO_COATED_VESICLE_MEMBRANE	0.261964592	-0.01278042	1.53053115	0.16293386	0.34052045	-5.42100529
hetero after UVB irradiation VS hetero before UVB irradiation	GO_COATED_VESICLE_MEMBRANE1	0.314403266	0.011791382	2.214451878	0.05829504	0.31824803	-4.18744377
WT after UVB irradiation VS WT before UVB irradiation	GO_COATED_VESICLE_MEMBRANE2	0.221208817	0.009708188	1.50792873	0.16955322	0.67524339	-4.94716252
hetero after UVB irradiation VS hetero before UVB irradiation	GO_COATED_VESICLE1	0.306066885	0.009049274	2.362090414	0.04637968	0.2879169	-3.97307157
WT after UVB irradiation VS WT before UVB irradiation	GO_COATED_VESICLE2	0.226742006	-0.00510013	1.529502026	0.16421539	0.66887289	-4.9207095
homo after UVB irradiation VS homo before UVB irradiation	GO_COBALAMIN_BINDING	0.127449314	-0.02006016	0.450679308	0.66372167	0.79617466	-6.44074888
hetero after UVB irradiation VS hetero before UVB irradiation	GO_COBALAMIN_BINDING1	0.022371762	-0.0216195	0.113907501	0.91216949	0.96720392	-6.22445373
WT after UVB irradiation VS WT before UVB irradiation	GO_COBALAMIN_BINDING2	0.214609059	-0.01691717	0.666985186	0.52332871	0.86461731	-5.76792899
homo after UVB irradiation VS homo before UVB irradiation	GO_COBALAMIN_METABOLIC_PROCESS	0.19461718	-0.06439593	1.22705256	0.2533527	0.44834222	-5.79260453
hetero after UVB irradiation VS hetero before UVB irradiation	GO_COBALAMIN_METABOLIC_PROCESS1	-0.00333087	0.026922341	-0.01874295	0.98551363	0.99461317	-6.2312936
WT after UVB irradiation VS WT before UVB irradiation	GO_COBALAMIN_METABOLIC_PROCESS2	0.134838784	0.008556485	0.628986817	0.54668742	0.87364475	-5.7925536
homo after UVB irradiation VS homo before UVB irradiation	GO_COBALAMIN_TRANSPORT	0.507853148	0.05450245	2.55069581	0.03306913	0.1295064	-3.93060311
hetero after UVB irradiation VS hetero before UVB irradiation	GO_COBALAMIN_TRANSPORT1	0.234591569	0.128476719	0.850842555	0.42004297	0.7220868	-5.85659027
WT after UVB irradiation VS WT before UVB irradiation	GO_COBALAMIN_TRANSPORT2	0.453275578	-0.0160493	1.776632322	0.11307931	0.61801327	-4.60515776
homo after UVB irradiation VS homo before UVB irradiation	GO_COBALT_ION_TRANSPORT	0.347192415	0.039910883	1.689587208	0.12808234	0.29308593	-5.20804174
hetero after UVB irradiation VS hetero before UVB irradiation	GO_COBALT_ION_TRANSPORT1	0.199828346	0.018583397	0.70406387	0.501734	0.77390992	-5.97111083
WT after UVB irradiation VS WT before UVB irradiation	GO_COBALT_ION_TRANSPORT2	0.186654134	-0.05254896	0.831810465	0.42936124	0.82782832	-5.6466358
homo after UVB irradiation VS homo before UVB irradiation	GO_COCHLEA_DEVELOPMENT	0.111696873	-0.01318045	0.690709542	0.50853559	0.68117089	-6.29639367
hetero after UVB irradiation VS hetero before UVB irradiation	GO_COCHLEA_DEVELOPMENT1	0.102515199	-0.03049025	0.672247643	0.52071091	0.78691749	-5.99345562
WT after UVB irradiation VS WT before UVB irradiation	GO_COCHLEA_DEVELOPMENT2	-0.06579582	0.027287494	-0.3604326	0.72774457	0.93296251	-5.92809576
homo after UVB irradiation VS homo before UVB irradiation	GO_COCHLEA_MORPHOGENESIS	0.238645317	-0.02848444	1.201392197	0.26264342	0.45802753	-5.82155816
hetero after UVB irradiation VS hetero before UVB irradiation	GO_COCHLEA_MORPHOGENESIS1	0.074557108	-0.02444022	0.44465496	0.66856904	0.86440066	-6.1256379
WT after UVB irradiation VS WT before UVB irradiation	GO_COCHLEA_MORPHOGENESIS2	-0.14366038	0.050736708	-0.71423725	0.49515066	0.85459393	-5.73552498
homo after UVB irradiation VS homo before UVB irradiation	GO_COENZYME_A_BIOSYNTHETIC_PROCESS	0.005551053	-0.05831268	0.025588037	0.98018838	0.9901862	-6.55049739
hetero after UVB irradiation VS hetero before UVB irradiation	GO_COENZYME_A_BIOSYNTHETIC_PROCESS1	-0.22942306	-0.00237764	-1.01830956	0.33887513	0.65861414	-5.70457933
WT after UVB irradiation VS WT before UVB irradiation	GO_COENZYME_A_BIOSYNTHETIC_PROCESS2	-0.0308128	-0.03839422	-0.19112222	0.85313496	0.96605575	-5.97683878
homo after UVB irradiation VS homo before UVB irradiation	GO_COENZYME_A_METABOLIC_PROCESS	-0.18104863	-0.06706133	-0.95183174	0.36798218	0.55832954	-6.07936548
hetero after UVB irradiation VS hetero before UVB irradiation	GO_COENZYME_A_METABOLIC_PROCESS1	-0.20556786	-0.03057377	-1.05779131	0.32160214	0.64129204	-5.66569483
WT after UVB irradiation VS WT before UVB irradiation	GO_COENZYME_A_METABOLIC_PROCESS2	0.029617129	-0.01514799	0.144560123	0.88859141	0.97136322	-5.98505509
homo after UVB irradiation VS homo before UVB irradiation	GO_COGNITION	0.19990589	-0.00538981	1.584895843	0.15015786	0.32372589	-5.34944223
hetero after UVB irradiation VS hetero before UVB irradiation	GO_COGNITION1	0.045464505	0.011913206	0.302247766	0.77031359	0.91003052	-6.18223902
WT after UVB irradiation VS WT before UVB irradiation	GO_COGNITION2	0.029163634	-0.00279009	0.164875758	0.87308337	0.96914919	-5.98174849
homo after UVB irradiation VS homo before UVB irradiation	GO_COHESIN_COMPLEX	-0.55842186	0.001451344	-2.52730644	0.03431865	0.1326802	-3.96678802
hetero after UVB irradiation VS hetero before UVB irradiation	GO_COHESIN_COMPLEX1	-0.40204758	-0.00861733	-1.76630271	0.11603904	0.41924211	-4.81686453
WT after UVB irradiation VS WT before UVB irradiation	GO_COHESIN_COMPLEX2	-0.4723008	0.052690736	-1.90136118	0.09332827	0.59033229	-4.4386665
homo after UVB irradiation VS homo before UVB irradiation	GO_COHESIN_LOADING	-0.86967491	0.078699282	-5.38647744	0.0005731	0.01735126	0.133628414
hetero after UVB irradiation VS hetero before UVB irradiation	GO_COHESIN_LOADING1	-0.23280219	-0.00992581	-0.70850347	0.49912124	0.7722442	-5.96792071
WT after UVB irradiation VS WT before UVB irradiation	GO_COHESIN_LOADING2	-0.44307639	-0.0119288	-1.2103261	0.26029516	0.74056733	-5.28936441
homo after UVB irradiation VS homo before UVB irradiation	GO_COLLAGEN_ACTIVATED_SIGNALING_PATHWAY	0.427733726	0.085705807	1.946436127	0.08603765	0.22868471	-4.84431735
hetero after UVB irradiation VS hetero before UVB irradiation	GO_COLLAGEN_ACTIVATED_SIGNALING_PATHWAY1	0.323172433	0.04350108	1.381528652	0.20514795	0.52992741	-5.30903209
WT after UVB irradiation VS WT before UVB irradiation	GO_COLLAGEN_ACTIVATED_SIGNALING_PATHWAY2	0.200631647	0.018804439	0.919333808	0.38448809	0.80629399	-5.573196
homo after UVB irradiation VS homo before UVB irradiation	GO_COLLAGEN_ACTIVATED_TYROSINE_KINASE_RECEPTOR_SIGNALING_PATHWAY	0.366917234	0.050691138	1.644610746	0.13717464	0.3062223	-5.2693415
hetero after UVB irradiation VS hetero before UVB irradiation	GO_COLLAGEN_ACTIVATED_TYROSINE_KINASE_RECEPTOR_SIGNALING_PATHWAY1	0.1769619	0.016862085	0.691129759	0.50939528	0.77892609	-5.98030438
WT after UVB irradiation VS WT before UVB irradiation	GO_COLLAGEN_ACTIVATED_TYROSINE_KINASE_RECEPTOR_SIGNALING_PATHWAY2	0.066681065	0.008287893	0.3024469	0.76993456	0.94145237	-5.94808729
homo after UVB irradiation VS homo before UVB irradiation	GO_COLLAGEN_BINDING	0.650773429	-0.00200286	4.227431146	0.00263566	0.03390967	-1.40123182
homo after UVB irradiation VS homo before UVB irradiation	GO_COLLAGEN_BINDING_INVOLVED_IN_CELL_MATRIX_ADHESION	0.659756773	-0.03396105	3.07460641	0.01452126	0.08204067	-3.11775617
hetero after UVB irradiation VS hetero before UVB irradiation	GO_COLLAGEN_BINDING_INVOLVED_IN_CELL_MATRIX_ADHESION1	0.918477779	0.009909662	6.355840659	0.00023723	0.07788488	1.016147114
WT after UVB irradiation VS WT before UVB irradiation	GO_COLLAGEN_BINDING_INVOLVED_IN_CELL_MATRIX_ADHESION2	0.439683136	0.028447378	1.410447356	0.19564531	0.69389529	-5.06413173
hetero after UVB irradiation VS hetero before UVB irradiation	GO_COLLAGEN_BINDING1	0.316520636	0.03261738	1.260154485	0.24377066	0.57159915	-5.45007373
WT after UVB irradiation VS WT before UVB irradiation	GO_COLLAGEN_BINDING2	0.315267185	0.013727958	1.197719416	0.26490648	0.74156847	-5.30280428
homo after UVB irradiation VS homo before UVB irradiation	GO_COLLAGEN_BIOSYNTHETIC_PROCESS	0.429072852	-0.00652965	2.973892633	0.0169835	0.08900813	-3.27367397
hetero after UVB irradiation VS hetero before UVB irradiation	GO_COLLAGEN_BIOSYNTHETIC_PROCESS1	0.105461872	-0.00066512	0.426299406	0.68133462	0.87175352	-6.13409543
WT after UVB irradiation VS WT before UVB irradiation	GO_COLLAGEN_BIOSYNTHETIC_PROCESS2	0.100084524	-0.01484497	0.395950266	0.70236989	0.92532318	-5.91418195
homo after UVB irradiation VS homo before UVB irradiation	GO_COLLAGEN_CATABOLIC_PROCESS	0.826774092	-0.01222841	6.550543784	0.00014906	0.01016637	1.465931363
hetero after UVB irradiation VS hetero before UVB irradiation	GO_COLLAGEN_CATABOLIC_PROCESS1	0.510540683	0.085479579	2.255672342	0.05469029	0.30967379	-4.12780917
WT after UVB irradiation VS WT before UVB irradiation	GO_COLLAGEN_CATABOLIC_PROCESS2	0.480887744	0.024536528	2.058022117	0.07316335	0.55377265	-4.22447817
homo after UVB irradiation VS homo before UVB irradiation	GO_COLLAGEN_CONTAINING_EXTRACELLULAR_MATRIX	0.559561009	-0.00405761	4.246066777	0.0025678	0.03359312	-1.37489864
hetero after UVB irradiation VS hetero before UVB irradiation	GO_COLLAGEN_CONTAINING_EXTRACELLULAR_MATRIX1	0.234968768	0.024497566	1.103290738	0.30256058	0.62459204	-5.61953544
WT after UVB irradiation VS WT before UVB irradiation	GO_COLLAGEN_CONTAINING_EXTRACELLULAR_MATRIX2	0.279260253	0.014644047	1.234159018	0.25175865	0.73564768	-5.26369938
homo after UVB irradiation VS homo before UVB irradiation	GO_COLLAGEN_FIBRIL_ORGANIZATION	0.403071503	-0.01526192	2.43062378	0.04000972	0.14370574	-4.11596128
hetero after UVB irradiation VS hetero before UVB irradiation	GO_COLLAGEN_FIBRIL_ORGANIZATION1	0.064403689	-0.01245748	0.250630669	0.80853255	0.92568169	-6.19755928

WT after UVB irradiation VS WT before UVB irradiation	GO_COLLAGEN_FIBRIL_ORGANIZATION2	0.117602932	-0.01827763	0.493121133	0.63502065	0.90549535	-5.86976198
homo after UVB irradiation VS homo before UVB irradiation	GO_COLLAGEN_METABOLIC_PROCESS	0.619495982	-0.01224908	4.548998591	0.00169251	0.02787404	-0.95425657
hetero after UVB irradiation VS hetero before UVB irradiation	GO_COLLAGEN_METABOLIC_PROCESS1	0.365627851	0.054052214	1.563807977	0.15719666	0.47630266	-5.08339263
WT after UVB irradiation VS WT before UVB irradiation	GO_COLLAGEN_METABOLIC_PROCESS2	0.321720937	0.015925226	1.32008337	0.22290043	0.71788338	-5.16848894
homo after UVB irradiation VS homo before UVB irradiation	GO_COLLAGEN_RECEPTOR_ACTIVITY	0.516215272	0.212680949	1.820780816	0.10464944	0.2579011	-5.02493139
hetero after UVB irradiation VS hetero before UVB irradiation	GO_COLLAGEN_RECEPTOR_ACTIVITY1	0.25006467	0.01349593	0.881025481	0.40446207	0.71132442	-5.8308072
WT after UVB irradiation VS WT before UVB irradiation	GO_COLLAGEN_RECEPTOR_ACTIVITY2	0.247121775	-0.05988668	1.079450292	0.31148665	0.76876416	-5.42408185
homo after UVB irradiation VS homo before UVB irradiation	GO_COLLAGEN_TRIMER	0.597685793	-0.01917556	4.379670439	0.00213304	0.03092226	-1.18764469
hetero after UVB irradiation VS hetero before UVB irradiation	GO_COLLAGEN_TRIMER1	0.20200864	0.020748803	0.942388528	0.37407809	0.6871463	-5.776161
WT after UVB irradiation VS WT before UVB irradiation	GO_COLLAGEN_TRIMER2	0.216263339	0.000774869	0.874177485	0.40719916	0.81717677	-5.61183416
homo after UVB irradiation VS homo before UVB irradiation	GO_COLLAGEN_TYPE_IV_TRIMER	-0.04558126	0.04116226	-0.18529002	0.85743425	0.92157008	-6.53205389
hetero after UVB irradiation VS hetero before UVB irradiation	GO_COLLAGEN_TYPE_IV_TRIMER1	0.187617101	-0.0647646	0.768858929	0.46447089	0.75191798	-5.92282995
WT after UVB irradiation VS WT before UVB irradiation	GO_COLLAGEN_TYPE_IV_TRIMER2	-0.05964788	0.068802712	-0.25439299	0.80553302	0.95404687	-5.96206331
homo after UVB irradiation VS homo before UVB irradiation	GO_COLLATERAL_SPROUTING	0.126010062	-0.00334812	0.820061127	0.43501878	0.61811126	-6.19620028
homo after UVB irradiation VS homo before UVB irradiation	GO_COLLATERAL_SPROUTING_IN_ABSENCE_OF_INJURY	-0.14943786	-0.00499965	-0.54246592	0.60170611	0.7508352	-6.39219872
hetero after UVB irradiation VS hetero before UVB irradiation	GO_COLLATERAL_SPROUTING_IN_ABSENCE_OF_INJURY1	-0.72616727	0.011431373	-3.96386374	0.00431234	0.12634511	-1.69094757
WT after UVB irradiation VS WT before UVB irradiation	GO_COLLATERAL_SPROUTING_IN_ABSENCE_OF_INJURY2	-0.23025988	0.097129746	-0.71639899	0.49388498	0.8538002	-5.73399598
hetero after UVB irradiation VS hetero before UVB irradiation	GO_COLLATERAL_SPROUTING1	-0.10108274	-0.000333	-0.49837519	0.63187643	0.8484405	-6.09895212
WT after UVB irradiation VS WT before UVB irradiation	GO_COLLATERAL_SPROUTING2	0.004520781	-0.00817105	0.028405707	0.97802618	0.99433096	-5.99564094
homo after UVB irradiation VS homo before UVB irradiation	GO_COLLECTING_DUCT_DEVELOPMENT	-0.32239049	0.030177915	-2.27902691	0.05089603	0.16653498	-4.34805907
hetero after UVB irradiation VS hetero before UVB irradiation	GO_COLLECTING_DUCT_DEVELOPMENT1	-0.48194716	0.062158204	-3.04172554	0.01637688	0.18894229	-2.97895604
WT after UVB irradiation VS WT before UVB irradiation	GO_COLLECTING_DUCT_DEVELOPMENT2	-0.44491749	0.098079901	-1.80094166	0.10894401	0.61315287	-4.57303648
homo after UVB irradiation VS homo before UVB irradiation	GO_COLUMNAR_CUBOIDAL_EPITHELIAL_CELL_DEVELOPMENT	0.038286111	0.005399954	0.290039896	0.77887561	0.87261401	-6.5049244
hetero after UVB irradiation VS hetero before UVB irradiation	GO_COLUMNAR_CUBOIDAL_EPITHELIAL_CELL_DEVELOPMENT1	0.119008611	0.000355056	0.844315264	0.42346754	0.7252313	-5.86206885
WT after UVB irradiation VS WT before UVB irradiation	GO_COLUMNAR_CUBOIDAL_EPITHELIAL_CELL_DEVELOPMENT2	-0.02907618	-0.01196248	-0.18703793	0.85623222	0.96638003	-5.97764994
homo after UVB irradiation VS homo before UVB irradiation	GO_COLUMNAR_CUBOIDAL_EPITHELIAL_CELL_DIFFERENTIATION	-0.08234196	0.038964386	-0.57976775	0.57741291	0.73379971	-6.37007871
hetero after UVB irradiation VS hetero before UVB irradiation	GO_COLUMNAR_CUBOIDAL_EPITHELIAL_CELL_DIFFERENTIATION1	-0.0441407	0.007278854	-0.34306623	0.74055173	0.89887154	-6.16814897
WT after UVB irradiation VS WT before UVB irradiation	GO_COLUMNAR_CUBOIDAL_EPITHELIAL_CELL_DIFFERENTIATION2	-0.14913012	0.023570992	-0.85288577	0.41823361	0.8230207	-5.62950197
homo after UVB irradiation VS homo before UVB irradiation	GO_COLUMNAR_CUBOIDAL_EPITHELIAL_CELL_MATURATION	-0.45053813	0.029945339	-2.07450502	0.07034974	0.20337589	-4.65590099
hetero after UVB irradiation VS hetero before UVB irradiation	GO_COLUMNAR_CUBOIDAL_EPITHELIAL_CELL_MATURATION1	0.032478824	-0.00321582	0.143284542	0.88967391	0.95951091	-6.22036535
WT after UVB irradiation VS WT before UVB irradiation	GO_COLUMNAR_CUBOIDAL_EPITHELIAL_CELL_MATURATION2	-0.15645841	0.001984824	-0.62247331	0.55075276	0.87546015	-5.7966443
homo after UVB irradiation VS homo before UVB irradiation	GO_COMMA_SHAPED_BODY_MORPHOGENESIS	0.222836277	0.055999196	0.868491081	0.40945058	0.59617782	-6.15493068
hetero after UVB irradiation VS hetero before UVB irradiation	GO_COMMA_SHAPED_BODY_MORPHOGENESIS1	0.471337588	0.025252587	2.202003951	0.05942932	0.3212231	-4.2054133
WT after UVB irradiation VS WT before UVB irradiation	GO_COMMA_SHAPED_BODY_MORPHOGENESIS2	0.113481046	0.008516855	0.473390896	0.64843361	0.90907607	-5.87952476
homo after UVB irradiation VS homo before UVB irradiation	GO_COMMISSURAL_NEURON_AXON_GUIDANCE	0.050069896	0.038705	0.235083471	0.81982066	0.89881824	-6.52062873
hetero after UVB irradiation VS hetero before UVB irradiation	GO_COMMISSURAL_NEURON_AXON_GUIDANCE1	-0.0688847	0.060327191	-0.34379773	0.74002243	0.89874658	-6.16788068
WT after UVB irradiation VS WT before UVB irradiation	GO_COMMISSURAL_NEURON_AXON_GUIDANCE2	-0.09282245	0.027258811	-0.43311039	0.67623993	0.91685973	-5.89828504
homo after UVB irradiation VS homo before UVB irradiation	GO_COMMITMENT_COMPLEX	-1.08561568	-0.00201605	-7.26243529	7.09E-05	0.00942429	2.186019583
hetero after UVB irradiation VS hetero before UVB irradiation	GO_COMMITMENT_COMPLEX1	-0.93466968	-0.15926375	-3.22903538	0.01237902	0.17183889	-2.70894175
WT after UVB irradiation VS WT before UVB irradiation	GO_COMMITMENT_COMPLEX2	-0.36984436	-0.00218263	-0.96096119	0.36438371	0.79568799	-5.53620691
homo after UVB irradiation VS homo before UVB irradiation	GO_COMMITMENT_OF_NEURONAL_CELL_TO_SPECIFIC_NEURON_TYPE_IN_FOREBRAIN	0.364807764	0.016899047	1.999168452	0.07920774	0.21718109	-4.76721615
hetero after UVB irradiation VS hetero before UVB irradiation	GO_COMMITMENT_OF_NEURONAL_CELL_TO_SPECIFIC_NEURON_TYPE_IN_FOREBRAIN1	0.298098856	0.088434507	1.226754221	0.25542284	0.58262782	-5.48742956
WT after UVB irradiation VS WT before UVB irradiation	GO_COMMITMENT_OF_NEURONAL_CELL_TO_SPECIFIC_NEURON_TYPE_IN_FOREBRAIN2	0.34259284	0.015589312	1.756154613	0.11667664	0.62406566	-4.63208274
homo after UVB irradiation VS homo before UVB irradiation	GO_COMMON_BILE_DUCT_DEVELOPMENT	-0.02373562	-0.03357138	-0.07972385	0.93833917	0.96724442	-6.54736983
hetero after UVB irradiation VS hetero before UVB irradiation	GO_COMMON_BILE_DUCT_DEVELOPMENT1	0.231012157	-0.07764356	0.712516839	0.49676679	0.77015247	-5.96502177
WT after UVB irradiation VS WT before UVB irradiation	GO_COMMON_BILE_DUCT_DEVELOPMENT2	-0.08464425	0.021741008	-0.36427356	0.72498253	0.93154998	-5.92665186
homo after UVB irradiation VS homo before UVB irradiation	GO_COMMON_MYELOID_PROGENITOR_CELL_PROLIFERATION	0.241445462	0.004799671	1.005839281	0.34281558	0.53550646	-6.02744834
hetero after UVB irradiation VS hetero before UVB irradiation	GO_COMMON_MYELOID_PROGENITOR_CELL_PROLIFERATION1	0.053955739	0.083029369	0.197585575	0.8483879	0.94253966	-6.2103668
WT after UVB irradiation VS WT before UVB irradiation	GO_COMMON_MYELOID_PROGENITOR_CELL_PROLIFERATION2	0.14709714	-0.00408307	0.573742429	0.58172299	0.88904835	-5.82602029
homo after UVB irradiation VS homo before UVB irradiation	GO_COMMON_PARTNER_SMAD_PROTEIN_PHOSPHORYLATION	0.024009735	-0.06653752	0.090337785	0.93015353	0.96223454	-6.54638007
hetero after UVB irradiation VS hetero before UVB irradiation	GO_COMMON_PARTNER_SMAD_PROTEIN_PHOSPHORYLATION1	-0.06730121	-0.02568335	-0.31211115	0.76308235	0.90723412	-6.17899333
WT after UVB irradiation VS WT before UVB irradiation	GO_COMMON_PARTNER_SMAD_PROTEIN_PHOSPHORYLATION2	-0.06515476	0.023067138	-0.24383097	0.81342577	0.955326	-5.96481753
homo after UVB irradiation VS homo before UVB irradiation	GO_COMPACT_MYELIN	0.321102948	-0.031135	1.934203416	0.08770085	0.23146841	-4.86209942
hetero after UVB irradiation VS hetero before UVB irradiation	GO_COMPACT_MYELIN1	0.063100009	-0.00953713	0.245573615	0.8123086	0.92731128	-6.19890913
WT after UVB irradiation VS WT before UVB irradiation	GO_COMPACT_MYELIN2	-0.07234843	-0.08870344	-0.27047301	0.7935624	0.95040958	-5.95764988
homo after UVB irradiation VS homo before UVB irradiation	GO_COMPARTMENT_PATTERN_SPECIFICATION	0.002875536	0.005002284	0.01323988	0.98974806	0.99473624	-6.55076051
hetero after UVB irradiation VS hetero before UVB irradiation	GO_COMPARTMENT_PATTERN_SPECIFICATION1	0.250944519	0.043032165	1.167964599	0.27705667	0.60400502	-5.55154243
WT after UVB irradiation VS WT before UVB irradiation	GO_COMPARTMENT_PATTERN_SPECIFICATION2	-0.06109452	-0.09232305	-0.22231886	0.82957052	0.95961989	-5.97007126
homo after UVB irradiation VS homo before UVB irradiation	GO_COMPLEMENT_ACTIVATION	0.606359806	0.002449453	3.210386009	0.01177554	0.0728941	-2.90844661
homo after UVB irradiation VS homo before UVB irradiation	GO_COMPLEMENT_ACTIVATION_ALTERNATIVE_PATHWAY	0.738053742	-0.02468033	3.557262125	0.00695925	0.05494793	-2.38061077
hetero after UVB irradiation VS hetero before UVB irradiation	GO_COMPLEMENT_ACTIVATION_ALTERNATIVE_PATHWAY1	0.168216305	0.056854215	0.725236826	0.48935171	0.76616077	-5.95573951
WT after UVB irradiation VS WT before UVB irradiation	GO_COMPLEMENT_ACTIVATION_ALTERNATIVE_PATHWAY2	0.285056318	0.001785751	1.004124197	0.34437688	0.78369331	-5.49650847
homo after UVB irradiation VS homo before UVB irradiation	GO_COMPLEMENT_ACTIVATION_LECTIN_PATHWAY	0.691421903	-0.02176771	3.789229217	0.0049377	0.04588513	-2.03486732
hetero after UVB irradiation VS hetero before UVB irradiation	GO_COMPLEMENT_ACTIVATION_LECTIN_PATHWAY1	0.342935807	0.082650841	1.697347016	0.12877448	0.43632521	-4.90924662
WT after UVB irradiation VS WT before UVB irradiation	GO_COMPLEMENT_ACTIVATION_LECTIN_PATHWAY2	0.071874978	0.046892244	0.322613949	0.75516	0.93835001	-5.94152009
hetero after UVB irradiation VS hetero before UVB irradiation	GO_COMPLEMENT_ACTIVATION1	0.387826216	0.069427773	2.012375747	0.07965432	0.36339704	-4.47634543
WT after UVB irradiation VS WT before UVB irradiation	GO_COMPLEMENT_ACTIVATION2	0.280316025	-0.02240829	1.069550328	0.31566386	0.77149112	-5.4338225
homo after UVB irradiation VS homo before UVB irradiation	GO_COMPLEMENT_BINDING	0.536422926	0.0093655	2.987776266	0.01661982	0.08834967	-3.2521539
hetero after UVB irradiation VS hetero before UVB irradiation	GO_COMPLEMENT_BINDING1	0.233824474	-0.00430528	1.073177966	0.31505991	0.63556206	-5.6502441
WT after UVB irradiation VS WT before UVB irradiation	GO_COMPLEMENT_BINDING2	0.14098406	-0.05325125	0.449155351	0.66509731	0.9126723	-5.89100163
homo after UVB irradiation VS homo before UVB irradiation	GO_COMPLEMENT_COMPONENT_C1Q_COMPLEX_BINDING	0.239088913	0.047788683	1.60059905	0.14663969	0.31945112	-5.32852564
hetero after UVB irradiation VS hetero before UVB irradiation	GO_COMPLEMENT_COMPONENT_C1Q_COMPLEX_BINDING1	-0.06902233	-0.04902753	-0.29919528	0.7725564	0.91147696	-6.18322288
WT after UVB irradiation VS WT before UVB irradiation	GO_COMPLEMENT_COMPONENT_C1Q_COMPLEX_BINDING2	-0.01546052	-0.09902926	-0.04940462	0.96179372	0.99047885	-5.99477897
homo after UVB irradiation VS homo before UVB irradiation	GO_COMPLEMENT_COMPONENT_C3B_BINDING	0.578439598	-0.02901909	2.299710343	0.0492527	0.16292806	-4.31655406
hetero after UVB irradiation VS hetero before UVB irradiation	GO_COMPLEMENT_COMPONENT_C3B_BINDING1	0.703404125	-0.04728932	2.569723515	0.03364557	0.25343238	-3.66912514
WT after UVB irradiation VS WT before UVB irradiation	GO_COMPLEMENT_COMPONENT_C3B_BINDING2	0.536795728	-0.09742078	1.630553793	0.14117512	0.65097221	-4.7943084
homo after UVB irradiation VS homo before UVB irradiation	GO_COMPLEMENT_DEPENDENT_CYTOTOXICITY	0.311528537	-0.01549883	1.180576434	0.27038373	0.46562895	-5.84473123
hetero after UVB irradiation VS hetero before UVB irradiation	GO_COMPLEMENT_DEPENDENT_CYTOTOXICITY1	0.038629701	0.078535732	0.155993924	0.87997333	0.95631805	-6.2183087
WT after UVB irradiation VS WT before UVB irradiation	GO_COMPLEMENT_DEPENDENT_CYTOTOXICITY2	0.298114543	0.007796292	1.185500819	0.26943971	0.74431614	-5.31573925
homo after UVB irradiation VS homo before UVB irradiation	GO_COMPLEMENT_RECEPTOR_ACTIVITY	0.952074929	0.056141679	6.554336087	0.00014845	0.01016637	1.469948724
hetero after UVB irradiation VS hetero before UVB irradiation	GO_COMPLEMENT_RECEPTOR_ACTIVITY1	0.882087478	0.096491881	5.682274889	0.00049615	0.07788488	0.349161378
WT after UVB irradiation VS WT before UVB irradiation	GO_COMPLEMENT_RECEPTOR_ACTIVITY2	0.722808358	0.138888984	2.715845932	0.02612609	0.43720841	-3.29523125
homo after UVB irradiation VS homo before UVB irradiation	GO_COMPLEMENT_RECEPTOR_MEDIATED_SIGNALING_PATHWAY	0.91940135	0.040539717	5.685511062	0.00039874	0.0149358	0.495331247
hetero after UVB irradiation VS hetero before UVB irradiation	GO_COMPLEMENT_RECEPTOR_MEDIATED_SIGNALING_PATHWAY1	0.702742378	0.078531287	4.449668132	0.00224063	0.10650321	-1.06340116
WT after UVB irradiation VS WT before UVB irradiation	GO_COMPLEMENT_RECEPTOR_MEDIATED_SIGNALING_PATHWAY2	0.598656249	0.138099752	2.825295854	0.02204193	0.41488864	-3.13989879
homo after UVB irradiation VS homo before UVB irradiation	GO_COMPLEX_OF_COLLAGEN_TRIMERS	0.413061841	-0.01348682	2.226178393	0.0553456	0.17476921	-4.42828194
hetero after UVB irradiation VS hetero before UVB irradiation	GO_COMPLEX_OF_COLLAGEN_TRIMERS1	0.215144644	0.023886143	0.97969503	0.35645108	0.67198476	-5.74152221
WT after UVB irradiation VS WT before UVB irradiation	GO_COMPLEX_OF_COLLAGEN_TRIMERS2	0.203423196	-0.00118512	0.855211395	0.4170182	0.82238909	-5.62758958
homo after UVB irradiation VS homo before UVB irradiation	GO_CONDENSED_CHROMOSOME	-0.51410826	-0.01283327	-3.14865822	0.01294969	0.07691649	-3.0034547
homo after UVB irradiation VS homo before UVB irradiation	GO_CONDENSED_CHROMOSOME_CENTROMERIC_REGION	-0.53888769	-0.0277608	-2.99700173	0.01638263	0.08759365	-3.23785835
hetero after UVB irradiation VS hetero before UVB irradiation	GO_CONDENSED_CHROMOSOME_CENTROMERIC_REGION1	0.121406845	-0.01301682	0.432955896	0.67669232	0.86987298	-6.13106757
WT after UVB irradiation VS WT before UVB irradiation	GO_CONDENSED_CHROMOSOME_CENTROMERIC_REGION2	2.04E-05	0.002525236	7.43E-05	0.99994251	0.99999694	-5.99606666
homo after UVB irradiation VS homo before UVB irradiation	GO_CONDENSED_CHROMOSOME_INNER_KINETOCHORE	-0.52604619	0.029784949	-1.63201659	0.13982456	0.30972546	-5.28636016
hetero after UVB irradiation VS hetero before UVB irradiation	GO_CONDENSED_CHROMOSOME_INNER_KINETOCHORE1	-0.12721241	-0.06738817	-0.28974653	0.77951324	0.91401375	-6.18620648
WT after UVB irradiation VS WT before UVB irradiation	GO_CONDENSED_CHROMOSOME_INNER_KINETOCHORE2	-0.1336209	0.022680786	-0.35841902	0.72919423	0.93319914	-5.92884679
homo after UVB irradiation VS homo before UVB irradiation	GO_CONDENSED_CHROMOSOME_OUTER_KINETOCHORE	-0.60316171	-0.03551949	-2.38483837	0.04302663	0.14999859	-4.18632696
hetero after UVB irradiation VS hetero before UVB irradiation	GO_CONDENSED_CHROMOSOME_OUTER_KINETOCHORE1	0.273340339	-0.06552252	0.76344933	0.46750993	0.7530936	-5.92700076
WT after UVB irradiation VS WT before UVB irradiation	GO_CONDENSED_CHROMOSOME_OUTER_KINETOCHORE2	-0.0477142	0.014088892	-0.1450676	0.88820337	0.97119273	-5.98497774
hetero after UVB irradiation VS hetero before UVB irradiation	GO_CONDENSED_CHROMOSOME1	0.006300077	-0.0280367	0.025042618	0.98064564	0.99313593	-6.23114403
WT after UVB irradiation VS WT before UVB irradiation	GO_CONDENSED_CHROMOSOME2	-0.0469707	0.009287963	-0.19019678	0.85383651	0.9663792	-5.97702409
homo after UVB irradiation VS homo before UVB irradiation	GO_CONDENSED_NUCLEAR_CHROMOSOME	-0.4428829	0.006548931	-2.4613707	0.0381037	0.1400489	-4.06859997
homo after UVB irradiation VS homo before UVB irradiation	GO_CONDENSED_NUCLEAR_CHROMOSOME_CENTROMERIC_REGION	-0.4848376	-0.04624193	-1.99558921	0.07965434	0.21799886	-4.77247163
hetero after UVB irradiation VS hetero before UVB irradiation	GO_CONDENSED_NUCLEAR_CHROMOSOME_CENTROMERIC_REGION1	0.140023357	0.016447825	0.499569396	0.63107248	0.84801601	-6.09832643
WT after UVB irradiation VS WT before UVB irradiation	GO_CONDENSED_NUCLEAR_CHROMOSOME_CENTROMERIC_REGION2	-0.06952843	-0.02274174	-0.24027271	0.81608994	0.95613748	-5.96571952
homo after UVB irradiation VS homo before UVB irradiation	GO_CONDENSED_NUCLEAR_CHROMOSOME_KINETOCHORE	-0.66484965	-0.05931541	-2.68399354	0.02677855	0.11575014	-3.72389842
hetero after UVB irradiation VS hetero before UVB irradiation	GO_CONDENSED_NUCLEAR_CHROMOSOME_KINETOCHORE1	0.107667386	-0.01976195	0.336509314	0.7453028	0.90030853	-6.17052911
WT after UVB irradiation VS WT before UVB irradiation	GO_CONDENSED_NUCLEAR_CHROMOSOME_KINETOCHORE2	-0.11275954	-0.02876673	-0.34639294	0.73787622	0.93478445	-5.93324755
hetero after UVB irradiation VS hetero before UVB irradiation	GO_CONDENSED_NUCLEAR_CHROMOSOME1	-0.04940537	-0.02268819	-0.20603429	0.84200533	0.93969374	-6.2085274
WT after UVB irradiation VS WT before UVB irradiation	GO_CONDENSED_NUCLEAR_CHROMOSOME2	-0.08953931	0.000553718	-0.4076208	0.69411601	0.92191472	-5.90933619
homo after UVB irradiation VS homo before UVB irradiation	GO_CONDENSIN_COMPLEX	-0.84171849	0.012686008	-3.05415601	0.01498937	0.08315721	-3.14937698
hetero after UVB irradiation VS hetero before UVB irradiation	GO_CONDENSIN_COMPLEX1	0.054083958	0.004517735	0.139672865	0.89243427	0.96070914	-6.2209181
WT after UVB irradiation VS WT before UVB irradiation	GO_CONDENSIN_COMPLEX2	-0.04062939	-0.0313021	-0.11851706	0.9085453	0.97832631	-5.98866194
homo after UVB irradiation VS homo before UVB irradiation	GO_CONDITIONED_TASTE_AVERSION	0.605218978	0.086987441	1.94554781	0.0861574	0.22880916	-4.84561
hetero after UVB irradiation VS hetero before UVB irradiation	GO_CONDITIONED_TASTE_AVERSION1	0.371691574	0.09224218	2.063436078	0.07362537	0.35156244	-4.40397881
WT after UVB irradiation VS WT before UVB irradiation	GO_CONDITIONED_TASTE_AVERSION2	0.17132227	0.058334454	0.602222568	0.56350476	0.88099977	-5.80911621
homo after UVB irradiation VS homo before UVB irradiation	GO_CONNECTIVE_TISSUE_DEVELOPMENT	0.122921542	-0.00649941	0.998915319	0.34596745	0.53809461	-6.03423003
hetero after UVB irradiation VS hetero before UVB irradiation	GO_CONNECTIVE_TISSUE_DEVELOPMENT1	-0.09550309	-0.00764927	-0.58534963	0.57474361	0.81620465	-6.04975951
WT after UVB irradiation VS WT before UVB irradiation	GO_CONNECTIVE_TISSUE_DEVELOPMENT2	-0.05786891	-0.03343076	-0.34207167	0.74100575	0.93520732	-5.93479332
homo after UVB irradiation VS homo before UVB irradiation	GO_CONNECTIVE_TISSUE_REPLACEMENT	0.599176532	-0.02354087	4.004594478	0.00361442	0.03927685	-1.72007036
hetero after UVB irradiation VS hetero before UVB irradiation	GO_CONNECTIVE_TISSUE_REPLACEMENT1	0.267731146	0.032691078	1.175128695	0.27434239	0.6010071	-5.54384456
WT after UVB irradiation VS WT before UVB irradiation	GO_CONNECTIVE_TISSUE_REPLACEMENT2	0.237702196	-0.06908561	0.758630315	0.46957646	0.84565091	-5.70332278
homo after UVB irradiation VS homo before UVB irradiation	GO_CONNEXIN_BINDING	-0.10610619	-0.03388236	-0.61497435	0.55499668	0.7166713	-6.34796187
hetero after UVB irradiation VS hetero before UVB irradiation	GO_CONNEXIN_BINDING1	-0.19487272	0.030373744	-1.00464571	0.34501694	0.66238225	-5.71777634
WT after UVB irradiation VS WT before UVB irradiation	GO_CONNEXIN_BINDING2	0.133217934	0.046576319	0.63980468	0.53997494	0.87025652	-5.78567495
homo after UVB irradiation VS homo before UVB irradiation	GO_CONNEXIN_COMPLEX	0.307288126	-0.02228381	1.957041857	0.08462012	0.22632153	-4.82886801
hetero after UVB irradiation VS hetero before UVB irradiation	GO_CONNEXIN_COMPLEX1	0.230412641	-0.02833608	1.576793926	0.15419981	0.47198468	-5.06675879
WT after UVB irradiation VS WT before UVB irradiation	GO_CONNEXIN_COMPLEX2	0.057411184	0.080343817	0.288924296	0.77989795	0.94424676	-5.95225902
homo after UVB irradiation VS homo before UVB irradiation	GO_CONSTITUTIVE_SECRETORY_PATHWAY	0.116814308	-0.08254003	0.383733352	0.7107862	0.827472	-6.4707623
hetero after UVB irradiation VS hetero before UVB irradiation	GO_CONSTITUTIVE_SECRETORY_PATHWAY1	0.120220629	0.017887043	0.424283933	0.6827431	0.87233156	-6.1350034
WT after UVB irradiation VS WT before UVB irradiation	GO_CONSTITUTIVE_SECRETORY_PATHWAY2	-0.09232472	-0.05789942	-0.48548961	0.64019211	0.90646829	-5.8735826
homo after UVB irradiation VS homo before UVB irradiation	GO_CONTRACTILE_FIBER	0.382516804	-0.02943138	2.367767497	0.04420883	0.1525701	-4.21250828
hetero after UVB irradiation VS hetero before UVB irradiation	GO_CONTRACTILE_FIBER1	-0.0672765	-0.02648189	-0.32289198	0.75520682	0.9047334	-6.17532961
WT after UVB irradiation VS WT before UVB irradiation	GO_CONTRACTILE_FIBER2	0.108648912	-0.02312631	0.447544273	0.66621223	0.91342263	-5.89174432
homo after UVB irradiation VS homo before UVB irradiation	GO_CONTRACTILE_RING	-0.3278531	-0.01592726	-1.25126874	0.24483567	0.43879523	-5.76489618
hetero after UVB irradiation VS hetero before UVB irradiation	GO_CONTRACTILE_RING1	0.43802228	-0.00155617	2.39034972	0.0443941	0.28194098	-3.93182815
WT after UVB irradiation VS WT before UVB irradiation	GO_CONTRACTILE_RING2	0.264026482	0.053348769	0.848196322	0.42069195	0.82383319	-5.63334508
homo after UVB irradiation VS homo before UVB irradiation	GO_CONVERGENT_EXTENSION	-0.51191814	-0.02304853	-2.81319624	0.02184419	0.1028573	-3.52315559
homo after UVB irradiation VS homo before UVB irradiation	GO_CONVERGENT_EXTENSION_INVOLVED_IN_AXIS_ELONGATION	-0.65112649	0.047306384	-3.05387232	0.01499598	0.08316258	-3.14981579
hetero after UVB irradiation VS hetero before UVB irradiation	GO_CONVERGENT_EXTENSION_INVOLVED_IN_AXIS_ELONGATION1	-0.48436233	-0.1016714	-1.81475863	0.10780831	0.40602721	-4.75106327
WT after UVB irradiation VS WT before UVB irradiation	GO_CONVERGENT_EXTENSION_INVOLVED_IN_AXIS_ELONGATION2	-0.62227708	-0.05695814	-2.86437417	0.02074834	0.40469982	-3.08457857
homo after UVB irradiation VS homo before UVB irradiation	GO_CONVERGENT_EXTENSION_INVOLVED_IN_GASTRULATION	-0.3242721	-0.01044698	-1.49473663	0.17186876	0.35152701	-5.46737292
hetero after UVB irradiation VS hetero before UVB irradiation	GO_CONVERGENT_EXTENSION_INVOLVED_IN_GASTRULATION1	-0.30833897	-0.11252466	-1.10370152	0.30239284	0.62458221	-5.61911228
WT after UVB irradiation VS WT before UVB irradiation	GO_CONVERGENT_EXTENSION_INVOLVED_IN_GASTRULATION2	-0.54874801	-0.03315343	-2.2389947	0.05512151	0.5232848	-3.97196444
homo after UVB irradiation VS homo before UVB irradiation	GO_CONVERGENT_EXTENSION_INVOLVED_IN_ORGANOGENESIS	-0.4856893	-0.07532025	-2.39001196	0.04267463	0.14926511	-4.17838626
hetero after UVB irradiation VS hetero before UVB irradiation	GO_CONVERGENT_EXTENSION_INVOLVED_IN_ORGANOGENESIS1	-0.22449808	-0.09447144	-1.16450577	0.27837496	0.60400502	-5.55524733
WT after UVB irradiation VS WT before UVB irradiation	GO_CONVERGENT_EXTENSION_INVOLVED_IN_ORGANOGENESIS2	-0.49764808	0.054435807	-2.29477015	0.05050444	0.51082527	-3.8933663
hetero after UVB irradiation VS hetero before UVB irradiation	GO_CONVERGENT_EXTENSION1	-0.29107467	-0.06603754	-1.44495587	0.18716403	0.51246893	-5.23226913
WT after UVB irradiation VS WT before UVB irradiation	GO_CONVERGENT_EXTENSION2	-0.46182387	-0.01966772	-2.53238306	0.03479405	0.46805456	-3.55626266
homo after UVB irradiation VS homo before UVB irradiation	GO_COP9_SIGNALOSOME	-0.14637048	0.006428738	-0.99560389	0.34748258	0.53941663	-6.03746048
homo after UVB irradiation VS homo before UVB irradiation	GO_COP9_SIGNALOSOME_ASSEMBLY	-0.51185647	-0.06035929	-2.5051332	0.03554722	0.1349601	-4.00106009
hetero after UVB irradiation VS hetero before UVB irradiation	GO_COP9_SIGNALOSOME_ASSEMBLY1	-0.04131678	-0.04213223	-0.17493578	0.86555632	0.94896716	-6.21492158
WT after UVB irradiation VS WT before UVB irradiation	GO_COP9_SIGNALOSOME_ASSEMBLY2	-0.3350392	0.006656076	-1.71529102	0.12417695	0.63137109	-4.6854304

hetero after UVB irradiation VS hetero before UVB irradiation	GO_COP9_SIGNALOSOME1	-0.19149491	-0.04312713	-1.25972607	0.24391724	0.57159915	-5.45055703
WT after UVB irradiation VS WT before UVB irradiation	GO_COP9_SIGNALOSOME2	-0.05492766	0.003575408	-0.28892155	0.77989998	0.94424676	-5.95225984
homo after UVB irradiation VS homo before UVB irradiation	GO_COPI_COATED_VESICLE	0.636671206	-0.06019225	4.325548363	0.00229878	0.03212411	-1.26317361
homo after UVB irradiation VS homo before UVB irradiation	GO_COPI_COATED_VESICLE_BUDDING	0.656646765	-0.12199363	2.887298016	0.01944563	0.09606938	-3.40804166
hetero after UVB irradiation VS hetero before UVB irradiation	GO_COPI_COATED_VESICLE_BUDDING1	0.623208932	0.042189199	2.107211186	0.06881357	0.3402756	-4.34156826
WT after UVB irradiation VS WT before UVB irradiation	GO_COPI_COATED_VESICLE_BUDDING2	0.573701991	-0.06513172	2.487355177	0.03733685	0.47615783	-3.62031747
homo after UVB irradiation VS homo before UVB irradiation	GO_COPI_COATED_VESICLE_MEMBRANE	0.704860154	-0.06703572	4.639788689	0.00149766	0.02621042	-0.83095899
hetero after UVB irradiation VS hetero before UVB irradiation	GO_COPI_COATED_VESICLE_MEMBRANE1	0.544613962	0.016167751	2.810989206	0.02323374	0.21753618	-3.31527935
WT after UVB irradiation VS WT before UVB irradiation	GO_COPI_COATED_VESICLE_MEMBRANE2	0.495350578	-0.04747396	3.05583124	0.01545705	0.37067217	-2.81507897
hetero after UVB irradiation VS hetero before UVB irradiation	GO_COPI_COATED_VESICLE1	0.581898035	0.009276355	3.551106951	0.0077273	0.14291796	-2.25363628
WT after UVB irradiation VS WT before UVB irradiation	GO_COPI_COATED_VESICLE2	0.515360187	-0.04953458	3.320354465	0.01035512	0.33453212	-2.44847907
homo after UVB irradiation VS homo before UVB irradiation	GO_COPI_VESICLE_COAT	0.659982921	-0.05883466	3.892446867	0.0042485	0.04225671	-1.88321032
hetero after UVB irradiation VS hetero before UVB irradiation	GO_COPI_VESICLE_COAT1	0.544068173	0.005648495	2.777586462	0.02444991	0.22374282	-3.36419681
WT after UVB irradiation VS WT before UVB irradiation	GO_COPI_VESICLE_COAT2	0.399916114	-0.04321264	2.537887428	0.03449549	0.4670931	-3.54842998
homo after UVB irradiation VS homo before UVB irradiation	GO_COPII_COATED_ER_TO_GOLGI_TRANSPORT_VESICLE	0.399770799	-0.03246579	2.559027577	0.03263523	0.12850951	-3.91770575
hetero after UVB irradiation VS hetero before UVB irradiation	GO_COPII_COATED_ER_TO_GOLGI_TRANSPORT_VESICLE1	0.327708146	0.032185924	2.008106529	0.08017964	0.3642644	-4.48237354
WT after UVB irradiation VS WT before UVB irradiation	GO_COPII_COATED_ER_TO_GOLGI_TRANSPORT_VESICLE2	0.298518139	-0.018801	1.554559406	0.15820525	0.66180255	-4.88974152
homo after UVB irradiation VS homo before UVB irradiation	GO_COPII_COATED_VESICLE_BUDDING	0.331037837	-0.02448763	2.109065183	0.06661518	0.19682548	-4.60441979
hetero after UVB irradiation VS hetero before UVB irradiation	GO_COPII_COATED_VESICLE_BUDDING1	0.38297207	0.025330265	2.644716717	0.02997574	0.24197049	-3.55907051
WT after UVB irradiation VS WT before UVB irradiation	GO_COPII_COATED_VESICLE_BUDDING2	0.360915585	-0.02428488	2.369222235	0.0449363	0.50160745	-3.78805314
homo after UVB irradiation VS homo before UVB irradiation	GO_COPII_COATED_VESICLE_CARGO_LOADING	0.386314152	0.032962354	2.056881338	0.0723317	0.20642022	-4.68205621
hetero after UVB irradiation VS hetero before UVB irradiation	GO_COPII_COATED_VESICLE_CARGO_LOADING1	0.393843492	0.066273815	1.439538882	0.18864379	0.51443012	-5.2389017
WT after UVB irradiation VS WT before UVB irradiation	GO_COPII_COATED_VESICLE_CARGO_LOADING2	0.35429331	-0.08256661	1.89295232	0.09454954	0.59322598	-4.45001522
homo after UVB irradiation VS homo before UVB irradiation	GO_COPII_VESICLE_COAT	0.201214593	-0.00022452	0.924609377	0.38117518	0.57147232	-6.10466643
hetero after UVB irradiation VS hetero before UVB irradiation	GO_COPII_VESICLE_COAT1	0.20851209	0.021386673	0.872661099	0.40873792	0.71337032	-5.83802585
WT after UVB irradiation VS WT before UVB irradiation	GO_COPII_VESICLE_COAT2	0.249398071	-0.04635411	1.498366025	0.17196853	0.67808426	-4.95882488
homo after UVB irradiation VS homo before UVB irradiation	GO_COPPER_ION_BINDING	0.234191574	-0.01970064	1.674898793	0.13098894	0.29701917	-5.22814918
hetero after UVB irradiation VS hetero before UVB irradiation	GO_COPPER_ION_BINDING1	0.201257003	0.034632381	1.349161475	0.21489345	0.54213845	-5.34742188
WT after UVB irradiation VS WT before UVB irradiation	GO_COPPER_ION_BINDING2	0.196676418	0.029169622	1.086406413	0.30857766	0.76668381	-5.41719844
homo after UVB irradiation VS homo before UVB irradiation	GO_COPPER_ION_HOMEOSTASIS	-0.00446268	-0.06473652	-0.02766691	0.97857926	0.98896686	-6.55043664
hetero after UVB irradiation VS hetero before UVB irradiation	GO_COPPER_ION_HOMEOSTASIS1	0.489852174	0.006917717	3.494038473	0.00839197	0.14794737	-2.33336541
WT after UVB irradiation VS WT before UVB irradiation	GO_COPPER_ION_HOMEOSTASIS2	0.240798199	-0.01730932	1.193799882	0.2663538	0.74288093	-5.30696344
homo after UVB irradiation VS homo before UVB irradiation	GO_COPPER_ION_IMPORT	-0.13841656	0.02443337	-0.72373173	0.48905513	0.66520422	-6.27224117
hetero after UVB irradiation VS hetero before UVB irradiation	GO_COPPER_ION_IMPORT1	0.057199849	-0.03134808	0.186574202	0.8567243	0.94570833	-6.21264969
WT after UVB irradiation VS WT before UVB irradiation	GO_COPPER_ION_IMPORT2	-0.17790625	-0.04847297	-0.55697112	0.59260356	0.8931505	-5.83562337
homo after UVB irradiation VS homo before UVB irradiation	GO_COPPER_ION_TRANSMEMBRANE_TRANSPORT	0.150407579	-0.04474118	0.566816881	0.58578498	0.73928239	-6.37791272
hetero after UVB irradiation VS hetero before UVB irradiation	GO_COPPER_ION_TRANSMEMBRANE_TRANSPORT1	0.423685566	0.038267804	1.518406958	0.16809347	0.48947934	-5.140996
WT after UVB irradiation VS WT before UVB irradiation	GO_COPPER_ION_TRANSMEMBRANE_TRANSPORT2	0.033178256	0.072964398	0.113015013	0.91277026	0.97884691	-5.98933294
homo after UVB irradiation VS homo before UVB irradiation	GO_COPPER_ION_TRANSMEMBRANE_TRANSPORTER_ACTIVITY	-0.08487867	-0.06380826	-0.31815439	0.75819874	0.85956021	-6.4956454
hetero after UVB irradiation VS hetero before UVB irradiation	GO_COPPER_ION_TRANSMEMBRANE_TRANSPORTER_ACTIVITY1	0.154898132	-0.04092839	0.478002289	0.64567145	0.85438728	-6.1094096
WT after UVB irradiation VS WT before UVB irradiation	GO_COPPER_ION_TRANSMEMBRANE_TRANSPORTER_ACTIVITY2	-0.13844275	-0.0068764	-0.47205819	0.64934462	0.9092481	-5.88017067
homo after UVB irradiation VS homo before UVB irradiation	GO_COPPER_ION_TRANSPORT	-0.0230551	-0.03609557	-0.17490749	0.86532946	0.92600919	-6.53409822
hetero after UVB irradiation VS hetero before UVB irradiation	GO_COPPER_ION_TRANSPORT1	0.138538143	-0.06132756	0.743163793	0.47902344	0.76045937	-5.94241561
WT after UVB irradiation VS WT before UVB irradiation	GO_COPPER_ION_TRANSPORT2	-0.02109018	0.010421874	-0.1068539	0.9175048	0.98021613	-5.99004658
homo after UVB irradiation VS homo before UVB irradiation	GO_COPULATION	-0.01653395	0.01085948	-0.12558249	0.90304085	0.94720121	-6.54220963
hetero after UVB irradiation VS hetero before UVB irradiation	GO_COPULATION1	0.149612304	0.015685943	1.015520128	0.34012209	0.65952819	-5.70728445
WT after UVB irradiation VS WT before UVB irradiation	GO_COPULATION2	0.187428827	0.020252651	1.031451288	0.33214906	0.77839029	-5.47068705
homo after UVB irradiation VS homo before UVB irradiation	GO_CORE_MEDIATOR_COMPLEX	-0.79058972	-0.00099315	-4.22155333	0.00265746	0.03401579	-1.40954826
hetero after UVB irradiation VS hetero before UVB irradiation	GO_CORE_MEDIATOR_COMPLEX1	-0.35051117	-0.06298124	-1.2738805	0.23911342	0.56769689	-5.43453318
WT after UVB irradiation VS WT before UVB irradiation	GO_CORE_MEDIATOR_COMPLEX2	-0.50798146	-0.05628335	-1.39409334	0.20035217	0.69758436	-5.08332128
homo after UVB irradiation VS homo before UVB irradiation	GO_CORE_PROMOTER_SEQUENCE_SPECIFIC_DNA_BINDING	-0.19179678	0.068816338	-0.98632589	0.35175438	0.54377115	-6.04646682
hetero after UVB irradiation VS hetero before UVB irradiation	GO_CORE_PROMOTER_SEQUENCE_SPECIFIC_DNA_BINDING1	0.008122585	-0.02353494	0.047633661	0.96319735	0.98664387	-6.23025382
WT after UVB irradiation VS WT before UVB irradiation	GO_CORE_PROMOTER_SEQUENCE_SPECIFIC_DNA_BINDING2	-0.04938429	-0.01833793	-0.24023033	0.81612168	0.95613748	-5.96573018
homo after UVB irradiation VS homo before UVB irradiation	GO_CORECEPTOR_ACTIVITY	0.281211774	-0.0407655	2.054801884	0.0725691	0.20669798	-4.68513784
homo after UVB irradiation VS homo before UVB irradiation	GO_CORECEPTOR_ACTIVITY_INVOLVED_IN_WNT_SIGNALING_PATHWAY	-0.54401909	0.005586598	-3.50693157	0.00750426	0.05752743	-2.45645878
homo after UVB irradiation VS homo before UVB irradiation	GO_CORECEPTOR_ACTIVITY_INVOLVED_IN_WNT_SIGNALING_PATHWAY_PLANAR_CELL_POLARITY_PATHWAY	-0.62344881	0.0423771	-3.29923508	0.0102774	0.06782811	-2.77219701
hetero after UVB irradiation VS hetero before UVB irradiation	GO_CORECEPTOR_ACTIVITY_INVOLVED_IN_WNT_SIGNALING_PATHWAY_PLANAR_CELL_POLARITY_PATHWAY1	-0.6531338	0.024097817	-3.46921123	0.00869988	0.14994695	-2.36818575
WT after UVB irradiation VS WT before UVB irradiation	GO_CORECEPTOR_ACTIVITY_INVOLVED_IN_WNT_SIGNALING_PATHWAY_PLANAR_CELL_POLARITY_PATHWAY2	-0.57521426	-0.0360138	-2.69407705	0.02702706	0.43942403	-3.32617817
hetero after UVB irradiation VS hetero before UVB irradiation	GO_CORECEPTOR_ACTIVITY_INVOLVED_IN_WNT_SIGNALING_PATHWAY1	-0.59240316	0.005067554	-3.06122239	0.0159038	0.18566181	-2.95070404
WT after UVB irradiation VS WT before UVB irradiation	GO_CORECEPTOR_ACTIVITY_INVOLVED_IN_WNT_SIGNALING_PATHWAY2	-0.58178041	0.006481325	-3.14993807	0.01339214	0.35652294	-2.68378417
hetero after UVB irradiation VS hetero before UVB irradiation	GO_CORECEPTOR_ACTIVITY1	0.221464515	0.023714651	1.21713423	0.25886392	0.58521976	-5.49806565
WT after UVB irradiation VS WT before UVB irradiation	GO_CORECEPTOR_ACTIVITY2	0.163298482	-0.02262762	0.734877856	0.48315043	0.85146732	-5.72076179
homo after UVB irradiation VS homo before UVB irradiation	GO_CORNEA_DEVELOPMENT_IN_CAMERA_TYPE_EYE	-0.37412887	0.003045695	-2.62659599	0.02932286	0.12154985	-3.81298722
hetero after UVB irradiation VS hetero before UVB irradiation	GO_CORNEA_DEVELOPMENT_IN_CAMERA_TYPE_EYE1	-0.21154352	-0.05501869	-1.1342474	0.29012662	0.6144112	-5.58733107
WT after UVB irradiation VS WT before UVB irradiation	GO_CORNEA_DEVELOPMENT_IN_CAMERA_TYPE_EYE2	-0.26547044	0.052164482	-1.27823495	0.23658579	0.72811189	-5.21537248
homo after UVB irradiation VS homo before UVB irradiation	GO_CORNIFICATION	-0.20710285	0.032810378	-1.08222304	0.30948598	0.50382804	-5.95025635
hetero after UVB irradiation VS hetero before UVB irradiation	GO_CORNIFICATION1	-0.05230928	0.011099687	-0.27774359	0.78838126	0.91738598	-6.18986152
WT after UVB irradiation VS WT before UVB irradiation	GO_CORNIFICATION2	-0.19701906	0.097940248	-0.683221	0.51353688	0.85974355	-5.7570157
homo after UVB irradiation VS homo before UVB irradiation	GO_CORNIFIED_ENVELOPE	-0.20849084	0.037329945	-1.05800691	0.31976803	0.51324959	-5.97519385
homo after UVB irradiation VS homo before UVB irradiation	GO_CORNIFIED_ENVELOPE_ASSEMBLY	1.004250857	-0.05172585	5.963351231	0.00028771	0.01259855	0.819116379
hetero after UVB irradiation VS hetero before UVB irradiation	GO_CORNIFIED_ENVELOPE_ASSEMBLY1	0.86331499	0.02959432	4.960811647	0.00116816	0.09106857	-0.44635768
WT after UVB irradiation VS WT before UVB irradiation	GO_CORNIFIED_ENVELOPE_ASSEMBLY2	0.795833982	-0.01940503	2.999536666	0.01684875	0.38017888	-2.89401852
hetero after UVB irradiation VS hetero before UVB irradiation	GO_CORNIFIED_ENVELOPE1	0.078347434	0.028329951	0.313742897	0.76188842	0.90682777	-6.17844658
WT after UVB irradiation VS WT before UVB irradiation	GO_CORNIFIED_ENVELOPE2	-0.13493433	0.111379172	-0.4119763	0.69104661	0.92129334	-5.9074932
homo after UVB irradiation VS homo before UVB irradiation	GO_CORONARY_ARTERY_MORPHOGENESIS	0.069236681	0.011699192	0.30694061	0.7664224	0.8649507	-6.49944695
hetero after UVB irradiation VS hetero before UVB irradiation	GO_CORONARY_ARTERY_MORPHOGENESIS1	0.163060617	-0.01958621	0.953853149	0.36859328	0.68175554	-5.76562812
WT after UVB irradiation VS WT before UVB irradiation	GO_CORONARY_ARTERY_MORPHOGENESIS2	-0.19221038	-0.0695724	-1.16349429	0.27776433	0.74917538	-5.33880632
homo after UVB irradiation VS homo before UVB irradiation	GO_CORONARY_VASCULATURE_DEVELOPMENT	0.235406141	-0.02654483	1.629668446	0.14032377	0.31064474	-5.28952597
hetero after UVB irradiation VS hetero before UVB irradiation	GO_CORONARY_VASCULATURE_DEVELOPMENT1	0.172593928	0.014888451	1.130267967	0.29170157	0.61588295	-5.59150636
WT after UVB irradiation VS WT before UVB irradiation	GO_CORONARY_VASCULATURE_DEVELOPMENT2	0.128122777	-0.018835	0.89244786	0.397897	0.81477652	-5.59639045
homo after UVB irradiation VS homo before UVB irradiation	GO_CORONARY_VASCULATURE_MORPHOGENESIS	0.405441723	-0.02137591	2.196015674	0.058055	0.18029306	-4.47387539
hetero after UVB irradiation VS hetero before UVB irradiation	GO_CORONARY_VASCULATURE_MORPHOGENESIS1	0.141225015	-0.01450333	0.759196319	0.46990848	0.75417736	-5.93026213
WT after UVB irradiation VS WT before UVB irradiation	GO_CORONARY_VASCULATURE_MORPHOGENESIS2	0.079396407	-0.07849517	0.489725788	0.63731889	0.9057274	-5.87146872
homo after UVB irradiation VS homo before UVB irradiation	GO_CORPUS_CALLOSUM_DEVELOPMENT	0.180714019	-0.00232158	1.135334297	0.2878465	0.48302829	-5.89410042
hetero after UVB irradiation VS hetero before UVB irradiation	GO_CORPUS_CALLOSUM_DEVELOPMENT1	0.117342254	-0.02728873	0.862101523	0.41418191	0.71775318	-5.84705864
WT after UVB irradiation VS WT before UVB irradiation	GO_CORPUS_CALLOSUM_DEVELOPMENT2	0.158785095	0.003031483	1.02809458	0.33363282	0.77844475	-5.47388703
homo after UVB irradiation VS homo before UVB irradiation	GO_CORPUS_CALLOSUM_MORPHOGENESIS	-0.18126691	0.011372682	-0.77923376	0.45740807	0.63795381	-6.22942821
hetero after UVB irradiation VS hetero before UVB irradiation	GO_CORPUS_CALLOSUM_MORPHOGENESIS1	0.343922745	-0.03838749	1.747110452	0.11945982	0.42435801	-4.8427305
WT after UVB irradiation VS WT before UVB irradiation	GO_CORPUS_CALLOSUM_MORPHOGENESIS2	-0.07716485	-0.04088295	-0.36594659	0.72378079	0.93154998	-5.92601831
homo after UVB irradiation VS homo before UVB irradiation	GO_CORTICAL_ACTIN_CYTOSKELETON	0.248741724	0.016869301	1.654499999	0.13512614	0.30336571	-5.2559325
homo after UVB irradiation VS homo before UVB irradiation	GO_CORTICAL_ACTIN_CYTOSKELETON_ORGANIZATION	-0.00554595	-0.02232229	-0.03410933	0.97359325	0.9864974	-6.55021824
hetero after UVB irradiation VS hetero before UVB irradiation	GO_CORTICAL_ACTIN_CYTOSKELETON_ORGANIZATION1	0.284125812	-0.01462459	1.887752642	0.0964473	0.38778648	-4.65069729
WT after UVB irradiation VS WT before UVB irradiation	GO_CORTICAL_ACTIN_CYTOSKELETON_ORGANIZATION2	0.250401941	-0.02549528	1.655095538	0.13604384	0.6442872	-4.7630298
hetero after UVB irradiation VS hetero before UVB irradiation	GO_CORTICAL_ACTIN_CYTOSKELETON1	0.183248808	0.002644316	1.256567267	0.24500032	0.57257282	-5.4541172
WT after UVB irradiation VS WT before UVB irradiation	GO_CORTICAL_ACTIN_CYTOSKELETON2	0.178346453	0.055563562	1.236731065	0.25085144	0.73564768	-5.26090977
homo after UVB irradiation VS homo before UVB irradiation	GO_CORTICAL_CYTOSKELETON	0.227096086	0.011265081	1.47045396	0.17817534	0.36003471	-5.49847418
homo after UVB irradiation VS homo before UVB irradiation	GO_CORTICAL_CYTOSKELETON_ORGANIZATION	0.13496423	-0.01835687	0.738432192	0.48053842	0.65788158	-6.26117014
hetero after UVB irradiation VS hetero before UVB irradiation	GO_CORTICAL_CYTOSKELETON_ORGANIZATION1	0.458860371	0.007016433	3.497593914	0.00834884	0.14771631	-2.32838549
WT after UVB irradiation VS WT before UVB irradiation	GO_CORTICAL_CYTOSKELETON_ORGANIZATION2	0.27430757	-0.03274433	1.603340002	0.14707181	0.65371647	-4.82873626
hetero after UVB irradiation VS hetero before UVB irradiation	GO_CORTICAL_CYTOSKELETON1	0.192036559	0.00772392	1.382555843	0.20484506	0.529684	-5.3078049
WT after UVB irradiation VS WT before UVB irradiation	GO_CORTICAL_CYTOSKELETON2	0.105276386	0.045814039	0.710013346	0.49762967	0.85541458	-5.73850088
homo after UVB irradiation VS homo before UVB irradiation	GO_CORTICAL_ENDOPLASMIC_RETICULUM	-0.72784667	0.07686247	-2.57956607	0.03159008	0.1259932	-3.88589709
hetero after UVB irradiation VS hetero before UVB irradiation	GO_CORTICAL_ENDOPLASMIC_RETICULUM1	-1.17841668	-0.03391963	-8.77810832	2.50E-05	0.07788488	2.90662428
WT after UVB irradiation VS WT before UVB irradiation	GO_CORTICAL_ENDOPLASMIC_RETICULUM2	-0.7826749	-0.03752947	-4.07852525	0.00345173	0.25923179	-1.45166027
homo after UVB irradiation VS homo before UVB irradiation	GO_CORTICOSPINAL_TRACT_MORPHOGENESIS	0.212480562	0.016268641	0.978758455	0.35526776	0.54691074	-6.05376355
hetero after UVB irradiation VS hetero before UVB irradiation	GO_CORTICOSPINAL_TRACT_MORPHOGENESIS1	0.149164189	0.000505192	0.961628287	0.36490786	0.67942316	-5.7584281
WT after UVB irradiation VS WT before UVB irradiation	GO_CORTICOSPINAL_TRACT_MORPHOGENESIS2	0.09267413	-0.07964981	0.419703853	0.68561581	0.91888815	-5.90417727
homo after UVB irradiation VS homo before UVB irradiation	GO_CORTICOSTEROID_HORMONE_SECRETION	0.049656033	-0.02195398	0.313623259	0.76151781	0.86198652	-6.49719752
hetero after UVB irradiation VS hetero before UVB irradiation	GO_CORTICOSTEROID_HORMONE_SECRETION1	-0.19410458	0.059838374	-0.87797584	0.40601731	0.71185533	-5.8334456
WT after UVB irradiation VS WT before UVB irradiation	GO_CORTICOSTEROID_HORMONE_SECRETION2	-0.17594286	-0.01378401	-0.68701663	0.51126423	0.85882537	-5.75443088
homo after UVB irradiation VS homo before UVB irradiation	GO_CORTICOSTEROID_RECEPTOR_SIGNALING_PATHWAY	0.130582363	0.000765732	0.896357739	0.39522825	0.5841461	-6.13029352
hetero after UVB irradiation VS hetero before UVB irradiation	GO_CORTICOSTEROID_RECEPTOR_SIGNALING_PATHWAY1	0.064092937	-0.03637905	0.264120852	0.798486	0.9219049	-6.19382616
WT after UVB irradiation VS WT before UVB irradiation	GO_CORTICOSTEROID_RECEPTOR_SIGNALING_PATHWAY2	-0.02974718	-0.06650336	-0.14277459	0.88995695	0.97195786	-5.98532507
homo after UVB irradiation VS homo before UVB irradiation	GO_CORTICOSTERONE_SECRETION	0.688319295	-0.03977452	4.170251484	0.00285616	0.0352706	-1.48235281
hetero after UVB irradiation VS hetero before UVB irradiation	GO_CORTICOSTERONE_SECRETION1	0.283495722	0.063369313	1.224438938	0.25624752	0.58351672	-5.48999449
WT after UVB irradiation VS WT before UVB irradiation	GO_CORTICOSTERONE_SECRETION2	0.303164701	0.049626071	1.000986641	0.34580253	0.78455558	-5.49943938
homo after UVB irradiation VS homo before UVB irradiation	GO_CORTICOTROPIN_RELEASING_HORMONE_RECEPTOR_BINDING	0.294698644	0.024054203	1.484066144	0.17461528	0.35507754	-5.48107555
hetero after UVB irradiation VS hetero before UVB irradiation	GO_CORTICOTROPIN_RELEASING_HORMONE_RECEPTOR_BINDING1	0.072806585	-0.00312443	0.213191531	0.83660812	0.93734913	-6.20690961
WT after UVB irradiation VS WT before UVB irradiation	GO_CORTICOTROPIN_RELEASING_HORMONE_RECEPTOR_BINDING2	0.242839244	-0.01815618	0.66779967	0.52283478	0.86447875	-5.76738707
homo after UVB irradiation VS homo before UVB irradiation	GO_CORTICOTROPIN_SECRETION	-0.61541257	0.072280537	-3.70412828	0.00559546	0.04938527	-2.16094568
hetero after UVB irradiation VS hetero before UVB irradiation	GO_CORTICOTROPIN_SECRETION1	-0.22977091	-0.00843218	-1.10842059	0.30047115	0.62370564	-5.61424289
WT after UVB irradiation VS WT before UVB irradiation	GO_CORTICOTROPIN_SECRETION2	-0.2825742	0.000710013	-1.0041653	0.34435823	0.78369331	-5.49647003
homo after UVB irradiation VS homo before UVB irradiation	GO_CORTISOL_METABOLIC_PROCESS	0.428626921	-0.04869862	1.846540493	0.10054784	0.25136108	-4.98829106
hetero after UVB irradiation VS hetero before UVB irradiation	GO_CORTISOL_METABOLIC_PROCESS1	0.233899148	0.042313	0.978281111	0.35710753	0.67273203	-5.74285408
WT after UVB irradiation VS WT before UVB irradiation	GO_CORTISOL_METABOLIC_PROCESS2	0.188483242	0.035851344	0.850224165	0.41962766	0.82348328	-5.63168536
homo after UVB irradiation VS homo before UVB irradiation	GO_CORTISOL_SECRETION	-0.80643031	-0.06686016	-4.09629229	0.00317119	0.03719976	-1.58799365
hetero after UVB irradiation VS hetero before UVB irradiation	GO_CORTISOL_SECRETION1	-0.45558101	0.123451229	-2.27077734	0.05342566	0.30663129	-4.10590987
WT after UVB irradiation VS WT before UVB irradiation	GO_CORTISOL_SECRETION2	-0.72355767	0.039422315	-2.98160613	0.01731893	0.38271751	-2.91921888
homo after UVB irradiation VS homo before UVB irradiation	GO_CORVET_COMPLEX	-0.20339059	0.054427699	-1.05996873	0.31892527	0.51234272	-5.97318944
hetero after UVB irradiation VS hetero before UVB irradiation	GO_CORVET_COMPLEX1	0.683643853	0.038514269	2.106533823	0.06888562	0.34030002	-4.3425364
WT after UVB irradiation VS WT before UVB irradiation	GO_CORVET_COMPLEX2	0.279119379	0.137698784	0.861559082	0.41371346	0.820663	-5.62234803
homo after UVB irradiation VS homo before UVB irradiation	GO_COSTAMERE	0.168319872	-0.0362372	1.126810109	0.291236	0.48595315	-5.90324617
hetero after UVB irradiation VS hetero before UVB irradiation	GO_COSTAMERE1	-0.11003209	-0.09222463	-0.50294031	0.62880598	0.8472681	-6.09655274
WT after UVB irradiation VS WT before UVB irradiation	GO_COSTAMERE2	-0.29236457	-0.04767986	-1.42892311	0.19044431	0.69285981	-5.04229794
homo after UVB irradiation VS homo before UVB irradiation	GO_COTRANSLATIONAL_PROTEIN_TARGETING_TO_MEMBRANE	-0.6995144	0.01841775	-3.78011293	0.00500405	0.04621075	-2.0483291
hetero after UVB irradiation VS hetero before UVB irradiation	GO_COTRANSLATIONAL_PROTEIN_TARGETING_TO_MEMBRANE1	0.011798165	0.009364061	0.039217172	0.96969592	0.98899869	-6.23065013
WT after UVB irradiation VS WT before UVB irradiation	GO_COTRANSLATIONAL_PROTEIN_TARGETING_TO_MEMBRANE2	-0.44335813	-0.00409321	-1.64844233	0.13741773	0.64563358	-4.77153055
homo after UVB irradiation VS homo before UVB irradiation	GO_COVALENT_CHROMATIN_MODIFICATION	-0.36456351	-0.00544389	-2.32167138	0.04756549	0.15985514	-4.28304154
hetero after UVB irradiation VS hetero before UVB irradiation	GO_COVALENT_CHROMATIN_MODIFICATION1	-0.12396934	-0.03764506	-0.79703743	0.44885489	0.74180859	-5.90069953
WT after UVB irradiation VS WT before UVB irradiation	GO_COVALENT_CHROMATIN_MODIFICATION2	-0.20967989	-0.01489106	-1.02741644	0.3339332	0.77844475	-5.47453256
homo after UVB irradiation VS homo before UVB irradiation	GO_CRANIAL_GANGLION_DEVELOPMENT	0.341795794	-0.08450559	1.913761773	0.09054892	0.23605121	-4.89172317
hetero after UVB irradiation VS hetero before UVB irradiation	GO_CRANIAL_GANGLION_DEVELOPMENT1	-0.03108217	-0.04789001	-0.15467154	0.88098168	0.95677024	-6.21853078
WT after UVB irradiation VS WT before UVB irradiation	GO_CRANIAL_GANGLION_DEVELOPMENT2	-0.03412097	0.107929482	-0.14252676	0.89014651	0.97201136	-5.98536227
homo after UVB irradiation VS homo before UVB irradiation	GO_CRANIAL_NERVE_DEVELOPMENT	-0.12400513	0.007614105	-0.96235969	0.36297139	0.55356139	-6.06942308
hetero after UVB irradiation VS hetero before UVB irradiation	GO_CRANIAL_NERVE_DEVELOPMENT1	-0.04762436	0.003416139	-0.34650218	0.73806681	0.8974795	-6.16688395
WT after UVB irradiation VS WT before UVB irradiation	GO_CRANIAL_NERVE_DEVELOPMENT2	-0.15334187	0.045284234	-0.95488377	0.36726921	0.7989027	-5.54168754
homo after UVB irradiation VS homo before UVB irradiation	GO_CRANIAL_NERVE_FORMATION	0.307726164	-0.01786032	1.507081646	0.16873914	0.3478742	-5.45145071
hetero after UVB irradiation VS hetero before UVB irradiation	GO_CRANIAL_NERVE_FORMATION1	0.486921715	0.005118098	2.834403578	0.02241869	0.2152311	-3.28101569
WT after UVB irradiation VS WT before UVB irradiation	GO_CRANIAL_NERVE_FORMATION2	0.56446136	0.040678092	2.019350511	0.07770915	0.56130885	-4.27779628
homo after UVB irradiation VS homo before UVB irradiation	GO_CRANIAL_NERVE_MORPHOGENESIS	-0.13733046	-0.04454062	-1.10177918	0.3013733	0.49594615	-5.92981013
hetero after UVB irradiation VS hetero before UVB irradiation	GO_CRANIAL_NERVE_MORPHOGENESIS1	-0.20522166	-0.01141617	-1.44035444	0.18842035	0.51421677	-5.23790403
WT after UVB irradiation VS WT before UVB irradiation	GO_CRANIAL_NERVE_MORPHOGENESIS2	-0.08369428	0.042942814	-0.46588129	0.65357515	0.90964994	-5.88314191

homo after UVB irradiation VS homo before UVB irradiation	GO_CRANIAL_NERVE_STRUCTURAL_ORGANIZATION	-0.50329328	0.028249029	-3.3976485	0.00884883	0.06268944	-2.62206917
hetero after UVB irradiation VS hetero before UVB irradiation	GO_CRANIAL_NERVE_STRUCTURAL_ORGANIZATION1	-0.60187989	-0.01962256	-2.96009838	0.01852306	0.19863829	-3.09755411
WT after UVB irradiation VS WT before UVB irradiation	GO_CRANIAL_NERVE_STRUCTURAL_ORGANIZATION2	-0.33884209	0.008284076	-1.98761066	0.08164358	0.56617212	-4.32135219
homo after UVB irradiation VS homo before UVB irradiation	GO_CRANIAL_SKELETAL_SYSTEM_DEVELOPMENT	-0.04796697	-0.00585505	-0.32891378	0.75033875	0.85431286	-6.49187286
hetero after UVB irradiation VS hetero before UVB irradiation	GO_CRANIAL_SKELETAL_SYSTEM_DEVELOPMENT1	0.018159715	-0.00486062	0.102739327	0.92074499	0.96983444	-6.22576384
WT after UVB irradiation VS WT before UVB irradiation	GO_CRANIAL_SKELETAL_SYSTEM_DEVELOPMENT2	-0.10335276	0.025187053	-0.72955924	0.48622449	0.85164239	-5.7246009
homo after UVB irradiation VS homo before UVB irradiation	GO_CRANIAL_SUTURE_MORPHOGENESIS	-0.16210783	0.063577354	-1.0285277	0.33264045	0.52574564	-6.00497173
hetero after UVB irradiation VS hetero before UVB irradiation	GO_CRANIAL_SUTURE_MORPHOGENESIS1	-0.4140443	2.62E-05	-2.6437665	0.03001958	0.24205953	-3.56046505
WT after UVB irradiation VS WT before UVB irradiation	GO_CRANIAL_SUTURE_MORPHOGENESIS2	-0.69662257	-0.01453822	-4.89923487	0.00115363	0.19353487	-0.48354923
homo after UVB irradiation VS homo before UVB irradiation	GO_CRANIOFACIAL_SUTURE_MORPHOGENESIS	-0.01551529	-0.02143561	-0.09620179	0.92563481	0.95983321	-6.54578038
hetero after UVB irradiation VS hetero before UVB irradiation	GO_CRANIOFACIAL_SUTURE_MORPHOGENESIS1	-0.08191803	-0.01723938	-0.38134645	0.71305699	0.88725884	-6.15336756
WT after UVB irradiation VS WT before UVB irradiation	GO_CRANIOFACIAL_SUTURE_MORPHOGENESIS2	-0.34028353	-0.02493641	-2.07927079	0.07077679	0.54758105	-4.19507271
homo after UVB irradiation VS homo before UVB irradiation	GO_CRD_MEDIATED_MRNA_STABILITY_COMPLEX	-0.20198669	0.006589961	-1.13561653	0.28773482	0.48298212	-5.89379675
hetero after UVB irradiation VS hetero before UVB irradiation	GO_CRD_MEDIATED_MRNA_STABILITY_COMPLEX1	0.127550488	-0.04415025	0.345726674	0.73862737	0.89786519	-6.16717054
WT after UVB irradiation VS WT before UVB irradiation	GO_CRD_MEDIATED_MRNA_STABILITY_COMPLEX2	0.029332551	0.021284664	0.109759257	0.91527171	0.97967529	-5.98971502
homo after UVB irradiation VS homo before UVB irradiation	GO_CRD_MEDIATED_MRNA_STABILIZATION	-0.19919431	0.01536064	-0.8257272	0.43197176	0.61607417	-6.19147491
hetero after UVB irradiation VS hetero before UVB irradiation	GO_CRD_MEDIATED_MRNA_STABILIZATION1	0.32975839	0.01764164	0.864257828	0.41306606	0.71682968	-5.84522143
WT after UVB irradiation VS WT before UVB irradiation	GO_CRD_MEDIATED_MRNA_STABILIZATION2	0.070117229	0.075114953	0.222941573	0.82910193	0.95957725	-5.9699259
homo after UVB irradiation VS homo before UVB irradiation	GO_CREATINE_METABOLIC_PROCESS	0.76253463	-0.09274405	3.298159201	0.0102943	0.06787889	-2.77384302
hetero after UVB irradiation VS hetero before UVB irradiation	GO_CREATINE_METABOLIC_PROCESS1	0.367449497	0.03806024	1.149772008	0.28404795	0.60954889	-5.57094369
WT after UVB irradiation VS WT before UVB irradiation	GO_CREATINE_METABOLIC_PROCESS2	0.305839735	0.033697991	0.831021795	0.42978161	0.82808294	-5.64727006
homo after UVB irradiation VS homo before UVB irradiation	GO_CRISTAE_FORMATION	-0.20738668	-0.0107947	-0.77571119	0.45937537	0.63958886	-6.23222687
hetero after UVB irradiation VS hetero before UVB irradiation	GO_CRISTAE_FORMATION1	-0.25601584	-0.08808406	-1.10344431	0.30249786	0.62458221	-5.61937726
WT after UVB irradiation VS WT before UVB irradiation	GO_CRISTAE_FORMATION2	-0.04725777	-0.07049547	-0.16911677	0.86985307	0.96830648	-5.98100381
homo after UVB irradiation VS homo before UVB irradiation	GO_CROSSOVER_JUNCTION_ENDODEOXYRIBONUCLEASE_ACTIVITY	-0.58668114	0.017153755	-2.14663007	0.06277516	0.18924786	-4.54819216
hetero after UVB irradiation VS hetero before UVB irradiation	GO_CROSSOVER_JUNCTION_ENDODEOXYRIBONUCLEASE_ACTIVITY1	0.032225361	-0.09176733	0.120615311	0.90702471	0.96555872	-6.22360216
WT after UVB irradiation VS WT before UVB irradiation	GO_CROSSOVER_JUNCTION_ENDODEOXYRIBONUCLEASE_ACTIVITY2	0.464387496	-0.04173069	1.45531112	0.18322662	0.68626273	-5.01083601
homo after UVB irradiation VS homo before UVB irradiation	GO_CTF18_RFC_LIKE_COMPLEX	-0.15708707	-0.01746418	-0.47234343	0.64881027	0.78554632	-6.43005364
hetero after UVB irradiation VS hetero before UVB irradiation	GO_CTF18_RFC_LIKE_COMPLEX1	0.257413778	0.069164549	0.866645344	0.41183306	0.71577484	-5.84318283
WT after UVB irradiation VS WT before UVB irradiation	GO_CTF18_RFC_LIKE_COMPLEX2	0.300069708	0.081015767	1.003304835	0.34474875	0.78401253	-5.49727455
homo after UVB irradiation VS homo before UVB irradiation	GO_CUL2_RING_UBIQUITIN_LIGASE_COMPLEX	-0.27857378	0.032296492	-1.01438278	0.33895659	0.53195601	-6.01903018
hetero after UVB irradiation VS hetero before UVB irradiation	GO_CUL2_RING_UBIQUITIN_LIGASE_COMPLEX1	-0.20107929	-0.06726157	-1.25911642	0.24412596	0.57161579	-5.4512446
WT after UVB irradiation VS WT before UVB irradiation	GO_CUL2_RING_UBIQUITIN_LIGASE_COMPLEX2	-0.11187696	0.028532085	-0.6797761	0.51560495	0.86062462	-5.75935071
homo after UVB irradiation VS homo before UVB irradiation	GO_CUL3_RING_UBIQUITIN_LIGASE_COMPLEX	0.062609496	-0.02817398	0.307613371	0.76592813	0.86465739	-6.49922264
hetero after UVB irradiation VS hetero before UVB irradiation	GO_CUL3_RING_UBIQUITIN_LIGASE_COMPLEX1	-0.08004782	-0.01959693	-0.35222527	0.73393508	0.89605454	-6.16474978
WT after UVB irradiation VS WT before UVB irradiation	GO_CUL3_RING_UBIQUITIN_LIGASE_COMPLEX2	-0.05313451	-0.0122895	-0.30538048	0.76777901	0.94123239	-5.94715768
homo after UVB irradiation VS homo before UVB irradiation	GO_CUL4_RING_E3_UBIQUITIN_LIGASE_COMPLEX	-0.31098701	0.015870854	-2.23783937	0.05433188	0.17284247	-4.41061666
hetero after UVB irradiation VS hetero before UVB irradiation	GO_CUL4_RING_E3_UBIQUITIN_LIGASE_COMPLEX1	0.083450498	-0.06790693	0.441190475	0.67096983	0.86592608	-6.12726014
WT after UVB irradiation VS WT before UVB irradiation	GO_CUL4_RING_E3_UBIQUITIN_LIGASE_COMPLEX2	-0.11663854	-0.01445818	-0.49637069	0.63282503	0.90455096	-5.86811817
homo after UVB irradiation VS homo before UVB irradiation	GO_CUL4A_RING_E3_UBIQUITIN_LIGASE_COMPLEX	-0.55143668	0.035064168	-3.44737713	0.00820795	0.06037312	-2.546558
hetero after UVB irradiation VS hetero before UVB irradiation	GO_CUL4A_RING_E3_UBIQUITIN_LIGASE_COMPLEX1	0.038315702	-0.04073129	0.18000524	0.86170671	0.94770397	-6.21394982
WT after UVB irradiation VS WT before UVB irradiation	GO_CUL4A_RING_E3_UBIQUITIN_LIGASE_COMPLEX2	-0.00133468	-0.0363841	-0.0054215	0.99580547	0.99875662	-5.99605116
homo after UVB irradiation VS homo before UVB irradiation	GO_CUL4B_RING_E3_UBIQUITIN_LIGASE_COMPLEX	-0.16039855	-0.00589	-0.88770533	0.39960584	0.58822777	-6.13801185
hetero after UVB irradiation VS hetero before UVB irradiation	GO_CUL4B_RING_E3_UBIQUITIN_LIGASE_COMPLEX1	0.571921951	0.018588873	2.215741675	0.05817875	0.31819855	-4.18558077
WT after UVB irradiation VS WT before UVB irradiation	GO_CUL4B_RING_E3_UBIQUITIN_LIGASE_COMPLEX2	0.128927162	0.096642774	0.384347018	0.71061797	0.92846994	-5.91886578
homo after UVB irradiation VS homo before UVB irradiation	GO_CUL5_RING_UBIQUITIN_LIGASE_COMPLEX	0.182461164	-0.06832935	0.620345069	0.55162192	0.71418536	-6.34448334
hetero after UVB irradiation VS hetero before UVB irradiation	GO_CUL5_RING_UBIQUITIN_LIGASE_COMPLEX1	0.126305002	-0.10304194	0.515444827	0.62043514	0.84361764	-6.08987588
WT after UVB irradiation VS WT before UVB irradiation	GO_CUL5_RING_UBIQUITIN_LIGASE_COMPLEX2	-0.22511909	0.007297623	-0.86202761	0.41347027	0.820663	-5.62195988
homo after UVB irradiation VS homo before UVB irradiation	GO_CULLIN_FAMILY_PROTEIN_BINDING	0.139515027	-0.02086011	0.795965236	0.44814079	0.62979726	-6.21598647
hetero after UVB irradiation VS hetero before UVB irradiation	GO_CULLIN_FAMILY_PROTEIN_BINDING1	0.20866348	-0.05754429	1.577755118	0.15398007	0.47198377	-5.06552488
WT after UVB irradiation VS WT before UVB irradiation	GO_CULLIN_FAMILY_PROTEIN_BINDING2	-0.10087727	-0.0479038	-0.59577534	0.56760009	0.88290486	-5.81300827
homo after UVB irradiation VS homo before UVB irradiation	GO_CULLIN_RING_UBIQUITIN_LIGASE_COMPLEX	-0.08284155	-0.00285767	-0.6072425	0.55987607	0.72010647	-6.35292121
hetero after UVB irradiation VS hetero before UVB irradiation	GO_CULLIN_RING_UBIQUITIN_LIGASE_COMPLEX1	-0.00675296	-0.0349292	-0.049272	0.96193267	0.98633024	-6.23016775
WT after UVB irradiation VS WT before UVB irradiation	GO_CULLIN_RING_UBIQUITIN_LIGASE_COMPLEX2	-0.02797094	-0.01567842	-0.16808417	0.87063935	0.96863879	-5.98118685
homo after UVB irradiation VS homo before UVB irradiation	GO_CUPROUS_ION_BINDING	-0.27730535	0.013658876	-1.29605834	0.22971211	0.42094394	-5.71269185
hetero after UVB irradiation VS hetero before UVB irradiation	GO_CUPROUS_ION_BINDING1	-0.45770597	-0.05154281	-2.67468221	0.02862645	0.23751402	-3.51509621
WT after UVB irradiation VS WT before UVB irradiation	GO_CUPROUS_ION_BINDING2	-0.4284764	0.004310525	-1.83707917	0.10305911	0.60538498	-4.52498221
homo after UVB irradiation VS homo before UVB irradiation	GO_CUTICULAR_PLATE	-0.35726044	0.051001961	-1.29897663	0.22875463	0.4199194	-5.70924833
hetero after UVB irradiation VS hetero before UVB irradiation	GO_CUTICULAR_PLATE1	-0.50545618	0.040772458	-2.36022753	0.04651366	0.28809638	-3.97578844
WT after UVB irradiation VS WT before UVB irradiation	GO_CUTICULAR_PLATE2	-0.08507157	0.158511282	-0.37760596	0.71542854	0.93029655	-5.92152537
homo after UVB irradiation VS homo before UVB irradiation	GO_CXCR_CHEMOKINE_RECEPTOR_BINDING	0.716671625	0.029042689	4.512768446	0.00177776	0.02848466	-1.00381904
hetero after UVB irradiation VS hetero before UVB irradiation	GO_CXCR_CHEMOKINE_RECEPTOR_BINDING1	0.85687223	0.052016408	5.184828524	0.00088856	0.08366654	-0.19001546
WT after UVB irradiation VS WT before UVB irradiation	GO_CXCR_CHEMOKINE_RECEPTOR_BINDING2	0.680757627	0.045187151	3.101030073	0.01442665	0.36090271	-2.75190935
homo after UVB irradiation VS homo before UVB irradiation	GO_CXCR3_CHEMOKINE_RECEPTOR_BINDING	0.977146301	-0.02043271	4.860253994	0.00111845	0.02251691	-0.53696528
hetero after UVB irradiation VS hetero before UVB irradiation	GO_CXCR3_CHEMOKINE_RECEPTOR_BINDING1	0.525868713	0.04525328	2.177639282	0.06171316	0.32651774	-4.24052886
WT after UVB irradiation VS WT before UVB irradiation	GO_CXCR3_CHEMOKINE_RECEPTOR_BINDING2	0.772411734	0.034768166	2.392321002	0.04333695	0.49624823	-3.75530601
homo after UVB irradiation VS homo before UVB irradiation	GO_CYCLASE_ACTIVITY	0.401255204	-0.04440841	2.222240153	0.05569215	0.17530269	-4.43424322
hetero after UVB irradiation VS hetero before UVB irradiation	GO_CYCLASE_ACTIVITY1	0.097513857	0.040094274	0.68024312	0.51590048	0.78454919	-5.98792614
WT after UVB irradiation VS WT before UVB irradiation	GO_CYCLASE_ACTIVITY2	0.175463471	-0.03937746	0.69518566	0.50639432	0.85763155	-5.74882496
homo after UVB irradiation VS homo before UVB irradiation	GO_CYCLASE_REGULATOR_ACTIVITY	0.097683338	0.006445275	0.455443029	0.66042888	0.79403202	-6.438438
hetero after UVB irradiation VS hetero before UVB irradiation	GO_CYCLASE_REGULATOR_ACTIVITY1	-0.4495976	0.020088433	-2.0451085	0.07573636	0.35489543	-4.43000986
WT after UVB irradiation VS WT before UVB irradiation	GO_CYCLASE_REGULATOR_ACTIVITY2	-0.2449776	-0.03396978	-1.02711044	0.33406881	0.77844475	-5.47482373
homo after UVB irradiation VS homo before UVB irradiation	GO_CYCLIC_NUCLEOTIDE_BINDING	0.214138219	0.002931698	1.558232568	0.15630665	0.33126774	-5.38470812
hetero after UVB irradiation VS hetero before UVB irradiation	GO_CYCLIC_NUCLEOTIDE_BINDING1	-0.02064746	-0.03451907	-0.09399549	0.92746676	0.97303232	-6.22669553
WT after UVB irradiation VS WT before UVB irradiation	GO_CYCLIC_NUCLEOTIDE_BINDING2	0.098478717	-0.04894527	0.359928165	0.72810763	0.93296251	-5.92828428
homo after UVB irradiation VS homo before UVB irradiation	GO_CYCLIC_NUCLEOTIDE_BIOSYNTHETIC_PROCESS	0.493168733	-0.0179605	2.977442691	0.01688973	0.08878367	-3.26817057
hetero after UVB irradiation VS hetero before UVB irradiation	GO_CYCLIC_NUCLEOTIDE_BIOSYNTHETIC_PROCESS1	0.052493825	0.076373742	0.287458453	0.78120111	0.91401375	-6.1869149
WT after UVB irradiation VS WT before UVB irradiation	GO_CYCLIC_NUCLEOTIDE_BIOSYNTHETIC_PROCESS2	0.119052393	-0.01650179	0.490901876	0.63652234	0.90563438	-5.87087879
homo after UVB irradiation VS homo before UVB irradiation	GO_CYCLIC_NUCLEOTIDE_CATABOLIC_PROCESS	0.574945148	-0.04057606	3.109858781	0.0137497	0.07934316	-3.06330212
hetero after UVB irradiation VS hetero before UVB irradiation	GO_CYCLIC_NUCLEOTIDE_CATABOLIC_PROCESS1	0.28715111	-0.04167077	1.208234741	0.26208149	0.58898731	-5.50785525
WT after UVB irradiation VS WT before UVB irradiation	GO_CYCLIC_NUCLEOTIDE_CATABOLIC_PROCESS2	0.070256359	-0.01565213	0.253949839	0.80586371	0.95404687	-5.96218117
homo after UVB irradiation VS homo before UVB irradiation	GO_CYCLIC_NUCLEOTIDE_DEPENDENT_PROTEIN_KINASE_ACTIVITY	0.341088274	0.002481721	1.695489749	0.12693116	0.29140061	-5.19993799
hetero after UVB irradiation VS hetero before UVB irradiation	GO_CYCLIC_NUCLEOTIDE_DEPENDENT_PROTEIN_KINASE_ACTIVITY1	0.269292103	-0.0289517	1.051024488	0.32451282	0.64439659	-5.67243761
WT after UVB irradiation VS WT before UVB irradiation	GO_CYCLIC_NUCLEOTIDE_DEPENDENT_PROTEIN_KINASE_ACTIVITY2	0.407471223	-0.15487837	1.687673553	0.12949726	0.63548424	-4.72118364
homo after UVB irradiation VS homo before UVB irradiation	GO_CYCLIC_NUCLEOTIDE_GATED_ION_CHANNEL_ACTIVITY	0.283365451	0.136501918	1.118689999	0.29449439	0.48912253	-5.91191158
hetero after UVB irradiation VS hetero before UVB irradiation	GO_CYCLIC_NUCLEOTIDE_GATED_ION_CHANNEL_ACTIVITY1	-0.13215061	-0.01628229	-0.69256934	0.50853895	0.77834257	-5.97928854
WT after UVB irradiation VS WT before UVB irradiation	GO_CYCLIC_NUCLEOTIDE_GATED_ION_CHANNEL_ACTIVITY2	0.313685593	-0.00616185	1.305687375	0.22753032	0.71944822	-5.18472416
homo after UVB irradiation VS homo before UVB irradiation	GO_CYCLIC_NUCLEOTIDE_MEDIATED_SIGNALING	0.399763281	-0.01168787	3.092284426	0.01412887	0.08087652	-3.09044025
hetero after UVB irradiation VS hetero before UVB irradiation	GO_CYCLIC_NUCLEOTIDE_MEDIATED_SIGNALING1	0.17524048	0.014850435	1.015704241	0.34003968	0.65952819	-5.70710607
WT after UVB irradiation VS WT before UVB irradiation	GO_CYCLIC_NUCLEOTIDE_MEDIATED_SIGNALING2	0.151353445	-0.0059372	0.768055064	0.46426075	0.84258958	-5.69627107
homo after UVB irradiation VS homo before UVB irradiation	GO_CYCLIC_NUCLEOTIDE_METABOLIC_PROCESS	0.422066131	-0.03370499	2.697743535	0.02620318	0.11423533	-3.70254397
hetero after UVB irradiation VS hetero before UVB irradiation	GO_CYCLIC_NUCLEOTIDE_METABOLIC_PROCESS1	0.09414	0.00158192	0.491403149	0.63658045	0.85090495	-6.10257698
WT after UVB irradiation VS WT before UVB irradiation	GO_CYCLIC_NUCLEOTIDE_METABOLIC_PROCESS2	-0.01812776	-0.01199209	-0.08091038	0.93747728	0.98368758	-5.99261389
homo after UVB irradiation VS homo before UVB irradiation	GO_CYCLIC_NUCLEOTIDE_PHOSPHODIESTERASE_ACTIVITY	0.409989512	-0.00848303	2.339002409	0.04627467	0.15709118	-4.25655196
hetero after UVB irradiation VS hetero before UVB irradiation	GO_CYCLIC_NUCLEOTIDE_PHOSPHODIESTERASE_ACTIVITY1	0.251059117	-0.02168148	1.126088974	0.29336292	0.61752684	-5.59587985
WT after UVB irradiation VS WT before UVB irradiation	GO_CYCLIC_NUCLEOTIDE_PHOSPHODIESTERASE_ACTIVITY2	0.132905174	-0.03769346	0.501739643	0.62920584	0.90390703	-5.86538007
homo after UVB irradiation VS homo before UVB irradiation	GO_CYCLIN_A2_CDK2_COMPLEX	-0.05163996	0.149539165	-0.19883716	0.84715816	0.91586175	-6.52921078
hetero after UVB irradiation VS hetero before UVB irradiation	GO_CYCLIN_A2_CDK2_COMPLEX1	0.727123785	0.110080625	3.105817442	0.01487477	0.18054811	-2.88620327
WT after UVB irradiation VS WT before UVB irradiation	GO_CYCLIN_A2_CDK2_COMPLEX2	0.365082598	0.042823218	1.022629481	0.33605947	0.77905119	-5.47908014
homo after UVB irradiation VS homo before UVB irradiation	GO_CYCLIN_BINDING	-0.24683746	-0.02975486	-1.79295898	0.10925596	0.26443991	-5.06426383
hetero after UVB irradiation VS hetero before UVB irradiation	GO_CYCLIN_BINDING1	-0.21921482	-0.05420038	-1.07511027	0.31424577	0.63499052	-5.64829216
WT after UVB irradiation VS WT before UVB irradiation	GO_CYCLIN_BINDING2	-0.22341267	-0.01274389	-1.06868258	0.31603208	0.77149112	-5.43467314
homo after UVB irradiation VS homo before UVB irradiation	GO_CYCLIN_CDK_POSITIVE_TRANSCRIPTION_ELONGATION_FACTOR_COMPLEX	-0.35381059	0.038823323	-1.35658983	0.21053394	0.40088379	-5.64024563
hetero after UVB irradiation VS hetero before UVB irradiation	GO_CYCLIN_CDK_POSITIVE_TRANSCRIPTION_ELONGATION_FACTOR_COMPLEX1	-0.11003902	-0.02184321	-0.39202184	0.70546645	0.88301492	-6.14897711
WT after UVB irradiation VS WT before UVB irradiation	GO_CYCLIN_CDK_POSITIVE_TRANSCRIPTION_ELONGATION_FACTOR_COMPLEX2	-0.61632067	-0.0423973	-2.3029894	0.04985738	0.50853601	-3.88176044
homo after UVB irradiation VS homo before UVB irradiation	GO_CYCLIN_DEPENDENT_PROTEIN_KINASE_ACTIVITY	-0.0457848	0.030467422	-0.25325765	0.80620822	0.89004375	-6.5157932
hetero after UVB irradiation VS hetero before UVB irradiation	GO_CYCLIN_DEPENDENT_PROTEIN_KINASE_ACTIVITY1	0.200838813	0.001532859	1.069112217	0.31677835	0.63701027	-5.65434274
WT after UVB irradiation VS WT before UVB irradiation	GO_CYCLIN_DEPENDENT_PROTEIN_KINASE_ACTIVITY2	-0.03814519	0.0104205	-0.18121168	0.86065506	0.9676681	-5.97877701
homo after UVB irradiation VS homo before UVB irradiation	GO_CYCLIN_DEPENDENT_PROTEIN_KINASE_HOLOENZYME_COMPLEX	-0.29477758	0.030269237	-1.94992483	0.08556887	0.22797251	-4.83923864
hetero after UVB irradiation VS hetero before UVB irradiation	GO_CYCLIN_DEPENDENT_PROTEIN_KINASE_HOLOENZYME_COMPLEX1	0.108642674	0.001200728	0.512616334	0.62232351	0.84401411	-6.09139956
WT after UVB irradiation VS WT before UVB irradiation	GO_CYCLIN_DEPENDENT_PROTEIN_KINASE_HOLOENZYME_COMPLEX2	-0.07870411	-0.02917878	-0.29157413	0.77794209	0.94371579	-5.95145624
homo after UVB irradiation VS homo before UVB irradiation	GO_CYCLIN_DEPENDENT_PROTEIN_SERINE_THREONINE_KINASE_ACTIVATOR_ACTIVITY	0.066670802	0.050607064	0.285644552	0.78212556	0.87477845	-6.50629915
hetero after UVB irradiation VS hetero before UVB irradiation	GO_CYCLIN_DEPENDENT_PROTEIN_SERINE_THREONINE_KINASE_ACTIVATOR_ACTIVITY1	0.298030632	0.050955964	0.943382751	0.37360005	0.68683569	-5.77525155
WT after UVB irradiation VS WT before UVB irradiation	GO_CYCLIN_DEPENDENT_PROTEIN_SERINE_THREONINE_KINASE_ACTIVATOR_ACTIVITY2	0.160487404	-0.01192077	0.79002721	0.45202418	0.83611442	-5.67954303
homo after UVB irradiation VS homo before UVB irradiation	GO_CYCLIN_DEPENDENT_PROTEIN_SERINE_THREONINE_KINASE_INHIBITOR_ACTIVITY	0.492429191	0.014212898	3.186878927	0.01220899	0.07425294	-2.94459599
hetero after UVB irradiation VS hetero before UVB irradiation	GO_CYCLIN_DEPENDENT_PROTEIN_SERINE_THREONINE_KINASE_INHIBITOR_ACTIVITY1	0.217989581	0.010216536	0.984752323	0.35411055	0.67047741	-5.73674624
WT after UVB irradiation VS WT before UVB irradiation	GO_CYCLIN_DEPENDENT_PROTEIN_SERINE_THREONINE_KINASE_INHIBITOR_ACTIVITY2	0.172267862	-0.06713851	1.025694377	0.33469691	0.7787514	-5.47617035
homo after UVB irradiation VS homo before UVB irradiation	GO_CYCLIN_DEPENDENT_PROTEIN_SERINE_THREONINE_KINASE_REGULATOR_ACTIVITY	-0.00286317	0.02477352	-0.01557579	0.98793947	0.99393392	-6.55072357
hetero after UVB irradiation VS hetero before UVB irradiation	GO_CYCLIN_DEPENDENT_PROTEIN_SERINE_THREONINE_KINASE_REGULATOR_ACTIVITY1	0.160445001	0.013035494	0.782876982	0.45665731	0.74655831	-5.91190521
WT after UVB irradiation VS WT before UVB irradiation	GO_CYCLIN_DEPENDENT_PROTEIN_SERINE_THREONINE_KINASE_REGULATOR_ACTIVITY2	0.048094881	-0.04251737	0.259768309	0.80152517	0.95295738	-5.96061752
homo after UVB irradiation VS homo before UVB irradiation	GO_CYCLOHYDROLASE_ACTIVITY	-0.75803451	-0.02344126	-4.13178229	0.00301561	0.03623886	-1.53720107
hetero after UVB irradiation VS hetero before UVB irradiation	GO_CYCLOHYDROLASE_ACTIVITY1	-0.39235775	-0.01962566	-2.62422752	0.03093573	0.24586983	-3.5891409
WT after UVB irradiation VS WT before UVB irradiation	GO_CYCLOHYDROLASE_ACTIVITY2	0.111831256	-0.19323361	0.284100085	0.783463	0.9456763	-5.95370212
homo after UVB irradiation VS homo before UVB irradiation	GO_CYCLOOXYGENASE_PATHWAY	0.317378404	0.021566873	2.160487728	0.06141395	0.18701727	-4.52738258
hetero after UVB irradiation VS hetero before UVB irradiation	GO_CYCLOOXYGENASE_PATHWAY1	0.361461681	0.089241348	2.137363207	0.0656802	0.33418445	-4.29840018
WT after UVB irradiation VS WT before UVB irradiation	GO_CYCLOOXYGENASE_PATHWAY2	0.139748167	0.090960078	0.748943266	0.47508181	0.84826802	-5.71049276
homo after UVB irradiation VS homo before UVB irradiation	GO_CYCLOSPORIN_A_BINDING	-0.16170679	-0.04431538	-0.84793808	0.42016962	0.60549024	-6.17268733
hetero after UVB irradiation VS hetero before UVB irradiation	GO_CYCLOSPORIN_A_BINDING1	-0.19393636	-0.04807076	-1.08361139	0.31068371	0.63155376	-5.6396742
WT after UVB irradiation VS WT before UVB irradiation	GO_CYCLOSPORIN_A_BINDING2	-0.21317269	-0.06575853	-1.29694713	0.23038095	0.72256251	-5.19452659
homo after UVB irradiation VS homo before UVB irradiation	GO_CYSTEINE_METABOLIC_PROCESS	-0.42863899	0.009856106	-2.29381946	0.0497153	0.16390722	-4.32553291
hetero after UVB irradiation VS hetero before UVB irradiation	GO_CYSTEINE_METABOLIC_PROCESS1	-0.51276441	-0.05964288	-2.05925948	0.07410132	0.35255978	-4.40991623
WT after UVB irradiation VS WT before UVB irradiation	GO_CYSTEINE_METABOLIC_PROCESS2	-0.49844387	-0.02044817	-2.37749728	0.04435665	0.49817448	-3.7763251
homo after UVB irradiation VS homo before UVB irradiation	GO_CYSTEINE_TYPE_ENDOPEPTIDASE_ACTIVITY	0.087432223	-0.02966797	0.698559347	0.50386134	0.67755438	-6.2907428
hetero after UVB irradiation VS hetero before UVB irradiation	GO_CYSTEINE_TYPE_ENDOPEPTIDASE_ACTIVITY_INVOLVED_IN_APOPTOTIC_PROCESS	0.71654307	-0.05620591	3.311726537	0.01008338	0.06714917	-2.75309341
WT after UVB irradiation VS WT before UVB irradiation	GO_CYSTEINE_TYPE_ENDOPEPTIDASE_ACTIVITY_INVOLVED_IN_APOPTOTIC_PROCESS1	0.855781337	0.016993228	4.843318768	0.00135226	0.09333375	-0.584226
homo after UVB irradiation VS homo before UVB irradiation	GO_CYSTEINE_TYPE_ENDOPEPTIDASE_ACTIVITY_INVOLVED_IN_APOPTOTIC_PROCESS2	0.901901576	0.023727272	6.152886791	0.00025995	0.18842845	0.764928633
hetero after UVB irradiation VS hetero before UVB irradiation	GO_CYSTEINE_TYPE_ENDOPEPTIDASE_ACTIVITY_INVOLVED_IN_APOPTOTIC_SIGNALING_PATHWAY	0.41583226	-0.05167679	1.528349	0.16346645	0.34113317	-5.42384943
WT after UVB irradiation VS WT before UVB irradiation	GO_CYSTEINE_TYPE_ENDOPEPTIDASE_ACTIVITY_INVOLVED_IN_APOPTOTIC_SIGNALING_PATHWAY1	0.680397509	-0.00624842	3.127504539	0.01439981	0.17916057	-2.854899
homo after UVB irradiation VS homo before UVB irradiation	GO_CYSTEINE_TYPE_ENDOPEPTIDASE_ACTIVITY_INVOLVED_IN_APOPTOTIC_SIGNALING_PATHWAY2	0.847772338	0.004201256	5.707118668	0.00043107	0.18842845	0.351616342
hetero after UVB irradiation VS hetero before UVB irradiation	GO_CYSTEINE_TYPE_ENDOPEPTIDASE_ACTIVITY_INVOLVED_IN_EXECUTION_PHASE_OF_APOPTOSIS	0.559794733	-0.04539683	2.411912957	0.04121614	0.1462339	-4.1447416
WT after UVB irradiation VS WT before UVB irradiation	GO_CYSTEINE_TYPE_ENDOPEPTIDASE_ACTIVITY_INVOLVED_IN_EXECUTION_PHASE_OF_APOPTOSIS1	0.712873748	0.051494859	3.283264256	0.01142424	0.16699924	-2.63141917
homo after UVB irradiation VS homo before UVB irradiation	GO_CYSTEINE_TYPE_ENDOPEPTIDASE_ACTIVITY_INVOLVED_IN_EXECUTION_PHASE_OF_APOPTOSIS2	0.888980777	0.00077918	6.04722268	0.00029239	0.18842845	0.669870545
hetero after UVB irradiation VS hetero before UVB irradiation	GO_CYSTEINE_TYPE_ENDOPEPTIDASE_ACTIVITY1	0.164843005	-0.00916556	1.25941824	0.24402261	0.57159915	-5.45090423
WT after UVB irradiation VS WT before UVB irradiation	GO_CYSTEINE_TYPE_ENDOPEPTIDASE_ACTIVITY2	0.09583053	0.007739209	0.591103242	0.57057844	0.88389944	-5.81580488
homo after UVB irradiation VS homo before UVB irradiation	GO_CYSTEINE_TYPE_ENDOPEPTIDASE_INHIBITOR_ACTIVITY	0.080156909	0.017103	0.483254865	0.64136228	0.7801981	-6.42448684
hetero after UVB irradiation VS hetero before UVB irradiation	GO_CYSTEINE_TYPE_ENDOPEPTIDASE_INHIBITOR_ACTIVITY_INVOLVED_IN_APOPTOTIC_PROCESS	-0.1762951	0.012199202	-0.88584904	0.40054952	0.58888593	-6.13965972
WT after UVB irradiation VS WT before UVB irradiation	GO_CYSTEINE_TYPE_ENDOPEPTIDASE_INHIBITOR_ACTIVITY_INVOLVED_IN_APOPTOTIC_PROCESS1	0.05869157	-0.02840193	0.363404794	0.72589089	0.89215613	-6.16048335
homo after UVB irradiation VS homo before UVB irradiation	GO_CYSTEINE_TYPE_ENDOPEPTIDASE_INHIBITOR_ACTIVITY_INVOLVED_IN_APOPTOTIC_PROCESS2	0.02499509	-0.06025402	0.125427059	0.90324358	0.97585222	-5.98777424
hetero after UVB irradiation VS hetero before UVB irradiation	GO_CYSTEINE_TYPE_ENDOPEPTIDASE_INHIBITOR_ACTIVITY1	0.196197405	0.013895237	1.526685486	0.1660571	0.48743686	-5.13055775
WT after UVB irradiation VS WT before UVB irradiation	GO_CYSTEINE_TYPE_ENDOPEPTIDASE_INHIBITOR_ACTIVITY2	0.16437766	0.028342336	1.095986403	0.30460655	0.76513806	-5.40766597
homo after UVB irradiation VS homo before UVB irradiation	GO_CYSTEINE_TYPE_ENDOPEPTIDASE_REGULATOR_ACTIVITY_INVOLVED_IN_APOPTOTIC_PROCESS	0.099440652	-0.01165422	0.71547502	0.4938808	0.66937904	-6.27837352
hetero after UVB irradiation VS hetero before UVB irradiation	GO_CYSTEINE_TYPE_ENDOPEPTIDASE_REGULATOR_ACTIVITY_INVOLVED_IN_APOPTOTIC_PROCESS1	0.260527641	-0.00437535	1.958446158	0.08654261	0.37240327	-4.5522186

WT after UVB irradiation VS WT before UVB irradiation	GO_CYSTEINE_TYPE_ENDOPEPTIDASE_REGULATOR_ACTIVITY_INVOLVED_IN_APOPTOTIC_PROCESS2	0.23807542	-0.0139513	1.48377963	0.1757118	0.68033133	-4.97653756
homo after UVB irradiation VS homo before UVB irradiation	GO_CYSTEINE_TYPE_EXOPEPTIDASE_ACTIVITY	-0.17385086	-0.02030414	-0.79363632	0.44942311	0.63085102	-6.21787214
hetero after UVB irradiation VS hetero before UVB irradiation	GO_CYSTEINE_TYPE_EXOPEPTIDASE_ACTIVITY1	-0.3259446	-0.09619306	-1.45690502	0.18393615	0.50848235	-5.21758941
WT after UVB irradiation VS WT before UVB irradiation	GO_CYSTEINE_TYPE_EXOPEPTIDASE_ACTIVITY2	0.011243176	-0.14282474	0.032186059	0.97510288	0.9934792	-5.99552009
homo after UVB irradiation VS homo before UVB irradiation	GO_CYSTEINE_TYPE_PEPTIDASE_ACTIVITY	0.023310048	-0.03267316	0.183025474	0.85915487	0.92253599	-6.53250975
hetero after UVB irradiation VS hetero before UVB irradiation	GO_CYSTEINE_TYPE_PEPTIDASE_ACTIVITY1	0.095910403	-0.00767689	0.676692498	0.51803327	0.78586398	-5.99038881
WT after UVB irradiation VS WT before UVB irradiation	GO_CYSTEINE_TYPE_PEPTIDASE_ACTIVITY2	0.060099433	0.012145853	0.372731014	0.71891587	0.93096927	-5.92342046
homo after UVB irradiation VS homo before UVB irradiation	GO_CYTIDINE_DEAMINASE_ACTIVITY	0.819700001	-0.01100011	4.740452306	0.00130959	0.02440107	-0.69576888
hetero after UVB irradiation VS hetero before UVB irradiation	GO_CYTIDINE_DEAMINASE_ACTIVITY1	0.420441994	0.075176906	2.34815624	0.04739137	0.29034796	-3.99338722
WT after UVB irradiation VS WT before UVB irradiation	GO_CYTIDINE_DEAMINASE_ACTIVITY2	0.439335655	-0.02078207	1.55275294	0.15863182	0.66223376	-4.89198267
homo after UVB irradiation VS homo before UVB irradiation	GO_CYTIDINE_TO_URIDINE_EDITING	0.765873916	0.022903132	5.100706778	0.00081999	0.01963943	-0.22510488
hetero after UVB irradiation VS hetero before UVB irradiation	GO_CYTIDINE_TO_URIDINE_EDITING1	0.609983955	0.023401164	3.506953043	0.00823641	0.14647152	-2.31528466
WT after UVB irradiation VS WT before UVB irradiation	GO_CYTIDINE_TO_URIDINE_EDITING2	0.690503269	0.018177195	2.480952258	0.0377134	0.47615783	-3.62942233
homo after UVB irradiation VS homo before UVB irradiation	GO_CYTIDYLYLTRANSFERASE_ACTIVITY	0.283060713	2.62E-05	1.323415648	0.22086807	0.41181029	-5.68021315
hetero after UVB irradiation VS hetero before UVB irradiation	GO_CYTIDYLYLTRANSFERASE_ACTIVITY1	0.157517452	0.003340096	1.007269828	0.34383084	0.66160279	-5.7152524
WT after UVB irradiation VS WT before UVB irradiation	GO_CYTIDYLYLTRANSFERASE_ACTIVITY2	0.129557273	0.030120954	0.5717373	0.58301796	0.88972038	-5.82718218
homo after UVB irradiation VS homo before UVB irradiation	GO_CYTOCHROME_B5_REDUCTASE_ACTIVITY_ACTING_ON_NAD_P_H	0.398208987	-0.01811653	1.736741791	0.11914886	0.27945762	-5.14293475
hetero after UVB irradiation VS hetero before UVB irradiation	GO_CYTOCHROME_B5_REDUCTASE_ACTIVITY_ACTING_ON_NAD_P_H1	-0.14976382	-0.09711333	-0.74549151	0.47769291	0.75973664	-5.94066495
WT after UVB irradiation VS WT before UVB irradiation	GO_CYTOCHROME_B5_REDUCTASE_ACTIVITY_ACTING_ON_NAD_P_H2	0.012284939	-0.04948889	0.051969547	0.95981213	0.98993204	-5.99464182
homo after UVB irradiation VS homo before UVB irradiation	GO_CYTOCHROME_COMPLEX	-0.06845578	0.031497581	-0.28278895	0.78423945	0.87681048	-6.50718128
homo after UVB irradiation VS homo before UVB irradiation	GO_CYTOCHROME_COMPLEX_ASSEMBLY	-0.12018225	-0.04413066	-0.52871646	0.61079739	0.75733693	-6.40000732
hetero after UVB irradiation VS hetero before UVB irradiation	GO_CYTOCHROME_COMPLEX_ASSEMBLY1	0.040087497	-0.04450497	0.216632321	0.83401672	0.93587458	-6.20611244
WT after UVB irradiation VS WT before UVB irradiation	GO_CYTOCHROME_COMPLEX_ASSEMBLY2	0.186311618	-0.05733561	0.743026771	0.47846501	0.84979972	-5.71483284
hetero after UVB irradiation VS hetero before UVB irradiation	GO_CYTOCHROME_COMPLEX1	0.014367824	-0.04184785	0.055793497	0.95689974	0.98459722	-6.2297963
WT after UVB irradiation VS WT before UVB irradiation	GO_CYTOCHROME_COMPLEX2	0.02281611	-0.03418708	0.079871668	0.938278	0.98368811	-5.99270193
homo after UVB irradiation VS homo before UVB irradiation	GO_CYTOKINE_ACTIVITY	0.288266489	0.019451047	2.02530341	0.07601913	0.21177327	-4.72874798
hetero after UVB irradiation VS hetero before UVB irradiation	GO_CYTOKINE_ACTIVITY1	0.139338901	0.022789886	0.916417202	0.38672637	0.69764483	-5.7996485
WT after UVB irradiation VS WT before UVB irradiation	GO_CYTOKINE_ACTIVITY2	0.137393831	0.035027653	0.708845743	0.49831633	0.85541458	-5.73932078
homo after UVB irradiation VS homo before UVB irradiation	GO_CYTOKINE_BINDING	0.441146694	-0.05148748	2.773401997	0.02325582	0.10657122	-3.58499418
hetero after UVB irradiation VS hetero before UVB irradiation	GO_CYTOKINE_BINDING1	0.198009094	0.000148381	0.917560073	0.38616325	0.69720115	-5.79862591
WT after UVB irradiation VS WT before UVB irradiation	GO_CYTOKINE_BINDING2	0.199127009	-0.02868733	0.786749982	0.45383541	0.83736549	-5.68206353
homo after UVB irradiation VS homo before UVB irradiation	GO_CYTOKINE_MEDIATED_SIGNALING_PATHWAY	0.392708172	-0.02388967	2.649327064	0.02828703	0.11915509	-3.77771718
hetero after UVB irradiation VS hetero before UVB irradiation	GO_CYTOKINE_MEDIATED_SIGNALING_PATHWAY1	0.39650481	0.019494099	2.732765389	0.02618597	0.230549	-3.42989292
WT after UVB irradiation VS WT before UVB irradiation	GO_CYTOKINE_MEDIATED_SIGNALING_PATHWAY2	0.323176064	-0.00149392	1.8507656	0.10090983	0.60045416	-4.50669159
homo after UVB irradiation VS homo before UVB irradiation	GO_CYTOKINE_METABOLIC_PROCESS	0.421656939	-0.02445481	2.341819014	0.04606821	0.15667864	-4.25224352
hetero after UVB irradiation VS hetero before UVB irradiation	GO_CYTOKINE_METABOLIC_PROCESS1	0.590344043	0.026245968	4.367048303	0.00249838	0.11070877	-1.16733753
WT after UVB irradiation VS WT before UVB irradiation	GO_CYTOKINE_METABOLIC_PROCESS2	0.296126881	0.005841491	1.244118437	0.24826093	0.73564768	-5.25287626
homo after UVB irradiation VS homo before UVB irradiation	GO_CYTOKINE_PRODUCTION	0.392062513	-0.01033685	2.704082786	0.02594219	0.11380168	-3.69269754
homo after UVB irradiation VS homo before UVB irradiation	GO_CYTOKINE_PRODUCTION_INVOLVED_IN_IMMUNE_RESPONSE	0.416627489	-0.02583854	2.614290509	0.02989963	0.12287072	-3.83207305
hetero after UVB irradiation VS hetero before UVB irradiation	GO_CYTOKINE_PRODUCTION_INVOLVED_IN_IMMUNE_RESPONSE1	0.260920284	0.033740812	1.602329109	0.14845814	0.46627003	-5.03385331
WT after UVB irradiation VS WT before UVB irradiation	GO_CYTOKINE_PRODUCTION_INVOLVED_IN_IMMUNE_RESPONSE2	0.223147414	-0.01437456	1.015500163	0.33924541	0.78064003	-5.48582317
homo after UVB irradiation VS homo before UVB irradiation	GO_CYTOKINE_PRODUCTION_INVOLVED_IN_INFLAMMATORY_RESPONSE	0.438074172	0.01536947	2.661474675	0.02774882	0.11789461	-3.75886201
hetero after UVB irradiation VS hetero before UVB irradiation	GO_CYTOKINE_PRODUCTION_INVOLVED_IN_INFLAMMATORY_RESPONSE1	0.341748753	0.042620792	2.514230995	0.03665399	0.26290844	-3.75051665
WT after UVB irradiation VS WT before UVB irradiation	GO_CYTOKINE_PRODUCTION_INVOLVED_IN_INFLAMMATORY_RESPONSE2	0.365245563	-0.00762529	1.759001634	0.11617017	0.62316363	-4.62834682
hetero after UVB irradiation VS hetero before UVB irradiation	GO_CYTOKINE_PRODUCTION1	0.35709427	0.036709104	2.2940273	0.05153591	0.30169597	-4.07215718
WT after UVB irradiation VS WT before UVB irradiation	GO_CYTOKINE_PRODUCTION2	0.304777724	-0.0004529	1.688278988	0.1293784	0.63548424	-4.72040255
homo after UVB irradiation VS homo before UVB irradiation	GO_CYTOKINE_RECEPTOR_ACTIVITY	0.396759527	-0.05642659	2.277167986	0.05104636	0.16660581	-4.35088773
hetero after UVB irradiation VS hetero before UVB irradiation	GO_CYTOKINE_RECEPTOR_ACTIVITY1	0.209994763	-0.01033489	0.911229109	0.38929019	0.69943586	-5.80427773
WT after UVB irradiation VS WT before UVB irradiation	GO_CYTOKINE_RECEPTOR_ACTIVITY2	0.212986999	-0.02872752	0.779533119	0.45784116	0.83880233	-5.6875826
homo after UVB irradiation VS homo before UVB irradiation	GO_CYTOKINE_RECEPTOR_BINDING	0.427360663	0.006217578	3.282269793	0.01054722	0.06883079	-2.79816386
hetero after UVB irradiation VS hetero before UVB irradiation	GO_CYTOKINE_RECEPTOR_BINDING1	0.383734036	0.025335582	2.549231656	0.03472605	0.25582508	-3.69918816
WT after UVB irradiation VS WT before UVB irradiation	GO_CYTOKINE_RECEPTOR_BINDING2	0.300593678	0.011730934	1.569433191	0.15473223	0.65981549	-4.87123909
homo after UVB irradiation VS homo before UVB irradiation	GO_CYTOKINE_SECRETION	0.607075692	-0.02718124	4.144660608	0.00296119	0.03594912	-1.51881534
hetero after UVB irradiation VS hetero before UVB irradiation	GO_CYTOKINE_SECRETION1	0.570077059	0.050072346	3.591097834	0.00729508	0.14137605	-2.19802941
WT after UVB irradiation VS WT before UVB irradiation	GO_CYTOKINE_SECRETION2	0.352462231	0.001868853	1.610556635	0.14548659	0.65365874	-4.81963339
homo after UVB irradiation VS homo before UVB irradiation	GO_CYTOKINESIS	-0.04602405	-0.01452769	-0.29221083	0.77727209	0.8714603	-6.50423781
hetero after UVB irradiation VS hetero before UVB irradiation	GO_CYTOKINESIS1	0.269573265	-4.58E-05	1.982499151	0.08340191	0.36912975	-4.51845368
WT after UVB irradiation VS WT before UVB irradiation	GO_CYTOKINESIS2	0.168138574	0.011319652	0.912529128	0.38785032	0.80976349	-5.5791186
homo after UVB irradiation VS homo before UVB irradiation	GO_CYTOKINETIC_PROCESS	-0.09693748	-0.01407784	-0.43513443	0.67452013	0.80373441	-6.44812866
hetero after UVB irradiation VS hetero before UVB irradiation	GO_CYTOKINETIC_PROCESS1	0.458405203	0.021450685	2.352198921	0.04709559	0.28948384	-3.98749459
WT after UVB irradiation VS WT before UVB irradiation	GO_CYTOKINETIC_PROCESS2	0.272137806	0.081504996	1.024078575	0.33541472	0.77905119	-5.4777052
homo after UVB irradiation VS homo before UVB irradiation	GO_CYTOLYSIS	0.594877443	-0.02048764	2.48436704	0.03673805	0.13742543	-4.03312686
homo after UVB irradiation VS homo before UVB irradiation	GO_CYTOLYSIS_IN_OTHER_ORGANISM	0.212727643	0.030815597	0.758486203	0.46907609	0.64820262	-6.24575407
hetero after UVB irradiation VS hetero before UVB irradiation	GO_CYTOLYSIS_IN_OTHER_ORGANISM1	0.238100241	0.150866587	1.127424339	0.29283122	0.61702377	-5.59448358
WT after UVB irradiation VS WT before UVB irradiation	GO_CYTOLYSIS_IN_OTHER_ORGANISM2	0.592128376	0.035685754	2.080154042	0.07067925	0.54758105	-4.19384881
hetero after UVB irradiation VS hetero before UVB irradiation	GO_CYTOLYSIS1	0.217654735	0.088734032	0.947619683	0.37156796	0.68518581	-5.77136741
WT after UVB irradiation VS WT before UVB irradiation	GO_CYTOLYSIS2	0.40367287	0.043267799	1.321804904	0.22235218	0.71742564	-5.16654006
homo after UVB irradiation VS homo before UVB irradiation	GO_CYTOLYTIC_GRANULE	-0.18559831	0.041104689	-0.69545213	0.50570834	0.67898676	-6.29298638
hetero after UVB irradiation VS hetero before UVB irradiation	GO_CYTOLYTIC_GRANULE1	0.222050224	-0.02203367	0.820455246	0.43615212	0.73478547	-5.88179728
WT after UVB irradiation VS WT before UVB irradiation	GO_CYTOLYTIC_GRANULE2	0.179274881	-0.15939026	0.598319708	0.56598185	0.88229844	-5.81147684
homo after UVB irradiation VS homo before UVB irradiation	GO_CYTOPLASM_ORGANIZATION	-0.7729815	0.052110782	-3.94045998	0.00396358	0.04117099	-1.81315323
hetero after UVB irradiation VS hetero before UVB irradiation	GO_CYTOPLASM_ORGANIZATION1	-0.31669296	-0.01066514	-1.42566893	0.19247985	0.51810155	-5.25581996
WT after UVB irradiation VS WT before UVB irradiation	GO_CYTOPLASM_ORGANIZATION2	-0.54589991	-0.01088021	-2.486331	0.03739683	0.47615783	-3.62177391
homo after UVB irradiation VS homo before UVB irradiation	GO_CYTOPLASMIC_DYNEIN_COMPLEX	-0.08301215	-0.03111138	-0.45209913	0.66273944	0.79546698	-6.44006254
hetero after UVB irradiation VS hetero before UVB irradiation	GO_CYTOPLASMIC_DYNEIN_COMPLEX1	0.051493769	-0.01286993	0.305814592	0.7676958	0.90915884	-6.18107704
WT after UVB irradiation VS WT before UVB irradiation	GO_CYTOPLASMIC_DYNEIN_COMPLEX2	-0.15208993	-0.00517416	-0.72777892	0.4872563	0.85164239	-5.72588057
homo after UVB irradiation VS homo before UVB irradiation	GO_CYTOPLASMIC_EXOSOME_RNASE_COMPLEX	-0.95135263	0.010402416	-7.55047013	5.33E-05	0.00930476	2.458782577
hetero after UVB irradiation VS hetero before UVB irradiation	GO_CYTOPLASMIC_EXOSOME_RNASE_COMPLEX1	-0.57731899	-0.02273831	-2.31387288	0.04997581	0.29759608	-4.0433065
WT after UVB irradiation VS WT before UVB irradiation	GO_CYTOPLASMIC_EXOSOME_RNASE_COMPLEX2	-0.27362743	-0.00589968	-0.8058367	0.44335543	0.83340629	-5.66725963
homo after UVB irradiation VS homo before UVB irradiation	GO_CYTOPLASMIC_MICROTUBULE	-0.16513849	0.012328524	-1.20009998	0.26311859	0.45842125	-5.82300498
homo after UVB irradiation VS homo before UVB irradiation	GO_CYTOPLASMIC_MICROTUBULE_ORGANIZATION	-0.10888503	-0.03919616	-0.88254702	0.40223207	0.58968197	-6.14258396
hetero after UVB irradiation VS hetero before UVB irradiation	GO_CYTOPLASMIC_MICROTUBULE_ORGANIZATION1	0.134470131	-0.04181644	0.841347444	0.42503109	0.72623423	-5.86454831
WT after UVB irradiation VS WT before UVB irradiation	GO_CYTOPLASMIC_MICROTUBULE_ORGANIZATION2	-0.01920941	-0.00308307	-0.11439264	0.91171211	0.97857624	-5.98916791
hetero after UVB irradiation VS hetero before UVB irradiation	GO_CYTOPLASMIC_MICROTUBULE1	-0.09483962	-0.01458041	-0.56287912	0.58922191	0.82529231	-6.06316658
WT after UVB irradiation VS WT before UVB irradiation	GO_CYTOPLASMIC_MICROTUBULE2	-0.01980926	0.000866488	-0.12561778	0.90309732	0.97576564	-5.98774903
homo after UVB irradiation VS homo before UVB irradiation	GO_CYTOPLASMIC_PATTERN_RECOGNITION_RECEPTOR_SIGNALING_PATHWAY	0.266486373	-0.03725555	1.81140139	0.10618163	0.26002693	-5.03821978
homo after UVB irradiation VS homo before UVB irradiation	GO_CYTOPLASMIC_PATTERN_RECOGNITION_RECEPTOR_SIGNALING_PATHWAY_IN_RESPONSE_TO_VIRUS	-0.16003444	-0.00462763	-1.04834107	0.32394553	0.51731027	-5.98502833
hetero after UVB irradiation VS hetero before UVB irradiation	GO_CYTOPLASMIC_PATTERN_RECOGNITION_RECEPTOR_SIGNALING_PATHWAY_IN_RESPONSE_TO_VIRUS1	0.289166637	0.002237952	1.825227872	0.10610405	0.40362759	-4.73675695
WT after UVB irradiation VS WT before UVB irradiation	GO_CYTOPLASMIC_PATTERN_RECOGNITION_RECEPTOR_SIGNALING_PATHWAY_IN_RESPONSE_TO_VIRUS2	0.159852214	0.028427208	0.836839667	0.42668732	0.82688157	-5.64257945
hetero after UVB irradiation VS hetero before UVB irradiation	GO_CYTOPLASMIC_PATTERN_RECOGNITION_RECEPTOR_SIGNALING_PATHWAY1	0.419043499	0.016940626	2.938909454	0.01912682	0.20116236	-3.12841637
WT after UVB irradiation VS WT before UVB irradiation	GO_CYTOPLASMIC_PATTERN_RECOGNITION_RECEPTOR_SIGNALING_PATHWAY2	0.178399055	-0.00313994	1.184217651	0.26991945	0.74431614	-5.3170924
homo after UVB irradiation VS homo before UVB irradiation	GO_CYTOPLASMIC_REGION	-0.09310931	0.00671319	-0.72512715	0.48824256	0.66472879	-6.27119866
hetero after UVB irradiation VS hetero before UVB irradiation	GO_CYTOPLASMIC_REGION1	-0.04439421	-0.00681191	-0.28166747	0.78547852	0.91632149	-6.1886833
WT after UVB irradiation VS WT before UVB irradiation	GO_CYTOPLASMIC_REGION2	-0.06032021	-0.02221815	-0.407692	0.69406578	0.92191472	-5.90930621
homo after UVB irradiation VS homo before UVB irradiation	GO_CYTOPLASMIC_SEQUESTERING_OF_NF_KAPPAB	0.29172572	-0.05141744	1.64265721	0.13758265	0.30656759	-5.27198562
hetero after UVB irradiation VS hetero before UVB irradiation	GO_CYTOPLASMIC_SEQUESTERING_OF_NF_KAPPAB1	0.351580346	-0.04173315	1.649342469	0.13839622	0.4518178	-4.97261202
WT after UVB irradiation VS WT before UVB irradiation	GO_CYTOPLASMIC_SEQUESTERING_OF_NF_KAPPAB2	0.316825833	0.00973277	1.359331849	0.21068485	0.70821783	-5.12367148
homo after UVB irradiation VS homo before UVB irradiation	GO_CYTOPLASMIC_SEQUESTERING_OF_PROTEIN	0.418280233	-0.08440795	2.395664849	0.0422933	0.14842385	-4.16970677
hetero after UVB irradiation VS hetero before UVB irradiation	GO_CYTOPLASMIC_SEQUESTERING_OF_PROTEIN1	0.510326618	0.004285711	3.964649791	0.00430765	0.12634511	-1.68990086
WT after UVB irradiation VS WT before UVB irradiation	GO_CYTOPLASMIC_SEQUESTERING_OF_PROTEIN2	0.406308219	0.002334441	2.305739643	0.04964272	0.50853601	-3.87787578
homo after UVB irradiation VS homo before UVB irradiation	GO_CYTOPLASMIC_SEQUESTERING_OF_TRANSCRIPTION_FACTOR	0.429426381	-0.08969558	2.468033668	0.03770284	0.13923445	-4.05832622
hetero after UVB irradiation VS hetero before UVB irradiation	GO_CYTOPLASMIC_SEQUESTERING_OF_TRANSCRIPTION_FACTOR1	0.6274253	-0.00828348	4.17485741	0.00323114	0.11632799	-1.4135759
WT after UVB irradiation VS WT before UVB irradiation	GO_CYTOPLASMIC_SEQUESTERING_OF_TRANSCRIPTION_FACTOR2	0.540632177	0.007590995	3.349023801	0.00991997	0.33044931	-2.40923829
homo after UVB irradiation VS homo before UVB irradiation	GO_CYTOPLASMIC_SIDE_OF_ENDOPLASMIC_RETICULUM_MEMBRANE	-0.27196049	-0.00492845	-1.36315505	0.20853868	0.39831806	-5.63226188
hetero after UVB irradiation VS hetero before UVB irradiation	GO_CYTOPLASMIC_SIDE_OF_ENDOPLASMIC_RETICULUM_MEMBRANE1	0.230708401	-0.00496145	0.823148256	0.43470739	0.73406477	-5.87959417
WT after UVB irradiation VS WT before UVB irradiation	GO_CYTOPLASMIC_SIDE_OF_ENDOPLASMIC_RETICULUM_MEMBRANE2	-0.1728737	-0.01879898	-1.07230515	0.31449709	0.77104985	-5.43111862
homo after UVB irradiation VS homo before UVB irradiation	GO_CYTOPLASMIC_SIDE_OF_LYSOSOMAL_MEMBRANE	-0.06417954	-0.03785184	-0.24715302	0.81077307	0.89281234	-6.51745699
hetero after UVB irradiation VS hetero before UVB irradiation	GO_CYTOPLASMIC_SIDE_OF_LYSOSOMAL_MEMBRANE1	-0.09057583	-0.0110615	-0.50157027	0.62972665	0.84752935	-6.09727497
WT after UVB irradiation VS WT before UVB irradiation	GO_CYTOPLASMIC_SIDE_OF_LYSOSOMAL_MEMBRANE2	-0.3961096	-0.03551416	-1.60276415	0.14719898	0.65388781	-4.82946178
homo after UVB irradiation VS homo before UVB irradiation	GO_CYTOPLASMIC_SIDE_OF_MEMBRANE	0.209531314	-0.01935089	1.49851898	0.17090445	0.35045005	-5.46250242
hetero after UVB irradiation VS hetero before UVB irradiation	GO_CYTOPLASMIC_SIDE_OF_MEMBRANE1	0.28902742	0.003666453	2.331730684	0.04861244	0.2936247	-4.01731611
WT after UVB irradiation VS WT before UVB irradiation	GO_CYTOPLASMIC_SIDE_OF_MEMBRANE2	0.094033176	-0.02938313	0.570984442	0.58350459	0.88980639	-5.82761747
homo after UVB irradiation VS homo before UVB irradiation	GO_CYTOPLASMIC_SIDE_OF_ROUGH_ENDOPLASMIC_RETICULUM_MEMBRANE	-0.34524356	-0.02948091	-0.88544081	0.40075726	0.58904132	-6.14002173
hetero after UVB irradiation VS hetero before UVB irradiation	GO_CYTOPLASMIC_SIDE_OF_ROUGH_ENDOPLASMIC_RETICULUM_MEMBRANE1	0.147323822	0.015824525	0.473742109	0.64857498	0.85558615	-6.11154447
WT after UVB irradiation VS WT before UVB irradiation	GO_CYTOPLASMIC_SIDE_OF_ROUGH_ENDOPLASMIC_RETICULUM_MEMBRANE2	-0.28628018	0.032045946	-0.91189522	0.38816463	0.80982312	-5.57966854
homo after UVB irradiation VS homo before UVB irradiation	GO_CYTOPLASMIC_STRESS_GRANULE	-0.37890134	0.015773013	-2.4815806	0.03690086	0.13772029	-4.03742726
hetero after UVB irradiation VS hetero before UVB irradiation	GO_CYTOPLASMIC_STRESS_GRANULE1	-0.1743047	-0.04853307	-0.91962366	0.38514801	0.69631227	-5.79677695
WT after UVB irradiation VS WT before UVB irradiation	GO_CYTOPLASMIC_STRESS_GRANULE2	-0.19707605	-0.01807133	-0.94672459	0.37116978	0.80078252	-5.54900257
homo after UVB irradiation VS homo before UVB irradiation	GO_CYTOPLASMIC_TRANSLATION	-0.84199176	0.014711741	-6.45073155	0.0001662	0.01043635	1.35947194
hetero after UVB irradiation VS hetero before UVB irradiation	GO_CYTOPLASMIC_TRANSLATION1	-0.3272029	-0.03943652	-1.16448141	0.27838426	0.60400502	-5.55527339
WT after UVB irradiation VS WT before UVB irradiation	GO_CYTOPLASMIC_TRANSLATION2	-0.50670617	-0.03083522	-2.08913266	0.0696951	0.54504008	-4.18140027
homo after UVB irradiation VS homo before UVB irradiation	GO_CYTOPLASMIC_TRANSLATIONAL_ELONGATION	-0.51315125	-0.0476691	-2.61983639	0.02963827	0.12234182	-3.82347208
hetero after UVB irradiation VS hetero before UVB irradiation	GO_CYTOPLASMIC_TRANSLATIONAL_ELONGATION1	-0.64368776	-0.09840362	-3.02136235	0.01688672	0.19058304	-3.00849574
WT after UVB irradiation VS WT before UVB irradiation	GO_CYTOPLASMIC_TRANSLATIONAL_ELONGATION2	-0.2035385	-0.07555636	-0.80793645	0.44221266	0.83246998	-5.66561277
homo after UVB irradiation VS homo before UVB irradiation	GO_CYTOPLASMIC_TRANSLATIONAL_INITIATION	-0.86985315	-0.0016756	-6.64792692	0.0001342	0.01007802	1.568461294
hetero after UVB irradiation VS hetero before UVB irradiation	GO_CYTOPLASMIC_TRANSLATIONAL_INITIATION1	-0.3671674	-0.06655134	-1.24433328	0.24923331	0.57689205	-5.46785068
WT after UVB irradiation VS WT before UVB irradiation	GO_CYTOPLASMIC_TRANSLATIONAL_INITIATION2	-0.49704699	-0.04451604	-1.68932489	0.1291733	0.63548424	-4.71905291
homo after UVB irradiation VS homo before UVB irradiation	GO_CYTOPLASMIC_UBIQUITIN_LIGASE_COMPLEX	0.023026506	0.0114961	0.125248549	0.90329718	0.94720457	-6.54225552
hetero after UVB irradiation VS hetero before UVB irradiation	GO_CYTOPLASMIC_UBIQUITIN_LIGASE_COMPLEX1	0.160968186	-0.03605443	0.630398636	0.54633679	0.7993465	-6.02144525
WT after UVB irradiation VS WT before UVB irradiation	GO_CYTOPLASMIC_UBIQUITIN_LIGASE_COMPLEX2	0.157438583	0.013010866	1.043502612	0.32686395	0.77548734	-5.45913394
homo after UVB irradiation VS homo before UVB irradiation	GO_CYTOSKELETAL_ANCHOR_ACTIVITY	0.668186679	-0.03125056	5.087385662	0.00083403	0.01971289	-0.24214192
hetero after UVB irradiation VS hetero before UVB irradiation	GO_CYTOSKELETAL_ANCHOR_ACTIVITY1	0.251755842	0.046222994	1.710575022	0.12623447	0.43339219	-4.89164534
WT after UVB irradiation VS WT before UVB irradiation	GO_CYTOSKELETAL_ANCHOR_ACTIVITY2	0.199797126	-0.00411702	0.964243021	0.36283258	0.79520373	-5.53323606
homo after UVB irradiation VS homo before UVB irradiation	GO_CYTOSKELETAL_PROTEIN_BINDING	0.108059702	-0.00505581	0.854334455	0.41681286	0.60285282	-6.16719926
hetero after UVB irradiation VS hetero before UVB irradiation	GO_CYTOSKELETAL_PROTEIN_BINDING1	0.041594548	-0.00800944	0.340395381	0.74248558	0.8995791	-6.16912385
WT after UVB irradiation VS WT before UVB irradiation	GO_CYTOSKELETAL_PROTEIN_BINDING2	-0.01924451	0.000460996	-0.13803649	0.89358243	0.97322079	-5.9860253
homo after UVB irradiation VS homo before UVB irradiation	GO_CYTOSKELETON_DEPENDENT_CYTOKINESIS	-0.02984019	-0.02476035	-0.19121061	0.85293956	0.91867959	-6.5308358
hetero after UVB irradiation VS hetero before UVB irradiation	GO_CYTOSKELETON_DEPENDENT_CYTOKINESIS1	0.380697538	-0.01547219	2.601521212	0.03203645	0.24829472	-3.62246474
WT after UVB irradiation VS WT before UVB irradiation	GO_CYTOSKELETON_DEPENDENT_CYTOKINESIS2	0.250509011	0.000673522	1.325997251	0.22102189	0.71573842	-5.16178749
homo after UVB irradiation VS homo before UVB irradiation	GO_CYTOSKELETON_DEPENDENT_INTRACELLULAR_TRANSPORT	-0.12371965	-0.01068116	-0.99181803	0.34922093	0.54115858	-6.04114348
hetero after UVB irradiation VS hetero before UVB irradiation	GO_CYTOSKELETON_DEPENDENT_INTRACELLULAR_TRANSPORT1	-0.14479223	-0.0217395	-1.03858822	0.32991572	0.65005158	-5.68474566
WT after UVB irradiation VS WT before UVB irradiation	GO_CYTOSKELETON_DEPENDENT_INTRACELLULAR_TRANSPORT2	-0.06701905	-0.02569728	-0.43146432	0.67738793	0.91685973	-5.899018
homo after UVB irradiation VS homo before UVB irradiation	GO_CYTOSKELETON_OF_PRESYNAPTIC_ACTIVE_ZONE	-0.63943801	0.03164077	-2.9272728	0.01826616	0.09285351	-3.34598605
hetero after UVB irradiation VS hetero before UVB irradiation	GO_CYTOSKELETON_OF_PRESYNAPTIC_ACTIVE_ZONE1	0.500023154	0.045885605	1.962339597	0.08602658	0.37204226	-4.54676157
WT after UVB irradiation VS WT before UVB irradiation	GO_CYTOSKELETON_OF_PRESYNAPTIC_ACTIVE_ZONE2	-0.13918605	0.11199024	-0.39186117	0.70527189	0.92644338	-5.91584785
homo after UVB irradiation VS homo before UVB irradiation	GO_CYTOSKELETON_ORGANIZATION	0.058538229	-0.00613583	0.464056503	0.6544949	0.78985781	-6.43420103
hetero after UVB irradiation VS hetero before UVB irradiation	GO_CYTOSKELETON_ORGANIZATION1	0.128102489	-0.0028789	1.024794389	0.33598984	0.65575193	-5.69826883
WT after UVB irradiation VS WT before UVB irradiation	GO_CYTOSKELETON_ORGANIZATION2	0.039201411	-0.0127479	0.279182149	0.78710291	0.94689038	-5.95514877
homo after UVB irradiation VS homo before UVB irradiation	GO_CYTOSOLIC_CALCIUM_ION_TRANSPORT	0.270905614	-0.02283283	1.982040786	0.0813669	0.22080384	-4.7923364
hetero after UVB irradiation VS hetero before UVB irradiation	GO_CYTOSOLIC_CALCIUM_ION_TRANSPORT1	0.114179427	0.011100768	0.693426753	0.50802935	0.77796039	-5.97868262
WT after UVB irradiation VS WT before UVB irradiation	GO_CYTOSOLIC_CALCIUM_ION_TRANSPORT2	0.12663527	-0.01124895	0.595979159	0.56747036	0.88290486	-5.81288581
homo after UVB irradiation VS homo before UVB irradiation	GO_CYTOSOLIC_LARGE_RIBOSOMAL_SUBUNIT	-0.95372996	0.016327709	-6.26344981	0.0002045	0.01125399	1.155909094

hetero after UVB irradiation VS hetero before UVB irradiation	GO_CYTOSOLIC_LARGE_RIBOSOMAL_SUBUNIT1	-0.21727035	-0.02261984	-0.64885525	0.53494301	0.79354737	-6.00929915
WT after UVB irradiation VS WT before UVB irradiation	GO_CYTOSOLIC_LARGE_RIBOSOMAL_SUBUNIT2	-0.65230529	-0.01841062	-2.5034003	0.03640987	0.47615783	-3.59749706
homo after UVB irradiation VS homo before UVB irradiation	GO_CYTOSOLIC_PROTEASOME_COMPLEX	-0.51941003	0.088365139	-1.69920823	0.12621084	0.29036478	-5.19482592
hetero after UVB irradiation VS hetero before UVB irradiation	GO_CYTOSOLIC_PROTEASOME_COMPLEX1	-0.04195778	-0.00793091	-0.14112232	0.89132628	0.96027811	-6.22069796
WT after UVB irradiation VS WT before UVB irradiation	GO_CYTOSOLIC_PROTEASOME_COMPLEX2	-0.16359448	0.019439376	-0.43642647	0.67392996	0.91622321	-5.89680039
homo after UVB irradiation VS homo before UVB irradiation	GO_CYTOSOLIC_RIBOSOME	-0.89753671	0.020562196	-5.84827017	0.00032895	0.01344677	0.686421169
hetero after UVB irradiation VS hetero before UVB irradiation	GO_CYTOSOLIC_RIBOSOME1	-0.11664291	-0.00911743	-0.36920411	0.7217322	0.89103348	-6.15821946
WT after UVB irradiation VS WT before UVB irradiation	GO_CYTOSOLIC_RIBOSOME2	-0.55156937	-0.01902076	-2.2193554	0.05684498	0.5232848	-3.99956717
homo after UVB irradiation VS homo before UVB irradiation	GO_CYTOSOLIC_SMALL_RIBOSOMAL_SUBUNIT	-0.88720806	0.010611404	-5.22990636	0.00069639	0.0185944	-0.06132277
hetero after UVB irradiation VS hetero before UVB irradiation	GO_CYTOSOLIC_SMALL_RIBOSOMAL_SUBUNIT1	0.027097336	0.038394406	0.085796315	0.93377549	0.97579263	-6.22749412
WT after UVB irradiation VS WT before UVB irradiation	GO_CYTOSOLIC_SMALL_RIBOSOMAL_SUBUNIT2	-0.53422084	-0.00610026	-2.1183962	0.06657957	0.53809776	-4.14074107
homo after UVB irradiation VS homo before UVB irradiation	GO_CYTOSOLIC_TRANSPORT	0.186334837	-0.01977627	1.187188845	0.267905	0.46304451	-5.83740085
hetero after UVB irradiation VS hetero before UVB irradiation	GO_CYTOSOLIC_TRANSPORT1	0.215377901	-0.0253709	1.540501715	0.16270802	0.48395878	-5.11307163
WT after UVB irradiation VS WT before UVB irradiation	GO_CYTOSOLIC_TRANSPORT2	0.128049849	0.003743903	0.754422759	0.47196252	0.84703593	-5.70644678
homo after UVB irradiation VS homo before UVB irradiation	GO_D_AMINO_ACID_TRANSPORT	0.32827737	0.03518941	2.129877331	0.0644602	0.19256021	-4.57330146
hetero after UVB irradiation VS hetero before UVB irradiation	GO_D_AMINO_ACID_TRANSPORT1	0.33192701	0.106201645	2.45186021	0.04036341	0.27269233	-3.84188945
WT after UVB irradiation VS WT before UVB irradiation	GO_D_AMINO_ACID_TRANSPORT2	0.205735184	-0.0085504	0.591905124	0.57006663	0.88382859	-5.81532632
homo after UVB irradiation VS homo before UVB irradiation	GO_D_GLUCOSE_TRANSMEMBRANE_TRANSPORTER_ACTIVITY	0.611073868	0.064754899	2.315345662	0.0480455	0.16066337	-4.29270087
hetero after UVB irradiation VS hetero before UVB irradiation	GO_D_GLUCOSE_TRANSMEMBRANE_TRANSPORTER_ACTIVITY1	0.361220111	0.10411498	1.379995639	0.20560071	0.53029961	-5.31086257
WT after UVB irradiation VS WT before UVB irradiation	GO_D_GLUCOSE_TRANSMEMBRANE_TRANSPORTER_ACTIVITY2	0.508169159	0.071799145	1.88467888	0.09576601	0.59686908	-4.46116474
homo after UVB irradiation VS homo before UVB irradiation	GO_D1_DOPAMINE_RECEPTOR_BINDING	0.310282398	-0.0217987	1.781951684	0.11113042	0.26731077	-5.07975386
hetero after UVB irradiation VS hetero before UVB irradiation	GO_D1_DOPAMINE_RECEPTOR_BINDING1	0.083025245	-0.00687878	0.474405231	0.64812261	0.85544745	-6.11121335
WT after UVB irradiation VS WT before UVB irradiation	GO_D1_DOPAMINE_RECEPTOR_BINDING2	0.463643916	-0.02497512	1.812777126	0.10698258	0.61215273	-4.55733732
homo after UVB irradiation VS homo before UVB irradiation	GO_D2_DOPAMINE_RECEPTOR_BINDING	0.320094692	0.006678781	1.87371708	0.09638547	0.24565808	-4.94941162
hetero after UVB irradiation VS hetero before UVB irradiation	GO_D2_DOPAMINE_RECEPTOR_BINDING1	0.011572219	0.014348767	0.065277485	0.94958426	0.98092529	-6.22917391
WT after UVB irradiation VS WT before UVB irradiation	GO_D2_DOPAMINE_RECEPTOR_BINDING2	0.348096178	0.022301035	1.670800485	0.13285074	0.6390086	-4.74290237
homo after UVB irradiation VS homo before UVB irradiation	GO_DAMAGED_DNA_BINDING	-0.56268689	-0.03346051	-3.77463606	0.00504437	0.04637942	-2.0564218
hetero after UVB irradiation VS hetero before UVB irradiation	GO_DAMAGED_DNA_BINDING1	-0.27390826	-0.07083152	-1.20518883	0.26319031	0.58968057	-5.51119472
WT after UVB irradiation VS WT before UVB irradiation	GO_DAMAGED_DNA_BINDING2	-0.15187294	0.016089574	-0.58452375	0.57478775	0.88586474	-5.81970913
homo after UVB irradiation VS homo before UVB irradiation	GO_DATP_METABOLIC_PROCESS	0.761262135	-0.09392432	3.698818872	0.00563947	0.04968458	-2.1688418
hetero after UVB irradiation VS hetero before UVB irradiation	GO_DATP_METABOLIC_PROCESS1	0.708836748	0.015893897	3.476161457	0.00861249	0.14984918	-2.35842992
WT after UVB irradiation VS WT before UVB irradiation	GO_DATP_METABOLIC_PROCESS2	0.548880505	0.020413042	3.015414395	0.01644349	0.37790033	-2.87172573
homo after UVB irradiation VS homo before UVB irradiation	GO_DE_NOVO_CENTRIOLE_ASSEMBLY	-0.4494616	-0.01837371	-2.21555001	0.05628578	0.17648478	-4.44436439
hetero after UVB irradiation VS hetero before UVB irradiation	GO_DE_NOVO_CENTRIOLE_ASSEMBLY1	0.116262007	-0.06138131	0.438615547	0.6727568	0.86692859	-6.12845804
WT after UVB irradiation VS WT before UVB irradiation	GO_DE_NOVO_CENTRIOLE_ASSEMBLY2	-0.1235832	0.007732914	-0.55504064	0.59386311	0.8931505	-5.83671191
homo after UVB irradiation VS homo before UVB irradiation	GO_DE_NOVO_IMP_BIOSYNTHETIC_PROCESS	-0.81172096	-0.01480941	-4.30783288	0.002356	0.03234184	-1.28799303
hetero after UVB irradiation VS hetero before UVB irradiation	GO_DE_NOVO_IMP_BIOSYNTHETIC_PROCESS1	-0.6554114	-0.04463248	-2.43145339	0.04165792	0.27500759	-3.87175008
WT after UVB irradiation VS WT before UVB irradiation	GO_DE_NOVO_IMP_BIOSYNTHETIC_PROCESS2	-0.1939072	0.004046748	-0.48943818	0.63751375	0.9057274	-5.87161279
homo after UVB irradiation VS homo before UVB irradiation	GO_DE_NOVO_NAD_BIOSYNTHETIC_PROCESS_FROM_TRYPTOPHAN	0.586552268	-0.01981552	3.77873176	0.00501419	0.04627539	-2.05036958
hetero after UVB irradiation VS hetero before UVB irradiation	GO_DE_NOVO_NAD_BIOSYNTHETIC_PROCESS_FROM_TRYPTOPHAN1	0.099452855	0.002082887	0.555428381	0.59406693	0.8285493	-6.06750563
WT after UVB irradiation VS WT before UVB irradiation	GO_DE_NOVO_NAD_BIOSYNTHETIC_PROCESS_FROM_TRYPTOPHAN2	0.095627057	-0.10395387	0.283988243	0.78354572	0.9456763	-5.9537353
homo after UVB irradiation VS homo before UVB irradiation	GO_DE_NOVO_PROTEIN_FOLDING	-0.43603598	0.0119007	-3.08306158	0.01433219	0.08162875	-3.10468916
hetero after UVB irradiation VS hetero before UVB irradiation	GO_DE_NOVO_PROTEIN_FOLDING1	-0.27789224	-0.01701237	-1.4423445	0.18787609	0.51348391	-5.23546824
WT after UVB irradiation VS WT before UVB irradiation	GO_DE_NOVO_PROTEIN_FOLDING2	-0.22161917	-0.04084824	-1.04149431	0.32774014	0.77639671	-5.46106619
homo after UVB irradiation VS homo before UVB irradiation	GO_DE_NOVO_PYRIMIDINE_NUCLEOBASE_BIOSYNTHETIC_PROCESS	-0.44507589	0.037241269	-1.39001038	0.20054562	0.38924462	-5.59935454
hetero after UVB irradiation VS hetero before UVB irradiation	GO_DE_NOVO_PYRIMIDINE_NUCLEOBASE_BIOSYNTHETIC_PROCESS1	-0.46032735	-0.06194433	-1.61313742	0.14608724	0.46297111	-5.0198483
WT after UVB irradiation VS WT before UVB irradiation	GO_DE_NOVO_PYRIMIDINE_NUCLEOBASE_BIOSYNTHETIC_PROCESS2	0.171765869	0.073944606	0.507138684	0.62557708	0.90205599	-5.86259884
homo after UVB irradiation VS homo before UVB irradiation	GO_DEACETYLASE_ACTIVITY	0.134676619	-0.02509161	1.004332228	0.34349975	0.53605578	-6.02892754
hetero after UVB irradiation VS hetero before UVB irradiation	GO_DEACETYLASE_ACTIVITY1	0.058807594	-0.01636457	0.384401402	0.71088128	0.88637166	-6.15212306
WT after UVB irradiation VS WT before UVB irradiation	GO_DEACETYLASE_ACTIVITY2	0.035839166	0.001922175	0.172035892	0.86763114	0.96790654	-5.98048033
homo after UVB irradiation VS homo before UVB irradiation	GO_DEAD_H_BOX_RNA_HELICASE_BINDING	-0.82372069	0.00283224	-4.10585005	0.00312846	0.03687438	-1.57429674
hetero after UVB irradiation VS hetero before UVB irradiation	GO_DEAD_H_BOX_RNA_HELICASE_BINDING1	-0.95428074	0.013890566	-5.71299337	0.00047913	0.07788488	0.381127717
WT after UVB irradiation VS WT before UVB irradiation	GO_DEAD_H_BOX_RNA_HELICASE_BINDING2	-0.80655163	0.055045785	-3.82292043	0.00495479	0.27432744	-1.77752974
homo after UVB irradiation VS homo before UVB irradiation	GO_DEADENYLATION_DEPENDENT_DECAPPING_OF_NUCLEAR_TRANSCRIBED_MRNA	-0.09225713	-0.03470824	-0.52138384	0.61567526	0.76112255	-6.40409513
hetero after UVB irradiation VS hetero before UVB irradiation	GO_DEADENYLATION_DEPENDENT_DECAPPING_OF_NUCLEAR_TRANSCRIBED_MRNA1	0.113000917	-0.01687273	0.475278516	0.6475271	0.85523965	-6.11077662
WT after UVB irradiation VS WT before UVB irradiation	GO_DEADENYLATION_DEPENDENT_DECAPPING_OF_NUCLEAR_TRANSCRIBED_MRNA2	-0.19717574	0.035623181	-0.74020615	0.48008344	0.85038327	-5.71689146
homo after UVB irradiation VS homo before UVB irradiation	GO_DEAMINASE_ACTIVITY	0.193533109	-0.00761577	1.18815144	0.26754571	0.46269207	-5.83633133
hetero after UVB irradiation VS hetero before UVB irradiation	GO_DEAMINASE_ACTIVITY1	0.090019866	0.036916765	0.516259232	0.61989198	0.84332543	-6.08943572
WT after UVB irradiation VS WT before UVB irradiation	GO_DEAMINASE_ACTIVITY2	0.29056817	-0.02153561	1.692042213	0.12864185	0.6349056	-4.71554475
homo after UVB irradiation VS homo before UVB irradiation	GO_DEATH_DOMAIN_BINDING	0.269159182	-0.03462406	1.788807458	0.10995943	0.26561978	-5.07011089
hetero after UVB irradiation VS hetero before UVB irradiation	GO_DEATH_DOMAIN_BINDING1	0.218486348	-0.07441386	1.557220879	0.15873694	0.47878838	-5.09180374
WT after UVB irradiation VS WT before UVB irradiation	GO_DEATH_DOMAIN_BINDING2	0.209331369	-0.06737201	1.291501915	0.23217218	0.72434776	-5.20061255
homo after UVB irradiation VS homo before UVB irradiation	GO_DEATH_INDUCING_SIGNALING_COMPLEX	0.591711828	-0.00610827	3.868006063	0.00440187	0.0428485	-1.91899286
homo after UVB irradiation VS homo before UVB irradiation	GO_DEATH_INDUCING_SIGNALING_COMPLEX_ASSEMBLY	0.964476402	-0.05979756	6.995823514	9.30E-05	0.00992256	1.924199052
hetero after UVB irradiation VS hetero before UVB irradiation	GO_DEATH_INDUCING_SIGNALING_COMPLEX_ASSEMBLY1	0.887455431	0.070611578	4.235052879	0.00297931	0.11447176	-1.33578612
WT after UVB irradiation VS WT before UVB irradiation	GO_DEATH_INDUCING_SIGNALING_COMPLEX_ASSEMBLY2	0.995043137	-0.00443006	6.314295388	0.00021778	0.18842845	0.906743485
hetero after UVB irradiation VS hetero before UVB irradiation	GO_DEATH_INDUCING_SIGNALING_COMPLEX1	0.651370614	0.016289445	3.369768753	0.01005884	0.15773452	-2.50844117
WT after UVB irradiation VS WT before UVB irradiation	GO_DEATH_INDUCING_SIGNALING_COMPLEX2	0.75702392	-0.01736034	4.872669556	0.00119345	0.19353487	-0.5129778
homo after UVB irradiation VS homo before UVB irradiation	GO_DEATH_RECEPTOR_ACTIVITY	0.818041352	0.001511867	6.61438914	0.00013912	0.01011342	1.533299225
hetero after UVB irradiation VS hetero before UVB irradiation	GO_DEATH_RECEPTOR_ACTIVITY1	0.381325719	0.019830505	1.979028858	0.08384814	0.36997436	-4.52333281
WT after UVB irradiation VS WT before UVB irradiation	GO_DEATH_RECEPTOR_ACTIVITY2	0.362730766	0.004593581	1.718076108	0.12365184	0.63133281	-4.68181109
homo after UVB irradiation VS homo before UVB irradiation	GO_DEATH_RECEPTOR_BINDING	0.221660754	0.007314863	1.40049419	0.19749799	0.38607469	-5.58640095
hetero after UVB irradiation VS hetero before UVB irradiation	GO_DEATH_RECEPTOR_BINDING1	0.263992287	0.008125383	1.759524476	0.11723652	0.42070868	-4.82601285
WT after UVB irradiation VS WT before UVB irradiation	GO_DEATH_RECEPTOR_BINDING2	0.391116005	-0.02678913	2.387770365	0.04364745	0.49660728	-3.76175981
homo after UVB irradiation VS homo before UVB irradiation	GO_DECIDUALIZATION	0.112473215	0.008906725	0.5049091	0.6267084	0.76915634	-6.41308443
hetero after UVB irradiation VS hetero before UVB irradiation	GO_DECIDUALIZATION1	0.195949377	0.015355405	1.28979989	0.23380643	0.56214284	-5.41637428
WT after UVB irradiation VS WT before UVB irradiation	GO_DECIDUALIZATION2	0.037544829	0.051181052	0.251404208	0.80776411	0.95404687	-5.96285433
homo after UVB irradiation VS homo before UVB irradiation	GO_DEDIFFERENTIATION	0.216094072	0.065932207	1.053787209	0.32158658	0.51518639	-5.9794956
hetero after UVB irradiation VS hetero before UVB irradiation	GO_DEDIFFERENTIATION1	0.243474	0.047952293	1.16649131	0.27761757	0.60400502	-5.55312146
WT after UVB irradiation VS WT before UVB irradiation	GO_DEDIFFERENTIATION2	0.350660883	0.004921724	2.039905985	0.07525977	0.55793508	-4.24948848
homo after UVB irradiation VS homo before UVB irradiation	GO_DEFENSE_RESPONSE	0.457902229	-0.01509723	3.361856736	0.00934258	0.06433347	-2.6765662
homo after UVB irradiation VS homo before UVB irradiation	GO_DEFENSE_RESPONSE_TO_BACTERIUM	0.694224549	-0.00094123	4.85785596	0.00112196	0.02252621	-0.54012168
hetero after UVB irradiation VS hetero before UVB irradiation	GO_DEFENSE_RESPONSE_TO_BACTERIUM1	0.629655426	0.041203963	4.212084255	0.00307281	0.11479066	-1.3653969
WT after UVB irradiation VS WT before UVB irradiation	GO_DEFENSE_RESPONSE_TO_BACTERIUM2	0.427021967	0.043983852	2.140118243	0.06435553	0.53678963	-4.11047894
homo after UVB irradiation VS homo before UVB irradiation	GO_DEFENSE_RESPONSE_TO_FUNGUS	0.744570587	-0.01756957	4.272355978	0.00247525	0.03315789	-1.33783913
hetero after UVB irradiation VS hetero before UVB irradiation	GO_DEFENSE_RESPONSE_TO_FUNGUS1	0.771455764	0.051786226	4.411646567	0.00235547	0.10774734	-1.11108838
WT after UVB irradiation VS WT before UVB irradiation	GO_DEFENSE_RESPONSE_TO_FUNGUS2	0.622149375	0.06865944	3.206857256	0.01228524	0.34717171	-2.6048196
homo after UVB irradiation VS homo before UVB irradiation	GO_DEFENSE_RESPONSE_TO_GRAM_NEGATIVE_BACTERIUM	0.540017139	-0.01709542	3.048967387	0.01511062	0.08342215	-3.15740317
hetero after UVB irradiation VS hetero before UVB irradiation	GO_DEFENSE_RESPONSE_TO_GRAM_NEGATIVE_BACTERIUM1	0.570126688	0.029423145	4.258814567	0.00288582	0.11333595	-1.30524581
WT after UVB irradiation VS WT before UVB irradiation	GO_DEFENSE_RESPONSE_TO_GRAM_NEGATIVE_BACTERIUM2	0.384435292	0.021844102	2.009909968	0.07885981	0.56283216	-4.29077134
homo after UVB irradiation VS homo before UVB irradiation	GO_DEFENSE_RESPONSE_TO_GRAM_POSITIVE_BACTERIUM	0.636108811	0.019563242	4.274010715	0.00246955	0.03315789	-1.33550995
hetero after UVB irradiation VS hetero before UVB irradiation	GO_DEFENSE_RESPONSE_TO_GRAM_POSITIVE_BACTERIUM1	0.589502522	0.085731132	3.486716775	0.00848155	0.14863874	-2.34362575
WT after UVB irradiation VS WT before UVB irradiation	GO_DEFENSE_RESPONSE_TO_GRAM_POSITIVE_BACTERIUM2	0.448214191	0.037325638	1.984311447	0.08206342	0.56748353	-4.32586844
homo after UVB irradiation VS homo before UVB irradiation	GO_DEFENSE_RESPONSE_TO_OTHER_ORGANISM	0.460162375	-0.01345065	3.220714681	0.01159021	0.07224597	-2.8925759
hetero after UVB irradiation VS hetero before UVB irradiation	GO_DEFENSE_RESPONSE_TO_OTHER_ORGANISM1	0.457942458	0.026363886	3.237434266	0.0122258	0.17108413	-2.6969145
WT after UVB irradiation VS WT before UVB irradiation	GO_DEFENSE_RESPONSE_TO_OTHER_ORGANISM2	0.39434598	0.016326557	2.470796971	0.03831851	0.47798151	-3.64386065
homo after UVB irradiation VS homo before UVB irradiation	GO_DEFENSE_RESPONSE_TO_VIRUS	0.385393484	-0.00974243	2.73399365	0.02474609	0.1107607	-3.64623001
hetero after UVB irradiation VS hetero before UVB irradiation	GO_DEFENSE_RESPONSE_TO_VIRUS1	0.425041383	0.029497542	3.135637615	0.01422582	0.17832833	-2.84317039
WT after UVB irradiation VS WT before UVB irradiation	GO_DEFENSE_RESPONSE_TO_VIRUS2	0.412578871	0.007706045	2.801356307	0.02287523	0.41742287	-3.17382891
hetero after UVB irradiation VS hetero before UVB irradiation	GO_DEFENSE_RESPONSE1	0.412784162	0.027810236	2.857922401	0.0216298	0.21196897	-3.24662369
WT after UVB irradiation VS WT before UVB irradiation	GO_DEFENSE_RESPONSE2	0.345378812	0.010556416	2.003616478	0.07963603	0.56435935	-4.29941179
homo after UVB irradiation VS homo before UVB irradiation	GO_DEFINITIVE_ERYTHROCYTE_DIFFERENTIATION	0.84436239	-0.00279691	4.369816222	0.00216223	0.03106914	-1.2013632
hetero after UVB irradiation VS hetero before UVB irradiation	GO_DEFINITIVE_ERYTHROCYTE_DIFFERENTIATION1	0.4373255	0.007018762	2.405395971	0.04337207	0.27866302	-3.90984757
WT after UVB irradiation VS WT before UVB irradiation	GO_DEFINITIVE_ERYTHROCYTE_DIFFERENTIATION2	0.69851864	-0.00442522	4.271726813	0.00264311	0.23639052	-1.2128592
homo after UVB irradiation VS homo before UVB irradiation	GO_DEFINITIVE_HEMOPOIESIS	0.529455402	-0.03358429	3.300567969	0.01025652	0.06775085	-2.77015794
hetero after UVB irradiation VS hetero before UVB irradiation	GO_DEFINITIVE_HEMOPOIESIS1	0.43458865	0.031373265	2.248639692	0.05528921	0.31091755	-4.13799692
WT after UVB irradiation VS WT before UVB irradiation	GO_DEFINITIVE_HEMOPOIESIS2	0.49837432	-0.0551332	2.592657847	0.03166273	0.45353809	-3.4704788
homo after UVB irradiation VS homo before UVB irradiation	GO_DEHYDROASCORBIC_ACID_TRANSMEMBRANE_TRANSPORTER_ACTIVITY	0.62850903	0.11850898	3.064544686	0.01474964	0.08257891	-3.13331107
hetero after UVB irradiation VS hetero before UVB irradiation	GO_DEHYDROASCORBIC_ACID_TRANSMEMBRANE_TRANSPORTER_ACTIVITY1	0.854292906	0.096357598	3.424224754	0.00928881	0.15362111	-2.43148207
WT after UVB irradiation VS WT before UVB irradiation	GO_DEHYDROASCORBIC_ACID_TRANSMEMBRANE_TRANSPORTER_ACTIVITY2	0.544997718	0.056916194	2.135155821	0.0648571	0.53678963	-4.11739821
homo after UVB irradiation VS homo before UVB irradiation	GO_DEHYDROASCORBIC_ACID_TRANSPORT	0.62850903	0.11850898	3.064544686	0.01474964	0.08257891	-3.13331107
hetero after UVB irradiation VS hetero before UVB irradiation	GO_DEHYDROASCORBIC_ACID_TRANSPORT1	0.854292906	0.096357598	3.424224754	0.00928881	0.15362111	-2.43148207
WT after UVB irradiation VS WT before UVB irradiation	GO_DEHYDROASCORBIC_ACID_TRANSPORT2	0.544997718	0.056916194	2.135155821	0.0648571	0.53678963	-4.11739821
homo after UVB irradiation VS homo before UVB irradiation	GO_DELAMINATION	-0.36055302	-0.0243425	-1.27239245	0.23760229	0.42983898	-5.74042798
hetero after UVB irradiation VS hetero before UVB irradiation	GO_DELAMINATION1	0.224330709	-0.05115732	0.806804033	0.44352666	0.73891789	-5.89287208
WT after UVB irradiation VS WT before UVB irradiation	GO_DELAMINATION2	0.129952624	0.105158637	0.390957217	0.70591411	0.92646087	-5.91621387
homo after UVB irradiation VS homo before UVB irradiation	GO_DELAYED_RECTIFIER_POTASSIUM_CHANNEL_ACTIVITY	-0.08043659	-0.02816582	-0.3757178	0.71651546	0.83165162	-6.47404521
hetero after UVB irradiation VS hetero before UVB irradiation	GO_DELAYED_RECTIFIER_POTASSIUM_CHANNEL_ACTIVITY1	-0.32779994	0.001213696	-1.61141495	0.14646274	0.46337998	-5.02208323
WT after UVB irradiation VS WT before UVB irradiation	GO_DELAYED_RECTIFIER_POTASSIUM_CHANNEL_ACTIVITY2	-0.33169619	0.02520671	-1.55530023	0.15803062	0.66180255	-4.88882206
homo after UVB irradiation VS homo before UVB irradiation	GO_DELTA_CATENIN_BINDING	0.61255503	-0.03720073	3.516120514	0.00740148	0.05700611	-2.4425904
hetero after UVB irradiation VS hetero before UVB irradiation	GO_DELTA_CATENIN_BINDING1	0.351049557	-0.06701575	1.435240978	0.1898252	0.51471162	-5.24415409
WT after UVB irradiation VS WT before UVB irradiation	GO_DELTA_CATENIN_BINDING2	0.388867877	0.096888858	1.389581765	0.20166788	0.69810294	-5.08859213
homo after UVB irradiation VS homo before UVB irradiation	GO_DEMETHYLASE_ACTIVITY	-0.49286416	-0.01853184	-2.65229545	0.02815454	0.11875136	-3.77311013
hetero after UVB irradiation VS hetero before UVB irradiation	GO_DEMETHYLASE_ACTIVITY1	-0.22953693	-0.04296303	-1.27580153	0.23846765	0.56736997	-5.43234955
WT after UVB irradiation VS WT before UVB irradiation	GO_DEMETHYLASE_ACTIVITY2	-0.3734984	-0.02310151	-1.70426875	0.12627553	0.63339498	-4.69972964
homo after UVB irradiation VS homo before UVB irradiation	GO_DEMETHYLATION	-0.39619333	-0.00831323	-2.40147833	0.0419047	0.14772032	-4.16077735
hetero after UVB irradiation VS hetero before UVB irradiation	GO_DEMETHYLATION1	-0.31728912	-0.03154886	-1.95041213	0.08761688	0.37420395	-4.56346849
WT after UVB irradiation VS WT before UVB irradiation	GO_DEMETHYLATION2	-0.38204222	-0.00202965	-2.0908266	0.06951094	0.54475458	-4.17905026
homo after UVB irradiation VS homo before UVB irradiation	GO_DENDRITE_ARBORIZATION	-0.13539742	0.02625763	-0.67994445	0.51498953	0.68595545	-6.30405073
hetero after UVB irradiation VS hetero before UVB irradiation	GO_DENDRITE_ARBORIZATION1	-0.53666147	-0.02379798	-3.26943768	0.01166005	0.16851317	-2.65115453
WT after UVB irradiation VS WT before UVB irradiation	GO_DENDRITE_ARBORIZATION2	-0.15850904	0.084582686	-0.96523308	0.3623656	0.79519319	-5.53233826
homo after UVB irradiation VS homo before UVB irradiation	GO_DENDRITE_CYTOPLASM	-0.10245431	0.015362252	-0.49751152	0.63169528	0.77277023	-6.41703267
hetero after UVB irradiation VS hetero before UVB irradiation	GO_DENDRITE_CYTOPLASM1	-0.00323048	-0.01940946	-0.01534514	0.98813953	0.99549307	-6.23135642
WT after UVB irradiation VS WT before UVB irradiation	GO_DENDRITE_CYTOPLASM2	-0.16854186	0.010443014	-0.78637017	0.45404564	0.83739988	-5.68235506
homo after UVB irradiation VS homo before UVB irradiation	GO_DENDRITE_DEVELOPMENT	0.124021424	-0.00912301	0.9141558	0.38633213	0.57593354	-6.11422436
hetero after UVB irradiation VS hetero before UVB irradiation	GO_DENDRITE_DEVELOPMENT1	-0.03013432	0.001982177	-0.22638964	0.82667996	0.93413454	-6.20378331
WT after UVB irradiation VS WT before UVB irradiation	GO_DENDRITE_DEVELOPMENT2	-0.068921	-0.02813043	-0.48247825	0.64223853	0.90737329	-5.87507479
homo after UVB irradiation VS homo before UVB irradiation	GO_DENDRITE_EXTENSION	0.183431871	0.026946657	1.45239185	0.18299804	0.36646792	-5.52141712
hetero after UVB irradiation VS hetero before UVB irradiation	GO_DENDRITE_EXTENSION1	-0.02985629	0.010369598	-0.21539913	0.83494525	0.93644494	-6.2063996
WT after UVB irradiation VS WT before UVB irradiation	GO_DENDRITE_EXTENSION2	-0.27917535	0.01880471	-1.66118745	0.13479695	0.64229853	-4.75523259
homo after UVB irradiation VS homo before UVB irradiation	GO_DENDRITE_MEMBRANE	-0.07674904	0.028591557	-0.55475439	0.59364264	0.74518411	-6.38506212
hetero after UVB irradiation VS hetero before UVB irradiation	GO_DENDRITE_MEMBRANE1	-0.33893384	-0.01975729	-1.89677129	0.09512566	0.3853041	-4.63820044
WT after UVB irradiation VS WT before UVB irradiation	GO_DENDRITE_MEMBRANE2	-0.25284911	0.021813391	-1.46609753	0.18034649	0.68278032	-4.99788353
homo after UVB irradiation VS homo before UVB irradiation	GO_DENDRITE_MORPHOGENESIS	0.166715615	-0.02299609	1.121756095	0.29326065	0.48806218	-5.90864497
hetero after UVB irradiation VS hetero before UVB irradiation	GO_DENDRITE_MORPHOGENESIS1	-0.05048455	-4.46E-05	-0.38521756	0.7103005	0.88599205	-6.15178896
WT after UVB irradiation VS WT before UVB irradiation	GO_DENDRITE_MORPHOGENESIS2	-0.13262929	-0.03177812	-0.91237322	0.38792761	0.80976349	-5.57925388
homo after UVB irradiation VS homo before UVB irradiation	GO_DENDRITE_SELF_AVOIDANCE	0.349434139	-0.00616376	2.037080842	0.07462307	0.20983472	-4.71136016
hetero after UVB irradiation VS hetero before UVB irradiation	GO_DENDRITE_SELF_AVOIDANCE1	0.273106618	0.076567802	1.296394799	0.23163739	0.56030965	-5.40880966
WT after UVB irradiation VS WT before UVB irradiation	GO_DENDRITE_SELF_AVOIDANCE2	0.027998781	0.036426491	0.142108655	0.89046634	0.97208254	-5.9854249
homo after UVB irradiation VS homo before UVB irradiation	GO_DENDRITE_TERMINUS	-0.30049736	0.028199571	-1.47253086	0.17762805	0.35922394	-5.49582553
hetero after UVB irradiation VS hetero before UVB irradiation	GO_DENDRITE_TERMINUS1	-0.25792965	-0.05919443	-1.55569171	0.15909646	0.47924721	-5.09375378
WT after UVB irradiation VS WT before UVB irradiation	GO_DENDRITE_TERMINUS2	-0.35368571	0.03138223	-1.52842655	0.16447788	0.66920322	-4.92203288

homo after UVB irradiation VS homo before UVB irradiation	GO_DENDRITIC_CELL_ANTIGEN_PROCESSING_AND_PRESENTATION	0.536087242	-0.06078701	2.44146587	0.03932692	0.14245149	-4.09926983
hetero after UVB irradiation VS hetero before UVB irradiation	GO_DENDRITIC_CELL_ANTIGEN_PROCESSING_AND_PRESENTATION1	0.326263119	-0.01148723	1.148011874	0.2847319	0.60984853	-5.5728095
WT after UVB irradiation VS WT before UVB irradiation	GO_DENDRITIC_CELL_ANTIGEN_PROCESSING_AND_PRESENTATION2	0.315578949	0.005023122	0.908735373	0.3897341	0.81049974	-5.58240527
homo after UVB irradiation VS homo before UVB irradiation	GO_DENDRITIC_CELL_APOPTOTIC_PROCESS	0.265723768	-0.01918893	1.111367148	0.29745768	0.49205007	-5.91968678
hetero after UVB irradiation VS hetero before UVB irradiation	GO_DENDRITIC_CELL_APOPTOTIC_PROCESS1	-0.00686947	0.024432677	-0.02805911	0.97831498	0.99256196	-6.23105717
WT after UVB irradiation VS WT before UVB irradiation	GO_DENDRITIC_CELL_APOPTOTIC_PROCESS2	-0.11163205	-0.01357834	-0.3095108	0.76474775	0.94102792	-5.94583401
homo after UVB irradiation VS homo before UVB irradiation	GO_DENDRITIC_CELL_CHEMOTAXIS	0.65725719	-0.01822617	4.018890121	0.00354117	0.03892545	-1.69940076
hetero after UVB irradiation VS hetero before UVB irradiation	GO_DENDRITIC_CELL_CHEMOTAXIS1	0.556974763	0.038557081	2.511235719	0.03682396	0.26300791	-3.75490777
WT after UVB irradiation VS WT before UVB irradiation	GO_DENDRITIC_CELL_CHEMOTAXIS2	0.302676994	-0.02201472	1.257198477	0.24372896	0.73330286	-5.23857539
homo after UVB irradiation VS homo before UVB irradiation	GO_DENDRITIC_CELL_CYTOKINE_PRODUCTION	0.142154641	-0.01325943	0.922055884	0.38243021	0.57262444	-6.10700928
hetero after UVB irradiation VS hetero before UVB irradiation	GO_DENDRITIC_CELL_CYTOKINE_PRODUCTION1	0.347898752	0.057224765	1.957338839	0.08668991	0.37240327	-4.55377
WT after UVB irradiation VS WT before UVB irradiation	GO_DENDRITIC_CELL_CYTOKINE_PRODUCTION2	0.084652877	-0.0076199	0.365020838	0.72444566	0.93154998	-5.92636923
homo after UVB irradiation VS homo before UVB irradiation	GO_DENDRITIC_CELL_DIFFERENTIATION	0.695831587	-0.04495303	5.283147762	0.0006515	0.01817776	0.005402398
hetero after UVB irradiation VS hetero before UVB irradiation	GO_DENDRITIC_CELL_DIFFERENTIATION1	0.486505201	0.030370063	2.232676331	0.05667305	0.31450454	-4.16110187
WT after UVB irradiation VS WT before UVB irradiation	GO_DENDRITIC_CELL_DIFFERENTIATION2	0.399577315	-0.01494736	1.447631259	0.18530194	0.68726847	-5.02002579
homo after UVB irradiation VS homo before UVB irradiation	GO_DENDRITIC_CELL_MIGRATION	0.63221875	-0.04238766	4.234994078	0.00260789	0.03384751	-1.39053871
hetero after UVB irradiation VS hetero before UVB irradiation	GO_DENDRITIC_CELL_MIGRATION1	0.55735445	0.036789725	2.780396686	0.0243451	0.22354468	-3.36007977
WT after UVB irradiation VS WT before UVB irradiation	GO_DENDRITIC_CELL_MIGRATION2	0.339129915	-0.04077676	1.524812497	0.16536273	0.66948086	-4.92647643
homo after UVB irradiation VS homo before UVB irradiation	GO_DENDRITIC_GROWTH_CONE	-0.45344312	0.017573051	-2.19626264	0.05803229	0.18029106	-4.47350268
hetero after UVB irradiation VS hetero before UVB irradiation	GO_DENDRITIC_GROWTH_CONE1	-0.37156044	-0.09252654	-2.35182947	0.04712254	0.28948384	-3.98803316
WT after UVB irradiation VS WT before UVB irradiation	GO_DENDRITIC_GROWTH_CONE2	-0.33515917	0.052907168	-1.30017992	0.22932307	0.72156087	-5.19090576
homo after UVB irradiation VS homo before UVB irradiation	GO_DENDRITIC_SHAFT	0.482712508	-0.01483876	3.888112907	0.00427527	0.04234489	-1.88954957
hetero after UVB irradiation VS hetero before UVB irradiation	GO_DENDRITIC_SHAFT1	0.307840309	0.044224967	1.835426311	0.10446848	0.40029539	-4.72279126
WT after UVB irradiation VS WT before UVB irradiation	GO_DENDRITIC_SHAFT2	0.35697494	0.038504475	2.166724952	0.06173057	0.53184019	-4.07332365
homo after UVB irradiation VS homo before UVB irradiation	GO_DENDRITIC_SPINE_DEVELOPMENT	0.187187142	-0.01572429	1.31794432	0.22261334	0.41394629	-5.68674393
hetero after UVB irradiation VS hetero before UVB irradiation	GO_DENDRITIC_SPINE_DEVELOPMENT1	0.028148881	-0.01401624	0.210307345	0.83878197	0.93850976	-6.20756811
WT after UVB irradiation VS WT before UVB irradiation	GO_DENDRITIC_SPINE_DEVELOPMENT2	-0.02878466	-0.01546591	-0.18979635	0.85414012	0.9663792	-5.977104
homo after UVB irradiation VS homo before UVB irradiation	GO_DENDRITIC_SPINE_HEAD	0.401741312	-0.02337925	2.394580959	0.04236615	0.14853821	-4.17137123
hetero after UVB irradiation VS hetero before UVB irradiation	GO_DENDRITIC_SPINE_HEAD1	0.313704613	-0.02058479	1.510417794	0.17007993	0.49267756	-5.15104115
WT after UVB irradiation VS WT before UVB irradiation	GO_DENDRITIC_SPINE_HEAD2	0.612656575	-0.00646711	3.198687341	0.01243803	0.34762883	-2.61613183
homo after UVB irradiation VS homo before UVB irradiation	GO_DENDRITIC_SPINE_MAINTENANCE	0.009813312	0.007771879	0.051660641	0.96001662	0.97912218	-6.54939225
hetero after UVB irradiation VS hetero before UVB irradiation	GO_DENDRITIC_SPINE_MAINTENANCE1	-0.02055767	-0.02175888	-0.12639794	0.90259328	0.9639333	-6.22282909
WT after UVB irradiation VS WT before UVB irradiation	GO_DENDRITIC_SPINE_MAINTENANCE2	-0.17564398	-0.01883367	-1.15113887	0.28252902	0.75292132	-5.35162581
homo after UVB irradiation VS homo before UVB irradiation	GO_DENDRITIC_SPINE_MEMBRANE	-0.06262728	0.000824684	-0.36547905	0.72386143	0.8368868	-6.47814124
hetero after UVB irradiation VS hetero before UVB irradiation	GO_DENDRITIC_SPINE_MEMBRANE1	-0.44755242	-0.04651116	-3.25890726	0.01184309	0.16933784	-2.66619919
WT after UVB irradiation VS WT before UVB irradiation	GO_DENDRITIC_SPINE_MEMBRANE2	-0.05610375	0.033174816	-0.37364791	0.71825942	0.93096927	-5.92306584
homo after UVB irradiation VS homo before UVB irradiation	GO_DENDRITIC_SPINE_MORPHOGENESIS	0.206007903	-0.02582087	1.423226508	0.19102768	0.37692143	-5.55811071
hetero after UVB irradiation VS hetero before UVB irradiation	GO_DENDRITIC_SPINE_MORPHOGENESIS1	0.068383123	0.009103346	0.449259713	0.66538437	0.86302584	-6.1234631
WT after UVB irradiation VS WT before UVB irradiation	GO_DENDRITIC_SPINE_MORPHOGENESIS2	0.017919159	-0.00673064	0.109005781	0.91585077	0.97967628	-5.98980186
homo after UVB irradiation VS homo before UVB irradiation	GO_DENDRITIC_SPINE_NECK	0.424553029	-0.02666925	3.205409717	0.01186594	0.07323882	-2.91609584
hetero after UVB irradiation VS hetero before UVB irradiation	GO_DENDRITIC_SPINE_NECK1	-0.00266701	-0.00910475	-0.01267448	0.99020359	0.99615986	-6.231397
WT after UVB irradiation VS WT before UVB irradiation	GO_DENDRITIC_SPINE_NECK2	0.288684633	-0.03759071	0.944236391	0.37236537	0.8012534	-5.55122351
homo after UVB irradiation VS homo before UVB irradiation	GO_DENDRITIC_TRANSPORT	-0.26223408	0.003177628	-1.01417934	0.33904809	0.53197847	-6.01923128
hetero after UVB irradiation VS hetero before UVB irradiation	GO_DENDRITIC_TRANSPORT1	-0.05990257	-0.04192491	-0.28404424	0.78372201	0.91561314	-6.18796177
WT after UVB irradiation VS WT before UVB irradiation	GO_DENDRITIC_TRANSPORT2	-0.25904762	-0.0514468	-0.9374824	0.37562505	0.80254393	-5.55722879
homo after UVB irradiation VS homo before UVB irradiation	GO_DENDRITIC_TREE	0.068574491	-0.00070925	0.553787949	0.59427466	0.74558021	-6.38562876
hetero after UVB irradiation VS hetero before UVB irradiation	GO_DENDRITIC_TREE1	-0.02196216	-0.00071477	-0.17440502	0.86595959	0.94912721	-6.21502173
WT after UVB irradiation VS WT before UVB irradiation	GO_DENDRITIC_TREE2	0.011627715	-0.00234739	0.081179055	0.93727017	0.98368758	-5.99259093
homo after UVB irradiation VS homo before UVB irradiation	GO_DENSE_BODY	-0.24174914	0.053848129	-0.94007157	0.37363973	0.56389871	-6.09036803
hetero after UVB irradiation VS hetero before UVB irradiation	GO_DENSE_BODY1	0.237380094	0.095542696	0.836968512	0.42734545	0.72827932	-5.86819347
WT after UVB irradiation VS WT before UVB irradiation	GO_DENSE_BODY2	0.089553862	-0.09073513	0.343257651	0.74014633	0.93506555	-5.93437095
homo after UVB irradiation VS homo before UVB irradiation	GO_DENSE_CORE_GRANULE	-0.21396805	0.016590796	-1.6853761	0.12890952	0.29415769	-5.21381505
homo after UVB irradiation VS homo before UVB irradiation	GO_DENSE_CORE_GRANULE_EXOCYTOSIS	0.237935244	-0.00346716	1.329388949	0.218976	0.40974393	-5.67306332
hetero after UVB irradiation VS hetero before UVB irradiation	GO_DENSE_CORE_GRANULE_EXOCYTOSIS1	-0.27263441	-0.0429016	-1.10835294	0.30049863	0.62370564	-5.6143128
WT after UVB irradiation VS WT before UVB irradiation	GO_DENSE_CORE_GRANULE_EXOCYTOSIS2	-0.23548016	-0.01005273	-1.1295183	0.29102543	0.75646944	-5.37382722
homo after UVB irradiation VS homo before UVB irradiation	GO_DENSE_CORE_GRANULE_MEMBRANE	-0.32315248	0.062135925	-1.73121148	0.12016585	0.28125099	-5.15061332
hetero after UVB irradiation VS hetero before UVB irradiation	GO_DENSE_CORE_GRANULE_MEMBRANE1	-0.0043449	-0.04612204	-0.01827559	0.9858748	0.99470555	-6.23130299
WT after UVB irradiation VS WT before UVB irradiation	GO_DENSE_CORE_GRANULE_MEMBRANE2	-0.10912108	0.081984208	-0.45760283	0.65926595	0.91105574	-5.88706605
homo after UVB irradiation VS homo before UVB irradiation	GO_DENSE_CORE_GRANULE_TRANSPORT	-0.30497836	0.031586467	-1.5719495	0.15311561	0.32734165	-5.36660523
hetero after UVB irradiation VS hetero before UVB irradiation	GO_DENSE_CORE_GRANULE_TRANSPORT1	0.007180599	-0.00115298	0.032036988	0.97524185	0.99170758	-6.23092754
WT after UVB irradiation VS WT before UVB irradiation	GO_DENSE_CORE_GRANULE_TRANSPORT2	-0.28880745	0.033855134	-1.46783849	0.1798854	0.68255081	-4.99578803
hetero after UVB irradiation VS hetero before UVB irradiation	GO_DENSE_CORE_GRANULE1	-0.24883389	-0.02405873	-1.55091676	0.16022385	0.48083448	-5.09983671
WT after UVB irradiation VS WT before UVB irradiation	GO_DENSE_CORE_GRANULE2	-0.41509276	0.048318115	-2.80255654	0.02283269	0.41742287	-3.17212711
homo after UVB irradiation VS homo before UVB irradiation	GO_DENTATE_GYRUS_DEVELOPMENT	0.225281071	0.058378095	1.307110921	0.22610359	0.41681894	-5.69962334
hetero after UVB irradiation VS hetero before UVB irradiation	GO_DENTATE_GYRUS_DEVELOPMENT1	-0.27709755	0.056386589	-1.23643933	0.25199705	0.57984434	-5.47666539
WT after UVB irradiation VS WT before UVB irradiation	GO_DENTATE_GYRUS_DEVELOPMENT2	-0.00697658	-0.00469133	-0.02862415	0.97785725	0.99433096	-5.99563436
homo after UVB irradiation VS homo before UVB irradiation	GO_DENTATE_GYRUS_MOSSY_FIBER	0.01482911	0.045362852	0.058069793	0.95506207	0.97631027	-6.54900642
hetero after UVB irradiation VS hetero before UVB irradiation	GO_DENTATE_GYRUS_MOSSY_FIBER1	-0.4859862	-0.05963378	-2.13486347	0.06593454	0.33426479	-4.30198428
WT after UVB irradiation VS WT before UVB irradiation	GO_DENTATE_GYRUS_MOSSY_FIBER2	-0.35021051	-0.10211287	-1.34755168	0.21428936	0.71210914	-5.13720711
homo after UVB irradiation VS homo before UVB irradiation	GO_DENTINOGENESIS	0.918478052	0.073311777	5.08529971	0.00083625	0.01971335	-0.24481229
hetero after UVB irradiation VS hetero before UVB irradiation	GO_DENTINOGENESIS1	0.628359986	0.00345133	2.465239752	0.03953684	0.27162031	-3.82230115
WT after UVB irradiation VS WT before UVB irradiation	GO_DENTINOGENESIS2	0.404975525	0.020626498	1.669455573	0.13312146	0.63954295	-4.74462935
homo after UVB irradiation VS homo before UVB irradiation	GO_DEOXYCYTIDINE_DEAMINASE_ACTIVITY	0.772885525	0.080000951	2.72353434	0.0251578	0.11168881	-3.6624803
hetero after UVB irradiation VS hetero before UVB irradiation	GO_DEOXYCYTIDINE_DEAMINASE_ACTIVITY1	0.919461063	0.040873947	6.004383609	0.00034618	0.07788488	0.676889701
WT after UVB irradiation VS WT before UVB irradiation	GO_DEOXYCYTIDINE_DEAMINASE_ACTIVITY2	0.639201085	0.009560069	2.304917107	0.04970682	0.50853601	-3.87903766
homo after UVB irradiation VS homo before UVB irradiation	GO_DEOXYRIBONUCLEASE_ACTIVITY	-0.30592948	-0.00138158	-2.06051186	0.07191903	0.20580189	-4.67667371
hetero after UVB irradiation VS hetero before UVB irradiation	GO_DEOXYRIBONUCLEASE_ACTIVITY1	0.05041837	-0.04354339	0.257950137	0.80307668	0.92371161	-6.19555763
WT after UVB irradiation VS WT before UVB irradiation	GO_DEOXYRIBONUCLEASE_ACTIVITY2	0.044269181	-0.00568343	0.187179534	0.8561248	0.96638003	-5.97762211
homo after UVB irradiation VS homo before UVB irradiation	GO_DEOXYRIBONUCLEASE_I_ACTIVITY	-0.27360388	-0.05465325	-1.47254481	0.17762438	0.35922394	-5.49580772
hetero after UVB irradiation VS hetero before UVB irradiation	GO_DEOXYRIBONUCLEASE_I_ACTIVITY1	-0.12754024	-0.00230518	-0.65085515	0.5337171	0.79279875	-6.00796419
WT after UVB irradiation VS WT before UVB irradiation	GO_DEOXYRIBONUCLEASE_I_ACTIVITY2	-0.52754948	-0.08356196	-2.70414892	0.02660631	0.43935696	-3.31185822
homo after UVB irradiation VS homo before UVB irradiation	GO_DEOXYRIBONUCLEOSIDE_DIPHOSPHATE_METABOLIC_PROCESS	0.20397882	-0.01426744	0.962713912	0.36280368	0.55343125	-6.06908705
hetero after UVB irradiation VS hetero before UVB irradiation	GO_DEOXYRIBONUCLEOSIDE_DIPHOSPHATE_METABOLIC_PROCESS1	0.080001676	-0.05766805	0.26167859	0.80030192	0.92259806	-6.19451625
WT after UVB irradiation VS WT before UVB irradiation	GO_DEOXYRIBONUCLEOSIDE_DIPHOSPHATE_METABOLIC_PROCESS2	0.536777859	0.016585611	2.430883984	0.04079319	0.48971155	-3.70057395
homo after UVB irradiation VS homo before UVB irradiation	GO_DEOXYRIBONUCLEOSIDE_METABOLIC_PROCESS	0.23024687	0.020889881	0.88328946	0.40185332	0.58948739	-6.14192724
hetero after UVB irradiation VS hetero before UVB irradiation	GO_DEOXYRIBONUCLEOSIDE_METABOLIC_PROCESS1	0.242002602	0.029870591	1.247529437	0.24812156	0.57549225	-5.46427125
WT after UVB irradiation VS WT before UVB irradiation	GO_DEOXYRIBONUCLEOSIDE_METABOLIC_PROCESS2	0.584321281	0.001382841	3.53656985	0.00751009	0.31411149	-2.15525994
homo after UVB irradiation VS homo before UVB irradiation	GO_DEOXYRIBONUCLEOSIDE_MONOPHOSPHATE_BIOSYNTHETIC_PROCESS	-0.25102558	-0.0210177	-0.89825529	0.39427283	0.58316971	-6.12859261
hetero after UVB irradiation VS hetero before UVB irradiation	GO_DEOXYRIBONUCLEOSIDE_MONOPHOSPHATE_BIOSYNTHETIC_PROCESS1	0.179279615	-0.03503597	0.816813673	0.43811101	0.73580311	-5.88476679
WT after UVB irradiation VS WT before UVB irradiation	GO_DEOXYRIBONUCLEOSIDE_MONOPHOSPHATE_BIOSYNTHETIC_PROCESS2	0.43331349	0.012147989	1.985480541	0.08191441	0.56741066	-4.32426833
homo after UVB irradiation VS homo before UVB irradiation	GO_DEOXYRIBONUCLEOSIDE_MONOPHOSPHATE_METABOLIC_PROCESS	-0.23879961	-0.03306478	-0.77452029	0.46004173	0.64015425	-6.23317055
hetero after UVB irradiation VS hetero before UVB irradiation	GO_DEOXYRIBONUCLEOSIDE_MONOPHOSPHATE_METABOLIC_PROCESS1	0.259354251	-0.07355215	1.596151168	0.14982909	0.46800892	-5.04183804
WT after UVB irradiation VS WT before UVB irradiation	GO_DEOXYRIBONUCLEOSIDE_MONOPHOSPHATE_METABOLIC_PROCESS2	0.451391239	0.04576195	2.18380729	0.06010109	0.52638053	-4.04942068
homo after UVB irradiation VS homo before UVB irradiation	GO_DEOXYRIBONUCLEOSIDE_TRIPHOSPHATE_BIOSYNTHETIC_PROCESS	-0.2279326	-0.03361	-1.09909489	0.30247682	0.49709577	-5.93263269
hetero after UVB irradiation VS hetero before UVB irradiation	GO_DEOXYRIBONUCLEOSIDE_TRIPHOSPHATE_BIOSYNTHETIC_PROCESS1	-0.31836416	-0.11458216	-2.18651376	0.06087141	0.32538394	-4.22774744
WT after UVB irradiation VS WT before UVB irradiation	GO_DEOXYRIBONUCLEOSIDE_TRIPHOSPHATE_BIOSYNTHETIC_PROCESS2	0.328419128	-0.07188608	1.2029035	0.26300215	0.74156847	-5.29728907
homo after UVB irradiation VS homo before UVB irradiation	GO_DEOXYRIBONUCLEOSIDE_TRIPHOSPHATE_CATABOLIC_PROCESS	-0.09816377	-0.02201502	-0.46552665	0.65348466	0.78909035	-6.43347033
hetero after UVB irradiation VS hetero before UVB irradiation	GO_DEOXYRIBONUCLEOSIDE_TRIPHOSPHATE_CATABOLIC_PROCESS1	-0.014365	0.018762705	-0.05558668	0.95705932	0.98468605	-6.22980879
WT after UVB irradiation VS WT before UVB irradiation	GO_DEOXYRIBONUCLEOSIDE_TRIPHOSPHATE_CATABOLIC_PROCESS2	0.321411825	-0.07480041	1.342403545	0.21588117	0.71317629	-5.14309998
homo after UVB irradiation VS homo before UVB irradiation	GO_DEOXYRIBONUCLEOSIDE_TRIPHOSPHATE_METABOLIC_PROCESS	-0.11837735	-0.00320118	-0.58934807	0.57126304	0.72888882	-6.36417874
hetero after UVB irradiation VS hetero before UVB irradiation	GO_DEOXYRIBONUCLEOSIDE_TRIPHOSPHATE_METABOLIC_PROCESS1	-0.08164623	-0.02790988	-0.46059616	0.65757497	0.8600227	-6.11801854
WT after UVB irradiation VS WT before UVB irradiation	GO_DEOXYRIBONUCLEOSIDE_TRIPHOSPHATE_METABOLIC_PROCESS2	0.236780266	-0.05312804	1.419900893	0.19296877	0.69334561	-5.05298026
homo after UVB irradiation VS homo before UVB irradiation	GO_DEOXYRIBONUCLEOTIDE_BINDING	-0.15230334	0.012398225	-0.633414	0.5434602	0.70834147	-6.33590404
hetero after UVB irradiation VS hetero before UVB irradiation	GO_DEOXYRIBONUCLEOTIDE_BINDING1	0.037706083	0.058932652	0.172874475	0.86712272	0.94962616	-6.21530884
WT after UVB irradiation VS WT before UVB irradiation	GO_DEOXYRIBONUCLEOTIDE_BINDING2	0.511359424	-0.11933139	2.224097518	0.05642401	0.5232848	-3.99290596
homo after UVB irradiation VS homo before UVB irradiation	GO_DEOXYRIBONUCLEOTIDE_BIOSYNTHETIC_PROCESS	-0.19504577	-0.03020573	-0.74637553	0.47597664	0.65406344	-6.25510694
hetero after UVB irradiation VS hetero before UVB irradiation	GO_DEOXYRIBONUCLEOTIDE_BIOSYNTHETIC_PROCESS1	0.167800721	-0.04834433	0.875847442	0.40710527	0.71234974	-5.83528258
WT after UVB irradiation VS WT before UVB irradiation	GO_DEOXYRIBONUCLEOTIDE_BIOSYNTHETIC_PROCESS2	0.192220045	-0.04228397	1.034864164	0.33064569	0.77726938	-5.46742548
homo after UVB irradiation VS homo before UVB irradiation	GO_DEOXYRIBONUCLEOTIDE_METABOLIC_PROCESS	-0.35125723	-0.061852	-2.20708156	0.05704615	0.17805204	-4.45716556
hetero after UVB irradiation VS hetero before UVB irradiation	GO_DEOXYRIBONUCLEOTIDE_METABOLIC_PROCESS1	-0.03138963	-0.04176444	-0.15728563	0.8789886	0.95595193	-6.21808996
WT after UVB irradiation VS WT before UVB irradiation	GO_DEOXYRIBONUCLEOTIDE_METABOLIC_PROCESS2	0.176700651	-0.00188885	0.939544692	0.3746275	0.80246519	-5.55539873
homo after UVB irradiation VS homo before UVB irradiation	GO_DEOXYRIBOSE_PHOSPHATE_BIOSYNTHETIC_PROCESS	-0.09537529	-0.03214268	-0.34845856	0.73613995	0.8445796	-6.48470773
hetero after UVB irradiation VS hetero before UVB irradiation	GO_DEOXYRIBOSE_PHOSPHATE_BIOSYNTHETIC_PROCESS1	0.070765188	-0.05085776	0.409872889	0.69285245	0.87721579	-6.14137585
WT after UVB irradiation VS WT before UVB irradiation	GO_DEOXYRIBOSE_PHOSPHATE_BIOSYNTHETIC_PROCESS2	0.178820916	0.003922677	1.03808947	0.32922978	0.77711658	-5.46433574
homo after UVB irradiation VS homo before UVB irradiation	GO_DEOXYRIBOSE_PHOSPHATE_CATABOLIC_PROCESS	-0.45346819	-0.05188863	-3.30788917	0.01014257	0.06732969	-2.7589606
hetero after UVB irradiation VS hetero before UVB irradiation	GO_DEOXYRIBOSE_PHOSPHATE_CATABOLIC_PROCESS1	-0.10734229	-0.00179084	-0.42391778	0.68299913	0.87258313	-6.13516792
WT after UVB irradiation VS WT before UVB irradiation	GO_DEOXYRIBOSE_PHOSPHATE_CATABOLIC_PROCESS2	0.046441398	0.00387068	0.191911306	0.85253687	0.96605575	-5.97668006
homo after UVB irradiation VS homo before UVB irradiation	GO_DEPHOSPHORYLATION	0.006243202	-0.01481228	0.049449218	0.96172658	0.97985357	-6.54951493
homo after UVB irradiation VS homo before UVB irradiation	GO_DEPHOSPHORYLATION_OF_RNA_POLYMERASE_II_C_TERMINAL_DOMAIN	-0.0829292	-0.11669905	-0.27264981	0.79176023	0.88161214	-6.51024312
hetero after UVB irradiation VS hetero before UVB irradiation	GO_DEPHOSPHORYLATION_OF_RNA_POLYMERASE_II_C_TERMINAL_DOMAIN1	0.028116239	-0.03918273	0.09057459	0.93009828	0.97440754	-6.22703756
WT after UVB irradiation VS WT before UVB irradiation	GO_DEPHOSPHORYLATION_OF_RNA_POLYMERASE_II_C_TERMINAL_DOMAIN2	-0.27886229	0.007799192	-0.73835674	0.48114653	0.8504347	-5.71823756
hetero after UVB irradiation VS hetero before UVB irradiation	GO_DEPHOSPHORYLATION1	-0.01669149	-0.00080534	-0.1347659	0.8961871	0.96152498	-6.22164659
WT after UVB irradiation VS WT before UVB irradiation	GO_DEPHOSPHORYLATION2	-0.03483907	-0.01010219	-0.25153449	0.80766682	0.95404687	-5.96282004
homo after UVB irradiation VS homo before UVB irradiation	GO_DEPYRIMIDINATION	-0.78356809	-0.06735188	-4.54738582	0.00169621	0.02787404	-0.95645846
hetero after UVB irradiation VS hetero before UVB irradiation	GO_DEPYRIMIDINATION1	-0.52063106	-0.10015914	-2.02049494	0.07866448	0.36149891	-4.4648713
WT after UVB irradiation VS WT before UVB irradiation	GO_DEPYRIMIDINATION2	-0.03096487	-0.00387426	-0.11269462	0.91301637	0.97884691	-5.98937103
homo after UVB irradiation VS homo before UVB irradiation	GO_DERLIN_1_RETROTRANSLOCATION_COMPLEX	0.443503068	0.01227597	3.065471569	0.01472845	0.08257891	-3.13187793
hetero after UVB irradiation VS hetero before UVB irradiation	GO_DERLIN_1_RETROTRANSLOCATION_COMPLEX1	0.05334709	0.001204588	0.177656498	0.8634898	0.94819913	-6.21440347
WT after UVB irradiation VS WT before UVB irradiation	GO_DERLIN_1_RETROTRANSLOCATION_COMPLEX2	0.453403175	-0.09071323	2.128627992	0.06552274	0.537335	-4.12649495
homo after UVB irradiation VS homo before UVB irradiation	GO_DERMATAN_SULFATE_METABOLIC_PROCESS	0.670356568	0.00341676	4.398775882	0.00207764	0.03080331	-1.16108955
hetero after UVB irradiation VS hetero before UVB irradiation	GO_DERMATAN_SULFATE_METABOLIC_PROCESS1	0.209409781	-0.0100371	0.770525716	0.46353717	0.75147819	-5.92153979
WT after UVB irradiation VS WT before UVB irradiation	GO_DERMATAN_SULFATE_METABOLIC_PROCESS2	0.388819868	-0.00242546	1.861046286	0.0993234	0.59874718	-4.4929207
homo after UVB irradiation VS homo before UVB irradiation	GO_DERMATAN_SULFATE_PROTEOGLYCAN_METABOLIC_PROCESS	0.594405186	-0.00446729	4.010625719	0.00358332	0.03908552	-1.71134646
hetero after UVB irradiation VS hetero before UVB irradiation	GO_DERMATAN_SULFATE_PROTEOGLYCAN_METABOLIC_PROCESS1	0.174507467	-0.0041892	0.663262962	0.52614946	0.78925093	-5.99959996
WT after UVB irradiation VS WT before UVB irradiation	GO_DERMATAN_SULFATE_PROTEOGLYCAN_METABOLIC_PROCESS2	0.280192091	-0.03442962	1.320987177	0.22261246	0.71742564	-5.16746597
homo after UVB irradiation VS homo before UVB irradiation	GO_DESMOSOME	-0.151557	0.010608403	-0.77573865	0.45936001	0.63958886	-6.23220509
homo after UVB irradiation VS homo before UVB irradiation	GO_DESMOSOME_ORGANIZATION	-0.15798545	-0.02038822	-0.52139212	0.61566974	0.76112255	-6.40409055
hetero after UVB irradiation VS hetero before UVB irradiation	GO_DESMOSOME_ORGANIZATION1	0.079707778	0.048446755	0.25059257	0.80856098	0.92568169	-6.19756955
WT after UVB irradiation VS WT before UVB irradiation	GO_DESMOSOME_ORGANIZATION2	-0.19807944	0.110385832	-0.50526301	0.62683653	0.90307787	-5.86356822
hetero after UVB irradiation VS hetero before UVB irradiation	GO_DESMOSOME1	0.076747982	0.011398854	0.29703768	0.77414307	0.91152166	-6.18391242
WT after UVB irradiation VS WT before UVB irradiation	GO_DESMOSOME2	-0.16165048	0.049814435	-0.51660388	0.61924152	0.90068273	-5.85765592
homo after UVB irradiation VS homo before UVB irradiation	GO_DETECTION_OF_ABIOTIC_STIMULUS	0.279297986	0.030077276	2.284940356	0.05042073	0.16560398	-4.33905762
hetero after UVB irradiation VS hetero before UVB irradiation	GO_DETECTION_OF_ABIOTIC_STIMULUS1	0.121486847	0.033586441	0.924995672	0.38251428	0.69452204	-5.79194812
WT after UVB irradiation VS WT before UVB irradiation	GO_DETECTION_OF_ABIOTIC_STIMULUS2	0.033955179	0.031122968	0.205948499	0.84191475	0.96348476	-5.97374844
homo after UVB irradiation VS homo before UVB irradiation	GO_DETECTION_OF_BACTERIAL_LIPOPROTEIN	0.988722517	-0.03746505	4.863395002	0.00111386	0.02245522	-0.53283231
hetero after UVB irradiation VS hetero before UVB irradiation	GO_DETECTION_OF_BACTERIAL_LIPOPROTEIN1	0.722888315	0.063428183	3.127694294	0.01439573	0.17916057	-2.85462529
WT after UVB irradiation VS WT before UVB irradiation	GO_DETECTION_OF_BACTERIAL_LIPOPROTEIN2	0.797377311	0.117527331	3.032462836	0.01601963	0.37691292	-2.84781348
homo after UVB irradiation VS homo before UVB irradiation	GO_DETECTION_OF_BIOTIC_STIMULUS	0.835359567	-0.02138477	6.059213944	0.00025767	0.0121036	0.92814142
hetero after UVB irradiation VS hetero before UVB irradiation	GO_DETECTION_OF_BIOTIC_STIMULUS1	0.664699454	0.059056834	3.835417469	0.00515717	0.12876333	-1.86329638
WT after UVB irradiation VS WT before UVB irradiation	GO_DETECTION_OF_BIOTIC_STIMULUS2	0.598106423	0.048647548	2.68358203	0.02747274	0.44019019	-3.3411025
homo after UVB irradiation VS homo before UVB irradiation	GO_DETECTION_OF_CALCIUM_ION	-0.24549632	0.033900024	-1.69320218	0.12737617	0.29214954	-5.20308024
hetero after UVB irradiation VS hetero before UVB irradiation	GO_DETECTION_OF_CALCIUM_ION1	-0.24491621	-0.02709395	-1.41863526	0.19445132	0.51906864	-5.26436387

WT after UVB irradiation VS WT before UVB irradiation	GO_DETECTION_OF_CALCIUM_ION2	-0.28327435	0.013177102	-1.35701903	0.21138838	0.70891132	-5.12633456
homo after UVB irradiation VS homo before UVB irradiation	GO_DETECTION_OF_CELL_DENSITY	0.201367174	-0.05074018	1.377455679	0.20424878	0.39365666	-5.61478807
hetero after UVB irradiation VS hetero before UVB irradiation	GO_DETECTION_OF_CELL_DENSITY1	0.243091696	0.000396663	1.354023114	0.21340454	0.54045743	-5.34169054
WT after UVB irradiation VS WT before UVB irradiation	GO_DETECTION_OF_CELL_DENSITY2	0.317121932	0.024492395	1.585837908	0.15098188	0.6585056	-4.85073093
homo after UVB irradiation VS homo before UVB irradiation	GO_DETECTION_OF_CHEMICAL_STIMULUS_INVOLVED_IN_SENSORY_PERCEPTION_OF_TASTE	0.403967926	0.008848031	2.53679601	0.03380604	0.13138681	-3.95211093
hetero after UVB irradiation VS hetero before UVB irradiation	GO_DETECTION_OF_CHEMICAL_STIMULUS_INVOLVED_IN_SENSORY_PERCEPTION_OF_TASTE1	0.292754224	0.031530641	1.492710308	0.17455806	0.49725459	-5.17320536
WT after UVB irradiation VS WT before UVB irradiation	GO_DETECTION_OF_CHEMICAL_STIMULUS_INVOLVED_IN_SENSORY_PERCEPTION_OF_TASTE2	-0.23284852	0.132102236	-1.17777441	0.27233894	0.74503458	-5.32387187
homo after UVB irradiation VS homo before UVB irradiation	GO_DETECTION_OF_EXTERNAL_BIOTIC_STIMULUS	0.996718953	0.000568177	7.076165058	8.56E-05	0.00991667	2.004063904
hetero after UVB irradiation VS hetero before UVB irradiation	GO_DETECTION_OF_EXTERNAL_BIOTIC_STIMULUS1	0.815543879	0.102115018	3.932648834	0.00450297	0.12742869	-1.73259301
WT after UVB irradiation VS WT before UVB irradiation	GO_DETECTION_OF_EXTERNAL_BIOTIC_STIMULUS2	0.747898239	0.088218606	3.105015848	0.01433929	0.36090271	-2.74634837
homo after UVB irradiation VS homo before UVB irradiation	GO_DETECTION_OF_LIGHT_STIMULUS	0.307144411	0.03636844	2.429830134	0.04006016	0.14370574	-4.11718269
homo after UVB irradiation VS homo before UVB irradiation	GO_DETECTION_OF_LIGHT_STIMULUS_INVOLVED_IN_SENSORY_PERCEPTION	0.409199958	0.040267202	3.358749418	0.00938679	0.06441548	-2.68130312
hetero after UVB irradiation VS hetero before UVB irradiation	GO_DETECTION_OF_LIGHT_STIMULUS_INVOLVED_IN_SENSORY_PERCEPTION1	0.2792441	0.014350842	1.367355845	0.20936701	0.53468667	-5.32590904
WT after UVB irradiation VS WT before UVB irradiation	GO_DETECTION_OF_LIGHT_STIMULUS_INVOLVED_IN_SENSORY_PERCEPTION2	-0.07815519	0.036814187	-0.4067289	0.69474529	0.9219193	-5.90971128
hetero after UVB irradiation VS hetero before UVB irradiation	GO_DETECTION_OF_LIGHT_STIMULUS1	0.148556603	0.036864408	1.14420904	0.28621416	0.61167612	-5.5768338
WT after UVB irradiation VS WT before UVB irradiation	GO_DETECTION_OF_LIGHT_STIMULUS2	0.025741781	0.006027585	0.156278373	0.87963954	0.9693507	-5.98320047
homo after UVB irradiation VS homo before UVB irradiation	GO_DETECTION_OF_MECHANICAL_STIMULUS	0.314877764	0.015164325	2.326694718	0.04718771	0.1590505	-4.27536739
homo after UVB irradiation VS homo before UVB irradiation	GO_DETECTION_OF_MECHANICAL_STIMULUS_INVOLVED_IN_SENSORY_PERCEPTION	0.276505799	0.028528085	2.068355884	0.07103521	0.20457974	-4.66503454
homo after UVB irradiation VS homo before UVB irradiation	GO_DETECTION_OF_MECHANICAL_STIMULUS_INVOLVED_IN_SENSORY_PERCEPTION_OF_PAIN	0.435754869	0.031259245	2.852302447	0.02054264	0.09940161	-3.46239608
hetero after UVB irradiation VS hetero before UVB irradiation	GO_DETECTION_OF_MECHANICAL_STIMULUS_INVOLVED_IN_SENSORY_PERCEPTION_OF_PAIN1	0.422551943	0.040465972	2.68484045	0.02818333	0.2365604	-3.50019097
WT after UVB irradiation VS WT before UVB irradiation	GO_DETECTION_OF_MECHANICAL_STIMULUS_INVOLVED_IN_SENSORY_PERCEPTION_OF_PAIN2	0.168745074	0.084857223	0.618047599	0.55352513	0.87677659	-5.79940186
homo after UVB irradiation VS homo before UVB irradiation	GO_DETECTION_OF_MECHANICAL_STIMULUS_INVOLVED_IN_SENSORY_PERCEPTION_OF_SOUND	-0.04623699	0.033190505	-0.29250323	0.7770562	0.8714603	-6.50414494
hetero after UVB irradiation VS hetero before UVB irradiation	GO_DETECTION_OF_MECHANICAL_STIMULUS_INVOLVED_IN_SENSORY_PERCEPTION_OF_SOUND1	-0.06992764	0.004567544	-0.39946785	0.7001928	0.88146568	-6.14584591
WT after UVB irradiation VS WT before UVB irradiation	GO_DETECTION_OF_MECHANICAL_STIMULUS_INVOLVED_IN_SENSORY_PERCEPTION_OF_SOUND2	-0.16284089	-0.00074534	-0.78712727	0.45362665	0.83718965	-5.68177381
hetero after UVB irradiation VS hetero before UVB irradiation	GO_DETECTION_OF_MECHANICAL_STIMULUS_INVOLVED_IN_SENSORY_PERCEPTION1	0.23574916	0.01702801	1.589993014	0.15120714	0.46910372	-5.04978228
WT after UVB irradiation VS WT before UVB irradiation	GO_DETECTION_OF_MECHANICAL_STIMULUS_INVOLVED_IN_SENSORY_PERCEPTION2	-0.03230789	0.032482535	-0.2111967	0.83795205	0.9621583	-5.97259982
hetero after UVB irradiation VS hetero before UVB irradiation	GO_DETECTION_OF_MECHANICAL_STIMULUS1	0.117864878	0.004122105	0.750722662	0.47471162	0.75777203	-5.93671341
WT after UVB irradiation VS WT before UVB irradiation	GO_DETECTION_OF_MECHANICAL_STIMULUS2	-0.03594174	0.020871288	-0.21063506	0.83837589	0.9623128	-5.9727241
homo after UVB irradiation VS homo before UVB irradiation	GO_DETECTION_OF_MOLECULE_OF_BACTERIAL_ORIGIN	1.107506244	-0.01223651	8.070864547	3.25E-05	0.00847131	2.926344057
hetero after UVB irradiation VS hetero before UVB irradiation	GO_DETECTION_OF_MOLECULE_OF_BACTERIAL_ORIGIN1	0.757140838	0.091967029	3.624243839	0.00695637	0.1390235	-2.15210879
WT after UVB irradiation VS WT before UVB irradiation	GO_DETECTION_OF_MOLECULE_OF_BACTERIAL_ORIGIN2	0.692235072	0.050680091	2.407047421	0.04234724	0.495137	-3.73441334
homo after UVB irradiation VS homo before UVB irradiation	GO_DETECTION_OF_OTHER_ORGANISM	0.948329537	-0.01286137	6.354515197	0.00018478	0.01080099	1.255516808
hetero after UVB irradiation VS hetero before UVB irradiation	GO_DETECTION_OF_OTHER_ORGANISM1	0.812566379	0.097088789	3.570677265	0.00751247	0.14177754	-2.22639646
WT after UVB irradiation VS WT before UVB irradiation	GO_DETECTION_OF_OTHER_ORGANISM2	0.709417739	0.097824333	3.174723653	0.01289781	0.35377874	-2.64935533
homo after UVB irradiation VS homo before UVB irradiation	GO_DETECTION_OF_STIMULUS	0.152766426	0.06203357	1.201735554	0.26251728	0.45802406	-5.82117355
homo after UVB irradiation VS homo before UVB irradiation	GO_DETECTION_OF_STIMULUS_INVOLVED_IN_SENSORY_PERCEPTION	0.104935384	0.087300196	0.746524713	0.47589123	0.65400698	-6.25499253
homo after UVB irradiation VS homo before UVB irradiation	GO_DETECTION_OF_STIMULUS_INVOLVED_IN_SENSORY_PERCEPTION_OF_PAIN	0.339231165	0.021429373	2.09989287	0.06758709	0.1984841	-4.61810692
hetero after UVB irradiation VS hetero before UVB irradiation	GO_DETECTION_OF_STIMULUS_INVOLVED_IN_SENSORY_PERCEPTION_OF_PAIN1	0.403993209	0.043584763	2.560087372	0.03414932	0.25492251	-3.68326292
WT after UVB irradiation VS WT before UVB irradiation	GO_DETECTION_OF_STIMULUS_INVOLVED_IN_SENSORY_PERCEPTION_OF_PAIN2	0.304767672	0.088023636	1.690668676	0.12891023	0.63495603	-4.71731834
hetero after UVB irradiation VS hetero before UVB irradiation	GO_DETECTION_OF_STIMULUS_INVOLVED_IN_SENSORY_PERCEPTION1	0.118629419	0.094329419	0.776829073	0.46001746	0.74838338	-5.91663917
WT after UVB irradiation VS WT before UVB irradiation	GO_DETECTION_OF_STIMULUS_INVOLVED_IN_SENSORY_PERCEPTION2	-0.03259263	0.075316235	-0.19817468	0.84779321	0.96522688	-5.97539727
hetero after UVB irradiation VS hetero before UVB irradiation	GO_DETECTION_OF_STIMULUS1	0.152792201	0.070840514	1.196039584	0.26654432	0.59300892	-5.5211915
WT after UVB irradiation VS WT before UVB irradiation	GO_DETECTION_OF_STIMULUS2	0.040342774	0.048157748	0.248368255	0.81003233	0.95468789	-5.96364843
homo after UVB irradiation VS homo before UVB irradiation	GO_DETECTION_OF_TEMPERATURE_STIMULUS	-0.08192898	0.027965328	-0.44621522	0.66681437	0.79845664	-6.44289342
homo after UVB irradiation VS homo before UVB irradiation	GO_DETECTION_OF_TEMPERATURE_STIMULUS_INVOLVED_IN_SENSORY_PERCEPTION	0.185235698	0.018170241	0.983438487	0.35309184	0.54532457	-6.04925615
hetero after UVB irradiation VS hetero before UVB irradiation	GO_DETECTION_OF_TEMPERATURE_STIMULUS_INVOLVED_IN_SENSORY_PERCEPTION1	0.362481506	0.06103747	1.897998287	0.09494719	0.38479559	-4.63649868
WT after UVB irradiation VS WT before UVB irradiation	GO_DETECTION_OF_TEMPERATURE_STIMULUS_INVOLVED_IN_SENSORY_PERCEPTION2	0.292633942	0.10301055	1.8201671	0.10577481	0.61027421	-4.54751534
hetero after UVB irradiation VS hetero before UVB irradiation	GO_DETECTION_OF_TEMPERATURE_STIMULUS1	0.121204796	0.056606111	0.635626258	0.54309502	0.79799154	-6.01803694
WT after UVB irradiation VS WT before UVB irradiation	GO_DETECTION_OF_TEMPERATURE_STIMULUS2	0.087037868	0.131427171	0.483325461	0.64166246	0.90690671	-5.87465586
homo after UVB irradiation VS homo before UVB irradiation	GO_DETECTION_OF_VIRUS	0.345191438	-0.07760119	1.367542298	0.20721442	0.39732367	-5.62691323
hetero after UVB irradiation VS hetero before UVB irradiation	GO_DETECTION_OF_VIRUS1	0.450187748	0.008896781	2.714319177	0.02693691	0.23196771	-3.45694531
WT after UVB irradiation VS WT before UVB irradiation	GO_DETECTION_OF_VIRUS2	0.477058285	0.031222456	2.766634429	0.02414195	0.42212473	-3.22308954
homo after UVB irradiation VS homo before UVB irradiation	GO_DETECTION_OF_VISIBLE_LIGHT	0.271423934	0.036866458	2.249655525	0.05332338	0.1710737	-4.39269513
hetero after UVB irradiation VS hetero before UVB irradiation	GO_DETECTION_OF_VISIBLE_LIGHT1	0.164041752	0.025195565	1.301069947	0.23011014	0.55823811	-5.40343234
WT after UVB irradiation VS WT before UVB irradiation	GO_DETECTION_OF_VISIBLE_LIGHT2	0.037807667	0.00470241	0.20984526	0.838972	0.9624308	-5.97289833
homo after UVB irradiation VS homo before UVB irradiation	GO_DETERMINATION_OF_ADULT_LIFESPAN	-0.1237637	-0.0041502	-0.65540475	0.52988927	0.69713619	-6.32110292
hetero after UVB irradiation VS hetero before UVB irradiation	GO_DETERMINATION_OF_ADULT_LIFESPAN1	0.225422819	-0.03718218	1.166711553	0.27753366	0.60400502	-5.5528855
WT after UVB irradiation VS WT before UVB irradiation	GO_DETERMINATION_OF_ADULT_LIFESPAN2	0.298799355	-0.00424688	1.564885266	0.15578678	0.66118652	-4.87690589
homo after UVB irradiation VS homo before UVB irradiation	GO_DETERMINATION_OF_DIGESTIVE_TRACT_LEFT_RIGHT_ASYMMETRY	0.064308018	-0.01420389	0.209768116	0.83888892	0.91059093	-6.52677198
hetero after UVB irradiation VS hetero before UVB irradiation	GO_DETERMINATION_OF_DIGESTIVE_TRACT_LEFT_RIGHT_ASYMMETRY1	-0.00319854	-0.0048914	-0.01147169	0.9911332	0.99624062	-6.23141275
WT after UVB irradiation VS WT before UVB irradiation	GO_DETERMINATION_OF_DIGESTIVE_TRACT_LEFT_RIGHT_ASYMMETRY2	-0.15976269	-0.0373209	-0.64313823	0.53791643	0.87025652	-5.78353404
homo after UVB irradiation VS homo before UVB irradiation	GO_DETERMINATION_OF_DORSAL_VENTRAL_ASYMMETRY	0.541041768	-0.00074589	2.658063032	0.02789891	0.11808674	-3.76415789
hetero after UVB irradiation VS hetero before UVB irradiation	GO_DETERMINATION_OF_DORSAL_VENTRAL_ASYMMETRY1	0.212918304	0.062729765	0.977126164	0.35764443	0.67298394	-5.7439409
WT after UVB irradiation VS WT before UVB irradiation	GO_DETERMINATION_OF_DORSAL_VENTRAL_ASYMMETRY2	0.006628904	0.023524772	0.034556314	0.97327019	0.99307736	-5.99543663
homo after UVB irradiation VS homo before UVB irradiation	GO_DETERMINATION_OF_LEFT_RIGHT_ASYMMETRY_IN_LATERAL_MESODERM	-0.88844892	0.05420133	-5.54777476	0.00047054	0.01610724	0.330450993
hetero after UVB irradiation VS hetero before UVB irradiation	GO_DETERMINATION_OF_LEFT_RIGHT_ASYMMETRY_IN_LATERAL_MESODERM1	-0.75233794	0.007917702	-4.28925712	0.00277066	0.11250952	-1.26625719
WT after UVB irradiation VS WT before UVB irradiation	GO_DETERMINATION_OF_LEFT_RIGHT_ASYMMETRY_IN_LATERAL_MESODERM2	-0.25993673	0.10065918	-1.12174909	0.29412823	0.75745298	-5.38173237
homo after UVB irradiation VS homo before UVB irradiation	GO_DETERMINATION_OF_LIVER_LEFT_RIGHT_ASYMMETRY	0.32298937	-0.01465306	1.376430528	0.20455375	0.39402162	-5.61604447
hetero after UVB irradiation VS hetero before UVB irradiation	GO_DETERMINATION_OF_LIVER_LEFT_RIGHT_ASYMMETRY1	-0.1548197	-0.01922757	-0.47898371	0.64500348	0.85438728	-6.10891523
WT after UVB irradiation VS WT before UVB irradiation	GO_DETERMINATION_OF_LIVER_LEFT_RIGHT_ASYMMETRY2	-0.20833957	-0.09116784	-0.70668288	0.49958989	0.85545885	-5.74083642
homo after UVB irradiation VS homo before UVB irradiation	GO_DETERMINATION_OF_PANCREATIC_LEFT_RIGHT_ASYMMETRY	0.067535333	-0.00291072	0.217095661	0.8333574	0.90712216	-6.52506487
hetero after UVB irradiation VS hetero before UVB irradiation	GO_DETERMINATION_OF_PANCREATIC_LEFT_RIGHT_ASYMMETRY1	-0.19130245	-0.0195182	-0.56805765	0.58586737	0.82335261	-6.06011947
WT after UVB irradiation VS WT before UVB irradiation	GO_DETERMINATION_OF_PANCREATIC_LEFT_RIGHT_ASYMMETRY2	-0.28231892	-0.09379661	-0.88106325	0.40367528	0.81613006	-5.60604421
homo after UVB irradiation VS homo before UVB irradiation	GO_DETOXIFICATION	0.44183557	-0.01589521	3.157246284	0.01277926	0.07647347	-2.99022075
homo after UVB irradiation VS homo before UVB irradiation	GO_DETOXIFICATION_OF_INORGANIC_COMPOUND	-0.06095249	-0.06253813	-0.28827112	0.78018291	0.87353256	-6.5054801
hetero after UVB irradiation VS hetero before UVB irradiation	GO_DETOXIFICATION_OF_INORGANIC_COMPOUND1	-0.55386767	-0.01428618	-3.52164435	0.00806316	0.14537603	-2.29474336
WT after UVB irradiation VS WT before UVB irradiation	GO_DETOXIFICATION_OF_INORGANIC_COMPOUND2	0.106579725	0.035120521	0.501003574	0.6297014	0.90411526	-5.86575709
homo after UVB irradiation VS homo before UVB irradiation	GO_DETOXIFICATION_OF_NITROGEN_COMPOUND	0.903133782	-0.00413735	4.437855537	0.0019691	0.03009606	-1.10694665
hetero after UVB irradiation VS hetero before UVB irradiation	GO_DETOXIFICATION_OF_NITROGEN_COMPOUND1	0.213669957	-0.07090156	0.47796572	0.64569635	0.85438728	-6.109428
WT after UVB irradiation VS WT before UVB irradiation	GO_DETOXIFICATION_OF_NITROGEN_COMPOUND2	0.400542308	0.06566867	1.021505565	0.33656019	0.77956659	-5.48014553
hetero after UVB irradiation VS hetero before UVB irradiation	GO_DETOXIFICATION1	0.286306164	0.022258899	1.816700549	0.10749024	0.4057626	-4.74841195
WT after UVB irradiation VS WT before UVB irradiation	GO_DETOXIFICATION2	0.311406304	0.017684465	1.610744225	0.14544559	0.65365874	-4.81939651
homo after UVB irradiation VS homo before UVB irradiation	GO_DEUTEROSOME	-0.44974331	-0.05495562	-1.94742766	0.08590416	0.22852769	-4.84287425
hetero after UVB irradiation VS hetero before UVB irradiation	GO_DEUTEROSOME1	0.459832124	0.010233604	1.784515574	0.11287819	0.41499637	-4.79221383
WT after UVB irradiation VS WT before UVB irradiation	GO_DEUTEROSOME2	-0.03549147	0.080222252	-0.14343248	0.88945376	0.9716774	-5.98522598
homo after UVB irradiation VS homo before UVB irradiation	GO_DEVELOPMENT_OF_PRIMARY_FEMALE_SEXUAL_CHARACTERISTICS	0.248567901	0.008510215	1.938906943	0.08705774	0.23038757	-4.85526691
hetero after UVB irradiation VS hetero before UVB irradiation	GO_DEVELOPMENT_OF_PRIMARY_FEMALE_SEXUAL_CHARACTERISTICS1	0.052394796	-0.01255883	0.362201557	0.72675496	0.89222406	-6.16094872
WT after UVB irradiation VS WT before UVB irradiation	GO_DEVELOPMENT_OF_PRIMARY_FEMALE_SEXUAL_CHARACTERISTICS2	0.060044741	0.02838009	0.41952233	0.68574316	0.91888815	-5.90425584
homo after UVB irradiation VS homo before UVB irradiation	GO_DEVELOPMENT_OF_PRIMARY_SEXUAL_CHARACTERISTICS	0.141527887	0.010271386	1.139746689	0.28610443	0.48081575	-5.88934664
hetero after UVB irradiation VS hetero before UVB irradiation	GO_DEVELOPMENT_OF_PRIMARY_SEXUAL_CHARACTERISTICS1	0.013365689	0.010385308	0.098838442	0.92374297	0.9711279	-6.2261897
WT after UVB irradiation VS WT before UVB irradiation	GO_DEVELOPMENT_OF_PRIMARY_SEXUAL_CHARACTERISTICS2	0.032159142	0.023927418	0.228560775	0.82487675	0.95860343	-5.96859609
homo after UVB irradiation VS homo before UVB irradiation	GO_DEVELOPMENT_OF_SECONDARY_FEMALE_SEXUAL_CHARACTERISTICS	-0.47142919	-0.05966034	-2.32299064	0.04746598	0.15966618	-4.28102641
hetero after UVB irradiation VS hetero before UVB irradiation	GO_DEVELOPMENT_OF_SECONDARY_FEMALE_SEXUAL_CHARACTERISTICS1	0.170047182	-0.10257102	0.725157875	0.48939752	0.76616077	-5.95579757
WT after UVB irradiation VS WT before UVB irradiation	GO_DEVELOPMENT_OF_SECONDARY_FEMALE_SEXUAL_CHARACTERISTICS2	0.088713947	-0.03606496	0.347671821	0.73695104	0.93478445	-5.93278647
homo after UVB irradiation VS homo before UVB irradiation	GO_DEVELOPMENT_OF_SECONDARY_SEXUAL_CHARACTERISTICS	0.070549167	-0.03666295	0.348670531	0.73598653	0.84455313	-6.48462781
hetero after UVB irradiation VS hetero before UVB irradiation	GO_DEVELOPMENT_OF_SECONDARY_SEXUAL_CHARACTERISTICS1	0.442473025	-0.09341913	1.963264371	0.08590445	0.37193478	-4.54546492
WT after UVB irradiation VS WT before UVB irradiation	GO_DEVELOPMENT_OF_SECONDARY_SEXUAL_CHARACTERISTICS2	0.276267605	-0.01323171	1.181607597	0.27089742	0.74431614	-5.31984171
homo after UVB irradiation VS homo before UVB irradiation	GO_DEVELOPMENTAL_CELL_GROWTH	0.125988287	-0.00706692	0.969176906	0.35975389	0.55111472	-6.06293864
hetero after UVB irradiation VS hetero before UVB irradiation	GO_DEVELOPMENTAL_CELL_GROWTH1	-0.0012935	0.001641594	-0.01010952	0.99218601	0.99657418	-6.23142869
WT after UVB irradiation VS WT before UVB irradiation	GO_DEVELOPMENTAL_CELL_GROWTH2	-0.01561243	-0.00220608	-0.11091712	0.91438199	0.97942476	-5.98958041
homo after UVB irradiation VS homo before UVB irradiation	GO_DEVELOPMENTAL_GROWTH	0.044361245	-0.00332251	0.355143194	0.73130788	0.8412777	-6.482165
homo after UVB irradiation VS homo before UVB irradiation	GO_DEVELOPMENTAL_GROWTH_INVOLVED_IN_MORPHOGENESIS	0.118787515	-0.00952889	0.936472267	0.375384	0.56532137	-6.09371349
hetero after UVB irradiation VS hetero before UVB irradiation	GO_DEVELOPMENTAL_GROWTH_INVOLVED_IN_MORPHOGENESIS1	0.063975755	0.004584415	0.504611954	0.62768358	0.84669911	-6.09566903
WT after UVB irradiation VS WT before UVB irradiation	GO_DEVELOPMENTAL_GROWTH_INVOLVED_IN_MORPHOGENESIS2	0.005588367	0.008167556	0.039242735	0.96964714	0.99229361	-5.99525417
hetero after UVB irradiation VS hetero before UVB irradiation	GO_DEVELOPMENTAL_GROWTH1	-0.00945304	0.007640441	-0.07359621	0.94317179	0.9788608	-6.2285478
WT after UVB irradiation VS WT before UVB irradiation	GO_DEVELOPMENTAL_GROWTH2	-0.04585555	-0.00658197	-0.32543553	0.75310123	0.93734883	-5.94056836
homo after UVB irradiation VS homo before UVB irradiation	GO_DEVELOPMENTAL_INDUCTION	-0.38246136	0.004665967	-2.57700979	0.03171829	0.12619774	-3.88985725
hetero after UVB irradiation VS hetero before UVB irradiation	GO_DEVELOPMENTAL_INDUCTION1	-0.3705313	0.008238831	-2.86307252	0.02146092	0.21125909	-3.23909611
WT after UVB irradiation VS WT before UVB irradiation	GO_DEVELOPMENTAL_INDUCTION2	-0.42405216	0.014893502	-2.46125133	0.03889623	0.48194089	-3.65742936
homo after UVB irradiation VS homo before UVB irradiation	GO_DEVELOPMENTAL_MATURATION	0.204647378	-0.00875092	1.699037433	0.12624385	0.29036478	-5.19506085
hetero after UVB irradiation VS hetero before UVB irradiation	GO_DEVELOPMENTAL_MATURATION1	0.12340682	0.00783922	0.923667345	0.38316428	0.69484725	-5.79314421
WT after UVB irradiation VS WT before UVB irradiation	GO_DEVELOPMENTAL_MATURATION2	0.119481745	0.009934461	0.823634307	0.43373309	0.82957538	-5.65318688
homo after UVB irradiation VS homo before UVB irradiation	GO_DEVELOPMENTAL_PIGMENTATION	-0.20208349	-0.00534572	-1.22039968	0.25573505	0.45131335	-5.80015184
hetero after UVB irradiation VS hetero before UVB irradiation	GO_DEVELOPMENTAL_PIGMENTATION1	0.012583098	-0.00425597	0.080499619	0.93785364	0.97722007	-6.22797136
WT after UVB irradiation VS WT before UVB irradiation	GO_DEVELOPMENTAL_PIGMENTATION2	-0.1916689	0.003440387	-1.05893021	0.3201936	0.77325115	-5.44419823
homo after UVB irradiation VS homo before UVB irradiation	GO_DEVELOPMENTAL_PROCESS_INVOLVED_IN_REPRODUCTION	0.012814396	0.006590118	0.10347078	0.92003742	0.95666517	-6.54498478
hetero after UVB irradiation VS hetero before UVB irradiation	GO_DEVELOPMENTAL_PROCESS_INVOLVED_IN_REPRODUCTION1	0.009206889	0.009860756	0.072035931	0.9443742	0.97941986	-6.22867094
WT after UVB irradiation VS WT before UVB irradiation	GO_DEVELOPMENTAL_PROCESS_INVOLVED_IN_REPRODUCTION2	-0.02126682	0.019578657	-0.1523957	0.88260365	0.96988473	-5.98383088
homo after UVB irradiation VS homo before UVB irradiation	GO_DIACYLGLYCEROL_BINDING	0.691298209	-0.0379558	5.148629924	0.00077156	0.01926545	-0.16404612
hetero after UVB irradiation VS hetero before UVB irradiation	GO_DIACYLGLYCEROL_BINDING1	0.264969637	0.070747267	1.112644165	0.29875953	0.62161528	-5.60987213
WT after UVB irradiation VS WT before UVB irradiation	GO_DIACYLGLYCEROL_BINDING2	0.27374268	-0.05865251	1.148548603	0.28353626	0.75292132	-5.35430125
homo after UVB irradiation VS homo before UVB irradiation	GO_DIACYLGLYCEROL_BIOSYNTHETIC_PROCESS	0.426585396	-0.08274922	1.902321472	0.09218113	0.23881967	-4.9082513
hetero after UVB irradiation VS hetero before UVB irradiation	GO_DIACYLGLYCEROL_BIOSYNTHETIC_PROCESS1	-0.02304989	-0.01825441	-0.08087737	0.93756273	0.97716313	-6.22793833
WT after UVB irradiation VS WT before UVB irradiation	GO_DIACYLGLYCEROL_BIOSYNTHETIC_PROCESS2	0.309438898	-0.05717129	0.903627291	0.39228099	0.8128181	-5.58681322
homo after UVB irradiation VS homo before UVB irradiation	GO_DIACYLGLYCEROL_KINASE_ACTIVITY	-0.11425705	0.000893153	-0.61370419	0.55579655	0.71701107	-6.3487805
hetero after UVB irradiation VS hetero before UVB irradiation	GO_DIACYLGLYCEROL_KINASE_ACTIVITY1	0.136258148	0.033956481	0.518087224	0.61867372	0.84254497	-6.08844539
WT after UVB irradiation VS WT before UVB irradiation	GO_DIACYLGLYCEROL_KINASE_ACTIVITY2	-0.3261966	0.047973798	-1.74282123	0.11907617	0.6266787	-4.64954643
homo after UVB irradiation VS homo before UVB irradiation	GO_DIACYLGLYCEROL_METABOLIC_PROCESS	0.292148937	-0.0045358	1.941434379	0.08671402	0.22978672	-4.851593
hetero after UVB irradiation VS hetero before UVB irradiation	GO_DIACYLGLYCEROL_METABOLIC_PROCESS1	-0.00686581	-0.00727126	-0.03156896	0.9756034	0.9917549	-6.23094368
WT after UVB irradiation VS WT before UVB irradiation	GO_DIACYLGLYCEROL_METABOLIC_PROCESS2	-0.06960838	-0.01552074	-0.30594984	0.7673609	0.94123239	-5.94697625
homo after UVB irradiation VS homo before UVB irradiation	GO_DIACYLGLYCEROL_O_ACYLTRANSFERASE_ACTIVITY	1.060188085	0.005612776	5.924490975	0.00030096	0.01276035	0.774530461
hetero after UVB irradiation VS hetero before UVB irradiation	GO_DIACYLGLYCEROL_O_ACYLTRANSFERASE_ACTIVITY1	0.812277932	0.067559768	2.406450959	0.0433013	0.2785349	-3.90830586
WT after UVB irradiation VS WT before UVB irradiation	GO_DIACYLGLYCEROL_O_ACYLTRANSFERASE_ACTIVITY2	0.784514025	0.083716981	2.658334724	0.02857596	0.44482637	-3.37701515
homo after UVB irradiation VS homo before UVB irradiation	GO_DIADENOSINE_POLYPHOSPHATE_CATABOLIC_PROCESS	0.009678743	-0.03164007	0.054599735	0.95774433	0.97788923	-6.54922091
hetero after UVB irradiation VS hetero before UVB irradiation	GO_DIADENOSINE_POLYPHOSPHATE_CATABOLIC_PROCESS1	0.008922018	-0.02539475	0.032980201	0.97451323	0.99133797	-6.23089429
WT after UVB irradiation VS WT before UVB irradiation	GO_DIADENOSINE_POLYPHOSPHATE_CATABOLIC_PROCESS2	0.05514982	-0.07871876	0.188850406	0.85485743	0.9663792	-5.97729211
homo after UVB irradiation VS homo before UVB irradiation	GO_DIADENOSINE_POLYPHOSPHATE_METABOLIC_PROCESS	-0.28145106	0.007907563	-1.80750578	0.10682418	0.2611238	-5.04373047
hetero after UVB irradiation VS hetero before UVB irradiation	GO_DIADENOSINE_POLYPHOSPHATE_METABOLIC_PROCESS1	-0.20679625	-0.04655408	-0.79780876	0.4484325	0.74180859	-5.90008426
WT after UVB irradiation VS WT before UVB irradiation	GO_DIADENOSINE_POLYPHOSPHATE_METABOLIC_PROCESS2	-0.06045078	-0.01492666	-0.27754162	0.78831836	0.9469715	-5.95562584
homo after UVB irradiation VS homo before UVB irradiation	GO_DIAPEDESIS	0.484492784	-0.00531078	2.414978519	0.04101601	0.14581385	-4.1400285
hetero after UVB irradiation VS hetero before UVB irradiation	GO_DIAPEDESIS1	0.596141914	-0.01575504	3.356278453	0.01025986	0.15907272	-2.52756189
WT after UVB irradiation VS WT before UVB irradiation	GO_DIAPEDESIS2	0.364148869	-0.1086592	1.273953253	0.23802523	0.72863458	-5.22011522
homo after UVB irradiation VS homo before UVB irradiation	GO_DIAPHRAGM_DEVELOPMENT	-0.23422134	0.091322833	-1.52709551	0.16377309	0.3413373	-5.42548218
hetero after UVB irradiation VS hetero before UVB irradiation	GO_DIAPHRAGM_DEVELOPMENT1	-0.18538441	0.042227143	-1.31709712	0.22493952	0.55375502	-5.38490598
WT after UVB irradiation VS WT before UVB irradiation	GO_DIAPHRAGM_DEVELOPMENT2	-0.21372284	0.092152386	-0.96587539	0.36206289	0.79519319	-5.53175542
homo after UVB irradiation VS homo before UVB irradiation	GO_DICARBOXYLIC_ACID_BIOSYNTHETIC_PROCESS	-0.15009513	0.054240494	-0.72264381	0.48968923	0.66562641	-6.27305272
hetero after UVB irradiation VS hetero before UVB irradiation	GO_DICARBOXYLIC_ACID_BIOSYNTHETIC_PROCESS1	0.046083242	-0.02602912	0.15902007	0.87766671	0.95526929	-6.21779342
WT after UVB irradiation VS WT before UVB irradiation	GO_DICARBOXYLIC_ACID_BIOSYNTHETIC_PROCESS2	0.072919001	-0.07299322	0.317980854	0.75854506	0.93908133	-5.94306534
homo after UVB irradiation VS homo before UVB irradiation	GO_DICARBOXYLIC_ACID_CATABOLIC_PROCESS	0.431416088	-0.01597431	2.433835813	0.0398062	0.14323827	-4.11101743
hetero after UVB irradiation VS hetero before UVB irradiation	GO_DICARBOXYLIC_ACID_CATABOLIC_PROCESS1	-0.07200244	0.014156955	-0.29090584	0.77865852	0.91378123	-6.18584545
WT after UVB irradiation VS WT before UVB irradiation	GO_DICARBOXYLIC_ACID_CATABOLIC_PROCESS2	0.101324852	-0.01597508	0.363685484	0.72540514	0.93175969	-5.92687389
homo after UVB irradiation VS homo before UVB irradiation	GO_DICARBOXYLIC_ACID_METABOLIC_PROCESS	-0.22832195	0.014547616	-1.36668012	0.20747409	0.39761686	-5.6279652

hetero after UVB irradiation VS hetero before UVB irradiation	GO_DICARBOXYLIC_ACID_METABOLIC_PROCESS1	-0.26313707	-0.02289165	-1.62660776	0.143181	0.45851089	-5.00233121
WT after UVB irradiation VS WT before UVB irradiation	GO_DICARBOXYLIC_ACID_METABOLIC_PROCESS2	-0.0798764	-0.02876566	-0.4545864	0.66134541	0.91169919	-5.88847934
homo after UVB irradiation VS homo before UVB irradiation	GO_DICARBOXYLIC_ACID_TRANSMEMBRANE_TRANSPORTER_ACTIVITY	0.216451661	0.018238127	1.563503277	0.15507348	0.32964843	-5.37776214
hetero after UVB irradiation VS hetero before UVB irradiation	GO_DICARBOXYLIC_ACID_TRANSMEMBRANE_TRANSPORTER_ACTIVITY1	-0.19665362	-0.03717376	-1.09704609	0.30511969	0.62689374	-5.62595427
WT after UVB irradiation VS WT before UVB irradiation	GO_DICARBOXYLIC_ACID_TRANSMEMBRANE_TRANSPORTER_ACTIVITY2	-0.00615348	0.00906528	-0.03704895	0.97134305	0.99295046	-5.99534246
homo after UVB irradiation VS homo before UVB irradiation	GO_DICARBOXYLIC_ACID_TRANSPORT	0.088236768	0.011555534	0.705538442	0.49972827	0.67390022	-6.28567118
hetero after UVB irradiation VS hetero before UVB irradiation	GO_DICARBOXYLIC_ACID_TRANSPORT1	-0.09216646	0.002449068	-0.64378475	0.5380588	0.79532113	-6.01266729
WT after UVB irradiation VS WT before UVB irradiation	GO_DICARBOXYLIC_ACID_TRANSPORT2	-0.06001844	-0.0094499	-0.36466315	0.72470261	0.93154998	-5.92650458
homo after UVB irradiation VS homo before UVB irradiation	GO_DICHOTOMOUS_SUBDIVISION_OF_AN_EPITHELIAL_TERMINAL_UNIT	0.043009052	-0.03494369	0.176524758	0.86409856	0.92516161	-6.53378747
hetero after UVB irradiation VS hetero before UVB irradiation	GO_DICHOTOMOUS_SUBDIVISION_OF_AN_EPITHELIAL_TERMINAL_UNIT1	-0.16935607	-0.04198921	-0.89836	0.39570325	0.70336462	-5.81566952
WT after UVB irradiation VS WT before UVB irradiation	GO_DICHOTOMOUS_SUBDIVISION_OF_AN_EPITHELIAL_TERMINAL_UNIT2	-0.26169968	-0.01151748	-1.25541411	0.2443431	0.73383326	-5.24053205
homo after UVB irradiation VS homo before UVB irradiation	GO_DICHOTOMOUS_SUBDIVISION_OF_TERMINAL_UNITS_INVOLVED_IN_SALIVARY_GLAND_BRANCHING	0.373776435	0.022382477	1.554849028	0.15710293	0.33250357	-5.38916046
hetero after UVB irradiation VS hetero before UVB irradiation	GO_DICHOTOMOUS_SUBDIVISION_OF_TERMINAL_UNITS_INVOLVED_IN_SALIVARY_GLAND_BRANCHING1	-0.20218738	-0.03850927	-0.81583707	0.43863738	0.73626814	-5.88556128
WT after UVB irradiation VS WT before UVB irradiation	GO_DICHOTOMOUS_SUBDIVISION_OF_TERMINAL_UNITS_INVOLVED_IN_SALIVARY_GLAND_BRANCHING2	0.180912725	0.046335449	0.592006995	0.57000163	0.88382859	-5.81526548
homo after UVB irradiation VS homo before UVB irradiation	GO_DIENCEPHALON_DEVELOPMENT	-0.05051025	0.024284234	-0.41198859	0.69074682	0.81462086	-6.458658
hetero after UVB irradiation VS hetero before UVB irradiation	GO_DIENCEPHALON_DEVELOPMENT1	0.028333258	0.065294672	0.213181906	0.83661538	0.93734913	-6.20691182
WT after UVB irradiation VS WT before UVB irradiation	GO_DIENCEPHALON_DEVELOPMENT2	0.003632717	0.029002338	0.022440134	0.98263998	0.996184	-5.99580097
homo after UVB irradiation VS homo before UVB irradiation	GO_DIET_INDUCED_THERMOGENESIS	0.100791849	-0.03473262	0.814314033	0.43812439	0.62107605	-6.20096504
hetero after UVB irradiation VS hetero before UVB irradiation	GO_DIET_INDUCED_THERMOGENESIS1	0.011916638	0.030104158	0.085375369	0.93409952	0.97579263	-6.22753315
WT after UVB irradiation VS WT before UVB irradiation	GO_DIET_INDUCED_THERMOGENESIS2	0.125895178	0.014933744	0.51111605	0.62291075	0.90175221	-5.86053217
homo after UVB irradiation VS homo before UVB irradiation	GO_DIGESTION	0.229223048	0.063671687	1.722369376	0.12180863	0.28388028	-5.16286686
hetero after UVB irradiation VS hetero before UVB irradiation	GO_DIGESTION1	0.066862845	0.040593978	0.476543949	0.64666466	0.85503438	-6.11014243
WT after UVB irradiation VS WT before UVB irradiation	GO_DIGESTION2	0.17626552	0.046967329	1.026957762	0.33413649	0.77844475	-5.47496899
homo after UVB irradiation VS homo before UVB irradiation	GO_DIGESTIVE_SYSTEM_DEVELOPMENT	-0.06800059	0.016290962	-0.48497956	0.64018892	0.77943601	-6.42359595
hetero after UVB irradiation VS hetero before UVB irradiation	GO_DIGESTIVE_SYSTEM_DEVELOPMENT1	-0.06405531	0.033968724	-0.4994882	0.63112713	0.84801601	-6.09836902
WT after UVB irradiation VS WT before UVB irradiation	GO_DIGESTIVE_SYSTEM_DEVELOPMENT2	-0.14251569	0.009864324	-1.01990326	0.33727504	0.78034487	-5.48166286
homo after UVB irradiation VS homo before UVB irradiation	GO_DIGESTIVE_SYSTEM_PROCESS	0.249152932	0.057104996	1.834868254	0.10238721	0.254365	-5.0049196
hetero after UVB irradiation VS hetero before UVB irradiation	GO_DIGESTIVE_SYSTEM_PROCESS1	0.097617195	0.033903048	0.733739447	0.48443522	0.76305786	-5.94945523
WT after UVB irradiation VS WT before UVB irradiation	GO_DIGESTIVE_SYSTEM_PROCESS2	0.165149948	0.046550756	0.945568071	0.37172514	0.80104807	-5.55003544
homo after UVB irradiation VS homo before UVB irradiation	GO_DIGESTIVE_TRACT_MORPHOGENESIS	-0.33601709	0.027700998	-2.17389823	0.06012423	0.18460927	-4.50721146
hetero after UVB irradiation VS hetero before UVB irradiation	GO_DIGESTIVE_TRACT_MORPHOGENESIS1	-0.271225	0.030566272	-2.006124	0.08042473	0.36432827	-4.48517162
WT after UVB irradiation VS WT before UVB irradiation	GO_DIGESTIVE_TRACT_MORPHOGENESIS2	-0.30093339	0.012053775	-2.00092959	0.07996967	0.56457304	-4.30309838
homo after UVB irradiation VS homo before UVB irradiation	GO_DIHYDROLIPOYL_DEHYDROGENASE_COMPLEX	-0.50759333	-0.0088746	-1.9632555	0.08380005	0.22492494	-4.81980289
hetero after UVB irradiation VS hetero before UVB irradiation	GO_DIHYDROLIPOYL_DEHYDROGENASE_COMPLEX1	-0.73736841	-0.07466633	-4.52865952	0.00202102	0.10427325	-0.96511755
WT after UVB irradiation VS WT before UVB irradiation	GO_DIHYDROLIPOYL_DEHYDROGENASE_COMPLEX2	-0.38197044	-0.03108942	-1.82948405	0.10427046	0.60721285	-4.53511117
homo after UVB irradiation VS homo before UVB irradiation	GO_DIOL_BIOSYNTHETIC_PROCESS	0.51541139	-0.05113744	3.471398798	0.00791607	0.05919815	-2.5101713
hetero after UVB irradiation VS hetero before UVB irradiation	GO_DIOL_BIOSYNTHETIC_PROCESS1	0.260499724	-0.01042006	1.232235347	0.25347933	0.58122808	-5.48134464
WT after UVB irradiation VS WT before UVB irradiation	GO_DIOL_BIOSYNTHETIC_PROCESS2	0.546504131	-0.04603259	3.152261001	0.01334498	0.35652294	-2.68055467
homo after UVB irradiation VS homo before UVB irradiation	GO_DIOL_METABOLIC_PROCESS	0.626525915	-0.03962431	4.424901623	0.00200439	0.03035667	-1.12486756
hetero after UVB irradiation VS hetero before UVB irradiation	GO_DIOL_METABOLIC_PROCESS1	0.378173637	-0.00271712	1.726020867	0.12332772	0.42961096	-4.87101901
WT after UVB irradiation VS WT before UVB irradiation	GO_DIOL_METABOLIC_PROCESS2	0.545653183	-0.02620051	2.673088781	0.02792587	0.44213442	-3.35602688
homo after UVB irradiation VS homo before UVB irradiation	GO_DIOXYGENASE_ACTIVITY	0.070946365	-0.01561584	0.512114323	0.62187061	0.7653832	-6.40918626
hetero after UVB irradiation VS hetero before UVB irradiation	GO_DIOXYGENASE_ACTIVITY1	-0.07405715	-0.01677086	-0.56944052	0.58497338	0.82303782	-6.05930144
WT after UVB irradiation VS WT before UVB irradiation	GO_DIOXYGENASE_ACTIVITY2	-0.01158712	0.002683098	-0.07784406	0.93984127	0.9842223	-5.99287053
homo after UVB irradiation VS homo before UVB irradiation	GO_DIPEPTIDASE_ACTIVITY	0.519009772	-0.04607178	2.795646221	0.02245558	0.10433784	-3.55042711
hetero after UVB irradiation VS hetero before UVB irradiation	GO_DIPEPTIDASE_ACTIVITY1	0.079752343	0.017837449	0.415702286	0.68875506	0.87516315	-6.13882347
WT after UVB irradiation VS WT before UVB irradiation	GO_DIPEPTIDASE_ACTIVITY2	0.005183177	-0.08412847	0.018616165	0.98559784	0.99685342	-5.99588381
homo after UVB irradiation VS homo before UVB irradiation	GO_DIPEPTIDE_TRANSMEMBRANE_TRANSPORT	0.101772743	0.129106514	0.255862486	0.80426279	0.88919592	-6.5150711
hetero after UVB irradiation VS hetero before UVB irradiation	GO_DIPEPTIDE_TRANSMEMBRANE_TRANSPORT1	0.003432031	-0.03305423	0.011186016	0.99135399	0.99624062	-6.23141626
WT after UVB irradiation VS WT before UVB irradiation	GO_DIPEPTIDE_TRANSMEMBRANE_TRANSPORT2	0.069348358	-0.01671736	0.270145389	0.79380573	0.95041937	-5.95774245
homo after UVB irradiation VS homo before UVB irradiation	GO_DIPEPTIDYL_PEPTIDASE_ACTIVITY	0.417000598	-0.1143929	1.885709151	0.09460114	0.2429144	-4.93218478
hetero after UVB irradiation VS hetero before UVB irradiation	GO_DIPEPTIDYL_PEPTIDASE_ACTIVITY1	0.723360038	-0.02527744	3.771177442	0.00564493	0.13201411	-1.95045158
WT after UVB irradiation VS WT before UVB irradiation	GO_DIPEPTIDYL_PEPTIDASE_ACTIVITY2	0.39910046	-0.08095559	1.489462165	0.17424497	0.67974963	-4.96964815
homo after UVB irradiation VS homo before UVB irradiation	GO_DIPHOSPHOINOSITOL_POLYPHOSPHATE_METABOLIC_PROCESS	0.412740729	-0.09194391	1.792535697	0.10932749	0.26449617	-5.06486026
hetero after UVB irradiation VS hetero before UVB irradiation	GO_DIPHOSPHOINOSITOL_POLYPHOSPHATE_METABOLIC_PROCESS1	0.382310487	0.03678307	1.287731011	0.23449042	0.56254359	-5.41874233
WT after UVB irradiation VS WT before UVB irradiation	GO_DIPHOSPHOINOSITOL_POLYPHOSPHATE_METABOLIC_PROCESS2	0.424242329	-0.09672231	1.626250772	0.14209288	0.65167133	-4.79977028
homo after UVB irradiation VS homo before UVB irradiation	GO_DIPHOSPHOTRANSFERASE_ACTIVITY	-0.33546286	-0.01576788	-1.76382211	0.11428345	0.27193337	-5.10517625
hetero after UVB irradiation VS hetero before UVB irradiation	GO_DIPHOSPHOTRANSFERASE_ACTIVITY1	-0.23895306	-0.00980528	-1.07736722	0.31329696	0.63433176	-5.64600903
WT after UVB irradiation VS WT before UVB irradiation	GO_DIPHOSPHOTRANSFERASE_ACTIVITY2	-0.19863238	0.013541412	-0.77593126	0.45984923	0.83943532	-5.69032091
homo after UVB irradiation VS homo before UVB irradiation	GO_DIRECT_OSSIFICATION	0.155222294	-0.08772931	0.44027554	0.6709398	0.80173169	-6.44571529
hetero after UVB irradiation VS hetero before UVB irradiation	GO_DIRECT_OSSIFICATION1	-0.22049024	-0.05364068	-0.69039333	0.5098337	0.77951571	-5.98082332
WT after UVB irradiation VS WT before UVB irradiation	GO_DIRECT_OSSIFICATION2	-0.19143827	-0.04204918	-0.61766445	0.55376553	0.87677659	-5.79963975
homo after UVB irradiation VS homo before UVB irradiation	GO_DISACCHARIDE_BIOSYNTHETIC_PROCESS	0.590912607	0.025740425	3.289227889	0.01043567	0.0683833	-2.78751096
hetero after UVB irradiation VS hetero before UVB irradiation	GO_DISACCHARIDE_BIOSYNTHETIC_PROCESS1	0.612766352	0.098922872	3.443943784	0.00902561	0.15065833	-2.40370548
WT after UVB irradiation VS WT before UVB irradiation	GO_DISACCHARIDE_BIOSYNTHETIC_PROCESS2	0.387647186	0.089018259	1.642141131	0.13873067	0.64780591	-4.77956709
homo after UVB irradiation VS homo before UVB irradiation	GO_DISACCHARIDE_METABOLIC_PROCESS	0.456595638	0.0259328	2.644928178	0.02848453	0.11958641	-3.78454391
hetero after UVB irradiation VS hetero before UVB irradiation	GO_DISACCHARIDE_METABOLIC_PROCESS1	0.427403927	0.094940574	2.790836805	0.02395978	0.22165943	-3.34478717
WT after UVB irradiation VS WT before UVB irradiation	GO_DISACCHARIDE_METABOLIC_PROCESS2	0.224167375	0.056897398	1.082349857	0.31027146	0.76749122	-5.42121651
homo after UVB irradiation VS homo before UVB irradiation	GO_DISORDERED_DOMAIN_SPECIFIC_BINDING	-0.24456662	0.00253952	-1.82626106	0.10376386	0.2567477	-5.01715403
hetero after UVB irradiation VS hetero before UVB irradiation	GO_DISORDERED_DOMAIN_SPECIFIC_BINDING1	-0.11436709	-0.04083539	-0.77594528	0.46050988	0.74849092	-5.91732833
WT after UVB irradiation VS WT before UVB irradiation	GO_DISORDERED_DOMAIN_SPECIFIC_BINDING2	-0.06765699	-0.02805159	-0.40468261	0.69619	0.92251627	-5.91056887
homo after UVB irradiation VS homo before UVB irradiation	GO_DISTAL_AXON	0.198399486	-0.00253837	1.590097541	0.14898409	0.32218028	-5.34252551
hetero after UVB irradiation VS hetero before UVB irradiation	GO_DISTAL_AXON1	0.067079305	0.006601006	0.484125785	0.64150932	0.85287918	-6.10630947
WT after UVB irradiation VS WT before UVB irradiation	GO_DISTAL_AXON2	0.061738614	0.003959038	0.405010842	0.69595817	0.92243778	-5.91043159
homo after UVB irradiation VS homo before UVB irradiation	GO_DISTAL_CONVOLUTED_TUBULE_DEVELOPMENT	0.277020019	0.163469382	1.783906884	0.11079528	0.26676553	-5.07700541
hetero after UVB irradiation VS hetero before UVB irradiation	GO_DISTAL_CONVOLUTED_TUBULE_DEVELOPMENT1	0.083972242	0.137377869	0.269933755	0.79416916	0.91962333	-6.19215834
WT after UVB irradiation VS WT before UVB irradiation	GO_DISTAL_CONVOLUTED_TUBULE_DEVELOPMENT2	0.408017789	0.025786488	1.802291675	0.10871858	0.61267537	-4.57124772
homo after UVB irradiation VS homo before UVB irradiation	GO_DISTAL_TUBULE_DEVELOPMENT	-0.0939888	0.032715056	-0.45687848	0.65943819	0.79329334	-6.43773714
hetero after UVB irradiation VS hetero before UVB irradiation	GO_DISTAL_TUBULE_DEVELOPMENT1	-0.14733082	0.037718083	-0.89737361	0.39619794	0.70369237	-5.81653729
WT after UVB irradiation VS WT before UVB irradiation	GO_DISTAL_TUBULE_DEVELOPMENT2	-0.16218171	0.036756086	-0.92839806	0.38004253	0.80359153	-5.56525225
homo after UVB irradiation VS homo before UVB irradiation	GO_DISULFIDE_OXIDOREDUCTASE_ACTIVITY	0.073637144	-0.04061276	0.394399418	0.70319251	0.82287974	-6.46629024
hetero after UVB irradiation VS hetero before UVB irradiation	GO_DISULFIDE_OXIDOREDUCTASE_ACTIVITY1	0.339345118	0.01871771	2.280011182	0.05266699	0.30522584	-4.09251114
WT after UVB irradiation VS WT before UVB irradiation	GO_DISULFIDE_OXIDOREDUCTASE_ACTIVITY2	0.361402703	-0.02857673	1.802437848	0.1086942	0.61267537	-4.57105401
homo after UVB irradiation VS homo before UVB irradiation	GO_DITERPENOID_BIOSYNTHETIC_PROCESS	0.273770506	0.019028918	1.079439692	0.31065444	0.50477071	-5.95314416
hetero after UVB irradiation VS hetero before UVB irradiation	GO_DITERPENOID_BIOSYNTHETIC_PROCESS1	0.300091011	0.08045167	1.328239505	0.2214037	0.54895092	-5.37194308
WT after UVB irradiation VS WT before UVB irradiation	GO_DITERPENOID_BIOSYNTHETIC_PROCESS2	0.050917225	0.082902835	0.165221624	0.87281984	0.96914919	-5.98168846
homo after UVB irradiation VS homo before UVB irradiation	GO_DIVALENT_INORGANIC_ANION_HOMEOSTASIS	0.075503155	0.05891473	0.436819825	0.67334543	0.80335101	-6.44734047
hetero after UVB irradiation VS hetero before UVB irradiation	GO_DIVALENT_INORGANIC_ANION_HOMEOSTASIS1	0.021117987	0.05801788	0.111191962	0.91425354	0.96745438	-6.22478467
WT after UVB irradiation VS WT before UVB irradiation	GO_DIVALENT_INORGANIC_ANION_HOMEOSTASIS2	0.051153773	0.136794258	0.279626875	0.78677353	0.9468616	-5.95501897
homo after UVB irradiation VS homo before UVB irradiation	GO_DIVALENT_INORGANIC_CATION_HOMEOSTASIS	0.268015128	-0.01526731	2.01271723	0.07753894	0.21431222	-4.747294
hetero after UVB irradiation VS hetero before UVB irradiation	GO_DIVALENT_INORGANIC_CATION_HOMEOSTASIS1	0.129161284	0.015061544	0.868755899	0.41074527	0.71481981	-5.84137688
WT after UVB irradiation VS WT before UVB irradiation	GO_DIVALENT_INORGANIC_CATION_HOMEOSTASIS2	0.10576584	-0.01194898	0.516585624	0.61925371	0.90068273	-5.85766553
homo after UVB irradiation VS homo before UVB irradiation	GO_DIVALENT_INORGANIC_CATION_TRANSMEMBRANE_TRANSPORTER_ACTIVITY	0.115285822	0.005710379	0.921949143	0.38248274	0.57262444	-6.1071071
hetero after UVB irradiation VS hetero before UVB irradiation	GO_DIVALENT_INORGANIC_CATION_TRANSMEMBRANE_TRANSPORTER_ACTIVITY1	-0.03098259	0.02695397	-0.21453205	0.83559826	0.93666051	-6.20660053
WT after UVB irradiation VS WT before UVB irradiation	GO_DIVALENT_INORGANIC_CATION_TRANSMEMBRANE_TRANSPORTER_ACTIVITY2	0.051272477	0.024047031	0.317261305	0.75907128	0.93913078	-5.94330337
homo after UVB irradiation VS homo before UVB irradiation	GO_DIVALENT_INORGANIC_CATION_TRANSPORT	0.23162336	-0.00503686	1.813944642	0.1057641	0.25934876	-5.03461943
hetero after UVB irradiation VS hetero before UVB irradiation	GO_DIVALENT_INORGANIC_CATION_TRANSPORT1	0.083478906	0.015665408	0.544682943	0.60109275	0.83337583	-6.07366949
WT after UVB irradiation VS WT before UVB irradiation	GO_DIVALENT_INORGANIC_CATION_TRANSPORT2	0.096616978	-0.00213254	0.502626487	0.62860905	0.90390703	-5.86492514
homo after UVB irradiation VS homo before UVB irradiation	GO_DIVALENT_METAL_ION_EXPORT	-0.37519452	0.008712775	-2.24601157	0.0536324	0.17145868	-4.39822414
hetero after UVB irradiation VS hetero before UVB irradiation	GO_DIVALENT_METAL_ION_EXPORT1	-0.26599217	-0.03432916	-2.0284391	0.07770756	0.35877746	-4.45363213
WT after UVB irradiation VS WT before UVB irradiation	GO_DIVALENT_METAL_ION_EXPORT2	-0.15835747	0.001129191	-0.96120888	0.36426647	0.79568799	-5.53598297
homo after UVB irradiation VS homo before UVB irradiation	GO_DNA_APURINIC_OR_APYRIMIDINIC_SITE_ENDONUCLEASE_ACTIVITY	-0.43289474	-0.02512403	-2.64687441	0.02839698	0.11935465	-3.78152359
hetero after UVB irradiation VS hetero before UVB irradiation	GO_DNA_APURINIC_OR_APYRIMIDINIC_SITE_ENDONUCLEASE_ACTIVITY1	-0.19547832	-0.07297738	-0.73287566	0.48493322	0.76337374	-5.95009656
WT after UVB irradiation VS WT before UVB irradiation	GO_DNA_APURINIC_OR_APYRIMIDINIC_SITE_ENDONUCLEASE_ACTIVITY2	-0.18304903	-0.01931864	-0.57734916	0.57939771	0.88805924	-5.82392096
homo after UVB irradiation VS homo before UVB irradiation	GO_DNA_BINDING_BENDING	-0.17698991	0.020469735	-0.79046019	0.45117589	0.63270954	-6.22043616
hetero after UVB irradiation VS hetero before UVB irradiation	GO_DNA_BINDING_BENDING1	-0.00350303	0.011067089	-0.02049145	0.98416242	0.9941334	-6.2312564
WT after UVB irradiation VS WT before UVB irradiation	GO_DNA_BINDING_BENDING2	-0.02557323	-0.02259237	-0.11397037	0.91203643	0.97857624	-5.98921871
homo after UVB irradiation VS homo before UVB irradiation	GO_DNA_BINDING_TRANSCRIPTION_ACTIVATOR_ACTIVITY	-0.02802432	0.027486255	-0.20494688	0.84253363	0.91293551	-6.52786356
hetero after UVB irradiation VS hetero before UVB irradiation	GO_DNA_BINDING_TRANSCRIPTION_ACTIVATOR_ACTIVITY1	-0.02898395	0.021471428	-0.21915152	0.83212078	0.9354456	-6.20552079
WT after UVB irradiation VS WT before UVB irradiation	GO_DNA_BINDING_TRANSCRIPTION_ACTIVATOR_ACTIVITY2	-0.0719299	0.022485481	-0.5102773	0.62347254	0.90175221	-5.86096925
homo after UVB irradiation VS homo before UVB irradiation	GO_DNA_BINDING_TRANSCRIPTION_FACTOR_ACTIVITY	-0.07056993	0.02354968	-0.46594768	0.65319548	0.78906399	-6.43326067
hetero after UVB irradiation VS hetero before UVB irradiation	GO_DNA_BINDING_TRANSCRIPTION_FACTOR_ACTIVITY1	-0.029312	0.009958277	-0.22643552	0.8266455	0.93413454	-6.20377212
WT after UVB irradiation VS WT before UVB irradiation	GO_DNA_BINDING_TRANSCRIPTION_FACTOR_ACTIVITY2	-0.10598233	0.01824531	-0.72592754	0.48833079	0.8517724	-5.72720844
homo after UVB irradiation VS homo before UVB irradiation	GO_DNA_BINDING_TRANSCRIPTION_FACTOR_BINDING	-0.06646361	0.006010562	-0.47645223	0.6460007	0.78383755	-6.42797153
hetero after UVB irradiation VS hetero before UVB irradiation	GO_DNA_BINDING_TRANSCRIPTION_FACTOR_BINDING1	-0.06455533	-0.01049708	-0.5161307	0.61997769	0.84332543	-6.08950523
WT after UVB irradiation VS WT before UVB irradiation	GO_DNA_BINDING_TRANSCRIPTION_FACTOR_BINDING2	-0.0469519	-0.01690931	-0.32485153	0.75352718	0.93734883	-5.94076601
homo after UVB irradiation VS homo before UVB irradiation	GO_DNA_BINDING_TRANSCRIPTION_REPRESSOR_ACTIVITY	-0.02838011	0.002807765	-0.19257485	0.85190468	0.91816808	-6.53054974
hetero after UVB irradiation VS hetero before UVB irradiation	GO_DNA_BINDING_TRANSCRIPTION_REPRESSOR_ACTIVITY1	0.05771705	0.008808739	0.450005616	0.66486918	0.86258134	-6.12310881
WT after UVB irradiation VS WT before UVB irradiation	GO_DNA_BINDING_TRANSCRIPTION_REPRESSOR_ACTIVITY2	-0.07728873	0.007794828	-0.50119957	0.62956943	0.90404372	-5.86565675
homo after UVB irradiation VS homo before UVB irradiation	GO_DNA_BIOSYNTHETIC_PROCESS	-0.27217828	-0.00121261	-1.91245177	0.09073442	0.23638209	-4.89361764
hetero after UVB irradiation VS hetero before UVB irradiation	GO_DNA_BIOSYNTHETIC_PROCESS1	-0.00272999	-0.03300953	-0.01579914	0.98778866	0.99527548	-6.23134875
WT after UVB irradiation VS WT before UVB irradiation	GO_DNA_BIOSYNTHETIC_PROCESS2	0.00770731	0.022535852	0.038897991	0.96991363	0.99229361	-5.99526838
homo after UVB irradiation VS homo before UVB irradiation	GO_DNA_CATABOLIC_PROCESS	-0.07349196	0.016049031	-0.42481375	0.68173401	0.80826068	-6.45289168
homo after UVB irradiation VS homo before UVB irradiation	GO_DNA_CATABOLIC_PROCESS_ENDONUCLEOLYTIC	-0.12270159	0.011095954	-0.67069305	0.52057619	0.69028151	-6.3105453
hetero after UVB irradiation VS hetero before UVB irradiation	GO_DNA_CATABOLIC_PROCESS_ENDONUCLEOLYTIC1	-0.11351009	-0.02296033	-0.86825779	0.41100181	0.71492968	-5.84180343
WT after UVB irradiation VS WT before UVB irradiation	GO_DNA_CATABOLIC_PROCESS_ENDONUCLEOLYTIC2	0.010976167	-0.02882094	0.068669269	0.9469183	0.98594946	-5.99357931
homo after UVB irradiation VS homo before UVB irradiation	GO_DNA_CATABOLIC_PROCESS_EXONUCLEOLYTIC	-0.01293857	0.016657727	-0.07358031	0.94308077	0.96981673	-6.54788635
hetero after UVB irradiation VS hetero before UVB irradiation	GO_DNA_CATABOLIC_PROCESS_EXONUCLEOLYTIC1	0.353132501	0.014968853	2.622985553	0.03099493	0.24615776	-3.59096365
WT after UVB irradiation VS WT before UVB irradiation	GO_DNA_CATABOLIC_PROCESS_EXONUCLEOLYTIC2	0.452630854	-0.04646852	3.024107713	0.01622591	0.37790033	-2.85952925
hetero after UVB irradiation VS hetero before UVB irradiation	GO_DNA_CATABOLIC_PROCESS1	0.046241145	0.011120457	0.365228745	0.72458188	0.89176195	-6.15977506
WT after UVB irradiation VS WT before UVB irradiation	GO_DNA_CATABOLIC_PROCESS2	0.126609766	-0.01825798	0.849746419	0.41987823	0.82348328	-5.63207667
homo after UVB irradiation VS homo before UVB irradiation	GO_DNA_CLAMP_LOADER_ACTIVITY	-0.35870199	-0.03704885	-1.42180302	0.19142735	0.37747156	-5.55989029
hetero after UVB irradiation VS hetero before UVB irradiation	GO_DNA_CLAMP_LOADER_ACTIVITY1	0.234993245	-0.0106106	0.828385765	0.43190711	0.73171313	-5.8752922
WT after UVB irradiation VS WT before UVB irradiation	GO_DNA_CLAMP_LOADER_ACTIVITY2	0.11232306	0.023775449	0.355101854	0.73158492	0.93368824	-5.93007517
homo after UVB irradiation VS homo before UVB irradiation	GO_DNA_CONFORMATION_CHANGE	-0.51786657	-0.02230513	-3.40100129	0.008804	0.06249189	-2.61697042
hetero after UVB irradiation VS hetero before UVB irradiation	GO_DNA_CONFORMATION_CHANGE1	-0.09057761	-0.03892469	-0.40033128	0.69958239	0.88146568	-6.14547917
WT after UVB irradiation VS WT before UVB irradiation	GO_DNA_CONFORMATION_CHANGE2	-0.09443574	-0.00036637	-0.38706623	0.70868135	0.92758951	-5.91778014
homo after UVB irradiation VS homo before UVB irradiation	GO_DNA_CYTOSINE_DEAMINATION	0.95053003	0.011588278	5.55248235	0.00046787	0.01609396	0.336134657
hetero after UVB irradiation VS hetero before UVB irradiation	GO_DNA_CYTOSINE_DEAMINATION1	0.606280831	0.067094446	3.387857654	0.00979581	0.15626693	-2.48283695
WT after UVB irradiation VS WT before UVB irradiation	GO_DNA_CYTOSINE_DEAMINATION2	0.603270057	-0.0050967	2.321720103	0.04841351	0.50722877	-3.85529193
homo after UVB irradiation VS homo before UVB irradiation	GO_DNA_DAMAGE_INDUCED_PROTEIN_PHOSPHORYLATION	-0.21232954	-0.04269973	-1.05287895	0.32197906	0.51562073	-5.98041982
hetero after UVB irradiation VS hetero before UVB irradiation	GO_DNA_DAMAGE_INDUCED_PROTEIN_PHOSPHORYLATION1	-0.05295492	-0.00013809	-0.32513448	0.75357244	0.90404186	-6.17455232
WT after UVB irradiation VS WT before UVB irradiation	GO_DNA_DAMAGE_INDUCED_PROTEIN_PHOSPHORYLATION2	-0.14708305	0.058435282	-0.83521592	0.42754936	0.8274908	-5.64389132
homo after UVB irradiation VS homo before UVB irradiation	GO_DNA_DAMAGE_RESPONSE_DETECTION_OF_DNA_DAMAGE	-0.38013846	0.008079376	-2.04016143	0.07426201	0.20940535	-4.70680683
hetero after UVB irradiation VS hetero before UVB irradiation	GO_DNA_DAMAGE_RESPONSE_DETECTION_OF_DNA_DAMAGE1	0.24006148	-0.00152256	1.402218906	0.19912183	0.52337757	-5.28421043
WT after UVB irradiation VS WT before UVB irradiation	GO_DNA_DAMAGE_RESPONSE_DETECTION_OF_DNA_DAMAGE2	0.042261012	0.017197289	0.162831547	0.87464132	0.96914919	-5.98210072
homo after UVB irradiation VS homo before UVB irradiation	GO_DNA_DAMAGE_RESPONSE_SIGNAL_TRANSDUCTION_BY_P53_CLASS_MEDIATOR	-0.38070793	-0.00826634	-2.39513399	0.04232897	0.1484431	-4.17052199
hetero after UVB irradiation VS hetero before UVB irradiation	GO_DNA_DAMAGE_RESPONSE_SIGNAL_TRANSDUCTION_BY_P53_CLASS_MEDIATOR1	0.084558838	0.001613613	0.418256018	0.68696352	0.8742128	-6.13769448
WT after UVB irradiation VS WT before UVB irradiation	GO_DNA_DAMAGE_RESPONSE_SIGNAL_TRANSDUCTION_BY_P53_CLASS_MEDIATOR2	-0.14948	0.027705925	-0.68089514	0.51493259	0.86008604	-5.75859334
homo after UVB irradiation VS homo before UVB irradiation	GO_DNA_DAMAGE_RESPONSE_SIGNAL_TRANSDUCTION_RESULTING_IN_TRANSCRIPTION	-0.61928218	-0.02049622	-4.12119439	0.00306115	0.03652486	-1.55233505
hetero after UVB irradiation VS hetero before UVB irradiation	GO_DNA_DAMAGE_RESPONSE_SIGNAL_TRANSDUCTION_RESULTING_IN_TRANSCRIPTION1	-0.00411186	-0.04885154	-0.01687856	0.98695445	0.99497847	-6.23132962
WT after UVB irradiation VS WT before UVB irradiation	GO_DNA_DAMAGE_RESPONSE_SIGNAL_TRANSDUCTION_RESULTING_IN_TRANSCRIPTION2	-0.28451716	0.003992368	-1.00009998	0.34620622	0.78466675	-5.50026638

homo after UVB irradiation VS homo before UVB irradiation	GO_DNA_DEALKYLATION	-0.13393803	-0.01887	-1.0592715	0.31922459	0.51271193	-5.97390213
homo after UVB irradiation VS homo before UVB irradiation	GO_DNA_DEALKYLATION_INVOLVED_IN_DNA_REPAIR	0.089343194	-0.04692153	0.454757783	0.66090206	0.79440675	-6.43877183
hetero after UVB irradiation VS hetero before UVB irradiation	GO_DNA_DEALKYLATION_INVOLVED_IN_DNA_REPAIR1	0.35400448	0.009201456	1.971863761	0.08477678	0.37147425	-4.53339865
WT after UVB irradiation VS WT before UVB irradiation	GO_DNA_DEALKYLATION_INVOLVED_IN_DNA_REPAIR2	0.266070505	0.006324559	1.411240687	0.19541946	0.69389529	-5.06319755
hetero after UVB irradiation VS hetero before UVB irradiation	GO_DNA_DEALKYLATION1	-0.12044258	-0.00792423	-0.75609613	0.47166202	0.75543741	-5.93262963
WT after UVB irradiation VS WT before UVB irradiation	GO_DNA_DEALKYLATION2	-0.05611505	0.002741701	-0.31306556	0.76214235	0.94022192	-5.94468091
homo after UVB irradiation VS homo before UVB irradiation	GO_DNA_DEAMINATION	0.006092643	0.044235431	0.037318039	0.97111037	0.98524868	-6.55009248
hetero after UVB irradiation VS hetero before UVB irradiation	GO_DNA_DEAMINATION1	-0.17998618	0.104061237	-0.92745558	0.38131268	0.69355746	-5.78972947
WT after UVB irradiation VS WT before UVB irradiation	GO_DNA_DEAMINATION2	0.152097543	0.030229569	0.651788999	0.53259648	0.86897754	-5.77793179
homo after UVB irradiation VS homo before UVB irradiation	GO_DNA_DEMETHYLASE_ACTIVITY	0.270026764	-0.05354849	0.979929052	0.35472257	0.54644568	-6.05263772
hetero after UVB irradiation VS hetero before UVB irradiation	GO_DNA_DEMETHYLASE_ACTIVITY1	0.617532247	0.018832727	2.347107652	0.04746839	0.29051993	-3.99491544
WT after UVB irradiation VS WT before UVB irradiation	GO_DNA_DEMETHYLASE_ACTIVITY2	0.150071809	-0.03133251	0.795663838	0.44892041	0.83490988	-5.67518717
homo after UVB irradiation VS homo before UVB irradiation	GO_DNA_DEMETHYLATION	-0.15167714	-0.01784652	-1.25305043	0.24421855	0.43810847	-5.76284306
hetero after UVB irradiation VS hetero before UVB irradiation	GO_DNA_DEMETHYLATION1	-0.22566821	-0.00783535	-1.43936081	0.18869261	0.51443012	-5.2391195
WT after UVB irradiation VS WT before UVB irradiation	GO_DNA_DEMETHYLATION2	-0.21393882	-0.01069613	-1.26430345	0.2412964	0.73118354	-5.23076652
homo after UVB irradiation VS homo before UVB irradiation	GO_DNA_DEPENDENT_ATPASE_ACTIVITY	-0.66771789	-0.01337713	-3.94632781	0.00393018	0.0410237	-1.80461306
hetero after UVB irradiation VS hetero before UVB irradiation	GO_DNA_DEPENDENT_ATPASE_ACTIVITY1	-0.26978644	-0.06734795	-1.12545095	0.29361723	0.61756748	-5.59654656
WT after UVB irradiation VS WT before UVB irradiation	GO_DNA_DEPENDENT_ATPASE_ACTIVITY2	-0.18396053	-0.01341473	-0.69260437	0.50792998	0.85790577	-5.75060266
homo after UVB irradiation VS homo before UVB irradiation	GO_DNA_DEPENDENT_DNA_REPLICATION	-0.48533577	-0.04119881	-2.5044405	0.03558631	0.13499928	-4.00213022
homo after UVB irradiation VS homo before UVB irradiation	GO_DNA_DEPENDENT_DNA_REPLICATION_MAINTENANCE_OF_FIDELITY	-0.44639732	-0.03090665	-2.12920823	0.06452841	0.19256021	-4.57430322
hetero after UVB irradiation VS hetero before UVB irradiation	GO_DNA_DEPENDENT_DNA_REPLICATION_MAINTENANCE_OF_FIDELITY1	0.070333396	0.012705769	0.249882605	0.80909078	0.92574486	-6.19776066
WT after UVB irradiation VS WT before UVB irradiation	GO_DNA_DEPENDENT_DNA_REPLICATION_MAINTENANCE_OF_FIDELITY2	0.040822784	0.008448985	0.176532697	0.86421077	0.96790654	-5.97965655
hetero after UVB irradiation VS hetero before UVB irradiation	GO_DNA_DEPENDENT_DNA_REPLICATION1	0.046517804	-0.0098745	0.18918359	0.85474702	0.94469681	-6.21212043
WT after UVB irradiation VS WT before UVB irradiation	GO_DNA_DEPENDENT_DNA_REPLICATION2	0.053335935	0.02239997	0.212742238	0.83678601	0.96182256	-5.97225612
homo after UVB irradiation VS homo before UVB irradiation	GO_DNA_DIRECTED_DNA_POLYMERASE_ACTIVITY	-0.04372088	-0.02110883	-0.21651485	0.83379549	0.90730696	-6.5252023
hetero after UVB irradiation VS hetero before UVB irradiation	GO_DNA_DIRECTED_DNA_POLYMERASE_ACTIVITY1	0.153244754	-0.00218563	0.882396815	0.40376412	0.71080211	-5.82961837
WT after UVB irradiation VS WT before UVB irradiation	GO_DNA_DIRECTED_DNA_POLYMERASE_ACTIVITY2	0.304880646	-0.03470422	1.678253481	0.13135971	0.63700751	-4.7333209
homo after UVB irradiation VS homo before UVB irradiation	GO_DNA_DOUBLE_STRAND_BREAK_PROCESSING	-0.62237887	-0.01419375	-3.40470751	0.00875473	0.06226195	-2.61133547
hetero after UVB irradiation VS hetero before UVB irradiation	GO_DNA_DOUBLE_STRAND_BREAK_PROCESSING1	-0.20073466	-0.06342857	-0.80207922	0.44609888	0.74052375	-5.89666881
WT after UVB irradiation VS WT before UVB irradiation	GO_DNA_DOUBLE_STRAND_BREAK_PROCESSING2	-0.29361695	-0.01102114	-1.08626617	0.3086361	0.76668381	-5.41733753
homo after UVB irradiation VS homo before UVB irradiation	GO_DNA_ENDOREDUPLICATION	-0.97564248	0.001169399	-5.17586831	0.00074543	0.01907069	-0.12950382
hetero after UVB irradiation VS hetero before UVB irradiation	GO_DNA_ENDOREDUPLICATION1	0.077573956	0.059776433	0.175709702	0.86496838	0.94881427	-6.21477501
WT after UVB irradiation VS WT before UVB irradiation	GO_DNA_ENDOREDUPLICATION2	-0.3426625	0.001405635	-0.79433418	0.44965127	0.83496965	-5.67621706
homo after UVB irradiation VS homo before UVB irradiation	GO_DNA_GEOMETRIC_CHANGE	-0.64296436	-0.03070273	-3.90727086	0.00415827	0.04194363	-1.86154667
hetero after UVB irradiation VS hetero before UVB irradiation	GO_DNA_GEOMETRIC_CHANGE1	-0.18938548	-0.05651492	-0.78894097	0.45330489	0.74497075	-5.90712738
WT after UVB irradiation VS WT before UVB irradiation	GO_DNA_GEOMETRIC_CHANGE2	-0.14880487	-0.00777036	-0.5812408	0.57689461	0.8871242	-5.82164229
homo after UVB irradiation VS homo before UVB irradiation	GO_DNA_HELICASE_ACTIVITY	-0.67816358	-0.02425838	-3.81937982	0.00472483	0.04478121	-1.99042163
hetero after UVB irradiation VS hetero before UVB irradiation	GO_DNA_HELICASE_ACTIVITY1	-0.29391651	-0.07475528	-1.15559795	0.28179363	0.60750762	-5.56475376
WT after UVB irradiation VS WT before UVB irradiation	GO_DNA_HELICASE_ACTIVITY2	-0.13920912	-0.00126877	-0.48639517	0.63957736	0.90634429	-5.87313217
homo after UVB irradiation VS homo before UVB irradiation	GO_DNA_HELICASE_COMPLEX	-0.69982245	-0.01336981	-4.75127176	0.00129095	0.02423739	-0.68133372
hetero after UVB irradiation VS hetero before UVB irradiation	GO_DNA_HELICASE_COMPLEX1	-0.40676305	-0.00767806	-1.92562774	0.09101255	0.37831177	-4.5980818
WT after UVB irradiation VS WT before UVB irradiation	GO_DNA_HELICASE_COMPLEX2	0.056855254	0.049360838	0.192615427	0.85200327	0.96605575	-5.97653788
homo after UVB irradiation VS homo before UVB irradiation	GO_DNA_INSERTION_OR_DELETION_BINDING	-0.83520912	-0.03974683	-3.96563745	0.00382239	0.04047966	-1.77654301
hetero after UVB irradiation VS hetero before UVB irradiation	GO_DNA_INSERTION_OR_DELETION_BINDING1	-0.325762	-0.06726123	-1.06482347	0.31859903	0.63839403	-5.65865378
WT after UVB irradiation VS WT before UVB irradiation	GO_DNA_INSERTION_OR_DELETION_BINDING2	-0.25852653	-0.02099758	-0.71740261	0.49329807	0.85343701	-5.73328475
homo after UVB irradiation VS homo before UVB irradiation	GO_DNA_INTEGRATION	-0.13953944	-0.03929172	-0.51790722	0.61799511	0.76269068	-6.40601463
hetero after UVB irradiation VS hetero before UVB irradiation	GO_DNA_INTEGRATION1	0.409166081	0.010010017	1.528943383	0.16550557	0.48677492	-5.12770568
WT after UVB irradiation VS WT before UVB irradiation	GO_DNA_INTEGRATION2	0.037077498	0.048869103	0.154329425	0.88112716	0.9693507	-5.98351888
homo after UVB irradiation VS homo before UVB irradiation	GO_DNA_INTEGRITY_CHECKPOINT	-0.42318365	-0.01596587	-2.87380621	0.01986123	0.0971147	-3.42899423
hetero after UVB irradiation VS hetero before UVB irradiation	GO_DNA_INTEGRITY_CHECKPOINT1	-0.00214632	-0.02358216	-0.01075483	0.99168725	0.99634623	-6.23142139
WT after UVB irradiation VS WT before UVB irradiation	GO_DNA_INTEGRITY_CHECKPOINT2	-0.10990334	-0.01636181	-0.49238328	0.63551973	0.90550211	-5.87013382
homo after UVB irradiation VS homo before UVB irradiation	GO_DNA_LIGATION	-0.34226376	-0.00448558	-1.85496959	0.09923898	0.24918661	-4.97625636
homo after UVB irradiation VS homo before UVB irradiation	GO_DNA_LIGATION_INVOLVED_IN_DNA_REPAIR	-0.37833608	0.049629784	-1.45477495	0.18235523	0.36587574	-5.51839952
hetero after UVB irradiation VS hetero before UVB irradiation	GO_DNA_LIGATION_INVOLVED_IN_DNA_REPAIR1	-0.36973907	-0.07520643	-1.3476794	0.21534913	0.54268132	-5.3491666
WT after UVB irradiation VS WT before UVB irradiation	GO_DNA_LIGATION_INVOLVED_IN_DNA_REPAIR2	-0.46694906	-0.00701413	-1.4445191	0.18614882	0.68829819	-5.0237421
hetero after UVB irradiation VS hetero before UVB irradiation	GO_DNA_LIGATION1	-0.04567574	-0.10774029	-0.22354012	0.82882073	0.93461067	-6.20447398
WT after UVB irradiation VS WT before UVB irradiation	GO_DNA_LIGATION2	0.003010909	0.034193929	0.010233545	0.99208259	0.9985657	-5.99601141
homo after UVB irradiation VS homo before UVB irradiation	GO_DNA_METABOLIC_PROCESS	-0.37828788	-0.00958014	-2.81059602	0.02193369	0.10304852	-3.52719605
hetero after UVB irradiation VS hetero before UVB irradiation	GO_DNA_METABOLIC_PROCESS1	-0.04033401	-0.03020427	-0.22800292	0.82546862	0.93364073	-6.20338846
WT after UVB irradiation VS WT before UVB irradiation	GO_DNA_METABOLIC_PROCESS2	-0.02655679	0.007360513	-0.13174407	0.89840133	0.97448415	-5.98691889
homo after UVB irradiation VS homo before UVB irradiation	GO_DNA_METHYLATION	-0.2957001	0.028775887	-1.9805129	0.08156223	0.22099577	-4.79457372
homo after UVB irradiation VS homo before UVB irradiation	GO_DNA_METHYLATION_DEPENDENT_HETEROCHROMATIN_ASSEMBLY	-0.24780458	-0.02550729	-1.03075574	0.33165383	0.52468006	-6.00274364
hetero after UVB irradiation VS hetero before UVB irradiation	GO_DNA_METHYLATION_DEPENDENT_HETEROCHROMATIN_ASSEMBLY1	0.1374316	-0.0401371	0.647083409	0.53603056	0.79430276	-6.01047881
WT after UVB irradiation VS WT before UVB irradiation	GO_DNA_METHYLATION_DEPENDENT_HETEROCHROMATIN_ASSEMBLY2	-0.03690426	-0.0455388	-0.14927816	0.88498506	0.97059594	-5.98432565
homo after UVB irradiation VS homo before UVB irradiation	GO_DNA_METHYLATION_INVOLVED_IN_GAMETE_GENERATION	-0.12415346	0.097233883	-0.58019483	0.57713797	0.73363985	-6.3698176
hetero after UVB irradiation VS hetero before UVB irradiation	GO_DNA_METHYLATION_INVOLVED_IN_GAMETE_GENERATION1	-0.21551209	0.017634781	-1.27743047	0.23792122	0.56685999	-5.43049629
WT after UVB irradiation VS WT before UVB irradiation	GO_DNA_METHYLATION_INVOLVED_IN_GAMETE_GENERATION2	-0.12334173	0.015577807	-0.65140858	0.53282976	0.86902762	-5.77817956
homo after UVB irradiation VS homo before UVB irradiation	GO_DNA_METHYLATION_ON_CYTOSINE	0.465579964	-0.07407833	1.957871546	0.08451017	0.22621379	-4.82765815
hetero after UVB irradiation VS hetero before UVB irradiation	GO_DNA_METHYLATION_ON_CYTOSINE1	0.80072845	0.051678436	3.402408707	0.00958951	0.15528522	-2.46226941
WT after UVB irradiation VS WT before UVB irradiation	GO_DNA_METHYLATION_ON_CYTOSINE2	0.533374361	0.011735228	3.280895615	0.01098721	0.33778795	-2.50265694
homo after UVB irradiation VS homo before UVB irradiation	GO_DNA_METHYLATION_OR_DEMETHYLATION	-0.2684676	0.016717638	-2.01556263	0.07719281	0.21360102	-4.74310452
hetero after UVB irradiation VS hetero before UVB irradiation	GO_DNA_METHYLATION_OR_DEMETHYLATION1	-0.14939658	-0.01342899	-0.95722669	0.36699082	0.68091512	-5.76250973
WT after UVB irradiation VS WT before UVB irradiation	GO_DNA_METHYLATION_OR_DEMETHYLATION2	-0.12517769	-0.00227733	-0.71258955	0.49611675	0.85517988	-5.73668768
hetero after UVB irradiation VS hetero before UVB irradiation	GO_DNA_METHYLATION1	-0.15023696	-0.00407661	-0.90754421	0.3911187	0.70111625	-5.80755291
WT after UVB irradiation VS WT before UVB irradiation	GO_DNA_METHYLATION2	-0.10318694	0.004603302	-0.58197538	0.57642281	0.88690141	-5.82121059
homo after UVB irradiation VS homo before UVB irradiation	GO_DNA_METHYLTRANSFERASE_ACTIVITY	0.143900952	-0.01388398	0.456239511	0.6598791	0.79369434	-6.43804937
hetero after UVB irradiation VS hetero before UVB irradiation	GO_DNA_METHYLTRANSFERASE_ACTIVITY1	0.346198134	0.008625465	1.524733339	0.16653528	0.48782273	-5.13302184
WT after UVB irradiation VS WT before UVB irradiation	GO_DNA_METHYLTRANSFERASE_ACTIVITY2	0.490053454	-0.04881856	2.179920773	0.06046806	0.52788521	-4.05486219
homo after UVB irradiation VS homo before UVB irradiation	GO_DNA_MODIFICATION	-0.30170835	0.010517055	-2.43427637	0.03977837	0.14323827	-4.11033926
hetero after UVB irradiation VS hetero before UVB irradiation	GO_DNA_MODIFICATION1	-0.18653959	-0.02057096	-1.14917076	0.28428143	0.60954889	-5.57158126
WT after UVB irradiation VS WT before UVB irradiation	GO_DNA_MODIFICATION2	-0.05334163	0.00427344	-0.31532627	0.76048706	0.93919119	-5.9439409
homo after UVB irradiation VS homo before UVB irradiation	GO_DNA_N_GLYCOSYLASE_ACTIVITY	-0.53828777	-0.03284292	-3.5345512	0.00719981	0.05603773	-2.41480159
hetero after UVB irradiation VS hetero before UVB irradiation	GO_DNA_N_GLYCOSYLASE_ACTIVITY1	-0.383285	-0.08055893	-1.45607977	0.18415748	0.50859587	-5.2186054
WT after UVB irradiation VS WT before UVB irradiation	GO_DNA_N_GLYCOSYLASE_ACTIVITY2	0.084848988	0.011018876	0.360521384	0.72768068	0.93296251	-5.92806255
homo after UVB irradiation VS homo before UVB irradiation	GO_DNA_PACKAGING	-0.47850691	-0.00875843	-3.31879962	0.00997522	0.06669883	-2.7422823
homo after UVB irradiation VS homo before UVB irradiation	GO_DNA_PACKAGING_COMPLEX	-0.71694732	0.000463405	-3.58783599	0.00664876	0.05379154	-2.33467394
hetero after UVB irradiation VS hetero before UVB irradiation	GO_DNA_PACKAGING_COMPLEX1	-0.11423121	-0.08844937	-0.41013519	0.69266785	0.87711377	-6.14126174
WT after UVB irradiation VS WT before UVB irradiation	GO_DNA_PACKAGING_COMPLEX2	-0.22388105	-0.01882365	-0.69901732	0.50412017	0.85763155	-5.74617543
hetero after UVB irradiation VS hetero before UVB irradiation	GO_DNA_PACKAGING1	-0.04447153	-0.02408322	-0.1993687	0.84703983	0.941822	-6.20998494
WT after UVB irradiation VS WT before UVB irradiation	GO_DNA_PACKAGING2	-0.06765161	-0.00619603	-0.28220027	0.78486845	0.94603477	-5.9542639
homo after UVB irradiation VS homo before UVB irradiation	GO_DNA_POLYMERASE_ACTIVITY	-0.16414284	-0.03179028	-0.82947576	0.42996401	0.61421182	-6.18833359
hetero after UVB irradiation VS hetero before UVB irradiation	GO_DNA_POLYMERASE_ACTIVITY1	0.056071357	-0.01966235	0.279360961	0.78718436	0.91722367	-6.18937783
WT after UVB irradiation VS WT before UVB irradiation	GO_DNA_POLYMERASE_ACTIVITY2	0.208710355	-0.01395336	1.162285429	0.27822761	0.74917538	-5.34006478
homo after UVB irradiation VS homo before UVB irradiation	GO_DNA_POLYMERASE_BINDING	-0.65446954	-0.04662849	-2.77874174	0.02306109	0.10619627	-3.57669633
hetero after UVB irradiation VS hetero before UVB irradiation	GO_DNA_POLYMERASE_BINDING1	-0.11050275	-0.01909025	-0.43674042	0.67405951	0.86749613	-6.12932618
WT after UVB irradiation VS WT before UVB irradiation	GO_DNA_POLYMERASE_BINDING2	0.040816434	0.007183121	0.157139873	0.87898213	0.9693507	-5.98305845
homo after UVB irradiation VS homo before UVB irradiation	GO_DNA_POLYMERASE_COMPLEX	-0.31756939	-0.01078459	-1.21956836	0.25603404	0.45157097	-5.80109294
hetero after UVB irradiation VS hetero before UVB irradiation	GO_DNA_POLYMERASE_COMPLEX1	0.157792042	-0.05090295	0.741927336	0.47973119	0.76097951	-5.94334362
WT after UVB irradiation VS WT before UVB irradiation	GO_DNA_POLYMERASE_COMPLEX2	0.295635022	-0.0114907	1.539722784	0.16173952	0.66526114	-4.90810893
homo after UVB irradiation VS homo before UVB irradiation	GO_DNA_PROTECTION	-0.64929293	0.006895556	-3.79574244	0.00489086	0.04559347	-2.025256
hetero after UVB irradiation VS hetero before UVB irradiation	GO_DNA_PROTECTION1	-0.47941813	-0.06674913	-1.59032313	0.15113297	0.46900487	-5.04935679
WT after UVB irradiation VS WT before UVB irradiation	GO_DNA_PROTECTION2	-0.45746729	-0.01637904	-1.32193066	0.22231218	0.71742564	-5.16639764
homo after UVB irradiation VS homo before UVB irradiation	GO_DNA_RECOMBINASE_MEDIATOR_COMPLEX	-0.3824763	0.014343161	-2.24815298	0.05345058	0.17120964	-4.39497521
hetero after UVB irradiation VS hetero before UVB irradiation	GO_DNA_RECOMBINASE_MEDIATOR_COMPLEX1	-0.24446431	-0.07699505	-1.02702495	0.33500179	0.6548759	-5.69609126
WT after UVB irradiation VS WT before UVB irradiation	GO_DNA_RECOMBINASE_MEDIATOR_COMPLEX2	-0.35913325	-0.13237686	-2.31030295	0.04928857	0.50780915	-3.87142886
homo after UVB irradiation VS homo before UVB irradiation	GO_DNA_RECOMBINATION	-0.34381476	-0.0284031	-2.30937003	0.04850337	0.16155008	-4.30182099
hetero after UVB irradiation VS hetero before UVB irradiation	GO_DNA_RECOMBINATION1	0.039977555	-0.03028745	0.219693487	0.83171305	0.9354456	-6.20539262
WT after UVB irradiation VS WT before UVB irradiation	GO_DNA_RECOMBINATION2	0.128224311	0.011682703	0.689713694	0.50965317	0.85879879	-5.75258651
homo after UVB irradiation VS homo before UVB irradiation	GO_DNA_REPAIR	-0.4346744	-0.01837968	-3.1096822	0.01375346	0.07934316	-3.0635747
homo after UVB irradiation VS homo before UVB irradiation	GO_DNA_REPAIR_COMPLEX	-0.49289461	-0.03011352	-3.47038233	0.0079282	0.05920872	-2.51170979
homo after UVB irradiation VS homo before UVB irradiation	GO_DNA_REPAIR_COMPLEX_ASSEMBLY	-0.71522731	-0.02883739	-2.98115137	0.01679234	0.08852811	-3.26242177
hetero after UVB irradiation VS hetero before UVB irradiation	GO_DNA_REPAIR_COMPLEX_ASSEMBLY1	0.033761224	0.069354947	0.118647148	0.90853378	0.96579492	-6.22385705
WT after UVB irradiation VS WT before UVB irradiation	GO_DNA_REPAIR_COMPLEX_ASSEMBLY2	-0.3874092	-0.0216996	-1.54363916	0.16079975	0.66503175	-4.90326926
hetero after UVB irradiation VS hetero before UVB irradiation	GO_DNA_REPAIR_COMPLEX1	-0.17958425	-0.06842014	-0.85518457	0.41777575	0.72088233	-5.85292661
WT after UVB irradiation VS WT before UVB irradiation	GO_DNA_REPAIR_COMPLEX2	-0.1180016	0.046722685	-0.42333551	0.68307021	0.91771427	-5.90259859
hetero after UVB irradiation VS hetero before UVB irradiation	GO_DNA_REPAIR1	-0.02850103	-0.03265732	-0.15416294	0.88136956	0.95684882	-6.2186157
WT after UVB irradiation VS WT before UVB irradiation	GO_DNA_REPAIR2	-0.02098583	0.014134417	-0.09335317	0.92789146	0.98214275	-5.99147091
homo after UVB irradiation VS homo before UVB irradiation	GO_DNA_REPLICATION	-0.50306668	-0.03724535	-2.99610825	0.01640545	0.08765213	-3.23924271
homo after UVB irradiation VS homo before UVB irradiation	GO_DNA_REPLICATION_CHECKPOINT	-0.48468072	-0.03965187	-2.21307243	0.0565072	0.17699482	-4.44811078
hetero after UVB irradiation VS hetero before UVB irradiation	GO_DNA_REPLICATION_CHECKPOINT1	0.002880643	-0.00590485	0.008729341	0.99325277	0.99676536	-6.23144279
WT after UVB irradiation VS WT before UVB irradiation	GO_DNA_REPLICATION_CHECKPOINT2	-0.03498594	-0.01575454	-0.13761286	0.89390671	0.97322079	-5.98608676
homo after UVB irradiation VS homo before UVB irradiation	GO_DNA_REPLICATION_DEPENDENT_NUCLEOSOME_ORGANIZATION	-0.69834168	-0.03654001	-2.2461813	0.05361796	0.17144968	-4.39796665
hetero after UVB irradiation VS hetero before UVB irradiation	GO_DNA_REPLICATION_DEPENDENT_NUCLEOSOME_ORGANIZATION1	-0.23657468	-0.02043631	-0.68817932	0.51115318	0.78040107	-5.98238053
WT after UVB irradiation VS WT before UVB irradiation	GO_DNA_REPLICATION_DEPENDENT_NUCLEOSOME_ORGANIZATION2	0.132413063	0.026388019	0.435994705	0.67423052	0.91622321	-5.89699431
homo after UVB irradiation VS homo before UVB irradiation	GO_DNA_REPLICATION_FACTOR_A_COMPLEX	-0.37223024	-0.05019598	-1.93555041	0.08751621	0.2311161	-4.86014335
hetero after UVB irradiation VS hetero before UVB irradiation	GO_DNA_REPLICATION_FACTOR_A_COMPLEX1	-0.02618425	-0.03596681	-0.12995153	0.89987183	0.96269412	-6.22233614
WT after UVB irradiation VS WT before UVB irradiation	GO_DNA_REPLICATION_FACTOR_A_COMPLEX2	0.036804388	0.05041405	0.110259044	0.91488765	0.97967529	-5.98965709
homo after UVB irradiation VS homo before UVB irradiation	GO_DNA_REPLICATION_FACTOR_C_COMPLEX	-0.05547811	0.001693308	-0.16670585	0.87157762	0.92946038	-6.53563042
hetero after UVB irradiation VS hetero before UVB irradiation	GO_DNA_REPLICATION_FACTOR_C_COMPLEX1	0.797175454	-0.00990187	3.36491036	0.01013075	0.15834979	-2.51532479
WT after UVB irradiation VS WT before UVB irradiation	GO_DNA_REPLICATION_FACTOR_C_COMPLEX2	0.122497948	0.058745489	0.402147003	0.69798199	0.92319802	-5.91162578
homo after UVB irradiation VS homo before UVB irradiation	GO_DNA_REPLICATION_INDEPENDENT_NUCLEOSOME_ORGANIZATION	-0.58707742	-0.02815463	-2.48065495	0.03695511	0.1377543	-4.03885571
hetero after UVB irradiation VS hetero before UVB irradiation	GO_DNA_REPLICATION_INDEPENDENT_NUCLEOSOME_ORGANIZATION1	-0.08647231	-0.02206107	-0.29640956	0.7746052	0.91152166	-6.18411224
WT after UVB irradiation VS WT before UVB irradiation	GO_DNA_REPLICATION_INDEPENDENT_NUCLEOSOME_ORGANIZATION2	0.022797264	0.029045998	0.075860725	0.94137067	0.98458464	-5.99303126
homo after UVB irradiation VS homo before UVB irradiation	GO_DNA_REPLICATION_INITIATION	-0.51369928	-0.04460277	-2.00487119	0.07850116	0.21600627	-4.75883623
hetero after UVB irradiation VS hetero before UVB irradiation	GO_DNA_REPLICATION_INITIATION1	0.116924759	-0.01497208	0.421788112	0.68448911	0.87335247	-6.13612208
WT after UVB irradiation VS WT before UVB irradiation	GO_DNA_REPLICATION_INITIATION2	0.100946215	-0.02069208	0.379093936	0.71436552	0.92987478	-5.9209422
homo after UVB irradiation VS homo before UVB irradiation	GO_DNA_REPLICATION_ORIGIN_BINDING	-0.49573975	-0.04447966	-1.82534052	0.10391212	0.25697245	-5.0184611
hetero after UVB irradiation VS hetero before UVB irradiation	GO_DNA_REPLICATION_ORIGIN_BINDING1	0.024338213	-0.01782146	0.083509491	0.93553596	0.97618448	-6.22770389
WT after UVB irradiation VS WT before UVB irradiation	GO_DNA_REPLICATION_ORIGIN_BINDING2	0.014831641	-0.00782514	0.053311638	0.95877539	0.98927761	-5.99456729
homo after UVB irradiation VS homo before UVB irradiation	GO_DNA_REPLICATION_PREINITIATION_COMPLEX	-0.1911086	-0.13982152	-0.61334549	0.55602256	0.71711456	-6.3490114
hetero after UVB irradiation VS hetero before UVB irradiation	GO_DNA_REPLICATION_PREINITIATION_COMPLEX1	0.033980125	-0.09615798	0.109006391	0.91593138	0.96799239	-6.22504525
WT after UVB irradiation VS WT before UVB irradiation	GO_DNA_REPLICATION_PREINITIATION_COMPLEX2	0.416231359	0.042890288	1.519557826	0.16665687	0.67150908	-4.9329274
homo after UVB irradiation VS homo before UVB irradiation	GO_DNA_REPLICATION_SYNTHESIS_OF_RNA_PRIMER	-0.34994946	-0.00693198	-1.08204132	0.30956216	0.50382804	-5.95044506
hetero after UVB irradiation VS hetero before UVB irradiation	GO_DNA_REPLICATION_SYNTHESIS_OF_RNA_PRIMER1	0.301786988	0.044530146	0.832108508	0.42992436	0.7308714	-5.87222062
WT after UVB irradiation VS WT before UVB irradiation	GO_DNA_REPLICATION_SYNTHESIS_OF_RNA_PRIMER2	0.403676496	-0.02608154	1.2489027	0.24659518	0.73559835	-5.24765679
hetero after UVB irradiation VS hetero before UVB irradiation	GO_DNA_REPLICATION1	-0.020109	-0.02792848	-0.08938826	0.93101108	0.97454005	-6.22715321
WT after UVB irradiation VS WT before UVB irradiation	GO_DNA_REPLICATION2	-0.00458503	0.020239253	-0.01858723	0.98562022	0.99685342	-5.99588437
homo after UVB irradiation VS homo before UVB irradiation	GO_DNA_SECONDARY_STRUCTURE_BINDING	-0.55241747	-0.01368597	-2.97974141	0.0168293	0.08860149	-3.26460729
hetero after UVB irradiation VS hetero before UVB irradiation	GO_DNA_SECONDARY_STRUCTURE_BINDING1	-0.17377686	-0.04533499	-0.74446094	0.47828168	0.759973	-5.94144062
WT after UVB irradiation VS WT before UVB irradiation	GO_DNA_SECONDARY_STRUCTURE_BINDING2	-0.25277125	-0.05859556	-0.96897982	0.36060247	0.79382707	-5.52893418
homo after UVB irradiation VS homo before UVB irradiation	GO_DNA_STRAND_ELONGATION	-0.64482107	-0.08595579	-2.43455132	0.03976101	0.14323827	-4.10991601
homo after UVB irradiation VS homo before UVB irradiation	GO_DNA_STRAND_ELONGATION_INVOLVED_IN_DNA_REPLICATION	-0.57359595	-0.0709268	-2.16222869	0.061245	0.1866954	-4.52476576

hetero after UVB irradiation VS hetero before UVB irradiation	GO_DNA_STRAND_ELONGATION_INVOLVED_IN_DNA_REPLICATION1	0.274348385	-0.00079658	1.036569339	0.33079937	0.65066323	-5.6867334
WT after UVB irradiation VS WT before UVB irradiation	GO_DNA_STRAND_ELONGATION_INVOLVED_IN_DNA_REPLICATION2	0.165231204	0.093636666	0.565129682	0.58729668	0.89177209	-5.83098458
hetero after UVB irradiation VS hetero before UVB irradiation	GO_DNA_STRAND_ELONGATION1	0.098747123	-0.04354047	0.360870561	0.72771126	0.89256855	-6.16146178
WT after UVB irradiation VS WT before UVB irradiation	GO_DNA_STRAND_ELONGATION2	0.05749973	0.089855222	0.186571307	0.85658625	0.96655783	-5.97774151
homo after UVB irradiation VS homo before UVB irradiation	GO_DNA_STRAND_RENATURATION	-0.20387806	-0.02657398	-0.73249542	0.48396629	0.66104133	-6.26566466
hetero after UVB irradiation VS hetero before UVB irradiation	GO_DNA_STRAND_RENATURATION1	0.027171889	-0.03545808	0.112266872	0.91342851	0.96745438	-6.22465463
WT after UVB irradiation VS WT before UVB irradiation	GO_DNA_STRAND_RENATURATION2	0.18314393	0.029420976	0.634307126	0.54338001	0.87168809	-5.78918381
homo after UVB irradiation VS homo before UVB irradiation	GO_DNA_SYNTHESIS_INVOLVED_IN_DNA_REPAIR	-0.11443508	0.02103097	-0.68557095	0.51161	0.68324243	-6.30006205
hetero after UVB irradiation VS hetero before UVB irradiation	GO_DNA_SYNTHESIS_INVOLVED_IN_DNA_REPAIR1	0.361530667	-0.02842956	2.208449701	0.05883926	0.32014495	-4.19611074
WT after UVB irradiation VS WT before UVB irradiation	GO_DNA_SYNTHESIS_INVOLVED_IN_DNA_REPAIR2	0.260819415	0.013435385	1.309937129	0.22615512	0.71906685	-5.17994302
homo after UVB irradiation VS homo before UVB irradiation	GO_DNA_TEMPLATED_TRANSCRIPTION_ELONGATION	-0.69972033	0.007581599	-5.25695582	0.00067318	0.01827706	-0.027367
hetero after UVB irradiation VS hetero before UVB irradiation	GO_DNA_TEMPLATED_TRANSCRIPTION_ELONGATION1	-0.36005063	-0.06365129	-1.70722396	0.12687348	0.43356764	-4.89610987
WT after UVB irradiation VS WT before UVB irradiation	GO_DNA_TEMPLATED_TRANSCRIPTION_ELONGATION2	-0.3312676	-0.02239856	-1.18898046	0.2681423	0.74379247	-5.3120648
homo after UVB irradiation VS homo before UVB irradiation	GO_DNA_TEMPLATED_TRANSCRIPTION_INITIATION	-0.45046095	0.023645401	-3.10471858	0.01385949	0.07983003	-3.07123769
hetero after UVB irradiation VS hetero before UVB irradiation	GO_DNA_TEMPLATED_TRANSCRIPTION_INITIATION1	-0.28250836	-0.04878485	-1.92381609	0.09126567	0.37849236	-4.60060634
WT after UVB irradiation VS WT before UVB irradiation	GO_DNA_TEMPLATED_TRANSCRIPTION_INITIATION2	-0.25609761	-0.0244561	-1.29260644	0.23180789	0.72404374	-5.19937936
homo after UVB irradiation VS homo before UVB irradiation	GO_DNA_TEMPLATED_TRANSCRIPTION_TERMINATION	-0.73520247	0.002151456	-5.32397518	0.00061921	0.01780829	0.056267231
hetero after UVB irradiation VS hetero before UVB irradiation	GO_DNA_TEMPLATED_TRANSCRIPTION_TERMINATION1	-0.28928767	-0.08811659	-1.36426471	0.21029715	0.53576394	-5.32957617
WT after UVB irradiation VS WT before UVB irradiation	GO_DNA_TEMPLATED_TRANSCRIPTION_TERMINATION2	-0.22076548	-0.00304258	-0.73250659	0.48451942	0.85146975	-5.72247641
homo after UVB irradiation VS homo before UVB irradiation	GO_DNA_TOPOISOMERASE_ACTIVITY	-0.45933799	-0.09868584	-1.84721503	0.1004425	0.25122542	-4.9873288
hetero after UVB irradiation VS hetero before UVB irradiation	GO_DNA_TOPOISOMERASE_ACTIVITY1	-0.18769332	-0.17183983	-0.5687641	0.58541057	0.82332428	-6.0597018
WT after UVB irradiation VS WT before UVB irradiation	GO_DNA_TOPOISOMERASE_ACTIVITY2	-0.31118021	0.002706617	-0.89699433	0.39560612	0.81453088	-5.59250717
homo after UVB irradiation VS homo before UVB irradiation	GO_DNA_TOPOISOMERASE_BINDING	-0.75847584	0.028791359	-3.61232579	0.00641064	0.05293893	-2.29795558
hetero after UVB irradiation VS hetero before UVB irradiation	GO_DNA_TOPOISOMERASE_BINDING1	0.012552346	-0.03493058	0.033323389	0.97424813	0.99125262	-6.23088195
WT after UVB irradiation VS WT before UVB irradiation	GO_DNA_TOPOISOMERASE_BINDING2	-0.31111695	-0.03164426	-0.76603391	0.46539733	0.84295719	-5.6977896
homo after UVB irradiation VS homo before UVB irradiation	GO_DNA_TOPOLOGICAL_CHANGE	-0.50364421	-0.09830276	-1.91600312	0.09023239	0.23550749	-4.88848071
hetero after UVB irradiation VS hetero before UVB irradiation	GO_DNA_TOPOLOGICAL_CHANGE1	-0.15341375	-0.14504358	-0.46031335	0.65776925	0.86007701	-6.11815593
WT after UVB irradiation VS WT before UVB irradiation	GO_DNA_TOPOLOGICAL_CHANGE2	-0.26402501	0.035535027	-0.77056249	0.46285328	0.84111321	-5.69438243
homo after UVB irradiation VS homo before UVB irradiation	GO_DNA_TRANSLOCASE_ACTIVITY	-0.42046117	-0.01728686	-1.79795074	0.1084157	0.26316187	-5.05722573
hetero after UVB irradiation VS hetero before UVB irradiation	GO_DNA_TRANSLOCASE_ACTIVITY1	0.058768747	-0.00834655	0.177057696	0.86394452	0.94829797	-6.21451818
WT after UVB irradiation VS WT before UVB irradiation	GO_DNA_TRANSLOCASE_ACTIVITY2	0.124754755	0.015276503	0.32082358	0.75646743	0.93854182	-5.9421198
homo after UVB irradiation VS homo before UVB irradiation	GO_DNA_UNWINDING_INVOLVED_IN_DNA_REPLICATION	-0.68300716	-0.07275239	-2.89081274	0.01933885	0.09580645	-3.40258397
hetero after UVB irradiation VS hetero before UVB irradiation	GO_DNA_UNWINDING_INVOLVED_IN_DNA_REPLICATION1	-0.20171583	-0.02594864	-0.65869851	0.52892569	0.79049147	-6.00269328
WT after UVB irradiation VS WT before UVB irradiation	GO_DNA_UNWINDING_INVOLVED_IN_DNA_REPLICATION2	-0.16185002	0.018439265	-0.5693787	0.58454326	0.89002321	-5.82854411
homo after UVB irradiation VS homo before UVB irradiation	GO_DOLICHOL_METABOLIC_PROCESS	0.693442706	-0.00337633	2.739538399	0.02453063	0.11006341	-3.63761483
hetero after UVB irradiation VS hetero before UVB irradiation	GO_DOLICHOL_METABOLIC_PROCESS1	0.561180677	0.031448091	1.828974994	0.10550031	0.40257629	-4.73162901
WT after UVB irradiation VS WT before UVB irradiation	GO_DOLICHOL_METABOLIC_PROCESS2	0.556848802	0.025389458	2.000750881	0.07999191	0.56457304	-4.30334353
homo after UVB irradiation VS homo before UVB irradiation	GO_DOLICHYL_DIPHOSPHATE_BIOSYNTHETIC_PROCESS	0.636811464	-0.04312034	3.300727059	0.01025403	0.06775085	-2.76991458
hetero after UVB irradiation VS hetero before UVB irradiation	GO_DOLICHYL_DIPHOSPHATE_BIOSYNTHETIC_PROCESS1	0.640706703	0.050318184	2.356272373	0.04679943	0.28875901	-3.98155587
WT after UVB irradiation VS WT before UVB irradiation	GO_DOLICHYL_DIPHOSPHATE_BIOSYNTHETIC_PROCESS2	0.969473345	0.029097913	6.689161892	0.00014612	0.18842845	1.220802235
homo after UVB irradiation VS homo before UVB irradiation	GO_DOLICHYL_DIPHOSPHOOLIGOSACCHARIDE_PROTEIN_GLYCOTRANSFERASE_ACTIVITY	0.0962388	-0.0675066	0.434379695	0.67504648	0.80407304	-6.44848067
hetero after UVB irradiation VS hetero before UVB irradiation	GO_DOLICHYL_DIPHOSPHOOLIGOSACCHARIDE_PROTEIN_GLYCOTRANSFERASE_ACTIVITY1	0.177027882	-0.11739486	0.448982865	0.66557564	0.86302584	-6.12359445
WT after UVB irradiation VS WT before UVB irradiation	GO_DOLICHYL_DIPHOSPHOOLIGOSACCHARIDE_PROTEIN_GLYCOTRANSFERASE_ACTIVITY2	0.341723471	-0.01042522	1.001785036	0.34543933	0.78446163	-5.49869424
homo after UVB irradiation VS homo before UVB irradiation	GO_DOLICHYL_PHOSPHATE_MANNOSE_PROTEIN_MANNOSYLTRANSFERASE_ACTIVITY	-0.50105013	-0.04237291	-2.5114604	0.03519219	0.13455639	-3.99128375
hetero after UVB irradiation VS hetero before UVB irradiation	GO_DOLICHYL_PHOSPHATE_MANNOSE_PROTEIN_MANNOSYLTRANSFERASE_ACTIVITY1	-0.17960615	-0.07059321	-0.56849905	0.58558193	0.82335261	-6.05985856
WT after UVB irradiation VS WT before UVB irradiation	GO_DOLICHYL_PHOSPHATE_MANNOSE_PROTEIN_MANNOSYLTRANSFERASE_ACTIVITY2	-0.40833728	-0.01645729	-2.07417215	0.07134243	0.54915836	-4.20213534
homo after UVB irradiation VS homo before UVB irradiation	GO_DOPAMINE_BINDING	-0.03958345	0.074311495	-0.13819961	0.8933655	0.94187288	-6.54038696
hetero after UVB irradiation VS hetero before UVB irradiation	GO_DOPAMINE_BINDING1	0.127032708	0.163345771	0.571455159	0.58367233	0.82191128	-6.05810643
WT after UVB irradiation VS WT before UVB irradiation	GO_DOPAMINE_BINDING2	-0.01280816	0.067272325	-0.03938511	0.96953708	0.99229361	-5.99524826
homo after UVB irradiation VS homo before UVB irradiation	GO_DOPAMINE_BIOSYNTHETIC_PROCESS	0.15485347	0.005892625	0.575934047	0.57988421	0.73548357	-6.37241473
hetero after UVB irradiation VS hetero before UVB irradiation	GO_DOPAMINE_BIOSYNTHETIC_PROCESS1	-0.14112008	0.05548633	-0.60180692	0.56426912	0.81069215	-6.0396365
WT after UVB irradiation VS WT before UVB irradiation	GO_DOPAMINE_BIOSYNTHETIC_PROCESS2	0.14928771	0.006164711	0.570394502	0.58388607	0.89000397	-5.82795819
homo after UVB irradiation VS homo before UVB irradiation	GO_DOPAMINE_METABOLIC_PROCESS	0.173802716	-0.01610747	0.872178157	0.40754825	0.59452249	-6.15170784
hetero after UVB irradiation VS hetero before UVB irradiation	GO_DOPAMINE_METABOLIC_PROCESS1	0.073687153	0.060999818	0.515341498	0.62050407	0.84361764	-6.08993168
WT after UVB irradiation VS WT before UVB irradiation	GO_DOPAMINE_METABOLIC_PROCESS2	0.235760993	0.034795278	1.054456275	0.32211696	0.77346379	-5.44854628
homo after UVB irradiation VS homo before UVB irradiation	GO_DOPAMINE_NEUROTRANSMITTER_RECEPTOR_ACTIVITY	0.121141521	0.025677757	0.335826308	0.74530513	0.85087945	-6.48938468
hetero after UVB irradiation VS hetero before UVB irradiation	GO_DOPAMINE_NEUROTRANSMITTER_RECEPTOR_ACTIVITY1	-0.09490266	0.094675648	-0.35092466	0.73487324	0.89625316	-6.16523775
WT after UVB irradiation VS WT before UVB irradiation	GO_DOPAMINE_NEUROTRANSMITTER_RECEPTOR_ACTIVITY2	0.011217432	0.019409966	0.027995437	0.97834346	0.99444926	-5.99565314
homo after UVB irradiation VS homo before UVB irradiation	GO_DOPAMINE_RECEPTOR_BINDING	0.316191751	-0.01798248	2.152274144	0.06221722	0.18825461	-4.53972089
hetero after UVB irradiation VS hetero before UVB irradiation	GO_DOPAMINE_RECEPTOR_BINDING1	0.192940095	0.004732168	1.250967267	0.24693038	0.57435447	-5.46041447
WT after UVB irradiation VS WT before UVB irradiation	GO_DOPAMINE_RECEPTOR_BINDING2	0.299680486	-0.05417487	1.66352857	0.13432057	0.64200725	-4.75223268
homo after UVB irradiation VS homo before UVB irradiation	GO_DOPAMINE_RECEPTOR_SIGNALING_PATHWAY	0.192741856	-0.01257107	1.477155486	0.17641472	0.35770731	-5.48992003
hetero after UVB irradiation VS hetero before UVB irradiation	GO_DOPAMINE_RECEPTOR_SIGNALING_PATHWAY1	0.252687649	0.016776737	1.93341359	0.0899324	0.37657277	-4.58722343
WT after UVB irradiation VS WT before UVB irradiation	GO_DOPAMINE_RECEPTOR_SIGNALING_PATHWAY2	0.309471864	-0.01315605	1.613350211	0.14487714	0.65365874	-4.81610441
homo after UVB irradiation VS homo before UVB irradiation	GO_DOPAMINE_TRANSPORT	0.030467641	-0.03564088	0.166540462	0.87170371	0.92946038	-6.53566057
hetero after UVB irradiation VS hetero before UVB irradiation	GO_DOPAMINE_TRANSPORT1	-0.12064116	0.002484045	-0.72474969	0.48963436	0.76629239	-5.95609762
WT after UVB irradiation VS WT before UVB irradiation	GO_DOPAMINE_TRANSPORT2	-0.04770484	0.029915779	-0.22802294	0.82528089	0.95860343	-5.96872479
homo after UVB irradiation VS homo before UVB irradiation	GO_DOPAMINERGIC_NEURON_AXON_GUIDANCE	-0.52097377	0.016796802	-1.57462532	0.15250001	0.32662448	-5.36306399
hetero after UVB irradiation VS hetero before UVB irradiation	GO_DOPAMINERGIC_NEURON_AXON_GUIDANCE1	-0.53129223	-0.12666179	-3.42081808	0.0093351	0.15390918	-2.43628574
WT after UVB irradiation VS WT before UVB irradiation	GO_DOPAMINERGIC_NEURON_AXON_GUIDANCE2	-0.47302629	0.009928577	-2.17557135	0.06088136	0.52823908	-4.06094961
homo after UVB irradiation VS homo before UVB irradiation	GO_DOPAMINERGIC_NEURON_DIFFERENTIATION	-0.29126464	0.043087584	-2.31585978	0.04800631	0.16064152	-4.291916
hetero after UVB irradiation VS hetero before UVB irradiation	GO_DOPAMINERGIC_NEURON_DIFFERENTIATION1	-0.0895391	0.022548152	-0.5999364	0.56545407	0.81102647	-6.04079994
WT after UVB irradiation VS WT before UVB irradiation	GO_DOPAMINERGIC_NEURON_DIFFERENTIATION2	-0.30487519	0.003012095	-2.05155234	0.07390544	0.55617051	-4.23341654
homo after UVB irradiation VS homo before UVB irradiation	GO_DOPAMINERGIC_SYNAPSE	-0.3339469	0.005366899	-1.85173309	0.09973963	0.25014597	-4.98087989
hetero after UVB irradiation VS hetero before UVB irradiation	GO_DOPAMINERGIC_SYNAPSE1	-0.07921137	0.043853883	-0.60625809	0.56145515	0.80941342	-6.03685473
WT after UVB irradiation VS WT before UVB irradiation	GO_DOPAMINERGIC_SYNAPSE2	-0.53421294	0.032416568	-2.08357789	0.07030237	0.54682668	-4.18910328
homo after UVB irradiation VS homo before UVB irradiation	GO_DORSAL_AORTA_DEVELOPMENT	0.317396891	0.006945201	1.339627473	0.21576502	0.40617992	-5.66076023
hetero after UVB irradiation VS hetero before UVB irradiation	GO_DORSAL_AORTA_DEVELOPMENT1	0.237697517	-0.02659104	1.524366669	0.16662524	0.48782273	-5.13348449
WT after UVB irradiation VS WT before UVB irradiation	GO_DORSAL_AORTA_DEVELOPMENT2	0.491908178	0.067620622	2.636473076	0.02956779	0.45008842	-3.40812103
homo after UVB irradiation VS homo before UVB irradiation	GO_DORSAL_AORTA_MORPHOGENESIS	0.578160157	-2.17E-05	2.985025223	0.01669124	0.08835006	-3.25641751
hetero after UVB irradiation VS hetero before UVB irradiation	GO_DORSAL_AORTA_MORPHOGENESIS1	0.50435564	0.019551731	3.027093136	0.01674158	0.1902572	-3.00017915
WT after UVB irradiation VS WT before UVB irradiation	GO_DORSAL_AORTA_MORPHOGENESIS2	0.665873985	0.06922437	2.400447378	0.04278797	0.49624823	-3.74377832
homo after UVB irradiation VS homo before UVB irradiation	GO_DORSAL_ROOT_GANGLION_DEVELOPMENT	0.046537653	0.010573539	0.255818807	0.8042954	0.88919592	-6.51508326
hetero after UVB irradiation VS hetero before UVB irradiation	GO_DORSAL_ROOT_GANGLION_DEVELOPMENT1	-0.38769871	-0.01624405	-2.37607625	0.04538606	0.28450159	-3.95266652
WT after UVB irradiation VS WT before UVB irradiation	GO_DORSAL_ROOT_GANGLION_DEVELOPMENT2	-0.24971976	0.072397918	-1.50874739	0.16934786	0.67524339	-4.94616228
homo after UVB irradiation VS homo before UVB irradiation	GO_DORSAL_SPINAL_CORD_DEVELOPMENT	0.167323524	0.044994874	0.889100603	0.39889759	0.58771283	-6.13677137
hetero after UVB irradiation VS hetero before UVB irradiation	GO_DORSAL_SPINAL_CORD_DEVELOPMENT1	0.113087975	0.063505729	0.480118459	0.64423158	0.85438728	-6.10834245
WT after UVB irradiation VS WT before UVB irradiation	GO_DORSAL_SPINAL_CORD_DEVELOPMENT2	-0.01850119	0.018730875	-0.07704098	0.94046052	0.98449214	-5.99293611
homo after UVB irradiation VS homo before UVB irradiation	GO_DORSAL_VENTRAL_AXIS_SPECIFICATION	-0.45852977	0.042297608	-1.81010133	0.10639566	0.26024876	-5.04005939
hetero after UVB irradiation VS hetero before UVB irradiation	GO_DORSAL_VENTRAL_AXIS_SPECIFICATION1	-0.27169947	-0.07383742	-0.96509658	0.36327282	0.67787867	-5.75520164
WT after UVB irradiation VS WT before UVB irradiation	GO_DORSAL_VENTRAL_AXIS_SPECIFICATION2	-0.29439384	0.025061548	-1.05823376	0.32049242	0.77336431	-5.44487598
homo after UVB irradiation VS homo before UVB irradiation	GO_DORSAL_VENTRAL_NEURAL_TUBE_PATTERNING	-0.2459706	-0.01141254	-1.63322844	0.13956756	0.30935713	-5.28472541
hetero after UVB irradiation VS hetero before UVB irradiation	GO_DORSAL_VENTRAL_NEURAL_TUBE_PATTERNING1	-0.08525368	0.011119303	-0.43882941	0.67260829	0.86681615	-6.1283588
WT after UVB irradiation VS WT before UVB irradiation	GO_DORSAL_VENTRAL_NEURAL_TUBE_PATTERNING2	-0.22597339	0.011996901	-1.24006426	0.24967982	0.73564768	-5.25728893
homo after UVB irradiation VS homo before UVB irradiation	GO_DORSAL_VENTRAL_PATTERN_FORMATION	-0.15255036	0.035603348	-1.18649227	0.26816524	0.46338577	-5.83817441
hetero after UVB irradiation VS hetero before UVB irradiation	GO_DORSAL_VENTRAL_PATTERN_FORMATION1	-0.09812696	0.054083984	-0.66165055	0.52712915	0.78995796	-6.00069486
WT after UVB irradiation VS WT before UVB irradiation	GO_DORSAL_VENTRAL_PATTERN_FORMATION2	-0.13035534	0.030221004	-0.86966119	0.40952229	0.8186051	-5.61561159
homo after UVB irradiation VS homo before UVB irradiation	GO_DOSAGE_COMPENSATION	-0.53957985	0.006698142	-2.53461517	0.03392316	0.13177257	-3.95548441
hetero after UVB irradiation VS hetero before UVB irradiation	GO_DOSAGE_COMPENSATION1	-0.20775168	-0.03088631	-0.9140045	0.38791712	0.69807916	-5.80180392
WT after UVB irradiation VS WT before UVB irradiation	GO_DOSAGE_COMPENSATION2	-0.48938517	-0.07198816	-1.79359685	0.11017821	0.61521034	-4.58275933
homo after UVB irradiation VS homo before UVB irradiation	GO_DOUBLE_STRAND_BREAK_REPAIR	-0.39124962	-0.01580188	-2.54830245	0.03319485	0.12986649	-3.93430726
homo after UVB irradiation VS homo before UVB irradiation	GO_DOUBLE_STRAND_BREAK_REPAIR_INVOLVED_IN_MEIOTIC_RECOMBINATION	-0.6415309	-0.06829512	-2.08626528	0.06905662	0.20104262	-4.6384106
hetero after UVB irradiation VS hetero before UVB irradiation	GO_DOUBLE_STRAND_BREAK_REPAIR_INVOLVED_IN_MEIOTIC_RECOMBINATION1	-0.12782568	-0.00613363	-0.38723582	0.70886517	0.88509177	-6.15095985
WT after UVB irradiation VS WT before UVB irradiation	GO_DOUBLE_STRAND_BREAK_REPAIR_INVOLVED_IN_MEIOTIC_RECOMBINATION2	-0.31192352	-0.02810313	-1.33305377	0.21879811	0.71478521	-5.15376707
homo after UVB irradiation VS homo before UVB irradiation	GO_DOUBLE_STRAND_BREAK_REPAIR_VIA_BREAK_INDUCED_REPLICATION	-0.49702001	-0.10350624	-1.69155343	0.1276978	0.29261436	-5.20534375
hetero after UVB irradiation VS hetero before UVB irradiation	GO_DOUBLE_STRAND_BREAK_REPAIR_VIA_BREAK_INDUCED_REPLICATION1	0.438661715	0.028334378	1.555494811	0.15914281	0.47924721	-5.09400481
WT after UVB irradiation VS WT before UVB irradiation	GO_DOUBLE_STRAND_BREAK_REPAIR_VIA_BREAK_INDUCED_REPLICATION2	0.277351434	0.056758302	1.134369837	0.28910122	0.75486529	-5.36887118
homo after UVB irradiation VS homo before UVB irradiation	GO_DOUBLE_STRAND_BREAK_REPAIR_VIA_CLASSICAL_NONHOMOLOGOUS_END_JOINING	-0.75463496	-0.00200337	-3.49091728	0.00768699	0.05835229	-2.48064996
hetero after UVB irradiation VS hetero before UVB irradiation	GO_DOUBLE_STRAND_BREAK_REPAIR_VIA_CLASSICAL_NONHOMOLOGOUS_END_JOINING1	-0.64046978	-0.05796082	-2.43413406	0.0414855	0.27438818	-3.86782871
WT after UVB irradiation VS WT before UVB irradiation	GO_DOUBLE_STRAND_BREAK_REPAIR_VIA_CLASSICAL_NONHOMOLOGOUS_END_JOINING2	-0.42047073	-0.00847832	-1.35747091	0.21125076	0.70891132	-5.12581446
homo after UVB irradiation VS homo before UVB irradiation	GO_DOUBLE_STRAND_BREAK_REPAIR_VIA_SINGLE_STRAND_ANNEALING	-0.51507021	-0.04334566	-2.10305594	0.06725037	0.19800754	-4.61338885
hetero after UVB irradiation VS hetero before UVB irradiation	GO_DOUBLE_STRAND_BREAK_REPAIR_VIA_SINGLE_STRAND_ANNEALING1	-0.1467062	-0.08457605	-0.48340354	0.64199953	0.85314707	-6.10667705
WT after UVB irradiation VS WT before UVB irradiation	GO_DOUBLE_STRAND_BREAK_REPAIR_VIA_SINGLE_STRAND_ANNEALING2	-0.04906528	0.080232413	-0.17189668	0.86773707	0.96790654	-5.9805055
homo after UVB irradiation VS homo before UVB irradiation	GO_DOUBLE_STRAND_BREAK_REPAIR_VIA_SYNTHESIS_DEPENDENT_STRAND_ANNEALING	-0.71581846	-0.03069345	-3.64108131	0.00614254	0.05183233	-2.25493093
hetero after UVB irradiation VS hetero before UVB irradiation	GO_DOUBLE_STRAND_BREAK_REPAIR_VIA_SYNTHESIS_DEPENDENT_STRAND_ANNEALING1	-0.06577987	0.066977986	-0.2094721	0.83941178	0.93872655	-6.20775715
WT after UVB irradiation VS WT before UVB irradiation	GO_DOUBLE_STRAND_BREAK_REPAIR_VIA_SYNTHESIS_DEPENDENT_STRAND_ANNEALING2	-0.20541567	-0.03594704	-0.79603866	0.44871453	0.83490988	-5.67489658
hetero after UVB irradiation VS hetero before UVB irradiation	GO_DOUBLE_STRAND_BREAK_REPAIR1	0.043408524	-0.01620916	0.222843575	0.82934427	0.93464793	-6.20464149
WT after UVB irradiation VS WT before UVB irradiation	GO_DOUBLE_STRAND_BREAK_REPAIR2	0.081827064	0.022700124	0.376211273	0.71642552	0.93059023	-5.92206996
homo after UVB irradiation VS homo before UVB irradiation	GO_DOUBLE_STRANDED_METHYLATED_DNA_BINDING	-0.3963099	0.043300747	-1.52819362	0.16350443	0.34113317	-5.42405186
hetero after UVB irradiation VS hetero before UVB irradiation	GO_DOUBLE_STRANDED_METHYLATED_DNA_BINDING1	-0.85067493	0.017155867	-5.46031305	0.00064082	0.08125048	0.113629948
WT after UVB irradiation VS WT before UVB irradiation	GO_DOUBLE_STRANDED_METHYLATED_DNA_BINDING2	-0.55029508	0.030537528	-2.82712745	0.02197947	0.41478923	-3.13730405
homo after UVB irradiation VS homo before UVB irradiation	GO_DOUBLE_STRANDED_RNA_ADENOSINE_DEAMINASE_ACTIVITY	-0.04870592	0.09197176	-0.21537646	0.83465434	0.90762824	-6.5254706
hetero after UVB irradiation VS hetero before UVB irradiation	GO_DOUBLE_STRANDED_RNA_ADENOSINE_DEAMINASE_ACTIVITY1	-0.30974477	0.051694577	-1.23028426	0.25416973	0.58165765	-5.48351272
WT after UVB irradiation VS WT before UVB irradiation	GO_DOUBLE_STRANDED_RNA_ADENOSINE_DEAMINASE_ACTIVITY2	-0.01193703	-0.01822608	-0.04968832	0.96157453	0.99043451	-5.99476414
homo after UVB irradiation VS homo before UVB irradiation	GO_DOUBLE_STRANDED_RNA_BINDING	-0.21617474	-0.02992215	-1.68433479	0.12911482	0.29434647	-5.21524159
hetero after UVB irradiation VS hetero before UVB irradiation	GO_DOUBLE_STRANDED_RNA_BINDING1	-0.03252301	-0.01305233	-0.17386028	0.86637352	0.94936935	-6.2151242
WT after UVB irradiation VS WT before UVB irradiation	GO_DOUBLE_STRANDED_RNA_BINDING2	-0.00843242	-0.01707678	-0.04282174	0.96688072	0.99147098	-5.99509923
homo after UVB irradiation VS homo before UVB irradiation	GO_DOUBLE_STRANDED_TELOMERIC_DNA_BINDING	-0.76409863	-0.059698	-3.95136054	0.00390178	0.04086482	-1.79729209
hetero after UVB irradiation VS hetero before UVB irradiation	GO_DOUBLE_STRANDED_TELOMERIC_DNA_BINDING1	-0.57586353	-0.08307896	-2.16372002	0.06305665	0.32971788	-4.26055469
WT after UVB irradiation VS WT before UVB irradiation	GO_DOUBLE_STRANDED_TELOMERIC_DNA_BINDING2	-0.15635346	0.066340859	-0.41509479	0.68885268	0.92035234	-5.90616213
homo after UVB irradiation VS homo before UVB irradiation	GO_DRINKING_BEHAVIOR	0.229605751	-0.00598324	1.233662521	0.25100391	0.44552138	-5.78507803
hetero after UVB irradiation VS hetero before UVB irradiation	GO_DRINKING_BEHAVIOR1	0.214580933	0.073493278	1.139340469	0.28812093	0.61313996	-5.58197222
WT after UVB irradiation VS WT before UVB irradiation	GO_DRINKING_BEHAVIOR2	0.299317086	0.043075494	1.183887616	0.27004295	0.74431614	-5.31744027
homo after UVB irradiation VS homo before UVB irradiation	GO_DRUG_BINDING	-0.01840556	-0.01220524	-0.12718415	0.90181165	0.94665205	-6.54198788
hetero after UVB irradiation VS hetero before UVB irradiation	GO_DRUG_BINDING1	-0.03007458	0.007068137	-0.2023585	0.8447807	0.94052476	-6.20933703
WT after UVB irradiation VS WT before UVB irradiation	GO_DRUG_BINDING2	0.049064573	-0.0166228	0.287732002	0.78077854	0.94457689	-5.95261789
homo after UVB irradiation VS homo before UVB irradiation	GO_DRUG_CATABOLIC_PROCESS	-0.09662174	0.054772536	-0.51161699	0.62220391	0.76558114	-6.40945699
hetero after UVB irradiation VS hetero before UVB irradiation	GO_DRUG_CATABOLIC_PROCESS1	-0.14986764	0.010117352	-0.5590365	0.59171792	0.82683615	-6.06541103
WT after UVB irradiation VS WT before UVB irradiation	GO_DRUG_CATABOLIC_PROCESS2	-0.18919889	0.046236464	-0.77919265	0.45803073	0.83880233	-5.6878419
homo after UVB irradiation VS homo before UVB irradiation	GO_DRUG_METABOLIC_PROCESS	0.010187771	0.025220735	0.055346101	0.95716736	0.97763834	-6.54917589
hetero after UVB irradiation VS hetero before UVB irradiation	GO_DRUG_METABOLIC_PROCESS1	-0.29203466	-0.01615227	-1.46482947	0.1818228	0.50686486	-5.20781714
WT after UVB irradiation VS WT before UVB irradiation	GO_DRUG_METABOLIC_PROCESS2	-0.09055584	0.001299347	-0.36828075	0.7221055	0.93154998	-5.92512973
homo after UVB irradiation VS homo before UVB irradiation	GO_DRUG_TRANSMEMBRANE_TRANSPORT	0.556643427	-0.05273123	3.063759714	0.01476762	0.08257891	-3.13452482
hetero after UVB irradiation VS hetero before UVB irradiation	GO_DRUG_TRANSMEMBRANE_TRANSPORT1	-0.30007089	-0.0324415	-2.22173265	0.05764159	0.31725449	-4.17692472
WT after UVB irradiation VS WT before UVB irradiation	GO_DRUG_TRANSMEMBRANE_TRANSPORT2	0.396559707	-0.05870776	1.402376641	0.19795595	0.69702831	-5.07361801
homo after UVB irradiation VS homo before UVB irradiation	GO_DRUG_TRANSPORT	0.428393254	-0.00507144	2.677654625	0.02704812	0.11633608	-3.7337417
hetero after UVB irradiation VS hetero before UVB irradiation	GO_DRUG_TRANSPORT1	-0.36779041	-0.05947391	-2.23425192	0.05653495	0.31421081	-4.15882268
WT after UVB irradiation VS WT before UVB irradiation	GO_DRUG_TRANSPORT2	0.51051603	-0.03354553	2.152090877	0.06316103	0.53593918	-4.09377123
homo after UVB irradiation VS homo before UVB irradiation	GO_DTTP_DIPHOSPHATASE_ACTIVITY	-0.13056199	0.045413749	-0.43489126	0.6746897	0.80384271	-6.44824213
hetero after UVB irradiation VS hetero before UVB irradiation	GO_DTTP_DIPHOSPHATASE_ACTIVITY1	0.051328496	-0.06222097	0.201703193	0.84527572	0.94077349	-6.20947986
WT after UVB irradiation VS WT before UVB irradiation	GO_DTTP_DIPHOSPHATASE_ACTIVITY2	-0.18687874	-0.13052128	-0.84371457	0.42305086	0.82491361	-5.63700161
homo after UVB irradiation VS homo before UVB irradiation	GO_DUCTUS_ARTERIOSUS_CLOSURE	-0.36418432	-0.01559033	-1.42029553	0.19185141	0.37788991	-5.5617737

hetero after UVB irradiation VS hetero before UVB irradiation	GO_DUCTUS_ARTERIOSUS_CLOSURE1	-0.82247459	0.027236153	-4.50280111	0.00209022	0.10552127	-0.997174
WT after UVB irradiation VS WT before UVB irradiation	GO_DUCTUS_ARTERIOSUS_CLOSURE2	-0.76159646	0.030456013	-4.0717081	0.00348474	0.25923179	-1.46020623
homo after UVB irradiation VS homo before UVB irradiation	GO_DUMP_METABOLIC_PROCESS	-0.74438557	-0.03665306	-3.00895131	0.01608061	0.08661851	-3.21934682
hetero after UVB irradiation VS hetero before UVB irradiation	GO_DUMP_METABOLIC_PROCESS1	0.135595808	-0.0669216	0.595843659	0.56805177	0.81261967	-6.04333407
WT after UVB irradiation VS WT before UVB irradiation	GO_DUMP_METABOLIC_PROCESS2	0.54347239	0.036887742	2.10425945	0.06806724	0.54090914	-4.16039904
homo after UVB irradiation VS homo before UVB irradiation	GO_DYNACTIN_BINDING	-0.08723727	0.008842353	-0.43168006	0.67693073	0.80508276	-6.44973502
hetero after UVB irradiation VS hetero before UVB irradiation	GO_DYNACTIN_BINDING1	-0.2053971	0.013911258	-0.9907494	0.35135021	0.66793657	-5.73105822
WT after UVB irradiation VS WT before UVB irradiation	GO_DYNACTIN_BINDING2	-0.10585766	0.089503266	-0.48962089	0.63738996	0.9057274	-5.87152128
homo after UVB irradiation VS homo before UVB irradiation	GO_DYNACTIN_COMPLEX	0.29064999	-0.01052314	1.162570321	0.27722817	0.47318131	-5.8645452
hetero after UVB irradiation VS hetero before UVB irradiation	GO_DYNACTIN_COMPLEX1	-0.11901512	0.034666131	-0.60225025	0.56398849	0.81055567	-6.03936028
WT after UVB irradiation VS WT before UVB irradiation	GO_DYNACTIN_COMPLEX2	0.097980353	0.054917864	0.412879013	0.69041122	0.92104951	-5.90710888
homo after UVB irradiation VS homo before UVB irradiation	GO_DYNAMIN_FAMILY_PROTEIN_POLYMERIZATION_INVOLVED_IN_MEMBRANE_FISSION	0.385987242	-0.02342914	1.800640652	0.10796542	0.26283307	-5.05342963
hetero after UVB irradiation VS hetero before UVB irradiation	GO_DYNAMIN_FAMILY_PROTEIN_POLYMERIZATION_INVOLVED_IN_MEMBRANE_FISSION1	0.212674514	0.014655032	0.711818557	0.49717593	0.77054175	-5.96552718
WT after UVB irradiation VS WT before UVB irradiation	GO_DYNAMIN_FAMILY_PROTEIN_POLYMERIZATION_INVOLVED_IN_MEMBRANE_FISSION2	0.134065323	-0.15924593	0.486486534	0.63951536	0.90634429	-5.87308668
homo after UVB irradiation VS homo before UVB irradiation	GO_DYNEIN_COMPLEX	-0.12458387	0.009159118	-0.76608408	0.46478061	0.64394346	-6.23981967
homo after UVB irradiation VS homo before UVB irradiation	GO_DYNEIN_COMPLEX_BINDING	-0.45892125	0.030004732	-3.44253465	0.00826815	0.06041217	-2.55390029
hetero after UVB irradiation VS hetero before UVB irradiation	GO_DYNEIN_COMPLEX_BINDING1	-0.0863667	-0.009309	-0.42857913	0.67974307	0.87119134	-6.13306348
WT after UVB irradiation VS WT before UVB irradiation	GO_DYNEIN_COMPLEX_BINDING2	-0.20186356	0.056240943	-1.01723848	0.33846647	0.78064003	-5.48418234
hetero after UVB irradiation VS hetero before UVB irradiation	GO_DYNEIN_COMPLEX1	-0.16336434	-0.02036187	-0.902328	0.39371775	0.70256648	-5.81217091
WT after UVB irradiation VS WT before UVB irradiation	GO_DYNEIN_COMPLEX2	-0.19804726	0.007480208	-1.15541496	0.28087257	0.75166417	-5.3471999
homo after UVB irradiation VS homo before UVB irradiation	GO_DYNEIN_HEAVY_CHAIN_BINDING	-0.40505746	0.008815748	-3.11181949	0.01370806	0.07925813	-3.06027553
hetero after UVB irradiation VS hetero before UVB irradiation	GO_DYNEIN_HEAVY_CHAIN_BINDING1	-0.31939236	-0.00259274	-1.34655166	0.21569642	0.54299403	-5.35049342
WT after UVB irradiation VS WT before UVB irradiation	GO_DYNEIN_HEAVY_CHAIN_BINDING2	-0.32177463	-0.03431905	-1.58674269	0.15077745	0.6585056	-4.84959678
homo after UVB irradiation VS homo before UVB irradiation	GO_DYNEIN_INTERMEDIATE_CHAIN_BINDING	-0.33222613	0.036631261	-2.66351734	0.02765935	0.11759406	-3.75569103
hetero after UVB irradiation VS hetero before UVB irradiation	GO_DYNEIN_INTERMEDIATE_CHAIN_BINDING1	-0.42568126	-0.01445841	-2.14379663	0.06503005	0.33405943	-4.28917175
WT after UVB irradiation VS WT before UVB irradiation	GO_DYNEIN_INTERMEDIATE_CHAIN_BINDING2	-0.30556306	0.002135557	-1.86440846	0.09880974	0.59874718	-4.48841129
homo after UVB irradiation VS homo before UVB irradiation	GO_DYNEIN_LIGHT_CHAIN_BINDING	-0.67333019	-0.03774853	-4.04763855	0.00339862	0.03831433	-1.65792217
hetero after UVB irradiation VS hetero before UVB irradiation	GO_DYNEIN_LIGHT_CHAIN_BINDING1	-0.9451042	0.045466258	-5.70447648	0.00048379	0.07788488	0.372280067
WT after UVB irradiation VS WT before UVB irradiation	GO_DYNEIN_LIGHT_CHAIN_BINDING2	-0.5024495	-0.03193553	-2.19151621	0.0593797	0.52638053	-4.03862201
homo after UVB irradiation VS homo before UVB irradiation	GO_DYNEIN_LIGHT_INTERMEDIATE_CHAIN_BINDING	-0.21689489	0.018920586	-1.68424835	0.12913187	0.29434647	-5.21536
hetero after UVB irradiation VS hetero before UVB irradiation	GO_DYNEIN_LIGHT_INTERMEDIATE_CHAIN_BINDING1	-0.30818211	0.010030749	-1.56019652	0.15803945	0.47777713	-5.08800634
WT after UVB irradiation VS WT before UVB irradiation	GO_DYNEIN_LIGHT_INTERMEDIATE_CHAIN_BINDING2	-0.40783368	-0.00893613	-2.72288558	0.02584137	0.43581842	-3.28522657
homo after UVB irradiation VS homo before UVB irradiation	GO_DYSTROGLYCAN_BINDING	0.100735057	0.050152772	0.414894431	0.68870012	0.81391709	-6.45736635
hetero after UVB irradiation VS hetero before UVB irradiation	GO_DYSTROGLYCAN_BINDING1	-0.04536142	0.035910261	-0.17414238	0.86615915	0.94927395	-6.21507118
WT after UVB irradiation VS WT before UVB irradiation	GO_DYSTROGLYCAN_BINDING2	-0.25576221	0.045566757	-1.02422017	0.33535177	0.77905119	-5.47757077
homo after UVB irradiation VS homo before UVB irradiation	GO_DYSTROGLYCAN_COMPLEX	0.702289654	0.001716811	4.295946613	0.00239525	0.03263777	-1.30467239
hetero after UVB irradiation VS hetero before UVB irradiation	GO_DYSTROGLYCAN_COMPLEX1	-0.10148801	0.04566198	-0.29259712	0.77741216	0.91343573	-6.18531622
WT after UVB irradiation VS WT before UVB irradiation	GO_DYSTROGLYCAN_COMPLEX2	0.117332432	-0.05246546	0.335611085	0.74569413	0.93591138	-5.93706924
homo after UVB irradiation VS homo before UVB irradiation	GO_E_BOX_BINDING	0.186707152	-0.00519306	1.302300147	0.2276683	0.41875871	-5.70532048
hetero after UVB irradiation VS hetero before UVB irradiation	GO_E_BOX_BINDING1	0.049562824	0.023379405	0.296807289	0.77431257	0.91152166	-6.18398576
WT after UVB irradiation VS WT before UVB irradiation	GO_E_BOX_BINDING2	0.053703184	-0.04745996	0.338543722	0.74356454	0.93561604	-5.93604135
homo after UVB irradiation VS homo before UVB irradiation	GO_EAR_DEVELOPMENT	-0.0874455	0.007245612	-0.69374968	0.5067221	0.67962332	-6.29421188
hetero after UVB irradiation VS hetero before UVB irradiation	GO_EAR_DEVELOPMENT1	-0.04978326	0.000955715	-0.37006563	0.72111524	0.89103348	-6.15788019
WT after UVB irradiation VS WT before UVB irradiation	GO_EAR_DEVELOPMENT2	-0.15265433	0.009970685	-1.02168082	0.33648208	0.77956659	-5.47997946
homo after UVB irradiation VS homo before UVB irradiation	GO_EAR_MORPHOGENESIS	-0.02419906	0.016762841	-0.20163217	0.84504179	0.91444465	-6.52859948
hetero after UVB irradiation VS hetero before UVB irradiation	GO_EAR_MORPHOGENESIS1	-0.05887015	0.006972286	-0.39521869	0.70320017	0.88183935	-6.14763969
WT after UVB irradiation VS WT before UVB irradiation	GO_EAR_MORPHOGENESIS2	-0.14422721	0.020714094	-0.93351103	0.37755155	0.80338597	-5.560744
homo after UVB irradiation VS homo before UVB irradiation	GO_EARLY_ENDOSOME	0.274527703	-0.01911555	2.019989308	0.07665728	0.21283753	-4.73658299
homo after UVB irradiation VS homo before UVB irradiation	GO_EARLY_ENDOSOME_MEMBRANE	0.336907146	-0.01282854	2.320404857	0.04766121	0.15995827	-4.28497591
hetero after UVB irradiation VS hetero before UVB irradiation	GO_EARLY_ENDOSOME_MEMBRANE1	0.40447389	0.03148721	3.108462896	0.01481596	0.18054811	-2.88238241
WT after UVB irradiation VS WT before UVB irradiation	GO_EARLY_ENDOSOME_MEMBRANE2	0.301086949	0.027461633	2.154120573	0.0629607	0.53593918	-4.0909369
homo after UVB irradiation VS homo before UVB irradiation	GO_EARLY_ENDOSOME_TO_GOLGI_TRANSPORT	0.301964843	-0.05163354	1.985650056	0.08090725	0.21996099	-4.78704893
hetero after UVB irradiation VS hetero before UVB irradiation	GO_EARLY_ENDOSOME_TO_GOLGI_TRANSPORT1	0.517400308	-0.01474092	2.776568715	0.02448798	0.22374282	-3.36568789
WT after UVB irradiation VS WT before UVB irradiation	GO_EARLY_ENDOSOME_TO_GOLGI_TRANSPORT2	0.303246624	0.013449297	1.37545279	0.20583698	0.70285634	-5.10503385
hetero after UVB irradiation VS hetero before UVB irradiation	GO_EARLY_ENDOSOME1	0.34664977	0.012335477	2.750621178	0.0254796	0.22788198	-3.40371414
WT after UVB irradiation VS WT before UVB irradiation	GO_EARLY_ENDOSOME2	0.303160002	0.002406389	2.126724764	0.06571807	0.537335	-4.12914602
homo after UVB irradiation VS homo before UVB irradiation	GO_EARLY_PHAGOSOME	0.493369399	-0.062263	2.615164892	0.02985826	0.12287072	-3.83071707
hetero after UVB irradiation VS hetero before UVB irradiation	GO_EARLY_PHAGOSOME1	0.584383697	-0.01690299	3.774377326	0.0056195	0.13201411	-1.94609544
WT after UVB irradiation VS WT before UVB irradiation	GO_EARLY_PHAGOSOME2	0.441121836	0.012070392	2.385986902	0.04376975	0.49685404	-3.76428884
homo after UVB irradiation VS homo before UVB irradiation	GO_EARP_COMPLEX	0.063299454	-0.08285827	0.213527645	0.83604967	0.90838106	-6.52590336
hetero after UVB irradiation VS hetero before UVB irradiation	GO_EARP_COMPLEX1	0.535975142	0.080407948	1.864853091	0.09988252	0.39250771	-4.68233604
WT after UVB irradiation VS WT before UVB irradiation	GO_EARP_COMPLEX2	0.265004046	0.04364447	0.720405378	0.49154475	0.85299376	-5.7311516
homo after UVB irradiation VS homo before UVB irradiation	GO_EATING_BEHAVIOR	0.040791413	0.063923575	0.230408298	0.82333304	0.90124605	-6.52181524
hetero after UVB irradiation VS hetero before UVB irradiation	GO_EATING_BEHAVIOR1	0.2564008	0.042109025	1.6404465	0.14025066	0.45467738	-4.9842638
WT after UVB irradiation VS WT before UVB irradiation	GO_EATING_BEHAVIOR2	0.235636268	0.024913798	1.59545881	0.14882097	0.65641891	-4.83865493
homo after UVB irradiation VS homo before UVB irradiation	GO_ECTODERM_DEVELOPMENT	0.295564273	-0.04489799	1.435217681	0.18768973	0.37283776	-5.54307777
hetero after UVB irradiation VS hetero before UVB irradiation	GO_ECTODERM_DEVELOPMENT1	0.388126032	-0.00954923	2.272122825	0.05331444	0.30635423	-4.10395804
WT after UVB irradiation VS WT before UVB irradiation	GO_ECTODERM_DEVELOPMENT2	0.194728457	0.062353881	1.049228117	0.32437596	0.77346379	-5.45361001
homo after UVB irradiation VS homo before UVB irradiation	GO_ECTODERMAL_CELL_DIFFERENTIATION	0.358840508	-0.08910489	2.096229872	0.06797907	0.19919918	-4.62356816
hetero after UVB irradiation VS hetero before UVB irradiation	GO_ECTODERMAL_CELL_DIFFERENTIATION1	0.601008042	0.013731825	4.518908201	0.00204682	0.1045081	-0.97719273
WT after UVB irradiation VS WT before UVB irradiation	GO_ECTODERMAL_CELL_DIFFERENTIATION2	0.271541264	-0.01218107	1.556797525	0.15767819	0.66180255	-4.88696304
homo after UVB irradiation VS homo before UVB irradiation	GO_ECTODERMAL_PLACODE_DEVELOPMENT	0.100187206	0.031908147	0.546240796	0.59922285	0.74919356	-6.39002224
hetero after UVB irradiation VS hetero before UVB irradiation	GO_ECTODERMAL_PLACODE_DEVELOPMENT1	0.329367788	-0.01096692	2.006158828	0.08042042	0.36432827	-4.48512247
WT after UVB irradiation VS WT before UVB irradiation	GO_ECTODERMAL_PLACODE_DEVELOPMENT2	0.05548744	0.069711534	0.316158736	0.75987786	0.93915558	-5.9436671
homo after UVB irradiation VS homo before UVB irradiation	GO_ECTOPIC_GERM_CELL_PROGRAMMED_CELL_DEATH	-0.10082015	-0.00314722	-0.50774909	0.62479925	0.76777105	-6.41155413
hetero after UVB irradiation VS hetero before UVB irradiation	GO_ECTOPIC_GERM_CELL_PROGRAMMED_CELL_DEATH1	0.369679614	0.04286725	1.463224527	0.18224906	0.50688348	-5.20979868
WT after UVB irradiation VS WT before UVB irradiation	GO_ECTOPIC_GERM_CELL_PROGRAMMED_CELL_DEATH2	0.026285257	-0.03585351	0.084177254	0.93495939	0.98322796	-5.99232957
homo after UVB irradiation VS homo before UVB irradiation	GO_EFFLUX_TRANSMEMBRANE_TRANSPORTER_ACTIVITY	0.65854238	-0.06159884	4.935063893	0.00101458	0.02163603	-0.43895536
hetero after UVB irradiation VS hetero before UVB irradiation	GO_EFFLUX_TRANSMEMBRANE_TRANSPORTER_ACTIVITY1	0.15403758	0.023797172	0.85972324	0.41541512	0.71912037	-5.84908063
WT after UVB irradiation VS WT before UVB irradiation	GO_EFFLUX_TRANSMEMBRANE_TRANSPORTER_ACTIVITY2	0.358272114	0.022454839	1.827207087	0.10463623	0.60768077	-4.53814476
homo after UVB irradiation VS homo before UVB irradiation	GO_EGG_ACTIVATION	0.333391474	0.111397883	2.014457332	0.07732709	0.21393247	-4.74473215
hetero after UVB irradiation VS hetero before UVB irradiation	GO_EGG_ACTIVATION1	-0.29390208	-0.00466806	-1.16647003	0.27762568	0.60400502	-5.55314426
WT after UVB irradiation VS WT before UVB irradiation	GO_EGG_ACTIVATION2	-0.47060845	0.04624535	-1.6907617	0.12889204	0.63495603	-4.71719824
homo after UVB irradiation VS homo before UVB irradiation	GO_EKC_KEOPS_COMPLEX	-0.32866899	-0.02295065	-1.79758811	0.10847653	0.26320095	-5.05773729
hetero after UVB irradiation VS hetero before UVB irradiation	GO_EKC_KEOPS_COMPLEX1	-0.49645483	-0.06561655	-1.94504445	0.08834178	0.37512841	-4.57097673
WT after UVB irradiation VS WT before UVB irradiation	GO_EKC_KEOPS_COMPLEX2	-0.33213025	-0.01656363	-1.2718053	0.23875012	0.7291361	-5.22249061
homo after UVB irradiation VS homo before UVB irradiation	GO_ELASTIC_FIBER_ASSEMBLY	0.381379079	-0.03906327	2.244608132	0.05375189	0.1716919	-4.40035307
hetero after UVB irradiation VS hetero before UVB irradiation	GO_ELASTIC_FIBER_ASSEMBLY1	0.077019858	0.046466737	0.327716199	0.75169247	0.9028876	-6.17365097
WT after UVB irradiation VS WT before UVB irradiation	GO_ELASTIC_FIBER_ASSEMBLY2	0.203697421	0.024653762	0.853992072	0.41765512	0.82297693	-5.62859278
homo after UVB irradiation VS homo before UVB irradiation	GO_ELASTIN_CATABOLIC_PROCESS	1.193289507	-0.00550811	6.959174786	9.66E-05	0.00997194	1.887486751
hetero after UVB irradiation VS hetero before UVB irradiation	GO_ELASTIN_CATABOLIC_PROCESS1	0.861689826	0.179474847	3.17213657	0.01347177	0.17646302	-2.79061256
WT after UVB irradiation VS WT before UVB irradiation	GO_ELASTIN_CATABOLIC_PROCESS2	0.774575939	0.068643625	2.49534276	0.03687243	0.47615783	-3.60895777
homo after UVB irradiation VS homo before UVB irradiation	GO_ELASTIN_METABOLIC_PROCESS	0.490760363	0.061009165	2.796514943	0.02242491	0.10429384	-3.54907714
hetero after UVB irradiation VS hetero before UVB irradiation	GO_ELASTIN_METABOLIC_PROCESS1	0.473608058	0.033742628	3.432035071	0.0091836	0.15277703	-2.42047444
WT after UVB irradiation VS WT before UVB irradiation	GO_ELASTIN_METABOLIC_PROCESS2	0.340123961	0.006720843	1.297558525	0.23018057	0.72256251	-5.19384225
homo after UVB irradiation VS homo before UVB irradiation	GO_ELECTRON_TRANSFER_ACTIVITY	0.211108622	-0.00563957	0.940074572	0.37363828	0.56389871	-6.09036524
hetero after UVB irradiation VS hetero before UVB irradiation	GO_ELECTRON_TRANSFER_ACTIVITY1	-0.04758954	0.008206473	-0.27305613	0.7918535	0.91874432	-6.19124777
WT after UVB irradiation VS WT before UVB irradiation	GO_ELECTRON_TRANSFER_ACTIVITY2	0.139097229	-0.05354804	0.538747774	0.60455138	0.89591658	-5.84575985
homo after UVB irradiation VS homo before UVB irradiation	GO_ELECTRON_TRANSPORT_CHAIN	0.141587076	-0.00729004	0.581495935	0.5763008	0.73314404	-6.369021
hetero after UVB irradiation VS hetero before UVB irradiation	GO_ELECTRON_TRANSPORT_CHAIN1	-0.18124491	-0.00228656	-0.89244154	0.39867819	0.70580628	-5.82086473
WT after UVB irradiation VS WT before UVB irradiation	GO_ELECTRON_TRANSPORT_CHAIN2	0.142297651	-0.04535814	0.541884766	0.60248548	0.89591106	-5.84403716
homo after UVB irradiation VS homo before UVB irradiation	GO_ELONGATOR_HOLOENZYME_COMPLEX	-0.98898999	0.004256797	-5.91121963	0.00030564	0.01277723	0.759251914
hetero after UVB irradiation VS hetero before UVB irradiation	GO_ELONGATOR_HOLOENZYME_COMPLEX1	-0.86735972	-0.08425472	-4.95013784	0.0011837	0.09106857	-0.45878497
WT after UVB irradiation VS WT before UVB irradiation	GO_ELONGATOR_HOLOENZYME_COMPLEX2	-0.10056418	-0.0967684	-0.29270183	0.77711024	0.94369781	-5.95111242
homo after UVB irradiation VS homo before UVB irradiation	GO_ELONGIN_COMPLEX	-0.12867532	0.150784556	-0.33796909	0.74374738	0.84956112	-6.48860316
hetero after UVB irradiation VS hetero before UVB irradiation	GO_ELONGIN_COMPLEX1	0.338489456	0.040504757	0.777484355	0.45965259	0.74830469	-5.91612776
WT after UVB irradiation VS WT before UVB irradiation	GO_ELONGIN_COMPLEX2	0.360736901	-0.08139865	1.227070305	0.25427307	0.73589193	-5.27136781
homo after UVB irradiation VS homo before UVB irradiation	GO_EMBRYO_DEVELOPMENT	-0.02811606	0.007387214	-0.21868924	0.8321557	0.90629924	-6.52468594
homo after UVB irradiation VS homo before UVB irradiation	GO_EMBRYO_DEVELOPMENT_ENDING_IN_BIRTH_OR_EGG_HATCHING	-0.05869797	0.00524398	-0.45206535	0.6627628	0.79546698	-6.44007889
hetero after UVB irradiation VS hetero before UVB irradiation	GO_EMBRYO_DEVELOPMENT_ENDING_IN_BIRTH_OR_EGG_HATCHING1	0.028001238	0.010646086	0.213116345	0.83666477	0.93734913	-6.20692689
WT after UVB irradiation VS WT before UVB irradiation	GO_EMBRYO_DEVELOPMENT_ENDING_IN_BIRTH_OR_EGG_HATCHING2	-0.02437781	0.007567611	-0.16447344	0.87338995	0.96914919	-5.98181815
hetero after UVB irradiation VS hetero before UVB irradiation	GO_EMBRYO_DEVELOPMENT1	0.028442931	0.004913721	0.229543308	0.82431248	0.93321249	-6.20300887
WT after UVB irradiation VS WT before UVB irradiation	GO_EMBRYO_DEVELOPMENT2	-0.03802998	0.006853111	-0.26876922	0.79482809	0.95079231	-5.95813009
homo after UVB irradiation VS homo before UVB irradiation	GO_EMBRYO_IMPLANTATION	0.441907115	0.04870162	3.416076978	0.00860539	0.06164548	-2.59405763
hetero after UVB irradiation VS hetero before UVB irradiation	GO_EMBRYO_IMPLANTATION1	0.160490586	-0.00762268	0.955212667	0.36794687	0.68165913	-5.76437246
WT after UVB irradiation VS WT before UVB irradiation	GO_EMBRYO_IMPLANTATION2	0.244999429	0.00228332	1.549841729	0.15932144	0.66303059	-4.8955916
homo after UVB irradiation VS homo before UVB irradiation	GO_EMBRYONIC_APPENDAGE_MORPHOGENESIS	-0.03950484	0.005354519	-0.32069711	0.7563385	0.85836247	-6.49476489
hetero after UVB irradiation VS hetero before UVB irradiation	GO_EMBRYONIC_APPENDAGE_MORPHOGENESIS1	-0.10699825	0.001837288	-0.78348506	0.45632039	0.74625552	-5.91142751
WT after UVB irradiation VS WT before UVB irradiation	GO_EMBRYONIC_APPENDAGE_MORPHOGENESIS2	-0.11768308	-0.00216849	-0.76138008	0.46802141	0.84422343	-5.70127308
homo after UVB irradiation VS homo before UVB irradiation	GO_EMBRYONIC_AXIS_SPECIFICATION	-0.16930077	0.017220777	-1.03518095	0.32970094	0.52295665	-5.99830733
hetero after UVB irradiation VS hetero before UVB irradiation	GO_EMBRYONIC_AXIS_SPECIFICATION1	-0.0384624	0.021278334	-0.2121815	0.83736923	0.93778236	-6.20714122
WT after UVB irradiation VS WT before UVB irradiation	GO_EMBRYONIC_AXIS_SPECIFICATION2	-0.05951363	0.086889115	-0.30469585	0.76828188	0.94123239	-5.94737542
homo after UVB irradiation VS homo before UVB irradiation	GO_EMBRYONIC_BODY_MORPHOGENESIS	0.26444751	-0.01298668	1.912732651	0.09069461	0.23637944	-4.89321149
hetero after UVB irradiation VS hetero before UVB irradiation	GO_EMBRYONIC_BODY_MORPHOGENESIS1	0.063873191	-0.0024049	0.297333618	0.77392538	0.91152166	-6.18381813
WT after UVB irradiation VS WT before UVB irradiation	GO_EMBRYONIC_BODY_MORPHOGENESIS2	-0.30500747	0.012032449	-1.43597292	0.18849196	0.69085152	-5.03392425
homo after UVB irradiation VS homo before UVB irradiation	GO_EMBRYONIC_BRAIN_DEVELOPMENT	0.110520162	0.033105189	0.546268817	0.59920444	0.74919356	-6.39000603
hetero after UVB irradiation VS hetero before UVB irradiation	GO_EMBRYONIC_BRAIN_DEVELOPMENT1	-0.01273472	-0.0353653	-0.08850567	0.93169024	0.97492875	-6.22723827
WT after UVB irradiation VS WT before UVB irradiation	GO_EMBRYONIC_BRAIN_DEVELOPMENT2	-0.15854105	0.023216591	-0.77791729	0.45874128	0.8390123	-5.68881237
homo after UVB irradiation VS homo before UVB irradiation	GO_EMBRYONIC_CAMERA_TYPE_EYE_DEVELOPMENT	-0.29287878	0.042697989	-1.96179487	0.08399213	0.22519475	-4.82193471
hetero after UVB irradiation VS hetero before UVB irradiation	GO_EMBRYONIC_CAMERA_TYPE_EYE_DEVELOPMENT1	-0.07063612	0.016621211	-0.39265567	0.70501687	0.88289792	-6.14871277
WT after UVB irradiation VS WT before UVB irradiation	GO_EMBRYONIC_CAMERA_TYPE_EYE_DEVELOPMENT2	-0.123135	0.020379038	-0.81011264	0.44103041	0.83220385	-5.66390215
homo after UVB irradiation VS homo before UVB irradiation	GO_EMBRYONIC_CAMERA_TYPE_EYE_FORMATION	-0.28595941	0.076093412	-1.75596294	0.11567607	0.27401687	-5.11616128
hetero after UVB irradiation VS hetero before UVB irradiation	GO_EMBRYONIC_CAMERA_TYPE_EYE_FORMATION1	-0.29349224	0.021762938	-1.64687043	0.13890925	0.45248233	-4.97585277
WT after UVB irradiation VS WT before UVB irradiation	GO_EMBRYONIC_CAMERA_TYPE_EYE_FORMATION2	-0.22549979	0.114335137	-1.3342035	0.2184376	0.71478521	-5.15245782
homo after UVB irradiation VS homo before UVB irradiation	GO_EMBRYONIC_CAMERA_TYPE_EYE_MORPHOGENESIS	-0.3621532	0.039282783	-2.57794932	0.03167111	0.12617996	-3.88840178
hetero after UVB irradiation VS hetero before UVB irradiation	GO_EMBRYONIC_CAMERA_TYPE_EYE_MORPHOGENESIS1	-0.18107009	0.008780916	-1.09631185	0.30542172	0.62717218	-5.62670726
WT after UVB irradiation VS WT before UVB irradiation	GO_EMBRYONIC_CAMERA_TYPE_EYE_MORPHOGENESIS2	-0.27350471	-0.00328159	-1.8363533	0.10317431	0.60538498	-4.5259509
homo after UVB irradiation VS homo before UVB irradiation	GO_EMBRYONIC_CLEAVAGE	-0.35432088	0.045889695	-0.96287265	0.36272855	0.55337384	-6.06893643
hetero after UVB irradiation VS hetero before UVB irradiation	GO_EMBRYONIC_CLEAVAGE1	-0.20952738	-0.01190333	-0.60687502	0.56106577	0.80919173	-6.03646771
WT after UVB irradiation VS WT before UVB irradiation	GO_EMBRYONIC_CLEAVAGE2	-0.17472341	0.080290503	-0.47369842	0.64822348	0.90907607	-5.87937547
homo after UVB irradiation VS homo before UVB irradiation	GO_EMBRYONIC_CRANIAL_SKELETON_MORPHOGENESIS	0.055281492	1.65E-05	0.371667655	0.71941763	0.83370866	-6.47567856
hetero after UVB irradiation VS hetero before UVB irradiation	GO_EMBRYONIC_CRANIAL_SKELETON_MORPHOGENESIS1	0.039152999	3.06E-05	0.201546471	0.84539412	0.94077349	-6.20951395
WT after UVB irradiation VS WT before UVB irradiation	GO_EMBRYONIC_CRANIAL_SKELETON_MORPHOGENESIS2	0.017914332	0.04521958	0.125667729	0.90305902	0.97576564	-5.98774242
homo after UVB irradiation VS homo before UVB irradiation	GO_EMBRYONIC_DIGESTIVE_TRACT_DEVELOPMENT	0.043543114	0.01631421	0.295624047	0.77475327	0.86971294	-6.50314818
hetero after UVB irradiation VS hetero before UVB irradiation	GO_EMBRYONIC_DIGESTIVE_TRACT_DEVELOPMENT1	0.159786454	0.048050943	1.151788819	0.28326591	0.60884576	-5.56880335
WT after UVB irradiation VS WT before UVB irradiation	GO_EMBRYONIC_DIGESTIVE_TRACT_DEVELOPMENT2	0.101791819	-0.00596361	0.567367278	0.58584579	0.89090338	-5.82970148
homo after UVB irradiation VS homo before UVB irradiation	GO_EMBRYONIC_DIGESTIVE_TRACT_MORPHOGENESIS	-0.12744736	0.021562319	-0.85074479	0.41869436	0.60416041	-6.17028343
hetero after UVB irradiation VS hetero before UVB irradiation	GO_EMBRYONIC_DIGESTIVE_TRACT_MORPHOGENESIS1	0.009321784	0.047746639	0.056538512	0.9563249	0.98427357	-6.22975093
WT after UVB irradiation VS WT before UVB irradiation	GO_EMBRYONIC_DIGESTIVE_TRACT_MORPHOGENESIS2	0.002646561	-0.00305172	0.012174527	0.99058098	0.99848757	-5.99598846
homo after UVB irradiation VS homo before UVB irradiation	GO_EMBRYONIC_DIGIT_MORPHOGENESIS	-0.22821367	0.008287458	-1.5881213	0.14942905	0.32260376	-5.34515472
hetero after UVB irradiation VS hetero before UVB irradiation	GO_EMBRYONIC_DIGIT_MORPHOGENESIS1	-0.09666054	0.011264268	-0.4824724	0.6426318	0.85352609	-6.10715018
WT after UVB irradiation VS WT before UVB irradiation	GO_EMBRYONIC_DIGIT_MORPHOGENESIS2	-0.19230667	-0.01432484	-1.22644173	0.25449704	0.73589193	-5.27204638

homo after UVB irradiation VS homo before UVB irradiation	GO_EMBRYONIC_EYE_MORPHOGENESIS	-0.22394086	0.048642004	-1.67363671	0.13124149	0.29723769	-5.22987294
hetero after UVB irradiation VS hetero before UVB irradiation	GO_EMBRYONIC_EYE_MORPHOGENESIS1	-0.02791587	0.01975612	-0.20461539	0.84307638	0.93982285	-6.20884164
WT after UVB irradiation VS WT before UVB irradiation	GO_EMBRYONIC_EYE_MORPHOGENESIS2	-0.12929948	-0.00658911	-0.81984177	0.43577139	0.83019343	-5.65620726
homo after UVB irradiation VS homo before UVB irradiation	GO_EMBRYONIC_FORELIMB_MORPHOGENESIS	0.183819764	0.020983652	1.247420705	0.24617293	0.44011487	-5.76932373
hetero after UVB irradiation VS hetero before UVB irradiation	GO_EMBRYONIC_FORELIMB_MORPHOGENESIS1	-0.20717957	0.001649887	-1.2599277	0.24384824	0.57159915	-5.45032958
WT after UVB irradiation VS WT before UVB irradiation	GO_EMBRYONIC_FORELIMB_MORPHOGENESIS2	-0.04333943	0.04009573	-0.25600025	0.804334	0.95374177	-5.96163412
homo after UVB irradiation VS homo before UVB irradiation	GO_EMBRYONIC_HEART_TUBE_DEVELOPMENT	-0.01835275	0.026989708	-0.12308108	0.90496114	0.94793523	-6.54255036
hetero after UVB irradiation VS hetero before UVB irradiation	GO_EMBRYONIC_HEART_TUBE_DEVELOPMENT1	-0.05002175	0.007987343	-0.34114783	0.74194057	0.89944672	-6.16884995
WT after UVB irradiation VS WT before UVB irradiation	GO_EMBRYONIC_HEART_TUBE_DEVELOPMENT2	-0.16872081	-0.02696615	-1.15605834	0.28062402	0.75150204	-5.34653298
homo after UVB irradiation VS homo before UVB irradiation	GO_EMBRYONIC_HEART_TUBE_MORPHOGENESIS	-0.01866704	0.02905361	-0.11735203	0.90936168	0.95052063	-6.543305
hetero after UVB irradiation VS hetero before UVB irradiation	GO_EMBRYONIC_HEART_TUBE_MORPHOGENESIS1	-0.02147742	0.008236677	-0.14100361	0.89141701	0.96027811	-6.22071607
WT after UVB irradiation VS WT before UVB irradiation	GO_EMBRYONIC_HEART_TUBE_MORPHOGENESIS2	-0.13903406	-0.02719719	-0.9245269	0.38193651	0.80483179	-5.56865245
homo after UVB irradiation VS homo before UVB irradiation	GO_EMBRYONIC_HEMOPOIESIS	0.026769341	-0.03492406	0.172036221	0.86751573	0.92712717	-6.53464293
hetero after UVB irradiation VS hetero before UVB irradiation	GO_EMBRYONIC_HEMOPOIESIS1	0.08594576	-0.02366716	0.469143683	0.65171626	0.85695287	-6.11382864
WT after UVB irradiation VS WT before UVB irradiation	GO_EMBRYONIC_HEMOPOIESIS2	-0.0295491	-0.04798834	-0.13479558	0.89606383	0.97377562	-5.98649073
homo after UVB irradiation VS homo before UVB irradiation	GO_EMBRYONIC_HINDLIMB_MORPHOGENESIS	-0.36212593	0.013049303	-2.32813297	0.04708009	0.1588025	-4.2731696
hetero after UVB irradiation VS hetero before UVB irradiation	GO_EMBRYONIC_HINDLIMB_MORPHOGENESIS1	-0.45441136	0.008062602	-3.68643289	0.00636537	0.13584504	-2.06637335
WT after UVB irradiation VS WT before UVB irradiation	GO_EMBRYONIC_HINDLIMB_MORPHOGENESIS2	-0.37092911	-0.01246583	-2.34067507	0.04699484	0.50583727	-3.82847976
homo after UVB irradiation VS homo before UVB irradiation	GO_EMBRYONIC_MORPHOGENESIS	0.039809021	0.009832194	0.317370208	0.75877278	0.86000076	-6.49591557
hetero after UVB irradiation VS hetero before UVB irradiation	GO_EMBRYONIC_MORPHOGENESIS1	0.018360778	0.008960575	0.134586389	0.89632444	0.96152498	-6.22167275
WT after UVB irradiation VS WT before UVB irradiation	GO_EMBRYONIC_MORPHOGENESIS2	-0.04157717	0.008585154	-0.28230407	0.78479164	0.94601945	-5.9542333
homo after UVB irradiation VS homo before UVB irradiation	GO_EMBRYONIC_NEUROCRANIUM_MORPHOGENESIS	0.131292767	0.003220792	0.541133501	0.60258393	0.75167634	-6.39296359
hetero after UVB irradiation VS hetero before UVB irradiation	GO_EMBRYONIC_NEUROCRANIUM_MORPHOGENESIS1	0.373431584	0.033567348	2.214434762	0.05829659	0.31824803	-4.18746849
WT after UVB irradiation VS WT before UVB irradiation	GO_EMBRYONIC_NEUROCRANIUM_MORPHOGENESIS2	0.539170601	0.104577298	2.535166838	0.03464273	0.46729689	-3.55230141
homo after UVB irradiation VS homo before UVB irradiation	GO_EMBRYONIC_ORGAN_DEVELOPMENT	-0.05571368	0.009131976	-0.46714384	0.65237425	0.78840604	-6.43266403
hetero after UVB irradiation VS hetero before UVB irradiation	GO_EMBRYONIC_ORGAN_DEVELOPMENT1	-0.00149582	0.00160341	-0.01117374	0.99136348	0.99624062	-6.2314164
WT after UVB irradiation VS WT before UVB irradiation	GO_EMBRYONIC_ORGAN_DEVELOPMENT2	-0.0551419	0.005993178	-0.38146177	0.71267528	0.92947342	-5.92000966
homo after UVB irradiation VS homo before UVB irradiation	GO_EMBRYONIC_ORGAN_MORPHOGENESIS	-0.05740888	0.011639603	-0.47032226	0.65019452	0.78653337	-6.4310716
hetero after UVB irradiation VS hetero before UVB irradiation	GO_EMBRYONIC_ORGAN_MORPHOGENESIS1	-0.0369547	0.018812256	-0.24108675	0.81566329	0.92926938	-6.2000841
WT after UVB irradiation VS WT before UVB irradiation	GO_EMBRYONIC_ORGAN_MORPHOGENESIS2	-0.08419157	0.007349109	-0.56171274	0.58951609	0.89215883	-5.83293494
homo after UVB irradiation VS homo before UVB irradiation	GO_EMBRYONIC_PATTERN_SPECIFICATION	-0.23498253	0.0060042	-1.62501617	0.14131763	0.31204912	-5.29579149
hetero after UVB irradiation VS hetero before UVB irradiation	GO_EMBRYONIC_PATTERN_SPECIFICATION1	-0.26031011	0.017227195	-1.85001157	0.10217097	0.3967998	-4.70276878
WT after UVB irradiation VS WT before UVB irradiation	GO_EMBRYONIC_PATTERN_SPECIFICATION2	-0.25194378	0.020709732	-1.616276	0.14424135	0.65263518	-4.8124053
homo after UVB irradiation VS homo before UVB irradiation	GO_EMBRYONIC_PLACENTA_DEVELOPMENT	0.024700465	0.000314446	0.173705049	0.86624488	0.92645135	-6.53432743
hetero after UVB irradiation VS hetero before UVB irradiation	GO_EMBRYONIC_PLACENTA_DEVELOPMENT1	0.192527653	0.003031516	1.424126926	0.19291055	0.51844079	-5.25769512
WT after UVB irradiation VS WT before UVB irradiation	GO_EMBRYONIC_PLACENTA_DEVELOPMENT2	0.013699015	0.015409372	0.093896179	0.9274734	0.98214275	-5.99141732
homo after UVB irradiation VS homo before UVB irradiation	GO_EMBRYONIC_PLACENTA_MORPHOGENESIS	-0.17623551	0.024127832	-0.9540545	0.36692001	0.5572199	-6.07727361
hetero after UVB irradiation VS hetero before UVB irradiation	GO_EMBRYONIC_PLACENTA_MORPHOGENESIS1	-0.23012156	-0.01589002	-1.49841585	0.17310376	0.49569554	-5.16607903
WT after UVB irradiation VS WT before UVB irradiation	GO_EMBRYONIC_PLACENTA_MORPHOGENESIS2	-0.27440127	0.047370215	-1.49645611	0.17245459	0.67849973	-4.96114942
homo after UVB irradiation VS homo before UVB irradiation	GO_EMBRYONIC_PROCESS_INVOLVED_IN_FEMALE_PREGNANCY	-0.38071833	-0.04345209	-2.36001029	0.0447567	0.15377757	-4.22439495
hetero after UVB irradiation VS hetero before UVB irradiation	GO_EMBRYONIC_PROCESS_INVOLVED_IN_FEMALE_PREGNANCY1	-0.08968378	0.018756047	-0.33451259	0.74675194	0.90058797	-6.17124506
WT after UVB irradiation VS WT before UVB irradiation	GO_EMBRYONIC_PROCESS_INVOLVED_IN_FEMALE_PREGNANCY2	-0.30161424	0.004174309	-1.27903661	0.2363171	0.72774029	-5.21448338
homo after UVB irradiation VS homo before UVB irradiation	GO_EMBRYONIC_RETINA_MORPHOGENESIS_IN_CAMERA_TYPE_EYE	-0.05672761	0.063922304	-0.23872824	0.81708539	0.89649605	-6.51968743
hetero after UVB irradiation VS hetero before UVB irradiation	GO_EMBRYONIC_RETINA_MORPHOGENESIS_IN_CAMERA_TYPE_EYE1	-0.49431284	-0.00677507	-3.57057696	0.00751356	0.14177754	-2.22653593
WT after UVB irradiation VS WT before UVB irradiation	GO_EMBRYONIC_RETINA_MORPHOGENESIS_IN_CAMERA_TYPE_EYE2	-0.3581527	0.058950944	-1.13619652	0.28837938	0.75467539	-5.36700125
homo after UVB irradiation VS homo before UVB irradiation	GO_EMBRYONIC_SKELETAL_JOINT_DEVELOPMENT	0.006334239	-0.02364097	0.031224946	0.97582543	0.98746869	-6.55032165
hetero after UVB irradiation VS hetero before UVB irradiation	GO_EMBRYONIC_SKELETAL_JOINT_DEVELOPMENT1	-0.22413047	-0.03900495	-1.01201453	0.34169419	0.66014434	-5.71067611
WT after UVB irradiation VS WT before UVB irradiation	GO_EMBRYONIC_SKELETAL_JOINT_DEVELOPMENT2	0.093482023	0.02712517	0.445015901	0.66796371	0.91409156	-5.89290476
homo after UVB irradiation VS homo before UVB irradiation	GO_EMBRYONIC_SKELETAL_JOINT_MORPHOGENESIS	-0.21503018	-0.0187618	-1.07216213	0.31372594	0.50770772	-5.96066851
hetero after UVB irradiation VS hetero before UVB irradiation	GO_EMBRYONIC_SKELETAL_JOINT_MORPHOGENESIS1	-0.43117372	-0.05347635	-1.91681725	0.0922499	0.37946684	-4.61035202
WT after UVB irradiation VS WT before UVB irradiation	GO_EMBRYONIC_SKELETAL_JOINT_MORPHOGENESIS2	0.05619385	0.028387786	0.280060122	0.78645269	0.94673513	-5.95489232
homo after UVB irradiation VS homo before UVB irradiation	GO_EMBRYONIC_SKELETAL_SYSTEM_DEVELOPMENT	-0.02703999	-0.00782767	-0.20526378	0.84229394	0.91287688	-6.52779259
hetero after UVB irradiation VS hetero before UVB irradiation	GO_EMBRYONIC_SKELETAL_SYSTEM_DEVELOPMENT1	-0.02033606	0.005265784	-0.10695671	0.9175053	0.96850334	-6.22528493
WT after UVB irradiation VS WT before UVB irradiation	GO_EMBRYONIC_SKELETAL_SYSTEM_DEVELOPMENT2	-0.00321488	0.034275458	-0.01924816	0.98510897	0.99679464	-5.99587118
homo after UVB irradiation VS homo before UVB irradiation	GO_EMBRYONIC_SKELETAL_SYSTEM_MORPHOGENESIS	-0.07231746	-0.00951626	-0.57518704	0.58036644	0.73584221	-6.37286825
hetero after UVB irradiation VS hetero before UVB irradiation	GO_EMBRYONIC_SKELETAL_SYSTEM_MORPHOGENESIS1	-0.0480076	0.004403589	-0.23668184	0.81896066	0.93071163	-6.20121686
WT after UVB irradiation VS WT before UVB irradiation	GO_EMBRYONIC_SKELETAL_SYSTEM_MORPHOGENESIS2	0.026131893	0.020042461	0.155777695	0.88002166	0.9693507	-5.98328264
homo after UVB irradiation VS homo before UVB irradiation	GO_EMBRYONIC_VISCEROCRANIUM_MORPHOGENESIS	0.554483825	0.011888905	2.456298426	0.03841173	0.14064386	-4.07641859
hetero after UVB irradiation VS hetero before UVB irradiation	GO_EMBRYONIC_VISCEROCRANIUM_MORPHOGENESIS1	0.423712207	0.063576198	1.72120448	0.12422734	0.43060808	-4.87745924
WT after UVB irradiation VS WT before UVB irradiation	GO_EMBRYONIC_VISCEROCRANIUM_MORPHOGENESIS2	0.332134302	0.071799573	1.467696657	0.17992293	0.68255081	-4.9959588
homo after UVB irradiation VS homo before UVB irradiation	GO_ENAMEL_MINERALIZATION	0.051357024	-0.0034968	0.26230117	0.79946023	0.88630695	-6.513255
hetero after UVB irradiation VS hetero before UVB irradiation	GO_ENAMEL_MINERALIZATION1	0.143537591	0.048112486	0.796960524	0.44889701	0.74180859	-5.90076084
WT after UVB irradiation VS WT before UVB irradiation	GO_ENAMEL_MINERALIZATION2	-0.05851825	-0.02977745	-0.3024041	0.76996602	0.94145237	-5.94810079
homo after UVB irradiation VS homo before UVB irradiation	GO_ENDOCANNABINOID_SIGNALING_PATHWAY	-0.19163481	-0.02884879	-0.59336995	0.56869237	0.72710056	-6.36167543
hetero after UVB irradiation VS hetero before UVB irradiation	GO_ENDOCANNABINOID_SIGNALING_PATHWAY1	-0.5641164	0.046491787	-2.89360273	0.02048754	0.20617329	-3.19450009
WT after UVB irradiation VS WT before UVB irradiation	GO_ENDOCANNABINOID_SIGNALING_PATHWAY2	-0.37329352	0.051507703	-1.81308872	0.10693139	0.61209718	-4.55692348
homo after UVB irradiation VS homo before UVB irradiation	GO_ENDOCARDIAL_CELL_DIFFERENTIATION	0.01580982	0.002847309	0.062431193	0.95169169	0.97427085	-6.54871811
hetero after UVB irradiation VS hetero before UVB irradiation	GO_ENDOCARDIAL_CELL_DIFFERENTIATION1	0.348374101	-0.01303344	1.519530331	0.16781583	0.48938545	-5.1395813
WT after UVB irradiation VS WT before UVB irradiation	GO_ENDOCARDIAL_CELL_DIFFERENTIATION2	-0.19679312	0.001550236	-0.99539341	0.3483551	0.7859549	-5.50464682
homo after UVB irradiation VS homo before UVB irradiation	GO_ENDOCARDIAL_CUSHION_DEVELOPMENT	-0.13772142	0.002554775	-0.9589588	0.36458448	0.55471751	-6.07264435
hetero after UVB irradiation VS hetero before UVB irradiation	GO_ENDOCARDIAL_CUSHION_DEVELOPMENT1	-0.08357355	-0.01586587	-0.54382293	0.60165701	0.83354152	-6.074158
WT after UVB irradiation VS WT before UVB irradiation	GO_ENDOCARDIAL_CUSHION_DEVELOPMENT2	-0.18189512	0.000501348	-1.19254864	0.2668172	0.74323208	-5.30828923
homo after UVB irradiation VS homo before UVB irradiation	GO_ENDOCARDIAL_CUSHION_FORMATION	-0.25176121	-0.02141154	-1.41685151	0.19282329	0.37904532	-5.56607208
hetero after UVB irradiation VS hetero before UVB irradiation	GO_ENDOCARDIAL_CUSHION_FORMATION1	-0.01447653	0.012167784	-0.08769565	0.93231361	0.97492875	-6.22731559
WT after UVB irradiation VS WT before UVB irradiation	GO_ENDOCARDIAL_CUSHION_FORMATION2	-0.16843313	0.027024126	-1.12775398	0.29172774	0.75646944	-5.37562581
homo after UVB irradiation VS homo before UVB irradiation	GO_ENDOCARDIAL_CUSHION_MORPHOGENESIS	-0.08747426	0.006621985	-0.54939787	0.59715026	0.74805997	-6.3881912
hetero after UVB irradiation VS hetero before UVB irradiation	GO_ENDOCARDIAL_CUSHION_MORPHOGENESIS1	0.060693442	-0.01216144	0.376841235	0.71627067	0.88877312	-6.15518542
WT after UVB irradiation VS WT before UVB irradiation	GO_ENDOCARDIAL_CUSHION_MORPHOGENESIS2	-0.07057592	-0.00090341	-0.44694992	0.66662375	0.9135561	-5.89201766
homo after UVB irradiation VS homo before UVB irradiation	GO_ENDOCARDIUM_DEVELOPMENT	-0.00630347	-0.05270134	-0.04274783	0.9669096	0.98282718	-6.54985393
hetero after UVB irradiation VS hetero before UVB irradiation	GO_ENDOCARDIUM_DEVELOPMENT1	0.289440388	-0.03491649	1.793965647	0.11127029	0.41109274	-4.77938418
WT after UVB irradiation VS WT before UVB irradiation	GO_ENDOCARDIUM_DEVELOPMENT2	-0.06798919	0.007932657	-0.39861478	0.70048169	0.92463338	-5.9130875
homo after UVB irradiation VS homo before UVB irradiation	GO_ENDOCARDIUM_MORPHOGENESIS	0.465715718	-0.06919642	2.509526781	0.03530031	0.13468829	-3.99427172
hetero after UVB irradiation VS hetero before UVB irradiation	GO_ENDOCARDIUM_MORPHOGENESIS1	0.252047189	-0.02467179	0.955019537	0.36803865	0.68165913	-5.76455092
WT after UVB irradiation VS WT before UVB irradiation	GO_ENDOCARDIUM_MORPHOGENESIS2	0.21269142	0.084154238	0.95542617	0.367011	0.79878577	-5.5411995
homo after UVB irradiation VS homo before UVB irradiation	GO_ENDOCHONDRAL_BONE_MORPHOGENESIS	-0.00952661	-0.00646023	-0.07623105	0.94103462	0.96839943	-6.54766856
hetero after UVB irradiation VS hetero before UVB irradiation	GO_ENDOCHONDRAL_BONE_MORPHOGENESIS1	-0.13247799	-0.00896699	-0.80585401	0.44404304	0.73903666	-5.893637
WT after UVB irradiation VS WT before UVB irradiation	GO_ENDOCHONDRAL_BONE_MORPHOGENESIS2	-0.10196979	-0.00986048	-0.5982615	0.56601884	0.88229844	-5.81151194
homo after UVB irradiation VS homo before UVB irradiation	GO_ENDOCRINE_HORMONE_SECRETION	0.257283884	-0.00927406	1.718041955	0.12262018	0.28502179	-5.16885335
hetero after UVB irradiation VS hetero before UVB irradiation	GO_ENDOCRINE_HORMONE_SECRETION1	0.033081569	0.034740766	0.22342319	0.82890862	0.93461067	-6.20450213
WT after UVB irradiation VS WT before UVB irradiation	GO_ENDOCRINE_HORMONE_SECRETION2	0.001584698	0.020149288	0.008447825	0.99346411	0.9985657	-5.99602901
homo after UVB irradiation VS homo before UVB irradiation	GO_ENDOCRINE_PANCREAS_DEVELOPMENT	-0.06143673	0.048072258	-0.34570543	0.73813371	0.84616167	-6.48574134
hetero after UVB irradiation VS hetero before UVB irradiation	GO_ENDOCRINE_PANCREAS_DEVELOPMENT1	0.005058166	0.026318923	0.039642248	0.96936765	0.98899869	-6.23063196
WT after UVB irradiation VS WT before UVB irradiation	GO_ENDOCRINE_PANCREAS_DEVELOPMENT2	-0.13192616	-0.02827038	-0.7958925	0.4487948	0.83490988	-5.67500991
homo after UVB irradiation VS homo before UVB irradiation	GO_ENDOCRINE_PROCESS	0.197482407	-0.0144246	1.414891771	0.19337822	0.37982664	-5.56851517
hetero after UVB irradiation VS hetero before UVB irradiation	GO_ENDOCRINE_PROCESS1	0.099729192	0.0282193	0.784637891	0.45568209	0.74613056	-5.91052101
WT after UVB irradiation VS WT before UVB irradiation	GO_ENDOCRINE_PROCESS2	0.043223234	-0.02105421	0.239524599	0.8166504	0.95637726	-5.96590749
homo after UVB irradiation VS homo before UVB irradiation	GO_ENDOCRINE_SYSTEM_DEVELOPMENT	0.013360853	0.040162907	0.095387435	0.92626218	0.96009349	-6.54586591
hetero after UVB irradiation VS hetero before UVB irradiation	GO_ENDOCRINE_SYSTEM_DEVELOPMENT1	-0.00093536	0.037872256	-0.00721811	0.99442083	0.99766616	-6.23145586
WT after UVB irradiation VS WT before UVB irradiation	GO_ENDOCRINE_SYSTEM_DEVELOPMENT2	0.022799285	0.011066953	0.161825428	0.87540832	0.9693507	-5.98227248
homo after UVB irradiation VS homo before UVB irradiation	GO_ENDOCYTIC_RECYCLING	0.189701983	-0.0404747	1.026624693	0.33348491	0.5264481	-6.00687185
hetero after UVB irradiation VS hetero before UVB irradiation	GO_ENDOCYTIC_RECYCLING1	0.338798586	-0.01777949	2.752252712	0.02541604	0.22784879	-3.40132253
WT after UVB irradiation VS WT before UVB irradiation	GO_ENDOCYTIC_RECYCLING2	0.394419838	0.002846092	2.726164888	0.02570983	0.43459329	-3.28056658
homo after UVB irradiation VS homo before UVB irradiation	GO_ENDOCYTIC_VESICLE	0.370200429	-0.02129198	2.544254233	0.0334086	0.1304261	-3.94057185
homo after UVB irradiation VS homo before UVB irradiation	GO_ENDOCYTIC_VESICLE_LUMEN	0.011123655	0.033980836	0.064300829	0.95024719	0.97359676	-6.54858814
hetero after UVB irradiation VS hetero before UVB irradiation	GO_ENDOCYTIC_VESICLE_LUMEN1	0.294275143	0.054181865	1.483009224	0.17705589	0.50051267	-5.18528863
WT after UVB irradiation VS WT before UVB irradiation	GO_ENDOCYTIC_VESICLE_LUMEN2	0.134196521	0.023262551	0.597968105	0.56620532	0.88240489	-5.81168882
homo after UVB irradiation VS homo before UVB irradiation	GO_ENDOCYTIC_VESICLE_MEMBRANE	0.39402362	-0.03883153	2.928546901	0.01822981	0.09274941	-3.34400888
hetero after UVB irradiation VS hetero before UVB irradiation	GO_ENDOCYTIC_VESICLE_MEMBRANE1	0.305801144	0.010465653	2.14258719	0.06515179	0.33409111	-4.29090709
WT after UVB irradiation VS WT before UVB irradiation	GO_ENDOCYTIC_VESICLE_MEMBRANE2	0.305005998	0.019116444	1.945625426	0.08714424	0.5790399	-4.37865971
hetero after UVB irradiation VS hetero before UVB irradiation	GO_ENDOCYTIC_VESICLE1	0.362261733	0.019662025	2.571357007	0.03356093	0.2531992	-3.66672841
WT after UVB irradiation VS WT before UVB irradiation	GO_ENDOCYTIC_VESICLE2	0.290255447	-0.00400626	1.957458193	0.0855589	0.57584638	-4.36254567
homo after UVB irradiation VS homo before UVB irradiation	GO_ENDOCYTOSIS	0.372405105	-0.01128892	2.697293099	0.02622183	0.11427917	-3.70324358
homo after UVB irradiation VS homo before UVB irradiation	GO_ENDOCYTOSIS_INVOLVED_IN_VIRAL_ENTRY_INTO_HOST_CELL	0.478774511	-0.02345234	2.131918501	0.06425255	0.19221059	-4.57024497
hetero after UVB irradiation VS hetero before UVB irradiation	GO_ENDOCYTOSIS_INVOLVED_IN_VIRAL_ENTRY_INTO_HOST_CELL1	0.373477754	0.085732365	1.943701558	0.08852403	0.37525442	-4.57285413
WT after UVB irradiation VS WT before UVB irradiation	GO_ENDOCYTOSIS_INVOLVED_IN_VIRAL_ENTRY_INTO_HOST_CELL2	0.167455184	-0.07169973	0.740493926	0.47991815	0.85038327	-5.71668173
hetero after UVB irradiation VS hetero before UVB irradiation	GO_ENDOCYTOSIS1	0.329927013	0.00903159	2.349657555	0.04728131	0.29009686	-3.99119904
WT after UVB irradiation VS WT before UVB irradiation	GO_ENDOCYTOSIS2	0.27685817	-0.00064248	1.779705028	0.11254858	0.6175316	-4.60110699
homo after UVB irradiation VS homo before UVB irradiation	GO_ENDODEOXYRIBONUCLEASE_ACTIVITY	-0.44796942	-0.02387629	-2.52486416	0.03445184	0.13291656	-3.97056447
homo after UVB irradiation VS homo before UVB irradiation	GO_ENDODEOXYRIBONUCLEASE_ACTIVITY_PRODUCING_3_PHOSPHOMONOESTERS	-0.70618326	0.009499143	-4.9135156	0.00104338	0.02188549	-0.4670951
hetero after UVB irradiation VS hetero before UVB irradiation	GO_ENDODEOXYRIBONUCLEASE_ACTIVITY_PRODUCING_3_PHOSPHOMONOESTERS1	-0.24888574	-0.11868022	-1.19157193	0.26819488	0.5949314	-5.52605426
WT after UVB irradiation VS WT before UVB irradiation	GO_ENDODEOXYRIBONUCLEASE_ACTIVITY_PRODUCING_3_PHOSPHOMONOESTERS2	0.182303678	-0.08955432	0.642269693	0.53845231	0.87025652	-5.7840928
homo after UVB irradiation VS homo before UVB irradiation	GO_ENDODEOXYRIBONUCLEASE_ACTIVITY_PRODUCING_5_PHOSPHOMONOESTERS	-0.58449591	-0.03843731	-2.74965463	0.02414248	0.10908467	-3.62189596
hetero after UVB irradiation VS hetero before UVB irradiation	GO_ENDODEOXYRIBONUCLEASE_ACTIVITY_PRODUCING_5_PHOSPHOMONOESTERS1	-0.03352101	-0.04985694	-0.12665795	0.90239411	0.96386011	-6.22279348
WT after UVB irradiation VS WT before UVB irradiation	GO_ENDODEOXYRIBONUCLEASE_ACTIVITY_PRODUCING_5_PHOSPHOMONOESTERS2	-0.23402078	-0.01307533	-0.76148138	0.46796419	0.84422341	-5.70119745
hetero after UVB irradiation VS hetero before UVB irradiation	GO_ENDODEOXYRIBONUCLEASE_ACTIVITY1	-0.10118518	-0.08860802	-0.47839332	0.64540527	0.85438728	-6.10921274
WT after UVB irradiation VS WT before UVB irradiation	GO_ENDODEOXYRIBONUCLEASE_ACTIVITY2	-0.02624772	-0.03080719	-0.10878296	0.91602202	0.97967628	-5.98982743
homo after UVB irradiation VS homo before UVB irradiation	GO_ENDODEOXYRIBONUCLEASE_COMPLEX	-0.51357162	-0.04173597	-2.40061856	0.04196195	0.14772032	-4.16209816
hetero after UVB irradiation VS hetero before UVB irradiation	GO_ENDODEOXYRIBONUCLEASE_COMPLEX1	0.173442873	-0.10152011	0.604063837	0.56284131	0.80987677	-6.03822836
WT after UVB irradiation VS WT before UVB irradiation	GO_ENDODEOXYRIBONUCLEASE_COMPLEX2	-0.2370303	-0.11863141	-0.6650537	0.52450118	0.86518284	-5.76921177
homo after UVB irradiation VS homo before UVB irradiation	GO_ENDODERM_DEVELOPMENT	0.34608921	0.015042362	1.971879247	0.08267451	0.2229583	-4.80720506
hetero after UVB irradiation VS hetero before UVB irradiation	GO_ENDODERM_DEVELOPMENT1	0.388043771	0.065143191	2.762878495	0.0250061	0.22609685	-3.38574844
WT after UVB irradiation VS WT before UVB irradiation	GO_ENDODERM_DEVELOPMENT2	0.299301121	0.035416906	1.85401475	0.10040587	0.60005033	-4.50234229
homo after UVB irradiation VS homo before UVB irradiation	GO_ENDODERM_FORMATION	0.447362936	0.014912347	3.014418225	0.01594437	0.08618384	-3.21087987
hetero after UVB irradiation VS hetero before UVB irradiation	GO_ENDODERM_FORMATION1	0.484507296	0.064343278	3.583190889	0.00737845	0.14158176	-2.20900642
WT after UVB irradiation VS WT before UVB irradiation	GO_ENDODERM_FORMATION2	0.433009518	0.012239965	2.629935815	0.02987115	0.45106473	-3.41742384
homo after UVB irradiation VS homo before UVB irradiation	GO_ENDODERMAL_CELL_DIFFERENTIATION	0.62723555	0.013445922	4.157331702	0.00290868	0.03557259	-1.5007492
hetero after UVB irradiation VS hetero before UVB irradiation	GO_ENDODERMAL_CELL_DIFFERENTIATION1	0.639325871	0.068082038	4.310726883	0.00269243	0.11212131	-1.23885403
WT after UVB irradiation VS WT before UVB irradiation	GO_ENDODERMAL_CELL_DIFFERENTIATION2	0.549772282	0.015506517	3.100255336	0.01444369	0.36090271	-2.75299045
homo after UVB irradiation VS homo before UVB irradiation	GO_ENDODERMAL_CELL_FATE_COMMITMENT	-0.09991107	0.028441002	-0.63776891	0.54075643	0.70612713	-6.33300918
hetero after UVB irradiation VS hetero before UVB irradiation	GO_ENDODERMAL_CELL_FATE_COMMITMENT1	0.255530594	0.034313918	1.24912955	0.24756654	0.5747451	-5.462477
WT after UVB irradiation VS WT before UVB irradiation	GO_ENDODERMAL_CELL_FATE_COMMITMENT2	-0.13634458	0.01025816	-0.78411096	0.45529747	0.83838592	-5.68408672
homo after UVB irradiation VS homo before UVB irradiation	GO_ENDODERMAL_CELL_FATE_SPECIFICATION	0.213064846	0.097544085	1.153907394	0.28057072	0.47568728	-5.87400043
hetero after UVB irradiation VS hetero before UVB irradiation	GO_ENDODERMAL_CELL_FATE_SPECIFICATION1	0.786426888	0.066538237	6.075715416	0.00032023	0.07788488	0.747266457
WT after UVB irradiation VS WT before UVB irradiation	GO_ENDODERMAL_CELL_FATE_SPECIFICATION2	0.147570566	-0.08783017	0.586967715	0.57322215	0.8851145	-5.81826354
homo after UVB irradiation VS homo before UVB irradiation	GO_ENDOGENOUS_LIPID_ANTIGEN_BINDING	1.017022863	-0.03439778	3.974981182	0.00377137	0.04021247	-1.76297883
hetero after UVB irradiation VS hetero before UVB irradiation	GO_ENDOGENOUS_LIPID_ANTIGEN_BINDING1	0.281100553	0.050266029	0.637876879	0.54170291	0.79722035	-6.0165618
WT after UVB irradiation VS WT before UVB irradiation	GO_ENDOGENOUS_LIPID_ANTIGEN_BINDING2	0.608778586	-0.04872385	1.705449281	0.1260492	0.63290509	-4.69820001
homo after UVB irradiation VS homo before UVB irradiation	GO_ENDOLYSOSOME	0.902647245	-0.03839065	6.732417706	0.00012262	0.01007802	1.656359236
homo after UVB irradiation VS homo before UVB irradiation	GO_ENDOLYSOSOME_LUMEN	1.121894031	0.009863385	7.264291472	7.08E-05	0.00942429	2.187810484
hetero after UVB irradiation VS hetero before UVB irradiation	GO_ENDOLYSOSOME_LUMEN1	0.918947962	0.066021331	4.244214914	0.00294288	0.11368267	-1.32399918
WT after UVB irradiation VS WT before UVB irradiation	GO_ENDOLYSOSOME_LUMEN2	0.858614919	0.023700896	4.45402101	0.00206504	0.21016409	-0.99364447
homo after UVB irradiation VS homo before UVB irradiation	GO_ENDOLYSOSOME_MEMBRANE	0.824089969	-0.04941029	6.422718661	0.00017139	0.01062663	1.329341497

hetero after UVB irradiation VS hetero before UVB irradiation	GO_ENDOLYSOSOME_MEMBRANE1	0.90312854	0.069062926	5.785964952	0.00044123	0.07788488	0.456456602
WT after UVB irradiation VS WT before UVB irradiation	GO_ENDOLYSOSOME_MEMBRANE2	0.883309339	0.017545601	5.117588593	0.00087622	0.19353487	-0.24647424
hetero after UVB irradiation VS hetero before UVB irradiation	GO_ENDOLYSOSOME1	0.922619559	0.058924004	5.734019413	0.00046786	0.07788488	0.402920437
WT after UVB irradiation VS WT before UVB irradiation	GO_ENDOLYSOSOME2	0.878908976	0.016885912	5.265243841	0.0007303	0.18842845	-0.09098374
homo after UVB irradiation VS homo before UVB irradiation	GO_ENDOMEMBRANE_SYSTEM_ORGANIZATION	0.208085168	-0.02700008	1.406979386	0.19563294	0.3833938	-5.57835827
hetero after UVB irradiation VS hetero before UVB irradiation	GO_ENDOMEMBRANE_SYSTEM_ORGANIZATION1	0.226802583	-0.00176401	1.769378433	0.11549946	0.4188523	-4.81270868
WT after UVB irradiation VS WT before UVB irradiation	GO_ENDOMEMBRANE_SYSTEM_ORGANIZATION2	0.133764245	-0.01190412	0.933589903	0.37751322	0.80338597	-5.5606743
homo after UVB irradiation VS homo before UVB irradiation	GO_ENDONUCLEASE_ACTIVITY	-0.36970938	0.000326679	-2.79312118	0.02254498	0.10458857	-3.55435095
homo after UVB irradiation VS homo before UVB irradiation	GO_ENDONUCLEASE_ACTIVITY_ACTIVE_WITH_EITHER_RIBO_OR_DEOXYRIBONUCLEIC_ACIDS_AND_PRODUCING_3_PHOSPHOMONOESTERS	-0.377457	-0.01703233	-2.5286619	0.03424495	0.13253416	-3.96469192
hetero after UVB irradiation VS hetero before UVB irradiation	GO_ENDONUCLEASE_ACTIVITY_ACTIVE_WITH_EITHER_RIBO_OR_DEOXYRIBONUCLEIC_ACIDS_AND_PRODUCING_3_PHOSPHOMONOESTERS1	-0.24806103	-0.08610373	-1.83180947	0.10504577	0.40146476	-4.72774744
WT after UVB irradiation VS WT before UVB irradiation	GO_ENDONUCLEASE_ACTIVITY_ACTIVE_WITH_EITHER_RIBO_OR_DEOXYRIBONUCLEIC_ACIDS_AND_PRODUCING_3_PHOSPHOMONOESTERS2	0.095165537	-0.03185597	0.45280263	0.66257657	0.9121786	-5.88931093
homo after UVB irradiation VS homo before UVB irradiation	GO_ENDONUCLEASE_ACTIVITY_ACTIVE_WITH_EITHER_RIBO_OR_DEOXYRIBONUCLEIC_ACIDS_AND_PRODUCING_5_PHOSPHOMONOESTERS	-0.58463818	-0.01709678	-3.52844164	0.00726601	0.05631538	-2.42400915
hetero after UVB irradiation VS hetero before UVB irradiation	GO_ENDONUCLEASE_ACTIVITY_ACTIVE_WITH_EITHER_RIBO_OR_DEOXYRIBONUCLEIC_ACIDS_AND_PRODUCING_5_PHOSPHOMONOESTERS1	-0.32177102	-0.09855602	-1.57910636	0.15367164	0.47184575	-5.06378961
WT after UVB irradiation VS WT before UVB irradiation	GO_ENDONUCLEASE_ACTIVITY_ACTIVE_WITH_EITHER_RIBO_OR_DEOXYRIBONUCLEIC_ACIDS_AND_PRODUCING_5_PHOSPHOMONOESTERS2	-0.23186576	-0.01291716	-0.81583279	0.43793317	0.83056493	-5.65938735
hetero after UVB irradiation VS hetero before UVB irradiation	GO_ENDONUCLEASE_ACTIVITY1	-0.11911051	-0.07912377	-0.66255604	0.52657884	0.78971179	-6.00008028
WT after UVB irradiation VS WT before UVB irradiation	GO_ENDONUCLEASE_ACTIVITY2	-0.0399968	0.004701861	-0.18044547	0.8612371	0.9676681	-5.9789226
homo after UVB irradiation VS homo before UVB irradiation	GO_ENDONUCLEASE_COMPLEX	-0.65789617	-0.01911975	-4.68938163	0.0014016	0.02523054	-0.76415641
hetero after UVB irradiation VS hetero before UVB irradiation	GO_ENDONUCLEASE_COMPLEX1	-0.16341036	-0.08542031	-0.76466749	0.46682443	0.75303308	-5.92606375
WT after UVB irradiation VS WT before UVB irradiation	GO_ENDONUCLEASE_COMPLEX2	-0.26760387	-0.02179961	-1.14880448	0.28343663	0.75292132	-5.35403714
homo after UVB irradiation VS homo before UVB irradiation	GO_ENDONUCLEOLYTIC_CLEAVAGE_IN_5_ETS_OF_TRICISTRONIC_RRNA_TRANSCRIPT_SSU_RRNA_5_8S_RRNA_LSU_RRNA	-0.73066358	0.019701094	-3.41447667	0.00862624	0.06173419	-2.5964888
hetero after UVB irradiation VS hetero before UVB irradiation	GO_ENDONUCLEOLYTIC_CLEAVAGE_IN_5_ETS_OF_TRICISTRONIC_RRNA_TRANSCRIPT_SSU_RRNA_5_8S_RRNA_LSU_RRNA1	-0.93095112	-0.08059358	-5.44064672	0.00065573	0.08158876	0.09237192
WT after UVB irradiation VS WT before UVB irradiation	GO_ENDONUCLEOLYTIC_CLEAVAGE_IN_5_ETS_OF_TRICISTRONIC_RRNA_TRANSCRIPT_SSU_RRNA_5_8S_RRNA_LSU_RRNA2	-0.29016616	0.001356855	-0.82851954	0.43111723	0.82885935	-5.64927911
homo after UVB irradiation VS homo before UVB irradiation	TIC_CLEAVAGE_IN_ITS1_TO_SEPARATE_SSU_RRNA_FROM_5_8S_RRNA_AND_LSU_RRNA_FROM_TRICISTRONIC_RRNA_TRANSCRIPT_SSU_RRNA_5_	-1.01578081	0.049411803	-7.35185063	6.48E-05	0.00942429	2.271797311
hetero after UVB irradiation VS hetero before UVB irradiation	IC_CLEAVAGE_IN_ITS1_TO_SEPARATE_SSU_RRNA_FROM_5_8S_RRNA_AND_LSU_RRNA_FROM_TRICISTRONIC_RRNA_TRANSCRIPT_SSU_RRNA_5_	-0.87207672	-0.08423558	-3.98184885	0.00420646	0.12588577	-1.66702315
WT after UVB irradiation VS WT before UVB irradiation	IC_CLEAVAGE_IN_ITS1_TO_SEPARATE_SSU_RRNA_FROM_5_8S_RRNA_AND_LSU_RRNA_FROM_TRICISTRONIC_RRNA_TRANSCRIPT_SSU_RRNA_5_	-0.35994591	-0.00823925	-1.03642724	0.32995891	0.77726938	-5.465929
homo after UVB irradiation VS homo before UVB irradiation	GO_ENDONUCLEOLYTIC_CLEAVAGE_INVOLVED_IN_RRNA_PROCESSING	-0.88317394	0.028758227	-5.35275417	0.00059749	0.01760084	0.091964098
hetero after UVB irradiation VS hetero before UVB irradiation	GO_ENDONUCLEOLYTIC_CLEAVAGE_INVOLVED_IN_RRNA_PROCESSING1	-0.71778843	-0.07956032	-3.30677017	0.01103484	0.16397794	-2.59791699
WT after UVB irradiation VS WT before UVB irradiation	GO_ENDONUCLEOLYTIC_CLEAVAGE_INVOLVED_IN_RRNA_PROCESSING2	-0.42070922	-0.00963118	-1.18555809	0.26941832	0.74431614	-5.31567883
homo after UVB irradiation VS homo before UVB irradiation	GO_ENDOPEPTIDASE_ACTIVATOR_ACTIVITY	0.380723521	-0.00935184	2.196900706	0.05797367	0.18018481	-4.47253972
hetero after UVB irradiation VS hetero before UVB irradiation	GO_ENDOPEPTIDASE_ACTIVATOR_ACTIVITY1	0.435369859	0.036008963	3.341766842	0.01048087	0.16126769	-2.54815435
WT after UVB irradiation VS WT before UVB irradiation	GO_ENDOPEPTIDASE_ACTIVATOR_ACTIVITY2	0.662157439	-0.0100737	3.314640032	0.01044424	0.33505574	-2.4563131
homo after UVB irradiation VS homo before UVB irradiation	GO_ENDOPEPTIDASE_ACTIVITY	0.241525034	-0.00450312	1.888331271	0.09421519	0.24234741	-4.9284124
hetero after UVB irradiation VS hetero before UVB irradiation	GO_ENDOPEPTIDASE_ACTIVITY1	0.209487528	0.017225312	1.619442246	0.1447203	0.46094742	-5.01165798
WT after UVB irradiation VS WT before UVB irradiation	GO_ENDOPEPTIDASE_ACTIVITY2	0.217508131	0.019996286	1.547029054	0.15999028	0.66392783	-4.89907512
homo after UVB irradiation VS homo before UVB irradiation	GO_ENDOPEPTIDASE_COMPLEX	-0.38025083	0.003470398	-1.60809644	0.14498646	0.31723301	-5.31850149
hetero after UVB irradiation VS hetero before UVB irradiation	GO_ENDOPEPTIDASE_COMPLEX1	-0.09733045	-0.03609083	-0.43193061	0.67740641	0.87018447	-6.13153685
WT after UVB irradiation VS WT before UVB irradiation	GO_ENDOPEPTIDASE_COMPLEX2	-0.06854486	-0.03195966	-0.22342129	0.82874099	0.95957725	-5.96981365
homo after UVB irradiation VS homo before UVB irradiation	GO_ENDOPEPTIDASE_REGULATOR_ACTIVITY	0.310327701	0.005220402	2.085173861	0.06917565	0.20114779	-4.64003503
hetero after UVB irradiation VS hetero before UVB irradiation	GO_ENDOPEPTIDASE_REGULATOR_ACTIVITY1	0.188852784	0.018623808	1.125239479	0.29370156	0.61756748	-5.59676748
WT after UVB irradiation VS WT before UVB irradiation	GO_ENDOPEPTIDASE_REGULATOR_ACTIVITY2	0.234174321	0.033865807	1.401134666	0.19831363	0.69702831	-5.07507502
homo after UVB irradiation VS homo before UVB irradiation	GO_ENDOPLASMIC_RETICULUM	0.299214177	-0.02366588	2.441720053	0.03931106	0.14244246	-4.09887839
homo after UVB irradiation VS homo before UVB irradiation	GO_ENDOPLASMIC_RETICULUM_CALCIUM_ION_HOMEOSTASIS	0.579883213	-0.07717016	3.400679851	0.00880829	0.06249226	-2.6174592
hetero after UVB irradiation VS hetero before UVB irradiation	GO_ENDOPLASMIC_RETICULUM_CALCIUM_ION_HOMEOSTASIS1	0.293741905	0.009401725	1.273105091	0.23937449	0.56769689	-5.43541398
WT after UVB irradiation VS WT before UVB irradiation	GO_ENDOPLASMIC_RETICULUM_CALCIUM_ION_HOMEOSTASIS2	0.384364806	-0.06400485	1.748839523	0.11798744	0.62616955	-4.64167054
homo after UVB irradiation VS homo before UVB irradiation	GO_ENDOPLASMIC_RETICULUM_CHAPERONE_COMPLEX	0.052277461	-0.05290036	0.232090419	0.8220688	0.90054705	-6.52139104
hetero after UVB irradiation VS hetero before UVB irradiation	GO_ENDOPLASMIC_RETICULUM_CHAPERONE_COMPLEX1	0.504606458	-0.0246271	2.048606785	0.07532891	0.35411506	-4.42504587
WT after UVB irradiation VS WT before UVB irradiation	GO_ENDOPLASMIC_RETICULUM_CHAPERONE_COMPLEX2	0.480872032	-0.10318409	1.760063306	0.11598183	0.62283254	-4.62695305
homo after UVB irradiation VS homo before UVB irradiation	GO_ENDOPLASMIC_RETICULUM_EXIT_SITE	0.518066957	0.039594105	2.918789226	0.01851014	0.0934377	-3.35915223
hetero after UVB irradiation VS hetero before UVB irradiation	GO_ENDOPLASMIC_RETICULUM_EXIT_SITE1	0.366940817	-0.01001201	1.351735127	0.21410414	0.54114961	-5.34438938
WT after UVB irradiation VS WT before UVB irradiation	GO_ENDOPLASMIC_RETICULUM_EXIT_SITE2	0.321569858	-0.05049338	1.627496845	0.14182655	0.6516606	-4.79818932
homo after UVB irradiation VS homo before UVB irradiation	GO_ENDOPLASMIC_RETICULUM_GOLGI_INTERMEDIATE_COMPARTMENT	0.443780808	-0.03803466	3.089633519	0.014187	0.08105207	-3.0945353
homo after UVB irradiation VS homo before UVB irradiation	GO_ENDOPLASMIC_RETICULUM_GOLGI_INTERMEDIATE_COMPARTMENT_MEMBRANE	0.380155641	-0.03578898	2.587223582	0.03120915	0.12525249	-3.87403231
hetero after UVB irradiation VS hetero before UVB irradiation	GO_ENDOPLASMIC_RETICULUM_GOLGI_INTERMEDIATE_COMPARTMENT_MEMBRANE1	0.220953839	0.001348953	1.516838857	0.16848172	0.48995083	-5.14296984
WT after UVB irradiation VS WT before UVB irradiation	GO_ENDOPLASMIC_RETICULUM_GOLGI_INTERMEDIATE_COMPARTMENT_MEMBRANE2	0.205906965	0.000677054	1.191364346	0.26725641	0.74336868	-5.30954321
hetero after UVB irradiation VS hetero before UVB irradiation	GO_ENDOPLASMIC_RETICULUM_GOLGI_INTERMEDIATE_COMPARTMENT1	0.275453481	0.010042555	1.902230556	0.09433407	0.38408634	-4.63062595
WT after UVB irradiation VS WT before UVB irradiation	GO_ENDOPLASMIC_RETICULUM_GOLGI_INTERMEDIATE_COMPARTMENT2	0.238516552	-0.02454958	1.307378993	0.22698207	0.71906685	-5.18282219
homo after UVB irradiation VS homo before UVB irradiation	GO_ENDOPLASMIC_RETICULUM_LOCALIZATION	0.39947524	-0.024395	2.197179741	0.05794805	0.18014313	-4.47211857
hetero after UVB irradiation VS hetero before UVB irradiation	GO_ENDOPLASMIC_RETICULUM_LOCALIZATION1	0.392484473	-0.05028147	1.457476133	0.18378311	0.50846076	-5.2168861
WT after UVB irradiation VS WT before UVB irradiation	GO_ENDOPLASMIC_RETICULUM_LOCALIZATION2	0.375099929	0.005659195	1.941780388	0.08766545	0.57920555	-4.38388949
homo after UVB irradiation VS homo before UVB irradiation	GO_ENDOPLASMIC_RETICULUM_LUMEN	0.531002354	-0.00481554	3.934043919	0.00400044	0.04131177	-1.82249676
hetero after UVB irradiation VS hetero before UVB irradiation	GO_ENDOPLASMIC_RETICULUM_LUMEN1	0.341111144	0.027892653	1.865115395	0.09984252	0.39245806	-4.6819744
WT after UVB irradiation VS WT before UVB irradiation	GO_ENDOPLASMIC_RETICULUM_LUMEN2	0.317421369	0.02062591	1.588139265	0.1504624	0.65844472	-4.84784554
homo after UVB irradiation VS homo before UVB irradiation	GO_ENDOPLASMIC_RETICULUM_MANNOSE_TRIMMING	0.448026172	-0.04187889	2.537166473	0.03378619	0.13134424	-3.95153784
hetero after UVB irradiation VS hetero before UVB irradiation	GO_ENDOPLASMIC_RETICULUM_MANNOSE_TRIMMING1	0.253787512	-0.00245067	1.233480459	0.25303956	0.5810538	-5.47995986
WT after UVB irradiation VS WT before UVB irradiation	GO_ENDOPLASMIC_RETICULUM_MANNOSE_TRIMMING2	0.340577361	0.00582225	2.085900187	0.07004786	0.5460625	-4.18588345
homo after UVB irradiation VS homo before UVB irradiation	GO_ENDOPLASMIC_RETICULUM_MEMBRANE_ORGANIZATION	0.237355254	-0.02961813	1.11933704	0.29423369	0.4889647	-5.91122277
hetero after UVB irradiation VS hetero before UVB irradiation	GO_ENDOPLASMIC_RETICULUM_MEMBRANE_ORGANIZATION1	-0.1286212	-0.07762974	-0.39186485	0.70557782	0.88307942	-6.14904251
WT after UVB irradiation VS WT before UVB irradiation	GO_ENDOPLASMIC_RETICULUM_MEMBRANE_ORGANIZATION2	0.409575007	0.072395372	2.057776117	0.07319143	0.55377265	-4.22481816
homo after UVB irradiation VS homo before UVB irradiation	GO_ENDOPLASMIC_RETICULUM_ORGANIZATION	0.1149115	0.005475168	0.785152655	0.45411515	0.63511284	-6.22470112
hetero after UVB irradiation VS hetero before UVB irradiation	GO_ENDOPLASMIC_RETICULUM_ORGANIZATION1	0.132808726	-0.01627805	0.889613776	0.40010527	0.7076422	-5.82333715
WT after UVB irradiation VS WT before UVB irradiation	GO_ENDOPLASMIC_RETICULUM_ORGANIZATION2	0.105954172	-0.03713968	0.707319403	0.49921487	0.85542427	-5.7403908
homo after UVB irradiation VS homo before UVB irradiation	GO_ENDOPLASMIC_RETICULUM_PALMITOYLTRANSFERASE_COMPLEX	0.426667856	0.000883305	2.389588437	0.04270334	0.14931871	-4.17903641
hetero after UVB irradiation VS hetero before UVB irradiation	GO_ENDOPLASMIC_RETICULUM_PALMITOYLTRANSFERASE_COMPLEX1	0.164658186	0.015299845	1.017580911	0.33920052	0.65910481	-5.70528649
WT after UVB irradiation VS WT before UVB irradiation	GO_ENDOPLASMIC_RETICULUM_PALMITOYLTRANSFERASE_COMPLEX2	0.044684557	0.154603125	0.139615423	0.89237397	0.97322079	-5.98579457
homo after UVB irradiation VS homo before UVB irradiation	GO_ENDOPLASMIC_RETICULUM_PLASMA_MEMBRANE_TETHERING	0.79695054	-0.04357412	5.322473315	0.00062036	0.01780829	0.054400774
hetero after UVB irradiation VS hetero before UVB irradiation	GO_ENDOPLASMIC_RETICULUM_PLASMA_MEMBRANE_TETHERING1	0.400784817	-0.00520719	1.063174331	0.31930132	0.63860264	-5.6603081
WT after UVB irradiation VS WT before UVB irradiation	GO_ENDOPLASMIC_RETICULUM_PLASMA_MEMBRANE_TETHERING2	0.466555535	0.034816359	1.662381593	0.13455377	0.64200725	-4.75370265
homo after UVB irradiation VS homo before UVB irradiation	GO_ENDOPLASMIC_RETICULUM_QUALITY_CONTROL_COMPARTMENT	0.510419643	-0.01498184	3.303147656	0.01021621	0.06769675	-2.76621194
hetero after UVB irradiation VS hetero before UVB irradiation	GO_ENDOPLASMIC_RETICULUM_QUALITY_CONTROL_COMPARTMENT1	0.333927399	-0.01578551	1.742650826	0.12026814	0.42502498	-4.84872431
WT after UVB irradiation VS WT before UVB irradiation	GO_ENDOPLASMIC_RETICULUM_QUALITY_CONTROL_COMPARTMENT2	0.345381034	-0.01309349	2.032213285	0.07616753	0.55880099	-4.26009132
homo after UVB irradiation VS homo before UVB irradiation	GO_ENDOPLASMIC_RETICULUM_SUBCOMPARTMENT	-0.12551929	-0.0148022	-0.97108528	0.35885702	0.55065541	-6.06111692
hetero after UVB irradiation VS hetero before UVB irradiation	GO_ENDOPLASMIC_RETICULUM_SUBCOMPARTMENT1	-0.23488746	-0.05507516	-1.50270992	0.1720164	0.49496277	-5.16070609
WT after UVB irradiation VS WT before UVB irradiation	GO_ENDOPLASMIC_RETICULUM_SUBCOMPARTMENT2	-0.27941235	-0.0544166	-1.62486705	0.14238916	0.65167133	-4.80152522
homo after UVB irradiation VS homo before UVB irradiation	GO_ENDOPLASMIC_RETICULUM_TO_CYTOSOL_TRANSPORT	0.345645715	-0.06492206	1.527011358	0.1637937	0.3413373	-5.42559177
hetero after UVB irradiation VS hetero before UVB irradiation	GO_ENDOPLASMIC_RETICULUM_TO_CYTOSOL_TRANSPORT1	0.279194525	0.011309196	1.581357778	0.15315898	0.47172072	-5.0608967
WT after UVB irradiation VS WT before UVB irradiation	GO_ENDOPLASMIC_RETICULUM_TO_CYTOSOL_TRANSPORT2	0.256288411	-0.03144965	1.329548653	0.2199003	0.71478521	-5.15775425
homo after UVB irradiation VS homo before UVB irradiation	GO_ENDOPLASMIC_RETICULUM_TO_GOLGI_VESICLE_MEDIATED_TRANSPORT	0.422569803	-0.02863187	2.965569934	0.01720546	0.08971634	-3.2865779
hetero after UVB irradiation VS hetero before UVB irradiation	GO_ENDOPLASMIC_RETICULUM_TO_GOLGI_VESICLE_MEDIATED_TRANSPORT1	0.39152843	0.017737022	2.894990114	0.02044441	0.20617329	-3.1924747
WT after UVB irradiation VS WT before UVB irradiation	GO_ENDOPLASMIC_RETICULUM_TO_GOLGI_VESICLE_MEDIATED_TRANSPORT2	0.343011284	-0.02392793	2.207889639	0.05787571	0.52490436	-4.01566285
homo after UVB irradiation VS homo before UVB irradiation	GO_ENDOPLASMIC_RETICULUM_TUBULAR_NETWORK	-0.15542468	-0.01156872	-1.03572489	0.32946151	0.52278101	-5.99776102
homo after UVB irradiation VS homo before UVB irradiation	GO_ENDOPLASMIC_RETICULUM_TUBULAR_NETWORK_MEMBRANE	-0.2529585	-0.00399854	-0.74538926	0.4765415	0.65441308	-6.25586284
hetero after UVB irradiation VS hetero before UVB irradiation	GO_ENDOPLASMIC_RETICULUM_TUBULAR_NETWORK_MEMBRANE1	-0.20552451	-0.0610211	-1.20156283	0.26451536	0.59120563	-5.51516277
WT after UVB irradiation VS WT before UVB irradiation	GO_ENDOPLASMIC_RETICULUM_TUBULAR_NETWORK_MEMBRANE2	-0.59419934	-0.1207309	-3.10672835	0.01430193	0.36090271	-2.74395956
homo after UVB irradiation VS homo before UVB irradiation	GO_ENDOPLASMIC_RETICULUM_TUBULAR_NETWORK_ORGANIZATION	-0.1962848	0.060466541	-1.01469552	0.33881596	0.53179186	-6.01872099
hetero after UVB irradiation VS hetero before UVB irradiation	GO_ENDOPLASMIC_RETICULUM_TUBULAR_NETWORK_ORGANIZATION1	-0.06121714	-0.06575361	-0.42893584	0.67949419	0.87104651	-6.13290153
WT after UVB irradiation VS WT before UVB irradiation	GO_ENDOPLASMIC_RETICULUM_TUBULAR_NETWORK_ORGANIZATION2	-0.34815484	-0.02157489	-1.53561348	0.16273089	0.66595112	-4.91318024
hetero after UVB irradiation VS hetero before UVB irradiation	GO_ENDOPLASMIC_RETICULUM_TUBULAR_NETWORK1	-0.15859558	-0.0789868	-1.18853077	0.26932324	0.59632699	-5.52935728
WT after UVB irradiation VS WT before UVB irradiation	GO_ENDOPLASMIC_RETICULUM_TUBULAR_NETWORK2	-0.32541184	-0.0509809	-1.85900405	0.09963665	0.59874718	-4.4956584
homo after UVB irradiation VS homo before UVB irradiation	GO_ENDOPLASMIC_RETICULUM_UNFOLDED_PROTEIN_RESPONSE	0.263172949	-0.01799434	1.616956807	0.14305453	0.31471014	-5.30662404
hetero after UVB irradiation VS hetero before UVB irradiation	GO_ENDOPLASMIC_RETICULUM_UNFOLDED_PROTEIN_RESPONSE1	0.304633735	-0.0038788	1.880676394	0.09749658	0.38954886	-4.66048835
WT after UVB irradiation VS WT before UVB irradiation	GO_ENDOPLASMIC_RETICULUM_UNFOLDED_PROTEIN_RESPONSE2	0.269735335	-0.02842726	1.493013516	0.1733338	0.67927579	-4.96533537
hetero after UVB irradiation VS hetero before UVB irradiation	GO_ENDOPLASMIC_RETICULUM1	0.177962584	-0.00234719	1.165098426	0.27814871	0.60400502	-5.55461305
WT after UVB irradiation VS WT before UVB irradiation	GO_ENDOPLASMIC_RETICULUM2	0.165101478	-0.01850365	1.037955556	0.32928847	0.77711658	-5.46446416
homo after UVB irradiation VS homo before UVB irradiation	GO_ENDOPOLYPHOSPHATASE_ACTIVITY	0.412740729	-0.09194391	1.792535697	0.10932749	0.26449617	-5.06486026
hetero after UVB irradiation VS hetero before UVB irradiation	GO_ENDOPOLYPHOSPHATASE_ACTIVITY1	0.382310487	0.03678307	1.287731011	0.23449042	0.56254359	-5.41874233
WT after UVB irradiation VS WT before UVB irradiation	GO_ENDOPOLYPHOSPHATASE_ACTIVITY2	0.424242329	-0.09672231	1.626250772	0.14209288	0.65167133	-4.79977028
homo after UVB irradiation VS homo before UVB irradiation	GO_ENDORIBONUCLEASE_ACTIVITY	-0.23790829	0.005108465	-1.76427047	0.11420448	0.27182507	-5.10454891
homo after UVB irradiation VS homo before UVB irradiation	GO_ENDORIBONUCLEASE_ACTIVITY_PRODUCING_3_PHOSPHOMONOESTERS	0.224061876	-0.04891755	0.987234838	0.35133414	0.54323532	-6.0455874
hetero after UVB irradiation VS hetero before UVB irradiation	GO_ENDORIBONUCLEASE_ACTIVITY_PRODUCING_3_PHOSPHOMONOESTERS1	-0.14914532	-0.04067755	-0.59200775	0.57049266	0.81405954	-6.04569484
WT after UVB irradiation VS WT before UVB irradiation	GO_ENDORIBONUCLEASE_ACTIVITY_PRODUCING_3_PHOSPHOMONOESTERS2	-0.00759354	0.049745658	-0.02575198	0.98007849	0.99512555	-5.99571676
homo after UVB irradiation VS homo before UVB irradiation	GO_ENDORIBONUCLEASE_ACTIVITY_PRODUCING_5_PHOSPHOMONOESTERS	-0.64312388	-0.0134538	-4.28460788	0.00243335	0.0330029	-1.32060334
hetero after UVB irradiation VS hetero before UVB irradiation	GO_ENDORIBONUCLEASE_ACTIVITY_PRODUCING_5_PHOSPHOMONOESTERS1	-0.38807012	-0.09375573	-1.85031662	0.10212343	0.3967998	-4.7023494
WT after UVB irradiation VS WT before UVB irradiation	GO_ENDORIBONUCLEASE_ACTIVITY_PRODUCING_5_PHOSPHOMONOESTERS2	-0.25618876	-0.02014188	-0.8914278	0.39841229	0.81477652	-5.59725952
hetero after UVB irradiation VS hetero before UVB irradiation	GO_ENDORIBONUCLEASE_ACTIVITY1	-0.14283209	-0.05366495	-0.78972622	0.45287199	0.74457706	-5.90650639
WT after UVB irradiation VS WT before UVB irradiation	GO_ENDORIBONUCLEASE_ACTIVITY2	0.00287412	0.006455446	0.012950731	0.9899805	0.99848757	-5.99597817
homo after UVB irradiation VS homo before UVB irradiation	GO_ENDORIBONUCLEASE_COMPLEX	-0.66638693	-0.02652967	-4.68345286	0.00141272	0.02530056	-0.77212216
hetero after UVB irradiation VS hetero before UVB irradiation	GO_ENDORIBONUCLEASE_COMPLEX1	-0.24989101	-0.05168683	-1.16748376	0.27723963	0.60400502	-5.55205793
WT after UVB irradiation VS WT before UVB irradiation	GO_ENDORIBONUCLEASE_COMPLEX2	-0.26251677	-0.01261408	-1.22615696	0.25459856	0.73589193	-5.27235372
homo after UVB irradiation VS homo before UVB irradiation	GO_ENDOSOMAL_TRANSPORT	0.347687655	-0.02786513	2.353619439	0.04521316	0.15476285	-4.23418286
hetero after UVB irradiation VS hetero before UVB irradiation	GO_ENDOSOMAL_TRANSPORT1	0.344964304	-0.00245567	2.696029539	0.02770339	0.23528578	-3.48377476
WT after UVB irradiation VS WT before UVB irradiation	GO_ENDOSOMAL_TRANSPORT2	0.28453679	-0.00010359	1.880217093	0.09642821	0.59686908	-4.46717072
homo after UVB irradiation VS homo before UVB irradiation	GO_ENDOSOMAL_VESICLE_FUSION	0.598327709	-0.06150169	3.218633331	0.01162731	0.07233734	-2.8957734
hetero after UVB irradiation VS hetero before UVB irradiation	GO_ENDOSOMAL_VESICLE_FUSION1	0.51205412	0.155905118	1.814572351	0.10783886	0.40602721	-4.75131754
WT after UVB irradiation VS WT before UVB irradiation	GO_ENDOSOMAL_VESICLE_FUSION2	0.676716588	-0.05047408	3.41864883	0.00894157	0.31908946	-2.31438668
homo after UVB irradiation VS homo before UVB irradiation	GO_ENDOSOME	0.307847244	-0.02088812	2.216824158	0.05617225	0.17618147	-4.44243735
homo after UVB irradiation VS homo before UVB irradiation	GO_ENDOSOME_LUMEN	0.44180279	-0.03546764	3.151232893	0.01289835	0.07677679	-2.9994867
hetero after UVB irradiation VS hetero before UVB irradiation	GO_ENDOSOME_LUMEN1	0.310059474	-0.01273207	1.485996469	0.17628336	0.49975739	-5.18157237
WT after UVB irradiation VS WT before UVB irradiation	GO_ENDOSOME_LUMEN2	0.044260136	0.004355112	0.199041067	0.84713755	0.96522688	-5.97521661
homo after UVB irradiation VS homo before UVB irradiation	GO_ENDOSOME_MEMBRANE	0.379181738	-0.01518083	2.653315907	0.02810913	0.11867493	-3.77152629
hetero after UVB irradiation VS hetero before UVB irradiation	GO_ENDOSOME_MEMBRANE1	0.420547319	0.009989063	3.251563007	0.01197254	0.1697279	-2.67669895
WT after UVB irradiation VS WT before UVB irradiation	GO_ENDOSOME_MEMBRANE2	0.363439665	-0.00983788	2.565080213	0.03305823	0.4606613	-3.50973021
homo after UVB irradiation VS homo before UVB irradiation	GO_ENDOSOME_ORGANIZATION	0.301861228	-0.02522222	1.544655853	0.15952386	0.33550924	-5.40254189
hetero after UVB irradiation VS hetero before UVB irradiation	GO_ENDOSOME_ORGANIZATION1	0.351884172	0.026157937	2.574573384	0.03339491	0.25309171	-3.66200909
WT after UVB irradiation VS WT before UVB irradiation	GO_ENDOSOME_ORGANIZATION2	0.207124275	0.025535771	1.035246731	0.3304775	0.77726938	-5.46705937
homo after UVB irradiation VS homo before UVB irradiation	GO_ENDOSOME_TO_LYSOSOME_TRANSPORT	0.185162796	-0.03004282	1.307183379	0.22608009	0.41681894	-5.69953742
homo after UVB irradiation VS homo before UVB irradiation	GO_ENDOSOME_TO_LYSOSOME_TRANSPORT_VIA_MULTIVESICULAR_BODY_SORTING_PATHWAY	0.639120108	-0.10820892	2.965352957	0.01721129	0.08971634	-3.28691434
hetero after UVB irradiation VS hetero before UVB irradiation	GO_ENDOSOME_TO_LYSOSOME_TRANSPORT_VIA_MULTIVESICULAR_BODY_SORTING_PATHWAY1	0.309491098	-0.01749281	1.267263104	0.24134924	0.56981589	-5.44203893
WT after UVB irradiation VS WT before UVB irradiation	GO_ENDOSOME_TO_LYSOSOME_TRANSPORT_VIA_MULTIVESICULAR_BODY_SORTING_PATHWAY2	0.361193284	0.005419631	1.493884839	0.1731109	0.67927579	-4.96427639
hetero after UVB irradiation VS hetero before UVB irradiation	GO_ENDOSOME_TO_LYSOSOME_TRANSPORT1	0.127841827	0.024302798	0.745834457	0.47749708	0.7595751	-5.94040662
WT after UVB irradiation VS WT before UVB irradiation	GO_ENDOSOME_TO_LYSOSOME_TRANSPORT2	0.058435199	-0.00237789	0.376561762	0.71617492	0.93049774	-5.92193329
homo after UVB irradiation VS homo before UVB irradiation	GO_ENDOSOME_TO_PLASMA_MEMBRANE_PROTEIN_TRANSPORT	0.618737469	-0.05121018	4.475546146	0.0018702	0.02923568	-1.05495168
hetero after UVB irradiation VS hetero before UVB irradiation	GO_ENDOSOME_TO_PLASMA_MEMBRANE_PROTEIN_TRANSPORT1	0.437047571	0.014154077	2.19776694	0.05982038	0.32192177	-4.21152536
WT after UVB irradiation VS WT before UVB irradiation	GO_ENDOSOME_TO_PLASMA_MEMBRANE_PROTEIN_TRANSPORT2	0.424129909	-0.06521897	2.492976482	0.0370094	0.47615783	-3.61232319
hetero after UVB irradiation VS hetero before UVB irradiation	GO_ENDOSOME1	0.331293457	0.005926949	2.571898081	0.03353294	0.2531992	-3.66593451
WT after UVB irradiation VS WT before UVB irradiation	GO_ENDOSOME2	0.279861886	-0.00940499	1.976563954	0.08305753	0.56902503	-4.33646534
homo after UVB irradiation VS homo before UVB irradiation	GO_ENDOTHELIAL_CELL_ACTIVATION	-0.05719605	-0.00912139	-0.27206524	0.79219455	0.88180348	-6.51041631
hetero after UVB irradiation VS hetero before UVB irradiation	GO_ENDOTHELIAL_CELL_ACTIVATION1	0.104511877	0.079617986	0.620731044	0.55236212	0.80286203	-6.02768153
WT after UVB irradiation VS WT before UVB irradiation	GO_ENDOTHELIAL_CELL_ACTIVATION2	0.302745727	-0.01518658	1.390472658	0.20140747	0.6976925	-5.08755209
homo after UVB irradiation VS homo before UVB irradiation	GO_ENDOTHELIAL_CELL_APOPTOTIC_PROCESS	0.364107378	-0.00270408	2.815811155	0.02175457	0.10273032	-3.51909234
hetero after UVB irradiation VS hetero before UVB irradiation	GO_ENDOTHELIAL_CELL_APOPTOTIC_PROCESS1	0.338424842	0.026889055	2.286110904	0.05217173	0.30344742	-4.08365555
WT after UVB irradiation VS WT before UVB irradiation	GO_ENDOTHELIAL_CELL_APOPTOTIC_PROCESS2	0.11565031	0.000685881	0.557805091	0.59205988	0.89299138	-5.83515204
homo after UVB irradiation VS homo before UVB irradiation	GO_ENDOTHELIAL_CELL_CHEMOTAXIS	0.351429922	-0.03732117	2.164093525	0.06106453	0.18632593	-4.52196215

hetero after UVB irradiation VS hetero before UVB irradiation	GO_ENDOTHELIAL_CELL_CHEMOTAXIS1	0.047664342	0.005764268	0.211900452	0.83758104	0.93787721	-6.20720547
WT after UVB irradiation VS WT before UVB irradiation	GO_ENDOTHELIAL_CELL_CHEMOTAXIS2	0.125442419	-0.02381737	0.572201342	0.58271813	0.88953878	-5.82691362
homo after UVB irradiation VS homo before UVB irradiation	GO_ENDOTHELIAL_CELL_DEVELOPMENT	0.35816903	-0.02486937	2.051774068	0.07291612	0.2071267	-4.68962317
hetero after UVB irradiation VS hetero before UVB irradiation	GO_ENDOTHELIAL_CELL_DEVELOPMENT1	0.336020871	0.015651123	2.046619077	0.07556016	0.35452009	-4.42786666
WT after UVB irradiation VS WT before UVB irradiation	GO_ENDOTHELIAL_CELL_DEVELOPMENT2	0.24156643	-0.03296287	1.334421476	0.21836931	0.71478521	-5.15220952
homo after UVB irradiation VS homo before UVB irradiation	GO_ENDOTHELIAL_CELL_FATE_COMMITMENT	0.510386258	-0.03968293	2.424287035	0.04041428	0.14458023	-4.12571186
hetero after UVB irradiation VS hetero before UVB irradiation	GO_ENDOTHELIAL_CELL_FATE_COMMITMENT1	0.56601299	0.011424908	1.999100846	0.08129884	0.36518896	-4.49507755
WT after UVB irradiation VS WT before UVB irradiation	GO_ENDOTHELIAL_CELL_FATE_COMMITMENT2	-0.10189544	0.088976857	-0.33678881	0.74483862	0.93574017	-5.93665749
homo after UVB irradiation VS homo before UVB irradiation	GO_ENDOTHELIAL_CELL_MATRIX_ADHESION	0.471784443	-0.04959418	2.521426357	0.03464021	0.13332959	-3.97587967
homo after UVB irradiation VS homo before UVB irradiation	GO_ENDOTHELIAL_CELL_MATRIX_ADHESION_VIA_FIBRONECTIN	0.229617054	-0.03092504	0.847960824	0.42015765	0.60549024	-6.17266788
hetero after UVB irradiation VS hetero before UVB irradiation	GO_ENDOTHELIAL_CELL_MATRIX_ADHESION_VIA_FIBRONECTIN1	0.234392832	0.098473366	0.899270621	0.39524695	0.7033063	-5.81486771
WT after UVB irradiation VS WT before UVB irradiation	GO_ENDOTHELIAL_CELL_MATRIX_ADHESION_VIA_FIBRONECTIN2	0.270772033	-0.0939461	1.086962074	0.30834622	0.76668326	-5.41664719
hetero after UVB irradiation VS hetero before UVB irradiation	GO_ENDOTHELIAL_CELL_MATRIX_ADHESION1	0.351845906	0.029266398	1.910053261	0.0932108	0.38184856	-4.61975961
WT after UVB irradiation VS WT before UVB irradiation	GO_ENDOTHELIAL_CELL_MATRIX_ADHESION2	0.419869161	-0.06729766	1.78936411	0.11089543	0.61576748	-4.58835559
homo after UVB irradiation VS homo before UVB irradiation	GO_ENDOTHELIAL_CELL_MIGRATION	0.454224456	-0.02793878	3.677492461	0.00582	0.05041635	-2.20059323
hetero after UVB irradiation VS hetero before UVB irradiation	GO_ENDOTHELIAL_CELL_MIGRATION1	0.329535727	0.014374174	1.904488624	0.09400851	0.38351936	-4.62749085
WT after UVB irradiation VS WT before UVB irradiation	GO_ENDOTHELIAL_CELL_MIGRATION2	0.233010428	-0.01476966	1.203003547	0.26296551	0.74156847	-5.29718248
homo after UVB irradiation VS homo before UVB irradiation	GO_ENDOTHELIAL_CELL_MORPHOGENESIS	0.424962031	-0.01063035	2.353882877	0.04519425	0.1547405	-4.23377948
hetero after UVB irradiation VS hetero before UVB irradiation	GO_ENDOTHELIAL_CELL_MORPHOGENESIS1	0.400125707	0.029293579	2.1129066	0.06821065	0.33946589	-4.33342506
WT after UVB irradiation VS WT before UVB irradiation	GO_ENDOTHELIAL_CELL_MORPHOGENESIS2	0.321037153	-0.02875039	1.421244399	0.192591	0.69334561	-5.05139199
homo after UVB irradiation VS homo before UVB irradiation	GO_ENDOTHELIAL_CELL_PROLIFERATION	0.388560383	-0.02159265	3.130510077	0.01331767	0.07817376	-3.03143616
hetero after UVB irradiation VS hetero before UVB irradiation	GO_ENDOTHELIAL_CELL_PROLIFERATION1	0.350915101	0.013538924	2.074075209	0.07242644	0.34858227	-4.38884042
WT after UVB irradiation VS WT before UVB irradiation	GO_ENDOTHELIAL_CELL_PROLIFERATION2	0.219557963	-0.0166069	1.076218115	0.31284563	0.77011405	-5.42726924
homo after UVB irradiation VS homo before UVB irradiation	GO_ENDOTHELIN_RECEPTOR_SIGNALING_PATHWAY	0.071062994	-0.03934032	0.254701065	0.80513002	0.88968218	-6.51539396
hetero after UVB irradiation VS hetero before UVB irradiation	GO_ENDOTHELIN_RECEPTOR_SIGNALING_PATHWAY1	0.319626852	-0.05846075	1.416067893	0.19517534	0.51964455	-5.26747645
WT after UVB irradiation VS WT before UVB irradiation	GO_ENDOTHELIN_RECEPTOR_SIGNALING_PATHWAY2	0.444684923	-0.01797528	1.646185058	0.13788674	0.64637696	-4.7744111
homo after UVB irradiation VS homo before UVB irradiation	GO_ENDOTHELIUM_DEVELOPMENT	0.32303028	-0.02883749	2.157029767	0.06175089	0.18761225	-4.53257857
hetero after UVB irradiation VS hetero before UVB irradiation	GO_ENDOTHELIUM_DEVELOPMENT1	0.294365982	0.010552591	1.886268508	0.09666647	0.38819802	-4.65275186
WT after UVB irradiation VS WT before UVB irradiation	GO_ENDOTHELIUM_DEVELOPMENT2	0.134004631	-0.0132757	0.769811238	0.46327467	0.84131729	-5.69494884
homo after UVB irradiation VS homo before UVB irradiation	GO_ENERGY_COUPLED_PROTON_TRANSMEMBRANE_TRANSPORT_AGAINST_ELECTROCHEMICAL_GRADIENT	0.254555997	-0.02746024	0.629237424	0.54606075	0.71016013	-6.33866347
hetero after UVB irradiation VS hetero before UVB irradiation	GO_ENERGY_COUPLED_PROTON_TRANSMEMBRANE_TRANSPORT_AGAINST_ELECTROCHEMICAL_GRADIENT1	-0.25899013	-0.03805147	-0.63877481	0.54114809	0.79694618	-6.01597195
WT after UVB irradiation VS WT before UVB irradiation	GO_ENERGY_COUPLED_PROTON_TRANSMEMBRANE_TRANSPORT_AGAINST_ELECTROCHEMICAL_GRADIENT2	0.367357094	0.033920093	0.923181208	0.38259651	0.80508768	-5.56983179
homo after UVB irradiation VS homo before UVB irradiation	GO_ENERGY_DERIVATION_BY_OXIDATION_OF_ORGANIC_COMPOUNDS	0.01445383	0.007105407	0.070021679	0.94582845	0.97136109	-6.54816664
hetero after UVB irradiation VS hetero before UVB irradiation	GO_ENERGY_DERIVATION_BY_OXIDATION_OF_ORGANIC_COMPOUNDS1	-0.23800929	-0.02708416	-1.32316663	0.22300752	0.5517604	-5.37785318
WT after UVB irradiation VS WT before UVB irradiation	GO_ENERGY_DERIVATION_BY_OXIDATION_OF_ORGANIC_COMPOUNDS2	0.001666242	-0.0613065	0.007072163	0.99452841	0.9985657	-5.99604028
homo after UVB irradiation VS homo before UVB irradiation	GO_ENERGY_HOMEOSTASIS	-0.17835936	0.007035569	-1.32245152	0.22117477	0.41205354	-5.68136524
hetero after UVB irradiation VS hetero before UVB irradiation	GO_ENERGY_HOMEOSTASIS1	0.000200729	0.015498368	0.001464814	0.99886778	0.99940583	-6.23148295
WT after UVB irradiation VS WT before UVB irradiation	GO_ENERGY_HOMEOSTASIS2	-0.25837713	0.0131403	-1.66815503	0.13338373	0.64011176	-4.74629876
homo after UVB irradiation VS homo before UVB irradiation	GO_ENERGY_RESERVE_METABOLIC_PROCESS	0.114395763	-0.01254359	0.838739998	0.42502968	0.60989528	-6.18051863
hetero after UVB irradiation VS hetero before UVB irradiation	GO_ENERGY_RESERVE_METABOLIC_PROCESS1	-0.22579651	-0.02512959	-1.40457441	0.19844569	0.52299779	-5.28137095
WT after UVB irradiation VS WT before UVB irradiation	GO_ENERGY_RESERVE_METABOLIC_PROCESS2	-0.09229824	-0.07607332	-0.43437166	0.67536091	0.91684204	-5.89772163
homo after UVB irradiation VS homo before UVB irradiation	GO_ENGULFMENT_OF_APOPTOTIC_CELL	0.645829987	-0.00530182	4.889000276	0.00107723	0.02224337	-0.49919911
hetero after UVB irradiation VS hetero before UVB irradiation	GO_ENGULFMENT_OF_APOPTOTIC_CELL1	0.701507143	-0.00079867	4.901355247	0.00125764	0.09106857	-0.51582953
WT after UVB irradiation VS WT before UVB irradiation	GO_ENGULFMENT_OF_APOPTOTIC_CELL2	0.695121205	0.031650694	3.796969073	0.0051427	0.27446335	-1.81122734
homo after UVB irradiation VS homo before UVB irradiation	GO_ENONE_REDUCTASE_ACTIVITY	0.898001973	0.001919964	4.091149967	0.00319444	0.03726176	-1.59536841
hetero after UVB irradiation VS hetero before UVB irradiation	GO_ENONE_REDUCTASE_ACTIVITY1	0.532894344	0.016786111	1.795815681	0.11095805	0.41045301	-4.77686945
WT after UVB irradiation VS WT before UVB irradiation	GO_ENONE_REDUCTASE_ACTIVITY2	0.337103565	0.075245502	1.061779384	0.3189734	0.77323848	-5.44142212
homo after UVB irradiation VS homo before UVB irradiation	GO_ENOYL_COA_HYDRATASE_ACTIVITY	-0.38471379	0.025790752	-1.38250511	0.20275234	0.39188164	-5.60859118
hetero after UVB irradiation VS hetero before UVB irradiation	GO_ENOYL_COA_HYDRATASE_ACTIVITY1	-0.71837609	0.005035426	-3.25687111	0.01187883	0.16938831	-2.6691096
WT after UVB irradiation VS WT before UVB irradiation	GO_ENOYL_COA_HYDRATASE_ACTIVITY2	-0.67267153	-0.0506898	-3.41904527	0.0089363	0.31908946	-2.31384846
homo after UVB irradiation VS homo before UVB irradiation	GO_ENSHEATHMENT_OF_NEURONS	0.023015177	-0.02351279	0.177941485	0.86302061	0.92461124	-6.53351292
hetero after UVB irradiation VS hetero before UVB irradiation	GO_ENSHEATHMENT_OF_NEURONS1	-0.05160801	-0.00159105	-0.37378657	0.71845308	0.89013381	-6.15640615
WT after UVB irradiation VS WT before UVB irradiation	GO_ENSHEATHMENT_OF_NEURONS2	-0.06471369	-0.01799756	-0.42558323	0.68149681	0.91716791	-5.901615
homo after UVB irradiation VS homo before UVB irradiation	GO_ENTERIC_NERVOUS_SYSTEM_DEVELOPMENT	0.192416061	0.027874221	1.20022586	0.26307227	0.45839621	-5.82286409
hetero after UVB irradiation VS hetero before UVB irradiation	GO_ENTERIC_NERVOUS_SYSTEM_DEVELOPMENT1	0.217642584	0.001720366	1.149036384	0.28433364	0.60954889	-5.57172372
WT after UVB irradiation VS WT before UVB irradiation	GO_ENTERIC_NERVOUS_SYSTEM_DEVELOPMENT2	0.416521045	0.091685588	2.72858848	0.02561305	0.43395158	-3.27712282
homo after UVB irradiation VS homo before UVB irradiation	GO_ENTEROENDOCRINE_CELL_DIFFERENTIATION	-0.10590782	0.042731311	-0.52860986	0.61086816	0.75733693	-6.40006713
hetero after UVB irradiation VS hetero before UVB irradiation	GO_ENTEROENDOCRINE_CELL_DIFFERENTIATION1	0.022028597	0.000237036	0.142507265	0.89026784	0.95987083	-6.2204855
WT after UVB irradiation VS WT before UVB irradiation	GO_ENTEROENDOCRINE_CELL_DIFFERENTIATION2	-0.13284791	-0.0282226	-0.65912045	0.52811286	0.86664792	-5.77313157
homo after UVB irradiation VS homo before UVB irradiation	GO_ENTRAINMENT_OF_CIRCADIAN_CLOCK	-0.33642205	0.038771517	-1.81967062	0.10482971	0.25821599	-5.02650576
hetero after UVB irradiation VS hetero before UVB irradiation	GO_ENTRAINMENT_OF_CIRCADIAN_CLOCK1	-0.33648141	-0.02153205	-2.05108041	0.0750421	0.35391027	-4.4215345
WT after UVB irradiation VS WT before UVB irradiation	GO_ENTRAINMENT_OF_CIRCADIAN_CLOCK2	-0.29468857	0.001305266	-1.7855793	0.11154046	0.61576748	-4.59335528
homo after UVB irradiation VS homo before UVB irradiation	GO_ENTRY_INTO_HOST	0.249126139	0.013593847	1.694183034	0.12718518	0.29189296	-5.20173317
hetero after UVB irradiation VS hetero before UVB irradiation	GO_ENTRY_INTO_HOST1	0.360400591	0.023260761	2.73627551	0.02604553	0.22986115	-3.42474602
WT after UVB irradiation VS WT before UVB irradiation	GO_ENTRY_INTO_HOST2	0.270270808	-0.03589534	1.821611849	0.1055402	0.60971039	-4.5455934
homo after UVB irradiation VS homo before UVB irradiation	GO_ENTRY_OF_BACTERIUM_INTO_HOST_CELL	0.140624497	-0.05387708	0.481787072	0.64236171	0.78096323	-6.42524268
hetero after UVB irradiation VS hetero before UVB irradiation	GO_ENTRY_OF_BACTERIUM_INTO_HOST_CELL1	0.272477033	-0.00132276	1.230658342	0.25403724	0.58163375	-5.48309721
WT after UVB irradiation VS WT before UVB irradiation	GO_ENTRY_OF_BACTERIUM_INTO_HOST_CELL2	0.191777231	-0.01996063	0.808854954	0.4417134	0.83231427	-5.66489124
homo after UVB irradiation VS homo before UVB irradiation	GO_ENUCLEATE_ERYTHROCYTE_DIFFERENTIATION	-0.14600842	0.010755448	-0.75397399	0.47163939	0.65017117	-6.24925408
hetero after UVB irradiation VS hetero before UVB irradiation	GO_ENUCLEATE_ERYTHROCYTE_DIFFERENTIATION1	0.171260084	-0.00092087	0.75859519	0.47024816	0.75448318	-5.93072184
WT after UVB irradiation VS WT before UVB irradiation	GO_ENUCLEATE_ERYTHROCYTE_DIFFERENTIATION2	-0.32755524	-0.02057659	-1.30663266	0.22722381	0.71940395	-5.18366152
homo after UVB irradiation VS homo before UVB irradiation	GO_ENVELOPE	-0.13474467	-0.0144331	-0.96754293	0.36052313	0.55166319	-6.06449617
hetero after UVB irradiation VS hetero before UVB irradiation	GO_ENVELOPE1	-0.09249421	-0.02833033	-0.72448849	0.48978597	0.7663988	-5.95628956
WT after UVB irradiation VS WT before UVB irradiation	GO_ENVELOPE2	-0.00774812	-0.03115123	-0.05348429	0.95864202	0.98927761	-5.99455757
homo after UVB irradiation VS homo before UVB irradiation	GO_ENZYME_ACTIVATOR_ACTIVITY	0.17384191	-0.01299283	1.31597985	0.22324283	0.41417456	-5.68908453
hetero after UVB irradiation VS hetero before UVB irradiation	GO_ENZYME_ACTIVATOR_ACTIVITY1	0.248820983	0.003535127	2.001262808	0.08102878	0.36443021	-4.49202923
WT after UVB irradiation VS WT before UVB irradiation	GO_ENZYME_ACTIVATOR_ACTIVITY2	0.146965157	-0.03081481	0.98964931	0.35099138	0.78816886	-5.50997139
homo after UVB irradiation VS homo before UVB irradiation	GO_ENZYME_DIRECTED_RRNA_PSEUDOURIDINE_SYNTHESIS	-0.63275904	0.076161661	-4.29313094	0.00240465	0.03270547	-1.3086266
hetero after UVB irradiation VS hetero before UVB irradiation	GO_ENZYME_DIRECTED_RRNA_PSEUDOURIDINE_SYNTHESIS1	-0.34615414	-0.1280015	-1.10345741	0.30249251	0.62458221	-5.61936376
WT after UVB irradiation VS WT before UVB irradiation	GO_ENZYME_DIRECTED_RRNA_PSEUDOURIDINE_SYNTHESIS2	-0.31343389	0.034229936	-0.8711516	0.4087546	0.81822963	-5.61436677
homo after UVB irradiation VS homo before UVB irradiation	GO_ENZYME_INHIBITOR_ACTIVITY	0.149093436	-0.00117813	1.156999029	0.27937411	0.4747494	-5.87063185
hetero after UVB irradiation VS hetero before UVB irradiation	GO_ENZYME_INHIBITOR_ACTIVITY1	0.023175631	-0.0010158	0.17197594	0.86780572	0.95000402	-6.21547622
WT after UVB irradiation VS WT before UVB irradiation	GO_ENZYME_INHIBITOR_ACTIVITY2	0.01203374	0.00568812	0.084140359	0.93498782	0.98322796	-5.99233284
homo after UVB irradiation VS homo before UVB irradiation	GO_ENZYME_LINKED_RECEPTOR_PROTEIN_SIGNALING_PATHWAY	0.196277375	-0.0138499	1.465076078	0.17959942	0.36226745	-5.50532252
hetero after UVB irradiation VS hetero before UVB irradiation	GO_ENZYME_LINKED_RECEPTOR_PROTEIN_SIGNALING_PATHWAY1	0.160219011	0.007873316	1.168423139	0.27688229	0.60400502	-5.5510507
WT after UVB irradiation VS WT before UVB irradiation	GO_ENZYME_LINKED_RECEPTOR_PROTEIN_SIGNALING_PATHWAY2	0.079817536	-0.00264168	0.547507824	0.59879188	0.89476186	-5.84092609
homo after UVB irradiation VS homo before UVB irradiation	GO_ENZYME_REGULATOR_ACTIVITY	0.117991927	-0.00743702	0.93957535	0.37387985	0.56414571	-6.09082987
hetero after UVB irradiation VS hetero before UVB irradiation	GO_ENZYME_REGULATOR_ACTIVITY1	0.119327907	-0.00186174	0.957207097	0.36700011	0.68091512	-5.76252787
WT after UVB irradiation VS WT before UVB irradiation	GO_ENZYME_REGULATOR_ACTIVITY2	0.077247464	-0.01065927	0.550114305	0.59708394	0.89380885	-5.83947393
homo after UVB irradiation VS homo before UVB irradiation	GO_EOSINOPHIL_ACTIVATION	0.685451768	-0.09969972	3.774102386	0.00504832	0.04637942	-2.05721058
hetero after UVB irradiation VS hetero before UVB irradiation	GO_EOSINOPHIL_ACTIVATION1	0.218138287	0.018751469	0.610977704	0.55848038	0.80712929	-6.03388488
WT after UVB irradiation VS WT before UVB irradiation	GO_EOSINOPHIL_ACTIVATION2	0.273330376	0.033148523	0.892792192	0.39772316	0.81477652	-5.59609691
homo after UVB irradiation VS homo before UVB irradiation	GO_EOSINOPHIL_CHEMOTAXIS	0.217255298	-0.02193244	0.896073647	0.39537144	0.58426761	-6.13054792
hetero after UVB irradiation VS hetero before UVB irradiation	GO_EOSINOPHIL_CHEMOTAXIS1	0.095556273	0.04919847	0.538680396	0.60503701	0.83482123	-6.07706425
WT after UVB irradiation VS WT before UVB irradiation	GO_EOSINOPHIL_CHEMOTAXIS2	0.081997497	0.042470566	0.377769171	0.71531191	0.93029655	-5.92146151
homo after UVB irradiation VS homo before UVB irradiation	GO_EOSINOPHIL_DIFFERENTIATION	-0.15742131	-0.05747876	-0.7518886	0.47282716	0.65125167	-6.25086553
hetero after UVB irradiation VS hetero before UVB irradiation	GO_EOSINOPHIL_DIFFERENTIATION1	-0.59237174	0.017986114	-1.88690239	0.09657281	0.38807961	-4.65187441
WT after UVB irradiation VS WT before UVB irradiation	GO_EOSINOPHIL_DIFFERENTIATION2	-0.27926255	-0.00113838	-0.85490298	0.41717924	0.82259674	-5.62784344
homo after UVB irradiation VS homo before UVB irradiation	GO_EOSINOPHIL_MIGRATION	0.233998758	-0.01037516	1.063083971	0.31759058	0.51108878	-5.9700008
hetero after UVB irradiation VS hetero before UVB irradiation	GO_EOSINOPHIL_MIGRATION1	0.196566216	0.053956216	1.303141409	0.2294362	0.55747992	-5.40104586
WT after UVB irradiation VS WT before UVB irradiation	GO_EOSINOPHIL_MIGRATION2	0.122869189	0.015083116	0.525692379	0.61318972	0.89975394	-5.85282957
homo after UVB irradiation VS homo before UVB irradiation	GO_EPHRIN_RECEPTOR_ACTIVITY	-0.14582079	0.059532854	-0.77442578	0.46009465	0.64016752	-6.23324539
hetero after UVB irradiation VS hetero before UVB irradiation	GO_EPHRIN_RECEPTOR_ACTIVITY1	-0.09586431	0.046341429	-0.41967932	0.68596594	0.87376352	-6.13706238
WT after UVB irradiation VS WT before UVB irradiation	GO_EPHRIN_RECEPTOR_ACTIVITY2	-0.08658309	0.039083226	-0.38135114	0.71275421	0.92947342	-5.92005335
homo after UVB irradiation VS homo before UVB irradiation	GO_EPHRIN_RECEPTOR_BINDING	0.182295362	-0.01879504	0.889907227	0.39848854	0.58728607	-6.1360535
hetero after UVB irradiation VS hetero before UVB irradiation	GO_EPHRIN_RECEPTOR_BINDING1	0.477026783	-0.02461205	3.371511897	0.01003317	0.15749936	-2.50597208
WT after UVB irradiation VS WT before UVB irradiation	GO_EPHRIN_RECEPTOR_BINDING2	0.364156223	-0.00719107	2.190219563	0.05950044	0.52638053	-4.04043885
homo after UVB irradiation VS homo before UVB irradiation	GO_EPHRIN_RECEPTOR_SIGNALING_PATHWAY	0.356641462	-0.02905253	1.907867294	0.09138643	0.23742773	-4.90024372
hetero after UVB irradiation VS hetero before UVB irradiation	GO_EPHRIN_RECEPTOR_SIGNALING_PATHWAY1	0.457269432	-0.00337853	2.978432022	0.0180167	0.19634745	-3.07087481
WT after UVB irradiation VS WT before UVB irradiation	GO_EPHRIN_RECEPTOR_SIGNALING_PATHWAY2	0.36355273	0.014994084	2.039100249	0.07535436	0.55793508	-4.25059952
homo after UVB irradiation VS homo before UVB irradiation	GO_EPIBOLY	0.652317323	-0.01930701	4.426683497	0.0019995	0.03035653	-1.12240092
hetero after UVB irradiation VS hetero before UVB irradiation	GO_EPIBOLY1	0.725255355	0.002981507	5.752114215	0.00045839	0.07788488	0.421618287
WT after UVB irradiation VS WT before UVB irradiation	GO_EPIBOLY2	0.541368782	0.010149125	3.56930317	0.00715736	0.31015346	-2.11144303
homo after UVB irradiation VS homo before UVB irradiation	GO_EPIDERMAL_CELL_DIFFERENTIATION	-0.30847747	0.031320326	-1.9158356	0.09025601	0.23552748	-4.88872309
hetero after UVB irradiation VS hetero before UVB irradiation	GO_EPIDERMAL_CELL_DIFFERENTIATION1	-0.14610345	0.013463764	-0.79637714	0.44921668	0.74180859	-5.90122581
WT after UVB irradiation VS WT before UVB irradiation	GO_EPIDERMAL_CELL_DIFFERENTIATION2	-0.29161093	0.051660891	-1.24754349	0.24706747	0.73564768	-5.24914098
homo after UVB irradiation VS homo before UVB irradiation	GO_EPIDERMAL_GROWTH_FACTOR_RECEPTOR_BINDING	0.44119307	-0.02826861	2.457630694	0.03833059	0.14042811	-4.07436518
hetero after UVB irradiation VS hetero before UVB irradiation	GO_EPIDERMAL_GROWTH_FACTOR_RECEPTOR_BINDING1	0.703449334	-0.00056283	4.709812371	0.0016008	0.09569098	-0.74375798
WT after UVB irradiation VS WT before UVB irradiation	GO_EPIDERMAL_GROWTH_FACTOR_RECEPTOR_BINDING2	0.383522745	0.055265159	2.141299596	0.06423669	0.53678963	-4.10883124
homo after UVB irradiation VS homo before UVB irradiation	GO_EPIDERMAL_GROWTH_FACTOR_RECEPTOR_SIGNALING_PATHWAY	0.317861183	-0.03091249	1.741521917	0.11827626	0.27806321	-5.13628883
hetero after UVB irradiation VS hetero before UVB irradiation	GO_EPIDERMAL_GROWTH_FACTOR_RECEPTOR_SIGNALING_PATHWAY1	0.451201197	0.002631437	3.551718777	0.00772049	0.14291796	-2.25278389
WT after UVB irradiation VS WT before UVB irradiation	GO_EPIDERMAL_GROWTH_FACTOR_RECEPTOR_SIGNALING_PATHWAY2	0.273160024	-0.00709272	1.873579141	0.09742147	0.59779444	-4.47609703
homo after UVB irradiation VS homo before UVB irradiation	GO_EPIDERMIS_DEVELOPMENT	-0.23564871	0.023085666	-1.5783004	0.15165816	0.32571962	-5.35819511
hetero after UVB irradiation VS hetero before UVB irradiation	GO_EPIDERMIS_DEVELOPMENT1	-0.06815215	0.026053745	-0.41461077	0.68952144	0.87560916	-6.13930401
WT after UVB irradiation VS WT before UVB irradiation	GO_EPIDERMIS_DEVELOPMENT2	-0.20226242	0.059100059	-0.93993772	0.37443761	0.80243622	-5.55504959
homo after UVB irradiation VS homo before UVB irradiation	GO_EPIDERMIS_MORPHOGENESIS	-0.32952594	0.003257559	-2.53353032	0.03398157	0.13189531	-3.95716245
hetero after UVB irradiation VS hetero before UVB irradiation	GO_EPIDERMIS_MORPHOGENESIS1	-0.17036834	0.031426484	-0.93372459	0.37826305	0.69105479	-5.78405418
WT after UVB irradiation VS WT before UVB irradiation	GO_EPIDERMIS_MORPHOGENESIS2	-0.25487534	0.116389791	-1.49873851	0.17187388	0.67808426	-4.95837134
homo after UVB irradiation VS homo before UVB irradiation	GO_EPIDIDYMIS_DEVELOPMENT	0.090670714	-0.06676366	0.426897611	0.68027463	0.80717317	-6.45193878
hetero after UVB irradiation VS hetero before UVB irradiation	GO_EPIDIDYMIS_DEVELOPMENT1	0.072584258	-0.09366091	0.244473768	0.81313054	0.92796243	-6.19919912
WT after UVB irradiation VS WT before UVB irradiation	GO_EPIDIDYMIS_DEVELOPMENT2	0.161019612	0.106937705	0.578775323	0.5784797	0.88761996	-5.82308753
homo after UVB irradiation VS homo before UVB irradiation	GO_EPINEPHRINE_BINDING	0.279624726	-0.00183677	1.404155956	0.19644303	0.3845728	-5.58186255
hetero after UVB irradiation VS hetero before UVB irradiation	GO_EPINEPHRINE_BINDING1	-0.40849612	0.016374369	-2.17466465	0.06199787	0.32684552	-4.24481072
WT after UVB irradiation VS WT before UVB irradiation	GO_EPINEPHRINE_BINDING2	-0.05587137	-0.00093596	-0.19221822	0.85230428	0.96605575	-5.97661815
homo after UVB irradiation VS homo before UVB irradiation	GO_EPINEPHRINE_TRANSPORT	0.365457971	0.060684438	2.502185448	0.03571386	0.13520509	-4.00561381
hetero after UVB irradiation VS hetero before UVB irradiation	GO_EPINEPHRINE_TRANSPORT1	-0.12773902	-0.03408901	-0.59665223	0.56753802	0.81259373	-6.04283467
WT after UVB irradiation VS WT before UVB irradiation	GO_EPINEPHRINE_TRANSPORT2	-0.09940269	0.035476307	-0.42527749	0.68171073	0.91716791	-5.90174908
homo after UVB irradiation VS homo before UVB irradiation	GO_EPITHELIAL_CELL_APOPTOTIC_PROCESS	0.143462873	-0.00859635	1.154091009	0.28049953	0.47567589	-5.87380054
hetero after UVB irradiation VS hetero before UVB irradiation	GO_EPITHELIAL_CELL_APOPTOTIC_PROCESS1	0.146737599	0.010334013	1.092893282	0.30683107	0.627591	-5.63020834
WT after UVB irradiation VS WT before UVB irradiation	GO_EPITHELIAL_CELL_APOPTOTIC_PROCESS2	0.011422326	0.022843322	0.065795636	0.94913596	0.98620317	-5.99378308
homo after UVB irradiation VS homo before UVB irradiation	GO_EPITHELIAL_CELL_CELL_ADHESION	0.346852442	0.005313651	1.579576003	0.15136694	0.32547311	-5.35650374
hetero after UVB irradiation VS hetero before UVB irradiation	GO_EPITHELIAL_CELL_CELL_ADHESION1	0.390780752	-0.00553278	2.556121792	0.03435886	0.25538558	-3.68908063
WT after UVB irradiation VS WT before UVB irradiation	GO_EPITHELIAL_CELL_CELL_ADHESION2	0.170401983	-0.03216609	0.6910332	0.50886613	0.85830167	-5.75168185
homo after UVB irradiation VS homo before UVB irradiation	GO_EPITHELIAL_CELL_DEVELOPMENT	0.137825896	-0.00615555	0.885314961	0.40082131	0.5890768	-6.1401333
hetero after UVB irradiation VS hetero before UVB irradiation	GO_EPITHELIAL_CELL_DEVELOPMENT1	0.195240144	0.004259295	1.574627038	0.15469624	0.47324093	-5.06953914
WT after UVB irradiation VS WT before UVB irradiation	GO_EPITHELIAL_CELL_DEVELOPMENT2	0.040264622	-0.00686375	0.275721741	0.7896674	0.94771648	-5.95615184
homo after UVB irradiation VS homo before UVB irradiation	GO_EPITHELIAL_CELL_DIFFERENTIATION	-0.0408737	0.023294064	-0.2866011	0.7814179	0.87418573	-6.50600172
homo after UVB irradiation VS homo before UVB irradiation	GO_EPITHELIAL_CELL_DIFFERENTIATION_INVOLVED_IN_KIDNEY_DEVELOPMENT	0.206980997	0.026857082	1.380847081	0.20324266	0.39262364	-5.61062754
hetero after UVB irradiation VS hetero before UVB irradiation	GO_EPITHELIAL_CELL_DIFFERENTIATION_INVOLVED_IN_KIDNEY_DEVELOPMENT1	0.068868704	0.020558831	0.392353894	0.7052309	0.88289792	-6.14883867
WT after UVB irradiation VS WT before UVB irradiation	GO_EPITHELIAL_CELL_DIFFERENTIATION_INVOLVED_IN_KIDNEY_DEVELOPMENT2	0.118846788	0.011583839	0.774716745	0.46052766	0.8397644	-5.69124181
homo after UVB irradiation VS homo before UVB irradiation	GO_EPITHELIAL_CELL_DIFFERENTIATION_INVOLVED_IN_PROSTATE_GLAND_DEVELOPMENT	-0.22704017	0.009691486	-1.08532685	0.30818705	0.50275536	-5.94702949
hetero after UVB irradiation VS hetero before UVB irradiation	GO_EPITHELIAL_CELL_DIFFERENTIATION_INVOLVED_IN_PROSTATE_GLAND_DEVELOPMENT1	-0.53497979	0.021974026	-2.72084482	0.02666875	0.23166634	-3.44737425

WT after UVB irradiation VS WT before UVB irradiation	GO_EPITHELIAL_CELL_DIFFERENTIATION_INVOLVED_IN_PROSTATE_GLAND_DEVELOPMENT2	-0.24308498	0.076024932	-0.86860547	0.41006669	0.81881631	-5.61649228
hetero after UVB irradiation VS hetero before UVB irradiation	GO_EPITHELIAL_CELL_DIFFERENTIATION1	0.008872058	0.023158725	0.070978939	0.94518885	0.97953855	-6.22875287
WT after UVB irradiation VS WT before UVB irradiation	GO_EPITHELIAL_CELL_DIFFERENTIATION2	-0.11263275	0.03598367	-0.73275126	0.48437805	0.85146732	-5.72229972
homo after UVB irradiation VS homo before UVB irradiation	GO_EPITHELIAL_CELL_FATE_COMMITMENT	0.221352143	0.062946224	1.284353216	0.23358656	0.42493058	-5.72645237
hetero after UVB irradiation VS hetero before UVB irradiation	GO_EPITHELIAL_CELL_FATE_COMMITMENT1	0.35491777	0.001386891	1.90875205	0.09339675	0.38229192	-4.62156812
WT after UVB irradiation VS WT before UVB irradiation	GO_EPITHELIAL_CELL_FATE_COMMITMENT2	-0.14796075	0.106833949	-0.7197247	0.49194185	0.85299376	-5.73163584
homo after UVB irradiation VS homo before UVB irradiation	GO_EPITHELIAL_CELL_MATURATION	-0.29294701	0.025187768	-1.83756731	0.10195908	0.25368575	-5.00107828
hetero after UVB irradiation VS hetero before UVB irradiation	GO_EPITHELIAL_CELL_MATURATION1	0.170044329	0.053238633	0.843235961	0.42403569	0.72563714	-5.86297139
WT after UVB irradiation VS WT before UVB irradiation	GO_EPITHELIAL_CELL_MATURATION2	-0.15339314	0.026794492	-0.80290264	0.44495564	0.83340629	-5.66955481
homo after UVB irradiation VS homo before UVB irradiation	GO_EPITHELIAL_CELL_MORPHOGENESIS	0.09464306	-0.02201174	0.536855529	0.60540699	0.75385967	-6.39540753
hetero after UVB irradiation VS hetero before UVB irradiation	GO_EPITHELIAL_CELL_MORPHOGENESIS1	0.042302307	-0.00603906	0.296082397	0.77484594	0.91162707	-6.18421616
WT after UVB irradiation VS WT before UVB irradiation	GO_EPITHELIAL_CELL_MORPHOGENESIS2	-0.03444944	-0.02211055	-0.18798315	0.85551519	0.9663792	-5.97746376
homo after UVB irradiation VS homo before UVB irradiation	GO_EPITHELIAL_CELL_PROLIFERATION	0.185846258	-0.00501538	1.450459285	0.18352079	0.36729748	-5.5238621
homo after UVB irradiation VS homo before UVB irradiation	GO_EPITHELIAL_CELL_PROLIFERATION_INVOLVED_IN_LUNG_MORPHOGENESIS	-0.42849236	0.049321467	-3.2521067	0.01104544	0.07037888	-2.84438986
hetero after UVB irradiation VS hetero before UVB irradiation	GO_EPITHELIAL_CELL_PROLIFERATION_INVOLVED_IN_LUNG_MORPHOGENESIS1	-0.06270554	0.014103919	-0.33687221	0.74503954	0.90030853	-6.17039854
WT after UVB irradiation VS WT before UVB irradiation	GO_EPITHELIAL_CELL_PROLIFERATION_INVOLVED_IN_LUNG_MORPHOGENESIS2	-0.34654804	0.037179539	-2.01577637	0.07814286	0.56140246	-4.28271052
homo after UVB irradiation VS homo before UVB irradiation	GO_EPITHELIAL_CELL_PROLIFERATION_INVOLVED_IN_PROSTATE_GLAND_DEVELOPMENT	-0.29933072	0.027027254	-2.2234466	0.05558576	0.17512363	-4.43241729
hetero after UVB irradiation VS hetero before UVB irradiation	GO_EPITHELIAL_CELL_PROLIFERATION_INVOLVED_IN_PROSTATE_GLAND_DEVELOPMENT1	-0.42959239	0.032281036	-1.90471663	0.09397569	0.38349151	-4.62717421
WT after UVB irradiation VS WT before UVB irradiation	GO_EPITHELIAL_CELL_PROLIFERATION_INVOLVED_IN_PROSTATE_GLAND_DEVELOPMENT2	-0.53950902	0.071276577	-2.38051056	0.04414744	0.49769531	-3.77205345
homo after UVB irradiation VS homo before UVB irradiation	GO_EPITHELIAL_CELL_PROLIFERATION_INVOLVED_IN_SALIVARY_GLAND_MORPHOGENESIS	-0.32779946	-0.00094904	-1.48480322	0.17442432	0.35474896	-5.48013083
hetero after UVB irradiation VS hetero before UVB irradiation	GO_EPITHELIAL_CELL_PROLIFERATION_INVOLVED_IN_SALIVARY_GLAND_MORPHOGENESIS1	-0.03943194	0.056393455	-0.15127713	0.88357105	0.95768873	-6.21909224
WT after UVB irradiation VS WT before UVB irradiation	GO_EPITHELIAL_CELL_PROLIFERATION_INVOLVED_IN_SALIVARY_GLAND_MORPHOGENESIS2	-0.11744677	0.018506707	-0.7930045	0.45038296	0.83548728	-5.67724551
hetero after UVB irradiation VS hetero before UVB irradiation	GO_EPITHELIAL_CELL_PROLIFERATION1	0.19210769	0.008784454	1.424471911	0.19281412	0.51844079	-5.2572757
WT after UVB irradiation VS WT before UVB irradiation	GO_EPITHELIAL_CELL_PROLIFERATION2	0.031472776	-0.00100801	0.201072647	0.8456006	0.96472824	-5.97478994
homo after UVB irradiation VS homo before UVB irradiation	GO_EPITHELIAL_CILIUM_MOVEMENT_INVOLVED_IN_DETERMINATION_OF_LEFT_RIGHT_ASYMMETRY	-0.37259576	-0.0137624	-1.51823356	0.16595569	0.34434873	-5.43700429
hetero after UVB irradiation VS hetero before UVB irradiation	GO_EPITHELIAL_CILIUM_MOVEMENT_INVOLVED_IN_DETERMINATION_OF_LEFT_RIGHT_ASYMMETRY1	-0.73903485	-0.00323159	-3.9222782	0.0045683	0.12742869	-1.7464632
WT after UVB irradiation VS WT before UVB irradiation	GO_EPITHELIAL_CILIUM_MOVEMENT_INVOLVED_IN_DETERMINATION_OF_LEFT_RIGHT_ASYMMETRY2	-0.55301687	-0.01805418	-2.53990319	0.03438681	0.4670931	-3.54556148
homo after UVB irradiation VS homo before UVB irradiation	GO_EPITHELIAL_FLUID_TRANSPORT	0.258824182	-0.07070039	1.738548843	0.11881828	0.2789869	-5.14042334
hetero after UVB irradiation VS hetero before UVB irradiation	GO_EPITHELIAL_FLUID_TRANSPORT1	0.166277337	-0.05021775	1.164969081	0.27819808	0.60400502	-5.55475149
WT after UVB irradiation VS WT before UVB irradiation	GO_EPITHELIAL_FLUID_TRANSPORT2	0.198840584	-0.03367223	1.199532153	0.2642393	0.74156847	-5.30087759
homo after UVB irradiation VS homo before UVB irradiation	GO_EPITHELIAL_MESENCHYMAL_CELL_SIGNALING	-0.74928516	0.077074033	-4.78474916	0.00123508	0.02360887	-0.63678604
hetero after UVB irradiation VS hetero before UVB irradiation	GO_EPITHELIAL_MESENCHYMAL_CELL_SIGNALING1	-0.62380335	0.06012133	-3.07014719	0.01569205	0.1846506	-2.93778194
WT after UVB irradiation VS WT before UVB irradiation	GO_EPITHELIAL_MESENCHYMAL_CELL_SIGNALING2	-0.53454841	0.060048829	-2.34816948	0.04644546	0.50545399	-3.818718
homo after UVB irradiation VS homo before UVB irradiation	GO_EPITHELIAL_STRUCTURE_MAINTENANCE	0.327514012	0.040047183	2.421197406	0.04061301	0.14499783	-4.13046465
hetero after UVB irradiation VS hetero before UVB irradiation	GO_EPITHELIAL_STRUCTURE_MAINTENANCE1	0.060406565	-0.02186452	0.341369042	0.74178037	0.8993586	-6.16876931
WT after UVB irradiation VS WT before UVB irradiation	GO_EPITHELIAL_STRUCTURE_MAINTENANCE2	-0.01252408	0.01089619	-0.06382143	0.95065978	0.98658866	-5.99391803
homo after UVB irradiation VS homo before UVB irradiation	GO_EPITHELIAL_TO_MESENCHYMAL_TRANSITION	0.008762098	0.00443344	0.064292233	0.95025384	0.97359676	-6.54858875
homo after UVB irradiation VS homo before UVB irradiation	GO_EPITHELIAL_TO_MESENCHYMAL_TRANSITION_INVOLVED_IN_ENDOCARDIAL_CUSHION_FORMATION	-0.30676596	-0.03005072	-1.74423316	0.11778397	0.27699412	-5.13251565
hetero after UVB irradiation VS hetero before UVB irradiation	GO_EPITHELIAL_TO_MESENCHYMAL_TRANSITION_INVOLVED_IN_ENDOCARDIAL_CUSHION_FORMATION1	0.040139781	0.007182859	0.194276253	0.8508912	0.94304872	-6.21106652
WT after UVB irradiation VS WT before UVB irradiation	GO_EPITHELIAL_TO_MESENCHYMAL_TRANSITION_INVOLVED_IN_ENDOCARDIAL_CUSHION_FORMATION2	-0.16247992	0.025875678	-0.88429487	0.402029	0.81601572	-5.60331413
hetero after UVB irradiation VS hetero before UVB irradiation	GO_EPITHELIAL_TO_MESENCHYMAL_TRANSITION1	0.020096835	0.019018214	0.153253976	0.88206286	0.95722623	-6.21876677
WT after UVB irradiation VS WT before UVB irradiation	GO_EPITHELIAL_TO_MESENCHYMAL_TRANSITION2	-0.0255286	0.024257113	-0.17350427	0.86651393	0.96790654	-5.98021366
homo after UVB irradiation VS homo before UVB irradiation	GO_EPITHELIAL_TUBE_BRANCHING_INVOLVED_IN_LUNG_MORPHOGENESIS	-0.35072136	0.047895942	-2.15633034	0.06181926	0.1876716	-4.53362928
hetero after UVB irradiation VS hetero before UVB irradiation	GO_EPITHELIAL_TUBE_BRANCHING_INVOLVED_IN_LUNG_MORPHOGENESIS1	-0.29079075	0.03212118	-1.68157904	0.13186431	0.44121703	-4.93014954
WT after UVB irradiation VS WT before UVB irradiation	GO_EPITHELIAL_TUBE_BRANCHING_INVOLVED_IN_LUNG_MORPHOGENESIS2	-0.32822643	-0.00160311	-1.75443348	0.11698382	0.62452834	-4.63434007
homo after UVB irradiation VS homo before UVB irradiation	GO_EPITHELIAL_TUBE_FORMATION	-0.05015348	0.010580239	-0.35904075	0.72849631	0.83994218	-6.48066081
hetero after UVB irradiation VS hetero before UVB irradiation	GO_EPITHELIAL_TUBE_FORMATION1	-0.034532	-0.00290274	-0.21536726	0.83496924	0.93644494	-6.20640699
WT after UVB irradiation VS WT before UVB irradiation	GO_EPITHELIAL_TUBE_FORMATION2	-0.02965539	0.00627609	-0.20216823	0.84477205	0.96467526	-5.97455807
homo after UVB irradiation VS homo before UVB irradiation	GO_EPITHELIAL_TUBE_MORPHOGENESIS	0.062754555	0.006635436	0.469234085	0.65094038	0.7870347	-6.43161794
hetero after UVB irradiation VS hetero before UVB irradiation	GO_EPITHELIAL_TUBE_MORPHOGENESIS1	0.033371799	0.011401424	0.236160644	0.81935107	0.93071163	-6.20134952
WT after UVB irradiation VS WT before UVB irradiation	GO_EPITHELIAL_TUBE_MORPHOGENESIS2	-0.02468222	-0.00166862	-0.17431958	0.86589374	0.96790654	-5.98006461
homo after UVB irradiation VS homo before UVB irradiation	GO_EPITHELIUM_DEVELOPMENT	-0.04222016	0.01176993	-0.3098278	0.76430203	0.86390334	-6.49848091
hetero after UVB irradiation VS hetero before UVB irradiation	GO_EPITHELIUM_DEVELOPMENT1	0.030298413	0.019644869	0.229784428	0.82413154	0.93321249	-6.20294922
WT after UVB irradiation VS WT before UVB irradiation	GO_EPITHELIUM_DEVELOPMENT2	-0.06779769	0.022377017	-0.4500971	0.66444601	0.91232901	-5.89056632
homo after UVB irradiation VS homo before UVB irradiation	GO_EPOXIDE_HYDROLASE_ACTIVITY	0.37117292	0.026768171	1.655999567	0.13481797	0.30281716	-5.25389575
hetero after UVB irradiation VS hetero before UVB irradiation	GO_EPOXIDE_HYDROLASE_ACTIVITY1	0.274479163	-0.02767101	1.336897749	0.21868922	0.54565069	-5.36182378
WT after UVB irradiation VS WT before UVB irradiation	GO_EPOXIDE_HYDROLASE_ACTIVITY2	0.424781535	0.025328539	1.380471042	0.20434774	0.70090667	-5.09920552
homo after UVB irradiation VS homo before UVB irradiation	GO_EPOXIDE_METABOLIC_PROCESS	0.250980465	-0.0083616	0.803349842	0.44409113	0.62664495	-6.20997609
hetero after UVB irradiation VS hetero before UVB irradiation	GO_EPOXIDE_METABOLIC_PROCESS1	0.247187981	-0.00527608	1.600768643	0.14880334	0.46658352	-5.03587155
WT after UVB irradiation VS WT before UVB irradiation	GO_EPOXIDE_METABOLIC_PROCESS2	0.22880902	0.071900954	1.118217431	0.2955474	0.75850312	-5.38531298
homo after UVB irradiation VS homo before UVB irradiation	GO_EPOXYGENASE_P450_PATHWAY	-0.00599286	0.078034942	-0.03165408	0.97549332	0.98734985	-6.55030684
hetero after UVB irradiation VS hetero before UVB irradiation	GO_EPOXYGENASE_P450_PATHWAY1	-0.11206518	-0.01555721	-0.56660165	0.58680946	0.82407264	-6.06097879
WT after UVB irradiation VS WT before UVB irradiation	GO_EPOXYGENASE_P450_PATHWAY2	-0.13089823	0.034063715	-0.48089216	0.64331768	0.90762491	-5.87585721
homo after UVB irradiation VS homo before UVB irradiation	GO_EPSILON_DNA_POLYMERASE_COMPLEX	-0.02201847	-0.06611045	-0.07442791	0.94242645	0.9694937	-6.54781755
hetero after UVB irradiation VS hetero before UVB irradiation	GO_EPSILON_DNA_POLYMERASE_COMPLEX1	0.042324279	-0.06316517	0.270791013	0.79353317	0.91941572	-6.19190936
WT after UVB irradiation VS WT before UVB irradiation	GO_EPSILON_DNA_POLYMERASE_COMPLEX2	0.45611627	-0.0118686	2.148228545	0.06354397	0.53678963	-4.0991632
homo after UVB irradiation VS homo before UVB irradiation	GO_EQUATORIAL_MICROTUBULE_ORGANIZING_CENTER	-0.26684319	0.049136373	-0.88849677	0.39920399	0.58787079	-6.1373084
hetero after UVB irradiation VS hetero before UVB irradiation	GO_EQUATORIAL_MICROTUBULE_ORGANIZING_CENTER1	0.026280804	-0.1038904	0.092921561	0.92829278	0.97341446	-6.22680427
WT after UVB irradiation VS WT before UVB irradiation	GO_EQUATORIAL_MICROTUBULE_ORGANIZING_CENTER2	0.672152938	-0.12150348	2.653748698	0.02878116	0.44654821	-3.38353977
homo after UVB irradiation VS homo before UVB irradiation	GO_EQUILIBRIOCEPTION	0.5758536	0.071183422	3.014776829	0.01593547	0.08618384	-3.21032453
hetero after UVB irradiation VS hetero before UVB irradiation	GO_EQUILIBRIOCEPTION1	0.276710688	0.086676931	1.017072133	0.33942786	0.65919947	-5.70578005
WT after UVB irradiation VS WT before UVB irradiation	GO_EQUILIBRIOCEPTION2	0.014217255	0.011968712	0.070126133	0.94579419	0.9855442	-5.99347269
homo after UVB irradiation VS homo before UVB irradiation	GO_ER_ASSOCIATED_MISFOLDED_PROTEIN_CATABOLIC_PROCESS	-0.23264444	-0.08321486	-1.23233055	0.25147576	0.44600426	-5.78659692
hetero after UVB irradiation VS hetero before UVB irradiation	GO_ER_ASSOCIATED_MISFOLDED_PROTEIN_CATABOLIC_PROCESS1	-0.20778197	-0.01814787	-0.98572638	0.35366109	0.6701712	-5.73582419
WT after UVB irradiation VS WT before UVB irradiation	GO_ER_ASSOCIATED_MISFOLDED_PROTEIN_CATABOLIC_PROCESS2	0.105208721	0.011027151	0.435761435	0.67439293	0.91622321	-5.897099
homo after UVB irradiation VS homo before UVB irradiation	GO_ER_MEMBRANE_INSERTION_COMPLEX	-0.20302413	-0.02127565	-1.3207035	0.22173175	0.41272646	-5.68345266
hetero after UVB irradiation VS hetero before UVB irradiation	GO_ER_MEMBRANE_INSERTION_COMPLEX1	0.030735999	0.004739694	0.111864124	0.91373762	0.96745438	-6.2247035
WT after UVB irradiation VS WT before UVB irradiation	GO_ER_MEMBRANE_INSERTION_COMPLEX2	-0.23793913	0.112775374	-0.69208151	0.5082414	0.85808011	-5.75096204
homo after UVB irradiation VS homo before UVB irradiation	GO_ER_MEMBRANE_PROTEIN_COMPLEX	-0.21476508	-0.02136304	-0.87939723	0.40384174	0.59098499	-6.14536498
hetero after UVB irradiation VS hetero before UVB irradiation	GO_ER_MEMBRANE_PROTEIN_COMPLEX1	-0.23813039	-0.05373968	-0.77943276	0.45856885	0.74772822	-5.91460498
WT after UVB irradiation VS WT before UVB irradiation	GO_ER_MEMBRANE_PROTEIN_COMPLEX2	-0.02361751	-0.05113777	-0.09179777	0.92908908	0.98241384	-5.9916227
homo after UVB irradiation VS homo before UVB irradiation	GO_ER_NUCLEUS_SIGNALING_PATHWAY	0.034659864	-0.00825907	0.215427691	0.83461569	0.90762824	-6.52545855
hetero after UVB irradiation VS hetero before UVB irradiation	GO_ER_NUCLEUS_SIGNALING_PATHWAY1	0.056304009	-0.00274397	0.379392003	0.7144504	0.88776082	-6.15415874
WT after UVB irradiation VS WT before UVB irradiation	GO_ER_NUCLEUS_SIGNALING_PATHWAY2	0.023488386	-0.00142921	0.128960673	0.90053438	0.9752088	-5.98730093
homo after UVB irradiation VS homo before UVB irradiation	GO_ER_OVERLOAD_RESPONSE	0.115709439	-0.02208937	0.631542516	0.54462457	0.7093183	-6.33714256
hetero after UVB irradiation VS hetero before UVB irradiation	GO_ER_OVERLOAD_RESPONSE1	0.087217987	-0.02657143	0.450449517	0.66456267	0.86248812	-6.12289771
WT after UVB irradiation VS WT before UVB irradiation	GO_ER_OVERLOAD_RESPONSE2	0.238695786	-0.13793749	0.832291269	0.4291051	0.82774962	-5.64624888
homo after UVB irradiation VS homo before UVB irradiation	GO_ER_RETENTION_SEQUENCE_BINDING	0.660017227	-0.08340375	3.203898743	0.01189353	0.07326259	-2.91841877
hetero after UVB irradiation VS hetero before UVB irradiation	GO_ER_RETENTION_SEQUENCE_BINDING1	0.462707291	0.003586136	2.007474601	0.08025769	0.3642825	-4.48326551
WT after UVB irradiation VS WT before UVB irradiation	GO_ER_RETENTION_SEQUENCE_BINDING2	0.750567645	-0.00586306	3.480061977	0.00816312	0.31868555	-2.2312684
homo after UVB irradiation VS homo before UVB irradiation	GO_ER_TO_GOLGI_TRANSPORT_VESICLE_MEMBRANE	0.266286363	-0.03212621	1.477001042	0.17645512	0.35773433	-5.49011742
hetero after UVB irradiation VS hetero before UVB irradiation	GO_ER_TO_GOLGI_TRANSPORT_VESICLE_MEMBRANE1	0.197641069	0.017568521	1.019859027	0.338184	0.65799619	-5.70307427
WT after UVB irradiation VS WT before UVB irradiation	GO_ER_TO_GOLGI_TRANSPORT_VESICLE_MEMBRANE2	0.234926608	0.004431765	1.166606192	0.2765746	0.74788357	-5.33556256
homo after UVB irradiation VS homo before UVB irradiation	GO_ER_UBIQUITIN_LIGASE_COMPLEX	0.078417284	-0.02992944	0.347082394	0.73713627	0.84539914	-6.48522538
hetero after UVB irradiation VS hetero before UVB irradiation	GO_ER_UBIQUITIN_LIGASE_COMPLEX1	0.276970508	0.011872532	1.174297942	0.27465602	0.60122003	-5.54473887
WT after UVB irradiation VS WT before UVB irradiation	GO_ER_UBIQUITIN_LIGASE_COMPLEX2	0.120245668	-0.02489023	0.777245062	0.45911609	0.8390123	-5.68932334
homo after UVB irradiation VS homo before UVB irradiation	GO_ERAD_PATHWAY	0.110561713	-0.03744996	0.724770967	0.48844988	0.66488322	-6.27146493
hetero after UVB irradiation VS hetero before UVB irradiation	GO_ERAD_PATHWAY1	0.0804925	0.005383592	0.47058095	0.65073363	0.85686396	-6.11311696
WT after UVB irradiation VS WT before UVB irradiation	GO_ERAD_PATHWAY2	0.104085233	-0.02295752	0.669784186	0.5216325	0.86373437	-5.76606419
homo after UVB irradiation VS homo before UVB irradiation	GO_ERBB_3_CLASS_RECEPTOR_BINDING	0.128842764	0.035549239	0.357598555	0.72953616	0.84061989	-6.48121925
hetero after UVB irradiation VS hetero before UVB irradiation	GO_ERBB_3_CLASS_RECEPTOR_BINDING1	0.11611296	-0.0087037	0.542005022	0.60285069	0.83402069	-6.07518829
WT after UVB irradiation VS WT before UVB irradiation	GO_ERBB_3_CLASS_RECEPTOR_BINDING2	0.234235334	0.110477452	0.747915985	0.47566811	0.84846534	-5.71124846
homo after UVB irradiation VS homo before UVB irradiation	GO_ERBB_SIGNALING_PATHWAY	0.275559551	-0.02302914	1.577411392	0.15186142	0.32594116	-5.35937345
hetero after UVB irradiation VS hetero before UVB irradiation	GO_ERBB_SIGNALING_PATHWAY1	0.384933949	0.007889214	3.088325648	0.01526982	0.1825855	-2.9114824
WT after UVB irradiation VS WT before UVB irradiation	GO_ERBB_SIGNALING_PATHWAY2	0.224518628	-0.00228824	1.512796672	0.16833537	0.67379949	-4.94121069
homo after UVB irradiation VS homo before UVB irradiation	GO_ERBB2_SIGNALING_PATHWAY	-0.09793028	-0.0177605	-0.57154529	0.58272055	0.73730201	-6.37507142
hetero after UVB irradiation VS hetero before UVB irradiation	GO_ERBB2_SIGNALING_PATHWAY1	-0.04500409	0.034295559	-0.31544263	0.76064546	0.90682777	-6.17787411
WT after UVB irradiation VS WT before UVB irradiation	GO_ERBB2_SIGNALING_PATHWAY2	-0.20581226	0.005308929	-1.21279079	0.2594014	0.73996638	-5.28672576
homo after UVB irradiation VS homo before UVB irradiation	GO_ERK1_AND_ERK2_CASCADE	0.252200281	-0.0035482	1.831402935	0.10293938	0.25530696	-5.00984811
hetero after UVB irradiation VS hetero before UVB irradiation	GO_ERK1_AND_ERK2_CASCADE1	0.229678598	0.03185538	1.453785909	0.18477393	0.50935615	-5.22142777
WT after UVB irradiation VS WT before UVB irradiation	GO_ERK1_AND_ERK2_CASCADE2	0.157136137	0.003351899	0.843720643	0.42304766	0.82491361	-5.63699666
homo after UVB irradiation VS homo before UVB irradiation	GO_ERROR_FREE_TRANSLESION_SYNTHESIS	-0.25122329	0.025927273	-1.08219597	0.30949733	0.50382804	-5.95028446
hetero after UVB irradiation VS hetero before UVB irradiation	GO_ERROR_FREE_TRANSLESION_SYNTHESIS1	0.117315404	-0.02988232	0.559617287	0.59134029	0.82654328	-6.0650727
WT after UVB irradiation VS WT before UVB irradiation	GO_ERROR_FREE_TRANSLESION_SYNTHESIS2	-0.11230695	0.020777076	-0.38297962	0.71159267	0.92903533	-5.91940893
homo after UVB irradiation VS homo before UVB irradiation	GO_ERROR_PRONE_TRANSLESION_SYNTHESIS	-0.04641426	0.013755133	-0.19459635	0.85037177	0.91763048	-6.53012216
hetero after UVB irradiation VS hetero before UVB irradiation	GO_ERROR_PRONE_TRANSLESION_SYNTHESIS1	0.349474074	0.008219953	2.24667078	0.05545806	0.31104873	-4.1408482
WT after UVB irradiation VS WT before UVB irradiation	GO_ERROR_PRONE_TRANSLESION_SYNTHESIS2	0.041970713	-0.02564815	0.208314927	0.84012736	0.96316991	-5.97323406
homo after UVB irradiation VS homo before UVB irradiation	GO_ERYTHROCYTE_DEVELOPMENT	0.027039589	0.002954061	0.153770618	0.88145113	0.93599873	-6.53789838
hetero after UVB irradiation VS hetero before UVB irradiation	GO_ERYTHROCYTE_DEVELOPMENT1	0.077496357	-0.02259528	0.620837396	0.55229562	0.80284446	-6.02761339
WT after UVB irradiation VS WT before UVB irradiation	GO_ERYTHROCYTE_DEVELOPMENT2	0.130037475	-0.02356378	0.855917967	0.41664943	0.82220893	-5.62700772
homo after UVB irradiation VS homo before UVB irradiation	GO_ERYTHROCYTE_HOMEOSTASIS	0.096879702	-0.02784548	0.715979595	0.49358503	0.66910107	-6.27800054
hetero after UVB irradiation VS hetero before UVB irradiation	GO_ERYTHROCYTE_HOMEOSTASIS1	0.191227052	-0.02314553	1.401458768	0.19934045	0.52365051	-5.28512616
WT after UVB irradiation VS WT before UVB irradiation	GO_ERYTHROCYTE_HOMEOSTASIS2	0.116702937	-0.02053587	0.793387393	0.45017218	0.83530628	-5.67694951
homo after UVB irradiation VS homo before UVB irradiation	GO_ERYTHROCYTE_MATURATION	-0.00517946	-0.00541018	-0.0241123	0.98133072	0.99086538	-6.55053764
hetero after UVB irradiation VS hetero before UVB irradiation	GO_ERYTHROCYTE_MATURATION1	0.35420191	-0.01597778	2.193290855	0.06023627	0.32333444	-4.21797983
WT after UVB irradiation VS WT before UVB irradiation	GO_ERYTHROCYTE_MATURATION2	0.053627136	-0.00033356	0.23498435	0.82005416	0.95747601	-5.96703593
homo after UVB irradiation VS homo before UVB irradiation	GO_ESC_E_Z_COMPLEX	-0.66205502	-0.00189919	-4.04956935	0.00338927	0.03823813	-1.65514062
hetero after UVB irradiation VS hetero before UVB irradiation	GO_ESC_E_Z_COMPLEX1	-0.24383888	-0.05070027	-1.02055484	0.33787399	0.65765319	-5.70239784
WT after UVB irradiation VS WT before UVB irradiation	GO_ESC_E_Z_COMPLEX2	-0.35332471	-0.00768948	-1.36632955	0.20856853	0.7053907	-5.11559748
homo after UVB irradiation VS homo before UVB irradiation	GO_ESCRT_COMPLEX	0.42372844	-0.02442557	1.896036258	0.09308973	0.24041992	-4.91731584
homo after UVB irradiation VS homo before UVB irradiation	GO_ESCRT_COMPLEX_DISASSEMBLY	0.444225767	-0.0246069	1.448802671	0.18396994	0.36791496	-5.52595645
hetero after UVB irradiation VS hetero before UVB irradiation	GO_ESCRT_COMPLEX_DISASSEMBLY1	0.480766228	-0.00444955	2.118595787	0.06761358	0.33813693	-4.32528565
WT after UVB irradiation VS WT before UVB irradiation	GO_ESCRT_COMPLEX_DISASSEMBLY2	0.317262323	0.055289568	1.013239037	0.34026068	0.7811501	-5.4879543
hetero after UVB irradiation VS hetero before UVB irradiation	GO_ESCRT_COMPLEX1	0.222147952	0.004676717	0.978080322	0.35720083	0.67273203	-5.74304309
WT after UVB irradiation VS WT before UVB irradiation	GO_ESCRT_COMPLEX2	0.272450835	0.010838245	1.091182589	0.30659275	0.76587333	-5.41245355
homo after UVB irradiation VS homo before UVB irradiation	GO_ESCRT_I_COMPLEX	0.541677564	-0.01666921	2.368332359	0.0441692	0.15254035	-4.21164246
hetero after UVB irradiation VS hetero before UVB irradiation	GO_ESCRT_I_COMPLEX1	0.354092222	-0.01857452	1.117497251	0.29680244	0.62080891	-5.60483525
WT after UVB irradiation VS WT before UVB irradiation	GO_ESCRT_I_COMPLEX2	0.156205641	-0.05737329	0.678782998	0.51620209	0.8610867	-5.7600219
homo after UVB irradiation VS homo before UVB irradiation	GO_ESCRT_III_COMPLEX	0.458636159	-0.03894645	1.690224127	0.12795766	0.29298235	-5.20716794
hetero after UVB irradiation VS hetero before UVB irradiation	GO_ESCRT_III_COMPLEX1	0.137758556	0.005124253	0.518643156	0.61830347	0.84244562	-6.08814356
WT after UVB irradiation VS WT before UVB irradiation	GO_ESCRT_III_COMPLEX2	0.404218033	-0.00155849	1.450071696	0.18464022	0.68726847	-5.01710848
homo after UVB irradiation VS homo before UVB irradiation	GO_ESTABLISHMENT_OF_BLOOD_BRAIN_BARRIER	-0.17863423	-0.02901672	-1.13227182	0.28906059	0.4840264	-5.89739197
hetero after UVB irradiation VS hetero before UVB irradiation	GO_ESTABLISHMENT_OF_BLOOD_BRAIN_BARRIER1	-0.34092735	-0.00931132	-2.24864615	0.05528866	0.31091755	-4.13798757
WT after UVB irradiation VS WT before UVB irradiation	GO_ESTABLISHMENT_OF_BLOOD_BRAIN_BARRIER2	-0.15822705	0.02781355	-0.92363121	0.38237571	0.80508768	-5.56943757
homo after UVB irradiation VS homo before UVB irradiation	GO_ESTABLISHMENT_OF_CELL_POLARITY	-0.17815265	-0.0084286	-1.06311903	0.31757558	0.51108878	-5.96996488
hetero after UVB irradiation VS hetero before UVB irradiation	GO_ESTABLISHMENT_OF_CELL_POLARITY1	-0.02416574	-0.02043137	-0.16887885	0.87016081	0.95109523	-6.21604652
WT after UVB irradiation VS WT before UVB irradiation	GO_ESTABLISHMENT_OF_CELL_POLARITY2	-0.15533836	0.002954544	-0.90877965	0.38971208	0.81049974	-5.58236698
homo after UVB irradiation VS homo before UVB irradiation	GO_ESTABLISHMENT_OF_CENTROSOME_LOCALIZATION	-0.98454475	-0.00462528	-4.79113657	0.00122472	0.02353767	-0.62830661
hetero after UVB irradiation VS hetero before UVB irradiation	GO_ESTABLISHMENT_OF_CENTROSOME_LOCALIZATION1	-0.6073253	-0.08069934	-1.89918276	0.09477522	0.38472677	-4.63485553
WT after UVB irradiation VS WT before UVB irradiation	GO_ESTABLISHMENT_OF_CENTROSOME_LOCALIZATION2	-0.65626574	-0.07306157	-3.01797632	0.01637905	0.37790033	-2.86813074
homo after UVB irradiation VS homo before UVB irradiation	GO_ESTABLISHMENT_OF_ENDOTHELIAL_BARRIER	0.366613744	-0.01388732	2.031616677	0.07526766	0.21070815	-4.71943126
hetero after UVB irradiation VS hetero before UVB irradiation	GO_ESTABLISHMENT_OF_ENDOTHELIAL_BARRIER1	0.367631154	0.012754505	2.451743352	0.0403707	0.27269233	-3.8420605

WT after UVB irradiation VS WT before UVB irradiation	GO_ESTABLISHMENT_OF_ENDOTHELIAL_BARRIER2	0.262373087	-0.03841802	1.642055245	0.13874864	0.64780591	-4.77967653
homo after UVB irradiation VS homo before UVB irradiation	GO_ESTABLISHMENT_OF_ENDOTHELIAL_BLOOD_BRAIN_BARRIER	-0.0475297	-0.02355784	-0.19244371	0.85200414	0.91820822	-6.53057732
hetero after UVB irradiation VS hetero before UVB irradiation	GO_ESTABLISHMENT_OF_ENDOTHELIAL_BLOOD_BRAIN_BARRIER1	-0.11662139	0.065697201	-0.69387745	0.50776161	0.77771749	-5.97836386
WT after UVB irradiation VS WT before UVB irradiation	GO_ESTABLISHMENT_OF_ENDOTHELIAL_BLOOD_BRAIN_BARRIER2	0.378731185	0.058606286	2.157108651	0.06266692	0.535481	-4.08676326
homo after UVB irradiation VS homo before UVB irradiation	GO_ESTABLISHMENT_OF_ENDOTHELIAL_INTESTINAL_BARRIER	-0.06865712	0.056298612	-0.28975198	0.77908836	0.87262033	-6.50501509
hetero after UVB irradiation VS hetero before UVB irradiation	GO_ESTABLISHMENT_OF_ENDOTHELIAL_INTESTINAL_BARRIER1	-0.1955018	-0.00493236	-0.96743352	0.36217424	0.67684951	-5.75302255
WT after UVB irradiation VS WT before UVB irradiation	GO_ESTABLISHMENT_OF_ENDOTHELIAL_INTESTINAL_BARRIER2	-0.11501037	-0.03583146	-0.45802389	0.65897593	0.91105574	-5.88686806
homo after UVB irradiation VS homo before UVB irradiation	GO_ESTABLISHMENT_OF_EPITHELIAL_CELL_APICAL_BASAL_POLARITY	-0.02903067	0.013674359	-0.10614802	0.91797701	0.95521227	-6.5446772
hetero after UVB irradiation VS hetero before UVB irradiation	GO_ESTABLISHMENT_OF_EPITHELIAL_CELL_APICAL_BASAL_POLARITY1	-0.03070783	-0.00717476	-0.18306912	0.85938199	0.94669956	-6.21334917
WT after UVB irradiation VS WT before UVB irradiation	GO_ESTABLISHMENT_OF_EPITHELIAL_CELL_APICAL_BASAL_POLARITY2	0.02419382	-0.0512241	0.135451089	0.89556184	0.97368307	-5.98639748
homo after UVB irradiation VS homo before UVB irradiation	GO_ESTABLISHMENT_OF_EPITHELIAL_CELL_POLARITY	-0.14194377	0.031110776	-0.56319027	0.58814136	0.74102985	-6.38007716
hetero after UVB irradiation VS hetero before UVB irradiation	GO_ESTABLISHMENT_OF_EPITHELIAL_CELL_POLARITY1	0.002100079	-0.02240985	0.01180886	0.99087261	0.99624062	-6.23140849
WT after UVB irradiation VS WT before UVB irradiation	GO_ESTABLISHMENT_OF_EPITHELIAL_CELL_POLARITY2	-0.16745959	-0.00946205	-0.8277563	0.4315252	0.82894128	-5.64989091
homo after UVB irradiation VS homo before UVB irradiation	GO_ESTABLISHMENT_OF_GOLGI_LOCALIZATION	0.32104933	-0.03377137	1.153059392	0.28089965	0.47602621	-5.87492326
hetero after UVB irradiation VS hetero before UVB irradiation	GO_ESTABLISHMENT_OF_GOLGI_LOCALIZATION1	0.654073288	0.03685142	5.028366325	0.00107485	0.089745	-0.36815625
WT after UVB irradiation VS WT before UVB irradiation	GO_ESTABLISHMENT_OF_GOLGI_LOCALIZATION2	0.394381824	-0.09917363	2.111970246	0.06725181	0.54024768	-4.19568041
homo after UVB irradiation VS homo before UVB irradiation	GO_ESTABLISHMENT_OF_LYMPHOCYTE_POLARITY	0.571432484	-0.02071945	3.021874287	0.01576048	0.08564125	-3.19933434
hetero after UVB irradiation VS hetero before UVB irradiation	GO_ESTABLISHMENT_OF_LYMPHOCYTE_POLARITY1	0.206530294	-0.00042576	0.782711178	0.45674921	0.74662582	-5.91203541
WT after UVB irradiation VS WT before UVB irradiation	GO_ESTABLISHMENT_OF_LYMPHOCYTE_POLARITY2	0.244854579	0.064764926	1.203124501	0.26292122	0.74156847	-5.2970536
homo after UVB irradiation VS homo before UVB irradiation	GO_ESTABLISHMENT_OF_MEIOTIC_SPINDLE_LOCALIZATION	0.544771629	-0.04481557	3.128308234	0.01336305	0.07828445	-3.03483246
hetero after UVB irradiation VS hetero before UVB irradiation	GO_ESTABLISHMENT_OF_MEIOTIC_SPINDLE_LOCALIZATION1	0.495615112	0.020043078	1.995945104	0.08169461	0.36587869	-4.49952538
WT after UVB irradiation VS WT before UVB irradiation	GO_ESTABLISHMENT_OF_MEIOTIC_SPINDLE_LOCALIZATION2	0.708663661	0.096021603	3.78618868	0.00522298	0.27446335	-1.82525789
homo after UVB irradiation VS homo before UVB irradiation	GO_ESTABLISHMENT_OF_MITOCHONDRION_LOCALIZATION	-0.27304071	0.000736302	-2.07607306	0.07017597	0.20305623	-4.65357061
hetero after UVB irradiation VS hetero before UVB irradiation	GO_ESTABLISHMENT_OF_MITOCHONDRION_LOCALIZATION1	-0.31206608	0.000645747	-2.17910775	0.06157308	0.32651774	-4.23841463
WT after UVB irradiation VS WT before UVB irradiation	GO_ESTABLISHMENT_OF_MITOCHONDRION_LOCALIZATION2	-0.41195391	-0.00915653	-2.80751094	0.02265794	0.41742287	-3.16510305
homo after UVB irradiation VS homo before UVB irradiation	GO_ESTABLISHMENT_OF_MITOTIC_SPINDLE_LOCALIZATION	-0.55610958	-0.03174964	-3.60286894	0.00650151	0.05353946	-2.31212631
hetero after UVB irradiation VS hetero before UVB irradiation	GO_ESTABLISHMENT_OF_MITOTIC_SPINDLE_LOCALIZATION1	0.095102006	-0.02212126	0.429617191	0.67901894	0.87087984	-6.13259184
WT after UVB irradiation VS WT before UVB irradiation	GO_ESTABLISHMENT_OF_MITOTIC_SPINDLE_LOCALIZATION2	-0.10234578	-0.02336771	-0.44007316	0.67139393	0.91526653	-5.89515529
homo after UVB irradiation VS homo before UVB irradiation	GO_ESTABLISHMENT_OF_ORGANELLE_LOCALIZATION	0.041119502	-0.00864458	0.317469914	0.75869978	0.85998407	-6.49588126
hetero after UVB irradiation VS hetero before UVB irradiation	GO_ESTABLISHMENT_OF_ORGANELLE_LOCALIZATION1	0.18287144	-0.00460798	1.398901993	0.20007735	0.52469029	-5.28820417
WT after UVB irradiation VS WT before UVB irradiation	GO_ESTABLISHMENT_OF_ORGANELLE_LOCALIZATION2	0.161538184	0.00058315	1.016289215	0.33889166	0.78064003	-5.48507863
homo after UVB irradiation VS homo before UVB irradiation	GO_ESTABLISHMENT_OF_PIGMENT_GRANULE_LOCALIZATION	-0.17106475	-0.01368369	-1.10507061	0.30002454	0.49458313	-5.9263422
hetero after UVB irradiation VS hetero before UVB irradiation	GO_ESTABLISHMENT_OF_PIGMENT_GRANULE_LOCALIZATION1	-0.14096751	-0.00635511	-0.87698574	0.40652315	0.71194505	-5.83430059
WT after UVB irradiation VS WT before UVB irradiation	GO_ESTABLISHMENT_OF_PIGMENT_GRANULE_LOCALIZATION2	-0.02370919	0.016241628	-0.12874903	0.90069661	0.9752088	-5.98732965
homo after UVB irradiation VS homo before UVB irradiation	GO_ESTABLISHMENT_OF_PLANAR_POLARITY_INVOLVED_IN_NEURAL_TUBE_CLOSURE	0.074355516	-0.00083603	0.301902859	0.77012713	0.86700993	-6.5011114
hetero after UVB irradiation VS hetero before UVB irradiation	GO_ESTABLISHMENT_OF_PLANAR_POLARITY_INVOLVED_IN_NEURAL_TUBE_CLOSURE1	0.002443271	-0.0261617	0.01129888	0.99126676	0.99624062	-6.23141488
WT after UVB irradiation VS WT before UVB irradiation	GO_ESTABLISHMENT_OF_PLANAR_POLARITY_INVOLVED_IN_NEURAL_TUBE_CLOSURE2	-0.20487952	-0.00456741	-0.83692631	0.42664136	0.82688157	-5.64250939
homo after UVB irradiation VS homo before UVB irradiation	GO_ESTABLISHMENT_OF_PLANAR_POLARITY_OF_EMBRYONIC_EPITHELIUM	-0.03062644	0.002744206	-0.13093359	0.89893521	0.94511163	-6.54145781
hetero after UVB irradiation VS hetero before UVB irradiation	GO_ESTABLISHMENT_OF_PLANAR_POLARITY_OF_EMBRYONIC_EPITHELIUM1	0.021722723	-0.01756254	0.097997501	0.92438944	0.97152219	-6.22627935
WT after UVB irradiation VS WT before UVB irradiation	GO_ESTABLISHMENT_OF_PLANAR_POLARITY_OF_EMBRYONIC_EPITHELIUM2	-0.23467536	-0.02049624	-0.94824451	0.37044085	0.79991157	-5.54764364
homo after UVB irradiation VS homo before UVB irradiation	GO_ESTABLISHMENT_OF_PROTEIN_LOCALIZATION_TO_CHROMATIN	-0.52323649	-0.02863374	-1.98340801	0.08119248	0.22049272	-4.79033384
hetero after UVB irradiation VS hetero before UVB irradiation	GO_ESTABLISHMENT_OF_PROTEIN_LOCALIZATION_TO_CHROMATIN1	-0.18994484	-0.05817298	-0.84303656	0.42414071	0.72563714	-5.86313802
WT after UVB irradiation VS WT before UVB irradiation	GO_ESTABLISHMENT_OF_PROTEIN_LOCALIZATION_TO_CHROMATIN2	-0.2791419	-0.14491927	-1.12917535	0.29116184	0.75646944	-5.37417699
homo after UVB irradiation VS homo before UVB irradiation	GO_ESTABLISHMENT_OF_PROTEIN_LOCALIZATION_TO_CHROMOSOME	-0.82631575	-0.0049036	-6.64133834	0.00013515	0.01007802	1.561565862
hetero after UVB irradiation VS hetero before UVB irradiation	GO_ESTABLISHMENT_OF_PROTEIN_LOCALIZATION_TO_CHROMOSOME1	-0.44976737	-0.06114439	-1.75036736	0.11887273	0.42318117	-4.83834919
WT after UVB irradiation VS WT before UVB irradiation	GO_ESTABLISHMENT_OF_PROTEIN_LOCALIZATION_TO_CHROMOSOME2	-0.28395411	-0.00689133	-0.91757308	0.38535603	0.80708189	-5.57473186
homo after UVB irradiation VS homo before UVB irradiation	GO_ESTABLISHMENT_OF_PROTEIN_LOCALIZATION_TO_ENDOPLASMIC_RETICULUM	-0.60795687	0.009907292	-3.01433213	0.0159465	0.08618384	-3.21101321
hetero after UVB irradiation VS hetero before UVB irradiation	GO_ESTABLISHMENT_OF_PROTEIN_LOCALIZATION_TO_ENDOPLASMIC_RETICULUM1	0.024171712	0.015017897	0.08347144	0.93556526	0.97618448	-6.22770733
WT after UVB irradiation VS WT before UVB irradiation	GO_ESTABLISHMENT_OF_PROTEIN_LOCALIZATION_TO_ENDOPLASMIC_RETICULUM2	-0.4242895	-0.01034602	-1.61105537	0.14537761	0.65365874	-4.81900357
homo after UVB irradiation VS homo before UVB irradiation	GO_ESTABLISHMENT_OF_PROTEIN_LOCALIZATION_TO_MEMBRANE	-0.20211752	-0.00957313	-1.30264921	0.22755446	0.4186015	-5.70490757
hetero after UVB irradiation VS hetero before UVB irradiation	GO_ESTABLISHMENT_OF_PROTEIN_LOCALIZATION_TO_MEMBRANE1	0.028027144	-0.01888035	0.163451324	0.87429133	0.95321437	-6.21702114
WT after UVB irradiation VS WT before UVB irradiation	GO_ESTABLISHMENT_OF_PROTEIN_LOCALIZATION_TO_MEMBRANE2	-0.18607293	-0.01878436	-1.0718904	0.31467254	0.77104985	-5.43152602
homo after UVB irradiation VS homo before UVB irradiation	GO_ESTABLISHMENT_OF_PROTEIN_LOCALIZATION_TO_MITOCHONDRIAL_MEMBRANE	-0.06971352	-0.00924038	-0.46127349	0.65640936	0.79117114	-6.43557823
hetero after UVB irradiation VS hetero before UVB irradiation	GO_ESTABLISHMENT_OF_PROTEIN_LOCALIZATION_TO_MITOCHONDRIAL_MEMBRANE1	0.029813437	-0.03463887	0.23598735	0.81948089	0.93071163	-6.20139357
WT after UVB irradiation VS WT before UVB irradiation	GO_ESTABLISHMENT_OF_PROTEIN_LOCALIZATION_TO_MITOCHONDRIAL_MEMBRANE2	0.118306482	-0.02650293	0.703747852	0.50132141	0.85681918	-5.74288668
homo after UVB irradiation VS homo before UVB irradiation	GO_ESTABLISHMENT_OF_PROTEIN_LOCALIZATION_TO_ORGANELLE	-0.23798215	-0.01584915	-1.66340766	0.13330499	0.30037895	-5.24382062
hetero after UVB irradiation VS hetero before UVB irradiation	GO_ESTABLISHMENT_OF_PROTEIN_LOCALIZATION_TO_ORGANELLE1	-0.02282046	-0.01302554	-0.14640117	0.88729321	0.95898643	-6.21987709
WT after UVB irradiation VS WT before UVB irradiation	GO_ESTABLISHMENT_OF_PROTEIN_LOCALIZATION_TO_ORGANELLE2	-0.10821934	-0.02672041	-0.64993572	0.53373351	0.8692322	-5.77913761
homo after UVB irradiation VS homo before UVB irradiation	GO_ESTABLISHMENT_OF_PROTEIN_LOCALIZATION_TO_PLASMA_MEMBRANE	0.111417471	-0.05158196	0.772338962	0.46126397	0.64106916	-6.23489583
hetero after UVB irradiation VS hetero before UVB irradiation	GO_ESTABLISHMENT_OF_PROTEIN_LOCALIZATION_TO_PLASMA_MEMBRANE1	0.028793706	-0.04186598	0.202596354	0.84460104	0.94052476	-6.20928508
WT after UVB irradiation VS WT before UVB irradiation	GO_ESTABLISHMENT_OF_PROTEIN_LOCALIZATION_TO_PLASMA_MEMBRANE2	-0.10433336	-0.05487038	-0.69709912	0.50525784	0.85763155	-5.74750343
homo after UVB irradiation VS homo before UVB irradiation	GO_ESTABLISHMENT_OF_PROTEIN_LOCALIZATION_TO_POSTSYNAPTIC_MEMBRANE	0.328658318	-0.04671046	2.028525657	0.07563468	0.21119036	-4.72399395
hetero after UVB irradiation VS hetero before UVB irradiation	GO_ESTABLISHMENT_OF_PROTEIN_LOCALIZATION_TO_POSTSYNAPTIC_MEMBRANE1	0.078879698	-0.02883222	0.359212545	0.72890323	0.89295432	-6.16209833
WT after UVB irradiation VS WT before UVB irradiation	GO_ESTABLISHMENT_OF_PROTEIN_LOCALIZATION_TO_POSTSYNAPTIC_MEMBRANE2	0.150056605	-0.01215059	0.870824171	0.40892316	0.81822963	-5.6146404
homo after UVB irradiation VS homo before UVB irradiation	GO_ESTABLISHMENT_OF_PROTEIN_LOCALIZATION_TO_TELOMERE	-0.97091295	-0.02091307	-7.80306304	4.18E-05	0.00847131	2.689682537
hetero after UVB irradiation VS hetero before UVB irradiation	GO_ESTABLISHMENT_OF_PROTEIN_LOCALIZATION_TO_TELOMERE1	-0.55569301	-0.06109074	-2.07635021	0.07217256	0.34803961	-4.38560078
WT after UVB irradiation VS WT before UVB irradiation	GO_ESTABLISHMENT_OF_PROTEIN_LOCALIZATION_TO_TELOMERE2	-0.33400338	0.0051276	-1.01001466	0.34171247	0.78172749	-5.49098707
homo after UVB irradiation VS homo before UVB irradiation	GO_ESTABLISHMENT_OF_PROTEIN_LOCALIZATION_TO_VACUOLE	0.20471269	-0.02169801	1.139414344	0.28623535	0.48092623	-5.88970516
hetero after UVB irradiation VS hetero before UVB irradiation	GO_ESTABLISHMENT_OF_PROTEIN_LOCALIZATION_TO_VACUOLE1	0.28630317	0.012667246	1.936374058	0.08952493	0.37636178	-4.583091
WT after UVB irradiation VS WT before UVB irradiation	GO_ESTABLISHMENT_OF_PROTEIN_LOCALIZATION_TO_VACUOLE2	0.333152398	0.031168184	1.916680176	0.09114202	0.58423903	-4.41794935
homo after UVB irradiation VS homo before UVB irradiation	GO_ESTABLISHMENT_OF_RNA_LOCALIZATION	-0.67220925	-0.00626764	-5.00133836	0.00093128	0.02088233	-0.35287019
hetero after UVB irradiation VS hetero before UVB irradiation	GO_ESTABLISHMENT_OF_RNA_LOCALIZATION1	-0.2335463	-0.05573393	-1.1308489	0.29147122	0.6158825	-5.59089748
WT after UVB irradiation VS WT before UVB irradiation	GO_ESTABLISHMENT_OF_RNA_LOCALIZATION2	-0.2999186	-0.04559793	-1.17251244	0.27432796	0.74597065	-5.32938957
homo after UVB irradiation VS homo before UVB irradiation	GO_ESTABLISHMENT_OF_SERTOLI_CELL_BARRIER	0.229977692	0.04145229	0.95093127	0.36841313	0.55881698	-6.08021181
hetero after UVB irradiation VS hetero before UVB irradiation	GO_ESTABLISHMENT_OF_SERTOLI_CELL_BARRIER1	0.322707787	0.001136615	1.522437263	0.1670993	0.4885405	-5.13591796
WT after UVB irradiation VS WT before UVB irradiation	GO_ESTABLISHMENT_OF_SERTOLI_CELL_BARRIER2	0.336554477	-0.0278144	2.029242691	0.07652091	0.55925897	-4.26418286
homo after UVB irradiation VS homo before UVB irradiation	GO_ESTABLISHMENT_OF_SISTER_CHROMATID_COHESION	-0.55339764	0.03810192	-2.38356495	0.04311372	0.15020364	-4.18828105
hetero after UVB irradiation VS hetero before UVB irradiation	GO_ESTABLISHMENT_OF_SISTER_CHROMATID_COHESION1	-0.09249098	-0.00088056	-0.34853227	0.73660016	0.89665665	-6.16613079
WT after UVB irradiation VS WT before UVB irradiation	GO_ESTABLISHMENT_OF_SISTER_CHROMATID_COHESION2	-0.36735507	-0.03225838	-1.18981172	0.26783311	0.74336868	-5.31118591
homo after UVB irradiation VS homo before UVB irradiation	GO_ESTABLISHMENT_OF_SPINDLE_ORIENTATION	-0.41821452	-0.01732196	-2.4398225	0.03942966	0.14264856	-4.10180047
hetero after UVB irradiation VS hetero before UVB irradiation	GO_ESTABLISHMENT_OF_SPINDLE_ORIENTATION1	0.067532891	-0.01627552	0.30232448	0.77025726	0.91003052	-6.18221417
WT after UVB irradiation VS WT before UVB irradiation	GO_ESTABLISHMENT_OF_SPINDLE_ORIENTATION2	-0.02921557	0.008512576	-0.12417966	0.90420028	0.97631401	-5.98793819
homo after UVB irradiation VS homo before UVB irradiation	GO_ESTABLISHMENT_OF_TISSUE_POLARITY	-0.18902328	-0.0076438	-0.9896682	0.35021097	0.54223726	-6.04323
hetero after UVB irradiation VS hetero before UVB irradiation	GO_ESTABLISHMENT_OF_TISSUE_POLARITY1	0.044796942	0.00601454	0.21862134	0.83251969	0.9354456	-6.20564587
WT after UVB irradiation VS WT before UVB irradiation	GO_ESTABLISHMENT_OF_TISSUE_POLARITY2	-0.03553715	-0.01300546	-0.15591111	0.87991983	0.9693507	-5.98326077
homo after UVB irradiation VS homo before UVB irradiation	GO_ESTABLISHMENT_OF_VIRAL_LATENCY	-0.36589631	-0.05621826	-1.36679067	0.20744078	0.39761054	-5.62783034
hetero after UVB irradiation VS hetero before UVB irradiation	GO_ESTABLISHMENT_OF_VIRAL_LATENCY1	-0.31664734	-0.04324622	-1.16974639	0.27637956	0.60324846	-5.54963092
WT after UVB irradiation VS WT before UVB irradiation	GO_ESTABLISHMENT_OF_VIRAL_LATENCY2	0.077536875	0.012213486	0.258324765	0.80260087	0.95333317	-5.9610087
homo after UVB irradiation VS homo before UVB irradiation	GO_ESTABLISHMENT_OR_MAINTENANCE_OF_ACTIN_CYTOSKELETON_POLARITY	-0.10082685	0.006105487	-0.35617089	0.73056611	0.84100098	-6.48176992
hetero after UVB irradiation VS hetero before UVB irradiation	GO_ESTABLISHMENT_OR_MAINTENANCE_OF_ACTIN_CYTOSKELETON_POLARITY1	0.560331205	-0.01548818	3.1608985	0.01369936	0.17665328	-2.80678157
WT after UVB irradiation VS WT before UVB irradiation	GO_ESTABLISHMENT_OR_MAINTENANCE_OF_ACTIN_CYTOSKELETON_POLARITY2	-0.07475774	-0.07173142	-0.42714747	0.68040283	0.91716791	-5.90092757
homo after UVB irradiation VS homo before UVB irradiation	GO_ESTABLISHMENT_OR_MAINTENANCE_OF_BIPOLAR_CELL_POLARITY	-0.07687798	0.025335919	-0.37906994	0.71411712	0.8299068	-6.47268042
hetero after UVB irradiation VS hetero before UVB irradiation	GO_ESTABLISHMENT_OR_MAINTENANCE_OF_BIPOLAR_CELL_POLARITY1	-0.25119773	0.006498274	-1.33960729	0.21784565	0.54539029	-5.35864873
WT after UVB irradiation VS WT before UVB irradiation	GO_ESTABLISHMENT_OR_MAINTENANCE_OF_BIPOLAR_CELL_POLARITY2	-0.14084302	0.020113533	-0.69074694	0.50903681	0.85830167	-5.75187824
homo after UVB irradiation VS homo before UVB irradiation	GO_ESTABLISHMENT_OR_MAINTENANCE_OF_CELL_POLARITY	-0.00705993	-0.00774455	-0.04588012	0.96448679	0.98169567	-6.54970161
hetero after UVB irradiation VS hetero before UVB irradiation	GO_ESTABLISHMENT_OR_MAINTENANCE_OF_CELL_POLARITY1	0.086679226	-0.00654315	0.673249054	0.5201069	0.78674499	-5.99276624
WT after UVB irradiation VS WT before UVB irradiation	GO_ESTABLISHMENT_OR_MAINTENANCE_OF_CELL_POLARITY2	-0.03039605	-0.0021666	-0.19700026	0.84868217	0.96522688	-5.9756409
homo after UVB irradiation VS homo before UVB irradiation	GO_ESTABLISHMENT_OR_MAINTENANCE_OF_CYTOSKELETON_POLARITY	-0.09629635	-0.02184647	-0.49814764	0.63126567	0.77244045	-6.41669531
hetero after UVB irradiation VS hetero before UVB irradiation	GO_ESTABLISHMENT_OR_MAINTENANCE_OF_CYTOSKELETON_POLARITY1	0.349335889	-0.03194011	2.414556476	0.04276149	0.27754618	-3.89645867
WT after UVB irradiation VS WT before UVB irradiation	GO_ESTABLISHMENT_OR_MAINTENANCE_OF_CYTOSKELETON_POLARITY2	0.050572501	-0.04209321	0.276410568	0.7891567	0.94742978	-5.95595315
homo after UVB irradiation VS homo before UVB irradiation	GO_ESTABLISHMENT_OR_MAINTENANCE_OF_MONOPOLAR_CELL_POLARITY	0.177289869	-0.02088686	0.733893148	0.48315783	0.66012037	-6.26460935
hetero after UVB irradiation VS hetero before UVB irradiation	GO_ESTABLISHMENT_OR_MAINTENANCE_OF_MONOPOLAR_CELL_POLARITY1	0.144082405	0.003131264	0.890821308	0.39949541	0.7069986	-5.82228214
WT after UVB irradiation VS WT before UVB irradiation	GO_ESTABLISHMENT_OR_MAINTENANCE_OF_MONOPOLAR_CELL_POLARITY2	0.050291852	-0.0195259	0.24220167	0.81464536	0.95586033	-5.96523216
homo after UVB irradiation VS homo before UVB irradiation	GO_ESTABLISHMENT_OR_MAINTENANCE_OF_TRANSMEMBRANE_ELECTROCHEMICAL_GRADIENT	0.410242482	0.041666378	1.961064631	0.08408832	0.22541169	-4.8230003
hetero after UVB irradiation VS hetero before UVB irradiation	GO_ESTABLISHMENT_OR_MAINTENANCE_OF_TRANSMEMBRANE_ELECTROCHEMICAL_GRADIENT1	0.182408335	0.001965956	1.135959362	0.28945118	0.61410673	-5.58553166
WT after UVB irradiation VS WT before UVB irradiation	GO_ESTABLISHMENT_OR_MAINTENANCE_OF_TRANSMEMBRANE_ELECTROCHEMICAL_GRADIENT2	0.233217734	0.071928656	1.43757941	0.18804953	0.69085152	-5.03201283
homo after UVB irradiation VS homo before UVB irradiation	GO_ESTRADIOL_17_BETA_DEHYDROGENASE_ACTIVITY	-0.01999937	-0.03425888	-0.08519902	0.9341156	0.96498032	-6.54687467
hetero after UVB irradiation VS hetero before UVB irradiation	GO_ESTRADIOL_17_BETA_DEHYDROGENASE_ACTIVITY1	-0.35979403	0.014127151	-1.84126906	0.10354228	0.3994431	-4.71477734
WT after UVB irradiation VS WT before UVB irradiation	GO_ESTRADIOL_17_BETA_DEHYDROGENASE_ACTIVITY2	-0.31687637	-0.0079419	-1.55311246	0.15854684	0.66223376	-4.89153674
homo after UVB irradiation VS homo before UVB irradiation	GO_ESTROGEN_16_ALPHA_HYDROXYLASE_ACTIVITY	-0.09074427	0.065922167	-0.38433288	0.71035845	0.82731841	-6.47051407
hetero after UVB irradiation VS hetero before UVB irradiation	GO_ESTROGEN_16_ALPHA_HYDROXYLASE_ACTIVITY1	-0.0941573	0.02744182	-0.39106461	0.70614565	0.8835375	-6.14937554
WT after UVB irradiation VS WT before UVB irradiation	GO_ESTROGEN_16_ALPHA_HYDROXYLASE_ACTIVITY2	-0.31492391	0.11352851	-1.74489377	0.11870019	0.62626976	-4.64683543
homo after UVB irradiation VS homo before UVB irradiation	GO_ESTROGEN_2_HYDROXYLASE_ACTIVITY	-0.19840699	0.00923621	-0.78678367	0.45321054	0.6344436	-6.22339311
hetero after UVB irradiation VS hetero before UVB irradiation	GO_ESTROGEN_2_HYDROXYLASE_ACTIVITY1	-0.09715504	-0.01295837	-0.31208504	0.76310146	0.90723412	-6.17900206
WT after UVB irradiation VS WT before UVB irradiation	GO_ESTROGEN_2_HYDROXYLASE_ACTIVITY2	-0.48785057	0.079236551	-2.01890177	0.07776348	0.56140246	-4.2784134
homo after UVB irradiation VS homo before UVB irradiation	GO_ESTROGEN_BIOSYNTHETIC_PROCESS	-0.06325237	0.001241922	-0.23404113	0.82060339	0.8994091	-6.5208953
hetero after UVB irradiation VS hetero before UVB irradiation	GO_ESTROGEN_BIOSYNTHETIC_PROCESS1	-0.29202416	-0.00470717	-1.54518842	0.16158588	0.48268789	-5.10712163
WT after UVB irradiation VS WT before UVB irradiation	GO_ESTROGEN_BIOSYNTHETIC_PROCESS2	-0.39787344	-0.02353511	-1.88314262	0.09599352	0.59686908	-4.46323325
homo after UVB irradiation VS homo before UVB irradiation	GO_ESTROGEN_METABOLIC_PROCESS	0.006525493	0.014583924	0.04000926	0.96902817	0.983964	-6.54997828
hetero after UVB irradiation VS hetero before UVB irradiation	GO_ESTROGEN_METABOLIC_PROCESS1	-0.13196793	-0.03231134	-0.62465524	0.54991164	0.80098311	-6.02516062
WT after UVB irradiation VS WT before UVB irradiation	GO_ESTROGEN_METABOLIC_PROCESS2	-0.32273201	0.016435105	-1.95256318	0.08621134	0.57723262	-4.36921538
homo after UVB irradiation VS homo before UVB irradiation	GO_ESTROGEN_RECEPTOR_ACTIVITY	-0.15484858	-0.01225698	-0.57748356	0.57888465	0.73491059	-6.37147227
hetero after UVB irradiation VS hetero before UVB irradiation	GO_ESTROGEN_RECEPTOR_ACTIVITY1	-0.48899149	0.032359651	-2.30553458	0.05062546	0.29923261	-4.0554327
WT after UVB irradiation VS WT before UVB irradiation	GO_ESTROGEN_RECEPTOR_ACTIVITY2	-0.27655665	0.093919753	-1.19974958	0.26415937	0.74156847	-5.30064636
homo after UVB irradiation VS homo before UVB irradiation	GO_ESTROGEN_RECEPTOR_BINDING	-0.17412472	0.003068041	-1.22513281	0.25403826	0.44928603	-5.79478527
hetero after UVB irradiation VS hetero before UVB irradiation	GO_ESTROGEN_RECEPTOR_BINDING1	-0.12878416	-0.02558763	-0.77866871	0.45899363	0.74772822	-5.91520251
WT after UVB irradiation VS WT before UVB irradiation	GO_ESTROGEN_RECEPTOR_BINDING2	-0.00534955	0.028935161	-0.03089194	0.97610356	0.99395255	-5.99556315
homo after UVB irradiation VS homo before UVB irradiation	GO_ESTROUS_CYCLE	-0.00132081	-0.01524504	-0.00684387	0.99470051	0.99697176	-6.55083101
hetero after UVB irradiation VS hetero before UVB irradiation	GO_ESTROUS_CYCLE1	-0.29782639	0.052664468	-1.96707417	0.08540308	0.37183255	-4.54012116
WT after UVB irradiation VS WT before UVB irradiation	GO_ESTROUS_CYCLE2	-0.17888906	0.042644281	-0.89121543	0.39851963	0.81477652	-5.59744036
homo after UVB irradiation VS homo before UVB irradiation	GO_ETHANOL_CATABOLIC_PROCESS	0.009906849	0.131706015	0.045635289	0.96467615	0.98169567	-6.5497139
hetero after UVB irradiation VS hetero before UVB irradiation	GO_ETHANOL_CATABOLIC_PROCESS1	-0.52054152	0.048539213	-2.6199292	0.03114109	0.24648003	-3.59544924
WT after UVB irradiation VS WT before UVB irradiation	GO_ETHANOL_CATABOLIC_PROCESS2	0.030462966	0.064728716	0.145451456	0.88790988	0.97111575	-5.98491907
homo after UVB irradiation VS homo before UVB irradiation	GO_ETHANOL_METABOLIC_PROCESS	0.002164482	0.04202817	0.009004783	0.99302727	0.99634161	-6.55081222
hetero after UVB irradiation VS hetero before UVB irradiation	GO_ETHANOL_METABOLIC_PROCESS1	-0.3482119	-0.01488069	-1.56855556	0.15609495	0.4750128	-5.07731936
WT after UVB irradiation VS WT before UVB irradiation	GO_ETHANOL_METABOLIC_PROCESS2	-0.12347428	-0.00725146	-0.55309556	0.59513367	0.8931505	-5.83780517
homo after UVB irradiation VS homo before UVB irradiation	GO_ETHANOL_OXIDATION	-0.16945264	0.031218514	-0.59957209	0.56474103	0.72386723	-6.35778448
hetero after UVB irradiation VS hetero before UVB irradiation	GO_ETHANOL_OXIDATION1	-0.43527265	-0.03866724	-1.93175954	0.09016083	0.37720291	-4.58953139
WT after UVB irradiation VS WT before UVB irradiation	GO_ETHANOL_OXIDATION2	-0.28447506	-0.05879048	-1.36800024	0.20806598	0.70487366	-5.11366614
homo after UVB irradiation VS homo before UVB irradiation	GO_ETHANOLAMINE_CONTAINING_COMPOUND_METABOLIC_PROCESS	0.129308759	0.030989343	0.798655183	0.44666277	0.62876933	-6.2138026
hetero after UVB irradiation VS hetero before UVB irradiation	GO_ETHANOLAMINE_CONTAINING_COMPOUND_METABOLIC_PROCESS1	-0.02446199	-0.01912161	-0.14384752	0.88924378	0.95932748	-6.22027793
WT after UVB irradiation VS WT before UVB irradiation	GO_ETHANOLAMINE_CONTAINING_COMPOUND_METABOLIC_PROCESS2	-0.02434513	-0.08394095	-0.09258417	0.92848355	0.98233894	-5.99154627
homo after UVB irradiation VS homo before UVB irradiation	GO_ETHER_BIOSYNTHETIC_PROCESS	0.198271037	-0.04731434	0.884845368	0.40106041	0.58920105	-6.14054951
hetero after UVB irradiation VS hetero before UVB irradiation	GO_ETHER_BIOSYNTHETIC_PROCESS1	-0.00936395	-0.0270304	-0.06778501	0.9476509	0.98040642	-6.22899307
WT after UVB irradiation VS WT before UVB irradiation	GO_ETHER_BIOSYNTHETIC_PROCESS2	-0.03878217	0.009038427	-0.17966524	0.86182988	0.9676681	-5.97907023
homo after UVB irradiation VS homo before UVB irradiation	GO_ETHER_METABOLIC_PROCESS	0.092594019	-0.0137809	0.618720078	0.55264174	0.7149824	-6.34553873
hetero after UVB irradiation VS hetero before UVB irradiation	GO_ETHER_METABOLIC_PROCESS1	-0.07693041	-0.01892507	-0.58230854	0.57669121	0.81749064	-6.05160208
WT after UVB irradiation VS WT before UVB irradiation	GO_ETHER_METABOLIC_PROCESS2	0.017030806	-0.00663132	0.084329527	0.93484204	0.98322796	-5.99231604
homo after UVB irradiation VS homo before UVB irradiation	GO_EUCHROMATIN	-0.51299507	0.044825054	-3.36145603	0.00934827	0.06433347	-2.67717701
hetero after UVB irradiation VS hetero before UVB irradiation	GO_EUCHROMATIN1	-0.48771747	-0.0399007	-2.84278304	0.02213425	0.21467833	-3.2687593
WT after UVB irradiation VS WT before UVB irradiation	GO_EUCHROMATIN2	-0.46428492	-0.00803007	-2.97763963	0.01742477	0.38271751	-2.92479712
homo after UVB irradiation VS homo before UVB irradiation	GO_EUKARYOTIC_48S_PREINITIATION_COMPLEX	-1.06541749	0.004440477	-8.14203275	3.04E-05	0.00847131	2.987868488

hetero after UVB irradiation VS hetero before UVB irradiation	GO_EUKARYOTIC_48S_PREINITIATION_COMPLEX1	-0.61062886	-0.08066753	-1.92478842	0.09112973	0.37831177	-4.59925148
WT after UVB irradiation VS WT before UVB irradiation	GO_EUKARYOTIC_48S_PREINITIATION_COMPLEX2	-0.61026555	-0.08717964	-1.85112344	0.10085421	0.60045416	-4.50621272
homo after UVB irradiation VS homo before UVB irradiation	GO_EUKARYOTIC_INITIATION_FACTOR_4E_BINDING	-0.45284301	0.029919053	-2.90527954	0.01890564	0.09455708	-3.38012275
hetero after UVB irradiation VS hetero before UVB irradiation	GO_EUKARYOTIC_INITIATION_FACTOR_4E_BINDING1	-0.1327612	-0.00275961	-0.53731302	0.60593746	0.83508108	-6.0778327
WT after UVB irradiation VS WT before UVB irradiation	GO_EUKARYOTIC_INITIATION_FACTOR_4E_BINDING2	-0.0992473	-0.00354603	-0.65267681	0.53205231	0.86897754	-5.77735305
homo after UVB irradiation VS homo before UVB irradiation	GO_EUKARYOTIC_TRANSLATION_INITIATION_FACTOR_2_COMPLEX	-1.09161004	0.016113022	-8.44475142	2.31E-05	0.00847131	3.243349704
hetero after UVB irradiation VS hetero before UVB irradiation	GO_EUKARYOTIC_TRANSLATION_INITIATION_FACTOR_2_COMPLEX1	-0.76872824	-0.11355875	-2.36094839	0.04646177	0.28809638	-3.97473714
WT after UVB irradiation VS WT before UVB irradiation	GO_EUKARYOTIC_TRANSLATION_INITIATION_FACTOR_2_COMPLEX2	-0.72000212	-0.09873299	-2.12866305	0.06551915	0.537335	-4.12644611
homo after UVB irradiation VS homo before UVB irradiation	GO_EUKARYOTIC_TRANSLATION_INITIATION_FACTOR_2B_COMPLEX	-0.98521107	0.027178792	-7.31234619	6.74E-05	0.00942429	2.234023887
hetero after UVB irradiation VS hetero before UVB irradiation	GO_EUKARYOTIC_TRANSLATION_INITIATION_FACTOR_2B_COMPLEX1	-0.76756676	-0.13007197	-2.30935439	0.05032681	0.29860291	-4.0498784
WT after UVB irradiation VS WT before UVB irradiation	GO_EUKARYOTIC_TRANSLATION_INITIATION_FACTOR_2B_COMPLEX2	-0.42361442	-0.00510917	-1.16309885	0.2779158	0.74917538	-5.33921809
homo after UVB irradiation VS homo before UVB irradiation	GO_EUKARYOTIC_TRANSLATION_INITIATION_FACTOR_3_COMPLEX	-1.07933997	0.00910897	-8.18984224	2.91E-05	0.00847131	3.02888302
homo after UVB irradiation VS homo before UVB irradiation	GO_EUKARYOTIC_TRANSLATION_INITIATION_FACTOR_3_COMPLEX_EIF3M	-1.05621794	0.032176922	-7.05635488	8.74E-05	0.00991667	1.984449506
hetero after UVB irradiation VS hetero before UVB irradiation	GO_EUKARYOTIC_TRANSLATION_INITIATION_FACTOR_3_COMPLEX_EIF3M1	-0.44568272	-0.04739502	-1.3891644	0.20290572	0.52731188	-5.29989683
WT after UVB irradiation VS WT before UVB irradiation	GO_EUKARYOTIC_TRANSLATION_INITIATION_FACTOR_3_COMPLEX_EIF3M2	-0.50342159	-0.02606928	-1.47079077	0.17910589	0.68213098	-4.99223142
hetero after UVB irradiation VS hetero before UVB irradiation	GO_EUKARYOTIC_TRANSLATION_INITIATION_FACTOR_3_COMPLEX1	-0.60874614	-0.07538297	-1.92745627	0.09075776	0.37799082	-4.59553295
WT after UVB irradiation VS WT before UVB irradiation	GO_EUKARYOTIC_TRANSLATION_INITIATION_FACTOR_3_COMPLEX2	-0.69340284	-0.10991221	-2.32067518	0.04849295	0.50722877	-3.85676923
homo after UVB irradiation VS homo before UVB irradiation	GO_EUKARYOTIC_TRANSLATION_INITIATION_FACTOR_4F_COMPLEX	-0.38384358	-0.01395653	-2.37395141	0.04377688	0.15168242	-4.20302772
hetero after UVB irradiation VS hetero before UVB irradiation	GO_EUKARYOTIC_TRANSLATION_INITIATION_FACTOR_4F_COMPLEX1	0.060195465	0.00516167	0.214680039	0.8354868	0.93666051	-6.20656629
WT after UVB irradiation VS WT before UVB irradiation	GO_EUKARYOTIC_TRANSLATION_INITIATION_FACTOR_4F_COMPLEX2	-0.32766687	-0.03471827	-1.79006207	0.11077686	0.61576748	-4.58743314
homo after UVB irradiation VS homo before UVB irradiation	GO_EXCITATORY_CHEMICAL_SYNAPTIC_TRANSMISSION	0.240252672	0.097398683	0.824910058	0.43241028	0.61641021	-6.19215809
hetero after UVB irradiation VS hetero before UVB irradiation	GO_EXCITATORY_CHEMICAL_SYNAPTIC_TRANSMISSION1	0.092865291	0.078104958	0.396997459	0.70194055	0.88151888	-6.14689102
WT after UVB irradiation VS WT before UVB irradiation	GO_EXCITATORY_CHEMICAL_SYNAPTIC_TRANSMISSION2	0.421193061	-0.00511416	1.345578174	0.21489838	0.71229004	-5.13946773
homo after UVB irradiation VS homo before UVB irradiation	GO_EXCITATORY_EXTRACELLULAR_LIGAND_GATED_ION_CHANNEL_ACTIVITY	0.12913645	-0.01697698	0.667006562	0.52281267	0.69169325	-6.31311105
hetero after UVB irradiation VS hetero before UVB irradiation	GO_EXCITATORY_EXTRACELLULAR_LIGAND_GATED_ION_CHANNEL_ACTIVITY1	-0.18902689	0.001673385	-1.11147549	0.29923235	0.62216043	-5.61108272
WT after UVB irradiation VS WT before UVB irradiation	GO_EXCITATORY_EXTRACELLULAR_LIGAND_GATED_ION_CHANNEL_ACTIVITY2	-0.34314967	0.00111024	-1.65731067	0.13558922	0.64375376	-4.76019609
homo after UVB irradiation VS homo before UVB irradiation	GO_EXCITATORY_SYNAPSE	0.130298656	0.016710768	1.002442295	0.34435919	0.53672461	-6.03078009
homo after UVB irradiation VS homo before UVB irradiation	GO_EXCITATORY_SYNAPSE_ASSEMBLY	0.043992632	0.014528614	0.260421782	0.80086113	0.88690691	-6.51378969
hetero after UVB irradiation VS hetero before UVB irradiation	GO_EXCITATORY_SYNAPSE_ASSEMBLY1	0.183112838	0.015983025	1.319909853	0.22404242	0.55258552	-5.38164006
WT after UVB irradiation VS WT before UVB irradiation	GO_EXCITATORY_SYNAPSE_ASSEMBLY2	0.12033445	-0.01382818	0.685804524	0.5119893	0.8596457	-5.7552577
hetero after UVB irradiation VS hetero before UVB irradiation	GO_EXCITATORY_SYNAPSE1	-0.23858957	-0.00119382	-1.52387647	0.16674557	0.48800031	-5.13410291
WT after UVB irradiation VS WT before UVB irradiation	GO_EXCITATORY_SYNAPSE2	-0.28179751	-0.0322267	-1.56043204	0.15682567	0.66180255	-4.88244674
homo after UVB irradiation VS homo before UVB irradiation	GO_EXCRETION	0.357298089	0.015616589	2.837315913	0.02103173	0.10066974	-3.48567878
hetero after UVB irradiation VS hetero before UVB irradiation	GO_EXCRETION1	0.24070036	0.032595576	1.306820995	0.22824321	0.5567791	-5.39680083
WT after UVB irradiation VS WT before UVB irradiation	GO_EXCRETION2	0.162013745	0.035080727	0.900450914	0.39387079	0.81358	-5.58954414
homo after UVB irradiation VS homo before UVB irradiation	GO_EXECUTION_PHASE_OF_APOPTOSIS	0.190329371	-0.01474339	1.374211079	0.20521536	0.39488369	-5.61876257
hetero after UVB irradiation VS hetero before UVB irradiation	GO_EXECUTION_PHASE_OF_APOPTOSIS1	0.276571301	0.01235652	2.184420996	0.06106888	0.32556646	-4.23076247
WT after UVB irradiation VS WT before UVB irradiation	GO_EXECUTION_PHASE_OF_APOPTOSIS2	0.33721384	0.008657239	2.408206509	0.04227032	0.495137	-3.73276846
homo after UVB irradiation VS homo before UVB irradiation	GO_EXIT_FROM_MITOSIS	-0.33197391	-0.03048546	-2.45977018	0.03820063	0.14015001	-4.0710673
hetero after UVB irradiation VS hetero before UVB irradiation	GO_EXIT_FROM_MITOSIS1	-0.06186984	-0.0141335	-0.38087894	0.71339019	0.88743767	-6.15355717
WT after UVB irradiation VS WT before UVB irradiation	GO_EXIT_FROM_MITOSIS2	-0.0349731	0.004799451	-0.17426947	0.86593185	0.96790654	-5.98007379
homo after UVB irradiation VS homo before UVB irradiation	GO_EXOCRINE_PANCREAS_DEVELOPMENT	0.434207155	-0.02889089	2.526713452	0.03435094	0.13268146	-3.96770498
hetero after UVB irradiation VS hetero before UVB irradiation	GO_EXOCRINE_PANCREAS_DEVELOPMENT1	-0.13280763	0.059218038	-0.68598937	0.51246044	0.78158824	-5.98391649
WT after UVB irradiation VS WT before UVB irradiation	GO_EXOCRINE_PANCREAS_DEVELOPMENT2	-0.19853918	-0.04720316	-0.79347007	0.45012668	0.83530628	-5.67688558
homo after UVB irradiation VS homo before UVB irradiation	GO_EXOCRINE_SYSTEM_DEVELOPMENT	0.096421175	-0.00276384	0.567295794	0.5854742	0.73899508	-6.37762593
hetero after UVB irradiation VS hetero before UVB irradiation	GO_EXOCRINE_SYSTEM_DEVELOPMENT1	0.0277102	0.031201643	0.179743812	0.86190514	0.94785156	-6.21400061
WT after UVB irradiation VS WT before UVB irradiation	GO_EXOCRINE_SYSTEM_DEVELOPMENT2	-0.07540495	0.051720819	-0.50155617	0.62932935	0.90390703	-5.86547409
homo after UVB irradiation VS homo before UVB irradiation	GO_EXOCYST	0.569293445	-0.04546937	2.9540858	0.01751667	0.09069947	-3.30438752
homo after UVB irradiation VS homo before UVB irradiation	GO_EXOCYST_LOCALIZATION	0.832983132	-0.05558643	5.229436863	0.0006968	0.0185944	-0.06191316
hetero after UVB irradiation VS hetero before UVB irradiation	GO_EXOCYST_LOCALIZATION1	0.650260131	0.021725723	2.952904814	0.01872578	0.19947197	-3.10802845
WT after UVB irradiation VS WT before UVB irradiation	GO_EXOCYST_LOCALIZATION2	0.830967982	0.036297307	4.384328794	0.00226807	0.21875705	-1.07674541
hetero after UVB irradiation VS hetero before UVB irradiation	GO_EXOCYST1	0.574290348	-0.00754803	3.375850079	0.00996958	0.15702536	-2.49982884
WT after UVB irradiation VS WT before UVB irradiation	GO_EXOCYST2	0.445925848	-0.07430751	2.982321122	0.01729992	0.38271751	-2.91821349
homo after UVB irradiation VS homo before UVB irradiation	GO_EXOCYTIC_INSERTION_OF_NEUROTRANSMITTER_RECEPTOR_TO_POSTSYNAPTIC_MEMBRANE	-0.29500042	0.033243607	-1.35271528	0.21171915	0.4020261	-5.64494596
hetero after UVB irradiation VS hetero before UVB irradiation	GO_EXOCYTIC_INSERTION_OF_NEUROTRANSMITTER_RECEPTOR_TO_POSTSYNAPTIC_MEMBRANE1	-0.4184203	0.002550385	-2.57604272	0.03331934	0.25304179	-3.65985313
WT after UVB irradiation VS WT before UVB irradiation	GO_EXOCYTIC_INSERTION_OF_NEUROTRANSMITTER_RECEPTOR_TO_POSTSYNAPTIC_MEMBRANE2	-0.40594227	0.080606948	-2.29351257	0.05060418	0.5111334	-3.89514154
homo after UVB irradiation VS homo before UVB irradiation	GO_EXOCYTIC_PROCESS	0.291445357	-0.02052463	2.109109334	0.06661054	0.19682548	-4.60435387
hetero after UVB irradiation VS hetero before UVB irradiation	GO_EXOCYTIC_PROCESS1	0.150223678	0.007255389	1.052872377	0.32371593	0.64333364	-5.67059947
WT after UVB irradiation VS WT before UVB irradiation	GO_EXOCYTIC_PROCESS2	0.145942617	-0.00296684	1.002677262	0.34503378	0.78422102	-5.49786098
homo after UVB irradiation VS homo before UVB irradiation	GO_EXOCYTIC_VESICLE	0.257969353	-0.01907074	2.039260451	0.07436744	0.20955513	-4.70813876
hetero after UVB irradiation VS hetero before UVB irradiation	GO_EXOCYTIC_VESICLE1	0.168643645	0.007348276	1.201256692	0.26462749	0.59120563	-5.51549742
WT after UVB irradiation VS WT before UVB irradiation	GO_EXOCYTIC_VESICLE2	0.149790031	-0.00456255	0.908128223	0.3900362	0.8107852	-5.58293025
homo after UVB irradiation VS homo before UVB irradiation	GO_EXOCYTOSIS	0.531340085	-0.02412347	4.037016194	0.00345056	0.03854651	-1.67323453
hetero after UVB irradiation VS hetero before UVB irradiation	GO_EXOCYTOSIS1	0.531206559	0.019417626	3.910765788	0.00464201	0.12761654	-1.7618803
WT after UVB irradiation VS WT before UVB irradiation	GO_EXOCYTOSIS2	0.417308923	0.005160338	2.70046094	0.02675959	0.43942403	-3.31710139
homo after UVB irradiation VS homo before UVB irradiation	GO_EXODEOXYRIBONUCLEASE_ACTIVITY	-0.04203838	-0.02066821	-0.29585019	0.77458649	0.86970804	-6.50307555
hetero after UVB irradiation VS hetero before UVB irradiation	GO_EXODEOXYRIBONUCLEASE_ACTIVITY1	0.311589933	0.009179884	1.764173546	0.11641394	0.4194277	-4.81973971
WT after UVB irradiation VS WT before UVB irradiation	GO_EXODEOXYRIBONUCLEASE_ACTIVITY2	0.250879199	-0.00526057	1.180732067	0.27122612	0.74431614	-5.32076302
homo after UVB irradiation VS homo before UVB irradiation	GO_EXOGENOUS_PROTEIN_BINDING	0.239410864	0.031426467	1.503024224	0.16976211	0.34916185	-5.45669198
hetero after UVB irradiation VS hetero before UVB irradiation	GO_EXOGENOUS_PROTEIN_BINDING1	0.234507803	0.018892376	1.657134367	0.13679054	0.44981284	-4.96238256
WT after UVB irradiation VS WT before UVB irradiation	GO_EXOGENOUS_PROTEIN_BINDING2	0.159483923	-0.05138184	1.013816304	0.34000126	0.78105961	-5.48741057
homo after UVB irradiation VS homo before UVB irradiation	GO_EXON_EXON_JUNCTION_COMPLEX	-0.77361663	-0.04709734	-5.12020201	0.0007999	0.01944661	-0.20022216
hetero after UVB irradiation VS hetero before UVB irradiation	GO_EXON_EXON_JUNCTION_COMPLEX1	-0.52236402	-0.04079498	-2.00832363	0.08015285	0.36426243	-4.48206708
WT after UVB irradiation VS WT before UVB irradiation	GO_EXON_EXON_JUNCTION_COMPLEX2	-0.47794365	-0.04461574	-1.57061812	0.15445854	0.65981549	-4.86976129
homo after UVB irradiation VS homo before UVB irradiation	GO_EXONUCLEASE_ACTIVITY	-0.44021072	-0.01270218	-3.44353789	0.00825564	0.06041217	-2.55237895
homo after UVB irradiation VS homo before UVB irradiation	GO_EXONUCLEASE_ACTIVITY_ACTIVE_WITH_EITHER_RIBO_OR_DEOXYRIBONUCLEIC_ACIDS_AND_PRODUCING_5_PHOSPHOMONOESTERS	-0.4297263	-0.018717	-3.09849587	0.01399363	0.08035173	-3.08084656
hetero after UVB irradiation VS hetero before UVB irradiation	GO_EXONUCLEASE_ACTIVITY_ACTIVE_WITH_EITHER_RIBO_OR_DEOXYRIBONUCLEIC_ACIDS_AND_PRODUCING_5_PHOSPHOMONOESTERS1	-0.02119119	-0.02556942	-0.0960359	0.92589763	0.9721464	-6.22648551
WT after UVB irradiation VS WT before UVB irradiation	GO_EXONUCLEASE_ACTIVITY_ACTIVE_WITH_EITHER_RIBO_OR_DEOXYRIBONUCLEIC_ACIDS_AND_PRODUCING_5_PHOSPHOMONOESTERS2	-0.19289177	0.000690836	-0.73864625	0.48098001	0.8504347	-5.71802703
hetero after UVB irradiation VS hetero before UVB irradiation	GO_EXONUCLEASE_ACTIVITY1	-0.05165602	-0.04245492	-0.25328678	0.80655139	0.92458203	-6.19683946
WT after UVB irradiation VS WT before UVB irradiation	GO_EXONUCLEASE_ACTIVITY2	-0.17950345	-0.01064073	-0.70325802	0.50161075	0.85694257	-5.74322813
homo after UVB irradiation VS homo before UVB irradiation	GO_EXOPEPTIDASE_ACTIVITY	0.472233818	-0.04731106	3.395274621	0.00888071	0.0627921	-2.62567989
hetero after UVB irradiation VS hetero before UVB irradiation	GO_EXOPEPTIDASE_ACTIVITY1	0.364491033	0.017647715	2.574247827	0.03341167	0.25309171	-3.66248678
WT after UVB irradiation VS WT before UVB irradiation	GO_EXOPEPTIDASE_ACTIVITY2	0.276281545	-0.00420137	1.435157077	0.18871699	0.69085152	-5.03489449
homo after UVB irradiation VS homo before UVB irradiation	GO_EXOPOLYPHOSPHATASE_ACTIVITY	0.955828269	-0.09198163	7.018521848	9.09E-05	0.00991667	1.946848231
hetero after UVB irradiation VS hetero before UVB irradiation	GO_EXOPOLYPHOSPHATASE_ACTIVITY1	0.540854407	0.000603451	3.399256519	0.00963381	0.15528522	-2.46672274
WT after UVB irradiation VS WT before UVB irradiation	GO_EXOPOLYPHOSPHATASE_ACTIVITY2	0.310211814	-0.03196461	1.007884695	0.34267409	0.78172749	-5.49298643
homo after UVB irradiation VS homo before UVB irradiation	GO_EXORIBONUCLEASE_ACTIVITY	-0.58614039	-0.0313787	-4.41670608	0.00202706	0.03051518	-1.13621895
hetero after UVB irradiation VS hetero before UVB irradiation	GO_EXORIBONUCLEASE_ACTIVITY1	-0.07374423	-0.0457744	-0.33343836	0.747532	0.90061257	-6.17162852
WT after UVB irradiation VS WT before UVB irradiation	GO_EXORIBONUCLEASE_ACTIVITY2	-0.31132907	0.001212377	-1.20594416	0.26189044	0.74156847	-5.29404671
homo after UVB irradiation VS homo before UVB irradiation	GO_EXORIBONUCLEASE_COMPLEX	-0.76436549	0.022493714	-5.269659	0.00066256	0.01818244	-0.01146023
hetero after UVB irradiation VS hetero before UVB irradiation	GO_EXORIBONUCLEASE_COMPLEX1	-0.4123184	-0.06863686	-1.98983725	0.08246591	0.36807092	-4.50812824
WT after UVB irradiation VS WT before UVB irradiation	GO_EXORIBONUCLEASE_COMPLEX2	-0.28712933	-0.00480091	-1.06977967	0.31556659	0.77149112	-5.43359759
homo after UVB irradiation VS homo before UVB irradiation	GO_EXPLORATION_BEHAVIOR	0.09051276	0.014006117	0.724237904	0.48876028	0.66488322	-6.27186322
hetero after UVB irradiation VS hetero before UVB irradiation	GO_EXPLORATION_BEHAVIOR1	-0.10664715	-0.02049426	-0.71547461	0.49503618	0.76903628	-5.96287615
WT after UVB irradiation VS WT before UVB irradiation	GO_EXPLORATION_BEHAVIOR2	-0.14469242	0.008676644	-0.74198402	0.47906291	0.85010295	-5.71559468
homo after UVB irradiation VS homo before UVB irradiation	GO_EXPORT_ACROSS_PLASMA_MEMBRANE	0.118168331	0.012738725	0.849177729	0.41951759	0.60504506	-6.17162641
hetero after UVB irradiation VS hetero before UVB irradiation	GO_EXPORT_ACROSS_PLASMA_MEMBRANE1	-0.22109918	0.00770728	-1.240642	0.25052249	0.57806649	-5.47197711
WT after UVB irradiation VS WT before UVB irradiation	GO_EXPORT_ACROSS_PLASMA_MEMBRANE2	-0.08131574	0.048156787	-0.49062201	0.63671185	0.90563438	-5.87101929
homo after UVB irradiation VS homo before UVB irradiation	GO_EXTERNAL_ENCAPSULATING_STRUCTURE	-0.55025347	0.11750641	-2.51015821	0.03526496	0.13465351	-3.99329601
hetero after UVB irradiation VS hetero before UVB irradiation	GO_EXTERNAL_ENCAPSULATING_STRUCTURE1	-0.51480074	-0.02724363	-1.62026992	0.14454172	0.46084809	-5.01058166
WT after UVB irradiation VS WT before UVB irradiation	GO_EXTERNAL_ENCAPSULATING_STRUCTURE2	-0.4111412	0.150897894	-1.48011173	0.17666442	0.68058088	-4.9809769
homo after UVB irradiation VS homo before UVB irradiation	GO_EXTERNAL_SIDE_OF_APICAL_PLASMA_MEMBRANE	-0.04299521	-0.02581715	-0.17668525	0.86397643	0.92516161	-6.53375647
hetero after UVB irradiation VS hetero before UVB irradiation	GO_EXTERNAL_SIDE_OF_APICAL_PLASMA_MEMBRANE1	0.278670405	0.051058069	1.493147383	0.17444627	0.49708871	-5.17265996
WT after UVB irradiation VS WT before UVB irradiation	GO_EXTERNAL_SIDE_OF_APICAL_PLASMA_MEMBRANE2	-0.15819578	-0.07505108	-0.42743984	0.68019844	0.91716791	-5.90079882
homo after UVB irradiation VS homo before UVB irradiation	GO_EXTERNAL_SIDE_OF_PLASMA_MEMBRANE	0.541256094	-0.01747304	3.989746	0.00369222	0.03971292	-1.74156981
hetero after UVB irradiation VS hetero before UVB irradiation	GO_EXTERNAL_SIDE_OF_PLASMA_MEMBRANE1	0.314447432	0.027707512	1.765044662	0.11626042	0.41924211	-4.81856354
WT after UVB irradiation VS WT before UVB irradiation	GO_EXTERNAL_SIDE_OF_PLASMA_MEMBRANE2	0.284444806	-0.00774731	1.156764867	0.28035129	0.75145468	-5.34580032
homo after UVB irradiation VS homo before UVB irradiation	GO_EXTRACELLULAR_EXOSOME_ASSEMBLY	-0.18902887	0.012145072	-0.67707743	0.51671687	0.68711649	-6.30607191
hetero after UVB irradiation VS hetero before UVB irradiation	GO_EXTRACELLULAR_EXOSOME_ASSEMBLY1	0.132466954	0.037048173	0.826883279	0.43270915	0.73223121	-5.87652863
WT after UVB irradiation VS WT before UVB irradiation	GO_EXTRACELLULAR_EXOSOME_ASSEMBLY2	-0.28714102	-0.05934768	-1.26301115	0.24173733	0.73130745	-5.23218897
homo after UVB irradiation VS homo before UVB irradiation	GO_EXTRACELLULAR_LIGAND_GATED_ION_CHANNEL_ACTIVITY	-0.1389412	0.055834574	-0.85169864	0.41819383	0.60383798	-6.16946496
hetero after UVB irradiation VS hetero before UVB irradiation	GO_EXTRACELLULAR_LIGAND_GATED_ION_CHANNEL_ACTIVITY1	-0.40029026	0.031441561	-2.51559142	0.03657706	0.26251512	-3.74852217
WT after UVB irradiation VS WT before UVB irradiation	GO_EXTRACELLULAR_LIGAND_GATED_ION_CHANNEL_ACTIVITY2	-0.34012137	0.024471747	-1.78404628	0.11180273	0.61576748	-4.59537922
homo after UVB irradiation VS homo before UVB irradiation	GO_EXTRACELLULAR_MATRIX	0.500525456	-0.00943113	3.844945231	0.004552	0.04377704	-1.95282856
homo after UVB irradiation VS homo before UVB irradiation	GO_EXTRACELLULAR_MATRIX_ASSEMBLY	0.169189923	-0.0192195	1.158235087	0.27889685	0.47420864	-5.86928326
hetero after UVB irradiation VS hetero before UVB irradiation	GO_EXTRACELLULAR_MATRIX_ASSEMBLY1	0.13232406	0.030192919	0.686709603	0.51203027	0.78133596	-5.98341182
WT after UVB irradiation VS WT before UVB irradiation	GO_EXTRACELLULAR_MATRIX_ASSEMBLY2	0.070888059	-0.00779679	0.35210261	0.73374917	0.93423996	-5.93117631
homo after UVB irradiation VS homo before UVB irradiation	GO_EXTRACELLULAR_MATRIX_BINDING	0.603791917	0.000633827	4.664389357	0.00144915	0.02567235	-0.79777301
hetero after UVB irradiation VS hetero before UVB irradiation	GO_EXTRACELLULAR_MATRIX_BINDING1	0.401014179	0.026568739	1.97120168	0.08486309	0.37147425	-4.53432822
WT after UVB irradiation VS WT before UVB irradiation	GO_EXTRACELLULAR_MATRIX_BINDING2	0.474302961	0.035356939	2.301231905	0.04999504	0.50853601	-3.88424255
homo after UVB irradiation VS homo before UVB irradiation	GO_EXTRACELLULAR_MATRIX_CELL_SIGNALING	0.75206725	-0.07848443	4.191207655	0.00277313	0.03485781	-1.45256588
hetero after UVB irradiation VS hetero before UVB irradiation	GO_EXTRACELLULAR_MATRIX_CELL_SIGNALING1	0.358278023	-0.06608615	1.446310633	0.18679556	0.51240846	-5.23060817
WT after UVB irradiation VS WT before UVB irradiation	GO_EXTRACELLULAR_MATRIX_CELL_SIGNALING2	0.362182851	0.059261497	1.190952557	0.26740926	0.74336868	-5.30997903
homo after UVB irradiation VS homo before UVB irradiation	GO_EXTRACELLULAR_MATRIX_CONSTITUENT_CONFERRING_ELASTICITY	0.734833731	-0.02815965	3.29637709	0.01032235	0.06798529	-2.77656971
hetero after UVB irradiation VS hetero before UVB irradiation	GO_EXTRACELLULAR_MATRIX_CONSTITUENT_CONFERRING_ELASTICITY1	0.692980775	0.030914403	2.732920454	0.02617975	0.230549	-3.42966554
WT after UVB irradiation VS WT before UVB irradiation	GO_EXTRACELLULAR_MATRIX_CONSTITUENT_CONFERRING_ELASTICITY2	0.775911048	0.04923988	2.990825556	0.01707549	0.38237184	-2.90625818
homo after UVB irradiation VS homo before UVB irradiation	GO_EXTRACELLULAR_MATRIX_CONSTITUENT_SECRETION	0.258792901	-0.0039249	1.020908546	0.33603135	0.52924231	-6.01256299
hetero after UVB irradiation VS hetero before UVB irradiation	GO_EXTRACELLULAR_MATRIX_CONSTITUENT_SECRETION1	-0.09173804	-0.01331099	-0.35051327	0.73517008	0.89626925	-6.16539174
WT after UVB irradiation VS WT before UVB irradiation	GO_EXTRACELLULAR_MATRIX_CONSTITUENT_SECRETION2	-0.25299482	-0.06937386	-0.97671916	0.35698087	0.79119413	-5.52187018
homo after UVB irradiation VS homo before UVB irradiation	GO_EXTRACELLULAR_MATRIX_DISASSEMBLY	0.813042356	-0.02370005	6.653599249	0.00013338	0.01007802	1.574393014
hetero after UVB irradiation VS hetero before UVB irradiation	GO_EXTRACELLULAR_MATRIX_DISASSEMBLY1	0.619766864	0.068283144	3.522263293	0.00805595	0.14537603	-2.29387859
WT after UVB irradiation VS WT before UVB irradiation	GO_EXTRACELLULAR_MATRIX_DISASSEMBLY2	0.503454078	0.026259682	2.610972074	0.03076926	0.45318201	-3.44441244
homo after UVB irradiation VS homo before UVB irradiation	GO_EXTRACELLULAR_MATRIX_PROTEIN_BINDING	1.110965854	0.10047063	7.849474818	4.00E-05	0.00847131	2.731288903
hetero after UVB irradiation VS hetero before UVB irradiation	GO_EXTRACELLULAR_MATRIX_PROTEIN_BINDING1	0.894159692	0.073648937	3.626614951	0.0069328	0.1390235	-2.14882978
WT after UVB irradiation VS WT before UVB irradiation	GO_EXTRACELLULAR_MATRIX_PROTEIN_BINDING2	0.6101355	0.002490888	1.772807256	0.11374327	0.61911707	-4.61019653
homo after UVB irradiation VS homo before UVB irradiation	GO_EXTRACELLULAR_MATRIX_STRUCTURAL_CONSTITUENT	0.626407001	-0.01514523	4.803489237	0.00120495	0.02347563	-0.61192649
homo after UVB irradiation VS homo before UVB irradiation	GO_EXTRACELLULAR_MATRIX_STRUCTURAL_CONSTITUENT_CONFERRING_COMPRESSION_RESISTANCE	0.420393694	0.07299833	2.2888384	0.05010982	0.16480291	-4.33312141
hetero after UVB irradiation VS hetero before UVB irradiation	GO_EXTRACELLULAR_MATRIX_STRUCTURAL_CONSTITUENT_CONFERRING_COMPRESSION_RESISTANCE1	0.073280068	0.038997255	0.308102413	0.76601837	0.90804684	-6.18032472
WT after UVB irradiation VS WT before UVB irradiation	GO_EXTRACELLULAR_MATRIX_STRUCTURAL_CONSTITUENT_CONFERRING_COMPRESSION_RESISTANCE2	0.125862057	0.005559176	0.470881165	0.65014972	0.9092481	-5.88073969
homo after UVB irradiation VS homo before UVB irradiation	GO_EXTRACELLULAR_MATRIX_STRUCTURAL_CONSTITUENT_CONFERRING_TENSILE_STRENGTH	0.566810868	0.005980712	3.891865072	0.00425208	0.04225671	-1.88406116
hetero after UVB irradiation VS hetero before UVB irradiation	GO_EXTRACELLULAR_MATRIX_STRUCTURAL_CONSTITUENT_CONFERRING_TENSILE_STRENGTH1	0.227135002	0.035495707	1.138674876	0.28838241	0.61324588	-5.58267351
WT after UVB irradiation VS WT before UVB irradiation	GO_EXTRACELLULAR_MATRIX_STRUCTURAL_CONSTITUENT_CONFERRING_TENSILE_STRENGTH2	0.230085878	-0.00026772	1.018977028	0.33768879	0.78058796	-5.48253915
hetero after UVB irradiation VS hetero before UVB irradiation	GO_EXTRACELLULAR_MATRIX_STRUCTURAL_CONSTITUENT1	0.329255741	0.039076833	1.525185223	0.16642448	0.48782273	-5.1324516
WT after UVB irradiation VS WT before UVB irradiation	GO_EXTRACELLULAR_MATRIX_STRUCTURAL_CONSTITUENT2	0.34743439	0.018678629	1.416864847	0.19382484	0.69334561	-5.05656626
hetero after UVB irradiation VS hetero before UVB irradiation	GO_EXTRACELLULAR_MATRIX1	0.183092382	0.017998222	0.901553905	0.39410452	0.703023	-5.81285441
WT after UVB irradiation VS WT before UVB irradiation	GO_EXTRACELLULAR_MATRIX2	0.225625319	0.005621785	1.023702909	0.33558177	0.77905119	-5.47806179
homo after UVB irradiation VS homo before UVB irradiation	GO_EXTRACELLULAR_MEMBRANE_BOUNDED_ORGANELLE	0.85436719	-0.01740799	6.951002254	9.74E-05	0.00997194	1.879275895
hetero after UVB irradiation VS hetero before UVB irradiation	GO_EXTRACELLULAR_MEMBRANE_BOUNDED_ORGANELLE1	0.223885799	0.045882555	0.740072124	0.4807944	0.76122341	-5.94473353
WT after UVB irradiation VS WT before UVB irradiation	GO_EXTRACELLULAR_MEMBRANE_BOUNDED_ORGANELLE2	0.682223347	-0.04578272	2.497175252	0.03676671	0.47615783	-3.60635144
homo after UVB irradiation VS homo before UVB irradiation	GO_EXTRACELLULAR_REGULATION_OF_SIGNAL_TRANSDUCTION	0.279681289	-0.0722302	1.426899897	0.18999966	0.37569676	-5.5535135
hetero after UVB irradiation VS hetero before UVB irradiation	GO_EXTRACELLULAR_REGULATION_OF_SIGNAL_TRANSDUCTION1	0.091454524	0.032205208	0.362228699	0.72673546	0.89222406	-6.16093824
WT after UVB irradiation VS WT before UVB irradiation	GO_EXTRACELLULAR_REGULATION_OF_SIGNAL_TRANSDUCTION2	0.188456545	-0.04438416	0.552474022	0.59553997	0.89338502	-5.83815376

homo after UVB irradiation VS homo before UVB irradiation	GO_EXTRACELLULAR_STRUCTURE_ORGANIZATION	0.581328916	-0.00745042	4.755647654	0.0012835	0.02415887	-0.67550074
hetero after UVB irradiation VS hetero before UVB irradiation	GO_EXTRACELLULAR_STRUCTURE_ORGANIZATION1	0.35478949	0.02785171	1.781287583	0.11343243	0.4153372	-4.79659014
WT after UVB irradiation VS WT before UVB irradiation	GO_EXTRACELLULAR_STRUCTURE_ORGANIZATION2	0.293205093	0.009751599	1.351073205	0.21320632	0.71037453	-5.13316828
homo after UVB irradiation VS homo before UVB irradiation	GO_EXTRACELLULAR_TRANSPORT	-0.09187646	0.024966229	-0.4975554	0.63166564	0.77277023	-6.41700941
hetero after UVB irradiation VS hetero before UVB irradiation	GO_EXTRACELLULAR_TRANSPORT1	-0.44717864	-0.02692122	-3.241965	0.01214397	0.170826	-2.69042955
WT after UVB irradiation VS WT before UVB irradiation	GO_EXTRACELLULAR_TRANSPORT2	-0.13843127	-0.02030038	-0.78506364	0.45476931	0.83813114	-5.68335702
homo after UVB irradiation VS homo before UVB irradiation	GO_EXTRACELLULAR_VESICLE_BIOGENESIS	0.325890459	-0.01007454	1.350972589	0.21225409	0.40232993	-5.64705731
hetero after UVB irradiation VS hetero before UVB irradiation	GO_EXTRACELLULAR_VESICLE_BIOGENESIS1	0.363292111	0.021334941	2.178745521	0.06160761	0.32651774	-4.23893617
WT after UVB irradiation VS WT before UVB irradiation	GO_EXTRACELLULAR_VESICLE_BIOGENESIS2	0.174229266	0.008552117	0.984559818	0.35333981	0.78942569	-5.51466935
homo after UVB irradiation VS homo before UVB irradiation	GO_EXTRAEMBRYONIC_MEMBRANE_DEVELOPMENT	-0.14536957	0.003664874	-0.54042224	0.60305281	0.75204606	-6.39337118
hetero after UVB irradiation VS hetero before UVB irradiation	GO_EXTRAEMBRYONIC_MEMBRANE_DEVELOPMENT1	0.352124237	-0.02776797	2.05384304	0.07472304	0.35351495	-4.41761157
WT after UVB irradiation VS WT before UVB irradiation	GO_EXTRAEMBRYONIC_MEMBRANE_DEVELOPMENT2	-0.0889078	0.033896959	-0.41927823	0.68591443	0.91892342	-5.90436144
homo after UVB irradiation VS homo before UVB irradiation	GO_EXTRINSIC_APOPTOTIC_SIGNALING_PATHWAY	0.203584935	-0.0139632	1.339770869	0.21572034	0.40614762	-5.66058749
homo after UVB irradiation VS homo before UVB irradiation	GO_EXTRINSIC_APOPTOTIC_SIGNALING_PATHWAY_VIA_DEATH_DOMAIN_RECEPTORS	0.154925882	-0.01698859	1.033120835	0.33060899	0.52384741	-6.00037443
hetero after UVB irradiation VS hetero before UVB irradiation	GO_EXTRINSIC_APOPTOTIC_SIGNALING_PATHWAY_VIA_DEATH_DOMAIN_RECEPTORS1	0.36046748	0.017595996	2.465131532	0.03954346	0.27162031	-3.82245962
WT after UVB irradiation VS WT before UVB irradiation	GO_EXTRINSIC_APOPTOTIC_SIGNALING_PATHWAY_VIA_DEATH_DOMAIN_RECEPTORS2	0.248921345	-0.03994815	1.585627713	0.1510294	0.6585056	-4.85099436
hetero after UVB irradiation VS hetero before UVB irradiation	GO_EXTRINSIC_APOPTOTIC_SIGNALING_PATHWAY1	0.229707735	0.003700933	1.770677345	0.1152723	0.41843252	-4.81095275
WT after UVB irradiation VS WT before UVB irradiation	GO_EXTRINSIC_APOPTOTIC_SIGNALING_PATHWAY2	0.13260211	-0.0300184	0.894133866	0.39704634	0.81477652	-5.59495224
homo after UVB irradiation VS homo before UVB irradiation	GO_EXTRINSIC_COMPONENT_OF_CYTOPLASMIC_SIDE_OF_PLASMA_MEMBRANE	0.396281256	-0.02904051	2.869000051	0.02001148	0.09751233	-3.43645897
hetero after UVB irradiation VS hetero before UVB irradiation	GO_EXTRINSIC_COMPONENT_OF_CYTOPLASMIC_SIDE_OF_PLASMA_MEMBRANE1	0.46375372	0.019995577	3.574276012	0.00747366	0.14177754	-2.2213931
WT after UVB irradiation VS WT before UVB irradiation	GO_EXTRINSIC_COMPONENT_OF_CYTOPLASMIC_SIDE_OF_PLASMA_MEMBRANE2	0.212093162	-0.01668054	1.136586173	0.28822559	0.75467539	-5.3666021
homo after UVB irradiation VS homo before UVB irradiation	GO_EXTRINSIC_COMPONENT_OF_ENDOPLASMIC_RETICULUM_MEMBRANE	0.013683527	-0.05633051	0.049076676	0.96201466	0.98001176	-6.54953507
hetero after UVB irradiation VS hetero before UVB irradiation	GO_EXTRINSIC_COMPONENT_OF_ENDOPLASMIC_RETICULUM_MEMBRANE1	0.353241331	-0.01023194	1.96030402	0.086296	0.37240327	-4.54961504
WT after UVB irradiation VS WT before UVB irradiation	GO_EXTRINSIC_COMPONENT_OF_ENDOPLASMIC_RETICULUM_MEMBRANE2	0.526436552	-0.00969382	3.381141011	0.0094553	0.32224447	-2.36540449
homo after UVB irradiation VS homo before UVB irradiation	GO_EXTRINSIC_COMPONENT_OF_ENDOSOME_MEMBRANE	0.499907113	-0.03794196	3.046386113	0.01517132	0.08353847	-3.1613966
hetero after UVB irradiation VS hetero before UVB irradiation	GO_EXTRINSIC_COMPONENT_OF_ENDOSOME_MEMBRANE1	0.702877604	-0.03065052	3.910820189	0.00464166	0.12761654	-1.7618074
WT after UVB irradiation VS WT before UVB irradiation	GO_EXTRINSIC_COMPONENT_OF_ENDOSOME_MEMBRANE2	0.274039737	-0.0911992	1.137880098	0.28771537	0.75467539	-5.36527594
homo after UVB irradiation VS homo before UVB irradiation	GO_EXTRINSIC_COMPONENT_OF_EXTERNAL_SIDE_OF_PLASMA_MEMBRANE	0.114902008	-0.00994496	0.646760562	0.53519928	0.70172686	-6.32697538
hetero after UVB irradiation VS hetero before UVB irradiation	GO_EXTRINSIC_COMPONENT_OF_EXTERNAL_SIDE_OF_PLASMA_MEMBRANE1	0.15351021	0.016403712	0.691614624	0.50910676	0.778727	-5.97996244
WT after UVB irradiation VS WT before UVB irradiation	GO_EXTRINSIC_COMPONENT_OF_EXTERNAL_SIDE_OF_PLASMA_MEMBRANE2	-0.05216286	-0.03949177	-0.15150159	0.8832865	0.97008693	-5.98397382
homo after UVB irradiation VS homo before UVB irradiation	GO_EXTRINSIC_COMPONENT_OF_MEMBRANE	0.204536099	-0.01825043	1.444813838	0.18505538	0.36928496	-5.53099349
hetero after UVB irradiation VS hetero before UVB irradiation	GO_EXTRINSIC_COMPONENT_OF_MEMBRANE1	0.219350009	0.007037645	1.723132337	0.12386651	0.43057108	-4.87488232
WT after UVB irradiation VS WT before UVB irradiation	GO_EXTRINSIC_COMPONENT_OF_MEMBRANE2	0.110552374	-0.00382941	0.753912988	0.47225214	0.84709188	-5.70682426
homo after UVB irradiation VS homo before UVB irradiation	GO_EXTRINSIC_COMPONENT_OF_MITOCHONDRIAL_INNER_MEMBRANE	-0.49219092	-0.00217813	-2.22845749	0.05514601	0.17448321	-4.42483098
hetero after UVB irradiation VS hetero before UVB irradiation	GO_EXTRINSIC_COMPONENT_OF_MITOCHONDRIAL_INNER_MEMBRANE1	-0.43708082	-0.09887829	-2.62998087	0.03066303	0.24470723	-3.5806971
WT after UVB irradiation VS WT before UVB irradiation	GO_EXTRINSIC_COMPONENT_OF_MITOCHONDRIAL_INNER_MEMBRANE2	-0.21154096	-0.05582441	-0.91733116	0.3854754	0.80721732	-5.5749427
homo after UVB irradiation VS homo before UVB irradiation	GO_EXTRINSIC_COMPONENT_OF_ORGANELLE_MEMBRANE	0.204912529	-0.03386819	1.30900313	0.22549064	0.41646626	-5.69737874
hetero after UVB irradiation VS hetero before UVB irradiation	GO_EXTRINSIC_COMPONENT_OF_ORGANELLE_MEMBRANE1	0.293763622	-0.03243459	2.031218276	0.07737548	0.3585269	-4.44969738
WT after UVB irradiation VS WT before UVB irradiation	GO_EXTRINSIC_COMPONENT_OF_ORGANELLE_MEMBRANE2	0.302932006	-0.01970464	2.027697867	0.0767053	0.55925897	-4.26630999
homo after UVB irradiation VS homo before UVB irradiation	GO_EXTRINSIC_COMPONENT_OF_PLASMA_MEMBRANE	0.275982377	-0.01422571	2.09824666	0.06776298	0.19884236	-4.62056163
hetero after UVB irradiation VS hetero before UVB irradiation	GO_EXTRINSIC_COMPONENT_OF_PLASMA_MEMBRANE1	0.249835963	0.023453776	1.919569775	0.09186161	0.37905759	-4.6065206
WT after UVB irradiation VS WT before UVB irradiation	GO_EXTRINSIC_COMPONENT_OF_PLASMA_MEMBRANE2	0.091045552	-0.0009956	0.564871329	0.58746432	0.89180239	-5.83113242
homo after UVB irradiation VS homo before UVB irradiation	GO_EXTRINSIC_COMPONENT_OF_POSTSYNAPTIC_DENSITY_MEMBRANE	0.164318035	-0.01977194	0.598418064	0.56547507	0.72443055	-6.35851127
hetero after UVB irradiation VS hetero before UVB irradiation	GO_EXTRINSIC_COMPONENT_OF_POSTSYNAPTIC_DENSITY_MEMBRANE1	-0.12856309	-0.09069602	-0.44657318	0.66724151	0.86365959	-6.12473452
WT after UVB irradiation VS WT before UVB irradiation	GO_EXTRINSIC_COMPONENT_OF_POSTSYNAPTIC_DENSITY_MEMBRANE2	-0.40011008	0.112090412	-1.34717908	0.21440423	0.71210914	-5.13763408
homo after UVB irradiation VS homo before UVB irradiation	GO_EXTRINSIC_COMPONENT_OF_POSTSYNAPTIC_MEMBRANE	0.219762202	-0.00437445	0.964287821	0.36205921	0.55296645	-6.06759276
hetero after UVB irradiation VS hetero before UVB irradiation	GO_EXTRINSIC_COMPONENT_OF_POSTSYNAPTIC_MEMBRANE1	-0.0274609	-0.02030614	-0.12008296	0.90743284	0.96555872	-6.22367151
WT after UVB irradiation VS WT before UVB irradiation	GO_EXTRINSIC_COMPONENT_OF_POSTSYNAPTIC_MEMBRANE2	0.001300403	-0.06452993	0.00720184	0.99442808	0.9985657	-5.9960393
homo after UVB irradiation VS homo before UVB irradiation	GO_EXTRINSIC_COMPONENT_OF_POSTSYNAPTIC_SPECIALIZATION_MEMBRANE	-0.11727438	0.006710596	-0.52554403	0.61290528	0.75926236	-6.40178244
hetero after UVB irradiation VS hetero before UVB irradiation	GO_EXTRINSIC_COMPONENT_OF_POSTSYNAPTIC_SPECIALIZATION_MEMBRANE1	-0.11290738	-0.0895131	-0.56226474	0.58962059	0.82554715	-6.06352638
WT after UVB irradiation VS WT before UVB irradiation	GO_EXTRINSIC_COMPONENT_OF_POSTSYNAPTIC_SPECIALIZATION_MEMBRANE2	-0.33575192	0.023621415	-1.44719325	0.18542092	0.68726847	-5.02054909
homo after UVB irradiation VS homo before UVB irradiation	GO_EXTRINSIC_COMPONENT_OF_PRESYNAPTIC_MEMBRANE	0.516486996	-0.04195744	2.736988283	0.02462949	0.11040623	-3.64157712
hetero after UVB irradiation VS hetero before UVB irradiation	GO_EXTRINSIC_COMPONENT_OF_PRESYNAPTIC_MEMBRANE1	0.06511757	0.036406951	0.252496795	0.80714048	0.92477404	-6.19705433
WT after UVB irradiation VS WT before UVB irradiation	GO_EXTRINSIC_COMPONENT_OF_PRESYNAPTIC_MEMBRANE2	0.053151115	-0.01105196	0.208955113	0.83964399	0.9631268	-5.97309391
homo after UVB irradiation VS homo before UVB irradiation	GO_EXTRINSIC_COMPONENT_OF_SYNAPTIC_MEMBRANE	0.369604669	-0.02866673	2.000757812	0.0790102	0.21694294	-4.76488145
hetero after UVB irradiation VS hetero before UVB irradiation	GO_EXTRINSIC_COMPONENT_OF_SYNAPTIC_MEMBRANE1	0.085928153	0.010054573	0.474870644	0.6478052	0.85523965	-6.11098069
WT after UVB irradiation VS WT before UVB irradiation	GO_EXTRINSIC_COMPONENT_OF_SYNAPTIC_MEMBRANE2	0.042333701	-0.0061358	0.217724612	0.83302995	0.96075976	-5.97113127
homo after UVB irradiation VS homo before UVB irradiation	GO_EYE_MORPHOGENESIS	-0.02412391	0.023014835	-0.18159469	0.86024241	0.92317629	-6.5327949
hetero after UVB irradiation VS hetero before UVB irradiation	GO_EYE_MORPHOGENESIS1	-0.07894395	0.020732309	-0.62302891	0.55092643	0.80132778	-6.02620711
WT after UVB irradiation VS WT before UVB irradiation	GO_EYE_MORPHOGENESIS2	-0.09634655	0.000802494	-0.58589633	0.57390818	0.88547579	-5.81889794
homo after UVB irradiation VS homo before UVB irradiation	GO_EYE_PHOTORECEPTOR_CELL_DEVELOPMENT	0.10246015	0.043743634	0.563286811	0.58807857	0.74102985	-6.38001971
hetero after UVB irradiation VS hetero before UVB irradiation	GO_EYE_PHOTORECEPTOR_CELL_DEVELOPMENT1	-0.08788732	0.031905474	-0.54316154	0.60209114	0.83357281	-6.07453321
WT after UVB irradiation VS WT before UVB irradiation	GO_EYE_PHOTORECEPTOR_CELL_DEVELOPMENT2	-0.06210525	0.002341637	-0.3336856	0.74709363	0.93606982	-5.93773939
homo after UVB irradiation VS homo before UVB irradiation	GO_EYE_PHOTORECEPTOR_CELL_DIFFERENTIATION	0.124522199	0.042299281	0.81752165	0.4363892	0.61938976	-6.1983092
hetero after UVB irradiation VS hetero before UVB irradiation	GO_EYE_PHOTORECEPTOR_CELL_DIFFERENTIATION1	-0.19492976	0.026999597	-1.18775098	0.26961319	0.59632699	-5.53020328
WT after UVB irradiation VS WT before UVB irradiation	GO_EYE_PHOTORECEPTOR_CELL_DIFFERENTIATION2	-0.03239927	-0.00032408	-0.17105487	0.86837772	0.96790654	-5.98065725
homo after UVB irradiation VS homo before UVB irradiation	GO_EYE_PIGMENTATION	-0.58184885	0.012882667	-3.30843385	0.01013415	0.06731392	-2.75812773
hetero after UVB irradiation VS hetero before UVB irradiation	GO_EYE_PIGMENTATION1	-0.36284864	0.048523791	-1.15877784	0.28056934	0.6064276	-5.561366
WT after UVB irradiation VS WT before UVB irradiation	GO_EYE_PIGMENTATION2	-0.4680213	0.079894418	-2.73367608	0.02541112	0.43286805	-3.26989431
homo after UVB irradiation VS homo before UVB irradiation	GO_EYELID_DEVELOPMENT_IN_CAMERA_TYPE_EYE	-0.04118978	0.077908123	-0.18791774	0.85543869	0.92002833	-6.53151795
hetero after UVB irradiation VS hetero before UVB irradiation	GO_EYELID_DEVELOPMENT_IN_CAMERA_TYPE_EYE1	0.026997906	0.064156183	0.151371858	0.88349877	0.95768873	-6.21907674
WT after UVB irradiation VS WT before UVB irradiation	GO_EYELID_DEVELOPMENT_IN_CAMERA_TYPE_EYE2	-0.29288283	0.104940035	-1.66191466	0.13464881	0.64200725	-4.75430095
homo after UVB irradiation VS homo before UVB irradiation	GO_F_ACTIN_CAPPING_PROTEIN_COMPLEX	0.692006682	0.002117447	3.921486328	0.00407366	0.04160203	-1.84080043
hetero after UVB irradiation VS hetero before UVB irradiation	GO_F_ACTIN_CAPPING_PROTEIN_COMPLEX1	1.101013617	0.035522995	7.576683827	7.11E-05	0.07788488	2.058678724
WT after UVB irradiation VS WT before UVB irradiation	GO_F_ACTIN_CAPPING_PROTEIN_COMPLEX2	0.568359009	0.123679995	3.447657988	0.0085645	0.31908946	-2.27505965
homo after UVB irradiation VS homo before UVB irradiation	GO_FACE_DEVELOPMENT	-0.0288206	0.011783761	-0.21943911	0.8315904	0.90593382	-6.52450668
hetero after UVB irradiation VS hetero before UVB irradiation	GO_FACE_DEVELOPMENT1	-0.10313088	0.053168538	-0.73259706	0.48509391	0.76337374	-5.95030326
WT after UVB irradiation VS WT before UVB irradiation	GO_FACE_DEVELOPMENT2	-0.0334789	0.071101913	-0.22326548	0.82885821	0.95957725	-5.96985014
homo after UVB irradiation VS homo before UVB irradiation	GO_FACIAL_NERVE_MORPHOGENESIS	-0.24499475	0.009383058	-1.67794015	0.13038217	0.29609874	-5.22399269
hetero after UVB irradiation VS hetero before UVB irradiation	GO_FACIAL_NERVE_MORPHOGENESIS1	-0.48529274	-0.0156751	-2.22621514	0.05724291	0.31564886	-4.17044543
WT after UVB irradiation VS WT before UVB irradiation	GO_FACIAL_NERVE_MORPHOGENESIS2	-0.35187121	-0.01641107	-2.13086796	0.06529358	0.53678963	-4.12337416
homo after UVB irradiation VS homo before UVB irradiation	GO_FACIAL_NERVE_STRUCTURAL_ORGANIZATION	-0.4790129	0.027470611	-3.44320984	0.00825973	0.06041217	-2.5528764
hetero after UVB irradiation VS hetero before UVB irradiation	GO_FACIAL_NERVE_STRUCTURAL_ORGANIZATION1	-0.51442152	-0.05734532	-2.41017582	0.04305238	0.27795897	-3.90286199
WT after UVB irradiation VS WT before UVB irradiation	GO_FACIAL_NERVE_STRUCTURAL_ORGANIZATION2	-0.26343301	-0.0082181	-1.38198105	0.20390146	0.70041289	-5.09744931
homo after UVB irradiation VS homo before UVB irradiation	GO_FACIT_COLLAGEN_TRIMER	0.564342975	0.107863048	2.9774999	0.01688823	0.08878367	-3.26808188
hetero after UVB irradiation VS hetero before UVB irradiation	GO_FACIT_COLLAGEN_TRIMER1	-0.05050393	0.090960317	-0.30475661	0.76847195	0.90949407	-6.18142309
WT after UVB irradiation VS WT before UVB irradiation	GO_FACIT_COLLAGEN_TRIMER2	0.341962906	-0.01108942	1.124854172	0.29288499	0.75699426	-5.37857761
homo after UVB irradiation VS homo before UVB irradiation	GO_FAD_BINDING	-0.23652524	-0.03124952	-1.5458525	0.15923793	0.3352172	-5.40097343
hetero after UVB irradiation VS hetero before UVB irradiation	GO_FAD_BINDING1	-0.28308495	-0.05023332	-1.5712574	0.1554711	0.47419007	-5.07385897
WT after UVB irradiation VS WT before UVB irradiation	GO_FAD_BINDING2	-0.22355428	-0.07453746	-1.04999888	0.32404215	0.77346379	-5.45286467
homo after UVB irradiation VS homo before UVB irradiation	GO_FANCONI_ANAEMIA_NUCLEAR_COMPLEX	-0.15071737	-0.00488699	-0.74769138	0.47522369	0.65339383	-6.25409708
hetero after UVB irradiation VS hetero before UVB irradiation	GO_FANCONI_ANAEMIA_NUCLEAR_COMPLEX1	0.458662444	-0.03472589	2.56771905	0.03374973	0.25357971	-3.67206613
WT after UVB irradiation VS WT before UVB irradiation	GO_FANCONI_ANAEMIA_NUCLEAR_COMPLEX2	0.353523029	-0.00277214	1.519426629	0.16668929	0.67150908	-4.93308832
homo after UVB irradiation VS homo before UVB irradiation	GO_FARNESYL_DIPHOSPHATE_METABOLIC_PROCESS	-0.64686426	-0.05172366	-3.37178273	0.00920281	0.06393677	-2.66144064
hetero after UVB irradiation VS hetero before UVB irradiation	GO_FARNESYL_DIPHOSPHATE_METABOLIC_PROCESS1	-0.42828695	0.102092956	-1.28067714	0.23683529	0.56567523	-5.42679801
WT after UVB irradiation VS WT before UVB irradiation	GO_FARNESYL_DIPHOSPHATE_METABOLIC_PROCESS2	-0.51052322	0.049888564	-1.7195499	0.12337479	0.63133281	-4.67989486
homo after UVB irradiation VS homo before UVB irradiation	GO_FAS_SIGNALING_PATHWAY	0.19016585	0.057472457	0.713116707	0.49526467	0.67040481	-6.28011368
hetero after UVB irradiation VS hetero before UVB irradiation	GO_FAS_SIGNALING_PATHWAY1	-0.15028584	0.002139034	-0.42066215	0.68527747	0.87367844	-6.1366247
WT after UVB irradiation VS WT before UVB irradiation	GO_FAS_SIGNALING_PATHWAY2	0.253305586	-0.12751155	1.614470974	0.14463329	0.65365874	-4.81468779
homo after UVB irradiation VS homo before UVB irradiation	GO_FASCIA_ADHERENS	0.774843278	-0.05543169	4.700072397	0.00138177	0.02505013	-0.74980656
hetero after UVB irradiation VS hetero before UVB irradiation	GO_FASCIA_ADHERENS1	0.411316056	0.007283865	2.263762477	0.05400929	0.30816437	-4.116083
WT after UVB irradiation VS WT before UVB irradiation	GO_FASCIA_ADHERENS2	0.261094659	0.002212352	1.12038296	0.29467655	0.75784975	-5.38311839
homo after UVB irradiation VS homo before UVB irradiation	GO_FAT_CELL_DIFFERENTIATION	0.145904751	-0.02633667	1.128078294	0.29072972	0.48550634	-5.90188869
hetero after UVB irradiation VS hetero before UVB irradiation	GO_FAT_CELL_DIFFERENTIATION1	0.071657343	0.00200525	0.432705277	0.67686684	0.86994575	-6.13118238
WT after UVB irradiation VS WT before UVB irradiation	GO_FAT_CELL_DIFFERENTIATION2	0.011925375	-0.00418476	0.072070178	0.94429437	0.98531933	-5.99332692
homo after UVB irradiation VS homo before UVB irradiation	GO_FAT_CELL_PROLIFERATION	0.082571584	-0.06019363	0.459720844	0.65747861	0.79171047	-6.43634316
hetero after UVB irradiation VS hetero before UVB irradiation	GO_FAT_CELL_PROLIFERATION1	0.134265657	-0.0429087	0.545845392	0.60033052	0.83297985	-6.07300804
WT after UVB irradiation VS WT before UVB irradiation	GO_FAT_CELL_PROLIFERATION2	0.177293482	-0.04893669	0.764157816	0.466454	0.84342918	-5.69919608
homo after UVB irradiation VS homo before UVB irradiation	GO_FAT_PAD_DEVELOPMENT	-0.03269408	0.04410828	-0.13891138	0.89282023	0.94176096	-6.54027896
hetero after UVB irradiation VS hetero before UVB irradiation	GO_FAT_PAD_DEVELOPMENT1	-0.39953662	-0.05624419	-2.24780593	0.05536065	0.31097412	-4.13920438
WT after UVB irradiation VS WT before UVB irradiation	GO_FAT_PAD_DEVELOPMENT2	0.019770503	-0.00169565	0.101374459	0.92171845	0.98043563	-5.99064776
homo after UVB irradiation VS homo before UVB irradiation	GO_FAT_SOLUBLE_VITAMIN_BIOSYNTHETIC_PROCESS	0.253733018	0.025016094	1.109721795	0.29812674	0.49254997	-5.9214286
hetero after UVB irradiation VS hetero before UVB irradiation	GO_FAT_SOLUBLE_VITAMIN_BIOSYNTHETIC_PROCESS1	0.665067258	0.016049878	3.863299427	0.00495972	0.12780541	-1.82566541
WT after UVB irradiation VS WT before UVB irradiation	GO_FAT_SOLUBLE_VITAMIN_BIOSYNTHETIC_PROCESS2	0.221737477	0.006582098	1.127561237	0.29180454	0.75646944	-5.37582218
homo after UVB irradiation VS homo before UVB irradiation	GO_FAT_SOLUBLE_VITAMIN_METABOLIC_PROCESS	0.413724559	0.020455181	2.943221535	0.01781645	0.09160881	-3.32123995
hetero after UVB irradiation VS hetero before UVB irradiation	GO_FAT_SOLUBLE_VITAMIN_METABOLIC_PROCESS1	0.514881015	0.056499099	3.696729475	0.0062728	0.13548627	-2.05223214
WT after UVB irradiation VS WT before UVB irradiation	GO_FAT_SOLUBLE_VITAMIN_METABOLIC_PROCESS2	0.333424027	0.051284668	2.135791474	0.06479264	0.53678963	-4.11651209
homo after UVB irradiation VS homo before UVB irradiation	GO_FATTY_ACID_ALPHA_OXIDATION	0.488280129	0.007070167	3.310160837	0.01010748	0.06724729	-2.75548715
hetero after UVB irradiation VS hetero before UVB irradiation	GO_FATTY_ACID_ALPHA_OXIDATION1	0.184002558	-0.02070703	0.935213079	0.37754161	0.69053619	-5.7827022
WT after UVB irradiation VS WT before UVB irradiation	GO_FATTY_ACID_ALPHA_OXIDATION2	0.222721642	-0.03151837	0.825193786	0.43289686	0.82957538	-5.65194152
homo after UVB irradiation VS homo before UVB irradiation	GO_FATTY_ACID_BETA_OXIDATION	-0.0930469	-0.03412796	-0.45606019	0.66000286	0.79377837	-6.43813693
homo after UVB irradiation VS homo before UVB irradiation	GO_FATTY_ACID_BETA_OXIDATION_USING_ACYL_COA_DEHYDROGENASE	0.02797548	-0.04159986	0.11165139	0.91374368	0.95240256	-6.54402032
hetero after UVB irradiation VS hetero before UVB irradiation	GO_FATTY_ACID_BETA_OXIDATION_USING_ACYL_COA_DEHYDROGENASE1	-0.59899728	0.010579619	-2.29393249	0.05154348	0.30169597	-4.07229492
WT after UVB irradiation VS WT before UVB irradiation	GO_FATTY_ACID_BETA_OXIDATION_USING_ACYL_COA_DEHYDROGENASE2	-0.31917613	0.032869935	-1.01162666	0.34098607	0.78168336	-5.48947178
homo after UVB irradiation VS homo before UVB irradiation	GO_FATTY_ACID_BETA_OXIDATION_USING_ACYL_COA_OXIDASE	0.004751255	-0.05916146	0.020045979	0.98447861	0.99218493	-6.55063618
hetero after UVB irradiation VS hetero before UVB irradiation	GO_FATTY_ACID_BETA_OXIDATION_USING_ACYL_COA_OXIDASE1	-0.27063512	-0.02476874	-1.01208678	0.34166173	0.66014434	-5.71060629
WT after UVB irradiation VS WT before UVB irradiation	GO_FATTY_ACID_BETA_OXIDATION_USING_ACYL_COA_OXIDASE2	-0.25557717	-0.07189879	-1.14374903	0.28541027	0.75392834	-5.35924747
hetero after UVB irradiation VS hetero before UVB irradiation	GO_FATTY_ACID_BETA_OXIDATION1	-0.4334309	-0.02520683	-2.24778441	0.0553625	0.31097412	-4.13923555
WT after UVB irradiation VS WT before UVB irradiation	GO_FATTY_ACID_BETA_OXIDATION2	-0.1874137	-0.03359486	-0.80203579	0.44542917	0.83340629	-5.67023156
homo after UVB irradiation VS homo before UVB irradiation	GO_FATTY_ACID_BINDING	-0.00137974	0.056291801	-0.00744196	0.9942374	0.99675584	-6.55082632
hetero after UVB irradiation VS hetero before UVB irradiation	GO_FATTY_ACID_BINDING1	-0.08941274	0.014656467	-0.47954448	0.64462197	0.85438728	-6.10863234
WT after UVB irradiation VS WT before UVB irradiation	GO_FATTY_ACID_BINDING2	-0.04313861	0.032620733	-0.20804545	0.84033085	0.96316991	-5.97329293
homo after UVB irradiation VS homo before UVB irradiation	GO_FATTY_ACID_BIOSYNTHETIC_PROCESS	0.025555242	-0.00801828	0.163259536	0.87420595	0.93091768	-6.53625245
hetero after UVB irradiation VS hetero before UVB irradiation	GO_FATTY_ACID_BIOSYNTHETIC_PROCESS1	-0.02820641	-0.00784674	-0.16946207	0.86971721	0.95091488	-6.21593991
WT after UVB irradiation VS WT before UVB irradiation	GO_FATTY_ACID_BIOSYNTHETIC_PROCESS2	0.026614975	0.036414029	0.152885368	0.88222972	0.96983492	-5.98375224
homo after UVB irradiation VS homo before UVB irradiation	GO_FATTY_ACID_CATABOLIC_PROCESS	-0.08596198	-0.02888868	-0.4712416	0.64956471	0.78596478	-6.43060909
hetero after UVB irradiation VS hetero before UVB irradiation	GO_FATTY_ACID_CATABOLIC_PROCESS1	-0.4269298	-0.01647156	-2.44560064	0.0407561	0.27317438	-3.85105101
WT after UVB irradiation VS WT before UVB irradiation	GO_FATTY_ACID_CATABOLIC_PROCESS2	-0.17101827	-0.02887413	-0.75866039	0.46955943	0.84565091	-5.70330039
homo after UVB irradiation VS homo before UVB irradiation	GO_FATTY_ACID_DERIVATIVE_BINDING	0.013622813	-0.00201027	0.091591943	0.92918686	0.96156059	-6.54625498
hetero after UVB irradiation VS hetero before UVB irradiation	GO_FATTY_ACID_DERIVATIVE_BINDING1	-0.20237089	0.013048016	-1.32907415	0.22114077	0.54852054	-5.37096936
WT after UVB irradiation VS WT before UVB irradiation	GO_FATTY_ACID_DERIVATIVE_BINDING2	-0.03038007	0.008882672	-0.17160691	0.86795758	0.96790654	-5.98055782
homo after UVB irradiation VS homo before UVB irradiation	GO_FATTY_ACID_DERIVATIVE_BIOSYNTHETIC_PROCESS	-0.03272321	0.008230888	-0.18854186	0.85496488	0.91978689	-6.53138956
hetero after UVB irradiation VS hetero before UVB irradiation	GO_FATTY_ACID_DERIVATIVE_BIOSYNTHETIC_PROCESS1	0.026721722	-0.01788378	0.164787817	0.87327383	0.95275627	-6.21678408
WT after UVB irradiation VS WT before UVB irradiation	GO_FATTY_ACID_DERIVATIVE_BIOSYNTHETIC_PROCESS2	0.018559585	0.025205607	0.103217679	0.92030073	0.98043563	-5.99044905
homo after UVB irradiation VS homo before UVB irradiation	GO_FATTY_ACID_DERIVATIVE_CATABOLIC_PROCESS	-0.18446132	0.013770406	-0.99684494	0.34691416	0.53882877	-6.03625076
hetero after UVB irradiation VS hetero before UVB irradiation	GO_FATTY_ACID_DERIVATIVE_CATABOLIC_PROCESS1	-0.09467052	0.06311556	-0.4593456	0.65843427	0.86007701	-6.11862544
WT after UVB irradiation VS WT before UVB irradiation	GO_FATTY_ACID_DERIVATIVE_CATABOLIC_PROCESS2	-0.16762156	0.052145874	-1.04374819	0.32675693	0.77543801	-5.45889747
homo after UVB irradiation VS homo before UVB irradiation	GO_FATTY_ACID_DERIVATIVE_METABOLIC_PROCESS	0.088005472	0.011452277	0.548533857	0.59771709	0.74819842	-6.38869329
hetero after UVB irradiation VS hetero before UVB irradiation	GO_FATTY_ACID_DERIVATIVE_METABOLIC_PROCESS1	0.073429489	0.006186934	0.447108133	0.66687152	0.86352197	-6.12448192
WT after UVB irradiation VS WT before UVB irradiation	GO_FATTY_ACID_DERIVATIVE_METABOLIC_PROCESS2	0.034259896	0.024603782	0.189351835	0.85447718	0.9663792	-5.97719252
homo after UVB irradiation VS homo before UVB irradiation	GO_FATTY_ACID_DERIVATIVE_TRANSPORT	0.324543379	0.048067539	2.460584894	0.03815126	0.14008414	-4.06981138
hetero after UVB irradiation VS hetero before UVB irradiation	GO_FATTY_ACID_DERIVATIVE_TRANSPORT1	0.279267361	0.043562978	1.86284238	0.10018967	0.39308661	-4.68510764

WT after UVB irradiation VS WT before UVB irradiation	GO_FATTY_ACID_DERIVATIVE_TRANSPORT2	0.193989625	-0.00244978	0.89412603	0.39705029	0.81477652	-5.59495893
homo after UVB irradiation VS homo before UVB irradiation	GO_FATTY_ACID_ELONGATION	0.18485154	-0.06249377	0.734280518	0.48293393	0.6599908	-6.26431657
homo after UVB irradiation VS homo before UVB irradiation	GO_FATTY_ACID_ELONGATION_SATURATED_FATTY_ACID	-0.05212036	-0.00407433	-0.15492969	0.88056548	0.93512556	-6.53770258
hetero after UVB irradiation VS hetero before UVB irradiation	GO_FATTY_ACID_ELONGATION_SATURATED_FATTY_ACID1	-0.13897606	-0.10812771	-0.4541682	0.6619976	0.86164211	-6.12112146
WT after UVB irradiation VS WT before UVB irradiation	GO_FATTY_ACID_ELONGATION_SATURATED_FATTY_ACID2	-0.3134251	-0.10279086	-0.97697216	0.35686294	0.79119413	-5.52163852
hetero after UVB irradiation VS hetero before UVB irradiation	GO_FATTY_ACID_ELONGATION1	0.018295346	-0.04419027	0.063362826	0.95106075	0.98125104	-6.2293074
WT after UVB irradiation VS WT before UVB irradiation	GO_FATTY_ACID_ELONGATION2	-0.07364326	-0.08010066	-0.26682842	0.79627062	0.95157276	-5.95867347
homo after UVB irradiation VS homo before UVB irradiation	GO_FATTY_ACID_HOMEOSTASIS	0.201091675	-0.00267186	1.109000513	0.29842042	0.49281448	-5.92219157
hetero after UVB irradiation VS hetero before UVB irradiation	GO_FATTY_ACID_HOMEOSTASIS1	-0.171303	-0.00398805	-1.02326392	0.33666908	0.65647312	-5.69976088
WT after UVB irradiation VS WT before UVB irradiation	GO_FATTY_ACID_HOMEOSTASIS2	0.055453693	0.009005048	0.270877296	0.79326217	0.95025325	-5.9575355
homo after UVB irradiation VS homo before UVB irradiation	GO_FATTY_ACID_LIGASE_ACTIVITY	-0.05886915	-0.00317228	-0.30833198	0.76540031	0.86419375	-6.49898251
hetero after UVB irradiation VS hetero before UVB irradiation	GO_FATTY_ACID_LIGASE_ACTIVITY1	-0.1841413	0.008628883	-1.09551248	0.30575081	0.6274085	-5.62752664
WT after UVB irradiation VS WT before UVB irradiation	GO_FATTY_ACID_LIGASE_ACTIVITY2	-0.17649544	0.080539666	-0.66760165	0.52295484	0.86453012	-5.76751888
homo after UVB irradiation VS homo before UVB irradiation	GO_FATTY_ACID_METABOLIC_PROCESS	-0.00117305	-0.01420748	-0.00727427	0.99436724	0.99679104	-6.55082768
hetero after UVB irradiation VS hetero before UVB irradiation	GO_FATTY_ACID_METABOLIC_PROCESS1	-0.17144244	-0.01209017	-1.07097496	0.31599013	0.63620732	-5.65246635
WT after UVB irradiation VS WT before UVB irradiation	GO_FATTY_ACID_METABOLIC_PROCESS2	-0.06283036	0.0046219	-0.33413335	0.7467681	0.93606982	-5.93758389
homo after UVB irradiation VS homo before UVB irradiation	GO_FATTY_ACID_OMEGA_OXIDATION	0.222220364	-0.03139527	0.910630059	0.38808282	0.57748696	-6.11742808
hetero after UVB irradiation VS hetero before UVB irradiation	GO_FATTY_ACID_OMEGA_OXIDATION1	-0.1568494	-0.01143686	-0.57522959	0.58123918	0.82056159	-6.05585715
WT after UVB irradiation VS WT before UVB irradiation	GO_FATTY_ACID_OMEGA_OXIDATION2	-0.02633834	0.026214542	-0.11830004	0.90871189	0.97832631	-5.98868901
homo after UVB irradiation VS homo before UVB irradiation	GO_FATTY_ACID_SYNTHASE_ACTIVITY	-0.01387376	-0.04038592	-0.04756679	0.96318231	0.98080968	-6.54961513
hetero after UVB irradiation VS hetero before UVB irradiation	GO_FATTY_ACID_SYNTHASE_ACTIVITY1	0.026338169	-0.04696171	0.09119036	0.92962453	0.97425707	-6.22697693
WT after UVB irradiation VS WT before UVB irradiation	GO_FATTY_ACID_SYNTHASE_ACTIVITY2	0.070248381	-0.00958122	0.251360946	0.80779642	0.95404687	-5.96286572
homo after UVB irradiation VS homo before UVB irradiation	GO_FATTY_ACID_TRANSMEMBRANE_TRANSPORT	0.20024847	-0.02141677	1.070149621	0.3145795	0.50851678	-5.96274252
hetero after UVB irradiation VS hetero before UVB irradiation	GO_FATTY_ACID_TRANSMEMBRANE_TRANSPORT1	0.008634583	-0.05092661	0.036209145	0.97201911	0.99014423	-6.23077315
WT after UVB irradiation VS WT before UVB irradiation	GO_FATTY_ACID_TRANSMEMBRANE_TRANSPORT2	-0.03077239	-0.04015315	-0.15534949	0.88034848	0.9693507	-5.98335272
homo after UVB irradiation VS homo before UVB irradiation	GO_FATTY_ACID_TRANSMEMBRANE_TRANSPORTER_ACTIVITY	0.117121539	0.01498455	0.617416264	0.55346078	0.71549898	-6.34638369
hetero after UVB irradiation VS hetero before UVB irradiation	GO_FATTY_ACID_TRANSMEMBRANE_TRANSPORTER_ACTIVITY1	-0.08279567	-0.01771772	-0.28600406	0.78227463	0.91453371	-6.18736234
WT after UVB irradiation VS WT before UVB irradiation	GO_FATTY_ACID_TRANSMEMBRANE_TRANSPORTER_ACTIVITY2	-0.10915495	0.042847784	-0.44037495	0.67118426	0.91526653	-5.89501857
homo after UVB irradiation VS homo before UVB irradiation	GO_FATTY_ACID_TRANSPORT	0.177007101	0.019202181	1.406526458	0.1957627	0.38350574	-5.5789207
hetero after UVB irradiation VS hetero before UVB irradiation	GO_FATTY_ACID_TRANSPORT1	0.082860309	0.021131728	0.527869859	0.61217557	0.83884085	-6.08309023
WT after UVB irradiation VS WT before UVB irradiation	GO_FATTY_ACID_TRANSPORT2	-0.02228578	-0.01243509	-0.12011942	0.90731544	0.97767462	-5.98846055
homo after UVB irradiation VS homo before UVB irradiation	GO_FATTY_ACYL_COA_BIOSYNTHETIC_PROCESS	-0.12209727	-0.04606282	-0.46114211	0.6564998	0.79117114	-6.43564305
hetero after UVB irradiation VS hetero before UVB irradiation	GO_FATTY_ACYL_COA_BIOSYNTHETIC_PROCESS1	-0.30985728	-0.03972944	-1.61744679	0.14515165	0.46133513	-5.01425183
WT after UVB irradiation VS WT before UVB irradiation	GO_FATTY_ACYL_COA_BIOSYNTHETIC_PROCESS2	-0.10287864	-0.02971694	-0.43244309	0.67670521	0.91685973	-5.89858249
homo after UVB irradiation VS homo before UVB irradiation	GO_FATTY_ACYL_COA_METABOLIC_PROCESS	-0.1280422	-0.0501094	-0.50547703	0.62632637	0.76894329	-6.41277905
hetero after UVB irradiation VS hetero before UVB irradiation	GO_FATTY_ACYL_COA_METABOLIC_PROCESS1	-0.23994458	-0.04424286	-1.2123109	0.26060368	0.58691513	-5.50337738
WT after UVB irradiation VS WT before UVB irradiation	GO_FATTY_ACYL_COA_METABOLIC_PROCESS2	-0.06010794	-0.02861575	-0.28974751	0.77929015	0.94411282	-5.95201039
homo after UVB irradiation VS homo before UVB irradiation	GO_FATTY_ACYL_COA_SYNTHASE_ACTIVITY	-0.23675571	-0.00361259	-1.04928915	0.32353392	0.51682075	-5.98406675
hetero after UVB irradiation VS hetero before UVB irradiation	GO_FATTY_ACYL_COA_SYNTHASE_ACTIVITY1	-0.22525424	-0.02634698	-1.32032629	0.22390986	0.55258552	-5.38115617
WT after UVB irradiation VS WT before UVB irradiation	GO_FATTY_ACYL_COA_SYNTHASE_ACTIVITY2	-0.06570283	0.054889956	-0.2277307	0.82550051	0.95860343	-5.96879459
homo after UVB irradiation VS homo before UVB irradiation	GO_FATZ_BINDING	0.381286769	-0.00702968	1.116554552	0.29535609	0.48994715	-5.91418281
hetero after UVB irradiation VS hetero before UVB irradiation	GO_FATZ_BINDING1	-0.21363863	-0.07288801	-0.62582032	0.54918535	0.80045598	-6.02440941
WT after UVB irradiation VS WT before UVB irradiation	GO_FATZ_BINDING2	0.214472585	-0.05508713	0.548033466	0.59844723	0.89476186	-5.84063375
homo after UVB irradiation VS homo before UVB irradiation	GO_FC_EPSILON_RECEPTOR_SIGNALING_PATHWAY	-0.00524318	-0.01003376	-0.02385664	0.98152863	0.9909974	-6.55054437
hetero after UVB irradiation VS hetero before UVB irradiation	GO_FC_EPSILON_RECEPTOR_SIGNALING_PATHWAY1	0.216549073	-0.01511919	1.175681493	0.27413386	0.60088269	-5.54324923
WT after UVB irradiation VS WT before UVB irradiation	GO_FC_EPSILON_RECEPTOR_SIGNALING_PATHWAY2	0.199496121	0.002220583	1.107721758	0.29979728	0.76190626	-5.39590644
homo after UVB irradiation VS homo before UVB irradiation	GO_FC_RECEPTOR_MEDIATED_STIMULATORY_SIGNALING_PATHWAY	0.561668397	-0.01996456	3.791709885	0.0049198	0.04577651	-2.03120605
hetero after UVB irradiation VS hetero before UVB irradiation	GO_FC_RECEPTOR_MEDIATED_STIMULATORY_SIGNALING_PATHWAY1	0.598575929	0.031327668	4.223166471	0.00302731	0.11447176	-1.35109888
WT after UVB irradiation VS WT before UVB irradiation	GO_FC_RECEPTOR_MEDIATED_STIMULATORY_SIGNALING_PATHWAY2	0.494856838	0.005853594	3.029903946	0.01608251	0.37691292	-2.85140096
homo after UVB irradiation VS homo before UVB irradiation	GO_FC_RECEPTOR_SIGNALING_PATHWAY	0.191097228	-0.02571445	1.027680596	0.33301615	0.52604574	-6.00581788
hetero after UVB irradiation VS hetero before UVB irradiation	GO_FC_RECEPTOR_SIGNALING_PATHWAY1	0.360269498	4.42E-05	2.376739598	0.04533947	0.28437748	-3.95169838
WT after UVB irradiation VS WT before UVB irradiation	GO_FC_RECEPTOR_SIGNALING_PATHWAY2	0.355436501	0.005351477	2.244114594	0.05468078	0.52261933	-3.96476191
homo after UVB irradiation VS homo before UVB irradiation	GO_FEAR_RESPONSE	0.117834638	-0.00642387	0.942189459	0.37261617	0.56292964	-6.08839468
hetero after UVB irradiation VS hetero before UVB irradiation	GO_FEAR_RESPONSE1	-0.14534702	-0.00789892	-0.76243263	0.46808257	0.75313005	-5.92778182
WT after UVB irradiation VS WT before UVB irradiation	GO_FEAR_RESPONSE2	-0.0877272	0.003489293	-0.4480718	0.66584707	0.91327309	-5.89150141
homo after UVB irradiation VS homo before UVB irradiation	GO_FEEDING_BEHAVIOR	0.000856418	0.026007075	0.006287426	0.99513138	0.99719147	-6.55083502
hetero after UVB irradiation VS hetero before UVB irradiation	GO_FEEDING_BEHAVIOR1	0.09008716	0.052841152	0.683391195	0.51401408	0.78258272	-5.98573315
WT after UVB irradiation VS WT before UVB irradiation	GO_FEEDING_BEHAVIOR2	-0.0202084	0.039605376	-0.1309047	0.89904449	0.9748126	-5.98703496
homo after UVB irradiation VS homo before UVB irradiation	GO_FEMALE_GAMETE_GENERATION	-0.14706288	-0.00684982	-1.03833649	0.32831377	0.52163923	-5.99513498
hetero after UVB irradiation VS hetero before UVB irradiation	GO_FEMALE_GAMETE_GENERATION1	-0.03831773	0.002627141	-0.25838434	0.80275339	0.92371161	-6.19543711
WT after UVB irradiation VS WT before UVB irradiation	GO_FEMALE_GAMETE_GENERATION2	-0.17229142	0.043492919	-1.15110364	0.2825427	0.75292132	-5.35166223
homo after UVB irradiation VS homo before UVB irradiation	GO_FEMALE_GENITALIA_DEVELOPMENT	0.212170311	0.017178157	1.218951337	0.25625614	0.45169277	-5.80179116
hetero after UVB irradiation VS hetero before UVB irradiation	GO_FEMALE_GENITALIA_DEVELOPMENT1	0.032197739	-0.03648945	0.201948021	0.84509077	0.94071812	-6.20942655
WT after UVB irradiation VS WT before UVB irradiation	GO_FEMALE_GENITALIA_DEVELOPMENT2	0.017911279	-0.00374238	0.106649331	0.91766206	0.98030542	-5.99006959
homo after UVB irradiation VS homo before UVB irradiation	GO_FEMALE_MATING_BEHAVIOR	-0.47476226	0.0731032	-2.49209792	0.0362901	0.13654994	-4.02119257
hetero after UVB irradiation VS hetero before UVB irradiation	GO_FEMALE_MATING_BEHAVIOR1	-0.64740602	0.099347119	-2.96028452	0.01851785	0.19863829	-3.09728313
WT after UVB irradiation VS WT before UVB irradiation	GO_FEMALE_MATING_BEHAVIOR2	-0.47934174	-0.02128084	-2.82428128	0.0220766	0.41501192	-3.14133617
homo after UVB irradiation VS homo before UVB irradiation	GO_FEMALE_MEIOSIS_CHROMOSOME_SEGREGATION	-0.78194879	-0.0222825	-2.82906301	0.02130617	0.10126093	-3.49850143
hetero after UVB irradiation VS hetero before UVB irradiation	GO_FEMALE_MEIOSIS_CHROMOSOME_SEGREGATION1	0.160416646	0.063410811	0.617051412	0.5546657	0.80503221	-6.03003229
WT after UVB irradiation VS WT before UVB irradiation	GO_FEMALE_MEIOSIS_CHROMOSOME_SEGREGATION2	0.083223707	0.035101425	0.228024624	0.82527963	0.95860343	-5.96872438
homo after UVB irradiation VS homo before UVB irradiation	GO_FEMALE_MEIOSIS_I	0.13014598	-0.00577769	0.397463128	0.70101775	0.82163107	-6.46498387
hetero after UVB irradiation VS hetero before UVB irradiation	GO_FEMALE_MEIOSIS_I1	0.357669065	0.027572628	2.250855131	0.05509984	0.31051949	-4.13478813
WT after UVB irradiation VS WT before UVB irradiation	GO_FEMALE_MEIOSIS_I2	0.383590691	0.031482679	1.432089703	0.18956518	0.69190481	-5.03853958
homo after UVB irradiation VS homo before UVB irradiation	GO_FEMALE_MEIOTIC_NUCLEAR_DIVISION	-0.25709846	0.023540404	-1.25470884	0.2436453	0.43738494	-5.76093021
hetero after UVB irradiation VS hetero before UVB irradiation	GO_FEMALE_MEIOTIC_NUCLEAR_DIVISION1	0.284234652	0.004729915	1.54547288	0.161518	0.48265686	-5.1067602
WT after UVB irradiation VS WT before UVB irradiation	GO_FEMALE_MEIOTIC_NUCLEAR_DIVISION2	0.182198814	0.06338079	0.763780659	0.46666661	0.84342918	-5.69947847
homo after UVB irradiation VS homo before UVB irradiation	GO_FEMALE_PRONUCLEUS	-0.45041462	-0.01166576	-2.02588238	0.07594991	0.21171002	-4.72789396
hetero after UVB irradiation VS hetero before UVB irradiation	GO_FEMALE_PRONUCLEUS1	-0.19194535	-0.02958416	-0.62392356	0.55036806	0.80124226	-6.02563174
WT after UVB irradiation VS WT before UVB irradiation	GO_FEMALE_PRONUCLEUS2	-0.35444407	-0.00164653	-1.39386262	0.20041927	0.69758436	-5.08359107
homo after UVB irradiation VS homo before UVB irradiation	GO_FEMALE_SEX_DIFFERENTIATION	0.231483642	0.006390196	1.765081202	0.1140618	0.27164551	-5.10341436
hetero after UVB irradiation VS hetero before UVB irradiation	GO_FEMALE_SEX_DIFFERENTIATION1	0.047421652	-0.01345802	0.335673638	0.74590917	0.90054138	-6.17082925
WT after UVB irradiation VS WT before UVB irradiation	GO_FEMALE_SEX_DIFFERENTIATION2	0.04902374	0.024827551	0.338572557	0.74354361	0.93561604	-5.9360312
homo after UVB irradiation VS homo before UVB irradiation	GO_FERMENTATION	-0.04512689	-0.04531827	-0.14467624	0.88840615	0.93938541	-6.53938393
hetero after UVB irradiation VS hetero before UVB irradiation	GO_FERMENTATION1	0.002657237	-0.06578666	0.00617106	0.99523013	0.99792976	-6.23146346
WT after UVB irradiation VS WT before UVB irradiation	GO_FERMENTATION2	0.528695777	-0.01632752	1.927320422	0.08965243	0.58325853	-4.40352813
homo after UVB irradiation VS homo before UVB irradiation	GO_FERRIC_IRON_BINDING	0.43598941	-0.12825714	1.449324981	0.18382823	0.36773121	-5.52529628
hetero after UVB irradiation VS hetero before UVB irradiation	GO_FERRIC_IRON_BINDING1	0.601644389	0.068561782	2.782785257	0.02425638	0.22305048	-3.35658068
WT after UVB irradiation VS WT before UVB irradiation	GO_FERRIC_IRON_BINDING2	0.73683422	0.027071739	3.276479942	0.01106043	0.33861197	-2.50873169
homo after UVB irradiation VS homo before UVB irradiation	GO_FERROUS_IRON_BINDING	0.511405096	-0.04204059	2.656922444	0.02794927	0.11824755	-3.76592835
hetero after UVB irradiation VS hetero before UVB irradiation	GO_FERROUS_IRON_BINDING1	0.188690637	0.012338153	0.760242126	0.46931793	0.75397445	-5.92946162
WT after UVB irradiation VS WT before UVB irradiation	GO_FERROUS_IRON_BINDING2	0.171676968	-0.04754951	0.651940109	0.53250383	0.86897754	-5.77783334
homo after UVB irradiation VS homo before UVB irradiation	GO_FERTILIZATION	0.061553331	0.035398078	0.461265169	0.65641509	0.79117114	-6.43558234
hetero after UVB irradiation VS hetero before UVB irradiation	GO_FERTILIZATION1	-0.08190847	0.02109374	-0.57957268	0.57844651	0.8187705	-6.05325221
WT after UVB irradiation VS WT before UVB irradiation	GO_FERTILIZATION2	0.028627456	0.041548124	0.188986033	0.85475457	0.9663792	-5.9772652
homo after UVB irradiation VS homo before UVB irradiation	GO_FEVER_GENERATION	0.662317663	0.055264471	4.440597219	0.00196172	0.03006141	-1.10315703
hetero after UVB irradiation VS hetero before UVB irradiation	GO_FEVER_GENERATION1	0.624295398	0.031320282	4.228359138	0.00300624	0.11447176	-1.34440648
WT after UVB irradiation VS WT before UVB irradiation	GO_FEVER_GENERATION2	0.546357965	0.000404553	2.482353881	0.03763065	0.47615783	-3.62742934
homo after UVB irradiation VS homo before UVB irradiation	GO_FHF_COMPLEX	-0.67443757	-0.01756662	-2.79600921	0.02244276	0.10433784	-3.54986304
hetero after UVB irradiation VS hetero before UVB irradiation	GO_FHF_COMPLEX1	-0.35079598	-0.04300111	-0.92788845	0.38110153	0.69340742	-5.78933857
WT after UVB irradiation VS WT before UVB irradiation	GO_FHF_COMPLEX2	-0.49809251	0.096333919	-1.62760143	0.14180422	0.6516606	-4.7980566
homo after UVB irradiation VS homo before UVB irradiation	GO_FIBRILLAR_CENTER	-0.42449356	0.00766931	-3.04360777	0.01523693	0.08371643	-3.16569528
hetero after UVB irradiation VS hetero before UVB irradiation	GO_FIBRILLAR_CENTER1	-0.18103015	-0.03973642	-0.98612535	0.35347712	0.66999466	-5.73544631
WT after UVB irradiation VS WT before UVB irradiation	GO_FIBRILLAR_CENTER2	-0.15362173	-0.00906997	-0.69777196	0.5048586	0.85763155	-5.74703798
homo after UVB irradiation VS homo before UVB irradiation	GO_FIBRILLAR_COLLAGEN_TRIMER	0.477024628	0.018048829	2.118120231	0.06566908	0.19471138	-4.59089113
hetero after UVB irradiation VS hetero before UVB irradiation	GO_FIBRILLAR_COLLAGEN_TRIMER1	0.141233321	0.049218851	0.563699967	0.58868947	0.82518303	-6.06268529
WT after UVB irradiation VS WT before UVB irradiation	GO_FIBRILLAR_COLLAGEN_TRIMER2	0.180317261	-0.00070547	0.697353687	0.50510677	0.85763155	-5.74732738
homo after UVB irradiation VS homo before UVB irradiation	GO_FIBRINOGEN_BINDING	0.825232835	0.048709809	4.787070857	0.0012313	0.0235672	-0.63370318
hetero after UVB irradiation VS hetero before UVB irradiation	GO_FIBRINOGEN_BINDING1	0.706094284	0.032239116	2.721596238	0.02663805	0.23166634	-3.44627222
WT after UVB irradiation VS WT before UVB irradiation	GO_FIBRINOGEN_BINDING2	0.620304589	0.051022504	1.737956542	0.11996306	0.62875505	-4.6559045
homo after UVB irradiation VS homo before UVB irradiation	GO_FIBRINOGEN_COMPLEX	0.137898874	0.007876622	0.399149632	0.69982184	0.82086248	-6.46426059
hetero after UVB irradiation VS hetero before UVB irradiation	GO_FIBRINOGEN_COMPLEX1	0.134570996	-0.00634974	0.509058211	0.62470324	0.84482633	-6.09330518
WT after UVB irradiation VS WT before UVB irradiation	GO_FIBRINOGEN_COMPLEX2	0.309766505	0.050534704	0.858542548	0.41528162	0.8213754	-5.62484288
homo after UVB irradiation VS homo before UVB irradiation	GO_FIBRINOLYSIS	0.531018141	0.041112471	3.562006968	0.00691008	0.05473538	-2.37347478
hetero after UVB irradiation VS hetero before UVB irradiation	GO_FIBRINOLYSIS1	0.574279361	0.058914591	3.116281165	0.01464359	0.18000301	-2.87109408
WT after UVB irradiation VS WT before UVB irradiation	GO_FIBRINOLYSIS2	0.456014687	0.035332646	1.80800001	0.10777023	0.61228673	-4.5636786
homo after UVB irradiation VS homo before UVB irradiation	GO_FIBROBLAST_ACTIVATION	-0.02935178	-0.0480272	-0.12058948	0.90687454	0.94926568	-6.54288296
hetero after UVB irradiation VS hetero before UVB irradiation	GO_FIBROBLAST_ACTIVATION1	0.004551945	0.003829653	0.025946795	0.97994701	0.99279767	-6.23111903
WT after UVB irradiation VS WT before UVB irradiation	GO_FIBROBLAST_ACTIVATION2	-0.16689952	-0.00102458	-0.71685125	0.49362044	0.85370213	-5.73367558
homo after UVB irradiation VS homo before UVB irradiation	GO_FIBROBLAST_APOPTOTIC_PROCESS	0.030775381	0.027357664	0.174596562	0.86556615	0.92607714	-6.53415764
hetero after UVB irradiation VS hetero before UVB irradiation	GO_FIBROBLAST_APOPTOTIC_PROCESS1	0.325608234	-0.01571072	2.289054108	0.05193443	0.3026637	-4.07938131
WT after UVB irradiation VS WT before UVB irradiation	GO_FIBROBLAST_APOPTOTIC_PROCESS2	0.142443926	-0.06513969	0.726454377	0.48802487	0.8517724	-5.72683087
homo after UVB irradiation VS homo before UVB irradiation	GO_FIBROBLAST_GROWTH_FACTOR_ACTIVATED_RECEPTOR_ACTIVITY	-0.23139667	-0.02196595	-1.02740539	0.33313828	0.52612602	-6.00609267
hetero after UVB irradiation VS hetero before UVB irradiation	GO_FIBROBLAST_GROWTH_FACTOR_ACTIVATED_RECEPTOR_ACTIVITY1	-0.68706257	-0.00124064	-2.80864661	0.02331695	0.21817689	-3.31870865
WT after UVB irradiation VS WT before UVB irradiation	GO_FIBROBLAST_GROWTH_FACTOR_ACTIVATED_RECEPTOR_ACTIVITY2	-0.55743441	-0.11959274	-2.26165931	0.05319683	0.51991848	-3.94006079
homo after UVB irradiation VS homo before UVB irradiation	GO_FIBROBLAST_GROWTH_FACTOR_BINDING	0.245488174	-0.04567288	1.94575735	0.08612914	0.22880916	-4.8453051
hetero after UVB irradiation VS hetero before UVB irradiation	GO_FIBROBLAST_GROWTH_FACTOR_BINDING1	-0.02857182	-0.00453383	-0.17920512	0.86231404	0.94810134	-6.21410503
WT after UVB irradiation VS WT before UVB irradiation	GO_FIBROBLAST_GROWTH_FACTOR_BINDING2	0.037563707	-0.03451647	0.171181193	0.86828158	0.96790654	-5.98063452
homo after UVB irradiation VS homo before UVB irradiation	GO_FIBROBLAST_GROWTH_FACTOR_PRODUCTION	0.398800245	-0.01723474	2.436495784	0.03963846	0.14303473	-4.10692259
hetero after UVB irradiation VS hetero before UVB irradiation	GO_FIBROBLAST_GROWTH_FACTOR_PRODUCTION1	0.286698703	0.051870545	1.66517182	0.1351523	0.44685353	-4.95180775
WT after UVB irradiation VS WT before UVB irradiation	GO_FIBROBLAST_GROWTH_FACTOR_PRODUCTION2	0.138753065	-0.04453878	0.459170739	0.65818631	0.91105574	-5.88632794
homo after UVB irradiation VS homo before UVB irradiation	GO_FIBROBLAST_GROWTH_FACTOR_RECEPTOR_BINDING	-0.24614838	0.034849888	-1.46250834	0.1802829	0.36295153	-5.50858722
hetero after UVB irradiation VS hetero before UVB irradiation	GO_FIBROBLAST_GROWTH_FACTOR_RECEPTOR_BINDING1	-0.24755551	0.018150579	-1.33867619	0.21813522	0.54557898	-5.35974024
WT after UVB irradiation VS WT before UVB irradiation	GO_FIBROBLAST_GROWTH_FACTOR_RECEPTOR_BINDING2	-0.39541431	-0.00122507	-2.09678429	0.06886698	0.54354218	-4.17078156
homo after UVB irradiation VS homo before UVB irradiation	GO_FIBROBLAST_GROWTH_FACTOR_RECEPTOR_SIGNALING_PATHWAY	-0.3306987	0.017219061	-2.46523745	0.03787055	0.13950933	-4.0626382
hetero after UVB irradiation VS hetero before UVB irradiation	GO_FIBROBLAST_GROWTH_FACTOR_RECEPTOR_SIGNALING_PATHWAY1	-0.11542799	-0.00554822	-0.78651423	0.45464449	0.74570481	-5.90904317
WT after UVB irradiation VS WT before UVB irradiation	GO_FIBROBLAST_GROWTH_FACTOR_RECEPTOR_SIGNALING_PATHWAY2	-0.17547947	0.014286342	-1.03597905	0.33015572	0.77726938	-5.46635827
homo after UVB irradiation VS homo before UVB irradiation	GO_FIBROBLAST_MIGRATION	0.433200623	-0.02697928	3.433812946	0.00837775	0.06088353	-2.56713031
hetero after UVB irradiation VS hetero before UVB irradiation	GO_FIBROBLAST_MIGRATION1	0.303057012	0.014558418	1.849714392	0.1022173	0.3967998	-4.70317732
WT after UVB irradiation VS WT before UVB irradiation	GO_FIBROBLAST_MIGRATION2	0.385223313	-0.03225879	2.407199412	0.04233715	0.495137	-3.73419765
homo after UVB irradiation VS homo before UVB irradiation	GO_FIBROBLAST_PROLIFERATION	-0.01420352	0.011807725	-0.11471777	0.9113862	0.95151443	-6.54363996
hetero after UVB irradiation VS hetero before UVB irradiation	GO_FIBROBLAST_PROLIFERATION1	0.098851994	0.00945886	0.659553431	0.52840502	0.79043843	-6.00211535
WT after UVB irradiation VS WT before UVB irradiation	GO_FIBROBLAST_PROLIFERATION2	0.117819151	0.022294293	0.735494884	0.48279462	0.85139062	-5.72031483
homo after UVB irradiation VS homo before UVB irradiation	GO_FIBRONECTIN_BINDING	0.485196313	-0.03387676	2.923915658	0.01836231	0.09298992	-3.35119597
hetero after UVB irradiation VS hetero before UVB irradiation	GO_FIBRONECTIN_BINDING1	0.281248648	0.021245855	0.989132049	0.35209303	0.66862242	-5.73259484
WT after UVB irradiation VS WT before UVB irradiation	GO_FIBRONECTIN_BINDING2	0.204938913	-0.04995252	0.673171024	0.51958457	0.86297237	-5.76379848
homo after UVB irradiation VS homo before UVB irradiation	GO_FICOLIN_1_RICH_GRANULE	0.331250572	-0.02030404	2.133621273	0.06407982	0.19184946	-4.56769459
homo after UVB irradiation VS homo before UVB irradiation	GO_FICOLIN_1_RICH_GRANULE_MEMBRANE	0.844303476	-0.01875917	5.89735355	0.00031062	0.01287021	0.743260455
hetero after UVB irradiation VS hetero before UVB irradiation	GO_FICOLIN_1_RICH_GRANULE_MEMBRANE1	0.864097089	0.02582394	6.21945219	0.00027422	0.07788488	0.886711169
WT after UVB irradiation VS WT before UVB irradiation	GO_FICOLIN_1_RICH_GRANULE_MEMBRANE2	0.693526786	0.031893656	4.006581983	0.00381776	0.26532716	-1.5422544
hetero after UVB irradiation VS hetero before UVB irradiation	GO_FICOLIN_1_RICH_GRANULE1	0.474412421	0.021157852	3.687028777	0.00635997	0.13584504	-2.06555454
WT after UVB irradiation VS WT before UVB irradiation	GO_FICOLIN_1_RICH_GRANULE2	0.375734984	0.014479576	2.223321903	0.05649265	0.5232848	-3.99399562
homo after UVB irradiation VS homo before UVB irradiation	GO_FILAMENTOUS_ACTIN	0.249391848	-0.04272819	2.012614333	0.07755149	0.21431222	-4.74744547

hetero after UVB irradiation VS hetero before UVB irradiation	GO_FILAMENTOUS_ACTIN1	0.041730039	-0.01700373	0.234412554	0.82066088	0.93122669	-6.20179239
WT after UVB irradiation VS WT before UVB irradiation	GO_FILAMENTOUS_ACTIN2	-0.02745324	0.007290629	-0.13265698	0.89770192	0.97409039	-5.98679183
homo after UVB irradiation VS homo before UVB irradiation	GO_FILAMIN_BINDING	0.683993499	-0.03376954	4.94777792	0.00099799	0.02151484	-0.4223868
hetero after UVB irradiation VS hetero before UVB irradiation	GO_FILAMIN_BINDING1	0.358806762	0.016086383	1.394211808	0.20143533	0.5260849	-5.29384199
WT after UVB irradiation VS WT before UVB irradiation	GO_FILAMIN_BINDING2	0.412601857	0.050152475	2.133192633	0.06505659	0.53678963	-4.1201346
homo after UVB irradiation VS homo before UVB irradiation	GO_FILOPODIUM	0.091167045	-0.01388296	0.657018526	0.52890148	0.69678636	-6.31999882
homo after UVB irradiation VS homo before UVB irradiation	GO_FILOPODIUM_ASSEMBLY	0.21281389	-0.02601558	1.369149298	0.20673117	0.39678463	-5.6249514
hetero after UVB irradiation VS hetero before UVB irradiation	GO_FILOPODIUM_ASSEMBLY1	0.131668871	-0.00852855	1.015144314	0.34029036	0.65952819	-5.70764847
WT after UVB irradiation VS WT before UVB irradiation	GO_FILOPODIUM_ASSEMBLY2	0.126550492	-0.02198987	0.699362575	0.50391557	0.85763155	-5.74593606
homo after UVB irradiation VS homo before UVB irradiation	GO_FILOPODIUM_MEMBRANE	0.081053393	0.017994189	0.607786948	0.55953168	0.71975322	-6.35257387
hetero after UVB irradiation VS hetero before UVB irradiation	GO_FILOPODIUM_MEMBRANE1	-0.10568867	-0.05430688	-0.48610655	0.64016588	0.8522738	-6.10529873
WT after UVB irradiation VS WT before UVB irradiation	GO_FILOPODIUM_MEMBRANE2	0.051864523	-0.0266993	0.246361047	0.81153301	0.95508327	-5.96416824
homo after UVB irradiation VS homo before UVB irradiation	GO_FILOPODIUM_TIP	-0.42364419	0.008929936	-2.97412076	0.01697746	0.08900813	-3.2733203
hetero after UVB irradiation VS hetero before UVB irradiation	GO_FILOPODIUM_TIP1	-0.2313239	-0.01398159	-1.07006721	0.31637405	0.63665578	-5.65338105
WT after UVB irradiation VS WT before UVB irradiation	GO_FILOPODIUM_TIP2	-0.24295099	0.011874555	-1.01834888	0.33796961	0.78064003	-5.48313309
hetero after UVB irradiation VS hetero before UVB irradiation	GO_FILOPODIUM1	0.062217155	-0.00782094	0.463844373	0.65534554	0.85889323	-6.11643487
WT after UVB irradiation VS WT before UVB irradiation	GO_FILOPODIUM2	0.12681152	-0.01669929	0.910715158	0.38875022	0.80982312	-5.58069148
homo after UVB irradiation VS homo before UVB irradiation	GO_FILTRATION_DIAPHRAGM	0.164123295	-0.08846102	0.611387731	0.55725704	0.71801642	-6.3502695
hetero after UVB irradiation VS hetero before UVB irradiation	GO_FILTRATION_DIAPHRAGM1	0.469121796	0.000509596	2.552685004	0.03454152	0.25577582	-3.69412236
WT after UVB irradiation VS WT before UVB irradiation	GO_FILTRATION_DIAPHRAGM2	0.294362478	-0.12302299	1.300187321	0.22932065	0.72156087	-5.19089746
homo after UVB irradiation VS homo before UVB irradiation	GO_FK506_BINDING	0.116000651	0.020565803	0.776431349	0.45897271	0.63926949	-6.23165559
hetero after UVB irradiation VS hetero before UVB irradiation	GO_FK506_BINDING1	-0.27791156	-0.00421573	-1.65946458	0.1363137	0.44922439	-4.95931911
WT after UVB irradiation VS WT before UVB irradiation	GO_FK506_BINDING2	0.249478527	-0.00383144	1.376318609	0.20557937	0.70257868	-5.10402916
homo after UVB irradiation VS homo before UVB irradiation	GO_FLAP_ENDONUCLEASE_ACTIVITY	-0.43225254	-0.09113607	-1.52892348	0.16332609	0.3410379	-5.42310089
hetero after UVB irradiation VS hetero before UVB irradiation	GO_FLAP_ENDONUCLEASE_ACTIVITY1	0.204809304	-0.02464148	0.703791415	0.50189463	0.77390992	-5.97130603
WT after UVB irradiation VS WT before UVB irradiation	GO_FLAP_ENDONUCLEASE_ACTIVITY2	0.213341804	0.017582964	0.686439298	0.5116095	0.85920353	-5.75482486
homo after UVB irradiation VS homo before UVB irradiation	GO_FLAVIN_ADENINE_DINUCLEOTIDE_BINDING	-0.06679114	-0.03974555	-0.40399983	0.69638746	0.81857718	-6.46216411
hetero after UVB irradiation VS hetero before UVB irradiation	GO_FLAVIN_ADENINE_DINUCLEOTIDE_BINDING1	-0.28459599	-0.02362756	-1.8919955	0.09582335	0.38611944	-4.64482061
WT after UVB irradiation VS WT before UVB irradiation	GO_FLAVIN_ADENINE_DINUCLEOTIDE_BINDING2	-0.15604183	-0.04837418	-0.77935686	0.45793929	0.83880233	-5.68771685
homo after UVB irradiation VS homo before UVB irradiation	GO_FLAVIN_CONTAINING_COMPOUND_METABOLIC_PROCESS	-0.6165962	0.021639032	-3.12627325	0.01340514	0.0783444	-3.03797165
hetero after UVB irradiation VS hetero before UVB irradiation	GO_FLAVIN_CONTAINING_COMPOUND_METABOLIC_PROCESS1	-0.00516641	-0.07320881	-0.0195526	0.98488795	0.99440528	-6.23127679
WT after UVB irradiation VS WT before UVB irradiation	GO_FLAVIN_CONTAINING_COMPOUND_METABOLIC_PROCESS2	-0.30745616	-0.02502726	-1.10647341	0.30030598	0.76236287	-5.39716165
homo after UVB irradiation VS homo before UVB irradiation	GO_FLAVONE_METABOLIC_PROCESS	-0.7556849	0.144219974	-3.17611616	0.01241298	0.07504233	-2.96116021
hetero after UVB irradiation VS hetero before UVB irradiation	GO_FLAVONE_METABOLIC_PROCESS1	-1.06799172	-0.06031056	-5.03407006	0.00106735	0.089745	-0.36158921
WT after UVB irradiation VS WT before UVB irradiation	GO_FLAVONE_METABOLIC_PROCESS2	-1.00327709	0.000603445	-5.02293982	0.00098635	0.19353487	-0.34818712
homo after UVB irradiation VS homo before UVB irradiation	GO_FLAVONOID_GLUCURONIDATION	-0.8336524	0.104787956	-4.44177555	0.00195856	0.03004413	-1.10152867
hetero after UVB irradiation VS hetero before UVB irradiation	GO_FLAVONOID_GLUCURONIDATION1	-0.40034397	0.046375675	-2.05225865	0.07490586	0.35387991	-4.41986158
WT after UVB irradiation VS WT before UVB irradiation	GO_FLAVONOID_GLUCURONIDATION2	-0.50025837	-0.02859109	-2.21920545	0.05685834	0.5232848	-3.99977778
homo after UVB irradiation VS homo before UVB irradiation	GO_FLAVONOID_METABOLIC_PROCESS	-0.50639571	0.177945395	-3.53207907	0.00722652	0.05615677	-2.41852678
hetero after UVB irradiation VS hetero before UVB irradiation	GO_FLAVONOID_METABOLIC_PROCESS1	-0.51456854	-0.01652817	-2.77082782	0.02470389	0.22472139	-3.37409941
WT after UVB irradiation VS WT before UVB irradiation	GO_FLAVONOID_METABOLIC_PROCESS2	-0.46829058	-0.04583265	-1.87113034	0.09779035	0.59808854	-4.4793873
homo after UVB irradiation VS homo before UVB irradiation	GO_FLEMMING_BODY	-0.44354906	0.037238569	-3.20763593	0.01182541	0.07310747	-2.91267363
hetero after UVB irradiation VS hetero before UVB irradiation	GO_FLEMMING_BODY1	0.122942825	-0.02075901	0.631736325	0.54550617	0.79902732	-6.02057551
WT after UVB irradiation VS WT before UVB irradiation	GO_FLEMMING_BODY2	-0.1007062	-0.01258964	-0.399561	0.69981169	0.92425542	-5.91269714
homo after UVB irradiation VS homo before UVB irradiation	GO_FLIPPASE_ACTIVITY	-0.25684512	-0.04971521	-0.93885493	0.37422867	0.56438349	-6.09150003
hetero after UVB irradiation VS hetero before UVB irradiation	GO_FLIPPASE_ACTIVITY1	-0.25261187	-0.0536639	-1.09494438	0.30598486	0.6274085	-5.62810869
WT after UVB irradiation VS WT before UVB irradiation	GO_FLIPPASE_ACTIVITY2	0.00875843	-0.11656351	0.036593891	0.97169486	0.99299885	-5.99536014
homo after UVB irradiation VS homo before UVB irradiation	GO_FLOOR_PLATE_DEVELOPMENT	-0.3132839	0.039340702	-1.81060868	0.10631209	0.26012561	-5.03934154
hetero after UVB irradiation VS hetero before UVB irradiation	GO_FLOOR_PLATE_DEVELOPMENT1	-0.21056152	0.035798728	-1.15627814	0.28153139	0.60726167	-5.56402964
WT after UVB irradiation VS WT before UVB irradiation	GO_FLOOR_PLATE_DEVELOPMENT2	-0.05205261	0.058094921	-0.17518681	0.86523416	0.96790654	-5.97990532
homo after UVB irradiation VS homo before UVB irradiation	GO_FLOPPASE_ACTIVITY	0.348172624	-0.03296341	1.62053022	0.14228203	0.31342826	-5.30182441
hetero after UVB irradiation VS hetero before UVB irradiation	GO_FLOPPASE_ACTIVITY1	0.428453719	0.034153963	1.873951965	0.09850381	0.39052906	-4.66978083
WT after UVB irradiation VS WT before UVB irradiation	GO_FLOPPASE_ACTIVITY2	0.660080841	-0.02183166	2.470964453	0.03830845	0.47798151	-3.64362256
homo after UVB irradiation VS homo before UVB irradiation	GO_FLOTILLIN_COMPLEX	0.304161768	-0.00772931	0.975643779	0.35672145	0.54828105	-6.05675393
hetero after UVB irradiation VS hetero before UVB irradiation	GO_FLOTILLIN_COMPLEX1	0.566866248	0.024420892	2.821073954	0.02287899	0.21601721	-3.3005189
WT after UVB irradiation VS WT before UVB irradiation	GO_FLOTILLIN_COMPLEX2	0.633832931	0.068945977	4.099515517	0.00335218	0.25923179	-1.42539775
homo after UVB irradiation VS homo before UVB irradiation	GO_FLUID_TRANSPORT	0.413027054	-0.00016572	3.442299661	0.00827108	0.06041217	-2.55425664
hetero after UVB irradiation VS hetero before UVB irradiation	GO_FLUID_TRANSPORT1	0.1421152	0.015965227	0.971816798	0.36012041	0.67478563	-5.74892428
WT after UVB irradiation VS WT before UVB irradiation	GO_FLUID_TRANSPORT2	0.163531273	0.057744641	1.067588116	0.31649699	0.77149112	-5.4357453
homo after UVB irradiation VS homo before UVB irradiation	GO_FMN_BINDING	0.131250912	0.009904138	0.646160403	0.53556913	0.7017802	-6.32738049
hetero after UVB irradiation VS hetero before UVB irradiation	GO_FMN_BINDING1	0.037751277	-0.0227774	0.257904504	0.80311066	0.92371161	-6.19557029
WT after UVB irradiation VS WT before UVB irradiation	GO_FMN_BINDING2	0.159752287	-0.00273703	1.053185854	0.32266476	0.77346379	-5.44977847
homo after UVB irradiation VS homo before UVB irradiation	GO_FOAM_CELL_DIFFERENTIATION	0.492489415	0.002552633	3.148526129	0.01295233	0.07691649	-3.00365828
hetero after UVB irradiation VS hetero before UVB irradiation	GO_FOAM_CELL_DIFFERENTIATION1	0.265836995	0.0540309	1.409310044	0.19709244	0.52148036	-5.27565394
WT after UVB irradiation VS WT before UVB irradiation	GO_FOAM_CELL_DIFFERENTIATION2	0.210529575	-0.00684541	0.897401193	0.39540157	0.81453088	-5.59215888
homo after UVB irradiation VS homo before UVB irradiation	GO_FOCAL_ADHESION_ASSEMBLY	0.411248859	-0.02617051	2.603159346	0.03043127	0.12393687	-3.8493324
hetero after UVB irradiation VS hetero before UVB irradiation	GO_FOCAL_ADHESION_ASSEMBLY1	0.392271222	0.007258763	2.642315767	0.03008664	0.24233539	-3.56259415
WT after UVB irradiation VS WT before UVB irradiation	GO_FOCAL_ADHESION_ASSEMBLY2	0.260424528	-0.01814479	1.763784844	0.11532388	0.62065447	-4.62206473
homo after UVB irradiation VS homo before UVB irradiation	GO_FOLATE_IMPORT_ACROSS_PLASMA_MEMBRANE	0.216869106	0.087163527	1.721117843	0.12204283	0.28408433	-5.16459893
hetero after UVB irradiation VS hetero before UVB irradiation	GO_FOLATE_IMPORT_ACROSS_PLASMA_MEMBRANE1	-0.1553995	-0.06695845	-0.72534903	0.48928662	0.76616077	-5.955657
WT after UVB irradiation VS WT before UVB irradiation	GO_FOLATE_IMPORT_ACROSS_PLASMA_MEMBRANE2	0.368871324	0.011827065	1.839303528	0.10270688	0.60494124	-4.52201288
homo after UVB irradiation VS homo before UVB irradiation	GO_FOLATE_TRANSMEMBRANE_TRANSPORT	0.178271805	0.042109062	1.08243936	0.30939531	0.50379753	-5.95003168
hetero after UVB irradiation VS hetero before UVB irradiation	GO_FOLATE_TRANSMEMBRANE_TRANSPORT1	-0.12962986	-0.06647376	-0.78303215	0.45657132	0.74650043	-5.91178334
WT after UVB irradiation VS WT before UVB irradiation	GO_FOLATE_TRANSMEMBRANE_TRANSPORT2	0.061881118	-0.04982968	0.321748486	0.75579191	0.93854182	-5.94181039
homo after UVB irradiation VS homo before UVB irradiation	GO_FOLIC_ACID_BINDING	-0.08557647	0.066915542	-0.50180177	0.62880068	0.77069864	-6.41474955
hetero after UVB irradiation VS hetero before UVB irradiation	GO_FOLIC_ACID_BINDING1	-0.03056623	-0.04821742	-0.13565212	0.89550911	0.96152498	-6.22151694
WT after UVB irradiation VS WT before UVB irradiation	GO_FOLIC_ACID_BINDING2	0.411862353	-0.0184891	2.290377909	0.05085365	0.51295095	-3.89956598
homo after UVB irradiation VS homo before UVB irradiation	GO_FOLIC_ACID_CONTAINING_COMPOUND_BIOSYNTHETIC_PROCESS	-0.24179108	0.01801723	-0.91137855	0.38771068	0.57714865	-6.1167488
hetero after UVB irradiation VS hetero before UVB irradiation	GO_FOLIC_ACID_CONTAINING_COMPOUND_BIOSYNTHETIC_PROCESS1	-0.12987537	-0.06476424	-0.74166854	0.47987941	0.76097951	-5.94353769
WT after UVB irradiation VS WT before UVB irradiation	GO_FOLIC_ACID_CONTAINING_COMPOUND_BIOSYNTHETIC_PROCESS2	0.450110315	-0.00211759	1.452029723	0.18411082	0.68726847	-5.01476586
homo after UVB irradiation VS homo before UVB irradiation	GO_FOLIC_ACID_CONTAINING_COMPOUND_METABOLIC_PROCESS	-0.12234248	0.001222427	-0.6862635	0.51119498	0.68287357	-6.29956907
hetero after UVB irradiation VS hetero before UVB irradiation	GO_FOLIC_ACID_CONTAINING_COMPOUND_METABOLIC_PROCESS1	-0.0307791	-0.04931467	-0.21539364	0.83494938	0.93644494	-6.20640087
WT after UVB irradiation VS WT before UVB irradiation	GO_FOLIC_ACID_CONTAINING_COMPOUND_METABOLIC_PROCESS2	0.160664211	-0.051109	0.693691391	0.50728293	0.85779469	-5.74985475
homo after UVB irradiation VS homo before UVB irradiation	GO_FOLIC_ACID_METABOLIC_PROCESS	-0.20561192	-0.01617092	-1.03614434	0.32927696	0.52276924	-5.9973396
hetero after UVB irradiation VS hetero before UVB irradiation	GO_FOLIC_ACID_METABOLIC_PROCESS1	-0.03859922	-0.06206001	-0.22007611	0.83142523	0.9354456	-6.20530195
WT after UVB irradiation VS WT before UVB irradiation	GO_FOLIC_ACID_METABOLIC_PROCESS2	0.11805421	-0.05792964	0.501714942	0.62922247	0.90390703	-5.86539273
homo after UVB irradiation VS homo before UVB irradiation	GO_FOLIC_ACID_TRANSPORT	0.153983437	0.037667383	0.997038863	0.3468254	0.53880434	-6.03606163
hetero after UVB irradiation VS hetero before UVB irradiation	GO_FOLIC_ACID_TRANSPORT1	-0.10044146	-0.04969157	-0.50621546	0.6266079	0.84625903	-6.09481876
WT after UVB irradiation VS WT before UVB irradiation	GO_FOLIC_ACID_TRANSPORT2	0.0917207	-0.0501166	0.577115718	0.57954806	0.88805924	-5.8240572
homo after UVB irradiation VS homo before UVB irradiation	GO_FOLLICLE_STIMULATING_HORMONE_SECRETION	0.228190674	0.023761456	0.861984949	0.41282269	0.59901744	-6.16058995
hetero after UVB irradiation VS hetero before UVB irradiation	GO_FOLLICLE_STIMULATING_HORMONE_SECRETION1	0.035176635	0.035546986	0.178341836	0.86296943	0.94819913	-6.21427171
WT after UVB irradiation VS WT before UVB irradiation	GO_FOLLICLE_STIMULATING_HORMONE_SECRETION2	0.356300974	0.098715936	2.179585106	0.06049986	0.52788521	-4.05533207
homo after UVB irradiation VS homo before UVB irradiation	GO_FOREBRAIN_CELL_MIGRATION	0.286732096	0.002818496	2.126111054	0.06484507	0.19300497	-4.57893907
hetero after UVB irradiation VS hetero before UVB irradiation	GO_FOREBRAIN_CELL_MIGRATION1	0.271389237	0.032547923	1.595669783	0.14993639	0.46804769	-5.04245959
WT after UVB irradiation VS WT before UVB irradiation	GO_FOREBRAIN_CELL_MIGRATION2	0.232118338	0.033736279	1.531898991	0.16363171	0.66797045	-4.91775831
homo after UVB irradiation VS homo before UVB irradiation	GO_FOREBRAIN_DEVELOPMENT	0.021106685	0.023763446	0.167268298	0.87114882	0.92940595	-6.53552768
hetero after UVB irradiation VS hetero before UVB irradiation	GO_FOREBRAIN_DEVELOPMENT1	0.057203303	0.029070654	0.464968177	0.65457507	0.85834087	-6.11588451
WT after UVB irradiation VS WT before UVB irradiation	GO_FOREBRAIN_DEVELOPMENT2	0.00778701	0.019199339	0.055370649	0.95718499	0.98841297	-5.99444926
homo after UVB irradiation VS homo before UVB irradiation	GO_FOREBRAIN_DORSAL_VENTRAL_PATTERN_FORMATION	-0.68433052	0.050569713	-3.47154985	0.00791427	0.05919815	-2.50994269
hetero after UVB irradiation VS hetero before UVB irradiation	GO_FOREBRAIN_DORSAL_VENTRAL_PATTERN_FORMATION1	0.16062052	0.06070279	0.678912865	0.5166989	0.78479751	-5.98885013
WT after UVB irradiation VS WT before UVB irradiation	GO_FOREBRAIN_DORSAL_VENTRAL_PATTERN_FORMATION2	-0.14372112	0.132208064	-0.69486031	0.50658772	0.85763155	-5.74904935
homo after UVB irradiation VS homo before UVB irradiation	GO_FOREBRAIN_GENERATION_OF_NEURONS	-0.08030528	0.040430326	-0.60274739	0.56272417	0.72241153	-6.3557781
hetero after UVB irradiation VS hetero before UVB irradiation	GO_FOREBRAIN_GENERATION_OF_NEURONS1	0.165820075	0.074046921	1.197369996	0.26605442	0.592678	-5.51974105
WT after UVB irradiation VS WT before UVB irradiation	GO_FOREBRAIN_GENERATION_OF_NEURONS2	-0.08458106	0.031867712	-0.50402705	0.62766714	0.90339257	-5.86420515
homo after UVB irradiation VS homo before UVB irradiation	GO_FOREBRAIN_MORPHOGENESIS	0.079364303	0.10245354	0.365582592	0.723787	0.83686631	-6.47810037
hetero after UVB irradiation VS hetero before UVB irradiation	GO_FOREBRAIN_MORPHOGENESIS1	0.179505157	0.043392946	0.70435363	0.50156321	0.7738974	-5.97090315
WT after UVB irradiation VS WT before UVB irradiation	GO_FOREBRAIN_MORPHOGENESIS2	-0.1606126	-0.02349891	-0.90999574	0.38910754	0.81008789	-5.58131459
homo after UVB irradiation VS homo before UVB irradiation	GO_FOREBRAIN_NEUROBLAST_DIVISION	-0.60862607	0.037387845	-2.34494822	0.04583991	0.15618519	-4.24745581
hetero after UVB irradiation VS hetero before UVB irradiation	GO_FOREBRAIN_NEUROBLAST_DIVISION1	-0.73202365	-0.07381659	-4.09402306	0.00360616	0.11882956	-1.51898178
WT after UVB irradiation VS WT before UVB irradiation	GO_FOREBRAIN_NEUROBLAST_DIVISION2	-0.51714972	-0.05998754	-2.41223727	0.04200389	0.495137	-3.72704784
homo after UVB irradiation VS homo before UVB irradiation	GO_FOREBRAIN_NEURON_DEVELOPMENT	-0.19093323	0.031852907	-1.32076773	0.22171126	0.41272646	-5.68337599
hetero after UVB irradiation VS hetero before UVB irradiation	GO_FOREBRAIN_NEURON_DEVELOPMENT1	0.06951117	0.079401611	0.470757444	0.65061301	0.85686396	-6.11302943
WT after UVB irradiation VS WT before UVB irradiation	GO_FOREBRAIN_NEURON_DEVELOPMENT2	-0.26461413	0.023952841	-1.5235508	0.16567264	0.67018396	-4.92802643
homo after UVB irradiation VS homo before UVB irradiation	GO_FOREBRAIN_NEURON_DIFFERENTIATION	0.034856588	0.049968451	0.269392	0.79418167	0.88313181	-6.51120363
hetero after UVB irradiation VS hetero before UVB irradiation	GO_FOREBRAIN_NEURON_DIFFERENTIATION1	0.265169894	0.076989302	1.86511281	0.09984291	0.39245806	-4.68197796
WT after UVB irradiation VS WT before UVB irradiation	GO_FOREBRAIN_NEURON_DIFFERENTIATION2	-0.05171816	0.041929731	-0.29949311	0.7721071	0.94219669	-5.94901445
homo after UVB irradiation VS homo before UVB irradiation	GO_FOREBRAIN_NEURON_FATE_COMMITMENT	0.071734871	0.041078686	0.42430196	0.68209265	0.80855019	-6.45312503
hetero after UVB irradiation VS hetero before UVB irradiation	GO_FOREBRAIN_NEURON_FATE_COMMITMENT1	0.084472597	0.09247867	0.322432529	0.75554184	0.90493306	-6.17548822
WT after UVB irradiation VS WT before UVB irradiation	GO_FOREBRAIN_NEURON_FATE_COMMITMENT2	0.081179411	0.080253614	0.449645789	0.66475809	0.9126277	-5.89077504
homo after UVB irradiation VS homo before UVB irradiation	GO_FOREBRAIN_RADIAL_GLIAL_CELL_DIFFERENTIATION	-0.28222432	0.094036649	-0.9586378	0.36473701	0.55471751	-6.07294793
hetero after UVB irradiation VS hetero before UVB irradiation	GO_FOREBRAIN_RADIAL_GLIAL_CELL_DIFFERENTIATION1	0.20348147	0.051747778	1.036674258	0.3307534	0.65066323	-5.68663017
WT after UVB irradiation VS WT before UVB irradiation	GO_FOREBRAIN_RADIAL_GLIAL_CELL_DIFFERENTIATION2	-0.32699788	0.080265723	-1.01323813	0.34026108	0.7811501	-5.48795516
homo after UVB irradiation VS homo before UVB irradiation	GO_FOREBRAIN_REGIONALIZATION	-0.52110332	0.104752658	-3.38250999	0.00905423	0.0633504	-2.64510447
hetero after UVB irradiation VS hetero before UVB irradiation	GO_FOREBRAIN_REGIONALIZATION1	-0.30345001	0.042611559	-1.90355967	0.09414231	0.38395908	-4.62878076
WT after UVB irradiation VS WT before UVB irradiation	GO_FOREBRAIN_REGIONALIZATION2	-0.43274255	0.056355401	-2.30593376	0.0496276	0.50853601	-3.87760158
homo after UVB irradiation VS homo before UVB irradiation	GO_FOREBRAIN_VENTRICULAR_ZONE_PROGENITOR_CELL_DIVISION	0.227420099	0.047189431	1.097007414	0.30333718	0.49781436	-5.93482416
hetero after UVB irradiation VS hetero before UVB irradiation	GO_FOREBRAIN_VENTRICULAR_ZONE_PROGENITOR_CELL_DIVISION1	-0.16585879	0.015451686	-0.85526897	0.41773177	0.72088233	-5.85285525
WT after UVB irradiation VS WT before UVB irradiation	GO_FOREBRAIN_VENTRICULAR_ZONE_PROGENITOR_CELL_DIVISION2	0.035084894	-0.03956671	0.123447965	0.90476153	0.97663418	-5.9880336
homo after UVB irradiation VS homo before UVB irradiation	GO_FOREGUT_MORPHOGENESIS	-0.39980754	0.069900872	-1.42884443	0.18945743	0.37512723	-5.55107706
hetero after UVB irradiation VS hetero before UVB irradiation	GO_FOREGUT_MORPHOGENESIS1	-0.41148161	0.062305519	-2.15170414	0.06423963	0.33225374	-4.27782054
WT after UVB irradiation VS WT before UVB irradiation	GO_FOREGUT_MORPHOGENESIS2	-0.48305592	0.027906885	-2.63847567	0.02947549	0.44934895	-3.40527135
homo after UVB irradiation VS homo before UVB irradiation	GO_FORELIMB_MORPHOGENESIS	0.008246501	0.01145534	0.054936852	0.95748372	0.97771218	-6.54920065
hetero after UVB irradiation VS hetero before UVB irradiation	GO_FORELIMB_MORPHOGENESIS1	-0.2076326	0.004985992	-1.31604396	0.22527621	0.55420419	-5.3861277
WT after UVB irradiation VS WT before UVB irradiation	GO_FORELIMB_MORPHOGENESIS2	-0.13878977	0.034886868	-0.84562638	0.42204348	0.82449435	-5.63544377
homo after UVB irradiation VS homo before UVB irradiation	GO_FORMATE_METABOLIC_PROCESS	0.3731497	-0.01426421	1.581807966	0.1508586	0.3246639	-5.35354257
hetero after UVB irradiation VS hetero before UVB irradiation	GO_FORMATE_METABOLIC_PROCESS1	0.322430298	-0.05830657	1.323716694	0.22283314	0.55142139	-5.377213
WT after UVB irradiation VS WT before UVB irradiation	GO_FORMATE_METABOLIC_PROCESS2	0.236018304	-0.00111071	1.559099851	0.15713767	0.66180255	-4.88410275
homo after UVB irradiation VS homo before UVB irradiation	GO_FORMATION_OF_ANATOMICAL_BOUNDARY	-0.26535836	0.140409947	-0.97392957	0.3575234	0.54912291	-6.05839651
hetero after UVB irradiation VS hetero before UVB irradiation	GO_FORMATION_OF_ANATOMICAL_BOUNDARY1	-0.19621742	0.036765897	-0.59757292	0.56695336	0.81234334	-6.04226528
WT after UVB irradiation VS WT before UVB irradiation	GO_FORMATION_OF_ANATOMICAL_BOUNDARY2	0.10124736	0.138819534	0.302467811	0.76991918	0.94145237	-5.94808069
homo after UVB irradiation VS homo before UVB irradiation	GO_FORMATION_OF_CYTOPLASMIC_TRANSLATION_INITIATION_COMPLEX	-1.05588539	-0.00337199	-7.95805111	3.61E-05	0.00847131	2.827649436
hetero after UVB irradiation VS hetero before UVB irradiation	GO_FORMATION_OF_CYTOPLASMIC_TRANSLATION_INITIATION_COMPLEX1	-0.59626545	-0.08671038	-1.81321339	0.10806204	0.40604091	-4.75317222
WT after UVB irradiation VS WT before UVB irradiation	GO_FORMATION_OF_CYTOPLASMIC_TRANSLATION_INITIATION_COMPLEX2	-0.66226794	-0.10643454	-2.09381935	0.06918672	0.54423691	-4.17489729
homo after UVB irradiation VS homo before UVB irradiation	GO_FORMATION_OF_EXTRACHROMOSOMAL_CIRCULAR_DNA	-0.46993843	-0.07994022	-2.33504758	0.04656612	0.15783849	-4.26259991
hetero after UVB irradiation VS hetero before UVB irradiation	GO_FORMATION_OF_EXTRACHROMOSOMAL_CIRCULAR_DNA1	-0.12439221	-0.06044479	-0.4174201	0.68754972	0.87456009	-6.13806476
WT after UVB irradiation VS WT before UVB irradiation	GO_FORMATION_OF_EXTRACHROMOSOMAL_CIRCULAR_DNA2	0.092654146	0.01922937	0.36037471	0.72778623	0.93296251	-5.9281174
homo after UVB irradiation VS homo before UVB irradiation	GO_FORMATION_OF_PRIMARY_GERM_LAYER	0.163694842	0.018405519	1.097943626	0.30295108	0.49729132	-5.93384169
hetero after UVB irradiation VS hetero before UVB irradiation	GO_FORMATION_OF_PRIMARY_GERM_LAYER1	0.189620316	0.036671078	1.285358478	0.2352769	0.56350364	-5.42145496
WT after UVB irradiation VS WT before UVB irradiation	GO_FORMATION_OF_PRIMARY_GERM_LAYER2	0.076681867	0.025082622	0.490251444	0.63696281	0.9057274	-5.87120521
homo after UVB irradiation VS homo before UVB irradiation	GO_FORMATION_OF_TRANSLATION_PREINITIATION_COMPLEX	-0.967169	-0.00518166	-6.9131883	0.00010136	0.00997194	1.841169545
hetero after UVB irradiation VS hetero before UVB irradiation	GO_FORMATION_OF_TRANSLATION_PREINITIATION_COMPLEX1	-0.28500508	-0.02414439	-0.8115563	0.44094977	0.73724702	-5.8890344
WT after UVB irradiation VS WT before UVB irradiation	GO_FORMATION_OF_TRANSLATION_PREINITIATION_COMPLEX2	-0.58283282	-0.10180566	-2.00182438	0.07985841	0.56457304	-4.30187081

homo after UVB irradiation VS homo before UVB irradiation	GO_FOUR_WAY_JUNCTION_DNA_BINDING	-0.8176575	0.02557901	-4.17622783	0.00283221	0.03518388	-1.47385147
hetero after UVB irradiation VS hetero before UVB irradiation	GO_FOUR_WAY_JUNCTION_DNA_BINDING1	-0.19535886	-0.05785365	-0.80822142	0.44275701	0.7384454	-5.89172946
WT after UVB irradiation VS WT before UVB irradiation	GO_FOUR_WAY_JUNCTION_DNA_BINDING2	-0.26319758	-0.05106909	-0.99744323	0.34741798	0.78529818	-5.50274098
homo after UVB irradiation VS homo before UVB irradiation	GO_FOUR_WAY_JUNCTION_HELICASE_ACTIVITY	-0.82215926	0.00546881	-3.92952969	0.0040266	0.04132163	-1.8290741
hetero after UVB irradiation VS hetero before UVB irradiation	GO_FOUR_WAY_JUNCTION_HELICASE_ACTIVITY1	-0.54306811	-0.11185865	-1.58366139	0.15263606	0.47093079	-5.05793463
WT after UVB irradiation VS WT before UVB irradiation	GO_FOUR_WAY_JUNCTION_HELICASE_ACTIVITY2	-0.52636335	-0.14285554	-1.50765058	0.16962304	0.67524339	-4.94750229
homo after UVB irradiation VS homo before UVB irradiation	GO_FREE_UBIQUITIN_CHAIN_POLYMERIZATION	0.416991915	0.003493462	2.774521708	0.02321485	0.10652412	-3.58325417
hetero after UVB irradiation VS hetero before UVB irradiation	GO_FREE_UBIQUITIN_CHAIN_POLYMERIZATION1	0.734542978	0.014905318	3.94618647	0.0044192	0.12651442	-1.71451275
WT after UVB irradiation VS WT before UVB irradiation	GO_FREE_UBIQUITIN_CHAIN_POLYMERIZATION2	0.6440951	-0.00973427	3.316604668	0.01041351	0.33479777	-2.45361928
homo after UVB irradiation VS homo before UVB irradiation	GO_FRIZZLED_BINDING	-0.44227829	0.034485774	-3.21497876	0.01169275	0.07253044	-2.90138855
hetero after UVB irradiation VS hetero before UVB irradiation	GO_FRIZZLED_BINDING1	-0.4758028	0.008650852	-3.16049988	0.01370751	0.17665328	-2.80735532
WT after UVB irradiation VS WT before UVB irradiation	GO_FRIZZLED_BINDING2	-0.43079115	0.02376082	-2.48588056	0.03742323	0.47615783	-3.62241446
homo after UVB irradiation VS homo before UVB irradiation	GO_FRUCTOSE_1_6_BISPHOSPHATE_METABOLIC_PROCESS	0.347729149	0.058624346	1.752202418	0.116348	0.27506367	-5.12140977
hetero after UVB irradiation VS hetero before UVB irradiation	GO_FRUCTOSE_1_6_BISPHOSPHATE_METABOLIC_PROCESS1	0.337562928	0.013177191	1.651336752	0.13798361	0.45118267	-4.96999596
WT after UVB irradiation VS WT before UVB irradiation	GO_FRUCTOSE_1_6_BISPHOSPHATE_METABOLIC_PROCESS2	0.06594638	0.076891335	0.276390324	0.78917171	0.94742978	-5.955959
homo after UVB irradiation VS homo before UVB irradiation	GO_FRUCTOSE_2_6_BISPHOSPHATE_2_PHOSPHATASE_ACTIVITY	0.41537031	-0.0294785	2.710109705	0.02569651	0.11319506	-3.68333558
hetero after UVB irradiation VS hetero before UVB irradiation	GO_FRUCTOSE_2_6_BISPHOSPHATE_2_PHOSPHATASE_ACTIVITY1	0.325735849	-0.06226928	1.45445489	0.18459396	0.50920822	-5.22060491
WT after UVB irradiation VS WT before UVB irradiation	GO_FRUCTOSE_2_6_BISPHOSPHATE_2_PHOSPHATASE_ACTIVITY2	-0.00863127	-0.03900647	-0.02882575	0.97770134	0.99433096	-5.99562825
homo after UVB irradiation VS homo before UVB irradiation	GO_FRUCTOSE_2_6_BISPHOSPHATE_METABOLIC_PROCESS	0.41537031	-0.0294785	2.710109705	0.02569651	0.11319506	-3.68333558
hetero after UVB irradiation VS hetero before UVB irradiation	GO_FRUCTOSE_2_6_BISPHOSPHATE_METABOLIC_PROCESS1	0.325735849	-0.06226928	1.45445489	0.18459396	0.50920822	-5.22060491
WT after UVB irradiation VS WT before UVB irradiation	GO_FRUCTOSE_2_6_BISPHOSPHATE_METABOLIC_PROCESS2	-0.00863127	-0.03900647	-0.02882575	0.97770134	0.99433096	-5.99562825
homo after UVB irradiation VS homo before UVB irradiation	GO_FRUCTOSE_6_PHOSPHATE_BINDING	0.921409918	-0.0473207	4.826348496	0.00116927	0.0231735	-0.58167803
hetero after UVB irradiation VS hetero before UVB irradiation	GO_FRUCTOSE_6_PHOSPHATE_BINDING1	0.329810907	0.076369044	1.555769394	0.15907818	0.47924721	-5.09365475
WT after UVB irradiation VS WT before UVB irradiation	GO_FRUCTOSE_6_PHOSPHATE_BINDING2	0.581613183	0.052119495	1.890165646	0.09495762	0.59504681	-4.45377245
homo after UVB irradiation VS homo before UVB irradiation	GO_FRUCTOSE_6_PHOSPHATE_METABOLIC_PROCESS	0.515397353	-0.01576161	2.677988906	0.02703383	0.11630986	-3.73322264
hetero after UVB irradiation VS hetero before UVB irradiation	GO_FRUCTOSE_6_PHOSPHATE_METABOLIC_PROCESS1	0.42480661	0.040879895	2.070207505	0.07286007	0.34990725	-4.39434605
WT after UVB irradiation VS WT before UVB irradiation	GO_FRUCTOSE_6_PHOSPHATE_METABOLIC_PROCESS2	0.344602523	0.074436513	1.923808432	0.0901415	0.58325853	-4.40829091
homo after UVB irradiation VS homo before UVB irradiation	GO_FRUCTOSE_BINDING	0.624354045	-0.0758453	2.773079915	0.02326762	0.10657122	-3.58549468
hetero after UVB irradiation VS hetero before UVB irradiation	GO_FRUCTOSE_BINDING1	0.035915803	-0.01625216	0.097878837	0.92448066	0.97152219	-6.22629194
WT after UVB irradiation VS WT before UVB irradiation	GO_FRUCTOSE_BINDING2	0.30437741	0.044562537	1.075942875	0.31296158	0.77011405	-5.42754035
homo after UVB irradiation VS homo before UVB irradiation	GO_FRUCTOSE_CATABOLIC_PROCESS_TO_HYDROXYACETONE_PHOSPHATE_AND_GLYCERALDEHYDE_3_PHOSPHATE	0.242628502	-0.04590004	1.142162412	0.28515426	0.47986854	-5.88673835
hetero after UVB irradiation VS hetero before UVB irradiation	GO_FRUCTOSE_CATABOLIC_PROCESS_TO_HYDROXYACETONE_PHOSPHATE_AND_GLYCERALDEHYDE_3_PHOSPHATE1	-0.42557908	0.018566901	-1.76365041	0.11650623	0.41961581	-4.82044593
WT after UVB irradiation VS WT before UVB irradiation	GO_FRUCTOSE_CATABOLIC_PROCESS_TO_HYDROXYACETONE_PHOSPHATE_AND_GLYCERALDEHYDE_3_PHOSPHATE2	0.19090454	0.094371458	1.00968596	0.34186073	0.78172749	-5.49129583
homo after UVB irradiation VS homo before UVB irradiation	GO_FRUCTOSE_METABOLIC_PROCESS	0.423094889	-0.00534707	2.975565601	0.01693925	0.0888949	-3.27108043
hetero after UVB irradiation VS hetero before UVB irradiation	GO_FRUCTOSE_METABOLIC_PROCESS1	0.077390714	-0.00838746	0.368806092	0.72201731	0.89103348	-6.15837594
WT after UVB irradiation VS WT before UVB irradiation	GO_FRUCTOSE_METABOLIC_PROCESS2	0.040734168	0.034555616	0.216434285	0.83400226	0.96098784	-5.97142505
homo after UVB irradiation VS homo before UVB irradiation	GO_FRUCTOSE_TRANSMEMBRANE_TRANSPORT	0.78392493	0.09556454	3.765027339	0.00511595	0.04651635	-2.07062911
hetero after UVB irradiation VS hetero before UVB irradiation	GO_FRUCTOSE_TRANSMEMBRANE_TRANSPORT1	0.709267767	0.095498937	2.564323033	0.03392695	0.25425399	-3.6770487
WT after UVB irradiation VS WT before UVB irradiation	GO_FRUCTOSE_TRANSMEMBRANE_TRANSPORT2	0.489147368	0.12189111	1.703338116	0.12645422	0.63364512	-4.70093514
homo after UVB irradiation VS homo before UVB irradiation	GO_FUCOSE_BINDING	0.550339625	0.110512499	3.484830175	0.00775767	0.05855549	-2.48985229
hetero after UVB irradiation VS hetero before UVB irradiation	GO_FUCOSE_BINDING1	0.876319368	0.011123548	3.789025697	0.00550463	0.13120925	-1.92617371
WT after UVB irradiation VS WT before UVB irradiation	GO_FUCOSE_BINDING2	0.482605047	-0.02687117	1.611187516	0.14534875	0.65365874	-4.81883668
homo after UVB irradiation VS homo before UVB irradiation	GO_FUCOSE_CATABOLIC_PROCESS	-0.05136404	0.006584968	-0.24238088	0.81434686	0.89494464	-6.51872976
hetero after UVB irradiation VS hetero before UVB irradiation	GO_FUCOSE_CATABOLIC_PROCESS1	0.057414341	-0.04843483	0.386573352	0.70933616	0.88545507	-6.15123245
WT after UVB irradiation VS WT before UVB irradiation	GO_FUCOSE_CATABOLIC_PROCESS2	0.072312791	-0.02947301	0.399465461	0.69987932	0.92425542	-5.9127366
homo after UVB irradiation VS homo before UVB irradiation	GO_FUCOSE_METABOLIC_PROCESS	0.245988847	-0.0058229	1.011480534	0.34026377	0.5332136	-6.02189605
hetero after UVB irradiation VS hetero before UVB irradiation	GO_FUCOSE_METABOLIC_PROCESS1	0.318050565	-0.01020439	1.511782505	0.16973912	0.49222887	-5.14932721
WT after UVB irradiation VS WT before UVB irradiation	GO_FUCOSE_METABOLIC_PROCESS2	0.165883683	-0.05053557	0.816304665	0.43767834	0.83056493	-5.65901371
homo after UVB irradiation VS homo before UVB irradiation	GO_FUCOSYLATION	0.327525898	-0.00262314	1.577884233	0.15175328	0.32584299	-5.35874676
hetero after UVB irradiation VS hetero before UVB irradiation	GO_FUCOSYLATION1	0.346153556	0.001084008	1.312048498	0.22655746	0.55569846	-5.39075714
WT after UVB irradiation VS WT before UVB irradiation	GO_FUCOSYLATION2	0.29759574	-0.05257591	1.549944678	0.15929701	0.66303059	-4.89546404
homo after UVB irradiation VS homo before UVB irradiation	GO_FUCOSYLTRANSFERASE_ACTIVITY	0.200436797	-0.00545099	0.915152878	0.38583808	0.57553953	-6.11331652
hetero after UVB irradiation VS hetero before UVB irradiation	GO_FUCOSYLTRANSFERASE_ACTIVITY1	0.313920329	-0.00844003	1.444753421	0.18721915	0.51246893	-5.23251726
WT after UVB irradiation VS WT before UVB irradiation	GO_FUCOSYLTRANSFERASE_ACTIVITY2	0.130488962	-0.06348227	0.661702545	0.52653923	0.86617234	-5.77142956
homo after UVB irradiation VS homo before UVB irradiation	GO_FUNGIFORM_PAPILLA_DEVELOPMENT	0.039261169	0.021075696	0.194544668	0.85041095	0.91763048	-6.53013314
hetero after UVB irradiation VS hetero before UVB irradiation	GO_FUNGIFORM_PAPILLA_DEVELOPMENT1	0.004243871	-0.00878403	0.016489371	0.98725522	0.99514571	-6.23133666
WT after UVB irradiation VS WT before UVB irradiation	GO_FUNGIFORM_PAPILLA_DEVELOPMENT2	-0.29189093	0.075902438	-1.12166247	0.29416297	0.75745298	-5.38182029
homo after UVB irradiation VS homo before UVB irradiation	GO_FUNGIFORM_PAPILLA_MORPHOGENESIS	0.235470789	-0.0302093	1.322636988	0.22111574	0.41200329	-5.68114366
hetero after UVB irradiation VS hetero before UVB irradiation	GO_FUNGIFORM_PAPILLA_MORPHOGENESIS1	0.218027894	-0.02399874	0.784664629	0.45566729	0.74613056	-5.91049997
WT after UVB irradiation VS WT before UVB irradiation	GO_FUNGIFORM_PAPILLA_MORPHOGENESIS2	-0.09406494	0.080354509	-0.34805296	0.73667539	0.93474796	-5.93264874
homo after UVB irradiation VS homo before UVB irradiation	GO_FUSION_OF_SPERM_TO_EGG_PLASMA_MEMBRANE_INVOLVED_IN_SINGLE_FERTILIZATION	-0.05781371	0.083538652	-0.32347366	0.7543091	0.85684762	-6.4937956
hetero after UVB irradiation VS hetero before UVB irradiation	GO_FUSION_OF_SPERM_TO_EGG_PLASMA_MEMBRANE_INVOLVED_IN_SINGLE_FERTILIZATION1	0.010939811	0.01294098	0.071467722	0.94481213	0.97953855	-6.22871513
WT after UVB irradiation VS WT before UVB irradiation	GO_FUSION_OF_SPERM_TO_EGG_PLASMA_MEMBRANE_INVOLVED_IN_SINGLE_FERTILIZATION2	0.018914027	0.103367827	0.092425173	0.92860597	0.98233894	-5.99156178
homo after UVB irradiation VS homo before UVB irradiation	GO_G_PROTEIN_ALPHA_SUBUNIT_BINDING	0.464379223	-0.02145966	3.440547739	0.00829298	0.06049409	-2.55691357
hetero after UVB irradiation VS hetero before UVB irradiation	GO_G_PROTEIN_ALPHA_SUBUNIT_BINDING1	0.230067034	0.003641912	1.213039712	0.26034018	0.5868591	-5.50257568
WT after UVB irradiation VS WT before UVB irradiation	GO_G_PROTEIN_ALPHA_SUBUNIT_BINDING2	0.018261482	-0.0472221	0.073367374	0.94329372	0.98531933	-5.99322744
homo after UVB irradiation VS homo before UVB irradiation	GO_G_PROTEIN_BETA_GAMMA_SUBUNIT_COMPLEX	0.432867611	0.020310784	1.753531376	0.11611013	0.27472136	-5.11955554
hetero after UVB irradiation VS hetero before UVB irradiation	GO_G_PROTEIN_BETA_GAMMA_SUBUNIT_COMPLEX_BINDING	0.368087974	-0.01773492	2.046271642	0.07355088	0.20828829	-4.6977691
WT after UVB irradiation VS WT before UVB irradiation	GO_G_PROTEIN_BETA_GAMMA_SUBUNIT_COMPLEX_BINDING1	0.326324202	-0.00522101	1.905134927	0.09391552	0.38339979	-4.62659329
homo after UVB irradiation VS homo before UVB irradiation	GO_G_PROTEIN_BETA_GAMMA_SUBUNIT_COMPLEX_BINDING2	0.19474176	-0.00142582	0.746850202	0.47627689	0.84863777	-5.71203153
hetero after UVB irradiation VS hetero before UVB irradiation	GO_G_PROTEIN_BETA_GAMMA_SUBUNIT_COMPLEX1	0.534025571	0.011626271	2.771277922	0.02468689	0.22472139	-3.37343989
WT after UVB irradiation VS WT before UVB irradiation	GO_G_PROTEIN_BETA_GAMMA_SUBUNIT_COMPLEX2	0.360405913	-0.07153259	1.331817664	0.21918626	0.71478521	-5.15517392
homo after UVB irradiation VS homo before UVB irradiation	GO_G_PROTEIN_BETA_SUBUNIT_BINDING	0.317116031	0.026594601	1.599457598	0.14689288	0.31967117	-5.33004966
hetero after UVB irradiation VS hetero before UVB irradiation	GO_G_PROTEIN_BETA_SUBUNIT_BINDING1	0.252092853	0.003682865	1.504973272	0.17144573	0.49447611	-5.15787078
WT after UVB irradiation VS WT before UVB irradiation	GO_G_PROTEIN_BETA_SUBUNIT_BINDING2	0.192131901	-0.01722086	1.014905308	0.33951228	0.78076714	-5.48638417
homo after UVB irradiation VS homo before UVB irradiation	GO_G_PROTEIN_COUPLED_ACETYLCHOLINE_RECEPTOR_ACTIVITY	0.119147196	-0.00211157	0.796216198	0.44800276	0.62979726	-6.21578299
hetero after UVB irradiation VS hetero before UVB irradiation	GO_G_PROTEIN_COUPLED_ACETYLCHOLINE_RECEPTOR_ACTIVITY1	-0.23594191	-0.00407609	-1.11359061	0.29837705	0.62161528	-5.60889108
WT after UVB irradiation VS WT before UVB irradiation	GO_G_PROTEIN_COUPLED_ACETYLCHOLINE_RECEPTOR_ACTIVITY2	-0.23396996	-0.02064553	-1.21895812	0.25717607	0.73855744	-5.28010737
homo after UVB irradiation VS homo before UVB irradiation	GO_G_PROTEIN_COUPLED_ACETYLCHOLINE_RECEPTOR_SIGNALING_PATHWAY	0.475789633	-0.03146987	3.469249798	0.00794173	0.05921693	-2.51342406
hetero after UVB irradiation VS hetero before UVB irradiation	GO_G_PROTEIN_COUPLED_ACETYLCHOLINE_RECEPTOR_SIGNALING_PATHWAY1	0.601943393	0.015875672	4.611559878	0.00181544	0.09780003	-0.86311877
WT after UVB irradiation VS WT before UVB irradiation	GO_G_PROTEIN_COUPLED_ACETYLCHOLINE_RECEPTOR_SIGNALING_PATHWAY2	0.337209438	0.025328408	1.679426837	0.13112639	0.63700751	-4.73181072
homo after UVB irradiation VS homo before UVB irradiation	GO_G_PROTEIN_COUPLED_ADENOSINE_RECEPTOR_ACTIVITY	0.487641183	-0.051313	2.063329872	0.07160029	0.20541559	-4.6724938
hetero after UVB irradiation VS hetero before UVB irradiation	GO_G_PROTEIN_COUPLED_ADENOSINE_RECEPTOR_ACTIVITY1	0.291244473	0.011051023	1.488048757	0.17575436	0.49901327	-5.1790169
WT after UVB irradiation VS WT before UVB irradiation	GO_G_PROTEIN_COUPLED_ADENOSINE_RECEPTOR_ACTIVITY2	0.440057804	-0.06219462	1.3979614	0.19923008	0.69740523	-5.0787943
homo after UVB irradiation VS homo before UVB irradiation	GO_G_PROTEIN_COUPLED_AMINE_RECEPTOR_ACTIVITY	0.235917246	0.034543432	1.68781603	0.12842965	0.29342567	-5.21047081
hetero after UVB irradiation VS hetero before UVB irradiation	GO_G_PROTEIN_COUPLED_AMINE_RECEPTOR_ACTIVITY1	-0.12237592	-0.00486146	-0.7424123	0.47945351	0.76097838	-5.9429798
WT after UVB irradiation VS WT before UVB irradiation	GO_G_PROTEIN_COUPLED_AMINE_RECEPTOR_ACTIVITY2	0.146617706	0.022367146	0.619125881	0.55284892	0.8765866	-5.79873164
homo after UVB irradiation VS homo before UVB irradiation	GO_G_PROTEIN_COUPLED_CHEMOATTRACTANT_RECEPTOR_ACTIVITY	0.349809853	-0.02387142	1.633486308	0.13951293	0.30931374	-5.28437748
hetero after UVB irradiation VS hetero before UVB irradiation	GO_G_PROTEIN_COUPLED_CHEMOATTRACTANT_RECEPTOR_ACTIVITY1	0.21630026	0.005716628	1.119212146	0.29611334	0.62021979	-5.60305167
WT after UVB irradiation VS WT before UVB irradiation	GO_G_PROTEIN_COUPLED_CHEMOATTRACTANT_RECEPTOR_ACTIVITY2	0.118129344	-0.02471551	0.387048442	0.70869401	0.92758951	-5.91778726
homo after UVB irradiation VS homo before UVB irradiation	GO_G_PROTEIN_COUPLED_GLUTAMATE_RECEPTOR_ACTIVITY	-0.33844689	0.033874185	-1.70373837	0.1253384	0.28900294	-5.1885909
hetero after UVB irradiation VS hetero before UVB irradiation	GO_G_PROTEIN_COUPLED_GLUTAMATE_RECEPTOR_ACTIVITY1	-0.29968163	0.048593364	-1.53896374	0.16307779	0.48457024	-5.11502214
WT after UVB irradiation VS WT before UVB irradiation	GO_G_PROTEIN_COUPLED_GLUTAMATE_RECEPTOR_ACTIVITY2	-0.27879879	0.17693584	-1.64002836	0.13917346	0.64810012	-4.78225854
homo after UVB irradiation VS homo before UVB irradiation	GO_G_PROTEIN_COUPLED_GLUTAMATE_RECEPTOR_BINDING	0.207435545	-0.10505205	0.964257337	0.36207362	0.55296645	-6.06762172
hetero after UVB irradiation VS hetero before UVB irradiation	GO_G_PROTEIN_COUPLED_GLUTAMATE_RECEPTOR_BINDING1	0.508900896	0.010841646	2.989306453	0.0177232	0.19475684	-3.05506126
WT after UVB irradiation VS WT before UVB irradiation	GO_G_PROTEIN_COUPLED_GLUTAMATE_RECEPTOR_BINDING2	0.034618178	-0.01578198	0.245472986	0.81219722	0.95510355	-5.96439689
homo after UVB irradiation VS homo before UVB irradiation	GO_G_PROTEIN_COUPLED_GLUTAMATE_RECEPTOR_SIGNALING_PATHWAY	-0.13852079	0.029484959	-0.92162536	0.38264211	0.57262444	-6.10740377
hetero after UVB irradiation VS hetero before UVB irradiation	GO_G_PROTEIN_COUPLED_GLUTAMATE_RECEPTOR_SIGNALING_PATHWAY1	-0.17319546	0.068936095	-1.21914213	0.25814252	0.58489002	-5.49585028
WT after UVB irradiation VS WT before UVB irradiation	GO_G_PROTEIN_COUPLED_GLUTAMATE_RECEPTOR_SIGNALING_PATHWAY2	-0.09867439	0.052377299	-0.54540328	0.60017285	0.89567499	-5.84209395
homo after UVB irradiation VS homo before UVB irradiation	GO_G_PROTEIN_COUPLED_NEUROTRANSMITTER_RECEPTOR_ACTIVITY	0.166192697	-0.01537437	1.182022139	0.2698402	0.46529935	-5.84313102
hetero after UVB irradiation VS hetero before UVB irradiation	GO_G_PROTEIN_COUPLED_NEUROTRANSMITTER_RECEPTOR_ACTIVITY1	-0.40666322	0.027157729	-2.22764594	0.05711623	0.31518588	-4.16837675
WT after UVB irradiation VS WT before UVB irradiation	GO_G_PROTEIN_COUPLED_NEUROTRANSMITTER_RECEPTOR_ACTIVITY2	-0.1896462	0.021253582	-0.75410731	0.47214173	0.84703593	-5.7066804
homo after UVB irradiation VS homo before UVB irradiation	GO_G_PROTEIN_COUPLED_PURINERGIC_NUCLEOTIDE_RECEPTOR_ACTIVITY	0.325763394	-0.0378226	1.676295403	0.13070998	0.29661498	-5.22624094
hetero after UVB irradiation VS hetero before UVB irradiation	GO_G_PROTEIN_COUPLED_PURINERGIC_NUCLEOTIDE_RECEPTOR_ACTIVITY1	0.584255781	0.006475399	3.69798007	0.00626166	0.13548627	-2.05051564
WT after UVB irradiation VS WT before UVB irradiation	GO_G_PROTEIN_COUPLED_PURINERGIC_NUCLEOTIDE_RECEPTOR_ACTIVITY2	0.549553776	0.049047483	2.375274004	0.04451165	0.4987535	-3.77947649
homo after UVB irradiation VS homo before UVB irradiation	GO_G_PROTEIN_COUPLED_PURINERGIC_NUCLEOTIDE_RECEPTOR_SIGNALING_PATHWAY	0.325763394	-0.0378226	1.676295403	0.13070998	0.29661498	-5.22624094
hetero after UVB irradiation VS hetero before UVB irradiation	GO_G_PROTEIN_COUPLED_PURINERGIC_NUCLEOTIDE_RECEPTOR_SIGNALING_PATHWAY1	0.584255781	0.006475399	3.69798007	0.00626166	0.13548627	-2.05051564
WT after UVB irradiation VS WT before UVB irradiation	GO_G_PROTEIN_COUPLED_PURINERGIC_NUCLEOTIDE_RECEPTOR_SIGNALING_PATHWAY2	0.549553776	0.049047483	2.375274004	0.04451165	0.4987535	-3.77947649
homo after UVB irradiation VS homo before UVB irradiation	GO_G_PROTEIN_COUPLED_PURINERGIC_RECEPTOR_SIGNALING_PATHWAY	0.600614438	-0.04069047	3.834499952	0.00462178	0.04425934	-1.96817755
hetero after UVB irradiation VS hetero before UVB irradiation	GO_G_PROTEIN_COUPLED_PURINERGIC_RECEPTOR_SIGNALING_PATHWAY1	0.575892612	-0.00503382	3.840843238	0.00511809	0.12822163	-1.85596407
WT after UVB irradiation VS WT before UVB irradiation	GO_G_PROTEIN_COUPLED_PURINERGIC_RECEPTOR_SIGNALING_PATHWAY2	0.345420187	0.003036858	1.844789396	0.10184304	0.60239077	-4.51468421
homo after UVB irradiation VS homo before UVB irradiation	GO_G_PROTEIN_COUPLED_RECEPTOR_ACTIVITY	0.232234654	0.045446329	1.910081321	0.09107099	0.23678027	-4.89704447
hetero after UVB irradiation VS hetero before UVB irradiation	GO_G_PROTEIN_COUPLED_RECEPTOR_ACTIVITY1	0.118791581	0.061497861	0.942462022	0.37404274	0.6871463	-5.7760938
WT after UVB irradiation VS WT before UVB irradiation	GO_G_PROTEIN_COUPLED_RECEPTOR_ACTIVITY2	0.097851205	0.05159261	0.536238118	0.60620688	0.89704906	-5.84713136
homo after UVB irradiation VS homo before UVB irradiation	GO_G_PROTEIN_COUPLED_RECEPTOR_BINDING	0.124544779	-0.00362942	0.872064583	0.40760675	0.59454906	-6.15180729
hetero after UVB irradiation VS hetero before UVB irradiation	GO_G_PROTEIN_COUPLED_RECEPTOR_BINDING1	0.097104169	0.013834027	0.681227796	0.51530998	0.78382785	-5.98724116
WT after UVB irradiation VS WT before UVB irradiation	GO_G_PROTEIN_COUPLED_RECEPTOR_BINDING2	0.023126618	0.004199677	0.145869233	0.88759048	0.97109821	-5.98485502
homo after UVB irradiation VS homo before UVB irradiation	GO_G_PROTEIN_COUPLED_RECEPTOR_COMPLEX	0.477945738	-0.04373007	2.372317647	0.04389059	0.15200503	-4.20553284
hetero after UVB irradiation VS hetero before UVB irradiation	GO_G_PROTEIN_COUPLED_RECEPTOR_COMPLEX1	-0.08967891	0.058525068	-0.44547843	0.66799899	0.86396644	-6.12525054
WT after UVB irradiation VS WT before UVB irradiation	GO_G_PROTEIN_COUPLED_RECEPTOR_COMPLEX2	-0.12174132	-0.05584508	-0.40426043	0.69648822	0.92259918	-5.91074529
homo after UVB irradiation VS homo before UVB irradiation	GO_G_PROTEIN_COUPLED_RECEPTOR_DIMERIC_COMPLEX	0.477211553	-0.04683285	2.487599427	0.03655008	0.13689581	-4.0281375
hetero after UVB irradiation VS hetero before UVB irradiation	GO_G_PROTEIN_COUPLED_RECEPTOR_DIMERIC_COMPLEX1	-0.07728315	0.059751245	-0.33064565	0.74956143	0.90157552	-6.17261983
WT after UVB irradiation VS WT before UVB irradiation	GO_G_PROTEIN_COUPLED_RECEPTOR_DIMERIC_COMPLEX2	-0.20033624	-0.07306982	-0.64365416	0.53759825	0.87025652	-5.7832018
homo after UVB irradiation VS homo before UVB irradiation	GO_G_PROTEIN_COUPLED_RECEPTOR_INTERNALIZATION	0.668111419	0.007459217	4.688736783	0.0014028	0.02523054	-0.76502254
hetero after UVB irradiation VS hetero before UVB irradiation	GO_G_PROTEIN_COUPLED_RECEPTOR_INTERNALIZATION1	0.571750343	0.074679651	3.880050792	0.004845	0.12780541	-1.80311482
WT after UVB irradiation VS WT before UVB irradiation	GO_G_PROTEIN_COUPLED_RECEPTOR_INTERNALIZATION2	0.512753937	0.035059283	2.719558761	0.02597553	0.43662303	-3.28995442
homo after UVB irradiation VS homo before UVB irradiation	GO_G_PROTEIN_COUPLED_RECEPTOR_KINASE_ACTIVITY	0.122580369	-0.00693105	0.57241008	0.58216105	0.73691462	-6.37454941
hetero after UVB irradiation VS hetero before UVB irradiation	GO_G_PROTEIN_COUPLED_RECEPTOR_KINASE_ACTIVITY1	0.786070482	0.058303332	4.258797811	0.00288588	0.11333595	-1.30526731
WT after UVB irradiation VS WT before UVB irradiation	GO_G_PROTEIN_COUPLED_RECEPTOR_KINASE_ACTIVITY2	0.521325848	-0.07849356	1.940908329	0.08778407	0.57920555	-4.38507516
homo after UVB irradiation VS homo before UVB irradiation	GO_G_PROTEIN_COUPLED_RECEPTOR_SIGNALING_PATHWAY	0.255479542	0.021202644	2.071585733	0.07067436	0.2039385	-4.66023813
hetero after UVB irradiation VS hetero before UVB irradiation	GO_G_PROTEIN_COUPLED_RECEPTOR_SIGNALING_PATHWAY_COUPLED_TO_CYCLIC_NUCLEOTIDE_SECOND_MESSENGER	0.329363463	-0.0057376	2.670173268	0.02736984	0.11721876	-3.74535777
WT after UVB irradiation VS WT before UVB irradiation	GO_G_PROTEIN_COUPLED_RECEPTOR_SIGNALING_PATHWAY_COUPLED_TO_CYCLIC_NUCLEOTIDE_SECOND_MESSENGER1	0.141720755	0.02583751	0.96739644	0.36219165	0.67684951	-5.75305715
homo after UVB irradiation VS homo before UVB irradiation	GO_G_PROTEIN_COUPLED_RECEPTOR_SIGNALING_PATHWAY_COUPLED_TO_CYCLIC_NUCLEOTIDE_SECOND_MESSENGER2	0.115971688	0.027268925	0.616221913	0.55467116	0.87730786	-5.80053422
hetero after UVB irradiation VS hetero before UVB irradiation	GO_G_PROTEIN_COUPLED_RECEPTOR_SIGNALING_PATHWAY_INVOLVED_IN_HEART_PROCESS	0.551242923	0.026119052	3.315456664	0.01002619	0.06691833	-2.74739142
WT after UVB irradiation VS WT before UVB irradiation	GO_G_PROTEIN_COUPLED_RECEPTOR_SIGNALING_PATHWAY_INVOLVED_IN_HEART_PROCESS1	0.189843395	0.007244508	0.787890895	0.45388422	0.74508172	-5.90795697
homo after UVB irradiation VS homo before UVB irradiation	GO_G_PROTEIN_COUPLED_RECEPTOR_SIGNALING_PATHWAY_INVOLVED_IN_HEART_PROCESS2	0.087189453	-0.01104005	0.425958939	0.68123398	0.91716791	-5.90145011
hetero after UVB irradiation VS hetero before UVB irradiation	GO_G_PROTEIN_COUPLED_RECEPTOR_SIGNALING_PATHWAY1	0.143804864	0.046429436	1.091490031	0.30741107	0.62806003	-5.63164316
WT after UVB irradiation VS WT before UVB irradiation	GO_G_PROTEIN_COUPLED_RECEPTOR_SIGNALING_PATHWAY2	0.088291086	0.03037257	0.492883829	0.63518114	0.90549535	-5.86988163
homo after UVB irradiation VS homo before UVB irradiation	GO_G_PROTEIN_COUPLED_SEROTONIN_RECEPTOR_BINDING	0.170970832	-0.02080613	0.871062037	0.40812344	0.5950376	-6.15268463
hetero after UVB irradiation VS hetero before UVB irradiation	GO_G_PROTEIN_COUPLED_SEROTONIN_RECEPTOR_BINDING1	0.19120862	-0.01750534	0.736235018	0.48299831	0.76250381	-5.9475987
WT after UVB irradiation VS WT before UVB irradiation	GO_G_PROTEIN_COUPLED_SEROTONIN_RECEPTOR_BINDING2	0.44577567	3.75E-05	1.969905895	0.08392111	0.57149232	-4.34556245
homo after UVB irradiation VS homo before UVB irradiation	GO_G_PROTEIN_GAMMA_SUBUNIT_BINDING	0.605763733	-0.06274805	4.077025549	0.00325924	0.03754616	-1.61564436
hetero after UVB irradiation VS hetero before UVB irradiation	GO_G_PROTEIN_GAMMA_SUBUNIT_BINDING1	1.142664903	0.008677118	8.953516063	2.17E-05	0.07788488	3.01769277
WT after UVB irradiation VS WT before UVB irradiation	GO_G_PROTEIN_GAMMA_SUBUNIT_BINDING2	0.222085732	-0.15310558	0.743837618	0.47800042	0.84935006	-5.71423979
homo after UVB irradiation VS homo before UVB irradiation	GO_G_QUADRUPLEX_DNA_BINDING	-0.64371965	-0.04491606	-2.59124798	0.03101082	0.12503461	-3.8677957
hetero after UVB irradiation VS hetero before UVB irradiation	GO_G_QUADRUPLEX_DNA_BINDING1	-0.09981172	-0.10037321	-0.3666007	0.7235979	0.89156735	-6.15924004
WT after UVB irradiation VS WT before UVB irradiation	GO_G_QUADRUPLEX_DNA_BINDING2	-0.36752479	-0.05756401	-1.45732266	0.18268644	0.68598062	-5.00842453
homo after UVB irradiation VS homo before UVB irradiation	GO_G_QUADRUPLEX_DNA_UNWINDING	-0.86196179	-0.08082224	-4.00606759	0.0036068	0.0392229	-1.7179391
hetero after UVB irradiation VS hetero before UVB irradiation	GO_G_QUADRUPLEX_DNA_UNWINDING1	-0.09513314	-0.07355044	-0.26800626	0.79559975	0.92055751	-6.19271532
WT after UVB irradiation VS WT before UVB irradiation	GO_G_QUADRUPLEX_DNA_UNWINDING2	-0.46542514	-0.03848558	-1.47116513	0.17900726	0.68213098	-4.99178015
homo after UVB irradiation VS homo before UVB irradiation	GO_G_QUADRUPLEX_RNA_BINDING	-0.48264318	-0.08447895	-1.69812319	0.12642064	0.29063551	-5.19631816
hetero after UVB irradiation VS hetero before UVB irradiation	GO_G_QUADRUPLEX_RNA_BINDING1	-0.17282911	0.10009922	-0.4420411	0.67037999	0.86556131	-6.12686295

WT after UVB irradiation VS WT before UVB irradiation	GO_G_QUADRUPLEX_RNA_BINDING2	-0.21421049	-0.03288123	-0.64661358	0.53577537	0.86990942	-5.78129145
homo after UVB irradiation VS homo before UVB irradiation	GO_G_RICH_STRAND_TELOMERIC_DNA_BINDING	-0.67474733	-0.0842617	-3.38926001	0.00896203	0.06300973	-2.63483068
hetero after UVB irradiation VS hetero before UVB irradiation	GO_G_RICH_STRAND_TELOMERIC_DNA_BINDING1	-0.0738295	-0.06249299	-0.27993321	0.78676102	0.91698304	-6.18920604
WT after UVB irradiation VS WT before UVB irradiation	GO_G_RICH_STRAND_TELOMERIC_DNA_BINDING2	0.005890752	0.083052364	0.016446573	0.98727613	0.99740162	-5.99592394
homo after UVB irradiation VS homo before UVB irradiation	GO_G0_TO_G1_TRANSITION	-0.37059615	-0.04957204	-2.01608361	0.07712959	0.21360102	-4.74233724
hetero after UVB irradiation VS hetero before UVB irradiation	GO_G0_TO_G1_TRANSITION1	-0.1913982	-0.04802858	-1.27366671	0.23918537	0.56769689	-5.43477605
WT after UVB irradiation VS WT before UVB irradiation	GO_G0_TO_G1_TRANSITION2	-0.19190601	-0.04813791	-0.92598672	0.38122147	0.8044018	-5.56737156
homo after UVB irradiation VS homo before UVB irradiation	GO_G1_TO_G0_TRANSITION	-0.34304516	0.012089755	-1.540463	0.16052937	0.33663946	-5.40803233
hetero after UVB irradiation VS hetero before UVB irradiation	GO_G1_TO_G0_TRANSITION1	-0.3556382	-0.01575795	-1.66014012	0.13617575	0.44903003	-4.95843064
WT after UVB irradiation VS WT before UVB irradiation	GO_G1_TO_G0_TRANSITION2	-0.0836997	0.038722384	-0.32518514	0.75328385	0.93734883	-5.94065315
homo after UVB irradiation VS homo before UVB irradiation	GO_G2_DNA_DAMAGE_CHECKPOINT	-0.32610001	-0.00063765	-1.9319189	0.08801485	0.23190623	-4.86541581
hetero after UVB irradiation VS hetero before UVB irradiation	GO_G2_DNA_DAMAGE_CHECKPOINT1	0.071288618	-0.02048225	0.303099381	0.76968828	0.91003052	-6.1819628
WT after UVB irradiation VS WT before UVB irradiation	GO_G2_DNA_DAMAGE_CHECKPOINT2	0.138712156	-0.0220082	0.676680441	0.51746776	0.86168548	-5.76144007
homo after UVB irradiation VS homo before UVB irradiation	GO_GABA_ERGIC_SYNAPSE	-0.10596138	0.024285524	-0.67329586	0.51900068	0.68874937	-6.30872614
hetero after UVB irradiation VS hetero before UVB irradiation	GO_GABA_ERGIC_SYNAPSE1	-0.27649039	0.00415443	-1.86679875	0.09958616	0.39223254	-4.6796531
WT after UVB irradiation VS WT before UVB irradiation	GO_GABA_ERGIC_SYNAPSE2	-0.20250488	-0.04351517	-1.27166909	0.23879615	0.7291361	-5.22264115
homo after UVB irradiation VS homo before UVB irradiation	GO_GABA_GATED_CHLORIDE_ION_CHANNEL_ACTIVITY	-0.24706625	0.144750862	-0.97824905	0.35550521	0.54719217	-6.05425314
hetero after UVB irradiation VS hetero before UVB irradiation	GO_GABA_GATED_CHLORIDE_ION_CHANNEL_ACTIVITY1	-0.75938697	-0.02302964	-3.41529504	0.00941067	0.15459076	-2.44407673
WT after UVB irradiation VS WT before UVB irradiation	GO_GABA_GATED_CHLORIDE_ION_CHANNEL_ACTIVITY2	-0.53602669	0.082799983	-2.20324485	0.05829849	0.52586237	-4.02217902
homo after UVB irradiation VS homo before UVB irradiation	GO_GABA_RECEPTOR_ACTIVITY	-0.29304572	0.130996605	-1.52185526	0.16506059	0.34321532	-5.43229993
hetero after UVB irradiation VS hetero before UVB irradiation	GO_GABA_RECEPTOR_ACTIVITY1	-0.83062715	0.003978126	-5.87127578	0.00040105	0.07788488	0.543448014
WT after UVB irradiation VS WT before UVB irradiation	GO_GABA_RECEPTOR_ACTIVITY2	-0.71177653	0.039948803	-3.94587529	0.00415909	0.26635361	-1.6193914
homo after UVB irradiation VS homo before UVB irradiation	GO_GABA_RECEPTOR_BINDING	-0.13645441	-0.010374	-0.6405381	0.53904133	0.70494797	-6.33115906
hetero after UVB irradiation VS hetero before UVB irradiation	GO_GABA_RECEPTOR_BINDING1	-0.4229052	-0.02774386	-1.94867945	0.08785024	0.37433387	-4.56589285
WT after UVB irradiation VS WT before UVB irradiation	GO_GABA_RECEPTOR_BINDING2	-0.21241172	-0.09291888	-1.30881229	0.22651842	0.71906685	-5.18120945
homo after UVB irradiation VS homo before UVB irradiation	GO_GABA_RECEPTOR_COMPLEX	-0.24775164	0.152127155	-1.27386228	0.2371057	0.42926866	-5.73871524
hetero after UVB irradiation VS hetero before UVB irradiation	GO_GABA_RECEPTOR_COMPLEX1	-0.80376425	0.022669547	-5.10639229	0.0009771	0.08582157	-0.27880043
WT after UVB irradiation VS WT before UVB irradiation	GO_GABA_RECEPTOR_COMPLEX2	-0.67355801	0.050293466	-3.5655564	0.00719683	0.31015346	-2.11645044
homo after UVB irradiation VS homo before UVB irradiation	GO_GABAERGIC_NEURON_DIFFERENTIATION	0.275902975	-0.00239228	1.29960054	0.22855036	0.41967638	-5.70851147
hetero after UVB irradiation VS hetero before UVB irradiation	GO_GABAERGIC_NEURON_DIFFERENTIATION1	0.309170663	0.114931532	2.256287697	0.05463819	0.30961641	-4.12691749
WT after UVB irradiation VS WT before UVB irradiation	GO_GABAERGIC_NEURON_DIFFERENTIATION2	0.460978175	0.102578055	3.102896743	0.01438567	0.36090271	-2.74930476
homo after UVB irradiation VS homo before UVB irradiation	GO_GALACTOLIPID_BIOSYNTHETIC_PROCESS	0.183255771	-0.0733072	0.656137148	0.52944083	0.69702546	-6.32060214
hetero after UVB irradiation VS hetero before UVB irradiation	GO_GALACTOLIPID_BIOSYNTHETIC_PROCESS1	0.053957056	0.017202025	0.169805628	0.86945592	0.95080444	-6.21587694
WT after UVB irradiation VS WT before UVB irradiation	GO_GALACTOLIPID_BIOSYNTHETIC_PROCESS2	0.184916827	-0.02564916	0.574192151	0.58143277	0.88889863	-5.82575919
homo after UVB irradiation VS homo before UVB irradiation	GO_GALACTOLIPID_METABOLIC_PROCESS	0.326866805	-0.00841887	1.564940663	0.1547387	0.32926451	-5.37586571
hetero after UVB irradiation VS hetero before UVB irradiation	GO_GALACTOLIPID_METABOLIC_PROCESS1	0.120876802	-0.00869288	0.506533411	0.62639472	0.84604856	-6.09464986
WT after UVB irradiation VS WT before UVB irradiation	GO_GALACTOLIPID_METABOLIC_PROCESS2	0.304158896	0.004848058	1.205266329	0.26213793	0.74156847	-5.29476998
homo after UVB irradiation VS homo before UVB irradiation	GO_GALACTOSE_CATABOLIC_PROCESS	0.510228066	0.013600951	3.482528092	0.00778458	0.05861189	-2.49333354
hetero after UVB irradiation VS hetero before UVB irradiation	GO_GALACTOSE_CATABOLIC_PROCESS1	0.454829762	0.018128532	2.60372262	0.031928	0.24829472	-3.61923404
WT after UVB irradiation VS WT before UVB irradiation	GO_GALACTOSE_CATABOLIC_PROCESS2	0.271464745	-0.02516227	1.308681376	0.22656073	0.71906685	-5.1813568
homo after UVB irradiation VS homo before UVB irradiation	GO_GALACTOSE_METABOLIC_PROCESS	0.544127279	0.013063815	4.024231548	0.00351421	0.0388749	-1.69168519
hetero after UVB irradiation VS hetero before UVB irradiation	GO_GALACTOSE_METABOLIC_PROCESS1	0.45741827	0.012983176	2.479125376	0.03869714	0.26921969	-3.801964
WT after UVB irradiation VS WT before UVB irradiation	GO_GALACTOSE_METABOLIC_PROCESS2	0.312063137	-0.0475617	1.354050691	0.21229427	0.70980553	-5.12974843
homo after UVB irradiation VS homo before UVB irradiation	GO_GALACTOSIDASE_ACTIVITY	0.321094004	-0.03538723	2.263656011	0.05215237	0.16895993	-4.37143386
hetero after UVB irradiation VS hetero before UVB irradiation	GO_GALACTOSIDASE_ACTIVITY1	0.675399853	-0.01899473	2.620875822	0.03109574	0.24642792	-3.59405995
WT after UVB irradiation VS WT before UVB irradiation	GO_GALACTOSIDASE_ACTIVITY2	0.058594108	-0.09637465	0.188932842	0.85479491	0.9663792	-5.97727576
homo after UVB irradiation VS homo before UVB irradiation	GO_GALACTOSIDE_BINDING	0.699107603	-0.00376678	4.321940869	0.00231031	0.03212973	-1.26822385
hetero after UVB irradiation VS hetero before UVB irradiation	GO_GALACTOSIDE_BINDING1	0.873661458	-0.00780432	3.517411023	0.00811268	0.14545647	-2.30065942
WT after UVB irradiation VS WT before UVB irradiation	GO_GALACTOSIDE_BINDING2	0.451420163	-0.05928974	1.662225349	0.13458556	0.64200725	-4.75390286
homo after UVB irradiation VS homo before UVB irradiation	GO_GALACTOSYLTRANSFERASE_ACTIVITY	0.439449165	0.017118425	2.916631534	0.01857273	0.09355293	-3.36250121
hetero after UVB irradiation VS hetero before UVB irradiation	GO_GALACTOSYLTRANSFERASE_ACTIVITY1	0.318694797	0.008574503	1.384349474	0.20431712	0.52911607	-5.30566077
WT after UVB irradiation VS WT before UVB irradiation	GO_GALACTOSYLTRANSFERASE_ACTIVITY2	0.401622722	-0.03913578	1.465232344	0.18057602	0.68278032	-4.9989244
homo after UVB irradiation VS homo before UVB irradiation	GO_GAMETE_GENERATION	-0.06596799	0.007969471	-0.53872512	0.60417237	0.75295766	-6.39434168
hetero after UVB irradiation VS hetero before UVB irradiation	GO_GAMETE_GENERATION1	-0.03786993	0.007135317	-0.27749838	0.78856277	0.91738598	-6.1899346
WT after UVB irradiation VS WT before UVB irradiation	GO_GAMETE_GENERATION2	-0.08325756	0.011715757	-0.59129741	0.57045449	0.883893	-5.81568905
homo after UVB irradiation VS homo before UVB irradiation	GO_GAMMA_AMINOBUTYRIC_ACID_METABOLIC_PROCESS	-0.33755078	0.054713086	-1.66732034	0.1325122	0.29922696	-5.23849045
hetero after UVB irradiation VS hetero before UVB irradiation	GO_GAMMA_AMINOBUTYRIC_ACID_METABOLIC_PROCESS1	-0.23145354	0.009817082	-0.87002795	0.41009064	0.714323	-5.84028669
WT after UVB irradiation VS WT before UVB irradiation	GO_GAMMA_AMINOBUTYRIC_ACID_METABOLIC_PROCESS2	-0.16658481	0.029687898	-0.97269761	0.35885932	0.79181584	-5.52554626
homo after UVB irradiation VS homo before UVB irradiation	GO_GAMMA_AMINOBUTYRIC_ACID_SECRETION	-0.16481971	0.08902883	-0.87639854	0.40537845	0.59249239	-6.14800497
hetero after UVB irradiation VS hetero before UVB irradiation	GO_GAMMA_AMINOBUTYRIC_ACID_SECRETION1	0.160426733	0.008327122	0.795146407	0.44989156	0.74207367	-5.90220579
WT after UVB irradiation VS WT before UVB irradiation	GO_GAMMA_AMINOBUTYRIC_ACID_SECRETION2	-0.06749476	-0.05401185	-0.27994203	0.78654013	0.94673513	-5.95492686
homo after UVB irradiation VS homo before UVB irradiation	GO_GAMMA_AMINOBUTYRIC_ACID_SIGNALING_PATHWAY	-0.07703408	0.07489399	-0.42914616	0.67870151	0.80595462	-6.45090557
hetero after UVB irradiation VS hetero before UVB irradiation	GO_GAMMA_AMINOBUTYRIC_ACID_SIGNALING_PATHWAY1	-0.62820014	0.002534779	-4.11129826	0.00352224	0.11758852	-1.49636495
WT after UVB irradiation VS WT before UVB irradiation	GO_GAMMA_AMINOBUTYRIC_ACID_SIGNALING_PATHWAY2	-0.30604844	0.013596516	-1.31853059	0.22339593	0.71863455	-5.17024544
homo after UVB irradiation VS homo before UVB irradiation	GO_GAMMA_AMINOBUTYRIC_ACID_TRANSMEMBRANE_TRANSPORTER_ACTIVITY	-0.06726766	0.093326252	-0.271768	0.79241542	0.88186131	-6.51050423
hetero after UVB irradiation VS hetero before UVB irradiation	GO_GAMMA_AMINOBUTYRIC_ACID_TRANSMEMBRANE_TRANSPORTER_ACTIVITY1	-0.27532651	-0.00394532	-1.11444027	0.29803402	0.62160331	-5.60800983
WT after UVB irradiation VS WT before UVB irradiation	GO_GAMMA_AMINOBUTYRIC_ACID_TRANSMEMBRANE_TRANSPORTER_ACTIVITY2	0.023697631	-0.03233212	0.122166832	0.90574438	0.97708947	-5.98819931
homo after UVB irradiation VS homo before UVB irradiation	GO_GAMMA_AMINOBUTYRIC_ACID_TRANSPORT	-0.06499407	0.097623347	-0.34925224	0.73556557	0.84444499	-6.48440828
hetero after UVB irradiation VS hetero before UVB irradiation	GO_GAMMA_AMINOBUTYRIC_ACID_TRANSPORT1	0.073485391	0.03739595	0.368067514	0.72254648	0.8911687	-6.15866588
WT after UVB irradiation VS WT before UVB irradiation	GO_GAMMA_AMINOBUTYRIC_ACID_TRANSPORT2	-0.08966908	-0.04698791	-0.48388152	0.64128451	0.90671989	-5.87438052
homo after UVB irradiation VS homo before UVB irradiation	GO_GAMMA_CATENIN_BINDING	0.200500679	-0.02335458	1.095481921	0.30396714	0.49823871	-5.9364237
hetero after UVB irradiation VS hetero before UVB irradiation	GO_GAMMA_CATENIN_BINDING1	0.087116974	-0.02290166	0.422623708	0.68390432	0.87298375	-6.13574825
WT after UVB irradiation VS WT before UVB irradiation	GO_GAMMA_CATENIN_BINDING2	-0.09798224	0.087005474	-0.36735398	0.72277048	0.93154998	-5.92548319
homo after UVB irradiation VS homo before UVB irradiation	GO_GAMMA_DELTA_T_CELL_ACTIVATION	0.370928067	-0.00081855	2.349679728	0.04549685	0.15527136	-4.24021441
hetero after UVB irradiation VS hetero before UVB irradiation	GO_GAMMA_DELTA_T_CELL_ACTIVATION1	0.227256339	-0.01041636	1.188300682	0.26940877	0.59632699	-5.52960694
WT after UVB irradiation VS WT before UVB irradiation	GO_GAMMA_DELTA_T_CELL_ACTIVATION2	0.139914365	-0.00669843	0.709470218	0.49794901	0.85541458	-5.73888241
homo after UVB irradiation VS homo before UVB irradiation	GO_GAMMA_DELTA_T_CELL_DIFFERENTIATION	0.45787692	-0.02102805	3.060961956	0.01483187	0.08275003	-3.13885107
hetero after UVB irradiation VS hetero before UVB irradiation	GO_GAMMA_DELTA_T_CELL_DIFFERENTIATION1	0.428643804	0.018640659	2.348369906	0.04737569	0.29034796	-3.99307581
WT after UVB irradiation VS WT before UVB irradiation	GO_GAMMA_DELTA_T_CELL_DIFFERENTIATION2	0.286175151	0.019714224	1.678549614	0.13130079	0.63700751	-4.7329398
homo after UVB irradiation VS homo before UVB irradiation	GO_GAMMA_SECRETASE_COMPLEX	0.953997729	-0.02763626	5.284804552	0.00065015	0.01817776	0.007471615
hetero after UVB irradiation VS hetero before UVB irradiation	GO_GAMMA_SECRETASE_COMPLEX1	1.074284871	0.045229969	7.268309888	9.50E-05	0.07788488	1.814005806
WT after UVB irradiation VS WT before UVB irradiation	GO_GAMMA_SECRETASE_COMPLEX2	0.888690596	-0.0740651	3.424585913	0.00886299	0.31908946	-2.30632853
homo after UVB irradiation VS homo before UVB irradiation	GO_GAMMA_TUBULIN_BINDING	-0.06619903	-0.02283585	-0.39261371	0.70446142	0.82368569	-6.46704718
hetero after UVB irradiation VS hetero before UVB irradiation	GO_GAMMA_TUBULIN_BINDING1	0.134031829	-0.02791238	0.797053568	0.44884604	0.74180859	-5.90068666
WT after UVB irradiation VS WT before UVB irradiation	GO_GAMMA_TUBULIN_BINDING2	0.029467412	-0.04363745	0.163275071	0.87430325	0.96914919	-5.98202467
homo after UVB irradiation VS homo before UVB irradiation	GO_GAMMA_TUBULIN_COMPLEX	-0.183047	-0.02652545	-0.83972641	0.42450662	0.60943249	-6.17968222
hetero after UVB irradiation VS hetero before UVB irradiation	GO_GAMMA_TUBULIN_COMPLEX1	0.188964204	-0.02009675	0.812207526	0.44059745	0.73724211	-5.88850703
WT after UVB irradiation VS WT before UVB irradiation	GO_GAMMA_TUBULIN_COMPLEX2	0.059999707	-0.04336214	0.265260696	0.79743648	0.95180107	-5.95910957
homo after UVB irradiation VS homo before UVB irradiation	GO_GAMMA_TUBULIN_LARGE_COMPLEX	-0.36164062	0.010628735	-2.30224277	0.04905515	0.1626026	-4.31269274
hetero after UVB irradiation VS hetero before UVB irradiation	GO_GAMMA_TUBULIN_LARGE_COMPLEX1	0.278171406	-0.02699453	1.106244115	0.30135623	0.6237902	-5.61649055
WT after UVB irradiation VS WT before UVB irradiation	GO_GAMMA_TUBULIN_LARGE_COMPLEX2	0.060878169	0.019311482	0.182503841	0.85967369	0.96750403	-5.9785301
homo after UVB irradiation VS homo before UVB irradiation	GO_GANGLION_DEVELOPMENT	-0.03126835	0.001702084	-0.21831466	0.83243812	0.90645583	-6.52477526
hetero after UVB irradiation VS hetero before UVB irradiation	GO_GANGLION_DEVELOPMENT1	-0.35112995	-0.01996237	-2.47009812	0.03924096	0.27101991	-3.81518637
WT after UVB irradiation VS WT before UVB irradiation	GO_GANGLION_DEVELOPMENT2	-0.18931295	0.040468323	-1.24588922	0.2476433	0.73564768	-5.25094592
homo after UVB irradiation VS homo before UVB irradiation	GO_GANGLIOSIDE_BIOSYNTHETIC_PROCESS	0.392296921	0.009011446	1.814687796	0.10564239	0.25921478	-5.03356699
hetero after UVB irradiation VS hetero before UVB irradiation	GO_GANGLIOSIDE_BIOSYNTHETIC_PROCESS1	0.214987385	0.033919785	1.350211848	0.21457101	0.54178812	-5.34618466
WT after UVB irradiation VS WT before UVB irradiation	GO_GANGLIOSIDE_BIOSYNTHETIC_PROCESS2	0.047607411	-0.00500022	0.262187089	0.79972375	0.95240527	-5.95995726
homo after UVB irradiation VS homo before UVB irradiation	GO_GANGLIOSIDE_CATABOLIC_PROCESS	0.318601689	-0.05812758	0.97035078	0.35920201	0.55078388	-6.06181841
hetero after UVB irradiation VS hetero before UVB irradiation	GO_GANGLIOSIDE_CATABOLIC_PROCESS1	0.662583579	-0.07654642	3.28489363	0.01139679	0.16699924	-2.62909488
WT after UVB irradiation VS WT before UVB irradiation	GO_GANGLIOSIDE_CATABOLIC_PROCESS2	0.348508559	-0.03772543	1.173120202	0.27409762	0.74597065	-5.32875314
homo after UVB irradiation VS homo before UVB irradiation	GO_GANGLIOSIDE_METABOLIC_PROCESS	0.440233061	-0.01874653	2.052594953	0.07282188	0.20697852	-4.68840733
hetero after UVB irradiation VS hetero before UVB irradiation	GO_GANGLIOSIDE_METABOLIC_PROCESS1	0.464196281	0.014394115	3.291403204	0.01128779	0.16606447	-2.61981198
WT after UVB irradiation VS WT before UVB irradiation	GO_GANGLIOSIDE_METABOLIC_PROCESS2	0.237824215	0.028879456	1.358442879	0.21095502	0.7088031	-5.1246954
homo after UVB irradiation VS homo before UVB irradiation	GO_GAP_JUNCTION	0.085753362	-0.019121	0.660038397	0.52705602	0.69525035	-6.31792616
hetero after UVB irradiation VS hetero before UVB irradiation	GO_GAP_JUNCTION_ASSEMBLY	0.259915611	-0.03934521	1.096159885	0.30368704	0.49795735	-5.93571304
WT after UVB irradiation VS WT before UVB irradiation	GO_GAP_JUNCTION_ASSEMBLY1	0.276777627	-0.01437882	1.61822739	0.14498277	0.46116703	-5.01323733
homo after UVB irradiation VS homo before UVB irradiation	GO_GAP_JUNCTION_ASSEMBLY2	0.052065127	0.056011915	0.304218621	0.76863247	0.94123239	-5.94752691
hetero after UVB irradiation VS hetero before UVB irradiation	GO_GAP_JUNCTION_CHANNEL_ACTIVITY	0.307288126	-0.02228381	1.957041857	0.08462012	0.22632153	-4.82886801
WT after UVB irradiation VS WT before UVB irradiation	GO_GAP_JUNCTION_CHANNEL_ACTIVITY_INVOLVED_IN_CELL_COMMUNICATION_BY_ELECTRICAL_COUPLING	0.018231514	0.006659639	0.084480088	0.93467006	0.96531194	-6.54694156
homo after UVB irradiation VS homo before UVB irradiation	GO_GAP_JUNCTION_CHANNEL_ACTIVITY_INVOLVED_IN_CELL_COMMUNICATION_BY_ELECTRICAL_COUPLING1	0.166044643	-0.05420733	0.823723023	0.43439948	0.73391329	-5.87912318
hetero after UVB irradiation VS hetero before UVB irradiation	GO_GAP_JUNCTION_CHANNEL_ACTIVITY_INVOLVED_IN_CELL_COMMUNICATION_BY_ELECTRICAL_COUPLING2	-0.06246269	0.048918074	-0.33775624	0.74413615	0.93566956	-5.93631822
WT after UVB irradiation VS WT before UVB irradiation	GO_GAP_JUNCTION_CHANNEL_ACTIVITY1	0.230412641	-0.02833608	1.576793926	0.15419981	0.47198468	-5.06675879
homo after UVB irradiation VS homo before UVB irradiation	GO_GAP_JUNCTION_CHANNEL_ACTIVITY2	0.057411184	0.080343817	0.288924296	0.77989795	0.94424676	-5.95225902
hetero after UVB irradiation VS hetero before UVB irradiation	GO_GAP_JUNCTION1	0.148997779	-0.02682471	0.964891406	0.3633694	0.67787867	-5.75539276
WT after UVB irradiation VS WT before UVB irradiation	GO_GAP_JUNCTION2	-0.126881	0.073766469	-0.63046514	0.54576721	0.87342878	-5.79161982
homo after UVB irradiation VS homo before UVB irradiation	GO_GARP_COMPLEX	-0.45401756	0.015237138	-1.94419419	0.08634019	0.22902532	-4.84757935
hetero after UVB irradiation VS hetero before UVB irradiation	GO_GARP_COMPLEX1	0.141650116	0.088982869	0.39793873	0.7012744	0.88146568	-6.14649355
WT after UVB irradiation VS WT before UVB irradiation	GO_GARP_COMPLEX2	0.07321021	0.086427026	0.188253358	0.85531024	0.9663792	-5.97741036
homo after UVB irradiation VS homo before UVB irradiation	GO_GAS_HOMEOSTASIS	0.076517964	-0.06716182	0.392240869	0.70472648	0.82386506	-6.46720481
hetero after UVB irradiation VS hetero before UVB irradiation	GO_GAS_HOMEOSTASIS1	0.024513377	0.043818008	0.112100983	0.91355583	0.96745438	-6.22467478
WT after UVB irradiation VS WT before UVB irradiation	GO_GAS_HOMEOSTASIS2	0.020417775	0.031615916	0.137037678	0.89434704	0.97322079	-5.98616991
homo after UVB irradiation VS homo before UVB irradiation	GO_GAS_TRANSPORT	0.127170537	-0.02418965	0.615097105	0.55491941	0.7166713	-6.34788267
hetero after UVB irradiation VS hetero before UVB irradiation	GO_GAS_TRANSPORT1	0.231596884	0.052794938	0.767098115	0.46545864	0.75236145	-5.92419031
WT after UVB irradiation VS WT before UVB irradiation	GO_GAS_TRANSPORT2	0.340710564	0.088005078	1.21695601	0.25789674	0.73923843	-5.28225837
homo after UVB irradiation VS homo before UVB irradiation	GO_GASTRIC_ACID_SECRETION	-0.1305442	-0.02944576	-0.71939679	0.49158493	0.66755488	-6.2754685
hetero after UVB irradiation VS hetero before UVB irradiation	GO_GASTRIC_ACID_SECRETION1	-0.18285349	-0.03464493	-1.16233793	0.27920381	0.60489376	-5.55756551
WT after UVB irradiation VS WT before UVB irradiation	GO_GASTRIC_ACID_SECRETION2	-0.19289232	0.085717378	-1.17690532	0.27266664	0.74503727	-5.32478437
homo after UVB irradiation VS homo before UVB irradiation	GO_GASTRIC_EMPTYING	-0.11869249	0.111852717	-0.46627782	0.65296877	0.7888547	-6.43309614
hetero after UVB irradiation VS hetero before UVB irradiation	GO_GASTRIC_EMPTYING1	-0.33238918	-0.03841493	-1.8741418	0.09847524	0.39052906	-4.66951866
WT after UVB irradiation VS WT before UVB irradiation	GO_GASTRIC_EMPTYING2	0.320177927	-0.01333675	1.065682483	0.31730775	0.77246704	-5.43761018
homo after UVB irradiation VS homo before UVB irradiation	GO_GASTRIC_MOTILITY	0.262560324	0.158992792	1.340455764	0.21550702	0.40587302	-5.65976228
hetero after UVB irradiation VS hetero before UVB irradiation	GO_GASTRIC_MOTILITY1	-0.09686884	0.044606901	-0.63502584	0.54346677	0.79809106	-6.01842969
WT after UVB irradiation VS WT before UVB irradiation	GO_GASTRIC_MOTILITY2	0.451351852	-0.0088702	2.19176021	0.059357	0.52638053	-4.0382801
homo after UVB irradiation VS homo before UVB irradiation	GO_GASTRO_INTESTINAL_SYSTEM_SMOOTH_MUSCLE_CONTRACTION	0.298436295	-0.0049634	2.223615343	0.0555709	0.17511418	-4.43216188
hetero after UVB irradiation VS hetero before UVB irradiation	GO_GASTRO_INTESTINAL_SYSTEM_SMOOTH_MUSCLE_CONTRACTION1	0.145990512	0.006185862	0.942486038	0.37403118	0.6871463	-5.77607184
WT after UVB irradiation VS WT before UVB irradiation	GO_GASTRO_INTESTINAL_SYSTEM_SMOOTH_MUSCLE_CONTRACTION2	-0.06031639	0.034514466	-0.33312293	0.74750278	0.93606982	-5.93793452
homo after UVB irradiation VS homo before UVB irradiation	GO_GASTRULATION	0.15996665	0.014948444	1.127721402	0.29087212	0.48567548	-5.90227082
hetero after UVB irradiation VS hetero before UVB irradiation	GO_GASTRULATION_WITH_MOUTH_FORMING_SECOND	-0.04445512	0.020648899	-0.17811237	0.86289061	0.92457891	-6.53347965
WT after UVB irradiation VS WT before UVB irradiation	GO_GASTRULATION_WITH_MOUTH_FORMING_SECOND1	-0.17898915	0.035313159	-0.83212205	0.42991715	0.7308714	-5.87220942
homo after UVB irradiation VS homo before UVB irradiation	GO_GASTRULATION_WITH_MOUTH_FORMING_SECOND2	-0.20613503	0.102680581	-1.08127157	0.31072293	0.76780613	-5.42228273
hetero after UVB irradiation VS hetero before UVB irradiation	GO_GASTRULATION1	0.126265219	0.016666948	0.920712232	0.38461324	0.69593538	-5.79580025
WT after UVB irradiation VS WT before UVB irradiation	GO_GASTRULATION2	0.047939272	0.026868341	0.320811814	0.75647603	0.93854182	-5.94212373
homo after UVB irradiation VS homo before UVB irradiation	GO_GATED_CHANNEL_ACTIVITY	0.116254703	0.021901919	0.816737196	0.43681312	0.61975302	-6.19895953
hetero after UVB irradiation VS hetero before UVB irradiation	GO_GATED_CHANNEL_ACTIVITY1	-0.14660686	0.01082546	-0.98772349	0.35274093	0.6691564	-5.73393151
WT after UVB irradiation VS WT before UVB irradiation	GO_GATED_CHANNEL_ACTIVITY2	-0.14287069	0.001100112	-0.77322022	0.46136454	0.84025946	-5.69237484
homo after UVB irradiation VS homo before UVB irradiation	GO_GATOR1_COMPLEX	-0.68306348	0.000667278	-4.72087344	0.00134407	0.02466963	-0.72193777
hetero after UVB irradiation VS hetero before UVB irradiation	GO_GATOR1_COMPLEX1	-0.10702272	-0.10842129	-0.3319151	0.74863868	0.90120022	-6.17217023
WT after UVB irradiation VS WT before UVB irradiation	GO_GATOR1_COMPLEX2	-0.21020923	0.006707828	-0.64500465	0.53676595	0.86992784	-5.78233102
homo after UVB irradiation VS homo before UVB irradiation	GO_GATOR2_COMPLEX	-0.29788654	-0.01325671	-1.06927461	0.31495118	0.5088389	-5.96364335
hetero after UVB irradiation VS hetero before UVB irradiation	GO_GATOR2_COMPLEX1	0.041660187	0.080012684	0.149079717	0.88524811	0.95825763	-6.21944911
WT after UVB irradiation VS WT before UVB irradiation	GO_GATOR2_COMPLEX2	-0.34259029	0.047829312	-1.09980008	0.303037	0.76462443	-5.40385435
homo after UVB irradiation VS homo before UVB irradiation	GO_GDP_BINDING	0.246866157	-0.04099285	1.702217288	0.12563072	0.28937916	-5.1906853

hetero after UVB irradiation VS hetero before UVB irradiation	GO_GDP_BINDING1	0.254582911	0.001271074	1.545155225	0.1615938	0.48268789	-5.10716381
WT after UVB irradiation VS WT before UVB irradiation	GO_GDP_BINDING2	0.086341487	0.012665319	0.583576646	0.57539512	0.88606346	-5.82026785
homo after UVB irradiation VS homo before UVB irradiation	GO_GDP_DISSOCIATION_INHIBITOR_ACTIVITY	-0.43403214	-0.01564909	-3.46211943	0.0080275	0.05953233	-2.52422012
hetero after UVB irradiation VS hetero before UVB irradiation	GO_GDP_DISSOCIATION_INHIBITOR_ACTIVITY1	-0.16950687	-0.02791731	-0.85081771	0.42005597	0.7220868	-5.85661119
WT after UVB irradiation VS WT before UVB irradiation	GO_GDP_DISSOCIATION_INHIBITOR_ACTIVITY2	-0.25930242	-0.03694916	-0.9424391	0.37323075	0.8017852	-5.55282488
homo after UVB irradiation VS homo before UVB irradiation	GO_GDP_MANNOSE_METABOLIC_PROCESS	0.707402953	-0.04525609	2.710256611	0.02569055	0.11319506	-3.68310737
hetero after UVB irradiation VS hetero before UVB irradiation	GO_GDP_MANNOSE_METABOLIC_PROCESS1	0.742966919	-0.00441439	4.429739245	0.00230005	0.10650321	-1.08836556
WT after UVB irradiation VS WT before UVB irradiation	GO_GDP_MANNOSE_METABOLIC_PROCESS2	0.92809784	-0.05901998	4.701504426	0.00148872	0.2011505	-0.70565551
homo after UVB irradiation VS homo before UVB irradiation	GO_GDP_METABOLIC_PROCESS	-0.10113029	-0.03508224	-0.57629482	0.5796514	0.7353779	-6.37219551
hetero after UVB irradiation VS hetero before UVB irradiation	GO_GDP_METABOLIC_PROCESS1	0.148454962	0.009018976	0.551720793	0.59648601	0.8307454	-6.06964495
WT after UVB irradiation VS WT before UVB irradiation	GO_GDP_METABOLIC_PROCESS2	-0.22771452	0.069683741	-1.08291102	0.31003672	0.76739547	-5.42066133
homo after UVB irradiation VS homo before UVB irradiation	GO_GEMINI_OF_COILED_BODIES	-0.75550768	-0.03722366	-4.05302341	0.00337261	0.03819619	-1.65016593
hetero after UVB irradiation VS hetero before UVB irradiation	GO_GEMINI_OF_COILED_BODIES1	-0.40412573	-0.06233195	-1.29699946	0.23143937	0.56003926	-5.40811488
WT after UVB irradiation VS WT before UVB irradiation	GO_GEMINI_OF_COILED_BODIES2	-0.15542145	-0.04416336	-0.55912759	0.59119829	0.89248172	-5.83440329
homo after UVB irradiation VS homo before UVB irradiation	GO_GENE_SILENCING	-0.42808784	0.000939665	-2.75238771	0.02403869	0.10888244	-3.61764909
hetero after UVB irradiation VS hetero before UVB irradiation	GO_GENE_SILENCING1	-0.12979728	-0.03806218	-0.72341444	0.49040966	0.76673925	-5.95707815
WT after UVB irradiation VS WT before UVB irradiation	GO_GENE_SILENCING2	-0.15823594	-0.01709426	-0.69838258	0.50449645	0.85763155	-5.74661522
homo after UVB irradiation VS homo before UVB irradiation	GO_GENERAL_TRANSCRIPTION_INITIATION_FACTOR_ACTIVITY	-0.52356618	0.024738519	-3.00123383	0.016275	0.08728108	-3.23130156
hetero after UVB irradiation VS hetero before UVB irradiation	GO_GENERAL_TRANSCRIPTION_INITIATION_FACTOR_ACTIVITY1	-0.30952817	-0.0561434	-1.69408284	0.12940854	0.4371686	-4.91358083
WT after UVB irradiation VS WT before UVB irradiation	GO_GENERAL_TRANSCRIPTION_INITIATION_FACTOR_ACTIVITY2	-0.14566921	-0.0263167	-0.55020201	0.59702651	0.89380885	-5.83942495
homo after UVB irradiation VS homo before UVB irradiation	GO_GENERAL_TRANSCRIPTION_INITIATION_FACTOR_BINDING	-0.44237452	0.008817591	-2.71667128	0.02543175	0.1125846	-3.67314244
hetero after UVB irradiation VS hetero before UVB irradiation	GO_GENERAL_TRANSCRIPTION_INITIATION_FACTOR_BINDING1	-0.36132635	-0.07856537	-1.93856041	0.08922515	0.37595306	-4.58003785
WT after UVB irradiation VS WT before UVB irradiation	GO_GENERAL_TRANSCRIPTION_INITIATION_FACTOR_BINDING2	-0.21055661	-0.04005633	-0.80684754	0.44280504	0.8332247	-5.66646727
homo after UVB irradiation VS homo before UVB irradiation	GO_GENERATION_OF_PRECURSOR_METABOLITES_AND_ENERGY	0.060194332	-0.00646916	0.34221181	0.74066673	0.84752947	-6.48704149
hetero after UVB irradiation VS hetero before UVB irradiation	GO_GENERATION_OF_PRECURSOR_METABOLITES_AND_ENERGY1	-0.11326056	-0.0128918	-0.73462277	0.4839263	0.76282518	-5.94879873
WT after UVB irradiation VS WT before UVB irradiation	GO_GENERATION_OF_PRECURSOR_METABOLITES_AND_ENERGY2	0.0958702	-0.03896604	0.51077978	0.62313595	0.90175221	-5.86070748
homo after UVB irradiation VS homo before UVB irradiation	GO_GENETIC_IMPRINTING	-0.14744964	-0.01313938	-0.82581902	0.43192251	0.61607417	-6.19139811
hetero after UVB irradiation VS hetero before UVB irradiation	GO_GENETIC_IMPRINTING1	-0.15402211	-0.01521648	-0.8371542	0.42724713	0.72827932	-5.86803923
WT after UVB irradiation VS WT before UVB irradiation	GO_GENETIC_IMPRINTING2	-0.15119596	-0.06722118	-1.04108156	0.32792044	0.77649582	-5.46146297
homo after UVB irradiation VS homo before UVB irradiation	GO_GENITALIA_DEVELOPMENT	-0.01770095	0.011318719	-0.12817626	0.90105038	0.94612213	-6.54184912
hetero after UVB irradiation VS hetero before UVB irradiation	GO_GENITALIA_DEVELOPMENT1	-0.14064667	0.01333799	-1.01459907	0.3405346	0.65956586	-5.70817643
WT after UVB irradiation VS WT before UVB irradiation	GO_GENITALIA_DEVELOPMENT2	-0.25711485	0.047653732	-1.66929702	0.13315341	0.63954295	-4.7448329
homo after UVB irradiation VS homo before UVB irradiation	GO_GENITALIA_MORPHOGENESIS	-0.08387745	0.01574667	-0.610014	0.5581242	0.7185064	-6.35115011
hetero after UVB irradiation VS hetero before UVB irradiation	GO_GENITALIA_MORPHOGENESIS1	-0.31406497	0.001903491	-1.9001728	0.0946317	0.38467916	-4.63348186
WT after UVB irradiation VS WT before UVB irradiation	GO_GENITALIA_MORPHOGENESIS2	-0.37684409	0.054559498	-2.33960572	0.04707376	0.50594864	-3.82999304
homo after UVB irradiation VS homo before UVB irradiation	GO_GERM_CELL_DEVELOPMENT	-0.22243286	0.002901557	-1.8181433	0.10507818	0.25865533	-5.02867101
hetero after UVB irradiation VS hetero before UVB irradiation	GO_GERM_CELL_DEVELOPMENT1	-0.14337737	-0.01251084	-0.98307991	0.35488326	0.67085029	-5.73832772
WT after UVB irradiation VS WT before UVB irradiation	GO_GERM_CELL_DEVELOPMENT2	-0.19803575	0.009663757	-1.36740402	0.2082452	0.70505869	-5.11435553
homo after UVB irradiation VS homo before UVB irradiation	GO_GERM_CELL_MIGRATION	-0.55382005	-0.05736842	-1.98034747	0.08158341	0.22099577	-4.79481592
hetero after UVB irradiation VS hetero before UVB irradiation	GO_GERM_CELL_MIGRATION1	-0.37404208	-0.11896027	-1.85236511	0.10180477	0.39668105	-4.69953249
WT after UVB irradiation VS WT before UVB irradiation	GO_GERM_CELL_MIGRATION2	-0.59016977	-0.13053953	-3.06686456	0.01519864	0.36889188	-2.7996409
homo after UVB irradiation VS homo before UVB irradiation	GO_GERM_CELL_NUCLEUS	-0.06347174	0.031537869	-0.36941056	0.72103707	0.83461883	-6.47658138
hetero after UVB irradiation VS hetero before UVB irradiation	GO_GERM_CELL_NUCLEUS1	-0.08323248	0.001554236	-0.3772163	0.71600289	0.88852194	-6.15503487
WT after UVB irradiation VS WT before UVB irradiation	GO_GERM_CELL_NUCLEUS2	0.142163035	0.056805343	0.932368092	0.37810733	0.80338597	-5.56175346
homo after UVB irradiation VS homo before UVB irradiation	GO_GERM_CELL_PROLIFERATION	0.127858447	-0.01714302	0.514253509	0.62043801	0.76418391	-6.40801894
hetero after UVB irradiation VS hetero before UVB irradiation	GO_GERM_CELL_PROLIFERATION1	0.613255304	0.019913977	4.105894622	0.00354826	0.1181675	-1.50343417
WT after UVB irradiation VS WT before UVB irradiation	GO_GERM_CELL_PROLIFERATION2	0.280395982	-0.10383549	0.919846362	0.3842357	0.80622298	-5.57274846
homo after UVB irradiation VS homo before UVB irradiation	GO_GERMINAL_CENTER_B_CELL_DIFFERENTIATION	0.35759746	0.025221156	1.316980935	0.22292185	0.41407206	-5.68789205
hetero after UVB irradiation VS hetero before UVB irradiation	GO_GERMINAL_CENTER_B_CELL_DIFFERENTIATION1	-0.46276258	0.046434014	-1.56260935	0.15747593	0.47646636	-5.08492449
WT after UVB irradiation VS WT before UVB irradiation	GO_GERMINAL_CENTER_B_CELL_DIFFERENTIATION2	0.039665119	-0.01791495	0.190404545	0.853679	0.9663792	-5.97698257
homo after UVB irradiation VS homo before UVB irradiation	GO_GERMINAL_CENTER_FORMATION	0.571267589	0.006632526	2.688827264	0.02657483	0.11519864	-3.71639184
hetero after UVB irradiation VS hetero before UVB irradiation	GO_GERMINAL_CENTER_FORMATION1	0.172515329	0.025992527	0.88177081	0.40408262	0.71110831	-5.83016125
WT after UVB irradiation VS WT before UVB irradiation	GO_GERMINAL_CENTER_FORMATION2	0.221795753	-0.01469759	0.973189239	0.35862928	0.79181584	-5.52509749
homo after UVB irradiation VS homo before UVB irradiation	GO_GLAND_DEVELOPMENT	0.091313292	0.010087107	0.732174943	0.48415178	0.66112677	-6.26590638
hetero after UVB irradiation VS hetero before UVB irradiation	GO_GLAND_DEVELOPMENT1	0.064148929	0.006829911	0.513909411	0.62145985	0.84379983	-6.09070396
WT after UVB irradiation VS WT before UVB irradiation	GO_GLAND_DEVELOPMENT2	0.000108533	0.024265784	0.000771139	0.99940338	0.99997598	-5.99606635
homo after UVB irradiation VS homo before UVB irradiation	GO_GLAND_MORPHOGENESIS	0.09069518	-0.02609696	0.668483524	0.52191594	0.69112648	-6.31208462
hetero after UVB irradiation VS hetero before UVB irradiation	GO_GLAND_MORPHOGENESIS1	0.035089199	0.018578826	0.245614334	0.81227817	0.92731128	-6.19889836
WT after UVB irradiation VS WT before UVB irradiation	GO_GLAND_MORPHOGENESIS2	-0.10360255	0.053891094	-0.73324114	0.48409508	0.85146732	-5.72194578
homo after UVB irradiation VS homo before UVB irradiation	GO_GLANDULAR_EPITHELIAL_CELL_DEVELOPMENT	-0.08541258	0.018171453	-0.54931089	0.59720731	0.74806793	-6.38824178
hetero after UVB irradiation VS hetero before UVB irradiation	GO_GLANDULAR_EPITHELIAL_CELL_DEVELOPMENT1	-0.02162109	-0.02678554	-0.13531318	0.8957684	0.96152498	-6.22156663
WT after UVB irradiation VS WT before UVB irradiation	GO_GLANDULAR_EPITHELIAL_CELL_DEVELOPMENT2	-0.09478229	-0.0402586	-0.55556122	0.59352331	0.8931505	-5.83641872
homo after UVB irradiation VS homo before UVB irradiation	GO_GLANDULAR_EPITHELIAL_CELL_DIFFERENTIATION	-0.25468603	0.029574744	-1.64061821	0.1380097	0.3072272	-5.27474374
hetero after UVB irradiation VS hetero before UVB irradiation	GO_GLANDULAR_EPITHELIAL_CELL_DIFFERENTIATION1	-0.24883006	0.022089465	-1.70593015	0.127121	0.43374076	-4.89783256
WT after UVB irradiation VS WT before UVB irradiation	GO_GLANDULAR_EPITHELIAL_CELL_DIFFERENTIATION2	-0.31031168	0.011947251	-1.58245997	0.15174731	0.65936135	-4.8549624
homo after UVB irradiation VS homo before UVB irradiation	GO_GLIAL_CELL_ACTIVATION	0.592694957	-0.00851724	3.773831	0.00505033	0.04637942	-2.0576117
hetero after UVB irradiation VS hetero before UVB irradiation	GO_GLIAL_CELL_ACTIVATION1	0.510898934	0.026593125	3.375749846	0.00997105	0.15702536	-2.49997075
WT after UVB irradiation VS WT before UVB irradiation	GO_GLIAL_CELL_ACTIVATION2	0.330165564	-0.02487984	1.322355252	0.22217716	0.71742564	-5.1659167
homo after UVB irradiation VS homo before UVB irradiation	GO_GLIAL_CELL_APOPTOTIC_PROCESS	0.206879604	0.011230526	1.207073697	0.26056257	0.45610204	-5.81518408
hetero after UVB irradiation VS hetero before UVB irradiation	GO_GLIAL_CELL_APOPTOTIC_PROCESS1	0.437578064	-0.01741164	2.308132162	0.05042218	0.29894137	-4.05165576
WT after UVB irradiation VS WT before UVB irradiation	GO_GLIAL_CELL_APOPTOTIC_PROCESS2	0.208961598	-0.01452122	0.951114634	0.36906728	0.79984535	-5.54507285
homo after UVB irradiation VS homo before UVB irradiation	GO_GLIAL_CELL_DERIVED_NEUROTROPHIC_FACTOR_RECEPTOR_ACTIVITY	0.160105038	-0.01377951	0.573462072	0.58148084	0.73691462	-6.37391342
hetero after UVB irradiation VS hetero before UVB irradiation	GO_GLIAL_CELL_DERIVED_NEUROTROPHIC_FACTOR_RECEPTOR_ACTIVITY1	0.138468563	0.00664401	0.604157955	0.56278181	0.80987677	-6.03816953
WT after UVB irradiation VS WT before UVB irradiation	GO_GLIAL_CELL_DERIVED_NEUROTROPHIC_FACTOR_RECEPTOR_ACTIVITY2	0.162159193	-0.01626163	0.675842323	0.51797281	0.86191073	-5.7620043
homo after UVB irradiation VS homo before UVB irradiation	GO_GLIAL_CELL_DERIVED_NEUROTROPHIC_FACTOR_RECEPTOR_SIGNALING_PATHWAY	0.099276042	-0.0783203	0.559582574	0.59049062	0.74293376	-6.38221756
hetero after UVB irradiation VS hetero before UVB irradiation	GO_GLIAL_CELL_DERIVED_NEUROTROPHIC_FACTOR_RECEPTOR_SIGNALING_PATHWAY1	0.0300698	-0.02109443	0.150435082	0.88421362	0.95796301	-6.21922961
WT after UVB irradiation VS WT before UVB irradiation	GO_GLIAL_CELL_DERIVED_NEUROTROPHIC_FACTOR_RECEPTOR_SIGNALING_PATHWAY2	0.070991903	0.014490191	0.30328364	0.76931952	0.94123239	-5.94782303
homo after UVB irradiation VS homo before UVB irradiation	GO_GLIAL_CELL_DEVELOPMENT	0.143136339	-0.0216916	1.137084001	0.28715468	0.48226101	-5.89221694
hetero after UVB irradiation VS hetero before UVB irradiation	GO_GLIAL_CELL_DEVELOPMENT1	0.040775283	0.009050644	0.24114256	0.81562154	0.92926938	-6.20006962
WT after UVB irradiation VS WT before UVB irradiation	GO_GLIAL_CELL_DEVELOPMENT2	-0.04217041	-0.00949011	-0.25793151	0.802894	0.95333317	-5.9611149
homo after UVB irradiation VS homo before UVB irradiation	GO_GLIAL_CELL_DIFFERENTIATION	0.105971589	0.00338582	0.865581754	0.41095606	0.59739994	-6.15746569
hetero after UVB irradiation VS hetero before UVB irradiation	GO_GLIAL_CELL_DIFFERENTIATION1	0.033940142	0.00694507	0.229060399	0.82467488	0.93326961	-6.20312814
WT after UVB irradiation VS WT before UVB irradiation	GO_GLIAL_CELL_DIFFERENTIATION2	-0.03637582	0.008057141	-0.23174485	0.82248526	0.95836402	-5.96782807
homo after UVB irradiation VS homo before UVB irradiation	GO_GLIAL_CELL_FATE_COMMITMENT	0.353013701	0.058178897	1.919422736	0.08975148	0.23466204	-4.88353097
hetero after UVB irradiation VS hetero before UVB irradiation	GO_GLIAL_CELL_FATE_COMMITMENT1	0.416365447	0.016532742	2.417553939	0.04256358	0.27740933	-3.89207657
WT after UVB irradiation VS WT before UVB irradiation	GO_GLIAL_CELL_FATE_COMMITMENT2	0.436182058	-0.00097702	1.938835003	0.08806672	0.58017886	-4.38789345
homo after UVB irradiation VS homo before UVB irradiation	GO_GLIAL_CELL_FATE_SPECIFICATION	0.457495963	0.092059939	1.262706383	0.24089658	0.4338362	-5.75168177
hetero after UVB irradiation VS hetero before UVB irradiation	GO_GLIAL_CELL_FATE_SPECIFICATION1	0.472936328	0.055696877	1.715070827	0.12538187	0.43239729	-4.8856498
WT after UVB irradiation VS WT before UVB irradiation	GO_GLIAL_CELL_FATE_SPECIFICATION2	0.751156549	0.130239663	2.267311694	0.05272732	0.51991848	-3.93209654
homo after UVB irradiation VS homo before UVB irradiation	GO_GLIAL_CELL_MIGRATION	0.326286393	-0.04708397	2.369038807	0.04411969	0.15244069	-4.21055957
hetero after UVB irradiation VS hetero before UVB irradiation	GO_GLIAL_CELL_MIGRATION1	0.363546307	0.00774993	1.843403378	0.10320589	0.39866653	-4.71184758
WT after UVB irradiation VS WT before UVB irradiation	GO_GLIAL_CELL_MIGRATION2	0.293051352	0.006974444	1.644623334	0.1382121	0.64683525	-4.77640298
homo after UVB irradiation VS homo before UVB irradiation	GO_GLIAL_CELL_PROJECTION	0.05758577	0.02367015	0.409520189	0.69248753	0.8158913	-6.45974837
hetero after UVB irradiation VS hetero before UVB irradiation	GO_GLIAL_CELL_PROJECTION1	-0.05441663	0.040581455	-0.40345565	0.69737553	0.88005213	-6.14414574
WT after UVB irradiation VS WT before UVB irradiation	GO_GLIAL_CELL_PROJECTION2	-0.18248822	0.01492198	-0.95074192	0.36924544	0.79984535	-5.54540703
homo after UVB irradiation VS homo before UVB irradiation	GO_GLIAL_CELL_PROLIFERATION	0.026568673	0.007650159	0.205421917	0.84217433	0.91287688	-6.52775713
hetero after UVB irradiation VS hetero before UVB irradiation	GO_GLIAL_CELL_PROLIFERATION1	0.185679102	0.001015234	1.381056903	0.20528718	0.52992939	-5.3095955
WT after UVB irradiation VS WT before UVB irradiation	GO_GLIAL_CELL_PROLIFERATION2	0.057444599	0.000525917	0.385947393	0.70947791	0.92792392	-5.91822772
homo after UVB irradiation VS homo before UVB irradiation	GO_GLIOGENESIS	0.103425248	0.001474604	0.842410293	0.42308572	0.60834804	-6.17740231
hetero after UVB irradiation VS hetero before UVB irradiation	GO_GLIOGENESIS1	0.106319692	0.00161142	0.735664721	0.48332643	0.76253233	-5.94802345
WT after UVB irradiation VS WT before UVB irradiation	GO_GLIOGENESIS2	0.011515978	0.00546149	0.076628446	0.94077863	0.98454508	-5.99296954
homo after UVB irradiation VS homo before UVB irradiation	GO_GLOBAL_GENOME_NUCLEOTIDE_EXCISION_REPAIR	-0.30071014	0.024438078	-1.78124771	0.11125132	0.26755797	-5.08074314
hetero after UVB irradiation VS hetero before UVB irradiation	GO_GLOBAL_GENOME_NUCLEOTIDE_EXCISION_REPAIR1	0.164883754	-0.00626899	0.914757853	0.38754503	0.69797552	-5.80113139
WT after UVB irradiation VS WT before UVB irradiation	GO_GLOBAL_GENOME_NUCLEOTIDE_EXCISION_REPAIR2	-0.16084041	0.031575096	-0.5538636	0.5946318	0.8931505	-5.83737391
homo after UVB irradiation VS homo before UVB irradiation	GO_GLOMERULAR_BASEMENT_MEMBRANE_DEVELOPMENT	0.249912493	-0.04347643	1.533162968	0.16229358	0.3395189	-5.41757209
hetero after UVB irradiation VS hetero before UVB irradiation	GO_GLOMERULAR_BASEMENT_MEMBRANE_DEVELOPMENT1	0.104088824	0.011944336	0.314986088	0.76097924	0.90682777	-6.17802817
WT after UVB irradiation VS WT before UVB irradiation	GO_GLOMERULAR_BASEMENT_MEMBRANE_DEVELOPMENT2	-0.07202492	0.006298653	-0.34298019	0.74034736	0.93506555	-5.93446989
homo after UVB irradiation VS homo before UVB irradiation	GO_GLOMERULAR_EPITHELIAL_CELL_DEVELOPMENT	-0.00750189	0.006762412	-0.03686952	0.97145742	0.98531737	-6.55011073
hetero after UVB irradiation VS hetero before UVB irradiation	GO_GLOMERULAR_EPITHELIAL_CELL_DEVELOPMENT1	0.007028776	0.014856805	0.031541737	0.97562443	0.9917549	-6.23094462
WT after UVB irradiation VS WT before UVB irradiation	GO_GLOMERULAR_EPITHELIAL_CELL_DEVELOPMENT2	0.069924413	-0.01489791	0.409247453	0.69296897	0.92156983	-5.90865008
homo after UVB irradiation VS homo before UVB irradiation	GO_GLOMERULAR_EPITHELIAL_CELL_DIFFERENTIATION	0.247213642	0.026491171	1.663540182	0.13327807	0.30036415	-5.24364019
hetero after UVB irradiation VS hetero before UVB irradiation	GO_GLOMERULAR_EPITHELIAL_CELL_DIFFERENTIATION1	0.096017835	0.012753109	0.505701085	0.62695286	0.84641504	-6.09509178
WT after UVB irradiation VS WT before UVB irradiation	GO_GLOMERULAR_EPITHELIAL_CELL_DIFFERENTIATION2	0.034809068	-0.00099781	0.235927195	0.81934699	0.95710469	-5.96680335
homo after UVB irradiation VS homo before UVB irradiation	GO_GLOMERULAR_EPITHELIUM_DEVELOPMENT	0.163758984	0.019885497	0.936050543	0.37558877	0.5654523	-6.0941048
hetero after UVB irradiation VS hetero before UVB irradiation	GO_GLOMERULAR_EPITHELIUM_DEVELOPMENT1	0.024017458	0.01305542	0.11882434	0.90839791	0.96574426	-6.22383428
WT after UVB irradiation VS WT before UVB irradiation	GO_GLOMERULAR_EPITHELIUM_DEVELOPMENT2	-0.03357595	-0.0095113	-0.22399086	0.82831249	0.95952649	-5.96968006
homo after UVB irradiation VS homo before UVB irradiation	GO_GLOMERULAR_MESANGIAL_CELL_DEVELOPMENT	0.699608069	-0.04672783	3.143534046	0.0130525	0.07713852	-3.01135319
hetero after UVB irradiation VS hetero before UVB irradiation	GO_GLOMERULAR_MESANGIAL_CELL_DEVELOPMENT1	0.373138851	0.06554094	1.258195387	0.24444157	0.57199173	-5.45228293
WT after UVB irradiation VS WT before UVB irradiation	GO_GLOMERULAR_MESANGIAL_CELL_DEVELOPMENT2	0.402658597	0.039931027	1.613848202	0.14476875	0.65365874	-4.81547501
homo after UVB irradiation VS homo before UVB irradiation	GO_GLOMERULAR_MESANGIAL_CELL_DIFFERENTIATION	0.419070086	-0.035554	2.576407402	0.03174858	0.12619838	-3.89079041
hetero after UVB irradiation VS hetero before UVB irradiation	GO_GLOMERULAR_MESANGIAL_CELL_DIFFERENTIATION1	0.144953853	0.082066046	0.472520931	0.64940846	0.85597778	-6.11215311
WT after UVB irradiation VS WT before UVB irradiation	GO_GLOMERULAR_MESANGIAL_CELL_DIFFERENTIATION2	0.126744262	0.009408073	0.51590516	0.61970807	0.90087565	-5.85802372
homo after UVB irradiation VS homo before UVB irradiation	GO_GLOMERULAR_MESANGIAL_CELL_PROLIFERATION	0.112962752	0.045769108	0.60404909	0.56189856	0.72185023	-6.3549528
hetero after UVB irradiation VS hetero before UVB irradiation	GO_GLOMERULAR_MESANGIAL_CELL_PROLIFERATION1	-0.45114056	-0.00747756	-2.24809874	0.05533555	0.31097412	-4.13878034
WT after UVB irradiation VS WT before UVB irradiation	GO_GLOMERULAR_MESANGIAL_CELL_PROLIFERATION2	-0.11822384	-0.03637994	-0.52825015	0.6114922	0.89914213	-5.85145718
homo after UVB irradiation VS homo before UVB irradiation	GO_GLOMERULAR_MESANGIUM_DEVELOPMENT	0.122201904	0.010466678	0.724235836	0.48876148	0.66488322	-6.27186476
hetero after UVB irradiation VS hetero before UVB irradiation	GO_GLOMERULAR_MESANGIUM_DEVELOPMENT1	-0.25864373	0.021096626	-1.15014647	0.28390262	0.60942163	-5.57054649
WT after UVB irradiation VS WT before UVB irradiation	GO_GLOMERULAR_MESANGIUM_DEVELOPMENT2	-0.10134776	-0.03915759	-0.46710339	0.65273708	0.90932135	-5.88255698
homo after UVB irradiation VS homo before UVB irradiation	GO_GLOMERULUS_DEVELOPMENT	0.141049809	-0.00931899	0.941440657	0.37297782	0.56318773	-6.08909279
hetero after UVB irradiation VS hetero before UVB irradiation	GO_GLOMERULUS_DEVELOPMENT1	0.058936326	0.009224427	0.333165998	0.74772983	0.90069399	-6.17172556
WT after UVB irradiation VS WT before UVB irradiation	GO_GLOMERULUS_DEVELOPMENT2	0.030079791	-0.02648193	0.169630704	0.8694618	0.96830456	-5.98091229
homo after UVB irradiation VS homo before UVB irradiation	GO_GLOMERULUS_MORPHOGENESIS	0.086393548	0.053253217	0.614544922	0.55526703	0.7166713	-6.34823881
hetero after UVB irradiation VS hetero before UVB irradiation	GO_GLOMERULUS_MORPHOGENESIS1	0.411179446	0.0433857	3.024873018	0.01679765	0.1902572	-3.00340071
WT after UVB irradiation VS WT before UVB irradiation	GO_GLOMERULUS_MORPHOGENESIS2	0.367325188	-0.00289309	1.954310275	0.08597793	0.57619266	-4.36683545
homo after UVB irradiation VS homo before UVB irradiation	GO_GLOMERULUS_VASCULATURE_MORPHOGENESIS	0.039614101	0.080823706	0.238254225	0.81744097	0.89680889	-6.51981066
hetero after UVB irradiation VS hetero before UVB irradiation	GO_GLOMERULUS_VASCULATURE_MORPHOGENESIS1	0.356500139	0.060466535	2.090197162	0.07064599	0.34366035	-4.36586346
WT after UVB irradiation VS WT before UVB irradiation	GO_GLOMERULUS_VASCULATURE_MORPHOGENESIS2	0.353948716	-0.02372956	1.406363665	0.19681151	0.69565208	-5.06893561
homo after UVB irradiation VS homo before UVB irradiation	GO_GLOSSOPHARYNGEAL_NERVE_DEVELOPMENT	-0.67623181	0.008301412	-4.08730852	0.00321193	0.03738045	-1.60088006
hetero after UVB irradiation VS hetero before UVB irradiation	GO_GLOSSOPHARYNGEAL_NERVE_DEVELOPMENT1	-0.65584033	-0.05726944	-3.72467509	0.00602877	0.13438331	-2.01393031
WT after UVB irradiation VS WT before UVB irradiation	GO_GLOSSOPHARYNGEAL_NERVE_DEVELOPMENT2	-0.43731512	0.001237456	-1.76649947	0.11484616	0.6200371	-4.61849641
homo after UVB irradiation VS homo before UVB irradiation	GO_GLUCOCORTICOID_BIOSYNTHETIC_PROCESS	0.244171413	-0.02761092	1.356314887	0.21061786	0.40094108	-5.64057945
hetero after UVB irradiation VS hetero before UVB irradiation	GO_GLUCOCORTICOID_BIOSYNTHETIC_PROCESS1	0.293194481	0.032210868	1.838910831	0.10391517	0.40004492	-4.71801301
WT after UVB irradiation VS WT before UVB irradiation	GO_GLUCOCORTICOID_BIOSYNTHETIC_PROCESS2	0.052148811	0.040084423	0.297505898	0.77356992	0.94274743	-5.94963321
homo after UVB irradiation VS homo before UVB irradiation	GO_GLUCOCORTICOID_METABOLIC_PROCESS	0.296966421	-0.00646042	2.053910672	0.07267108	0.20682876	-4.68645827
hetero after UVB irradiation VS hetero before UVB irradiation	GO_GLUCOCORTICOID_METABOLIC_PROCESS1	0.285371386	0.046254302	1.869782414	0.09913333	0.3913075	-4.6755369
WT after UVB irradiation VS WT before UVB irradiation	GO_GLUCOCORTICOID_METABOLIC_PROCESS2	0.062873475	0.041240035	0.371013399	0.72014624	0.93131173	-5.92408251
homo after UVB irradiation VS homo before UVB irradiation	GO_GLUCOCORTICOID_RECEPTOR_BINDING	0.207078361	-0.0184068	1.043172366	0.32619667	0.51968856	-5.99025901
hetero after UVB irradiation VS hetero before UVB irradiation	GO_GLUCOCORTICOID_RECEPTOR_BINDING1	-0.05172447	0.028309775	-0.19810864	0.84799239	0.94241286	-6.21025514
WT after UVB irradiation VS WT before UVB irradiation	GO_GLUCOCORTICOID_RECEPTOR_BINDING2	0.027548049	-0.00499009	0.104443664	0.91935792	0.98043563	-5.99031491
homo after UVB irradiation VS homo before UVB irradiation	GO_GLUCOCORTICOID_SECRETION	-0.12323287	-0.03084069	-0.68464159	0.51216726	0.6834918	-6.3007229
hetero after UVB irradiation VS hetero before UVB irradiation	GO_GLUCOCORTICOID_SECRETION1	-0.21694243	0.055604875	-1.00503066	0.34484275	0.66234164	-5.7174064
WT after UVB irradiation VS WT before UVB irradiation	GO_GLUCOCORTICOID_SECRETION2	-0.21723647	-0.04797099	-0.90185659	0.39316666	0.81282718	-5.58833656

homo after UVB irradiation VS homo before UVB irradiation	GO_GLUCOSAMINE_CONTAINING_COMPOUND_CATABOLIC_PROCESS	0.737820997	-0.0150476	4.873379681	0.00109942	0.02245522	-0.5197048
hetero after UVB irradiation VS hetero before UVB irradiation	GO_GLUCOSAMINE_CONTAINING_COMPOUND_CATABOLIC_PROCESS1	0.76547469	0.039538109	6.0537555	0.00032798	0.07788488	0.725684207
WT after UVB irradiation VS WT before UVB irradiation	GO_GLUCOSAMINE_CONTAINING_COMPOUND_CATABOLIC_PROCESS2	0.651320557	0.011319354	4.02594039	0.00371534	0.26532716	-1.51778945
homo after UVB irradiation VS homo before UVB irradiation	GO_GLUCOSAMINE_CONTAINING_COMPOUND_METABOLIC_PROCESS	0.627599972	-0.01573295	4.911913155	0.00104556	0.02188549	-0.46919066
hetero after UVB irradiation VS hetero before UVB irradiation	GO_GLUCOSAMINE_CONTAINING_COMPOUND_METABOLIC_PROCESS1	0.511994725	0.031571358	3.111366765	0.01475169	0.18019533	-2.87818905
WT after UVB irradiation VS WT before UVB irradiation	GO_GLUCOSAMINE_CONTAINING_COMPOUND_METABOLIC_PROCESS2	0.435172025	-0.00665974	2.142499055	0.06411625	0.53678963	-4.10715808
homo after UVB irradiation VS homo before UVB irradiation	GO_GLUCOSE_6_PHOSPHATE_METABOLIC_PROCESS	0.113931652	-0.00360786	0.61307556	0.55619267	0.71727128	-6.34918508
hetero after UVB irradiation VS hetero before UVB irradiation	GO_GLUCOSE_6_PHOSPHATE_METABOLIC_PROCESS1	0.344288858	0.029047264	2.577529588	0.03324305	0.25298323	-3.65767141
WT after UVB irradiation VS WT before UVB irradiation	GO_GLUCOSE_6_PHOSPHATE_METABOLIC_PROCESS2	0.427324293	0.006903439	2.022087296	0.07737863	0.56116995	-4.27403176
homo after UVB irradiation VS homo before UVB irradiation	GO_GLUCOSE_BINDING	0.873064711	-0.01122066	5.012365243	0.00091815	0.02088233	-0.33861482
hetero after UVB irradiation VS hetero before UVB irradiation	GO_GLUCOSE_BINDING1	0.836026822	0.014970165	4.55427847	0.00195491	0.10229299	-0.9334708
WT after UVB irradiation VS WT before UVB irradiation	GO_GLUCOSE_BINDING2	0.714809453	0.01702933	3.059732789	0.01536515	0.37067217	-2.80961854
homo after UVB irradiation VS homo before UVB irradiation	GO_GLUCOSE_CATABOLIC_PROCESS	0.586694292	-0.02047119	3.884779281	0.00429599	0.04234489	-1.89442737
hetero after UVB irradiation VS hetero before UVB irradiation	GO_GLUCOSE_CATABOLIC_PROCESS1	0.405832346	0.016815788	1.901389059	0.09445568	0.38417529	-4.63179397
WT after UVB irradiation VS WT before UVB irradiation	GO_GLUCOSE_CATABOLIC_PROCESS2	0.394880079	0.008836917	2.015119872	0.07822278	0.56140246	-4.28361291
homo after UVB irradiation VS homo before UVB irradiation	GO_GLUCOSE_IMPORT	0.274189947	-0.00175194	2.219181129	0.05596282	0.1758721	-4.43887194
homo after UVB irradiation VS homo before UVB irradiation	GO_GLUCOSE_IMPORT_ACROSS_PLASMA_MEMBRANE	0.887324346	0.038029985	4.936666193	0.00101247	0.02162235	-0.43686588
hetero after UVB irradiation VS hetero before UVB irradiation	GO_GLUCOSE_IMPORT_ACROSS_PLASMA_MEMBRANE1	0.511846362	0.118909343	2.138194153	0.06559587	0.33412348	-4.29720857
WT after UVB irradiation VS WT before UVB irradiation	GO_GLUCOSE_IMPORT_ACROSS_PLASMA_MEMBRANE2	0.595726684	0.090858533	2.402090518	0.04267782	0.49624823	-3.74144703
hetero after UVB irradiation VS hetero before UVB irradiation	GO_GLUCOSE_IMPORT1	0.018759674	0.021247307	0.109028514	0.91591439	0.96799239	-6.22504263
WT after UVB irradiation VS WT before UVB irradiation	GO_GLUCOSE_IMPORT2	0.016834933	-0.02030413	0.094981119	0.92663819	0.98193306	-5.99130932
homo after UVB irradiation VS homo before UVB irradiation	GO_GLUCOSE_METABOLIC_PROCESS	0.196415461	-0.01772442	1.538526581	0.16099566	0.33742551	-5.41056527
hetero after UVB irradiation VS hetero before UVB irradiation	GO_GLUCOSE_METABOLIC_PROCESS1	0.039994257	0.00090428	0.281227779	0.78580361	0.91645441	-6.18881613
WT after UVB irradiation VS WT before UVB irradiation	GO_GLUCOSE_METABOLIC_PROCESS2	0.126651395	-0.00887467	0.862428287	0.41326238	0.820663	-5.62162781
homo after UVB irradiation VS homo before UVB irradiation	GO_GLUCOSE_SODIUM_SYMPORTER_ACTIVITY	0.30217792	0.057842849	0.921110403	0.38289568	0.57282964	-6.10787543
hetero after UVB irradiation VS hetero before UVB irradiation	GO_GLUCOSE_SODIUM_SYMPORTER_ACTIVITY1	0.054983263	0.119562506	0.199826938	0.84669348	0.94158791	-6.20988625
WT after UVB irradiation VS WT before UVB irradiation	GO_GLUCOSE_SODIUM_SYMPORTER_ACTIVITY2	0.277461433	0.142816941	1.310686179	0.22591346	0.71906685	-5.1790993
homo after UVB irradiation VS homo before UVB irradiation	GO_GLUCOSIDASE_ACTIVITY	0.180367368	0.00097068	1.039473499	0.32781505	0.52112106	-5.9939901
hetero after UVB irradiation VS hetero before UVB irradiation	GO_GLUCOSIDASE_ACTIVITY1	0.334195375	-0.01567936	1.220724928	0.25757504	0.58452006	-5.49410222
WT after UVB irradiation VS WT before UVB irradiation	GO_GLUCOSIDASE_ACTIVITY2	0.296377262	-0.02985629	1.633452072	0.14056003	0.65051831	-4.79062578
homo after UVB irradiation VS homo before UVB irradiation	GO_GLUCOSYLCERAMIDE_METABOLIC_PROCESS	0.353351948	-0.03126929	1.305675008	0.22656967	0.4173622	-5.70132525
hetero after UVB irradiation VS hetero before UVB irradiation	GO_GLUCOSYLCERAMIDE_METABOLIC_PROCESS1	0.592222654	0.061254064	4.260632947	0.0028788	0.11333595	-1.30291259
WT after UVB irradiation VS WT before UVB irradiation	GO_GLUCOSYLCERAMIDE_METABOLIC_PROCESS2	0.472668708	-0.05640706	2.42409293	0.04123	0.49225821	-3.71021741
homo after UVB irradiation VS homo before UVB irradiation	GO_GLUCOSYLTRANSFERASE_ACTIVITY	0.442021321	-0.05971806	2.853716847	0.02049709	0.09927523	-3.46019886
hetero after UVB irradiation VS hetero before UVB irradiation	GO_GLUCOSYLTRANSFERASE_ACTIVITY1	0.587914309	-0.03340462	3.085258044	0.01534022	0.18284357	-2.91591845
WT after UVB irradiation VS WT before UVB irradiation	GO_GLUCOSYLTRANSFERASE_ACTIVITY2	0.452935601	-0.03110459	2.07889257	0.0708186	0.54758105	-4.19559677
homo after UVB irradiation VS homo before UVB irradiation	GO_GLUCURONOSYL_N_ACETYLGALACTOSAMINYL_PROTEOGLYCAN_4_BETA_N_ACETYLGALACTOSAMINYLTRANSFERASE_ACTIVITY	0.186498749	-0.00985029	0.94497268	0.37127418	0.561362	-6.08579599
hetero after UVB irradiation VS hetero before UVB irradiation	GO_GLUCURONOSYL_N_ACETYLGALACTOSAMINYL_PROTEOGLYCAN_4_BETA_N_ACETYLGALACTOSAMINYLTRANSFERASE_ACTIVITY1	0.14904321	0.100032672	0.704923304	0.50122754	0.77357112	-5.97049464
WT after UVB irradiation VS WT before UVB irradiation	GO_GLUCURONOSYL_N_ACETYLGALACTOSAMINYL_PROTEOGLYCAN_4_BETA_N_ACETYLGALACTOSAMINYLTRANSFERASE_ACTIVITY2	0.017850561	-0.08082086	0.04984332	0.96145477	0.99043451	-5.99475601
homo after UVB irradiation VS homo before UVB irradiation	GO_GLUCURONOSYLTRANSFERASE_ACTIVITY	-0.15462366	0.056171634	-0.72911524	0.48592502	0.66252932	-6.26820947
hetero after UVB irradiation VS hetero before UVB irradiation	GO_GLUCURONOSYLTRANSFERASE_ACTIVITY1	0.09068361	0.041653154	0.586596558	0.57394613	0.81567458	-6.04900148
WT after UVB irradiation VS WT before UVB irradiation	GO_GLUCURONOSYLTRANSFERASE_ACTIVITY2	0.111602746	0.001763311	0.51808471	0.61825335	0.90020288	-5.85687488
homo after UVB irradiation VS homo before UVB irradiation	GO_GLUTAMATE_BINDING	-0.40380545	0.03432113	-1.95493171	0.08490036	0.22680568	-4.83194422
hetero after UVB irradiation VS hetero before UVB irradiation	GO_GLUTAMATE_BINDING1	-0.43081995	-0.01144309	-2.58777313	0.0327223	0.25073401	-3.64264011
WT after UVB irradiation VS WT before UVB irradiation	GO_GLUTAMATE_BINDING2	-0.23132515	0.049479115	-1.14580369	0.2846068	0.75320386	-5.35713182
homo after UVB irradiation VS homo before UVB irradiation	GO_GLUTAMATE_BIOSYNTHETIC_PROCESS	0.13556045	-0.0673611	0.68924924	0.50940811	0.68167999	-6.29743864
hetero after UVB irradiation VS hetero before UVB irradiation	GO_GLUTAMATE_BIOSYNTHETIC_PROCESS1	0.16957924	-0.04847103	0.540432034	0.60388458	0.83419966	-6.0760772
WT after UVB irradiation VS WT before UVB irradiation	GO_GLUTAMATE_BIOSYNTHETIC_PROCESS2	0.402419959	-0.01652664	1.375288275	0.20588597	0.70285634	-5.1052247
homo after UVB irradiation VS homo before UVB irradiation	GO_GLUTAMATE_CATABOLIC_PROCESS	0.345661541	0.002864535	1.328957729	0.21911212	0.40991856	-5.67358017
hetero after UVB irradiation VS hetero before UVB irradiation	GO_GLUTAMATE_CATABOLIC_PROCESS1	-0.2261272	0.021987692	-0.75305647	0.47338552	0.75680588	-5.93494281
WT after UVB irradiation VS WT before UVB irradiation	GO_GLUTAMATE_CATABOLIC_PROCESS2	-0.13581148	-0.02055227	-0.3982868	0.700714	0.92463338	-5.9132226
homo after UVB irradiation VS homo before UVB irradiation	GO_GLUTAMATE_GATED_CALCIUM_ION_CHANNEL_ACTIVITY	-0.19782104	0.090829529	-0.64103089	0.53873646	0.70473663	-6.33082907
hetero after UVB irradiation VS hetero before UVB irradiation	GO_GLUTAMATE_GATED_CALCIUM_ION_CHANNEL_ACTIVITY1	0.019362981	0.055905878	0.08382882	0.93529011	0.97617349	-6.22767493
WT after UVB irradiation VS WT before UVB irradiation	GO_GLUTAMATE_GATED_CALCIUM_ION_CHANNEL_ACTIVITY2	0.145065876	-0.0428673	0.380133778	0.71362304	0.92947354	-5.92053336
homo after UVB irradiation VS homo before UVB irradiation	GO_GLUTAMATE_METABOLIC_PROCESS	0.049562653	-0.00764783	0.3353356	0.74566203	0.85110444	-6.48956297
hetero after UVB irradiation VS hetero before UVB irradiation	GO_GLUTAMATE_METABOLIC_PROCESS1	-0.27371757	-0.02991156	-1.81289583	0.10811425	0.40604091	-4.75360555
WT after UVB irradiation VS WT before UVB irradiation	GO_GLUTAMATE_METABOLIC_PROCESS2	0.034815499	-0.00705675	0.18755684	0.85583857	0.9663792	-5.97754784
homo after UVB irradiation VS homo before UVB irradiation	GO_GLUTAMATE_RECEPTOR_ACTIVITY	-0.32282969	0.030728252	-2.03115578	0.07532228	0.21070815	-4.72011174
hetero after UVB irradiation VS hetero before UVB irradiation	GO_GLUTAMATE_RECEPTOR_ACTIVITY1	-0.24488901	0.033292926	-1.54171651	0.16241648	0.48367662	-5.11153027
WT after UVB irradiation VS WT before UVB irradiation	GO_GLUTAMATE_RECEPTOR_ACTIVITY2	0.01632577	0.064310683	0.08338717	0.93556826	0.98322796	-5.99239936
homo after UVB irradiation VS homo before UVB irradiation	GO_GLUTAMATE_RECEPTOR_BINDING	0.365963106	-0.02235799	2.68502622	0.02673489	0.11563462	-3.72229475
hetero after UVB irradiation VS hetero before UVB irradiation	GO_GLUTAMATE_RECEPTOR_BINDING1	0.139208602	0.02947188	0.830997415	0.43051547	0.73139858	-5.87313856
WT after UVB irradiation VS WT before UVB irradiation	GO_GLUTAMATE_RECEPTOR_BINDING2	-0.0384715	-0.00165962	-0.2396748	0.81653786	0.95637428	-5.9658698
homo after UVB irradiation VS homo before UVB irradiation	GO_GLUTAMATE_RECEPTOR_SIGNALING_PATHWAY	0.147096018	0.015274614	1.020880355	0.33604394	0.52924231	-6.012591
hetero after UVB irradiation VS hetero before UVB irradiation	GO_GLUTAMATE_RECEPTOR_SIGNALING_PATHWAY1	-0.01936918	0.019543314	-0.14699423	0.88684032	0.95896443	-6.21978299
WT after UVB irradiation VS WT before UVB irradiation	GO_GLUTAMATE_RECEPTOR_SIGNALING_PATHWAY2	-9.49E-05	0.036936303	-0.00049738	0.99961519	0.99997598	-5.99606654
homo after UVB irradiation VS homo before UVB irradiation	GO_GLUTAMATE_SECRETION	-0.02691008	0.040483021	-0.19368954	0.85105933	0.91780487	-6.53031451
hetero after UVB irradiation VS hetero before UVB irradiation	GO_GLUTAMATE_SECRETION1	0.003295022	0.023833005	0.020852413	0.98388349	0.99402728	-6.23124831
WT after UVB irradiation VS WT before UVB irradiation	GO_GLUTAMATE_SECRETION2	-0.18280828	0.000701274	-1.13114031	0.29038097	0.75615882	-5.37217194
homo after UVB irradiation VS homo before UVB irradiation	GO_GLUTAMATERGIC_SYNAPSE	0.103607605	-0.00047793	0.83358816	0.42776879	0.61221841	-6.18487355
hetero after UVB irradiation VS hetero before UVB irradiation	GO_GLUTAMATERGIC_SYNAPSE1	0.043437113	0.001167972	0.341839172	0.74143995	0.89935341	-6.16859777
WT after UVB irradiation VS WT before UVB irradiation	GO_GLUTAMATERGIC_SYNAPSE2	0.045119676	-0.00757489	0.290152365	0.7789913	0.94403996	-5.95188786
homo after UVB irradiation VS homo before UVB irradiation	GO_GLUTAMINE_FAMILY_AMINO_ACID_BIOSYNTHETIC_PROCESS	0.107427877	0.027035698	0.734226472	0.48296516	0.6599908	-6.26435742
hetero after UVB irradiation VS hetero before UVB irradiation	GO_GLUTAMINE_FAMILY_AMINO_ACID_BIOSYNTHETIC_PROCESS1	0.013903241	-0.0150768	0.079650064	0.93850793	0.97752686	-6.22804508
WT after UVB irradiation VS WT before UVB irradiation	GO_GLUTAMINE_FAMILY_AMINO_ACID_BIOSYNTHETIC_PROCESS2	0.242062877	-0.02274555	1.446809855	0.18552513	0.68726847	-5.02100708
homo after UVB irradiation VS homo before UVB irradiation	GO_GLUTAMINE_FAMILY_AMINO_ACID_CATABOLIC_PROCESS	0.233834285	-0.03196387	1.602931115	0.14612363	0.31879751	-5.32541021
hetero after UVB irradiation VS hetero before UVB irradiation	GO_GLUTAMINE_FAMILY_AMINO_ACID_CATABOLIC_PROCESS1	0.016493758	-0.0566453	0.080656093	0.93773313	0.97722007	-6.22795769
WT after UVB irradiation VS WT before UVB irradiation	GO_GLUTAMINE_FAMILY_AMINO_ACID_CATABOLIC_PROCESS2	-0.15680298	-0.06499276	-0.60166897	0.56385575	0.88099977	-5.8094519
homo after UVB irradiation VS homo before UVB irradiation	GO_GLUTAMINE_FAMILY_AMINO_ACID_METABOLIC_PROCESS	-0.00649837	0.008266562	-0.0483091	0.96260825	0.98048108	-6.54957608
hetero after UVB irradiation VS hetero before UVB irradiation	GO_GLUTAMINE_FAMILY_AMINO_ACID_METABOLIC_PROCESS1	-0.06767614	-0.02067469	-0.51395951	0.6214264	0.84379983	-6.09067698
WT after UVB irradiation VS WT before UVB irradiation	GO_GLUTAMINE_FAMILY_AMINO_ACID_METABOLIC_PROCESS2	0.016844265	-0.02078911	0.105541906	0.91851346	0.98043563	-5.9901934
homo after UVB irradiation VS homo before UVB irradiation	GO_GLUTAMINE_METABOLIC_PROCESS	-0.21530354	-0.01168147	-1.3433979	0.21459273	0.40511001	-5.65621435
hetero after UVB irradiation VS hetero before UVB irradiation	GO_GLUTAMINE_METABOLIC_PROCESS1	-0.13124166	-0.0331255	-0.74935953	0.4754873	0.75835393	-5.9377454
WT after UVB irradiation VS WT before UVB irradiation	GO_GLUTAMINE_METABOLIC_PROCESS2	0.105155351	0.00378671	0.619483819	0.55262455	0.87641918	-5.79850893
homo after UVB irradiation VS homo before UVB irradiation	GO_GLUTAMINE_TRANSPORT	-0.5277823	0.051272872	-3.6020994	0.00650896	0.05354108	-2.31327989
hetero after UVB irradiation VS hetero before UVB irradiation	GO_GLUTAMINE_TRANSPORT1	-0.37267075	0.010939702	-2.03636517	0.07676412	0.35710322	-4.44240656
WT after UVB irradiation VS WT before UVB irradiation	GO_GLUTAMINE_TRANSPORT2	-0.42992766	-0.05398623	-2.59709574	0.03144383	0.45353809	-3.46416231
homo after UVB irradiation VS homo before UVB irradiation	GO_GLUTATHIONE_CATABOLIC_PROCESS	-0.10894237	0.0319402	-0.63850141	0.54030244	0.70584659	-6.33252049
hetero after UVB irradiation VS hetero before UVB irradiation	GO_GLUTATHIONE_CATABOLIC_PROCESS1	0.180809992	0.026589365	1.178906715	0.2729198	0.59910814	-5.53977202
WT after UVB irradiation VS WT before UVB irradiation	GO_GLUTATHIONE_CATABOLIC_PROCESS2	-0.13526794	0.019113405	-0.74817434	0.47552062	0.84841709	-5.71105849
homo after UVB irradiation VS homo before UVB irradiation	GO_GLUTATHIONE_DERIVATIVE_METABOLIC_PROCESS	0.300699221	-0.03991239	1.096060721	0.303728	0.49795735	-5.935817
hetero after UVB irradiation VS hetero before UVB irradiation	GO_GLUTATHIONE_DERIVATIVE_METABOLIC_PROCESS1	0.146386632	0.002996229	0.659141571	0.52865582	0.79049147	-6.00239385
WT after UVB irradiation VS WT before UVB irradiation	GO_GLUTATHIONE_DERIVATIVE_METABOLIC_PROCESS2	0.220739648	-0.03162723	0.750373052	0.47426658	0.84774103	-5.70943947
homo after UVB irradiation VS homo before UVB irradiation	GO_GLUTATHIONE_DISULFIDE_OXIDOREDUCTASE_ACTIVITY	-0.06000412	-0.02104515	-0.18582808	0.85702555	0.921265	-6.53194476
hetero after UVB irradiation VS hetero before UVB irradiation	GO_GLUTATHIONE_DISULFIDE_OXIDOREDUCTASE_ACTIVITY1	-0.03552916	0.027588151	-0.16547753	0.87274883	0.95256767	-6.216661
WT after UVB irradiation VS WT before UVB irradiation	GO_GLUTATHIONE_DISULFIDE_OXIDOREDUCTASE_ACTIVITY2	0.30411274	-0.02111103	1.976131046	0.08311342	0.5691437	-4.33705711
homo after UVB irradiation VS homo before UVB irradiation	GO_GLUTATHIONE_HYDROLASE_ACTIVITY	0.175611057	0.013697659	0.686924997	0.51079876	0.68259144	-6.29909778
hetero after UVB irradiation VS hetero before UVB irradiation	GO_GLUTATHIONE_HYDROLASE_ACTIVITY1	0.422357052	0.047948834	2.183987512	0.06110987	0.32556646	-4.23138691
WT after UVB irradiation VS WT before UVB irradiation	GO_GLUTATHIONE_HYDROLASE_ACTIVITY2	0.360633332	-0.00266622	1.94591322	0.08710535	0.5790399	-4.37826814
homo after UVB irradiation VS homo before UVB irradiation	GO_GLUTATHIONE_METABOLIC_PROCESS	0.196834638	-0.02608741	1.226922823	0.25339898	0.44837036	-5.79275198
hetero after UVB irradiation VS hetero before UVB irradiation	GO_GLUTATHIONE_METABOLIC_PROCESS1	0.272428973	0.002396708	1.787213733	0.11241688	0.41398142	-4.78855344
WT after UVB irradiation VS WT before UVB irradiation	GO_GLUTATHIONE_METABOLIC_PROCESS2	0.088110015	0.057744363	0.468726706	0.65162467	0.90930021	-5.88177778
homo after UVB irradiation VS homo before UVB irradiation	GO_GLUTATHIONE_PEROXIDASE_ACTIVITY	0.570143486	-0.01354809	2.339451016	0.04624173	0.15705517	-4.25586581
hetero after UVB irradiation VS hetero before UVB irradiation	GO_GLUTATHIONE_PEROXIDASE_ACTIVITY1	0.329426046	0.052247219	1.331357323	0.2204229	0.54746446	-5.3683038
WT after UVB irradiation VS WT before UVB irradiation	GO_GLUTATHIONE_PEROXIDASE_ACTIVITY2	0.346479713	0.005912397	1.126903668	0.2920667	0.75675183	-5.37649193
homo after UVB irradiation VS homo before UVB irradiation	GO_GLUTATHIONE_TRANSFERASE_ACTIVITY	0.451165472	-0.03747493	1.592457771	0.14845424	0.32150562	-5.3393832
hetero after UVB irradiation VS hetero before UVB irradiation	GO_GLUTATHIONE_TRANSFERASE_ACTIVITY1	0.392184323	0.031552438	1.748884855	0.11913963	0.42351828	-4.84034392
WT after UVB irradiation VS WT before UVB irradiation	GO_GLUTATHIONE_TRANSFERASE_ACTIVITY2	0.356342879	-0.01018663	1.222984266	0.25573187	0.73742118	-5.27577468
homo after UVB irradiation VS homo before UVB irradiation	GO_GLUTATHIONE_TRANSPORT	-0.16893201	0.109718718	-0.53543613	0.60634522	0.75432781	-6.39621442
hetero after UVB irradiation VS hetero before UVB irradiation	GO_GLUTATHIONE_TRANSPORT1	0.352234419	0.01897113	1.271837539	0.2398018	0.5680482	-5.43685307
WT after UVB irradiation VS WT before UVB irradiation	GO_GLUTATHIONE_TRANSPORT2	0.321368754	0.068753807	1.260100276	0.24273298	0.73251751	-5.23538955
homo after UVB irradiation VS homo before UVB irradiation	GO_GLYCERALDEHYDE_3_PHOSPHATE_DEHYDROGENASE_NADPLUS_NON_PHOSPHORYLATING_ACTIVITY	-0.10074872	0.010458998	-0.57571257	0.58002716	0.73553844	-6.37254925
hetero after UVB irradiation VS hetero before UVB irradiation	GO_GLYCERALDEHYDE_3_PHOSPHATE_DEHYDROGENASE_NADPLUS_NON_PHOSPHORYLATING_ACTIVITY1	-0.21711257	-0.06108196	-0.80695584	0.44344418	0.73891789	-5.89274978
WT after UVB irradiation VS WT before UVB irradiation	GO_GLYCERALDEHYDE_3_PHOSPHATE_DEHYDROGENASE_NADPLUS_NON_PHOSPHORYLATING_ACTIVITY2	0.05593467	-0.079338	0.305747099	0.76750977	0.94123239	-5.94704089
homo after UVB irradiation VS homo before UVB irradiation	GO_GLYCERALDEHYDE_3_PHOSPHATE_METABOLIC_PROCESS	0.030195388	-0.0731673	0.189716682	0.85407317	0.91916261	-6.53114674
hetero after UVB irradiation VS hetero before UVB irradiation	GO_GLYCERALDEHYDE_3_PHOSPHATE_METABOLIC_PROCESS1	-0.26145051	-0.00248353	-1.09116756	0.30754448	0.62806003	-5.6319727
WT after UVB irradiation VS WT before UVB irradiation	GO_GLYCERALDEHYDE_3_PHOSPHATE_METABOLIC_PROCESS2	0.043462123	0.07459806	0.178760075	0.8625177	0.9676681	-5.9792407
homo after UVB irradiation VS homo before UVB irradiation	GO_GLYCEROL_3_PHOSPHATE_METABOLIC_PROCESS	-0.08468486	0.010552591	-0.31546482	0.76016822	0.86127258	-6.49656932
hetero after UVB irradiation VS hetero before UVB irradiation	GO_GLYCEROL_3_PHOSPHATE_METABOLIC_PROCESS1	-0.36243279	0.04190547	-1.14630625	0.28539594	0.61082284	-5.57461561
WT after UVB irradiation VS WT before UVB irradiation	GO_GLYCEROL_3_PHOSPHATE_METABOLIC_PROCESS2	-0.02142482	0.012658603	-0.08725348	0.93258915	0.98322796	-5.99205157
homo after UVB irradiation VS homo before UVB irradiation	GO_GLYCEROL_CHANNEL_ACTIVITY	0.607591355	-0.00366824	3.421453914	0.00853569	0.06138462	-2.58589089
hetero after UVB irradiation VS hetero before UVB irradiation	GO_GLYCEROL_CHANNEL_ACTIVITY1	0.322625061	0.03641815	1.310272502	0.22712898	0.55615899	-5.39281212
WT after UVB irradiation VS WT before UVB irradiation	GO_GLYCEROL_CHANNEL_ACTIVITY2	0.191799794	0.021094964	0.893694784	0.39726775	0.81477652	-5.595327
homo after UVB irradiation VS homo before UVB irradiation	GO_GLYCEROL_ETHER_METABOLIC_PROCESS	0.051240301	-0.0277074	0.383922093	0.71065152	0.82744868	-6.47068419
hetero after UVB irradiation VS hetero before UVB irradiation	GO_GLYCEROL_ETHER_METABOLIC_PROCESS1	-0.15414445	-0.0281077	-1.01575482	0.34001704	0.65952819	-5.70705706
WT after UVB irradiation VS WT before UVB irradiation	GO_GLYCEROL_ETHER_METABOLIC_PROCESS2	-0.02756329	-0.0201297	-0.11445893	0.9116612	0.97857624	-5.98915992
homo after UVB irradiation VS homo before UVB irradiation	GO_GLYCEROL_TRANSMEMBRANE_TRANSPORTER_ACTIVITY	0.847806218	0.026922926	6.648551703	0.00013411	0.01007802	1.569114863
hetero after UVB irradiation VS hetero before UVB irradiation	GO_GLYCEROL_TRANSMEMBRANE_TRANSPORTER_ACTIVITY1	0.423375764	0.051155251	2.547049484	0.03484318	0.25592132	-3.70238912
WT after UVB irradiation VS WT before UVB irradiation	GO_GLYCEROL_TRANSMEMBRANE_TRANSPORTER_ACTIVITY2	0.430325394	0.017682472	2.134447062	0.06492905	0.53678963	-4.11838618
homo after UVB irradiation VS homo before UVB irradiation	GO_GLYCEROL_TRANSPORT	0.847806218	0.026922926	6.648551703	0.00013411	0.01007802	1.569114863
hetero after UVB irradiation VS hetero before UVB irradiation	GO_GLYCEROL_TRANSPORT1	0.423375764	0.051155251	2.547049484	0.03484318	0.25592132	-3.70238912
WT after UVB irradiation VS WT before UVB irradiation	GO_GLYCEROL_TRANSPORT2	0.430325394	0.017682472	2.134447062	0.06492905	0.53678963	-4.11838618
homo after UVB irradiation VS homo before UVB irradiation	GO_GLYCEROLIPID_BIOSYNTHETIC_PROCESS	0.224500951	-0.02257509	1.779442356	0.11156193	0.26788398	-5.08327935
hetero after UVB irradiation VS hetero before UVB irradiation	GO_GLYCEROLIPID_BIOSYNTHETIC_PROCESS1	0.093570693	0.002132154	0.662717573	0.52648071	0.78966755	-5.99997057
WT after UVB irradiation VS WT before UVB irradiation	GO_GLYCEROLIPID_BIOSYNTHETIC_PROCESS2	0.159519292	0.004654692	1.020562955	0.33698058	0.78029538	-5.48103837
homo after UVB irradiation VS homo before UVB irradiation	GO_GLYCEROLIPID_CATABOLIC_PROCESS	0.008264401	0.032563355	0.058144646	0.95500422	0.97631027	-6.54900165
hetero after UVB irradiation VS hetero before UVB irradiation	GO_GLYCEROLIPID_CATABOLIC_PROCESS1	-0.16598002	0.030360036	-1.26926379	0.24067141	0.56885269	-5.43977232
WT after UVB irradiation VS WT before UVB irradiation	GO_GLYCEROLIPID_CATABOLIC_PROCESS2	-0.05252533	0.018732139	-0.32721902	0.75180098	0.93734883	-5.93996263
homo after UVB irradiation VS homo before UVB irradiation	GO_GLYCEROLIPID_METABOLIC_PROCESS	0.140723163	-0.0173069	1.120948535	0.2935852	0.48830057	-5.90950597
hetero after UVB irradiation VS hetero before UVB irradiation	GO_GLYCEROLIPID_METABOLIC_PROCESS1	-0.00974776	0.000280813	-0.07093847	0.94522004	0.97953855	-6.22875598
WT after UVB irradiation VS WT before UVB irradiation	GO_GLYCEROLIPID_METABOLIC_PROCESS2	0.04306414	0.005613015	0.278848176	0.7873503	0.94689038	-5.95524612
homo after UVB irradiation VS homo before UVB irradiation	GO_GLYCEROPHOSPHOLIPID_CATABOLIC_PROCESS	0.269548881	0.007045893	1.904288285	0.09189854	0.23833861	-4.90541244
hetero after UVB irradiation VS hetero before UVB irradiation	GO_GLYCEROPHOSPHOLIPID_CATABOLIC_PROCESS1	0.166861341	0.005325037	1.181937835	0.27178282	0.59794652	-5.53649812
WT after UVB irradiation VS WT before UVB irradiation	GO_GLYCEROPHOSPHOLIPID_CATABOLIC_PROCESS2	0.206391285	-0.00749873	1.426718039	0.19105861	0.69325102	-5.0449123
homo after UVB irradiation VS homo before UVB irradiation	GO_GLYCEROPHOSPHOLIPID_METABOLIC_PROCESS	0.192490078	-0.02387942	1.574953326	0.1524247	0.32651057	-5.36262968
hetero after UVB irradiation VS hetero before UVB irradiation	GO_GLYCEROPHOSPHOLIPID_METABOLIC_PROCESS1	0.051284534	-0.0020462	0.388265929	0.70813306	0.88462677	-6.15053507
WT after UVB irradiation VS WT before UVB irradiation	GO_GLYCEROPHOSPHOLIPID_METABOLIC_PROCESS2	0.096029718	0.002426551	0.648425129	0.53466137	0.86957641	-5.7801182
homo after UVB irradiation VS homo before UVB irradiation	GO_GLYCINE_BINDING	-0.30720157	-0.02932938	-1.45873509	0.18129142	0.36438538	-5.51337859
hetero after UVB irradiation VS hetero before UVB irradiation	GO_GLYCINE_BINDING1	-0.28995272	-0.00298651	-1.51913448	0.16791362	0.48947715	-5.14007986
WT after UVB irradiation VS WT before UVB irradiation	GO_GLYCINE_BINDING2	-0.22183598	0.025859214	-1.37204871	0.20685254	0.70344592	-5.10898026
homo after UVB irradiation VS homo before UVB irradiation	GO_GLYCINE_METABOLIC_PROCESS	-0.41679532	0.065796147	-2.06562345	0.07134188	0.20504264	-4.66909051
hetero after UVB irradiation VS hetero before UVB irradiation	GO_GLYCINE_METABOLIC_PROCESS1	-0.61278418	-0.01889833	-3.3951131	0.00969236	0.15539797	-2.47257832

WT after UVB irradiation VS WT before UVB irradiation	GO_GLYCINE_METABOLIC_PROCESS2	-0.19050717	-0.01494237	-1.12290557	0.2936647	0.7573105	-5.38055812
homo after UVB irradiation VS homo before UVB irradiation	GO_GLYCINE_N_ACYLTRANSFERASE_ACTIVITY	0.410037574	-0.06316139	1.17678757	0.27181242	0.46700849	-5.84891846
hetero after UVB irradiation VS hetero before UVB irradiation	GO_GLYCINE_N_ACYLTRANSFERASE_ACTIVITY1	-0.22788001	0.14396771	-0.61065613	0.55868277	0.80719896	-6.0340879
WT after UVB irradiation VS WT before UVB irradiation	GO_GLYCINE_N_ACYLTRANSFERASE_ACTIVITY2	0.102052525	-0.0789561	0.244426064	0.81298046	0.955326	-5.9646654
homo after UVB irradiation VS homo before UVB irradiation	GO_GLYCINE_TRANSMEMBRANE_TRANSPORTER_ACTIVITY	0.143482193	-0.02157314	0.780421382	0.45674607	0.63761282	-6.22848219
hetero after UVB irradiation VS hetero before UVB irradiation	GO_GLYCINE_TRANSMEMBRANE_TRANSPORTER_ACTIVITY1	-0.06737607	-0.0174038	-0.46008273	0.6579277	0.86007701	-6.1182679
WT after UVB irradiation VS WT before UVB irradiation	GO_GLYCINE_TRANSMEMBRANE_TRANSPORTER_ACTIVITY2	0.597189347	0.029010637	3.732840355	0.00564047	0.27931663	-1.89496658
homo after UVB irradiation VS homo before UVB irradiation	GO_GLYCINE_TRANSPORT	0.082990307	-0.02857284	0.402905752	0.69716152	0.81910928	-6.46263915
hetero after UVB irradiation VS hetero before UVB irradiation	GO_GLYCINE_TRANSPORT1	-0.1775004	0.00463846	-0.86806894	0.4110991	0.71492968	-5.84196509
WT after UVB irradiation VS WT before UVB irradiation	GO_GLYCINE_TRANSPORT2	0.172838916	-0.0193162	1.124717531	0.29293961	0.75699426	-5.37871657
homo after UVB irradiation VS homo before UVB irradiation	GO_GLYCINERGIC_SYNAPSE	-0.26288581	0.03676096	-1.30453972	0.22693874	0.41778132	-5.70266998
hetero after UVB irradiation VS hetero before UVB irradiation	GO_GLYCINERGIC_SYNAPSE1	-0.01303362	-0.01588943	-0.0475043	0.96329721	0.98664387	-6.23026049
WT after UVB irradiation VS WT before UVB irradiation	GO_GLYCINERGIC_SYNAPSE2	-0.21543864	0.030187324	-0.72952181	0.48624618	0.85164239	-5.72462784
homo after UVB irradiation VS homo before UVB irradiation	GO_GLYCOGEN_BINDING	0.317792971	-0.006493	1.603180814	0.14606847	0.31877143	-5.3250765
hetero after UVB irradiation VS hetero before UVB irradiation	GO_GLYCOGEN_BINDING1	0.03763386	-0.0571133	0.247300986	0.81101819	0.92677026	-6.19845109
WT after UVB irradiation VS WT before UVB irradiation	GO_GLYCOGEN_BINDING2	0.088663951	0.005804849	0.32109802	0.75626696	0.93854182	-5.94202808
homo after UVB irradiation VS homo before UVB irradiation	GO_GLYCOGEN_BIOSYNTHETIC_PROCESS	0.234350839	-0.02578877	1.968704543	0.08308716	0.22370321	-4.81184499
hetero after UVB irradiation VS hetero before UVB irradiation	GO_GLYCOGEN_BIOSYNTHETIC_PROCESS1	-0.13010429	-0.0393067	-0.76436095	0.46699686	0.75303308	-5.92629967
WT after UVB irradiation VS WT before UVB irradiation	GO_GLYCOGEN_BIOSYNTHETIC_PROCESS2	-0.0396949	-0.07822033	-0.22716455	0.82592603	0.95879279	-5.96892956
homo after UVB irradiation VS homo before UVB irradiation	GO_GLYCOGEN_GRANULE	0.304339909	-0.01976995	1.643175582	0.13747428	0.30649326	-5.27128415
hetero after UVB irradiation VS hetero before UVB irradiation	GO_GLYCOGEN_GRANULE1	0.182935296	-0.04057549	1.150915495	0.28360433	0.60906208	-5.56973049
WT after UVB irradiation VS WT before UVB irradiation	GO_GLYCOGEN_GRANULE2	0.421899708	-0.10105533	1.910068869	0.09207948	0.58696194	-4.42689702
homo after UVB irradiation VS homo before UVB irradiation	GO_GLYCOLIPID_BINDING	0.494217155	-0.04082663	2.990632373	0.01654601	0.08811602	-3.24772778
hetero after UVB irradiation VS hetero before UVB irradiation	GO_GLYCOLIPID_BINDING1	0.411435174	0.009526341	2.550260878	0.03467095	0.25582508	-3.69767839
WT after UVB irradiation VS WT before UVB irradiation	GO_GLYCOLIPID_BINDING2	0.525080853	-0.00216161	2.708487052	0.02642716	0.43935696	-3.30569123
homo after UVB irradiation VS homo before UVB irradiation	GO_GLYCOLIPID_BIOSYNTHETIC_PROCESS	0.13619159	-0.01214776	0.876290474	0.4054339	0.59249239	-6.14809996
hetero after UVB irradiation VS hetero before UVB irradiation	GO_GLYCOLIPID_BIOSYNTHETIC_PROCESS1	0.047128703	0.004852369	0.348890519	0.73634145	0.89641568	-6.16599744
WT after UVB irradiation VS WT before UVB irradiation	GO_GLYCOLIPID_BIOSYNTHETIC_PROCESS2	0.028813711	-0.00090368	0.198690273	0.847403	0.96522688	-5.97528985
homo after UVB irradiation VS homo before UVB irradiation	GO_GLYCOLIPID_CATABOLIC_PROCESS	0.422874041	-0.05979867	1.755823244	0.11570096	0.27401687	-5.11635634
hetero after UVB irradiation VS hetero before UVB irradiation	GO_GLYCOLIPID_CATABOLIC_PROCESS1	0.711389836	-0.01735078	4.296670347	0.00274337	0.11244771	-1.25678646
WT after UVB irradiation VS WT before UVB irradiation	GO_GLYCOLIPID_CATABOLIC_PROCESS2	0.530996391	-0.0128793	2.513421032	0.03584278	0.47437837	-3.58324205
homo after UVB irradiation VS homo before UVB irradiation	GO_GLYCOLIPID_MANNOSYLTRANSFERASE_ACTIVITY	-0.30141333	-0.02468292	-1.3841913	0.20225474	0.39130266	-5.6065187
hetero after UVB irradiation VS hetero before UVB irradiation	GO_GLYCOLIPID_MANNOSYLTRANSFERASE_ACTIVITY1	0.255904519	-0.01050775	1.135537562	0.28961748	0.61410673	-5.58597518
WT after UVB irradiation VS WT before UVB irradiation	GO_GLYCOLIPID_MANNOSYLTRANSFERASE_ACTIVITY2	0.100946107	0.014348895	0.467215408	0.65266029	0.90930021	-5.88250329
homo after UVB irradiation VS homo before UVB irradiation	GO_GLYCOLIPID_TRANSPORT	0.892361117	-0.04112601	6.095667675	0.00024716	0.01196149	0.96924324
hetero after UVB irradiation VS hetero before UVB irradiation	GO_GLYCOLIPID_TRANSPORT1	0.947817232	-0.00915293	6.474049132	0.0002096	0.07788488	1.126103211
WT after UVB irradiation VS WT before UVB irradiation	GO_GLYCOLIPID_TRANSPORT2	0.760952199	-0.02967144	4.217957414	0.00284537	0.24078388	-1.27865602
homo after UVB irradiation VS homo before UVB irradiation	GO_GLYCOLYTIC_FERMENTATION	-0.39819983	-0.02215312	-1.22199436	0.25516233	0.45073384	-5.79834534
hetero after UVB irradiation VS hetero before UVB irradiation	GO_GLYCOLYTIC_FERMENTATION1	-0.12016917	-0.03257092	-0.27980553	0.78685547	0.91698304	-6.1892444
WT after UVB irradiation VS WT before UVB irradiation	GO_GLYCOLYTIC_FERMENTATION2	0.528358146	-0.04230148	2.158077754	0.06257193	0.53546755	-4.0854094
homo after UVB irradiation VS homo before UVB irradiation	GO_GLYCOLYTIC_PROCESS_THROUGH_FRUCTOSE_6_PHOSPHATE	0.665469393	-0.02408178	4.329317816	0.0022868	0.03201732	-1.25789876
hetero after UVB irradiation VS hetero before UVB irradiation	GO_GLYCOLYTIC_PROCESS_THROUGH_FRUCTOSE_6_PHOSPHATE1	0.485433105	0.010089375	2.406089436	0.04332554	0.27856717	-3.90883418
WT after UVB irradiation VS WT before UVB irradiation	GO_GLYCOLYTIC_PROCESS_THROUGH_FRUCTOSE_6_PHOSPHATE2	0.444281675	0.028686346	2.330908152	0.04772058	0.50650586	-3.84229848
homo after UVB irradiation VS homo before UVB irradiation	GO_GLYCOPROTEIN_BIOSYNTHETIC_PROCESS	0.313915104	-0.01671279	2.555524016	0.03281698	0.12900379	-3.92312963
hetero after UVB irradiation VS hetero before UVB irradiation	GO_GLYCOPROTEIN_BIOSYNTHETIC_PROCESS1	0.20701027	-0.01108629	1.225840626	0.25574799	0.58309908	-5.48844205
WT after UVB irradiation VS WT before UVB irradiation	GO_GLYCOPROTEIN_BIOSYNTHETIC_PROCESS2	0.166552266	-0.0157074	0.973564695	0.35845368	0.79175288	-5.52475466
homo after UVB irradiation VS homo before UVB irradiation	GO_GLYCOPROTEIN_CATABOLIC_PROCESS	0.660861895	-0.05138228	5.008043346	0.00092327	0.02088233	-0.34419979
hetero after UVB irradiation VS hetero before UVB irradiation	GO_GLYCOPROTEIN_CATABOLIC_PROCESS1	0.367856194	0.005500641	2.014542999	0.07938892	0.3626016	-4.47328391
WT after UVB irradiation VS WT before UVB irradiation	GO_GLYCOPROTEIN_CATABOLIC_PROCESS2	0.548685733	0.013666718	3.083039429	0.01482791	0.36591226	-2.77702945
homo after UVB irradiation VS homo before UVB irradiation	GO_GLYCOPROTEIN_COMPLEX	-0.06245457	0.008155537	-0.29791749	0.77306238	0.86885618	-6.50240907
hetero after UVB irradiation VS hetero before UVB irradiation	GO_GLYCOPROTEIN_COMPLEX1	-0.54310209	-0.01938233	-2.2612304	0.05422152	0.30876256	-4.11975383
WT after UVB irradiation VS WT before UVB irradiation	GO_GLYCOPROTEIN_COMPLEX2	-0.33603747	-0.09393758	-1.32925612	0.2199925	0.71478521	-5.15808672
homo after UVB irradiation VS homo before UVB irradiation	GO_GLYCOPROTEIN_METABOLIC_PROCESS	0.337803118	-0.01563769	2.822729888	0.02151927	0.10187996	-3.50834173
hetero after UVB irradiation VS hetero before UVB irradiation	GO_GLYCOPROTEIN_METABOLIC_PROCESS1	0.223543968	-0.00682711	1.31394527	0.22594844	0.55487024	-5.38856049
WT after UVB irradiation VS WT before UVB irradiation	GO_GLYCOPROTEIN_METABOLIC_PROCESS2	0.215037435	-0.00880937	1.290090923	0.23263825	0.72451161	-5.20218693
homo after UVB irradiation VS homo before UVB irradiation	GO_GLYCOSAMINOGLYCAN_BINDING	0.372910676	-0.01086876	2.586174209	0.03126107	0.12530445	-3.8756584
hetero after UVB irradiation VS hetero before UVB irradiation	GO_GLYCOSAMINOGLYCAN_BINDING1	0.112043188	0.010848049	0.600160993	0.56531172	0.81102647	-6.04066042
WT after UVB irradiation VS WT before UVB irradiation	GO_GLYCOSAMINOGLYCAN_BINDING2	0.219213802	0.008852514	1.107792785	0.29976836	0.76190626	-5.39583499
homo after UVB irradiation VS homo before UVB irradiation	GO_GLYCOSIDE_CATABOLIC_PROCESS	0.231166185	-0.05846862	1.363942883	0.20830034	0.39822021	-5.63130219
hetero after UVB irradiation VS hetero before UVB irradiation	GO_GLYCOSIDE_CATABOLIC_PROCESS1	0.453668303	-0.05119485	2.412503179	0.04289759	0.27767067	-3.89946017
WT after UVB irradiation VS WT before UVB irradiation	GO_GLYCOSIDE_CATABOLIC_PROCESS2	0.354686613	-0.05131098	1.610253805	0.1455528	0.65365874	-4.82001576
homo after UVB irradiation VS homo before UVB irradiation	GO_GLYCOSIDE_METABOLIC_PROCESS	0.239890281	0.009376573	1.333712028	0.21761527	0.40825686	-5.66787587
hetero after UVB irradiation VS hetero before UVB irradiation	GO_GLYCOSIDE_METABOLIC_PROCESS1	0.425013617	0.005158765	2.716627904	0.02684172	0.23196771	-3.45355904
WT after UVB irradiation VS WT before UVB irradiation	GO_GLYCOSIDE_METABOLIC_PROCESS2	0.348675482	0.025040676	1.767363877	0.11469443	0.61998013	-4.6173597
homo after UVB irradiation VS homo before UVB irradiation	GO_GLYCOSPHINGOLIPID_BINDING	0.742929536	-0.02838015	4.236946732	0.00260077	0.03384751	-1.38777927
hetero after UVB irradiation VS hetero before UVB irradiation	GO_GLYCOSPHINGOLIPID_BINDING1	0.492485058	0.067706128	2.301815566	0.05091793	0.30023856	-4.06083918
WT after UVB irradiation VS WT before UVB irradiation	GO_GLYCOSPHINGOLIPID_BINDING2	0.412724354	-0.03245259	1.274002982	0.23800847	0.72863458	-5.22006019
homo after UVB irradiation VS homo before UVB irradiation	GO_GLYCOSPHINGOLIPID_BIOSYNTHETIC_PROCESS	0.333419002	-0.03335085	1.907177255	0.09148495	0.23764186	-4.90124054
hetero after UVB irradiation VS hetero before UVB irradiation	GO_GLYCOSPHINGOLIPID_BIOSYNTHETIC_PROCESS1	0.144367611	0.000698835	0.767501222	0.46523239	0.75235709	-5.92387911
WT after UVB irradiation VS WT before UVB irradiation	GO_GLYCOSPHINGOLIPID_BIOSYNTHETIC_PROCESS2	0.009551809	-0.0429849	0.04526903	0.96498935	0.99087638	-5.9949855
homo after UVB irradiation VS homo before UVB irradiation	GO_GLYCOSPHINGOLIPID_METABOLIC_PROCESS	0.449082878	-0.05788115	2.327969514	0.04709231	0.15880741	-4.27341939
hetero after UVB irradiation VS hetero before UVB irradiation	GO_GLYCOSPHINGOLIPID_METABOLIC_PROCESS1	0.4403985	-0.01017868	2.647234542	0.0298599	0.24142332	-3.55537538
WT after UVB irradiation VS WT before UVB irradiation	GO_GLYCOSPHINGOLIPID_METABOLIC_PROCESS2	0.318763126	-0.00871983	1.873824311	0.09738462	0.59779444	-4.47576754
homo after UVB irradiation VS homo before UVB irradiation	GO_GLYCOSYL_COMPOUND_BIOSYNTHETIC_PROCESS	-0.1820922	-0.04433278	-0.92001681	0.38343457	0.57325814	-6.10887637
hetero after UVB irradiation VS hetero before UVB irradiation	GO_GLYCOSYL_COMPOUND_BIOSYNTHETIC_PROCESS1	0.05926369	-0.08550262	0.294989757	0.77565014	0.91233219	-6.1845624
WT after UVB irradiation VS WT before UVB irradiation	GO_GLYCOSYL_COMPOUND_BIOSYNTHETIC_PROCESS2	0.240424032	-0.02720148	1.162308524	0.27821875	0.74917538	-5.34004075
homo after UVB irradiation VS homo before UVB irradiation	GO_GLYCOSYL_COMPOUND_CATABOLIC_PROCESS	0.198630226	-0.02287359	1.389417762	0.20071911	0.3894009	-5.60008499
hetero after UVB irradiation VS hetero before UVB irradiation	GO_GLYCOSYL_COMPOUND_CATABOLIC_PROCESS1	0.23036266	-0.00543799	1.592931457	0.15054815	0.46844437	-5.04599345
WT after UVB irradiation VS WT before UVB irradiation	GO_GLYCOSYL_COMPOUND_CATABOLIC_PROCESS2	0.173426941	-0.0434327	0.926717523	0.38086389	0.80406414	-5.56672972
homo after UVB irradiation VS homo before UVB irradiation	GO_GLYCOSYL_COMPOUND_METABOLIC_PROCESS	-0.01580799	-0.02818524	-0.11392549	0.91199523	0.9516556	-6.54373922
hetero after UVB irradiation VS hetero before UVB irradiation	GO_GLYCOSYL_COMPOUND_METABOLIC_PROCESS1	0.07259393	-0.01632825	0.50706864	0.62603595	0.84577078	-6.09436533
WT after UVB irradiation VS WT before UVB irradiation	GO_GLYCOSYL_COMPOUND_METABOLIC_PROCESS2	0.179658163	-0.0049387	1.106140813	0.30044163	0.76247487	-5.3974959
homo after UVB irradiation VS homo before UVB irradiation	GO_GLYCOSYLATION	0.291118309	-0.0126826	2.369976473	0.04405405	0.15235659	-4.20912217
hetero after UVB irradiation VS hetero before UVB irradiation	GO_GLYCOSYLATION1	0.226696946	-0.00804768	1.305332628	0.22872512	0.55721087	-5.39851882
WT after UVB irradiation VS WT before UVB irradiation	GO_GLYCOSYLATION2	0.185480532	-0.01610675	1.06045724	0.31953918	0.77323848	-5.44271105
homo after UVB irradiation VS homo before UVB irradiation	GO_GLYCOSYLCERAMIDE_BIOSYNTHETIC_PROCESS	0.35931037	-0.07143912	2.107409832	0.06678957	0.19720162	-4.6068912
hetero after UVB irradiation VS hetero before UVB irradiation	GO_GLYCOSYLCERAMIDE_BIOSYNTHETIC_PROCESS1	0.271022616	-0.01911258	1.220966496	0.25748852	0.58452006	-5.4938353
WT after UVB irradiation VS WT before UVB irradiation	GO_GLYCOSYLCERAMIDE_BIOSYNTHETIC_PROCESS2	0.244682742	-0.06198269	1.146648961	0.28427679	0.75306903	-5.35626067
homo after UVB irradiation VS homo before UVB irradiation	GO_GLYCOSYLCERAMIDE_CATABOLIC_PROCESS	0.378963787	-0.0996362	1.499453193	0.17066702	0.35032188	-5.46129837
hetero after UVB irradiation VS hetero before UVB irradiation	GO_GLYCOSYLCERAMIDE_CATABOLIC_PROCESS1	0.74523435	0.047709083	4.675982704	0.00167141	0.09676143	-0.7846684
WT after UVB irradiation VS WT before UVB irradiation	GO_GLYCOSYLCERAMIDE_CATABOLIC_PROCESS2	0.603175096	-0.0084917	2.934780465	0.01861222	0.38933605	-2.98514996
homo after UVB irradiation VS homo before UVB irradiation	GO_GLYCOSYLCERAMIDE_METABOLIC_PROCESS	0.493099669	-0.04738598	2.702148303	0.02602155	0.11394659	-3.69570235
hetero after UVB irradiation VS hetero before UVB irradiation	GO_GLYCOSYLCERAMIDE_METABOLIC_PROCESS1	0.519882622	0.013030144	3.520604955	0.00807529	0.14537603	-2.29619569
WT after UVB irradiation VS WT before UVB irradiation	GO_GLYCOSYLCERAMIDE_METABOLIC_PROCESS2	0.346540607	-0.05507893	1.584421197	0.15130247	0.6586344	-4.85250615
homo after UVB irradiation VS homo before UVB irradiation	GO_GLYCOSYLPHOSPHATIDYLINOSITOL_N_ACETYLGLUCOSAMINYLTRANSFERASE_GPI_GNT_COMPLEX	-0.30239042	-0.05663726	-1.36367268	0.20838206	0.39822507	-5.63163137
hetero after UVB irradiation VS hetero before UVB irradiation	GO_GLYCOSYLPHOSPHATIDYLINOSITOL_N_ACETYLGLUCOSAMINYLTRANSFERASE_GPI_GNT_COMPLEX1	-0.49236047	0.060147698	-3.30998945	0.01098261	0.16390046	-2.59333353
WT after UVB irradiation VS WT before UVB irradiation	GO_GLYCOSYLPHOSPHATIDYLINOSITOL_N_ACETYLGLUCOSAMINYLTRANSFERASE_GPI_GNT_COMPLEX2	-0.14542402	0.008208192	-0.70694904	0.49943305	0.85545885	-5.74065013
homo after UVB irradiation VS homo before UVB irradiation	GO_GLYOXYLATE_METABOLIC_PROCESS	0.07427772	-0.00445245	0.25622752	0.80399028	0.88912504	-6.51496932
hetero after UVB irradiation VS hetero before UVB irradiation	GO_GLYOXYLATE_METABOLIC_PROCESS1	-0.13073795	0.044969782	-0.4509177	0.66423947	0.86231261	-6.12267484
WT after UVB irradiation VS WT before UVB irradiation	GO_GLYOXYLATE_METABOLIC_PROCESS2	-0.16507965	-0.04460263	-0.74450075	0.47762069	0.84915768	-5.71375438
homo after UVB irradiation VS homo before UVB irradiation	GO_GMP_METABOLIC_PROCESS	-0.02945922	-0.03742353	-0.24259656	0.81418524	0.89494464	-6.51867277
hetero after UVB irradiation VS hetero before UVB irradiation	GO_GMP_METABOLIC_PROCESS1	0.023964948	-0.03478327	0.131236232	0.89888831	0.96269412	-6.22215458
WT after UVB irradiation VS WT before UVB irradiation	GO_GMP_METABOLIC_PROCESS2	0.0058009	0.04323132	0.031225169	0.97584588	0.99381675	-5.99555223
homo after UVB irradiation VS homo before UVB irradiation	GO_GOLGI_APPARATUS	0.300729348	-0.02203849	2.39236682	0.04251536	0.14895002	-4.17477098
hetero after UVB irradiation VS hetero before UVB irradiation	GO_GOLGI_APPARATUS1	0.214585113	0.002203052	1.553696871	0.15956658	0.47983787	-5.09629621
WT after UVB irradiation VS WT before UVB irradiation	GO_GOLGI_APPARATUS2	0.190397026	-0.00480316	1.305804509	0.22749232	0.71944822	-5.18459251
homo after UVB irradiation VS homo before UVB irradiation	GO_GOLGI_ASSOCIATED_VESICLE	0.320522794	-0.02907154	1.993656519	0.0798965	0.21823112	-4.77530813
homo after UVB irradiation VS homo before UVB irradiation	GO_GOLGI_ASSOCIATED_VESICLE_MEMBRANE	0.288898557	-0.01391156	1.593395615	0.14824418	0.32122587	-5.33813391
hetero after UVB irradiation VS hetero before UVB irradiation	GO_GOLGI_ASSOCIATED_VESICLE_MEMBRANE1	0.234498743	0.021954699	1.512140395	0.16964984	0.49210794	-5.14887761
WT after UVB irradiation VS WT before UVB irradiation	GO_GOLGI_ASSOCIATED_VESICLE_MEMBRANE2	0.27001882	0.011668894	1.754581827	0.11695731	0.62452834	-4.63414554
hetero after UVB irradiation VS hetero before UVB irradiation	GO_GOLGI_ASSOCIATED_VESICLE1	0.365761131	0.011110149	2.824291073	0.02276701	0.21590616	-3.29581108
WT after UVB irradiation VS WT before UVB irradiation	GO_GOLGI_ASSOCIATED_VESICLE2	0.327814117	-0.01245639	2.25394543	0.05384429	0.51991848	-3.95092482
homo after UVB irradiation VS homo before UVB irradiation	GO_GOLGI_CIS_CISTERNA	-0.1527438	0.01790325	-0.72427092	0.48874105	0.66488322	-6.27183856
hetero after UVB irradiation VS hetero before UVB irradiation	GO_GOLGI_CIS_CISTERNA1	0.01472197	-0.041921	0.070481213	0.94557248	0.9795751	-6.22879102
WT after UVB irradiation VS WT before UVB irradiation	GO_GOLGI_CIS_CISTERNA2	0.023206695	0.05491367	0.092730454	0.92837091	0.98233894	-5.99153199
homo after UVB irradiation VS homo before UVB irradiation	GO_GOLGI_CISTERNA	0.266110825	-0.00679472	1.980819267	0.08152303	0.22098371	-4.79412514
homo after UVB irradiation VS homo before UVB irradiation	GO_GOLGI_CISTERNA_MEMBRANE	0.219929024	-0.01940838	1.671674109	0.13163512	0.2978683	-5.23255224
hetero after UVB irradiation VS hetero before UVB irradiation	GO_GOLGI_CISTERNA_MEMBRANE1	0.141772064	-0.01641158	0.913693336	0.38807089	0.69816801	-5.80208157
WT after UVB irradiation VS WT before UVB irradiation	GO_GOLGI_CISTERNA_MEMBRANE2	0.14044523	-0.04339341	0.853850093	0.41772933	0.82297693	-5.62870952
hetero after UVB irradiation VS hetero before UVB irradiation	GO_GOLGI_CISTERNA1	0.220745936	-0.01564116	1.436743486	0.18941145	0.51444052	-5.24231891
WT after UVB irradiation VS WT before UVB irradiation	GO_GOLGI_CISTERNA2	0.192489592	-0.02341101	1.29239231	0.23187848	0.72404374	-5.19961849
homo after UVB irradiation VS homo before UVB irradiation	GO_GOLGI_DISASSEMBLY	-0.4385962	0.082954222	-1.96506113	0.08356317	0.22449331	-4.81716676
hetero after UVB irradiation VS hetero before UVB irradiation	GO_GOLGI_DISASSEMBLY1	0.394090501	0.071619745	1.687335342	0.13072843	0.43970412	-4.92252861
WT after UVB irradiation VS WT before UVB irradiation	GO_GOLGI_DISASSEMBLY2	-0.08580998	0.015080748	-0.39151177	0.7055201	0.92646087	-5.91598942
homo after UVB irradiation VS homo before UVB irradiation	GO_GOLGI_LOCALIZATION	0.132845864	-0.01171212	0.557170092	0.59206443	0.74395022	-6.38364175
hetero after UVB irradiation VS hetero before UVB irradiation	GO_GOLGI_LOCALIZATION1	0.210427125	-0.01431032	1.275570907	0.23854509	0.56742432	-5.43261181
WT after UVB irradiation VS WT before UVB irradiation	GO_GOLGI_LOCALIZATION2	0.162503099	-0.07184321	1.047591074	0.32508582	0.77400637	-5.45519172
homo after UVB irradiation VS homo before UVB irradiation	GO_GOLGI_LUMEN	0.407800394	-0.01100811	3.147319249	0.01297647	0.07696355	-3.00551845
hetero after UVB irradiation VS hetero before UVB irradiation	GO_GOLGI_LUMEN1	0.132290187	0.033203642	0.659697091	0.52831756	0.79043843	-6.00201817
WT after UVB irradiation VS WT before UVB irradiation	GO_GOLGI_LUMEN2	0.23109647	0.050472225	1.202489097	0.26315396	0.74156847	-5.29773053
homo after UVB irradiation VS homo before UVB irradiation	GO_GOLGI_MEDIAL_CISTERNA	0.564661345	-0.04062116	3.492720743	0.00766618	0.05825424	-2.47792429
hetero after UVB irradiation VS hetero before UVB irradiation	GO_GOLGI_MEDIAL_CISTERNA1	0.815590375	0.028732621	4.086269413	0.00364453	0.11924328	-1.52914881
WT after UVB irradiation VS WT before UVB irradiation	GO_GOLGI_MEDIAL_CISTERNA2	0.595095938	-0.03653849	2.798577961	0.02297402	0.41742287	-3.17776858
homo after UVB irradiation VS homo before UVB irradiation	GO_GOLGI_MEMBRANE	0.345404516	-0.02652884	2.689507543	0.02654628	0.11515093	-3.71533536
hetero after UVB irradiation VS hetero before UVB irradiation	GO_GOLGI_MEMBRANE1	0.241151136	0.001550045	1.771111509	0.11519647	0.41843252	-4.81036571
WT after UVB irradiation VS WT before UVB irradiation	GO_GOLGI_MEMBRANE2	0.212235697	-0.01602688	1.47955824	0.17680857	0.68058088	-4.98164629
homo after UVB irradiation VS homo before UVB irradiation	GO_GOLGI_ORGANIZATION	0.048695003	-0.0281255	0.274510422	0.79037835	0.8806295	-6.50968947
hetero after UVB irradiation VS hetero before UVB irradiation	GO_GOLGI_ORGANIZATION1	0.140381834	-0.00728982	1.058512424	0.32129317	0.64110913	-5.6649744
WT after UVB irradiation VS WT before UVB irradiation	GO_GOLGI_ORGANIZATION2	0.029049125	-0.04260741	0.19537066	0.84991607	0.96560255	-5.9759766
homo after UVB irradiation VS homo before UVB irradiation	GO_GOLGI_RIBBON_FORMATION	-0.17801825	-0.06594033	-0.70688952	0.49893063	0.67319369	-6.28468422
hetero after UVB irradiation VS hetero before UVB irradiation	GO_GOLGI_RIBBON_FORMATION1	-0.23970628	-0.06256475	-1.25404128	0.24586934	0.5734231	-5.45695997
WT after UVB irradiation VS WT before UVB irradiation	GO_GOLGI_RIBBON_FORMATION2	-0.24661016	-0.05722363	-1.29315118	0.23162841	0.72404374	-5.19877093
homo after UVB irradiation VS homo before UVB irradiation	GO_GOLGI_STACK	0.27185468	-0.00902865	2.046448782	0.07353036	0.20827015	-4.69750696
hetero after UVB irradiation VS hetero before UVB irradiation	GO_GOLGI_STACK1	0.276664733	-0.00913757	2.060504341	0.07395915	0.35255978	-4.40814687
WT after UVB irradiation VS WT before UVB irradiation	GO_GOLGI_STACK2	0.224816887	-0.0177307	1.577432103	0.15289314	0.65981549	-4.86125248
homo after UVB irradiation VS homo before UVB irradiation	GO_GOLGI_TO_ENDOSOME_TRANSPORT	-0.370042	-0.06665668	-2.35654621	0.04500355	0.15433821	-4.22970094
hetero after UVB irradiation VS hetero before UVB irradiation	GO_GOLGI_TO_ENDOSOME_TRANSPORT1	0.078801866	-0.02757369	0.51157332	0.6230206	0.84440275	-6.09195945
WT after UVB irradiation VS WT before UVB irradiation	GO_GOLGI_TO_ENDOSOME_TRANSPORT2	-0.15046498	-0.06286982	-0.64602344	0.53613858	0.86990942	-5.78167302
homo after UVB irradiation VS homo before UVB irradiation	GO_GOLGI_TO_LYSOSOME_TRANSPORT	0.069730112	-0.0179667	0.464528153	0.65417072	0.78972491	-6.43396685
hetero after UVB irradiation VS hetero before UVB irradiation	GO_GOLGI_TO_LYSOSOME_TRANSPORT1	0.435353368	-0.03998923	2.910316379	0.01997421	0.20525028	-3.1701077
WT after UVB irradiation VS WT before UVB irradiation	GO_GOLGI_TO_LYSOSOME_TRANSPORT2	0.262936027	0.021548061	1.007781945	0.34272053	0.78172749	-5.4930828
homo after UVB irradiation VS homo before UVB irradiation	GO_GOLGI_TO_PLASMA_MEMBRANE_PROTEIN_TRANSPORT	-0.02098052	-0.04379312	-0.12948947	0.9000429	0.94566985	-6.54166379

hetero after UVB irradiation VS hetero before UVB irradiation	GO_GOLGI_TO_PLASMA_MEMBRANE_PROTEIN_TRANSPORT1	0.008049822	-0.02462571	0.057903571	0.95527173	0.98346401	-6.22966625
WT after UVB irradiation VS WT before UVB irradiation	GO_GOLGI_TO_PLASMA_MEMBRANE_PROTEIN_TRANSPORT2	-0.24047373	-0.04255994	-1.57830736	0.15269311	0.65981549	-4.86015821
homo after UVB irradiation VS homo before UVB irradiation	GO_GOLGI_TO_PLASMA_MEMBRANE_TRANSPORT	-0.03405801	-0.04134891	-0.20823239	0.84004944	0.91138138	-6.52712241
hetero after UVB irradiation VS hetero before UVB irradiation	GO_GOLGI_TO_PLASMA_MEMBRANE_TRANSPORT1	0.023209775	-0.02601822	0.165076611	0.87305399	0.95272776	-6.21673261
WT after UVB irradiation VS WT before UVB irradiation	GO_GOLGI_TO_PLASMA_MEMBRANE_TRANSPORT2	-0.20363482	-0.04367207	-1.26924149	0.23961777	0.72963866	-5.22532251
homo after UVB irradiation VS homo before UVB irradiation	GO_GOLGI_TO_VACUOLE_TRANSPORT	0.183733169	-0.03355979	1.342850645	0.21476254	0.40524712	-5.65687466
hetero after UVB irradiation VS hetero before UVB irradiation	GO_GOLGI_TO_VACUOLE_TRANSPORT1	0.539994671	0.006957049	3.445962178	0.00899913	0.15055678	-2.40086511
WT after UVB irradiation VS WT before UVB irradiation	GO_GOLGI_TO_VACUOLE_TRANSPORT2	0.262988764	0.024343656	1.097412514	0.30401887	0.76505447	-5.40624175
homo after UVB irradiation VS homo before UVB irradiation	GO_GOLGI_TRANS_CISTERNA	0.821930926	-0.07988756	6.218542965	0.00021506	0.01130622	1.106349726
hetero after UVB irradiation VS hetero before UVB irradiation	GO_GOLGI_TRANS_CISTERNA1	0.62357175	0.040739541	2.428976719	0.04181785	0.27534456	-3.8753727
WT after UVB irradiation VS WT before UVB irradiation	GO_GOLGI_TRANS_CISTERNA2	0.448762776	-0.02980055	1.755272355	0.11683401	0.62424536	-4.63323997
homo after UVB irradiation VS homo before UVB irradiation	GO_GOLGI_TRANSPORT_COMPLEX	0.258410689	-0.01822238	0.906009445	0.39038584	0.57975902	-6.1216114
hetero after UVB irradiation VS hetero before UVB irradiation	GO_GOLGI_TRANSPORT_COMPLEX1	0.293938202	-0.05087579	1.195766305	0.26664504	0.59300892	-5.52148931
WT after UVB irradiation VS WT before UVB irradiation	GO_GOLGI_TRANSPORT_COMPLEX2	0.121456746	-0.02087265	0.44902046	0.66519063	0.9126723	-5.89106391
homo after UVB irradiation VS homo before UVB irradiation	GO_GOLGI_VESICLE_BUDDING	0.348925051	-0.03424119	2.083554553	0.06935262	0.20134405	-4.6424447
hetero after UVB irradiation VS hetero before UVB irradiation	GO_GOLGI_VESICLE_BUDDING1	0.37937138	0.017726416	2.538088429	0.03532839	0.25845596	-3.7155328
WT after UVB irradiation VS WT before UVB irradiation	GO_GOLGI_VESICLE_BUDDING2	0.347852866	-0.0344772	2.226703641	0.05619397	0.5232848	-3.98924412
homo after UVB irradiation VS homo before UVB irradiation	GO_GOLGI_VESICLE_TRANSPORT	0.236419308	-0.03416687	1.648979639	0.13626617	0.304956	-5.26342255
hetero after UVB irradiation VS hetero before UVB irradiation	GO_GOLGI_VESICLE_TRANSPORT1	0.311783924	0.002606183	2.51816744	0.03643183	0.26240867	-3.74474539
WT after UVB irradiation VS WT before UVB irradiation	GO_GOLGI_VESICLE_TRANSPORT2	0.211805173	-0.01384591	1.33219882	0.21906651	0.71478521	-5.1547402
homo after UVB irradiation VS homo before UVB irradiation	GO_GONADAL_MESODERM_DEVELOPMENT	0.796881768	0.092743797	2.317245028	0.04790087	0.1603978	-4.28980108
hetero after UVB irradiation VS hetero before UVB irradiation	GO_GONADAL_MESODERM_DEVELOPMENT1	-0.90139032	-0.01567195	-2.80332039	0.02350731	0.21954039	-3.32650646
WT after UVB irradiation VS WT before UVB irradiation	GO_GONADAL_MESODERM_DEVELOPMENT2	-0.35083219	-0.11660909	-0.93097673	0.37878473	0.80338597	-5.56298102
homo after UVB irradiation VS homo before UVB irradiation	GO_GONADOTROPIN_SECRETION	0.065792191	0.016465699	0.274730078	0.79021526	0.88059007	-6.50962386
hetero after UVB irradiation VS hetero before UVB irradiation	GO_GONADOTROPIN_SECRETION1	-0.00301271	-0.00640676	-0.0139114	0.9892476	0.99613332	-6.23137916
WT after UVB irradiation VS WT before UVB irradiation	GO_GONADOTROPIN_SECRETION2	-0.00093087	0.077616262	-0.00454644	0.99648249	0.99898723	-5.99605576
homo after UVB irradiation VS homo before UVB irradiation	GO_GPI_ANCHOR_BINDING	0.377878265	-0.05241162	2.307910185	0.04861588	0.16176427	-4.30404834
hetero after UVB irradiation VS hetero before UVB irradiation	GO_GPI_ANCHOR_BINDING1	0.182945908	-0.01590607	0.809249682	0.44219923	0.73804258	-5.89089947
WT after UVB irradiation VS WT before UVB irradiation	GO_GPI_ANCHOR_BINDING2	0.474610366	-0.01447727	2.020274725	0.07759738	0.56130885	-4.27652515
homo after UVB irradiation VS homo before UVB irradiation	GO_GPI_ANCHOR_METABOLIC_PROCESS	-0.07448907	-0.03560988	-0.48429686	0.64065325	0.77978556	-6.42394896
hetero after UVB irradiation VS hetero before UVB irradiation	GO_GPI_ANCHOR_METABOLIC_PROCESS1	-0.1000346	-0.03895381	-0.5908906	0.57120466	0.81424167	-6.04637977
WT after UVB irradiation VS WT before UVB irradiation	GO_GPI_ANCHOR_METABOLIC_PROCESS2	0.039958112	0.020316634	0.191436802	0.85289651	0.96605575	-5.97677558
homo after UVB irradiation VS homo before UVB irradiation	GO_GPI_ANCHOR_TRANSAMIDASE_COMPLEX	0.513711504	-0.07222869	2.095122234	0.06809803	0.19937164	-4.62521902
hetero after UVB irradiation VS hetero before UVB irradiation	GO_GPI_ANCHOR_TRANSAMIDASE_COMPLEX1	0.384504506	0.057851872	1.764848305	0.11629501	0.41926439	-4.81882868
WT after UVB irradiation VS WT before UVB irradiation	GO_GPI_ANCHOR_TRANSAMIDASE_COMPLEX2	0.814112906	0.01062675	3.423435816	0.00887816	0.31908946	-2.30788913
homo after UVB irradiation VS homo before UVB irradiation	GO_GPI_LINKED_EPHRIN_RECEPTOR_ACTIVITY	-0.27941927	0.092546478	-1.27388011	0.23709969	0.42926866	-5.73869446
hetero after UVB irradiation VS hetero before UVB irradiation	GO_GPI_LINKED_EPHRIN_RECEPTOR_ACTIVITY1	-0.45739245	-0.00255285	-2.53655701	0.03541199	0.25868552	-3.71777885
WT after UVB irradiation VS WT before UVB irradiation	GO_GPI_LINKED_EPHRIN_RECEPTOR_ACTIVITY2	-0.07036382	-0.00781955	-0.37438575	0.71773135	0.9309626	-5.92277986
homo after UVB irradiation VS homo before UVB irradiation	GO_GRANULAR_COMPONENT	-0.94844777	0.040831996	-5.6383969	0.00042185	0.01537777	0.439261021
hetero after UVB irradiation VS hetero before UVB irradiation	GO_GRANULAR_COMPONENT1	-0.83082492	-0.05896182	-3.60538473	0.00714698	0.13963559	-2.17821738
WT after UVB irradiation VS WT before UVB irradiation	GO_GRANULAR_COMPONENT2	-0.8035357	-0.04700282	-3.29373653	0.01077717	0.33609774	-2.48500492
homo after UVB irradiation VS homo before UVB irradiation	GO_GRANULOCYTE_CHEMOTAXIS	0.48502577	-0.02006746	3.223805892	0.01153534	0.07209952	-2.8878276
hetero after UVB irradiation VS hetero before UVB irradiation	GO_GRANULOCYTE_CHEMOTAXIS1	0.476421324	0.011378485	3.163474029	0.01364685	0.17665328	-2.80307489
WT after UVB irradiation VS WT before UVB irradiation	GO_GRANULOCYTE_CHEMOTAXIS2	0.327798676	0.017266354	1.481805449	0.17622396	0.68058088	-4.97892769
homo after UVB irradiation VS homo before UVB irradiation	GO_GRANULOCYTE_COLONY_STIMULATING_FACTOR_PRODUCTION	-0.08011451	-0.06477159	-0.3095025	0.76454083	0.86390334	-6.4985902
hetero after UVB irradiation VS hetero before UVB irradiation	GO_GRANULOCYTE_COLONY_STIMULATING_FACTOR_PRODUCTION1	0.240573567	0.01677442	0.663955828	0.52572882	0.78916946	-5.99912875
WT after UVB irradiation VS WT before UVB irradiation	GO_GRANULOCYTE_COLONY_STIMULATING_FACTOR_PRODUCTION2	0.058257261	-0.04475891	0.181382232	0.86052551	0.9676681	-5.97874452
homo after UVB irradiation VS homo before UVB irradiation	GO_GRANULOCYTE_DIFFERENTIATION	0.368762521	-0.01334522	2.412248752	0.04119417	0.14620067	-4.14422538
hetero after UVB irradiation VS hetero before UVB irradiation	GO_GRANULOCYTE_DIFFERENTIATION1	0.338592782	0.01775203	1.690111543	0.13018387	0.43858291	-4.91884902
WT after UVB irradiation VS WT before UVB irradiation	GO_GRANULOCYTE_DIFFERENTIATION2	0.387860747	0.027474521	1.985084774	0.08196482	0.56741066	-4.32481003
homo after UVB irradiation VS homo before UVB irradiation	GO_GRANULOCYTE_MACROPHAGE_COLONY_STIMULATING_FACTOR_PRODUCTION	0.729472183	-0.00526874	4.73497374	0.00131914	0.02448624	-0.70308536
hetero after UVB irradiation VS hetero before UVB irradiation	GO_GRANULOCYTE_MACROPHAGE_COLONY_STIMULATING_FACTOR_PRODUCTION1	0.639799463	0.031897045	2.947375321	0.01888317	0.19988522	-3.1160821
WT after UVB irradiation VS WT before UVB irradiation	GO_GRANULOCYTE_MACROPHAGE_COLONY_STIMULATING_FACTOR_PRODUCTION2	0.570655138	0.050990837	2.320305116	0.04852112	0.50722877	-3.85729241
homo after UVB irradiation VS homo before UVB irradiation	GO_GRANULOCYTE_MIGRATION	0.488624004	-0.01542846	3.225671197	0.01150236	0.07198524	-2.88496272
hetero after UVB irradiation VS hetero before UVB irradiation	GO_GRANULOCYTE_MIGRATION1	0.518914534	0.014111285	3.576580206	0.00744893	0.14177754	-2.21819051
WT after UVB irradiation VS WT before UVB irradiation	GO_GRANULOCYTE_MIGRATION2	0.37572471	0.012749782	1.765156082	0.11508234	0.62050687	-4.62026253
homo after UVB irradiation VS homo before UVB irradiation	GO_GRANZYME_MEDIATED_APOPTOTIC_SIGNALING_PATHWAY	0.815004282	-0.03664426	3.521880413	0.00733782	0.05666366	-2.43390195
hetero after UVB irradiation VS hetero before UVB irradiation	GO_GRANZYME_MEDIATED_APOPTOTIC_SIGNALING_PATHWAY1	1.179530875	-0.02350064	5.934333731	0.00037395	0.07788488	0.607009669
WT after UVB irradiation VS WT before UVB irradiation	GO_GRANZYME_MEDIATED_APOPTOTIC_SIGNALING_PATHWAY2	0.427352576	-0.00664247	1.344265031	0.21530443	0.71254709	-5.14097079
homo after UVB irradiation VS homo before UVB irradiation	GO_GROOMING_BEHAVIOR	-0.22494631	0.053698031	-1.36875239	0.20685043	0.39683981	-5.62543608
hetero after UVB irradiation VS hetero before UVB irradiation	GO_GROOMING_BEHAVIOR1	-0.05482152	0.072478159	-0.30018498	0.77182897	0.91120156	-6.18290496
WT after UVB irradiation VS WT before UVB irradiation	GO_GROOMING_BEHAVIOR2	0.079184509	0.034261387	0.465917432	0.65355036	0.90964994	-5.88312463
homo after UVB irradiation VS homo before UVB irradiation	GO_GROWTH	0.017744352	-0.00122934	0.143461231	0.88933613	0.94010984	-6.53957558
homo after UVB irradiation VS homo before UVB irradiation	GO_GROWTH_CONE_MEMBRANE	-0.02045752	-0.03481729	-0.07303161	0.94350438	0.97012919	-6.54793047
hetero after UVB irradiation VS hetero before UVB irradiation	GO_GROWTH_CONE_MEMBRANE1	-0.07072463	0.024710595	-0.34451802	0.73950138	0.89840955	-6.16761595
WT after UVB irradiation VS WT before UVB irradiation	GO_GROWTH_CONE_MEMBRANE2	-0.10239545	-0.00590606	-0.45456699	0.66135879	0.91169919	-5.8884884
homo after UVB irradiation VS homo before UVB irradiation	GO_GROWTH_FACTOR_ACTIVITY	0.31734192	0.015056978	2.458648405	0.03826871	0.14023625	-4.07279649
hetero after UVB irradiation VS hetero before UVB irradiation	GO_GROWTH_FACTOR_ACTIVITY1	0.147709599	0.009488681	0.783091344	0.45653852	0.74650043	-5.91173685
WT after UVB irradiation VS WT before UVB irradiation	GO_GROWTH_FACTOR_ACTIVITY2	0.107906757	0.006683314	0.518599261	0.61791018	0.90020288	-5.85660301
homo after UVB irradiation VS homo before UVB irradiation	GO_GROWTH_FACTOR_BINDING	0.329962772	-0.02268667	2.551851526	0.0330086	0.12942723	-3.92881432
hetero after UVB irradiation VS hetero before UVB irradiation	GO_GROWTH_FACTOR_BINDING1	0.139193772	-0.00235301	0.774403915	0.46136951	0.74911625	-5.91852866
WT after UVB irradiation VS WT before UVB irradiation	GO_GROWTH_FACTOR_BINDING2	0.121809371	-0.01349937	0.671628946	0.52051641	0.86304554	-5.76483135
homo after UVB irradiation VS homo before UVB irradiation	GO_GROWTH_FACTOR_COMPLEX	0.382348743	-0.09758429	1.809303453	0.10652721	0.26048419	-5.04118812
hetero after UVB irradiation VS hetero before UVB irradiation	GO_GROWTH_FACTOR_COMPLEX1	0.062512233	-0.00748553	0.174059515	0.86622213	0.94927395	-6.21508676
WT after UVB irradiation VS WT before UVB irradiation	GO_GROWTH_FACTOR_COMPLEX2	0.191475786	-0.03490091	0.620388415	0.55205777	0.87627369	-5.79794557
homo after UVB irradiation VS homo before UVB irradiation	GO_GROWTH_FACTOR_RECEPTOR_BINDING	0.439240445	-0.00692858	3.329054002	0.00982058	0.06617454	-2.72661642
hetero after UVB irradiation VS hetero before UVB irradiation	GO_GROWTH_FACTOR_RECEPTOR_BINDING1	0.471861224	0.016842116	3.205138547	0.01282613	0.17315837	-2.74320243
WT after UVB irradiation VS WT before UVB irradiation	GO_GROWTH_FACTOR_RECEPTOR_BINDING2	0.380717714	0.017607099	2.411018644	0.04208426	0.495137	-3.72877744
homo after UVB irradiation VS homo before UVB irradiation	GO_GROWTH_HORMONE_RECEPTOR_BINDING	1.022184845	-0.06574351	4.607792465	0.00156341	0.02682706	-0.87426392
hetero after UVB irradiation VS hetero before UVB irradiation	GO_GROWTH_HORMONE_RECEPTOR_BINDING1	0.655803915	0.053303824	2.693941875	0.0277923	0.23528578	-3.48683756
WT after UVB irradiation VS WT before UVB irradiation	GO_GROWTH_HORMONE_RECEPTOR_BINDING2	0.585178978	-0.07719154	1.936979219	0.08832046	0.58080792	-4.39041523
homo after UVB irradiation VS homo before UVB irradiation	GO_GROWTH_HORMONE_RECEPTOR_SIGNALING_PATHWAY_VIA_JAK_STAT	0.83764873	-0.07985186	5.571934099	0.00045699	0.01594272	0.35958315
hetero after UVB irradiation VS hetero before UVB irradiation	GO_GROWTH_HORMONE_RECEPTOR_SIGNALING_PATHWAY_VIA_JAK_STAT1	0.575081033	-0.03812462	2.164713393	0.06295982	0.32964008	-4.2591264
WT after UVB irradiation VS WT before UVB irradiation	GO_GROWTH_HORMONE_RECEPTOR_SIGNALING_PATHWAY_VIA_JAK_STAT2	0.384502098	-0.10745317	1.257994062	0.24345555	0.73311008	-5.2377024
homo after UVB irradiation VS homo before UVB irradiation	GO_GROWTH_HORMONE_SECRETION	0.131943649	0.031562763	0.459234708	0.65781356	0.79180554	-6.43658216
hetero after UVB irradiation VS hetero before UVB irradiation	GO_GROWTH_HORMONE_SECRETION1	0.241149294	0.024189162	1.183709789	0.27111996	0.59721184	-5.53458157
WT after UVB irradiation VS WT before UVB irradiation	GO_GROWTH_HORMONE_SECRETION2	-0.04575822	-0.09654788	-0.19778209	0.84809035	0.96522688	-5.97547887
homo after UVB irradiation VS homo before UVB irradiation	GO_GROWTH_INVOLVED_IN_HEART_MORPHOGENESIS	-0.06937295	-0.03667139	-0.20389706	0.8433278	0.91347513	-6.52809793
hetero after UVB irradiation VS hetero before UVB irradiation	GO_GROWTH_INVOLVED_IN_HEART_MORPHOGENESIS1	0.56813793	0.004240055	2.459878257	0.03986599	0.27221772	-3.83015158
WT after UVB irradiation VS WT before UVB irradiation	GO_GROWTH_INVOLVED_IN_HEART_MORPHOGENESIS2	0.342046622	0.139331976	1.137546238	0.28784695	0.75467539	-5.36561822
homo after UVB irradiation VS homo before UVB irradiation	GO_GROWTH_PLATE_CARTILAGE_CHONDROCYTE_DEVELOPMENT	0.346814441	0.057480052	1.779901623	0.11148284	0.26787208	-5.08263426
hetero after UVB irradiation VS hetero before UVB irradiation	GO_GROWTH_PLATE_CARTILAGE_CHONDROCYTE_DEVELOPMENT1	0.340841063	-0.0284199	1.438268998	0.18899218	0.51443012	-5.24045453
WT after UVB irradiation VS WT before UVB irradiation	GO_GROWTH_PLATE_CARTILAGE_CHONDROCYTE_DEVELOPMENT2	0.337700675	-0.09659476	2.259572848	0.05337119	0.51991848	-3.94299987
homo after UVB irradiation VS homo before UVB irradiation	GO_GROWTH_PLATE_CARTILAGE_CHONDROCYTE_DIFFERENTIATION	0.297688793	0.056753634	1.333252755	0.21775948	0.40847506	-5.66842748
hetero after UVB irradiation VS hetero before UVB irradiation	GO_GROWTH_PLATE_CARTILAGE_CHONDROCYTE_DIFFERENTIATION1	0.383370238	-0.01731703	1.772706059	0.11491835	0.41808067	-4.8082092
WT after UVB irradiation VS WT before UVB irradiation	GO_GROWTH_PLATE_CARTILAGE_CHONDROCYTE_DIFFERENTIATION2	0.31428017	0.030691602	1.940494023	0.08784048	0.57920555	-4.38563841
homo after UVB irradiation VS homo before UVB irradiation	GO_GROWTH_PLATE_CARTILAGE_DEVELOPMENT	0.110571814	0.026430506	0.787425502	0.4528549	0.63429617	-6.22287775
hetero after UVB irradiation VS hetero before UVB irradiation	GO_GROWTH_PLATE_CARTILAGE_DEVELOPMENT1	0.041691271	0.003807272	0.250541991	0.80859872	0.92568169	-6.19758318
WT after UVB irradiation VS WT before UVB irradiation	GO_GROWTH_PLATE_CARTILAGE_DEVELOPMENT2	0.019676586	-0.03493494	0.116666677	0.90996585	0.97842738	-5.98889115
hetero after UVB irradiation VS hetero before UVB irradiation	GO_GROWTH1	-0.02747393	0.005259688	-0.21803362	0.83296196	0.93554473	-6.20578417
WT after UVB irradiation VS WT before UVB irradiation	GO_GROWTH2	-0.04434245	-0.00900441	-0.32009511	0.75699964	0.93867697	-5.94236288
homo after UVB irradiation VS homo before UVB irradiation	GO_GTP_BIOSYNTHETIC_PROCESS	-0.19793091	-0.03120629	-1.00711045	0.3422393	0.53517277	-6.02619932
hetero after UVB irradiation VS hetero before UVB irradiation	GO_GTP_BIOSYNTHETIC_PROCESS1	-0.3545878	-0.09895728	-1.45377097	0.18477795	0.50935615	-5.22144614
WT after UVB irradiation VS WT before UVB irradiation	GO_GTP_BIOSYNTHETIC_PROCESS2	0.170121009	-0.03188344	1.052040221	0.32315937	0.77346379	-5.45088867
homo after UVB irradiation VS homo before UVB irradiation	GO_GTP_DEPENDENT_PROTEIN_BINDING	0.17415947	-0.04789291	0.987938594	0.35100903	0.5429876	-6.04490608
hetero after UVB irradiation VS hetero before UVB irradiation	GO_GTP_DEPENDENT_PROTEIN_BINDING1	0.373594415	0.008483087	1.938011534	0.08930032	0.37595306	-4.58080443
WT after UVB irradiation VS WT before UVB irradiation	GO_GTP_DEPENDENT_PROTEIN_BINDING2	0.248448249	0.010682521	1.424889636	0.19156929	0.69334561	-5.04707834
homo after UVB irradiation VS homo before UVB irradiation	GO_GTP_METABOLIC_PROCESS	-0.13518513	-0.02976694	-1.06503264	0.3167579	0.51008254	-5.96800263
hetero after UVB irradiation VS hetero before UVB irradiation	GO_GTP_METABOLIC_PROCESS1	-0.07174017	-0.06228892	-0.40151932	0.69874287	0.88080043	-6.1449733
WT after UVB irradiation VS WT before UVB irradiation	GO_GTP_METABOLIC_PROCESS2	0.034915217	-0.05284477	0.183901508	0.85861249	0.96699569	-5.97826107
homo after UVB irradiation VS homo before UVB irradiation	GO_GTP_RHO_BINDING	0.040803374	-0.03125025	0.292134876	0.77732818	0.8714603	-6.50426191
hetero after UVB irradiation VS hetero before UVB irradiation	GO_GTP_RHO_BINDING1	-0.01725896	-0.06045369	-0.08695447	0.93288405	0.9752585	-6.22738572
WT after UVB irradiation VS WT before UVB irradiation	GO_GTP_RHO_BINDING2	0.012037228	-0.03605825	0.066174468	0.94884358	0.9862012	-5.99375671
homo after UVB irradiation VS homo before UVB irradiation	GO_GTPASE_ACTIVATING_PROTEIN_BINDING	-0.16330854	-0.01449046	-1.04985127	0.32329006	0.51654303	-5.9834963
hetero after UVB irradiation VS hetero before UVB irradiation	GO_GTPASE_ACTIVATING_PROTEIN_BINDING1	0.178869987	-0.01644659	1.11124067	0.29932743	0.62222329	-5.61132586
WT after UVB irradiation VS WT before UVB irradiation	GO_GTPASE_ACTIVATING_PROTEIN_BINDING2	0.211788516	0.029024788	1.269574925	0.23950478	0.72963866	-5.22495441
homo after UVB irradiation VS homo before UVB irradiation	GO_GTPASE_ACTIVITY	0.139710558	-0.0167738	1.107955199	0.29884644	0.49313171	-5.92329666
hetero after UVB irradiation VS hetero before UVB irradiation	GO_GTPASE_ACTIVITY1	0.239508197	-0.0113656	1.942738101	0.08865502	0.37537832	-4.57420083
WT after UVB irradiation VS WT before UVB irradiation	GO_GTPASE_ACTIVITY2	0.115902907	-0.01456473	0.792509992	0.45065527	0.83557229	-5.67762762
homo after UVB irradiation VS homo before UVB irradiation	GO_GTPASE_BINDING	0.031623709	-0.0225994	0.22832914	0.82489643	0.90177025	-6.52233534
hetero after UVB irradiation VS hetero before UVB irradiation	GO_GTPASE_BINDING1	0.168240856	-0.02420954	1.306496992	0.22834804	0.55686647	-5.39717493
WT after UVB irradiation VS WT before UVB irradiation	GO_GTPASE_BINDING2	0.034260223	-0.01719628	0.226923646	0.82610711	0.95885344	-5.9689869
homo after UVB irradiation VS homo before UVB irradiation	GO_GTPASE_INHIBITOR_ACTIVITY	0.170478582	0.021080864	1.11030132	0.29789095	0.49244815	-5.92081531
hetero after UVB irradiation VS hetero before UVB irradiation	GO_GTPASE_INHIBITOR_ACTIVITY1	-0.11993782	-0.05109475	-0.75902293	0.47000644	0.75417736	-5.93039476
WT after UVB irradiation VS WT before UVB irradiation	GO_GTPASE_INHIBITOR_ACTIVITY2	0.190381342	-0.05517659	1.084215937	0.30949138	0.76728607	-5.4193695
homo after UVB irradiation VS homo before UVB irradiation	GO_GTPASE_REGULATOR_ACTIVITY	0.104295287	-0.02021275	0.754404036	0.4713947	0.65012818	-6.24892129
hetero after UVB irradiation VS hetero before UVB irradiation	GO_GTPASE_REGULATOR_ACTIVITY1	0.160699188	-0.01324642	1.235764572	0.25223447	0.5800182	-5.47741714
WT after UVB irradiation VS WT before UVB irradiation	GO_GTPASE_REGULATOR_ACTIVITY2	0.047445919	-0.04063672	0.291129355	0.77827027	0.94371579	-5.95159148
homo after UVB irradiation VS homo before UVB irradiation	GO_GUANOSINE_CONTAINING_COMPOUND_BIOSYNTHETIC_PROCESS	-0.23534729	-0.04464845	-1.15636927	0.27961752	0.4748896	-5.87131854
hetero after UVB irradiation VS hetero before UVB irradiation	GO_GUANOSINE_CONTAINING_COMPOUND_BIOSYNTHETIC_PROCESS1	-0.23626833	-0.06352224	-0.99318302	0.35023473	0.66713765	-5.72874244
WT after UVB irradiation VS WT before UVB irradiation	GO_GUANOSINE_CONTAINING_COMPOUND_BIOSYNTHETIC_PROCESS2	0.215777147	-0.00902732	1.005304437	0.34384176	0.78348998	-5.49540415
homo after UVB irradiation VS homo before UVB irradiation	GO_GUANOSINE_CONTAINING_COMPOUND_METABOLIC_PROCESS	-0.07789374	-0.0435198	-0.62865599	0.54642336	0.71025526	-6.33904631
hetero after UVB irradiation VS hetero before UVB irradiation	GO_GUANOSINE_CONTAINING_COMPOUND_METABOLIC_PROCESS1	-0.04808106	-0.03760363	-0.32073685	0.75677876	0.90565384	-6.17607169
WT after UVB irradiation VS WT before UVB irradiation	GO_GUANOSINE_CONTAINING_COMPOUND_METABOLIC_PROCESS2	-0.03363936	0.001554058	-0.18192186	0.86011567	0.9676681	-5.97864152
homo after UVB irradiation VS homo before UVB irradiation	GO_GUANYL_NUCLEOTIDE_BINDING	0.025151318	-0.00866661	0.208465598	0.83987318	0.91132416	-6.52706936
hetero after UVB irradiation VS hetero before UVB irradiation	GO_GUANYL_NUCLEOTIDE_BINDING1	0.138709955	-0.01448389	1.093683641	0.30650478	0.627591	-5.62939961
WT after UVB irradiation VS WT before UVB irradiation	GO_GUANYL_NUCLEOTIDE_BINDING2	0.084191859	-0.02794619	0.570546929	0.58378749	0.89000397	-5.82787018
homo after UVB irradiation VS homo before UVB irradiation	GO_GUANYL_NUCLEOTIDE_EXCHANGE_FACTOR_ACTIVITY	0.068035858	-0.02565234	0.519651808	0.61683043	0.76191549	-6.40505292
hetero after UVB irradiation VS hetero before UVB irradiation	GO_GUANYL_NUCLEOTIDE_EXCHANGE_FACTOR_ACTIVITY1	0.154040329	-0.01259179	1.223364307	0.25663104	0.58385284	-5.4911839
WT after UVB irradiation VS WT before UVB irradiation	GO_GUANYL_NUCLEOTIDE_EXCHANGE_FACTOR_ACTIVITY2	0.012087062	-0.0376756	0.080890161	0.93749286	0.98368758	-5.99261561
homo after UVB irradiation VS homo before UVB irradiation	GO_GUANYL_NUCLEOTIDE_EXCHANGE_FACTOR_COMPLEX	-0.04579114	0.010105086	-0.38327632	0.71111235	0.82752819	-6.47095128
hetero after UVB irradiation VS hetero before UVB irradiation	GO_GUANYL_NUCLEOTIDE_EXCHANGE_FACTOR_COMPLEX1	0.068499039	-0.04589507	0.368842472	0.72199125	0.89103348	-6.15836164
WT after UVB irradiation VS WT before UVB irradiation	GO_GUANYL_NUCLEOTIDE_EXCHANGE_FACTOR_COMPLEX2	-0.34896435	-0.06619886	-2.02261818	0.07731468	0.56116995	-4.27330135
homo after UVB irradiation VS homo before UVB irradiation	GO_GUANYLATE_CYCLASE_ACTIVITY	0.384107418	-0.01522205	2.408332658	0.04145111	0.14677108	-4.15024493
hetero after UVB irradiation VS hetero before UVB irradiation	GO_GUANYLATE_CYCLASE_ACTIVITY1	-0.15133315	0.084327742	-0.85264162	0.41910253	0.72166655	-5.85507414
WT after UVB irradiation VS WT before UVB irradiation	GO_GUANYLATE_CYCLASE_ACTIVITY2	-0.15009306	-0.04459482	-0.5017171	0.62922102	0.90390703	-5.86539163
homo after UVB irradiation VS homo before UVB irradiation	GO_GUANYLATE_CYCLASE_REGULATOR_ACTIVITY	0.227584693	0.004824904	0.855273242	0.41632177	0.60237871	-6.16639088
hetero after UVB irradiation VS hetero before UVB irradiation	GO_GUANYLATE_CYCLASE_REGULATOR_ACTIVITY1	-0.34446226	0.026233224	-1.34360855	0.21660505	0.54366799	-5.35395288
WT after UVB irradiation VS WT before UVB irradiation	GO_GUANYLATE_CYCLASE_REGULATOR_ACTIVITY2	-0.04413984	-0.0114984	-0.18232053	0.8598129	0.96753538	-5.97856523
homo after UVB irradiation VS homo before UVB irradiation	GO_GUANYLATE_KINASE_ACTIVITY	0.019687356	-0.06275938	0.124468934	0.90389563	0.94744292	-6.54236216
hetero after UVB irradiation VS hetero before UVB irradiation	GO_GUANYLATE_KINASE_ACTIVITY1	0.185177431	0.009683845	0.720212639	0.49227198	0.76753004	-5.95942295
WT after UVB irradiation VS WT before UVB irradiation	GO_GUANYLATE_KINASE_ACTIVITY2	-0.22691853	0.050988055	-1.1113215	0.29833423	0.75997207	-5.39228123

homo after UVB irradiation VS homo before UVB irradiation	GO_GUANYLYLTRANSFERASE_ACTIVITY	0.085853335	-0.09260785	0.438621087	0.67209102	0.802431	-6.44649488
hetero after UVB irradiation VS hetero before UVB irradiation	GO_GUANYLYLTRANSFERASE_ACTIVITY1	0.231658258	-0.02972724	0.834836163	0.42847562	0.72933283	-5.8699628
WT after UVB irradiation VS WT before UVB irradiation	GO_GUANYLYLTRANSFERASE_ACTIVITY2	0.006562553	-0.06952005	0.025675363	0.98013774	0.99512555	-5.99571884
homo after UVB irradiation VS homo before UVB irradiation	GO_H3_HISTONE_ACETYLTRANSFERASE_ACTIVITY	-0.50252595	0.084021966	-1.94726224	0.08592642	0.22852769	-4.84311502
hetero after UVB irradiation VS hetero before UVB irradiation	GO_H3_HISTONE_ACETYLTRANSFERASE_ACTIVITY1	-0.35765806	-0.05983269	-1.54669271	0.1612272	0.48224836	-5.10520992
WT after UVB irradiation VS WT before UVB irradiation	GO_H3_HISTONE_ACETYLTRANSFERASE_ACTIVITY2	-0.10516883	0.010821045	-0.38100213	0.71300325	0.92947342	-5.92019112
homo after UVB irradiation VS homo before UVB irradiation	GO_H3_HISTONE_ACETYLTRANSFERASE_COMPLEX	-0.75060283	0.008586238	-4.73066537	0.0013267	0.02450673	-0.7088424
hetero after UVB irradiation VS hetero before UVB irradiation	GO_H3_HISTONE_ACETYLTRANSFERASE_COMPLEX1	-0.59345462	-0.11507138	-2.23124307	0.05679897	0.31461576	-4.16317492
WT after UVB irradiation VS WT before UVB irradiation	GO_H3_HISTONE_ACETYLTRANSFERASE_COMPLEX2	-0.26659725	0.024745142	-0.80368801	0.44452692	0.83340629	-5.66894114
homo after UVB irradiation VS homo before UVB irradiation	GO_H4_H2A_HISTONE_ACETYLTRANSFERASE_COMPLEX	-0.47599532	0.01790465	-2.94785694	0.0176879	0.09113838	-3.31404912
hetero after UVB irradiation VS hetero before UVB irradiation	GO_H4_H2A_HISTONE_ACETYLTRANSFERASE_COMPLEX1	-0.14646367	-0.02235927	-0.67486201	0.51913494	0.78616125	-5.99165396
WT after UVB irradiation VS WT before UVB irradiation	GO_H4_H2A_HISTONE_ACETYLTRANSFERASE_COMPLEX2	-0.14724536	-0.00917124	-0.60948776	0.55891032	0.879729	-5.80468477
homo after UVB irradiation VS homo before UVB irradiation	GO_H4_HISTONE_ACETYLTRANSFERASE_ACTIVITY	-0.47038223	-0.02544117	-2.60059062	0.03055532	0.12408458	-3.8533146
hetero after UVB irradiation VS hetero before UVB irradiation	GO_H4_HISTONE_ACETYLTRANSFERASE_ACTIVITY1	-0.13768605	-0.05538669	-0.82016135	0.43630999	0.73478547	-5.88203735
WT after UVB irradiation VS WT before UVB irradiation	GO_H4_HISTONE_ACETYLTRANSFERASE_ACTIVITY2	-0.18501353	-0.04209559	-0.81660231	0.43751765	0.83056493	-5.65877794
homo after UVB irradiation VS homo before UVB irradiation	GO_H4_HISTONE_ACETYLTRANSFERASE_COMPLEX	-0.48943585	-0.0080159	-2.92430948	0.01835101	0.09296457	-3.35058479
hetero after UVB irradiation VS hetero before UVB irradiation	GO_H4_HISTONE_ACETYLTRANSFERASE_COMPLEX1	-0.2354274	-0.07144033	-1.07773839	0.31314113	0.63410327	-5.64563322
WT after UVB irradiation VS WT before UVB irradiation	GO_H4_HISTONE_ACETYLTRANSFERASE_COMPLEX2	-0.30105956	-0.0383906	-1.20561052	0.26201224	0.74156847	-5.29440275
homo after UVB irradiation VS homo before UVB irradiation	GO_HAIR_CELL_DIFFERENTIATION	-0.0551132	0.028625137	-0.36448428	0.72457679	0.83712359	-6.47853337
hetero after UVB irradiation VS hetero before UVB irradiation	GO_HAIR_CELL_DIFFERENTIATION1	0.057435483	0.027798419	0.396131041	0.70255397	0.88151888	-6.14725609
WT after UVB irradiation VS WT before UVB irradiation	GO_HAIR_CELL_DIFFERENTIATION2	-0.13139301	0.030208524	-0.71956648	0.49203418	0.85299376	-5.73174833
homo after UVB irradiation VS homo before UVB irradiation	GO_HAIR_CYCLE_PHASE	-0.28293339	0.022528512	-1.22027429	0.25578013	0.45133892	-5.80029381
hetero after UVB irradiation VS hetero before UVB irradiation	GO_HAIR_CYCLE_PHASE1	-0.05657309	0.017135929	-0.31795358	0.75881066	0.90635732	-6.17702289
WT after UVB irradiation VS WT before UVB irradiation	GO_HAIR_CYCLE_PHASE2	-0.46804805	0.07219122	-1.8418594	0.10230356	0.60387743	-4.51859942
homo after UVB irradiation VS homo before UVB irradiation	GO_HAIR_FOLLICLE_MATURATION	-0.31743953	0.022646635	-1.86370505	0.09789957	0.24760581	-4.96376122
hetero after UVB irradiation VS hetero before UVB irradiation	GO_HAIR_FOLLICLE_MATURATION1	0.138871201	0.009860254	0.630435592	0.54631384	0.7993465	-6.02142124
WT after UVB irradiation VS WT before UVB irradiation	GO_HAIR_FOLLICLE_MATURATION2	-0.14332715	0.047266028	-0.63815932	0.5409927	0.87025652	-5.78672796
homo after UVB irradiation VS homo before UVB irradiation	GO_HAIR_FOLLICLE_PLACODE_FORMATION	-0.32058729	0.115096153	-0.86375194	0.41190493	0.5981495	-6.15905646
hetero after UVB irradiation VS hetero before UVB irradiation	GO_HAIR_FOLLICLE_PLACODE_FORMATION1	0.046108135	0.026419105	0.109308015	0.9156998	0.96799239	-6.22500959
WT after UVB irradiation VS WT before UVB irradiation	GO_HAIR_FOLLICLE_PLACODE_FORMATION2	0.044790585	0.022913696	0.105809927	0.9183074	0.98043563	-5.99016356
homo after UVB irradiation VS homo before UVB irradiation	GO_HAPTOGLOBIN_BINDING	-0.22905767	-0.05923034	-0.66716015	0.52271937	0.69163183	-6.31300441
hetero after UVB irradiation VS hetero before UVB irradiation	GO_HAPTOGLOBIN_BINDING1	0.472435016	0.105398447	1.09258084	0.30696014	0.627591	-5.63052793
WT after UVB irradiation VS WT before UVB irradiation	GO_HAPTOGLOBIN_BINDING2	0.437958222	0.004828507	1.176066271	0.27298332	0.74517478	-5.32566489
homo after UVB irradiation VS homo before UVB irradiation	GO_HAPTOGLOBIN_HEMOGLOBIN_COMPLEX	0.19507837	-0.16341198	0.614034997	0.55558816	0.71686758	-6.34856744
hetero after UVB irradiation VS hetero before UVB irradiation	GO_HAPTOGLOBIN_HEMOGLOBIN_COMPLEX1	0.367946844	0.194994874	0.925550372	0.38224308	0.69435363	-5.79144823
WT after UVB irradiation VS WT before UVB irradiation	GO_HAPTOGLOBIN_HEMOGLOBIN_COMPLEX2	0.370919916	0.006108599	1.070865496	0.31510642	0.77130626	-5.43253229
homo after UVB irradiation VS homo before UVB irradiation	GO_HARD_PALATE_DEVELOPMENT	0.066142177	0.020331183	0.206598571	0.84128454	0.91211784	-6.52749243
hetero after UVB irradiation VS hetero before UVB irradiation	GO_HARD_PALATE_DEVELOPMENT1	0.16066206	0.021301856	0.560542773	0.59073881	0.82629572	-6.06453291
WT after UVB irradiation VS WT before UVB irradiation	GO_HARD_PALATE_DEVELOPMENT2	0.067762717	0.138932027	0.301327407	0.7707577	0.94187408	-5.94843973
homo after UVB irradiation VS homo before UVB irradiation	GO_HAUS_COMPLEX	-0.2174385	-0.05368879	-0.83264942	0.42826921	0.61269668	-6.18566464
hetero after UVB irradiation VS hetero before UVB irradiation	GO_HAUS_COMPLEX1	0.270125128	0.002206182	0.988404119	0.35242775	0.66877878	-5.7332858
WT after UVB irradiation VS WT before UVB irradiation	GO_HAUS_COMPLEX2	0.425475642	-0.08719384	2.257822433	0.05351791	0.51991848	-3.94546526
homo after UVB irradiation VS homo before UVB irradiation	GO_HEAD_DEVELOPMENT	-0.03511238	0.013045846	-0.29394114	0.77599485	0.87080945	-6.50368697
hetero after UVB irradiation VS hetero before UVB irradiation	GO_HEAD_DEVELOPMENT1	-0.011183	0.014276929	-0.09021997	0.93037113	0.97446978	-6.22707229
WT after UVB irradiation VS WT before UVB irradiation	GO_HEAD_DEVELOPMENT2	-0.06349408	-0.00173194	-0.45170152	0.6633371	0.91232901	-5.88982271
homo after UVB irradiation VS homo before UVB irradiation	GO_HEAD_MORPHOGENESIS	-0.11356547	-0.00650352	-0.83569982	0.4266446	0.6113803	-6.18309126
hetero after UVB irradiation VS hetero before UVB irradiation	GO_HEAD_MORPHOGENESIS1	-0.13671673	0.06047392	-0.92903675	0.3805418	0.69296098	-5.7883009
WT after UVB irradiation VS WT before UVB irradiation	GO_HEAD_MORPHOGENESIS2	-0.14916358	0.052127055	-0.95786041	0.36585381	0.79730157	-5.5390066
homo after UVB irradiation VS homo before UVB irradiation	GO_HEART_DEVELOPMENT	0.095771875	-0.01093469	0.785571494	0.45388273	0.63499692	-6.22436545
hetero after UVB irradiation VS hetero before UVB irradiation	GO_HEART_DEVELOPMENT1	-0.02420535	-0.00277133	-0.17791094	0.86329659	0.94819913	-6.21435461
WT after UVB irradiation VS WT before UVB irradiation	GO_HEART_DEVELOPMENT2	0.006440276	-0.02141854	0.041652088	0.96778476	0.99191629	-5.99515135
homo after UVB irradiation VS homo before UVB irradiation	GO_HEART_FIELD_SPECIFICATION	-0.17430759	-0.01382106	-1.03032227	0.3318456	0.52470418	-6.0031774
hetero after UVB irradiation VS hetero before UVB irradiation	GO_HEART_FIELD_SPECIFICATION1	-0.20900558	-0.02776962	-1.12788524	0.29264789	0.61681363	-5.59400139
WT after UVB irradiation VS WT before UVB irradiation	GO_HEART_FIELD_SPECIFICATION2	-0.34390458	0.060219754	-1.75973739	0.11603961	0.62291618	-4.62738095
homo after UVB irradiation VS homo before UVB irradiation	GO_HEART_FORMATION	-0.1391602	0.011213957	-1.05082028	0.32287003	0.51631913	-5.9825124
hetero after UVB irradiation VS hetero before UVB irradiation	GO_HEART_FORMATION1	-0.31499467	-0.05967087	-2.41256725	0.04289333	0.27767067	-3.89936652
WT after UVB irradiation VS WT before UVB irradiation	GO_HEART_FORMATION2	-0.38585787	0.029269666	-2.26209818	0.05316023	0.51991848	-3.93944253
homo after UVB irradiation VS homo before UVB irradiation	GO_HEART_GROWTH	-0.05185038	0.002921669	-0.37017604	0.72048769	0.83429353	-6.47627579
hetero after UVB irradiation VS hetero before UVB irradiation	GO_HEART_GROWTH1	-0.14298833	-0.00477075	-1.09484156	0.30602724	0.6274085	-5.62821401
WT after UVB irradiation VS WT before UVB irradiation	GO_HEART_GROWTH2	-0.09835121	-0.00753551	-0.65322245	0.53171803	0.86886037	-5.77699702
homo after UVB irradiation VS homo before UVB irradiation	GO_HEART_INDUCTION	-0.56429771	-0.01963087	-4.12918527	0.00302671	0.03631314	-1.54091164
hetero after UVB irradiation VS hetero before UVB irradiation	GO_HEART_INDUCTION1	-0.20045305	-0.05258569	-1.39001717	0.20265664	0.52703584	-5.29887475
WT after UVB irradiation VS WT before UVB irradiation	GO_HEART_INDUCTION2	-0.53250728	0.020813911	-1.8265784	0.10473744	0.60802924	-4.53898212
homo after UVB irradiation VS homo before UVB irradiation	GO_HEART_MORPHOGENESIS	0.110966371	-0.00226485	0.902740412	0.39202115	0.58123743	-6.12456056
hetero after UVB irradiation VS hetero before UVB irradiation	GO_HEART_MORPHOGENESIS1	-0.0064644	-0.00365546	-0.04631726	0.96421359	0.98681757	-6.23032087
WT after UVB irradiation VS WT before UVB irradiation	GO_HEART_MORPHOGENESIS2	-0.01528765	-0.01242752	-0.09725533	0.92488776	0.98118223	-5.99107892
homo after UVB irradiation VS homo before UVB irradiation	GO_HEART_PROCESS	0.283560727	-0.01059041	2.069897713	0.07086273	0.20430335	-4.66274516
hetero after UVB irradiation VS hetero before UVB irradiation	GO_HEART_PROCESS1	-0.04053075	0.003859928	-0.22947356	0.82436482	0.93321249	-6.20302611
WT after UVB irradiation VS WT before UVB irradiation	GO_HEART_PROCESS2	0.059056446	-0.01079876	0.284369767	0.78326356	0.94564513	-5.95362208
homo after UVB irradiation VS homo before UVB irradiation	GO_HEART_TRABECULA_FORMATION	0.666575033	-0.04269108	4.254719319	0.00253694	0.03339355	-1.36268981
hetero after UVB irradiation VS hetero before UVB irradiation	GO_HEART_TRABECULA_FORMATION1	0.323527143	0.064089859	1.44820909	0.18628029	0.5116875	-5.22827917
WT after UVB irradiation VS WT before UVB irradiation	GO_HEART_TRABECULA_FORMATION2	0.569311386	0.022572747	2.337096996	0.04725942	0.50650586	-3.83354297
homo after UVB irradiation VS homo before UVB irradiation	GO_HEART_TRABECULA_MORPHOGENESIS	0.19733171	-0.06759711	1.442066536	0.18580624	0.37029697	-5.53445798
hetero after UVB irradiation VS hetero before UVB irradiation	GO_HEART_TRABECULA_MORPHOGENESIS1	0.17377905	0.024378987	1.241214345	0.25032223	0.57795624	-5.47133782
WT after UVB irradiation VS WT before UVB irradiation	GO_HEART_TRABECULA_MORPHOGENESIS2	0.310172031	0.010956487	1.681917094	0.13063246	0.63684485	-4.72860408
homo after UVB irradiation VS homo before UVB irradiation	GO_HEART_VALVE_DEVELOPMENT	0.150486127	0.001422658	1.005025338	0.34318496	0.53576349	-6.02824745
hetero after UVB irradiation VS hetero before UVB irradiation	GO_HEART_VALVE_DEVELOPMENT1	-0.01347372	-0.0200095	-0.08861564	0.93160561	0.97492875	-6.22722772
WT after UVB irradiation VS WT before UVB irradiation	GO_HEART_VALVE_DEVELOPMENT2	-0.03428718	0.023257534	-0.21545232	0.8347424	0.96139028	-5.97164747
homo after UVB irradiation VS homo before UVB irradiation	GO_HEART_VALVE_FORMATION	0.439017125	0.033154379	2.435180901	0.03972129	0.14321208	-4.10894684
hetero after UVB irradiation VS hetero before UVB irradiation	GO_HEART_VALVE_FORMATION1	0.190240497	0.054014898	0.922099382	0.38393259	0.69547131	-5.79455433
WT after UVB irradiation VS WT before UVB irradiation	GO_HEART_VALVE_FORMATION2	0.229737593	0.056443938	1.032405313	0.33172828	0.7781676	-5.46977613
homo after UVB irradiation VS homo before UVB irradiation	GO_HEAT_ACCLIMATION	-0.60239641	0.086229109	-3.05087046	0.01506603	0.08330064	-3.15445919
hetero after UVB irradiation VS hetero before UVB irradiation	GO_HEAT_ACCLIMATION1	-0.54481195	0.079572111	-1.4831222	0.17702662	0.50051267	-5.18514815
WT after UVB irradiation VS WT before UVB irradiation	GO_HEAT_ACCLIMATION2	-0.38998433	0.016937411	-1.44647424	0.18561639	0.68726847	-5.02140794
homo after UVB irradiation VS homo before UVB irradiation	GO_HEAT_GENERATION	0.623437078	0.064014968	3.737761547	0.005325	0.0477116	-2.11100799
hetero after UVB irradiation VS hetero before UVB irradiation	GO_HEAT_GENERATION1	0.407040594	0.030818931	2.464298564	0.03959442	0.27162031	-3.82367935
WT after UVB irradiation VS WT before UVB irradiation	GO_HEAT_GENERATION2	0.372159656	0.03255099	1.68721129	0.12958809	0.63548424	-4.72177993
homo after UVB irradiation VS homo before UVB irradiation	GO_HEAT_SHOCK_PROTEIN_BINDING	-0.19952608	-0.01106803	-1.48685942	0.17389258	0.35409587	-5.47749394
hetero after UVB irradiation VS hetero before UVB irradiation	GO_HEAT_SHOCK_PROTEIN_BINDING1	-0.10243284	-0.03428622	-0.62352578	0.55061628	0.80124226	-6.02588766
WT after UVB irradiation VS WT before UVB irradiation	GO_HEAT_SHOCK_PROTEIN_BINDING2	-0.14888101	-0.00491336	-0.93976453	0.37452128	0.80246519	-5.55520345
homo after UVB irradiation VS homo before UVB irradiation	GO_HELICASE_ACTIVITY	-0.63683373	-0.00725737	-4.36465957	0.00217768	0.03119996	-1.20854795
hetero after UVB irradiation VS hetero before UVB irradiation	GO_HELICASE_ACTIVITY1	-0.21680814	-0.0593151	-0.97962142	0.35648523	0.67198476	-5.74159158
WT after UVB irradiation VS WT before UVB irradiation	GO_HELICASE_ACTIVITY2	-0.11800213	0.001612663	-0.43912902	0.67205009	0.91558199	-5.89558246
homo after UVB irradiation VS homo before UVB irradiation	GO_HEMATOPOIETIC_PROGENITOR_CELL_DIFFERENTIATION	-0.09509448	-0.00050103	-0.62990442	0.54564495	0.70987239	-6.3382239
hetero after UVB irradiation VS hetero before UVB irradiation	GO_HEMATOPOIETIC_PROGENITOR_CELL_DIFFERENTIATION1	0.083416494	-0.01176597	0.600173645	0.5653037	0.81102647	-6.04065256
WT after UVB irradiation VS WT before UVB irradiation	GO_HEMATOPOIETIC_PROGENITOR_CELL_DIFFERENTIATION2	0.072412257	0.030223341	0.394203162	0.70360917	0.92570885	-5.91489575
homo after UVB irradiation VS homo before UVB irradiation	GO_HEMATOPOIETIC_STEM_CELL_DIFFERENTIATION	-0.22331631	0.023185488	-1.11270482	0.29691462	0.491558	-5.91826928
hetero after UVB irradiation VS hetero before UVB irradiation	GO_HEMATOPOIETIC_STEM_CELL_DIFFERENTIATION1	0.038172865	-0.02353432	0.209064529	0.83971915	0.93884647	-6.20784912
WT after UVB irradiation VS WT before UVB irradiation	GO_HEMATOPOIETIC_STEM_CELL_DIFFERENTIATION2	-0.01502823	-0.00916396	-0.06476296	0.94993302	0.98654836	-5.99385418
homo after UVB irradiation VS homo before UVB irradiation	GO_HEMATOPOIETIC_STEM_CELL_HOMEOSTASIS	0.676180346	0.060551727	4.099373486	0.00315735	0.03715551	-1.58357664
hetero after UVB irradiation VS hetero before UVB irradiation	GO_HEMATOPOIETIC_STEM_CELL_HOMEOSTASIS1	0.683165999	0.044003133	2.972325044	0.01818375	0.19726134	-3.07975919
WT after UVB irradiation VS WT before UVB irradiation	GO_HEMATOPOIETIC_STEM_CELL_HOMEOSTASIS2	0.774014322	-0.01431857	4.215397728	0.0028554	0.24078388	-1.2818011
homo after UVB irradiation VS homo before UVB irradiation	GO_HEMATOPOIETIC_STEM_CELL_MIGRATION	0.68490424	-0.07092694	3.461410931	0.00803608	0.05953233	-2.52529315
hetero after UVB irradiation VS hetero before UVB irradiation	GO_HEMATOPOIETIC_STEM_CELL_MIGRATION1	0.510053763	0.024126787	2.515797002	0.03656545	0.26251512	-3.74822077
WT after UVB irradiation VS WT before UVB irradiation	GO_HEMATOPOIETIC_STEM_CELL_MIGRATION2	0.351561266	-0.03646555	1.857142547	0.09992298	0.59874718	-4.49815289
homo after UVB irradiation VS homo before UVB irradiation	GO_HEMATOPOIETIC_STEM_CELL_PROLIFERATION	-0.17249724	-0.0141971	-1.06110582	0.31843759	0.5119584	-5.97202638
hetero after UVB irradiation VS hetero before UVB irradiation	GO_HEMATOPOIETIC_STEM_CELL_PROLIFERATION1	0.013211893	-0.01885344	0.077980732	0.93979373	0.97787938	-6.22818766
WT after UVB irradiation VS WT before UVB irradiation	GO_HEMATOPOIETIC_STEM_CELL_PROLIFERATION2	-0.05563091	-0.00844676	-0.29852217	0.7728217	0.94264488	-5.94931727
homo after UVB irradiation VS homo before UVB irradiation	GO_HEME_METABOLIC_PROCESS	0.352961483	-0.018404	2.067282535	0.07115552	0.20472647	-4.66662799
hetero after UVB irradiation VS hetero before UVB irradiation	GO_HEME_METABOLIC_PROCESS1	0.368630128	0.040077611	2.556078226	0.03436117	0.25538558	-3.68914454
WT after UVB irradiation VS WT before UVB irradiation	GO_HEME_METABOLIC_PROCESS2	0.299809599	0.012650502	1.710624111	0.12506152	0.63170411	-4.69148959
homo after UVB irradiation VS homo before UVB irradiation	GO_HEME_TRANSMEMBRANE_TRANSPORTER_ACTIVITY	0.419100792	0.055315339	1.968926241	0.08305828	0.22370321	-4.81152105
hetero after UVB irradiation VS hetero before UVB irradiation	GO_HEME_TRANSMEMBRANE_TRANSPORTER_ACTIVITY1	0.197081377	0.083742084	1.063526111	0.31915141	0.63860264	-5.65995537
WT after UVB irradiation VS WT before UVB irradiation	GO_HEME_TRANSMEMBRANE_TRANSPORTER_ACTIVITY2	0.053724698	0.075907322	0.301037401	0.77097099	0.94190073	-5.94853082
homo after UVB irradiation VS homo before UVB irradiation	GO_HEME_TRANSPORT	0.419100792	0.055315339	1.968926241	0.08305828	0.22370321	-4.81152105
hetero after UVB irradiation VS hetero before UVB irradiation	GO_HEME_TRANSPORT1	0.197081377	0.083742084	1.063526111	0.31915141	0.63860264	-5.65995537
WT after UVB irradiation VS WT before UVB irradiation	GO_HEME_TRANSPORT2	0.053724698	0.075907322	0.301037401	0.77097099	0.94190073	-5.94853082
homo after UVB irradiation VS homo before UVB irradiation	GO_HEMIDESMOSOME	0.574474047	-0.12415063	1.87164411	0.09669712	0.24603489	-4.95238515
homo after UVB irradiation VS homo before UVB irradiation	GO_HEMIDESMOSOME_ASSEMBLY	1.10112573	-0.01160974	6.857466978	0.00010745	0.01007802	1.784670861
hetero after UVB irradiation VS hetero before UVB irradiation	GO_HEMIDESMOSOME_ASSEMBLY1	0.883769124	0.105702949	3.039794897	0.01642452	0.18934388	-2.98175531
WT after UVB irradiation VS WT before UVB irradiation	GO_HEMIDESMOSOME_ASSEMBLY2	1.034156238	0.019326071	7.142589763	9.21E-05	0.18842845	1.573604464
hetero after UVB irradiation VS hetero before UVB irradiation	GO_HEMIDESMOSOME1	-0.01683825	-0.01789162	-0.06547784	0.94942977	0.98092529	-6.22915971
WT after UVB irradiation VS WT before UVB irradiation	GO_HEMIDESMOSOME2	0.249543443	0.001982589	0.976393269	0.35713282	0.79119413	-5.52216852
homo after UVB irradiation VS homo before UVB irradiation	GO_HEMOGLOBIN_ALPHA_BINDING	-0.52812293	-0.01857604	-1.48758402	0.17370553	0.35384625	-5.4765642
hetero after UVB irradiation VS hetero before UVB irradiation	GO_HEMOGLOBIN_ALPHA_BINDING1	0.16864377	0.018963625	0.372424752	0.71942692	0.89052082	-6.15694728
WT after UVB irradiation VS WT before UVB irradiation	GO_HEMOGLOBIN_ALPHA_BINDING2	0.300162723	0.06694246	0.862923632	0.41300547	0.820663	-5.6212171
homo after UVB irradiation VS homo before UVB irradiation	GO_HEMOGLOBIN_BINDING	-0.24080211	-0.01219493	-0.65538278	0.52990272	0.69713619	-6.32111794
hetero after UVB irradiation VS hetero before UVB irradiation	GO_HEMOGLOBIN_BINDING1	-0.35828397	0.04871203	-1.06704232	0.31765605	0.63754495	-5.65642499
WT after UVB irradiation VS WT before UVB irradiation	GO_HEMOGLOBIN_BINDING2	0.003660408	0.088662617	0.012827647	0.99007571	0.99848757	-5.99597984
homo after UVB irradiation VS homo before UVB irradiation	GO_HEMOGLOBIN_BIOSYNTHETIC_PROCESS	0.306454781	-0.01490416	1.527389113	0.16370122	0.3413373	-5.42509981
hetero after UVB irradiation VS hetero before UVB irradiation	GO_HEMOGLOBIN_BIOSYNTHETIC_PROCESS1	0.113699461	0.019235878	0.467917724	0.65255499	0.85713917	-6.11443406
WT after UVB irradiation VS WT before UVB irradiation	GO_HEMOGLOBIN_BIOSYNTHETIC_PROCESS2	0.289029245	0.050993477	1.367701785	0.20815568	0.70501569	-5.11401126
homo after UVB irradiation VS homo before UVB irradiation	GO_HEMOGLOBIN_COMPLEX	-0.22905767	-0.05923034	-0.66716015	0.52271937	0.69163183	-6.31300441
hetero after UVB irradiation VS hetero before UVB irradiation	GO_HEMOGLOBIN_COMPLEX1	0.472435016	0.105398447	1.09258084	0.30696014	0.627591	-5.63052793
WT after UVB irradiation VS WT before UVB irradiation	GO_HEMOGLOBIN_COMPLEX2	0.437958222	0.004828507	1.176066271	0.27298332	0.74517478	-5.32566489
homo after UVB irradiation VS homo before UVB irradiation	GO_HEMOGLOBIN_METABOLIC_PROCESS	0.527877186	-0.01665607	3.61313021	0.00640298	0.05290523	-2.29675067
hetero after UVB irradiation VS hetero before UVB irradiation	GO_HEMOGLOBIN_METABOLIC_PROCESS1	0.340732661	0.065943716	1.503316219	0.17186337	0.49483236	-5.15994679
WT after UVB irradiation VS WT before UVB irradiation	GO_HEMOGLOBIN_METABOLIC_PROCESS2	0.327943079	0.063403764	1.388878994	0.2018735	0.69830181	-5.08941227
homo after UVB irradiation VS homo before UVB irradiation	GO_HEPARAN_SULFATE_GLUCOSAMINE_3_SULFOTRANSFERASE_1_ACTIVITY	-0.22411155	0.105904239	-1.32643621	0.21990956	0.41088951	-5.67660023
hetero after UVB irradiation VS hetero before UVB irradiation	GO_HEPARAN_SULFATE_GLUCOSAMINE_3_SULFOTRANSFERASE_1_ACTIVITY1	-0.40454675	0.024492674	-1.68537619	0.131114	0.44000869	-4.92512368
WT after UVB irradiation VS WT before UVB irradiation	GO_HEPARAN_SULFATE_GLUCOSAMINE_3_SULFOTRANSFERASE_1_ACTIVITY2	-0.29602736	0.091049173	-1.74024089	0.11954583	0.62863012	-4.65291981
homo after UVB irradiation VS homo before UVB irradiation	GO_HEPARAN_SULFATE_PROTEOGLYCAN_BINDING	0.797255599	-0.01022972	5.599898769	0.00044183	0.01560266	0.393191198
hetero after UVB irradiation VS hetero before UVB irradiation	GO_HEPARAN_SULFATE_PROTEOGLYCAN_BINDING1	0.302979949	0.059734413	1.275873153	0.2384436	0.56736997	-5.43226809
WT after UVB irradiation VS WT before UVB irradiation	GO_HEPARAN_SULFATE_PROTEOGLYCAN_BINDING2	0.266768283	-0.02329347	1.336514266	0.21771459	0.71478521	-5.14982434
homo after UVB irradiation VS homo before UVB irradiation	GO_HEPARAN_SULFATE_PROTEOGLYCAN_BIOSYNTHETIC_PROCESS	0.368312331	-0.08080792	2.140684084	0.06336823	0.19041437	-4.55711017
homo after UVB irradiation VS homo before UVB irradiation	GO_HEPARAN_SULFATE_PROTEOGLYCAN_BIOSYNTHETIC_PROCESS_ENZYMATIC_MODIFICATION	0.108161971	-0.05866252	0.405977299	0.69498935	0.8175335	-6.46130238
hetero after UVB irradiation VS hetero before UVB irradiation	GO_HEPARAN_SULFATE_PROTEOGLYCAN_BIOSYNTHETIC_PROCESS_ENZYMATIC_MODIFICATION1	0.192564141	0.027977201	0.929097173	0.38051237	0.69296098	-5.78824628
WT after UVB irradiation VS WT before UVB irradiation	GO_HEPARAN_SULFATE_PROTEOGLYCAN_BIOSYNTHETIC_PROCESS_ENZYMATIC_MODIFICATION2	-0.2822445	0.13006875	-1.36905734	0.20774855	0.70428803	-5.11244341
homo after UVB irradiation VS homo before UVB irradiation	GO_HEPARAN_SULFATE_PROTEOGLYCAN_BIOSYNTHETIC_PROCESS_POLYSACCHARIDE_CHAIN_BIOSYNTHETIC_PROCESS	0.518255995	-0.04785434	2.405208064	0.04165727	0.14720698	-4.15504678
hetero after UVB irradiation VS hetero before UVB irradiation	GO_HEPARAN_SULFATE_PROTEOGLYCAN_BIOSYNTHETIC_PROCESS_POLYSACCHARIDE_CHAIN_BIOSYNTHETIC_PROCESS1	0.697021044	0.025611346	4.018981354	0.00399661	0.12441764	-1.61779225
WT after UVB irradiation VS WT before UVB irradiation	GO_HEPARAN_SULFATE_PROTEOGLYCAN_BIOSYNTHETIC_PROCESS_POLYSACCHARIDE_CHAIN_BIOSYNTHETIC_PROCESS2	0.523485412	0.01385877	3.6552464	0.00631242	0.29981324	-1.99716381
hetero after UVB irradiation VS hetero before UVB irradiation	GO_HEPARAN_SULFATE_PROTEOGLYCAN_BIOSYNTHETIC_PROCESS1	0.283120372	-0.0075066	1.580906039	0.15326172	0.47184018	-5.06147732

WT after UVB irradiation VS WT before UVB irradiation	GO_HEPARAN_SULFATE_PROTEOGLYCAN_BIOSYNTHETIC_PROCESS2	0.27046501	0.032077443	1.706722865	0.12580545	0.63290509	-4.69654931
homo after UVB irradiation VS homo before UVB irradiation	GO_HEPARAN_SULFATE_PROTEOGLYCAN_METABOLIC_PROCESS	0.417206738	-0.05644255	2.733717266	0.02475688	0.11077539	-3.64665943
hetero after UVB irradiation VS hetero before UVB irradiation	GO_HEPARAN_SULFATE_PROTEOGLYCAN_METABOLIC_PROCESS1	0.330269927	-0.00361259	1.958143522	0.08658284	0.37240327	-4.55264263
WT after UVB irradiation VS WT before UVB irradiation	GO_HEPARAN_SULFATE_PROTEOGLYCAN_METABOLIC_PROCESS2	0.373153378	0.020246562	2.286358206	0.05117533	0.5144376	-3.90523839
homo after UVB irradiation VS homo before UVB irradiation	GO_HEPARAN_SULFATE_SULFOTRANSFERASE_ACTIVITY	0.245266115	0.021733734	1.662106099	0.13356968	0.30088349	-5.24559236
hetero after UVB irradiation VS hetero before UVB irradiation	GO_HEPARAN_SULFATE_SULFOTRANSFERASE_ACTIVITY1	0.132803663	0.040365455	0.668270962	0.52311375	0.7882475	-5.9961842
WT after UVB irradiation VS WT before UVB irradiation	GO_HEPARAN_SULFATE_SULFOTRANSFERASE_ACTIVITY2	-0.0672634	0.097046498	-0.35934743	0.72852569	0.93296251	-5.92850102
homo after UVB irradiation VS homo before UVB irradiation	GO_HEPARIN_BINDING	0.312334832	-0.01335591	2.236950083	0.05440854	0.17300297	-4.41196458
hetero after UVB irradiation VS hetero before UVB irradiation	GO_HEPARIN_BINDING1	0.041760222	0.013944956	0.221894591	0.83005768	0.93498711	-6.20486889
WT after UVB irradiation VS WT before UVB irradiation	GO_HEPARIN_BINDING2	0.137265079	0.005227438	0.682304993	0.51408628	0.85974355	-5.7576376
homo after UVB irradiation VS homo before UVB irradiation	GO_HEPARIN_METABOLIC_PROCESS	0.314997317	0.001849945	1.614284338	0.14363476	0.31546632	-5.31021004
hetero after UVB irradiation VS hetero before UVB irradiation	GO_HEPARIN_METABOLIC_PROCESS1	0.238493714	-0.00515363	1.070972846	0.31599102	0.63620732	-5.65246848
WT after UVB irradiation VS WT before UVB irradiation	GO_HEPARIN_METABOLIC_PROCESS2	0.240709719	-0.00672437	1.646956703	0.13772625	0.64618714	-4.77342659
homo after UVB irradiation VS homo before UVB irradiation	GO_HEPATIC_STELLATE_CELL_ACTIVATION	-0.00266155	-0.06325879	-0.01239883	0.99039927	0.99491641	-6.55077235
hetero after UVB irradiation VS hetero before UVB irradiation	GO_HEPATIC_STELLATE_CELL_ACTIVATION1	-0.01179821	0.013836801	-0.04739872	0.96337871	0.98664387	-6.23026592
WT after UVB irradiation VS WT before UVB irradiation	GO_HEPATIC_STELLATE_CELL_ACTIVATION2	-0.07757947	-0.06711966	-0.35431036	0.73215581	0.93383088	-5.93036664
homo after UVB irradiation VS homo before UVB irradiation	GO_HEPATICOBILIARY_SYSTEM_DEVELOPMENT	-0.03498992	0.010612447	-0.28777572	0.78054919	0.87375402	-6.50563515
hetero after UVB irradiation VS hetero before UVB irradiation	GO_HEPATICOBILIARY_SYSTEM_DEVELOPMENT1	0.090548877	0.005499831	0.659318434	0.52854811	0.79043843	-6.00227427
WT after UVB irradiation VS WT before UVB irradiation	GO_HEPATICOBILIARY_SYSTEM_DEVELOPMENT2	-0.07518874	0.022916475	-0.52021426	0.61683373	0.90020288	-5.85574805
homo after UVB irradiation VS homo before UVB irradiation	GO_HEPATOCYTE_APOPTOTIC_PROCESS	-0.44699506	-0.03462703	-2.71735242	0.02540442	0.11251292	-3.67208429
hetero after UVB irradiation VS hetero before UVB irradiation	GO_HEPATOCYTE_APOPTOTIC_PROCESS1	-0.16286455	-0.0318525	-0.92547411	0.38228035	0.69435363	-5.79151696
WT after UVB irradiation VS WT before UVB irradiation	GO_HEPATOCYTE_APOPTOTIC_PROCESS2	-0.05938196	-0.01804986	-0.30621087	0.76716924	0.94123239	-5.94689296
homo after UVB irradiation VS homo before UVB irradiation	GO_HEPATOCYTE_DIFFERENTIATION	-0.28257876	-0.00947041	-1.60278422	0.14615609	0.31882119	-5.32560653
hetero after UVB irradiation VS hetero before UVB irradiation	GO_HEPATOCYTE_DIFFERENTIATION1	-0.06830067	-0.034943	-0.28010043	0.78663733	0.91694608	-6.18915578
WT after UVB irradiation VS WT before UVB irradiation	GO_HEPATOCYTE_DIFFERENTIATION2	-0.14514574	0.127890024	-0.66717556	0.52321324	0.86461731	-5.76780238
homo after UVB irradiation VS homo before UVB irradiation	GO_HEPATOCYTE_GROWTH_FACTOR_RECEPTOR_SIGNALING_PATHWAY	0.647817066	-0.03609783	5.166083297	0.0007547	0.01908163	-0.14189915
hetero after UVB irradiation VS hetero before UVB irradiation	GO_HEPATOCYTE_GROWTH_FACTOR_RECEPTOR_SIGNALING_PATHWAY1	0.581845145	0.032008973	3.734260596	0.00594742	0.134025	-2.0008191
WT after UVB irradiation VS WT before UVB irradiation	GO_HEPATOCYTE_GROWTH_FACTOR_RECEPTOR_SIGNALING_PATHWAY2	0.499815679	-0.02195882	2.577092841	0.03244283	0.45650945	-3.49263275
homo after UVB irradiation VS homo before UVB irradiation	GO_HEPOXILIN_METABOLIC_PROCESS	0.369483271	-0.0752078	1.40547005	0.19606563	0.38393585	-5.5802321
hetero after UVB irradiation VS hetero before UVB irradiation	GO_HEPOXILIN_METABOLIC_PROCESS1	0.492938513	0.031703319	2.694393794	0.02777303	0.23528578	-3.48617455
WT after UVB irradiation VS WT before UVB irradiation	GO_HEPOXILIN_METABOLIC_PROCESS2	0.454277079	0.065644663	2.600468394	0.0312785	0.45353809	-3.45936201
homo after UVB irradiation VS homo before UVB irradiation	GO_HETEROCHROMATIN	-0.28732932	9.99E-05	-1.62251167	0.14185532	0.3128713	-5.2991607
homo after UVB irradiation VS homo before UVB irradiation	GO_HETEROCHROMATIN_ORGANIZATION	-0.1505358	-0.01537238	-0.70516591	0.49994835	0.6740738	-6.28594303
hetero after UVB irradiation VS hetero before UVB irradiation	GO_HETEROCHROMATIN_ORGANIZATION1	0.13138074	-0.02283232	0.725581755	0.48915164	0.76616077	-5.95548581
WT after UVB irradiation VS WT before UVB irradiation	GO_HETEROCHROMATIN_ORGANIZATION2	-0.05352499	-0.01810389	-0.24109611	0.81547321	0.95612327	-5.96551195
hetero after UVB irradiation VS hetero before UVB irradiation	GO_HETEROCHROMATIN1	0.04212904	-0.01749869	0.222583314	0.82953991	0.93468067	-6.20470395
WT after UVB irradiation VS WT before UVB irradiation	GO_HETEROCHROMATIN2	-0.23574181	-0.00784514	-1.02908757	0.33319336	0.77844475	-5.47294122
homo after UVB irradiation VS homo before UVB irradiation	GO_HETEROPHILIC_CELL_CELL_ADHESION_VIA_PLASMA_MEMBRANE_CELL_ADHESION_MOLECULES	0.210504481	-0.00996314	1.43779488	0.18697903	0.37175474	-5.53983702
hetero after UVB irradiation VS hetero before UVB irradiation	GO_HETEROPHILIC_CELL_CELL_ADHESION_VIA_PLASMA_MEMBRANE_CELL_ADHESION_MOLECULES1	0.124279006	0.023177098	0.654350406	0.53157864	0.79183076	-6.00562226
WT after UVB irradiation VS WT before UVB irradiation	GO_HETEROPHILIC_CELL_CELL_ADHESION_VIA_PLASMA_MEMBRANE_CELL_ADHESION_MOLECULES2	0.052869149	-0.02892287	0.252048886	0.80728271	0.95404687	-5.96268449
homo after UVB irradiation VS homo before UVB irradiation	GO_HETEROTRIMERIC_G_PROTEIN_COMPLEX	0.450079702	-0.01146914	2.258461366	0.05258385	0.16967531	-4.37932583
hetero after UVB irradiation VS hetero before UVB irradiation	GO_HETEROTRIMERIC_G_PROTEIN_COMPLEX1	0.57451443	0.022643875	3.91298611	0.00462769	0.12761654	-1.75890528
WT after UVB irradiation VS WT before UVB irradiation	GO_HETEROTRIMERIC_G_PROTEIN_COMPLEX2	0.243739823	-0.02851816	1.076865674	0.31257299	0.77011405	-5.42663122
homo after UVB irradiation VS homo before UVB irradiation	GO_HETEROTYPIC_CELL_CELL_ADHESION	0.64787387	-0.02342323	5.086570975	0.0008349	0.01971289	-0.24318478
hetero after UVB irradiation VS hetero before UVB irradiation	GO_HETEROTYPIC_CELL_CELL_ADHESION1	0.429811468	0.032275801	2.552600335	0.03454603	0.25577582	-3.69424656
WT after UVB irradiation VS WT before UVB irradiation	GO_HETEROTYPIC_CELL_CELL_ADHESION2	0.36613937	0.003255911	1.573619265	0.1537673	0.65981549	-4.86601593
homo after UVB irradiation VS homo before UVB irradiation	GO_HEXOKINASE_ACTIVITY	0.976007578	-0.00337657	4.604464395	0.00157043	0.02685377	-0.87877749
hetero after UVB irradiation VS hetero before UVB irradiation	GO_HEXOKINASE_ACTIVITY1	0.637077056	-0.00911584	2.114523153	0.06804047	0.33919095	-4.33111281
WT after UVB irradiation VS WT before UVB irradiation	GO_HEXOKINASE_ACTIVITY2	0.701713427	0.021065685	2.641754313	0.02932501	0.44931137	-3.40060598
homo after UVB irradiation VS homo before UVB irradiation	GO_HEXOSAMINIDASE_ACTIVITY	0.467034495	0.040900839	3.243938222	0.01118456	0.07086755	-2.85692092
hetero after UVB irradiation VS hetero before UVB irradiation	GO_HEXOSAMINIDASE_ACTIVITY1	0.435484861	-0.01064524	2.869620903	0.02124817	0.20972443	-3.22952668
WT after UVB irradiation VS WT before UVB irradiation	GO_HEXOSAMINIDASE_ACTIVITY2	0.370024033	-0.03302726	2.359379448	0.04563567	0.503699	-3.80199774
homo after UVB irradiation VS homo before UVB irradiation	GO_HEXOSE_CATABOLIC_PROCESS	0.507678723	-0.02243883	3.474237595	0.00788231	0.05910532	-2.50587519
hetero after UVB irradiation VS hetero before UVB irradiation	GO_HEXOSE_CATABOLIC_PROCESS1	0.371773584	0.009158033	2.097369786	0.06986773	0.34285607	-4.35562707
WT after UVB irradiation VS WT before UVB irradiation	GO_HEXOSE_CATABOLIC_PROCESS2	0.351371074	0.004566356	1.859903893	0.09949851	0.59874718	-4.49445225
homo after UVB irradiation VS homo before UVB irradiation	GO_HEXOSE_PHOSPHATE_TRANSPORT	0.150908873	0.088636845	0.689425999	0.50930245	0.68167999	-6.29731226
hetero after UVB irradiation VS hetero before UVB irradiation	GO_HEXOSE_PHOSPHATE_TRANSPORT1	-0.58406439	0.064674079	-3.62404291	0.00695838	0.1390235	-2.1523867
WT after UVB irradiation VS WT before UVB irradiation	GO_HEXOSE_PHOSPHATE_TRANSPORT2	0.105027251	0.107020658	0.336194248	0.74527047	0.93583699	-5.93686553
homo after UVB irradiation VS homo before UVB irradiation	GO_HEXOSE_TRANSMEMBRANE_TRANSPORTER_ACTIVITY	0.531722323	0.029240608	2.480310464	0.03697532	0.1377543	-4.03938731
hetero after UVB irradiation VS hetero before UVB irradiation	GO_HEXOSE_TRANSMEMBRANE_TRANSPORTER_ACTIVITY1	0.314220299	0.072890433	1.47878933	0.17815235	0.50216872	-5.19053145
WT after UVB irradiation VS WT before UVB irradiation	GO_HEXOSE_TRANSMEMBRANE_TRANSPORTER_ACTIVITY2	0.474953984	0.075858181	2.165230837	0.06187515	0.53241488	-4.07541259
homo after UVB irradiation VS homo before UVB irradiation	GO_HFE_TRANSFERRIN_RECEPTOR_COMPLEX	0.328897151	-0.01484733	1.345786376	0.21385293	0.40438592	-5.65333044
hetero after UVB irradiation VS hetero before UVB irradiation	GO_HFE_TRANSFERRIN_RECEPTOR_COMPLEX1	-0.01615195	0.015734879	-0.06945441	0.94636396	0.97985397	-6.2288689
WT after UVB irradiation VS WT before UVB irradiation	GO_HFE_TRANSFERRIN_RECEPTOR_COMPLEX2	0.150360241	-0.08205678	0.671066086	0.52085679	0.86304554	-5.76520783
homo after UVB irradiation VS homo before UVB irradiation	GO_HIGH_DENSITY_LIPOPROTEIN_PARTICLE	0.405338131	0.065825258	2.868794025	0.02001795	0.09751233	-3.43677897
homo after UVB irradiation VS homo before UVB irradiation	GO_HIGH_DENSITY_LIPOPROTEIN_PARTICLE_ASSEMBLY	0.777396538	-0.0244799	5.610856191	0.00043604	0.01557794	0.406326946
hetero after UVB irradiation VS hetero before UVB irradiation	GO_HIGH_DENSITY_LIPOPROTEIN_PARTICLE_ASSEMBLY1	0.358163057	0.071906323	1.599872256	0.14900196	0.46700785	-5.03703048
WT after UVB irradiation VS WT before UVB irradiation	GO_HIGH_DENSITY_LIPOPROTEIN_PARTICLE_ASSEMBLY2	0.498460337	0.042545502	1.795734787	0.10981761	0.61471744	-4.57993076
homo after UVB irradiation VS homo before UVB irradiation	GO_HIGH_DENSITY_LIPOPROTEIN_PARTICLE_BINDING	0.688689152	0.075797574	4.65728008	0.00146299	0.02585552	-0.80735354
hetero after UVB irradiation VS hetero before UVB irradiation	GO_HIGH_DENSITY_LIPOPROTEIN_PARTICLE_BINDING1	0.297154579	0.036369912	2.022056291	0.0784755	0.36108029	-4.4626633
WT after UVB irradiation VS WT before UVB irradiation	GO_HIGH_DENSITY_LIPOPROTEIN_PARTICLE_BINDING2	0.36998738	-0.03041315	1.494827022	0.17287015	0.67864986	-4.96313092
homo after UVB irradiation VS homo before UVB irradiation	GO_HIGH_DENSITY_LIPOPROTEIN_PARTICLE_CLEARANCE	0.582717618	-0.02883581	2.729580866	0.02491894	0.11097478	-3.65308617
hetero after UVB irradiation VS hetero before UVB irradiation	GO_HIGH_DENSITY_LIPOPROTEIN_PARTICLE_CLEARANCE1	0.241472627	0.042487229	1.412324463	0.19623526	0.52060431	-5.27200906
WT after UVB irradiation VS WT before UVB irradiation	GO_HIGH_DENSITY_LIPOPROTEIN_PARTICLE_CLEARANCE2	0.459446899	-0.00719844	1.979503768	0.08267895	0.56864787	-4.33244573
homo after UVB irradiation VS homo before UVB irradiation	GO_HIGH_DENSITY_LIPOPROTEIN_PARTICLE_REMODELING	0.230623862	0.003176798	1.29134879	0.23126443	0.42232015	-5.71823827
hetero after UVB irradiation VS hetero before UVB irradiation	GO_HIGH_DENSITY_LIPOPROTEIN_PARTICLE_REMODELING1	0.043794034	0.0349926	0.225417008	0.8274105	0.93421603	-6.20402003
WT after UVB irradiation VS WT before UVB irradiation	GO_HIGH_DENSITY_LIPOPROTEIN_PARTICLE_REMODELING2	0.196891452	-0.01658881	1.087050056	0.30830958	0.76668326	-5.41655989
hetero after UVB irradiation VS hetero before UVB irradiation	GO_HIGH_DENSITY_LIPOPROTEIN_PARTICLE1	0.144686729	0.09557298	0.823839156	0.43433728	0.73389223	-5.87902798
WT after UVB irradiation VS WT before UVB irradiation	GO_HIGH_DENSITY_LIPOPROTEIN_PARTICLE2	0.159647145	0.063694459	0.687010433	0.51126793	0.85882537	-5.75443512
homo after UVB irradiation VS homo before UVB irradiation	GO_HIGH_VOLTAGE_GATED_CALCIUM_CHANNEL_ACTIVITY	0.543824134	-0.03337292	2.451244346	0.03872115	0.14136763	-4.08420706
hetero after UVB irradiation VS hetero before UVB irradiation	GO_HIGH_VOLTAGE_GATED_CALCIUM_CHANNEL_ACTIVITY1	0.08108502	-0.03914156	0.289148463	0.7799543	0.91401375	-6.18639218
WT after UVB irradiation VS WT before UVB irradiation	GO_HIGH_VOLTAGE_GATED_CALCIUM_CHANNEL_ACTIVITY2	0.391444309	0.011587852	1.255707378	0.24424207	0.73383326	-5.24021058
homo after UVB irradiation VS homo before UVB irradiation	GO_HINDBRAIN_DEVELOPMENT	-0.13393001	0.030274467	-1.11087599	0.29765728	0.4922152	-5.92020693
hetero after UVB irradiation VS hetero before UVB irradiation	GO_HINDBRAIN_DEVELOPMENT1	-0.11353139	0.017040837	-0.82425847	0.43411276	0.73359687	-5.87868417
WT after UVB irradiation VS WT before UVB irradiation	GO_HINDBRAIN_DEVELOPMENT2	-0.11570766	0.013106236	-0.77033409	0.46298136	0.84114789	-5.69455468
homo after UVB irradiation VS homo before UVB irradiation	GO_HINDBRAIN_MORPHOGENESIS	-0.35642982	0.03350484	-2.77825883	0.02307864	0.10619627	-3.57744676
hetero after UVB irradiation VS hetero before UVB irradiation	GO_HINDBRAIN_MORPHOGENESIS1	-0.18695533	-0.00011979	-1.41302438	0.1960367	0.52060431	-5.27116211
WT after UVB irradiation VS WT before UVB irradiation	GO_HINDBRAIN_MORPHOGENESIS2	-0.31963952	0.017618948	-1.88416688	0.09584178	0.59686908	-4.46185419
homo after UVB irradiation VS homo before UVB irradiation	GO_HINDBRAIN_RADIAL_GLIA_GUIDED_CELL_MIGRATION	0.384847583	0.03186951	2.584960783	0.03132123	0.12542568	-3.87753865
hetero after UVB irradiation VS hetero before UVB irradiation	GO_HINDBRAIN_RADIAL_GLIA_GUIDED_CELL_MIGRATION1	-0.08033887	0.039673833	-0.34912241	0.73617402	0.8963204	-6.16591105
WT after UVB irradiation VS WT before UVB irradiation	GO_HINDBRAIN_RADIAL_GLIA_GUIDED_CELL_MIGRATION2	0.131939043	-0.04137319	0.496586662	0.63267923	0.90455096	-5.86800855
homo after UVB irradiation VS homo before UVB irradiation	GO_HINDGUT_DEVELOPMENT	-0.59130005	-0.01235052	-3.00886154	0.01608286	0.08661851	-3.21948586
hetero after UVB irradiation VS hetero before UVB irradiation	GO_HINDGUT_DEVELOPMENT1	-0.47007575	-0.03017016	-2.19898666	0.05970754	0.32192177	-4.2097661
WT after UVB irradiation VS WT before UVB irradiation	GO_HINDGUT_DEVELOPMENT2	-0.61600965	0.007041663	-3.46054096	0.00840248	0.31908946	-2.2576318
homo after UVB irradiation VS homo before UVB irradiation	GO_HINDLIMB_MORPHOGENESIS	-0.38243771	0.006933504	-2.59008149	0.03106817	0.12509852	-3.86960349
hetero after UVB irradiation VS hetero before UVB irradiation	GO_HINDLIMB_MORPHOGENESIS1	-0.42148647	0.03078288	-3.26492018	0.01173821	0.16890793	-2.65760713
WT after UVB irradiation VS WT before UVB irradiation	GO_HINDLIMB_MORPHOGENESIS2	-0.40283186	-0.01264413	-2.49706786	0.0367729	0.47615783	-3.60650418
homo after UVB irradiation VS homo before UVB irradiation	GO_HIPPO_SIGNALING	-0.11044314	0.023272862	-0.57790718	0.57861154	0.73477617	-6.37121421
hetero after UVB irradiation VS hetero before UVB irradiation	GO_HIPPO_SIGNALING1	-0.11516105	-0.04404485	-0.75493802	0.47231819	0.75607802	-5.9335119
WT after UVB irradiation VS WT before UVB irradiation	GO_HIPPO_SIGNALING2	-0.1812965	-0.03267371	-1.13006064	0.29080982	0.75624915	-5.37327395
homo after UVB irradiation VS homo before UVB irradiation	GO_HIPPOCAMPAL_MOSSY_FIBER_TO_CA3_SYNAPSE	-0.10458185	-0.01328645	-0.75570052	0.4706575	0.64941494	-6.247917
hetero after UVB irradiation VS hetero before UVB irradiation	GO_HIPPOCAMPAL_MOSSY_FIBER_TO_CA3_SYNAPSE1	-0.17594731	-0.00719587	-1.24055109	0.25055431	0.57806649	-5.47207864
WT after UVB irradiation VS WT before UVB irradiation	GO_HIPPOCAMPAL_MOSSY_FIBER_TO_CA3_SYNAPSE2	-0.22888694	-0.04587776	-1.557842	0.15743277	0.66180255	-4.88566571
homo after UVB irradiation VS homo before UVB irradiation	GO_HIPPOCAMPUS_DEVELOPMENT	-0.09961163	0.028433801	-0.78056208	0.45666769	0.63761282	-6.22837004
hetero after UVB irradiation VS hetero before UVB irradiation	GO_HIPPOCAMPUS_DEVELOPMENT1	-0.15938893	0.021165214	-1.15366614	0.28253952	0.60810077	-5.56680868
WT after UVB irradiation VS WT before UVB irradiation	GO_HIPPOCAMPUS_DEVELOPMENT2	-0.14414841	0.040885666	-1.02439903	0.33527226	0.77905119	-5.47740094
homo after UVB irradiation VS homo before UVB irradiation	GO_HIS_PURKINJE_SYSTEM_DEVELOPMENT	0.109991034	-0.09093456	0.760797223	0.46776679	0.64669613	-6.24395446
hetero after UVB irradiation VS hetero before UVB irradiation	GO_HIS_PURKINJE_SYSTEM_DEVELOPMENT1	-0.58800431	-0.09541151	-2.96801786	0.01830253	0.19771025	-3.08602678
WT after UVB irradiation VS WT before UVB irradiation	GO_HIS_PURKINJE_SYSTEM_DEVELOPMENT2	-0.58861317	-0.10178943	-2.58399061	0.03209474	0.45445578	-3.48281505
homo after UVB irradiation VS homo before UVB irradiation	GO_HISTAMINE_METABOLIC_PROCESS	0.624810273	0.072097806	2.524298893	0.03448275	0.13297782	-3.97143849
hetero after UVB irradiation VS hetero before UVB irradiation	GO_HISTAMINE_METABOLIC_PROCESS1	0.561638836	0.078313652	2.154747796	0.06393792	0.33147829	-4.27344898
WT after UVB irradiation VS WT before UVB irradiation	GO_HISTAMINE_METABOLIC_PROCESS2	0.287713938	0.014877629	0.840776882	0.42460209	0.82612337	-5.63938968
homo after UVB irradiation VS homo before UVB irradiation	GO_HISTAMINE_PRODUCTION_INVOLVED_IN_INFLAMMATORY_RESPONSE	0.741495412	-0.0343403	4.228407202	0.00263206	0.033899	-1.39985131
hetero after UVB irradiation VS hetero before UVB irradiation	GO_HISTAMINE_PRODUCTION_INVOLVED_IN_INFLAMMATORY_RESPONSE1	0.572848508	0.060482157	4.19545389	0.00314248	0.11536387	-1.38689143
WT after UVB irradiation VS WT before UVB irradiation	GO_HISTAMINE_PRODUCTION_INVOLVED_IN_INFLAMMATORY_RESPONSE2	0.770545866	0.082750406	5.018370446	0.00099203	0.19353487	-0.35313813
homo after UVB irradiation VS homo before UVB irradiation	GO_HISTAMINE_RECEPTOR_ACTIVITY	0.2846223	0.030654772	0.992420274	0.34894395	0.54095661	-6.04055833
hetero after UVB irradiation VS hetero before UVB irradiation	GO_HISTAMINE_RECEPTOR_ACTIVITY1	-0.06430805	-0.02818531	-0.22135203	0.83046564	0.93512809	-6.20499846
WT after UVB irradiation VS WT before UVB irradiation	GO_HISTAMINE_RECEPTOR_ACTIVITY2	0.147914904	0.02966612	0.51342208	0.62136754	0.90107688	-5.85932704
homo after UVB irradiation VS homo before UVB irradiation	GO_HISTAMINE_SECRETION	0.500997603	-0.02570882	2.673750114	0.02721554	0.11674993	-3.73980429
hetero after UVB irradiation VS hetero before UVB irradiation	GO_HISTAMINE_SECRETION1	0.372627605	0.001358354	2.437210463	0.04128852	0.27382175	-3.86332801
WT after UVB irradiation VS WT before UVB irradiation	GO_HISTAMINE_SECRETION2	0.512208093	-0.02387943	2.960489182	0.0178902	0.3854098	-2.94893084
homo after UVB irradiation VS homo before UVB irradiation	GO_HISTAMINE_TRANSPORT	0.577381701	-0.05623184	3.663086826	0.0059454	0.05080272	-2.22207218
hetero after UVB irradiation VS hetero before UVB irradiation	GO_HISTAMINE_TRANSPORT1	0.241409164	0.010822443	1.592860599	0.15056401	0.46844437	-5.04608486
WT after UVB irradiation VS WT before UVB irradiation	GO_HISTAMINE_TRANSPORT2	0.46295333	-0.02033068	2.4643545	0.03870746	0.48068087	-3.65301865
homo after UVB irradiation VS homo before UVB irradiation	GO_HISTIDINE_METABOLIC_PROCESS	0.124862422	-0.01396126	0.730437647	0.48515811	0.66211766	-6.26721511
hetero after UVB irradiation VS hetero before UVB irradiation	GO_HISTIDINE_METABOLIC_PROCESS1	-0.09814762	-0.05521004	-0.50797783	0.62542675	0.84551508	-6.09388134
WT after UVB irradiation VS WT before UVB irradiation	GO_HISTIDINE_METABOLIC_PROCESS2	-0.04391138	-0.08325831	-0.17752291	0.863458	0.96779961	-5.97947232
homo after UVB irradiation VS homo before UVB irradiation	GO_HISTONE_ACETYLTRANSFERASE_ACTIVITY_H3_K23_SPECIFIC	-0.60010357	0.050313723	-2.33210362	0.04678426	0.15827496	-4.26710076
hetero after UVB irradiation VS hetero before UVB irradiation	GO_HISTONE_ACETYLTRANSFERASE_ACTIVITY_H3_K23_SPECIFIC1	-0.57889802	-0.04625179	-2.60158237	0.03203343	0.24829472	-3.62237499
WT after UVB irradiation VS WT before UVB irradiation	GO_HISTONE_ACETYLTRANSFERASE_ACTIVITY_H3_K23_SPECIFIC2	-0.32429839	0.031987092	-1.17507256	0.27335876	0.74578577	-5.32670715
homo after UVB irradiation VS homo before UVB irradiation	GO_HISTONE_ACETYLTRANSFERASE_ACTIVITY_H4_K16_SPECIFIC	-0.5619409	0.004760092	-2.5886813	0.03113716	0.12512052	-3.87177337
hetero after UVB irradiation VS hetero before UVB irradiation	GO_HISTONE_ACETYLTRANSFERASE_ACTIVITY_H4_K16_SPECIFIC1	-0.39286761	-0.01155975	-1.60256648	0.1484057	0.46627003	-5.03354622
WT after UVB irradiation VS WT before UVB irradiation	GO_HISTONE_ACETYLTRANSFERASE_ACTIVITY_H4_K16_SPECIFIC2	-0.25755596	-0.03974534	-0.91077618	0.38871992	0.80982312	-5.58063861
homo after UVB irradiation VS homo before UVB irradiation	GO_HISTONE_ACETYLTRANSFERASE_BINDING	-0.26886985	-0.01882624	-1.5441526	0.15964425	0.33553843	-5.40320131
hetero after UVB irradiation VS hetero before UVB irradiation	GO_HISTONE_ACETYLTRANSFERASE_BINDING1	0.129995298	0.001178821	0.931920558	0.37913879	0.69214054	-5.78569046
WT after UVB irradiation VS WT before UVB irradiation	GO_HISTONE_ACETYLTRANSFERASE_BINDING2	-0.07978381	-0.01554789	-0.5292372	0.61083779	0.89907573	-5.85092592
homo after UVB irradiation VS homo before UVB irradiation	GO_HISTONE_ARGININE_METHYLATION	-0.31267473	-0.00120442	-1.62716672	0.14085741	0.31121917	-5.29289633
hetero after UVB irradiation VS hetero before UVB irradiation	GO_HISTONE_ARGININE_METHYLATION1	-0.59292479	-0.01318774	-3.177113	0.01337227	0.17586555	-2.78345655
WT after UVB irradiation VS WT before UVB irradiation	GO_HISTONE_ARGININE_METHYLATION2	0.146691717	-0.00898106	0.607464937	0.56018735	0.88015308	-5.80592344
homo after UVB irradiation VS homo before UVB irradiation	GO_HISTONE_ARGININE_N_METHYLTRANSFERASE_ACTIVITY	-0.55680033	-0.02249903	-3.09529718	0.0140631	0.08059386	-3.08578672
hetero after UVB irradiation VS hetero before UVB irradiation	GO_HISTONE_ARGININE_N_METHYLTRANSFERASE_ACTIVITY1	-0.84622099	0.012442195	-5.52037383	0.00059757	0.08125048	0.178157237
WT after UVB irradiation VS WT before UVB irradiation	GO_HISTONE_ARGININE_N_METHYLTRANSFERASE_ACTIVITY2	0.058043322	0.008276538	0.202623	0.84442818	0.96450382	-5.97446146
homo after UVB irradiation VS homo before UVB irradiation	GO_HISTONE_BINDING	-0.45102695	-0.01097133	-2.9855544	0.01667748	0.08835006	-3.25559736
hetero after UVB irradiation VS hetero before UVB irradiation	GO_HISTONE_BINDING1	-0.18214589	-0.04893237	-1.04459696	0.32729656	0.64661776	-5.6788125
WT after UVB irradiation VS WT before UVB irradiation	GO_HISTONE_BINDING2	-0.23336518	-0.02238189	-1.0956679	0.30473792	0.76513806	-5.40798387
homo after UVB irradiation VS homo before UVB irradiation	GO_HISTONE_CITRULLINATION	-0.9929345	0.102808824	-5.51860047	0.0004875	0.01621654	0.295151089
hetero after UVB irradiation VS hetero before UVB irradiation	GO_HISTONE_CITRULLINATION1	-0.57873014	-0.0136528	-2.25987589	0.05433539	0.30886472	-4.12171724
WT after UVB irradiation VS WT before UVB irradiation	GO_HISTONE_CITRULLINATION2	-0.5014128	0.07301306	-2.39463714	0.04317977	0.49624823	-3.7520208
homo after UVB irradiation VS homo before UVB irradiation	GO_HISTONE_DEACETYLASE_ACTIVITY_H3_K14_SPECIFIC	0.054682827	0.033967787	0.266671604	0.79620548	0.88454226	-6.511997
hetero after UVB irradiation VS hetero before UVB irradiation	GO_HISTONE_DEACETYLASE_ACTIVITY_H3_K14_SPECIFIC1	0.155398267	0.001977013	0.507985175	0.62542183	0.84551508	-6.09387743
WT after UVB irradiation VS WT before UVB irradiation	GO_HISTONE_DEACETYLASE_ACTIVITY_H3_K14_SPECIFIC2	0.106019881	0.002389525	0.381080373	0.71294742	0.92947342	-5.92016024
homo after UVB irradiation VS homo before UVB irradiation	GO_HISTONE_DEACETYLASE_BINDING	-0.23667648	-0.00097809	-1.80404614	0.10739788	0.26217874	-5.04862026

hetero after UVB irradiation VS hetero before UVB irradiation	GO_HISTONE_DEACETYLASE_BINDING1	0.085726361	-0.038279	0.602766133	0.56366203	0.81050447	-6.03903861
WT after UVB irradiation VS WT before UVB irradiation	GO_HISTONE_DEACETYLASE_BINDING2	-0.08629459	-0.00191303	-0.50677267	0.62582274	0.90232206	-5.86278827
homo after UVB irradiation VS homo before UVB irradiation	GO_HISTONE_DEACETYLASE_COMPLEX	-0.30781821	0.006093393	-1.97329141	0.08249159	0.22258702	-4.80514032
hetero after UVB irradiation VS hetero before UVB irradiation	GO_HISTONE_DEACETYLASE_COMPLEX1	-0.0242573	-0.0293348	-0.1418388	0.89077868	0.96007086	-6.2205883
WT after UVB irradiation VS WT before UVB irradiation	GO_HISTONE_DEACETYLASE_COMPLEX2	-0.07497093	0.004636742	-0.32629046	0.75247784	0.93734883	-5.9402784
homo after UVB irradiation VS homo before UVB irradiation	GO_HISTONE_DEACETYLATION	0.006415766	-0.00183952	0.043619069	0.96623566	0.98227746	-6.54981264
hetero after UVB irradiation VS hetero before UVB irradiation	GO_HISTONE_DEACETYLATION1	-0.01873714	-0.03795574	-0.14406684	0.88907622	0.95932215	-6.22024378
WT after UVB irradiation VS WT before UVB irradiation	GO_HISTONE_DEACETYLATION2	0.047495251	-0.02507704	0.306900887	0.76666268	0.94123239	-5.94667245
homo after UVB irradiation VS homo before UVB irradiation	GO_HISTONE_DEMETHYLASE_ACTIVITY_H3_K27_SPECIFIC	-0.90632764	0.002858552	-4.401304	0.00207043	0.03080331	-1.15757986
hetero after UVB irradiation VS hetero before UVB irradiation	GO_HISTONE_DEMETHYLASE_ACTIVITY_H3_K27_SPECIFIC1	-0.57473642	-0.06227162	-1.58756421	0.15175382	0.46990313	-5.0529114
WT after UVB irradiation VS WT before UVB irradiation	GO_HISTONE_DEMETHYLASE_ACTIVITY_H3_K27_SPECIFIC2	-0.87389876	-0.02248905	-4.56309965	0.0017857	0.20370459	-0.86534327
homo after UVB irradiation VS homo before UVB irradiation	GO_HISTONE_DEMETHYLASE_ACTIVITY_H3_K36_SPECIFIC	-0.62520688	-0.04824302	-2.18072383	0.05947804	0.18316308	-4.4969327
hetero after UVB irradiation VS hetero before UVB irradiation	GO_HISTONE_DEMETHYLASE_ACTIVITY_H3_K36_SPECIFIC1	-0.4112257	-0.07271904	-1.59240814	0.15066532	0.46844437	-5.04666847
WT after UVB irradiation VS WT before UVB irradiation	GO_HISTONE_DEMETHYLASE_ACTIVITY_H3_K36_SPECIFIC2	-0.31242912	-0.00464604	-0.94438278	0.37229495	0.8012534	-5.55109297
homo after UVB irradiation VS homo before UVB irradiation	GO_HISTONE_DEMETHYLASE_ACTIVITY_H3_K4_SPECIFIC	-0.8847176	-0.01554103	-5.41160631	0.00055564	0.01718452	0.164558736
hetero after UVB irradiation VS hetero before UVB irradiation	GO_HISTONE_DEMETHYLASE_ACTIVITY_H3_K4_SPECIFIC1	-0.3774255	-0.10462953	-1.1233476	0.29445687	0.6179563	-5.59874257
WT after UVB irradiation VS WT before UVB irradiation	GO_HISTONE_DEMETHYLASE_ACTIVITY_H3_K4_SPECIFIC2	-0.43545891	0.006610537	-1.38684024	0.20247105	0.69852129	-5.09179015
homo after UVB irradiation VS homo before UVB irradiation	GO_HISTONE_DEMETHYLASE_ACTIVITY_H3_K9_SPECIFIC	-0.4386222	0.015188616	-1.26876654	0.23883104	0.43133394	-5.7446475
hetero after UVB irradiation VS hetero before UVB irradiation	GO_HISTONE_DEMETHYLASE_ACTIVITY_H3_K9_SPECIFIC1	-0.46523024	-0.00672865	-1.7308168	0.12243797	0.42855169	-4.86459859
WT after UVB irradiation VS WT before UVB irradiation	GO_HISTONE_DEMETHYLASE_ACTIVITY_H3_K9_SPECIFIC2	-0.50219991	0.010776177	-2.00112766	0.07994503	0.56457304	-4.30282666
homo after UVB irradiation VS homo before UVB irradiation	GO_HISTONE_DEPHOSPHORYLATION	0.156127162	0.026018505	0.826588328	0.43150996	0.61566204	-6.19075434
hetero after UVB irradiation VS hetero before UVB irradiation	GO_HISTONE_DEPHOSPHORYLATION1	-0.07331876	-0.0291744	-0.44694984	0.66698099	0.86352197	-6.1245567
WT after UVB irradiation VS WT before UVB irradiation	GO_HISTONE_DEPHOSPHORYLATION2	0.119176034	-0.04571524	0.646772971	0.5356773	0.86990942	-5.78118834
homo after UVB irradiation VS homo before UVB irradiation	GO_HISTONE_DEUBIQUITINATION	-0.34311692	-0.01630089	-2.44293336	0.03923541	0.14232962	-4.09700983
hetero after UVB irradiation VS hetero before UVB irradiation	GO_HISTONE_DEUBIQUITINATION1	0.160414043	-0.04409174	0.894916247	0.3974323	0.70478441	-5.81869581
WT after UVB irradiation VS WT before UVB irradiation	GO_HISTONE_DEUBIQUITINATION2	-0.11349327	-0.01917123	-0.57335181	0.58197514	0.88904835	-5.82624693
homo after UVB irradiation VS homo before UVB irradiation	GO_HISTONE_EXCHANGE	-0.57317261	-0.01564001	-2.65453972	0.02805479	0.11859194	-3.76962677
hetero after UVB irradiation VS hetero before UVB irradiation	GO_HISTONE_EXCHANGE1	-0.10757007	-0.04747157	-0.41354941	0.69026702	0.87576597	-6.13977011
WT after UVB irradiation VS WT before UVB irradiation	GO_HISTONE_EXCHANGE2	0.001976641	0.014977466	0.007534472	0.99417073	0.9985657	-5.99603671
homo after UVB irradiation VS homo before UVB irradiation	GO_HISTONE_H2A_ACETYLATION	-0.48527203	0.015285438	-2.62511301	0.02939177	0.1216646	-3.81528762
hetero after UVB irradiation VS hetero before UVB irradiation	GO_HISTONE_H2A_ACETYLATION1	-0.25841317	-0.05445977	-1.08234624	0.31121179	0.63195954	-5.64095988
WT after UVB irradiation VS WT before UVB irradiation	GO_HISTONE_H2A_ACETYLATION2	-0.08559693	-0.00682346	-0.27785036	0.78808957	0.9469715	-5.95553627
homo after UVB irradiation VS homo before UVB irradiation	GO_HISTONE_H2A_K119_MONOUBIQUITINATION	-0.44111401	-0.00195311	-2.12630259	0.06482544	0.19300497	-4.57865243
hetero after UVB irradiation VS hetero before UVB irradiation	GO_HISTONE_H2A_K119_MONOUBIQUITINATION1	-0.4924253	0.016681437	-2.21676158	0.05808695	0.31809313	-4.18410746
WT after UVB irradiation VS WT before UVB irradiation	GO_HISTONE_H2A_K119_MONOUBIQUITINATION2	-0.43014495	0.00939189	-2.1639911	0.06199536	0.5331384	-4.07714566
homo after UVB irradiation VS homo before UVB irradiation	GO_HISTONE_H2A_K63_LINKED_UBIQUITINATION	-0.75571183	-0.082363	-4.14339449	0.00296649	0.03594912	-1.52062185
hetero after UVB irradiation VS hetero before UVB irradiation	GO_HISTONE_H2A_K63_LINKED_UBIQUITINATION1	-0.00323001	-0.01404584	-0.00987858	0.99236451	0.99657418	-6.23143119
WT after UVB irradiation VS WT before UVB irradiation	GO_HISTONE_H2A_K63_LINKED_UBIQUITINATION2	-0.26491974	0.097743198	-0.6369369	0.54174959	0.87032935	-5.78750873
homo after UVB irradiation VS homo before UVB irradiation	GO_HISTONE_H2A_MONOUBIQUITINATION	-0.46203798	-0.02198994	-2.80620767	0.02208558	0.10340002	-3.53401518
hetero after UVB irradiation VS hetero before UVB irradiation	GO_HISTONE_H2A_MONOUBIQUITINATION1	-0.16646012	-0.04102255	-0.70168498	0.50313758	0.77390992	-5.97281297
WT after UVB irradiation VS WT before UVB irradiation	GO_HISTONE_H2A_MONOUBIQUITINATION2	-0.33925983	0.014791748	-1.23505196	0.25144338	0.73564768	-5.26273134
homo after UVB irradiation VS homo before UVB irradiation	GO_HISTONE_H2A_PHOSPHORYLATION	-0.07627615	-0.07391511	-0.1927029	0.85180756	0.91816808	-6.53052278
hetero after UVB irradiation VS hetero before UVB irradiation	GO_HISTONE_H2A_PHOSPHORYLATION1	0.180552275	0.011271582	0.518070368	0.61868495	0.84254497	-6.08845454
WT after UVB irradiation VS WT before UVB irradiation	GO_HISTONE_H2A_PHOSPHORYLATION2	-0.17757892	-0.03208337	-0.9686571	0.36075408	0.7938634	-5.52922779
homo after UVB irradiation VS homo before UVB irradiation	GO_HISTONE_H2A_UBIQUITINATION	-0.45319587	-0.02773324	-2.63831109	0.02878428	0.12035506	-3.79481195
hetero after UVB irradiation VS hetero before UVB irradiation	GO_HISTONE_H2A_UBIQUITINATION1	-0.15556787	-0.02367105	-0.63922368	0.54087087	0.79694618	-6.01567681
WT after UVB irradiation VS WT before UVB irradiation	GO_HISTONE_H2A_UBIQUITINATION2	-0.35749145	0.023447809	-1.34640701	0.21464242	0.71222466	-5.13851856
homo after UVB irradiation VS homo before UVB irradiation	GO_HISTONE_H2B_UBIQUITINATION	-0.4114727	-0.02724547	-1.50277385	0.16982542	0.34916236	-5.45701514
hetero after UVB irradiation VS hetero before UVB irradiation	GO_HISTONE_H2B_UBIQUITINATION1	-0.09796165	-0.02524536	-0.36925735	0.72169407	0.89103348	-6.15819851
WT after UVB irradiation VS WT before UVB irradiation	GO_HISTONE_H2B_UBIQUITINATION2	-0.31894565	-0.01262093	-1.07895533	0.31169445	0.76904783	-5.42457041
homo after UVB irradiation VS homo before UVB irradiation	GO_HISTONE_H3_ACETYLATION	-0.39047159	-0.00662363	-2.67171258	0.02730333	0.11705846	-3.74296784
hetero after UVB irradiation VS hetero before UVB irradiation	GO_HISTONE_H3_ACETYLATION1	-0.25572931	-0.0720824	-1.84366323	0.10316501	0.39866653	-4.71149081
WT after UVB irradiation VS WT before UVB irradiation	GO_HISTONE_H3_ACETYLATION2	-0.17156375	-0.03433645	-0.77321858	0.46136545	0.84025946	-5.69237608
homo after UVB irradiation VS homo before UVB irradiation	GO_HISTONE_H3_DEACETYLATION	-0.11479007	0.014036874	-0.51500115	0.61993773	0.76376866	-6.40760989
hetero after UVB irradiation VS hetero before UVB irradiation	GO_HISTONE_H3_DEACETYLATION1	0.063018995	-0.03771569	0.276105496	0.78959411	0.91807191	-6.19034859
WT after UVB irradiation VS WT before UVB irradiation	GO_HISTONE_H3_DEACETYLATION2	-0.22448652	-0.02329332	-0.83700809	0.42659797	0.82688157	-5.64244325
homo after UVB irradiation VS homo before UVB irradiation	GO_HISTONE_H3_K14_ACETYLATION	-0.71501597	0.017938494	-4.49653186	0.00181746	0.02890877	-1.02609691
hetero after UVB irradiation VS hetero before UVB irradiation	GO_HISTONE_H3_K14_ACETYLATION1	-0.61878379	-0.08584855	-3.15372156	0.01384682	0.17734093	-2.81711394
WT after UVB irradiation VS WT before UVB irradiation	GO_HISTONE_H3_K14_ACETYLATION2	-0.31855306	-0.0242859	-1.12785361	0.29168804	0.75646944	-5.3755243
homo after UVB irradiation VS homo before UVB irradiation	GO_HISTONE_H3_K23_ACETYLATION	-0.60010357	0.050313723	-2.33210362	0.04678426	0.15827496	-4.26710076
hetero after UVB irradiation VS hetero before UVB irradiation	GO_HISTONE_H3_K23_ACETYLATION1	-0.57889802	-0.04625179	-2.60158237	0.03203343	0.24829472	-3.62237499
WT after UVB irradiation VS WT before UVB irradiation	GO_HISTONE_H3_K23_ACETYLATION2	-0.32429839	0.031987092	-1.17507256	0.27335876	0.74578577	-5.32670715
homo after UVB irradiation VS homo before UVB irradiation	GO_HISTONE_H3_K27_DEMETHYLATION	-0.90632764	0.002858552	-4.401304	0.00207043	0.03080331	-1.15757986
hetero after UVB irradiation VS hetero before UVB irradiation	GO_HISTONE_H3_K27_DEMETHYLATION1	-0.57473642	-0.06227162	-1.58756421	0.15175382	0.46990313	-5.0529114
WT after UVB irradiation VS WT before UVB irradiation	GO_HISTONE_H3_K27_DEMETHYLATION2	-0.87389876	-0.02248905	-4.56309965	0.0017857	0.20370459	-0.86534327
homo after UVB irradiation VS homo before UVB irradiation	GO_HISTONE_H3_K27_METHYLATION	-0.49381005	-0.00030471	-2.47154448	0.03749333	0.13884294	-4.05291142
hetero after UVB irradiation VS hetero before UVB irradiation	GO_HISTONE_H3_K27_METHYLATION1	-0.09749068	-0.00387179	-0.48742229	0.63927427	0.85216517	-6.1046252
WT after UVB irradiation VS WT before UVB irradiation	GO_HISTONE_H3_K27_METHYLATION2	-0.38003021	-0.00231691	-1.55619521	0.15781988	0.66180255	-4.88771098
homo after UVB irradiation VS homo before UVB irradiation	GO_HISTONE_H3_K27_TRIMETHYLATION	-0.9192337	-0.02098548	-4.45175753	0.00193198	0.0297852	-1.08774297
hetero after UVB irradiation VS hetero before UVB irradiation	GO_HISTONE_H3_K27_TRIMETHYLATION1	-0.47710098	0.116209924	-1.88124656	0.09741164	0.38932993	-4.6596999
WT after UVB irradiation VS WT before UVB irradiation	GO_HISTONE_H3_K27_TRIMETHYLATION2	-0.72189907	0.002308501	-2.99442529	0.01698141	0.380843	-2.90119957
homo after UVB irradiation VS homo before UVB irradiation	GO_HISTONE_H3_K36_DEMETHYLATION	-0.62520688	-0.04824302	-2.18072383	0.05947804	0.18316308	-4.4969327
hetero after UVB irradiation VS hetero before UVB irradiation	GO_HISTONE_H3_K36_DEMETHYLATION1	-0.4112257	-0.07271904	-1.59240814	0.15066532	0.46844437	-5.04666847
WT after UVB irradiation VS WT before UVB irradiation	GO_HISTONE_H3_K36_DEMETHYLATION2	-0.31242912	-0.00464604	-0.94438278	0.37229495	0.8012534	-5.55109297
homo after UVB irradiation VS homo before UVB irradiation	GO_HISTONE_H3_K36_METHYLATION	-0.59601784	-0.03545558	-3.35071554	0.00950212	0.06491798	-2.6935544
hetero after UVB irradiation VS hetero before UVB irradiation	GO_HISTONE_H3_K36_METHYLATION1	-0.46981103	-0.11247907	-2.012074	0.07969134	0.36339704	-4.47677162
WT after UVB irradiation VS WT before UVB irradiation	GO_HISTONE_H3_K36_METHYLATION2	-0.44544328	-0.07441743	-1.64500955	0.13813157	0.64683525	-4.77591046
homo after UVB irradiation VS homo before UVB irradiation	GO_HISTONE_H3_K36_TRIMETHYLATION	-0.63245472	0.037059357	-2.63935682	0.0287367	0.12020053	-3.79318935
hetero after UVB irradiation VS hetero before UVB irradiation	GO_HISTONE_H3_K36_TRIMETHYLATION1	-0.65795598	-0.03193668	-2.03980501	0.07635816	0.35638773	-4.43753109
WT after UVB irradiation VS WT before UVB irradiation	GO_HISTONE_H3_K36_TRIMETHYLATION2	-0.48865329	0.026533257	-1.81189871	0.107127	0.61228673	-4.55850382
homo after UVB irradiation VS homo before UVB irradiation	GO_HISTONE_H3_K4_DEMETHYLATION	-0.88253442	-0.00822121	-5.63803245	0.00042204	0.01537777	0.43882596
homo after UVB irradiation VS homo before UVB irradiation	GO_HISTONE_H3_K4_DEMETHYLATION_TRIMETHYL_H3_K4_SPECIFIC	-0.88145359	0.036895762	-5.46817673	0.0005184	0.01662816	0.233828334
hetero after UVB irradiation VS hetero before UVB irradiation	GO_HISTONE_H3_K4_DEMETHYLATION_TRIMETHYL_H3_K4_SPECIFIC1	-0.2956987	-0.06943739	-0.87737046	0.40632654	0.71194505	-5.83396846
WT after UVB irradiation VS WT before UVB irradiation	GO_HISTONE_H3_K4_DEMETHYLATION_TRIMETHYL_H3_K4_SPECIFIC2	-0.37328976	-0.01052343	-1.21254015	0.25949217	0.73996638	-5.28699425
hetero after UVB irradiation VS hetero before UVB irradiation	GO_HISTONE_H3_K4_DEMETHYLATION1	-0.37743033	-0.10282794	-1.11852205	0.29639049	0.62039917	-5.60376964
WT after UVB irradiation VS WT before UVB irradiation	GO_HISTONE_H3_K4_DEMETHYLATION2	-0.42466026	0.006806065	-1.30751399	0.22693836	0.71906685	-5.18267034
homo after UVB irradiation VS homo before UVB irradiation	GO_HISTONE_H3_K4_DIMETHYLATION	-0.8047614	-0.01347985	-2.91634032	0.0185812	0.09355293	-3.36295321
hetero after UVB irradiation VS hetero before UVB irradiation	GO_HISTONE_H3_K4_DIMETHYLATION1	-0.9664846	-0.01525261	-7.2303356	9.85E-05	0.07788488	1.783044279
WT after UVB irradiation VS WT before UVB irradiation	GO_HISTONE_H3_K4_DIMETHYLATION2	-0.44564987	0.019008253	-1.05483245	0.3219549	0.77346379	-5.44818121
homo after UVB irradiation VS homo before UVB irradiation	GO_HISTONE_H3_K4_METHYLATION	-0.52545641	-0.00951705	-2.91043538	0.01875368	0.09415099	-3.37211901
hetero after UVB irradiation VS hetero before UVB irradiation	GO_HISTONE_H3_K4_METHYLATION1	-0.21234938	-0.05098011	-1.11282049	0.29868824	0.62161528	-5.6096894
WT after UVB irradiation VS WT before UVB irradiation	GO_HISTONE_H3_K4_METHYLATION2	-0.25302332	0.000813488	-0.91178661	0.3882185	0.80982312	-5.57976274
homo after UVB irradiation VS homo before UVB irradiation	GO_HISTONE_H3_K4_MONOMETHYLATION	-0.76211804	0.040616987	-2.63430541	0.0289673	0.12072024	-3.80102707
hetero after UVB irradiation VS hetero before UVB irradiation	GO_HISTONE_H3_K4_MONOMETHYLATION1	-0.81753996	0.020008454	-4.24418679	0.00294299	0.11368267	-1.32403534
WT after UVB irradiation VS WT before UVB irradiation	GO_HISTONE_H3_K4_MONOMETHYLATION2	-0.49237621	-0.05222683	-1.35632759	0.2115991	0.70907106	-5.12713019
homo after UVB irradiation VS homo before UVB irradiation	GO_HISTONE_H3_K4_TRIMETHYLATION	-0.58285246	0.011482878	-2.14271732	0.06316481	0.18991914	-4.55406139
hetero after UVB irradiation VS hetero before UVB irradiation	GO_HISTONE_H3_K4_TRIMETHYLATION1	-0.39183482	-0.04167238	-2.03740358	0.07664135	0.35706231	-4.44093501
WT after UVB irradiation VS WT before UVB irradiation	GO_HISTONE_H3_K4_TRIMETHYLATION2	-0.33350157	0.017242184	-0.8921093	0.39806797	0.81477652	-5.59667899
homo after UVB irradiation VS homo before UVB irradiation	GO_HISTONE_H3_K9_ACETYLATION	-0.60166501	0.030830866	-4.03979668	0.00343689	0.03849136	-1.66922485
hetero after UVB irradiation VS hetero before UVB irradiation	GO_HISTONE_H3_K9_ACETYLATION1	-0.33792773	-0.10990142	-2.25110717	0.05507833	0.31051949	-4.13442305
WT after UVB irradiation VS WT before UVB irradiation	GO_HISTONE_H3_K9_ACETYLATION2	-0.04750218	-0.06642669	-0.21050949	0.83847066	0.9623128	-5.97275185
homo after UVB irradiation VS homo before UVB irradiation	GO_HISTONE_H3_K9_DEMETHYLATION	-0.36295876	-0.00566063	-1.0161134	0.33817894	0.53112671	-6.01731823
hetero after UVB irradiation VS hetero before UVB irradiation	GO_HISTONE_H3_K9_DEMETHYLATION1	-0.3136509	0.002291942	-1.21882319	0.258257	0.58493328	-5.49620234
WT after UVB irradiation VS WT before UVB irradiation	GO_HISTONE_H3_K9_DEMETHYLATION2	-0.4362119	0.017992477	-1.80726689	0.10789159	0.61228673	-4.5646512
homo after UVB irradiation VS homo before UVB irradiation	GO_HISTONE_H3_K9_DIMETHYLATION	-0.42743908	0.018155348	-1.46387122	0.17991984	0.36236909	-5.50685483
hetero after UVB irradiation VS hetero before UVB irradiation	GO_HISTONE_H3_K9_DIMETHYLATION1	-0.01059655	-0.06356098	-0.05353681	0.9586411	0.98503151	-6.22993005
WT after UVB irradiation VS WT before UVB irradiation	GO_HISTONE_H3_K9_DIMETHYLATION2	-0.42441651	-0.02182759	-2.13638718	0.06473229	0.53678963	-4.1156816
homo after UVB irradiation VS homo before UVB irradiation	GO_HISTONE_H3_K9_METHYLATION	-0.41244467	0.019053696	-1.80879006	0.10661194	0.26064819	-5.04191429
hetero after UVB irradiation VS hetero before UVB irradiation	GO_HISTONE_H3_K9_METHYLATION1	-0.16740104	-0.0559993	-1.00235291	0.34605584	0.66286618	-5.71997751
WT after UVB irradiation VS WT before UVB irradiation	GO_HISTONE_H3_K9_METHYLATION2	-0.33924731	0.000436983	-1.38861841	0.20194979	0.69830181	-5.08971631
homo after UVB irradiation VS homo before UVB irradiation	GO_HISTONE_H3_K9_MODIFICATION	-0.44233813	0.039697442	-2.17406533	0.06010833	0.18460927	-4.50695991
hetero after UVB irradiation VS hetero before UVB irradiation	GO_HISTONE_H3_K9_MODIFICATION1	-0.2392206	-0.06649317	-1.45885477	0.18341416	0.50787377	-5.21518773
WT after UVB irradiation VS WT before UVB irradiation	GO_HISTONE_H3_K9_MODIFICATION2	-0.28323765	-0.01642797	-1.19854828	0.26460125	0.74156847	-5.30192356
homo after UVB irradiation VS homo before UVB irradiation	GO_HISTONE_H3_K9_TRIMETHYLATION	-0.44019016	0.011717637	-2.06556211	0.07134878	0.20504264	-4.66918155
hetero after UVB irradiation VS hetero before UVB irradiation	GO_HISTONE_H3_K9_TRIMETHYLATION1	-0.28596163	-0.03142746	-1.66113679	0.13597246	0.44861237	-4.9571195
WT after UVB irradiation VS WT before UVB irradiation	GO_HISTONE_H3_K9_TRIMETHYLATION2	-0.4804592	-0.00549684	-2.09701852	0.06884178	0.54354218	-4.17045635
homo after UVB irradiation VS homo before UVB irradiation	GO_HISTONE_H3_S10_PHOSPHORYLATION	0.094792557	-0.07192427	0.361932234	0.72641328	0.83837647	-6.47953462
hetero after UVB irradiation VS hetero before UVB irradiation	GO_HISTONE_H3_S10_PHOSPHORYLATION1	0.224098459	-0.07323488	0.637632166	0.54185417	0.79726618	-6.01672242
WT after UVB irradiation VS WT before UVB irradiation	GO_HISTONE_H3_S10_PHOSPHORYLATION2	0.035056364	-0.0401805	0.109969958	0.9151098	0.97967529	-5.98969063
homo after UVB irradiation VS homo before UVB irradiation	GO_HISTONE_H4_ACETYLATION	-0.49969067	-0.01363561	-3.21724566	0.01165211	0.07243059	-2.8979054
hetero after UVB irradiation VS hetero before UVB irradiation	GO_HISTONE_H4_ACETYLATION1	-0.20208674	-0.05108813	-1.04554926	0.32688295	0.64640644	-5.67786984
WT after UVB irradiation VS WT before UVB irradiation	GO_HISTONE_H4_ACETYLATION2	-0.28413129	-0.03324764	-1.23492158	0.25148939	0.73564768	-5.26287272
homo after UVB irradiation VS homo before UVB irradiation	GO_HISTONE_H4_DEACETYLATION	0.418087849	-0.00710435	1.659547246	0.13409146	0.30159849	-5.24907359
hetero after UVB irradiation VS hetero before UVB irradiation	GO_HISTONE_H4_DEACETYLATION1	0.322916942	-0.02819792	1.102066667	0.30306086	0.62502639	-5.62079572
WT after UVB irradiation VS WT before UVB irradiation	GO_HISTONE_H4_DEACETYLATION2	-0.09557207	0.018390793	-0.31937658	0.75752472	0.9389209	-5.94260212
homo after UVB irradiation VS homo before UVB irradiation	GO_HISTONE_H4_K12_ACETYLATION	-0.44736021	-0.02773917	-2.01657756	0.07706971	0.21354103	-4.74160971
hetero after UVB irradiation VS hetero before UVB irradiation	GO_HISTONE_H4_K12_ACETYLATION1	-0.27706787	-0.02479924	-1.72323695	0.12384696	0.43057108	-4.87474245
WT after UVB irradiation VS WT before UVB irradiation	GO_HISTONE_H4_K12_ACETYLATION2	-0.48432925	-0.03316401	-1.50287922	0.17082481	0.6778332	-4.95332564
homo after UVB irradiation VS homo before UVB irradiation	GO_HISTONE_H4_K16_ACETYLATION	-0.41479784	-0.02300886	-1.99547615	0.07966849	0.21799886	-4.77263758
hetero after UVB irradiation VS hetero before UVB irradiation	GO_HISTONE_H4_K16_ACETYLATION1	-0.08874472	-0.03133555	-0.37215736	0.7196182	0.89052082	-6.15705331
WT after UVB irradiation VS WT before UVB irradiation	GO_HISTONE_H4_K16_ACETYLATION2	-0.15028576	-0.01502695	-0.53427017	0.60750672	0.89725083	-5.84820268
homo after UVB irradiation VS homo before UVB irradiation	GO_HISTONE_H4_K20_METHYLATION	-0.90043916	0.006539236	-3.7967752	0.00488348	0.04555642	-2.02373251
hetero after UVB irradiation VS hetero before UVB irradiation	GO_HISTONE_H4_K20_METHYLATION1	-0.53644092	-0.08976992	-1.93148179	0.09019925	0.37720291	-4.58991889
WT after UVB irradiation VS WT before UVB irradiation	GO_HISTONE_H4_K20_METHYLATION2	-0.3179799	-0.0152592	-0.89863252	0.394783	0.81423482	-5.59110404
homo after UVB irradiation VS homo before UVB irradiation	GO_HISTONE_H4_K20_TRIMETHYLATION	-0.77051549	-0.02539903	-3.4306282	0.00841815	0.06101507	-2.57196318
hetero after UVB irradiation VS hetero before UVB irradiation	GO_HISTONE_H4_K20_TRIMETHYLATION1	-0.64157433	-0.09904079	-2.75567038	0.02528344	0.22748928	-3.39631295
WT after UVB irradiation VS WT before UVB irradiation	GO_HISTONE_H4_K20_TRIMETHYLATION2	-0.34399283	-0.02989895	-0.99159928	0.35009475	0.78758749	-5.5081665
homo after UVB irradiation VS homo before UVB irradiation	GO_HISTONE_H4_K5_ACETYLATION	-0.50177977	-0.03353851	-2.23333755	0.05472104	0.17369341	-4.41743898
hetero after UVB irradiation VS hetero before UVB irradiation	GO_HISTONE_H4_K5_ACETYLATION1	-0.30706338	-0.04347806	-1.61455909	0.14577798	0.46276998	-5.0180028
WT after UVB irradiation VS WT before UVB irradiation	GO_HISTONE_H4_K5_ACETYLATION2	-0.43623813	-0.03322591	-1.51511859	0.16775724	0.67272632	-4.93836822
homo after UVB irradiation VS homo before UVB irradiation	GO_HISTONE_H4_R3_METHYLATION	-0.72037687	-0.05289461	-3.39300247	0.00891134	0.06286429	-2.62913638
hetero after UVB irradiation VS hetero before UVB irradiation	GO_HISTONE_H4_R3_METHYLATION1	-0.66086716	-0.05778278	-2.27463356	0.05310751	0.30612069	-4.10031536
WT after UVB irradiation VS WT before UVB irradiation	GO_HISTONE_H4_R3_METHYLATION2	-0.14023766	-0.06539841	-0.45497706	0.66107591	0.91169919	-5.8882968
homo after UVB irradiation VS homo before UVB irradiation	GO_HISTONE_KINASE_ACTIVITY	-0.34696811	-0.01456491	-1.68029496	0.12991414	0.2954906	-5.22077199
hetero after UVB irradiation VS hetero before UVB irradiation	GO_HISTONE_KINASE_ACTIVITY1	0.339199796	-0.01965383	1.183634466	0.27114811	0.59721184	-5.53466308
WT after UVB irradiation VS WT before UVB irradiation	GO_HISTONE_KINASE_ACTIVITY2	0.002195076	-0.03681549	0.007184294	0.99444166	0.9985657	-5.99603943
homo after UVB irradiation VS homo before UVB irradiation	GO_HISTONE_LYSINE_N_METHYLTRANSFERASE_ACTIVITY	-0.5201224	-0.03951356	-2.50767908	0.03540394	0.13468829	-3.99712669
hetero after UVB irradiation VS hetero before UVB irradiation	GO_HISTONE_LYSINE_N_METHYLTRANSFERASE_ACTIVITY1	-0.39488145	-0.06812027	-2.15457599	0.06395491	0.33147829	-4.27369578
WT after UVB irradiation VS WT before UVB irradiation	GO_HISTONE_LYSINE_N_METHYLTRANSFERASE_ACTIVITY2	-0.32011413	-0.04443363	-1.2869775	0.23366947	0.72488129	-5.20565701
homo after UVB irradiation VS homo before UVB irradiation	GO_HISTONE_METHYLATION	-0.45805755	0.006118612	-2.60876277	0.03016245	0.12333258	-3.84064467
hetero after UVB irradiation VS hetero before UVB irradiation	GO_HISTONE_METHYLATION1	-0.29107845	-0.04653157	-1.6690686	0.13436458	0.44563334	-4.94667247
WT after UVB irradiation VS WT before UVB irradiation	GO_HISTONE_METHYLATION2	-0.28660439	-0.02162477	-1.24097717	0.24935973	0.73564768	-5.25629612

homo after UVB irradiation VS homo before UVB irradiation	GO_HISTONE_METHYLTRANSFERASE_ACTIVITY	-0.52170031	-0.00840813	-2.70139373	0.02605257	0.11395879	-3.69687439
homo after UVB irradiation VS homo before UVB irradiation	GO_HISTONE_METHYLTRANSFERASE_ACTIVITY_H3_K27_SPECIFIC	-0.40718759	0.060609316	-1.50534047	0.16917746	0.34845505	-5.45370089
hetero after UVB irradiation VS hetero before UVB irradiation	GO_HISTONE_METHYLTRANSFERASE_ACTIVITY_H3_K27_SPECIFIC1	-0.12062009	-0.0095335	-0.39881121	0.70065718	0.88146568	-6.14612431
WT after UVB irradiation VS WT before UVB irradiation	GO_HISTONE_METHYLTRANSFERASE_ACTIVITY_H3_K27_SPECIFIC2	-0.24017959	0.033600831	-0.98903421	0.35127458	0.78816886	-5.51054017
homo after UVB irradiation VS homo before UVB irradiation	GO_HISTONE_METHYLTRANSFERASE_ACTIVITY_H3_K36_SPECIFIC	-0.44520178	-0.02202017	-1.9321446	0.08798378	0.23190623	-4.86508824
hetero after UVB irradiation VS hetero before UVB irradiation	GO_HISTONE_METHYLTRANSFERASE_ACTIVITY_H3_K36_SPECIFIC1	-0.38938117	-0.0938959	-1.67288392	0.13359745	0.44450147	-4.9416393
WT after UVB irradiation VS WT before UVB irradiation	GO_HISTONE_METHYLTRANSFERASE_ACTIVITY_H3_K36_SPECIFIC2	-0.42774128	-0.09146804	-1.72055352	0.12318647	0.63133281	-4.67858954
homo after UVB irradiation VS homo before UVB irradiation	GO_HISTONE_METHYLTRANSFERASE_ACTIVITY_H3_K4_SPECIFIC	-0.72627861	-0.02655815	-3.39498602	0.00888459	0.0627921	-2.62611889
hetero after UVB irradiation VS hetero before UVB irradiation	GO_HISTONE_METHYLTRANSFERASE_ACTIVITY_H3_K4_SPECIFIC1	-0.48065939	-0.08511835	-2.27302269	0.05324018	0.30616216	-4.10265255
WT after UVB irradiation VS WT before UVB irradiation	GO_HISTONE_METHYLTRANSFERASE_ACTIVITY_H3_K4_SPECIFIC2	-0.43930807	-0.06198442	-1.35685981	0.21143688	0.70891132	-5.12651779
homo after UVB irradiation VS homo before UVB irradiation	GO_HISTONE_METHYLTRANSFERASE_ACTIVITY_H3_K9_SPECIFIC	-0.1619517	-0.0374271	-0.57300078	0.58177905	0.73691462	-6.37419243
hetero after UVB irradiation VS hetero before UVB irradiation	GO_HISTONE_METHYLTRANSFERASE_ACTIVITY_H3_K9_SPECIFIC1	0.276654362	0.017564382	1.217049673	0.25889434	0.58521976	-5.4981589
WT after UVB irradiation VS WT before UVB irradiation	GO_HISTONE_METHYLTRANSFERASE_ACTIVITY_H3_K9_SPECIFIC2	-0.02181511	0.017372996	-0.08587189	0.93365358	0.98322796	-5.99217766
homo after UVB irradiation VS homo before UVB irradiation	GO_HISTONE_METHYLTRANSFERASE_ACTIVITY_H4_K20_SPECIFIC	-0.75459219	-0.01221715	-2.52809914	0.03427553	0.13254822	-3.9655622
hetero after UVB irradiation VS hetero before UVB irradiation	GO_HISTONE_METHYLTRANSFERASE_ACTIVITY_H4_K20_SPECIFIC1	-0.44110921	-0.04165798	-1.30284618	0.22953214	0.55752697	-5.40138613
WT after UVB irradiation VS WT before UVB irradiation	GO_HISTONE_METHYLTRANSFERASE_ACTIVITY_H4_K20_SPECIFIC2	-0.00363702	0.057262057	-0.01081688	0.9916313	0.9985657	-5.99600493
hetero after UVB irradiation VS hetero before UVB irradiation	GO_HISTONE_METHYLTRANSFERASE_ACTIVITY1	-0.39661552	-0.05500344	-2.46029413	0.03984036	0.27216866	-3.82954269
WT after UVB irradiation VS WT before UVB irradiation	GO_HISTONE_METHYLTRANSFERASE_ACTIVITY2	-0.24234874	-0.02884203	-1.01599914	0.33902168	0.78064003	-5.48535239
homo after UVB irradiation VS homo before UVB irradiation	GO_HISTONE_METHYLTRANSFERASE_BINDING	-0.96826518	0.046448784	-6.18692555	0.00022286	0.0115395	1.071281093
hetero after UVB irradiation VS hetero before UVB irradiation	GO_HISTONE_METHYLTRANSFERASE_BINDING1	-0.66027749	-0.11857282	-2.29075555	0.05179774	0.30233295	-4.07691004
WT after UVB irradiation VS WT before UVB irradiation	GO_HISTONE_METHYLTRANSFERASE_BINDING2	-0.64710491	-0.05632041	-2.12706833	0.06568277	0.537335	-4.12866749
homo after UVB irradiation VS homo before UVB irradiation	GO_HISTONE_METHYLTRANSFERASE_COMPLEX	-0.71366393	-0.00836429	-5.18832096	0.0007338	0.01903666	-0.11375111
hetero after UVB irradiation VS hetero before UVB irradiation	GO_HISTONE_METHYLTRANSFERASE_COMPLEX1	-0.4267343	-0.06925752	-2.36959999	0.04584347	0.28591137	-3.96211695
WT after UVB irradiation VS WT before UVB irradiation	GO_HISTONE_METHYLTRANSFERASE_COMPLEX2	-0.36788027	-0.04947098	-1.3896357	0.2016521	0.69810294	-5.08852918
homo after UVB irradiation VS homo before UVB irradiation	GO_HISTONE_MONOUBIQUITINATION	-0.4414672	-0.0201876	-2.44794809	0.0389243	0.1417912	-4.08928553
hetero after UVB irradiation VS hetero before UVB irradiation	GO_HISTONE_MONOUBIQUITINATION1	-0.05916163	-0.06208017	-0.24080252	0.81587593	0.92926938	-6.20015781
WT after UVB irradiation VS WT before UVB irradiation	GO_HISTONE_MONOUBIQUITINATION2	-0.36190847	-0.01248748	-1.33182287	0.21918462	0.71478521	-5.15516799
homo after UVB irradiation VS homo before UVB irradiation	GO_HISTONE_MRNA_CATABOLIC_PROCESS	-0.31925233	-0.04148673	-1.770586	0.11309745	0.27028002	-5.09570482
hetero after UVB irradiation VS hetero before UVB irradiation	GO_HISTONE_MRNA_CATABOLIC_PROCESS1	0.101877387	-0.04039175	0.521494843	0.61640609	0.84094751	-6.08659058
WT after UVB irradiation VS WT before UVB irradiation	GO_HISTONE_MRNA_CATABOLIC_PROCESS2	-0.24250027	-0.00364838	-0.89335023	0.39744155	0.81477652	-5.59562098
homo after UVB irradiation VS homo before UVB irradiation	GO_HISTONE_MRNA_METABOLIC_PROCESS	-0.52231516	-0.03346772	-3.57711225	0.00675596	0.05419269	-2.35077414
hetero after UVB irradiation VS hetero before UVB irradiation	GO_HISTONE_MRNA_METABOLIC_PROCESS1	-0.02126171	-0.03138304	-0.10467493	0.91925791	0.9695904	-6.22554642
WT after UVB irradiation VS WT before UVB irradiation	GO_HISTONE_MRNA_METABOLIC_PROCESS2	-0.16599222	0.019798055	-0.57901789	0.57832364	0.88761996	-5.82294558
homo after UVB irradiation VS homo before UVB irradiation	GO_HISTONE_PHOSPHORYLATION	-0.07182102	-0.00490468	-0.47771223	0.64514032	0.78317996	-6.42732961
hetero after UVB irradiation VS hetero before UVB irradiation	GO_HISTONE_PHOSPHORYLATION1	0.324483556	-0.00956249	1.780636392	0.11354455	0.41535726	-4.79747261
WT after UVB irradiation VS WT before UVB irradiation	GO_HISTONE_PHOSPHORYLATION2	0.082228999	-0.03096292	0.394339695	0.70351229	0.92570885	-5.91484008
homo after UVB irradiation VS homo before UVB irradiation	GO_HISTONE_PRE_MRNA_3_END_PROCESSING_COMPLEX	-0.37546062	-0.04356642	-1.64374623	0.13735507	0.30641704	-5.27051181
hetero after UVB irradiation VS hetero before UVB irradiation	GO_HISTONE_PRE_MRNA_3_END_PROCESSING_COMPLEX1	0.149125174	-0.03676853	0.723029153	0.49063352	0.76680371	-5.95736079
WT after UVB irradiation VS WT before UVB irradiation	GO_HISTONE_PRE_MRNA_3_END_PROCESSING_COMPLEX2	0.259652852	0.00637961	0.927789864	0.38033963	0.80359153	-5.5657872
homo after UVB irradiation VS homo before UVB irradiation	GO_HISTONE_SERINE_KINASE_ACTIVITY	-0.10498079	0.016686324	-0.3962362	0.70188833	0.82204493	-6.4655082
hetero after UVB irradiation VS hetero before UVB irradiation	GO_HISTONE_SERINE_KINASE_ACTIVITY1	0.20511769	-0.00274157	0.744576006	0.47821592	0.759973	-5.94135406
WT after UVB irradiation VS WT before UVB irradiation	GO_HISTONE_SERINE_KINASE_ACTIVITY2	-0.01971254	-0.02692663	-0.06001274	0.95360018	0.98732041	-5.99416677
homo after UVB irradiation VS homo before UVB irradiation	GO_HISTONE_SERINE_PHOSPHORYLATION	0.175414278	-0.03095656	0.701655241	0.50202529	0.67594774	-6.28849856
hetero after UVB irradiation VS hetero before UVB irradiation	GO_HISTONE_SERINE_PHOSPHORYLATION1	0.249486209	-0.04824345	0.915579374	0.38713956	0.69764483	-5.8003975
WT after UVB irradiation VS WT before UVB irradiation	GO_HISTONE_SERINE_PHOSPHORYLATION2	-0.0209561	-0.05431665	-0.07991241	0.9382466	0.98368811	-5.9926985
homo after UVB irradiation VS homo before UVB irradiation	GO_HISTONE_THREONINE_KINASE_ACTIVITY	-0.46951597	-0.03437931	-2.77836596	0.02307474	0.10619627	-3.57728028
hetero after UVB irradiation VS hetero before UVB irradiation	GO_HISTONE_THREONINE_KINASE_ACTIVITY1	0.383737952	-0.00546898	1.430762021	0.19106332	0.51579406	-5.2496183
WT after UVB irradiation VS WT before UVB irradiation	GO_HISTONE_THREONINE_KINASE_ACTIVITY2	0.178768725	-0.06253423	0.766895072	0.46491284	0.84273676	-5.69714301
homo after UVB irradiation VS homo before UVB irradiation	GO_HISTONE_THREONINE_PHOSPHORYLATION	-0.46951597	-0.03437931	-2.77836596	0.02307474	0.10619627	-3.57728028
hetero after UVB irradiation VS hetero before UVB irradiation	GO_HISTONE_THREONINE_PHOSPHORYLATION1	0.383737952	-0.00546898	1.430762021	0.19106332	0.51579406	-5.2496183
WT after UVB irradiation VS WT before UVB irradiation	GO_HISTONE_THREONINE_PHOSPHORYLATION2	0.178768725	-0.06253423	0.766895072	0.46491284	0.84273676	-5.69714301
homo after UVB irradiation VS homo before UVB irradiation	GO_HISTONE_UBIQUITINATION	-0.49224774	-0.00100304	-2.90556637	0.01889716	0.09455708	-3.37967747
hetero after UVB irradiation VS hetero before UVB irradiation	GO_HISTONE_UBIQUITINATION1	-0.13623652	-0.04346403	-0.59004871	0.57174157	0.81424167	-6.04689516
WT after UVB irradiation VS WT before UVB irradiation	GO_HISTONE_UBIQUITINATION2	-0.35610256	-0.0091814	-1.30498583	0.22775802	0.71951396	-5.18551249
homo after UVB irradiation VS homo before UVB irradiation	GO_HMG_BOX_DOMAIN_BINDING	-0.01929819	0.062168396	-0.08664029	0.93300415	0.96403461	-6.54673887
hetero after UVB irradiation VS hetero before UVB irradiation	GO_HMG_BOX_DOMAIN_BINDING1	0.069502713	-0.00297786	0.3059583	0.76759039	0.90910698	-6.18102995
WT after UVB irradiation VS WT before UVB irradiation	GO_HMG_BOX_DOMAIN_BINDING2	0.02589239	0.026128934	0.104134351	0.91959577	0.98043563	-5.9903489
homo after UVB irradiation VS homo before UVB irradiation	GO_HOLLIDAY_JUNCTION_RESOLVASE_COMPLEX	-0.54056308	0.001305092	-3.09663535	0.01403399	0.08048956	-3.08371994
hetero after UVB irradiation VS hetero before UVB irradiation	GO_HOLLIDAY_JUNCTION_RESOLVASE_COMPLEX1	0.246411689	-0.08135157	0.896571955	0.39660032	0.7038269	-5.81724198
WT after UVB irradiation VS WT before UVB irradiation	GO_HOLLIDAY_JUNCTION_RESOLVASE_COMPLEX2	-0.0650058	-0.09412493	-0.1879207	0.85556256	0.9663792	-5.97747609
homo after UVB irradiation VS homo before UVB irradiation	GO_HOMEOSTASIS_OF_NUMBER_OF_CELLS	0.116361255	-0.02247113	0.939163485	0.37407924	0.56427347	-6.09121305
homo after UVB irradiation VS homo before UVB irradiation	GO_HOMEOSTASIS_OF_NUMBER_OF_CELLS_WITHIN_A_TISSUE	0.375042009	-0.05422443	2.590467433	0.03104919	0.12509779	-3.86900537
hetero after UVB irradiation VS hetero before UVB irradiation	GO_HOMEOSTASIS_OF_NUMBER_OF_CELLS_WITHIN_A_TISSUE1	0.190008379	-0.0036549	1.123977947	0.29420504	0.61784413	-5.59808475
WT after UVB irradiation VS WT before UVB irradiation	GO_HOMEOSTASIS_OF_NUMBER_OF_CELLS_WITHIN_A_TISSUE2	0.253497106	0.001802224	1.32059979	0.22273585	0.71766657	-5.16790449
hetero after UVB irradiation VS hetero before UVB irradiation	GO_HOMEOSTASIS_OF_NUMBER_OF_CELLS1	0.192871476	-0.00230432	1.528627812	0.16558255	0.48681732	-5.12810443
WT after UVB irradiation VS WT before UVB irradiation	GO_HOMEOSTASIS_OF_NUMBER_OF_CELLS2	0.111867572	-0.01429537	0.724373694	0.48923378	0.8517724	-5.72832063
homo after UVB irradiation VS homo before UVB irradiation	GO_HOMEOSTATIC_PROCESS	0.160183318	-0.00720211	1.320435637	0.2218172	0.41278139	-5.68377237
hetero after UVB irradiation VS hetero before UVB irradiation	GO_HOMEOSTATIC_PROCESS1	0.102898314	0.000473897	0.812493301	0.4404429	0.7372023	-5.88827549
WT after UVB irradiation VS WT before UVB irradiation	GO_HOMEOSTATIC_PROCESS2	0.084054505	-0.00729195	0.550671712	0.59671903	0.89380878	-5.83916255
homo after UVB irradiation VS homo before UVB irradiation	GO_HOMOCYSTEINE_METABOLIC_PROCESS	-0.36752924	0.010235824	-1.57560633	0.15227488	0.3263582	-5.3617649
hetero after UVB irradiation VS hetero before UVB irradiation	GO_HOMOCYSTEINE_METABOLIC_PROCESS1	-0.27724508	-0.0295688	-1.64051559	0.14023617	0.45467738	-4.98417342
WT after UVB irradiation VS WT before UVB irradiation	GO_HOMOCYSTEINE_METABOLIC_PROCESS2	-0.28509577	-0.06194182	-1.59556377	0.14879755	0.65641891	-4.83852299
homo after UVB irradiation VS homo before UVB irradiation	GO_HOMOLOGOUS_CHROMOSOME_PAIRING_AT_MEIOSIS	-0.20398765	0.006571029	-0.98039039	0.35450787	0.54644568	-6.05219373
hetero after UVB irradiation VS hetero before UVB irradiation	GO_HOMOLOGOUS_CHROMOSOME_PAIRING_AT_MEIOSIS1	0.032958374	0.04668503	0.217095401	0.83366812	0.93574283	-6.20600419
WT after UVB irradiation VS WT before UVB irradiation	GO_HOMOLOGOUS_CHROMOSOME_PAIRING_AT_MEIOSIS2	-0.09834835	0.022275959	-0.62453201	0.54946592	0.87530826	-5.79535552
homo after UVB irradiation VS homo before UVB irradiation	GO_HOMOLOGOUS_CHROMOSOME_SEGREGATION	-0.29323306	0.004180031	-1.37887587	0.20382693	0.39339184	-5.61304657
hetero after UVB irradiation VS hetero before UVB irradiation	GO_HOMOLOGOUS_CHROMOSOME_SEGREGATION1	0.106683111	0.049853689	0.627096152	0.54839066	0.80031215	-6.02358535
WT after UVB irradiation VS WT before UVB irradiation	GO_HOMOLOGOUS_CHROMOSOME_SEGREGATION2	-0.03346028	0.014635416	-0.19782906	0.84805479	0.96522688	-5.97546912
homo after UVB irradiation VS homo before UVB irradiation	GO_HOMOLOGOUS_RECOMBINATION	-0.51291511	-0.02028365	-3.03802611	0.01536964	0.08414192	-3.17433242
hetero after UVB irradiation VS hetero before UVB irradiation	GO_HOMOLOGOUS_RECOMBINATION1	0.027162287	-0.00830817	0.129437258	0.90026558	0.96275837	-6.22240832
WT after UVB irradiation VS WT before UVB irradiation	GO_HOMOLOGOUS_RECOMBINATION2	-0.11010918	-0.00308214	-0.51569415	0.619849	0.90087565	-5.8581347
homo after UVB irradiation VS homo before UVB irradiation	GO_HOMOPHILIC_CELL_ADHESION_VIA_PLASMA_MEMBRANE_ADHESION_MOLECULES	0.451058817	-0.00983821	3.558105078	0.00695049	0.05493753	-2.37934283
hetero after UVB irradiation VS hetero before UVB irradiation	GO_HOMOPHILIC_CELL_ADHESION_VIA_PLASMA_MEMBRANE_ADHESION_MOLECULES1	0.362077349	0.043810455	2.481106198	0.03857884	0.26884499	-3.79906224
WT after UVB irradiation VS WT before UVB irradiation	GO_HOMOPHILIC_CELL_ADHESION_VIA_PLASMA_MEMBRANE_ADHESION_MOLECULES2	0.244533339	0.016711393	1.220023486	0.25679326	0.73829991	-5.27896182
homo after UVB irradiation VS homo before UVB irradiation	GO_HOMOSERINE_METABOLIC_PROCESS	-0.44076926	-0.01655132	-1.5086561	0.16834366	0.34730147	-5.44941474
hetero after UVB irradiation VS hetero before UVB irradiation	GO_HOMOSERINE_METABOLIC_PROCESS1	-0.84656589	-0.03655166	-3.71405203	0.00612032	0.13491028	-2.02847657
WT after UVB irradiation VS WT before UVB irradiation	GO_HOMOSERINE_METABOLIC_PROCESS2	-0.70483316	0.044921808	-2.65206616	0.02885683	0.44654821	-3.38593364
homo after UVB irradiation VS homo before UVB irradiation	GO_HOMOTYPIC_CELL_CELL_ADHESION	0.408728891	-0.01741122	2.90375412	0.01895085	0.09463276	-3.3824909
hetero after UVB irradiation VS hetero before UVB irradiation	GO_HOMOTYPIC_CELL_CELL_ADHESION1	0.353318842	-0.00791992	2.379641267	0.04513623	0.2841637	-3.94746308
WT after UVB irradiation VS WT before UVB irradiation	GO_HOMOTYPIC_CELL_CELL_ADHESION2	0.231632372	-0.00807107	1.377639768	0.20518681	0.70190963	-5.10249538
homo after UVB irradiation VS homo before UVB irradiation	GO_HOPS_COMPLEX	-0.39813367	-0.04198108	-2.43770484	0.03956244	0.14295371	-4.10506114
hetero after UVB irradiation VS hetero before UVB irradiation	GO_HOPS_COMPLEX1	0.171637725	0.001378718	0.533059452	0.60874312	0.83691545	-6.0802116
WT after UVB irradiation VS WT before UVB irradiation	GO_HOPS_COMPLEX2	-0.12590648	-0.00776425	-0.43762779	0.67309403	0.91558199	-5.89625989
homo after UVB irradiation VS homo before UVB irradiation	GO_HORMONE_ACTIVITY	0.296743792	0.045896622	1.998456106	0.07929643	0.21730314	-4.76826235
hetero after UVB irradiation VS hetero before UVB irradiation	GO_HORMONE_ACTIVITY1	0.08051172	0.051487514	0.517664767	0.61895516	0.84259225	-6.08867455
WT after UVB irradiation VS WT before UVB irradiation	GO_HORMONE_ACTIVITY2	0.145797806	0.029958118	0.740504617	0.47991201	0.85038327	-5.71667394
homo after UVB irradiation VS homo before UVB irradiation	GO_HORMONE_BINDING	0.229277223	-0.00105358	1.842476588	0.10118468	0.25231887	-4.99408544
hetero after UVB irradiation VS hetero before UVB irradiation	GO_HORMONE_BINDING1	0.058121101	0.012333226	0.387032308	0.70900985	0.8851975	-6.15104364
WT after UVB irradiation VS WT before UVB irradiation	GO_HORMONE_BINDING2	-0.07653371	-0.02927494	-0.41585801	0.68831621	0.91993413	-5.9058349
homo after UVB irradiation VS homo before UVB irradiation	GO_HORMONE_BIOSYNTHETIC_PROCESS	0.278742713	0.019102049	2.074309357	0.07037145	0.20337589	-4.65619174
hetero after UVB irradiation VS hetero before UVB irradiation	GO_HORMONE_BIOSYNTHETIC_PROCESS1	0.115866798	0.039821758	0.770892349	0.46333196	0.75131059	-5.92125568
WT after UVB irradiation VS WT before UVB irradiation	GO_HORMONE_BIOSYNTHETIC_PROCESS2	0.01307453	0.042125743	0.079745583	0.9383752	0.98370412	-5.99271254
homo after UVB irradiation VS homo before UVB irradiation	GO_HORMONE_CATABOLIC_PROCESS	0.627970418	-0.00903399	3.392105199	0.00892346	0.06291616	-2.6305015
hetero after UVB irradiation VS hetero before UVB irradiation	GO_HORMONE_CATABOLIC_PROCESS1	0.431990763	0.048206357	2.748932506	0.02554555	0.22788198	-3.40618958
WT after UVB irradiation VS WT before UVB irradiation	GO_HORMONE_CATABOLIC_PROCESS2	0.242184331	0.001438548	1.228922782	0.25361398	0.73589193	-5.26936667
homo after UVB irradiation VS homo before UVB irradiation	GO_HORMONE_MEDIATED_APOPTOTIC_SIGNALING_PATHWAY	-0.19816384	0.088560109	-0.58070795	0.57680773	0.73347278	-6.36950363
hetero after UVB irradiation VS hetero before UVB irradiation	GO_HORMONE_MEDIATED_APOPTOTIC_SIGNALING_PATHWAY1	-0.82674918	0.043711352	-2.94921644	0.01883061	0.19947197	-3.11340031
WT after UVB irradiation VS WT before UVB irradiation	GO_HORMONE_MEDIATED_APOPTOTIC_SIGNALING_PATHWAY2	-0.2201337	0.058223495	-1.18633957	0.26912651	0.74431614	-5.31485421
homo after UVB irradiation VS homo before UVB irradiation	GO_HORMONE_MEDIATED_SIGNALING_PATHWAY	-0.03113349	0.013693676	-0.24354152	0.81347724	0.89465522	-6.51842246
hetero after UVB irradiation VS hetero before UVB irradiation	GO_HORMONE_MEDIATED_SIGNALING_PATHWAY1	-0.13033422	0.012229687	-0.99462069	0.34957703	0.66645461	-5.72737234
WT after UVB irradiation VS WT before UVB irradiation	GO_HORMONE_MEDIATED_SIGNALING_PATHWAY2	-0.12687476	0.021581363	-0.86414965	0.41237009	0.82049743	-5.62019974
homo after UVB irradiation VS homo before UVB irradiation	GO_HORMONE_METABOLIC_PROCESS	0.198875232	0.015330687	1.472990691	0.17750708	0.35912694	-5.49523881
hetero after UVB irradiation VS hetero before UVB irradiation	GO_HORMONE_METABOLIC_PROCESS1	0.031828728	0.029276342	0.213220279	0.83658646	0.93734913	-6.206903
WT after UVB irradiation VS WT before UVB irradiation	GO_HORMONE_METABOLIC_PROCESS2	-0.00360558	0.038506506	-0.02238379	0.98268357	0.996184	-5.9958023
homo after UVB irradiation VS homo before UVB irradiation	GO_HORMONE_RECEPTOR_BINDING	-0.15927301	-0.00092256	-1.08688014	0.30753862	0.50211734	-5.94541202
hetero after UVB irradiation VS hetero before UVB irradiation	GO_HORMONE_RECEPTOR_BINDING1	-0.19545419	-0.00997454	-1.54744005	0.16104927	0.48216946	-5.1042598
WT after UVB irradiation VS WT before UVB irradiation	GO_HORMONE_RECEPTOR_BINDING2	-0.10460893	0.002954024	-0.73359286	0.48389198	0.85146732	-5.72169154
homo after UVB irradiation VS homo before UVB irradiation	GO_HORMONE_TRANSPORT	0.115673339	0.01171963	0.925839815	0.38057149	0.57091527	-6.10353562
hetero after UVB irradiation VS hetero before UVB irradiation	GO_HORMONE_TRANSPORT1	0.022790529	0.02696103	0.164418308	0.87355512	0.95292225	-6.21684982
WT after UVB irradiation VS WT before UVB irradiation	GO_HORMONE_TRANSPORT2	-0.01764781	0.010327805	-0.10902075	0.91583926	0.97967628	-5.98980014
homo after UVB irradiation VS homo before UVB irradiation	GO_HOST_CELL_CYTOPLASM	0.718384401	-0.03383787	4.58522542	0.00161166	0.02721194	-0.90490371
hetero after UVB irradiation VS hetero before UVB irradiation	GO_HOST_CELL_CYTOPLASM1	0.549297804	0.055161117	2.234262406	0.05653403	0.31421081	-4.1588075
WT after UVB irradiation VS WT before UVB irradiation	GO_HOST_CELL_CYTOPLASM2	0.507831575	-0.04701848	1.956274585	0.08571622	0.57599199	-4.36415886
homo after UVB irradiation VS homo before UVB irradiation	GO_HOST_CELL_NUCLEAR_PART	0.0368542	-0.05739787	0.203797608	0.84340304	0.91347513	-6.52812006
hetero after UVB irradiation VS hetero before UVB irradiation	GO_HOST_CELL_NUCLEAR_PART1	0.43988257	-0.06716765	1.445222999	0.18709132	0.51246214	-5.23194169
WT after UVB irradiation VS WT before UVB irradiation	GO_HOST_CELL_NUCLEAR_PART2	0.13589517	-0.02979378	0.470210548	0.65060865	0.9092481	-5.8810633
homo after UVB irradiation VS homo before UVB irradiation	GO_HOST_CELL_NUCLEUS	-0.31467124	-0.04410032	-1.55454562	0.15717451	0.33258163	-5.38955946
hetero after UVB irradiation VS hetero before UVB irradiation	GO_HOST_CELL_NUCLEUS1	0.232748046	-0.03124657	0.73026153	0.48644237	0.76418009	-5.95203344
WT after UVB irradiation VS WT before UVB irradiation	GO_HOST_CELL_NUCLEUS2	-0.05495066	0.005519306	-0.23154791	0.82263312	0.95836402	-5.96787588
homo after UVB irradiation VS homo before UVB irradiation	GO_HOST_CELL_PART	0.262830368	-0.04567766	1.661974445	0.13359648	0.30089793	-5.24577153
hetero after UVB irradiation VS hetero before UVB irradiation	GO_HOST_CELL_PART1	0.436102934	-0.00616878	3.02698204	0.01674438	0.1902572	-3.00034035
WT after UVB irradiation VS WT before UVB irradiation	GO_HOST_CELL_PART2	0.285433738	-0.0804866	1.192508012	0.26683225	0.74323208	-5.30833226
homo after UVB irradiation VS homo before UVB irradiation	GO_HOST_CELLULAR_COMPONENT	-0.28313753	-0.02732467	-1.62168982	0.14203216	0.31315832	-5.30026573
hetero after UVB irradiation VS hetero before UVB irradiation	GO_HOST_CELLULAR_COMPONENT1	0.084827658	-0.02355219	0.498092396	0.63206688	0.8484405	-6.09910007
WT after UVB irradiation VS WT before UVB irradiation	GO_HOST_CELLULAR_COMPONENT2	0.108390693	-0.03244422	0.447777321	0.6660509	0.91342234	-5.89163704
homo after UVB irradiation VS homo before UVB irradiation	GO_HOST_INTRACELLULAR_ORGANELLE	-0.26194873	-0.05293164	-1.35849522	0.20995319	0.40028005	-5.63793104
hetero after UVB irradiation VS hetero before UVB irradiation	GO_HOST_INTRACELLULAR_ORGANELLE1	0.182273689	-0.04178184	0.70768066	0.49960482	0.77248814	-5.96851327
WT after UVB irradiation VS WT before UVB irradiation	GO_HOST_INTRACELLULAR_ORGANELLE2	-0.10489634	-0.01711315	-0.42536538	0.68164923	0.91716791	-5.90171055
homo after UVB irradiation VS homo before UVB irradiation	GO_HRD1P_UBIQUITIN_LIGASE_ERAD_L_COMPLEX	0.065427581	-0.02773133	0.342899449	0.7401679	0.84744616	-6.4867866
hetero after UVB irradiation VS hetero before UVB irradiation	GO_HRD1P_UBIQUITIN_LIGASE_ERAD_L_COMPLEX1	0.309021031	0.016356371	1.048252849	0.32571093	0.64521291	-5.67519012
WT after UVB irradiation VS WT before UVB irradiation	GO_HRD1P_UBIQUITIN_LIGASE_ERAD_L_COMPLEX2	0.177890803	-0.03978208	1.049008364	0.32447118	0.77346379	-5.45382244
homo after UVB irradiation VS homo before UVB irradiation	GO_HSP70_PROTEIN_BINDING	-0.26846458	-0.01915674	-2.03719705	0.07460942	0.20983472	-4.71118844
hetero after UVB irradiation VS hetero before UVB irradiation	GO_HSP70_PROTEIN_BINDING1	-0.09704775	-0.03219507	-0.62542542	0.54943146	0.80065284	-6.02466417
WT after UVB irradiation VS WT before UVB irradiation	GO_HSP70_PROTEIN_BINDING2	-0.2346924	-0.01974026	-1.44652753	0.18560189	0.68726847	-5.02134429
homo after UVB irradiation VS homo before UVB irradiation	GO_HSP90_PROTEIN_BINDING	0.003162682	0.016736128	0.020739807	0.98394147	0.99200337	-6.55062065
hetero after UVB irradiation VS hetero before UVB irradiation	GO_HSP90_PROTEIN_BINDING1	-0.00064828	-0.02428026	-0.00369521	0.99714381	0.99876826	-6.23147671
WT after UVB irradiation VS WT before UVB irradiation	GO_HSP90_PROTEIN_BINDING2	-0.02132394	-0.01723659	-0.12830851	0.90103429	0.97526754	-5.98738927
homo after UVB irradiation VS homo before UVB irradiation	GO_HULC_COMPLEX	-0.50919513	0.101122616	-1.44777909	0.18424795	0.36822141	-5.52724979
hetero after UVB irradiation VS hetero before UVB irradiation	GO_HULC_COMPLEX1	-0.25275418	0.089926816	-0.73201159	0.48543171	0.76356788	-5.95073743
WT after UVB irradiation VS WT before UVB irradiation	GO_HULC_COMPLEX2	-0.66165442	-0.01135387	-2.25531404	0.05372885	0.51991848	-3.94899773
homo after UVB irradiation VS homo before UVB irradiation	GO_HUMORAL_IMMUNE_RESPONSE	0.579888416	0.006626155	3.767179955	0.00509982	0.0464877	-2.06744526
homo after UVB irradiation VS homo before UVB irradiation	GO_HUMORAL_IMMUNE_RESPONSE_MEDIATED_BY_CIRCULATING_IMMUNOGLOBULIN	0.423375391	0.048417985	2.091869866	0.06844852	0.19998459	-4.630065

hetero after UVB irradiation VS hetero before UVB irradiation	GO_HUMORAL_IMMUNE_RESPONSE_MEDIATED_BY_CIRCULATING_IMMUNOGLOBULIN1	0.168956652	0.052408405	0.996564261	0.34868938	0.66574648	-5.7255177
WT after UVB irradiation VS WT before UVB irradiation	GO_HUMORAL_IMMUNE_RESPONSE_MEDIATED_BY_CIRCULATING_IMMUNOGLOBULIN2	0.213595833	-0.02206341	0.83314224	0.42865203	0.82753247	-5.64556362
hetero after UVB irradiation VS hetero before UVB irradiation	GO_HUMORAL_IMMUNE_RESPONSE1	0.484399912	0.052288099	3.02715335	0.01674006	0.1902572	-3.00009178
WT after UVB irradiation VS WT before UVB irradiation	GO_HUMORAL_IMMUNE_RESPONSE2	0.347303449	0.035774415	1.629108977	0.14148267	0.65141238	-4.79614308
homo after UVB irradiation VS homo before UVB irradiation	GO_HYALOID_VASCULAR_PLEXUS_REGRESSION	0.366761124	0.110486068	2.044939491	0.07370535	0.2084857	-4.69974023
hetero after UVB irradiation VS hetero before UVB irradiation	GO_HYALOID_VASCULAR_PLEXUS_REGRESSION1	0.7261315	0.157245492	3.955318119	0.00436365	0.12650291	-1.70233334
WT after UVB irradiation VS WT before UVB irradiation	GO_HYALOID_VASCULAR_PLEXUS_REGRESSION2	0.745624431	0.051769676	3.118096397	0.01405648	0.36090271	-2.72810948
homo after UVB irradiation VS homo before UVB irradiation	GO_HYALURONAN_BIOSYNTHETIC_PROCESS	0.32168956	0.060831301	1.776895141	0.11200156	0.26857443	-5.08685586
hetero after UVB irradiation VS hetero before UVB irradiation	GO_HYALURONAN_BIOSYNTHETIC_PROCESS1	0.516778779	0.036158302	2.59014798	0.03260276	0.25034181	-3.63915512
WT after UVB irradiation VS WT before UVB irradiation	GO_HYALURONAN_BIOSYNTHETIC_PROCESS2	0.627122669	0.023456039	3.568226294	0.00716868	0.31015346	-2.11288202
homo after UVB irradiation VS homo before UVB irradiation	GO_HYALURONAN_CATABOLIC_PROCESS	0.531321989	-0.00117586	3.683271739	0.00577048	0.05035773	-2.19198335
hetero after UVB irradiation VS hetero before UVB irradiation	GO_HYALURONAN_CATABOLIC_PROCESS1	0.463387617	-0.04624202	2.903272552	0.0201889	0.20610175	-3.18038573
WT after UVB irradiation VS WT before UVB irradiation	GO_HYALURONAN_CATABOLIC_PROCESS2	0.571622488	-0.0951974	2.971708822	0.01758428	0.38386276	-2.93314019
homo after UVB irradiation VS homo before UVB irradiation	GO_HYALURONAN_METABOLIC_PROCESS	0.492975454	0.025768368	3.488547604	0.00771442	0.05842479	-2.48423191
hetero after UVB irradiation VS hetero before UVB irradiation	GO_HYALURONAN_METABOLIC_PROCESS1	0.474868488	0.0013153	3.234629635	0.01227674	0.17108413	-2.70092992
WT after UVB irradiation VS WT before UVB irradiation	GO_HYALURONAN_METABOLIC_PROCESS2	0.570249052	-0.04813074	3.217322147	0.01209239	0.34615945	-2.59034075
homo after UVB irradiation VS homo before UVB irradiation	GO_HYALURONIC_ACID_BINDING	0.310443713	0.034710665	1.915605195	0.09028851	0.23557063	-4.88905647
hetero after UVB irradiation VS hetero before UVB irradiation	GO_HYALURONIC_ACID_BINDING1	0.135896741	0.052466492	0.979843725	0.3563821	0.67198476	-5.74138206
WT after UVB irradiation VS WT before UVB irradiation	GO_HYALURONIC_ACID_BINDING2	0.404205262	-0.02764495	2.21442048	0.05728636	0.523456	-4.00649662
homo after UVB irradiation VS homo before UVB irradiation	GO_HYALURONONGLUCOSAMINIDASE_ACTIVITY	-0.06405477	0.031418627	-0.24225509	0.81444112	0.89497727	-6.51876298
hetero after UVB irradiation VS hetero before UVB irradiation	GO_HYALURONONGLUCOSAMINIDASE_ACTIVITY1	0.087675111	-0.03093832	0.403160437	0.69758392	0.88005213	-6.14427216
WT after UVB irradiation VS WT before UVB irradiation	GO_HYALURONONGLUCOSAMINIDASE_ACTIVITY2	-0.13700106	0.048311746	-0.5803747	0.57745116	0.88725702	-5.82215062
homo after UVB irradiation VS homo before UVB irradiation	GO_HYDRO_LYASE_ACTIVITY	0.063400897	-0.02917818	0.325048456	0.75315897	0.85633618	-6.49324222
hetero after UVB irradiation VS hetero before UVB irradiation	GO_HYDRO_LYASE_ACTIVITY1	-0.19343421	-0.02606064	-1.12355444	0.29437422	0.61789273	-5.59852674
WT after UVB irradiation VS WT before UVB irradiation	GO_HYDRO_LYASE_ACTIVITY2	-0.03432855	0.010289542	-0.17674625	0.86404841	0.96790654	-5.9796169
homo after UVB irradiation VS homo before UVB irradiation	GO_HYDROGEN_PEROXIDE_BIOSYNTHETIC_PROCESS	0.243618728	-0.00411959	1.774042501	0.11249583	0.26923466	-5.09085857
hetero after UVB irradiation VS hetero before UVB irradiation	GO_HYDROGEN_PEROXIDE_BIOSYNTHETIC_PROCESS1	0.049927955	0.051858733	0.317226139	0.75934205	0.90649879	-6.17727017
WT after UVB irradiation VS WT before UVB irradiation	GO_HYDROGEN_PEROXIDE_BIOSYNTHETIC_PROCESS2	0.204844047	0.052228098	0.929770026	0.37937294	0.80338597	-5.56404447
homo after UVB irradiation VS homo before UVB irradiation	GO_HYDROGEN_PEROXIDE_CATABOLIC_PROCESS	0.399656547	-0.04087729	2.170495779	0.06044892	0.18514833	-4.5123322
hetero after UVB irradiation VS hetero before UVB irradiation	GO_HYDROGEN_PEROXIDE_CATABOLIC_PROCESS1	0.322084592	0.019720104	1.389452583	0.20282152	0.52718589	-5.29955147
WT after UVB irradiation VS WT before UVB irradiation	GO_HYDROGEN_PEROXIDE_CATABOLIC_PROCESS2	0.290456035	0.056797208	1.207979897	0.26114832	0.74128665	-5.29187285
homo after UVB irradiation VS homo before UVB irradiation	GO_HYDROGEN_PEROXIDE_METABOLIC_PROCESS	0.330849468	-0.03189524	2.171698537	0.06033395	0.18502679	-4.51052226
hetero after UVB irradiation VS hetero before UVB irradiation	GO_HYDROGEN_PEROXIDE_METABOLIC_PROCESS1	0.256886036	0.041923153	1.387871339	0.20328392	0.52745868	-5.30144591
WT after UVB irradiation VS WT before UVB irradiation	GO_HYDROGEN_PEROXIDE_METABOLIC_PROCESS2	0.246068987	0.037430449	1.19587699	0.265586	0.74237377	-5.30476049
homo after UVB irradiation VS homo before UVB irradiation	GO_HYDROGEN_SULFIDE_BIOSYNTHETIC_PROCESS	-0.94688303	0.048312133	-6.75106247	0.00012021	0.01007802	1.675624598
hetero after UVB irradiation VS hetero before UVB irradiation	GO_HYDROGEN_SULFIDE_BIOSYNTHETIC_PROCESS1	-0.59621551	-0.05560416	-1.92563995	0.09101085	0.37831177	-4.59806477
WT after UVB irradiation VS WT before UVB irradiation	GO_HYDROGEN_SULFIDE_BIOSYNTHETIC_PROCESS2	-0.66636702	-0.01612913	-2.15447591	0.0629257	0.53593918	-4.09044064
homo after UVB irradiation VS homo before UVB irradiation	GO_HYDROGEN_SULFIDE_METABOLIC_PROCESS	-0.72967027	0.026988384	-6.08996194	0.00024877	0.01196149	0.962822917
hetero after UVB irradiation VS hetero before UVB irradiation	GO_HYDROGEN_SULFIDE_METABOLIC_PROCESS1	-0.19872294	0.002722759	-0.69334426	0.50807836	0.77796039	-5.97874095
WT after UVB irradiation VS WT before UVB irradiation	GO_HYDROGEN_SULFIDE_METABOLIC_PROCESS2	-0.50597496	0.00276085	-1.91718862	0.09107031	0.58419798	-4.41726082
homo after UVB irradiation VS homo before UVB irradiation	GO_HYDROLASE_ACTIVITY_ACTING_ON_ACID_ANHYDRIDES	-0.18140601	-0.00429904	-1.52694793	0.16380923	0.3413373	-5.42567436
hetero after UVB irradiation VS hetero before UVB irradiation	GO_HYDROLASE_ACTIVITY_ACTING_ON_ACID_ANHYDRIDES1	-0.02768187	-0.02813346	-0.19230172	0.85238568	0.94335882	-6.21147843
WT after UVB irradiation VS WT before UVB irradiation	GO_HYDROLASE_ACTIVITY_ACTING_ON_ACID_ANHYDRIDES2	0.000669578	-0.01081629	0.004180613	0.99676552	0.99906689	-5.99605744
homo after UVB irradiation VS homo before UVB irradiation	GO_HYDROLASE_ACTIVITY_ACTING_ON_CARBON_NITROGEN_BUT_NOT_PEPTIDE_BONDS	0.060142105	-0.0207472	0.495159048	0.63328534	0.7738814	-6.41827674
homo after UVB irradiation VS homo before UVB irradiation	GO_HYDROLASE_ACTIVITY_ACTING_ON_CARBON_NITROGEN_BUT_NOT_PEPTIDE_BONDS_IN_CYCLIC_AMIDES	-0.0123676	0.027051474	-0.05441076	0.95789042	0.97797073	-6.54923221
hetero after UVB irradiation VS hetero before UVB irradiation	GO_HYDROLASE_ACTIVITY_ACTING_ON_CARBON_NITROGEN_BUT_NOT_PEPTIDE_BONDS_IN_CYCLIC_AMIDES1	-0.37983339	0.050315333	-1.43655692	0.18946278	0.51448524	-5.24254685
WT after UVB irradiation VS WT before UVB irradiation	GO_HYDROLASE_ACTIVITY_ACTING_ON_CARBON_NITROGEN_BUT_NOT_PEPTIDE_BONDS_IN_CYCLIC_AMIDES2	-0.40700078	0.060528403	-2.18377896	0.06010376	0.52638053	-4.04946035
homo after UVB irradiation VS homo before UVB irradiation	GO_HYDROLASE_ACTIVITY_ACTING_ON_CARBON_NITROGEN_BUT_NOT_PEPTIDE_BONDS_IN_CYCLIC_AMIDINES	0.03020395	0.018080205	0.177600206	0.86328025	0.92468801	-6.53357925
hetero after UVB irradiation VS hetero before UVB irradiation	GO_HYDROLASE_ACTIVITY_ACTING_ON_CARBON_NITROGEN_BUT_NOT_PEPTIDE_BONDS_IN_CYCLIC_AMIDINES1	0.04572797	0.040486322	0.277299653	0.78870989	0.91738598	-6.1899938
WT after UVB irradiation VS WT before UVB irradiation	GO_HYDROLASE_ACTIVITY_ACTING_ON_CARBON_NITROGEN_BUT_NOT_PEPTIDE_BONDS_IN_CYCLIC_AMIDINES2	0.222759357	-0.04612913	1.066990355	0.31675113	0.77149112	-5.43633054
homo after UVB irradiation VS homo before UVB irradiation	GO_HYDROLASE_ACTIVITY_ACTING_ON_CARBON_NITROGEN_BUT_NOT_PEPTIDE_BONDS_IN_LINEAR_AMIDES	0.143986839	-0.03578889	1.146137939	0.2835961	0.47861706	-5.88243724
hetero after UVB irradiation VS hetero before UVB irradiation	GO_HYDROLASE_ACTIVITY_ACTING_ON_CARBON_NITROGEN_BUT_NOT_PEPTIDE_BONDS_IN_LINEAR_AMIDES1	0.049282699	-0.02354159	0.287361856	0.78127239	0.91401375	-6.18694469
WT after UVB irradiation VS WT before UVB irradiation	GO_HYDROLASE_ACTIVITY_ACTING_ON_CARBON_NITROGEN_BUT_NOT_PEPTIDE_BONDS_IN_LINEAR_AMIDES2	0.137015679	-0.02287108	0.764120228	0.46647519	0.84342918	-5.69922423
homo after UVB irradiation VS homo before UVB irradiation	GO_HYDROLASE_ACTIVITY_ACTING_ON_CARBON_NITROGEN_BUT_NOT_PEPTIDE_BONDS_IN_LINEAR_AMIDINES	-0.38387171	0.009047942	-1.73792104	0.11893303	0.27911812	-5.14129598
hetero after UVB irradiation VS hetero before UVB irradiation	GO_HYDROLASE_ACTIVITY_ACTING_ON_CARBON_NITROGEN_BUT_NOT_PEPTIDE_BONDS_IN_LINEAR_AMIDINES1	-0.02269144	0.005962978	-0.10675861	0.91765744	0.96852537	-6.22530786
WT after UVB irradiation VS WT before UVB irradiation	GO_HYDROLASE_ACTIVITY_ACTING_ON_CARBON_NITROGEN_BUT_NOT_PEPTIDE_BONDS_IN_LINEAR_AMIDINES2	-0.29176706	0.086785159	-1.46585257	0.18041145	0.68278032	-4.99817826
hetero after UVB irradiation VS hetero before UVB irradiation	GO_HYDROLASE_ACTIVITY_ACTING_ON_CARBON_NITROGEN_BUT_NOT_PEPTIDE_BONDS1	0.025924467	-0.00072789	0.196152837	0.84947145	0.94258929	-6.21067117
WT after UVB irradiation VS WT before UVB irradiation	GO_HYDROLASE_ACTIVITY_ACTING_ON_CARBON_NITROGEN_BUT_NOT_PEPTIDE_BONDS2	0.103137429	0.002167814	0.70752585	0.49909328	0.85541458	-5.74024619
homo after UVB irradiation VS homo before UVB irradiation	GO_HYDROLASE_ACTIVITY_ACTING_ON_ESTER_BONDS	-0.06825146	-0.00944523	-0.56256702	0.58854684	0.74135959	-6.38044784
hetero after UVB irradiation VS hetero before UVB irradiation	GO_HYDROLASE_ACTIVITY_ACTING_ON_ESTER_BONDS1	-0.04234864	-0.00889217	-0.33446379	0.74678736	0.90058797	-6.1712625
WT after UVB irradiation VS WT before UVB irradiation	GO_HYDROLASE_ACTIVITY_ACTING_ON_ESTER_BONDS2	0.000150565	-0.00968892	0.001050371	0.99918734	0.99997598	-5.99606608
homo after UVB irradiation VS homo before UVB irradiation	GO_HYDROLASE_ACTIVITY_ACTING_ON_ETHER_BONDS	-0.09325686	0.007469062	-0.55445923	0.59383563	0.74536296	-6.38523528
hetero after UVB irradiation VS hetero before UVB irradiation	GO_HYDROLASE_ACTIVITY_ACTING_ON_ETHER_BONDS1	0.042647644	-0.00677959	0.218516412	0.83259865	0.9354456	-6.20567058
WT after UVB irradiation VS WT before UVB irradiation	GO_HYDROLASE_ACTIVITY_ACTING_ON_ETHER_BONDS2	0.128531706	0.025909347	0.542670256	0.60196878	0.89585048	-5.84360436
homo after UVB irradiation VS homo before UVB irradiation	GO_HYDROLASE_ACTIVITY_ACTING_ON_GLYCOSYL_BONDS	0.218728857	-0.02017685	1.588914978	0.14925021	0.3224963	-5.34409901
hetero after UVB irradiation VS hetero before UVB irradiation	GO_HYDROLASE_ACTIVITY_ACTING_ON_GLYCOSYL_BONDS1	0.249490279	-0.00160144	1.731791482	0.12225788	0.42830895	-4.86329284
WT after UVB irradiation VS WT before UVB irradiation	GO_HYDROLASE_ACTIVITY_ACTING_ON_GLYCOSYL_BONDS2	0.218120432	-0.00014596	1.415978323	0.19407544	0.69334561	-5.05761254
homo after UVB irradiation VS homo before UVB irradiation	GO_HYDROLASE_ACTIVITY_HYDROLYZING_N_GLYCOSYL_COMPOUNDS	0.392352552	-0.00943189	2.940390899	0.01789542	0.09169538	-3.32563141
hetero after UVB irradiation VS hetero before UVB irradiation	GO_HYDROLASE_ACTIVITY_HYDROLYZING_N_GLYCOSYL_COMPOUNDS1	0.280091457	-0.02060531	1.813516521	0.10801222	0.40604091	-4.75275856
WT after UVB irradiation VS WT before UVB irradiation	GO_HYDROLASE_ACTIVITY_HYDROLYZING_N_GLYCOSYL_COMPOUNDS2	0.354341388	0.008501427	2.193183421	0.05922481	0.52638053	-4.03628564
homo after UVB irradiation VS homo before UVB irradiation	GO_HYDROLASE_ACTIVITY_HYDROLYZING_O_GLYCOSYL_COMPOUNDS	0.244169292	-0.01825098	1.66950347	0.13207173	0.29846593	-5.23551376
hetero after UVB irradiation VS hetero before UVB irradiation	GO_HYDROLASE_ACTIVITY_HYDROLYZING_O_GLYCOSYL_COMPOUNDS1	0.323624756	-0.00762992	2.131675634	0.0662603	0.33502634	-4.30655361
WT after UVB irradiation VS WT before UVB irradiation	GO_HYDROLASE_ACTIVITY_HYDROLYZING_O_GLYCOSYL_COMPOUNDS2	0.22412774	0.001000099	1.441343064	0.18701659	0.68957923	-5.02753008
homo after UVB irradiation VS homo before UVB irradiation	GO_HYDROXYMETHYL_FORMYL_AND_RELATED_TRANSFERASE_ACTIVITY	-0.01620377	-0.02380397	-0.07462418	0.94227494	0.96940541	-6.5478015
hetero after UVB irradiation VS hetero before UVB irradiation	GO_HYDROXYMETHYL_FORMYL_AND_RELATED_TRANSFERASE_ACTIVITY1	-0.25953679	-0.00177019	-0.93077443	0.37969594	0.69230116	-5.78672872
WT after UVB irradiation VS WT before UVB irradiation	GO_HYDROXYMETHYL_FORMYL_AND_RELATED_TRANSFERASE_ACTIVITY2	-0.12939281	0.002902413	-0.51072859	0.62317024	0.90175221	-5.86073416
homo after UVB irradiation VS homo before UVB irradiation	GO_HYPEROSMOTIC_RESPONSE	-0.00260015	0.022299703	-0.01391006	0.98922916	0.99468893	-6.55075053
hetero after UVB irradiation VS hetero before UVB irradiation	GO_HYPEROSMOTIC_RESPONSE1	0.064540261	-0.01081427	0.401721218	0.69860025	0.88069863	-6.14488719
WT after UVB irradiation VS WT before UVB irradiation	GO_HYPEROSMOTIC_RESPONSE2	0.124391985	-0.03266722	0.716719502	0.4936975	0.85370213	-5.73376894
homo after UVB irradiation VS homo before UVB irradiation	GO_HYPEROSMOTIC_SALINITY_RESPONSE	-0.4259737	0.037601088	-1.99031059	0.08031741	0.21892205	-4.7802166
hetero after UVB irradiation VS hetero before UVB irradiation	GO_HYPEROSMOTIC_SALINITY_RESPONSE1	-0.17093043	0.001174891	-0.94397715	0.37331448	0.6866793	-5.77470747
WT after UVB irradiation VS WT before UVB irradiation	GO_HYPEROSMOTIC_SALINITY_RESPONSE2	-0.06871656	-0.00724184	-0.42131025	0.68448928	0.91852399	-5.90348057
homo after UVB irradiation VS homo before UVB irradiation	GO_HYPERSENSITIVITY	0.6627939	-0.0039917	2.725051574	0.02509764	0.11153947	-3.6601231
hetero after UVB irradiation VS hetero before UVB irradiation	GO_HYPERSENSITIVITY1	0.492851719	0.032444385	2.141149979	0.06529675	0.33409111	-4.29296897
WT after UVB irradiation VS WT before UVB irradiation	GO_HYPERSENSITIVITY2	0.630996751	0.043761329	3.516562979	0.00773473	0.31443913	-2.18211847
homo after UVB irradiation VS homo before UVB irradiation	GO_HYPOGLYCIN_A_GAMMA_GLUTAMYL_TRANSPEPTIDASE_ACTIVITY	0.175611057	0.013697659	0.686924997	0.51079876	0.68259144	-6.29909778
hetero after UVB irradiation VS hetero before UVB irradiation	GO_HYPOGLYCIN_A_GAMMA_GLUTAMYL_TRANSPEPTIDASE_ACTIVITY1	0.422357052	0.047948834	2.183987512	0.06110987	0.32556646	-4.23138691
WT after UVB irradiation VS WT before UVB irradiation	GO_HYPOGLYCIN_A_GAMMA_GLUTAMYL_TRANSPEPTIDASE_ACTIVITY2	0.360633332	-0.00266622	1.94591322	0.08710535	0.5790399	-4.37826814
homo after UVB irradiation VS homo before UVB irradiation	GO_HYPOTHALAMUS_CELL_DIFFERENTIATION	0.067919874	0.048856322	0.266185318	0.79656742	0.88476181	-6.51213798
hetero after UVB irradiation VS hetero before UVB irradiation	GO_HYPOTHALAMUS_CELL_DIFFERENTIATION1	-0.16630997	-0.01244558	-0.89867	0.39554787	0.70336462	-5.81539663
WT after UVB irradiation VS WT before UVB irradiation	GO_HYPOTHALAMUS_CELL_DIFFERENTIATION2	-0.02759075	0.030922194	-0.1156141	0.91077407	0.97857624	-5.98901993
homo after UVB irradiation VS homo before UVB irradiation	GO_HYPOTHALAMUS_DEVELOPMENT	0.044838114	0.065226536	0.2421794	0.81449785	0.89497727	-6.51878296
hetero after UVB irradiation VS hetero before UVB irradiation	GO_HYPOTHALAMUS_DEVELOPMENT1	0.092812691	0.066202821	0.549412292	0.59799495	0.83214008	-6.0709703
WT after UVB irradiation VS WT before UVB irradiation	GO_HYPOTHALAMUS_DEVELOPMENT2	0.066181899	0.035620198	0.324147711	0.75404063	0.93735807	-5.94100375
homo after UVB irradiation VS homo before UVB irradiation	GO_HYPOTONIC_RESPONSE	0.245056002	-0.01460975	1.121157076	0.29350136	0.48826562	-5.90928367
hetero after UVB irradiation VS hetero before UVB irradiation	GO_HYPOTONIC_RESPONSE1	-0.15380974	-0.02670889	-1.16969174	0.2764003	0.60324846	-5.54968957
WT after UVB irradiation VS WT before UVB irradiation	GO_HYPOTONIC_RESPONSE2	0.219155783	-0.03078584	1.093288842	0.30572064	0.76523073	-5.41035629
homo after UVB irradiation VS homo before UVB irradiation	GO_HYPOXIA_INDUCIBLE_FACTOR_1ALPHA_SIGNALING_PATHWAY	0.399268729	0.02924644	1.45757736	0.18160185	0.36474633	-5.51484726
hetero after UVB irradiation VS hetero before UVB irradiation	GO_HYPOXIA_INDUCIBLE_FACTOR_1ALPHA_SIGNALING_PATHWAY1	0.509977299	0.146163335	2.219939483	0.05780185	0.31756518	-4.17951602
WT after UVB irradiation VS WT before UVB irradiation	GO_HYPOXIA_INDUCIBLE_FACTOR_1ALPHA_SIGNALING_PATHWAY2	-0.06310903	0.01981804	-0.37553169	0.71691153	0.93079917	-5.92233462
homo after UVB irradiation VS homo before UVB irradiation	GO_I_BAND	0.332892989	-0.01057405	2.061695106	0.07178503	0.20559149	-4.67491883
hetero after UVB irradiation VS hetero before UVB irradiation	GO_I_BAND1	-0.0731049	-0.02128102	-0.38603273	0.70972062	0.88571021	-6.15145458
WT after UVB irradiation VS WT before UVB irradiation	GO_I_BAND2	0.082658144	-0.02049737	0.337004045	0.74468231	0.93574017	-5.93658209
homo after UVB irradiation VS homo before UVB irradiation	GO_I_KAPPAB_KINASE_NF_KAPPAB_SIGNALING	0.35381766	-0.01209173	2.463601579	0.03796901	0.13972786	-4.06516053
hetero after UVB irradiation VS hetero before UVB irradiation	GO_I_KAPPAB_KINASE_NF_KAPPAB_SIGNALING1	0.353837908	0.004080554	2.550427075	0.03466206	0.25582508	-3.69743459
WT after UVB irradiation VS WT before UVB irradiation	GO_I_KAPPAB_KINASE_NF_KAPPAB_SIGNALING2	0.264878192	-0.02826636	1.655344329	0.13599271	0.64425182	-4.76271162
homo after UVB irradiation VS homo before UVB irradiation	GO_I_KAPPAB_PHOSPHORYLATION	0.700053639	-0.01243945	5.276208408	0.00065716	0.01817776	-0.00326908
hetero after UVB irradiation VS hetero before UVB irradiation	GO_I_KAPPAB_PHOSPHORYLATION1	0.750044495	0.073117103	4.346862244	0.00256613	0.11202264	-1.19290793
WT after UVB irradiation VS WT before UVB irradiation	GO_I_KAPPAB_PHOSPHORYLATION2	0.819512127	0.024692532	4.798073889	0.00131346	0.19402435	-0.59629603
homo after UVB irradiation VS homo before UVB irradiation	GO_I_SMAD_BINDING	-0.65091332	0.00854628	-3.52086038	0.00734905	0.05672071	-2.43544034
hetero after UVB irradiation VS hetero before UVB irradiation	GO_I_SMAD_BINDING1	-0.64216079	-0.04264482	-4.72021965	0.00157973	0.09569098	-0.73121193
WT after UVB irradiation VS WT before UVB irradiation	GO_I_SMAD_BINDING2	-0.49456989	-0.0184035	-2.23571106	0.05540602	0.5232848	-3.97658237
homo after UVB irradiation VS homo before UVB irradiation	GO_ICOSANOID_BINDING	0.626240453	0.043173352	2.898984988	0.01909289	0.09505862	-3.3898951
hetero after UVB irradiation VS hetero before UVB irradiation	GO_ICOSANOID_BINDING1	0.943665157	0.011533563	5.291489444	0.00078197	0.08366654	-0.07094658
WT after UVB irradiation VS WT before UVB irradiation	GO_ICOSANOID_BINDING2	0.486375778	0.003403943	2.092757014	0.06930164	0.54456032	-4.17637163
homo after UVB irradiation VS homo before UVB irradiation	GO_ICOSANOID_BIOSYNTHETIC_PROCESS	0.172781698	0.019227653	1.009774715	0.34103387	0.53402342	-6.02357752
hetero after UVB irradiation VS hetero before UVB irradiation	GO_ICOSANOID_BIOSYNTHETIC_PROCESS1	0.307340724	0.031665647	2.200608546	0.05955783	0.32149178	-4.20742648
WT after UVB irradiation VS WT before UVB irradiation	GO_ICOSANOID_BIOSYNTHETIC_PROCESS2	0.187635083	0.0747153	1.290878718	0.23237794	0.72451161	-5.20130805
homo after UVB irradiation VS homo before UVB irradiation	GO_ICOSANOID_CATABOLIC_PROCESS	0.215109454	0.025463391	0.871336693	0.40798185	0.59494022	-6.15244436
hetero after UVB irradiation VS hetero before UVB irradiation	GO_ICOSANOID_CATABOLIC_PROCESS1	0.079533306	0.055338107	0.33519961	0.7462532	0.90058797	-6.17099918
WT after UVB irradiation VS WT before UVB irradiation	GO_ICOSANOID_CATABOLIC_PROCESS2	0.078456974	0.102484613	0.300165079	0.77161267	0.94202287	-5.9488043
homo after UVB irradiation VS homo before UVB irradiation	GO_ICOSANOID_METABOLIC_PROCESS	0.235324237	0.037057781	1.578633645	0.15158203	0.3256035	-5.35775332
hetero after UVB irradiation VS hetero before UVB irradiation	GO_ICOSANOID_METABOLIC_PROCESS1	0.23626909	0.035070927	1.579088082	0.15367581	0.47184575	-5.06381309
WT after UVB irradiation VS WT before UVB irradiation	GO_ICOSANOID_METABOLIC_PROCESS2	0.094051293	0.039344191	0.555720721	0.59341922	0.8931505	-5.83632883
homo after UVB irradiation VS homo before UVB irradiation	GO_ICOSANOID_RECEPTOR_ACTIVITY	0.559852291	-0.02492629	3.242904906	0.01120229	0.07091898	-2.85850648
hetero after UVB irradiation VS hetero before UVB irradiation	GO_ICOSANOID_RECEPTOR_ACTIVITY1	0.288433395	0.045914676	1.191720705	0.26813978	0.5948986	-5.52589252
WT after UVB irradiation VS WT before UVB irradiation	GO_ICOSANOID_RECEPTOR_ACTIVITY2	0.335495132	0.001787475	1.347036286	0.21444826	0.71210914	-5.13779768
homo after UVB irradiation VS homo before UVB irradiation	GO_ICOSATETRAENOIC_ACID_BINDING	0.645795377	0.050587004	3.574745616	0.00677987	0.0542279	-2.35432907
hetero after UVB irradiation VS hetero before UVB irradiation	GO_ICOSATETRAENOIC_ACID_BINDING1	0.807609552	0.043037217	3.628443734	0.00691467	0.1390235	-2.14630129
WT after UVB irradiation VS WT before UVB irradiation	GO_ICOSATETRAENOIC_ACID_BINDING2	0.308947415	0.045012143	1.112578172	0.29782482	0.75951483	-5.39101367
homo after UVB irradiation VS homo before UVB irradiation	GO_IDENTICAL_PROTEIN_BINDING	0.067589909	-0.00831075	0.567506031	0.5853378	0.73897082	-6.37749997
hetero after UVB irradiation VS hetero before UVB irradiation	GO_IDENTICAL_PROTEIN_BINDING1	0.109133747	0.002133366	0.878857908	0.40556703	0.71185533	-5.83268324
WT after UVB irradiation VS WT before UVB irradiation	GO_IDENTICAL_PROTEIN_BINDING2	0.112565663	-0.0089731	0.803767357	0.44448362	0.83340629	-5.66887911
homo after UVB irradiation VS homo before UVB irradiation	GO_IGE_BINDING	0.429627821	-0.01915215	1.507149342	0.16872212	0.3478742	-5.4513632
hetero after UVB irradiation VS hetero before UVB irradiation	GO_IGE_BINDING1	0.268288164	-0.00161141	1.43410502	0.19013854	0.5147346	-5.24554085
WT after UVB irradiation VS WT before UVB irradiation	GO_IGE_BINDING2	0.040990905	-0.03082097	0.231029926	0.82302205	0.95856757	-5.96800143
homo after UVB irradiation VS homo before UVB irradiation	GO_IGG_BINDING	1.103356122	-0.02930392	7.084076884	8.50E-05	0.00991667	2.011883287
hetero after UVB irradiation VS hetero before UVB irradiation	GO_IGG_BINDING1	1.074466613	0.085886505	7.02735558	0.00011991	0.07788488	1.614362284
WT after UVB irradiation VS WT before UVB irradiation	GO_IGG_BINDING2	0.921827446	0.011242473	3.318544638	0.01038326	0.33455411	-2.45095975
homo after UVB irradiation VS homo before UVB irradiation	GO_IKAPPAB_KINASE_COMPLEX	-0.00167032	-0.01243583	-0.00543002	0.9957953	0.99755286	-6.55084054
hetero after UVB irradiation VS hetero before UVB irradiation	GO_IKAPPAB_KINASE_COMPLEX1	0.361278932	0.028636093	1.352162735	0.21397323	0.54111209	-5.3438852
WT after UVB irradiation VS WT before UVB irradiation	GO_IKAPPAB_KINASE_COMPLEX2	-0.082325	0.087192773	-0.26671245	0.79635685	0.95157276	-5.95870581
homo after UVB irradiation VS homo before UVB irradiation	GO_IMIDAZOLE_CONTAINING_COMPOUND_CATABOLIC_PROCESS	0.46518083	0.005171919	2.171261169	0.06037573	0.18507782	-4.51118046
hetero after UVB irradiation VS hetero before UVB irradiation	GO_IMIDAZOLE_CONTAINING_COMPOUND_CATABOLIC_PROCESS1	0.046776394	-0.05491732	0.224869471	0.82782183	0.93446596	-6.20415284
WT after UVB irradiation VS WT before UVB irradiation	GO_IMIDAZOLE_CONTAINING_COMPOUND_CATABOLIC_PROCESS2	-0.0522679	-0.06456692	-0.20884833	0.8397246	0.96314441	-5.97311731
homo after UVB irradiation VS homo before UVB irradiation	GO_IMIDAZOLE_CONTAINING_COMPOUND_METABOLIC_PROCESS	0.209093786	-0.0064427	1.045009989	0.32539495	0.51883882	-5.98840161
hetero after UVB irradiation VS hetero before UVB irradiation	GO_IMIDAZOLE_CONTAINING_COMPOUND_METABOLIC_PROCESS1	-0.08009289	-0.03610909	-0.44576148	0.66780311	0.86386453	-6.12511724
WT after UVB irradiation VS WT before UVB irradiation	GO_IMIDAZOLE_CONTAINING_COMPOUND_METABOLIC_PROCESS2	-0.03784966	-0.0504358	-0.16161564	0.87556827	0.9693507	-5.98230816
homo after UVB irradiation VS homo before UVB irradiation	GO_IMMATURE_B_CELL_DIFFERENTIATION	-0.1005823	-0.03972593	-0.55403461	0.59411332	0.7455246	-6.38548423
hetero after UVB irradiation VS hetero before UVB irradiation	GO_IMMATURE_B_CELL_DIFFERENTIATION1	0.287823494	-0.07901381	1.044975833	0.32713196	0.64655226	-5.67843754
WT after UVB irradiation VS WT before UVB irradiation	GO_IMMATURE_B_CELL_DIFFERENTIATION2	-0.41647745	0.032989337	-1.89498642	0.09425272	0.59237113	-4.4472715
homo after UVB irradiation VS homo before UVB irradiation	GO_IMMATURE_T_CELL_PROLIFERATION	-0.02745821	0.011597338	-0.12544827	0.90314387	0.94720457	-6.54222809

homo after UVB irradiation VS homo before UVB irradiation	GO_IMMATURE_T_CELL_PROLIFERATION_IN_THYMUS	-0.35423693	0.057563062	-1.73843377	0.11883931	0.2789869	-5.14058329
hetero after UVB irradiation VS hetero before UVB irradiation	GO_IMMATURE_T_CELL_PROLIFERATION_IN_THYMUS1	-0.03914706	-0.04372721	-0.14082181	0.89155597	0.96027811	-6.22074378
WT after UVB irradiation VS WT before UVB irradiation	GO_IMMATURE_T_CELL_PROLIFERATION_IN_THYMUS2	-0.05237551	0.008212594	-0.29720052	0.7737948	0.94274743	-5.94972794
hetero after UVB irradiation VS hetero before UVB irradiation	GO_IMMATURE_T_CELL_PROLIFERATION1	0.083400178	-0.01993707	0.333965746	0.74714899	0.90061257	-6.17144041
WT after UVB irradiation VS WT before UVB irradiation	GO_IMMATURE_T_CELL_PROLIFERATION2	-0.04308661	-0.00505277	-0.20689546	0.84119938	0.96318896	-5.9735433
homo after UVB irradiation VS homo before UVB irradiation	GO_IMMUNE_EFFECTOR_PROCESS	0.548360412	-0.01560429	3.953170949	0.00389161	0.04081797	-1.79465939
hetero after UVB irradiation VS hetero before UVB irradiation	GO_IMMUNE_EFFECTOR_PROCESS1	0.551614778	0.027711571	4.004865674	0.00407502	0.12527297	-1.63648093
WT after UVB irradiation VS WT before UVB irradiation	GO_IMMUNE_EFFECTOR_PROCESS2	0.479615493	0.005841557	3.00095229	0.0168122	0.37993508	-2.89203007
homo after UVB irradiation VS homo before UVB irradiation	GO_IMMUNE_RECEPTOR_ACTIVITY	0.389168365	-0.05429068	2.205895974	0.05715341	0.17819836	-4.4589568
hetero after UVB irradiation VS hetero before UVB irradiation	GO_IMMUNE_RECEPTOR_ACTIVITY1	0.219312986	-0.00086166	1.001843167	0.34628713	0.66300933	-5.72046636
WT after UVB irradiation VS WT before UVB irradiation	GO_IMMUNE_RECEPTOR_ACTIVITY2	0.227990715	-0.02123277	0.805559364	0.44350652	0.83340629	-5.66747688
homo after UVB irradiation VS homo before UVB irradiation	GO_IMMUNE_RESPONSE_INHIBITING_CELL_SURFACE_RECEPTOR_SIGNALING_PATHWAY	1.062685949	-0.01241883	7.244117755	7.22E-05	0.00942728	2.168322836
hetero after UVB irradiation VS hetero before UVB irradiation	GO_IMMUNE_RESPONSE_INHIBITING_CELL_SURFACE_RECEPTOR_SIGNALING_PATHWAY1	0.992983273	0.083540273	4.272060379	0.00283509	0.11250952	-1.28826239
WT after UVB irradiation VS WT before UVB irradiation	GO_IMMUNE_RESPONSE_INHIBITING_CELL_SURFACE_RECEPTOR_SIGNALING_PATHWAY2	0.790793157	-0.00170036	2.479214238	0.03781627	0.47615783	-3.63189358
homo after UVB irradiation VS homo before UVB irradiation	GO_IMMUNE_RESPONSE_INHIBITING_SIGNAL_TRANSDUCTION	1.122068997	0.00701149	7.276484505	6.99E-05	0.00942429	2.199563891
hetero after UVB irradiation VS hetero before UVB irradiation	GO_IMMUNE_RESPONSE_INHIBITING_SIGNAL_TRANSDUCTION1	0.860708142	0.06914104	3.280882223	0.0114645	0.16699924	-2.63481766
WT after UVB irradiation VS WT before UVB irradiation	GO_IMMUNE_RESPONSE_INHIBITING_SIGNAL_TRANSDUCTION2	0.811087325	0.002103091	3.393900644	0.00927712	0.32212347	-2.34802779
homo after UVB irradiation VS homo before UVB irradiation	GO_IMMUNE_RESPONSE_REGULATING_SIGNALING_PATHWAY	0.314007587	-0.02322425	2.107140875	0.06681795	0.19720649	-4.60729269
hetero after UVB irradiation VS hetero before UVB irradiation	GO_IMMUNE_RESPONSE_REGULATING_SIGNALING_PATHWAY1	0.382470541	0.011243248	2.876371027	0.02103116	0.20848775	-3.21966473
WT after UVB irradiation VS WT before UVB irradiation	GO_IMMUNE_RESPONSE_REGULATING_SIGNALING_PATHWAY2	0.326654327	0.008832881	2.057583576	0.07321342	0.55377265	-4.22508427
homo after UVB irradiation VS homo before UVB irradiation	GO_IMMUNE_RESPONSE_TO_TUMOR_CELL	0.223144338	-0.03987647	1.154254326	0.28043623	0.47567589	-5.87362274
hetero after UVB irradiation VS hetero before UVB irradiation	GO_IMMUNE_RESPONSE_TO_TUMOR_CELL1	0.154092517	-0.00767976	0.674939706	0.51908815	0.78616125	-5.99160032
WT after UVB irradiation VS WT before UVB irradiation	GO_IMMUNE_RESPONSE_TO_TUMOR_CELL2	-0.12802816	-0.05019557	-0.44311174	0.6692842	0.91458718	-5.89377459
homo after UVB irradiation VS homo before UVB irradiation	GO_IMMUNE_SYSTEM_DEVELOPMENT	0.180083021	-0.01683458	1.434381236	0.1879209	0.37318649	-5.54412883
hetero after UVB irradiation VS hetero before UVB irradiation	GO_IMMUNE_SYSTEM_DEVELOPMENT1	0.224722952	0.004458422	1.781843144	0.11333686	0.41533559	-4.79583717
WT after UVB irradiation VS WT before UVB irradiation	GO_IMMUNE_SYSTEM_DEVELOPMENT2	0.193680066	0.004429912	1.341003725	0.21631575	0.71342248	-5.14469992
homo after UVB irradiation VS homo before UVB irradiation	GO_IMMUNOGLOBULIN_BINDING	0.894596613	-0.05677337	4.452713092	0.00192946	0.02978347	-1.08642409
hetero after UVB irradiation VS hetero before UVB irradiation	GO_IMMUNOGLOBULIN_BINDING1	0.896140914	0.034698533	6.256089985	0.00026369	0.07788488	0.921754509
WT after UVB irradiation VS WT before UVB irradiation	GO_IMMUNOGLOBULIN_BINDING2	0.71091147	0.019891047	3.242070922	0.01164887	0.34267286	-2.55614931
homo after UVB irradiation VS homo before UVB irradiation	GO_IMMUNOGLOBULIN_COMPLEX	0.352309761	0.049299812	1.155513655	0.27994851	0.47517829	-5.87225109
homo after UVB irradiation VS homo before UVB irradiation	GO_IMMUNOGLOBULIN_COMPLEX_CIRCULATING	0.17449306	0.11518916	0.496493063	0.63238342	0.77298157	-6.41757194
hetero after UVB irradiation VS hetero before UVB irradiation	GO_IMMUNOGLOBULIN_COMPLEX_CIRCULATING1	-0.02745875	0.053990724	-0.06843143	0.94715256	0.98026641	-6.22894534
WT after UVB irradiation VS WT before UVB irradiation	GO_IMMUNOGLOBULIN_COMPLEX_CIRCULATING2	-0.33776988	-0.14803698	-1.05145581	0.32341191	0.77346379	-5.45145467
hetero after UVB irradiation VS hetero before UVB irradiation	GO_IMMUNOGLOBULIN_COMPLEX1	0.263977835	0.047872123	0.996424681	0.34875307	0.66574648	-5.72565098
WT after UVB irradiation VS WT before UVB irradiation	GO_IMMUNOGLOBULIN_COMPLEX2	-0.05042251	-0.03176151	-0.15612877	0.87975371	0.9693507	-5.98322505
homo after UVB irradiation VS homo before UVB irradiation	GO_IMMUNOGLOBULIN_PRODUCTION	0.266166997	-0.02367136	2.026658512	0.07585722	0.21153156	-4.72674899
homo after UVB irradiation VS homo before UVB irradiation	GO_IMMUNOGLOBULIN_PRODUCTION_INVOLVED_IN_IMMUNOGLOBULIN_MEDIATED_IMMUNE_RESPONSE	0.039069172	-0.02782709	0.253947453	0.8056929	0.88980767	-6.51560268
hetero after UVB irradiation VS hetero before UVB irradiation	GO_IMMUNOGLOBULIN_PRODUCTION_INVOLVED_IN_IMMUNOGLOBULIN_MEDIATED_IMMUNE_RESPONSE1	0.084212255	0.003888677	0.623843893	0.55041776	0.80124226	-6.025683
WT after UVB irradiation VS WT before UVB irradiation	GO_IMMUNOGLOBULIN_PRODUCTION_INVOLVED_IN_IMMUNOGLOBULIN_MEDIATED_IMMUNE_RESPONSE2	0.262613194	-0.00230321	1.603649803	0.14700344	0.65371647	-4.82834589
hetero after UVB irradiation VS hetero before UVB irradiation	GO_IMMUNOGLOBULIN_PRODUCTION1	0.194696203	0.005921462	1.456875428	0.18394408	0.50848235	-5.21762584
WT after UVB irradiation VS WT before UVB irradiation	GO_IMMUNOGLOBULIN_PRODUCTION2	0.308545414	0.013677992	1.934301146	0.08868786	0.58115751	-4.39405308
homo after UVB irradiation VS homo before UVB irradiation	GO_IMMUNOGLOBULIN_RECEPTOR_ACTIVITY	0.822327395	-0.05093711	4.387767687	0.00210937	0.03091156	-1.17638328
hetero after UVB irradiation VS hetero before UVB irradiation	GO_IMMUNOGLOBULIN_RECEPTOR_ACTIVITY1	0.729398247	-0.01125585	2.676250492	0.02855757	0.23751402	-3.51279499
WT after UVB irradiation VS WT before UVB irradiation	GO_IMMUNOGLOBULIN_RECEPTOR_ACTIVITY2	0.521434961	0.016919024	1.860817527	0.09935844	0.59874718	-4.49322742
homo after UVB irradiation VS homo before UVB irradiation	GO_IMMUNOGLOBULIN_RECEPTOR_BINDING	0.717182434	0.036138722	3.463107535	0.00801556	0.05953233	-2.52272373
hetero after UVB irradiation VS hetero before UVB irradiation	GO_IMMUNOGLOBULIN_RECEPTOR_BINDING1	0.732946708	0.023173586	3.533891234	0.0079217	0.1443985	-2.27764191
WT after UVB irradiation VS WT before UVB irradiation	GO_IMMUNOGLOBULIN_RECEPTOR_BINDING2	0.366232424	0.03956699	1.152700844	0.28192304	0.75259375	-5.35001043
homo after UVB irradiation VS homo before UVB irradiation	GO_IMMUNOGLOBULIN_SECRETION	0.823892559	0.017965512	6.068363208	0.00025498	0.01206025	0.938475703
hetero after UVB irradiation VS hetero before UVB irradiation	GO_IMMUNOGLOBULIN_SECRETION1	0.397338553	0.028013819	1.607137852	0.14739899	0.4651453	-5.0276279
WT after UVB irradiation VS WT before UVB irradiation	GO_IMMUNOGLOBULIN_SECRETION2	0.536240806	0.070615613	2.153339958	0.06303768	0.53593918	-4.09202704
homo after UVB irradiation VS homo before UVB irradiation	GO_IMMUNOGLOBULIN_V_D_J_RECOMBINATION	-0.24142278	0.070719487	-0.88194499	0.40253938	0.58989807	-6.14311614
hetero after UVB irradiation VS hetero before UVB irradiation	GO_IMMUNOGLOBULIN_V_D_J_RECOMBINATION1	-0.34242066	0.032202388	-0.90733738	0.39122151	0.70111625	-5.80773642
WT after UVB irradiation VS WT before UVB irradiation	GO_IMMUNOGLOBULIN_V_D_J_RECOMBINATION2	-0.48685888	-0.0608474	-1.48430951	0.17557456	0.68033133	-4.97589574
homo after UVB irradiation VS homo before UVB irradiation	GO_IMMUNOLOGICAL_MEMORY_FORMATION_PROCESS	-0.34129563	-0.01621759	-1.91729572	0.09005032	0.23521905	-4.88661011
hetero after UVB irradiation VS hetero before UVB irradiation	GO_IMMUNOLOGICAL_MEMORY_FORMATION_PROCESS1	-0.26738001	-0.01400044	-0.93400716	0.37812601	0.69088998	-5.78379765
WT after UVB irradiation VS WT before UVB irradiation	GO_IMMUNOLOGICAL_MEMORY_FORMATION_PROCESS2	-0.1726336	-0.0356172	-0.64806094	0.53488521	0.86957641	-5.7803543
homo after UVB irradiation VS homo before UVB irradiation	GO_IMMUNOLOGICAL_MEMORY_PROCESS	-0.14664917	-0.04026329	-0.71606917	0.49353254	0.66910107	-6.27793431
hetero after UVB irradiation VS hetero before UVB irradiation	GO_IMMUNOLOGICAL_MEMORY_PROCESS1	-0.11742099	-0.01686439	-0.47306797	0.64903503	0.8557563	-6.11188065
WT after UVB irradiation VS WT before UVB irradiation	GO_IMMUNOLOGICAL_MEMORY_PROCESS2	0.002532277	-0.02765592	0.009025175	0.99301744	0.9985657	-5.99602369
homo after UVB irradiation VS homo before UVB irradiation	GO_IMMUNOLOGICAL_SYNAPSE	0.252475167	-0.03813586	1.305132882	0.22674584	0.41753038	-5.70196748
homo after UVB irradiation VS homo before UVB irradiation	GO_IMMUNOLOGICAL_SYNAPSE_FORMATION	-0.19697956	-0.04393043	-0.96820818	0.3602098	0.55141244	-6.0638623
hetero after UVB irradiation VS hetero before UVB irradiation	GO_IMMUNOLOGICAL_SYNAPSE_FORMATION1	-0.07801674	-0.01102141	-0.33458347	0.74670047	0.90058797	-6.17121971
WT after UVB irradiation VS WT before UVB irradiation	GO_IMMUNOLOGICAL_SYNAPSE_FORMATION2	-0.08430438	-0.03110051	-0.54558622	0.60005274	0.89567499	-5.84199259
hetero after UVB irradiation VS hetero before UVB irradiation	GO_IMMUNOLOGICAL_SYNAPSE1	0.40604955	0.028816964	2.463446878	0.0396466	0.27176547	-3.82492645
WT after UVB irradiation VS WT before UVB irradiation	GO_IMMUNOLOGICAL_SYNAPSE2	0.270642407	-0.05265695	1.32865834	0.22018102	0.71479746	-5.15876599
homo after UVB irradiation VS homo before UVB irradiation	GO_IMP_BIOSYNTHETIC_PROCESS	-0.39100932	-0.02392407	-1.82472831	0.10401083	0.25705708	-5.01933022
hetero after UVB irradiation VS hetero before UVB irradiation	GO_IMP_BIOSYNTHETIC_PROCESS1	-0.21160439	-0.05045782	-0.86269857	0.41387274	0.71760062	-5.84655032
WT after UVB irradiation VS WT before UVB irradiation	GO_IMP_BIOSYNTHETIC_PROCESS2	0.05351389	0.006599602	0.194591071	0.85050652	0.96583938	-5.97613621
homo after UVB irradiation VS homo before UVB irradiation	GO_IMP_METABOLIC_PROCESS	-0.26145597	-0.00163386	-1.20818626	0.26015669	0.45554078	-5.81393345
hetero after UVB irradiation VS hetero before UVB irradiation	GO_IMP_METABOLIC_PROCESS1	-0.05148311	-0.02247617	-0.21620361	0.83433948	0.93604273	-6.20621245
WT after UVB irradiation VS WT before UVB irradiation	GO_IMP_METABOLIC_PROCESS2	0.231030042	0.025502966	0.962677831	0.36357174	0.79550655	-5.53465392
homo after UVB irradiation VS homo before UVB irradiation	GO_IMPORT_ACROSS_PLASMA_MEMBRANE	0.166051123	0.02186841	1.211486392	0.25895578	0.45433485	-5.81021905
hetero after UVB irradiation VS hetero before UVB irradiation	GO_IMPORT_ACROSS_PLASMA_MEMBRANE1	-0.12873158	0.025911333	-0.91626808	0.38679989	0.69764483	-5.79978185
WT after UVB irradiation VS WT before UVB irradiation	GO_IMPORT_ACROSS_PLASMA_MEMBRANE2	-0.00592749	0.019531994	-0.03865215	0.97010368	0.99229361	-5.99527844
homo after UVB irradiation VS homo before UVB irradiation	GO_IMPORT_INTO_CELL	0.197848056	0.009580821	1.520217638	0.16546479	0.3436684	-5.43442786
hetero after UVB irradiation VS hetero before UVB irradiation	GO_IMPORT_INTO_CELL1	-0.06441875	0.018051607	-0.49302024	0.63548782	0.85025939	-6.10174049
WT after UVB irradiation VS WT before UVB irradiation	GO_IMPORT_INTO_CELL2	0.003262338	0.021864533	0.019698973	0.98476026	0.99670828	-5.99586192
homo after UVB irradiation VS homo before UVB irradiation	GO_IMPORT_INTO_NUCLEUS	-0.26940905	-0.01835291	-1.76680896	0.1137583	0.27141977	-5.10099575
hetero after UVB irradiation VS hetero before UVB irradiation	GO_IMPORT_INTO_NUCLEUS1	0.043485876	-0.02326102	0.262597198	0.79961875	0.92259806	-6.19425743
WT after UVB irradiation VS WT before UVB irradiation	GO_IMPORT_INTO_NUCLEUS2	-0.04712709	-0.03659489	-0.23083243	0.82317035	0.95856757	-5.96804922
homo after UVB irradiation VS homo before UVB irradiation	GO_IMPORTIN_ALPHA_FAMILY_PROTEIN_BINDING	-0.19566205	-0.01345268	-0.83771635	0.42557296	0.61029342	-6.18138573
hetero after UVB irradiation VS hetero before UVB irradiation	GO_IMPORTIN_ALPHA_FAMILY_PROTEIN_BINDING1	0.221166054	0.048351136	0.786273485	0.45477753	0.74571588	-5.90923295
WT after UVB irradiation VS WT before UVB irradiation	GO_IMPORTIN_ALPHA_FAMILY_PROTEIN_BINDING2	-0.095209	0.011036837	-0.32896839	0.75052643	0.93718509	-5.93936536
homo after UVB irradiation VS homo before UVB irradiation	GO_IN_UTERO_EMBRYONIC_DEVELOPMENT	-0.07381864	0.007181201	-0.51919321	0.61713647	0.76222656	-6.40530601
hetero after UVB irradiation VS hetero before UVB irradiation	GO_IN_UTERO_EMBRYONIC_DEVELOPMENT1	0.041652953	-0.00046456	0.325368543	0.75340192	0.90398429	-6.17447089
WT after UVB irradiation VS WT before UVB irradiation	GO_IN_UTERO_EMBRYONIC_DEVELOPMENT2	-0.03604033	0.004380138	-0.23918816	0.81690248	0.9565564	-5.96599184
homo after UVB irradiation VS homo before UVB irradiation	GO_INACTIVATION_OF_MAPK_ACTIVITY	-0.07471374	0.008489767	-0.38351927	0.71093897	0.827472	-6.47085085
hetero after UVB irradiation VS hetero before UVB irradiation	GO_INACTIVATION_OF_MAPK_ACTIVITY1	-0.0670374	-0.02245523	-0.46082716	0.6574163	0.86000309	-6.11790626
WT after UVB irradiation VS WT before UVB irradiation	GO_INACTIVATION_OF_MAPK_ACTIVITY2	-0.2795024	-0.0022403	-1.8289634	0.10435399	0.60721285	-4.53580495
homo after UVB irradiation VS homo before UVB irradiation	GO_INCLUSION_BODY	-0.07113613	0.001149842	-0.42286943	0.68309695	0.80941559	-6.45377675
homo after UVB irradiation VS homo before UVB irradiation	GO_INCLUSION_BODY_ASSEMBLY	-0.34486334	0.016082877	-1.70294112	0.12549153	0.28917529	-5.18968875
hetero after UVB irradiation VS hetero before UVB irradiation	GO_INCLUSION_BODY_ASSEMBLY1	-0.25062946	-0.03266781	-1.13762502	0.28879523	0.61351317	-5.58377908
WT after UVB irradiation VS WT before UVB irradiation	GO_INCLUSION_BODY_ASSEMBLY2	-0.36602928	-0.01993549	-1.81926821	0.10592103	0.6103384	-4.54871085
hetero after UVB irradiation VS hetero before UVB irradiation	GO_INCLUSION_BODY1	0.058376485	0.006522453	0.388269526	0.70813051	0.88462677	-6.15053359
WT after UVB irradiation VS WT before UVB irradiation	GO_INCLUSION_BODY2	-0.12657119	-0.02529469	-0.82325242	0.43393804	0.82957538	-5.65349154
homo after UVB irradiation VS homo before UVB irradiation	GO_INDOLALKYLAMINE_METABOLIC_PROCESS	0.164932526	-0.0277374	1.089244926	0.30655348	0.50103109	-5.94294623
hetero after UVB irradiation VS hetero before UVB irradiation	GO_INDOLALKYLAMINE_METABOLIC_PROCESS1	-0.12925472	-0.04261758	-0.64566096	0.53690461	0.7947523	-6.01142375
WT after UVB irradiation VS WT before UVB irradiation	GO_INDOLALKYLAMINE_METABOLIC_PROCESS2	-0.05727881	-0.0369474	-0.21603769	0.83430117	0.96118216	-5.971515
homo after UVB irradiation VS homo before UVB irradiation	GO_INDOLE_CONTAINING_COMPOUND_BIOSYNTHETIC_PROCESS	0.221655793	-0.03499464	0.945548662	0.37099691	0.56128782	-6.08525743
hetero after UVB irradiation VS hetero before UVB irradiation	GO_INDOLE_CONTAINING_COMPOUND_BIOSYNTHETIC_PROCESS1	-0.31126468	-0.0799431	-1.12157256	0.29516695	0.6189404	-5.60059352
WT after UVB irradiation VS WT before UVB irradiation	GO_INDOLE_CONTAINING_COMPOUND_BIOSYNTHETIC_PROCESS2	0.23677297	-0.02768043	0.685093937	0.51241466	0.85974355	-5.75574181
homo after UVB irradiation VS homo before UVB irradiation	GO_INDOLE_CONTAINING_COMPOUND_CATABOLIC_PROCESS	0.299937919	-0.00362902	1.602551374	0.14620755	0.31888631	-5.32591767
hetero after UVB irradiation VS hetero before UVB irradiation	GO_INDOLE_CONTAINING_COMPOUND_CATABOLIC_PROCESS1	-0.0093336	-0.02133493	-0.04688355	0.96377641	0.98664387	-6.23029226
WT after UVB irradiation VS WT before UVB irradiation	GO_INDOLE_CONTAINING_COMPOUND_CATABOLIC_PROCESS2	-0.0854124	-0.05005949	-0.30642582	0.76701143	0.94123239	-5.94682432
homo after UVB irradiation VS homo before UVB irradiation	GO_INDOLE_CONTAINING_COMPOUND_METABOLIC_PROCESS	0.143630088	0.004989447	0.981720599	0.35388938	0.54609909	-6.05091264
hetero after UVB irradiation VS hetero before UVB irradiation	GO_INDOLE_CONTAINING_COMPOUND_METABOLIC_PROCESS1	-0.07467357	-0.00679285	-0.46335583	0.65568062	0.85903418	-6.11667373
WT after UVB irradiation VS WT before UVB irradiation	GO_INDOLE_CONTAINING_COMPOUND_METABOLIC_PROCESS2	0.00250996	0.00269164	0.012142157	0.99060602	0.99848757	-5.99598887
homo after UVB irradiation VS homo before UVB irradiation	GO_INDUCTION_OF_BACTERIAL_AGGLUTINATION	0.505797621	0.054730434	2.015962244	0.07714432	0.21360102	-4.74251598
hetero after UVB irradiation VS hetero before UVB irradiation	GO_INDUCTION_OF_BACTERIAL_AGGLUTINATION1	0.072700194	0.100267558	0.198628301	0.84759951	0.94216033	-6.21014391
WT after UVB irradiation VS WT before UVB irradiation	GO_INDUCTION_OF_BACTERIAL_AGGLUTINATION2	0.175288322	0.157345557	1.042642611	0.32723893	0.77600271	-5.45996172
homo after UVB irradiation VS homo before UVB irradiation	GO_INDUCTION_OF_POSITIVE_CHEMOTAXIS	0.74959379	-0.01189163	4.599240239	0.00158151	0.02693236	-0.88586607
hetero after UVB irradiation VS hetero before UVB irradiation	GO_INDUCTION_OF_POSITIVE_CHEMOTAXIS1	0.550805139	0.028866749	2.614962093	0.03138013	0.24669433	-3.60273907
WT after UVB irradiation VS WT before UVB irradiation	GO_INDUCTION_OF_POSITIVE_CHEMOTAXIS2	0.570221779	0.034155068	1.847850169	0.10136407	0.60153	-4.51059186
homo after UVB irradiation VS homo before UVB irradiation	GO_INFLAMMASOME_COMPLEX	0.736421934	0.009464085	5.236505781	0.00069065	0.0185307	-0.05302773
hetero after UVB irradiation VS hetero before UVB irradiation	GO_INFLAMMASOME_COMPLEX1	0.861571023	0.036851435	5.586902531	0.00055337	0.08125048	0.248942911
WT after UVB irradiation VS WT before UVB irradiation	GO_INFLAMMASOME_COMPLEX2	0.523643396	-0.05902911	2.094068096	0.06915984	0.54423691	-4.17455205
homo after UVB irradiation VS homo before UVB irradiation	GO_INFLAMMATORY_CELL_APOPTOTIC_PROCESS	0.412433763	-0.0185205	2.659883504	0.02781872	0.11799966	-3.76133202
hetero after UVB irradiation VS hetero before UVB irradiation	GO_INFLAMMATORY_CELL_APOPTOTIC_PROCESS1	0.444307907	0.046100478	3.032061881	0.01661679	0.19011944	-2.9929705
WT after UVB irradiation VS WT before UVB irradiation	GO_INFLAMMATORY_CELL_APOPTOTIC_PROCESS2	0.162431359	-0.03789475	0.803950791	0.44438354	0.83340629	-5.6687357
homo after UVB irradiation VS homo before UVB irradiation	GO_INFLAMMATORY_RESPONSE	0.524281208	-0.01353536	3.965024556	0.00382576	0.04047966	-1.77743316
homo after UVB irradiation VS homo before UVB irradiation	GO_INFLAMMATORY_RESPONSE_TO_ANTIGENIC_STIMULUS	0.521283572	-0.03734142	3.215606327	0.01168149	0.0724961	-2.90042424
hetero after UVB irradiation VS hetero before UVB irradiation	GO_INFLAMMATORY_RESPONSE_TO_ANTIGENIC_STIMULUS1	0.3975098	0.038144239	2.368763754	0.04590287	0.28616087	-3.96333701
WT after UVB irradiation VS WT before UVB irradiation	GO_INFLAMMATORY_RESPONSE_TO_ANTIGENIC_STIMULUS2	0.401095166	0.025149003	2.02279954	0.07729284	0.56116995	-4.27305182
homo after UVB irradiation VS homo before UVB irradiation	GO_INFLAMMATORY_RESPONSE_TO_WOUNDING	0.830489271	-0.0141282	5.152144615	0.00076813	0.01924501	-0.15958238
hetero after UVB irradiation VS hetero before UVB irradiation	GO_INFLAMMATORY_RESPONSE_TO_WOUNDING1	0.732466054	0.07220327	4.280916049	0.00280171	0.11250952	-1.27692433
WT after UVB irradiation VS WT before UVB irradiation	GO_INFLAMMATORY_RESPONSE_TO_WOUNDING2	0.751726397	0.060604021	3.390411068	0.0093255	0.32213413	-2.35277791
hetero after UVB irradiation VS hetero before UVB irradiation	GO_INFLAMMATORY_RESPONSE1	0.437461138	0.032401172	2.830346714	0.02255776	0.21561659	-3.28695066
WT after UVB irradiation VS WT before UVB irradiation	GO_INFLAMMATORY_RESPONSE2	0.329676156	0.009820501	1.623910707	0.14259426	0.65167133	-4.80273772
homo after UVB irradiation VS homo before UVB irradiation	GO_INHIBIN_BINDING	0.238430612	-0.07907409	0.866740149	0.41035617	0.59673097	-6.15645718
hetero after UVB irradiation VS hetero before UVB irradiation	GO_INHIBIN_BINDING1	0.015519437	0.081101187	0.05890905	0.95449604	0.98300834	-6.22960258
WT after UVB irradiation VS WT before UVB irradiation	GO_INHIBIN_BINDING2	0.402726774	0.122099124	1.797561121	0.10951044	0.61466507	-4.57751343
homo after UVB irradiation VS homo before UVB irradiation	GO_INHIBITION_OF_CYSTEINE_TYPE_ENDOPEPTIDASE_ACTIVITY_INVOLVED_IN_APOPTOTIC_PROCESS	0.070199821	0.065277746	0.388744801	0.70721399	0.82533466	-6.46867582
hetero after UVB irradiation VS hetero before UVB irradiation	GO_INHIBITION_OF_CYSTEINE_TYPE_ENDOPEPTIDASE_ACTIVITY_INVOLVED_IN_APOPTOTIC_PROCESS1	-0.13946814	-0.03680349	-0.54058325	0.60378514	0.83419966	-6.07599185
WT after UVB irradiation VS WT before UVB irradiation	GO_INHIBITION_OF_CYSTEINE_TYPE_ENDOPEPTIDASE_ACTIVITY_INVOLVED_IN_APOPTOTIC_PROCESS2	0.28223934	0.012792911	1.263383495	0.24161022	0.73118354	-5.23177922
homo after UVB irradiation VS homo before UVB irradiation	GO_INHIBITION_OF_NEUROEPITHELIAL_CELL_DIFFERENTIATION	-0.33045175	0.185384256	-0.94842251	0.36961574	0.55994999	-6.08256636
hetero after UVB irradiation VS hetero before UVB irradiation	GO_INHIBITION_OF_NEUROEPITHELIAL_CELL_DIFFERENTIATION1	0.077412954	0.169718712	0.49862779	0.63170633	0.8484405	-6.09881988
WT after UVB irradiation VS WT before UVB irradiation	GO_INHIBITION_OF_NEUROEPITHELIAL_CELL_DIFFERENTIATION2	-0.41302534	0.120138332	-1.44189424	0.18686574	0.68957923	-5.02687304
homo after UVB irradiation VS homo before UVB irradiation	GO_INHIBITORY_EXTRACELLULAR_LIGAND_GATED_ION_CHANNEL_ACTIVITY	-0.22216715	0.126390145	-0.83392907	0.42758716	0.61201782	-6.18458607
hetero after UVB irradiation VS hetero before UVB irradiation	GO_INHIBITORY_EXTRACELLULAR_LIGAND_GATED_ION_CHANNEL_ACTIVITY1	-0.82461759	-0.00428798	-4.48333889	0.002144	0.1055457	-1.02137656
WT after UVB irradiation VS WT before UVB irradiation	GO_INHIBITORY_EXTRACELLULAR_LIGAND_GATED_ION_CHANNEL_ACTIVITY2	-0.536676	0.10443807	-2.76555471	0.02418248	0.42212473	-3.2246222
homo after UVB irradiation VS homo before UVB irradiation	GO_INHIBITORY_POSTSYNAPTIC_POTENTIAL	0.120247715	-0.03357177	0.465565629	0.65345789	0.78909035	-6.43345093
hetero after UVB irradiation VS hetero before UVB irradiation	GO_INHIBITORY_POSTSYNAPTIC_POTENTIAL1	0.229995405	0.005096101	1.318460683	0.22450424	0.55314486	-5.38332326
WT after UVB irradiation VS WT before UVB irradiation	GO_INHIBITORY_POSTSYNAPTIC_POTENTIAL2	-0.10078238	0.008764711	-0.50224631	0.62886485	0.90390703	-5.86512026
homo after UVB irradiation VS homo before UVB irradiation	GO_INHIBITORY_SYNAPSE	0.336349153	0.006790771	2.50820338	0.0353745	0.13468829	-3.9963166
homo after UVB irradiation VS homo before UVB irradiation	GO_INHIBITORY_SYNAPSE_ASSEMBLY	-0.00326124	0.03226226	-0.01776092	0.98624768	0.99306612	-6.5506836
hetero after UVB irradiation VS hetero before UVB irradiation	GO_INHIBITORY_SYNAPSE_ASSEMBLY1	-0.1139609	0.015358482	-0.55066618	0.59717509	0.83131279	-6.07025106
WT after UVB irradiation VS WT before UVB irradiation	GO_INHIBITORY_SYNAPSE_ASSEMBLY2	-0.03153574	-0.02143803	-0.1579771	0.87834333	0.9693507	-5.9829197
hetero after UVB irradiation VS hetero before UVB irradiation	GO_INHIBITORY_SYNAPSE1	0.266392393	0.0167771	1.458592064	0.18348441	0.50789011	-5.21551143
WT after UVB irradiation VS WT before UVB irradiation	GO_INHIBITORY_SYNAPSE2	0.32517339	-0.02360829	1.879423705	0.09654642	0.59686908	-4.46823819
homo after UVB irradiation VS homo before UVB irradiation	GO_INNATE_IMMUNE_RESPONSE	0.471097641	-0.01775582	3.278475849	0.01060857	0.06902586	-2.80397412
homo after UVB irradiation VS homo before UVB irradiation	GO_INNATE_IMMUNE_RESPONSE_ACTIVATING_SIGNAL_TRANSDUCTION	0.088445195	-0.00485736	0.394518756	0.70310775	0.82287974	-6.46623954
hetero after UVB irradiation VS hetero before UVB irradiation	GO_INNATE_IMMUNE_RESPONSE_ACTIVATING_SIGNAL_TRANSDUCTION1	0.251881961	0.018116443	1.276187854	0.23833796	0.56736997	-5.43191016

WT after UVB irradiation VS WT before UVB irradiation	GO_INNATE_IMMUNE_RESPONSE_ACTIVATING_SIGNAL_TRANSDUCTION2	0.205347688	-0.00260938	1.011052887	0.34124449	0.78168336	-5.49001134
homo after UVB irradiation VS homo before UVB irradiation	GO_INNATE_IMMUNE_RESPONSE_IN_MUCOSA	0.532624294	0.09987786	2.59414117	0.03086903	0.1247153	-3.86331166
hetero after UVB irradiation VS hetero before UVB irradiation	GO_INNATE_IMMUNE_RESPONSE_IN_MUCOSA1	0.520995369	0.065192351	2.687385345	0.02807342	0.2360407	-3.49645704
WT after UVB irradiation VS WT before UVB irradiation	GO_INNATE_IMMUNE_RESPONSE_IN_MUCOSA2	0.325148954	0.114345644	1.183377194	0.27023405	0.74431614	-5.31797815
hetero after UVB irradiation VS hetero before UVB irradiation	GO_INNATE_IMMUNE_RESPONSE1	0.46502776	0.025321204	3.171851886	0.01347749	0.17646302	-2.79102201
WT after UVB irradiation VS WT before UVB irradiation	GO_INNATE_IMMUNE_RESPONSE2	0.400805235	0.016205764	2.474562594	0.038093	0.47719723	-3.63850721
homo after UVB irradiation VS homo before UVB irradiation	GO_INNER_ACROSOMAL_MEMBRANE	-0.24702857	0.020254478	-1.39601327	0.19879564	0.38763573	-5.59194477
hetero after UVB irradiation VS hetero before UVB irradiation	GO_INNER_ACROSOMAL_MEMBRANE1	0.040521303	-0.00704523	0.178395742	0.8629285	0.94819913	-6.21426133
WT after UVB irradiation VS WT before UVB irradiation	GO_INNER_ACROSOMAL_MEMBRANE2	0.081127204	0.073990871	0.23824213	0.81761143	0.9565564	-5.9662284
homo after UVB irradiation VS homo before UVB irradiation	GO_INNER_CELL_MASS_CELL_DIFFERENTIATION	0.11647169	-0.01384771	0.609814029	0.5582505	0.7186063	-6.35127814
hetero after UVB irradiation VS hetero before UVB irradiation	GO_INNER_CELL_MASS_CELL_DIFFERENTIATION1	-0.0634171	0.031366332	-0.39860841	0.70080062	0.88146568	-6.14621021
WT after UVB irradiation VS WT before UVB irradiation	GO_INNER_CELL_MASS_CELL_DIFFERENTIATION2	-0.29795605	0.011501037	-1.11799239	0.29563801	0.75860375	-5.38554087
homo after UVB irradiation VS homo before UVB irradiation	GO_INNER_CELL_MASS_CELL_PROLIFERATION	-0.22144473	-0.01332005	-1.2889008	0.2320748	0.42327621	-5.72111601
hetero after UVB irradiation VS hetero before UVB irradiation	GO_INNER_CELL_MASS_CELL_PROLIFERATION1	0.246172819	-0.02969389	1.099183981	0.30424163	0.62604791	-5.62375971
WT after UVB irradiation VS WT before UVB irradiation	GO_INNER_CELL_MASS_CELL_PROLIFERATION2	0.160115328	0.027064274	0.660070558	0.52753349	0.8664124	-5.77250599
homo after UVB irradiation VS homo before UVB irradiation	GO_INNER_DYNEIN_ARM_ASSEMBLY	-0.2311946	-0.01339942	-1.16429195	0.27656774	0.47248323	-5.86266009
hetero after UVB irradiation VS hetero before UVB irradiation	GO_INNER_DYNEIN_ARM_ASSEMBLY1	-0.41243833	-0.00193486	-2.15802841	0.06361429	0.33110844	-4.26873562
WT after UVB irradiation VS WT before UVB irradiation	GO_INNER_DYNEIN_ARM_ASSEMBLY2	-0.23337089	-0.01480069	-1.51707451	0.16727162	0.67227268	-4.93597204
homo after UVB irradiation VS homo before UVB irradiation	GO_INNER_EAR_AUDITORY_RECEPTOR_CELL_DIFFERENTIATION	-0.09781816	0.018430491	-0.66536676	0.52380937	0.69242529	-6.31424826
hetero after UVB irradiation VS hetero before UVB irradiation	GO_INNER_EAR_AUDITORY_RECEPTOR_CELL_DIFFERENTIATION1	0.168981921	0.027319092	1.0928707	0.3068404	0.627591	-5.63023144
WT after UVB irradiation VS WT before UVB irradiation	GO_INNER_EAR_AUDITORY_RECEPTOR_CELL_DIFFERENTIATION2	-0.13758791	0.001256627	-0.75282805	0.47286894	0.84737425	-5.70762691
homo after UVB irradiation VS homo before UVB irradiation	GO_INNER_EAR_MORPHOGENESIS	0.037518731	0.016250423	0.308365432	0.76537574	0.86419375	-6.49897132
hetero after UVB irradiation VS hetero before UVB irradiation	GO_INNER_EAR_MORPHOGENESIS1	-0.05243839	-0.00624276	-0.34217525	0.74119663	0.89913659	-6.168475
WT after UVB irradiation VS WT before UVB irradiation	GO_INNER_EAR_MORPHOGENESIS2	-0.17485964	0.015750881	-1.0700579	0.31544863	0.77149112	-5.4333247
homo after UVB irradiation VS homo before UVB irradiation	GO_INNER_EAR_RECEPTOR_CELL_DEVELOPMENT	0.102100514	0.024465245	0.725885905	0.48780109	0.66431161	-6.27063106
hetero after UVB irradiation VS hetero before UVB irradiation	GO_INNER_EAR_RECEPTOR_CELL_DEVELOPMENT1	0.087028956	0.025523911	0.485998338	0.64023924	0.8522738	-6.10535405
WT after UVB irradiation VS WT before UVB irradiation	GO_INNER_EAR_RECEPTOR_CELL_DEVELOPMENT2	-0.14408276	-0.00020232	-0.88106689	0.40367343	0.81613006	-5.60604114
homo after UVB irradiation VS homo before UVB irradiation	GO_INNER_EAR_RECEPTOR_CELL_STEREOCILIUM_ORGANIZATION	0.118039544	0.024085754	0.825814016	0.43192519	0.61607417	-6.19140229
hetero after UVB irradiation VS hetero before UVB irradiation	GO_INNER_EAR_RECEPTOR_CELL_STEREOCILIUM_ORGANIZATION1	0.047416154	0.00327145	0.204976313	0.8428039	0.9398031	-6.20876191
WT after UVB irradiation VS WT before UVB irradiation	GO_INNER_EAR_RECEPTOR_CELL_STEREOCILIUM_ORGANIZATION2	-0.31134671	0.028907863	-1.81771608	0.10617396	0.61036582	-4.55077464
homo after UVB irradiation VS homo before UVB irradiation	GO_INNER_MITOCHONDRIAL_MEMBRANE_ORGANIZATION	-0.30168774	0.01008866	-1.19441303	0.26521816	0.46039765	-5.82935936
hetero after UVB irradiation VS hetero before UVB irradiation	GO_INNER_MITOCHONDRIAL_MEMBRANE_ORGANIZATION1	-0.27924716	-0.05495481	-1.37350954	0.20752596	0.5319221	-5.31859393
WT after UVB irradiation VS WT before UVB irradiation	GO_INNER_MITOCHONDRIAL_MEMBRANE_ORGANIZATION2	-0.16961346	-0.0721216	-0.78916887	0.45249809	0.83625615	-5.68020403
homo after UVB irradiation VS homo before UVB irradiation	GO_INNER_MITOCHONDRIAL_MEMBRANE_PROTEIN_COMPLEX	-0.06836826	-0.02889572	-0.2390877	0.81681577	0.89645622	-6.51959382
hetero after UVB irradiation VS hetero before UVB irradiation	GO_INNER_MITOCHONDRIAL_MEMBRANE_PROTEIN_COMPLEX1	-0.35142659	-0.0537646	-1.6514617	0.13795779	0.45118267	-4.96983202
WT after UVB irradiation VS WT before UVB irradiation	GO_INNER_MITOCHONDRIAL_MEMBRANE_PROTEIN_COMPLEX2	-0.07182744	-0.07728522	-0.25064818	0.80832878	0.95404687	-5.96305297
homo after UVB irradiation VS homo before UVB irradiation	GO_INNERVATION	0.190112501	-0.0098139	0.992683716	0.34882285	0.54082568	-6.04030228
hetero after UVB irradiation VS hetero before UVB irradiation	GO_INNERVATION1	-0.1061332	-0.01281506	-0.58608395	0.5742739	0.81590465	-6.04931328
WT after UVB irradiation VS WT before UVB irradiation	GO_INNERVATION2	0.060937828	0.021487994	0.383683484	0.71109088	0.92872685	-5.91912958
homo after UVB irradiation VS homo before UVB irradiation	GO_INO80_TYPE_COMPLEX	-0.66746145	0.003766276	-4.52971914	0.00173731	0.02811012	-0.98060518
hetero after UVB irradiation VS hetero before UVB irradiation	GO_INO80_TYPE_COMPLEX1	-0.31264482	-0.02060962	-1.50747564	0.17081678	0.49326269	-5.15473342
WT after UVB irradiation VS WT before UVB irradiation	GO_INO80_TYPE_COMPLEX2	0.037963495	0.036901912	0.133273421	0.8972297	0.97406155	-5.98670553
homo after UVB irradiation VS homo before UVB irradiation	GO_INORGANIC_ANION_EXCHANGER_ACTIVITY	0.072305212	0.004097729	0.345020146	0.7386303	0.84640219	-6.48599738
hetero after UVB irradiation VS hetero before UVB irradiation	GO_INORGANIC_ANION_EXCHANGER_ACTIVITY1	-0.4262384	0.023540641	-2.99915963	0.01746158	0.19358605	-3.04074011
WT after UVB irradiation VS WT before UVB irradiation	GO_INORGANIC_ANION_EXCHANGER_ACTIVITY2	-0.3946043	0.02412055	-2.0082916	0.07905871	0.56285824	-4.29299394
homo after UVB irradiation VS homo before UVB irradiation	GO_INORGANIC_ANION_TRANSMEMBRANE_TRANSPORT	0.101479421	0.029586486	0.791360895	0.45067836	0.63231228	-6.21970994
hetero after UVB irradiation VS hetero before UVB irradiation	GO_INORGANIC_ANION_TRANSMEMBRANE_TRANSPORT1	-0.26327509	-0.00761125	-1.98433075	0.08316733	0.36861389	-4.51587749
WT after UVB irradiation VS WT before UVB irradiation	GO_INORGANIC_ANION_TRANSMEMBRANE_TRANSPORT2	-0.25408418	0.03041644	-1.69517448	0.12803175	0.63467098	-4.71149783
homo after UVB irradiation VS homo before UVB irradiation	GO_INORGANIC_ANION_TRANSMEMBRANE_TRANSPORTER_ACTIVITY	0.046656637	0.02666798	0.349688514	0.73524992	0.84423304	-6.48424339
hetero after UVB irradiation VS hetero before UVB irradiation	GO_INORGANIC_ANION_TRANSMEMBRANE_TRANSPORTER_ACTIVITY1	-0.2988939	-0.01662565	-2.33239286	0.04856261	0.29346252	-4.01635187
WT after UVB irradiation VS WT before UVB irradiation	GO_INORGANIC_ANION_TRANSMEMBRANE_TRANSPORTER_ACTIVITY2	-0.29151461	0.032863182	-2.0283006	0.07663331	0.55925897	-4.26548012
homo after UVB irradiation VS homo before UVB irradiation	GO_INORGANIC_ANION_TRANSPORT	0.063736848	0.022471176	0.519647978	0.61683299	0.76191549	-6.40505503
hetero after UVB irradiation VS hetero before UVB irradiation	GO_INORGANIC_ANION_TRANSPORT1	-0.24233938	-0.01329702	-1.92848784	0.09061432	0.37792676	-4.59409466
WT after UVB irradiation VS WT before UVB irradiation	GO_INORGANIC_ANION_TRANSPORT2	-0.21473937	0.027885957	-1.51199532	0.16853531	0.67400422	-4.94219116
homo after UVB irradiation VS homo before UVB irradiation	GO_INORGANIC_ION_IMPORT_ACROSS_PLASMA_MEMBRANE	0.190657258	0.015725139	1.290919949	0.23140622	0.42247452	-5.71874266
hetero after UVB irradiation VS hetero before UVB irradiation	GO_INORGANIC_ION_IMPORT_ACROSS_PLASMA_MEMBRANE1	-0.10118028	0.024969666	-0.73126019	0.48586548	0.76391974	-5.95129421
WT after UVB irradiation VS WT before UVB irradiation	GO_INORGANIC_ION_IMPORT_ACROSS_PLASMA_MEMBRANE2	-0.01391167	0.034845276	-0.0840043	0.93509267	0.98322796	-5.9923449
homo after UVB irradiation VS homo before UVB irradiation	GO_INORGANIC_ION_TRANSMEMBRANE_TRANSPORT	0.122209783	0.007521429	0.917519593	0.38466721	0.57425736	-6.1111584
hetero after UVB irradiation VS hetero before UVB irradiation	GO_INORGANIC_ION_TRANSMEMBRANE_TRANSPORT1	-0.05923711	0.008139136	-0.43902439	0.67247291	0.86681615	-6.12826828
WT after UVB irradiation VS WT before UVB irradiation	GO_INORGANIC_ION_TRANSMEMBRANE_TRANSPORT2	-0.05655592	0.004311314	-0.34300629	0.74032844	0.93506555	-5.93446059
homo after UVB irradiation VS homo before UVB irradiation	GO_INORGANIC_PHOSPHATE_TRANSMEMBRANE_TRANSPORTER_ACTIVITY	0.379565092	0.019797135	2.449429822	0.03883284	0.14167028	-4.08700277
hetero after UVB irradiation VS hetero before UVB irradiation	GO_INORGANIC_PHOSPHATE_TRANSMEMBRANE_TRANSPORTER_ACTIVITY1	0.337230762	0.03840345	2.307799483	0.05044817	0.29894137	-4.05213952
WT after UVB irradiation VS WT before UVB irradiation	GO_INORGANIC_PHOSPHATE_TRANSMEMBRANE_TRANSPORTER_ACTIVITY2	-0.21950408	-0.05231477	-0.73670082	0.4820997	0.85127391	-5.71944036
homo after UVB irradiation VS homo before UVB irradiation	GO_INOSITOL_1_3_4_5_TETRAKISPHOSPHATE_5_PHOSPHATASE_ACTIVITY	1.169859565	-0.03592646	8.113401426	3.12E-05	0.00847131	2.963184916
hetero after UVB irradiation VS hetero before UVB irradiation	GO_INOSITOL_1_3_4_5_TETRAKISPHOSPHATE_5_PHOSPHATASE_ACTIVITY1	0.502360528	0.118240156	1.588891385	0.15145487	0.46960877	-5.05120184
WT after UVB irradiation VS WT before UVB irradiation	GO_INOSITOL_1_3_4_5_TETRAKISPHOSPHATE_5_PHOSPHATASE_ACTIVITY2	0.772501111	0.032082073	2.20612364	0.0580361	0.52490436	-4.01814065
homo after UVB irradiation VS homo before UVB irradiation	GO_INOSITOL_1_3_4_5_TETRAKISPHOSPHATE_BINDING	0.088940783	-0.00799726	0.380515954	0.71308358	0.8292336	-6.47208808
hetero after UVB irradiation VS hetero before UVB irradiation	GO_INOSITOL_1_3_4_5_TETRAKISPHOSPHATE_BINDING1	-0.1070561	0.011939639	-0.37907621	0.71467565	0.88776082	-6.15428621
WT after UVB irradiation VS WT before UVB irradiation	GO_INOSITOL_1_3_4_5_TETRAKISPHOSPHATE_BINDING2	-0.12542351	0.000433905	-0.5498746	0.5972409	0.89386246	-5.83960774
homo after UVB irradiation VS homo before UVB irradiation	GO_INOSITOL_1_4_5_TRISPHOSPHATE_BINDING	0.629430075	-0.03377647	4.846600971	0.00113862	0.02277031	-0.55494822
hetero after UVB irradiation VS hetero before UVB irradiation	GO_INOSITOL_1_4_5_TRISPHOSPHATE_BINDING1	0.209061276	0.014991968	1.011161404	0.34207762	0.66041641	-5.71150016
WT after UVB irradiation VS WT before UVB irradiation	GO_INOSITOL_1_4_5_TRISPHOSPHATE_BINDING2	0.264566826	-0.02212186	1.029008865	0.33322818	0.77844475	-5.47301621
homo after UVB irradiation VS homo before UVB irradiation	GO_INOSITOL_HEXAKISPHOSPHATE_5_KINASE_ACTIVITY	-0.51320581	0.035878106	-1.74710606	0.11726442	0.27616837	-5.12851462
hetero after UVB irradiation VS hetero before UVB irradiation	GO_INOSITOL_HEXAKISPHOSPHATE_5_KINASE_ACTIVITY1	-0.81091761	-0.08364745	-3.65031776	0.00670174	0.13773103	-2.11609503
WT after UVB irradiation VS WT before UVB irradiation	GO_INOSITOL_HEXAKISPHOSPHATE_5_KINASE_ACTIVITY2	-0.29300223	-0.03142139	-0.98346281	0.35384755	0.78942569	-5.51567952
homo after UVB irradiation VS homo before UVB irradiation	GO_INOSITOL_HEXAKISPHOSPHATE_KINASE_ACTIVITY	-0.18700457	-0.04725541	-0.76557599	0.46506704	0.64423114	-6.24021812
hetero after UVB irradiation VS hetero before UVB irradiation	GO_INOSITOL_HEXAKISPHOSPHATE_KINASE_ACTIVITY1	-0.39079806	0.008357289	-1.71784736	0.12485801	0.43161534	-4.88194371
WT after UVB irradiation VS WT before UVB irradiation	GO_INOSITOL_HEXAKISPHOSPHATE_KINASE_ACTIVITY2	-0.23643728	0.00240308	-0.92553515	0.38144255	0.80459516	-5.56776795
homo after UVB irradiation VS homo before UVB irradiation	GO_INOSITOL_LIPID_MEDIATED_SIGNALING	0.284973091	-0.00782444	2.082879055	0.06942657	0.2014408	-4.64344974
hetero after UVB irradiation VS hetero before UVB irradiation	GO_INOSITOL_LIPID_MEDIATED_SIGNALING1	0.16339179	0.020323597	0.994978665	0.34941341	0.66628241	-5.72703095
WT after UVB irradiation VS WT before UVB irradiation	GO_INOSITOL_LIPID_MEDIATED_SIGNALING2	0.141900534	0.014088339	0.82386391	0.4336099	0.82957538	-5.65300365
homo after UVB irradiation VS homo before UVB irradiation	GO_INOSITOL_METABOLIC_PROCESS	-0.01938187	-0.02824073	-0.08142644	0.93702555	0.96657812	-6.54721937
hetero after UVB irradiation VS hetero before UVB irradiation	GO_INOSITOL_METABOLIC_PROCESS1	0.401389437	0.016587782	2.328755061	0.048837	0.29457705	-4.02164871
WT after UVB irradiation VS WT before UVB irradiation	GO_INOSITOL_METABOLIC_PROCESS2	0.239947804	-0.0216376	1.334046446	0.21848682	0.71478521	-5.1526367
homo after UVB irradiation VS homo before UVB irradiation	GO_INOSITOL_PHOSPHATE_BIOSYNTHETIC_PROCESS	0.331901998	-0.03201052	1.793926804	0.10909257	0.26417444	-5.06289991
hetero after UVB irradiation VS hetero before UVB irradiation	GO_INOSITOL_PHOSPHATE_BIOSYNTHETIC_PROCESS1	0.171783371	0.008548577	1.148075712	0.28470707	0.60984853	-5.57274186
WT after UVB irradiation VS WT before UVB irradiation	GO_INOSITOL_PHOSPHATE_BIOSYNTHETIC_PROCESS2	0.266107314	0.002357392	1.624226623	0.14252648	0.65167133	-4.80233722
homo after UVB irradiation VS homo before UVB irradiation	GO_INOSITOL_PHOSPHATE_CATABOLIC_PROCESS	0.557469418	-0.02460009	3.066514736	0.01470464	0.08257891	-3.13026505
hetero after UVB irradiation VS hetero before UVB irradiation	GO_INOSITOL_PHOSPHATE_CATABOLIC_PROCESS1	0.369578819	0.077965882	2.175399303	0.06192744	0.32672921	-4.24375332
WT after UVB irradiation VS WT before UVB irradiation	GO_INOSITOL_PHOSPHATE_CATABOLIC_PROCESS2	0.334670215	0.005596638	1.807586533	0.10783866	0.61228673	-4.56422716
homo after UVB irradiation VS homo before UVB irradiation	GO_INOSITOL_PHOSPHATE_MEDIATED_SIGNALING	0.280140761	0.002138558	2.100775019	0.06749302	0.19836889	-4.6167913
hetero after UVB irradiation VS hetero before UVB irradiation	GO_INOSITOL_PHOSPHATE_MEDIATED_SIGNALING1	0.133170728	-0.00964289	0.739511589	0.48111594	0.76129872	-5.94515289
WT after UVB irradiation VS WT before UVB irradiation	GO_INOSITOL_PHOSPHATE_MEDIATED_SIGNALING2	0.220585526	-0.00980856	1.17011943	0.27523642	0.74692779	-5.33189325
homo after UVB irradiation VS homo before UVB irradiation	GO_INOSITOL_PHOSPHATE_METABOLIC_PROCESS	0.394508424	-0.02235203	2.511148155	0.03520962	0.13457354	-3.99176627
hetero after UVB irradiation VS hetero before UVB irradiation	GO_INOSITOL_PHOSPHATE_METABOLIC_PROCESS1	0.211411877	0.041598805	1.607787958	0.14725633	0.46496154	-5.02678558
WT after UVB irradiation VS WT before UVB irradiation	GO_INOSITOL_PHOSPHATE_METABOLIC_PROCESS2	0.24933885	0.014463953	1.587179477	0.15067885	0.65844472	-4.84904915
homo after UVB irradiation VS homo before UVB irradiation	GO_INOSITOL_PHOSPHATE_PHOSPHATASE_ACTIVITY	0.742025723	-0.02551261	3.979174396	0.00374871	0.0401449	-1.75689555
hetero after UVB irradiation VS hetero before UVB irradiation	GO_INOSITOL_PHOSPHATE_PHOSPHATASE_ACTIVITY1	0.295692361	0.051462413	1.253571272	0.24603132	0.5737102	-5.45748851
WT after UVB irradiation VS WT before UVB irradiation	GO_INOSITOL_PHOSPHATE_PHOSPHATASE_ACTIVITY2	0.438136095	0.012503035	2.140904778	0.06427638	0.53678963	-4.10938193
homo after UVB irradiation VS homo before UVB irradiation	GO_INOSITOL_POLYPHOSPHATE_5_PHOSPHATASE_ACTIVITY	1.198112318	-0.01496828	8.40006833	2.41E-05	0.00847131	3.20626207
hetero after UVB irradiation VS hetero before UVB irradiation	GO_INOSITOL_POLYPHOSPHATE_5_PHOSPHATASE_ACTIVITY1	0.38275008	0.148488294	1.179447909	0.27271651	0.59910746	-5.5391879
WT after UVB irradiation VS WT before UVB irradiation	GO_INOSITOL_POLYPHOSPHATE_5_PHOSPHATASE_ACTIVITY2	0.50543796	0.008228185	1.687984021	0.1294363	0.63548424	-4.72078311
homo after UVB irradiation VS homo before UVB irradiation	GO_INOSITOL_TETRAKISPHOSPHATE_PHOSPHATASE_ACTIVITY	0.856388905	-0.00474194	5.409158351	0.00055731	0.01718452	0.161549964
hetero after UVB irradiation VS hetero before UVB irradiation	GO_INOSITOL_TETRAKISPHOSPHATE_PHOSPHATASE_ACTIVITY1	0.287701718	0.089427446	1.052180424	0.32401415	0.64380615	-5.67128805
WT after UVB irradiation VS WT before UVB irradiation	GO_INOSITOL_TETRAKISPHOSPHATE_PHOSPHATASE_ACTIVITY2	0.611748981	0.043032606	2.123808119	0.06601851	0.537335	-4.13320772
homo after UVB irradiation VS homo before UVB irradiation	GO_INOSITOL_TRISPHOSPHATE_BIOSYNTHETIC_PROCESS	0.785114368	0.010005078	4.033117286	0.00346984	0.03870326	-1.67885892
hetero after UVB irradiation VS hetero before UVB irradiation	GO_INOSITOL_TRISPHOSPHATE_BIOSYNTHETIC_PROCESS1	0.807482816	-0.02990905	4.954158744	0.00117782	0.09106857	-0.45410123
WT after UVB irradiation VS WT before UVB irradiation	GO_INOSITOL_TRISPHOSPHATE_BIOSYNTHETIC_PROCESS2	0.976381648	-0.00347866	6.06539241	0.00028651	0.18842845	0.686343158
homo after UVB irradiation VS homo before UVB irradiation	GO_INOSITOL_TRISPHOSPHATE_KINASE_ACTIVITY	0.576970965	-0.00102947	2.022443207	0.07636196	0.21233308	-4.73296583
hetero after UVB irradiation VS hetero before UVB irradiation	GO_INOSITOL_TRISPHOSPHATE_KINASE_ACTIVITY1	0.006849278	0.082439288	0.024309092	0.98121242	0.99326558	-6.23116366
WT after UVB irradiation VS WT before UVB irradiation	GO_INOSITOL_TRISPHOSPHATE_KINASE_ACTIVITY2	0.088760428	-0.00226302	0.276131892	0.7893633	0.94758271	-5.95603359
homo after UVB irradiation VS homo before UVB irradiation	GO_INOSITOL_TRISPHOSPHATE_METABOLIC_PROCESS	0.677006306	-0.00844365	2.848794473	0.02065607	0.09963465	-3.46784572
hetero after UVB irradiation VS hetero before UVB irradiation	GO_INOSITOL_TRISPHOSPHATE_METABOLIC_PROCESS1	0.601476222	-0.0184069	3.566178097	0.00756129	0.14187436	-2.23265418
WT after UVB irradiation VS WT before UVB irradiation	GO_INOSITOL_TRISPHOSPHATE_METABOLIC_PROCESS2	0.735929426	0.000647633	4.190322541	0.00295575	0.24418663	-1.31267234
homo after UVB irradiation VS homo before UVB irradiation	GO_INOSITOL_TRISPHOSPHATE_PHOSPHATASE_ACTIVITY	1.141602084	-0.0113402	8.270621631	2.71E-05	0.00847131	3.097609459
hetero after UVB irradiation VS hetero before UVB irradiation	GO_INOSITOL_TRISPHOSPHATE_PHOSPHATASE_ACTIVITY1	0.395057943	0.127066672	1.2329724	0.25321893	0.58110396	-5.48052502
WT after UVB irradiation VS WT before UVB irradiation	GO_INOSITOL_TRISPHOSPHATE_PHOSPHATASE_ACTIVITY2	0.631982784	0.041472074	2.546811491	0.03401695	0.46566619	-3.53573033
homo after UVB irradiation VS homo before UVB irradiation	GO_INSEMINATION	0.098811211	-0.01283321	0.565706713	0.58650576	0.73987566	-6.37857666
hetero after UVB irradiation VS hetero before UVB irradiation	GO_INSEMINATION1	0.118184637	0.01226681	0.54224838	0.60269082	0.83402069	-6.07505056
WT after UVB irradiation VS WT before UVB irradiation	GO_INSEMINATION2	0.183881523	0.001705061	0.738804468	0.48088902	0.8504347	-5.71791195
homo after UVB irradiation VS homo before UVB irradiation	GO_INSULIN_BINDING	0.191981074	-0.00014485	0.713906558	0.49480091	0.67007222	-6.27953142
hetero after UVB irradiation VS hetero before UVB irradiation	GO_INSULIN_BINDING1	-0.23725054	-0.05702242	-0.73340334	0.48462895	0.76328154	-5.94970485
WT after UVB irradiation VS WT before UVB irradiation	GO_INSULIN_BINDING2	0.025620368	-0.06406474	0.114709129	0.91146904	0.97857624	-5.98912972
homo after UVB irradiation VS homo before UVB irradiation	GO_INSULIN_LIKE_GROWTH_FACTOR_BINDING	0.543130259	-0.01501941	3.844700111	0.00455362	0.04377704	-1.95318859
hetero after UVB irradiation VS hetero before UVB irradiation	GO_INSULIN_LIKE_GROWTH_FACTOR_BINDING1	0.215135937	-0.00909801	1.013994298	0.34080567	0.65970464	-5.70876178
WT after UVB irradiation VS WT before UVB irradiation	GO_INSULIN_LIKE_GROWTH_FACTOR_BINDING2	0.339526552	0.011915309	1.724436168	0.12246041	0.63133281	-4.67353673
homo after UVB irradiation VS homo before UVB irradiation	GO_INSULIN_LIKE_GROWTH_FACTOR_I_BINDING	0.625025125	-0.06369746	4.1218053	0.0030585	0.03652486	-1.5514614
hetero after UVB irradiation VS hetero before UVB irradiation	GO_INSULIN_LIKE_GROWTH_FACTOR_I_BINDING1	0.047652162	-0.0308907	0.186322223	0.85691529	0.94581809	-6.21270042
WT after UVB irradiation VS WT before UVB irradiation	GO_INSULIN_LIKE_GROWTH_FACTOR_I_BINDING2	0.264374038	-0.00853272	1.338948358	0.21695521	0.71409876	-5.14704729
homo after UVB irradiation VS homo before UVB irradiation	GO_INSULIN_LIKE_GROWTH_FACTOR_II_BINDING	0.505054664	-0.06093533	2.89304848	0.01927124	0.09562395	-3.39911244
hetero after UVB irradiation VS hetero before UVB irradiation	GO_INSULIN_LIKE_GROWTH_FACTOR_II_BINDING1	-0.20647353	-0.00306389	-0.99402958	0.34984734	0.66688378	-5.72793585
WT after UVB irradiation VS WT before UVB irradiation	GO_INSULIN_LIKE_GROWTH_FACTOR_II_BINDING2	-0.0927938	-0.03806285	-0.4050904	0.69590199	0.92243778	-5.9103983
homo after UVB irradiation VS homo before UVB irradiation	GO_INSULIN_LIKE_GROWTH_FACTOR_RECEPTOR_BINDING	0.954554398	-0.05179202	6.509588947	0.00015584	0.01016637	1.422418077
hetero after UVB irradiation VS hetero before UVB irradiation	GO_INSULIN_LIKE_GROWTH_FACTOR_RECEPTOR_BINDING1	0.287210958	0.060511184	1.217821776	0.25861671	0.58521976	-5.49730734
WT after UVB irradiation VS WT before UVB irradiation	GO_INSULIN_LIKE_GROWTH_FACTOR_RECEPTOR_BINDING2	0.329107179	-0.01569205	1.168685542	0.27578195	0.74751947	-5.33339175
homo after UVB irradiation VS homo before UVB irradiation	GO_INSULIN_LIKE_GROWTH_FACTOR_RECEPTOR_SIGNALING_PATHWAY	0.245700799	-0.03613672	1.841642519	0.10131586	0.25242582	-4.99527403
hetero after UVB irradiation VS hetero before UVB irradiation	GO_INSULIN_LIKE_GROWTH_FACTOR_RECEPTOR_SIGNALING_PATHWAY1	-0.02083936	-0.00565681	-0.11398911	0.91210687	0.96720392	-6.22444366
WT after UVB irradiation VS WT before UVB irradiation	GO_INSULIN_LIKE_GROWTH_FACTOR_RECEPTOR_SIGNALING_PATHWAY2	-0.07370291	0.019739713	-0.40222454	0.69792717	0.92319802	-5.91159356
homo after UVB irradiation VS homo before UVB irradiation	GO_INSULIN_METABOLIC_PROCESS	0.18926585	0.046801608	0.935023044	0.376088	0.56585784	-6.09505759
hetero after UVB irradiation VS hetero before UVB irradiation	GO_INSULIN_METABOLIC_PROCESS1	0.634535336	-0.00396235	3.015757503	0.01702995	0.19095279	-3.016632
WT after UVB irradiation VS WT before UVB irradiation	GO_INSULIN_METABOLIC_PROCESS2	0.014787076	0.080983458	0.08234379	0.9363724	0.9832831	-5.99249052
homo after UVB irradiation VS homo before UVB irradiation	GO_INSULIN_RECEPTOR_BINDING	0.297663766	-0.06726257	1.871399065	0.09673403	0.24603489	-4.95273656
hetero after UVB irradiation VS hetero before UVB irradiation	GO_INSULIN_RECEPTOR_BINDING1	0.372150994	-6.37E-05	2.366812815	0.04604175	0.28690545	-3.96618322
WT after UVB irradiation VS WT before UVB irradiation	GO_INSULIN_RECEPTOR_BINDING2	0.042049259	-0.07168792	0.179308585	0.86210088	0.9676681	-5.9791375
homo after UVB irradiation VS homo before UVB irradiation	GO_INSULIN_RECEPTOR_SIGNALING_PATHWAY	0.145231165	-0.01826202	0.905301539	0.39073955	0.57998745	-6.12225078
hetero after UVB irradiation VS hetero before UVB irradiation	GO_INSULIN_RECEPTOR_SIGNALING_PATHWAY1	0.002459115	-0.00634872	0.01900147	0.98531385	0.99454759	-6.23128831
WT after UVB irradiation VS WT before UVB irradiation	GO_INSULIN_RECEPTOR_SIGNALING_PATHWAY2	-0.00663123	-0.0175951	-0.04554199	0.9647784	0.99087638	-5.99497243
homo after UVB irradiation VS homo before UVB irradiation	GO_INSULIN_RECEPTOR_SUBSTRATE_BINDING	0.136442652	-0.0120291	0.516047063	0.6192382	0.76342593	-6.40703671
hetero after UVB irradiation VS hetero before UVB irradiation	GO_INSULIN_RECEPTOR_SUBSTRATE_BINDING1	0.141934966	0.032134225	0.55225489	0.5961372	0.83041636	-6.06933758
WT after UVB irradiation VS WT before UVB irradiation	GO_INSULIN_RECEPTOR_SUBSTRATE_BINDING2	0.125175727	-0.05015369	0.539122055	0.6043047	0.89591658	-5.8455548
homo after UVB irradiation VS homo before UVB irradiation	GO_INSULIN_RESPONSIVE_COMPARTMENT	0.570141653	-0.08406329	3.768569454	0.00508944	0.04644702	-2.06539042
hetero after UVB irradiation VS hetero before UVB irradiation	GO_INSULIN_RESPONSIVE_COMPARTMENT1	0.529373073	0.038068984	2.536639214	0.0354075	0.25868552	-3.71765829

WT after UVB irradiation VS WT before UVB irradiation	GO_INSULIN_RESPONSIVE_COMPARTMENT2	0.444017324	-0.03983983	1.729360152	0.12154534	0.63058669	-4.66712188
homo after UVB irradiation VS homo before UVB irradiation	GO_INSULIN_SECRETION	0.098707724	0.001929114	0.807195987	0.44199179	0.62475794	-6.20682693
homo after UVB irradiation VS homo before UVB irradiation	GO_INSULIN_SECRETION_INVOLVED_IN_CELLULAR_RESPONSE_TO_GLUCOSE_STIMULUS	0.204717359	-0.03314609	1.427817537	0.1897436	0.37541612	-5.55236398
hetero after UVB irradiation VS hetero before UVB irradiation	GO_INSULIN_SECRETION_INVOLVED_IN_CELLULAR_RESPONSE_TO_GLUCOSE_STIMULUS1	0.085906972	0.009654191	0.641175545	0.53966639	0.79624851	-6.01439126
WT after UVB irradiation VS WT before UVB irradiation	GO_INSULIN_SECRETION_INVOLVED_IN_CELLULAR_RESPONSE_TO_GLUCOSE_STIMULUS2	0.056822411	-0.0097006	0.357161646	0.73010006	0.93327298	-5.92931371
hetero after UVB irradiation VS hetero before UVB irradiation	GO_INSULIN_SECRETION1	0.005690449	0.017465203	0.039514913	0.96946599	0.98899869	-6.23063742
WT after UVB irradiation VS WT before UVB irradiation	GO_INSULIN_SECRETION2	-0.05480938	-0.00763089	-0.33144551	0.74872304	0.93642732	-5.93851433
homo after UVB irradiation VS homo before UVB irradiation	GO_INTEGRAL_COMPONENT_OF_CYTOPLASMIC_SIDE_OF_ENDOPLASMIC_RETICULUM_MEMBRANE	0.205897151	0.032348764	0.647466103	0.53476468	0.70128165	-6.3264987
hetero after UVB irradiation VS hetero before UVB irradiation	GO_INTEGRAL_COMPONENT_OF_CYTOPLASMIC_SIDE_OF_ENDOPLASMIC_RETICULUM_MEMBRANE1	0.555017707	-0.02578414	1.922004832	0.09151941	0.37872731	-4.60312958
WT after UVB irradiation VS WT before UVB irradiation	GO_INTEGRAL_COMPONENT_OF_CYTOPLASMIC_SIDE_OF_ENDOPLASMIC_RETICULUM_MEMBRANE2	0.276055595	-0.09259104	0.975476467	0.35756054	0.79138181	-5.52300738
homo after UVB irradiation VS homo before UVB irradiation	GO_INTEGRAL_COMPONENT_OF_PRESYNAPTIC_ACTIVE_ZONE_MEMBRANE	0.191841996	-0.04525977	0.969981488	0.35937556	0.5509355	-6.06217094
hetero after UVB irradiation VS hetero before UVB irradiation	GO_INTEGRAL_COMPONENT_OF_PRESYNAPTIC_ACTIVE_ZONE_MEMBRANE1	0.301248655	-0.05363351	1.492172249	0.17469577	0.49736582	-5.17387666
WT after UVB irradiation VS WT before UVB irradiation	GO_INTEGRAL_COMPONENT_OF_PRESYNAPTIC_ACTIVE_ZONE_MEMBRANE2	0.189290697	-0.01940476	1.112901276	0.29769396	0.75950789	-5.39068761
homo after UVB irradiation VS homo before UVB irradiation	GO_INTEGRAL_COMPONENT_OF_SYNAPTIC_VESICLE_MEMBRANE	-0.11296826	-0.00266024	-0.69660593	0.505022	0.67862043	-6.29215431
hetero after UVB irradiation VS hetero before UVB irradiation	GO_INTEGRAL_COMPONENT_OF_SYNAPTIC_VESICLE_MEMBRANE1	-0.31785663	0.002497286	-1.87483474	0.09837101	0.39052906	-4.66856158
WT after UVB irradiation VS WT before UVB irradiation	GO_INTEGRAL_COMPONENT_OF_SYNAPTIC_VESICLE_MEMBRANE2	-0.23976248	-0.01630955	-1.27379201	0.23807959	0.72863458	-5.22029363
homo after UVB irradiation VS homo before UVB irradiation	GO_INTEGRATOR_COMPLEX	-0.61753815	0.026099163	-3.35894471	0.00938401	0.06441548	-2.68100538
hetero after UVB irradiation VS hetero before UVB irradiation	GO_INTEGRATOR_COMPLEX1	-0.20593749	-0.06987856	-0.8033323	0.4454157	0.73977264	-5.8956637
WT after UVB irradiation VS WT before UVB irradiation	GO_INTEGRATOR_COMPLEX2	-0.44707987	-0.01931841	-1.54373891	0.16077587	0.66503175	-4.90314591
homo after UVB irradiation VS homo before UVB irradiation	GO_INTEGRIN_ACTIVATION	0.523073357	-0.00606795	3.696324002	0.00566028	0.04978246	-2.17255335
hetero after UVB irradiation VS hetero before UVB irradiation	GO_INTEGRIN_ACTIVATION1	0.419052086	0.025430415	2.822738391	0.02282098	0.21591374	-3.29808317
WT after UVB irradiation VS WT before UVB irradiation	GO_INTEGRIN_ACTIVATION2	0.425641562	-0.02562303	1.999498224	0.08014796	0.56457304	-4.30506174
homo after UVB irradiation VS homo before UVB irradiation	GO_INTEGRIN_BINDING	0.585137018	-0.0248167	4.4798086	0.00185935	0.02922084	-1.04908539
hetero after UVB irradiation VS hetero before UVB irradiation	GO_INTEGRIN_BINDING1	0.424797776	0.02599528	2.258542388	0.05444773	0.30913068	-4.12364999
WT after UVB irradiation VS WT before UVB irradiation	GO_INTEGRIN_BINDING2	0.361192451	0.019110359	1.611900247	0.14519318	0.65365874	-4.81793643
homo after UVB irradiation VS homo before UVB irradiation	GO_INTEGRIN_BIOSYNTHETIC_PROCESS	0.242685848	-0.04593489	1.339519648	0.21579863	0.40619137	-5.66089011
hetero after UVB irradiation VS hetero before UVB irradiation	GO_INTEGRIN_BIOSYNTHETIC_PROCESS1	-0.06414271	-0.02725155	-0.28018899	0.78657182	0.91694216	-6.18912914
WT after UVB irradiation VS WT before UVB irradiation	GO_INTEGRIN_BIOSYNTHETIC_PROCESS2	-0.00787453	-0.06066712	-0.02128154	0.98353613	0.99634469	-5.9958277
homo after UVB irradiation VS homo before UVB irradiation	GO_INTEGRIN_MEDIATED_SIGNALING_PATHWAY	0.671676493	-0.03466411	5.052850309	0.00087167	0.02025696	-0.28644193
hetero after UVB irradiation VS hetero before UVB irradiation	GO_INTEGRIN_MEDIATED_SIGNALING_PATHWAY1	0.499390789	0.031047726	2.717467988	0.02680717	0.23196771	-3.45232689
WT after UVB irradiation VS WT before UVB irradiation	GO_INTEGRIN_MEDIATED_SIGNALING_PATHWAY2	0.43252566	0.019283146	2.132149027	0.06516288	0.53678963	-4.12158902
homo after UVB irradiation VS homo before UVB irradiation	GO_INTERACTION_WITH_HOST	0.240063971	0.003324789	1.59783092	0.14725439	0.320162	-5.33222056
hetero after UVB irradiation VS hetero before UVB irradiation	GO_INTERACTION_WITH_HOST1	0.333674531	0.010785669	2.630983987	0.03061574	0.24470723	-3.57922489
WT after UVB irradiation VS WT before UVB irradiation	GO_INTERACTION_WITH_HOST2	0.294806945	-0.0354969	1.962007575	0.08495678	0.57516172	-4.35634238
homo after UVB irradiation VS homo before UVB irradiation	GO_INTERACTION_WITH_SYMBIONT	0.052006921	0.012204067	0.357310462	0.72974395	0.84061989	-6.48133055
hetero after UVB irradiation VS hetero before UVB irradiation	GO_INTERACTION_WITH_SYMBIONT1	0.208735581	0.021640157	1.621801915	0.14421172	0.46030459	-5.00858874
WT after UVB irradiation VS WT before UVB irradiation	GO_INTERACTION_WITH_SYMBIONT2	0.134067237	-0.02495545	0.764314836	0.4663655	0.84342918	-5.69907848
homo after UVB irradiation VS homo before UVB irradiation	GO_INTERCALATED_DISC	0.493388615	-0.02752457	3.64245329	0.00613004	0.05181046	-2.2528806
hetero after UVB irradiation VS hetero before UVB irradiation	GO_INTERCALATED_DISC1	0.161179177	-0.01408645	1.072061221	0.31553118	0.63580201	-5.65137102
WT after UVB irradiation VS WT before UVB irradiation	GO_INTERCALATED_DISC2	0.178152596	0.008191529	0.848177998	0.42070158	0.82383319	-5.63336006
homo after UVB irradiation VS homo before UVB irradiation	GO_INTERCELLULAR_BRIDGE	-0.19126812	-0.01119698	-1.38679281	0.2014891	0.39036034	-5.60331814
hetero after UVB irradiation VS hetero before UVB irradiation	GO_INTERCELLULAR_BRIDGE1	0.025349841	-0.00662619	0.151022824	0.8837651	0.95769271	-6.21913381
WT after UVB irradiation VS WT before UVB irradiation	GO_INTERCELLULAR_BRIDGE2	-0.01765618	0.011019907	-0.09720868	0.92492367	0.98118223	-5.9910837
homo after UVB irradiation VS homo before UVB irradiation	GO_INTERCELLULAR_CANALICULUS	-0.18197063	-0.06821073	-0.97992041	0.35472659	0.54644568	-6.05264603
hetero after UVB irradiation VS hetero before UVB irradiation	GO_INTERCELLULAR_CANALICULUS1	-0.71924306	-0.08955146	-2.84625095	0.02201763	0.21402646	-3.26368783
WT after UVB irradiation VS WT before UVB irradiation	GO_INTERCELLULAR_CANALICULUS2	-0.305157	-0.07360212	-1.15682094	0.28032965	0.75145468	-5.34574216
homo after UVB irradiation VS homo before UVB irradiation	GO_INTERCELLULAR_TRANSPORT	-0.34180758	-0.02355534	-1.19647207	0.2644564	0.4596003	-5.8270611
hetero after UVB irradiation VS hetero before UVB irradiation	GO_INTERCELLULAR_TRANSPORT1	0.130837431	0.106113132	0.494180698	0.63470432	0.84991504	-6.10113863
WT after UVB irradiation VS WT before UVB irradiation	GO_INTERCELLULAR_TRANSPORT2	-0.60790431	0.085289441	-2.70543178	0.0265532	0.43935696	-3.31003449
homo after UVB irradiation VS homo before UVB irradiation	GO_INTERCHROMATIN_GRANULE	-0.34175676	0.075030098	-1.32598042	0.22005397	0.4109512	-5.67714575
hetero after UVB irradiation VS hetero before UVB irradiation	GO_INTERCHROMATIN_GRANULE1	-0.03482173	-0.05668395	-0.10334815	0.92027722	0.96983444	-6.22569589
WT after UVB irradiation VS WT before UVB irradiation	GO_INTERCHROMATIN_GRANULE2	-0.18119528	-0.09575801	-0.74964405	0.47468213	0.84774103	-5.70997672
homo after UVB irradiation VS homo before UVB irradiation	GO_INTERFERON_ALPHA_PRODUCTION	0.665382358	-0.02284298	4.396048283	0.00208546	0.03080331	-1.16487728
hetero after UVB irradiation VS hetero before UVB irradiation	GO_INTERFERON_ALPHA_PRODUCTION1	0.649357177	0.049313152	3.459569255	0.00882268	0.14994695	-2.38173028
WT after UVB irradiation VS WT before UVB irradiation	GO_INTERFERON_ALPHA_PRODUCTION2	0.536499832	-0.03075777	3.241610523	0.01165696	0.34267286	-2.55678472
homo after UVB irradiation VS homo before UVB irradiation	GO_INTERFERON_BETA_PRODUCTION	0.388068776	0.012679956	2.269133325	0.0517012	0.1680887	-4.36310824
hetero after UVB irradiation VS hetero before UVB irradiation	GO_INTERFERON_BETA_PRODUCTION1	0.603764765	0.048486529	3.571075492	0.00750817	0.14177754	-2.22584271
WT after UVB irradiation VS WT before UVB irradiation	GO_INTERFERON_BETA_PRODUCTION2	0.426810578	-0.00993766	2.727837793	0.02564299	0.43396053	-3.27818948
homo after UVB irradiation VS homo before UVB irradiation	GO_INTERFERON_GAMMA_MEDIATED_SIGNALING_PATHWAY	0.551046039	-0.01755839	3.173047909	0.01247178	0.07521295	-2.96588382
hetero after UVB irradiation VS hetero before UVB irradiation	GO_INTERFERON_GAMMA_MEDIATED_SIGNALING_PATHWAY1	0.421543773	0.043466817	1.923062131	0.09137121	0.37870619	-4.60165676
WT after UVB irradiation VS WT before UVB irradiation	GO_INTERFERON_GAMMA_MEDIATED_SIGNALING_PATHWAY2	0.410309646	0.030369685	1.723572948	0.12262148	0.63133281	-4.67466052
homo after UVB irradiation VS homo before UVB irradiation	GO_INTERFERON_GAMMA_PRODUCTION	0.58191974	-0.01773623	4.004042105	0.00361728	0.03927904	-1.7208696
hetero after UVB irradiation VS hetero before UVB irradiation	GO_INTERFERON_GAMMA_PRODUCTION1	0.433916661	0.037872227	2.173065085	0.06215151	0.32753845	-4.24711273
WT after UVB irradiation VS WT before UVB irradiation	GO_INTERFERON_GAMMA_PRODUCTION2	0.372048267	0.018539798	1.719896016	0.12330982	0.63133281	-4.67944473
homo after UVB irradiation VS homo before UVB irradiation	GO_INTERFERON_RECEPTOR_ACTIVITY	0.640609803	-0.06202872	2.610953396	0.03005801	0.1231444	-3.83724792
hetero after UVB irradiation VS hetero before UVB irradiation	GO_INTERFERON_RECEPTOR_ACTIVITY1	0.805506793	-0.03602001	3.321751494	0.010794	0.16309248	-2.57659734
WT after UVB irradiation VS WT before UVB irradiation	GO_INTERFERON_RECEPTOR_ACTIVITY2	0.538424103	-0.11179222	2.047051219	0.07442604	0.55789253	-4.23963087
homo after UVB irradiation VS homo before UVB irradiation	GO_INTERKINETIC_NUCLEAR_MIGRATION	0.034026708	0.012693216	0.114107678	0.91185517	0.9516556	-6.54371645
hetero after UVB irradiation VS hetero before UVB irradiation	GO_INTERKINETIC_NUCLEAR_MIGRATION1	-0.30768737	-0.0505211	-1.29583	0.23182247	0.5605067	-5.40945847
WT after UVB irradiation VS WT before UVB irradiation	GO_INTERKINETIC_NUCLEAR_MIGRATION2	-0.33751307	-0.00653647	-1.2779159	0.2366928	0.72828932	-5.21572624
homo after UVB irradiation VS homo before UVB irradiation	GO_INTERLEUKIN_1_ALPHA_PRODUCTION	0.438101033	-0.06453804	1.78447645	0.11069783	0.26661789	-5.07620452
hetero after UVB irradiation VS hetero before UVB irradiation	GO_INTERLEUKIN_1_ALPHA_PRODUCTION1	0.536702671	0.036825447	2.311548844	0.05015604	0.29806785	-4.04668691
WT after UVB irradiation VS WT before UVB irradiation	GO_INTERLEUKIN_1_ALPHA_PRODUCTION2	0.399964625	0.066112537	1.326807772	0.22076548	0.71553618	-5.16086759
homo after UVB irradiation VS homo before UVB irradiation	GO_INTERLEUKIN_1_BETA_PRODUCTION	0.559872642	-0.01737812	3.580056108	0.00672635	0.0540636	-2.34635304
hetero after UVB irradiation VS hetero before UVB irradiation	GO_INTERLEUKIN_1_BETA_PRODUCTION1	0.568791472	0.057322803	3.565675948	0.00756676	0.14187436	-2.23335277
WT after UVB irradiation VS WT before UVB irradiation	GO_INTERLEUKIN_1_BETA_PRODUCTION2	0.344184241	-0.020243	1.305700362	0.2275261	0.71944822	-5.18470956
homo after UVB irradiation VS homo before UVB irradiation	GO_INTERLEUKIN_1_BINDING	0.356682251	0.00699841	0.990874046	0.34965539	0.54160434	-6.0420601
hetero after UVB irradiation VS hetero before UVB irradiation	GO_INTERLEUKIN_1_BINDING1	0.437268858	-0.01889105	1.695629542	0.12910774	0.4368396	-4.91152755
WT after UVB irradiation VS WT before UVB irradiation	GO_INTERLEUKIN_1_BINDING2	-0.01765657	0.045716916	-0.07605651	0.94121968	0.98458464	-5.99301558
homo after UVB irradiation VS homo before UVB irradiation	GO_INTERLEUKIN_1_MEDIATED_SIGNALING_PATHWAY	0.063305164	-0.01596248	0.264912322	0.79751515	0.88534547	-6.51250585
hetero after UVB irradiation VS hetero before UVB irradiation	GO_INTERLEUKIN_1_MEDIATED_SIGNALING_PATHWAY1	0.263287614	0.006110627	1.580441396	0.15336746	0.47184575	-5.06207443
WT after UVB irradiation VS WT before UVB irradiation	GO_INTERLEUKIN_1_MEDIATED_SIGNALING_PATHWAY2	0.088574389	-0.01264445	0.426827755	0.68062637	0.91716791	-5.90106827
homo after UVB irradiation VS homo before UVB irradiation	GO_INTERLEUKIN_1_PRODUCTION	0.545875847	-0.02332403	3.589288963	0.00663438	0.05379154	-2.33249352
hetero after UVB irradiation VS hetero before UVB irradiation	GO_INTERLEUKIN_1_PRODUCTION1	0.551914136	0.053969575	3.558201559	0.00764868	0.14237942	-2.24375527
WT after UVB irradiation VS WT before UVB irradiation	GO_INTERLEUKIN_1_PRODUCTION2	0.317420502	-0.01648575	1.245243143	0.2478685	0.73564768	-5.25165042
homo after UVB irradiation VS homo before UVB irradiation	GO_INTERLEUKIN_1_RECEPTOR_ACTIVITY	0.422512496	-0.02102619	1.709923883	0.12415615	0.28735451	-5.18006493
hetero after UVB irradiation VS hetero before UVB irradiation	GO_INTERLEUKIN_1_RECEPTOR_ACTIVITY1	0.237646808	0.011875274	0.655290258	0.53100452	0.79162851	-6.00499062
WT after UVB irradiation VS WT before UVB irradiation	GO_INTERLEUKIN_1_RECEPTOR_ACTIVITY2	-0.05009495	0.078958716	-0.17431179	0.86589966	0.96790654	-5.98006604
homo after UVB irradiation VS homo before UVB irradiation	GO_INTERLEUKIN_1_RECEPTOR_BINDING	0.904576455	-0.01673989	6.389672365	0.00017774	0.01066218	1.293654208
hetero after UVB irradiation VS hetero before UVB irradiation	GO_INTERLEUKIN_1_RECEPTOR_BINDING1	0.866222555	0.058779593	5.988113021	0.00035242	0.07788488	0.660726678
WT after UVB irradiation VS WT before UVB irradiation	GO_INTERLEUKIN_1_RECEPTOR_BINDING2	0.744122574	0.03296515	5.268657223	0.00072726	0.18842845	-0.08743484
homo after UVB irradiation VS homo before UVB irradiation	GO_INTERLEUKIN_10_PRODUCTION	0.532350576	-0.01689567	3.504431207	0.00753248	0.05756439	-2.46023402
hetero after UVB irradiation VS hetero before UVB irradiation	GO_INTERLEUKIN_10_PRODUCTION1	0.427973785	0.055918846	2.323345867	0.04924788	0.29581029	-4.02952279
WT after UVB irradiation VS WT before UVB irradiation	GO_INTERLEUKIN_10_PRODUCTION2	0.380139199	0.01629393	1.548751439	0.15958041	0.66317523	-4.89694232
homo after UVB irradiation VS homo before UVB irradiation	GO_INTERLEUKIN_12_PRODUCTION	0.586718457	0.004497227	3.469075767	0.00794382	0.05921693	-2.5136875
hetero after UVB irradiation VS hetero before UVB irradiation	GO_INTERLEUKIN_12_PRODUCTION1	0.582678909	0.045683583	2.689100634	0.0279996	0.23585808	-3.49394038
WT after UVB irradiation VS WT before UVB irradiation	GO_INTERLEUKIN_12_PRODUCTION2	0.410900929	-0.01784361	1.449015961	0.18492622	0.68726847	-5.01837084
homo after UVB irradiation VS homo before UVB irradiation	GO_INTERLEUKIN_12_RECEPTOR_BINDING	0.419030725	-0.08485236	2.200553061	0.05763923	0.17933421	-4.46702625
hetero after UVB irradiation VS hetero before UVB irradiation	GO_INTERLEUKIN_12_RECEPTOR_BINDING1	0.313696834	-0.05176408	0.787922917	0.45386654	0.74508172	-5.90793169
WT after UVB irradiation VS WT before UVB irradiation	GO_INTERLEUKIN_12_RECEPTOR_BINDING2	0.299925476	-0.06788504	0.808814956	0.44173513	0.83231427	-5.66492267
homo after UVB irradiation VS homo before UVB irradiation	GO_INTERLEUKIN_13_PRODUCTION	0.275795627	0.009597206	1.510294729	0.16793294	0.34669648	-5.44729452
hetero after UVB irradiation VS hetero before UVB irradiation	GO_INTERLEUKIN_13_PRODUCTION1	0.1487025	0.006384168	0.691282474	0.5093044	0.77886783	-5.9801967
WT after UVB irradiation VS WT before UVB irradiation	GO_INTERLEUKIN_13_PRODUCTION2	0.396411245	0.06098374	2.50525246	0.03630437	0.47615783	-3.59486242
homo after UVB irradiation VS homo before UVB irradiation	GO_INTERLEUKIN_17_PRODUCTION	0.547678689	-0.04817937	3.265426338	0.01082246	0.06966773	-2.82396793
hetero after UVB irradiation VS hetero before UVB irradiation	GO_INTERLEUKIN_17_PRODUCTION1	0.35933016	0.045681887	1.578872169	0.15372505	0.47184575	-5.06409041
WT after UVB irradiation VS WT before UVB irradiation	GO_INTERLEUKIN_17_PRODUCTION2	0.340033349	0.022055524	1.406373623	0.19680866	0.69565208	-5.0689239
homo after UVB irradiation VS homo before UVB irradiation	GO_INTERLEUKIN_17_RECEPTOR_ACTIVITY	0.659139743	-0.04138408	4.752432611	0.00128897	0.024231	-0.67978604
hetero after UVB irradiation VS hetero before UVB irradiation	GO_INTERLEUKIN_17_RECEPTOR_ACTIVITY1	0.206369458	0.00681376	0.848245877	0.42140299	0.72363813	-5.85877392
WT after UVB irradiation VS WT before UVB irradiation	GO_INTERLEUKIN_17_RECEPTOR_ACTIVITY2	0.393979364	-0.00207001	1.814684966	0.10666953	0.61130963	-4.55480305
homo after UVB irradiation VS homo before UVB irradiation	GO_INTERLEUKIN_18_MEDIATED_SIGNALING_PATHWAY	1.094400606	-0.02157035	9.087980462	1.32E-05	0.00847131	3.754441216
hetero after UVB irradiation VS hetero before UVB irradiation	GO_INTERLEUKIN_18_MEDIATED_SIGNALING_PATHWAY1	1.017729785	0.078358807	5.218540376	0.00085324	0.08366654	-0.15217506
WT after UVB irradiation VS WT before UVB irradiation	GO_INTERLEUKIN_18_MEDIATED_SIGNALING_PATHWAY2	0.965545955	0.109872532	4.099626975	0.00335166	0.25923179	-1.42525851
homo after UVB irradiation VS homo before UVB irradiation	GO_INTERLEUKIN_18_PRODUCTION	0.604305935	-0.00353992	2.553301313	0.03293282	0.12933702	-3.92657027
hetero after UVB irradiation VS hetero before UVB irradiation	GO_INTERLEUKIN_18_PRODUCTION1	0.750666334	0.067714026	5.091063666	0.0009955	0.08692098	-0.29627322
WT after UVB irradiation VS WT before UVB irradiation	GO_INTERLEUKIN_18_PRODUCTION2	0.279935573	-0.04954868	1.113930245	0.29727751	0.7587209	-5.38964875
homo after UVB irradiation VS homo before UVB irradiation	GO_INTERLEUKIN_2_PRODUCTION	0.252123237	-0.02729006	1.409252873	0.19498276	0.38232269	-5.57553344
hetero after UVB irradiation VS hetero before UVB irradiation	GO_INTERLEUKIN_2_PRODUCTION1	0.220127755	0.03237764	1.376699055	0.20657727	0.53105475	-5.31479476
WT after UVB irradiation VS WT before UVB irradiation	GO_INTERLEUKIN_2_PRODUCTION2	0.265507207	-0.01034734	1.308449991	0.22663554	0.71906685	-5.18161721
homo after UVB irradiation VS homo before UVB irradiation	GO_INTERLEUKIN_2_RECEPTOR_BINDING	-0.48153972	0.030767148	-2.20103212	0.05759551	0.17923595	-4.46630292
hetero after UVB irradiation VS hetero before UVB irradiation	GO_INTERLEUKIN_2_RECEPTOR_BINDING1	-0.74154168	0.051776603	-3.60724057	0.00712798	0.13963559	-2.17564593
WT after UVB irradiation VS WT before UVB irradiation	GO_INTERLEUKIN_2_RECEPTOR_BINDING2	-0.12783477	0.005089415	-0.74012732	0.48012872	0.85038327	-5.71694889
homo after UVB irradiation VS homo before UVB irradiation	GO_INTERLEUKIN_21_MEDIATED_SIGNALING_PATHWAY	1.035248441	-0.02645102	6.782153459	0.00011632	0.01007802	1.707645513
hetero after UVB irradiation VS hetero before UVB irradiation	GO_INTERLEUKIN_21_MEDIATED_SIGNALING_PATHWAY1	0.94570067	0.146658713	5.158587416	0.00091716	0.08366654	-0.21960294
WT after UVB irradiation VS WT before UVB irradiation	GO_INTERLEUKIN_21_MEDIATED_SIGNALING_PATHWAY2	0.883630449	0.020941431	3.464588498	0.00835224	0.31908946	-2.25216114
homo after UVB irradiation VS homo before UVB irradiation	GO_INTERLEUKIN_21_PRODUCTION	0.374834327	0.003893307	0.868539834	0.40942539	0.59617782	-6.15488814
hetero after UVB irradiation VS hetero before UVB irradiation	GO_INTERLEUKIN_21_PRODUCTION1	-0.09361737	0.045752852	-0.25687712	0.80387578	0.92374885	-6.19585461
WT after UVB irradiation VS WT before UVB irradiation	GO_INTERLEUKIN_21_PRODUCTION2	-0.13348262	-0.07285771	-0.37688221	0.71594584	0.93033952	-5.92180822
homo after UVB irradiation VS homo before UVB irradiation	GO_INTERLEUKIN_23_MEDIATED_SIGNALING_PATHWAY	0.599784119	-0.01241319	2.942784848	0.01782861	0.09163941	-3.32191741
hetero after UVB irradiation VS hetero before UVB irradiation	GO_INTERLEUKIN_23_MEDIATED_SIGNALING_PATHWAY1	0.639761429	-0.01456431	2.200517999	0.05956618	0.32149178	-4.2075571
WT after UVB irradiation VS WT before UVB irradiation	GO_INTERLEUKIN_23_MEDIATED_SIGNALING_PATHWAY2	0.623831511	-0.03067793	2.119175944	0.06649844	0.53809776	-4.13965593
homo after UVB irradiation VS homo before UVB irradiation	GO_INTERLEUKIN_23_PRODUCTION	0.549443033	0.064508595	2.052673436	0.07281288	0.20697852	-4.68829108
hetero after UVB irradiation VS hetero before UVB irradiation	GO_INTERLEUKIN_23_PRODUCTION1	0.696355993	0.039582568	2.764861444	0.02493036	0.22571948	-3.38284242
WT after UVB irradiation VS WT before UVB irradiation	GO_INTERLEUKIN_23_PRODUCTION2	0.363245289	-0.01641541	1.161764385	0.27842749	0.74920864	-5.34060693
homo after UVB irradiation VS homo before UVB irradiation	GO_INTERLEUKIN_27_MEDIATED_SIGNALING_PATHWAY	0.977835363	0.023590607	7.003467088	9.23E-05	0.00992256	1.93183362
hetero after UVB irradiation VS hetero before UVB irradiation	GO_INTERLEUKIN_27_MEDIATED_SIGNALING_PATHWAY1	0.763177037	0.064219089	3.27709102	0.0115289	0.16744133	-2.64022792
WT after UVB irradiation VS WT before UVB irradiation	GO_INTERLEUKIN_27_MEDIATED_SIGNALING_PATHWAY2	0.742728463	-0.02679981	2.886448261	0.02005257	0.39829087	-3.05337003
homo after UVB irradiation VS homo before UVB irradiation	GO_INTERLEUKIN_35_MEDIATED_SIGNALING_PATHWAY	0.766800248	0.054921811	4.422195122	0.00201185	0.03035667	-1.1286151
hetero after UVB irradiation VS hetero before UVB irradiation	GO_INTERLEUKIN_35_MEDIATED_SIGNALING_PATHWAY1	0.634569848	0.071173752	2.364906656	0.04617785	0.28703971	-3.96896384
WT after UVB irradiation VS WT before UVB irradiation	GO_INTERLEUKIN_35_MEDIATED_SIGNALING_PATHWAY2	0.433252912	-0.06701437	1.396637665	0.19961346	0.69758436	-5.08034435
homo after UVB irradiation VS homo before UVB irradiation	GO_INTERLEUKIN_4_MEDIATED_SIGNALING_PATHWAY	0.78666198	-0.0142847	4.287963019	0.00242201	0.03291121	-1.31588734
hetero after UVB irradiation VS hetero before UVB irradiation	GO_INTERLEUKIN_4_MEDIATED_SIGNALING_PATHWAY1	0.845965184	0.0302648	4.732001607	0.00155625	0.09569098	-0.71703114
WT after UVB irradiation VS WT before UVB irradiation	GO_INTERLEUKIN_4_MEDIATED_SIGNALING_PATHWAY2	0.667788676	-0.02025707	2.410454179	0.04212154	0.495137	-3.72957857
homo after UVB irradiation VS homo before UVB irradiation	GO_INTERLEUKIN_4_PRODUCTION	0.133692141	0.011792721	0.798809143	0.44657827	0.62871165	-6.21367741
hetero after UVB irradiation VS hetero before UVB irradiation	GO_INTERLEUKIN_4_PRODUCTION1	0.008303831	-0.03456779	0.045083053	0.96516645	0.98745294	-6.23038203
WT after UVB irradiation VS WT before UVB irradiation	GO_INTERLEUKIN_4_PRODUCTION2	-0.00769262	0.055351972	-0.03217681	0.97511002	0.9934792	-5.9955204
homo after UVB irradiation VS homo before UVB irradiation	GO_INTERLEUKIN_5_PRODUCTION	0.249481978	0.015747998	1.196409238	0.26447962	0.4596003	-5.82713128
hetero after UVB irradiation VS hetero before UVB irradiation	GO_INTERLEUKIN_5_PRODUCTION1	0.246457733	0.027291352	1.167917801	0.27707447	0.60400502	-5.55159261
WT after UVB irradiation VS WT before UVB irradiation	GO_INTERLEUKIN_5_PRODUCTION2	0.458096644	0.050370062	2.58648777	0.03196966	0.45445578	-3.47926078
homo after UVB irradiation VS homo before UVB irradiation	GO_INTERLEUKIN_6_MEDIATED_SIGNALING_PATHWAY	0.265921464	-0.00561757	1.445621246	0.18483522	0.36904523	-5.52997457
hetero after UVB irradiation VS hetero before UVB irradiation	GO_INTERLEUKIN_6_MEDIATED_SIGNALING_PATHWAY1	0.302195091	0.050547167	2.167050234	0.06273261	0.32880724	-4.25576588
WT after UVB irradiation VS WT before UVB irradiation	GO_INTERLEUKIN_6_MEDIATED_SIGNALING_PATHWAY2	0.354717882	-0.00697243	2.341713813	0.04691831	0.50583727	-3.82700969
homo after UVB irradiation VS homo before UVB irradiation	GO_INTERLEUKIN_6_PRODUCTION	0.645791683	-0.01115132	4.423311507	0.00200877	0.03035667	-1.12706917

hetero after UVB irradiation VS hetero before UVB irradiation	GO_INTERLEUKIN_6_PRODUCTION1	0.624288982	0.050326239	3.679698823	0.00642669	0.13614289	-2.07563019
WT after UVB irradiation VS WT before UVB irradiation	GO_INTERLEUKIN_6_PRODUCTION2	0.50979962	0.032629734	2.654726404	0.02873729	0.44654821	-3.38214874
homo after UVB irradiation VS homo before UVB irradiation	GO_INTERLEUKIN_6_RECEPTOR_BINDING	0.769487555	0.014531982	4.933949314	0.00101605	0.02163608	-0.44040907
hetero after UVB irradiation VS hetero before UVB irradiation	GO_INTERLEUKIN_6_RECEPTOR_BINDING1	0.259871699	0.023969561	0.959747339	0.36579689	0.68021041	-5.76017411
WT after UVB irradiation VS WT before UVB irradiation	GO_INTERLEUKIN_6_RECEPTOR_BINDING2	0.418687838	-0.01457822	1.492581533	0.17344441	0.67927579	-4.96586027
homo after UVB irradiation VS homo before UVB irradiation	GO_INTERLEUKIN_7_MEDIATED_SIGNALING_PATHWAY	0.375568901	-0.02941514	2.399418637	0.04204197	0.1479163	-4.1639414
hetero after UVB irradiation VS hetero before UVB irradiation	GO_INTERLEUKIN_7_MEDIATED_SIGNALING_PATHWAY1	0.095321499	0.0107989	0.455181552	0.66129944	0.86118917	-6.12063504
WT after UVB irradiation VS WT before UVB irradiation	GO_INTERLEUKIN_7_MEDIATED_SIGNALING_PATHWAY2	0.104615203	-0.12907325	0.341308423	0.74155903	0.93520732	-5.93506439
homo after UVB irradiation VS homo before UVB irradiation	GO_INTERLEUKIN_8_PRODUCTION	0.633544062	0.030094626	4.271455689	0.00247836	0.03315789	-1.33910653
hetero after UVB irradiation VS hetero before UVB irradiation	GO_INTERLEUKIN_8_PRODUCTION1	0.587886076	0.056089641	3.519475554	0.00808849	0.14537603	-2.29777394
WT after UVB irradiation VS WT before UVB irradiation	GO_INTERLEUKIN_8_PRODUCTION2	0.399350187	0.010945801	1.79166512	0.11050499	0.61576748	-4.58531397
homo after UVB irradiation VS homo before UVB irradiation	GO_INTERMEDIATE_DENSITY_LIPOPROTEIN_PARTICLE	0.666811234	0.094248183	2.566751242	0.03223815	0.12747543	-3.90574638
hetero after UVB irradiation VS hetero before UVB irradiation	GO_INTERMEDIATE_DENSITY_LIPOPROTEIN_PARTICLE1	0.715863348	0.010797389	3.263250194	0.01176724	0.16890793	-2.65999303
WT after UVB irradiation VS WT before UVB irradiation	GO_INTERMEDIATE_DENSITY_LIPOPROTEIN_PARTICLE2	0.596564696	0.152854126	2.254904198	0.0537634	0.51991848	-3.94957483
homo after UVB irradiation VS homo before UVB irradiation	GO_INTERMEDIATE_FILAMENT_BASED_PROCESS	0.149275443	0.010708001	0.690882454	0.50843233	0.6810944	-6.29626981
hetero after UVB irradiation VS hetero before UVB irradiation	GO_INTERMEDIATE_FILAMENT_BASED_PROCESS1	-0.01319471	0.038739265	-0.08526767	0.93418242	0.97579263	-6.22754311
WT after UVB irradiation VS WT before UVB irradiation	GO_INTERMEDIATE_FILAMENT_BASED_PROCESS2	-0.04136048	0.04699734	-0.23474786	0.82023157	0.95751521	-5.96709413
homo after UVB irradiation VS homo before UVB irradiation	GO_INTERMEDIATE_FILAMENT_BINDING	0.021010559	-0.00160371	0.118587844	0.90841215	0.94999916	-6.54314525
hetero after UVB irradiation VS hetero before UVB irradiation	GO_INTERMEDIATE_FILAMENT_BINDING1	-0.21569348	0.000489	-0.98444045	0.35425455	0.6705208	-5.73704132
WT after UVB irradiation VS WT before UVB irradiation	GO_INTERMEDIATE_FILAMENT_BINDING2	-0.35138879	0.000497773	-1.94443519	0.08730526	0.5790399	-4.38027893
homo after UVB irradiation VS homo before UVB irradiation	GO_INTERMEDIATE_FILAMENT_BUNDLE_ASSEMBLY	0.365437962	0.024841008	1.087406823	0.30731899	0.50189314	-5.94486319
hetero after UVB irradiation VS hetero before UVB irradiation	GO_INTERMEDIATE_FILAMENT_BUNDLE_ASSEMBLY1	0.16432892	0.032602452	0.649167529	0.53475148	0.79344322	-6.00909094
WT after UVB irradiation VS WT before UVB irradiation	GO_INTERMEDIATE_FILAMENT_BUNDLE_ASSEMBLY2	-0.13009982	0.05455372	-0.45890475	0.65836941	0.91105574	-5.88645332
homo after UVB irradiation VS homo before UVB irradiation	GO_INTERMEDIATE_FILAMENT_CYTOSKELETON	-0.38198129	0.011633444	-2.68835546	0.02659464	0.11522522	-3.71712456
hetero after UVB irradiation VS hetero before UVB irradiation	GO_INTERMEDIATE_FILAMENT_CYTOSKELETON1	-0.31640659	-0.01870337	-2.06948977	0.07294082	0.34990725	-4.39536745
WT after UVB irradiation VS WT before UVB irradiation	GO_INTERMEDIATE_FILAMENT_CYTOSKELETON2	-0.34515353	0.01686603	-1.70381869	0.12636191	0.6336129	-4.70031265
homo after UVB irradiation VS homo before UVB irradiation	GO_INTERMEDIATE_FILAMENT_ORGANIZATION	0.253301471	0.037190974	1.065710036	0.31646885	0.50989527	-5.96730738
hetero after UVB irradiation VS hetero before UVB irradiation	GO_INTERMEDIATE_FILAMENT_ORGANIZATION1	-0.0476318	0.022371613	-0.26702922	0.79632522	0.92104081	-6.19299615
WT after UVB irradiation VS WT before UVB irradiation	GO_INTERMEDIATE_FILAMENT_ORGANIZATION2	-0.07473268	0.017507601	-0.43616403	0.67411265	0.91622321	-5.89691828
homo after UVB irradiation VS homo before UVB irradiation	GO_INTERMEMBRANE_LIPID_TRANSFER	0.418870664	0.021474341	3.287374836	0.01046526	0.06852046	-2.7903476
hetero after UVB irradiation VS hetero before UVB irradiation	GO_INTERMEMBRANE_LIPID_TRANSFER1	0.355302639	0.02948987	2.662404369	0.0291716	0.2392555	-3.53311307
WT after UVB irradiation VS WT before UVB irradiation	GO_INTERMEMBRANE_LIPID_TRANSFER2	0.145155814	0.005281607	0.958893815	0.36536338	0.79687664	-5.53807433
homo after UVB irradiation VS homo before UVB irradiation	GO_INTERPHOTORECEPTOR_MATRIX	0.731071029	0.003896171	2.55991953	0.03258912	0.12845023	-3.9163248
hetero after UVB irradiation VS hetero before UVB irradiation	GO_INTERPHOTORECEPTOR_MATRIX1	0.360718104	0.157097803	1.280826842	0.23678532	0.56564743	-5.42662734
WT after UVB irradiation VS WT before UVB irradiation	GO_INTERPHOTORECEPTOR_MATRIX2	0.219607073	-0.01532786	0.652250198	0.53231375	0.86897754	-5.77763124
homo after UVB irradiation VS homo before UVB irradiation	GO_INTERSTITIAL_MATRIX	0.228558997	-0.05055129	1.115030749	0.29597221	0.49036285	-5.91580155
hetero after UVB irradiation VS hetero before UVB irradiation	GO_INTERSTITIAL_MATRIX1	-0.07466044	-0.01695018	-0.3579745	0.72979379	0.89333777	-6.1625718
WT after UVB irradiation VS WT before UVB irradiation	GO_INTERSTITIAL_MATRIX2	0.043263859	0.008599614	0.146013672	0.88748005	0.97106404	-5.98483284
homo after UVB irradiation VS homo before UVB irradiation	GO_INTERSTRAND_CROSS_LINK_REPAIR	-0.3673436	-0.01405099	-1.97216129	0.08263795	0.22290042	-4.80679272
hetero after UVB irradiation VS hetero before UVB irradiation	GO_INTERSTRAND_CROSS_LINK_REPAIR1	0.090828875	-0.0225691	0.419270744	0.68625224	0.87376352	-6.13724404
WT after UVB irradiation VS WT before UVB irradiation	GO_INTERSTRAND_CROSS_LINK_REPAIR2	0.162946229	0.007987757	0.630847954	0.54552907	0.87323707	-5.79137769
homo after UVB irradiation VS homo before UVB irradiation	GO_INTESTINAL_ABSORPTION	0.226586366	0.0977052	1.557068863	0.15658011	0.33172638	-5.38624
hetero after UVB irradiation VS hetero before UVB irradiation	GO_INTESTINAL_ABSORPTION1	0.064949569	0.070705903	0.43088799	0.67813293	0.87043576	-6.13201299
WT after UVB irradiation VS WT before UVB irradiation	GO_INTESTINAL_ABSORPTION2	0.157512166	0.066223364	0.911532179	0.38834471	0.80982312	-5.57998336
homo after UVB irradiation VS homo before UVB irradiation	GO_INTESTINAL_EPITHELIAL_CELL_DEVELOPMENT	-0.00981762	0.021112685	-0.05618321	0.95652028	0.97711268	-6.54912467
hetero after UVB irradiation VS hetero before UVB irradiation	GO_INTESTINAL_EPITHELIAL_CELL_DEVELOPMENT1	0.262591472	0.064172887	1.690144997	0.13017732	0.43858291	-4.91880466
WT after UVB irradiation VS WT before UVB irradiation	GO_INTESTINAL_EPITHELIAL_CELL_DEVELOPMENT2	0.181174247	0.004240998	1.060235568	0.31963411	0.77323848	-5.44292703
homo after UVB irradiation VS homo before UVB irradiation	GO_INTESTINAL_EPITHELIAL_CELL_DIFFERENTIATION	0.202441978	0.028498984	1.118897491	0.29441077	0.48911887	-5.91169072
hetero after UVB irradiation VS hetero before UVB irradiation	GO_INTESTINAL_EPITHELIAL_CELL_DIFFERENTIATION1	0.088612554	0.061949279	0.581159348	0.57742815	0.81810176	-6.05229608
WT after UVB irradiation VS WT before UVB irradiation	GO_INTESTINAL_EPITHELIAL_CELL_DIFFERENTIATION2	0.048427961	0.045212585	0.219413144	0.83175805	0.96027645	-5.97074422
homo after UVB irradiation VS homo before UVB irradiation	GO_INTESTINAL_EPITHELIAL_STRUCTURE_MAINTENANCE	0.232414388	0.0692198	0.888677754	0.39911213	0.58782975	-6.13714748
hetero after UVB irradiation VS hetero before UVB irradiation	GO_INTESTINAL_EPITHELIAL_STRUCTURE_MAINTENANCE1	0.200668389	0.018917058	1.002898447	0.34580843	0.66286618	-5.71945412
WT after UVB irradiation VS WT before UVB irradiation	GO_INTESTINAL_EPITHELIAL_STRUCTURE_MAINTENANCE2	-0.27018827	0.057670691	-1.28028433	0.23589942	0.72774029	-5.21309883
homo after UVB irradiation VS homo before UVB irradiation	GO_INTESTINAL_HEXOSE_ABSORPTION	-0.00035686	-0.0055384	-0.00205083	0.99841195	0.99888577	-6.55085441
hetero after UVB irradiation VS hetero before UVB irradiation	GO_INTESTINAL_HEXOSE_ABSORPTION1	-0.04707787	0.091586085	-0.19340935	0.85154727	0.94321939	-6.21124788
WT after UVB irradiation VS WT before UVB irradiation	GO_INTESTINAL_HEXOSE_ABSORPTION2	0.020093173	0.062898671	0.06573907	0.94917962	0.98620317	-5.993787
homo after UVB irradiation VS homo before UVB irradiation	GO_INTESTINAL_LIPID_ABSORPTION	0.384699687	0.164345225	2.847004877	0.02071418	0.09969096	-3.47062592
hetero after UVB irradiation VS hetero before UVB irradiation	GO_INTESTINAL_LIPID_ABSORPTION1	0.129816581	0.037196661	0.590271837	0.57159924	0.81424167	-6.04675863
WT after UVB irradiation VS WT before UVB irradiation	GO_INTESTINAL_LIPID_ABSORPTION2	0.068603347	0.092850444	0.311291159	0.76344246	0.94088195	-5.9452581
homo after UVB irradiation VS homo before UVB irradiation	GO_INTESTINE_SMOOTH_MUSCLE_CONTRACTION	0.226397284	-0.01106788	1.344224191	0.21433655	0.40497208	-5.65521703
hetero after UVB irradiation VS hetero before UVB irradiation	GO_INTESTINE_SMOOTH_MUSCLE_CONTRACTION1	-0.03854898	0.009696201	-0.17098667	0.86855783	0.95035143	-6.2156595
WT after UVB irradiation VS WT before UVB irradiation	GO_INTESTINE_SMOOTH_MUSCLE_CONTRACTION2	-0.24068768	-0.01404086	-1.36154046	0.2100149	0.7066673	-5.12112585
homo after UVB irradiation VS homo before UVB irradiation	GO_INTRA_GOLGI_VESICLE_MEDIATED_TRANSPORT	0.258755871	-0.02539927	1.544287035	0.15961208	0.33552965	-5.40302517
hetero after UVB irradiation VS hetero before UVB irradiation	GO_INTRA_GOLGI_VESICLE_MEDIATED_TRANSPORT1	0.31833866	0.016766809	1.602332769	0.14845733	0.46627003	-5.03384857
WT after UVB irradiation VS WT before UVB irradiation	GO_INTRA_GOLGI_VESICLE_MEDIATED_TRANSPORT2	0.114442884	-0.03463491	0.6821688	0.51416799	0.85974355	-5.75773
homo after UVB irradiation VS homo before UVB irradiation	GO_INTRA_S_DNA_DAMAGE_CHECKPOINT	-0.31621309	-0.01089696	-1.69838862	0.12636929	0.29056272	-5.19595315
hetero after UVB irradiation VS hetero before UVB irradiation	GO_INTRA_S_DNA_DAMAGE_CHECKPOINT1	-0.08783326	-0.04008059	-0.32919845	0.75061392	0.90240011	-6.17313035
WT after UVB irradiation VS WT before UVB irradiation	GO_INTRA_S_DNA_DAMAGE_CHECKPOINT2	-0.16573523	-0.03861513	-0.57888696	0.57840787	0.88761996	-5.82302221
homo after UVB irradiation VS homo before UVB irradiation	GO_INTRACELLULAR_CALCIUM_ACTIVATED_CHLORIDE_CHANNEL_ACTIVITY	0.017252474	-0.00330842	0.084443519	0.93469826	0.96531194	-6.54694495
hetero after UVB irradiation VS hetero before UVB irradiation	GO_INTRACELLULAR_CALCIUM_ACTIVATED_CHLORIDE_CHANNEL_ACTIVITY1	-0.05743668	-0.05591056	-0.27535928	0.79014681	0.918108	-6.19056954
WT after UVB irradiation VS WT before UVB irradiation	GO_INTRACELLULAR_CALCIUM_ACTIVATED_CHLORIDE_CHANNEL_ACTIVITY2	-0.13196672	0.030848536	-0.62313762	0.55033732	0.87546015	-5.79622886
homo after UVB irradiation VS homo before UVB irradiation	GO_INTRACELLULAR_CAMP_ACTIVATED_CATION_CHANNEL_ACTIVITY	0.194348145	0.169523196	0.760713519	0.46781417	0.64670105	-6.24401972
hetero after UVB irradiation VS hetero before UVB irradiation	GO_INTRACELLULAR_CAMP_ACTIVATED_CATION_CHANNEL_ACTIVITY1	-0.27159877	-0.00995906	-1.45932597	0.18328821	0.50777048	-5.21460704
WT after UVB irradiation VS WT before UVB irradiation	GO_INTRACELLULAR_CAMP_ACTIVATED_CATION_CHANNEL_ACTIVITY2	0.241650517	-0.01437568	1.071454267	0.31485711	0.77104985	-5.43195431
homo after UVB irradiation VS homo before UVB irradiation	GO_INTRACELLULAR_CGMP_ACTIVATED_CATION_CHANNEL_ACTIVITY	0.197539567	0.167457952	1.00316665	0.3440296	0.53638574	-6.03007038
hetero after UVB irradiation VS hetero before UVB irradiation	GO_INTRACELLULAR_CGMP_ACTIVATED_CATION_CHANNEL_ACTIVITY1	-0.22081653	-0.04255856	-1.07501844	0.31428443	0.63499052	-5.64838499
WT after UVB irradiation VS WT before UVB irradiation	GO_INTRACELLULAR_CGMP_ACTIVATED_CATION_CHANNEL_ACTIVITY2	0.180824232	-0.02992492	0.648408401	0.53467165	0.86957641	-5.78012905
homo after UVB irradiation VS homo before UVB irradiation	GO_INTRACELLULAR_DISTRIBUTION_OF_MITOCHONDRIA	-0.61894726	-0.00523325	-4.07296794	0.00327812	0.03764174	-1.62147449
hetero after UVB irradiation VS hetero before UVB irradiation	GO_INTRACELLULAR_DISTRIBUTION_OF_MITOCHONDRIA1	-0.53278012	-0.03787844	-2.45793184	0.03998616	0.27243899	-3.83300126
WT after UVB irradiation VS WT before UVB irradiation	GO_INTRACELLULAR_DISTRIBUTION_OF_MITOCHONDRIA2	-0.33003526	-0.107639	-1.18647936	0.26907434	0.74431614	-5.31470666
homo after UVB irradiation VS homo before UVB irradiation	GO_INTRACELLULAR_ESTROGEN_RECEPTOR_SIGNALING_PATHWAY	-0.0304251	0.016422438	-0.19340403	0.85127583	0.91782548	-6.53037489
hetero after UVB irradiation VS hetero before UVB irradiation	GO_INTRACELLULAR_ESTROGEN_RECEPTOR_SIGNALING_PATHWAY1	-0.05722534	-0.00173886	-0.36619691	0.72388744	0.89166733	-6.1593977
WT after UVB irradiation VS WT before UVB irradiation	GO_INTRACELLULAR_ESTROGEN_RECEPTOR_SIGNALING_PATHWAY2	0.081691391	0.043998674	0.467364638	0.65255799	0.90930021	-5.88243175
homo after UVB irradiation VS homo before UVB irradiation	GO_INTRACELLULAR_LIGAND_GATED_ION_CHANNEL_ACTIVITY	0.484988122	-0.0041501	2.803383239	0.02218391	0.10369593	-3.53840414
hetero after UVB irradiation VS hetero before UVB irradiation	GO_INTRACELLULAR_LIGAND_GATED_ION_CHANNEL_ACTIVITY1	-0.02023389	-0.00747198	-0.10882877	0.91606775	0.96799239	-6.2250662
WT after UVB irradiation VS WT before UVB irradiation	GO_INTRACELLULAR_LIGAND_GATED_ION_CHANNEL_ACTIVITY2	0.047249201	0.024974114	0.228214384	0.82513703	0.95860343	-5.96867901
homo after UVB irradiation VS homo before UVB irradiation	GO_INTRACELLULAR_LIPID_TRANSPORT	0.056933467	-0.0327089	0.315311898	0.76028026	0.86127258	-6.49662162
hetero after UVB irradiation VS hetero before UVB irradiation	GO_INTRACELLULAR_LIPID_TRANSPORT1	-0.0189253	-0.03242024	-0.10250156	0.92092769	0.96983444	-6.22579026
WT after UVB irradiation VS WT before UVB irradiation	GO_INTRACELLULAR_LIPID_TRANSPORT2	-0.01195521	0.010804291	-0.08447904	0.93472683	0.98322796	-5.99230274
homo after UVB irradiation VS homo before UVB irradiation	GO_INTRACELLULAR_MRNA_LOCALIZATION	-0.77152134	0.028581032	-5.39825958	0.00056484	0.01723042	0.148142984
hetero after UVB irradiation VS hetero before UVB irradiation	GO_INTRACELLULAR_MRNA_LOCALIZATION1	-0.53384421	-0.00721984	-3.60607346	0.00713992	0.13963559	-2.17726302
WT after UVB irradiation VS WT before UVB irradiation	GO_INTRACELLULAR_MRNA_LOCALIZATION2	-0.46882723	0.002870316	-2.36153631	0.04548149	0.50274932	-3.79894256
homo after UVB irradiation VS homo before UVB irradiation	GO_INTRACELLULAR_PROTEIN_TRANSPORT	-0.11902205	-0.01261447	-0.87405898	0.40658026	0.59357982	-6.15005947
hetero after UVB irradiation VS hetero before UVB irradiation	GO_INTRACELLULAR_PROTEIN_TRANSPORT1	0.029674485	-0.01795285	0.21697178	0.83376118	0.93574283	-6.20603311
WT after UVB irradiation VS WT before UVB irradiation	GO_INTRACELLULAR_PROTEIN_TRANSPORT2	-0.03922985	-0.02337025	-0.24404782	0.8132635	0.955326	-5.96476214
homo after UVB irradiation VS homo before UVB irradiation	GO_INTRACELLULAR_RECEPTOR_SIGNALING_PATHWAY	0.059619291	-0.00276331	0.455826556	0.66016412	0.79377837	-6.43825095
hetero after UVB irradiation VS hetero before UVB irradiation	GO_INTRACELLULAR_RECEPTOR_SIGNALING_PATHWAY1	0.042907696	0.012603386	0.331174039	0.7491773	0.90145676	-6.1724329
WT after UVB irradiation VS WT before UVB irradiation	GO_INTRACELLULAR_RECEPTOR_SIGNALING_PATHWAY2	-0.02558179	0.012920061	-0.17467665	0.86562215	0.96790654	-5.97999912
homo after UVB irradiation VS homo before UVB irradiation	GO_INTRACELLULAR_STEROID_HORMONE_RECEPTOR_SIGNALING_PATHWAY	-0.05457005	0.013923666	-0.3899651	0.7063453	0.82484273	-6.4681638
hetero after UVB irradiation VS hetero before UVB irradiation	GO_INTRACELLULAR_STEROID_HORMONE_RECEPTOR_SIGNALING_PATHWAY1	-0.01781989	0.00075149	-0.13569052	0.89547974	0.96152498	-6.22151131
WT after UVB irradiation VS WT before UVB irradiation	GO_INTRACELLULAR_STEROID_HORMONE_RECEPTOR_SIGNALING_PATHWAY2	-0.03548626	0.02764369	-0.22224158	0.82962869	0.95961989	-5.97008927
homo after UVB irradiation VS homo before UVB irradiation	GO_INTRACELLULAR_STEROL_TRANSPORT	0.095801752	-0.01508849	0.769208882	0.46302156	0.64248323	-6.23736416
hetero after UVB irradiation VS hetero before UVB irradiation	GO_INTRACELLULAR_STEROL_TRANSPORT1	0.043073662	-0.02175629	0.298770995	0.77286833	0.91148058	-6.18335886
WT after UVB irradiation VS WT before UVB irradiation	GO_INTRACELLULAR_STEROL_TRANSPORT2	0.108550904	0.031036651	0.708165295	0.49871678	0.85541458	-5.73979804
homo after UVB irradiation VS homo before UVB irradiation	GO_INTRACELLULAR_TRANSPORT	-0.03381953	-0.01626141	-0.25845721	0.80232634	0.88808354	-6.51434457
hetero after UVB irradiation VS hetero before UVB irradiation	GO_INTRACELLULAR_TRANSPORT1	0.069809164	-0.01430607	0.53414249	0.60802809	0.83639997	-6.07960755
WT after UVB irradiation VS WT before UVB irradiation	GO_INTRACELLULAR_TRANSPORT2	0.027297878	-0.01868211	0.17066141	0.86867719	0.96790797	-5.98072792
homo after UVB irradiation VS homo before UVB irradiation	GO_INTRACILIARY_RETROGRADE_TRANSPORT	-0.42669637	-0.02500101	-1.79924806	0.10819831	0.26307774	-5.0553952
hetero after UVB irradiation VS hetero before UVB irradiation	GO_INTRACILIARY_RETROGRADE_TRANSPORT1	-0.3465839	0.012774883	-1.1965895	0.26634173	0.59287051	-5.5205921
WT after UVB irradiation VS WT before UVB irradiation	GO_INTRACILIARY_RETROGRADE_TRANSPORT2	-0.43725078	0.022269869	-1.99718138	0.08043736	0.56526951	-4.30823885
homo after UVB irradiation VS homo before UVB irradiation	GO_INTRACILIARY_TRANSPORT	-0.31314941	-0.02473046	-1.85710793	0.09890952	0.24883069	-4.97319985
hetero after UVB irradiation VS hetero before UVB irradiation	GO_INTRACILIARY_TRANSPORT_INVOLVED_IN_CILIUM_ASSEMBLY	-0.2127691	-0.01928269	-1.11528509	0.2958693	0.49035751	-5.91553148
hetero after UVB irradiation VS hetero before UVB irradiation	GO_INTRACILIARY_TRANSPORT_INVOLVED_IN_CILIUM_ASSEMBLY1	-0.37731035	-0.02734148	-1.92281301	0.09140611	0.37870619	-4.60200381
WT after UVB irradiation VS WT before UVB irradiation	GO_INTRACILIARY_TRANSPORT_INVOLVED_IN_CILIUM_ASSEMBLY2	-0.44617859	-0.02385889	-2.79496321	0.02310321	0.41742287	-3.1828948
homo after UVB irradiation VS homo before UVB irradiation	GO_INTRACILIARY_TRANSPORT_PARTICLE	-0.20362028	-0.02286152	-0.93729502	0.37498476	0.56522948	-6.09294967
hetero after UVB irradiation VS hetero before UVB irradiation	GO_INTRACILIARY_TRANSPORT_PARTICLE_A	0.050704553	0.022694869	0.151222929	0.88339843	0.93692042	-6.53832362
hetero after UVB irradiation VS hetero before UVB irradiation	GO_INTRACILIARY_TRANSPORT_PARTICLE_A1	-0.17922703	-0.01897394	-0.64362487	0.53815723	0.79532113	-6.01277311
WT after UVB irradiation VS WT before UVB irradiation	GO_INTRACILIARY_TRANSPORT_PARTICLE_A2	-0.34934399	0.029480938	-1.24322547	0.24857287	0.73564768	-5.25384899
homo after UVB irradiation VS homo before UVB irradiation	GO_INTRACILIARY_TRANSPORT_PARTICLE_B	-0.23633127	-0.01467359	-1.12213024	0.29311038	0.48792215	-5.9082459
hetero after UVB irradiation VS hetero before UVB irradiation	GO_INTRACILIARY_TRANSPORT_PARTICLE_B_BINDING	-0.36260045	-0.01414322	-1.14678942	0.28334141	0.47838417	-5.88173138
hetero after UVB irradiation VS hetero before UVB irradiation	GO_INTRACILIARY_TRANSPORT_PARTICLE_B_BINDING1	-0.28101833	-0.03015456	-0.79002999	0.4527046	0.74446775	-5.90626603
WT after UVB irradiation VS WT before UVB irradiation	GO_INTRACILIARY_TRANSPORT_PARTICLE_B_BINDING2	-0.07880209	0.043425436	-0.23320003	0.82139296	0.95812946	-5.96747359
hetero after UVB irradiation VS hetero before UVB irradiation	GO_INTRACILIARY_TRANSPORT_PARTICLE_B1	-0.44398337	-0.01833274	-2.43645531	0.04133678	0.27389564	-3.86443283
WT after UVB irradiation VS WT before UVB irradiation	GO_INTRACILIARY_TRANSPORT_PARTICLE_B2	-0.23501446	-0.04139951	-1.12869206	0.29135416	0.75646944	-5.37466975
hetero after UVB irradiation VS hetero before UVB irradiation	GO_INTRACILIARY_TRANSPORT_PARTICLE1	-0.33944802	-0.00570955	-1.71488247	0.12541749	0.43239729	-4.88590114
WT after UVB irradiation VS WT before UVB irradiation	GO_INTRACILIARY_TRANSPORT_PARTICLE2	-0.29985929	-0.02112405	-1.38495622	0.20302461	0.6993544	-5.09398572
hetero after UVB irradiation VS hetero before UVB irradiation	GO_INTRACILIARY_TRANSPORT1	-0.38706994	-0.02474829	-2.15677193	0.06373805	0.33116853	-4.27054102
WT after UVB irradiation VS WT before UVB irradiation	GO_INTRACILIARY_TRANSPORT2	-0.30319491	-0.0530563	-1.87277874	0.0975419	0.59779444	-4.47717264
homo after UVB irradiation VS homo before UVB irradiation	GO_INTRAMEMBRANE_LIPID_TRANSPORTER_ACTIVITY	0.274147478	-0.04235141	1.451466728	0.18324812	0.36686915	-5.52258778
hetero after UVB irradiation VS hetero before UVB irradiation	GO_INTRAMEMBRANE_LIPID_TRANSPORTER_ACTIVITY1	0.198257831	0.024625826	1.145354017	0.28576722	0.61112769	-5.57562313
WT after UVB irradiation VS WT before UVB irradiation	GO_INTRAMEMBRANE_LIPID_TRANSPORTER_ACTIVITY2	0.334704078	-0.00190775	1.677942313	0.13142165	0.63700751	-4.73372131
homo after UVB irradiation VS homo before UVB irradiation	GO_INTRAMOLECULAR_OXIDOREDUCTASE_ACTIVITY	0.200913505	-0.03596646	1.371808792	0.20593355	0.3956981	-5.62170152
homo after UVB irradiation VS homo before UVB irradiation	GO_INTRAMOLECULAR_OXIDOREDUCTASE_ACTIVITY_INTERCONVERTING_ALDOSES_AND_KETOSES	0.329088387	-0.04482098	1.753192391	0.11617076	0.27482076	-5.12002857
hetero after UVB irradiation VS hetero before UVB irradiation	GO_INTRAMOLECULAR_OXIDOREDUCTASE_ACTIVITY_INTERCONVERTING_ALDOSES_AND_KETOSES1	0.465043871	-0.03641756	2.242526565	0.05581513	0.3120771	-4.14684825
WT after UVB irradiation VS WT before UVB irradiation	GO_INTRAMOLECULAR_OXIDOREDUCTASE_ACTIVITY_INTERCONVERTING_ALDOSES_AND_KETOSES2	0.91865192	0.055584997	5.29292919	0.00070602	0.18842845	-0.06225836
homo after UVB irradiation VS homo before UVB irradiation	GO_INTRAMOLECULAR_OXIDOREDUCTASE_ACTIVITY_TRANSPOSING_C_C_BONDS	0.206573236	-0.05668788	0.890315645	0.39828155	0.58709827	-6.13568982
hetero after UVB irradiation VS hetero before UVB irradiation	GO_INTRAMOLECULAR_OXIDOREDUCTASE_ACTIVITY_TRANSPOSING_C_C_BONDS1	0.116571899	0.006716166	0.457652245	0.6595987	0.86040243	-6.11944476
WT after UVB irradiation VS WT before UVB irradiation	GO_INTRAMOLECULAR_OXIDOREDUCTASE_ACTIVITY_TRANSPOSING_C_C_BONDS2	0.062025731	0.076240701	0.199809164	0.84655638	0.96504408	-5.9750558
hetero after UVB irradiation VS hetero before UVB irradiation	GO_INTRAMOLECULAR_OXIDOREDUCTASE_ACTIVITY1	0.312845373	-0.00998123	1.830787114	0.1052095	0.40177831	-4.72914773
WT after UVB irradiation VS WT before UVB irradiation	GO_INTRAMOLECULAR_OXIDOREDUCTASE_ACTIVITY2	0.342825929	0.02894377	2.151770951	0.06319267	0.53593918	-4.09421794
homo after UVB irradiation VS homo before UVB irradiation	GO_INTRAMOLECULAR_TRANSFERASE_ACTIVITY	-0.43573312	0.009885975	-2.6862402	0.02668366	0.1154753	-3.72040951
homo after UVB irradiation VS homo before UVB irradiation	GO_INTRAMOLECULAR_TRANSFERASE_ACTIVITY_PHOSPHOTRANSFERASES	0.342796612	-0.02417573	2.217773691	0.05608778	0.1760786	-4.44100109
hetero after UVB irradiation VS hetero before UVB irradiation	GO_INTRAMOLECULAR_TRANSFERASE_ACTIVITY_PHOSPHOTRANSFERASES1	0.059442149	0.021726671	0.369291695	0.72166947	0.89103348	-6.158185
WT after UVB irradiation VS WT before UVB irradiation	GO_INTRAMOLECULAR_TRANSFERASE_ACTIVITY_PHOSPHOTRANSFERASES2	-0.25502807	-0.01580946	-1.43508844	0.18873593	0.69085152	-5.03497611
hetero after UVB irradiation VS hetero before UVB irradiation	GO_INTRAMOLECULAR_TRANSFERASE_ACTIVITY1	-0.32073014	-0.04778058	-1.25277512	0.24630591	0.57380644	-5.45838351
WT after UVB irradiation VS WT before UVB irradiation	GO_INTRAMOLECULAR_TRANSFERASE_ACTIVITY2	-0.46356183	-0.02101205	-2.1418319	0.06418321	0.53678963	-4.10808874
homo after UVB irradiation VS homo before UVB irradiation	GO_INTRASPECIES_INTERACTION_BETWEEN_ORGANISMS	0.258798958	-0.01873054	1.906570975	0.0915716	0.23782188	-4.90211625
hetero after UVB irradiation VS hetero before UVB irradiation	GO_INTRASPECIES_INTERACTION_BETWEEN_ORGANISMS1	-0.00483955	-0.02369532	-0.03019373	0.97666582	0.99212405	-6.23098974
WT after UVB irradiation VS WT before UVB irradiation	GO_INTRASPECIES_INTERACTION_BETWEEN_ORGANISMS2	0.00395409	0.027358419	0.021515812	0.98335493	0.99634469	-5.99582241
homo after UVB irradiation VS homo before UVB irradiation	GO_INTRINSIC_APOPTOTIC_SIGNALING_PATHWAY	0.067583527	-0.01793679	0.491999004	0.63542442	0.77610976	-6.41993914
homo after UVB irradiation VS homo before UVB irradiation	GO_INTRINSIC_APOPTOTIC_SIGNALING_PATHWAY_BY_P53_CLASS_MEDIATOR	-0.31068755	-0.02370117	-2.61429834	0.02989925	0.12287072	-3.83206091
hetero after UVB irradiation VS hetero before UVB irradiation	GO_INTRINSIC_APOPTOTIC_SIGNALING_PATHWAY_BY_P53_CLASS_MEDIATOR1	-0.16976769	-0.00092043	-1.18414907	0.27095584	0.59719558	-5.53410614

WT after UVB irradiation VS WT before UVB irradiation	GO_INTRINSIC_APOPTOTIC_SIGNALING_PATHWAY_BY_P53_CLASS_MEDIATOR2	-0.31763647	0.028690868	-1.87108747	0.09779682	0.59808854	-4.47944487
homo after UVB irradiation VS homo before UVB irradiation	GO_INTRINSIC_APOPTOTIC_SIGNALING_PATHWAY_IN_RESPONSE_TO_DNA_DAMAGE	0.044004486	-0.01832803	0.319172398	0.75745377	0.85909962	-6.4952937
homo after UVB irradiation VS homo before UVB irradiation	GO_INTRINSIC_APOPTOTIC_SIGNALING_PATHWAY_IN_RESPONSE_TO_DNA_DAMAGE_BY_P53_CLASS_MEDIATOR	-0.33747965	-0.00974969	-2.59700637	0.03072926	0.12447754	-3.85887064
hetero after UVB irradiation VS hetero before UVB irradiation	GO_INTRINSIC_APOPTOTIC_SIGNALING_PATHWAY_IN_RESPONSE_TO_DNA_DAMAGE_BY_P53_CLASS_MEDIATOR1	-0.14899024	-0.00865324	-0.98058683	0.3560375	0.67191321	-5.74068139
WT after UVB irradiation VS WT before UVB irradiation	GO_INTRINSIC_APOPTOTIC_SIGNALING_PATHWAY_IN_RESPONSE_TO_DNA_DAMAGE_BY_P53_CLASS_MEDIATOR2	-0.35545503	0.018408528	-1.79772364	0.10948315	0.61466507	-4.57729828
hetero after UVB irradiation VS hetero before UVB irradiation	GO_INTRINSIC_APOPTOTIC_SIGNALING_PATHWAY_IN_RESPONSE_TO_DNA_DAMAGE1	0.152022761	-0.01125515	1.136108395	0.28939244	0.61407461	-5.58537492
WT after UVB irradiation VS WT before UVB irradiation	GO_INTRINSIC_APOPTOTIC_SIGNALING_PATHWAY_IN_RESPONSE_TO_DNA_DAMAGE2	0.066655775	-0.00207483	0.458754887	0.65847257	0.91105574	-5.88652394
homo after UVB irradiation VS homo before UVB irradiation	GO_INTRINSIC_APOPTOTIC_SIGNALING_PATHWAY_IN_RESPONSE_TO_ENDOPLASMIC_RETICULUM_STRESS	0.246984181	-0.03057434	1.452772331	0.18289528	0.36646792	-5.52093553
hetero after UVB irradiation VS hetero before UVB irradiation	GO_INTRINSIC_APOPTOTIC_SIGNALING_PATHWAY_IN_RESPONSE_TO_ENDOPLASMIC_RETICULUM_STRESS1	0.350659497	0.024609516	2.139858217	0.0654273	0.33409179	-4.29482193
WT after UVB irradiation VS WT before UVB irradiation	GO_INTRINSIC_APOPTOTIC_SIGNALING_PATHWAY_IN_RESPONSE_TO_ENDOPLASMIC_RETICULUM_STRESS2	0.314743042	-0.03886045	1.548346719	0.15967663	0.66317523	-4.89744359
homo after UVB irradiation VS homo before UVB irradiation	GO_INTRINSIC_APOPTOTIC_SIGNALING_PATHWAY_IN_RESPONSE_TO_HYDROGEN_PEROXIDE	0.475003607	-0.09924127	3.621367216	0.00632503	0.05261813	-2.2844171
hetero after UVB irradiation VS hetero before UVB irradiation	GO_INTRINSIC_APOPTOTIC_SIGNALING_PATHWAY_IN_RESPONSE_TO_HYDROGEN_PEROXIDE1	-0.09223407	0.005972281	-0.43235539	0.67711052	0.86994602	-6.13134256
WT after UVB irradiation VS WT before UVB irradiation	GO_INTRINSIC_APOPTOTIC_SIGNALING_PATHWAY_IN_RESPONSE_TO_HYDROGEN_PEROXIDE2	0.187094759	-0.0598688	0.647982283	0.53493357	0.86957641	-5.78040528
homo after UVB irradiation VS homo before UVB irradiation	GO_INTRINSIC_APOPTOTIC_SIGNALING_PATHWAY_IN_RESPONSE_TO_HYPOXIA	0.555204475	0.003246547	2.654203516	0.02806971	0.11859514	-3.77014861
hetero after UVB irradiation VS hetero before UVB irradiation	GO_INTRINSIC_APOPTOTIC_SIGNALING_PATHWAY_IN_RESPONSE_TO_HYPOXIA1	0.521011421	0.073719469	2.167795556	0.06266031	0.32880724	-4.25469391
WT after UVB irradiation VS WT before UVB irradiation	GO_INTRINSIC_APOPTOTIC_SIGNALING_PATHWAY_IN_RESPONSE_TO_HYPOXIA2	0.609839711	-0.00572553	2.620304885	0.03032387	0.45106473	-3.4311299
homo after UVB irradiation VS homo before UVB irradiation	GO_INTRINSIC_APOPTOTIC_SIGNALING_PATHWAY_IN_RESPONSE_TO_OSMOTIC_STRESS	0.515643936	0.034697714	2.742428949	0.02441908	0.10984126	-3.6331235
hetero after UVB irradiation VS hetero before UVB irradiation	GO_INTRINSIC_APOPTOTIC_SIGNALING_PATHWAY_IN_RESPONSE_TO_OSMOTIC_STRESS1	0.784447654	6.31E-05	5.291728063	0.00078175	0.08366654	-0.07068234
WT after UVB irradiation VS WT before UVB irradiation	GO_INTRINSIC_APOPTOTIC_SIGNALING_PATHWAY_IN_RESPONSE_TO_OSMOTIC_STRESS2	0.492074231	0.070995194	1.864203599	0.09884096	0.59874718	-4.48868613
homo after UVB irradiation VS homo before UVB irradiation	GO_INTRINSIC_APOPTOTIC_SIGNALING_PATHWAY_IN_RESPONSE_TO_OXIDATIVE_STRESS	0.165919726	-0.04496259	1.19513434	0.2649511	0.46016977	-5.82855456
hetero after UVB irradiation VS hetero before UVB irradiation	GO_INTRINSIC_APOPTOTIC_SIGNALING_PATHWAY_IN_RESPONSE_TO_OXIDATIVE_STRESS1	0.187786148	-0.01077817	1.444225614	0.18736291	0.51265105	-5.23316407
WT after UVB irradiation VS WT before UVB irradiation	GO_INTRINSIC_APOPTOTIC_SIGNALING_PATHWAY_IN_RESPONSE_TO_OXIDATIVE_STRESS2	0.139548371	-0.03296947	0.84044142	0.42477948	0.82621204	-5.63966194
hetero after UVB irradiation VS hetero before UVB irradiation	GO_INTRINSIC_APOPTOTIC_SIGNALING_PATHWAY1	0.121688418	0.005386612	0.990312983	0.35155053	0.66814524	-5.73147304
WT after UVB irradiation VS WT before UVB irradiation	GO_INTRINSIC_APOPTOTIC_SIGNALING_PATHWAY2	0.103616146	-0.01398412	0.722996623	0.49003493	0.85196106	-5.72930456
homo after UVB irradiation VS homo before UVB irradiation	GO_INTRINSIC_COMPONENT_OF_ENDOPLASMIC_RETICULUM_MEMBRANE	0.218972734	-0.04156647	1.593089882	0.14831263	0.32129323	-5.33854122
hetero after UVB irradiation VS hetero before UVB irradiation	GO_INTRINSIC_COMPONENT_OF_ENDOPLASMIC_RETICULUM_MEMBRANE1	0.09549783	-0.02923353	0.543943696	0.60157775	0.83350999	-6.07408945
WT after UVB irradiation VS WT before UVB irradiation	GO_INTRINSIC_COMPONENT_OF_ENDOPLASMIC_RETICULUM_MEMBRANE2	0.209687839	-0.01685848	1.251708067	0.24562278	0.73462815	-5.24459014
homo after UVB irradiation VS homo before UVB irradiation	GO_INTRINSIC_COMPONENT_OF_EXTERNAL_SIDE_OF_PLASMA_MEMBRANE	0.748933997	0.005433699	4.974957406	0.00096351	0.02123375	-0.38705336
hetero after UVB irradiation VS hetero before UVB irradiation	GO_INTRINSIC_COMPONENT_OF_EXTERNAL_SIDE_OF_PLASMA_MEMBRANE1	0.567470738	0.060391842	2.993601247	0.01760867	0.19405039	-3.0488181
WT after UVB irradiation VS WT before UVB irradiation	GO_INTRINSIC_COMPONENT_OF_EXTERNAL_SIDE_OF_PLASMA_MEMBRANE2	0.498314693	0.050701275	2.566257804	0.03299738	0.460248	-3.50805419
homo after UVB irradiation VS homo before UVB irradiation	GO_INTRINSIC_COMPONENT_OF_GOLGI_MEMBRANE	0.312897956	-0.01503244	2.409366672	0.04138311	0.14666445	-4.14865566
hetero after UVB irradiation VS hetero before UVB irradiation	GO_INTRINSIC_COMPONENT_OF_GOLGI_MEMBRANE1	0.18202972	-0.00461652	1.014177997	0.34072331	0.65970464	-5.70858401
WT after UVB irradiation VS WT before UVB irradiation	GO_INTRINSIC_COMPONENT_OF_GOLGI_MEMBRANE2	0.253724325	-0.05478886	1.301750092	0.22881074	0.72133305	-5.18914506
homo after UVB irradiation VS homo before UVB irradiation	GO_INTRINSIC_COMPONENT_OF_MITOCHONDRIAL_INNER_MEMBRANE	-0.15773251	-0.02295587	-0.66760327	0.52245027	0.69152247	-6.31269661
hetero after UVB irradiation VS hetero before UVB irradiation	GO_INTRINSIC_COMPONENT_OF_MITOCHONDRIAL_INNER_MEMBRANE1	-0.31287701	-0.05989771	-1.80606149	0.10924378	0.40782387	-4.76292433
WT after UVB irradiation VS WT before UVB irradiation	GO_INTRINSIC_COMPONENT_OF_MITOCHONDRIAL_INNER_MEMBRANE2	-0.03223196	-0.08930622	-0.16123213	0.87586068	0.9693507	-5.98237327
homo after UVB irradiation VS homo before UVB irradiation	GO_INTRINSIC_COMPONENT_OF_MITOCHONDRIAL_MEMBRANE	-0.16255889	-0.01398273	-0.83134947	0.42896285	0.6133325	-6.18675889
hetero after UVB irradiation VS hetero before UVB irradiation	GO_INTRINSIC_COMPONENT_OF_MITOCHONDRIAL_MEMBRANE1	-0.26899601	-0.04988231	-1.65246849	0.13774996	0.45099587	-4.96851073
WT after UVB irradiation VS WT before UVB irradiation	GO_INTRINSIC_COMPONENT_OF_MITOCHONDRIAL_MEMBRANE2	-0.0974385	-0.07034371	-0.49876713	0.6312083	0.90411526	-5.86689942
homo after UVB irradiation VS homo before UVB irradiation	GO_INTRINSIC_COMPONENT_OF_MITOCHONDRIAL_OUTER_MEMBRANE	-0.06612937	0.00191003	-0.42417098	0.68218445	0.80859405	-6.4531847
hetero after UVB irradiation VS hetero before UVB irradiation	GO_INTRINSIC_COMPONENT_OF_MITOCHONDRIAL_OUTER_MEMBRANE1	-0.15928759	-0.02351619	-0.99598376	0.34895432	0.66595577	-5.72607193
WT after UVB irradiation VS WT before UVB irradiation	GO_INTRINSIC_COMPONENT_OF_MITOCHONDRIAL_OUTER_MEMBRANE2	-0.3444423	-0.04758911	-2.05856306	0.07310163	0.55377265	-4.22373051
homo after UVB irradiation VS homo before UVB irradiation	GO_INTRINSIC_COMPONENT_OF_NUCLEAR_INNER_MEMBRANE	0.166687888	-0.00348733	0.936126392	0.37555193	0.5654523	-6.09403443
hetero after UVB irradiation VS hetero before UVB irradiation	GO_INTRINSIC_COMPONENT_OF_NUCLEAR_INNER_MEMBRANE1	0.018616313	0.027964794	0.090154047	0.93042185	0.97446978	-6.22707873
WT after UVB irradiation VS WT before UVB irradiation	GO_INTRINSIC_COMPONENT_OF_NUCLEAR_INNER_MEMBRANE2	-0.11981426	-0.07704437	-0.70283379	0.50186144	0.85706548	-5.74352368
homo after UVB irradiation VS homo before UVB irradiation	GO_INTRINSIC_COMPONENT_OF_ORGANELLE_MEMBRANE	0.114357361	-0.02417489	0.918755929	0.38405659	0.57369401	-6.11002923
hetero after UVB irradiation VS hetero before UVB irradiation	GO_INTRINSIC_COMPONENT_OF_ORGANELLE_MEMBRANE1	-0.01046434	-0.02906211	-0.0644886	0.95019258	0.98092529	-6.22922939
WT after UVB irradiation VS WT before UVB irradiation	GO_INTRINSIC_COMPONENT_OF_ORGANELLE_MEMBRANE2	0.075912123	-0.03288929	0.468550372	0.65174546	0.90930021	-5.88186254
homo after UVB irradiation VS homo before UVB irradiation	GO_INTRINSIC_COMPONENT_OF_PEROXISOMAL_MEMBRANE	-0.05017872	-0.04092429	-0.27584938	0.78938439	0.8801753	-6.50928876
hetero after UVB irradiation VS hetero before UVB irradiation	GO_INTRINSIC_COMPONENT_OF_PEROXISOMAL_MEMBRANE1	-0.35198819	-0.11989079	-1.9300469	0.09039794	0.37777176	-4.59192043
WT after UVB irradiation VS WT before UVB irradiation	GO_INTRINSIC_COMPONENT_OF_PEROXISOMAL_MEMBRANE2	-0.07233322	-0.01507507	-0.36407105	0.72512805	0.93154998	-5.92672836
homo after UVB irradiation VS homo before UVB irradiation	GO_INTRINSIC_COMPONENT_OF_PLASMA_MEMBRANE	0.315396939	0.003870875	2.605861857	0.03030132	0.12366244	-3.84514251
hetero after UVB irradiation VS hetero before UVB irradiation	GO_INTRINSIC_COMPONENT_OF_PLASMA_MEMBRANE1	0.140131597	0.019896787	0.956963493	0.36711565	0.68091512	-5.76275333
WT after UVB irradiation VS WT before UVB irradiation	GO_INTRINSIC_COMPONENT_OF_PLASMA_MEMBRANE2	0.116086584	0.004124005	0.632168567	0.54470803	0.87280307	-5.7905414
homo after UVB irradiation VS homo before UVB irradiation	GO_INTRINSIC_COMPONENT_OF_POSTSYNAPTIC_DENSITY_MEMBRANE	-0.15729535	0.027384287	-0.85744252	0.41518857	0.60138769	-6.16452011
hetero after UVB irradiation VS hetero before UVB irradiation	GO_INTRINSIC_COMPONENT_OF_POSTSYNAPTIC_DENSITY_MEMBRANE1	-0.45165882	0.019335389	-3.37785869	0.00994029	0.15702536	-2.49698523
WT after UVB irradiation VS WT before UVB irradiation	GO_INTRINSIC_COMPONENT_OF_POSTSYNAPTIC_DENSITY_MEMBRANE2	-0.11456472	-0.02596804	-0.60968169	0.55878797	0.879729	-5.80456581
homo after UVB irradiation VS homo before UVB irradiation	GO_INTRINSIC_COMPONENT_OF_POSTSYNAPTIC_MEMBRANE	0.005385392	0.007367097	0.032312207	0.97498399	0.98709878	-6.55028374
hetero after UVB irradiation VS hetero before UVB irradiation	GO_INTRINSIC_COMPONENT_OF_POSTSYNAPTIC_MEMBRANE1	-0.28851609	0.009640465	-1.96919884	0.0851247	0.37178552	-4.53713965
WT after UVB irradiation VS WT before UVB irradiation	GO_INTRINSIC_COMPONENT_OF_POSTSYNAPTIC_MEMBRANE2	-0.10689268	-0.00808707	-0.53706139	0.60566354	0.89673691	-5.8466821
homo after UVB irradiation VS homo before UVB irradiation	GO_INTRINSIC_COMPONENT_OF_POSTSYNAPTIC_SPECIALIZATION_MEMBRANE	0.021554632	0.010154391	0.127597185	0.90149471	0.94652134	-6.54193024
hetero after UVB irradiation VS hetero before UVB irradiation	GO_INTRINSIC_COMPONENT_OF_POSTSYNAPTIC_SPECIALIZATION_MEMBRANE1	-0.39337283	-0.01347113	-2.60929694	0.03165507	0.24789178	-3.61105328
WT after UVB irradiation VS WT before UVB irradiation	GO_INTRINSIC_COMPONENT_OF_POSTSYNAPTIC_SPECIALIZATION_MEMBRANE2	-0.14030534	-0.03295518	-0.69907525	0.50408583	0.85763155	-5.74613527
homo after UVB irradiation VS homo before UVB irradiation	GO_INTRINSIC_COMPONENT_OF_PRESYNAPTIC_ACTIVE_ZONE_MEMBRANE	0.028608364	-0.03833977	0.179552007	0.86179558	0.92396959	-6.53319816
hetero after UVB irradiation VS hetero before UVB irradiation	GO_INTRINSIC_COMPONENT_OF_PRESYNAPTIC_ACTIVE_ZONE_MEMBRANE1	0.062219411	-0.0346564	0.314540604	0.76130499	0.90682777	-6.17817829
WT after UVB irradiation VS WT before UVB irradiation	GO_INTRINSIC_COMPONENT_OF_PRESYNAPTIC_ACTIVE_ZONE_MEMBRANE2	0.060432989	-0.03502713	0.365862938	0.72384085	0.93154998	-5.92605006
homo after UVB irradiation VS homo before UVB irradiation	GO_INTRINSIC_COMPONENT_OF_PRESYNAPTIC_MEMBRANE	0.126191119	0.006702724	0.809689214	0.44063453	0.62327429	-6.20477868
hetero after UVB irradiation VS hetero before UVB irradiation	GO_INTRINSIC_COMPONENT_OF_PRESYNAPTIC_MEMBRANE1	-0.03124819	0.023915842	-0.22230962	0.82974566	0.934707	-6.20476955
WT after UVB irradiation VS WT before UVB irradiation	GO_INTRINSIC_COMPONENT_OF_PRESYNAPTIC_MEMBRANE2	-0.08934075	-0.01098835	-0.50230784	0.62882345	0.90390703	-5.86508869
homo after UVB irradiation VS homo before UVB irradiation	GO_INTRINSIC_COMPONENT_OF_SYNAPTIC_MEMBRANE	0.073046955	0.009410202	0.505004627	0.62664413	0.76914143	-6.41303309
hetero after UVB irradiation VS hetero before UVB irradiation	GO_INTRINSIC_COMPONENT_OF_SYNAPTIC_MEMBRANE1	-0.17814383	0.028427734	-1.25128937	0.24681902	0.57435447	-5.46005275
WT after UVB irradiation VS WT before UVB irradiation	GO_INTRINSIC_COMPONENT_OF_SYNAPTIC_MEMBRANE2	-0.10437379	-0.00839202	-0.58342973	0.57548937	0.88611191	-5.82035445
homo after UVB irradiation VS homo before UVB irradiation	GO_INTRINSIC_COMPONENT_OF_SYNAPTIC_VESICLE_MEMBRANE	0.001487146	-0.00696884	0.008611674	0.99333166	0.99634161	-6.55081602
hetero after UVB irradiation VS hetero before UVB irradiation	GO_INTRINSIC_COMPONENT_OF_SYNAPTIC_VESICLE_MEMBRANE1	-0.12133383	0.003982628	-0.73574836	0.4832783	0.76253233	-5.94796117
WT after UVB irradiation VS WT before UVB irradiation	GO_INTRINSIC_COMPONENT_OF_SYNAPTIC_VESICLE_MEMBRANE2	-0.126766	0.014792897	-0.74716004	0.47609985	0.84846534	-5.71180398
homo after UVB irradiation VS homo before UVB irradiation	GO_INTRINSIC_COMPONENT_OF_THE_CYTOPLASMIC_SIDE_OF_THE_PLASMA_MEMBRANE	0.610408447	-0.04649862	3.453472966	0.00813282	0.06012827	-2.53731875
hetero after UVB irradiation VS hetero before UVB irradiation	GO_INTRINSIC_COMPONENT_OF_THE_CYTOPLASMIC_SIDE_OF_THE_PLASMA_MEMBRANE1	0.032125991	0.027391656	0.155231644	0.88055456	0.95666787	-6.21843695
WT after UVB irradiation VS WT before UVB irradiation	GO_INTRINSIC_COMPONENT_OF_THE_CYTOPLASMIC_SIDE_OF_THE_PLASMA_MEMBRANE2	0.341981847	-0.03170315	1.512368629	0.16844214	0.67381423	-4.94173444
homo after UVB irradiation VS homo before UVB irradiation	GO_INTRINSIC_COMPONENT_OF_VACUOLAR_MEMBRANE	0.277658005	-0.04899574	1.206372368	0.2608187	0.45621651	-5.81597203
hetero after UVB irradiation VS hetero before UVB irradiation	GO_INTRINSIC_COMPONENT_OF_VACUOLAR_MEMBRANE1	-0.04837601	0.012523575	-0.16010954	0.87683659	0.95484139	-6.2176055
WT after UVB irradiation VS WT before UVB irradiation	GO_INTRINSIC_COMPONENT_OF_VACUOLAR_MEMBRANE2	0.123697764	-0.02076323	0.819961398	0.43570699	0.83017793	-5.65611217
homo after UVB irradiation VS homo before UVB irradiation	GO_INTRONIC_TRANSCRIPTION_REGULATORY_REGION_SEQUENCE_SPECIFIC_DNA_BINDING	-0.15966163	-0.00384273	-0.65461718	0.53037174	0.69735468	-6.32164087
hetero after UVB irradiation VS hetero before UVB irradiation	GO_INTRONIC_TRANSCRIPTION_REGULATORY_REGION_SEQUENCE_SPECIFIC_DNA_BINDING1	-0.33761628	-0.01693222	-1.33310763	0.21987393	0.54721871	-5.36625845
WT after UVB irradiation VS WT before UVB irradiation	GO_INTRONIC_TRANSCRIPTION_REGULATORY_REGION_SEQUENCE_SPECIFIC_DNA_BINDING2	-0.45599246	-0.00123683	-2.60386612	0.03111284	0.45353809	-3.45452607
homo after UVB irradiation VS homo before UVB irradiation	GO_INVADOPODIUM	0.369400007	0.011293348	1.690896505	0.12782616	0.29281755	-5.20624533
homo after UVB irradiation VS homo before UVB irradiation	GO_INVADOPODIUM_MEMBRANE	-0.12871874	0.000136263	-0.67171605	0.51995661	0.68964584	-6.30983105
hetero after UVB irradiation VS hetero before UVB irradiation	GO_INVADOPODIUM_MEMBRANE1	0.279013171	-0.02864647	1.217640981	0.2586817	0.58521976	-5.49750677
WT after UVB irradiation VS WT before UVB irradiation	GO_INVADOPODIUM_MEMBRANE2	0.077590203	-0.07112498	0.314807016	0.76086714	0.93919119	-5.94411133
hetero after UVB irradiation VS hetero before UVB irradiation	GO_INVADOPODIUM1	0.529213276	0.009839552	3.902404675	0.00469635	0.1277306	-1.77309035
WT after UVB irradiation VS WT before UVB irradiation	GO_INVADOPODIUM2	0.394882595	-0.068	2.548321466	0.03393665	0.46529347	-3.53358143
homo after UVB irradiation VS homo before UVB irradiation	GO_INWARD_RECTIFIER_POTASSIUM_CHANNEL_ACTIVITY	0.189305661	0.012536459	1.118106159	0.29472978	0.48940333	-5.91253286
hetero after UVB irradiation VS hetero before UVB irradiation	GO_INWARD_RECTIFIER_POTASSIUM_CHANNEL_ACTIVITY1	-0.01389172	0.000822423	-0.08026531	0.93803409	0.97724026	-6.22799177
WT after UVB irradiation VS WT before UVB irradiation	GO_INWARD_RECTIFIER_POTASSIUM_CHANNEL_ACTIVITY2	-0.06732591	-0.00555136	-0.29237218	0.77735338	0.94371579	-5.95121306
homo after UVB irradiation VS homo before UVB irradiation	GO_IODIDE_TRANSPORT	0.22653303	0.070669068	0.825664257	0.43200553	0.61607417	-6.19152756
hetero after UVB irradiation VS hetero before UVB irradiation	GO_IODIDE_TRANSPORT1	-0.60213969	-0.0567957	-3.50104376	0.0083072	0.14740706	-2.32355508
WT after UVB irradiation VS WT before UVB irradiation	GO_IODIDE_TRANSPORT2	-0.36977132	0.027007166	-1.32134659	0.22249803	0.71742564	-5.16705906
homo after UVB irradiation VS homo before UVB irradiation	GO_ION_CHANNEL_BINDING	0.326704299	-0.00027441	2.596321443	0.03076261	0.12454428	-3.8599323
hetero after UVB irradiation VS hetero before UVB irradiation	GO_ION_CHANNEL_BINDING1	0.186936369	0.007640914	1.010342132	0.34244615	0.6607997	-5.71229101
WT after UVB irradiation VS WT before UVB irradiation	GO_ION_CHANNEL_BINDING2	0.124254563	-0.01440082	0.659990924	0.52758204	0.8664124	-5.77255845
homo after UVB irradiation VS homo before UVB irradiation	GO_ION_CHANNEL_REGULATOR_ACTIVITY	0.279614664	0.001171169	1.898030104	0.09280057	0.23996111	-4.91444154
hetero after UVB irradiation VS hetero before UVB irradiation	GO_ION_CHANNEL_REGULATOR_ACTIVITY1	0.038956677	0.025593539	0.239785555	0.81663692	0.92947858	-6.20042085
WT after UVB irradiation VS WT before UVB irradiation	GO_ION_CHANNEL_REGULATOR_ACTIVITY2	0.047454063	-0.00545547	0.245008447	0.81254473	0.95514231	-5.96451617
homo after UVB irradiation VS homo before UVB irradiation	GO_ION_GATED_CHANNEL_ACTIVITY	0.312457266	0.024900551	1.962270984	0.08392947	0.22510855	-4.82123987
hetero after UVB irradiation VS hetero before UVB irradiation	GO_ION_GATED_CHANNEL_ACTIVITY1	0.009073488	-0.02592009	0.050251885	0.96117633	0.98584962	-6.23011488
WT after UVB irradiation VS WT before UVB irradiation	GO_ION_GATED_CHANNEL_ACTIVITY2	-0.10377791	0.003969165	-0.54310934	0.60168005	0.89567499	-5.84336217
homo after UVB irradiation VS homo before UVB irradiation	GO_ION_HOMEOSTASIS	0.225217026	-0.00999489	1.704594821	0.12517409	0.28875942	-5.18741124
hetero after UVB irradiation VS hetero before UVB irradiation	GO_ION_HOMEOSTASIS1	0.105300078	0.011327366	0.796711662	0.44903336	0.74180859	-5.90095922
WT after UVB irradiation VS WT before UVB irradiation	GO_ION_HOMEOSTASIS2	0.095537169	-0.00562779	0.533957942	0.60771309	0.89725083	-5.84837232
homo after UVB irradiation VS homo before UVB irradiation	GO_ION_TRANSMEMBRANE_TRANSPORT	0.152211334	0.004445409	1.20477346	0.2614034	0.4568909	-5.81776724
hetero after UVB irradiation VS hetero before UVB irradiation	GO_ION_TRANSMEMBRANE_TRANSPORT1	-0.0464741	0.007402676	-0.3415299	0.74166388	0.89935341	-6.16871065
WT after UVB irradiation VS WT before UVB irradiation	GO_ION_TRANSMEMBRANE_TRANSPORT2	-0.05008392	0.002692499	-0.30189338	0.77034152	0.94172976	-5.94826171
homo after UVB irradiation VS homo before UVB irradiation	GO_ION_TRANSMEMBRANE_TRANSPORTER_ACTIVITY	0.109891352	0.016964727	0.86952399	0.40891702	0.59575363	-6.15402897
homo after UVB irradiation VS homo before UVB irradiation	GO_ION_TRANSMEMBRANE_TRANSPORTER_ACTIVITY_PHOSPHORYLATIVE_MECHANISM	0.047502802	-0.0010891	0.255126461	0.80481235	0.88968218	-6.51527587
hetero after UVB irradiation VS hetero before UVB irradiation	GO_ION_TRANSMEMBRANE_TRANSPORTER_ACTIVITY_PHOSPHORYLATIVE_MECHANISM1	-0.0567289	0.022808056	-0.29396908	0.77640163	0.9128751	-6.1848847
WT after UVB irradiation VS WT before UVB irradiation	GO_ION_TRANSMEMBRANE_TRANSPORTER_ACTIVITY_PHOSPHORYLATIVE_MECHANISM2	-0.03938012	0.005688299	-0.2567847	0.80374899	0.95360379	-5.96142368
hetero after UVB irradiation VS hetero before UVB irradiation	GO_ION_TRANSMEMBRANE_TRANSPORTER_ACTIVITY1	-0.07568714	0.015128787	-0.58933292	0.57219827	0.81455335	-6.04733282
WT after UVB irradiation VS WT before UVB irradiation	GO_ION_TRANSMEMBRANE_TRANSPORTER_ACTIVITY2	-0.0776325	0.01629725	-0.49464059	0.63399352	0.90505644	-5.86899461
homo after UVB irradiation VS homo before UVB irradiation	GO_ION_TRANSPORT	0.164921406	0.006880472	1.295086698	0.23003165	0.42137252	-5.71383723
hetero after UVB irradiation VS hetero before UVB irradiation	GO_ION_TRANSPORT1	0.002656551	0.009308949	0.01963665	0.98482299	0.99440528	-6.231275
WT after UVB irradiation VS WT before UVB irradiation	GO_ION_TRANSPORT2	0.001145581	0.003363063	0.006926634	0.994641	0.9985657	-5.99604135
homo after UVB irradiation VS homo before UVB irradiation	GO_IONOTROPIC_GLUTAMATE_RECEPTOR_ACTIVITY	-0.41183224	0.020366198	-2.39872621	0.04208822	0.1479163	-4.16500498
hetero after UVB irradiation VS hetero before UVB irradiation	GO_IONOTROPIC_GLUTAMATE_RECEPTOR_ACTIVITY1	-0.35959461	0.008096052	-1.91142295	0.09301545	0.38136592	-4.61785549
WT after UVB irradiation VS WT before UVB irradiation	GO_IONOTROPIC_GLUTAMATE_RECEPTOR_ACTIVITY2	-0.01745471	0.053140784	-0.08310089	0.9357889	0.98322796	-5.99242449
homo after UVB irradiation VS homo before UVB irradiation	GO_IONOTROPIC_GLUTAMATE_RECEPTOR_BINDING	0.34747091	0.012851868	2.055015855	0.07254464	0.206691	-4.68482079
hetero after UVB irradiation VS hetero before UVB irradiation	GO_IONOTROPIC_GLUTAMATE_RECEPTOR_BINDING1	0.022062335	0.039752478	0.113408234	0.9125526	0.96720392	-6.22451517
WT after UVB irradiation VS WT before UVB irradiation	GO_IONOTROPIC_GLUTAMATE_RECEPTOR_BINDING2	-0.03674152	0.005328943	-0.22925063	0.82435844	0.95860343	-5.96843058
homo after UVB irradiation VS homo before UVB irradiation	GO_IONOTROPIC_GLUTAMATE_RECEPTOR_SIGNALING_PATHWAY	-0.23344759	0.027942096	-1.55204578	0.15776541	0.33335396	-5.39284526
hetero after UVB irradiation VS hetero before UVB irradiation	GO_IONOTROPIC_GLUTAMATE_RECEPTOR_SIGNALING_PATHWAY1	-0.08110856	0.023523936	-0.45317658	0.66268114	0.86198891	-6.12159647
WT after UVB irradiation VS WT before UVB irradiation	GO_IONOTROPIC_GLUTAMATE_RECEPTOR_SIGNALING_PATHWAY2	0.049143021	0.042495453	0.265246895	0.79744674	0.95180107	-5.9591134
homo after UVB irradiation VS homo before UVB irradiation	GO_IPAF_INFLAMMASOME_COMPLEX	1.074095762	0.004269816	7.797958429	4.20E-05	0.00847131	2.685091111
hetero after UVB irradiation VS hetero before UVB irradiation	GO_IPAF_INFLAMMASOME_COMPLEX1	1.094755842	0.05639834	7.5376331	7.37E-05	0.07788488	2.028348546
WT after UVB irradiation VS WT before UVB irradiation	GO_IPAF_INFLAMMASOME_COMPLEX2	1.085482263	0.11699399	4.553603345	0.00180832	0.20370459	-0.87642735
homo after UVB irradiation VS homo before UVB irradiation	GO_IRE1_MEDIATED_UNFOLDED_PROTEIN_RESPONSE	0.321362969	-0.01365893	1.785246639	0.11056618	0.26647478	-5.07512135
hetero after UVB irradiation VS hetero before UVB irradiation	GO_IRE1_MEDIATED_UNFOLDED_PROTEIN_RESPONSE1	0.367387414	-0.00272455	2.082824717	0.07145479	0.34570185	-4.37637612
WT after UVB irradiation VS WT before UVB irradiation	GO_IRE1_MEDIATED_UNFOLDED_PROTEIN_RESPONSE2	0.404713836	-0.02102894	2.108088073	0.06766116	0.54059322	-4.15507806
homo after UVB irradiation VS homo before UVB irradiation	GO_IRES_DEPENDENT_VIRAL_TRANSLATIONAL_INITIATION	-0.96233639	0.035205314	-6.37011591	0.00018162	0.0106863	1.272461722
hetero after UVB irradiation VS hetero before UVB irradiation	GO_IRES_DEPENDENT_VIRAL_TRANSLATIONAL_INITIATION1	-0.20386138	0.002336021	-0.60120754	0.56464866	0.81083323	-6.04000967
WT after UVB irradiation VS WT before UVB irradiation	GO_IRES_DEPENDENT_VIRAL_TRANSLATIONAL_INITIATION2	-0.41977299	-0.07365061	-1.4045539	0.19733027	0.69698487	-5.07106197
homo after UVB irradiation VS homo before UVB irradiation	GO_IRIS_MORPHOGENESIS	-0.13750175	0.027091647	-0.496875	0.63212532	0.77297591	-6.41736983
hetero after UVB irradiation VS hetero before UVB irradiation	GO_IRIS_MORPHOGENESIS1	-0.13315938	0.035810735	-0.48196355	0.64297745	0.85383147	-6.10740838
WT after UVB irradiation VS WT before UVB irradiation	GO_IRIS_MORPHOGENESIS2	-0.50061685	0.048820751	-2.44196764	0.04009025	0.48732442	-3.68483081
homo after UVB irradiation VS homo before UVB irradiation	GO_IRON_COORDINATION_ENTITY_TRANSPORT	0.587093124	0.046781003	3.819438186	0.00472443	0.04478121	-1.99033571
hetero after UVB irradiation VS hetero before UVB irradiation	GO_IRON_COORDINATION_ENTITY_TRANSPORT1	0.256068666	0.070246731	1.338692599	0.21813011	0.54557898	-5.35972101
WT after UVB irradiation VS WT before UVB irradiation	GO_IRON_COORDINATION_ENTITY_TRANSPORT2	0.090732132	0.078006762	0.495726463	0.63326	0.90473597	-5.86844486
homo after UVB irradiation VS homo before UVB irradiation	GO_IRON_IMPORT_INTO_CELL	0.32961927	0.031042862	1.715387292	0.12312051	0.28585421	-5.17252232
hetero after UVB irradiation VS hetero before UVB irradiation	GO_IRON_IMPORT_INTO_CELL1	0.558775121	0.019274161	3.132071918	0.01430183	0.17869412	-2.84831169
WT after UVB irradiation VS WT before UVB irradiation	GO_IRON_IMPORT_INTO_CELL2	0.208582277	-0.08422947	0.71858621	0.49260649	0.85303943	-5.73244486
homo after UVB irradiation VS homo before UVB irradiation	GO_IRON_ION_BINDING	0.222395726	-0.00183984	1.668653461	0.13224307	0.29876162	-5.23667297
hetero after UVB irradiation VS hetero before UVB irradiation	GO_IRON_ION_BINDING1	0.146446111	0.015674899	0.874519118	0.40778532	0.71286342	-5.83642718
WT after UVB irradiation VS WT before UVB irradiation	GO_IRON_ION_BINDING2	0.11728757	-0.00911927	0.57349536	0.58188247	0.88904835	-5.82616366
homo after UVB irradiation VS homo before UVB irradiation	GO_IRON_ION_HOMEOSTASIS	0.123077396	0.013723225	0.828038753	0.43073291	0.61479603	-6.18953923
hetero after UVB irradiation VS hetero before UVB irradiation	GO_IRON_ION_HOMEOSTASIS1	0.127805558	0.019476113	0.882701772	0.40360903	0.7106602	-5.82935379

WT after UVB irradiation VS WT before UVB irradiation	GO_IRON_ION_HOMEOSTASIS2	0.136270707	-0.0082364	0.781258927	0.4568811	0.83880233	-5.68626673
homo after UVB irradiation VS homo before UVB irradiation	GO_IRON_ION_IMPORT_ACROSS_PLASMA_MEMBRANE	0.239629774	-0.01460859	0.993305987	0.34853692	0.54046073	-6.03969726
hetero after UVB irradiation VS hetero before UVB irradiation	GO_IRON_ION_IMPORT_ACROSS_PLASMA_MEMBRANE1	0.522494621	-0.05241853	2.535359546	0.03547751	0.25868552	-3.71953507
WT after UVB irradiation VS WT before UVB irradiation	GO_IRON_ION_IMPORT_ACROSS_PLASMA_MEMBRANE2	-0.07592059	-0.08711336	-0.2378263	0.81792311	0.9565994	-5.96633208
homo after UVB irradiation VS homo before UVB irradiation	GO_IRON_ION_TRANSMEMBRANE_TRANSPORT	-0.15151341	0.012326508	-1.02524184	0.33409959	0.52701746	-6.0082509
hetero after UVB irradiation VS hetero before UVB irradiation	GO_IRON_ION_TRANSMEMBRANE_TRANSPORT1	0.074327509	-0.06018268	0.336976614	0.74496381	0.90030853	-6.17036095
WT after UVB irradiation VS WT before UVB irradiation	GO_IRON_ION_TRANSMEMBRANE_TRANSPORT2	-0.20462496	-0.08697425	-0.78250646	0.45618794	0.83880233	-5.68531399
homo after UVB irradiation VS homo before UVB irradiation	GO_IRON_ION_TRANSMEMBRANE_TRANSPORTER_ACTIVITY	-0.27288087	0.027041957	-1.67944037	0.13008381	0.29578534	-5.22194106
hetero after UVB irradiation VS hetero before UVB irradiation	GO_IRON_ION_TRANSMEMBRANE_TRANSPORTER_ACTIVITY1	-0.08652579	-0.03114148	-0.41234975	0.69111018	0.87620446	-6.14029558
WT after UVB irradiation VS WT before UVB irradiation	GO_IRON_ION_TRANSMEMBRANE_TRANSPORTER_ACTIVITY2	-0.12924444	-0.09974174	-0.50405507	0.6276483	0.90339257	-5.86419072
homo after UVB irradiation VS homo before UVB irradiation	GO_IRON_ION_TRANSPORT	0.268398381	-0.01441785	1.610175193	0.14453109	0.31677489	-5.31571789
hetero after UVB irradiation VS hetero before UVB irradiation	GO_IRON_ION_TRANSPORT1	0.294182526	0.005181014	2.250698424	0.05511321	0.31051949	-4.13501512
WT after UVB irradiation VS WT before UVB irradiation	GO_IRON_ION_TRANSPORT2	0.215147987	-0.01728615	1.466269815	0.18030082	0.68278032	-4.99767622
homo after UVB irradiation VS homo before UVB irradiation	GO_IRON_SULFUR_CLUSTER_ASSEMBLY	-0.15426774	-0.01379664	-0.76342746	0.46627957	0.64540332	-6.24190048
hetero after UVB irradiation VS hetero before UVB irradiation	GO_IRON_SULFUR_CLUSTER_ASSEMBLY1	0.1723989	-0.05669497	1.00344335	0.34556145	0.66286618	-5.71893113
WT after UVB irradiation VS WT before UVB irradiation	GO_IRON_SULFUR_CLUSTER_ASSEMBLY2	0.126797564	-0.05508424	0.713718576	0.49545464	0.85463754	-5.73589123
homo after UVB irradiation VS homo before UVB irradiation	GO_ISG15_PROTEIN_CONJUGATION	0.698598937	0.04296645	2.744475914	0.02434039	0.10960975	-3.62994289
hetero after UVB irradiation VS hetero before UVB irradiation	GO_ISG15_PROTEIN_CONJUGATION1	0.57203697	0.067864156	2.364964828	0.04617369	0.28703971	-3.96887899
WT after UVB irradiation VS WT before UVB irradiation	GO_ISG15_PROTEIN_CONJUGATION2	0.430143736	-0.02863572	1.73802042	0.11995137	0.62875505	-4.65582106
homo after UVB irradiation VS homo before UVB irradiation	GO_ISOCITRATE_DEHYDROGENASE_ACTIVITY	0.27023449	-0.0414225	0.83437057	0.42735202	0.61193044	-6.18421362
hetero after UVB irradiation VS hetero before UVB irradiation	GO_ISOCITRATE_DEHYDROGENASE_ACTIVITY1	-0.21687287	-0.07317458	-0.6653855	0.52486152	0.78912283	-5.99815505
WT after UVB irradiation VS WT before UVB irradiation	GO_ISOCITRATE_DEHYDROGENASE_ACTIVITY2	-0.25238462	-0.08776368	-0.80911191	0.4415738	0.83231427	-5.66468926
homo after UVB irradiation VS homo before UVB irradiation	GO_ISOCITRATE_METABOLIC_PROCESS	0.20758992	-0.01975599	0.620856312	0.5513013	0.71418536	-6.34415078
hetero after UVB irradiation VS hetero before UVB irradiation	GO_ISOCITRATE_METABOLIC_PROCESS1	-0.30837109	-0.05607461	-0.93157301	0.37930768	0.69228283	-5.7860054
WT after UVB irradiation VS WT before UVB irradiation	GO_ISOCITRATE_METABOLIC_PROCESS2	-0.16906912	-0.07677317	-0.53204037	0.60898132	0.89786566	-5.84941213
homo after UVB irradiation VS homo before UVB irradiation	GO_ISOLEUCINE_METABOLIC_PROCESS	0.262522812	-0.01701236	0.77956401	0.45722392	0.63795381	-6.22916527
hetero after UVB irradiation VS hetero before UVB irradiation	GO_ISOLEUCINE_METABOLIC_PROCESS1	-0.38741604	0.090782421	-1.42647893	0.19225395	0.51768236	-5.2548345
WT after UVB irradiation VS WT before UVB irradiation	GO_ISOLEUCINE_METABOLIC_PROCESS2	-0.06619915	-0.11664442	-0.28401457	0.78352625	0.9456763	-5.95372749
homo after UVB irradiation VS homo before UVB irradiation	GO_ISOMERASE_ACTIVITY	-0.02005642	-0.02475469	-0.15305605	0.88199722	0.93624176	-6.53801836
hetero after UVB irradiation VS hetero before UVB irradiation	GO_ISOMERASE_ACTIVITY1	-0.00741355	-0.01996103	-0.04782553	0.96304923	0.98664387	-6.23024389
WT after UVB irradiation VS WT before UVB irradiation	GO_ISOMERASE_ACTIVITY2	0.059825911	0.007993692	0.394990235	0.70305076	0.92548867	-5.91457456
homo after UVB irradiation VS homo before UVB irradiation	GO_ISOPENTENYL_DIPHOSPHATE_BIOSYNTHETIC_PROCESS	0.40247739	0.015554919	1.056807366	0.32028418	0.51379863	-5.97641804
hetero after UVB irradiation VS hetero before UVB irradiation	GO_ISOPENTENYL_DIPHOSPHATE_BIOSYNTHETIC_PROCESS1	0.596649079	-0.07942867	1.435534509	0.18974431	0.51465757	-5.24379566
WT after UVB irradiation VS WT before UVB irradiation	GO_ISOPENTENYL_DIPHOSPHATE_BIOSYNTHETIC_PROCESS2	0.504862217	0.017901934	1.378809935	0.20483966	0.7016757	-5.10113617
homo after UVB irradiation VS homo before UVB irradiation	GO_ISOPEPTIDASE_ACTIVITY	-0.60977358	-0.00845622	-3.59257732	0.00660194	0.05370718	-2.32755971
hetero after UVB irradiation VS hetero before UVB irradiation	GO_ISOPEPTIDASE_ACTIVITY1	-0.19533667	-0.04532225	-0.78796362	0.45384408	0.74508172	-5.90789955
WT after UVB irradiation VS WT before UVB irradiation	GO_ISOPEPTIDASE_ACTIVITY2	-0.2506166	-0.0137689	-0.9103815	0.38891591	0.8098285	-5.58098051
homo after UVB irradiation VS homo before UVB irradiation	GO_ISOPRENOID_BINDING	0.280155055	0.100097082	1.787791941	0.11013215	0.26577598	-5.07154027
hetero after UVB irradiation VS hetero before UVB irradiation	GO_ISOPRENOID_BINDING1	-0.01403755	0.070246587	-0.1022272	0.92113851	0.96983444	-6.22582068
WT after UVB irradiation VS WT before UVB irradiation	GO_ISOPRENOID_BINDING2	0.053879685	0.091831771	0.27974043	0.78668943	0.94683761	-5.95498579
homo after UVB irradiation VS homo before UVB irradiation	GO_ISOPRENOID_BIOSYNTHETIC_PROCESS	0.02866314	-0.0056783	0.1446803	0.88840305	0.93938541	-6.53938329
hetero after UVB irradiation VS hetero before UVB irradiation	GO_ISOPRENOID_BIOSYNTHETIC_PROCESS1	0.05738646	-0.00239843	0.252149066	0.80739982	0.92499935	-6.1971487
WT after UVB irradiation VS WT before UVB irradiation	GO_ISOPRENOID_BIOSYNTHETIC_PROCESS2	0.020777722	0.026486727	0.080601192	0.93771562	0.98368811	-5.99264021
homo after UVB irradiation VS homo before UVB irradiation	GO_ISOPRENOID_CATABOLIC_PROCESS	0.467404474	0.009481863	2.45439996	0.03852767	0.1409402	-4.07934442
hetero after UVB irradiation VS hetero before UVB irradiation	GO_ISOPRENOID_CATABOLIC_PROCESS1	0.487786881	0.079613252	2.971378701	0.01820978	0.19726134	-3.08113615
WT after UVB irradiation VS WT before UVB irradiation	GO_ISOPRENOID_CATABOLIC_PROCESS2	0.306690022	0.022572539	1.943598495	0.08741863	0.57914913	-4.38141701
homo after UVB irradiation VS homo before UVB irradiation	GO_ISOPRENOID_METABOLIC_PROCESS	0.263231022	0.030257535	1.878470007	0.09567448	0.24426788	-4.94258905
hetero after UVB irradiation VS hetero before UVB irradiation	GO_ISOPRENOID_METABOLIC_PROCESS1	0.068798688	0.02921247	0.430333928	0.67851916	0.87062858	-6.13226557
WT after UVB irradiation VS WT before UVB irradiation	GO_ISOPRENOID_METABOLIC_PROCESS2	0.12031684	0.031215672	0.590512307	0.57095578	0.8839005	-5.81615717
homo after UVB irradiation VS homo before UVB irradiation	GO_ISOTYPE_SWITCHING_TO_IGA_ISOTYPES	-0.34093267	-0.06032885	-2.3845831	0.04304408	0.15002396	-4.18671869
hetero after UVB irradiation VS hetero before UVB irradiation	GO_ISOTYPE_SWITCHING_TO_IGA_ISOTYPES1	-0.18287042	-0.01744042	-0.62652212	0.54874813	0.80041738	-6.0239563
WT after UVB irradiation VS WT before UVB irradiation	GO_ISOTYPE_SWITCHING_TO_IGA_ISOTYPES2	0.090871506	-0.08806706	0.372331458	0.719202	0.93096927	-5.92357473
homo after UVB irradiation VS homo before UVB irradiation	GO_ISOTYPE_SWITCHING_TO_IGG_ISOTYPES	0.049688755	-0.02163292	0.249282245	0.80918002	0.89186839	-6.51688122
hetero after UVB irradiation VS hetero before UVB irradiation	GO_ISOTYPE_SWITCHING_TO_IGG_ISOTYPES1	-0.08141425	0.007292209	-0.56837827	0.58566003	0.82335261	-6.05992997
WT after UVB irradiation VS WT before UVB irradiation	GO_ISOTYPE_SWITCHING_TO_IGG_ISOTYPES2	0.307849085	-0.04652824	1.652439874	0.13659071	0.64480779	-4.7664248
homo after UVB irradiation VS homo before UVB irradiation	GO_ISWI_TYPE_COMPLEX	-0.57315839	-0.04977074	-3.23429297	0.0113512	0.07143268	-2.87172419
hetero after UVB irradiation VS hetero before UVB irradiation	GO_ISWI_TYPE_COMPLEX1	-0.15517073	-0.00584736	-0.59137711	0.57089453	0.81424167	-6.04608164
WT after UVB irradiation VS WT before UVB irradiation	GO_ISWI_TYPE_COMPLEX2	-0.22224862	0.051649474	-0.86853754	0.41010174	0.81881631	-5.61654892
homo after UVB irradiation VS homo before UVB irradiation	GO_JNK_CASCADE	0.105009635	-0.01229415	0.769745248	0.46272006	0.64224605	-6.2369418
hetero after UVB irradiation VS hetero before UVB irradiation	GO_JNK_CASCADE1	0.126146754	-0.00452437	0.999234348	0.34747273	0.66476179	-5.72296527
WT after UVB irradiation VS WT before UVB irradiation	GO_JNK_CASCADE2	-0.02244491	-0.00270723	-0.15624033	0.87966858	0.9693507	-5.98320672
homo after UVB irradiation VS homo before UVB irradiation	GO_JUN_KINASE_BINDING	-0.2300406	-0.03795081	-0.88195118	0.40253622	0.58989807	-6.14311067
hetero after UVB irradiation VS hetero before UVB irradiation	GO_JUN_KINASE_BINDING1	-0.34428608	-0.07365042	-1.5691497	0.15595757	0.47498806	-5.07655867
WT after UVB irradiation VS WT before UVB irradiation	GO_JUN_KINASE_BINDING2	-0.33780126	-0.09316416	-1.45751988	0.18263356	0.68598062	-5.008188
homo after UVB irradiation VS homo before UVB irradiation	GO_JUN_KINASE_KINASE_KINASE_ACTIVITY	0.307936967	-0.01704409	1.901053412	0.09236376	0.23917147	-4.91008101
hetero after UVB irradiation VS hetero before UVB irradiation	GO_JUN_KINASE_KINASE_KINASE_ACTIVITY1	0.474782088	0.018006076	3.128107679	0.01438683	0.17916057	-2.85402901
WT after UVB irradiation VS WT before UVB irradiation	GO_JUN_KINASE_KINASE_KINASE_ACTIVITY2	0.324638844	0.020730528	1.8777882	0.09679053	0.59686908	-4.4704382
homo after UVB irradiation VS homo before UVB irradiation	GO_JUNCTIONAL_MEMBRANE_COMPLEX	0.63417312	-0.00259644	3.362005767	0.00934046	0.06433347	-2.67633903
hetero after UVB irradiation VS hetero before UVB irradiation	GO_JUNCTIONAL_MEMBRANE_COMPLEX1	-0.24920767	-0.04997602	-0.92159033	0.38418227	0.69552909	-5.79501173
WT after UVB irradiation VS WT before UVB irradiation	GO_JUNCTIONAL_MEMBRANE_COMPLEX2	0.041688622	0.014419342	0.126453693	0.90245633	0.97569904	-5.98763808
homo after UVB irradiation VS homo before UVB irradiation	GO_JUNCTIONAL_SARCOPLASMIC_RETICULUM_MEMBRANE	0.569039392	0.034087581	3.025840957	0.01566355	0.08529409	-3.19319308
hetero after UVB irradiation VS hetero before UVB irradiation	GO_JUNCTIONAL_SARCOPLASMIC_RETICULUM_MEMBRANE1	-0.27978801	-0.08661658	-1.21191586	0.2607466	0.5870578	-5.5038118
WT after UVB irradiation VS WT before UVB irradiation	GO_JUNCTIONAL_SARCOPLASMIC_RETICULUM_MEMBRANE2	0.123192329	-0.03793042	0.354053211	0.73234133	0.93397019	-5.9304612
homo after UVB irradiation VS homo before UVB irradiation	GO_JUXTAPARANODE_REGION_OF_AXON	0.381399969	-0.0440363	1.803768714	0.10744401	0.26224797	-5.04901219
hetero after UVB irradiation VS hetero before UVB irradiation	GO_JUXTAPARANODE_REGION_OF_AXON1	0.068802183	-0.03719488	0.228583653	0.82503269	0.93360295	-6.20324565
WT after UVB irradiation VS WT before UVB irradiation	GO_JUXTAPARANODE_REGION_OF_AXON2	0.009440014	-0.01840029	0.029443267	0.97722381	0.99426998	-5.99560927
homo after UVB irradiation VS homo before UVB irradiation	GO_K48_LINKED_POLYUBIQUITIN_MODIFICATION_DEPENDENT_PROTEIN_BINDING	-0.12547262	0.04471749	-0.56814921	0.58492061	0.73867546	-6.37711433
hetero after UVB irradiation VS hetero before UVB irradiation	GO_K48_LINKED_POLYUBIQUITIN_MODIFICATION_DEPENDENT_PROTEIN_BINDING1	0.101791785	-0.03231044	0.390219892	0.70674525	0.88394075	-6.14972636
WT after UVB irradiation VS WT before UVB irradiation	GO_K48_LINKED_POLYUBIQUITIN_MODIFICATION_DEPENDENT_PROTEIN_BINDING2	0.37828416	-0.00176841	1.210913313	0.26008199	0.74045261	-5.28873607
homo after UVB irradiation VS homo before UVB irradiation	GO_K63_LINKED_POLYUBIQUITIN_MODIFICATION_DEPENDENT_PROTEIN_BINDING	0.321392729	-0.05797394	1.872197891	0.09661377	0.24602751	-4.95159092
hetero after UVB irradiation VS hetero before UVB irradiation	GO_K63_LINKED_POLYUBIQUITIN_MODIFICATION_DEPENDENT_PROTEIN_BINDING1	0.306238092	0.047949691	1.623768209	0.14378918	0.45985114	-5.00602955
WT after UVB irradiation VS WT before UVB irradiation	GO_K63_LINKED_POLYUBIQUITIN_MODIFICATION_DEPENDENT_PROTEIN_BINDING2	0.076594982	-0.00027892	0.444304198	0.66845711	0.91431526	-5.89323028
homo after UVB irradiation VS homo before UVB irradiation	GO_KAINATE_SELECTIVE_GLUTAMATE_RECEPTOR_ACTIVITY	-0.91000241	-0.00372107	-5.03260792	0.00089458	0.02059491	-0.31249567
hetero after UVB irradiation VS hetero before UVB irradiation	GO_KAINATE_SELECTIVE_GLUTAMATE_RECEPTOR_ACTIVITY1	-0.45392789	0.041530123	-1.40949794	0.19703891	0.52143224	-5.27542688
WT after UVB irradiation VS WT before UVB irradiation	GO_KAINATE_SELECTIVE_GLUTAMATE_RECEPTOR_ACTIVITY2	-0.06858005	0.121146663	-0.2516637	0.80757033	0.95404687	-5.96278602
homo after UVB irradiation VS homo before UVB irradiation	GO_KERATAN_SULFATE_BIOSYNTHETIC_PROCESS	0.392195821	-0.03168366	2.658124391	0.0278962	0.11808674	-3.76406265
hetero after UVB irradiation VS hetero before UVB irradiation	GO_KERATAN_SULFATE_BIOSYNTHETIC_PROCESS1	0.1308323	0.036901704	0.593611195	0.56947162	0.8137684	-6.04470971
WT after UVB irradiation VS WT before UVB irradiation	GO_KERATAN_SULFATE_BIOSYNTHETIC_PROCESS2	0.279983455	-0.014315	1.15305801	0.28178462	0.75255145	-5.34964084
homo after UVB irradiation VS homo before UVB irradiation	GO_KERATAN_SULFATE_CATABOLIC_PROCESS	0.241099879	-0.06508295	0.917028807	0.3849098	0.57444518	-6.1116063
hetero after UVB irradiation VS hetero before UVB irradiation	GO_KERATAN_SULFATE_CATABOLIC_PROCESS1	0.009238216	-0.04688893	0.03352181	0.97409485	0.99122431	-6.23087476
WT after UVB irradiation VS WT before UVB irradiation	GO_KERATAN_SULFATE_CATABOLIC_PROCESS2	0.082588533	-0.07362873	0.263559859	0.79870193	0.95209758	-5.95957985
homo after UVB irradiation VS homo before UVB irradiation	GO_KERATAN_SULFATE_METABOLIC_PROCESS	0.484918133	-0.03494307	3.348598571	0.00953276	0.06506705	-2.69678365
hetero after UVB irradiation VS hetero before UVB irradiation	GO_KERATAN_SULFATE_METABOLIC_PROCESS1	0.264304133	0.029672055	1.245476279	0.24883525	0.57651327	-5.46657131
WT after UVB irradiation VS WT before UVB irradiation	GO_KERATAN_SULFATE_METABOLIC_PROCESS2	0.322905747	-0.02789258	1.271192502	0.23895726	0.72928346	-5.22316782
homo after UVB irradiation VS homo before UVB irradiation	GO_KERATIN_FILAMENT	-0.63373792	0.018572967	-4.126356	0.00303886	0.03639967	-1.54495517
hetero after UVB irradiation VS hetero before UVB irradiation	GO_KERATIN_FILAMENT_BINDING	0.119200336	-0.01737533	0.381592882	0.71231426	0.8287308	-6.47164551
WT after UVB irradiation VS WT before UVB irradiation	GO_KERATIN_FILAMENT_BINDING1	-0.20424944	0.014953158	-1.06330949	0.31924372	0.63860264	-5.66017259
homo after UVB irradiation VS homo before UVB irradiation	GO_KERATIN_FILAMENT_BINDING2	-0.25533438	-0.00445241	-1.06984241	0.31553999	0.77149112	-5.43353606
hetero after UVB irradiation VS hetero before UVB irradiation	GO_KERATIN_FILAMENT1	-0.47758058	-0.03465429	-1.95824275	0.08656965	0.37240327	-4.5525036
WT after UVB irradiation VS WT before UVB irradiation	GO_KERATIN_FILAMENT2	-0.5318798	-0.00631397	-1.88357256	0.0959298	0.59686908	-4.4626544
homo after UVB irradiation VS homo before UVB irradiation	GO_KERATINIZATION	-0.42647228	0.021684369	-2.27663493	0.05108955	0.16668771	-4.35169878
hetero after UVB irradiation VS hetero before UVB irradiation	GO_KERATINIZATION1	-0.22964876	-0.00639599	-1.07529681	0.31416727	0.63499052	-5.6481036
WT after UVB irradiation VS WT before UVB irradiation	GO_KERATINIZATION2	-0.34604987	0.056350593	-1.2248071	0.25508023	0.73617995	-5.27380993
homo after UVB irradiation VS homo before UVB irradiation	GO_KERATINOCYTE_APOPTOTIC_PROCESS	-0.22815158	0.000653653	-0.96395605	0.36221605	0.55300173	-6.06790791
hetero after UVB irradiation VS hetero before UVB irradiation	GO_KERATINOCYTE_APOPTOTIC_PROCESS1	-0.35846622	0.028491706	-1.62766215	0.14295576	0.45818529	-5.00095715
WT after UVB irradiation VS WT before UVB irradiation	GO_KERATINOCYTE_APOPTOTIC_PROCESS2	-0.59250948	0.118806303	-2.31665577	0.04879974	0.50722877	-3.86245107
homo after UVB irradiation VS homo before UVB irradiation	GO_KERATINOCYTE_DEVELOPMENT	-0.16430839	-0.00506294	-0.6554393	0.52986811	0.69713619	-6.32107931
hetero after UVB irradiation VS hetero before UVB irradiation	GO_KERATINOCYTE_DEVELOPMENT1	0.122135736	-0.03046363	0.468025646	0.65248114	0.85713917	-6.11438082
WT after UVB irradiation VS WT before UVB irradiation	GO_KERATINOCYTE_DEVELOPMENT2	-0.15016404	0.062320684	-0.57097181	0.58351276	0.88980639	-5.82762477
homo after UVB irradiation VS homo before UVB irradiation	GO_KERATINOCYTE_DIFFERENTIATION	-0.32222442	0.021801647	-1.88929956	0.09407304	0.24220208	-4.92701884
hetero after UVB irradiation VS hetero before UVB irradiation	GO_KERATINOCYTE_DIFFERENTIATION1	-0.16438676	0.006984435	-0.84529028	0.42295475	0.7249295	-5.8612527
WT after UVB irradiation VS WT before UVB irradiation	GO_KERATINOCYTE_DIFFERENTIATION2	-0.30180949	0.0583032	-1.18736181	0.26874519	0.74431614	-5.31377499
homo after UVB irradiation VS homo before UVB irradiation	GO_KERATINOCYTE_MIGRATION	0.370953688	-0.0027402	2.125046923	0.06495421	0.19328792	-4.58053143
hetero after UVB irradiation VS hetero before UVB irradiation	GO_KERATINOCYTE_MIGRATION1	0.637651045	0.019058137	4.220539993	0.00303802	0.11447176	-1.35448565
WT after UVB irradiation VS WT before UVB irradiation	GO_KERATINOCYTE_MIGRATION2	0.451090207	0.042190392	2.803980716	0.02278231	0.41742287	-3.17010787
homo after UVB irradiation VS homo before UVB irradiation	GO_KERATINOCYTE_PROLIFERATION	0.022018685	0.038818285	0.138473267	0.89315585	0.94187288	-6.5403455
hetero after UVB irradiation VS hetero before UVB irradiation	GO_KERATINOCYTE_PROLIFERATION1	0.142677696	0.034735609	1.092460422	0.30700989	0.627591	-5.63065108
WT after UVB irradiation VS WT before UVB irradiation	GO_KERATINOCYTE_PROLIFERATION2	-0.04285661	0.079714532	-0.23068042	0.82328451	0.95856757	-5.96808599
homo after UVB irradiation VS homo before UVB irradiation	GO_KETONE_BIOSYNTHETIC_PROCESS	-0.06188234	-0.01157041	-0.36456596	0.72451803	0.83712125	-6.47850121
hetero after UVB irradiation VS hetero before UVB irradiation	GO_KETONE_BIOSYNTHETIC_PROCESS1	-0.31306022	-0.03634008	-2.30935177	0.05032702	0.29860291	-4.04988221
WT after UVB irradiation VS WT before UVB irradiation	GO_KETONE_BIOSYNTHETIC_PROCESS2	-0.28231832	0.02192119	-1.45346836	0.1837227	0.68707449	-5.01304353
homo after UVB irradiation VS homo before UVB irradiation	GO_KETONE_BODY_BIOSYNTHETIC_PROCESS	0.008529437	-0.05484376	0.04199408	0.96749268	0.98314898	-6.54988898
hetero after UVB irradiation VS hetero before UVB irradiation	GO_KETONE_BODY_BIOSYNTHETIC_PROCESS1	-0.11321397	-0.0522777	-0.62701955	0.54843836	0.80031215	-6.02363487
WT after UVB irradiation VS WT before UVB irradiation	GO_KETONE_BODY_BIOSYNTHETIC_PROCESS2	-0.15837286	-0.05319542	-0.66676978	0.52345939	0.86471827	-5.76807222
homo after UVB irradiation VS homo before UVB irradiation	GO_KETONE_BODY_METABOLIC_PROCESS	-0.21806491	-0.00110274	-0.99900928	0.34592453	0.53808457	-6.03413825
hetero after UVB irradiation VS hetero before UVB irradiation	GO_KETONE_BODY_METABOLIC_PROCESS1	-0.36506061	-0.05304812	-2.33548894	0.04833029	0.29314231	-4.01184294
WT after UVB irradiation VS WT before UVB irradiation	GO_KETONE_BODY_METABOLIC_PROCESS2	-0.36764081	0.002460686	-1.81598123	0.10645733	0.61116853	-4.55308059
homo after UVB irradiation VS homo before UVB irradiation	GO_KETONE_CATABOLIC_PROCESS	0.235835057	0.043797967	1.372983707	0.20558203	0.3952285	-5.62026453
hetero after UVB irradiation VS hetero before UVB irradiation	GO_KETONE_CATABOLIC_PROCESS1	0.191119833	0.039522585	1.129612099	0.29196181	0.61588295	-5.59219351
WT after UVB irradiation VS WT before UVB irradiation	GO_KETONE_CATABOLIC_PROCESS2	0.145260016	0.047916423	0.519583874	0.61725379	0.90020288	-5.85608206
homo after UVB irradiation VS homo before UVB irradiation	GO_KETOSTEROID_MONOOXYGENASE_ACTIVITY	0.41005295	0.085603472	1.708912313	0.12434879	0.28761992	-5.18146024
hetero after UVB irradiation VS hetero before UVB irradiation	GO_KETOSTEROID_MONOOXYGENASE_ACTIVITY1	0.27784739	0.10786371	1.146144227	0.28545909	0.61082284	-5.57478708
WT after UVB irradiation VS WT before UVB irradiation	GO_KETOSTEROID_MONOOXYGENASE_ACTIVITY2	0.014983608	0.053707486	0.068849373	0.94677933	0.98594946	-5.99356625
homo after UVB irradiation VS homo before UVB irradiation	GO_KICSTOR_COMPLEX	-0.22071375	-0.03836459	-0.948734	0.36946626	0.55990004	-6.08227428
hetero after UVB irradiation VS hetero before UVB irradiation	GO_KICSTOR_COMPLEX1	-0.18815303	-0.0659483	-0.63638756	0.54262388	0.7977485	-6.01753848
WT after UVB irradiation VS WT before UVB irradiation	GO_KICSTOR_COMPLEX2	-0.514852	-0.02379691	-1.61597016	0.14430769	0.65263518	-4.81279212
homo after UVB irradiation VS homo before UVB irradiation	GO_KIDNEY_EPITHELIUM_DEVELOPMENT	-0.10660966	0.019676321	-0.84746207	0.42042018	0.60563652	-6.17309437
hetero after UVB irradiation VS hetero before UVB irradiation	GO_KIDNEY_EPITHELIUM_DEVELOPMENT1	-0.21494107	0.022599032	-1.35680867	0.21255546	0.5391931	-5.33840105
WT after UVB irradiation VS WT before UVB irradiation	GO_KIDNEY_EPITHELIUM_DEVELOPMENT2	-0.21694614	0.022479809	-1.47245555	0.17866765	0.68213098	-4.99022414
homo after UVB irradiation VS homo before UVB irradiation	GO_KIDNEY_MESENCHYME_DEVELOPMENT	-0.20291614	0.067076765	-1.58937015	0.14914773	0.32243964	-5.34349344
hetero after UVB irradiation VS hetero before UVB irradiation	GO_KIDNEY_MESENCHYME_DEVELOPMENT1	-0.30194685	0.023254577	-1.69220346	0.12977492	0.4380052	-4.91607462
WT after UVB irradiation VS WT before UVB irradiation	GO_KIDNEY_MESENCHYME_DEVELOPMENT2	-0.1269021	0.032637669	-0.73191722	0.48486007	0.85146975	-5.72290184
homo after UVB irradiation VS homo before UVB irradiation	GO_KIDNEY_MORPHOGENESIS	-0.08661932	0.022403051	-0.62916366	0.54610674	0.71016013	-6.33871206
hetero after UVB irradiation VS hetero before UVB irradiation	GO_KIDNEY_MORPHOGENESIS1	-0.17712676	0.018805469	-1.10393923	0.3022958	0.62456971	-5.61886736
WT after UVB irradiation VS WT before UVB irradiation	GO_KIDNEY_MORPHOGENESIS2	-0.11397339	0.046308161	-0.75922113	0.46924205	0.84559836	-5.70288291
homo after UVB irradiation VS homo before UVB irradiation	GO_KILLING_OF_CELLS_IN_OTHER_ORGANISM_INVOLVED_IN_SYMBIOTIC_INTERACTION	0.474674895	-0.03880601	2.600140397	0.03057711	0.12413382	-3.85401253
hetero after UVB irradiation VS hetero before UVB irradiation	GO_KILLING_OF_CELLS_IN_OTHER_ORGANISM_INVOLVED_IN_SYMBIOTIC_INTERACTION1	0.567331533	0.049964194	3.163048582	0.01365551	0.17665328	-2.80368715
WT after UVB irradiation VS WT before UVB irradiation	GO_KILLING_OF_CELLS_IN_OTHER_ORGANISM_INVOLVED_IN_SYMBIOTIC_INTERACTION2	0.464222582	0.072159928	2.396673594	0.04304204	0.49624823	-3.74913206
homo after UVB irradiation VS homo before UVB irradiation	GO_KILLING_OF_CELLS_OF_OTHER_ORGANISM	0.519854595	-0.01310195	2.954292942	0.01751101	0.09069947	-3.30406624
hetero after UVB irradiation VS hetero before UVB irradiation	GO_KILLING_OF_CELLS_OF_OTHER_ORGANISM1	0.661302462	0.040581193	4.684131267	0.00165409	0.09676143	-0.77479633
WT after UVB irradiation VS WT before UVB irradiation	GO_KILLING_OF_CELLS_OF_OTHER_ORGANISM2	0.252709969	0.040157632	1.270351769	0.2392417	0.72928936	-5.22409658
homo after UVB irradiation VS homo before UVB irradiation	GO_KINASE_ACTIVATOR_ACTIVITY	0.091265144	0.00762562	0.517834596	0.61804362	0.76269068	-6.4060546

hetero after UVB irradiation VS hetero before UVB irradiation	GO_KINASE_ACTIVATOR_ACTIVITY1	0.29506176	0.028596956	2.131978788	0.06622925	0.33502634	-4.30611914
WT after UVB irradiation VS WT before UVB irradiation	GO_KINASE_ACTIVATOR_ACTIVITY2	0.109645798	-0.03251596	0.69422447	0.50696581	0.85773353	-5.7494876
homo after UVB irradiation VS homo before UVB irradiation	GO_KINASE_ACTIVITY	0.040020577	-0.01408652	0.308936702	0.76495623	0.86409149	-6.49878001
hetero after UVB irradiation VS hetero before UVB irradiation	GO_KINASE_ACTIVITY1	0.063711406	-0.00396852	0.503467857	0.62845166	0.84712092	-6.09627415
WT after UVB irradiation VS WT before UVB irradiation	GO_KINASE_ACTIVITY2	-0.01392866	-0.01700765	-0.09845407	0.92396529	0.98116508	-5.99095529
homo after UVB irradiation VS homo before UVB irradiation	GO_KINASE_BINDING	0.08839912	-0.01691498	0.670225216	0.52085969	0.69034726	-6.3108716
hetero after UVB irradiation VS hetero before UVB irradiation	GO_KINASE_BINDING1	0.180519624	-0.00374983	1.474263437	0.17933504	0.50330437	-5.19614542
WT after UVB irradiation VS WT before UVB irradiation	GO_KINASE_BINDING2	0.052551998	-0.02331208	0.373891322	0.71808519	0.93096927	-5.92297155
homo after UVB irradiation VS homo before UVB irradiation	GO_KINASE_INHIBITOR_ACTIVITY	0.312612168	-0.01240895	2.272569927	0.0514201	0.16755924	-4.35788237
hetero after UVB irradiation VS hetero before UVB irradiation	GO_KINASE_INHIBITOR_ACTIVITY1	0.289276086	0.002304694	2.32665127	0.04899639	0.29497788	-4.02471146
WT after UVB irradiation VS WT before UVB irradiation	GO_KINASE_INHIBITOR_ACTIVITY2	0.184930831	-0.06805222	1.167906809	0.27607859	0.74766532	-5.33420505
homo after UVB irradiation VS homo before UVB irradiation	GO_KINASE_REGULATOR_ACTIVITY	0.1262245	0.005616671	0.909285137	0.38875214	0.57801666	-6.11864751
hetero after UVB irradiation VS hetero before UVB irradiation	GO_KINASE_REGULATOR_ACTIVITY1	0.234757094	0.008523422	1.782826789	0.11316783	0.41521928	-4.79450377
WT after UVB irradiation VS WT before UVB irradiation	GO_KINASE_REGULATOR_ACTIVITY2	0.124818454	-0.03009476	0.814999002	0.4383837	0.83056493	-5.6600471
homo after UVB irradiation VS homo before UVB irradiation	GO_KINESIN_BINDING	-0.12258651	0.020329699	-0.7994779	0.44621137	0.62835051	-6.2131334
hetero after UVB irradiation VS hetero before UVB irradiation	GO_KINESIN_BINDING1	-0.25066317	-0.03364274	-1.7750562	0.1145096	0.41722066	-4.80502942
WT after UVB irradiation VS WT before UVB irradiation	GO_KINESIN_BINDING2	-0.25891846	-0.00274193	-1.75541413	0.11680871	0.62424536	-4.63305403
homo after UVB irradiation VS homo before UVB irradiation	GO_KINESIN_COMPLEX	-0.1577005	0.016543804	-0.89344884	0.39669612	0.58540447	-6.13289525
hetero after UVB irradiation VS hetero before UVB irradiation	GO_KINESIN_COMPLEX1	0.019694183	-0.05211058	0.102497076	0.92093113	0.96983444	-6.22579076
WT after UVB irradiation VS WT before UVB irradiation	GO_KINESIN_COMPLEX2	-0.05749774	-0.00082802	-0.23701869	0.81852854	0.95674608	-5.96653295
homo after UVB irradiation VS homo before UVB irradiation	GO_KINETOCHORE	-0.52448619	-0.03405757	-3.29532916	0.01033888	0.0679874	-2.77817322
homo after UVB irradiation VS homo before UVB irradiation	GO_KINETOCHORE_ASSEMBLY	-0.72591471	-0.07205073	-4.12457152	0.00304654	0.03646216	-1.54750613
hetero after UVB irradiation VS hetero before UVB irradiation	GO_KINETOCHORE_ASSEMBLY1	0.059335315	-0.03645682	0.193831913	0.85122746	0.94316582	-6.21115958
WT after UVB irradiation VS WT before UVB irradiation	GO_KINETOCHORE_ASSEMBLY2	0.087954534	0.036567126	0.292060466	0.77758331	0.94371579	-5.95130812
homo after UVB irradiation VS homo before UVB irradiation	GO_KINETOCHORE_BINDING	-0.38539544	-0.04568885	-1.46391356	0.17990858	0.36236909	-5.506801
hetero after UVB irradiation VS hetero before UVB irradiation	GO_KINETOCHORE_BINDING1	0.135703386	0.019598225	0.342307334	0.74110102	0.89909452	-6.16842672
WT after UVB irradiation VS WT before UVB irradiation	GO_KINETOCHORE_BINDING2	0.184393796	0.075514303	0.57568624	0.58046916	0.888473	-5.82489038
homo after UVB irradiation VS homo before UVB irradiation	GO_KINETOCHORE_MICROTUBULE	-0.42218556	-0.01326634	-3.14185787	0.01308632	0.0772459	-3.01393725
hetero after UVB irradiation VS hetero before UVB irradiation	GO_KINETOCHORE_MICROTUBULE1	0.158678313	0.004542404	0.493654504	0.63505953	0.84997174	-6.1014117
WT after UVB irradiation VS WT before UVB irradiation	GO_KINETOCHORE_MICROTUBULE2	-0.09663849	0.042501762	-0.36283669	0.72601529	0.93220288	-5.92719374
homo after UVB irradiation VS homo before UVB irradiation	GO_KINETOCHORE_ORGANIZATION	-0.65285796	-0.08812706	-2.8827394	0.01958504	0.09649966	-3.41512072
hetero after UVB irradiation VS hetero before UVB irradiation	GO_KINETOCHORE_ORGANIZATION1	0.118486686	-0.01933954	0.368179995	0.72246588	0.8911687	-6.15862176
WT after UVB irradiation VS WT before UVB irradiation	GO_KINETOCHORE_ORGANIZATION2	0.163895487	0.044466827	0.548082132	0.59841533	0.89476186	-5.84060667
hetero after UVB irradiation VS hetero before UVB irradiation	GO_KINETOCHORE1	0.169106337	-0.01137491	0.646315942	0.53650204	0.79463142	-6.01098887
WT after UVB irradiation VS WT before UVB irradiation	GO_KINETOCHORE2	-0.01138979	0.016107723	-0.04112935	0.96818881	0.99191629	-5.99517418
homo after UVB irradiation VS homo before UVB irradiation	GO_KINOCILIUM	-0.6552131	0.057821428	-4.11395932	0.00309269	0.03668719	-1.56268603
hetero after UVB irradiation VS hetero before UVB irradiation	GO_KINOCILIUM1	-0.63806447	-0.01833339	-3.01076577	0.01715857	0.1913886	-3.02388024
WT after UVB irradiation VS WT before UVB irradiation	GO_KINOCILIUM2	-0.77994522	0.047921847	-3.91403685	0.00435106	0.26635361	-1.66009742
homo after UVB irradiation VS homo before UVB irradiation	GO_KYNURENINE_METABOLIC_PROCESS	0.005635949	-0.01451692	0.02691031	0.97916488	0.98935543	-6.5504593
hetero after UVB irradiation VS hetero before UVB irradiation	GO_KYNURENINE_METABOLIC_PROCESS1	0.092424464	-0.00517835	0.506064764	0.62670895	0.84631805	-6.09489877
WT after UVB irradiation VS WT before UVB irradiation	GO_KYNURENINE_METABOLIC_PROCESS2	-0.16745593	-0.00796267	-0.8013962	0.44577877	0.83354755	-5.67073049
homo after UVB irradiation VS homo before UVB irradiation	GO_L_ALANINE_TRANSMEMBRANE_TRANSPORTER_ACTIVITY	-0.58927953	-0.11556536	-2.79817585	0.02236638	0.10412594	-3.54649615
hetero after UVB irradiation VS hetero before UVB irradiation	GO_L_ALANINE_TRANSMEMBRANE_TRANSPORTER_ACTIVITY1	-0.54347731	-0.03454847	-2.2815244	0.05254369	0.30489864	-4.09031459
WT after UVB irradiation VS WT before UVB irradiation	GO_L_ALANINE_TRANSMEMBRANE_TRANSPORTER_ACTIVITY2	-0.13270595	-0.0382885	-0.49976409	0.63053632	0.90411526	-5.86639078
homo after UVB irradiation VS homo before UVB irradiation	GO_L_ALANINE_TRANSPORT	-0.3945479	-0.12063921	-1.85957036	0.0985314	0.24859426	-4.96967836
hetero after UVB irradiation VS hetero before UVB irradiation	GO_L_ALANINE_TRANSPORT1	-0.40167298	-0.01199887	-1.75454753	0.11812317	0.42214228	-4.83272097
WT after UVB irradiation VS WT before UVB irradiation	GO_L_ALANINE_TRANSPORT2	0.014936775	0.022749155	0.062191385	0.95191812	0.98676072	-5.99402635
homo after UVB irradiation VS homo before UVB irradiation	GO_L_ALPHA_AMINO_ACID_TRANSMEMBRANE_TRANSPORT	-0.03402858	0.021658138	-0.25683986	0.8035332	0.88881939	-6.51479828
hetero after UVB irradiation VS hetero before UVB irradiation	GO_L_ALPHA_AMINO_ACID_TRANSMEMBRANE_TRANSPORT1	-0.23874115	0.021966513	-1.83296202	0.10486149	0.40086427	-4.72616848
WT after UVB irradiation VS WT before UVB irradiation	GO_L_ALPHA_AMINO_ACID_TRANSMEMBRANE_TRANSPORT2	-0.08692638	0.005187454	-0.5503845	0.59690703	0.89380878	-5.83932303
homo after UVB irradiation VS homo before UVB irradiation	GO_L_AMINO_ACID_TRANSMEMBRANE_TRANSPORTER_ACTIVITY	-0.07457353	0.019290771	-0.5810857	0.57656468	0.7333533	-6.36927234
hetero after UVB irradiation VS hetero before UVB irradiation	GO_L_AMINO_ACID_TRANSMEMBRANE_TRANSPORTER_ACTIVITY1	-0.21803733	0.016877551	-1.45844663	0.18352332	0.50789011	-5.21569062
WT after UVB irradiation VS WT before UVB irradiation	GO_L_AMINO_ACID_TRANSMEMBRANE_TRANSPORTER_ACTIVITY2	-0.1365042	-0.0104809	-0.9004065	0.39389305	0.81358	-5.58958227
homo after UVB irradiation VS homo before UVB irradiation	GO_L_AMINO_ACID_TRANSPORT	0.001460777	0.021506792	0.011169069	0.99135146	0.99546635	-6.55078825
hetero after UVB irradiation VS hetero before UVB irradiation	GO_L_AMINO_ACID_TRANSPORT1	-0.12440031	0.014633954	-0.97406491	0.35907045	0.67375936	-5.74681677
WT after UVB irradiation VS WT before UVB irradiation	GO_L_AMINO_ACID_TRANSPORT2	-0.04667282	0.001480808	-0.29126741	0.7781684	0.94371579	-5.95154953
homo after UVB irradiation VS homo before UVB irradiation	GO_L_ARGININE_IMPORT_ACROSS_PLASMA_MEMBRANE	-0.02894517	0.001045653	-0.1508009	0.88372109	0.93712754	-6.53839338
hetero after UVB irradiation VS hetero before UVB irradiation	GO_L_ARGININE_IMPORT_ACROSS_PLASMA_MEMBRANE1	-0.35348192	0.024063885	-1.76208634	0.11678257	0.42020083	-4.82255687
WT after UVB irradiation VS WT before UVB irradiation	GO_L_ARGININE_IMPORT_ACROSS_PLASMA_MEMBRANE2	-0.0160336	-0.0023894	-0.06653415	0.94856598	0.98614246	-5.99373154
homo after UVB irradiation VS homo before UVB irradiation	GO_L_ARGININE_TRANSMEMBRANE_TRANSPORT	-0.05277007	-0.00433675	-0.27386259	0.7908594	0.88100794	-6.50988266
hetero after UVB irradiation VS hetero before UVB irradiation	GO_L_ARGININE_TRANSMEMBRANE_TRANSPORT1	-0.35940654	0.001598674	-1.95792485	0.08661192	0.37240327	-4.552949
WT after UVB irradiation VS WT before UVB irradiation	GO_L_ARGININE_TRANSMEMBRANE_TRANSPORT2	-0.09547537	-0.02185115	-0.46700623	0.65280369	0.9093283	-5.88260353
homo after UVB irradiation VS homo before UVB irradiation	GO_L_ARGININE_TRANSMEMBRANE_TRANSPORTER_ACTIVITY	0.111889601	-0.01542572	0.584895752	0.57411649	0.73124781	-6.36693173
hetero after UVB irradiation VS hetero before UVB irradiation	GO_L_ARGININE_TRANSMEMBRANE_TRANSPORTER_ACTIVITY1	-0.1487026	0.008562419	-0.69549241	0.50680295	0.77689638	-5.97722016
WT after UVB irradiation VS WT before UVB irradiation	GO_L_ARGININE_TRANSMEMBRANE_TRANSPORTER_ACTIVITY2	0.090163239	0.031694202	0.38941683	0.70700907	0.92691191	-5.91683574
homo after UVB irradiation VS homo before UVB irradiation	GO_L_ARGININE_TRANSPORT	0.046213001	-0.04398041	0.214538746	0.8352865	0.90774894	-6.52566714
hetero after UVB irradiation VS hetero before UVB irradiation	GO_L_ARGININE_TRANSPORT1	-0.10369864	0.031620925	-0.54115753	0.60340761	0.83419966	-6.07566752
WT after UVB irradiation VS WT before UVB irradiation	GO_L_ARGININE_TRANSPORT2	-0.0596071	0.012221058	-0.34245735	0.74072622	0.93520732	-5.93465612
homo after UVB irradiation VS homo before UVB irradiation	GO_L_ASCORBIC_ACID_BINDING	0.823984615	-0.01161653	6.512208237	0.0001554	0.01016637	1.425208017
hetero after UVB irradiation VS hetero before UVB irradiation	GO_L_ASCORBIC_ACID_BINDING1	0.536209188	0.041656248	2.091091869	0.07054845	0.34357966	-4.36458705
WT after UVB irradiation VS WT before UVB irradiation	GO_L_ASCORBIC_ACID_BINDING2	0.579029981	0.04237129	2.599153423	0.03134285	0.45353809	-3.46123361
homo after UVB irradiation VS homo before UVB irradiation	GO_L_ASCORBIC_ACID_METABOLIC_PROCESS	0.484603635	-0.01137426	2.029057313	0.07557143	0.21109363	-4.72320933
hetero after UVB irradiation VS hetero before UVB irradiation	GO_L_ASCORBIC_ACID_METABOLIC_PROCESS1	0.554119483	0.04053123	3.005083345	0.01730623	0.19229726	-3.03213367
WT after UVB irradiation VS WT before UVB irradiation	GO_L_ASCORBIC_ACID_METABOLIC_PROCESS2	0.353939779	-0.01495547	1.823515262	0.10523186	0.60922187	-4.54306043
homo after UVB irradiation VS homo before UVB irradiation	GO_L_ASPARTATE_TRANSMEMBRANE_TRANSPORT	-0.27991687	0.035783153	-2.3138238	0.0481617	0.16094253	-4.295024
hetero after UVB irradiation VS hetero before UVB irradiation	GO_L_ASPARTATE_TRANSMEMBRANE_TRANSPORT1	-0.51150553	0.069653445	-2.69131009	0.02790479	0.23570907	-3.49069873
WT after UVB irradiation VS WT before UVB irradiation	GO_L_ASPARTATE_TRANSMEMBRANE_TRANSPORT2	0.001079044	0.001165236	0.007012881	0.99457427	0.9985657	-5.99604072
homo after UVB irradiation VS homo before UVB irradiation	GO_L_ASPARTATE_TRANSMEMBRANE_TRANSPORTER_ACTIVITY	-0.2235928	0.053249615	-1.69039193	0.12792483	0.29295263	-5.20693771
hetero after UVB irradiation VS hetero before UVB irradiation	GO_L_ASPARTATE_TRANSMEMBRANE_TRANSPORTER_ACTIVITY1	-0.55118099	0.053927628	-2.5837379	0.03292644	0.25148162	-3.64856151
WT after UVB irradiation VS WT before UVB irradiation	GO_L_ASPARTATE_TRANSMEMBRANE_TRANSPORTER_ACTIVITY2	0.092902978	0.000652767	0.553022918	0.59518114	0.8931505	-5.83784592
homo after UVB irradiation VS homo before UVB irradiation	GO_L_GLUTAMATE_IMPORT	0.109283527	0.053719571	0.744742023	0.47691244	0.65473968	-6.25635843
hetero after UVB irradiation VS hetero before UVB irradiation	GO_L_GLUTAMATE_IMPORT1	0.273859615	0.034721519	1.920771288	0.09169261	0.37878391	-4.60484756
WT after UVB irradiation VS WT before UVB irradiation	GO_L_GLUTAMATE_IMPORT2	-0.01806976	-0.04070315	-0.08615879	0.93343253	0.98322796	-5.99215164
homo after UVB irradiation VS homo before UVB irradiation	GO_L_GLUTAMATE_TRANSMEMBRANE_TRANSPORT	0.13799988	0.027866051	1.074337736	0.31280525	0.50674845	-5.95842314
hetero after UVB irradiation VS hetero before UVB irradiation	GO_L_GLUTAMATE_TRANSMEMBRANE_TRANSPORT1	0.085989614	0.006774901	0.456283476	0.66054065	0.86073729	-6.12010493
WT after UVB irradiation VS WT before UVB irradiation	GO_L_GLUTAMATE_TRANSMEMBRANE_TRANSPORT2	0.135565163	-0.03893917	0.671460345	0.52061835	0.86304554	-5.76494415
homo after UVB irradiation VS homo before UVB irradiation	GO_L_GLUTAMINE_TRANSMEMBRANE_TRANSPORTER_ACTIVITY	-0.43983654	0.033191343	-2.97760996	0.01688533	0.08878367	-3.26791128
hetero after UVB irradiation VS hetero before UVB irradiation	GO_L_GLUTAMINE_TRANSMEMBRANE_TRANSPORTER_ACTIVITY1	-0.23434993	0.045041088	-1.30973931	0.22730081	0.55615899	-5.39342873
WT after UVB irradiation VS WT before UVB irradiation	GO_L_GLUTAMINE_TRANSMEMBRANE_TRANSPORTER_ACTIVITY2	-0.34359081	-0.01406967	-1.93642806	0.08839595	0.58080792	-4.39116404
homo after UVB irradiation VS homo before UVB irradiation	GO_L_HISTIDINE_TRANSMEMBRANE_TRANSPORT	-0.21695564	0.024620527	-1.24646504	0.24650598	0.44044676	-5.77042189
hetero after UVB irradiation VS hetero before UVB irradiation	GO_L_HISTIDINE_TRANSMEMBRANE_TRANSPORT1	-0.37293035	-0.01450065	-2.07549136	0.0722683	0.3482662	-4.3868239
WT after UVB irradiation VS WT before UVB irradiation	GO_L_HISTIDINE_TRANSMEMBRANE_TRANSPORT2	0.127429107	0.032324415	0.461153823	0.656822	0.91071336	-5.88539096
homo after UVB irradiation VS homo before UVB irradiation	GO_L_HISTIDINE_TRANSMEMBRANE_TRANSPORTER_ACTIVITY	-0.13204534	0.04414005	-0.87094944	0.4081815	0.5950376	-6.15278311
hetero after UVB irradiation VS hetero before UVB irradiation	GO_L_HISTIDINE_TRANSMEMBRANE_TRANSPORTER_ACTIVITY1	-0.12362659	-0.0257374	-0.61909265	0.55338711	0.80411425	-6.02872979
WT after UVB irradiation VS WT before UVB irradiation	GO_L_HISTIDINE_TRANSMEMBRANE_TRANSPORTER_ACTIVITY2	0.273211072	0.096193151	1.146319927	0.28440522	0.75306903	-5.35659983
homo after UVB irradiation VS homo before UVB irradiation	GO_L_KYNURENINE_METABOLIC_PROCESS	-0.39941468	0.032177643	-1.70958552	0.12422056	0.28745849	-5.1805317
hetero after UVB irradiation VS hetero before UVB irradiation	GO_L_KYNURENINE_METABOLIC_PROCESS1	0.150642185	0.048519477	0.904383202	0.39269222	0.70228655	-5.81035396
WT after UVB irradiation VS WT before UVB irradiation	GO_L_KYNURENINE_METABOLIC_PROCESS2	-0.260738	-0.03125974	-1.24774481	0.24699746	0.73564768	-5.24892121
homo after UVB irradiation VS homo before UVB irradiation	GO_L_LACTATE_DEHYDROGENASE_ACTIVITY	0.140914157	0.016824384	0.412020202	0.69072454	0.81462086	-6.45864399
hetero after UVB irradiation VS hetero before UVB irradiation	GO_L_LACTATE_DEHYDROGENASE_ACTIVITY1	0.127123705	0.041773184	0.491007796	0.63684773	0.85094067	-6.1027811
WT after UVB irradiation VS WT before UVB irradiation	GO_L_LACTATE_DEHYDROGENASE_ACTIVITY2	-0.01899267	-0.05384596	-0.06144467	0.9524946	0.98676072	-5.99407504
homo after UVB irradiation VS homo before UVB irradiation	GO_L_LEUCINE_TRANSMEMBRANE_TRANSPORTER_ACTIVITY	-0.64699309	-0.09597629	-2.10654508	0.06688085	0.19727785	-4.60818203
hetero after UVB irradiation VS hetero before UVB irradiation	GO_L_LEUCINE_TRANSMEMBRANE_TRANSPORTER_ACTIVITY1	-0.66716892	-0.04968274	-2.22453975	0.0573916	0.31611438	-4.17286742
WT after UVB irradiation VS WT before UVB irradiation	GO_L_LEUCINE_TRANSMEMBRANE_TRANSPORTER_ACTIVITY2	-0.47171587	-0.05204853	-1.48959046	0.17421198	0.67974963	-4.96949245
homo after UVB irradiation VS homo before UVB irradiation	GO_L_LYSINE_TRANSMEMBRANE_TRANSPORT	-0.5831144	0.001092357	-3.85756819	0.00446915	0.04321062	-1.9342989
hetero after UVB irradiation VS hetero before UVB irradiation	GO_L_LYSINE_TRANSMEMBRANE_TRANSPORT1	-0.47140447	-0.04378561	-2.80114465	0.02358553	0.21999243	-3.32969216
WT after UVB irradiation VS WT before UVB irradiation	GO_L_LYSINE_TRANSMEMBRANE_TRANSPORT2	-0.30838042	-0.07493788	-1.8032684	0.10855576	0.61267537	-4.56995325
homo after UVB irradiation VS homo before UVB irradiation	GO_L_LYSINE_TRANSMEMBRANE_TRANSPORTER_ACTIVITY	-0.64683396	0.014773729	-3.62387281	0.00630152	0.0525376	-2.28066696
hetero after UVB irradiation VS hetero before UVB irradiation	GO_L_LYSINE_TRANSMEMBRANE_TRANSPORTER_ACTIVITY1	-0.33253778	-0.05540794	-1.37589906	0.20681487	0.53120282	-5.31574816
WT after UVB irradiation VS WT before UVB irradiation	GO_L_LYSINE_TRANSMEMBRANE_TRANSPORTER_ACTIVITY2	-0.2043599	-0.08385041	-0.90192476	0.39313254	0.81282718	-5.58827795
homo after UVB irradiation VS homo before UVB irradiation	GO_L_METHIONINE_SALVAGE_FROM_METHYLTHIOADENOSINE	0.395194568	-0.02254739	1.436342286	0.18737931	0.37231137	-5.54166404
hetero after UVB irradiation VS hetero before UVB irradiation	GO_L_METHIONINE_SALVAGE_FROM_METHYLTHIOADENOSINE1	0.044326077	0.02494819	0.163571454	0.87419986	0.95321437	-6.21699991
WT after UVB irradiation VS WT before UVB irradiation	GO_L_METHIONINE_SALVAGE_FROM_METHYLTHIOADENOSINE2	0.320660861	-0.08796467	1.03214488	0.33184311	0.7781676	-5.47002486
homo after UVB irradiation VS homo before UVB irradiation	GO_L_ORNITHINE_TRANSMEMBRANE_TRANSPORTER_ACTIVITY	-0.48401255	0.001181539	-2.34660587	0.04571943	0.15581563	-4.24491915
hetero after UVB irradiation VS hetero before UVB irradiation	GO_L_ORNITHINE_TRANSMEMBRANE_TRANSPORTER_ACTIVITY1	-0.33447526	-0.01428927	-1.75545421	0.11796118	0.4219719	-4.83149949
WT after UVB irradiation VS WT before UVB irradiation	GO_L_ORNITHINE_TRANSMEMBRANE_TRANSPORTER_ACTIVITY2	-0.22993448	-0.01170017	-1.16611453	0.27676229	0.74802923	-5.33607545
homo after UVB irradiation VS homo before UVB irradiation	GO_L_PHENYLALANINE_METABOLIC_PROCESS	0.433997607	0.043537329	2.057305038	0.07228343	0.20638289	-4.68142819
hetero after UVB irradiation VS hetero before UVB irradiation	GO_L_PHENYLALANINE_METABOLIC_PROCESS1	-0.37406147	-0.03987141	-1.49008378	0.17523121	0.49811439	-5.17648105
WT after UVB irradiation VS WT before UVB irradiation	GO_L_PHENYLALANINE_METABOLIC_PROCESS2	0.44271932	0.013329535	1.678060547	0.13139811	0.63700751	-4.73356917
homo after UVB irradiation VS homo before UVB irradiation	GO_L_PROLINE_TRANSMEMBRANE_TRANSPORTER_ACTIVITY	-0.15424767	-0.05702537	-0.5892038	0.57135538	0.72888882	-6.36426825
hetero after UVB irradiation VS hetero before UVB irradiation	GO_L_PROLINE_TRANSMEMBRANE_TRANSPORTER_ACTIVITY1	-0.1119015	0.032694627	-0.52383021	0.61485453	0.83965967	-6.08531288
WT after UVB irradiation VS WT before UVB irradiation	GO_L_PROLINE_TRANSMEMBRANE_TRANSPORTER_ACTIVITY2	0.181209927	0.02109635	0.777572009	0.45893377	0.8390123	-5.68907487
homo after UVB irradiation VS homo before UVB irradiation	GO_L_SERINE_BIOSYNTHETIC_PROCESS	0.258348202	-0.03107691	1.49476916	0.17186045	0.35152701	-5.46733106
hetero after UVB irradiation VS hetero before UVB irradiation	GO_L_SERINE_BIOSYNTHETIC_PROCESS1	0.623592854	0.028790503	3.767449682	0.00567471	0.13201411	-1.95552829
WT after UVB irradiation VS WT before UVB irradiation	GO_L_SERINE_BIOSYNTHETIC_PROCESS2	0.34724967	-0.00292339	1.032295702	0.33177661	0.7781676	-5.46988082
homo after UVB irradiation VS homo before UVB irradiation	GO_L_SERINE_METABOLIC_PROCESS	-0.13582439	0.008908589	-0.68275952	0.51329695	0.68438048	-6.30205877
hetero after UVB irradiation VS hetero before UVB irradiation	GO_L_SERINE_METABOLIC_PROCESS1	0.000316768	0.026123832	0.001969233	0.99847789	0.99922277	-6.23148201
WT after UVB irradiation VS WT before UVB irradiation	GO_L_SERINE_METABOLIC_PROCESS2	-0.05781168	0.000153306	-0.17195378	0.86769362	0.96790654	-5.98049518
homo after UVB irradiation VS homo before UVB irradiation	GO_L_SERINE_TRANSMEMBRANE_TRANSPORTER_ACTIVITY	0.363424655	-0.0463443	2.207607265	0.05699866	0.17797909	-4.45637123
hetero after UVB irradiation VS hetero before UVB irradiation	GO_L_SERINE_TRANSMEMBRANE_TRANSPORTER_ACTIVITY1	0.152021905	0.024576179	0.752754393	0.47355703	0.75680588	-5.93517226
WT after UVB irradiation VS WT before UVB irradiation	GO_L_SERINE_TRANSMEMBRANE_TRANSPORTER_ACTIVITY2	0.413139438	0.019376758	2.393074504	0.04328575	0.49624823	-3.75423728
homo after UVB irradiation VS homo before UVB irradiation	GO_L_TYPE_VOLTAGE_GATED_CALCIUM_CHANNEL_COMPLEX	0.501518219	-0.0505779	2.053939372	0.07266779	0.20682876	-4.68641575
hetero after UVB irradiation VS hetero before UVB irradiation	GO_L_TYPE_VOLTAGE_GATED_CALCIUM_CHANNEL_COMPLEX1	-0.18839065	-0.03404009	-0.70913989	0.49874741	0.77200427	-5.96746196
WT after UVB irradiation VS WT before UVB irradiation	GO_L_TYPE_VOLTAGE_GATED_CALCIUM_CHANNEL_COMPLEX2	0.342699261	0.045802978	1.212830557	0.259387	0.73996638	-5.28668315
homo after UVB irradiation VS homo before UVB irradiation	GO_LABYRINTHINE_LAYER_BLOOD_VESSEL_DEVELOPMENT	0.083351683	-0.03577919	0.479864381	0.64367204	0.7819553	-6.42622947
hetero after UVB irradiation VS hetero before UVB irradiation	GO_LABYRINTHINE_LAYER_BLOOD_VESSEL_DEVELOPMENT1	0.352510066	-0.01086213	2.587358303	0.03274322	0.25073401	-3.64324884
WT after UVB irradiation VS WT before UVB irradiation	GO_LABYRINTHINE_LAYER_BLOOD_VESSEL_DEVELOPMENT2	0.220645861	0.03351778	1.347248638	0.21438278	0.71210914	-5.13755437
homo after UVB irradiation VS homo before UVB irradiation	GO_LABYRINTHINE_LAYER_DEVELOPMENT	0.05448151	0.000353818	0.329198541	0.75013114	0.85420829	-6.49177135
hetero after UVB irradiation VS hetero before UVB irradiation	GO_LABYRINTHINE_LAYER_DEVELOPMENT1	0.194874085	0.023505675	1.375127145	0.20704435	0.53151528	-5.31666779
WT after UVB irradiation VS WT before UVB irradiation	GO_LABYRINTHINE_LAYER_DEVELOPMENT2	0.054773241	0.021695672	0.317996134	0.75853388	0.93908133	-5.94306028
homo after UVB irradiation VS homo before UVB irradiation	GO_LABYRINTHINE_LAYER_MORPHOGENESIS	-0.26458427	0.018621284	-1.61333821	0.14384069	0.3158716	-5.31147886
hetero after UVB irradiation VS hetero before UVB irradiation	GO_LABYRINTHINE_LAYER_MORPHOGENESIS1	-0.132103	-0.01062015	-0.74462477	0.47818805	0.759973	-5.94131737
WT after UVB irradiation VS WT before UVB irradiation	GO_LABYRINTHINE_LAYER_MORPHOGENESIS2	-0.3015942	0.03718405	-1.79688378	0.10962427	0.61466507	-4.57841007
homo after UVB irradiation VS homo before UVB irradiation	GO_LACRIMAL_GLAND_DEVELOPMENT	-0.4383989	-0.02209096	-2.12729975	0.06472335	0.19283717	-4.57716004
hetero after UVB irradiation VS hetero before UVB irradiation	GO_LACRIMAL_GLAND_DEVELOPMENT1	0.094200427	-0.05120463	0.403406312	0.69741036	0.88005213	-6.14416687
WT after UVB irradiation VS WT before UVB irradiation	GO_LACRIMAL_GLAND_DEVELOPMENT2	0.124524394	0.045511897	0.415816514	0.68834537	0.91993413	-5.90585271
homo after UVB irradiation VS homo before UVB irradiation	GO_LACTATE_DEHYDROGENASE_ACTIVITY	0.457237477	0.019835402	1.821880108	0.10447123	0.25776324	-5.02337209
hetero after UVB irradiation VS hetero before UVB irradiation	GO_LACTATE_DEHYDROGENASE_ACTIVITY1	0.222335003	0.018893425	0.769491741	0.46411624	0.75170107	-5.92234041
WT after UVB irradiation VS WT before UVB irradiation	GO_LACTATE_DEHYDROGENASE_ACTIVITY2	0.080221599	-0.06426284	0.241972113	0.81481723	0.95586033	-5.96529036
homo after UVB irradiation VS homo before UVB irradiation	GO_LACTATE_METABOLIC_PROCESS	0.223999145	-0.01304836	1.501461092	0.17015769	0.34964689	-5.45870907
hetero after UVB irradiation VS hetero before UVB irradiation	GO_LACTATE_METABOLIC_PROCESS1	0.142067891	-0.03707109	0.587832971	0.57315599	0.8149441	-6.04824838
WT after UVB irradiation VS WT before UVB irradiation	GO_LACTATE_METABOLIC_PROCESS2	0.051541483	-0.02771123	0.228869092	0.82464509	0.95860343	-5.96852218

homo after UVB irradiation VS homo before UVB irradiation	GO_LACTATE_TRANSMEMBRANE_TRANSPORT	-0.03920057	0.014999326	-0.20191497	0.84482773	0.91438874	-6.52853716
hetero after UVB irradiation VS hetero before UVB irradiation	GO_LACTATE_TRANSMEMBRANE_TRANSPORT1	-0.08893609	-0.04973292	-0.4737204	0.6485898	0.85558615	-6.11155531
WT after UVB irradiation VS WT before UVB irradiation	GO_LACTATE_TRANSMEMBRANE_TRANSPORT2	0.278455593	-0.07935112	1.326010047	0.22101784	0.71573842	-5.16177297
homo after UVB irradiation VS homo before UVB irradiation	GO_LACTATE_TRANSMEMBRANE_TRANSPORTER_ACTIVITY	-0.21677095	0.02085482	-1.16865205	0.27490086	0.47056165	-5.85787706
hetero after UVB irradiation VS hetero before UVB irradiation	GO_LACTATE_TRANSMEMBRANE_TRANSPORTER_ACTIVITY1	-0.23810363	-0.0501949	-1.35512464	0.21306843	0.53985493	-5.34039023
WT after UVB irradiation VS WT before UVB irradiation	GO_LACTATE_TRANSMEMBRANE_TRANSPORTER_ACTIVITY2	0.106447959	-0.04499051	0.53585125	0.60646229	0.89704906	-5.84734226
homo after UVB irradiation VS homo before UVB irradiation	GO_LACTATION	0.236204871	0.000700486	1.364791462	0.20804389	0.39809436	-5.63026813
hetero after UVB irradiation VS hetero before UVB irradiation	GO_LACTATION1	0.10450314	-0.01359144	0.61590656	0.55538357	0.80533165	-6.03076112
WT after UVB irradiation VS WT before UVB irradiation	GO_LACTATION2	0.080719913	0.013890849	0.550573098	0.59678358	0.89380878	-5.83921766
homo after UVB irradiation VS homo before UVB irradiation	GO_LAMELLAR_BODY	0.559026339	0.038898646	2.819442485	0.02163074	0.10224372	-3.51344979
hetero after UVB irradiation VS hetero before UVB irradiation	GO_LAMELLAR_BODY1	0.724637329	0.048793123	5.371256933	0.00071143	0.08366654	0.016854592
WT after UVB irradiation VS WT before UVB irradiation	GO_LAMELLAR_BODY2	0.534220606	0.034055616	2.857015616	0.02098579	0.40609596	-3.0949888
homo after UVB irradiation VS homo before UVB irradiation	GO_LAMELLIPODIUM	0.460024037	-0.03491072	3.257210035	0.01095944	0.06998201	-2.83656365
homo after UVB irradiation VS homo before UVB irradiation	GO_LAMELLIPODIUM_ASSEMBLY	0.430248627	-0.04683829	3.118626497	0.01356452	0.07877672	-3.04976993
hetero after UVB irradiation VS hetero before UVB irradiation	GO_LAMELLIPODIUM_ASSEMBLY1	0.414983219	0.007856827	2.924115559	0.01956048	0.2025582	-3.14998102
WT after UVB irradiation VS WT before UVB irradiation	GO_LAMELLIPODIUM_ASSEMBLY2	0.252592754	-0.01465563	1.776769199	0.11305562	0.61801327	-4.60497737
homo after UVB irradiation VS homo before UVB irradiation	GO_LAMELLIPODIUM_MEMBRANE	0.223043967	-0.01003001	1.729094458	0.12055729	0.28199241	-5.15354974
hetero after UVB irradiation VS hetero before UVB irradiation	GO_LAMELLIPODIUM_MEMBRANE1	0.326623379	-0.04900771	2.49599023	0.03770155	0.26657756	-3.77725364
WT after UVB irradiation VS WT before UVB irradiation	GO_LAMELLIPODIUM_MEMBRANE2	0.152408547	0.00484065	1.050180425	0.32396357	0.77346379	-5.45268905
homo after UVB irradiation VS homo before UVB irradiation	GO_LAMELLIPODIUM_MORPHOGENESIS	0.673788534	-0.0697849	4.401431309	0.00207007	0.03080331	-1.15740314
hetero after UVB irradiation VS hetero before UVB irradiation	GO_LAMELLIPODIUM_MORPHOGENESIS1	0.648895502	0.007195894	3.27180259	0.01161936	0.16825902	-2.64777748
WT after UVB irradiation VS WT before UVB irradiation	GO_LAMELLIPODIUM_MORPHOGENESIS2	0.69692053	-0.00314679	4.094550046	0.00337545	0.25923179	-1.43160349
homo after UVB irradiation VS homo before UVB irradiation	GO_LAMELLIPODIUM_ORGANIZATION	0.487870565	-0.04170855	3.486235821	0.00774129	0.05849368	-2.48772693
hetero after UVB irradiation VS hetero before UVB irradiation	GO_LAMELLIPODIUM_ORGANIZATION1	0.46214499	0.00706516	3.160779077	0.0137018	0.17665328	-2.80695346
WT after UVB irradiation VS WT before UVB irradiation	GO_LAMELLIPODIUM_ORGANIZATION2	0.357328428	0.000335385	2.516873069	0.0356495	0.47351907	-3.5783309
hetero after UVB irradiation VS hetero before UVB irradiation	GO_LAMELLIPODIUM1	0.451586383	0.005081059	3.24767699	0.01204164	0.1700349	-2.68225698
WT after UVB irradiation VS WT before UVB irradiation	GO_LAMELLIPODIUM2	0.3375838	-0.01896873	2.343665814	0.04677483	0.50583727	-3.82424696
homo after UVB irradiation VS homo before UVB irradiation	GO_LAMIN_BINDING	0.088295274	-0.02641549	0.484874388	0.64026044	0.77943601	-6.42365036
hetero after UVB irradiation VS hetero before UVB irradiation	GO_LAMIN_BINDING1	0.120436586	0.033631899	0.902514217	0.39362475	0.70256648	-5.81200642
WT after UVB irradiation VS WT before UVB irradiation	GO_LAMIN_BINDING2	-0.14396829	-0.0181281	-0.69961711	0.50376476	0.85763155	-5.74575952
homo after UVB irradiation VS homo before UVB irradiation	GO_LAMIN_FILAMENT	0.213293834	-0.03660299	1.343952855	0.21442064	0.40499238	-5.65554457
hetero after UVB irradiation VS hetero before UVB irradiation	GO_LAMIN_FILAMENT1	0.145803846	-0.02397682	0.772954426	0.46217888	0.7502653	-5.91965559
WT after UVB irradiation VS WT before UVB irradiation	GO_LAMIN_FILAMENT2	0.280880349	0.030715826	1.29305421	0.23166035	0.72404374	-5.19887925
homo after UVB irradiation VS homo before UVB irradiation	GO_LAMININ_1_BINDING	0.243290894	0.07143386	1.02834987	0.33271929	0.52574564	-6.00514941
hetero after UVB irradiation VS hetero before UVB irradiation	GO_LAMININ_1_BINDING1	-0.00365774	0.036667964	-0.01417337	0.98904513	0.99599741	-6.23137517
WT after UVB irradiation VS WT before UVB irradiation	GO_LAMININ_1_BINDING2	-0.03156951	0.007131057	-0.15556669	0.8801827	0.9693507	-5.9833172
homo after UVB irradiation VS homo before UVB irradiation	GO_LAMININ_BINDING	0.506149744	0.020707767	2.966450954	0.01718182	0.08965776	-3.2852118
hetero after UVB irradiation VS hetero before UVB irradiation	GO_LAMININ_BINDING1	0.418985624	0.012888268	2.571683521	0.03354404	0.2531992	-3.66624933
WT after UVB irradiation VS WT before UVB irradiation	GO_LAMININ_BINDING2	0.456301787	0.042571059	2.995918888	0.01694253	0.380843	-2.89910096
homo after UVB irradiation VS homo before UVB irradiation	GO_LAMININ_COMPLEX	0.333476325	-0.0372358	1.64745982	0.13658158	0.30539644	-5.26548247
hetero after UVB irradiation VS hetero before UVB irradiation	GO_LAMININ_COMPLEX1	0.024192804	-0.01544844	0.164516271	0.87348054	0.95291134	-6.2168324
WT after UVB irradiation VS WT before UVB irradiation	GO_LAMININ_COMPLEX2	0.159444764	-0.06451071	0.676391372	0.51764192	0.86168548	-5.76163475
homo after UVB irradiation VS homo before UVB irradiation	GO_LARGE_RIBOSOMAL_SUBUNIT	-0.66572948	0.015568646	-3.80611601	0.00481725	0.04526889	-2.00995951
homo after UVB irradiation VS homo before UVB irradiation	GO_LARGE_RIBOSOMAL_SUBUNIT_RRNA_BINDING	-1.16147297	-0.03306565	-7.59943683	5.08E-05	0.00916572	2.504140067
hetero after UVB irradiation VS hetero before UVB irradiation	GO_LARGE_RIBOSOMAL_SUBUNIT_RRNA_BINDING1	-0.51344701	-0.17500126	-1.19894211	0.26547648	0.59226139	-5.51802568
WT after UVB irradiation VS WT before UVB irradiation	GO_LARGE_RIBOSOMAL_SUBUNIT_RRNA_BINDING2	-0.95760768	-0.09926996	-3.9407758	0.00418921	0.26635361	-1.62589966
hetero after UVB irradiation VS hetero before UVB irradiation	GO_LARGE_RIBOSOMAL_SUBUNIT1	-0.17287179	-0.03997065	-0.6796375	0.51626388	0.78459792	-5.988347
WT after UVB irradiation VS WT before UVB irradiation	GO_LARGE_RIBOSOMAL_SUBUNIT2	-0.3004149	-0.02433912	-1.24343089	0.24850109	0.73564768	-5.25362527
homo after UVB irradiation VS homo before UVB irradiation	GO_LATE_ENDOSOME	0.315885685	-0.0261647	2.094732269	0.06813996	0.1993866	-4.62580018
homo after UVB irradiation VS homo before UVB irradiation	GO_LATE_ENDOSOME_LUMEN	0.567869287	0.001129412	2.613408715	0.0299414	0.12296765	-3.83344049
hetero after UVB irradiation VS hetero before UVB irradiation	GO_LATE_ENDOSOME_LUMEN1	0.427744478	-0.04377423	1.438417618	0.18895138	0.51443012	-5.24027283
WT after UVB irradiation VS WT before UVB irradiation	GO_LATE_ENDOSOME_LUMEN2	0.0028643	-0.06993051	0.009253253	0.99284099	0.9985657	-5.99602149
homo after UVB irradiation VS homo before UVB irradiation	GO_LATE_ENDOSOME_MEMBRANE	0.431470651	-0.02827916	2.948905489	0.01765895	0.09105282	-3.31242263
hetero after UVB irradiation VS hetero before UVB irradiation	GO_LATE_ENDOSOME_MEMBRANE1	0.408641597	0.01827719	2.714983155	0.0269095	0.23196771	-3.45597143
WT after UVB irradiation VS WT before UVB irradiation	GO_LATE_ENDOSOME_MEMBRANE2	0.427215655	-0.00269043	2.919728388	0.01904892	0.39193477	-3.00637833
homo after UVB irradiation VS homo before UVB irradiation	GO_LATE_ENDOSOME_TO_GOLGI_TRANSPORT	-0.22618826	0.046210102	-0.83163249	0.42881177	0.6131759	-6.18652078
hetero after UVB irradiation VS hetero before UVB irradiation	GO_LATE_ENDOSOME_TO_GOLGI_TRANSPORT1	0.063258005	0.023227996	0.208529571	0.84012263	0.939013	-6.20796958
WT after UVB irradiation VS WT before UVB irradiation	GO_LATE_ENDOSOME_TO_GOLGI_TRANSPORT2	-0.08033238	-0.02353109	-0.3795075	0.71407018	0.92981831	-5.92077973
homo after UVB irradiation VS homo before UVB irradiation	GO_LATE_ENDOSOME_TO_LYSOSOME_TRANSPORT	0.345623592	-0.0790741	1.886092205	0.09454467	0.24289498	-4.93163381
hetero after UVB irradiation VS hetero before UVB irradiation	GO_LATE_ENDOSOME_TO_LYSOSOME_TRANSPORT1	0.341398144	-0.01296903	1.777766036	0.11403999	0.41652826	-4.80136088
WT after UVB irradiation VS WT before UVB irradiation	GO_LATE_ENDOSOME_TO_LYSOSOME_TRANSPORT2	0.341261489	-0.13944509	1.163901886	0.27760826	0.74917538	-5.33838179
homo after UVB irradiation VS homo before UVB irradiation	GO_LATE_ENDOSOME_TO_VACUOLE_TRANSPORT	0.248477218	-0.04074025	0.96675642	0.36089384	0.55200149	-6.06524514
homo after UVB irradiation VS homo before UVB irradiation	GO_LATE_ENDOSOME_TO_VACUOLE_TRANSPORT_VIA_MULTIVESICULAR_BODY_SORTING_PATHWAY	0.124930603	-0.06095901	0.538280231	0.60446604	0.75315547	-6.39459563
hetero after UVB irradiation VS hetero before UVB irradiation	GO_LATE_ENDOSOME_TO_VACUOLE_TRANSPORT_VIA_MULTIVESICULAR_BODY_SORTING_PATHWAY1	0.045308474	0.01775413	0.177336957	0.86373245	0.9482357	-6.21446473
WT after UVB irradiation VS WT before UVB irradiation	GO_LATE_ENDOSOME_TO_VACUOLE_TRANSPORT_VIA_MULTIVESICULAR_BODY_SORTING_PATHWAY2	0.48267803	0.062987841	1.977350233	0.08295611	0.56867582	-4.33539043
hetero after UVB irradiation VS hetero before UVB irradiation	GO_LATE_ENDOSOME_TO_VACUOLE_TRANSPORT1	0.043618644	0.01854817	0.175658389	0.86500735	0.94881427	-6.21478475
WT after UVB irradiation VS WT before UVB irradiation	GO_LATE_ENDOSOME_TO_VACUOLE_TRANSPORT2	0.375567166	0.021628262	1.419614968	0.19304925	0.69334561	-5.05331817
hetero after UVB irradiation VS hetero before UVB irradiation	GO_LATE_ENDOSOME1	0.317985424	0.007703662	2.452189759	0.04034284	0.27269233	-3.84140707
WT after UVB irradiation VS WT before UVB irradiation	GO_LATE_ENDOSOME2	0.301150047	-0.01122823	2.041079495	0.07512222	0.55793508	-4.24787011
homo after UVB irradiation VS homo before UVB irradiation	GO_LATE_NUCLEOPHAGY	0.055745841	0.005120931	0.149705153	0.88455893	0.93767949	-6.5385736
hetero after UVB irradiation VS hetero before UVB irradiation	GO_LATE_NUCLEOPHAGY1	0.299031189	0.027650601	1.195952034	0.26657658	0.59300892	-5.52128692
WT after UVB irradiation VS WT before UVB irradiation	GO_LATE_NUCLEOPHAGY2	0.105262383	0.123298401	0.307055479	0.76654921	0.94123239	-5.94662298
homo after UVB irradiation VS homo before UVB irradiation	GO_LATERAL_ELEMENT	-0.45005016	0.023403326	-1.85463829	0.09929012	0.2492302	-4.97672979
hetero after UVB irradiation VS hetero before UVB irradiation	GO_LATERAL_ELEMENT1	-0.11175796	0.066159407	-0.44866901	0.6657925	0.86317077	-6.12374327
WT after UVB irradiation VS WT before UVB irradiation	GO_LATERAL_ELEMENT2	-0.08479315	0.082204386	-0.49500017	0.63375057	0.90499876	-5.86881269
homo after UVB irradiation VS homo before UVB irradiation	GO_LATERAL_MESODERM_DEVELOPMENT	-0.61020878	-0.00706519	-4.62790227	0.00152173	0.02648139	-0.84702774
hetero after UVB irradiation VS hetero before UVB irradiation	GO_LATERAL_MESODERM_DEVELOPMENT1	-0.60656214	0.040813577	-3.8239407	0.00524088	0.1288742	-1.87882084
WT after UVB irradiation VS WT before UVB irradiation	GO_LATERAL_MESODERM_DEVELOPMENT2	-0.20265006	0.058183655	-1.01359587	0.3401003	0.78110554	-5.48761823
homo after UVB irradiation VS homo before UVB irradiation	GO_LATERAL_PLASMA_MEMBRANE	0.078586433	-0.0110529	0.462700457	0.6554274	0.79065987	-6.43487306
hetero after UVB irradiation VS hetero before UVB irradiation	GO_LATERAL_PLASMA_MEMBRANE1	0.00835145	0.020784729	0.048022034	0.96289754	0.98664387	-6.23023368
WT after UVB irradiation VS WT before UVB irradiation	GO_LATERAL_PLASMA_MEMBRANE2	-0.05921642	0.074508663	-0.32037016	0.75679868	0.93865171	-5.94227116
homo after UVB irradiation VS homo before UVB irradiation	GO_LATERAL_SPROUTING_FROM_AN_EPITHELIUM	-0.63147002	-0.00052514	-3.83837947	0.00459573	0.04412439	-1.96247504
hetero after UVB irradiation VS hetero before UVB irradiation	GO_LATERAL_SPROUTING_FROM_AN_EPITHELIUM1	-0.56500892	-0.04295715	-3.24498402	0.01208977	0.17038844	-2.68610958
WT after UVB irradiation VS WT before UVB irradiation	GO_LATERAL_SPROUTING_FROM_AN_EPITHELIUM2	-0.50585875	0.012603189	-3.18419591	0.01271398	0.35163556	-2.63621532
homo after UVB irradiation VS homo before UVB irradiation	GO_LATERAL_VENTRICLE_DEVELOPMENT	0.009006167	0.040565436	0.071041296	0.94504111	0.97096509	-6.54808775
hetero after UVB irradiation VS hetero before UVB irradiation	GO_LATERAL_VENTRICLE_DEVELOPMENT1	-0.2603942	-0.00124031	-1.62709502	0.14307687	0.45836784	-5.00169628
WT after UVB irradiation VS WT before UVB irradiation	GO_LATERAL_VENTRICLE_DEVELOPMENT2	-0.1158031	0.062485893	-0.59451345	0.56840363	0.88290486	-5.81376558
homo after UVB irradiation VS homo before UVB irradiation	GO_LAYER_FORMATION_IN_CEREBRAL_CORTEX	0.11374756	-0.00576157	0.517045972	0.6185705	0.76284847	-6.40648828
hetero after UVB irradiation VS hetero before UVB irradiation	GO_LAYER_FORMATION_IN_CEREBRAL_CORTEX1	0.251315375	0.056008275	1.173562156	0.27493404	0.60165011	-5.54553059
WT after UVB irradiation VS WT before UVB irradiation	GO_LAYER_FORMATION_IN_CEREBRAL_CORTEX2	0.145519488	0.031700468	0.714915533	0.4947533	0.85444593	-5.73504566
homo after UVB irradiation VS homo before UVB irradiation	GO_LBD_DOMAIN_BINDING	-0.05305714	-0.05003148	-0.25589889	0.80423561	0.88919592	-6.51506095
hetero after UVB irradiation VS hetero before UVB irradiation	GO_LBD_DOMAIN_BINDING1	-0.10040266	-0.01854569	-0.33968716	0.7429987	0.8995791	-6.16938112
WT after UVB irradiation VS WT before UVB irradiation	GO_LBD_DOMAIN_BINDING2	-0.24522107	0.022653509	-1.00056541	0.34599427	0.78459186	-5.49983234
homo after UVB irradiation VS homo before UVB irradiation	GO_LEADING_EDGE_MEMBRANE	0.229719314	-0.01364794	1.705618126	0.12497802	0.28848752	-5.18600141
hetero after UVB irradiation VS hetero before UVB irradiation	GO_LEADING_EDGE_MEMBRANE1	0.177747968	-0.00623377	1.315481729	0.22545613	0.55437938	-5.38677967
WT after UVB irradiation VS WT before UVB irradiation	GO_LEADING_EDGE_MEMBRANE2	0.090933751	0.011081497	0.603102812	0.56294695	0.88086185	-5.80858187
homo after UVB irradiation VS homo before UVB irradiation	GO_LEARNED_VOCALIZATION_BEHAVIOR_OR_VOCAL_LEARNING	-0.36824597	0.006017115	-2.03348285	0.07504691	0.21037182	-4.71667551
hetero after UVB irradiation VS hetero before UVB irradiation	GO_LEARNED_VOCALIZATION_BEHAVIOR_OR_VOCAL_LEARNING1	-0.4047598	-0.03860098	-2.26001161	0.05432397	0.30886472	-4.12152051
WT after UVB irradiation VS WT before UVB irradiation	GO_LEARNED_VOCALIZATION_BEHAVIOR_OR_VOCAL_LEARNING2	-0.08119674	0.025933137	-0.3723036	0.71922195	0.93096927	-5.92358548
homo after UVB irradiation VS homo before UVB irradiation	GO_LEARNING	0.248518924	-0.0040364	2.007221308	0.07821175	0.21564353	-4.75538055
hetero after UVB irradiation VS hetero before UVB irradiation	GO_LEARNING1	0.084582225	0.004338021	0.544145105	0.60144559	0.83350999	-6.07397508
WT after UVB irradiation VS WT before UVB irradiation	GO_LEARNING2	0.073574539	-0.00947879	0.376021233	0.71656142	0.93059023	-5.92214402
homo after UVB irradiation VS homo before UVB irradiation	GO_LEFT_RIGHT_AXIS_SPECIFICATION	-0.48957737	0.023517636	-3.38212741	0.00905948	0.0633504	-2.64568689
hetero after UVB irradiation VS hetero before UVB irradiation	GO_LEFT_RIGHT_AXIS_SPECIFICATION1	-0.6323899	-0.03075126	-4.13784043	0.00339737	0.11632799	-1.46171132
WT after UVB irradiation VS WT before UVB irradiation	GO_LEFT_RIGHT_AXIS_SPECIFICATION2	-0.64745899	-0.02277242	-3.50132936	0.00791055	0.3154082	-2.2026079
homo after UVB irradiation VS homo before UVB irradiation	GO_LEFT_RIGHT_PATTERN_FORMATION	-0.25169167	0.012498759	-1.90811894	0.09135052	0.23742773	-4.89988017
hetero after UVB irradiation VS hetero before UVB irradiation	GO_LEFT_RIGHT_PATTERN_FORMATION1	-0.40624728	-0.04799891	-2.40669212	0.04328514	0.2785349	-3.90795343
WT after UVB irradiation VS WT before UVB irradiation	GO_LEFT_RIGHT_PATTERN_FORMATION2	-0.48783025	-0.0323094	-2.55108702	0.03379008	0.46471589	-3.52964561
homo after UVB irradiation VS homo before UVB irradiation	GO_LENS_DEVELOPMENT_IN_CAMERA_TYPE_EYE	-0.28152275	-0.00421593	-2.1883794	0.05876139	0.18190514	-4.48539447
hetero after UVB irradiation VS hetero before UVB irradiation	GO_LENS_DEVELOPMENT_IN_CAMERA_TYPE_EYE1	-0.2143672	-0.00608575	-1.46087487	0.18287473	0.50719609	-5.21269753
WT after UVB irradiation VS WT before UVB irradiation	GO_LENS_DEVELOPMENT_IN_CAMERA_TYPE_EYE2	-0.25975271	0.015637046	-1.64700311	0.1377166	0.64618714	-4.77336737
homo after UVB irradiation VS homo before UVB irradiation	GO_LENS_FIBER_CELL_DEVELOPMENT	-0.01067816	-0.05747884	-0.06584789	0.94905207	0.97335416	-6.54847769
hetero after UVB irradiation VS hetero before UVB irradiation	GO_LENS_FIBER_CELL_DEVELOPMENT1	-0.51679011	0.032601616	-2.62784749	0.03076386	0.24498194	-3.58382812
WT after UVB irradiation VS WT before UVB irradiation	GO_LENS_FIBER_CELL_DEVELOPMENT2	-0.12325081	-0.02708107	-0.48874235	0.63798534	0.90589211	-5.871961
homo after UVB irradiation VS homo before UVB irradiation	GO_LENS_FIBER_CELL_DIFFERENTIATION	-0.20343479	-0.00706898	-1.473091	0.1774807	0.35912694	-5.49511082
hetero after UVB irradiation VS hetero before UVB irradiation	GO_LENS_FIBER_CELL_DIFFERENTIATION1	-0.14540095	-0.00671918	-0.9412304	0.37463551	0.68770888	-5.77721943
WT after UVB irradiation VS WT before UVB irradiation	GO_LENS_FIBER_CELL_DIFFERENTIATION2	-0.08479737	-0.01356807	-0.44703848	0.66656243	0.9135561	-5.89197696
homo after UVB irradiation VS homo before UVB irradiation	GO_LENS_FIBER_CELL_MORPHOGENESIS	-0.4928586	-0.01920314	-2.8362592	0.02106667	0.10070646	-3.48732057
hetero after UVB irradiation VS hetero before UVB irradiation	GO_LENS_FIBER_CELL_MORPHOGENESIS1	-0.62802737	-0.0325428	-2.29066414	0.05180508	0.30233295	-4.07704281
WT after UVB irradiation VS WT before UVB irradiation	GO_LENS_FIBER_CELL_MORPHOGENESIS2	-0.6210555	-0.05176363	-1.79109112	0.11060227	0.61576748	-4.58607286
homo after UVB irradiation VS homo before UVB irradiation	GO_LENS_MORPHOGENESIS_IN_CAMERA_TYPE_EYE	-0.48079343	0.009170326	-3.19982353	0.01196828	0.07355929	-2.92468472
hetero after UVB irradiation VS hetero before UVB irradiation	GO_LENS_MORPHOGENESIS_IN_CAMERA_TYPE_EYE1	-0.17810379	0.037244924	-1.09801793	0.3047203	0.62665244	-5.62495706
WT after UVB irradiation VS WT before UVB irradiation	GO_LENS_MORPHOGENESIS_IN_CAMERA_TYPE_EYE2	-0.42929507	0.001069813	-2.0446479	0.07470546	0.55789253	-4.24294749
homo after UVB irradiation VS homo before UVB irradiation	GO_LEPTIN_MEDIATED_SIGNALING_PATHWAY	0.116399595	-0.00439065	0.533124365	0.60787495	0.75565754	-6.39752433
hetero after UVB irradiation VS hetero before UVB irradiation	GO_LEPTIN_MEDIATED_SIGNALING_PATHWAY1	-0.05469072	-0.06159501	-0.34581668	0.7385623	0.89786006	-6.16713731
WT after UVB irradiation VS WT before UVB irradiation	GO_LEPTIN_MEDIATED_SIGNALING_PATHWAY2	-0.01135107	-0.05901011	-0.04604944	0.96438626	0.99075605	-5.99494791
homo after UVB irradiation VS homo before UVB irradiation	GO_LEUCINE_BINDING	-0.31859909	0.027195661	-1.30874625	0.22557377	0.41650725	-5.69768359
hetero after UVB irradiation VS hetero before UVB irradiation	GO_LEUCINE_BINDING1	-0.39348616	-0.07118466	-1.71336006	0.12570567	0.43268299	-4.88793205
WT after UVB irradiation VS WT before UVB irradiation	GO_LEUCINE_BINDING2	-0.56472404	-0.0543547	-2.48168799	0.03766994	0.47615783	-3.62837619
homo after UVB irradiation VS homo before UVB irradiation	GO_LEUCINE_CATABOLIC_PROCESS	-0.33533418	0.019911965	-1.13575705	0.28767922	0.48296727	-5.89364553
hetero after UVB irradiation VS hetero before UVB irradiation	GO_LEUCINE_CATABOLIC_PROCESS1	-0.65691009	-0.03981879	-4.37732566	0.00246463	0.11054122	-1.15434536
WT after UVB irradiation VS WT before UVB irradiation	GO_LEUCINE_CATABOLIC_PROCESS2	-0.02226543	-0.14191659	-0.06652967	0.94856944	0.98614246	-5.99373186
homo after UVB irradiation VS homo before UVB irradiation	GO_LEUCINE_IMPORT_ACROSS_PLASMA_MEMBRANE	-0.34723114	-0.00144623	-1.16469197	0.27641447	0.47248323	-5.86222179
hetero after UVB irradiation VS hetero before UVB irradiation	GO_LEUCINE_IMPORT_ACROSS_PLASMA_MEMBRANE1	-0.785532	-0.01673004	-4.00617162	0.0040677	0.12527297	-1.63475055
WT after UVB irradiation VS WT before UVB irradiation	GO_LEUCINE_IMPORT_ACROSS_PLASMA_MEMBRANE2	-0.627913	-0.03940413	-2.81565342	0.02237374	0.41688033	-3.15356176
homo after UVB irradiation VS homo before UVB irradiation	GO_LEUCINE_METABOLIC_PROCESS	-0.00830614	-0.0472747	-0.02840337	0.97800923	0.98873983	-6.55041398
hetero after UVB irradiation VS hetero before UVB irradiation	GO_LEUCINE_METABOLIC_PROCESS1	-0.49187786	-0.07649092	-2.49395893	0.03782008	0.26663787	-3.78023043
WT after UVB irradiation VS WT before UVB irradiation	GO_LEUCINE_METABOLIC_PROCESS2	0.078415862	-0.09943467	0.250503523	0.80843683	0.95404687	-5.96309091
homo after UVB irradiation VS homo before UVB irradiation	GO_LEUCINE_TRANSPORT	-0.67534153	-0.01937997	-2.82030609	0.0216014	0.10213774	-3.51210789
hetero after UVB irradiation VS hetero before UVB irradiation	GO_LEUCINE_TRANSPORT1	-0.89819219	-0.01754825	-5.748077	0.00046048	0.07788488	0.417451068
WT after UVB irradiation VS WT before UVB irradiation	GO_LEUCINE_TRANSPORT2	-0.78783715	-0.0399212	-4.01285695	0.00378423	0.26532716	-1.5343171
homo after UVB irradiation VS homo before UVB irradiation	GO_LEUCINE_ZIPPER_DOMAIN_BINDING	-0.09649668	-0.00942454	-0.43227518	0.67651515	0.80497776	-6.44945915
hetero after UVB irradiation VS hetero before UVB irradiation	GO_LEUCINE_ZIPPER_DOMAIN_BINDING1	0.23345254	0.00098096	0.992849561	0.35038742	0.66713765	-5.72906001
WT after UVB irradiation VS WT before UVB irradiation	GO_LEUCINE_ZIPPER_DOMAIN_BINDING2	-0.04236311	0.036271962	-0.16425626	0.87355545	0.96914919	-5.98185569
homo after UVB irradiation VS homo before UVB irradiation	GO_LEUKEMIA_INHIBITORY_FACTOR_SIGNALING_PATHWAY	0.65916254	-0.07278997	2.970682265	0.01706877	0.08922557	-3.27865119
hetero after UVB irradiation VS hetero before UVB irradiation	GO_LEUKEMIA_INHIBITORY_FACTOR_SIGNALING_PATHWAY1	0.268187943	-0.00449376	0.983263912	0.35479818	0.67083786	-5.73815382
WT after UVB irradiation VS WT before UVB irradiation	GO_LEUKEMIA_INHIBITORY_FACTOR_SIGNALING_PATHWAY2	0.392994746	-0.01855649	1.191245771	0.26730041	0.74336868	-5.30966871
homo after UVB irradiation VS homo before UVB irradiation	GO_LEUKOCYTE_ACTIVATION_INVOLVED_IN_INFLAMMATORY_RESPONSE	0.652068468	-0.00447578	4.329611525	0.00228587	0.03201732	-1.25748784
hetero after UVB irradiation VS hetero before UVB irradiation	GO_LEUKOCYTE_ACTIVATION_INVOLVED_IN_INFLAMMATORY_RESPONSE1	0.573831469	0.036029971	3.842700901	0.00510479	0.1281058	-1.85345469
WT after UVB irradiation VS WT before UVB irradiation	GO_LEUKOCYTE_ACTIVATION_INVOLVED_IN_INFLAMMATORY_RESPONSE2	0.358314803	-0.02844258	1.340606712	0.21643914	0.71342248	-5.14515351
homo after UVB irradiation VS homo before UVB irradiation	GO_LEUKOCYTE_ADHESION_TO_ARTERIAL_ENDOTHELIAL_CELL	0.28301824	0.080344004	1.245849976	0.24672053	0.44071059	-5.77112836
hetero after UVB irradiation VS hetero before UVB irradiation	GO_LEUKOCYTE_ADHESION_TO_ARTERIAL_ENDOTHELIAL_CELL1	0.269762479	0.042774102	1.189217986	0.26906792	0.59622699	-5.52861139

WT after UVB irradiation VS WT before UVB irradiation	GO_LEUKOCYTE_ADHESION_TO_ARTERIAL_ENDOTHELIAL_CELL2	0.268161041	0.144344247	1.081796318	0.31050316	0.76760753	-5.42176395
homo after UVB irradiation VS homo before UVB irradiation	GO_LEUKOCYTE_ADHESION_TO_VASCULAR_ENDOTHELIAL_CELL	0.669138648	0.014666499	4.429495915	0.0019918	0.03030201	-1.11850869
hetero after UVB irradiation VS hetero before UVB irradiation	GO_LEUKOCYTE_ADHESION_TO_VASCULAR_ENDOTHELIAL_CELL1	0.545996749	0.024051076	3.498390465	0.0083392	0.14771631	-2.32727004
WT after UVB irradiation VS WT before UVB irradiation	GO_LEUKOCYTE_ADHESION_TO_VASCULAR_ENDOTHELIAL_CELL2	0.293030511	0.012004492	1.252477158	0.24535675	0.73450731	-5.24374863
homo after UVB irradiation VS homo before UVB irradiation	GO_LEUKOCYTE_AGGREGATION	0.485533269	-0.01030359	2.700751463	0.02607901	0.11400535	-3.697872
hetero after UVB irradiation VS hetero before UVB irradiation	GO_LEUKOCYTE_AGGREGATION1	0.558487378	0.065620454	2.695113386	0.02774237	0.23528578	-3.48511884
WT after UVB irradiation VS WT before UVB irradiation	GO_LEUKOCYTE_AGGREGATION2	0.401597952	0.001175954	1.521206343	0.16624989	0.67086399	-4.93090483
homo after UVB irradiation VS homo before UVB irradiation	GO_LEUKOCYTE_APOPTOTIC_PROCESS	0.283093515	-0.00435412	1.926782133	0.0887248	0.23284936	-4.87286758
hetero after UVB irradiation VS hetero before UVB irradiation	GO_LEUKOCYTE_APOPTOTIC_PROCESS1	0.262586745	0.012199002	1.598439678	0.1493199	0.46730953	-5.03888198
WT after UVB irradiation VS WT before UVB irradiation	GO_LEUKOCYTE_APOPTOTIC_PROCESS2	0.166685574	-0.06471066	0.781080334	0.45698038	0.83880233	-5.68640301
homo after UVB irradiation VS homo before UVB irradiation	GO_LEUKOCYTE_CELL_CELL_ADHESION	0.38553623	-0.02104327	2.593097906	0.03092008	0.1248393	-3.86492861
hetero after UVB irradiation VS hetero before UVB irradiation	GO_LEUKOCYTE_CELL_CELL_ADHESION1	0.333545735	0.016540975	2.134199325	0.06600228	0.33426479	-4.30293636
WT after UVB irradiation VS WT before UVB irradiation	GO_LEUKOCYTE_CELL_CELL_ADHESION2	0.188161952	-0.01788767	0.952794298	0.36826519	0.79942934	-5.54356552
homo after UVB irradiation VS homo before UVB irradiation	GO_LEUKOCYTE_CHEMOTAXIS	0.605138434	-0.02065437	4.416170461	0.00202856	0.03051518	-1.13696117
homo after UVB irradiation VS homo before UVB irradiation	GO_LEUKOCYTE_CHEMOTAXIS_INVOLVED_IN_INFLAMMATORY_RESPONSE	0.790986385	-0.07895623	3.61750056	0.0063615	0.05279898	-2.2902058
hetero after UVB irradiation VS hetero before UVB irradiation	GO_LEUKOCYTE_CHEMOTAXIS_INVOLVED_IN_INFLAMMATORY_RESPONSE1	0.537057942	0.076707687	1.872600181	0.09870748	0.39071492	-4.67164746
WT after UVB irradiation VS WT before UVB irradiation	GO_LEUKOCYTE_CHEMOTAXIS_INVOLVED_IN_INFLAMMATORY_RESPONSE2	0.607098774	0.027186687	1.88457511	0.09578136	0.59686908	-4.46130448
hetero after UVB irradiation VS hetero before UVB irradiation	GO_LEUKOCYTE_CHEMOTAXIS1	0.528670204	0.028386673	3.486869948	0.00847967	0.14863874	-2.34341103
WT after UVB irradiation VS WT before UVB irradiation	GO_LEUKOCYTE_CHEMOTAXIS2	0.420939415	0.008692485	1.896155954	0.09408247	0.59186437	-4.44569352
homo after UVB irradiation VS homo before UVB irradiation	GO_LEUKOCYTE_DIFFERENTIATION	0.387918134	-0.02106437	2.969506906	0.01710009	0.0893365	-3.2804735
hetero after UVB irradiation VS hetero before UVB irradiation	GO_LEUKOCYTE_DIFFERENTIATION1	0.31583094	0.02705573	2.01448599	0.07939589	0.3626016	-4.47336445
WT after UVB irradiation VS WT before UVB irradiation	GO_LEUKOCYTE_DIFFERENTIATION2	0.273351376	0.001052856	1.491428528	0.17373994	0.67974963	-4.96726087
homo after UVB irradiation VS homo before UVB irradiation	GO_LEUKOCYTE_HOMEOSTASIS	0.361596084	-0.02586833	2.862476515	0.02021729	0.09814032	-3.44659172
hetero after UVB irradiation VS hetero before UVB irradiation	GO_LEUKOCYTE_HOMEOSTASIS1	0.420598532	0.023670598	2.656853601	0.02942154	0.24078997	-3.54125886
WT after UVB irradiation VS WT before UVB irradiation	GO_LEUKOCYTE_HOMEOSTASIS2	0.274429415	-0.04204586	1.18644037	0.26908889	0.74431614	-5.31474782
homo after UVB irradiation VS homo before UVB irradiation	GO_LEUKOCYTE_MEDIATED_CYTOTOXICITY	0.516022631	-0.02296896	3.319377681	0.00996644	0.0666703	-2.74139894
hetero after UVB irradiation VS hetero before UVB irradiation	GO_LEUKOCYTE_MEDIATED_CYTOTOXICITY1	0.414945967	0.03491651	2.253911465	0.05483964	0.31016242	-4.13036054
WT after UVB irradiation VS WT before UVB irradiation	GO_LEUKOCYTE_MEDIATED_CYTOTOXICITY2	0.26489068	-0.04132859	1.197557515	0.26496613	0.74156847	-5.30297626
homo after UVB irradiation VS homo before UVB irradiation	GO_LEUKOCYTE_MEDIATED_IMMUNITY	0.596528691	-0.02205307	4.381963315	0.00212631	0.03092226	-1.1844548
hetero after UVB irradiation VS hetero before UVB irradiation	GO_LEUKOCYTE_MEDIATED_IMMUNITY1	0.577134279	0.02813764	4.1590624	0.00330097	0.11632799	-1.43408737
WT after UVB irradiation VS WT before UVB irradiation	GO_LEUKOCYTE_MEDIATED_IMMUNITY2	0.502944346	0.005559766	3.062828642	0.01529264	0.37040629	-2.80528673
homo after UVB irradiation VS homo before UVB irradiation	GO_LEUKOCYTE_MIGRATION	0.550718032	-0.01719394	3.990751071	0.0036869	0.0396846	-1.74011357
homo after UVB irradiation VS homo before UVB irradiation	GO_LEUKOCYTE_MIGRATION_INVOLVED_IN_INFLAMMATORY_RESPONSE	0.665475169	-0.05282161	3.248482649	0.01110693	0.07061828	-2.84994878
hetero after UVB irradiation VS hetero before UVB irradiation	GO_LEUKOCYTE_MIGRATION_INVOLVED_IN_INFLAMMATORY_RESPONSE1	0.666045182	0.055478881	3.610342231	0.00709634	0.13963559	-2.17134935
WT after UVB irradiation VS WT before UVB irradiation	GO_LEUKOCYTE_MIGRATION_INVOLVED_IN_INFLAMMATORY_RESPONSE2	0.632030892	0.057289966	2.113001989	0.06714343	0.54021113	-4.14824553
hetero after UVB irradiation VS hetero before UVB irradiation	GO_LEUKOCYTE_MIGRATION1	0.548666258	0.030762638	3.536644564	0.00789026	0.14436002	-2.27379997
WT after UVB irradiation VS WT before UVB irradiation	GO_LEUKOCYTE_MIGRATION2	0.413888616	0.012264545	1.991106958	0.08120092	0.5656709	-4.31656381
homo after UVB irradiation VS homo before UVB irradiation	GO_LEUKOCYTE_PROLIFERATION	0.366595829	-0.0249661	2.615423091	0.02984606	0.12285588	-3.83031665
hetero after UVB irradiation VS hetero before UVB irradiation	GO_LEUKOCYTE_PROLIFERATION1	0.316799918	0.013917871	2.109460273	0.06857486	0.33988857	-4.33835317
WT after UVB irradiation VS WT before UVB irradiation	GO_LEUKOCYTE_PROLIFERATION2	0.187275434	-0.01624518	0.932509713	0.37803843	0.80338597	-5.56162843
homo after UVB irradiation VS homo before UVB irradiation	GO_LEUKOCYTE_TETHERING_OR_ROLLING	0.651124878	0.006192196	4.281783252	0.00244294	0.0330029	-1.32457498
hetero after UVB irradiation VS hetero before UVB irradiation	GO_LEUKOCYTE_TETHERING_OR_ROLLING1	0.577272794	-0.00258603	3.922987359	0.0045638	0.12742869	-1.74551419
WT after UVB irradiation VS WT before UVB irradiation	GO_LEUKOCYTE_TETHERING_OR_ROLLING2	0.256142946	-0.0151192	1.051487938	0.32339802	0.77346379	-5.45142356
homo after UVB irradiation VS homo before UVB irradiation	GO_LEUKOTRIENE_B4_20_MONOOXYGENASE_ACTIVITY	0.648037894	0.047924222	2.533122878	0.03400353	0.13191117	-3.95779264
hetero after UVB irradiation VS hetero before UVB irradiation	GO_LEUKOTRIENE_B4_20_MONOOXYGENASE_ACTIVITY1	0.444659382	-0.00626035	1.707865878	0.12675084	0.43339219	-4.89525495
WT after UVB irradiation VS WT before UVB irradiation	GO_LEUKOTRIENE_B4_20_MONOOXYGENASE_ACTIVITY2	0.453021926	0.116109532	1.390526537	0.20139173	0.6976925	-5.08748918
homo after UVB irradiation VS homo before UVB irradiation	GO_LEUKOTRIENE_B4_METABOLIC_PROCESS	0.648037894	0.047924222	2.533122878	0.03400353	0.13191117	-3.95779264
hetero after UVB irradiation VS hetero before UVB irradiation	GO_LEUKOTRIENE_B4_METABOLIC_PROCESS1	0.444659382	-0.00626035	1.707865878	0.12675084	0.43339219	-4.89525495
WT after UVB irradiation VS WT before UVB irradiation	GO_LEUKOTRIENE_B4_METABOLIC_PROCESS2	0.453021926	0.116109532	1.390526537	0.20139173	0.6976925	-5.08748918
homo after UVB irradiation VS homo before UVB irradiation	GO_LEUKOTRIENE_BIOSYNTHETIC_PROCESS	0.522307493	0.043587065	2.94983455	0.01763335	0.0910162	-3.31098151
hetero after UVB irradiation VS hetero before UVB irradiation	GO_LEUKOTRIENE_BIOSYNTHETIC_PROCESS1	0.604536019	0.004957686	3.924546305	0.00455392	0.12742869	-1.74342827
WT after UVB irradiation VS WT before UVB irradiation	GO_LEUKOTRIENE_BIOSYNTHETIC_PROCESS2	0.407578385	0.035616184	2.17390231	0.06104069	0.52862529	-4.06328496
homo after UVB irradiation VS homo before UVB irradiation	GO_LEUKOTRIENE_C4_SYNTHASE_ACTIVITY	0.363868467	-0.04128606	1.446647425	0.18455573	0.36863683	-5.52867908
hetero after UVB irradiation VS hetero before UVB irradiation	GO_LEUKOTRIENE_C4_SYNTHASE_ACTIVITY1	0.586105097	-0.00522361	3.029474912	0.01668164	0.19018719	-2.99672343
WT after UVB irradiation VS WT before UVB irradiation	GO_LEUKOTRIENE_C4_SYNTHASE_ACTIVITY2	0.229078902	0.059965496	0.697348361	0.50510993	0.85763155	-5.74733106
homo after UVB irradiation VS homo before UVB irradiation	GO_LEUKOTRIENE_D4_METABOLIC_PROCESS	0.175611057	0.013697659	0.686924997	0.51079876	0.68259144	-6.29909778
hetero after UVB irradiation VS hetero before UVB irradiation	GO_LEUKOTRIENE_D4_METABOLIC_PROCESS1	0.422357052	0.047948834	2.183987512	0.06110987	0.32556646	-4.23138691
WT after UVB irradiation VS WT before UVB irradiation	GO_LEUKOTRIENE_D4_METABOLIC_PROCESS2	0.360633332	-0.00266622	1.94591322	0.08710535	0.5790399	-4.37826814
homo after UVB irradiation VS homo before UVB irradiation	GO_LEUKOTRIENE_METABOLIC_PROCESS	0.659146991	0.059787272	4.554038776	0.001681	0.02787404	-0.94737788
hetero after UVB irradiation VS hetero before UVB irradiation	GO_LEUKOTRIENE_METABOLIC_PROCESS1	0.634167711	0.021389317	4.621714381	0.00179187	0.09780003	-0.85070591
WT after UVB irradiation VS WT before UVB irradiation	GO_LEUKOTRIENE_METABOLIC_PROCESS2	0.48171372	0.05752497	2.447302842	0.03975625	0.48645468	-3.67725102
homo after UVB irradiation VS homo before UVB irradiation	GO_LEUKOTRIENE_SIGNALING_PATHWAY	0.834544399	-0.00176884	6.081301195	0.00025124	0.01199868	0.953068337
hetero after UVB irradiation VS hetero before UVB irradiation	GO_LEUKOTRIENE_SIGNALING_PATHWAY1	0.453548288	-0.0248493	1.515585941	0.16879251	0.49058543	-5.14454618
WT after UVB irradiation VS WT before UVB irradiation	GO_LEUKOTRIENE_SIGNALING_PATHWAY2	0.494213519	0.049900919	2.230803605	0.05583395	0.5232848	-3.98348184
homo after UVB irradiation VS homo before UVB irradiation	GO_LEWY_BODY	0.074184495	-0.0467363	0.301530856	0.77040095	0.86711973	-6.50123324
hetero after UVB irradiation VS hetero before UVB irradiation	GO_LEWY_BODY1	-0.12131485	-0.00167059	-0.51799506	0.61873512	0.84254497	-6.0884954
WT after UVB irradiation VS WT before UVB irradiation	GO_LEWY_BODY2	-0.33647266	-0.05263949	-1.30422324	0.22800574	0.71987179	-5.18636911
homo after UVB irradiation VS homo before UVB irradiation	GO_LEYDIG_CELL_DIFFERENTIATION	0.000730681	-0.07566062	0.003743723	0.99710106	0.9982238	-6.55084903
hetero after UVB irradiation VS hetero before UVB irradiation	GO_LEYDIG_CELL_DIFFERENTIATION1	0.036907436	0.000644651	0.135673877	0.89549247	0.96152498	-6.22151375
WT after UVB irradiation VS WT before UVB irradiation	GO_LEYDIG_CELL_DIFFERENTIATION2	0.022481112	0.012352859	0.125661797	0.90306357	0.97576564	-5.9877432
homo after UVB irradiation VS homo before UVB irradiation	GO_LIGAND_ACTIVATED_TRANSCRIPTION_FACTOR_ACTIVITY	0.018902017	0.011826401	0.111922389	0.91353529	0.95237327	-6.54398712
hetero after UVB irradiation VS hetero before UVB irradiation	GO_LIGAND_ACTIVATED_TRANSCRIPTION_FACTOR_ACTIVITY1	-0.36715145	0.014582562	-2.36526002	0.04615259	0.28703971	-3.9684484
WT after UVB irradiation VS WT before UVB irradiation	GO_LIGAND_ACTIVATED_TRANSCRIPTION_FACTOR_ACTIVITY2	-0.22138168	0.034793107	-1.0436709	0.32679061	0.77543801	-5.4589719
homo after UVB irradiation VS homo before UVB irradiation	GO_LIGAND_GATED_ANION_CHANNEL_ACTIVITY	-0.25517357	0.09401033	-1.15919941	0.27852498	0.47396138	-5.86823043
hetero after UVB irradiation VS hetero before UVB irradiation	GO_LIGAND_GATED_ANION_CHANNEL_ACTIVITY1	-0.69085705	-0.00319919	-3.96573655	0.00430118	0.12634511	-1.68845389
WT after UVB irradiation VS WT before UVB irradiation	GO_LIGAND_GATED_ANION_CHANNEL_ACTIVITY2	-0.54319001	0.057935034	-2.71572082	0.02613118	0.43720841	-3.29540907
homo after UVB irradiation VS homo before UVB irradiation	GO_LIGAND_GATED_CALCIUM_CHANNEL_ACTIVITY	0.507133545	-0.04302712	3.500426628	0.00757792	0.05782181	-2.46628184
hetero after UVB irradiation VS hetero before UVB irradiation	GO_LIGAND_GATED_CALCIUM_CHANNEL_ACTIVITY1	0.140822861	0.028692102	0.762077444	0.46828273	0.75313005	-5.92805448
WT after UVB irradiation VS WT before UVB irradiation	GO_LIGAND_GATED_CALCIUM_CHANNEL_ACTIVITY2	0.142640883	0.0291782	0.668108852	0.52264736	0.8642657	-5.7671812
homo after UVB irradiation VS homo before UVB irradiation	GO_LIGAND_GATED_CATION_CHANNEL_ACTIVITY	0.172650988	0.006293648	1.147605616	0.28302259	0.47835965	-5.88084664
hetero after UVB irradiation VS hetero before UVB irradiation	GO_LIGAND_GATED_CATION_CHANNEL_ACTIVITY1	-0.09231037	0.010973221	-0.57144052	0.58368178	0.82191128	-6.05811512
WT after UVB irradiation VS WT before UVB irradiation	GO_LIGAND_GATED_CATION_CHANNEL_ACTIVITY2	-0.09652074	0.011689999	-0.5133874	0.62139073	0.90107688	-5.8593452
homo after UVB irradiation VS homo before UVB irradiation	GO_LIGAND_GATED_ION_CHANNEL_ACTIVITY	0.105654988	0.028915453	0.69505459	0.50594495	0.67911857	-6.29327279
hetero after UVB irradiation VS hetero before UVB irradiation	GO_LIGAND_GATED_ION_CHANNEL_ACTIVITY1	-0.17906649	0.003557204	-1.1356248	0.28958308	0.61410673	-5.58588346
WT after UVB irradiation VS WT before UVB irradiation	GO_LIGAND_GATED_ION_CHANNEL_ACTIVITY2	-0.16089098	0.017887971	-0.87175492	0.40844413	0.81816343	-5.61386238
homo after UVB irradiation VS homo before UVB irradiation	GO_LIGAND_GATED_SODIUM_CHANNEL_ACTIVITY	-0.11022485	-0.06067188	-0.50858183	0.62424002	0.76737172	-6.41110389
hetero after UVB irradiation VS hetero before UVB irradiation	GO_LIGAND_GATED_SODIUM_CHANNEL_ACTIVITY1	-0.09321363	0.043302979	-0.42075582	0.68521186	0.87367844	-6.13658294
WT after UVB irradiation VS WT before UVB irradiation	GO_LIGAND_GATED_SODIUM_CHANNEL_ACTIVITY2	-0.37524312	-0.03112705	-1.44163733	0.18693604	0.68957923	-5.02717931
homo after UVB irradiation VS homo before UVB irradiation	GO_LIGASE_ACTIVITY	-0.28559256	0.001819772	-1.75135101	0.11650063	0.27520407	-5.12259737
homo after UVB irradiation VS homo before UVB irradiation	GO_LIGASE_ACTIVITY_FORMING_CARBON_CARBON_BONDS	-0.35565799	-0.02838023	-0.94166589	0.37286901	0.56308105	-6.08888285
hetero after UVB irradiation VS hetero before UVB irradiation	GO_LIGASE_ACTIVITY_FORMING_CARBON_CARBON_BONDS1	-0.91024731	-0.03327588	-5.92236972	0.00037893	0.07788488	0.594998071
WT after UVB irradiation VS WT before UVB irradiation	GO_LIGASE_ACTIVITY_FORMING_CARBON_CARBON_BONDS2	0.141290304	-0.05314626	0.571351204	0.5832675	0.88973314	-5.82740548
homo after UVB irradiation VS homo before UVB irradiation	GO_LIGASE_ACTIVITY_FORMING_CARBON_NITROGEN_BONDS	-0.20035873	-0.01124677	-1.36887287	0.20681423	0.39682194	-5.62528897
hetero after UVB irradiation VS hetero before UVB irradiation	GO_LIGASE_ACTIVITY_FORMING_CARBON_NITROGEN_BONDS1	-0.21235133	-0.03870104	-1.56458335	0.15701625	0.47604927	-5.08240137
WT after UVB irradiation VS WT before UVB irradiation	GO_LIGASE_ACTIVITY_FORMING_CARBON_NITROGEN_BONDS2	0.136809314	0.025707927	0.674960377	0.5185046	0.86195476	-5.76259736
homo after UVB irradiation VS homo before UVB irradiation	GO_LIGASE_ACTIVITY_FORMING_CARBON_OXYGEN_BONDS	-0.71715461	-0.02382892	-4.17497446	0.00283722	0.03518388	-1.47563394
hetero after UVB irradiation VS hetero before UVB irradiation	GO_LIGASE_ACTIVITY_FORMING_CARBON_OXYGEN_BONDS1	-0.42610652	-0.08350079	-2.2774588	0.05287562	0.30597357	-4.09621562
WT after UVB irradiation VS WT before UVB irradiation	GO_LIGASE_ACTIVITY_FORMING_CARBON_OXYGEN_BONDS2	-0.12430317	-0.01207551	-0.4555135	0.66070595	0.91158787	-5.8880459
homo after UVB irradiation VS homo before UVB irradiation	GO_LIGASE_ACTIVITY_FORMING_CARBON_SULFUR_BONDS	-0.21001019	0.013723018	-1.23523073	0.25044932	0.44512593	-5.78328831
hetero after UVB irradiation VS hetero before UVB irradiation	GO_LIGASE_ACTIVITY_FORMING_CARBON_SULFUR_BONDS1	-0.05824304	-0.00408135	-0.41617562	0.68842284	0.87487346	-6.13861471
WT after UVB irradiation VS WT before UVB irradiation	GO_LIGASE_ACTIVITY_FORMING_CARBON_SULFUR_BONDS2	-0.16736361	0.006322029	-0.91564199	0.38630959	0.80838451	-5.57641362
homo after UVB irradiation VS homo before UVB irradiation	GO_LIGASE_ACTIVITY_FORMING_PHOSPHORIC_ESTER_BONDS	-0.60558493	0.014018383	-2.50510972	0.03554855	0.1349601	-4.00109636
hetero after UVB irradiation VS hetero before UVB irradiation	GO_LIGASE_ACTIVITY_FORMING_PHOSPHORIC_ESTER_BONDS1	-0.12095934	-0.15472292	-0.36238546	0.72662287	0.89222406	-6.16087769
WT after UVB irradiation VS WT before UVB irradiation	GO_LIGASE_ACTIVITY_FORMING_PHOSPHORIC_ESTER_BONDS2	-0.05565613	0.123681021	-0.14554186	0.88784076	0.97111575	-5.98490522
hetero after UVB irradiation VS hetero before UVB irradiation	GO_LIGASE_ACTIVITY1	-0.21851885	-0.03625105	-1.64506279	0.13928551	0.45310781	-4.97822109
WT after UVB irradiation VS WT before UVB irradiation	GO_LIGASE_ACTIVITY2	0.02818258	-0.00587499	0.173459811	0.86654775	0.96790654	-5.98022176
homo after UVB irradiation VS homo before UVB irradiation	GO_LIM_DOMAIN_BINDING	0.941976911	0.007892161	7.031144713	8.97E-05	0.00991667	1.959414557
hetero after UVB irradiation VS hetero before UVB irradiation	GO_LIM_DOMAIN_BINDING1	0.549455435	0.035693737	1.805039907	0.10941357	0.40805964	-4.76431612
WT after UVB irradiation VS WT before UVB irradiation	GO_LIM_DOMAIN_BINDING2	0.586369582	-0.02882178	2.48218257	0.03764075	0.47615783	-3.62767293
homo after UVB irradiation VS homo before UVB irradiation	GO_LIMB_BUD_FORMATION	-0.4223277	0.021894965	-2.34066523	0.04615268	0.15689358	-4.25400853
hetero after UVB irradiation VS hetero before UVB irradiation	GO_LIMB_BUD_FORMATION1	-0.43233599	0.074716732	-2.05674844	0.07438891	0.35272584	-4.41348441
WT after UVB irradiation VS WT before UVB irradiation	GO_LIMB_BUD_FORMATION2	-0.21263598	0.069135334	-1.03624974	0.33003684	0.77726938	-5.46609902
homo after UVB irradiation VS homo before UVB irradiation	GO_LIMBIC_SYSTEM_DEVELOPMENT	-0.05728195	0.042283726	-0.44778148	0.6657285	0.7976742	-6.4421433
hetero after UVB irradiation VS hetero before UVB irradiation	GO_LIMBIC_SYSTEM_DEVELOPMENT1	-0.05415584	0.050950001	-0.42664035	0.68109648	0.87173733	-6.13394143
WT after UVB irradiation VS WT before UVB irradiation	GO_LIMBIC_SYSTEM_DEVELOPMENT2	-0.04448864	0.037354421	-0.30410523	0.76871578	0.94123239	-5.94756287
homo after UVB irradiation VS homo before UVB irradiation	GO_LINOLEIC_ACID_METABOLIC_PROCESS	0.079427265	-0.04681252	0.362022796	0.72634808	0.83837647	-6.47949921
hetero after UVB irradiation VS hetero before UVB irradiation	GO_LINOLEIC_ACID_METABOLIC_PROCESS1	0.434871577	0.035448864	2.697920347	0.02762312	0.23528578	-3.48100083
WT after UVB irradiation VS WT before UVB irradiation	GO_LINOLEIC_ACID_METABOLIC_PROCESS2	0.263115092	0.016396946	1.47090995	0.17907448	0.68213098	-4.99208776
homo after UVB irradiation VS homo before UVB irradiation	GO_LIPASE_ACTIVATOR_ACTIVITY	0.402462393	0.023611947	2.002889385	0.07874602	0.21642485	-4.76174931
hetero after UVB irradiation VS hetero before UVB irradiation	GO_LIPASE_ACTIVATOR_ACTIVITY1	0.443847344	-0.0003651	2.567952717	0.03373757	0.25357971	-3.67172329
WT after UVB irradiation VS WT before UVB irradiation	GO_LIPASE_ACTIVATOR_ACTIVITY2	0.195447561	-0.03948349	0.820875005	0.43521542	0.83017793	-5.65538555
homo after UVB irradiation VS homo before UVB irradiation	GO_LIPASE_ACTIVITY	0.112950709	0.008098806	0.791778428	0.45044785	0.63204896	-6.21937305
hetero after UVB irradiation VS hetero before UVB irradiation	GO_LIPASE_ACTIVITY1	-0.08414038	0.028644949	-0.64553795	0.53698024	0.7947523	-6.01150539
WT after UVB irradiation VS WT before UVB irradiation	GO_LIPASE_ACTIVITY2	-0.02376488	0.018500053	-0.15858609	0.87787874	0.9693507	-5.98281831
homo after UVB irradiation VS homo before UVB irradiation	GO_LIPASE_BINDING	0.01052283	-0.06824213	0.033383931	0.97415461	0.9867252	-6.55024511
hetero after UVB irradiation VS hetero before UVB irradiation	GO_LIPASE_BINDING1	0.120351055	0.027153637	0.603666198	0.56309272	0.81004002	-6.0384768
WT after UVB irradiation VS WT before UVB irradiation	GO_LIPASE_BINDING2	-0.39034117	-0.08307203	-1.37466635	0.20607122	0.7028684	-5.1059461
homo after UVB irradiation VS homo before UVB irradiation	GO_LIPASE_INHIBITOR_ACTIVITY	0.549891122	0.049711721	2.755635475	0.02391596	0.1086214	-3.6126024
hetero after UVB irradiation VS hetero before UVB irradiation	GO_LIPASE_INHIBITOR_ACTIVITY1	0.184955722	0.036372414	1.393867467	0.20153534	0.52625296	-5.29425547
WT after UVB irradiation VS WT before UVB irradiation	GO_LIPASE_INHIBITOR_ACTIVITY2	0.135281632	0.044229698	0.709941731	0.49767177	0.85541458	-5.7385512
homo after UVB irradiation VS homo before UVB irradiation	GO_LIPID_ANTIGEN_BINDING	1.264233129	-0.02333193	8.092666695	3.18E-05	0.00847131	2.945252036
hetero after UVB irradiation VS hetero before UVB irradiation	GO_LIPID_ANTIGEN_BINDING1	0.62129893	-0.03503426	1.893907289	0.09554347	0.38572694	-4.64217119
WT after UVB irradiation VS WT before UVB irradiation	GO_LIPID_ANTIGEN_BINDING2	0.88757374	0.066794515	2.673554697	0.02790558	0.44213442	-3.35536416
homo after UVB irradiation VS homo before UVB irradiation	GO_LIPID_BINDING	0.319852206	-0.00574675	2.566237227	0.03226442	0.12754515	-3.90654238
hetero after UVB irradiation VS hetero before UVB irradiation	GO_LIPID_BINDING1	0.244845756	0.01876512	1.80288329	0.10977284	0.40890644	-4.76725329
WT after UVB irradiation VS WT before UVB irradiation	GO_LIPID_BINDING2	0.189035757	0.006723612	1.27352001	0.23817129	0.72863458	-5.22059454
homo after UVB irradiation VS homo before UVB irradiation	GO_LIPID_BIOSYNTHETIC_PROCESS	0.159038204	-0.01580387	1.243450863	0.24755888	0.44185129	-5.77388174
hetero after UVB irradiation VS hetero before UVB irradiation	GO_LIPID_BIOSYNTHETIC_PROCESS1	0.071576963	0.001434277	0.485920771	0.64029182	0.8522738	-6.1053937
WT after UVB irradiation VS WT before UVB irradiation	GO_LIPID_BIOSYNTHETIC_PROCESS2	0.096931075	0.006960872	0.611390116	0.55771088	0.87953066	-5.80351645
homo after UVB irradiation VS homo before UVB irradiation	GO_LIPID_CATABOLIC_PROCESS	0.086665926	-0.00525389	0.548588541	0.59768121	0.74819842	-6.38866153
hetero after UVB irradiation VS hetero before UVB irradiation	GO_LIPID_CATABOLIC_PROCESS1	-0.125522	4.05E-05	-0.89703465	0.39636804	0.7038269	-5.81683531
WT after UVB irradiation VS WT before UVB irradiation	GO_LIPID_CATABOLIC_PROCESS2	-0.01343737	0.008686345	-0.07539553	0.94172943	0.98463518	-5.99306836
homo after UVB irradiation VS homo before UVB irradiation	GO_LIPID_DIGESTION	0.442440639	0.191337066	3.551238085	0.00702222	0.0552029	-2.38967425
hetero after UVB irradiation VS hetero before UVB irradiation	GO_LIPID_DIGESTION1	0.190601226	0.047824798	0.770004418	0.46382906	0.75155229	-5.92194355
WT after UVB irradiation VS WT before UVB irradiation	GO_LIPID_DIGESTION2	0.215787028	0.117581287	1.017157748	0.33850261	0.78064003	-5.48425858
homo after UVB irradiation VS homo before UVB irradiation	GO_LIPID_DROPLET	0.177022824	-0.01399858	1.196574793	0.26441845	0.4596003	-5.82694637
homo after UVB irradiation VS homo before UVB irradiation	GO_LIPID_DROPLET_FORMATION	0.035308617	0.005417849	0.202722595	0.84421648	0.91402073	-6.5283587
hetero after UVB irradiation VS hetero before UVB irradiation	GO_LIPID_DROPLET_FORMATION1	-0.15150746	0.012811284	-1.12388921	0.29424048	0.61784413	-5.59817738
WT after UVB irradiation VS WT before UVB irradiation	GO_LIPID_DROPLET_FORMATION2	-0.111212	0.02758259	-0.43076068	0.67787894	0.91685973	-5.8993305
homo after UVB irradiation VS homo before UVB irradiation	GO_LIPID_DROPLET_ORGANIZATION	0.125730812	0.0173004	0.840875816	0.4238977	0.60899134	-6.17870657
hetero after UVB irradiation VS hetero before UVB irradiation	GO_LIPID_DROPLET_ORGANIZATION1	-0.27660251	-0.0009096	-1.54926974	0.1606144	0.48161472	-5.10193269
WT after UVB irradiation VS WT before UVB irradiation	GO_LIPID_DROPLET_ORGANIZATION2	-0.25382148	-0.05427986	-1.5028918	0.17082163	0.6778332	-4.9533103

hetero after UVB irradiation VS hetero before UVB irradiation	GO_LIPID_DROPLET1	-0.05638454	-0.02915942	-0.35901845	0.72904282	0.89295432	-6.16217266
WT after UVB irradiation VS WT before UVB irradiation	GO_LIPID_DROPLET2	-0.04751425	-0.03415543	-0.31848394	0.75817722	0.93908133	-5.9428986
homo after UVB irradiation VS homo before UVB irradiation	GO_LIPID_EXPORT_FROM_CELL	0.249935391	0.01739236	1.862173762	0.09813312	0.24805591	-4.96595324
hetero after UVB irradiation VS hetero before UVB irradiation	GO_LIPID_EXPORT_FROM_CELL1	0.15483936	0.060391663	1.118799783	0.29627892	0.62036115	-5.60348073
WT after UVB irradiation VS WT before UVB irradiation	GO_LIPID_EXPORT_FROM_CELL2	0.016837795	-0.00278001	0.074086393	0.94273912	0.98504656	-5.99317154
homo after UVB irradiation VS homo before UVB irradiation	GO_LIPID_GLYCOSYLATION	0.492316155	0.013289057	2.810803591	0.02192653	0.1030477	-3.52687351
hetero after UVB irradiation VS hetero before UVB irradiation	GO_LIPID_GLYCOSYLATION1	0.583520019	0.001859812	3.020467612	0.0169095	0.19058304	-3.00979442
WT after UVB irradiation VS WT before UVB irradiation	GO_LIPID_GLYCOSYLATION2	0.538460301	0.034343386	3.807383033	0.00506639	0.27446335	-1.79769168
homo after UVB irradiation VS homo before UVB irradiation	GO_LIPID_HOMEOSTASIS	0.173086666	-0.01230761	1.429091157	0.18938872	0.3750983	-5.55076779
hetero after UVB irradiation VS hetero before UVB irradiation	GO_LIPID_HOMEOSTASIS1	-0.00809237	0.018485524	-0.05814304	0.95508698	0.98334241	-6.22965119
WT after UVB irradiation VS WT before UVB irradiation	GO_LIPID_HOMEOSTASIS2	0.078480741	0.001180501	0.446284337	0.66708475	0.91378165	-5.89232337
homo after UVB irradiation VS homo before UVB irradiation	GO_LIPID_HYDROXYLATION	0.175696478	0.197704212	0.749802171	0.47401748	0.6524784	-6.2524739
hetero after UVB irradiation VS hetero before UVB irradiation	GO_LIPID_HYDROXYLATION1	-0.08469119	0.13667032	-0.39722587	0.70177888	0.88151888	-6.14679466
WT after UVB irradiation VS WT before UVB irradiation	GO_LIPID_HYDROXYLATION2	-0.27773375	0.195335455	-1.05566211	0.32159768	0.77346379	-5.44737572
homo after UVB irradiation VS homo before UVB irradiation	GO_LIPID_IMPORT_INTO_CELL	0.258149662	0.032656431	1.811135089	0.10622544	0.26007394	-5.03859664
hetero after UVB irradiation VS hetero before UVB irradiation	GO_LIPID_IMPORT_INTO_CELL1	0.159488836	-0.01398214	0.663707336	0.52587966	0.78916946	-5.9992978
WT after UVB irradiation VS WT before UVB irradiation	GO_LIPID_IMPORT_INTO_CELL2	-0.04930626	5.88E-06	-0.19722082	0.84851521	0.96522688	-5.97559526
homo after UVB irradiation VS homo before UVB irradiation	GO_LIPID_KINASE_ACTIVITY	-0.21855247	0.034279954	-0.55704253	0.59214771	0.74395022	-6.3837169
hetero after UVB irradiation VS hetero before UVB irradiation	GO_LIPID_KINASE_ACTIVITY1	-0.09802913	-0.01847753	-0.39704681	0.70190562	0.88151888	-6.14687021
WT after UVB irradiation VS WT before UVB irradiation	GO_LIPID_KINASE_ACTIVITY2	0.385101273	-0.04354112	1.720693776	0.12316017	0.63133281	-4.6784071
homo after UVB irradiation VS homo before UVB irradiation	GO_LIPID_LOCALIZATION	0.295678258	4.14E-05	2.432916272	0.03986436	0.14330974	-4.11243285
hetero after UVB irradiation VS hetero before UVB irradiation	GO_LIPID_LOCALIZATION1	0.125065171	0.023180283	0.910159793	0.38982016	0.69986449	-5.80522924
WT after UVB irradiation VS WT before UVB irradiation	GO_LIPID_LOCALIZATION2	0.09466318	0.018158761	0.570407209	0.58387785	0.89000397	-5.82795085
homo after UVB irradiation VS homo before UVB irradiation	GO_LIPID_METABOLIC_PROCESS	0.161074053	-0.01399982	1.250465147	0.24511443	0.43909376	-5.76582156
hetero after UVB irradiation VS hetero before UVB irradiation	GO_LIPID_METABOLIC_PROCESS1	0.04548523	0.000897921	0.314302323	0.76147925	0.90682777	-6.1782585
WT after UVB irradiation VS WT before UVB irradiation	GO_LIPID_METABOLIC_PROCESS2	0.070463292	0.007984059	0.431895573	0.67708708	0.91685973	-5.89882623
homo after UVB irradiation VS homo before UVB irradiation	GO_LIPID_MODIFICATION	0.189332033	-0.02763276	1.321642754	0.22143232	0.41248129	-5.68233126
hetero after UVB irradiation VS hetero before UVB irradiation	GO_LIPID_MODIFICATION1	-0.05185244	-0.00721991	-0.28839694	0.78050865	0.91401375	-6.186625
WT after UVB irradiation VS WT before UVB irradiation	GO_LIPID_MODIFICATION2	0.012684335	-0.00732396	0.067004799	0.94820276	0.98614246	-5.9936984
homo after UVB irradiation VS homo before UVB irradiation	GO_LIPID_OXIDATION	0.065376331	-0.04556295	0.357191167	0.72983	0.84061989	-6.48137661
hetero after UVB irradiation VS hetero before UVB irradiation	GO_LIPID_OXIDATION1	-0.24880483	-0.02517383	-1.27119826	0.24001755	0.56816205	-5.43757852
WT after UVB irradiation VS WT before UVB irradiation	GO_LIPID_OXIDATION2	-0.0675877	-0.02587501	-0.31025826	0.76419965	0.94098943	-5.94559261
homo after UVB irradiation VS homo before UVB irradiation	GO_LIPID_PHOSPHATASE_ACTIVITY	0.628251513	-0.00209289	3.940393064	0.00396396	0.04117099	-1.81325065
hetero after UVB irradiation VS hetero before UVB irradiation	GO_LIPID_PHOSPHATASE_ACTIVITY1	0.23744791	-0.05066649	0.902520389	0.39362166	0.70256648	-5.81200097
WT after UVB irradiation VS WT before UVB irradiation	GO_LIPID_PHOSPHATASE_ACTIVITY2	0.343073306	0.01929644	0.97721778	0.35674848	0.79119413	-5.52141358
homo after UVB irradiation VS homo before UVB irradiation	GO_LIPID_PHOSPHORYLATION	0.123697022	-0.03811364	0.910151613	0.38832083	0.57760815	-6.11786205
hetero after UVB irradiation VS hetero before UVB irradiation	GO_LIPID_PHOSPHORYLATION1	0.028377917	-0.01471082	0.187266014	0.85619997	0.94531139	-6.21251008
WT after UVB irradiation VS WT before UVB irradiation	GO_LIPID_PHOSPHORYLATION2	-0.01360683	-0.01048272	-0.08573753	0.9337571	0.98322796	-5.99218981
homo after UVB irradiation VS homo before UVB irradiation	GO_LIPID_STORAGE	0.394259431	0.011341884	3.156848453	0.01278711	0.07648939	-2.99083369
hetero after UVB irradiation VS hetero before UVB irradiation	GO_LIPID_STORAGE1	0.136706905	0.007599062	0.899452689	0.39515577	0.7033063	-5.81470732
WT after UVB irradiation VS WT before UVB irradiation	GO_LIPID_STORAGE2	0.091548404	-0.02652044	0.493800054	0.63456161	0.9052809	-5.86941938
homo after UVB irradiation VS homo before UVB irradiation	GO_LIPID_TRANSFER_ACTIVITY	0.447802379	0.029093786	3.431443145	0.0084078	0.06100162	-2.5707264
hetero after UVB irradiation VS hetero before UVB irradiation	GO_LIPID_TRANSFER_ACTIVITY1	0.350538697	0.03223747	2.618784008	0.03119604	0.24648003	-3.59712995
WT after UVB irradiation VS WT before UVB irradiation	GO_LIPID_TRANSFER_ACTIVITY2	0.181042071	0.005644589	1.218901691	0.25719636	0.73855744	-5.28016803
homo after UVB irradiation VS homo before UVB irradiation	GO_LIPID_TRANSLOCATION	0.403297462	-0.01884629	2.32252728	0.04750091	0.15971085	-4.2817342
hetero after UVB irradiation VS hetero before UVB irradiation	GO_LIPID_TRANSLOCATION1	0.368683733	0.046802348	2.01018219	0.07992381	0.36366772	-4.47944317
WT after UVB irradiation VS WT before UVB irradiation	GO_LIPID_TRANSLOCATION2	0.355748675	0.013417311	2.219501807	0.05683193	0.5232848	-3.99936156
homo after UVB irradiation VS homo before UVB irradiation	GO_LIPID_TRANSPORT_ACROSS_BLOOD_BRAIN_BARRIER	0.467115534	0.032089812	1.977328879	0.08197075	0.22167902	-4.79923425
hetero after UVB irradiation VS hetero before UVB irradiation	GO_LIPID_TRANSPORT_ACROSS_BLOOD_BRAIN_BARRIER1	-0.19767854	-0.02446914	-1.17727877	0.27353205	0.60018422	-5.54152797
WT after UVB irradiation VS WT before UVB irradiation	GO_LIPID_TRANSPORT_ACROSS_BLOOD_BRAIN_BARRIER2	-0.31771525	0.127551221	-1.11138639	0.29830791	0.75997207	-5.3922158
homo after UVB irradiation VS homo before UVB irradiation	GO_LIPID_TRANSPORTER_ACTIVITY	0.30581547	0.010891469	2.299489598	0.04926996	0.16294862	-4.3168906
hetero after UVB irradiation VS hetero before UVB irradiation	GO_LIPID_TRANSPORTER_ACTIVITY1	0.164434408	0.0209844	1.176201552	0.2739378	0.60071721	-5.54268898
WT after UVB irradiation VS WT before UVB irradiation	GO_LIPID_TRANSPORTER_ACTIVITY2	0.139402321	0.033394193	0.873973731	0.40730376	0.81717677	-5.61200492
homo after UVB irradiation VS homo before UVB irradiation	GO_LIPOPEPTIDE_BINDING	1.154913769	0.006482717	8.10814564	3.14E-05	0.00847131	2.958643882
hetero after UVB irradiation VS hetero before UVB irradiation	GO_LIPOPEPTIDE_BINDING1	0.969567628	0.015581528	6.104160727	0.00031049	0.07788488	0.775112431
WT after UVB irradiation VS WT before UVB irradiation	GO_LIPOPEPTIDE_BINDING2	0.813188725	0.034269823	3.384675489	0.00940558	0.32218592	-2.36058889
homo after UVB irradiation VS homo before UVB irradiation	GO_LIPOPHAGY	0.451899971	-0.066123	1.596467606	0.14755799	0.32041102	-5.3340391
hetero after UVB irradiation VS hetero before UVB irradiation	GO_LIPOPHAGY1	0.607110532	-0.02101615	2.730545367	0.02627519	0.23092121	-3.43314831
WT after UVB irradiation VS WT before UVB irradiation	GO_LIPOPHAGY2	0.611506403	0.009215807	3.267486341	0.01121115	0.33971849	-2.52111171
homo after UVB irradiation VS homo before UVB irradiation	GO_LIPOPOLYSACCHARIDE_BINDING	0.643425996	-0.00713725	4.045913122	0.003407	0.03837948	-1.66040831
hetero after UVB irradiation VS hetero before UVB irradiation	GO_LIPOPOLYSACCHARIDE_BINDING1	0.65288388	0.040571736	4.491617808	0.00212094	0.1055457	-1.01107327
WT after UVB irradiation VS WT before UVB irradiation	GO_LIPOPOLYSACCHARIDE_BINDING2	0.600872282	0.038273614	3.804606489	0.00508662	0.27446335	-1.80129879
homo after UVB irradiation VS homo before UVB irradiation	GO_LIPOPOLYSACCHARIDE_IMMUNE_RECEPTOR_ACTIVITY	1.144573769	0.015470436	8.296520068	2.64E-05	0.00847131	3.119492642
hetero after UVB irradiation VS hetero before UVB irradiation	GO_LIPOPOLYSACCHARIDE_IMMUNE_RECEPTOR_ACTIVITY1	1.006084358	0.059589531	5.431805038	0.00066255	0.08158876	0.082793816
WT after UVB irradiation VS WT before UVB irradiation	GO_LIPOPOLYSACCHARIDE_IMMUNE_RECEPTOR_ACTIVITY2	0.743345925	-0.00760486	2.425108545	0.04116438	0.49187265	-3.70877534
homo after UVB irradiation VS homo before UVB irradiation	GO_LIPOPOLYSACCHARIDE_MEDIATED_SIGNALING_PATHWAY	0.560530768	-0.01236465	4.000493083	0.00363573	0.03936353	-1.72600576
hetero after UVB irradiation VS hetero before UVB irradiation	GO_LIPOPOLYSACCHARIDE_MEDIATED_SIGNALING_PATHWAY1	0.638367059	0.036695462	4.180739123	0.00320555	0.11632799	-1.40594846
WT after UVB irradiation VS WT before UVB irradiation	GO_LIPOPOLYSACCHARIDE_MEDIATED_SIGNALING_PATHWAY2	0.381104618	-0.00412636	1.772121802	0.11386263	0.61911707	-4.61109902
homo after UVB irradiation VS homo before UVB irradiation	GO_LIPOPOLYSACCHARIDE_RECEPTOR_COMPLEX	0.725441944	0.019891043	5.041235428	0.00088474	0.02043198	-0.30138336
hetero after UVB irradiation VS hetero before UVB irradiation	GO_LIPOPOLYSACCHARIDE_RECEPTOR_COMPLEX1	0.558357438	-0.00600915	3.418349653	0.00936879	0.15412029	-2.43976732
WT after UVB irradiation VS WT before UVB irradiation	GO_LIPOPOLYSACCHARIDE_RECEPTOR_COMPLEX2	0.570192727	0.00545523	2.7538405	0.0246267	0.42639272	-3.24125353
homo after UVB irradiation VS homo before UVB irradiation	GO_LIPOPROTEIN_BIOSYNTHETIC_PROCESS	0.059920469	-0.01012248	0.427836314	0.67961769	0.80665313	-6.45150807
hetero after UVB irradiation VS hetero before UVB irradiation	GO_LIPOPROTEIN_BIOSYNTHETIC_PROCESS1	0.089195927	-0.00950947	0.629720679	0.54675805	0.79959978	-6.02188541
WT after UVB irradiation VS WT before UVB irradiation	GO_LIPOPROTEIN_BIOSYNTHETIC_PROCESS2	0.108033817	0.007623033	0.66654342	0.52359674	0.86476967	-5.76822268
homo after UVB irradiation VS homo before UVB irradiation	GO_LIPOPROTEIN_CATABOLIC_PROCESS	-0.21511363	0.032104659	-1.2033596	0.26192132	0.45741691	-5.8193533
hetero after UVB irradiation VS hetero before UVB irradiation	GO_LIPOPROTEIN_CATABOLIC_PROCESS1	-0.06686055	-0.0159478	-0.29459509	0.77594069	0.91242095	-6.18468715
WT after UVB irradiation VS WT before UVB irradiation	GO_LIPOPROTEIN_CATABOLIC_PROCESS2	0.007557247	0.057750814	0.040328742	0.96880765	0.99220588	-5.99520858
homo after UVB irradiation VS homo before UVB irradiation	GO_LIPOPROTEIN_LIPASE_ACTIVITY	-0.40737487	0.005339563	-2.20722093	0.05703356	0.17805039	-4.45695499
hetero after UVB irradiation VS hetero before UVB irradiation	GO_LIPOPROTEIN_LIPASE_ACTIVITY1	-0.49825475	-0.02383985	-3.44514934	0.00900978	0.15056439	-2.40200892
WT after UVB irradiation VS WT before UVB irradiation	GO_LIPOPROTEIN_LIPASE_ACTIVITY2	-0.22376907	-0.13108754	-0.82820149	0.4312872	0.82885935	-5.64953411
homo after UVB irradiation VS homo before UVB irradiation	GO_LIPOPROTEIN_LOCALIZATION	0.701649529	0.003126715	5.311436382	0.00062893	0.01788158	0.040673665
hetero after UVB irradiation VS hetero before UVB irradiation	GO_LIPOPROTEIN_LOCALIZATION1	0.536502332	0.047521433	2.5336322	0.03557223	0.25880535	-3.72206835
WT after UVB irradiation VS WT before UVB irradiation	GO_LIPOPROTEIN_LOCALIZATION2	0.387003697	-0.00094069	1.685805528	0.12986464	0.63604796	-4.72359286
homo after UVB irradiation VS homo before UVB irradiation	GO_LIPOPROTEIN_METABOLIC_PROCESS	0.0843449	-0.00638793	0.63085298	0.54505395	0.70954139	-6.33759805
hetero after UVB irradiation VS hetero before UVB irradiation	GO_LIPOPROTEIN_METABOLIC_PROCESS1	0.107053239	0.011245782	0.739658557	0.48103162	0.76127227	-5.94504296
WT after UVB irradiation VS WT before UVB irradiation	GO_LIPOPROTEIN_METABOLIC_PROCESS2	0.16531287	0.009784328	1.134708125	0.28896743	0.75486529	-5.36852504
homo after UVB irradiation VS homo before UVB irradiation	GO_LIPOPROTEIN_PARTICLE_RECEPTOR_ACTIVITY	0.278435588	0.020424443	1.109234497	0.29832513	0.49278121	-5.92194411
hetero after UVB irradiation VS hetero before UVB irradiation	GO_LIPOPROTEIN_PARTICLE_RECEPTOR_ACTIVITY1	0.14538511	0.011891972	1.076669153	0.31359018	0.63451494	-5.64671557
WT after UVB irradiation VS WT before UVB irradiation	GO_LIPOPROTEIN_PARTICLE_RECEPTOR_ACTIVITY2	0.380007679	-0.05110549	1.807479034	0.10785645	0.61228673	-4.56436977
homo after UVB irradiation VS homo before UVB irradiation	GO_LIPOPROTEIN_PARTICLE_RECEPTOR_BINDING	0.300683821	0.016724615	1.603055463	0.14609616	0.31878471	-5.32524403
hetero after UVB irradiation VS hetero before UVB irradiation	GO_LIPOPROTEIN_PARTICLE_RECEPTOR_BINDING1	0.565289749	0.013100578	4.368391572	0.00249394	0.11070877	-1.16563841
WT after UVB irradiation VS WT before UVB irradiation	GO_LIPOPROTEIN_PARTICLE_RECEPTOR_BINDING2	0.324377598	0.001794684	2.145109703	0.06385486	0.53678963	-4.10351576
homo after UVB irradiation VS homo before UVB irradiation	GO_LIPOSACCHARIDE_METABOLIC_PROCESS	0.32644544	-0.03845014	1.960939002	0.08410488	0.22541513	-4.82318361
hetero after UVB irradiation VS hetero before UVB irradiation	GO_LIPOSACCHARIDE_METABOLIC_PROCESS1	0.280375028	-0.00445541	1.902289961	0.09432549	0.38408634	-4.63054349
WT after UVB irradiation VS WT before UVB irradiation	GO_LIPOSACCHARIDE_METABOLIC_PROCESS2	0.253881829	-0.00894581	1.636777568	0.13985729	0.64922117	-4.78639658
homo after UVB irradiation VS homo before UVB irradiation	GO_LIPOXIN_METABOLIC_PROCESS	0.46544924	-0.02499777	2.327701767	0.04711233	0.15883863	-4.27382855
hetero after UVB irradiation VS hetero before UVB irradiation	GO_LIPOXIN_METABOLIC_PROCESS1	0.45026055	0.090026975	3.381986566	0.00988037	0.15671485	-2.49114292
WT after UVB irradiation VS WT before UVB irradiation	GO_LIPOXIN_METABOLIC_PROCESS2	0.280384027	0.093784569	1.15100203	0.28258216	0.75292132	-5.35176725
homo after UVB irradiation VS homo before UVB irradiation	GO_LIPOXYGENASE_PATHWAY	0.511636915	-0.02132528	2.233325981	0.05472205	0.17369341	-4.41745651
hetero after UVB irradiation VS hetero before UVB irradiation	GO_LIPOXYGENASE_PATHWAY1	0.408558519	0.071616605	2.24562771	0.05554772	0.31118532	-4.14235855
WT after UVB irradiation VS WT before UVB irradiation	GO_LIPOXYGENASE_PATHWAY2	0.280913846	0.102200411	1.382184681	0.20384134	0.70036941	-5.09721239
homo after UVB irradiation VS homo before UVB irradiation	GO_LIVER_MORPHOGENESIS	0.086885643	-0.02547528	0.5299182	0.6099999	0.75683388	-6.39933228
hetero after UVB irradiation VS hetero before UVB irradiation	GO_LIVER_MORPHOGENESIS1	-0.10181802	-0.02045934	-0.51035129	0.62383786	0.84454808	-6.09261408
WT after UVB irradiation VS WT before UVB irradiation	GO_LIVER_MORPHOGENESIS2	-0.25912682	-0.01625034	-1.55957302	0.15702679	0.66180255	-4.88351464
homo after UVB irradiation VS homo before UVB irradiation	GO_LIVER_REGENERATION	-0.16348428	0.038092445	-1.05184593	0.3224259	0.51598131	-5.98147025
hetero after UVB irradiation VS hetero before UVB irradiation	GO_LIVER_REGENERATION1	0.282175669	0.041963165	1.378334782	0.20609221	0.53054722	-5.31284435
WT after UVB irradiation VS WT before UVB irradiation	GO_LIVER_REGENERATION2	-0.14945169	0.000292464	-0.85363476	0.4178419	0.82297693	-5.62888654
homo after UVB irradiation VS homo before UVB irradiation	GO_LOBAR_BRONCHUS_DEVELOPMENT	-0.27700764	0.105888698	-1.08437461	0.3085851	0.50312566	-5.94802021
hetero after UVB irradiation VS hetero before UVB irradiation	GO_LOBAR_BRONCHUS_DEVELOPMENT1	-0.42031712	0.087429434	-2.75286955	0.02539206	0.22777214	-3.40041836
WT after UVB irradiation VS WT before UVB irradiation	GO_LOBAR_BRONCHUS_DEVELOPMENT2	-0.42619865	0.042365618	-1.14319162	0.28562856	0.75392834	-5.35982097
homo after UVB irradiation VS homo before UVB irradiation	GO_LOBAR_BRONCHUS_EPITHELIUM_DEVELOPMENT	-0.25170786	0.091097781	-0.92639232	0.38030065	0.57062497	-6.10302746
hetero after UVB irradiation VS hetero before UVB irradiation	GO_LOBAR_BRONCHUS_EPITHELIUM_DEVELOPMENT1	-0.22959308	0.095238693	-1.54438841	0.16177693	0.48300268	-5.10813794
WT after UVB irradiation VS WT before UVB irradiation	GO_LOBAR_BRONCHUS_EPITHELIUM_DEVELOPMENT2	-0.46964841	0.013471675	-1.36975665	0.20753878	0.70428803	-5.11163421
homo after UVB irradiation VS homo before UVB irradiation	GO_LOCALIZATION_WITHIN_MEMBRANE	0.225814555	-0.0310612	1.526217519	0.16398818	0.34156297	-5.42662538
hetero after UVB irradiation VS hetero before UVB irradiation	GO_LOCALIZATION_WITHIN_MEMBRANE1	0.10111332	0.019294626	0.523780334	0.61488764	0.83965967	-6.08534023
WT after UVB irradiation VS WT before UVB irradiation	GO_LOCALIZATION_WITHIN_MEMBRANE2	0.179683512	-0.01417359	1.053403736	0.32257076	0.77346379	-5.44956722
homo after UVB irradiation VS homo before UVB irradiation	GO_LOCOMOTION_INVOLVED_IN_LOCOMOTORY_BEHAVIOR	-0.24375416	-0.0006246	-1.16454187	0.27647197	0.47248323	-5.86238627
hetero after UVB irradiation VS hetero before UVB irradiation	GO_LOCOMOTION_INVOLVED_IN_LOCOMOTORY_BEHAVIOR1	-0.02046358	0.060707159	-0.11374494	0.91229423	0.96720392	-6.22447377
WT after UVB irradiation VS WT before UVB irradiation	GO_LOCOMOTION_INVOLVED_IN_LOCOMOTORY_BEHAVIOR2	-0.2897866	0.027010664	-1.36689156	0.20839936	0.70519361	-5.11494795
homo after UVB irradiation VS homo before UVB irradiation	GO_LOCOMOTOR_RHYTHM	-0.25975558	0.019334159	-1.19567529	0.26475096	0.45995186	-5.82795078
hetero after UVB irradiation VS hetero before UVB irradiation	GO_LOCOMOTOR_RHYTHM1	-0.43769903	-0.02830758	-2.61886465	0.03119216	0.24648003	-3.59701159
WT after UVB irradiation VS WT before UVB irradiation	GO_LOCOMOTOR_RHYTHM2	-0.35901184	0.033352712	-2.12102329	0.06630664	0.537335	-4.1370847
homo after UVB irradiation VS homo before UVB irradiation	GO_LOCOMOTORY_BEHAVIOR	0.023524372	0.023195402	0.167818636	0.8707293	0.92920628	-6.53542682
hetero after UVB irradiation VS hetero before UVB irradiation	GO_LOCOMOTORY_BEHAVIOR1	-0.06220335	0.025162772	-0.4638952	0.65531069	0.85889323	-6.11641001
WT after UVB irradiation VS WT before UVB irradiation	GO_LOCOMOTORY_BEHAVIOR2	0.008282986	0.017888736	0.047328058	0.96339822	0.99054347	-5.99488493
homo after UVB irradiation VS homo before UVB irradiation	GO_LOCOMOTORY_EXPLORATION_BEHAVIOR	0.423427645	-0.01871348	2.444078821	0.03916412	0.14224587	-4.09524564
hetero after UVB irradiation VS hetero before UVB irradiation	GO_LOCOMOTORY_EXPLORATION_BEHAVIOR1	-0.13955925	-0.02974586	-0.48186502	0.64304439	0.85384352	-6.10745834
WT after UVB irradiation VS WT before UVB irradiation	GO_LOCOMOTORY_EXPLORATION_BEHAVIOR2	-0.15472473	-0.060154	-0.67146956	0.52061278	0.86304554	-5.76493799
homo after UVB irradiation VS homo before UVB irradiation	GO_LONG_CHAIN_FATTY_ACID_BINDING	0.416056222	0.077374466	2.507999272	0.03538596	0.13468829	-3.99663197
hetero after UVB irradiation VS hetero before UVB irradiation	GO_LONG_CHAIN_FATTY_ACID_BINDING1	0.385180952	0.044740794	1.92159982	0.09157624	0.37872731	-4.60369369
WT after UVB irradiation VS WT before UVB irradiation	GO_LONG_CHAIN_FATTY_ACID_BINDING2	0.281864328	0.034818201	1.163396333	0.27780184	0.74917538	-5.33890833
homo after UVB irradiation VS homo before UVB irradiation	GO_LONG_CHAIN_FATTY_ACID_BIOSYNTHETIC_PROCESS	0.248990628	-0.04280263	1.353677936	0.21142414	0.40176229	-5.64377893
hetero after UVB irradiation VS hetero before UVB irradiation	GO_LONG_CHAIN_FATTY_ACID_BIOSYNTHETIC_PROCESS1	0.278061282	0.028707609	1.423892483	0.19297611	0.51844079	-5.25798012
WT after UVB irradiation VS WT before UVB irradiation	GO_LONG_CHAIN_FATTY_ACID_BIOSYNTHETIC_PROCESS2	0.128971391	0.055615361	0.667084897	0.52326823	0.86461731	-5.76786268
homo after UVB irradiation VS homo before UVB irradiation	GO_LONG_CHAIN_FATTY_ACID_CATABOLIC_PROCESS	0.08651782	0.028298421	0.399221261	0.69977106	0.82086248	-6.46422981
hetero after UVB irradiation VS hetero before UVB irradiation	GO_LONG_CHAIN_FATTY_ACID_CATABOLIC_PROCESS1	-0.02806831	0.014235174	-0.15568569	0.88020834	0.95650301	-6.21836064
WT after UVB irradiation VS WT before UVB irradiation	GO_LONG_CHAIN_FATTY_ACID_CATABOLIC_PROCESS2	-0.12951387	0.024643671	-0.59256008	0.56964879	0.88366521	-5.814935
homo after UVB irradiation VS homo before UVB irradiation	GO_LONG_CHAIN_FATTY_ACID_COA_LIGASE_ACTIVITY	0.013394057	-0.00794004	0.061125287	0.95270076	0.9748216	-6.54880663
hetero after UVB irradiation VS hetero before UVB irradiation	GO_LONG_CHAIN_FATTY_ACID_COA_LIGASE_ACTIVITY1	-0.00213411	-0.01418887	-0.00931532	0.99279986	0.99672424	-6.23143705
WT after UVB irradiation VS WT before UVB irradiation	GO_LONG_CHAIN_FATTY_ACID_COA_LIGASE_ACTIVITY2	-0.19132862	0.029334864	-0.73065393	0.48559075	0.85147728	-5.7238127
homo after UVB irradiation VS homo before UVB irradiation	GO_LONG_CHAIN_FATTY_ACID_IMPORT_ACROSS_PLASMA_MEMBRANE	0.474605075	0.003090859	3.468947458	0.00794535	0.05921693	-2.51388173
hetero after UVB irradiation VS hetero before UVB irradiation	GO_LONG_CHAIN_FATTY_ACID_IMPORT_ACROSS_PLASMA_MEMBRANE1	0.38048	0.026068745	1.471331538	0.18010493	0.50429379	-5.19977716
WT after UVB irradiation VS WT before UVB irradiation	GO_LONG_CHAIN_FATTY_ACID_IMPORT_ACROSS_PLASMA_MEMBRANE2	0.283628184	0.002157679	1.006771552	0.34317746	0.78236826	-5.49403003
homo after UVB irradiation VS homo before UVB irradiation	GO_LONG_CHAIN_FATTY_ACID_METABOLIC_PROCESS	0.03839266	-0.00767941	0.222035768	0.82963365	0.90467034	-6.52388127
hetero after UVB irradiation VS hetero before UVB irradiation	GO_LONG_CHAIN_FATTY_ACID_METABOLIC_PROCESS1	0.003625131	-0.00873541	0.021272493	0.98355887	0.99386391	-6.23123871
WT after UVB irradiation VS WT before UVB irradiation	GO_LONG_CHAIN_FATTY_ACID_METABOLIC_PROCESS2	-0.0790395	0.021512981	-0.42882301	0.67923191	0.91716791	-5.90018857
homo after UVB irradiation VS homo before UVB irradiation	GO_LONG_CHAIN_FATTY_ACID_TRANSPORT	0.127934155	0.011594796	0.902172057	0.39230597	0.58130929	-6.12507241
hetero after UVB irradiation VS hetero before UVB irradiation	GO_LONG_CHAIN_FATTY_ACID_TRANSPORT1	0.014472589	0.015651748	0.082122171	0.93660415	0.97657793	-6.22782839
WT after UVB irradiation VS WT before UVB irradiation	GO_LONG_CHAIN_FATTY_ACID_TRANSPORT2	-0.09692151	-0.02065665	-0.50212903	0.62894378	0.90390703	-5.86518042
homo after UVB irradiation VS homo before UVB irradiation	GO_LONG_CHAIN_FATTY_ACID_TRANSPORTER_ACTIVITY	0.218562316	-0.01458456	1.031149815	0.33147956	0.52451682	-6.00234916
hetero after UVB irradiation VS hetero before UVB irradiation	GO_LONG_CHAIN_FATTY_ACID_TRANSPORTER_ACTIVITY1	-0.00252182	0.011505715	-0.0119035	0.99079946	0.99624062	-6.23140727
WT after UVB irradiation VS WT before UVB irradiation	GO_LONG_CHAIN_FATTY_ACID_TRANSPORTER_ACTIVITY2	-0.01830054	0.007943571	-0.07555361	0.94160752	0.98463518	-5.99305578

homo after UVB irradiation VS homo before UVB irradiation	GO_LONG_CHAIN_FATTY_ACYL_COA_BINDING	-0.28303961	-0.03430376	-1.23919616	0.24905148	0.44354796	-5.77875585
hetero after UVB irradiation VS hetero before UVB irradiation	GO_LONG_CHAIN_FATTY_ACYL_COA_BINDING1	-0.37995988	0.012130532	-1.73727917	0.12124856	0.42657805	-4.85593548
WT after UVB irradiation VS WT before UVB irradiation	GO_LONG_CHAIN_FATTY_ACYL_COA_BINDING2	0.215143779	0.029095784	0.998778629	0.34680849	0.78506717	-5.50149777
homo after UVB irradiation VS homo before UVB irradiation	GO_LONG_CHAIN_FATTY_ACYL_COA_BIOSYNTHETIC_PROCESS	-0.01776089	-0.04388371	-0.06611932	0.9488424	0.97335416	-6.54845804
hetero after UVB irradiation VS hetero before UVB irradiation	GO_LONG_CHAIN_FATTY_ACYL_COA_BIOSYNTHETIC_PROCESS1	-0.20354253	-0.05708984	-0.79132529	0.45199131	0.74404104	-5.90524022
WT after UVB irradiation VS WT before UVB irradiation	GO_LONG_CHAIN_FATTY_ACYL_COA_BIOSYNTHETIC_PROCESS2	-0.20466553	-0.0432942	-0.7587169	0.46952744	0.84565091	-5.70325834
homo after UVB irradiation VS homo before UVB irradiation	GO_LONG_CHAIN_FATTY_ACYL_COA_METABOLIC_PROCESS	-0.01081997	-0.05611833	-0.04055491	0.96860604	0.98360925	-6.54995416
hetero after UVB irradiation VS hetero before UVB irradiation	GO_LONG_CHAIN_FATTY_ACYL_COA_METABOLIC_PROCESS1	-0.15739025	-0.06112286	-0.64409736	0.53786639	0.79532113	-6.01246032
WT after UVB irradiation VS WT before UVB irradiation	GO_LONG_CHAIN_FATTY_ACYL_COA_METABOLIC_PROCESS2	-0.1826069	-0.03473472	-0.73614864	0.48241781	0.85130284	-5.71984092
homo after UVB irradiation VS homo before UVB irradiation	GO_LONG_TERM_MEMORY	0.203334034	-0.01295588	1.254656269	0.24366345	0.43738494	-5.76099087
hetero after UVB irradiation VS hetero before UVB irradiation	GO_LONG_TERM_MEMORY1	-0.13117212	0.00186121	-0.63603746	0.54284051	0.79782695	-6.01776777
WT after UVB irradiation VS WT before UVB irradiation	GO_LONG_TERM_MEMORY2	-0.08829399	-0.004494	-0.45337078	0.66218431	0.9121786	-5.88904639
homo after UVB irradiation VS homo before UVB irradiation	GO_LONG_TERM_SYNAPTIC_DEPRESSION	0.338656703	-0.01346068	1.896173518	0.09306979	0.24041992	-4.91711801
hetero after UVB irradiation VS hetero before UVB irradiation	GO_LONG_TERM_SYNAPTIC_DEPRESSION1	0.076215358	0.050351748	0.428149567	0.68004283	0.87122001	-6.13325833
WT after UVB irradiation VS WT before UVB irradiation	GO_LONG_TERM_SYNAPTIC_DEPRESSION2	0.136456841	-0.00754484	0.733308758	0.48405603	0.85146732	-5.72189692
homo after UVB irradiation VS homo before UVB irradiation	GO_LONG_TERM_SYNAPTIC_POTENTIATION	0.085189324	-0.03041073	0.631061606	0.54492402	0.70951431	-6.33746028
hetero after UVB irradiation VS hetero before UVB irradiation	GO_LONG_TERM_SYNAPTIC_POTENTIATION1	-0.05610732	0.011915938	-0.27011334	0.79403591	0.9195412	-6.19210624
WT after UVB irradiation VS WT before UVB irradiation	GO_LONG_TERM_SYNAPTIC_POTENTIATION2	-0.05953645	-0.01149008	-0.35556341	0.73125209	0.9334623	-5.92990492
homo after UVB irradiation VS homo before UVB irradiation	GO_LOOP_OF_HENLE_DEVELOPMENT	0.354143294	0.087332218	1.334977676	0.21721826	0.40782314	-5.66635512
hetero after UVB irradiation VS hetero before UVB irradiation	GO_LOOP_OF_HENLE_DEVELOPMENT1	0.390188872	0.027187038	1.335279892	0.21919426	0.54626422	-5.36371771
WT after UVB irradiation VS WT before UVB irradiation	GO_LOOP_OF_HENLE_DEVELOPMENT2	-0.18118865	0.073054583	-0.75044974	0.47422288	0.84774103	-5.70938293
homo after UVB irradiation VS homo before UVB irradiation	GO_LOW_DENSITY_LIPOPROTEIN_PARTICLE	0.382193251	0.021488411	2.664775559	0.02760438	0.11746189	-3.75373775
homo after UVB irradiation VS homo before UVB irradiation	GO_LOW_DENSITY_LIPOPROTEIN_PARTICLE_BINDING	0.800716493	0.015017341	4.949918356	0.00099523	0.02151484	-0.41959997
hetero after UVB irradiation VS hetero before UVB irradiation	GO_LOW_DENSITY_LIPOPROTEIN_PARTICLE_BINDING1	0.491068506	0.089265032	2.917835487	0.01974765	0.20405905	-3.15913936
WT after UVB irradiation VS WT before UVB irradiation	GO_LOW_DENSITY_LIPOPROTEIN_PARTICLE_BINDING2	0.448214823	0.03553999	1.630552932	0.1411753	0.65097221	-4.79430949
homo after UVB irradiation VS homo before UVB irradiation	GO_LOW_DENSITY_LIPOPROTEIN_PARTICLE_CLEARANCE	0.537356546	-0.02716291	3.264945615	0.01083042	0.06967066	-2.82470474
hetero after UVB irradiation VS hetero before UVB irradiation	GO_LOW_DENSITY_LIPOPROTEIN_PARTICLE_CLEARANCE1	0.561205007	0.018910768	4.028700456	0.00394357	0.12418963	-1.60494318
WT after UVB irradiation VS WT before UVB irradiation	GO_LOW_DENSITY_LIPOPROTEIN_PARTICLE_CLEARANCE2	0.617654921	0.018166733	3.789795842	0.00519597	0.27446335	-1.82056111
homo after UVB irradiation VS homo before UVB irradiation	GO_LOW_DENSITY_LIPOPROTEIN_PARTICLE_RECEPTOR_ACTIVITY	0.266926608	0.025229272	1.043753859	0.32594281	0.51946824	-5.98967153
hetero after UVB irradiation VS hetero before UVB irradiation	GO_LOW_DENSITY_LIPOPROTEIN_PARTICLE_RECEPTOR_ACTIVITY1	0.147523118	0.021995302	0.858866323	0.41586009	0.71964718	-5.84980804
WT after UVB irradiation VS WT before UVB irradiation	GO_LOW_DENSITY_LIPOPROTEIN_PARTICLE_RECEPTOR_ACTIVITY2	0.437600695	-0.07935965	2.022935867	0.07727643	0.56116995	-4.27286425
homo after UVB irradiation VS homo before UVB irradiation	GO_LOW_DENSITY_LIPOPROTEIN_PARTICLE_RECEPTOR_BINDING	0.206060635	-0.0093574	1.031610551	0.3312759	0.52441949	-6.00188782
hetero after UVB irradiation VS hetero before UVB irradiation	GO_LOW_DENSITY_LIPOPROTEIN_PARTICLE_RECEPTOR_BINDING1	0.609628613	0.005498804	4.452803072	0.00223144	0.10650321	-1.05948026
WT after UVB irradiation VS WT before UVB irradiation	GO_LOW_DENSITY_LIPOPROTEIN_PARTICLE_RECEPTOR_BINDING2	0.295324621	-0.00134689	1.95529703	0.08584636	0.57599199	-4.36549099
homo after UVB irradiation VS homo before UVB irradiation	GO_LOW_DENSITY_LIPOPROTEIN_PARTICLE_RECEPTOR_BIOSYNTHETIC_PROCESS	0.565695133	-0.01578508	2.727780493	0.02498982	0.11117719	-3.65588335
hetero after UVB irradiation VS hetero before UVB irradiation	GO_LOW_DENSITY_LIPOPROTEIN_PARTICLE_RECEPTOR_BIOSYNTHETIC_PROCESS1	0.40786145	0.021114627	2.059526771	0.07407077	0.35255978	-4.40953635
WT after UVB irradiation VS WT before UVB irradiation	GO_LOW_DENSITY_LIPOPROTEIN_PARTICLE_RECEPTOR_BIOSYNTHETIC_PROCESS2	0.471093704	0.03914104	2.720531639	0.02593622	0.43662303	-3.2885718
homo after UVB irradiation VS homo before UVB irradiation	GO_LOW_DENSITY_LIPOPROTEIN_PARTICLE_RECEPTOR_CATABOLIC_PROCESS	0.798840122	-0.0195402	5.285542607	0.00064955	0.01817776	0.008393257
hetero after UVB irradiation VS hetero before UVB irradiation	GO_LOW_DENSITY_LIPOPROTEIN_PARTICLE_RECEPTOR_CATABOLIC_PROCESS1	0.756104024	0.029454037	4.745128114	0.00153054	0.09569098	-0.70126013
WT after UVB irradiation VS WT before UVB irradiation	GO_LOW_DENSITY_LIPOPROTEIN_PARTICLE_RECEPTOR_CATABOLIC_PROCESS2	0.976811703	0.028267441	6.688781941	0.00014617	0.18842845	1.220494438
homo after UVB irradiation VS homo before UVB irradiation	GO_LOW_DENSITY_LIPOPROTEIN_PARTICLE_REMODELING	0.313745097	0.026554911	1.074543751	0.31271817	0.50667348	-5.95821034
hetero after UVB irradiation VS hetero before UVB irradiation	GO_LOW_DENSITY_LIPOPROTEIN_PARTICLE_REMODELING1	-0.14246691	0.02874992	-0.56324977	0.58898145	0.82529231	-6.06294933
WT after UVB irradiation VS WT before UVB irradiation	GO_LOW_DENSITY_LIPOPROTEIN_PARTICLE_REMODELING2	0.010871742	0.045591673	0.065927277	0.94903436	0.98620317	-5.99377394
hetero after UVB irradiation VS hetero before UVB irradiation	GO_LOW_DENSITY_LIPOPROTEIN_PARTICLE1	0.310157048	0.065459218	1.931398998	0.0902107	0.37720291	-4.59003438
WT after UVB irradiation VS WT before UVB irradiation	GO_LOW_DENSITY_LIPOPROTEIN_PARTICLE2	0.386160946	0.011710746	1.585206776	0.15112462	0.6585056	-4.85152187
homo after UVB irradiation VS homo before UVB irradiation	GO_LOW_DENSITY_LIPOPROTEIN_RECEPTOR_PARTICLE_METABOLIC_PROCESS	0.69372335	-0.03436207	4.368112772	0.00216732	0.03111198	-1.20373616
hetero after UVB irradiation VS hetero before UVB irradiation	GO_LOW_DENSITY_LIPOPROTEIN_RECEPTOR_PARTICLE_METABOLIC_PROCESS1	0.584274633	0.039020408	3.164397203	0.01362808	0.17665328	-2.80174642
WT after UVB irradiation VS WT before UVB irradiation	GO_LOW_DENSITY_LIPOPROTEIN_RECEPTOR_PARTICLE_METABOLIC_PROCESS2	0.749349452	0.033479059	4.577408406	0.00175221	0.20370459	-0.848673
homo after UVB irradiation VS homo before UVB irradiation	GO_LRR_DOMAIN_BINDING	-0.12316116	0.02359651	-0.53346795	0.60764747	0.75550204	-6.39732998
hetero after UVB irradiation VS hetero before UVB irradiation	GO_LRR_DOMAIN_BINDING1	0.129234222	-0.00633232	0.613116606	0.55713527	0.80617348	-6.0325321
WT after UVB irradiation VS WT before UVB irradiation	GO_LRR_DOMAIN_BINDING2	-0.1391005	0.029967117	-0.46406338	0.65482278	0.91006679	-5.88400933
homo after UVB irradiation VS homo before UVB irradiation	GO_LSM1_7_PAT1_COMPLEX	-0.41681648	-0.05840715	-1.57527308	0.15235132	0.32640077	-5.36220625
hetero after UVB irradiation VS hetero before UVB irradiation	GO_LSM1_7_PAT1_COMPLEX1	0.258406066	-0.10590869	0.734444433	0.48402902	0.76282518	-5.94893133
WT after UVB irradiation VS WT before UVB irradiation	GO_LSM1_7_PAT1_COMPLEX2	0.173811601	-0.05189632	0.476657692	0.64620317	0.90879826	-5.87793422
homo after UVB irradiation VS homo before UVB irradiation	GO_LSM2_8_COMPLEX	-0.5407008	-0.03009225	-1.73319313	0.11980052	0.28061845	-5.14786318
hetero after UVB irradiation VS hetero before UVB irradiation	GO_LSM2_8_COMPLEX1	-0.20998169	-0.03472313	-0.63838607	0.54138825	0.79711883	-6.0162274
WT after UVB irradiation VS WT before UVB irradiation	GO_LSM2_8_COMPLEX2	-0.0246553	-0.04872346	-0.07985186	0.93829327	0.98368811	-5.9927036
homo after UVB irradiation VS homo before UVB irradiation	GO_LUMENAL_SIDE_OF_ENDOPLASMIC_RETICULUM_MEMBRANE	0.186937736	-0.03554054	0.62091432	0.55126493	0.71418536	-6.34411303
hetero after UVB irradiation VS hetero before UVB irradiation	GO_LUMENAL_SIDE_OF_ENDOPLASMIC_RETICULUM_MEMBRANE1	0.147745122	-0.04130736	0.632863301	0.54480696	0.79870659	-6.01984149
WT after UVB irradiation VS WT before UVB irradiation	GO_LUMENAL_SIDE_OF_ENDOPLASMIC_RETICULUM_MEMBRANE2	0.320558478	-0.02627508	1.135758566	0.28855231	0.75478015	-5.36744977
homo after UVB irradiation VS homo before UVB irradiation	GO_LUMENAL_SIDE_OF_MEMBRANE	0.13789182	-0.02617041	0.581945029	0.576012	0.73290301	-6.36874566
hetero after UVB irradiation VS hetero before UVB irradiation	GO_LUMENAL_SIDE_OF_MEMBRANE1	0.198357775	-0.00897283	1.040967914	0.32887649	0.64895713	-5.68239896
WT after UVB irradiation VS WT before UVB irradiation	GO_LUMENAL_SIDE_OF_MEMBRANE2	0.296469333	-0.01098968	1.127587791	0.29179396	0.75646944	-5.37579512
homo after UVB irradiation VS homo before UVB irradiation	GO_LUNG_ALVEOLUS_DEVELOPMENT	-0.12080285	0.02585164	-0.62205772	0.55054828	0.71378137	-6.3433683
hetero after UVB irradiation VS hetero before UVB irradiation	GO_LUNG_ALVEOLUS_DEVELOPMENT1	-0.04870677	0.011537595	-0.26557794	0.79740322	0.92163248	-6.19341144
WT after UVB irradiation VS WT before UVB irradiation	GO_LUNG_ALVEOLUS_DEVELOPMENT2	-0.07791304	0.030586873	-0.49619769	0.63294182	0.90455096	-5.86820593
homo after UVB irradiation VS homo before UVB irradiation	GO_LUNG_ASSOCIATED_MESENCHYME_DEVELOPMENT	-0.56424321	0.035838508	-3.56985158	0.00682958	0.05432461	-2.36168245
hetero after UVB irradiation VS hetero before UVB irradiation	GO_LUNG_ASSOCIATED_MESENCHYME_DEVELOPMENT1	-0.46584496	-0.00913498	-2.43985406	0.04112001	0.27359812	-3.85946012
WT after UVB irradiation VS WT before UVB irradiation	GO_LUNG_ASSOCIATED_MESENCHYME_DEVELOPMENT2	-0.16045557	0.031128906	-0.98386411	0.35366175	0.78942569	-5.51531008
homo after UVB irradiation VS homo before UVB irradiation	GO_LUNG_CELL_DIFFERENTIATION	0.013256261	0.01526246	0.075572289	0.94154308	0.96878757	-6.5477234
hetero after UVB irradiation VS hetero before UVB irradiation	GO_LUNG_CELL_DIFFERENTIATION1	-0.35388994	0.014467638	-2.41872489	0.04248652	0.27740933	-3.89036458
WT after UVB irradiation VS WT before UVB irradiation	GO_LUNG_CELL_DIFFERENTIATION2	-0.27857685	-0.0091252	-1.41917368	0.19317352	0.69334561	-5.05383961
homo after UVB irradiation VS homo before UVB irradiation	GO_LUNG_EPITHELIUM_DEVELOPMENT	-0.18458644	0.022284552	-1.13206192	0.28914395	0.48408411	-5.89761734
hetero after UVB irradiation VS hetero before UVB irradiation	GO_LUNG_EPITHELIUM_DEVELOPMENT1	-0.41498552	-0.0178966	-2.96250966	0.01845562	0.19863829	-3.09404389
WT after UVB irradiation VS WT before UVB irradiation	GO_LUNG_EPITHELIUM_DEVELOPMENT2	-0.4175857	-0.01688437	-2.32504185	0.04816185	0.50693606	-3.85059514
homo after UVB irradiation VS homo before UVB irradiation	GO_LUNG_GROWTH	0.631849002	-0.00642036	2.157336186	0.06172096	0.18761225	-4.53211823
hetero after UVB irradiation VS hetero before UVB irradiation	GO_LUNG_GROWTH1	0.239030912	0.030395636	0.872992092	0.4085681	0.71330228	-5.83774126
WT after UVB irradiation VS WT before UVB irradiation	GO_LUNG_GROWTH2	0.337074775	-0.08167145	1.036641602	0.32986481	0.77726938	-5.46572364
homo after UVB irradiation VS homo before UVB irradiation	GO_LUNG_LOBE_DEVELOPMENT	0.065281771	-0.00358718	0.46038727	0.65701956	0.79128684	-6.43601514
hetero after UVB irradiation VS hetero before UVB irradiation	GO_LUNG_LOBE_DEVELOPMENT1	-0.40049845	-0.0272065	-1.67651125	0.13287189	0.44318662	-4.93684937
WT after UVB irradiation VS WT before UVB irradiation	GO_LUNG_LOBE_DEVELOPMENT2	-0.2831917	-0.07260242	-1.75050904	0.11768707	0.62576396	-4.63948376
homo after UVB irradiation VS homo before UVB irradiation	GO_LUNG_MORPHOGENESIS	-0.13791517	0.051536928	-0.82523089	0.43223807	0.61628379	-6.19188993
hetero after UVB irradiation VS hetero before UVB irradiation	GO_LUNG_MORPHOGENESIS1	-0.17485396	0.042611391	-1.16540131	0.27803314	0.60400502	-5.5542888
WT after UVB irradiation VS WT before UVB irradiation	GO_LUNG_MORPHOGENESIS2	-0.12743974	0.025355806	-0.69865701	0.50433374	0.85763155	-5.74642512
homo after UVB irradiation VS homo before UVB irradiation	GO_LUNG_SACCULE_DEVELOPMENT	-0.34151206	0.013223668	-1.49655855	0.17140366	0.35101358	-5.46502772
hetero after UVB irradiation VS hetero before UVB irradiation	GO_LUNG_SACCULE_DEVELOPMENT1	-0.3196885	-0.00494188	-1.62014165	0.14456938	0.46084809	-5.01074848
WT after UVB irradiation VS WT before UVB irradiation	GO_LUNG_SACCULE_DEVELOPMENT2	-0.05165998	-0.0458723	-0.20353255	0.84374055	0.96450382	-5.97426758
homo after UVB irradiation VS homo before UVB irradiation	GO_LUNG_SECRETORY_CELL_DIFFERENTIATION	-0.26658008	-0.00914443	-1.56803628	0.15401993	0.32840226	-5.37177832
hetero after UVB irradiation VS hetero before UVB irradiation	GO_LUNG_SECRETORY_CELL_DIFFERENTIATION1	-0.32499908	0.059519354	-1.87946261	0.09767766	0.3897511	-4.66216653
WT after UVB irradiation VS WT before UVB irradiation	GO_LUNG_SECRETORY_CELL_DIFFERENTIATION2	-0.14305779	-0.0034814	-0.54995207	0.59719017	0.89386246	-5.8395645
homo after UVB irradiation VS homo before UVB irradiation	GO_LUNG_VASCULATURE_DEVELOPMENT	-0.28218631	0.019517353	-0.88068951	0.40318078	0.59031859	-6.14422499
hetero after UVB irradiation VS hetero before UVB irradiation	GO_LUNG_VASCULATURE_DEVELOPMENT1	-0.42884304	0.037645643	-2.26307406	0.05406691	0.30816437	-4.11708109
WT after UVB irradiation VS WT before UVB irradiation	GO_LUNG_VASCULATURE_DEVELOPMENT2	-0.5350441	-0.03487787	-2.40941386	0.04219033	0.495137	-3.73105502
homo after UVB irradiation VS homo before UVB irradiation	GO_LUTEINIZATION	0.743198188	-0.02219829	5.609607302	0.0004367	0.01557794	0.404830714
hetero after UVB irradiation VS hetero before UVB irradiation	GO_LUTEINIZATION1	0.638156751	0.003709334	3.90475863	0.00468098	0.1277306	-1.76993321
WT after UVB irradiation VS WT before UVB irradiation	GO_LUTEINIZATION2	0.646599748	0.069323377	3.821702919	0.00496344	0.27432744	-1.7791082
homo after UVB irradiation VS homo before UVB irradiation	GO_LUTEINIZING_HORMONE_SECRETION	-0.44709213	0.036999258	-2.20391063	0.05733346	0.17864651	-4.46195584
hetero after UVB irradiation VS hetero before UVB irradiation	GO_LUTEINIZING_HORMONE_SECRETION1	-0.40327803	0.028817166	-1.4439749	0.18743124	0.51274293	-5.23347127
WT after UVB irradiation VS WT before UVB irradiation	GO_LUTEINIZING_HORMONE_SECRETION2	-0.44916777	0.047113679	-1.80663492	0.1079963	0.61228673	-4.5654895
homo after UVB irradiation VS homo before UVB irradiation	GO_LUTEOLYSIS	0.254709356	-0.02789878	1.210816604	0.25919914	0.45459969	-5.81097348
hetero after UVB irradiation VS hetero before UVB irradiation	GO_LUTEOLYSIS1	0.592980814	-0.01696104	3.973897693	0.00425292	0.12601617	-1.67759368
WT after UVB irradiation VS WT before UVB irradiation	GO_LUTEOLYSIS2	0.469093403	-0.04428824	2.668285198	0.02813584	0.44434881	-3.36285971
homo after UVB irradiation VS homo before UVB irradiation	GO_LYASE_ACTIVITY	0.086747321	-0.03493823	0.543086412	0.60129754	0.75064274	-6.39184192
hetero after UVB irradiation VS hetero before UVB irradiation	GO_LYASE_ACTIVITY1	-0.03693435	-0.02540592	-0.25914759	0.80218519	0.92357057	-6.19522477
WT after UVB irradiation VS WT before UVB irradiation	GO_LYASE_ACTIVITY2	0.094807479	-0.00373167	0.586122237	0.57376349	0.88547483	-5.81876426
homo after UVB irradiation VS homo before UVB irradiation	GO_LYMPH_NODE_DEVELOPMENT	0.344097506	-0.02002339	1.819444096	0.10486652	0.25822047	-5.02682695
hetero after UVB irradiation VS hetero before UVB irradiation	GO_LYMPH_NODE_DEVELOPMENT1	0.233183207	-0.01178043	1.518326405	0.1681134	0.48947934	-5.14109742
WT after UVB irradiation VS WT before UVB irradiation	GO_LYMPH_NODE_DEVELOPMENT2	0.364528321	-0.02386388	1.828101025	0.10449248	0.60732398	-4.53695393
homo after UVB irradiation VS homo before UVB irradiation	GO_LYMPH_VESSEL_DEVELOPMENT	0.405632558	-0.05631928	3.016950748	0.01588166	0.08600836	-3.20695804
hetero after UVB irradiation VS hetero before UVB irradiation	GO_LYMPH_VESSEL_DEVELOPMENT1	0.166486725	-0.03304082	0.845091969	0.42305902	0.7249295	-5.86141876
WT after UVB irradiation VS WT before UVB irradiation	GO_LYMPH_VESSEL_DEVELOPMENT2	0.104593722	-0.00551244	0.473750379	0.64818798	0.90907607	-5.87935024
homo after UVB irradiation VS homo before UVB irradiation	GO_LYMPH_VESSEL_MORPHOGENESIS	0.250205287	-0.0508204	1.688881973	0.12822053	0.29326571	-5.20900907
hetero after UVB irradiation VS hetero before UVB irradiation	GO_LYMPH_VESSEL_MORPHOGENESIS1	0.038311541	-0.05355986	0.168808054	0.87021466	0.95109523	-6.21605944
WT after UVB irradiation VS WT before UVB irradiation	GO_LYMPH_VESSEL_MORPHOGENESIS2	0.015404102	0.004751011	0.061874512	0.95216275	0.98676072	-5.99404709
homo after UVB irradiation VS homo before UVB irradiation	GO_LYMPHANGIOGENESIS	0.188821933	-0.03776125	1.375351717	0.20487511	0.3944347	-5.617366
hetero after UVB irradiation VS hetero before UVB irradiation	GO_LYMPHANGIOGENESIS1	-0.06439532	-0.06739117	-0.2999547	0.77199821	0.9113251	-6.18297902
WT after UVB irradiation VS WT before UVB irradiation	GO_LYMPHANGIOGENESIS2	-0.13489491	0.028649204	-0.58894428	0.57195773	0.88409386	-5.81709039
homo after UVB irradiation VS homo before UVB irradiation	GO_LYMPHATIC_ENDOTHELIAL_CELL_DIFFERENTIATION	0.420028869	-0.04196877	1.727887915	0.12078091	0.28228761	-5.15522254
hetero after UVB irradiation VS hetero before UVB irradiation	GO_LYMPHATIC_ENDOTHELIAL_CELL_DIFFERENTIATION1	0.196334061	-0.07321621	0.730264242	0.4864408	0.76418009	-5.95203143
WT after UVB irradiation VS WT before UVB irradiation	GO_LYMPHATIC_ENDOTHELIAL_CELL_DIFFERENTIATION2	0.183716792	0.093885164	0.661829184	0.52646212	0.86617234	-5.77134593
homo after UVB irradiation VS homo before UVB irradiation	GO_LYMPHOCYTE_ACTIVATION	0.36274439	-0.02090804	2.730126202	0.02489751	0.11097478	-3.65223889
homo after UVB irradiation VS homo before UVB irradiation	GO_LYMPHOCYTE_ACTIVATION_INVOLVED_IN_IMMUNE_RESPONSE	0.304070267	-0.01634363	2.159902785	0.06147082	0.18707463	-4.52826168
hetero after UVB irradiation VS hetero before UVB irradiation	GO_LYMPHOCYTE_ACTIVATION_INVOLVED_IN_IMMUNE_RESPONSE1	0.317127139	0.026346806	2.027897055	0.07777249	0.35896492	-4.45439939
WT after UVB irradiation VS WT before UVB irradiation	GO_LYMPHOCYTE_ACTIVATION_INVOLVED_IN_IMMUNE_RESPONSE2	0.278138975	-0.00309718	1.537392494	0.16230103	0.66583874	-4.91098561
hetero after UVB irradiation VS hetero before UVB irradiation	GO_LYMPHOCYTE_ACTIVATION1	0.339343077	0.019497188	2.243928128	0.05569412	0.31172928	-4.14481926
WT after UVB irradiation VS WT before UVB irradiation	GO_LYMPHOCYTE_ACTIVATION2	0.263048088	-0.00587208	1.510305707	0.16895757	0.67508951	-4.94425755
homo after UVB irradiation VS homo before UVB irradiation	GO_LYMPHOCYTE_AGGREGATION	0.733783041	-0.0305663	3.078497938	0.01443392	0.08192359	-3.11174153
hetero after UVB irradiation VS hetero before UVB irradiation	GO_LYMPHOCYTE_AGGREGATION1	0.665797589	0.113065053	2.84079509	0.02220139	0.21500017	-3.27166673
WT after UVB irradiation VS WT before UVB irradiation	GO_LYMPHOCYTE_AGGREGATION2	0.878633906	0.045620199	3.587935279	0.00696448	0.30956262	-2.086573
homo after UVB irradiation VS homo before UVB irradiation	GO_LYMPHOCYTE_ANERGY	-0.91073867	0.030295457	-4.02942727	0.00348819	0.03873229	-1.68418398
hetero after UVB irradiation VS hetero before UVB irradiation	GO_LYMPHOCYTE_ANERGY1	-0.33350503	-0.07569309	-0.83010708	0.43098954	0.73171313	-5.87387339
WT after UVB irradiation VS WT before UVB irradiation	GO_LYMPHOCYTE_ANERGY2	-0.24154525	-0.03656645	-0.68355112	0.51333896	0.85974355	-5.75679139
homo after UVB irradiation VS homo before UVB irradiation	GO_LYMPHOCYTE_APOPTOTIC_PROCESS	0.237058917	-0.00099376	1.514009488	0.16700514	0.345537	-5.44248323
hetero after UVB irradiation VS hetero before UVB irradiation	GO_LYMPHOCYTE_APOPTOTIC_PROCESS1	0.248900985	-0.0088215	1.509451019	0.17032174	0.49289421	-5.15225483
WT after UVB irradiation VS WT before UVB irradiation	GO_LYMPHOCYTE_APOPTOTIC_PROCESS2	0.135001451	-0.05120997	0.66039279	0.52733709	0.8664124	-5.77229363
homo after UVB irradiation VS homo before UVB irradiation	GO_LYMPHOCYTE_CHEMOTAXIS	0.370184341	-0.01635311	2.185901909	0.05899238	0.18231425	-4.48912956
hetero after UVB irradiation VS hetero before UVB irradiation	GO_LYMPHOCYTE_CHEMOTAXIS1	0.391162613	0.017957215	2.639634093	0.030211	0.242735	-3.5665298
WT after UVB irradiation VS WT before UVB irradiation	GO_LYMPHOCYTE_CHEMOTAXIS2	0.337756942	0.004375975	1.331970111	0.21913836	0.71478521	-5.15500046
homo after UVB irradiation VS homo before UVB irradiation	GO_LYMPHOCYTE_COSTIMULATION	0.318929674	-0.02158788	2.005078643	0.07847557	0.21597613	-4.75853125
hetero after UVB irradiation VS hetero before UVB irradiation	GO_LYMPHOCYTE_COSTIMULATION1	0.262375782	0.014383988	1.345127511	0.21613569	0.54343701	-5.352168
WT after UVB irradiation VS WT before UVB irradiation	GO_LYMPHOCYTE_COSTIMULATION2	0.111706893	-0.00847953	0.558844127	0.59138291	0.89248172	-5.83456391
homo after UVB irradiation VS homo before UVB irradiation	GO_LYMPHOCYTE_DIFFERENTIATION	0.313909744	-0.02108806	2.373292092	0.04382273	0.15180565	-4.20403871
hetero after UVB irradiation VS hetero before UVB irradiation	GO_LYMPHOCYTE_DIFFERENTIATION1	0.222726216	0.011760031	1.48071483	0.1776513	0.50135768	-5.1881402
WT after UVB irradiation VS WT before UVB irradiation	GO_LYMPHOCYTE_DIFFERENTIATION2	0.195938823	-0.00753231	1.081922016	0.31045053	0.76760753	-5.42163966
homo after UVB irradiation VS homo before UVB irradiation	GO_LYMPHOCYTE_HOMEOSTASIS	0.372817428	-0.03247953	2.771946277	0.0233092	0.10659248	-3.58725633
hetero after UVB irradiation VS hetero before UVB irradiation	GO_LYMPHOCYTE_HOMEOSTASIS1	0.458613957	0.023413884	3.011658046	0.01713551	0.1913886	-3.02258447
WT after UVB irradiation VS WT before UVB irradiation	GO_LYMPHOCYTE_HOMEOSTASIS2	0.328080673	-0.02938558	1.498360009	0.17197006	0.67808426	-4.9588322
homo after UVB irradiation VS homo before UVB irradiation	GO_LYMPHOCYTE_MEDIATED_IMMUNITY	0.407911426	-0.00557229	2.703282839	0.02597498	0.11389189	-3.69394009
hetero after UVB irradiation VS hetero before UVB irradiation	GO_LYMPHOCYTE_MEDIATED_IMMUNITY1	0.270469151	0.03463864	1.648287267	0.138615	0.45202273	-4.97399562
WT after UVB irradiation VS WT before UVB irradiation	GO_LYMPHOCYTE_MEDIATED_IMMUNITY2	0.280623824	-0.01516001	1.334722236	0.21827512	0.71478521	-5.15186688
homo after UVB irradiation VS homo before UVB irradiation	GO_LYMPHOCYTE_MIGRATION	0.507746649	-0.02094697	3.040021509	0.01532206	0.08400296	-3.17124454
homo after UVB irradiation VS homo before UVB irradiation	GO_LYMPHOCYTE_MIGRATION_INTO_LYMPHOID_ORGANS	0.794040117	-0.03153524	2.989580497	0.01657315	0.08822873	-3.24935784

hetero after UVB irradiation VS hetero before UVB irradiation	GO_LYMPHOCYTE_MIGRATION_INTO_LYMPHOID_ORGANS1	0.476345442	0.025755548	1.678476127	0.13248039	0.44226904	-4.93425279
WT after UVB irradiation VS WT before UVB irradiation	GO_LYMPHOCYTE_MIGRATION_INTO_LYMPHOID_ORGANS2	0.517767758	-0.00340619	2.680607195	0.02760043	0.44102721	-3.34533331
hetero after UVB irradiation VS hetero before UVB irradiation	GO_LYMPHOCYTE_MIGRATION1	0.466957675	0.02468361	2.791129057	0.02394909	0.22165943	-3.34435913
WT after UVB irradiation VS WT before UVB irradiation	GO_LYMPHOCYTE_MIGRATION2	0.440561522	0.00678986	2.14160995	0.0642055	0.53678963	-4.10839834
homo after UVB irradiation VS homo before UVB irradiation	GO_LYMPHOID_PROGENITOR_CELL_DIFFERENTIATION	-0.2543007	-0.01394154	-1.52750676	0.16367243	0.3413373	-5.42494659
hetero after UVB irradiation VS hetero before UVB irradiation	GO_LYMPHOID_PROGENITOR_CELL_DIFFERENTIATION1	-0.0609765	-0.00583289	-0.45911471	0.65859298	0.86007701	-6.11873733
WT after UVB irradiation VS WT before UVB irradiation	GO_LYMPHOID_PROGENITOR_CELL_DIFFERENTIATION2	-0.24447679	0.031560275	-1.33636021	0.21776273	0.71478521	-5.14999999
homo after UVB irradiation VS homo before UVB irradiation	GO_LYS48_SPECIFIC_DEUBIQUITINASE_ACTIVITY	0.051951731	-0.08621503	0.287239574	0.78094566	0.87393718	-6.50580265
hetero after UVB irradiation VS hetero before UVB irradiation	GO_LYS48_SPECIFIC_DEUBIQUITINASE_ACTIVITY1	0.190521448	-0.06363345	0.883320823	0.40329433	0.7106602	-5.82881648
WT after UVB irradiation VS WT before UVB irradiation	GO_LYS48_SPECIFIC_DEUBIQUITINASE_ACTIVITY2	0.010823741	-0.0191662	0.061448823	0.9524914	0.98676072	-5.99407477
homo after UVB irradiation VS homo before UVB irradiation	GO_LYS63_SPECIFIC_DEUBIQUITINASE_ACTIVITY	-0.27603363	-0.050278	-1.72738147	0.12087489	0.28246252	-5.15592454
hetero after UVB irradiation VS hetero before UVB irradiation	GO_LYS63_SPECIFIC_DEUBIQUITINASE_ACTIVITY1	-0.1013267	-0.05329395	-0.47575757	0.64720054	0.85521824	-6.11053672
WT after UVB irradiation VS WT before UVB irradiation	GO_LYS63_SPECIFIC_DEUBIQUITINASE_ACTIVITY2	-0.27665995	-0.00185647	-1.55744448	0.15752613	0.66180255	-4.88615951
homo after UVB irradiation VS homo before UVB irradiation	GO_LYSINE_ACETYLATED_HISTONE_BINDING	-0.54613358	-0.03961835	-2.75641779	0.02388649	0.10852615	-3.61138676
hetero after UVB irradiation VS hetero before UVB irradiation	GO_LYSINE_ACETYLATED_HISTONE_BINDING1	0.032550217	-0.04220857	0.135499427	0.89562592	0.96152498	-6.22153934
WT after UVB irradiation VS WT before UVB irradiation	GO_LYSINE_ACETYLATED_HISTONE_BINDING2	-0.51675982	-0.04430336	-2.66150871	0.02843481	0.44441257	-3.37249968
homo after UVB irradiation VS homo before UVB irradiation	GO_LYSINE_METABOLIC_PROCESS	0.088011833	-0.00505865	0.376688643	0.71582051	0.83110648	-6.47365114
hetero after UVB irradiation VS hetero before UVB irradiation	GO_LYSINE_METABOLIC_PROCESS1	-0.02377197	0.013761367	-0.15079527	0.88393875	0.95780586	-6.21917094
WT after UVB irradiation VS WT before UVB irradiation	GO_LYSINE_METABOLIC_PROCESS2	0.006844534	-0.00860872	0.035618335	0.97244908	0.99307736	-5.99539731
homo after UVB irradiation VS homo before UVB irradiation	GO_LYSINE_N_ACETYLTRANSFERASE_ACTIVITY_ACTING_ON_ACETYL_PHOSPHATE_AS_DONOR	-0.26251518	-0.01543914	-0.99575141	0.34741497	0.53937957	-6.03731675
hetero after UVB irradiation VS hetero before UVB irradiation	GO_LYSINE_N_ACETYLTRANSFERASE_ACTIVITY_ACTING_ON_ACETYL_PHOSPHATE_AS_DONOR1	-0.26056398	0.016192712	-1.03969596	0.32943164	0.6493579	-5.68365377
WT after UVB irradiation VS WT before UVB irradiation	GO_LYSINE_N_ACETYLTRANSFERASE_ACTIVITY_ACTING_ON_ACETYL_PHOSPHATE_AS_DONOR2	-0.23398652	-0.0796184	-1.50015311	0.17151484	0.67808426	-4.95664841
homo after UVB irradiation VS homo before UVB irradiation	GO_LYSINE_N_METHYLTRANSFERASE_ACTIVITY	-0.44542738	-0.04500263	-2.90931138	0.0187867	0.09420375	-3.37386381
hetero after UVB irradiation VS hetero before UVB irradiation	GO_LYSINE_N_METHYLTRANSFERASE_ACTIVITY1	-0.3865591	-0.05188883	-2.40708354	0.04325893	0.2785349	-3.90738141
WT after UVB irradiation VS WT before UVB irradiation	GO_LYSINE_N_METHYLTRANSFERASE_ACTIVITY2	-0.26852907	-0.04328817	-1.26582859	0.2407769	0.73118354	-5.22908657
homo after UVB irradiation VS homo before UVB irradiation	GO_LYSOPHOSPHATIDIC_ACID_BINDING	0.683199292	-0.07298618	3.155514161	0.01281345	0.07652714	-2.99288951
hetero after UVB irradiation VS hetero before UVB irradiation	GO_LYSOPHOSPHATIDIC_ACID_BINDING1	0.556444663	0.073019414	2.727667043	0.02639134	0.23118466	-3.43736918
WT after UVB irradiation VS WT before UVB irradiation	GO_LYSOPHOSPHATIDIC_ACID_BINDING2	0.681332356	0.060187155	2.390654507	0.0434504	0.49624823	-3.7576696
homo after UVB irradiation VS homo before UVB irradiation	GO_LYSOPHOSPHATIDIC_ACID_RECEPTOR_ACTIVITY	0.233451754	0.072236293	1.137069258	0.2871605	0.48226101	-5.89223282
hetero after UVB irradiation VS hetero before UVB irradiation	GO_LYSOPHOSPHATIDIC_ACID_RECEPTOR_ACTIVITY1	0.101559369	-0.00793852	0.478513093	0.64532375	0.85438728	-6.10915241
WT after UVB irradiation VS WT before UVB irradiation	GO_LYSOPHOSPHATIDIC_ACID_RECEPTOR_ACTIVITY2	-0.12042513	0.04929767	-0.51727238	0.61879533	0.90054858	-5.85730359
homo after UVB irradiation VS homo before UVB irradiation	GO_LYSOPHOSPHOLIPASE_ACTIVITY	-0.37923035	0.002525377	-2.03340232	0.07505642	0.21037182	-4.71679446
hetero after UVB irradiation VS hetero before UVB irradiation	GO_LYSOPHOSPHOLIPASE_ACTIVITY1	-0.28949486	-0.03622954	-1.62978441	0.14250341	0.45781209	-4.99819021
WT after UVB irradiation VS WT before UVB irradiation	GO_LYSOPHOSPHOLIPASE_ACTIVITY2	-0.40320298	-0.03139193	-2.31106184	0.04922992	0.50780915	-3.87035658
homo after UVB irradiation VS homo before UVB irradiation	GO_LYSOPHOSPHOLIPID_ACYLTRANSFERASE_ACTIVITY	0.123832721	-0.00743915	0.727106118	0.48709166	0.66365171	-6.26971717
hetero after UVB irradiation VS hetero before UVB irradiation	GO_LYSOPHOSPHOLIPID_ACYLTRANSFERASE_ACTIVITY1	0.049871977	-0.01872923	0.308653025	0.76561486	0.90794023	-6.18014285
WT after UVB irradiation VS WT before UVB irradiation	GO_LYSOPHOSPHOLIPID_ACYLTRANSFERASE_ACTIVITY2	0.347356431	0.01944323	2.12210207	0.06619488	0.537335	-4.13558298
homo after UVB irradiation VS homo before UVB irradiation	GO_LYSOSOMAL_LUMEN	0.531509592	-0.02702834	3.531013091	0.00723807	0.05618735	-2.42013329
homo after UVB irradiation VS homo before UVB irradiation	GO_LYSOSOMAL_LUMEN_ACIDIFICATION	0.188460761	-0.06440089	0.763000935	0.46652053	0.64563851	-6.24223398
hetero after UVB irradiation VS hetero before UVB irradiation	GO_LYSOSOMAL_LUMEN_ACIDIFICATION1	0.231949961	0.012184584	1.630812016	0.14228485	0.45755083	-4.99684984
WT after UVB irradiation VS WT before UVB irradiation	GO_LYSOSOMAL_LUMEN_ACIDIFICATION2	0.613721421	-0.04918671	2.80426342	0.02277233	0.41742287	-3.16970706
hetero after UVB irradiation VS hetero before UVB irradiation	GO_LYSOSOMAL_LUMEN1	0.367877957	0.008144976	1.806757181	0.10912829	0.40782387	-4.76197635
WT after UVB irradiation VS WT before UVB irradiation	GO_LYSOSOMAL_LUMEN2	0.48871617	0.026599619	2.416137679	0.04174769	0.495137	-3.72151148
homo after UVB irradiation VS homo before UVB irradiation	GO_LYSOSOMAL_MICROAUTOPHAGY	-0.17000879	-0.05565038	-0.57658918	0.57946148	0.73526338	-6.37201655
hetero after UVB irradiation VS hetero before UVB irradiation	GO_LYSOSOMAL_MICROAUTOPHAGY1	-0.08060051	0.005962576	-0.30679858	0.76697418	0.90866119	-6.18075414
WT after UVB irradiation VS WT before UVB irradiation	GO_LYSOSOMAL_MICROAUTOPHAGY2	-0.22037039	0.031302039	-0.66487609	0.52460907	0.86518284	-5.76932956
homo after UVB irradiation VS homo before UVB irradiation	GO_LYSOSOMAL_PROTEIN_CATABOLIC_PROCESS	0.343939626	-0.04868355	2.317345255	0.04789325	0.1603978	-4.28964805
hetero after UVB irradiation VS hetero before UVB irradiation	GO_LYSOSOMAL_PROTEIN_CATABOLIC_PROCESS1	0.261472951	-0.04158702	1.364978959	0.21008191	0.53574509	-5.32872927
WT after UVB irradiation VS WT before UVB irradiation	GO_LYSOSOMAL_PROTEIN_CATABOLIC_PROCESS2	0.480452255	-0.07859567	3.006311441	0.01667457	0.37941205	-2.88450392
homo after UVB irradiation VS homo before UVB irradiation	GO_LYSOSOMAL_TRANSPORT	0.19255958	-0.02178193	1.262905542	0.24082846	0.43382637	-5.75145095
hetero after UVB irradiation VS hetero before UVB irradiation	GO_LYSOSOMAL_TRANSPORT1	0.224677311	0.026511774	1.500670948	0.17253195	0.49537636	-5.16325836
WT after UVB irradiation VS WT before UVB irradiation	GO_LYSOSOMAL_TRANSPORT2	0.207884397	-0.0083258	1.284048471	0.23464312	0.72622244	-5.2089167
homo after UVB irradiation VS homo before UVB irradiation	GO_LYSOSOME_LOCALIZATION	0.540478257	-0.03330062	3.773793915	0.0050506	0.04637942	-2.05766652
hetero after UVB irradiation VS hetero before UVB irradiation	GO_LYSOSOME_LOCALIZATION1	0.461280291	0.021762337	3.087817724	0.01528145	0.1825855	-2.91221685
WT after UVB irradiation VS WT before UVB irradiation	GO_LYSOSOME_LOCALIZATION2	0.523584784	-0.00100832	3.391093597	0.00931601	0.32213413	-2.3518487
homo after UVB irradiation VS homo before UVB irradiation	GO_LYSOZYME_ACTIVITY	-0.19979886	0.040044159	-0.74216717	0.47838994	0.65603572	-6.25832627
hetero after UVB irradiation VS hetero before UVB irradiation	GO_LYSOZYME_ACTIVITY1	0.146468519	0.080620307	0.59822111	0.56654195	0.81219207	-6.04186393
WT after UVB irradiation VS WT before UVB irradiation	GO_LYSOZYME_ACTIVITY2	-0.03063779	0.122471757	-0.15445458	0.88103161	0.9693507	-5.98349855
homo after UVB irradiation VS homo before UVB irradiation	GO_LYTIC_VACUOLE_ORGANIZATION	0.323051242	-0.04375681	2.505280147	0.03553894	0.1349601	-4.00083306
hetero after UVB irradiation VS hetero before UVB irradiation	GO_LYTIC_VACUOLE_ORGANIZATION1	0.341561698	0.020081712	2.504998221	0.03718048	0.26452997	-3.76405118
WT after UVB irradiation VS WT before UVB irradiation	GO_LYTIC_VACUOLE_ORGANIZATION2	0.459052526	-0.02380893	3.086883042	0.01474122	0.36438213	-2.77166001
homo after UVB irradiation VS homo before UVB irradiation	GO_M_BAND	0.176249454	0.01399551	0.645113431	0.5362147	0.70237177	-6.32808638
hetero after UVB irradiation VS hetero before UVB irradiation	GO_M_BAND1	-0.47896847	-0.02128277	-1.69549326	0.12913422	0.4368396	-4.9117085
WT after UVB irradiation VS WT before UVB irradiation	GO_M_BAND2	-0.29657929	-0.03890067	-1.14670843	0.28425359	0.75306903	-5.35619936
homo after UVB irradiation VS homo before UVB irradiation	GO_M7G_5_PPPN_DIPHOSPHATASE_ACTIVITY	0.241926234	-0.04117259	1.199933561	0.26317984	0.45842125	-5.82319123
hetero after UVB irradiation VS hetero before UVB irradiation	GO_M7G_5_PPPN_DIPHOSPHATASE_ACTIVITY1	0.432059459	0.084944916	1.744658006	0.1199037	0.42488104	-4.84602741
WT after UVB irradiation VS WT before UVB irradiation	GO_M7G_5_PPPN_DIPHOSPHATASE_ACTIVITY2	0.515592841	-0.03882301	2.287021451	0.05112212	0.51425294	-3.90430254
homo after UVB irradiation VS homo before UVB irradiation	GO_MACROAUTOPHAGY	0.195497371	-0.0246215	1.245270741	0.24692272	0.44092917	-5.77179346
hetero after UVB irradiation VS hetero before UVB irradiation	GO_MACROAUTOPHAGY1	0.161872626	-0.01487457	1.235337421	0.25238487	0.58027285	-5.47789289
WT after UVB irradiation VS WT before UVB irradiation	GO_MACROAUTOPHAGY2	0.141562371	-0.01542852	0.88249546	0.40294508	0.81613006	-5.60483528
homo after UVB irradiation VS homo before UVB irradiation	GO_MACROLIDE_BINDING	0.121709908	0.030895752	0.709815894	0.49720574	0.67191072	-6.28254077
hetero after UVB irradiation VS hetero before UVB irradiation	GO_MACROLIDE_BINDING1	-0.15488157	0.013773425	-0.88826437	0.40078756	0.70818121	-5.82451474
WT after UVB irradiation VS WT before UVB irradiation	GO_MACROLIDE_BINDING2	0.330576552	0.029047121	1.653909948	0.13628774	0.64462785	-4.76454577
homo after UVB irradiation VS homo before UVB irradiation	GO_MACROMOLECULE_CATABOLIC_PROCESS	-0.15143395	-0.00768527	-1.10574226	0.29974991	0.49434615	-5.9256336
hetero after UVB irradiation VS hetero before UVB irradiation	GO_MACROMOLECULE_CATABOLIC_PROCESS1	0.053728931	-0.00985877	0.390757553	0.70636358	0.88361199	-6.14950314
WT after UVB irradiation VS WT before UVB irradiation	GO_MACROMOLECULE_CATABOLIC_PROCESS2	-0.05544651	-0.01055666	-0.32827278	0.75103314	0.93734883	-5.93960323
homo after UVB irradiation VS homo before UVB irradiation	GO_MACROMOLECULE_DEACYLATION	-0.06434081	-0.00492388	-0.4642286	0.6543766	0.78977963	-6.4341156
hetero after UVB irradiation VS hetero before UVB irradiation	GO_MACROMOLECULE_DEACYLATION1	-0.07239719	-0.02622332	-0.54624181	0.60007071	0.83297985	-6.07278218
WT after UVB irradiation VS WT before UVB irradiation	GO_MACROMOLECULE_DEACYLATION2	-0.01688431	-0.00894801	-0.11393509	0.91206353	0.97857624	-5.98922294
homo after UVB irradiation VS homo before UVB irradiation	GO_MACROMOLECULE_DEPALMITOYLATION	-0.25915507	-0.0260823	-1.41945197	0.19208906	0.37814444	-5.56282711
hetero after UVB irradiation VS hetero before UVB irradiation	GO_MACROMOLECULE_DEPALMITOYLATION1	-0.22248926	-0.04789574	-0.82862749	0.43177818	0.73171313	-5.87509311
WT after UVB irradiation VS WT before UVB irradiation	GO_MACROMOLECULE_DEPALMITOYLATION2	0.050089928	0.041442966	0.26427984	0.79816617	0.95180107	-5.95938114
homo after UVB irradiation VS homo before UVB irradiation	GO_MACROMOLECULE_TRANSMEMBRANE_TRANSPORTER_ACTIVITY	-0.22254088	0.021234987	-0.87377233	0.40672768	0.59367758	-6.15031089
hetero after UVB irradiation VS hetero before UVB irradiation	GO_MACROMOLECULE_TRANSMEMBRANE_TRANSPORTER_ACTIVITY1	0.172490916	-0.02664318	0.785005531	0.45547866	0.74613056	-5.91023168
WT after UVB irradiation VS WT before UVB irradiation	GO_MACROMOLECULE_TRANSMEMBRANE_TRANSPORTER_ACTIVITY2	0.019480986	-0.08047451	0.084255791	0.93489887	0.98322796	-5.9923226
homo after UVB irradiation VS homo before UVB irradiation	GO_MACROPHAGE_ACTIVATION	0.575782438	-0.01891626	4.228362717	0.00263222	0.033899	-1.39991422
homo after UVB irradiation VS homo before UVB irradiation	GO_MACROPHAGE_ACTIVATION_INVOLVED_IN_IMMUNE_RESPONSE	0.379322471	-0.0296395	2.0442809	0.07378184	0.20858211	-4.70071457
hetero after UVB irradiation VS hetero before UVB irradiation	GO_MACROPHAGE_ACTIVATION_INVOLVED_IN_IMMUNE_RESPONSE1	0.493240468	0.054913398	2.52126633	0.03625791	0.26141901	-3.74020177
WT after UVB irradiation VS WT before UVB irradiation	GO_MACROPHAGE_ACTIVATION_INVOLVED_IN_IMMUNE_RESPONSE2	0.03285731	-0.04616994	0.117399544	0.90940318	0.97832631	-5.9888008
hetero after UVB irradiation VS hetero before UVB irradiation	GO_MACROPHAGE_ACTIVATION1	0.489565342	0.024220005	3.123294138	0.01449076	0.17916057	-2.86097317
WT after UVB irradiation VS WT before UVB irradiation	GO_MACROPHAGE_ACTIVATION2	0.293882172	-0.0154301	1.216851385	0.25793445	0.73923843	-5.28237071
homo after UVB irradiation VS homo before UVB irradiation	GO_MACROPHAGE_APOPTOTIC_PROCESS	0.694885137	-0.08562881	3.903557178	0.00418068	0.0420834	-1.866971
hetero after UVB irradiation VS hetero before UVB irradiation	GO_MACROPHAGE_APOPTOTIC_PROCESS1	0.485849262	0.111916087	2.33334822	0.0484908	0.29337035	-4.01496063
WT after UVB irradiation VS WT before UVB irradiation	GO_MACROPHAGE_APOPTOTIC_PROCESS2	0.46225724	-0.01826354	2.23110248	0.05580779	0.5232848	-3.98306172
homo after UVB irradiation VS homo before UVB irradiation	GO_MACROPHAGE_CHEMOTAXIS	0.511964313	-0.04172436	3.484513114	0.00776137	0.05855549	-2.49033173
hetero after UVB irradiation VS hetero before UVB irradiation	GO_MACROPHAGE_CHEMOTAXIS1	0.50409438	0.016527546	3.44891899	0.00896047	0.15043564	-2.39670511
WT after UVB irradiation VS WT before UVB irradiation	GO_MACROPHAGE_CHEMOTAXIS2	0.549386524	0.01813349	3.188668561	0.01262813	0.35109482	-2.63001424
homo after UVB irradiation VS homo before UVB irradiation	GO_MACROPHAGE_COLONY_STIMULATING_FACTOR_PRODUCTION	-0.08011451	-0.06477159	-0.3095025	0.76454083	0.86390334	-6.4985902
hetero after UVB irradiation VS hetero before UVB irradiation	GO_MACROPHAGE_COLONY_STIMULATING_FACTOR_PRODUCTION1	0.240573567	0.01677442	0.663955828	0.52572882	0.78916946	-5.99912875
WT after UVB irradiation VS WT before UVB irradiation	GO_MACROPHAGE_COLONY_STIMULATING_FACTOR_PRODUCTION2	0.058257261	-0.04475891	0.181382232	0.86052551	0.9676681	-5.97874452
homo after UVB irradiation VS homo before UVB irradiation	GO_MACROPHAGE_COLONY_STIMULATING_FACTOR_SIGNALING_PATHWAY	0.678602886	-0.05969487	3.60210201	0.00650893	0.05354108	-2.31327599
hetero after UVB irradiation VS hetero before UVB irradiation	GO_MACROPHAGE_COLONY_STIMULATING_FACTOR_SIGNALING_PATHWAY1	0.721925872	0.014139515	3.308458811	0.01100741	0.16390046	-2.59551263
WT after UVB irradiation VS WT before UVB irradiation	GO_MACROPHAGE_COLONY_STIMULATING_FACTOR_SIGNALING_PATHWAY2	0.723489934	-0.02857639	3.116881562	0.01408249	0.36090271	-2.72980266
homo after UVB irradiation VS homo before UVB irradiation	GO_MACROPHAGE_CYTOKINE_PRODUCTION	0.358037549	-0.00463606	1.597061177	0.14742574	0.32035953	-5.33324743
hetero after UVB irradiation VS hetero before UVB irradiation	GO_MACROPHAGE_CYTOKINE_PRODUCTION1	0.263635803	0.030610598	1.138236836	0.2885546	0.61335518	-5.58313488
WT after UVB irradiation VS WT before UVB irradiation	GO_MACROPHAGE_CYTOKINE_PRODUCTION2	0.076115049	-0.03270505	0.266009231	0.79687975	0.95173422	-5.95890166
homo after UVB irradiation VS homo before UVB irradiation	GO_MACROPHAGE_DIFFERENTIATION	0.55105068	-0.00925118	3.950631182	0.00390588	0.04087878	-1.79835284
hetero after UVB irradiation VS hetero before UVB irradiation	GO_MACROPHAGE_DIFFERENTIATION1	0.415661557	0.035459131	2.23348478	0.05660215	0.3143834	-4.15993243
WT after UVB irradiation VS WT before UVB irradiation	GO_MACROPHAGE_DIFFERENTIATION2	0.370993835	-0.02669301	1.631287446	0.14101919	0.65097221	-4.79337649
homo after UVB irradiation VS homo before UVB irradiation	GO_MACROPHAGE_FUSION	0.978029335	0.036602205	6.308317212	0.00019451	0.01093793	1.205134116
hetero after UVB irradiation VS hetero before UVB irradiation	GO_MACROPHAGE_FUSION1	0.97391077	-0.00269304	5.669455878	0.00050345	0.07819845	0.33577666
WT after UVB irradiation VS WT before UVB irradiation	GO_MACROPHAGE_FUSION2	0.4387033	-0.01440505	1.441199074	0.18705602	0.68957923	-5.0277017
homo after UVB irradiation VS homo before UVB irradiation	GO_MACROPHAGE_INFLAMMATORY_PROTEIN_1_ALPHA_PRODUCTION	0.522868815	-0.11534029	2.247817606	0.05347902	0.17122501	-4.39548408
hetero after UVB irradiation VS hetero before UVB irradiation	GO_MACROPHAGE_INFLAMMATORY_PROTEIN_1_ALPHA_PRODUCTION1	0.936707171	-0.01653572	6.675392851	0.00017038	0.07788488	1.308734691
WT after UVB irradiation VS WT before UVB irradiation	GO_MACROPHAGE_INFLAMMATORY_PROTEIN_1_ALPHA_PRODUCTION2	0.524708239	-0.01822306	1.980346821	0.08257069	0.56864787	-4.3312927
homo after UVB irradiation VS homo before UVB irradiation	GO_MACROPHAGE_MIGRATION	0.597155987	-0.03874735	4.341672007	0.002248	0.03175393	-1.24062585
hetero after UVB irradiation VS hetero before UVB irradiation	GO_MACROPHAGE_MIGRATION1	0.541838922	0.021715025	3.46797488	0.00871553	0.14994695	-2.36992184
WT after UVB irradiation VS WT before UVB irradiation	GO_MACROPHAGE_MIGRATION2	0.577049355	0.025121295	3.215648078	0.01212303	0.34615945	-2.59265609
homo after UVB irradiation VS homo before UVB irradiation	GO_MACROPHAGE_PROLIFERATION	0.395821353	-0.0099376	3.064583048	0.01474877	0.08257891	-3.13325175
hetero after UVB irradiation VS hetero before UVB irradiation	GO_MACROPHAGE_PROLIFERATION1	0.404654438	0.03651989	2.526142078	0.03598596	0.26055388	-3.73305239
WT after UVB irradiation VS WT before UVB irradiation	GO_MACROPHAGE_PROLIFERATION2	0.441331192	0.071258038	2.61568117	0.0305437	0.45178212	-3.43771033
homo after UVB irradiation VS homo before UVB irradiation	GO_MACROPINOCYTOSIS	-0.304737	-0.02499726	-1.54350421	0.15979948	0.33573878	-5.40405075
hetero after UVB irradiation VS hetero before UVB irradiation	GO_MACROPINOCYTOSIS1	-0.02499968	-0.04022141	-0.13836658	0.89343303	0.96101082	-6.22111457
WT after UVB irradiation VS WT before UVB irradiation	GO_MACROPINOCYTOSIS2	-0.12243114	0.046866504	-0.55595694	0.59326508	0.8931505	-5.83619567
homo after UVB irradiation VS homo before UVB irradiation	GO_MAGNESIUM_DEPENDENT_PROTEIN_SERINE_THREONINE_PHOSPHATASE_ACTIVITY	0.068210903	0.018302595	0.345881362	0.73800624	0.846147	-6.48567553
hetero after UVB irradiation VS hetero before UVB irradiation	GO_MAGNESIUM_DEPENDENT_PROTEIN_SERINE_THREONINE_PHOSPHATASE_ACTIVITY1	0.01078747	-0.02635987	0.047190961	0.9635391	0.98664387	-6.23027658
WT after UVB irradiation VS WT before UVB irradiation	GO_MAGNESIUM_DEPENDENT_PROTEIN_SERINE_THREONINE_PHOSPHATASE_ACTIVITY2	0.267840063	-0.0212094	1.420182144	0.19288963	0.69334561	-5.05264785
homo after UVB irradiation VS homo before UVB irradiation	GO_MAGNESIUM_ION_BINDING	-0.07268262	-0.00486209	-0.60196531	0.56322054	0.72270814	-6.35627318
hetero after UVB irradiation VS hetero before UVB irradiation	GO_MAGNESIUM_ION_BINDING1	0.019840048	-0.00268615	0.158175025	0.8783107	0.95570774	-6.2179383
WT after UVB irradiation VS WT before UVB irradiation	GO_MAGNESIUM_ION_BINDING2	0.026166316	-0.00999522	0.177395728	0.86355467	0.96783446	-5.97949604
homo after UVB irradiation VS homo before UVB irradiation	GO_MAGNESIUM_ION_HOMEOSTASIS	-0.50390756	0.038732302	-3.48381274	0.00776955	0.05858725	-2.4913908
hetero after UVB irradiation VS hetero before UVB irradiation	GO_MAGNESIUM_ION_HOMEOSTASIS1	-0.412231	-0.01252336	-2.17535662	0.06193153	0.32672921	-4.24381476
WT after UVB irradiation VS WT before UVB irradiation	GO_MAGNESIUM_ION_HOMEOSTASIS2	-0.93753196	-0.04135348	-6.08099459	0.00028156	0.18842845	0.700445911
homo after UVB irradiation VS homo before UVB irradiation	GO_MAGNESIUM_ION_TRANSMEMBRANE_TRANSPORTER_ACTIVITY	-0.09686713	-0.04341343	-0.45076781	0.66366042	0.79617466	-6.44070616
hetero after UVB irradiation VS hetero before UVB irradiation	GO_MAGNESIUM_ION_TRANSMEMBRANE_TRANSPORTER_ACTIVITY1	0.226240742	0.002292594	1.03152693	0.33301444	0.6526842	-5.69168536
WT after UVB irradiation VS WT before UVB irradiation	GO_MAGNESIUM_ION_TRANSMEMBRANE_TRANSPORTER_ACTIVITY2	-0.34522206	-0.05243455	-1.92574376	0.08987168	0.58325853	-4.40566667
homo after UVB irradiation VS homo before UVB irradiation	GO_MAGNESIUM_ION_TRANSPORT	-0.0978344	-0.0437018	-0.51370255	0.62080682	0.76452026	-6.40832002
hetero after UVB irradiation VS hetero before UVB irradiation	GO_MAGNESIUM_ION_TRANSPORT1	0.221432579	-0.00163349	0.9987312	0.34770175	0.66500541	-5.72344664
WT after UVB irradiation VS WT before UVB irradiation	GO_MAGNESIUM_ION_TRANSPORT2	-0.27911874	-0.06731102	-1.40031616	0.19854967	0.6971205	-5.07603483
homo after UVB irradiation VS homo before UVB irradiation	GO_MAIN_AXON	0.261463134	-0.00431909	1.464346292	0.17979344	0.36234698	-5.50625073
hetero after UVB irradiation VS hetero before UVB irradiation	GO_MAIN_AXON1	0.117762775	0.009303952	0.57409127	0.58197239	0.82095894	-6.05653693
WT after UVB irradiation VS WT before UVB irradiation	GO_MAIN_AXON2	0.042579935	-0.00972622	0.178467659	0.86273992	0.9676681	-5.97929559
homo after UVB irradiation VS homo before UVB irradiation	GO_MAINTENANCE_OF_ANIMAL_ORGAN_IDENTITY	-0.32780583	-0.04200274	-2.21157834	0.05664114	0.17727536	-4.45036954
hetero after UVB irradiation VS hetero before UVB irradiation	GO_MAINTENANCE_OF_ANIMAL_ORGAN_IDENTITY1	-0.49488816	0.018505179	-2.36066108	0.04648245	0.28809638	-3.97515616
WT after UVB irradiation VS WT before UVB irradiation	GO_MAINTENANCE_OF_ANIMAL_ORGAN_IDENTITY2	-0.7803452	0.000412968	-4.54083367	0.00183921	0.20396123	-0.89135791
homo after UVB irradiation VS homo before UVB irradiation	GO_MAINTENANCE_OF_APICAL_BASAL_CELL_POLARITY	-0.29586543	-0.00560699	-2.10125965	0.06744139	0.19836889	-4.61606846
hetero after UVB irradiation VS hetero before UVB irradiation	GO_MAINTENANCE_OF_APICAL_BASAL_CELL_POLARITY1	-0.20184956	0.031604786	-1.05505467	0.32277681	0.64215917	-5.66842561
WT after UVB irradiation VS WT before UVB irradiation	GO_MAINTENANCE_OF_APICAL_BASAL_CELL_POLARITY2	-0.16605892	0.046009755	-0.71803857	0.4929264	0.85306847	-5.73283361
homo after UVB irradiation VS homo before UVB irradiation	GO_MAINTENANCE_OF_BLOOD_BRAIN_BARRIER	0.174646864	0.014441482	1.21268225	0.25852174	0.45393151	-5.80887134
hetero after UVB irradiation VS hetero before UVB irradiation	GO_MAINTENANCE_OF_BLOOD_BRAIN_BARRIER1	0.06236933	-0.02094833	0.433875497	0.67605214	0.86927722	-6.13064576
WT after UVB irradiation VS WT before UVB irradiation	GO_MAINTENANCE_OF_BLOOD_BRAIN_BARRIER2	-0.01233656	-0.0018832	-0.07247917	0.94397887	0.98531933	-5.99329575
homo after UVB irradiation VS homo before UVB irradiation	GO_MAINTENANCE_OF_CELL_NUMBER	-0.23585078	0.016210995	-1.62417018	0.14149904	0.31235623	-5.29692985

hetero after UVB irradiation VS hetero before UVB irradiation	GO_MAINTENANCE_OF_CELL_NUMBER1	-0.11916817	-0.0246418	-0.85814911	0.41623277	0.71987	-5.85041641
WT after UVB irradiation VS WT before UVB irradiation	GO_MAINTENANCE_OF_CELL_NUMBER2	-0.15793362	0.004847549	-0.84063349	0.42467791	0.82612337	-5.63950607
homo after UVB irradiation VS homo before UVB irradiation	GO_MAINTENANCE_OF_CELL_POLARITY	0.063042889	-0.01736245	0.397440411	0.70103387	0.82163107	-6.46499359
hetero after UVB irradiation VS hetero before UVB irradiation	GO_MAINTENANCE_OF_CELL_POLARITY1	0.112467203	0.024781573	0.750225805	0.47499425	0.75784786	-5.93708975
WT after UVB irradiation VS WT before UVB irradiation	GO_MAINTENANCE_OF_CELL_POLARITY2	0.112420392	0.039435434	0.60057174	0.56455176	0.88150126	-5.81011641
homo after UVB irradiation VS homo before UVB irradiation	GO_MAINTENANCE_OF_CENTROSOME_LOCATION	-0.8154799	-0.00029014	-4.49170079	0.00182946	0.02899815	-1.03273343
hetero after UVB irradiation VS hetero before UVB irradiation	GO_MAINTENANCE_OF_CENTROSOME_LOCATION1	-0.47600524	-0.04727074	-1.49938932	0.17285672	0.49554869	-5.16486169
WT after UVB irradiation VS WT before UVB irradiation	GO_MAINTENANCE_OF_CENTROSOME_LOCATION2	0.008203814	0.00243743	0.028320188	0.97809231	0.99433096	-5.99564349
homo after UVB irradiation VS homo before UVB irradiation	GO_MAINTENANCE_OF_DNA_METHYLATION	-0.60297652	-0.02101891	-2.35487325	0.04512324	0.1545691	-4.23226296
hetero after UVB irradiation VS hetero before UVB irradiation	GO_MAINTENANCE_OF_DNA_METHYLATION1	0.093051302	-0.05849882	0.266809509	0.79648839	0.92108015	-6.19305917
WT after UVB irradiation VS WT before UVB irradiation	GO_MAINTENANCE_OF_DNA_METHYLATION2	-0.1252236	0.027195371	-0.40047729	0.69916314	0.92389099	-5.91231829
homo after UVB irradiation VS homo before UVB irradiation	GO_MAINTENANCE_OF_DNA_REPEAT_ELEMENTS	-0.6286034	-0.04637357	-4.32252575	0.00230843	0.03212973	-1.26740492
hetero after UVB irradiation VS hetero before UVB irradiation	GO_MAINTENANCE_OF_DNA_REPEAT_ELEMENTS1	-0.06220752	-0.08706106	-0.27742401	0.78861783	0.91738598	-6.18995676
WT after UVB irradiation VS WT before UVB irradiation	GO_MAINTENANCE_OF_DNA_REPEAT_ELEMENTS2	-0.30392694	0.064432193	-0.98905775	0.35126374	0.78816886	-5.51051841
homo after UVB irradiation VS homo before UVB irradiation	GO_MAINTENANCE_OF_GASTROINTESTINAL_EPITHELIUM	0.453595061	0.073444461	3.500779426	0.00757391	0.05782107	-2.46574897
hetero after UVB irradiation VS hetero before UVB irradiation	GO_MAINTENANCE_OF_GASTROINTESTINAL_EPITHELIUM1	0.189175582	-0.00077867	0.971080641	0.36046473	0.67521718	-5.74961357
WT after UVB irradiation VS WT before UVB irradiation	GO_MAINTENANCE_OF_GASTROINTESTINAL_EPITHELIUM2	0.004352438	0.010809132	0.02126981	0.98354521	0.99634469	-5.99582796
homo after UVB irradiation VS homo before UVB irradiation	GO_MAINTENANCE_OF_LOCATION	0.365556428	-0.02184709	3.047949327	0.01513453	0.08349167	-3.15897815
[truncated: 6,115,293 more chars]
